# Global burden of 292 causes of death in 204 countries and territories and 660 subnational locations, 1990–2023: a systematic analysis for the Global Burden of Disease Study 2023

**DOI:** 10.1016/S0140-6736(25)01917-8

**Published:** 2025-10-18

**Authors:** GBD 2023 Causes of Death Collaborators, Mohsen Naghavi, Hmwe Hmwe Kyu, Bhoomadevi A, Mohammad Amin Aalipour, Hasan Aalruz, Hazim S Ababneh, Bedru J Abafita, Ukachukwu O Abaraogu, Cristiana Abbafati, Madineh Abbasi, Faezeh Abbaspour, Hedayat Abbastabar, Abdallah H A Abd Al Magied, Samar Abd ElHafeez, Ashraf Nabiel Abdalla, Mohammed Altigani Abdalla, Emad M Abdallah, Barkhad Aden Abdeeq, Nadin M I Abdel Razeq, Ahmed Abdelrahman Abdelgalil, Reda Abdel-Hameed, Michael Abdelmasseh, Mahmoud Abdelnabi, Wael M Abdel-Rahman, Arman Abdous, Mostafa M Abdrabou, Jeza Muhamad Abdul Aziz, Deldar Morad Abdulah, Auwal Abdullahi, Toufik Abdul-Rahman, Habtamu Abebe Getahun, Aidin Abedi, Armita Abedi, Parisa Abedi, Asrat Agalu Abejew, Roberto Ariel Abeldaño Zuñiga, Shehab Uddin Al Abid, Syed Hani Abidi, Alemwork Abie, Olugbenga Olusola Abiodun, Richard Gyan Aboagye, Shady Abohashem, Hassan Abolhassani, Ulric Sena Abonie, Nagah M Abourashed, Mohamed Abouzid, Dmitry Abramov, Lucas Guimarães Abreu, Dariush Abtahi, Rana Kamal Abu Farha, Fuad Hamdi A Abuadas, Aminu Kende Abubakar, Nermeen Abu-Elala, Eman Abu-Gharbieh, Sawsan Abuhammad, Ahmad Y Abuhelwa, Hana J Abukhadijah, Niveen ME Abu-Rmeileh, Salahdein Aburuz, Dina Abushanab, Manfred Mario Kokou Accrombessi, Anirudh Balakrishna Acharya, Apurba Acharya, Ousman Adal, Lisa C Adams, Abdu A Adamu, Isaac Yeboah Addo, Oluwafemi Atanda Adeagbo, Tajudeen Adesanmi Adebisi, Isaac Akinkunmi Adedeji, Kamoru Ademola Adedokun, Oluwatobi E Adegbile, Nurudeen A Adegoke, Olumide Thomas Adeleke, Bulcha Guye Adema, Bashir Aden, Isaac Ayodeji Adesina, Miracle Ayomikun Adesina, Juliana Bunmi Adetunji, Habeeb Omoponle Adewuyi, Temitayo Esther Adeyeoluwa, Mache Tsadik Adhana, Ripon Kumar Adhikary, Usha Adiga, Tanin Adl Parvar, Mohd Adnan, Qorinah Estiningtyas Sakilah Adnani, Prince Owusu Adoma, Leticia Akua Adzigbli, David Adzrago, Giuseppina Affinito, Ahmed M Afifi, Clifford Afoakwah, Aanuoluwapo Adeyimika Afolabi, Rotimi Felix Afolabi, Vlad-Adrian Afrăsânie, Saira Afzal, Gizachew Beykaso Agafari, Suneth Buddhika Agampodi, Thilini Chanchala Agampodi, Navidha Aggarwal, Mahdi Aghaalikhani, Sepehr Aghajanian, Seyed Mohammad Kazem Aghamir, Feleke Doyore Agide, César Agostinis Sobrinho, Anurag Agrawal, Williams Agyemang-Duah, Mahsa Ahadi, Bright Opoku Ahinkorah, Aqeel Ahmad, Danish Ahmad, Faisal Ahmad, Ijaz Ahmad, Khabir Ahmad, Khurshid Ahmad, Sajjad Ahmad, Tauseef Ahmad, Waqas Ahmad, Negar Sadat Ahmadi, Ali Ahmed, Ayman Ahmed, Gasha Salih Ahmed, Haroon Ahmed, Junaid Ahmed, Luai A Ahmed, MD Faisal Ahmed, Mehrunnisha Sharif Ahmed, Meqdad Saleh Ahmed, Muktar Beshir Ahmed, Mushood Ahmed, Shabbir Ahmed, Sindew Mahmud Ahmed, Syed Anees Ahmed, Gulzhanat Aimagambetova, Marjan Ajami, Budi Aji, Hossein Akbarialiabad, Saeid Akbarifard, Oluwasefunmi Akeju, Roland Eghoghosoa Akhigbe, Ruslan Akhmedullin, Olufemi Ambrose Akinkuotu, Mohammed Ahmed Akkaif, Wole Akosile, Ashley E Akrami, Ralph Kwame Akyea, Alaa Al Amiry, Salah Al Awaidy, Syed Mahfuz Al Hasan, Ammar Al Homsi, Mohammad Khaled Al Nawayseh, Omar Al Omari, Zain Al Ta'ani, Yazan Al Thaher, Omar Ali Mohammed Al Zaabi, Mohammad Ahmmad Mahmoud Al Zoubi, Mousa Ali Al-Abbadi, Tariq A Alalwan, Ziyad Al-Aly, Khurshid Alam, Manjurul Alam, Mohammad Khursheed Alam, Mostafa Alam, Rasmieh Mustafa Al-Amer, Abebaw Alamrew, Amani Alansari, Turki M Alanzi, Fahmi Y Al-Ashwal, Mohammed Albashtawy, Wafa A Aldhaleei, Mohammed S Aldossary, Robert W Aldridge, Shereen M Aleidi, Bezawit Abeje Alemayehu, Fentahun Alemnew, Melaku Birhanu Alemu, Kefyalew Addis Alene, Ayman Al-Eyadhy, Ali M Alfalki, Fahad D Algahtani, Abdelazeem M Algammal, Khairat Al-Habbal, Nma Bida Alhaji, Samar Al-Hajj, Fadwa Naji Alhalaiqa, Mohammed Khaled Al-Hanawi, Khalid A Alhasan, Ashraf Alhumaidi, Fahad A Alhumaydhi, Amjad Ali, Haroon Muhammad Ali, Irfan Ali, Liaqat Ali, Maratab Ali, Mohammad Daud Ali, Mohammed Usman Ali, Rafat Ali, Shahid Ali, Syed Shujait Ali, Waad Ali, Gianfranco Alicandro, Montaha Al-Iede, Sheikh Mohammad Alif, Hamid Alinejad Rokny, Morteza Alipour, Samah W Al-Jabi, Mohamad Aljofan, Moath Saleh Aljohani, Syed Mohamed Aljunid, Ahmad Alkhatib, Mustafa Alkhawam, Peter Allebeck, Khaled S Allemailem, Mohammed Z Allouh, Wesam Taher Almagharbeh, Sabah Al-Marwani, Nihad A Almasri, Joseph Uy Almazan, Hesham M Al-Mekhlafi, Omar Almidani, Amr Almobayed, Khaldoon Aied Alnawafleh, Hasan Yaser Alniss, Margret Beaula Alocious Sukumar, Mahmoud A Alomari, Mohammad R Alosta, Jaber S Alqahtani, Saleh A Alqahtani, Mohammad R Alqudimat, Ahmad Rajeh Al-Qudimat, Intima Alrimawi, Sahel Majed Alrousan, Salman Khalifah Al-Sabah, Mohammed A Alsabri, Zaid Altaany, Awais Altaf, Alaa B Al-Tammemi, Jaffar A Al-Tawfiq, Malik A Althobiani, Khalid A Altirkawi, Javier Alvarez-Galvez, Nelson Alvis-Guzman, Nelson J Alvis-Zakzuk, Hassan Alwafi, Mohammad Al-Wardat, Yaser Mohammed Al-Worafi, Hany Aly, Mohammad Sharif Ibrahim Alyahya, Hosam Alzahrani, Karem H Alzoubi, Adel Sharaf Al-Zubairi, Ekiyor Joseph Amafah, Joy Amafah, Reza Amani-Beni, Faten Amer, Bardia Amidi, Amr Amin, Tarek Tawfik Amin, Alireza Amindarolzarbi, Saeed Amini, Ehsan Amini-Salehi, Majid Aminzare, Sohrab Amiri, Joanne O Amlag, Dickson A Amugsi, Ganiyu Adeniyi Amusa, Filippos Anagnostakis, Roshan A Ananda, Nazanin Anaraki, Robert Ancuceanu, Deanna Anderlini, David B Anderson, Nguyen Hoang Anh, Abdul-Azeez Adeyemi Anjorin, Samuel Egyakwa Ankomah, Kabilan Annadurai, Sumbul Ansari, Alireza Ansari-Moghaddam, Catherine M Antony, Ernoiz Antriyandarti, Boluwatife Stephen Anuoluwa, Iyadunni Adesola Anuoluwa, Saeid Anvari, Saleha Anwar, Sumadi Lukman Anwar, Razique Anwer, Shahnawaz Anwer, Anayochukwu Edward Anyasodor, Francis Appiah, Juan Pablo Arab, Hossein Arabi, Jalal Arabloo, Mosab Arafat, Daniel T Araki, Aleksandr Y Aravkin, Demelash Areda, Getnet Mesfin Aregu, Jorge Arias de la Torre, Ghazal Arjmand, Benedetta Armocida, Johan Ärnlöv, Jesu Arockiaraj, Mahwish Arooj, Anton A Artamonov, Ashokan Arumugam, Deepavalli Arumuganainar, Umesh Raj Aryal, Nurila Aryntayeva, Mahsa Asadi Anar, Muhammad Asaduzzaman, Syed Mohammed Basheeruddin Asdaq, Mulusew Andualem A Asemahagn, Mulu Tiruneh Asemu, Saeed Asgary, Mohammad Asghari-Jafarabadi, Syed Amir Ashraf, Tahira Ashraf, Mitra Ashrafi, Milad Ashrafizadeh, Bernard Kwadwo Yeboah Asiamah-Asare, Saeed Aslani, Yuni Asri, Batyrbek Assembekov, Seyyed Shamsadin Athari, Alok Atreya, Julie Alaere Atta, Zeenah A Atwan, Khursheed Aurangzeb, Marcel Ausloos, Abolfazl Avan, Núbia Carelli Pereira Avelar, Sana Javaid Awan, Adedapo Wasiu Awotidebe, Lemessa Assefa A Ayana, Haleh Ayatollahi, Yusuf Oloruntoyin Ayipo, Seyed Mohammad Ayyoubzadeh, Sina Azadnajafabad, Arian Azadnia, James Mba Azam, Alireza Azarboo, Zelalem Nigussie Azene, Gulrez Shah Azhar, Amirali Azimi, Farya Azimi, Mohd Yusmaidie Aziz, Sadat Abdulla Aziz, Amin Azizan, Ahmed Y Azzam, Giridhara Rathnaiah Babu, Youngoh Bae, Arvind Bagga, Nasser Bagheri, Sara Bagheri, Elahe Baghizadeh, Fereshteh Baghizadeh, Sana Baghizadeh, Khlood K Baghlaf, Najmeh Bahmanziari, Ruhai Bai, Mohamed Ibrahem Baklola, Abdulaziz T Bako, Wondu Feyisa Balcha, Maher Balkis, Jose Balmori-de-la-Miyar, Mohammadreza Balooch Hasankhani, Ovidiu Constantin Baltatu, Soham Bandyopadhyay, Palash Chandra Banik, Noel C Barengo, Suzanne Lyn Barker-Collo, Hiba Jawdat Barqawi, Amadou Barrow, Sandra Barteit, Lingkan Barua, MD Abu Bashar, Shahid Bashir, Guido Basile, Rehana Basri, Quique Bassat, Mohammad-Mahdi Bastan, Abdul-Monim Batiha, Kavita Batra, Matteo Bauckneht, Mahdis Bayat, Mohammad Amin Bayat Tork, Thomas Beaney, Neeraj Bedi, Narasimha M Beeraka, Massimiliano Beghi, Jina Behjati, Bezawit K Bekele, Almaz Nibret Belay, Demeke Mesfin Belay, Asnake Gashaw Belayneh, Melesse Belayneh, Abel Cherkos Belete, Gokce Belge Bilgin, Muhammad Bashir Bello, Olorunjuwon Omolaja Bello, Umar Muhammad Bello, Luis Belo, Apostolos Beloukas, Riyad Bendardaf, Isabela M Bensenor, Samiun Nazrin Bente Kamal Tune, Maria Bergami, Alemshet Yirga Berhie, Abiye Assefa Berihun, Amiel Nazer C Bermudez, Robert S Bernstein, Gregory J Bertolacci, Paola Bertuccio, Paulo J G Bettencourt, Ajeet Singh Bhadoria, Akshaya Srikanth Bhagavathula, Neeraj Bhala, Buna Bhandari, Kayleigh Bhangdia, Charmi Bhanushali, Nikha Bhardwaj, Pankaj Bhardwaj, Ashish Bhargava, Sonu Bhaskar, Anup Bhat, Priyadarshini Bhattacharjee, Shuvarthi Bhattacharjee, Gurjit Kaur Bhatti, Jasvinder Singh Bhatti, Mohiuddin Ahmed Bhuiyan, Zulfiqar A Bhutta, Soumitra S Bhuyan, Haoran Bi, Sibhatu Kassa Biadgilign, Raluca Bievel-Radulescu, Naif Kandash Binsaleh, Catherine Bisignano, Atanu Biswas, Bijit Biswas, Mohammad Shahangir Biswas, Ahmad Naoras Bitar, Molalegne Bitew, Bruno Bizzozero-Peroni, Tone Bjørge, Virginia Bodolica, Eyob Ketema Bogale, Lucimere Bohn, Obasanjo Afolabi Bolarinwa, Paria Bolourinejad, Aime Bonny, Sri Harsha Boppana, Hamed Borhany, Mina Borran, Sudipta Bose, Samuel Adolf Bosoka, Alejandro Botero Carvajal, Soufiane Boufous, Christopher Boxe, Dejana Braithwaite, Luisa C Brant, Michael Brauer, Nicholas J K Breitborde, Susanne Breitner, Hermann Brenner, Edmond D Brewer, Maria L Bringas Vega, Julie Brown, Annie J Browne, Traolach Brugha, Raffaele Bugiardini, Norma B Bulamu, Tsion Samuel Bunare, Danilo Buonsenso, Richard A Burns, Akeem Olayinka Busari, Felix Busch, Yasser Bustanji, Nadeem Shafique Butt, Zahid A Butt, Sanjay C J, Tianji Cai, Rose Cairns, Mehtap Çakmak Barsbay, Daniela Calina, Luis Alberto Cámera, Luciana Aparecida Campos, Ismael Campos-Nonato, Fan Cao, Si Cao, Angelo Capodici, Rosario Cárdenas, Giulia Carreras, Juan Jesus Carrero, Andrea Carugno, Andre F Carvalho, Felix Carvalho, Márcia Carvalho, Ana Paula Carvalho-e-Silva, Joao Mauricio Castaldelli-Maia, Carlos A Castañeda-Orjuela, Giulio Castelpietra, Ferrán Catalá-López, Alberico L Catapano, Maria Sofia Cattaruzza, Luca Cegolon, Francieli Cembranel, Muthia Cenderadewi, Kelly M Cercy, Ester Cerin, Pamela Roxana Chacón-Uscamaita, Chiranjib Chakraborty, Sandip Chakraborty, Joht Singh Chandan, Rama Mohan Chandika, Miyuru Chandradasa, Baskaran Chandrasekaran, Vijay Kumar Chattu, Victoria Chatzimavridou-Grigoriadou, Anis Ahmad Chaudhary, Sirshendu Chaudhuri, Akhilanand Chaurasia, An-Tian Chen, Catherine S Chen, Guangjin Chen, Haiyan Chen, Hana Chen, Haowei Chen, Hui Chen, Rucheng Chen, Shanquan Chen, Simiao Chen, Xiang Chen, Haojin Cheng, Ka Ching Cheung, Nicholas WS Chew, Gerald Chi, Fatemeh Chichagi, Izumi Chihara, Odgerel Chimed-Ochir, Patrick R Ching, Jesus Lorenzo Chirinos-Caceres, Daniel Youngwhan Cho, William C S Cho, Bryan Chong, Yuen Yu Chong, Hou In Chou, Enayet Karim Chowdhury, Sreshtha Chowdhury, Hanne Christensen, Ting-Wu Chuang, Isaac Sunday Chukwu, Erin Chung, Sheng-Chia Chung, Sunghyun Chung, Muhammad Chutiyami, Arrigo Francesco Giuseppe Cicero, Cain C T Clark, Fred Cohen, Alyssa Columbus, Joao Conde, Stephen E Congly, Nathalie Conrad, Leslie Trumbull Cooper, Alexandru Corlateanu, Samuele Cortese, Paolo Angelo Cortesi, Claudia Cosma, Ewerton Cousin, Emma Johnson Cowart, Michael H Criqui, Andrew Crist, Jessica A Cruz, Natalia Cruz-Martins, Xiaolin Cui, Garland T Culbreth, Patricia Cullen, Matthew Cunningham, Nour Dababo, Ali Dabbagh, Omid Dadras, Tukur Dahiru, Xiaochen Dai, Zhaoli Dai, Mayank Dalakoti, Koustuv Dalal, Gloria Dalla Costa, Emanuele D'Amico, Roy Arokiam Daniel, Lucio D'Anna, Pojsakorn Danpanichkul, Samuel E Danso, Samuel Demissie Darcho, Latefa Ali Dardas, Chengetai Dare, Bahar Darouei, Reza Darvishi Cheshmeh Soltani, Sayan Kumar Das, Claudio Alberto Dávila-Cervantes, Nicole Davis Weaver, Dimash Davletov, Kairat Davletov, Fernando Pio De la Hoz, Alejandro de la Torre-Luque, Edward Christopher Dee, Sindhura Deekonda, Amanda Deen, Louisa Degenhardt, Paria Dehesh, Pouria Delbari, Laura Delgado-Ortiz, Mohammad Delsoz, Andreas K Demetriades, Edgar Denova-Gutiérrez, Tadios Niguss Derese, Ismail Dergaa, Kebede Deribe, Hunegnaw Almaw Derseh, Nikolaos Dervenis, Emina Dervišević, Hardik Dineshbhai Desai, Abraham Aregay Desta, Vinoth Gnana Chellaiyan Devanbu, Pradeep Kumar Devarakonda, Syed Masudur Rahman Dewan, Arkadeep Dhali, Kuldeep Dhama, Sreedhar Dharmagadda, Mandira Lamichhane Dhimal, Meghnath Dhimal, Bibha Dhungel, Marcello Di Pumpo, Diana Dias da Silva, Daniel Diaz, Luis Antonio Diaz, Kimia Didehvar, Elangovan Dilipan, Lauren K Dillard, Xueting Ding, Saeid Doaei, Sushil Dohare, Klara Georgieva Dokova, Mario D'Oria, Fariba Dorostkar, E Ray Dorsey, Ojas Prakashbhai Doshi, Leila Doshmangir, Robert Kokou Dowou, Menayit Tamrat Dresse, Tim Robert Driscoll, Ashel Chelsea Dsouza, Jiang Du, John Dube, Judy R Dubno, Emeka W Dumbili, Samuel C Dumith, Bruce B Duncan, Andre Rodrigues Duraes, Oyewole Christopher Durojaiye, Ashit Kumar Dutta, Siddhartha Dutta, Sulagna Dutta, Osamudiamen Ebohon, Ejemai Eboreime, Lamiaa Labieb Mahmoud Ebraheim, Alireza Ebrahimi, Mohammad Hossein Ebrahimi, Abdelaziz Ed-Dra, David Edvardsson, Ferry Efendi, Behrad Eftekhari, Foolad Eghbali, Ashkan Eighaei Sedeh, Terje Andreas Eikemo, Ebrahim Eini, Michael Ekholuenetale, Temitope Cyrus Ekundayo, Rabie Adel El Arab, Abdelfatteh EL Omri, Maysaa El Sayed Zaki, Mohamed Ahmed Eladl, Reza Elahi, Said El-Ashker, Rana Elbeshbeishy, Noha Mousaad Elemam, Ghada Metwally Tawfik ElGohary, Muhammed Elhadi, Mohamed Elhoumed, Waseem El-Huneidi, Omar Abdelsadek Abdou Elmeligy, Mohamed A Elmonem, Rami Elmorsi, Mohamed Hassan Elnaem, Gihan ELNahas, Mohammed Elshaer, Ibrahim Elsohaby, Abdelgawad Salah Eltahawy, Tadele Emagneneh, Misganu Endriyas, Ryenchindorj Erkhembayar, Christopher Imokhuede Esezobor, Derese Eshetu, Majid Eslami, Narges Eslami, Rafaela Cavalheiro do Espírito Santo, Kara Estep, Oghenowede Eyawo, Ugochukwu Anthony Eze, Elochukwu Ezenwankwo, Heidar Fadavian, Adeniyi Francis Fagbamigbe, Omotayo Francis Fagbule, Ayesha Fahim, Saman Fahimi, Aamir Fahira, Ildar Ravisovich Fakhradiyev, Aliasghar Fakhri-Demeshghieh, Luca Falzone, Qiping Fan, Mohammad Farahmand, Ali Faramarzi, Mohammad Fareed, Zaki Farhana, Liliana Faria, Carla Sofia e Sá Farinha, MoezAlIslam Ezzat Mahmoud Faris, Andre Faro, Syed Muhammad Yousaf Farooq, Hossein Farrokhpour, Fatemeh Farshad, Farima Farsi, Folorunso Oludayo Fasina, Modupe Margaret Fasina, Ali Fatehizadeh, Davood Fathi, Zareen Fatima, Mohammad Fayaz, Pooria Fazeli, Valery L Feigin, Alireza Feizkhah, Gelana Fekadu, Ginenus Fekadu, Ulrich Membe Femoe, Talukdar Raian Ferdous, Seyed-Mohammad Fereshtehnejad, Rodrigo Fernandez-Jimenez, Pietro Ferrara, Alize J Ferrari, Nuno Ferreira, Getahun Fetensa, Bikila Regassa Feyisa, Alexander Finnemore, Claudio Fiorilla, Florian Fischer, Ida Fitriana, Federica Fogacci, Morenike Oluwatoyin Folayan, Artem Alekseevich Fomenkov, Marco Fonzo, Lisa M Force, Daniela Fortuna, Matteo Foschi, Maryam Fotouhi, Kayode Raphael Fowobaje, Richard Charles Franklin, Alberto Freitas, Jinming Fu, Takeshi Fukumoto, Ami Fukunaga, John E Fuller, Sridevi G, Peter Andras Gaal, Muktar A Gadanya, Dominic Dormenyo Gadeka, Lebo Francina Gafane-Matemane, Márió Gajdács, Yaseen Galali, Dinara Galiyeva, Silvano Gallus, Dhanraj Ganapathy, Balasankar Ganesan, Shivaprakash Gangachannaiah, Xiang Gao, Yijie Gao, Bashiru Garba, Miguel Garcia-Argibay, David Garcia-Azorin, William M Gardner, Wendy Paola Gastélum Espinoza, Zisis Gatzioufas, Prem Gautam, Rupesh K Gautam, Bamba Gaye, Hong-Han Ge, Feven Sahle Gebre, Miglas Welay Gebregergis, Mesfin Gebrehiwot, Miesa Gelchu, Stefano Gelibter, Nsikakabasi Samuel George, Lemma Getacher, Genanew K Getahun, Kalab Yigermal Gete, Peter W Gething, Keyghobad Ghadiri, Fataneh Ghadirian, Amir Ghaffari Jolfayi, Arin Ghamkhar, Shakiba Ghasemi Assl, Fariba Ghassemi, Ramy Mohamed Ghazy, Sama Ghoba, Maryam Gholamalizadeh, Zainab Gholami, Nasim Gholizadeh, Zeinab Ghorbani, Elena Ghotbi, Arun Ghuge, Alessandro Gialluisi, Konstantinos Giannakis, Syed Abdullah Gilani, Tiffany K Gill, Bikash Ranjan Giri, Alem Abera Girmay, Alessandro Girombelli, Laszlo Göbölös, Anil Kumar Goel, Archit Goel, Rajesh Kumar Goel, Lay Hoon Goh, Kimiya Gohari, Mahaveer Golechha, Ali Golestani, Davide Golinelli, Melika Golmohammadi, Wenping Gong, Alessandra C Goulart, Ayman Grada, Simon Matthew Graham, Michal Grivna, Shi-Yang Guan, Giovanni Guarducci, Mohammed Ibrahim Mohialdeen Gubari, Mesay Dechasa Gudeta, Avirup Guha, Stefano Guicciardi, Sheffali Gulati, Sasidhar Gunturu, Cui Guo, Xingzhi Guo, Zhaoyu Guo, Zhifeng Guo, Bhawna Gupta, Gaurav Gupta, Lalit Gupta, Rajeev Gupta, Reyna Alma Gutiérrez, Roberth Steven Gutiérrez-Murillo, Jose Guzman-Esquivel, Abrham Tesfaye Habteyes, Awoke Derbie Habteyohannes, Tesfahun Simon Hadaro, Najah R Hadi, Zahra Hadian, Abdul Hafiz, Sarah Hafsia, Faraidoon Haghdoost, Arian Haghtalab, Nguyen Hai Nam, Addisalem Haile, Demewoz Haile, Pritam Halder, Sebastian Haller, Rabih Halwani, Kosar Hikmat Hama Aziz, Islam M Hamad, Randah R Hamadeh, Samer Hamidi, Erin B Hamilton, Ahmad Hammoud, Chieh Han, Hannah Han, Asif Hanif, Nasrin Hanifi, Graeme J Hankey, Fahad Hanna, Ashanul Haque, Md Nuruzzaman Haque, Obaid I Haque, Arief Hargono, Andy Martahan Andreas Hariandja, Josep Maria Haro, Ashley Ann Harris, Ahmed I Hasaballah, Faizul Hasan, Md Kamrul Hasan, Towhid Hasan, Hamidreza Hasani, Ali Hasanpour- Dehkordi, Mohammad Hashem Hashempur, Nada Tawfig Hashim, Ammarah Hasnain, Abbas M Hassan, Amr Hassan, Ibrahim Nagmeldin Hassan, Ikrama Hassan, Nageeb Hassan, Omed Hassan Ahmed, Yusuf Hassan Wada, Mahgol Sadat Hassan Zadeh Tabatabaei, Soheil Hassanipour, Lasanthi Wathsala Hathagoda, Johannes Haubold, Rasmus J Havmoeller, Simon I Hay, Youssef Hbid, Jiawei He, Jeffrey J Hebert, Golnaz Heidari, Mohammad Heidari, Mojtaba Heydari, Kamal Hezam, Yuta Hiraike, Nobuyuki Horita, Alamgir Hossain, Lubna Hossain, Md Belal Hossain, Md Mahbub Hossain, Md Sabbir Hossain, Mohammad Bellal Hossain, Fatemeh Sadat Hosseini, Mehdi Hosseinzadeh, Mihaela Hostiuc, Sorin Hostiuc, Peter J Hotez, Priya Hotwani, Hanno Hoven, Chengxi Hu, Yifei Hu, Junjie Huang, Weijun Huang, Yefei Huang, Yuting Huang, Zhenyao Huang, Mega Hasanul Huda, Ayesha Humayun, Waqar Husain, Kiavash Hushmandi, Javid Hussain, Nawfal R Hussein, Mohamed Ibrahim Husseiny, Luigi Francesco Iannone, Segun Emmanuel Ibitoye, Khalid S Ibrahim, Ramzi Ibrahim, Reem Ibrahim, Umar Idris Ibrahim, Anel Ibrayeva, Fidelia Ida, Kevin S Ikuta, Olayinka Stephen Ilesanmi, Irena M Ilic, Milena D Ilic, Muhammad Hamza Ilyas, Mohammad Tarique Imam, Masoud Imani, Lucius Chidiebere Imoh, Arit Inok, Meesha Iqbal, Mujahid Iqbal, Lalu Muhammad Irham, Mustafa Alhaji Isa, Benni Iskandar, Teresa R Iskander, Md Rabiul Islam, Md Shahinul Islam, Md Shariful Islam, Sheikh Mohammed Shariful Islam, Farhad Islami, Faisal Ismail, Nahlah Elkudssiah Ismail, Yerlan Ismoldayev, Gaetano Isola, Masao Iwagami, Ihoghosa Osamuyi Iyamu, Vinothini J, Jalil Jaafari, Louis Jacob, Kathryn H Jacobsen, Ali Jadidi, Farhad Jadidi-Niaragh, Mohammadsadegh Jafari, Morteza Jafarinia, Abdollah Jafarzadeh, Shabbar Jaffar, Haitham Jahrami, Ammar Abdulrahman Jairoun, Vikash Jaiswal, Sanobar Jaka, Mihajlo Jakovljevic, Reza Jalilzadeh Yengejeh, Mohamed Jalloh, Armaan Jamal, Qazi Mohammad Sajid Jamal, Jazlan Jamaluddin, Jerin James, Hasan Jamil, Safayet Jamil, Roland Dominic G Jamora, Masoud Jamshidi, Shaghayegh JamshidiRastabi, Rajiv Janardhanan, Chinmay T Jani, Esmaeil Jarrahi, Tahereh Javaheri, Syed Sarmad Javaid, Anita Javanmardi, Javad Javidnia, Talha Jawaid, Qassim Jawell Odah Abed, Sathish Kumar Jayapal, Shubha Jayaram, Ruwan Duminda Jayasinghe, Yovanthi Anurangi Jayasinghe, Sun Ha Jee, Jayakumar Jeganathan, Diptismita Jena, Seogsong Jeong, Bijay Mukesh Jeswani, Vivekanand Jha, John S Ji, Min Jiang, Wenyi Jin, Nabi Jomehzadeh, Jost B Jonas, Tamas Joo, Abu Jor, Abel Joseph, Nitin Joseph, Meha Joshi, George Joy, Jacek Jerzy Jozwiak, Mikk Jürisson, Vaishali K, Billingsley Kaambwa, Ali Kabir, Zubair Kabir, Rajendra Kadel, Dler H Hussein Kadir, Ashish Kumar Kakkar, Pradnya Vishal Kakodkar, Rizwan Kalani, Khalil Kalavani, Feroze Kaliyadan, Sanjay Kalra, Md Moustafa Kamal, Mehnaz Kamal, Sivesh Kathir Kamarajah, Rajesh Kamath, Saltanat Kamenova, Arun Kamireddy, Ramat T Kamorudeen, Devanish Narasimhasanth Kamtam, Naser Kamyari, Oleksandr Kamyshnyi, Mona Kanaan, Saddam Fuad Kanaan, Jiseung Kang, Kehinde Kazeem Kanmodi, Suthanthira Kannan S, Rami S Kantar, Debasish Kar, Sujita Kumar Kar, Paschalis Karakasis, Jafar Karami, Reema A Karasneh, Mohammad Amin Karimi, Salah Eddin Karimi, Arman Karimi Behnagh, Mohmed Isaqali Karobari, Tomasz M Karpiński, Adarsh Katamreddy, Joonas H Kauppila, Kanica Kaushal, Foad Kazemi, Nastaran Kazemi Rad, Sina Kazemian, Hafte Kahsay Kebede, Yabets Tesfaye Kebede, Tibebeselassie S Keflie, Swetha N Kempegowda, Salima Kerai, Jessica A Kerr, Vikash Ranjan Keshri, Kamyab Keshtkar, Emmanuelle Kesse-Guyot, Reza Khademi, Yousef Saleh Khader, Sidra Khalid, Hazim O Khalifa, Anas Husam Khalifeh, Anees Ahmed Khalil, Anita Khalili, Pantea Khalili, Alireza Khalilian, Ghazaleh Khalili-Tanha, Mohamed Khalis, Faham Khamesipour, Ajmal Khan, Fayaz Khan, Gulfaraz Khan, Iman Waheed Khan, Maseer Khan, Md Abdullah Saeed Khan, Mohammad Jobair Khan, Muhammad Hamza Khan, Muhammad Mueed Khan, Muhammad Umair Khan, Muhammad Umer Khan, Salman Ali Khan, Serab Khan, Sumaiya Khan, Ubaid Khan, Yusuf Saleem Khan, Zahid Khan, Vishnu Khanal, Shaghayegh Khanmohammadi, Sameer Uttamaro Khasbage, Zenith Khashim, Khaled Khatab, Haitham Khatatbeh, Moawiah Mohammad Khatatbeh, Mahalaqua Nazli Khatib, Kavin Khatri, Hamid Reza Khayat Kashani, Khalid A Kheirallah, Sunil Kumar Khokhar, Najmaddin Salih Husen Khoshnaw, Atulya Aman Khosla, Ardeshir Khosravi, Farbod Khosravi, Sepehr Khosravi, Mahmood Khosrowjerdi, P Ratan Khuman, Zemene Demelash Kifle, Hye Jun Kim, Jinho Kim, Kwanghyun Kim, Min Seo Kim, Yun Jin Kim, Ruth W Kimokoti, Tadele Kinati, Yohannes Kinfu, Sanjay Kini B, Mary Kirk, Adnan Kisa, Sezer Kisa, Katarzyna Kissimova-Skarbek, Tegene Atamenta Kitaw, Mika Kivimäki, Abdul Basith KM, Shivakumar KM, Ann Kristin Skrindo Knudsen, Nazarii Kobyliak, Jonathan M Kocarnik, Sonali Kochhar, Prakash Babu Kodali, Michail Kokkorakis, Ali-Asghar Kolahi, Diana Gladys Kolieghu Tcheumeni, Kairi Kolves, Joyce Komesuor, Farzad Kompani, Aida Kondybayeva, Isaac Koomson, Gerbrand Koren, Tapos Kormoker, Vladimir Andreevich Korshunov, Oleksii Korzh, Soewarta Kosen, Karel Kostev, Parvaiz A Koul, Irene Akwo Kretchy, James-Paul Kretchy, Kewal Krishan, Chong-Han Kua, Ananya Kuanar, Barthelemy Kuate Defo, Mohammed Kuddus, Ilari Kuitunen, Mukhtar Kulimbet, Shweta Kulshreshtha, Dewesh Kumar, Dhasarathi Kumar, Jogender Kumar, Kamal Kumar, Mukesh Kumar, Nitesh Kumar, Nithin Kumar, Tarun Kumar, Tushar Kumar, Vijay Kumar, Vikash Kumar, Subramanian Kumaran, Jibin Kunjavara, Setor K Kunutsor, Almagul Kurmanova, Om P Kurmi, Maria Dyah Kurniasari, Krishna Prasad Kurpad, Asep Kusnali, Christina Yeni Kustanti, Dian Kusuma, Tezer Kutluk, Assylkhan Kuttybayev, Evans F Kyei, Grace Kwakyewaa Kyei, Frank Kyei-Arthur, Ville Kytö, Pallavi L C, Adriano La Vecchia, Carlo La Vecchia, Alessio Lachi, Muhammad Awwal Ladan, Abraham K Lagat, Chandrakant Lahariya, Daphne Teck Ching Lai, Balzhan Lakanova, Anita Lakhani, Tea Lallukka, Judit Lám, Iván Landires, Berthold Langguth, Ariane Laplante-Lévesque, Laura Lara-Castor, Savita Lasrado, Kamaluddin Latief, Areeba Latif, Mahrukh Latif, Jerrald Lau, Paolo Lauriola, Aliyu Lawan, Teniola Lawanson, Harriet L S Lawford, Eilean Rathinasamy Lazarus, Dai Quang Le, Duc Tin Le, Thao Thi Thu Le, Caterina Ledda, Ivan Lee, Paul H Lee, Seung Won Lee, Yo Han Lee, James Leigh, Vasileios Leivaditis, Matthew J Lennon, Matilde Leonardi, Elvynna Leong, Negin Letafatkar, Chengfeng Li, Hui Li, Jiaying Li, Jie Li, Ming-Chieh Li, Si Li, Wei Li, Weilong Li, Zhaolong Adrian Li, Zhengrui Li, Yanxue Lian, Chen Liao, Stephen S Lim, Jialing Lin, Queran Lin, Shuzhi Lin, Daniel Lindholm, Christine Linehan, Yuewei Ling, Shai Linn, Haipeng Liu, Jue Liu, Xianliang Liu, Xiaofeng Liu, Xuefeng Liu, Zhe Liu, Zhenyu Liu, Erand Llanaj, Michael J Loftus, Valerie Lohner, José Francisco López-Gil, Platon D Lopukhov, Stefan Lorkowski, Rafael Lozano, Shanjie Luan, Jailos Lubinda, Taraneh Lucas, Giancarlo Lucchetti, Alessandra Lugo, Raimundas Lunevicius, Huaxia Luo, Lisha Luo, Susu Luo, Lei Lv, Miltiadis D Lytras, Ellina Lytvyak, Kevin Sheng-Kai Ma, Zheng Feei Ma, Raymond Saa-Eru Maalman, Kelsey Lynn Maass, Mahmoud Mabrok, Nikolaos Machairas, Monika Machoy, Seyed Ataollah Madinezad, Aurea Marilia Madureira-Carvalho, Pasquale Maffia, Sasikumar Mahalingam, Samatar Abshir Mahamed, Nozad Hussein Mahmood, Shakeel Ahmed Ibne Mahmood, Alireza Mahmoudi, My Tra Mai, Hao Mai Xuan, Rituparna Maiti, Marek Majdan, Abdelrahman M Makram, Omar M Makram, Mohammad-Reza Malekpour, Reza Malekzadeh, Hardeep Singh Malhotra, Ahmad Azam Malik, Farihah Malik, Deborah Carvalho Malta, Mustapha Mangdow, Jyothsna Manikkath, Yosef Manla, Fahmida Mannan, Farheen Mansoor, Marjan Mansourian, Mohammad Ali Mansournia, Lorenzo Giovanni Mantovani, Changkun Mao, Tahir Maqbool, Bishnu P Marasini, Hamid Reza Marateb, Joemer C Maravilla, Adilson Marques, Bernardo Alfonso Martinez-Guerra, Ramon Martinez-Piedra, Daniela Martini, Santi Martini, Francisco Rogerlândio Martins-Melo, Miquel Martorell, Winfried März, Roy Rillera Marzo, Sammer Marzouk, Sugeng Mashudi, Stefano Masi, Yasith Mathangasinghe, Stephanie Mathieson, Alexander G Mathioudakis, Medha Mathur, Neeta Mathur, Rita Mattiello, Richard James Maude, Pallab K Maulik, Miranda L May, Mahsa Mayeli, Mohsen Mazidi, Antonio Mazzotti, Ikechukwu Innocent Mbachu, Martin McKee, Michael A McPhail, Steven M McPhail, Rishi P Mediratta, Jitendra Meena, Medhin Mehari, Riffat Mehboob, Ravi Mehrotra, Vini Mehta, Tesfahun Mekene Meto, Hadush Negash Meles, Addisu Melese, Satish Melwani, Aishe Memetova, Walter Mendoza, Godfred Antony Menezes, Ritesh G Menezes, Emiru Ayalew Mengistie, George A Mensah, Sultan Ayoub Meo, Michelangelo Mercogliano, Atte Meretoja, Tuomo J Meretoja, Tomislav Mestrovic, Chamila Dinushi Kukulege Mettananda, Sachith Mettananda, Mohamed M M Metwally, Adquate Mhlanga, Tomasz Miazgowski, Irmina Maria Michalek, Andrea Michelerio, Hiwot Soboksa Mideksa, Kebadnew Mulatu Mihretie, Ted R Miller, Giuseppe Minervini, Wai-kit Ming, GK Mini, Mojgan Mirghafourvand, Andreea Mirica, Alireza Mirkheshti, Seyed Ali Mirshahvalad, Mizan Kiros Mirutse, Maryam Mirzaei, Archana Mishra, Ashim Mishra, Vinaytosh Mishra, Philip B Mitchell, Sayan Mitra, Chaitanya Mittal, Mohammadreza Mobayen, Madeline E Moberg, Shivani Modi, Ahmed Ismail Mohamed, Heba M Mohamed, Jama Mohamed, Mona Gamal Mohamed, Nouh Saad Mohamed, Khabab Abbasher Hussien Mohamed Ahmed, Taj Mohammad, Abdolreza Mohammadi, Mohammad Reza Mohammadi, Abdollah Mohammadian-Hafshejani, Ibrahim Mohammadzadeh, Abdulwase Mohammed, Ammas Siraj Mohammed, Hussen Mohammed, Omer Mohammed, Shafiu Mohammed, Suleiman Mohammed, Yahaya Mohammed, Mohammad Mohseni, Tsz-ngai Mok, Amin Mokari-Yamchi, Ali H Mokdad, Sabrina Molinaro, Amirabbas Mollaei, Shaher Momani, Lorenzo Monasta, Amirabbas Monazzami, Himel Mondal, Marco Montalti, Yousef Moradi, Mohammad Moradi-Joo, Maziar Moradi-Lakeh, Paula Moraga, Lidia Morawska, Rafael Silveira Moreira, Mahmoud M Morsy, Reza Mosaddeghi Heris, Jonathan F Mosser, Elias Mossialos, Maha Motavvef, Vincent Mougin, Asma Mousavi, Seyede Zohre Mousavi, Amin Mousavi Khaneghah, Seyed Mohamad Sadegh Mousavi Kiasary, Amanda Movo, Hagar Lotfy Mowafy, Kimia Mozahheb Yousefi, Matías Mrejen, Rabia Mubarak, Sumaira Mubarik, Steward Mudenda, Faraz Mughal, Syed Aun Muhammad, Muhammad Solihuddin Muhtar, Oscar J Mujica, Sukhes Mukherjee, Sumoni Mukherjee, Amartya Mukhopadhyay, M A Muktadir, Sileshi Mulatu, Francesk Mulita, Chalie Mulugeta, Damaris Felistus Mulwa, Javier Muñoz Laguna, Anjana Munshi, Efren Murillo-Zamora, Ali Mushtaq, Mubarak Taiwo Mustapha, Sathish Muthu, Saravanan Muthupandian, Claude Mambo Muvunyi, Woojae Myung, Amin Nabavi, Fatemehzahra Naddafi, Ayoub Nafei, Ahamarshan Jayaraman Nagarajan, Ganesh R Naik, Gurudatta Naik, Firzan Nainu, Sanjeev Nair, Hastyar Hama Rashid Najmuldeen, Noureddin Nakhostin Ansari, Gopal Nambi, Ni Gusti Ayu Nanditha, Vinay Nangia, Jobert Richie Nansseu, Ibrahim A Naqid, Aparna Ichalangod Narayana, Shumaila Nargus, Delaram Narimani Davani, Yvonne Nartey, Bruno Ramos Nascimento, Gustavo G Nascimento, Abdallah Y Naser, Abdulqadir J Nashwan, Hamide Nasiri, Mahmoud Nassar, Zuhair S Natto, Javaid Nauman, Samidi Nirasha Kumari Navaratna, Biswa Prakash Nayak, Shalini Ganesh Nayak, Vinod C Nayak, Shumaila Naz, Athare Nazri-Panjaki, G Takop Nchanji, Sabina Onyinye Nduaguba, Amanuel Tebabal Nega, Meti T Negassa, Chernet Tafere Negesse, Ionut Negoi, Ruxandra Irina Negoi, Alina Gabriela Negru, Chakib Nejjari, Samata Nepal, Olivia D Nesbit, Henok Biresaw Netsere, Marie Ng, Georges Nguefack-Tsague, Josephine W Ngunjiri, Cuong Tat Nguyen, Dang Nguyen, Huong-Dung Thi Nguyen, Nghia Phu Nguyen, The Phuong Nguyen, Van Thanh Nguyen, Ambe Marius Ngwa, Robina Khan Niazi, Luciano Nieddu, Yeshambel T Nigatu, Ali Nikoobar, Vikram Niranjan, Abebe Melis Nisro, Chukwudi A Nnaji, Shuhei Nomura, Syed Toukir Ahmed Noor, Mohammadamin Noorafrooz, Mamoona Noreen, Masoud Noroozi, Jean Jacques Noubiap, Mehran Nouri, Taylor Noyes, Valentine C Nriagu, Chisom Adaobi Nri-Ezedi, Jean Claude Nshimiyimana, Fred Nugen, Mengistu H Nunemo, Nurfatimah Nurfatimah, Dieta Nurrika, Sylvester Dodzi Nyadanu, Felix Kwasi Nyande, Bogdan Oancea, Ramez M Odat, Fabio Massimo Oddi, Ismail A Odetokun, Oluwakemi Ololade Odukoya, Joseph Kojo Oduro, Michael Safo Oduro, Oluwafunmilayo Tosin Ogundeko-Olugbami, Abiola Ogunkoya, Oluwafunmbi Ebenezer Ogunmiluyi, In-Hwan Oh, Sarah Oh, Hassan Okati-Aliabad, Sylvester Reuben Okeke, Deborah Oluwatosin Okeke-Obayemi, Akinkunmi Paul Okekunle, Olalekan John Okesanya, Osaretin Christabel Okonji, Bolanle Adeyemi Ola, Oluwaseyi Isaiah Olabisi, Oladotun Victor Olalusi, Matthew Idowu Olatubi, Arão Belitardo Oliveira, Gláucia Maria Moraes Oliveira, Abdulhakeem Abayomi Olorukooba, Oluseye Olalekan Oludoye, Ronald Olum, Bolajoko Olubukunola Olusanya, Jacob Olusegun Olusanya, Oluwafemi G Oluwole, Folorunsho Bright Omage, Goran Latif Omer, Abidemi E Omonisi, Kanyin Liane Ong, Sandersan Onie, Obinna E Onwujekwe, Oluwaseyi Aina Gbolade Opesemowo, John Nelson Opio, Marcel Opitz, Aksoltan Shyhdurdyevna Oradova, Michal Ordak, Verner N Orish, Raffaele Ornello, Atakan Orscelik, Alberto Ortiz, Esteban Ortiz-Prado, Augustus Osborne, Samuel M Ostroff, John W Ostrominski, Uchechukwu Levi Osuagwu, Olayinka Osuolale, Elham H Othman, Adrian Otoiu, Abdu Oumer, Jerry John Ouner, Amel Ouyahia, Mayowa O Owolabi, Irene Amoakoh Owusu, Oladayo Ayobami Oyebanji, Kolapo Oyebola, Tope Oyelade, Kehinde Adewole Oyeniran, Oyetunde T Oyeyemi, Ilker Ozsahin, Mahesh P A, Kevin Pacheco-Barrios, Alicia Padron-Monedero, Jagadish Rao Padubidri, Dimpal Manilal Paija, Anton Pak, Yeganeh Pakbaz, Pramod Kumar Pal, Tamás Palicz, Raffaele Palladino, Tejasri Paluvai, Feng Pan, Sujogya Kumar Panda, Songhomitra Panda-Jonas, Deepshikha Pande Katare, Seithikurippu R Pandi-Perumal, Victoria Pando-Robles, Apurvakumar Pandya, Helena Ullyartha Pangaribuan, Georgios D Panos, Leonidas D Panos, Ioannis Pantazopoulos, Anca Pantea Stoian, Giovanni Paolino, Mario Virgilio Papa, Ilias Papadimopoulos, Paraskevi Papadopoulou, Peyvand Parhizkar Roudsari, Romil R Parikh, Chulwoo Park, Seoyeon Park, Arpit Parmar, Roberto Passera, Jay Patel, Mitesh Patel, Neel Navinkumar Patel, Sangram Kishor Patel, Satyananda Patel, Bharat Smita Umakant Patil, Shankargouda Patil, Dimitrios Patoulias, Apurba Patra, Venkata Suresh Patthipati, Shrikant Pawar, Shubhadarshini Pawar, Hamidreza Pazoki Toroudi, Neil Pearce, Amy E Peden, Paolo Pedersini, Jarmila Pekarcikova, Louise Penberthy, Veincent Christian Filipino Pepito, Emmanuel K Peprah, Prince Peprah, João Perdigão, Gavin Pereira, Gladymar Perez Chacon, Arokiasamy Perianayagam, Norberto Perico, Simone Perna, Konrad Pesudovs, Pavlo Petakh, Ionela-Roxana Petcu, Olumuyiwa James Peter, Fanny Emily Petermann-Rocha, William A Petri, Hoang Nhat Pham, Hoang Tran Pham, Tung Thanh Pham, Anil K Philip, Michael R Phillips, Zahra Zahid Piracha, Edoardo Pirera, Moein Piroozkhkah, Saeed Pirouzpanah, Enrico Pisoni, Evgenii Plotnikov, Indrashis Podder, Dimitri Poddighe, Roman V Polibin, Ramesh Poluru, Arjun Pon Avudaiappan, Ville T Ponkilainen, Ion Popa, Djordje S Popovic, Thantrira Porntaveetus, Sajjad Pourasghary, Reza Pourbabaki, Farzad Pourghazi, Naeimeh Pourtaheri, Sergio I Prada, Jalandhar Pradhan, Rifky Octavia Pradipta, Akila Prashant, Elton Junio Sady Prates, Natalie Pritchett, Harsh Priya, Nicola Riccardo Pugliese, Jagadeesh Puvvula, Nameer Hashim Qasim, Ibrahim Qattea, Xiang Qi, Zhipeng Qi, Yanan Qiao, Zahiruddin Syed Quazi, Navid Rabiee, Reza Rabiei, Basuki Rachmat, Raghu Anekal Radhakrishnan, Venkatraman Radhakrishnan, Maja R Radojčić, Negar Radpour, Hadi Raeisi Shahraki, Lida Rafati, Ibrar Rafique, Pracheth Raghuveer, Fakher Rahim, Hawbash Mohammed-Amin Rahim, Sajjad Rahimi, Vafa Rahimi-Movaghar, Fryad Majeed Rahman, Mahbubur Rahman, Md Mosfequr Rahman, Mohammad Hifz Ur Rahman, Mohammad Meshbahur Rahman, Mosiur Rahman, Amir Masoud Rahmani, Saeed Rahmani, Masoud Rahmati, Ghasem Rahmatpour Rokni, Hakim Rahmoune, Diego Raimondo, Ivano Raimondo, Sunil Kumar Raina, Jeffrey Pradeep Raj, Adarsh Raja, Sathish Rajaa, Erta Rajabi, Gunaseelan Rajendran, Judah Rajendran, Vinoth Rajendran, Shaman Rajindrajith, Pushp Lata Rajpoot, Prashant Rajput, Mahmoud Mohammed Ramadan, Majed Ramadan, Kadar Ramadhan, Chitra Ramasamy, Shakthi Kumaran Ramasamy, Zahra Ramezani, Marzieh Ramezani Farani, Robinson Ramírez-Vélez, Juwel Rana, Kirtan Rana, Shailendra Singh Rana, Chhabi Lal Ranabhat, Nemanja Rancic, Smitha Rani, Fatemeh - Ranjbar Noei, Chythra R Rao, Kumuda Rao, Mithun Rao, Davide Rasella, Sina Rashedi, Vahid Rashedi, Mamunur Rashid, Mohammad-Mahdi Rashidi, Mohammad Aziz Rasouli, Ashkan Rasouli-Saravani, Azad Rasul, Devarajan Rathish, Abdur Rauf, Santosh Kumar Rauniyar, Ilari Rautalin, Ramin Ravangard, David Laith Rawaf, Lal Rawal, Reza Rawassizadeh, Bahman Razi, C Mahony Reategui-Rivera, Elrashdy Redwan, Aqeeb Ur Rehman, Faizan Ur Rehman, Wajiha Rehman, Lennart Reifels, Rainer Reile, Giuseppe Remuzzi, Bhageerathy Reshmi, Stefano Restaino, Luis Felipe Reyes, Mina Rezaei, Nazila Rezaei, Nima Rezaei, Mohsen Rezaeian, Donya Rezazadeh Eidgahi, Taeho Gregory Rhee, Yohanes Andy Rias, Antonio Luiz P Ribeiro, Tércia Moreira Ribeiro da Silva, Jennifer Rickard, Moattar Raza Rizvi, Hannah Elizabeth Robinson-Oden, Hermano Alexandre Lima Rocha, João Rocha Rocha-Gomes, Mónica Rodrigues, Thales Philipe Rodrigues da Silva, Jefferson Antonio Buendia Rodriguez, Leonardo Roever, Peter Rohloff, Iftitakhur Rohmah, Susanne Röhr, David Rojas-Rueda, Megan L Rolfzen, Debby Syahru Romadlon, Michele Romoli, Luca Ronfani, Kevin T Root, Emily Rosenblad, Amirhossein Roshanshad, Morteza Rostamian, Gregory A Roth, Kunle Rotimi, Himanshu Sekhar Rout, Hanieh Rouzbahani, Reza Rouzbahani, Jemma V Rowlands, Adrija Roy, Bedanta Roy, Priyanka Roy, Sharmistha Roy, Shubhanjali Roy, Simanta Roy, Parameswari Royapuram Parthasarathy, Enrico Rubagotti, Susan Fred Rumisha, Michele Russo, Godfrey Mutashambara Rwegerera, Aly M A Saad, Michela Sabbatucci, Maha Mohamed Saber-Ayad, Siamak Sabour, Perminder S Sachdev, Seyed Kiarash Sadat Rafiei, Basema Ahmad Saddik, Bashdar Abuzed Sadee, Tarannom Sadegh, Ehsan Sadeghi, Erfan Sadeghi, Fatemeh Sadeghi-Ghyassi, Mohd Saeed, Umar Saeed, Maryam Saeedi, Mahdi Safdarian, Sare Safi, Sher Zaman Safi, Rajesh Sagar, Mastooreh Sagharichi, Amene Saghazadeh, Dominic Sagoe, Indranil Saha, Nondo Saha, Fatemeh Saheb Sharif-Askari, Narjes Saheb Sharif-Askari, Amirhossein Sahebkar, Kirti Sundar Sahu, Zahra Saif, S Mohammad Sajadi, Md Refat Uz Zaman Sajib, Mirza Rizwan Sajid, Dorsa Salabat, Payman Salamati, Luciane B Salaroli, Mohamed A Saleh, Leili Salehi, Mahdi Salehi, Marwa Rashad Salem, Mohammed Z Y Salem, Aanuoluwa James Salemcity, Dauda Salihu, Sohrab Salimi, Malik Sallam, Hossein Samadi Kafil, Jayami Eshana Samaranayake, Saad Samargandy, Waqas Sami, Yoseph Leonardo Samodra, Abdallah M Samy, Sandeep G Sangle, Elaheh Sanjari, Sathish Sankar, Francesco Sanmarchi, Francesca Sanna, Damian F Santomauro, Itamar S Santos, Lucas H C C Santos, Milena M Santric-Milicevic, Adekunle Sanyaolu, Bruno Piassi Sao Jose, Krishna Prasad Sapkota, Sivan Yegnanarayana Iyer Saraswathy, Yaser Sarikhani, Hemen Sarma, Mohammad Sarmadi, Gargi Sachin Sarode, Sachin C Sarode, Benn Sartorius, Arash Sarveazad, Michele Sassano, Mukesh Kumar Sathya Narayanan, Maheswar Satpathy, Reza Sattarpour, Davide Sattin, Mehrdad Savabi Far, Monika Sawhney, Sangeeta Gopal Saxena, Ganesh Kumar Saya, Abu Sayeed, Christophe Schinckus, Jurgen Carlo Schmidt, Maria Inês Schmidt, Rachel D Schneider, Art Schuermans, Austin E Schumacher, Aletta Elisabeth Schutte, Ghil Schwarz, David C Schwebel, Falk Schwendicke, Sneha Annie Sebastian, Amin Sedigh, Soraya Seedat, Mario Šekerija, Muthamizh Selvamani, Vimalraj Selvaraj, Yuliya Semenova, Mohammad H Semreen, Fikadu Waltengus Sendeku, Pallav Sengupta, Yigit Can Senol, Subramanian Senthilkumaran, Sadaf G Sepanlou, Edson Serván-Mori, Yashendra Sethi, Seyed Mohammad Seyed Alshohadaei, Allen Seylani, Abubakar Sha'aban, Mahan Shafie, Arezoo Shafieioun, Shazlin Shaharudin, Muhammad Shahbaz, Samiah Shahid, Syed Ahsan Shahid, Endrit Shahini, Fatemeh Shahrahmani, Hamid R Shahsavari, Moyad Jamal Shahwan, Masood Ali Shaikh, Alireza Shakeri, Ali Shakerimoghaddam, Ali S Shalash, Muhammad Aaqib Shamim, Farzane Shams, Mehran Shams-Beyranvand, Anas Shamsi, Alfiya Shamsutdinova, Dan Shan, Shan Shan, Mohd Shanawaz, Amin Sharifan, Javad Sharifi Rad, Avimanu Sharma, Bhoopesh Kumar Sharma, Bunty Sharma, Gaurav Sharma, Kamal Sharma, Kamlesh Sharma, Manoj Sharma, Ravi Kumar Sharma, Ujjawal Sharma, Vishal Sharma, Shamee Shastry, Maryam Shayan, Babangida Shehu Bappah, Fateme Sheida, Ali Sheidaei, Ali Sheikhy, Rekha Raghuveer Shenoy, Samendra P Sherchan, B Suresh Kumar Shetty, Shiran Shetty, Fanchao Shi, Fang Shi, Amir Shiani, Belayneh Fentahun Shibesh, Kenji Shibuya, Desalegn Shiferaw, Tariku Shimels, Md Monir Hossain Shimul, Min-Jeong Shin, Rahman Shiri, Reza Shirkoohi, Aminu Shittu, Abdul-karim Olayinka Shitu, Ivy Shiue, Velizar Shivarov, Nathan A Shlobin, Ambreen Shoaib, Shayan Shojaei, Sina Shool, Seyed Afshin Shorofi, Sunil Shrestha, Suleiman Adeiza Shuaibu, Kerem Shuval, Zahra Siavashpour, Nicole Remaliah Samantha Sibuyi, Emmanuel Edwar Siddig, Ahmed Kamal Siddiqi, Diego Augusto Santos Silva, João Pedro Silva, Luís Manuel Lopes Rodrigues Silva, Padam Prasad Simkhada, Biagio Simonetti, Abhinav Singh, Amit Singh, Balbir Bagicha Singh, Baljinder Singh, Bhim Pratap Singh, Harmanjit Singh, Harpreet Singh, Jasvinder A Singh, Jawahar Singh, Kalpana Singh, Mayank Singh, Narinder Pal Singh, Paramdeep Singh, Poornima Suryanath Singh, Puneetpal Singh, Rakesh K Singh, Samer Singh, Satwinder Singh, Surendra Singh, Surjit Singh, Mukesh Kumar Sinha, Robert Sinto, Sarah Brooke Sirota, Dagne Feleke Siyoum, Natia Skhvitaridze, Anna Aleksandrovna Skryabina, David A Sleet, Mahdieh SobhZahedi, Marzieh Soheili, MdSalman Sohel, Somaye Sohrabi, Shipra Solanki, Lencho Kajela Solbana, Solikhah Solikhah, Sameh S M Soliman, Weiyi Song, Aayushi Sood, Prashant Sood, Soroush Soraneh, Reed J D Sorensen, Joan B Soriano, Fernando Sousa, Marco Aurelio Sousa, Ireneous N Soyiri, Ceren Soylu, Michael Spartalis, Chandrashekhar T Sreeramareddy, Suresh Kumar Srinivasamurthy, Shyamkumar Sriram, Prateek Srivastav, Devin Bailey Srivastava, Lauryn K Stafford, Jeffrey D Stanaway, Muhammad Haroon Stanikzai, Nadine Steckling-Muschack, Dan J Stein, Caitlyn Steiner, Jaimie D Steinmetz, Paschalis Steiropoulos, Blossom Christa Maree Stephan, Aleksandar Stevanović, Leo Stockfelt, Sebastian Straube, Jacob L Stubbs, Peter Stubbs, Omer Subasi, Narayan Subedi, Alisha Suhag, Hasnat Sujon, Thitiporn Sukaew, Surajo Kamilu Sulaiman, Auwal Garba Suleiman, Muritala Suleiman Odidi, Muhammad Suleman, Mark J M Sullman, Anusha Sultan Meo, Haitong Zhe Sun, Jing Sun, Mao-ling Sun, Xiaodong Sun, Xiaohui Sun, Zhong Sun, Zhuanlan Sun, Suraj Sundaragiri, Thanigaivel Sundaram, Johan Sundström, David Sunkersing, Sumam Sunny, Vinay Suresh, Hani Susianti, Chandan Kumar Swain, Vivianne M Swart, Dayinta Annisa Syaiful, Tasmin L Symons, Lukasz Szarpak, Mindy D Szeto, Sree Sudha T Y, Payam Tabaee Damavandi, Rafael Tabarés-Seisdedos, Fatemeh Sadat Tabatabaei, Seyed Shahaboddin Tabatabaei, Seyyed Mohammad Tabatabaei, Seyed-Amir Tabatabaeizadeh, Shima Tabatabai, Celine Tabche, Mohammad Tabish, Takahiro Tabuchi, Getu Ferenji Tadesse, Farzad Taghizadeh-Hesary, Zanan Mohammed-Ameen Taha, Jabeen Taiba, Shima Tajabadi, Iman M Talaat, Mircea Tampa, Jacques Lukenze Tamuzi, Ker-Kan Tan, Mohammad Tanashat, Haosu Tang, Mohsan Tanveer, Abiyu Abadi Tareke, Sarvenaz Taridashti, Ingan Ukur Tarigan, Mengistie Kassahun Tariku, Saba Tariq, Aigul Yelgondiyevna Tazhiyeva, Tarilate Temedie-Asogwa, Mohamad-Hani Temsah, Masayuki Teramoto, Azimeraw Arega Tesfu, Nahom Worku Teshager, Gizachew A Tessema, Jay Tewari, Alireza Teymouri, Chandan Kumar Thakur, Kavumpurathu Raman Thankappan, Rekha Thapar, Ismaeel Tharwat, Samar Tharwat, Rasiah Thayakaran, Muthu Thiruvengadam, Manuel Sebastian Thomas, Wei Tian, Jansje Henny Vera Ticoalu, Madi Tleshev, Sojit Tomo, Marcello Tonelli, Roman Topor-Madry, Mathilde Touvier, Marcos Roberto Tovani-Palone, Khaled Trabelsi, Quynh Thuy Huong Tran, Tam Quoc Minh Tran, Thang Huu Tran, Nguyen Tran Minh Duc, Domenico Trico, Indang Trihandini, Manjari Tripathi, Tulika Tripathi, Samuel Joseph Tromans, Quynh Xuan Nguyen Truong, Thien Tan Tri Tai Truyen, Gary Tse, Vasilis-Spyridon Tseriotis, Evangelia Eirini Tsermpini, Lorainne Tudor Car, Munkhtuya Tumurkhuu, Zhouting Tuo, Biruk Shalmeno Tusa, Sok Cin Tye, Stefanos Tyrovolas, Aniefiok John Udoakang, Atta Ullah, Himayat Ullah, Saeed Ullah, Muhammad Umair, Hauwa Onozasi Umar, Lawan Umar, Muhammad Umar, Muhammad Umar, Shehu Salihu Umar, Eduardo A Undurraga, Bhaskaran Unnikrishnan, Dinesh Upadhya, Era Upadhyay, Dipan Uppal, Daniele Urso, Jibrin Sammani Usman, Kelechi Julian Uzor, Hande Uzunçıbuk, Pratyusha Vadagam, Asokan Govindaraj Vaithinathan, Pascual R Valdez, Mario Valenti, Zahir Vally, Jef Van den Eynde, Javad Varasteh, Joe Varghese, Pavani Varma, Tommi Juhani Vasankari, Sampara Vasishta, Srivatsa Surya Vasudevan, Alireza Vaysi, Siavash Vaziri, Narayanaswamy Venketasubramanian, Madhur Verma, Megan Verma, Poonam Verma, Massimiliano Veroux, Georgios-Ioannis Verras, Simone Vidale, Mathavaswami Vijayageetha, Simone Villa, Jorge Hugo Villafañe, Leonardo Villani, David Villarreal-Zegarra, Francesco S Violante, Senthil Visaga Ambi, Luciano Magalhães Vitorino, Vasily Vlassov, Stein Emil Vollset, Avina Vongpradith, Theo Vos, Mehdi Vosoughi, Elpida Vounzoulaki, Linh Vu, Isidora S Vujcic, Krishna Dhavan Vyas, Henok Toga Wada, Yasir Waheed, Mohd Wahid, Mugi Wahidin, Mandaras Tariku Walde, Megha Walia, Jin-Yi Wan, Arvinder Wander, Fang Wang, Fulin Wang, Junshi Wang, Liang Wang, Qingzhi Wang, Ruixuan Wang, Shu Wang, Wanzhou Wang, Xing Wang, Xuequan Wang, Yan Wang, Yanzhong Wang, Yichen Wang, Youxin Wang, Yuan-Pang Wang, Zhihua Wang, Tanveer A Wani, Mary Njeri Wanjau, Ahmed Bilal Waqar, Muhammad Waqas, John W Ward, Paul Ward, Toyiba Hiyaru Wassie, Stefanie Watson, Ishanka Weerasekara, Fei-Long Wei, Xueying Wei, Robert G Weintraub, Daniel J Weiss, Eli J Weiss, Katherine M Wells, Andrea Werdecker, Ronny Westerman, Taweewat Wiangkham, Yohanes Cakrapradipta Wibowo, Dakshitha Praneeth Wickramasinghe, Nuwan Darshana Wickramasinghe, Samuel Wiebe, Angga Wilandika, Peter Willeit, Shadrach Wilson, Andrew Awuah Wireko, Charles Shey Wiysonge, Abay Tadesse Woday, Bogdan Wojtyniak, Nathnael Abera Woldehana, Dawit Habte Woldeyes, Axel Walter Wolf, Tewodros Eshete Wonde, Yen Jun Wong, Daniel Tarekegn Worede, Abdulhalik Workicho, Minichil Chanie Worku, Ai-Min Wu, Chenkai Wu, Felicia Wu, James Fan Wu, Jinyi Wu, Peng Wu, Zenghong Wu, Yihun Miskir Wubie, Ratna Dwi Wulandari, Zhijia Xia, Guangqin Xiao, Lishun Xiao, Na Xiao, Wanqing Xie, Site Xu, Suowen Xu, Xiaoyue Xu, Yvonne Yiru Xu, Mukesh Kumar Yadav, Vikas Yadav, Mahnaz Yadollahi, Saba Yahoo (Syed), Galal Yahya, Kazumasa Yamagishi, Guangcan Yan, Haibo Yang, Yuichiro Yano, Haiqiang Yao, Laiang Yao, Amir Yarahmadi, Habib Yaribeygi, Haya Yasin, Mohamed A Yassin, Yuichi Yasufuku, Sanni Yaya, Pengpeng Ye, Meghdad Yeganeh, Ali Cem Yekdeş, Mohammad Hossein YektaKooshali, Kuanysh A Yergaliyev, Subah Abderehim Yesuf, Saber Yezli, Siyan Yi, Dehui Yin, Paul Yip, Malede Berihun Yismaw, Yazachew Engida Yismaw, Dong Keon Yon, Naohiro Yonemoto, Seok-Jun Yoon, Mustafa Z Younis, Saideh Yousefi, Abdilahi Yousuf, Chuanhua Yu, Yong Yu, Hui Yuan, Faith H Yuh, Ghazala Yunus, Umar Yunusa, Siddhesh Zadey, Vesna Zadnik, Mubashir Zafar, Manijeh Zaghampour, Emilia Zainal Abidin, Fathiah Zakham, Nazar Zaki, Giulia Zamagni, Nelson Zamora, Hussaini Zandam, Aurora Zanghì, Heather J Zar, Kourosh Zarea, Mohammed Zawiah, Mohammed G M Zeariya, Abay Mulu Zenebe, Sebastian Zensen, Nejimu Biza Zepro, Eyael M Zeru, Tiansong Zhan, Yongle Zhan, Beijian Zhang, Casper J P Zhang, Haijun Zhang, Julio Min Fei Zhang, Kexin Zhang, Liqun Zhang, Meixin Zhang, Xiaoyi Zhang, Xiu-Hang Zhang, Yunquan Zhang, Zhiqiang Zhang, Sholpan Bolatovna Zhangelova, Hanqing Zhao, Jianhui Zhao, Jiefeng Zhao, Yang Zhao, Zhongyi Zhao, Anthony Zhong, Claire Chenwen Zhong, Jiayan Zhou, Juexiao Zhou, Bin Zhu, Abzal Zhumagaliuly, Magdalena Zielińska, Ghazal Zoghi, Mohamed Ali Zoromba, Zhiyong Zou, Rafat Mohammad Zrieq, Liesl J Zuhlke, Lilik Zuhriyah, Alimuddin Zumla, Ahed H Zyoud, Sa'ed H Zyoud, Shaher H Zyoud, Eve E Wool, Christopher J L Murray

**Affiliations:** a Institute for Health Metrics and Evaluation, University of Washington, Seattle, WA, USA; b Department of Health Metrics Sciences, School of Medicine, University of Washington, Seattle, WA, USA; c Amity Institute of Public Health, Amity University, Uttar Pradesh, India; d Shahid Beheshti University of Medical Sciences, Tehran, Iran; e Department of Nursing, Al Zaytoonah University of Jordan, Amman, Jordan; f Department of Radiation Oncology, Massachusetts General Hospital, Boston, MA, USA; g Menzies Institute for Medical Research, University of Tasmania, Hobart, TAS, Australia; h School of Health & Life Scinces, University of the West of Scotland, Paisley, UK; i Department of Medical Rehabilitation, University of Nigeria Nsukka, Enugu, Nigeria; j Department of Legal and Economic Studies, La Sapienza University, Rome, Italy; k Infectious and Tropical Research Center, Tabriz University of Medical Sciences, Tabriz, Iran; l Department of Medicine, University of California San Francisco, San Francisco, CA, USA; m Advanced Diagnostic and Interventional Radiology Research Center, Tehran University of Medical Sciences, Tehran, Iran; n College of Pharmacy, Ajman University, Ajman, United Arab Emirates; o Department of Epidemiology, Alexandria University, Alexandria, Egypt; p College of Pharmacy, Umm Al-Qura University, Makka, Saudi Arabia; q Hull York Medical School, University of Hull, Hull, UK; r Department of Biology, Qassim University, Buraydah, Saudi Arabia; s Department of Health and Nutrition, Save the Children, Hargeisa, Somalia; t School of Nursing, The University of Jordan, Amman, Jordan; u College of Pharmacy, King Saud University, Riyadh, Saudi Arabia; v Basic Science Department, University of Hail, Hail, Saudi Arabia; w Chemistry Department, Al-Azhar University, Cairo, Egypt; x Department of Surgery, Marshall University, Huntington, WV, USA; y Department of Cardiovascular Medicine, Mayo Clinic, Phoenix, AZ, USA; z Department of Medical Laboratory Science, University of Sharjah, Sharjah, United Arab Emirates; aa Faculty of Veterinary Medicine, Islamic Azad University, Karaj, Iran; bb Department of Cardiovascular Medicine, Cairo University, Cairo, Egypt; cc Komar University of Science and Technology, Sulaymaniyah, Iraq; dd Baxshin Hospital, Baxshin Research Center, Sulaymaniyah, Iraq; ee Community and Maternity Nursing Unit, University of Duhok, Duhok, Iraq; ff Department of Physiotherapy, Bayero University Kano, Kano, Nigeria; gg Department of Physiotherapy, Federal University Wukari, Wukari, Nigeria; hh Department of Research, Toufik's World Medical Association, Sumy, Ukraine; ii Department of Epidemiology and Biostatistics, University of Gondar, Gondar, Ethiopia; jj Department of Neurosurgery, University of Southern California, Los Angeles, CA, USA; kk Keck School of Medicine, University of Southern California, Los Angeles, CA, USA; ll Department of Emergency Medicine, Zanjan University of Medical Sciences, Zanjan, Iran; mm Yale School of Medicine, Yale University, New Haven, CT, USA; nn Neuroendocrine Unit, Harvard University, Boston, MA, USA; oo School of Pharmacy, Bahir Dar University, Bahir Dar, Ethiopia; pp Postgraduate Department, University of Sierra Sur, Miahuatlan de Porfirio Diaz, Mexico; qq Yhteiskuntadatatieteen keskus (Centre for Social Data Science), University of Helsinki, Helsinki, Finland; rr Nuffield Department of Population Health, University of Oxford, Oxford, UK; ss National Heart Foundation Hospital and Research Institute, Dhaka, Bangladesh; tt Department of Biomedical Sciences, Nazarbayev University School of Medicine, Astana, Kazakhstan; uu Department of Midwifery, Bahir Dar University, Bahir Dar, Ethiopia; vv Department of Internal Medicine, Federal Medical Centre, Abuja, Nigeria; ww Department of Family and Community Health, University of Health and Allied Sciences, Ho, Ghana; xx School of Population Health, University of New South Wales, Sydney, NSW, Australia; yy Cardiovascular Research Center, Massachusetts General Hospital, Boston, MA, USA; zz Department of Radiology, Harvard University, Boston, MA, USA; aaa Research Center for Immunodeficiencies, Tehran University of Medical Sciences, Tehran, Iran; bbb Department of Medical Biochemistry and Biophysics, Karolinska Institutet (Karolinska Institute), Stockholm, Sweden; ccc Department of Sport, Exercise and Rehabilitation, Northumbria University, Newcastle, UK; ddd Zoology Department, Benha University, Benha, Egypt; eee Department of Physical Pharmacy and Pharmacokinetics, Poznan University of Medical Sciences, Poznan, Poland; fff Department of Cardiovascular Disease, Loma Linda University Medical Center, Loma Linda, CA, USA; ggg Department of Pediatric Dentistry, Universidade Federal de Minas Gerais (Federal University of Minas Gerais), Belo Horizonte, Brazil; hhh Department of Anesthesiology, Shahid Beheshti University of Medical Sciences, Tehran, Iran; iii Clinical Pharmacy and Therapeutics Department, Applied Science Private University, Amman, Jordan; jjj Community Health Nursing Department, Jouf University, Sakaka, Saudi Arabia; kkk Graduate School of Public Health, St. Luke's International University, Tokyo, Japan; lll Division of Population Data Science, National Cancer Center, Tokyo, Japan; mmm Faculty of Veterinary Medicine, Cairo University, Cairo, Egypt; nnn Faculty of Veterinary Medicine, King Salman International University, Rus Sudr, Egypt; ooo Clinical Sciences Department, University of Sharjah, Sharjah, United Arab Emirates; ppp Department of Biopharmaceutics and Clinical Pharmacy, University of Jordan, Amman, Jordan; qqq Department of Nursing, University of Sharjah, Sharjah, United Arab Emirates; rrr Department of Maternal and Child Health Nursing, Jordan University of Science and Technology, irbid, Jordan; sss Department of Pharmacy Practice and Pharmacotherapeutics, University of Sharjah, Sharjah, United Arab Emirates; ttt Medical Research Center, Hamad Medical Corporation, Doha, Qatar; uuu College of Health Sciences, Qatar University, Doha, Qatar; vvv Birzeit University, Ramallah, Palestine; www Department of Pharmacology and Therapeutics, United Arab Emirates University, Al Ain, United Arab Emirates; xxx College of Pharmacy, University of Jordan, Amman, Jordan; yyy Department of Pharmacy, Hamad Medical Corporation, Doha, Qatar; zzz Department of Disease Control, London School of Hygiene & Tropical Medicine, London, UK; aaaa Department of Clinical Research, Clinical Research Institute of Benin (IRCB), Abomey-Calavi, Benin; bbbb Department of Restorative Dentistry, University of Sharjah, Sharjah, United Arab Emirates; cccc Department of Forensic Medicine and Toxicology, Karnali Academy of Health Sciences (KAHS), Jumla, Nepal; dddd Department of Emergency and Critical Care Nursing, Bahir Dar University, Bahir Dar, Ethiopia; eeee Department of Diagnostic and Interventional Radiology, Technical University of Munich, Munich, Germany; ffff Stanford University, Palo Alto, CA, USA; gggg Department of Global Health, Stellenbosch University, Cape Town, South Africa; hhhh Cochrane South Africa, South African Medical Research Council, Cape Town, South Africa; iiii School of Medicine, University of Sydney, Sydney, NSW, Australia; jjjj Centre for Social Research in Health, University of New South Wales, Sydney, NSW, Australia; kkkk Department of Health Promotion, Education and Behavior, University of South Carolina, Columbia, SC, USA; llll Department of Public Health, University of KwaZulu-Natal, Durban, South Africa; mmmm Department of Microbiology, Ladoke Akintola University, Osogbo, Nigeria; nnnn NMC Healthcare, Independent Consultant, Sharjah, United Arab Emirates; oooo Department of Sociology, Olabisi Onabanjo University, Ago-Iwoye, Nigeria; pppp Department of Immunology, Roswell Park Comprehensive Cancer Center, Buffalo, NY, USA; qqqq Graduate Program Division, University at Buffalo, Buffalo, NY, USA; rrrr Department of Pediatrics, East Tennessee State University, Johnson City, TN, USA; ssss Center for Cardiovascular Risk Research, Johnson City, TN, USA; tttt Translational Research Team, The University of Sydney, Sydney, NSW, Australia; uuuu Melanoma Institute Australia, The University of Sydney, Sydney, NSW, Australia; vvvv Department of Family Medicine, Bowen University, Iwo, Nigeria; wwww Department of Family Medicine, Bowen University Teaching Hospital, Ogbomoso, Nigeria; xxxx Department of Nursing, Wolaita Sodo University, Woliata Sodo, Ethiopia; yyyy United Arab Emirates University, Al Ain, United Arab Emirates; zzzz Institute of Public Health, Walden University, Al Ain, United Arab Emirates; aaaaa Department of Microbiology, University of Medical Sciences, Ondo, Ondo City, Nigeria; bbbbb Slum and Rural Health Initiative Research Academy, Slum and Rural Health Initiative, Ibadan, Nigeria; ccccc Department of Physiotherapy, University of Ibadan, Ibadan, Nigeria; ddddd Department of Biochemistry, Osun State University, Osogbo, Nigeria; eeeee Department of Educational Counselling and Developmental Psychology, University of Ibadan, Ibadan, Nigeria; fffff Department of Educational Psychology, University of Johannesburg, Johannesburg, South Africa; ggggg Department of Pharmacology and Therapeutics, University of Medical Sciences, Ondo, Ondo, Nigeria; hhhhh Department of Veterinary Medicine, University of Ibadan, Ibadan, Nigeria; iiiii School of Public Health, Mekelle University, Mekelle, Ethiopia; jjjjj Department of Fisheries and Marine Bioscience, Jashore University of Science and Technology, Jashore, Bangladesh; kkkkk Research School of Population Health, Australian National University, Canberra, ACT, Australia; lllll Apollo Institute Of Medical Sciences & Research Chittoor, Apollo Hospital, Chittoor, India; mmmmm Tehran University School of Medicine, Tehran University of Medical Sciences, Tehran, Iran; nnnnn Department of Biology, University of Hail, Hail, Saudi Arabia; ooooo Department of Public Health, Universitas Padjadjaran (Padjadjaran University), Bandung, Indonesia; ppppp Department of Health Administration and Education, University of Education Winneba, Winneba, Ghana; qqqqq Department of Epidemiology and Biostatistics, University of Health and Allied Sciences, Ho, Ghana; rrrrr School of Public Health, University of Texas Health Science Center at Houston, Houston, TX, USA; sssss Department of Public Health and Preventive Medicine, University of Naples “”Federico II“”, Naples, Italy; ttttt Department of Surgery, University of Toledo, Toledo, OH, USA; uuuuu Australian Centre for Health Services Innovation, Queensland University of Technology, Kelvin Grove, QLD, Australia; vvvvv Jamieson Trauma Institute, Metro North Health, Herston, QLD, Australia; wwwww Technical Services Directorate, MSI Nigeria Reproductive Choices, Abuja, Nigeria; xxxxx Department of Epidemiology and Medical Statistics, University of Ibadan, Ibadan, Nigeria; yyyyy Medical Oncology Department, University of Medicine and Pharmacy “Gr. T. Popa” Iasi in Romania, Iasi, Romania; zzzzz Medical Oncology Department, Regional Institute of Oncology, Iasi, Romania; aaaaaa Department of Community Medicine, King Edward Memorial Hospital, Lahore, Pakistan; bbbbbb Department of Public Health, Public Health Institute, Lahore, Pakistan; cccccc Department of Public Health, Wachemo University, Hossana, Ethiopia; dddddd Department of New Initiatives, International Vaccine Institute, Seoul, South Korea; eeeeee Department of Community Medicine, Rajarata University of Sri Lanka, Anuradhapura, Sri Lanka; ffffff MM College of Pharmacy, Maharishi Markandeshwar (Deemed to be University), Ambala, India; gggggg Department of Orthopedic Surgery and Sports Medicine, Boston Children's Hospital, Boston, MA, USA; hhhhhh Department of Neurosurgery, Alborz University of Medical Sciences, Karaj, Iran; iiiiii Neuroscience Research Center, Iran University of Medical Sciences, Tehran, Iran; jjjjjj Urology Research Center, Tehran University of Medical Sciences, Tehran, Iran; kkkkkk Department of Health Education and Health Promotion, Wachemo University, Hossana, Ethiopia; llllll Health Research and Innovation Sciences Center, Klaipeda University, Klaipeda, Lithuania; mmmmmm SPRINT Sport Physical Activity and Health Research & Innovation Center, Polytechnic Institute of Guarda, Guarda, Portugal; nnnnnn Trivedi School of Biosciences, Ashoka University, Sonipat, India; oooooo Department of Public Health Sciences, Queen's University, Kingston, ON, Canada; pppppp Rajaie Trauma Research Center, Shiraz University of Medical Sciences, Shiraz, Iran; qqqqqq School of Public Health, University of Technology Sydney, Sydney, NSW, Australia; rrrrrr College of Medicine, Shaqra University, Shaqra, Saudi Arabia; ssssss School of Medicine and Psychology, Australian National University, Canberra, ACT, Australia; tttttt Health Research Institute, University of Canberra, Canberra, NSW, Australia; uuuuuu Biological Production Unit, National Institute of Health, Islamabad, Pakistan; vvvvvv World Health Organization (WHO), Islamabad, Pakistan; wwwwww College of Veterinary Sciences, The University of Agriculture, Peshawar, Peshawar, Pakistan; xxxxxx Department of Research, King Khaled Eye Specialist Hospital & Research Center, Riyadh, Saudi Arabia; yyyyyy Department of Health Informatics, Qassim University, Buraidha, Saudi Arabia; zzzzzz Department of Health and Biological Sciences, Abasyn University, Peshawar, Pakistan; aaaaaaa Department of Natural Sciences, Lebanese American University, Beirut, Lebanon; bbbbbbb School of Public Health, Zhejiang University, Hangzhou, China; ccccccc Department of Community Health Sciences, Sohail University, Karachi, Pakistan; ddddddd College of Medicine, University of Cincinnati, Cincinnati, OH, USA; eeeeeee School of Medicine, Tehran University of Medical Sciences, Tehran, Iran; fffffff Department of Pharmacy Practice, Riphah Institute of Pharmaceutical Sciences, Islamabad, Pakistan; ggggggg Division of Infectious Diseases and Global Public Health (IDGPH), University of California San Diego, San Diego, CA, USA; hhhhhhh Institute of Endemic Diseases, University of Khartoum, Khartoum, Sudan; iiiiiii Swiss Tropical and Public Health Institute, University of Basel, Basel, Switzerland; jjjjjjj Medical Laboratory Science Department, University of Human Development, Sulaymaniyah, Iraq; kkkkkkk Department of Biosciences, COMSATS Institute of Information Technology, Islamabad, Pakistan; lllllll Manipal College of Dental Sciences, Mangalore, Manipal Academy of Higher Education, Mangalore, India; mmmmmmm Institute of Public Health, United Arab Emirates University, Al Ain, United Arab Emirates; nnnnnnn Department of Health Sciences and Informatics, Bangladesh Institute of Innovative Health Research, Dhaka, Bangladesh; ooooooo College of Nursing, Majmaah University, Al Majmaah, Saudi Arabia; ppppppp Department of Pathology and Microbiology, University of Duhok, Duhok, Iraq; qqqqqqq College of Medicine and Public Health, Flinders University, Adelaide, SA, Australia; rrrrrrr Faculty of Public Health, Jimma University, Jimma, Ethiopia; sssssss Department of Medicine, Rawalpindi Medical University, Rawalpindi, Pakistan; ttttttt Maternal and Child Health Division (MCHD), International Centre for Diarrhoeal Disease Research, Bangladesh, Dhaka, Bangladesh; uuuuuuu Department of Public Health Epidemiology, Debre Berhan University, Deberbirhan, Ethiopia; vvvvvvv Menelik II Medical and Health Science College, EpiMetrics, Inc., Addis Ababa, Ethiopia; wwwwwww Brody School of Medicine, East Carolina University, Greenville, NC, USA; xxxxxxx School of Medicine, Nazarbayev University, Astana, Kazakhstan; yyyyyyy Clinical Academic Department of Women's Health, University Medical Center, NU Medicine, Astana, Kazakhstan; zzzzzzz National Nutrition and Food Technology Research Institute, Shahid Beheshti University of Medical Sciences, Tehran, Iran; aaaaaaaa Faculty of Medicine and Public Health, Jenderal Soedirman University, Purwokerto, Indonesia; bbbbbbbb St George and Sutherland Clinical School, University of New South Wales, Sydney, NSW, Australia; cccccccc Department of Water Engineering, Graduate University of Advanced Technology, Kerman, Iran; dddddddd Oxford Vaccine Group, University of Oxford, Oxford, UK; eeeeeeee Department of Physiology, Ladoke Akintola University, Ogbomoso, Nigeria; ffffffff Department of Medicine, Nazarbayev University School of Medicine, Astana, Kazakhstan; gggggggg School of Veterinary Medicine, Texas Tech University, Amarillo, TX, USA; hhhhhhhh Department of Cardiology, Fudan University, Shanghai, China; iiiiiiii Faculty of Health and Behavioural Sciences, The University of Queensland, Brisbane, QLD, Australia; jjjjjjjj Chicago College of Osteopathic Medicine, Midwestern University, Downers Grove, IL, USA; kkkkkkkk Feinberg School of Medicine, Northwestern University, Chicago, IL, USA; llllllll Centre for Academic Primary Care, University of Nottingham, Nottingham, UK; mmmmmmmm College of Pharmacy and Health Sciences, Ajman University, Ajman, United Arab Emirates; nnnnnnnn Centre of Medical and Bio-allied Health Sciences Research, Ajman University, Ajman, United Arab Emirates; oooooooo Department of Communicable Diseases, Ministry of Health, Muscat, Oman; pppppppp Middle East, Eurasia, and Africa Influenza Stakeholders Network, Muscat, Oman; qqqqqqqq Division of Public Health Sciences, Washington University in St. Louis, St. Louis, MO, USA; rrrrrrrr Department of Urology, Cleveland Clinic Abu Dhabi, Abu Dhabi, United Arab Emirates; ssssssss The University of Jordan, Jordanian Public Health Society, Amman, Jordan; tttttttt American University in the Emirates, Dubai, United Arab Emirates; uuuuuuuu Fundamentals and Administration Department, Sultan Qaboos University, Muscat, Oman; vvvvvvvv Jordan Medical Association, Amman, Jordan; wwwwwwww Faculty of Pharmacy, Philadelphia University, Amman, Jordan; xxxxxxxx School of Pharmacy, Cardiff University, Cardiff, UK; yyyyyyyy Department of Adult Health and Critical Care, Sultan Qaboos University, Muscat, Oman; zzzzzzzz School of Public Health, University of Texas, Houston, TX, USA; aaaaaaaaa The University of Jordan School of Medicine, University of Jordan, Amman, Jordan; bbbbbbbbb Department of Biology, University of Bahrain, Zallaq, Bahrain; ccccccccc Department of Research and Development, Washington University in St. Louis, St. Louis, MO, USA; ddddddddd Clinical Epidemiology Center, US Department of Veterans Affairs (VA), St. Louis, MO, USA; eeeeeeeee Murdoch Business School, Murdoch University, Perth, WA, Australia; fffffffff Department of Bioengineering, George Mason University, Fairfax, VA, USA; ggggggggg Preventive Dentistry Department, Jouf University, Sakaka, Saudi Arabia; hhhhhhhhh Department of Oral and Maxillofacial Surgery, Shahid Beheshti University of Medical Sciences, Tehran, Iran; iiiiiiiii School of Nursing, Yarmouk University, Irbid, Jordan; jjjjjjjjj School of Nursing and Midwifery, Western Sydney University, Sydney, NSW, Australia; kkkkkkkkk Department of Nursing and Midwifery, Woldia University, Woldia, Ethiopia; lllllllll Department of Surgery, Hamad Medical Corporation, Doha, Qatar; mmmmmmmmm Department of Health Information Management and Technology, Imam Abdulrahman Bin Faisal University, Dammam, Saudi Arabia; nnnnnnnnn Department of Clinical Pharmacy, Al-Ayen Iraqi University, Thi-Qar, Iraq; ooooooooo Department of Clinical Pharmacy and Pharmacy Practice, University of Science and Technology, Sana'a, Yemen; ppppppppp Department of Community and Mental Health, Al al-Bayt University, Mafraq, Jordan; qqqqqqqqq Division of Gastroenterology and Hepatology, Mayo Clinic, Rochester, MN, USA; rrrrrrrrr General Directorate of Research and Studies, Ministry of Health, Riyadh, Saudi Arabia; sssssssss Institute of Health Informatics, University College London, London, UK; ttttttttt College of Pharmacy, University of Sharjah, Sharjah, United Arab Emirates; uuuuuuuuu School of Pharmacy, The University of Jordan, Amman, Jordan; vvvvvvvvv Curtin School of Population Health, Curtin University, Perth, WA, Australia; wwwwwwwww Department of Health Systems and Policy, University of Gondar, Gondar, Ethiopia; xxxxxxxxx Faculty of Health Sciences, Curtin University, Perth, WA, Australia; yyyyyyyyy Geospatial and Tuberculosis Research Team, Telethon Kids Institute, Perth, WA, Australia; zzzzzzzzz Pediatric Intensive Care Unit, King Saud University, Riyadh, Saudi Arabia; aaaaaaaaaa Department of Epidemiology and Biostatistics, University of South Carolina, Columbia, SC, USA; bbbbbbbbbb Department of Public Health, University of Hail, Hail, Saudi Arabia; cccccccccc Department of Bacteriology, Immunology, and Mycology, Suez Canal University, Ismailia, Egypt; dddddddddd College of Medicine and Health Sciences, Khalifa University, Abu Dhabi, United Arab Emirates; eeeeeeeeee Africa Center of Excellence for Mycotoxin and Food Safety, Minna, Nigeria; ffffffffff Faculty of Health Sciences, Epidemiology and Population Health Department, American University of Beirut, Beirut, Lebanon; gggggggggg British Columbia Injury Research Prevention Unit, British Columbia Children's Hospital Research Institute, Vancouver, BC, Canada; hhhhhhhhhh College of Nursing, Qatar University, Doha, Qatar; iiiiiiiiii Department of Health Services and Hospital Administration, King Abdulaziz University, Jeddah, Saudi Arabia; jjjjjjjjjj Health Economics Research Group, King Abdulaziz University, Jeddah, Saudi Arabia; kkkkkkkkkk Pediatric Department, King Saud University, Riyadh, Saudi Arabia; llllllllll Kidney and Pancreas Health Center, King Faisal Specialist Hospital & Research Center, Riyadh, Saudi Arabia; mmmmmmmmmm Faculty of Dentistry, Ibn Al-Nafis University for Medical Sciences, Sana'a, Yemen; nnnnnnnnnn College of Applied Medical Sciences, Qassim University, Buraydah, Saudi Arabia; oooooooooo Department of Biotechnology and Genetic Engineering, Hazara University Mansehra, Mansehra, Pakistan; pppppppppp Department of Biotechnology, University of Malakand, Chakdara, Pakistan; qqqqqqqqqq Department of Statistics and Operations Research, Aligarh Muslim University, Aligarh, India; rrrrrrrrrr Department of Biological Sciences, National University of Medical Sciences (NUMS), Rawalpindi, Pakistan; ssssssssss School of Food and Agricultural Sciences, University of Management and Technology, Lahore, Pakistan; tttttttttt Department of Pharmacy, Mohammed Al-Mana College for Medical Sciences, Dammam, Saudi Arabia; uuuuuuuuuu Department of Medical Rehabilitation (Physiotherapy), University of Maiduguri, Maiduguri, Nigeria; vvvvvvvvvv Nethersole School of Nursing, The Chinese University of Hong Kong, Hong Kong, China; wwwwwwwwww Department of Biosciences, Jamia Millia Islamia, New Delhi, India; xxxxxxxxxx Center for Biotechnology and Microbiology, University of Swat, Charbagh, Pakistan; yyyyyyyyyy Center for Biotechnology and Microbiology, University of Swat, Swat, Pakistan; zzzzzzzzzz Department of Geography, Sultan Qaboos University, Muscat, Oman; aaaaaaaaaaa Department of Pathophysiology and Transplantation, Università degli Studi di Milano (University of Milan), Milan, Italy; bbbbbbbbbbb Cystic Fibrosis Center, Fondazione IRCCS Ospedale Maggiore Policlinico (IRCCS “”Ca' Granda Maggiore Policlinico“” Hospital Foundation), Milan, Italy; ccccccccccc The School of Medicine, The University of Jordan, Amman, Jordan; ddddddddddd Institute of Health and Wellbeing, Federation University Australia, Melbourne, VIC, Australia; eeeeeeeeeee School of Public Health and Preventive Medicine, Monash University, Melbourne, VIC, Australia; fffffffffff The Graduate School of Biomedical Engineering, University of New South Wales, Sydney, NSW, Australia; ggggggggggg Biomedical Physics Group, University of Hamburg, Hamburg, Germany; hhhhhhhhhhh Department of Clinical and Community Pharmacy, An-Najah National University, Nablus, Palestine; iiiiiiiiiii Department of Biomedical Sciences, Nazarbayev University, Astana, Kazakhstan; jjjjjjjjjjj Family and Community Medicine Department, Qassim University, Al Qassim, Saudi Arabia; kkkkkkkkkkk Department of Public Health and Community Medicine, International Medical University, Kuala Lumpur, Malaysia; lllllllllll International Centre for Casemix and Clinical Coding, National University of Malaysia, Bandar Tun Razak, Malaysia; mmmmmmmmmmm College of Life Sciences, Birmingham City University, Birmingham, UK; nnnnnnnnnnn Cardiovascular Divivsion, University of Alabama, Birmingham, AL, USA; ooooooooooo Department of Global Public Health, Karolinska Institutet (Karolinska Institute), Stockholm, Sweden; ppppppppppp Department of Medical Laboratories, College of Applied Medical Sciences, Qassim University, Buraydah, Saudi Arabia; qqqqqqqqqqq College of Medicine and Health Sciences, United Arab Emirates University, Al Ain, United Arab Emirates; rrrrrrrrrrr Faculty of Medicine, Jordan University of Science and Technology, Irbid, Jordan; sssssssssss Faculty of Nursing, University of Tabuk, Tabuk, Saudi Arabia; ttttttttttt Independent Consultant, Amman, Jordan; uuuuuuuuuuu Rehabilitation Sciences Department, Qatar University, Doha, Qatar; vvvvvvvvvvv Department of Medicine, Nazarbayev University, Astana, Kazakhstan; wwwwwwwwwww Department of Parasitology, University of Malaya, Kuala Lumpur, Malaysia; xxxxxxxxxxx Department of Parasitology, Sana'a University, Sana'a, Yemen; yyyyyyyyyyy Nuffield Department of Surgical Sciences, University of Oxford, Oxford, UK; zzzzzzzzzzz Ophthalmology Department, University of Miami, Miami, FL, USA; aaaaaaaaaaaa Nursing Faculty, University of Tabuk, Tabuk, Saudi Arabia; bbbbbbbbbbbb School of Public Health, SRM Institute of Science and Technology, Chennai, India; cccccccccccc Department of Physical Therapy and Rehabilitation Sciences, Jordan University of Science and Technology, Irbid, Jordan; dddddddddddd Department of Rehabilitation Sciences and Physical Therapy, Jordan University of Science and Technology, Irbid, Jordan; eeeeeeeeeeee Faculty of Nursing, Zarqa University, Zarqa, Jordan; ffffffffffff Department of Respiratory Care, Prince Sultan Military College of Health Sciences, Dammam, Saudi Arabia; gggggggggggg Liver, Digestive, and Lifestyle Health Research Section, King Faisal Specialist Hospital & Research Center, Riyadh, Saudi Arabia; hhhhhhhhhhhh Division of Gastroenterology and Hepatology, Weill Cornell Medicine, New York, NY, USA; iiiiiiiiiiii American University of the Middle East, Egaila, Kuwait; jjjjjjjjjjjj Surgical Research Section, Hamad Medical Corporation, Doha, Qatar; kkkkkkkkkkkk Department of Nursing, Georgetown University, Washington, DC, USA; llllllllllll Macro-Fiscal Policy Department, Ministry of Finance, Dubai, United Arab Emirates; mmmmmmmmmmmm Department of Surgery, Kuwait University, Kuwait, Kuwait; nnnnnnnnnnnn Jaber Al Ahmad Al Sabah Hospital, Ministry of Health, Kuwait, Kuwait; oooooooooooo Department of Emergency Medicine, Sana'a University, Sanaa, Yemen; pppppppppppp Pediatric Emergency Medicine Department, Drexel University, Philadelphia, PA, USA; qqqqqqqqqqqq Department of Basic Sciences, Yarmouk University, Irbid, Jordan; rrrrrrrrrrrr Health Science Division, Higher Colleges of Technology, Sharjah, United Arab Emirates; ssssssssssss Institute of Molecular Biology and Biotechnology, The University of Lahore, Lahore, Pakistan; tttttttttttt Faculty of Health Sciences, Equator University of Science and Technology, Uganda, Masaka, Uganda; uuuuuuuuuuuu Research, Policy, and Training Directorate, Jordan Center for Disease Control, Amman, Jordan; vvvvvvvvvvvv Applied Science Research Center, Applied Science Private University, Amman, Jordan; wwwwwwwwwwww Department of Specialty Internal Medicine, Johns Hopkins Aramco Healthcare, Dhahran, Saudi Arabia; xxxxxxxxxxxx Department of Medicine, Indiana University School of Medicine, Indianapolis, IN, USA; yyyyyyyyyyyy Department of Respiratory Therapy, King Abdulaziz University, Jeddah, Saudi Arabia; zzzzzzzzzzzz Respiratory Therapy Unit, King Abdulaziz University, Jeddah, Saudi Arabia; aaaaaaaaaaaaa University of Sharjah, Sharjah, United Arab Emirates; bbbbbbbbbbbbb Faculty of Health Sciences, University of Cadiz, Cadiz, Spain; ccccccccccccc Research Group in Health Economics, Universidad de Cartagena (University of Cartagena), Cartagena, Colombia; ddddddddddddd Research Group in Hospital Management and Health Policies, Universidad de la Costa (University of the Coast), Barranquilla, Colombia; eeeeeeeeeeeee Department of Economic Sciences, Universidad de la Costa (University of the Coast), Barranquilla, Colombia; fffffffffffff National Health Observatory, National Institute of Health, Bogota, Colombia; ggggggggggggg Department of Clinical Pharmacology and Toxicology, Umm Al-Qura University, Makkah, Saudi Arabia; hhhhhhhhhhhhh Department of Rehabilitation Sciences, Jordan University of Science and Technology, Irbid, Jordan; iiiiiiiiiiiii Department of Medical Sciences, Azal University for Human Development, Sana'a, Yemen; jjjjjjjjjjjjj Department of Clinical Sciences, University of Science and Technology of Fujairah, Fujairah, United Arab Emirates; kkkkkkkkkkkkk Department of Pediatrics, Cleveland Clinic, Cleveland, OH, USA; lllllllllllll Department of Physiotherapy, Taif University, Taif, Saudi Arabia; mmmmmmmmmmmmm Department of Pharmaceutical Sciences, Qatar University, Doha, Qatar; nnnnnnnnnnnnn Department of Clinical Pharmacy, Jordan University of Science and Technology, Irbid, Jordan; ooooooooooooo Department of Biochemistry and Molecular Biology, Sana'a University, Sana'a, Yemen; ppppppppppppp Laboratory Medicine Department, Al-Baha University, Al-Aqiq, Saudi Arabia; qqqqqqqqqqqqq London School of Hygiene and Tropical Medicine, University of London, London, UK; rrrrrrrrrrrrr Global Health Advocacy Incubator (GHAI), University of Central Nicaragua, Washington, DC, USA; sssssssssssss Isfahan Cardiovascular Research Institute, Heart Failure Research Center., Isfahan University of Medical Sciences, Isfahan, Iran; ttttttttttttt Department of Pharmacy, An-Najah National University, Nablus, Palestine; uuuuuuuuuuuuu Student Research Committee, Lorestan University of Medical Sciences, Khorramabad, Iran; vvvvvvvvvvvvv Health Policy Research Center, Shiraz University of Medical Sciences, Shiraz, Iran; wwwwwwwwwwwww College of Medicine, University of Sharjah, Sharjah, United Arab Emirates; xxxxxxxxxxxxx Summer Program, University of Chicago, Chicago, IL, USA; yyyyyyyyyyyyy Public Health and Community Medicine Department, Cairo University, Cairo, Egypt; zzzzzzzzzzzzz Department of Radiology and Radiological Science, University of Maryland, Baltimore, MD, USA; aaaaaaaaaaaaaa Khomein University of Medical Sciences, Khomein, Iran; bbbbbbbbbbbbbb Gastrointestinal and Liver Diseases Research Center, Guilan University of Medical Sciences, Rasht, Iran; cccccccccccccc Department of Food Safety and Hygiene, Zanjan University of Medical Sciences, Zanjan, Iran; dddddddddddddd Spiritual Health Research Center, Baqiyatallah University of Medical Sciences, Tehran, Iran; eeeeeeeeeeeeee Department of Health and Wellbeing, African Population and Health Research Center, Nairobi, Kenya; ffffffffffffff Department of Medicine, University of Jos, Jos, Nigeria; gggggggggggggg Department of Internal Medicine, Jos University Teaching Hospital, Jos, Nigeria; hhhhhhhhhhhhhh Center for Biomedical Image Computing & Analytics, University of Pennsylvania, Philadelphia, PA, USA; iiiiiiiiiiiiii University of Bologna, Bologna, Italy; jjjjjjjjjjjjjj Department of General Medicine, Eastern Health, Box Hill, VIC, Australia; kkkkkkkkkkkkkk Orthopedic Department, Tehran University of Medical Sciences, Tehran, Iran; llllllllllllll Faculty of Pharmacy, Carol Davila University of Medicine and Pharmacy, Bucharest, Romania; mmmmmmmmmmmmmm Centre for Sensorimotor Performance, The University of Queensland, Brisbane, QLD, Australia; nnnnnnnnnnnnnn Neurology Department, Royal Brisbane and Women's Hospital, Brisbane, QLD, Australia; oooooooooooooo Faculty of Medicine and Health, University of Sydney, Sydney, NSW, Australia; pppppppppppppp Sydney Musculoskeletal Health, University of Sydney, Sydney, NSW, Australia; qqqqqqqqqqqqqq Department of General Medicine, Thai Binh University of Medicine and Pharmacy in Vietnam, Thai Binh City, Viet Nam; rrrrrrrrrrrrrr Department of Microbiology, Lagos State University, Ojo, Nigeria; ssssssssssssss Department of Management, University of Cape Coast, Cape Coast, Ghana; tttttttttttttt Department of Public Health, The Apollo University, Chittoor, India; uuuuuuuuuuuuuu Department of Physiotherapy, Galgotias University, Greater Noida, India; vvvvvvvvvvvvvv Department of Epidemiology and Biostatistics, Zahedan University of Medical Sciences, Zahedan, Iran; wwwwwwwwwwwwww Agribusiness Study Program, Sebelas Maret University, Surakarta, Indonesia; xxxxxxxxxxxxxx Department of Environmental and Occupational Health, University of Medical Sciences, Ondo, Ondo, Nigeria; yyyyyyyyyyyyyy Department of Microbiology, University of Medical Sciences, Ondo, Ondo, Nigeria; zzzzzzzzzzzzzz Regenerative Medicine, Organ Procurement and Transplantation Multi-disciplinary Center, Guilan University of Medical Sciences, Rasht, Iran; aaaaaaaaaaaaaaa Centre for Interdisciplinary Research in Basic Sciences (CIRBSc), Jamia Millia Islamia, New Delhi, India; bbbbbbbbbbbbbbb School of Chemical and Life Sciences (SCLS), Jamia Hamdard, New Delhi, India; ccccccccccccccc Department of Surgery, Gadjah Mada University, Yogyakarta, Indonesia; ddddddddddddddd Department of Pathology, Imam Mohammad Ibn Saud Islamic University, Riyadh, Saudi Arabia; eeeeeeeeeeeeeee Department of Rehabilitation Sciences, Hong Kong Polytechnic University, Kowloon, Hong Kong, China; fffffffffffffff Rural Health Research Institute, Charles Sturt University, Orange, NSW, Australia; ggggggggggggggg Department of Social Sciences, Berekum College of Education, Berekum, Ghana; hhhhhhhhhhhhhhh School of Public Health, Kwame Nkrumah University of Science and Technology, Kumasi, Ghana; iiiiiiiiiiiiiii Division of Gastroenterology, Hepatology, and Nutrition, Virginia Commonwealth University, Richmond, VA, USA; jjjjjjjjjjjjjjj Gastroenterology Department, Pontificia Universidad Catolica de Chile (Pontifical Catholic University of Chile), Santiago, Chile; kkkkkkkkkkkkkkk Geneva University Hospital, University of Geneva, Geneva, Switzerland; lllllllllllllll Health Management and Economics Research Center, Iran University of Medical Sciences, Tehran, Iran; mmmmmmmmmmmmmmm College of Pharmacy, Al Ain University, Abu Dhabi, United Arab Emirates; nnnnnnnnnnnnnnn University of Texas Health Science Center at Houston, Houston, TX, USA; ooooooooooooooo Department of Applied Mathematics, University of Washington, Seattle, WA, USA; ppppppppppppppp College of Art and Science, Ottawa University, Surprise, AZ, USA; qqqqqqqqqqqqqqq School of Life Sciences, Arizona State University, Tempe, AZ, USA; rrrrrrrrrrrrrrr Department of Medical Laboratory Science, Bahir Dar University, Bahir Dar, Ethiopia; sssssssssssssss Care in Long Term Conditions Research Division, King's College London, London, UK; ttttttttttttttt CIBER Epidemiology and Public Health (CIBERESP), Madrid, Spain; uuuuuuuuuuuuuuu School of Medicine, Shahid Beheshti University of Medical Sciences, Tehran, Iran; vvvvvvvvvvvvvvv Department of Cardiovascular, Endocrine-Metabolic Diseases and Aging, Istituto Superiore di Sanità (ISS), Rome, Italy; wwwwwwwwwwwwwww Department of Neurobiology, Care Sciences and Society, Karolinska Institutet (Karolinska Institute), Stockholm, Sweden; xxxxxxxxxxxxxxx School of Health and Social Studies, Dalarna University, Falun, Sweden; yyyyyyyyyyyyyyy Department of Biotechnology, Sri Ramaswamy Memorial Institute of Science and Technology, Kattankulathur, India; zzzzzzzzzzzzzzz University College of Medicine & Dentistry, The University of Lahore, Lahore, Pakistan; aaaaaaaaaaaaaaaa Institute for Biomedical Problems, Russian Academy of Sciences, Moscow, Russia; bbbbbbbbbbbbbbbb Department of Physiotherapy, University of Sharjah, Sharjah, United Arab Emirates; cccccccccccccccc Department of Physiotherapy, Manipal Academy of Higher Education, Manipal, India; dddddddddddddddd Department of Periodontics, Saveetha University, Chennai, India; eeeeeeeeeeeeeeee Department of Research, Nepal Health Research Council, Kathmandu, Nepal; ffffffffffffffff Department of Public Health, Kazakh National Medical University, Almaty, Kazakhstan; gggggggggggggggg Department of Clinical Disciplines, Al Farabi Kazakh National University, Almaty, Kazakhstan; hhhhhhhhhhhhhhhh College of Medicine, University of Arizona, Tucson, AZ, USA; iiiiiiiiiiiiiiii Department of Community Medicine and Global Health, University of Oslo, Oslo, Norway; jjjjjjjjjjjjjjjj Department of Pharmacy Practice, AlMaarefa University, Riyadh, Saudi Arabia; kkkkkkkkkkkkkkkk School of Public Health, Bahir Dar University, Bahir Dar, Ethiopia; llllllllllllllll Department of Public Health, Debre Tabor University, Debre Tabor, Ethiopia; mmmmmmmmmmmmmmmm Research Institute of Dental Sciences, Shahid Beheshti University of Medical Sciences, Tehran, Iran; nnnnnnnnnnnnnnnn National Agency for Strategic Research in Medical Education (NASRME), Ministry of Health and Medical Education, Tehran, Iran; oooooooooooooooo Cabrini Research, Cabrini Health, Malvern, VIC, Australia; pppppppppppppppp College of Applied Medical Science, University of Hail, Hail, Saudi Arabia; qqqqqqqqqqqqqqqq Pioneer Journal of Biostatistics and Medical Research (PJBMR), Pakistan, Pakistan; rrrrrrrrrrrrrrrr School of Medicine, Zanjan University of Medical Sciences, Zanjan, Iran; ssssssssssssssss Department of Radiation Oncology, Shandong University, Shandong, China; tttttttttttttttt Deakin Health Economics/School of Health and Social Development, Deakin University, Melbourne, VIC, Australia; uuuuuuuuuuuuuuuu Faculty of Medicine, Nursing, and Health Sciences, Monash University, Melbourne, VIC, Australia; vvvvvvvvvvvvvvvv Nursing Department, Institute of Technology and Health Science RS dr Soepraoen, Malang, Indonesia; wwwwwwwwwwwwwwww Faculty of Health Science, Institute of Technology and Health Science RS dr Soepraoen, Malang, Indonesia; xxxxxxxxxxxxxxxx Atchabar Scientific Research Institute, Kazakh National Medical University, Almaty, Kazakhstan; yyyyyyyyyyyyyyyy Department of Immunology, Zanjan University of Medical Sciences, Zanjan, Iran; zzzzzzzzzzzzzzzz Department of Forensic Medicine, Lumbini Medical College, Palpa, Nepal; aaaaaaaaaaaaaaaaa Management Policy and Community Health, University of Texas, Houston, TX, USA; bbbbbbbbbbbbbbbbb College of Medicine, University of Basrah, Basrah, Iraq; ccccccccccccccccc Department of Computer Engineering, King Saud University, Riyadh, Saudi Arabia; ddddddddddddddddd School of Business, University of Leicester, Leicester, UK; eeeeeeeeeeeeeeeee Department of Statistics and Econometrics, Bucharest University of Economic Studies, Bucharest, Romania; fffffffffffffffff Robarts Research Institute, The University of Western Ontario, London, ON, Canada; ggggggggggggggggg Departament of Physiotherapy, Federal University of Santa Catarina, Araranguá, Brazil; hhhhhhhhhhhhhhhhh Institute of Molecular Biology and Biotechnology, The University of Lahore, lahore, Pakistan; iiiiiiiiiiiiiiiii School of Nursing and Public Health, University of KwaZulu-Natal, Durban, South Africa; jjjjjjjjjjjjjjjjj Department of Public Health, Wollega University, Nekemte, Ethiopia; kkkkkkkkkkkkkkkkk Department of Health Behavior and Society, Jimma University, Jimma, Ethiopia; lllllllllllllllll Department of Health Information Management, Iran University of Medical Sciences, Tehran, Iran; mmmmmmmmmmmmmmmmm Medicinal Chemistry Unit, Kwara State University, Malete, Ilorin, Nigeria; nnnnnnnnnnnnnnnnn Centre for Drug Research, Universiti Sains Malaysia, Pinang, Malaysia; ooooooooooooooooo Department of Health Information Management, Tehran University of Medical Sciences, Tehran, Iran; ppppppppppppppppp Department of Surgery, Washington University in St. Louis, St. Louis, MO, USA; qqqqqqqqqqqqqqqqq Research and Technology Deputy, Kurdistan University of Medical Sciences, Sanandaj, Iran; rrrrrrrrrrrrrrrrr Department of Infectious Disease Epidemiology, London School of Hygiene & Tropical Medicine, London, UK; sssssssssssssssss Department of Applied Mathematics, Stellenbosch University, Stellenbosch, South Africa; ttttttttttttttttt Department of Reproductive Health, University of Gondar, Gondar, Ethiopia; uuuuuuuuuuuuuuuuu The World Bank, Washington, DC, USA; vvvvvvvvvvvvvvvvv Department of Medicine, Tehran University of Medical Sciences, Tehran, Iran; wwwwwwwwwwwwwwwww Department of Psychiatry, University of Social Welfare and Rehabilitation Sciences, Tehran, Iran; xxxxxxxxxxxxxxxxx Advanced Medical & Dental Institute, Universiti Sains Malaysia, Penang, Malaysia; yyyyyyyyyyyyyyyyy Department of Anesthesia, Cihan University -Sulaimaniya, Sulaymaniyah, Iraq; zzzzzzzzzzzzzzzzz Department of Basic Sciences, University of Sulaimani, Sulaymaniyah, Iraq; aaaaaaaaaaaaaaaaaa Rheumatology Research Center, Tehran University of Medical Sciences, Tehran, Iran; bbbbbbbbbbbbbbbbbb Tehran University of Medical Sciences, Tehran, Iran; cccccccccccccccccc ASIDE Healthcare, Lewes, DE, USA; dddddddddddddddddd Faculty of Medicine, October 6 University, 6th of October City, Egypt; eeeeeeeeeeeeeeeeee Department of Population Medicine, Qatar University, Doha, Qatar; ffffffffffffffffff Department of Precision Medicine, Sungkyunkwan University, Seongnam, South Korea; gggggggggggggggggg Department of Pediatrics, All India Institute of Medical Sciences, New Delhi, India; hhhhhhhhhhhhhhhhhh School of Medical Education and Learning Technologies, Shahid Beheshti University of Medical Sciences, Tehran, Iran; iiiiiiiiiiiiiiiiii Shahid Rajii Hospital, Shahid Beheshti University of Medical Sciences, Tehran, Iran; jjjjjjjjjjjjjjjjjj Anesthesiology Research Center, Shahid Beheshti University of Medical Sciences, Tehran, Iran; kkkkkkkkkkkkkkkkkk Dental Material Research Center, Islamic Azad University, Tehran, Iran; llllllllllllllllll Pediatric Dentistry Department, King Abdulaziz University, Jeddah, Saudi Arabia; mmmmmmmmmmmmmmmmmm School of Public Health, Tehran University of Medical Sciences, Tehran, Iran; nnnnnnnnnnnnnnnnnn Clinical Research Center, Nanjing Children's Hospital, Nanjing, China; oooooooooooooooooo Faculty of Medicine, Mansoura University, Mansoura, Egypt; pppppppppppppppppp TIRR Memorial Hermann, Houston, TX, USA; qqqqqqqqqqqqqqqqqq Lerner College of Medicine, Cleveland Clinic, Cleveland, OH, USA; rrrrrrrrrrrrrrrrrr Chen Senior Medical Center, Tamarac, FL, USA; ssssssssssssssssss Anahuac Business School, Universidad Anahuac Mexico, Mexico City, Mexico; tttttttttttttttttt Department of Epidemiology and Biostatistics, Kerman University of Medical Sciences, Kerman, Iran; uuuuuuuuuuuuuuuuuu College of Medicine, Alfaisal University, Riyadh, Saudi Arabia; vvvvvvvvvvvvvvvvvv Center of Innovation, Technology and Education (CITE), Anhembi Morumbi University, São José dos Campos, Brazil; wwwwwwwwwwwwwwwwww Department of Neurosurgery, University of Southampton, Southampton, UK; xxxxxxxxxxxxxxxxxx Department of Non-communicable Diseases, Bangladesh University of Health Sciences, Dhaka, Bangladesh; yyyyyyyyyyyyyyyyyy Department of Translational Medicine, Florida International University, Miami, FL, USA; zzzzzzzzzzzzzzzzzz School of Psychology, University of Auckland, Auckland, New Zealand; aaaaaaaaaaaaaaaaaaa Department of Public and Environmental Health, University of The Gambia, Banjul, The Gambia; bbbbbbbbbbbbbbbbbbb Department of Epidemiology, University of Florida, Gainesville, FL, USA; ccccccccccccccccccc Heidelberg Institute of Global Health (HIGH), Heidelberg University Hospital, Heidelberg, Germany; ddddddddddddddddddd Department of Community and Family Medicine, All India Institute of Medical Sciences, Gorakhpur, India; eeeeeeeeeeeeeeeeeee University Institute of Food Science and Technology, The University of Lahore, Lahore, Pakistan; fffffffffffffffffff Department of General Surgery and Medical-Surgical Specialties, University of Catania, Catania, Italy; ggggggggggggggggggg College of Medicine, Jouf University, Sakaka, Saudi Arabia; hhhhhhhhhhhhhhhhhhh Barcelona Institute for Global Health, ISGlobal Instituto de Salud Global de Barcelona (Barcelona Institute for Global Health), Barcelona, Spain; iiiiiiiiiiiiiiiiiii Catalan Institution for Research and Advanced Studies (ICREA), Barcelona, Spain; jjjjjjjjjjjjjjjjjjj Non-communicable Diseases Research Center, Tehran University of Medical Sciences, Tehran, Iran; kkkkkkkkkkkkkkkkkkk School of Medicine, Iran University of Medical Sciences, Tehran, Iran; lllllllllllllllllll Faculty of Nursing, King Khalid University, Mahyil Asir, Saudi Arabia; mmmmmmmmmmmmmmmmmmm Department of Medical Education, University of Nevada Las Vegas, Las Vegas, NV, USA; nnnnnnnnnnnnnnnnnnn Department of Health Sciences (DISSAL), University of Genoa, Genova, Italy; ooooooooooooooooooo Cancer Research Center, Shahid Beheshti University of Medical Sciences, Tehran, Iran; ppppppppppppppppppp Pastor Institute, Tehran University of Medical Sciences, Tehran, Iran; qqqqqqqqqqqqqqqqqqq Biological Science Division, University of Chicago, Chicago, IL, USA; rrrrrrrrrrrrrrrrrrr The George Institute for Global Health, Imperial College London, London, UK; sssssssssssssssssss School of Public Health, Dr. D. Y. Patil University, Mumbai, India; ttttttttttttttttttt Jazan University, Jazan, Saudi Arabia; uuuuuuuuuuuuuuuuuuu Department of Human Anatomy and Histology, I.M. Sechenov First Moscow State Medical University, Moscow, Russia; vvvvvvvvvvvvvvvvvvv Department of Mental Health, AUSL Romagna, Ravenna, Italy; wwwwwwwwwwwwwwwwwww Milken Institute of Public Health, George Washington University, Washington, DC, USA; xxxxxxxxxxxxxxxxxxx College of Medicine and Health Science, Bahir Dar University, Bahir dar, Ethiopia; yyyyyyyyyyyyyyyyyyy Menzies School of Health Research, Charles Darwin University, Darwin, NT, Australia; zzzzzzzzzzzzzzzzzzz College of Health Science, Debre Tabor University, Debre Tabor, Ethiopia; aaaaaaaaaaaaaaaaaaaa Department of Public Health, Bahir Dar University, Bahir Dar, Ethiopia; bbbbbbbbbbbbbbbbbbbb Department of Public Health, University of South Africa, Pretoria, South Africa; cccccccccccccccccccc Department of Midwifery, Arba Minch University, Arba Minch, Ethiopia; dddddddddddddddddddd Department of Radiology, Mayo Clinic, Rochester, MN, USA; eeeeeeeeeeeeeeeeeeee Infectious Disease Research Department, King Abdullah International Medical Research Center, Riyadh, Saudi Arabia; ffffffffffffffffffff Department of Veterinary Microbiology, Usmanu Danfodiyo University, Sokoto, Sokoto, Nigeria; gggggggggggggggggggg Department of Physiotherapy and Paramedicine, Glasgow Caledonian University, Glasgow, UK; hhhhhhhhhhhhhhhhhhhh Department of Biological Sciences, University of Porto, Porto, Portugal; iiiiiiiiiiiiiiiiiiii Research Unit on Applied Molecular Biosciences (UCIBIO), University of Porto, Porto, Portugal; jjjjjjjjjjjjjjjjjjjj Department of Biomedical Sciences, University of West Attica, Athens, Greece; kkkkkkkkkkkkkkkkkkkk National AIDS Reference Center of Southern Greece, University of West Attica, Athens, Greece; llllllllllllllllllll Center of Excellence of Cancer Research, University of Sharjah, Sharjah, United Arab Emirates; mmmmmmmmmmmmmmmmmmmm Department of Internal Medicine, Universidade de São Paulo (University of São Paulo), São Paulo, Brazil; nnnnnnnnnnnnnnnnnnnn BRAC James P Grant School of Public Health, BRAC University, Dhaka, Bangladesh; oooooooooooooooooooo Dipartimento di Scienze Mediche e Chirurgiche, University of Bologna, Bologna, Italy; pppppppppppppppppppp Department of Nursing, Bahir Dar University, Bahir Dar, Ethiopia; qqqqqqqqqqqqqqqqqqqq School of Public Health, Johns Hopkins University, Baltimore, MD, USA; rrrrrrrrrrrrrrrrrrrr Department of Epidemiology and Biostatistics, University of the Philippines Manila, Manila, Philippines; ssssssssssssssssssss Hubert Department of Global Health, Emory University, Atlanta, GA, USA; tttttttttttttttttttt Department of Global Health, George Washington University, Washington, DC, USA; uuuuuuuuuuuuuuuuuuuu Department of Public Health, Experimental and Forensic Medicine, University of Pavia, Pavia, Italy; vvvvvvvvvvvvvvvvvvvv Faculty of Medicine, Universidade Católica Portuguesa (Catholic University of Portugal), Sintra, Portugal; wwwwwwwwwwwwwwwwwwww Center for Interdisciplinary Research in Health (CIIS), Universidade Católica Portuguesa (Catholic University of Portugal), Lisbon, Portugal; xxxxxxxxxxxxxxxxxxxx Department of Community and Family Medicine, All India Institute of Medical Sciences, Rishikesh, India; yyyyyyyyyyyyyyyyyyyy Community Health Department, University of South Wales, South Wales, UK; zzzzzzzzzzzzzzzzzzzz Department of Public Health, North Dakota State University, Fargo, ND, USA; aaaaaaaaaaaaaaaaaaaaa Institute of Applied Health Research, University of Nottingham, Nottingham, UK; bbbbbbbbbbbbbbbbbbbbb Institute of Applied Health Research, University of Birmingham, Birmingham, UK; ccccccccccccccccccccc Dwyer School of Health Sciences, Indiana University South Bend, South Bend, IN, USA; ddddddddddddddddddddd Department of Global Health and Population, Harvard University, Boston, MA, USA; eeeeeeeeeeeeeeeeeeeee Department of Internal Medicine, Saint Vincent Hospital, Worcester, Worcester, MA, USA; fffffffffffffffffffff Department of Anatomy, All India Institute of Medical Sciences, Jodhpur, India; ggggggggggggggggggggg Department of Community Medicine and Family Medicine, All India Institute of Medical Sciences, Jodhpur, India; hhhhhhhhhhhhhhhhhhhhh School of Public Health, All India Institute of Medical Sciences, Jodhpur, India; iiiiiiiiiiiiiiiiiiiii Department of Internal Medicine, Wayne State University, Gross Pointe Woods, MI, USA; jjjjjjjjjjjjjjjjjjjjj Global Health Neurology Lab, NSW Brain Clot Bank, Sydney, NSW, Australia; kkkkkkkkkkkkkkkkkkkkk Division of Cerebrovascular Medicine and Neurology, National Cerebral and Cardiovascular Center, Suita, Japan; lllllllllllllllllllll Manipal College of Health Professions, Manipal Academy of Higher Education, Udupi, India; mmmmmmmmmmmmmmmmmmmmm Translational and Clinical Research Institute, Newcastle University, Newcastle upon Tyne, UK; nnnnnnnnnnnnnnnnnnnnn School of Sport & Health Sciences, University of Brighton, Brighton, UK; ooooooooooooooooooooo Department of Public Health Research, Bengal Rural Welfare Service (BRWS), Kolkata, India; ppppppppppppppppppppp Department of Medical Lab Technology, Chandigarh University, Mohali, India; qqqqqqqqqqqqqqqqqqqqq Laboratory of Translational Medicine and Nanotherapeutics, Central University of Punjab, Bathinda, India; rrrrrrrrrrrrrrrrrrrrr Department of Pharmacy, University of Asia Pacific, Dhaka, Bangladesh; sssssssssssssssssssss Centre for Global Child Health, University of Toronto, Toronto, ON, Canada; ttttttttttttttttttttt Institute for Global Health & Development, Aga Khan University, Karachi, Pakistan; uuuuuuuuuuuuuuuuuuuuu Department of Health Administration, Rutgers University, New Brunswick, NJ, USA; vvvvvvvvvvvvvvvvvvvvv School of Public Health, Xuzhou Medical University, Xuzhou, China; wwwwwwwwwwwwwwwwwwwww Independent Consultant, Addis Ababa, Ethiopia; xxxxxxxxxxxxxxxxxxxxx Fondazione Banca Degli Occhi Del Veneto, Carol Davila University of Medicine and Pharmacy, Venice, Italy; yyyyyyyyyyyyyyyyyyyyy Department of Medical Laboratory Sciences, University of Hail, Hail, Saudi Arabia; zzzzzzzzzzzzzzzzzzzzz Department of Neurology, Institute of Post-Graduate Medical Education and Research and Seth Sukhlal Karnani Memorial Hospital, Kolkata, India; aaaaaaaaaaaaaaaaaaaaaa Department of Community Medicine and Family Medicine, All India Institute of Medical Sciences, Deoghar, India; bbbbbbbbbbbbbbbbbbbbbb Department of Biochemistry and Biotechnology, University of Science and Technology Chittagong, Chittagong, Bangladesh; cccccccccccccccccccccc Department of Clinical Pharmacy, Universiti Sultan Zainal Abidin, Besut, Malaysia; dddddddddddddddddddddd Health Biotechnology Directorate at Bio and Emerging Technology Institute, Addis Ababa University, Addis Ababa, Ethiopia; eeeeeeeeeeeeeeeeeeeeee Department of Physical Education and Health, Universidad de la República, Rivera, Uruguay; ffffffffffffffffffffff Department of Global Public Health and Primary Care, University of Bergen, Bergen, Norway; gggggggggggggggggggggg Cancer Registry of Norway, Oslo, Norway; hhhhhhhhhhhhhhhhhhhhhh School of Business Administration, American University of Sharjah, Sharjah, United Arab Emirates; iiiiiiiiiiiiiiiiiiiiii Department of Health Promotion and Behavioural Science, Bahir Dar University, Bahir Dar, Ethiopia; jjjjjjjjjjjjjjjjjjjjjj Faculty of Psychology, Education, and Sport, University Lusofona, Porto, Portugal; kkkkkkkkkkkkkkkkkkkkkk Research Centre for Physical Activity, Health, and Leisure, University of Porto, Porto, Portugal; llllllllllllllllllllll Global healthcare management, York University, London, UK; mmmmmmmmmmmmmmmmmmmmmm Demography and Population Studies, University of the Witwatersrand, Johannesburg, South Africa; nnnnnnnnnnnnnnnnnnnnnn Ophthalmology Department, Isfahan University of Medical Sciences, Isfahan, Iran; oooooooooooooooooooooo Faculty of Medicine and Pharmaceutical Sciences, University of Douala, Douala, Cameroon; pppppppppppppppppppppp Department of Cardiology, Centre Hospitalier Montfermeil (Montfermeil Hospital Center), Montfermeil, France; qqqqqqqqqqqqqqqqqqqqqq Department of Anesthesia and Critical Care Medicine, Johns Hopkins University, Baltimore, MD, USA; rrrrrrrrrrrrrrrrrrrrrr Internal Medicine Department, Shahid Beheshti University of Medical Sciences, Tehran, Iran; ssssssssssssssssssssss Department of Internal Medicine, Shahrekord University of Medical Sciences, Shahrekord, Iran; tttttttttttttttttttttt College of Human and Social Futures, University of Newcastle, Sydney, NSW, Australia; uuuuuuuuuuuuuuuuuuuuuu Disease Surveillance Department, Ghana Health Service, Ho, Ghana; vvvvvvvvvvvvvvvvvvvvvv Facultad de Salud (Faculty of Health), Universidad Santiago de Cali, Cali, Colombia; wwwwwwwwwwwwwwwwwwwwww Transport and Road Safety (TARS) Research Centre, University of New South Wales, Sydney, NSW, Australia; xxxxxxxxxxxxxxxxxxxxxx Department of Earth, Environment, and Equity, Howard University, Washington, DC, USA; yyyyyyyyyyyyyyyyyyyyyy Cancer Population Sciences Program, University of Florida Health Cancer Center, Gainesville, FL, USA; zzzzzzzzzzzzzzzzzzzzzz Department of Internal Medicine, Universidade Federal de Minas Gerais (Federal University of Minas Gerais), Belo Horizonte, Brazil; aaaaaaaaaaaaaaaaaaaaaaa School of Population and Public Health, University of British Columbia, Vancouver, BC, Canada; bbbbbbbbbbbbbbbbbbbbbbb Department of Psychiatry and Behavioral Health, Ohio State University, Columbus, OH, USA; ccccccccccccccccccccccc Department of Psychology, Ohio State University, Columbus, OH, USA; ddddddddddddddddddddddd Institute for Medical Information Processing, Biometry, and Epidemiology, LMU Munich, Neuherberg, Germany; eeeeeeeeeeeeeeeeeeeeeee Institute of Epidemiology, Helmholtz Zentrum München (German Research Center for Environmental Health), Neuherberg, Germany; fffffffffffffffffffffff Division of Clinical Epidemiology and Aging Research, German Cancer Research Center, Heidelberg, Germany; ggggggggggggggggggggggg Joint China-Cuba Lab for Neurotechnology, University of Electronic Sciences and Technology of China UESTC, Chengdu, China; hhhhhhhhhhhhhhhhhhhhhhh Neuroinformatics Department, Cuban Neuroscience Center, Havana, Cuba; iiiiiiiiiiiiiiiiiiiiiii Department of Injury, The George Institute for Global Health, Newtown, NSW, Australia; jjjjjjjjjjjjjjjjjjjjjjj Faculty of Medicine, University of New South Wales, Kensington, NSW, Australia; kkkkkkkkkkkkkkkkkkkkkkk The Malaria Atlas Project, Telethon Kids Institute, Nedlands, WA, Australia; lllllllllllllllllllllll Department of Health Sciences, University of Leicester, Leicester, UK; mmmmmmmmmmmmmmmmmmmmmmm Department of Medical and Surgical Sciences, University of Bologna, Bologna, Italy; nnnnnnnnnnnnnnnnnnnnnnn Flinders Health and Medical Research Institute, Flinders University, Adelaide, SA, Australia; ooooooooooooooooooooooo Department of Environmental Health, Bahir Dar University, Bahir Dar, Ethiopia; ppppppppppppppppppppppp Department of Woman and Child Health and Public Health, Fondazione Policlinico Universitario A. Gemelli IRCCS (Agostino Gemelli University Polyclinic IRCCS), Rome, Italy; qqqqqqqqqqqqqqqqqqqqqqq Global Health Research Institute, Università Cattolica del Sacro Cuore (Catholic University of the Sacred Heart), Rome, Italy; rrrrrrrrrrrrrrrrrrrrrrr National Centre for Epidemiology and Population Health, Australian National University, Canberra, ACT, Australia; sssssssssssssssssssssss Department of Medical Laboratory Science, Ladoke Akintola University, Ogbomoso, Nigeria; ttttttttttttttttttttttt School of Medicine and Health, Technical University of Munich, Munich, Germany; uuuuuuuuuuuuuuuuuuuuuuu Department of Radiology, University of Cambridge, Cambridge, UK; vvvvvvvvvvvvvvvvvvvvvvv Department of Basic Biomedical Sciences, University of Sharjah, Sharjah, United Arab Emirates; wwwwwwwwwwwwwwwwwwwwwww Department of Family and Community Medicine, King Abdulaziz University, Jeddah, Saudi Arabia; xxxxxxxxxxxxxxxxxxxxxxx School of Public Health Sciences, University of Waterloo, Waterloo, ON, Canada; yyyyyyyyyyyyyyyyyyyyyyy Al Shifa School of Public Health, Al Shifa Trust Eye Hospital, Rawalpindi, Pakistan; zzzzzzzzzzzzzzzzzzzzzzz JSS Dental College & Hospital, Jagadguru Sri Shivarathreeswara University, Mysore, India; aaaaaaaaaaaaaaaaaaaaaaaa Department of Sociology, University of Macau, Macau, China; bbbbbbbbbbbbbbbbbbbbbbbb The Children's Hospital at Westmead, New South Wales Poisons Information Centre, Sydney, NSW, Australia; cccccccccccccccccccccccc Faculty of Health Sciences Healthcare Management Department, Ankara University, Ankara, Türkiye; dddddddddddddddddddddddd Department of Clinical Pharmacy, University of Medicine and Pharmacy of Craiova, Craiova, Romania; eeeeeeeeeeeeeeeeeeeeeeee Department of Internal and Geriatric Medicine, Hospital Italiano de Buenos Aires (Italian Hospital of Buenos Aires), Buenos Aires, Argentina; ffffffffffffffffffffffff Board of Directors, Argentine Society of Medicine, Buenos Aires, Argentina; gggggggggggggggggggggggg Center of Innovation, Technology and Education (CITE), Anhembi Morumbi University, Sao Jose dos Campos, Brazil; hhhhhhhhhhhhhhhhhhhhhhhh Center for Nutrition and Health Research, National Institute of Public Health, Cuernavaca, Mexico; iiiiiiiiiiiiiiiiiiiiiiii Department of Ophthalmology, Beijing Institute of Ophthalmology, Beijing, China; jjjjjjjjjjjjjjjjjjjjjjjj Department of Anesthesiology, Third Xiangya Hospital of Central South University, Changsha, China; kkkkkkkkkkkkkkkkkkkkkkkk Unit of Hygiene and Public Health, Romagna Local Health Authority, Forlì-Cesena, Italy; llllllllllllllllllllllll Interdisciplinary Research Center for Health Science, Sant'Anna School of Advanced Studies, Pisa, Italy; mmmmmmmmmmmmmmmmmmmmmmmm Department of Health Care, Metropolitan Autonomous University, Mexico City, Mexico; nnnnnnnnnnnnnnnnnnnnnnnn Institute for Cancer Research, Prevention and Clinical Network, Florence, Italy; oooooooooooooooooooooooo Department of Medical Epidemiology and Biostatistics, Karolinska Institutet (Karolinska Institute), Stockholm, Sweden; pppppppppppppppppppppppp Department of Medicine and Surgery, University of Insubria, Varese, Italy; qqqqqqqqqqqqqqqqqqqqqqqq IMPInstitute for Mental and Physical Health and Clinical Translation (IMPACT), Deakin University, Geelong, VIC, Australia; rrrrrrrrrrrrrrrrrrrrrrrr Faculty of Health Sciences, University Fernando Pessoa, Porto, Portugal; ssssssssssssssssssssssss Associated Laboratory for Green Chemistry (LAQV), University of Porto, Porto, Portugal; tttttttttttttttttttttttt School of Health Science, University of Sydney, Sydney, NSW, Australia; uuuuuuuuuuuuuuuuuuuuuuuu Education Center of Australia, Health Science College, Sydney, NSW, Australia; vvvvvvvvvvvvvvvvvvvvvvvv Department of Psychiatry, Universidade de São Paulo (University of São Paulo), São Paulo, Brazil; wwwwwwwwwwwwwwwwwwwwwwww Public Health Department, National University of Colombia, Bogota, Colombia; xxxxxxxxxxxxxxxxxxxxxxxx Epidemiology and Public Health Evaluation Group, National University of Colombia, Bogota, Colombia; yyyyyyyyyyyyyyyyyyyyyyyy Division of Country Health Policies and Systems (CPS), World Health Organization (WHO), -, Italy; zzzzzzzzzzzzzzzzzzzzzzzz Mental Health Flagship, World Health Organization (WHO), Copenhagen, Denmark; aaaaaaaaaaaaaaaaaaaaaaaaa Institute of Public Goods and Policies (IPP), Spanish National Research Council, Madrid, Spain; bbbbbbbbbbbbbbbbbbbbbbbbb Centre for Biomedical Research in Mental Health Network (CIBERSAM), Institute of Health Carlos III, Madrid, Spain; ccccccccccccccccccccccccc Department of Pharmacological and Biomolecular Sciences, Università degli Studi di Milano (University of Milan), Milan, Italy; ddddddddddddddddddddddddd MultiMedica Sesto San Giovanni IRCCS, Sesto San Giovanni, Italy; eeeeeeeeeeeeeeeeeeeeeeeee Department of Public Health and Infectious Diseases, La Sapienza University, Rome, Italy; fffffffffffffffffffffffff Department of Medical, Surgical, and Health Sciences, University of Trieste, Trieste, Italy; ggggggggggggggggggggggggg Public Health Unit, University Health Agency Giuliano-Isontina (ASUGI), Trieste, Italy; hhhhhhhhhhhhhhhhhhhhhhhhh Department of Nutrition, Federal University of Santa Catarina, Florianópolis, Brazil; iiiiiiiiiiiiiiiiiiiiiiiii College of Public Health, Medical, and Veterinary Sciences, James Cook University, Townsville, QLD, Australia; jjjjjjjjjjjjjjjjjjjjjjjjj Department of Public Health, University of Mataram, Mataram, Indonesia; kkkkkkkkkkkkkkkkkkkkkkkkk Mary MacKillop Institute for Health Research, Australian Catholic University, Melbourne, VIC, Australia; lllllllllllllllllllllllll School of Public Health, University of Hong Kong, Hong Kong, China; mmmmmmmmmmmmmmmmmmmmmmmmm Posgrado de Medicina, Facultad de Ciencias de la Salud, Universidad Científica del Sur (University of the South), Lima, Peru; nnnnnnnnnnnnnnnnnnnnnnnnn Department of Biotechnology, Adamas University, Kolkata, India; ooooooooooooooooooooooooo Institute for Skeletal Aging & Orthopedic Surgery, Hallym University, Chuncheon, South Korea; ppppppppppppppppppppppppp State Disease Investigation Laboratory, Animal Resources Development Department, Agartala, India; qqqqqqqqqqqqqqqqqqqqqqqqq Department of Applied Health Sciences, University of Birmingham, Birmingham, UK; rrrrrrrrrrrrrrrrrrrrrrrrr Clinical Nutrition Department, Jazan University, Jazan, Saudi Arabia; sssssssssssssssssssssssss Department of Psychiatry, University of Kelaniya, Ragama, Sri Lanka; ttttttttttttttttttttttttt University Psychiatry Unit, Colombo North Teaching Hospital, Ragama, Sri Lanka; uuuuuuuuuuuuuuuuuuuuuuuuu Manipal College of Health Professions, Manipal Academy of Higher Education, Karnataka, India; vvvvvvvvvvvvvvvvvvvvvvvvv Department of Epidemiology and Biostatistics, Semey Medical University (SMU), Semey, Kazakhstan; wwwwwwwwwwwwwwwwwwwwwwwww Department of Community Medicine, Datta Meghe Institute of Medical Sciences, Sawangi, India; xxxxxxxxxxxxxxxxxxxxxxxxx Department of Endocrinology, University of Manchester, Manchester, UK; yyyyyyyyyyyyyyyyyyyyyyyyy Department of Endocrinology, Christie Hospital NHS Foundation Trust, Manchester, UK; zzzzzzzzzzzzzzzzzzzzzzzzz Department of Biology, Al-Imam Mohammad Ibn Saud Islamic University, Riyadh, Saudi Arabia; aaaaaaaaaaaaaaaaaaaaaaaaaa Department of Public Health, Indian Institute of Public Health, Hyderabad, India; bbbbbbbbbbbbbbbbbbbbbbbbbb Department of Oral Medicine and Radiology, King George's Medical University, Lucknow, India; cccccccccccccccccccccccccc Peking Union Medical College Hospital, Chinese Academy of Medical Sciences, Beijing, China; dddddddddddddddddddddddddd Hospital of Stomatology, Sun Yat-sen University, Guangzhou, China; eeeeeeeeeeeeeeeeeeeeeeeeee Clinical Project Management Office, National Clinical Research Centerfor Infectious Diseases, Shenzhen, Shenzhen, China; ffffffffffffffffffffffffff Faculty of Humanities and Health Sciences, Curtin University, Miri, Malaysia; gggggggggggggggggggggggggg Clinical Research Center, Zhujiang Hospital of Southern Medical University, Guangzhou, China; hhhhhhhhhhhhhhhhhhhhhhhhhh Science and Technology Department, Northern Jiangsu People's Hospital, Yangzhou, China; iiiiiiiiiiiiiiiiiiiiiiiiii School of Public Health, Zhejiang Chinese Medical University, Hangzhou, China; jjjjjjjjjjjjjjjjjjjjjjjjjj Faculty of Epidemiology and Population Health, London School of Hygiene & Tropical Medicine, London, UK; kkkkkkkkkkkkkkkkkkkkkkkkkk Heidelberg Institute of Global Health (HIGH), Heidelberg University, Heidelberg, Germany; llllllllllllllllllllllllll Department of Computer, Electrical and Mathematical Sciences and Engineering, King Abdullah University of Science and Technology, Thuwal, Saudi Arabia; mmmmmmmmmmmmmmmmmmmmmmmmmm School of Chinese Medicine (Teaching and Research Division), Hong Kong Baptist University, Hong Kong, China; nnnnnnnnnnnnnnnnnnnnnnnnnn Department of Rehabilitation Sciences, Hong Kong Polytechnic University, Hong Kong, China; oooooooooooooooooooooooooo Yong Loo Lin School of Medicine, National University of Singapore, Singapore, Singapore; pppppppppppppppppppppppppp Division of Cardiovascular Medicine, Harvard University, Boston, MA, USA; qqqqqqqqqqqqqqqqqqqqqqqqqq Department of Scientific Research, Tehran University of Medical Sciences, Tehran, Iran; rrrrrrrrrrrrrrrrrrrrrrrrrr Department of Public Health and Health Policy, Hiroshima University, Hiroshima, Japan; ssssssssssssssssssssssssss Division of Infectious Diseases, Virginia Commonwealth University, Richmond, VA, USA; tttttttttttttttttttttttttt Department of Public Health, Administration, and Social Sciences, Cayetano Heredia University, Lima, Peru; uuuuuuuuuuuuuuuuuuuuuuuuuu Division of Plastic Surgery, University of Wisconsin–Madison, Madison, WI, USA; vvvvvvvvvvvvvvvvvvvvvvvvvv Department of Clinical Oncology, Queen Elizabeth Hospital, Hong Kong, China; wwwwwwwwwwwwwwwwwwwwwwwwww Department of Medicine, National University of Singapore, Singapore, Singapore; xxxxxxxxxxxxxxxxxxxxxxxxxx The Nethersole School of Nursing, The Chinese University of Hong Kong, Hong Kong, China; yyyyyyyyyyyyyyyyyyyyyyyyyy Department of Medicine, University of Hong Kong, Hong Kong, China; zzzzzzzzzzzzzzzzzzzzzzzzzz School of Public Health, Curtin University, Perth, WA, Australia; aaaaaaaaaaaaaaaaaaaaaaaaaaa Department of Epidemiology and Preventative Medicine, Monash University, Melbourne, VIC, Australia; bbbbbbbbbbbbbbbbbbbbbbbbbbb Robert Stemple College of Public Health and Social Work, Florida International University, Miami, FL, USA; ccccccccccccccccccccccccccc Bispebjerg Hospital, University of Copenhagen, Copenhagen, Denmark; ddddddddddddddddddddddddddd Department of Molecular Parasitology and Tropical Diseases, Taipei Medical University, Taipei, Taiwan; eeeeeeeeeeeeeeeeeeeeeeeeeee Department of Paediatric Surgery, Federal Medical Centre, Umuahia, Nigeria; fffffffffffffffffffffffffff Department of Pediatrics, University of Washington, Seattle, WA, USA; ggggggggggggggggggggggggggg Department of Health Informatics, University College London, London, UK; hhhhhhhhhhhhhhhhhhhhhhhhhhh Health Data Research UK, London, UK; iiiiiiiiiiiiiiiiiiiiiiiiiii Department of Health Behavior, Texas A&M University, College Station, TX, USA; jjjjjjjjjjjjjjjjjjjjjjjjjjj School of Nursing and Midwifery, University of Technology Sydney, Sydney, NSW, Australia; kkkkkkkkkkkkkkkkkkkkkkkkkkk The David S. and Ruth L. Gottesman Center for Headache Treatment and Translational Research, Icahn School of Medicine at Mount Sinai, New York, NY, USA; lllllllllllllllllllllllllll Department of Medicine, Icahn School of Medicine at Mount Sinai, New York, NY, USA; mmmmmmmmmmmmmmmmmmmmmmmmmmm Department of Biostatistics, Johns Hopkins University, Baltimore, MD, USA; nnnnnnnnnnnnnnnnnnnnnnnnnnn Nova Medical School, NOVA University of Lisbon, Lisbon, Portugal; ooooooooooooooooooooooooooo Department of Medicine, University of Calgary, Calgary, AB, Canada; ppppppppppppppppppppppppppp Department of Cardiovascular Sciences, Katholieke Universiteit Leuven, Leuven, Belgium; qqqqqqqqqqqqqqqqqqqqqqqqqqq Department of Cardiovascular Medicine, Mayo Clinic, Jacksonville, FL, USA; rrrrrrrrrrrrrrrrrrrrrrrrrrr Department of Respiratory Medicine and Allergology, Nicolae Testemitanu State University of Medicine and Pharmacy, Chisinau, Moldova; sssssssssssssssssssssssssss School of Psychology, University of Southampton, Southampton, UK; ttttttttttttttttttttttttttt Department of Child and Adolescent Psychiatry, New York University, New York, NY, USA; uuuuuuuuuuuuuuuuuuuuuuuuuuu Research Center on Public Health (CESP), University of Milan Bicocca, Monza, Italy; vvvvvvvvvvvvvvvvvvvvvvvvvvv Laboratory of Public Health, Instituto Auxologico Italiano IRCCS (Italian Auxological Institute), Milan, Italy; wwwwwwwwwwwwwwwwwwwwwwwwwww Department of Health Sciences, University of Florence, Florence, Italy; xxxxxxxxxxxxxxxxxxxxxxxxxxx Department of Family Medicine and Public Health, University of California San Diego, La Jolla, CA, USA; yyyyyyyyyyyyyyyyyyyyyyyyyyy Life and Health Sciences Research Institute (ICVS), University of Minho, Braga, Portugal; zzzzzzzzzzzzzzzzzzzzzzzzzzz Institute for Research and Innovation in Health (i3S), University of Porto, Porto, Portugal; aaaaaaaaaaaaaaaaaaaaaaaaaaaa School of Medicine, The Chinese University of Hong Kong, Shenzhen, Shenzhen, China; bbbbbbbbbbbbbbbbbbbbbbbbbbbb School of Population Health, University of New South Wales, Kensington, NSW, Australia; cccccccccccccccccccccccccccc Global Women's Health Program, The George Institute for Global Health, Newtown, NSW, Australia; dddddddddddddddddddddddddddd Research Department, Cleveland Clinic Abu Dhabi, Abu Dhabi, United Arab Emirates; eeeeeeeeeeeeeeeeeeeeeeeeeeee Faculty of Medicine, University of Aleppo, Aleppo, Syria; ffffffffffffffffffffffffffff Department of Anesthesia, Critical Care and Pain Medicine, Shahid Beheshti University of Medical Sciences, Tehran, Iran; gggggggggggggggggggggggggggg Research Center for Child Psychiatry, University of Turku, Turku, Finland; hhhhhhhhhhhhhhhhhhhhhhhhhhhh Health Statistics and Informatics, Public Health Division, Northern Territory Government, Darwin, WA, Australia; iiiiiiiiiiiiiiiiiiiiiiiiiiii Department of Community Medicine, Ahmadu Bello University, Zaria, Nigeria; jjjjjjjjjjjjjjjjjjjjjjjjjjjj School of Pharmacy and Charles Perkins Centre, University of Sydney, Sydney, NSW, Australia; kkkkkkkkkkkkkkkkkkkkkkkkkkkk Department of Public Health and Primary Care, University of Cambridge, Cambridge, UK; llllllllllllllllllllllllllll Cardiovascular Metabolic Translational Research Program, National University of Singapore, Singapore, Singapore; mmmmmmmmmmmmmmmmmmmmmmmmmmmm Institute for Health Sciences, Mid Sweden University, Sundsvall, Sweden; nnnnnnnnnnnnnnnnnnnnnnnnnnnn Nutrition Department, Harvard University, Boston, MA, USA; oooooooooooooooooooooooooooo Department of Medical and Surgical Sciences and Advanced Technologies “”GF Ingrassia“”, University of Catania, Catania, Italy; pppppppppppppppppppppppppppp Department of Community Medicine, Employees' State Insurance Model Hospital, Chennai, India; qqqqqqqqqqqqqqqqqqqqqqqqqqqq Department of Brain Sciences, Imperial College London, London, UK; rrrrrrrrrrrrrrrrrrrrrrrrrrrr Department of Internal Medicine, Texas Tech University, Lubbock, TX, USA; ssssssssssssssssssssssssssss Ga East Municipal Hospital, Ghana Health Service, Accra, Ghana; tttttttttttttttttttttttttttt Department of Public Health, Haramaya University, Harar, Ethiopia; uuuuuuuuuuuuuuuuuuuuuuuuuuuu The University of Jordan, Amman, Jordan; vvvvvvvvvvvvvvvvvvvvvvvvvvvv School of Public Health, University of the Witwatersrand, Johannesburg, South Africa; wwwwwwwwwwwwwwwwwwwwwwwwwwww Heart Failure Research Center, Isfahan University of Medical Sciences, Isfahan, Iran; xxxxxxxxxxxxxxxxxxxxxxxxxxxx Department of Environmental Health, Arak University of Medical Sciences, Arak, Iran; yyyyyyyyyyyyyyyyyyyyyyyyyyyy Department of Pharmacology, Apollo Institute of Medical Sciences and Research, Chittoor, India; zzzzzzzzzzzzzzzzzzzzzzzzzzzz Department of Population and Development, Latin American Faculty of Social Sciences Mexico, Mexico City, Mexico; aaaaaaaaaaaaaaaaaaaaaaaaaaaaa Atchabarov Scientific-Research Institute of Fundamental and Applied Medicine, Kazakh National Medical University, Almaty, Kazakhstan; bbbbbbbbbbbbbbbbbbbbbbbbbbbbb Population Health Research Center, Kazakh National Medical University, Almaty, Kazakhstan; ccccccccccccccccccccccccccccc Department of Public Health, National University of Colombia, Bogota, Colombia; ddddddddddddddddddddddddddddd Department of Legal Medicine, Psychiatry and Pathology, Universidad Complutense de Madrid (Complutense University of Madrid), Madrid, Spain; eeeeeeeeeeeeeeeeeeeeeeeeeeeee Memorial Sloan Kettering Cancer Center, New York, NY, USA; fffffffffffffffffffffffffffff Department of Pediatrics, Brookdale University Hospital Medical Center, Brooklyn, NY, USA; ggggggggggggggggggggggggggggg National Drug and Alcohol Research Centre, University of New South Wales, Sydney, NSW, Australia; hhhhhhhhhhhhhhhhhhhhhhhhhhhhh Department of Biostatistics and Epidemiology, Kerman University of Medical Sciences, Kerman, Iran; iiiiiiiiiiiiiiiiiiiiiiiiiiiii Department of Neurosurgery, Tehran University of Medical Sciences, Tehran, Iran; jjjjjjjjjjjjjjjjjjjjjjjjjjjjj NCDs and Environment Programme, ISGlobal Instituto de Salud Global de Barcelona (Barcelona Institute for Global Health), Barcelona, Spain; kkkkkkkkkkkkkkkkkkkkkkkkkkkkk Department of Experimental and Health Sciences, Pompeu Fabra University, Barcelona, Spain; lllllllllllllllllllllllllllll Ophthalmology Department, University of Tennessee, Memphis, TN, USA; mmmmmmmmmmmmmmmmmmmmmmmmmmmmm Department of Neurosurgery, University of Edinburgh, Edinburgh, UK; nnnnnnnnnnnnnnnnnnnnnnnnnnnnn Department of Neurosurgery, National Health Service (NHS) Scotland, Edinburgh, UK; ooooooooooooooooooooooooooooo Dirección de Nutrición, Instituto Nacional de Nutrición Salvador Zubirán (Salvador Zubiran National Institute of Medical Sciences and Nutrition), Mexico City, Mexico; ppppppppppppppppppppppppppppp Research and Training Directorate, Eka Kotebe General Hospital, Addis Ababa, Ethiopia; qqqqqqqqqqqqqqqqqqqqqqqqqqqqq Department of Biological Sciences, University of Manouba, Manouba, Tunisia; rrrrrrrrrrrrrrrrrrrrrrrrrrrrr Department of Social Sciences, University of Jendouba, El Kef, Tunisia; sssssssssssssssssssssssssssss Wellcome Trust Brighton and Sussex Centre for Global Health Research, Brighton and Sussex Medical School, Brighton, UK; ttttttttttttttttttttttttttttt School of Public Health, Addis Ababa University, Addis Ababa, Ethiopia; uuuuuuuuuuuuuuuuuuuuuuuuuuuuu Department of Nutrition and Dietetics, Bahir Dar University, Bahir Dar, Ethiopia; vvvvvvvvvvvvvvvvvvvvvvvvvvvvv St Paul's Eye Unit, Royal Liverpool University Hospital, Liverpool, UK; wwwwwwwwwwwwwwwwwwwwwwwwwwwww Department of Forensic Medicine, University of Sarajevo, Sarajevo, Bosnia and Herzegovina; xxxxxxxxxxxxxxxxxxxxxxxxxxxxx Clinical and Public Health Research, Independent Consultant, Ahmedabad, India; yyyyyyyyyyyyyyyyyyyyyyyyyyyyy Department of Statistics, Computer Science, Applications “”G. Parenti“” (DiSIA), University of Florence and University of Palermo, Florence, Italy; zzzzzzzzzzzzzzzzzzzzzzzzzzzzz Chettinad Hospital & Research Institute, Chettinad Academy of Research and Education, Chennai, India; aaaaaaaaaaaaaaaaaaaaaaaaaaaaaa Department of Cardiology, Icahn School of Medicine at Mount Sinai, New York, NY, USA; bbbbbbbbbbbbbbbbbbbbbbbbbbbbbb Department of Pharmacy, United International University, Dhaka, Bangladesh; cccccccccccccccccccccccccccccc Pharmacology Division, Center for Life Sciences Research Bangladesh, Dhaka, Bangladesh; dddddddddddddddddddddddddddddd Sheffield Teaching Hospitals NHS Foundation Trust, Sheffield, UK; eeeeeeeeeeeeeeeeeeeeeeeeeeeeee Division of Pathology, ICAR-Indian Veterinary Research Institute, Bareilly, India; ffffffffffffffffffffffffffffff Manipal College of Pharmaceutical Sciences, Manipal Academy of Higher Education, Manipal, India; gggggggggggggggggggggggggggggg Research Department, Planetary Health Research Centre (PHRC), Kathmandu, Nepal; hhhhhhhhhhhhhhhhhhhhhhhhhhhhhh Institute of Occupational, Social and Environmental Medicine, Goethe University Frankfurt, Frankfurt am Main, Germany; iiiiiiiiiiiiiiiiiiiiiiiiiiiiii Research Department, Nepal Health Research Council, Kathmandu, Nepal; jjjjjjjjjjjjjjjjjjjjjjjjjjjjjj Population Interventions Unit, University of Melbourne, Melbourne, VIC, Australia; kkkkkkkkkkkkkkkkkkkkkkkkkkkkkk Department of Life Science and Public Health, Università Cattolica del Sacro Cuore (Catholic University of the Sacred Heart), Rome, Italy; llllllllllllllllllllllllllllll Escola Superior de Saúde (Higher School of Health), Instituto Politécnico do Porto (Polytechnic Institute of Porto), Porto, Portugal; mmmmmmmmmmmmmmmmmmmmmmmmmmmmmm Public Health Intelligence Unit, National Institute of Public Health, Cuernavaca, Mexico; nnnnnnnnnnnnnnnnnnnnnnnnnnnnnn Department of Gastroenterology, Pontificia Universidad Catolica de Chile (Pontifical Catholic University of Chile), Santiago, Chile; oooooooooooooooooooooooooooooo University of California San Diego, La Jolla, CA, USA; pppppppppppppppppppppppppppppp Department of Anesthesiology, Rutgers University, Newark, NJ, USA; qqqqqqqqqqqqqqqqqqqqqqqqqqqqqq Department of Physiology, Saveetha University, Chennai, India; rrrrrrrrrrrrrrrrrrrrrrrrrrrrrr Department of Otolaryngology - Head and Neck Surgery, Medical University of South Carolina, Charleston, SC, USA; ssssssssssssssssssssssssssssss Joe C. Wen School of Population & Public Health, University of California Irvine, Irvine, CA, USA; tttttttttttttttttttttttttttttt School of Health, Guilan University of Medical Sciences, Rasht, Iran; uuuuuuuuuuuuuuuuuuuuuuuuuuuuuu Department of Community Nutrition, Shahid Beheshti University of Medical Sciences, Tehran, Iran; vvvvvvvvvvvvvvvvvvvvvvvvvvvvvv Department of Public Health, Jazan University, Jazan, Saudi Arabia; wwwwwwwwwwwwwwwwwwwwwwwwwwwwww Department of Social Medicine and Health Care Organisation, Medical University of Varna, Varna, Bulgaria; xxxxxxxxxxxxxxxxxxxxxxxxxxxxxx Cardio-Thoraco-Vascular Department, Azienda Sanitaria Universitaria Giuliano Isontina, Trieste, Italy; yyyyyyyyyyyyyyyyyyyyyyyyyyyyyy Department of Medical Laboratory Sciences, Iran University of Medical Sciences, Tehran, Iran; zzzzzzzzzzzzzzzzzzzzzzzzzzzzzz University of Rochester, Rochester, NY, USA; aaaaaaaaaaaaaaaaaaaaaaaaaaaaaaa Independent Consultant, Bridgewater, NJ, USA; bbbbbbbbbbbbbbbbbbbbbbbbbbbbbbb Department of Health Policy and Management, Tabriz University of Medical Sciences, Tabriz, Iran; ccccccccccccccccccccccccccccccc Department of Epidemiology, University of Pittsburgh, Pittsburgh, PA, USA; ddddddddddddddddddddddddddddddd Department of Psychiatry, University of Pittsburgh Medical Center, Pittsburgh, PA, USA; eeeeeeeeeeeeeeeeeeeeeeeeeeeeeee School of Public Health, University of Sydney, Sydney, NSW, Australia; fffffffffffffffffffffffffffffff Department of Medicine, Bangalore Medical College and Research Institute, Bangalore, India; ggggggggggggggggggggggggggggggg Department of Pathology, China Medical University, Liaoning, China; hhhhhhhhhhhhhhhhhhhhhhhhhhhhhhh Office of Institutional Analysis, University of Windsor, Windsor, ON, Canada; iiiiiiiiiiiiiiiiiiiiiiiiiiiiiii School of Sociology, University College Dublin, Dublin, Ireland; jjjjjjjjjjjjjjjjjjjjjjjjjjjjjjj Postgraduate Program in Health Sciences, Federal University of Rio Grande do Sul, Rio Grande, Brazil; kkkkkkkkkkkkkkkkkkkkkkkkkkkkkkk Postgraduate Program in Epidemiology, Federal University of Rio Grande do Sul, Porto Alegre, Brazil; lllllllllllllllllllllllllllllll School of Medicine, Federal University of Bahia, Salvador, Brazil; mmmmmmmmmmmmmmmmmmmmmmmmmmmmmmm Department of Internal Medicine, Escola Bahiana de Medicina e Saúde Pública (Bahiana School of Medicine and Public Health), Salvador, Brazil; nnnnnnnnnnnnnnnnnnnnnnnnnnnnnnn Department of Infection and Tropical Medicine, University of Sheffield, Sheffield, UK; ooooooooooooooooooooooooooooooo AlMaarefa University, Riyadh, Saudi Arabia; ppppppppppppppppppppppppppppppp Department of Pharmacology, All India Institute of Medical Sciences, Rajkot, India; qqqqqqqqqqqqqqqqqqqqqqqqqqqqqqq College of Medicine, Ajman University, Ajman, United Arab Emirates; rrrrrrrrrrrrrrrrrrrrrrrrrrrrrrr Department of Microbiology and Immunology, Northwestern University, Chicago, IL, USA; sssssssssssssssssssssssssssssss Department of Biological and Chemical Sciences, Michael and Cecilia Ibru University, Delta State, Nigeria; ttttttttttttttttttttttttttttttt Department of Psychiatry, Dalhousie University, Halifax, NS, Canada; uuuuuuuuuuuuuuuuuuuuuuuuuuuuuuu Department of Psychiatry, University of Alberta, Edmonton, AB, Canada; vvvvvvvvvvvvvvvvvvvvvvvvvvvvvvv Histology Department, Zagazig University, Zagazig, Egypt; wwwwwwwwwwwwwwwwwwwwwwwwwwwwwww Fred Hutchinson Cancer Research Center, Seattle, WA, USA; xxxxxxxxxxxxxxxxxxxxxxxxxxxxxxx Environmental and Occupational Health Research Center, Shahroud University of Medical Sciences, Shahroud, Iran; yyyyyyyyyyyyyyyyyyyyyyyyyyyyyyy Higher School of Technology, Sultan Moulay Slimane University, Beni Mellal, Morocco; zzzzzzzzzzzzzzzzzzzzzzzzzzzzzzz School of Nursing and Midwifery, La Trobe University, Melbourne, VIC, Australia; aaaaaaaaaaaaaaaaaaaaaaaaaaaaaaaa Advanced Nursing Department, Universitas Airlangga (Airlangga University), Surabaya, Indonesia; bbbbbbbbbbbbbbbbbbbbbbbbbbbbbbbb La Trobe University, Melbourne, VIC, Australia; cccccccccccccccccccccccccccccccc Gastrointestinal and Liver Disease Research Center, Guilan University of Medical Sciences, Rasht, Iran; dddddddddddddddddddddddddddddddd Iran University of Medical Sciences, Tehran, Iran; eeeeeeeeeeeeeeeeeeeeeeeeeeeeeeee Isenberg School of Management, University of Massachusetts Amherst, Amherst, MA, USA; ffffffffffffffffffffffffffffffff Massachusetts General Hospital, Boston, MA, USA; gggggggggggggggggggggggggggggggg Centre for Global Health Inequalities Research (CHAIN), Norwegian University of Science and Technology, Trondheim, Norway; hhhhhhhhhhhhhhhhhhhhhhhhhhhhhhhh Private Orthodontist, Ahvaz, Iran; iiiiiiiiiiiiiiiiiiiiiiiiiiiiiiii Faculty of Science and Health, University of Portsmouth, Hampshire, UK; jjjjjjjjjjjjjjjjjjjjjjjjjjjjjjjj Almoosa College of Health Sciences, Al Ahsa, Saudi Arabia; kkkkkkkkkkkkkkkkkkkkkkkkkkkkkkkk Clinical Pathology Department, Mansoura University, Mansoura, Egypt; llllllllllllllllllllllllllllllll Department of Basic Medical Sciences, University of Sharjah, Sharjah, United Arab Emirates; mmmmmmmmmmmmmmmmmmmmmmmmmmmmmmmm Department of Anatomy and Embryology, Mansoura University, Mansoura, Egypt; nnnnnnnnnnnnnnnnnnnnnnnnnnnnnnnn Department of Radiology, Tehran University of Medical Sciences, Tehran, Iran; oooooooooooooooooooooooooooooooo Deanship of Preparatory Year and Supporting Studies, Imam Abdulrahman Bin Faisal University, Dammam, Saudi Arabia; pppppppppppppppppppppppppppppppp College of Medicine, RAK Medical and Health Sciences University, Ras Al Khaimah, United Arab Emirates; qqqqqqqqqqqqqqqqqqqqqqqqqqqqqqqq Faculty of Medicine, Ain Shams University, Cairo, Egypt; rrrrrrrrrrrrrrrrrrrrrrrrrrrrrrrr Sharjah Institute for Medical Research, University of Sharjah, Sharjah, United Arab Emirates; ssssssssssssssssssssssssssssssss Department of Internal Medicine, Ain Shams University, Cairo, Egypt; tttttttttttttttttttttttttttttttt Section of Adult Hematology, King Saud University, Riyadh, Saudi Arabia; uuuuuuuuuuuuuuuuuuuuuuuuuuuuuuuu College of Medicine, Korea University, Seoul, South Korea; vvvvvvvvvvvvvvvvvvvvvvvvvvvvvvvv Houston Methodist Hospital, Houston, TX, USA; wwwwwwwwwwwwwwwwwwwwwwwwwwwwwwww National Institute of Public Health Research, Ministry of Health, Nouakchott, Mauritania; xxxxxxxxxxxxxxxxxxxxxxxxxxxxxxxx Pediatric Dentistry and Dental Public Health Department, Alexandria University, Alexandria, Egypt; yyyyyyyyyyyyyyyyyyyyyyyyyyyyyyyy Department of Clinical and Chemical Pathology, Cairo University, Cairo, Egypt; zzzzzzzzzzzzzzzzzzzzzzzzzzzzzzzz MD Anderson Cancer Center Department of Plastic Surgery, University of Texas, Houston, TX, USA; aaaaaaaaaaaaaaaaaaaaaaaaaaaaaaaaa School of Pharmacy and Pharmaceutical Sciences, Ulster University, Coleraine, UK; bbbbbbbbbbbbbbbbbbbbbbbbbbbbbbbbb Department of Neuropsychiatry, Ain Shams University, Cairo, Egypt; ccccccccccccccccccccccccccccccccc Executive Committee, International Association for Women Mental Health, Potomac, MD, USA; ddddddddddddddddddddddddddddddddd Department of Clinical Pathology, Mansoura University, Mansoura, Egypt; eeeeeeeeeeeeeeeeeeeeeeeeeeeeeeeee Department of Infectious Diseases and Public Health, City University of Hong Kong, Hong Kong, China; fffffffffffffffffffffffffffffffff Department of Animal Medicine, Zagazig University, Zagazig, Egypt; ggggggggggggggggggggggggggggggggg Faculty of Veterinary Medicine, Damanhour University, Damanhur, Egypt; hhhhhhhhhhhhhhhhhhhhhhhhhhhhhhhhh Department of Midwifery, Woldia University, Addis Ababa, Ethiopia; iiiiiiiiiiiiiiiiiiiiiiiiiiiiiiiii Health Research and Technology Transfer Directorate, South Ethiopia Region Public Health Institute, Jinka, Ethiopia; jjjjjjjjjjjjjjjjjjjjjjjjjjjjjjjjj Department of Public Health, Hawassa University, Hawassa, Ethiopia; kkkkkkkkkkkkkkkkkkkkkkkkkkkkkkkkk Evidence-Based Medical Research Institute of Mongolia, Ulaanbaatar, Mongolia; lllllllllllllllllllllllllllllllll Department of Paediatrics, University of Lagos, Lagos, Nigeria; mmmmmmmmmmmmmmmmmmmmmmmmmmmmmmmmm Department of Paediatrics, Lagos University Teaching Hospital, Lagos, Nigeria; nnnnnnnnnnnnnnnnnnnnnnnnnnnnnnnnn Goba College of Medicine and Health Sciences, Madda Walabu University, Robe, Ethiopia; ooooooooooooooooooooooooooooooooo Department of Bacteriology and Virology, Semnan University of Medical Sciences, Semnan, Iran; ppppppppppppppppppppppppppppppppp Cancer Research Center, Semnan University of Medical Sciences, Semnan, Iran; qqqqqqqqqqqqqqqqqqqqqqqqqqqqqqqqq Faculty of Health, York University, Toronto, ON, Canada; rrrrrrrrrrrrrrrrrrrrrrrrrrrrrrrrr Department of Ophthalmology, Federal Medical Centre, Asaba, Nigeria; sssssssssssssssssssssssssssssssss Postgraduate School, University of Edinburgh, Edinburgh, UK; ttttttttttttttttttttttttttttttttt Drexel Dornsife School of Public Health, Drexel University, Philadelphia, PA, USA; uuuuuuuuuuuuuuuuuuuuuuuuuuuuuuuuu Department of Electrical and Computer Engineering (ECE), Tarbiat Modares University, Tehran, Iran; vvvvvvvvvvvvvvvvvvvvvvvvvvvvvvvvv Research Centre for Healthcare and Community, Coventry University, Coventry, UK; wwwwwwwwwwwwwwwwwwwwwwwwwwwwwwwww Department of Periodontology and Community Dentistry, University of Ibadan, Ibadan, Nigeria; xxxxxxxxxxxxxxxxxxxxxxxxxxxxxxxxx School of Public Health, University of Nevada Reno, Reno, NV, USA; yyyyyyyyyyyyyyyyyyyyyyyyyyyyyyyyy Department of Oral Biology, Riphah International University, Islamabad, Pakistan; zzzzzzzzzzzzzzzzzzzzzzzzzzzzzzzzz Digestive Diseases Research Institute (DDRI), Tehran University of Medical Sciences, Tehran, Iran; aaaaaaaaaaaaaaaaaaaaaaaaaaaaaaaaaa Key Laboratory of Computer-Aided Drug Design, Guangdong Medical University, Dongguan, China; bbbbbbbbbbbbbbbbbbbbbbbbbbbbbbbbbb Director of the Scientific and Technological Park, Kazakh National Medical University, Almaty, Kazakhstan; cccccccccccccccccccccccccccccccccc Department of Medicine, Korea University, Seoul, South Korea; dddddddddddddddddddddddddddddddddd Department of Food Hygiene and Quality Control, University of Tehran, Tehran, Iran; eeeeeeeeeeeeeeeeeeeeeeeeeeeeeeeeee Department of Biomedical and Biotechnological Sciences, University of Catania, Catania, Italy; ffffffffffffffffffffffffffffffffff Epidemiology and Biostatistics Unit, IRCCS Pascale, Naples, Italy; gggggggggggggggggggggggggggggggggg Department of Public Health Sciences, Clemson University, Clemson, SC, USA; hhhhhhhhhhhhhhhhhhhhhhhhhhhhhhhhhh Pediatric Infectious Disease Research Center, Tehran University of Medical Sciences, Tehran, Iran; iiiiiiiiiiiiiiiiiiiiiiiiiiiiiiiiii Student Research Committee, Shiraz University of Medical Sciences, Shiraz, Iran; jjjjjjjjjjjjjjjjjjjjjjjjjjjjjjjjjj Department of Otolaryngology, Shiraz University of Medical Sciences, Shiraz, Iran; kkkkkkkkkkkkkkkkkkkkkkkkkkkkkkkkkk College of Medicine, AlMaarefa University, Riyadh, Saudi Arabia; llllllllllllllllllllllllllllllllll Saveetha Medical College and Hospital, Saveetha Institute of Medical and Technical Sciences (SIMATS), Chennai, India; mmmmmmmmmmmmmmmmmmmmmmmmmmmmmmmmmm Division of Statistics, Bangladesh Bank, Sylhet, Bangladesh; nnnnnnnnnnnnnnnnnnnnnnnnnnnnnnnnnn School of Human and Social Sciences (FCHS), University of Algarve, Faro, Portugal; oooooooooooooooooooooooooooooooooo University Research Center in Psychology, Faro, Portugal; pppppppppppppppppppppppppppppppppp Environmental Statistics Unit, National Institute of Statistics, Lisbon, Portugal; qqqqqqqqqqqqqqqqqqqqqqqqqqqqqqqqqq Ecological Economics and Environmental Management, NOVA University of Lisbon, Lisbon, Portugal; rrrrrrrrrrrrrrrrrrrrrrrrrrrrrrrrrr Department of Clinical Nutrition and Dietetics, Applied Science Private University, Amman, Jordan; ssssssssssssssssssssssssssssssssss Department of Psychology, Federal University of Sergipe, São Cristóvão, Brazil; tttttttttttttttttttttttttttttttttt Department of Radiography and Imaging Technology, Green International University, Lahore, Pakistan; uuuuuuuuuuuuuuuuuuuuuuuuuuuuuuuuuu Endocrinology and Metabolism Research Institute, Non-Communicable Diseases Research Center (NCDRC), Tehran, Iran; vvvvvvvvvvvvvvvvvvvvvvvvvvvvvvvvvv Dentistry Research Institute, Tehran University of Medical Sciences, Tehran, Iran; wwwwwwwwwwwwwwwwwwwwwwwwwwwwwwwwww Obesity and Eating Habits Research Center, Tehran University of Medical Sciences, Tehran, Iran; xxxxxxxxxxxxxxxxxxxxxxxxxxxxxxxxxx Department of Veterinary Tropical Diseases, University of Pretoria, Pretoria, South Africa; yyyyyyyyyyyyyyyyyyyyyyyyyyyyyyyyyy Animal Production and Health Division (EMPRES), Food and Agriculture Organization of the United Nations, Rome, Italy; zzzzzzzzzzzzzzzzzzzzzzzzzzzzzzzzzz Charité Universitätsmedizin Berlin (Charité University Medical Center Berlin), Berlin, Germany; aaaaaaaaaaaaaaaaaaaaaaaaaaaaaaaaaaa School of Engineering, Edith Cowan University, Joondalup, WA, Australia; bbbbbbbbbbbbbbbbbbbbbbbbbbbbbbbbbbb University Institute of Radiological Sciences and Medical Imaging Technology, The University of Lahore, Lahore, Pakistan; ccccccccccccccccccccccccccccccccccc Biostatistics Unit, Shahed University, Tehran, Iran; ddddddddddddddddddddddddddddddddddd Trauma Research Center, Shiraz University of Medical Sciences, Shiraz, Iran; eeeeeeeeeeeeeeeeeeeeeeeeeeeeeeeeeee Department of Medical Immunology, Shiraz University of Medical Sciences, Shiraz, Iran; fffffffffffffffffffffffffffffffffff National Institute for Stroke and Applied Neurosciences, Auckland University of Technology, Auckland, New Zealand; ggggggggggggggggggggggggggggggggggg Research Center of Neurology, Moscow, Russia; hhhhhhhhhhhhhhhhhhhhhhhhhhhhhhhhhhh Department of Social Medicine and Epidemiology, Guilan University of Medical Sciences, Rasht, Iran; iiiiiiiiiiiiiiiiiiiiiiiiiiiiiiiiiii School of Nursing, Haramaya University, Harar, Ethiopia; jjjjjjjjjjjjjjjjjjjjjjjjjjjjjjjjjjj Department of Pharmacy, Wollega University, Nekemte, Ethiopia; kkkkkkkkkkkkkkkkkkkkkkkkkkkkkkkkkkk Department of Pathobiology, University of Pennsylvania, Philadelphia, PA, USA; lllllllllllllllllllllllllllllllllll Department of Microbiology and Physiology, Tulane University, New Orleans, LA, USA; mmmmmmmmmmmmmmmmmmmmmmmmmmmmmmmmmmm Department of Biomedical Engineering, University of Houston, Houston, TX, USA; nnnnnnnnnnnnnnnnnnnnnnnnnnnnnnnnnnn Division of Neurology, University of Toronto, Toronto, ON, Canada; ooooooooooooooooooooooooooooooooooo Department of Neurobiology, Care Sciences, and Society, Karolinska Institutet (Karolinska Institute), Stockholm, Sweden; ppppppppppppppppppppppppppppppppppp Cardiovascular Health and Imaging Laboratory, Centro Nacional de Investigaciones Cardiovasculares (CNIC) (National Centre for Cardiovascular Disease Research), Madrid, Spain; qqqqqqqqqqqqqqqqqqqqqqqqqqqqqqqqqqq Department of Cardiology, Hospital Clinico San Carlos, IdISSC, Madrid, Spain; rrrrrrrrrrrrrrrrrrrrrrrrrrrrrrrrrrr Center for Public Health Research, University of Milan Bicocca, Monza, Italy; sssssssssssssssssssssssssssssssssss Laboratory of Public Health, IRCCS Istituto Auxologico Italiano, Milan, Italy; ttttttttttttttttttttttttttttttttttt School of Public Health, The University of Queensland, Brisbane, QLD, Australia; uuuuuuuuuuuuuuuuuuuuuuuuuuuuuuuuuuu Queensland Centre for Mental Health Research, Wacol, QLD, Australia; vvvvvvvvvvvvvvvvvvvvvvvvvvvvvvvvvvv Department of Social Sciences, University of Nicosia, Nicosia, Cyprus; wwwwwwwwwwwwwwwwwwwwwwwwwwwwwwwwwww Department of Nursing, Wollega University, Nekemte, Ethiopia; xxxxxxxxxxxxxxxxxxxxxxxxxxxxxxxxxxx Institute of Health Sciences, Wollega University, Nekemte, Ethiopia; yyyyyyyyyyyyyyyyyyyyyyyyyyyyyyyyyyy Jimma University, Jimma, Ethiopia; zzzzzzzzzzzzzzzzzzzzzzzzzzzzzzzzzzz Medical School, Universidad de Navarra, Pamplona, Spain; aaaaaaaaaaaaaaaaaaaaaaaaaaaaaaaaaaaa Department of Public Health, University of Naples “”Federico II“”, Naples, Italy; bbbbbbbbbbbbbbbbbbbbbbbbbbbbbbbbbbbb Institute of Public Health, Charité Universitätsmedizin Berlin (Charité University Medical Center Berlin), Berlin, Germany; cccccccccccccccccccccccccccccccccccc Department of Pharmacology, Gadjah Mada University, Yogyakarta, Indonesia; dddddddddddddddddddddddddddddddddddd Department of Child Dental Health, Obafemi Awolowo University, Ile-Ife, Nigeria; eeeeeeeeeeeeeeeeeeeeeeeeeeeeeeeeeeee Clinical Science Department, Nigerian Institute of Medical Research, Lagos, Nigeria; ffffffffffffffffffffffffffffffffffff Department of Cell Biology and Biotechnology, K.A. Timiryazev Institute of Plant Physiology, Moscow, Russia; gggggggggggggggggggggggggggggggggggg Department of Cardiac, Thoracic, Vascular Sciences and Public Health, University of Padova, Italy, Padova, Italy; hhhhhhhhhhhhhhhhhhhhhhhhhhhhhhhhhhhh Division of Pediatric Hematology-Oncology, St. Jude Children's Research Hospital, Seattle, WA, USA; iiiiiiiiiiiiiiiiiiiiiiiiiiiiiiiiiiii Innovation in Healthcare and Social Services Department, Emilia-Romagna Region, Bologna, Italy; jjjjjjjjjjjjjjjjjjjjjjjjjjjjjjjjjjjj Department of Neuroscience, Multiple Sclerosis Research Center, Ravenna, Italy; kkkkkkkkkkkkkkkkkkkkkkkkkkkkkkkkkkkk Department of Biotechnological and Applied Clinical Sciences, University of L'Aquila, L'Aquila, Italy; llllllllllllllllllllllllllllllllllll Department of Radiology, University of Southern California, Los Angeles, CA, USA; mmmmmmmmmmmmmmmmmmmmmmmmmmmmmmmmmmmm Department of Medicine, Iran University of Medical Sciences, Tehran, Iran; nnnnnnnnnnnnnnnnnnnnnnnnnnnnnnnnnnnn Clinical Epidemiology Division (KEP), Karolinska Institutet (Karolinska Institute), Stockholms, Sweden; oooooooooooooooooooooooooooooooooooo College of Medicine, Dentistry and Public Health, James Cook University, Townsville, QLD, Australia; pppppppppppppppppppppppppppppppppppp Department of Community Medicine Department of Community Medicine Information and Decision-Making in Health (MEDCIDS), University of Porto, Porto, Portugal; qqqqqqqqqqqqqqqqqqqqqqqqqqqqqqqqqqqq Center for Health Technology and Services Research (CINTESIS), Porto, Portugal; rrrrrrrrrrrrrrrrrrrrrrrrrrrrrrrrrrrr Department of Biostatistics, School of Public Health, Xuzhou Medical University, Xuzhou, China; ssssssssssssssssssssssssssssssssssss Department of Dermatology, Kyoto Prefectural University of Medicine, Kyoto, Japan; tttttttttttttttttttttttttttttttttttt Department of Community Medicine and Family Medicine, All India Institute of Medical Sciences, Gorakhpur, India; uuuuuuuuuuuuuuuuuuuuuuuuuuuuuuuuuuuu Health Services Management Training Centre, Semmelweis University, Budapest, Hungary; vvvvvvvvvvvvvvvvvvvvvvvvvvvvvvvvvvvv Department of Applied Social Sciences, Sapientia Hungarian University of Transylvania, Târgu-Mureş, Romania; wwwwwwwwwwwwwwwwwwwwwwwwwwwwwwwwwwww Department of Community Medicine, Bayero University Kano, Kano, Nigeria; xxxxxxxxxxxxxxxxxxxxxxxxxxxxxxxxxxxx Department of Community Medicine, Aminu Kano Teaching Hospital, Kano, Nigeria; yyyyyyyyyyyyyyyyyyyyyyyyyyyyyyyyyyyy School of Public Health, University of Ghana, Legon, Ghana; zzzzzzzzzzzzzzzzzzzzzzzzzzzzzzzzzzzz Hypertension in Africa Research Team, North-West University, Potchefstroom, South Africa; aaaaaaaaaaaaaaaaaaaaaaaaaaaaaaaaaaaaa Department of Public Health, University of Szeged, Szeged, Hungary; bbbbbbbbbbbbbbbbbbbbbbbbbbbbbbbbbbbbb Department of Food Technology, Salahaddin University-Erbil, Erbil, Iraq; ccccccccccccccccccccccccccccccccccccc Department of Nutrition and Dietetics, Cihan University-Erbil, Erbil, Iraq; ddddddddddddddddddddddddddddddddddddd Department of Medical Epidemiology, Mario Negri Institute for Pharmacological Research, Milan, Italy; eeeeeeeeeeeeeeeeeeeeeeeeeeeeeeeeeeeee Department of Prosthodontics, Saveetha University, Chennai, India; fffffffffffffffffffffffffffffffffffff School of American Education, Institute of Health & Management, Australia, Melbourne, VIC, Australia; ggggggggggggggggggggggggggggggggggggg Swinburne University of Technology, School of Engineering, Melbourne, VIC, Australia; hhhhhhhhhhhhhhhhhhhhhhhhhhhhhhhhhhhhh Department of Pharmacology, Manipal Academy of Higher Education, Manipal, India; iiiiiiiiiiiiiiiiiiiiiiiiiiiiiiiiiiiii Department of Biostatistics, Xuzhou Medical University, Xuzhou, China; jjjjjjjjjjjjjjjjjjjjjjjjjjjjjjjjjjjjj Key Lab of Environment and Health, Xuzhou Medical University, Xuzhou, China; kkkkkkkkkkkkkkkkkkkkkkkkkkkkkkkkkkkkk Department of Joint Surgery and Sports Medicine, Institute of Science Tokyo, Tokyo, Japan; lllllllllllllllllllllllllllllllllllll Department of Veterinary Public Health and Preventive Medicine, Usmanu Danfodiyo University, Sokoto, Sokoto, Nigeria; mmmmmmmmmmmmmmmmmmmmmmmmmmmmmmmmmmmmm Department of Public Health, SIMAD University, Mogadishu, Somalia; nnnnnnnnnnnnnnnnnnnnnnnnnnnnnnnnnnnnn Centre for Innovation in Mental Health, University of Southampton, Southampton, UK; ooooooooooooooooooooooooooooooooooooo School of Medicine, Orebro University, Orebro, Sweden; ppppppppppppppppppppppppppppppppppppp Department of Medicine, University of Valladolid, Valladolid, Spain; qqqqqqqqqqqqqqqqqqqqqqqqqqqqqqqqqqqqq Department of Neurology, Hospital Universitario Rio Hortega, Valladolid, Spain; rrrrrrrrrrrrrrrrrrrrrrrrrrrrrrrrrrrrr Human Nutrition Laboratory, Autonomous University of Sinaloa, Culiacán, Mexico; sssssssssssssssssssssssssssssssssssss Department of Health Sciences, Autonomous University of Occident, Culiacán, Mexico; ttttttttttttttttttttttttttttttttttttt Department of Ophthalmology, University of Basel, Basel, Switzerland; uuuuuuuuuuuuuuuuuuuuuuuuuuuuuuuuuuuuu Professional Services Division, Texas State Board of Pharmacy, Austin, TX, USA; vvvvvvvvvvvvvvvvvvvvvvvvvvvvvvvvvvvvv Department of Pharmacology, IES Institute of Pharmacy, Bhopal, India; wwwwwwwwwwwwwwwwwwwwwwwwwwwwwwwwwwwww Institute of Health and Development (ISED), Alliance for Medical Research in Africa (AMedRA), Dakar, Senegal; xxxxxxxxxxxxxxxxxxxxxxxxxxxxxxxxxxxxx Alliance for Medical Research in Africa (AMedRA), Dakar, Senegal; yyyyyyyyyyyyyyyyyyyyyyyyyyyyyyyyyyyyy School of Public Health, Shandong First Medical University and Shandong Academy of Medical Sciences, Jinan, China; zzzzzzzzzzzzzzzzzzzzzzzzzzzzzzzzzzzzz College of Health Sciences, Addis Ababa University, Addis Ababa, Ethiopia; aaaaaaaaaaaaaaaaaaaaaaaaaaaaaaaaaaaaaa Department of Midwifery, Adigrat University, Adigrat, Ethiopia; bbbbbbbbbbbbbbbbbbbbbbbbbbbbbbbbbbbbbb Environmental Pollution Monitoring and Study Desk, Ethiopian Environmental Protection Authority, Addis Ababa, Ethiopia; cccccccccccccccccccccccccccccccccccccc School of Public Health, Bule Hora University, Bule Hora, Ethiopia; dddddddddddddddddddddddddddddddddddddd Neurology and Stroke Unit, ASST Grande Ospedale Metropolitano Niguarda, Milan, Italy; eeeeeeeeeeeeeeeeeeeeeeeeeeeeeeeeeeeeee Institute of Public Health, Jagiellonian University Medical College, Krakow, Poland; ffffffffffffffffffffffffffffffffffffff School of Medicine and Population Health, University of Sheffield, Sheffield, UK; gggggggggggggggggggggggggggggggggggggg Department of Public Health, Debre Berhan University, Debre Berhan, Ethiopia; hhhhhhhhhhhhhhhhhhhhhhhhhhhhhhhhhhhhhh Department of Public Health, Menelik II Medical and Health Science College, Addis Ababa, Ethiopia; iiiiiiiiiiiiiiiiiiiiiiiiiiiiiiiiiiiiii College of Medicine and Health Science, Bahir Dar University, Bahir Dar, Ethiopia; jjjjjjjjjjjjjjjjjjjjjjjjjjjjjjjjjjjjjj School of Population Health, Curtin University, Perth, WA, Australia; kkkkkkkkkkkkkkkkkkkkkkkkkkkkkkkkkkkkkk Child Health Analytics Research Program, Telethon Kids Institute, Perth, WA, Australia; llllllllllllllllllllllllllllllllllllll Infectious Disease Research Center, Kermanshah University of Medical Sciences, Kermanshah, Iran; mmmmmmmmmmmmmmmmmmmmmmmmmmmmmmmmmmmmmm Pediatric Department, Kermanshah University of Medical Sciences, Kermanshah, Iran; nnnnnnnnnnnnnnnnnnnnnnnnnnnnnnnnnnnnnn School of Nursing and Midwifery, Shahid Beheshti University of Medical Sciences, Tehran, Iran; oooooooooooooooooooooooooooooooooooooo Department of Cardiology, Iran University of Medical Sciences, Tehran, Iran; pppppppppppppppppppppppppppppppppppppp Faculty of Medicine, Shahid Beheshti University of Medical Sciences, Tehran, Iran; qqqqqqqqqqqqqqqqqqqqqqqqqqqqqqqqqqqqqq Research Committee of Qom University of Medical Sciences, Qom University of Medical Sciences, Qom, Iran; rrrrrrrrrrrrrrrrrrrrrrrrrrrrrrrrrrrrrr Department of Global Health Sciences, University of California San Francisco, San Francisco, CA, USA; ssssssssssssssssssssssssssssssssssssss Department of Ophthalmology, Tehran University of Medical Sciences, Tehran, Iran; tttttttttttttttttttttttttttttttttttttt Tropical Health Department, Alexandria University, Alexandria, Egypt; uuuuuuuuuuuuuuuuuuuuuuuuuuuuuuuuuuuuuu Family and Community Medicine Department, King Khalid University, Abha, Saudi Arabia; vvvvvvvvvvvvvvvvvvvvvvvvvvvvvvvvvvvvvv Department of Physics, University of Zanjan, Zanjan, Iran; wwwwwwwwwwwwwwwwwwwwwwwwwwwwwwwwwwwwww Department of Dermatology, Mazandaran University of Medical Sciences, Sari, Iran; xxxxxxxxxxxxxxxxxxxxxxxxxxxxxxxxxxxxxx Cardiovascular Diseases Research Center, Guilan University of Medical Sciences, Rasht, Iran; yyyyyyyyyyyyyyyyyyyyyyyyyyyyyyyyyyyyyy Obstetrics and Gynecology Department, Shahid Beheshti University of Medical Sciences, Tehran, Iran; zzzzzzzzzzzzzzzzzzzzzzzzzzzzzzzzzzzzzz Department of Biology, Government Institute of Science, Nagpur, India; aaaaaaaaaaaaaaaaaaaaaaaaaaaaaaaaaaaaaaa Department of Clinical Research, National Institute For Research In Reproductive and Child Health, Mumbai, India; bbbbbbbbbbbbbbbbbbbbbbbbbbbbbbbbbbbbbbb Department of Epidemiology and Prevention, IRCCS Neuromed, Pozzilli, Italy; ccccccccccccccccccccccccccccccccccccccc GBD Collaborating Unit, Norwegian Institute of Public Health, Bergen, Norway; ddddddddddddddddddddddddddddddddddddddd Department of Biological Sciences and Chemistry (DBSC), University of Nizwa, Nizwa, Oman; eeeeeeeeeeeeeeeeeeeeeeeeeeeeeeeeeeeeeee Adelaide Medical School, University of Adelaide, Adelaide, SA, Australia; fffffffffffffffffffffffffffffffffffffff Department of Zoology, KKS Women's College, Balasore, India; ggggggggggggggggggggggggggggggggggggggg Department of Nursing, Aksum University, Aksum, Ethiopia; hhhhhhhhhhhhhhhhhhhhhhhhhhhhhhhhhhhhhhh Department of Anesthesiology and Critical Care Medicine, Ospedale SS Annunziata Savigliano, Savigliano, Italy; iiiiiiiiiiiiiiiiiiiiiiiiiiiiiiiiiiiiiii Department of Cardiac Surgery, Cleveland Clinic Abu Dhabi, Abu Dhabi, United Arab Emirates; jjjjjjjjjjjjjjjjjjjjjjjjjjjjjjjjjjjjjjj Lerner College of Medicine, Case Western Reserve University, Cleveland, OH, USA; kkkkkkkkkkkkkkkkkkkkkkkkkkkkkkkkkkkkkkk Department of Radiation Oncology, All India Institute of Medical Sciences, Bathinda, India; lllllllllllllllllllllllllllllllllllllll Department of Medicine, All India Institute of Medical Sciences, Bathinda, India; mmmmmmmmmmmmmmmmmmmmmmmmmmmmmmmmmmmmmmm Department of Pharmaceutical Sciences and Drug Research, Punjabi University Patiala, Patiala, India; nnnnnnnnnnnnnnnnnnnnnnnnnnnnnnnnnnnnnnn Department of Biostatistics, Tarbiat Modares University, Tehran, Iran; ooooooooooooooooooooooooooooooooooooooo Quantitative Department, Non-Communicable Diseases Research Center (NCDRC), Tehran, Iran; ppppppppppppppppppppppppppppppppppppppp Department of Health Systems and Policy Research, Indian Institute of Public Health, Gandhinagar, India; qqqqqqqqqqqqqqqqqqqqqqqqqqqqqqqqqqqqqqq Non-Communicable Diseases Research Center (NCDRC), Tehran, Iran; rrrrrrrrrrrrrrrrrrrrrrrrrrrrrrrrrrrrrrr Department of Life Sciences, Health and Healthcare Professions, Link Campus University, Rome, Italy; sssssssssssssssssssssssssssssssssssssss Health Services Research, Evaluation and Policy Unit, AUSL della Romagna, Ravenna, Italy; ttttttttttttttttttttttttttttttttttttttt Research Institute for Endocrine Sciences, Tehran, Iran; uuuuuuuuuuuuuuuuuuuuuuuuuuuuuuuuuuuuuuu Senior Department of Tuberculosis, The Eighth Medical Center of PLA General Hospital, Beijing, China; vvvvvvvvvvvvvvvvvvvvvvvvvvvvvvvvvvvvvvv Department of Epidemiology, Universidade de São Paulo (University of São Paulo), São Paulo, Brazil; wwwwwwwwwwwwwwwwwwwwwwwwwwwwwwwwwwwwwww Department of Dermatology, Case Western Reserve University, Libertyville, IL, USA; xxxxxxxxxxxxxxxxxxxxxxxxxxxxxxxxxxxxxxx Nuffield Department of Orthopaedics, Rheumatology, and Musculoskeletal Sciences, University of Oxford, Oxford, UK; yyyyyyyyyyyyyyyyyyyyyyyyyyyyyyyyyyyyyyy Liverpool Orthopaedic and Trauma Service, University of Liverpool, Liverpool, UK; zzzzzzzzzzzzzzzzzzzzzzzzzzzzzzzzzzzzzzz Department of Public Health and Preventive Medicine, Charles University, Prague, Czech Republic; aaaaaaaaaaaaaaaaaaaaaaaaaaaaaaaaaaaaaaaa Department of Epidemiology and Biostatistics, Anhui Medical University, Hefei, China; bbbbbbbbbbbbbbbbbbbbbbbbbbbbbbbbbbbbbbbb Health Directorate, Local Health Authority of Ferrara, Ferrara, Italy; cccccccccccccccccccccccccccccccccccccccc Department of Clinical Science, University of Sulaimani, Sulaimani, Iraq; dddddddddddddddddddddddddddddddddddddddd Department of Clinical Pharmacy, Haramaya University, Harar, Ethiopia; eeeeeeeeeeeeeeeeeeeeeeeeeeeeeeeeeeeeeeee Harrington Heart and Vascular Institute, Case Western Reserve University, Cleveland, OH, USA; ffffffffffffffffffffffffffffffffffffffff Division of Cardiovascular Medicine, Ohio State University, Columbus, OH, USA; gggggggggggggggggggggggggggggggggggggggg Department of the Health Directorate, Local Health Authority of Bologna, Bologna, Italy; hhhhhhhhhhhhhhhhhhhhhhhhhhhhhhhhhhhhhhhh Department of Biomedical and Neuromotor Sciences, University of Bologna, Bologna, Italy; iiiiiiiiiiiiiiiiiiiiiiiiiiiiiiiiiiiiiiii Department of Psychiatry, Bronxcare Health System, Bronx, NY, USA; jjjjjjjjjjjjjjjjjjjjjjjjjjjjjjjjjjjjjjjj Department of Psychiatry, Icahn School of Medicine at Mount Sinai, New York, NY, USA; kkkkkkkkkkkkkkkkkkkkkkkkkkkkkkkkkkkkkkkk Department of Urban Planning and Design, University of Hong Kong, Hong Kong, China; llllllllllllllllllllllllllllllllllllllll Department of Geriatric Neurology, Shaanxi Provincial People’s Hospital, Xi'an, China; mmmmmmmmmmmmmmmmmmmmmmmmmmmmmmmmmmmmmmmm Big Data Institute, Nuffield Department of Population Health, University of Oxford, Oxford, UK; nnnnnnnnnnnnnnnnnnnnnnnnnnnnnnnnnnnnnnnn Nanyang Maternal and Child Health Care Hospital, Nanyang Central Hospital, Nanyang, China; oooooooooooooooooooooooooooooooooooooooo Department of Public Health, Torrens University Australia, Melbourne, VIC, Australia; pppppppppppppppppppppppppppppppppppppppp Department of Pharmacology, Chitkara University, Rajpura, India; qqqqqqqqqqqqqqqqqqqqqqqqqqqqqqqqqqqqqqqq Department of Anaesthesia, Maulana Azad Medical College, New Delhi, India; rrrrrrrrrrrrrrrrrrrrrrrrrrrrrrrrrrrrrrrr Department of Preventive Cardiology & Medicine, Eternal Heart Care Centre & Research Institute, Jaipur, India; ssssssssssssssssssssssssssssssssssssssss Department of Medicine, Mahatma Gandhi University Medical Sciences, Jaipur, India; tttttttttttttttttttttttttttttttttttttttt Department of Epidemiology and Psychosocial Research, Ramón de la Fuente Muñiz National Institute of Psychiatry, Mexico City, Mexico; uuuuuuuuuuuuuuuuuuuuuuuuuuuuuuuuuuuuuuuu Doctoral Program in Biomedical Gerontology, Pontifical Catholic University of Rio Grande do Sul, Porto Alegre, Brazil; vvvvvvvvvvvvvvvvvvvvvvvvvvvvvvvvvvvvvvvv Research Unit in Epidemiology Clinic, Mexican Institute of Social Security, Colima, Mexico; wwwwwwwwwwwwwwwwwwwwwwwwwwwwwwwwwwwwwwww College of Health Science, Dilla University, Dilla, Ethiopia; xxxxxxxxxxxxxxxxxxxxxxxxxxxxxxxxxxxxxxxx Department of Medical Microbiology, Bahir Dar University, Bahir Dar, Ethiopia; yyyyyyyyyyyyyyyyyyyyyyyyyyyyyyyyyyyyyyyy Department of Clinical Pharmacology and Medicine, University of Kufa, Najaf, Iraq; zzzzzzzzzzzzzzzzzzzzzzzzzzzzzzzzzzzzzzzz Department of Microbiology and Parasitology, Umm Al-Qura University, Makkah, Saudi Arabia; aaaaaaaaaaaaaaaaaaaaaaaaaaaaaaaaaaaaaaaaa Epidemiology Programme, London School of Hygiene & Tropical Medicine, London, UK; bbbbbbbbbbbbbbbbbbbbbbbbbbbbbbbbbbbbbbbbb Malaria Atlas Project, Perth, WA, Australia; ccccccccccccccccccccccccccccccccccccccccc The George Institute for Global health, University of New South Wales, Sydney, NSW, Australia; ddddddddddddddddddddddddddddddddddddddddd School of Medicine, Urmia University of Medical Sciences, Urmia, Iran; eeeeeeeeeeeeeeeeeeeeeeeeeeeeeeeeeeeeeeeee School of Medicine, Hamedan University of Medical Sciences, Hamedan, Iran; fffffffffffffffffffffffffffffffffffffffff Department of Liver Tumor, Cancer Center, Cho Ray Hospital, Ho Chi Minh City, Viet Nam; ggggggggggggggggggggggggggggggggggggggggg Liver Transplant Unit, Cho Ray Hospital, Ho Chi Minh City, Viet Nam; hhhhhhhhhhhhhhhhhhhhhhhhhhhhhhhhhhhhhhhhh Department of Nursing and Midwifery, Arba Minch University, Arba Minch, Ethiopia; iiiiiiiiiiiiiiiiiiiiiiiiiiiiiiiiiiiiiiiii Department of Community Medicine, Post Graduate Institute of Medical Education and Research, Chandigarh, India; jjjjjjjjjjjjjjjjjjjjjjjjjjjjjjjjjjjjjjjjj Centre for Community Medicine, All India Institute of Medical Sciences, New Delhi, India; kkkkkkkkkkkkkkkkkkkkkkkkkkkkkkkkkkkkkkkkk Department of Infectious Disease Epidemiology, Robert Koch Institute, Berlin, Germany; lllllllllllllllllllllllllllllllllllllllll Department of Public Health, Charité Insitute of Public Health, Berlin, Germany; mmmmmmmmmmmmmmmmmmmmmmmmmmmmmmmmmmmmmmmmm College of Science, University of Sulaimani, Sulaymaniyah, Iraq; nnnnnnnnnnnnnnnnnnnnnnnnnnnnnnnnnnnnnnnnn Department of Pharmacy, American University of Madaba, Amman, Jordan; ooooooooooooooooooooooooooooooooooooooooo Department of Family and Community Medicine, Arabian Gulf University, Manama, Bahrain; ppppppppppppppppppppppppppppppppppppppppp School of Health and Environmental Studies, Hamdan Bin Mohammed Smart University, Dubai, United Arab Emirates; qqqqqqqqqqqqqqqqqqqqqqqqqqqqqqqqqqqqqqqqq Department of Medical and Technical Information Technology, Bauman Moscow State Technical University, Moscow, Russia; rrrrrrrrrrrrrrrrrrrrrrrrrrrrrrrrrrrrrrrrr Sakarya University, Sakarya, Türkiye; sssssssssssssssssssssssssssssssssssssssss Department of Critical Care and Emergency Nursing, Zanjan University of Medical Sciences, Zanjan, Iran; ttttttttttttttttttttttttttttttttttttttttt Centre for Neuromuscular and Neurological Disorders (Perron Institute), The University of Western Australia, Perth, WA, Australia; uuuuuuuuuuuuuuuuuuuuuuuuuuuuuuuuuuuuuuuuu Stroke Research Centre, Perron Institute for Neurological and Translational Science, Perth, WA, Australia; vvvvvvvvvvvvvvvvvvvvvvvvvvvvvvvvvvvvvvvvv Department of Health and Education, Torrens University Australia, Melbourne, VIC, Australia; wwwwwwwwwwwwwwwwwwwwwwwwwwwwwwwwwwwwwwwww Department of Chemistry, University of Hail, Hail, Saudi Arabia; xxxxxxxxxxxxxxxxxxxxxxxxxxxxxxxxxxxxxxxxx Department of Population Science and Human Resource Development, University of Rajshahi, Rajshahi, Bangladesh; yyyyyyyyyyyyyyyyyyyyyyyyyyyyyyyyyyyyyyyyy Department of Medicine, MedStar Health, Baltimore, MD, USA; zzzzzzzzzzzzzzzzzzzzzzzzzzzzzzzzzzzzzzzzz Department of Epidemiology Population Biostatistics and Health Promotion, Universitas Airlangga (Airlangga University), Surabaya, Indonesia; aaaaaaaaaaaaaaaaaaaaaaaaaaaaaaaaaaaaaaaaaa Directorate General of Health Human Resources, Ministry of Health, Jakarta, Indonesia; bbbbbbbbbbbbbbbbbbbbbbbbbbbbbbbbbbbbbbbbbb Research Unit, Parc Sanitari Sant Joan de Deu, Barcelona, Spain; cccccccccccccccccccccccccccccccccccccccccc Department of Mental Health, Biomedical Research Networking Center for Mental Health Network (CiberSAM), Madrid, Spain; dddddddddddddddddddddddddddddddddddddddddd Department of Zoology and Entomology, Al-Azhar University, Cairo, Egypt; eeeeeeeeeeeeeeeeeeeeeeeeeeeeeeeeeeeeeeeeee Faculty of Nursing, Chulalongkorn University, Bangkok, Thailand; ffffffffffffffffffffffffffffffffffffffffff Department of Health Research Methods, Evidence, and Impact, McMaster University, Hamilton, ON, Canada; gggggggggggggggggggggggggggggggggggggggggg Department of Biochemistry and Molecular Biology, Tejgaon College, Dhaka, Bangladesh; hhhhhhhhhhhhhhhhhhhhhhhhhhhhhhhhhhhhhhhhhh Department of Food Technology and Nutrition Science, Noakhali Science and Technology University, Noakhali, Bangladesh; iiiiiiiiiiiiiiiiiiiiiiiiiiiiiiiiiiiiiiiiii Department of Ophthalmology, Iran University of Medical Sciences, Tehran, Iran; jjjjjjjjjjjjjjjjjjjjjjjjjjjjjjjjjjjjjjjjjj Department of Medical Surgical, Shahroud University of Medical Sciences, Shahrekord, Iran; kkkkkkkkkkkkkkkkkkkkkkkkkkkkkkkkkkkkkkkkkk Research Center for Traditional Medicine and History of Medicine, Shiraz University of Medical Sciences, Shiraz, Iran; llllllllllllllllllllllllllllllllllllllllll Department of Periodontics, RAK Medical and Health Sciences University, Ras Al Khaimah, United Arab Emirates; mmmmmmmmmmmmmmmmmmmmmmmmmmmmmmmmmmmmmmmmmm Department of Oral Rehabilitation, University of Khartoum, Khartoum, Sudan; nnnnnnnnnnnnnnnnnnnnnnnnnnnnnnnnnnnnnnnnnn Department of Biotechnology, Lahore University of Biological and Applied Sciences, Lahore, Pakistan; oooooooooooooooooooooooooooooooooooooooooo Department of Plastic Surgery, University of Texas, Houston, TX, USA; pppppppppppppppppppppppppppppppppppppppppp Department of Neurology, Cairo University, Cairo, Egypt; qqqqqqqqqqqqqqqqqqqqqqqqqqqqqqqqqqqqqqqqqq Department of Medicine, University of Khartoum, Khartoum, Sudan; rrrrrrrrrrrrrrrrrrrrrrrrrrrrrrrrrrrrrrrrrr Department of Community Medicine, Federal University Teaching Hospital, Lafia, Nigeria; ssssssssssssssssssssssssssssssssssssssssss Department of Epidemiology and Community Medicine, Federal University of Lafia, Lafia, Nigeria; tttttttttttttttttttttttttttttttttttttttttt Ajman University, Ajman, United Arab Emirates; uuuuuuuuuuuuuuuuuuuuuuuuuuuuuuuuuuuuuuuuuu Institute of Research and Development, Duy Tan University, Da Nang, Viet Nam; vvvvvvvvvvvvvvvvvvvvvvvvvvvvvvvvvvvvvvvvvv Centre for Research Impact & Outcome, Chitkara University, Rajpura, India; wwwwwwwwwwwwwwwwwwwwwwwwwwwwwwwwwwwwwwwwww Department of Health Policy and Financing, Society for Family Health, Abuja, Nigeria; xxxxxxxxxxxxxxxxxxxxxxxxxxxxxxxxxxxxxxxxxx Sina Trauma and Surgery Research Center, Tehran University of Medical Sciences, Tehran, Iran; yyyyyyyyyyyyyyyyyyyyyyyyyyyyyyyyyyyyyyyyyy Caspian Digestive Disease Research Center, Guilan University of Medical Sciences, Rasht, Iran; zzzzzzzzzzzzzzzzzzzzzzzzzzzzzzzzzzzzzzzzzz Department of Paediatrics, University of Colombo, Colombo, Sri Lanka; aaaaaaaaaaaaaaaaaaaaaaaaaaaaaaaaaaaaaaaaaaa Paediatric Professorial Unit, Lady Ridgeway Hospital for Children, Colombo, Sri Lanka; bbbbbbbbbbbbbbbbbbbbbbbbbbbbbbbbbbbbbbbbbbb Institute of Diagnostic and Interventional Radiology and Neuroradiology, University Hospital Essen, Essen, Germany; ccccccccccccccccccccccccccccccccccccccccccc Institute of Artificial Intelligence in Medicine, University Hospital Essen, Essen, Germany; ddddddddddddddddddddddddddddddddddddddddddd Skaane University Hospital, Skaane County Council, Malmö, Sweden; eeeeeeeeeeeeeeeeeeeeeeeeeeeeeeeeeeeeeeeeeee UK Dementia Research Institute Care Research & Technology Centre, Imperial College London, London, UK; fffffffffffffffffffffffffffffffffffffffffff School of Public Health, Imperial College London, London, UK; ggggggggggggggggggggggggggggggggggggggggggg Faculty of Kinesiology, University of New Brunswick, Fredericton, NB, Canada; hhhhhhhhhhhhhhhhhhhhhhhhhhhhhhhhhhhhhhhhhhh School of Allied Health, Murdoch University, Murdoch, WA, Australia; iiiiiiiiiiiiiiiiiiiiiiiiiiiiiiiiiiiiiiiiiii Independent Consultant, Santa Clara, CA, USA; jjjjjjjjjjjjjjjjjjjjjjjjjjjjjjjjjjjjjjjjjjj Community-Oriented Nursing Midwifery Research Center, Shahrekord University of Medical Sciences, Shahrekord, Iran; kkkkkkkkkkkkkkkkkkkkkkkkkkkkkkkkkkkkkkkkkkk Poostchi Ophthalmology Research Center, Shiraz University of Medical Sciences, Shiraz, Iran; lllllllllllllllllllllllllllllllllllllllllll Department of Microbiology, Taiz University, Taiz, Yemen; mmmmmmmmmmmmmmmmmmmmmmmmmmmmmmmmmmmmmmmmmmm School of Medicine, Nankai University, Tianjin, China; nnnnnnnnnnnnnnnnnnnnnnnnnnnnnnnnnnnnnnnnnnn Graduate School of Medicine, University of Tokyo, Tokyo, Japan; ooooooooooooooooooooooooooooooooooooooooooo Department of Pulmonology, Yokohama City University, Yokohama, Japan; ppppppppppppppppppppppppppppppppppppppppppp National Human Genome Research Institute (NHGRI), National Institutes of Health, Bethesda, MD, USA; qqqqqqqqqqqqqqqqqqqqqqqqqqqqqqqqqqqqqqqqqqq Department of Physics, University of Rajshahi, Rajshahi, Bangladesh; rrrrrrrrrrrrrrrrrrrrrrrrrrrrrrrrrrrrrrrrrrr Centre for Advancing Health Outcomes, Vancouver, BC, Canada; sssssssssssssssssssssssssssssssssssssssssss Department of Decision and Information Sciences, University of Houston, Houston, TX, USA; ttttttttttttttttttttttttttttttttttttttttttt Public Health Research Group, Nature Study Society of Bangladesh, Khulna, Bangladesh; uuuuuuuuuuuuuuuuuuuuuuuuuuuuuuuuuuuuuuuuuuu Department of Statistics, Shahjalal University of Science and Technology, Sylhet, Bangladesh; vvvvvvvvvvvvvvvvvvvvvvvvvvvvvvvvvvvvvvvvvvv Department of Population Sciences, University of Dhaka, Dhaka, Bangladesh; wwwwwwwwwwwwwwwwwwwwwwwwwwwwwwwwwwwwwwwwwww Department of Pharmacoeconomics and Pharmaceutical Administration, Tehran University of Medical Sciences, Tehran, Iran; xxxxxxxxxxxxxxxxxxxxxxxxxxxxxxxxxxxxxxxxxxx School of Engineering and Technology, Duy Tan University, Da Nang, Viet Nam; yyyyyyyyyyyyyyyyyyyyyyyyyyyyyyyyyyyyyyyyyyy Jadara Research Center, Jadara University, Irbid, Jordan; zzzzzzzzzzzzzzzzzzzzzzzzzzzzzzzzzzzzzzzzzzz Department of Internal Medicine, Carol Davila University of Medicine and Pharmacy, Bucharest, Romania; aaaaaaaaaaaaaaaaaaaaaaaaaaaaaaaaaaaaaaaaaaaa Department of Legal Medicine and Bioethics, Carol Davila University of Medicine and Pharmacy, Bucharest, Romania; bbbbbbbbbbbbbbbbbbbbbbbbbbbbbbbbbbbbbbbbbbbb Department of Clinical Legal Medicine, National Institute of Legal Medicine Mina Minovici, Bucharest, Romania; cccccccccccccccccccccccccccccccccccccccccccc National School of Tropical Medicine, Baylor College of Medicine, Houston, TX, USA; dddddddddddddddddddddddddddddddddddddddddddd Internal Medicine Department, Parkview Health, Fort Wayne, IN, USA; eeeeeeeeeeeeeeeeeeeeeeeeeeeeeeeeeeeeeeeeeeee Department of Medicine, Liaquat University Of Medical and Health Sciences, Jamshoro, Pakistan; ffffffffffffffffffffffffffffffffffffffffffff Institute for Occupational and Maritime Medicine (ZfAM), University Medical Center Hamburg-Eppendorf (UKE), Hamburg, Germany; gggggggggggggggggggggggggggggggggggggggggggg Department of Psychological and Cognitive Sciences, Tsinghua University, Beijing, China; hhhhhhhhhhhhhhhhhhhhhhhhhhhhhhhhhhhhhhhhhhhh Maternal Care and Child Health Department, Capital Medical University, Beijing, China; iiiiiiiiiiiiiiiiiiiiiiiiiiiiiiiiiiiiiiiiiiii Faculty of Medicine, The Chinese University of Hong Kong, Hong Kong, China; jjjjjjjjjjjjjjjjjjjjjjjjjjjjjjjjjjjjjjjjjjjj Department of Otorhinolaryngology Head and Neck Surgery, Shanghai Jiao Tong University, Shanghai, China; kkkkkkkkkkkkkkkkkkkkkkkkkkkkkkkkkkkkkkkkkkkk Division of Gastroenterology and Hepatology, Mayo Clinic, Jacksonville, FL, USA; llllllllllllllllllllllllllllllllllllllllllll Pediatric Nursing department, University of Indonesia, Depok, Indonesia; mmmmmmmmmmmmmmmmmmmmmmmmmmmmmmmmmmmmmmmmmmmm Department of Public Health and Community Medicine, Shaikh Zayed Postgraduate Medical Institute, Lahore, Pakistan; nnnnnnnnnnnnnnnnnnnnnnnnnnnnnnnnnnnnnnnnnnnn Department of Humanities, COMSATS University Islamabad, Islamabad, Pakistan; oooooooooooooooooooooooooooooooooooooooooooo Nephrology and Urology Research Center, Baqiyatallah University of Medical Sciences, Tehran, Iran; pppppppppppppppppppppppppppppppppppppppppppp Department of Biomolecular Sciences, University of Zakho, Zakho, Iraq; qqqqqqqqqqqqqqqqqqqqqqqqqqqqqqqqqqqqqqqqqqqq Artur Riggs Diabetes & Metabolism Research Institute, Cancer Prevention and Research Institute, Duarte, CA, USA; rrrrrrrrrrrrrrrrrrrrrrrrrrrrrrrrrrrrrrrrrrrr Zagazig University, Zagazig, Egypt; ssssssssssssssssssssssssssssssssssssssssssss Department of Biomedical, Metabolic, and Neural Science, University of Modena and Reggio Emilia, Modena, Italy; tttttttttttttttttttttttttttttttttttttttttttt Department of Health Promotion and Education, University of Ibadan, Ibadan, Nigeria; uuuuuuuuuuuuuuuuuuuuuuuuuuuuuuuuuuuuuuuuuuuu Department of Biology, University of Zakho, Zakho, Iraq; vvvvvvvvvvvvvvvvvvvvvvvvvvvvvvvvvvvvvvvvvvvv Genetics and Molecular Biology Department, Abu Dhabi University, Abu Dhabi, United Arab Emirates; wwwwwwwwwwwwwwwwwwwwwwwwwwwwwwwwwwwwwwwwwwww Faculty of Pharmacy, Sultan Zainal Abidin University, Terengganu, Malaysia; xxxxxxxxxxxxxxxxxxxxxxxxxxxxxxxxxxxxxxxxxxxx Science and Technology Park, Kazakh National Medical University, Almaty, Kazakhstan; yyyyyyyyyyyyyyyyyyyyyyyyyyyyyyyyyyyyyyyyyyyy Pharmacoepidemiology Department, Sanofi, Cambridge, MA, USA; zzzzzzzzzzzzzzzzzzzzzzzzzzzzzzzzzzzzzzzzzzzz Division of Infectious Diseases, Veterans Affairs Greater Los Angeles, Los Angeles, CA, USA; aaaaaaaaaaaaaaaaaaaaaaaaaaaaaaaaaaaaaaaaaaaaa West Africa RCC, Africa Centre for Disease Control and Prevention, Abuja, Nigeria; bbbbbbbbbbbbbbbbbbbbbbbbbbbbbbbbbbbbbbbbbbbbb Department of Community Medicine, University College Hospital, Ibadan, Ibadan, Nigeria; ccccccccccccccccccccccccccccccccccccccccccccc Faculty of Medicine, University of Belgrade, Belgrade, Serbia; ddddddddddddddddddddddddddddddddddddddddddddd Faculty of Medical Sciences, University of Kragujevac, Kragujevac, Serbia; eeeeeeeeeeeeeeeeeeeeeeeeeeeeeeeeeeeeeeeeeeeee Department of Orthopaedic Surgery, Massachusetts General Hospital, Boston, MA, USA; fffffffffffffffffffffffffffffffffffffffffffff Department of Clinical Pharmacy, Prince Sattam bin Abdulaziz University, Al Kharj, Saudi Arabia; ggggggggggggggggggggggggggggggggggggggggggggg Department of Biostatistics, Iran University of Medical Sciences, Tehran, Iran; hhhhhhhhhhhhhhhhhhhhhhhhhhhhhhhhhhhhhhhhhhhhh Department of Chemical Pathology, University of Jos, Jos, Nigeria; iiiiiiiiiiiiiiiiiiiiiiiiiiiiiiiiiiiiiiiiiiiii Department of Chemical Pathology, Jos University Teaching Hospital, Jos, Nigeria; jjjjjjjjjjjjjjjjjjjjjjjjjjjjjjjjjjjjjjjjjjjjj Faculty of Health and Life Sciences, University of Exeter, Exeter, UK; kkkkkkkkkkkkkkkkkkkkkkkkkkkkkkkkkkkkkkkkkkkkk Department of Psychology, Wuhan University, Wuhan, China; lllllllllllllllllllllllllllllllllllllllllllll Faculty of Pharmacy, Universitas Ahmad Dahlan, Yogyakarta, Indonesia; mmmmmmmmmmmmmmmmmmmmmmmmmmmmmmmmmmmmmmmmmmmmm Department of Microbiology, University of Maiduguri, Maiduguri, Nigeria; nnnnnnnnnnnnnnnnnnnnnnnnnnnnnnnnnnnnnnnnnnnnn Department of Biotechnology, Sharda University, Greater Noida, India; ooooooooooooooooooooooooooooooooooooooooooooo School of Pharmacy, Taipei Medical University, Taipei, Taiwan; ppppppppppppppppppppppppppppppppppppppppppppp Department of Pharmaceutical Technology, Sekolah Tinggi Ilmu Farmasi Riau, Pekanbaru, Indonesia; qqqqqqqqqqqqqqqqqqqqqqqqqqqqqqqqqqqqqqqqqqqqq Independent Researcher, Cairo, Egypt; rrrrrrrrrrrrrrrrrrrrrrrrrrrrrrrrrrrrrrrrrrrrr School of Pharmacy, BRAC University, Dhaka, Bangladesh; sssssssssssssssssssssssssssssssssssssssssssss Journal of Biosciences and Public Health, Published by 4-Green Research Society, Journal of Biological Sciences and Public Health, Dhaka, Bangladesh; ttttttttttttttttttttttttttttttttttttttttttttt Institute for Physical Activity and Nutrition, Deakin University, Burwood, VIC, Australia; uuuuuuuuuuuuuuuuuuuuuuuuuuuuuuuuuuuuuuuuuuuuu Department of Surveillance and Health Equity Science, American Cancer Society, Atlanta, GA, USA; vvvvvvvvvvvvvvvvvvvvvvvvvvvvvvvvvvvvvvvvvvvvv Clinical Laboratory Department, Tobruk University, Tobruk, Libya; wwwwwwwwwwwwwwwwwwwwwwwwwwwwwwwwwwwwwwwwwwwww Department of Blood Transmitted Diseases, National Centre for Disease Control (NCDC), Tobruk, Libya; xxxxxxxxxxxxxxxxxxxxxxxxxxxxxxxxxxxxxxxxxxxxx Department of Clinical Pharmacy & Pharmacy Practice, Asian Institute of Medicine, Science and Technology, Bedong, Malaysia; yyyyyyyyyyyyyyyyyyyyyyyyyyyyyyyyyyyyyyyyyyyyy Malaysian Academy of Pharmacy, Puchong, Malaysia; zzzzzzzzzzzzzzzzzzzzzzzzzzzzzzzzzzzzzzzzzzzzz Department of Urology, Kazakh National Medical University, Almaty, Kazakhstan; aaaaaaaaaaaaaaaaaaaaaaaaaaaaaaaaaaaaaaaaaaaaaa Department of Health Services Research, University of Tsukuba, Tsukuba, Japan; bbbbbbbbbbbbbbbbbbbbbbbbbbbbbbbbbbbbbbbbbbbbbb Department of Non-Communicable Disease Epidemiology, London School of Hygiene & Tropical Medicine, London, UK; cccccccccccccccccccccccccccccccccccccccccccccc Knowledge Translation Program, Centre for Health Evaluation and Outcome Sciences, Vancouver, BC, Canada; dddddddddddddddddddddddddddddddddddddddddddddd Department of Environmental Health Engineering, Guilan University of Medical Sciences, Rasht, Iran; eeeeeeeeeeeeeeeeeeeeeeeeeeeeeeeeeeeeeeeeeeeeee Department of Physical Medicine and Rehabilitation, Université Paris Cité, Paris, France; ffffffffffffffffffffffffffffffffffffffffffffff Research and Development Unit, Biomedical Research Networking Center for Mental Health Network (CiberSAM), Barcelona, Spain; gggggggggggggggggggggggggggggggggggggggggggggg Department of Health Studies, University of Richmond, Richmond, VA, USA; hhhhhhhhhhhhhhhhhhhhhhhhhhhhhhhhhhhhhhhhhhhhhh Department of Nursing, Arak University of Medical Sciences, Arak, Iran; iiiiiiiiiiiiiiiiiiiiiiiiiiiiiiiiiiiiiiiiiiiiii Department of Immunology, Tabriz University of Medical Sciences, Tabriz, Iran; jjjjjjjjjjjjjjjjjjjjjjjjjjjjjjjjjjjjjjjjjjjjjj School of Medicine, Volgograd State Medical University, Volgograd, Russia; kkkkkkkkkkkkkkkkkkkkkkkkkkkkkkkkkkkkkkkkkkkkkk Shiraz Neuroscience Research Center, Shiraz University of Medical Sciences, Shiraz, Iran; llllllllllllllllllllllllllllllllllllllllllllll Department of Immunology, Kerman University of Medical Sciences, Kerman, Iran; mmmmmmmmmmmmmmmmmmmmmmmmmmmmmmmmmmmmmmmmmmmmmm Department of Immunology, Rafsanjan University of Medical Sciences, Rafsanjan, Iran; nnnnnnnnnnnnnnnnnnnnnnnnnnnnnnnnnnnnnnnnnnnnnn UCL Institute for Global Health, University of London, London, UK; oooooooooooooooooooooooooooooooooooooooooooooo College of Medicine and Health Sciences, Arabian Gulf University, Manama, Bahrain; pppppppppppppppppppppppppppppppppppppppppppppp Government Hospitals, Manama, Bahrain; qqqqqqqqqqqqqqqqqqqqqqqqqqqqqqqqqqqqqqqqqqqqqq Department of Health and Safety, Dubai Municipality, Dubai, United Arab Emirates; rrrrrrrrrrrrrrrrrrrrrrrrrrrrrrrrrrrrrrrrrrrrrr Department of Research and Academic Affairs, Larkin Community Hospital, South Miami, FL, USA; ssssssssssssssssssssssssssssssssssssssssssssss Department of Medicine, AMA School of Medicine, Makati, Philippines; tttttttttttttttttttttttttttttttttttttttttttttt Department of Behavioral Health, Nassau University Medical center, East Meadow, NY, USA; uuuuuuuuuuuuuuuuuuuuuuuuuuuuuuuuuuuuuuuuuuuuuu School of Global Public Health, New York University, New York, NY, USA; vvvvvvvvvvvvvvvvvvvvvvvvvvvvvvvvvvvvvvvvvvvvvv UNESCO-TWAS Section of Economic & Social Sciences, Humanities & Arts, The World Academy of Sciences UNESCO-TWAS, Trieste, Italy; wwwwwwwwwwwwwwwwwwwwwwwwwwwwwwwwwwwwwwwwwwwwww Shaanxi University of Technology, Hanzhong, China; xxxxxxxxxxxxxxxxxxxxxxxxxxxxxxxxxxxxxxxxxxxxxx Department of Environmental Engineering, Islamic Azad University, Ahvaz, Iran; yyyyyyyyyyyyyyyyyyyyyyyyyyyyyyyyyyyyyyyyyyyyyy Department of Neurosurgery, Medical College of Wisconsin, Milwaukee, WI, USA; zzzzzzzzzzzzzzzzzzzzzzzzzzzzzzzzzzzzzzzzzzzzzz Department of Public Health Sciences, University of Chicago, Chicago, IL, USA; aaaaaaaaaaaaaaaaaaaaaaaaaaaaaaaaaaaaaaaaaaaaaaa Department of Health Informatics, Qassim University, Buraydah, Saudi Arabia; bbbbbbbbbbbbbbbbbbbbbbbbbbbbbbbbbbbbbbbbbbbbbbb Department of Primary Care Medicine, Universiti Malaya, Kuala Lumpur, Malaysia; ccccccccccccccccccccccccccccccccccccccccccccccc SRM Medical College Hospital and Research Centre, Sri Ramaswamy Memorial Institute of Science and Technology, Kattankulathur, India; ddddddddddddddddddddddddddddddddddddddddddddddd Department of Public Health, Daffodil International University, Dhaka, Bangladesh; eeeeeeeeeeeeeeeeeeeeeeeeeeeeeeeeeeeeeeeeeeeeeee Department of Public and Community Health, Frontier University Garowe, Puntland, Somalia; fffffffffffffffffffffffffffffffffffffffffffffff Department of Neurosciences, University of the Philippines Manila, Manila, Philippines; ggggggggggggggggggggggggggggggggggggggggggggggg Institute for Neurosciences, St. Luke's Medical Center, Bonifacio Global City, Philippines; hhhhhhhhhhhhhhhhhhhhhhhhhhhhhhhhhhhhhhhhhhhhhhh Institute for Musculoskeletal Health, University of Sydney, Sydney, NSW, Australia; iiiiiiiiiiiiiiiiiiiiiiiiiiiiiiiiiiiiiiiiiiiiiii Shahrekord University of Medical Sciences, Shahrekord, Iran; jjjjjjjjjjjjjjjjjjjjjjjjjjjjjjjjjjjjjjjjjjjjjjj Division of Medical Research, Sri Ramaswamy Memorial Institute of Science and Technology, Kattankulathur, India; kkkkkkkkkkkkkkkkkkkkkkkkkkkkkkkkkkkkkkkkkkkkkkk Department of Internal Medicine, Harvard University, Cambridge, MA, USA; lllllllllllllllllllllllllllllllllllllllllllllll Department of Stem Cells and Developmental Biology, Royan Institution, Tehran, Iran; mmmmmmmmmmmmmmmmmmmmmmmmmmmmmmmmmmmmmmmmmmmmmmm Health Informatics Lab, Boston University, Boston, MA, USA; nnnnnnnnnnnnnnnnnnnnnnnnnnnnnnnnnnnnnnnnnnnnnnn Department of Medicine, University of Mississippi Medical Center, Jackson, MS, USA; ooooooooooooooooooooooooooooooooooooooooooooooo Department of Medicine, Jinnah Sindh Medical University, Karachi, Pakistan; ppppppppppppppppppppppppppppppppppppppppppppppp Karolinska Institutet Campus Solna, Karolinska Institutet (Karolinska Institute), Stockholm, Sweden; qqqqqqqqqqqqqqqqqqqqqqqqqqqqqqqqqqqqqqqqqqqqqqq Invasive Fungi Research Center, Mazandaran University of Medical Sciences, Sari, Iran; rrrrrrrrrrrrrrrrrrrrrrrrrrrrrrrrrrrrrrrrrrrrrrr Department of Medical Mycology, Mazandaran University of Medical Sciences, Sari, Iran; sssssssssssssssssssssssssssssssssssssssssssssss Department of Pharmacology, Imam Mohammad Ibn Saud Islamic University, Riyadh, Saudi Arabia; ttttttttttttttttttttttttttttttttttttttttttttttt Department of Nursing, Middle Technical University of Kut Technical Institute, Baghdad, Iraq; uuuuuuuuuuuuuuuuuuuuuuuuuuuuuuuuuuuuuuuuuuuuuuu The Medical City for Military and Security Services School, The Medical City for Military and Security Services School, Oman, Muscat, Oman; vvvvvvvvvvvvvvvvvvvvvvvvvvvvvvvvvvvvvvvvvvvvvvv Department of Biochemistry, Government Medical College, Mysuru, India; wwwwwwwwwwwwwwwwwwwwwwwwwwwwwwwwwwwwwwwwwwwwwww Department of Oral Medicine and Periodontology, University of Peradeniya, Peradeniya, Sri Lanka; xxxxxxxxxxxxxxxxxxxxxxxxxxxxxxxxxxxxxxxxxxxxxxx Department of Oral Medicine and Periodontology, Saveetha University, Chennai, India; yyyyyyyyyyyyyyyyyyyyyyyyyyyyyyyyyyyyyyyyyyyyyyy Department of Research, University of Puthisastra, Phnom Penh, Cambodia; zzzzzzzzzzzzzzzzzzzzzzzzzzzzzzzzzzzzzzzzzzzzzzz Department of Epidemiology and Health Promotion, Yonsei University, Seoul, South Korea; aaaaaaaaaaaaaaaaaaaaaaaaaaaaaaaaaaaaaaaaaaaaaaaa Department of General Medicine, Manipal Academy of Higher Education, Mangalore, India; bbbbbbbbbbbbbbbbbbbbbbbbbbbbbbbbbbbbbbbbbbbbbbbb Graphic Era Deemed to be University, Graphic Era (Deemed to be University), Dehradun, India; cccccccccccccccccccccccccccccccccccccccccccccccc Department of Internal Medicine, GCS Medical College, Hospital & Research Centre, Ahmedabad, India; dddddddddddddddddddddddddddddddddddddddddddddddd The George Institute for Global Health, New Delhi, India; eeeeeeeeeeeeeeeeeeeeeeeeeeeeeeeeeeeeeeeeeeeeeeee Manipal Academy of Higher Education, Manipal, India; ffffffffffffffffffffffffffffffffffffffffffffffff Vanke School of Public Health, Tsinghua University, Beijing, China; gggggggggggggggggggggggggggggggggggggggggggggggg Xuzhou Medical University, Xuzhou, China; hhhhhhhhhhhhhhhhhhhhhhhhhhhhhhhhhhhhhhhhhhhhhhhh Department of Orthopedics, Wuhan University, Wuhan, China; iiiiiiiiiiiiiiiiiiiiiiiiiiiiiiiiiiiiiiiiiiiiiiii Department of Biomedical Sciences, City University of Hong Kong, Hong Kong, China; jjjjjjjjjjjjjjjjjjjjjjjjjjjjjjjjjjjjjjjjjjjjjjjj Department of Microbiology, Faculty of Medicine, Ahvaz Jundishapur University of Medical Sciences, Ahvaz, Iran; kkkkkkkkkkkkkkkkkkkkkkkkkkkkkkkkkkkkkkkkkkkkkkkk Department of Microbiology, Abadan School of Medical Sciences, Abadan, Iran; llllllllllllllllllllllllllllllllllllllllllllllll Rothschild Foundation Hospital, Institut Français de Myopie, Paris, France; mmmmmmmmmmmmmmmmmmmmmmmmmmmmmmmmmmmmmmmmmmmmmmmm Singapore Eye Research Institute, Singapore, Singapore; nnnnnnnnnnnnnnnnnnnnnnnnnnnnnnnnnnnnnnnnnnnnnnnn Hungarian Health Management Association, Budapest, Hungary; oooooooooooooooooooooooooooooooooooooooooooooooo Department of Biomedical Engineering, Hong Kong Polytechnic University, Hong Kong, China; pppppppppppppppppppppppppppppppppppppppppppppppp Department of Gastroenterology and Hepatology, Stanford University, Stanford, CA, USA; qqqqqqqqqqqqqqqqqqqqqqqqqqqqqqqqqqqqqqqqqqqqqqqq Department of Community Medicine, Manipal Academy of Higher Education, Mangalore, India; rrrrrrrrrrrrrrrrrrrrrrrrrrrrrrrrrrrrrrrrrrrrrrrr Department of Management, Indira Gandhi Delhi Technical University for Women, Delhi, India; ssssssssssssssssssssssssssssssssssssssssssssssss Nursing & Midwifery Research Department (NMRD), Hamad Medical Corporation, Doha, Qatar; tttttttttttttttttttttttttttttttttttttttttttttttt Department of Family Medicine and Public Health, University of Opole, Opole, Poland; uuuuuuuuuuuuuuuuuuuuuuuuuuuuuuuuuuuuuuuuuuuuuuuu Institute of Family Medicine and Public Health, University of Tartu, Tartu, Estonia; vvvvvvvvvvvvvvvvvvvvvvvvvvvvvvvvvvvvvvvvvvvvvvvv Health Economics Unit, Flinders University, Adelaide, SA, Australia; wwwwwwwwwwwwwwwwwwwwwwwwwwwwwwwwwwwwwwwwwwwwwwww Minimally Invasive Surgery Research Center, Iran University of Medical Sciences, Tehran, Iran; xxxxxxxxxxxxxxxxxxxxxxxxxxxxxxxxxxxxxxxxxxxxxxxx Research Department, TobaccoFree Research Institute Ireland, Dublin, Ireland; yyyyyyyyyyyyyyyyyyyyyyyyyyyyyyyyyyyyyyyyyyyyyyyy School of Public Health, University College Cork, Cork, Ireland; zzzzzzzzzzzzzzzzzzzzzzzzzzzzzzzzzzzzzzzzzzzzzzzz Policy, Research, and International Development Directorate, Public Health Wales, Cardiff, UK; aaaaaaaaaaaaaaaaaaaaaaaaaaaaaaaaaaaaaaaaaaaaaaaaa Department of Statistics, Salahaddin University-Erbil, Erbil, Iraq; bbbbbbbbbbbbbbbbbbbbbbbbbbbbbbbbbbbbbbbbbbbbbbbbb Department of Business Administrations, Cihan University-Erbil, Erbil, Iraq; ccccccccccccccccccccccccccccccccccccccccccccccccc Department of Pharmacology, Post Graduate Institute of Medical Education and Research, Chandigarh, India; ddddddddddddddddddddddddddddddddddddddddddddddddd Independent Consultant, Pune, India; eeeeeeeeeeeeeeeeeeeeeeeeeeeeeeeeeeeeeeeeeeeeeeeee Department of Neurology, University of Washington, Seattle, WA, USA; fffffffffffffffffffffffffffffffffffffffffffffffff Department of Health, Khoy Medical Sciences, Khoy, Iran; ggggggggggggggggggggggggggggggggggggggggggggggggg Department of Dermatology, King Faisal University, Hofuf, Saudi Arabia; hhhhhhhhhhhhhhhhhhhhhhhhhhhhhhhhhhhhhhhhhhhhhhhhh Department of Endocrinology, Bharti Hospital Karnal, Karnal, India; iiiiiiiiiiiiiiiiiiiiiiiiiiiiiiiiiiiiiiiiiiiiiiiii University Centre for Research and Development, Chandigarh University, Mohali, India; jjjjjjjjjjjjjjjjjjjjjjjjjjjjjjjjjjjjjjjjjjjjjjjjj Canberra Business School, University of Canberra, Hawker, ACT, Australia; kkkkkkkkkkkkkkkkkkkkkkkkkkkkkkkkkkkkkkkkkkkkkkkkk College of Pharmacy, Prince Sattam bin Abdulaziz University, Al Kharj, Saudi Arabia; lllllllllllllllllllllllllllllllllllllllllllllllll NIHR Global Health Research Unit on Global Surgery, University of Birmingham, Birmingham, UK; mmmmmmmmmmmmmmmmmmmmmmmmmmmmmmmmmmmmmmmmmmmmmmmmm Prasanna School of Public Health, Manipal Academy of Higher Education, Manipal, India; nnnnnnnnnnnnnnnnnnnnnnnnnnnnnnnnnnnnnnnnnnnnnnnnn Care and Public Health Research Institute (CAPHRI), Maastricht University, Maastricht, Netherlands; ooooooooooooooooooooooooooooooooooooooooooooooooo Department of General Medical Practice No. 2, Kazakh National Medical University, Almaty, Kazakhstan; ppppppppppppppppppppppppppppppppppppppppppppppppp Russell H. Morgan Department of Radiology and Radiological Science, Johns Hopkins University, Baltimore, MD, USA; qqqqqqqqqqqqqqqqqqqqqqqqqqqqqqqqqqqqqqqqqqqqqqqqq Department of Public Health, South Wales University, Treforest, UK; rrrrrrrrrrrrrrrrrrrrrrrrrrrrrrrrrrrrrrrrrrrrrrrrr Department of Cardiothoracic Surgery, Stanford University, Palo Alto, CA, USA; sssssssssssssssssssssssssssssssssssssssssssssssss Department of Biostatistics and Epidemiology, Abadan University of Medical Sciences, Abadan, Iran; ttttttttttttttttttttttttttttttttttttttttttttttttt Microbiology, Virology and Immunology Department, I. Horbachevsky Ternopil National Medical University, Ternopil, Ukraine; uuuuuuuuuuuuuuuuuuuuuuuuuuuuuuuuuuuuuuuuuuuuuuuuu Department of Health Sciences, University of York, York, UK; vvvvvvvvvvvvvvvvvvvvvvvvvvvvvvvvvvvvvvvvvvvvvvvvv Department of Rehabilitation Sciences, Qatar University, Doha, Qatar; wwwwwwwwwwwwwwwwwwwwwwwwwwwwwwwwwwwwwwwwwwwwwwwww School of Health and Environmental Science, Korea University, Seoul, South Korea; xxxxxxxxxxxxxxxxxxxxxxxxxxxxxxxxxxxxxxxxxxxxxxxxx Department of Anesthesia, Critical Care and Pain Medicine, Massachusetts General Hospital, Boston, MA, USA; yyyyyyyyyyyyyyyyyyyyyyyyyyyyyyyyyyyyyyyyyyyyyyyyy Office of the Executive Director, Cephas Health Research Initiative Inc, Ibadan, Nigeria; zzzzzzzzzzzzzzzzzzzzzzzzzzzzzzzzzzzzzzzzzzzzzzzzz Department of Community Medicine, ESIC Medical College and Hospital Chennai, Chennai, India; aaaaaaaaaaaaaaaaaaaaaaaaaaaaaaaaaaaaaaaaaaaaaaaaaa The Hansjörg Wyss Department of Plastic and Reconstructive Surgery, NYU Langone Health, New York, NY, USA; bbbbbbbbbbbbbbbbbbbbbbbbbbbbbbbbbbbbbbbbbbbbbbbbbb Cleft Lip and Palate Surgery Division, Global Smile Foundation, Norwood, MA, USA; cccccccccccccccccccccccccccccccccccccccccccccccccc Community and Primary Care Research Group, Plymouth University, Plymouth, UK; dddddddddddddddddddddddddddddddddddddddddddddddddd Department of Psychiatry, King George's Medical University, Lucknow, India; eeeeeeeeeeeeeeeeeeeeeeeeeeeeeeeeeeeeeeeeeeeeeeeeee 2nd Department of Cardiology, Aristotle University of Thessaloniki, Thessaloniki, Greece; ffffffffffffffffffffffffffffffffffffffffffffffffff Laboratory Science Department, Khomein University of Medical Sciences, Khomein, Iran; gggggggggggggggggggggggggggggggggggggggggggggggggg Department of Immunology, Tehran University of Medical Sciences, Tehran, Iran; hhhhhhhhhhhhhhhhhhhhhhhhhhhhhhhhhhhhhhhhhhhhhhhhhh Department of Basic Medical Sciences, Yarmouk University, Irbid, Jordan; iiiiiiiiiiiiiiiiiiiiiiiiiiiiiiiiiiiiiiiiiiiiiiiiii Department of General Medicine, Shahid Beheshti University of Medical Sciences, Tehran, Iran; jjjjjjjjjjjjjjjjjjjjjjjjjjjjjjjjjjjjjjjjjjjjjjjjjj Social Determinants of Health Research Center, Tabriz University of Medical Sciences, Tabriz, Iran; kkkkkkkkkkkkkkkkkkkkkkkkkkkkkkkkkkkkkkkkkkkkkkkkkk Endocrine Research Center, Iran University of Medical Sciences, Tehran, Iran; llllllllllllllllllllllllllllllllllllllllllllllllll Department of Echocardiography, Iran University of Medical Sciences, Tehran, Iran; mmmmmmmmmmmmmmmmmmmmmmmmmmmmmmmmmmmmmmmmmmmmmmmmmm Saveetha Medical College and Hospital, Saveetha University, Chennai, India; nnnnnnnnnnnnnnnnnnnnnnnnnnnnnnnnnnnnnnnnnnnnnnnnnn Chair and Department of Medical Microbiology, Poznan University of Medical Sciences, Poznan, Poland; oooooooooooooooooooooooooooooooooooooooooooooooooo Department of Medicine, Jacobi Medical Center, New York, NY, USA; pppppppppppppppppppppppppppppppppppppppppppppppppp Surgery Research Unit, University of Oulu, Oulu, Finland; qqqqqqqqqqqqqqqqqqqqqqqqqqqqqqqqqqqqqqqqqqqqqqqqqq Department of Molecular Medicine and Surgery, Karolinska Institutet (Karolinska Institute), Stockholm, Sweden; rrrrrrrrrrrrrrrrrrrrrrrrrrrrrrrrrrrrrrrrrrrrrrrrrr Department of Clinical Research and Epidemiology, Institute of Liver and Biliary Sciences, New Delhi, India; ssssssssssssssssssssssssssssssssssssssssssssssssss Department of Neurosurgery, Johns Hopkins University, Baltimore, MD, USA; tttttttttttttttttttttttttttttttttttttttttttttttttt Cardiac Primary Prevention Research Center, Tehran University of Medical Sciences, Tehran, Iran; uuuuuuuuuuuuuuuuuuuuuuuuuuuuuuuuuuuuuuuuuuuuuuuuuu Department of Cardiac Electrophysiology, Tehran University of Medical Sciences, Tehran, Iran; vvvvvvvvvvvvvvvvvvvvvvvvvvvvvvvvvvvvvvvvvvvvvvvvvv School of Pharmacy, Jimma University, Jimma, Ethiopia; wwwwwwwwwwwwwwwwwwwwwwwwwwwwwwwwwwwwwwwwwwwwwwwwww Department of Internal Medicine, Yale New Haven Health—Bridgeport Hospital, Bridgeport, CT, USA; xxxxxxxxxxxxxxxxxxxxxxxxxxxxxxxxxxxxxxxxxxxxxxxxxx Institute of Biological Chemistry and Nutrition, University Hohenheim, Stuttgart, Germany; yyyyyyyyyyyyyyyyyyyyyyyyyyyyyyyyyyyyyyyyyyyyyyyyyy Department of Biochemistry, JSS Medical College, Mysuru, India; zzzzzzzzzzzzzzzzzzzzzzzzzzzzzzzzzzzzzzzzzzzzzzzzzz Center of Global Child Health, The Hospital for Sick Children, Toronto, ON, Canada; aaaaaaaaaaaaaaaaaaaaaaaaaaaaaaaaaaaaaaaaaaaaaaaaaaa Centre for Adolescent Health, Murdoch Childrens Research Institute, Parkville, VIC, Australia; bbbbbbbbbbbbbbbbbbbbbbbbbbbbbbbbbbbbbbbbbbbbbbbbbbb Department of Psychological Medicine, University of Otago, Christchurch, New Zealand; ccccccccccccccccccccccccccccccccccccccccccccccccccc Jindal School of Public Health and Human Development, O. P. Jindal Global University, Sonipat, India; ddddddddddddddddddddddddddddddddddddddddddddddddddd Department of Biomedical Informatics, Arizona State University, Phoenix, AZ, USA; eeeeeeeeeeeeeeeeeeeeeeeeeeeeeeeeeeeeeeeeeeeeeeeeeee Department of Human Nutrition of INRAE, National Research Institute for Agriculture, Food and Environment, Paris, France; fffffffffffffffffffffffffffffffffffffffffffffffffff Sorbonne Paris Nord University, Bobigny, France; ggggggggggggggggggggggggggggggggggggggggggggggggggg Faculty of Medicine, Mashhad University of Medical Sciences, Mashhad, Iran; hhhhhhhhhhhhhhhhhhhhhhhhhhhhhhhhhhhhhhhhhhhhhhhhhhh Department of Public Health, Jordan University of Science and Technology, Irbid, Jordan; iiiiiiiiiiiiiiiiiiiiiiiiiiiiiiiiiiiiiiiiiiiiiiiiiii Lahore Medical Research Center, Lahore, Pakistan; jjjjjjjjjjjjjjjjjjjjjjjjjjjjjjjjjjjjjjjjjjjjjjjjjjj Department of Veterinary Medicine, United Arab Emirates University, Al Ain, United Arab Emirates; kkkkkkkkkkkkkkkkkkkkkkkkkkkkkkkkkkkkkkkkkkkkkkkkkkk Faculty of Veterinary Medicine, Kafrelsheikh University, Kafrelsheikh, Egypt; lllllllllllllllllllllllllllllllllllllllllllllllllll Department of Nursing, Zarqa University, Zarqa, Jordan; mmmmmmmmmmmmmmmmmmmmmmmmmmmmmmmmmmmmmmmmmmmmmmmmmmm University Institute of Diet and Nutritional Sciences, The University of Lahore, Lahore, Pakistan; nnnnnnnnnnnnnnnnnnnnnnnnnnnnnnnnnnnnnnnnnnnnnnnnnnn Department of Medicine, Guilan University of Medical Sciences, Rasht, Iran; ooooooooooooooooooooooooooooooooooooooooooooooooooo Department of Obstetrics & Gynecology, Iran University of Medical Sciences, Tehran, Iran; ppppppppppppppppppppppppppppppppppppppppppppppppppp Department of Biostatistics, Mazandaran University of Medical Sciences, Sari, Iran; qqqqqqqqqqqqqqqqqqqqqqqqqqqqqqqqqqqqqqqqqqqqqqqqqqq Department of Medical Genetics and Molecular Medicine, Mashhad University of Medical Sciences, Mashhad, Iran; rrrrrrrrrrrrrrrrrrrrrrrrrrrrrrrrrrrrrrrrrrrrrrrrrrr Department of Public Health, Mohammed VI Center for Research and Innovation, Rabat, Morocco; sssssssssssssssssssssssssssssssssssssssssssssssssss Higher Institute of Nursing Professions and Health Techniques, Rabat, Morocco; ttttttttttttttttttttttttttttttttttttttttttttttttttt Food and Drug Research Center, Iran Food and Drug Administration, Tehran, Iran; uuuuuuuuuuuuuuuuuuuuuuuuuuuuuuuuuuuuuuuuuuuuuuuuuuu Natural and Medical Sciences Research Center, University of Nizwa, Nizwa, Oman; vvvvvvvvvvvvvvvvvvvvvvvvvvvvvvvvvvvvvvvvvvvvvvvvvvv Department of Physical Therapy, King Abdulaziz University, Jeddah, Saudi Arabia; wwwwwwwwwwwwwwwwwwwwwwwwwwwwwwwwwwwwwwwwwwwwwwwwwww Internal Medicine Department, Reading Hospital Tower Health, Reading, PA, USA; xxxxxxxxxxxxxxxxxxxxxxxxxxxxxxxxxxxxxxxxxxxxxxxxxxx Epidemiology Program, Jazan University, Jazan, Saudi Arabia; yyyyyyyyyyyyyyyyyyyyyyyyyyyyyyyyyyyyyyyyyyyyyyyyyyy Department of Community Medicine, National Institute of Preventive and Social Medicine, Dhaka, Bangladesh; zzzzzzzzzzzzzzzzzzzzzzzzzzzzzzzzzzzzzzzzzzzzzzzzzzz BDStatistics Center for Research, Dhaka, Bangladesh; aaaaaaaaaaaaaaaaaaaaaaaaaaaaaaaaaaaaaaaaaaaaaaaaaaaa Karachi Medical and Dental College, Karachi, Pakistan; bbbbbbbbbbbbbbbbbbbbbbbbbbbbbbbbbbbbbbbbbbbbbbbbbbbb Center for Atmospheric Particle Studies (CAPS), Carnegie Mellon University, Pittsburgh, PA, USA; cccccccccccccccccccccccccccccccccccccccccccccccccccc Department of Mechanical Engineering (MechE), Carnegie Mellon University, Pittsburgh, PA, USA; dddddddddddddddddddddddddddddddddddddddddddddddddddd Aston Pharmacy School, Aston University, Birmingham, UK; eeeeeeeeeeeeeeeeeeeeeeeeeeeeeeeeeeeeeeeeeeeeeeeeeeee Joint Doctoral School, Silesian University of Technology, Gliwice, Poland; ffffffffffffffffffffffffffffffffffffffffffffffffffff Dr. Panjwani Center for Molecular Medicine & Drug Research, University of Karachi, Karachi, Pakistan; gggggggggggggggggggggggggggggggggggggggggggggggggggg International Center for Chemical and Biological Sciences, Karachi, Pakistan; hhhhhhhhhhhhhhhhhhhhhhhhhhhhhhhhhhhhhhhhhhhhhhhhhhhh Department of Medicine, University of Maryland, Baltimore, MD, USA; iiiiiiiiiiiiiiiiiiiiiiiiiiiiiiiiiiiiiiiiiiiiiiiiiiii College of Medicine, University of Hail, Hail, Saudi Arabia; jjjjjjjjjjjjjjjjjjjjjjjjjjjjjjjjjjjjjjjjjjjjjjjjjjjj Department of Cardiology, University of South Wales, Treforest, UK; kkkkkkkkkkkkkkkkkkkkkkkkkkkkkkkkkkkkkkkkkkkkkkkkkkkk Department of Cardiology, University of Buckingham, Buckingham, UK; llllllllllllllllllllllllllllllllllllllllllllllllllll Department of Health, Nepal Development Society, Chitwan, Nepal; mmmmmmmmmmmmmmmmmmmmmmmmmmmmmmmmmmmmmmmmmmmmmmmmmmmm Department of Preventable Non Communicable Disease, Menzies School of Health Research, Alice Springs, NT, Australia; nnnnnnnnnnnnnnnnnnnnnnnnnnnnnnnnnnnnnnnnnnnnnnnnnnnn Department of Epidemiology, Non-Communicable Diseases Research Center (NCDRC), Tehran, Iran; oooooooooooooooooooooooooooooooooooooooooooooooooooo Department of Pharmacology, All India Institute of Medical Sciences, Raipur, India; pppppppppppppppppppppppppppppppppppppppppppppppppppp Department of Physiology and Biomedical Engineering, Mayo Clinic, Rochester, MN, USA; qqqqqqqqqqqqqqqqqqqqqqqqqqqqqqqqqqqqqqqqqqqqqqqqqqqq College of Health, Wellbeing and Life Sciences, Sheffield Hallam University, Sheffield, UK; rrrrrrrrrrrrrrrrrrrrrrrrrrrrrrrrrrrrrrrrrrrrrrrrrrrr College of Arts and Sciences, Ohio University, Zanesville, OH, USA; ssssssssssssssssssssssssssssssssssssssssssssssssssss Faculty of Nursing, Yarmouk University, Irbid, Jordan; tttttttttttttttttttttttttttttttttttttttttttttttttttt Global Consortium for Public Health Research, Datta Meghe Institute of Higher Education and Research, Wardha, India; uuuuuuuuuuuuuuuuuuuuuuuuuuuuuuuuuuuuuuuuuuuuuuuuuuuu Department of Orthopaedics, Postgraduate Medical Institute, Sangrur, India; vvvvvvvvvvvvvvvvvvvvvvvvvvvvvvvvvvvvvvvvvvvvvvvvvvvv Department of Neurosurgery, Shahid Beheshti University of Medical Sciences, Tehran, Iran; wwwwwwwwwwwwwwwwwwwwwwwwwwwwwwwwwwwwwwwwwwwwwwwwwwww Penn Medicine, University of Pennsylvania, Philadelphia, PA, USA; xxxxxxxxxxxxxxxxxxxxxxxxxxxxxxxxxxxxxxxxxxxxxxxxxxxx University of Sulaimani College of Medicine, Sulaimani Polytechnic University, Sulaymaniyah, Iraq; yyyyyyyyyyyyyyyyyyyyyyyyyyyyyyyyyyyyyyyyyyyyyyyyyyyy Department of Internal Medicine, Corewell Health East William Beaumont University Hospital, Royal Oak, MI, USA; zzzzzzzzzzzzzzzzzzzzzzzzzzzzzzzzzzzzzzzzzzzzzzzzzzzz Department of Medical Oncology, Miami Cancer Institute, Miami, FL, USA; aaaaaaaaaaaaaaaaaaaaaaaaaaaaaaaaaaaaaaaaaaaaaaaaaaaaa Deputy for Public Health, Ministry of Health and Medical Education, Tehran, Iran; bbbbbbbbbbbbbbbbbbbbbbbbbbbbbbbbbbbbbbbbbbbbbbbbbbbbb Health Equity Research Center, Tehran University of Medical Sciences, Tehran, Iran; ccccccccccccccccccccccccccccccccccccccccccccccccccccc Department of Radiology, University of Washington, Seattle, WA, USA; ddddddddddddddddddddddddddddddddddddddddddddddddddddd Cardiothoracic Imaging Section, University of Washington, Seattle, WA, USA; eeeeeeeeeeeeeeeeeeeeeeeeeeeeeeeeeeeeeeeeeeeeeeeeeeeee Department of Epidemiology and Biostatistics, Non-Communicable Diseases Research Center (NCDRC), Tehran, Iran; fffffffffffffffffffffffffffffffffffffffffffffffffffff Department of Clinical Research, Icahn School of Medicine at Mount Sinai, New York City, NY, USA; ggggggggggggggggggggggggggggggggggggggggggggggggggggg Research Department, University of Inland Norway, Elverum, Norway; hhhhhhhhhhhhhhhhhhhhhhhhhhhhhhhhhhhhhhhhhhhhhhhhhhhhh Ashok & Rita Patel Institute of Physiotherapy, Charotar University of Science and Technology, Changa, India; iiiiiiiiiiiiiiiiiiiiiiiiiiiiiiiiiiiiiiiiiiiiiiiiiiiii Department of Pharmacology, University of Gondar, Gondar, Ethiopia; jjjjjjjjjjjjjjjjjjjjjjjjjjjjjjjjjjjjjjjjjjjjjjjjjjjjj Department of Biomedical Sciences, Seoul National University, Seoul, South Korea; kkkkkkkkkkkkkkkkkkkkkkkkkkkkkkkkkkkkkkkkkkkkkkkkkkkkk Department of Health Policy and Management, Korea University, Seoul, South Korea; lllllllllllllllllllllllllllllllllllllllllllllllllllll School of Medicine, Creighton University, Omaha, NE, USA; mmmmmmmmmmmmmmmmmmmmmmmmmmmmmmmmmmmmmmmmmmmmmmmmmmmmm Cardiovascular Disease Initiative, Broad Institute of MIT and Harvard, Cambridge, MA, USA; nnnnnnnnnnnnnnnnnnnnnnnnnnnnnnnnnnnnnnnnnnnnnnnnnnnnn School of Traditional Chinese Medicine, Xiamen University Malaysia, Sepang, Malaysia; ooooooooooooooooooooooooooooooooooooooooooooooooooooo Health and Healing Research, Education, and Service, Inc., Boston, MA, USA; ppppppppppppppppppppppppppppppppppppppppppppppppppppp Millennium Prevention, Inc., Westwood, MA, USA; qqqqqqqqqqqqqqqqqqqqqqqqqqqqqqqqqqqqqqqqqqqqqqqqqqqqq Department of Nursing, Salale University, Fitche, Ethiopia; rrrrrrrrrrrrrrrrrrrrrrrrrrrrrrrrrrrrrrrrrrrrrrrrrrrrr The Pacific Community, Noumea, New Caledonia; sssssssssssssssssssssssssssssssssssssssssssssssssssss College of Medicine, Qatar University, Doha, Qatar; ttttttttttttttttttttttttttttttttttttttttttttttttttttt Department of Community Medicine, Manipal Academy of Higher Education, Manipal, India; uuuuuuuuuuuuuuuuuuuuuuuuuuuuuuuuuuuuuuuuuuuuuuuuuuuuu School of Health Sciences, Kristiania University College, Oslo, Norway; vvvvvvvvvvvvvvvvvvvvvvvvvvvvvvvvvvvvvvvvvvvvvvvvvvvvv Department of International Health and Sustainable Development, Tulane University, New Orleans, LA, USA; wwwwwwwwwwwwwwwwwwwwwwwwwwwwwwwwwwwwwwwwwwwwwwwwwwwww Department of Nursing and Health Promotion, Oslo Metropolitan University, Oslo, Norway; xxxxxxxxxxxxxxxxxxxxxxxxxxxxxxxxxxxxxxxxxxxxxxxxxxxxx Department of Health Economics and Social Security, Jagiellonian University Medical College, Krakow, Poland; yyyyyyyyyyyyyyyyyyyyyyyyyyyyyyyyyyyyyyyyyyyyyyyyyyyyy College of Health Sciences, Woldia University, Woldia, Ethiopia; zzzzzzzzzzzzzzzzzzzzzzzzzzzzzzzzzzzzzzzzzzzzzzzzzzzzz Department of Brain Sciences, University College London, London, UK; aaaaaaaaaaaaaaaaaaaaaaaaaaaaaaaaaaaaaaaaaaaaaaaaaaaaaa Department of Public Health, University of Helsinki, Helsinki, Finland; bbbbbbbbbbbbbbbbbbbbbbbbbbbbbbbbbbbbbbbbbbbbbbbbbbbbbb Department of Public Health Dentistry, Krishna Vishwa Vidyapeeth (Deemed to be University), Karad, India; cccccccccccccccccccccccccccccccccccccccccccccccccccccc Centre for Disease Burden, Norwegian Institute of Public Health, Bergen, Norway; dddddddddddddddddddddddddddddddddddddddddddddddddddddd Endocrinology Department, Bogomolets National Medical University, Kyiv, Ukraine; eeeeeeeeeeeeeeeeeeeeeeeeeeeeeeeeeeeeeeeeeeeeeeeeeeeeee Scientific Department, Medical Laboratory CSD, Kyiv, Ukraine; ffffffffffffffffffffffffffffffffffffffffffffffffffffff Department of Global Health, University of Washington, Seattle, WA, USA; gggggggggggggggggggggggggggggggggggggggggggggggggggggg Global Healthcare Consulting, New Delhi, India; hhhhhhhhhhhhhhhhhhhhhhhhhhhhhhhhhhhhhhhhhhhhhhhhhhhhhh Department of Public Health and Community Medicine, Central University of Kerala, Kasaragod, India; iiiiiiiiiiiiiiiiiiiiiiiiiiiiiiiiiiiiiiiiiiiiiiiiiiiiii Center for Tobacco Control Research and Education, University of California San Francisco, San Francisco, CA, USA; jjjjjjjjjjjjjjjjjjjjjjjjjjjjjjjjjjjjjjjjjjjjjjjjjjjjjj Department of Medicine, Harvard University, Boston, MA, USA; kkkkkkkkkkkkkkkkkkkkkkkkkkkkkkkkkkkkkkkkkkkkkkkkkkkkkk Social Determinants of Health Research Center, Shahid Beheshti University of Medical Sciences, Tehran, Iran; llllllllllllllllllllllllllllllllllllllllllllllllllllll Mycobacteriology Unit, Center for Health Promotion and Research, Bamenda, Cameroon; mmmmmmmmmmmmmmmmmmmmmmmmmmmmmmmmmmmmmmmmmmmmmmmmmmmmmm Australian Institute for Suicide Research and Prevention, Griffith University, Mount Gravatt, QLD, Australia; nnnnnnnnnnnnnnnnnnnnnnnnnnnnnnnnnnnnnnnnnnnnnnnnnnnnnn Department of Population and Behavioural Sciences, University of Health and Allied Sciences, Hohoe, Ghana; oooooooooooooooooooooooooooooooooooooooooooooooooooooo Children's Medical Center, Tehran University of Medical Sciences, Tehran, Iran; pppppppppppppppppppppppppppppppppppppppppppppppppppppp Scientific and Educational Center for Neurology and Applied Neuroscience, Kazakh National Medical University, Almaty, Kazakhstan; qqqqqqqqqqqqqqqqqqqqqqqqqqqqqqqqqqqqqqqqqqqqqqqqqqqqqq Centre for the Business and Economics of Health, The University of Queensland, Brisbane, QLD, Australia; rrrrrrrrrrrrrrrrrrrrrrrrrrrrrrrrrrrrrrrrrrrrrrrrrrrrrr Copernicus Institute of Sustainable Development, Utrecht University, Utrecht, Netherlands; ssssssssssssssssssssssssssssssssssssssssssssssssssssss Department of Science and Environmental Studies, The Education University of Hong Kong, Tai Po, New Territories, Hong Kong, China; tttttttttttttttttttttttttttttttttttttttttttttttttttttt Department of Epidemiology and Evidence-Based Medicine, I.M. Sechenov First Moscow State Medical University, Moscow, Russia; uuuuuuuuuuuuuuuuuuuuuuuuuuuuuuuuuuuuuuuuuuuuuuuuuuuuuu Department of General Practice and Family Medicine, Kharkiv National Medical University, Kharkiv, Ukraine; vvvvvvvvvvvvvvvvvvvvvvvvvvvvvvvvvvvvvvvvvvvvvvvvvvvvvv Independent Consultant, Jakarta, Indonesia; wwwwwwwwwwwwwwwwwwwwwwwwwwwwwwwwwwwwwwwwwwwwwwwwwwwwww Department of Epidemiology, IQVIA, Frankfurt am Main, Germany; xxxxxxxxxxxxxxxxxxxxxxxxxxxxxxxxxxxxxxxxxxxxxxxxxxxxxx University Hospital Marburg, Marburg, Germany; yyyyyyyyyyyyyyyyyyyyyyyyyyyyyyyyyyyyyyyyyyyyyyyyyyyyyy Department of Internal and Pulmonary Medicine, Sheri Kashmir Institute of Medical Sciences, Srinagar, India; zzzzzzzzzzzzzzzzzzzzzzzzzzzzzzzzzzzzzzzzzzzzzzzzzzzzzz School of Pharmacy, University of Ghana, Legon, Ghana; aaaaaaaaaaaaaaaaaaaaaaaaaaaaaaaaaaaaaaaaaaaaaaaaaaaaaaa Department of Public Health, Central University, Accra, Ghana; bbbbbbbbbbbbbbbbbbbbbbbbbbbbbbbbbbbbbbbbbbbbbbbbbbbbbbb Central University, Accra, Ghana; ccccccccccccccccccccccccccccccccccccccccccccccccccccccc Department of Anthropology, Panjab University, Chandigarh, India; ddddddddddddddddddddddddddddddddddddddddddddddddddddddd School of Applied Science, Republic Polytechnic, Singapore, Singapore; eeeeeeeeeeeeeeeeeeeeeeeeeeeeeeeeeeeeeeeeeeeeeeeeeeeeeee Centre for Biotechnology, Siksha ‘O’ Anusandhan Deemed to be University, Bhubaneswar, India; fffffffffffffffffffffffffffffffffffffffffffffffffffffff Department of Demography, University of Montreal, Montreal, QC, Canada; ggggggggggggggggggggggggggggggggggggggggggggggggggggggg Department of Social and Preventive Medicine, University of Montreal, Montreal, QC, Canada; hhhhhhhhhhhhhhhhhhhhhhhhhhhhhhhhhhhhhhhhhhhhhhhhhhhhhhh Department of Biochemistry, University of Hail, Hail, Saudi Arabia; iiiiiiiiiiiiiiiiiiiiiiiiiiiiiiiiiiiiiiiiiiiiiiiiiiiiiii Department of Pediatrics, Kuopio University Hospital, Kuopio, Finland; jjjjjjjjjjjjjjjjjjjjjjjjjjjjjjjjjjjjjjjjjjjjjjjjjjjjjjj Institute of Clinical Medicine, University of Eastern Finland, Kuopio, Finland; kkkkkkkkkkkkkkkkkkkkkkkkkkkkkkkkkkkkkkkkkkkkkkkkkkkkkkk Research and Publication Activity Division, Kazakh National Medical University, Almaty, Kazakhstan; lllllllllllllllllllllllllllllllllllllllllllllllllllllll Center of Medicine and Public Health, Asfendiyarov Kazakh National Medical University, Almaty, Kazakhstan; mmmmmmmmmmmmmmmmmmmmmmmmmmmmmmmmmmmmmmmmmmmmmmmmmmmmmmm Amity Centre for Water Studies and Research, Amity University Rajasthan, Jaipur, India; nnnnnnnnnnnnnnnnnnnnnnnnnnnnnnnnnnnnnnnnnnnnnnnnnnnnnnn Department of Community Medicine, Rajendra Institute of Medical Sciences, Ranchi, India; ooooooooooooooooooooooooooooooooooooooooooooooooooooooo SRM Centre for Clinical Trials and Research (CCTR), Sri Ramaswamy Memorial Institute of Science and Technology, Chennai, India; ppppppppppppppppppppppppppppppppppppppppppppppppppppppp Department of Pediatrics, Post Graduate Institute of Medical Education and Research, Chandigarh, India; qqqqqqqqqqqqqqqqqqqqqqqqqqqqqqqqqqqqqqqqqqqqqqqqqqqqqqq Department of Mathematics, Amity University Haryana, Gurugram, India; rrrrrrrrrrrrrrrrrrrrrrrrrrrrrrrrrrrrrrrrrrrrrrrrrrrrrrr Department of Community Medicine, Vardhman Mahavir Medical College and Safdarjung Hospital, Delhi, India; sssssssssssssssssssssssssssssssssssssssssssssssssssssss Department of Pharmacology and Toxicology, National Institute of Pharmaceutical Education and Research, Hajipur, Hajipur, India; ttttttttttttttttttttttttttttttttttttttttttttttttttttttt Department of Pharmacology, Regional Institute of Medical Sciences, Imphal, India; uuuuuuuuuuuuuuuuuuuuuuuuuuuuuuuuuuuuuuuuuuuuuuuuuuuuuuu Department of Anaesthesiology, Rajendra Institute of Medical Sciences, Ranchi, India; vvvvvvvvvvvvvvvvvvvvvvvvvvvvvvvvvvvvvvvvvvvvvvvvvvvvvvv Department of Economics, Manipal University, Jaipur, Jaipur, India; wwwwwwwwwwwwwwwwwwwwwwwwwwwwwwwwwwwwwwwwwwwwwwwwwwwwwww Department of Gastroenterology & Hepatology, Creighton University, Phoenix, AZ, USA; xxxxxxxxxxxxxxxxxxxxxxxxxxxxxxxxxxxxxxxxxxxxxxxxxxxxxxx IITM Pravartak Technologies Foundation, Chennai, India; yyyyyyyyyyyyyyyyyyyyyyyyyyyyyyyyyyyyyyyyyyyyyyyyyyyyyyy Section of Cardiology, University of Manitoba, Winnipeg, MB, Canada; zzzzzzzzzzzzzzzzzzzzzzzzzzzzzzzzzzzzzzzzzzzzzzzzzzzzzzz Department of Translational Health Sciences, University of Bristol, Bristol, UK; aaaaaaaaaaaaaaaaaaaaaaaaaaaaaaaaaaaaaaaaaaaaaaaaaaaaaaaa Department of Clinical Subjects, Al Farabi Kazakh National University, Almaty, Kazakhstan; bbbbbbbbbbbbbbbbbbbbbbbbbbbbbbbbbbbbbbbbbbbbbbbbbbbbbbbb Faculty of Health and Life Sciences, Coventry University, Coventry, UK; cccccccccccccccccccccccccccccccccccccccccccccccccccccccc Department of Medicine, McMaster University, Hamilton, ON, Canada; dddddddddddddddddddddddddddddddddddddddddddddddddddddddd Faculty of Medicine and Health Science, Universitas Kristen Satya Wacana (Satya Wacana Christian University), Salatiga, Indonesia; eeeeeeeeeeeeeeeeeeeeeeeeeeeeeeeeeeeeeeeeeeeeeeeeeeeeeeee School of Nursing, Taipei Medical University, Taipei, Taiwan; ffffffffffffffffffffffffffffffffffffffffffffffffffffffff Division of Cardiology, University of Illinois, Champaign, IL, USA; gggggggggggggggggggggggggggggggggggggggggggggggggggggggg National Research and Innovation Agency (BRIN), Jakarta, Indonesia; hhhhhhhhhhhhhhhhhhhhhhhhhhhhhhhhhhhhhhhhhhhhhhhhhhhhhhhh Institute for Health Sciences, STIKES Bethesda Yakkum Yogyakarta Indonesia, Yogyakarta, Indonesia; iiiiiiiiiiiiiiiiiiiiiiiiiiiiiiiiiiiiiiiiiiiiiiiiiiiiiiii Department of Public Health and Epidemiology, Khalifa University of Science and Technology, Abu Dhabi, United Arab Emirates; jjjjjjjjjjjjjjjjjjjjjjjjjjjjjjjjjjjjjjjjjjjjjjjjjjjjjjjj Faculty of Public Health, University of Indonesia, Depok, Indonesia; kkkkkkkkkkkkkkkkkkkkkkkkkkkkkkkkkkkkkkkkkkkkkkkkkkkkkkkk Department of Pediatric Oncology, Medicana Health International, Istanbul, Türkiye; llllllllllllllllllllllllllllllllllllllllllllllllllllllll Department of Pediatric Oncology, Hacettepe University, Ankara, Türkiye; mmmmmmmmmmmmmmmmmmmmmmmmmmmmmmmmmmmmmmmmmmmmmmmmmmmmmmmm Department of Nursing, University of Massachusetts Boston, Boston, MA, USA; nnnnnnnnnnnnnnnnnnnnnnnnnnnnnnnnnnnnnnnnnnnnnnnnnnnnnnnn Department of Environment and Public Health, University of Environment and Sustainable Development, Somanya, Ghana; oooooooooooooooooooooooooooooooooooooooooooooooooooooooo Clinical Research Center, Turku University Hospital, Turku, Finland; pppppppppppppppppppppppppppppppppppppppppppppppppppppppp Heart Center, University of Turku, Turku, Finland; qqqqqqqqqqqqqqqqqqqqqqqqqqqqqqqqqqqqqqqqqqqqqqqqqqqqqqqq Kasturba Medical College, Manipal, Manipal Academy of Higher Education, Manipal, India; rrrrrrrrrrrrrrrrrrrrrrrrrrrrrrrrrrrrrrrrrrrrrrrrrrrrrrrr Department of Medicine and Surgery, University of Milano - Bicocca, Milan, Italy; ssssssssssssssssssssssssssssssssssssssssssssssssssssssss Pediatric Emergency Department, Fondazione IRCCS Ospedale Maggiore Policlinico (IRCCS “”Ca' Granda Maggiore Policlinico“” Hospital Foundation), Milan, Italy; tttttttttttttttttttttttttttttttttttttttttttttttttttttttt Department of Clinical Sciences and Community Health, Università degli Studi di Milano (University of Milan), Milan, Italy; uuuuuuuuuuuuuuuuuuuuuuuuuuuuuuuuuuuuuuuuuuuuuuuuuuuuuuuu Department of Medicine, UniCamillus University, Rome, Italy; vvvvvvvvvvvvvvvvvvvvvvvvvvvvvvvvvvvvvvvvvvvvvvvvvvvvvvvv Department of Nursing Science, Bayero University Kano, Kano, Nigeria; wwwwwwwwwwwwwwwwwwwwwwwwwwwwwwwwwwwwwwwwwwwwwwwwwwwwwwww Genetic Resource Program, International Maize and Wheat Improvement Center (CIMMYT), Nairobi, Kenya; xxxxxxxxxxxxxxxxxxxxxxxxxxxxxxxxxxxxxxxxxxxxxxxxxxxxxxxx Department of Basic Sciences, Jomo Kenyatta University of Agriculture and Technology, Nairobi, Kenya; yyyyyyyyyyyyyyyyyyyyyyyyyyyyyyyyyyyyyyyyyyyyyyyyyyyyyyyy Division of Evidence Synthesis, Foundation for People-centric Health Systems, New Delhi, India; zzzzzzzzzzzzzzzzzzzzzzzzzzzzzzzzzzzzzzzzzzzzzzzzzzzzzzzz Division of Lifestyle Medicine, Centre for Health: The Specialty Practice, New Delhi, India; aaaaaaaaaaaaaaaaaaaaaaaaaaaaaaaaaaaaaaaaaaaaaaaaaaaaaaaaa School of Digital Science, Universiti Brunei Darussalam (University of Brunei Darussalam), Bandar Seri Begawan, Brunei; bbbbbbbbbbbbbbbbbbbbbbbbbbbbbbbbbbbbbbbbbbbbbbbbbbbbbbbbb Institute of Applied Data Analytics, Universiti Brunei Darussalam (University of Brunei Darussalam), Bandar Seri Begawan, Brunei; ccccccccccccccccccccccccccccccccccccccccccccccccccccccccc Department of Research, Kazakh National Medical University, Almaty, Kazakhstan; ddddddddddddddddddddddddddddddddddddddddddddddddddddddddd Department of Chemistry, Dayalbagh Educational Institute, Agra, India; eeeeeeeeeeeeeeeeeeeeeeeeeeeeeeeeeeeeeeeeeeeeeeeeeeeeeeeee NEVES Society for Patient Safety, Budapest, Hungary; fffffffffffffffffffffffffffffffffffffffffffffffffffffffff Unidad de Genética y Salud Pública, Instituto de Ciencias Médicas, Las Tablas, Panama; ggggggggggggggggggggggggggggggggggggggggggggggggggggggggg Ministry of Health, Hospital Joaquín Pablo Franco Sayas, Las Tablas, Panama; hhhhhhhhhhhhhhhhhhhhhhhhhhhhhhhhhhhhhhhhhhhhhhhhhhhhhhhhh Department of Psychiatry and Psychotherapy, University of Regensburg, Regensburg, Germany; iiiiiiiiiiiiiiiiiiiiiiiiiiiiiiiiiiiiiiiiiiiiiiiiiiiiiiiii Department of Behavioural Sciences and Learning, Linköping University, Linköping, Sweden; jjjjjjjjjjjjjjjjjjjjjjjjjjjjjjjjjjjjjjjjjjjjjjjjjjjjjjjjj Department of Otorhinolaryngology, Father Muller Medical College, Mangalore, India; kkkkkkkkkkkkkkkkkkkkkkkkkkkkkkkkkkkkkkkkkkkkkkkkkkkkkkkkk Centre for Family Welfare, University of Indonesia, Depok, Indonesia; lllllllllllllllllllllllllllllllllllllllllllllllllllllllll Department of Global Health and Health Security, Taipei Medical University, Taipei, Taiwan; mmmmmmmmmmmmmmmmmmmmmmmmmmmmmmmmmmmmmmmmmmmmmmmmmmmmmmmmm University of Management and Technology, Lahore, Pakistan; nnnnnnnnnnnnnnnnnnnnnnnnnnnnnnnnnnnnnnnnnnnnnnnnnnnnnnnnn Department of Surgery, National University of Singapore, Singapore, Singapore; ooooooooooooooooooooooooooooooooooooooooooooooooooooooooo International Society of Doctors for the Environment, Arezzo, Italy; ppppppppppppppppppppppppppppppppppppppppppppppppppppppppp School of Physical Therapy, The University of Western Ontario, London, ON, Canada; qqqqqqqqqqqqqqqqqqqqqqqqqqqqqqqqqqqqqqqqqqqqqqqqqqqqqqqqq Centre for Clinical Trials, Research, and Implementation Science (CCTRIS), Lagos, Nigeria; rrrrrrrrrrrrrrrrrrrrrrrrrrrrrrrrrrrrrrrrrrrrrrrrrrrrrrrrr UQ Centre for Clinical Research, The University of Queensland, Brisbane, QLD, Australia; sssssssssssssssssssssssssssssssssssssssssssssssssssssssss College of Nursing, Sultan Qaboos University, Muscat, Oman; ttttttttttttttttttttttttttttttttttttttttttttttttttttttttt Nam Can Tho University, Faculty of Medicine, Can Tho, Viet Nam; uuuuuuuuuuuuuuuuuuuuuuuuuuuuuuuuuuuuuuuuuuuuuuuuuuuuuuuuu Vascular Surgery Department, Cho Ray Hospital, Ho Chi Minh City, Viet Nam; vvvvvvvvvvvvvvvvvvvvvvvvvvvvvvvvvvvvvvvvvvvvvvvvvvvvvvvvv Department of Thoracic and Vascular Surgery, Nam Can Tho University, Vietnam, Can Tho City, Viet Nam; wwwwwwwwwwwwwwwwwwwwwwwwwwwwwwwwwwwwwwwwwwwwwwwwwwwwwwwww University of Medicine and Pharmacy at Ho Chi Minh City, Ho Chi Minh City, Viet Nam; xxxxxxxxxxxxxxxxxxxxxxxxxxxxxxxxxxxxxxxxxxxxxxxxxxxxxxxxx Department of Clinical and Experimental Medicine, University of Catania, Catania, Italy; yyyyyyyyyyyyyyyyyyyyyyyyyyyyyyyyyyyyyyyyyyyyyyyyyyyyyyyyy STEM, University of South Australia, Adelaide, SA, Australia; zzzzzzzzzzzzzzzzzzzzzzzzzzzzzzzzzzzzzzzzzzzzzzzzzzzzzzzzz Southampton Clinical Trials Unit, University of Southampton, Southampton, UK; aaaaaaaaaaaaaaaaaaaaaaaaaaaaaaaaaaaaaaaaaaaaaaaaaaaaaaaaaa Department of Precision Medicine, Sungkyunkwan University, Suwon-si, South Korea; bbbbbbbbbbbbbbbbbbbbbbbbbbbbbbbbbbbbbbbbbbbbbbbbbbbbbbbbbb Department of Preventive Medicine, Korea University, Seoul, South Korea; cccccccccccccccccccccccccccccccccccccccccccccccccccccccccc Asbestos and Dust Diseases Research Institute, University of Sydney, Sydney, NSW, Australia; dddddddddddddddddddddddddddddddddddddddddddddddddddddddddd Department of Cardiothoracic and Vascular Surgery, Westpfalz Klinikum, Kaiserslautern, Germany; eeeeeeeeeeeeeeeeeeeeeeeeeeeeeeeeeeeeeeeeeeeeeeeeeeeeeeeeee Department of Cardiothoracic Surgery, University of Patras, Patras, Greece; ffffffffffffffffffffffffffffffffffffffffffffffffffffffffff Centre for Healthy Brain Ageing, University of New South Wales, Sydney, NSW, Australia; gggggggggggggggggggggggggggggggggggggggggggggggggggggggggg SC Neurologia, Salute Pubblica e Disabilità (Neurology, Public Health, Disability Unit), Fondazione IRCCS Istituto Neurologico Carlo Besta (IRCCS Foundation Carlo Besta Neurological Institute), Milan, Italy; hhhhhhhhhhhhhhhhhhhhhhhhhhhhhhhhhhhhhhhhhhhhhhhhhhhhhhhhhh Faculty of Science, Universiti Brunei Darussalam (University of Brunei Darussalam), Bandar Seri Begawan, Brunei; iiiiiiiiiiiiiiiiiiiiiiiiiiiiiiiiiiiiiiiiiiiiiiiiiiiiiiiiii Nutrition & Health Innovation Research Institute, Edith Cowan University, Perth, WA, Australia; jjjjjjjjjjjjjjjjjjjjjjjjjjjjjjjjjjjjjjjjjjjjjjjjjjjjjjjjjj Department of Rheumatology and Immunology, The People’s Hospital of Baoan Shenzhen, Shenzhen, China; kkkkkkkkkkkkkkkkkkkkkkkkkkkkkkkkkkkkkkkkkkkkkkkkkkkkkkkkkk Global Health Research Center, Guangdong Academy of Medical Sciences and General Hospital, Guangzhou, China; llllllllllllllllllllllllllllllllllllllllllllllllllllllllll Department of Health Promotion and Health Education, National Taiwan Normal University, Taipei, Taiwan; mmmmmmmmmmmmmmmmmmmmmmmmmmmmmmmmmmmmmmmmmmmmmmmmmmmmmmmmmm Department of Pulmonary Critical Care Medicine, Mayo Clinic, Phoenix, AZ, USA; nnnnnnnnnnnnnnnnnnnnnnnnnnnnnnnnnnnnnnnnnnnnnnnnnnnnnnnnnn Population Studies Center, University of Pennsylvania, Philadelphia, PA, USA; oooooooooooooooooooooooooooooooooooooooooooooooooooooooooo Department of Psychiatry, Washington University in St. Louis, St. Louis, MO, USA; pppppppppppppppppppppppppppppppppppppppppppppppppppppppppp School of Medicine, Shanghai Jiao Tong University, Shanghai, China; qqqqqqqqqqqqqqqqqqqqqqqqqqqqqqqqqqqqqqqqqqqqqqqqqqqqqqqqqq Discipline of Physiology, National University of Ireland - Galway, Galway, Ireland; rrrrrrrrrrrrrrrrrrrrrrrrrrrrrrrrrrrrrrrrrrrrrrrrrrrrrrrrrr Cardiovascular Medicine Department, The Second Affiliated Hospital of Nanchang University, NanChang, China; ssssssssssssssssssssssssssssssssssssssssssssssssssssssssss International Centre for Future Health Systems, University of New South Wales, Sydney, NSW, Australia; tttttttttttttttttttttttttttttttttttttttttttttttttttttttttt WHO Collaborating Centre for Public Health Education and Training, Imperial College London, London, UK; uuuuuuuuuuuuuuuuuuuuuuuuuuuuuuuuuuuuuuuuuuuuuuuuuuuuuuuuuu Department of Food Science and Human Nutrition, Iowa State University, Ames, IA, USA; vvvvvvvvvvvvvvvvvvvvvvvvvvvvvvvvvvvvvvvvvvvvvvvvvvvvvvvvvv The Center for Drug Safety and Policy Research, Xi'an Jiaotong University, Xi'an, China; wwwwwwwwwwwwwwwwwwwwwwwwwwwwwwwwwwwwwwwwwwwwwwwwwwwwwwwwww Department of Medical Sciences, Uppsala University, Uppsala, Sweden; xxxxxxxxxxxxxxxxxxxxxxxxxxxxxxxxxxxxxxxxxxxxxxxxxxxxxxxxxx Department of Medicine, Norrtälje Hospital (Tiohundra), Norrtälje, Sweden; yyyyyyyyyyyyyyyyyyyyyyyyyyyyyyyyyyyyyyyyyyyyyyyyyyyyyyyyyy UCD Centre for Disability Studies, University College Dublin, Dublin, Ireland; zzzzzzzzzzzzzzzzzzzzzzzzzzzzzzzzzzzzzzzzzzzzzzzzzzzzzzzzzz Management Science and Engineering, Stanford University, Stanford, CA, USA; aaaaaaaaaaaaaaaaaaaaaaaaaaaaaaaaaaaaaaaaaaaaaaaaaaaaaaaaaaa School of Public Health, Zefat Academic College, Haifa, Israel; bbbbbbbbbbbbbbbbbbbbbbbbbbbbbbbbbbbbbbbbbbbbbbbbbbbbbbbbbbb Centre for Intelligent Healthcare, Coventry University, Coventry, UK; ccccccccccccccccccccccccccccccccccccccccccccccccccccccccccc Department of Epidemiology and Biostatistics, Peking University, Beijing, China; ddddddddddddddddddddddddddddddddddddddddddddddddddddddddddd School of Nursing and Health Sciences, Hong Kong Metropolitan University, Hong Kong, China; eeeeeeeeeeeeeeeeeeeeeeeeeeeeeeeeeeeeeeeeeeeeeeeeeeeeeeeeeee Department of Radiology and Biomedical Imaging, Yale University, New Haven, CT, USA; fffffffffffffffffffffffffffffffffffffffffffffffffffffffffff Department of Radiology, Massachusetts General Hospital, Boston, MA, USA; ggggggggggggggggggggggggggggggggggggggggggggggggggggggggggg Lerner Research Institute, Cleveland Clinic, Cleveland, OH, USA; hhhhhhhhhhhhhhhhhhhhhhhhhhhhhhhhhhhhhhhhhhhhhhhhhhhhhhhhhhh Department of Quantitative Health Science, Case Western Reserve University, Cleveland, OH, USA; iiiiiiiiiiiiiiiiiiiiiiiiiiiiiiiiiiiiiiiiiiiiiiiiiiiiiiiiiii College of Mathematics and Computer, Xinyu University, Xinyu, China; jjjjjjjjjjjjjjjjjjjjjjjjjjjjjjjjjjjjjjjjjjjjjjjjjjjjjjjjjjj Department of Urology, General Hospital of Central Theater Command, Wuhan, China; kkkkkkkkkkkkkkkkkkkkkkkkkkkkkkkkkkkkkkkkkkkkkkkkkkkkkkkkkkk School of Medicine, Wuhan University of Science and Technology, Wuhan, China; lllllllllllllllllllllllllllllllllllllllllllllllllllllllllll Department of Molecular Epidemiology, German Institute of Human Nutrition Potsdam-Rehbrücke, Potsdam, Germany; mmmmmmmmmmmmmmmmmmmmmmmmmmmmmmmmmmmmmmmmmmmmmmmmmmmmmmmmmmm German Center for Diabetes Research (DZD), München-Neuherberg, Germany; nnnnnnnnnnnnnnnnnnnnnnnnnnnnnnnnnnnnnnnnnnnnnnnnnnnnnnnnnnn Department of Infectious Diseases, Monash University, Melbourne, VIC, Australia; ooooooooooooooooooooooooooooooooooooooooooooooooooooooooooo Department of Infectious Diseases, Alfred Health, Melbourne, VIC, Australia; ppppppppppppppppppppppppppppppppppppppppppppppppppppppppppp Department of Cardiology, University of Cologne, Cologne, Germany; qqqqqqqqqqqqqqqqqqqqqqqqqqqqqqqqqqqqqqqqqqqqqqqqqqqqqqqqqqq School of Medicine, Universidad Espíritu Santo, Samborondón, Ecuador; rrrrrrrrrrrrrrrrrrrrrrrrrrrrrrrrrrrrrrrrrrrrrrrrrrrrrrrrrrr Vicerrectoría de Investigación y Postgrado, Universidad de Los Lagos, Osorno, Chile; sssssssssssssssssssssssssssssssssssssssssssssssssssssssssss Institute of Nutritional Sciences, Friedrich Schiller University Jena, Jena, Germany; ttttttttttttttttttttttttttttttttttttttttttttttttttttttttttt Competence Cluster for Nutrition and Cardiovascular Health (nutriCARD), Jena, Germany; uuuuuuuuuuuuuuuuuuuuuuuuuuuuuuuuuuuuuuuuuuuuuuuuuuuuuuuuuuu School of Medicine, National Autonomous University of Mexico, Mexico City, Mexico; vvvvvvvvvvvvvvvvvvvvvvvvvvvvvvvvvvvvvvvvvvvvvvvvvvvvvvvvvvv Department of Spine Surgery, Qingdao Municipal Hospital Group, Qingdao, China; wwwwwwwwwwwwwwwwwwwwwwwwwwwwwwwwwwwwwwwwwwwwwwwwwwwwwwwwwww Geospatial Health and Development Team-Child Health Analytics, Telethon Kids Institute, Perth, WA, Australia; xxxxxxxxxxxxxxxxxxxxxxxxxxxxxxxxxxxxxxxxxxxxxxxxxxxxxxxxxxx Scientific Research and Surveillance Systems, Macha Research Trust, Choma, Zambia; yyyyyyyyyyyyyyyyyyyyyyyyyyyyyyyyyyyyyyyyyyyyyyyyyyyyyyyyyyy Injury Prevention and Safety Promotion Research Center, Shahid Beheshti University of Medical Sciences, Tehran, Iran; zzzzzzzzzzzzzzzzzzzzzzzzzzzzzzzzzzzzzzzzzzzzzzzzzzzzzzzzzzz School of Medicine, Federal University of Juiz de Fora, Juiz de Fora, Brazil; aaaaaaaaaaaaaaaaaaaaaaaaaaaaaaaaaaaaaaaaaaaaaaaaaaaaaaaaaaaa Department of Emergency General and Trauma Surgery, NHS University Hospitals of Liverpool Group, Aintree Hospital, Liverpool, UK; bbbbbbbbbbbbbbbbbbbbbbbbbbbbbbbbbbbbbbbbbbbbbbbbbbbbbbbbbbbb Peking University First Hospital, Peking University, Beijing, China; cccccccccccccccccccccccccccccccccccccccccccccccccccccccccccc Center for Evidence-Based and Translational Medicine, Wuhan University, Wuhan, China; dddddddddddddddddddddddddddddddddddddddddddddddddddddddddddd The Third Department of Hepatic Surgery, Eastern Hepatobiliary Surgery Hospital, Shanghai, China; eeeeeeeeeeeeeeeeeeeeeeeeeeeeeeeeeeeeeeeeeeeeeeeeeeeeeeeeeeee Moores Cancer Center, University of California San Diego, San Diego, CA, USA; ffffffffffffffffffffffffffffffffffffffffffffffffffffffffffff Department of Clinical Data Science and Evidence, Novo Nordisk, Plainsboro, NJ, USA; gggggggggggggggggggggggggggggggggggggggggggggggggggggggggggg College of Engineering, Effat University, Jeddah, Saudi Arabia; hhhhhhhhhhhhhhhhhhhhhhhhhhhhhhhhhhhhhhhhhhhhhhhhhhhhhhhhhhhh Management of Information Systems Department, The American College of Greece, Aghia Paraskevi, Greece; iiiiiiiiiiiiiiiiiiiiiiiiiiiiiiiiiiiiiiiiiiiiiiiiiiiiiiiiiiii Department of Medicine, University of Alberta, Edmonton, AB, Canada; jjjjjjjjjjjjjjjjjjjjjjjjjjjjjjjjjjjjjjjjjjjjjjjjjjjjjjjjjjjj Tulane University, New Orleans, LA, USA; kkkkkkkkkkkkkkkkkkkkkkkkkkkkkkkkkkkkkkkkkkkkkkkkkkkkkkkkkkkk Center for Global Health, Perelman School of Medicine, University of Pennsylvania, Philadelphia, PA, USA; llllllllllllllllllllllllllllllllllllllllllllllllllllllllllll Centre for Public Health and Wellbeing, University of the West of England, Bristol, UK; mmmmmmmmmmmmmmmmmmmmmmmmmmmmmmmmmmmmmmmmmmmmmmmmmmmmmmmmmmmm School of Medicine, University of Health and Allied Sciences, Ho, Ghana; nnnnnnnnnnnnnnnnnnnnnnnnnnnnnnnnnnnnnnnnnnnnnnnnnnnnnnnnnnnn Faculty of Veterinary Medicine, Suez Canal University, Ismailia, Egypt; oooooooooooooooooooooooooooooooooooooooooooooooooooooooooooo Department of Microbiology and Parasitology, King Salman International University, South of Sinai, Egypt; pppppppppppppppppppppppppppppppppppppppppppppppppppppppppppp 2nd Department of Propaedeutic Surgery, University of Athens, Athens, Greece; qqqqqqqqqqqqqqqqqqqqqqqqqqqqqqqqqqqqqqqqqqqqqqqqqqqqqqqqqqqq Department of Periodontology, Pomeranian Medical University, Szczecin, Poland; rrrrrrrrrrrrrrrrrrrrrrrrrrrrrrrrrrrrrrrrrrrrrrrrrrrrrrrrrrrr Associate Laboratory i4HB, University Institute of Health Sciences - CESPU, Gandra, Portugal; ssssssssssssssssssssssssssssssssssssssssssssssssssssssssssss UCIBIO Research Unit on Applied Molecular Biosciences, University Institute of Health Sciences, Gandra, Portugal; tttttttttttttttttttttttttttttttttttttttttttttttttttttttttttt School of Infection & Immunity, University of Glasgow, Glasgow, UK; uuuuuuuuuuuuuuuuuuuuuuuuuuuuuuuuuuuuuuuuuuuuuuuuuuuuuuuuuuuu Department of Pharmacy, University of Naples Federico II, Naples, Italy; vvvvvvvvvvvvvvvvvvvvvvvvvvvvvvvvvvvvvvvvvvvvvvvvvvvvvvvvvvvv Department of Emergency Medicine, Sri Lakshmi Narayana Institute of Medical Science, Puducherry, Pondicherry, India; wwwwwwwwwwwwwwwwwwwwwwwwwwwwwwwwwwwwwwwwwwwwwwwwwwwwwwwwwwww Department of One Health in Tropical Infectiousness Diseases, Jigjiga University, Jigjiga, Ethiopia; xxxxxxxxxxxxxxxxxxxxxxxxxxxxxxxxxxxxxxxxxxxxxxxxxxxxxxxxxxxx College of Health Science, Amoud University, Borama, Somalia; yyyyyyyyyyyyyyyyyyyyyyyyyyyyyyyyyyyyyyyyyyyyyyyyyyyyyyyyyyyy Research Center, Cihan University -Sulaimaniya, Sulaymaniyah, Iraq; zzzzzzzzzzzzzzzzzzzzzzzzzzzzzzzzzzzzzzzzzzzzzzzzzzzzzzzzzzzz Institute of Health Science, Nam Can Tho University, Can Tho, Viet Nam; aaaaaaaaaaaaaaaaaaaaaaaaaaaaaaaaaaaaaaaaaaaaaaaaaaaaaaaaaaaaa Smart Healthcare Management, National Taipei University, New Taipei City, Taiwan; bbbbbbbbbbbbbbbbbbbbbbbbbbbbbbbbbbbbbbbbbbbbbbbbbbbbbbbbbbbbb Department of Pharmacology, All India Institute of Medical Sciences, Bhubaneswar, India; ccccccccccccccccccccccccccccccccccccccccccccccccccccccccccccc Department of Public Health, Trnava University, Trnava, Slovakia; ddddddddddddddddddddddddddddddddddddddddddddddddddddddddddddd The Orthopaedic Department, October 6 University, 6th of October City, Egypt; eeeeeeeeeeeeeeeeeeeeeeeeeeeeeeeeeeeeeeeeeeeeeeeeeeeeeeeeeeeee Department of Medicine, Medical College of Georgia at Augusta University, Augusta, GA, USA; fffffffffffffffffffffffffffffffffffffffffffffffffffffffffffff Department of Cardiology, October 6 University, 6th of October City, Egypt; ggggggggggggggggggggggggggggggggggggggggggggggggggggggggggggg Non-communicable Disease Research Center, Shiraz University of Medical Sciences, Shiraz, Iran; hhhhhhhhhhhhhhhhhhhhhhhhhhhhhhhhhhhhhhhhhhhhhhhhhhhhhhhhhhhhh Department of Neurology, King George's Medical University, Lucknow, India; iiiiiiiiiiiiiiiiiiiiiiiiiiiiiiiiiiiiiiiiiiiiiiiiiiiiiiiiiiiii Rabigh Faculty of Medicine, King Abdulaziz University, Jeddah, Saudi Arabia; jjjjjjjjjjjjjjjjjjjjjjjjjjjjjjjjjjjjjjjjjjjjjjjjjjjjjjjjjjjjj University Institute of Public Health, The University of Lahore, Lahore, Pakistan; kkkkkkkkkkkkkkkkkkkkkkkkkkkkkkkkkkkkkkkkkkkkkkkkkkkkkkkkkkkkk Department of Maternal-Child Nursing and Public Health, Universidade Federal de Minas Gerais (Federal University of Minas Gerais), Belo Horizonte, Brazil; lllllllllllllllllllllllllllllllllllllllllllllllllllllllllllll University of Kansas Medical Center, A.T. Still University, Kansas City, KS, USA; mmmmmmmmmmmmmmmmmmmmmmmmmmmmmmmmmmmmmmmmmmmmmmmmmmmmmmmmmmmmm Internal Medicine Department, Eisenhower Health, Palm Desert, CA, USA; nnnnnnnnnnnnnnnnnnnnnnnnnnnnnnnnnnnnnnnnnnnnnnnnnnnnnnnnnnnnn Department of Cardiovascular Science, University of Manchester, Manchester, UK; ooooooooooooooooooooooooooooooooooooooooooooooooooooooooooooo Population Health Research Institute (PHRI), McMaster University, Hamilton, ON, Canada; ppppppppppppppppppppppppppppppppppppppppppppppppppppppppppppp International Center for Chemical and Biological Sciences, University of Karachi, Karachi, Pakistan; qqqqqqqqqqqqqqqqqqqqqqqqqqqqqqqqqqqqqqqqqqqqqqqqqqqqqqqqqqqqq Department of Epidemiology and Biostatistics, Isfahan University of Medical Sciences, Isfahan, Iran; rrrrrrrrrrrrrrrrrrrrrrrrrrrrrrrrrrrrrrrrrrrrrrrrrrrrrrrrrrrrr Biomedical Engineering Research Center (CREB), Universitat Politècnica de Catalunya (Barcelona Tech - UPC), Barcelona, Spain; sssssssssssssssssssssssssssssssssssssssssssssssssssssssssssss Department of Epidemiology and Biostatistics, Tehran University of Medical Sciences, Tehran, Iran; ttttttttttttttttttttttttttttttttttttttttttttttttttttttttttttt School of Medicine and Surgery, University of Milan Bicocca, Monza, Italy; uuuuuuuuuuuuuuuuuuuuuuuuuuuuuuuuuuuuuuuuuuuuuuuuuuuuuuuuuuuuu Department of Urology, Anhui Medical University, Hefei, China; vvvvvvvvvvvvvvvvvvvvvvvvvvvvvvvvvvvvvvvvvvvvvvvvvvvvvvvvvvvvv Department of Biotechnology, Tribhuvan University, Kathmandu, Nepal; wwwwwwwwwwwwwwwwwwwwwwwwwwwwwwwwwwwwwwwwwwwwwwwwwwwwwwwwwwwww Department of Biomedical Engineering, University of Isfahan, Isfahan, Iran; xxxxxxxxxxxxxxxxxxxxxxxxxxxxxxxxxxxxxxxxxxxxxxxxxxxxxxxxxxxxx Automatic Control Department, Universitat Politècnica de Catalunya (Barcelona Tech - UPC), Barcelona, Spain; yyyyyyyyyyyyyyyyyyyyyyyyyyyyyyyyyyyyyyyyyyyyyyyyyyyyyyyyyyyyy Far Eastern University, Manila, Philippines; zzzzzzzzzzzzzzzzzzzzzzzzzzzzzzzzzzzzzzzzzzzzzzzzzzzzzzzzzzzzz Faculty of Human Kinetics, Universidade de Lisboa (University of Lisbon), Lisbon, Portugal; aaaaaaaaaaaaaaaaaaaaaaaaaaaaaaaaaaaaaaaaaaaaaaaaaaaaaaaaaaaaaa Department of Infectious Diseases, Instituto Nacional de Nutrición Salvador Zubirán (Salvador Zubiran National Institute of Medical Sciences and Nutrition), Mexico City, Mexico; bbbbbbbbbbbbbbbbbbbbbbbbbbbbbbbbbbbbbbbbbbbbbbbbbbbbbbbbbbbbbb Department of Non-communicable Diseases and Mental Health, Pan American Health Organization, Washington, DC, USA; cccccccccccccccccccccccccccccccccccccccccccccccccccccccccccccc Department of Food, Environmental and Nutritional Sciences, Università degli Studi di Milano (University of Milan), Milan, Italy; dddddddddddddddddddddddddddddddddddddddddddddddddddddddddddddd Faculty of Public Health, Universitas Airlangga (Airlangga University), Surabaya, Indonesia; eeeeeeeeeeeeeeeeeeeeeeeeeeeeeeeeeeeeeeeeeeeeeeeeeeeeeeeeeeeeee Indonesian Public Health Association, Surabaya, Indonesia; ffffffffffffffffffffffffffffffffffffffffffffffffffffffffffffff Campus Fortaleza, Federal Institute of Education, Science and Technology of Ceará, Fortaleza, Brazil; gggggggggggggggggggggggggggggggggggggggggggggggggggggggggggggg Department of Nutrition and Dietetics, University of Concepción, Concepción, Chile; hhhhhhhhhhhhhhhhhhhhhhhhhhhhhhhhhhhhhhhhhhhhhhhhhhhhhhhhhhhhhh Centre for Healthy Living, University of Concepción, Concepción, Chile; iiiiiiiiiiiiiiiiiiiiiiiiiiiiiiiiiiiiiiiiiiiiiiiiiiiiiiiiiiiiii Clinical Institute of Medical and Chemical Laboratory Diagnostics, Medical University of Graz, Graz, Austria; jjjjjjjjjjjjjjjjjjjjjjjjjjjjjjjjjjjjjjjjjjjjjjjjjjjjjjjjjjjjjj Medical Clinic V, Heidelberg University, Mannheim, Germany; kkkkkkkkkkkkkkkkkkkkkkkkkkkkkkkkkkkkkkkkkkkkkkkkkkkkkkkkkkkkkk Faculty of Humanities and Health Sciences, Curtin University, Sarawak, Malaysia; llllllllllllllllllllllllllllllllllllllllllllllllllllllllllllll Jeffrey Cheah School of Medicine and Health Sciences, Monash University, Subang Jaya, Malaysia; mmmmmmmmmmmmmmmmmmmmmmmmmmmmmmmmmmmmmmmmmmmmmmmmmmmmmmmmmmmmmm Medical Scientist Training Program, Northwestern University, Chicago, IL, USA; nnnnnnnnnnnnnnnnnnnnnnnnnnnnnnnnnnnnnnnnnnnnnnnnnnnnnnnnnnnnnn Department of Nursing, Muhammadiyah University of Surakarta, Ponorogo, Indonesia; oooooooooooooooooooooooooooooooooooooooooooooooooooooooooooooo Department of Clinical and Experimental Medicine, University of Pisa, Pisa, Italy; pppppppppppppppppppppppppppppppppppppppppppppppppppppppppppppp Department of Anatomy and Developmental Biology, Monash University, Clayton, VIC, Australia; qqqqqqqqqqqqqqqqqqqqqqqqqqqqqqqqqqqqqqqqqqqqqqqqqqqqqqqqqqqqqq Department of Anatomy, Genetics and Biomedical Informatics, University of Colombo, Colombo, Sri Lanka; rrrrrrrrrrrrrrrrrrrrrrrrrrrrrrrrrrrrrrrrrrrrrrrrrrrrrrrrrrrrrr University of Sydney, Sydney, NSW, Australia; ssssssssssssssssssssssssssssssssssssssssssssssssssssssssssssss Division of Immunology, Immunity to Infection and Respiratory Medicine, University of Manchester, Manchester, UK; tttttttttttttttttttttttttttttttttttttttttttttttttttttttttttttt North West Lung Centre, Manchester University NHS Foundation Trust, Manchester, UK; uuuuuuuuuuuuuuuuuuuuuuuuuuuuuuuuuuuuuuuuuuuuuuuuuuuuuuuuuuuuuu Department of Community Medicine, Geetanjali Medical College and Hospital, Udaipur, India; vvvvvvvvvvvvvvvvvvvvvvvvvvvvvvvvvvvvvvvvvvvvvvvvvvvvvvvvvvvvvv Department of Community Medicine, Apollo Institute of Medical Sciences and Research, Hyderabad, India; wwwwwwwwwwwwwwwwwwwwwwwwwwwwwwwwwwwwwwwwwwwwwwwwwwwwwwwwwwwwww Department of Social Medicine, Federal University of Rio Grande do Sul, Porto Alegre, Brazil; xxxxxxxxxxxxxxxxxxxxxxxxxxxxxxxxxxxxxxxxxxxxxxxxxxxxxxxxxxxxxx Department of Epidemiology, Mahidol-Oxford Tropical Medicine Research Unit, Bangkok, Thailand; yyyyyyyyyyyyyyyyyyyyyyyyyyyyyyyyyyyyyyyyyyyyyyyyyyyyyyyyyyyyyy Nuffield Department of Medicine, University of Oxford, Oxford, UK; zzzzzzzzzzzzzzzzzzzzzzzzzzzzzzzzzzzzzzzzzzzzzzzzzzzzzzzzzzzzzz Research Division, The George Institute for Global Health, New Delhi, India; aaaaaaaaaaaaaaaaaaaaaaaaaaaaaaaaaaaaaaaaaaaaaaaaaaaaaaaaaaaaaaa School of Medicine, University of New South Wales, Sydney, NSW, Australia; bbbbbbbbbbbbbbbbbbbbbbbbbbbbbbbbbbbbbbbbbbbbbbbbbbbbbbbbbbbbbbb Nuffield Department of Population Health, University of Oxford, London, UK; ccccccccccccccccccccccccccccccccccccccccccccccccccccccccccccccc Orthopedic Trauma Pathology Department, IRCCS, Bologna, Italy; ddddddddddddddddddddddddddddddddddddddddddddddddddddddddddddddd Department of Obstetrics and Gynaecology, Nnamdi Azikiwe University, Awka, Nigeria; eeeeeeeeeeeeeeeeeeeeeeeeeeeeeeeeeeeeeeeeeeeeeeeeeeeeeeeeeeeeeee Department of Health Services Research and Policy, London School of Hygiene & Tropical Medicine, London, UK; fffffffffffffffffffffffffffffffffffffffffffffffffffffffffffffff The Malaria Atlas Project, Telethon Kids Institute, Perth, WA, Australia; ggggggggggggggggggggggggggggggggggggggggggggggggggggggggggggggg Digital Health and Informatics Directorate, Queensland Health, Brisbane, QLD, Australia; hhhhhhhhhhhhhhhhhhhhhhhhhhhhhhhhhhhhhhhhhhhhhhhhhhhhhhhhhhhhhhh Division of Pediatric Hospital Medicine, Stanford University, Palo Alto, CA, USA; iiiiiiiiiiiiiiiiiiiiiiiiiiiiiiiiiiiiiiiiiiiiiiiiiiiiiiiiiiiiiii Department of Epidemiology, Adigrat University, Adigrat, Ethiopia; jjjjjjjjjjjjjjjjjjjjjjjjjjjjjjjjjjjjjjjjjjjjjjjjjjjjjjjjjjjjjjj National Heart, Lung and Blood Institute, National Heart, Lung, and Blood Institute, Bethesda, MD, USA; kkkkkkkkkkkkkkkkkkkkkkkkkkkkkkkkkkkkkkkkkkkkkkkkkkkkkkkkkkkkkkk Research and Development Department, Lahore Medical Research Center, Lahore, Pakistan; lllllllllllllllllllllllllllllllllllllllllllllllllllllllllllllll Centre for Health Innovation and Policy, Noida, India; mmmmmmmmmmmmmmmmmmmmmmmmmmmmmmmmmmmmmmmmmmmmmmmmmmmmmmmmmmmmmmm Department of Dental Research Cell, Dr. D. Y. Patil University, Pune, India; nnnnnnnnnnnnnnnnnnnnnnnnnnnnnnnnnnnnnnnnnnnnnnnnnnnnnnnnnnnnnnn Department of Public Health, Arba Minch University, Arba Minch, Ethiopia; ooooooooooooooooooooooooooooooooooooooooooooooooooooooooooooooo Department of Medical Laboratory Sciences, Adigrat University, Adigrat, Ethiopia; ppppppppppppppppppppppppppppppppppppppppppppppppppppppppppppppp Department of General Practice, Monash University, Melbourne, VIC, Australia; qqqqqqqqqqqqqqqqqqqqqqqqqqqqqqqqqqqqqqqqqqqqqqqqqqqqqqqqqqqqqqq Health Care Authority, Olympia, WA, USA; rrrrrrrrrrrrrrrrrrrrrrrrrrrrrrrrrrrrrrrrrrrrrrrrrrrrrrrrrrrrrrr Dirección General de Investigación, Desarrollo e Innovación (DGIDI), Universidad Científica del Sur (University of the South), Lima, Peru; sssssssssssssssssssssssssssssssssssssssssssssssssssssssssssssss Department of Medical Microbiology and Immunology, Trinity Medical Sciences University, St. Vincent, Saint Vincent and the Grenadines; ttttttttttttttttttttttttttttttttttttttttttttttttttttttttttttttt Kasturba Medical College, Manipal Academy of Higher Education, Mangalore, India; uuuuuuuuuuuuuuuuuuuuuuuuuuuuuuuuuuuuuuuuuuuuuuuuuuuuuuuuuuuuuuu Department of Pathology - Forensic Medicine Division, Imam Abdulrahman Bin Faisal University, Dammam, Saudi Arabia; vvvvvvvvvvvvvvvvvvvvvvvvvvvvvvvvvvvvvvvvvvvvvvvvvvvvvvvvvvvvvvv Department of Adult Health Nursing, Bahir Dar University, Bahir Dar, Ethiopia; wwwwwwwwwwwwwwwwwwwwwwwwwwwwwwwwwwwwwwwwwwwwwwwwwwwwwwwwwwwwwww Center for Translation Research and Implementation Science, National Institutes of Health, Bethesda, MD, USA; xxxxxxxxxxxxxxxxxxxxxxxxxxxxxxxxxxxxxxxxxxxxxxxxxxxxxxxxxxxxxxx Department of Medicine, University of Cape Town, Cape Town, South Africa; yyyyyyyyyyyyyyyyyyyyyyyyyyyyyyyyyyyyyyyyyyyyyyyyyyyyyyyyyyyyyyy Department of Physiology, King Saud University, Riyadh, Saudi Arabia; zzzzzzzzzzzzzzzzzzzzzzzzzzzzzzzzzzzzzzzzzzzzzzzzzzzzzzzzzzzzzzz Department of Public Health, University “Federico II” of Naples, Naples, Italy; aaaaaaaaaaaaaaaaaaaaaaaaaaaaaaaaaaaaaaaaaaaaaaaaaaaaaaaaaaaaaaaa General Administration Department, Helsinki University Hospital, Helsinki, Finland; bbbbbbbbbbbbbbbbbbbbbbbbbbbbbbbbbbbbbbbbbbbbbbbbbbbbbbbbbbbbbbbb School of Health Sciences, University of Melbourne, Melbourne, VIC, Australia; cccccccccccccccccccccccccccccccccccccccccccccccccccccccccccccccc Comprehensive Cancer Center, Helsinki University Hospital, Helsinki, Finland; dddddddddddddddddddddddddddddddddddddddddddddddddddddddddddddddd University of Helsinki, Helsinki, Finland; eeeeeeeeeeeeeeeeeeeeeeeeeeeeeeeeeeeeeeeeeeeeeeeeeeeeeeeeeeeeeeee University Centre Varazdin, University North, Varazdin, Croatia; ffffffffffffffffffffffffffffffffffffffffffffffffffffffffffffffff Department of Pharmacology, University of Kelaniya, Ragama, Sri Lanka; gggggggggggggggggggggggggggggggggggggggggggggggggggggggggggggggg Clinical Medicine Department, Colombo North Teaching Hospital, Ragama, Sri Lanka; hhhhhhhhhhhhhhhhhhhhhhhhhhhhhhhhhhhhhhhhhhhhhhhhhhhhhhhhhhhhhhhh Department of Paediatrics, University of Kelaniya, Ragama, Sri Lanka; iiiiiiiiiiiiiiiiiiiiiiiiiiiiiiiiiiiiiiiiiiiiiiiiiiiiiiiiiiiiiiii University Paediatrics Unit, Colombo North Teaching Hospital, Ragama, Sri Lanka; jjjjjjjjjjjjjjjjjjjjjjjjjjjjjjjjjjjjjjjjjjjjjjjjjjjjjjjjjjjjjjjj Department of Pathology, Zagazig University, Zagazig, Egypt; kkkkkkkkkkkkkkkkkkkkkkkkkkkkkkkkkkkkkkkkkkkkkkkkkkkkkkkkkkkkkkkk Stritch School of Medicine, Loyola University Chicago, Chicago, IL, USA; llllllllllllllllllllllllllllllllllllllllllllllllllllllllllllllll Department of Propedeutics of Internal Diseases & Arterial Hypertension, Pomeranian Medical University, Szczecin, Poland; mmmmmmmmmmmmmmmmmmmmmmmmmmmmmmmmmmmmmmmmmmmmmmmmmmmmmmmmmmmmmmmm Department of Pathology, Maria Sklodowska-Curie National Research Institute of Oncology, Warsaw, Poland; nnnnnnnnnnnnnnnnnnnnnnnnnnnnnnnnnnnnnnnnnnnnnnnnnnnnnnnnnnnnnnnn Dermatology Unit, Fondazione IRCCS Policlinico San Matteo, Pavia, Italy; oooooooooooooooooooooooooooooooooooooooooooooooooooooooooooooooo Department of Oncology, Addis Ababa University, Addis Abeba, Ethiopia; pppppppppppppppppppppppppppppppppppppppppppppppppppppppppppppppp Department of Epidemiology and Biostatistics, Bahir Dar University, Bahir Dar, Ethiopia; qqqqqqqqqqqqqqqqqqqqqqqqqqqqqqqqqqqqqqqqqqqqqqqqqqqqqqqqqqqqqqqq College of Human Medicine, Michigan State University, Flint, MI, USA; rrrrrrrrrrrrrrrrrrrrrrrrrrrrrrrrrrrrrrrrrrrrrrrrrrrrrrrrrrrrrrrr Multidisciplinary Department of Medical-Surgical and Dental Specialties, University of Campania Luigi Vanvitelli, Naples, Italy; ssssssssssssssssssssssssssssssssssssssssssssssssssssssssssssssss Saveetha Dental College and Hospitals, Saveetha University, Chennai, India; tttttttttttttttttttttttttttttttttttttttttttttttttttttttttttttttt Department of Public Health Dentistry, Saveetha Institute of Medical and Technical Sciences (SIMATS), Chennai, India; uuuuuuuuuuuuuuuuuuuuuuuuuuuuuuuuuuuuuuuuuuuuuuuuuuuuuuuuuuuuuuuu Global Institute of Public Health, Ananthapuri Hospitals and Research Institute, Trivandrum, India; vvvvvvvvvvvvvvvvvvvvvvvvvvvvvvvvvvvvvvvvvvvvvvvvvvvvvvvvvvvvvvvv Faculty of Nursing and Midwifery, Tabriz University of Medical Sciences, Tabriz, Iran; wwwwwwwwwwwwwwwwwwwwwwwwwwwwwwwwwwwwwwwwwwwwwwwwwwwwwwwwwwwwwwww University Health Network, University of Toronto, Toronto, ON, Canada; xxxxxxxxxxxxxxxxxxxxxxxxxxxxxxxxxxxxxxxxxxxxxxxxxxxxxxxxxxxxxxxx Department of Radiology, Health Sciences North, Sudbury, ON, Canada; yyyyyyyyyyyyyyyyyyyyyyyyyyyyyyyyyyyyyyyyyyyyyyyyyyyyyyyyyyyyyyyy Bergen Center for Ethics and Priority Setting, University of Bergen, Bergen, Norway; zzzzzzzzzzzzzzzzzzzzzzzzzzzzzzzzzzzzzzzzzzzzzzzzzzzzzzzzzzzzzzzz Department of Physiotherapy, School of Rehabilitation Sciences, Kermanshah University of Medical Sciences, Kermanshah, Iran; aaaaaaaaaaaaaaaaaaaaaaaaaaaaaaaaaaaaaaaaaaaaaaaaaaaaaaaaaaaaaaaaa Department of Forensic Medicine and Toxicology, Rohilkhand Medical College, Bareilly, India; bbbbbbbbbbbbbbbbbbbbbbbbbbbbbbbbbbbbbbbbbbbbbbbbbbbbbbbbbbbbbbbbb Thumbay College of Management and AI in Healthcare, Gulf Medical University, Ajman, United Arab Emirates; ccccccccccccccccccccccccccccccccccccccccccccccccccccccccccccccccc Research and Development Department, Panacea Institute of Interdisciplinary Research and Education, Varanasi, India; ddddddddddddddddddddddddddddddddddddddddddddddddddddddddddddddddd Discipline of Psychiatry and Mental Health, University of New South Wales, Sydney, NSW, Australia; eeeeeeeeeeeeeeeeeeeeeeeeeeeeeeeeeeeeeeeeeeeeeeeeeeeeeeeeeeeeeeeee Central Clinical School, Faculty of Medicine and Health, University of Sydney, Sydney, NSW, Australia; fffffffffffffffffffffffffffffffffffffffffffffffffffffffffffffffff Department of Forensic Medicine and Toxicology, All India Institute of Medical Sciences, Patna, India; ggggggggggggggggggggggggggggggggggggggggggggggggggggggggggggggggg Burn and Regenerative Medicine Research Center, Guilan University of Medical Sciences, Rasht, Iran; hhhhhhhhhhhhhhhhhhhhhhhhhhhhhhhhhhhhhhhhhhhhhhhhhhhhhhhhhhhhhhhhh Department of Internal Medicine, Albert Einstein Hospital, Philadelphia, PA, USA; iiiiiiiiiiiiiiiiiiiiiiiiiiiiiiiiiiiiiiiiiiiiiiiiiiiiiiiiiiiiiiiii College of Health Science, University of Hargeisa, Hargeisa, Somalia; jjjjjjjjjjjjjjjjjjjjjjjjjjjjjjjjjjjjjjjjjjjjjjjjjjjjjjjjjjjjjjjjj Institute of Health Science, Jimma University, Jimma, Ethiopia; kkkkkkkkkkkkkkkkkkkkkkkkkkkkkkkkkkkkkkkkkkkkkkkkkkkkkkkkkkkkkkkkk Higher Colleges of Technology-Health Sciences Division-Pharmacy Program, Higher Colleges of Technology, Dubai, United Arab Emirates; lllllllllllllllllllllllllllllllllllllllllllllllllllllllllllllllll Faculty of Pharmacy, Cairo University, Cairo, Egypt; mmmmmmmmmmmmmmmmmmmmmmmmmmmmmmmmmmmmmmmmmmmmmmmmmmmmmmmmmmmmmmmmm College of Applied and Natural Science, University of Hargeisa, Hargeisa, Somalia; nnnnnnnnnnnnnnnnnnnnnnnnnnnnnnnnnnnnnnnnnnnnnnnnnnnnnnnnnnnnnnnnn RAK College of Nursing, RAK Medical and Health Sciences University, Ras Alkhima, United Arab Emirates; ooooooooooooooooooooooooooooooooooooooooooooooooooooooooooooooooo Nursing College, Sohag University, Sohag, Egypt; ppppppppppppppppppppppppppppppppppppppppppppppppppppppppppppppppp Molecular Biology Unit, Sirius Training and Research Centre, Khartoum, Sudan; qqqqqqqqqqqqqqqqqqqqqqqqqqqqqqqqqqqqqqqqqqqqqqqqqqqqqqqqqqqqqqqqq Bio-Statistical and Molecular Biology Department, Sirius Training and Research Centre, Khartoum, Sudan; rrrrrrrrrrrrrrrrrrrrrrrrrrrrrrrrrrrrrrrrrrrrrrrrrrrrrrrrrrrrrrrrr Faculty of Medicine, University of Khartoum, Khartoum, Sudan; sssssssssssssssssssssssssssssssssssssssssssssssssssssssssssssssss Department of Biophysics, All India Institute of Medical Sciences, New Delhi, India; ttttttttttttttttttttttttttttttttttttttttttttttttttttttttttttttttt Centre For Interdisciplinary Research In Basic Sciences (CIRBSc), Jamia Millia Islamia, New Delhi, India; uuuuuuuuuuuuuuuuuuuuuuuuuuuuuuuuuuuuuuuuuuuuuuuuuuuuuuuuuuuuuuuuu Urology Department, Tehran University of Medical Sciences, Tehran, Iran; vvvvvvvvvvvvvvvvvvvvvvvvvvvvvvvvvvvvvvvvvvvvvvvvvvvvvvvvvvvvvvvvv Department of Bacteriology, Tarbiat Modares University, Tehran, Iran; wwwwwwwwwwwwwwwwwwwwwwwwwwwwwwwwwwwwwwwwwwwwwwwwwwwwwwwwwwwwwwwww Modeling in Health Research Center, Shahrekord University of Medical Sciences, Shahrekord, Iran; xxxxxxxxxxxxxxxxxxxxxxxxxxxxxxxxxxxxxxxxxxxxxxxxxxxxxxxxxxxxxxxxx Skull Base Research Center, Shahid Beheshti University of Medical Sciences, Tehran, Iran; yyyyyyyyyyyyyyyyyyyyyyyyyyyyyyyyyyyyyyyyyyyyyyyyyyyyyyyyyyyyyyyyy University of Gondar, Gondar, Ethiopia; zzzzzzzzzzzzzzzzzzzzzzzzzzzzzzzzzzzzzzzzzzzzzzzzzzzzzzzzzzzzzzzzz School of Pharmacy, Haramaya University, Harar, Ethiopia; aaaaaaaaaaaaaaaaaaaaaaaaaaaaaaaaaaaaaaaaaaaaaaaaaaaaaaaaaaaaaaaaaa Department of Public Health, Dire Dawa University, Dire Dawa, Ethiopia; bbbbbbbbbbbbbbbbbbbbbbbbbbbbbbbbbbbbbbbbbbbbbbbbbbbbbbbbbbbbbbbbbb Department of Medicine, Government Medical College Kozhikode, Kozhikode, India; cccccccccccccccccccccccccccccccccccccccccccccccccccccccccccccccccc Health Systems and Policy Research Unit, Ahmadu Bello University, Zaria, Nigeria; dddddddddddddddddddddddddddddddddddddddddddddddddddddddddddddddddd Department of Health Sciences, Azare, National Institute for Research in Tribal Health, Bauchi, Nigeria; eeeeeeeeeeeeeeeeeeeeeeeeeeeeeeeeeeeeeeeeeeeeeeeeeeeeeeeeeeeeeeeeee Medical Microbiology Department, Usmanu Danfodiyo University, Sokoto, Sokoto, Nigeria; ffffffffffffffffffffffffffffffffffffffffffffffffffffffffffffffffff Medical Microbiology Department, Usmanu Danfodiyo University Teaching Hospital, Sokoto, Nigeria; gggggggggggggggggggggggggggggggggggggggggggggggggggggggggggggggggg Department of Health Services Management, Iran University of Medical Sciences, Iran, Iran; hhhhhhhhhhhhhhhhhhhhhhhhhhhhhhhhhhhhhhhhhhhhhhhhhhhhhhhhhhhhhhhhhh Department of Health Services Management, Isfahan University of Medical Sciences, Isfahan, Iran; iiiiiiiiiiiiiiiiiiiiiiiiiiiiiiiiiiiiiiiiiiiiiiiiiiiiiiiiiiiiiiiiii Department of Infectious Disease and Public Health, Jockey Club College of Veterinary Medicine and Life Sciences, City University of Hong Kong, Hong Kong, China; jjjjjjjjjjjjjjjjjjjjjjjjjjjjjjjjjjjjjjjjjjjjjjjjjjjjjjjjjjjjjjjjjj Maternal and Childhood Obesity Research Center, Urmia University of Medical Sciences, Urmia, Iran; kkkkkkkkkkkkkkkkkkkkkkkkkkkkkkkkkkkkkkkkkkkkkkkkkkkkkkkkkkkkkkkkkk Institute of Clinical Physiology, National Research Council, Pisa, Italy; llllllllllllllllllllllllllllllllllllllllllllllllllllllllllllllllll Department Medical-Surgical Nursing, Golestan University of Medical Sciences, Gorgan, Iran; mmmmmmmmmmmmmmmmmmmmmmmmmmmmmmmmmmmmmmmmmmmmmmmmmmmmmmmmmmmmmmmmmm Department of Mathematics, The University of Jordan, Amman, Jordan; nnnnnnnnnnnnnnnnnnnnnnnnnnnnnnnnnnnnnnnnnnnnnnnnnnnnnnnnnnnnnnnnnn Nonlinear Dynamics Research Center (NDRC), Ajman University, Ajman, United Arab Emirates; oooooooooooooooooooooooooooooooooooooooooooooooooooooooooooooooooo Clinical Epidemiology and Public Health Research Unit, Burlo Garofolo Institute for Maternal and Child Health, Trieste, Italy; pppppppppppppppppppppppppppppppppppppppppppppppppppppppppppppppppp Department of Sport Physiology, Razi University, Kermanshah, Iran; qqqqqqqqqqqqqqqqqqqqqqqqqqqqqqqqqqqqqqqqqqqqqqqqqqqqqqqqqqqqqqqqqq Department of Physiology, All India Institute of Medical Sciences, Deoghar, India; rrrrrrrrrrrrrrrrrrrrrrrrrrrrrrrrrrrrrrrrrrrrrrrrrrrrrrrrrrrrrrrrrr Department of Collective Prevention and Public Health, General Directorate for Personal Care, Health, and Welfare, Bologna, Italy; ssssssssssssssssssssssssssssssssssssssssssssssssssssssssssssssssss Department of Epidemiology and Biostatistics, Kurdistan University of Medical Sciences, Sanandaj, Iran; tttttttttttttttttttttttttttttttttttttttttttttttttttttttttttttttttt Social Determinants of Health Research Center, Yasuj University of Medical Sciences, Yasuj, Iran; uuuuuuuuuuuuuuuuuuuuuuuuuuuuuuuuuuuuuuuuuuuuuuuuuuuuuuuuuuuuuuuuuu Gastrointestinal and Liver Diseases Research Center, Iran University of Medical Sciences, Tehran, Iran; vvvvvvvvvvvvvvvvvvvvvvvvvvvvvvvvvvvvvvvvvvvvvvvvvvvvvvvvvvvvvvvvvv Preventive Medicine and Public Health Research Center, Iran University of Medical Sciences, Tehran, Iran; wwwwwwwwwwwwwwwwwwwwwwwwwwwwwwwwwwwwwwwwwwwwwwwwwwwwwwwwwwwwwwwwww Computer, Electrical, and Mathematical Sciences and Engineering Division, King Abdullah University of Science and Technology, Thuwal, Saudi Arabia; xxxxxxxxxxxxxxxxxxxxxxxxxxxxxxxxxxxxxxxxxxxxxxxxxxxxxxxxxxxxxxxxxx International Laboratory for Air Quality and Health, Queensland University of Technology, Brisbane, QLD, Australia; yyyyyyyyyyyyyyyyyyyyyyyyyyyyyyyyyyyyyyyyyyyyyyyyyyyyyyyyyyyyyyyyyy Department of Public Health, Oswaldo Cruz Foundation, Recife, Brazil; zzzzzzzzzzzzzzzzzzzzzzzzzzzzzzzzzzzzzzzzzzzzzzzzzzzzzzzzzzzzzzzzzz Department of Public Health, Federal University of Pernambuco, Recife, Brazil; aaaaaaaaaaaaaaaaaaaaaaaaaaaaaaaaaaaaaaaaaaaaaaaaaaaaaaaaaaaaaaaaaaa Faculty of Medicine, October 6 University, Giza, Egypt; bbbbbbbbbbbbbbbbbbbbbbbbbbbbbbbbbbbbbbbbbbbbbbbbbbbbbbbbbbbbbbbbbbb Neurosciences Research Center (NSRC), Tabriz University of Medical Sciences, Tabriz, Iran; ccccccccccccccccccccccccccccccccccccccccccccccccccccccccccccccccccc Student Research Committee, Tabriz University of Medical Sciences, Tabriz, Iran; ddddddddddddddddddddddddddddddddddddddddddddddddddddddddddddddddddd Department of Health Policy, London School of Economics and Political Science, London, UK; eeeeeeeeeeeeeeeeeeeeeeeeeeeeeeeeeeeeeeeeeeeeeeeeeeeeeeeeeeeeeeeeeee Department of Surgery and Cancer, Imperial College London, London, UK; fffffffffffffffffffffffffffffffffffffffffffffffffffffffffffffffffff Department of Psychiatry, Tehran University of Medical Sciences, Tehran, Iran; ggggggggggggggggggggggggggggggggggggggggggggggggggggggggggggggggggg Tehran Heart Center, Cardiovascular Diseases Research Institute, Tehran University of Medical Sciences, Tehran, Iran; hhhhhhhhhhhhhhhhhhhhhhhhhhhhhhhhhhhhhhhhhhhhhhhhhhhhhhhhhhhhhhhhhhh Department of Audiology, School of Rehabilitation, Shahid Beheshti University of Medical Sciences, Tehran, Iran; iiiiiiiiiiiiiiiiiiiiiiiiiiiiiiiiiiiiiiiiiiiiiiiiiiiiiiiiiiiiiiiiiii Faculty of Biotechnologies (BioTech), ITMO University, Saint Petersburg, Russia; jjjjjjjjjjjjjjjjjjjjjjjjjjjjjjjjjjjjjjjjjjjjjjjjjjjjjjjjjjjjjjjjjjj Department of Physical and Environmental Sciences, Texas A&M University, Corpus Christi, TX, USA; kkkkkkkkkkkkkkkkkkkkkkkkkkkkkkkkkkkkkkkkkkkkkkkkkkkkkkkkkkkkkkkkkkk Shiraz University of Medical Sciences, Shiraz, Iran; lllllllllllllllllllllllllllllllllllllllllllllllllllllllllllllllllll Medical Microbiology and Immunology Department, Cairo University, Cairo, Egypt; mmmmmmmmmmmmmmmmmmmmmmmmmmmmmmmmmmmmmmmmmmmmmmmmmmmmmmmmmmmmmmmmmmm Antimicrobial Resistance Research Center, Iran University of Medical Sciences, Tehran, Iran; nnnnnnnnnnnnnnnnnnnnnnnnnnnnnnnnnnnnnnnnnnnnnnnnnnnnnnnnnnnnnnnnnnn Hazrat-e Rasool General Hospital, Iran University of Medical Sciences, Tehran, Iran; ooooooooooooooooooooooooooooooooooooooooooooooooooooooooooooooooooo René Rachou Institute, Oswaldo Cruz Foundation, Belo Horizonte, Brazil; ppppppppppppppppppppppppppppppppppppppppppppppppppppppppppppppppppp PMAS Arid Agriculture University Rawalpindi, Rawalpindi, Pakistan; qqqqqqqqqqqqqqqqqqqqqqqqqqqqqqqqqqqqqqqqqqqqqqqqqqqqqqqqqqqqqqqqqqq Unit of Pharmacotherapy, Epidemiology and Economics, Rijksuniversiteit Groningen (University of Groningen), Groningen, Netherlands; rrrrrrrrrrrrrrrrrrrrrrrrrrrrrrrrrrrrrrrrrrrrrrrrrrrrrrrrrrrrrrrrrrr Department of Epidemiology and Biostatistics, Wuhan University, Wuhan, China; sssssssssssssssssssssssssssssssssssssssssssssssssssssssssssssssssss Department of Pharmacy, University of Zambia, Lusaka, Zambia; ttttttttttttttttttttttttttttttttttttttttttttttttttttttttttttttttttt School of Medicine, Keele University, Keele, UK; uuuuuuuuuuuuuuuuuuuuuuuuuuuuuuuuuuuuuuuuuuuuuuuuuuuuuuuuuuuuuuuuuuu Division of Psychology and Mental Health, University of Manchester, Manchester, UK; vvvvvvvvvvvvvvvvvvvvvvvvvvvvvvvvvvvvvvvvvvvvvvvvvvvvvvvvvvvvvvvvvvv Institute of Molecular Biology and Biotechnology, Bahauddin Zakariya University Multan, Multan, Pakistan; wwwwwwwwwwwwwwwwwwwwwwwwwwwwwwwwwwwwwwwwwwwwwwwwwwwwwwwwwwwwwwwwwww International Ph.D. Program in Biotech and Healthcare Management, Taipei Medical University, Taipei, Taiwan; xxxxxxxxxxxxxxxxxxxxxxxxxxxxxxxxxxxxxxxxxxxxxxxxxxxxxxxxxxxxxxxxxxx Department of Evidence and Intelligence for Action in Health, Pan American Health Organization, Washington, DC, USA; yyyyyyyyyyyyyyyyyyyyyyyyyyyyyyyyyyyyyyyyyyyyyyyyyyyyyyyyyyyyyyyyyyy Department of Biochemistry, All India Institute of Medical Sciences, Bhopal, India; zzzzzzzzzzzzzzzzzzzzzzzzzzzzzzzzzzzzzzzzzzzzzzzzzzzzzzzzzzzzzzzzzzz Knowledge Management Department, Prahlad Omkarwati Foundation (POF), Mumbai, India; aaaaaaaaaaaaaaaaaaaaaaaaaaaaaaaaaaaaaaaaaaaaaaaaaaaaaaaaaaaaaaaaaaaa Changescape Consulting, Independent Consultant, New Delhi, India; bbbbbbbbbbbbbbbbbbbbbbbbbbbbbbbbbbbbbbbbbbbbbbbbbbbbbbbbbbbbbbbbbbbb Department of Medicine, National University Health System, Singapore, Singapore; cccccccccccccccccccccccccccccccccccccccccccccccccccccccccccccccccccc Department of Mechanical Engineering, North Carolina Agricultural and Technical State University, Greensboro, NC, USA; dddddddddddddddddddddddddddddddddddddddddddddddddddddddddddddddddddd Department of Pediatrics and Child Health Nursing, Bahir Dar University, Bahir Dar, Ethiopia; eeeeeeeeeeeeeeeeeeeeeeeeeeeeeeeeeeeeeeeeeeeeeeeeeeeeeeeeeeeeeeeeeeee Department of Surgery, General University Hospital of Patras, Patras, Greece; ffffffffffffffffffffffffffffffffffffffffffffffffffffffffffffffffffff Faculty of Medicine, University of Thessaly, Larissa, Greece; gggggggggggggggggggggggggggggggggggggggggggggggggggggggggggggggggggg College of Health Science, Woldia University, Woldia, Ethiopia; hhhhhhhhhhhhhhhhhhhhhhhhhhhhhhhhhhhhhhhhhhhhhhhhhhhhhhhhhhhhhhhhhhhh Statistics and Actuarial Sciences Department, Jomo Kenyatta University of Agriculture and Technology, Nairobi, Kenya; iiiiiiiiiiiiiiiiiiiiiiiiiiiiiiiiiiiiiiiiiiiiiiiiiiiiiiiiiiiiiiiiiiii Department of Animal and Human Health, Jomo Kenyatta University of Agriculture and Technology, Nairobi, Kenya; jjjjjjjjjjjjjjjjjjjjjjjjjjjjjjjjjjjjjjjjjjjjjjjjjjjjjjjjjjjjjjjjjjjj Epidemiology, Biostatistics and Prevention Institute (EBPI), University of Zürich, Zurich, Switzerland; kkkkkkkkkkkkkkkkkkkkkkkkkkkkkkkkkkkkkkkkkkkkkkkkkkkkkkkkkkkkkkkkkkkk Department of Human Genetics and Molecular Medicine, Central University of Punjab, Bathinda, India; llllllllllllllllllllllllllllllllllllllllllllllllllllllllllllllllllll Clinical Epidemiology Research Unit, Mexican Institute of Social Security, Villa de Alvarez, Mexico; mmmmmmmmmmmmmmmmmmmmmmmmmmmmmmmmmmmmmmmmmmmmmmmmmmmmmmmmmmmmmmmmmmmm Postgraduate in Medical Sciences, Universidad de Colima, Colima, Mexico; nnnnnnnnnnnnnnnnnnnnnnnnnnnnnnnnnnnnnnnnnnnnnnnnnnnnnnnnnnnnnnnnnnnn Department of Internal Medicine, Cleveland Clinic, Cleveland, OH, USA; oooooooooooooooooooooooooooooooooooooooooooooooooooooooooooooooooooo Operational Research Center in Healthcare, Near East University, Nicosia, Cyprus; pppppppppppppppppppppppppppppppppppppppppppppppppppppppppppppppppppp Department of Research Methods, Orthopaedic Research Group, Coimbatore, India; qqqqqqqqqqqqqqqqqqqqqqqqqqqqqqqqqqqqqqqqqqqqqqqqqqqqqqqqqqqqqqqqqqqq Central Research Laboratory, Meenakshi Medical College Hospital and Research Institute, Chennai, India; rrrrrrrrrrrrrrrrrrrrrrrrrrrrrrrrrrrrrrrrrrrrrrrrrrrrrrrrrrrrrrrrrrrr University of Tabuk, Tabuk, Saudi Arabia; ssssssssssssssssssssssssssssssssssssssssssssssssssssssssssssssssssss Prince Fahad bin Sultan Chair for Biomedical Research, University of Tabuk, Tabuk, Saudi Arabia; tttttttttttttttttttttttttttttttttttttttttttttttttttttttttttttttttttt Director General, Rwanda Biomedical Centre, Kigali, Rwanda; uuuuuuuuuuuuuuuuuuuuuuuuuuuuuuuuuuuuuuuuuuuuuuuuuuuuuuuuuuuuuuuuuuuu College of Medicine and Health Sciences, University of Rwanda, Kigali, Rwanda; vvvvvvvvvvvvvvvvvvvvvvvvvvvvvvvvvvvvvvvvvvvvvvvvvvvvvvvvvvvvvvvvvvvv Department of Psychiatry, Seoul National University, Seoul, South Korea; wwwwwwwwwwwwwwwwwwwwwwwwwwwwwwwwwwwwwwwwwwwwwwwwwwwwwwwwwwwwwwwwwwww Department of Neuropsychiatry, Seoul National University Bundang Hospital, Seongnam, South Korea; xxxxxxxxxxxxxxxxxxxxxxxxxxxxxxxxxxxxxxxxxxxxxxxxxxxxxxxxxxxxxxxxxxxx Department of Ophthalmology, University of Tennessee, Memphis, TN, USA; yyyyyyyyyyyyyyyyyyyyyyyyyyyyyyyyyyyyyyyyyyyyyyyyyyyyyyyyyyyyyyyyyyyy Department of Geriatric Health, Tabriz University of Medical Sciences, Tabriz, Iran; zzzzzzzzzzzzzzzzzzzzzzzzzzzzzzzzzzzzzzzzzzzzzzzzzzzzzzzzzzzzzzzzzzzz Department of Health Education & Promotion, Gonabad University of Medical Sciences, Gonabad, Iran; aaaaaaaaaaaaaaaaaaaaaaaaaaaaaaaaaaaaaaaaaaaaaaaaaaaaaaaaaaaaaaaaaaaaa Elderly Health Research Center, Research and Academic Institution, Tehran, Iran; bbbbbbbbbbbbbbbbbbbbbbbbbbbbbbbbbbbbbbbbbbbbbbbbbbbbbbbbbbbbbbbbbbbbb Research and Analytics Department, Initiative for Financing Health and Human Development, Chennai, India; ccccccccccccccccccccccccccccccccccccccccccccccccccccccccccccccccccccc Department of Research and Analytics, Bioinsilico Technologies, Chennai, India; ddddddddddddddddddddddddddddddddddddddddddddddddddddddddddddddddddddd Department of Computer Science and IT, Torrens University Australia, Adelaide, SA, Australia; eeeeeeeeeeeeeeeeeeeeeeeeeeeeeeeeeeeeeeeeeeeeeeeeeeeeeeeeeeeeeeeeeeeee Department of Health Services Research, University of Alabama at Birmingham, Birmingham, AL, USA; fffffffffffffffffffffffffffffffffffffffffffffffffffffffffffffffffffff Faculty of Pharmacy, Hasanuddin University, Makassar, Indonesia; ggggggggggggggggggggggggggggggggggggggggggggggggggggggggggggggggggggg Department of Pulmonary Medicine, Government Medical College, Thrissur, Thrissur, India; hhhhhhhhhhhhhhhhhhhhhhhhhhhhhhhhhhhhhhhhhhhhhhhhhhhhhhhhhhhhhhhhhhhhh Health Action by People, Trivandrum, India; iiiiiiiiiiiiiiiiiiiiiiiiiiiiiiiiiiiiiiiiiiiiiiiiiiiiiiiiiiiiiiiiiiiii College of Health Sciences, Cihan University -Sulaimaniya, Sulaymaniyah, Iraq; jjjjjjjjjjjjjjjjjjjjjjjjjjjjjjjjjjjjjjjjjjjjjjjjjjjjjjjjjjjjjjjjjjjjj University of Sulaimani, Sulaymaniyah, Iraq; kkkkkkkkkkkkkkkkkkkkkkkkkkkkkkkkkkkkkkkkkkkkkkkkkkkkkkkkkkkkkkkkkkkkk Department of Physiotherapy, Tehran University of Medical Sciences, Tehran, Iran; lllllllllllllllllllllllllllllllllllllllllllllllllllllllllllllllllllll Research Center for War-affected People, Tehran University of Medical Sciences, Tehran, Iran; mmmmmmmmmmmmmmmmmmmmmmmmmmmmmmmmmmmmmmmmmmmmmmmmmmmmmmmmmmmmmmmmmmmmm Department of Health and Rehabilitation Sciences, Prince Sattam bin Abdulaziz University, Al Kharj, Saudi Arabia; nnnnnnnnnnnnnnnnnnnnnnnnnnnnnnnnnnnnnnnnnnnnnnnnnnnnnnnnnnnnnnnnnnnnn Suraj Eye Institute, Nagpur, India; ooooooooooooooooooooooooooooooooooooooooooooooooooooooooooooooooooooo Department for the Control of Disease, Epidemics, and Pandemics, Ministry of Public Health, Yaoundé, Cameroon; ppppppppppppppppppppppppppppppppppppppppppppppppppppppppppppppppppppp Department of Public Heath, University of Yaoundé I, Yaoundé, Cameroon; qqqqqqqqqqqqqqqqqqqqqqqqqqqqqqqqqqqqqqqqqqqqqqqqqqqqqqqqqqqqqqqqqqqqq Department of Biomedical Sciences, University of Zakho, Zakho, Iraq; rrrrrrrrrrrrrrrrrrrrrrrrrrrrrrrrrrrrrrrrrrrrrrrrrrrrrrrrrrrrrrrrrrrrr Manipal College of Dental Sciences, Manipal Academy of Higher Education, Manipal, India; sssssssssssssssssssssssssssssssssssssssssssssssssssssssssssssssssssss Bristol Medical School, University of Bristol, Bristol, UK; ttttttttttttttttttttttttttttttttttttttttttttttttttttttttttttttttttttt Department of Clinical Medicine, Universidade Federal de Minas Gerais (Federal University of Minas Gerais), Belo Horizonte, Brazil; uuuuuuuuuuuuuuuuuuuuuuuuuuuuuuuuuuuuuuuuuuuuuuuuuuuuuuuuuuuuuuuuuuuuu Clinical Hospital, Universidade Federal de Minas Gerais (Federal University of Minas Gerais), Belo Horizonte, Brazil; vvvvvvvvvvvvvvvvvvvvvvvvvvvvvvvvvvvvvvvvvvvvvvvvvvvvvvvvvvvvvvvvvvvvv National Dental Research Institute Singapore, Duke-NUS Medical School, Singapore, Singapore; wwwwwwwwwwwwwwwwwwwwwwwwwwwwwwwwwwwwwwwwwwwwwwwwwwwwwwwwwwwwwwwwwwwww Department of Applied Pharmaceutical Sciences and Clinical Pharmacy, Isra University, Amman, Jordan; xxxxxxxxxxxxxxxxxxxxxxxxxxxxxxxxxxxxxxxxxxxxxxxxxxxxxxxxxxxxxxxxxxxxx Division of Endocrinology and Diabetes, University of Vermont, South Burlington, VT, USA; yyyyyyyyyyyyyyyyyyyyyyyyyyyyyyyyyyyyyyyyyyyyyyyyyyyyyyyyyyyyyyyyyyyyy Department of Dental Public Health, King Abdulaziz University, Jeddah, Saudi Arabia; zzzzzzzzzzzzzzzzzzzzzzzzzzzzzzzzzzzzzzzzzzzzzzzzzzzzzzzzzzzzzzzzzzzzz Department of Health Policy and Oral Epidemiology, Harvard University, Boston, MA, USA; aaaaaaaaaaaaaaaaaaaaaaaaaaaaaaaaaaaaaaaaaaaaaaaaaaaaaaaaaaaaaaaaaaaaaa Department of Circulation and Medical Imaging, Norwegian University of Science and Technology, Trondheim, Norway; bbbbbbbbbbbbbbbbbbbbbbbbbbbbbbbbbbbbbbbbbbbbbbbbbbbbbbbbbbbbbbbbbbbbbb Department of Community Medicine, University of Peradeniya, Kandy, Sri Lanka; cccccccccccccccccccccccccccccccccccccccccccccccccccccccccccccccccccccc Postgraduate Institute of Medicine, University of Colombo, Colombo, Sri Lanka; dddddddddddddddddddddddddddddddddddddddddddddddddddddddddddddddddddddd Amity Institute of Forensic Sciences, Amity University, Noida, India; eeeeeeeeeeeeeeeeeeeeeeeeeeeeeeeeeeeeeeeeeeeeeeeeeeeeeeeeeeeeeeeeeeeeee Manipal College of Nursing, Manipal Academy of Higher Education, Manipal, India; ffffffffffffffffffffffffffffffffffffffffffffffffffffffffffffffffffffff Department of Forensic Medicine, Manipal Academy of Higher Education, Manipal, India; gggggggggggggggggggggggggggggggggggggggggggggggggggggggggggggggggggggg Department of Health Promotion, Zahedan University of Medical Sciences, Zahedan, Iran; hhhhhhhhhhhhhhhhhhhhhhhhhhhhhhhhhhhhhhhhhhhhhhhhhhhhhhhhhhhhhhhhhhhhhh Department of Research, TroDDIVaT Initiative, Buea, Cameroon; iiiiiiiiiiiiiiiiiiiiiiiiiiiiiiiiiiiiiiiiiiiiiiiiiiiiiiiiiiiiiiiiiiiiii Department of Microbiology and Parasitology, University of Buea, Buea, Cameroon; jjjjjjjjjjjjjjjjjjjjjjjjjjjjjjjjjjjjjjjjjjjjjjjjjjjjjjjjjjjjjjjjjjjjjj School of Pharmacy, West Virginia University, Morgantown, WV, USA; kkkkkkkkkkkkkkkkkkkkkkkkkkkkkkkkkkkkkkkkkkkkkkkkkkkkkkkkkkkkkkkkkkkkkk Department of Pharmacy, Bahir Dar University, Bahir Dar, Ethiopia; llllllllllllllllllllllllllllllllllllllllllllllllllllllllllllllllllllll Department of General Surgery, Carol Davila University of Medicine and Pharmacy, Bucharest, Romania; mmmmmmmmmmmmmmmmmmmmmmmmmmmmmmmmmmmmmmmmmmmmmmmmmmmmmmmmmmmmmmmmmmmmmm Department of General Surgery, Emergency University Hospital of Bucharest, Bucharest, Romania; nnnnnnnnnnnnnnnnnnnnnnnnnnnnnnnnnnnnnnnnnnnnnnnnnnnnnnnnnnnnnnnnnnnnnn Department of Anatomy and Embryology, Carol Davila University of Medicine and Pharmacy, Bucharest, Romania; oooooooooooooooooooooooooooooooooooooooooooooooooooooooooooooooooooooo Department of Cardiology, Cardio-Aid, Bucharest, Romania; pppppppppppppppppppppppppppppppppppppppppppppppppppppppppppppppppppppp Department of Cardiology, University of Medicine and Pharmacy “”Victor Babes“”, Timisoara, Romania; qqqqqqqqqqqqqqqqqqqqqqqqqqqqqqqqqqqqqqqqqqqqqqqqqqqqqqqqqqqqqqqqqqqqqq Rocordis Heart Center, Cardiology and Cardiovascular Surgery Hospital, Timisoara, Romania; rrrrrrrrrrrrrrrrrrrrrrrrrrrrrrrrrrrrrrrrrrrrrrrrrrrrrrrrrrrrrrrrrrrrrr Euromed Research Center, Euromed University of Fes, Fez, Morocco; ssssssssssssssssssssssssssssssssssssssssssssssssssssssssssssssssssssss Faculty of Medicine, Pharmacy, and Dentistry, University Sidi Mohammed Ben Abdellah, Fez, Morocco; tttttttttttttttttttttttttttttttttttttttttttttttttttttttttttttttttttttt Department of Community Medicine, Lumbini Medical College, Palpa, Nepal; uuuuuuuuuuuuuuuuuuuuuuuuuuuuuuuuuuuuuuuuuuuuuuuuuuuuuuuuuuuuuuuuuuuuuu School of Nursing, University of Gondar, Gondar, Ethiopia; vvvvvvvvvvvvvvvvvvvvvvvvvvvvvvvvvvvvvvvvvvvvvvvvvvvvvvvvvvvvvvvvvvvvvv College of Medicine and Health Sciences, Bahir Dar University, Bahir Dar, Ethiopia; wwwwwwwwwwwwwwwwwwwwwwwwwwwwwwwwwwwwwwwwwwwwwwwwwwwwwwwwwwwwwwwwwwwwww Department of Public Health, University of Yaoundé I, Yaoundé, Cameroon; xxxxxxxxxxxxxxxxxxxxxxxxxxxxxxxxxxxxxxxxxxxxxxxxxxxxxxxxxxxxxxxxxxxxxx Department of Biological Sciences, University of Embu, Embu, Kenya; yyyyyyyyyyyyyyyyyyyyyyyyyyyyyyyyyyyyyyyyyyyyyyyyyyyyyyyyyyyyyyyyyyyyyy Institute for Global Health Innovations, Duy Tan University, Hanoi, Viet Nam; zzzzzzzzzzzzzzzzzzzzzzzzzzzzzzzzzzzzzzzzzzzzzzzzzzzzzzzzzzzzzzzzzzzzzz Harvard T.H. Chan School of Public Health, Harvard University, Cambridge, MA, USA; aaaaaaaaaaaaaaaaaaaaaaaaaaaaaaaaaaaaaaaaaaaaaaaaaaaaaaaaaaaaaaaaaaaaaaa Department of Medical Engineering, University of South Florida, Tampa, FL, USA; bbbbbbbbbbbbbbbbbbbbbbbbbbbbbbbbbbbbbbbbbbbbbbbbbbbbbbbbbbbbbbbbbbbbbbb Faculty of Medicine, Nam Can Tho University, Can Tho, Viet Nam; ccccccccccccccccccccccccccccccccccccccccccccccccccccccccccccccccccccccc International Medical Faculty, Nam Can Tho University, Can Tho, Viet Nam; ddddddddddddddddddddddddddddddddddddddddddddddddddddddddddddddddddddddd Cardiovascular Research Department, Methodist Hospitals, Merrillville, IN, USA; eeeeeeeeeeeeeeeeeeeeeeeeeeeeeeeeeeeeeeeeeeeeeeeeeeeeeeeeeeeeeeeeeeeeeee Hitotsubashi Institute for Advanced Study (HIAS), Hitotsubashi University, Tokyo, Japan; fffffffffffffffffffffffffffffffffffffffffffffffffffffffffffffffffffffff Institute for Cancer Control, National Cancer Center, Chuo-ku, Japan; ggggggggggggggggggggggggggggggggggggggggggggggggggggggggggggggggggggggg Tuberculosis Group, Oxford University Clinical Research Unit, Vietnam, Ho Chi Minh City, Viet Nam; hhhhhhhhhhhhhhhhhhhhhhhhhhhhhhhhhhhhhhhhhhhhhhhhhhhhhhhhhhhhhhhhhhhhhhh Department of General Medicine, University of Medicine and Pharmacy at Ho Chi Minh City, Ho Chi Minh City, Viet Nam; iiiiiiiiiiiiiiiiiiiiiiiiiiiiiiiiiiiiiiiiiiiiiiiiiiiiiiiiiiiiiiiiiiiiiii Department of Public Health, University of Bamenda, Bamenda, Cameroon; jjjjjjjjjjjjjjjjjjjjjjjjjjjjjjjjjjjjjjjjjjjjjjjjjjjjjjjjjjjjjjjjjjjjjjj International Islamic University Islamabad, Islamabad, Pakistan; kkkkkkkkkkkkkkkkkkkkkkkkkkkkkkkkkkkkkkkkkkkkkkkkkkkkkkkkkkkkkkkkkkkkkkk Department of Humanities and Social Science, University for International Studies in Rome, Rome, Italy; lllllllllllllllllllllllllllllllllllllllllllllllllllllllllllllllllllllll Institute for Mental Health Policy Research, Centre for Addiction and Mental Health, Toronto, ON, Canada; mmmmmmmmmmmmmmmmmmmmmmmmmmmmmmmmmmmmmmmmmmmmmmmmmmmmmmmmmmmmmmmmmmmmmmm School of Medicine, University of Limerick, Limerick, Ireland; nnnnnnnnnnnnnnnnnnnnnnnnnnnnnnnnnnnnnnnnnnnnnnnnnnnnnnnnnnnnnnnnnnnnnnn Department of Public Health, UNICAF, Larnaca, Cyprus; ooooooooooooooooooooooooooooooooooooooooooooooooooooooooooooooooooooooo Department of Pathology, Hawassa University, Hawassa, Ethiopia; ppppppppppppppppppppppppppppppppppppppppppppppppppppppppppppppppppppppp Technical Department, University of Cape Town, Cape Town, South Africa; qqqqqqqqqqqqqqqqqqqqqqqqqqqqqqqqqqqqqqqqqqqqqqqqqqqqqqqqqqqqqqqqqqqqqqq School of Public Health and Family Medicine, University of Cape Town, Cape Town, South Africa; rrrrrrrrrrrrrrrrrrrrrrrrrrrrrrrrrrrrrrrrrrrrrrrrrrrrrrrrrrrrrrrrrrrrrrr Global Research Institute, Keio University, Tokyo, Japan; sssssssssssssssssssssssssssssssssssssssssssssssssssssssssssssssssssssss Family Health Research Institute, Tehran University of Medical Sciences, Tehran, Iran; ttttttttttttttttttttttttttttttttttttttttttttttttttttttttttttttttttttttt Department of Microbiology and Molecular Genetics, The Women University Multan, Multan, Pakistan; uuuuuuuuuuuuuuuuuuuuuuuuuuuuuuuuuuuuuuuuuuuuuuuuuuuuuuuuuuuuuuuuuuuuuuu School of Biomedical Engineering, Science and Health Systems, Drexel University, Philadelphia, PA, USA; vvvvvvvvvvvvvvvvvvvvvvvvvvvvvvvvvvvvvvvvvvvvvvvvvvvvvvvvvvvvvvvvvvvvvvv Division of Cardiology, University of California San Francisco, San Francisco, CA, USA; wwwwwwwwwwwwwwwwwwwwwwwwwwwwwwwwwwwwwwwwwwwwwwwwwwwwwwwwwwwwwwwwwwwwwww Health Research Institute, Babol University of Medical Sciences, Babol, Iran; xxxxxxxxxxxxxxxxxxxxxxxxxxxxxxxxxxxxxxxxxxxxxxxxxxxxxxxxxxxxxxxxxxxxxxx Internal Medicine Department, Maimonides Medical Center, Brooklyn, NY, USA; yyyyyyyyyyyyyyyyyyyyyyyyyyyyyyyyyyyyyyyyyyyyyyyyyyyyyyyyyyyyyyyyyyyyyyy Department of Paediatrics, Nnamdi Azikiwe University, Awka, Nigeria; zzzzzzzzzzzzzzzzzzzzzzzzzzzzzzzzzzzzzzzzzzzzzzzzzzzzzzzzzzzzzzzzzzzzzzz Global Health Department, Euclid University, Banqui, Central African Republic; aaaaaaaaaaaaaaaaaaaaaaaaaaaaaaaaaaaaaaaaaaaaaaaaaaaaaaaaaaaaaaaaaaaaaaaa School of Information, University of California Berkeley, Berkeley, CA, USA; bbbbbbbbbbbbbbbbbbbbbbbbbbbbbbbbbbbbbbbbbbbbbbbbbbbbbbbbbbbbbbbbbbbbbbbb Midwifery Department, Poltekkes Kemenkes Palu, Palu, Indonesia; cccccccccccccccccccccccccccccccccccccccccccccccccccccccccccccccccccccccc Department of Public Health, Banten School of Health Science, South Tangerang, Indonesia; dddddddddddddddddddddddddddddddddddddddddddddddddddddddddddddddddddddddd Ministry of Research, Technology and Higher Education, Higher Education Service Institutions (LL-DIKTI) Region IV, Bandung, Indonesia; eeeeeeeeeeeeeeeeeeeeeeeeeeeeeeeeeeeeeeeeeeeeeeeeeeeeeeeeeeeeeeeeeeeeeeee Department of Nursing, University of Health and Allied Sciences, Ho, Ghana; ffffffffffffffffffffffffffffffffffffffffffffffffffffffffffffffffffffffff Department of Applied Economics and Quantitative Analysis, University of Bucharest, Bucharest, Romania; gggggggggggggggggggggggggggggggggggggggggggggggggggggggggggggggggggggggg Bioinformatics Department, National Institute of Research and Development for Biological Sciences, Bucharest, Romania; hhhhhhhhhhhhhhhhhhhhhhhhhhhhhhhhhhhhhhhhhhhhhhhhhhhhhhhhhhhhhhhhhhhhhhhh Department of Biomedicine and Prevention, University of Rome “”Tor Vergata“”, Rome, Italy; iiiiiiiiiiiiiiiiiiiiiiiiiiiiiiiiiiiiiiiiiiiiiiiiiiiiiiiiiiiiiiiiiiiiiiii Department of Veterinary Public Health and Preventive Medicine, University of Ilorin, Ilorin, Nigeria; jjjjjjjjjjjjjjjjjjjjjjjjjjjjjjjjjjjjjjjjjjjjjjjjjjjjjjjjjjjjjjjjjjjjjjjj Department of Community Health and Primary Care, University of Lagos, Idi Araba, Nigeria; kkkkkkkkkkkkkkkkkkkkkkkkkkkkkkkkkkkkkkkkkkkkkkkkkkkkkkkkkkkkkkkkkkkkkkkk Department of Family and Preventive Medicine, University of Utah, Salt Lake City, UT, USA; llllllllllllllllllllllllllllllllllllllllllllllllllllllllllllllllllllllll Department of Population and Health, University of Cape Coast, Cape Coast, Ghana; mmmmmmmmmmmmmmmmmmmmmmmmmmmmmmmmmmmmmmmmmmmmmmmmmmmmmmmmmmmmmmmmmmmmmmmm PSSM Data Sciences, Pfizer Research & Development, Pfizer Inc., Groton, CT, USA; nnnnnnnnnnnnnnnnnnnnnnnnnnnnnnnnnnnnnnnnnnnnnnnnnnnnnnnnnnnnnnnnnnnnnnnn Technical Unit, Malaria Consortium, London, UK; oooooooooooooooooooooooooooooooooooooooooooooooooooooooooooooooooooooooo Department of Physiology, University of Medical Sciences, Ondo, Nigeria; pppppppppppppppppppppppppppppppppppppppppppppppppppppppppppppppppppppppp Department of Preventive Medicine, University of Ulsan, Seoul, South Korea; qqqqqqqqqqqqqqqqqqqqqqqqqqqqqqqqqqqqqqqqqqqqqqqqqqqqqqqqqqqqqqqqqqqqqqqq Institute for Global Engagement & Empowerment, Yonsei University, Seoul, South Korea; rrrrrrrrrrrrrrrrrrrrrrrrrrrrrrrrrrrrrrrrrrrrrrrrrrrrrrrrrrrrrrrrrrrrrrrr Health Promotion Research Center, Zahedan University of Medical Sciences, Zahedan, Iran; ssssssssssssssssssssssssssssssssssssssssssssssssssssssssssssssssssssssss Counselling and Human Development Studies, University of Ibadan, Ibadan, Nigeria; tttttttttttttttttttttttttttttttttttttttttttttttttttttttttttttttttttttttt Department of Food and Nutrition, Seoul National University, Seoul, South Korea; uuuuuuuuuuuuuuuuuuuuuuuuuuuuuuuuuuuuuuuuuuuuuuuuuuuuuuuuuuuuuuuuuuuuuuuu College of Medicine, University of Ibadan, Ibadan, Nigeria; vvvvvvvvvvvvvvvvvvvvvvvvvvvvvvvvvvvvvvvvvvvvvvvvvvvvvvvvvvvvvvvvvvvvvvvv Faculty of Medicine, University of Thessaly, Volos, Greece; wwwwwwwwwwwwwwwwwwwwwwwwwwwwwwwwwwwwwwwwwwwwwwwwwwwwwwwwwwwwwwwwwwwwwwww Department of Medical Laboratory Science, Federal Neuropsychiatric Hospital, Abeokuta, Nigeria; xxxxxxxxxxxxxxxxxxxxxxxxxxxxxxxxxxxxxxxxxxxxxxxxxxxxxxxxxxxxxxxxxxxxxxxx School of Pharmacy, University of the Western Cape, Cape Town, South Africa; yyyyyyyyyyyyyyyyyyyyyyyyyyyyyyyyyyyyyyyyyyyyyyyyyyyyyyyyyyyyyyyyyyyyyyyy Department of Psychiatry, Griffith University, Gold Coast, QLD, Australia; zzzzzzzzzzzzzzzzzzzzzzzzzzzzzzzzzzzzzzzzzzzzzzzzzzzzzzzzzzzzzzzzzzzzzzzz College of Health Sciences, Bowen University, Iwo, Nigeria; aaaaaaaaaaaaaaaaaaaaaaaaaaaaaaaaaaaaaaaaaaaaaaaaaaaaaaaaaaaaaaaaaaaaaaaaa Department of Neurology, University College Hospital, Ibadan, Ibadan, Nigeria; bbbbbbbbbbbbbbbbbbbbbbbbbbbbbbbbbbbbbbbbbbbbbbbbbbbbbbbbbbbbbbbbbbbbbbbbb Department of Medicine, University of Ibadan, Ibadan, Nigeria; ccccccccccccccccccccccccccccccccccccccccccccccccccccccccccccccccccccccccc Department of Nursing Science, Bowen University Iwo, Iwo, Nigeria; ddddddddddddddddddddddddddddddddddddddddddddddddddddddddddddddddddddddddd Cumming School of Medicine, University of Calgary, Calgary, AB, Canada; eeeeeeeeeeeeeeeeeeeeeeeeeeeeeeeeeeeeeeeeeeeeeeeeeeeeeeeeeeeeeeeeeeeeeeeee Center for Clinical and Epidemiological Research, Universidade de São Paulo (University of São Paulo), São Paulo, Brazil; fffffffffffffffffffffffffffffffffffffffffffffffffffffffffffffffffffffffff Associação Brasileira de Cefaleia em Salvas e Enxaqueca (ABRACES), São Paulo, Brazil; ggggggggggggggggggggggggggggggggggggggggggggggggggggggggggggggggggggggggg Cardiology Department, Federal University of Rio de Janeiro, Rio de Janeiro, Brazil; hhhhhhhhhhhhhhhhhhhhhhhhhhhhhhhhhhhhhhhhhhhhhhhhhhhhhhhhhhhhhhhhhhhhhhhhh School of Health and Life Sciences, Teesside University, Middlesbrough, UK; iiiiiiiiiiiiiiiiiiiiiiiiiiiiiiiiiiiiiiiiiiiiiiiiiiiiiiiiiiiiiiiiiiiiiiiii Department of Epidemiology, Johns Hopkins University, Baltimore, MD, USA; jjjjjjjjjjjjjjjjjjjjjjjjjjjjjjjjjjjjjjjjjjjjjjjjjjjjjjjjjjjjjjjjjjjjjjjjj School of Public Health, Makerere University, Kampala, Uganda; kkkkkkkkkkkkkkkkkkkkkkkkkkkkkkkkkkkkkkkkkkkkkkkkkkkkkkkkkkkkkkkkkkkkkkkkk Centre for Healthy Start Initiative, Lagos, Nigeria; lllllllllllllllllllllllllllllllllllllllllllllllllllllllllllllllllllllllll Research Policy & Administration, Centre for Healthy Start Initiative, Lagos, Nigeria; mmmmmmmmmmmmmmmmmmmmmmmmmmmmmmmmmmmmmmmmmmmmmmmmmmmmmmmmmmmmmmmmmmmmmmmmm Department of Pharmacology and Therapeutics, Olabisi Onabanjo University, Sagamu, Nigeria; nnnnnnnnnnnnnnnnnnnnnnnnnnnnnnnnnnnnnnnnnnnnnnnnnnnnnnnnnnnnnnnnnnnnnnnnn Institute of Infectious Disease and Molecular Medicine, University of Cape Town, Cape Town, South Africa; ooooooooooooooooooooooooooooooooooooooooooooooooooooooooooooooooooooooooo Institute of Chemistry, Universidade Estadual de Campinas (State University of Campinas), Campinas, Brazil; ppppppppppppppppppppppppppppppppppppppppppppppppppppppppppppppppppppppppp Department of Computational Biology, Brazilian Agricultural Research Institute (EMBRAPA), Campinas, Brazil; qqqqqqqqqqqqqqqqqqqqqqqqqqqqqqqqqqqqqqqqqqqqqqqqqqqqqqqqqqqqqqqqqqqqqqqqq Surgery Department, Sulaimani University, Sulaimani, Iraq; rrrrrrrrrrrrrrrrrrrrrrrrrrrrrrrrrrrrrrrrrrrrrrrrrrrrrrrrrrrrrrrrrrrrrrrrr ENT Department, Tor Vergata University of Rome, Rome, Italy; sssssssssssssssssssssssssssssssssssssssssssssssssssssssssssssssssssssssss Department of Anatomic Pathology, Ekiti State University, Ado- Ekiti, Nigeria; ttttttttttttttttttttttttttttttttttttttttttttttttttttttttttttttttttttttttt Department of Anatomic Pathology, Ekiti State University Teaching Hospital, Ado Ekiti, Nigeria; uuuuuuuuuuuuuuuuuuuuuuuuuuuuuuuuuuuuuuuuuuuuuuuuuuuuuuuuuuuuuuuuuuuuuuuuu Department of Global Health and Social Medicine, Harvard University, Boston, MA, USA; vvvvvvvvvvvvvvvvvvvvvvvvvvvvvvvvvvvvvvvvvvvvvvvvvvvvvvvvvvvvvvvvvvvvvvvvv Wellspring Research, Wellspring Center Indonesia, Jakarta, Indonesia; wwwwwwwwwwwwwwwwwwwwwwwwwwwwwwwwwwwwwwwwwwwwwwwwwwwwwwwwwwwwwwwwwwwwwwwww Department of Pharmacology and Therapeutics, University of Nigeria Nsukka, Enugu, Nigeria; xxxxxxxxxxxxxxxxxxxxxxxxxxxxxxxxxxxxxxxxxxxxxxxxxxxxxxxxxxxxxxxxxxxxxxxxx Department of Mathematics, Science and Technology Education, University of Johannesburg, Johannesburg, South Africa; yyyyyyyyyyyyyyyyyyyyyyyyyyyyyyyyyyyyyyyyyyyyyyyyyyyyyyyyyyyyyyyyyyyyyyyyy Department of Health, Lira District Local Government, Lira, SA, Australia; zzzzzzzzzzzzzzzzzzzzzzzzzzzzzzzzzzzzzzzzzzzzzzzzzzzzzzzzzzzzzzzzzzzzzzzzz Scientific Laboratory “”Center for Collective Use“”, Kazakh National Medical University, Almaty, Kazakhstan; aaaaaaaaaaaaaaaaaaaaaaaaaaaaaaaaaaaaaaaaaaaaaaaaaaaaaaaaaaaaaaaaaaaaaaaaaa Department of Pharmacotherapy and Pharmaceutical Care, Medical University of Warsaw, Warsaw, Poland; bbbbbbbbbbbbbbbbbbbbbbbbbbbbbbbbbbbbbbbbbbbbbbbbbbbbbbbbbbbbbbbbbbbbbbbbbb Department of Microbiology and Immunology, University of Health and Allied Sciences, Ho, Ghana; cccccccccccccccccccccccccccccccccccccccccccccccccccccccccccccccccccccccccc Sickle Cell Unit, Ho Teaching Hospital, Ho, Ghana; dddddddddddddddddddddddddddddddddddddddddddddddddddddddddddddddddddddddddd Department of Neurology, ASL Avezzano-Sulmona-L'Aquila, L'Aquila, Italy; eeeeeeeeeeeeeeeeeeeeeeeeeeeeeeeeeeeeeeeeeeeeeeeeeeeeeeeeeeeeeeeeeeeeeeeeee Department of Neurosurgery, University of California San Francisco, San Francisco, CA, USA; ffffffffffffffffffffffffffffffffffffffffffffffffffffffffffffffffffffffffff Department of Nephrology and Hypertension, IIS-Fundacion Jimenez Diaz, Madrid, Spain; gggggggggggggggggggggggggggggggggggggggggggggggggggggggggggggggggggggggggg Department of Medicine, Universidad Autónoma de Madrid (Autonomous University of Madrid), Madrid, Spain; hhhhhhhhhhhhhhhhhhhhhhhhhhhhhhhhhhhhhhhhhhhhhhhhhhhhhhhhhhhhhhhhhhhhhhhhhh One Health Global Research Group, Universidad de las Americas (University of the Americas), Quito, Ecuador; iiiiiiiiiiiiiiiiiiiiiiiiiiiiiiiiiiiiiiiiiiiiiiiiiiiiiiiiiiiiiiiiiiiiiiiiii Department of Biological Sciences, Njala University, Freetown, Sierra Leone; jjjjjjjjjjjjjjjjjjjjjjjjjjjjjjjjjjjjjjjjjjjjjjjjjjjjjjjjjjjjjjjjjjjjjjjjjj Henry M Jackson School of International Studies, University of Washington, Seattle, WA, USA; kkkkkkkkkkkkkkkkkkkkkkkkkkkkkkkkkkkkkkkkkkkkkkkkkkkkkkkkkkkkkkkkkkkkkkkkkk Cardiovascular Division, Harvard University, Boston, MA, USA; llllllllllllllllllllllllllllllllllllllllllllllllllllllllllllllllllllllllll School of Medicine, Western Sydney University, Bathurst, NSW, Australia; mmmmmmmmmmmmmmmmmmmmmmmmmmmmmmmmmmmmmmmmmmmmmmmmmmmmmmmmmmmmmmmmmmmmmmmmmm Department of Optometry and Vision Science, University of KwaZulu-Natal, KwaZulu-Natal, South Africa; nnnnnnnnnnnnnnnnnnnnnnnnnnnnnnnnnnnnnnnnnnnnnnnnnnnnnnnnnnnnnnnnnnnnnnnnnn Department of Biological Sciences, Elizade University, Ilara-Mokin, Nigeria; oooooooooooooooooooooooooooooooooooooooooooooooooooooooooooooooooooooooooo Faculty of Nursing, Applied Science Private University, Amman, Jordan; pppppppppppppppppppppppppppppppppppppppppppppppppppppppppppppppppppppppppp School of Public Health, Haramaya University, Harar, Ethiopia; qqqqqqqqqqqqqqqqqqqqqqqqqqqqqqqqqqqqqqqqqqqqqqqqqqqqqqqqqqqqqqqqqqqqqqqqqq School of Nursing, University of California San Francisco, San Francisco, CA, USA; rrrrrrrrrrrrrrrrrrrrrrrrrrrrrrrrrrrrrrrrrrrrrrrrrrrrrrrrrrrrrrrrrrrrrrrrrr Faculty of Medicine, University Ferhat Abbas of Setif, Setif, Algeria; ssssssssssssssssssssssssssssssssssssssssssssssssssssssssssssssssssssssssss Division of Infectious Diseases, University Hospital of Setif, Setif, Algeria; tttttttttttttttttttttttttttttttttttttttttttttttttttttttttttttttttttttttttt Department of Medicine, University College Hospital, Ibadan, Ibadan, Nigeria; uuuuuuuuuuuuuuuuuuuuuuuuuuuuuuuuuuuuuuuuuuuuuuuuuuuuuuuuuuuuuuuuuuuuuuuuuu West African Center for Cell Biology of Infectious Pathogens, University of Ghana, Legon, Ghana; vvvvvvvvvvvvvvvvvvvvvvvvvvvvvvvvvvvvvvvvvvvvvvvvvvvvvvvvvvvvvvvvvvvvvvvvvv University Hospitals, Case Western Reserve University, Cleveland, OH, USA; wwwwwwwwwwwwwwwwwwwwwwwwwwwwwwwwwwwwwwwwwwwwwwwwwwwwwwwwwwwwwwwwwwwwwwwwww Department of Biochemistry and Nutrition, Nigerian Institute of Medical Research, Lagos, Nigeria; xxxxxxxxxxxxxxxxxxxxxxxxxxxxxxxxxxxxxxxxxxxxxxxxxxxxxxxxxxxxxxxxxxxxxxxxxx Division of Medicine, University College London, London, UK; yyyyyyyyyyyyyyyyyyyyyyyyyyyyyyyyyyyyyyyyyyyyyyyyyyyyyyyyyyyyyyyyyyyyyyyyyy Department of Biological Sciences, Bamidele Olumilua University of Education Science & Technology, Ikere-Ekiti, Nigeria; zzzzzzzzzzzzzzzzzzzzzzzzzzzzzzzzzzzzzzzzzzzzzzzzzzzzzzzzzzzzzzzzzzzzzzzzzz Plant Systems Biology, International Center for Genetic Engineering & Biotechnology (ICGEB), Cape Town, South Africa; aaaaaaaaaaaaaaaaaaaaaaaaaaaaaaaaaaaaaaaaaaaaaaaaaaaaaaaaaaaaaaaaaaaaaaaaaaa Department of Biosciences and Biotechnology, University of Medical Sciences, Ondo, Ondo, Nigeria; bbbbbbbbbbbbbbbbbbbbbbbbbbbbbbbbbbbbbbbbbbbbbbbbbbbbbbbbbbbbbbbbbbbbbbbbbbb Operational Research Center in Healthcare, Near East University, Nicosia, Türkiye; ccccccccccccccccccccccccccccccccccccccccccccccccccccccccccccccccccccccccccc Department of Mathematical Sciences, Saveetha School of Engineering (SIMATS), Chennai, India; ddddddddddddddddddddddddddddddddddddddddddddddddddddddddddddddddddddddddddd Department of Respiratory Medicine, Jagadguru Sri Shivarathreeswara University, Mysore, India; eeeeeeeeeeeeeeeeeeeeeeeeeeeeeeeeeeeeeeeeeeeeeeeeeeeeeeeeeeeeeeeeeeeeeeeeeee Department of Physical Medicine and Rehabilitation, Harvard University, Boston, MA, USA; fffffffffffffffffffffffffffffffffffffffffffffffffffffffffffffffffffffffffff Universidad San Ignacio de Loyola, Lima, Peru; ggggggggggggggggggggggggggggggggggggggggggggggggggggggggggggggggggggggggggg National School of Public Health, Institute of Health Carlos III, Madrid, Spain; hhhhhhhhhhhhhhhhhhhhhhhhhhhhhhhhhhhhhhhhhhhhhhhhhhhhhhhhhhhhhhhhhhhhhhhhhhh Department of Forensic Medicine and Toxicology, Manipal Academy of Higher Education, Mangalore, India; iiiiiiiiiiiiiiiiiiiiiiiiiiiiiiiiiiiiiiiiiiiiiiiiiiiiiiiiiiiiiiiiiiiiiiiiiii Ashok & Rita Patel Institute of Physiotherapy, Charotar University of Science and Technology, Anand, India; jjjjjjjjjjjjjjjjjjjjjjjjjjjjjjjjjjjjjjjjjjjjjjjjjjjjjjjjjjjjjjjjjjjjjjjjjjj Breast Health and Cancer Research Center, Iran University of Medical Sciences, Tehran, Iran; kkkkkkkkkkkkkkkkkkkkkkkkkkkkkkkkkkkkkkkkkkkkkkkkkkkkkkkkkkkkkkkkkkkkkkkkkkk Department of Neurology, National Institute of Mental Health and Neurosciences, Bangalore, India; lllllllllllllllllllllllllllllllllllllllllllllllllllllllllllllllllllllllllll Department of Primary Care and Public Health, Imperial College London, London, UK; mmmmmmmmmmmmmmmmmmmmmmmmmmmmmmmmmmmmmmmmmmmmmmmmmmmmmmmmmmmmmmmmmmmmmmmmmmm Primary Health Center, Directorate of Public Health and Family Welfare, Eluru District, India; nnnnnnnnnnnnnnnnnnnnnnnnnnnnnnnnnnnnnnnnnnnnnnnnnnnnnnnnnnnnnnnnnnnnnnnnnnn Department of Ophthalmology, Heidelberg University, Heidelberg, Germany; ooooooooooooooooooooooooooooooooooooooooooooooooooooooooooooooooooooooooooo Amity Institute of Biotechnology, Centre for Medical Biotechnology, Amity University Uttar Pradesh, Noida, India; ppppppppppppppppppppppppppppppppppppppppppppppppppppppppppppppppppppppppppp Centre for Research and Development, Chandigarh University, Punjab, India; qqqqqqqqqqqqqqqqqqqqqqqqqqqqqqqqqqqqqqqqqqqqqqqqqqqqqqqqqqqqqqqqqqqqqqqqqqq Division of Research and Development, Lovely Professional University, Phagwara, India; rrrrrrrrrrrrrrrrrrrrrrrrrrrrrrrrrrrrrrrrrrrrrrrrrrrrrrrrrrrrrrrrrrrrrrrrrrr Infectious Disease Research Center, National Institute of Public Health, Cuernavaca, Mexico; sssssssssssssssssssssssssssssssssssssssssssssssssssssssssssssssssssssssssss Department of Health Policy, Management and Behavioural Sciences, Indian Institute of Public Health, Gandhinagar, India; ttttttttttttttttttttttttttttttttttttttttttttttttttttttttttttttttttttttttttt National Institute of Health Research and Development, Ministry of Health Indonesia, Jakarta, Indonesia; uuuuuuuuuuuuuuuuuuuuuuuuuuuuuuuuuuuuuuuuuuuuuuuuuuuuuuuuuuuuuuuuuuuuuuuuuuu Division of Ophthalmology & Visual Sciences, University of Nottingham, Nottingham, UK; vvvvvvvvvvvvvvvvvvvvvvvvvvvvvvvvvvvvvvvvvvvvvvvvvvvvvvvvvvvvvvvvvvvvvvvvvvv First Department of Ophthalmology, Aristotle University of Thessaloniki, Thessaloniki, Greece; wwwwwwwwwwwwwwwwwwwwwwwwwwwwwwwwwwwwwwwwwwwwwwwwwwwwwwwwwwwwwwwwwwwwwwwwwww Department of Neurology, University of Bern, Biel/Bienne, Switzerland; xxxxxxxxxxxxxxxxxxxxxxxxxxxxxxxxxxxxxxxxxxxxxxxxxxxxxxxxxxxxxxxxxxxxxxxxxxx Department of Neurology, University of Cyprus, Nicosia, Cyprus; yyyyyyyyyyyyyyyyyyyyyyyyyyyyyyyyyyyyyyyyyyyyyyyyyyyyyyyyyyyyyyyyyyyyyyyyyyy Department of Emergency Medicine, University of Thessaly, Larissa, Greece; zzzzzzzzzzzzzzzzzzzzzzzzzzzzzzzzzzzzzzzzzzzzzzzzzzzzzzzzzzzzzzzzzzzzzzzzzzz Department of Emergency Medicine, University of Bern, Bern, Switzerland; aaaaaaaaaaaaaaaaaaaaaaaaaaaaaaaaaaaaaaaaaaaaaaaaaaaaaaaaaaaaaaaaaaaaaaaaaaaa Department of Diabetes, Nutrition and Metabolic Diseases, Carol Davila University of Medicine and Pharmacy, Bucharest, Romania; bbbbbbbbbbbbbbbbbbbbbbbbbbbbbbbbbbbbbbbbbbbbbbbbbbbbbbbbbbbbbbbbbbbbbbbbbbbb Unit of Dermatology, IRCCS Ospedale San Raffaele, Milano, Italy; cccccccccccccccccccccccccccccccccccccccccccccccccccccccccccccccccccccccccccc University of Padua, Padua, Italy; dddddddddddddddddddddddddddddddddddddddddddddddddddddddddddddddddddddddddddd Department of Medicine and Surgery, University of Bologna, Bologna, Italy; eeeeeeeeeeeeeeeeeeeeeeeeeeeeeeeeeeeeeeeeeeeeeeeeeeeeeeeeeeeeeeeeeeeeeeeeeeee Medical University of Vienna, Vienna, Austria; ffffffffffffffffffffffffffffffffffffffffffffffffffffffffffffffffffffffffffff Department of Science and Mathematics, Deree-The American College of Greece, Athens, Greece; gggggggggggggggggggggggggggggggggggggggggggggggggggggggggggggggggggggggggggg Department of Biophysics, University of Athens, Athens, Greece; hhhhhhhhhhhhhhhhhhhhhhhhhhhhhhhhhhhhhhhhhhhhhhhhhhhhhhhhhhhhhhhhhhhhhhhhhhhh Cardiac Research Center, Tehran University of Medical Sciences, Tehran, Iran; iiiiiiiiiiiiiiiiiiiiiiiiiiiiiiiiiiiiiiiiiiiiiiiiiiiiiiiiiiiiiiiiiiiiiiiiiiii Division of Health Policy and Management, University of Minnesota, Minneapolis, MN, USA; jjjjjjjjjjjjjjjjjjjjjjjjjjjjjjjjjjjjjjjjjjjjjjjjjjjjjjjjjjjjjjjjjjjjjjjjjjjj Department of Sociology, Anthropology, and Public Health, University of Maryland, Baltimore County, Baltimore, MD, USA; kkkkkkkkkkkkkkkkkkkkkkkkkkkkkkkkkkkkkkkkkkkkkkkkkkkkkkkkkkkkkkkkkkkkkkkkkkkk Department of Biomedical Data Science, Stanford University, Stanford, CA, USA; llllllllllllllllllllllllllllllllllllllllllllllllllllllllllllllllllllllllllll Department of Psychiatry, All India Institute of Medical Sciences, Bhubaneswar, India; mmmmmmmmmmmmmmmmmmmmmmmmmmmmmmmmmmmmmmmmmmmmmmmmmmmmmmmmmmmmmmmmmmmmmmmmmmmm Department of Medical Sciences, University of Torino, Torino, Italy; nnnnnnnnnnnnnnnnnnnnnnnnnnnnnnnnnnnnnnnnnnnnnnnnnnnnnnnnnnnnnnnnnnnnnnnnnnnn Department of Imaging, AOU Città della Salute e della Scienza di Torino (AOU City of Health and Science of Turin), Torino, Italy; oooooooooooooooooooooooooooooooooooooooooooooooooooooooooooooooooooooooooooo Faculty of Medicine and Health, University of Leeds, Leeds, UK; pppppppppppppppppppppppppppppppppppppppppppppppppppppppppppppppppppppppppppp Marwadi University Research and Development Cell, Marwadi University, Rajkot, India; qqqqqqqqqqqqqqqqqqqqqqqqqqqqqqqqqqqqqqqqqqqqqqqqqqqqqqqqqqqqqqqqqqqqqqqqqqqq Department of Cardiovascular Medicine, University of Tennessee, Nashville, TN, USA; rrrrrrrrrrrrrrrrrrrrrrrrrrrrrrrrrrrrrrrrrrrrrrrrrrrrrrrrrrrrrrrrrrrrrrrrrrrr Department of Research and Training, Population Council Institute, New Delhi, India; ssssssssssssssssssssssssssssssssssssssssssssssssssssssssssssssssssssssssssss Mahatma Gandhi Institute of Medical Sciences, Sevagram, Maharashtra University of Health Sciences, Wardha, India; tttttttttttttttttttttttttttttttttttttttttttttttttttttttttttttttttttttttttttt College of Dental Medicine, Roseman University of Health Sciences, South Jordan, UT, USA; uuuuuuuuuuuuuuuuuuuuuuuuuuuuuuuuuuuuuuuuuuuuuuuuuuuuuuuuuuuuuuuuuuuuuuuuuuuu Second Propedeutic Department of Internal Medicine, Aristotle University of Thessaloniki, Thessaloniki, Greece; vvvvvvvvvvvvvvvvvvvvvvvvvvvvvvvvvvvvvvvvvvvvvvvvvvvvvvvvvvvvvvvvvvvvvvvvvvvv Department of Human Anatomy, All India Institute of Medical Sciences, Bathinda, India; wwwwwwwwwwwwwwwwwwwwwwwwwwwwwwwwwwwwwwwwwwwwwwwwwwwwwwwwwwwwwwwwwwwwwwwwwwww Department of Internal Medicine, Advent Health, Palm Coast, FL, USA; xxxxxxxxxxxxxxxxxxxxxxxxxxxxxxxxxxxxxxxxxxxxxxxxxxxxxxxxxxxxxxxxxxxxxxxxxxxx Department of Hospital Medicine, Sound Physicians, Palm Coast, FL, USA; yyyyyyyyyyyyyyyyyyyyyyyyyyyyyyyyyyyyyyyyyyyyyyyyyyyyyyyyyyyyyyyyyyyyyyyyyyyy Department of Genetics, Yale University, New Haven, CT, USA; zzzzzzzzzzzzzzzzzzzzzzzzzzzzzzzzzzzzzzzzzzzzzzzzzzzzzzzzzzzzzzzzzzzzzzzzzzzz Department of Interventional Cardiology, Cedars Sinai Medical Center, Los Angeles, CA, USA; aaaaaaaaaaaaaaaaaaaaaaaaaaaaaaaaaaaaaaaaaaaaaaaaaaaaaaaaaaaaaaaaaaaaaaaaaaaaa Physiology Research Center, Iran University of Medical Sciences, Tehran, Iran; bbbbbbbbbbbbbbbbbbbbbbbbbbbbbbbbbbbbbbbbbbbbbbbbbbbbbbbbbbbbbbbbbbbbbbbbbbbbb Department of Physiology, Iran University of Medical Sciences, Tehran, Iran; ccccccccccccccccccccccccccccccccccccccccccccccccccccccccccccccccccccccccccccc Department of Medical Statistics, London School of Hygiene & Tropical Medicine, London, UK; ddddddddddddddddddddddddddddddddddddddddddddddddddddddddddddddddddddddddddddd IRCCS Fondazione Don Carlo Gnocchi, Milan, Italy; eeeeeeeeeeeeeeeeeeeeeeeeeeeeeeeeeeeeeeeeeeeeeeeeeeeeeeeeeeeeeeeeeeeeeeeeeeeee Department of Clinical and Experimental Sciences, University of Brescia, Brescia, Italy; fffffffffffffffffffffffffffffffffffffffffffffffffffffffffffffffffffffffffffff Center for Research and Innovation, Ateneo De Manila University, Pasig City, Philippines; ggggggggggggggggggggggggggggggggggggggggggggggggggggggggggggggggggggggggggggg Australian Institute of Health Innovation, Macquarie University, Sydney, NSW, Australia; hhhhhhhhhhhhhhhhhhhhhhhhhhhhhhhhhhhhhhhhhhhhhhhhhhhhhhhhhhhhhhhhhhhhhhhhhhhhh Research Institute for Medicines, Universidade de Lisboa (University of Lisbon), Lisbon, Portugal; iiiiiiiiiiiiiiiiiiiiiiiiiiiiiiiiiiiiiiiiiiiiiiiiiiiiiiiiiiiiiiiiiiiiiiiiiiiii School of Population Health, Curtin University, Bentley, WA, Australia; jjjjjjjjjjjjjjjjjjjjjjjjjjjjjjjjjjjjjjjjjjjjjjjjjjjjjjjjjjjjjjjjjjjjjjjjjjjjj Centre for Fertility and Health, Norwegian Institute of Public Health, Oslo, Norway; kkkkkkkkkkkkkkkkkkkkkkkkkkkkkkkkkkkkkkkkkkkkkkkkkkkkkkkkkkkkkkkkkkkkkkkkkkkkk Kirby Institute, University of New South Wales, Sydney, NSW, Australia; lllllllllllllllllllllllllllllllllllllllllllllllllllllllllllllllllllllllllllll Social and Economic Survey Research Institute (SESRI), Qatar University, Doha, Qatar; mmmmmmmmmmmmmmmmmmmmmmmmmmmmmmmmmmmmmmmmmmmmmmmmmmmmmmmmmmmmmmmmmmmmmmmmmmmmm Mario Negri Institute for Pharmacological Research, Bergamo, Italy; nnnnnnnnnnnnnnnnnnnnnnnnnnnnnnnnnnnnnnnnnnnnnnnnnnnnnnnnnnnnnnnnnnnnnnnnnnnnn Department of Food, Environmental and Nutritional Sciences, Università degli Studi di Milano (University of Milan), Milano, Italy; ooooooooooooooooooooooooooooooooooooooooooooooooooooooooooooooooooooooooooooo School of Optometry and Vision Science, University of New South Wales, Sydney, NSW, Australia; ppppppppppppppppppppppppppppppppppppppppppppppppppppppppppppppppppppppppppppp Department of Biochemistry and Pharmacology, Uzhhorod National University, Uzhhorod, Ukraine; qqqqqqqqqqqqqqqqqqqqqqqqqqqqqqqqqqqqqqqqqqqqqqqqqqqqqqqqqqqqqqqqqqqqqqqqqqqqq Mathematical and Computer Sciences, University of Medical Sciences, Ondo, Ondo, Nigeria; rrrrrrrrrrrrrrrrrrrrrrrrrrrrrrrrrrrrrrrrrrrrrrrrrrrrrrrrrrrrrrrrrrrrrrrrrrrrr Facultad de Medicina (Faculty of Medicine), Universidad Diego Portales (Diego Portales University), Santiago, Chile; sssssssssssssssssssssssssssssssssssssssssssssssssssssssssssssssssssssssssssss School of Cardiovascular and Metabolic Health, University of Glasgow, Glasgow, UK; ttttttttttttttttttttttttttttttttttttttttttttttttttttttttttttttttttttttttttttt School of Medicine, University of Virginia, Charlottesville, VA, USA; uuuuuuuuuuuuuuuuuuuuuuuuuuuuuuuuuuuuuuuuuuuuuuuuuuuuuuuuuuuuuuuuuuuuuuuuuuuuu Department of Internal Medicine, University of Arizona, Tucson, AZ, USA; vvvvvvvvvvvvvvvvvvvvvvvvvvvvvvvvvvvvvvvvvvvvvvvvvvvvvvvvvvvvvvvvvvvvvvvvvvvvv Department of Cardiovascular Medicine, Mayo Clinic, Rochester, MN, USA; wwwwwwwwwwwwwwwwwwwwwwwwwwwwwwwwwwwwwwwwwwwwwwwwwwwwwwwwwwwwwwwwwwwwwwwwwwwww Department of Internal Medicine, Weiss Memorial Hospital, Chicago, IL, USA; xxxxxxxxxxxxxxxxxxxxxxxxxxxxxxxxxxxxxxxxxxxxxxxxxxxxxxxxxxxxxxxxxxxxxxxxxxxxx College of Health Sciences, VinUniversity, Hanoi, Viet Nam; yyyyyyyyyyyyyyyyyyyyyyyyyyyyyyyyyyyyyyyyyyyyyyyyyyyyyyyyyyyyyyyyyyyyyyyyyyyyy Research Advancement Consortium in Health, Hanoi, Viet Nam; zzzzzzzzzzzzzzzzzzzzzzzzzzzzzzzzzzzzzzzzzzzzzzzzzzzzzzzzzzzzzzzzzzzzzzzzzzzzz School of Pharmacy, University of Nizwa, Nizwa, Oman; aaaaaaaaaaaaaaaaaaaaaaaaaaaaaaaaaaaaaaaaaaaaaaaaaaaaaaaaaaaaaaaaaaaaaaaaaaaaaa Shanghai Mental Health Center, Shanghai Jiao Tong University, Shanghai, China; bbbbbbbbbbbbbbbbbbbbbbbbbbbbbbbbbbbbbbbbbbbbbbbbbbbbbbbbbbbbbbbbbbbbbbbbbbbbbb Departments of Psychiatry and Epidemiology, Columbia University, New York, NY, USA; cccccccccccccccccccccccccccccccccccccccccccccccccccccccccccccccccccccccccccccc International Center of Medical Sciences Research, Islamabad, Pakistan; dddddddddddddddddddddddddddddddddddddddddddddddddddddddddddddddddddddddddddddd Riphah International University, Islamabad, Pakistan; eeeeeeeeeeeeeeeeeeeeeeeeeeeeeeeeeeeeeeeeeeeeeeeeeeeeeeeeeeeeeeeeeeeeeeeeeeeeee Department of Promoting Health, Maternal-Infant, Excellence and Internal and Specialized Medicine (PROMISE) G. D'Alessandro, University of Palermo, Palermo, Italy; ffffffffffffffffffffffffffffffffffffffffffffffffffffffffffffffffffffffffffffff Department of Bioinformatics, Tehran University of Medical Sciences, Tehran, Iran; gggggggggggggggggggggggggggggggggggggggggggggggggggggggggggggggggggggggggggggg Molecular Medicine Research Center, Tabriz University of Medical Sciences, Tabriz, Iran; hhhhhhhhhhhhhhhhhhhhhhhhhhhhhhhhhhhhhhhhhhhhhhhhhhhhhhhhhhhhhhhhhhhhhhhhhhhhhh Air and Climate Unit, European Commission, Ispra, Italy; iiiiiiiiiiiiiiiiiiiiiiiiiiiiiiiiiiiiiiiiiiiiiiiiiiiiiiiiiiiiiiiiiiiiiiiiiiiiii Mental Health Research Institute, Tomsk National Research Medical Center, Tomsk, Russia; jjjjjjjjjjjjjjjjjjjjjjjjjjjjjjjjjjjjjjjjjjjjjjjjjjjjjjjjjjjjjjjjjjjjjjjjjjjjjj Siberian State Medical University, Tomsk, Russia; kkkkkkkkkkkkkkkkkkkkkkkkkkkkkkkkkkkkkkkkkkkkkkkkkkkkkkkkkkkkkkkkkkkkkkkkkkkkkk Department of Dermatology, College of Medicine and Sagore Dutta Hospital, Kolkata, India; llllllllllllllllllllllllllllllllllllllllllllllllllllllllllllllllllllllllllllll College of Health Sciences (CHS), VinUniversity, Hanoi, Viet Nam; mmmmmmmmmmmmmmmmmmmmmmmmmmmmmmmmmmmmmmmmmmmmmmmmmmmmmmmmmmmmmmmmmmmmmmmmmmmmmm Department of Data Management and Analysis, The INCLEN Trust International, New Delhi, India; nnnnnnnnnnnnnnnnnnnnnnnnnnnnnnnnnnnnnnnnnnnnnnnnnnnnnnnnnnnnnnnnnnnnnnnnnnnnnn Department of Medical and Surgical Oncology, Miami Cancer Institute, Miami, FL, USA; oooooooooooooooooooooooooooooooooooooooooooooooooooooooooooooooooooooooooooooo Department of Ortopedics and Traumatology, University of Tampere, Tampere, Finland; pppppppppppppppppppppppppppppppppppppppppppppppppppppppppppppppppppppppppppppp Management Department, Bucharest University of Economic Studies, Bucharest, Romania; qqqqqqqqqqqqqqqqqqqqqqqqqqqqqqqqqqqqqqqqqqqqqqqqqqqqqqqqqqqqqqqqqqqqqqqqqqqqqq Academy of Romanian Scientists, Bucharest, Romania; rrrrrrrrrrrrrrrrrrrrrrrrrrrrrrrrrrrrrrrrrrrrrrrrrrrrrrrrrrrrrrrrrrrrrrrrrrrrrr Department of Internal Medicine, University of Novi Sad, Novi Sad, Serbia; ssssssssssssssssssssssssssssssssssssssssssssssssssssssssssssssssssssssssssssss Clinic for Endocrinology, Diabetes and Metabolic Disorders, Clinical Center of Vojvodina, Novi Sad, Serbia; tttttttttttttttttttttttttttttttttttttttttttttttttttttttttttttttttttttttttttttt Center of Excellence in Precision Medicine and Digital Health, Chulalongkorn University, Bangkok, Thailand; uuuuuuuuuuuuuuuuuuuuuuuuuuuuuuuuuuuuuuuuuuuuuuuuuuuuuuuuuuuuuuuuuuuuuuuuuuuuuu Department of Occupational Health and Safety Engineering, Shiraz University of Medical Sciences, Shiraz, Iran; vvvvvvvvvvvvvvvvvvvvvvvvvvvvvvvvvvvvvvvvvvvvvvvvvvvvvvvvvvvvvvvvvvvvvvvvvvvvvv Non-communicable Diseases Research Center, Bam University of Medical Sciences, Bam, Iran; wwwwwwwwwwwwwwwwwwwwwwwwwwwwwwwwwwwwwwwwwwwwwwwwwwwwwwwwwwwwwwwwwwwwwwwwwwwwww Centro de Investigaciones Clinicas (Clinical Research Center), Fundación Valle del Lili (Valle del Lili Foundation), Cali, Colombia; xxxxxxxxxxxxxxxxxxxxxxxxxxxxxxxxxxxxxxxxxxxxxxxxxxxxxxxxxxxxxxxxxxxxxxxxxxxxxx Centro PROESA, Universidad ICESI, Cali, Colombia; yyyyyyyyyyyyyyyyyyyyyyyyyyyyyyyyyyyyyyyyyyyyyyyyyyyyyyyyyyyyyyyyyyyyyyyyyyyyyy Department of Humanities and Social Sciences, National Institute of Technology Rourkela, Rourkela, India; zzzzzzzzzzzzzzzzzzzzzzzzzzzzzzzzzzzzzzzzzzzzzzzzzzzzzzzzzzzzzzzzzzzzzzzzzzzzzz Department of Fundamental Nursing, Universitas Airlangga (Airlangga University), Surabaya, Indonesia; aaaaaaaaaaaaaaaaaaaaaaaaaaaaaaaaaaaaaaaaaaaaaaaaaaaaaaaaaaaaaaaaaaaaaaaaaaaaaaa Research Center in Advancing Community Healthcare, Surabaya, Indonesia; bbbbbbbbbbbbbbbbbbbbbbbbbbbbbbbbbbbbbbbbbbbbbbbbbbbbbbbbbbbbbbbbbbbbbbbbbbbbbbb Department of Biochemistry, JSS Academy of Higher Education and Research, Mysuru, India; ccccccccccccccccccccccccccccccccccccccccccccccccccccccccccccccccccccccccccccccc Centre for Dental Education and Research, All India Institute of Medical Sciences, New Delhi, India; ddddddddddddddddddddddddddddddddddddddddddddddddddddddddddddddddddddddddddddddd Department of Biostatistics, Epidemiology, and Informatics, University of Pennsylvania, Philadelphia, PA, USA; eeeeeeeeeeeeeeeeeeeeeeeeeeeeeeeeeeeeeeeeeeeeeeeeeeeeeeeeeeeeeeeeeeeeeeeeeeeeeee Department of Medical Instrumentation Techniques Engineering, Al-Rafidain University College, Baghdad, Iraq; fffffffffffffffffffffffffffffffffffffffffffffffffffffffffffffffffffffffffffffff Department of Cybersecurity, Kyiv National University of Construction and Architecture, Kyiv, Ukraine; ggggggggggggggggggggggggggggggggggggggggggggggggggggggggggggggggggggggggggggggg Department of Neonatology, Case Western Reserve University, Akron, OH, USA; hhhhhhhhhhhhhhhhhhhhhhhhhhhhhhhhhhhhhhhhhhhhhhhhhhhhhhhhhhhhhhhhhhhhhhhhhhhhhhh Rory Meyers College of Nursing, New York University, New York, NY, USA; iiiiiiiiiiiiiiiiiiiiiiiiiiiiiiiiiiiiiiiiiiiiiiiiiiiiiiiiiiiiiiiiiiiiiiiiiiiiiii Department of Epidemiology, Shandong University, Jinan, China; jjjjjjjjjjjjjjjjjjjjjjjjjjjjjjjjjjjjjjjjjjjjjjjjjjjjjjjjjjjjjjjjjjjjjjjjjjjjjjj Department of Biomaterials, Saveetha University, Chennai, India; kkkkkkkkkkkkkkkkkkkkkkkkkkkkkkkkkkkkkkkkkkkkkkkkkkkkkkkkkkkkkkkkkkkkkkkkkkkkkkk Department of Health Information Technology and Management, Shahid Beheshti University of Medical Sciences, Tehran, Iran; lllllllllllllllllllllllllllllllllllllllllllllllllllllllllllllllllllllllllllllll Research Center for Public Health and Nutrition, National Research and Innovation Agency (BRIN), Jakarta, Indonesia; mmmmmmmmmmmmmmmmmmmmmmmmmmmmmmmmmmmmmmmmmmmmmmmmmmmmmmmmmmmmmmmmmmmmmmmmmmmmmmm Oman Dental College, Oman; nnnnnnnnnnnnnnnnnnnnnnnnnnnnnnnnnnnnnnnnnnnnnnnnnnnnnnnnnnnnnnnnnnnnnnnnnnnnnnn Department of Medical Oncology, Cancer Institute (W.I.A), Chennai, India; ooooooooooooooooooooooooooooooooooooooooooooooooooooooooooooooooooooooooooooooo Department of Nephrology and Urology, Shahid Beheshti University of Medical Sciences, Tehran, Iran; ppppppppppppppppppppppppppppppppppppppppppppppppppppppppppppppppppppppppppppppp Department of Epidemiology and Biostatistics, Shahrekord University of Medical Sciences, Shahrekord, Iran; qqqqqqqqqqqqqqqqqqqqqqqqqqqqqqqqqqqqqqqqqqqqqqqqqqqqqqqqqqqqqqqqqqqqqqqqqqqqqqq Deputy of Health, Hamadan University of Medical Sciences, Hamadan, Iran; rrrrrrrrrrrrrrrrrrrrrrrrrrrrrrrrrrrrrrrrrrrrrrrrrrrrrrrrrrrrrrrrrrrrrrrrrrrrrrr Health Research Institute (HRI), National Institutes of Health, Islamabad, Pakistan; sssssssssssssssssssssssssssssssssssssssssssssssssssssssssssssssssssssssssssssss Department of Epidemiology, National Institute of Mental Health and Neurosciences, Bengaluru, India; ttttttttttttttttttttttttttttttttttttttttttttttttttttttttttttttttttttttttttttttt Osh State University, Osh, Kyrgyzstan; uuuuuuuuuuuuuuuuuuuuuuuuuuuuuuuuuuuuuuuuuuuuuuuuuuuuuuuuuuuuuuuuuuuuuuuuuuuuuuu Director of Central Asia Research Collaboration Group, Asfendiyarov Kazakh National Medical University, Almaty, Kazakhstan; vvvvvvvvvvvvvvvvvvvvvvvvvvvvvvvvvvvvvvvvvvvvvvvvvvvvvvvvvvvvvvvvvvvvvvvvvvvvvvv Department of Environmental Health Engineering, Torbat Heydariyeh University of Medical Sciences, Torbat Heydariyeh, Iran; wwwwwwwwwwwwwwwwwwwwwwwwwwwwwwwwwwwwwwwwwwwwwwwwwwwwwwwwwwwwwwwwwwwwwwwwwwwwwww Health Science Research Centre, Torbat Heydariyeh University of Medical Sciences, Torbat Heydariyeh, Iran; xxxxxxxxxxxxxxxxxxxxxxxxxxxxxxxxxxxxxxxxxxxxxxxxxxxxxxxxxxxxxxxxxxxxxxxxxxxxxxx Faculty of Health Sciences, Qaiwan International University, Sulaymaniyah, Iraq; yyyyyyyyyyyyyyyyyyyyyyyyyyyyyyyyyyyyyyyyyyyyyyyyyyyyyyyyyyyyyyyyyyyyyyyyyyyyyyy Department of Epidemiology, Institute of Epidemiology, Disease Control and Research (IEDCR), Dhaka, Bangladesh; zzzzzzzzzzzzzzzzzzzzzzzzzzzzzzzzzzzzzzzzzzzzzzzzzzzzzzzzzzzzzzzzzzzzzzzzzzzzzzz Department of Pathobiology and Population Sciences (PPS), Royal Veterinary College (RVC), London, UK; aaaaaaaaaaaaaaaaaaaaaaaaaaaaaaaaaaaaaaaaaaaaaaaaaaaaaaaaaaaaaaaaaaaaaaaaaaaaaaaa College of Medicine and Health Sciences, National University of Science and Technology, Sohar, Oman; bbbbbbbbbbbbbbbbbbbbbbbbbbbbbbbbbbbbbbbbbbbbbbbbbbbbbbbbbbbbbbbbbbbbbbbbbbbbbbbb Department of Biostatistics, National Institute of Preventive and Social Medicine, Dhaka, Bangladesh; cccccccccccccccccccccccccccccccccccccccccccccccccccccccccccccccccccccccccccccccc Future Technology Research Center, National Yunlin University of Science and Technology, Yunlin, Taiwan; dddddddddddddddddddddddddddddddddddddddddddddddddddddddddddddddddddddddddddddddd Health Service Research and Quality of Life Center (CEReSS), Aix-Marseille University, Marseille, France; eeeeeeeeeeeeeeeeeeeeeeeeeeeeeeeeeeeeeeeeeeeeeeeeeeeeeeeeeeeeeeeeeeeeeeeeeeeeeeee Faculty of Medicine, University of Setif Algeria, Setif, Algeria; ffffffffffffffffffffffffffffffffffffffffffffffffffffffffffffffffffffffffffffffff LIRSSEI Research Lab, University of Setif Algeria, Setif, Algeria; gggggggggggggggggggggggggggggggggggggggggggggggggggggggggggggggggggggggggggggggg Division of Gynecology and Human Reproduction Physiopathology, IRCCS Azienda Ospedaliero-Universitaria di Bologna, Bologna, Italy; hhhhhhhhhhhhhhhhhhhhhhhhhhhhhhhhhhhhhhhhhhhhhhhhhhhhhhhhhhhhhhhhhhhhhhhhhhhhhhhh Department of Medical, Surgical and Experimental Sciences, University of Sassari, Sassari, Italy; iiiiiiiiiiiiiiiiiiiiiiiiiiiiiiiiiiiiiiiiiiiiiiiiiiiiiiiiiiiiiiiiiiiiiiiiiiiiiiii Gynecology and Breast Care Center, Mater Olbia Hospital, Olbia, Italy; jjjjjjjjjjjjjjjjjjjjjjjjjjjjjjjjjjjjjjjjjjjjjjjjjjjjjjjjjjjjjjjjjjjjjjjjjjjjjjjj Dr. Rajendra Prasad Government Medical College, Tanda, Kangra, India; kkkkkkkkkkkkkkkkkkkkkkkkkkkkkkkkkkkkkkkkkkkkkkkkkkkkkkkkkkkkkkkkkkkkkkkkkkkkkkkk Department of Cardiology, Dow University of Health Sciences, Karachi, Pakistan; llllllllllllllllllllllllllllllllllllllllllllllllllllllllllllllllllllllllllllllll Department of Infectious Diseases and Tropical Medicine, Tehran University of Medical Sciences, Tehran, Iran; mmmmmmmmmmmmmmmmmmmmmmmmmmmmmmmmmmmmmmmmmmmmmmmmmmmmmmmmmmmmmmmmmmmmmmmmmmmmmmmm Emergency Medicine Department, Sri Manakula Vinayagar Medical College and Hospital, Puducherry, India; nnnnnnnnnnnnnnnnnnnnnnnnnnnnnnnnnnnnnnnnnnnnnnnnnnnnnnnnnnnnnnnnnnnnnnnnnnnnnnnn Department of Cardiovascular Medicine, Cleveland Clinic, Cleveland, OH, USA; oooooooooooooooooooooooooooooooooooooooooooooooooooooooooooooooooooooooooooooooo Centre for Chronic Disease Control, New Delhi, India; pppppppppppppppppppppppppppppppppppppppppppppppppppppppppppppppppppppppppppppppp Department of Cardiology, Mansoura University, Mansoura, Egypt; qqqqqqqqqqqqqqqqqqqqqqqqqqqqqqqqqqqqqqqqqqqqqqqqqqqqqqqqqqqqqqqqqqqqqqqqqqqqqqqq Department of Population Health, King Saud bin Abdulaziz University for Health Sciences, Jeddah, Saudi Arabia; rrrrrrrrrrrrrrrrrrrrrrrrrrrrrrrrrrrrrrrrrrrrrrrrrrrrrrrrrrrrrrrrrrrrrrrrrrrrrrrr Department of Midwifery, Ministry of Health of the Republic of Indonesia, Palu, Indonesia; ssssssssssssssssssssssssssssssssssssssssssssssssssssssssssssssssssssssssssssssss Department of Anatomy, Govt. Siddhartha Medical College, Vijayawada, India; tttttttttttttttttttttttttttttttttttttttttttttttttttttttttttttttttttttttttttttttt Department of Radiology, Stanford University, Stanford, CA, USA; uuuuuuuuuuuuuuuuuuuuuuuuuuuuuuuuuuuuuuuuuuuuuuuuuuuuuuuuuuuuuuuuuuuuuuuuuuuuuuuu Department of Biological Science and Bioengineering, Inha University, Incheon, South Korea; vvvvvvvvvvvvvvvvvvvvvvvvvvvvvvvvvvvvvvvvvvvvvvvvvvvvvvvvvvvvvvvvvvvvvvvvvvvvvvvv The Navarra Medical Research Institute (IdiSNA), Universidad Pública de Navarra (Public University of Navarra), Pamplona, Spain; wwwwwwwwwwwwwwwwwwwwwwwwwwwwwwwwwwwwwwwwwwwwwwwwwwwwwwwwwwwwwwwwwwwwwwwwwwwwwwww South Asian Institute for Social Transformation (SAIST), Dhaka, Bangladesh; xxxxxxxxxxxxxxxxxxxxxxxxxxxxxxxxxxxxxxxxxxxxxxxxxxxxxxxxxxxxxxxxxxxxxxxxxxxxxxxx Department of Epidemiology, Biostatistics and Occupational Health, McGill University, Montreal, QC, Canada; yyyyyyyyyyyyyyyyyyyyyyyyyyyyyyyyyyyyyyyyyyyyyyyyyyyyyyyyyyyyyyyyyyyyyyyyyyyyyyyy Department of Community Medicine, NKP Salve Institute of Medical Sciences and Research Centre, Nagpur, India; zzzzzzzzzzzzzzzzzzzzzzzzzzzzzzzzzzzzzzzzzzzzzzzzzzzzzzzzzzzzzzzzzzzzzzzzzzzzzzzz Department of Dentistry, All India Institute of Medical Sciences, Bathinda, India; aaaaaaaaaaaaaaaaaaaaaaaaaaaaaaaaaaaaaaaaaaaaaaaaaaaaaaaaaaaaaaaaaaaaaaaaaaaaaaaaa Department of Research, Eastern Scientific LLC, Richmond, KY, USA; bbbbbbbbbbbbbbbbbbbbbbbbbbbbbbbbbbbbbbbbbbbbbbbbbbbbbbbbbbbbbbbbbbbbbbbbbbbbbbbbb Planetary Health Research Centre (PHRC), Kathmandu, Nepal; ccccccccccccccccccccccccccccccccccccccccccccccccccccccccccccccccccccccccccccccccc Centre for Clinical Pharmacology, University of Defence in Belgrade, Belgrade, Serbia; ddddddddddddddddddddddddddddddddddddddddddddddddddddddddddddddddddddddddddddddddd Centre for Clinical Pharmacology, Medical College of Georgia at Augusta University, Belgrade, Serbia; eeeeeeeeeeeeeeeeeeeeeeeeeeeeeeeeeeeeeeeeeeeeeeeeeeeeeeeeeeeeeeeeeeeeeeeeeeeeeeeee Department of Forensic Medicine and Toxicology, Jagadguru Sri Shivarathreeswara University, Mysore, India; fffffffffffffffffffffffffffffffffffffffffffffffffffffffffffffffffffffffffffffffff Department of Nursing and Midwifery, Golestan University of Medical Sciences, Gorgan, Iran; ggggggggggggggggggggggggggggggggggggggggggggggggggggggggggggggggggggggggggggggggg Department of Oral Medicine and Radiology, NITTE (Deemed to be University), Mangalore, India; hhhhhhhhhhhhhhhhhhhhhhhhhhhhhhhhhhhhhhhhhhhhhhhhhhhhhhhhhhhhhhhhhhhhhhhhhhhhhhhhh Kasturba Medical College Mangalore, Manipal Academy of Higher Education, Manipal, India; iiiiiiiiiiiiiiiiiiiiiiiiiiiiiiiiiiiiiiiiiiiiiiiiiiiiiiiiiiiiiiiiiiiiiiiiiiiiiiiii Institute of Collective Health, Federal University of Bahia, Salvador, Brazil; jjjjjjjjjjjjjjjjjjjjjjjjjjjjjjjjjjjjjjjjjjjjjjjjjjjjjjjjjjjjjjjjjjjjjjjjjjjjjjjjj Barcelona Institute for Global Health, Barcelona, Spain; kkkkkkkkkkkkkkkkkkkkkkkkkkkkkkkkkkkkkkkkkkkkkkkkkkkkkkkkkkkkkkkkkkkkkkkkkkkkkkkkk Brigham and Women's Hospital, Harvard Medical School, Boston, MA, USA; lllllllllllllllllllllllllllllllllllllllllllllllllllllllllllllllllllllllllllllllll Iranian Research Center on Aging, University of Social Welfare and Rehabilitation Sciences, Tehran, Iran; mmmmmmmmmmmmmmmmmmmmmmmmmmmmmmmmmmmmmmmmmmmmmmmmmmmmmmmmmmmmmmmmmmmmmmmmmmmmmmmmm Unit for Public Health Science, University of Gävle, Sweden, Stockholm, Sweden; nnnnnnnnnnnnnnnnnnnnnnnnnnnnnnnnnnnnnnnnnnnnnnnnnnnnnnnnnnnnnnnnnnnnnnnnnnnnnnnnn Epidemiology and Biostatistics, Kurdistan University of Medical Sciences, Sanandaj, Iran; ooooooooooooooooooooooooooooooooooooooooooooooooooooooooooooooooooooooooooooooooo Department of Immunology, Shahid Beheshti University of Medical Sciences, Tehran, Iran; ppppppppppppppppppppppppppppppppppppppppppppppppppppppppppppppppppppppppppppppppp Department of Geography, Soran University, Soran, Iraq; qqqqqqqqqqqqqqqqqqqqqqqqqqqqqqqqqqqqqqqqqqqqqqqqqqqqqqqqqqqqqqqqqqqqqqqqqqqqqqqqq Department of Family Medicine, Rajarata University of Sri Lanka, Anuradhapura, Sri Lanka; rrrrrrrrrrrrrrrrrrrrrrrrrrrrrrrrrrrrrrrrrrrrrrrrrrrrrrrrrrrrrrrrrrrrrrrrrrrrrrrrr University of Swabi, Swabi, Pakistan; sssssssssssssssssssssssssssssssssssssssssssssssssssssssssssssssssssssssssssssssss Department of Global Health Policy, University of Tokyo, Tokyo, Japan; ttttttttttttttttttttttttttttttttttttttttttttttttttttttttttttttttttttttttttttttttt Department of Neurosurgery, Helsinki University Hospital, Helsinki, Finland; uuuuuuuuuuuuuuuuuuuuuuuuuuuuuuuuuuuuuuuuuuuuuuuuuuuuuuuuuuuuuuuuuuuuuuuuuuuuuuuuu The National Institute for Stroke and Applied Neurosciences, Auckland University of Technology, Auckland, New Zealand; vvvvvvvvvvvvvvvvvvvvvvvvvvvvvvvvvvvvvvvvvvvvvvvvvvvvvvvvvvvvvvvvvvvvvvvvvvvvvvvvv Department of Health Services Management, Shiraz University of Medical Sciences, Shiraz, Iran; wwwwwwwwwwwwwwwwwwwwwwwwwwwwwwwwwwwwwwwwwwwwwwwwwwwwwwwwwwwwwwwwwwwwwwwwwwwwwwwww Inovus Medical, St Helens, UK; xxxxxxxxxxxxxxxxxxxxxxxxxxxxxxxxxxxxxxxxxxxxxxxxxxxxxxxxxxxxxxxxxxxxxxxxxxxxxxxxx School of Health, Medical and Applied Sciences, CQ University, Sydney, NSW, Australia; yyyyyyyyyyyyyyyyyyyyyyyyyyyyyyyyyyyyyyyyyyyyyyyyyyyyyyyyyyyyyyyyyyyyyyyyyyyyyyyyy Department of Computer Science, Boston University, Boston, MA, USA; zzzzzzzzzzzzzzzzzzzzzzzzzzzzzzzzzzzzzzzzzzzzzzzzzzzzzzzzzzzzzzzzzzzzzzzzzzzzzzzzz Department of Hematology, North Khorasan University of Medical Sciences, Bojnurd, Iran; aaaaaaaaaaaaaaaaaaaaaaaaaaaaaaaaaaaaaaaaaaaaaaaaaaaaaaaaaaaaaaaaaaaaaaaaaaaaaaaaaa Department of Hematology, Tarbiat Modares University, Tehran, Iran; bbbbbbbbbbbbbbbbbbbbbbbbbbbbbbbbbbbbbbbbbbbbbbbbbbbbbbbbbbbbbbbbbbbbbbbbbbbbbbbbbb Escuela de Posgrado, Universidad San Ignacio de Loyola, Lima, Peru; cccccccccccccccccccccccccccccccccccccccccccccccccccccccccccccccccccccccccccccccccc Department of Biological Sciences, King Abdulaziz University, Jeddah, Egypt; dddddddddddddddddddddddddddddddddddddddddddddddddddddddddddddddddddddddddddddddddd Department of Protein Research, Research and Academic Institution, Alexandria, Egypt; eeeeeeeeeeeeeeeeeeeeeeeeeeeeeeeeeeeeeeeeeeeeeeeeeeeeeeeeeeeeeeeeeeeeeeeeeeeeeeeeee Department of Internal Medicine, University of Alabama at Birmingham, Birmingham, AL, USA; ffffffffffffffffffffffffffffffffffffffffffffffffffffffffffffffffffffffffffffffffff Department of Internal Medicine, King Edward Medical University, Lahore, Pakistan; gggggggggggggggggggggggggggggggggggggggggggggggggggggggggggggggggggggggggggggggggg Human Capability Building, Saudi Authority for Data and Artificial Intelligence, Riyadh, Saudi Arabia; hhhhhhhhhhhhhhhhhhhhhhhhhhhhhhhhhhhhhhhhhhhhhhhhhhhhhhhhhhhhhhhhhhhhhhhhhhhhhhhhhh Institute of Center and Research Studies, Umm Al-Qura University, Makkah, Saudi Arabia; iiiiiiiiiiiiiiiiiiiiiiiiiiiiiiiiiiiiiiiiiiiiiiiiiiiiiiiiiiiiiiiiiiiiiiiiiiiiiiiiii The School of Pharmaceutical Sciences, University of Science Malaysia, Penang, Malaysia; jjjjjjjjjjjjjjjjjjjjjjjjjjjjjjjjjjjjjjjjjjjjjjjjjjjjjjjjjjjjjjjjjjjjjjjjjjjjjjjjjj Melbourne School of Population and Global Health, University of Melbourne, Melbourne, VIC, Australia; kkkkkkkkkkkkkkkkkkkkkkkkkkkkkkkkkkkkkkkkkkkkkkkkkkkkkkkkkkkkkkkkkkkkkkkkkkkkkkkkkk Department for Epidemiology and Biostatistics, National Institute for Health Development, Tallinn, Estonia; llllllllllllllllllllllllllllllllllllllllllllllllllllllllllllllllllllllllllllllllll Department of Health Information Management, Manipal Academy of Higher Education, Manipal, India; mmmmmmmmmmmmmmmmmmmmmmmmmmmmmmmmmmmmmmmmmmmmmmmmmmmmmmmmmmmmmmmmmmmmmmmmmmmmmmmmmm Department of Obstetrics and Gynecology, Azienda Sanitaria Universitaria Friuli Centrale, Udine, Italy; nnnnnnnnnnnnnnnnnnnnnnnnnnnnnnnnnnnnnnnnnnnnnnnnnnnnnnnnnnnnnnnnnnnnnnnnnnnnnnnnnn Unisabana Center for Translational Science, Universidad de La Sabana (Savannah University), Chia, Colombia; oooooooooooooooooooooooooooooooooooooooooooooooooooooooooooooooooooooooooooooooooo Critical Care Department, Clinica Universidad De La Sabana (Savannah University Clinic), Chia, Colombia; pppppppppppppppppppppppppppppppppppppppppppppppppppppppppppppppppppppppppppppppppp School of Environment, Tehran University, Tehran, Iran; qqqqqqqqqqqqqqqqqqqqqqqqqqqqqqqqqqqqqqqqqqqqqqqqqqqqqqqqqqqqqqqqqqqqqqqqqqqqqqqqqq Network of Immunity in Infection, Malignancy and Autoimmunity (NIIMA), Universal Scientific Education and Research Network (USERN), Tehran, Iran; rrrrrrrrrrrrrrrrrrrrrrrrrrrrrrrrrrrrrrrrrrrrrrrrrrrrrrrrrrrrrrrrrrrrrrrrrrrrrrrrrr Department of Epidemiology and Biostatistics, Rafsanjan University of Medical Sciences, Rafsanjan, Iran; ssssssssssssssssssssssssssssssssssssssssssssssssssssssssssssssssssssssssssssssssss Rasoul Akram Hospital, Islamic Azad University, Iran, Iran; tttttttttttttttttttttttttttttttttttttttttttttttttttttttttttttttttttttttttttttttttt Department of Public Health Sciences, University of Connecticut, Farmington, CT, USA; uuuuuuuuuuuuuuuuuuuuuuuuuuuuuuuuuuuuuuuuuuuuuuuuuuuuuuuuuuuuuuuuuuuuuuuuuuuuuuuuuu Department of Psychiatry, Yale University, New Haven, CT, USA; vvvvvvvvvvvvvvvvvvvvvvvvvvvvvvvvvvvvvvvvvvvvvvvvvvvvvvvvvvvvvvvvvvvvvvvvvvvvvvvvvv College of Nursing, Institut Ilmu Kesehatan Bhakti Wiyata Kediri (Bhakti Wiyata Kediri Institute of Health Sciences), Kediri, Indonesia; wwwwwwwwwwwwwwwwwwwwwwwwwwwwwwwwwwwwwwwwwwwwwwwwwwwwwwwwwwwwwwwwwwwwwwwwwwwwwwwwww College of Nursing, Taipei Medical University, Taipei, Taiwan; xxxxxxxxxxxxxxxxxxxxxxxxxxxxxxxxxxxxxxxxxxxxxxxxxxxxxxxxxxxxxxxxxxxxxxxxxxxxxxxxxx Centre of Telehealth, Universidade Federal de Minas Gerais (Federal University of Minas Gerais), Belo Horizonte, Brazil; yyyyyyyyyyyyyyyyyyyyyyyyyyyyyyyyyyyyyyyyyyyyyyyyyyyyyyyyyyyyyyyyyyyyyyyyyyyyyyyyyy Escola de Enfermagem da UFMG, Universidade Federal de Minas Gerais (Federal University of Minas Gerais), Belo Horizonte, Brazil; zzzzzzzzzzzzzzzzzzzzzzzzzzzzzzzzzzzzzzzzzzzzzzzzzzzzzzzzzzzzzzzzzzzzzzzzzzzzzzzzzz Department of Surgery, University of Minnesota, Minneapolis, MN, USA; aaaaaaaaaaaaaaaaaaaaaaaaaaaaaaaaaaaaaaaaaaaaaaaaaaaaaaaaaaaaaaaaaaaaaaaaaaaaaaaaaaa Department of Surgery, University Teaching Hospital of Kigali, Kigali, Rwanda; bbbbbbbbbbbbbbbbbbbbbbbbbbbbbbbbbbbbbbbbbbbbbbbbbbbbbbbbbbbbbbbbbbbbbbbbbbbbbbbbbbb Department of Physiology and Physiotherapy, DIT University, Delhi, India; ccccccccccccccccccccccccccccccccccccccccccccccccccccccccccccccccccccccccccccccccccc Community Health Department, Federal University of Ceará, Fortaleza, Brazil; ddddddddddddddddddddddddddddddddddddddddddddddddddddddddddddddddddddddddddddddddddd Faculty of Medicine, University of Porto, Porto, Portugal; eeeeeeeeeeeeeeeeeeeeeeeeeeeeeeeeeeeeeeeeeeeeeeeeeeeeeeeeeeeeeeeeeeeeeeeeeeeeeeeeeee Department of Geography and Demography, University of Coimbra, Coimbra, Portugal; fffffffffffffffffffffffffffffffffffffffffffffffffffffffffffffffffffffffffffffffffff Department of Nursing in Women's Health, Federal University of São Paulo, São Paulo, Brazil; ggggggggggggggggggggggggggggggggggggggggggggggggggggggggggggggggggggggggggggggggggg Vaccination Research Observatory, Universidade Federal de Minas Gerais (Federal University of Minas Gerais), Belo Horizonte, Brazil; hhhhhhhhhhhhhhhhhhhhhhhhhhhhhhhhhhhhhhhhhhhhhhhhhhhhhhhhhhhhhhhhhhhhhhhhhhhhhhhhhhh Department of Pharmacology and Toxicology, University of Antioquia, Medellin, Colombia; iiiiiiiiiiiiiiiiiiiiiiiiiiiiiiiiiiiiiiiiiiiiiiiiiiiiiiiiiiiiiiiiiiiiiiiiiiiiiiiiiii Warwick Medical School, University of Warwick, Coventry, UK; jjjjjjjjjjjjjjjjjjjjjjjjjjjjjjjjjjjjjjjjjjjjjjjjjjjjjjjjjjjjjjjjjjjjjjjjjjjjjjjjjjj Department of Clinical Research, Universidade de São Paulo (University of São Paulo), Ribeirão Preto, Brazil; kkkkkkkkkkkkkkkkkkkkkkkkkkkkkkkkkkkkkkkkkkkkkkkkkkkkkkkkkkkkkkkkkkkkkkkkkkkkkkkkkkk Gilbert and Rose-Marie Chagoury School of Medicine, Lebanese American University, Beirut, Lebanon; lllllllllllllllllllllllllllllllllllllllllllllllllllllllllllllllllllllllllllllllllll Division of Global Health Equity, Harvard University, Boston, MA, USA; mmmmmmmmmmmmmmmmmmmmmmmmmmmmmmmmmmmmmmmmmmmmmmmmmmmmmmmmmmmmmmmmmmmmmmmmmmmmmmmmmmm Center for Indigenous Health Research, Wuqu' Kawoq Maya Health Alliance, Tecpan, Guatemala; nnnnnnnnnnnnnnnnnnnnnnnnnnnnnnnnnnnnnnnnnnnnnnnnnnnnnnnnnnnnnnnnnnnnnnnnnnnnnnnnnnn Centre for Healthy Brain Ageing (CHeBA), University of New South Wales, Sydney, NSW, Australia; ooooooooooooooooooooooooooooooooooooooooooooooooooooooooooooooooooooooooooooooooooo Department of Environmental and Radiological Health Sciences, Colorado State University, Fort Collins, CO, USA; ppppppppppppppppppppppppppppppppppppppppppppppppppppppppppppppppppppppppppppppppppp Department of Anesthesiology, University of Nebraska Medical Center, Omaha, NE, USA; qqqqqqqqqqqqqqqqqqqqqqqqqqqqqqqqqqqqqqqqqqqqqqqqqqqqqqqqqqqqqqqqqqqqqqqqqqqqqqqqqqq Department of Neurosciences, Maurizio Bufalini Hospital, Cesena, Italy; rrrrrrrrrrrrrrrrrrrrrrrrrrrrrrrrrrrrrrrrrrrrrrrrrrrrrrrrrrrrrrrrrrrrrrrrrrrrrrrrrrr Centre for Global Epilepsy, University of Oxford, Oxford, UK; sssssssssssssssssssssssssssssssssssssssssssssssssssssssssssssssssssssssssssssssssss College of Medicine, University of Florida, Gainesville, FL, USA; ttttttttttttttttttttttttttttttttttttttttttttttttttttttttttttttttttttttttttttttttttt Department of Ophthalmology and Visual Sciences, University of Wisconsin–Madison, Madison, WI, USA; uuuuuuuuuuuuuuuuuuuuuuuuuuuuuuuuuuuuuuuuuuuuuuuuuuuuuuuuuuuuuuuuuuuuuuuuuuuuuuuuuuu School of Medicine, Gonabad University of Medical Sciences, Gonabad, Iran; vvvvvvvvvvvvvvvvvvvvvvvvvvvvvvvvvvvvvvvvvvvvvvvvvvvvvvvvvvvvvvvvvvvvvvvvvvvvvvvvvvv Division of Cardiology, University of Washington, Seattle, WA, USA; wwwwwwwwwwwwwwwwwwwwwwwwwwwwwwwwwwwwwwwwwwwwwwwwwwwwwwwwwwwwwwwwwwwwwwwwwwwwwwwwwww Department of Pharmacy Services, Alberta Health Services, Edmonton, AB, Canada; xxxxxxxxxxxxxxxxxxxxxxxxxxxxxxxxxxxxxxxxxxxxxxxxxxxxxxxxxxxxxxxxxxxxxxxxxxxxxxxxxxx West African Postgraduate College of Pharmacists, Lagos, Nigeria; yyyyyyyyyyyyyyyyyyyyyyyyyyyyyyyyyyyyyyyyyyyyyyyyyyyyyyyyyyyyyyyyyyyyyyyyyyyyyyyyyyy Department of Analytical and Applied Economics, Utkal University, Bhubaneswar, India; zzzzzzzzzzzzzzzzzzzzzzzzzzzzzzzzzzzzzzzzzzzzzzzzzzzzzzzzzzzzzzzzzzzzzzzzzzzzzzzzzzz RUSA Centre of Excellence in Public Policy and Governance, Utkal University, Bhubaneswar, India; aaaaaaaaaaaaaaaaaaaaaaaaaaaaaaaaaaaaaaaaaaaaaaaaaaaaaaaaaaaaaaaaaaaaaaaaaaaaaaaaaaaa Isfahan University of Medical Sciences, Islamic Azad University, Isfahan, Iran; bbbbbbbbbbbbbbbbbbbbbbbbbbbbbbbbbbbbbbbbbbbbbbbbbbbbbbbbbbbbbbbbbbbbbbbbbbbbbbbbbbbb Family and Prevention Medicine, Isfahan University of Medical Sciences, Isfahan, Iran; cccccccccccccccccccccccccccccccccccccccccccccccccccccccccccccccccccccccccccccccccccc Department of Community Medicine, RVM Medical College and Research Centre, Hyderabad, Hyderabad, India; dddddddddddddddddddddddddddddddddddddddddddddddddddddddddddddddddddddddddddddddddddd Achutha Menon Centre for Health Science Studies, Sree Chitra Tirunal Institute for Medical Sciences and Technology, Thiruvananthapuram, India; eeeeeeeeeeeeeeeeeeeeeeeeeeeeeeeeeeeeeeeeeeeeeeeeeeeeeeeeeeeeeeeeeeeeeeeeeeeeeeeeeeee Faculty of Medicine, Quest International University Perak, Ipoh, Malaysia; ffffffffffffffffffffffffffffffffffffffffffffffffffffffffffffffffffffffffffffffffffff Department of Labour, Government of West Bengal, Kolkata, India; gggggggggggggggggggggggggggggggggggggggggggggggggggggggggggggggggggggggggggggggggggg Department of Public Health, New Mexico State University, Las Cruces, NM, USA; hhhhhhhhhhhhhhhhhhhhhhhhhhhhhhhhhhhhhhhhhhhhhhhhhhhhhhhhhhhhhhhhhhhhhhhhhhhhhhhhhhhh Research Department, Indian Institute of Public Health, Delhi, India; iiiiiiiiiiiiiiiiiiiiiiiiiiiiiiiiiiiiiiiiiiiiiiiiiiiiiiiiiiiiiiiiiiiiiiiiiiiiiiiiiiii Department of Epidemiology, Florida International University, Miami, FL, USA; jjjjjjjjjjjjjjjjjjjjjjjjjjjjjjjjjjjjjjjjjjjjjjjjjjjjjjjjjjjjjjjjjjjjjjjjjjjjjjjjjjjj Department of Biochemistry, Saveetha University, Chennai, India; kkkkkkkkkkkkkkkkkkkkkkkkkkkkkkkkkkkkkkkkkkkkkkkkkkkkkkkkkkkkkkkkkkkkkkkkkkkkkkkkkkkk Departamento de Ciencias Básicas Médicas, Universidad ICESI, Cali, Colombia; llllllllllllllllllllllllllllllllllllllllllllllllllllllllllllllllllllllllllllllllllll Department of Health Statistics, National Institute for Medical Research, Dar es Salaam, Tanzania; mmmmmmmmmmmmmmmmmmmmmmmmmmmmmmmmmmmmmmmmmmmmmmmmmmmmmmmmmmmmmmmmmmmmmmmmmmmmmmmmmmmm Department of Cardiology, SS. Annunziata Hospital - ASL2 Abruzzo, Chieti, Italy; nnnnnnnnnnnnnnnnnnnnnnnnnnnnnnnnnnnnnnnnnnnnnnnnnnnnnnnnnnnnnnnnnnnnnnnnnnnnnnnnnnnn Department of Internal Medicine, Muhimbili University of Health and Allied Sciences, Dar es Salaam, Tanzania; oooooooooooooooooooooooooooooooooooooooooooooooooooooooooooooooooooooooooooooooooooo Department of Internal Medicine, University of Botswana, Gaborone, Botswana; pppppppppppppppppppppppppppppppppppppppppppppppppppppppppppppppppppppppppppppppppppp Cardiovascular Department, Zagazig University, Zagazig, Egypt; qqqqqqqqqqqqqqqqqqqqqqqqqqqqqqqqqqqqqqqqqqqqqqqqqqqqqqqqqqqqqqqqqqqqqqqqqqqqqqqqqqqq Department Infectious Diseases, National Institute of Health, Rome, Italy; rrrrrrrrrrrrrrrrrrrrrrrrrrrrrrrrrrrrrrrrrrrrrrrrrrrrrrrrrrrrrrrrrrrrrrrrrrrrrrrrrrrr Department for Health Prevention, Ministry of Health, Rome, Italy; ssssssssssssssssssssssssssssssssssssssssssssssssssssssssssssssssssssssssssssssssssss Department of Medical Pharmacology, Cairo University, Giza, Egypt; tttttttttttttttttttttttttttttttttttttttttttttttttttttttttttttttttttttttttttttttttttt Department of Epidemiology, Shahid Beheshti University of Medical Sciences, Tehran, Iran; uuuuuuuuuuuuuuuuuuuuuuuuuuuuuuuuuuuuuuuuuuuuuuuuuuuuuuuuuuuuuuuuuuuuuuuuuuuuuuuuuuuu School of Psychiatry, University of New South Wales, Sydney, NSW, Australia; vvvvvvvvvvvvvvvvvvvvvvvvvvvvvvvvvvvvvvvvvvvvvvvvvvvvvvvvvvvvvvvvvvvvvvvvvvvvvvvvvvvv Neuropsychiatric Institute, Prince of Wales Hospital, Randwick, NSW, Australia; wwwwwwwwwwwwwwwwwwwwwwwwwwwwwwwwwwwwwwwwwwwwwwwwwwwwwwwwwwwwwwwwwwwwwwwwwwwwwwwwwwww School of Public Health, Shahid Beheshti University of Medical Sciences, Tehran, Iran; xxxxxxxxxxxxxxxxxxxxxxxxxxxxxxxxxxxxxxxxxxxxxxxxxxxxxxxxxxxxxxxxxxxxxxxxxxxxxxxxxxxx Department of Public Health and Epidemiology, Khalifa University, Abu Dhabi, United Arab Emirates; yyyyyyyyyyyyyyyyyyyyyyyyyyyyyyyyyyyyyyyyyyyyyyyyyyyyyyyyyyyyyyyyyyyyyyyyyyyyyyyyyyyy Department of Computer, University of Science and Culture, Tehran, Iran; zzzzzzzzzzzzzzzzzzzzzzzzzzzzzzzzzzzzzzzzzzzzzzzzzzzzzzzzzzzzzzzzzzzzzzzzzzzzzzzzzzzz Research Center for Environmental Determinants of Health, Kermanshah University of Medical Sciences, Kermanshah, Iran; aaaaaaaaaaaaaaaaaaaaaaaaaaaaaaaaaaaaaaaaaaaaaaaaaaaaaaaaaaaaaaaaaaaaaaaaaaaaaaaaaaaaa Department of Biostatistics, Shiraz University of Medical Sciences, Shiraz, Iran; bbbbbbbbbbbbbbbbbbbbbbbbbbbbbbbbbbbbbbbbbbbbbbbbbbbbbbbbbbbbbbbbbbbbbbbbbbbbbbbbbbbbb Iranian Research Center for Evidence-based Medicine, Tabriz University of Medical Sciences, Tabriz, Iran; ccccccccccccccccccccccccccccccccccccccccccccccccccccccccccccccccccccccccccccccccccccc Department of Nursing and Midwifery, Saveh University of Medical Sciences, Saveh, Iran; ddddddddddddddddddddddddddddddddddddddddddddddddddddddddddddddddddddddddddddddddddddd Department of Neurology, Christian-Doppler University Hospital, Salzburg, Austria; eeeeeeeeeeeeeeeeeeeeeeeeeeeeeeeeeeeeeeeeeeeeeeeeeeeeeeeeeeeeeeeeeeeeeeeeeeeeeeeeeeeee Spinal Cord Injury and Tissue Regeneration Center Salzburg (SCI-TReCS), Paracelsus Medical University, Salzburg, Austria; fffffffffffffffffffffffffffffffffffffffffffffffffffffffffffffffffffffffffffffffffffff Ophthalmic Research Center, Shahid Beheshti University of Medical Sciences, Tehran, Iran; ggggggggggggggggggggggggggggggggggggggggggggggggggggggggggggggggggggggggggggggggggggg Faculty of Medicine, Bioscience and Nursing, MAHSA University, Selangor, Malaysia; hhhhhhhhhhhhhhhhhhhhhhhhhhhhhhhhhhhhhhhhhhhhhhhhhhhhhhhhhhhhhhhhhhhhhhhhhhhhhhhhhhhhh Interdisciplinary Research Centre in Biomedical Materials (IRCBM), COMSATS Institute of Information Technology, Lahore, Pakistan; iiiiiiiiiiiiiiiiiiiiiiiiiiiiiiiiiiiiiiiiiiiiiiiiiiiiiiiiiiiiiiiiiiiiiiiiiiiiiiiiiiiii Department of Psychiatry, All India Institute of Medical Sciences, New Delhi, India; jjjjjjjjjjjjjjjjjjjjjjjjjjjjjjjjjjjjjjjjjjjjjjjjjjjjjjjjjjjjjjjjjjjjjjjjjjjjjjjjjjjjj Department of Psychosocial Science, University of Bergen, Bergen, Norway; kkkkkkkkkkkkkkkkkkkkkkkkkkkkkkkkkkkkkkkkkkkkkkkkkkkkkkkkkkkkkkkkkkkkkkkkkkkkkkkkkkkkk ICMR - National Institute for Research in Bacterial Infections, Indian Council of Medical Research, Kolkata, India; lllllllllllllllllllllllllllllllllllllllllllllllllllllllllllllllllllllllllllllllllllll Sharjah Institute of Medical Sciences, University of Sharjah, Sharjah, United Arab Emirates; mmmmmmmmmmmmmmmmmmmmmmmmmmmmmmmmmmmmmmmmmmmmmmmmmmmmmmmmmmmmmmmmmmmmmmmmmmmmmmmmmmmmm Center for Global Health Research, Saveetha University, Chennai, India; nnnnnnnnnnnnnnnnnnnnnnnnnnnnnnnnnnnnnnnnnnnnnnnnnnnnnnnnnnnnnnnnnnnnnnnnnnnnnnnnnnnnn Biotechnology Research Center, Mashhad University of Medical Sciences, Mashhad, Iran; ooooooooooooooooooooooooooooooooooooooooooooooooooooooooooooooooooooooooooooooooooooo Canadian Red Cross, Red Cross, Ottawa, ON, Canada; ppppppppppppppppppppppppppppppppppppppppppppppppppppppppppppppppppppppppppppppppppppp Department of Psychiatry, Ministry of Health, Manama, Bahrain; qqqqqqqqqqqqqqqqqqqqqqqqqqqqqqqqqqqqqqqqqqqqqqqqqqqqqqqqqqqqqqqqqqqqqqqqqqqqqqqqqqqqq College of Pharmacy, Al-Hadba University, Mosul, Iraq; rrrrrrrrrrrrrrrrrrrrrrrrrrrrrrrrrrrrrrrrrrrrrrrrrrrrrrrrrrrrrrrrrrrrrrrrrrrrrrrrrrrrr Department of Health and Kinesiology, University of Illinois, Urbana-Champaign, IL, USA; sssssssssssssssssssssssssssssssssssssssssssssssssssssssssssssssssssssssssssssssssssss Department of Statistics, University of Gujrat, Gujrat, Pakistan; ttttttttttttttttttttttttttttttttttttttttttttttttttttttttttttttttttttttttttttttttttttt Non-Communicable Diseases Research Center (NCDRC), Tehran University of Medical Sciences, Tehran, Iran; uuuuuuuuuuuuuuuuuuuuuuuuuuuuuuuuuuuuuuuuuuuuuuuuuuuuuuuuuuuuuuuuuuuuuuuuuuuuuuuuuuuuu Department of Integrated Health Education, Federal University of Espirito Santo, Vitória, Brazil; vvvvvvvvvvvvvvvvvvvvvvvvvvvvvvvvvvvvvvvvvvvvvvvvvvvvvvvvvvvvvvvvvvvvvvvvvvvvvvvvvvvvv Faculty of Pharmacy, Mansoura University, Mansoura, Egypt; wwwwwwwwwwwwwwwwwwwwwwwwwwwwwwwwwwwwwwwwwwwwwwwwwwwwwwwwwwwwwwwwwwwwwwwwwwwwwwwwwwwww Department of Health Education & Promotion, A.C.S. Medical College and Hospital, Karaj, Iran; xxxxxxxxxxxxxxxxxxxxxxxxxxxxxxxxxxxxxxxxxxxxxxxxxxxxxxxxxxxxxxxxxxxxxxxxxxxxxxxxxxxxx Research Center for Health, Safety and Environment, Alborz University of Medical Sciences, Karaj, Iran; yyyyyyyyyyyyyyyyyyyyyyyyyyyyyyyyyyyyyyyyyyyyyyyyyyyyyyyyyyyyyyyyyyyyyyyyyyyyyyyyyyyyy Department of Endocrinology, Mayo Clinic, Rochester, MN, USA; zzzzzzzzzzzzzzzzzzzzzzzzzzzzzzzzzzzzzzzzzzzzzzzzzzzzzzzzzzzzzzzzzzzzzzzzzzzzzzzzzzzzz Student Research Committee, Kashan University of Medical Sciences, Kashan, Iran; aaaaaaaaaaaaaaaaaaaaaaaaaaaaaaaaaaaaaaaaaaaaaaaaaaaaaaaaaaaaaaaaaaaaaaaaaaaaaaaaaaaaaa Public Health and Community Medicine Department, Cairo University, Giza, Egypt; bbbbbbbbbbbbbbbbbbbbbbbbbbbbbbbbbbbbbbbbbbbbbbbbbbbbbbbbbbbbbbbbbbbbbbbbbbbbbbbbbbbbbb Technology Management Department, University College of Applied Sciences, Gaza, Palestine; cccccccccccccccccccccccccccccccccccccccccccccccccccccccccccccccccccccccccccccccccccccc School of Economics and Management, Universität Kassel (University of Kassel), Kassel, Germany; dddddddddddddddddddddddddddddddddddddddddddddddddddddddddddddddddddddddddddddddddddddd Department of Biochemistry, University of Medical Sciences, Ondo, Ondo city, Nigeria; eeeeeeeeeeeeeeeeeeeeeeeeeeeeeeeeeeeeeeeeeeeeeeeeeeeeeeeeeeeeeeeeeeeeeeeeeeeeeeeeeeeeee College of Nursing, Jouf University, Jouf, Saudi Arabia; ffffffffffffffffffffffffffffffffffffffffffffffffffffffffffffffffffffffffffffffffffffff Department of Pathology, Microbiology and Forensic Medicine, The University of Jordan, Amman, Jordan; gggggggggggggggggggggggggggggggggggggggggggggggggggggggggggggggggggggggggggggggggggggg Department of Clinical Laboratories and Forensic Medicine, The University of Jordan, Amman, Jordan; hhhhhhhhhhhhhhhhhhhhhhhhhhhhhhhhhhhhhhhhhhhhhhhhhhhhhhhhhhhhhhhhhhhhhhhhhhhhhhhhhhhhhh Drug Applied Research Center, Tabriz University of Medical Sciences, Tabriz, Iran; iiiiiiiiiiiiiiiiiiiiiiiiiiiiiiiiiiiiiiiiiiiiiiiiiiiiiiiiiiiiiiiiiiiiiiiiiiiiiiiiiiiiii Surgical Department, North Colombo Teaching Hospital, Ragama, Sri Lanka; jjjjjjjjjjjjjjjjjjjjjjjjjjjjjjjjjjjjjjjjjjjjjjjjjjjjjjjjjjjjjjjjjjjjjjjjjjjjjjjjjjjjjj Department of Community Medicine, King Abdulaziz University, Jeddah, Saudi Arabia; kkkkkkkkkkkkkkkkkkkkkkkkkkkkkkkkkkkkkkkkkkkkkkkkkkkkkkkkkkkkkkkkkkkkkkkkkkkkkkkkkkkkkk College of Nursing, Qatar University, Lusail, Qatar; llllllllllllllllllllllllllllllllllllllllllllllllllllllllllllllllllllllllllllllllllllll Institute of Epidemiology and Preventive Medicine, National Taiwan University, Taipei, Taiwan; mmmmmmmmmmmmmmmmmmmmmmmmmmmmmmmmmmmmmmmmmmmmmmmmmmmmmmmmmmmmmmmmmmmmmmmmmmmmmmmmmmmmmm Benang Merah Research Center (BMRC), Minahasa Utara, Indonesia; nnnnnnnnnnnnnnnnnnnnnnnnnnnnnnnnnnnnnnnnnnnnnnnnnnnnnnnnnnnnnnnnnnnnnnnnnnnnnnnnnnnnnn Department of Entomology, Ain Shams University, Cairo, Egypt; oooooooooooooooooooooooooooooooooooooooooooooooooooooooooooooooooooooooooooooooooooooo Medical Ain Shams Research Institute (MASRI), Ain Shams University, Cairo, Egypt; pppppppppppppppppppppppppppppppppppppppppppppppppppppppppppppppppppppppppppppppppppppp Department of Forensic Biology, Government Institute of Forensic Science Chhatrapati Sambhajinagar, Chhatrapati Sambhajinagar, India; qqqqqqqqqqqqqqqqqqqqqqqqqqqqqqqqqqqqqqqqqqqqqqqqqqqqqqqqqqqqqqqqqqqqqqqqqqqqqqqqqqqqqq Department of Microbiology, Saveetha University, Chennai, India; rrrrrrrrrrrrrrrrrrrrrrrrrrrrrrrrrrrrrrrrrrrrrrrrrrrrrrrrrrrrrrrrrrrrrrrrrrrrrrrrrrrrrr Primary Healthcare Department, Azienda USL di Bologna, Bologna, Italy; ssssssssssssssssssssssssssssssssssssssssssssssssssssssssssssssssssssssssssssssssssssss Center for Clinical and Epidemiological Research, University of São Paulo, São Paulo, Brazil; tttttttttttttttttttttttttttttttttttttttttttttttttttttttttttttttttttttttttttttttttttttt University of São Paulo City, São Paulo, Brazil; uuuuuuuuuuuuuuuuuuuuuuuuuuuuuuuuuuuuuuuuuuuuuuuuuuuuuuuuuuuuuuuuuuuuuuuuuuuuuuuuuuuuuu School of Public Health and Health Management, University of Belgrade, Belgrade, Serbia; vvvvvvvvvvvvvvvvvvvvvvvvvvvvvvvvvvvvvvvvvvvvvvvvvvvvvvvvvvvvvvvvvvvvvvvvvvvvvvvvvvvvvv Department of Osteopathic Medicine, D'Youville University, Buffalo, NY, USA; wwwwwwwwwwwwwwwwwwwwwwwwwwwwwwwwwwwwwwwwwwwwwwwwwwwwwwwwwwwwwwwwwwwwwwwwwwwwwwwwwwwwww Department of Infectious Diseases and Tropical Medicine, Universidade Federal de Minas Gerais (Federal University of Minas Gerais), Belo Horizonte, Brazil; xxxxxxxxxxxxxxxxxxxxxxxxxxxxxxxxxxxxxxxxxxxxxxxxxxxxxxxxxxxxxxxxxxxxxxxxxxxxxxxxxxxxxx Department of Sociology and Gerontology, Miami University, Oxford, OH, USA; yyyyyyyyyyyyyyyyyyyyyyyyyyyyyyyyyyyyyyyyyyyyyyyyyyyyyyyyyyyyyyyyyyyyyyyyyyyyyyyyyyyyyy Independent Consultant, Thiruvananthapuram, India; zzzzzzzzzzzzzzzzzzzzzzzzzzzzzzzzzzzzzzzzzzzzzzzzzzzzzzzzzzzzzzzzzzzzzzzzzzzzzzzzzzzzzz Department of Public Health, Jahrom University of Medical Sciences, Jahrom, Iran; aaaaaaaaaaaaaaaaaaaaaaaaaaaaaaaaaaaaaaaaaaaaaaaaaaaaaaaaaaaaaaaaaaaaaaaaaaaaaaaaaaaaaaa Botany Department, Bodoland University, Kokrajhar, India; bbbbbbbbbbbbbbbbbbbbbbbbbbbbbbbbbbbbbbbbbbbbbbbbbbbbbbbbbbbbbbbbbbbbbbbbbbbbbbbbbbbbbbb Faculty of Science, Queensland University of Technology, Brisbane, QLD, Australia; ccccccccccccccccccccccccccccccccccccccccccccccccccccccccccccccccccccccccccccccccccccccc Department of Oral Pathology and Microbiology, Dr. D. Y. Patil Vidyapeeth, Pune (Deemed to be University), Pune, India; ddddddddddddddddddddddddddddddddddddddddddddddddddddddddddddddddddddddddddddddddddddddd Faculty of Medicine, The University of Queensland, Brisbane, QLD, Australia; eeeeeeeeeeeeeeeeeeeeeeeeeeeeeeeeeeeeeeeeeeeeeeeeeeeeeeeeeeeeeeeeeeeeeeeeeeeeeeeeeeeeeee Colorectal Research Center, Iran University of Medical Sciences, Tehran, Iran; fffffffffffffffffffffffffffffffffffffffffffffffffffffffffffffffffffffffffffffffffffffff Department of Epidemiology, National Institute for Research in Tuberculosis, Chennai, India; ggggggggggggggggggggggggggggggggggggggggggggggggggggggggggggggggggggggggggggggggggggggg UGC Centre of Advanced Study in Psychology, Utkal University, Bhubaneswar, India; hhhhhhhhhhhhhhhhhhhhhhhhhhhhhhhhhhhhhhhhhhhhhhhhhhhhhhhhhhhhhhhhhhhhhhhhhhhhhhhhhhhhhhh Udyam-Global Association for Sustainable Development, Bhubaneswar, India; iiiiiiiiiiiiiiiiiiiiiiiiiiiiiiiiiiiiiiiiiiiiiiiiiiiiiiiiiiiiiiiiiiiiiiiiiiiiiiiiiiiiiii Maternal, Fetal, and Neonatal Research Center, Tehran University of Medical Sciences, Tehran, Iran; jjjjjjjjjjjjjjjjjjjjjjjjjjjjjjjjjjjjjjjjjjjjjjjjjjjjjjjjjjjjjjjjjjjjjjjjjjjjjjjjjjjjjjj Women's Reproductive Health Research Center, Tabriz University of Medical Sciences, Tabriz, Iran; kkkkkkkkkkkkkkkkkkkkkkkkkkkkkkkkkkkkkkkkkkkkkkkkkkkkkkkkkkkkkkkkkkkkkkkkkkkkkkkkkkkkkkk IRCCS Istituti Clinici Scientifici Maugeri (IRCCS Maugeri Scientific Clinical Institute), Milan, Italy; lllllllllllllllllllllllllllllllllllllllllllllllllllllllllllllllllllllllllllllllllllllll Precision Medicine Department, Università degli studi della Campania Luigi Vanvitelli (University of Campania Luigi Vanvitelli), Naples, Italy; mmmmmmmmmmmmmmmmmmmmmmmmmmmmmmmmmmmmmmmmmmmmmmmmmmmmmmmmmmmmmmmmmmmmmmmmmmmmmmmmmmmmmmm Department of Public Health Sciences, University of North Carolina at Charlotte, Charlotte, NC, USA; nnnnnnnnnnnnnnnnnnnnnnnnnnnnnnnnnnnnnnnnnnnnnnnnnnnnnnnnnnnnnnnnnnnnnnnnnnnnnnnnnnnnnnn Department of Public Health Sciences, Coastal Carolina University, Conway, SC, USA; ooooooooooooooooooooooooooooooooooooooooooooooooooooooooooooooooooooooooooooooooooooooo Harvard Extension School, Harvard University, Cambridge, MA, USA; ppppppppppppppppppppppppppppppppppppppppppppppppppppppppppppppppppppppppppppppppppppppp Department of Preventive and Social Medicine, Jawaharlal Institute of Postgraduate Medical Education and Research, Puducherry, India; qqqqqqqqqqqqqqqqqqqqqqqqqqqqqqqqqqqqqqqqqqqqqqqqqqqqqqqqqqqqqqqqqqqqqqqqqqqqqqqqqqqqqqq Department of Post-Harvest Technology and Marketing, Patuakhali Science and Technology University, Patuakhali, Bangladesh; rrrrrrrrrrrrrrrrrrrrrrrrrrrrrrrrrrrrrrrrrrrrrrrrrrrrrrrrrrrrrrrrrrrrrrrrrrrrrrrrrrrrrrr Faculty of Business and Computing, University of the Fraser Valley, Abbotsford, BC, Canada; sssssssssssssssssssssssssssssssssssssssssssssssssssssssssssssssssssssssssssssssssssssss Graduate School of Business, ESAN University, Lima, Peru; ttttttttttttttttttttttttttttttttttttttttttttttttttttttttttttttttttttttttttttttttttttttt Chief Data Officer Directorate, UK Department of Health and Social Care, London, UK; uuuuuuuuuuuuuuuuuuuuuuuuuuuuuuuuuuuuuuuuuuuuuuuuuuuuuuuuuuuuuuuuuuuuuuuuuuuuuuuuuuuuuuu Faculty of Medicine, Katholieke Universiteit Leuven, Leuven, Belgium; vvvvvvvvvvvvvvvvvvvvvvvvvvvvvvvvvvvvvvvvvvvvvvvvvvvvvvvvvvvvvvvvvvvvvvvvvvvvvvvvvvvvvvv The George Institute for Global Health, Sydney, NSW, Australia; wwwwwwwwwwwwwwwwwwwwwwwwwwwwwwwwwwwwwwwwwwwwwwwwwwwwwwwwwwwwwwwwwwwwwwwwwwwwwwwwwwwwwww Department of Psychology, University of Alabama at Birmingham, Birmingham, AL, USA; xxxxxxxxxxxxxxxxxxxxxxxxxxxxxxxxxxxxxxxxxxxxxxxxxxxxxxxxxxxxxxxxxxxxxxxxxxxxxxxxxxxxxxx Clinic for Conservative Dentistry and Periodontology, University Hospital of the Ludwig-Maximilians-University Munich, Munich, Germany; yyyyyyyyyyyyyyyyyyyyyyyyyyyyyyyyyyyyyyyyyyyyyyyyyyyyyyyyyyyyyyyyyyyyyyyyyyyyyyyyyyyyyyy Augusta Health, Fishersville, VA, USA; zzzzzzzzzzzzzzzzzzzzzzzzzzzzzzzzzzzzzzzzzzzzzzzzzzzzzzzzzzzzzzzzzzzzzzzzzzzzzzzzzzzzzzz Operating Room Department, Khomein University of Medical Sciences, Khomein, Iran; aaaaaaaaaaaaaaaaaaaaaaaaaaaaaaaaaaaaaaaaaaaaaaaaaaaaaaaaaaaaaaaaaaaaaaaaaaaaaaaaaaaaaaaa Department of Medical Education, Tehran University of Medical Sciences, Tehran, Iran; bbbbbbbbbbbbbbbbbbbbbbbbbbbbbbbbbbbbbbbbbbbbbbbbbbbbbbbbbbbbbbbbbbbbbbbbbbbbbbbbbbbbbbbb Department of Psychiatry, Stellenbosch University, Cape Town, South Africa; cccccccccccccccccccccccccccccccccccccccccccccccccccccccccccccccccccccccccccccccccccccccc Department of Medical Statistics, University of Zagreb, Zagreb, Croatia; dddddddddddddddddddddddddddddddddddddddddddddddddddddddddddddddddddddddddddddddddddddddd Department of Epidemiology and Prevention of Chronic Noncommunicable Diseases, Croatian Institute of Public Health, Zagreb, Croatia; eeeeeeeeeeeeeeeeeeeeeeeeeeeeeeeeeeeeeeeeeeeeeeeeeeeeeeeeeeeeeeeeeeeeeeeeeeeeeeeeeeeeeeee Department of Applied Mechanics and Biomedical Engineering, Indian Institute of Technology Delhi, Chennai, India; ffffffffffffffffffffffffffffffffffffffffffffffffffffffffffffffffffffffffffffffffffffffff Research Institute of Medical & Health Sciences, University of Sharjah, Sharjah, United Arab Emirates; gggggggggggggggggggggggggggggggggggggggggggggggggggggggggggggggggggggggggggggggggggggggg Department of Biomedical Sciences, Gulf Medical University, Ajman, United Arab Emirates; hhhhhhhhhhhhhhhhhhhhhhhhhhhhhhhhhhhhhhhhhhhhhhhhhhhhhhhhhhhhhhhhhhhhhhhhhhhhhhhhhhhhhhhh Emergency Department, Manian Medical Centre, Erode, India; iiiiiiiiiiiiiiiiiiiiiiiiiiiiiiiiiiiiiiiiiiiiiiiiiiiiiiiiiiiiiiiiiiiiiiiiiiiiiiiiiiiiiiii Center for Health Systems Research, National Institute of Public Health, Cuernavaca, Mexico; jjjjjjjjjjjjjjjjjjjjjjjjjjjjjjjjjjjjjjjjjjjjjjjjjjjjjjjjjjjjjjjjjjjjjjjjjjjjjjjjjjjjjjjj Department of Medicine, Swami Vivekanand Subharti University, Meerut, India; kkkkkkkkkkkkkkkkkkkkkkkkkkkkkkkkkkkkkkkkkkkkkkkkkkkkkkkkkkkkkkkkkkkkkkkkkkkkkkkkkkkkkkkk National Heart, Lung, and Blood Institute, National Institutes of Health, Rockville, MD, USA; llllllllllllllllllllllllllllllllllllllllllllllllllllllllllllllllllllllllllllllllllllllll Division of Population Medicine, Cardiff University, Cardiff, UK; mmmmmmmmmmmmmmmmmmmmmmmmmmmmmmmmmmmmmmmmmmmmmmmmmmmmmmmmmmmmmmmmmmmmmmmmmmmmmmmmmmmmmmmm Department of Neurology, Tehran University of Medical Sciences, Tehran, Iran; nnnnnnnnnnnnnnnnnnnnnnnnnnnnnnnnnnnnnnnnnnnnnnnnnnnnnnnnnnnnnnnnnnnnnnnnnnnnnnnnnnnnnnnn Department of Radiology, Northwestern University, Chicago, IL, USA; oooooooooooooooooooooooooooooooooooooooooooooooooooooooooooooooooooooooooooooooooooooooo School of Health Sciences, Universiti Sains Malaysia, Kota Bharu, Malaysia; pppppppppppppppppppppppppppppppppppppppppppppppppppppppppppppppppppppppppppppppppppppppp H.E.J. Research Institute of Chemistry, University of Karachi, Karachi, Pakistan; qqqqqqqqqqqqqqqqqqqqqqqqqqqqqqqqqqqqqqqqqqqqqqqqqqqqqqqqqqqqqqqqqqqqqqqqqqqqqqqqqqqqqqqq Research Centre for Health Sciences (RCHS), The University of Lahore, Lahore, Pakistan; rrrrrrrrrrrrrrrrrrrrrrrrrrrrrrrrrrrrrrrrrrrrrrrrrrrrrrrrrrrrrrrrrrrrrrrrrrrrrrrrrrrrrrrr Department of Biotechnology, Quaid-i-Azam University Islamabad, Islamabad, Pakistan; ssssssssssssssssssssssssssssssssssssssssssssssssssssssssssssssssssssssssssssssssssssssss Gastroenterology Unit, IRCCS, Castellana Grotte (Bari), Italy; tttttttttttttttttttttttttttttttttttttttttttttttttttttttttttttttttttttttttttttttttttttttt Department of Chemistry, Institute for Advanced Studies in Basic Sciences (IASBS), Zanjan, Iran; uuuuuuuuuuuuuuuuuuuuuuuuuuuuuuuuuuuuuuuuuuuuuuuuuuuuuuuuuuuuuuuuuuuuuuuuuuuuuuuuuuuuuuuu Center for Medical and Bio-Allied Health Sciences Research, Ajman University, Ajman, United Arab Emirates; vvvvvvvvvvvvvvvvvvvvvvvvvvvvvvvvvvvvvvvvvvvvvvvvvvvvvvvvvvvvvvvvvvvvvvvvvvvvvvvvvvvvvvvv Independent Consultant, Karachi, Pakistan; wwwwwwwwwwwwwwwwwwwwwwwwwwwwwwwwwwwwwwwwwwwwwwwwwwwwwwwwwwwwwwwwwwwwwwwwwwwwwwwwwwwwwwww Noncommunicable Diseases Research Center, Neyshabur University of Medical Sciences, Neyshabur, Iran; xxxxxxxxxxxxxxxxxxxxxxxxxxxxxxxxxxxxxxxxxxxxxxxxxxxxxxxxxxxxxxxxxxxxxxxxxxxxxxxxxxxxxxxx Neurology Department, Ain Shams University, Cairo, Egypt; yyyyyyyyyyyyyyyyyyyyyyyyyyyyyyyyyyyyyyyyyyyyyyyyyyyyyyyyyyyyyyyyyyyyyyyyyyyyyyyyyyyyyyyy Department of Pharmacology, All India Institute of Medical Sciences, Jodhpur, India; zzzzzzzzzzzzzzzzzzzzzzzzzzzzzzzzzzzzzzzzzzzzzzzzzzzzzzzzzzzzzzzzzzzzzzzzzzzzzzzzzzzzzzzz Division of Neurological Science, VETSUISSE, University of Bern, Bern, Switzerland; aaaaaaaaaaaaaaaaaaaaaaaaaaaaaaaaaaaaaaaaaaaaaaaaaaaaaaaaaaaaaaaaaaaaaaaaaaaaaaaaaaaaaaaaa School of Medicine, Alborz University of Medical Sciences, Karaj, Iran; bbbbbbbbbbbbbbbbbbbbbbbbbbbbbbbbbbbbbbbbbbbbbbbbbbbbbbbbbbbbbbbbbbbbbbbbbbbbbbbbbbbbbbbbb Department of Science, Kazakh National Medical University, Almaty, Kazakhstan; ccccccccccccccccccccccccccccccccccccccccccccccccccccccccccccccccccccccccccccccccccccccccc National University of Ireland - Galway, Galway, Ireland; ddddddddddddddddddddddddddddddddddddddddddddddddddddddddddddddddddddddddddddddddddddddddd Columbia University, New York, NY, USA; eeeeeeeeeeeeeeeeeeeeeeeeeeeeeeeeeeeeeeeeeeeeeeeeeeeeeeeeeeeeeeeeeeeeeeeeeeeeeeeeeeeeeeeee Department of Statistics, Harbin Institute of Technology, Harbin, China; fffffffffffffffffffffffffffffffffffffffffffffffffffffffffffffffffffffffffffffffffffffffff Department of Sociology, Zhejiang University, Hangzhou, China; ggggggggggggggggggggggggggggggggggggggggggggggggggggggggggggggggggggggggggggggggggggggggg College of Nursing and Health Sciences, Jazan University, Jazan, Saudi Arabia; hhhhhhhhhhhhhhhhhhhhhhhhhhhhhhhhhhhhhhhhhhhhhhhhhhhhhhhhhhhhhhhhhhhhhhhhhhhhhhhhhhhhhhhhh Department for Evidence-based Medicine and Evaluation, University for Continuing Education Krems, Krems, Austria; iiiiiiiiiiiiiiiiiiiiiiiiiiiiiiiiiiiiiiiiiiiiiiiiiiiiiiiiiiiiiiiiiiiiiiiiiiiiiiiiiiiiiiiii Universidad Espíritu Santo, Samborondón, Ecuador; jjjjjjjjjjjjjjjjjjjjjjjjjjjjjjjjjjjjjjjjjjjjjjjjjjjjjjjjjjjjjjjjjjjjjjjjjjjjjjjjjjjjjjjjj Amity Institute of Biotechnology, Amity University Rajasthan, Rajasthan, India; kkkkkkkkkkkkkkkkkkkkkkkkkkkkkkkkkkkkkkkkkkkkkkkkkkkkkkkkkkkkkkkkkkkkkkkkkkkkkkkkkkkkkkkkk Department of Forensic Science, Shree Guru Gobind Singh Tricentenary University, Gurugram, India; lllllllllllllllllllllllllllllllllllllllllllllllllllllllllllllllllllllllllllllllllllllllll Department of Biotechnology, Graphic Era (Deemed to be University), Dehradun, India; mmmmmmmmmmmmmmmmmmmmmmmmmmmmmmmmmmmmmmmmmmmmmmmmmmmmmmmmmmmmmmmmmmmmmmmmmmmmmmmmmmmmmmmmm Department of Biotechnology, Indian Institute of Technology Hyderabad, Kandi, India; nnnnnnnnnnnnnnnnnnnnnnnnnnnnnnnnnnnnnnnnnnnnnnnnnnnnnnnnnnnnnnnnnnnnnnnnnnnnnnnnnnnnnnnnn UN Mehta Institute of Cardiology and Research Center, B.J. Medical College, Ahmedabad, India; ooooooooooooooooooooooooooooooooooooooooooooooooooooooooooooooooooooooooooooooooooooooooo Department of Cardiology, Government Medical College, Ahmedabad, India; ppppppppppppppppppppppppppppppppppppppppppppppppppppppppppppppppppppppppppppppppppppppppp Department of Chemistry, Shree Guru Gobind Singh Tricentenary University, Gurugram, India; qqqqqqqqqqqqqqqqqqqqqqqqqqqqqqqqqqqqqqqqqqqqqqqqqqqqqqqqqqqqqqqqqqqqqqqqqqqqqqqqqqqqqqqqq Department of Social and Behavioral Health, University of Nevada Las Vegas, Las Vegas, NV, USA; rrrrrrrrrrrrrrrrrrrrrrrrrrrrrrrrrrrrrrrrrrrrrrrrrrrrrrrrrrrrrrrrrrrrrrrrrrrrrrrrrrrrrrrrr Department of University Institute of Biotechnology, Chandigarh University, Punjab, India; sssssssssssssssssssssssssssssssssssssssssssssssssssssssssssssssssssssssssssssssssssssssss Institute of Forensic Science & Criminology, Panjab University, Chandigarh, India; ttttttttttttttttttttttttttttttttttttttttttttttttttttttttttttttttttttttttttttttttttttttttt Department of Ophthalmology, Harvard University, Boston, MA, USA; uuuuuuuuuuuuuuuuuuuuuuuuuuuuuuuuuuuuuuuuuuuuuuuuuuuuuuuuuuuuuuuuuuuuuuuuuuuuuuuuuuuuuuuuu Ophthalmic Research Center (ORC), Shahid Beheshti University of Medical Sciences, Tehran, Iran; vvvvvvvvvvvvvvvvvvvvvvvvvvvvvvvvvvvvvvvvvvvvvvvvvvvvvvvvvvvvvvvvvvvvvvvvvvvvvvvvvvvvvvvvv Physiotherapy Department, Federal University of Health Sciences, Azare, Nigeria; wwwwwwwwwwwwwwwwwwwwwwwwwwwwwwwwwwwwwwwwwwwwwwwwwwwwwwwwwwwwwwwwwwwwwwwwwwwwwwwwwwwwwwwww Department of Endocrinology and Metabolism Population Sciences, Tehran University of Medical Sciences, Tehran, Iran; xxxxxxxxxxxxxxxxxxxxxxxxxxxxxxxxxxxxxxxxxxxxxxxxxxxxxxxxxxxxxxxxxxxxxxxxxxxxxxxxxxxxxxxxx School of Medicine, Non-Communicable Diseases Research Center (NCDRC), Tehran, Iran; yyyyyyyyyyyyyyyyyyyyyyyyyyyyyyyyyyyyyyyyyyyyyyyyyyyyyyyyyyyyyyyyyyyyyyyyyyyyyyyyyyyyyyyyy Department of Biology, Morgan State University, Baltimore, MD, USA; zzzzzzzzzzzzzzzzzzzzzzzzzzzzzzzzzzzzzzzzzzzzzzzzzzzzzzzzzzzzzzzzzzzzzzzzzzzzzzzzzzzzzzzzz Department of Environmental Health Sciences, Tulane University, New Orleans, LA, USA; aaaaaaaaaaaaaaaaaaaaaaaaaaaaaaaaaaaaaaaaaaaaaaaaaaaaaaaaaaaaaaaaaaaaaaaaaaaaaaaaaaaaaaaaaa Department of Gastroenterology and Hepatology, Manipal Academy of Higher Education, Udupi, India; bbbbbbbbbbbbbbbbbbbbbbbbbbbbbbbbbbbbbbbbbbbbbbbbbbbbbbbbbbbbbbbbbbbbbbbbbbbbbbbbbbbbbbbbbb Renji Hospital, Shanghai Jiao Tong University, Shanghai, China; cccccccccccccccccccccccccccccccccccccccccccccccccccccccccccccccccccccccccccccccccccccccccc Department of Epidemiology and Health Statistics, Wenzhou Medical University, Wenzhou, China; dddddddddddddddddddddddddddddddddddddddddddddddddddddddddddddddddddddddddddddddddddddddddd Department of Speech Therapy, Kermanshah University of Medical Sciences, Kermanshah, Iran; eeeeeeeeeeeeeeeeeeeeeeeeeeeeeeeeeeeeeeeeeeeeeeeeeeeeeeeeeeeeeeeeeeeeeeeeeeeeeeeeeeeeeeeeee Department of HIV/AIDS Prevention and Control, Amahara Regional Sate Health Bureau, Bahir Dar, Ethiopia; ffffffffffffffffffffffffffffffffffffffffffffffffffffffffffffffffffffffffffffffffffffffffff Tokyo Foundation for Policy Research, Tokyo, Japan; gggggggggggggggggggggggggggggggggggggggggggggggggggggggggggggggggggggggggggggggggggggggggg Department of Public Health, Dambi Dollo University, Dembi Dollo, Ethiopia; hhhhhhhhhhhhhhhhhhhhhhhhhhhhhhhhhhhhhhhhhhhhhhhhhhhhhhhhhhhhhhhhhhhhhhhhhhhhhhhhhhhhhhhhhh Department of Epidemiology, Jimma University, Jimma, Ethiopia; iiiiiiiiiiiiiiiiiiiiiiiiiiiiiiiiiiiiiiiiiiiiiiiiiiiiiiiiiiiiiiiiiiiiiiiiiiiiiiiiiiiiiiiiii Department of Pharmacology, Saint Paul's Hospital Millennium Medical College, Addis Ababa, Ethiopia; jjjjjjjjjjjjjjjjjjjjjjjjjjjjjjjjjjjjjjjjjjjjjjjjjjjjjjjjjjjjjjjjjjjjjjjjjjjjjjjjjjjjjjjjjj Korea University, Seoul, South Korea; kkkkkkkkkkkkkkkkkkkkkkkkkkkkkkkkkkkkkkkkkkkkkkkkkkkkkkkkkkkkkkkkkkkkkkkkkkkkkkkkkkkkkkkkkk Finnish Institute of Occupational Health, Helsinki, Finland; llllllllllllllllllllllllllllllllllllllllllllllllllllllllllllllllllllllllllllllllllllllllll Cancer Research Center, Tehran University of Medical Sciences, Tehran, Iran; mmmmmmmmmmmmmmmmmmmmmmmmmmmmmmmmmmmmmmmmmmmmmmmmmmmmmmmmmmmmmmmmmmmmmmmmmmmmmmmmmmmmmmmmmm Cancer Biology Research Center, Tehran University of Medical Sciences, Tehran, Iran; nnnnnnnnnnnnnnnnnnnnnnnnnnnnnnnnnnnnnnnnnnnnnnnnnnnnnnnnnnnnnnnnnnnnnnnnnnnnnnnnnnnnnnnnnn Department of Medicine, Ladoke Akintola University, Ogbomoso, Nigeria; oooooooooooooooooooooooooooooooooooooooooooooooooooooooooooooooooooooooooooooooooooooooooo Oulu Business School, University of Oulu, Oulu, Finland; pppppppppppppppppppppppppppppppppppppppppppppppppppppppppppppppppppppppppppppppppppppppppp Martti Ahtisaari Institute, University of Oulu, Oulu, Finland; qqqqqqqqqqqqqqqqqqqqqqqqqqqqqqqqqqqqqqqqqqqqqqqqqqqqqqqqqqqqqqqqqqqqqqqqqqqqqqqqqqqqqqqqqq Department of Experimental Research, Medical University Pleven, Pleven, Bulgaria; rrrrrrrrrrrrrrrrrrrrrrrrrrrrrrrrrrrrrrrrrrrrrrrrrrrrrrrrrrrrrrrrrrrrrrrrrrrrrrrrrrrrrrrrrr Department of Genetics, Sofia University “”St. Kliment Ohridiski“”, Sofia, Bulgaria; ssssssssssssssssssssssssssssssssssssssssssssssssssssssssssssssssssssssssssssssssssssssssss Department of Neurosurgery, Columbia University Medical Center, New York, NY, USA; tttttttttttttttttttttttttttttttttttttttttttttttttttttttttttttttttttttttttttttttttttttttttt Department of Clinical Practice, Jazan University, Jazan, Saudi Arabia; uuuuuuuuuuuuuuuuuuuuuuuuuuuuuuuuuuuuuuuuuuuuuuuuuuuuuuuuuuuuuuuuuuuuuuuuuuuuuuuuuuuuuuuuuu Student Scientific Research Center, Tehran University of Medical Sciences, Tehran, Iran; vvvvvvvvvvvvvvvvvvvvvvvvvvvvvvvvvvvvvvvvvvvvvvvvvvvvvvvvvvvvvvvvvvvvvvvvvvvvvvvvvvvvvvvvvv Center for Technology and Innovation in Cardiovascular Informatics, Iran University of Medical Sciences, Tehran, Iran; wwwwwwwwwwwwwwwwwwwwwwwwwwwwwwwwwwwwwwwwwwwwwwwwwwwwwwwwwwwwwwwwwwwwwwwwwwwwwwwwwwwwwwwwww Department of Medical-Surgical Nursing, Mazandaran University of Medical Sciences, Sari, Iran; xxxxxxxxxxxxxxxxxxxxxxxxxxxxxxxxxxxxxxxxxxxxxxxxxxxxxxxxxxxxxxxxxxxxxxxxxxxxxxxxxxxxxxxxxx Department of Nursing and Health Sciences, Flinders University, Adelaide, SA, Australia; yyyyyyyyyyyyyyyyyyyyyyyyyyyyyyyyyyyyyyyyyyyyyyyyyyyyyyyyyyyyyyyyyyyyyyyyyyyyyyyyyyyyyyyyyy Department of Research and Academics, Kathmandu Cancer Center, Bhaktapur, Nepal; zzzzzzzzzzzzzzzzzzzzzzzzzzzzzzzzzzzzzzzzzzzzzzzzzzzzzzzzzzzzzzzzzzzzzzzzzzzzzzzzzzzzzzzzzz Person-Centered Research, Monash University, Box Hill, VIC, Australia; aaaaaaaaaaaaaaaaaaaaaaaaaaaaaaaaaaaaaaaaaaaaaaaaaaaaaaaaaaaaaaaaaaaaaaaaaaaaaaaaaaaaaaaaaaa Clinical Pharmacy and Pharmacy Practice, Usmanu Danfodiyo University, Sokoto, Sokoto, Nigeria; bbbbbbbbbbbbbbbbbbbbbbbbbbbbbbbbbbbbbbbbbbbbbbbbbbbbbbbbbbbbbbbbbbbbbbbbbbbbbbbbbbbbbbbbbbb Kenneth H. Cooper Institute, Texas Tech University Health Sciences Center, Dallas, TX, USA; ccccccccccccccccccccccccccccccccccccccccccccccccccccccccccccccccccccccccccccccccccccccccccc Radio-Oncology Department of Shohadaye Tajrish Hospital, Shahid Beheshti University of Medical Sciences, Tehran, Iran; ddddddddddddddddddddddddddddddddddddddddddddddddddddddddddddddddddddddddddddddddddddddddddd Advanced Materials Division, Mintek, Randburg, South Africa; eeeeeeeeeeeeeeeeeeeeeeeeeeeeeeeeeeeeeeeeeeeeeeeeeeeeeeeeeeeeeeeeeeeeeeeeeeeeeeeeeeeeeeeeeee Department of Biotechnology, University of the Western Cape, Bellville, South Africa; fffffffffffffffffffffffffffffffffffffffffffffffffffffffffffffffffffffffffffffffffffffffffff Unit of Basic Medical Sciences, University of Khartoum, Khartoum, Sudan; ggggggggggggggggggggggggggggggggggggggggggggggggggggggggggggggggggggggggggggggggggggggggggg Department of Medical Microbiology and Infectious Diseases, Erasmus University, Rotterdam, Netherlands; hhhhhhhhhhhhhhhhhhhhhhhhhhhhhhhhhhhhhhhhhhhhhhhhhhhhhhhhhhhhhhhhhhhhhhhhhhhhhhhhhhhhhhhhhhh Department of Cardiothoracic Imaging, Emory University, Atlanta, GA, USA; iiiiiiiiiiiiiiiiiiiiiiiiiiiiiiiiiiiiiiiiiiiiiiiiiiiiiiiiiiiiiiiiiiiiiiiiiiiiiiiiiiiiiiiiiii Department of Physical Education, Federal University of Santa Catarina, Florianópolis, Brazil; jjjjjjjjjjjjjjjjjjjjjjjjjjjjjjjjjjjjjjjjjjjjjjjjjjjjjjjjjjjjjjjjjjjjjjjjjjjjjjjjjjjjjjjjjjj Sport Physical Activity and Health Research & Innovation Center (SPRINT), Polytechnic Institute of Guarda, Guarda, Portugal; kkkkkkkkkkkkkkkkkkkkkkkkkkkkkkkkkkkkkkkkkkkkkkkkkkkkkkkkkkkkkkkkkkkkkkkkkkkkkkkkkkkkkkkkkkk RISE Health, University of Beira Interior, Covilhã, Portugal; lllllllllllllllllllllllllllllllllllllllllllllllllllllllllllllllllllllllllllllllllllllllllll School of Human and Health Sciences, University of Huddersfield, Huddersfield, UK; mmmmmmmmmmmmmmmmmmmmmmmmmmmmmmmmmmmmmmmmmmmmmmmmmmmmmmmmmmmmmmmmmmmmmmmmmmmmmmmmmmmmmmmmmmm Department of Law, Economics, Management and Quantitative Methods, University of Sannio, Benevento, Italy; nnnnnnnnnnnnnnnnnnnnnnnnnnnnnnnnnnnnnnnnnnnnnnnnnnnnnnnnnnnnnnnnnnnnnnnnnnnnnnnnnnnnnnnnnnn WSB University in Gdańsk, Gdańsk, Poland; ooooooooooooooooooooooooooooooooooooooooooooooooooooooooooooooooooooooooooooooooooooooooooo Department of Dentistry, All India Institute of Medical Sciences, Bhopal, India; ppppppppppppppppppppppppppppppppppppppppppppppppppppppppppppppppppppppppppppppppppppppppppp Department of Microbiology, Central University of Punjab, Bathinda, India; qqqqqqqqqqqqqqqqqqqqqqqqqqqqqqqqqqqqqqqqqqqqqqqqqqqqqqqqqqqqqqqqqqqqqqqqqqqqqqqqqqqqqqqqqqq Department of Laboratory Medicine, All India Institute of Medical Sciences, New Delhi, India; rrrrrrrrrrrrrrrrrrrrrrrrrrrrrrrrrrrrrrrrrrrrrrrrrrrrrrrrrrrrrrrrrrrrrrrrrrrrrrrrrrrrrrrrrrr School of Public Health & Zoonoses, Guru Angad Dev Veterinary & Animal Sciences University, Ludhiana, India; sssssssssssssssssssssssssssssssssssssssssssssssssssssssssssssssssssssssssssssssssssssssssss School of Veterinary Science, University of Sydney, Sydney, NSW, Australia; ttttttttttttttttttttttttttttttttttttttttttttttttttttttttttttttttttttttttttttttttttttttttttt Department of Biochemistry, Central University of Punjab, Bathinda, India; uuuuuuuuuuuuuuuuuuuuuuuuuuuuuuuuuuuuuuuuuuuuuuuuuuuuuuuuuuuuuuuuuuuuuuuuuuuuuuuuuuuuuuuuuuu Department of Agriculture and Environmental Sceinces, National Institute of Food Technology Entrepreneurship and Management-Kundli (NIFTEM-K), Sonipat, India; vvvvvvvvvvvvvvvvvvvvvvvvvvvvvvvvvvvvvvvvvvvvvvvvvvvvvvvvvvvvvvvvvvvvvvvvvvvvvvvvvvvvvvvvvvv Department of Pharmacology, Government Medical College and Hospital, Chandigarh, India; wwwwwwwwwwwwwwwwwwwwwwwwwwwwwwwwwwwwwwwwwwwwwwwwwwwwwwwwwwwwwwwwwwwwwwwwwwwwwwwwwwwwwwwwwww School of Pharmaceutical Sciences, IFTM University, Moradabad, India; xxxxxxxxxxxxxxxxxxxxxxxxxxxxxxxxxxxxxxxxxxxxxxxxxxxxxxxxxxxxxxxxxxxxxxxxxxxxxxxxxxxxxxxxxxx School of Medicine, Baylor College of Medicine, Houston, TX, USA; yyyyyyyyyyyyyyyyyyyyyyyyyyyyyyyyyyyyyyyyyyyyyyyyyyyyyyyyyyyyyyyyyyyyyyyyyyyyyyyyyyyyyyyyyyy Department of Medicine Service, US Department of Veterans Affairs (VA), Houston, TX, USA; zzzzzzzzzzzzzzzzzzzzzzzzzzzzzzzzzzzzzzzzzzzzzzzzzzzzzzzzzzzzzzzzzzzzzzzzzzzzzzzzzzzzzzzzzzz Department of Psychiatry, All India Institute of Medical Sciences, Punjab, India; aaaaaaaaaaaaaaaaaaaaaaaaaaaaaaaaaaaaaaaaaaaaaaaaaaaaaaaaaaaaaaaaaaaaaaaaaaaaaaaaaaaaaaaaaaaa Research Department, Hamad Medical Corporation, Doha, Qatar; bbbbbbbbbbbbbbbbbbbbbbbbbbbbbbbbbbbbbbbbbbbbbbbbbbbbbbbbbbbbbbbbbbbbbbbbbbbbbbbbbbbbbbbbbbbb Medical Oncology Lab, All India Institute of Medical Sciences, New Delhi, India; cccccccccccccccccccccccccccccccccccccccccccccccccccccccccccccccccccccccccccccccccccccccccccc Faculty of Medicine and Health Sciences, Shree Guru Gobind Singh Tricentenary University, Gurugram, India; dddddddddddddddddddddddddddddddddddddddddddddddddddddddddddddddddddddddddddddddddddddddddddd Department of Radiodiagnosis, All India Institute of Medical Sciences, Bathinda, India; eeeeeeeeeeeeeeeeeeeeeeeeeeeeeeeeeeeeeeeeeeeeeeeeeeeeeeeeeeeeeeeeeeeeeeeeeeeeeeeeeeeeeeeeeeee Amity Institute of Public Health and Hospital Administration, Amity University, Noida, India; ffffffffffffffffffffffffffffffffffffffffffffffffffffffffffffffffffffffffffffffffffffffffffff Department of Human Genetics, Punjabi University Patiala, Patiala, India; gggggggggggggggggggggggggggggggggggggggggggggggggggggggggggggggggggggggggggggggggggggggggggg Department of Biochemistry, Banaras Hindu University, Varanasi, India; hhhhhhhhhhhhhhhhhhhhhhhhhhhhhhhhhhhhhhhhhhhhhhhhhhhhhhhhhhhhhhhhhhhhhhhhhhhhhhhhhhhhhhhhhhhh Institute of Medical Sciences, Banaras Hindu University, Varanasi, India; iiiiiiiiiiiiiiiiiiiiiiiiiiiiiiiiiiiiiiiiiiiiiiiiiiiiiiiiiiiiiiiiiiiiiiiiiiiiiiiiiiiiiiiiiiii Department of Computer Science & Engineering, Central University of Punjab, Bathinda, India; jjjjjjjjjjjjjjjjjjjjjjjjjjjjjjjjjjjjjjjjjjjjjjjjjjjjjjjjjjjjjjjjjjjjjjjjjjjjjjjjjjjjjjjjjjjj Department of Community Medicine, Veer Chandra Singh Garhwali Government Institute of Medical Science and Research, Srinagar Garhwal, India; kkkkkkkkkkkkkkkkkkkkkkkkkkkkkkkkkkkkkkkkkkkkkkkkkkkkkkkkkkkkkkkkkkkkkkkkkkkkkkkkkkkkkkkkkkkk Department of Internal Medicine, University of Indonesia, Jakarta, Indonesia; llllllllllllllllllllllllllllllllllllllllllllllllllllllllllllllllllllllllllllllllllllllllllll Department of Internal Medicine, Dr. Cipto Mangunkusumo National Hospital, Jakarta Pusat, Indonesia; mmmmmmmmmmmmmmmmmmmmmmmmmmmmmmmmmmmmmmmmmmmmmmmmmmmmmmmmmmmmmmmmmmmmmmmmmmmmmmmmmmmmmmmmmmmm Department of Anesthesiology, New York Medical College, Passaic, NJ, USA; nnnnnnnnnnnnnnnnnnnnnnnnnnnnnnnnnnnnnnnnnnnnnnnnnnnnnnnnnnnnnnnnnnnnnnnnnnnnnnnnnnnnnnnnnnnn Global and European Health Education and Study Institute, University of Georgia, Tbilisi, Georgia; oooooooooooooooooooooooooooooooooooooooooooooooooooooooooooooooooooooooooooooooooooooooooooo National Center for Disease Control and Public Health, Tbilisi, Georgia; pppppppppppppppppppppppppppppppppppppppppppppppppppppppppppppppppppppppppppppppppppppppppppp Department of Infectious Diseases and Epidemiology, Pirogov Russian National Research Medical University, Moscow, Russia; qqqqqqqqqqqqqqqqqqqqqqqqqqqqqqqqqqqqqqqqqqqqqqqqqqqqqqqqqqqqqqqqqqqqqqqqqqqqqqqqqqqqqqqqqqqq Division of Injury Prevention, The Bizzell Group, Atlanta, GA, USA; rrrrrrrrrrrrrrrrrrrrrrrrrrrrrrrrrrrrrrrrrrrrrrrrrrrrrrrrrrrrrrrrrrrrrrrrrrrrrrrrrrrrrrrrrrrr Rollins School of Public Health, Emory University, Atlanta, GA, USA; ssssssssssssssssssssssssssssssssssssssssssssssssssssssssssssssssssssssssssssssssssssssssssss Department of Biology, Guilan University of Medical Sciences, Rasht, Iran; tttttttttttttttttttttttttttttttttttttttttttttttttttttttttttttttttttttttttttttttttttttttttttt Department of Pharmacy and Pharmaceutical Sciences, Western New England University, Springfield, MA, USA; uuuuuuuuuuuuuuuuuuuuuuuuuuuuuuuuuuuuuuuuuuuuuuuuuuuuuuuuuuuuuuuuuuuuuuuuuuuuuuuuuuuuuuuuuuuu Department of Development Studies, Daffodil International University, Dhaka, Bangladesh; vvvvvvvvvvvvvvvvvvvvvvvvvvvvvvvvvvvvvvvvvvvvvvvvvvvvvvvvvvvvvvvvvvvvvvvvvvvvvvvvvvvvvvvvvvvv Department of Biochemistry, American University of Integrative Sciences, Bridgetown, Barbados; wwwwwwwwwwwwwwwwwwwwwwwwwwwwwwwwwwwwwwwwwwwwwwwwwwwwwwwwwwwwwwwwwwwwwwwwwwwwwwwwwwwwwwwwwwww School of Public Health, Wollega University, Nekemte, Ethiopia; xxxxxxxxxxxxxxxxxxxxxxxxxxxxxxxxxxxxxxxxxxxxxxxxxxxxxxxxxxxxxxxxxxxxxxxxxxxxxxxxxxxxxxxxxxxx Faculty of Public Health, Universitas Ahmad Dahlan, Yogyakarta, Indonesia; yyyyyyyyyyyyyyyyyyyyyyyyyyyyyyyyyyyyyyyyyyyyyyyyyyyyyyyyyyyyyyyyyyyyyyyyyyyyyyyyyyyyyyyyyyyy Department of Medicinal Chemistry, University of Sharjah, Sharjah, United Arab Emirates; zzzzzzzzzzzzzzzzzzzzzzzzzzzzzzzzzzzzzzzzzzzzzzzzzzzzzzzzzzzzzzzzzzzzzzzzzzzzzzzzzzzzzzzzzzzz Department of Endocrinology, Case Western Reserve University, Cleveland, OH, USA; aaaaaaaaaaaaaaaaaaaaaaaaaaaaaaaaaaaaaaaaaaaaaaaaaaaaaaaaaaaaaaaaaaaaaaaaaaaaaaaaaaaaaaaaaaaaa Independent Consultant, New Delhi, India; bbbbbbbbbbbbbbbbbbbbbbbbbbbbbbbbbbbbbbbbbbbbbbbbbbbbbbbbbbbbbbbbbbbbbbbbbbbbbbbbbbbbbbbbbbbbb School of Medicine, Babol University of Medical Sciences, Babol, Iran; ccccccccccccccccccccccccccccccccccccccccccccccccccccccccccccccccccccccccccccccccccccccccccccc Hospital Universitario de La Princesa, Universidad Autónoma de Madrid (Autonomous University of Madrid), Madrid, Spain; ddddddddddddddddddddddddddddddddddddddddddddddddddddddddddddddddddddddddddddddddddddddddddddd Centro de Investigación Biomédica en Red Enfermedades Respiratorias (CIBERES) (Center for Biomedical Research in Respiratory Diseases Network), Madrid, Spain; eeeeeeeeeeeeeeeeeeeeeeeeeeeeeeeeeeeeeeeeeeeeeeeeeeeeeeeeeeeeeeeeeeeeeeeeeeeeeeeeeeeeeeeeeeeee School of Primary and Allied Health Care, Monash University, Melbourne, VIC, Australia; fffffffffffffffffffffffffffffffffffffffffffffffffffffffffffffffffffffffffffffffffffffffffffff Universidade Federal de Minas Gerais (Federal University of Minas Gerais), Belo Horizonte, Brazil; ggggggggggggggggggggggggggggggggggggggggggggggggggggggggggggggggggggggggggggggggggggggggggggg Hull York Medical School, University of Hull, Hull City, UK; hhhhhhhhhhhhhhhhhhhhhhhhhhhhhhhhhhhhhhhhhhhhhhhhhhhhhhhhhhhhhhhhhhhhhhhhhhhhhhhhhhhhhhhhhhhhh Doheny Eye Institute, University of California Los Angeles, Pasadena, CA, USA; iiiiiiiiiiiiiiiiiiiiiiiiiiiiiiiiiiiiiiiiiiiiiiiiiiiiiiiiiiiiiiiiiiiiiiiiiiiiiiiiiiiiiiiiiiiii 3rd Department of Cardiology, University of Athens, Athens, Greece; jjjjjjjjjjjjjjjjjjjjjjjjjjjjjjjjjjjjjjjjjjjjjjjjjjjjjjjjjjjjjjjjjjjjjjjjjjjjjjjjjjjjjjjjjjjjj Medical and Diagnostic Research Centre, University of Hail, Hail, Saudi Arabia; kkkkkkkkkkkkkkkkkkkkkkkkkkkkkkkkkkkkkkkkkkkkkkkkkkkkkkkkkkkkkkkkkkkkkkkkkkkkkkkkkkkkkkkkkkkkk Department of Pharmacology, RAK Medical and Health Sciences University, Ras Al Khaimah, United Arab Emirates; lllllllllllllllllllllllllllllllllllllllllllllllllllllllllllllllllllllllllllllllllllllllllllll College of Health and Public Service, University of North Texas, Denton, TX, USA; mmmmmmmmmmmmmmmmmmmmmmmmmmmmmmmmmmmmmmmmmmmmmmmmmmmmmmmmmmmmmmmmmmmmmmmmmmmmmmmmmmmmmmmmmmmmm Manipal College of Health Professions, Manipal Academy of Higher Education, manipal, India; nnnnnnnnnnnnnnnnnnnnnnnnnnnnnnnnnnnnnnnnnnnnnnnnnnnnnnnnnnnnnnnnnnnnnnnnnnnnnnnnnnnnnnnnnnnnn Department of Public Health, Kandahar University, Kandahar, Afghanistan; ooooooooooooooooooooooooooooooooooooooooooooooooooooooooooooooooooooooooooooooooooooooooooooo Department of Health Professions, DHGS German University for Health and Sports, Berlin, Germany; ppppppppppppppppppppppppppppppppppppppppppppppppppppppppppppppppppppppppppppppppppppppppppppp SAMRC Unit on Risk and Resilience in Mental Disorders, University of Cape Town, Cape Town, South Africa; qqqqqqqqqqqqqqqqqqqqqqqqqqqqqqqqqqqqqqqqqqqqqqqqqqqqqqqqqqqqqqqqqqqqqqqqqqqqqqqqqqqqqqqqqqqqq Department of Medicine, Democritus University of Thrace, Alexandroupolis, Greece; rrrrrrrrrrrrrrrrrrrrrrrrrrrrrrrrrrrrrrrrrrrrrrrrrrrrrrrrrrrrrrrrrrrrrrrrrrrrrrrrrrrrrrrrrrrrr enAble Institute, Curtin University, Perth, WA, Australia; sssssssssssssssssssssssssssssssssssssssssssssssssssssssssssssssssssssssssssssssssssssssssssss Occupational and Environmental Medicine Department, University of Gothenburg, Gothenburg, Sweden; ttttttttttttttttttttttttttttttttttttttttttttttttttttttttttttttttttttttttttttttttttttttttttttt Division of Preventive Medicine, University of Alberta, Edmonton, AB, Canada; uuuuuuuuuuuuuuuuuuuuuuuuuuuuuuuuuuuuuuuuuuuuuuuuuuuuuuuuuuuuuuuuuuuuuuuuuuuuuuuuuuuuuuuuuuuuu School of Public Health, University of Alberta, Edmonton, AB, Canada; vvvvvvvvvvvvvvvvvvvvvvvvvvvvvvvvvvvvvvvvvvvvvvvvvvvvvvvvvvvvvvvvvvvvvvvvvvvvvvvvvvvvvvvvvvvvv Department of Medicine, University of British Columbia, Vancouver, BC, Canada; wwwwwwwwwwwwwwwwwwwwwwwwwwwwwwwwwwwwwwwwwwwwwwwwwwwwwwwwwwwwwwwwwwwwwwwwwwwwwwwwwwwwwwwwwwwww Discipline of Physiotherapy, University of Technology Sydney, Sydney, NSW, Australia; xxxxxxxxxxxxxxxxxxxxxxxxxxxxxxxxxxxxxxxxxxxxxxxxxxxxxxxxxxxxxxxxxxxxxxxxxxxxxxxxxxxxxxxxxxxxx Department of Orthopaedics, Massachusetts General Hospital, Boston, MA, USA; yyyyyyyyyyyyyyyyyyyyyyyyyyyyyyyyyyyyyyyyyyyyyyyyyyyyyyyyyyyyyyyyyyyyyyyyyyyyyyyyyyyyyyyyyyyyy Department of Orthopaedics, Harvard University, Cambridge, MA, USA; zzzzzzzzzzzzzzzzzzzzzzzzzzzzzzzzzzzzzzzzzzzzzzzzzzzzzzzzzzzzzzzzzzzzzzzzzzzzzzzzzzzzzzzzzzzzz Research Department, Nepal Development Society, Kathmandu, Nepal; aaaaaaaaaaaaaaaaaaaaaaaaaaaaaaaaaaaaaaaaaaaaaaaaaaaaaaaaaaaaaaaaaaaaaaaaaaaaaaaaaaaaaaaaaaaaaa School of Exercise and Nutrition Sciences, Deakin University, Melbourne, VIC, Australia; bbbbbbbbbbbbbbbbbbbbbbbbbbbbbbbbbbbbbbbbbbbbbbbbbbbbbbbbbbbbbbbbbbbbbbbbbbbbbbbbbbbbbbbbbbbbbb Integrative Epidemiology Unit, University of Bristol, Bristol, UK; cccccccccccccccccccccccccccccccccccccccccccccccccccccccccccccccccccccccccccccccccccccccccccccc Clinical Research Unit, Projahnmo Research Foundation, Dhaka, Bangladesh; dddddddddddddddddddddddddddddddddddddddddddddddddddddddddddddddddddddddddddddddddddddddddddddd Praboromarajchanok Institute, Ministry of Public Health, Nonthaburi, Thailand; eeeeeeeeeeeeeeeeeeeeeeeeeeeeeeeeeeeeeeeeeeeeeeeeeeeeeeeeeeeeeeeeeeeeeeeeeeeeeeeeeeeeeeeeeeeeee Department of Physiotherapy, Tishk International University, Erbil, Iraq; ffffffffffffffffffffffffffffffffffffffffffffffffffffffffffffffffffffffffffffffffffffffffffffff Department of Community Medicine, Ahmadu Bello University, Kaduna State, Nigeria; gggggggggggggggggggggggggggggggggggggggggggggggggggggggggggggggggggggggggggggggggggggggggggggg Department of Human Anatomy, Federal University, Dutse, Dutse, Nigeria; hhhhhhhhhhhhhhhhhhhhhhhhhhhhhhhhhhhhhhhhhhhhhhhhhhhhhhhhhhhhhhhhhhhhhhhhhhhhhhhhhhhhhhhhhhhhhh School of Life Sciences, Xiamen University, Xiamen, China; iiiiiiiiiiiiiiiiiiiiiiiiiiiiiiiiiiiiiiiiiiiiiiiiiiiiiiiiiiiiiiiiiiiiiiiiiiiiiiiiiiiiiiiiiiiiii Department of Life and Health Sciences, University of Nicosia, Nicosia, Cyprus; jjjjjjjjjjjjjjjjjjjjjjjjjjjjjjjjjjjjjjjjjjjjjjjjjjjjjjjjjjjjjjjjjjjjjjjjjjjjjjjjjjjjjjjjjjjjjj School of Medicine, Medical Sciences and Nutrition, University of Aberdeen, Aberdeen, UK; kkkkkkkkkkkkkkkkkkkkkkkkkkkkkkkkkkkkkkkkkkkkkkkkkkkkkkkkkkkkkkkkkkkkkkkkkkkkkkkkkkkkkkkkkkkkkk University Diabetes Center, King Saud University, Riyadh, Saudi Arabia; llllllllllllllllllllllllllllllllllllllllllllllllllllllllllllllllllllllllllllllllllllllllllllll Yusuf Hamied Department of Chemistry, University of Cambridge, Cambridgeshire, UK; mmmmmmmmmmmmmmmmmmmmmmmmmmmmmmmmmmmmmmmmmmmmmmmmmmmmmmmmmmmmmmmmmmmmmmmmmmmmmmmmmmmmmmmmmmmmmm Institute of Integrated Intelligence and Systems, Griffith University, Brisbane, QLD, Australia; nnnnnnnnnnnnnnnnnnnnnnnnnnnnnnnnnnnnnnnnnnnnnnnnnnnnnnnnnnnnnnnnnnnnnnnnnnnnnnnnnnnnnnnnnnnnnn The First Hospital of China Medical University, China Medical University, Shenyang, China; oooooooooooooooooooooooooooooooooooooooooooooooooooooooooooooooooooooooooooooooooooooooooooooo Department of Endocrinology and Metabolism, Affiliated Hospital of Shandong Second Medical University, Weifang, China; pppppppppppppppppppppppppppppppppppppppppppppppppppppppppppppppppppppppppppppppppppppppppppppp School of Clinical Medicine, Tsinghua University, Beijing, China; qqqqqqqqqqqqqqqqqqqqqqqqqqqqqqqqqqqqqqqqqqqqqqqqqqqqqqqqqqqqqqqqqqqqqqqqqqqqqqqqqqqqqqqqqqqqqq Department of Neurology, Tsinghua University, Beijing, China; rrrrrrrrrrrrrrrrrrrrrrrrrrrrrrrrrrrrrrrrrrrrrrrrrrrrrrrrrrrrrrrrrrrrrrrrrrrrrrrrrrrrrrrrrrrrrr Department of Biomedical Sciences, Universiti Putra Malaysia (University of Putra Malaysia), Selangor, Malaysia; ssssssssssssssssssssssssssssssssssssssssssssssssssssssssssssssssssssssssssssssssssssssssssssss High-Quality Development Evaluation Research Institute, Nanjing University of Posts and Telecommunications, Nanjing, China; tttttttttttttttttttttttttttttttttttttttttttttttttttttttttttttttttttttttttttttttttttttttttttttt Gandhi Medical College, Kaloji Narayana Rao University of Health Sciences (KNRUHS), Secunderabad, India; uuuuuuuuuuuuuuuuuuuuuuuuuuuuuuuuuuuuuuuuuuuuuuuuuuuuuuuuuuuuuuuuuuuuuuuuuuuuuuuuuuuuuuuuuuuuuu Department of Population Health Sciences, University College London, London, UK; vvvvvvvvvvvvvvvvvvvvvvvvvvvvvvvvvvvvvvvvvvvvvvvvvvvvvvvvvvvvvvvvvvvvvvvvvvvvvvvvvvvvvvvvvvvvvv Department of Psychiatry, University of Oxford, Oxford, UK; wwwwwwwwwwwwwwwwwwwwwwwwwwwwwwwwwwwwwwwwwwwwwwwwwwwwwwwwwwwwwwwwwwwwwwwwwwwwwwwwwwwwwwwwwwwwww Department of Clinical Pathology, Brawijaya University, Malang, Indonesia; xxxxxxxxxxxxxxxxxxxxxxxxxxxxxxxxxxxxxxxxxxxxxxxxxxxxxxxxxxxxxxxxxxxxxxxxxxxxxxxxxxxxxxxxxxxxxx Hospital Central Laboratory, Dr Saiful Anwar General Hospital, Malang, Indonesia; yyyyyyyyyyyyyyyyyyyyyyyyyyyyyyyyyyyyyyyyyyyyyyyyyyyyyyyyyyyyyyyyyyyyyyyyyyyyyyyyyyyyyyyyyyyyyy School of Population Health, Curtin University, Perth, VIC, Australia; zzzzzzzzzzzzzzzzzzzzzzzzzzzzzzzzzzzzzzzzzzzzzzzzzzzzzzzzzzzzzzzzzzzzzzzzzzzzzzzzzzzzzzzzzzzzzz Department of Clinical Research and Development, LUXMED Group, Warsaw, Poland; aaaaaaaaaaaaaaaaaaaaaaaaaaaaaaaaaaaaaaaaaaaaaaaaaaaaaaaaaaaaaaaaaaaaaaaaaaaaaaaaaaaaaaaaaaaaaaa Collegium Medicum, John Paul II Catholic University of Lublin, Lublin, Poland; bbbbbbbbbbbbbbbbbbbbbbbbbbbbbbbbbbbbbbbbbbbbbbbbbbbbbbbbbbbbbbbbbbbbbbbbbbbbbbbbbbbbbbbbbbbbbbb Northwestern University, Chicago, IL, USA; ccccccccccccccccccccccccccccccccccccccccccccccccccccccccccccccccccccccccccccccccccccccccccccccc Department of Pharmacology, All India Institute of Medical Sciences, Deoghar, India; ddddddddddddddddddddddddddddddddddddddddddddddddddddddddddddddddddddddddddddddddddddddddddddddd Department of Neurology, Neurocenter of Southern Switzerland (NSI), Lugano, Switzerland; eeeeeeeeeeeeeeeeeeeeeeeeeeeeeeeeeeeeeeeeeeeeeeeeeeeeeeeeeeeeeeeeeeeeeeeeeeeeeeeeeeeeeeeeeeeeeee Department of Medicine, University of Valencia, Valencia, Spain; fffffffffffffffffffffffffffffffffffffffffffffffffffffffffffffffffffffffffffffffffffffffffffffff Carlos III Health Institute, Biomedical Research Networking Center for Mental Health Network (CiberSAM), Madrid, Spain; ggggggggggggggggggggggggggggggggggggggggggggggggggggggggggggggggggggggggggggggggggggggggggggggg Neurology Department, Shahid Beheshti University of Medical Sciences, Tehran, Iran; hhhhhhhhhhhhhhhhhhhhhhhhhhhhhhhhhhhhhhhhhhhhhhhhhhhhhhhhhhhhhhhhhhhhhhhhhhhhhhhhhhhhhhhhhhhhhhh Department of Medical Informatics, Mashhad University of Medical Sciences, Mashhad, Iran; iiiiiiiiiiiiiiiiiiiiiiiiiiiiiiiiiiiiiiiiiiiiiiiiiiiiiiiiiiiiiiiiiiiiiiiiiiiiiiiiiiiiiiiiiiiiiii Applied Biomedical Research Center, Mashhad University of Medical Sciences, Mashhad, Iran; jjjjjjjjjjjjjjjjjjjjjjjjjjjjjjjjjjjjjjjjjjjjjjjjjjjjjjjjjjjjjjjjjjjjjjjjjjjjjjjjjjjjjjjjjjjjjjj Department of Basic Medical Sciences, Islamic Azad University, Mashhad, Iran; kkkkkkkkkkkkkkkkkkkkkkkkkkkkkkkkkkkkkkkkkkkkkkkkkkkkkkkkkkkkkkkkkkkkkkkkkkkkkkkkkkkkkkkkkkkkkkk Department of Internal Medicine, Islamic Azad University, Mashhad, Iran; lllllllllllllllllllllllllllllllllllllllllllllllllllllllllllllllllllllllllllllllllllllllllllllll Department of Medical Education, Shahid Beheshti University of Medical Sciences, Tehran, Iran; mmmmmmmmmmmmmmmmmmmmmmmmmmmmmmmmmmmmmmmmmmmmmmmmmmmmmmmmmmmmmmmmmmmmmmmmmmmmmmmmmmmmmmmmmmmmmmm Division of Epidemiology, Tohoku University, Sendai, Japan; nnnnnnnnnnnnnnnnnnnnnnnnnnnnnnnnnnnnnnnnnnnnnnnnnnnnnnnnnnnnnnnnnnnnnnnnnnnnnnnnnnnnnnnnnnnnnnn Department of Internal Medicine, Oncology Unit, St Paul's Hospital Millenium Medical College, Addis Ababa, Ethiopia; ooooooooooooooooooooooooooooooooooooooooooooooooooooooooooooooooooooooooooooooooooooooooooooooo The Five Senses Health Institute, Iran University of Medical Sciences, Tehran, Iran; ppppppppppppppppppppppppppppppppppppppppppppppppppppppppppppppppppppppppppppppppppppppppppppppp Duhok Research Centre, University of Duhok, Duhok, Iraq; qqqqqqqqqqqqqqqqqqqqqqqqqqqqqqqqqqqqqqqqqqqqqqqqqqqqqqqqqqqqqqqqqqqqqqqqqqqqqqqqqqqqqqqqqqqqqqq Department of Environmental, Agricultural and Occupational Health, University of Nebraska Medical Center, Omaha, NE, USA; rrrrrrrrrrrrrrrrrrrrrrrrrrrrrrrrrrrrrrrrrrrrrrrrrrrrrrrrrrrrrrrrrrrrrrrrrrrrrrrrrrrrrrrrrrrrrrr Department of Pathology, Alexandria University, Alexandria, Egypt; sssssssssssssssssssssssssssssssssssssssssssssssssssssssssssssssssssssssssssssssssssssssssssssss Department of Dermatology, Carol Davila University of Medicine and Pharmacy, Bucharest, Romania; ttttttttttttttttttttttttttttttttttttttttttttttttttttttttttttttttttttttttttttttttttttttttttttttt Department of Dermato-Venereology, Dr. Victor Babes Clinical Hospital of Infectious Diseases and Tropical Diseases, Bucharest, Romania; uuuuuuuuuuuuuuuuuuuuuuuuuuuuuuuuuuuuuuuuuuuuuuuuuuuuuuuuuuuuuuuuuuuuuuuuuuuuuuuuuuuuuuuuuuuuuuu Department of Epidemiology, Stellenbosch University, Cape Town, South Africa; vvvvvvvvvvvvvvvvvvvvvvvvvvvvvvvvvvvvvvvvvvvvvvvvvvvvvvvvvvvvvvvvvvvvvvvvvvvvvvvvvvvvvvvvvvvvvvv Department of Medicine, Northlands Medical Group, Omuthiya, Namibia; wwwwwwwwwwwwwwwwwwwwwwwwwwwwwwwwwwwwwwwwwwwwwwwwwwwwwwwwwwwwwwwwwwwwwwwwwwwwwwwwwwwwwwwwwwwwwww Faculty of Medicine, Yarmouk University, Irbid, Jordan; xxxxxxxxxxxxxxxxxxxxxxxxxxxxxxxxxxxxxxxxxxxxxxxxxxxxxxxxxxxxxxxxxxxxxxxxxxxxxxxxxxxxxxxxxxxxxxx State Key Laboratory of Numerical Modeling for Atmospheric Sciences and Geophysical Fluid Dynamics (LASG), Chinese Academy of Sciences, Beijing, China; yyyyyyyyyyyyyyyyyyyyyyyyyyyyyyyyyyyyyyyyyyyyyyyyyyyyyyyyyyyyyyyyyyyyyyyyyyyyyyyyyyyyyyyyyyyyyyy Department of Computer and Software Engineering, National University of Science and Technology (NUST), Islamabad, Pakistan; zzzzzzzzzzzzzzzzzzzzzzzzzzzzzzzzzzzzzzzzzzzzzzzzzzzzzzzzzzzzzzzzzzzzzzzzzzzzzzzzzzzzzzzzzzzzzzz Nossal Institute for Global Health, University of Melbourne, Melbourne, VIC, Australia; aaaaaaaaaaaaaaaaaaaaaaaaaaaaaaaaaaaaaaaaaaaaaaaaaaaaaaaaaaaaaaaaaaaaaaaaaaaaaaaaaaaaaaaaaaaaaaaa Department of Psychology, Montclair State University, Montclair, NJ, USA; bbbbbbbbbbbbbbbbbbbbbbbbbbbbbbbbbbbbbbbbbbbbbbbbbbbbbbbbbbbbbbbbbbbbbbbbbbbbbbbbbbbbbbbbbbbbbbbb Department of Public Health, Debre Markos University, Debre Markos, Ethiopia; cccccccccccccccccccccccccccccccccccccccccccccccccccccccccccccccccccccccccccccccccccccccccccccccc Department of Pharmacology and Therapeutics, The University of Faisalabad, Faisalabad, Pakistan; dddddddddddddddddddddddddddddddddddddddddddddddddddddddddddddddddddddddddddddddddddddddddddddddd Department of Metabolism and Systems Science, University of Birmingham, Birmingham, UK; eeeeeeeeeeeeeeeeeeeeeeeeeeeeeeeeeeeeeeeeeeeeeeeeeeeeeeeeeeeeeeeeeeeeeeeeeeeeeeeeeeeeeeeeeeeeeeee Department of Pharmaceutical Health Outcomes and Policy, University of Houston College of Pharmacy, Houston, TX, USA; ffffffffffffffffffffffffffffffffffffffffffffffffffffffffffffffffffffffffffffffffffffffffffffffff Research Chair for Evidence-Based Health Care and Knowledge Translation, King Saud University, Riyadh, Saudi Arabia; gggggggggggggggggggggggggggggggggggggggggggggggggggggggggggggggggggggggggggggggggggggggggggggggg Department of Preventive Medicine, Northwestern University, Chicago, IL, USA; hhhhhhhhhhhhhhhhhhhhhhhhhhhhhhhhhhhhhhhhhhhhhhhhhhhhhhhhhhhhhhhhhhhhhhhhhhhhhhhhhhhhhhhhhhhhhhhh Department of Pediatrics and Child Health, University of Gondar, Gondar, Ethiopia; iiiiiiiiiiiiiiiiiiiiiiiiiiiiiiiiiiiiiiiiiiiiiiiiiiiiiiiiiiiiiiiiiiiiiiiiiiiiiiiiiiiiiiiiiiiiiiii School of Public Health, University of Adelaide, Adelaide, SA, Australia; jjjjjjjjjjjjjjjjjjjjjjjjjjjjjjjjjjjjjjjjjjjjjjjjjjjjjjjjjjjjjjjjjjjjjjjjjjjjjjjjjjjjjjjjjjjjjjjj Internal Medicine Department, King George's Medical University, Lucknow, India; kkkkkkkkkkkkkkkkkkkkkkkkkkkkkkkkkkkkkkkkkkkkkkkkkkkkkkkkkkkkkkkkkkkkkkkkkkkkkkkkkkkkkkkkkkkkkkkk Department of Abdominal Surgery, Katholieke Universiteit Leuven, Leuven, Belgium; llllllllllllllllllllllllllllllllllllllllllllllllllllllllllllllllllllllllllllllllllllllllllllllll Department of Clinical Microbiology, Karnali Academy of Health Sciences (KAHS), Jumla, Nepal; mmmmmmmmmmmmmmmmmmmmmmmmmmmmmmmmmmmmmmmmmmmmmmmmmmmmmmmmmmmmmmmmmmmmmmmmmmmmmmmmmmmmmmmmmmmmmmmm Amrita Vishwa Vidyapeetham, Amrita Institute of Medical Sciences, Kochi, India; nnnnnnnnnnnnnnnnnnnnnnnnnnnnnnnnnnnnnnnnnnnnnnnnnnnnnnnnnnnnnnnnnnnnnnnnnnnnnnnnnnnnnnnnnnnnnnnn Department of Economics, The American University in Cairo, Cairo, Egypt; oooooooooooooooooooooooooooooooooooooooooooooooooooooooooooooooooooooooooooooooooooooooooooooooo Rheumatology and Immunology Unit, Mansoura University, Mansoura, Egypt; pppppppppppppppppppppppppppppppppppppppppppppppppppppppppppppppppppppppppppppppppppppppppppppppp Faculty of Medicine, University of Southampton, Southampton, UK; qqqqqqqqqqqqqqqqqqqqqqqqqqqqqqqqqqqqqqqqqqqqqqqqqqqqqqqqqqqqqqqqqqqqqqqqqqqqqqqqqqqqqqqqqqqqqqqq Department of Applied Bioscience, Konkuk University, Seoul, South Korea; rrrrrrrrrrrrrrrrrrrrrrrrrrrrrrrrrrrrrrrrrrrrrrrrrrrrrrrrrrrrrrrrrrrrrrrrrrrrrrrrrrrrrrrrrrrrrrrr Department of Conservative Dentistry and Endodontics, Manipal Academy of Higher Education, Mangalore, India; ssssssssssssssssssssssssssssssssssssssssssssssssssssssssssssssssssssssssssssssssssssssssssssssss School of Public Health, Harbin Medical University, Harbin, China; tttttttttttttttttttttttttttttttttttttttttttttttttttttttttttttttttttttttttttttttttttttttttttttttt Faculty of Public Health, Universitas Sam Ratulangi (Sam Ratulangi University), Manado, Indonesia; uuuuuuuuuuuuuuuuuuuuuuuuuuuuuuuuuuuuuuuuuuuuuuuuuuuuuuuuuuuuuuuuuuuuuuuuuuuuuuuuuuuuuuuuuuuuuuuu Department of Prosthetic Dentistry, Kazakh National Medical University, Almaty, Kazakhstan; vvvvvvvvvvvvvvvvvvvvvvvvvvvvvvvvvvvvvvvvvvvvvvvvvvvvvvvvvvvvvvvvvvvvvvvvvvvvvvvvvvvvvvvvvvvvvvvv Department of Biochemistry, All India Institute of Medical Sciences, Jodhpur, India; wwwwwwwwwwwwwwwwwwwwwwwwwwwwwwwwwwwwwwwwwwwwwwwwwwwwwwwwwwwwwwwwwwwwwwwwwwwwwwwwwwwwwwwwwwwwwwww Interdisciplinary Health Data Center, Jagiellonian University Medical College, Kraków, Poland; xxxxxxxxxxxxxxxxxxxxxxxxxxxxxxxxxxxxxxxxxxxxxxxxxxxxxxxxxxxxxxxxxxxxxxxxxxxxxxxxxxxxxxxxxxxxxxxx Nutritional Epidemiology Research Team (EREN), National Institute for Health and Medical Research (INSERM), Paris, France; yyyyyyyyyyyyyyyyyyyyyyyyyyyyyyyyyyyyyyyyyyyyyyyyyyyyyyyyyyyyyyyyyyyyyyyyyyyyyyyyyyyyyyyyyyyyyyyy Department of Health, Medicine and Human Biology, Sorbonne Paris Nord University, Bobigny, France; zzzzzzzzzzzzzzzzzzzzzzzzzzzzzzzzzzzzzzzzzzzzzzzzzzzzzzzzzzzzzzzzzzzzzzzzzzzzzzzzzzzzzzzzzzzzzzzz High Institute of Sport and Physical Education of Sfax, University of Sfax, Sfax, Tunisia; aaaaaaaaaaaaaaaaaaaaaaaaaaaaaaaaaaaaaaaaaaaaaaaaaaaaaaaaaaaaaaaaaaaaaaaaaaaaaaaaaaaaaaaaaaaaaaaaa Department of Movement Sciences and Sports Training, The University of Jordan, Amman, Jordan; bbbbbbbbbbbbbbbbbbbbbbbbbbbbbbbbbbbbbbbbbbbbbbbbbbbbbbbbbbbbbbbbbbbbbbbbbbbbbbbbbbbbbbbbbbbbbbbbb Second Department of Internal Medicine, Kansai Medical University, Hirakata, Japan; ccccccccccccccccccccccccccccccccccccccccccccccccccccccccccccccccccccccccccccccccccccccccccccccccc John T. Milliken Department of Medicine, Washington University in St. Louis, Saint Louis, MO, USA; ddddddddddddddddddddddddddddddddddddddddddddddddddddddddddddddddddddddddddddddddddddddddddddddddd Department of Internal Medicine, University of Medicine and Pharmacy at Ho Chi Minh City, Ho Chi Minh City, Viet Nam; eeeeeeeeeeeeeeeeeeeeeeeeeeeeeeeeeeeeeeeeeeeeeeeeeeeeeeeeeeeeeeeeeeeeeeeeeeeeeeeeeeeeeeeeeeeeeeeee Department of Business Analytics, University of Massachusetts Dartmouth, Dartmouth, MA, USA; fffffffffffffffffffffffffffffffffffffffffffffffffffffffffffffffffffffffffffffffffffffffffffffffff Molecular Neuroscience Research Center, Shiga University of Medical Science, Shiga, Japan; ggggggggggggggggggggggggggggggggggggggggggggggggggggggggggggggggggggggggggggggggggggggggggggggggg ALS Vietnam Research and Advocacy Initiative, ALS Vietnam, Quang Ngai, Viet Nam; hhhhhhhhhhhhhhhhhhhhhhhhhhhhhhhhhhhhhhhhhhhhhhhhhhhhhhhhhhhhhhhhhhhhhhhhhhhhhhhhhhhhhhhhhhhhhhhhh Department of Neurology, All India Institute of Medical Sciences, Delhi, India; iiiiiiiiiiiiiiiiiiiiiiiiiiiiiiiiiiiiiiiiiiiiiiiiiiiiiiiiiiiiiiiiiiiiiiiiiiiiiiiiiiiiiiiiiiiiiiiii Department of Studies in Economics and Planning, Central University of Gujarat, Gandhinagar, India; jjjjjjjjjjjjjjjjjjjjjjjjjjjjjjjjjjjjjjjjjjjjjjjjjjjjjjjjjjjjjjjjjjjjjjjjjjjjjjjjjjjjjjjjjjjjjjjjj Adult Learning Disability Service, Leicestershire Partnership National Health Service Trust, Leicester, UK; kkkkkkkkkkkkkkkkkkkkkkkkkkkkkkkkkkkkkkkkkkkkkkkkkkkkkkkkkkkkkkkkkkkkkkkkkkkkkkkkkkkkkkkkkkkkkkkkk College of Health Science, VinUniversity, Hanoi, Viet Nam; lllllllllllllllllllllllllllllllllllllllllllllllllllllllllllllllllllllllllllllllllllllllllllllllll Department of Cardiology, Tianjin Medical University, Tianjin, China; mmmmmmmmmmmmmmmmmmmmmmmmmmmmmmmmmmmmmmmmmmmmmmmmmmmmmmmmmmmmmmmmmmmmmmmmmmmmmmmmmmmmmmmmmmmmmmmmm Kent and Medway Medical School, Canterbury, UK; nnnnnnnnnnnnnnnnnnnnnnnnnnnnnnnnnnnnnnnnnnnnnnnnnnnnnnnnnnnnnnnnnnnnnnnnnnnnnnnnnnnnnnnnnnnnnnnnn Laboratory of Clinical Pharmacology, Aristotle University of Thessaloniki, Thessaloniki, Greece; ooooooooooooooooooooooooooooooooooooooooooooooooooooooooooooooooooooooooooooooooooooooooooooooooo Department of Epidemiology and Public Health, University College London, London, UK; ppppppppppppppppppppppppppppppppppppppppppppppppppppppppppppppppppppppppppppppppppppppppppppppppp Department of Internal Medicine, Wake Forest University, Winston-Salem, NC, USA; qqqqqqqqqqqqqqqqqqqqqqqqqqqqqqqqqqqqqqqqqqqqqqqqqqqqqqqqqqqqqqqqqqqqqqqqqqqqqqqqqqqqqqqqqqqqqqqqq Department of Urology, The Second Hospital of Tianjin Medical University, Tianjin, China; rrrrrrrrrrrrrrrrrrrrrrrrrrrrrrrrrrrrrrrrrrrrrrrrrrrrrrrrrrrrrrrrrrrrrrrrrrrrrrrrrrrrrrrrrrrrrrrrr Department of Epidemiology and Biostatistics, Haramaya University, Harar, Ethiopia; sssssssssssssssssssssssssssssssssssssssssssssssssssssssssssssssssssssssssssssssssssssssssssssssss Joslin Diabetes Center, Harvard University, Boston, MA, USA; ttttttttttttttttttttttttttttttttttttttttttttttttttttttttttttttttttttttttttttttttttttttttttttttttt Department of Nutrition and Food Studies, George Mason University, Fairfax, VA, USA; uuuuuuuuuuuuuuuuuuuuuuuuuuuuuuuuuuuuuuuuuuuuuuuuuuuuuuuuuuuuuuuuuuuuuuuuuuuuuuuuuuuuuuuuuuuuuuuuu School of Nursing, Hong Kong Polytechnic University, Hong Kong, China; vvvvvvvvvvvvvvvvvvvvvvvvvvvvvvvvvvvvvvvvvvvvvvvvvvvvvvvvvvvvvvvvvvvvvvvvvvvvvvvvvvvvvvvvvvvvvvvvv Hayatabad Medical Complex, Postgraduate Medical Institute, Peshawar, Pakistan; wwwwwwwwwwwwwwwwwwwwwwwwwwwwwwwwwwwwwwwwwwwwwwwwwwwwwwwwwwwwwwwwwwwwwwwwwwwwwwwwwwwwwwwwwwwwwwwww Department of Biology and Biochemistry, University of Houston, Houston, TX, USA; xxxxxxxxxxxxxxxxxxxxxxxxxxxxxxxxxxxxxxxxxxxxxxxxxxxxxxxxxxxxxxxxxxxxxxxxxxxxxxxxxxxxxxxxxxxxxxxxx Medical Genomics Research Department, King Abdullah International Medical Research Center, Riyadh, Saudi Arabia; yyyyyyyyyyyyyyyyyyyyyyyyyyyyyyyyyyyyyyyyyyyyyyyyyyyyyyyyyyyyyyyyyyyyyyyyyyyyyyyyyyyyyyyyyyyyyyyyy Department of Life Sciences, University of Management and Technology, Lahore, Pakistan; zzzzzzzzzzzzzzzzzzzzzzzzzzzzzzzzzzzzzzzzzzzzzzzzzzzzzzzzzzzzzzzzzzzzzzzzzzzzzzzzzzzzzzzzzzzzzzzzz Department of Human Anatomy, Federal University Dutse, Dutse, Nigeria; aaaaaaaaaaaaaaaaaaaaaaaaaaaaaaaaaaaaaaaaaaaaaaaaaaaaaaaaaaaaaaaaaaaaaaaaaaaaaaaaaaaaaaaaaaaaaaaaaa Department of Physiotherapy, Federal Ministry of Health, Azare, Nigeria; bbbbbbbbbbbbbbbbbbbbbbbbbbbbbbbbbbbbbbbbbbbbbbbbbbbbbbbbbbbbbbbbbbbbbbbbbbbbbbbbbbbbbbbbbbbbbbbbbb Federal University of Health Sciences Teaching Hospital Azare, Azare, Nigeria; cccccccccccccccccccccccccccccccccccccccccccccccccccccccccccccccccccccccccccccccccccccccccccccccccc Lahore Business School, The University of Lahore, Lahore, Pakistan; dddddddddddddddddddddddddddddddddddddddddddddddddddddddddddddddddddddddddddddddddddddddddddddddddd Department of Medicine, Khairpur Medical College, Khairpur, Pakistan; eeeeeeeeeeeeeeeeeeeeeeeeeeeeeeeeeeeeeeeeeeeeeeeeeeeeeeeeeeeeeeeeeeeeeeeeeeeeeeeeeeeeeeeeeeeeeeeeee Department of Oncology, Federal Medical Centre, Gusau, Nigeria; ffffffffffffffffffffffffffffffffffffffffffffffffffffffffffffffffffffffffffffffffffffffffffffffffff School of Government, Pontificia Universidad Catolica de Chile (Pontifical Catholic University of Chile), Santiago, Chile; gggggggggggggggggggggggggggggggggggggggggggggggggggggggggggggggggggggggggggggggggggggggggggggggggg Kasturba Medical College, Manipal Academy of Higher Education, Manipal, India; hhhhhhhhhhhhhhhhhhhhhhhhhhhhhhhhhhhhhhhhhhhhhhhhhhhhhhhhhhhhhhhhhhhhhhhhhhhhhhhhhhhhhhhhhhhhhhhhhh Amity Institute of Biotechnology, Amity University Rajasthan, Jaipur, India; iiiiiiiiiiiiiiiiiiiiiiiiiiiiiiiiiiiiiiiiiiiiiiiiiiiiiiiiiiiiiiiiiiiiiiiiiiiiiiiiiiiiiiiiiiiiiiiiii Section of Advanced Heart Failure and Transplant, Northwell Health / North Shore University Hospital, Manhasset, NY, USA; jjjjjjjjjjjjjjjjjjjjjjjjjjjjjjjjjjjjjjjjjjjjjjjjjjjjjjjjjjjjjjjjjjjjjjjjjjjjjjjjjjjjjjjjjjjjjjjjjj Center for Neurodegenerative Diseases and the Aging Brain, University of Bari, Tricase, Italy; kkkkkkkkkkkkkkkkkkkkkkkkkkkkkkkkkkkkkkkkkkkkkkkkkkkkkkkkkkkkkkkkkkkkkkkkkkkkkkkkkkkkkkkkkkkkkkkkkk Institute of Psychiatry, Psychology & Neuroscience, King's College London, London, UK; llllllllllllllllllllllllllllllllllllllllllllllllllllllllllllllllllllllllllllllllllllllllllllllllll Harvard Kennedy School, Harvard University, Cambridge, MA, USA; mmmmmmmmmmmmmmmmmmmmmmmmmmmmmmmmmmmmmmmmmmmmmmmmmmmmmmmmmmmmmmmmmmmmmmmmmmmmmmmmmmmmmmmmmmmmmmmmmm Department of Orthodontics, University of Trakya, Edirne, Türkiye; nnnnnnnnnnnnnnnnnnnnnnnnnnnnnnnnnnnnnnnnnnnnnnnnnnnnnnnnnnnnnnnnnnnnnnnnnnnnnnnnnnnnnnnnnnnnnnnnnn Johnson & Johnson, Duquesne University, Pittsburgh, PA, USA; oooooooooooooooooooooooooooooooooooooooooooooooooooooooooooooooooooooooooooooooooooooooooooooooooo College of Health and Sport Sciences, University of Bahrain, Zallaq, Bahrain; pppppppppppppppppppppppppppppppppppppppppppppppppppppppppppppppppppppppppppppppppppppppppppppppppp Sociedad Argentina de Medicina, Buenos Aires, Argentina; qqqqqqqqqqqqqqqqqqqqqqqqqqqqqqqqqqqqqqqqqqqqqqqqqqqqqqqqqqqqqqqqqqqqqqqqqqqqqqqqqqqqqqqqqqqqqqqqqq Hospital Vélez Sarsfield, Buenos Aires, Argentina; rrrrrrrrrrrrrrrrrrrrrrrrrrrrrrrrrrrrrrrrrrrrrrrrrrrrrrrrrrrrrrrrrrrrrrrrrrrrrrrrrrrrrrrrrrrrrrrrrr Department of Biomedical Sciences, Humanitas University, Milan, Italy; ssssssssssssssssssssssssssssssssssssssssssssssssssssssssssssssssssssssssssssssssssssssssssssssssss Dermatology Unit, IRCCS Humanitas Research Hospital, Milan, Italy; tttttttttttttttttttttttttttttttttttttttttttttttttttttttttttttttttttttttttttttttttttttttttttttttttt Department of Psychology, Zayed University, Abu Dhabi, United Arab Emirates; uuuuuuuuuuuuuuuuuuuuuuuuuuuuuuuuuuuuuuuuuuuuuuuuuuuuuuuuuuuuuuuuuuuuuuuuuuuuuuuuuuuuuuuuuuuuuuuuuu Faculty of Sciences, University of Guilan, Rasht, Iran; vvvvvvvvvvvvvvvvvvvvvvvvvvvvvvvvvvvvvvvvvvvvvvvvvvvvvvvvvvvvvvvvvvvvvvvvvvvvvvvvvvvvvvvvvvvvvvvvvv UKK Institute, Tampere, Finland; wwwwwwwwwwwwwwwwwwwwwwwwwwwwwwwwwwwwwwwwwwwwwwwwwwwwwwwwwwwwwwwwwwwwwwwwwwwwwwwwwwwwwwwwwwwwwwwwww Faculty of Medicine and Health Technology, Tampere University, Tampere, Finland; xxxxxxxxxxxxxxxxxxxxxxxxxxxxxxxxxxxxxxxxxxxxxxxxxxxxxxxxxxxxxxxxxxxxxxxxxxxxxxxxxxxxxxxxxxxxxxxxxx Department of Biochemistry, Apollo Institute of Medical Sciences and Research, Chittoor, India; yyyyyyyyyyyyyyyyyyyyyyyyyyyyyyyyyyyyyyyyyyyyyyyyyyyyyyyyyyyyyyyyyyyyyyyyyyyyyyyyyyyyyyyyyyyyyyyyyy Department of Otolaryngology Head and Neck Surgery, Louisiana State University Health Sciences Center, Shreveport, LA, USA; zzzzzzzzzzzzzzzzzzzzzzzzzzzzzzzzzzzzzzzzzzzzzzzzzzzzzzzzzzzzzzzzzzzzzzzzzzzzzzzzzzzzzzzzzzzzzzzzzz Biomedical Engineering Department, University of Texas, Arlington, TX, USA; aaaaaaaaaaaaaaaaaaaaaaaaaaaaaaaaaaaaaaaaaaaaaaaaaaaaaaaaaaaaaaaaaaaaaaaaaaaaaaaaaaaaaaaaaaaaaaaaaaa Department of Infectious Disease, Kermanshah University of Medical Sciences, Kermanshah, Iran; bbbbbbbbbbbbbbbbbbbbbbbbbbbbbbbbbbbbbbbbbbbbbbbbbbbbbbbbbbbbbbbbbbbbbbbbbbbbbbbbbbbbbbbbbbbbbbbbbbb Raffles Neuroscience Centre, Raffles Hospital, Singapore, Singapore; ccccccccccccccccccccccccccccccccccccccccccccccccccccccccccccccccccccccccccccccccccccccccccccccccccc Department of Community Medicine and Family Medicine, All India Institute of Medical Sciences, Bathinda, India; ddddddddddddddddddddddddddddddddddddddddddddddddddddddddddddddddddddddddddddddddddddddddddddddddddd Department of Surgery, University of Southampton, Southampton, UK; eeeeeeeeeeeeeeeeeeeeeeeeeeeeeeeeeeeeeeeeeeeeeeeeeeeeeeeeeeeeeeeeeeeeeeeeeeeeeeeeeeeeeeeeeeeeeeeeeee College of Medicine and Veterinary Medicine, University of Edinburgh, Edinburgh, UK; fffffffffffffffffffffffffffffffffffffffffffffffffffffffffffffffffffffffffffffffffffffffffffffffffff Department of Neurology, Infermi Hospital, Rimini, Italy; ggggggggggggggggggggggggggggggggggggggggggggggggggggggggggggggggggggggggggggggggggggggggggggggggggg Department of Neurology & Stroke Unit, Sant'Anna Hospital, Como, Italy; hhhhhhhhhhhhhhhhhhhhhhhhhhhhhhhhhhhhhhhhhhhhhhhhhhhhhhhhhhhhhhhhhhhhhhhhhhhhhhhhhhhhhhhhhhhhhhhhhhh Department of Community Medicine, All India Institute of Medical Sciences, Nagpur, India; iiiiiiiiiiiiiiiiiiiiiiiiiiiiiiiiiiiiiiiiiiiiiiiiiiiiiiiiiiiiiiiiiiiiiiiiiiiiiiiiiiiiiiiiiiiiiiiiiii Department of Biomedical Sciences for Health, Università degli Studi di Milano (University of Milan), Milano, Italy; jjjjjjjjjjjjjjjjjjjjjjjjjjjjjjjjjjjjjjjjjjjjjjjjjjjjjjjjjjjjjjjjjjjjjjjjjjjjjjjjjjjjjjjjjjjjjjjjjjj Department of Physiotherapy, Universidad Europea de Madrid (European University of Madrid), Villaviciosa de Odón, Spain; kkkkkkkkkkkkkkkkkkkkkkkkkkkkkkkkkkkkkkkkkkkkkkkkkkkkkkkkkkkkkkkkkkkkkkkkkkkkkkkkkkkkkkkkkkkkkkkkkkk Department of Health Science and Public Health, Università Cattolica del Sacro Cuore (Catholic University of the Sacred Heart), Rome, Italy; lllllllllllllllllllllllllllllllllllllllllllllllllllllllllllllllllllllllllllllllllllllllllllllllllll Saint Camillus International University of Health Sciences - UniCamillus, Rome, Italy; mmmmmmmmmmmmmmmmmmmmmmmmmmmmmmmmmmmmmmmmmmmmmmmmmmmmmmmmmmmmmmmmmmmmmmmmmmmmmmmmmmmmmmmmmmmmmmmmmmm Digital Health Research Center, Instituto Peruano de Orientación Psicológica, Lima, Peru; nnnnnnnnnnnnnnnnnnnnnnnnnnnnnnnnnnnnnnnnnnnnnnnnnnnnnnnnnnnnnnnnnnnnnnnnnnnnnnnnnnnnnnnnnnnnnnnnnnn Department of Biomedical Informatics, University of Utah, Salt Lake City, UT, USA; ooooooooooooooooooooooooooooooooooooooooooooooooooooooooooooooooooooooooooooooooooooooooooooooooooo Occupational Medicine Unit, Sant'Orsola Malpighi Hospital, Bologna, Italy; ppppppppppppppppppppppppppppppppppppppppppppppppppppppppppppppppppppppppppppppppppppppppppppppppppp Department of Bioengineering, School of Chemical and Biotechnology, SASTRA Deemed to be University, Thanjavur, India; qqqqqqqqqqqqqqqqqqqqqqqqqqqqqqqqqqqqqqqqqqqqqqqqqqqqqqqqqqqqqqqqqqqqqqqqqqqqqqqqqqqqqqqqqqqqqqqqqqq Faculty of Medicine of Itajubá, Brazil, Itajubá, Brazil; rrrrrrrrrrrrrrrrrrrrrrrrrrrrrrrrrrrrrrrrrrrrrrrrrrrrrrrrrrrrrrrrrrrrrrrrrrrrrrrrrrrrrrrrrrrrrrrrrrr Department of Health Care Administration and Economics, National Research University Higher School of Economics, Moscow, Russia; sssssssssssssssssssssssssssssssssssssssssssssssssssssssssssssssssssssssssssssssssssssssssssssssssss Department of Environmental Health Engineering, Ardabil University of Medical Science, Ardabil, Iran; ttttttttttttttttttttttttttttttttttttttttttttttttttttttttttttttttttttttttttttttttttttttttttttttttttt Diabetes Research Centre, University of Leicester, Leicester, UK; uuuuuuuuuuuuuuuuuuuuuuuuuuuuuuuuuuuuuuuuuuuuuuuuuuuuuuuuuuuuuuuuuuuuuuuuuuuuuuuuuuuuuuuuuuuuuuuuuuu Faculty of Public Health, VNU University of Medicine and Pharmacy, Hanoi, Viet Nam; vvvvvvvvvvvvvvvvvvvvvvvvvvvvvvvvvvvvvvvvvvvvvvvvvvvvvvvvvvvvvvvvvvvvvvvvvvvvvvvvvvvvvvvvvvvvvvvvvvv International Institute for Training and Research (INSTAR), VNU University of Medicine and Pharmacy, Hanoi, Viet Nam; wwwwwwwwwwwwwwwwwwwwwwwwwwwwwwwwwwwwwwwwwwwwwwwwwwwwwwwwwwwwwwwwwwwwwwwwwwwwwwwwwwwwwwwwwwwwwwwwwww Department of Biomedical Sciences, Arba Minch University, Arba Minch, Ethiopia; xxxxxxxxxxxxxxxxxxxxxxxxxxxxxxxxxxxxxxxxxxxxxxxxxxxxxxxxxxxxxxxxxxxxxxxxxxxxxxxxxxxxxxxxxxxxxxxxxxx Department of Medical Physiology, Addis Ababa University, Addis Ababa, Ethiopia; yyyyyyyyyyyyyyyyyyyyyyyyyyyyyyyyyyyyyyyyyyyyyyyyyyyyyyyyyyyyyyyyyyyyyyyyyyyyyyyyyyyyyyyyyyyyyyyyyyy NUST School of Health Sciences, National University of Science and Technology (NUST), Islamabad, Pakistan; zzzzzzzzzzzzzzzzzzzzzzzzzzzzzzzzzzzzzzzzzzzzzzzzzzzzzzzzzzzzzzzzzzzzzzzzzzzzzzzzzzzzzzzzzzzzzzzzzzz Széchenyi István University, Gyor, Hungary; aaaaaaaaaaaaaaaaaaaaaaaaaaaaaaaaaaaaaaaaaaaaaaaaaaaaaaaaaaaaaaaaaaaaaaaaaaaaaaaaaaaaaaaaaaaaaaaaaaaa Department of Nursing and Applied Health Sciences, Jazan University, Jazan, Saudi Arabia; bbbbbbbbbbbbbbbbbbbbbbbbbbbbbbbbbbbbbbbbbbbbbbbbbbbbbbbbbbbbbbbbbbbbbbbbbbbbbbbbbbbbbbbbbbbbbbbbbbbb Research Organization for Health, National Research and Innovation Agency (BRIN), Bogor, Indonesia; cccccccccccccccccccccccccccccccccccccccccccccccccccccccccccccccccccccccccccccccccccccccccccccccccccc Department of Psychiatry, Haramaya University, Harar, Ethiopia; dddddddddddddddddddddddddddddddddddddddddddddddddddddddddddddddddddddddddddddddddddddddddddddddddddd School of Chinese Medicine, Beijing University of Chinese Medicine, Beijing, China; eeeeeeeeeeeeeeeeeeeeeeeeeeeeeeeeeeeeeeeeeeeeeeeeeeeeeeeeeeeeeeeeeeeeeeeeeeeeeeeeeeeeeeeeeeeeeeeeeeee University of Chicago, Chicago, IL, USA; ffffffffffffffffffffffffffffffffffffffffffffffffffffffffffffffffffffffffffffffffffffffffffffffffffff Department of Pediatrics, All India Institute of Medical Sciences, Bathinda, India; gggggggggggggggggggggggggggggggggggggggggggggggggggggggggggggggggggggggggggggggggggggggggggggggggggg West China Hospital, Chengdu, China; hhhhhhhhhhhhhhhhhhhhhhhhhhhhhhhhhhhhhhhhhhhhhhhhhhhhhhhhhhhhhhhhhhhhhhhhhhhhhhhhhhhhhhhhhhhhhhhhhhhh Faculty of Health Sciences, University of Macau, Macau, China; iiiiiiiiiiiiiiiiiiiiiiiiiiiiiiiiiiiiiiiiiiiiiiiiiiiiiiiiiiiiiiiiiiiiiiiiiiiiiiiiiiiiiiiiiiiiiiiiiiii Department of Laboratory Medicine, Guangdong Provincial People's Hospital, Guangzhou, China; jjjjjjjjjjjjjjjjjjjjjjjjjjjjjjjjjjjjjjjjjjjjjjjjjjjjjjjjjjjjjjjjjjjjjjjjjjjjjjjjjjjjjjjjjjjjjjjjjjjj School of Biomedical Sciences, The University of Western Australia, Perth, WA, Australia; kkkkkkkkkkkkkkkkkkkkkkkkkkkkkkkkkkkkkkkkkkkkkkkkkkkkkkkkkkkkkkkkkkkkkkkkkkkkkkkkkkkkkkkkkkkkkkkkkkkk Department of Health Services Research, Management and Policy, University of Florida, Gainesville, FL, USA; llllllllllllllllllllllllllllllllllllllllllllllllllllllllllllllllllllllllllllllllllllllllllllllllllll Department of Neurosurgery, Capital Medical University, Beijing, China; mmmmmmmmmmmmmmmmmmmmmmmmmmmmmmmmmmmmmmmmmmmmmmmmmmmmmmmmmmmmmmmmmmmmmmmmmmmmmmmmmmmmmmmmmmmmmmmmmmmm Department of Neurosurgery, Beijing Tiantan Hospital, Beijing, China; nnnnnnnnnnnnnnnnnnnnnnnnnnnnnnnnnnnnnnnnnnnnnnnnnnnnnnnnnnnnnnnnnnnnnnnnnnnnnnnnnnnnnnnnnnnnnnnnnnnn National Institute of Health Data Science, Peking University, Beijing, China; oooooooooooooooooooooooooooooooooooooooooooooooooooooooooooooooooooooooooooooooooooooooooooooooooooo College of Agriculture, Northwest A&F University, Xianyang City, China; pppppppppppppppppppppppppppppppppppppppppppppppppppppppppppppppppppppppppppppppppppppppppppppppppppp Enze Medical Health Academy, Taizhou Hospital of Zhejiang Province, Taizhou, China; qqqqqqqqqqqqqqqqqqqqqqqqqqqqqqqqqqqqqqqqqqqqqqqqqqqqqqqqqqqqqqqqqqqqqqqqqqqqqqqqqqqqqqqqqqqqqqqqqqqq School of Public Health, Zhengzhou University, Zhengzhou, China; rrrrrrrrrrrrrrrrrrrrrrrrrrrrrrrrrrrrrrrrrrrrrrrrrrrrrrrrrrrrrrrrrrrrrrrrrrrrrrrrrrrrrrrrrrrrrrrrrrrr School of Life Course and Population Sciences, King's College London, London, UK; ssssssssssssssssssssssssssssssssssssssssssssssssssssssssssssssssssssssssssssssssssssssssssssssssssss School of Public Health, Peking University, Beijing, China; tttttttttttttttttttttttttttttttttttttttttttttttttttttttttttttttttttttttttttttttttttttttttttttttttttt Division of Life Sciences and Medicine, University of Science and Technology of China, Heifei, China; uuuuuuuuuuuuuuuuuuuuuuuuuuuuuuuuuuuuuuuuuuuuuuuuuuuuuuuuuuuuuuuuuuuuuuuuuuuuuuuuuuuuuuuuuuuuuuuuuuuu Department of Pharmaceutical Chemistry College of Pharmacy, King Saud University, Riyadh, Saudi Arabia; vvvvvvvvvvvvvvvvvvvvvvvvvvvvvvvvvvvvvvvvvvvvvvvvvvvvvvvvvvvvvvvvvvvvvvvvvvvvvvvvvvvvvvvvvvvvvvvvvvvv School of Medicine and Dentistry, Griffith University, Gold Coast, QLD, Australia; wwwwwwwwwwwwwwwwwwwwwwwwwwwwwwwwwwwwwwwwwwwwwwwwwwwwwwwwwwwwwwwwwwwwwwwwwwwwwwwwwwwwwwwwwwwwwwwwwwww School of Nursing Sciences, University of Nairobi, Nairobi, Kenya; xxxxxxxxxxxxxxxxxxxxxxxxxxxxxxxxxxxxxxxxxxxxxxxxxxxxxxxxxxxxxxxxxxxxxxxxxxxxxxxxxxxxxxxxxxxxxxxxxxxx Faculty of Sciences, The University of Lahore, Lahore, Pakistan; yyyyyyyyyyyyyyyyyyyyyyyyyyyyyyyyyyyyyyyyyyyyyyyyyyyyyyyyyyyyyyyyyyyyyyyyyyyyyyyyyyyyyyyyyyyyyyyyyyyy Coalition for Global Hepatitis Elimination, Task Force for Global Health, Decatur, GA, USA; zzzzzzzzzzzzzzzzzzzzzzzzzzzzzzzzzzzzzzzzzzzzzzzzzzzzzzzzzzzzzzzzzzzzzzzzzzzzzzzzzzzzzzzzzzzzzzzzzzzz Centre for Health Policy Research, Torrens University Australia, Adelaide, SA, Australia; aaaaaaaaaaaaaaaaaaaaaaaaaaaaaaaaaaaaaaaaaaaaaaaaaaaaaaaaaaaaaaaaaaaaaaaaaaaaaaaaaaaaaaaaaaaaaaaaaaaaa Institute of Health and Wellbeing, Federation University, Melbourne, VIC, Australia; bbbbbbbbbbbbbbbbbbbbbbbbbbbbbbbbbbbbbbbbbbbbbbbbbbbbbbbbbbbbbbbbbbbbbbbbbbbbbbbbbbbbbbbbbbbbbbbbbbbbb University of Adelaide, North Terrace, NSW, Australia; ccccccccccccccccccccccccccccccccccccccccccccccccccccccccccccccccccccccccccccccccccccccccccccccccccccc Department of Orthopaedics, General Hospital of Central Theater Command, Wuhan, China; ddddddddddddddddddddddddddddddddddddddddddddddddddddddddddddddddddddddddddddddddddddddddddddddddddddd Fourth Military Medical University, Xi'an, China; eeeeeeeeeeeeeeeeeeeeeeeeeeeeeeeeeeeeeeeeeeeeeeeeeeeeeeeeeeeeeeeeeeeeeeeeeeeeeeeeeeeeeeeeeeeeeeeeeeeee Department of Geriatrics, The Eighth Affiliated Hospital of Sun Yat-sen University, Shenzhen, China; fffffffffffffffffffffffffffffffffffffffffffffffffffffffffffffffffffffffffffffffffffffffffffffffffffff Cardiology Department, Royal Children's Hospital, Melbourne, VIC, Australia; ggggggggggggggggggggggggggggggggggggggggggggggggggggggggggggggggggggggggggggggggggggggggggggggggggggg Department of Critical Care and Neurosciences, Murdoch Childrens Research Institute, Parkville, VIC, Australia; hhhhhhhhhhhhhhhhhhhhhhhhhhhhhhhhhhhhhhhhhhhhhhhhhhhhhhhhhhhhhhhhhhhhhhhhhhhhhhhhhhhhhhhhhhhhhhhhhhhhh Demographic Change and Aging Research Area, Federal Institute for Population Research, Wiesbaden, Germany; iiiiiiiiiiiiiiiiiiiiiiiiiiiiiiiiiiiiiiiiiiiiiiiiiiiiiiiiiiiiiiiiiiiiiiiiiiiiiiiiiiiiiiiiiiiiiiiiiiiii Competence Center of Mortality-Follow-Up of the German National Cohort, Federal Institute for Population Research, Wiesbaden, Germany; jjjjjjjjjjjjjjjjjjjjjjjjjjjjjjjjjjjjjjjjjjjjjjjjjjjjjjjjjjjjjjjjjjjjjjjjjjjjjjjjjjjjjjjjjjjjjjjjjjjjj Department of Physical Therapy, Naresuan University, Phitsanulok, Thailand; kkkkkkkkkkkkkkkkkkkkkkkkkkkkkkkkkkkkkkkkkkkkkkkkkkkkkkkkkkkkkkkkkkkkkkkkkkkkkkkkkkkkkkkkkkkkkkkkkkkkk Department of Experimental Pharmacology, Heidelberg University, Mannheim, Germany; lllllllllllllllllllllllllllllllllllllllllllllllllllllllllllllllllllllllllllllllllllllllllllllllllllll Department of Surgery, University of Colombo, Colombo, Sri Lanka; mmmmmmmmmmmmmmmmmmmmmmmmmmmmmmmmmmmmmmmmmmmmmmmmmmmmmmmmmmmmmmmmmmmmmmmmmmmmmmmmmmmmmmmmmmmmmmmmmmmmm Department of Clinical Neurosciences, University of Calgary, Calgary, AB, Canada; nnnnnnnnnnnnnnnnnnnnnnnnnnnnnnnnnnnnnnnnnnnnnnnnnnnnnnnnnnnnnnnnnnnnnnnnnnnnnnnnnnnnnnnnnnnnnnnnnnnnn Department of Community Health Sciences, University of Calgary, Calgary, AB, Canada; ooooooooooooooooooooooooooooooooooooooooooooooooooooooooooooooooooooooooooooooooooooooooooooooooooooo Department of Nursing, Universitas Aisyiyah Bandung, Bandung, Indonesia; ppppppppppppppppppppppppppppppppppppppppppppppppppppppppppppppppppppppppppppppppppppppppppppppppppppp Institute of Clinical Epidemiology, Medical University Innsbruck, Innsbruck, Austria; qqqqqqqqqqqqqqqqqqqqqqqqqqqqqqqqqqqqqqqqqqqqqqqqqqqqqqqqqqqqqqqqqqqqqqqqqqqqqqqqqqqqqqqqqqqqqqqqqqqqq Research Organisation, Inter-Continental Omni-Research in Medicine Collaborative, Berlin, Germany; rrrrrrrrrrrrrrrrrrrrrrrrrrrrrrrrrrrrrrrrrrrrrrrrrrrrrrrrrrrrrrrrrrrrrrrrrrrrrrrrrrrrrrrrrrrrrrrrrrrrr Department of Public Health, Samara University, Samara, Ethiopia; sssssssssssssssssssssssssssssssssssssssssssssssssssssssssssssssssssssssssssssssssssssssssssssssssssss Department of Population Health Monitoring and Analysis, National Institute of Public Health, Warsaw, Poland; ttttttttttttttttttttttttttttttttttttttttttttttttttttttttttttttttttttttttttttttttttttttttttttttttttttt Division of Minimally Invasive Surgery, Johns Hopkins University, Baltimore, MD, USA; uuuuuuuuuuuuuuuuuuuuuuuuuuuuuuuuuuuuuuuuuuuuuuuuuuuuuuuuuuuuuuuuuuuuuuuuuuuuuuuuuuuuuuuuuuuuuuuuuuuuu Department of Surgery, MyungSung Medical College, Addis Ababa, Ethiopia; vvvvvvvvvvvvvvvvvvvvvvvvvvvvvvvvvvvvvvvvvvvvvvvvvvvvvvvvvvvvvvvvvvvvvvvvvvvvvvvvvvvvvvvvvvvvvvvvvvvvv Department of Anatomy, Histology, and Embryology, Bahir Dar University, Bahir Dar, Ethiopia; wwwwwwwwwwwwwwwwwwwwwwwwwwwwwwwwwwwwwwwwwwwwwwwwwwwwwwwwwwwwwwwwwwwwwwwwwwwwwwwwwwwwwwwwwwwwwwwwwwwww Institute of Health and Care Sciences, University of Gothenburg, Gothenburg, Sweden; xxxxxxxxxxxxxxxxxxxxxxxxxxxxxxxxxxxxxxxxxxxxxxxxxxxxxxxxxxxxxxxxxxxxxxxxxxxxxxxxxxxxxxxxxxxxxxxxxxxxx Faculty of Health Sciences, Oslo Metropolitan University, Oslo, Norway; yyyyyyyyyyyyyyyyyyyyyyyyyyyyyyyyyyyyyyyyyyyyyyyyyyyyyyyyyyyyyyyyyyyyyyyyyyyyyyyyyyyyyyyyyyyyyyyyyyyyy Faculty of Health, University of Technology Sydney, Australia, NSW, Australia; zzzzzzzzzzzzzzzzzzzzzzzzzzzzzzzzzzzzzzzzzzzzzzzzzzzzzzzzzzzzzzzzzzzzzzzzzzzzzzzzzzzzzzzzzzzzzzzzzzzzz School of Pharmacy, Monash University, Subang Jaya, Malaysia; aaaaaaaaaaaaaaaaaaaaaaaaaaaaaaaaaaaaaaaaaaaaaaaaaaaaaaaaaaaaaaaaaaaaaaaaaaaaaaaaaaaaaaaaaaaaaaaaaaaaaa Department of Theory and Empiricism of Healthcare, Universität Kassel (University of Kassel), Kassel, Germany; bbbbbbbbbbbbbbbbbbbbbbbbbbbbbbbbbbbbbbbbbbbbbbbbbbbbbbbbbbbbbbbbbbbbbbbbbbbbbbbbbbbbbbbbbbbbbbbbbbbbbb Department of Pharmacy, University of Gondar, Gondar, Ethiopia; cccccccccccccccccccccccccccccccccccccccccccccccccccccccccccccccccccccccccccccccccccccccccccccccccccccc The Second Affiliated Hospital, Wenzhou Medical University, Wenzhou, China; dddddddddddddddddddddddddddddddddddddddddddddddddddddddddddddddddddddddddddddddddddddddddddddddddddddd Global Health Research Center, Duke Kunshan University, Kunshan, China; eeeeeeeeeeeeeeeeeeeeeeeeeeeeeeeeeeeeeeeeeeeeeeeeeeeeeeeeeeeeeeeeeeeeeeeeeeeeeeeeeeeeeeeeeeeeeeeeeeeeee Duke Global Health Institute, Duke University, Durham, NC, USA; ffffffffffffffffffffffffffffffffffffffffffffffffffffffffffffffffffffffffffffffffffffffffffffffffffffff Department of Food Science and Human Nutrition, Michigan State University, East Lansing, MI, USA; gggggggggggggggggggggggggggggggggggggggggggggggggggggggggggggggggggggggggggggggggggggggggggggggggggggg Division of Hematology and Oncology, Medical College of Wisconsin, Milwaukee, WI, USA; hhhhhhhhhhhhhhhhhhhhhhhhhhhhhhhhhhhhhhhhhhhhhhhhhhhhhhhhhhhhhhhhhhhhhhhhhhhhhhhhhhhhhhhhhhhhhhhhhhhhhh Department of Public Health, Wuhan Fourth Hospital, Wuhan, China; iiiiiiiiiiiiiiiiiiiiiiiiiiiiiiiiiiiiiiiiiiiiiiiiiiiiiiiiiiiiiiiiiiiiiiiiiiiiiiiiiiiiiiiiiiiiiiiiiiiiii Shenzhen Institute of Advanced Technology, Chinese Academy of Sciences, Shenzhen, China; jjjjjjjjjjjjjjjjjjjjjjjjjjjjjjjjjjjjjjjjjjjjjjjjjjjjjjjjjjjjjjjjjjjjjjjjjjjjjjjjjjjjjjjjjjjjjjjjjjjjjj Division of Gastroenterology, Huazhong University of Science and Technology, Wuhan, China; kkkkkkkkkkkkkkkkkkkkkkkkkkkkkkkkkkkkkkkkkkkkkkkkkkkkkkkkkkkkkkkkkkkkkkkkkkkkkkkkkkkkkkkkkkkkkkkkkkkkkk Department of Health Policy and Administration, Universitas Airlangga (Airlangga University), Surabaya, Indonesia; llllllllllllllllllllllllllllllllllllllllllllllllllllllllllllllllllllllllllllllllllllllllllllllllllllll Western Institute of Digital-Intelligent Medicine, Chongqing Medical University, Chongqing, China; mmmmmmmmmmmmmmmmmmmmmmmmmmmmmmmmmmmmmmmmmmmmmmmmmmmmmmmmmmmmmmmmmmmmmmmmmmmmmmmmmmmmmmmmmmmmmmmmmmmmmm Tongji Medical College, Huazhong University of Science and Technology, Wuhan, China; nnnnnnnnnnnnnnnnnnnnnnnnnnnnnnnnnnnnnnnnnnnnnnnnnnnnnnnnnnnnnnnnnnnnnnnnnnnnnnnnnnnnnnnnnnnnnnnnnnnnnn School of Nursing and Rehabilitation, Shandong University, Jinan, China; oooooooooooooooooooooooooooooooooooooooooooooooooooooooooooooooooooooooooooooooooooooooooooooooooooooo Department of Intelligent Medical Engineering, Anhui Medical University, Anhui, China; pppppppppppppppppppppppppppppppppppppppppppppppppppppppppppppppppppppppppppppppppppppppppppppppppppppp Department of Surgery, The First Affiliated Hospital of Anhui Medical University, Hefei, Anhui, China; qqqqqqqqqqqqqqqqqqqqqqqqqqqqqqqqqqqqqqqqqqqqqqqqqqqqqqqqqqqqqqqqqqqqqqqqqqqqqqqqqqqqqqqqqqqqqqqqqqqqqq Ruijin Hospital, Shanghai Jiao Tong University, Shanghai, China; rrrrrrrrrrrrrrrrrrrrrrrrrrrrrrrrrrrrrrrrrrrrrrrrrrrrrrrrrrrrrrrrrrrrrrrrrrrrrrrrrrrrrrrrrrrrrrrrrrrrrr Department of Endocrinology, University of Science and Technology of China, Hefei, China; ssssssssssssssssssssssssssssssssssssssssssssssssssssssssssssssssssssssssssssssssssssssssssssssssssssss School of Medicine, University of Rochester, Rochester, NY, USA; tttttttttttttttttttttttttttttttttttttttttttttttttttttttttttttttttttttttttttttttttttttttttttttttttttttt Cardiovascular Program, The George Institute for Global Health, Sydney, NSW, Australia; uuuuuuuuuuuuuuuuuuuuuuuuuuuuuuuuuuuuuuuuuuuuuuuuuuuuuuuuuuuuuuuuuuuuuuuuuuuuuuuuuuuuuuuuuuuuuuuuuuuuuu Department of Environmental Health and Epidemiology, National Institute for Research in Environmental Health, Bhopal, India; vvvvvvvvvvvvvvvvvvvvvvvvvvvvvvvvvvvvvvvvvvvvvvvvvvvvvvvvvvvvvvvvvvvvvvvvvvvvvvvvvvvvvvvvvvvvvvvvvvvvvv Department of Microbiology and Immunology, Zagazig University, Zagazig, Egypt; wwwwwwwwwwwwwwwwwwwwwwwwwwwwwwwwwwwwwwwwwwwwwwwwwwwwwwwwwwwwwwwwwwwwwwwwwwwwwwwwwwwwwwwwwwwwwwwwwwwwww Department of Cells and Tissues, Molecular Biology Institute of Barcelona, Barcelona, Spain; xxxxxxxxxxxxxxxxxxxxxxxxxxxxxxxxxxxxxxxxxxxxxxxxxxxxxxxxxxxxxxxxxxxxxxxxxxxxxxxxxxxxxxxxxxxxxxxxxxxxxx Department of Public Health, Juntendo University, Tokyo, Japan; yyyyyyyyyyyyyyyyyyyyyyyyyyyyyyyyyyyyyyyyyyyyyyyyyyyyyyyyyyyyyyyyyyyyyyyyyyyyyyyyyyyyyyyyyyyyyyyyyyyyyy Department of Public Health Medicine, University of Tsukuba, Tsukuba, Japan; zzzzzzzzzzzzzzzzzzzzzzzzzzzzzzzzzzzzzzzzzzzzzzzzzzzzzzzzzzzzzzzzzzzzzzzzzzzzzzzzzzzzzzzzzzzzzzzzzzzzzz Department of Public Health Administration, Linyi People's Hospital, Linyi, China; aaaaaaaaaaaaaaaaaaaaaaaaaaaaaaaaaaaaaaaaaaaaaaaaaaaaaaaaaaaaaaaaaaaaaaaaaaaaaaaaaaaaaaaaaaaaaaaaaaaaaaa Faculty of Medicine, Juntendo University, Tokyo, Japan; bbbbbbbbbbbbbbbbbbbbbbbbbbbbbbbbbbbbbbbbbbbbbbbbbbbbbbbbbbbbbbbbbbbbbbbbbbbbbbbbbbbbbbbbbbbbbbbbbbbbbbb School of Traditional Chinese Medicine, Beijing University of Chinese Medicine, Beijing, China; ccccccccccccccccccccccccccccccccccccccccccccccccccccccccccccccccccccccccccccccccccccccccccccccccccccccc Pritzker School of Medicine, University of Chicago, Chicago, IL, USA; ddddddddddddddddddddddddddddddddddddddddddddddddddddddddddddddddddddddddddddddddddddddddddddddddddddddd School of Public Health and Primary Care, The Chinese University of Hong Kong, Hong Kong, China; eeeeeeeeeeeeeeeeeeeeeeeeeeeeeeeeeeeeeeeeeeeeeeeeeeeeeeeeeeeeeeeeeeeeeeeeeeeeeeeeeeeeeeeeeeeeeeeeeeeeeee Department of Medicine, Thomas Jefferson University, Philadelphia, PA, USA; fffffffffffffffffffffffffffffffffffffffffffffffffffffffffffffffffffffffffffffffffffffffffffffffffffffff Department of Medicine, Mashhad University of Medical Sciences, Mashhad, Iran; ggggggggggggggggggggggggggggggggggggggggggggggggggggggggggggggggggggggggggggggggggggggggggggggggggggggg Research Center of Physiology, Semnan University of Medical Sciences, Semnan, Iran; hhhhhhhhhhhhhhhhhhhhhhhhhhhhhhhhhhhhhhhhhhhhhhhhhhhhhhhhhhhhhhhhhhhhhhhhhhhhhhhhhhhhhhhhhhhhhhhhhhhhhhh Hematology Section, Hamad Medical Corporation, Doha, Qatar; iiiiiiiiiiiiiiiiiiiiiiiiiiiiiiiiiiiiiiiiiiiiiiiiiiiiiiiiiiiiiiiiiiiiiiiiiiiiiiiiiiiiiiiiiiiiiiiiiiiiiii Department of Biostatistics and Data Science, The University of Osaka, Suita, Japan; jjjjjjjjjjjjjjjjjjjjjjjjjjjjjjjjjjjjjjjjjjjjjjjjjjjjjjjjjjjjjjjjjjjjjjjjjjjjjjjjjjjjjjjjjjjjjjjjjjjjjjj National Center for Chronic and Noncommunicable Disease Control and Prevention, Chinese Center for Disease Control and Prevention, Beijing, China; kkkkkkkkkkkkkkkkkkkkkkkkkkkkkkkkkkkkkkkkkkkkkkkkkkkkkkkkkkkkkkkkkkkkkkkkkkkkkkkkkkkkkkkkkkkkkkkkkkkkkkk The George Institute for Global Health, University of New South Wales, Sydney, NSW, Australia; lllllllllllllllllllllllllllllllllllllllllllllllllllllllllllllllllllllllllllllllllllllllllllllllllllllll School of Biotechnology, University of Tehran, Tehran, Iran; mmmmmmmmmmmmmmmmmmmmmmmmmmmmmmmmmmmmmmmmmmmmmmmmmmmmmmmmmmmmmmmmmmmmmmmmmmmmmmmmmmmmmmmmmmmmmmmmmmmmmmm Department of Public Health, Trakya University, Edirne, Türkiye; nnnnnnnnnnnnnnnnnnnnnnnnnnnnnnnnnnnnnnnnnnnnnnnnnnnnnnnnnnnnnnnnnnnnnnnnnnnnnnnnnnnnnnnnnnnnnnnnnnnnnnn Medical Biotechnology Research Center, Guilan University of Medical Sciences, Rasht, Iran; ooooooooooooooooooooooooooooooooooooooooooooooooooooooooooooooooooooooooooooooooooooooooooooooooooooooo Department of Family Medicine, St. Paul's Hospital Millennium Medical College, Addis Ababa, Ethiopia; ppppppppppppppppppppppppppppppppppppppppppppppppppppppppppppppppppppppppppppppppppppppppppppppppppppppp Family Medicine Department, St. Peter's Specialized Hospital, Addis Ababa, Ethiopia; qqqqqqqqqqqqqqqqqqqqqqqqqqqqqqqqqqqqqqqqqqqqqqqqqqqqqqqqqqqqqqqqqqqqqqqqqqqqqqqqqqqqqqqqqqqqqqqqqqqqqqq Biostatics, Epidemiology, and Science Computing Department, King Faisal Specialist Hospital & Research Center, Riyadh, Saudi Arabia; rrrrrrrrrrrrrrrrrrrrrrrrrrrrrrrrrrrrrrrrrrrrrrrrrrrrrrrrrrrrrrrrrrrrrrrrrrrrrrrrrrrrrrrrrrrrrrrrrrrrrrr Saw Swee Hock School of Public Health, National University of Singapore, Singapore, Singapore; sssssssssssssssssssssssssssssssssssssssssssssssssssssssssssssssssssssssssssssssssssssssssssssssssssssss KHANA Center for Population Health Research, Phnom Penh, Cambodia; ttttttttttttttttttttttttttttttttttttttttttttttttttttttttttttttttttttttttttttttttttttttttttttttttttttttt Department of Epidemiology, Xuzhou Medical University, Xuzhou, China; uuuuuuuuuuuuuuuuuuuuuuuuuuuuuuuuuuuuuuuuuuuuuuuuuuuuuuuuuuuuuuuuuuuuuuuuuuuuuuuuuuuuuuuuuuuuuuuuuuuuuuu Centre for Suicide Research and Prevention, University of Hong Kong, Hong Kong, China; vvvvvvvvvvvvvvvvvvvvvvvvvvvvvvvvvvvvvvvvvvvvvvvvvvvvvvvvvvvvvvvvvvvvvvvvvvvvvvvvvvvvvvvvvvvvvvvvvvvvvvv Department of Social Work and Social Administration, University of Hong Kong, Hong Kong, China; wwwwwwwwwwwwwwwwwwwwwwwwwwwwwwwwwwwwwwwwwwwwwwwwwwwwwwwwwwwwwwwwwwwwwwwwwwwwwwwwwwwwwwwwwwwwwwwwwwwwwww Department of Pharmacology, Bahir Dar University, Bahir Dar, Ethiopia; xxxxxxxxxxxxxxxxxxxxxxxxxxxxxxxxxxxxxxxxxxxxxxxxxxxxxxxxxxxxxxxxxxxxxxxxxxxxxxxxxxxxxxxxxxxxxxxxxxxxxxx Pharmacy Department, Alkan Health Science, Business and Technology College, Bahir Dar, Ethiopia; yyyyyyyyyyyyyyyyyyyyyyyyyyyyyyyyyyyyyyyyyyyyyyyyyyyyyyyyyyyyyyyyyyyyyyyyyyyyyyyyyyyyyyyyyyyyyyyyyyyyyyy Department of Pediatrics, Kyung Hee University, Seoul, South Korea; zzzzzzzzzzzzzzzzzzzzzzzzzzzzzzzzzzzzzzzzzzzzzzzzzzzzzzzzzzzzzzzzzzzzzzzzzzzzzzzzzzzzzzzzzzzzzzzzzzzzzzz Department of Biostatistics, University of Toyama, Toyama, Japan; aaaaaaaaaaaaaaaaaaaaaaaaaaaaaaaaaaaaaaaaaaaaaaaaaaaaaaaaaaaaaaaaaaaaaaaaaaaaaaaaaaaaaaaaaaaaaaaaaaaaaaaa Department of Health Policy and Management, Jackson State University, Jackson, MS, USA; bbbbbbbbbbbbbbbbbbbbbbbbbbbbbbbbbbbbbbbbbbbbbbbbbbbbbbbbbbbbbbbbbbbbbbbbbbbbbbbbbbbbbbbbbbbbbbbbbbbbbbbb School of Business & Economics, Universiti Putra Malaysia (University of Putra Malaysia), Kuala Lumpur, Malaysia; cccccccccccccccccccccccccccccccccccccccccccccccccccccccccccccccccccccccccccccccccccccccccccccccccccccccc Department of Public Health, Sirjan School of Medical Sciences, Sirjan, Iran, Sirjan, Iran; dddddddddddddddddddddddddddddddddddddddddddddddddddddddddddddddddddddddddddddddddddddddddddddddddddddddd Department of Public Health, Jigjiga University, Jigjiga, Ethiopia; eeeeeeeeeeeeeeeeeeeeeeeeeeeeeeeeeeeeeeeeeeeeeeeeeeeeeeeeeeeeeeeeeeeeeeeeeeeeeeeeeeeeeeeeeeeeeeeeeeeeeeee School of Public Health, Hubei University of Medicine, Shiyan, China; ffffffffffffffffffffffffffffffffffffffffffffffffffffffffffffffffffffffffffffffffffffffffffffffffffffffff Southeast University Affiliated Xuzhou Central Hospital, Clinical Hospital, Xuzhou, China; gggggggggggggggggggggggggggggggggggggggggggggggggggggggggggggggggggggggggggggggggggggggggggggggggggggggg Department of Basic Science, University of Hail, Hail, Saudi Arabia; hhhhhhhhhhhhhhhhhhhhhhhhhhhhhhhhhhhhhhhhhhhhhhhhhhhhhhhhhhhhhhhhhhhhhhhhhhhhhhhhhhhhhhhhhhhhhhhhhhhhhhhh Department of Nursing Science, Bayero University, Kano, Nigeria; iiiiiiiiiiiiiiiiiiiiiiiiiiiiiiiiiiiiiiiiiiiiiiiiiiiiiiiiiiiiiiiiiiiiiiiiiiiiiiiiiiiiiiiiiiiiiiiiiiiiiiii Faculty of Nursing, University of Alberta, Edmonton, AB, Canada; jjjjjjjjjjjjjjjjjjjjjjjjjjjjjjjjjjjjjjjjjjjjjjjjjjjjjjjjjjjjjjjjjjjjjjjjjjjjjjjjjjjjjjjjjjjjjjjjjjjjjjjj Association for Socially Applicable Research (ASAR), Pune, India; kkkkkkkkkkkkkkkkkkkkkkkkkkkkkkkkkkkkkkkkkkkkkkkkkkkkkkkkkkkkkkkkkkkkkkkkkkkkkkkkkkkkkkkkkkkkkkkkkkkkkkkk Department of Emergency Medicine, Global Emergency Medicine Innovation and Implementation (GEMINI) Research Center, Durham, NC, USA; llllllllllllllllllllllllllllllllllllllllllllllllllllllllllllllllllllllllllllllllllllllllllllllllllllllll Epidemiology and Cancer Registry Sector, Institute of Oncology Ljubljana, Ljubljana, Slovenia; mmmmmmmmmmmmmmmmmmmmmmmmmmmmmmmmmmmmmmmmmmmmmmmmmmmmmmmmmmmmmmmmmmmmmmmmmmmmmmmmmmmmmmmmmmmmmmmmmmmmmmmm Family and Community Medicine Department, University of Hail, Hail, Saudi Arabia; nnnnnnnnnnnnnnnnnnnnnnnnnnnnnnnnnnnnnnnnnnnnnnnnnnnnnnnnnnnnnnnnnnnnnnnnnnnnnnnnnnnnnnnnnnnnnnnnnnnnnnnn Islamic Azad University, Tehran, Iran; oooooooooooooooooooooooooooooooooooooooooooooooooooooooooooooooooooooooooooooooooooooooooooooooooooooooo Department of Environmental and Occupational Health, Universiti Putra Malaysia (University of Putra Malaysia), UPM Serdang, Malaysia; pppppppppppppppppppppppppppppppppppppppppppppppppppppppppppppppppppppppppppppppppppppppppppppppppppppppp Faculty of Medicine and Health Sciences, Hodeidah University, Hodeidah, Yemen; qqqqqqqqqqqqqqqqqqqqqqqqqqqqqqqqqqqqqqqqqqqqqqqqqqqqqqqqqqqqqqqqqqqqqqqqqqqqqqqqqqqqqqqqqqqqqqqqqqqqqqqq Department of Computer Science and Software Engineering, United Arab Emirates University, Al Ain, United Arab Emirates; rrrrrrrrrrrrrrrrrrrrrrrrrrrrrrrrrrrrrrrrrrrrrrrrrrrrrrrrrrrrrrrrrrrrrrrrrrrrrrrrrrrrrrrrrrrrrrrrrrrrrrrr Health Investigation Center, Universidad Católica Boliviana San Pablo, Tarija, Bolivia; ssssssssssssssssssssssssssssssssssssssssssssssssssssssssssssssssssssssssssssssssssssssssssssssssssssssss San Pablo Catholic University Tarija Bolivia, Tarija, Bolivia; tttttttttttttttttttttttttttttttttttttttttttttttttttttttttttttttttttttttttttttttttttttttttttttttttttttttt The Heller School for Social Policy and Management, Brandeis University, Waltham, MA, USA; uuuuuuuuuuuuuuuuuuuuuuuuuuuuuuuuuuuuuuuuuuuuuuuuuuuuuuuuuuuuuuuuuuuuuuuuuuuuuuuuuuuuuuuuuuuuuuuuuuuuuuuu Sant'Elia Hospital, University of Catania, Caltanissetta, Italy; vvvvvvvvvvvvvvvvvvvvvvvvvvvvvvvvvvvvvvvvvvvvvvvvvvvvvvvvvvvvvvvvvvvvvvvvvvvvvvvvvvvvvvvvvvvvvvvvvvvvvvvv Department of Paediatrics and Child Health, University of Cape Town, Cape Town, South Africa; wwwwwwwwwwwwwwwwwwwwwwwwwwwwwwwwwwwwwwwwwwwwwwwwwwwwwwwwwwwwwwwwwwwwwwwwwwwwwwwwwwwwwwwwwwwwwwwwwwwwwwww Unit on Child & Adolescent Health, Medical Research Council South Africa, Cape Town, South Africa; xxxxxxxxxxxxxxxxxxxxxxxxxxxxxxxxxxxxxxxxxxxxxxxxxxxxxxxxxxxxxxxxxxxxxxxxxxxxxxxxxxxxxxxxxxxxxxxxxxxxxxxx Nursing Care Research Center in Chronic Diseases, Ahvaz Jundishapur University of Medical Sciences, Ahvaz, Iran; yyyyyyyyyyyyyyyyyyyyyyyyyyyyyyyyyyyyyyyyyyyyyyyyyyyyyyyyyyyyyyyyyyyyyyyyyyyyyyyyyyyyyyyyyyyyyyyyyyyyyyyy Department of Clinical Practice, Northern Border University, Rafha, Saudi Arabia; zzzzzzzzzzzzzzzzzzzzzzzzzzzzzzzzzzzzzzzzzzzzzzzzzzzzzzzzzzzzzzzzzzzzzzzzzzzzzzzzzzzzzzzzzzzzzzzzzzzzzzzz Institute of Diagnostic and Interventional Radiology and Neuroradiology, University of Duisburg-Essen, Essen, Germany; aaaaaaaaaaaaaaaaaaaaaaaaaaaaaaaaaaaaaaaaaaaaaaaaaaaaaaaaaaaaaaaaaaaaaaaaaaaaaaaaaaaaaaaaaaaaaaaaaaaaaaaaa Department of Epidemiology and Public Health, University of Basel, Basel, Switzerland; bbbbbbbbbbbbbbbbbbbbbbbbbbbbbbbbbbbbbbbbbbbbbbbbbbbbbbbbbbbbbbbbbbbbbbbbbbbbbbbbbbbbbbbbbbbbbbbbbbbbbbbbb Department of Nursing, Samara University, Semera, Ethiopia; ccccccccccccccccccccccccccccccccccccccccccccccccccccccccccccccccccccccccccccccccccccccccccccccccccccccccc Department of Psychiatry, Johns Hopkins University, Baltimore, MD, USA; ddddddddddddddddddddddddddddddddddddddddddddddddddddddddddddddddddddddddddddddddddddddddddddddddddddddddd Department of Surgery, University of Hong Kong, Hong Kong, China; eeeeeeeeeeeeeeeeeeeeeeeeeeeeeeeeeeeeeeeeeeeeeeeeeeeeeeeeeeeeeeeeeeeeeeeeeeeeeeeeeeeeeeeeeeeeeeeeeeeeeeeee Department of Cardiology, Zhongshan Hospital, Shanghai, China; fffffffffffffffffffffffffffffffffffffffffffffffffffffffffffffffffffffffffffffffffffffffffffffffffffffffff Department of International Health, Johns Hopkins University, Baltimore, MD, USA; ggggggggggggggggggggggggggggggggggggggggggggggggggggggggggggggggggggggggggggggggggggggggggggggggggggggggg Faculty of Medicine, Universidade de São Paulo (University of São Paulo), São Paulo, Brazil; hhhhhhhhhhhhhhhhhhhhhhhhhhhhhhhhhhhhhhhhhhhhhhhhhhhhhhhhhhhhhhhhhhhhhhhhhhhhhhhhhhhhhhhhhhhhhhhhhhhhhhhhh Department of Endocrinology and Metabolism, Shandong Second Medical University, Weifang, China; iiiiiiiiiiiiiiiiiiiiiiiiiiiiiiiiiiiiiiiiiiiiiiiiiiiiiiiiiiiiiiiiiiiiiiiiiiiiiiiiiiiiiiiiiiiiiiiiiiiiiiiii Medical Oncology Department of Gastrointestinal Cancer, Cancer Hospital of Dalian University of Technology, Shenyang, China; jjjjjjjjjjjjjjjjjjjjjjjjjjjjjjjjjjjjjjjjjjjjjjjjjjjjjjjjjjjjjjjjjjjjjjjjjjjjjjjjjjjjjjjjjjjjjjjjjjjjjjjjj School of Biomedical Engineering, Dalian University of Technology, Dalian, China; kkkkkkkkkkkkkkkkkkkkkkkkkkkkkkkkkkkkkkkkkkkkkkkkkkkkkkkkkkkkkkkkkkkkkkkkkkkkkkkkkkkkkkkkkkkkkkkkkkkkkkkkk Department of Internal Medicine, Jacobi Medical Center, Bronx, NY, USA; lllllllllllllllllllllllllllllllllllllllllllllllllllllllllllllllllllllllllllllllllllllllllllllllllllllllll Department of Internal Medicine, Albert Einstein College of Medicine, Bronx, NY, USA; mmmmmmmmmmmmmmmmmmmmmmmmmmmmmmmmmmmmmmmmmmmmmmmmmmmmmmmmmmmmmmmmmmmmmmmmmmmmmmmmmmmmmmmmmmmmmmmmmmmmmmmmm Burn Surgery Department, The First Hospital of Jilin University, Changchun, China; nnnnnnnnnnnnnnnnnnnnnnnnnnnnnnnnnnnnnnnnnnnnnnnnnnnnnnnnnnnnnnnnnnnnnnnnnnnnnnnnnnnnnnnnnnnnnnnnnnnnnnnnn School of Public Health, Wuhan University of Science and Technology, Wuhan, China; ooooooooooooooooooooooooooooooooooooooooooooooooooooooooooooooooooooooooooooooooooooooooooooooooooooooooo Hubei Province Key Laboratory of Occupational Hazard Identification and Control, Wuhan University of Science and Technology, Wuhan, China; ppppppppppppppppppppppppppppppppppppppppppppppppppppppppppppppppppppppppppppppppppppppppppppppppppppppppp Tianjin Medical University General Hospital, Tianjin Centers for Disease Control and Prevention, Tianjin, China; qqqqqqqqqqqqqqqqqqqqqqqqqqqqqqqqqqqqqqqqqqqqqqqqqqqqqqqqqqqqqqqqqqqqqqqqqqqqqqqqqqqqqqqqqqqqqqqqqqqqqqqqq Department of Internal Disease, Kazakh National Medical University, Almaty, Kazakhstan; rrrrrrrrrrrrrrrrrrrrrrrrrrrrrrrrrrrrrrrrrrrrrrrrrrrrrrrrrrrrrrrrrrrrrrrrrrrrrrrrrrrrrrrrrrrrrrrrrrrrrrrrr College of Traditional Chinese Medicine, Hebei University, Baoding, China; sssssssssssssssssssssssssssssssssssssssssssssssssssssssssssssssssssssssssssssssssssssssssssssssssssssssss Department of Epidemiology and Biostatistics, Zhejiang University, Hangzhou, China; ttttttttttttttttttttttttttttttttttttttttttttttttttttttttttttttttttttttttttttttttttttttttttttttttttttttttt The First Affiliated Hospital of Guizhou University of Traditional Chinese Medicine, Guiyang, China; uuuuuuuuuuuuuuuuuuuuuuuuuuuuuuuuuuuuuuuuuuuuuuuuuuuuuuuuuuuuuuuuuuuuuuuuuuuuuuuuuuuuuuuuuuuuuuuuuuuuuuuuu The First Affiliated Hospital of Jinan University, Jinan University, Guangzhou, China; vvvvvvvvvvvvvvvvvvvvvvvvvvvvvvvvvvvvvvvvvvvvvvvvvvvvvvvvvvvvvvvvvvvvvvvvvvvvvvvvvvvvvvvvvvvvvvvvvvvvvvvvv Department of Health Management, Shengjing Hospital of China Medical University, Shenyang, China; wwwwwwwwwwwwwwwwwwwwwwwwwwwwwwwwwwwwwwwwwwwwwwwwwwwwwwwwwwwwwwwwwwwwwwwwwwwwwwwwwwwwwwwwwwwwwwwwwwwwwwwww Harvard Medical School, Harvard University, Boston, MA, USA; xxxxxxxxxxxxxxxxxxxxxxxxxxxxxxxxxxxxxxxxxxxxxxxxxxxxxxxxxxxxxxxxxxxxxxxxxxxxxxxxxxxxxxxxxxxxxxxxxxxxxxxxx Jockey Club School of Public Health and Primary Care, The Chinese University of Hong Kong, Hong Kong, China; yyyyyyyyyyyyyyyyyyyyyyyyyyyyyyyyyyyyyyyyyyyyyyyyyyyyyyyyyyyyyyyyyyyyyyyyyyyyyyyyyyyyyyyyyyyyyyyyyyyyyyyyy School of Medicine, Stanford University, Palo Alto, CA, USA; zzzzzzzzzzzzzzzzzzzzzzzzzzzzzzzzzzzzzzzzzzzzzzzzzzzzzzzzzzzzzzzzzzzzzzzzzzzzzzzzzzzzzzzzzzzzzzzzzzzzzzzzz School of Data Science, The Chinese University of Hong Kong, Shenzhen, Shenzhen, China; aaaaaaaaaaaaaaaaaaaaaaaaaaaaaaaaaaaaaaaaaaaaaaaaaaaaaaaaaaaaaaaaaaaaaaaaaaaaaaaaaaaaaaaaaaaaaaaaaaaaaaaaaa School of Public Health and Emergency Management, Southern University of Science and Technology, Shenzhen, China; bbbbbbbbbbbbbbbbbbbbbbbbbbbbbbbbbbbbbbbbbbbbbbbbbbbbbbbbbbbbbbbbbbbbbbbbbbbbbbbbbbbbbbbbbbbbbbbbbbbbbbbbbb Department of Biochemistry and Pharmacogenomics, Medical University of Warsaw, Warsaw, Poland; cccccccccccccccccccccccccccccccccccccccccccccccccccccccccccccccccccccccccccccccccccccccccccccccccccccccccc Endocrinology and Metabolism Research Center, Hormozgan University of Medical Sciences, Bandar Abbas, Iran; dddddddddddddddddddddddddddddddddddddddddddddddddddddddddddddddddddddddddddddddddddddddddddddddddddddddddd College of Nursing, Prince Sattam bin Abdulaziz University, Al-Kharj, Saudi Arabia; eeeeeeeeeeeeeeeeeeeeeeeeeeeeeeeeeeeeeeeeeeeeeeeeeeeeeeeeeeeeeeeeeeeeeeeeeeeeeeeeeeeeeeeeeeeeeeeeeeeeeeeeee Faculty of Nursing, Mansoura University, Mansoura, Egypt; ffffffffffffffffffffffffffffffffffffffffffffffffffffffffffffffffffffffffffffffffffffffffffffffffffffffffff Institute of Child and Adolescent Health, Peking University, Beijing, China; gggggggggggggggggggggggggggggggggggggggggggggggggggggggggggggggggggggggggggggggggggggggggggggggggggggggggg Department of Medical-Surgical Nursing, University of Hail, Hail, Saudi Arabia; hhhhhhhhhhhhhhhhhhhhhhhhhhhhhhhhhhhhhhhhhhhhhhhhhhhhhhhhhhhhhhhhhhhhhhhhhhhhhhhhhhhhhhhhhhhhhhhhhhhhhhhhhh Department of Public Health, Universitas Brawijaya, Malang, Indonesia; iiiiiiiiiiiiiiiiiiiiiiiiiiiiiiiiiiiiiiiiiiiiiiiiiiiiiiiiiiiiiiiiiiiiiiiiiiiiiiiiiiiiiiiiiiiiiiiiiiiiiiiiii Center for Clinical Microbiology, University College London, London, UK; jjjjjjjjjjjjjjjjjjjjjjjjjjjjjjjjjjjjjjjjjjjjjjjjjjjjjjjjjjjjjjjjjjjjjjjjjjjjjjjjjjjjjjjjjjjjjjjjjjjjjjjjjj NIHR-Biomedical Research Centre (NIHR-BRC), University College London Hospitals, London, UK; kkkkkkkkkkkkkkkkkkkkkkkkkkkkkkkkkkkkkkkkkkkkkkkkkkkkkkkkkkkkkkkkkkkkkkkkkkkkkkkkkkkkkkkkkkkkkkkkkkkkkkkkkk Department of Chemistry, An-Najah National University, Nablus, Palestine; llllllllllllllllllllllllllllllllllllllllllllllllllllllllllllllllllllllllllllllllllllllllllllllllllllllllll Clinical Research Centre, An-Najah National University Hospital, Nablus, Palestine; mmmmmmmmmmmmmmmmmmmmmmmmmmmmmmmmmmmmmmmmmmmmmmmmmmmmmmmmmmmmmmmmmmmmmmmmmmmmmmmmmmmmmmmmmmmmmmmmmmmmmmmmmm Department of Building Engineering and Environment, Palestine Technical University (Kadoorie), Tulkarem, Palestine; nnnnnnnnnnnnnnnnnnnnnnnnnnnnnnnnnnnnnnnnnnnnnnnnnnnnnnnnnnnnnnnnnnnnnnnnnnnnnnnnnnnnnnnnnnnnnnnnnnnnnnnnnn Civil Engineering and Sustainable Structures, Palestine Technical University (Kadoorie), Tulkarem, Palestine

## Abstract

**Background:**

Timely and comprehensive analyses of causes of death stratified by age, sex, and location are essential for shaping effective health policies aimed at reducing global mortality. The Global Burden of Diseases, Injuries, and Risk Factors Study (GBD) 2023 provides cause-specific mortality estimates measured in counts, rates, and years of life lost (YLLs). GBD 2023 aimed to enhance our understanding of the relationship between age and cause of death by quantifying the probability of dying before age 70 years (70q0) and the mean age at death by cause and sex. This study enables comparisons of the impact of causes of death over time, offering a deeper understanding of how these causes affect global populations.

**Methods:**

GBD 2023 produced estimates for 292 causes of death disaggregated by age-sex-location-year in 204 countries and territories and 660 subnational locations for each year from 1990 until 2023. We used a modelling tool developed for GBD, the Cause of Death Ensemble model (CODEm), to estimate cause-specific death rates for most causes. We computed YLLs as the product of the number of deaths for each cause-age-sex-location-year and the standard life expectancy at each age. Probability of death was calculated as the chance of dying from a given cause in a specific age period, for a specific population. Mean age at death was calculated by first assigning the midpoint age of each age group for every death, followed by computing the mean of all midpoint ages across all deaths attributed to a given cause. We used GBD death estimates to calculate the observed mean age at death and to model the expected mean age across causes, sexes, years, and locations. The expected mean age reflects the expected mean age at death for individuals within a population, based on global mortality rates and the population's age structure. Comparatively, the observed mean age represents the actual mean age at death, influenced by all factors unique to a location-specific population, including its age structure. As part of the modelling process, uncertainty intervals (UIs) were generated using the 2·5th and 97·5th percentiles from a 250-draw distribution for each metric. Findings are reported as counts and age-standardised rates. Methodological improvements for cause-of-death estimates in GBD 2023 include a correction for the misclassification of deaths due to COVID-19, updates to the method used to estimate COVID-19, and updates to the CODEm modelling framework. This analysis used 55 761 data sources, including vital registration and verbal autopsy data as well as data from surveys, censuses, surveillance systems, and cancer registries, among others. For GBD 2023, there were 312 new country-years of vital registration cause-of-death data, 3 country-years of surveillance data, 51 country-years of verbal autopsy data, and 144 country-years of other data types that were added to those used in previous GBD rounds.

**Findings:**

The initial years of the COVID-19 pandemic caused shifts in long-standing rankings of the leading causes of global deaths: it ranked as the number one age-standardised cause of death at Level 3 of the GBD cause classification hierarchy in 2021. By 2023, COVID-19 dropped to the 20th place among the leading global causes, returning the rankings of the leading two causes to those typical across the time series (ie, ischaemic heart disease and stroke). While ischaemic heart disease and stroke persist as leading causes of death, there has been progress in reducing their age-standardised mortality rates globally. Four other leading causes have also shown large declines in global age-standardised mortality rates across the study period: diarrhoeal diseases, tuberculosis, stomach cancer, and measles. Other causes of death showed disparate patterns between sexes, notably for deaths from conflict and terrorism in some locations. A large reduction in age-standardised rates of YLLs occurred for neonatal disorders. Despite this, neonatal disorders remained the leading cause of global YLLs over the period studied, except in 2021, when COVID-19 was temporarily the leading cause. Compared to 1990, there has been a considerable reduction in total YLLs in many vaccine-preventable diseases, most notably diphtheria, pertussis, tetanus, and measles. In addition, this study quantified the mean age at death for all-cause mortality and cause-specific mortality and found noticeable variation by sex and location. The global all-cause mean age at death increased from 46·8 years (95% UI 46·6–47·0) in 1990 to 63·4 years (63·1–63·7) in 2023. For males, mean age increased from 45·4 years (45·1–45·7) to 61·2 years (60·7–61·6), and for females it increased from 48·5 years (48·1–48·8) to 65·9 years (65·5–66·3), from 1990 to 2023. The highest all-cause mean age at death in 2023 was found in the high-income super-region, where the mean age for females reached 80·9 years (80·9–81·0) and for males 74·8 years (74·8–74·9). By comparison, the lowest all-cause mean age at death occurred in sub-Saharan Africa, where it was 38·0 years (37·5–38·4) for females and 35·6 years (35·2–35·9) for males in 2023. Lastly, our study found that all-cause 70q0 decreased across each GBD super-region and region from 2000 to 2023, although with large variability between them. For females, we found that 70q0 notably increased from drug use disorders and conflict and terrorism. Leading causes that increased 70q0 for males also included drug use disorders, as well as diabetes. In sub-Saharan Africa, there was an increase in 70q0 for many non-communicable diseases (NCDs). Additionally, the mean age at death from NCDs was lower than the expected mean age at death for this super-region. By comparison, there was an increase in 70q0 for drug use disorders in the high-income super-region, which also had an observed mean age at death lower than the expected value.

**Interpretation:**

We examined global mortality patterns over the past three decades, highlighting—with enhanced estimation methods—the impacts of major events such as the COVID-19 pandemic, in addition to broader trends such as increasing NCDs in low-income regions that reflect ongoing shifts in the global epidemiological transition. This study also delves into premature mortality patterns, exploring the interplay between age and causes of death and deepening our understanding of where targeted resources could be applied to further reduce preventable sources of mortality. We provide essential insights into global and regional health disparities, identifying locations in need of targeted interventions to address both communicable and non-communicable diseases. There is an ever-present need for strengthened health-care systems that are resilient to future pandemics and the shifting burden of disease, particularly among ageing populations in regions with high mortality rates. Robust estimates of causes of death are increasingly essential to inform health priorities and guide efforts toward achieving global health equity. The need for global collaboration to reduce preventable mortality is more important than ever, as shifting burdens of disease are affecting all nations, albeit at different paces and scales.

**Funding:**

Gates Foundation.

## Introduction

Measuring causes of death is a foundational step towards developing effective strategies to improve human health. The Global Burden of Diseases, Injuries, and Risk Factors Study (GBD) provides comprehensive and systematic analyses of causes of death worldwide and across time. The utility of GBD cause of death estimates has been particularly valuable during the onset of COVID-19.^1^, ^2^, ^3^ However, GBD estimates have uses beyond informing preparation for stochastic events, such as a novel virus or new pandemic; these estimates are used integrally as tools for understanding public health trends, shaping health policy, and monitoring progress toward global health goals.^4^ As a global public good, GBD 2023 contributes freely available, updated, and comprehensive estimates of causes of death to the existing body of scientific literature. In addition to presenting the routinely updated estimates of causes of death, the current study expands our analysis to further explore the relationship between age and cause of death.

This study investigates important age patterns in mortality by estimating the probability of dying from any given cause before the age of 70 years (70q0). The probability of death measure is a fundamental indicator in public health because it can effectively capture improvements in survival within all age groups before age 70 years.^5^ In recent publications, it has become common practice to classify deaths occurring before 70 years of age as premature.^6^ Some studies, including the Global Health 2050 report from the *Lancet* Commission on Investing in Health, have shown that the probability of all-cause mortality before age 70 years has decreased globally and across major regions.^5^ The Global Health 2050 report concluded that further reductions, by as much as 50%, are attainable by mid-century with targeted health investments, a goal referred to as 50 by 50.^5^ To support progress towards 50 by 50, our study aims to address remaining questions, including which causes of death deviate from the broader improvements in premature mortality, and where disparities might exist in the likelihood of dying before age 70 years within specific populations. These are pressing concerns for policy makers and health-planning teams at both national and international levels.

Another primary objective of GBD 2023 was to calculate the mean age at the time of death across causes and locations. This metric allows for straightforward observations of national and regional disease burdens in relation to global values. Related studies find that the overall mortality rate from all causes has been decreasing over the past 75 years,^7^ and the mean age at the time of death has been trending upward for many countries.^8^ Estimates of mean age at death are influenced not only by a population's age distribution but also by disease characteristics, health-care access, socioeconomic status, comorbidities, and other risk factors.^8^, ^9^ Although some of this general upward trend can be attributed to shifts in age structure and sex distribution by location, in certain areas and for specific causes, the mean age is much higher or lower than expected.^8^ Quantifying the difference between the expected mean age at death (based only on population age structure and disease characteristics) and the observed mean age at death provides policy makers with additional population-level understanding beyond simply comparing age-standardised death rates between locations. These quantified differences could be linked to modifiable factors within a community, such as high blood pressure or the use of alcohol, tobacco, or drugs, which can be targeted through public health interventions.^10^

**Research in context**

**Evidence before this study**

The Global Burden of Diseases, Injuries, and Risk Factors Study (GBD) is a worldwide research initiative that provides comprehensive and timely assessments of mortality, morbidity, and risk factors disaggregated to granular levels that are meaningful for policy development. In the last iteration, the GBD 2021 causes-of-death publication marked a major advancement in the evidence base; the study delineated cause-specific mortality to provide insights on the primary causes of death influencing life expectancy across locations. It also identified several causes with shifting mortality trends that had important implications for targeted policy initiatives—causes that were once widespread across the globe but became increasingly localised and in need of tailored reduction strategies. The GBD 2021 causes-of-death analysis was also the first of its kind to publish worldwide estimates of deaths from the initial years of the COVID-19 pandemic, quantifying its effect on life expectancy and offering comparisons to deaths from other causes. While estimates from other studies are published periodically that assess specific causes of death among a subset of populations or across a narrower timeframe, GBD remains the only research effort to offer cause-specific estimates of mortality to this degree of time and location detail and to produce these assessments in peer-reviewed and GATHER-compliant publications.

**Added value of this study**

This study provides new and more robust evidence of mortality patterns across the globe, updating and extending the analysis from GBD 2021, and reanalysing the entire time series to supersede all previous GBD publications. We provide estimates of cause-specific mortality for 292 causes of death within 204 countries and territories and 660 subnational locations, disaggregated by age and sex, from 1990 to 2023. These estimates include 11 474 new sources compared with GBD 2021. This update advances mortality measurements in several ways. First, we present the probability of death before age 70 years (70q0) by sex and year to enable measurements of premature mortality by individual causes. We describe causes of death that are not following global improvements in 70q0 to highlight locations where disparities are occurring in the likelihood of dying before age 70 years. Second, we calculate the mean age at death by assigning the midpoint age of each age group for every death, followed by computing the overall mean across all deaths attributed to a given cause. Our analysis of mean age of death offers insights into a country's ability to manage different disease burdens relative to global benchmarks, independent of local population structure. Additionally, our study examines the correlation between mean age at death and the Socio-demographic Index (SDI) to evaluate whether countries at the higher end of the SDI exhibit older mean ages at death for a given cause compared with countries with a lower SDI value, while controlling for SDI's effect on population structure. This approach adds a novel dimension to understanding how sociodemographic factors influence both the risk and timing of mortality. This study also builds upon our estimates from GBD 2021 to include 2 additional years of COVID-19 analysis, providing a more comprehensive picture of COVID-19 mortality worldwide. Lastly, we report estimates for several newly disaggregated causes of death, including ulcerative colitis; Crohn's disease; thyroid disease; other endocrine, metabolic, and blood and immune disease; and electrocution.

**Implications of all the available evidence**

Our study offers a thorough analysis of causes of death over the past 34 years, including new findings into the full duration of the COVID-19 pandemic. We highlight causes of death that have declined in certain locations, which could lend insight for policy change and implementation. We also identify causes that persist as major sources of mortality across populations, signifying priority areas for future intervention. Additionally, our study investigated important age patterns in mortality by estimating the probability of dying from any given cause before age 70 years, thereby advancing our understanding of the relationship between age and cause of death. The Global Health 2050 report set a target to reduce the probability of premature deaths by 50% by 2050. We aim to complement and support this goal by offering an in-depth analysis of 70q0 across time, sex, and geographical locations. Lastly, our mean age of death analysis is a valuable metric for comparing observed mortality levels with expected patterns to help identify locations that are keeping pace with development trends and those that might be falling behind. Evidence from this study can be used to examine epidemiological patterns and trends across time and locations, and to gauge progress in global development goals. These findings can also guide future policy initiatives aimed at furthering reductions in cause-specific mortality and, in particular, achieving better pandemic preparedness within the context of specific locations. In aggregate, cyclical updates to GBD reflect improvements in data availability and enhanced methodology that reduce bias and improve transparency, supporting the development and implementation of new evidence-based health policies worldwide.

The timeframe of this analysis allows for important new insights into COVID-19, including two additional years of estimation since GBD 2021, new data collected, and improved methodology. As we mark 5 years since the onset of the COVID-19 pandemic—declared officially by WHO in March, 2020^11^—it is important to reflect on its impact. Substantial declines in deaths from COVID-19 were not noted until 2023, after a period of extraordinary global disruption.^7^ Since that time, countries with robust vital registration systems have been able to publish mortality data for the years with the highest number of COVID-19 deaths. In addition, localised studies revealed shifts in mortality patterns occurring for certain causes of death during the height of the pandemic.^12^ As additional vital registration data become available, a more comprehensive understanding of the long-term effects of COVID-19 on global mortality will continue to unfold. Key questions remain regarding the total number of deaths attributed to COVID-19, the populations most affected, and which causes of death—and to what extent—were affected by the COVID-19 pandemic.

This study provides new insights related to trends in 70q0 and the mean age at death, and identifies and delineates causes that most heavily affect mortality across populations. An updated understanding of how the COVID-19 pandemic interrupted or altered previous trajectories in mortality by cause, age, sex, or location is another important contribution of GBD 2023. At the same time, tracking changes in 70q0 and the mean age at death across causes, populations, and over time—alongside metrics such as the number of deaths, age-standardised mortality rates, and years of life lost (YLLs), offers more actionable insights to improve health at the population level. These patterns can be an essential guide for policy makers when shaping health priorities. This manuscript was produced as part of the GBD Collaborator Network and in accordance with the GBD Protocol.^13^

## Methods

### Overview

GBD 2023 produced estimates for each epidemiological quantity of interest for 292 causes of death by age-sex-location-year for 25 age groups from birth to 95 years and older; for males, females, and all sexes combined; in 204 countries and territories grouped into 21 regions and seven super-regions; and for every year from 1990 to 2023. GBD 2023 also includes subnational analyses for 20 countries and territories. This study drew on the expertise of a network of 14 410 international collaborators from more than 160 countries and territories who provide, review, and analyse the available data to generate these metrics.

GBD 2023 produced updated estimates of health loss around the world using the best available data. For each GBD round, newly available data and updated methods are used to update the full time series of estimates from 1990 to the latest year of analysis. Consequently, GBD 2023 estimates supersede all previous estimates. The methods used to generate estimates for GBD 2023 closely followed those for GBD 2021.^14^ These methods have been extensively peer reviewed over previous rounds of GBD^14^, ^15^, ^16^, ^17^, ^18^, ^19^ and concurrently as part of the peer review process for GBD 2023. Here, we provide an overview of the methods with an emphasis on the main methodological changes since GBD 2021; a comprehensive description of the analytical methods for GBD 2023 is provided in appendix 1.

The GBD 2023 cause-of-death estimates described here include cause-specific mortality, observed and expected mean ages at death, cause-specific probabilities of death before age 70 years, and the premature death metric YLLs. YLLs were calculated as the number of deaths for each cause-age-sex-location-year multiplied by the standard life expectancy at each age (appendix 1 section 6.3). Standard life expectancy is calculated from the lowest age-specific mortality rate between countries.^7^ In brief, cause-specific death rates for 214 causes were estimated using the Cause of Death Ensemble model (CODEm), while alternative strategies were used to model causes with very limited data, changes in reporting over the study period, or very specific epidemiology. The modelling strategy used for all cause of death estimates can be found in appendix 1 (table S8). CODEm is a modelling tool developed specifically for GBD that evaluates the out-of-sample predictive validity of different statistical models and covariate permutations and then combines the results from those evaluations to produce cause-specific fatal burden estimates.

Methodological improvements for cause-of-death estimates in the current round of estimation focused on several key areas. First, a method for the identification and correction of causes displaying excess mortality spikes due to misclassified COVID-19 deaths was applied to all vital registration data between the years of 2020 and 2023. Second, we added 312 new country-years of vital registration data on cause of death, 3 country-years of surveillance data, 51 country-years of verbal autopsy data, and 144 country-years of other data types. Third, all CODEms were fitted to mortality rates rather than cause fractions. Fourth, we updated the modelling framework for COVID-19 to incorporate pandemic-era vital registration data and preliminary vital registration reporting.

### The GBD disease and injury hierarchy

GBD classifies diseases and injuries into a hierarchy with four Levels that include both fatal and non-fatal causes. Level 1 causes include three broad aggregate categories (communicable, maternal, neonatal, and nutritional [CMNN] diseases; non-communicable diseases [NCDs]; and injuries); Level 2 disaggregates those categories into 22 clusters of causes, which are further disaggregated into Level 3 and Level 4 causes. At the most detailed Level, 292 fatal causes are estimated. For a full list of causes of death by Level, see appendix 1 (table S1). For GBD 2023, five causes of death were estimated for the first time: ulcerative colitis; Crohn's disease; thyroid disease; other endocrine, metabolic, blood, and immune disease; and electrocution.

### Data sources, processing, and assessing for completeness

The GBD 2023 cause-of-death database included data sources identified in previous rounds of estimation in addition to 11 474 new sources, for a total of 55 761 data sources—these sources are detailed in appendix 1 (table S3) and can be accessed through the Global Health Data Exchange (GHDx) website. Multiple data types were included to capture the widest array of information, including vital registration data for all 292 causes, as well as verbal autopsy, survey, census, surveillance, cancer registry, and police record data; open-source databases; and minimally invasive tissue sampling. To standardise these data so that they could be compared by cause, age, sex, location, and time, a set of data processing corrections were applied. First, deaths with insufficient or missing age and sex detail underwent a process of distribution via age and sex splitting (appendix 1 section 3.5). In addition, garbage codes, which are non-specific, implausible, or intermediate rather than underlying cause-of-death codes from the ICD, were redistributed to appropriate targets to assign the underlying cause of death.^20^ Data sources with more than 50% of all deaths assigned to major garbage codes (class 1 or class 2 garbage codes) in any location-year were excluded to mitigate the potential for bias from these sources (appendix 1 section 3.11).

Assessing data completeness illustrates the coverage from a data source on overall mortality for the country. Vital registration and verbal autopsy data completeness—a source-specific estimate of the percentage of total cause-specific deaths that are reported in a given location and year—was assessed by location-year, and sources with less than 50% completeness were excluded. We excluded 283 country-years of data due to insufficient completeness or excessive garbage coding. The estimated all-cause mortality for each age-sex-location-year was then multiplied by the cause fraction for the corresponding age-sex-location-year to adjust all included sources to 100% completeness. GBD assesses the quality of all vital registration and verbal autopsy data using a star ranking system of one to five stars, based on the percentage of completeness and percentage of garbage coding. Vital registration and verbal autopsy data availability, completeness, and five-star quality rating for each location-year are available in appendix 1 (figures S4 and S5). Full details on all data processing corrections can be found in appendix 1 (section 3.16).

### Presentation of cause-specific mortality estimates

Cause-specific mortality estimates for GBD 2023 are given in death counts and age-standardised rates per 100 000 population, calculated using the GBD standard population structure.^7^ For changes over time, we present percentage changes over the period 1990–2023, and annualised rates of change as the difference in the natural log of the values at the start and end of the time interval divided by the number of years in the interval. 95% uncertainty intervals (UIs) for all metrics are computed using the 2·5th and 97·5th percentiles from a 250-draw distribution for each metric (appendix 1 section 4.1.3). To reduce computing power and time, the number of computations per process was scaled back from 500 in GBD 2021 to 250 in GBD 2023, as simulation testing revealed that final estimates and their uncertainty were not affected by this reduction. See appendix 1 (section 4.1.3) for further details on this update.

### Measuring probability of premature death

In accordance with the GBD framework, a death that occurs at any age before the standard (expected) life expectancy is classified as premature. To inform discussions and debates in the literature on premature deaths before age 70 years, in alignment with studies from WHO,^6^, ^21^ the US National Institutes of Health,^22^ and the US Centers for Disease Control and Prevention,^23^ we computed the probability of death from birth to age 70 years (70q0).

### Calculation of the probability of premature death by cause

The probability-of-death metric represents the chance of dying from a given cause in a specific age period, for a specific population. Methods for calculating all-cause probability of premature death have been described elsewhere.^7^ For example, for males aged 0–70 years in Canada in 1990, a probability of death of 0·1 from ischaemic heart disease indicates a 10% chance of dying from this cause before age 70 years. Cause-specific probability of death can be calculated as follows:

$$q_{x,c}^{n}=deaths_{x,c}^{n}\times q_{x}^{n}$$

where

$$q_{x,c}^{n}$$

represents the probability of death for ages *x* to *x* + *n* in cause *c*,

$${\text{deaths}}_{x,c}^{n}$$

represents deaths in cause fraction space for age group *x* to *x* + *n* in cause *c*; and

$$q_{x}^{n}$$

represents the all cause probability of death for ages *x* to *x* + *n*.

### Socio-demographic Index

The Socio-demographic Index (SDI) is a composite measure of two demographic indicators (total fertility rate in people younger than 25 years and mean educational attainment for those aged 15 years and older) and an economic indicator (lag-distributed income per capita).^7^ Values are given as a range between 0·0 and 1·0. Additional details describing the calculation of SDI for GBD 2023 are provided in appendix 1 (section 5) of our companion publication.^7^

### Calculation of the observed mean age at death and the expected mean age at death based on the global age-specific rates

The calculation of mean age at death uses cause-specific GBD modelled death estimates. GBD produces cause-of-death estimates for every location-year-age-sex group, even when no reported cause-of-death data are available. GBD uses standard 5-year age groups (eg, 15–19, 20–24, and 25–29 years) from age 5 years to 94 years. The remaining non-standard age groups consist of 0–6 days, 7–27 days, 1–5 months, 6–11 months, 12–23 months, 2–4 years, and 95 years and older. For this calculation, each GBD age group is assigned a distinct age at death by taking the mean age of each age group. For example, the age group 15–19 years, which represents people from the day they turn 15 years of age to the day before they turn 20 years of age, was assigned to have a distinct age at death of 17·5 years. The only age group without a discernible mean is the 95 years and older group. For this age group, the distinct age at death was calculated by adding the life expectancy of the 95 years and older age group to 95 years by sex-year-location.

The observed mean age at death uses GBD estimates directly, and each death can be assigned a distinct age at death. Distinct ages are then summed together for a given demographic consisting of a given location-year-sex-cause. This value is then divided by the total number of deaths for the same demographic to quantify the mean age at death.

Expected deaths were calculated using cause-specific GBD global mortality rates by age and sex and applying them to each country's population. By multiplying the mortality rate on the population to calculate deaths, expected death estimates control for population structure. These expected death estimates are comparable to normal GBD estimates, consisting of the same age-sex groups. The same process used to calculate the observed mean age at death is then applied to the expected deaths to calculate the expected mean age at death.

The relationship between mean age at death and SDI was explored by running linear regressions of SDI against the observed mean age at death as well as running linear regressions of SDI against the difference of observed and expected mean ages at death. Appendix 2 (table S14) reports the resulting *r*^2^, slope, and p values of each regression. An individual regression was run for each cause–sex combination, in which each observation represents a country in 1990, 2010, 2019, 2021, or 2023. By observing the relationship between SDI and the difference between observed and expected values, we are able to measure the correlation of SDI with the mean age at death while accounting for differences in population structure that might also be correlated with SDI.

### Correction for the misclassification of COVID-19 deaths

GBD 2023 received 83 country-years of vital registration data from 2020, 67 from 2021, and 38 from 2022. During these years, there is evidence that deaths due to COVID-19 were misclassified as other causes of death.^24^, ^25^ Relative to smooth prepandemic mortality trends, these misclassified COVID-19 deaths contributed to mortality spikes in the other causes of death. To systematically identify these spikes in other causes, we developed a Support Vector Machine, a machine-learning algorithm for identifying deviations from the established time trends in the years 2020–22. After we identified causes with mortality spikes during the COVID-19 pandemic years, we ascertained, for each cause of interest, whether the spike was a result of COVID-19 misclassification or a true increase in mortality. To do this, we evaluated the correlation between the rate of excess mortality in the cause of interest and the observed mortality rate of COVID-19. Mortality spikes identified to contain COVID-19 misclassification were then considered eligible for correction.

When a mortality spike had been identified as being eligible for correction, we calculated the portion of excess mortality attributable to misclassified COVID-19. We first created an estimate of expected deaths absent of any pandemic effects using the mean of two counterfactual estimates: one calculated by a linear regression of the 5 years before the start of the pandemic (2015–19), and another calculated using a global relative rate of non-COVID-19 deaths, adapted from a previously published method used in the correction of misclassified HIV.^26^ Total excess mortality could then be estimated by subtracting the expected death count total from the observed death count total. Finally, total excess mortality was then scaled according to the level of correlation between COVID-19 rate and excess mortality rate to calculate the amount of excess attributable to COVID-19. The total excess attributable to COVID-19 was then subtracted from the cause of interest and reassigned to COVID-19. The full details regarding the identification and correction of misclassified COVID-19 can be found in appendix 1 (section 3.8), appendix 2 (table S2), and appendix 3. Detailed results of this process can be found in appendix 2 (table S3).

### Estimation of COVID-19 as a cause of death

For modelling COVID-19, we supplemented the corrected vital registration data described above with two other sources of data: 9 country-years of provisional vital registration data and 342 country-years of surveillance data that were reported during the pandemic (to 2022). We developed an analysis method using OneMod, a modelling tool that combines robust feature selection, correlated time-series splines, and covariate effect sizes across age groups, in addition to kernel regression for residual smoothing. It included the following candidate covariates: total COVID-19 infections and variant prevalence; COVID-19 vaccinations;^27^ COVID-19 infection detection rate;^27^ Healthcare Access and Quality Index;^28^ and prevalence of risk factors and comorbidities including obesity, smoking, cancer, cardiovascular disease, chronic kidney disease, chronic obstructive pulmonary disease, and diabetes.^29^, ^30^ In the first stage of this model pipeline, we used only the corrected vital registration data to estimate age patterns and sex ratios, which were then used to split the provisional vital registration by age and sex, and split the surveillance data that did not contain detailed age and sex information into the 25 granular GBD age groups. We then ran the models using the entire dataset, setting the infection detection rate to 100% for the corrected vital registration data. After fitting these models, we made predictions assuming that the infection detection rate was 100% in all locations. Details on the estimation of COVID-19 deaths can be found in appendix 1 (section 5).

### GBD research and reporting practices

This study used de-identified data and was approved by the University of Washington Institutional Review Board (study number 9060). GBD 2023 complies with the Guidelines for Accurate and Transparent Health Estimates Reporting (GATHER) statement (appendix 1 section 2.4).^31^ A completed GATHER checklist is provided in appendix 1 (table S13). Software packages used in the cause of death analysis for GBD 2023 were Python version 3.10.4, Stata version 13.1, and R version 4.4.0. Statistical code used for GBD estimation is publicly available online at the GHDx website.

### Role of the funding source

The funders of this study had no role in the study design, data collection, data analysis, data interpretation, or the writing of the report. The corresponding author had full access to the data in the study and final responsibility for the decision to submit for publication.

## Results

Detailed results for each cause of death in this analysis are available in downloadable form through the GBD Results tool and via visual exploration through the GBD Compare tool.

### Global all-cause mortality

Relative to the rate in 1990, the percentage change in annual age-standardised mortality rate from 1991–2019 globally for all causes of death fluctuated between a decrease of 2·9% (95% UI –3·5 to –2·3) and a slight increase of 0·1% (–0·7 to 1·0; appendix 2 figure S1). A notable increase occurred between 2019 and 2020 (6·5% [5·9 to 7·1]) and 2020 and 2021 (7·0% [6·5 to 7·6]), followed by a large decrease between 2021 and 2022 (–9·5% [–10·7 to –8·2]). The total number of global deaths for all sexes and all age groups increased from 47·9 million (47·6–48·3) in 1990 to 55·2 million (54·8–55·5) in 2019. An increase occurred during the initial years of the COVID-19 pandemic, with global deaths reaching 65·9 million (65·6–66·2) in 2021. As the pandemic subsided, the annual global death toll decreased to 60·0 million (59·0–61·2) in 2023. From 2000 to 2023, this represents an overall decline of 30·5% (29·0–31·7), in which the global age-standardised mortality rate for all sexes and age groups dropped from 1009·0 (1002·5–1015·2) deaths per 100 000 to 701·5 (690·2–714·9) deaths per 100 000 (table 1, appendix 2 table S7).

**Table 1:** Global death and YLL numbers, age-standardised rates per 100 000, and percentage change between 2000 and 2023 for all sexes combined for all GBD causes and Levels 1–4 of the cause hierarchy

			**All-age deaths, thousands**			**Age-standardised death rate per 100 000 population**			**All-age YLLs, thousands**			**Age-standardised YLL rate per 100 000 population**		
			2000	2023	Percentage change, 2000–23	2000	2023	Percentage change, 2000–23	2000	2023	Percentage change, 2000–23	2000	2023	Percentage change, 2000–23
**All causes**			**50 746·1 (50 430·8 to 51 060·8)**	**60 043·1 (59 045·4 to 61 239·8)**	18·3% (16·1 to 20·8)*	**1009·0 (1002·5 to 1015·2)**	**701·5 (690·2 to 714·9)**	–30·5% (−31·7 to −29·0)*	**2 058 941·2 (2 046 424·5 to 2 073 121·2)**	**1 808 856·0 (1 784 551·7 to 1 836 374·3)**	–12·1% (−13·3 to −10·7)*	**36 126·3 (35 903·4 to 36 364·3)**	**22 647·5 (22 371·3 to 22 958·5)**	–37·3% (−38·1 to −36·3)*
**Communicable, maternal, neonatal, and nutritional diseases**			**15 054·6 (14 495·6 to 15 946·2)**	**10 326·0 (9761·6 to 11 100·0)**	–31·1% (−34·7 to −27·2)*	**263·4 (252·2 to 280·2)**	**136·9 (129·9 to 146·0)**	–47·8% (−50·4 to −45·2)*	**1 005 458·1 (980 016·4 to 1 042 665·5)**	**553 137·9 (526 426·5 to 581 083·9)**	–44·9% (−47·2 to −42·7)*	**16 568·9 (16 142·1 to 17 230·8)**	**8024·1 (7654·4 to 8382·4)**	–51·5% (−53·6 to −49·6)*
	**HIV/AIDS and sexually transmitted infections**		**1578·2 (1462·9 to 1719·2)**	**917·2 (799·7 to 1072·2)**	–41·9% (−49·0 to −34·1)*	**26·1 (24·2 to 28·4)**	**11·2 (9·7 to 13·1)**	–57·1% (−62·5 to −51·3)*	**90 307·6 (83 037·7 to 100 135·0)**	**47 785·7 (40 824·0 to 56 588·0)**	–47·1% (−53·4 to −40·5)*	**1466·2 (1347·8 to 1623·3)**	**610·4 (519·2 to 732·2)**	–58·4% (−63·8 to −53·0)*
	HIV/AIDS		1489·3 (1387·8 to 1620·1)	833·4 (727·1 to 959·0)	−44·1% (−50·8 to −36·5)*	24·6 (22·9 to 26·8)	9·9 (8·6 to 11·3)	−59·9% (−64·7 to −54·7)*	82 763·4 (76 737·0 to 90 409·0)	40 794·5 (35 907·8 to 46 637·8)	−50·7% (−56·0 to −44·6)*	1343·9 (1246·5 to 1465·6)	497·8 (439·2 to 568·8)	−63·0% (−66·9 to −58·6)*
		HIV/AIDS and drug-susceptible tuberculosis co-infection	477·2 (340·6 to 569·7)	190·5 (125·2 to 252·6)	−60·1% (−68·6 to −49·1)*	7·9 (5·6 to 9·4)	2·3 (1·5 to 3·0)	−71·3% (−77·5 to −63·5)*	26 632·9 (18 980·6 to 31 911·6)	9365·2 (6135·8 to 12 463·7)	−64·8% (−72·6 to −55·4)*	432·4 (307·9 to 517·9)	114·5 (74·9 to 152·5)	−73·5% (−79·4 to −66·5)*
		HIV/AIDS and multidrug-resistant tuberculosis without extensive drug resistance co-infection	29·9 (7·8 to 81·5)	18·8 (6·5 to 38·9)	−37·2% (−73·3 to 82·7)	0·5 (0·1 to 1·4)	0·2 (0·1 to 0·5)	−55·0% (−80·8 to 31·4)	1656·9 (434·8 to 4546·7)	927·2 (319·3 to 1921·4)	−44·0% (−75·7 to 61·2)	26·9 (7·1 to 74·0)	11·3 (3·9 to 23·5)	−57·9% (−81·6 to 20·0)
		HIV/AIDS and extensively drug-resistant tuberculosis co-infection	0·4 (0·1 to 0·9)	0·8 (0·3 to 1·6)	125·1% (4·4 to 443·7)*	0·0 (0·0 to 0·0)	0·0 (0·0 to 0·0)	61·2% (−24·9 to 286·1)	19·0 (5·2 to 46·4)	39·3 (15·5 to 78·1)	107·0% (−3·4 to 402·6)	0·3 (0·1 to 0·8)	0·5 (0·2 to 0·9)	54·0% (−27·8 to 272·7)
		HIV/AIDS resulting in other diseases	981·9 (863·6 to 1174·0)	623·3 (519·2 to 770·0)	−36·5% (−48·1 to −24·5)*	16·2 (14·3 to 19·4)	7·4 (6·2 to 9·1)	−54·5% (−62·8 to −46·0)*	54 454·7 (47 620·4 to 64 665·7)	30 462·9 (25 561·4 to 37 382·8)	−44·1% (−53·5 to −33·4)*	884·3 (772·9 to 1048·7)	371·5 (311·5 to 454·2)	−58·0% (−65·0 to −50·2)*
	Sexually transmitted infections excluding HIV		88·9 (39·6 to 164·0)	83·7 (35·7 to 154·3)	−5·8% (−21·2 to 7·1)	1·5 (0·7 to 2·7)	1·3 (0·5 to 2·5)	−9·4% (−25·8 to 2·4)	7544·2 (3116·7 to 14 412·4)	6991·2 (2728·7 to 13 257·7)	−7·3% (−21·0 to 5·3)	122·2 (50·6 to 233·3)	112·6 (43·1 to 214·8)	−7·9% (−22·1 to 4·7)
		Syphilis	82·9 (33·2 to 159·9)	77·0 (29·5 to 147·5)	−7·1% (−21·1 to 6·4)	1·3 (0·5 to 2·6)	1·2 (0·5 to 2·4)	−8·0% (−22·1 to 4·5)	7283·1 (2827·8 to 14 218·5)	6725·4 (2488·9 to 12 994·1)	−7·7% (−21·8 to 6·1)	117·9 (45·8 to 230·1)	109·4 (40·2 to 211·6)	−7·2% (−21·9 to 6·7)
		Chlamydial infection	1·3 (0·7 to 2·6)	1·5 (0·9 to 2·6)	11·0% (−51·0 to 135·0)	0·0 (0·0 to 0·0)	0·0 (0·0 to 0·0)	−26·1% (−67·1 to 54·7)	63·0 (32·0 to 126·6)	62·8 (34·9 to 115·3)	−0·3% (−58·5 to 122·2)	1·0 (0·5 to 2·0)	0·8 (0·4 to 1·4)	−25·7% (−69·0 to 64·3)
		Gonococcal infection	0·6 (0·3 to 1·0)	0·6 (0·4 to 1·0)	7·8% (−41·6 to 91·3)	0·0 (0·0 to 0·0)	0·0 (0·0 to 0·0)	−29·0% (−61·0 to 25·3)	26·7 (14·7 to 49·0)	25·9 (15·5 to 45·2)	−3·0% (−51·3 to 80·2)	0·4 (0·2 to 0·8)	0·3 (0·2 to 0·6)	−27·4% (−63·7 to 35·7)
		Other sexually transmitted infections	4·0 (2·3 to 7·7)	4·6 (2·8 to 8·0)	15·0% (−46·8 to 127·4)	0·1 (0·0 to 0·1)	0·1 (0·0 to 0·1)	−27·4% (−66·0 to 40·7)	171·3 (91·5 to 335·0)	177·0 (101·3 to 317·3)	3·3% (−55·2 to 116·4)	2·9 (1·6 to 5·6)	2·1 (1·2 to 3·8)	−26·8% (−68·1 to 51·8)
	**Respiratory infections and tuberculosis**		**4446·2 (4000·3 to 4975·1)**	**4337·1 (3947·6 to 4723·1)**	**–1·4% (−13·0 to 13·0)**	**84·0 (75·8 to 93·8)**	**52·9 (48·1 to 57·6)**	–36·4% (−43·4 to −27·4)*	**233 209·3 (208 675·3 to 263 016·9)**	**156 730·3 (138 940·5 to 173 994·9)**	–32·5% (−41·5 to −20·9)*	**3962·0 (3548·2 to 4466·5)**	**2106·8 (1864·0 to 2361·4)**	–46·6% (−54·1 to −37·6)*
	Tuberculosis		1760·3 (1397·6 to 2165·4)	1010·5 (806·6 to 1244·7)	−42·6% (−57·8 to −20·2)*	32·7 (26·0 to 40·3)	11·6 (9·2 to 14·4)	−64·4% (−73·7 to −50·8)*	72 482·5 (56 508·1 to 91 015·3)	37 633·5 (29 040·2 to 46 853·0)	−48·1% (−62·7 to −28·3)*	1249·9 (977·5 to 1562·4)	455·2 (349·3 to 570·1)	−63·6% (−73·8 to −49·8)*
		Drug-susceptible tuberculosis	1629·6 (1241·5 to 2010·7)	908·3 (670·6 to 1140·9)	−44·3% (−61·0 to −18·8)*	30·3 (23·1 to 37·7)	10·5 (7·7 to 13·2)	−65·4% (−75·8 to −49·9)*	67 228·8 (50 572·5 to 84 499·1)	33 952·7 (24 886·5 to 43 347·3)	−49·5% (−65·7 to −27·8)*	1158·9 (874·0 to 1451·8)	411·2 (300·0 to 528·0)	−64·5% (−75·9 to −49·5)*
		Multidrug-resistant tuberculosis without extensive drug resistance	127·1 (35·2 to 305·7)	95·5 (28·9 to 224·1)	−24·9% (−76·0 to 147·9)	2·4 (0·7 to 5·7)	1·1 (0·3 to 2·6)	−53·8% (−85·3 to 51·1)	5112·2 (1376·5 to 12 610·0)	3448·2 (1093·5 to 7932·4)	−32·5% (−76·7 to 118·8)	88·6 (24·0 to 217·4)	41·3 (13·2 to 94·0)	−53·4% (−83·9 to 50·0)
		Extensively drug-resistant tuberculosis	3·6 (1·1 to 8·8)	6·8 (2·6 to 14·5)	86·6% (−21·9 to 400·0)	0·1 (0·0 to 0·2)	0·1 (0·0 to 0·2)	13·5% (−52·4 to 199·2)	141·5 (41·2 to 346·0)	232·6 (92·1 to 503·8)	64·5% (−30·6 to 336·4)	2·5 (0·7 to 6·0)	2·7 (1·1 to 5·9)	10·5% (−53·2 to 191·5)
	Lower respiratory infections		2646·6 (2369·3 to 2950·0)	2501·3 (2241·0 to 2812·2)	−5·5% (−19·0 to 9·4)	50·5 (45·6 to 55·7)	31·6 (28·3 to 35·3)	−37·5% (−46·0 to −28·1)*	158 286·0 (136 227·6 to 182 886·1)	98 421·5 (87 415·4 to 111 734·7)	−37·8% (−48·2 to −24·7)*	2671·2 (2305·3 to 3075·7)	1391·9 (1215·8 to 1601·7)	−47·9% (−56·6 to −36·7)*
	Upper respiratory infections		38·3 (7·1 to 93·9)	27·1 (6·1 to 77·3)	−29·4% (−68·7 to 48·4)	0·7 (0·1 to 1·7)	0·4 (0·1 to 1·1)	−46·9% (−76·8 to 6·8)	2389·4 (387·8 to 6402·0)	1727·4 (277·4 to 5097·2)	−27·7% (−69·3 to 65·7)	40·1 (6·6 to 106·4)	25·9 (3·9 to 76·6)	−35·4% (−72·7 to 47·4)
	Otitis media		1·0 (0·3 to 3·7)	0·7 (0·3 to 1·7)	−35·3% (−78·8 to 166·6)	0·0 (0·0 to 0·1)	0·0 (0·0 to 0·0)	−56·0% (−85·2 to 83·5)	51·3 (11·9 to 206·6)	31·9 (10·2 to 94·8)	−37·9% (−81·5 to 219·9)	0·9 (0·2 to 3·5)	0·4 (0·1 to 1·3)	−49·7% (−85·1 to 156·2)
	COVID-19		0·0 (0·0 to 0·0)	797·6 (722·9 to 857·0)	0·0% (0·0 to 0·0)	0·0 (0·0 to 0·0)	9·3 (8·4 to 10·0)	0·0% (0·0 to 0·0)	0·0 (0·0 to 0·0)	18 916·1 (17 699·8 to 19 764·2)	0·0% (0·0 to 0·0)	0·0 (0·0 to 0·0)	233·3 (219·0 to 244·0)	0·0% (0·0 to 0·0)
	**Enteric infections**		**2658·5 (2142·0 to 3452·1)**	**1268·6 (962·6 to 1683·3)**	–52·3% (−61·9 to −40·0)*	**48·4 (39·1 to 64·1)**	**16·4 (12·6 to 21·3)**	–66·1% (−72·4 to −57·5)*	**165 871·4 (133 972·2 to 201 264·5)**	**61 613·6 (48 551·8 to 78 598·2)**	–62·9% (−70·1 to −52·3)*	**2769·9 (2241·4 to 3358·4)**	**879·9 (694·4 to 1126·1)**	–68·2% (−74·7 to −59·0)*
	Diarrhoeal diseases		2336·2 (1838·9 to 3112·9)	1107·1 (810·5 to 1535·9)	−52·6% (−63·3 to −38·5)*	43·2 (33·8 to 59·0)	14·2 (10·6 to 19·2)	−67·2% (−74·1 to −57·7)*	140 734·9 (108 979·9 to 177 999·5)	49 534·6 (37 596·6 to 65 658·5)	−64·8% (−73·3 to −52·6)*	2365·9 (1833·8 to 2982·2)	706·0 (531·1 to 931·4)	−70·2% (−77·4 to −59·1)*
	Typhoid and paratyphoid		231·3 (122·0 to 378·2)	82·8 (43·4 to 135·4)	−64·2% (−68·2 to −58·6)*	3·7 (2·0 to 6·1)	1·1 (0·6 to 1·9)	−69·2% (−72·5 to −64·7)*	18 217·0 (9546·2 to 30 015·7)	6154·0 (3217·3 to 9994·0)	−66·2% (−70·0 to −61·0)*	291·2 (153·4 to 480·7)	86·9 (45·6 to 141·2)	−70·1% (−73·6 to −65·4)*
		Typhoid fever	196·9 (101·7 to 326·2)	72·0 (38·1 to 118·6)	−63·5% (−67·5 to −57·7)*	3·2 (1·6 to 5·2)	1·0 (0·5 to 1·6)	−68·5% (−72·0 to −63·9)*	15 503·9 (7911·3 to 25 488·6)	5352·6 (2773·7 to 8719·9)	−65·5% (−69·5 to −60·1)*	247·9 (125·7 to 407·8)	75·6 (39·0 to 122·4)	−69·5% (−73·1 to −64·8)*
		Paratyphoid fever	34·4 (16·0 to 64·7)	10·9 (5·2 to 20·9)	−68·5% (−72·5 to −62·5)*	0·6 (0·3 to 1·0)	0·1 (0·1 to 0·3)	−72·8% (−76·3 to −67·9)*	2713·1 (1252·9 to 5065·2)	801·4 (379·4 to 1567·5)	−70·5% (−74·4 to −64·7)*	43·3 (19·9 to 80·5)	11·3 (5·3 to 22·4)	−73·9% (−77·5 to −69·0)*
	Invasive non-typhoidal salmonella		89·5 (72·5 to 112·2)	76·7 (60·1 to 100·1)	−14·3% (−23·9 to −4·0)*	1·5 (1·2 to 1·8)	1·1 (0·8 to 1·4)	−25·9% (−35·3 to −16·8)*	6834·5 (5356·2 to 8716·2)	5843·9 (4441·4 to 7770·4)	−14·5% (−25·0 to −3·3)*	111·4 (87·2 to 142·0)	85·9 (64·3 to 115·6)	−22·8% (−32·5 to −12·5)*
	Other intestinal infectious diseases		1·5 (1·2 to 1·9)	1·9 (1·4 to 2·4)	27·5% (−13·5 to 82·5)	0·0 (0·0 to 0·0)	0·0 (0·0 to 0·0)	−14·9% (−41·8 to 23·6)	85·0 (62·3 to 111·7)	81·0 (59·0 to 105·1)	−4·7% (−41·4 to 46·5)	1·4 (1·0 to 1·8)	1·1 (0·8 to 1·4)	−24·5% (−53·6 to 16·2)
	**Neglected tropical diseases and malaria**		**1050·3 (652·3 to 1601·1)**	**804·9 (394·2 to 1392·4)**	–23·4% (−40·1 to −11·4)*	**17·5 (10·9 to 26·6)**	**11·3 (5·4 to 19·5)**	–35·5% (−50·0 to −25·6)*	**79 141·6 (47 827·7 to 122 595·4)**	**56 346·7 (26 007·0 to 98 093·7)**	–28·8% (−44·8 to −17·9)*	**1295·3 (783·7 to 2006·4)**	**829·8 (378·5 to 1443·5)**	–35·9% (−51·1 to −26·1)*
	Malaria		881·4 (478·4 to 1450·7)	670·0 (261·2 to 1257·9)	−24·0% (−46·2 to −11·3)*	14·6 (8·0 to 24·0)	9·6 (3·7 to 17·9)	−34·5% (−54·1 to −23·8)*	68 625·0 (36 825·2 to 112 650·3)	49 102·3 (18 837·4 to 91 563·7)	−28·4% (−49·6 to −16·1)*	1124·7 (604·9 to 1843·1)	732·1 (280·5 to 1358·1)	−34·9% (−54·2 to −23·9)*
	Chagas disease		10·6 (9·8 to 11·6)	8·4 (7·5 to 9·4)	−20·5% (−27·2 to −13·9)*	0·2 (0·2 to 0·2)	0·1 (0·1 to 0·1)	−57·0% (−60·5 to −53·3)*	295·2 (273·9 to 321·8)	190·7 (171·5 to 210·9)	−35·4% (−40·5 to −30·5)*	5·6 (5·2 to 6·1)	2·1 (1·9 to 2·3)	−62·8% (−65·8 to −59·9)*
	Leishmaniasis		10·1 (2·5 to 24·2)	4·6 (1·9 to 8·7)	−54·1% (−67·8 to −17·0)*	0·2 (0·0 to 0·4)	0·1 (0·0 to 0·1)	−61·8% (−74·1 to −27·2)*	738·5 (194·5 to 1743·9)	332·1 (141·3 to 610·1)	−55·0% (−69·8 to −17·1)*	11·9 (3·1 to 28·2)	4·6 (2·0 to 8·4)	−61·1% (−74·4 to −26·4)*
		Visceral leishmaniasis	10·1 (2·5 to 24·2)	4·6 (1·9 to 8·7)	−54·1% (−67·8 to −17·0)*	0·2 (0·0 to 0·4)	0·1 (0·0 to 0·1)	−61·8% (−74·1 to −27·2)*	738·5 (194·5 to 1743·9)	332·1 (141·3 to 610·1)	−55·0% (−69·8 to −17·1)*	11·9 (3·1 to 28·2)	4·6 (2·0 to 8·4)	−61·1% (−74·4 to −26·4)*
	African trypanosomiasis		26·5 (13·4 to 45·8)	1·4 (0·7 to 2·5)	−94·7% (−95·2 to −94·1)*	0·4 (0·2 to 0·7)	0·0 (0·0 to 0·0)	−95·9% (−96·3 to −95·5)*	1620·1 (825·8 to 2806·8)	84·2 (40·1 to 147·4)	−94·8% (−95·3 to −94·3)*	25·2 (12·8 to 43·8)	1·1 (0·5 to 1·9)	−95·8% (−96·2 to −95·4)*
	Schistosomiasis		21·6 (19·8 to 23·9)	13·5 (12·3 to 14·8)	−37·7% (−42·8 to −31·9)*	0·4 (0·4 to 0·4)	0·2 (0·1 to 0·2)	−59·4% (−62·7 to −56·0)*	987·8 (898·1 to 1115·3)	564·3 (508·2 to 623·0)	−42·9% (−47·7 to −37·9)*	16·5 (15·0 to 18·5)	6·8 (6·2 to 7·5)	−58·5% (−61·9 to −55·1)*
	Cysticercosis		2·4 (1·8 to 3·2)	1·5 (1·1 to 2·1)	−36·4% (−53·9 to −10·1)*	0·0 (0·0 to 0·1)	0·0 (0·0 to 0·0)	−53·4% (−66·4 to −34·2)*	135·9 (99·2 to 194·0)	80·1 (55·6 to 114·4)	−41·1% (−58·3 to −16·5)*	2·2 (1·6 to 3·1)	1·0 (0·7 to 1·4)	−54·0% (−67·8 to −35·4)*
	Cystic echinococcosis		3·0 (2·3 to 3·8)	1·4 (1·0 to 1·8)	−53·8% (−67·8 to −34·9)*	0·1 (0·0 to 0·1)	0·0 (0·0 to 0·0)	−68·1% (−77·5 to −55·3)*	179·3 (134·7 to 227·6)	61·8 (41·9 to 82·6)	−65·6% (−77·2 to −51·9)*	3·0 (2·2 to 3·8)	0·8 (0·5 to 1·1)	−73·4% (−83·0 to −62·5)*
	Dengue		24·0 (9·0 to 60·2)	52·7 (25·2 to 108·9)	119·6% (−18·1 to 616·4)	0·4 (0·2 to 1·0)	0·7 (0·3 to 1·3)	60·9% (−40·0 to 415·1)	1612·3 (601·4 to 4086·4)	2655·6 (1233·0 to 5361·4)	64·7% (−39·5 to 442·1)	26·2 (9·8 to 66·3)	35·1 (16·2 to 70·8)	34·0% (−50·9 to 335·2)
	Yellow fever		12·6 (4·5 to 26·7)	4·4 (1·5 to 9·3)	−65·0% (−71·0 to −58·8)*	0·2 (0·1 to 0·4)	0·1 (0·0 to 0·1)	−70·9% (−75·8 to −65·6)*	901·0 (318·6 to 1930·3)	310·0 (108·7 to 652·8)	−65·6% (−71·5 to −58·9)*	14·0 (5·0 to 30·1)	4·1 (1·4 to 8·7)	−70·5% (−75·7 to −64·7)*
	Rabies		26·5 (13·9 to 44·0)	15·8 (6·7 to 27·4)	−40·3% (−74·0 to 15·5)	0·4 (0·2 to 0·7)	0·2 (0·1 to 0·3)	−55·7% (−80·5 to −14·4)*	1681·5 (851·8 to 2850·0)	870·9 (348·4 to 1588·3)	−48·2% (−78·6 to 4·3)	27·0 (13·7 to 45·7)	11·3 (4·4 to 20·8)	−58·1% (−82·6 to −15·3)*
	Intestinal nematode infections		14·3 (10·9 to 18·9)	5·0 (3·8 to 6·1)	−65·3% (−69·5 to −59·6)*	0·2 (0·2 to 0·3)	0·1 (0·1 to 0·1)	−68·6% (−72·6 to −63·4)*	1224·7 (925·4 to 1619·0)	409·0 (309·6 to 510·2)	−66·6% (−70·9 to −61·0)*	20·2 (15·2 to 26·7)	6·2 (4·7 to 7·7)	−69·3% (−73·2 to −64·1)*
		Ascariasis	14·3 (10·9 to 18·9)	5·0 (3·8 to 6·1)	−65·3% (−69·5 to −59·6)*	0·2 (0·2 to 0·3)	0·1 (0·1 to 0·1)	−68·6% (−72·6 to −63·4)*	1224·7 (925·4 to 1619·0)	409·0 (309·6 to 510·2)	−66·6% (−70·9 to −61·0)*	20·2 (15·2 to 26·7)	6·2 (4·7 to 7·7)	−69·3% (−73·2 to −64·1)*
	Ebola virus disease		0·3 (0·3 to 0·4)	0·0 (0·0 to 0·0)	−100·0% (−100·0 to −100·0)*	0·0 (0·0 to 0·0)	0·0 (0·0 to 0·0)	−100·0% (−100·0 to −100·0)*	18·1 (14·9 to 21·4)	0·0 (0·0 to 0·0)	−100·0% (−100·0 to −100·0)*	0·3 (0·2 to 0·3)	0·0 (0·0 to 0·0)	−100·0% (−100·0 to −100·0)*
	Zika virus disease		0·0 (0·0 to 0·0)	0·0 (0·0 to 0·0)	0·0% (0·0 to 0·0)	0·0 (0·0 to 0·0)	0·0 (0·0 to 0·0)	0·0% (0·0 to 0·0)	0·0 (0·0 to 0·0)	0·0 (0·0 to 0·0)	0·0% (0·0 to 0·0)	0·0 (0·0 to 0·0)	0·0 (0·0 to 0·0)	0·0% (0·0 to 0·0)
	Other neglected tropical diseases		16·9 (9·9 to 29·1)	26·3 (13·0 to 48·3)	55·1% (−31·6 to 234·7)	0·3 (0·2 to 0·5)	0·4 (0·2 to 0·7)	22·0% (−46·2 to 168·5)	1121·9 (595·1 to 2077·8)	1685·7 (699·5 to 3352·0)	50·3% (−41·1 to 256·5)	18·5 (9·9 to 34·2)	24·6 (9·9 to 49·6)	32·6% (−48·4 to 216·0)
	**Other infectious diseases**		**1888·8 (1440·7 to 2382·5)**	**841·7 (682·3 to 1009·2)**	–55·4% (−62·5 to −44·9)*	**31·4 (24·1 to 39·5)**	**11·6 (9·3 to 14·1)**	–62·9% (−68·6 to −54·7)*	**148 686·9 (110 615·9 to 190 966·4)**	**56 632·6 (44 640·6 to 69 674·9)**	–61·9% (−68·3 to −52·4)*	**2429·6 (1806·0 to 3120·1)**	**826·9 (639·9 to 1034·9)**	–66·0% (−71·7 to −57·3)*
	Meningitis		428·9 (341·9 to 547·5)	258·8 (202·2 to 334·6)	−39·7% (−56·7 to −17·2)*	7·2 (5·7 to 9·1)	3·5 (2·7 to 4·6)	−51·3% (−65·6 to −32·1)*	31 766·9 (24 209·5 to 41 522·9)	16 918·5 (13 198·7 to 22 556·0)	−46·7% (−63·5 to −23·3)*	516·5 (393·3 to 676·7)	239·5 (185·0 to 326·9)	−53·6% (−68·3 to −34·4)*
	Encephalitis		82·0 (52·0 to 119·1)	76·5 (54·7 to 113·4)	−6·7% (−43·1 to 38·0)	1·4 (0·9 to 2·1)	1·0 (0·7 to 1·4)	−33·5% (−59·4 to −1·4)*	4995·7 (3183·0 to 7374·9)	3683·9 (2525·8 to 5765·2)	−26·3% (−56·1 to 14·0)	82·4 (52·5 to 121·5)	50·0 (34·1 to 79·1)	−39·3% (−64·0 to −5·9)*
	Diphtheria		23·3 (18·0 to 29·6)	4·0 (3·0 to 5·3)	−82·8% (−86·7 to −78·1)*	0·4 (0·3 to 0·5)	0·1 (0·0 to 0·1)	−84·5% (−88·1 to −80·2)*	1960·7 (1506·4 to 2513·8)	329·7 (242·6 to 440·7)	−83·2% (−87·2 to −78·5)*	32·0 (24·5 to 41·1)	4·9 (3·6 to 6·6)	−84·7% (−88·4 to −80·2)*
	Pertussis		205·9 (111·4 to 328·6)	115·2 (66·6 to 189·2)	−44·0% (−68·8 to −4·2)*	3·4 (1·8 to 5·4)	1·8 (1·0 to 2·9)	−46·8% (−70·4 to −9·1)*	17 890·3 (9682·8 to 28 542·9)	10 001·5 (5766·0 to 16 405·3)	−44·1% (−68·9 to −4·3)*	292·3 (158·3 to 466·3)	155·9 (89·8 to 256·0)	−46·7% (−70·3 to −8·9)*
	Tetanus		126·4 (91·1 to 179·2)	19·8 (11·7 to 31·0)	−84·4% (−90·8 to −74·3)*	2·1 (1·5 to 3·0)	0·3 (0·2 to 0·4)	−87·3% (−92·5 to −79·8)*	9792·2 (6895·6 to 13 888·7)	1239·8 (745·3 to 1917·7)	−87·3% (−92·8 to −79·6)*	158·6 (111·8 to 225·2)	17·8 (10·8 to 27·3)	−88·8% (−93·6 to −82·0)*
	Measles		762·8 (347·4 to 1338·5)	143·6 (58·6 to 255·2)	−81·2% (−84·4 to −78·3)*	12·5 (5·7 to 21·9)	2·2 (0·9 to 3·9)	−82·3% (−85·3 to −79·6)*	66 032·7 (30 137·0 to 115 712·6)	12 431·7 (5068·1 to 22 097·5)	−81·2% (−84·4 to −78·3)*	1081·2 (493·8 to 1893·1)	191·9 (78·2 to 341·2)	−82·2% (−85·3 to −79·5)*
	Varicella and herpes zoster		15·3 (14·0 to 16·5)	13·7 (12·2 to 14·8)	−10·4% (−17·9 to −2·5)*	0·3 (0·3 to 0·3)	0·2 (0·2 to 0·2)	−41·0% (−45·3 to −36·3)*	872·2 (789·1 to 966·6)	598·8 (534·3 to 662·1)	−31·4% (−37·6 to −23·8)*	14·7 (13·4 to 16·3)	8·6 (7·6 to 9·6)	−41·7% (−47·1 to −35·0)*
	Acute hepatitis		171·7 (124·5 to 227·8)	93·1 (65·4 to 123·4)	−45·8% (−64·5 to −19·6)*	2·9 (2·1 to 3·9)	1·2 (0·8 to 1·6)	−59·7% (−73·8 to −40·0)*	10 991·5 (7719·8 to 14 818·4)	5146·2 (3538·2 to 6952·0)	−53·2% (−71·5 to −28·7)*	179·1 (126·1 to 241·8)	69·0 (46·7 to 94·0)	−61·5% (−76·8 to −41·1)*
		Acute hepatitis A	103·8 (75·1 to 147·2)	35·6 (21·8 to 52·0)	−65·7% (−80·3 to −45·6)*	1·7 (1·3 to 2·5)	0·5 (0·3 to 0·7)	−73·2% (−84·7 to −57·2)*	7122·9 (4960·5 to 10 188·7)	2170·2 (1293·2 to 3222·5)	−69·5% (−82·9 to −50·7)*	115·5 (80·5 to 165·6)	30·0 (17·7 to 45·3)	−74·1% (−85·5 to −57·6)*
		Acute hepatitis B	54·1 (31·1 to 87·3)	45·9 (28·0 to 66·9)	−15·2% (−53·0 to 53·2)	0·9 (0·5 to 1·5)	0·6 (0·3 to 0·8)	−38·8% (−66·5 to 8·1)	3169·3 (1652·8 to 5272·4)	2403·0 (1395·3 to 3610·2)	−24·2% (−60·0 to 43·3)	52·0 (27·5 to 86·2)	31·6 (18·0 to 47·9)	−39·1% (−68·0 to 13·5)
		Acute hepatitis C	10·4 (5·0 to 18·1)	7·2 (3·8 to 11·9)	−30·5% (−65·7 to 27·1)	0·2 (0·1 to 0·3)	0·1 (0·0 to 0·1)	−55·0% (−77·6 to −17·2)*	483·3 (210·2 to 873·5)	312·1 (148·6 to 543·9)	−35·4% (−67·6 to 23·6)	8·2 (3·6 to 14·5)	3·8 (1·8 to 6·8)	−53·1% (−76·5 to −12·5)*
		Acute hepatitis E	3·5 (1·6 to 6·5)	4·4 (2·1 to 8·1)	26·0% (−45·0 to 173·8)	0·1 (0·0 to 0·1)	0·1 (0·0 to 0·1)	−4·3% (−57·7 to 116·7)	216·0 (93·2 to 443·9)	260·9 (119·4 to 490·9)	20·8% (−50·7 to 180·6)	3·5 (1·5 to 7·2)	3·6 (1·6 to 6·8)	1·7% (−58·5 to 144·7)
	Other unspecified infectious diseases		72·5 (41·4 to 115·1)	117·0 (65·3 to 191·9)	61·4% (3·4 to 144·9)*	1·3 (0·7 to 2·0)	1·5 (0·8 to 2·5)	18·2% (−24·2 to 81·2)	4384·6 (2323·4 to 7276·8)	6282·5 (3183·3 to 10 888·1)	43·3% (−15·8 to 131·4)	72·6 (38·8 to 120·2)	89·1 (44·2 to 156·6)	22·7% (−28·3 to 100·6)
	**Maternal and neonatal disorders**		**3007·2 (2877·3 to 3149·7)**	**1867·9 (1739·6 to 1993·0)**	–37·9% (−42·5 to −33·6)*	**48·2 (46·1 to 50·5)**	**29·7 (27·6 to 31·7)**	–38·5% (−43·0 to −34·0)*	**259 274·7 (247 935·5 to 271 583·0)**	**160 961·8 (149 602·1 to 171 949·9)**	–37·9% (−42·5 to −33·3)*	**4162·7 (3979·9 to 4360·5)**	**2581·6 (2402·3 to 2761·6)**	–38·0% (−42·6 to −33·3)*
	Maternal disorders		396·7 (355·3 to 438·6)	239·9 (207·8 to 280·3)	−39·5% (−48·1 to −27·9)*	6·1 (5·4 to 6·7)	3·0 (2·6 to 3·5)	−50·8% (−57·7 to −41·3)*	24 511·9 (21 945·7 to 27 102·5)	14 559·5 (12 630·9 to 16 968·2)	−40·6% (−48·8 to −29·1)*	374·3 (335·1 to 413·9)	182·7 (158·5 to 212·8)	−51·2% (−57·8 to −41·7)*
		Maternal haemorrhage	133·9 (102·0 to 165·4)	52·0 (35·8 to 70·0)	−61·2% (−74·3 to −43·7)*	2·1 (1·6 to 2·5)	0·6 (0·4 to 0·9)	−68·5% (−79·2 to −54·4)*	8217·9 (6262·5 to 10 169·5)	3127·0 (2160·9 to 4205·8)	−61·9% (−74·9 to −44·7)*	125·7 (95·8 to 155·5)	39·1 (27·1 to 52·7)	−68·9% (−79·5 to −54·8)*
		Maternal sepsis and other pregnancy-related infections	43·6 (30·6 to 61·2)	26·7 (19·0 to 37·1)	−38·7% (−58·2 to −8·0)*	0·7 (0·5 to 0·9)	0·3 (0·2 to 0·5)	−49·9% (−65·7 to −24·8)*	2719·9 (1902·2 to 3807·8)	1627·1 (1156·8 to 2253·8)	−40·2% (−59·2 to −10·2)*	41·4 (29·0 to 58·0)	20·4 (14·5 to 28·3)	−50·6% (−66·2 to −25·9)*
		Maternal hypertensive disorders	68·9 (53·9 to 85·1)	48·2 (37·3 to 59·9)	−30·0% (−49·1 to −5·6)*	1·1 (0·8 to 1·3)	0·6 (0·5 to 0·8)	−42·7% (−58·4 to −22·7)*	4299·7 (3367·0 to 5317·2)	2940·4 (2275·4 to 3662·6)	−31·6% (−50·3 to −8·0)*	65·5 (51·3 to 80·9)	37·0 (28·6 to 46·1)	−43·5% (−59·0 to −24·0)*
		Maternal obstructed labour and uterine rupture	22·1 (13·4 to 33·4)	12·2 (7·5 to 18·4)	−44·8% (−69·7 to 5·3)	0·3 (0·2 to 0·5)	0·2 (0·1 to 0·2)	−55·4% (−75·4 to −14·9)*	1342·8 (806·8 to 2039·5)	736·3 (452·0 to 1119·2)	−45·2% (−70·0 to 4·9)	20·6 (12·4 to 31·3)	9·2 (5·7 to 14·0)	−55·2% (−75·5 to −14·4)*
		Maternal abortive outcome	43·4 (29·1 to 64·0)	19·6 (12·2 to 30·8)	−54·8% (−71·2 to −19·8)*	0·7 (0·4 to 1·0)	0·2 (0·2 to 0·4)	−63·2% (−76·6 to −34·9)*	2675·5 (1786·7 to 3944·6)	1205·0 (748·7 to 1890·4)	−55·0% (−71·4 to −20·1)*	40·9 (27·4 to 60·3)	15·1 (9·4 to 23·7)	−63·0% (−76·5 to −34·3)*
		Ectopic pregnancy	9·9 (6·6 to 14·1)	12·7 (8·5 to 17·8)	28·6% (−22·4 to 99·5)	0·2 (0·1 to 0·2)	0·2 (0·1 to 0·2)	4·8% (−36·7 to 62·5)	613·2 (410·2 to 872·7)	776·7 (520·9 to 1079·9)	26·7% (−23·5 to 96·4)	9·4 (6·3 to 13·3)	9·8 (6·5 to 13·5)	4·3% (−37·0 to 61·6)
		Indirect maternal deaths	29·7 (19·6 to 42·7)	23·0 (16·3 to 32·7)	−22·5% (−47·7 to 20·4)	0·5 (0·3 to 0·7)	0·3 (0·2 to 0·4)	−36·5% (−57·1 to −1·3)*	1860·5 (1228·0 to 2667·9)	1409·4 (1002·3 to 2002·6)	−24·2% (−48·7 to 18·0)	28·3 (18·7 to 40·7)	17·7 (12·6 to 25·2)	−37·4% (−57·7 to −2·5)*
		Late maternal deaths	7·2 (6·1 to 8·9)	8·0 (6·8 to 9·9)	10·8% (−3·3 to 29·3)	0·1 (0·1 to 0·1)	0·1 (0·1 to 0·1)	−10·0% (−21·2 to 5·2)	447·0 (371·7 to 549·3)	484·1 (404·2 to 596·5)	8·3% (−6·1 to 26·5)	6·8 (5·7 to 8·4)	6·1 (5·0 to 7·5)	−11·1% (−22·5 to 3·9)
		Maternal deaths aggravated by HIV/AIDS	2·7 (1·7 to 3·6)	1·7 (1·0 to 2·3)	−38·9% (−49·5 to −26·4)*	0·0 (0·0 to 0·1)	0·0 (0·0 to 0·0)	−50·9% (−59·4 to −41·0)*	166·9 (105·8 to 223·3)	96·1 (60·2 to 134·9)	−42·4% (−52·4 to −31·0)*	2·6 (1·6 to 3·4)	1·2 (0·7 to 1·7)	−53·3% (−61·4 to −44·1)*
		Other direct maternal disorders	35·3 (23·1 to 51·0)	35·7 (25·2 to 51·8)	1·2% (−37·2 to 71·5)	0·5 (0·4 to 0·8)	0·4 (0·3 to 0·6)	−17·8% (−49·1 to 39·2)	2168·5 (1419·8 to 3142·4)	2157·5 (1520·0 to 3142·4)	−0·5% (−38·6 to 68·3)	33·2 (21·7 to 48·1)	27·0 (19·1 to 39·4)	−18·5% (−49·7 to 37·9)
	Neonatal disorders		2610·4 (2495·1 to 2736·7)	1628·0 (1506·0 to 1748·0)	−37·6% (−42·6 to −32·6)*	42·1 (40·3 to 44·2)	26·7 (24·7 to 28·6)	−36·7% (−41·7 to −31·5)*	234 762·8 (224 399·3 to 246 110·0)	146 402·3 (135 446·8 to 157 187·9)	−37·6% (−42·6 to −32·6)*	3788·5 (3620·9 to 3971·9)	2398·9 (2219·7 to 2575·4)	−36·7% (−41·7 to −31·5)*
		Neonatal preterm birth	1064·4 (922·5 to 1244·7)	623·3 (509·7 to 735·0)	−41·4% (−54·4 to −25·6)*	17·2 (14·9 to 20·1)	10·2 (8·4 to 12·0)	−40·5% (−53·7 to −24·4)*	95 728·4 (82 962·8 to 111 938·1)	56 052·0 (45 839·1 to 66 086·5)	−41·4% (−54·4 to −25·6)*	1544·6 (1338·6 to 1806·0)	918·4 (751·2 to 1082·6)	−40·5% (−53·7 to −24·4)*
		Neonatal encephalopathy due to birth asphyxia and trauma	825·2 (676·6 to 995·4)	562·1 (471·4 to 678·7)	−31·9% (−47·1 to −7·1)*	13·3 (10·9 to 16·1)	9·2 (7·7 to 11·1)	−30·8% (−46·2 to −5·6)*	74 223·5 (60 862·4 to 89 528·0)	50 562·7 (42 408·8 to 61 048·3)	−31·9% (−47·1 to −7·1)*	1196·9 (981·4 to 1444·0)	828·9 (695·3 to 1000·7)	−30·7% (−46·2 to −5·6)*
		Neonatal sepsis and other neonatal infections	324·7 (213·8 to 466·6)	223·2 (155·6 to 315·2)	−31·3% (−54·8 to 2·5)	5·2 (3·5 to 7·5)	3·7 (2·5 to 5·2)	−30·3% (−54·2 to 3·8)	29 199·1 (19 224·7 to 41 953·3)	20 069·1 (13 989·7 to 28 336·6)	−31·3% (−54·8 to 2·5)	471·9 (310·7 to 678·0)	328·7 (229·1 to 464·0)	−30·3% (−54·2 to 3·8)
		Haemolytic disease and other neonatal jaundice	99·2 (54·4 to 180·0)	30·3 (16·7 to 47·8)	−69·4% (−82·9 to −42·2)*	1·6 (0·9 to 2·9)	0·5 (0·3 to 0·8)	−69·0% (−82·7 to −41·4)*	8913·3 (4890·9 to 16 180·1)	2726·9 (1501·2 to 4296·3)	−69·4% (−82·9 to −42·2)*	144·1 (79·1 to 261·5)	44·7 (24·6 to 70·4)	−69·0% (−82·7 to −41·4)*
		Other neonatal disorders	296·9 (207·3 to 420·9)	189·0 (124·2 to 267·4)	−36·4% (−59·3 to 1·7)	4·8 (3·3 to 6·8)	3·1 (2·0 to 4·4)	−35·4% (−58·7 to 3·2)	26 698·5 (18 643·0 to 37 846·9)	16 991·7 (11 163·8 to 24 043·6)	−36·4% (−59·3 to 1·7)	431·0 (301·0 to 610·8)	278·3 (182·8 to 393·9)	−35·4% (−58·7 to 3·2)
	**Nutritional deficiencies**		**425·4 (355·6 to 499·8)**	**288·6 (231·1 to 350·3)**	–32·2% (−47·4 to −12·6)*	**7·7 (6·5 to 9·1)**	**3·8 (3·0 to 4·6)**	–51·5% (−62·4 to −37·5)*	**28 966·7 (23 792·6 to 34 568·4)**	**13 067·2 (10 013·5 to 16 919·1)**	–54·9% (−66·7 to −39·4)*	**483·1 (397·1 to 575·5)**	**188·6 (142·9 to 246·9)**	–61·0% (−71·2 to −47·6)*
	Protein-energy malnutrition		378·2 (316·6 to 448·9)	245·8 (195·9 to 300·8)	−35·1% (−50·6 to −15·6)*	6·8 (5·7 to 8·1)	3·3 (2·6 to 4·1)	−52·4% (−63·9 to −38·7)*	26 783·9 (21 969·1 to 32 265·2)	11 799·0 (8808·0 to 15 515·0)	−56·0% (−68·5 to −41·4)*	445·7 (365·8 to 536·0)	172·7 (127·5 to 229·0)	−61·3% (−72·3 to −48·2)*
	Other nutritional deficiencies		47·2 (36·0 to 61·1)	42·8 (30·1 to 58·5)	−9·4% (−36·5 to 34·5)	0·9 (0·7 to 1·2)	0·5 (0·4 to 0·7)	−45·2% (−61·9 to −19·1)*	2182·8 (1568·7 to 3015·5)	1268·2 (859·0 to 1769·6)	−41·9% (−62·0 to −7·4)*	37·4 (27·1 to 51·4)	15·9 (10·8 to 22·2)	−57·4% (−72·5 to −31·2)*
**Non-communicable diseases**			**31 130·5 (30 469·8 to 31 722·1)**	**44 842·6 (43 824·4 to 46 047·5)**	43·9% (39·5 to 48·6)*	**666·6 (653·0 to 678·2)**	**506·2 (494·7 to 519·3)**	–24·1% (−26·4 to −21·7)*	**813 992·9 (789 034·7 to 837 268·3)**	**1 035 074·3 (1 009 265·1 to 1 063 890·6)**	27·1% (22·2 to 32·4)*	**15 672·8 (15 242·2 to 16 077·7)**	**11 866·9 (11 560·1 to 12 221·0)**	–24·3% (−27·1 to −21·4)*
	**Neoplasms**		**7072·9 (6679·9 to 7356·8)**	**10 567·9 (9726·9 to 11 166·9)**	49·3% (41·2 to 57·8)*	**143·9 (135·8 to 150·0)**	**117·0 (107·7 to 123·5)**	–18·8% (−22·6 to −14·4)*	**197 152·6 (187 838·8 to 204 021·0)**	**267 421·9 (252 512·2 to 280 600·0)**	35·6% (29·5 to 43·6)*	**3734·1 (3556·7 to 3868·0)**	**2984·7 (2819·7 to 3129·3)**	–20·1% (−23·5 to −15·3)*
	Lip and oral cavity cancer		120·6 (108·1 to 131·0)	225·7 (198·6 to 262·3)	87·1% (60·6 to 124·1)*	2·4 (2·2 to 2·6)	2·5 (2·2 to 2·9)	3·0% (−11·5 to 23·0)	3545·0 (3170·0 to 3892·6)	6290·1 (5461·5 to 7446·1)	77·4% (51·0 to 114·4)*	67·0 (60·0 to 73·4)	69·5 (60·3 to 82·4)	3·7% (−11·8 to 25·5)
	Nasopharynx cancer		64·2 (56·3 to 71·5)	75·4 (63·3 to 89·1)	17·4% (−4·1 to 43·0)	1·2 (1·1 to 1·3)	0·8 (0·7 to 1·0)	−31·1% (−43·8 to −15·9)*	2273·6 (1967·4 to 2553·2)	2495·5 (2058·5 to 2957·0)	9·7% (−11·4 to 36·3)	40·7 (35·4 to 45·7)	28·0 (23·1 to 33·2)	−31·2% (−44·6 to −14·5)*
	Other pharynx cancer		54·2 (46·7 to 65·7)	113·9 (92·5 to 140·6)	110·2% (60·8 to 168·8)*	1·1 (0·9 to 1·3)	1·2 (1·0 to 1·5)	16·5% (−10·7 to 49·2)	1639·7 (1400·5 to 1993·3)	3273·8 (2635·2 to 4090·5)	99·6% (52·0 to 159·1)*	31·1 (26·6 to 37·9)	35·8 (28·8 to 44·7)	14·9% (−12·5 to 48·8)
	Oesophageal cancer		443·5 (380·3 to 481·6)	577·8 (505·7 to 643·2)	29·8% (15·0 to 51·9)*	9·0 (7·7 to 9·8)	6·3 (5·5 to 7·0)	−30·0% (−37·9 to −18·1)*	11 478·2 (9812·4 to 12 479·0)	13 899·3 (12 424·2 to 15 658·7)	20·8% (7·0 to 43·2)*	223·7 (191·7 to 243·5)	151·2 (135·0 to 170·7)	−32·6% (−40·3 to −20·4)*
	Stomach cancer		943·1 (808·3 to 1042·2)	935·9 (797·9 to 1083·5)	−0·9% (−12·2 to 15·3)	19·2 (16·5 to 21·3)	10·3 (8·8 to 11·9)	−46·6% (−52·7 to −38·1)*	24 561·0 (20 779·6 to 26 982·5)	22 182·4 (19 028·7 to 25 650·1)	−9·8% (−21·1 to 4·9)	474·3 (402·0 to 521·7)	243·2 (208·1 to 281·4)	−48·8% (−55·2 to −40·4)*
	Colon and rectum cancer		704·3 (655·8 to 742·9)	1107·1 (997·7 to 1214·9)	57·1% (46·5 to 68·9)*	14·9 (13·8 to 15·8)	12·3 (11·0 to 13·5)	−17·9% (−23·5 to −12·0)*	16 986·2 (15 891·8 to 17 911·6)	25 041·4 (22 808·4 to 27 266·0)	47·4% (35·7 to 59·8)*	333·9 (312·4 to 351·9)	275·5 (250·7 to 300·3)	−17·5% (−24·0 to −10·7)*
	Liver cancer		325·8 (297·8 to 357·9)	507·7 (442·4 to 570·0)	55·9% (34·6 to 79·0)*	6·4 (5·9 to 7·0)	5·6 (4·9 to 6·3)	−13·0% (−24·8 to −0·4)*	9798·0 (8817·8 to 10 941·2)	13 782·5 (11 945·8 to 15 939·9)	40·7% (18·6 to 65·0)*	182·9 (165·1 to 203·6)	153·3 (132·4 to 178·3)	−16·2% (−29·4 to −1·7)*
		Liver cancer due to hepatitis B	141·4 (123·5 to 162·0)	188·3 (161·3 to 220·6)	33·4% (13·6 to 55·5)*	2·7 (2·3 to 3·1)	2·1 (1·8 to 2·4)	−22·4% (−33·8 to −9·1)*	4851·2 (4219·9 to 5543·1)	5956·2 (5089·8 to 6886·3)	22·9% (3·2 to 44·1)*	87·8 (76·3 to 100·2)	66·3 (56·6 to 76·8)	−24·4% (−36·3 to −11·2)*
		Liver cancer due to hepatitis C	93·5 (82·0 to 107·7)	155·5 (131·1 to 186·4)	66·3% (45·9 to 87·6)*	2·0 (1·7 to 2·2)	1·7 (1·5 to 2·1)	−12·5% (−22·8 to −1·6)*	2179·6 (1920·9 to 2566·2)	3355·5 (2790·7 to 4121·1)	53·8% (34·1 to 76·2)*	43·5 (38·2 to 51·0)	36·6 (30·5 to 45·0)	−15·8% (−26·2 to −3·6)*
		Liver cancer due to alcohol use	51·1 (42·4 to 63·0)	95·1 (75·9 to 117·6)	86·1% (58·3 to 116·7)*	1·0 (0·8 to 1·3)	1·0 (0·8 to 1·3)	1·3% (−13·8 to 17·7)	1361·2 (1123·7 to 1695·5)	2413·0 (1899·3 to 3046·1)	77·3% (47·2 to 111·3)*	26·2 (21·7 to 32·6)	26·2 (20·5 to 33·1)	0·0% (−16·7 to 19·2)
		Liver cancer due to NASH	20·5 (16·3 to 25·4)	41·7 (31·6 to 52·6)	103·9% (69·0 to 138·6)*	0·4 (0·3 to 0·5)	0·5 (0·3 to 0·6)	10·8% (−7·6 to 29·5)	551·6 (439·7 to 686·3)	1045·7 (801·0 to 1337·1)	89·6% (53·9 to 125·3)*	10·5 (8·4 to 13·0)	11·5 (8·8 to 14·7)	9·6% (−10·9 to 30·4)
		Hepatoblastoma	3·9 (2·9 to 5·2)	3·5 (2·3 to 5·2)	−10·9% (−44·5 to 45·1)	0·1 (0·0 to 0·1)	0·1 (0·0 to 0·1)	−16·0% (−47·7 to 36·9)	343·5 (250·1 to 455·3)	306·0 (198·7 to 454·9)	−10·9% (−44·6 to 45·2)	5·6 (4·1 to 7·5)	4·7 (3·1 to 7·0)	−15·9% (−47·7 to 37·2)
		Liver cancer due to other causes	15·5 (13·0 to 18·5)	23·6 (18·4 to 29·7)	52·4% (27·4 to 76·4)*	0·3 (0·2 to 0·4)	0·3 (0·2 to 0·3)	−12·6% (−25·5 to 1·4)	510·9 (423·9 to 623·8)	706·0 (541·5 to 886·1)	38·2% (14·7 to 67·0)*	9·3 (7·7 to 11·3)	7·9 (6·1 to 9·9)	−14·7% (−29·6 to 2·6)
	Gallbladder and biliary tract cancer		118·6 (105·8 to 132·7)	184·1 (159·8 to 220·6)	55·2% (40·4 to 69·6)*	2·5 (2·3 to 2·8)	2·0 (1·8 to 2·4)	−19·5% (−27·0 to −12·0)*	2709·1 (2406·6 to 3056·5)	3948·6 (3411·9 to 4727·4)	45·7% (30·1 to 60·0)*	54·2 (48·3 to 60·8)	43·1 (37·3 to 51·6)	−20·5% (−28·9 to −12·5)*
	Pancreatic cancer		280·5 (262·5 to 294·3)	552·7 (501·8 to 588·7)	96·9% (85·9 to 108·2)*	5·9 (5·4 to 6·2)	6·1 (5·5 to 6·5)	3·1% (−2·3 to 8·8)	6706·1 (6336·4 to 7017·3)	12 167·8 (11 281·6 to 12 986·1)	81·3% (70·8 to 92·7)*	132·7 (125·0 to 139·0)	132·4 (122·7 to 141·4)	−0·4% (−5·9 to 5·7)
	Larynx cancer		87·9 (77·7 to 98·8)	130·8 (112·1 to 156·4)	48·7% (27·4 to 76·0)*	1·8 (1·6 to 2·0)	1·4 (1·2 to 1·7)	−19·5% (−31·0 to −4·7)*	2408·8 (2122·0 to 2724·1)	3434·1 (2894·3 to 4138·7)	42·5% (20·4 to 70·9)*	46·6 (41·0 to 52·6)	37·3 (31·4 to 45·0)	−19·9% (−32·3 to −4·0)*
	Tracheal, bronchus, and lung cancer		1322·0 (1243·4 to 1408·8)	2037·1 (1857·7 to 2212·9)	53·9% (41·9 to 65·1)*	27·0 (25·3 to 28·7)	22·2 (20·2 to 24·1)	−17·8% (−24·0 to −11·9)*	33 085·6 (30 779·3 to 35 405·5)	46 132·3 (41 948·2 to 50 238·8)	39·3% (28·5 to 49·9)*	647·1 (602·6 to 692·3)	499·5 (453·8 to 544·3)	−22·9% (−28·8 to −17·0)*
	Malignant skin melanoma		41·9 (38·7 to 45·5)	66·6 (59·9 to 75·2)	58·7% (47·4 to 70·1)*	0·9 (0·8 to 0·9)	0·7 (0·7 to 0·8)	−12·8% (−19·0 to −6·3)*	1212·0 (1107·5 to 1334·2)	1680·0 (1484·3 to 1954·4)	38·6% (26·3 to 50·2)*	22·7 (20·8 to 24·8)	18·8 (16·6 to 21·9)	−17·1% (−24·5 to −10·0)*
	Non-melanoma skin cancer		30·2 (27·2 to 33·7)	63·9 (54·4 to 71·6)	111·1% (83·2 to 138·5)*	0·7 (0·6 to 0·8)	0·7 (0·6 to 0·8)	6·5% (−7·2 to 20·1)	678·1 (604·1 to 763·1)	1239·4 (1044·5 to 1409·7)	82·5% (55·1 to 111·2)*	13·7 (12·2 to 15·3)	13·8 (11·6 to 15·7)	1·0% (−13·9 to 17·0)
		Non-melanoma skin cancer (squamous-cell carcinoma)	30·2 (27·2 to 33·7)	63·9 (54·4 to 71·6)	111·1% (83·2 to 138·5)*	0·7 (0·6 to 0·8)	0·7 (0·6 to 0·8)	6·5% (−7·2 to 20·1)	678·1 (604·1 to 763·1)	1239·4 (1044·5 to 1409·7)	82·5% (55·1 to 111·2)*	13·7 (12·2 to 15·3)	13·8 (11·6 to 15·7)	1·0% (−13·9 to 17·0)
	Soft tissue and other extraosseous sarcomas		37·9 (31·1 to 48·9)	60·9 (49·0 to 75·8)	60·8% (19·9 to 101·6)*	0·7 (0·6 to 0·9)	0·7 (0·6 to 0·9)	−1·8% (−26·4 to 22·9)	1535·8 (1215·1 to 2079·6)	2151·0 (1655·4 to 2783·7)	40·1% (−2·5 to 84·4)	26·4 (21·1 to 35·5)	25·9 (19·7 to 33·9)	−1·9% (−31·5 to 29·1)
	Malignant neoplasm of bone and articular cartilage		49·0 (40·5 to 61·5)	76·7 (60·8 to 96·4)	56·3% (16·7 to 98·9)*	0·9 (0·7 to 1·1)	0·9 (0·7 to 1·1)	−0·8% (−25·2 to 25·0)	2161·9 (1708·1 to 2857·2)	2942·0 (2216·9 to 3849·4)	36·0% (−4·8 to 81·7)	35·8 (28·6 to 46·8)	35·3 (26·2 to 46·5)	−1·6% (−31·3 to 31·8)
	Breast cancer		446·4 (408·7 to 481·8)	780·2 (685·7 to 871·0)	74·7% (55·2 to 95·7)*	9·0 (8·2 to 9·7)	8·7 (7·6 to 9·7)	−3·4% (−13·9 to 8·3)	13 394·8 (12 216·4 to 14 561·3)	23 024·3 (19 985·8 to 25 997·2)	71·9% (49·2 to 97·7)*	250·8 (229·4 to 271·8)	258·0 (223·9 to 291·7)	2·8% (−10·7 to 18·5)
	Cervical cancer		235·7 (190·6 to 300·7)	369·5 (291·7 to 474·5)	56·7% (20·6 to 105·5)*	4·5 (3·6 to 5·7)	4·1 (3·3 to 5·3)	−7·7% (−28·8 to 20·6)	8243·4 (6589·2 to 10 746·9)	12 876·8 (9852·7 to 16 768·3)	56·2% (16·4 to 107·0)*	147·9 (118·6 to 191·9)	146·1 (111·5 to 190·7)	−1·2% (−26·4 to 31·2)
	Uterine cancer		67·2 (57·6 to 74·9)	108·6 (93·2 to 126·2)	61·6% (37·3 to 85·8)*	1·4 (1·2 to 1·5)	1·2 (1·0 to 1·4)	−14·0% (−26·6 to −1·3)*	1741·3 (1464·8 to 1966·4)	2639·7 (2227·3 to 3105·3)	51·5% (26·0 to 79·4)*	33·7 (28·5 to 38·0)	28·8 (24·3 to 33·9)	−14·6% (−28·9 to 0·5)
	Ovarian cancer		129·9 (117·1 to 142·7)	221·0 (191·6 to 255·0)	70·1% (48·1 to 96·3)*	2·6 (2·4 to 2·9)	2·4 (2·1 to 2·8)	−7·1% (−19·3 to 6·9)	3699·3 (3315·2 to 4116·5)	6304·4 (5313·3 to 7450·7)	70·4% (43·9 to 102·3)*	70·0 (62·9 to 77·6)	70·1 (59·0 to 83·1)	0·0% (−15·9 to 18·7)
	Prostate cancer		274·4 (243·7 to 301·9)	473·0 (415·9 to 530·2)	72·4% (55·5 to 94·8)*	6·2 (5·5 to 6·9)	5·3 (4·6 to 5·9)	−15·4% (−23·3 to −4·7)*	4863·4 (4357·1 to 5362·3)	8010·1 (6965·5 to 9037·7)	64·7% (46·0 to 88·0)*	104·1 (93·0 to 114·6)	87·7 (76·4 to 99·0)	−15·7% (−25·0 to −3·7)*
	Testicular cancer		8·8 (7·2 to 10·9)	11·9 (9·6 to 14·7)	35·5% (2·0 to 76·8)*	0·2 (0·1 to 0·2)	0·1 (0·1 to 0·2)	−6·6% (−29·4 to 21·6)	422·9 (344·0 to 536·9)	540·0 (429·0 to 678·7)	27·7% (−6·6 to 70·5)	6·8 (5·6 to 8·6)	6·5 (5·2 to 8·2)	−4·4% (−29·7 to 27·6)
	Kidney cancer		101·1 (93·2 to 108·7)	165·4 (146·7 to 180·0)	63·5% (52·2 to 75·7)*	2·1 (1·9 to 2·2)	1·8 (1·6 to 2·0)	−12·0% (−17·9 to −5·6)*	2704·6 (2467·2 to 2955·4)	3899·7 (3438·2 to 4322·7)	44·1% (31·5 to 57·5)*	52·1 (47·6 to 56·6)	43·7 (38·4 to 48·5)	−16·1% (−23·4 to −8·3)*
	Bladder cancer		146·2 (135·1 to 156·8)	233·7 (208·5 to 257·6)	59·9% (48·2 to 75·5)*	3·2 (3·0 to 3·5)	2·6 (2·3 to 2·9)	−18·8% (−24·6 to −11·0)*	3017·5 (2768·9 to 3254·1)	4330·4 (3953·5 to 4822·8)	43·5% (30·9 to 60·7)*	61·9 (57·0 to 66·6)	47·6 (43·3 to 53·0)	−23·2% (−29·8 to −14·1)*
	Brain and central nervous system cancer		175·2 (150·9 to 200·1)	264·2 (230·5 to 313·2)	50·6% (37·4 to 63·0)*	3·3 (2·8 to 3·7)	3·0 (2·6 to 3·5)	−8·3% (−16·2 to −0·6)*	7154·4 (6034·0 to 8243·3)	9028·2 (7915·8 to 10 869·8)	26·1% (12·7 to 39·1)*	123·9 (105·1 to 142·8)	106·5 (93·2 to 128·1)	−14·1% (−23·4 to −5·1)*
	Eye cancer		9·5 (6·5 to 14·1)	10·1 (7·3 to 14·2)	6·2% (−33·5 to 68·4)	0·2 (0·1 to 0·3)	0·1 (0·1 to 0·2)	−27·7% (−53·6 to 12·4)	520·5 (299·7 to 898·3)	476·9 (279·7 to 796·8)	−8·4% (−51·7 to 77·4)	8·9 (5·2 to 15·1)	6·5 (3·6 to 11·5)	−26·4% (−62·8 to 41·6)
		Retinoblastoma	4·0 (1·8 to 8·4)	3·2 (1·3 to 7·0)	−18·9% (−71·3 to 130·7)	0·1 (0·0 to 0·1)	0·0 (0·0 to 0·1)	−24·8% (−73·3 to 114·0)	344·5 (156·1 to 726·0)	279·9 (110·1 to 608·4)	−18·7% (−71·2 to 131·0)	5·7 (2·6 to 12·0)	4·3 (1·7 to 9·3)	−24·6% (−73·3 to 114·5)
		Other eye cancers	5·5 (4·3 to 7·1)	6·9 (5·4 to 8·8)	24·2% (1·1 to 52·6)*	0·1 (0·1 to 0·1)	0·1 (0·1 to 0·1)	−29·5% (−42·7 to −13·7)*	175·9 (132·9 to 235·4)	197·0 (146·6 to 271·5)	12·0% (−15·5 to 42·5)	3·2 (2·4 to 4·2)	2·2 (1·7 to 3·1)	−29·6% (−47·0 to −10·3)*
	Neuroblastoma and other peripheral nervous cell tumours		4·2 (3·5 to 5·0)	6·0 (5·0 to 7·6)	42·9% (15·7 to 79·2)*	0·1 (0·1 to 0·1)	0·1 (0·1 to 0·1)	4·3% (−16·2 to 31·0)	263·2 (214·8 to 316·7)	324·3 (261·2 to 429·6)	23·2% (−4·9 to 59·2)	4·3 (3·6 to 5·2)	4·4 (3·5 to 5·9)	1·6% (−22·6 to 31·7)
	Thyroid cancer		28·8 (25·7 to 33·5)	52·2 (44·7 to 61·5)	81·2% (51·9 to 123·3)*	0·6 (0·5 to 0·7)	0·6 (0·5 to 0·7)	−0·7% (−16·7 to 21·7)	814·1 (710·2 to 975·7)	1415·2 (1192·5 to 1715·1)	73·8% (41·4 to 120·8)*	15·2 (13·3 to 18·1)	16·0 (13·4 to 19·4)	4·8% (−14·5 to 33·5)
	Mesothelioma		17·2 (15·6 to 19·2)	28·0 (24·8 to 30·9)	62·2% (43·5 to 82·1)*	0·4 (0·3 to 0·4)	0·3 (0·3 to 0·3)	−13·2% (−23·0 to −2·7)*	427·0 (385·1 to 476·7)	615·3 (544·3 to 685·3)	44·0% (26·5 to 63·2)*	8·3 (7·5 to 9·3)	6·8 (6·0 to 7·5)	−18·9% (−28·5 to −8·1)*
	Hodgkin lymphoma		28·8 (22·4 to 35·4)	27·2 (21·0 to 34·9)	−5·7% (−27·9 to 15·4)	0·5 (0·4 to 0·6)	0·3 (0·2 to 0·4)	−38·0% (−52·6 to −24·3)*	1310·0 (1006·9 to 1659·6)	1150·9 (837·0 to 1516·3)	−12·2% (−35·8 to 12·2)	21·7 (16·7 to 27·4)	14·0 (10·1 to 18·5)	−35·4% (−52·8 to −17·5)*
	Non-Hodgkin lymphoma		188·5 (173·9 to 209·5)	283·1 (247·5 to 320·5)	50·1% (26·9 to 71·3)*	3·8 (3·5 to 4·2)	3·2 (2·8 to 3·6)	−15·2% (−28·1 to −3·1)*	6038·6 (5446·5 to 6866·0)	8039·7 (6845·8 to 9375·2)	33·1% (8·9 to 58·4)*	109·6 (99·4 to 124·2)	93·5 (79·1 to 109·6)	−14·7% (−30·2 to 2·0)
		Burkitt lymphoma	5·0 (3·6 to 6·8)	6·7 (5·1 to 9·6)	34·0% (−8·9 to 94·4)	0·1 (0·1 to 0·1)	0·1 (0·1 to 0·1)	−3·2% (−34·5 to 40·3)	305·6 (212·8 to 434·6)	365·9 (257·0 to 567·1)	19·7% (−25·9 to 88·3)	4·9 (3·4 to 7·0)	4·8 (3·3 to 7·5)	−3·1% (−40·4 to 52·2)
		Other non-Hodgkin lymphoma	183·5 (169·0 to 203·9)	276·4 (241·6 to 312·8)	50·6% (27·6 to 72·0)*	3·7 (3·4 to 4·1)	3·1 (2·7 to 3·5)	−15·5% (−27·8 to −3·2)*	5733·1 (5235·3 to 6499·7)	7673·8 (6582·3 to 8895·1)	33·8% (10·3 to 60·3)*	104·6 (95·7 to 118·1)	88·7 (75·8 to 103·6)	−15·3% (−30·0 to 2·2)
	Multiple myeloma		68·2 (62·3 to 73·8)	125·1 (112·8 to 137·0)	83·3% (65·4 to 100·6)*	1·4 (1·3 to 1·6)	1·4 (1·2 to 1·5)	−4·9% (−14·1 to 3·8)	1551·0 (1408·1 to 1694·9)	2701·7 (2426·4 to 2995·3)	74·2% (53·6 to 95·2)*	31·0 (28·2 to 33·8)	29·5 (26·5 to 32·7)	−4·9% (−16·1 to 6·5)
	Leukaemia		291·9 (260·2 to 320·5)	342·0 (307·2 to 381·8)	17·1% (3·7 to 31·0)*	5·5 (4·9 to 6·0)	4·0 (3·6 to 4·4)	−27·5% (−35·5 to −19·0)*	12 723·9 (11 104·5 to 14 146·2)	11 905·5 (10 517·9 to 13 661·0)	−6·5% (−20·4 to 7·0)	215·3 (188·8 to 238·2)	145·4 (128·0 to 168·1)	−32·5% (−42·5 to −22·8)*
		Acute lymphoid leukaemia	87·3 (64·5 to 111·6)	77·5 (54·2 to 98·7)	−11·3% (−27·4 to 8·6)	1·5 (1·1 to 1·9)	1·0 (0·7 to 1·2)	−33·4% (−45·1 to −17·7)*	5511·5 (4114·6 to 6944·4)	4318·8 (3077·5 to 5438·2)	−21·7% (−37·2 to −3·0)*	88·0 (65·9 to 111·0)	56·7 (40·6 to 71·6)	−35·6% (−48·3 to −19·7)*
		Chronic lymphoid leukaemia	37·5 (33·5 to 41·6)	44·8 (39·6 to 51·6)	19·5% (6·3 to 33·5)*	0·8 (0·7 to 0·9)	0·5 (0·4 to 0·6)	−38·7% (−45·2 to −31·8)*	855·9 (734·5 to 969·5)	885·1 (779·6 to 1049·9)	3·3% (−10·8 to 22·1)	17·0 (14·8 to 19·1)	9·8 (8·6 to 11·7)	−42·3% (−50·1 to −32·5)*
		Acute myeloid leukaemia	91·9 (75·5 to 108·0)	131·6 (112·6 to 152·5)	43·2% (25·3 to 62·8)*	1·7 (1·4 to 2·0)	1·5 (1·3 to 1·7)	−13·3% (−23·6 to −2·1)*	3731·7 (2862·0 to 4654·8)	4161·7 (3387·0 to 5135·5)	11·5% (−7·1 to 33·5)	63·9 (50·0 to 78·5)	49·6 (39·9 to 62·1)	−22·5% (−35·9 to −7·8)*
		Chronic myeloid leukaemia	34·3 (28·1 to 40·9)	26·3 (21·2 to 32·7)	−23·2% (−39·3 to 1·6)	0·7 (0·6 to 0·8)	0·3 (0·2 to 0·4)	−55·5% (−64·7 to −41·9)*	1187·5 (919·7 to 1486·7)	779·0 (585·9 to 1037·8)	−34·4% (−51·5 to −9·6)*	21·0 (16·6 to 26·0)	9·1 (6·8 to 12·2)	−56·9% (−68·2 to −41·3)*
		Other leukaemia	41·1 (33·0 to 53·7)	61·7 (49·3 to 77·0)	50·1% (20·7 to 93·1)*	0·8 (0·6 to 1·0)	0·7 (0·6 to 0·9)	−12·7% (−28·7 to 10·8)	1437·2 (1103·7 to 1975·1)	1760·9 (1338·4 to 2323·2)	22·4% (−5·7 to 61·8)	25·4 (19·8 to 34·5)	20·3 (15·3 to 26·9)	−20·3% (−38·2 to 4·3)
	Other malignant neoplasms		157·2 (142·0 to 174·1)	226·0 (196·9 to 251·0)	43·6% (23·7 to 66·1)*	3·1 (2·8 to 3·4)	2·6 (2·2 to 2·8)	−17·5% (−28·9 to −5·2)*	5479·8 (4821·3 to 6210·0)	6520·7 (5597·1 to 7450·5)	18·9% (−1·3 to 40·6)	98·1 (86·8 to 110·8)	76·6 (65·7 to 87·9)	−22·0% (−35·3 to −7·4)*
	Other neoplasms		69·9 (61·2 to 79·1)	124·5 (106·4 to 145·3)	78·0% (59·2 to 96·0)*	1·5 (1·3 to 1·7)	1·4 (1·2 to 1·7)	−4·2% (−14·2 to 5·3)	2003·8 (1653·3 to 2380·4)	2957·9 (2467·6 to 3615·1)	47·5% (25·1 to 68·6)*	37·4 (31·3 to 43·8)	34·3 (28·4 to 42·1)	−8·4% (−21·7 to 5·3)
		Myelodysplastic, myeloproliferative, and other haemopoietic neoplasms	26·1 (23·0 to 29·0)	62·0 (51·7 to 71·9)	137·2% (111·0 to 163·5)*	0·6 (0·5 to 0·7)	0·7 (0·6 to 0·8)	16·1% (3·1 to 29·2)*	503·0 (433·2 to 564·6)	1058·9 (900·6 to 1244·8)	110·4% (86·3 to 135·2)*	10·5 (9·2 to 11·7)	11·9 (10·1 to 14·0)	13·4% (0·5 to 26·5)*
		Other benign and in-situ neoplasms	43·8 (35·8 to 52·8)	62·5 (49·1 to 79·0)	42·6% (18·6 to 70·1)*	0·9 (0·7 to 1·0)	0·7 (0·6 to 0·9)	−18·4% (−31·8 to −3·7)*	1500·8 (1140·1 to 1879·8)	1899·1 (1431·7 to 2501·8)	26·5% (0·3 to 55·0)*	26·9 (20·8 to 33·4)	22·3 (16·7 to 29·6)	−16·9% (−34·2 to 2·2)
	**Cardiovascular diseases**		**14 562·6 (13 675·2 to 15 351·9)**	**19 159·2 (17 364·3 to 20 420·7)**	31·4% (21·3 to 41·7)*	**322·0 (298·9 to 340·4)**	**215·2 (194·3 to 229·8)**	–33·2% (−37·9 to −28·3)*	**324 658·5 (305 331·0 to 346 523·1)**	**395 762·2 (364 152·2 to 424 474·4)**	21·8% (11·3 to 33·6)*	**6527·3 (6126·6 to 6937·0)**	**4411·0 (4053·4 to 4736·6)**	–32·5% (−38·1 to −26·3)*
	Rheumatic heart disease		437·4 (347·4 to 541·2)	388·9 (261·0 to 554·4)	−11·1% (−37·1 to 22·8)	8·5 (6·9 to 10·5)	4·4 (3·0 to 6·3)	−48·4% (−63·9 to −28·9)*	15 177·2 (11 748·8 to 19 150·7)	11 820·6 (7609·9 to 17 181·5)	−22·1% (−46·6 to 10·2)	269·8 (209·7 to 338·4)	136·6 (88·5 to 199·2)	−49·4% (−65·0 to −28·8)*
	Ischaemic heart disease		6286·6 (5802·6 to 6713·4)	8905·9 (8043·9 to 9659·6)	41·5% (30·2 to 53·6)*	140·1 (129·0 to 149·5)	99·8 (89·9 to 108·4)	−28·9% (−34·1 to −22·9)*	136 804·5 (126 478·8 to 147 444·0)	182 550·6 (167 559·7 to 199 538·9)	33·3% (20·3 to 46·9)*	2778·4 (2582·7 to 2973·5)	2020·0 (1852·7 to 2212·8)	−27·4% (−34·2 to −19·9)*
	Stroke		6059·2 (5579·8 to 6549·6)	6793·2 (6064·9 to 7467·6)	12·0% (0·1 to 24·2)*	133·0 (122·9 to 143·6)	75·9 (67·8 to 83·5)	−43·0% (−49·0 to −36·9)*	131 886·2 (120 831·0 to 143 533·8)	139 860·2 (124 616·6 to 154 296·3)	6·0% (−6·9 to 20·1)	2663·6 (2441·1 to 2894·2)	1552·5 (1381·1 to 1713·2)	−41·8% (−48·9 to −34·1)*
		Ischaemic stroke	2853·9 (2602·5 to 3179·8)	3279·0 (2869·9 to 3689·1)	14·7% (1·0 to 29·6)*	66·4 (60·5 to 73·8)	37·0 (32·4 to 41·7)	−44·4% (−50·8 to −37·4)*	50 456·2 (45 793·6 to 56 641·0)	54 733·9 (48 138·2 to 61 730·7)	8·3% (−6·5 to 24·7)	1084·8 (982·9 to 1212·7)	606·9 (532·2 to 685·6)	−44·2% (−51·6 to −35·8)*
		Intracerebral haemorrhage	2857·0 (2539·1 to 3198·6)	3156·7 (2752·8 to 3546·9)	10·4% (−4·4 to 30·3)	59·4 (52·7 to 66·2)	34·9 (30·4 to 39·3)	−41·3% (−49·1 to −31·1)*	71 650·8 (62 936·0 to 81 253·0)	75 460·6 (65 335·9 to 85 395·0)	5·3% (−11·6 to 26·3)	1394·1 (1228·3 to 1566·3)	836·2 (723·7 to 946·8)	−40·0% (−49·4 to −28·3)*
		Subarachnoid haemorrhage	348·4 (245·3 to 428·2)	357·5 (303·7 to 430·2)	2·5% (−18·1 to 43·5)	7·1 (5·0 to 8·8)	4·0 (3·4 to 4·8)	−44·1% (−55·3 to −21·3)*	9779·2 (7247·7 to 12 102·1)	9665·7 (8021·1 to 12 175·6)	−1·2% (−22·0 to 31·6)	184·6 (135·3 to 228·2)	109·4 (90·3 to 138·2)	−40·7% (−53·1 to −20·5)*
	Hypertensive heart disease		797·5 (643·5 to 953·1)	1485·0 (1179·4 to 1825·7)	86·1% (50·7 to 126·4)*	18·1 (14·5 to 21·6)	16·8 (13·4 to 20·7)	−7·2% (−24·6 to 12·6)	16 085·1 (12 843·2 to 19 689·6)	27 326·2 (21 762·9 to 34 181·3)	69·8% (34·7 to 110·8)*	333·9 (267·7 to 403·3)	303·6 (241·5 to 380·2)	−9·1% (−27·5 to 12·1)
	Non-rheumatic valvular heart disease		97·5 (85·8 to 107·2)	191·3 (157·0 to 214·6)	96·1% (76·6 to 116·1)*	2·4 (2·1 to 2·6)	2·2 (1·8 to 2·5)	−7·7% (−16·0 to 1·0)	1741·9 (1536·7 to 1934·4)	3035·5 (2581·4 to 3528·7)	74·3% (50·5 to 98·0)*	37·8 (33·0 to 42·0)	34·6 (29·4 to 40·3)	−8·3% (−19·7 to 4·1)
		Non-rheumatic calcific aortic valve disease	71·3 (61·2 to 78·4)	149·4 (120·1 to 166·0)	109·6% (89·9 to 129·6)*	1·8 (1·5 to 2·0)	1·7 (1·4 to 1·9)	−3·6% (−12·1 to 5·2)	1158·7 (1020·8 to 1275·0)	2169·7 (1837·7 to 2474·0)	87·3% (65·7 to 112·0)*	26·1 (22·9 to 28·7)	24·8 (20·9 to 28·3)	−5·0% (−15·6 to 7·2)
		Non-rheumatic degenerative mitral valve disease	25·3 (21·7 to 29·2)	39·7 (32·4 to 50·7)	57·0% (26·9 to 92·7)*	0·6 (0·5 to 0·7)	0·5 (0·4 to 0·6)	−21·5% (−35·9 to −3·9)*	558·2 (471·0 to 683·5)	815·6 (640·0 to 1100·6)	46·2% (9·3 to 88·8)*	11·2 (9·5 to 13·5)	9·3 (7·3 to 12·5)	−17·2% (−37·2 to 7·6)
		Other non-rheumatic valve diseases	0·9 (0·6 to 1·6)	2·1 (1·3 to 3·3)	133·0% (63·5 to 251·5)*	0·0 (0·0 to 0·0)	0·0 (0·0 to 0·0)	24·3% (−11·1 to 89·3)	25·0 (16·4 to 41·2)	50·2 (31·9 to 81·1)	100·9% (31·2 to 213·3)*	0·5 (0·3 to 0·8)	0·6 (0·4 to 0·9)	22·4% (−19·0 to 92·5)
	Cardiomyopathy and myocarditis		335·7 (295·6 to 388·6)	399·9 (338·4 to 465·1)	19·1% (−2·0 to 44·7)	7·1 (6·3 to 8·2)	4·6 (3·9 to 5·3)	−35·9% (−47·4 to −21·8)*	10 322·7 (8777·2 to 12 535·3)	11 541·4 (9460·2 to 13 920·1)	11·8% (−12·3 to 40·5)	191·5 (164·2 to 229·9)	136·0 (109·7 to 166·5)	−29·0% (−44·8 to −11·1)*
		Myocarditis	21·6 (14·5 to 33·6)	16·9 (11·3 to 24·1)	−21·9% (−46·1 to 20·7)	0·4 (0·3 to 0·6)	0·2 (0·1 to 0·3)	−50·9% (−65·6 to −25·6)*	1015·0 (656·8 to 1588·1)	624·3 (414·2 to 940·0)	−38·5% (−62·2 to −4·0)*	17·2 (11·1 to 26·8)	8·1 (5·3 to 12·2)	−53·0% (−71·2 to −27·5)*
		Alcoholic cardiomyopathy	76·5 (68·6 to 86·0)	62·3 (56·0 to 71·6)	−18·6% (−28·8 to −7·4)*	1·4 (1·3 to 1·6)	0·7 (0·6 to 0·8)	−52·3% (−58·3 to −45·7)*	2727·3 (2448·9 to 3040·3)	2108·8 (1889·5 to 2420·0)	−22·7% (−32·4 to −11·9)*	49·0 (44·0 to 54·7)	23·6 (21·2 to 27·2)	−51·8% (−57·8 to −45·1)*
		Other cardiomyopathy	237·6 (203·3 to 287·4)	320·7 (260·3 to 380·7)	35·0% (6·2 to 69·4)*	5·3 (4·5 to 6·3)	3·7 (3·0 to 4·4)	−30·2% (−44·5 to −13·6)*	6580·4 (5359·5 to 8558·1)	8808·3 (6840·4 to 10 925·2)	33·9% (−1·0 to 77·5)	125·3 (103·8 to 160·1)	104·3 (80·2 to 130·9)	−16·8% (−38·9 to 9·4)
	Pulmonary arterial hypertension		20·0 (15·0 to 27·0)	22·8 (17·5 to 29·8)	13·8% (−17·0 to 60·6)	0·4 (0·3 to 0·5)	0·3 (0·2 to 0·4)	−33·4% (−50·6 to −5·8)*	778·6 (532·5 to 1147·1)	682·9 (494·1 to 978·5)	−12·4% (−43·6 to 31·2)	13·7 (9·5 to 19·7)	8·5 (6·0 to 12·5)	−37·6% (−59·6 to −7·0)*
	Atrial fibrillation and flutter		160·4 (144·5 to 174·3)	377·7 (319·0 to 424·2)	135·2% (112·7 to 157·0)*	4·2 (3·8 to 4·6)	4·4 (3·7 to 5·0)	3·9% (−5·7 to 13·2)	2213·1 (2033·2 to 2395·7)	4863·5 (4236·4 to 5380·4)	119·6% (98·6 to 138·7)*	53·7 (49·0 to 58·3)	55·5 (48·2 to 61·5)	3·3% (−6·0 to 12·3)
	Aortic aneurysm		109·6 (101·9 to 119·8)	167·4 (147·1 to 187·3)	52·8% (37·7 to 68·0)*	2·4 (2·2 to 2·6)	1·9 (1·6 to 2·1)	−22·5% (−29·6 to −15·3)*	2303·7 (2122·4 to 2557·6)	3416·5 (3025·9 to 3838·3)	48·4% (30·4 to 64·9)*	46·8 (43·0 to 51·7)	37·8 (33·4 to 42·5)	−19·2% (−28·8 to −10·4)*
	Lower extremity peripheral arterial disease		52·9 (47·8 to 57·6)	74·9 (66·1 to 83·1)	41·5% (27·5 to 55·5)*	1·3 (1·2 to 1·4)	0·9 (0·7 to 0·9)	−34·4% (−40·7 to −27·9)*	871·1 (800·3 to 943·3)	1189·8 (1063·1 to 1332·8)	36·6% (21·1 to 53·5)*	19·5 (17·7 to 21·2)	13·2 (11·8 to 14·8)	−32·4% (−39·7 to −24·4)*
	Endocarditis		52·4 (44·2 to 62·6)	86·2 (74·2 to 100·8)	64·7% (33·7 to 100·9)*	1·1 (0·9 to 1·3)	1·0 (0·9 to 1·2)	−9·7% (−25·4 to 8·3)	1688·4 (1314·4 to 2173·5)	2302·6 (1918·6 to 2840·0)	36·4% (−1·1 to 83·6)	30·6 (24·4 to 38·7)	27·2 (22·5 to 33·8)	−11·0% (−34·6 to 18·2)
	Other cardiovascular and circulatory diseases		153·3 (129·8 to 185·3)	265·9 (217·1 to 317·9)	73·4% (37·2 to 116·3)*	3·2 (2·8 to 3·8)	3·0 (2·5 to 3·6)	−6·2% (−24·8 to 15·6)	4786·1 (3817·4 to 6161·9)	7172·3 (5626·5 to 8764·7)	49·9% (8·5 to 97·8)*	88·0 (71·0 to 111·7)	85·3 (66·0 to 105·5)	−3·1% (−29·5 to 27·4)
	**Chronic respiratory diseases**		**2885·3 (2366·5 to 3234·0)**	**4163·7 (3612·7 to 5138·2)**	44·0% (20·3 to 85·9)*	**63·4 (52·2 to 70·5)**	**46·8 (40·6 to 57·7)**	–26·4% (−38·1 to −5·5)*	**61 339·5 (50 077·3 to 68 952·7)**	**78 935·2 (69 624·7 to 96 907·6)**	28·5% (7·7 to 65·7)*	**1243·6 (1021·6 to 1394·8)**	**882·8 (774·9 to 1083·8)**	–29·1% (−40·9 to −9·1)*
	Chronic obstructive pulmonary disease		2404·0 (1975·9 to 2700·6)	3426·1 (2955·2 to 4079·8)	42·1% (19·5 to 84·7)*	53·5 (44·1 to 60·1)	38·4 (33·2 to 45·8)	−28·4% (−39·6 to −7·2)*	46 704·9 (38 738·6 to 52 933·2)	59 918·6 (51 344·7 to 71 728·7)	28·1% (7·8 to 67·1)*	972·5 (808·1 to 1097·6)	659·7 (563·7 to 788·5)	−32·3% (−42·9 to −12·1)*
	Pneumoconiosis		15·8 (12·3 to 22·4)	18·7 (14·1 to 24·9)	18·1% (−18·8 to 57·5)	0·3 (0·3 to 0·5)	0·2 (0·2 to 0·3)	−37·6% (−56·2 to −18·0)*	362·9 (268·6 to 542·3)	391·8 (288·8 to 521·1)	7·6% (−29·1 to 48·2)	7·2 (5·4 to 10·6)	4·3 (3·2 to 5·7)	−39·9% (−59·6 to −18·2)*
		Silicosis	8·7 (6·4 to 13·1)	11·0 (7·7 to 15·2)	26·1% (−21·0 to 81·5)	0·2 (0·1 to 0·3)	0·1 (0·1 to 0·2)	−32·8% (−57·1 to −5·5)*	205·9 (142·7 to 328·1)	241·5 (166·3 to 336·6)	16·8% (−29·7 to 81·1)	4·0 (2·8 to 6·3)	2·7 (1·8 to 3·7)	−34·3% (−60·0 to −0·4)*
		Asbestosis	1·8 (1·5 to 2·4)	3·0 (2·4 to 4·0)	63·1% (22·1 to 110·6)*	0·0 (0·0 to 0·1)	0·0 (0·0 to 0·0)	−16·2% (−36·8 to 7·4)	37·1 (28·9 to 50·5)	51·0 (40·0 to 71·9)	37·4% (0·3 to 88·5)*	0·8 (0·6 to 1·0)	0·6 (0·4 to 0·8)	−25·6% (−45·3 to 1·5)
		Coal worker pneumoconiosis	3·3 (2·5 to 5·1)	2·3 (1·7 to 3·1)	−30·5% (−57·8 to 3·8)	0·1 (0·1 to 0·1)	0·0 (0·0 to 0·0)	−63·9% (−77·5 to −47·1)*	71·1 (49·1 to 117·8)	45·6 (31·5 to 61·9)	−36·0% (−64·5 to 4·1)	1·4 (1·0 to 2·3)	0·5 (0·3 to 0·7)	−65·0% (−80·1 to −44·0)*
		Other pneumoconiosis	1·9 (1·4 to 2·9)	2·4 (1·6 to 3·6)	22·8% (−27·1 to 87·9)	0·0 (0·0 to 0·1)	0·0 (0·0 to 0·0)	−34·3% (−60·1 to 0·5)	48·9 (33·8 to 77·7)	53·8 (36·1 to 83·0)	10·0% (−35·9 to 72·1)	0·9 (0·7 to 1·5)	0·6 (0·4 to 0·9)	−36·8% (−63·0 to −1·6)*
	Asthma		342·7 (221·6 to 503·5)	441·9 (305·8 to 665·1)	29·0% (−16·8 to 97·0)	7·0 (4·6 to 10·4)	5·0 (3·5 to 7·5)	−29·1% (−53·8 to 7·6)	10 076·9 (6600·9 to 14 431·5)	11 758·2 (8323·7 to 16 923·2)	16·7% (−23·3 to 76·3)	187·6 (122·6 to 271·2)	135·7 (96·1 to 194·8)	−27·7% (−51·9 to 9·8)
	Interstitial lung disease and pulmonary sarcoidosis		76·3 (62·5 to 111·0)	213·2 (176·1 to 261·2)	179·6% (96·2 to 241·6)*	1·7 (1·4 to 2·4)	2·4 (2·0 to 2·9)	43·2% (2·3 to 73·7)*	1663·0 (1328·0 to 2584·8)	3997·3 (3268·2 to 5136·7)	140·4% (59·0 to 205·6)*	33·5 (27·0 to 51·1)	44·4 (36·3 to 57·1)	32·7% (−11·1 to 67·6)
	Other chronic respiratory diseases		46·5 (34·7 to 66·7)	63·6 (46·6 to 90·1)	36·8% (−15·1 to 94·6)	0·9 (0·7 to 1·2)	0·8 (0·6 to 1·1)	−9·4% (−41·9 to 28·4)	2531·7 (1808·6 to 3549·2)	2869·4 (2005·3 to 4285·7)	13·3% (−31·5 to 62·9)	42·9 (30·8 to 60·5)	38·7 (26·5 to 57·4)	−9·9% (−44·7 to 28·6)
	**Digestive diseases**		**2052·3 (1849·8 to 2264·3)**	**2416·3 (2151·6 to 2671·8)**	17·7% (0·6 to 38·0)*	**40·8 (36·9 to 44·9)**	**27·3 (24·2 to 30·2)**	–33·1% (−42·9 to −22·0)*	**68 187·2 (60 449·9 to 75 780·1)**	**69 999·8 (61 560·7 to 78 001·8)**	**2·7% (−13·8 to 21·9)**	**1235·1 (1100·2 to 1367·0)**	**805·7 (705·4 to 899·9)**	–34·8% (−45·2 to −22·4)*
	Cirrhosis and other chronic liver diseases		1127·0 (1003·3 to 1275·7)	1282·0 (1141·9 to 1430·1)	13·8% (−3·9 to 35·5)	21·4 (19·1 to 24·2)	14·3 (12·7 to 16·0)	−33·4% (−43·4 to −20·8)*	39 866·9 (35 267·0 to 45 423·6)	41 637·4 (36 506·1 to 47 053·8)	4·4% (−12·9 to 26·8)	713·2 (631·8 to 812·3)	473·2 (413·0 to 535·6)	−33·7% (−44·6 to −19·7)*
		Chronic hepatitis B including cirrhosis	391·2 (332·4 to 464·5)	394·2 (324·6 to 464·5)	0·8% (−16·9 to 24·3)	7·4 (6·3 to 8·9)	4·4 (3·6 to 5·2)	−41·1% (−51·8 to −27·6)*	13 496·9 (11 469·0 to 15 799·5)	12 747·5 (10 558·1 to 15 071·6)	−5·6% (−24·1 to 18·3)	242·7 (207·0 to 285·7)	143·8 (118·7 to 170·7)	−40·7% (−52·3 to −26·2)*
		Chronic hepatitis C including cirrhosis	283·6 (236·1 to 343·1)	334·5 (276·9 to 403·7)	18·0% (−1·9 to 40·1)	5·4 (4·5 to 6·5)	3·7 (3·1 to 4·5)	−31·6% (−43·1 to −18·8)*	9665·1 (7973·5 to 11 667·2)	10 741·2 (8791·1 to 12 988·0)	11·1% (−8·0 to 35·1)	174·6 (144·6 to 210·6)	121·1 (98·5 to 146·6)	−30·6% (−42·5 to −15·7)*
		Cirrhosis due to alcohol use	251·9 (215·4 to 291·7)	308·8 (259·4 to 358·2)	22·6% (6·3 to 41·5)*	4·8 (4·1 to 5·6)	3·4 (2·8 to 3·9)	−29·9% (−39·5 to −19·3)*	8268·1 (7071·2 to 9724·4)	9473·6 (7963·9 to 11 068·1)	14·6% (−1·8 to 33·0)	152·2 (130·0 to 178·2)	104·9 (87·8 to 122·8)	−31·1% (−41·2 to −20·0)*
		Non-alcoholic fatty liver disease including cirrhosis	52·3 (37·2 to 69·9)	89·8 (64·7 to 120·3)	71·8% (56·2 to 89·9)*	1·1 (0·7 to 1·4)	1·0 (0·7 to 1·3)	−5·7% (−13·6 to 4·5)	1524·5 (1048·0 to 2082·8)	2459·8 (1710·6 to 3299·7)	61·4% (47·5 to 78·1)*	28·7 (19·8 to 38·9)	27·2 (19·0 to 36·3)	−5·2% (−13·8 to 5·3)
		Cirrhosis due to other causes	148·1 (120·2 to 186·3)	154·7 (118·8 to 200·3)	4·5% (−15·7 to 28·9)	2·7 (2·1 to 3·4)	1·8 (1·4 to 2·3)	−32·6% (−44·5 to −17·2)*	6912·3 (5638·5 to 8562·7)	6215·3 (4872·2 to 7830·9)	−10·1% (−28·5 to 12·6)	115·1 (93·6 to 141·1)	76·1 (59·5 to 95·8)	−33·9% (−47·1 to −16·8)*
	Upper digestive system diseases		316·1 (265·6 to 370·2)	265·4 (220·0 to 328·5)	−16·1% (−34·2 to 7·7)	6·5 (5·5 to 7·6)	3·0 (2·5 to 3·7)	−53·6% (−63·2 to −40·5)*	9368·2 (7717·1 to 11 147·1)	6795·7 (5464·0 to 8582·5)	−27·5% (−45·3 to −3·4)*	173·5 (144·1 to 205·7)	77·7 (62·1 to 98·5)	−55·2% (−66·0 to −40·5)*
		Peptic ulcer disease	273·7 (230·0 to 325·8)	222·4 (180·2 to 281·7)	−18·8% (−38·0 to 7·4)	5·6 (4·7 to 6·6)	2·5 (2·0 to 3·2)	−55·2% (−65·8 to −40·6)*	8019·7 (6460·1 to 9888·4)	5642·5 (4413·6 to 7489·3)	−29·6% (−48·2 to −4·6)*	149·0 (121·6 to 182·8)	64·2 (49·9 to 85·8)	−56·9% (−68·2 to −41·1)*
		Gastritis and duodenitis	42·3 (29·3 to 57·6)	42·9 (27·1 to 58·5)	1·4% (−27·6 to 46·8)	0·9 (0·6 to 1·2)	0·5 (0·3 to 0·7)	−43·2% (−58·6 to −16·9)*	1348·4 (904·5 to 1906·6)	1153·2 (684·6 to 1724·0)	−14·5% (−43·1 to 30·2)	24·6 (16·6 to 34·1)	13·6 (8·0 to 20·4)	−44·7% (−63·2 to −15·3)*
	Appendicitis		33·2 (21·4 to 47·8)	31·0 (21·9 to 43·5)	−6·7% (−36·7 to 46·4)	0·6 (0·4 to 0·9)	0·4 (0·3 to 0·5)	−40·6% (−59·2 to −7·9)*	1498·4 (900·8 to 2229·2)	1174·6 (815·5 to 1725·4)	−21·6% (−49·0 to 30·6)	24·9 (15·3 to 36·8)	14·2 (9·9 to 21·0)	−43·0% (−62·6 to −5·9)*
	Paralytic ileus and intestinal obstruction		184·9 (152·8 to 221·6)	243·7 (201·6 to 286·2)	31·8% (3·5 to 72·6)*	3·8 (3·2 to 4·4)	2·9 (2·4 to 3·4)	−24·2% (−39·5 to −1·5)*	7095·3 (5679·0 to 8921·9)	7030·5 (5498·7 to 8614·0)	−0·9% (−26·5 to 37·0)	125·4 (101·3 to 156·0)	87·7 (68·4 to 107·3)	−30·1% (−47·6 to −3·7)*
	Inguinal, femoral, and abdominal hernia		39·0 (30·0 to 51·9)	51·1 (38·9 to 67·4)	31·2% (−10·1 to 86·1)	0·8 (0·6 to 1·1)	0·6 (0·4 to 0·8)	−28·5% (−50·2 to −0·2)*	1234·4 (906·7 to 1724·3)	1260·5 (923·4 to 1769·8)	2·1% (−34·7 to 57·5)	22·8 (16·9 to 31·3)	15·2 (11·1 to 21·5)	−33·2% (−56·6 to 1·9)
	Inflammatory bowel disease		27·5 (22·9 to 32·9)	46·5 (40·4 to 53·1)	69·4% (37·2 to 103·5)*	0·6 (0·5 to 0·7)	0·5 (0·5 to 0·6)	−12·5% (−28·4 to 4·5)	724·9 (569·0 to 893·6)	1031·8 (874·2 to 1235·1)	42·3% (9·1 to 78·3)*	13·9 (11·1 to 16·9)	11·9 (10·0 to 14·3)	−14·4% (−33·7 to 5·9)
		Ulcerative colitis	22·7 (18·5 to 27·6)	39·6 (34·1 to 45·8)	74·0% (36·1 to 112·0)*	0·5 (0·4 to 0·6)	0·5 (0·4 to 0·5)	−11·3% (−28·9 to 6·8)	580·6 (447·7 to 740·8)	836·6 (703·4 to 1019·5)	44·1% (7·1 to 84·4)*	11·2 (8·8 to 14·1)	9·6 (8·1 to 11·8)	−14·3% (−35·5 to 8·3)
		Crohn's disease	4·7 (3·8 to 5·8)	7·0 (5·9 to 8·5)	47·1% (17·0 to 88·1)*	0·1 (0·1 to 0·1)	0·1 (0·1 to 0·1)	−18·8% (−35·0 to 3·4)	144·2 (115·1 to 184·4)	195·2 (157·7 to 249·4)	35·3% (3·1 to 81·1)*	2·6 (2·1 to 3·3)	2·2 (1·8 to 2·9)	−15·2% (−35·4 to 13·1)
	Vascular intestinal disorders		67·8 (61·3 to 73·8)	92·6 (82·4 to 102·5)	36·7% (23·9 to 48·4)*	1·6 (1·4 to 1·7)	1·0 (0·9 to 1·2)	−33·4% (−39·3 to −27·9)*	1291·4 (1150·7 to 1428·0)	1670·7 (1519·1 to 1869·3)	29·4% (13·9 to 43·1)*	27·2 (24·3 to 29·9)	18·6 (16·9 to 20·9)	−31·5% (−39·4 to −23·9)*
	Gallbladder and biliary diseases		74·1 (61·1 to 87·8)	146·7 (121·8 to 171·4)	98·1% (62·0 to 132·6)*	1·7 (1·4 to 2·0)	1·7 (1·4 to 2·0)	−1·0% (−18·0 to 15·3)	1581·2 (1272·2 to 1894·0)	2682·4 (2235·9 to 3182·5)	69·7% (33·5 to 103·5)*	32·2 (26·1 to 38·2)	30·3 (25·2 to 35·9)	−6·0% (−25·9 to 12·1)
	Pancreatitis		88·7 (75·8 to 110·6)	124·5 (107·8 to 147·3)	40·2% (13·8 to 74·3)*	1·7 (1·5 to 2·1)	1·4 (1·2 to 1·7)	−18·6% (−34·0 to 0·7)	3037·6 (2554·9 to 3840·7)	3817·4 (3236·2 to 4664·5)	25·7% (0·2 to 59·2)*	54·1 (45·6 to 68·2)	43·6 (36·9 to 53·5)	−19·5% (−36·1 to 2·1)
	Other digestive diseases		94·1 (77·9 to 117·0)	132·9 (113·6 to 154·5)	41·1% (13·6 to 71·1)*	2·1 (1·7 to 2·5)	1·5 (1·3 to 1·8)	−27·1% (−40·3 to −12·6)*	2489·0 (1949·9 to 3230·7)	2898·9 (2418·3 to 3535·1)	16·5% (−13·8 to 53·1)	47·9 (38·2 to 61·4)	33·4 (27·8 to 40·7)	−30·4% (−47·6 to −8·8)*
	**Neurological disorders**		**1316·1 (623·0 to 2702·2)**	**3024·3 (1357·2 to 6102·8)**	129·3% (116·2 to 142·1)*	**32·8 (14·4 to 70·0)**	**35·2 (15·7 to 71·2)**	7·0% (0·1 to 17·0)*	**24 683·4 (14 830·2 to 43 397·8)**	**47 512·9 (25 997·7 to 86 959·8)**	92·2% (65·1 to 109·6)*	**526·2 (293·7 to 980·0)**	**553·8 (307·3 to 1002·1)**	**5·1% (−4·8 to 16·0)**
	Alzheimer's disease and other dementias		934·3 (230·7 to 2338·9)	2214·6 (549·4 to 5363·7)	136·4% (124·0 to 153·2)*	24·9 (6·1 to 62·5)	25·9 (6·4 to 62·7)	3·7% (−2·0 to 10·9)	12 473·3 (3072·0 to 31 556·2)	27 638·4 (6912·9 to 67 708·7)	121·0% (107·9 to 135·8)*	307·2 (75·6 to 765·4)	315·8 (78·8 to 771·3)	2·6% (−2·8 to 9·3)
	Parkinson's disease		190·7 (173·2 to 206·8)	427·1 (379·1 to 469·9)	123·8% (105·9 to 139·8)*	4·5 (4·1 to 4·9)	4·8 (4·3 to 5·3)	8·1% (−0·4 to 15·6)	3030·0 (2784·8 to 3284·1)	6345·3 (5691·2 to 6953·1)	109·3% (92·5 to 123·6)*	67·0 (61·3 to 72·7)	70·7 (63·4 to 77·3)	5·4% (−2·9 to 12·6)
	Idiopathic epilepsy		118·7 (90·3 to 150·2)	159·4 (125·4 to 202·9)	34·3% (−2·7 to 80·8)	2·0 (1·5 to 2·5)	2·0 (1·5 to 2·5)	−1·3% (−28·5 to 32·8)	6929·8 (5144·4 to 8895·3)	8186·6 (6217·1 to 10 738·7)	18·1% (−16·9 to 62·3)	110·6 (82·5 to 141·8)	105·3 (79·4 to 139·4)	−4·8% (−33·1 to 31·0)
	Multiple sclerosis		12·2 (11·2 to 13·4)	19·1 (17·4 to 21·7)	56·5% (41·2 to 75·0)*	0·2 (0·2 to 0·3)	0·2 (0·2 to 0·2)	−10·2% (−18·8 to 0·6)	409·2 (373·8 to 456·4)	551·3 (495·4 to 629·0)	34·7% (19·4 to 54·4)*	7·5 (6·8 to 8·3)	6·1 (5·5 to 7·0)	−18·1% (−27·1 to −5·9)*
	Motor neuron disease		22·6 (20·2 to 24·7)	44·6 (40·8 to 50·0)	97·4% (78·9 to 116·7)*	0·5 (0·4 to 0·5)	0·5 (0·4 to 0·6)	8·7% (−1·2 to 19·8)	680·0 (583·5 to 765·6)	1158·0 (1046·7 to 1347·7)	70·3% (49·4 to 95·4)*	12·7 (11·0 to 14·2)	13·1 (11·8 to 15·4)	3·3% (−9·2 to 17·6)
	Other neurological disorders		37·7 (33·9 to 41·5)	159·5 (137·4 to 180·4)	323·0% (270·6 to 373·9)*	0·8 (0·7 to 0·9)	1·8 (1·6 to 2·1)	128·7% (101·1 to 156·1)*	1161·1 (1036·5 to 1273·0)	3633·3 (3290·0 to 4022·3)	212·7% (173·9 to 258·7)*	21·3 (19·0 to 23·4)	42·7 (38·5 to 47·5)	100·7% (77·1 to 130·6)*
	**Mental disorders**		**0·2 (0·1 to 0·3)**	**0·2 (0·1 to 0·4)**	**8·6% (−20·1 to 42·2)**	**0·0 (0·0 to 0·0)**	**0·0 (0·0 to 0·0)**	**–15·4% (−37·7 to 10·8)**	**12·3 (5·3 to 18·2)**	**13·2 (6·5 to 21·0)**	**7·4% (−20·8 to 40·9)**	**0·2 (0·1 to 0·3)**	**0·2 (0·1 to 0·3)**	**–15·1% (−37·3 to 11·2)**
	Eating disorders		0·2 (0·1 to 0·3)	0·2 (0·1 to 0·4)	8·6% (−20·1 to 42·2)	0·0 (0·0 to 0·0)	0·0 (0·0 to 0·0)	−15·4% (−37·7 to 10·8)	12·3 (5·3 to 18·2)	13·2 (6·5 to 21·0)	7·4% (−20·8 to 40·9)	0·2 (0·1 to 0·3)	0·2 (0·1 to 0·3)	−15·1% (−37·3 to 11·2)
		Anorexia nervosa	0·2 (0·1 to 0·3)	0·2 (0·1 to 0·4)	8·6% (−20·1 to 42·2)	0·0 (0·0 to 0·0)	0·0 (0·0 to 0·0)	−15·4% (−37·7 to 10·8)	12·3 (5·3 to 18·2)	13·2 (6·5 to 21·0)	7·4% (−20·8 to 40·9)	0·2 (0·1 to 0·3)	0·2 (0·1 to 0·3)	−15·1% (−37·3 to 11·2)
	**Substance use disorders**		**254·5 (234·9 to 279·7)**	**344·1 (316·6 to 374·4)**	35·2% (19·4 to 50·6)*	**4·5 (4·1 to 4·9)**	**3·9 (3·6 to 4·3)**	–11·5% (−21·7 to −1·5)*	**11 302·6 (10 393·2 to 12 449·2)**	**14 426·8 (13 200·2 to 15 765·4)**	27·6% (12·1 to 43·2)*	**190·0 (175·0 to 209·1)**	**168·3 (153·9 to 184·1)**	–11·4% (−22·1 to −0·6)*
	Alcohol use disorders		170·2 (155·8 to 190·1)	171·6 (154·5 to 197·0)	0·8% (−14·9 to 19·9)	3·0 (2·8 to 3·4)	1·9 (1·7 to 2·2)	−36·7% (−46·6 to −24·5)*	7088·2 (6447·9 to 7992·7)	6460·2 (5727·4 to 7529·1)	−8·9% (−24·1 to 9·9)	122·0 (111·3 to 137·2)	73·6 (65·1 to 86·1)	−39·7% (−49·8 to −27·1)*
	Drug use disorders		84·3 (73·2 to 96·7)	172·5 (149·2 to 198·8)	104·5% (68·6 to 142·7)*	1·4 (1·2 to 1·6)	2·0 (1·7 to 2·3)	42·4% (17·6 to 69·2)*	4214·4 (3647·9 to 4824·1)	7966·6 (6900·5 to 9123·5)	89·0% (55·2 to 124·0)*	68·0 (58·9 to 77·9)	94·7 (82·1 to 108·4)	39·4% (14·5 to 64·9)*
		Opioid use disorders	57·0 (49·1 to 65·6)	125·9 (108·2 to 144·8)	120·9% (83·6 to 165·2)*	1·0 (0·8 to 1·1)	1·5 (1·3 to 1·7)	53·4% (27·7 to 83·6)*	2810·3 (2410·7 to 3260·9)	5833·0 (5027·1 to 6697·7)	107·6% (73·0 to 148·1)*	45·5 (39·1 to 52·6)	69·4 (59·9 to 79·7)	52·7% (27·5 to 82·7)*
		Cocaine use disorders	6·4 (4·9 to 8·3)	17·6 (13·9 to 21·7)	174·7% (91·8 to 280·3)*	0·1 (0·1 to 0·1)	0·2 (0·2 to 0·3)	90·0% (32·7 to 162·9)*	318·2 (245·3 to 412·1)	795·1 (632·4 to 972·2)	149·9% (75·8 to 245·5)*	5·2 (4·0 to 6·7)	9·4 (7·5 to 11·4)	81·5% (27·6 to 150·9)*
		Amphetamine use disorders	5·9 (3·9 to 9·6)	12·0 (9·6 to 15·0)	101·8% (21·8 to 221·3)*	0·1 (0·1 to 0·2)	0·1 (0·1 to 0·2)	43·0% (−13·7 to 126·9)	318·2 (207·1 to 514·5)	551·0 (441·8 to 685·2)	73·1% (5·3 to 173·8)*	5·0 (3·3 to 8·2)	6·5 (5·3 to 8·1)	29·6% (−21·3 to 105·4)
		Other drug use disorders	15·0 (11·6 to 20·1)	17·0 (14·7 to 20·6)	13·4% (−17·5 to 52·6)	0·3 (0·2 to 0·3)	0·2 (0·2 to 0·2)	−20·2% (−41·7 to 6·6)	767·7 (588·7 to 1029·3)	787·5 (679·6 to 948·9)	2·6% (−26·0 to 38·0)	12·3 (9·4 to 16·5)	9·4 (8·1 to 11·4)	−23·5% (−44·7 to 2·8)
	**Diabetes and kidney diseases**		**1719·5 (1560·3 to 1893·2)**	**3535·6 (3142·0 to 3888·8)**	105·5% (84·4 to 133·2)*	**36·3 (33·0 to 40·1)**	**39·5 (35·0 to 43·4)**	**8·5% (−2·7 to 22·2)**	**44 977·7 (40 396·5 to 49 071·0)**	**82 210·8 (74 339·9 to 91 232·8)**	82·7% (63·7 to 108·2)*	**863·5 (779·1 to 943·9)**	**919·4 (831·2 to 1020·6)**	**6·4% (−4·2 to 21·3)**
	Diabetes mellitus		911·2 (784·3 to 1036·0)	2004·5 (1693·2 to 2306·4)	119·8% (90·0 to 160·5)*	19·2 (16·5 to 21·9)	22·1 (18·7 to 25·5)	14·9% (−0·5 to 35·9)	22 415·9 (19 298·7 to 25 392·7)	45 981·4 (38 780·6 to 53 228·8)	105·0% (76·7 to 143·0)*	438·9 (377·6 to 497·5)	506·2 (426·9 to 586·5)	15·3% (−0·3 to 36·2)
		Type 1 diabetes mellitus	48·4 (40·6 to 60·3)	54·4 (42·3 to 71·6)	12·2% (−9·7 to 44·6)	0·8 (0·7 to 1·1)	0·6 (0·5 to 0·8)	−23·0% (−38·1 to −0·8)*	2430·9 (2003·6 to 2963·8)	2556·8 (1980·0 to 3271·3)	5·2% (−15·3 to 36·3)	39·9 (32·8 to 48·7)	31·7 (24·5 to 40·4)	−20·5% (−35·9 to 3·4)
		Type 2 diabetes mellitus	862·8 (740·0 to 985·7)	1950·1 (1646·3 to 2235·9)	125·9% (94·9 to 168·7)*	18·4 (15·8 to 21·0)	21·5 (18·1 to 24·7)	16·7% (0·8 to 38·4)*	19 985·0 (17 051·7 to 22 877·7)	43 424·6 (36 681·1 to 50 101·1)	117·2% (86·9 to 159·7)*	399·1 (341·0 to 456·2)	474·4 (401·0 to 548·0)	18·8% (2·3 to 41·7)*
	Chronic kidney disease		796·0 (701·6 to 902·0)	1520·1 (1331·1 to 1696·5)	90·9% (66·1 to 123·2)*	16·9 (14·7 to 19·1)	17·2 (15·1 to 19·2)	1·9% (−10·7 to 18·3)	22 063·1 (19 132·0 to 25 266·9)	35 905·8 (30 906·8 to 40 911·1)	62·7% (37·0 to 97·2)*	416·0 (362·4 to 475·7)	409·4 (351·1 to 466·8)	−1·6% (−16·7 to 18·7)
		Chronic kidney disease due to type 1 diabetes mellitus	45·0 (35·2 to 57·9)	76·5 (56·4 to 98·4)	70·1% (42·3 to 106·9)*	0·8 (0·6 to 1·1)	0·9 (0·6 to 1·1)	4·1% (−12·3 to 25·6)	1750·8 (1375·2 to 2246·8)	2871·7 (2147·2 to 3677·5)	64·0% (34·9 to 103·4)*	30·8 (24·1 to 40·0)	32·6 (24·3 to 41·7)	5·7% (−12·0 to 30·1)
		Chronic kidney disease due to type 2 diabetes mellitus	159·1 (123·4 to 194·4)	343·2 (271·1 to 414·0)	115·7% (88·8 to 150·2)*	3·4 (2·7 to 4·2)	3·8 (3·0 to 4·6)	10·3% (−3·0 to 28·1)	3440·0 (2713·0 to 4265·4)	6906·2 (5505·2 to 8292·3)	100·8% (72·7 to 135·2)*	70·2 (55·2 to 86·9)	75·2 (60·1 to 90·2)	7·1% (−7·2 to 25·5)
		Chronic kidney disease due to hypertension	189·4 (153·9 to 229·6)	442·3 (358·5 to 530·1)	133·5% (100·4 to 175·1)*	4·4 (3·6 to 5·4)	5·0 (4·1 to 6·0)	13·3% (−1·1 to 32·8)	3714·7 (2947·7 to 4580·8)	7829·3 (6387·7 to 9431·1)	110·8% (78·6 to 154·9)*	78·2 (63·1 to 96·4)	87·5 (71·6 to 105·0)	12·0% (−4·8 to 34·6)
		Chronic kidney disease due to glomerulonephritis	123·3 (101·2 to 145·5)	193·9 (161·9 to 228·9)	57·2% (34·8 to 84·7)*	2·4 (2·0 to 2·8)	2·2 (1·9 to 2·6)	−6·2% (−18·2 to 9·1)	4606·6 (3752·7 to 5506·5)	6352·6 (5165·6 to 7683·4)	37·9% (14·5 to 68·2)*	80·1 (65·4 to 95·7)	74·8 (60·6 to 89·8)	−6·5% (−22·2 to 14·5)
		Chronic kidney disease due to other and unspecified causes	279·2 (237·8 to 321·8)	464·1 (387·6 to 545·1)	66·2% (44·2 to 93·0)*	5·8 (4·9 to 6·7)	5·3 (4·4 to 6·2)	−8·8% (−20·0 to 6·1)	8550·9 (7197·2 to 10 086·6)	11 945·9 (9922·4 to 14 087·8)	39·7% (15·0 to 71·7)*	156·8 (133·8 to 183·6)	139·3 (115·7 to 163·9)	−11·1% (−25·5 to 8·6)
	Acute glomerulonephritis		12·3 (7·9 to 18·4)	11·1 (7·5 to 14·7)	−9·7% (−38·7 to 32·5)	0·2 (0·1 to 0·3)	0·1 (0·1 to 0·2)	−45·0% (−62·9 to −18·8)*	498·7 (308·1 to 729·6)	323·6 (190·1 to 466·4)	−35·1% (−55·7 to 2·4)	8·5 (5·3 to 12·6)	3·9 (2·2 to 5·6)	−54·9% (−69·9 to −27·5)*
	**Skin and subcutaneous diseases**		**66·0 (52·4 to 83·7)**	**161·1 (130·9 to 196·4)**	144·2% (86·6 to 231·0)*	**1·4 (1·2 to 1·8)**	**1·9 (1·5 to 2·3)**	30·2% (1·6 to 75·2)*	**2121·2 (1546·9 to 2956·1)**	**4110·4 (3136·7 to 5381·9)**	93·8% (35·9 to 193·5)*	**38·9 (29·0 to 53·1)**	**49·2 (37·0 to 65·4)**	**26·4% (−11·1 to 90·6)**
	Bacterial skin diseases		40·1 (28·2 to 52·9)	101·7 (82·5 to 128·7)	153·8% (82·3 to 276·5)*	0·8 (0·6 to 1·1)	1·2 (0·9 to 1·5)	43·4% (4·5 to 107·0)*	1508·6 (1004·4 to 2158·2)	2763·4 (2067·2 to 3755·8)	83·2% (21·8 to 204·5)*	26·8 (18·1 to 37·8)	33·6 (24·7 to 46·4)	25·2% (−16·7 to 108·4)
		Cellulitis	15·8 (10·4 to 23·6)	39·7 (28·9 to 55·5)	150·7% (60·4 to 311·7)*	0·3 (0·2 to 0·5)	0·5 (0·3 to 0·6)	39·4% (−9·9 to 126·4)	537·4 (323·8 to 878·1)	1083·6 (728·7 to 1661·9)	101·7% (17·8 to 273·4)*	9·7 (6·0 to 15·6)	12·9 (8·5 to 20·0)	32·3% (−22·4 to 145·7)
		Pyoderma	24·2 (17·1 to 34·4)	62·0 (49·0 to 79·0)	155·9% (76·4 to 271·7)*	0·5 (0·4 to 0·7)	0·7 (0·6 to 0·9)	46·1% (2·6 to 108·2)*	971·2 (591·4 to 1459·5)	1679·8 (1222·9 to 2353·8)	73·0% (9·3 to 193·7)*	17·1 (10·7 to 25·4)	20·7 (14·7 to 29·7)	21·2% (−22·8 to 107·2)
	Decubitus ulcer		21·2 (16·3 to 27·8)	48·1 (36·3 to 62·8)	127·0% (65·8 to 213·6)*	0·5 (0·4 to 0·6)	0·5 (0·4 to 0·7)	8·2% (−17·9 to 44·9)	446·2 (309·0 to 651·9)	1010·4 (702·8 to 1440·1)	126·4% (44·0 to 260·4)*	9·1 (6·6 to 12·9)	11·5 (7·9 to 16·5)	26·1% (−17·0 to 92·3)
	Other skin and subcutaneous diseases		4·7 (2·8 to 8·3)	11·3 (6·9 to 17·6)	138·8% (14·2 to 384·7)*	0·1 (0·1 to 0·2)	0·1 (0·1 to 0·2)	32·9% (−35·6 to 164·1)	166·5 (83·7 to 338·4)	336·6 (190·9 to 584·5)	102·2% (−20·3 to 392·7)	3·0 (1·5 to 5·9)	4·1 (2·3 to 7·4)	38·5% (−45·7 to 228·7)
	**Musculoskeletal disorders**		**80·2 (66·5 to 92·9)**	**131·7 (107·9 to 154·1)**	64·1% (37·0 to 88·7)*	**1·7 (1·4 to 1·9)**	**1·5 (1·2 to 1·7)**	**–11·5% (−26·0 to 1·6)**	**2299·8 (1845·6 to 2683·2)**	**3173·8 (2620·9 to 3764·7)**	38·0% (11·9 to 62·2)*	**42·5 (34·5 to 49·5)**	**36·4 (30·0 to 43·3)**	**–14·5% (−30·4 to 0·4)**
	Rheumatoid arthritis		28·8 (20·7 to 36·4)	45·9 (32·7 to 57·9)	59·2% (27·7 to 99·0)*	0·6 (0·4 to 0·8)	0·5 (0·4 to 0·6)	−17·6% (−33·9 to 3·3)	645·5 (447·7 to 820·4)	916·4 (640·7 to 1183·4)	41·9% (11·4 to 77·5)*	12·9 (9·0 to 16·3)	10·1 (7·1 to 13·1)	−21·2% (−38·0 to −1·8)*
	Other musculoskeletal disorders		51·4 (42·1 to 60·6)	85·9 (67·9 to 103·6)	66·8% (37·1 to 97·7)*	1·1 (0·9 to 1·3)	1·0 (0·8 to 1·2)	−8·0% (−23·2 to 8·5)	1654·3 (1330·9 to 1948·5)	2257·4 (1790·5 to 2757·1)	36·4% (11·3 to 63·9)*	29·7 (24·2 to 34·8)	26·2 (20·8 to 31·9)	−11·6% (−27·1 to 5·9)
	**Other non-communicable diseases**		**1121·0 (992·9 to 1256·6)**	**1338·5 (1191·5 to 1551·1)**	19·4% (0·0 to 38·5)*	**19·8 (17·7 to 22·1)**	**17·9 (15·8 to 21·0)**	**–9·4% (−23·9 to 5·5)**	**77 258·0 (66 414·6 to 88 483·4)**	**71 507·3 (59 687·1 to 85 787·3)**	**–7·4% (−26·5 to 13·9)**	**1271·3 (1094·4 to 1452·8)**	**1055·5 (866·7 to 1280·7)**	**–17·0% (−34·1 to 2·8)**
	Congenital birth defects		659·3 (547·7 to 792·5)	562·7 (441·3 to 709·6)	−14·7% (−37·6 to 11·6)	10·7 (8·9 to 12·8)	8·8 (6·9 to 11·1)	−17·8% (−40·1 to 7·7)	56 848·7 (47 133·0 to 68 446·9)	47 701·9 (37 217·6 to 60 416·6)	−16·1% (−39·0 to 10·1)	919·0 (761·9 to 1106·5)	753·7 (586·8 to 956·6)	−18·0% (−40·5 to 7·7)
		Neural tube defects	65·3 (39·4 to 107·4)	41·7 (24·6 to 69·1)	−36·2% (−66·1 to 18·1)	1·1 (0·6 to 1·7)	0·7 (0·4 to 1·1)	−36·6% (−66·4 to 17·4)	5791·5 (3488·4 to 9536·6)	3673·3 (2158·6 to 6101·7)	−36·6% (−66·4 to 17·5)	93·6 (56·4 to 154·2)	59·2 (34·7 to 98·4)	−36·7% (−66·5 to 17·3)
		Congenital heart anomalies	386·7 (307·5 to 471·0)	301·2 (233·4 to 390·5)	−22·1% (−43·5 to 2·5)	6·2 (5·0 to 7·6)	4·7 (3·6 to 6·1)	−25·3% (−45·9 to −1·6)*	33 161·2 (26 261·7 to 40 446·1)	25 455·6 (19 627·3 to 33 175·6)	−23·2% (−44·6 to 1·3)	535·7 (424·1 to 653·5)	399·5 (307·3 to 522·4)	−25·4% (−46·2 to −1·5)*
		Orofacial clefts	11·5 (4·1 to 30·6)	3·3 (0·7 to 10·1)	−71·4% (−89·5 to −29·7)*	0·2 (0·1 to 0·5)	0·1 (0·0 to 0·2)	−71·2% (−89·4 to −29·4)*	1027·7 (367·6 to 2745·2)	293·6 (63·7 to 901·6)	−71·4% (−89·5 to −29·7)*	16·7 (6·0 to 44·6)	4·8 (1·0 to 14·7)	−71·2% (−89·4 to −29·4)*
		Down syndrome	23·9 (15·4 to 34·4)	29·3 (17·8 to 43·7)	22·7% (−32·7 to 104·2)	0·4 (0·3 to 0·6)	0·4 (0·3 to 0·7)	10·1% (−39·8 to 84·2)	1951·1 (1233·5 to 2848·7)	2251·1 (1330·7 to 3487·0)	15·4% (−37·7 to 97·3)	31·7 (20·1 to 46·3)	34·3 (20·0 to 53·7)	8·1% (−42·1 to 85·6)
		Other chromosomal abnormalities	14·9 (10·1 to 22·1)	22·9 (15·4 to 36·0)	53·6% (−7·1 to 142·1)	0·2 (0·2 to 0·4)	0·4 (0·2 to 0·6)	49·4% (−10·0 to 135·6)	1291·8 (869·2 to 1924·2)	1956·0 (1300·4 to 3117·2)	51·4% (−9·4 to 139·4)	20·9 (14·1 to 31·1)	31·2 (20·6 to 49·8)	49·2% (−10·9 to 135·9)
		Congenital musculoskeletal and limb anomalies	10·0 (6·6 to 16·3)	9·7 (6·2 to 15·0)	−3·4% (−43·6 to 73·9)	0·2 (0·1 to 0·3)	0·1 (0·1 to 0·2)	−7·0% (−45·8 to 67·5)	853·2 (560·2 to 1387·9)	813·1 (513·6 to 1266·7)	−4·7% (−44·8 to 72·9)	13·8 (9·0 to 22·4)	12·8 (8·1 to 20·0)	−6·7% (−46·1 to 69·2)
		Urogenital congenital anomalies	12·9 (7·9 to 21·7)	16·9 (9·3 to 30·8)	30·7% (−32·1 to 167·1)	0·2 (0·1 to 0·4)	0·3 (0·1 to 0·5)	18·9% (−40·2 to 144·6)	1039·6 (610·4 to 1775·6)	1309·1 (675·8 to 2494·3)	25·9% (−38·8 to 177·0)	16·9 (10·0 to 28·9)	20·5 (10·4 to 39·6)	21·2% (−42·1 to 168·0)
		Digestive congenital anomalies	63·1 (40·9 to 97·6)	67·0 (42·0 to 97·1)	6·1% (−34·6 to 73·4)	1·0 (0·7 to 1·6)	1·1 (0·7 to 1·6)	5·6% (−35·0 to 72·6)	5606·3 (3633·0 to 8669·4)	5922·1 (3705·9 to 8592·1)	5·6% (−35·0 to 72·7)	90·9 (58·9 to 140·6)	96·0 (60·0 to 139·3)	5·6% (−35·2 to 72·8)
		Other congenital birth defects	71·1 (42·4 to 125·4)	70·9 (37·7 to 132·9)	−0·3% (−32·6 to 41·8)	1·1 (0·7 to 2·0)	1·1 (0·6 to 2·1)	−3·5% (−35·3 to 38·1)	6126·5 (3636·7 to 10 879·7)	6028·0 (3169·5 to 11 394·8)	−1·6% (−34·1 to 40·9)	98·8 (58·7 to 175·6)	95·4 (49·9 to 181·0)	−3·5% (−36·0 to 38·9)
	Urinary diseases and male infertility		180·8 (162·1 to 199·0)	396·2 (357·0 to 433·6)	119·2% (95·5 to 146·1)*	3·9 (3·5 to 4·3)	4·5 (4·1 to 5·0)	15·6% (4·0 to 28·8)*	5005·7 (4426·8 to 5604·2)	8495·7 (7768·6 to 9356·0)	69·8% (48·9 to 95·1)*	94·5 (83·5 to 105·4)	98·2 (89·7 to 108·1)	3·9% (−9·0 to 19·0)
		Urinary tract infections and interstitial nephritis	122·2 (106·7 to 137·0)	288·5 (258·0 to 318·6)	136·2% (108·0 to 171·5)*	2·7 (2·4 to 3·0)	3·3 (3·0 to 3·7)	21·7% (7·9 to 38·1)*	3223·8 (2739·6 to 3698·5)	5884·9 (5246·1 to 6590·9)	82·6% (54·7 to 118·5)*	61·7 (53·2 to 70·3)	67·9 (60·5 to 76·0)	10·1% (−6·7 to 30·3)
		Urolithiasis	14·6 (12·1 to 17·2)	24·9 (21·0 to 30·0)	71·0% (34·9 to 108·7)*	0·3 (0·2 to 0·3)	0·3 (0·2 to 0·3)	−1·4% (−22·2 to 20·4)	500·4 (406·5 to 604·3)	685·2 (565·5 to 833·1)	37·0% (4·8 to 76·4)*	8·9 (7·3 to 10·7)	8·0 (6·6 to 9·7)	−9·9% (−31·4 to 16·1)
		Other urinary diseases	44·0 (33·4 to 58·5)	82·8 (65·2 to 102·2)	88·1% (32·5 to 160·1)*	0·9 (0·7 to 1·2)	0·9 (0·7 to 1·2)	2·9% (−27·1 to 41·0)	1281·5 (965·0 to 1715·2)	1925·7 (1463·1 to 2497·9)	50·3% (4·8 to 116·0)*	23·9 (18·0 to 31·8)	22·3 (16·9 to 28·9)	−7·0% (−35·1 to 32·8)
	Gynaecological diseases		5·3 (3·4 to 8·7)	14·1 (7·1 to 25·2)	165·6% (61·5 to 478·3)*	0·1 (0·1 to 0·2)	0·2 (0·1 to 0·3)	61·9% (−2·4 to 251·5)	210·4 (130·8 to 352·3)	502·5 (259·0 to 893·5)	138·7% (41·4 to 424·3)*	3·6 (2·3 to 6·0)	5·9 (3·0 to 10·4)	62·6% (−3·5 to 253·5)
		Uterine fibroids	1·6 (0·9 to 2·9)	4·0 (2·0 to 7·4)	151·9% (38·4 to 474·7)*	0·0 (0·0 to 0·1)	0·0 (0·0 to 0·1)	61·6% (−11·9 to 266·8)	67·5 (38·8 to 123·6)	159·1 (77·5 to 311·2)	135·4% (24·8 to 439·1)*	1·1 (0·7 to 2·1)	1·9 (0·9 to 3·6)	62·0% (−14·1 to 269·5)
		Endometriosis	0·0 (0·0 to 0·1)	0·2 (0·0 to 0·5)	260·1% (25·3 to 1199·7)*	0·0 (0·0 to 0·0)	0·0 (0·0 to 0·0)	160·1% (−10·5 to 826·6)	2·2 (0·8 to 5·5)	7·6 (1·5 to 25·5)	245·5% (19·5 to 1118·1)*	0·0 (0·0 to 0·1)	0·1 (0·0 to 0·3)	154·3% (−11·5 to 787·1)
		Genital prolapse	0·6 (0·3 to 1·1)	1·6 (0·8 to 3·5)	181·4% (14·7 to 706·6)*	0·0 (0·0 to 0·0)	0·0 (0·0 to 0·0)	50·8% (−37·5 to 324·1)	17·2 (8·5 to 33·6)	43·4 (19·3 to 91·8)	152·4% (−9·2 to 616·6)	0·3 (0·2 to 0·6)	0·5 (0·2 to 1·1)	63·2% (−40·3 to 353·9)
		Other gynaecological diseases	3·1 (1·7 to 5·2)	8·3 (3·9 to 15·2)	168·4% (45·0 to 483·1)*	0·1 (0·0 to 0·1)	0·1 (0·0 to 0·2)	63·2% (−11·4 to 249·0)	123·5 (65·5 to 210·9)	292·5 (134·9 to 525·3)	136·8% (26·7 to 433·0)*	2·1 (1·1 to 3·6)	3·4 (1·6 to 6·1)	61·4% (−13·4 to 260·5)
	Haemoglobinopathies and haemolytic anaemias		121·9 (88·9 to 183·0)	132·4 (86·7 to 218·0)	8·5% (−33·1 to 57·4)	2·2 (1·7 to 3·3)	1·6 (1·1 to 2·8)	−26·4% (−54·4 to 6·3)	6345·0 (4403·3 to 9919·8)	6361·5 (3804·8 to 11 317·4)	0·2% (−42·3 to 55·8)	105·1 (73·4 to 163·8)	84·8 (49·8 to 153·8)	−19·4% (−53·9 to 25·2)
		Thalassaemias	17·9 (13·9 to 24·6)	13·5 (9·1 to 19·6)	−24·6% (−55·4 to 19·7)	0·3 (0·2 to 0·4)	0·2 (0·1 to 0·3)	−35·7% (−62·2 to 2·9)	1330·1 (1021·3 to 1846·9)	956·2 (629·0 to 1407·7)	−28·1% (−58·3 to 15·9)	21·3 (16·3 to 29·6)	13·7 (8·9 to 20·6)	−35·7% (−63·0 to 3·7)
		Sickle cell disorders	45·6 (26·5 to 85·7)	54·0 (27·0 to 110·5)	18·4% (−34·4 to 100·9)	0·7 (0·4 to 1·4)	0·7 (0·4 to 1·5)	0·4% (−44·5 to 71·2)	3290·0 (1894·7 to 6160·7)	3768·4 (1866·6 to 7680·2)	14·5% (−37·1 to 98·2)	51·9 (29·9 to 97·8)	51·9 (25·6 to 107·0)	0·0% (−45·3 to 73·7)
		G6PD deficiency	12·6 (8·5 to 18·3)	12·9 (7·6 to 21·1)	2·9% (−42·6 to 65·7)	0·2 (0·2 to 0·3)	0·1 (0·1 to 0·2)	−34·3% (−63·1 to 4·3)	553·6 (387·9 to 794·2)	516·6 (310·1 to 816·5)	−6·7% (−48·2 to 55·1)	9·4 (6·6 to 13·5)	6·2 (3·8 to 9·8)	−33·4% (−62·6 to 11·0)
		Other haemoglobinopathies and haemolytic anaemias	45·9 (36·7 to 60·3)	51·9 (39·7 to 71·8)	13·1% (−21·8 to 52·5)	1·0 (0·8 to 1·3)	0·6 (0·4 to 0·8)	−41·2% (−59·3 to −21·0)*	1171·3 (969·0 to 1485·7)	1120·4 (875·8 to 1457·0)	−4·4% (−32·6 to 27·7)	22·5 (18·5 to 29·0)	12·8 (10·1 to 16·6)	−42·9% (−59·7 to −25·1)*
	Endocrine, metabolic, blood, and immune disorders		96·9 (84·3 to 108·9)	207·6 (179·5 to 233·9)	114·1% (89·0 to 141·4)*	2·0 (1·7 to 2·2)	2·4 (2·1 to 2·7)	23·4% (9·9 to 39·9)*	3757·6 (3032·0 to 4410·9)	6149·5 (5122·8 to 7402·5)	63·6% (35·7 to 93·7)*	66·2 (53·9 to 77·1)	75·6 (62·2 to 92·1)	14·0% (−5·1 to 35·2)
		Thyroid diseases	19·6 (14·9 to 25·2)	32·4 (25·6 to 41·1)	65·0% (22·4 to 113·1)*	0·4 (0·3 to 0·5)	0·4 (0·3 to 0·5)	−8·0% (−30·6 to 18·2)	674·5 (479·3 to 924·3)	967·1 (712·2 to 1279·4)	43·4% (−3·4 to 101·0)	12·1 (8·8 to 16·3)	11·7 (8·5 to 15·6)	−3·4% (−34·4 to 35·5)
		Other endocrine, metabolic, blood, and immune disorders	77·3 (67·1 to 86·5)	175·2 (153·3 to 197·4)	126·6% (103·1 to 154·9)*	1·5 (1·4 to 1·7)	2·0 (1·8 to 2·3)	31·8% (19·3 to 48·4)*	3083·1 (2538·1 to 3609·7)	5182·4 (4384·7 to 6163·9)	68·0% (43·1 to 96·0)*	54·1 (45·0 to 62·9)	63·9 (53·4 to 77·1)	17·9% (0·2 to 39·7)*
		Sudden infant death syndrome	56·7 (36·3 to 91·0)	25·6 (15·8 to 40·1)	−54·9% (−73·0 to −24·0)*	0·9 (0·6 to 1·5)	0·4 (0·3 to 0·7)	−54·7% (−72·9 to −23·7)*	5090·6 (3253·1 to 8163·9)	2296·2 (1414·7 to 3601·8)	−54·9% (−73·0 to −24·0)*	82·9 (53·0 to 132·9)	37·5 (23·1 to 58·8)	−54·7% (−72·9 to −23·7)*
**Injuries**			**4561·0 (4198·4 to 4847·2)**	**4874·5 (4365·5 to 5278·9)**	**6·9% (−1·4 to 17·0)**	**79·0 (73·0 to 83·8)**	**58·4 (52·3 to 63·4)**	–26·1% (−31·9 to −19·3)*	**239 490·2 (219 550·6 to 255 837·6)**	**220 643·9 (194 754·8 to 240 020·8)**	**–7·9% (−15·4 to 0·3)**	**3884·7 (3557·3 to 4148·4)**	**2756·6 (2426·8 to 2997·9)**	–29·0% (−34·8 to −22·7)*
	**Transport injuries**		**1370·0 (1150·1 to 1577·2)**	**1425·8 (1126·9 to 1685·0)**	**4·0% (−15·6 to 30·5)**	**23·0 (19·4 to 26·5)**	**17·1 (13·5 to 20·2)**	–25·9% (−40·1 to −7·0)*	**73 477·8 (61 024·6 to 85 100·8)**	**70 704·2 (55 582·2 to 83 652·9)**	**–3·8% (−21·5 to 21·2)**	**1180·1 (981·8 to 1364·4)**	**875·7 (687·3 to 1039·1)**	–25·8% (−39·4 to −6·6)*
	Road injuries		1285·3 (1073·5 to 1485·5)	1343·7 (1044·8 to 1583·5)	4·5% (−16·0 to 31·7)	21·6 (18·1 to 25·0)	16·1 (12·5 to 19·0)	−25·5% (−40·1 to −6·3)*	69 027·2 (57 038·4 to 79 536·6)	66 726·5 (51 479·6 to 79 414·8)	−3·4% (−21·7 to 22·6)	1108·4 (917·5 to 1276·3)	826·9 (636·8 to 987·2)	−25·4% (−39·6 to −5·4)*
		Pedestrian road injuries	517·6 (403·1 to 627·3)	401·4 (294·8 to 531·9)	−22·5% (−44·5 to 10·0)	8·9 (7·0 to 10·7)	4·8 (3·5 to 6·4)	−46·5% (−61·8 to −24·8)*	26 512·5 (20 298·8 to 32 278·7)	18 879·3 (13 593·8 to 25 843·3)	−28·8% (−49·4 to −0·1)*	432·5 (331·6 to 526·7)	235·1 (168·4 to 321·0)	−45·6% (−61·6 to −24·1)*
		Cyclist road injuries	62·3 (47·0 to 82·2)	89·1 (61·6 to 127·5)	42·6% (−15·8 to 118·5)	1·1 (0·8 to 1·4)	1·0 (0·7 to 1·5)	−5·3% (−44·0 to 44·6)	3040·8 (2295·4 to 4061·4)	3647·8 (2434·3 to 5248·3)	19·7% (−28·2 to 83·2)	49·8 (37·6 to 66·3)	43·4 (29·1 to 62·5)	−13·1% (−48·1 to 34·1)
		Motorcyclist road injuries	216·7 (161·2 to 286·9)	319·3 (216·6 to 416·4)	47·3% (−5·7 to 123·1)	3·5 (2·6 to 4·6)	3·8 (2·6 to 5·0)	9·4% (−30·1 to 65·5)	12 274·6 (8987·1 to 16 339·8)	16 474·0 (11 327·3 to 21 440·9)	34·2% (−15·6 to 102·9)	193·0 (142·0 to 256·8)	202·2 (139·0 to 263·5)	4·7% (−34·3 to 58·1)
		Motor vehicle road injuries	475·6 (394·0 to 598·0)	518·6 (416·7 to 648·1)	9·0% (−17·7 to 45·1)	7·9 (6·6 to 9·9)	6·3 (5·0 to 7·9)	−20·3% (−40·1 to 6·2)	26 488·4 (21 757·2 to 33 135·5)	26 954·9 (21 588·6 to 34 098·7)	1·7% (−23·2 to 35·9)	421·6 (346·7 to 527·4)	336·5 (268·3 to 428·6)	−20·2% (−39·9 to 7·0)
		Other road injuries	13·1 (8·7 to 18·9)	15·4 (10·0 to 22·6)	17·3% (−41·2 to 108·8)	0·2 (0·1 to 0·3)	0·2 (0·1 to 0·3)	−16·2% (−58·0 to 49·8)	711·0 (467·5 to 1033·5)	770·5 (491·4 to 1136·7)	8·4% (−47·1 to 97·1)	11·5 (7·5 to 16·6)	9·7 (6·1 to 14·4)	−15·4% (−58·9 to 55·1)
	Other transport injuries		84·7 (60·9 to 110·7)	82·0 (55·8 to 112·2)	−3·2% (−37·0 to 47·6)	1·4 (1·0 to 1·9)	1·0 (0·7 to 1·3)	−31·6% (−55·3 to 3·4)	4450·6 (3151·6 to 5913·9)	3977·7 (2673·9 to 5532·3)	−10·6% (−43·2 to 36·8)	71·6 (50·9 to 94·8)	48·7 (32·7 to 68·1)	−32·0% (−56·8 to 3·5)
	**Unintentional injuries**		**1791·4 (1590·1 to 1985·5)**	**2078·4 (1786·5 to 2291·5)**	16·0% (5·5 to 29·4)*	**32·6 (28·9 to 35·9)**	**24·9 (21·4 to 27·5)**	–23·5% (−30·2 to −14·8)*	**92 652·4 (80 125·4 to 103 949·3)**	**81 163·1 (69 397·1 to 91 146·1)**	–12·4% (−21·6 to −2·5)*	**1531·5 (1327·0 to 1713·6)**	**1033·8 (881·6 to 1164·1)**	–32·5% (−39·5 to −24·7)*
	Falls		487·5 (426·9 to 562·8)	857·4 (723·5 to 1002·4)	75·8% (51·9 to 105·1)*	10·2 (8·9 to 11·7)	9·9 (8·3 to 11·6)	−3·2% (−16·3 to 12·3)	16 299·1 (14 045·6 to 19 638·5)	21 114·3 (17 999·1 to 24 778·3)	29·5% (7·5 to 54·6)*	293·0 (253·6 to 349·4)	250·4 (213·8 to 296·1)	−14·6% (−29·6 to 2·3)
	Drowning		432·5 (372·3 to 512·2)	291·1 (234·0 to 362·8)	−32·7% (−46·0 to −11·9)*	7·1 (6·2 to 8·4)	3·7 (3·0 to 4·6)	−47·9% (−58·7 to −32·2)*	29 119·6 (24 541·5 to 34 727·6)	16 898·5 (13 180·4 to 21 593·5)	−42·0% (−54·9 to −23·5)*	464·9 (392·5 to 554·2)	225·5 (174·9 to 289·3)	−51·5% (−62·6 to −36·0)*
	Fire, heat, and hot substances		134·6 (103·6 to 173·8)	150·7 (102·9 to 205·0)	11·9% (−13·8 to 54·5)	2·4 (1·8 to 3·0)	1·9 (1·3 to 2·6)	−21·6% (−39·4 to 9·1)	7268·4 (5293·8 to 9998·4)	7433·1 (4843·5 to 10 612·7)	2·3% (−24·7 to 47·5)	119·7 (87·9 to 163·7)	97·7 (63·0 to 141·3)	−18·4% (−39·5 to 18·6)
	Poisonings		79·0 (65·0 to 99·8)	69·9 (54·4 to 91·7)	−11·6% (−37·9 to 24·6)	1·4 (1·1 to 1·7)	0·8 (0·6 to 1·1)	−37·8% (−56·7 to −11·9)*	4281·2 (3412·7 to 5626·8)	3335·6 (2479·5 to 4592·5)	−22·1% (−48·5 to 18·3)	70·0 (55·9 to 91·7)	42·5 (30·9 to 59·8)	−39·3% (−60·0 to −6·7)*
		Poisoning by carbon monoxide	44·0 (37·4 to 52·6)	31·0 (24·4 to 39·9)	−29·7% (−43·1 to −12·7)*	0·8 (0·7 to 0·9)	0·4 (0·3 to 0·5)	−52·8% (−61·9 to −40·7)*	2185·4 (1806·8 to 2684·2)	1290·8 (1014·9 to 1743·1)	−41·0% (−54·4 to −21·2)*	36·1 (29·9 to 44·0)	15·8 (12·3 to 21·7)	−56·4% (−66·5 to −40·9)*
		Poisoning by other means	35·0 (22·7 to 52·1)	38·9 (24·5 to 55·8)	11·2% (−37·6 to 97·9)	0·6 (0·4 to 0·9)	0·5 (0·3 to 0·7)	−18·5% (−54·5 to 46·8)	2095·8 (1309·4 to 3230·7)	2044·8 (1279·7 to 3095·7)	−2·5% (−47·0 to 80·7)	33·9 (21·2 to 52·2)	26·8 (16·4 to 41·4)	−21·0% (−57·6 to 48·2)
	Exposure to mechanical forces		127·9 (98·2 to 170·3)	104·1 (74·0 to 148·7)	−18·7% (−41·8 to 34·5)	2·1 (1·7 to 2·8)	1·3 (0·9 to 1·8)	−41·6% (−58·1 to −2·9)*	7151·8 (5320·4 to 9989·7)	5170·1 (3634·1 to 7802·5)	−27·7% (−51·6 to 24·4)	115·1 (85·9 to 160·4)	64·5 (45·2 to 100·2)	−43·9% (−62·5 to −3·0)*
		Unintentional firearm injuries	23·2 (13·8 to 37·9)	16·2 (10·0 to 25·8)	−30·1% (−56·8 to 26·7)	0·4 (0·2 to 0·6)	0·2 (0·1 to 0·3)	−47·3% (−67·6 to −3·4)*	1340·5 (796·7 to 2191·8)	888·1 (539·5 to 1434·2)	−33·7% (−60·1 to 25·4)	21·0 (12·5 to 34·4)	11·1 (6·7 to 18·4)	−47·0% (−68·3 to 1·1)
		Other exposure to mechanical forces	104·7 (80·0 to 141·2)	87·9 (60·6 to 127·5)	−16·1% (−39·2 to 35·4)	1·8 (1·4 to 2·4)	1·1 (0·7 to 1·5)	−40·4% (−56·8 to −2·3)*	5811·2 (4271·2 to 8200·8)	4282·0 (2889·5 to 6483·0)	−26·4% (−49·5 to 24·3)	94·1 (69·5 to 132·4)	53·4 (35·7 to 82·7)	−43·3% (−61·0 to −2·9)*
		Adverse effects of medical treatment	99·3 (77·6 to 127·7)	102·5 (85·3 to 128·6)	3·2% (−23·6 to 39·0)	1·9 (1·5 to 2·4)	1·2 (1·0 to 1·5)	−35·4% (−51·7 to −12·3)*	4425·4 (3389·4 to 5958·3)	3802·5 (2940·0 to 5042·3)	−14·1% (−40·4 to 22·0)	75·8 (58·4 to 101·4)	49·0 (37·1 to 65·9)	−35·4% (−54·7 to −6·9)*
		Animal contact	102·2 (61·8 to 150·3)	103·1 (61·8 to 148·7)	0·9% (−35·2 to 67·1)	1·7 (1·1 to 2·6)	1·2 (0·7 to 1·8)	−28·5% (−53·8 to 18·6)	5869·1 (3456·8 to 8754·7)	5034·6 (3000·3 to 7390·9)	−14·2% (−45·0 to 43·4)	95·1 (56·1 to 141·9)	64·1 (38·0 to 94·8)	−32·6% (−56·7 to 12·6)
		Venomous animal contact	92·4 (55·5 to 137·8)	93·7 (56·3 to 137·1)	1·4% (−35·7 to 67·2)	1·6 (0·9 to 2·3)	1·1 (0·7 to 1·7)	−28·0% (−54·0 to 18·9)	5338·7 (3104·6 to 8048·5)	4557·1 (2701·9 to 6798·8)	−14·6% (−46·6 to 42·3)	86·3 (50·3 to 130·4)	57·8 (34·0 to 87·1)	−33·0% (−58·3 to 11·6)
		Non-venomous animal contact	9·8 (5·9 to 18·1)	9·5 (5·2 to 14·9)	−3·5% (−45·4 to 70·9)	0·2 (0·1 to 0·3)	0·1 (0·1 to 0·2)	−32·8% (−61·6 to 19·6)	530·3 (300·5 to 1064·2)	477·5 (242·7 to 779·7)	−9·9% (−51·6 to 67·8)	8·8 (5·0 to 17·5)	6·2 (3·1 to 10·4)	−29·0% (−61·7 to 33·1)
	Foreign body		102·9 (79·8 to 125·1)	119·8 (91·3 to 148·7)	16·5% (−3·4 to 35·3)	1·9 (1·5 to 2·3)	1·5 (1·1 to 1·9)	−19·1% (−32·2 to −4·5)*	6041·1 (4342·0 to 7516·9)	5517·9 (3934·6 to 7185·8)	−8·7% (−28·9 to 12·5)	100·7 (72·9 to 124·7)	77·0 (54·0 to 100·7)	−23·6% (−40·7 to −4·7)*
		Pulmonary aspiration and foreign body in airway	99·3 (77·0 to 119·7)	117·6 (90·0 to 144·7)	18·4% (−1·0 to 37·8)	1·8 (1·5 to 2·2)	1·5 (1·1 to 1·9)	−17·8% (−31·1 to −2·8)*	5818·3 (4173·9 to 7253·5)	5414·8 (3882·7 to 6977·6)	−6·9% (−27·9 to 15·4)	97·1 (70·1 to 120·4)	75·6 (53·3 to 98·9)	−22·1% (−39·9 to −1·9)*
		Foreign body in other body part	3·6 (1·5 to 5·6)	2·3 (1·3 to 3·8)	−36·6% (−63·1 to 3·7)	0·1 (0·0 to 0·1)	0·0 (0·0 to 0·0)	−55·6% (−74·0 to −29·3)*	222·8 (80·8 to 360·1)	103·0 (49·1 to 181·1)	−53·8% (−76·0 to −22·9)*	3·7 (1·3 to 5·9)	1·4 (0·6 to 2·4)	−62·6% (−80·7 to −39·4)*
	Electrocution		56·7 (29·5 to 88·4)	42·4 (22·0 to 57·4)	−25·2% (−45·3 to 12·6)	0·9 (0·5 to 1·4)	0·5 (0·3 to 0·7)	−41·8% (−57·5 to −11·6)*	3443·9 (1765·2 to 5304·9)	2525·8 (1306·1 to 3478·2)	−26·7% (−47·1 to 8·0)	54·1 (27·8 to 83·6)	32·6 (16·7 to 45·4)	−39·8% (−56·8 to −10·4)*
	Environmental heat and cold exposure		56·3 (42·6 to 69·6)	83·4 (71·5 to 95·7)	48·1% (20·8 to 80·9)*	1·0 (0·8 to 1·3)	0·9 (0·8 to 1·1)	−9·2% (−25·8 to 10·4)	2292·9 (1710·6 to 2936·1)	2650·0 (2235·1 to 3102·6)	15·6% (−5·8 to 43·1)	39·7 (29·7 to 50·5)	30·6 (25·6 to 36·1)	−22·9% (−37·2 to −5·4)*
	Exposure to forces of nature		9·0 (8·2 to 9·9)	88·6 (80·7 to 97·4)	882·0% (882·0 to 882·0)*	0·2 (0·1 to 0·2)	1·1 (1·0 to 1·2)	598·5% (598·5 to 598·5)*	545·5 (496·7 to 599·7)	4274·0 (3891·6 to 4698·5)	683·5% (683·5 to 683·5)*	8·8 (8·0 to 9·7)	56·8 (51·7 to 62·4)	543·4% (543·4 to 543·4)*
	Other unintentional injuries		103·4 (59·7 to 176·9)	65·2 (39·7 to 104·3)	−36·9% (−64·3 to 17·1)	1·7 (1·0 to 2·9)	0·8 (0·5 to 1·3)	−53·8% (−73·8 to −14·8)*	5914·5 (3294·4 to 10 442·9)	3406·8 (2026·0 to 5684·8)	−42·4% (−69·2 to 8·5)	94·5 (53·0 to 166·1)	43·1 (25·5 to 72·6)	−54·4% (−75·6 to −14·7)*
	**Self-harm and interpersonal violence**		**1399·6 (1273·1 to 1514·3)**	**1370·4 (1252·7 to 1496·8)**	**–2·0% (−10·9 to 7·7)**	**23·4 (21·3 to 25·3)**	**16·4 (15·0 to 17·9)**	–29·9% (−36·4 to −22·8)*	**73 359·9 (66 978·1 to 79 476·6)**	**68 776·6 (62 700·9 to 75 042·7)**	**–6·2% (−14·7 to 3·9)**	**1173·1 (1069·1 to 1271·3)**	**847·1 (771·6 to 925·4)**	–27·8% (−34·5 to −19·8)*
	Self-harm		819·5 (707·3 to 905·0)	766·7 (675·7 to 857·9)	−6·3% (−17·0 to 6·2)	14·1 (12·2 to 15·6)	9·0 (7·9 to 10·1)	−36·4% (−43·6 to −27·9)*	39 034·8 (33 419·7 to 43 263·2)	34 358·5 (29 835·6 to 38 613·6)	−11·9% (−23·2 to 0·1)	636·2 (544·4 to 704·3)	413·9 (358·2 to 465·7)	−34·9% (−43·2 to −26·4)*
		Self-harm by firearm	67·3 (50·2 to 98·4)	66·6 (51·5 to 86·1)	−1·1% (−24·1 to 35·0)	1·2 (0·9 to 1·7)	0·8 (0·6 to 1·0)	−32·8% (−47·6 to −9·7)*	3262·0 (2317·0 to 4873·2)	2910·7 (2156·2 to 3887·0)	−10·8% (−34·1 to 25·9)	53·0 (38·1 to 78·9)	34·9 (25·8 to 46·8)	−34·1% (−50·6 to −7·8)*
		Self-harm by other specified means	752·2 (642·7 to 839·1)	700·2 (609·7 to 787·9)	−6·8% (−18·3 to 6·6)	13·0 (11·1 to 14·5)	8·2 (7·1 to 9·3)	−36·7% (−44·5 to −27·8)*	35 772·8 (30 493·1 to 39 978·1)	31 447·9 (26 807·0 to 35 588·2)	−12·0% (−23·9 to 0·5)	583·2 (498·2 to 651·3)	378·9 (322·4 to 428·9)	−35·0% (−43·9 to −25·8)*
	Interpersonal violence		464·3 (420·2 to 539·4)	435·7 (384·1 to 507·9)	−6·2% (−21·5 to 12·1)	7·5 (6·8 to 8·7)	5·3 (4·7 to 6·2)	−28·9% (−40·6 to −15·0)*	26 564·8 (23 942·0 to 31 034·3)	24 148·6 (21 130·2 to 28 446·6)	−9·1% (−24·0 to 9·0)	418·9 (377·6 to 489·5)	302·4 (264·5 to 358·0)	−27·8% (−39·7 to −13·2)*
		Physical violence by firearm	162·0 (145·0 to 185·9)	185·6 (167·5 to 209·2)	14·6% (−3·7 to 34·1)	2·6 (2·3 to 2·9)	2·3 (2·1 to 2·6)	−11·1% (−25·5 to 3·8)	9524·1 (8520·1 to 10 959·9)	10 535·3 (9479·3 to 11 925·5)	10·6% (−7·5 to 29·9)	148·0 (132·4 to 170·4)	131·2 (118·0 to 148·6)	−11·4% (−25·9 to 3·9)
		Physical violence by sharp object	116·7 (94·2 to 140·4)	89·9 (67·3 to 125·8)	−23·0% (−42·0 to 7·1)	1·9 (1·5 to 2·3)	1·1 (0·8 to 1·5)	−42·2% (−56·5 to −19·5)*	6511·2 (5234·5 to 7903·5)	4848·9 (3572·3 to 6814·3)	−25·5% (−44·6 to 3·9)	102·7 (82·6 to 124·4)	60·0 (44·0 to 84·5)	−41·6% (−56·6 to −18·3)*
		Physical violence by other means	185·6 (155·8 to 236·6)	160·2 (125·3 to 204·7)	−13·7% (−35·1 to 16·4)	3·1 (2·6 to 3·9)	2·0 (1·5 to 2·5)	−35·7% (−51·5 to −13·1)*	10 529·4 (8776·0 to 13 612·8)	8764·4 (6699·7 to 11 310·6)	−16·8% (−37·8 to 14·9)	168·2 (140·5 to 216·7)	111·2 (84·4 to 144·2)	−33·9% (−50·5 to −8·5)*
	Conflict and terrorism		108·7 (94·2 to 145·1)	159·1 (125·6 to 208·6)	46·3% (31·7 to 54·5)*	1·7 (1·4 to 2·2)	2·0 (1·6 to 2·6)	19·3% (7·7 to 25·9)*	7348·6 (6362·3 to 9833·7)	9771·2 (7782·4 to 12 730·0)	33·0% (20·6 to 39·9)*	111·5 (96·5 to 149·2)	124·6 (99·5 to 162·0)	11·7% (1·6 to 17·4)*
		Police conflict and executions	7·1 (5·5 to 9·1)	8·9 (6·3 to 13·3)	25·8% (−19·5 to 74·9)	0·1 (0·1 to 0·1)	0·1 (0·1 to 0·2)	−3·3% (−38·1 to 36·2)	411·7 (325·1 to 527·2)	498·2 (349·4 to 747·7)	21·0% (−22·3 to 68·8)	6·4 (5·1 to 8·3)	6·2 (4·4 to 9·3)	−3·8% (−38·1 to 35·3)
**Total cancers**			**7002·9 (6615·8 to 7281·6)**	**10 443·4 (9608·6 to 11 041·7)**	49·0% (41·0 to 57·5)*	**142·5 (134·4 to 148·4)**	**115·6 (106·3 to 122·1)**	–18·9% (−22·7 to −14·5)*	**195 148·9 (185 805·2 to 202 090·3)**	**264 464·0 (249 927·7 to 277 556·8)**	35·4% (29·4 to 43·6)*	**3696·7 (3518·2 to 3830·4)**	**2950·4 (2789·1 to 3094·1)**	–20·2% (−23·7 to −15·4)*
**Total burden related to hepatitis B**			**586·6 (522·9 to 656·6)**	**628·4 (537·2 to 707·3)**	**7·1% (−7·8 to 24·1)**	**11·0 (9·9 to 12·4)**	**7·0 (6·0 to 7·9)**	–36·3% (−45·4 to −25·8)*	**21 517·4 (19 085·4 to 24 352·8)**	**21 106·8 (18 086·9 to 23 933·9)**	**–1·9% (−17·8 to 15·7)**	**382·5 (338·7 to 429·2)**	**241·8 (207·1 to 274·7)**	–36·8% (−47·0 to −25·3)*
**Total burden related to hepatitis C**			**387·4 (339·4 to 445·3)**	**497·2 (426·4 to 566·3)**	28·3% (12·2 to 48·2)*	**7·6 (6·6 to 8·7)**	**5·5 (4·7 to 6·3)**	–27·2% (−36·3 to −15·8)*	**12 328·0 (10 658·9 to 14 400·8)**	**14 408·7 (12 311·5 to 16 728·9)**	16·9% (0·1 to 36·2)*	**226·2 (196·2 to 261·9)**	**161·6 (138·3 to 187·6)**	–28·6% (−38·5 to −16·3)*
**Total burden related to non-alcoholic fatty liver disease**			**72·7 (57·5 to 91·2)**	**131·5 (103·4 to 164·9)**	80·8% (62·1 to 98·8)*	**1·5 (1·2 to 1·8)**	**1·4 (1·1 to 1·8)**	**–1·1% (−10·7 to 8·9)**	**2076·1 (1604·1 to 2619·0)**	**3505·5 (2755·4 to 4412·3)**	68·9% (52·9 to 86·0)*	**39·3 (30·4 to 49·7)**	**38·8 (30·6 to 48·9)**	**–1·2% (−11·1 to 9·6)**
**Total cancers excluding non-melanoma skin cancer**			**6972·7 (6588·6 to 7250·5)**	**10 379·5 (9553·2 to 10 971·7)**	48·7% (40·8 to 57·3)*	**141·8 (133·8 to 147·8)**	**114·9 (105·7 to 121·3)**	–19·0% (−22·9 to −14·6)*	**194 470·7 (185 196·6 to 201 427·7)**	**263 224·6 (248 820·8 to 276 246·5)**	35·3% (29·2 to 43·4)*	**3683·1 (3505·6 to 3817·3)**	**2936·6 (2777·0 to 3078·5)**	–20·3% (−23·8 to −15·5)*

Values in parentheses are 95% uncertainty intervals. G6PD=glucose-6-phosphate dehydrogenase. NASH=non-alcoholic steatohepatitis. YLLs=years of life lost.

* Statistically significant percentage changes.

### Causes of death

**Figure 1:**
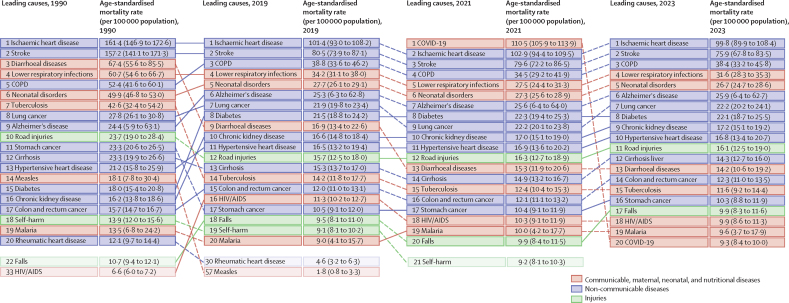
Leading Level 3 causes of global deaths and age-standardised mortality rate per 100 000 population for all sexes combined, 1990, 2019, 2021, and 2023 The 20 leading causes of death are shown in descending order. Causes are connected by lines between time periods; solid lines represent an increase or lateral shift in rank and dashed lines represent decreases in rank. Alzheimer's disease=Alzheimer's disease and other dementias. Cirrhosis=cirrhosis and other chronic liver diseases. COPD=chronic obstructive pulmonary disease. Lung cancer=tracheal, bronchus, and lung cancer.

Figure 1 shows the global rankings of the leading Level 3 causes of age-standardised mortality rates over the period studied. From 1990 to 2023, ischaemic heart disease and stroke consistently ranked as the first and second leading causes, respectively—except in 2021, when COVID-19 temporarily ranked as the leading cause of age-standardised deaths. In 2021, the rankings of the leading five Level 3 causes, in descending order, were COVID-19, ischaemic heart disease, stroke, chronic obstructive pulmonary disease (COPD), and lower respiratory infections. In 2023, COVID-19 dropped to the 20th leading cause of death, with ischaemic heart disease, stroke, COPD, lower respiratory infections, and neonatal disorders ranking as the leading five causes. Although the rankings of the leading two causes of death in 2023, ischaemic heart disease and stroke, were the same as they were in 1990, the age-standardised mortality rates for each have decreased: ischaemic heart disease decreased from 161·4 deaths (95% UI 146·9–172·6) per 100 000 population in 1990, to 99·8 deaths (89·9–108·4) per 100 000 in 2023; while stroke declined from 157·2 deaths (141·1–171·3) per 100 000 in 1990 to 75·9 deaths (67·8–83·5) per 100 000 in 2023. Other notable shifts in the rankings of leading causes of death have occurred over the past three decades. Four causes showed declines in age-standardised mortality rates between 1990 and 2023: diarrhoeal diseases (67·4 deaths [55·6–85·5] per 100 000 in 1990 to 14·2 deaths [10·6–19·2] per 100 000 in 2023), tuberculosis (42·6 deaths [32·4–54·2] per 100 000 in 1990 to 11·6 deaths [9·2–14·4] per 100 000 in 2023), stomach cancer (23·3 deaths [20·6–26·5] per 100 000 in 1990 to 10·3 deaths [8·8–11·9] per 100 000 in 2023), and measles (18·1 deaths [7·8–30·4] per 100 000 in 1990 to 2·2 deaths [0·9–3·9] per 100 000 in 2023). By contrast, some causes exhibited an increase in age-standardised mortality rates between 1990 and 2023, such as diabetes, chronic kidney disease, Alzheimer's disease and other dementias, and HIV/AIDS.

**Figure 2:**
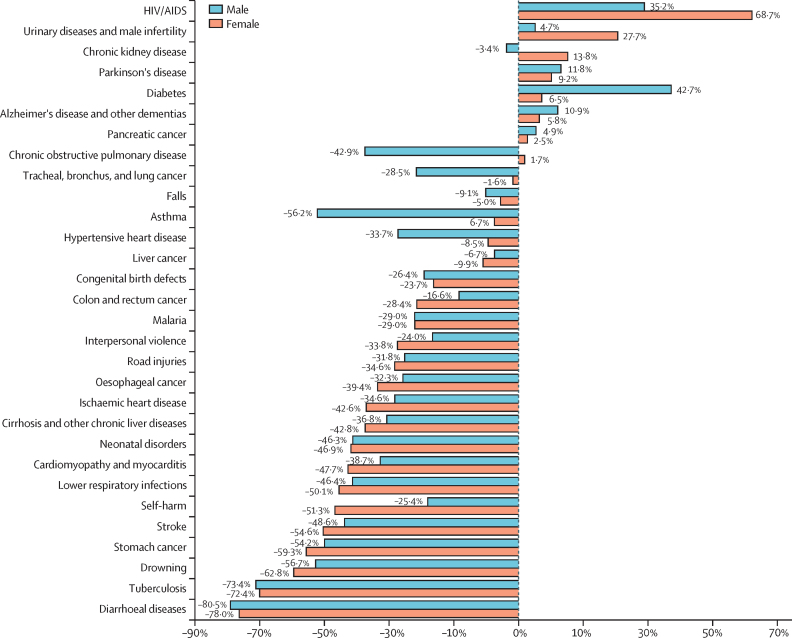
Percentage change in global age-standardised mortality rate from 1990 to 2023 among the leading 30 Level 3 causes of death, for males and females Figure shows the top 30 causes according to their global age-standardised mortality rate, sorted by percentage change from 1990 to 2023 in females, in descending order. COVID-19 and causes affecting only one sex (ie, cervical cancer) were omitted.

The percentage change among leading Level 3 causes of death varied over the study period between females and males at the global level (figure 2). The age-standardised mortality rates for HIV/AIDS, urinary diseases, chronic kidney disease, and COPD increased more among females than males. Likewise, those for falls, asthma, and hypertensive heart disease decreased more among males than females. Deaths from conflict and terrorism were particularly disparate by sex and location (appendix 2 table S18). Between 1995 and 2023, 51·3% (95% UI 45·9–53·9) of female deaths due to conflict and terrorism occurred in north Africa and the Middle East, despite only 7·2% of the global female population residing in this region. Eastern Europe contributed 7·5% (6·3–8·3) of female deaths from this cause, despite having only 3·4% of the global female population. Over the same time period, 52·0% (46·3–54·7) of male deaths due to conflict and terrorism occurred in north Africa and the Middle East, which accounts for only 7·7% of the global male population, and 8·0% (6·6–9·1) in eastern Europe, with only 2·9% of the global male population (appendix 2 table S18). In 2023, Palestine had the highest age-standardised mortality rate due to conflict and terrorism of any country in the world (385·8 deaths [351·3–424·1] per 100 000 population), more than five times that of the second-leading country, Ukraine (70·1 deaths [67·2–73·2] per 100 000). Sudan ranks as the third highest country in terms of age-standardised mortality rates for conflict and terrorism, while Russia and Burkina Faso follow as the fourth and fifth leading countries, in 2023 (appendix 2 table S7).

### Causes of YLLs

Over the study period, neonatal disorders remained the leading Level 3 cause of global YLLs, despite a decrease in age-standardised YLL rates, from 4487·8 YLLs (95% UI 4212·3–4761·6) per 100 000 population in 1990 to 2398·9 YLLs (2219·7–2575·4) per 100 000 in 2023, representing a decrease of 46·5% (–51·2 to –41·6; appendix 2 table S13). In 2021, however, COVID-19 temporarily surpassed neonatal disorders as the leading cause of global age-standardised YLLs, before dropping to the 25th position in 2023 (appendix figure S2). Since the year 2000, there has been a reduction of 37·6% (32·6–42·6) in total YLLs due to neonatal disorders, from 235 000 in 2000 to 146 000 in 2023 (table 1). In 1990, the total YLLs for vaccine-preventable diseases—including diphtheria, pertussis, tetanus, measles, varicella and herpes zoster, yellow fever, rabies, liver cancer due to hepatitis B, cervical cancer, chronic hepatitis B with cirrhosis, and acute hepatitis B—amounted to a sum of 178 million (95% UI 122–239) years (appendix 2 table S4). By 2023, this number had decreased by 66·5% (57·4–71·9) to 59·8 million (49·6–72·1) years. Similarly, for other preventable diseases that went through major international cooperation and large-scale interventions between 1990 and 2023, total YLLs also showed a decline. In 1990, total YLLs for these diseases were 1·07 billion (1·01–1·12) years; by 2023, they had decreased by 51·6% (48·8–54·4) to 516 million (488–543) years (appendix 2 table S4).

### COVID-19

Between 2020 and 2023, COVID-19 had an immense global impact, resulting in 18·0 million (95% UI 17·2–18·7) total deaths (appendix 2 table S5). Among these, 10·5 million (10·1–10·9) deaths were in males, and 7·53 million (6·98–7·96) deaths were in females. During 2020, the first year of the pandemic, there were 5·47 million (5·22–5·65) COVID-19 deaths. The highest number of COVID-19 deaths was recorded in 2021 (9·42 million [9·04–9·70] deaths) followed by 2·36 million (2·19–2·47) deaths in 2022, and the lowest number of deaths occurred in 2023 (797 000 deaths [723 000–857 000]). The age group most affected by COVID-19 varied over the years: the highest numbers of deaths occurred in the 70–74 years age group in 2020 and 2021, but in the 80–84 years age group in 2022, and in the 85–89 years age group in 2023 (appendix 2 figure S3). From 2020 to 2023, the five countries with the highest numbers of total COVID-19 deaths (in descending order) were India (3·08 million [2·92–3·28] deaths), the USA (1·21 million [1·10–1·27] deaths), Russia (1·06 million [1·00–1·09] deaths), Indonesia (849 000 deaths [780 000–920 000]), and Brazil (795 000 deaths [748 000–821 000]; appendix 2 table S5). In mortality rates, the countries with the highest burden (in descending order) were Tunisia (245·4 deaths [227·9–256·9] per 100 000), Bolivia (229·0 deaths [215·5–241·4] per 100 000), Peru (220·6 deaths [213·4–228·5] per 100 000), Montenegro (215·2 deaths [197·9–229·3] per 100 000), and Moldova (207·3 deaths [199·4–212·7] per 100 000; appendix 2 table S5).

### Changes in age-specific mortality rate from 2000 to 2023

Globally, between 2000 and 2023, all 5-year age groups from 10 years to 70 years showed decreases in age-specific mortality rates, ranging from –27·1% to –35·0% (table 2; appendix 2 table S19). However, this trend varied by country, region, sex, and cause of death. Among the 21 GBD regions, only four—high-income Asia Pacific, central Asia, east Asia, and south AsiA&Mdash;consistently showed decreases in death rates across all 5-year age groups by sex and Level 1 causes from 2000 to 2023. By contrast, the Caribbean region showed increases in death rates for 40 distinct Level 1 cause-sex-age groups; the largest of these increases was observed in females aged 30–34 years, in whom deaths from NCDs increased by 52·6%, and in females aged 25–29 years, in whom NCD deaths rose by 48·9%. Similarly, in high-income North America, there was an increase in death rates across 37 distinct Level 1 cause-sex-age groups. The most notable rise was in males aged 25–29 years, where NCD-related deaths rose by 130·5%, while injury-related deaths increased by 26·4% in males aged 60–64 years. Globally, we found the increase in NCD deaths was primarily due to drug use disorders, while the rise in injury-related deaths was linked to intentional injuries. Other notable injury-related increases around the globe include the conflicts in Palestine and Ukraine, as well as specific natural disasters such as the 2023 earthquake in Türkiye and the 2022–23 heatwaves in Europe (appendix 2 table S19).

**Table 2:** Percentage change in age-specific mortality rate between 2000 and 2023 for Level 1 causes

		**Age 10–14 years**	**Age 15–19 years**	**Age 20–24 years**	**Age 25–29 years**	**Age 30–34 years**	**Age 35–39 years**	**Age 40–44 years**	**Age 45–49 years**	**Age 50–54 years**	**Age 55–59 years**	**Age 60–64 years**	**Age 65–69 years**
**Communicable, maternal, neonatal, and nutritional diseases**													
Caribbean													
	Male	−32·0%	−22·6%	−33·9%	−46·5%	−50·7%	−53·6%	−53·5%	−49·7%	−39·8%	−32·1%	−29·3%	−26·5%
	Female	−23·6%	−16·3%	−24·6%	−38·1%	−36·2%	−35·2%	−38·5%	−36·6%	−37·9%	−35·7%	−33·2%	−26·8%
High-income North America													
	Male	−11·1%	−22·5%	−20·9%	−38·1%	−54·0%	−62·6%	−56·2%	−46·4%	−16·2%	13·8%	33·1%	37·1%
	Female	−23·7%	−21·6%	−18·5%	−20·0%	−24·4%	−26·6%	−23·8%	−12·4%	23·1%	34·1%	44·1%	47·7%
Central sub-Saharan Africa													
	Male	−59·9%	−66·8%	−67·2%	−65·6%	−64·5%	−61·5%	−61·0%	−57·0%	−53·6%	−52·5%	−50·0%	−49·2%
	Female	−56·6%	−52·3%	−56·3%	−60·4%	−61·1%	−59·9%	−56·3%	−53·0%	−52·2%	−52·3%	−49·8%	−47·5%
Oceania													
	Male	−34·2%	−30·9%	−18·5%	−18·6%	−20·0%	−16·9%	−16·4%	−15·9%	−19·1%	−19·2%	−26·2%	−26·1%
	Female	−32·1%	−27·4%	−13·1%	−5·1%	−0·7%	−4·8%	−7·9%	−10·8%	−11·7%	−10·2%	−15·2%	−12·9%
Western sub-Saharan Africa													
	Male	−48·0%	−43·3%	−46·4%	−53·9%	−59·6%	−59·0%	−57·3%	−54·2%	−50·6%	−48·9%	−46·6%	−46·8%
	Female	−44·8%	−48·6%	−56·7%	−58·2%	−56·6%	−53·7%	−51·6%	−48·6%	−48·9%	−48·4%	−49·7%	−48·1%
Southern sub-Saharan Africa													
	Male	−9·0%	37·4%	−42·0%	−72·8%	−75·8%	−73·9%	−63·8%	−58·0%	−50·0%	−42·7%	−41·7%	−40·5%
	Female	−16·9%	−37·9%	−71·6%	−81·3%	−75·6%	−68·3%	−55·9%	−52·4%	−51·9%	−51·2%	−46·5%	−39·2%
Southern Latin America													
	Male	−19·0%	−6·0%	−17·0%	−42·1%	−49·4%	−26·0%	−5·2%	16·8%	46·7%	49·7%	53·9%	59·5%
	Female	−20·8%	−13·5%	−17·5%	−25·0%	−21·5%	−1·6%	28·6%	47·0%	67·2%	83·6%	96·7%	110·6%
Tropical Latin America													
	Male	−20·8%	−8·7%	−10·6%	−27·0%	−42·0%	−40·7%	−33·5%	−26·7%	−17·7%	−10·4%	−4·7%	6·8%
	Female	−28·0%	−21·0%	−24·1%	−32·7%	−37·1%	−28·2%	−20·8%	−15·3%	−10·9%	−11·6%	−7·6%	6·1%
Eastern sub-Saharan Africa													
	Male	−63·2%	−61·4%	−61·9%	−71·3%	−76·1%	−78·4%	−76·1%	−71·7%	−63·4%	−59·5%	−57·1%	−57·9%
	Female	−64·5%	−64·6%	−72·2%	−78·8%	−79·0%	−78·6%	−73·3%	−68·3%	−62·8%	−60·7%	−59·2%	−57·8%
North Africa and Middle East													
	Male	−52·1%	−30·7%	−27·9%	−35·2%	−37·5%	−41·1%	−43·0%	−46·0%	−44·5%	−39·1%	−39·7%	−27·7%
	Female	−55·9%	−41·5%	−44·4%	−45·0%	−44·3%	−46·1%	−43·8%	−38·3%	−39·4%	−36·3%	−36·9%	−22·8%
Eastern Europe													
	Male	−40·5%	−57·4%	−78·1%	−62·0%	−35·7%	−30·7%	−36·1%	−50·2%	−59·0%	−56·0%	−47·0%	−21·4%
	Female	−33·3%	−41·3%	−44·9%	−2·3%	35·7%	45·0%	44·2%	14·7%	−3·6%	−6·1%	20·0%	53·1%
Central Latin America													
	Male	−33·7%	−22·7%	−30·0%	−31·5%	−30·5%	−28·8%	−20·9%	−15·9%	−11·2%	−8·7%	−11·6%	−13·2%
	Female	−35·2%	−26·8%	−28·7%	−27·7%	−22·1%	−23·0%	−18·1%	−11·6%	−15·4%	−10·7%	−13·6%	−15·8%
Central Europe													
	Male	−67·2%	−17·0%	−6·0%	−13·4%	−19·1%	−18·1%	−27·3%	−18·5%	4·4%	14·0%	36·9%	63·4%
	Female	−63·5%	−26·0%	−24·9%	−22·4%	−25·8%	−19·1%	−16·2%	−7·4%	19·7%	28·8%	51·8%	64·7%
Western Europe													
	Male	−46·9%	−51·5%	−56·4%	−68·3%	−73·7%	−74·3%	−63·3%	−46·0%	−30·3%	−21·1%	−10·7%	−11·7%
	Female	−43·6%	−49·9%	−42·2%	−52·3%	−57·0%	−55·5%	−44·9%	−34·5%	−23·7%	−18·3%	−9·8%	−9·7%
Southeast Asia													
	Male	−50·6%	−47·0%	−43·6%	−54·7%	−58·0%	−51·3%	−42·9%	−36·0%	−38·5%	−37·7%	−40·2%	−36·3%
	Female	−49·9%	−52·5%	−50·6%	−53·1%	−51·4%	−44·0%	−45·7%	−41·5%	−43·1%	−42·4%	−45·9%	−41·1%
Andean Latin America													
	Male	−39·1%	−39·2%	−39·2%	−40·5%	−40·0%	−35·9%	−29·6%	−25·3%	−18·9%	−3·7%	−3·7%	−1·3%
	Female	−36·7%	−47·2%	−46·4%	−46·7%	−45·6%	−42·1%	−36·7%	−30·9%	−28·6%	−18·4%	−19·9%	−14·4%
Australasia													
	Male	−29·1%	−50·8%	−44·1%	−66·6%	−62·6%	−66·8%	−57·2%	−46·0%	−35·1%	−30·4%	−29·9%	−29·7%
	Female	−42·0%	−50·7%	−38·6%	−45·4%	−49·7%	−40·4%	−21·0%	−17·5%	−17·0%	−15·1%	−21·3%	−27·1%
South Asia													
	Male	−74·2%	−71·2%	−71·8%	−73·1%	−70·3%	−70·2%	−65·8%	−68·1%	−68·4%	−65·7%	−68·1%	−69·1%
	Female	−70·6%	−75·6%	−76·2%	−74·3%	−71·0%	−70·6%	−67·7%	−65·3%	−67·5%	−59·9%	−67·3%	−67·6%
High-income Asia Pacific													
	Male	−54·1%	−59·7%	−50·2%	−49·5%	−58·8%	−64·0%	−64·0%	−57·1%	−46·3%	−45·3%	−38·0%	−39·2%
	Female	−57·1%	−52·2%	−45·3%	−51·3%	−58·3%	−49·4%	−49·3%	−47·2%	−44·0%	−47·1%	−46·4%	−49·9%
East Asia													
	Male	−60·6%	−56·6%	−38·2%	−22·9%	−24·3%	−24·0%	−24·2%	−24·4%	−30·2%	−40·6%	−45·2%	−42·4%
	Female	−69·8%	−71·9%	−72·5%	−69·7%	−67·5%	−56·1%	−46·4%	−38·3%	−43·4%	−51·8%	−52·8%	−46·9%
Central Asia													
	Male	−48·9%	−57·3%	−75·4%	−79·2%	−75·0%	−66·7%	−61·7%	−60·5%	−60·1%	−50·1%	−39·9%	−19·0%
	Female	−48·5%	−53·6%	−65·4%	−68·0%	−62·3%	−53·8%	−50·6%	−47·2%	−47·2%	−39·8%	−23·1%	−7·5%
**Non-communicable diseases**													
Caribbean													
	Male	−4·4%	2·1%	3·1%	14·9%	17·8%	19·3%	17·8%	9·8%	2·7%	5·1%	3·5%	3·7%
	Female	13·0%	8·0%	17·4%	48·9%	52·6%	44·6%	27·5%	13·1%	2·5%	4·1%	−0·9%	−1·9%
High-income North America													
	Male	−9·7%	29·5%	79·3%	130·5%	122·5%	70·4%	24·1%	−3·6%	−9·2%	−14·0%	−18·7%	−26·4%
	Female	−9·5%	16·3%	50·5%	67·0%	63·3%	30·4%	10·9%	−2·7%	−8·8%	−15·1%	−18·9%	−26·0%
Central sub-Saharan Africa													
	Male	−13·9%	−30·2%	−31·7%	−28·2%	−15·7%	14·5%	9·4%	1·5%	0·2%	−1·7%	5·6%	8·8%
	Female	−4·7%	−6·6%	−9·5%	30·0%	56·5%	81·1%	71·3%	68·9%	49·2%	26·2%	10·2%	7·3%
Oceania													
	Male	−12·8%	−18·5%	−1·1%	16·1%	10·0%	3·3%	−4·3%	−11·1%	−8·6%	−8·5%	−10·9%	−12·2%
	Female	−13·4%	−17·7%	0·9%	28·0%	46·9%	31·2%	14·8%	5·9%	4·1%	3·0%	−1·8%	−1·2%
Western sub-Saharan Africa													
	Male	−18·3%	−19·6%	−22·3%	−19·7%	−11·2%	11·4%	5·3%	−6·2%	−8·0%	−10·5%	−4·2%	−1·3%
	Female	−23·0%	−24·9%	−30·5%	1·2%	16·3%	38·2%	24·3%	28·2%	18·8%	4·1%	−5·0%	−7·3%
Southern sub-Saharan Africa													
	Male	−15·6%	−27·8%	−26·1%	−21·2%	−16·4%	−2·7%	0·0%	−19·1%	−32·2%	−24·0%	−19·3%	−9·7%
	Female	−21·8%	−13·5%	−5·1%	8·9%	23·9%	44·8%	54·1%	56·5%	27·4%	9·5%	−8·3%	−7·5%
Southern Latin America													
	Male	−23·9%	−14·4%	−6·9%	−10·1%	−20·6%	−22·1%	−30·1%	−36·3%	−37·3%	−35·7%	−31·5%	−29·7%
	Female	−23·5%	−12·4%	−9·6%	−8·8%	−13·8%	−11·7%	−19·8%	−26·1%	−28·9%	−26·0%	−21·9%	−21·3%
Tropical Latin America													
	Male	1·3%	12·9%	14·0%	−0·3%	−15·8%	−24·2%	−30·4%	−30·1%	−27·5%	−26·7%	−25·6%	−23·2%
	Female	0·4%	2·9%	7·0%	3·2%	−6·9%	−16·0%	−23·3%	−26·5%	−27·7%	−28·2%	−28·5%	−25·8%
Eastern sub-Saharan Africa													
	Male	−20·1%	−26·8%	−30·6%	−24·9%	−17·1%	2·4%	−2·3%	−5·3%	−8·7%	−6·7%	−0·3%	0·7%
	Female	−27·9%	−32·0%	−30·5%	−0·4%	12·6%	22·5%	18·3%	35·1%	23·6%	12·2%	1·1%	0·2%
North Africa and Middle East													
	Male	−28·3%	−16·5%	−7·0%	−5·6%	−12·2%	−26·4%	−34·5%	−37·9%	−35·7%	−25·4%	−25·4%	−21·5%
	Female	−33·7%	−25·8%	−11·9%	−6·2%	−2·7%	−12·0%	−19·4%	−22·6%	−29·6%	−21·1%	−27·4%	−23·9%
Eastern Europe													
	Male	−28·8%	−39·7%	−62·2%	−49·1%	−30·6%	−28·6%	−35·6%	−40·2%	−39·1%	−38·0%	−38·8%	−35·5%
	Female	−32·2%	−37·8%	−39·7%	−26·8%	−16·5%	−18·7%	−25·8%	−36·0%	−39·1%	−40·8%	−42·0%	−43·0%
Central Latin America													
	Male	0·0%	7·6%	5·4%	5·5%	3·7%	−2·6%	−3·6%	−3·3%	−3·2%	−6·6%	−8·1%	−11·2%
	Female	2·9%	1·5%	−0·5%	−1·4%	3·5%	−1·6%	−4·9%	−9·0%	−13·2%	−16·1%	−18·8%	−20·5%
Central Europe													
	Male	−45·4%	−31·8%	−23·1%	−19·1%	−20·2%	−29·2%	−43·0%	−45·8%	−39·8%	−34·2%	−30·0%	−28·2%
	Female	−30·7%	−25·4%	−22·4%	−22·0%	−22·4%	−31·0%	−41·1%	−43·4%	−38·9%	−34·0%	−29·5%	−33·6%
Western Europe													
	Male	−30·0%	−33·7%	−31·5%	−29·4%	−24·1%	−24·4%	−34·3%	−40·1%	−34·4%	−32·9%	−31·2%	−32·3%
	Female	−20·4%	−22·0%	−19·0%	−21·8%	−18·6%	−24·4%	−31·2%	−32·8%	−26·9%	−23·8%	−19·2%	−21·3%
Southeast Asia													
	Male	−23·7%	−22·7%	−13·8%	−6·8%	−10·5%	−9·3%	−3·6%	3·4%	−0·8%	−5·8%	−13·1%	−16·7%
	Female	−14·3%	−10·2%	−5·1%	0·1%	7·4%	9·3%	8·1%	4·9%	1·4%	−0·9%	−8·2%	−13·3%
Andean Latin America													
	Male	−6·5%	−5·1%	−11·0%	−5·8%	−10·2%	−13·1%	−14·2%	−18·4%	−16·9%	−12·8%	−14·6%	−13·9%
	Female	14·0%	−5·6%	−7·0%	−5·2%	−3·2%	−5·8%	−11·2%	−15·3%	−16·9%	−14·5%	−19·7%	−16·0%
Australasia													
	Male	−35·5%	−43·8%	−42·7%	−46·5%	−32·2%	−23·6%	−20·9%	−19·1%	−22·7%	−28·7%	−34·7%	−42·5%
	Female	−33·4%	−40·1%	−32·6%	−35·6%	−33·2%	−25·2%	−24·1%	−20·8%	−22·3%	−27·5%	−31·6%	−36·0%
South Asia													
	Male	−39·5%	−47·3%	−44·6%	−41·5%	−33·4%	−27·3%	−17·6%	−17·8%	−17·9%	−9·5%	−15·4%	−16·2%
	Female	−39·7%	−44·6%	−35·1%	−26·9%	−11·7%	−10·6%	−10·0%	−13·1%	−7·1%	−0·2%	−9·4%	−7·5%
High-income Asia Pacific													
	Male	−32·9%	−35·2%	−33·1%	−37·3%	−45·0%	−50·2%	−52·1%	−44·7%	−41·9%	−41·9%	−38·0%	−33·7%
	Female	−28·0%	−25·4%	−23·4%	−30·9%	−37·0%	−30·6%	−31·2%	−26·8%	−32·8%	−35·9%	−35·8%	−36·4%
East Asia													
	Male	−47·6%	−49·4%	−57·8%	−54·2%	−52·2%	−46·1%	−43·1%	−38·0%	−37·9%	−45·0%	−44·1%	−47·8%
	Female	−49·5%	−58·1%	−65·5%	−66·7%	−64·4%	−61·2%	−59·1%	−55·9%	−55·6%	−60·5%	−59·4%	−57·8%
Central Asia													
	Male	−19·6%	−32·9%	−46·0%	−50·4%	−44·6%	−37·5%	−36·3%	−38·7%	−42·5%	−33·6%	−33·4%	−30·8%
	Female	−16·0%	−24·9%	−38·2%	−41·4%	−36·0%	−31·8%	−34·5%	−39·1%	−44·0%	−40·3%	−39·4%	−35·8%
**Injuries**													
Caribbean													
	Male	−5·2%	31·0%	26·4%	35·3%	36·8%	38·1%	34·5%	27·0%	13·1%	14·9%	10·9%	9·1%
	Female	10·1%	11·6%	24·2%	45·7%	42·4%	33·4%	20·0%	9·3%	−3·6%	−0·9%	−3·4%	−6·9%
High-income North America													
	Male	−23·3%	−21·9%	−19·2%	1·6%	20·0%	12·8%	8·9%	2·1%	13·1%	18·9%	26·4%	19·8%
	Female	−18·9%	−31·0%	−7·5%	6·3%	14·6%	0·6%	1·7%	1·6%	11·4%	11·5%	13·1%	10·5%
Central sub-Saharan Africa													
	Male	−4·9%	−23·1%	−23·2%	−15·9%	2·8%	39·5%	32·2%	18·1%	13·1%	7·7%	13·8%	15·1%
	Female	2·3%	5·1%	4·3%	51·8%	66·9%	83·8%	75·3%	73·4%	57·2%	36·2%	21·3%	23·8%
Oceania													
	Male	−19·8%	−24·8%	−6·3%	8·5%	1·0%	−3·9%	−9·3%	−13·1%	−9·1%	−8·4%	−10·4%	−11·7%
	Female	−12·6%	−15·9%	9·4%	30·6%	45·8%	29·9%	18·9%	10·1%	13·4%	11·0%	9·5%	10·7%
Western sub-Saharan Africa													
	Male	−19·2%	−19·4%	−23·4%	−21·6%	−12·9%	17·6%	8·6%	−4·9%	−8·0%	−11·8%	−1·4%	−1·5%
	Female	−27·6%	−33·0%	−30·2%	−1·7%	7·1%	22·6%	10·8%	14·6%	6·3%	−5·0%	−15·1%	−11·6%
Southern sub-Saharan Africa													
	Male	−38·2%	−37·0%	−26·2%	−20·4%	−16·5%	−5·2%	−6·7%	−25·6%	−38·5%	−32·9%	−30·1%	−23·6%
	Female	−42·4%	−16·7%	−11·0%	−2·2%	0·4%	10·5%	15·8%	17·3%	1·8%	−14·0%	−27·1%	−25·7%
Southern Latin America													
	Male	−53·0%	−31·2%	−22·0%	−21·1%	−24·3%	−25·9%	−30·6%	−36·1%	−38·0%	−38·9%	−37·6%	−35·3%
	Female	−45·7%	−26·3%	−15·1%	−15·0%	−22·8%	−24·4%	−28·8%	−30·9%	−35·7%	−33·5%	−32·8%	−31·4%
Tropical Latin America													
	Male	−47·2%	−14·1%	−6·2%	−6·5%	−11·5%	−14·8%	−17·7%	−17·1%	−13·3%	−12·5%	−11·7%	−5·6%
	Female	−38·7%	−14·9%	2·8%	5·2%	−1·9%	−8·4%	−10·3%	−11·8%	−12·6%	−15·3%	−14·2%	−7·5%
Eastern sub-Saharan Africa													
	Male	−27·8%	−63·3%	−55·1%	−35·1%	−24·8%	−4·8%	−7·1%	−14·7%	−17·3%	−22·4%	−11·0%	−2·3%
	Female	−47·2%	−49·0%	−50·0%	−20·7%	−17·5%	−10·8%	−8·2%	1·4%	−2·5%	−4·2%	−12·1%	−8·1%
North Africa and Middle East													
	Male	−9·1%	−0·1%	15·1%	−5·7%	−14·0%	−25·6%	−32·7%	−33·6%	−30·9%	−20·7%	−30·3%	−14·6%
	Female	30·4%	24·2%	55·2%	45·4%	50·3%	13·2%	−2·4%	−8·4%	−5·7%	26·5%	−3·1%	64·6%
Eastern Europe													
	Male	−48·7%	9·2%	−2·3%	−11·4%	−21·6%	−38·0%	−44·4%	−50·7%	−55·5%	−53·9%	−49·7%	−44·0%
	Female	1·1%	−30·6%	−37·0%	−45·1%	−47·6%	−50·4%	−49·8%	−52·0%	−54·8%	−56·0%	−51·3%	−46·6%
Central Latin America													
	Male	−35·2%	−24·5%	−21·5%	−13·7%	−7·8%	−12·1%	−15·1%	−21·0%	−24·7%	−29·3%	−31·7%	−32·6%
	Female	−19·9%	−12·2%	−4·6%	−0·5%	3·4%	−6·7%	−12·4%	−20·5%	−27·6%	−30·2%	−33·2%	−35·3%
Central Europe													
	Male	−64·9%	−51·2%	−45·5%	−41·2%	−38·6%	−40·4%	−43·9%	−41·6%	−30·1%	−23·7%	−15·3%	−15·8%
	Female	−52·9%	−43·3%	−43·0%	−40·2%	−38·1%	−42·7%	−46·5%	−43·0%	−32·6%	−29·9%	−28·4%	−30·7%
Western Europe													
	Male	−57·2%	−51·2%	−47·6%	−41·5%	−31·8%	−23·0%	−16·7%	−8·0%	8·4%	13·5%	13·9%	5·7%
	Female	−51·4%	−49·7%	−38·7%	−34·5%	−29·0%	−28·3%	−24·0%	−16·0%	−4·1%	1·1%	3·4%	0·4%
Southeast Asia													
	Male	−43·0%	−36·2%	−35·1%	−34·7%	−32·6%	−27·6%	−20·1%	−14·0%	−11·1%	−11·9%	−17·2%	−19·4%
	Female	−39·2%	−27·6%	−22·5%	−21·9%	−15·2%	−14·3%	−12·8%	−17·6%	−9·7%	−9·3%	−15·3%	−21·1%
Andean Latin America													
	Male	−30·6%	−12·1%	1·6%	11·2%	1·4%	−6·2%	−11·7%	−19·4%	−20·2%	−17·0%	−20·6%	−20·9%
	Female	−2·4%	−4·2%	0·5%	4·8%	0·5%	−6·7%	−12·7%	−20·8%	−26·0%	−23·1%	−26·5%	−22·8%
Australasia													
	Male	−51·4%	−53·3%	−46·9%	−48·2%	−40·4%	−36·8%	−26·1%	−8·8%	−3·8%	2·2%	−1·4%	−8·3%
	Female	−47·0%	−45·1%	−29·2%	−34·3%	−35·7%	−30·4%	−24·2%	−9·5%	−6·9%	−13·0%	−21·8%	−25·2%
South Asia													
	Male	−46·2%	−42·2%	−33·0%	−24·3%	−15·7%	−12·0%	−4·2%	−13·4%	−17·4%	−16·2%	−23·3%	−28·4%
	Female	−47·3%	−48·5%	−46·3%	−36·4%	−28·3%	−24·4%	−19·4%	−22·7%	−23·8%	−16·9%	−26·8%	−25·7%
High-income Asia Pacific													
	Male	−51·6%	−48·3%	−35·5%	−32·5%	−38·9%	−40·3%	−41·2%	−39·8%	−41·1%	−44·7%	−37·9%	−33·9%
	Female	−35·5%	−13·8%	−1·8%	−6·1%	−17·1%	−14·4%	−16·0%	−14·5%	−26·3%	−37·6%	−43·3%	−44·3%
East Asia													
	Male	−67·3%	−68·8%	−73·6%	−70·7%	−70·5%	−67·4%	−63·0%	−54·2%	−50·1%	−54·0%	−51·2%	−51·3%
	Female	−66·1%	−71·2%	−78·3%	−79·7%	−78·6%	−75·6%	−71·5%	−66·3%	−63·8%	−64·9%	−61·4%	−60·6%
Central Asia													
	Male	−36·8%	−39·4%	−50·7%	−54·3%	−52·2%	−49·8%	−46·8%	−45·2%	−52·5%	−44·3%	−43·1%	−33·3%
	Female	−34·1%	−19·6%	−40·4%	−49·3%	−48·6%	−47·7%	−47·4%	−51·6%	−57·6%	−53·0%	−52·8%	−44·8%

Regions are ordered by the total number of cause-age combinations that showed an increase across all three Level 1 causes.

### Mean age at death

The mean age at death for all causes varied by sex and location (appendix 2 tables S15, S16, S17). The global mean age at death increased from 46·8 years (95% UI 46·6–47·0) in 1990 to 63·4 years (63·1–63·7) in 2023, for all sexes combined. For males, the mean age at death in 1990 was 45·4 years (45·1–45·7) and increased to 61·2 years (60·7–61·6) in 2023. For females, it was 48·5 years (48·1–48·8) in 1990 and 65·9 years (65·5–66·3) in 2023. The highest mean age at death observed in 2023 was found in the high-income super-region. Within this super-region, mean age at death for females reached 80·9 years (80·9–81·0), and was even higher in the high-income Asia Pacific region (85·1 years [85·1–85·2]), with Japan having the highest mean among all countries globally (86·0 years [86·0–86·1]). For males in the high-income super-region, the mean age at death was 74·8 years (74·8–74·9). In high-income Asia-Pacific, it was 78·6 years (78·5–78·6), and Japan also recorded the highest male mean age at death at 79·8 years (79·8–79·8).

At the other end of the spectrum, the lowest mean age at death in 2023 occurred in sub-Saharan Africa, where females had a mean age at death of 38·0 years (95% UI 37·5–38·4; appendix 2 table S16). For males, it was 35·6 years (35·2–35·9; appendix 2 table S17). Within this super-region, western sub-Saharan Africa had a mean age at death of 33·2 years (32·5–34·0) for females and 31·9 years (31·2–32·6) for males. Niger recorded the lowest mean age at death, with 21·5 years (20·3–22·8) for females and 21·8 years (20·6–23·2) for males.

### Mean age at death by cause

**Figure 3:**
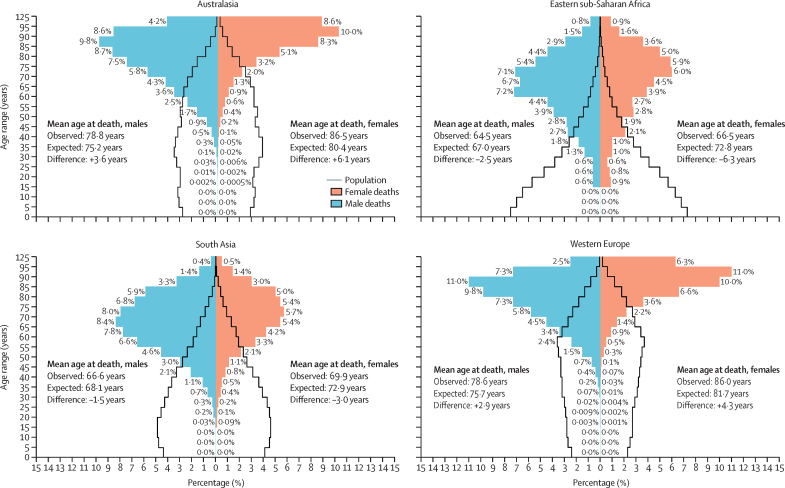
Comparison of age at death for ischaemic heart disease between four regions for males and females Graphs show the distribution of ischaemic heart disease deaths by age and sex within each region. Percentages represent the number of ischaemic heart disease deaths for a given age-sex group out of the total ischaemic heart disease deaths within a region (all ages and sexes combined), or the total number of individuals in a given age-sex group out of the total population in the region. The expected mean age at death is the result of calculating the mean age at death after applying the global mortality rate to a country's population by age and sex for a given cause; a positive difference indicates that the observed mean age at death is higher than expected.

In 2023, the gap between the observed and the expected mean age at death varied across causes, locations, and sexes (figure 3; appendix 2 figure S4, appendix 2 table S6). For the global leading cause of death, ischaemic heart disease, females in Switzerland died at the highest mean age of 88·4 years (95% UI 87·8–88·8), which is 6·8 years (6·1–7·5) higher than the expected age of 81·6 (80·7–82·4). By contrast, females in South Sudan died from the same cause at the lowest mean age of 61·2 years (58·9–63·5), which is 7·3 years (4·8–10·1) below the expected age of 68·5 years (67·1–70·2). For tracheal, bronchus, and lung cancer—the sixth-leading cause of death—females in Japan died at the highest mean age of 82·8 years (81·3–83·5), which is 4·2 years (3·6–4·6) later than the expected age of 78·6 (77·4–79·4). However, in Malawi, females died from this cause at the lowest mean age of 55·6 years (53·9–57·9), which is 12·8 years (10·4–14·6) earlier than the expected age of 68·4 (67·2–69·3). For the ninth-leading cause of death, chronic kidney disease, females in Spain died at the second highest mean age of 89·4 years (89·0–89·7), 9·8 years (8·8–11·1) later than the expected age of 79·6 (78·0–80·8). Meanwhile, in Angola, the same cause resulted in a mean age at death of just 46·1 years (42·9–50·3), which is 12·5 years (9·2–15·6) earlier than the expected age of 58·6 (56·1–61·2).

The observed mean age at death in 2023 shows a moderate relationship (*r*^2^≥0·50) with SDI across 96 of 141 Level 3 causes of death (appendix 2 table S14). Where SDI explains greater than 50% of the variance seen in the mean age at death, the relationship is positive: as SDI improves, the observed mean age at death increases. After controlling for any relationship SDI has with population structure by comparing observed and expected mean ages and examining their correlation with SDI, the results remain varied by sex.

When comparing observed and expected mean ages for females, a total of 118 causes show a positive correlation with SDI (appendix 2 table S14). Some causes, such as self-harm, exhibit a negative correlation—meaning that females in higher SDI countries died at younger ages than expected from self-harm compared to those in lower SDI countries. All of the causes that had a negative correlation with SDI were considered weak relationships (*r*^2^<0·50). For females, the difference between the observed and expected mean age at death in the following nine Level 3 causes showed a moderate positive correlation with SDI: ischaemic heart disease, stroke, breast cancer, pancreatic cancer, leukaemia, brain and central nervous system cancer, ovarian cancer, kidney cancer, and invasive non-typhoidal *Salmonella*.

When comparing observed and expected mean ages for males, a total of 110 causes show a positive correlation with SDI (appendix 2 table S14). Similar causes, such as drug use disorders, self-harm, and conflict and terrorism exhibited a negative correlation—indicating that males in higher SDI countries died at younger ages than expected from these causes compared to those in lower SDI countries. All negatively correlated causes were found to have weak relationships with SDI just as females did. For males, the difference between the observed and expected mean age at death in only six Level 3 causes showed a moderate correlation with SDI. These were leukaemia, brain and central nervous system cancer, other malignant neoplasms, other intestinal infectious diseases, kidney cancer, and invasive non-typhoidal *Salmonella*.

### All-cause 70q0

Across every GBD super-region and region, 70q0 from all causes combined decreased for both males and females between 2000 and 2023 (appendix 2 table S11). However, there was variation in these percentage changes between regions. For males, 70q0 in the Caribbean decreased 2·2%, and in high-income North America it decreased 9·6%. Conversely, in high-income Asia Pacific, this decrease was 36·0%, and in east Asia it was 43·8%. For females, 70q0 in Oceania decreased 2·6% and in the Caribbean 6·0%, while in eastern Europe the decrease was 35·6% and in east Asia it was 58·2%.

**Figure 4:**
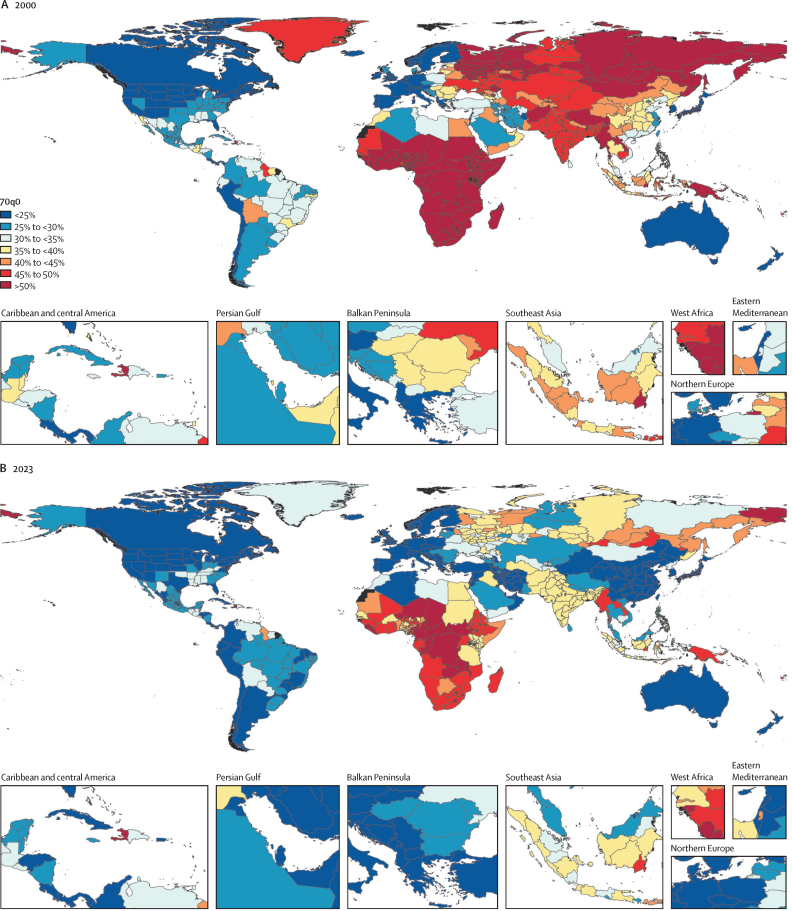
70q0 in males and females (A) 2000. (B) 2023. 70q0=probability of death before age 70 years.

National-level trends in 70q0 also varied (figure 4, appendix 2 table S11). For males, between 2000 and 2023, an increase in 70q0 occurred in six countries: Palestine (40·6%), Lebanon (19·4%), Guam (14·9%), Paraguay (14·6%), Dominican Republic (9·2%), and Venezuela (3·5%). For females, for the same period, 12 countries had an increase in 70q0: Libya (19·6%), Palestine (14·2%), Lebanon (13·7%), Venezuela (12·8%), Tonga (12·0%), Solomon Islands (7·8%), Samoa (6·6%), Guam (4·9%), Marshall Islands (4·7%), Paraguay (4·6%), Dominican Republic (1·2%), and Fiji (0·8%). For males, the primary cause of death driving these increases for Palestine and Lebanon was conflict and terrorism, while in Paraguay and Guam the primary driver was drug use disorders. Among females, the primary cause responsible for the increases remained conflict and terrorism for Palestine and Lebanon. Additionally, chronic kidney diseases contributed the most to the increases in Libya, and malaria the most to the increases in Venezuela (appendix 2 table S6)

### 70q0 by cause and location

Among the top 50 global causes of death for females, the 15 causes with the largest amount of increase in 70q0 between 2000 and 2023 were interstitial lung disease and pulmonary sarcoidosis (+74·8% change; 70q0 in 2023 0·1%); drug use disorders (+68·0%; 0·1%); lip and oral cavity cancer (+54·7%; 0·1%); atrial fibrillation and flutter (+52·2%; <0·1%); Alzheimer's disease and other dementias (+46·2%; 0·2%); diabetes (+42·7%; 0·8%); hypertensive heart disease (+40·9%; 0·4%); pancreatic cancer (+37·7%; 0·2%); Parkinson's disease (+36·7%; <0·1%); ovarian cancer (+36·5%; 0·3%); other cardiovascular and circulatory diseases (+35·9%; 0·1%); breast cancer (+33·6%; 1·0%); endocrine, metabolic, blood, and immune disorders (+29·9%; 0·1%); chronic kidney disease (+27·7%; 0·6%); and non-rheumatic valvular heart disease (+26·2%; <0·1%; appendix 2 table S6).

Among the top 50 global causes for males, the 15 causes with the largest amount of increase in 70q0 between 2000 and 2023 were diabetes (+75·6% change; 70q0 in 2023 1·0%); drug use disorders (+56·8%; 0·2%); atrial fibrillation and flutter (+51·1%; <0·1%); interstitial lung disease and pulmonary sarcoidosis (+48·2%; 0·1%); Alzheimer's disease and other dementias (+45·9%; 0·1%); Parkinson's disease (+44·4%; 0·1%); endocrine, metabolic, blood, and immune disorders (+41·3%; 0·1%); pancreatic cancer (+34·9%; 0·3%); lip and oral cavity cancer (+29·2%; 0·2%); prostate cancer (+24·7%; 0·2%); non-rheumatic valvular heart disease (+23·9%; <0·1%); chronic kidney disease (+18·6%; 0·7%); colon and rectum cancer (+18·0%; 0·6%); urinary diseases and male infertility (+18·0%; 0·1%); and other cardiovascular and circulatory diseases (+15·1%; 0·1%; appendix 2 table S6).

Among the top 50 causes for which a global increase in 70q0 occurred, we observed several notable declines at the super-region level between 2000 and 2023 (appendix 2 table S6). For example, 70q0 due to drug use disorders increased by 56·8% in males and 68·0% in females globally, yet decreased in southeast Asia, east Asia, and Oceania by 73·9% for males and 81·5% for females. For diabetes, 70q0 increased by 75·6% for males and 42·7% for females globally, but decreased by 9·0% for males and 34·6% for females in the high-income super-region. For ovarian cancer, there was a 36·5% increase in 70q0 in females globally, but a 25·5% decrease in the high-income super-region. For chronic kidney disease, 70q0 increased by 18·6% for males and 27·7% for females globally, but in central Europe, eastern Europe, and central Asia, it decreased by 10·4% for males and 15·3% for females.

Alternatively, some causes of death showed increased national 70q0 where there has otherwise been global progress to reduce 70q0. For example, a 47·4% decrease in 70q0 due to lower respiratory infections occurred among males globally, yet there were substantial increases in countries such as Poland (98·8%), Thailand (82·7%), and Argentina (66·0%). Similarly, for females, there was a 51·2% decrease in 70q0 due to lower respiratory infections globally, but notable increases in a number of countries, including Argentina (101·5%), Poland (48·2%), and Thailand (17·1%). Road injuries are another example: a 21·1% decline in 70q0 among males occurred globally, despite increases in many countries, most notably in Sierra Leone (259·8%), Uganda (180·5%), and Malawi (155·0%). For road injuries among females, there was a 20·3% decrease in 70q0 globally, with notable increases in the Democratic Republic of the Congo (158·5%), Sierra Leone (122·1%), and Pakistan (121·7%). Additionally, stroke showed global decreases in 70q0 of 15·6% among males and 20·7% among females, but increases in many countries for males (eg, Rwanda [97·0%], Burundi [85·7%], and Ethiopia [75·6%]) and females (eg, Ethiopia [128·4%], Zimbabwe [106·7%], and South Sudan [106·7%]).

### Joint examination of 70q0 and mean age at death in super-regions

**Figure 5:**
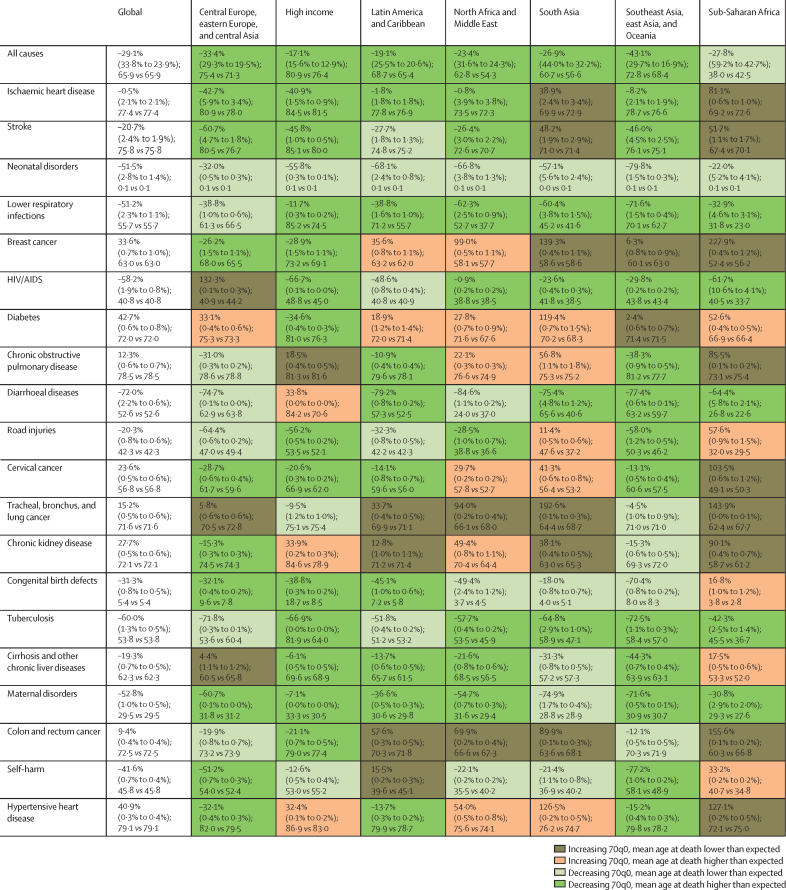
Change in 70q0 between 2000 and 2023 and the observed versus expected mean age at death in 2023 for females The contents of each cell are as follows: percentage change in 70q0 from 2000 to 2023 (70q0 in 2000 to 70q0 in 2023); observed *vs* expected mean age at death in years. 70q0=probability of death before age 70 years.

**Figure 6:**
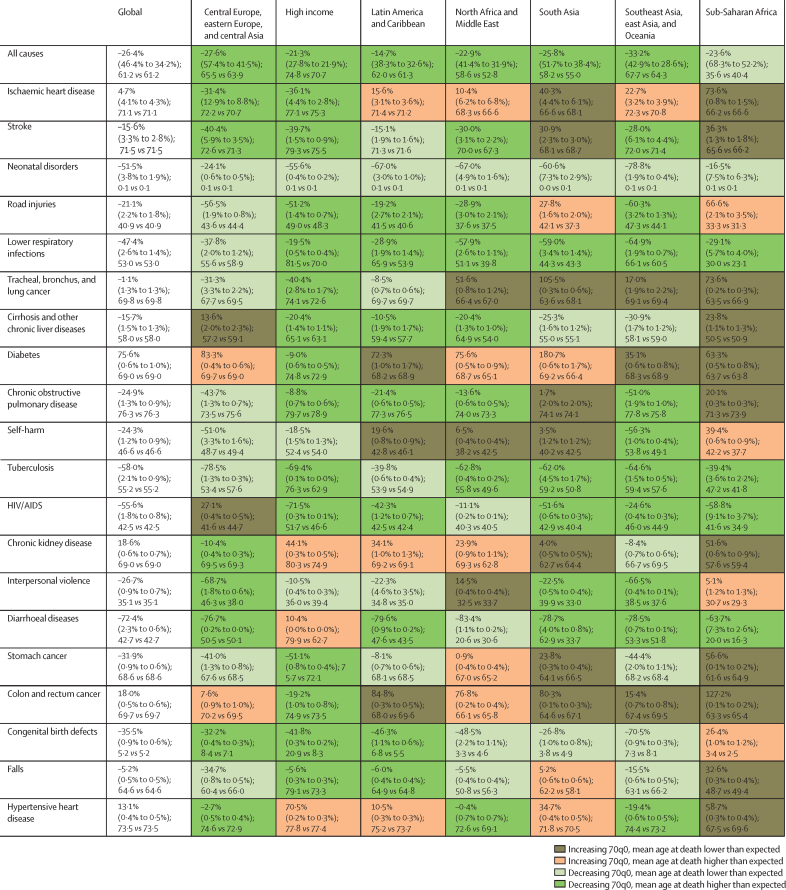
Change in 70q0 between 2000 and 2023 and the observed versus expected mean age at death in 2023 for males The contents of each cell are as follows: percentage change in 70q0 from 2000 to 2023 (70q0 in 2000 to 70q0 in 2023); observed *vs* expected mean age at death in years. 70q0=probability of death before age 70 years.

From 2000 to 2023, notable variation was observed between sexes when investigating the 70q0 and mean age at death metrics for the top 20 causes of death by super-region (figures 5, 6). For females in sub-Saharan Africa, several of the leading causes showed an increase in 70q0 and a mean age that was lower than expected: ischaemic heart disease (70q0 increased 81·1%, with mean age at death 3·4 years lower than expected); stroke (70q0 increased 51·7%, with mean age at death 2·7 years lower than expected); and breast cancer (70q0 increased 227·9%, with mean age at death 3·8 years lower than expected). There were six additional causes that showed this same pattern. For females in south Asia, 70q0 due to tracheal, bronchus, and lung cancer increased by 192·6%, with a mean age at death 4·3 years lower than expected (figure 5). In addition, ischaemic heart disease, stroke, breast cancer, chronic kidney disease, and colon and rectum cancer also had increasing 70q0 and lower mean age at death compared with expected age in south Asia. In this super-region, there were also five causes that had an increase in 70q0 only (with mean age at death not lower than expected), and five causes had a mean age at death lower than expected without an increased 70q0. In females in the high-income super-region, only COPD showed both increasing 70q0 and lower mean age at death than expected. Chronic kidney disease, diarrhoeal diseases, and hypertensive heart disease had increasing 70q0, but the mean age at death was not lower than expected. In females in southeast Asia, east Asia, and Oceania, there were only two causes among the leading 20 that had an increased 70q0, breast cancer and diabetes, but both of those causes had mean ages at death below the expected values (figure 5).

In males in sub-Saharan Africa, 11 of the top 20 causes of death showed an increase in 70q0 and a mean age that was lower than expected: ischaemic heart disease; stroke; tracheal, bronchus, and lung cancer; cirrhosis and other chronic liver diseases; diabetes; COPD; chronic kidney disease; stomach cancer; colon and rectum cancer; falls; and hypertensive heart disease (figure 6). Additionally, road injuries, self-harm, interpersonal violence, and congenital birth defects had an increase in 70q0, but the mean age at death was not lower than expected. In males in south Asia, eight of the top 20 causes of death showed an increase in 70q0 and a mean age at death that was lower than expected: ischaemic heart disease, stroke, tracheal, bronchus, and lung cancer, COPD, self-harm, chronic kidney disease, stomach cancer, and colon and rectum cancer. Additionally, road injuries, diabetes, falls, and hypertensive heart disease had an increase in 70q0 only. In males in the high-income super-region, 70q0 due to interpersonal violence decreased slightly but had a mean age at death that was more than 3 years lower than expected. Additionally, 70q0 for chronic kidney disease, diarrhoeal diseases, and hypertensive heart disease also increased.

## Discussion

### High-level overview on causes of death

This study offers valuable new insights into global causes of human mortality over the past several decades, building upon and expanding previous iterations of GBD research. In a post-COVID-19-pandemic world, our study highlights several encouraging patterns in global health. Across many leading causes of death, there were declines in overall mortality within the period studied, despite disrupted rankings during the height of the COVID-19 pandemic. The rate of total YLLs have also reduced considerably relative to 1990, and particularly for many of the vaccine-preventable diseases, illustrating successes in reductions to preventable causes of death. Patterns in 70q0 show improvements across the globe, with overall declines observed in every GBD super-region, region, and in most countries. Additionally, the all-cause global mean age at death has been rising, indicating that people are generally dying later in life. While these broad-level improvements show considerable promise—particularly in the aftermath of a pandemic—they sometimes conceal disparities occurring at local levels. Differences by sex, age, and location underscore the complexity of global health progress and the persistent challenges in addressing causes of death. We highlight some of these inequalities below.

### The role of international cooperation in reducing deaths and YLLs

Over the past three decades, we found large reductions in age-standardised rates of YLLs for four causes—respiratory infections and tuberculosis, nutritional deficiencies, other infectious diseases, and enteric infections—which had individual declines ranging from 58·9% to 79·0%. This achievement was facilitated by many years of sustained international cooperation with local governments. If we expand this scope to include maternal and neonatal disorders, neglected tropical diseases, malaria, HIV/AIDS, and sexually transmitted infections, and categorise them into two groups—vaccine-preventable diseases and other diseases under international intervention plans—we see that the first group had a 66·5% reduction in YLLs, while the second had a 51·6% decline. These findings highlight the profound impact of sustained funding, vaccination, international collaboration, and targeted support programmes with local governments in addressing public health challenges. Previous studies, including GBD 2021, have highlighted the importance of controlling infectious diseases as a key factor in improving life expectancy.^14^ Many of these diseases are concentrated in specific populations and locations, making their control an achievable goal.^14^ During a time of uncertainty regarding the future of global health funding, it is essential to maintain these efforts, as discontinuing them could jeopardise the gains made in public health.

### COVID-19: challenges to recording and lessons for preparedness

The COVID-19 pandemic produced a challenge to global health that has not been seen in recent history, including the difficulties associated with accurately recording and analysing deaths from a novel pandemic. GBD 2021 estimated COVID-19 mortality using confirmed COVID-19 death estimates and excess mortality, an approach which measured the total toll of the pandemic but had limitations for understanding how many deaths were directly attributable to COVID-19.^14^ GBD 2023 addresses the issues related to COVID-19 reporting by applying a method for identifying and correcting misclassified COVID-19 deaths in vital registration data in the years 2020–23. This has allowed us to more accurately quantify the COVID-19 pandemic, as well as to correct for inaccurate spikes in mortality from other causes that were instead misclassified COVID-19 deaths. The applied correction occurs after garbage code redistribution to ensure that any deaths from COVID-19 are correctly identified and that other changes in garbage code practices do not result in cause-specific excess deaths.

The additional 2 years of analysis since GBD 2021 allows for a more complete picture of COVID-19 mortality. We estimated a total of 18·0 million people died from COVID-19 globally between 2019 and 2023. This profound loss of life underscores shortcomings in global health systems and a need to fill critical gaps in preparedness for epidemic diseases. Necessary steps to better prepare for the next pandemic should include strengthening health-care infrastructure, enhancing global surveillance, improving vaccine development and delivery, ensuring equitable access to essential and preventive health services, and improving global collaboration to include data sharing and advanced disease monitoring.^32^, ^33^

In 2020 and 2021, the global mortality rate from COVID-19 for people younger than 70 years was several times higher than the rates for lower respiratory infections before the pandemic, in 2019. In 2023, the rate of COVID-19 deaths decreased to be lower than the sum of all other lower respiratory infections for those younger than 70 years. A similar pattern holds true for those aged 70 years and older. This suggests that COVID-19's impact on mortality could be comparable to other individual lower respiratory infections in future years, and that health-care systems should prepare for and expect future COVID-19 seasons to be endemic.^34^

Measuring the impact of a pandemic is inherently challenging; we recognise that future estimates might be subject to continued improvements as more data become available. The last pandemic of this nature to occur was the 1918 H1N1 influenza pandemic that killed between 21 million and 100 million people within 2 years.^35^, ^36^ The true death toll of the 1918 influenza pandemic still remains uncertain, and even with improved records, there will likely always be some ambiguity surrounding the COVID-19 estimates as well.

### Changes in age-specific mortality rate from 2000 to 2023

With all other factors held constant, the age-specific death rate for teenagers, young adults, and older adults (aged 10–69 years) should decline over time, reflecting improvements in health care, socioeconomic conditions, and public health measures.^7^ However, this trend has not been universal across all 21 GBD regions. Only four regions—high-income Asia Pacific, central Asia, east Asia, and south AsiA&Mdash;have had a declining age-specific death rate for both males and females across all 5-year age groups for the three Level 1 GBD causes: CMNN diseases, NCDs, and injuries.

In regions displaying an increase in NCDs, primary drivers vary by region; however, we commonly observed high rates of diabetes and kidney disease, cardiovascular disease, substance use disorders, and neoplasms. There were two regions where the increase in NCDs was primarily driven by an increase in drug use disorders: high-income North America and central Europe. The regions facing large increases in neoplasms were north Africa and the Middle East, southeast Asia, tropical Latin America, the Caribbean, and all four regions of sub-Saharan Africa. Diabetes and kidney disease largely contributed to the increase in central Europe, the Caribbean, central Latin America, and southern sub-Saharan Africa. Addressing these trends requires targeted public health interventions, improved health-care access, and socioeconomic policies to mitigate the underlying risk factors.

Among regions displaying an increase in injuries, there was similar variation by region, although common drivers included increases in self-harm, interpersonal violence, conflict and terrorism, environmental heat and cold exposure, and falls. In north Africa and the Middle East and eastern Europe, the rise in injury-related deaths was primarily due to collective and interpersonal violence, in addition to the earthquake in Türkiye. In central and eastern Europe, heatwaves have been occurring more frequently over the past decade. At the same time, increases in high-income North America, central Latin America, and tropical Latin America were all driven by increasing rates of self-harm.

### Deaths from violent causes

Our study shows several noteworthy patterns in deaths from violent causes occurring throughout the world. Trends of interpersonal violence showed global-level improvements with regional heterogeneity. Although global mortality from interpersonal violence has generally declined, the regions most heavily affected have seen worsening trends. The primary drivers associated with deaths from interpersonal violence are highly variable across locations.^37^ In some parts of the world, the drug trade fuels deaths from this form of violence by driving territorial conflicts, organised crime, and competition over illicit markets.^37^ Deaths from interpersonal violence can also be linked to several important social determinants of health, including adverse childhood experiences, alcohol and drug use disorders, and lack of social support, among others.^38^, ^39^

Deaths from conflict and terrorism are stochastic in nature and have fluctuated over the past three decades, displaying periods of declines and increases influenced by complex regional dynamics. In recent years, the area of conflict has begun to shift from north Africa and the Middle East to central Europe, eastern Europe, and central Asia, due to the war between Russia and Ukraine. Although the regional mortality rates of north Africa and the Middle East are no longer the highest, we found that Palestine had the highest mortality rate and 70q0 due to conflict and terrorism of any country in the world. These findings align with the recently reported number of fatalities in the Gaza Strip,^40^ and an estimated 30-year loss in life expectancy within the first 12 months of the war—a conservative estimate that nearly halves the pre-war life expectancy in Palestine.^41^

Global self-harm rates have been trending downwards since the early 1990s, but this progress conceals spikes in self-harm occurring in some locations.^42^ We observed an increase in self-harm in central Latin America, and more moderate increases occurring in Andean Latin America, high-income North America, high-income Asia Pacific, and tropical Latin America. There were, however, declines in self-harm in east Asia, particularly in China, where improved economic and social conditions, along with tailored and specified campaigns to reduce self-harm, have been useful in supporting population wellbeing.^43^, ^44^ We also observed that the mean age at death from self-harm has been increasing globally over the past three decades, a finding that potentially reflects both successes and failures with regard to self-harm prevention.^42^ While an increase in mean age at death due to self-harm could signal that intervention strategies tailored to younger groups have yielded improvements, increased deaths in older ages might indicate missed opportunities in addressing risk factors that are more relevant in older age groups, such as social isolation, economic insecurity, and increased chronic illness.^45^ Taken together, the findings that self-harm persists as a leading cause of death in young people in several regions while the global mean age at death due to self-harm increases suggest pivotal opportunities for further prevention strategies that must be carefully tailored to the intended demographic.

### Interpretation of mean age at death

The mean age at death measure provides a clear, easily interpretable metric for summarising the population affected by a given disease or injury. Interventions at both the individual and population levels vary depending on the age of those affected. For example, strategies to improve health outcomes for ischaemic heart disease in South Sudan, where the mean age at death due to this cause is 61·2 years for females, would be likely to focus on prevention strategies and early detection, whereas strategies to improve health outcomes for the same disease in Switzerland, where the mean age at death is 88·4 for females, might focus more on palliative care and limit treatment options. Identifying who is being affected by a disease using a single, interpretable measure could help policy makers to make informed decisions in complex situations.

There are some challenges when drawing comparisons in the observed mean age at death between populations with different age structures. An older population is likely to have an older mean age at death for a given cause than a population with a younger age structure. For this reason, comparing mean ages at death between locations with different population structures is not a good measure for how well a disease is being treated. To account for this, an evaluation of the expected mean age at death reflecting the given demographic's population structure is needed for comparison between locations.

The difference between the expected and observed mean age at death can reflect important factors and risks, beyond just the age distribution, that vary between and within different locations. When the observed mean age at death is lower than expected, it shows that people are dying younger than global rates would suggest. These differences across countries underscore notable inequalities. Causes of death that strongly correlate with SDI and differences between expected and observed mean ages at death are indicative of areas in which the global community has the capacity to improve health outcomes—yet resources and interventions remain unevenly distributed across locations. A lower mean age at death compared with the expected indicates weakness in public health, particularly in preventive measures, early diagnosis, and timely treatment that could delay or prevent deaths. Many examples of lower mean age at death are seen in cardiovascular diseases, cancers, and chronic respiratory diseases in low-income regions, suggesting challenges in both prevention and treatment.

### Probability of death

We included the probability of death between the ages of 0 and 70 years (70q0) in this study to assess the likelihood that an individual born today will die from a specific cause before reaching 70 years of age, assuming that current age-specific mortality rates remain unchanged. The 70q0 measure incorporates competing risks, acknowledging that individuals might die from other causes before reaching the high-risk ages for a particular cause. In other words, our goal was to provide a more comprehensive assessment of the 70q0 from a specific cause over the entire lifespan, assuming survival from other causes.

In 2024, the Global Health 2050 report set a target to cut global premature mortality in half by the year 2050, a goal referred to as 50 by 50.^5^ In support of this target, we aimed to better position GBD to provide the current state of national premature mortality estimates across causes and locations, which could be useful to track progress on future developments. We used an analysis of 70q0 to detail substantial sources of health loss that most contribute to premature death before age 70 years, providing a roadmap to help countries address their primary contributors to premature mortality.

Several studies suggest that probability of death is a valuable indicator, and providing 70q0 by cause of death highlights areas in which countries can improve the observed age at death for specific causes, drawing attention to locations and causes that have not kept pace with global progress in cause-specific mortality.^5^, ^46^ The probability of death measure also illustrates global and regional success stories in which, as a global health community, we have successfully reduced mortality rates for specific causes in people younger than 70 years. For instance, lower respiratory infections declined 51·2% among females and 47·4% among males between 2000 and 2023 due to reductions in the case-fatality rate and various risk factors,^47^ including reductions in household air pollution, a decrease in the prevalence of childhood wasting, and improved vaccine coverage, all of which were effective in reducing the burden of lower respiratory infections.^48^ Vaccines against *Haemophilus influenzae* type b and *Streptococcus pneumoniae* are particularly crucial in the reduction of lower respiratory infections in 70q0.^49^ Global improvements were also observed in diarrhoeal diseases, with declines of over 70% in 70q0 for males and females. There have been many multisectoral approaches that have contributed to a reduction in diarrhoeal deaths globally in 70q0, many of which have focused on diarrhoeal deaths in children younger than 5 years. These interventions include oral rehydration therapy, enhanced water, sanitation, and hygiene infrastructure, and the rollout of the rotavirus vaccination.^50^

The global reduction of 70q0 due to tuberculosis from 2000 to 2023 is another success story. There was an overall decline of 60·0% in females and 58·0% in males, and notable improvements in some super-regions, particularly central Europe, eastern Europe, and central Asia, where a 78·5% decrease among males and a 71·8% decrease among females occurred in that period. The analysis of 70q0 is more optimistic than other literature on tuberculosis, because the slowest progress in tuberculosis mortality has been in older adults.^47^ While we have seen success in reducing 70q0 from tuberculosis, with improvements in mortality for those aged 15 years and younger, more work is needed to reduce tuberculosis mortality in individuals aged 50 years and older,^47^, ^51^ and to reach WHO's End TB Strategy.^52^ Further reductions to tuberculosis-related risk factors, such as smoking, alongside early diagnosis and treatment, and the development of less toxic and shorter-duration tuberculosis treatments, are crucial for continued improvements to reach WHO targets by 2035.^47^ Lastly, improvements in 70q0 for neonatal disorders globally, decreasing by 51·5% for males and females, reflect the success of efforts to reduce mortality in those younger than 5 years across health sectors and multilaterally. This is generally cited as one of public health's biggest achievements of the 20th century.^53^ There are many lessons to be learned from this progress, including the importance of public health standards and measures adopted globally, such as the rollout of the *S pneumoniae* vaccine to prevent lower respiratory infections and the rotavirus vaccine for diarrhoea prevention. Sustainable Development Goal Target 3.2, which aims to end preventable deaths of newborns and children younger than 5 years by 2030, will build on the progress already made in reducing neonatal mortality and support ongoing efforts toward an 80% reduction in tuberculosis cases by 2030, as measured by WHO.^54^

Unfortunately, not all causes of death show an optimistic picture in terms of 70q0. There remain large disparities by cause and location. First, as the epidemiological transition continues, we see rising probabilities of deaths from NCDs in sub-Saharan Africa, Latin America and the Caribbean, south Asia, and southeast Asia, east Asia, and Oceania. With global progress in 70q0, there are outliers where increased mortality rates have occurred during this period. Ten countries saw an increase in 70q0 across all causes between 2000 and 2023; the largest increase was in Palestine at 33·1%, more than double that of the next-largest increase in Lebanon with 15·3%. The staggering increase in Palestine is driven almost entirely by the conflict between Israel and Palestine, with an increase in 70q0 of 8980% due to conflict and terrorism. Despite a substantial rise in 70q0 due to conflict and terrorism in many countries, including the remainder of the top ten countries (Sudan, Ukraine, Russia, Burkina Faso, Myanmar, Israel, Somalia, Syria, and Yemen), none of these countries had an overall increase in their all-cause 70q0 due to their increased risk of conflict deaths.

### Patterns in NCD mortality

Rising NCDs worldwide, especially in low-income areas, will represent a significant global health challenge moving forward.^55^, ^56^ Historically, low-income countries have been disproportionately affected by the burden of infectious diseases, but shifts towards more chronic conditions are a reflection of the ongoing global epidemiological transition.^57^ In 1990, the three regions with the highest overall mortality rates from all causes were western, eastern, and central sub-Saharan Africa, where 73·4% of deaths came from CMNN diseases. By 2023, CMNN diseases in these regions dropped to 51·4% of all deaths, representing a 30·0% decrease from 1990. Our findings also show that age-standardised mortality rates and 70q0 for both cardiovascular diseases and neoplasms are increasing in sub-Saharan Africa and in south Asia (appendix 2 figure S5). As further reductions in communicable diseases continue, it is likely that deaths from these NCDs could become the dominant sources of mortality in future years.

Findings from our study are in agreement with many studies drawing attention to the surge in NCDs occurring in low-income settings.^57^, ^58^ Although the concept of the epidemiological transition is not new, the speed and scale of the rise in NCDs in low-income regions is increasingly concerning.^57^ There are several important implications for health systems when disease burdens transition from communicable diseases to those from non-communicable sources.^10^, ^57^ Health-care infrastructure might face a range of growing challenges associated with increased care needs for chronic disease management requiring long-term care and ongoing treatment. Low-resourced locations remain poorly equipped to address the rising burden of NCDs, with health-care systems often underfunded and unable to provide adequate preventive care or treatment options.^57^ Collaborative and focused efforts—including coordinated policy initiatives and prevention programmes targeting key risk factors—are needed to alleviate immediate health challenges related to the rising burden of NCDs in low-income regions and to achieve long-term improvements in global health outcomes.

### Limitations

As with any study of this scope, there are several important limitations to consider. We provide cause-specific limitations for every GBD cause of death in detail in appendix 1 (section 3). Here, we highlight limitations with applicability across many causes. First, accuracy of cause-of-death estimates can be affected by data sparsity or unreliability from some regions, time periods, or age groups. In locations for which we have scarce or unreliable data, estimates are interpolated from neighbouring regional patterns by relying on predictive covariates. Second, the cause-of-death estimates rely on medically verifiable sources of cause-of-death data, for which quality can vary. Some datasets do not cover all deaths in a given age, sex, location, and year, and some have high levels of garbage-coded underlying causes of death, which require redistribution algorithms to correct. For transparency about data quality, we publish a star rating of the quality of all vital registration and verbal autopsy data (a 1–5 score compiled based on percentage completeness and percentage garbage). These scores are available in appendix 1 and in a publicly available visualisation tool. Third, for causes with limited data, it is preferable to provide estimates with appropriate uncertainty, rather than providing no information. Fourth, reporting lags in medically verifiable cause-of-death data are a factor in data availability for recent years, particularly 2023; therefore, estimates for these years rely more heavily on the modelling process. Fifth, there are several limitations that pertain to our COVID-19 estimates. While GBD 2023 reflects the most comprehensive set of COVID-19 estimates published by GBD to date, we still have a limited availability of time series for some locations from 2020 to 2023, particularly for 2023. Some location-cause-age-sex groups have a small enough sample size that their time series are stochastic by default, making the development of a counterfactual model difficult. To our knowledge, estimates from GBD 2023 reflect the best account of COVID-19 and miscoded COVID-19 to date. However, as we learn more about the virus and its presentation, it is possible our corrections will be updated to reflect new knowledge in the field. Sixth, mean age at death calculations also have limitations, as they are not standardised for different population age structures. Aggregate estimates are therefore influenced by the most populated areas. As a result, it can be unclear whether the increase in the mean age at death is attributed to a reduction of deaths in younger ages, or if it is simply a result of an ageing population. Our calculation of mean age at death is also limited by the granularity of GBD results. Here, each death is assigned an age group, whereas in reality, each death occurred at a specific age. This strategy does not capture effects within age groups, and it does not show how cohorts age from year to year. Seventh, 70q0 is a broad age group that does not capture improvements made in younger ages if the death occurs before age 70 years. For example, if the mean age at death improved from 30 years to 50 years in a period of time, but the overall mortality rate remained the same, 70q0 would not show this improvement. Lastly, data for stochastic events such as natural disasters and conflicts are generally reported without age and sex detail and instead leverage age-sex splitting using the available detailed data to split the deaths into the granular GBD age groups. These types of events are particularly subject to a lag in reporting, and these estimates will continue to be improved in the future.

### Conclusion

GBD cause of death studies are fundamental for understanding mortality trends and aligning them with public health decision making. While progress has been made in reducing deaths from infectious diseases on a global scale, the rising burden of NCDs presents new challenges, particularly for low-income nations. Patterns in premature mortality across the globe have been changing, signifying priority areas for public health intervention. Findings from GBD 2023 show a crucial need for continued investment in health care, improved data collection, and targeted interventions to address both emerging and persistent health issues. Tackling the global health challenges of the future will require sustained international collaboration in the prevention and treatment of both communicable and non-communicable diseases. Strengthening access and quality of health care in low-income and middle-income countries is needed for improving the prevention and treatment of NCDs in particular, which continue to rise as major health threats. A unified global effort will also be necessary to combat the growing number of deaths from drug use and violence, both of which require comprehensive strategies for prevention, treatment, and support. By fostering greater international cooperation and focusing on these key areas, we can make significant progress towards reducing global mortality rates and improving health outcomes for populations worldwide.

#### GBD 2023 Causes of Death Collaborators

#### Affiliations

#### Contributors

#### Data sharing

For detailed information on data sources and estimates, please visit the GHDx GBD 2023 website at http://ghdx.healthdata.org/gbd-2023.

## Declaration of interests

J Ärnlöv reports payment or honoraria for lectures, presentations, speakers bureaus, manuscript writing or educational events from AstraZeneca, Boehringer Ingelheim, and Novartis; participation on a Data Safety Monitoring Board or Advisory Board with AstraZeneca, Boehringer Ingelheim, and Astella; all outside the submitted work. D Abramov reports payment or honoraria for lectures, presentations, speakers bureaus, manuscript writing or educational events from AstraZeneca and Bayer; participation on a Data Safety Monitoring Board or Advisory Board with BridgeBio; all outside the submitted work. S Afzal reports support for the present manuscript from Institute of Public Health Lahore for study material, manuscripts, medical writings and library resources; grants or contracts from the Dean Institute of Public Health Lahore; payment or honoraria for lectures, presentations, speakers bureaus, manuscript writing or educational events from the Dean Institute of Public Health Lahore; support for attending meetings and/or travel from the Dean Institute of Public Health Lahore; participation on a Data Safety Monitoring Board or Advisory Board with Pakistan National Bioethics Committee as a Member, Institutional Review Board of Fatima Jinnah Medical University as a Member, Ethical Review Board and Data Monitoring Board Institute of Public Health Lahore Pakistan as a Member, Clinical Research Organization King Edward Medical University, Annals of King Edward Medical University Advisory Board as a Member; leadership or fiduciary roles in other board, society, committee or advocacy group, paid or unpaid, with Pakistan Higher Education Commission Research Committee as a Member, Pakistan Medical and Dental Commission Research and Journals Committee as a Member, Pakistan National Bioethics Committee as a Member, Pakistan Society of Internal Medicine as a Member, Pakistan Association of Medical Editors as a Member, Medical Microbiology and Infectious Diseases Society as a Member, Leads International as a Fellow, Faculty of Public Health UK as a Fellow, College of Physicians and Surgeons Pakistan as a Fellow; receipt of equipment, materials, drugs, medical writing, gifts or other services from Bergen University Norway; other financial or non-financial interests with Dean Institute of Public Health Birdwood Lahore; all outside the submitted work. C A Sobrinho reports grants or contracts from Fundação para a Ciência e Tecnologia (FCT) via grant CEECINST/00093/2021/CP2815/CT0001, outside the submitted work. R Ancuceanu reports consulting fees from Abbvie and Merck Romania; payment or honoraria for lectures, presentations, speakers bureaus, manuscript writing or educational events from Abbvie, Laropharm, Reckitt, Merck Romania, and MagnaPharm; support for attending meetings and/or travel from Merck Romania and Reckitt; all outside the submitted work. O C Baltatu reports support for the present manuscript from the National Council for Scientific and Technological Development Fellowship (CNPq, 304224/2022–7), the Anima Institute (AI) Research Professor Fellowship, and Alfaisal University; leadership or fiduciary roles in other board, society, committee or advocacy group, paid or unpaid, with VividiWise Analytics as Managing Partner and São José dos Campos Tech Park—CITE as Biotech Advisory Board Member; all outside the submitted work. S Barteit reports support for attending meetings and/or travel from Wellcome Trust, September 2023+January 2025; stock or stock options in Climate Change and Health Evaluation and Response System (€4,200 in shares); all outside the submitted work. A Beloukas reports grants or contracts from Gilead for a research grant and sponsorship to the University of West Attica, and from GSK/ViiV for a Research Sponsorship to the University of West Attica; payment or honoraria for lectures, presentations, speakers bureaus, manuscript writing or educational events from Gilead and GSK paid to the University of West Attica; support for attending meetings and/or travel from Gilead and GSK paid to the University of West Attica; receipt of equipment, materials, drugs, medical writing, gifts or other services from Cepheid in the form of FOC reagents for a research project; all outside the submitted work. P J G Bettencourt reports the following patents issued or pending: WO2020229805A1, BR112021022592A2, EP3965809A1, OA1202100511, US2023173050A1, EP4265271A2, EP4275700A2, EP4265271A3, EP4275700A3; all outside the submitted work. S Bhaskar reports grants or contracts from Japan Society for the Promotion of Science (JSPS), Japanese Ministry of Education, Culture, Sports, Science and Technology (MEXT), Grant-in-Aid for Scientific Research (KAKENHI; grant ID: 23KF0126), JSPS and the Australian Academy of Science, JSPS International Fellowship (grant ID P23712); leadership or fiduciary roles in other board, society, committee or advocacy group, paid or unpaid, with Rotary District 9675, Sydney, Australia as District Chair, Diversity, Equity, Inclusion & Belonging, with Global Health & Migration Hub Community, Global Health Hub Germany, Berlin, Germany as Chair, Founding Member and Manager, with PLOS One, BMC Neurology, Frontiers in Neurology, Frontiers in Stroke, Frontiers in Public Health, Journal of Aging Research, Neurology International, Diagnostics, & BMC Medical Research Methodology as an Editorial Board Member, with College of Reviewers, Canadian Institutes of Health Research (CIHR), Government of Canada as a Member, with World Headache Society, Bengaluru, India as Director of Research, with Cariplo Foundation, Milan, Italy as an Expert Adviser/Reviewer, with National Cerebral and Cardiovascular Center, Department of Neurology, Division of Cerebrovascular Medicine and Neurology, Suita, Osaka, Japan as Visiting Director, with Cardiff University Biobank, Cardiff, UK as a Member, Scientific Review Committee, with Rotary Reconciliation Action Plan as Chair, and with Japan Connect, Osaka, Japan as a Healthcare and Medical Adviser; all outside the submitted work. A Biswas reports consulting fees from LUPIN Pharmaceuticals Ltd, INTAS Pharmaceuticals Ltd, Alkem Laboratories Ltd, and Torrent Pharmaceuticals Ltd; all outside the submitted work. R Cairns reports grants or contracts from Reckitt for an untied educational grant to study poisoning; payment or honoraria for lectures, presentations, speakers bureaus, manuscript writing or educational events from The Pharmacy Guild of Australia and Reckitt; all outside the submitted work. M C D de Carvalho reports other financial or non-financial interests with LAQV/REQUIMTE, University of Porto, Porto, Portugal, and from FCT/MCTES under the scope of the project UIDP/50006/2020 (DOI 10.54499/UIDP/50006/2020); all outside the submitted work. A L Catapano reports grants or contracts from Chiesi, Amarin, and Ultragenyx; consulting fees from Amarin, Amgen, AstraZeneca, Chiesi, Daiichi Sankyo, Eli Lilly, Esperion, Ionis Pharmaceutical, Medscape, Menarini, MSD, Novartis, NovoNordisk, Regeneron, Sanofi, Ultragenyx, and Viatris; payment or honoraria for lectures, presentations, speakers bureaus, manuscript writing or educational events from Amarin, Amgen, AstraZeneca, Chiesi, Daiichi Sankyo, Eli Lilly, Esperion, Ionis Pharmaceutical, Medscape, Menarini, MSD, Novartis, NovoNordisk, Regeneron, Sanofi, Ultragenyx, and Viatris; participation on a Data Safety Monitoring Board or Advisory Board with Amarin, Amgen, AstraZeneca, Chiesi, Daiichi Sankyo, Eli Lilly, Esperion, Ionis Pharmaceutical, Medscape, Menarini, MSD, Novartis, NovoNordisk, Regeneron, Sanofi, Ultragenyx, and Viatris; all outside the submitted work. H Christensen reports grants or contracts from Velux Foundation, Novo Foundation, Br Hartman Fonden, Tværsfonden, and Lundbeck Foundation; participation on a Data Safety Monitoring Board or Advisory Board with Atricure: LEEAPS trial—DSMB; leadership or fiduciary roles in other board, society, committee or advocacy group, paid or unpaid, with Action Plan for Stroke in Europe as Past Chair; all outside the submitted work. F Cohen reports consulting fees from Abbvie and Pfizer; payment or honoraria for lectures, presentations, speakers bureaus, manuscript writing or educational events from Abbvie and Axsome; all outside the submitted work. J Conde reports grants or contracts from OncoNanoAI: Artificial intelligence to discover the next generation of personalised nanoparticles for triple-negative breast cancer therapy (2025–2027) (FCT grant LISBOA2030-FEDER-00862500-149983); patents issued or pending: “TRPV2 Antagonists” US Application (number US11273152B2), “Surfactant-based cellulose hydrogel methods and uses thereof” (PCT/IB2025/051694, 17/02/2025), “Self-immolative micelle, methods and uses thereof” (EP25165757, 24/03/2025); all outside the submitted work. S E Congly reports grants or contracts paid to their institution from AstraZeneca, Merck, Ipsen, Bausch Health, Oncoustics, Boehringer Ingelheim, and Gilead Sciences Canada; consulting fees paid to them from GSK and Boehringer Ingelheim; participation on a Data Safety Monitoring Board or Advisory Board with Boehringer Ingelheim, Gilead Sciences Canada, and AstraZeneca; leadership or fiduciary roles in other board, society, committee or advocacy group, paid or unpaid, with Canadian Association for the Study of the Liver as a Member of the Board of Directors and Alberta Society of Gastroenterology as Vice President; all outside the submitted work. N Conrad reports grants or contracts paid to their institution from Wellcome Trust Career Development Award (grant number 318034/Z/24/Z), Research Foundation Flanders (grant number 12ZU922N), and KU Leuven (internal funding); all outside the submitted work. S Cortese reports grants or contracts from the National Institute for Health and Care Research (NIHR) and the European Research Agency; payment or honoraria for lectures, presentations, speakers bureaus, manuscript writing or educational events from the Association for Child and Adolescent Mental Health (ACAMH), the British Association of Psychopharmacology (BAP), Medice; support for attending meetings and/or travel from the Association for Child and Adolescent Mental Health (ACAMH), the British Association of Psychopharmacology (BAP), Medice; leadership or fiduciary roles in other board, society, committee or advocacy group, paid or unpaid, with the European ADHD Guideline Group (EAGG); all outside the submitted work. E C Dee reports support for the present manuscript from Prostate Cancer Foundation Young Investigator Award and through the Cancer Center Support grant from the US National Cancer Institute (P30 CA008748). A K Demetriades reports non-fiduciary leadership roles in other board, society, committee or advocacy group with EANS (European Association of Neurosurgical Societies) as a Board member, AO SPINE as a Steering Committee Member for Knowledge Forum Degenerative, Global Neuro Foundation as a Board Member, AO SPINE as a Steering Committee Member for Knowledge Forum Degenerative; all outside the submitted work. X Ding reports grants or contracts from American Heart Association for a 2-year predoctoral fellowship (DOI: 10.58275/AHA.25PRE1373497.pc.gr.227106); quarterly payments made to their institution; all outside the submitted work. L L M Ebraheim reports support for the present manuscript from the Gates Foundation, and royalties or licenses from the Institute for Health Metrics and Evaluation outside the submitted work. A Faro reports support for the present manuscript from National Council for Scientific and Technological Development (CNPq, Brazil) for a personal grant “Researcher at CNPq—Level 1B”. A A Fomenkov reports support for the present manuscript from the Ministry of Science and Higher Education of the Russian Federation (theme number 122042600086–7). L M Force reports support for the present manuscript from Gates Foundation, St. Jude Children's Research Hospital; grants or contracts from St. Baldrick's Foundation, Conquer Cancer Foundation, NIH Loan Repayment Program; leadership or fiduciary roles in other board, society, committee or advocacy group, unpaid, with the *Lancet Oncology* International Advisory Board; all outside the submitted work. R C Franklin reports support for attending meetings and/or travel from ACTM—Annual Conference 2022–2024; leadership or fiduciary roles in other board, society, committee or advocacy group, paid or unpaid, with Australasian College of Tropical Medicine as President, Kidsafe Australia as President, Royal Life Saving Society Australia as a Board Member, and Auschem Training as a Board Member; all outside the submitted work. A Guha reports grants or contracts from American Heart Association and US Department of Defense; leadership or fiduciary roles in other board, society, committee or advocacy group, paid or unpaid, with ZERO Cancer health disparities working group; all outside the submitted work. A A Harris reports grants or contracts from the Gates Foundation and Gavi; all outside the submitted work. A Hassan reports consulting fees from Novartis, Sanofi Genzyme, Biologix, Astra Zeneca, Pfizer, Merz, Roche, Merck, Hikma Pharma, Janssen, Inspire Pharma, Future Pharma, and Elixir Pharma; payment or honoraria for lectures, presentations, speakers bureaus, manuscript writing or educational events from Novartis, Allergan, Abbvie, Merck, Biologix, Viatris, Pfizer, Eli Lilly, Janssen, Roche, Sanofi Genzyme, Bayer, AstraZeneca, Hikma Pharma, Al Andalus, Chemipharm, Lundbeck, Elixir, EvaPharma, Inspire Pharma, Future Pharma and Habib Scientific Office, and Everpharma; support for attending meetings and/or travel from Novartis, Allergan, Merz, Pfizer, Merck, Biologix, Roche, Sanofi Genzyme, Bayer, Hikma Pharma, Chemipharm, Al Andalus and Clavita Pharm; leadership or fiduciary roles in other board, society, committee or advocacy group, paid or unpaid, with MENA Headache Society as Vice President, Multiple Sclerosis Chapter of the Egyptian Society of Neurology as a Board Member, Headache Chapter of the Egyptian Society of Neurology as a Board Member, The International Headache Society (IHS) as a Member of the committee of education, the membership committee, and regional committee; all outside the submitted work. P J Hotez is a co-inventor on non-revenue generating patents for neglected tropical diseases owned by Baylor College of Medicine (BCM). He is also a co-inventor of a COVID-19 recombinant protein vaccine technology owned by BCM that was licensed by Baylor Ventures non-exclusively and with no patent restrictions to several companies committed to advance vaccines for low- and middle-income countries. The co-inventors have no involvement in license negotiations conducted by BCM. Similar to other research universities, a long-standing BCM policy provides its faculty and staff, who make discoveries and that result in a commercial license, a share of any royalty income. Any such distribution will be undertaken in accordance with BCM policy. P J Hotez is also the author of several books published by academic presses (ASM-Wiley) and Johns Hopkins University Press, and he receives modest royalty income from this activity. I M Ilic reports support for the present manuscript from Ministry of Science, Technological Development and Innovation of the Republic of Serbia; number 451–03–137/2025–03/200110. M D Ilic reports support for the present manuscript from Ministry of Science, Technological Development and Innovation of the Republic of Serbia number 451–03–47/2023–01/200111. N E Ismail reports leadership or fiduciary roles in other board, society, committee or advocacy group, unpaid, with Malaysian Academy of Pharmacy, Malaysia as the Bursar and Council Member and Malaysian Pharmacists Society Education Chapter Committee as a Committee Member; all outside the submitted work. I O Iyamu reports grants or contracts from Canadian Institutes for Health Research (CIHR) Health Systems Impact Fellowship (Funding Reference number IF8–196153), Michael Smith Health Research BC Trainee Award (award number HSIF-2024–04465), and CIHR Canadian HIV Trials Network (CTN+) post-doctoral fellowship; consulting fees from Excellence Community Education Welfare Scheme; support for attending meetings and/or travel from Pacific Public Health Foundation; leadership or fiduciary roles in other board, society, committee or advocacy group, paid or unpaid, with Public Health Association of British Columbia as Vice President; all outside the submitted work. V Jha reports consulting fees from Bayer, AstraZeneca, Boehringer Ingelheim, Baxter, Vera, Visterra, Otsuka, Novartis, Timberlyne, Biogen, Chinook, and Alpine; All payments to the George Institute; all outside the submitted work. T Joo reports support for the present manuscript from EU4Health Programme 2021–2027 under grant agreement 101126953 (The Joint Action on CARdiovascular diseases and DIabetes—JACARDI). The views and opinions expressed are those of the author(s) only and do not necessarily reflect those of the European Union or the European Health and Digital Executive Agency (HaDEA). Neither the European Union nor the granting authority can be held responsible for them; and National Research, Development and Innovation Office in Hungary (RRF-2.3.1-21-2022-00006, Data-Driven Health Division of National Laboratory for Health Security for funding of participation in the research project. J J Jozwiak reports payment or honoraria for lectures, presentations, speakers bureaus, manuscript writing or educational events from Novartis, Adamed, Amgen, Boehringer Ingelheim, Servier, Novo Nordisk; all outside the submitted work. R Kalani reports grants or contracts from the National Institutes of Health (NIH) (USA) 1R01NS138297; all outside the submitted work. M Kivimäki reports grants or contracts paid to their university from the Wellcome Trust (221854/Z/20/Z), Medical Research Council (MR/Y014154/1), National Institute on Aging (R01AG056477, R01AG062553) and Research Council of Finland (350426); all outside the submitted work. J M Kocarnik reports support for the present manuscript from Institute for Health Metrics and Evaluation as an employee, the Gates Foundation for funding to his institution, and American Lebanese Syrian Associated Charities for funding to his institution. K Krishan reports other financial or non-financial interests with non-financial support from the UGC Centre of Advanced Study, CAS II, awarded to the Department of Anthropology, Panjab University, Chandigarh, India, outside the submitted work. T Lallukka reports support for the present manuscript from the Research Council of Finland (330527), payments made to their institution. M-C Li reports grants or contracts from the National Science and Technology Council, Taiwan (NSTC 113–2314-B-003–002) and the “Higher Education Sprout Project” of National Taiwan Normal University; leadership or fiduciary roles in other board, society, committee or advocacy group, paid or unpaid, with *Journal of the American Heart Association* as Technical Editor; all outside the submitted work. D Lindholm reports stock or stock options in AstraZeneca during time of employment (>2.5 years ago); other financial or non-financial interests with AstraZeneca as a former employee (>2.5 years ago); all outside the submitted work. H Liu reports other financial or non-financial interests as a mentor of National Medical Research Association (NMRA, U.K.), a member of British Society for Cardiovascular Research (BSCR, U.K.), and a member of and Cardiovascular Analytics Group (CVAG, HKSAR of China), all are not-for-profit organisations; all outside the submitted work. J Liu reports support for the present manuscript from the National Natural Science Foundation (72474005) and Beijing Natural Science Foundation (L222027, Z240004); grants of contracts the National Natural Science Foundation (72474005) and Beijing Natural Science Foundation (L222027, Z240004), outside the submitted work. V Lohner reports support for the present manuscript from Marga and Walter Boll Foundation, Kerpen, Germany. S Lorkowski reports grants or contracts paid to their institution from dsm-firmenich (formerly DSM Nutritional Products); consulting fees from Danone, Novartis Pharma, and Swedish Orphan Biovitrum (SOBI); payment or honoraria for lectures, presentations, speakers bureaus, manuscript writing or educational events from AMARIN Germany, Amedes Holding, AMGEN, Berlin-Chemie, Boehringer Ingelheim Pharma, Daiichi Sankyo Deutschland, Danone, Hubert Burda Media Holding, Janssen-Cilag, Lilly Deutschland, Novartis Pharma, Novo Nordisk Pharma, Roche Pharma, Sanofi-Aventis, Swedish Orphan Biovitrum (SOBI), SYNLAB Holding Deutschland; support for attending meetings and/or travel from AMGEN; participation on a Data Safety Monitoring Board or Advisory Board with AMGEN, Daiichi Sankyo Deutschland, Novartis Pharma, Sanofi-Aventis; all outside the submitted work. K S-K Ma reports grants or contracts from the International Team for Implantology outside the submitted work. P Maffia reports grants or contracts from British Heart Foundation, NextGenerationEU PNRR, Heart Research UK, Italian Ministry of University, BBSRC International Partnerships Funding, and Scottish Founding Council; leadership or fiduciary roles in other board, society, committee or advocacy group, paid or unpaid, with Translational Section for the International Union of Basic and Clinical Pharmacology (IUPHAR) as Vice-Chair, the Translational Research Medical Review Panel for Heart Research UK (HRUK) as Chair, the European Society of Cardiology (ESC) Working Group on Atherosclerosis & Vascular Biology and Cell Biology of the Heart as a Nucleus Member, the British Atherosclerosis Society (BAS) as an Executive Committee Member, Immunotherapy Committee of the International Union of Immunological Societies (IUIS) as a Member, and the Translational Clinical Studies (TCS) Grant Panel for the Chief Scientist Office (CSO) as a Member; all outside the submitted work. H R Marateb reports grants or contracts from Universitat Politècnica de Catalunya . Barcelona Tech—UPC; all outside the submitted work. S Masi reports grants or contracts from Servier for personal contracts for consulting activities, lectures, presentations, manuscript writing and educational events, Tuscany Region for grants for research projects in the field of arterial hypertension and management of SARS-CoV2 infection, and Italian Ministry of University and Research for grants for research projects in the field of heart failure; consulting fees from Servier (2022-Present); payment or honoraria for lectures, presentations, speakers bureaus, manuscript writing or educational events from Servier (2018-Present); support for attending meetings and/or travel from Servier (2018-Present); participation on a Data Safety Monitoring Board or Advisory Board with Servier on advisory board for the lunch of new drugs (2024-Present); all outside the submitted work. R J Maude reports support for the present manuscript from Wellcome Trust. This research was supported in part by Wellcome Trust (grant number 220211) as it provides core funding for Mahidol Oxford Tropical Medicine Research and contributes to their salary. They are required by Wellcome to acknowledge this grant in all publications. S A Meo reports grants or contracts from the Ongoing Research Funding Program (ORF-2025–47), King Saud University, Riyadh, Saudi Arabia; all outside the submitted work. T R Miller reports grants or contracts from National Institute for Mental Health (USA), AB InBev Foundation, Santa Clara County Public Health Department (California); payment for expert testimony from lawyers representing state & local plaintiffs in opioid litigation; all outside the submitted work. H M Mohamed reports support for the present manuscript from Higher Colleges of Technology; participation on a Data Safety Monitoring Board or Advisory Board with FIP Technology Advisory Group as a Member; leadership or fiduciary roles in other board, society, committee or advocacy group, paid or unpaid, with ISPOR UAE chapter as Education Committee Member; all outside the submitted work. L Monasta reports support for the present manuscript from the Italian Ministry of Health (Ricerca Corrente 34/2017), payments made to the Institute for Maternal and Child Health IRCCS Burlo Garofolo. R da Silveira Moreira reports grants or contracts from CNPq (National Council for Scientific and Technological Development) for a CNPq Research Productivity Scholarship (scholarship registration number is 316607/2021–5); all outside the submitted work. J F Mosser reports support for the present manuscript from the Gates Foundation; grants or contracts from Gavi; honoraria for lectures, presentations, speakers bureaus, manuscript writing or educational events from Providence Medical Center for CME presentation; support for attending meetings and/or travel from the Gates Foundation; all outside the submitted work. F Mughal reports support for the present manuscript paid to their institution from the National Institute for Health and Care Research (NIHR) (USA) (300957). Views expressed in this manuscript are those of the authors and not of the NHS, NIHR, or DHSC. S Nomura reports support for the present manuscript from Ministry of Education, Culture, Sports, Science and Technology of Japan (24H00663) and the Japan Science and Technology Agency for Precursory Research for Embryonic Science and Technology (JPMJPR22R8). B OANCEA reports support for the present manuscript from Ministry of Research, Innovation and Digitalization through the Core Program of the National Research, Development and Innovation Plan 2022–2027, project number PN 23-02-0101, contract number 7N/2023; PNRR/2022/C9/MCID/I8 project 760096. R Olum reports grants or contracts from Gilead Sciences Inc. through the Gilead Research Scholars Program for Public Health; all outside the submitted work. S Onie reports support for the present manuscript from National Health and Medical Research Council, Australia for an Investigator Grant; consulting fees from WHO for the amount of USD$9000 from November 2023 to date; support for attending meetings and/or travel from Suicide Prevention Australia for travel and attendance fees for annual conference and International Association for Suicide Prevention for conference attendance fees; leadership or fiduciary roles in other board, society, committee or advocacy group, paid or unpaid, with International Association for Suicide Prevention as Vice President and Indonesian Association for Suicide Prevention as President; stock or stock options in Wellspring Indonesia, a local mental health clinic in Indonesia (not majority shareholder); all outside the submitted work. R Ornello reports consulting fees from Teva; payment or honoraria for lectures, presentations, speakers bureaus, manuscript writing or educational events from Novartis, Eli Lilly, Teva, AbbVie, Bayer, Pfizer, Lundbeck, Organon; support for attending meetings and/or travel from Teva and Novartis; participation on an Advisory Board with Eli Lilly and AbbVie; receipt of equipment, materials, drugs, medical writing, gifts or other services from Novartis; all outside the submitted work. A Ortiz reports grants or contracts from Sanofi paid to their institution The Fundación Jiménez Díaz Health Research Institute (IIS-FJD UAM) and as Director of the Catedra AstraZeneca-UAM of chronic kidney disease and electrolytes paid to their institution Universidad Autonoma de Madrid (UAM); consulting fees from Astellas, AstraZeneca, Bioporto, Boehringer Ingelheim, Fresenius Medical Care, GSK, Bayer, Sanofi-Genzyme, Lilly, Chiesi, Otsuka, Novo-Nordisk, and Sysmex; payment or honoraria for lectures, presentations, speakers bureaus, manuscript writing or educational events from Astellas, AstraZeneca, Bioporto, Boehringer Ingelheim, Fresenius Medical Care, GSK, Bayer, Sanofi-Genzyme, Sobi, Menarini, Lilly, Chiesi, Otsuka, Novo-Nordisk, Sysmex and Vifor Fresenius Medical Care Renal Pharma and Spafarma; support for attending meetings and/or travel from Astellas, AstraZeneca, Fresenius Medical Care, Boehringer-Ingelheim, Bayer, Sanofi-Genzyme, Chiesi, Sobi, and Bayer; participation on a Data Safety Monitoring Board or Advisory Board with Astellas, AstraZeneca, Boehringer-Ingelheim, Fresenius Medical Care, Bayer, Sanofi-Genzyme, Chiesi, Otsuka, Novo Nordisk, and Sysmex; leadership or fiduciary roles in other board, society, committee or advocacy group, unpaid, with Council ERA. SOMANE; all outside the submitted work. P K Pal reports grants or contracts paid to their institution from Indian Council of Medical Research (ICMR), Department of Science & Technology(DST)-Science and Engineering Research Board, Department of Biotechnology (DBT), DST-Cognitive Science Research Initiative, Wellcome Trust UK-India Alliance DBT, PACE scheme of BIRAC, Michael J. Fox Foundation, SKAN (Scientific Knowledge for Ageing and Neurological ailments)-Research Trust; payment or honoraria for lectures, presentations, speakers bureaus, manuscript writing or educational events from the International Parkinson and Movement Disorder Society, and Movement Disorder Societies of Korea, Taiwan and Bangladesh, Japanese Society of Neurology, Teva Pharmaceutical Industries and Elsevier Inc (payment of one-thirds of the honorarium to their institute); support for attending meetings and/or travel from the National Institute of Mental Health and Neurosciences (NIMHANS), International Parkinson and Movement Disorder Society, and Movement Disorder Societies of Korea, Taiwan and Bangladesh, Japanese Society of Neurology and Asian Oceanian Congress of Neurology.; leadership or fiduciary roles in other board, society, committee or advocacy group with Indian Academy of Neurology as Past President, Asian and Oceanian subsection of International Parkinson and Movement Disorder Society (MDS-AOS) as Past Secretary, *Annals of Movement Disorders* as Past Editor-in-Chief, the Parkinson Society of Karnataka as President, Infection Related Movement Disorders Study Group of MDS as Chair, Rare Movement Disorders Study Group of International Parkinson and Movement Disorder Society (IPMDS) as a Member, Education Committee of IAPRD as a Member, Rating Scales Education and Training Program Committee of IPMDS as a Member, Neurophysiology Study Group of IPMDS as a Member, Movement Disorders in Asia Study Group as a Member, Post-Stroke Movement Disorders as a Member, Ataxia Study Group of IPMDS as a Member, Ataxia Global Initiative as a Member, Movement Disorders Society of India as President, and the Education Committee of International Parkinson and Movement Disorder Society (IPMDS) as Chair—all unpaid posts except Annual Leadership stipend for 2023–2025, of which one-thirds to be paid to their institute; all outside the submitted work. S K Panda reports support for the present manuscript from Siksha ‘O’ Anusandhan (deemed to be university) in the form of a salary; grants or contracts from file number 17-59/2023-24/CCRH/Tech./Coll./ICMR-Diabetes/960 as co-investigator; all outside the submitted work. G D Panos reports support for attending meetings and/or travel (expenses covered without receiving direct payment) from Roche and Bayer AG; all outside the submitted work. R Passera reports participation on a Data Safety Monitoring Board or Advisory Board with the Data Safety Monitoring Board dello studio “Consolidation with ADCT-402 (loncastuximab tesirine) after immunochemotherapy: a phase II study in BTKi-treated/ineligible Relapse/Refractory Mantle Cell Lymphoma (MCL) patients”—FIL, Fondazione Italiana Linfomi, Alessandria (Italy), unpaid; leadership or fiduciary roles in other board, society, committee or advocacy group, paid or unpaid, with the EBMT Statistical Committee, European Society for Blood and Marrow Transplantation, Paris (France) as a member, and the IRB/IEC Comitato Etico AO SS. Antonio e Biagio Alessandria-ASL AL-VC (Italy) as a past Member (2020–2023); all outside the submitted work. A E Peden reports support for the present manuscript from the (Australian] National Health and Medical Research Council (grant number APP2009306). V C F Pepito reports grants or contracts from Sanofi Consumer Healthcare for study self-care in the Philippines, and Zuellig Family Foundation for health systems strengthening; all outside the submitted work. P Ionela-Roxana reports grants or contracts from the project ‘Societal and Economic Resilience within multi-hazards environment in Romania’ funded by European Union—NextgenerationEU and Romanian Government, under National Recovery and Resilience Plan for Romania, contract number 760050/ 23.05.2023, cod PNRR-C9-I8-CF 267/ 29.11.2022, through the Romanian Ministry of Research, Innovation and Digitalization, within Component 9, Investment I8; all outside the submitted work. L Ronfani reports support for the present manuscript from the Italian Ministry of Health (Ricerca Corrente 34/2017), payments made to the Institute for Maternal and Child Health IRCCS Burlo Garofolo. P S Sachdev reports grants or contracts from National Health and Medical Research Council of Australia, APP1169489 and National Institutes of Health, USA; grants 1RF1AG057531–01 and 2R01AG057531–02A1; payment or honoraria for lectures, presentations, speakers bureaus, manuscript writing or educational events from Alkem Labs for a lecture as part of the Frontiers of Psychiatry 2023 seminar, Mumbai, India, June 2023; participation on a Data Safety Monitoring Board or Advisory Board with Biogen Australia Medical Advisory committee in 2020 and 2021 Roche Australia Medical Advisory Committee in 2022, Eli Lilly, Expert Advisory Panel, 2025; leadership or fiduciary roles in other board, society, committee or advocacy group, unpaid, with International Neuropsychiatric Association as Executive Board Member and World Psychiatric Association as Planning Committee Member; all outside the submitted work. Y L Samodra reports grants or contracts from NSTC—NTU Institute of Epidemiology and Preventive Medicine, Taiwan for a post-doctoral fellow contract; leadership or fiduciary roles in other board, society, committee or advocacy group, paid or unpaid, with Benang Merah Research Center, Indonesia as Co-Founder; other financial or non-financial interests with Jago Beasiswa (idebeasiswa.com) as a scholarship mentor; all outside the submitted work. A E Schutte reports consulting fees from AstraZeneca, Medtronic, Sky Labs, Servier, and Roche; payment or honoraria for lectures, presentations, speakers bureaus, manuscript writing or educational events from AstraZeneca, Medtronic, Sky Labs, Servier, Omron, and Aktiia; support for attending meetings and/or travel from Medtronic, Servier; all outside the submitted work. M Šekerija reports consulting fees from Roche; payment or honoraria for lectures, presentations, speakers bureaus, manuscript writing or educational events from Astellas; all outside the submitted work. V Sharma reports other financial or non-financial interests with DFSS (MHA)'s research project (DFSS28(1)2019/EMR/6) at Institute of Forensic Science & Criminology, Panjab University, Chandigarh, India, outside the submitted work. V Shivarov reports one patent issued or pending with the Bulgarian Patent Office; other financial or non-financial interests with ICON plc. in the form of a salary; all outside the submitted work. J P Silva reports support for the present manuscript from Portuguese Foundation for Science and Technology for payment of a salary (contract with reference 2021.01789.CEECIND/CP1662/CT0014). L M L R Da Silva reports grants or contracts from SPRINT, Sport Physical Activity and Health Research e Innovation Center, Polytechnic of Guarda, 6300–559 6 Guarda, Portugal; and collaborate with RISE—UBI, Health Sciences Research Centre, University of Beira Interior, 6201–506 Covilhã, Portugal; all outside the submitted work. J A Singh reports consulting fees from ROMTech, Atheneum, Clearview healthcare partners, American College of Rheumatology, Yale, Hulio, Horizon Pharmaceuticals, DINORA, ANI/Exeltis, USA Inc., Frictionless Solutions, Schipher, Crealta/Horizon, Medisys, Fidia, PK Med, Two labs Inc., Adept Field Solutions, Clinical Care options, Putnam associates, Focus forward, Navigant consulting, Spherix, MedIQ, Jupiter Life Science, UBM LLC, Trio Health, Medscape, WebMD, and Practice Point communications; and the National Institutes of Health; Payment or honoraria for lectures, presentations, speakers bureaus, manuscript writing or educational events from Simply Speaking; Support for attending meetings and/or travel from Simply Speaking; Leadership or fiduciary role in other board, society, committee or advocacy group, paid or unpaid as a past steering committee member of the OMERACT, an international organisation that develops measures for clinical trials and receives arm's length funding from 12 pharmaceutical companies, and as a Chair of the Veterans Affairs Rheumatology Field Advisory Committee, and as editor and the Director of the UAB Cochrane Musculoskeletal Group Satellite Center on Network Meta-analysis; Stock or stock options in Atai life sciences, Kintara therapeutics, Intelligent Biosolutions, Acumen pharmaceutical, TPT Global Tech, Vaxart pharmaceuticals, Atyu biopharma, Adaptimmune Therapeutics, GeoVax Labs, Pieris Pharmaceuticals, Enzolytics Inc., Seres Therapeutics, Tonix Pharmaceuticals Holding Corp., Aebona Pharmaceuticals, and Charlotte's Web Holdings, Inc. and previously owned stock options in Amarin, Viking, and Moderna Pharmaceuticals; outside the submitted work. I N Soyiri reports leadership or fiduciary roles in board, society, committee or advocacy groups, unpaid as Trustee of the Citizens Advice Bureau for Hull & East Riding, United Kingdom; outside the submitted work. D J Stein reports consultancy honoraria from Discovery Vitality, Kanna, L’Oreal, Lundbeck, Orion, Servier, Seaport Therapeutics, Takeda, and Wellcome; all outside the submitted work. J Sundström reports direct or indirect stock ownership in companies (Anagram kommunikation AB, Sence Research AB, Symptoms Europe AB, MinForskning AB) providing services to companies and authorities in the health sector including Amgen, AstraZeneca, Bayer, Boehringer, Eli Lilly, Gilead, GSK, Göteborg University, Itrim, Ipsen, Janssen, Karolinska Institutet, LIF, Linköping University, Novo Nordisk, Parexel, Pfizer, Region Stockholm, Region Uppsala, Sanofi, STRAMA, Takeda, TLV, Uppsala University, Vifor Pharma, WeMind; all outside the submitted work. R Tabarés-Seisdedos reports grants or contracts from Valencian Regional Government's Ministry of Education (PROMETEO/CIPROM/2022/58) and the Spanish Ministry of Science, Innovation and Universities (PID2021–129099OB-I00). The funders were not involved in the design of the manuscript or decision to submit the manuscript for publication, nor will they be involved in any aspect of the study's conduct; all outside the submitted work. J H V Ticoalu reports leadership or fiduciary roles in other board, society, committee or advocacy group, paid or unpaid, with Benang Merah Research Center, Indonesia as Co-Founder; all outside the submitted work. D Trico reports payment or honoraria for lectures, presentations, speakers bureaus, manuscript writing or educational events from AstraZeneca, Eli Lilly, and Novo Nordisk; support for attending meetings and/or travel from AstraZeneca; participation on a Data Safety Monitoring Board or Advisory Board with Amarin, Boehringer Ingelheim, Novo Nordisk; leadership or fiduciary roles in other board, society, committee or advocacy group, paid or unpaid, with EASD Early Career Academy and EASD Committee on Clinical Affairs; receipt of equipment, materials, drugs, medical writing, gifts or other services from Abbott and PharmaNutra; all outside the submitted work. S J Tromans reports grants or contracts paid to University of Leicester, their institution, as part of the 2023/4 Adult Psychiatric Morbidity Survey team, collecting epidemiological data on community-based adults living in England (a contracted study from NHS Digital, via the Department of Health and Social Care. Contributions on chapters of the 2023/4 Adult Psychiatric Morbidity Survey report), as lead on a study funded by the National Institute for Health and Care Research Clinical Research Network, on optimising the survey design for people with learning disability and autism, as lead on a study from the National Institute for Health and Care Research related to reviewing a national training programme for health and social care professionals relating to learning disability and autism, and as Co-applicant on study funded by the National Institute for Health and Care Research related to Identification, recording, and reasonable adjustments for people with a learning disability and autistic people in NHS electronic clinical record systems; support for attending meetings and/or travel from the Royal College of Psychiatrists; leadership or fiduciary roles in board, society, committee or advocacy groups, paid or unpaid as Academic Secretary for the Neurodevelopmental Psychiatry Special Interest Group and Psychiatry of Intellectual Disability Faculty at the Royal College of Psychiatrists, as Editorial Board Member for *Progress in Neurology and Psychiatry, Advances in Mental Health and Intellectual Disability*, *Advances in Autism*, *BMC Psychiatry*, and *BJPsych Open*, and as Editor of *Psychiatry of Intellectual Disability Across Cultures* (Oxford University Press); outside the submitted work. V-S Tseriotis reports grants or contracts from the European Academy of Neurology, European Committee for Treatment and Research in Multiple Sclerosis; support for attending meetings and/or travel from Inovis, Genesis Pharma, and Novartis; all outside the submitted work. E Upadhyay reports patents issued or pending for “A system and method of reusable filters for anti-pollution mask” (Published); “A system and method for electricity generation through crop stubble by using microbial fuel cells” (Published); “A system for disposed personal protection equipment (PPE) into biofuel through pyrolysis and method” (Published); “A novel herbal pharmaceutical aid for formulation of gel and method thereof” (Published); “Herbal drug formulation for treating lung tissue degenerated by particulate matter exposure” (Published); “A method to transform cow dung into the wall paint by using natural materials and composition thereof” (Filed); “Biodegradable packaging composition and method of preparation thereof” (Filed); “Eco-friendly bio-shoe polish from banana and turmeric” (Filed); “Honey-based polyherbal syrup composition to treat air pollution-induced inflammation and preparation method thereof” (Filed); “Process for preparing a caffeine free, antioxidant and nutrient rich beverage” (Filed); leadership or fiduciary roles in other board, society, committee or advocacy group, paid or unpaid, with Meteorological Society, Jaipur (India) as Executive Council Member, Indian Chapter and DSTPURSE Program as member Secretary; all outside the submitted work. E Vounzoulaki reports grants or contracts from a National Institute for Health and Care Research (NIHR) Development and Skills Enhancement Award (DSE) until July 2026, outside the submitted work. Yichen Wang reports grants or contracts from Mayo Clinic Center for Digital Health and Mayo Clinic Office of Belonging (formerly the Office of Inclusion and Diversity) with support from Dalio Philanthropies, 2024 for an Artificial Intelligence-Machine Learning Award; support for attending meetings and/or travel from The International Foundation for Gastrointestinal Disorders and University of Kansas Health Center; a provisional patent, “A Method to Automate International Classification of Diseases Coding using Large Language Model”; all outside the submitted work. J W Ward reports grants or contracts from Abbott, Gilead, AbbVie, Merck, Siemens, GSK, Cepheid, Zydus Life, governmental agencies, and philanthropic organisations to the Task Force for Global Health for the general support of the Coalition for Global Hepatitis Elimination; all outside the submitted work. P Willeit reports consulting fees from Novartis Pharmaceuticals; outside the submitted work. J F Wu reports grants or contracts from the National Heart, Lung, and Blood Institute (R38HL167238) and prior funding from the American Society of Hematology Hematology Opportunities for the Next Generation of Research Scientists (HONORS) Award; all outside the submitted work. Y Yasufuku reports grants or contracts from Shionogi & Co, Ltd; their employment expenses are paid from the joint research fund provided by this pharmaceutical company to The University of Osaka, outside the submitted work. S Zadey reports payment or honoraria for lectures, presentations, speakers bureaus, manuscript writing or educational events from Think Global Health, The Hindu, and Harvard Public Health Magazine; leadership or fiduciary roles in other board, society, committee or advocacy group, paid or unpaid, with Association for Socially Applicable Research (ASAR) as Cofounding Director, Asia Working Group, The G4 Alliance as Chair, *Lancet* Citizens’ Commission as a Fellow, Duke GEMINI Research Center as Research Aide Sr., Maharashtra State Mental Health Policy as a Drafting Committee Member, and Dr D. Y. Patil University as Adjunct Research Faculty; all outside the submitted work. G Zamagni reports support for the present manuscript from the Italian Ministry of Health (Ricerca Corrente 34/2017), payments made to the Institute for Maternal and Child Health IRCCS Burlo Garofolo. M Zielińska reports other financial or non-financial interests with Alexion and AstraZeneca Rare Disease as an employee; all outside the submitted work.
