# Supplementary material for: Global burden of 292 causes of death in 204 countries and territories and 660 subnational locations, 1990–2023: a systematic analysis for the Global Burden of Disease Study 2023
Source: Lancet. 2025 Oct 18;406(10513):1811–72. doi: 10.1016/S0140-6736(25)01917-8 (PMC12535838; doi:10.1016/S0140-6736(25)01917-8)
Supplement: Supplementary appendix 1 [file mmc1.pdf]

THE LANCET

Supplementary appendix 1

This appendix formed part of the original submission and has been peer reviewed. We post it as supplied by the authors.

Supplement to: GBD 2023 Causes of Death Collaborators. Global burden of 292 causes of death in 204 countries and territories and 660 subnational locations, 1990–2023: a systematic analysis for the Global Burden of Disease Study 2023. *Lancet* 2025; published online Oct 12. https://doi.org/10.1016/S0140-6736(25)01917-8.

Appendix 1: methods appendix to "Global burden of 292 causes of death in 204 countries and territories and 660 subnational locations, 1990–2023: a systematic analysis for the Global Burden of Disease Study 2023"

This appendix provides further methodological detail for "Global burden of 292 causes of death in 204 countries and territories and 660 subnational locations, 1990–2023: a systematic analysis for the Global Burden of Disease Study 2023."

Preamble

This appendix provides further methodological detail for "Global burden of 292 causes of death in 204 countries and territories and 660 subnational locations, 1990–2023: a systematic analysis for the Global Burden of Disease Study 2023." This study complies with the Guidelines for Accurate and Transparent Health Estimates Reporting (GATHER) recommendations.[1] It includes detailed tables and information on data in an effort to maximise transparency in our estimation processes and provide a comprehensive description of analytical steps. We intend this appendix to be a living document, to be updated with each iteration of the Global Burden of Disease Study.

Portions of this appendix have been reproduced or adapted from appendices for GBD 2021 Causes of Death Collaborators,[2] and GBD 2021 Demographics Collaborators.[3] References are provided for reproduced or adapted sections.

Table of Contents

Preamble ... 2
Section 1: List of appendix figures and tables ... 5
Section 2: GBD overview ... 6
Section 2.1: Geographical locations of the analysis ... 6
Section 2.2: Time period of the analysis ... 6
Section 2.3: GBD cause list ... 6
Section 2.4: Statement of GATHER compliance ... 7
Section 2.5: Data input sources overview ... 7
Section 2.6: Funding sources ... 7
Section 2.7: Abbreviations ... 7
Section 3: GBD 2023 causes of death database ... 8
Section 3.1: CoD data identification[2] ... 8
Section 3.2: Verbal autopsy[2] ... 10
Section 3.3: Standardise input data (step 1)[2] ... 13
Section 3.4: Map to GBD cause list (step 2)[2] ... 15
Section 3.5: Age-sex splitting (step 3)[2] ... 16
Section 3.6: Correction for miscoding of Alzheimer's and other dementias, Parkinson's disease, and atrial fibrillation and flutter (step 4)[3] ... 17
Section 3.7: Redistribute (Step 5)[2] ... 19
Section 3.8: Correction for Misclassification of COVID-19 deaths ... 24
Section 3.9: HIV/AIDS misclassification correction (step 6)[2] ... 26
Section 3.9: Scale strata to province (step 7)[2] ... 26
Section 3.10: Restrictions post-redistribution (step 8)[2] ... 27
Section 3.11: Drop VR country-years or mark as non-representative (step 9)[2] ... 27
Section 3.12: Cause aggregation (step 10)[2] ... 27
Section 3.13: Remove shocks and HIV/AIDS maternal adjustments (step 11)[2] ... 28
Section 3.14: Noise reduction (step 12)[2] ... 32
Section 3.15: Cause of death database and outlier identification (step 13)[2] ... 35
Section 3.16: Causes of death data star-rating calculation[2] ... 35
Section 4: Causes of death modelling methods ... 37
Section 4.1: CODEm[2] ... 37
Section 4.2: Causes modelled outside of CODEm[2] ... 40
Section 5: COVID-19 estimation ... 43

Section 6: Central computation[2] 45
Section 6.1: Imported cases 45
Section 6.2: CoDCorrect 45
Section 6.3: Years of life lost calculation 46
Section 6.4: GBD world population age standard 46
Section 7: References 47
Section 8: Tables and figures 51
Section 9: CoD cause-specific modelling descriptions 312

Section 1: List of appendix figures and tables

See section 9 for all the appendix figures and tables listed here.

Appendix figures:

Appendix Figure S1. Analytical flowchart for the development of the GBD 2023 cause of death database and different strategies used to combine all medically verifiable cause of death data into a consistent set of cause-specific deaths for each location, age, sex, and year.

Appendix Figure S2: GBD 2021 causes of death estimation flowchart by modelling group.

Appendix Figure S3: Vital registration and verbal autopsy data availability by country, 1980−2023.

Appendix figure S4: Classification of national time series of vital registration and verbal autopsy data 1980−2023.

Appendix Figure S5: Classification of national time series of vital registration and verbal autopsy data 2010−2023.

Appendix Figure S6: Percent of vital registration deaths assigned to major garbage codes for all ages and sexes by country, 1980-2023.

Appendix Figure S7: Out-of-sample model performance for CODEm models for GBD 2023 and age-standardised cause-specific mortality rate by level 2 causes.

Appendix tables:

Appendix Table S1: GBD cause hierarchy with levels.

Appendix Table S2: List of International Classification of Diseases (ICD) codes mapped to the Global Burden of Disease cause list for causes of death.

Appendix Table S3: Total number of site years by cause and source type for GBD 2023.

Appendix Table S4: GBD location hierarchy with level.

Appendix Table S5: Underlying indicators for percent well-certified for data source with maximum percent well-certified in each 5-year time interval for 204 countries, 1980-2023.

Appendix Table S6: Restrictions on age and sex by cause for GBD 2023.

Appendix Table S7: HIV/AIDS-related garbage code redistribution packages.

Appendix Table S8: Modelling strategy for individual cause of death models in GBD 2023.

Appendix Table S9: Percent change before and after CoDCorrect by cause for all ages, both sexes, global, 2023.

Appendix Table S10: CODEm covariates used, level of covariate, and expected direction of covariate by cause, sex, and age.

Appendix Table S11: Comparison of GBD 2021 and GBD 2023 covariates and level of covariates used in cause of death modelling.

Appendix Table S12: CODEm predictive validity results by cause, model type, sex, and age.

Section 2: GBD overview

The Global Burden of Diseases, Injuries, and Risk Factors Study (GBD) is a collaborative research effort aimed at estimating morbidity and mortality from a comprehensive set of diseases, injuries, and risk factors. The GBD Collaborator Network draws on the expertise of over 14,000 contributors from around the world.

Section 2.1: Geographical locations of the analysis

We produced estimates for 204 countries and territories that were grouped into 21 regions and seven super-regions (table S1). The seven super-regions are central Europe, eastern Europe, and central Asia; high income; Latin America and the Caribbean; north Africa and the Middle East; south Asia; southeast Asia, east Asia, and Oceania; and sub-Saharan Africa. In GBD 2023 we continue to analyse at subnational levels countries that were added in previous cycles including Brazil, China, Ethiopia, India, Indonesia, Iran, Italy, Japan, Kenya, Mexico, New Zealand, Nigeria, Norway, Pakistan, Russia, the Philippines, Poland, South Africa, Sweden, the UK, and the USA. All analyses are at the first level of administrative organisation within each country except for New Zealand (by Māori ethnicity), Sweden (by Stockholm and non-Stockholm), the UK (by local government authorities), and the Philippines (by provinces). To meet data use requirements, in this publication we present subnational estimates for Brazil, Ethiopia, Indonesia, Iran, Japan, Kenya, Mexico, Norway, Pakistan, South Africa, Sweden, the UK, and the USA; given space constraints, these results are presented in Appendix 2 instead of the main text. Additionally, subnational estimates for China, India, Nigeria, and Russia are included in maps but are not reported in appendix tables. Subnational estimates for other countries will be released in separate publications, although please note that we only release estimates for a subset of these countries, per agreements with country partners.

At the most detailed spatial resolution, we generated estimates for 925 unique locations. As was done in GBD 2021, in GBD 2023 we continue to use the set of locations defined as standard locations and non-standard locations. Standard GBD locations are defined as the set of all subnationals belonging to countries where data quality is high and with populations over 200 million, in addition to all other countries. Standard locations include the subnationals for China, India, the USA, and Brazil, but not Indonesia; data for China, India, the USA, and Brazil are also included at the country level. All other countries with subnational estimates are defined as non-standard locations.

Section 2.2: Time period of the analysis

We estimated numbers and rates of incidence, prevalence, years lived with disability (YLDs), and disability-adjusted life-years (DALYs) for the years 1990–2023; we estimated deaths and years of life lost (YLLs) for 1980–2023.

Section 2.3: GBD cause list

The GBD cause and sequelae list for causes of death is organised hierarchically (see table S2) to accommodate different purposes and needs of various users.

The first two levels aggregate causes into general groupings. At Level 1 there are three cause groups: communicable, maternal, neonatal, and nutritional diseases (Group 1 diseases); non-communicable diseases (Group 2); and injuries (Group 3). These Level 1 aggregates are subdivided at Level 2 of the hierarchy into 22 cause groupings (eg, neonatal disorders, neurological disorders, and transport injuries). The disaggregation into Levels 3 and 4 contains the finest level of detail for causes captured in GBD 2023. The greatest detail available for some causes, such as anxiety disorders or rheumatoid arthritis, is at Level 3 of the hierarchy, while other specific causes are at Level 4 of the hierarchy with an aggregate category at Level 3 (for example, depressive disorders at Level 3, which encompasses major depressive disorders and dysthymia at Level 4). Sequelae of diseases and injuries are organised at Levels 5 and 6 of the hierarchy. In GBD, sequelae are defined as distinct, mutually exclusive categories of health consequences that can be directly attributed to a cause. For example, both neuropathy and blindness due to diabetic retinopathy are sequelae of diabetes; stroke and ischaemic heart disease are not, as these consequences cannot be categorically ascribed to diabetes in an individual despite good evidence for increased risk of these outcomes. The finest detail for all sequelae estimated in GBD is at Level 6 and is aggregated into summary sequelae categories (Level 5) for causes with large numbers of sequelae. Examples include the grouping of the infectious disease episodes and long-term sequelae of meningitis. For GBD 2023 there are 3499 mutually exclusive and

collectively exhaustive sequelae, 2089 cause sequelae, and 1410 injuries sequelae, and thus our YLD estimates at each level of the hierarchy sum to the total of the level above. Prevalence and incidence aggregation is estimated at the level of individuals who may have more than one sequela or disease and therefore are not additive.

The GBD cause list continues to evolve to reflect the policy relevance and public health and medical care importance of the causes of major losses of health. The cause and sequelae list expanded based on input from the Scientific Council and GBD Collaborator Network. For GBD 2023, the causes of death cause list has increased to 292 causes, from the 286 causes in GBD 2021. The non-fatal cause list has expanded from 364 causes in GBD 2019 to 365 causes in GBD 2023. The total number of fatal and non-fatal causes combined for GBD 2023 is 371. As in GBD 2021, we made no estimates for YLDs for just five causes, either because no disability is possible (as is the case with sudden infant death syndrome); because disability may occur rarely but at levels too low for accurate estimation given the data (as for aortic aneurysm); or because the disability is captured by the complicating causes that led to that cause of death (as for indirect maternal deaths, late maternal deaths, and maternal deaths aggravated by HIV/AIDS).

Section 2.4: Statement of GATHER compliance

This study complies with the Guidelines for Accurate and Transparent Health Estimates Reporting (GATHER) recommendations.[1] We have documented the steps in our analytical procedures and detailed the data sources used. See appendix table S4 for the GATHER checklist. The GATHER recommendations can be found at the GATHER website under GATHER Statement.

Section 2.5: Data input sources overview

GBD 2023 synthesises a large and growing number of data input sources including surveys, censuses, vital statistics, and other health-related data sources. The data from these sources are used to estimate morbidity; illness, and injury; and attributable risk for 204 countries and territories from 1990 to 2021; cause-specific mortality is estimated from 1980 to 2023. The input sources are accessible through an interactive citation tool available in the GHDx.

Citations for specific GBD components, causes and risks, and locations can be found through the GBD Sources Tool in the GHDx: http://ghdx.healthdata.org/gbd-2023/sources. This tool allows users to view and access GHDx records for input sources and export a comma-separated value (CSV) file that includes metadata, citations, and information about where the data were used in GBD, as data use agreements allow. As required by GATHER, additional metadata for input sources are available through the citation tool as well.

Section 2.6: Funding sources

This publication and the research it presents was funded by the Gates Foundation (OPP1152504); Queensland Department of Health, Australia; the National Health and Medical Research Council; the Norwegian Institute of Public Health; the New Zealand Ministry of Health, UK Department of Health and Social Care, St. Jude Children's Research Hospital. The funders of the study had no role in study design, data collection, data analysis, data interpretation, or writing of the report. All authors had full access to all data in the study and had final responsibility for the decision to submit for publication.

Section 2.7: Abbreviations

ART	antiretroviral therapy
BTL	basic tabulation list
CDC	United States Centers for Disease Control & Prevention
CHAMPS	Child Health and Mortality Prevention Surveillance
CoD	causes of death
CODEm	Cause of Death Ensemble modelling
CRVS	civil registration and vital statistics system
DALYs	disability-adjusted life-years

DHS	Demographic and Health Survey
DisMod-MR	disease model-Bayesian meta-regression
DSP	disease surveillance points
EMR	excess mortality rate
EUREG	European Registry
GATHER	Guidelines for Accurate and Transparent Health Estimates Reporting
GBD	Global Burden of Diseases, Injuries, and Risk Factors Study
GHDx	Global Health Data Exchange
GPR	Gaussian process regression
HDSS	Hierarchical Data Storage System
ICD	International Classification of Diseases
IHME	Institute for Health Metrics and Evaluation
INDEPTH	International Network for the Demographic Evaluation of Populations and their Health
InterVA	Interpreting Verbal Autopsy
LMER	linear mixed effects regression
MCCD	Medical Certification of Causes of Death
MITS	minimally invasive tissue sample
MMR	maternal mortality ratio
NORDCAN	database of cancer statistics for the Nordic countries
OPRM	other pandemic-related mortality
PAF	population attributable fraction
PCVA	Physician-Certified Verbal Autopsy
PHMRC	Population Health Metrics Research Consortium
PWC	piecewise continuous
RSME	root mean square error
SD	standard deviation
SRS	Sample Registration System
ST-GPR	spatiotemporal Gaussian process regression
UI	uncertainty interval
UNAIDS	Joint United Nations Programme on HIV and AIDS
UN	United Nations
USA	United States of America
USSR	Union of Soviet Socialist Republics
VA	verbal autopsy
VR	vital registration
WHO	World Health Organization
YLDs	years lived with disability
YLLs	years of life lost

Section 3: GBD 2023 causes of death database

All available data on causes of death (CoD) are standardised and pooled into a single database used to generate cause-specific mortality estimates by age, sex, year, and geography. Appendix figures 1 and 2 show the high-level view of data inputs, analytical steps, and outputs of the CoD analysis framework. Section 3 of this appendix provides details on each step in the development of the CoD database as illustrated in appendix figure 1.

Section 3.1: CoD data identification[2]

Section 3.1.1: Overview of data types

The CoD database contains eight types of data sources (table S5): vital registration (VR), verbal autopsy (VA), cancer registry, police records, sibling history, surveillance, survey/census, and minimally invasive tissue sample (MITS) diagnoses. In countries with VR systems with high completeness and low garbage coding, vital registration is often the

primary source of data for causes of death, but police data, surveillance data, and open-source databases are also used for select causes, such as injuries, violence, and maternal causes of death. Less than half of the world's population has deaths captured in a VR system; therefore, in countries with incomplete VR systems, vital statistics for causes of death may be supplemented with other data types (appendix figure 3).

Section 3.1.2: ICD detail

A majority of the CoD data are VR data obtained from the World Health Organization (WHO) Mortality Database; a compilation of data submitted to WHO by individual countries. VR is also obtained from country-specific mortality databases operated by official offices. Each cause is coded directly to the three- or four-digit ICD-coded cause of death, when possible, whereas cause codes in data tabulated by International Classification of Disease (ICD) are coded to aggregated cause groups. The CoD database contains 2608 country-years of detailed data from 1980 to 2018, which includes underlying CoD coded with three- to four-digit codes by country, year, sex, and age groups. Detailed causes are coded to one of the following ICD-detail coding systems: ICD-8, ICD-9, or ICD-10 (table S6). Each coding system has a similar cause hierarchy and cause list that has continually developed over time. ICD-10 is the current standard and the most exhaustive cause list. Within the cause lists, five-digit codes, where available, are truncated to four-digit codes to condense the lists. Updates to ICD-detail occur biannually as WHO releases new versions or as country collaborators provide additional data. Updates to data from WHO increasingly include ICD-10 CoD data as it is the most current classification of CoD, while updates to ICD-8 and ICD-9 detailed lists are less common. In the case of overlapping data, preference is given to data from pre-determined country collaborations, which are updated annually.

Section 3.1.3: ICD tabulations list

The ICD tabulation lists include the ICD-8 List A (ICD-8A and ICD-8B), ICD-9 Basic Tabulation List (BTL), ICD-10 Mortality Tabulation, Russia Tabulation; the former Union of Soviet Socialist Republics (USSR) Tabulation, Russia Tabulation from 1989-2001, Russia Tabulation after 2001, and India Medical Certification of Cause of Death (MCCD) and China Disease Surveillance Points (DSP) ICD-9 and ICD-10. These data sources make up 1096 country-years from 1980 to 2017 in the CoD database. All are condensed versions of the ICD-8, ICD-9, and ICD-10 detail lists with some differences in the format of cause lists depending on the data source. ICD-8A, ICD-9 BTL, and ICD-10 Mortality Tabulation CoD are assigned to subtotal groups (referred to as chapters) and cause groups respective to ICD-detail groups. Additionally, ICD-9 BTL includes ICD-9 detail codes for some cancers and a custom tabulation scheme for the former USSR countries. The Russia Tabulation lists, and India MCCD cause lists, each have custom nomenclatures based on ICD-detail cause codes.

Two of the drawbacks in using tabulation lists are discrepancies in the accuracy of death counts and lack of detail due to aggregated cause groups. There are instances where the sum of deaths in chapter subtotals are not equal to the sum of cause groups within the chapter. To account for any missing or duplicate deaths reported within the cause groupings, death counts are systematically adjusted by calculating the differences between subtotals and sub-causes within the cause groups. Any differences are assigned to a remainder cause group. To account for the lack of cause code detail, select cause groups are disaggregated (Step 1.1) to create a complete cause list. Updates to ICD tabulation lists obtained from WHO occur less frequently compared to ICD-detailed lists as more countries are reporting deaths in ICD-detail. In instances of overlapping data, preference is given first to detailed collaborator data, followed by detailed WHO data, then tabulated collaborator data, and finally tabulated WHO data.

Section 3.1.4: China Disease Surveillance Points /China Center for Disease Control and Prevention

The two primary sources of data for China are surveillance data from the China Disease Surveillance Points (DSP) system and VR data collected by the Chinese Center for Disease Control and Prevention (CDC). In the China DSP data, deaths were reported across 145 disease surveillance points used from 1991 to 2003, 161 disease surveillance points from 2004 to 2012, and 605 disease surveillance points from 2013 to 2017. While China DSP with ICD-10 coding is considered sample VR data, it provides national coverage and cause detail. Thus, it receives similar processing and treatment to the China CDC VR from 2008 to 2016. From 2008 to 2017, all of the deaths and CoD information from the DSP system and other system points throughout China were collected and reported via the Mortality Registration and Reporting System, an online reporting system of the Chinese CDC. For these years of DSP, we process using a country-specific cause map. For DSP ICD-9, which contains aggregated causes, we use a

tabulated cause map. For DSP ICD-10, we disaggregate causes using the ICD code detail in the CDC data. From 2018 to 2021, the format of the DSP data changed to aggregated causes and eastern, central, and western regions. These four years, we used previous years of formatted DSP data from 2010 to 2017 to split the aggregated causes and regions into ICD10 codes and provinces. The deaths in these data are reported at the strata level, a metric that is specific to China. Counties are stratified by urban and rural classification, but definitions of urbanity vary across counties. In Step 7, we use a method developed to scale up deaths from strata level to the province level.

Section 3.1.5: Sample registration system

Sample registration systems are expanding in several countries and are key sources of data in Indonesia and India. The Sample Registration System (SRS) is a dual-record system wherein a resident part-time enumerator continuously records births and deaths in each household within the sample unit every month. A full-time SRS supervisor thereafter independently collects the vital events along with other related details for each of the preceding six-month periods during the calendar year. Cause of death in SRS systems is recorded using either the detailed ICD-10 coding list, or the INDEPTH ICD-10 short list.[2]

Section 3.1.6: India Medical Certification of Cause of Death

The India MCCD has data for the urban parts of the majority of the states and union territories beginning in 1980. Deaths reported in this data source have been medically certified and are considered VR data. The CoD are reported in a tabulation list with a unique numbering scheme that conforms to ICD-9 and ICD-10 detail codes, which must be disaggregated. MCCD is state-split to fill in data gaps (Step 1.2 state splitting) prior to age-sex splitting. Because SRS is widely considered a more credible assessment of CoD in India, we chose to use MCCD data only in certain cases for modelling with Cause of Death Ensemble modelling (CODEm). We preserved MCCD data in the database for two primary reasons. First, where the three midpoint years of SRS data resulted in the loss of a clear time trend, as was the case for maternal mortality, we chose to preserve MCCD in addition to SRS. Second, MCCD has an advantage over SRS in cases where VA is not a valid instrument for ascertaining CoD, like encephalitis and dengue fever. In these cases, we kept MCCD over SRS.

Section 3.2: Verbal autopsy[2]

Section 3.2.1: Verbal autopsy coded to ICD-10 and other lists

In countries without VR systems, VA studies are a viable data source to inform CoD. Data are obtained by trained interviewers who use a standardised questionnaire to ask relatives about the signs, symptoms, and demographic characteristics of family members deceased within a year. CoD is assigned based on the answers to the questionnaires using a variety of methods, explained in more detailed in section 3.2.2.

VA data are highly heterogeneous: studies use different instruments, different cause lists (from single causes to full ICD cause lists), different methods for assigning CoD, different recall periods, and different age groups. Cultural differences may also affect the interpretation of specific questions. VAs are likely accurate in assigning CoD to road injury or homicide but less accurate for causes requiring medical certification, such as cardiovascular causes. Studies may also occur as standalone assessments or as part of an extended network, such as the International Network for the Demographic Evaluation of Populations and their Health (INDEPTH) Network[2]— a continuous surveillance source with several Demographic Surveillance Systems sites that collect data coded to ICD-detail causes.

Section 3.2.2: Methods of ascertaining cause of death from VA questionnaires

Verbal autopsy (VA) is one of the most common tools used to obtain data on causes of death in countries that lack a functional civil registration and vital statistics (CRVS) system. As such, a wide variety of methods have been developed for ascertaining cause of death based on a VA interview. The historical standard and still one of the most popular methods is Physician-Certified Verbal Autopsy (PCVA), in which two physicians review the interview responses and formulate the most likely cause of death independently. A third physician may arbitrate the result if there is disagreement. This method has some important advantages, including incorporating the knowledge and expertise of local physicians, but is often expensive and slow, includes the variability inherent to human judgement, and has a high opportunity cost in terms of physicians' time spent delivering health care.

In response to these challenges, a variety of automated CoD ascertainment methods have been developed based on algorithmic or probabilistic approaches.[4,5] Two popular automated methods are Tariff 2.0/SmartVA and Interpreting Verbal Autopsy (InterVA). The Tariff method is an algorithm designed and validated by the Population Health Metrics Research Consortium (PHMRC) and IHME. It is based on the association between individual signs and symptoms and causes of death and is trained on the PHMRC gold standard validation study database, a database of VAs collected for deaths that are linked to hospital records in India, the Philippines, Mexico, and Tanzania, where the hospital records provide the true cause of death.[6] The InterVA (Interpreting Verbal Autopsy) method is a Bayesian method, based on the probability of answering yes to a given line item conditional on the true cause of death. These conditional probabilities are determined using a mix of expert opinion and reference data.[7]

A 2014 study by the PHMRC and IHME found that InterVA showed markedly lower performance in predicting causes of death in the PHMRC gold standard validation database when compared to three other automated CoD ascertainment methods, including Tariff.[8] As a result, InterVA modelled VA data are vetted with special care and frequently excluded for causes other than injuries and maternal causes, which are easier for most VA CoD ascertainment methods to diagnose.

Each GBD round, a systematic review of verbal autopsy literature data is conducted to ascertain and include new VA studies using PubMed and Google Scholar. The PubMed search typically returns fewer articles, while Google Scholar returns more articles but tends to be less precise for capturing raw VA data. For GBD 2023, African Journals Online was also included in the literature search with the goal of addressing the dearth of VA sources from African regions, with a review of sources in French.

The search strings for the literature search are below.

Google Scholar search strings:

"verbal autopsy" AND ("cause of death" OR "causes of death" OR "reason for death" OR "reasons for death" OR "cause-specific mortality"), 2021-2021,2022-2022, 2023-2023

PubMed search strings:

("verbal autopsy") AND ("2019/04/24 2021/05/15"[Date - Publication] : "3000"[Date - Publication])

AJOL search strings:

("verbal autopsy")

Inclusion criteria:

- Mentions verbal autopsy
- Mentions causes of death or cause fractions
- References data collection or raw/crude data
- Data must provide a viable, non-biased CoD distribution at an acceptable GBD location level: for example, we do not use global or regional data.

Exclusion criteria:

- Studies already included in previous rounds of GBD
- Non-representative demographics, aka “sub-populations” (eg, HIV-positive people, ICU/NICU patients)
    - This does not include non-representative locations, eg, certain villages, cities, provinces, states, etc. within a GBD location
- Hospital-based sampling
- Methods/theory papers
- Review papers
    - We never extract from review papers, but instead trace the underlying studies and extract the dataset from those
- Papers published by the GBD

- Protocols
- Papers that only report on stillbirths
- Papers that cover exclusively pre-1980
- VA that uses the InterVA assignment method (except for injury and maternal-related deaths)
- Small sample sizes (<50 deaths)
    - Some exceptions have been made for studies from data-sparse countries
- Deaths for one cause without a denominator, for example, only cancer deaths without total deaths
- National level where we model subnationally (eg, South Africa, India, Indonesia)
- Locations reported that are higher than country level (eg, results for several countries together cannot be used)
- Adjusted/modelled estimates (ie, not raw counts) – if the numbers have uncertainty intervals, this is a good sign that they are actually modelled estimates
- Studies where the denominator is not “all deaths due to all causes,” eg, datasets where the denominator is all tuberculosis deaths

Figure A. PRISMA diagram for systematic review of verbal autopsy data included in GBD 2023

PRISMA Flow Diagram

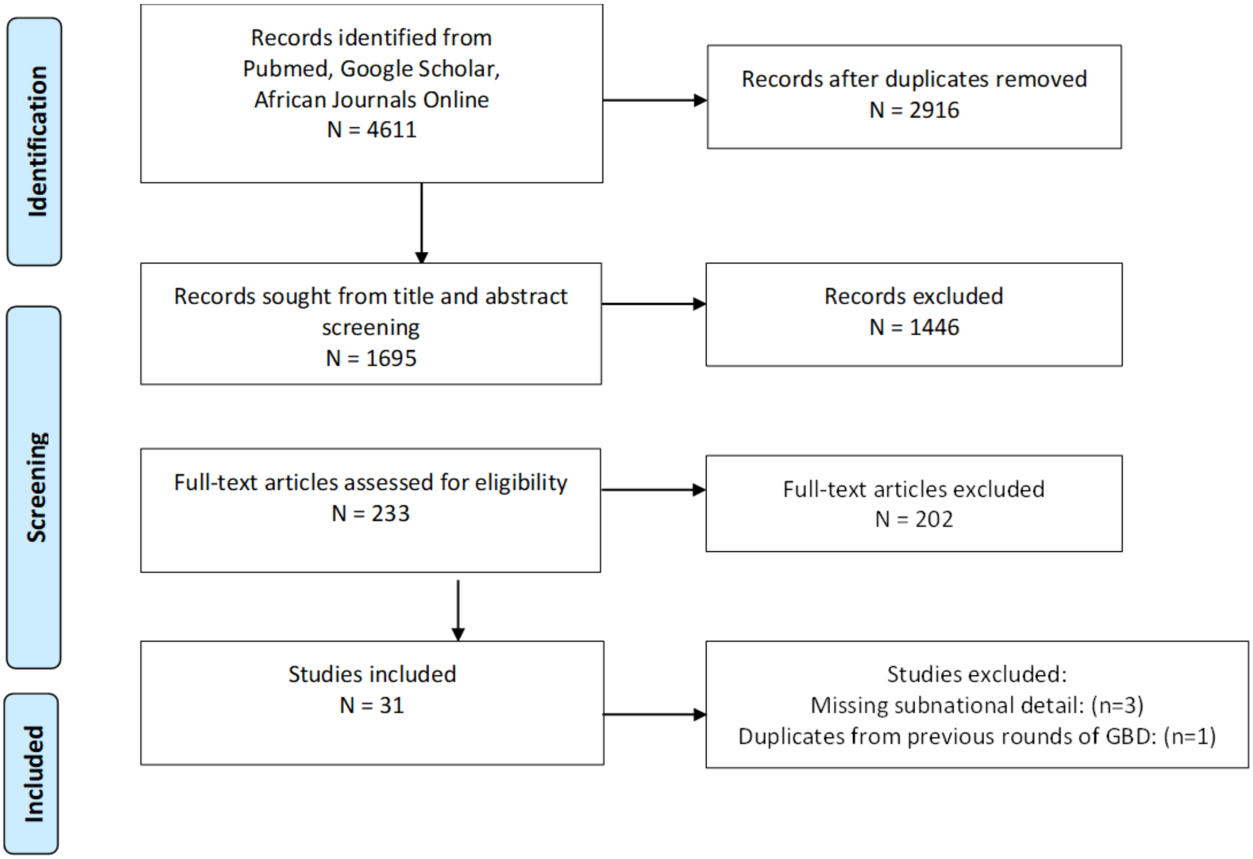

Section 3.2.3: Other data types

Section: 3.2.3.1: Maternal mortality data

In locations with low-quality or no VR, maternal mortality metrics can be found in surveillance, surveys, census, and sibling history data sources. The best data have death counts due to maternal causes and the total number of deaths for

women within the reproductive ages of 10–54 by year. If a data source is missing these components, creating a complete cause list is necessary by using livebirths and all-cause mortality deaths.[3] The livebirths are used to scale down the envelope to match the coverage of the raw data. Though death counts are the preferred metric, maternal mortality is often measured by using the maternal mortality ratio (MMR), which is easily converted to deaths by using livebirths. The China Maternal and Child Surveillance data are adjusted by scaling data from the strata to the province level (Step 7).

Section: 3.2.3.2: Surveys and censuses reporting fraction of deaths due to selected injuries

Surveys and censuses are often used in countries with less-developed VR systems; in countries with adequate VR, surveys and censuses are supplementary. Much like VAs, the CoD validity is a concern because of lack of medical certification at the time of death. For these data sources, we keep only causes related to maternal mortality and injuries. The remaining causes are accounted for as a remainder of total deaths in the sample size. One example of these surveys is DHS sibling history. In these surveys, females are interviewed regarding their sisters and survival status. Further questions are asked regarding the deceased siblings such as whether the sibling was pregnant or was within six weeks post-partum at the time of death. From these results, we can discern maternal mortality after applying correction factors including the Gakidou-King weights[9] and removing incidental HIV deaths.

Section 3.2.4: Police records

In most countries, police and crime reports are an important source of information for some types of injury deaths, notably road injuries and interpersonal violence. Our police data come from reports on road traffic and crime trends. The police reports used in this analysis were obtained from published studies, national agencies, and institutional surveys such as the United Nations (UN) Crime Trends survey and the UN Office on Drugs and Crime Global Study on Homicides. We assessed whether police reports were likely to be complete and to cover the entire country by comparing police trends with those seen in VR. Data are excluded in instances where police data for road traffic injuries are significantly lower than the VR. Inclusion methods for police data are based on data quality and completeness. For countries with high-quality data, with a star rating of 4 or 5, we utilise police records if the death counts provided are higher than those in the VR. For countries with lower-quality data, with a star rating of 0–3 stars, we always include police records. Police data that meet our inclusion criteria and provide complete coverage are uploaded to the database for use in road injuries and interpersonal violence deaths estimation.

Section 3.2.5: Population-based cancer registries

Section 3.2.5.1: Cancer registries with incidence

Data on cancer incidence were sought from population-based cancer registries as well as from databases that include multiple registries, including Cancer Incidence in Five Continents, NORDCAN, and EUREG. Cancer registries were identified through the membership list of the International Association of Cancer Registries, through the GBD Collaborator Network, or through publications. Registries were excluded if they were not representative of the coverage population, if the data were limited to years prior to 1980, if the source did not provide details on the population covered, or if the list of cancer types included was not comprehensive for the age group covered. Beginning in GBD 2019, childhood cancer-specific population-based cancer registry data were sought and included.

Section 3.2.5.2: Cancer registries with incidence and high-quality mortality data

In addition to incidence, some high-quality cancer registries also report cancer mortality data. These data were also extracted and used as inputs to the mortality-to-incidence model.

Section 3.3: Standardise input data (step 1)[2]

The input data to the CoD database are received in various formats and must be standardised to run through central CoD machinery to then upload to the database. Raw data inputs come from data sources such as mortality databases, literature reviews, or reports. Usable data sources must have a clear sample size of the number of deaths in the population and ideally, exhaustive cause lists. The complexity of the data cleaning process varies drastically across data sources. For VR microdata with the location, age, sex, year, and ICD-coded cause of every death, very little effort is necessary to standardise it into a consistent structure. Other sources such as VA, sibling history, and surveillance data may require careful review to accurately extract scans of hardcover CoD reports into spreadsheets that can be transformed and standardised based on the standard process described below.

At this point, data are assigned source identifiers so that they can be linked to the GHDx and cited appropriately. Any aggregate age and sex categories are flagged for age-sex splitting. The methods of cause-of-death assignment and data collection are reviewed to determine which source type to assign; for example, we distinguish sibling history data from surveys with a VA module. Only data at the most detailed level of the GBD location hierarchy are used. Documentation from the source is reviewed to determine if the population is representative of the location or only a subset of the population in that location. Data sources representing a subset of the population are flagged as non-representative; this flag is used by CODEm to increase the variance associated with such datapoints.

Finally, diagnostics are reviewed at this stage to avoid sending cleaning errors downstream. We review cause-specific deaths for each demographic group to ensure the data are reasonable. For example, it is unlikely that male breast cancer deaths are higher than female breast cancer deaths or deaths from neonatal causes occur in age groups over 1 year. All death totals are compared with the sum of cause-specific deaths to ensure the observed deaths are accounted for and sample size is complete.

Section 3.3.1: Disaggregation (step 1.1)

Causes of death in tabulated code systems are specified using tabulated codes, which consist of ranges of detailed codes from the corresponding detailed code systems. These tabulated codes represent groups of related causes, some of which are too general to be mapped directly to estimated GBD causes.

To correct for this, we use a set of age-, sex-, and super-region-specific proportions during data preparation to split tabulated codes into GBD causes and garbage codes that can then be mapped to a GBD cause. These proportions were calculated in GBD 2016 based on detailed VR from code systems ICD-10, ICD-9 detail, and ICD-8 detail. The target causes and garbage codes for a given tabulated code were determined based on the detailed codes that were missing from the tabulated cause list. For any cause and demographic group where we lacked ICD detail, global proportions were used.

As an example, the ICD-10 tabulated list includes a generic tabulated code for “Other intestinal infectious diseases.” In order to make this cause coding compatible with standard GBD cause mapping, it requires splitting onto the detailed causes it represents. To do this, we first define the list of target causes for which the tabulated code will be split. This includes diarrhoea, typhoid fever, and paratyphoid fever in this example. Next, detailed ICD-10 data are used to generate the proportions by which the tabulated code will be split across the target causes. Proportions are generated using all years of available detailed ICD-10 data by super-region, age, and sex. For example, in ICD-10 detail data, in Latin America and the Caribbean, for all-age males, 94.5% of deaths within “Other intestinal infectious diseases” were coded to diarrhoea. Thus, in ICD-10 tabulation data, 94.5% of the “Other intestinal infectious diseases” code were assigned to diarrhoea for countries within this super-region and demographic group.

Section 3.3.2: State splitting (step 1.2)

Two sources for CoD estimation in India are the MCCD report, which reports medically certified deaths from health facilities in mostly urban areas,[10] and the SRS, which collects information via VA about one-half of 1% of the total population in India, including both urban and rural areas, from 8853 sampling units as of 2014.[11] For MCCD, missing data impedes estimation of trends at the state level. We used a first-order, log-linear model of the four-way contingency table of deaths by sex, age, state, and year to estimate the missing state-years. We fit the model to all available data for MCCD separately for each cause, including state-specific all-age measurements and age-specific national measurements. From this, we produced estimates for each combination of sex, age, state, and year. We then used these estimates wherever the raw data did not include sex-specific, age-specific, and state-specific death counts.

For MCCD, the model was fit separately for ICD-10-based and ICD-9-based reports by using the tabulated cause list present in the data.

Section 3.3.3: Calculate non-maternal deaths (step 1.3)

In cases when maternal mortality metrics do not include both deaths due to maternal causes and deaths due to non-maternal causes for women of reproductive age, livebirths and all-cause mortality estimates can be used to calculate deaths, which is the same method used in sibling history data. Many studies report maternal deaths as the MMR.

MMR is the number of maternal deaths per 100 000 livebirths and can be used to calculate deaths when it has been derived from primary data and not estimated. Maternal deaths were calculated by using MMR and livebirths; if livebirths were missing, we substituted livebirth estimates and used the following equation:

$$Maternal\ deaths\ = \frac{MMR}{100{,}000} \times Live\ births$$

If a study was non-representative, we extracted sample size and livebirths from that study. After maternal deaths were calculated, we used the difference from all-cause mortality estimates to determine non-maternal deaths.

A more accurate and data-inclusive method of calculating maternal and non-maternal deaths incorporates coverage and splits deaths for a range of years into individual years. If there were livebirths in the study, we adjusted the coverage.

$$Coverage = \frac{Live\ births}{GBD\ estimated\ live\ births}$$

After coverage was calculated, totals deaths were scaled to be more representative. This gives a more accurate death count since the envelope assumes representative coverage. We then calculated non-maternal deaths by using all-cause mortality as an all-cause total.

$$Maternal\ envelope\ with\ coverage\ = Maternal\ envelope \times Coverage$$

An additional adjustment can be applied to maternal data spanning over a range of consecutive years, which allows for more data inclusion. The years within specified year ranges are separated into individual years, and total deaths within the year range were split between each individual year by using the fixed proportions of maternal deaths from VR in that particular country. We used only VR data to inform the proportions because it was both high-quality and representative.

Section 3.4: Map to GBD cause list (step 2)[2]

In GBD 2023, we used 439 maps to translate causes found in the input data to the GBD 2023 cause list (table S2). This included 31 maps for VR data, 314 for VA data sources, and 98 for other data types. The largest and most universal maps used were those for ICD-9 and ICD-10 VR data. The input data causes varied from three- to four-digit ICD codes to custom cause lists with cause names such as "cholera" or "hepatitis". Our mapping process enabled us to compare these various data sources across demographic groups, by mapping all ICD codes to the mutually exclusive and collectively exhaustive list of GBD causes.

A crucial aspect of enhancing the comparability of data for cause of death is to deal with uninformative, so-called garbage codes. Garbage codes are codes to which deaths were assigned that cannot or should not be considered as the underlying cause of death, for example: heart failure, ill-defined cancer site, senility, ill-defined external causes of injuries, and septicaemia. Additionally, any codes not specific enough to assign to a detailed cause of death is considered a garbage code, such as unspecified infectious disease or unspecified injury. In GBD 2019, we developed additional maps to translate ICD codes found in the input data that are non-underlying causes to appropriate target codes based on the levels of the GBD cause list. These garbage codes were mapped to Class 1–4 of the GBD cause list according to the following criteria:

Class 1 includes all garbage codes for which a Level 1 GBD causes (communicable, maternal, neonatal, and nutritional diseases; injuries; and non-communicable diseases) cannot be directly assigned. For example, the underlying causes of "sepsis" or "peritonitis", if not specified in the data, could be an injury, a non-communicable disease, or a type of communicable disease. In these cases, deaths will be redistributed across all three of these Level

1 causes. In addition, deaths coded to impossible or ill-defined causes of death (including "senility" and "unspecified causes") fall into this category, as they will be redistributed onto all causes.

Class 2 includes all garbage codes that can be assigned to one Level 1 cause in the GBD cause list. This would include deaths coded to "unspecified injuries" (X59), which are redistributed onto all injuries.

Class 3 includes all garbage codes for which we know the Level 2 CoD and can redistribute onto one Level 3 cause. This includes deaths coded to causes such as "unspecified cardiovascular disease", which falls within the Level 2 cause "cardiovascular diseases", as well as those coded to "unspecified cancer site", which falls within the Level 2 cause "neoplasms".

Class 4 includes all garbage codes for underlying causes of death that can be redistributed within a Level 4 cause. This includes garbage codes such as "unspecified stroke" or "unspecified road injuries."

Section 3.5: Age-sex splitting (step 3)[2]

Different sources, particularly VA studies, report deaths for a wide range of age groups with varying intervals. For the analysis of CoD, we mapped these different age intervals to the GBD standard set of age groups. While the proportions informing this mapping were updated in GBD 2023, the approach to undertake this mapping was the same as in the prior GBD studies (GBD 2021, GBD 2019, GBD 2017, GBD 2016, GBD 2015, GBD 2013, and GBD 2010).

In the process of assembling a consolidated demographic database, we found that the aggregation of age groups is perhaps the strongest source of inconsistency. By convention, such data are reported in broad age groupings such as 0–4, 5–14, and 15–49, or with both sexes together. The issue of comparability between age-sex groups arose when assembling the GBD CoD database. We developed a tool called age-sex splitting that takes aggregated age groupings and the "both sexes combined" grouping and divides them into what their constituent age groups would likely have been if respective cause-specific and country-specific age distributions had been used. The analytical framework for GBD includes six age categories for infants and children under 5: early neonatal (0–6 days), late neonatal (7–27 days), 1–5 months, 6–11 months, 12–23 months, and 2–4 years, and 19 age categories for those over 5: 5–9 years, 10–14 years, and so forth, proceeding in five-year age groups until the terminal age group of 95 years and older. We treat unknown ages and sexes in the same manner we treated the "all ages combined" age category and "both sexes combined" sex group. Through this process, we were able to directly compare all data sources on even terms.

The approach to age splitting is based on the following formula. The key assumption underlying this formula is that the relative risk of death by age group compared to a reference age group is invariant across populations. Although this assumption is likely violated in specific cases, a strong biologically based pattern of the relative risk of death for a cause by age is observed for most causes. The basic formula is as follows:

$$D_a = R_a N_a \left(\frac{D_a^{a+x}}{\sum_a^{a+x}(R_a N_a)}\right)$$

Where:

D_a= the number of deaths from a cause in age group *a*

R_a= global cause-specific mortality rate of age group *a*

N_a= the country-year-sex-specific population in age group *a*

D_a^{a+x}= the number of deaths in the age group *a* to *a*+*x*

With the assumption of invariant relative risks of death by age with respect to a reference age group, this equation can be used, along with population distribution by age, to split an aggregate number of deaths for the age groups *a* to *a*+*x*

into specific deaths for each age group within the aggregate interval.

$$D_{as} = R_{as}N_{as}(\frac{D_{as}^{a+x,s}}{\sum_a^{a+x}(R_{as}N_{as})})$$

Where:

D_{as}= the number of deaths from a cause in age group *a,* sex *s*

R_{as}= global cause-specific mortality rate of age group *a,* sex *s*

N_{as}= the country-year-sex-specific population in age group *a* for sex *s*

$D_{a,s}^{a+x,s}$= the number of deaths in the age group *a* to *a*+*x* for sex *s*

In some cases, deaths are reported for an aggregate age group for both sexes combined. The task in this case is more complicated, but the same principle can be applied. In this case, we assumed that the relative risks of death by age and sex are constant.

This equation can be used to split data aggregated by age and sex. The assumption, however, of invariant relative risks across age and sex is a stronger assumption. Fortunately, data pooled across sexes are less common in the published or unpublished CoD data.

The relative risk of death in a particular age group for a given sex is derived from the global distribution of cause-specific mortality rates found in available VR data. Location-years from the following code systems are used, provided they report the requisite age detail and sex detail: ICD-7, ICD-8, ICD-9 BTL, ICD-10 tabulated, ICD-9, and ICD-10. Upon compiling these data, we mapped them to GBD causes and retained any observation coded to either cause Level 3 or a most detailed cause. Cause aggregation was then performed to ensure accurate death totals at all levels of the cause hierarchy. Thus, age distributions were generated for most detailed causes, as well as all Level 1, 2, and 3 causes. In the event that a cause does not have a distribution, it will be split using the distribution of its nearest parent cause.

We next adjusted separately for estimated adult and child VR completeness. Location-year-age-sex-cause-specific deaths and population were then aggregated across all location-years to produce cause-specific mortality rates by age and sex. These were used to determine the risk of death at any age relative to any reference age group, as shown in the above equations.

Section 3.5.1: Remap unmodelled age-sex combinations

Occasionally, data sources include deaths by a cause for which consensus exists that death is rare for the sex and/or age. For example, some number of deaths may be attributed to cervical cancer in males, or to maternal causes in children younger than 10 years. We have constructed a list of age-sex-cause combinations we do not model. Some sex/cause combinations cannot be modelled because the input data for causes of death is limited to binary male and female sex groups and thus constrains the combinations of cause and gender that can be modelled for the GBD. Because of these limitations to our input data, when we encounter a death in an unmodelled age-sex-cause combination, cause/sex combinations occur, we reassign them to a related cause or garbage package. For example, IBD may be remapped to other digestive disorders. All unmodelled combinations are included in table S7.

Section 3.6: Correction for miscoding of Alzheimer's and other dementias, Parkinson's disease, and atrial fibrillation and flutter (step 4)[3]

Section 3.6.1: Objective

For certain causes of death, mortality rates reported in VR systems are impossible to reconcile with observed trends in disease prevalence and excess mortality. For dementia,[12–20] Parkinson's disease,[21,22] and atrial fibrillation and flutter, these disparities can largely be attributed to death certification practices which lead to under-coding or over-coding in many country-years. We sought to address the known bias in CoD data by first identifying the proportion of all deaths that should be assigned to these causes and next determining the GBD causes and garbage groups to which these deaths are being incorrectly assigned.

In past GBD iterations, we estimated Alzheimer's disease and other dementias, Parkinson's disease, and atrial fibrillation and flutter on the basis of longitudinal prevalence and excess mortality data to help account for changing patterns in death certification and corresponding implausible time trends in many VR sources. This method was first implemented for Alzheimer's disease and other dementias in GBD 2013. We added atrial fibrillation and flutter to the causes modelled in GBD 2015 and Parkinson's disease to the causes modelled in GBD 2016 by using this strategy. All of these causes were processed in CoDCorrect in a manner that was agnostic to the likely targets of misclassification, which inappropriately led to changes in mortality estimates for causes unrelated to these three in GBD 2015. For GBD 2016, we improved this process by completing a literature review to identify the causes of death most closely associated with Parkinson's and Alzheimer's diseases[3,10,11,17] and limiting the CoDCorrect adjustments to include only those causes. For GBD 2017, we refined this approach further by using multiple CoD data to determine the GBD causes and garbage codes from which we move deaths as well as the pattern of misclassification.

Section 3.6.2: Correction process

Changes in coding practices for Alzheimer's diseases and other dementias, Parkinson's disease, and atrial fibrillation and flutter result in spatiotemporal mortality trends that are incompatible with prevalence and case-fatality trends.[23] These changes in coding practices are believed to be the result of shifting consensus in cause of death certification, meaning there is a bias in vital registration (VR) data that needs correction. For Parkinson's disease and atrial fibrillation and flutter, we first estimated excess mortality from prevalence and CoD data in countries with the highest ratio of cause-specific mortality to prevalence, which represents the greatest willingness to code to an under-coded cause. Then, using DisMod-MR 2.1 (see Section 4.2.3), we derived estimates of cause-specific mortality rates from available prevalence surveys as well as the estimates of excess mortality rate, applied across all countries and over time. We divide this value by the all-cause mortality rate to determine the fraction of overall mortality to attribute to each under-coded cause. For dementia, the modelling process was redesigned in 2019 to no longer depend on vital registration data from the highest dementia mortality locations. Instead, we used relative risk data from cohort studies to calculate total number of excess deaths due to dementia, and end-stage disease proportions from linked hospital to death records to subset these deaths to the proportion of excess deaths with end-stage conditions, which we attributed to dementia. Finally, we used log-linear interpolation to interpolate final estimates of death due to dementia for the entire time series and saved as a custom CoD model.

To ascertain the causes from which we would move deaths to under-coded causes, we leveraged multiple CoD data from the USA—by looking to the combinations of intermediate and immediate causes (ie, chain causes) present on death certificates with an under-coded cause listed as underlying and identifying other death certificates with similar or identical chain causes, we can determine the expected pattern of miscoded deaths.

The first stage in this process is to parse out country-years of data where we believe coding practices to be relatively stable. For dementia, this "gold standard" dataset features USA 2010–2015; for Parkinson's, USA 2005–2015; and for atrial fibrillation and flutter, USA 2014–2015. We then collect all deaths in those years with the under-coded cause listed as underlying and remove any mention of the under-coded cause from the death certificate. Next, for each unique chain identified in this manner, we search the entire time series of USA data (1980–2015) to identify the distribution of underlying causes that share that chain. The premise here is that if the diagnosis of dementia, Parkinson's, or atrial fibrillation and flutter were missed, the other causes listed on the death certificate would have been the basis for certification. We then reallocate the under-coded cause deaths in the gold standard years by chain based on that alternative underlying cause distributions from the full time series.

Upon iterating through all unique chains, we are left with a counterfactual dataset excluding under-coded causes of death. Each remaining cause can be subdivided into correctly coded deaths and deaths that have been recoded from

an under-coded cause by the process described (although not all causes are necessarily targeted by the recoding algorithm). We then calculate the ratio of recoded deaths to total deaths by cause, age, and sex in our counterfactual dataset. This ratio represents the proportion of each cause that we believe to be miscoded Parkinson's disease, Alzheimer's disease and other dementia, or atrial fibrillation and flutter in a counterfactual scenario of 100% under-reporting for these causes.

We multiply the ratios derived from the multiple cause data by the cause-specific deaths in each VR dataset to determine the local pattern of miscoding. In this way, the method is sensitive to the observed epidemiology of a given country and year. Then, we calculate the deficit in under-coded cause mortality for each location, year, age, and sex by taking the difference in the expected cause fraction for a given under-coded cause based on prevalence and excess mortality compared to the proportion of deaths actually certified by the VR system. Finally, we scale the cause-specific miscoded deaths to match the deficit and then move them accordingly. We assumed that misclassification of actual dementia and Parkinson's deaths in past years occurred only for reported causes of deaths that were plausibly the direct result of dementia or resulted from misdiagnosis of other organic brain diseases based on clinical expert judgement. A similar assumption is used for atrial fibrillation and flutter, for which only cardiovascular causes and ill-defined garbage codes are considered. Finally, while we assumed that Parkinson's disease and atrial fibrillation and flutter are strictly under-coded in the VR, we assumed that Alzheimer's disease and other dementias can be under- or over-coded. Anywhere where our expected cause fraction for Alzheimer's disease and other dementia was lower than the observed, we removed deaths from this cause and redistributed them as non-specific garbage.

Because the deaths being reallocated vary by location-year, we need a mechanism to ensure plausible limits to how many deaths are extracted from each GBD cause and garbage code. To achieve this, we first run the above-mentioned algorithm on all 5-star VR data (see Section 3.16 for an explanation of the star data quality rating system). Then, we determine the 95th percentile of the proportion of deaths moved to an under-coded cause for each GBD cause and garbage code group by age and sex across location-years among these data. Those values are subsequently stored and applied as the limits for deaths moved by this process for all other VR data.

Section 3.7: Redistribute (Step 5)[2]

A crucial aspect of enhancing the comparability of data for CoD is to deal with uninformative, so-called garbage codes. Garbage codes, ie, codes that are not specific enough, are an immediate or intermediate CoD, or impossible CoD, should not be considered as the underlying cause of death—for example: heart failure, ill-defined cancer site, senility, ill-defined external causes of injuries, and septicaemia. The methods for redistributing these garbage-coded deaths are outlined in detail in Johnson et al,[24] and the primary algorithm for redistributing deaths assigned to these codes has not changed since GBD 2013.

Section 3.7.1: Redistribute HIV-related garbage codes (step 5.1)

Because of the disparate nature of HIV/AIDS mortality across space and time, dynamic redistribution of HIV/AIDS-related garbage codes was needed (table S8). To inform this redistribution, we generated target proportions for each garbage group by age band (under 1 month, 1–59 months, 5–19 years, 20–49 years, 50–59 years, 60–69 years, 70–79 years, and 80 years and older), five-year time interval, and sex. The garbage groups either target HIV or a remainder target. The allotment of deaths to either of these is based on the regional increase in the mortality rate of all codes in the group relative to the rates seen from 1980 to 1984—an increase greater than 5% is assumed to be HIV/AIDS-related, and the proportion of those deaths exceeding 5% are redistributed to HIV/AIDS. Any increase less than or equal to 5% is then assigned to the remainder target.

Section 3.7.2: Regress garbage codes versus non-garbage codes (step 5.2)

For each redistribution package, we defined the "universe" of data as all deaths coded to either the package's garbage codes or the package's redistribution targets for each country, year, age, and sex. We then ran a regression based on the following equation separately for each target group and sex:

$$TG_{crt} = \alpha + \beta_1 Gar_{crt} + \beta_2 Age_{crt} Gar_{crt} + \theta_r Gar_{crt} + \gamma_r + \varepsilon_{ct}$$

Where:

TG_{crt} = percentage of deaths within the given garbage code's universe that were coded to a given target group, by country

Gar_{crt} = percentage of deaths within the given garbage code's universe that were coded to a given set of garbage codes

Age_{crt} = age interaction term for the fixed effect on the interaction of garbage and age

α = constant

β_1 = slope coefficient describing the association between Gar_{crt} and TG_{crt}

β_2 = slope coefficient describing the association between the interaction $Age_{crt}Gar_{crt}$ and G_{crt}

γ_r = region-specific random intercept (or super-region if the random effect on region is not significant)

θ_r = region-specific random slope (or super-region if the random effect on region is not significant)

ε_{ct} = standard error, normally distributed and calculated by bootstrapping

This regression was adjusted from GBD 2013 to include fixed effects on the interaction of garbage and age to ensure smooth age patterns. We made this decision after investigating diagnostic visualisations that showed unlikely gaps between proportions assigned to different age groups.

Once proportions were produced for each country, sex, age, and target group, certain adjustments were made to conform our packages to the best medical evidence available. In some cases, we implemented restrictions on the proportions that the regressions could yield. For example, we did not allow any redistribution onto "Chagas disease" outside of Latin America and the Caribbean or "self-harm" under the age of 15 years. In other cases, we capped the proportion for some targets to the level that would be produced from proportional redistribution; for example, "haemoglobinopathy" and "haemolytic anaemia" were restricted to the level of proportional redistribution in the redistribution of "left heart failure". Occasionally, further adjustments were made on a case-by-case basis per country, age, sex, and target group to suppress the impact of outliers based on existing epidemiological evidence and expert judgment.

In GBD 2019, we updated the regressions for stroke and diabetes. We dropped the proportion of garbage from the regression formula and ran regression on high-quality, low proportion garbage data (4/5 stars, <50% GC). We also included all covariates included in the CODEm models for both stroke and diabetes.

Section 3.7.3: Development of an algorithm for redistribution of garbage codes based on multiple CoD data

Multiple CoD data are a form of individual record causes of death data that include an underlying CoD along with other causes in the death chain, including intermediate and immediate causes. By analysing this type of data, we can sometimes find the true underlying CoD in other CoD data where the underlying cause is a garbage code or a mis-assigned CoD.

As of GBD 2019, this method has been expanded and used in redistribution of the following intermediate causes: sepsis, embolism (pulmonary and arterial), heart failure (left, right, and unspecified), acute kidney injury, hepatic failure, acute respiratory failure, pneumonitis, and unspecified central nervous system disorders. Using multiple CoD records for the USA, Mexico, Brazil, Taiwan*, Italy, Canada, New Zealand, Austria, South

Africa, and Colombia, we identified the fraction of deaths where the underlying cause of death and the intermediate cause was in the causal chain. Using a mixed effect linear regression, we estimated the fraction of intermediate-cause-related deaths by underlying GBD cause. These fractions were multiplied by the GBD 2023 CoDCorrect result to calculate the number of deaths intermediate cause-related deaths for each GBD cause. Lastly, we calculated the "intermediate cause fraction", with total intermediate-cause-related deaths as the denominator, by age, sex, location, year, and GBD cause. These fractions were used to redistribute the intermediate-cause-related deaths to a GBD cause. An example is given below for sepsis, where a, s, l, y, c denotes a given age group, sex, location, year, and underlying cause of death:

1. $sepsis\ fraction = \beta_{HAQ\ Index} + \beta_{age\ group} + \beta_{sex} + Y_{cause} + \varepsilon$
2. $sepsis\ deaths_{a,s,l,y,c} = sepsis\ fraction_{a,s,l,y,c} * GBD\ deaths_{a,s,l,y,c}$
3. $total\ sepsis\ deaths_{a,s,l,y} = \sum_c sepsis\ deaths_{a,s,l,y,c}$
4. $fraction\ of\ sepsis\ to\ redistribute_{a,s,l,y} = \frac{sepsis\ deaths_{a,s,l,y,c}}{total\ sepsis\ deaths_{a,s,l,y}}$

To redistribute X59 and Y34 (unspecified injuries) deaths, we used a multi-step approach that utilised the pattern of nature of injury codes in the causal chain in the multiple CoD data. First, we looked at deaths where X59, Y34, and GBD injuries causes were the underlying cause of death and got the pattern of nature of injury codes in the chain. We then derived a cause-specific redistribution proportion based on the probability of a given pattern being coded to X59/Y34 or a GBD injuries cause and summing up these proportions for all patterns. An example below is given for X59:

5. $P_{(pattern_j|UCoD\ X59)} = \frac{\#\ of\ pattern_j\ deaths\ |\ UCoD\ X59}{\sum_{j=0}^{m}(\#\ of\ pattern_j\ deaths\ |UCoD\ X59)}$
6. $P_{(GBD\ injuries\ cause_i|pattern_j)} = \frac{\#\ of\ UCoD\ GBD\ injuries\ cause_i\ deaths\ |\ pattern_j}{\sum_{i=0}^{n}(\#\ of\ UCoD\ GBD\ injuries\ cause_i\ deaths\ |pattern_j)}$
7. $redistribution\ proportion_{GBD\ injuries\ cause_i} = \sum_{j=0}^{m}\left(P\left(pattern_j \middle| UCoDX59\right) * P(GBD\ injuries\ cause_i|pattern_j)\right)$

Where:

$pattern_j$ = a given nature of injury code pattern in the chain of the multiple CoD data

UCoD X59 = a death with X59 coded as the underlying cause of death (UCoD)

UCoD GBD injuries $cause_i$ = a death with a GBD injuries causes coded as the UCoD

We applied these cause-specific redistribution proportions on the data where X59/Y34 were the underlying cause of death to get the number of X59/Y34 deaths "attributable" to each GBD injuries cause. Then, for each GBD injuries cause in the multiple CoD data, we calculated the fraction of redistributed X59/Y34 deaths over the fraction of total injuries death for that cause and modelled this intermediate cause fraction using a mixed effects linear regression similar to the one mentioned above. Like mentioned above, these fractions were then multiplied by GBD 2023 CoDCorrect results, and the cause fractions for X59 and Y34 were calculated by age, sex, location, year, and GBD injuries cause, and then used to redistribute X59 and Y34 deaths to GBD injuries causes.

Additionally, multiple CoD data were used in the correction of the mis-assignment of deaths due to drug overdoses

to unintentional other poisoning. More than 90% of these types of poisonings are due to exposure to narcotics, psychodysleptics, and other drugs, specified or unspecified. More than 97% of these poisonings by substance or drug occurred in ages 15–65 years. These are clearly not cases of accidental ingestion of substances but rather deliberate ingestion and unintentional poisoning. Using multiple CoD records from the USA, Mexico, Brazil, Taiwan*, Italy, Colombia, Australia, and various European countries from 1980 to 2017, we selected all deaths with underlying causes coded to X40–X44 (Table A below). Table B shows the combination of other potential causes that can be found in the multiple CoD data for these underlying causes, and table A shows the ICD-10 codes corresponding to these causes. On the basis of Table B, we proportionally redistributed mis-assigned unintentional poisoning deaths to one of these causes. The main assumption behind this algorithm is the predominance of the fatality of some substances when a combination of drugs is considered. Given the combination of different drugs and substances in these codes, opium is the main cause of fatality.[25,26] Other substances, like cocaine, methamphetamine, and alcohol in combination with cannabis are less likely to be dominant in fatality.[27]

For example, if the multiple CoD data show that 40% of deaths include opioid use disorders as an intermediate cause where the underlying cause is X40–X44, the redistribution proportion for opioid use disorders will be exactly 40% due to the dominance of the fatality of opioid use disorders compared to other drugs in the above table. Additionally, in our final results, cannabis and psychoactive and psychedelic drug use disorder deaths were mapped to other drug use disorders.

Table A. ICD-10 codes for substances or drugs used to assign deaths coded to an underlying cause of unintentional poisoning by using multiple CoD data

Accidental poisoning codes	All X40, X41, X42, X43, X44 codes
Opioid codes	T40.0, T40.1, T40.2, T40.3, T40.4, T40.6, F11.0, F11.1, F11.2, F11.3, F11.4, F11.5, F11.6, F11.7, F11.8, F11.9
Amphetamine codes	T43.6, F15.0, F15.1, F15.2, F15.3, F15.4, F15.5, F15.6, F15.7, F15.8, F15.9
Cocaine codes	T40.5, F14.0, F14.1, F14.2, F14.3, F14.4, F14.5, F14.6, F14.7, F14.8, F14.9
Psychoactive and psychedelic drug codes	T40.8, T40.9, F16.0, F16.1, F16.2, F16.3, F16.4, F16.5, F16.6, F16.7, F16.8, F16.9
Alcohol codes	T51.0, T51.1, T51.2, T51.3, T51.8, T51.9, F10.0, F10.1, F10.2, F10.3, F10.4, F10.5, F10.6, F10.7, F10.8, F10.9
Cannabis codes	T40.7, F12.0, F12.1, F12.2, F12.3, F12.4, F12.5, F12.6, F12.7, F12.8, F12.9

Table B. Multiple cause of death selection algorithm used for redistributing unintentional poisoning causes of death to substance or drug use cause of death

Selection algorithm						
	Opioids	Cannabis	Cocaine	Amphetamines	Alcohol	Psychoactive and psychedelic drugs
Opioids	Opioids	Opioids	Opioids	Opioids	Opioids	Opioids
Cannabis	Opioids	Cannabis	Cocaine	Amphetamines	Alcohol	Psychoactive and psychedelic drugs
Cocaine	Opioids	Cocaine	Cocaine	Amphetamines + cocaine	Cocaine + alcohol	Cocaine

Amphetamines	Opioids	Amphetamines	Amphetamines + cocaine	Amphetamines	Amphetamines + alcohol	Amphetamines
Alcohol	Opioids	Alcohol	Cocaine + alcohol	Amphetamines + alcohol	Alcohol	Psychoactive and psychedelic drugs
Psychoactive and psychedelic drugs	Opioids	Psychoactive and psychedelic drugs	Cocaine	Amphetamines	Psychoactive and psychedelic drugs	Psychoactive and psychedelic drugs

Multiple CoD data were only available to us for the USA, Mexico, Brazil, Taiwan*, Italy, Colombia, Australia, and various European countries. Because of this limited sample, we applied the result from the multiple CoD analysis from each country to its respective super-region and used global proportions for sub-Saharan Africa. We hope for increased availability of multiple CoD data in future analyses to achieve a more precise distribution for more locations.

Section 3.7.4: Verbal autopsy anaemia adjustment (step 5.3)

To compensate for the over-representative cause fractions from anaemia found in VA studies, we redistributed these deaths based on the causal attribution of severe anaemia from GBD 2023. The proportions were country-year-age-sex-specific.

Section 3.7.5: Calculate redistribution uncertainty (step 5.4)

We categorised garbage codes into four levels in order of increasing specificity (see Section 3.4). Some garbage codes are redistributed on all causes (eg, unspecified causes of death) and others are only redistributed onto specific causes (eg, unspecified cancer). Major garbage refers to garbage codes in Levels 1 or 2. Because of the variation in redistribution, estimating uncertainty from garbage redistribution for CODEm modelling was an important goal for GBD 2019.

We assigned redistribution variance to each datapoint in the CoD database by calculating residual variance from a regression predicting the percentage of garbage-coded deaths redistributed to a cause, given the proportion of garbage codes we observed for that location, year, age, sex, cause, and the age-standardised relative rate of major garbage codes across all causes. If there is a cause that has greater residual variance, we assume greater redistribution uncertainty.

The two model inputs are the observed percentage of Levels 1, 2, and 3 garbage codes (by cause, age, sex, location, and year) in redistributed CoD data and the percentage of garbage codes in the raw data (calculated as the age-standardised mortality rate ratio of major garbage-coded deaths to all deaths in the raw data by location, year, and sex). Level 4 garbage codes were excluded from the model to avoid overestimating uncertainty in countries with high percentages of major garbage codes. Additionally, the classification of Level 4 garbage codes is not stable between successive GBD rounds—for example, “unspecified diabetes” was not a garbage code in GBD 2016, and in GBD 2017 was reclassified as a Level 4 garbage code to permit estimation of diabetes by type. These deaths are still taken into account later in the uncertainty estimation process. The model predicts the percentage of garbage-coded deaths redistributed to a cause, given the proportion of garbage codes we observed for that location, year, age, sex, cause, and the age-standardised relative rate of major garbage codes across all causes. From this model, we calculate residual variance. It is important to note that the variance here is a measurement of uncertainty of redistribution, not of the level of miscoding in the raw CoD data for a given demographic.

To calculate variance, a dataset was generated that contained percentage garbage by location, year, age, sex, and cause, where percentage garbage is determined by the equation:

$$pct_{garbage} = \frac{deaths_{redistributed} - deaths_{raw}}{deaths_{redistributed}}$$

A mixed-effect linear regression model was then fit to predict the logit percentage of deaths from redistribution by age-standardised relative rate of major garbage codes.

$$logit\left(pct_{garbage_{ij}}\right) = \beta_0 + \beta_1 * \log\left(ASR_{majorgarbage_{ij}}\right) + \beta_2 * 15yearage_{ij} + \gamma_{1j} * \log\left(ASR_{majorgarbage_{ij}}\right) + u_j + e_{ij},\ \ \theta_{\{i\}} \sim N(0, \sigma^2)$$

Where:

i indexes dataset-location-year-age-sex-cause datapoints nested within j groups by GBD region

$ASR_{majorgarbage_{ij}}$ is age-standardised relative rate of major garbage

Residual variance, as estimated by the mean absolute deviation, was calculated for each cause, sex, and age.

The next step was to use the residual variance to calculate uncertainty around each datapoint in the CoD database. First, we calculated the percentage garbage of each datapoint by treating all deaths that could not be directly mapped to a GBD cause as garbage, including Level 4 garbage codes. Percentage garbage was calculated as

$$pct_{garbage} = \frac{deaths_{redistributed} - deaths_{corrected}}{deaths_{corrected}}$$

Where:

$deaths_{corrected}$: deaths post misdiagnosis correction (Section 3.6)

$deaths_{redistributed}$: deaths post redistribution (Section 3.7)

Residual variance was matched to each datapoint, and 100 draws were sampled from a normal distribution by using the cause-age-sex-specific residual variance and mean of 0. The logit transformed percentage garbage was added to each value in the distribution. Each draw was then transformed out of logit space, and the post-redistribution deaths were calculated as:

$$deaths = \frac{deaths_{corrected}}{1 - pct\_garbage}$$

Draws of deaths were processed through noise reduction before calculating the final redistribution variance passed to CODEm, which was added to the total data variance. The mean of the draws was not used as the final estimate because it was found that the logit transformation biased the distribution of cause fractions higher. Instead, only point estimates were used.

Section 3.8: Correction for Misclassification of COVID-19 deaths

In GBD 2023, we received 83 Country years of data in 2020, 67 in 2021, and 38 in 2022. During these years, there is evidence that COVID-19 deaths were misclassified as other causes of death.[28,29] These misclassified COVID-19 deaths then manifest as spikes in the other causes of death that they are miscoded to. For identifying miscoded deaths we looked at 3 age bands, under 15, 15 to 54, and 55+ as well as limiting our analysis to the country level and level 3 causes. To systematically identify these spikes caused by misscoded COVID-19 in other causes, we developed a Support Vector Machine (SVM) that identified deviations from established time trends in the years 2020-2022. This SVM model looked at the data available from 2015 to 2019 for a given time series and provided a classification for the target year. Normalized features were created to inform the SVM about the nature of the data.

These features included the target year compared to every other year, the standard deviation of the data from 2015-2019, and whether the target year was the highest observed year.

After identifying spikes in mortality during the pandemic years through the use of the SVM, we had to determine if a given spike is miscoded COVID-19 or if it is a true increase in the cause. To do this, we first used a similar strategy that excess mortality estimates used where we generated a counterfactual estimate to represent a COVID-19 free world. In this analysis, the counterfactual consists of a linear regression based on the data available from 2015 to 2019. Using the counterfactual estimates, we ran a regression for each cause-location-age band where we compared the excess numbers of deaths against the observed known COVID-19 deaths to return a Pearson correlation coefficient as well as a p-value. This regression shows the relationship of how much of the spike occurred along with the presence of COVID-19. If a location-cause-age band has a positive Pearson correlation as well as a P value of under .05 then we believe that there is miscoded COVID-19, and the observation is eligible for correction.

Once a country, cause, year, sex, age band combination with excess mortality has been identified through the SVM and regressions, we then calculate an estimate for the portion of excess attributable to the misassignment of COVID-19. It is important to note that while the identification step is done using national aggregates and the age bands mentioned above, the calculation of excess deaths attributable to COVID-19 is done at the most granular level – detailed GBD age groups and subnational locations where possible. To calculate the amount of excess mortality attributable to COVID-19, we first create an estimate of expected deaths absent of any pandemic effects using a combination of two counterfactuals. Total excess is then scaled to determine the amount of total excess that is attributable to COVID-19. The details of these steps are below.

The first of the two counterfactual estimates is determined by a linear regression of pre-pandemic data. For each location, age, sex, cause combination identified, a linear regression between year and observed deaths is ran for the five years prior to the pandemic (2015 to 2019). The resulting linear equation is then used to predict forward an estimated death total independent of COVID-19 or any excess mortality for the years 2020, 2021, and 2022.

The second of the two counterfactuals is determined using a global relative rates approach, adapted from a similar method used in the correction of misclassified HIV.[30] This approach involves generating relative rates, which are simply ratios between observed mortality rates in a given age group and the mortality rate in age groups believed to be relatively free of COVID-19. For the years 2020, 2021, and 2022, we can generate expected death totals by comparing these ratios to those observed in pre-pandemic data. The details of this process are as followed:

1. From the five years prior to the pandemic (2015 to 2019), compute global, all year, cause specific death rates by age and sex.
2. Compute death rates for each location, year, age, sex, cause observation identified to contain excess mortality in 2020, 2021, and 2022.
3. Compute two sets of "reference death rates", for both the global data from step 1, and the granular data from step 2. Reference death rates are calculated as the average death rate from age groups relatively absent of COVID-19. The reference age groups are 5-9, 10-14, and 15-19.

$$GlobalReferenceDR_{s,c} = Average\left(DR_{5-9,s,c}\,;\,DR_{10-14,s,c}\,;\,DR_{15-19,s,c}\right)$$

$$ObservationReferenceDR_{l,y,s,c} = Average\left(DR_{5-9,l,y,s,c}\,;\,DR_{10-14,l,y,s,c}\,;\,DR_{15-19,l,y,s,c}\right)$$

4. For the global rates calculated in step 1, use their respective reference death rates to calculate a "relative death rate" for each age, sex, cause combination. This is simply the ratio between the age specific rate and the reference death rate calculated in step 3.

$$RelativeDR_{a,s,c} = \frac{DR_{a,s,c}}{ReferenceDR_{s,c}}$$

5. Finally, generate a counterfactual estimate such that the relative death rate of the observation would be equivalent to the global, COVID-19 free relative death rate. This can be done by multiplying the reference death rate of the observation by population, and then scaling to the global relative death rate.

$$Counterfactual_{l,y,a,s,c} = \left(ReferenceDR_{l,y,s,c} * Population_{l,y,a,s}\right) * GlobalRR_{a,s,c}$$

The final estimate for expected deaths is then calculated as the mean of the individual counterfactuals. Total excess mortality can then be estimated by subtracting the expected death total from the observed death total. The final step of the correction is then to determine what portion of the excess mortality should be attributed to COVID-19 misclassification. To do this, we use the regression coefficient from the linear regression ran during the identification step. This coefficient describes for a one unit increase in COVID-19, the associated increase in excess mortality. Thus, we can then multiply total excess by this coefficient to estimate the amount of total excess attributable to miscoded COVID-19. This total is then subtracted from the cause of interest and re-assigned to COVID-19.

Section 3.9: HIV/AIDS misclassification correction (step 6)[2]

In many location-years, certain causes of death known to be comorbid with HIV/AIDS (eg, tuberculosis, other infectious diseases) are seen to have age patterns that diverge from those observed in location-years without widespread HIV epidemics and are in fact more reflective of HIV mortality trends. To identify these instances, a global relative age pattern is generated by using all VR deaths in countries with observed HIV prevalence less than 1% in 2010 by using the following equation:

$$RR_{asc} = \frac{R_{asc}}{\bar{x}(R_{65sc}, R_{70sc}, R_{75sc})}$$

Where:

RR_{asc} is the relative death rate for age group a, sex s, and cause c;

R_{asc} is the rate for that age group

$\bar{x}(R_{65sc}, R_{70sc}, R_{75sc})$ is the mean of the rates in ages 65–69, 60–74, and 75–79 for that sex and cause.

This is preferable to comparing mortality rates because we are able to isolate divergence in age pattern while accounting for varying levels of overall mortality by fixing death rates to age groups that are unlikely to be confounded by the presence of HIV. Expected deaths for an identified cause were then determined by the equation:

$$ED_{lyasc} = \bar{x}\left(R_{ly65sc}, R_{ly70sc}, R_{ly75sc}\right) \times p_{lyas} \times RR_{asc}$$

Where:

ED_{lyasc} are deaths for location l, year y, age group a, sex s, and cause c;

$\bar{x}(R_{l65sc}, R_{l70sc}, R_{l75sc})$ is the mean of the rates for ages 65–69, 60–74, and 75–79 for that location-year-sex-cause;

p_{lyas} is the population for that location-year-age-sex

RR_{asc} is the global standard relative rate determined in the previous step for that age-sex-cause.

The expected deaths remain attributed to that particular cause, while the difference between observed and expected are reallocated to HIV/AIDS.

Section 3.9: Scale strata to province (step 7)[2]

Over time, a higher proportion of deaths have been registered in China through the expansion of the DSP system and provincial/county efforts to increase CoD registration. With the expansion of coverage, it is possible that province aggregates do not accurately represent the population distribution between urban and rural areas in each year. For this reason, we stratified the data preparation by urban and rural status for each county within each province.

Stratification was based on the median level of urbanisation across counties within each province as recorded in the 2010 China census. In the provinces of Tibet and Hainan, all counties were placed into one stratum based on largely homogeneous urbanisation levels within each province. This yielded a total of 62 analytical province-strata. Macao and Hong Kong were not included in this stratification system as the VR systems there are independent from that on the mainland; no weighting scheme needs to be carried out in these complete VR systems with quality CoD data.

Within each province-strata, a larger proportion of deaths in-hospital might be reported than that of deaths outside of hospital because of the internet hospital reporting system. To avoid bias, we reweighted in-hospital and out-of-hospital deaths based on the age-sex-province-specific fraction of deaths in and out of hospital in the DSP system. DSP data have been used to establish these percentages because in these communities, there is a concerted effort to identify all out-of-hospital deaths. Province-strata death rates are combined to produce overall province death rates by weighting each stratum by population in each age-sex-year group. Province death rates are rescaled so that all-cause mortality equals the estimated death rate in each age-sex-year estimated in the life-table analysis. The Bayesian noise reduction algorithm was used to deal with zero counts and small number issues for rare causes.[2]

Section 3.10: Restrictions post-redistribution (step 8)[2]

Some causes of death can only be reliably assigned through an autopsy by a trained physician. For example, a VA would be unlikely to reliably distinguish between ischaemic and haemorrhagic stroke.

This step ensures that the detail of the cause list at this point in the data prep process is reasonable given the detail of the original data source and the methods by which the CoD was assigned. A "bridge map" is applied over a certain set of sources to ensure that these sources do not contain causes that could not reliably be determined by the methods used. These causes, identified to be too detailed, are then aggregated to their parent cause. This correction is applied to ICD-9 detail, ICD-9 BTL, ICD-10 tabulated, ICD-8 detail, ICD-8 A, China DSP (tabulated ICD-9), India MCCD, India SRS, USSR tabulated ICD-9, the Philippine Vital Statistics Reports, Iran ICD-10 VR from the Ministry of Health and Medical Education, and all VA. An example of this would be the aggregation of all sub-types of lower respiratory infection to lower respiratory infection in ICD-9 BTL.

Section 3.11: Drop VR country-years or mark as non-representative (step 9)[2]

Lozano and colleagues[31] describe the negative impact that low-completeness VR data could have on CoD modelling for GBD 2010. In particular, in settings where a data source does not capture all deaths in a population, the cause composition of deaths captured might be different from those that are not. However, a completeness sensitivity test found that low-completeness VR data had little impact on the cause-specific mortality trends at the global level.

For GBD 2019, we investigated the impact of these data at the country and subnational levels and determined that these data produced unlikely trends in the models affected. Despite the minimal impact on global trends, better models were produced by eliminating or marking as non-representative data with extremely low completeness. VR completeness was estimated as the number of deaths registered divided by the number of deaths estimated in the GBD mortality envelope.

For this round, VR location-years with completeness less than 50% were dropped, while location-years with completeness between 50% and 69% were marked as non-representative.

In addition, any country-year with a number of deaths registered to major garbage codes greater than 50% of the deaths registered was dropped. Major garbage coding refers to garbage codes redistributed across Levels 1 and 2 of the cause hierarchy. When we redistribute garbage codes across Levels 1 and 2 of the cause hierarchy, this is because we do not have enough information to distribute them to more detailed levels [3 and 4].

Section 3.12: Cause aggregation (step 10)[2]

The cause list is organised in a top-down hierarchical format containing four levels. The first group, or Level 1, sums all causes. Following all-cause mortality are Level 2 causes, which include three broad groupings of causes of deaths: "communicable, maternal, neonatal, and nutritional diseases"; "non-communicable diseases"; and "injuries". Within

those Level 2 groupings are finer levels used for modelling. Level 3, or parent causes, are aggregated; the mortality estimate for a parent cause in the hierarchy represents the sum of the causes under that rubric. Sub-causes within Level 3 causes—Level 4—are more detailed. For example, the parent cause “intestinal infectious diseases” contains the three sub-causes: “typhoid fever”, “paratyphoid fever”, and “other intestinal infectious diseases”. Included in the parent cause estimate are deaths mapped directly to the parent and any Level 4 sub-causes. In data where there was not enough information to assign a Level 4 cause, we aggregated to the Level 3 parent cause. Exceptions to aggregating the Level 4 sub-causes to the parent are instances when certain sub-causes are not present. The United Nations Crime Trends police data only identify homicides, and aggregating homicides to injuries would not accurately represent all injuries.

Section 3.13: Remove shocks and HIV/AIDS maternal adjustments (step 11)[2]

Prior to GBD 2023, CODEm models used an HIV/AIDS- and shock-free envelope. To be comparable, cause fractions in the causes of death data were also made to be HIV/AIDS- and shock-free. New to GBD2023, CODEm models now use an envelope inclusive of HIV/AIDS, but still free of shocks. Cause fractions were uploaded to the CoD database as the number of deaths due to the cause over an adjusted sample in which the number of deaths due to “conflict and terrorism” and “exposure to forces of nature” were removed.

Section 3.13.1: Remove shocks from denominator where cause list includes shocks (step 11.1)

The first step to generate shock-free cause fractions was to remove any deaths from the sample that were directly coded to “conflict and terrorism”, or “exposure to forces of nature”. The cause fraction uploaded to the database can be calculated by a simple equation:

$$CF_{l,t,a,x,c} = \frac{D_{l,t,a,x,c}}{D_{l,t,a,x} - D_{l,t,a,x,war} - D_{l,t,a,x,disaster}}$$

Where:

$CF_{l,t,a,x,c}$ is the cause fraction for a location *l*, year *t*, age *a*, sex *x*, and cause *c*

$D_{l,t,a,x,c}$ is the number of deaths observed for cause *c* in location *l*, year *t*, age *a*, and sex *x*

$D_{l,t,a,x}$ is the total number of deaths due to all causes observed in location *l*, year *t*, age *a*, and sex *x*

$D_{l,t,a,x,war}$, and $D_{l,t,a,x,disaster}$ are the numbers of deaths observed in location *l*, year *t*, age *a*, and sex *x* for causes “conflict and terrorism”, and “exposure to forces of nature”, respectively

Cause fractions for shock causes were also uploaded to the database for use in separate estimation processes described by Wang et al.[32] In this case, cause fractions followed the standard equation, with variables following the same explanation.

$$CF_{l,t,a,x,c} = \frac{D_{l,t,a,x,c}}{D_{l,t,a,x}}$$

$$CF_{l,t,a,x,mat} = \frac{D_{l,t,a,x,maternal}}{D_{l,t,a,x,maternal} + \frac{E[D_{l,t,a,x,hiv\_shock\_free}]}{E[D_{l,t,a,x}]} D_{l,t,a,x,non-maternal}}$$

$CF_{l,t,a,x,mat}$

$D_{l,t,a,x,mat}$

$D_{l,t,a,x,non-maternal}$

$E[D_{l,t,a,x}]$

$$E[D_{l,t,a,x,hiv\_shock\_free}]$$

Section 3.13.2: HIV/AIDS correction of sibling history, census, and survey data (step 11.3)

As described in our analysis from GBD 2013, many studies have failed to find increased mortality in HIV+ pregnant mothers, but those who have advanced HIV are known to have increased baseline mortality. Prior to GBD 2013, we did not distinguish between deaths in HIV+ women that were caused by pregnancy and those for whom the pregnancy was incidental to their death. To more explicitly quantify the contribution of pregnancy to death in HIV+ women, and therefore more accurately estimate the maternal death count, we completed two additional analyses for GBD 2013 and all subsequent GBD analyses. First, we determined the population attributable fraction (PAF) of HIV/AIDS to pregnancy-related death. Second, we determined the proportion of pregnancy-related deaths in HIV+ pregnant mothers that are aggravated by pregnancy and are therefore by definition maternal deaths.

$$PAF = \frac{P(RR-1)}{1+P(RR-1)}$$

Where:

PAF is the population attributable fraction

P denotes the prevalence of HIV in pregnancy

RR is relative risk of mortality in HIV+ vs HIV- pregnant mothers.

To recap our analysis for GBD 2013, we used the paper published by Calvert and Ronsmans[33] to identify sources that could inform Step 1 of our HIV-correction analysis. We independently reviewed each of the component studies in Calvert and Ronsmans' review and extracted data directly, not from the systematic review paper. We identified only one additional study that was not used in Calvert and Ronsmans' analysis. We have, however, not used all the studies included in that review. Specific details are as follows:

1) Figueroa-Damian et al.[34] was excluded for not including any postpartum deaths at all.

2) In the case of Ryder et al.[35] and Zvandasara et al.,[36] we excluded those deaths that occurred more than 12 months after delivery.

3) We excluded the results from Chilongozi et al.[37] from the site that did not include any HIV- patients.

4) Leroy et al.[38] was not in the bibliography. We could not locate it for review, so it was excluded.

5) Kourtis et al.[39] was extracted with adjustment of the denominator based on the average number of hospitalisations per delivery in each group.

6) Ticconi et al.[40] was excluded for being both non-representative and including subgroup data from mothers with malaria infection.

A total of 21 sources were included in our analysis of the increased mortality risk of HIV+ versus HIV- women in pregnancy.[41] We performed DerSimonian-Laird random effects meta-analysis to derive a pooled estimate of RR of death during pregnancy given HIV positivity.[42] The pooled effect size was 6·40 (95% uncertainty interval [UI] 3·98–10·29), which was then used to calculate an HIV PAF for each country, age group, and year. To determine the proportion of those HIV-related deaths that were attributable to maternal causes, we performed a second systematic literature review. This time we sought evidence for the excess mortality risk of pregnancy in those women who are

already HIV+. Most studies have failed to find such an effect, but most also did not stratify their study population by stage of HIV or ART (antiretroviral therapy) status. Only two studies did this stratification, with a pooled effect size of 1·13 (95% UI 0·73–1·77).[43,44]

An updated literature review to inform the relative risk of mortality in pregnancy in HIV+ versus HIV- women had 14 non-usable sources. We completed this search on May 10, 2019, using the following search strings:

((HIV[Title/Abstract] OR "Acquired Immunodeficiency Syndrome"[Title/Abstract] OR AIDS[Title/Abstract]) AND (“pregnant”[Title/Abstract] OR “pregnancy”[Title/Abstract] OR “postpartum”[Title/Abstract] OR "post partum"[Title/Abstract]) AND (“mortality”[Title/Abstract] OR “death”[Title/Abstract]) NOT "case report" NOT (animals[MeSH] NOT humans[MeSH])

AND (2016/08/15[PDat] : 3000/12/31[PDat]))

Prevalence of HIV in pregnant women was calculated by using the Joint United Nations Programme on HIV and AIDS (UNAIDS) Spectrum model,[45] a compartmental HIV progression model used to generate age-specific incidence, prevalence, and death rates from pre-calculated incidence curves and assumptions about intervention scale-up and local variation in epidemiology. For each location, we used UNAIDS’ age-specific ratios of fertility in women living with HIV to fertility in women not living with HIV. In most locations, this ratio is assumed to be greater than one in women aged 15–24 years and less than one and decreasing as age increases beyond 24 years. Since Spectrum assumes fertile ages of 15–49 years, we used the ratio of HIV prevalence in pregnant women to HIV prevalence in the general population at either end of that range to extend estimates to age bands 10–14 years and 50–54 years.

Unlike GBD 2013, when we applied the PAF correction to the envelope of maternal deaths predicted by CODEm, we instead applied country-year-age-group-specific PAF to maternal mortality input data prior to modelling in CODEm. This ensured that both the numerator and denominator of all CF data were internally consistent in their exclusion of background HIV/AIDS mortality. The cause fractions for maternal deaths in sibling history, survey, and census data were therefore adjusted as follows:

$$CF_{l,t,a,x,mat_{adj}} = CF_{l,t,a,x,mat} \times \left(1 - ProP_{hiv_{l,t,a,x}}\right)$$

$$ProP_{hiv_{l,t,a,x}} = PAF_{l,t,a,x,hivpos} \times (1 - rr_{mat})$$

$$CF_{l,t,a,x,mat_{hiv}} = CF_{l,t,a,x,mat} \times ProP_{maternalhiv_{l,t,a,x}}$$

$$ProP_{maternalhiv_{l,t,a,x}} = PAF_{l,t,a,x,hivpos} \times rr_{mat}$$

Where:

$CF_{l,t,a,x,mat}$ = The proportion of deaths due to all maternal causes before HIV/AIDS correction for the location, year, age, and sex.

$CF_{l,t,a,x,mat_{adj}}$= The proportion of deaths due to maternal causes after the adjustment for the location, year, age, and sex.

$CF_{l,t,a,x,mat_{hiv}}$ = The proportion of deaths due to maternal deaths aggravated by HIV/AIDS after the adjustment for the location, year, age, and sex.

$PAF_{l,t,a,x,hivpos}$ = The PAF that describes the percentage of all maternal deaths that were HIV-related for the location, year, age, and sex.

$ProP_{hiv_{l,t,a,x}}$ = The proportion of deaths in pregnancy for the location, year, age, and sex that are estimated to be incidental deaths due to HIV/AIDS and therefore not a maternal CoD.

$ProP_{maternalhiv_{l,t,a,x}}$ =The proportion of deaths in pregnancy for the location, year, age, and sex that are estimated to be HIV+ and maternal deaths that are aggravated by HIV/AIDS.

rr_{mat} = 0.13/1.13 = The proportion of HIV/AIDS deaths during pregnancy that were exacerbated by the pregnancy.

Section 3.13.3: HIV/AIDS correction of other maternal mortality data (step 11.4)

Although a specific subset of codes in ICD-10 corresponds to HIV/AIDS deaths aggravated by pregnancy, these codes are sparsely used and unreliable. We therefore adapted the method described to also correct VR and VA sources for the systematic exclusion of HIV-related maternal deaths. This correction was calculated in the same manner, by using the same input data as above, with the only difference being that HIV correction of VR and VA sources resulted in a net increase in the maternal correction factor. Maternal deaths aggravated by HIV/AIDS are calculated in the following way:

$$CF_{l,t,a,x,mat_{hiv}} = CF_{l,t,a,x,mat} \times ProP_{maternalhiv_{l,t,a,x}}$$

$$ProP_{maternalhiv_{l,t,a,x}} = \frac{PAF_{l,t,a,x,hivpos} \times rr_{mat}}{1 - PAF_{l,t,a,x,hivpos} \times rr_{mat}}$$

Section 3.13.4: Crosswalk by code system

Over time, the International Classification of Diseases (ICD) is continually updated to support more detailed and accurate record keeping. When a new ICD version is released, it is not always directly compatible with the previous version. During this time of adoption, disjoints within the time series may develop due to the incompatibility. (source on challenges of ICD versions). For example, when ICD10 is adopted, many of the deaths that would be considered garbage codes in ICD9 are now mapped directly to an ICD code that maps to a real underlying GBD cause. This creates a disjoint at the year of adoption in both the target cause as well as related causes. In these scenarios, we trust ICD10 over ICD9 unless there is evidence supporting ICD9's performance. We also believe that ICD9 captures the trend over time of a given cause even if it has not accurately captured all the deaths. To correct for this disjoint between code-systems and utilize an older ICD's time trend we developed an adjustment factor using a method called a crosswalk,[46] by code-system where we compare the first 3 years of the more trusted code-system and the last 3 years of the code-system we want to adjust. This comparison produces a factor that we can apply to the entire code system we want to adjust that brings the average of the last 3 years to be equal to the average of the first 3 years of the trusted code-system.

$$\overline{CSO}_{l,a,s,c} * factor = \overline{CST}_{l,a,s,c}$$

Where:

$\overline{CSO} = average\ deaths\ of\ the\ last\ 3\ years\ of\ the\ old\ code\ system$

$\overline{CST} = average\ deaths\ of\ the\ first\ 3\ years\ of\ the\ trusted\ code\ system$

$l, a, s, c = Location, Age, Sex, Cause\ specific$

This adjustment is done for specific location, age, sex, level 2 causes and produces a continuous time trend through the year of ICD adoption that conserves the pattern within the adjusted code system.

This correction to a target cause requires deaths to be added or removed from other related causes. After the initial correction has been applied to a target level 3 cause, deaths are proportionally balanced between all level 3 causes within the related level 2 parent. This ensures that no deaths are added or lost during the correction and that deaths are only being moved from causes we believe are affected by the change in code-system. We applied this correction to time series that were identified to have disjoints at the year of code-system adoption. Corrections were only kept if they did not introduce disjoints within other level 3 causes during the balancing process. In general, we did not perform crosswalk by code systems if a country-cause's level 2 cause has a disjoint present. If there is a disjoint at the level 2 cause then balancing will move the disjoint from the target cause to a child cause.

Location	Level 2 Cause	Years Adjusted
Bulgaria	Cardiovascular diseases	1990-2004
Canada	Cardiovascular diseases	1986-1999
Spain	Cardiovascular diseases	1980-1998
France	Cardiovascular diseases	1980-1999
United Kingdom	Cardiovascular diseases	1980-1999
Croatia	Cardiovascular diseases	1985-1994
Hungary	Cardiovascular diseases	1980-1995
Italy	Cardiovascular diseases	1980-2002
Mexico	Cardiovascular diseases	1980-1997
Mauritius	Cardiovascular diseases	1980-2004
United States of America	Cardiovascular diseases	1980-1998
Japan	Diabetes and kidney diseases	1980-1994
Mauritius	Diabetes and kidney diseases	1980-2004
New Zealand	Diabetes and kidney diseases	1980-1999

Section 3.14: Noise reduction (step 12)[2]

To deal with problems of zero counts and stochastic variation in VR, VA, MITS diagnosed data, and maternal surveys, censuses, surveillance, and sibling histories, we use a Bayesian noise-reduction algorithm. We estimate a prior for a given series of data by running a Poisson regression to estimate the number of deaths due to each respective cause and sex with dummy variables for country, age, and year. With several notable exceptions (detailed below), these regressions are sex-, cause-, and GBD region-specific, so borrowing strength over age, year, and country is only within a given data type, GBD region, cause, and sex. The posterior estimate for each data is a weighted average between the prior and data:

$$posterior\ cause\ fraction = X \times weight + \mu \times (1 - weight)$$

Where:

X is the mean of the data in units of cause fractions

μ is the mean of the prior in units of cause fractions

The weight is determined by a function of the mean deaths per year in a given location-age-sex-cause for vital registration data:

$$VR\ weight_{l,a,s,c} = 1 - \frac{a}{b \times mean_y\left(deaths_{l,y,a,s,c}\right) + 1}$$

In this equation, a and b are hyper-parameters set to 0.99 and 0.1, respectively. This value of a was chosen to give the data only 1% weight when data are extremely sparse and therefore average approximately zero deaths per year for a given location, age, sex, and cause. This value of b was chosen based on a grid search to determine the optimal value of b that gives as much weight to the data as possible while still resulting in optimal smoothing of small numbers.

For non-VR, the weight is instead based on the variance of the prior, τ^2, and the variance of the data, σ^2. τ^2 is estimated from the Poisson regression, taking into account the variance-covariance matrix of the regression coefficients. For the data variance, σ^2, we use the Wilson approximation which provides an estimate of σ^2 even in cases with a zero count of cause-specific deaths. The weight used for non-VR is:

$$non-VR\ weight = \frac{\tau^2}{\tau^2 + \sigma^2}$$

This approach to noise reduction adjusts zero counts to non-zero values, mitigating the problem that zero counts in a log rate model or a logit cause fraction CODEm must be dropped from the regression, leading to upward bias in the estimates. This is particularly important in three settings: small high-income countries with low death counts overall; rare causes with small numbers of cause-specific deaths; and the analysis of sibling history, verbal autopsy, and survey data with small samples. For example, in sibling history data, for any given age group in any given year the number of deaths reported in the survey that are pregnancy-related or the number of deaths from all causes in that age group may be small.

There are several important exceptions to the data pooling and regression specifications outlined above. First, for country-series of VR from subnationally estimated countries, regressions are run on data from only that country with a fixed effect on subnational unit. Third, for VA data, all VA data for a given super-region are pooled together and a random effect on study-location is added to the regressions, allowing for different studies and surveillance sites to borrow strength from one another within a super-region. Unless the data are part of a time series (ie, the Matlab Health and Demographic Surveillance System and India Sample Registration System), the regression has no year component. Fourth, for VA data on malaria specifically, data are pooled into regional groups defined by the endemicity of malaria, as defined by the *Plasmodium falciparum* parasitaemia rate (PFPR). We include groups for hyper- (PFPR ≥0.4), meso- (PFPR 0.05 – <0.04), and hypo- (PFPR <0.05) endemic regions, separately for India; sub-Saharan Africa not including South Africa, Cabo Verde, and Mauritius, and including Yemen; and all remaining locations. Fifth, we run a separate regression for each cause in MITS diagnosed data from the Child Health and Mortality Prevention Surveillance (CHAMPS) study with fixed effects on age group and sex and a random effect on study-location. We include data from both sexes in one model and borrow strength across sex in order to combat data sparsity, and because CHAMPS includes only children under 5, an age demographic where sex differences are less extreme. Sixth, several individual VA studies are excepted from the above data pooling rules and regressed in their own separate models due to sample sizes that are too large and therefore dominate super-region-level models: Indonesia Sample Registration System (SRS), India verbal autopsy studies, and the Nepal Burden of Disease VA Study. Seventh, several VA study groups are excepted from the above regression specifications and run with individualised specifications: malaria-specific VA data from SSA are regressed with fixed effects on age group and study-location-year; VA data from the southeast Asia, east Asia, and Oceania super-region are regressed with fixed effects on age group and study-location; Matlab HDSS data are regressed with fixed effects on age group and year; and India SRS data are regressed with fixed effects on age group, year, and subnational location.

We also employ several strategies to combat data sparsity and failure to converge in noise reduction models. First, for models run on GBD regions of VR data, if a given year has only one country of data in that region, we bin this year with the preceding year in the Poisson regression. Second, for VA data, if a given cause/super-region/sex group has six or fewer observations or zero non-zero observations, we include all VA globally available for that cause/sex in a regression with fixed effects on age group and study-location and use the predictions from this global model as the prior for that cause/super-region/sex group. Third, for VA and CHAMPS data, if a model that includes random effects fails to converge, we rerun the model with the corresponding fixed effects. Fourth, after this, for all noise

reduction models, if the model fails to converge, we use a weighted average of the observed cause fractions within age groups in the model input data as the prior in noise reduction. This is essentially pooling information across study, location, and year within a grouping of data with similar data type and geography.

During noise reduction, all datapoints with cause fractions of zero are raised to a non-zero value through the Bayesian average with a non-zero prior. However, some of these values can be extremely small in magnitude (eg, on the order of 10^{-50} or smaller), to the point that they would be unobservable in the raw data. Cause fractions of this magnitude would become extremely large in absolute value when log- or logit-transformed during the CODEm process, leading to poor model fit in other ranges of the input data. However, these datapoints must remain non-zero in order to include them in a log- or logit-transformed model at all and avoid upwardly biasing estimates. To address this problem, we enforce a set of non-zero cause-, age- and sex-specific minimums, known as the "non-zero floor," on all cause fractions in the cause of death database. These floor values are chosen to be high enough to be relatively close to the bulk of the remaining data in log- or logit-space, but low enough to avoid significantly upwardly biasing the distribution of the data.

Floor values are determined based on VR data for cause/age/sex groups where we have enough data to do so accurately. We first consider VR data from all countries with greater than 50 million person-years represented in the database, after cause mapping and age-sex splitting but before any further processing. We calculate all-year cause fractions for each country, cause, age, and sex in this dataset. If the minimum cause fraction across country for a given cause/age/sex is non-zero, then we consider this cause/age/sex group to have sufficient data to determine a data-driven floor value. Otherwise, we consider this cause/age/sex to be too rare to have sufficient data to determine a minimum observable non-zero rate.

For cause/age/sex groups that have sufficient data, we set floor values based on all national-level noise-reduced VR datapoints in the CoD database that are non-zero before noise reduction. The floor value for a given cause/age/sex (c, a, s) is the minimum cause fraction across country-years (l, y) in this non-zero subset:

$$floor_{c,a,s} = \min_{l,y}\left(post\ NR\ VR\ cause\ fraction_{c,a,s,l,y}\right),$$

$$\text{where } cause\ fraction_{c,a,s,ly} \neq 0\ before\ NR$$

For all other cause/age/sex groups, which have insufficient data to inform the above calculation, we set a $floor_{c,a,s}$ value such that: (a) all floor values for a given cause add up to one death globally per year and (b) the age/sex distribution of the floor values in each cause follows the age/sex distribution of the global cause-specific mortality rates calculated in Section 2.6 for the purposes of age/sex splitting:

$$floor_{c,a,s} \times mortality\ envelope_{a,s} = \frac{R_{c,a,s} \times pop_{a,s}}{\sum_{a,s} R_{c,a,s} \times pop_{a,s}}$$

$floor_{c,a,s}$= floor value for cause c, age group a, sex s

$mortality\ envelope_{a,s}$= total estimated global deaths in 2018 for age group a, sex s

$pop_{a,s}$= total estimated global population in 2018 for age group a, sex s

$R_{c,a,s}$= global cause-specific mortality rate of cause c, age group a, sex s

This ensures that we add at most one death in the world per cause per year for causes where we have insufficient data to determine a plausible minimum observable cause fraction from the data.

Section 3.15: Cause of death database and outlier identification (step 13)[2]

Death rates for different causes of death generally have a stable age pattern. In large populations, these patterns will not change very rapidly over time. We can assume a relatively stable pattern in death rates for all causes except for some epidemic diseases and specific types of injuries. Rare causes in large populations and prevalent causes in small populations usually have stochastic patterns. To correct for these stochastic patterns, we implemented a noise-reduction process, explained in Step 12.

In VR data, we infrequently find one or more datapoints for specific geography/age/sex/year combinations that lie very far from the stable pattern of death rates. In these situations, the model usually ignores the datapoint(s). If the model fails to ignore these data, dramatic jumps or drops can occur in the death rates. When no logical explanation exists for variation in the death rates to this degree, we regard the datapoint(s) as outlier(s). The selection of datapoints to regard as outliers occurs after data have been prepped for modelling, as well as during preliminary reviews of the models.

In non-VR sources, data-collection methods and data quality can vary widely from source to source. Where datapoints in each age-sex-geography-year are very sparse, extreme datapoints can have a bad effect on regional estimation. In these situations, we investigate the study's methods and consider lower-quality datapoints as outliers.

Identifying outliers in the CoD data occurs prior to finalisation of models for each cause. We do not automate the selection of outliers but investigate the source of the offending data as well as reviewing other data sources for the same cause, geography, and year. Ultimately, outliers are identified based on the judgement of the modeller and senior faculty. Outlier decisions are reversible and may be revisited.

Section 3.16: Causes of death data star-rating calculation[2]

GBD estimates are most accurate when computed with a full time series of complete VR with a low percentage of garbage codes. We have developed a simple star-rating system from 0 to 5 to give a picture of the quality of data available in a given country over the full time series used in GBD estimates. Countries improve in the star rating as they increase availability, completeness, and detail of their mortality data and reduce the percentage of deaths coded to ill-defined garbage codes or highly aggregated causes (figures 5a and 5b). Underlying indicators for the percentage well-certified calculation are listed in table S9.

We assign star ratings to rate the quality of data for any given location year. Two dimensions determine this star rating: (I) the percentage of total deaths determined to be major garbage (such as ill-defined). Causes such as "injuries" or "cancer" will also be included in major garbage percentage because this percentage includes use of highly aggregated causes; and (II) the level of completeness of death registration (percentage of total deaths captured by the death registration). These two values were used to create a "percentage well-certified" value between 0 and 1, determined as:

$$pct_{wellcertified} = Completeness \times (1 - pct_{majgarbage})$$

The mapping of percentage well certified to star rating is as followed:

0 star: $0\% = pct_{wellcertified}$

1 star: $0\% < pct_{wellcertified} < 10\%$

2 star: $10\% <= pct_{wellcertified} < 35\%$

3 star: $35\% <= pct_{wellcertified} < 65\%$

4 star: $65\% <= pct_{wellcertified} < 85\%$

5 star: $pct_{wellcertified} \geq 85\%$

While stars are calculated for each five-year time interval as well as the full time series from 1980 to 2020, stars in the main text are presented for the full time series only.

In the case of VA, all garbage codes are considered ill-defined because redistribution for VA is highly imprecise.

For each VA data source, percentage well-certified is:

$$pct_{wellcertified} = VerbalAutopsyAdjustment \times (1 - pct_{majgarbage})$$

Where:

$$VerbalAutopsyAdjustment = SubAdj \times RegAdj \times AgeSexCoverage$$

SubAdj is 10% for subnationally representative studies; 100% for nationally representative studies. This adjustment, while arbitrary in its specific value, reflects the bias that can be associated with studies that only cover a potentially non-representative sample of a country's population.

RegAdj is 64% for all VA data sources. This accounts for the inaccuracy of VA in assigning CoD compared to medically verified VR. The specific multiplier 0·64 is based on the chance-corrected concordance of Physician Certified Verbal Autopsy (PCVA) versus medical certification by the Population Health Metrics Research Consortium.[47]

Age-sex coverage is the number of deaths estimated in the GBD mortality envelope for the ages and sexes in the study for the country and year divided by the number of deaths estimated in the GBD mortality envelope for the country and year. Studies that only cover children under 5 years or maternal mortality, for example, will be highly discounted by this multiplier.

Once percentage well-certified is calculated for each location-year of VR and each VA study-year, we then combine these into one measurement for each five-year time interval and the full time series 1980–2020. For each five-year time interval, we take the maximum percentage well-certified. Then for 1980–2020, we take the average of the maximum percentages well-certified for the seven five-year time intervals. Any five-year time interval in which no data were available were given a percentage well-certified value of zero.

Prior to GBD 2019, the causes of death team used an all-ages, both-sex cause fraction to estimate the percentage of garbage-coded deaths in a given location-year. Thus, the percentage of garbage for a given location year was determined as:

$$CF_G = \frac{D_G}{D}$$

Where:

CF_G represents the cause fraction of percentage garbage

D_G represents total garbage-coded deaths

D represents the total deaths in a given location/year.

In GBD 2019, we moved to calculating the percentage of garbage-coded deaths using an age-standardised cause fraction. The steps for creating these age-standardised cause fractions, in the case of garbage, are as follows:

1. Create both-sex, age-specific cause fractions of garbage for each age group.

2. Scale these cause fractions by a set of both-sex age weights, determined by global mortality estimates from 2010 to present. That is, weights for each GBD age group were determined as:

$$W_a = \frac{D_a}{D}$$

Where:

W_a is the weight for given age group "a"

D_a is the total both-sex, global deaths from 2010 to present in age group "a"

D is the total both-sex, global deaths from 2010 to present across all ages.

3. Sum these weighted cause fractions across all age groups to produce the age-standardised cause fraction.

In the case of percentage garbage for a given location-year, the formula to calculate percentage garbage would be given as the sum of the weighted age-specific cause fractions across all age groups "a":

$$CF_G = \Sigma_a\left(\frac{G_a}{D_a} \times W_a\right)$$

Where:

G_a represents the total both-sex garbage deaths in age group "a"

D_a represents the total both-sex deaths in age group "a"

W_a represents the weight generated from mortality estimates for age group "a"

ICD-10 and ICD-9 codes assigned to Level 1 or 2 garbage can be found in table S6.

In GBD 2023, a buffer system was implemented to prevent frequent star rating changes due to small fluctuations in completeness and percentage of garbage round over round. If a location-year decreases in star rating, as long as its PWC value remains within 3% of the cut-off for its former star rating, it will retain the former star rating.

Section 4: Causes of death modelling methods

Section 4.1: CODEm[2]

Section 4.1.1: Overview of methods

Cause of death ensemble modelling (CODEm) is the framework used to model most cause-specific death rates in the GBD.[48] It relies on four key components:

First, all available data are identified and gathered to be used in the modelling process. Although the data may vary in quality, they all contain some signal of the true epidemiological process.

Second, a diverse set of plausible models are developed to capture well-documented associations in the estimates. Using a wide variety of individual models to create an ensemble predictive model has been shown to outperform techniques using only a single model both in CoD estimation[48] and in more general prediction applications.[49,50]

Third, the out-of-sample predictive validity is assessed for all individual models, which are then ranked for use in the ensemble modelling stage.

Finally, differently weighted combinations of individual models are evaluated to select the ensemble model with the highest out-of-sample predictive validity.

For some causes (eg, lower respiratory infections), evidence exists that the relationship between covariates and death rates might differ between children and adults. Separate models are therefore run for different age ranges, when applicable. Additionally, separate models are developed for countries with extensive, complete, and representative VR for every cause to ensure that uncertainty can better reflect the more complete data in these locations.

In order to ensure the addition of subnational locations is not driving changes in estimates, we run a global model that excludes data from non-standard locations; the resulting covariate betas are then used as priors for the true global model.

In addition to CoD modelling, we also estimate fatal discontinuities. Fatal discontinuities are events that are stochastic in nature, that cannot be modelled because they do not have a predictable time trend. The fatal discontinuities by cause are aggregated by age and sex and added to the estimated number of deaths in CoD modelling for those causes during CoDCorrect. Details on their methods can be found in Section 3.4.

Section 4.1.2: Model pool development

Because many factors may co-vary with any given CoD, a range of plausible statistical models are developed for each cause. In the CODEm framework, four families of statistical models are used: linear mixed effects regression (LMER) models of the natural log of the cause-specific death rate, LMER models of the logit of the cause fraction, spatiotemporal Gaussian process regression (ST-GPR) models of the natural logarithm of the cause-specific death rate, and ST-GPR models of the logit of the cause fraction (see the 2x2 table in Foreman et al).[48] For more on ST-GPR, see section 4.3.3. For each family of models, all plausible relationships between covariates and the response variable are identified. Because all possible combinations of selected covariates are considered for each family of models, multi-collinearity between covariates may produce implausible signs on coefficients or unstable coefficients. Each combination is therefore tested for statistical significance (covariate coefficients must have a coefficient with p-value <0·05) and plausibility (the coefficients must have the directions expected on the basis of the literature). Only covariate combinations meeting these criteria are retained. This selection process is run for both cause fractions and death rates, then ST-GPR and LMER-only models are created for each set of covariates. For a detailed explanation of the covariate selection algorithm, see Foreman et al.[48]

Section 4.1.3: Data variance and uncertainty estimation

The families of models that go through ST-GPR described in Section 4.1.2 incorporate information about data variance. The main inputs for a Gaussian process regression (GPR) are a mean function, a covariance function, and data variance for each datapoint. These inputs are described in detail in Foreman et al.[48] For GBD 2019, we updated this calculation to incorporate garbage code redistribution uncertainty.

Three components of data variance are now used in CODEm: sampling variance, non-sampling variance, and garbage code redistribution variance. The computation of sampling variance and non-sampling variance has not changed since previous iterations of the GBD and is also described in Foreman et al.[48] Garbage code redistribution variance is computed in the CoD database process described in Section 2.7 of this appendix. Since variance is additive, we calculate total data variance as the sum of sampling variance, non-sampling variance, and redistribution variance. Increased data variance in GPR results in the GPR draws not following the datapoint as closely.

Section 4.1.4: Testing model pool on 15% sample

The performance of all models (individual and ensemble) is evaluated by means of out-of-sample predictive validity tests. 30% of the data are randomly excluded from the initial model fits. These individual model fits are evaluated and ranked by using half of the excluded data (15% of the total), then used to construct the ensembles on the basis of their performance. Data are held out from the analysis on the basis of the cause-specific missingness patterns for ages and years across locations. Out-of-sample predictive validity testing is repeated 20 times for each model, which has been shown to produce stable results.[48] These performance tests include the root mean square error (RMSE) for

the log of the cause-specific death rate, the direction of the predicted versus actual trend in the data, and the coverage of the predicted 95% UI.

Section 4.1.5: Ensemble development and testing

The component models are weighted on the basis of their predictive validity rank to determine their contribution to the ensemble estimate. The relative weights are determined both by the model ranks and by a parameter ψ, whose value determines how quickly the weights taper off as rank decreases. The distribution of ψ is described in more detail in Foreman et al.[48] A set of ensemble models is then created by using the weights constructed from the combinations of ranks and ψ values. These ensembles are tested by using the predictive validity metrics described in Section 4.1.4 on the remaining 15% of the data, and the ensemble with the best performance in out-of-sample trend and RMSE is chosen as the final model.

Section 4.1.6 Final estimation

Once a weighting scheme has been chosen, 250 draws are created for the final ensemble, and the number of draws contributed by each model is proportional to its weight. The mean of the draws is used as the final estimate for the CODEm process, and a 95% UI is created from the 0·025 and 0·975 quantiles of the draws. The validity of the UI can be checked via its coverage of the out-of-sample data; ideally, the 95% UI would capture 95% of these data. Higher coverage suggests that the UIs are too large, and lower coverage suggests overfitting.

To reduce computing power and time, we reduced the number of draws (or computations) per process to 250, from 500 in GBD 2021. Based on simulation testing, we determined that a change in the number of draws did not impact final mean estimates, nor lead to inappropriately narrow uncertainty estimates.

A simple R simulation illustrates how the estimated UI is only weakly dependent on the number of draws (500 or 250). Correctly using draws gives intervals of about the same width, and which interval is wider depends on random variation (as it should). Some code to illustrate this is given below. Running the example below (which simulates 250 draws and 500 draws around a simple normal distribution) provides an uncertainty width estimated from 250 draws and 500 draws. Because of the random nature of this code (and because we are not setting a seed), the values will change and are random each time. Running this repeatedly, the reader will see the widths remain similar depending on whether there are 250 draws or 500 draws, and the option that leads to the narrower UI width changes each time it is run.

```
# Sample 250 draws from N(0,1)
sample_250 <- rnorm(250, mean = 0, sd = 1)
ci_250 <- quantile(sample_250, probs = c(0.025, 0.975))
width_250 <- diff(ci_250)

# Sample 500 draws from N(0,1)
sample_500 <- rnorm(500, mean = 0, sd = 1)
ci_500 <- quantile(sample_500, probs = c(0.025, 0.975))
width_500 <- diff(ci_500)

# Create a simple table with the widths
```

```
ci_widths <- data.frame(Sample_Size = c(250, 500), CI_Width = c(width_250, width_500))
# Print the table
print(ci_widths)
```

Other notes

Section 4.1.7: Selection of causes for which CODEm is used

CODEm is used to model 235 causes, described in detail in Section 3.3. However, it is unsuitable for use in modelling certain causes, including those with very low death counts, those where cause-specific death record availability is inadequate, or those for which there are marked biases or variability for CoD certification over time that cannot be fully accounted for with the current garbage code redistribution algorithms. Criteria for causes where CODEm is not used are discussed in further detail in Section 3.2.

Section 4.1.8: Model-specific covariates

Modellers select covariates to be used in CODEm, but those covariates may not be significant or in the direction specified during the covariate selection step of CODEm and will therefore not be used in the model. These covariates are listed with a '—' for number of draws. Additionally, covariates may be selected by CODEm but only exist in submodels that perform poorly and may end up with zero draws included in the final ensemble. Finally, all other covariates are listed with the number of draws in the final ensemble from submodels that had the covariate.

Section 4.2: Causes modelled outside of CODEm[2]

Section 4.2.1: Overview

A number of causes required alternative modelling strategies to those used for CODEm because they were not compatible with CODEm estimation infrastructure and processes. Such unsuitability included having very low death counts; inadequate availability of cause-specific death records; and marked biases or variability for CoD certification over time that could not be fully accounted for with current garbage code redistribution algorithms. The inclusion of these causes in CODEm often renders its out-of-sample predictive validity testing unstable, but the validity of this type of testing is a key advantage of using CODEm for CoD estimation. Alternately, CODEm simply fails to generate plausible mortality rates in the absence of enough VR or VA data when these causes are included. For GBD 2023, we used alternative modelling approaches for these causes, including negative binomial models, natural history models, sub-cause proportion models, and prevalence-based models (table S10).

Section 4.2.2: Negative binomial models

"For 78 rare causes of death, too few observed deaths were included in the CoD database to produce stable estimates. For these causes, we estimated mortality by fitting count models - either negative binomial or Poisson - to available CoD data. Descriptions of the modelling process for each of these causes follows in the next sections.

Section 4.2.3: DisMod-MR 2.1

Until GBD 2010, non-fatal estimates were based on a single data source on prevalence, incidence, remission, or a mortality risk selected by the researcher as most relevant to a particular location and time. For GBD 2010, we set a more ambitious goal: to evaluate all available information on a disease that passes a minimum quality standard. That required a different analytical tool that would be able to pool disparate information presented in varying age groupings and from data sources by using different methods. The DisMod-MR 1.0 tool used in GBD 2010 evaluated and pooled all available data, adjusted data for systematic bias associated with methods that varied from the reference and produced estimates with UIs by world regions. For GBD 2013, the improved DisMod-MR 2.0 had increased computational speed, allowing computations that were consistent between all disease parameters at the country rather than the region level. The hundred-fold increase in speed of DisMod-MR 2.0 was partly due to a more efficient rewrite of the code in C++ but also to changing to a model specification using log rates rather than a negative binomial model used in DisMod-MR 1.0. In cross-validation tests, the log rates specification worked as well as or better than the negative binomial specification.[51] For GBD 2015, the computational engine (DisMod-MR 2.1) remained substantively unchanged, but we rewrote the wrapper code that organised the flow of data and settings

at each level of the analytical cascade. The sequence of estimation occurred at five levels: global, super-region, region, country, and, where applicable, subnational locations (see flow diagram of DisMod-MR 2.1 cascade that follows). The super-region priors were generated at the global level with mixed-effects, non-linear regression by using all available data; the super-region fit, in turn, informed the region fit, and so on down the cascade. The wrapper gave analysts the choice to branch the cascade in terms of time and sex at different levels depending on data density. The default used in most models was to branch by sex after the global fit but to retain all years of data until the lowest level in the cascade. For GBD 2023, we generated fits for the years 1990, 1995, 2000, 2005, 2010, 2015, 2020, 2022, 2023, and 2024.

In updating the wrapper, we consolidated the code base into a single language, Python, to make the code more transparent and efficient and to better deal with subnational estimation. The computational engine is limited to three levels of random effects; we differentiated estimates at the super-region, region, and country levels. In GBD 2013, the subnational units of China, Mexico, and the UK were treated as countries, such that a random effect was estimated for every location with contributing data. However, the lack of a hierarchy between country and subnational units meant that the fit to country data contributed as much to the estimation of a subnational unit as the fits for all other countries in the region. We found inconsistency between the country fit and the aggregation of subnational estimates when the country's epidemiology varied from the average of the region. Adding an additional level of random effects required a prohibitively comprehensive rewrite of the underlying DisMod-MR engine. Instead, we added a fifth layer to the cascade, with subnational estimation informed by the country fit and country covariates, plus an adjustment based on the average of the residuals between the subnational unit's available data and its prior. This procedure mimicked the impact of a random effect on estimates between subnationals.

For GBD 2015, we improved how country covariates differentiate non-fatal estimates for diseases with sparse data. The coefficients for country covariates were re-estimated at each level of the cascade. For a given location, country coefficients were calculated by using both data and prior information available for that location. In the absence of data, the coefficient of its parent location was chosen to utilise the predictive power of our covariates in data-sparse situations.

Beginning in GBD 2017, the DisMod-MR 2.1 tool was used.

Section 4.2.4: DisMod-MR 2.1 likelihood estimation

Analysts have the choice of using a Gaussian, log-Gaussian, Laplace, or log-Laplace likelihood function in DisMod-MR 2.1. The default log-Gaussian equation for the data likelihood is as follows:

$$-log\left[p\left(y_j\middle|\Phi\right)\right] = \log\left(\sqrt{2\pi}\right) + \log\left(\delta_j + s_j\right) + \frac{1}{2}\left(\frac{\log\left(a_j + \eta_j\right) - \log\left(m_j + \eta_j\right)}{\delta_j + s_j}\right)^2$$

Where:

y_j is a measurement value (ie, datapoint)

Φ denotes all model random variables

η_j is the offset value, *eta*, for a particular integrand (prevalence, incidence, remission, excess mortality rate, with-condition mortality rate, cause-specific mortality rate, relative risk, or standardised mortality ratio)

a_j is the adjusted measurement for datapoint j, defined by:

$$a_j = e^{(-u_j - c_j)} y_j$$

Where:

u_j is the total area effect (ie, the sum of the random effects at three levels of the cascade: super-region, region, and country)

c_j is the total covariate effect (ie, the mean combined fixed effects for sex, study-level, and country-level covariates), defined by:

$$c_j = \sum_{k=0}^{K[I(j)]-1} \beta_{I(j),k}\hat{X}_{k,j}$$

with standard deviation (SD)

$$s_j = \sum_{l=0}^{L[I(j)]-1} \zeta_{I(j),l}\hat{Z}_{k,j}$$

Where:

k denotes the mean value of each datapoint in relation to a covariate (also called x-covariate)

$I(j)$ denotes a datapoint for a particular integrand, j

$\beta_{I(j),k}$ is the multiplier of the kth x-covariate for the ith integrand

$\hat{X}_{k,j}$ is the covariate value corresponding to the datapoint j for covariate k

l denotes the SD of each datapoint in relation to a covariate (also called z-covariate)

$\zeta_{I(j),k}$ is the multiplier of the lth z-covariate for the ith integrand

δ_j is the SD for adjusted measurement j, defined by

$$\delta_j = log\left[y_j + e^{(-u_j-c_j)}\eta_j + c_j\right] - log\left[y_j + e^{(-u_j-c_j)}\eta_j\right]$$

Where mj denotes the model for the jth measurement, not counting effects or measurement noise and defined by:

$$m_j = \frac{1}{B(j)-A(j)}\int_{A(j)}^{B(j)} I_j(\text{a})\,\text{da}$$

Where:

$A(j)$ is the lower bound of the age range for a datapoint j

$B(j)$ is the upper bound of the age range for a datapoint j

$I(j)$ denotes the function of age corresponding to the integrand for datapoint j

The source code for DisMod-MR 2.1 as well as the wrapper code is available at https://github.com/ihmeuw/ihme-modelling/tree/master/gbd_2017/shared_code/central_comp/nonfatal/dismod.

Section 4.2.5: Natural history models

For some causes for which CoD data may be systematically biased either owing to misclassification or because the disease exists in focal communities without VR or VA studies, we have developed natural history models. In natural history models, incidence and case-fatality ratios are modelled separately and then combined to produce estimates of cause-specific mortality.

Section 4.2.6: Prevalence-based models

The modelling strategies for atrial fibrillation and flutter are distinct from those used for other causes modelled as natural history models. These models use prevalence estimates and excess mortality rates (EMR) generated through DisMod-MR 2.1 rather than incidence and case-fatality rates. This approach allows us to adjust estimates to more accurately reflect the number of deaths for which atrial fibrillation was the true underlying cause of death. Further details are provided in the CoD cause-specific modelling description for atrial fibrillation and flutter.

Section 4.2.7: Sub-cause proportion models

For certain sub-causes for which accurate diagnoses are known to be very difficult, we first modelled the parent cause in the GBD hierarchy with CODEm and then allocated deaths to specific causes by using proportions of the parent cause for each age-sex-location-year for each sub-cause. For these causes, we identified no significant predictors in negative binomial regressions. This approach was taken because the available data on these specific causes may come from sources other than VR, such as end-stage renal disease registries, or may come from too few places to model the death rates directly. Details for each cluster of causes analysed in this way follow.

Section 5: COVID-19 estimation

In GBD 2021, COVID-19 deaths were treated as shock deaths, and thus were not part of the HIV/AIDS- and shock-free envelope or the cause fractions used in CODEm. Like HIV/AIDS, COVID-19 was included in the shock-free envelope for GBD 2023. Modelling of COVID-19 mortality was done using a tool developed at IHME called OneMod, described in detail in Appendix 3.

Three types of data were used in the model. Corrected vital registration data, as described in Section 3.8, was included where available. For select countries (Bulgaria, Czechia, Denmark, Philippines, Türkiye, USA), we also identified recent provisional releases of vital registration data that were not complete enough for inclusion in the cause of death database but did include counts of COVID-19 deaths. Lastly, we included surveillance data that was reported during the COVID-19 pandemic (through 2022 for most countries, into 2023 for China) for location-years not covered by these prior sources. Country totals by year can be found in the table below.

	2020	2021	2022	2023
**Corrected VR**	75	64	36	1
**Provisional VR**	1	1	4	3
**Surveillance**	104	114	123	1

The provisional VR and surveillance data were not age- and sex-specific, so we first ran OneMod using only the corrected VR and used the out-of-sample predictions for each location-year to split these data by age and sex, based on the below formula, where the age- and sex- specific rate of the data, $d_{l,y,a,s}$, was equal to the all age rate, $d_{l,y}$, multiplied by the proportion of all deaths predicted by the model – the model rate, $m_{l,y,a,s}$, multiplied by the population, $p_{l,y,a,s}$ – in each age and sex. All models were fit using the binomial likelihood.

$$d_{l,y,a,s} = d_{l,y} \frac{m_{l,y,a,s} p_{l,y,a,s}}{\sum_{a=0}^{A} \sum_{s=1}^{2} m_{l,y,a,s} p_{l,y,a,s}}$$

The covariate pool provided to Rover is listed in table below. COVID-19 infection rate, variant prevalence, vaccination rate, and infection-detection rate (IDR) were all estimated by the IHME COVID-19 Forecasting Team.[52]

The value of IDR for all corrected VR data was set to 100%. All of these except for vaccination rate were included as fixed covariates, meaning they were automatically selected. Other covariates (risk factors, comorbidities, and Healthcare Access and Quality [HAQ]) were estimated as part of the Global Burden of Disease study. Covariates were selected by the Rover module based on an absolute t-statistic inclusion threshold of 0.5. Covariate effect sizes were estimated at the global level.

	**Selected for males**	**Selected for females**	**Coefficient directional constraint**
**Chronic kidney disease**	X	-	Positive
**Cardiovascular disease**	X	X	Positive
**Diabetes**	-	X	Positive
**Healthcare Access and Quality**	X	X	Negative
**Obesity**	X	X	Positive
**Smoking**	-	-	Positive
**Cancer**	-	-	Positive
**Chronic obstructive pulmonary disease**	-	-	Positive
**Total COVID-19 infections**	X	X	Positive
**Prevalence of alpha, beta, gamma, and delta variants**	X	X	-
**Prevalence of omicron and BA5 variants**	X	X	-
**Infection-detection rate (IDR; natural log)**	X	X	Positive

After estimating effect sizes in SpXMod, we then modified the data and predictions before running KReg. First, we calculated an empirical effect size for IDR in countries with corrected VR early in the pandemic and surveillance data based on the overlapping years, weighted by the total VR deaths in each year:

$$\beta_{l,a,s} = \sum_{y=2020}^{Y} \frac{d_{l,y}^{VR}}{d_l^{VR}} \frac{logit(r_{l,y,a,s}^{VR}) - logit(r_{l,y,a,s}^{surv})}{-\ln(IDR_{l,y})}$$

Where *r* is the rate and *d* represents deaths (because the covariate was in natural log space, $\ln(IDR_{max}) == \ln(1) == 0$),. Using this empirical effect size for countries with corrected VR and the modelled global effect size everywhere else, we then removed the IDR effect from rates in both the predictions and the data before running KReg, making a counterfactual as if the IDR was 100% in all location-years (this would not affect the corrected VR, as the IDR in those observations was already 100%):

$$logit(r_{l,y,a,s}^{couterfactual}) = logit(r_{l,y,a,s}^{original}) - \beta_{l,a,s} \ln(IDR_{l,y})$$

We generated draws from KReg and, in an effort to eliminate extreme outlier draws, resampled the 2.5% lowest and highest draws by location (as defined by cumulative all-age, both-sexes deaths over all years). We then raked the draws for subnational locations for which we only had parent-level data: New Zealand, Iran, India urban/rural, Indonesia, Ethiopia, Kenya, and Nigeria.

Two of the above-described pipelines were run on the full age- and sex-split dataset – one fitting age and time effects (by sex) at each level of the hierarchy (global, super region, region, and country), and one fitting only global- and then country-level effects. All countries in regions featuring at least one location with corrected VR used the full location hierarchy model, while countries in regions without any corrected VR used the model with only global and country effects (the latter group is comprised of South Asia, East Asia, and all regions in sub-Saharan Africa). This

was due to the fact that, while the super region and region effects captured important contextual variation in groups of locations with sufficient differentiability in the covariates, in the absence of any corrected data the IDR effect was absorbed by the location effects for these regions (most of which have estimated IDRs below 1%), leading to implausibly low counterfactual estimates. Were VR to become available in all super regions, we would explore using a single model that refit the IDR effect at a level below global. Because covariate selection was done at the global level (by sex), that process was the same for both model runs. The lambdas (on both the intercept and the spline) for each location level were as follows: global 1.0, super region 2.5, region 5.0, location 10.0. The spline was linear with knots at each year.

Section 6: Central computation[2]

Section 6.1: Imported cases

Imported cases are fatalities that occur in a geographic area where a particular CoD is known to be eradicated in a specific time period or where infection cannot occur. We apply space-time restrictions to these causes in the modelling strategy for that location and time period. However, in some rare cases, deaths from these causes occur outside of restricted locations and time periods. These deaths are referred to as imported cases.

Illustrating this concept, some diseases are transmitted only in certain regions of the world. For those diseases, we restricted our models to only endemic locations, excluding non-endemic countries from the analysis. In some cases, however, deaths for these geographically restricted diseases may occur in non-endemic countries. For example, individuals may become infected with a disease while travelling or residing in an endemic location. Through travel or migration, however, they may move to a non-endemic location prior to dying from that disease. Imported cases account for these kinds of deaths.

To calculate these imported cases, we find all cases from the VRs of data-rich countries for any CoD that is otherwise geographically or temporally restricted. We then create a beta distribution from that datapoint by using the sample size of the VR for that datapoint and upload these draws as a custom CoD model. This model is then used as an input to CoDCorrect.

Section 6.2: CoDCorrect

Section 6.2.1: Objective of CoDCorrect

As mentioned in the main text, the CoD models are cause-specific. As such, there is no guarantee that the sum of these models will equal the results of the all-cause mortality estimates or that model results of child causes add up to the parent model results. The CoDCorrect process is used to make the CoD and all-cause mortality estimates internally consistent by using a very simple algorithm.

Section 6.2.2: Algorithm and levels

The core algorithm remains the same as it did in GBD 2013. The equation can be written as follows:

$$CD_{lyasjd} = D_{lyasjd}\left(\frac{PD_{lyasjd}}{\sum_{j=1}^{j=k} D_{lyasjd}}\right)$$

Where:

CD_{lyasjd} is the corrected number of deaths for a location l, year y, age a, sex s, cause j, and draw d

PD_{lyasjd} is the parent CoD for a location l, year y, age a, sex s, cause j, and draw d

D_{lyasjd} is the uncorrected number of deaths estimated from a cause-specific model for a l, year y, age a, sex s, cause j, and draw d

The CoDCorrect process starts by rescaling the Level 1 causes to match the all-cause mortality estimates (used for PD_{lyasjd} in the previous equation). Level 2 causes are then rescaled to their corrected parent causes. This process continues until all levels of the hierarchy have been rescaled. Causes and their levels within the CoDCorrect hierarchy can be found in table S3.

Section 6.2.3: Diagnostic results of CoDCorrect by cause and location

For more detail on diagnostic results of CoDCorrect by cause see table S11.

Section 6.3: Years of life lost calculation

Years of life lost (YLLs) owing to premature mortality were computed for 925locations and 45 years. First, we used the lowest observed age-specific mortality rates by location and sex across all estimation years from locations with total populations greater than 5 million in the most recent year of estimation to establish a theoretical minimum risk reference life table. By convention, we also exclude years before 2010 with the reasoning that the previous decade has the lowest overall mortality than any other point in history. Additionally, years 2020-2021 were excluded from consideration for the TMRLT due to the COVID-19 pandemic.

The YLL is a metric that is computed by multiplying the number of estimated deaths by the predicted life expectancy by age, sex, location, and year. The metric therefore highlights premature deaths by applying a larger weight to deaths that occur in younger age groups. We propagated uncertainty from CoDCorrected deaths for all demographics. The predicted life expectancy is calculated with two main components, a global, sex-agnostic, all-time theoretical "best" life expectancy (assuming that males and females in all countries in the world could theoretically have this life expectancy) and the average age of death from with-shock life tables for each location, sex, age group, and year.[3]

Section 6.4: GBD world population age standard

Age-standardised populations in the GBD were calculated by using the GBD world population age standard. For GBD 2013, GBD 2015, and GBD 2016, the age-specific proportional distributions of all national locations from the UN Population Division World Population Prospects 2012 revision for all years from 2010 to 2035 were used to generate a standard population age structure by using the non-weighted mean across all the aforementioned country-years. For GBD 2017, we used the non-weighted mean of 2017 age-specific proportional distributions from the GBD 2017 population estimates for all national locations with a population greater than 5 million people in 2017 to generate an updated standard population age structure.[55] For GBD 2023, we have continued to use this method using GBD 2023 population estimates.[3]

Section 7: References

1 Stevens GA, Alkema L, Black RE, *et al.* Guidelines for Accurate and Transparent Health Estimates Reporting: the GATHER statement. *Lancet* 2016; **388**: e19–23.

2 GBD 2021 Causes of Death Collaborators. Global burden of 288 causes of death and life expectancy decomposition in 204 countries and territories and 811 subnational locations, 1990-2021: a systematic analysis for the Global Burden of Disease Study 2021. *Lancet* 2024; **403**: 2100–32.

3 GBD 2021 Demographics Collaborators. Global age-sex-specific mortality, life expectancy, and population estimates in 204 countries and territories and 811 subnational locations, 1950-2021, and the impact of the COVID-19 pandemic: a comprehensive demographic analysis for the Global Burden of Disease Study 2021. *Lancet* 2024; **403**: 1989–2056.

4 Uneke CJ, Uro-Chukwu HC, Chukwu OE. Validation of verbal autopsy methods for assessment of child mortality in sub-Saharan Africa and the policy implication: a rapid review. *Pan Afr Med J* 2019; **33**: 318.

5 Thomas L-M, D'Ambruoso L, Balabanova D. Verbal autopsy in health policy and systems: a literature review. *BMJ Glob Health* 2018; **3**: e000639.

6 Serina P, Riley I, Stewart A, *et al.* Improving performance of the Tariff Method for assigning causes of death to verbal autopsies. *BMC Med* 2015; **13**: 291.

7 Byass P, Hussain-Alkhateeb L, D'Ambruoso L, *et al.* An integrated approach to processing WHO-2016 verbal autopsy data: the InterVA-5 model. *BMC Med* 2019; **17**: 102.

8 Murray CJ, Lozano R, Flaxman AD, *et al.* Using verbal autopsy to measure causes of death: the comparative performance of existing methods. *BMC Med* 2014; **12**: 5.

9 Gakidou E, King G. Death by survey: estimating adult mortality without selection bias from sibling survival data. *Demography* 2006; **43**: 569–85.

10 Government of India. Medical certification of cause of death. 2023; published online Aug 11. https://censusindia.gov.in/census.website/node/381 (accessed Sept 29, 2023).

11 Government of India. ORGI SRS. 2023; published online Aug 11. https://censusindia.gov.in/census.website/node/294 (accessed Oct 16, 2023).

12 Ganguli M, Rodriguez EG. Reporting of dementia on death certificates: a community study. *J Am Geriatr Soc* 1999; **47**: 842–9.

13 Wachterman M, Kiely DK, Mitchell SL. Reporting dementia on the death certificates of nursing home residents dying with end-stage dementia. *JAMA* 2008; **300**: 2608–10.

14 Macera CA, Sun RK, Yeager KK, Brandes DA. Sensitivity and specificity of death certificate diagnoses for dementing illnesses, 1988-1990. *J Am Geriatr Soc* 1992; **40**: 479–81.

15 Olichney JM, Hofstetter CR, Galasko D, Thal LJ, Katzman R. Death certificate reporting of dementia and mortality in an Alzheimer's disease research center cohort. *J Am Geriatr Soc* 1995; **43**: 890–3.

16 GBD 2019 Collaborators. Global mortality from dementia: application of a new method and results from the Global Burden of Disease Study 2019. *Alzheimers Dement N Y N* 2021; **7**: e12200.

17 Todd S, Barr S, Passmore AP. Cause of death in Alzheimer's disease: a cohort study. *QJM* 2013; **106**: 747–53.

18 Brunnström HR, Englund EM. Cause of death in patients with dementia disorders. *Eur J Neurol* 2009; **16**: 488–92.

19 Gao L, Calloway R, Zhao E, Brayne C, Matthews FE, Medical Research Council Cognitive Function and Ageing Collaboration. Accuracy of death certification of dementia in population-based samples of older people: analysis over time. *Age Ageing* 2018; **47**: 589–94.

20 Romero JP, Benito-León J, Mitchell AJ, Trincado R, Bermejo-Pareja F. Under reporting of dementia deaths on death certificates using data from a population-based study (NEDICES). *J Alzheimers Dis JAD* 2014; **39**: 741–8.

21 Moscovich M, Boschetti G, Moro A, Teive HAG, Hassan A, Munhoz RP. Death certificate data and causes of death in patients with parkinsonism. *Parkinsonism Relat Disord* 2017; **41**: 99–103.

22 Pressley JC, Tang M-X, Marder K, Cote LJ, Mayeux R. Disparities in the recording of Parkinson's disease on death certificates. *Mov Disord* 2005; **20**: 315–21.

23 Duncan ME, Pitcher A, Goldacre MJ. Atrial fibrillation as a cause of death increased steeply in England between 1995 and 2010. *EP Eur* 2014; **16**: 797–802.

24 Johnson SC, Cunningham M, Dippenaar IN, *et al.* Public health utility of cause of death data: applying empirical algorithms to improve data quality. *BMC Med Inform Decis Mak* 2021; **21**: 175.

25 Barker B, Degenhardt L. Accidental drug-induced deaths in Australia 1997 - 2001. Sydney: National Drug and Alcohol Research Centre, 2003.

26 Roxburgh, A., Burns, L. Accidental drug-induced deaths due to opioids in Australia, 2013. Sydney: National Drug and Alcohol Research Centre, 2013.

27 Roxburgh A, Burns L. Cocaine and methamphetamine related drug-induced deaths in Australia, 2011. Sydney: National Drug and Alcohol Research Centre, 2015.

28 França EB, Ishitani LH, Abreu DMX de, *et al.* Measuring misclassification of Covid-19 as garbage codes: Results of investigating 1,365 deaths and implications for vital statistics in Brazil. *PLOS Glob Public Health* 2022; **2**: e0000199.

29 CDC. Excess deaths associated with COVID-19. 2023; published online Sept 28. https://www.cdc.gov/nchs/nvss/vsrr/covid19/excess_deaths.htm (accessed Jan 13, 2025).

30 Birnbaum JK, Murray CJ, Lozano R. Exposing misclassified HIV/AIDS deaths in South Africa. *Bull World Health Organ* 2011; **89**: 278–85.

31 Lozano R, Naghavi M, Foreman K, *et al.* Global and regional mortality from 235 causes of death for 20 age groups in 1990 and 2010: a systematic analysis for the Global Burden of Disease Study 2010. *Lancet* 2012; **380**: 2095–128.

32 Wang H, Abajobir AA, Abate KH, *et al.* Global, regional, and national under-5 mortality, adult mortality, age-specific mortality, and life expectancy, 1970–2016: a systematic analysis for the Global Burden of Disease Study 2016. *Lancet* 2017; **390**: 1084–150.

33 Calvert C, Ronsmans C. The contribution of HIV to pregnancy-related mortality: a systematic review and meta-analysis. *AIDS Lond Engl* 2013; **27**: 1631–9.

34 Figueroa-Damián R. Pregnancy outcome in women infected with the human immunodeficiency virus. *Salud Publica Mex* 1999; **41**: 362–7.

35 Ryder RW, Nsuami M, Nsa W, *et al.* Mortality in HIV-1-seropositive women, their spouses and their newly born children during 36 months of follow-up in Kinshasa, Zaïre. *AIDS* 1994; **8**: 667–72.

36 Zvandasara P, Saungweme G, Mlambo JT, Moyo J. Post Caesarean section infective morbidity in HIV-positive women at a tertiary training hospital in Zimbabwe. *Cent Afr J Med* 2007; **53**: 43–7.

37 Chilongozi D, Wang L, Brown L, *et al.* Morbidity and mortality among a cohort of human immunodeficiency virus type 1-infected and uninfected pregnant women and their infants from Malawi, Zambia, and Tanzania. *Pediatr Infect Dis J* 2008; **27**: 808–14.

38 Leroy V, Ladner J, Nyiraziraje M, *et al.* Effect of HIV-1 infection on pregnancy outcome in women in Kigali, Rwanda, 1992-1994. Pregnancy and HIV Study Group. *AIDS* 1998; **12**: 643–50.

39 Kourtis AP, Bansil P, McPheeters M, Meikle SF, Posner SF, Jamieson DJ. Hospitalizations of pregnant HIV-infected women in the USA prior to and during the era of HAART, 1994-2003. *AIDS* 2006; **20**: 1823–31.

40 Ticconi C, Mapfumo M, Dorrucci M, *et al.* Effect of maternal HIV and malaria infection on pregnancy and perinatal outcome in Zimbabwe. *J Acquir Immune Defic Syndr 1999* 2003; **34**: 289–94.

41 Brown T, Peerapatanapokin W. The Asian Epidemic Model: a process model for exploring HIV policy and programme alternatives in Asia. *Sex Transm Infect* 2004; **80**: i19–24.

42 DerSimonian R, Laird N. Meta-analysis in clinical trials. *Control Clin Trials* 1986; **7**: 177–88.

43 Matthews LT, Kaida A, Kanters S, *et al.* HIV-infected women on antiretroviral treatment have increased mortality during pregnant and postpartum periods. *AIDS* 2013; **27 Suppl 1**: S105-112.

44 Westreich D, Maskew M, Evans D, Firnhaber C, Majuba P, Sanne I. Incident pregnancy and time to death or AIDS among HIV-positive women receiving antiretroviral therapy. *PLOS ONE* 2013; **8**: e58117.

45 Stover J. AIM: A computer program for making HIV/AIDS projections and examining the social and economic impact of AIDS. Glastonbury, CT: USAID, 2005.

46 Zheng P, Barber R, Sorensen RJD, Murray CJL, Aravkin AY. Trimmed constrained mixed effects models: formulations and algorithms. *J Comput Graph Stat* 2021; **30**: 544–56.

47 Lozano R, Lopez AD, Atkinson C, Naghavi M, Flaxman AD, Murray CJ. Performance of physician-certified verbal autopsies: multisite validation study using clinical diagnostic gold standards. *Popul Health Metr* 2011; **9**: 32.

48 Foreman KJ, Lozano R, Lopez AD, Murray CJ. Modeling causes of death: an integrated approach using CODEm. *Popul Health Metr* 2012; **10**: 1.

49 Bell RM, Koren Y. Lessons from the Netflix prize challenge. *ACM SIGKDD Explor Newsl* 2007; **9**: 75–9.

50 Bell RM, Koren Y, Volinsky C. All together now: A perspective on the NETFLIX PRIZE. *CHANCE* 2010; **23**: 24–24.

51 Flaxman AD, Vos T, Murray CJL. An integrative metaregression framework for descriptive epidemiology. Univ. Wash. Press. 2015; published online Oct. https://uwapress.uw.edu/book/9780295991849/an-integrative-metaregression-framework-for-descriptive-epidemiology (accessed July 7, 2023).

52 COVID-19 Forecasting Team. Forecasting the trajectory of the COVID-19 pandemic into 2023 under plausible variant and intervention scenarios: a global modelling study. 2023; : 2023.03.07.23286952.

53 Fragoso TM, Bertoli W, Louzada F. Bayesian model averaging: a systematic review and conceptual classification. *Int Stat Rev* 2018; **86**: 1–28.

54 COVID-19 Excess Mortality Collaborators. Estimating excess mortality due to the COVID-19 pandemic: a systematic analysis of COVID-19-related mortality, 2020-21. *Lancet* 2022; **399**: 1513–36.

55 GBD 2017 Population and Fertility Collaborators. Population and fertility by age and sex for 195 countries and territories, 1950-2017: a systematic analysis for the Global Burden of Disease Study 2017. *Lancet* 2018; **392**: 1995–2051.

Section 8: Tables and figures

Figure S1. Analytical flowchart for the development of the GBD 2023 cause of death database and different strategies used to combine all medically verifiable cause of death data into a consistent set of cause-specific deaths for each location, age, sex, and year.

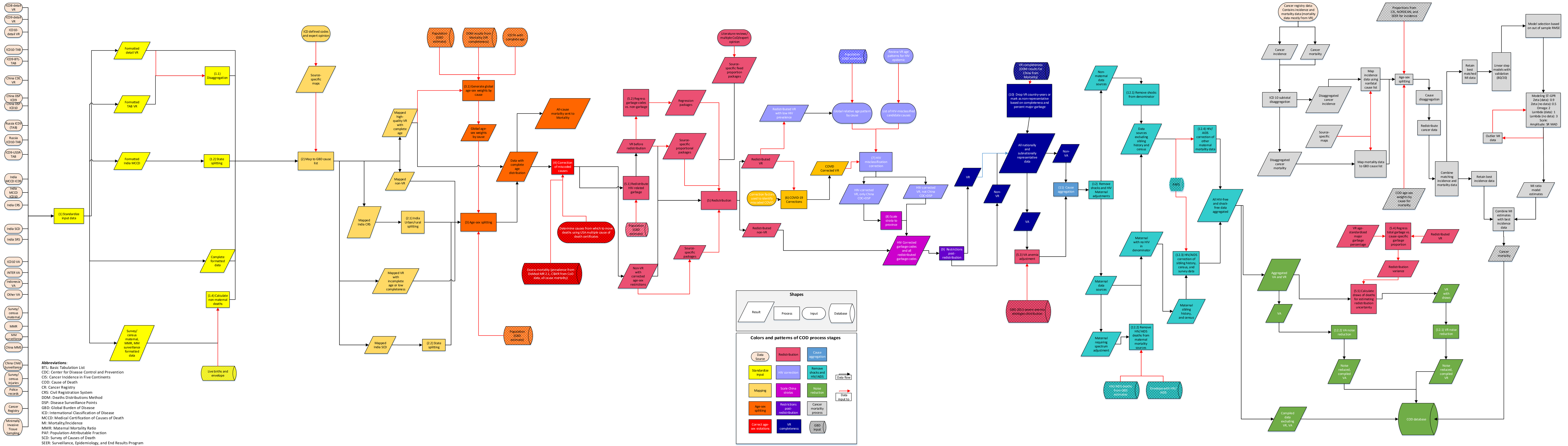

Abbreviations:
BTL: Basic Tabulation List
CDC: Center for Disease Control and Prevention
CI5: Cancer Incidence in Five Continents
COD: Cause of Death
CR: Cancer Registry
CRS: Civil Registration System
DDM: Deaths Distributions Method
DSP: Disease Surveillance Points
GBD: Global Burden of Disease
ICD: International Classification of Disease
MCCD: Medical Certification of Causes of Death
MI: Mortality/Incidence
MMR: Maternal Mortality Ratio
PAF: Population-Attributable Fraction
SCD: Survey of Causes of Death
SEER: Surveillance, Epidemiology, and End Results Program
SRS: Sample Registration System
ST-GPR: Spatiotemporal Gaussian Process Regression
TAB: Tabulation List
VA: Verbal Autopsy
VR: Vital Registration

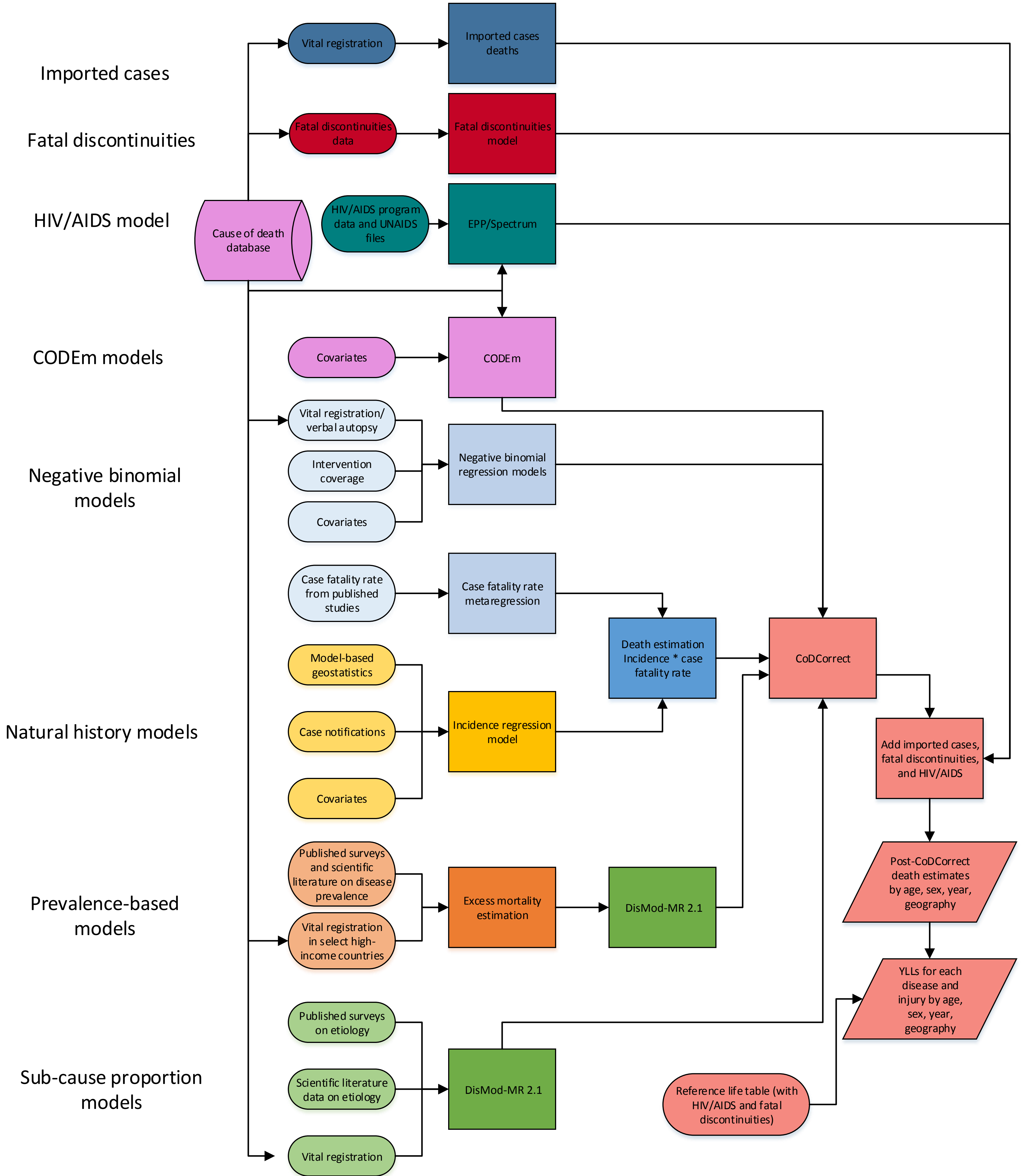

Appendix Figure S2: GBD 2021 causes of death estimation flowchart by modelling group

Abbreviations:
CODEm: Cause of death ensemble model
GBD: Global Burden of Disease
YLL: years of life lost

Figure 2. Analytical flowchart for the development of the GBD 2023 cause of death database and different strategies used to combine all medically verifiable cause of death data into a consistent set of cause-specific deaths for each location, age, sex, and year.

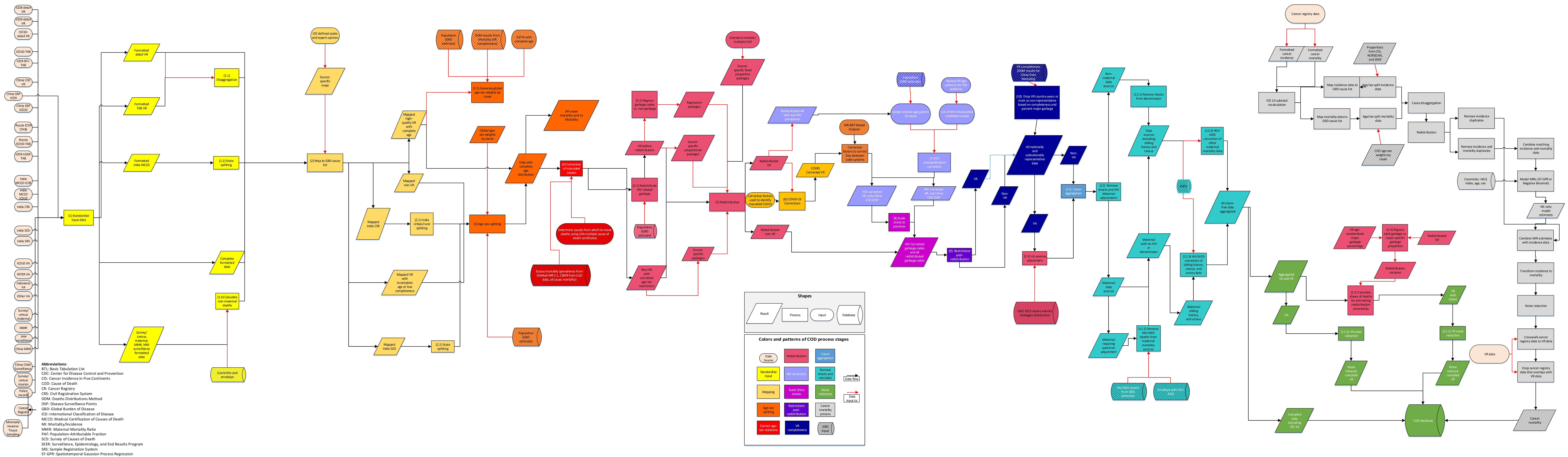

Abbreviations:
BTL: Basic Tabulation List
CDC: Center for Disease Control and Prevention
CI5: Cancer Incidence in Five Continents
COD: Cause of Death
CR: Cancer Registry
CRS: Civil Registration System
DDM: Deaths Distributions Method
DSP: Disease Surveillance Points
GBD: Global Burden of Disease
ICD: International Classification of Disease
MCCD: Medical Certification of Causes of Death
MI: Mortality/Incidence
MMR: Maternal Mortality Ratio
PAF: Population-Attributable Fraction
SCD: Survey of Causes of Death
SEER: Surveillance, Epidemiology, and End Results Program
SRS: Sample Registration System
ST-GPR: Spatiotemporal Gaussian Process Regression
TAB: Tabulation List
VA: Verbal Autopsy
VR: Vital Registration

Appendix Figure S3: Vital Registration and Verbal Autopsy data availability by country, 1980−2023

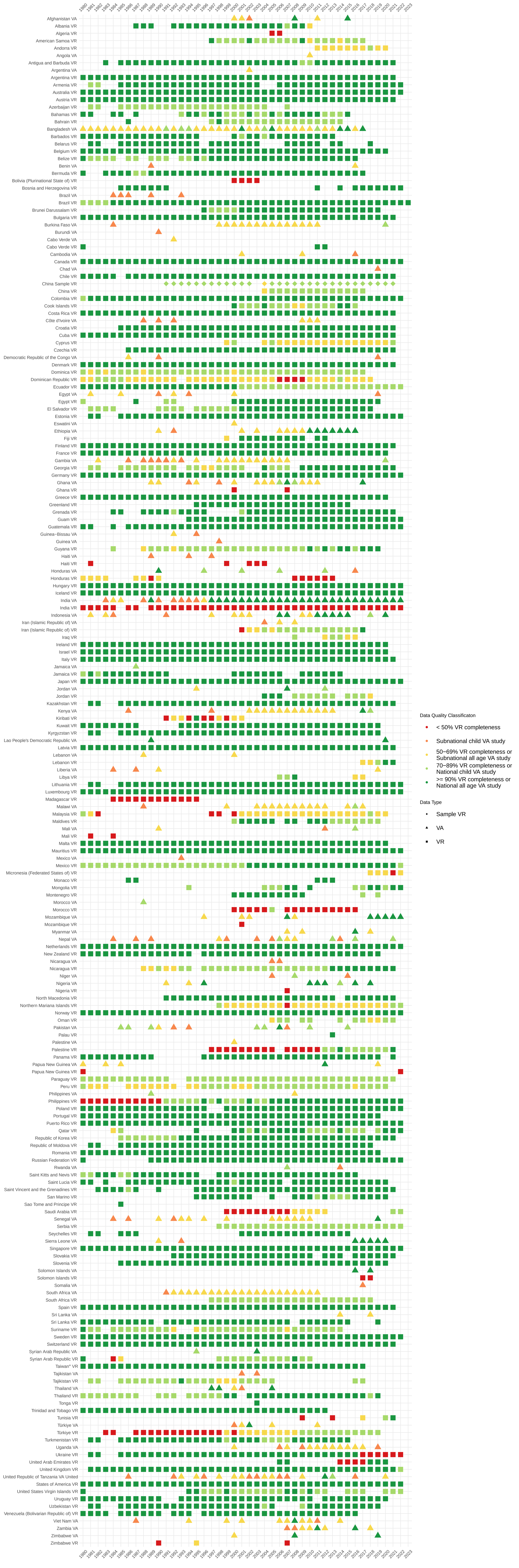

Appendix figure S4: Classification of national time series of vital registration and verbal autopsy data 1980−2023

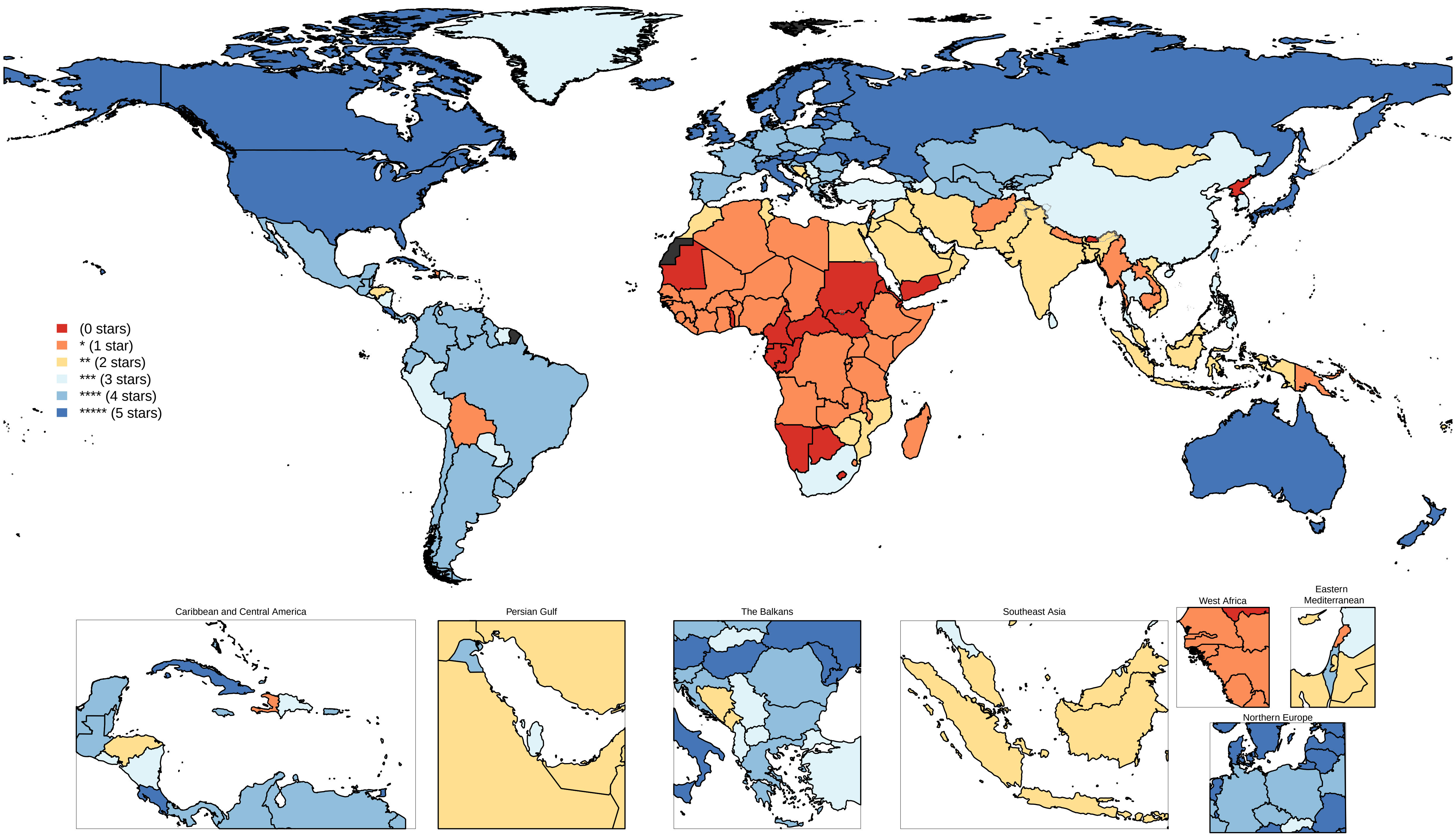

Appendix Figure S5: Classification of national time series of vital registration and verbal autopsy data 2010−2023

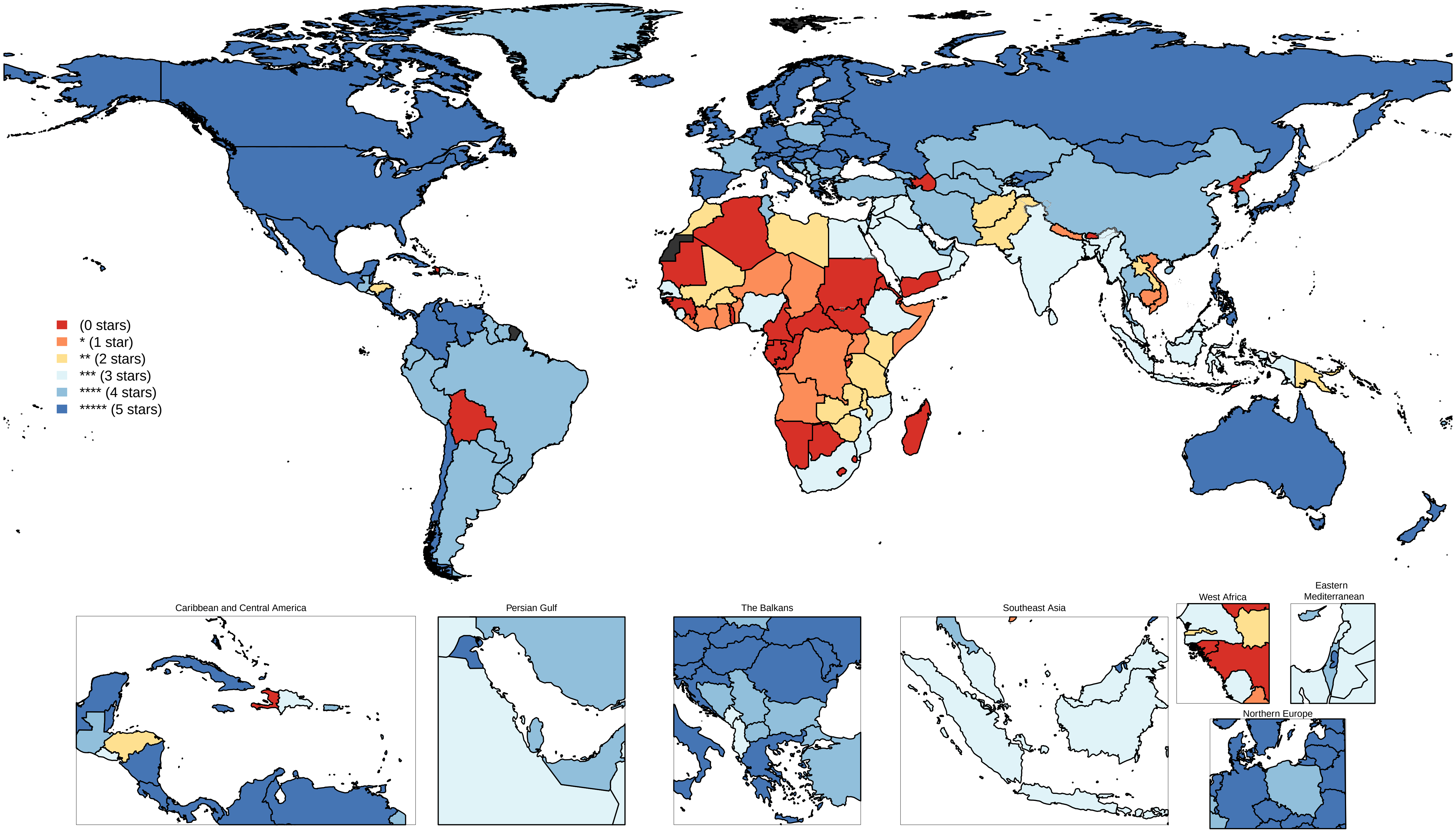

Appendix Figure S6: Percent of vital registration deaths assigned to major garbage codes for all ages and sexes by country, 1980-2023

Country	1980	1981	1982	1983	1984	1985	1986	1987	1988	1989	1990	1991	1992	1993	1994	1995	1996	1997	1998	1999	2000	2001	2002	2003	2004	2005	2006	2007	2008	2009	2010	2011	2012	2013	2014	2015	2016	2017	2018	2019	2020	2021	2022	2023
Albania								38	36	35			36	33	35	30	29	26	28	25	27	28	27	27	27	30	29	27	27	27	23													
Algeria																										52	50																	
American Samoa																		27	25	20	17	16	18	15	24	15	30	25	13	20	18	24	21	21	19	15	13	18						
Andorra																																23	19	17	19	17	15	17	23	17	16			
Antigua and Barbuda				30		38	41	39	34	31	26	25	26	25	25	29	20	27	22	20	19	23	22	17	19	28	27	18	21	20	22	30	28	26	21	25	24	22	26	32	35	29		
Argentina	25	24	28	29	30	31	30	30	29	30	30	31	31	31	31	31	31	32	32	32	32	32	33	32	31	31	31	32	33	32	32	32	31	31	29	27	27	26	28	28	25	25		
Armenia		11	13			13	12	12	32	14	13	13	14	17	19	16	15	14	14	13	13	13	14	15			10	11	10	10	11	11	12	12	12	11	11	15	21	22	16	17	19	
Australia	7	7	7	7	7	7	6	7	6	7	7	6	6	7	6	8	7	7	8	8	8	7	8	8	9	9	8	9	9	9	9	9	9	9	9	9	10	9	9	9	10	10	11	
Austria	9	9	9	17	8	8	7	7	8	7	7	8	8	8	8	8	8	8	8	9	9	9	6	5	6	6	7	7	7	9	9	9	10	9	9	11	11	11	12	12	11	12		
Azerbaijan		13	12			13	11	12	12	14	13	12	11	11	17	14	15	14	14	15	15	8	8	9	11			45																
Bahrain							25											33	43	42	39	40	42	39	42	39	40	38	40	44	47	44	41	37	34									
Barbados	25	26	25	25	25	25	23	23	24	23	23	24	21	23	22	21					22	22	22	25	28	27	18	18	20	18	18	18	20	20										
Belarus		14	14			13	12	12	12	14	20	23	23	23	22	23		22	20	20	20	20	18	19				19	20	18	18	20		19	17				11					
Belgium	21	20	20	21	21	22	21	20	21	21	20	21	18	16	16	16	14	14	14	15	14	14	14	15	14	14	14	14	14	15	17	17	18	17	17	17	17	19	18	18	16			
Belize	33	26	36	39	33		30	25		33	41	38		37	40	34	38	31	25	26	30	24	24	27	25	22	20	21	17	13	13	13	13	14	13	13	15							
Bermuda	11			19	8	10	20	8	10	9	11	9	10	11	14	20	5	5	7	10	8	12	7	8	12	12	14	12	11	20	14	11	11	9	7	10	11	10						
Bolivia																					74	72	71	68																				
Bosnia and Herzegovina						42	42	38	38	38	36	37																				34			27		32	31	32	32	28	28	31	
Brazil	38	38	38	38	39	38	37	36	36	35	34	34	34	34	33	32	30	30	30	30	28	28	27	26	25	23	20	20	19	19	19	18	18	17	17	18	18	17	17	17	17	16	18	19
Brunei																	29	20	15	19	18	19	18	19	19	18	18	18	18	24	24	16	14	15	16	18	18	15	16	14				
Bulgaria	22	22	21	21	20	20	20	22	22	22	20	20	20	19	20	22	24	26	27	27	27	27	27	28	28	25	27	26	26	30	32	34	28	30	29	31	32	33	33	30	28	26		
Canada	11	10	11	11	11	12	9	9	10	9	11	10	10	10	10	10	10	12	10	10	10	10	10	10	10	10	10	10	10	9	10	10	10	10	10	10	12	12	15	14	14	16	18	
Cape Verde	50																															30	30											
Chile	28	26	26	25	26		25	25	24	24	21	21	20	20	17	16	16	16	15	15	12	11	10	9	9	9	9	9	10	10	10	10	10	10	10	10	10	11	12	12	11	13		
China												23	21	18	14	16	16	17	15	15	17	27	25		5	5	9	8	14	13	12	12	11	10	8	7	7	7						
Colombia	29	31	28	25	25	24	24	23	22	22	22	22	21	19	18	19	18	17	14	12	11	11	10	11	11	11	11	11	11	11	11	12	11	10	9	9	9	9	9	9	8	7	8	
Cook Islands																					40	27	36	30	37	37	30	41	32	23	23	30	25	25	25	20	22							
Costa Rica	24	26	21	20	19	19	18	18	18	17	22	20	17	17	18	18	18	11	10	9	8	9	9	7	8	8	10	9	10	10	9	9	9	10	9	9	10	8	8	8	7	8		
Croatia						18	18	18	17	15	14	15	17	18	18	19	18	19	17	15	14	14	15	14	12	12	12	12	11	10	8	8	8	7	6	7	6	8	6	5	6	6		
Cuba	15	15	15	15	14	15	14	15	13	13	14	14	15	15	15	16	15	10	10	10	11	10	9	9	9	9	8	8	8	8	8	8	8	8	8	8	8	8	9	9	9	10		
Cyprus																				61	63				28	24	22	19	20	20	19	15	16	14	15	14	13	13	13	13	14	12		
Czechia							9	10	10	10	10	10	11	11	12	14	16	18	16	15	14	14	16	16	16	15	15	14	15	15	16	14	14	12	12	13	14	15	14	15	15	15		
Denmark	17	16	17	17	17	18	18	19	19	20	21	22	23	23	15	14	17	14	13	12	12	13	15	15	14	13	13	16	16	18	17	16	15	16	16	16	13	14	17	15	16	18		
Dominica	34	28	31	29	30	40	38	38	32	38	35	41	37	35	44	38	41	39	37	33	32	34	35	34	30	13	11	19	18	18	17	15	21	16	29	21	23	22						
Dominican Republic	51	48	45	41	38	37	36	35	35	36	34	36	35		34	34	32	30	28	28	26	25	24	23	24	22	19	19	18	16	17	19	22	19	28	28	31	32	36					
Ecuador	38	37	36	35	35	34	33	33	33	33	32	31	30	30	35	35	30	34	35	35	35	34	35	34	31	30	28	27	28	25	26	25	25	22	20	17	15	16	16	16	15	16	15	
Egypt	67							65				59	59								61	62	60	60	60	60	60	61	61	61	60	56	54	59	54	57	56	57	58	61				
El Salvador		44	47	50	49						40	39	39	39		37	33	36	36	33	31	32	33	34	32	31	31	33	33	33	34	33	33	36	37	40	40	41	44					
Estonia		9	9			9	9	9	9	11	15	15	19	18	6	7	7	7	7	7	8	8	8	9	9	9	9	10	7	7	8	8	8	9	9	9	11	8	7	8	8	9	10	
Federated States of Micronesia																																							27	26	25	30	24	
Fiji																				47		56	48	47	47	46	45	44	42	41		35	35											
Finland	17	19	18	18	18	19	19	9	9	10	10	10	9	9	9	9	5	4	4	4	4	4	4	5	5	5	5	5	5	5	5	5	6	5	6	5	5	6	6	5	6	6		
France	24	24	23	23	22	21	21	21	20	20	19	19	19	18	18	18	18	18	18	18	17	18	18	18	17	17	17	17	18	18	19	19	20	20	19	20	20	20	21	21	20			
Georgia		10	12			11	12	12	9	8	8	9	10		10	10	12	15	14	14	13	12			23	35	41	40		47	69	53	48	49	38	40	43	39	29	26	20	20		
Germany	21	21	21	21	21	20	21	20	20	18	18	16	15	15	15	14	14	14	14	14	15	15	15	15	15	15	14	15	15	15	15	14	14	15	15	15	15	15	15	15	16	16	16	
Ghana																					34							36																
Greece	19	20	18	18	17	16	20	25	25	24	24	23	23	23	22	21	22	21	23	23	22	21	21	21	20	21	20	17	21	16	18	18	18	17	18	18	17	17	16	15	14			
Greenland																16	19	15	15	19	14	12	17	16	15	14	19	15	16	14	18	24	23	22	36	40								
Grenada					26	44			36	34	36	37	51	35	35	35	31					24	20	26	23	20	23	23	26	18	15	12	13	13	18	17	13	15	12	13	14	15		
Guam															7	9	9	9	7	8	11	9	10	9	7	9	7	6	7	8	11	10	11	7	11	10	11	12	11	14	12	13	12	
Guatemala	34	36			31		34	33	34	35	36	36	38	38	35	33	34	32	32	31	33	33	31	31	31	31	31	30	29	25	24	23	22	22	22	24	26	26	28	28	27	26	30	
Guyana					37				23	26	25	27	25	25	24	24	25	24	27	22	17	21	19	16	17	16	17	18	18	21	19	19	21	20	20	20	16	18	17	15				
Haiti		70																		61			47	48	52																			
Honduras	56	55	65	77				39	36	43	38																		13	13	13	13	10	11										
Hungary	9	9	10	10	11	11	11	10	10	10	10	10	10	10	10	9	9	9	9	9	9	8	8	7	7	7	6	6	6	6	6	5	6	5	5	6	7	7	6	6	7	6	7	
Iceland	15	10	7	8	8	7	9	9	7	7	7	5	6	4	6	6	5	6	7	6	8	7	6	8	6	8	9	8	7	9	8	9	9	11	9	11	10	11	14	13	14	12	13	
India	31	36	40	39	35		29	38		44	42	42	43	40	41	42	42	43	41	43	42	41	40	38	39	41	41	36	43	45	44	38	37	55	42	43	44	43	47	49	41	36	46	
Iran																						43	39	35	27	23	20	19	18	17	18	17	16	18	18	19	14	14						
Iraq																													46				39	40	40	40	39							
Ireland	10	9	9	9	9	8	9	8	8	8	8	8	8	7	8	8	8	8	8	8	8	8	8	8	8	8	11	7	7	7	8	7	7	7	7	7	7	7	7	8	8			
Israel	20	20	19	18	19	18	17	17	18	17	18	18	20	16	16	16	17	19	19	19	18	18	17	18	19	19	19	19	19	18	18	19	19	19	20	21	21	22	21	21	19			
Italy	11	11	11	11	11	12	12	11	11	11	11	11	10	10	10	11	11	11	11	11	11	11	11	10	10	10	10	10	10	10	10	10	11	11	11	12	11	11	11	12	12	13		
Jamaica	35	36	31	29	29	28	28	27	25	27	31	29									21	27	30	22	16	20	13			12	13	11	12	12	12									
Japan	18	18	18	18	18	18	18	18	19	19	19	19	19	19	17	11	12	12	12	12	12	12	12	12	13	13	13	13	14	14	15	15	15	16	16	16	17	17	18	18	19	19	20	
Jordan																									23	21	19		19	18	18	20	21	22		22	24	21	22					
Kazakhstan		19	18			18	16	16	16	15	15	11	12	12	12	12	12	13	13	17	15	15	15	16	17	17	18	18	19	24	29	35	36	18	18	17	16	20	18	19	22	21		
Kiribati												50	45	46	49	44	55	41	35	48	45	54																						
Kuwait	27	26	32	29	27	25	28	22						24	27	31	24	28	22	29	25	21	24	23	20	19	20	19	22	18	16	16	18	22	18	19	14	17	13	16				
Kyrgyzstan		19	18			17	15	15	14	15	18	20	21	22	24	24	23	21	20	20	11	9	8	8	8	8	6	7	6	5	6	6	6	6	6	7	7	7	7	7				
Latvia	10	10	9	8	8	8	9	9	9	9	11	15	16	16	18	19	10	9	9	8	9	10	11	11	13	13	13	11	12	11	11	9	8	7	7	6	6	7	6	6	7	7		
Lebanon																																						44	42	41	42	38		
Libya																											88	91	90								51	52						
Lithuania		10	9			8	8	7	7	7	7	12	13	11	8	6	6	5	7	7	8	7	7	7	7	7	7	8	7	7	7	8	7	6	7	8	8	7	8	7	8	7	7	
Luxembourg	16	16	15	14	13	13	13	13	16	14	14	19	13	14	13	13	15	14	17	17	15	15	17	18	17	18	19	18	18	19	17	19	15	15	14	14	13	14	14	13	13	13	14	
Madagascar					54	51	53	50	54	53	50	52	52	53	52	50																												
Malaysia	42	41	27															33	33		38	40	38	39	40	39	38	37	36	27	27	26	26	26	26	26	26	25	24	25	23			
Maldives																					66	60	57	59	61	81		54	51		47	39	46	42	43	47	48	42	38	39				
Mali		72			75																																							
Malta	19	20	18	18	18	20	25	26	20	12	11	11	10	10	11	10	9	10	10	12	10	8	9	11	8	8	9	8	7	6	7	8	11	7	7	7	6	6	7	7	7			
Mauritius	39	33	27	28	25	25	26	23	22	23	24	24	26	28	27	28	31	30	23	23	21	18	18	18	18	16	17	15	16	16	16	15	16	15	13	14	15	15	15	16	18	17	16	
Mexico	30	29	27	25	25	23	23	22	20	19	18	18	17	17	16	15	15	14	14	13	13	13	13	13	12	12	12	13	13	13	13	13	12	13	12	12	11	11	11	11	9	9	11	
Moldova		10	10			8	8	9	10	11	20	21	23	25	24	20	14	11	10	9	8	5	4	4	3	3	3	3	3	3	3	3	3	3	3	4	3	4	4					
Monaco							24	25																								30	24	20										
Mongolia															25									76	77	77	75	74			8						7	8	9	8	10	7	9	
Montenegro																					31	35	34	33	32	24	30	25	28	31								25		30				
Morocco																					57	58	60	61	57	58		58	58	56	57	60	54	51	51	52	50							
Mozambique																						38																						
Netherlands	12	11	13	14	13	13	13	12	12	13	14	14	14	15	14	15	14	14	15	15	16	16	16	16	15	15	15	14	14	14	14	14	14	15	14	14	14	14	14	14	13	15	16	
New Zealand	5	5	5	5	5	5	5	5	5	5	5	5	5	5	4		4	4	4	3	3	3	3	3	3	3	3	3	4	4	4	4	4	4	4	4	5	5	5	5				
Nicaragua									28	29	28	26	26	27	27		24	19	17	16	17	14	14	13	13	13	14	14	14	12	11	10	10	10	10	12	9	10	10	11	8	9		
Nigeria																												97																
North Macedonia												21	21	21	19	20	20	19	18	19	17	17	19	17	31	31	21	21	21	20	21	23	22	23		28	25	27	31	33	31	27		
Northern Mariana Islands																			20	35	26	18	31	20	20	29	15	13	24	19	23	32	21	13	13	11	11	16	16	20	12	14	18	
Norway	11	10	10	10	9	9	9	9	9	9	9	10	10	11	11	11	9	9	10	11	11	12	12	13	13	14	14	15	15	15	16	15	16	16	16	15	15	14	15	16	15	13	14	
Oman																									18	17	18		46	59					56		49	63	74	70	62	60		
Palau																																		52										
Palestine																		43	36	34	35	38	40	39	34	35		35	36	32	31	30	31	31	28	33	32	29	10	10	11	8		
Panama	26	27	25	26	25	25	23	22	25	25							26	25	18	17	15	15	13	15	16	14	17	14	14	14	16	17	16	17	15	16	18	17	15	17		15		
Papua New Guinea	28																																										30	
Paraguay	37	37	39	40	41	40	36	37	37	34	33	32			26	28	27	35	33	35	34	31	34	34	29	28	25	26	25	25	22	21	22	21	21	19	17	16	17	16	16	14		
Peru	28	26	28	28			48	50	46	47	49	50	45		48	40	38	41	38	35	31	33	32	29	29	61	63	23	25	27	25	25	25	24	23	19	20	28	18	20	12			
Philippines	99	99	99	99	99	99	99	99	99	99	99	82	81	83	83	83	83	84	83	53	53	52	47	51	47	47	16	16	16	16	16	16	18	17	18	16	15	14	14	14	14	15	16	
Poland	38	37	38	37	37	37	37	37	37	37	37	38	37	37	37	36	36			27	25	25	25	25	25	25	25	25	25	27	26	25	25	26	27	27	27	25	26	25	23	18	19	
Portugal	24	23	24	24	23	23	24	24	24	23	23	23	23	23	23	22	23	24	24	25	25	22	18	19	18	24	26	22	21	21	21	19	20	19	14	15	14	14	14	14				
Puerto Rico	24	22	26	25	25	25	24	24	24	23	23	22	22	22	16	16	16	16	17	16	17	16	16	17	15	17	17	16	16	17	16	15	15	15	17	17	16	17	18	17	16	16	17	
Qatar					82	80										27					100	37	100	100	41	34	34	35	41	31	36	38	41	38	41	36	29	29		29	26	32	37	

**Romania**	26	26	26	26	25	25	24	23	23	22	23	23	24	18	18	18	18	17	17	14	14	14	14	14	13	13	13	13	13	13	13	14	14	13	15	15	15	15	15	16			
**Russia**	7									8	10	11	12	13	14	14	14	14	13	13	13	13	13	13	13	13	12	11	11	11	12	12	13	14	15	15	15	15	15	15	15	13	12
**Saint Kitts and Nevis**	27	30	32	29	33	29	36	33	35	39	44	39	30	34	28	20			19	31	28	39	23	30	34	21	15	13	14	14	11	16	10	11	10	9	8						
**Saint Lucia**	44	40		32			38	34	34	39	32	29	28	28	28	29	30	27	29	32	24	19	23	23	20	29	26		27	16	19	17	14	17	14	17	14	14	14	14	13		
**Saint Vincent and the Grenadines**			37	32	47	36	32	40			37					20	23	19	15	16	16	17	15	17	16	15	12	12	20	28	20	18	20	15	12	17	28	16	17	17	18	16	
**San Marino**																25	32	25	30	30	26	24	23			24			33	20	26	15	19	20	18	16	15	17	14	14	16		
**Saudi Arabia**																				46	47	46	49	50	50	46	47	50	52	52	53	54	56									23	23
**Serbia**																			22	23	23	23	23	22	24	19	18	18	18	18	18	20	19	20	21	22	21	21	21	23	23	22	23
**Seychelles**		29	30			34	35	28														23	23	24	23	23	24	24	22	26	25	21	23	23	24	22							
**Singapore**	13	12	10	9	10	8	13	8	8	10	8	7	6	6	5	5	5	4	5	5	6	5	5	5	5	5	5	6	6	6	7	6	4	3	3	4	4	4	5	5	4	4	5
**Slovakia**													18	18	16	18	18	19	18	17	15	15	15	15	15	16	27	22	11	11	11		8	8	9		11	13	12	14	11	11	
**Slovenia**						8	9	11	10	15	13	8	7	8	9	10	10	9	10	10	10	11	12	11	11	14	12	12	10	11	10	10	10	12	11	11	12	13	11	12	10		
**Solomon Islands**																																						14	16				
**South Africa**																		39	38	34	34	33	33	32	32	32	33	32	32	32	32	32	32	31	31	31	32	32	32				
**South Korea**						39	37	39	38	35	33	33	29	25	26	27	28	29	28	26	23	20	17	17	18	19	19	18	19	18	18	18	17	17	16	16	17	17	18	19	19	20	
**Spain**	22	22	22	22	21	22	21	19	19	19	18	17	17	16	15	14	14	14	14	15	14	14	14	14	14	14	14	15	15	14	13	13	13	12	12	13	12	12	13	13	11	12	
**Sri Lanka**	54	55	60	56	57	56	55	55	54	55		56	56	59	60	57	58	51	50	50	45	44	44	42	46	42	38	37		37	36	37	37	38	37	38				37			
**Suriname**	49	45	38		37	34	33	31	34	35	31	33	32			32	34	33	31	31	28	25	24	23	25	26	20	21	24	21	21	23	23	24	24								
**Sweden**	8	9	9	9	10	10	10	9	10	10	10	10	10	10	10	11	11	10	11	11	11	11	11	11	11	11	12	12	13	13	16	15	14	13	13	15	14	13	14	14	13	14	14
**Switzerland**	25	25	25	24	24	24	24	25	24	25	25	26	26	26	26	13	13	13	13	13	13	13	14	14	14	13	12	12	12	12	12	12	12	13	13	13	14	13	14	15	15	15	
**Syria**	71				78	78													54	47	47	49	48	42	34	41	38	32	33	26	42												
**São Tomé and Príncipe**						39																																					
**Taiwan***	19	21	21	21	21	21	21	21	21	22	23	22	23	23	22	20	19	18	18	17	17	17	16	16	15	15	15	15	18	17	17	17	15	15	14	15	15	15					
**Tajikistan**		16	18			24	23	24	21	20	21	21	27	34	32	32	35	37	40	36	38	36	32	33	34	35											32	33					
**Thailand**	72	71	71	72	71	71	72	70			67	66	66		66	67	66	67	66	64	63		57	52	56	56	56	55	55	55	53	48	47	43	40	38	36	36	36	33			
**The Bahamas**	26	27			22	24		18						24	18	18	25	17	16	12	13	12	24	15	15	16	14	15	14	13	14	12	15	14	15	15							
**Tonga**																								47																			
**Trinidad and Tobago**	22	20	19	18	19	19	17	16	16	15	16	16	15	15	14	16	15	14	14	11	10	11	10	10	10	11	11	12	13	12	12	10	10										
**Tunisia**																														26				35				35			32	23	
**Turkey**				64	65			61	61	60	61	61	60	59	60	60	60	60	57	60	56	55	55	55	62	60	60	60	61	21	21	21	23	15	16	16	17	18	21	24			
**Turkmenistan**		12	12			12	12	16	13	16	18	13	13	12	20	20	22	27	29	9	12	10	12	11	11	13	15	16	16	16	17	19	19	21	22	23							
**UK**		8	7	7	6	6	6	6	6	6	6	6	6	7	7	8	8	8	8	9	8	8	8	8	8	8	8	8	9	9	8	8	8	8	8	9	9	9	9	9	9	9	10
**USA**	9	10	10	10	10	11	11	11	11	10	10	10	10	10	10	10	10	10	11	11	11	12	12	12	12	12	12	12	12	13	13	13	13	13	13	14	14	14	14	15	14	14	15
**Ukraine**		12	12			11	12	13	15	17	21	22	22	20	18	18	19	17	17	16	16	16	17	18	18	13	13	12	12	11	10	10	10	8	10	9	10	6	9	8	8	8	8
**United Arab Emirates**																											49	47							34	32	36	38	26	61	43		
**Uruguay**	23	23	22	23	24	23	24	23	23	22	23			23	22	23	23	18	20	21	19	19	20	20	21	20	20	21	21	22	22		23	22	21	20	20	20	21	20	21		
**Uzbekistan**		14	13			13	14	14	14	13	13	14	14	15	16	16	18	19	18	18	17	18	22	24	13	12				10	10	11	11	12	15	14	14	17	16	17			
**Venezuela**	32	31	31	22		33	33	29	27	28	19		19	19	18		13	13	13	12	11	9	9	10	10	9	9	9	9	10	9	9	9	9	9	10	11						
**Virgin Islands**	21														10	10	12	10	12	10	11	11	13	12	9	13	11	11	10	12	15	13	17			18	11	16			18	15	15
**Zimbabwe**											35					42												26															

Appendix Figure S7: Out-of-sample model performance for CODEm models for GBD 2023 and age-standardized cause-specific mortality rate by level 2 causes

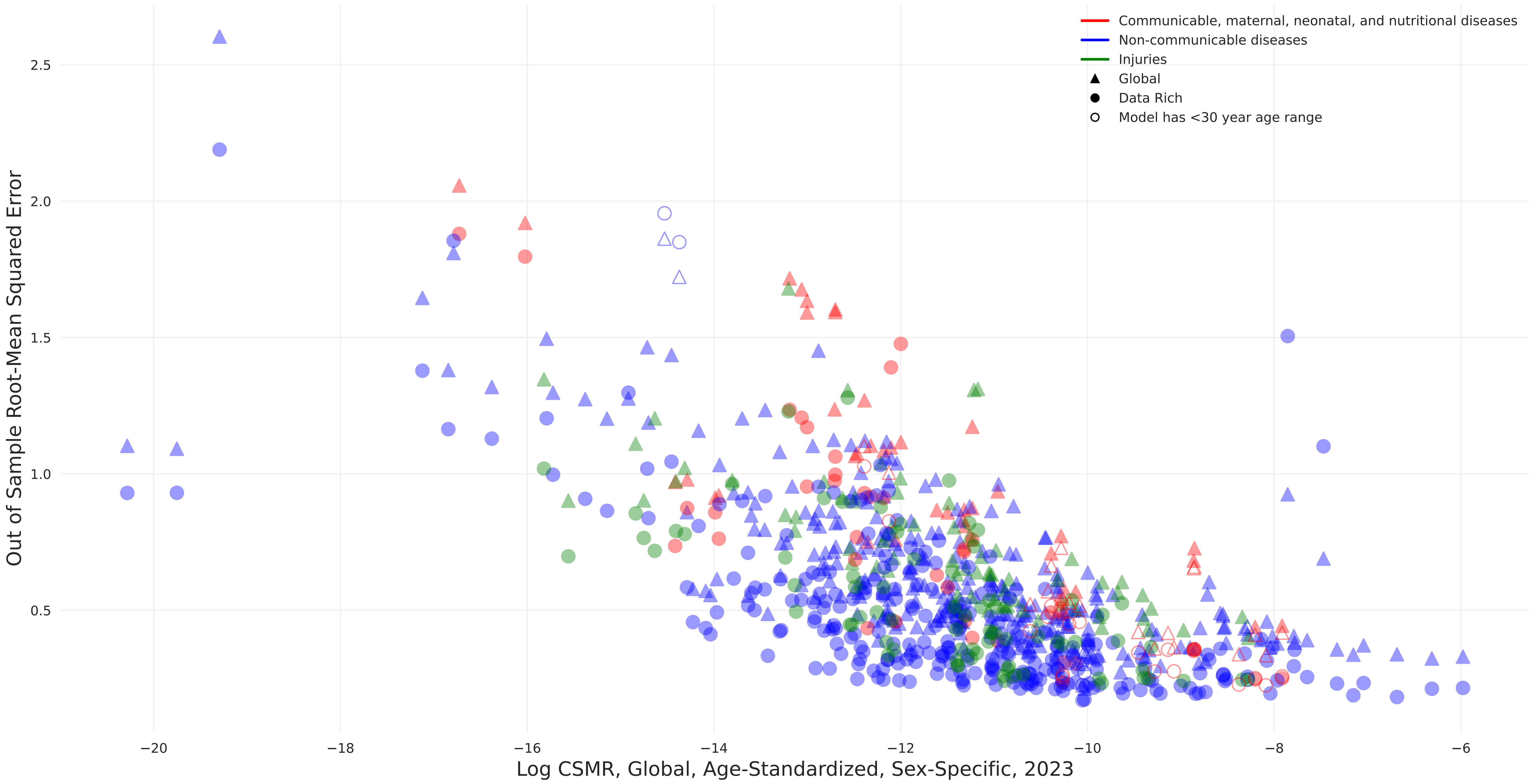

Appendix Table S1: GBD cause hierarchy with levels

Cause Name	Level
All causes	0
Communicable, maternal, neonatal, and nutritional diseases	1
HIV/AIDS and sexually transmitted infections	2
HIV/AIDS	3
HIV/AIDS - Drug-susceptible Tuberculosis	4
HIV/AIDS - Multidrug-resistant Tuberculosis without extensive drug resistance	4
HIV/AIDS - Extensively drug-resistant Tuberculosis	4
HIV/AIDS resulting in other diseases	4
Sexually transmitted infections excluding HIV	3
Syphilis	4
Chlamydial infection	4
Gonococcal infection	4
Trichomoniasis	4
Genital herpes	4
Other sexually transmitted infections	4
Respiratory infections and tuberculosis	2
Tuberculosis	3
Latent tuberculosis infection	4
Drug-susceptible tuberculosis	4
Multidrug-resistant tuberculosis without extensive drug resistance	4
Extensively drug-resistant tuberculosis	4
Lower respiratory infections	3
Upper respiratory infections	3
Otitis media	3
COVID-19	3
Enteric infections	2
Diarrheal diseases	3
Typhoid and paratyphoid	3
Typhoid fever	4
Paratyphoid fever	4
Invasive Non-typhoidal Salmonella (iNTS)	3
Other intestinal infectious diseases	3
Neglected tropical diseases and malaria	2
Malaria	3
Chagas disease	3
Leishmaniasis	3
Visceral leishmaniasis	4
Cutaneous and mucocutaneous leishmaniasis	4
African trypanosomiasis	3
Schistosomiasis	3
Cysticercosis	3
Cystic echinococcosis	3
Lymphatic filariasis	3
Onchocerciasis	3

GBD cause hierarchy with levels

Cause Name	Level
Trachoma	3
Dengue	3
Yellow fever	3
Rabies	3
Intestinal nematode infections	3
Ascariasis	4
Trichuriasis	4
Hookworm disease	4
Food-borne trematodiases	3
Leprosy	3
Ebola	3
Zika virus	3
Guinea worm disease	3
Other neglected tropical diseases	3
Other infectious diseases	2
Meningitis	3
Encephalitis	3
Diphtheria	3
Pertussis	3
Tetanus	3
Measles	3
Varicella and herpes zoster	3
Acute hepatitis	3
Acute hepatitis A	4
Acute hepatitis B	4
Acute hepatitis C	4
Acute hepatitis E	4
Other unspecified infectious diseases	3
Maternal and neonatal disorders	2
Maternal disorders	3
Maternal hemorrhage	4
Maternal sepsis and other maternal infections	4
Maternal hypertensive disorders	4
Maternal obstructed labor and uterine rupture	4
Maternal abortion and miscarriage	4
Ectopic pregnancy	4
Indirect maternal deaths	4
Late maternal deaths	4
Maternal deaths aggravated by HIV/AIDS	4
Other direct maternal disorders	4
Neonatal disorders	3
Neonatal preterm birth	4
Neonatal encephalopathy due to birth asphyxia and trauma	4
Neonatal sepsis and other neonatal infections	4
Hemolytic disease and other neonatal jaundice	4

GBD cause hierarchy with levels

Cause Name	Level
Other neonatal disorders	4
Nutritional deficiencies	2
Protein-energy malnutrition	3
Iodine deficiency	3
Vitamin A deficiency	3
Dietary iron deficiency	3
Other nutritional deficiencies	3
Non-communicable diseases	1
Neoplasms	2
Lip and oral cavity cancer	3
Nasopharynx cancer	3
Other pharynx cancer	3
Esophageal cancer	3
Stomach cancer	3
Colon and rectum cancer	3
Liver cancer	3
Liver cancer due to hepatitis B	4
Liver cancer due to hepatitis C	4
Liver cancer due to alcohol use	4
Liver cancer due to NASH	4
Hepatoblastoma	4
Liver cancer due to other causes	4
Gallbladder and biliary tract cancer	3
Pancreatic cancer	3
Larynx cancer	3
Tracheal, bronchus, and lung cancer	3
Malignant skin melanoma	3
Non-melanoma skin cancer	3
Non-melanoma skin cancer (squamous-cell carcinoma)	4
Non-melanoma skin cancer (basal-cell carcinoma)	4
Soft tissue and other extraosseous sarcomas	3
Malignant neoplasm of bone and articular cartilage	3
Breast cancer	3
Cervical cancer	3
Uterine cancer	3
Ovarian cancer	3
Prostate cancer	3
Testicular cancer	3
Kidney cancer	3
Bladder cancer	3
Brain and central nervous system cancer	3
Eye cancer	3
Retinoblastoma	4
Other eye cancers	4
Neuroblastoma and other peripheral nervous cell tumors	3

GBD cause hierarchy with levels	
**Cause Name**	**Level**
Thyroid cancer	3
Mesothelioma	3
Hodgkin lymphoma	3
Non-Hodgkin lymphoma	3
Burkitt lymphoma	4
Other non-Hodgkin lymphoma	4
Multiple myeloma	3
Leukemia	3
Acute lymphoid leukemia	4
Chronic lymphoid leukemia	4
Acute myeloid leukemia	4
Chronic myeloid leukemia	4
Other leukemia	4
Other malignant neoplasms	3
Other neoplasms	3
Myelodysplastic, myeloproliferative, and other hematopoietic neoplasms	4
Benign and in situ intestinal neoplasms	4
Benign and in situ cervical and uterine neoplasms	4
Other benign and in situ neoplasms	4
Cardiovascular diseases	2
Rheumatic heart disease	3
Ischemic heart disease	3
Stroke	3
Ischemic stroke	4
Intracerebral hemorrhage	4
Subarachnoid hemorrhage	4
Hypertensive heart disease	3
Non-rheumatic valvular heart disease	3
Non-rheumatic calcific aortic valve disease	4
Non-rheumatic degenerative mitral valve disease	4
Other non-rheumatic valve diseases	4
Cardiomyopathy and myocarditis	3
Myocarditis	4
Alcoholic cardiomyopathy	4
Other cardiomyopathy	4
Pulmonary Arterial Hypertension	3
Atrial fibrillation and flutter	3
Aortic aneurysm	3
Lower extremity peripheral arterial disease	3
Endocarditis	3
Other cardiovascular and circulatory diseases	3
Chronic respiratory diseases	2
Chronic obstructive pulmonary disease	3
Pneumoconiosis	3
Silicosis	4

GBD cause hierarchy with levels

Cause Name	Level
Asbestosis	4
Coal workers pneumoconiosis	4
Other pneumoconiosis	4
Asthma	3
Interstitial lung disease and pulmonary sarcoidosis	3
Other chronic respiratory diseases	3
Digestive diseases	2
Cirrhosis and other chronic liver diseases	3
Chronic hepatitis B including cirrhosis	4
Chronic hepatitis C including cirrhosis	4
Cirrhosis due to alcohol	4
Nonalcoholic fatty liver disease including cirrhosis	4
Cirrhosis due to other causes	4
Upper digestive system diseases	3
Peptic ulcer disease	4
Gastritis and duodenitis	4
Gastroesophageal reflux disease	4
Appendicitis	3
Paralytic ileus and intestinal obstruction	3
Inguinal, femoral, and abdominal hernia	3
Inflammatory bowel disease	3
Ulcerative colitis	4
Crohn's disease	4
Vascular intestinal disorders	3
Gallbladder and biliary diseases	3
Pancreatitis	3
Other digestive diseases	3
Neurological disorders	2
Alzheimer's disease and other dementias	3
Parkinson's disease	3
Idiopathic epilepsy	3
Multiple sclerosis	3
Motor neuron disease	3
Headache disorders	3
Migraine	4
Tension-type headache	4
Other neurological disorders	3
Mental disorders	2
Schizophrenia	3
Depressive disorders	3
Major depressive disorder	4
Dysthymia	4
Bipolar disorder	3
Anxiety disorders	3
Eating disorders	3

GBD cause hierarchy with levels

Cause Name	Level
Anorexia nervosa	4
Bulimia nervosa	4
Autism spectrum disorders	3
Attention-deficit/hyperactivity disorder	3
Conduct disorder	3
Idiopathic developmental intellectual disability	3
Other mental disorders	3
Substance use disorders	2
Alcohol use disorders	3
Drug use disorders	3
Opioid use disorders	4
Cocaine use disorders	4
Amphetamine use disorders	4
Cannabis use disorders	4
Other drug use disorders	4
Diabetes and kidney diseases	2
Diabetes mellitus	3
Diabetes mellitus type 1	4
Diabetes mellitus type 2	4
Chronic kidney disease	3
Chronic kidney disease due to diabetes mellitus type 1	4
Chronic kidney disease due to diabetes mellitus type 2	4
Chronic kidney disease due to hypertension	4
Chronic kidney disease due to glomerulonephritis	4
Chronic kidney disease due to other and unspecified causes	4
Acute glomerulonephritis	3
Skin and subcutaneous diseases	2
Dermatitis	3
Atopic dermatitis	4
Contact dermatitis	4
Seborrhoeic dermatitis	4
Psoriasis	3
Bacterial skin diseases	3
Cellulitis	4
Pyoderma	4
Scabies	3
Fungal skin diseases	3
Viral skin diseases	3
Acne vulgaris	3
Alopecia areata	3
Pruritus	3
Urticaria	3
Decubitus ulcer	3
Other skin and subcutaneous diseases	3
Sense organ diseases	2

GBD cause hierarchy with levels

Cause Name	Level
Blindness and vision loss	3
Glaucoma	4
Cataract	4
Age-related macular degeneration	4
Refraction disorders	4
Near vision loss	4
Other vision loss	4
Age-related and other hearing loss	3
Other sense organ diseases	3
Musculoskeletal disorders	2
Rheumatoid arthritis	3
Osteoarthritis	3
Osteoarthritis hip	4
Osteoarthritis knee	4
Osteoarthritis hand	4
Osteoarthritis other	4
Low back pain	3
Neck pain	3
Gout	3
Other musculoskeletal disorders	3
Other non-communicable diseases	2
Congenital birth defects	3
Neural tube defects	4
Congenital heart anomalies	4
Orofacial clefts	4
Down syndrome	4
Turner syndrome	4
Klinefelter syndrome	4
Other chromosomal abnormalities	4
Congenital musculoskeletal and limb anomalies	4
Urogenital congenital anomalies	4
Digestive congenital anomalies	4
Other congenital birth defects	4
Urinary diseases and male infertility	3
Urinary tract infections and interstitial nephritis	4
Urolithiasis	4
Benign prostatic hyperplasia	4
Male infertility	4
Other urinary diseases	4
Gynecological diseases	3
Uterine fibroids	4
Polycystic ovarian syndrome	4
Female infertility	4
Endometriosis	4
Genital prolapse	4

GBD cause hierarchy with levels

Cause Name	Level
Premenstrual syndrome	4
Other gynecological diseases	4
Hemoglobinopathies and hemolytic anemias	3
Thalassemias	4
Thalassemias trait	4
Sickle cell disorders	4
Sickle cell trait	4
G6PD deficiency	4
G6PD trait	4
Other hemoglobinopathies and hemolytic anemias	4
Endocrine, metabolic, blood, and immune disorders	3
Thyroid diseases	4
Other endocrine, metabolic, blood, and immune disorders	4
Oral disorders	3
Caries of deciduous teeth	4
Caries of permanent teeth	4
Periodontal diseases	4
Edentulism	4
Other oral disorders	4
Sudden infant death syndrome	3
Injuries	1
Transport injuries	2
Road injuries	3
Pedestrian road injuries	4
Cyclist road injuries	4
Motorcyclist road injuries	4
Motor vehicle road injuries	4
Other road injuries	4
Other transport injuries	3
Unintentional injuries	2
Falls	3
Drowning	3
Fire, heat, and hot substances	3
Poisonings	3
Poisoning by carbon monoxide	4
Poisoning by other means	4
Exposure to mechanical forces	3
Unintentional firearm injuries	4
Other exposure to mechanical forces	4
Adverse effects of medical treatment	3
Animal contact	3
Venomous animal contact	4
Non-venomous animal contact	4
Foreign body	3
Pulmonary aspiration and foreign body in airway	4

GBD cause hierarchy with levels

Cause Name	Level
Foreign body in eyes	4
Foreign body in other body part	4
Electrocution	3
Environmental heat and cold exposure	3
Exposure to forces of nature	3
Other unintentional injuries	3
Self-harm and interpersonal violence	2
Self-harm	3
Self-harm by firearm	4
Self-harm by other specified means	4
Interpersonal violence	3
Physical violence by firearm	4
Physical violence by sharp object	4
Sexual violence	4
Physical violence by other means	4
Conflict and terrorism	3
Police conflict and executions	3
Total cancers	1
Total burden related to hepatitis B	1
Total burden related to hepatitis C	1
Total burden related to Non-alcoholic fatty liver disease (NAFLD)	1
Total Cancers excluding Non-melanoma skin cancer	1

List of International Classification of Diseases (ICD) codes mapped to the Global Burden of Disease cause list for causes of death		
**Cause**	**ICD10**	**ICD9**
Communicable, maternal, neonatal, and nutritional diseases	A00-A00.9, A01.0-A14, A15-A28.9, A32-A39.9, A48.1-A48.2, A48.4-A48.5, A50-A58, A60-A60.9, A63-A63.8, A65-A65.0, A68-A70, A74, A74.8-A75.9, A77-A96.9, A98-A98.8, B00-B06.9, B10-B10.8, B15-B16.2, B17.0, B17.2, B19.1, B20-B27.9, B29.4, B33-B33.1, B33.3-B33.8, B34.2, B47-B48.8, B50-B53.8, B55.0, B56-B57.5, B60-B60.8, B63, B65-B67.9, B69-B72.0, B74.3-B75, B77-B78.9, B83-B83.8, B90-B91, B94.1, B95-B95.5, B97.2, B97.4-B97.6, C58-C58.0, D50.1-D50.8, D51-D52.0, D52.8-D53.9, D70.3, D89.3, E00-E02, E40-E46.9, E51-E61.9, E63-E64.0, E64.2-E64.9, F02.1, F02.4, F07.1, G00.0-G00.8, G02.8-G03.8, G04-G05.8, G14-G14.6, G21.3, H70-H70.9, I00, I02, I02.9, I41.2, I98.0-I98.1, J00-J02.8, J03-J03.8, J04-J04.2, J05-J05.1, J06.0-J06.8, J09-J15.8, J16-J16.9, J20-J21.9, J36-J36.0, J91.0, K23.0-K23.1, K52.1-K52.3, K67.0-K67.8, K75.3, K76.3, K77.0, K93.0-K93.1, M03.1, M12.1, M49.0-M49.1, M73.0-M73.1, M89.6, N74.0-N74.1, N96, N98-N98.9, O00-O07.9, O09-O16.9, O20-O26.9, O28-O36.9, O40-O48.1, O60-O77.9, O80-O92.7, O96-O98.6, O98.8-P04, P04.2, P04.5-P05.9, P07-P08.2, P10-P15.9, P20-P22.9, P23.0-P23.4, P24-P29.9, P35-P39.9, P50-P61.9, P70-P70.1, P70.4-P72.0, P72.2-P72.9, P76-P78.9, P83-P84, P90-P91.9, P94, P94.1-P94.9, P96, P96.3, P96.8, R19.7, U04-U04.9, U06-U07.2, U82-U89.9, Z16-Z16.3	001-001.9, 002.0-029, 032-034.9, 036-037.9, 040, 040.1-041.0, 042-066.9, 070.0-070.2, 071-074.1, 074.3-075.9, 078.3-078.7, 079-079.7, 080-084.9, 085.0, 086-088, 088.8-088.9, 090-101.6, 104-104.9, 120-124.9, 125.4-125.9, 127-127.2, 128-129.0, 136-136.2, 137-139.0, 181-181.9, 244.2, 260-263.9, 265-269.9, 281.0-281.9, 320.0-320.8, 321-323.9, 381-383.9, 390-390.9, 392, 392.9, 425.6, 460-464.4, 464.8-464.9, 465.0-465.8, 466-469, 475-475.9, 476.9, 480-482.8, 483.0-483.9, 484.0-484.7, 487-489, 558.2-558.9, 630-636.9, 638-638.9, 640-679.1, 716.0, 730.4-730.7, 760-760.6, 760.8-763.4, 763.6-768, 768.2-774.9, 775.0, 776-779.2, 979.5, V09-V09.9
HIV/AIDS and sexually transmitted infections	A50-A58, A60-A60.9, A63-A63.8, B20-B24.9, B63, F02.4, I98.0, K67.0-K67.2, M03.1, M73.0-M73.1	042-044.9, 054.1, 090-099.9
HIV/AIDS	B20-B24.9, F02.4	042-044.9
HIV/AIDS - Drug-susceptible Tuberculosis	B20.0	N/A
HIV/AIDS - Multidrug-resistant Tuberculosis without extensive drug resistance	N/A	N/A
HIV/AIDS - Extensively drug-resistant Tuberculosis	N/A	N/A
HIV/AIDS resulting in other diseases	B20, B20.1-B24.9, F02.4	042-044.9
Sexually transmitted infections excluding HIV	A50-A58, A60-A60.9, A63-A63.8, B63, I98.0, K67.0-K67.2, M03.1, M73.0-M73.1	054.1, 090-099.9
Syphilis	A50-A53.9, I98.0, K67.2, M03.1, M73.1	090-097.9
Chlamydial infection	A55-A56.8, K67.0	N/A
Gonococcal infection	A54-A54.9, K67.1, M73.0	098-098.9
Other sexually transmitted infections	A57-A58, A63-A63.8, B63	099-099.9
Respiratory infections and tuberculosis	A10-A14, A15-A19.9, A48.1, A70, B34.2, B90-B90.9, B97.2, B97.4-B97.6, H70-H70.9, J00-J02.8, J03-J03.8, J04-J04.2, J05-J05.1, J06.0-J06.8, J09-J15.8, J16-J16.9, J20-J21.9, J36-J36.0, J91.0, K23.0, K67.3, K93.0, M49.0, N74.0-N74.1, P23.0-P23.4, P37.0, U04-U04.9, U07-U07.2, U84.3	010-019.9, 034.0, 079.6, 137-137.9, 138.0-138.9, 320.4, 381-383.9, 460-464.4, 464.8-464.9, 465.0-465.8, 466-469, 475-475.9, 476.9, 480-482.8, 483.0-483.9, 484.1-484.2, 484.6-484.7, 487-489, 730.4-730.6, 770.0
Tuberculosis	A10-A14, A15-A19.9, B90-B90.9, K23.0, K67.3, K93.0, M49.0, N74.0-N74.1, P37.0, U84.3	010-019.9, 137-137.9, 138.0-138.9, 320.4, 730.4-730.6
Drug-susceptible tuberculosis	A10-A14, A15-A19.9, B90-B90.9, K23.0, K67.3, K93.0, M49.0, N74.0-N74.1, P37.0	010-019.9, 137-137.9, 138.0-138.9, 320.4, 730.4-730.6
Multidrug-resistant tuberculosis without extensive drug resistance	U84.3	N/A
Extensively drug-resistant tuberculosis	N/A	N/A
Lower respiratory infections	A48.1, A70, B97.4-B97.6, J09-J15.8, J16-J16.9, J20-J21.9, J91.0, P23.0-P23.4, U04-U04.9	079.6, 466-469, 480-480.2, 480.8-482.8, 483.0-483.9, 484.1-484.2, 484.6-484.7, 487-489, 770.0
Influenza	J09-J11.8, U04-U04.9	487-489
Pneumococcal pneumonia	J13-J13.9, J15.3-J15.4, J15.6	481-481.9
H influenzae type B pneumonia	J14-J14.0	482.2
Respiratory syncytial virus pneumonia	B97.4-B97.6, J12.1	079.6, 480.1
Other lower respiratory infections	A48.1, A70, J12-J12.0, J12.2-J12.9, J15-J15.2, J15.5, J15.7-J15.8, J16-J16.9, J20-J21.9, J91.0, P23.0-P23.4	466-469, 480-480.0, 480.2, 480.8-480.9, 482-482.1, 482.3-482.8, 483.0-483.9, 484.1-484.2, 484.6-484.7, 770.0
Upper respiratory infections	J00-J02.8, J03-J03.8, J04-J04.2, J05-J05.1, J06.0-J06.8, J36-J36.0	034.0, 460-464.4, 464.8-464.9, 465.0-465.8, 475-475.9, 476.9
Otitis media	H70-H70.9	381-383.9
COVID-19	B34.2, B97.2, U07-U07.2	480.3
Enteric infections	A00-A00.9, A01.0-A06.3, A06.9-A09.9, A80-A80.9, B91, G14-G14.6, K52.1-K52.3, M89.6, R19.7	001-001.9, 002.0-006.2, 006.9-009.9, 045-045.9, 138, 558.2-558.9, 730.7, 979.5
Diarrheal diseases	A00-A00.9, A02-A02.0, A02.8-A06.3, A06.9-A07, A07.2-A07.4, A08-A09.9, K52.1-K52.3, R19.7	001-001.9, 003.8-006.2, 006.9, 007.4-007.8, 008.2-009.9, 558.2-558.9
Leprosy	A30-A30.9	030-030.9
Typhoid and paratyphoid	A01.0-A01.4	002.0-002.9
Typhoid fever	A01.0	002.0
Paratyphoid fever	A01.1-A01.4	002.1-002.9
Invasive Non-typhoidal Salmonella (iNTS)	A02.1-A02.2	003-003.7
Other intestinal infectious diseases	A07.0-A07.1, A07.8-A07.9, A80-A80.9, B91, G14-G14.6, M89.6	007-007.3, 007.9-008.1, 045-045.9, 138, 730.7, 979.5

List of International Classification of Diseases (ICD) codes mapped to the Global Burden of Disease cause list for causes of death		
**Cause**	**ICD10**	**ICD9**
Neglected tropical diseases and malaria	A68-A68.9, A69.5-A69.9, A75-A75.9, A77-A77.3, A77.8-A79.9, A82-A82.9, A90-A96.9, A98-A98.8, B50-B53.8, B55.0, B56-B57.5, B60-B60.8, B65-B67.9, B69-B72.0, B74.3-B75, B77-B78.9, B83-B83.8, G02.8, G05.2, I41.2, I98.1, K23.1, K93.1, P35.4, P37.3-P37.4, U06-U06.9	060-061.8, 065-066.9, 071-071.9, 080-082.3, 082.5-084.9, 085.0, 086-088, 088.8-088.9, 120-124.9, 125.4-125.9, 127-127.2, 128-129.0, 425.6
Malaria	B50-B53.8, P37.3-P37.4	084-084.9
Chagas disease	B57-B57.5, G02.8, G05.2, I41.2, I98.1, K23.1, K93.1	086-086.2, 086.9, 425.6
Leishmaniasis	B55.0	085.0
Visceral leishmaniasis	B55.0	085.0
African trypanosomiasis	B56-B56.9	086.3-086.5
Schistosomiasis	B65-B65.9	120-120.9
Cysticercosis	B69-B69.9	123.1
Cystic echinococcosis	B67-B67.4, B67.8-B67.9	122-122.4, 122.8-122.9
Dengue	A90-A91.9	061-061.8
Yellow fever	A95-A95.9	060-060.9
Rabies	A82-A82.9	071-071.9
Intestinal nematode infections	B77-B77.9	127.0
Ascariasis	B77-B77.9	127.0
Ebola	A98.4	N/A
Zika virus	A92.5, P35.4, U06-U06.9	N/A
Other neglected tropical diseases	A68-A68.9, A69.5-A69.9, A75-A75.9, A77-A77.3, A77.8-A79.9, A92-A92.4, A92.8-A94.0, A96-A96.9, A98-A98.3, A98.5-A98.8, B60-B60.8, B67.5-B67.7, B70-B71.9, B74.3-B75, B78-B78.9, B83-B83.8	065-066.9, 080-082.3, 082.5-083.9, 087-088, 088.8-088.9, 122.5-122.7, 123-123.0, 123.2-124.9, 125.4-125.6, 125.9, 127, 127.1-127.2, 128-129.0
Other infectious diseases	A06.4-A06.8, A20-A28.9, A32-A39.9, A48.2, A48.4-A48.5, A65-A65.0, A69-A69.2, A74, A74.8-A74.9, A77.4, A81-A81.9, A83-A89.9, B00-B06.9, B10-B10.8, B15-B16.2, B17.0, B17.2, B19.1, B25-B27.9, B29.4, B33-B33.1, B33.3-B33.8, B47-B48.8, B94.1, B95-B95.5, D70.3, D89.3, F02.1, F07.1, G00.0-G00.8, G03-G03.8, G04-G05.1, G05.3-G05.8, G21.3, I00, I02, I02.9, K67.8, K75.3, K76.3, K77.0, M49.1, P35-P35.3, P35.8-P35.9, P37, P37.1, P37.5-P37.9, U82-U84, U85-U89.9, Z16-Z16.3	006.3-006.8, 020-029, 032-034, 034.1-034.9, 036-037.9, 040, 040.1-041.0, 046-054.0, 054.2-059.9, 062-064.9, 070.0-070.2, 072-074.1, 074.3-075.9, 078.3-078.7, 079-079.5, 079.7, 082.4, 100-101.6, 104-104.9, 136-136.2, 139-139.0, 320.0-320.3, 320.5-320.8, 321-323.9, 390-390.9, 392, 392.9, 484.0, 484.3-484.5, 771.0-771.3, V09-V09.9
Meningitis	A32.1, A39-A39.9, A87-A87.9, G00.0-G00.8, G03-G03.8	036-036.9, 047-049.9, 320.0-320.3, 320.5-320.8, 321-322.9
Encephalitis	A83-A86.4, B94.1, F07.1, G04-G05.1, G05.3-G05.8, G21.3	062-064.9, 139.0, 323, 323.4-323.9
Diphtheria	A36-A36.9	032-032.9
Pertussis	A37-A37.9	033-033.9, 484.3
Tetanus	A33-A35.0	037-037.9, 771.3
Measles	B05-B05.9	055-055.9, 484.0
Varicella and herpes zoster	B01-B02.9, P35.8	052-053.9
Acute hepatitis	B15-B16.2, B17.0, B17.2, B19.1, P35.3	070.0-070.2
Acute hepatitis A	B15-B15.9	070.0-070.1
Acute hepatitis B	B16-B16.2, B17.0, B19.1, P35.3	070.2
Acute hepatitis C	N/A	N/A
Acute hepatitis E	B17.2	N/A
Other unspecified infectious diseases	A06.4-A06.8, A20-A28.9, A32-A32.0, A32.7-A32.9, A38-A38.9, A48.2, A48.4-A48.5, A65-A65.0, A69-A69.2, A74, A74.8-A74.9, A77.4, A81-A81.9, A88-A89.9, B00-B00.9, B03-B04, B06-B06.9, B10-B10.8, B25-B27.9, B29.4, B33-B33.1, B33.3-B33.8, B47-B48.8, B95-B95.5, D70.3, D89.3, F02.1, I00, I02, I02.9, K67.8, K75.3, K76.3, K77.0, M49.1, P35-P35.2, P35.9, P37, P37.1, P37.5-P37.9, U82-U84, U85-U89.9, Z16-Z16.3	006.3-006.8, 020-029, 034, 034.1-034.9, 040, 040.1-041.0, 046-046.9, 050-051.9, 054-054.0, 054.2-054.9, 056-059.9, 072-074.1, 074.3-075.9, 078.3-078.7, 079-079.5, 079.7, 082.4, 100-101.6, 104-104.9, 136-136.2, 139, 323.0-323.3, 390-390.9, 392, 392.9, 484.4-484.5, 771.0-771.2, V09-V09.9
Maternal and neonatal disorders	C58-C58.0, N96, N98-N98.9, O00-O07.9, O09-O16.9, O20-O26.9, O28-O36.9, O40-O48.1, O60-O77.9, O80-O92.7, O96-O98.6, O98.8-P04, P04.2, P04.5-P05.9, P07-P08.2, P10-P15.9, P20-P22.9, P24-P29.9, P36-P36.9, P37.2, P38-P39.9, P50-P61.9, P70-P70.1, P70.4-P72.0, P72.2-P72.9, P76-P78.9, P83-P84, P90-P91.9, P94, P94.1-P94.9, P96, P96.3, P96.8	181-181.9, 630-636.9, 638-638.9, 640-679.1, 760-760.6, 760.8-763.4, 763.6-768, 768.2-770, 770.1-771, 771.4-774.9, 775.0, 776-779.2
Maternal disorders	C58-C58.0, N96, N98-N98.9, O00-O07.9, O09-O16.9, O20-O26.9, O28-O36.9, O40-O48.1, O60-O77.9, O80-O92.7, O96-O98.6, O98.8-O99.9	181-181.9, 630-636.9, 638-638.9, 640-679.1
Maternal hemorrhage	O20-O20.9, O43.2, O44-O46.9, O62-O62.9, O67-O67.9, O70, O72-O72.3	640-641.9, 661-661.9, 666-666.9
Maternal sepsis and other maternal infections	O23-O23.9, O41.1, O85-O86.8, O91-O91.2	658.4, 659.3, 670-670.9, 672-672.0, 675-675.9
Maternal hypertensive disorders	O10-O16.9	642-642.9
Maternal obstructed labor and uterine rupture	O32-O33.9, O64-O66.9, O71-O71.9	652-653.9, 660-660.9, 664-665.9
Maternal abortion and miscarriage	N96, O01-O07.9	630-632.9, 634-636.9, 638-638.9, 646.3
Ectopic pregnancy	O00-O00.9	633-633.9
Indirect maternal deaths	O24-O24.3, O24.8-O25.3, O98-O98.6, O98.8-O99.9	646-646.2, 646.4-648.7, 649-649.9
Late maternal deaths	O96-O97.9	N/A
Maternal deaths aggravated by HIV/AIDS	N/A	N/A

List of International Classification of Diseases (ICD) codes mapped to the Global Burden of Disease cause list for causes of death		
**Cause**	**ICD10**	**ICD9**
Other direct maternal disorders (internal)	C58-C58.0, N98-N98.9, O09-O09.9, O21-O22.9, O24.4, O26-O26.9, O28-O31.8, O34-O36.9, O40-O41.0, O41.8-O43.1, O43.8-O43.9, O47-O48.1, O60-O61.9, O63-O63.9, O68-O69.9, O70.0-O70.9, O73-O77.9, O80-O84.9, O87-O90.9, O92-O92.7	181-181.9, 643-645.2, 648.8-648.9, 650-651.9, 654-658.3, 658.8-659.2, 659.4-659.9, 662-663.9, 667-669.9, 671-671.9, 673-674.9, 676-679.1
Neonatal disorders	P00-P04, P04.2, P04.5-P05.9, P07-P08.2, P10-P15.9, P20-P22.9, P24-P29.9, P36-P36.9, P37.2, P38-P39.9, P50-P61.9, P70-P70.1, P70.4-P72.0, P72.2-P72.9, P76-P78.9, P83-P84, P90-P91.9, P94, P94.1-P94.9, P96, P96.3, P96.8	760-760.6, 760.8-763.4, 763.6-768, 768.2-770, 770.1-771, 771.4-774.9, 775.0, 776-779.2
Neonatal preterm birth	P01.0-P01.1, P05-P05.9, P07-P07.3, P22-P22.9, P25-P28.9, P52-P52.9, P61.2, P77-P78.9	761.0-761.1, 764-765.9, 769-769.9, 770.2-770.9, 772.1-772.9, 776.6, 777.0-777.9
Neonatal encephalopathy due to birth asphyxia and trauma	P01.7, P02-P03.9, P10-P15.9, P20-P21.9, P24-P24.9, P60-P60.0, P90-P91.9	761.7-763.4, 763.6-763.9, 767-768, 768.2-768.9, 770.1, 779.0-779.2
Neonatal sepsis and other neonatal infections	P36-P36.9, P37.2, P38-P39.9	771, 771.4-771.9
Hemolytic disease and other neonatal jaundice	P55-P59.9	773-774.9
Other neonatal disorders	P00-P01, P01.2-P01.6, P01.8-P01.9, P04, P04.2, P04.5-P04.9, P08-P08.2, P29-P29.9, P50-P51.9, P53-P54.9, P61-P61.1, P61.3-P61.9, P70-P70.1, P70.4-P72.0, P72.2-P72.9, P76-P76.9, P83-P84, P94, P94.1-P94.9, P96, P96.3, P96.8	760-760.6, 760.8-761, 761.2-761.6, 766-766.9, 770, 772-772.0, 775.0, 776-776.5, 776.7-777, 778-779
Nutritional deficiencies	D50.1-D50.8, D51-D52.0, D52.8-D53.9, E00-E02, E40-E46.9, E51-E61.9, E63-E64.0, E64.2-E64.9, M12.1	244.2, 260-263.9, 265-269.9, 281.0-281.9, 716.0
Protein-energy malnutrition	E40-E46.9, E64.0	260-263.9
Other nutritional deficiencies	D51-D52.0, D52.8-D53.9, E00-E02, E51-E61.9, E63-E64, E64.2-E64.9, M12.1	244.2, 265-269.9, 281.0-281.9, 716.0
Non-communicable diseases	A46-A46.0, A66-A67.9, B18-B18.9, B33.2, B86, C00-C13.9, C15-C22.8, C23-C25.9, C30-C34.9, C37-C38.8, C40-C41.9, C43-C45.9, C47-C54.9, C56-C57.8, C60-C63.8, C64-C67.9, C68.0-C68.8, C69.0-C69.8, C70-C73.9, C75-C75.8, C81-C82.9, C83.0-C83.8, C84-C85.0, C85.2-C85.8, C86-C86.6, C88-C91.0, C91.2-C91.3, C91.6, C92-C92.6, C93-C93.1, C93.3, C93.8, C94-C94.5, C94.7-C96.9, D00.1-D00.2, D01.0-D01.3, D02.0-D02.3, D03-D06.9, D07.0-D07.2, D07.4-D07.5, D09.0, D09.2-D09.3, D09.8, D10.0-D10.7, D11-D12.9, D13.0-D13.7, D14.0-D14.3, D15-D16.9, D22-D27.9, D28.0-D28.7, D29.0-D29.8, D30.0-D30.8, D31-D36, D36.1-D36.7, D37.1-D37.5, D38.0-D38.5, D39.1-D39.2, D39.8, D40.0-D40.8, D41.0-D41.8, D42-D43.9, D44.0-D44.8, D45-D47.9, D48.0-D48.6, D49.2-D49.4, D49.6, D55-D59, D59.1, D59.3-D59.5, D59.8-D61.9, D63.1, D64.0, D66-D67, D68.0-D69.4, D69.6-D69.8, D70-D70.0, D70.4-D75.8, D76-D77, D86-D86.9, D89-D89.2, E03-E03.1, E03.3-E06.3, E06.5-E07.1, E10-E11.9, E16.1-E16.9, E20-E23.0, E23.2-E24.1, E24.3-E27.2, E27.4-E34, E34.1-E34.8, E65-E66.0, E66.2-E68, E70-E85.2, E88-E88.2, E88.4-E88.9, F00-F02.0, F02.2-F02.3, F02.8-F03.9, F10-F16.9, F18-F18.9, F50.0-F50.5, G10-G13.8, G20-G20.9, G23-G24, G24.1-G25.0, G25.2-G25.3, G25.5, G25.8-G26.0, G30-G31.9, G35-G37.9, G40-G41.9, G45-G46.8, G47.3, G61-G61.9, G62.1, G70-G72, G72.1-G73.7, G90-G90.9, G95-G95.9, H05.0-H05.1, I01-I01.9, I02.0, I05-I09.9, I11-I13.9, I20-I25.9, I27.0-I27.2, I28-I28.9, I30-I31.1, I31.8-I37.8, I38-I41.1, I41.8-I41.9, I42.1-I42.8, I43-I43.9, I47-I48.9, I51.0-I51.4, I60-I63.9, I65-I66.9, I67.0-I67.3, I67.5-I67.7, I68.0-I68.2, I69.0-I69.3, I70.2-I70.7, I71-I73.9, I77-I89.9, I98, I98.2, J30-J35.9, J37-J39.9, J41-J46.9, J60-J63.8, J66-J68.9, J70, J70.8-J70.9, J82, J84-J84.9, J91, J91.8-J92.9, K20-K20.9, K22-K22.6, K22.8-K23, K23.8-K29.9, K31-K31.8, K35-K38.9, K40-K42.9, K44-K46.9, K50-K52, K52.8-K52.9, K55-K62.6, K62.8-K62.9, K63.5, K64-K64.9, K66.8, K67, K68, K70-K70.3, K71.7, K73-K75, K75.1-K75.2, K75.4-K76.2, K76.4-K77, K77.8, K80-K83.9, K85-K87.1, K90-K90.9, K92.8, K93.8, L00-L05.9, L08-L08.9, L10-L14.0, L51-L51.9, L88-L89.9, L93-L93.2, L97-L98.4, M00-M03.0, M03.2-M03.6, M05-M09.8, M30-M36.8, M40-M43.1, M65-M65.0, M71.0-M71.1, M72.5-M72.6, M80-M82.8, M86.3-M86.4, M87-M87.0, M88-M89.0, M89.5, M89.7-M89.9, N00-N08.8, N10-N12.9, N13.6, N15-N16.8, N18-N18.9, N20-N23.0, N25-N28.1, N29-N30.3, N30.8-N32.0, N32.3-N32.4, N34-N34.3, N36-N36.9, N39-N39.2, N41-N41.9, N44-N44.0, N45-N45.9, N49-N49.9, N60-N60.9, N72-N72.0, N75-N77.8, N80-N81.9, N83-	035-035.9, 074.2, 102-103.9, 133-133.6, 135-135.9, 140-148.9, 150-155.1, 155.3-158.9, 160-164.9, 170-175.9, 180-180.9, 182-183.8, 184.0-184.4, 184.8, 185-186.9, 187.1-187.8, 188-188.9, 189.0-189.8, 190-190.8, 191-193.9, 194.1-194.8, 200-202.8, 203-204.0, 204.2, 205-205.3, 206-206.1, 207-208.9, 209.0-209.1, 209.4-209.5, 210.0-210.9, 211.0-211.8, 212.0-212.8, 213-213.9, 217-220.9, 221.0-221.8, 222.0-222.8, 223.0-223.8, 224-228.9, 229.0, 229.8, 230.1-230.8, 231.0-231.2, 232-232.9, 233.0-233.2, 233.4-233.5, 233.7, 234.0-234.8, 235.0, 235.4, 235.6-235.8, 236.0-236.2, 236.4-236.5, 236.7, 237.0-237.1, 237.3, 237.5-237.9, 238.0-238.9, 239.2-239.4, 239.6, 240-243.9, 245-246.9, 251-251.2, 251.4-253.6, 253.8-259.1, 259.3-259.9, 270-273.9, 275-276, 277-277.2, 277.4-277.9, 278.0-278.8, 282-284.9, 286-286.5, 286.7-289.0, 289.4-289.7, 290-292.9, 294.1-294.9, 303-303.9, 304.0-304.8, 305.0, 305.2-305.8, 307.1, 327.2-327.8, 330-331.2, 331.5-332.0, 333-337.9, 340-341.9, 345-345.9, 349, 349.2-349.8, 353.8-353.9, 356-356.9, 357.0-357.1, 357.3-357.5, 357.7, 358-359.9, 376.0-376.1, 391-391.9, 392.0, 393-398.9, 402-404.9, 410-414.9, 416.0-416.1, 417-417.9, 420-423, 423.1-423.9, 424.0-424.3, 424.8, 425.0-425.5, 425.7-425.8, 427.0-427.3, 427.6-427.8, 429.0, 430-435.9, 437.0-437.2, 437.4-437.8, 440.2, 440.4, 441-443.9, 446-457, 457.1-457.9, 459, 459.1-459.3, 470-474.9, 476-476.1, 477-479, 491-493.9, 495-504.9, 506-506.9, 508-509, 515, 516-517.8, 518.6, 518.9, 519.1-519.4, 530-530.0, 530.2-530.6, 531-536.1, 537-537.6, 537.8, 538, 540-543.9, 550-551.1, 551.3-552.1, 552.3-553.1, 553.3-558.0, 560-560.3, 560.8-560.9, 562-562.1, 564-564.1, 564.5-564.7, 565-566.9, 569.0-569.5, 569.7, 571-571.9, 572.2-573.0, 573.4-577.9, 579-579.2, 579.4-583.9, 585-585.9, 588-590.9, 592-593.8, 594-598.1, 598.8-599.6, 599.8, 601-602.9, 604-604.9, 608.2, 610-610.9, 615-618.9, 620-620.9, 621.0, 621.4-621.9, 622.1-622.7, 629-629.8, 680-689, 694-695.5, 707-707.9, 710-711.9, 714-714.3, 714.8-714.9, 730.1, 732-732.9, 733.0-733.1, 740-758.9, 759.0-759.8, 760.7, 775.1-775.3, 788.0, 790.3, 798-798.0, E850-E850.29, E860-E860.19
Neoplasms	C00-C13.9, C15-C22.8, C23-C25.9, C30-C34.9, C37-C38.8, C40-C41.9, C43-C45.9, C47-C54.9, C56-C57.8, C60-C63.8, C64-C67.9, C68.0-C68.8, C69.0-C69.8, C70-C73.9, C75-C75.8, C81-C82.9, C83.0-C83.8, C84-C85.0, C85.2-C85.8, C86-C86.6, C88-C91.0, C91.2-C91.3, C91.6, C92-C92.6, C93-C93.1, C93.3, C93.8, C94-C94.5, C94.7-C96.9, D00.1-D00.2, D01.0-D01.3, D02.0-D02.3, D03-D06.9, D07.0-D07.2, D07.4-D07.5, D09.0, D09.2-D09.3, D09.8, D10.0-D10.7, D11-D12.9, D13.0-D13.7, D14.0-D14.3, D15-D16.9, D22-D24.9, D26.0-D27.9, D28.0-D28.1, D28.7, D29.0-D29.8, D30.0-D30.8, D31-D36, D36.1-D36.7, D37.1-D37.5, D38.0-D38.5, D39.1-D39.2, D39.8, D40.0-D40.8, D41.0-D41.8, D42-D43.9, D44.0-D44.8, D45-D47.9, D48.0-D48.6, D49.2-D49.4, D49.6	140-148.9, 150-155.1, 155.3-158.9, 160-164.9, 170-175.9, 180-180.9, 182-183.8, 184.0-184.4, 184.8, 185-186.9, 187.1-187.8, 188-188.9, 189.0-189.8, 190-190.8, 191-193.9, 194.1-194.8, 200-202.8, 203-204.0, 204.2, 205-205.3, 206-206.1, 207-208.9, 209.0-209.1, 209.4-209.5, 210.0-210.9, 211.0-211.8, 212.0-212.8, 213-213.9, 217-217.8, 219.0, 220-220.9, 221.0-221.8, 222.0-222.8, 223.0-223.8, 224-228.9, 229.0, 229.8, 230.1-230.8, 231.0-231.2, 232-232.9, 233.0-233.2, 233.4-233.5, 233.7, 234.0-234.8, 235.0, 235.4, 235.6-235.8, 236.1-236.2, 236.4-236.5, 236.7, 237.0-237.1, 237.3, 237.5-237.9, 238.0-238.9, 239.2-239.4, 239.6, 569.0, 610-610.9, 622.7
Lip and oral cavity cancer	C00-C08.9, D10.0-D10.5, D11-D11.9, D22.0, D23.0	140-145.9, 210.1-210.6, 235.0
Nasopharynx cancer	C11-C11.9, D10.6	147-147.9, 210.9
Other pharynx cancer	C09-C10.9, C12-C13.9, D10.7	146-146.9, 148-148.9, 210.7-210.8
Esophageal cancer	C15-C15.9, D00.1, D13.0	150-150.9, 211.0, 230.1
Stomach cancer	C16-C16.9, D00.2, D13.1, D37.1	151-151.9, 211.1, 230.2
Colon and rectum cancer	C18-C21.9, D01.0-D01.3, D12-D12.9, D37.3-D37.5	153-154.9, 209.1, 209.5, 211.3-211.4, 230.3-230.6, 569.0
Liver cancer	C22-C22.8, D13.4	155-155.1, 155.3-155.9, 211.5

List of International Classification of Diseases (ICD) codes mapped to the Global Burden of Disease cause list for causes of death		
**Cause**	**ICD10**	**ICD9**
Liver cancer due to hepatitis B	N/A	N/A
Liver cancer due to hepatitis C	N/A	N/A
Liver cancer due to alcohol use	N/A	N/A
Liver cancer due to NASH	N/A	N/A
Hepatoblastoma	C22.2	N/A
Liver cancer due to other causes	N/A	N/A
Gallbladder and biliary tract cancer	C23-C24.9, D13.5	156-156.9
Pancreatic cancer	C25-C25.9, D13.6-D13.7	157-157.9, 211.6-211.7
Larynx cancer	C32-C32.9, D02.0, D14.1, D38.0	161-161.9, 212.1, 231.0, 235.6
Tracheal, bronchus, and lung cancer	C33-C34.9, D02.1-D02.3, D14.2-D14.3, D38.1	162-162.9, 212.2-212.3, 231.1-231.2, 235.7
Malignant skin melanoma	C43-C43.9, D03-D03.9, D22, D22.1-D23, D23.1-D23.9, D48.5	172-172.9
Non-melanoma skin cancer	C44-C44.9, D04-D04.9, D49.2	173-173.9, 210.0, 222.4, 232-232.9, 238.2
Non-melanoma skin cancer (squamous-cell carcinoma)	C44-C44.9, D04-D04.9, D49.2	173, 173.1-173.9, 210.0, 222.4, 232-232.9, 238.2
Soft tissue and other extraosseous sarcomas	C49-C49.9	171-171.9
Malignant neoplasm of bone and articular cartilage	C40-C41.9	170-170.9
Breast cancer	C50-C50.9, D05-D05.9, D24-D24.9, D48.6, D49.3	174-175.9, 217-217.8, 233.0, 238.3, 239.3, 610-610.9
Cervical cancer	C53-C53.9, D06-D06.9, D26.0	180-180.9, 219.0, 233.1, 622.7
Uterine cancer	C54-C54.9, D07.0-D07.2, D26.1-D26.9	182-182.9, 233.2
Ovarian cancer	C56-C56.9, D27-D27.9, D39.1	183-183.0, 220-220.9, 236.2
Prostate cancer	C61-C61.9, D07.5, D29.1, D40.0	185-185.9, 222.2, 236.5
Testicular cancer	C62-C62.9, D29.2-D29.8, D40.1-D40.8	186-186.9, 222.0, 222.3, 236.4
Kidney cancer	C64-C65.9, D30.0-D30.1, D41.0-D41.1	189.0-189.1, 189.5-189.6, 223.0-223.1
Bladder cancer	C67-C67.9, D09.0, D30.3, D41.4-D41.8, D49.4	188-188.9, 223.3, 233.7, 236.7, 239.4
Brain and central nervous system cancer	C70-C72.9, C75.1-C75.3	191-192.9, 194.3-194.4
Eye cancer	C69.0-C69.8	190-190.8
Retinoblastoma	C69.2	190.5
Other eye cancers	C69.0-C69.1, C69.3-C69.8	190-190.4, 190.6-190.8
Neuroblastoma and other peripheral nervous cell tumors	C47-C47.9	N/A
Thyroid cancer	C73-C73.9, D09.3, D09.8, D34-D34.9, D44.0	193-193.9, 226-226.9
Mesothelioma	C45-C45.9	N/A
Hodgkin lymphoma	C81-C81.9	201-201.9
Non-Hodgkin lymphoma	C82-C82.9, C83.0-C83.8, C84-C85.0, C85.2-C85.8, C86-C86.6, C96-C96.9	200-200.9, 202-202.8
Burkitt lymphoma	C83.7	200.2
Other non-Hodgkin lymphoma	C82-C82.9, C83.0-C83.6, C83.8, C84-C85.0, C85.2-C85.8, C86-C86.6, C96-C96.9	200-200.1, 200.3-200.9, 202-202.8
Multiple myeloma	C88-C90.9	203-203.9
Leukemia	C91-C91.0, C91.2-C91.3, C91.6, C92-C92.6, C93-C93.1, C93.3, C93.8, C94-C94.5, C94.7-C95.9	204-204.0, 204.2, 205-205.3, 206-206.1, 207-208.9
Acute lymphoid leukemia	C91.0, C91.2-C91.3, C91.6	204.0, 204.2
Chronic lymphoid leukemia	N/A	N/A
Acute myeloid leukemia	C92.0, C92.3-C92.6, C93.0, C94.0, C94.2, C94.4-C94.5	205.0, 205.2-205.3, 206.0, 207.0, 207.2-207.8
Chronic myeloid leukemia	C92.1-C92.2	205.1
Other leukemia	C93.1, C93.3, C93.8, C94.1, C94.3, C94.7-C94.8	206.1, 207.1, 207.9
Other malignant neoplasms	C17-C17.9, C30-C31.9, C37-C38.8, C48-C48.9, C4A, C51-C52.9, C57-C57.8, C60-C60.9, C63-C63.8, C66-C66.9, C68.0-C68.8, C75-C75.0, C75.4-C75.8, D07.4, D09.2, D13.2-D13.3, D14.0, D15-D16.9, D28.0-D28.1, D28.7, D29.0, D30.2, D30.4-D30.8, D31-D31.9, D35-D35.1, D35.5-D36, D36.1-D36.7, D37.2, D38.2-D38.5, D39.2, D39.8, D41.2-D41.3, D44.1-D44.2, D44.5-D44.8, D48.0-D48.4	152-152.9, 158-158.9, 160-160.9, 163-164.9, 183.2-183.8, 184.0-184.4, 184.8, 187.1-187.8, 189.2-189.4, 189.8, 194.1, 194.5-194.8, 209.0, 209.4, 211.2, 211.8, 212.0, 212.4-212.8, 213-213.9, 221.0-221.8, 222.1, 222.8, 223.2, 223.8, 224-224.9, 227-227.1, 227.5-228.9, 229.0, 229.8, 230.7-230.8, 233.4-233.5, 234.0-234.8, 235.4, 235.8, 236.1, 237.3, 238.0-238.1, 239.2
Other neoplasms	D32-D33.9, D35.2-D35.4, D42-D43.9, D44.3-D44.4, D45-D47.9, D49.6	225-225.9, 227.3-227.4, 237.0-237.1, 237.5-237.9, 238.4-238.9, 239.6
Myelodysplastic, myeloproliferative, and other hematopoietic neoplasms	D45-D47.9	238.4-238.9
Non-malignant brain and other central nervous system neoplasms	D32-D33.9, D35.2-D35.4, D42-D43.9, D44.3-D44.4, D49.6	225-225.9, 227.3-227.4, 237.0-237.1, 237.5-237.9, 239.6
Other benign and in situ neoplasms (internal)	N/A	N/A
Cardiovascular diseases	B33.2, G45-G46.8, I01-I01.9, I02.0, I05-I09.9, I11-I11.9, I20-I25.9, I27.0, I27.2, I28-I28.9, I30-I31.1, I31.8-I37.8, I38-I41.1, I41.8-I41.9, I42.1-I42.8, I43-I43.9, I47-I48.9, I51.0-I51.4, I60-I63.9, I65-I66.9, I67.0-I67.3, I67.5-I67.6, I68.0-I68.2, I69.0-I69.3, I70.2-I70.7, I71-I73.9, I77-I83.9, I86-I89.0, I89.9, I98, K75.1	074.2, 391-391.9, 392.0, 393-398.9, 402-402.9, 410-414.9, 416.0, 417-417.9, 420-423, 423.1-423.9, 424.0-424.3, 424.8, 425.0-425.5, 425.7-425.8, 427.0-427.3, 427.6-427.8, 429.0, 430-435.9, 437.0-437.2, 437.5-437.8, 440.2, 440.4, 441-443.9, 447-454.9, 456, 456.3-457, 457.1, 457.8-457.9, 459, 459.1-459.3
Rheumatic heart disease	I01-I01.9, I02.0, I05-I09.9	391-391.9, 392.0, 393-398.9
Ischemic heart disease	I20-I25.9	410-414.9
Stroke	G45-G46.8, I60-I63.9, I65-I66.9, I67.0-I67.3, I67.5-I67.6, I68.1-I68.2, I69.0-I69.3	430-435.9, 437.0-437.2, 437.5-437.8
Ischemic stroke	G45-G46.8, I63-I63.9, I65-I66.9, I67.2-I67.3, I67.5-I67.6, I69.3	433-435.9, 437.0-437.1, 437.5-437.8
Intracerebral hemorrhage	I61-I62, I62.1-I62.9, I68.1-I68.2, I69.1-I69.2	431-432.9, 437.2

List of International Classification of Diseases (ICD) codes mapped to the Global Burden of Disease cause list for causes of death

Cause	ICD10	ICD9
Subarachnoid hemorrhage	I60-I60.9, I62.0, I67.0-I67.1, I69.0	430-430.9
Hypertensive heart disease	I11-I11.9	402-402.9
Non-rheumatic valvular heart disease	I34-I37.8	424.0-424.3, 424.8
Non-rheumatic calcific aortic valve disease	I35-I35.9	424.1
Non-rheumatic degenerative mitral valve disease	I34-I34.9	424.0
Other non-rheumatic valve diseases	I36-I37.8	424.2-424.3, 424.8
Cardiomyopathy and myocarditis	B33.2, I40-I41.1, I41.8-I41.9, I42.1-I42.8, I43-I43.9, I51.4	074.2, 422-422.9, 425.0-425.5, 425.7-425.8, 429.0
Myocarditis	B33.2, I40-I41.1, I41.8-I41.9	074.2, 422-422.9
Alcoholic cardiomyopathy	I42.6	425.5
Other cardiomyopathy	I42.1-I42.5, I42.7-I42.8, I43-I43.9, I51.4	425.0-425.4, 425.7-425.8, 429.0
Pulmonary Arterial Hypertension	I27.0	416.0
Atrial fibrillation and flutter	I48-I48.9	427.3
Aortic aneurysm	I71-I71.9	441-441.9
Lower extremity peripheral arterial disease	I70.2-I70.7, I73-I73.9	440.2, 440.4, 443.0-443.9
Endocarditis	I33-I33.9, I38-I39.9	421-421.9
Other cardiovascular and circulatory diseases	I27.2, I28-I28.9, I30-I31.1, I31.8-I32.8, I47-I47.9, I51.0-I51.3, I68.0, I72-I72.9, I77-I83.9, I86-I89.0, I89.9, I98, K75.1	417-417.9, 420-420.9, 423, 423.1-423.9, 427.0-427.2, 427.6-427.8, 442-443, 447-454.9, 456, 456.3-457, 457.1, 457.8-457.9, 459, 459.1-
Chronic respiratory diseases	D86-D86.2, D86.9, G47.3, J30-J35.9, J37-J39.9, J41-J46.9, J60-J63.8, J66-J68.9, J70, J70.8-J70.9, J82, J84-J84.9, J91, J91.8-J92.9	135-135.9, 327.2-327.8, 470-474.9, 476-476.1, 477-479, 491-493.9, 495-504.9, 506-506.9, 508-509, 515, 516-517.8, 518.6, 518.9, 519.1-
Chronic obstructive pulmonary disease	J41-J44.9	491-492.9, 496-499
Pneumoconiosis	J60-J63.8, J92.0	500-504.9
Silicosis	J62-J62.9	502-502.9, 503.0, 503.9
Asbestosis	J61-J61.0, J92.0	501
Coal workers pneumoconiosis	J60-J60.0	500-500.9, 501.0-501.9
Other pneumoconiosis	J63-J63.8	503, 503.1, 504-504.9
Asthma	J45-J46.9	493-493.9
Interstitial lung disease and pulmonary sarcoidosis	D86-D86.2, D86.9, J84-J84.9	135-135.9, 515, 516-516.9
Other chronic respiratory diseases	G47.3, J30-J35.9, J37-J39.9, J66-J68.9, J70, J70.8-J70.9, J82, J91, J91.8-J92, J92.9	327.2-327.8, 470-474.9, 476-476.1, 477-479, 495-495.9, 506-506.9, 508-509, 517-517.8, 518.6, 518.9, 519.1-519.4
Digestive diseases	B18-B18.9, I84-I85.9, I98.2, K20-K20.9, K22-K22.6, K22.8-K23, K23.8-K29.9, K31-K31.8, K35-K38.9, K40-K42.9, K44-K46.9, K50-K52, K52.8-K52.9, K55-K62.6, K62.8-K62.9, K63.5, K64-K64.9, K66.8, K67, K68, K70-K70.3, K71.7, K73-K75, K75.2, K75.4-K76.2, K76.4-K77, K77.8, K80-K83.9, K85-K87.1, K90-K90.9, K92.8, K93.8, M07.4-M07.5, M09.1-M09.2	455-455.9, 456.0-456.2, 530-530.0, 530.2-530.6, 531-536.1, 537-537.6, 537.8, 538, 540-543.9, 550-551.1, 551.3-552.1, 552.3-553.1, 553.3-558.0, 560-560.3, 560.8-560.9, 562-562.1, 564-564.1, 564.5-564.7, 565-566.9, 569.1-569.5, 569.7, 571-571.9, 572.2-573.0, 573.4-577.9, 579-579.2, 579.4-579.9
Cirrhosis and other chronic liver diseases	B18-B18.9, I85-I85.9, I98.2, K70-K70.3, K71.7, K73-K75, K75.2, K75.4-K76.2, K76.4-K76.9, K77.8	456.0-456.2, 571-571.9, 572.2-573.0, 573.4-573.9
Chronic hepatitis B including cirrhosis	N/A	N/A
Chronic hepatitis C including cirrhosis	N/A	N/A
Cirrhosis due to alcohol	N/A	N/A
Nonalcoholic fatty liver disease including cirrhosis	N/A	N/A
Cirrhosis due to other causes	N/A	N/A
Upper digestive system diseases	K25-K29.9	531-535.9
Peptic ulcer disease	K25-K28.9	531-534.9
Gastritis and duodenitis	K29-K29.9	535-535.9
Appendicitis	K35-K37.9	540-542.9
Paralytic ileus and intestinal obstruction	K56-K56.9	560-560.3, 560.8-560.9
Inguinal, femoral, and abdominal hernia	K40-K42.9, K44-K46.9	550-551.1, 551.3-552.1, 552.3-553.1, 553.3-553.9
Inflammatory bowel disease	K50-K52, K52.8-K52.9, M07.4-M07.5, M09.1-M09.2	555-556.9, 558-558.0, 569.5
Ulcerative colitis	K51-K52, K52.8-K52.9, M07.5, M09.2	556-556.9, 558.0
Crohn's disease	K50-K50.9, M07.4, M09.1	555-555.9
Vascular intestinal disorders	K55-K55.9	557-557.9
Gallbladder and biliary diseases	K80-K83.9, K87-K87.1	574-576.9
Pancreatitis	K85-K86.9	577-577.9, 579.4
Diverticular disease of intestines	K57-K57.9	562-562.1
Other digestive diseases (internal)	I84-I84.9, K20-K20.9, K22-K22.6, K22.8-K23, K23.8-K24, K31-K31.8, K38-K38.9, K58-K62.6, K62.8-K62.9, K63.5, K64-K64.9, K66.8, K67, K68, K77, K90-K90.9, K92.8, K93.8	455-455.9, 530-530.0, 530.2-530.6, 536-536.1, 537-537.6, 537.8, 538, 543-543.9, 564-564.1, 564.5-564.7, 565-566.9, 569.1-569.4, 569.7, 579-579.2, 579.8-579.9
Neurological disorders	F00-F02.0, F02.2-F02.3, F02.8-F03.9, G10-G13.8, G20-G20.9, G23-G24, G24.1-G25.0, G25.2-G25.3, G25.5, G25.8-G26.0, G30-G31.1, G31.8-G31.9, G35-G37.9, G40-G41.9, G61-G61.9, G70-G71.1, G71.3-G72, G72.2-G73.7, G90-G90.9, G95-G95.9, M33-M33.9, P94.0	290-290.9, 294.1-294.9, 330-331.2, 331.5-332.0, 333-337.9, 340-341.9, 345-345.9, 349, 349.2-349.8, 353.8-353.9, 356-356.9, 357.0-357.1, 357.3-357.4, 357.7, 358-359.9, 775.2
Alzheimer's disease and other dementias	F00-F02.0, F02.8-F03.9, G30-G30.9	290-290.9, 294.1-294.9, 331.0
Parkinson's disease	F02.3, G20-G20.9	332-332.0
Idiopathic epilepsy	G40-G41.9	345-345.9
Multiple sclerosis	G35-G35.9	340-340.9
Motor neuron disease	G12-G12.9	335-335.2, 335.8-335.9

List of International Classification of Diseases (ICD) codes mapped to the Global Burden of Disease cause list for causes of death		
**Cause**	**ICD10**	**ICD9**
Other neurological disorders	F02.2, G10-G11.9, G13-G13.8, G23-G24, G24.1-G25.0, G25.2-G25.3, G25.5, G25.8-G26.0, G31-G31.1, G31.8-G31.9, G36-G37.9, G61-G61.9, G70-G71.1, G71.3-G72, G72.2-G73.7, G90-G90.9, G95-G95.9, M33-M33.9, P94.0	330-331, 331.1-331.2, 331.5-331.9, 333-334.9, 335.3, 336-337.9, 341-341.9, 349, 349.2-349.8, 353.8-353.9, 356-356.9, 357.0-357.1, 357.3-357.4, 357.7, 358-359.9, 775.2
Mental disorders	F50.0-F50.5	307.1
Eating disorders	F50.0-F50.5	307.1
Anorexia nervosa	F50.0-F50.5	307.1
Substance use disorders	E24.4, F10-F16.9, F18-F18.9, G31.2, G62.1, G72.1, P04.3-P04.4, P96.1, Q86.0, R78.0-R78.5, X45-X45.9, X65-X65.9, Y15-Y15.9, Y90-Y91.9	291-292.9, 303-303.9, 304.0-304.8, 305.0, 305.2-305.8, 357.5, 760.7, 790.3, E850-E850.29, E860-E860.19
Alcohol use disorders	E24.4, F10-F10.9, G31.2, G62.1, G72.1, P04.3, Q86.0, R78.0, X45-X45.9, X65-X65.9, Y15-Y15.9, Y90-Y91.9	291-291.9, 303-303.9, 305.0, 357.5, 790.3, E860-E860.19
Drug use disorders	F11-F16.9, F18-F18.9, P04.4, P96.1, R78.1-R78.5	292-292.9, 304.0-304.8, 305.2-305.8, 760.7, E850-E850.29
Opioid use disorders	F11-F11.9, P96.1, R78.1	304.0, 305.5, E850.0-E850.29
Cocaine use disorders	F14-F14.9, R78.2	304.2, 305.6
Amphetamine use disorders	F15-F15.9	304.4, 305.7
Other drug use disorders	F13-F13.9, F16-F16.9, F18-F18.9, P04.4, R78.3-R78.5	292-292.9, 304.1, 304.5-304.8, 305.3-305.4, 305.8, 760.7
Diabetes and kidney diseases	D63.1, E10-E11.9, I12-I13.9, N00-N08.8, N15.0, N18-N18.9, P70.2	403-404.9, 580-583.9, 585-585.9, 589-589.9, 775.1
Diabetes mellitus	E10-E10.1, E10.3-E11.1, E11.3-E11.9, P70.2	775.1
Diabetes mellitus type 1	E10-E10.1, E10.3-E10.9, P70.2	775.1
Diabetes mellitus type 2	E11-E11.1, E11.3-E11.9	N/A
Chronic kidney disease	D63.1, E10.2, E11.2, I12-I13.9, N02-N08.8, N15.0, N18-N18.9	403-404.9, 581-583.9, 585-585.9, 589-589.9
Chronic kidney disease due to diabetes mellitus type 1	E10.2	N/A
Chronic kidney disease due to diabetes mellitus type 2	E11.2	N/A
Chronic kidney disease due to hypertension	I12-I13.9	403-404.9
Chronic kidney disease due to glomerulonephritis	N03-N06.9	581-583.9
Chronic kidney disease due to other and unspecified causes	N02-N02.9, N07-N08.8, N15.0	589-589.9
Acute glomerulonephritis	N00-N01.9	580-580.9
Skin and subcutaneous diseases	A46-A46.0, A66-A67.9, B86, D86.3, H05.0-H05.1, I89.1-I89.8, L00-L05.9, L08-L08.9, L10-L14.0, L51-L51.9, L88-L89.9, L97-L98.4, M07.0-M07.3, M09.0, M72.5-M72.6	035-035.9, 102-103.9, 133-133.6, 376.0-376.1, 457.2-457.3, 680-689, 694-695.3, 707-707.9
Bacterial skin diseases	A46-A46.0, A66-A67.9, H05.0-H05.1, I89.1-I89.8, L00-L05.9, L08-L08.9, L88, L97-L98.4, M72.5-M72.6	035-035.9, 102-103.9, 376.0-376.1, 457.2-457.3, 680-689
Cellulitis	H05.0, L03-L03.9, M72.5-M72.6	681-682.9
Pyoderma	A46-A46.0, A66-A67.9, H05.1, I89.1-I89.8, L00-L02.9, L04-L05.9, L08-L08.9, L88, L97-L98.4	035-035.9, 102-103.9, 376.0-376.1, 457.2-457.3, 680-680.9, 683-689
Decubitus ulcer	L89-L89.9	707-707.9
Other skin and subcutaneous diseases	D86.3, L10-L14.0, L51-L51.9	694-695.3
Musculoskeletal disorders	I27.1, I67.7, L93-L93.2, M00-M03.0, M03.2-M03.6, M05-M07, M07.6-M09, M09.8, M30-M32.9, M34-M36.8, M40-M43.1, M65-M65.0, M71.0-M71.1, M80-M82.8, M86.3-M86.4, M87-M87.0, M88-M89.0, M89.5, M89.7-M89.9	416.1, 437.4, 446-446.9, 695.4-695.5, 710-711.9, 714-714.3, 714.8-714.9, 730.1, 732-732.9, 733.0-733.1
Rheumatoid arthritis	M05-M06.9, M08.0-M08.4	714-714.3, 714.8-714.9
Other musculoskeletal disorders	I27.1, I67.7, L93-L93.2, M00-M03.0, M03.2-M03.6, M07, M07.6-M08, M08.8-M09, M09.8, M30-M32.9, M34-M36.8, M40-M43.1, M65-M65.0, M71.0-M71.1, M80-M82.8, M86.3-M86.4, M87-M87.0, M88-M89.0, M89.5, M89.7-M89.9	416.1, 437.4, 446-446.9, 695.4-695.5, 710-711.9, 730.1, 732-732.9, 733.0-733.1
Other non-communicable diseases	D25-D26, D28.2, D55-D59, D59.1, D59.3-D59.5, D59.8-D61.9, D64.0, D66-D67, D68.0-D69.4, D69.6-D69.8, D70-D70.0, D70.4-D75.8, D76-D77, D86.8, D89-D89.2, E03-E03.1, E03.3-E06.3, E06.5-E07.1, E16.1-E16.9, E20-E23.0, E23.2-E24.1, E24.3, E24.8-E27.2, E27.4-E34, E34.1-E34.8, E65-E66.0, E66.2-E68, E70-E85.2, E88-E88.2, E88.4-E88.9, G71.2, N10-N12.9, N13.6, N15, N15.1-N16.8, N20-N23.0, N25-N28.1, N29-N30.3, N30.8-N32.0, N32.3-N32.4, N34-N34.3, N36-N36.9, N39-N39.2, N41-N41.9, N44-N44.0, N45-N45.9, N49-N49.9, N60-N60.9, N72-N72.0, N75-N77.8, N80-N81.9, N83-N83.9, N84.0-N84.1, N87-N87.9, P72.1, P96.0, Q00-Q07.9, Q10.4-Q18.9, Q20-Q28.9, Q30-Q45.9, Q50-Q56.4, Q60-Q86, Q86.8-Q87.8, Q89-Q89.8, Q90-Q93.9, Q95-Q99.8, R95-R95.9	218-219, 219.1-219.9, 236.0, 240-243.9, 245-246.9, 251-251.2, 251.4-253.6, 253.8-259.1, 259.3-259.9, 270-273.9, 275-276, 277-277.2, 277.4-277.9, 278.0-278.8, 282-284.9, 286-286.5, 286.7-289.0, 289.4-289.7, 588-588.9, 590-590.9, 592-593.8, 594-598.1, 598.8-599.6, 599.8, 601-602.9, 604-604.9, 608.2, 615-618.9, 620-620.9, 621.0, 621.4-621.9, 622.1-622.6, 629-629.8, 740-758.9, 759.0-759.8, 775.3, 788.0, 798-798.0
Congenital birth defects	G71.2, P96.0, Q00-Q07.9, Q10.4-Q18.9, Q20-Q28.9, Q30-Q45.9, Q50-Q56.4, Q60-Q86, Q86.8-Q87.8, Q89-Q89.8, Q90-Q93.9, Q95-Q99.8	740-758.9, 759.0-759.8
Neural tube defects	Q00-Q01.9, Q05-Q05.9	740-741.9, 742.0
Congenital heart anomalies	Q20-Q28.9	745-747.9
Orofacial clefts	Q35-Q37.9	749-749.9
Down syndrome	Q90-Q90.9	758.0
Other chromosomal abnormalities	Q87-Q87.8, Q91-Q93.9, Q95-Q95.9, Q97-Q97.9, Q99-Q99.8	758, 758.1-758.5, 758.8-758.9
Congenital musculoskeletal and limb anomalies	Q65-Q78.9, Q79.6-Q79.9	754-756.5, 756.8-756.9
Urogenital congenital anomalies	P96.0, Q50-Q56.4, Q60-Q64.9	752.0-753.9
Digestive congenital anomalies	Q38-Q45.9, Q79-Q79.5	750-751.9, 756.6-756.7
Other congenital birth defects	G71.2, Q02-Q04.9, Q06-Q07.9, Q10.4-Q18.9, Q30-Q34.9, Q80-Q86, Q86.8, Q89-Q89.8	742, 742.1-744.9, 748-748.9, 752, 757-757.9, 759.0-759.8

List of International Classification of Diseases (ICD) codes mapped to the Global Burden of Disease cause list for causes of death		
**Cause**	**ICD10**	**ICD9**
Urinary diseases and male infertility	N10-N12.9, N13.6, N15, N15.1-N16.8, N20-N23.0, N25-N28.1, N29-N30.3, N30.8-N32.0, N32.3-N32.4, N34-N34.3, N36-N36.9, N39-N39.2, N41-N41.9, N44-N44.0, N45-N45.9, N49-N49.9	588-588.9, 590-590.9, 592-593.8, 594-598.1, 598.8-599.6, 599.8, 601-602.9, 604-604.9, 608.2, 788.0
Urinary tract infections and interstitial nephritis	N10-N12.9, N13.6, N15, N15.1-N16.8, N30-N30.3, N30.8-N30.9, N34-N34.3, N39.0-N39.2	590-590.9, 595-595.9, 597-597.9, 599.0
Urolithiasis	N20-N23.0	592-592.9, 594-594.9, 788.0
Garbage Code (GBD Level 1)	A40-A41.9, A48.0, A48.3, A49.0-A49.1, A59-A59.9, A71-A71.9, A74.0, B07-B07.9, B30-B30.9, B35-B36.9, B85-B85.4, B87-B88.9, B94.0, D50-D50.0, D50.9, D62-D63.0, D63.8-D64, D64.1-D65.9, D68, D69.9, E15-E16, E50-E50.9, E64.1, E85.3-E87.6, E87.8-E87.9, F19-F19.9, G06-G08.0, G32-G32.8, G43-G44.2, G44.4-G44.8, G47-G47.2, G47.4-G47.9, G50-G60.9, G62-G62.0, G62.2-G65.2, G80-G83.9, G89-G89.4, G91-G91.2, G91.4-G93, G93.1-G93.2, G93.4-G93.6, G94.0-G94.8, G99-H05, H05.2-H69.9, H71-H99, I26-I26.9, I31.2-I31.4, I46-I46.9, I50.0-I50.4, I76, I91-I91.1, I95-I95.1, I95.8-I95.9, J69-J69.9, J80-J80.9, J81.0, J85-J85.3, J86-J86.9, J90-J90.0, J93-J93.1, J93.8-J94.9, J96-J96.9, J98.1-J98.3, K00-K19, K30, K65-K66.1, K66.9, K68.1-K68.9, K71-K71.6, K71.8-K72.9, K75.0, L20-L30.9, L40-L50.9, L52-L54.8, L56-L56.2, L56.4-L56.5, L57-L57.9, L59-L68.9, L70-L76.8, L80-L87.9, L90-L92.9, L94-L96, L98.5-L99.8, M04, M10-M12.0, M12.2-M29, M37-M39, M43.2-M49, M49.2-M64, M65.1-M71, M71.2-M72.4, M72.8-M73, M73.8-M79.9, M83-M86.2, M86.5-M86.9, M87.2-M87.9, M89.1-M89.4, M90-M99.9, N17-N17.9, N19-N19.9, N32.1-N32.2, N32.8-N33.8, N35-N35.9, N37-N37.8, N39.3-N39.8, N42-N43.4, N44.1-N44.8, N46-N48.9, N50-N53.9, N61-N64.9, N82-N82.9, N91-N91.5, N95, N95.1-N95.9, N97-N97.9, R02-R02.9, R03.1-R04, R04.1-R04.9, R07.0, R08-R12.0, R14-R19.6, R19.8-R23, R23.1-R30.9, R32-R50.1, R50.8-R57.9, R58.0-R72.9, R74-R78, R78.6-R94.8, R96-R99.9, U05, U08-U49, U51-U81, U90-U99, X40-X44.9, X46-X46.9, X49-X49.9, Y10-	038-038.9, 040.0, 041.1, 076-078.2, 110-111.9, 125-125.3, 126-126.9, 127.3-127.9, 131-132.9, 133.8-134.9, 136.6, 139.1, 139.9, 247-248, 264-264.9, 274-274.9, 276.0-276.9, 277.3, 280-281, 285-285.9, 286.6, 289.1-289.3, 293, 294-294.0, 305, 305.9, 324-327.1, 328-329, 338-339.1, 339.3-339.8, 342-344.9, 346-346.9, 350-353.6, 354-355.9, 360-362, 362.1-376, 376.2-380.9, 384-389.9, 415-415.9, 423.0, 424, 424.4-424.5, 424.9, 427.5, 427.9-428.9, 437.3, 458-458.9, 459.0, 507-507.9, 510-513.9, 518.1-518.3, 520-529.9, 536.3, 536.8-536.9, 537.7, 537.9, 564.8-564.9, 567-568.9, 570-570.9, 572-572.1, 573.1-573.3, 584-584.9, 586-587.9, 603-603.9, 605-608.1, 608.3-609, 611-612.1, 619-619.9, 621, 621.1-621.3, 622-622.0, 622.8-623.6, 623.8-624.5, 624.8-628.9, 629.9, 690-693.9, 695.8-706.9, 708-709.9, 712-713.8, 715-716, 716.2-721.6, 721.8-730.0, 730.2-730.3, 730.8-731.9, 733, 733.2-734.2, 737-738, 738.2-739.9, 780-782.4, 782.6-784.6, 784.9, 785.4-786, 786.6, 786.8, 787, 787.3-788, 788.3-790.1, 790.4-796.1, 796.3-797.9, 798.1-799.9, E850.3, E850.4, E850.5, E850.6, E850.7, E850.8, E850.9, E851-E851.0, E852-E852.0, E852.1, E852.2, E852.3, E852.4, E852.5, E852.8, E852.9, E853-E853.0, E853.1, E853.2, E853.8, E853.9, E854-E854.0, E854.1, E854.2, E854.3, E854.8-E855.0, E855.1, E855.2, E855.3, E855.4, E855.5, E855.6, E855.8, E855.9, E858-E858.0, E858.1, E858.2, E858.3, E858.4, E858.5, E858.6, E858.7, E858.8, E858.9, E866-E866.0, E866.1, E866.2, E866.3, E866.4, E866.5, E866.6, E866.7, E866.8, E866.9, E980-E982.9, V01-V08, V10-uns
Garbage Code (GBD Level 2)	A14.9, A29-A30.9, A45-A45.9, A47-A48, A48.8-A49, A49.3-A49.9, A61-A62, A72-A73, A76, A97, B08-B09, B11-B14, B28-B29, B31-B32.4, B34-B34.1, B34.3-B34.9, B61-B62, B68-B68.9, B73-B74.2, B76-B76.9, B79-B81.8, B84, B92-B94, B94.8-B94.9, B95.6-B97.1, B97.3, B97.7-B99.9, F07.2, F17-F17.9, G44.3, G91.3, G93.0, G93.3, I10-I10.9, I15-I15.9, I27, I27.8-I27.9, I50, I50.8-I50.9, I67.4, I70-I70.1, I70.8-I70.9, I74-I75.8, J81, J81.1, K92.0-K92.2, N70-N71.9, N73-N74, N74.2-N74.8, R03-R03.0, R04.0, R05-R06.9, R13-R13.9, R23.0, R58, S00-T99.9, U50, W47-W48, W63, W71-W72, W82, W95-W97, W98, X07, X55-X56, X59-X59.9, Y20-Y34.9, Y86-Y87, Y87.2, Y89, Y89.9, Y92-Y99.9	000-000.9, 030-030.9, 041.2-041.9, 067-069, 078.8-078.9, 079.8-079.9, 089-089.9, 105-109.9, 119, 136.8-136.9, 139.8, 304, 304.9, 305.1, 339.2, 401-401.9, 405-405.9, 416, 416.2-416.9, 440-440.1, 440.3, 440.8-440.9, 444-445.8, 490-490.9, 494-494.9, 514-514.9, 515.0-515.9, 518-518.0, 518.4-518.5, 518.8, 536.2, 578-578.9, 599.7, 613-614.9, 714.4, 716.1, 721.7, 735-736.9, 738.0-738.1, 784.7-784.8, 786.3, 787.0-787.2, 796.2, 800-979.4, 979.6-E80, E83, E839, E85, E859, E87, E877, E88, E887-E887.0, E928.9, E929, E929.8-E929.9, E983-E985.7, E988-E989
Garbage Code (GBD Level 3)	A01, A31-A31.9, A42-A44.9, A49.2, A64-A64.0, A99-A99.0, B17, B17.1, B17.8-B17.9, B19-B19.0, B19.2-B19.9, B37-B46.9, B49-B49.9, B55, B55.1-B55.9, B58-B59.9, B89, B94.2, C14-C14.9, C22.9, C26-C29, C35-C36, C39-C39.9, C42, C46-C46.9, C55-C55.9, C57.9, C59, C63.9, C68, C68.9, C74-C74.9, C75.9-C80.9, C83, C83.9, C85.1, C85.9, C87, C94.6, C97-D00.0, D01, D01.4-D02, D02.4-D02.9, D07, D07.3, D07.6-D09, D09.1, D09.7, D09.9-D10, D10.9, D13, D13.9-D14, D14.4, D17-D21.9, D28, D28.9-D29, D29.9-D30, D30.9, D36.0, D36.9-D37.0, D37.6-D38, D38.6-D39.0, D39.7, D39.9-D40, D40.9-D41, D41.9, D44, D44.9, D48, D48.7-D49.1, D49.5, D49.7-D49.9, D54, D75.9, D79-D85, D87-D88, D89.8-D99, E07.8-E08.9, E17-E19, E34.0, E34.9-E35.8, E37-E39, E47-E49., E62, E69, E87.7, E90-E998, F04-F07.0, F07.8-F09.9, F20-F50, F50.8-F99.0, G09-G09.9, G15-G19, G21, G21.2, G21.4-G22.0, G27-G29, G33-G34, G38-G39., G42, G48-G49, G66-G69, G74-G79, G84-G88, G93.8-G94, G96-G96.9, G98-G98.9, I00.0, I03-I04., I14-I14., I16-I19, I29-I29.9, I44-I45.9, I49-I49.9, I51, I51.6-I59, I90, I91.9-I94, I96-I96.9, I98.4-I98.8, I99-ID5.9, J02.9, J03.9, J04.3, J06, J06.9, J40-J40.9, J47-J59, J65-J65.0, J71-J79, J81.9, J83, J85.9, J87-J89, J90.9, J93.6, J97-J98.0, J98.4-J99.8, K21-K21.9, K22.7, K31.9-K34, K39, K47-K49, K53-K54, K63-K63.4, K63.8-K63.9, K69, K70.4-K70.9, K78-K79, K84, K88-K89, K92, K92.9-K93, K96-K99, L06-L07, L09, L15-L19, L31-L39, L69, L77-L79, N09, N13-N13.5, N13.7-N13.9, N24, N28.8-N28.9, N38, N39.9-N40.9, N54-N59, N66-N69, N78-N79, N84, N84.2-N86, N88-N90.9, N92-N94.9, N95.0, O08-O08.9, O17-O19, O27, O37-O39, O49-O59, O78-O79, O93-O95.9, P06, P09, P16-P19.9, P30-P34.2, P40-P49, P62-P69, P73-P75.0, P79-P82, P85-P89, P92-P92.9, P96.9-P99.9, Q08-Q10.3, Q19, Q29-Q29., Q46-Q49, Q57, Q88, Q89.9, Q94, Q99.9-R01.2, R07, R07.1-R07.9, R31-R31.9,	002, 031-031.9, 039-039.9, 070, 070.4-070.9, 085, 085.1-085.9, 088.0-088.7, 112-118.9, 130-130.9, 136.3-136.5, 149-149.9, 155.2, 159-159.9, 165-169, 176-179.9, 183.9-184, 184.5, 184.9, 187, 187.9, 189, 189.9, 190.9, 195-199.9, 202.9, 209, 209.2-209.3, 209.6-210, 211, 211.9-212, 212.9, 214-216.9, 221, 221.9-222, 222.9-223, 223.9, 229, 229.1, 229.9-230.0, 230.9-231, 231.8-231.9, 233, 233.3, 233.6, 233.9-234, 234.9-235, 235.1-235.3, 235.5, 235.9-236, 236.3, 236.6, 236.9, 239-239.1, 239.5, 239.7-239.9, 249-249.9, 259.2, 278, 279-279.9, 293.0-293.9, 295-302.9, 306-307.0, 307.2-307.4, 307.6-319.9, 331.3-331.4, 332.1-332.9, 347-348.9, 349.9, 357, 357.8-357.9, 399-400.0, 406-409.4, 418-419.9, 426-427, 427.4, 429, 429.2-429.9, 459.5-459.9, 464.5, 465, 465.9, 505-505.9, 519, 519.8-519.9, 530.1, 530.7-530.9, 544-549, 559-559.0, 560.4-560.7, 561, 562.2-563, 569, 569.8-569.9, 591-591.9, 593.9, 599.9-600.9, 623.7, 624.6, 637-637.9, 639-639.9, 759, 759.9, 775, 775.4-775.9, 779.3, 779.7-779.9, 782.5, 785-785.3, 786.0-786.2, 786.4-786.5, 786.7, 786.9, 788.1-788.2, E950, E950.9, E958, E958.9-E959, E986-E987.9
Other urinary diseases	N25-N28.1, N29-N29.8, N31-N32.0, N32.3-N32.4, N36-N36.9, N39, N41-N41.9, N44-N44.0, N45-N45.9, N49-N49.9	588-588.9, 593-593.8, 596-596.9, 598-598.1, 598.8-599, 599.1-599.6, 599.8, 601-602.9, 604-604.9, 608.2
Garbage Code (GBD Level 4)	B16.9, B54-B54.0, B64, B82-B82.9, B83.9, C69, C69.9, C91.1, C91.4-C91.5, C91.7-C91.9, C92.7-C92.9, C93.2, C93.5-C93.7, C93.9, E12-E14.9, G00, G00.9-G02.1, G03.9, I37.9, I42-I42.0, I42.9, I51.5, I64-I64.9, I67, I67.8-I68, I68.8-I69, I69.4-I69.9, J07-J08, J15.9, J17-J19.6, J22-J29, J64-J64.9, P23, P23.5-P23.9, R73-R73.9, V87-V87.1, V87.4-V88.1, V88.4-V89.9, V99-V99.0, X84-X84.9, Y09-Y09.9, Y85-Y85.9	070.3, 194-194.0, 194.9, 204.1, 204.5-204.9, 205.8-205.9, 206.2-206.9, 237, 237.2, 237.4, 238, 244, 244.9, 250-250.9, 289.8-289.9, 307.5, 320, 320.9, 357.2, 362.0, 425, 425.9, 429.1, 436-437, 437.9-439.6, 482.9-483, 484, 484.8-486.9, 790.2, E800.8-E800.9, E801.8-E801.9, E802.8-E802.9, E803.8-E803.9, E804.8-E804.9, E805.8-E805.9, E806.8-E806.9, E807.8-E810, E810.8-E811, E811.8-E812, E812.8-E813, E813.8-E814, E814.8-E815, E815.8-E816, E816.8-E817, E817.8-E818, E818.8-E819, E819.8-E820, E820.8-E821, E821.8-E822, E822.8-E823, E823.8-E824, E824.8-E825, E825.8-E826, E826.8-E827, E827.8-E828, E828.8-E829, E829.8-E829.9,
Gynecological diseases	D25-D26, D28.2, E28.2, N60-N60.9, N72-N72.0, N75-N77.8, N80-N81.9, N83-N83.9, N84.0-N84.1, N87-N87.9	218-219, 219.1-219.9, 236.0, 256.4, 615-618.9, 620-620.9, 621.0, 621.4-621.9, 622.1-622.6, 629-629.8
Uterine fibroids	D25-D26, D28.2	218-219, 219.1-219.9, 236.0

List of International Classification of Diseases (ICD) codes mapped to the Global Burden of Disease cause list for causes of death		
**Cause**	**ICD10**	**ICD9**
Endometriosis	N80-N80.9	617-617.9
Genital prolapse	N81-N81.9	618-618.9
Other gynecological diseases	N60-N60.9, N72-N72.0, N75-N77.8, N83-N83.9, N84.0-N84.1, N87-N87.9	615-616.9, 620-620.9, 621.0, 621.4-621.9, 622.1-622.6, 629-629.8
Hemoglobinopathies and hemolytic anemias	D55-D59, D59.1, D59.3-D59.5, D59.8-D61.9, D64.0	282-284.9
Thalassemias	D56-D56.9	282.4
Sickle cell disorders	D57-D57.2, D57.4-D57.8	282.6
G6PD deficiency	D55-D55.2	282.2-282.3
Other hemoglobinopathies and hemolytic anemias	D55.3-D55.9, D58-D59, D59.1, D59.3-D59.5, D59.8-D61.9, D64.0	282-282.1, 282.7-284.9
Endocrine, metabolic, blood, and immune disorders	D66-D67, D68.0-D69.4, D69.6-D69.8, D70-D70.0, D70.4-D75.8, D76-D77, D86.8, D89-D89.2, E03-E03.1, E03.3-E06.3, E06.5-E07.1, E16.1-E16.9, E20-E23.0, E23.2-E24.1, E24.3, E24.8-E27.2, E27.4-E28.1, E28.3-E34, E34.1-E34.8, E65-E66.0, E66.2-E68, E70-E85.2, E88-E88.2, E88.4-E88.9, P72.1	240-243.9, 245-246.9, 251-251.2, 251.4-253.6, 253.8-256.3, 256.8-259.1, 259.3-259.9, 270-273.9, 275-276, 277-277.2, 277.4-277.9, 278.0-278.8, 286-286.5, 286.7-289.0, 289.4-289.7, 775.3
Thyroid diseases	E03-E03.1, E03.3-E06.3, E06.5-E07, E07.1, P72.1	240-243.9, 245-246.9, 775.3
Other endocrine, metabolic, blood, and immune disorders	D66-D67, D68.0-D69.4, D69.6-D69.8, D70-D70.0, D70.4-D75.8, D76-D77, D86.8, D89-D89.2, E07.0, E16.1-E16.9, E20-E23.0, E23.2-E24.1, E24.3, E24.8-E27.2, E27.4-E28.1, E28.3-E34, E34.1-E34.8, E65-E66.0, E66.2-E68, E70-E85.2, E88-E88.2, E88.4-E88.9	251-251.2, 251.4-253.6, 253.8-256.3, 256.8-259.1, 259.3-259.9, 270-273.9, 275-276, 277-277.2, 277.4-277.9, 278.0-278.8, 286-286.5, 286.7-289.0, 289.4-289.7
Sudden infant death syndrome	R95-R95.9	798-798.0
Injuries	D52.1, D59.0, D59.2, D59.6, D69.5, D70.1-D70.2, D78-D78.8, E03.2, E06.4, E09-E09.9, E16.0, E23.1, E24.2, E27.3, E36-E36.8, E66.1, E88.3, E89-E89.9, G21.0-G21.1, G24.0, G25.1, G25.4, G25.6-G25.7, G72.0, G93.7, G97-G97.9, I95.2-I95.3, I97-I97.9, I98.9, J70.0-J70.5, J95-J95.9, K43-K43.9, K52.0, K62.7, K91-K91.9, K94-K95.8, L55-L55.9, L56.3, L56.8-L56.9, L58-L58.9, M87.1, N14-N14.4, N30.4, N65-N65.1, N99-N99.9, P04.0-P04.1, P70.3, P93-P93.8, P96.2, P96.5, Q86.1-Q86.2, R50.2, U00-U03, V00-V86.9, V87.2-V87.3, V88.2-V88.3, V90-V98.8, W00-W46.2, W49-W62.9, W64-W70.9, W73-W81.9, W83-W94.9, W97.9, W99-X06.9, X08-X39.9, X47-X48.9, X50-X54.9, X57-X58.9, X60-X63.9, X66-X68.9, X70-X83.9, X85-Y08.9, Y35-Y84.9, Y87.0-Y87.1, Y88-Y88.3, Y89.0-Y89.1	244.0-244.1, 244.3-244.8, 251.3, 253.7, 349.0-349.1, 357.6, 457.0, 518.7, 519.0, 536.4, 539-539.9, 551.2, 552.2, 553.2, 558.1, 564.2-564.4, 569.6, 579.3, 598.2, 763.5, 779.4-779.5, E800-E800.3, E801-E801.3, E802-E802.3, E803-E803.3, E804-E804.3, E805-E805.3, E806-E806.3, E807-E807.3, E810.0-E810.7, E811.0-E811.7, E812.0-E812.7, E813.0-E813.7, E814.0-E814.7, E815.0-E815.7, E816.0-E816.7, E817.0-E817.7, E818.0-E818.7, E819.0-E819.7, E820.0-E820.7, E821.0-E821.7, E822.0-E822.7, E823.0-E823.7, E824.0-E824.7, E825.0-E825.7, E826.0-E826.4, E827.0-E827.4, E828.0-E828.4, E829.0-E829.4, E830-E838.9, E840-E849.9, E856-E857.09, E860.2-E865.99, E867-E869.99, E870-E876.9, E878-E879.9, E880-E886.99, E888-E928.89, E929.1-E929.5, E930-E949.9, E950.0-E950.8, E951-E957.9, E958.0-E958.8, E960-E979.9, E990-E999.1
Transport injuries	V00-V86.9, V87.2-V87.3, V88.2-V88.3, V90-V98.8	E800-E800.3, E801-E801.3, E802-E802.3, E803-E803.3, E804-E804.3, E805-E805.3, E806-E806.3, E807-E807.3, E810.0-E810.7, E811.0-E811.7, E812.0-E812.7, E813.0-E813.7, E814.0-E814.7, E815.0-E815.7, E816.0-E816.7, E817.0-E817.7, E818.0-E818.7, E819.0-E819.7, E820.0-E820.7, E821.0-E821.7, E822.0-E822.7, E823.0-E823.7, E824.0-E824.7, E825.0-E825.7, E826.0-E826.4, E827.0-E827.4, E828.0-E828.4, E829.0-E829.4, E830-E838.9, E840-
Road injuries	V01-V04.9, V06-V80.9, V82-V82.9, V87.2-V87.3	E800.3, E801.3, E802.3, E803.3, E804.3, E805.3, E806.3, E807.3, E810.0-E810.7, E811.0-E811.7, E812.0-E812.7, E813.0-E813.7, E814.0-E814.7, E815.0-E815.7, E816.0-E816.7, E817.0-E817.7, E818.0-E818.7, E819.0-E819.7, E820.0-E820.6, E821.0-E821.6, E822.0-E822.7, E823.0-E823.7, E824.0-E824.7, E825.0-E825.7, E826.0-E826.1, E826.3-E826.4, E827.0, E827.3-E827.4, E828.0,
Pedestrian road injuries	V01-V04.9, V06-V09.9	E810.7, E811.7, E812.7, E813.7, E814.7, E815.7, E816.7, E817.7, E818.7, E819.7, E822.7, E823.7, E824.7, E825.7, E826.0, E827.0, E828.0, E829.0
Cyclist road injuries	V10-V19.9	E800.3, E801.3, E802.3, E803.3, E804.3, E805.3, E806.3, E807.3, E810.6, E811.6, E812.6, E813.6, E814.6, E815.6, E816.6, E817.6, E818.6, E819.6, E820.6, E821.6, E822.6, E823.6, E824.6, E825.6, E826.1
Motorcyclist road injuries	V20-V29.9	E810.2-E810.3, E811.2-E811.3, E812.2-E812.3, E813.2-E813.3, E814.2-E814.3, E815.2-E815.3, E816.2-E816.3, E817.2-E817.3, E818.2-E818.3, E819.2-E819.3, E820.2-E820.3, E821.2-E821.3, E822.2-E822.3, E823.2-E823.3, E824.2-E824.3, E825.2-E825.3
Motor vehicle road injuries	V30-V79.9, V87.2-V87.3	E810.0-E810.1, E811.0-E811.1, E812.0-E812.1, E813.0-E813.1, E814.0-E814.1, E815.0-E815.1, E816.0-E816.1, E817.0-E817.1, E818.0-E818.1, E819.0-E819.1, E820.0-E820.1, E821.0-E821.1, E822.0-E822.1, E823.0-E823.1, E824.0-E824.1, E825.0-E825.1
Other road injuries	V80-V80.9, V82-V82.9	E810.4-E810.5, E811.4-E811.5, E812.4-E812.5, E813.4-E813.5, E814.4-E814.5, E815.4-E815.5, E816.4-E816.5, E817.4-E817.5, E818.4-E818.5, E819.4-E819.5, E820.4-E820.5, E821.4-E821.5, E822.4-E822.5, E823.4-E823.5, E824.4-E824.5, E825.4-E825.5, E826.3-E826.4, E827.3-E827.4, E828.4, E829.4
Other transport injuries	V00-V00.8, V05-V05.9, V81-V81.9, V83-V86.9, V88.2-V88.3, V90-V98.8	E800-E800.2, E801-E801.2, E802-E802.2, E803-E803.2, E804-E804.2, E805-E805.2, E806-E806.2, E807-E807.2, E820.7, E821.7, E826.2, E827.2, E828.2, E830-E838.9, E840-E849.9, E929.1
Unintentional injuries	D52.1, D59.0, D59.2, D59.6, D69.5, D70.1-D70.2, D78-D78.8, E03.2, E06.4, E09-E09.9, E16.0, E23.1, E24.2, E27.3, E36-E36.8, E66.1, E88.3, E89-E89.9, G21.0-G21.1, G24.0, G25.1, G25.4, G25.6-G25.7, G72.0, G93.7, G97-G97.9, I95.2-I95.3, I97-I97.9, I98.9, J70.0-J70.5, J95-J95.9, K43-K43.9, K52.0, K62.7, K91-K91.9, K94-K95.8, L55-L55.9, L56.3, L56.8-L56.9, L58-L58.9, M87.1, N14-N14.4, N30.4, N65-N65.1, N99-N99.9, P04.0-P04.1, P70.3, P93-P93.8, P96.2, P96.5, Q86.1-Q86.2, R50.2, W00-W46.2, W49-W62.9, W64-W70.9, W73-W81.9, W83-W94.9, W97.9, W99-X06.9, X08-X39.9, X47-X48.9, X50-X54.9, X57-X58.9, Y40-Y84.9, Y88-Y88.3	244.0-244.1, 244.3-244.8, 251.3, 253.7, 349.0-349.1, 357.6, 457.0, 518.7, 519.0, 536.4, 539-539.9, 551.2, 552.2, 553.2, 558.1, 564.2-564.4, 569.6, 579.3, 598.2, 763.5, 779.4-779.5, E856-E857.09, E860.2-E865.99, E867-E869.99, E870-E876.9, E878-E879.9, E880-E886.99, E888-E928.89, E929.2-E929.5, E930-E949.9
Falls	W00-W19.9	E880-E886.99, E888-E888.9, E929.3
Drowning	W65-W70.9, W73-W74.9	E910-E910.99

List of International Classification of Diseases (ICD) codes mapped to the Global Burden of Disease cause list for causes of death		
**Cause**	**ICD10**	**ICD9**
Fire, heat, and hot substances	W92-W92.9, X00-X06.9, X08-X19.9	E890-E899.09, E924-E924.99, E929.4
Poisonings	X47-X48.9	E856-E857.09, E860.2-E865.99, E867-E869.99, E929.2
Poisoning by carbon monoxide	X47-X47.9	E868-E869
Poisoning by other means	X48-X48.9	E856-E857.09, E860.2-E865.99, E867-E867.09, E869.0-E869.99, E929.2
Exposure to mechanical forces	W20-W38.9, W40-W43.9, W45.0-W45.2, W46-W46.2, W49-W52	E916-E922.99, E928.1-E928.7
Unintentional firearm injuries	W32-W34.9	E922-E922.99, E928.7
Other exposure to mechanical forces	W20-W31.9, W35-W38.9, W40-W43.9, W45.0-W45.2, W46-W46.2, W49-W52	E916-E921.99, E928.1-E928.6
Adverse effects of medical treatment	D52.1, D59.0, D59.2, D59.6, D69.5, D70.1-D70.2, D78-D78.8, E03.2, E06.4, E09-E09.9, E16.0, E23.1, E24.2, E27.3, E36-E36.8, E66.1, E88.3, E89-E89.9, G21.0-G21.1, G24.0, G25.1, G25.4, G25.6-G25.7, G72.0, G93.7, G97-G97.9, I95.2-I95.3, I97-I97.9, I98.9, J70.0-J70.5, J95-J95.9, K43-K43.9, K52.0, K62.7, K91-K91.9, K94-K95.8, M87.1, N14-N14.4, N30.4, N65-N65.1, N99-N99.9, P04.0-P04.1, P70.3, P93-P93.8, P96.2, P96.5, Q86.1-Q86.2, R50.2, Y40-Y84.9, Y88-Y88.3	244.0-244.1, 244.3-244.8, 251.3, 253.7, 349.0-349.1, 357.6, 457.0, 518.7, 519.0, 536.4, 539-539.9, 551.2, 552.2, 553.2, 558.1, 564.2-564.4, 569.6, 579.3, 598.2, 763.5, 779.4-779.5, E870-E876.9, E878-E879.9, E930-E949.9
Animal contact	W52.0-W62.9, W64-W64.9, X20-X29.9	E905-E906.99
Venomous animal contact	X20-X29.9	E905-E905.99
Non-venomous animal contact	W52.0-W62.9, W64-W64.9	E906-E906.99
Foreign body	W44-W45, W45.3-W45.9, W75-W76.9, W78-W80.9, W83-W84.9	E911-E913.19, E913.8-E915.09
Pulmonary aspiration and foreign body in airway	W75-W76.9, W78-W80.9, W83-W84.9	E911-E913.19, E913.8-E913.99
Foreign body in other body part	W44-W45, W45.3-W45.9	E914-E915.09
Electrocution	W85-W87.9	E925-E925.99
Environmental heat and cold exposure	L55-L55.9, L56.3, L56.8-L56.9, L58-L58.9, W88-W91.9, W93.2-W94.9, W97.9, W99-W99.9, X30-X32.9, X39-X39.9	E900-E902.99, E926-E926.99, E929.5
Exposure to forces of nature	X33-X38.9	E907-E909.9
Other unintentional injuries	W39-W39.9, W77-W77.9, W81-W81.9, W93-W93.1, X50-X54.9, X57-X58.9	E903-E904.99, E913.2-E913.39, E923-E923.99, E927-E928.09, E928.8-E928.89
Self-harm and interpersonal violence	U00-U03, X60-X63.9, X66-X68.9, X70-X83.9, X85-Y08.9, Y35-Y38.9, Y87.0-Y87.1, Y89.0-Y89.1	E950.0-E950.8, E951-E957.9, E958.0-E958.8, E960-E979.9, E990-E999.1
Self-harm	X60-X63.9, X66-X68.9, X70-X83.9, Y87.0	E950.0-E950.8, E951-E957.9, E958.0-E958.8
Self-harm by hanging, strangulation, and suffocation	X70-X70.9	E953-E953.9
Self-harm by fire, heat, and hot substances	X76-X77.9	E958.1
Self-harm by firearm	X72-X74.9	E955-E955.9
Self-harm by poisoning pesticides	X68-X68.9	E950.6
Self-harm by non pesticide substance and gas	X60-X63.9, X66-X67.9	E950.0-E950.5, E950.7-E950.8, E951-E952.9
Self-harm by other specified means (internal)	X71-X71.9, X75-X75.9, X78-X83.9, Y87.0	E954, E956-E957.9, E958.0, E958.2-E958.8
Interpersonal violence	X85-Y08.9, Y87.1	E960-E969
Physical violence by firearm	X93-X95.9	E965-E965.4
Physical violence by sharp object	X99-X99.9	E966
Physical violence by other means	X85-X92.9, X96-X98.9, Y00-Y04.9, Y06-Y08.9, Y87.1	E961-E964, E965.5-E965.9, E967-E969
Conflict and terrorism	U00-U03, Y36-Y38.9, Y89.1	E979-E979.9, E990-E999.1
Police conflict and executions	Y35-Y35.9, Y89.0	E970-E978
Still Born	P95-P95.9, P96.4	768.0-768.1, 779.6

Appendix Table S3: Total number of site years by cause and source type for GBD2023

Cause	Level	Vital Registration	Vital Registration - Sample	Verbal Autopsy	Surveillance	Survey/Census	Sibling History	MITS diagnosed	Cancer Registry	Police or Forensic Medicine Report
All causes	0	21174	953	3629	4815	1597	7137	10	2631	1616
Communicable, maternal, neonatal, and nutritional diseases	1	21174	953	3564	3399	1570	7137	10		
HIV/AIDS and sexually transmitted infections	2	20251	953	931	416			9		
HIV/AIDS	3	20251	953	913	416			9		
HIV/AIDS - Drug-susceptible Tuberculosis	4	11703	576		11			1		
HIV/AIDS - Multidrug-resistant Tuberculosis without extensive drug resistance	4	11699	576							
HIV/AIDS - Extensively drug-resistant Tuberculosis	4	11699	576							
HIV/AIDS resulting in other diseases	4	16515	576		58			9		
Sexually transmitted infections excluding HIV	3	19742	953	386	352			6		
Syphilis	4	19239	953		352			6		
Chlamydial infection	4	17455	953							
Gonococcal infection	4	18920	953							
Other sexually transmitted infections	4	18674	953							
Respiratory infections and tuberculosis	2	20261	953	2217	681			10		
Tuberculosis	3	20261	953	2140	676			9		
Drug-susceptible tuberculosis	4	12272	576		675			9		
Multidrug-resistant tuberculosis without extensive drug [illegible]	4	11699	576							
Lower respiratory infections	3	20261	953	2177	681			10		
Influenza	4	16067	576					3		
Pneumococcal pneumonia	4	16060	576					7		
H influenzae type B pneumonia	4	16060	576					1		
Respiratory syncytial virus pneumonia	4	16060	576					5		
Other lower respiratory infections	4	16060	576					10		
Upper respiratory infections	3	17473	953					2		
Otitis media	3	17970	953							
COVID-19	3	1324	64	63				1		
Enteric infections	2	20261	953	2176	575			10		
Diarrheal diseases	3	20261	953	2171	575			10		
Typhoid and paratyphoid	3	20217	953	434						
Typhoid fever	4	17382	576							
Paratyphoid fever	4	17304	576							
Invasive Non-typhoidal Salmonella (iNTS)	3	17130	953					2		
Other intestinal infectious diseases	3	17127	576							
Neglected tropical diseases and malaria	2	20265	953	1891	384			7		
Malaria	3	20168	953	1800	1			7		
Chagas disease	3	3576								
Leishmaniasis	3	18889	953	187						
Visceral leishmaniasis	4	18398	953	187						
African trypanosomiasis	3	16640	576							
Schistosomiasis	3	17483	576							
Cysticercosis	3	17578	953							
Cystic echinococcosis	3	18038	953							
Dengue	3	19285	953	618	1					
Yellow fever	3	16963	576	150						
Rabies	3	19913	953	1202	352			3		
Intestinal nematode infections	3	18630	953	338				2		
Ascariasis	4	17156	953					2		
Ebola	3	494	72		30					
Zika virus	3	11333	953							
Other neglected tropical diseases	3	17528	953					1		
Other infectious diseases	2	20261	953	2093	1011			10		
Meningitis	3	19747	953	1846	611			9		
Encephalitis	3	19455	953	687				5		
Diphtheria	3	19712	953							
Pertussis	3	19752	953	589				5		
Tetanus	3	19754	953	1389	396					
Measles	3	19748	953	1483	585			3		
Varicella and herpes zoster	3	18620	953	575				1		
Acute hepatitis	3	19136	953	1453						
Acute hepatitis A	4	16519	576							
Acute hepatitis B	4	16519	576							
Acute hepatitis C	4	11699	576							
Acute hepatitis E	4	16512	576							
Other unspecified infectious diseases	3	20257	953	1451	989			10		
Maternal and neonatal disorders	2	21167	953	3350	3017	1570	7137	10		
Maternal disorders	3	21165	953	2834	2335	1569	7137			
Maternal hemorrhage	4	18685	953	1150	990	23				
Maternal sepsis and other maternal infections	4	18684	953	856	875	19				
Maternal hypertensive disorders	4	18692	953	1021	980	21				
Maternal obstructed labor and uterine rupture	4	18685	953	878	849	12				
Maternal abortion and miscarriage	4	18704	953	378	849	15				
Ectopic pregnancy	4	18084	953		829	2				
Indirect maternal deaths	4	18685	953	944	1051	20				
Late maternal deaths	4	13027	953		152					
Maternal deaths aggravated by HIV/AIDS	4	21165	953	2834	2335	1569	7137			
Other direct maternal disorders	4	18685	953	349	930	16				
Neonatal disorders	3	19704	951	2055	682	1		10		
Neonatal preterm birth	4	18082	951	963	681			10		
Neonatal encephalopathy due to birth asphyxia and trauma	4	18083	951	959	681			10		
Neonatal sepsis and other neonatal infections	4	18070	951		634			10		
Hemolytic disease and other neonatal jaundice	4	18066	951					8		
Other neonatal disorders	4	18069	951	427	664			7		
Nutritional deficiencies	2	19407	953	1723				10		
Protein-energy malnutrition	3	18616	953	1713				10		
Dietary iron deficiency	3			741						
Other nutritional deficiencies	3	18073	953							
Non-communicable diseases	1	19716	953	2181	756			10	2631	64
Neoplasms	2	19696	953	1795	723			7	2631	
Lip and oral cavity cancer	3	18901	953	404					1408	
Nasopharynx cancer	3	18901	953						1322	
Other pharynx cancer	3	18901	953	186					1206	
Esophageal cancer	3	19187	953	508					1219	
Stomach cancer	3	19189	953	187					1204	
Colon and rectum cancer	3	19189	953	486					1206	
Liver cancer	3	18902	953	187				2	1737	
Liver cancer due to hepatitis B	4	16060	576							
Liver cancer due to hepatitis C	4	16060	576							
Liver cancer due to alcohol use	4	16060	576							
Liver cancer due to NASH	4	16060	576							

Total number of site years by cause and source type for GBD2023										
Cause	Level	Vital Registration	Vital Registration - Sample	Verbal Autopsy	Surveillance	Survey/Census	Sibling History	MITS diagnosed	Cancer Registry	Police or Forensic Medicine Report
Hepatoblastoma	4	18889	952	131				2	699	
Liver cancer due to other causes	4	16060	576							
Gallbladder and biliary tract cancer	3	18302	953						1265	
Pancreatic cancer	3	18902	953						1231	
Larynx cancer	3	19187	953	183					1194	
Tracheal, bronchus, and lung cancer	3	19691	953	501					1204	
Malignant skin melanoma	3	18901	953						1213	
Non-melanoma skin cancer	3	18901	953							
Non-melanoma skin cancer (squamous-cell carcinoma)	4	18303	953							
Soft tissue and other extraosseous sarcomas	3	16060	576					3	1815	
Malignant neoplasm of bone and articular cartilage	3	17676	953						1664	
Breast cancer	3	19691	953	525	1				1453	
Cervical cancer	3	19189	953	186					1216	
Uterine cancer	3	19188	953	183					1286	
Ovarian cancer	3	18901	953						1251	
Prostate cancer	3	19188	953						1185	
Testicular cancer	3	18133	953						1273	
Kidney cancer	3	18901	953					1	1383	
Bladder cancer	3	18901	953						1202	
Brain and central nervous system cancer	3	18902	953	184					1392	
Eye cancer	3	16591	576						1475	
Retinoblastoma	4	16501	576						1362	
Other eye cancers	4	16512	576						1367	
Neuroblastoma and other peripheral nervous cell tumors	3	16512	576						1437	
Thyroid cancer	3	18900	953						1374	
Mesothelioma	3	12575	576						1323	
Hodgkin lymphoma	3	18900	953						1415	
Non-Hodgkin lymphoma	3	18902	953						1809	
Burkitt lymphoma	4	16512	576						1799	
Other non-Hodgkin lymphoma	4	16512	576						1793	
Multiple myeloma	3	18282	953						1305	
Leukemia	3	19690	953	187	650			5	1882	
Acute lymphoid leukemia	4	15734	576					3	1249	
Chronic lymphoid leukemia	4	15511	576						1172	
Acute myeloid leukemia	4	15730	576					4	1250	
Chronic myeloid leukemia	4	15686	576						1233	
Other leukemia	4	15690	576						1099	
Other malignant neoplasms	3	18902	953		72			2	1455	
Other neoplasms	3	16178	576						163	
Myelodysplastic, myeloproliferative, and other [illegible]	4	16060	576							
Low-grade brain and other central nervous system [illegible]	4	16057	576						163	
Other benign and in situ neoplasms	4	4363								
Cardiovascular diseases	2	19710	953	1897	1			4		
Rheumatic heart disease	3	18904	953	187						
Ischemic heart disease	3	19203	953	1600						
Stroke	3	19705	953	1431	1					
Ischemic stroke	4	17395	953							
Intracerebral hemorrhage	4	17388	953							
Subarachnoid hemorrhage	4	17388	953							
Hypertensive heart disease	3	18264	953							
Non-rheumatic valvular heart disease	3	17558	953							
Non-rheumatic calcific aortic valve disease	4	16936	576							
Non-rheumatic degenerative mitral valve disease	4	16936	576							
Other non-rheumatic valve diseases	4	16827	576							
Cardiomyopathy and myocarditis	3	18265	953					4		
Myocarditis	4	17106	576					4		
Alcoholic cardiomyopathy	4	17106	576							
Other cardiomyopathy	4	17106	576					2		
Pulmonary Arterial Hypertension	3	16060	576							
Atrial fibrillation and flutter	3	16591	576							
Aortic aneurysm	3	17475	576							
Lower extremity peripheral arterial disease	3	16039	576							
Endocarditis	3	17468	953							
Other cardiovascular and circulatory diseases	3	18264	953							
Chronic respiratory diseases	2	18804	953	1739				6		
Chronic obstructive pulmonary disease	3	17474	953							
Pneumoconiosis	3	17269	576							
Silicosis	4	17146	576							
Asbestosis	4	17146	576							
Coal workers pneumoconiosis	4	17137	576							
Other pneumoconiosis	4	17129	576							
Asthma	3	17467	576					3		
Interstitial lung disease and pulmonary sarcoidosis	3	17467	576					1		
Other chronic respiratory diseases	3	17474	953					4		
Digestive diseases	2	19191	953	1746				8		
Cirrhosis and other chronic liver diseases	3	19191	953	1614				6		
Chronic hepatitis B including cirrhosis	4	16060	576							
Chronic hepatitis C including cirrhosis	4	16060	576							
Cirrhosis due to alcohol	4	16060	576							
Nonalcoholic fatty liver disease including cirrhosis	4	16060	576							
Cirrhosis due to other causes	4	16060	576							
Upper digestive system diseases	3	19190	953	625						
Peptic ulcer disease	4	17797	576							
Gastritis and duodenitis	4	17786	576							
Appendicitis	3	19189	953	438						
Paralytic ileus and intestinal obstruction	3	18902	953	657				4		
Inguinal, femoral, and abdominal hernia	3	18684	576	708						
Inflammatory bowel disease	3	17474	576							
Ulcerative colitis	4	16060	576							
Crohn's disease	4	16060	576							
Vascular intestinal disorders	3	17388	576							
Gallbladder and biliary diseases	3	18977	953							
Pancreatitis	3	17793	576							
Diverticular disease of intestines	3	16136	576							
Other digestive diseases	3	18862	953							
Neurological disorders	2	19188	953	1274				3		
Alzheimer's disease and other dementias	3	18900	953							
Parkinson's disease	3	18093	953							
Idiopathic epilepsy	3	18979	953	1227				3		
Multiple sclerosis	3	18309	894							

Total number of site years by cause and source type for GBD2023										
Cause	Level	Vital Registration	Vital Registration - Sample	Verbal Autopsy	Surveillance	Survey/Census	Sibling History	MITS diagnosed	Cancer Registry	Police or Forensic Medicine Report
Motor neuron disease	3	17208	953					2		
Other neurological disorders	3	18302	953							
Mental disorders	2	15733	504	10						
Eating disorders	3	15733	504							
Anorexia nervosa	4	14673	504							
Substance use disorders	2	19141	953	618						64
Alcohol use disorders	3	19141	953	599						
Drug use disorders	3	19136	953	539						64
Opioid use disorders	4	16930	576							
Cocaine use disorders	4	16927	576							
Amphetamine use disorders	4	16927	576							
Other drug use disorders	4	16932	576							
Diabetes and kidney diseases	2	19693	953	1841				2		
Diabetes mellitus	3	19191	953	1629				2		
Diabetes mellitus type 1	4	17419	576					2		
Diabetes mellitus type 2	4	17419	576							
Chronic kidney disease	3	19407	953	1485						
Chronic kidney disease due to diabetes mellitus type 1	4	11699	576							
Chronic kidney disease due to diabetes mellitus type 2	4	11699	576							
Chronic kidney disease due to hypertension	4	16060	576							
Chronic kidney disease due to glomerulonephritis	4	16060	576							
Chronic kidney disease due to other and unspecified [illegible]	4	16060	576							
Acute glomerulonephritis	3	17465	953							
Skin and subcutaneous diseases	2	19179	953	397				6		
Bacterial skin diseases	3	19179	953	373				6		
Cellulitis	4	16055	576					1		
Pyoderma	4	16060	576					6		
Decubitus ulcer	3	17503	953	194						
Other skin and subcutaneous diseases	3	16586	953							
Musculoskeletal disorders	2	18303	953	562						
Rheumatoid arthritis	3	17383	953							
Other musculoskeletal disorders	3	17475	953							
Other non-communicable diseases	2	19716	953	1828	683			10		
Congenital birth defects	3	19716	953	1743	683			10		
Neural tube defects	4	16146	953		590			10		
Congenital heart anomalies	4	16765	953		680			10		
Orofacial clefts	4	15904	576					4		
Down syndrome	4	16067	953		593			6		
Other chromosomal abnormalities	4	16058	576					10		
Congenital musculoskeletal and limb anomalies	4	16058	576					8		
Urogenital congenital anomalies	4	16058	576		500			7		
Digestive congenital anomalies	4	16060	576					8		
Other congenital birth defects	4	19389	953					10		
Urinary diseases and male infertility	3	19187	953	672						
Urinary tract infections and interstitial nephritis	4	18567	953	172						
Urolithiasis	4	17464	953	150						
Other urinary diseases	4	16591	953							
Gynecological diseases	3	18900	953	918						
Uterine fibroids	4	16512	576	102						
Endometriosis	4	15858	576							
Genital prolapse	4	16188	576							
Other gynecological diseases	4	16682	576							
Hemoglobinopathies and hemolytic anemias	3	19186	953	1275				4		
Thalassemias	4	17793	953	158						
Sickle cell disorders	4	17474	953	357				3		
G6PD deficiency	4	14930	504							
Other hemoglobinopathies and hemolytic anemias	4	17474	953					1		
Endocrine, metabolic, blood, and immune disorders	3	19188	953	988				5		
Thyroid diseases	4	16067	576							
Other endocrine, metabolic, blood, and immune disorders	4	16067	576					5		
Sudden infant death syndrome	3	13304	36							
Injuries	1	19693	953	2037	2094	33		8		1616
Transport injuries	2	19094	953	1252		12		2		60
Road injuries	3	18553	953	188		12		2		60
Pedestrian road injuries	4	16591	576	188				2		55
Cyclist road injuries	4	16590	576	185						33
Motorcyclist road injuries	4	16591	576	187		2				35
Motor vehicle road injuries	4	16591	576	187		8				46
Other road injuries	4	16591	576	185						34
Other transport injuries	3	17380	576	186						
Unintentional injuries	2	19691	953	1682	678	12		8		223
Falls	3	19189	953	1301	654	3				
Drowning	3	19690	953	1391	647	3		2		223
Fire, heat, and hot substances	3	19188	953	1395		1		4		191
Poisonings	3	19188	953	506	582			5		188
Poisoning by carbon monoxide	4	18329	953	108				2		188
Poisoning by other means	4	16371	953					5		
Exposure to mechanical forces	3	18901	953	402	669	8		2		
Unintentional firearm injuries	4	17189	953	314		1				
Other exposure to mechanical forces	4	16591	953	389	669	4		2		
Adverse effects of medical treatment	3	18900	953	187						
Animal contact	3	18621	953	1257		1				
Venomous animal contact	4	16917	953	525						
Non-venomous animal contact	4	16591	953							
Foreign body	3	18303	953	396				7		
Pulmonary aspiration and foreign body in airway	4	16592	953	186				6		
Foreign body in other body part	4	16591	953							
Electrocution	3	16590	576	19						
Environmental heat and cold exposure	3	19179	953	300						
Exposure to forces of nature	3	18859	953	330		5				
Other unintentional injuries (internal)	3	18902	953	397		1				
Self-harm and interpersonal violence	2	19693	953	1646	1416	12		4		1333
Self-harm	3	19191	953	1592	12	3				
Self-harm by hanging, strangulation, and suffocation	4	16126	576							
Self-harm by fire, heat, and hot substances	4	16123	576							
Self-harm by firearm	4	16127	576							
Self-harm by poisoning pesticides	4	16044	576							
Self-harm by non pesticide substance and gas	4	18033	576	272						
Self-harm by other specified means	4	16127	576							
Interpersonal violence	3	19447	953	1430		7		4		1333

Total number of site years by cause and source type for GBD2023

Cause	Level	Vital Registration	Vital Registration - Sample	Verbal Autopsy	Surveillance	Survey/Census	Sibling History	MITS diagnosed	Cancer Registry	Police or Forensic Medicine Report
Physical violence by firearm	4	16347	576	383		3		2		33
Physical violence by sharp object	4	16347	576	395						33
Physical violence by other means	4	16347	576	397				2		33
Conflict and terrorism	3	18054	953	125		3				
Police conflict and executions	3	17452	953	125	1404					

Appendix Table S4: GBD location hierarchy with levels

Geography	level
Global	0
Central Europe, eastern Europe, and central Asia	1
Central Asia	2
Armenia	3
Azerbaijan	3
Georgia	3
Kazakhstan	3
Kyrgyzstan	3
Mongolia	3
Tajikistan	3
Turkmenistan	3
Uzbekistan	3
Central Europe	2
Albania	3
Bosnia and Herzegovina	3
Bulgaria	3
Croatia	3
Czechia	3
Hungary	3
Montenegro	3
North Macedonia	3
Poland	3
Romania	3
Serbia	3
Slovakia	3
Slovenia	3
Eastern Europe	2
Belarus	3
Estonia	3
Latvia	3
Lithuania	3
Moldova	3
Russia	3
Ukraine	3
High income	1
Australasia	2
Australia	3
New Zealand	3
High-income Asia Pacific	2
Brunei	3
Japan	3
Singapore	3
South Korea	3
High-income North America	2
Canada	3
Greenland	3
USA	3
Southern Latin America	2

Argentina	3
Chile	3
Uruguay	3
Western Europe	2
Andorra	3
Austria	3
Belgium	3
Cyprus	3
Denmark	3
Finland	3
France	3
Germany	3
Greece	3
Iceland	3
Ireland	3
Israel	3
Italy	3
Luxembourg	3
Malta	3
Monaco	3
Netherlands	3
Norway	3
Portugal	3
San Marino	3
Spain	3
Sweden	3
Switzerland	3
UK	3
England	4
Northern Ireland	4
Scotland	4
Wales	4
Latin America and Caribbean	1
Andean Latin America	2
Bolivia	3
Ecuador	3
Peru	3
Caribbean	2
Antigua and Barbuda	3
Barbados	3
Belize	3
Bermuda	3
Cuba	3
Dominica	3
Dominican Republic	3
Grenada	3
Guyana	3
Haiti	3
Jamaica	3
Puerto Rico	3

Saint Kitts and Nevis	3
Saint Lucia	3
Saint Vincent and the Grenadines	3
Suriname	3
The Bahamas	3
Trinidad and Tobago	3
Virgin Islands	3
Central Latin America	2
Colombia	3
Costa Rica	3
El Salvador	3
Guatemala	3
Honduras	3
Mexico	3
Nicaragua	3
Panama	3
Venezuela	3
Tropical Latin America	2
Brazil	3
Paraguay	3
North Africa and Middle East	1
North Africa and Middle East	2
Afghanistan	3
Algeria	3
Bahrain	3
Egypt	3
Iran	3
Iraq	3
Jordan	3
Kuwait	3
Lebanon	3
Libya	3
Morocco	3
Oman	3
Palestine	3
Qatar	3
Saudi Arabia	3
Sudan	3
Syria	3
Tunisia	3
Türkiye	3
United Arab Emirates	3
Yemen	3
South Asia	1
South Asia	2
Bangladesh	3
Bhutan	3
India	3
Nepal	3
Pakistan	3

Southeast Asia, east Asia, and Oceania	1
East Asia	2
China	3
North Korea	3
Taiwan*	3
Oceania	2
American Samoa	3
Cook Islands	3
Federated States of Micronesia	3
Fiji	3
Guam	3
Kiribati	3
Marshall Islands	3
Nauru	3
Niue	3
Northern Mariana Islands	3
Palau	3
Papua New Guinea	3
Samoa	3
Solomon Islands	3
Tokelau	3
Tonga	3
Tuvalu	3
Vanuatu	3
Southeast Asia	2
Cambodia	3
Indonesia	3
Laos	3
Malaysia	3
Maldives	3
Mauritius	3
Myanmar	3
Philippines	3
Seychelles	3
Sri Lanka	3
Thailand	3
Timor-Leste	3
Viet Nam	3
Sub-Saharan Africa	1
Central sub-Saharan Africa	2
Angola	3
Central African Republic	3
Congo (Brazzaville)	3
DR Congo	3
Equatorial Guinea	3
Gabon	3
Eastern sub-Saharan Africa	2
Burundi	3
Comoros	3
Djibouti	3

Eritrea	3
Ethiopia	3
Kenya	3
Madagascar	3
Malawi	3
Mozambique	3
Rwanda	3
Somalia	3
South Sudan	3
Uganda	3
Tanzania	3
Zambia	3
Southern sub-Saharan Africa	2
Botswana	3
Eswatini	3
Lesotho	3
Namibia	3
South Africa	3
Zimbabwe	3
Western sub-Saharan Africa	2
Benin	3
Burkina Faso	3
Cabo Verde	3
Cameroon	3
Chad	3
Côte d'Ivoire	3
Ghana	3
Guinea	3
Guinea-Bissau	3
Liberia	3
Mali	3
Mauritania	3
Niger	3
Nigeria	3
São Tomé and Príncipe	3
Senegal	3
Sierra Leone	3
The Gambia	3
Togo	3

Appendix Table S5: Underlying indicators for percent well-certified for data source with maximum percent well certified in each 5-year time interval for 204 countries, 1980-2023								
Location	Time Window	Stars	Percent Well-Certified [PWC] (%)	Max PWC Data Year	Max PWC Data Source	Completeness (%)	Percent Major Garbage (%)	Verbal Autopsy Adjustment (None for VR) (%)
Afghanistan	1980-1984	0						
Afghanistan	1985-1989	0						
Afghanistan	1990-1994	0						
Afghanistan	1995-1999	0						
Afghanistan	2000-2004	1	3.4	2001	Afghanistan - Badghis Nutrition and Health Survey 2002		47.3	6.4
Afghanistan	2005-2009	2	28.2	2008	Afghanistan Special Demographic and Health Survey 2010		55.9	64.0
Afghanistan	2010-2024	2	25.2	2015	Afghanistan Health Survey 2015		8.2	27.5
Albania	1980-1984	0						
Albania	1985-1989	4	65.3	1989	Vital Registration	100.0	34.7	
Albania	1990-1994	4	67.5	1993	Vital Registration	100.0	32.5	
Albania	1995-1999	4	71.4	1997	Vital Registration	96.2	25.9	
Albania	2000-2004	4	73.4	2004	Vital Registration	100.0	26.6	
Albania	2005-2009	4	71.2	2008	Vital Registration	97.2	26.7	
Albania	2010-2024	3	53.7	2010	Vital Registration	69.4	22.6	
Algeria	1980-1984	0						
Algeria	1985-1989	0						
Algeria	1990-1994	0						
Algeria	1995-1999	0						
Algeria	2000-2004	0						
Algeria	2005-2009	2	14.8	2006	Vital Registration	29.5	49.8	
Algeria	2010-2024	0						
American Samoa	1980-1984	0						
American Samoa	1985-1989	0						
American Samoa	1990-1994	0						
American Samoa	1995-1999	4	67.2	1997	Vital Registration	91.6	26.7	
American Samoa	2000-2004	4	76.2	2002	Vital Registration	93.0	18.1	
American Samoa	2005-2009	4	73.0	2009	Vital Registration	91.5	20.3	
American Samoa	2010-2024	4	69.4	2015	Vital Registration	81.7	15.0	
Andorra	1980-1984	0						
Andorra	1985-1989	0						
Andorra	1990-1994	0						
Andorra	1995-1999	0						
Andorra	2000-2004	0						
Andorra	2005-2009	0						
Andorra	2010-2024	3	57.2	2020	Vital Registration	68.3	16.2	
Angola	1980-1984	0						
Angola	1985-1989	0						
Angola	1990-1994	0						
Angola	1995-1999	0						
Angola	2000-2004	0						
Angola	2005-2009	0						
Angola	2010-2024	1	2.7	2010	Angola - Dande Health and Demographic Surveillance System		58.2	6.4
Antigua and Barbuda	1980-1984	3	64.1	1983	Vital Registration	91.1	29.6	
Antigua and Barbuda	1985-1989	4	69.2	1989	Vital Registration	100.0	30.8	
Antigua and Barbuda	1990-1994	4	75.3	1994	Vital Registration	100.0	24.7	
Antigua and Barbuda	1995-1999	4	79.6	1999	Vital Registration	100.0	20.4	
Antigua and Barbuda	2000-2004	4	82.9	2003	Vital Registration	100.0	17.1	
Antigua and Barbuda	2005-2009	4	81.6	2007	Vital Registration	100.0	18.4	
Antigua and Barbuda	2010-2024	4	78.9	2014	Vital Registration	100.0	21.1	
Argentina	1980-1984	4	75.6	1981	Vital Registration	100.0	24.4	
Argentina	1985-1989	4	70.5	1988	Vital Registration	100.0	29.5	
Argentina	1990-1994	4	69.6	1990	Vital Registration	100.0	30.4	
Argentina	1995-1999	4	68.9	1996	Vital Registration	99.8	31.0	
Argentina	2000-2004	4	68.6	2004	Vital Registration	100.0	31.4	
Argentina	2005-2009	4	68.7	2005	Vital Registration	100.0	31.3	
Argentina	2010-2024	4	75.2	2021	Vital Registration	100.0	24.8	
Armenia	1980-1984	4	76.8	1982	Vital Registration	88.2	12.9	
Armenia	1985-1989	4	82.4	1986	Vital Registration	93.6	12.0	
Armenia	1990-1994	4	83.2	1992	Vital Registration	96.5	13.8	
Armenia	1995-1999	5	85.1	1999	Vital Registration	98.1	13.3	
Armenia	2000-2004	5	86.0	2002	Vital Registration	100.0	14.0	
Armenia	2005-2009	5	90.0	2006	Vital Registration	100.0	10.0	
Armenia	2010-2024	5	88.9	2016	Vital Registration	100.0	11.1	
Australia	1980-1984	5	93.2	1982	Vital Registration	100.0	6.8	
Australia	1985-1989	5	93.6	1988	Vital Registration	100.0	6.4	
Australia	1990-1994	5	93.6	1994	Vital Registration	100.0	6.4	
Australia	1995-1999	5	93.5	1996	Vital Registration	100.0	6.5	
Australia	2000-2004	5	92.7	2001	Vital Registration	99.9	7.3	
Australia	2005-2009	5	91.9	2006	Vital Registration	100.0	8.1	
Australia	2010-2024	5	91.2	2011	Vital Registration	100.0	8.8	
Austria	1980-1984	5	91.3	1984	Vital Registration	99.1	7.9	
Austria	1985-1989	5	93.1	1987	Vital Registration	100.0	6.9	
Austria	1990-1994	5	92.7	1990	Vital Registration	100.0	7.3	
Austria	1995-1999	5	92.2	1996	Vital Registration	100.0	7.8	
Austria	2000-2004	5	94.6	2003	Vital Registration	100.0	5.4	
Austria	2005-2009	5	93.5	2005	Vital Registration	100.0	6.5	
Austria	2010-2024	5	91.1	2010	Vital Registration	100.0	8.9	
Azerbaijan	1980-1984	4	74.0	1981	Vital Registration	84.6	12.5	
Azerbaijan	1985-1989	4	73.1	1988	Vital Registration	83.2	12.2	
Azerbaijan	1990-1994	4	73.2	1993	Vital Registration	82.3	11.1	
Azerbaijan	1995-1999	4	68.8	1995	Vital Registration	80.2	14.2	
Azerbaijan	2000-2004	4	74.9	2003	Vital Registration	82.0	8.7	
Azerbaijan	2005-2009	3	45.6	2007	Vital Registration	83.0	45.1	
Azerbaijan	2010-2024	0						
Bahrain	1980-1984	0						
Bahrain	1985-1989	4	75.0	1986	Vital Registration	100.0	25.0	
Bahrain	1990-1994	0						
Bahrain	1995-1999	3	55.8	1997	Vital Registration	83.8	33.4	
Bahrain	2000-2004	3	54.2	2000	Vital Registration	88.7	38.9	
Bahrain	2005-2009	3	50.5	2006	Vital Registration	83.7	39.7	
Bahrain	2010-2024	3	54.8	2014	Vital Registration	83.2	34.1	
Bangladesh	1980-1984	1	2.2	1983	Bangladesh - Matlab Health and Demographic Surveillance System		66.2	6.4
Bangladesh	1985-1989	1	3.2	1989	Bangladesh - Matlab Health and Demographic Surveillance System		49.3	6.4
Bangladesh	1990-1994	2	30.1	1991	Causes of childhood deaths in Bangladesh: an update		1.7	30.7
Bangladesh	1995-1999	1	3.9	1998	Bangladesh - Matlab Health and Demographic Surveillance System		39.7	6.4
Bangladesh	2000-2004	2	20.1	2004	Bangladesh Demographic and Health Survey 2004		4.2	21.0
Bangladesh	2005-2009	2	24.1	2005	Bangladesh Child Health and Injury Survey 2005		1.7	24.5
Bangladesh	2010-2024	3	51.9	2015	Verbal Autopsy		1.7	52.8
Barbados	1980-1984	4	75.1	1983	Vital Registration	100.0	24.9	
Barbados	1985-1989	4	77.3	1986	Vital Registration	100.0	22.7	
Barbados	1990-1994	4	79.2	1992	Vital Registration	100.0	20.8	
Barbados	1995-1999	4	79.2	1995	Vital Registration	100.0	20.8	
Barbados	2000-2004	4	76.9	2000	Vital Registration	99.2	22.5	
Barbados	2005-2009	4	78.9	2009	Vital Registration	96.7	18.3	
Barbados	2010-2024	4	78.8	2011	Vital Registration	96.3	18.2	
Belarus	1980-1984	5	84.9	1982	Vital Registration	98.9	14.2	
Belarus	1985-1989	5	88.1	1986	Vital Registration	100.0	11.9	
Belarus	1990-1994	4	79.8	1990	Vital Registration	100.0	20.2	
Belarus	1995-1999	4	80.5	1999	Vital Registration	100.0	19.5	
Belarus	2000-2004	4	81.5	2002	Vital Registration	99.8	18.3	
Belarus	2005-2009	4	82.1	2009	Vital Registration	100.0	17.9	
Belarus	2010-2024	5	89.2	2018	Vital Registration	100.0	10.8	
Belgium	1980-1984	4	79.9	1981	Vital Registration	100.0	20.1	
Belgium	1985-1989	4	80.4	1987	Vital Registration	100.0	19.6	
Belgium	1990-1994	4	84.0	1993	Vital Registration	100.0	16.0	
Belgium	1995-1999	5	86.3	1998	Vital Registration	100.0	13.7	
Belgium	2000-2004	5	86.2	2000	Vital Registration	100.0	13.8	
Belgium	2005-2009	5	86.3	2008	Vital Registration	100.0	13.7	
Belgium	2010-2024	4	84.1	2020	Vital Registration	100.0	15.9	
Belize	1980-1984	3	61.7	1980	Vital Registration	91.8	32.8	
Belize	1985-1989	3	64.2	1987	Vital Registration	85.8	25.1	
Belize	1990-1994	3	55.4	1993	Vital Registration	87.6	36.7	
Belize	1995-1999	4	74.6	1998	Vital Registration	100.0	25.4	
Belize	2000-2004	4	73.2	2002	Vital Registration	96.9	24.4	
Belize	2005-2009	5	87.3	2009	Vital Registration	100.0	12.7	
Belize	2010-2024	5	87.4	2011	Vital Registration	100.0	12.6	
Benin	1980-1984	0						
Benin	1985-1989	1	2.1	1989	Incidence de décès de 0 à 1 an dans une cohorte de 802 enfants en milieu rural au sud du Bénin		5.6	2.3
Benin	1990-1994	0						
Benin	1995-1999	0						
Benin	2000-2004	0						
Benin	2005-2009	0						
Benin	2010-2024	1	1.2	2016	A population-based cardiovascular cohort in Subsaharian Africa: The pilot project Tanve Health Study (Tahes) in Benin		54.8	2.8
Bermuda	1980-1984	5	89.5	1980	Vital Registration	100.0	10.5	
Bermuda	1985-1989	5	88.8	1989	Vital Registration	97.4	8.9	
Bermuda	1990-1994	5	90.6	1991	Vital Registration	99.4	8.8	
Bermuda	1995-1999	5	94.8	1996	Vital Registration	100.0	5.2	
Bermuda	2000-2004	5	92.9	2002	Vital Registration	100.0	7.1	
Bermuda	2005-2009	5	89.0	2008	Vital Registration	100.0	11.0	
Bermuda	2010-2024	5	92.9	2014	Vital Registration	100.0	7.1	
Bhutan	1980-1984	0						
Bhutan	1985-1989	0						
Bhutan	1990-1994	0						
Bhutan	1995-1999	0						
Bhutan	2000-2004	0						
Bhutan	2005-2009	0						
Bhutan	2010-2024	0						
Bolivia	1980-1984	0						
Bolivia	1985-1989	0						
Bolivia	1990-1994	0						
Bolivia	1995-1999	0						
Bolivia	2000-2004	2	12.3	2003	Vital Registration	37.8	67.5	
Bolivia	2005-2009	0						
Bolivia	2010-2024	0						
Bosnia and Herzegovina	1980-1984	0						
Bosnia and Herzegovina	1985-1989	3	62.3	1988	Vital Registration	100.0	37.7	
Bosnia and Herzegovina	1990-1994	4	63.6	1990	Vital Registration	100.0	36.4	
Bosnia and Herzegovina	1995-1999	0						
Bosnia and Herzegovina	2000-2004	0						
Bosnia and Herzegovina	2005-2009	0						
Bosnia and Herzegovina	2010-2024	4	72.4	2014	Vital Registration	99.6	27.2	
Botswana	1980-1984	0						
Botswana	1985-1989	0						
Botswana	1990-1994	0						
Botswana	1995-1999	0						
Botswana	2000-2004	0						
Botswana	2005-2009	0						
Botswana	2010-2024	0						
Brazil	1980-1984	3	56.6	1984	Vital Registration	92.6	38.9	
Brazil	1985-1989	3	60.9	1988	Vital Registration	95.2	36.0	
Brazil	1990-1994	3	64.3	1994	Vital Registration	95.9	33.0	
Brazil	1995-1999	4	67.4	1999	Vital Registration	95.6	29.5	
Brazil	2000-2004	4	72.4	2004	Vital Registration	96.7	25.2	
Brazil	2005-2009	4	78.4	2009	Vital Registration	96.8	19.0	
Brazil	2010-2024	4	83.7	2021	Vital Registration	99.2	15.6	
Brunei	1980-1984	0						
Brunei	1985-1989	0						
Brunei	1990-1994	0						
Brunei	1995-1999	4	73.8	1998	Vital Registration	86.7	14.9	
Brunei	2000-2004	4	79.8	2002	Vital Registration	97.8	18.4	
Brunei	2005-2009	4	81.9	2006	Vital Registration	100.0	18.1	
Brunei	2010-2024	5	85.8	2019	Vital Registration	100.0	14.2	
Bulgaria	1980-1984	4	78.9	1982	Vital Registration	100.0	21.1	
Bulgaria	1985-1989	4	80.1	1985	Vital Registration	100.0	19.9	
Bulgaria	1990-1994	4	80.8	1993	Vital Registration	100.0	19.1	

Underlying indicators for percent well-certified for data source with maximum percent well certified in each 5-year time interval for 204 countries, 1980-2023								
Location	Time Window	Stars	Percent Well-Certified [PWC] (%)	Max PWC Data Year	Max PWC Data Source	Completeness (%)	Percent Major Garbage (%)	Verbal Autopsy Adjustment (None for VR) (%)
Bulgaria	1995-1999	4	77.8	1995	Vital Registration	99.4	21.8	
Bulgaria	2000-2004	4	73.4	2002	Vital Registration	100.0	26.6	
Bulgaria	2005-2009	4	74.5	2005	Vital Registration	100.0	25.5	
Bulgaria	2010-2024	4	74.1	2021	Vital Registration	100.0	25.9	
Burkina Faso	1980-1984	1	3.6	1984	The burden of malaria mortality among African children in the year 2000		9.6	4.0
Burkina Faso	1985-1989	0						
Burkina Faso	1990-1994	0						
Burkina Faso	1995-1999	1	4.6	1998	Measuring the local burden of disease. A study of years of life lost in sub-Saharan Africa		27.6	6.4
Burkina Faso	2000-2004	1	6.3	2000	Burkina Faso - Nouna Health and Demographic Surveillance System		0.9	6.4
Burkina Faso	2005-2009	1	2.4	2009	An improved method for physician-certified verbal autopsy reduces the rate of discrepancy: experiences in the Nouna Health and Demographic Surveillance Site (NHDSS), Burkina Faso		63.1	6.4
Burkina Faso	2010-2024	2	13.0	2020	Effect of Neonatal Azithromycin on All-Cause and Cause-Specific Infant Mortality: A Randomized Controlled Trial		0.6	13.1
Burundi	1980-1984	0						
Burundi	1985-1989	0						
Burundi	1990-1994	1	3.0	1990	Mortality and morbidity at young ages in a stable hyperendemic malaria region, community Nyanza-Lac, Imbo South, Burundi		4.0	3.2
Burundi	1995-1999	0						
Burundi	2000-2004	0						
Burundi	2005-2009	0						
Burundi	2010-2024	0						
Cabo Verde	1980-1984	3	47.1	1980	Vital Registration	94.6	50.2	
Cabo Verde	1985-1989	0						
Cabo Verde	1990-1994	1	0.3	1992	Deaths among women of reproductive age in Cape Verde: causes and avoidability		6.5	0.3
Cabo Verde	1995-1999	0						
Cabo Verde	2000-2004	0						
Cabo Verde	2005-2009	0						
Cabo Verde	2010-2024	4	69.3	2012	Vital Registration	99.4	30.3	
Cambodia	1980-1984	0						
Cambodia	1985-1989	0						
Cambodia	1990-1994	0						
Cambodia	1995-1999	0						
Cambodia	2000-2004	1	0.8	2001	Community-based surveillance: a pilot study from rural Cambodia		86.9	6.4
Cambodia	2005-2009	1	4.1	2009	Mortality in Cambodia An 18-Month Prospective Community-based Surveillance of All-age Deaths Using Verbal Autopsies		35.5	6.4
Cambodia	2010-2024	1	0.3	2016	Why did our baby die soon after birth?"-Lessons on neonatal death in rural Cambodia from the perspective of caregivers		0.6	0.3
Cameroon	1980-1984	0						
Cameroon	1985-1989	0						
Cameroon	1990-1994	0						
Cameroon	1995-1999	0						
Cameroon	2000-2004	0						
Cameroon	2005-2009	0						
Cameroon	2010-2024	0						
Canada	1980-1984	5	90.4	1981	Vital Registration	100.0	9.6	
Canada	1985-1989	5	90.9	1986	Vital Registration	100.0	9.1	
Canada	1990-1994	5	90.2	1993	Vital Registration	100.0	9.8	
Canada	1995-1999	5	90.4	1999	Vital Registration	100.0	9.6	
Canada	2000-2004	5	90.2	2001	Vital Registration	100.0	9.8	
Canada	2005-2009	5	90.5	2009	Vital Registration	100.0	9.5	
Canada	2010-2024	5	90.3	2013	Vital Registration	100.0	9.7	
Central African Republic	1980-1984	0						
Central African Republic	1985-1989	0						
Central African Republic	1990-1994	0						
Central African Republic	1995-1999	0						
Central African Republic	2000-2004	0						
Central African Republic	2005-2009	0						
Central African Republic	2010-2024	0						
Chad	1980-1984	0						
Chad	1985-1989	0						
Chad	1990-1994	0						
Chad	1995-1999	0						
Chad	2000-2004	0						
Chad	2005-2009	0						
Chad	2010-2024	1	1.0	2019	Validation of elimination of maternal and neonatal tetanus in Chad - Weekly Epidemiological Record 2019		0.4	1.0
Chile	1980-1984	4	75.1	1983	Vital Registration	100.0	24.9	
Chile	1985-1989	4	75.6	1988	Vital Registration	100.0	24.4	
Chile	1990-1994	4	82.3	1994	Vital Registration	99.6	17.4	
Chile	1995-1999	5	85.0	1999	Vital Registration	100.0	15.0	
Chile	2000-2004	5	91.2	2004	Vital Registration	100.0	8.8	
Chile	2005-2009	5	91.2	2005	Vital Registration	100.0	8.8	
Chile	2010-2024	5	90.4	2012	Vital Registration	100.0	9.6	
China	1980-1984	0						
China	1985-1989	1	0.7	1986	Infant mortality among various nationalities in the middle part of Guizhou, China		1.4	0.7
China	1990-1994	4	69.9	1994	Vital Registration - Sample	81.5	14.3	
China	1995-1999	4	73.6	1998	Vital Registration - Sample	86.4	14.9	
China	2000-2004	4	66.5	2000	Vital Registration - Sample	80.0	16.8	
China	2005-2009	4	72.1	2009	Vital Registration - Sample	76.9	6.2	
China	2010-2024	4	71.8	2010	Vital Registration - Sample	76.6	6.2	
Colombia	1980-1984	4	69.3	1983	Vital Registration	92.9	25.4	
Colombia	1985-1989	4	71.8	1987	Vital Registration	93.5	23.3	
Colombia	1990-1994	4	74.9	1994	Vital Registration	91.4	18.0	
Colombia	1995-1999	5	88.1	1999	Vital Registration	100.0	11.9	
Colombia	2000-2004	5	89.6	2002	Vital Registration	100.0	10.4	
Colombia	2005-2009	5	87.4	2008	Vital Registration	98.0	10.9	
Colombia	2010-2024	5	92.4	2020	Vital Registration	100.0	7.6	
Comoros	1980-1984	0						
Comoros	1985-1989	0						
Comoros	1990-1994	0						
Comoros	1995-1999	0						
Comoros	2000-2004	0						
Comoros	2005-2009	0						
Comoros	2010-2024	0						
Congo (Brazzaville)	1980-1984	0						
Congo (Brazzaville)	1985-1989	0						
Congo (Brazzaville)	1990-1994	0						
Congo (Brazzaville)	1995-1999	0						
Congo (Brazzaville)	2000-2004	0						
Congo (Brazzaville)	2005-2009	0						
Congo (Brazzaville)	2010-2024	0						
Cook Islands	1980-1984	0						
Cook Islands	1985-1989	0						
Cook Islands	1990-1994	0						
Cook Islands	1995-1999	0						
Cook Islands	2000-2004	3	60.5	2000	Vital Registration	100.0	39.5	
Cook Islands	2005-2009	3	57.6	2006	Vital Registration	82.4	30.1	
Cook Islands	2010-2024	4	74.7	2014	Vital Registration	100.0	25.3	
Costa Rica	1980-1984	4	81.3	1984	Vital Registration	100.0	18.7	
Costa Rica	1985-1989	4	82.7	1989	Vital Registration	100.0	17.3	
Costa Rica	1990-1994	4	82.7	1993	Vital Registration	100.0	17.3	
Costa Rica	1995-1999	5	91.3	1999	Vital Registration	100.0	8.7	
Costa Rica	2000-2004	5	92.7	2003	Vital Registration	100.0	7.3	
Costa Rica	2005-2009	5	91.5	2005	Vital Registration	100.0	8.5	
Costa Rica	2010-2024	5	93.0	2020	Vital Registration	100.0	7.0	
Croatia	1980-1984	0						
Croatia	1985-1989	4	84.6	1989	Vital Registration	100.0	15.4	
Croatia	1990-1994	5	85.6	1990	Vital Registration	100.0	14.4	
Croatia	1995-1999	4	84.6	1999	Vital Registration	100.0	15.4	
Croatia	2000-2004	5	87.3	2004	Vital Registration	99.3	12.1	
Croatia	2005-2009	5	89.8	2009	Vital Registration	100.0	10.2	
Croatia	2010-2024	5	94.0	2020	Vital Registration	100.0	6.0	
Cuba	1980-1984	5	85.1	1983	Vital Registration	100.0	14.9	
Cuba	1985-1989	5	86.8	1988	Vital Registration	100.0	13.2	
Cuba	1990-1994	5	86.4	1990	Vital Registration	100.0	13.6	
Cuba	1995-1999	5	89.7	1997	Vital Registration	99.9	10.2	
Cuba	2000-2004	5	91.3	2004	Vital Registration	100.0	8.7	
Cuba	2005-2009	5	91.9	2008	Vital Registration	100.0	8.1	
Cuba	2010-2024	5	92.2	2011	Vital Registration	100.0	7.8	
Cyprus	1980-1984	0						
Cyprus	1985-1989	0						
Cyprus	1990-1994	0						
Cyprus	1995-1999	2	26.9	1999	Vital Registration	69.7	61.4	
Cyprus	2000-2004	3	49.3	2004	Vital Registration	69.0	28.5	
Cyprus	2005-2009	3	56.8	2007	Vital Registration	70.0	18.9	
Cyprus	2010-2024	4	62.7	2021	Vital Registration	71.1	11.7	
Czechia	1980-1984	0						
Czechia	1985-1989	5	91.1	1986	Vital Registration	100.0	8.9	
Czechia	1990-1994	5	90.2	1990	Vital Registration	100.0	9.7	
Czechia	1995-1999	5	85.7	1995	Vital Registration	100.0	14.3	
Czechia	2000-2004	5	86.2	2000	Vital Registration	99.8	13.6	
Czechia	2005-2009	5	86.3	2007	Vital Registration	100.0	13.7	
Czechia	2010-2024	5	87.9	2013	Vital Registration	100.0	12.1	
Côte d'Ivoire	1980-1984	0						
Côte d'Ivoire	1985-1989	1	3.5	1988	[Effect of hygiene and water sanitation and oral rehydration on diarrhoea and mortality of children under five in rural area of Côte d'Ivoire]		7.5	3.8
Côte d'Ivoire	1990-1994	1	3.3	1990	[Effect of hygiene and water sanitation and oral rehydration on diarrhoea and mortality of children under five in rural area of Côte d'Ivoire]		7.8	3.6
Côte d'Ivoire	1995-1999	0						
Côte d'Ivoire	2000-2004	0						
Côte d'Ivoire	2005-2009	1	0.2	2009	Africa, Asia, Oceania - INDEPTH Network Cause-Specific Mortality - Release 2014		96.9	6.4
Côte d'Ivoire	2010-2024	1	0.2	2010	Africa, Asia, Oceania - INDEPTH Network Cause-Specific Mortality - Release 2014		96.1	6.4
DR Congo	1980-1984	0						
DR Congo	1985-1989	1	0.8	1986	Etude de la mortalité globale et de la mortalité liée au paludisme dans le Kivu montagneux, Zaïre		87.0	6.4
DR Congo	1990-1994	1	3.5	1990	Influence of nutritional status on child mortality in rural Zaire		1.9	3.6
DR Congo	1995-1999	0						
DR Congo	2000-2004	0						
DR Congo	2005-2009	0						
DR Congo	2010-2024	1	0.8	2019	Validation of maternal and neonatal tetanus elimination in the Democratic Republic of the Congo - Weekly Epidemiological Record 2019		0.6	0.8
Denmark	1980-1984	4	83.6	1981	Vital Registration	100.0	16.4	
Denmark	1985-1989	4	82.3	1985	Vital Registration	100.0	17.7	
Denmark	1990-1994	5	84.8	1994	Vital Registration	99.3	14.5	
Denmark	1995-1999	5	88.3	1999	Vital Registration	100.0	11.7	
Denmark	2000-2004	5	87.4	2000	Vital Registration	99.7	12.3	
Denmark	2005-2009	5	87.0	2005	Vital Registration	99.7	12.8	
Denmark	2010-2024	5	87.5	2016	Vital Registration	100.0	12.5	
Djibouti	1980-1984	0						
Djibouti	1985-1989	0						
Djibouti	1990-1994	0						
Djibouti	1995-1999	0						
Djibouti	2000-2004	0						
Djibouti	2005-2009	0						
Djibouti	2010-2024	0						
Dominica	1980-1984	3	53.0	1984	Vital Registration	76.1	30.3	
Dominica	1985-1989	3	50.9	1986	Vital Registration	81.8	37.8	
Dominica	1990-1994	3	51.9	1993	Vital Registration	79.7	34.9	
Dominica	1995-1999	3	58.5	1999	Vital Registration	86.8	32.5	
Dominica	2000-2004	3	55.6	2004	Vital Registration	79.5	30.0	
Dominica	2005-2009	4	68.2	2006	Vital Registration	76.9	11.3	
Dominica	2010-2024	4	70.9	2011	Vital Registration	83.2	14.8	
Dominican Republic	1980-1984	3	47.6	1984	Vital Registration	77.0	38.2	
Dominican Republic	1985-1989	3	47.8	1985	Vital Registration	75.4	36.7	
Dominican Republic	1990-1994	3	42.7	1990	Vital Registration	65.1	34.5	
Dominican Republic	1995-1999	3	45.8	1999	Vital Registration	63.6	27.9	
Dominican Republic	2000-2004	3	45.9	2004	Vital Registration	60.0	23.6	

Underlying indicators for percent well-certified for data source with maximum percent well certified in each 5-year time interval for 204 countries, 1980-2023								
Location	Time Window	Stars	Percent Well-Certified [PWC] (%)	Max PWC Data Year	Max PWC Data Source	Completeness (%)	Percent Major Garbage (%)	Verbal Autopsy Adjustment (None for VR) (%)
Dominican Republic	2005-2009	3	45.0	2005	Vital Registration	57.3	21.5	
Dominican Republic	2010-2024	3	50.7	2014	Vital Registration	70.5	28.1	
Ecuador	1980-1984	4	64.8	1984	Vital Registration	100.0	35.2	
Ecuador	1985-1989	4	67.5	1988	Vital Registration	100.0	32.5	
Ecuador	1990-1994	4	68.7	1992	Vital Registration	98.7	30.4	
Ecuador	1995-1999	4	65.0	1996	Vital Registration	93.3	30.3	
Ecuador	2000-2004	3	60.0	2000	Vital Registration	91.7	34.5	
Ecuador	2005-2009	3	56.7	2009	Vital Registration	75.6	25.1	
Ecuador	2010-2024	4	68.7	2022	Vital Registration	81.1	15.2	
Egypt	1980-1984	2	26.2	1980	Vital Registration	79.2	66.9	
Egypt	1985-1989	2	31.5	1987	Vital Registration	90.4	65.2	
Egypt	1990-1994	3	35.9	1992	Vital Registration	87.8	59.1	
Egypt	1995-1999	0						
Egypt	2000-2004	3	38.4	2003	Vital Registration	95.5	59.8	
Egypt	2005-2009	3	37.9	2005	Vital Registration	94.1	59.7	
Egypt	2010-2024	3	44.9	2012	Vital Registration	98.5	54.5	
El Salvador	1980-1984	3	48.8	1981	Vital Registration	86.7	43.8	
El Salvador	1985-1989	0						
El Salvador	1990-1994	3	52.5	1993	Vital Registration	85.3	38.5	
El Salvador	1995-1999	3	59.4	1999	Vital Registration	88.1	32.6	
El Salvador	2000-2004	4	64.1	2001	Vital Registration	94.6	32.2	
El Salvador	2005-2009	4	64.1	2005	Vital Registration	93.1	31.1	
El Salvador	2010-2024	3	64.8	2012	Vital Registration	96.6	32.9	
Equatorial Guinea	1980-1984	0						
Equatorial Guinea	1985-1989	0						
Equatorial Guinea	1990-1994	0						
Equatorial Guinea	1995-1999	0						
Equatorial Guinea	2000-2004	0						
Equatorial Guinea	2005-2009	0						
Equatorial Guinea	2010-2024	0						
Eritrea	1980-1984	0						
Eritrea	1985-1989	0						
Eritrea	1990-1994	0						
Eritrea	1995-1999	0						
Eritrea	2000-2004	0						
Eritrea	2005-2009	0						
Eritrea	2010-2024	0						
Estonia	1980-1984	5	90.9	1981	Vital Registration	99.8	8.9	
Estonia	1985-1989	5	91.4	1988	Vital Registration	100.0	8.6	
Estonia	1990-1994	5	93.7	1994	Vital Registration	100.0	6.3	
Estonia	1995-1999	5	92.9	1995	Vital Registration	99.7	6.9	
Estonia	2000-2004	5	92.2	2000	Vital Registration	100.0	7.8	
Estonia	2005-2009	5	92.8	2009	Vital Registration	100.0	7.2	
Estonia	2010-2024	5	93.3	2018	Vital Registration	100.0	6.7	
Eswatini	1980-1984	0						
Eswatini	1985-1989	0						
Eswatini	1990-1994	0						
Eswatini	1995-1999	0						
Eswatini	2000-2004	1	1.1	2000	Effect of HIV infection on pregnancy-related mortality in sub-Saharan Africa: secondary analyses of pooled community-based data from the network for Analysing Longitudinal Population-based HIV/AIDS data on Africa (ALPHA)		12.7	1.3
Eswatini	2005-2009	0						
Eswatini	2010-2024	0						
Ethiopia	1980-1984	0						
Ethiopia	1985-1989	0						
Ethiopia	1990-1994	1	3.3	1992	Patterns of childhood mortality in three districts of north Gondar Administrative Zone. A community based study using the verbal autopsy method		1.9	3.3
Ethiopia	1995-1999	0						
Ethiopia	2000-2004	1	3.2	2001	HIV/AIDS-Related Mortality In Addis Ababa City Administration		50.2	6.4
Ethiopia	2005-2009	1	1.9	2006	Ethiopia - Addis Ababa Mortality Surveillance Program 2006		41.4	3.3
Ethiopia	2010-2024	3	45.6	2010	Ethiopia Demographic Surveillance Verbal Autopsy Data 2009-2016		28.8	64.0
Federated States of Micronesia	1980-1984	0						
Federated States of Micronesia	1985-1989	0						
Federated States of Micronesia	1990-1994	0						
Federated States of Micronesia	1995-1999	0						
Federated States of Micronesia	2000-2004	0						
Federated States of Micronesia	2005-2009	0						
Federated States of Micronesia	2010-2024	3	48.1	2022	Vital Registration	63.3	24.0	
Fiji	1980-1984	0						
Fiji	1985-1989	0						
Fiji	1990-1994	0						
Fiji	1995-1999	2	32.5	1999	Vital Registration	60.9	46.7	
Fiji	2000-2004	3	52.6	2003	Vital Registration	100.0	47.4	
Fiji	2005-2009	3	58.1	2009	Vital Registration	98.5	41.0	
Fiji	2010-2024	4	64.0	2012	Vital Registration	98.2	34.9	
Finland	1980-1984	4	82.6	1980	Vital Registration	100.0	17.4	
Finland	1985-1989	5	91.5	1987	Vital Registration	100.0	8.5	
Finland	1990-1994	5	91.4	1992	Vital Registration	100.0	8.6	
Finland	1995-1999	5	96.3	1998	Vital Registration	100.0	3.7	
Finland	2000-2004	5	95.9	2000	Vital Registration	100.0	4.1	
Finland	2005-2009	5	95.4	2005	Vital Registration	100.0	4.6	
Finland	2010-2024	5	95.0	2010	Vital Registration	100.0	5.0	
France	1980-1984	4	77.4	1984	Vital Registration	99.3	22.1	
France	1985-1989	4	80.3	1989	Vital Registration	100.0	19.7	
France	1990-1994	4	82.0	1993	Vital Registration	100.0	18.0	
France	1995-1999	4	82.4	1999	Vital Registration	100.0	17.6	
France	2000-2004	4	82.5	2000	Vital Registration	100.0	17.5	
France	2005-2009	4	82.6	2005	Vital Registration	100.0	17.4	
France	2010-2024	4	80.9	2010	Vital Registration	100.0	19.1	
Gabon	1980-1984	0						
Gabon	1985-1989	0						
Gabon	1990-1994	0						
Gabon	1995-1999	0						
Gabon	2000-2004	0						
Gabon	2005-2009	0						
Gabon	2010-2024	0						
Georgia	1980-1984	4	78.2	1981	Vital Registration	87.2	10.3	
Georgia	1985-1989	4	77.0	1988	Vital Registration	84.6	9.0	
Georgia	1990-1994	4	75.4	1990	Vital Registration	81.8	7.7	
Georgia	1995-1999	4	65.1	1995	Vital Registration	72.5	10.1	
Georgia	2000-2004	4	72.0	2004	Vital Registration	93.7	23.2	
Georgia	2005-2009	3	53.5	2005	Vital Registration	82.9	35.4	
Georgia	2010-2024	4	80.2	2021	Vital Registration	100.0	19.8	
Germany	1980-1984	4	79.2	1984	Vital Registration	99.8	20.6	
Germany	1985-1989	4	82.2	1989	Vital Registration	100.0	17.8	
Germany	1990-1994	5	85.2	1994	Vital Registration	100.0	14.8	
Germany	1995-1999	5	86.0	1998	Vital Registration	100.0	14.0	
Germany	2000-2004	5	84.9	2002	Vital Registration	99.6	14.7	
Germany	2005-2009	5	85.6	2006	Vital Registration	99.9	14.3	
Germany	2010-2024	5	85.3	2011	Vital Registration	99.5	14.2	
Ghana	1980-1984	0						
Ghana	1985-1989	1	0.4	1989	Maternal mortality among the Kassena-Nankana of northern Ghana		10.5	0.5
Ghana	1990-1994	1	2.2	1990	Vitamin A supplementation in northern Ghana: effects on clinic attendances, hospital admissions, and child mortality		1.7	2.2
Ghana	1995-1999	1	0.8	1998	Trend and causes of neonatal mortality in the Kassena-Nankana district of northern Ghana, 1995-2002		0.3	0.8
Ghana	2000-2004	1	8.0	2000	Vital Registration	12.2	34.3	
Ghana	2005-2009	2	19.7	2006	Ghana Child Verbal Autopsy Study 2008		0.7	19.8
Ghana	2010-2024	1	7.1	2017	Ghana Special Demographic and Health Survey 2017		0.7	7.1
Greece	1980-1984	4	82.2	1983	Vital Registration	100.0	17.8	
Greece	1985-1989	4	83.6	1985	Vital Registration	100.0	16.4	
Greece	1990-1994	4	77.9	1994	Vital Registration	99.7	21.9	
Greece	1995-1999	4	78.8	1995	Vital Registration	100.0	21.2	
Greece	2000-2004	4	79.6	2004	Vital Registration	100.0	20.4	
Greece	2005-2009	4	84.0	2009	Vital Registration	100.0	16.0	
Greece	2010-2024	5	85.2	2020	Vital Registration	99.4	14.3	
Greenland	1980-1984	0						
Greenland	1985-1989	0						
Greenland	1990-1994	0						
Greenland	1995-1999	5	85.2	1998	Vital Registration	100.0	14.8	
Greenland	2000-2004	5	87.7	2001	Vital Registration	100.0	12.3	
Greenland	2005-2009	5	86.4	2005	Vital Registration	100.0	13.6	
Greenland	2010-2024	4	82.2	2010	Vital Registration	100.0	17.8	
Grenada	1980-1984	4	70.6	1984	Vital Registration	96.0	26.4	
Grenada	1985-1989	4	66.4	1989	Vital Registration	100.0	33.6	
Grenada	1990-1994	4	65.4	1994	Vital Registration	100.0	34.6	
Grenada	1995-1999	3	64.8	1995	Vital Registration	100.0	35.2	
Grenada	2000-2004	4	79.9	2002	Vital Registration	100.0	20.1	
Grenada	2005-2009	4	79.9	2005	Vital Registration	100.0	20.1	
Grenada	2010-2024	5	88.2	2018	Vital Registration	100.0	11.8	
Guam	1980-1984	0						
Guam	1985-1989	0						
Guam	1990-1994	5	92.5	1994	Vital Registration	100.0	7.5	
Guam	1995-1999	5	93.5	1998	Vital Registration	100.0	6.5	
Guam	2000-2004	5	93.0	2004	Vital Registration	100.0	7.0	
Guam	2005-2009	5	93.6	2007	Vital Registration	100.0	6.4	
Guam	2010-2024	5	90.3	2015	Vital Registration	100.0	9.7	
Guatemala	1980-1984	4	68.5	1984	Vital Registration	100.0	31.5	
Guatemala	1985-1989	4	66.3	1987	Vital Registration	98.6	32.8	
Guatemala	1990-1994	4	65.1	1994	Vital Registration	100.0	34.9	
Guatemala	1995-1999	4	68.3	1998	Vital Registration	100.0	31.7	
Guatemala	2000-2004	4	69.2	2002	Vital Registration	99.6	30.5	
Guatemala	2005-2009	4	74.9	2009	Vital Registration	100.0	25.1	
Guatemala	2010-2024	4	78.4	2014	Vital Registration	100.0	21.6	
Guinea	1980-1984	0						
Guinea	1985-1989	0						
Guinea	1990-1994	0						
Guinea	1995-1999	1	3.4	1998	Guinea - Mandiana Mortality Study 1998-1999		0.6	3.4
Guinea	2000-2004	0						
Guinea	2005-2009	0						
Guinea	2010-2024	0						
Guinea-Bissau	1980-1984	0						
Guinea-Bissau	1985-1989	0						
Guinea-Bissau	1990-1994	1	0.4	1992	Maternal mortality in Guinea-Bissau: the use of verbal autopsy in a multi-ethnic population		11.2	0.4
Guinea-Bissau	1995-1999	1	2.3	1995	BCG vaccination scar associated with better childhood survival in Guinea-Bissau		2.5	2.4
Guinea-Bissau	2000-2004	0						
Guinea-Bissau	2005-2009	0						
Guinea-Bissau	2010-2024	0						
Guyana	1980-1984	3	47.9	1984	Vital Registration	75.9	36.9	
Guyana	1985-1989	3	60.8	1989	Vital Registration	82.0	25.9	
Guyana	1990-1994	3	57.6	1993	Vital Registration	76.7	24.8	
Guyana	1995-1999	4	65.5	1995	Vital Registration	85.6	23.5	
Guyana	2000-2004	4	70.3	2004	Vital Registration	85.0	17.2	
Guyana	2005-2009	4	74.8	2005	Vital Registration	88.9	15.8	
Guyana	2010-2024	4	82.0	2019	Vital Registration	96.3	14.8	
Haiti	1980-1984	2	14.9	1981	Vital Registration	49.9	70.1	
Haiti	1985-1989	1	1.8	1989	The utility of verbal autopsies for identifying HIV-1-related deaths in Haitian children		2.7	1.9
Haiti	1990-1994	1	1.5	1994	Survey on Infant mortality in Mirebalais, Haiti		3.9	1.6
Haiti	1995-1999	2	9.9	1999	Vital Registration	25.1	60.7	
Haiti	2000-2004	1	4.5	2003	Vital Registration	8.6	48.0	
Haiti	2005-2009	0						
Haiti	2010-2024	0						
Honduras	1980-1984	2	29.4	1980	Vital Registration	67.5	56.5	
Honduras	1985-1989	3	38.2	1988	Vital Registration	60.1	36.5	
Honduras	1990-1994	3	35.5	1990	Vital Registration	57.3	38.0	
Honduras	1995-1999	2	15.2	1996	Honduras Demographic and Health Survey 2005-2006		3.2	15.7

Underlying indicators for percent well-certified for data source with maximum percent well certified in each 5-year time interval for 204 countries, 1980-2023								
Location	Time Window	Stars	Percent Well-Certified [PWC] (%)	Max PWC Data Year	Max PWC Data Source	Completeness (%)	Percent Major Garbage (%)	Verbal Autopsy Adjustment (None for VR) (%)
Honduras	2000-2004	2	12.1	2001	Honduras Demographic and Health Survey 2005-2006		3.2	12.4
Honduras	2005-2009	2	11.8	2008	Vital Registration	13.7	13.5	
Honduras	2010-2024	2	12.4	2013	Vital Registration	14.0	11.4	
Hungary	1980-1984	5	90.9	1980	Vital Registration	100.0	9.1	
Hungary	1985-1989	5	90.1	1988	Vital Registration	100.0	9.9	
Hungary	1990-1994	5	89.9	1993	Vital Registration	99.8	10.0	
Hungary	1995-1999	5	91.4	1997	Vital Registration	99.9	8.5	
Hungary	2000-2004	5	92.9	2004	Vital Registration	99.6	6.7	
Hungary	2005-2009	5	94.1	2008	Vital Registration	99.9	5.8	
Hungary	2010-2024	5	94.5	2013	Vital Registration	100.0	5.5	
Iceland	1980-1984	5	92.9	1982	Vital Registration	100.0	7.1	
Iceland	1985-1989	5	93.2	1988	Vital Registration	100.0	6.8	
Iceland	1990-1994	5	96.0	1993	Vital Registration	100.0	4.0	
Iceland	1995-1999	5	94.8	1996	Vital Registration	100.0	5.2	
Iceland	2000-2004	5	93.9	2004	Vital Registration	99.7	5.8	
Iceland	2005-2009	5	92.5	2008	Vital Registration	100.0	7.5	
Iceland	2010-2024	5	91.6	2010	Vital Registration	99.5	8.0	
India	1980-1984	1	2.7	1983	Morbidity and mortality in diarrhea in rural Haryana Indian		1.5	2.7
India	1985-1989	1	4.6	1989	Maternal mortality in seven districts of Uttar Pradesh - an ICMR task force study		11.2	5.2
India	1990-1994	1	2.8	1991	Vital Registration	4.8	42.4	
India	1995-1999	1	5.0	1999	Vital Registration	8.8	42.5	
India	2000-2004	1	6.3	2003	India Study on Causes of Death by Verbal Autopsy 2003		1.8	6.4
India	2005-2009	3	49.8	2005	Verbal Autopsy		22.2	64.0
India	2010-2024	3	46.3	2016	Non-communicable diseases as a major contributor to deaths in 12 tribal districts in India		16.4	55.4
Indonesia	1980-1984	1	1.7	1984	The pattern of the causes of death in children in rural swampy area of South Sumatra, Indonesia		0.8	1.7
Indonesia	1985-1989	0						
Indonesia	1990-1994	1	1.7	1991	Care-seeking for fatal illnesses in young children in Indramayu, West Java, Indonesia		2.8	1.8
Indonesia	1995-1999	1	0.6	1997	Age- and cause-specific childhood mortality in Lombok, Indonesia, as a factor for determining the appropriateness of introducing Haemophilus influenzae type b and pneumococcal vaccines		1.7	0.6
Indonesia	2000-2004	1	0.3	2002	Africa, Asia, Oceania - INDEPTH Network Cause-Specific Mortality - Release 2014		95.7	6.4
Indonesia	2005-2009	3	54.0	2006	Indonesia Basic Health Research 2007-2008		2.1	55.2
Indonesia	2010-2024	3	63.4	2012	Indonesia Sample Registration System - Deaths 2012		1.0	64.0
Iran	1980-1984	0						
Iran	1985-1989	0						
Iran	1990-1994	0						
Iran	1995-1999	0						
Iran	2000-2004	3	51.9	2004	Vital Registration	70.8	26.6	
Iran	2005-2009	3	62.4	2007	Vital Registration	76.8	18.7	
Iran	2010-2024	4	78.3	2017	Vital Registration	91.4	14.3	
Iraq	1980-1984	0						
Iraq	1985-1989	0						
Iraq	1990-1994	0						
Iraq	1995-1999	0						
Iraq	2000-2004	0						
Iraq	2005-2009	3	38.9	2008	Vital Registration	71.9	46.0	
Iraq	2010-2024	3	44.9	2014	Vital Registration	74.5	39.8	
Ireland	1980-1984	5	90.9	1983	Vital Registration	100.0	9.1	
Ireland	1985-1989	5	92.1	1988	Vital Registration	99.9	7.8	
Ireland	1990-1994	5	92.7	1993	Vital Registration	100.0	7.3	
Ireland	1995-1999	5	92.1	1997	Vital Registration	100.0	7.8	
Ireland	2000-2004	5	92.1	2000	Vital Registration	100.0	7.9	
Ireland	2005-2009	5	92.9	2008	Vital Registration	100.0	7.1	
Ireland	2010-2024	5	92.9	2017	Vital Registration	100.0	7.1	
Israel	1980-1984	4	82.3	1983	Vital Registration	100.0	17.7	
Israel	1985-1989	4	82.9	1987	Vital Registration	100.0	17.1	
Israel	1990-1994	4	83.8	1994	Vital Registration	100.0	16.2	
Israel	1995-1999	4	83.7	1995	Vital Registration	100.0	16.3	
Israel	2000-2004	4	82.8	2002	Vital Registration	100.0	17.2	
Israel	2005-2009	4	81.2	2009	Vital Registration	99.4	18.4	
Israel	2010-2024	4	82.4	2010	Vital Registration	100.0	17.6	
Italy	1980-1984	5	89.3	1980	Vital Registration	100.0	10.7	
Italy	1985-1989	5	89.2	1989	Vital Registration	99.8	10.6	
Italy	1990-1994	5	90.0	1994	Vital Registration	100.0	10.0	
Italy	1995-1999	5	89.2	1995	Vital Registration	100.0	10.8	
Italy	2000-2004	5	89.8	2003	Vital Registration	100.0	10.2	
Italy	2005-2009	5	90.3	2008	Vital Registration	100.0	9.7	
Italy	2010-2024	5	90.0	2010	Vital Registration	100.0	10.0	
Jamaica	1980-1984	4	71.2	1983	Vital Registration	100.0	28.8	
Jamaica	1985-1989	4	71.1	1987	Vital Registration	96.8	26.5	
Jamaica	1990-1994	4	65.8	1991	Vital Registration	92.4	28.7	
Jamaica	1995-1999	0						
Jamaica	2000-2004	4	81.4	2004	Vital Registration	97.3	16.3	
Jamaica	2005-2009	4	83.0	2006	Vital Registration	94.9	12.5	
Jamaica	2010-2024	5	88.1	2014	Vital Registration	100.0	11.9	
Japan	1980-1984	4	82.5	1982	Vital Registration	100.0	17.5	
Japan	1985-1989	4	81.7	1985	Vital Registration	100.0	18.3	
Japan	1990-1994	4	83.3	1994	Vital Registration	100.0	16.7	
Japan	1995-1999	5	89.0	1995	Vital Registration	100.0	11.0	
Japan	2000-2004	5	87.9	2001	Vital Registration	100.0	12.1	
Japan	2005-2009	5	87.0	2005	Vital Registration	100.0	13.0	
Japan	2010-2024	5	85.4	2011	Vital Registration	100.0	14.6	
Jordan	1980-1984	0						
Jordan	1985-1989	0						
Jordan	1990-1994	0						
Jordan	1995-1999	1	1.6	1995	Mortality and causes of death in Jordan 1995-96: assessment by verbal autopsy		74.2	6.4
Jordan	2000-2004	4	74.1	2004	Vital Registration	95.8	22.6	
Jordan	2005-2009	4	81.1	2006	Vital Registration	100.0	18.9	
Jordan	2010-2024	3	61.4	2011	Vital Registration	76.8	20.0	
Kazakhstan	1980-1984	4	82.5	1982	Vital Registration	100.0	17.5	
Kazakhstan	1985-1989	4	83.0	1988	Vital Registration	99.3	16.3	
Kazakhstan	1990-1994	5	88.4	1993	Vital Registration	100.0	11.6	
Kazakhstan	1995-1999	5	88.0	1996	Vital Registration	100.0	12.0	
Kazakhstan	2000-2004	4	83.9	2003	Vital Registration	99.4	15.5	
Kazakhstan	2005-2009	4	81.7	2005	Vital Registration	98.5	17.0	
Kazakhstan	2010-2024	4	83.7	2016	Vital Registration	100.0	16.3	
Kenya	1980-1984	0						
Kenya	1985-1989	1	3.4	1986	Mortality patterns in a rural Kenyan community		1.2	3.4
Kenya	1990-1994	0						
Kenya	1995-1999	1	2.1	1997	The burden of malaria mortality among African children in the year 2000		8.4	2.3
Kenya	2000-2004	1	4.4	2003	Kenya - Nairobi Urban Health and Demographic Surveillance System		30.5	6.3
Kenya	2005-2009	1	5.1	2006	Kenya - Nairobi Urban Health and Demographic Surveillance System		20.7	6.4
Kenya	2010-2024	2	19.2	2017	Ascribing Causes of Maternal Mortality in Homa Bay County Kenya		0.6	19.3
Kiribati	1980-1984	0						
Kiribati	1985-1989	0						
Kiribati	1990-1994	3	32.2	1993	Vital Registration	59.4	45.9	
Kiribati	1995-1999	3	55.9	1995	Vital Registration	100.0	44.1	
Kiribati	2000-2004	2	30.7	2000	Vital Registration	56.1	45.2	
Kiribati	2005-2009	0						
Kiribati	2010-2024	0						
Kuwait	1980-1984	4	73.3	1981	Vital Registration	98.8	25.7	
Kuwait	1985-1989	4	77.3	1987	Vital Registration	99.1	22.1	
Kuwait	1990-1994	4	74.7	1993	Vital Registration	97.9	23.6	
Kuwait	1995-1999	4	78.3	1998	Vital Registration	100.0	21.7	
Kuwait	2000-2004	4	80.4	2004	Vital Registration	100.0	19.6	
Kuwait	2005-2009	4	82.0	2009	Vital Registration	100.0	18.0	
Kuwait	2010-2024	5	85.5	2016	Vital Registration	100.0	14.5	
Kyrgyzstan	1980-1984	4	75.3	1981	Vital Registration	93.1	19.1	
Kyrgyzstan	1985-1989	4	82.3	1988	Vital Registration	95.5	13.8	
Kyrgyzstan	1990-1994	4	74.2	1990	Vital Registration	90.8	18.3	
Kyrgyzstan	1995-1999	4	77.7	1998	Vital Registration	97.6	20.4	
Kyrgyzstan	2000-2004	5	90.1	2002	Vital Registration	97.8	8.0	
Kyrgyzstan	2005-2009	5	94.6	2009	Vital Registration	100.0	5.4	
Kyrgyzstan	2010-2024	5	94.2	2011	Vital Registration	100.0	5.8	
Laos	1980-1984	0						
Laos	1985-1989	1	4.0	1989	The Lao People's Democratic Republic: maternal mortality and female mortality: determining causes of deaths		9.9	4.4
Laos	1990-1994	0						
Laos	1995-1999	0						
Laos	2000-2004	0						
Laos	2005-2009	0						
Laos	2010-2024	2	28.6	2020	Assessment of the underlying causes of adult deaths using a short version of verbal autopsy in Xaiyabouli Province, Lao People's Democratic Republic		45.0	52.1
Latvia	1980-1984	5	91.5	1983	Vital Registration	99.9	8.4	
Latvia	1985-1989	5	91.6	1985	Vital Registration	100.0	8.4	
Latvia	1990-1994	5	88.8	1990	Vital Registration	100.0	11.2	
Latvia	1995-1999	5	91.8	1999	Vital Registration	100.0	8.2	
Latvia	2000-2004	5	91.2	2000	Vital Registration	100.0	8.8	
Latvia	2005-2009	5	89.2	2007	Vital Registration	100.0	10.8	
Latvia	2010-2024	5	94.2	2016	Vital Registration	100.0	5.8	
Lebanon	1980-1984	0						
Lebanon	1985-1989	1	3.9	1988	Non-communicable disease mortality rates using the verbal autopsy in a cohort of middle aged and older populations in Beirut during wartime, 1983-93		13.4	4.5
Lebanon	1990-1994	0						
Lebanon	1995-1999	0						
Lebanon	2000-2004	1	0.2	2000	Facility-based audit of maternal mortality in Lebanon: A feasibility study		12.6	0.3
Lebanon	2005-2009	0						
Lebanon	2010-2024	3	58.3	2020	Vital Registration	100.0	41.7	
Lesotho	1980-1984	0						
Lesotho	1985-1989	0						
Lesotho	1990-1994	0						
Lesotho	1995-1999	0						
Lesotho	2000-2004	0						
Lesotho	2005-2009	0						
Lesotho	2010-2024	0						
Liberia	1980-1984	1	4.1	1984	Infant and child mortality in two counties of Liberia: results of a survey in 1988 and trends since 1984		5.3	4.3
Liberia	1985-1989	1	4.0	1987	Infant and child mortality in two counties of Liberia: results of a survey in 1988 and trends since 1984		5.8	4.3
Liberia	1990-1994	1	4.5	1990	Application of the verbal autopsy during a clinical trial		9.5	5.0
Liberia	1995-1999	0						
Liberia	2000-2004	0						
Liberia	2005-2009	0						
Liberia	2010-2024	1	0.7	2019	Verbal autopsy analysis of maternal mortality in Bong County, Liberia: a retrospective mixed methods study		0.0	0.7
Libya	1980-1984	0						
Libya	1985-1989	0						
Libya	1990-1994	0						
Libya	1995-1999	0						
Libya	2000-2004	0						
Libya	2005-2009	2	10.8	2006	Vital Registration	86.3	87.5	
Libya	2010-2024	2	24.9	2016	Vital Registration	51.4	51.5	
Lithuania	1980-1984	5	90.3	1981	Vital Registration	100.0	9.7	
Lithuania	1985-1989	5	92.7	1988	Vital Registration	100.0	7.3	
Lithuania	1990-1994	5	93.0	1990	Vital Registration	100.0	7.0	
Lithuania	1995-1999	5	94.8	1997	Vital Registration	100.0	5.2	
Lithuania	2000-2004	5	93.2	2003	Vital Registration	100.0	6.8	
Lithuania	2005-2009	5	92.8	2009	Vital Registration	100.0	7.2	
Lithuania	2010-2024	5	93.6	2013	Vital Registration	100.0	6.4	
Luxembourg	1980-1984	5	86.3	1984	Vital Registration	99.5	13.3	
Luxembourg	1985-1989	5	87.1	1987	Vital Registration	100.0	12.9	
Luxembourg	1990-1994	5	87.0	1992	Vital Registration	100.0	13.0	
Luxembourg	1995-1999	5	86.9	1995	Vital Registration	99.4	12.6	
Luxembourg	2000-2004	5	85.1	2000	Vital Registration	100.0	14.9	
Luxembourg	2005-2009	4	81.7	2007	Vital Registration	100.0	18.3	

Underlying indicators for percent well-certified for data source with maximum percent well certified in each 5-year time interval for 204 countries, 1980-2023

Location	Time Window	Stars	Percent Well-Certified [PWC] (%)	Max PWC Data Year	Max PWC Data Source	Completeness (%)	Percent Major Garbage (%)	Verbal Autopsy Adjustment (None for VR) (%)
Luxembourg	2010-2024	5	87.5	2020	Vital Registration	100.0	12.5	
Madagascar	1980-1984	1	2.3	1984	Vital Registration	5.1	54.2	
Madagascar	1985-1989	1	2.9	1986	Vital Registration	6.2	52.8	
Madagascar	1990-1994	1	2.1	1990	Vital Registration	4.3	49.8	
Madagascar	1995-1999	1	2.2	1995	Vital Registration	4.4	50.0	
Madagascar	2000-2004	0						
Madagascar	2005-2009	0						
Madagascar	2010-2024	0						
Malawi	1980-1984	0						
Malawi	1985-1989	1	3.8	1988	Infant and second-year mortality in rural Malawi: causes and descriptive epidemiology		4.5	4.0
Malawi	1990-1994	0						
Malawi	1995-1999	1	1.5	1999	Estimation of AIDS adult mortality by verbal autopsy in rural Malawi		3.0	1.6
Malawi	2000-2004	1	2.5	2004	Declining child mortality in northern Malawi despite high rates of infection with HIV		3.2	2.6
Malawi	2005-2009	1	3.2	2008	Rates and causes of death in Chiradzulu District, Malawi, 2008: a key informant study		49.6	6.4
Malawi	2010-2024	2	12.7	2016	Cost-Effectiveness of Mass Treatment with Azithromycin for Reducing Child Mortality in Malawi: Secondary Analysis from the MORDOR Trial		1.9	13.0
Malaysia	1980-1984	3	42.0	1980	Vital Registration	72.1	41.7	
Malaysia	1985-1989	0						
Malaysia	1990-1994	0						
Malaysia	1995-1999	2	30.8	1997	Vital Registration	45.8	32.8	
Malaysia	2000-2004	3	34.7	2004	Vital Registration	57.7	39.8	
Malaysia	2005-2009	3	39.3	2008	Vital Registration	61.3	35.8	
Malaysia	2010-2024	3	53.5	2018	Vital Registration	70.2	23.9	
Maldives	1980-1984	0						
Maldives	1985-1989	0						
Maldives	1990-1994	0						
Maldives	1995-1999	0						
Maldives	2000-2004	3	42.6	2002	Vital Registration	98.0	56.5	
Maldives	2005-2009	3	46.4	2007	Vital Registration	99.8	53.5	
Maldives	2010-2024	3	55.8	2011	Vital Registration	91.0	38.7	
Mali	1980-1984	1	2.2	1981	Vital Registration	7.9	71.8	
Mali	1985-1989	0						
Mali	1990-1994	1	0.3	1990	Assessment of maternal mortality and late maternal mortality among a cohort of pregnant women in Bamako, Mali		10.8	0.3
Mali	1995-1999	0						
Mali	2000-2004	0						
Mali	2005-2009	0						
Mali	2010-2024	2	12.1	2016	[Contribution of verbal autopsy to the measurement of infant and child mortality in rural area in Niger]		1.1	12.2
Malta	1980-1984	4	82.4	1984	Vital Registration	99.9	17.6	
Malta	1985-1989	5	84.8	1989	Vital Registration	96.8	12.4	
Malta	1990-1994	5	89.8	1992	Vital Registration	100.0	10.2	
Malta	1995-1999	5	90.3	1998	Vital Registration	100.0	9.7	
Malta	2000-2004	5	91.3	2002	Vital Registration	100.0	8.7	
Malta	2005-2009	5	94.4	2009	Vital Registration	100.0	5.6	
Malta	2010-2024	5	93.8	2017	Vital Registration	100.0	6.2	
Marshall Islands	1980-1984	0						
Marshall Islands	1985-1989	0						
Marshall Islands	1990-1994	0						
Marshall Islands	1995-1999	0						
Marshall Islands	2000-2004	0						
Marshall Islands	2005-2009	0						
Marshall Islands	2010-2024	0						
Mauritania	1980-1984	0						
Mauritania	1985-1989	0						
Mauritania	1990-1994	0						
Mauritania	1995-1999	0						
Mauritania	2000-2004	0						
Mauritania	2005-2009	0						
Mauritania	2010-2024	0						
Mauritius	1980-1984	4	74.5	1984	Vital Registration	100.0	25.5	
Mauritius	1985-1989	4	78.5	1988	Vital Registration	100.0	21.5	
Mauritius	1990-1994	4	76.3	1991	Vital Registration	100.0	23.7	
Mauritius	1995-1999	4	76.9	1998	Vital Registration	100.0	23.1	
Mauritius	2000-2004	4	82.1	2002	Vital Registration	100.0	17.9	
Mauritius	2005-2009	5	84.3	2009	Vital Registration	100.0	15.7	
Mauritius	2010-2024	5	87.0	2014	Vital Registration	100.0	13.0	
Mexico	1980-1984	3	63.9	1984	Vital Registration	84.9	24.8	
Mexico	1985-1989	4	70.7	1989	Vital Registration	87.1	18.8	
Mexico	1990-1994	4	71.8	1990	Vital Registration	87.6	18.0	
Mexico	1995-1999	4	77.0	1999	Vital Registration	88.8	13.3	
Mexico	2000-2004	4	80.1	2004	Vital Registration	91.2	12.2	
Mexico	2005-2009	4	81.1	2007	Vital Registration	93.5	13.2	
Mexico	2010-2024	5	91.4	2020	Vital Registration	100.0	8.6	
Moldova	1980-1984	5	89.9	1981	Vital Registration	100.0	10.1	
Moldova	1985-1989	5	91.6	1985	Vital Registration	100.0	8.4	
Moldova	1990-1994	4	78.5	1991	Vital Registration	100.0	21.5	
Moldova	1995-1999	5	86.4	1996	Vital Registration	99.9	13.5	
Moldova	2000-2004	5	89.7	2003	Vital Registration	93.4	3.9	
Moldova	2005-2009	5	89.5	2005	Vital Registration	92.7	3.4	
Moldova	2010-2024	5	90.1	2012	Vital Registration	92.9	2.9	
Monaco	1980-1984	0						
Monaco	1985-1989	4	76.4	1986	Vital Registration	100.0	23.6	
Monaco	1990-1994	0						
Monaco	1995-1999	0						
Monaco	2000-2004	0						
Monaco	2005-2009	0						
Monaco	2010-2024	4	80.4	2013	Vital Registration	100.0	19.6	
Mongolia	1980-1984	0						
Mongolia	1985-1989	0						
Mongolia	1990-1994	3	59.1	1994	Vital Registration	79.2	25.3	
Mongolia	1995-1999	0						
Mongolia	2000-2004	2	21.0	2004	Vital Registration	88.3	76.3	
Mongolia	2005-2009	2	24.1	2008	Vital Registration	93.5	74.2	
Mongolia	2010-2024	5	93.1	2021	Vital Registration	100.0	6.9	
Montenegro	1980-1984	0						
Montenegro	1985-1989	0						
Montenegro	1990-1994	0						
Montenegro	1995-1999	0						
Montenegro	2000-2004	4	69.5	2000	Vital Registration	100.0	30.5	
Montenegro	2005-2009	4	72.4	2007	Vital Registration	97.0	25.4	
Montenegro	2010-2024	3	58.8	2017	Vital Registration	78.5	25.1	
Morocco	1980-1984	0						
Morocco	1985-1989	2	22.1	1988	Morocco National Survey on Causes and Circumstances of Infant and Child Deaths 1988-1989		2.6	22.7
Morocco	1990-1994	0						
Morocco	1995-1999	0						
Morocco	2000-2004	2	11.1	2004	Vital Registration	26.1	57.4	
Morocco	2005-2009	2	30.8	2005	Vital Registration	73.7	58.2	
Morocco	2010-2024	2	13.7	2014	Vital Registration	28.0	51.2	
Mozambique	1980-1984	0						
Mozambique	1985-1989	0						
Mozambique	1990-1994	0						
Mozambique	1995-1999	1	0.5	1996	Quality of registration of maternal deaths in Mozambique: a community-based study in rural and urban areas		11.2	0.5
Mozambique	2000-2004	2	11.0	2001	Vital Registration	17.7	37.6	
Mozambique	2005-2009	3	63.3	2007	Mozambique National Survey on the Causes of Death 2007-2008		1.0	64.0
Mozambique	2010-2024	3	43.7	2018	Mozambique Countrywide Mortality Surveillance for Action Data 2018-2020		31.7	64.0
Myanmar	1980-1984	0						
Myanmar	1985-1989	0						
Myanmar	1990-1994	0						
Myanmar	1995-1999	0						
Myanmar	2000-2004	0						
Myanmar	2005-2009	1	2.8	2007	Cause of Death Verification Study in Myanmar		55.7	6.2
Myanmar	2010-2024	3	48.9	2016	Myanmar National Mortality Survey 2016		23.6	64.0
Namibia	1980-1984	0						
Namibia	1985-1989	0						
Namibia	1990-1994	0						
Namibia	1995-1999	0						
Namibia	2000-2004	0						
Namibia	2005-2009	0						
Namibia	2010-2024	0						
Nauru	1980-1984	0						
Nauru	1985-1989	0						
Nauru	1990-1994	0						
Nauru	1995-1999	0						
Nauru	2000-2004	0						
Nauru	2005-2009	0						
Nauru	2010-2024	0						
Nepal	1980-1984	1	3.4	1984	Impact of a pilot acute respiratory infection (ARI) control programme in a rural community of the hill region of Nepal		2.5	3.5
Nepal	1985-1989	1	3.2	1987	Reduction in total under-five mortality in western Nepal through community-based antimicrobial treatment of pneumonia		1.8	3.3
Nepal	1990-1994	0						
Nepal	1995-1999	1	1.1	1999	Evaluation of neonatal verbal autopsy using physician review versus algorithm-based cause-of-death assignment in rural Nepal		2.0	1.2
Nepal	2000-2004	1	1.0	2003	Effect of daily zinc supplementation on child mortality in southern Nepal: a community-based, cluster randomised, placebo-controlled trial		1.8	1.0
Nepal	2005-2009	2	16.5	2006	Nepal Demographic and Health Survey 2006		4.2	17.3
Nepal	2010-2024	1	8.7	2013	Causes and age of neonatal death and associations with maternal and newborn care characteristics in Nepal: a verbal autopsy study		0.0	8.7
Netherlands	1980-1984	5	88.8	1981	Vital Registration	100.0	11.2	
Netherlands	1985-1989	5	87.6	1988	Vital Registration	100.0	12.4	
Netherlands	1990-1994	5	86.2	1991	Vital Registration	100.0	13.8	
Netherlands	1995-1999	5	85.9	1996	Vital Registration	100.0	14.1	
Netherlands	2000-2004	4	84.4	2004	Vital Registration	99.8	15.4	
Netherlands	2005-2009	5	86.0	2009	Vital Registration	100.0	14.0	
Netherlands	2010-2024	5	86.5	2020	Vital Registration	100.0	13.5	
New Zealand	1980-1984	5	95.1	1980	Vital Registration	100.0	4.9	
New Zealand	1985-1989	5	95.1	1988	Vital Registration	100.0	4.9	
New Zealand	1990-1994	5	95.3	1992	Vital Registration	100.0	4.7	
New Zealand	1995-1999	5	96.8	1999	Vital Registration	100.0	3.2	
New Zealand	2000-2004	5	97.1	2004	Vital Registration	100.0	2.9	
New Zealand	2005-2009	5	96.6	2006	Vital Registration	100.0	3.4	
New Zealand	2010-2024	5	96.2	2012	Vital Registration	100.0	3.8	
Nicaragua	1980-1984	0						
Nicaragua	1985-1989	3	50.2	1988	Vital Registration	69.4	27.6	
Nicaragua	1990-1994	3	53.8	1994	Vital Registration	73.6	26.9	
Nicaragua	1995-1999	4	70.1	1998	Vital Registration	84.4	17.0	
Nicaragua	2000-2004	4	70.4	2003	Vital Registration	80.8	12.9	
Nicaragua	2005-2009	4	74.5	2009	Vital Registration	84.4	11.7	
Nicaragua	2010-2024	5	92.3	2020	Vital Registration	100.0	7.7	
Niger	1980-1984	0						
Niger	1985-1989	0						
Niger	1990-1994	0						
Niger	1995-1999	0						
Niger	2000-2004	0						
Niger	2005-2009	3	39.3	2008	Direct estimates of national neonatal and child cause-specific mortality proportions in Niger by expert algorithm and physician-coded analysis of verbal autopsy interviews		0.9	39.6
Niger	2010-2024	1	3.7	2015	Cause-specific mortality of children younger than 5 years in communities receiving biannual mass azithromycin treatment in Niger: verbal autopsy results from a cluster-randomised controlled trial		0.9	3.7
Nigeria	1980-1984	0						
Nigeria	1985-1989	0						
Nigeria	1990-1994	1	3.8	1991	Community-based surveillance of paediatric deaths in Cross River State, Nigeria		2.1	3.9
Nigeria	1995-1999	1	3.7	1996	Using verbal autopsy to identify and proportionally assign cause of death in Ibadan, southwest Nigeria		85.5	25.7
Nigeria	2000-2004	0						
Nigeria	2005-2009	1	0.1	2007	Vital Registration	1.4	96.5	

Underlying indicators for percent well-certified for data source with maximum percent well certified in each 5-year time interval for 204 countries, 1980-2023								
Location	Time Window	Stars	Percent Well-Certified [PWC] (%)	Max PWC Data Year	Max PWC Data Source	Completeness (%)	Percent Major Garbage (%)	Verbal Autopsy Adjustment (None for VR) (%)
Nigeria	2010-2024	3	36.1	2016	Paediatric brought-in-dead at a tertiary health facility in South western Nigeria: Patterns and drivers		3.0	37.2
Niue	1980-1984	0						
Niue	1985-1989	0						
Niue	1990-1994	0						
Niue	1995-1999	0						
Niue	2000-2004	0						
Niue	2005-2009	0						
Niue	2010-2024	0						
North Korea	1980-1984	0						
North Korea	1985-1989	0						
North Korea	1990-1994	0						
North Korea	1995-1999	0						
North Korea	2000-2004	0						
North Korea	2005-2009	0						
North Korea	2010-2024	0						
North Macedonia	1980-1984	0						
North Macedonia	1985-1989	0						
North Macedonia	1990-1994	4	79.1	1992	Vital Registration	100.0	20.9	
North Macedonia	1995-1999	4	79.8	1998	Vital Registration	96.8	17.6	
North Macedonia	2000-2004	4	82.5	2003	Vital Registration	99.3	16.9	
North Macedonia	2005-2009	4	80.0	2009	Vital Registration	99.6	19.7	
North Macedonia	2010-2024	4	78.6	2010	Vital Registration	99.4	21.0	
Northern Mariana Islands	1980-1984	0						
Northern Mariana Islands	1985-1989	0						
Northern Mariana Islands	1990-1994	0						
Northern Mariana Islands	1995-1999	3	57.8	1998	Vital Registration	71.9	19.7	
Northern Mariana Islands	2000-2004	3	50.1	2004	Vital Registration	62.9	20.3	
Northern Mariana Islands	2005-2009	3	53.1	2006	Vital Registration	62.3	14.8	
Northern Mariana Islands	2010-2024	3	60.5	2021	Vital Registration	70.3	14.0	
Norway	1980-1984	5	90.6	1984	Vital Registration	100.0	9.4	
Norway	1985-1989	5	91.2	1988	Vital Registration	100.0	8.8	
Norway	1990-1994	5	90.6	1990	Vital Registration	100.0	9.4	
Norway	1995-1999	5	90.6	1997	Vital Registration	100.0	9.4	
Norway	2000-2004	5	88.4	2000	Vital Registration	99.8	11.4	
Norway	2005-2009	5	85.8	2005	Vital Registration	100.0	14.2	
Norway	2010-2024	5	86.5	2021	Vital Registration	99.9	13.4	
Oman	1980-1984	0						
Oman	1985-1989	0						
Oman	1990-1994	0						
Oman	1995-1999	0						
Oman	2000-2004	0						
Oman	2005-2009	3	61.5	2007	Vital Registration	75.4	18.4	
Oman	2010-2024	3	37.4	2016	Vital Registration	73.6	49.2	
Pakistan	1980-1984	0						
Pakistan	1985-1989	2	30.6	1986	Acute respiratory infections in children: a case management intervention in Abbottabad District, Pakistan		1.6	31.1
Pakistan	1990-1994	1	2.2	1990	Time to focus child survival programmes on the newborn: assessment of levels and causes of infant mortality in rural Pakistan		2.3	2.2
Pakistan	1995-1999	0						
Pakistan	2000-2004	2	11.4	2003	Impact of a community-based perinatal and newborn preventive care package on perinatal and neonatal mortality in a remote mountainous district in Northern Pakistan		0.0	11.4
Pakistan	2005-2009	2	20.2	2006	Pakistan Demographic and Health Survey 2006-2007		3.3	20.9
Pakistan	2010-2024	2	20.0	2010	Cause of Death in under 5 Children in a Demographic Surveillance Site in Pakistan		2.5	20.5
Palau	1980-1984	0						
Palau	1985-1989	0						
Palau	1990-1994	0						
Palau	1995-1999	0						
Palau	2000-2004	0						
Palau	2005-2009	0						
Palau	2010-2024	3	48.2	2013	Vital Registration	100.0	51.8	
Palestine	1980-1984	0						
Palestine	1985-1989	0						
Palestine	1990-1994	0						
Palestine	1995-1999	2	27.8	1999	Vital Registration	42.2	34.1	
Palestine	2000-2004	2	27.8	2000	Vital Registration	42.7	34.9	
Palestine	2005-2009	2	30.1	2009	Vital Registration	44.0	31.5	
Palestine	2010-2024	5	92.3	2021	Vital Registration	100.0	7.7	
Panama	1980-1984	4	73.3	1983	Vital Registration	99.0	26.0	
Panama	1985-1989	4	76.8	1987	Vital Registration	99.0	22.5	
Panama	1990-1994	0						
Panama	1995-1999	4	81.3	1999	Vital Registration	97.6	16.7	
Panama	2000-2004	5	85.0	2002	Vital Registration	98.1	13.4	
Panama	2005-2009	5	86.5	2007	Vital Registration	100.0	13.5	
Panama	2010-2024	5	85.1	2021	Vital Registration	100.0	14.9	
Papua New Guinea	1980-1984	1	9.0	1980	Vital Registration	12.5	28.2	
Papua New Guinea	1985-1989	1	3.6	1985	Mortality rates and the utilization of health services during terminal illness in the Asaro Valley, Eastern Highlands Province, Papua New Guinea		43.2	6.4
Papua New Guinea	1990-1994	0						
Papua New Guinea	1995-1999	0						
Papua New Guinea	2000-2004	0						
Papua New Guinea	2005-2009	0						
Papua New Guinea	2010-2024	2	25.9	2012	Epidemiological transition in Papua New Guinea: new evidence from verbal autopsy studies		39.5	42.8
Paraguay	1980-1984	3	51.1	1980	Vital Registration	81.6	37.4	
Paraguay	1985-1989	3	50.9	1989	Vital Registration	77.1	34.0	
Paraguay	1990-1994	3	58.6	1994	Vital Registration	79.5	26.3	
Paraguay	1995-1999	3	58.7	1996	Vital Registration	80.9	27.4	
Paraguay	2000-2004	3	59.2	2004	Vital Registration	83.0	28.6	
Paraguay	2005-2009	4	63.2	2009	Vital Registration	83.9	24.6	
Paraguay	2010-2024	4	73.9	2021	Vital Registration	86.3	14.3	
Peru	1980-1984	3	51.4	1980	Vital Registration	71.2	27.8	
Peru	1985-1989	2	34.9	1989	Vital Registration	65.5	46.8	
Peru	1990-1994	3	35.2	1992	Vital Registration	63.9	44.9	
Peru	1995-1999	3	46.2	1999	Vital Registration	71.5	35.4	
Peru	2000-2004	3	55.9	2004	Vital Registration	79.2	29.4	
Peru	2005-2009	3	58.2	2008	Vital Registration	77.1	24.6	
Peru	2010-2024	4	73.6	2020	Vital Registration	83.6	11.9	
Philippines	1980-1984	1	0.3	1980	Vital Registration	36.9	99.1	
Philippines	1985-1989	2	12.1	1989	Effect of not breastfeeding on the risk of diarrheal and respiratory mortality in children under 2 years of age in Metro Cebu, The Philippines		5.3	12.7
Philippines	1990-1994	2	16.7	1992	Vital Registration	89.1	81.3	
Philippines	1995-1999	3	42.0	1999	Vital Registration	88.5	52.5	
Philippines	2000-2004	3	48.0	2002	Vital Registration	90.9	47.2	
Philippines	2005-2009	4	78.4	2009	Vital Registration	93.3	16.0	
Philippines	2010-2024	5	85.1	2021	Vital Registration	100.0	14.9	
Poland	1980-1984	3	63.3	1984	Vital Registration	100.0	36.7	
Poland	1985-1989	3	63.4	1986	Vital Registration	100.0	36.6	
Poland	1990-1994	3	63.2	1994	Vital Registration	100.0	36.8	
Poland	1995-1999	4	72.9	1999	Vital Registration	100.0	27.1	
Poland	2000-2004	4	75.3	2002	Vital Registration	99.9	24.7	
Poland	2005-2009	4	75.4	2006	Vital Registration	100.0	24.6	
Poland	2010-2024	4	81.8	2021	Vital Registration	100.0	18.2	
Portugal	1980-1984	4	77.1	1984	Vital Registration	100.0	22.9	
Portugal	1985-1989	4	77.5	1985	Vital Registration	100.0	22.5	
Portugal	1990-1994	4	77.1	1991	Vital Registration	100.0	22.9	
Portugal	1995-1999	4	77.5	1995	Vital Registration	100.0	22.5	
Portugal	2000-2004	4	82.1	2002	Vital Registration	100.0	17.9	
Portugal	2005-2009	4	79.1	2009	Vital Registration	100.0	20.9	
Portugal	2010-2024	5	85.6	2016	Vital Registration	100.0	14.4	
Puerto Rico	1980-1984	4	77.6	1981	Vital Registration	100.0	22.4	
Puerto Rico	1985-1989	4	76.7	1989	Vital Registration	100.0	23.3	
Puerto Rico	1990-1994	4	84.3	1994	Vital Registration	100.0	15.7	
Puerto Rico	1995-1999	4	83.8	1997	Vital Registration	100.0	16.2	
Puerto Rico	2000-2004	4	84.5	2004	Vital Registration	100.0	15.5	
Puerto Rico	2005-2009	4	83.8	2008	Vital Registration	100.0	16.2	
Puerto Rico	2010-2024	4	84.7	2012	Vital Registration	100.0	15.3	
Qatar	1980-1984	2	11.8	1984	Vital Registration	66.3	82.2	
Qatar	1985-1989	2	16.3	1985	Vital Registration	81.2	79.9	
Qatar	1990-1994	0						
Qatar	1995-1999	4	67.1	1995	Vital Registration	92.4	27.3	
Qatar	2000-2004	3	58.2	2001	Vital Registration	91.8	36.6	
Qatar	2005-2009	4	66.2	2006	Vital Registration	100.0	33.8	
Qatar	2010-2024	4	74.4	2020	Vital Registration	99.9	25.5	
Romania	1980-1984	4	74.5	1984	Vital Registration	99.7	25.3	
Romania	1985-1989	4	77.6	1989	Vital Registration	100.0	22.4	
Romania	1990-1994	4	81.9	1994	Vital Registration	99.3	17.5	
Romania	1995-1999	5	85.7	1999	Vital Registration	100.0	14.3	
Romania	2000-2004	5	86.8	2004	Vital Registration	99.9	13.1	
Romania	2005-2009	5	87.2	2007	Vital Registration	100.0	12.8	
Romania	2010-2024	5	86.9	2013	Vital Registration	100.0	13.1	
Russia	1980-1984	5	92.5	1980	Vital Registration	100.0	7.5	
Russia	1985-1989	5	85.8	1989	Vital Registration	93.5	8.2	
Russia	1990-1994	5	90.1	1990	Vital Registration	100.0	9.9	
Russia	1995-1999	5	86.7	1999	Vital Registration	100.0	13.3	
Russia	2000-2004	5	87.2	2004	Vital Registration	100.0	12.8	
Russia	2005-2009	5	88.8	2009	Vital Registration	100.0	11.2	
Russia	2010-2024	5	88.4	2010	Vital Registration	100.0	11.6	
Rwanda	1980-1984	0						
Rwanda	1985-1989	0						
Rwanda	1990-1994	0						
Rwanda	1995-1999	0						
Rwanda	2000-2004	0						
Rwanda	2005-2009	2	26.2	2007	Rwanda Child Verbal Autopsy Study 2008		1.0	26.4
Rwanda	2010-2024	1	0.6	2014	Four delays of child mortality in Rwanda: a mixed methods analysis of verbal social autopsies		2.5	0.6
Saint Kitts and Nevis	1980-1984	4	65.9	1982	Vital Registration	96.7	31.8	
Saint Kitts and Nevis	1985-1989	4	67.4	1987	Vital Registration	100.0	32.6	
Saint Kitts and Nevis	1990-1994	4	72.2	1994	Vital Registration	100.0	27.8	
Saint Kitts and Nevis	1995-1999	4	81.2	1998	Vital Registration	100.0	18.8	
Saint Kitts and Nevis	2000-2004	4	75.6	2002	Vital Registration	97.6	22.6	
Saint Kitts and Nevis	2005-2009	5	85.6	2008	Vital Registration	100.0	14.4	
Saint Kitts and Nevis	2010-2024	5	91.7	2016	Vital Registration	100.0	8.3	
Saint Lucia	1980-1984	4	66.8	1983	Vital Registration	98.3	32.0	
Saint Lucia	1985-1989	4	65.9	1987	Vital Registration	100.0	34.1	
Saint Lucia	1990-1994	4	72.3	1994	Vital Registration	100.0	27.7	
Saint Lucia	1995-1999	4	73.4	1997	Vital Registration	100.0	26.6	
Saint Lucia	2000-2004	4	80.9	2001	Vital Registration	99.4	18.6	
Saint Lucia	2005-2009	4	82.6	2009	Vital Registration	98.1	15.7	
Saint Lucia	2010-2024	5	86.5	2014	Vital Registration	100.0	13.5	
Saint Vincent and the Grenadines	1980-1984	4	68.0	1983	Vital Registration	100.0	32.0	
Saint Vincent and the Grenadines	1985-1989	3	60.9	1986	Vital Registration	89.7	32.1	
Saint Vincent and the Grenadines	1990-1994	3	58.5	1990	Vital Registration	93.0	37.0	
Saint Vincent and the Grenadines	1995-1999	5	85.4	1998	Vital Registration	100.0	14.6	
Saint Vincent and the Grenadines	2000-2004	5	85.5	2002	Vital Registration	100.0	14.5	
Saint Vincent and the Grenadines	2005-2009	5	88.0	2006	Vital Registration	100.0	12.0	
Saint Vincent and the Grenadines	2010-2024	5	87.9	2014	Vital Registration	100.0	12.1	
Samoa	1980-1984	0						
Samoa	1985-1989	0						
Samoa	1990-1994	0						
Samoa	1995-1999	0						
Samoa	2000-2004	0						
Samoa	2005-2009	0						
Samoa	2010-2024	0						
San Marino	1980-1984	0						
San Marino	1985-1989	0						
San Marino	1990-1994	0						
San Marino	1995-1999	4	74.9	1997	Vital Registration	100.0	25.1	
San Marino	2000-2004	4	76.5	2002	Vital Registration	100.0	23.5	

Underlying indicators for percent well-certified for data source with maximum percent well certified in each 5-year time interval for 204 countries, 1980-2023								
Location	Time Window	Stars	Percent Well-Certified [PWC] (%)	Max PWC Data Year	Max PWC Data Source	Completeness (%)	Percent Major Garbage (%)	Verbal Autopsy Adjustment (None for VR) (%)
San Marino	2005-2009	4	79.7	2009	Vital Registration	100.0	20.3	
San Marino	2010-2024	5	86.0	2019	Vital Registration	100.0	14.0	
Saudi Arabia	1980-1984	0						
Saudi Arabia	1985-1989	0						
Saudi Arabia	1990-1994	0						
Saudi Arabia	1995-1999	2	19.9	1999	Vital Registration	37.1	46.2	
Saudi Arabia	2000-2004	2	22.6	2004	Vital Registration	44.8	49.5	
Saudi Arabia	2005-2009	2	25.0	2008	Vital Registration	51.9	51.9	
Saudi Arabia	2010-2024	3	63.0	2021	Vital Registration	82.3	23.4	
Senegal	1980-1984	1	2.8	1984	Senegal - Risk of Death Associated with Different Nutritional States in Children of Preschool age: Study Conducted in Niakhar (Senegal) 1983-1986		1.6	2.8
Senegal	1985-1989	1	3.5	1986	International differences in clinical patterns of diarrhoeal deaths: a comparison of children from Brazil, Senegal, Bangladesh, and India		4.9	3.6
Senegal	1990-1994	1	3.5	1990	Childhood mortality and probable causes of death using verbal autopsy in Niakhar, Senegal, 1989-2000		3.7	3.7
Senegal	1995-1999	1	3.4	1996	Childhood mortality and probable causes of death using verbal autopsy in Niakhar, Senegal, 1989-2000		2.5	3.5
Senegal	2000-2004	0						
Senegal	2005-2009	1	0.1	2006	Africa, Asia, Oceania - INDEPTH Network Cause-Specific Mortality - Release 2014		98.8	6.4
Senegal	2010-2024	3	37.7	2019	Enhancing the value of death registration with verbal autopsy data: a pilot study in the Senegalese urban population in 2019		41.1	64.0
Serbia	1980-1984	0						
Serbia	1985-1989	0						
Serbia	1990-1994	0						
Serbia	1995-1999	4	66.0	1998	Vital Registration	85.1	22.4	
Serbia	2000-2004	4	66.5	2003	Vital Registration	85.6	22.3	
Serbia	2005-2009	4	70.9	2009	Vital Registration	86.2	17.7	
Serbia	2010-2024	4	71.4	2010	Vital Registration	86.6	17.6	
Seychelles	1980-1984	4	71.3	1981	Vital Registration	100.0	28.7	
Seychelles	1985-1989	4	71.7	1987	Vital Registration	99.8	28.2	
Seychelles	1990-1994	0						
Seychelles	1995-1999	0						
Seychelles	2000-2004	4	76.8	2002	Vital Registration	100.0	23.2	
Seychelles	2005-2009	4	77.3	2008	Vital Registration	99.1	22.1	
Seychelles	2010-2024	4	78.7	2011	Vital Registration	100.0	21.3	
Sierra Leone	1980-1984	0						
Sierra Leone	1985-1989	0						
Sierra Leone	1990-1994	1	4.1	1990	Malaria in a rural area of Sierra Leone. I. Initial results		1.1	4.2
Sierra Leone	1995-1999	0						
Sierra Leone	2000-2004	0						
Sierra Leone	2005-2009	0						
Sierra Leone	2010-2024	3	47.3	2017	PRT_WFS_1979_1980_EXT_Y2008M10D10.DTA		8.6	51.8
Singapore	1980-1984	5	91.0	1983	Vital Registration	100.0	9.0	
Singapore	1985-1989	5	92.0	1988	Vital Registration	100.0	8.0	
Singapore	1990-1994	5	94.5	1994	Vital Registration	100.0	5.5	
Singapore	1995-1999	5	95.5	1997	Vital Registration	100.0	4.5	
Singapore	2000-2004	5	95.1	2003	Vital Registration	100.0	4.9	
Singapore	2005-2009	5	95.1	2005	Vital Registration	100.0	4.9	
Singapore	2010-2024	5	96.8	2014	Vital Registration	100.0	3.2	
Slovakia	1980-1984	0						
Slovakia	1985-1989	0						
Slovakia	1990-1994	4	83.6	1994	Vital Registration	100.0	16.4	
Slovakia	1995-1999	4	83.1	1999	Vital Registration	99.6	16.6	
Slovakia	2000-2004	5	85.4	2003	Vital Registration	100.0	14.6	
Slovakia	2005-2009	5	88.6	2008	Vital Registration	99.1	10.6	
Slovakia	2010-2024	5	91.2	2012	Vital Registration	99.1	8.0	
Slovenia	1980-1984	0						
Slovenia	1985-1989	5	91.7	1985	Vital Registration	100.0	8.3	
Slovenia	1990-1994	5	92.8	1992	Vital Registration	100.0	7.2	
Slovenia	1995-1999	5	91.3	1997	Vital Registration	100.0	8.7	
Slovenia	2000-2004	5	89.7	2000	Vital Registration	100.0	10.3	
Slovenia	2005-2009	5	89.7	2008	Vital Registration	100.0	10.3	
Slovenia	2010-2024	5	90.3	2020	Vital Registration	100.0	9.7	
Solomon Islands	1980-1984	0						
Solomon Islands	1985-1989	0						
Solomon Islands	1990-1994	0						
Solomon Islands	1995-1999	0						
Solomon Islands	2000-2004	0						
Solomon Islands	2005-2009	0						
Solomon Islands	2010-2024	3	51.8	2018	Diversity of epidemiological transition in the Pacific: Findings from the application of verbal autopsy in Papua New Guinea and the Solomon Islands		9.8	57.4
Somalia	1980-1984	0						
Somalia	1985-1989	0						
Somalia	1990-1994	0						
Somalia	1995-1999	0						
Somalia	2000-2004	0						
Somalia	2005-2009	0						
Somalia	2010-2024	1	3.0	2017	Use of verbal autopsy for establishing causes of child mortality in camps for internally displaced people in Mogadishu, Somalia: a population-based, prospective, cohort study		3.2	3.1
South Africa	1980-1984	0						
South Africa	1985-1989	0						
South Africa	1990-1994	1	1.3	1991	'A bothersome death' -- narrative accounts of infant mortality in Cape Town, South Africa		1.8	1.3
South Africa	1995-1999	3	51.8	1999	Vital Registration	78.8	34.2	
South Africa	2000-2004	3	55.1	2003	Vital Registration	81.5	32.4	
South Africa	2005-2009	3	56.9	2009	Vital Registration	83.9	32.2	
South Africa	2010-2024	3	59.8	2014	Vital Registration	86.4	30.8	
South Korea	1980-1984	0						
South Korea	1985-1989	3	54.3	1985	Vital Registration	88.3	38.5	
South Korea	1990-1994	4	71.9	1994	Vital Registration	97.2	26.1	
South Korea	1995-1999	4	73.6	1999	Vital Registration	100.0	26.4	
South Korea	2000-2004	4	83.5	2002	Vital Registration	100.0	16.5	
South Korea	2005-2009	4	82.3	2007	Vital Registration	100.0	17.7	
South Korea	2010-2024	4	83.6	2015	Vital Registration	100.0	16.4	
South Sudan	1980-1984	0						
South Sudan	1985-1989	0						
South Sudan	1990-1994	0						
South Sudan	1995-1999	0						
South Sudan	2000-2004	0						
South Sudan	2005-2009	0						
South Sudan	2010-2024	0						
Spain	1980-1984	4	78.7	1984	Vital Registration	99.7	21.0	
Spain	1985-1989	4	81.3	1989	Vital Registration	100.0	18.7	
Spain	1990-1994	5	84.8	1994	Vital Registration	99.7	14.9	
Spain	1995-1999	5	86.5	1998	Vital Registration	100.0	13.5	
Spain	2000-2004	5	86.0	2002	Vital Registration	100.0	14.0	
Spain	2005-2009	5	86.2	2009	Vital Registration	100.0	13.8	
Spain	2010-2024	5	88.5	2020	Vital Registration	100.0	11.5	
Sri Lanka	1980-1984	3	45.7	1980	Vital Registration	100.0	54.3	
Sri Lanka	1985-1989	3	46.2	1988	Vital Registration	100.0	53.8	
Sri Lanka	1990-1994	3	43.7	1992	Vital Registration	100.0	56.3	
Sri Lanka	1995-1999	3	50.3	1999	Vital Registration	100.0	49.7	
Sri Lanka	2000-2004	3	58.3	2003	Vital Registration	100.0	41.7	
Sri Lanka	2005-2009	4	63.4	2009	Vital Registration	100.0	36.6	
Sri Lanka	2010-2024	3	64.5	2010	Vital Registration	100.0	35.5	
Sudan	1980-1984	0						
Sudan	1985-1989	0						
Sudan	1990-1994	0						
Sudan	1995-1999	0						
Sudan	2000-2004	0						
Sudan	2005-2009	0						
Sudan	2010-2024	0						
Suriname	1980-1984	3	54.6	1982	Vital Registration	88.3	38.2	
Suriname	1985-1989	3	58.0	1989	Vital Registration	88.8	34.7	
Suriname	1990-1994	3	54.0	1991	Vital Registration	80.6	33.0	
Suriname	1995-1999	3	60.0	1999	Vital Registration	87.5	31.4	
Suriname	2000-2004	4	67.0	2002	Vital Registration	88.0	23.9	
Suriname	2005-2009	4	67.3	2008	Vital Registration	88.3	23.8	
Suriname	2010-2024	4	68.8	2010	Vital Registration	87.4	21.3	
Sweden	1980-1984	5	92.2	1980	Vital Registration	100.0	7.8	
Sweden	1985-1989	5	90.5	1988	Vital Registration	100.0	9.5	
Sweden	1990-1994	5	90.2	1990	Vital Registration	100.0	9.8	
Sweden	1995-1999	5	89.6	1997	Vital Registration	99.8	10.3	
Sweden	2000-2004	5	88.9	2003	Vital Registration	100.0	11.1	
Sweden	2005-2009	5	88.7	2005	Vital Registration	100.0	11.3	
Sweden	2010-2024	5	87.3	2020	Vital Registration	100.0	12.7	
Switzerland	1980-1984	4	75.9	1983	Vital Registration	100.0	24.1	
Switzerland	1985-1989	4	75.8	1986	Vital Registration	100.0	24.2	
Switzerland	1990-1994	4	75.1	1990	Vital Registration	100.0	24.9	
Switzerland	1995-1999	5	87.2	1997	Vital Registration	100.0	12.8	
Switzerland	2000-2004	5	86.8	2001	Vital Registration	100.0	13.2	
Switzerland	2005-2009	5	87.9	2007	Vital Registration	100.0	12.1	
Switzerland	2010-2024	5	87.8	2011	Vital Registration	100.0	12.2	
Syria	1980-1984	2	28.6	1980	Vital Registration	98.5	71.0	
Syria	1985-1989	2	12.7	1985	Vital Registration	57.6	78.0	
Syria	1990-1994	0						
Syria	1995-1999	3	45.8	1999	Vital Registration	86.4	47.0	
Syria	2000-2004	3	55.0	2004	Vital Registration	83.1	33.9	
Syria	2005-2009	4	62.8	2009	Vital Registration	84.7	25.9	
Syria	2010-2024	3	50.1	2010	Vital Registration	87.0	42.4	
São Tomé and Príncipe	1980-1984	0						
São Tomé and Príncipe	1985-1989	3	61.3	1985	Vital Registration	100.0	38.7	
São Tomé and Príncipe	1990-1994	0						
São Tomé and Príncipe	1995-1999	0						
São Tomé and Príncipe	2000-2004	0						
São Tomé and Príncipe	2005-2009	0						
São Tomé and Príncipe	2010-2024	0						
Taiwan*	1980-1984	4	80.5	1980	Vital Registration	100.0	19.5	
Taiwan*	1985-1989	4	79.2	1986	Vital Registration	100.0	20.8	
Taiwan*	1990-1994	4	77.9	1994	Vital Registration	100.0	22.1	
Taiwan*	1995-1999	4	83.1	1999	Vital Registration	100.0	16.9	
Taiwan*	2000-2004	4	84.6	2004	Vital Registration	100.0	15.4	
Taiwan*	2005-2009	5	85.0	2005	Vital Registration	100.0	15.0	
Taiwan*	2010-2024	5	85.6	2014	Vital Registration	100.0	14.4	
Tajikistan	1980-1984	4	74.9	1981	Vital Registration	89.6	16.4	
Tajikistan	1985-1989	4	70.7	1988	Vital Registration	89.8	21.3	
Tajikistan	1990-1994	4	66.2	1993	Vital Registration	100.0	33.8	
Tajikistan	1995-1999	3	52.0	1995	Vital Registration	76.1	31.6	
Tajikistan	2000-2004	3	49.5	2002	Vital Registration	72.5	31.7	
Tajikistan	2005-2009	3	49.6	2005	Vital Registration	76.2	34.9	
Tajikistan	2010-2024	3	59.9	2016	Vital Registration	87.9	31.9	
Tanzania	1980-1984	0						
Tanzania	1985-1989	1	3.6	1986	Risk factors for deaths in children under 5 years old in Bagamoyo district, Tanzania		2.4	3.7
Tanzania	1990-1994	1	2.9	1993	Community based studies on childhood mortality in a malaria holoendemic area on the Tanzanian coast		6.3	3.1
Tanzania	1995-1999	1	2.3	1995	The Policy Implications of Tanzania's Mortality Burden		64.6	6.4
Tanzania	2000-2004	1	6.3	2000	Tanzania - Rufiji Health and Demographic Surveillance System		0.9	6.4
Tanzania	2005-2009	1	2.8	2009	Pattern of all-causes and cause-specific mortality in an area with progressively declining malaria burden in Korogwe district, north-eastern Tanzania		56.8	6.4
Tanzania	2010-2024	2	21.4	2013	Verbal/social autopsy analysis of causes and determinants of under-5 mortality in Tanzania from 2010 to 2016		1.7	21.8
Thailand	1980-1984	2	24.5	1983	Vital Registration	86.4	71.6	
Thailand	1985-1989	2	25.2	1987	Vital Registration	83.7	69.9	
Thailand	1990-1994	2	29.7	1994	Vital Registration	86.6	65.7	
Thailand	1995-1999	3	62.6	1998	Thailand Burden of Disease and Injuries 1998-1999		2.2	64.0
Thailand	2000-2004	3	44.1	2003	Vital Registration	92.6	52.4	
Thailand	2005-2009	3	61.6	2005	Thailand Verbal Autopsy Study 2005		3.8	64.0
Thailand	2010-2024	4	62.9	2016	Vital Registration	98.5	36.1	
The Bahamas	1980-1984	4	73.6	1980	Vital Registration	100.0	26.4	

Underlying indicators for percent well-certified for data source with maximum percent well certified in each 5-year time interval for 204 countries, 1980-2023

Location	Time Window	Stars	Percent Well-Certified [PWC] (%)	Max PWC Data Year	Max PWC Data Source	Completeness (%)	Percent Major Garbage (%)	Verbal Autopsy Adjustment (None for VR) (%)
The Bahamas	1985-1989	4	82.2	1987	Vital Registration	100.0	17.8	
The Bahamas	1990-1994	4	75.1	1994	Vital Registration	92.1	18.5	
The Bahamas	1995-1999	5	83.8	1998	Vital Registration	100.0	16.2	
The Bahamas	2000-2004	4	78.5	2001	Vital Registration	89.5	12.3	
The Bahamas	2005-2009	4	81.7	2009	Vital Registration	94.0	13.1	
The Bahamas	2010-2024	5	82.2	2011	Vital Registration	93.6	12.2	
The Gambia	1980-1984	1	4.0	1982	Deaths in infancy and early childhood in a well-vaccinated, rural, West African population		1.6	4.0
The Gambia	1985-1989	1	3.5	1989	Changes in the pattern of infant and childhood mortality in upper river division, The Gambia, from 1989 to 1993		3.6	3.6
The Gambia	1990-1994	1	3.4	1990	Changes in the pattern of infant and childhood mortality in upper river division, The Gambia, from 1989 to 1993		4.5	3.6
The Gambia	1995-1999	1	2.8	1999	Reaching millennium development goal 4 - the Gambia		7.0	3.0
The Gambia	2000-2004	1	2.5	2002	Reaching millennium development goal 4 - the Gambia		7.5	2.7
The Gambia	2005-2009	1	2.3	2006	Preventive measures in infancy to reduce under-five mortality: a case-control study in The Gambia		6.0	2.4
The Gambia	2010-2024	2	13.9	2020	Verbal autopsy analysis of childhood deaths in rural Gambia		1.9	14.2
Timor-Leste	1980-1984	0						
Timor-Leste	1985-1989	0						
Timor-Leste	1990-1994	0						
Timor-Leste	1995-1999	0						
Timor-Leste	2000-2004	0						
Timor-Leste	2005-2009	0						
Timor-Leste	2010-2024	0						
Togo	1980-1984	0						
Togo	1985-1989	0						
Togo	1990-1994	0						
Togo	1995-1999	0						
Togo	2000-2004	0						
Togo	2005-2009	0						
Togo	2010-2024	0						
Tokelau	1980-1984	0						
Tokelau	1985-1989	0						
Tokelau	1990-1994	0						
Tokelau	1995-1999	0						
Tokelau	2000-2004	0						
Tokelau	2005-2009	0						
Tokelau	2010-2024	0						
Tonga	1980-1984	0						
Tonga	1985-1989	0						
Tonga	1990-1994	0						
Tonga	1995-1999	0						
Tonga	2000-2004	3	52.5	2003	Vital Registration	100.0	47.5	
Tonga	2005-2009	0						
Tonga	2010-2024	0						
Trinidad and Tobago	1980-1984	4	78.1	1983	Vital Registration	94.8	17.6	
Trinidad and Tobago	1985-1989	4	81.0	1989	Vital Registration	95.5	15.2	
Trinidad and Tobago	1990-1994	5	84.1	1994	Vital Registration	98.4	14.5	
Trinidad and Tobago	1995-1999	5	89.2	1999	Vital Registration	100.0	10.8	
Trinidad and Tobago	2000-2004	5	90.3	2002	Vital Registration	100.0	9.7	
Trinidad and Tobago	2005-2009	5	88.8	2005	Vital Registration	100.0	11.2	
Trinidad and Tobago	2010-2024	5	89.9	2012	Vital Registration	100.0	10.1	
Tunisia	1980-1984	0						
Tunisia	1985-1989	0						
Tunisia	1990-1994	0						
Tunisia	1995-1999	0						
Tunisia	2000-2004	0						
Tunisia	2005-2009	2	28.7	2009	Vital Registration	38.6	25.5	
Tunisia	2010-2024	4	72.8	2021	Vital Registration	94.3	22.8	
Turkmenistan	1980-1984	5	88.5	1981	Vital Registration	100.0	11.5	
Turkmenistan	1985-1989	5	88.0	1986	Vital Registration	100.0	12.0	
Turkmenistan	1990-1994	5	88.3	1993	Vital Registration	100.0	11.7	
Turkmenistan	1995-1999	4	82.2	1999	Vital Registration	89.8	8.5	
Turkmenistan	2000-2004	4	82.3	2002	Vital Registration	93.3	11.7	
Turkmenistan	2005-2009	4	80.3	2009	Vital Registration	95.8	16.2	
Turkmenistan	2010-2024	4	83.2	2010	Vital Registration	99.8	16.7	
Tuvalu	1980-1984	0						
Tuvalu	1985-1989	0						
Tuvalu	1990-1994	0						
Tuvalu	1995-1999	0						
Tuvalu	2000-2004	0						
Tuvalu	2005-2009	0						
Tuvalu	2010-2024	0						
Türkiye	1980-1984	2	12.3	1983	Vital Registration	34.6	64.4	
Türkiye	1985-1989	2	15.6	1989	Vital Registration	39.3	60.4	
Türkiye	1990-1994	2	17.2	1993	Vital Registration	41.6	58.6	
Türkiye	1995-1999	2	20.6	1999	Vital Registration	51.2	59.7	
Türkiye	2000-2004	3	59.0	2002	Turkey Verbal Autopsy Survey 2003		7.8	64.0
Türkiye	2005-2009	3	59.9	2009	Vital Registration	75.7	20.9	
Türkiye	2010-2024	4	72.1	2016	Vital Registration	86.5	16.7	
UK	1980-1984	5	93.4	1983	Vital Registration	100.0	6.6	
UK	1985-1989	5	94.0	1985	Vital Registration	100.0	6.0	
UK	1990-1994	5	94.0	1991	Vital Registration	100.0	6.0	
UK	1995-1999	5	92.3	1995	Vital Registration	100.0	7.7	
UK	2000-2004	5	91.8	2002	Vital Registration	100.0	8.2	
UK	2005-2009	5	91.6	2005	Vital Registration	100.0	8.4	
UK	2010-2024	5	91.8	2012	Vital Registration	100.0	8.2	
USA	1980-1984	5	90.7	1980	Vital Registration	100.0	9.3	
USA	1985-1989	5	89.9	1989	Vital Registration	100.0	10.1	
USA	1990-1994	5	90.4	1990	Vital Registration	100.0	9.6	
USA	1995-1999	5	90.0	1995	Vital Registration	100.0	10.0	
USA	2000-2004	5	88.7	2000	Vital Registration	100.0	11.3	
USA	2005-2009	5	87.9	2005	Vital Registration	100.0	12.1	
USA	2010-2024	5	87.4	2010	Vital Registration	100.0	12.6	
Uganda	1980-1984	0						
Uganda	1985-1989	0						
Uganda	1990-1994	0						
Uganda	1995-1999	0						
Uganda	2000-2004	1	0.8	2000	Effect of HIV infection on pregnancy-related mortality in sub-Saharan Africa: secondary analyses of pooled community-based data from the network for Analysing Longitudinal Population-based HIV/AIDS data on Africa (ALPHA)		12.8	0.9
Uganda	2005-2009	1	2.9	2006	Uganda Child Verbal Autopsy Study 2007		1.2	3.0
Uganda	2010-2024	1	3.0	2010	Under 10 mortality patterns, risk factors, and mechanisms in low resource settings of Eastern Uganda: An analysis of event history demographic and verbal social autopsy data		0.4	3.0
Ukraine	1980-1984	5	87.6	1981	Vital Registration	99.4	11.9	
Ukraine	1985-1989	5	88.9	1985	Vital Registration	100.0	11.1	
Ukraine	1990-1994	4	81.6	1994	Vital Registration	100.0	18.4	
Ukraine	1995-1999	4	83.2	1998	Vital Registration	99.9	16.7	
Ukraine	2000-2004	4	82.9	2000	Vital Registration	98.5	15.8	
Ukraine	2005-2009	5	89.0	2009	Vital Registration	100.0	11.0	
Ukraine	2010-2024	5	91.8	2013	Vital Registration	100.0	8.2	
United Arab Emirates	1980-1984	0						
United Arab Emirates	1985-1989	0						
United Arab Emirates	1990-1994	0						
United Arab Emirates	1995-1999	0						
United Arab Emirates	2000-2004	0						
United Arab Emirates	2005-2009	3	53.3	2007	Vital Registration	100.0	46.7	
United Arab Emirates	2010-2024	4	74.2	2018	Vital Registration	100.0	25.8	
Uruguay	1980-1984	4	77.1	1980	Vital Registration	100.0	22.9	
Uruguay	1985-1989	4	77.3	1989	Vital Registration	99.4	22.3	
Uruguay	1990-1994	4	77.4	1990	Vital Registration	100.0	22.6	
Uruguay	1995-1999	4	80.4	1997	Vital Registration	98.6	18.4	
Uruguay	2000-2004	4	80.8	2001	Vital Registration	100.0	19.2	
Uruguay	2005-2009	4	80.5	2005	Vital Registration	100.0	19.5	
Uruguay	2010-2024	4	80.4	2015	Vital Registration	100.0	19.6	
Uzbekistan	1980-1984	5	86.5	1982	Vital Registration	100.0	13.5	
Uzbekistan	1985-1989	5	87.0	1985	Vital Registration	100.0	13.0	
Uzbekistan	1990-1994	5	85.6	1992	Vital Registration	100.0	14.4	
Uzbekistan	1995-1999	4	82.2	1995	Vital Registration	97.8	16.0	
Uzbekistan	2000-2004	4	79.6	2000	Vital Registration	96.2	17.2	
Uzbekistan	2005-2009	4	81.2	2005	Vital Registration	92.1	11.9	
Uzbekistan	2010-2024	4	82.6	2011	Vital Registration	92.4	10.6	
Vanuatu	1980-1984	0						
Vanuatu	1985-1989	0						
Vanuatu	1990-1994	0						
Vanuatu	1995-1999	0						
Vanuatu	2000-2004	0						
Vanuatu	2005-2009	0						
Vanuatu	2010-2024	0						
Venezuela	1980-1984	4	77.9	1983	Vital Registration	100.0	22.1	
Venezuela	1985-1989	4	72.7	1988	Vital Registration	100.0	27.3	
Venezuela	1990-1994	4	82.3	1994	Vital Registration	100.0	17.7	
Venezuela	1995-1999	5	88.3	1999	Vital Registration	100.0	11.7	
Venezuela	2000-2004	5	90.6	2001	Vital Registration	100.0	9.4	
Venezuela	2005-2009	5	91.0	2007	Vital Registration	100.0	9.0	
Venezuela	2010-2024	5	91.5	2014	Vital Registration	100.0	8.5	
Viet Nam	1980-1984	0						
Viet Nam	1985-1989	1	1.3	1987	Are there social inequities in child morbidity and mortality in rural Vietnam		4.3	1.4
Viet Nam	1990-1994	1	0.9	1994	Maternal mortality in Vietnam in 1994-95		10.2	1.0
Viet Nam	1995-1999	1	1.2	1999	Applying verbal autopsy to determine cause of death in rural Vietnam		81.6	6.4
Viet Nam	2000-2004	1	1.4	2001	Socio-economic status inequality and major causes of death in adults: a 5-year follow-up study in rural Vietnam		74.8	5.6
Viet Nam	2005-2009	3	62.0	2008	Mortality measures from sample-based surveillance: evidence of the epidemiological transition in Viet Nam. Unpublished data		3.1	64.0
Viet Nam	2010-2024	1	4.8	2010	The causes of deaths in Chililab between 2008-2010 based on verbal autopsy method		25.5	6.4
Virgin Islands	1980-1984	4	78.0	1980	Vital Registration	98.4	20.7	
Virgin Islands	1985-1989	0						
Virgin Islands	1990-1994	4	83.0	1994	Vital Registration	91.7	9.5	
Virgin Islands	1995-1999	5	87.8	1995	Vital Registration	98.1	10.4	
Virgin Islands	2000-2004	4	76.1	2000	Vital Registration	86.0	11.5	
Virgin Islands	2005-2009	4	82.6	2008	Vital Registration	91.9	10.0	
Virgin Islands	2010-2024	4	77.3	2010	Vital Registration	90.8	14.9	
Yemen	1980-1984	0						
Yemen	1985-1989	0						
Yemen	1990-1994	0						
Yemen	1995-1999	0						
Yemen	2000-2004	0						
Yemen	2005-2009	0						
Yemen	2010-2024	0						
Zambia	1980-1984	0						
Zambia	1985-1989	0						
Zambia	1990-1994	0						
Zambia	1995-1999	0						
Zambia	2000-2004	0						
Zambia	2005-2009	1	6.4	2009	Zambia Sample Vital Registration with Verbal Autopsy (SAVVY) Data 2010		0.7	6.4
Zambia	2010-2024	2	32.7	2016	Zambia Sample Vital Registration with Verbal Autopsy (SAVVY) 2015-2016		48.9	64.0
Zimbabwe	1980-1984	0						
Zimbabwe	1985-1989	0						
Zimbabwe	1990-1994	2	27.9	1990	Vital Registration	42.8	35.0	
Zimbabwe	1995-1999	3	32.4	1995	Vital Registration	55.9	42.0	
Zimbabwe	2000-2004	1	1.5	2000	Effect of HIV infection on pregnancy-related mortality in sub-Saharan Africa: secondary analyses of pooled community-based data from the network for Analysing Longitudinal Population-based HIV/AIDS data on Africa (ALPHA)		12.9	1.7
Zimbabwe	2005-2009	2	24.7	2007	Vital Registration	33.1	25.6	
Zimbabwe	2010-2024	2	10.3	2019	Changes in causes of pregnancy-related and maternal mortality in Zimbabwe 2007-08 to 2018-19: findings from two reproductive age mortality surveys		5.0	10.8

Appendix Table S6: Restrictions on age and sex by cause for GBD 2023			
**Cause**	**Minimum Age**	**Maximum Age**	**Sex Restrictions**
HIV/AIDS and sexually transmitted infections			
HIV/AIDS	1 month		
HIV/AIDS - Drug-susceptible Tuberculosis	1 month		
HIV/AIDS - Multidrug-resistant Tuberculosis without extensive drug resista	1 month		
HIV/AIDS - Extensively drug-resistant Tuberculosis	1 month		
HIV/AIDS resulting in other diseases	1 month		
Sexually transmitted infections excluding HIV			
Syphilis			
Chlamydial infection	10		
Gonococcal infection	10		
Other sexually transmitted infections	10		
Respiratory infections and tuberculosis			
Tuberculosis	1 month		
Drug-susceptible tuberculosis	1 month		
Multidrug-resistant tuberculosis without extensive drug resistance	1 month		
Extensively drug-resistant tuberculosis	1 month		
Lower respiratory infections			
Upper respiratory infections			
Otitis media			
COVID-19			
Enteric infections			
Diarrhoeal diseases			
Typhoid and paratyphoid	7 days		
Typhoid fever	7 days		
Paratyphoid fever	7 days		
iNTS	7 days		
Other intestinal infectious diseases	7 days		
Neglected tropical diseases and malaria			
Malaria			
Chagas disease	1 month		
Leishmaniasis	1 month		
Visceral leishmaniasis	1 month		
African trypanosomiasis	2		
Schistosomiasis	6 months		
Cysticercosis	2		
Cystic echinococcosis	2		
Dengue	7 days		
Yellow fever	7 days		
Rabies	1 month		
Intestinal nematode infections	1 month		
Ascariasis	1 month		
Ebola virus disease			
Zika virus disease			
Other neglected tropical diseases			
Other infectious diseases			
Meningitis			
Encephalitis			
Diphtheria	1 month	59	

Pertussis	1 month	59	
Tetanus			
Measles	6 months	64	
Varicella and herpes zoster			
Acute hepatitis	1 month		
Acute hepatitis A	1 month		
Acute hepatitis B	1 month		
Acute hepatitis C	1 month		
Acute hepatitis E	1 month		
Other unspecified infectious diseases			
Maternal and neonatal disorders		54	
Maternal disorders	10	54	Females Only
Maternal haemorrhage	10	54	Females Only
Maternal sepsis and other pregnancy related infections	10	54	Females Only
Maternal hypertensive disorders	10	54	Females Only
Maternal obstructed labour and uterine rupture	10	54	Females Only
Maternal abortive outcome	10	54	Females Only
Ectopic pregnancy	10	54	Females Only
Indirect maternal deaths	10	54	Females Only
Late maternal deaths	10	54	Females Only
Maternal deaths aggravated by HIV/AIDS	10	54	Females Only
Other maternal disorders	10	54	Females Only
Neonatal disorders		4	
Neonatal preterm birth		4	
Neonatal encephalopathy due to birth asphyxia and trauma		4	
Neonatal sepsis and other neonatal infections		4	
Hemolytic disease and other neonatal jaundice		4	
Other neonatal disorders		4	
Nutritional deficiencies	1 month		
Protein-energy malnutrition	1 month		
Other nutritional deficiencies	1 month		
Neoplasms			
Lip and oral cavity cancer	15		
Nasopharynx cancer	5		
Other pharynx cancer	20		
Oesophageal cancer	20		
Stomach cancer	15		
Colon and rectum cancer	15		
Liver cancer			
Liver cancer due to hepatitis B	10		
Liver cancer due to hepatitis C	10		
Liver cancer due to alcohol use	15		
Liver cancer due to NASH	15		
Hepatoblastoma		9	
Liver cancer due to other causes	10		
Gallbladder and biliary tract cancer	20		
Pancreatic cancer	15		
Larynx cancer	20		
Tracheal, bronchus, and lung cancer	15		
Malignant skin melanoma	15		

Non-melanoma skin cancer	20		
Non-melanoma skin cancer (squamous-cell carcinoma)	20		
Soft tissue and other extraosseous sarcomas			
Malignant neoplasm of bone and articular cartilage	12 months		
Breast cancer	15		
Cervical cancer	15		Females Only
Uterine cancer	20		Females Only
Ovarian cancer	15		Females Only
Prostate cancer	20		Males Only
Testicular cancer	15		Males Only
Kidney cancer			
Bladder cancer	15		
Brain and nervous system cancer			
Eye cancer			
Retinoblastoma		9	
Other eye cancers	10		
Neuroblastoma and other peripheral nervous cell tumours			
Thyroid cancer	5		
Mesothelioma	20		
Hodgkin lymphoma	2		
Non-Hodgkin's lymphoma	12 months		
Burkitt lymphoma	12 months		
Other non-Hodgkin lymphoma	12 months		
Multiple myeloma	20		
Leukaemia			
Acute lymphoid leukaemia			
Chronic lymphoid leukaemia	20		
Acute myeloid leukaemia			
Chronic myeloid leukaemia			
Other leukaemia			
Other malignant cancers			
Other neoplasms			
Myelodysplastic, myeloproliferative, and other hematopoietic neoplasms			
Non-malignant brain and other central nervous system neoplasms	1 month		
Other benign and in situ neoplasms	1 month		
Cardiovascular diseases			
Rheumatic heart disease	12 months		
Ischaemic heart disease	15		
Stroke			
Ischaemic stroke			
Intracerebral hemorrhage			
Subarachnoid hemorrhage			
Hypertensive heart disease	15		
Non-rheumatic valvular heart disease	15		
Non-rheumatic calcific aortic valve disease	15		
Non-rheumatic degenerative mitral valve disease	15		
Other non-rheumatic valve diseases	15		
Cardiomyopathy and myocarditis			
Myocarditis			
Alcoholic cardiomyopathy	15		

Other cardiomyopathy			
Pulmonary Arterial Hypertension			
Atrial fibrillation and flutter	30		
Aortic aneurysm	15		
Lower extremity peripheral arterial disease	40		
Endocarditis			
Other cardiovascular and circulatory diseases			
Chronic respiratory diseases			
Chronic obstructive pulmonary disease	15		
Pneumoconiosis	15		
Silicosis	15		
Asbestosis	15		
Coal workers pneumoconiosis	15		
Other pneumoconiosis	15		
Asthma	12 months		
Interstitial lung disease and pulmonary sarcoidosis	2		
Other chronic respiratory diseases			
Digestive diseases			
Cirrhosis and other chronic liver diseases	12 months		
Chronic hepatitis B including cirrhosis	12 months		
Chronic hepatitis C including cirrhosis	12 months		
Cirrhosis due to alcohol	15		
Nonalcoholic fatty liver disease including cirrhosis	15		
Cirrhosis due to other causes	12 months		
Upper digestive system diseases	6 months		
Peptic ulcer disease	6 months		
Gastritis and duodenitis	6 months		
Appendicitis	12 months		
Paralytic ileus and intestinal obstruction			
Inguinal, femoral, and abdominal hernia			
Inflammatory bowel disease	2		
Ulcerative colitis	2		
Crohn's disease	2		
Vascular intestinal disorders	2		
Gallbladder and biliary diseases	2		
Pancreatitis	2		
Diverticular disease of intestines	2		
Other digestive diseases	12 months		
Neurological disorders			
Alzheimer's disease and other dementias	40		
Parkinson's disease	20		
Idiopathic epilepsy			
Multiple sclerosis	5		
Motor neuron disease			
Other neurological disorders			
Mental disorders	5		
Eating disorders	5	49	
Anorexia nervosa	5	49	
Substance use disorders			
Alcohol use disorders			

Drug use disorders			
Opioid use disorders			
Cocaine use disorders	10		
Amphetamine use disorders	10		
Other drug use disorders	10		
Diabetes and kidney diseases			
Diabetes mellitus	1 month		
Diabetes mellitus type 1	1 month		
Diabetes mellitus type 2	15		
Chronic kidney disease			
Chronic kidney disease due to diabetes mellitus type 1			
Chronic kidney disease due to diabetes mellitus type 2	15		
Chronic kidney disease due to hypertension	15		
Chronic kidney disease due to glomerulonephritis			
Chronic kidney disease due to other and unspecified causes			
Acute glomerulonephritis			
Skin and subcutaneous diseases			
Bacterial skin diseases			
Cellulitis			
Pyoderma			
Decubitus ulcer	2		
Other skin and subcutaneous diseases			
Musculoskeletal disorders	1 month		
Rheumatoid arthritis	5		
Other musculoskeletal disorders	1 month		
Other non-communicable diseases			
Congenital anomalies		69	
Neural tube defects		69	
Congenital heart anomalies		69	
Orofacial clefts		4	
Down's syndrome		69	
Other chromosomal abnormalities		69	
Congenital musculoskeletal and limb anomalies		69	
Urogenital congenital anomalies		69	
Digestive congenital anomalies		69	
Other congenital anomalies		69	
Urinary diseases and male infertility			
Urinary tract infections and interstitial nephritis			
Urolithiasis	2		
Other urinary diseases			
Gynecological diseases	10		Females Only
Uterine fibroids	10		Females Only
Endometriosis	10	54	Females Only
Genital prolapse	10		Females Only
Other gynecological diseases	10		Females Only
Hemoglobinopathies and hemolytic anaemias			
Thalassemias			
Sickle cell disorders			
G6PD deficiency			
Other hemoglobinopathies and hemolytic anaemias			

Endocrine, metabolic, blood, and immune disorders			
Thyroid diseases			
Other endocrine, metabolic, blood, and immune disorders			
Sudden infant death syndrome	7 days	23 months	
Transport injuries			
Road injuries			
Pedestrian road injuries			
Cyclist road injuries	12 months	94	
Motorcyclist road injuries		94	
Motor vehicle road injuries			
Other road injuries			
Other transport injuries			
Unintentional injuries			
Falls			
Drowning			
Fire, heat, and hot substances			
Poisonings			
Poisoning by carbon monoxide			
Poisoning by other means			
Exposure to mechanical forces			
Unintentional firearm injuries			
Other exposure to mechanical forces			
Adverse effects of medical treatment			
Animal contact			
Venomous animal contact			
Non-venomous animal contact			
Foreign body			
Pulmonary aspiration and foreign body in airway			
Foreign body in other body part			
Electrocution			
Environmental heat and cold exposure			
Exposure to forces of nature			
Other unintentional injuries			
Self-harm and interpersonal violence			
Self-harm	10		
Self-harm by hanging, strangulation, and suffocation	10		
Self-harm by fire, heat, and hot substances	10		
Self-harm by firearm	10		
Self-harm by poisoning pesticides	10		
Self-harm by non pesticide substance and gas	10		
Self-harm by other specified means	10		
Interpersonal violence			
Assault by firearm			
Assault by sharp object			
Assault by other means			
Conflict and terrorism			
Executions and police conflict	1 month		

Appendix Table S7: HIV/AIDS-related garbage code redistribution packages

Package Name	ICD10 codes	ICD9 codes
Actinomycosis	A42-A42.9	039-039.9, 113-113.6
Bartonellosis	A44-A44.9	088.0-088.7
Urogenital Candidiasis	B37.3-B37.4	112.1-112.2
Candidiasis	B37-B37.2, B37.5-B37.9	112-112.0, 112.3-112.9
Coccidioidomycosis	B38-B38.9	114-114.9
Histoplasmosis	B39-B39.9	115-115.9
Blastomycosis	B40-B40.9	116-116.0, 116.2-116.9
Paracoccidioidomycosis	B41-B41.9	116.1
Sporotrichosis and Chromomycosis	B42-B43.9	117.1
Zygomycosis	B46-B46.9	117.3
Aspergillosis	B44-B44.9	117.7
Toxoplasmosis	B58-B58.9	130-130.9
Pneumocystosis	B59-B59.9	136.3-136.5
Cryptococcosis	B45-B45.9	117.5
Nocardiosis	A43-A43.9	117.2
Unspecified mycosis	B49-B49.9	117-117.0, 117.4, 117.6, 117.8-118.9
Cutaneous leishmaniasis	B55, B55.1-B55.9	085.1-085.5
Mycobacterial skin infection	A31.1-A31.2	031.1
Other Mycobacterial infection	A31-A31.0, A31.8-A31.9	031-031.0, 031.2-031.9
Immunodeficiency cell	D81-D82.9	279.2-279.4
Immunodeficiency antibody	D80-D80.9	279.0-279.1
Immunodeficiency other	D83-D84.9, D89.8-D89.9	279, 279.5-279.9
Kaposi's sarcoma	C46-C46.9	176-176.9

Appendix Table S8: Modeling strategy for individual cause of death models in GBD 2023		
**Cause Name**	**Level**	**Model type**
Communicable, maternal, neonatal, and nutritional disea	Aggregate	
HIV/AIDS and sexually transmitted infections	Aggregate	
HIV/AIDS	3	EPP-ASM, Spectrum
HIV-AIDS - Drug-susceptible Tuberculosis	4	Data proportion
HIV/AIDS - Multidrug-resistant Tuberculosis without exte	4	Data proportion
HIV/AIDS - Extensively drug-resistant Tuberculosis	4	Data proportion
HIV/AIDS resulting in other diseases	4	Data proportion
Sexually transmitted infections excluding HIV	3	CODEm; natural history model (congenital syphilis)
Syphilis	4	Data proportion (age/sex-specific VR); CODEm (data rich congeital syphilis); natural history model (congenital syphilis)
Chlamydial infection	4	Data proportion (age/sex-specific VR)
Gonococcal infection	4	Data proportion (age/sex-specific VR)
Other sexually transmitted diseases	4	Data proportion (age/sex-specific VR)
Respiratory infections and tuberculosis	Aggregate	
Tuberculosis	3	CODEm
Drug-susceptible tuberculosis	4	Spatio-temporal Gaussian process regression proportion
Multidrug-resistant tuberculosis without extensive drug re	4	Spatio-temporal Gaussian process regression proportion
Extensively drug-resistant tuberculosis	4	Spatio-temporal Gaussian process regression proportion
Lower respiratory infections	3	CODEm
Upper respiratory infections	3	CODEm
Otitis media	3	CODEm
Enteric infections	Aggregate	
Diarrheal diseases	3	CODEm; Fatal Discontinuity

Typhoid and paratyphoid	Aggregate	
Typhoid fever	4	CODEm (data rich countries); natural history model (non-data rich countries)
Paratyphoid fever	4	CODEm (data rich countries); natural history model (non-data rich countries)
Invasive Non-typhoidal Salmonella (iNTS)	3	CODEm (data rich countries); natural history model (non-data rich countries)
Other intestinal infectious diseases	3	Negative binomial regression
Neglected tropical diseases and malaria	Aggregate	
Malaria	3	CODEm (Fatal age pattern); Natural history- Bayesian mixed-effects model (P. falciparum inside Africa); Natural history- INLA mixed-effects model (P. falciparum outside Africa); negative binomial regression (P. vivax)
Chagas disease	3	CODEm
Leishmaniasis	Aggregate	
Visceral leishmaniasis	4	Natural history model
African trypanosomiasis	3	Natural history model
Schistosomiasis	3	Negative binomial regression
Cysticercosis	3	Negative binomial regression
Cystic echinococcosis	3	Mixed effects poisson model
Dengue	3	CODEm, Fatal Discontinuity
Yellow fever	3	Natural history model
Rabies	3	CODEm
Intestinal nematode infections	Aggregate	
Ascariasis	4	Negative binomial regression
Ebola	3	Fatal Discontinuity
Zika virus	3	Natural history model
Other neglected tropical diseases	3	CODEm
Other infectious diseases	Aggregate	

Meningitis	3	CODEm, Fatal Discontinuity
Encephalitis	3	CODEm
Diphtheria	3	CODEm (data rich countries); negative binomial regression (non-data rich countries)
Whooping cough	3	CODEm (data rich countries); natural history model (non-data rich countries)
Tetanus	3	CODEm
Measles	3	CODEm (data rich countries); natural history model (non-data rich countries), Fatal Discontinuity
Varicella and herpes zoster	3	CODEm (data rich countries); negative binomial regression (non-data rich countries)
Acute hepatitis	3	CODEm
Acute hepatitis A	4	CODEm
Acute hepatitis B	4	CODEm
Acute hepatitis C	4	CODEm
Acute hepatitis E	4	CODEm
Other unspecified infectious diseases	3	CODEm, Fatal Discontinuity
Maternal and neonatal disorders	Aggregate	
Maternal disorders	3	CODEm
Maternal hemorrhage	4	Spatio-temporal Gaussian process regression
Maternal sepsis and other maternal infections	4	Spatio-temporal Gaussian process regression
Maternal hypertensive disorders	4	Spatio-temporal Gaussian process regression
Maternal obstructed labor and uterine rupture	4	Spatio-temporal Gaussian process regression

Maternal abortion and miscarriage	4	Spatio-temporal Gaussian process regression
Ectopic pregnancy	4	Spatio-temporal Gaussian process regression proportion
Indirect maternal deaths	4	Spatio-temporal Gaussian process regression
Late maternal deaths	4	DisMod MR-2.1 proportion model
Maternal deaths aggravated by HIV/AIDS	4	Spatio-temporal Gaussian process regression
Other maternal disorders	4	Spatio-temporal Gaussian process regression
Neonatal disorders	3	CODEm
Neonatal preterm birth	4	CODEm
Neonatal encephalopathy due to birth asphyxia and trau	4	CODEm
Neonatal sepsis and other neonatal infections	4	CODEm
Hemolytic disease and other neonatal jaundice	4	CODEm
Other neonatal disorders	4	CODEm
Nutritional deficiencies	2	CODEm
Protein-energy malnutrition	3	CODEm; Fatal Discontinuity
Other nutritional deficiencies	3	CODEm
Non-communicable diseases	Aggregate	
Neoplasms	Aggregate	
Lip and oral cavity cancer	3	CODEm
Nasopharynx cancer	3	CODEm
Other pharynx cancer	3	CODEm
Esophageal cancer	3	CODEm
Stomach cancer	3	CODEm
Colon and rectum cancer	3	CODEm
Liver cancer	3	CODEm
Liver cancer due to hepatitis B	4	DisMod MR-2.1 proportion model

Liver cancer due to hepatitis C	4	DisMod MR-2.1 proportion model
Liver cancer due to alcohol use	4	DisMod MR-2.1 proportion model
Liver cancer due to NASH	4	DisMod MR-2.1 proportion model
Liver cancer due to other causes	4	DisMod MR-2.1 proportion model
Gallbladder and biliary tract cancer	3	CODEm
Pancreatic cancer	3	CODEm
Larynx cancer	3	CODEm
Tracheal, bronchus, and lung cancer	3	CODEm
Malignant skin melanoma	3	CODEm
Non-melanoma skin cancer	Aggregate	
Non-melanoma skin cancer (squamous-cell carcinoma)	4	CODEm
Breast cancer	3	CODEm
Cervical cancer	3	CODEm
Uterine cancer	3	CODEm
Ovarian cancer	3	CODEm
Prostate cancer	3	CODEm
Testicular cancer	3	CODEm
Kidney cancer	3	CODEm
Bladder cancer	3	CODEm
Brain and nervous system cancer	3	CODEm
Thyroid cancer	3	CODEm
Mesothelioma	3	CODEm
Hodgkin lymphoma	3	CODEm
Non-Hodgkin lymphoma	3	CODEm
Multiple myeloma	3	CODEm
Leukemia	3	CODEm
Acute lymphoid leukemia	4	CODEm
Chronic lymphoid leukemia	4	CODEm
Acute myeloid leukemia	4	CODEm
Chronic myeloid leukemia	4	CODEm

Other leukemia	4	CODEm
Other malignant neoplasms	3	CODEm
Other neoplasms	Aggregate	
Myelodysplastic, myeloproliferative, and other hematopo	4	CODEm
Other benign and in situ neoplasms	4	CODEm
Cardiovascular diseases	2	CODEm
Rheumatic heart disease	3	CODEm
Ischemic heart disease	3	CODEm
Stroke	3	CODEm
Ischemic stroke	4	CODEm
Intracerebral hemorrhage	4	CODEm
Subarachnoid hemorrhage	4	CODEm
Hypertensive heart disease	3	CODEm
Non-rheumatic valvular heart disease	3	CODEm
Non-rheumatic calcific aortic valve disease	4	CODEm
Non-rheumatic degenerative mitral valve disease	4	CODEm
Other non-rheumatic valve diseases	4	CODEm
Cardiomyopathy and myocarditis	3	CODEm
Myocarditis	4	CODEm
Alcoholic cardiomyopathy	4	CODEm
Other cardiomyopathy	4	CODEm
Pulmonary arterial hypertension	3	CODEm
Atrial fibrillation and flutter	3	CODEm
Aortic aneurysm	3	CODEm
Peripheral artery disease	3	CODEm
Endocarditis	3	CODEm
Other cardiovascular and circulatory diseases	3	CODEm
Chronic respiratory diseases	2	CODEm
Chronic obstructive pulmonary disease	3	CODEm
Pneumoconiosis	3	CODEm

Silicosis	4	CODEm
Asbestosis	4	CODEm
Coal workers pneumoconiosis	4	CODEm
Other pneumoconiosis	4	CODEm
Asthma	3	CODEm
Interstitial lung disease and pulmonary sarcoidosis	3	CODEm
Other chronic respiratory diseases	3	CODEm
Digestive diseases	2	CODEm
Cirrhosis and other chronic liver diseases	3	CODEm
Chronic hepatitis B including cirrhosis	4	DisMod MR-2.1 proportion model
Chronic hepatitis C including cirrhosis	4	DisMod MR-2.1 proportion model
Cirrhosis due to alcohol use	4	DisMod MR-2.1 proportion model
Nonalcoholic fatty liver disease including cirrhosis	4	DisMod MR-2.1 proportion model
Cirrhosis due to other causes	4	DisMod MR-2.1 proportion model
Upper digestive system diseases	3	CODEm
Peptic ulcer disease	4	CODEm
Gastritis and duodenitis	4	CODEm
Appendicitis	3	CODEm
Paralytic ileus and intestinal obstruction	3	CODEm
Inguinal, femoral, and abdominal hernia	3	CODEm
Inflammatory bowel disease	3	CODEm
Crohn's disease	4	CODEm
Ulcerative colitis	4	CODEm
Vascular intestinal disorders	3	CODEm
Gallbladder and biliary diseases	3	CODEm
Pancreatitis	3	CODEm
Other digestive diseases	3	CODEm
Neurological disorders	Aggregate	
Alzheimer's disease and other dementias	3	DisMod MR-2.1; custom excess mortality analysis

Parkinson's disease	3	CODEm
Epilepsy	3	CODEm
Multiple sclerosis	3	CODEm
Motor neuron disease	3	CODEm
Other neurological disorders	3	CODEm
Mental disorders	Aggregate	
Eating disorders	3	CODEm
Anorexia nervosa	4	CODEm
Substance use disorders	Aggregate	
Alcohol use disorders	3	CODEm, imported cases
Drug use disorders	3	CODEm
Opioid use disorders	4	CODEm, imported cases
Cocaine use disorders	4	CODEm
Amphetamine use disorders	4	CODEm
Other drug use disorders	4	CODEm
Diabetes and kidney diseases	Aggregate	
Diabetes mellitus	3	CODEm
Diabetes mellitus type 1	4	CODEm
Diabetes mellitus type 2	4	CODEm
Chronic kidney disease	3	CODEm
Chronic kidney disease due to diabetes mellitus type 1	4	DisMod MR-2.1 proportion model
Chronic kidney disease due to diabetes mellitus type 2	4	DisMod MR-2.1 proportion model
Chronic kidney disease due to hypertension	4	DisMod MR-2.1 proportion model
Chronic kidney disease due to glomerulonephritis	4	DisMod MR-2.1 proportion model
Chronic kidney disease due to other causes	4	DisMod MR-2.1 proportion model
Acute glomerulonephritis	3	CODEm
Skin and subcutaneous diseases	2	CODEm
Bacterial skin diseases	3	CODEm
Cellulitis	4	CODEm
Pyoderma	4	CODEm

Decubitus ulcer	3	CODEm
Other skin and subcutaneous diseases	3	CODEm
Musculoskeletal disorders	2	CODEm
Rheumatoid arthritis	3	CODEm
Other musculoskeletal disorders	3	CODEm
Other non-communicable diseases	Aggregate	
Congenital birth defects	3	CODEm
Neural tube defects	4	CODEm
Congenital heart anomalies	4	CODEm
Orofacial clefts	4	CODEm
Down syndrome	4	CODEm
Other chromosomal abnormalities	4	CODEm
Congenital musculoskeletal and limb anomalies	4	CODEm
Urogenital congenital anomalies	4	CODEm
Digestive congenital anomalies	4	CODEm
Other congenital birth defects	4	CODEm
Urinary diseases and male infertility	3	CODEm
Urinary tract infections	4	CODEm
Urolithiasis	4	CODEm
Other urinary diseases	4	CODEm
Gynecological diseases	3	CODEm
Uterine fibroids	4	CODEm
Endometriosis	4	CODEm
Genital prolapse	4	CODEm
Other gynecological diseases	4	CODEm
Hemoglobinopathies and hemolytic anemias	3	CODEm
Thalassemias	4	DisMod MR-2.1 cause-specific mortality model
Sickle cell disorders	4	DisMod MR-2.1 cause-specific mortality model

G6PD deficiency	4	DisMod MR-2.1 cause-specific mortality model
Other hemoglobinopathies and hemolytic anemias	4	Data proportion
Endocrine, metabolic, blood, and immune disorders	3	CODEm
Thyroid disorders	4	CODEm
Other endocrine, metabolic, blood, and immune disorde	4	CODEm
Diverticular disease	3	CODEm
Sudden infant death syndrome	3	CODEm
Injuries	Aggregate	
Transport injuries	2	CODEm
Road injuries	3	CODEm
Pedestrian road injuries	4	CODEm
Cyclist road injuries	4	CODEm
Motorcyclist road injuries	4	CODEm
Motor vehicle road injuries	4	CODEm
Other road injuries	4	CODEm
Other transport injuries	3	CODEm; Fatal Discontinuity
Unintentional injuries	Aggregate	
Falls	3	CODEm
Drowning	3	CODEm
Fire, heat, and hot substances	3	CODEm; Fatal Discontinuity
Poisonings	3	CODEm
Poisoning by carbon monoxide	4	CODEm
Poisoning by other means	4	CODEm, Fatal Discontinuity
Exposure to mechanical forces	Aggregate	
Unintentional firearm injuries	4	CODEm
Other exposure to mechanical forces	4	CODEm; Fatal Discontinuity
Adverse effects of medical treatment	3	CODEm
Animal contact	3	CODEm
Venomous animal contact	4	CODEm

Non-venomous animal contact	4	CODEm, Fatal Discontinuity
Foreign body	Aggregate	
Pulmonary aspiration and foreign body in airway	4	CODEm
Foreign body in other body part	4	CODEm
Environmental heat and cold exposure	3	CODEm; Fatal Discontinuity
Exposure to forces of nature	3	Fatal Discontinuity
Other unintentional injuries	3	CODEm
Self-harm and interpersonal violence	Aggregate	
Self-harm	3	CODEm
Self-harm by firearm	4	CODEm
Self-harm by other specified means	4	CODEm
Interpersonal violence	3	CODEm
Physical violence by firearm	4	CODEm, Fatal Discontinuity
Physical violence by sharp object	4	CODEm, Fatal Discontinuity
Physical violence by other means	4	CODEm, Fatal Discontinuity
Conflict and terrorism	3	Fatal Discontinuity
Executions and police conflict	3	CODEm, Fatal Discontinuity
Pulmonary arterial hypertension	3	CODEm
Hepatoblastoma	4	CODEm (age split)
Burkitt lymphoma	4	CODEm
Other non-Hodgkin lymphoma	4	CODEm
Eye cancer	Aggregate	
Retinoblastoma	4	CODEm
Other eye cancers	4	CODEm
Soft tissue and other extraosseous sarcomas	3	CODEm
Malignant neoplasm of bone and articular cartilage	3	CODEm
Neuroblastoma and other peripheral nervous cell tumors	3	CODEm

Appendix Table S9: Percent change before and after CoDCorrect by cause for all ages, both sexes, global, 20		
**Cause name**	**CoDCorrect level**	**Percent change**
All causes	0	3.32<br>-4.38 to 8.32
Communicable, maternal, neonatal, and nutritional disorders	1	4.7<br>-1.79 to 10.04
Tuberculosis	3	6.06<br>-1.3 to 12.82
HIV/AIDS	3	-4.36<br>-11.14 to 3.14
HIV/AIDS resulting in other diseases	4	-4.87<br>-11.78 to 2.6
Diarrhoeal diseases	3	6.06<br>-2.79 to 14.08
Typhoid fever	4	-4.84<br>-12.21 to 2.58
Paratyphoid fever	4	-6.31<br>-14.25 to 1.57
Other intestinal infectious diseases	3	7.88<br>0.55 to 12.92
Lower respiratory infections	3	5.69<br>-1.45 to 11.61
Upper respiratory infections	3	6.13<br>-4.82 to 16.97
Otitis media	3	4.97<br>-4.46 to 13.8
Meningitis	3	9.78<br>-1.48 to 21.31
Encephalitis	3	5.98<br>0.08 to 11.64
Diphtheria	3	25.08<br>-1.1 to 51.76
Pertussis	3	0.24<br>-14.99 to 18.78
Tetanus	3	1.94<br>-5.85 to 9.45
Measles	3	-0.69<br>-18.06 to 22.79
Varicella and herpes zoster	3	6.35<br>-2.7 to 14.45
Neglected tropical diseases and malaria	2	6.02<br>-8.92 to 23.18
Malaria	3	5.39<br>-10.45 to 27.47
Chagas disease	3	-1.94<br>-8.44 to 1.6
Leishmaniasis	3	9.86<br>-3.68 to 37.73
Visceral leishmaniasis	4	9.86<br>-3.68 to 37.73
African trypanosomiasis	3	17.75<br>2.93 to 35.44
Schistosomiasis	3	10.83<br>1.63 to 19.77
Cysticercosis	3	15.28<br>6.34 to 23.83
Cystic echinococcosis	3	10.89<br>3.03 to 18.7
Dengue	3	7.71<br>-1.02 to 17.54
Yellow fever	3	26.11<br>10.89 to 44.36

Appendix Table S9: Percent change before and after CoDCorrect by cause for all ages, both sexes, global, 20		
**Cause name**	**CoDCorrect level**	**Percent change**
Rabies	3	7.32<br>-1.21 to 13.74
Intestinal nematode infections	3	13.63<br>-7.16 to 35.47
Ascariasis	4	13.63<br>-7.16 to 35.47
Other neglected tropical diseases	3	12.65<br>-2.51 to 26.72
Maternal disorders	3	16.19<br>6.89 to 26.79
Maternal haemorrhage	4	16.1<br>6.79 to 26.9
Maternal sepsis and other pregnancy related infections	4	14.98<br>3.0 to 30.69
Maternal hypertensive disorders	4	15.93<br>6.76 to 26.85
Maternal obstructed labour and uterine rupture	4	13.11<br>3.77 to 24.82
Ectopic pregnancy	4	15.85<br>5.82 to 28.31
Indirect maternal deaths	4	19.09<br>10.88 to 26.92
Late maternal deaths	4	14.06<br>7.3 to 20.89
Neonatal disorders	3	5.42<br>-2.64 to 14.59
Neonatal preterm birth	4	9.47<br>-3.87 to 23.82
Neonatal encephalopathy due to birth asphyxia and trauma	4	14.25<br>-2.12 to 33.51
Neonatal sepsis and other neonatal infections	4	1.16<br>-13.22 to 20.42
Hemolytic disease and other neonatal jaundice	4	7.43<br>-12.89 to 31.33
Other neonatal disorders	4	-1.03<br>-15.9 to 16.48
Nutritional deficiencies	2	9.21<br>-0.47 to 17.71
Protein-energy malnutrition	3	-3.01<br>-24.44 to 26.67
Other nutritional deficiencies	3	5.4<br>-21.15 to 39.7
Sexually transmitted infections excluding HIV	3	4.18<br>-4.05 to 14.02
Syphilis	4	3.57<br>-4.98 to 14.24
Chlamydial infection	4	12.1<br>4.6 to 19.76
Gonococcal infection	4	11.06<br>4.78 to 18.04
Other sexually transmitted infections	4	10.52<br>3.43 to 18.03
Acute hepatitis	3	5.94<br>-0.79 to 12.12
Acute hepatitis A	4	426.3<br>227.43 to 747.77
Acute hepatitis B	4	158.06<br>44.33 to 387.3
Acute hepatitis C	4	124.21<br>-13.44 to 533.89

Appendix Table S9: Percent change before and after CoDCorrect by cause for all ages, both sexes, global, 20		
**Cause name**	**CoDCorrect level**	**Percent change**
Acute hepatitis E	4	253.68<br>84.66 to 617.48
Other unspecified infectious diseases	3	5.28<br>-1.1 to 10.59
Non-communicable diseases	1	2.11<br>-5.62 to 7.59
Neoplasms	2	1.9<br>-4.04 to 6.06
Oesophageal cancer	3	0.54<br>-5.12 to 5.08
Stomach cancer	3	1.3<br>-5.01 to 5.95
Liver cancer	3	1.49<br>-4.42 to 6.32
Liver cancer due to hepatitis B	4	1.04<br>-4.69 to 5.51
Liver cancer due to hepatitis C	4	1.57<br>-6.47 to 7.01
Liver cancer due to alcohol use	4	2.04<br>-3.39 to 6.32
Liver cancer due to other causes	4	0.97<br>-4.47 to 5.86
Larynx cancer	3	1.44<br>-4.02 to 6.59
Tracheal, bronchus, and lung cancer	3	-0.04<br>-6.18 to 3.94
Breast cancer	3	3.88<br>-2.42 to 10.05
Cervical cancer	3	3.91<br>-2.29 to 11.22
Uterine cancer	3	2.63<br>-5.4 to 8.78
Prostate cancer	3	1.95<br>-5.15 to 7.29
Colon and rectum cancer	3	2.03<br>-5.07 to 6.44
Lip and oral cavity cancer	3	3.84<br>-1.84 to 8.79
Nasopharynx cancer	3	2.51<br>-2.37 to 7.14
Other pharynx cancer	3	4.21<br>-1.26 to 9.17
Gallbladder and biliary tract cancer	3	4.22<br>-4.3 to 9.98
Pancreatic cancer	3	1.29<br>-6.15 to 5.81
Malignant skin melanoma	3	1.02<br>-4.98 to 4.66
Non-melanoma skin cancer	3	2.38<br>-6.62 to 7.54
Ovarian cancer	3	3.75<br>-3.06 to 10.75
Testicular cancer	3	5.06<br>0.58 to 9.88
Kidney cancer	3	1.71<br>-5.02 to 5.56
Bladder cancer	3	1.76<br>-6.18 to 6.6
Brain and nervous system cancer	3	0.9<br>-3.72 to 3.91

Appendix Table S9: Percent change before and after CoDCorrect by cause for all ages, both sexes, global, 20		
**Cause name**	**CoDCorrect level**	**Percent change**
Thyroid cancer	3	5.6<br>-1.09 to 11.27
Mesothelioma	3	1.8<br>-4.91 to 5.45
Hodgkin lymphoma	3	6.36<br>0.23 to 10.57
Non-Hodgkin's lymphoma	3	3.74<br>-3.05 to 8.21
Multiple myeloma	3	2.1<br>-5.05 to 6.65
Leukaemia	3	3.28<br>-2.48 to 7.07
Other malignant cancers	3	3.45<br>-3.16 to 7.83
Other neoplasms	3	3.6<br>-5.49 to 9.02
Cardiovascular diseases	2	1.7<br>-5.93 to 7.38
Rheumatic heart disease	3	3.09<br>-10.33 to 18.17
Ischaemic heart disease	3	1.09<br>-7.18 to 8.71
Stroke	3	1.99<br>-8.52 to 10.59
Ischaemic stroke	4	-15.23<br>-26.47 to -2.18
Intracerebral hemorrhage	4	-13.2<br>-25.38 to 0.64
Subarachnoid hemorrhage	4	-6.17<br>-17.34 to 5.94
Hypertensive heart disease	3	2.96<br>-8.74 to 13.5
Cardiomyopathy and myocarditis	3	3.79<br>-5.05 to 12.45
Atrial fibrillation and flutter	3	4.25<br>-11.11 to 14.27
Aortic aneurysm	3	4.19<br>-5.5 to 11.17
Lower extremity peripheral arterial disease	3	3.09<br>-8.38 to 10.47
Endocarditis	3	4.65<br>-7.01 to 14.04
Non-rheumatic valvular heart disease	3	4.75<br>-11.15 to 14.09
Other cardiovascular and circulatory diseases	3	5.42<br>-4.16 to 16.26
Chronic respiratory diseases	2	2.86<br>-6.16 to 8.94
Chronic obstructive pulmonary disease	3	35.65<br>12.7 to 74.43
Pneumoconiosis	3	9.45<br>-8.43 to 39.11
Silicosis	4	16.2<br>-18.46 to 78.89
Asbestosis	4	11.44<br>-9.99 to 44.03
Coal workers pneumoconiosis	4	27.37<br>-1.23 to 76.24
Other pneumoconiosis	4	51.33<br>6.91 to 133.51

Appendix Table S9: Percent change before and after CoDCorrect by cause for all ages, both sexes, global, 20		
**Cause name**	**CoDCorrect level**	**Percent change**
Asthma	3	60.03<br>11.45 to 122.58
Interstitial lung disease and pulmonary sarcoidosis	3	27.73<br>6.96 to 54.04
Other chronic respiratory diseases	3	64.59<br>25.1 to 125.53
Cirrhosis and other chronic liver diseases	3	2.25<br>-11.52 to 18.3
Chronic hepatitis B including cirrhosis	4	1.07<br>-14.84 to 19.29
Chronic hepatitis C including cirrhosis	4	5.62<br>-10.16 to 24.09
Cirrhosis due to alcohol	4	1.03<br>-10.65 to 14.51
Cirrhosis due to other causes	4	6.45<br>-9.75 to 24.47
Digestive diseases	2	3.64<br>-2.58 to 8.32
Peptic ulcer disease	4	-9.93<br>-29.31 to 15.28
Gastritis and duodenitis	4	2.87<br>-16.61 to 29.64
Appendicitis	3	6.75<br>-9.73 to 25.86
Paralytic ileus and intestinal obstruction	3	5.8<br>-7.92 to 22.35
Inguinal, femoral, and abdominal hernia	3	6.31<br>-7.97 to 27.07
Inflammatory bowel disease	3	3.21<br>-8.0 to 13.16
Vascular intestinal disorders	3	3.07<br>-7.72 to 11.31
Gallbladder and biliary diseases	3	5.4<br>-7.67 to 16.24
Pancreatitis	3	4.41<br>-6.27 to 18.51
Neurological disorders	2	0.54<br>-12.33 to 11.6
Alzheimer's disease and other dementias	3	-0.65<br>-13.49 to 14.63
Parkinson's disease	3	3.33<br>-6.81 to 9.63
Idiopathic epilepsy	3	8.91<br>2.43 to 13.7
Multiple sclerosis	3	0.32<br>-5.09 to 3.42
Motor neuron disease	3	0.64<br>-5.37 to 4.17
Other neurological disorders	3	2.27<br>-7.64 to 8.19
Mental disorders	2	-4.94<br>-7.62 to -1.06
Alcohol use disorders	3	1.17<br>-1.9 to 4.06
Drug use disorders	3	-1.16<br>-5.7 to 2.8
Opioid use disorders	4	-4.24<br>-19.34 to 12.43
Cocaine use disorders	4	0.88<br>-17.21 to 21.74

Appendix Table S9: Percent change before and after CoDCorrect by cause for all ages, both sexes, global, 20

Cause name	CoDCorrect level	Percent change
Amphetamine use disorders	4	0.22<br>-15.0 to 19.78
Other drug use disorders	4	13.59<br>-1.83 to 31.89
Eating disorders	3	-4.94<br>-7.62 to -1.06
Anorexia nervosa	4	-4.94<br>-7.62 to -1.06
Diabetes mellitus	3	3.94<br>-2.35 to 9.19
Acute glomerulonephritis	3	2.37<br>-5.15 to 7.63
Chronic kidney disease	3	3.25<br>-4.64 to 9.11
Chronic kidney disease due to hypertension	4	3.37<br>-6.83 to 10.34
Chronic kidney disease due to glomerulonephritis	4	4.46<br>-1.75 to 8.98
Chronic kidney disease due to other and unspecified causes	4	2.67<br>-5.05 to 8.4
Urinary diseases and male infertility	3	5.69<br>-3.56 to 11.53
Urinary tract infections and interstitial nephritis	4	29.95<br>17.61 to 43.03
Urolithiasis	4	38.0<br>20.87 to 59.15
Other urinary diseases	4	108.12<br>67.69 to 155.56
Gynecological diseases	3	9.02<br>0.89 to 16.74
Uterine fibroids	4	102.6<br>14.04 to 252.61
Endometriosis	4	173.69<br>21.88 to 437.47
Genital prolapse	4	43.24<br>-32.16 to 193.47
Other gynecological diseases	4	174.43<br>39.21 to 411.0
Hemoglobinopathies and hemolytic anaemias	3	5.94<br>-2.69 to 13.84
Thalassemias	4	11.15<br>4.29 to 18.39
Sickle cell disorders	4	9.44<br>-3.2 to 25.57
G6PD deficiency	4	10.03<br>-0.1 to 30.24
Other hemoglobinopathies and hemolytic anaemias	4	9.56<br>-3.06 to 35.57
Endocrine, metabolic, blood, and immune disorders	3	2.43<br>-5.22 to 6.95
Other endocrine, metabolic, blood, and immune disorders	4	-9.37<br>-21.69 to 3.79
Musculoskeletal disorders	2	5.23<br>-3.6 to 11.98
Rheumatoid arthritis	3	32.81<br>1.19 to 66.36
Other musculoskeletal disorders	3	21.92<br>-2.8 to 49.68
Other non-communicable diseases	2	4.37<br>-1.37 to 9.19

Appendix Table S9: Percent change before and after CoDCorrect by cause for all ages, both sexes, global, 20		
**Cause name**	**CoDCorrect level**	**Percent change**
Congenital anomalies	3	3.86<br>-2.76 to 10.74
Neural tube defects	4	31.39<br>-6.91 to 83.25
Congenital heart anomalies	4	23.82<br>-11.61 to 62.43
Orofacial clefts	4	10.75<br>-30.44 to 70.43
Down's syndrome	4	14.15<br>-11.89 to 50.84
Other chromosomal abnormalities	4	25.84<br>-1.47 to 59.79
Congenital musculoskeletal and limb anomalies	4	44.27<br>7.54 to 92.12
Urogenital congenital anomalies	4	26.52<br>-2.24 to 62.38
Digestive congenital anomalies	4	24.63<br>-9.7 to 68.52
Other congenital anomalies	4	-4.92<br>-37.78 to 43.05
Skin and subcutaneous diseases	2	5.28<br>-3.11 to 10.71
Cellulitis	4	57.96<br>19.32 to 107.68
Pyoderma	4	29.0<br>-0.16 to 63.33
Decubitus ulcer	3	49.06<br>5.19 to 95.37
Other skin and subcutaneous diseases	3	178.13<br>77.16 to 326.11
Sudden infant death syndrome	3	0.73<br>-8.74 to 11.77
Injuries	1	12.48<br>7.02 to 17.22
Transport injuries	2	7.33<br>1.6 to 12.01
Road injuries	3	-1.15<br>-24.24 to 29.63
Pedestrian road injuries	4	15.77<br>-8.78 to 46.12
Cyclist road injuries	4	10.22<br>-15.4 to 41.19
Motorcyclist road injuries	4	8.85<br>-13.3 to 38.36
Motor vehicle road injuries	4	15.64<br>-6.7 to 41.87
Other road injuries	4	13.36<br>-9.14 to 39.67
Other transport injuries	3	8.76<br>-17.67 to 41.5
Unintentional injuries	2	12.87<br>6.56 to 17.84
Falls	3	5.4<br>-2.7 to 11.35
Drowning	3	4.72<br>-1.17 to 9.98
Fire, heat, and hot substances	3	8.94<br>1.99 to 14.62
Poisonings	3	4.84<br>-1.06 to 9.36

Appendix Table S9: Percent change before and after CoDCorrect by cause for all ages, both sexes, global, 20		
**Cause name**	**CoDCorrect level**	**Percent change**
Poisoning by carbon monoxide	4	15.89<br>-4.74 to 59.5
Poisoning by other means	4	113.24<br>42.77 to 229.6
Exposure to mechanical forces	3	6.84<br>1.18 to 12.27
Unintentional firearm injuries	4	-5.69<br>-34.85 to 38.33
Other exposure to mechanical forces	4	1.4<br>-26.58 to 43.37
Adverse effects of medical treatment	3	6.37<br>-0.1 to 11.52
Animal contact	3	8.15<br>1.47 to 13.51
Venomous animal contact	4	34.49<br>-22.22 to 99.54
Non-venomous animal contact	4	36.79<br>-20.66 to 124.49
Foreign body	3	3.81<br>-3.41 to 8.56
Pulmonary aspiration and foreign body in airway	4	1.24<br>-23.35 to 32.85
Foreign body in other body part	4	1.81<br>-32.85 to 56.73
Other unintentional injuries	3	7.52<br>2.15 to 12.83
Self-harm and interpersonal violence	2	17.85<br>13.65 to 23.3
Self-harm	3	5.1<br>1.05 to 8.79
Self-harm by firearm	4	20.23<br>1.8 to 40.98
Interpersonal violence	3	4.08<br>-0.37 to 8.18
Assault by firearm	4	-0.24<br>-11.61 to 12.16
Assault by sharp object	4	-0.75<br>-24.77 to 28.01
Assault by other means	4	4.74<br>-19.93 to 34.8
Maternal deaths aggravated by HIV/AIDS	4	5.51<br>-4.23 to 15.95
Environmental heat and cold exposure	3	147.0<br>115.62 to 206.95
Acute lymphoid leukaemia	4	90.78<br>51.06 to 142.79
Chronic lymphoid leukaemia	4	52.08<br>33.91 to 74.58
Acute myeloid leukaemia	4	64.21<br>46.1 to 83.49
Chronic myeloid leukaemia	4	113.52<br>81.95 to 154.79
Non-melanoma skin cancer (squamous-cell carcinoma)	4	2.38<br>-6.62 to 7.54
Executions and police conflict	3	2.7<br>-3.39 to 10.3
Drug-susceptible tuberculosis	4	6.06<br>-1.18 to 13.39
Zika virus disease	3	-0.28<br>-2.74 to 2.16

Appendix Table S9: Percent change before and after CoDCorrect by cause for all ages, both sexes, global, 20		
**Cause name**	**CoDCorrect level**	**Percent change**
Alcoholic cardiomyopathy	4	5.08<br>-5.27 to 16.92
Electrocution	3	3.05<br>-3.81 to 8.16
Myocarditis	4	4.89<br>-21.0 to 41.98
Other leukaemia	4	107.44<br>75.3 to 150.0
Other cardiomyopathy	4	-3.82<br>-26.75 to 25.13
Multidrug-resistant tuberculosis without extensive drug resistance	4	6.45<br>-1.91 to 13.95
Extensively drug-resistant tuberculosis	4	5.11<br>0.29 to 9.65
HIV/AIDS - Drug-susceptible Tuberculosis	4	-2.64<br>-9.75 to 4.34
HIV/AIDS - Multidrug-resistant Tuberculosis without extensive drug resistance	4	-4.62<br>-13.3 to 4.64
HIV/AIDS - Extensively drug-resistant Tuberculosis	4	1.86<br>-3.53 to 7.33
HIV/AIDS and sexually transmitted infections	2	-3.66<br>-10.35 to 3.26
Respiratory infections and tuberculosis	2	4.86<br>-2.27 to 10.42
Enteric infections	2	5.82<br>-2.93 to 13.88
Typhoid and paratyphoid	3	-5.03<br>-12.46 to 2.37
iNTS	3	16.61<br>-6.5 to 38.54
Other infectious diseases	2	4.94<br>-5.52 to 15.86
Maternal and neonatal disorders	2	6.7<br>-1.12 to 15.1
Myelodysplastic, myeloproliferative, and other hematopoietic neoplasms	4	3.15<br>-8.46 to 10.01
Non-rheumatic calcific aortic valve disease	4	5.43<br>-13.12 to 18.72
Non-rheumatic degenerative mitral valve disease	4	6.32<br>-12.66 to 27.68
Other non-rheumatic valve diseases	4	12.66<br>-11.38 to 52.42
Nonalcoholic fatty liver disease including cirrhosis	4	2.48<br>-6.1 to 11.57
Substance use disorders	2	-0.02<br>-3.07 to 3.2
Diabetes and kidney diseases	2	3.64<br>-3.26 to 9.08
Diabetes mellitus type 1	4	45.38<br>16.2 to 76.37
Diabetes mellitus type 2	4	21.69<br>-4.42 to 54.07
Bacterial skin diseases	3	33.16<br>4.68 to 68.31
Upper digestive system diseases	3	0.28<br>-12.79 to 16.52
Maternal abortive outcome	4	14.17<br>3.76 to 27.5

Appendix Table S9: Percent change before and after CoDCorrect by cause for all ages, both sexes, global, 20		
**Cause name**	**CoDCorrect level**	**Percent change**
Liver cancer due to NASH	4	1.59<br>-4.52 to 7.12
Chronic kidney disease due to diabetes mellitus type 1	4	4.84<br>-0.54 to 9.75
Chronic kidney disease due to diabetes mellitus type 2	4	2.54<br>-5.6 to 8.73
Pulmonary Arterial Hypertension	3	7.3<br>-4.01 to 19.46
Hepatoblastoma	4	10.61<br>-6.44 to 27.8
Burkitt lymphoma	4	15.12<br>-12.61 to 49.8
Other non-Hodgkin lymphoma	4	6.7<br>-9.78 to 23.35
Eye cancer	3	4.29<br>-4.83 to 12.28
Retinoblastoma	4	8.3<br>-12.54 to 26.2
Other eye cancers	4	2.47<br>-3.83 to 7.05
Soft tissue and other extraosseous sarcomas	3	4.76<br>-0.43 to 8.74
Malignant neoplasm of bone and articular cartilage	3	4.4<br>-0.72 to 8.82
Neuroblastoma and other peripheral nervous cell tumours	3	1.9<br>-3.05 to 6.09
Ulcerative colitis	4	11.3<br>-6.42 to 32.35
Crohn's disease	4	13.85<br>-1.21 to 34.58
Total burden related to hepatitis B	1	5.83<br>-6.33 to 18.94
Total burden related to hepatitis C	1	5.12<br>-6.32 to 18.45
Total burden related to Non-alcoholic fatty liver disease (NAFLD)	1	2.2<br>-4.84 to 9.23
Total cancers	1	3.36<br>-2.75 to 7.7
Thyroid diseases	4	-17.67<br>-39.41 to 10.69
COVID-19	3	1.03<br>-8.18 to 7.21
Total Cancers excluding Non-melanoma skin cancer	1	3.36<br>-2.74 to 7.71
Other maternal disorders	4	19.19<br>10.38 to 28.19
Other digestive diseases	3	3.73<br>-7.38 to 15.54
Self-harm by other specified means	4	33.46<br>5.92 to 58.27
Other benign and in situ neoplasms	4	4.06<br>-2.92 to 8.74

Appendix Table S10: CODEm covariates used, level of covariate, and expected direction of covariate by cause, sex, and age								
**Cause**	**Sex**	**Age Start**	**Age End**	**Model Version Type**	**Direction**	**Level**	**Covariate Name**	**Number of Draws**
Sexually transmitted infections excluding HIV	Female	10-14 years	95+ years	Data Rich	1	1	Syphilis prevalence (proportion)	--
Sexually transmitted infections excluding HIV	Female	10-14 years	95+ years	Data Rich	-1	2	Education (years per capita)	151
Sexually transmitted infections excluding HIV	Female	10-14 years	95+ years	Data Rich	-1	2	Maternal care and immunization	151
Sexually transmitted infections excluding HIV	Female	10-14 years	95+ years	Data Rich	-1	2	Legality of Abortion	329
Sexually transmitted infections excluding HIV	Female	10-14 years	95+ years	Data Rich	-1	2	Healthcare access and quality index	460
Sexually transmitted infections excluding HIV	Female	10-14 years	95+ years	Data Rich	1	2	Age-Specific Fertility Rate	--
Sexually transmitted infections excluding HIV	Female	10-14 years	95+ years	Data Rich	1	2	Total Fertility Rate	--
Sexually transmitted infections excluding HIV	Female	10-14 years	95+ years	Data Rich	-1	3	Antenatal Care (1 visit) Coverage (proportion)	88
Sexually transmitted infections excluding HIV	Female	10-14 years	95+ years	Data Rich	-1	3	Antenatal Care (4 visits) Coverage (proportion)	118
Sexually transmitted infections excluding HIV	Female	10-14 years	95+ years	Data Rich	-1	3	LDI (I$ per capita)	--
Sexually transmitted infections excluding HIV	Female	10-14 years	95+ years	Global	1	1	Syphilis prevalence (proportion)	--
Sexually transmitted infections excluding HIV	Female	10-14 years	95+ years	Global	-1	2	Healthcare access and quality index	205
Sexually transmitted infections excluding HIV	Female	10-14 years	95+ years	Global	-1	2	Maternal care and immunization	376
Sexually transmitted infections excluding HIV	Female	10-14 years	95+ years	Global	-1	2	Education (years per capita)	537
Sexually transmitted infections excluding HIV	Female	10-14 years	95+ years	Global	-1	2	Legality of Abortion	708
Sexually transmitted infections excluding HIV	Female	10-14 years	95+ years	Global	1	2	Age-Specific Fertility Rate	--
Sexually transmitted infections excluding HIV	Female	10-14 years	95+ years	Global	1	2	Total Fertility Rate	--
Sexually transmitted infections excluding HIV	Female	10-14 years	95+ years	Global	-1	3	Antenatal Care (4 visits) Coverage (proportion)	250
Sexually transmitted infections excluding HIV	Female	10-14 years	95+ years	Global	-1	3	Antenatal Care (1 visit) Coverage (proportion)	292
Sexually transmitted infections excluding HIV	Female	10-14 years	95+ years	Global	-1	3	LDI (I$ per capita)	--
Sexually transmitted infections excluding HIV	Male	10-14 years	95+ years	Data Rich	1	1	Syphilis prevalence (proportion)	--
Sexually transmitted infections excluding HIV	Male	10-14 years	95+ years	Data Rich	-1	2	Education (years per capita)	273
Sexually transmitted infections excluding HIV	Male	10-14 years	95+ years	Data Rich	-1	2	Maternal care and immunization	363
Sexually transmitted infections excluding HIV	Male	10-14 years	95+ years	Data Rich	-1	2	Healthcare access and quality index	476
Sexually transmitted infections excluding HIV	Male	10-14 years	95+ years	Data Rich	-1	2	Legality of Abortion	839
Sexually transmitted infections excluding HIV	Male	10-14 years	95+ years	Data Rich	1	2	Age-Specific Fertility Rate	--
Sexually transmitted infections excluding HIV	Male	10-14 years	95+ years	Data Rich	1	2	Total Fertility Rate	--
Sexually transmitted infections excluding HIV	Male	10-14 years	95+ years	Data Rich	-1	3	Antenatal Care (4 visits) Coverage (proportion)	52
Sexually transmitted infections excluding HIV	Male	10-14 years	95+ years	Data Rich	-1	3	Antenatal Care (1 visit) Coverage (proportion)	69
Sexually transmitted infections excluding HIV	Male	10-14 years	95+ years	Data Rich	-1	3	LDI (I$ per capita)	--
Sexually transmitted infections excluding HIV	Male	10-14 years	95+ years	Global	1	1	Syphilis prevalence (proportion)	--
Sexually transmitted infections excluding HIV	Male	10-14 years	95+ years	Global	-1	2	Maternal care and immunization	174
Sexually transmitted infections excluding HIV	Male	10-14 years	95+ years	Global	-1	2	Healthcare access and quality index	225
Sexually transmitted infections excluding HIV	Male	10-14 years	95+ years	Global	-1	2	Legality of Abortion	270
Sexually transmitted infections excluding HIV	Male	10-14 years	95+ years	Global	-1	2	Education (years per capita)	--
Sexually transmitted infections excluding HIV	Male	10-14 years	95+ years	Global	1	2	Age-Specific Fertility Rate	--
Sexually transmitted infections excluding HIV	Male	10-14 years	95+ years	Global	1	2	Total Fertility Rate	--
Sexually transmitted infections excluding HIV	Male	10-14 years	95+ years	Global	-1	3	Antenatal Care (4 visits) Coverage (proportion)	124
Sexually transmitted infections excluding HIV	Male	10-14 years	95+ years	Global	-1	3	Antenatal Care (1 visit) Coverage (proportion)	189
Sexually transmitted infections excluding HIV	Male	10-14 years	95+ years	Global	-1	3	LDI (I$ per capita)	--
Lower respiratory infections	Female	0-6 days	2-4 years	Data Rich	-1	1	Hib3 lagged five year coverage, COVID-inclusive (proportion)	76
Lower respiratory infections	Female	0-6 days	2-4 years	Data Rich	-1	1	Antibiotics for LRI	288
Lower respiratory infections	Female	0-6 days	2-4 years	Data Rich	-1	1	PCV3 lagged five year coverage, COVID-inclusive (proportion)	391
Lower respiratory infections	Female	0-6 days	2-4 years	Data Rich	1	1	Age- and sex-specific SEV for Child wasting	147
Lower respiratory infections	Female	0-6 days	2-4 years	Data Rich	1	1	Log-transformed SEV scalar: LRI	350
Lower respiratory infections	Female	0-6 days	2-4 years	Data Rich	1	1	Age- and sex-specific SEV for Child underweight	508
Lower respiratory infections	Female	0-6 days	2-4 years	Data Rich	1	1	Age- and sex-specific SEV for Child stunting	562
Lower respiratory infections	Female	0-6 days	2-4 years	Data Rich	1	1	Indoor Air Pollution (All Cooking Fuels)	626
Lower respiratory infections	Female	0-6 days	2-4 years	Data Rich	-1	2	DTP3 lagged five year coverage, COVID-inclusive (proportion)	258
Lower respiratory infections	Female	0-6 days	2-4 years	Data Rich	-1	2	Mask use	647
Lower respiratory infections	Female	0-6 days	2-4 years	Data Rich	-1	2	Healthcare access and quality index	827
Lower respiratory infections	Female	0-6 days	2-4 years	Data Rich	1	2	Age- and sex-specific SEV for Household air pollution	61
Lower respiratory infections	Female	0-6 days	2-4 years	Data Rich	1	2	Age-standardized SEV for Handwashing	204
Lower respiratory infections	Female	0-6 days	2-4 years	Data Rich	1	2	Secondhand smoke	404
Lower respiratory infections	Female	0-6 days	2-4 years	Data Rich	1	2	Outdoor Air Pollution (PM2.5)	535
Lower respiratory infections	Female	0-6 days	2-4 years	Data Rich	1	2	Age- and sex-specific SEV for Ambient particulate matter	585
Lower respiratory infections	Female	0-6 days	2-4 years	Data Rich	1	2	Zinc deficiency	642
Lower respiratory infections	Female	0-6 days	2-4 years	Data Rich	-1	3	Socio-demographic Index	459
Lower respiratory infections	Female	0-6 days	2-4 years	Data Rich	-1	3	Maternal Education (years per capita)	581
Lower respiratory infections	Female	0-6 days	2-4 years	Data Rich	1	3	Population Density (over 1000 ppl/sqkm, proportion)	0
Lower respiratory infections	Female	0-6 days	2-4 years	Data Rich	1	3	Age- and sex-specific SEV for Unsafe sanitation	209
Lower respiratory infections	Female	0-6 days	2-4 years	Global	-1	1	Antibiotics for LRI	598
Lower respiratory infections	Female	0-6 days	2-4 years	Global	-1	1	Hib3 lagged five year coverage, COVID-inclusive (proportion)	611
Lower respiratory infections	Female	0-6 days	2-4 years	Global	-1	1	PCV3 lagged five year coverage, COVID-inclusive (proportion)	618
Lower respiratory infections	Female	0-6 days	2-4 years	Global	1	1	Age- and sex-specific SEV for Child underweight	27
Lower respiratory infections	Female	0-6 days	2-4 years	Global	1	1	Age- and sex-specific SEV for Child wasting	68
Lower respiratory infections	Female	0-6 days	2-4 years	Global	1	1	Log-transformed SEV scalar: LRI	389
Lower respiratory infections	Female	0-6 days	2-4 years	Global	1	1	Indoor Air Pollution (All Cooking Fuels)	736
Lower respiratory infections	Female	0-6 days	2-4 years	Global	1	1	Age- and sex-specific SEV for Child stunting	776
Lower respiratory infections	Female	0-6 days	2-4 years	Global	-1	2	DTP3 lagged five year coverage, COVID-inclusive (proportion)	927
Lower respiratory infections	Female	0-6 days	2-4 years	Global	-1	2	Healthcare access and quality index	1000
Lower respiratory infections	Female	0-6 days	2-4 years	Global	-1	2	Mask use	1000
Lower respiratory infections	Female	0-6 days	2-4 years	Global	1	2	Age- and sex-specific SEV for Ambient particulate matter	0
Lower respiratory infections	Female	0-6 days	2-4 years	Global	1	2	Age-standardized SEV for Handwashing	0
Lower respiratory infections	Female	0-6 days	2-4 years	Global	1	2	Zinc deficiency	0
Lower respiratory infections	Female	0-6 days	2-4 years	Global	1	2	Outdoor Air Pollution (PM2.5)	473
Lower respiratory infections	Female	0-6 days	2-4 years	Global	1	2	Age- and sex-specific SEV for Household air pollution	780
Lower respiratory infections	Female	0-6 days	2-4 years	Global	1	2	Secondhand smoke	1000
Lower respiratory infections	Female	0-6 days	2-4 years	Global	-1	3	Maternal Education (years per capita)	121
Lower respiratory infections	Female	0-6 days	2-4 years	Global	-1	3	Socio-demographic Index	677
Lower respiratory infections	Female	0-6 days	2-4 years	Global	1	3	Age- and sex-specific SEV for Unsafe sanitation	645
Lower respiratory infections	Female	0-6 days	2-4 years	Global	1	3	Population Density (over 1000 ppl/sqkm, proportion)	670
Lower respiratory infections	Female	5-9 years	95+ years	Data Rich	1	1	Indoor Air Pollution (All Cooking Fuels)	0
Lower respiratory infections	Female	5-9 years	95+ years	Data Rich	1	1	Log-transformed SEV scalar: LRI	47
Lower respiratory infections	Female	5-9 years	95+ years	Data Rich	1	1	Secondhand smoke	255
Lower respiratory infections	Female	5-9 years	95+ years	Data Rich	1	1	Smoking Prevalence	457
Lower respiratory infections	Female	5-9 years	95+ years	Data Rich	1	1	Outdoor Air Pollution (PM2.5)	714

Appendix Table S10: CODEm covariates used, level of covariate, and expected direction of covariate by cause, sex, and age

Cause	Sex	Age Start	Age End	Model Version Type	Direction	Level	Covariate Name	Number of Draws
Lower respiratory infections	Female	5-9 years	95+ years	Data Rich	-1	2	DTP3 lagged five year coverage, COVID-inclusive (proportion)	216
Lower respiratory infections	Female	5-9 years	95+ years	Data Rich	-1	2	PCV3 lagged five year coverage, COVID-inclusive (proportion)	353
Lower respiratory infections	Female	5-9 years	95+ years	Data Rich	-1	2	Healthcare access and quality index	736
Lower respiratory infections	Female	5-9 years	95+ years	Data Rich	-1	2	Mask use	750
Lower respiratory infections	Female	5-9 years	95+ years	Data Rich	1	2	No access to handwashing facility	3
Lower respiratory infections	Female	5-9 years	95+ years	Data Rich	1	2	Age-standardized proportion adult underweight	523
Lower respiratory infections	Female	5-9 years	95+ years	Data Rich	-1	3	LDI (I$ per capita)	3
Lower respiratory infections	Female	5-9 years	95+ years	Data Rich	-1	3	Socio-demographic Index	104
Lower respiratory infections	Female	5-9 years	95+ years	Data Rich	-1	3	Education (years per capita)	138
Lower respiratory infections	Female	5-9 years	95+ years	Data Rich	1	3	Age- and sex-specific SEV for Unsafe sanitation	17
Lower respiratory infections	Female	5-9 years	95+ years	Global	1	1	Log-transformed SEV scalar: LRI	352
Lower respiratory infections	Female	5-9 years	95+ years	Global	1	1	Secondhand smoke	458
Lower respiratory infections	Female	5-9 years	95+ years	Global	1	1	Smoking Prevalence	678
Lower respiratory infections	Female	5-9 years	95+ years	Global	1	1	Indoor Air Pollution (All Cooking Fuels)	707
Lower respiratory infections	Female	5-9 years	95+ years	Global	1	1	Outdoor Air Pollution (PM2.5)	--
Lower respiratory infections	Female	5-9 years	95+ years	Global	-1	2	PCV3 lagged five year coverage, COVID-inclusive (proportion)	117
Lower respiratory infections	Female	5-9 years	95+ years	Global	-1	2	DTP3 lagged five year coverage, COVID-inclusive (proportion)	126
Lower respiratory infections	Female	5-9 years	95+ years	Global	-1	2	Healthcare access and quality index	186
Lower respiratory infections	Female	5-9 years	95+ years	Global	-1	2	Mask use	290
Lower respiratory infections	Female	5-9 years	95+ years	Global	1	2	No access to handwashing facility	40
Lower respiratory infections	Female	5-9 years	95+ years	Global	1	2	Age-standardized proportion adult underweight	259
Lower respiratory infections	Female	5-9 years	95+ years	Global	-1	3	Socio-demographic Index	222
Lower respiratory infections	Female	5-9 years	95+ years	Global	-1	3	LDI (I$ per capita)	400
Lower respiratory infections	Female	5-9 years	95+ years	Global	-1	3	Education (years per capita)	475
Lower respiratory infections	Female	5-9 years	95+ years	Global	1	3	Age- and sex-specific SEV for Unsafe sanitation	129
Lower respiratory infections	Male	0-6 days	2-4 years	Data Rich	-1	1	PCV3 lagged five year coverage, COVID-inclusive (proportion)	91
Lower respiratory infections	Male	0-6 days	2-4 years	Data Rich	-1	1	Hib3 lagged five year coverage, COVID-inclusive (proportion)	101
Lower respiratory infections	Male	0-6 days	2-4 years	Data Rich	-1	1	Antibiotics for LRI	723
Lower respiratory infections	Male	0-6 days	2-4 years	Data Rich	1	1	Log-transformed SEV scalar: LRI	97
Lower respiratory infections	Male	0-6 days	2-4 years	Data Rich	1	1	Age- and sex-specific SEV for Child wasting	210
Lower respiratory infections	Male	0-6 days	2-4 years	Data Rich	1	1	Indoor Air Pollution (All Cooking Fuels)	212
Lower respiratory infections	Male	0-6 days	2-4 years	Data Rich	1	1	Age- and sex-specific SEV for Child underweight	258
Lower respiratory infections	Male	0-6 days	2-4 years	Data Rich	1	1	Age- and sex-specific SEV for Child stunting	606
Lower respiratory infections	Male	0-6 days	2-4 years	Data Rich	-1	2	Healthcare access and quality index	836
Lower respiratory infections	Male	0-6 days	2-4 years	Data Rich	-1	2	DTP3 lagged five year coverage, COVID-inclusive (proportion)	939
Lower respiratory infections	Male	0-6 days	2-4 years	Data Rich	-1	2	Mask use	994
Lower respiratory infections	Male	0-6 days	2-4 years	Data Rich	1	2	Secondhand smoke	458
Lower respiratory infections	Male	0-6 days	2-4 years	Data Rich	1	2	Age- and sex-specific SEV for Household air pollution	464
Lower respiratory infections	Male	0-6 days	2-4 years	Data Rich	1	2	Age- and sex-specific SEV for Ambient particulate matter	523
Lower respiratory infections	Male	0-6 days	2-4 years	Data Rich	1	2	Age-standardized SEV for Handwashing	575
Lower respiratory infections	Male	0-6 days	2-4 years	Data Rich	1	2	Outdoor Air Pollution (PM2.5)	598
Lower respiratory infections	Male	0-6 days	2-4 years	Data Rich	1	2	Zinc deficiency	816
Lower respiratory infections	Male	0-6 days	2-4 years	Data Rich	-1	3	Socio-demographic Index	113
Lower respiratory infections	Male	0-6 days	2-4 years	Data Rich	-1	3	Maternal Education (years per capita)	641
Lower respiratory infections	Male	0-6 days	2-4 years	Data Rich	1	3	Population Density (over 1000 ppl/sqkm, proportion)	125
Lower respiratory infections	Male	0-6 days	2-4 years	Data Rich	1	3	Age- and sex-specific SEV for Unsafe sanitation	205
Lower respiratory infections	Male	0-6 days	2-4 years	Global	-1	1	PCV3 lagged five year coverage, COVID-inclusive (proportion)	329
Lower respiratory infections	Male	0-6 days	2-4 years	Global	-1	1	Hib3 lagged five year coverage, COVID-inclusive (proportion)	833
Lower respiratory infections	Male	0-6 days	2-4 years	Global	-1	1	Antibiotics for LRI	915
Lower respiratory infections	Male	0-6 days	2-4 years	Global	1	1	Age- and sex-specific SEV for Child wasting	48
Lower respiratory infections	Male	0-6 days	2-4 years	Global	1	1	Age- and sex-specific SEV for Child underweight	55
Lower respiratory infections	Male	0-6 days	2-4 years	Global	1	1	Log-transformed SEV scalar: LRI	171
Lower respiratory infections	Male	0-6 days	2-4 years	Global	1	1	Age- and sex-specific SEV for Child stunting	621
Lower respiratory infections	Male	0-6 days	2-4 years	Global	1	1	Indoor Air Pollution (All Cooking Fuels)	990
Lower respiratory infections	Male	0-6 days	2-4 years	Global	-1	2	DTP3 lagged five year coverage, COVID-inclusive (proportion)	993
Lower respiratory infections	Male	0-6 days	2-4 years	Global	-1	2	Healthcare access and quality index	1000
Lower respiratory infections	Male	0-6 days	2-4 years	Global	-1	2	Mask use	1000
Lower respiratory infections	Male	0-6 days	2-4 years	Global	1	2	Age-standardized SEV for Handwashing	0
Lower respiratory infections	Male	0-6 days	2-4 years	Global	1	2	Zinc deficiency	0
Lower respiratory infections	Male	0-6 days	2-4 years	Global	1	2	Age- and sex-specific SEV for Ambient particulate matter	273
Lower respiratory infections	Male	0-6 days	2-4 years	Global	1	2	Outdoor Air Pollution (PM2.5)	580
Lower respiratory infections	Male	0-6 days	2-4 years	Global	1	2	Age- and sex-specific SEV for Household air pollution	897
Lower respiratory infections	Male	0-6 days	2-4 years	Global	1	2	Secondhand smoke	1000
Lower respiratory infections	Male	0-6 days	2-4 years	Global	-1	3	Maternal Education (years per capita)	2
Lower respiratory infections	Male	0-6 days	2-4 years	Global	-1	3	Socio-demographic Index	470
Lower respiratory infections	Male	0-6 days	2-4 years	Global	1	3	Population Density (over 1000 ppl/sqkm, proportion)	218
Lower respiratory infections	Male	0-6 days	2-4 years	Global	1	3	Age- and sex-specific SEV for Unsafe sanitation	308
Lower respiratory infections	Male	5-9 years	95+ years	Data Rich	1	1	Indoor Air Pollution (All Cooking Fuels)	0
Lower respiratory infections	Male	5-9 years	95+ years	Data Rich	1	1	Log-transformed SEV scalar: LRI	2
Lower respiratory infections	Male	5-9 years	95+ years	Data Rich	1	1	Secondhand smoke	236
Lower respiratory infections	Male	5-9 years	95+ years	Data Rich	1	1	Smoking Prevalence	701
Lower respiratory infections	Male	5-9 years	95+ years	Data Rich	1	1	Outdoor Air Pollution (PM2.5)	732
Lower respiratory infections	Male	5-9 years	95+ years	Data Rich	-1	2	DTP3 lagged five year coverage, COVID-inclusive (proportion)	167
Lower respiratory infections	Male	5-9 years	95+ years	Data Rich	-1	2	PCV3 lagged five year coverage, COVID-inclusive (proportion)	420
Lower respiratory infections	Male	5-9 years	95+ years	Data Rich	-1	2	Mask use	631
Lower respiratory infections	Male	5-9 years	95+ years	Data Rich	-1	2	Healthcare access and quality index	736
Lower respiratory infections	Male	5-9 years	95+ years	Data Rich	1	2	No access to handwashing facility	0
Lower respiratory infections	Male	5-9 years	95+ years	Data Rich	1	2	Age-standardized proportion adult underweight	437
Lower respiratory infections	Male	5-9 years	95+ years	Data Rich	-1	3	LDI (I$ per capita)	14
Lower respiratory infections	Male	5-9 years	95+ years	Data Rich	-1	3	Socio-demographic Index	39
Lower respiratory infections	Male	5-9 years	95+ years	Data Rich	-1	3	Education (years per capita)	198
Lower respiratory infections	Male	5-9 years	95+ years	Data Rich	1	3	Age- and sex-specific SEV for Unsafe sanitation	80
Lower respiratory infections	Male	5-9 years	95+ years	Global	1	1	Log-transformed SEV scalar: LRI	404
Lower respiratory infections	Male	5-9 years	95+ years	Global	1	1	Smoking Prevalence	478
Lower respiratory infections	Male	5-9 years	95+ years	Global	1	1	Indoor Air Pollution (All Cooking Fuels)	641
Lower respiratory infections	Male	5-9 years	95+ years	Global	1	1	Secondhand smoke	752
Lower respiratory infections	Male	5-9 years	95+ years	Global	1	1	Outdoor Air Pollution (PM2.5)	--
Lower respiratory infections	Male	5-9 years	95+ years	Global	-1	2	Healthcare access and quality index	186
Lower respiratory infections	Male	5-9 years	95+ years	Global	-1	2	PCV3 lagged five year coverage, COVID-inclusive (proportion)	447
Lower respiratory infections	Male	5-9 years	95+ years	Global	-1	2	DTP3 lagged five year coverage, COVID-inclusive (proportion)	587
Lower respiratory infections	Male	5-9 years	95+ years	Global	-1	2	Mask use	746
Lower respiratory infections	Male	5-9 years	95+ years	Global	1	2	No access to handwashing facility	122
Lower respiratory infections	Male	5-9 years	95+ years	Global	1	2	Age-standardized proportion adult underweight	608
Lower respiratory infections	Male	5-9 years	95+ years	Global	-1	3	Socio-demographic Index	1
Lower respiratory infections	Male	5-9 years	95+ years	Global	-1	3	Education (years per capita)	333
Lower respiratory infections	Male	5-9 years	95+ years	Global	-1	3	LDI (I$ per capita)	359
Lower respiratory infections	Male	5-9 years	95+ years	Global	1	3	Age- and sex-specific SEV for Unsafe sanitation	274
Upper respiratory infections	Female	0-6 days	95+ years	Data Rich	1	1	Smoking Prevalence	1000
Upper respiratory infections	Female	0-6 days	95+ years	Data Rich	-1	2	Healthcare access and quality index	--

Appendix Table S10: CODEm covariates used, level of covariate, and expected direction of covariate by cause, sex, and age

Cause	Sex	Age Start	Age End	Model Version Type	Direction	Level	Covariate Name	Number of Draws
Upper respiratory infections	Female	0-6 days	95+ years	Data Rich	1	2	Indoor Air Pollution (All Cooking Fuels)	142
Upper respiratory infections	Female	0-6 days	95+ years	Data Rich	1	2	Outdoor Air Pollution (PM2.5)	507
Upper respiratory infections	Female	0-6 days	95+ years	Data Rich	-1	3	Socio-demographic Index	243
Upper respiratory infections	Female	0-6 days	95+ years	Data Rich	-1	3	Education (years per capita)	321
Upper respiratory infections	Female	0-6 days	95+ years	Data Rich	-1	3	LDI (I$ per capita)	--
Upper respiratory infections	Female	0-6 days	95+ years	Global	1	1	Smoking Prevalence	1000
Upper respiratory infections	Female	0-6 days	95+ years	Global	-1	2	Healthcare access and quality index	--
Upper respiratory infections	Female	0-6 days	95+ years	Global	1	2	Outdoor Air Pollution (PM2.5)	488
Upper respiratory infections	Female	0-6 days	95+ years	Global	1	2	Indoor Air Pollution (All Cooking Fuels)	--
Upper respiratory infections	Female	0-6 days	95+ years	Global	-1	3	Education (years per capita)	293
Upper respiratory infections	Female	0-6 days	95+ years	Global	-1	3	Socio-demographic Index	428
Upper respiratory infections	Female	0-6 days	95+ years	Global	-1	3	LDI (I$ per capita)	--
Upper respiratory infections	Male	0-6 days	95+ years	Data Rich	1	1	Smoking Prevalence	1000
Upper respiratory infections	Male	0-6 days	95+ years	Data Rich	-1	2	Healthcare access and quality index	--
Upper respiratory infections	Male	0-6 days	95+ years	Data Rich	1	2	Outdoor Air Pollution (PM2.5)	463
Upper respiratory infections	Male	0-6 days	95+ years	Data Rich	1	2	Indoor Air Pollution (All Cooking Fuels)	--
Upper respiratory infections	Male	0-6 days	95+ years	Data Rich	-1	3	Socio-demographic Index	243
Upper respiratory infections	Male	0-6 days	95+ years	Data Rich	-1	3	Education (years per capita)	321
Upper respiratory infections	Male	0-6 days	95+ years	Data Rich	-1	3	LDI (I$ per capita)	--
Upper respiratory infections	Male	0-6 days	95+ years	Global	1	1	Smoking Prevalence	1000
Upper respiratory infections	Male	0-6 days	95+ years	Global	-1	2	Healthcare access and quality index	--
Upper respiratory infections	Male	0-6 days	95+ years	Global	1	2	Outdoor Air Pollution (PM2.5)	513
Upper respiratory infections	Male	0-6 days	95+ years	Global	1	2	Indoor Air Pollution (All Cooking Fuels)	--
Upper respiratory infections	Male	0-6 days	95+ years	Global	-1	3	Education (years per capita)	318
Upper respiratory infections	Male	0-6 days	95+ years	Global	-1	3	Socio-demographic Index	403
Upper respiratory infections	Male	0-6 days	95+ years	Global	-1	3	LDI (I$ per capita)	--
Otitis media	Female	0-6 days	95+ years	Data Rich	1	1	Smoking Prevalence	465
Otitis media	Female	0-6 days	95+ years	Data Rich	1	1	Log-transformed SEV scalar: Otitis	868
Otitis media	Female	0-6 days	95+ years	Data Rich	-1	2	Healthcare access and quality index	456
Otitis media	Female	0-6 days	95+ years	Data Rich	1	2	Indoor Air Pollution (All Cooking Fuels)	304
Otitis media	Female	0-6 days	95+ years	Data Rich	1	2	Outdoor Air Pollution (PM2.5)	344
Otitis media	Female	0-6 days	95+ years	Data Rich	-1	3	Education (years per capita)	179
Otitis media	Female	0-6 days	95+ years	Data Rich	-1	3	Socio-demographic Index	218
Otitis media	Female	0-6 days	95+ years	Data Rich	-1	3	LDI (I$ per capita)	--
Otitis media	Female	0-6 days	95+ years	Global	1	1	Smoking Prevalence	641
Otitis media	Female	0-6 days	95+ years	Global	1	1	Log-transformed SEV scalar: Otitis	693
Otitis media	Female	0-6 days	95+ years	Global	-1	2	Healthcare access and quality index	567
Otitis media	Female	0-6 days	95+ years	Global	1	2	Indoor Air Pollution (All Cooking Fuels)	435
Otitis media	Female	0-6 days	95+ years	Global	1	2	Outdoor Air Pollution (PM2.5)	606
Otitis media	Female	0-6 days	95+ years	Global	-1	3	Socio-demographic Index	204
Otitis media	Female	0-6 days	95+ years	Global	-1	3	Education (years per capita)	291
Otitis media	Female	0-6 days	95+ years	Global	-1	3	LDI (I$ per capita)	--
Otitis media	Male	0-6 days	95+ years	Data Rich	1	1	Smoking Prevalence	1000
Otitis media	Male	0-6 days	95+ years	Data Rich	1	1	Log-transformed SEV scalar: Otitis	--
Otitis media	Male	0-6 days	95+ years	Data Rich	-1	2	Healthcare access and quality index	472
Otitis media	Male	0-6 days	95+ years	Data Rich	1	2	Outdoor Air Pollution (PM2.5)	160
Otitis media	Male	0-6 days	95+ years	Data Rich	1	2	Indoor Air Pollution (All Cooking Fuels)	--
Otitis media	Male	0-6 days	95+ years	Data Rich	-1	3	Socio-demographic Index	260
Otitis media	Male	0-6 days	95+ years	Data Rich	-1	3	Education (years per capita)	--
Otitis media	Male	0-6 days	95+ years	Data Rich	-1	3	LDI (I$ per capita)	--
Otitis media	Male	0-6 days	95+ years	Global	1	1	Log-transformed SEV scalar: Otitis	500
Otitis media	Male	0-6 days	95+ years	Global	1	1	Smoking Prevalence	1000
Otitis media	Male	0-6 days	95+ years	Global	-1	2	Healthcare access and quality index	449
Otitis media	Male	0-6 days	95+ years	Global	1	2	Indoor Air Pollution (All Cooking Fuels)	192
Otitis media	Male	0-6 days	95+ years	Global	1	2	Outdoor Air Pollution (PM2.5)	449
Otitis media	Male	0-6 days	95+ years	Global	-1	3	Socio-demographic Index	213
Otitis media	Male	0-6 days	95+ years	Global	-1	3	Education (years per capita)	--
Otitis media	Male	0-6 days	95+ years	Global	-1	3	LDI (I$ per capita)	--
Diarrhoeal diseases	Female	0-6 days	2-4 years	Data Rich	-1	1	Improved Water Source (proportion with access)	77
Diarrhoeal diseases	Female	0-6 days	2-4 years	Data Rich	-1	1	RotaC lagged five year coverage, COVID-inclusive (proportion)	283
Diarrhoeal diseases	Female	0-6 days	2-4 years	Data Rich	-1	1	Sanitation (proportion with access)	614
Diarrhoeal diseases	Female	0-6 days	2-4 years	Data Rich	-1	1	ORS (oral rehydration)	837
Diarrhoeal diseases	Female	0-6 days	2-4 years	Data Rich	-1	2	Zinc treatment for diarrhea	4
Diarrhoeal diseases	Female	0-6 days	2-4 years	Data Rich	-1	2	Healthcare access and quality index	--
Diarrhoeal diseases	Female	0-6 days	2-4 years	Data Rich	1	2	Zinc deficiency	559
Diarrhoeal diseases	Female	0-6 days	2-4 years	Data Rich	-1	3	LDI (I$ per capita)	0
Diarrhoeal diseases	Female	0-6 days	2-4 years	Data Rich	-1	3	Socio-demographic Index	36
Diarrhoeal diseases	Female	0-6 days	2-4 years	Data Rich	-1	3	Maternal Education (years per capita)	768
Diarrhoeal diseases	Female	0-6 days	2-4 years	Data Rich	1	3	No access to handwashing facility	85
Diarrhoeal diseases	Female	0-6 days	2-4 years	Global	-1	1	Improved Water Source (proportion with access)	265
Diarrhoeal diseases	Female	0-6 days	2-4 years	Global	-1	1	RotaC lagged five year coverage, COVID-inclusive (proportion)	706
Diarrhoeal diseases	Female	0-6 days	2-4 years	Global	-1	1	Sanitation (proportion with access)	954
Diarrhoeal diseases	Female	0-6 days	2-4 years	Global	-1	1	ORS (oral rehydration)	1000
Diarrhoeal diseases	Female	0-6 days	2-4 years	Global	-1	2	Zinc treatment for diarrhea	1
Diarrhoeal diseases	Female	0-6 days	2-4 years	Global	-1	2	Healthcare access and quality index	550
Diarrhoeal diseases	Female	0-6 days	2-4 years	Global	1	2	Zinc deficiency	0
Diarrhoeal diseases	Female	0-6 days	2-4 years	Global	-1	3	LDI (I$ per capita)	1
Diarrhoeal diseases	Female	0-6 days	2-4 years	Global	-1	3	Socio-demographic Index	358
Diarrhoeal diseases	Female	0-6 days	2-4 years	Global	-1	3	Maternal Education (years per capita)	604
Diarrhoeal diseases	Female	0-6 days	2-4 years	Global	1	3	No access to handwashing facility	137
Diarrhoeal diseases	Female	5-9 years	95+ years	Data Rich	-1	1	Sanitation (proportion with access)	441
Diarrhoeal diseases	Female	5-9 years	95+ years	Data Rich	-1	1	Improved Water Source (proportion with access)	1000
Diarrhoeal diseases	Female	5-9 years	95+ years	Data Rich	-1	2	ORS (oral rehydration)	512
Diarrhoeal diseases	Female	5-9 years	95+ years	Data Rich	-1	2	Healthcare access and quality index	--
Diarrhoeal diseases	Female	5-9 years	95+ years	Data Rich	-1	2	RotaC lagged five year coverage, COVID-inclusive (proportion)	--
Diarrhoeal diseases	Female	5-9 years	95+ years	Data Rich	-1	3	Education (years per capita)	--
Diarrhoeal diseases	Female	5-9 years	95+ years	Data Rich	-1	3	LDI (I$ per capita)	--
Diarrhoeal diseases	Female	5-9 years	95+ years	Data Rich	-1	3	Socio-demographic Index	--
Diarrhoeal diseases	Female	5-9 years	95+ years	Data Rich	1	3	No access to handwashing facility	193
Diarrhoeal diseases	Female	5-9 years	95+ years	Data Rich	1	3	Age-standardized proportion adult underweight	--
Diarrhoeal diseases	Female	5-9 years	95+ years	Global	-1	1	Improved Water Source (proportion with access)	648
Diarrhoeal diseases	Female	5-9 years	95+ years	Global	-1	1	Sanitation (proportion with access)	828
Diarrhoeal diseases	Female	5-9 years	95+ years	Global	-1	2	Healthcare access and quality index	143
Diarrhoeal diseases	Female	5-9 years	95+ years	Global	-1	2	ORS (oral rehydration)	385
Diarrhoeal diseases	Female	5-9 years	95+ years	Global	-1	2	RotaC lagged five year coverage, COVID-inclusive (proportion)	--
Diarrhoeal diseases	Female	5-9 years	95+ years	Global	-1	3	Education (years per capita)	57
Diarrhoeal diseases	Female	5-9 years	95+ years	Global	-1	3	LDI (I$ per capita)	--
Diarrhoeal diseases	Female	5-9 years	95+ years	Global	-1	3	Socio-demographic Index	--
Diarrhoeal diseases	Female	5-9 years	95+ years	Global	1	3	No access to handwashing facility	167
Diarrhoeal diseases	Female	5-9 years	95+ years	Global	1	3	Age-standardized proportion adult underweight	--
Diarrhoeal diseases	Male	0-6 days	2-4 years	Data Rich	-1	1	Improved Water Source (proportion with access)	137
Diarrhoeal diseases	Male	0-6 days	2-4 years	Data Rich	-1	1	RotaC lagged five year coverage, COVID-inclusive (proportion)	161
Diarrhoeal diseases	Male	0-6 days	2-4 years	Data Rich	-1	1	Sanitation (proportion with access)	574
Diarrhoeal diseases	Male	0-6 days	2-4 years	Data Rich	-1	1	ORS (oral rehydration)	927
Diarrhoeal diseases	Male	0-6 days	2-4 years	Data Rich	-1	2	Zinc treatment for diarrhea	0
Diarrhoeal diseases	Male	0-6 days	2-4 years	Data Rich	-1	2	Healthcare access and quality index	--
Diarrhoeal diseases	Male	0-6 days	2-4 years	Data Rich	1	2	Zinc deficiency	368
Diarrhoeal diseases	Male	0-6 days	2-4 years	Data Rich	-1	3	LDI (I$ per capita)	0
Diarrhoeal diseases	Male	0-6 days	2-4 years	Data Rich	-1	3	Socio-demographic Index	214
Diarrhoeal diseases	Male	0-6 days	2-4 years	Data Rich	-1	3	Maternal Education (years per capita)	770

Appendix Table S10: CODEm covariates used, level of covariate, and expected direction of covariate by cause, sex, and age

Cause	Sex	Age Start	Age End	Model Version Type	Direction	Level	Covariate Name	Number of Draws
Diarrhoeal diseases	Male	0-6 days	2-4 years	Data Rich	1	3	No access to handwashing facility	518
Diarrhoeal diseases	Male	0-6 days	2-4 years	Global	-1	1	Improved Water Source (proportion with access)	83
Diarrhoeal diseases	Male	0-6 days	2-4 years	Global	-1	1	RotaC lagged five year coverage, COVID-inclusive (proportion)	877
Diarrhoeal diseases	Male	0-6 days	2-4 years	Global	-1	1	Sanitation (proportion with access)	984
Diarrhoeal diseases	Male	0-6 days	2-4 years	Global	-1	1	ORS (oral rehydration)	1000
Diarrhoeal diseases	Male	0-6 days	2-4 years	Global	-1	2	Zinc treatment for diarrhea	0
Diarrhoeal diseases	Male	0-6 days	2-4 years	Global	-1	2	Healthcare access and quality index	651
Diarrhoeal diseases	Male	0-6 days	2-4 years	Global	1	2	Zinc deficiency	10
Diarrhoeal diseases	Male	0-6 days	2-4 years	Global	-1	3	LDI (I$ per capita)	0
Diarrhoeal diseases	Male	0-6 days	2-4 years	Global	-1	3	Socio-demographic Index	349
Diarrhoeal diseases	Male	0-6 days	2-4 years	Global	-1	3	Maternal Education (years per capita)	498
Diarrhoeal diseases	Male	0-6 days	2-4 years	Global	1	3	No access to handwashing facility	149
Diarrhoeal diseases	Male	5-9 years	95+ years	Data Rich	-1	1	Sanitation (proportion with access)	441
Diarrhoeal diseases	Male	5-9 years	95+ years	Data Rich	-1	1	Improved Water Source (proportion with access)	1000
Diarrhoeal diseases	Male	5-9 years	95+ years	Data Rich	-1	2	ORS (oral rehydration)	512
Diarrhoeal diseases	Male	5-9 years	95+ years	Data Rich	-1	2	Healthcare access and quality index	--
Diarrhoeal diseases	Male	5-9 years	95+ years	Data Rich	-1	2	RotaC lagged five year coverage, COVID-inclusive (proportion)	--
Diarrhoeal diseases	Male	5-9 years	95+ years	Data Rich	-1	3	Education (years per capita)	--
Diarrhoeal diseases	Male	5-9 years	95+ years	Data Rich	-1	3	LDI (I$ per capita)	--
Diarrhoeal diseases	Male	5-9 years	95+ years	Data Rich	-1	3	Socio-demographic Index	--
Diarrhoeal diseases	Male	5-9 years	95+ years	Data Rich	1	3	No access to handwashing facility	193
Diarrhoeal diseases	Male	5-9 years	95+ years	Data Rich	1	3	Age-standardized proportion adult underweight	--
Diarrhoeal diseases	Male	5-9 years	95+ years	Global	-1	1	Improved Water Source (proportion with access)	1000
Diarrhoeal diseases	Male	5-9 years	95+ years	Global	-1	1	Sanitation (proportion with access)	--
Diarrhoeal diseases	Male	5-9 years	95+ years	Global	-1	2	Healthcare access and quality index	795
Diarrhoeal diseases	Male	5-9 years	95+ years	Global	-1	2	ORS (oral rehydration)	795
Diarrhoeal diseases	Male	5-9 years	95+ years	Global	-1	2	RotaC lagged five year coverage, COVID-inclusive (proportion)	--
Diarrhoeal diseases	Male	5-9 years	95+ years	Global	-1	3	Education (years per capita)	--
Diarrhoeal diseases	Male	5-9 years	95+ years	Global	-1	3	LDI (I$ per capita)	--
Diarrhoeal diseases	Male	5-9 years	95+ years	Global	-1	3	Socio-demographic Index	--
Diarrhoeal diseases	Male	5-9 years	95+ years	Global	1	3	Age-standardized proportion adult underweight	244
Diarrhoeal diseases	Male	5-9 years	95+ years	Global	1	3	No access to handwashing facility	430
Rabies	Female	1-5 months	95+ years	Data Rich	-1	1	Antenatal Care (4 visits) Coverage (proportion)	376
Rabies	Female	1-5 months	95+ years	Data Rich	-1	1	In-Facility Delivery (proportion)	607
Rabies	Female	1-5 months	95+ years	Data Rich	-1	1	Socio-demographic Index	650
Rabies	Female	1-5 months	95+ years	Data Rich	-1	2	Healthcare access and quality index	303
Rabies	Female	1-5 months	95+ years	Data Rich	-1	2	Maternal care and immunization	313
Rabies	Female	1-5 months	95+ years	Data Rich	-1	2	Skilled Birth Attendance (proportion)	325
Rabies	Female	1-5 months	95+ years	Data Rich	1	3	Population Density (500-1000 ppl/sqkm, proportion)	--
Rabies	Female	1-5 months	95+ years	Data Rich	1	3	Population Density (under 150 ppl/sqkm, proportion)	--
Rabies	Female	1-5 months	95+ years	Global	-1	1	In-Facility Delivery (proportion)	291
Rabies	Female	1-5 months	95+ years	Global	-1	1	Antenatal Care (4 visits) Coverage (proportion)	415
Rabies	Female	1-5 months	95+ years	Global	-1	1	Socio-demographic Index	709
Rabies	Female	1-5 months	95+ years	Global	-1	2	Skilled Birth Attendance (proportion)	457
Rabies	Female	1-5 months	95+ years	Global	-1	2	Healthcare access and quality index	--
Rabies	Female	1-5 months	95+ years	Global	1	3	Population Density (500-1000 ppl/sqkm, proportion)	--
Rabies	Female	1-5 months	95+ years	Global	1	3	Population Density (under 150 ppl/sqkm, proportion)	--
Rabies	Male	1-5 months	95+ years	Data Rich	-1	1	In-Facility Delivery (proportion)	73
Rabies	Male	1-5 months	95+ years	Data Rich	-1	1	Antenatal Care (4 visits) Coverage (proportion)	703
Rabies	Male	1-5 months	95+ years	Data Rich	-1	1	Socio-demographic Index	866
Rabies	Male	1-5 months	95+ years	Data Rich	-1	2	Healthcare access and quality index	87
Rabies	Male	1-5 months	95+ years	Data Rich	-1	2	Skilled Birth Attendance (proportion)	289
Rabies	Male	1-5 months	95+ years	Data Rich	-1	2	Maternal care and immunization	313
Rabies	Male	1-5 months	95+ years	Data Rich	1	3	Population Density (500-1000 ppl/sqkm, proportion)	--
Rabies	Male	1-5 months	95+ years	Data Rich	1	3	Population Density (under 150 ppl/sqkm, proportion)	--
Rabies	Male	1-5 months	95+ years	Global	-1	1	Socio-demographic Index	431
Rabies	Male	1-5 months	95+ years	Global	-1	1	In-Facility Delivery (proportion)	569
Rabies	Male	1-5 months	95+ years	Global	-1	1	Antenatal Care (4 visits) Coverage (proportion)	--
Rabies	Male	1-5 months	95+ years	Global	-1	2	Healthcare access and quality index	362
Rabies	Male	1-5 months	95+ years	Global	-1	2	Skilled Birth Attendance (proportion)	--
Rabies	Male	1-5 months	95+ years	Global	1	3	Population Density (500-1000 ppl/sqkm, proportion)	--
Rabies	Male	1-5 months	95+ years	Global	1	3	Population Density (under 150 ppl/sqkm, proportion)	--
Other neglected tropical diseases	Female	0-6 days	95+ years	Data Rich	-1	1	Healthcare access and quality index	781
Other neglected tropical diseases	Female	0-6 days	95+ years	Data Rich	1	1	Latitude Under 15 (proportion)	369
Other neglected tropical diseases	Female	0-6 days	95+ years	Data Rich	-1	2	Sanitation (proportion with access)	222
Other neglected tropical diseases	Female	0-6 days	95+ years	Data Rich	1	2	Rainfall Quintile 5 (proportion)	--
Other neglected tropical diseases	Female	0-6 days	95+ years	Data Rich	-1	3	Education (years per capita)	87
Other neglected tropical diseases	Female	0-6 days	95+ years	Data Rich	-1	3	Socio-demographic Index	212
Other neglected tropical diseases	Female	0-6 days	95+ years	Data Rich	-1	3	LDI (I$ per capita)	--
Other neglected tropical diseases	Female	0-6 days	95+ years	Global	-1	1	Healthcare access and quality index	428
Other neglected tropical diseases	Female	0-6 days	95+ years	Global	1	1	Latitude Under 15 (proportion)	1000
Other neglected tropical diseases	Female	0-6 days	95+ years	Global	-1	2	Sanitation (proportion with access)	336
Other neglected tropical diseases	Female	0-6 days	95+ years	Global	1	2	Rainfall Quintile 5 (proportion)	--
Other neglected tropical diseases	Female	0-6 days	95+ years	Global	-1	3	Socio-demographic Index	160
Other neglected tropical diseases	Female	0-6 days	95+ years	Global	-1	3	Education (years per capita)	493
Other neglected tropical diseases	Female	0-6 days	95+ years	Global	-1	3	LDI (I$ per capita)	--
Other neglected tropical diseases	Male	0-6 days	95+ years	Data Rich	-1	1	Healthcare access and quality index	781
Other neglected tropical diseases	Male	0-6 days	95+ years	Data Rich	1	1	Latitude Under 15 (proportion)	344
Other neglected tropical diseases	Male	0-6 days	95+ years	Data Rich	-1	2	Sanitation (proportion with access)	222
Other neglected tropical diseases	Male	0-6 days	95+ years	Data Rich	1	2	Rainfall Quintile 5 (proportion)	--
Other neglected tropical diseases	Male	0-6 days	95+ years	Data Rich	-1	3	Education (years per capita)	180
Other neglected tropical diseases	Male	0-6 days	95+ years	Data Rich	-1	3	Socio-demographic Index	191
Other neglected tropical diseases	Male	0-6 days	95+ years	Data Rich	-1	3	LDI (I$ per capita)	--
Other neglected tropical diseases	Male	0-6 days	95+ years	Global	-1	1	Healthcare access and quality index	269
Other neglected tropical diseases	Male	0-6 days	95+ years	Global	1	1	Latitude Under 15 (proportion)	731
Other neglected tropical diseases	Male	0-6 days	95+ years	Global	-1	2	Sanitation (proportion with access)	405
Other neglected tropical diseases	Male	0-6 days	95+ years	Global	1	2	Rainfall Quintile 5 (proportion)	--
Other neglected tropical diseases	Male	0-6 days	95+ years	Global	-1	3	Education (years per capita)	193
Other neglected tropical diseases	Male	0-6 days	95+ years	Global	-1	3	Socio-demographic Index	244
Other neglected tropical diseases	Male	0-6 days	95+ years	Global	-1	3	LDI (I$ per capita)	--
Meningitis	Female	0-6 days	2-4 years	Data Rich	-1	1	PCV3 lagged five year coverage, COVID-inclusive (proportion)	536
Meningitis	Female	0-6 days	2-4 years	Data Rich	-1	1	Hib3 transformed: population-level coverage, including indirect effects (proportion)	550
Meningitis	Female	0-6 days	2-4 years	Data Rich	-1	1	Proportion of total population covered by menafrivac initiative (meningitis meningococcal type A vaccine)	--
Meningitis	Female	0-6 days	2-4 years	Data Rich	1	1	meningitis belt (proportion)	--
Meningitis	Female	0-6 days	2-4 years	Data Rich	-1	2	Maternal care and immunization	296
Meningitis	Female	0-6 days	2-4 years	Data Rich	-1	2	Healthcare access and quality index	570
Meningitis	Female	0-6 days	2-4 years	Data Rich	-1	2	Improved Water Source (proportion with access)	--
Meningitis	Female	0-6 days	2-4 years	Data Rich	1	2	Age- and sex-specific SEV for Child underweight	570
Meningitis	Female	0-6 days	2-4 years	Data Rich	-1	3	Sanitation (proportion with access)	58
Meningitis	Female	0-6 days	2-4 years	Data Rich	-1	3	Socio-demographic Index	451
Meningitis	Female	0-6 days	2-4 years	Data Rich	-1	3	Maternal Education (years per capita)	508
Meningitis	Female	0-6 days	2-4 years	Data Rich	-1	3	LDI (I$ per capita)	--

Appendix Table S10: CODEm covariates used, level of covariate, and expected direction of covariate by cause, sex, and age

Cause	Sex	Age Start	Age End	Model Version Type	Direction	Level	Covariate Name	Number of Draws
Meningitis	Female	0-6 days	2-4 years	Global	-1	1	Proportion of total population covered by menafrivac initiative (meningitis meningococcal type A vaccine)	119
Meningitis	Female	0-6 days	2-4 years	Global	-1	1	Hib3 transformed: population-level coverage, including indirect effects (proportion)	474
Meningitis	Female	0-6 days	2-4 years	Global	-1	1	PCV3 lagged five year coverage, COVID-inclusive (proportion)	697
Meningitis	Female	0-6 days	2-4 years	Global	1	1	meningitis belt (proportion)	1
Meningitis	Female	0-6 days	2-4 years	Global	-1	2	Maternal care and immunization	679
Meningitis	Female	0-6 days	2-4 years	Global	-1	2	Healthcare access and quality index	799
Meningitis	Female	0-6 days	2-4 years	Global	-1	2	Improved Water Source (proportion with access)	--
Meningitis	Female	0-6 days	2-4 years	Global	1	2	Age- and sex-specific SEV for Child underweight	729
Meningitis	Female	0-6 days	2-4 years	Global	-1	3	Maternal Education (years per capita)	119
Meningitis	Female	0-6 days	2-4 years	Global	-1	3	Socio-demographic Index	292
Meningitis	Female	0-6 days	2-4 years	Global	-1	3	Sanitation (proportion with access)	295
Meningitis	Female	0-6 days	2-4 years	Global	-1	3	LDI (I$ per capita)	--
Meningitis	Female	5-9 years	95+ years	Data Rich	-1	1	Hib3 transformed: population-level coverage, including indirect effects (proportion)	347
Meningitis	Female	5-9 years	95+ years	Data Rich	-1	1	PCV3 lagged five year coverage, COVID-inclusive (proportion)	758
Meningitis	Female	5-9 years	95+ years	Data Rich	-1	1	Proportion of total population covered by menafrivac initiative (meningitis meningococcal type A vaccine)	--
Meningitis	Female	5-9 years	95+ years	Data Rich	1	1	meningitis belt (proportion)	--
Meningitis	Female	5-9 years	95+ years	Data Rich	-1	2	Maternal care and immunization	106
Meningitis	Female	5-9 years	95+ years	Data Rich	-1	2	Healthcare access and quality index	680
Meningitis	Female	5-9 years	95+ years	Data Rich	-1	2	Improved Water Source (proportion with access)	--
Meningitis	Female	5-9 years	95+ years	Data Rich	-1	3	Sanitation (proportion with access)	9
Meningitis	Female	5-9 years	95+ years	Data Rich	-1	3	Socio-demographic Index	353
Meningitis	Female	5-9 years	95+ years	Data Rich	-1	3	Maternal Education (years per capita)	416
Meningitis	Female	5-9 years	95+ years	Data Rich	-1	3	LDI (I$ per capita)	--
Meningitis	Female	5-9 years	95+ years	Global	-1	1	Proportion of total population covered by menafrivac initiative (meningitis meningococcal type A vaccine)	2
Meningitis	Female	5-9 years	95+ years	Global	-1	1	Hib3 transformed: population-level coverage, including indirect effects (proportion)	178
Meningitis	Female	5-9 years	95+ years	Global	-1	1	PCV3 lagged five year coverage, COVID-inclusive (proportion)	575
Meningitis	Female	5-9 years	95+ years	Global	1	1	meningitis belt (proportion)	588
Meningitis	Female	5-9 years	95+ years	Global	-1	2	Maternal care and immunization	399
Meningitis	Female	5-9 years	95+ years	Global	-1	2	Healthcare access and quality index	618
Meningitis	Female	5-9 years	95+ years	Global	-1	2	Improved Water Source (proportion with access)	--
Meningitis	Female	5-9 years	95+ years	Global	-1	3	Maternal Education (years per capita)	157
Meningitis	Female	5-9 years	95+ years	Global	-1	3	Sanitation (proportion with access)	199
Meningitis	Female	5-9 years	95+ years	Global	-1	3	Socio-demographic Index	263
Meningitis	Female	5-9 years	95+ years	Global	-1	3	LDI (I$ per capita)	--
Meningitis	Male	0-6 days	2-4 years	Data Rich	-1	1	PCV3 lagged five year coverage, COVID-inclusive (proportion)	356
Meningitis	Male	0-6 days	2-4 years	Data Rich	-1	1	Hib3 transformed: population-level coverage, including indirect effects (proportion)	822
Meningitis	Male	0-6 days	2-4 years	Data Rich	-1	1	Proportion of total population covered by menafrivac initiative (meningitis meningococcal type A vaccine)	--
Meningitis	Male	0-6 days	2-4 years	Data Rich	1	1	meningitis belt (proportion)	--
Meningitis	Male	0-6 days	2-4 years	Data Rich	-1	2	Maternal care and immunization	120
Meningitis	Male	0-6 days	2-4 years	Data Rich	-1	2	Healthcare access and quality index	510
Meningitis	Male	0-6 days	2-4 years	Data Rich	-1	2	Improved Water Source (proportion with access)	--
Meningitis	Male	0-6 days	2-4 years	Data Rich	1	2	Age- and sex-specific SEV for Child underweight	557
Meningitis	Male	0-6 days	2-4 years	Data Rich	-1	3	Sanitation (proportion with access)	48
Meningitis	Male	0-6 days	2-4 years	Data Rich	-1	3	Socio-demographic Index	490
Meningitis	Male	0-6 days	2-4 years	Data Rich	-1	3	Maternal Education (years per capita)	505
Meningitis	Male	0-6 days	2-4 years	Data Rich	-1	3	LDI (I$ per capita)	--
Meningitis	Male	0-6 days	2-4 years	Global	-1	1	Proportion of total population covered by menafrivac initiative (meningitis meningococcal type A vaccine)	112
Meningitis	Male	0-6 days	2-4 years	Global	-1	1	PCV3 lagged five year coverage, COVID-inclusive (proportion)	612
Meningitis	Male	0-6 days	2-4 years	Global	-1	1	Hib3 transformed: population-level coverage, including indirect effects (proportion)	647
Meningitis	Male	0-6 days	2-4 years	Global	1	1	meningitis belt (proportion)	17
Meningitis	Male	0-6 days	2-4 years	Global	-1	2	Healthcare access and quality index	748
Meningitis	Male	0-6 days	2-4 years	Global	-1	2	Maternal care and immunization	748
Meningitis	Male	0-6 days	2-4 years	Global	-1	2	Improved Water Source (proportion with access)	--
Meningitis	Male	0-6 days	2-4 years	Global	1	2	Age- and sex-specific SEV for Child underweight	748
Meningitis	Male	0-6 days	2-4 years	Global	-1	3	Maternal Education (years per capita)	81
Meningitis	Male	0-6 days	2-4 years	Global	-1	3	Sanitation (proportion with access)	328
Meningitis	Male	0-6 days	2-4 years	Global	-1	3	Socio-demographic Index	330
Meningitis	Male	0-6 days	2-4 years	Global	-1	3	LDI (I$ per capita)	--
Meningitis	Male	5-9 years	95+ years	Data Rich	-1	1	Proportion of total population covered by menafrivac initiative (meningitis meningococcal type A vaccine)	318
Meningitis	Male	5-9 years	95+ years	Data Rich	-1	1	PCV3 lagged five year coverage, COVID-inclusive (proportion)	527
Meningitis	Male	5-9 years	95+ years	Data Rich	-1	1	Hib3 transformed: population-level coverage, including indirect effects (proportion)	1000
Meningitis	Male	5-9 years	95+ years	Data Rich	1	1	meningitis belt (proportion)	--
Meningitis	Male	5-9 years	95+ years	Data Rich	-1	2	Maternal care and immunization	76
Meningitis	Male	5-9 years	95+ years	Data Rich	-1	2	Healthcare access and quality index	545
Meningitis	Male	5-9 years	95+ years	Data Rich	-1	2	Improved Water Source (proportion with access)	--
Meningitis	Male	5-9 years	95+ years	Data Rich	-1	3	Sanitation (proportion with access)	9
Meningitis	Male	5-9 years	95+ years	Data Rich	-1	3	Maternal Education (years per capita)	257
Meningitis	Male	5-9 years	95+ years	Data Rich	-1	3	Socio-demographic Index	373
Meningitis	Male	5-9 years	95+ years	Data Rich	-1	3	LDI (I$ per capita)	--
Meningitis	Male	5-9 years	95+ years	Global	-1	1	Hib3 transformed: population-level coverage, including indirect effects (proportion)	365
Meningitis	Male	5-9 years	95+ years	Global	-1	1	PCV3 lagged five year coverage, COVID-inclusive (proportion)	726
Meningitis	Male	5-9 years	95+ years	Global	-1	1	Proportion of total population covered by menafrivac initiative (meningitis meningococcal type A vaccine)	--
Meningitis	Male	5-9 years	95+ years	Global	1	1	meningitis belt (proportion)	96
Meningitis	Male	5-9 years	95+ years	Global	-1	2	Maternal care and immunization	351
Meningitis	Male	5-9 years	95+ years	Global	-1	2	Healthcare access and quality index	681
Meningitis	Male	5-9 years	95+ years	Global	-1	2	Improved Water Source (proportion with access)	--
Meningitis	Male	5-9 years	95+ years	Global	-1	3	Sanitation (proportion with access)	48
Meningitis	Male	5-9 years	95+ years	Global	-1	3	Maternal Education (years per capita)	91
Meningitis	Male	5-9 years	95+ years	Global	-1	3	Socio-demographic Index	235
Meningitis	Male	5-9 years	95+ years	Global	-1	3	LDI (I$ per capita)	--
Encephalitis	Female	0-6 days	95+ years	Data Rich	1	1	Age- and sex-specific SEV for Child underweight	1000
Encephalitis	Female	0-6 days	95+ years	Data Rich	1	1	Japanese encelphalitis endemic area (binary)	--
Encephalitis	Female	0-6 days	95+ years	Data Rich	-1	2	Maternal care and immunization	92
Encephalitis	Female	0-6 days	95+ years	Data Rich	-1	2	Healthcare access and quality index	273
Encephalitis	Female	0-6 days	95+ years	Data Rich	-1	2	LDI (I$ per capita)	--

Appendix Table S10: CODEm covariates used, level of covariate, and expected direction of covariate by cause, sex, and age

Cause	Sex	Age Start	Age End	Model Version Type	Direction	Level	Covariate Name	Number of Draws
Encephalitis	Female	0-6 days	95+ years	Data Rich	-1	3	Maternal Education (years per capita)	90
Encephalitis	Female	0-6 days	95+ years	Data Rich	-1	3	DTP3 Coverage, COVID-free (proportion)	325
Encephalitis	Female	0-6 days	95+ years	Data Rich	-1	3	Improved Water Source (proportion with access)	449
Encephalitis	Female	0-6 days	95+ years	Data Rich	-1	3	In-Facility Delivery (proportion)	--
Encephalitis	Female	0-6 days	95+ years	Data Rich	-1	3	Sanitation (proportion with access)	--
Encephalitis	Female	0-6 days	95+ years	Data Rich	-1	3	Socio-demographic Index	--
Encephalitis	Female	0-6 days	95+ years	Global	1	1	Age- and sex-specific SEV for Child underweight	410
Encephalitis	Female	0-6 days	95+ years	Global	1	1	Japanese encelphalitis endemic area (binary)	1000
Encephalitis	Female	0-6 days	95+ years	Global	-1	2	Maternal care and immunization	225
Encephalitis	Female	0-6 days	95+ years	Global	-1	2	Healthcare access and quality index	426
Encephalitis	Female	0-6 days	95+ years	Global	-1	2	LDI (I$ per capita)	--
Encephalitis	Female	0-6 days	95+ years	Global	-1	3	Maternal Education (years per capita)	242
Encephalitis	Female	0-6 days	95+ years	Global	-1	3	DTP3 Coverage, COVID-free (proportion)	345
Encephalitis	Female	0-6 days	95+ years	Global	-1	3	Improved Water Source (proportion with access)	--
Encephalitis	Female	0-6 days	95+ years	Global	-1	3	In-Facility Delivery (proportion)	--
Encephalitis	Female	0-6 days	95+ years	Global	-1	3	Sanitation (proportion with access)	--
Encephalitis	Female	0-6 days	95+ years	Global	-1	3	Socio-demographic Index	--
Encephalitis	Male	0-6 days	95+ years	Data Rich	1	1	Age- and sex-specific SEV for Child underweight	1000
Encephalitis	Male	0-6 days	95+ years	Data Rich	1	1	Japanese encelphalitis endemic area (binary)	1000
Encephalitis	Male	0-6 days	95+ years	Data Rich	-1	2	Maternal care and immunization	243
Encephalitis	Male	0-6 days	95+ years	Data Rich	-1	2	Healthcare access and quality index	--
Encephalitis	Male	0-6 days	95+ years	Data Rich	-1	2	LDI (I$ per capita)	--
Encephalitis	Male	0-6 days	95+ years	Data Rich	-1	3	Improved Water Source (proportion with access)	142
Encephalitis	Male	0-6 days	95+ years	Data Rich	-1	3	DTP3 Coverage, COVID-free (proportion)	321
Encephalitis	Male	0-6 days	95+ years	Data Rich	-1	3	In-Facility Delivery (proportion)	--
Encephalitis	Male	0-6 days	95+ years	Data Rich	-1	3	Maternal Education (years per capita)	--
Encephalitis	Male	0-6 days	95+ years	Data Rich	-1	3	Sanitation (proportion with access)	--
Encephalitis	Male	0-6 days	95+ years	Data Rich	-1	3	Socio-demographic Index	--
Encephalitis	Male	0-6 days	95+ years	Global	1	1	Japanese encelphalitis endemic area (binary)	1000
Encephalitis	Male	0-6 days	95+ years	Global	1	1	Age- and sex-specific SEV for Child underweight	--
Encephalitis	Male	0-6 days	95+ years	Global	-1	2	Healthcare access and quality index	--
Encephalitis	Male	0-6 days	95+ years	Global	-1	2	LDI (I$ per capita)	--
Encephalitis	Male	0-6 days	95+ years	Global	-1	2	Maternal care and immunization	--
Encephalitis	Male	0-6 days	95+ years	Global	-1	3	Socio-demographic Index	137
Encephalitis	Male	0-6 days	95+ years	Global	-1	3	Maternal Education (years per capita)	139
Encephalitis	Male	0-6 days	95+ years	Global	-1	3	DTP3 Coverage, COVID-free (proportion)	425
Encephalitis	Male	0-6 days	95+ years	Global	-1	3	Improved Water Source (proportion with access)	--
Encephalitis	Male	0-6 days	95+ years	Global	-1	3	In-Facility Delivery (proportion)	--
Encephalitis	Male	0-6 days	95+ years	Global	-1	3	Sanitation (proportion with access)	--
Tetanus	Female	0-6 days	6-11 months	Data Rich	-1	1	DTP3 lagged five year coverage, COVID-inclusive (proportion)	357
Tetanus	Female	0-6 days	6-11 months	Data Rich	-1	1	tetanus toxoid maternal protection at birth	790
Tetanus	Female	0-6 days	6-11 months	Data Rich	-1	2	In-Facility Delivery (proportion)	306
Tetanus	Female	0-6 days	6-11 months	Data Rich	-1	2	Skilled Birth Attendance (proportion)	474
Tetanus	Female	0-6 days	6-11 months	Data Rich	-1	2	Healthcare access and quality index	556
Tetanus	Female	0-6 days	6-11 months	Data Rich	-1	3	Socio-demographic Index	647
Tetanus	Female	0-6 days	6-11 months	Data Rich	-1	3	Education (years per capita)	746
Tetanus	Female	0-6 days	6-11 months	Data Rich	-1	3	LDI (I$ per capita)	--
Tetanus	Female	0-6 days	6-11 months	Global	-1	1	tetanus toxoid maternal protection at birth	714
Tetanus	Female	0-6 days	6-11 months	Global	-1	1	DTP3 lagged five year coverage, COVID-inclusive (proportion)	754
Tetanus	Female	0-6 days	6-11 months	Global	-1	2	In-Facility Delivery (proportion)	18
Tetanus	Female	0-6 days	6-11 months	Global	-1	2	Skilled Birth Attendance (proportion)	778
Tetanus	Female	0-6 days	6-11 months	Global	-1	2	Healthcare access and quality index	796
Tetanus	Female	0-6 days	6-11 months	Global	-1	3	Education (years per capita)	381
Tetanus	Female	0-6 days	6-11 months	Global	-1	3	Socio-demographic Index	485
Tetanus	Female	0-6 days	6-11 months	Global	-1	3	LDI (I$ per capita)	--
Tetanus	Female	12-23 months	95+ years	Data Rich	-1	1	DTP3 lagged five year coverage, COVID-inclusive (proportion)	1000
Tetanus	Female	12-23 months	95+ years	Data Rich	-1	2	Healthcare access and quality index	666
Tetanus	Female	12-23 months	95+ years	Data Rich	-1	3	Education (years per capita)	616
Tetanus	Female	12-23 months	95+ years	Data Rich	-1	3	Sanitation (proportion with access)	616
Tetanus	Female	12-23 months	95+ years	Data Rich	-1	3	Socio-demographic Index	616
Tetanus	Female	12-23 months	95+ years	Data Rich	-1	3	LDI (I$ per capita)	--
Tetanus	Female	12-23 months	95+ years	Global	-1	1	DTP3 lagged five year coverage, COVID-inclusive (proportion)	1000
Tetanus	Female	12-23 months	95+ years	Global	-1	2	Healthcare access and quality index	702
Tetanus	Female	12-23 months	95+ years	Global	-1	3	Education (years per capita)	208
Tetanus	Female	12-23 months	95+ years	Global	-1	3	Socio-demographic Index	275
Tetanus	Female	12-23 months	95+ years	Global	-1	3	Sanitation (proportion with access)	424
Tetanus	Female	12-23 months	95+ years	Global	-1	3	LDI (I$ per capita)	--
Tetanus	Male	0-6 days	6-11 months	Data Rich	-1	1	tetanus toxoid maternal protection at birth	499
Tetanus	Male	0-6 days	6-11 months	Data Rich	-1	1	DTP3 lagged five year coverage, COVID-inclusive (proportion)	564
Tetanus	Male	0-6 days	6-11 months	Data Rich	-1	2	In-Facility Delivery (proportion)	258
Tetanus	Male	0-6 days	6-11 months	Data Rich	-1	2	Skilled Birth Attendance (proportion)	507
Tetanus	Male	0-6 days	6-11 months	Data Rich	-1	2	Healthcare access and quality index	680
Tetanus	Male	0-6 days	6-11 months	Data Rich	-1	3	Education (years per capita)	437
Tetanus	Male	0-6 days	6-11 months	Data Rich	-1	3	Socio-demographic Index	891
Tetanus	Male	0-6 days	6-11 months	Data Rich	-1	3	LDI (I$ per capita)	--
Tetanus	Male	0-6 days	6-11 months	Global	-1	1	DTP3 lagged five year coverage, COVID-inclusive (proportion)	660
Tetanus	Male	0-6 days	6-11 months	Global	-1	1	tetanus toxoid maternal protection at birth	903
Tetanus	Male	0-6 days	6-11 months	Global	-1	2	In-Facility Delivery (proportion)	118
Tetanus	Male	0-6 days	6-11 months	Global	-1	2	Skilled Birth Attendance (proportion)	738
Tetanus	Male	0-6 days	6-11 months	Global	-1	2	Healthcare access and quality index	821
Tetanus	Male	0-6 days	6-11 months	Global	-1	3	Education (years per capita)	310
Tetanus	Male	0-6 days	6-11 months	Global	-1	3	Socio-demographic Index	435
Tetanus	Male	0-6 days	6-11 months	Global	-1	3	LDI (I$ per capita)	--
Tetanus	Male	12-23 months	95+ years	Data Rich	-1	1	DTP3 lagged five year coverage, COVID-inclusive (proportion)	1000
Tetanus	Male	12-23 months	95+ years	Data Rich	-1	2	Healthcare access and quality index	577
Tetanus	Male	12-23 months	95+ years	Data Rich	-1	3	Sanitation (proportion with access)	125
Tetanus	Male	12-23 months	95+ years	Data Rich	-1	3	Education (years per capita)	370
Tetanus	Male	12-23 months	95+ years	Data Rich	-1	3	Socio-demographic Index	613
Tetanus	Male	12-23 months	95+ years	Data Rich	-1	3	LDI (I$ per capita)	--
Tetanus	Male	12-23 months	95+ years	Global	-1	1	DTP3 lagged five year coverage, COVID-inclusive (proportion)	1000
Tetanus	Male	12-23 months	95+ years	Global	-1	2	Healthcare access and quality index	684
Tetanus	Male	12-23 months	95+ years	Global	-1	3	Socio-demographic Index	343
Tetanus	Male	12-23 months	95+ years	Global	-1	3	Sanitation (proportion with access)	375
Tetanus	Male	12-23 months	95+ years	Global	-1	3	Education (years per capita)	549
Tetanus	Male	12-23 months	95+ years	Global	-1	3	LDI (I$ per capita)	--
Acute hepatitis	Female	1-5 months	95+ years	Data Rich	1	1	Hepatitis A Seroprevalence (anti-HAV) age standardized	196
Acute hepatitis	Female	1-5 months	95+ years	Data Rich	1	1	Hepatitis E Seroprevalence (anti-HEV) age standardized	207
Acute hepatitis	Female	1-5 months	95+ years	Data Rich	1	1	Log-transformed SEV scalar: Hep	451
Acute hepatitis	Female	1-5 months	95+ years	Data Rich	1	1	Hepatitis C Seroprevalence (anti-HCV) age standardized	674
Acute hepatitis	Female	1-5 months	95+ years	Data Rich	1	1	Vaccine adjusted HbSAg serporevalence age standardized	724
Acute hepatitis	Female	1-5 months	95+ years	Data Rich	-1	2	Healthcare access and quality index	0
Acute hepatitis	Female	1-5 months	95+ years	Data Rich	-1	2	Socio-demographic Index	0

Appendix Table S10: CODEm covariates used, level of covariate, and expected direction of covariate by cause, sex, and age

Cause	Sex	Age Start	Age End	Model Version Type	Direction	Level	Covariate Name	Number of Draws
Acute hepatitis	Female	1-5 months	95+ years	Data Rich	-1	2	Hepatitis B vaccine coverage, aged through time, COVID-free (proportion)	60
Acute hepatitis	Female	1-5 months	95+ years	Data Rich	1	2	Age- and sex-specific SEV for Unsafe sanitation	184
Acute hepatitis	Female	1-5 months	95+ years	Data Rich	1	2	Age- and sex-specific SEV for Unsafe water	480
Acute hepatitis	Female	1-5 months	95+ years	Data Rich	1	2	Intravenous drug use (proportion by age)	--
Acute hepatitis	Female	1-5 months	95+ years	Data Rich	-1	3	Education (years per capita)	0
Acute hepatitis	Female	1-5 months	95+ years	Data Rich	-1	3	LDI (I$ per capita)	--
Acute hepatitis	Female	1-5 months	95+ years	Global	1	1	Hepatitis E Seroprevalence (anti-HEV) age standardized	226
Acute hepatitis	Female	1-5 months	95+ years	Global	1	1	Hepatitis C Seroprevalence (anti-HCV) age standardized	427
Acute hepatitis	Female	1-5 months	95+ years	Global	1	1	Hepatitis A Seroprevalence (anti-HAV) age standardized	508
Acute hepatitis	Female	1-5 months	95+ years	Global	1	1	Log-transformed SEV scalar: Hep	562
Acute hepatitis	Female	1-5 months	95+ years	Global	1	1	Vaccine adjusted HbSAg serporevalence age standardized	980
Acute hepatitis	Female	1-5 months	95+ years	Global	-1	2	Socio-demographic Index	19
Acute hepatitis	Female	1-5 months	95+ years	Global	-1	2	Healthcare access and quality index	156
Acute hepatitis	Female	1-5 months	95+ years	Global	-1	2	Hepatitis B vaccine coverage, aged through time, COVID-free (proportion)	409
Acute hepatitis	Female	1-5 months	95+ years	Global	1	2	Age- and sex-specific SEV for Unsafe water	276
Acute hepatitis	Female	1-5 months	95+ years	Global	1	2	Age- and sex-specific SEV for Unsafe sanitation	705
Acute hepatitis	Female	1-5 months	95+ years	Global	1	2	Intravenous drug use (proportion by age)	--
Acute hepatitis	Female	1-5 months	95+ years	Global	-1	3	Education (years per capita)	326
Acute hepatitis	Female	1-5 months	95+ years	Global	-1	3	LDI (I$ per capita)	--
Acute hepatitis	Male	1-5 months	95+ years	Data Rich	1	1	Hepatitis C Seroprevalence (anti-HCV) age standardized	95
Acute hepatitis	Male	1-5 months	95+ years	Data Rich	1	1	Hepatitis E Seroprevalence (anti-HEV) age standardized	358
Acute hepatitis	Male	1-5 months	95+ years	Data Rich	1	1	Log-transformed SEV scalar: Hep	533
Acute hepatitis	Male	1-5 months	95+ years	Data Rich	1	1	Vaccine adjusted HbSAg serporevalence age standardized	564
Acute hepatitis	Male	1-5 months	95+ years	Data Rich	1	1	Hepatitis A Seroprevalence (anti-HAV) age standardized	579
Acute hepatitis	Male	1-5 months	95+ years	Data Rich	-1	2	Healthcare access and quality index	0
Acute hepatitis	Male	1-5 months	95+ years	Data Rich	-1	2	Socio-demographic Index	0
Acute hepatitis	Male	1-5 months	95+ years	Data Rich	-1	2	Hepatitis B vaccine coverage, aged through time, COVID-free (proportion)	181
Acute hepatitis	Male	1-5 months	95+ years	Data Rich	1	2	Age- and sex-specific SEV for Unsafe sanitation	289
Acute hepatitis	Male	1-5 months	95+ years	Data Rich	1	2	Age- and sex-specific SEV for Unsafe water	511
Acute hepatitis	Male	1-5 months	95+ years	Data Rich	1	2	Intravenous drug use (proportion by age)	--
Acute hepatitis	Male	1-5 months	95+ years	Data Rich	-1	3	Education (years per capita)	1
Acute hepatitis	Male	1-5 months	95+ years	Data Rich	-1	3	LDI (I$ per capita)	--
Acute hepatitis	Male	1-5 months	95+ years	Global	1	1	Log-transformed SEV scalar: Hep	198
Acute hepatitis	Male	1-5 months	95+ years	Global	1	1	Hepatitis C Seroprevalence (anti-HCV) age standardized	408
Acute hepatitis	Male	1-5 months	95+ years	Global	1	1	Hepatitis E Seroprevalence (anti-HEV) age standardized	557
Acute hepatitis	Male	1-5 months	95+ years	Global	1	1	Vaccine adjusted HbSAg serporevalence age standardized	591
Acute hepatitis	Male	1-5 months	95+ years	Global	1	1	Hepatitis A Seroprevalence (anti-HAV) age standardized	854
Acute hepatitis	Male	1-5 months	95+ years	Global	-1	2	Healthcare access and quality index	27
Acute hepatitis	Male	1-5 months	95+ years	Global	-1	2	Socio-demographic Index	146
Acute hepatitis	Male	1-5 months	95+ years	Global	-1	2	Hepatitis B vaccine coverage, aged through time, COVID-free (proportion)	537
Acute hepatitis	Male	1-5 months	95+ years	Global	1	2	Age- and sex-specific SEV for Unsafe water	293
Acute hepatitis	Male	1-5 months	95+ years	Global	1	2	Age- and sex-specific SEV for Unsafe sanitation	980
Acute hepatitis	Male	1-5 months	95+ years	Global	1	2	Intravenous drug use (proportion by age)	--
Acute hepatitis	Male	1-5 months	95+ years	Global	-1	3	Education (years per capita)	125
Acute hepatitis	Male	1-5 months	95+ years	Global	-1	3	LDI (I$ per capita)	--
Acute hepatitis A	Female	1-5 months	95+ years	Data Rich	1	1	Hepatitis A Seroprevalence (anti-HAV) age standardized	1000
Acute hepatitis A	Female	1-5 months	95+ years	Data Rich	1	1	Log-transformed SEV scalar: Hep	--
Acute hepatitis A	Female	1-5 months	95+ years	Data Rich	-1	2	Socio-demographic Index	271
Acute hepatitis A	Female	1-5 months	95+ years	Data Rich	-1	2	Healthcare access and quality index	--
Acute hepatitis A	Female	1-5 months	95+ years	Data Rich	1	2	Age- and sex-specific SEV for Unsafe sanitation	284
Acute hepatitis A	Female	1-5 months	95+ years	Data Rich	1	2	Age- and sex-specific SEV for Unsafe water	--
Acute hepatitis A	Female	1-5 months	95+ years	Data Rich	-1	3	Education (years per capita)	327
Acute hepatitis A	Female	1-5 months	95+ years	Data Rich	-1	3	LDI (I$ per capita)	--
Acute hepatitis A	Female	1-5 months	95+ years	Global	1	1	Log-transformed SEV scalar: Hep	602
Acute hepatitis A	Female	1-5 months	95+ years	Global	1	1	Hepatitis A Seroprevalence (anti-HAV) age standardized	1000
Acute hepatitis A	Female	1-5 months	95+ years	Global	-1	2	Healthcare access and quality index	647
Acute hepatitis A	Female	1-5 months	95+ years	Global	-1	2	Socio-demographic Index	647
Acute hepatitis A	Female	1-5 months	95+ years	Global	1	2	Age- and sex-specific SEV for Unsafe sanitation	249
Acute hepatitis A	Female	1-5 months	95+ years	Global	1	2	Age- and sex-specific SEV for Unsafe water	--
Acute hepatitis A	Female	1-5 months	95+ years	Global	-1	3	Education (years per capita)	348
Acute hepatitis A	Female	1-5 months	95+ years	Global	-1	3	LDI (I$ per capita)	--
Acute hepatitis A	Male	1-5 months	95+ years	Data Rich	1	1	Hepatitis A Seroprevalence (anti-HAV) age standardized	565
Acute hepatitis A	Male	1-5 months	95+ years	Data Rich	1	1	Log-transformed SEV scalar: Hep	732
Acute hepatitis A	Male	1-5 months	95+ years	Data Rich	-1	2	Healthcare access and quality index	64
Acute hepatitis A	Male	1-5 months	95+ years	Data Rich	-1	2	Socio-demographic Index	252
Acute hepatitis A	Male	1-5 months	95+ years	Data Rich	1	2	Age- and sex-specific SEV for Unsafe sanitation	175
Acute hepatitis A	Male	1-5 months	95+ years	Data Rich	1	2	Age- and sex-specific SEV for Unsafe water	354
Acute hepatitis A	Male	1-5 months	95+ years	Data Rich	-1	3	Education (years per capita)	90
Acute hepatitis A	Male	1-5 months	95+ years	Data Rich	-1	3	LDI (I$ per capita)	--
Acute hepatitis A	Male	1-5 months	95+ years	Global	1	1	Hepatitis A Seroprevalence (anti-HAV) age standardized	821
Acute hepatitis A	Male	1-5 months	95+ years	Global	1	1	Log-transformed SEV scalar: Hep	1000
Acute hepatitis A	Male	1-5 months	95+ years	Global	-1	2	Healthcare access and quality index	427
Acute hepatitis A	Male	1-5 months	95+ years	Global	-1	2	Socio-demographic Index	498
Acute hepatitis A	Male	1-5 months	95+ years	Global	1	2	Age- and sex-specific SEV for Unsafe sanitation	387
Acute hepatitis A	Male	1-5 months	95+ years	Global	1	2	Age- and sex-specific SEV for Unsafe water	741
Acute hepatitis A	Male	1-5 months	95+ years	Global	-1	3	Education (years per capita)	485
Acute hepatitis A	Male	1-5 months	95+ years	Global	-1	3	LDI (I$ per capita)	--
Acute hepatitis B	Female	1-5 months	95+ years	Data Rich	-1	1	Hepatitis B vaccine coverage, aged through time, COVID-free (proportion)	840
Acute hepatitis B	Female	1-5 months	95+ years	Data Rich	1	1	Vaccine adjusted HbSAg serporevalence age standardized	361
Acute hepatitis B	Female	1-5 months	95+ years	Data Rich	1	1	Intravenous drug use (proportion by age)	--
Acute hepatitis B	Female	1-5 months	95+ years	Data Rich	-1	2	Healthcare access and quality index	42
Acute hepatitis B	Female	1-5 months	95+ years	Data Rich	-1	2	Socio-demographic Index	65
Acute hepatitis B	Female	1-5 months	95+ years	Data Rich	1	2	Log-transformed SEV scalar: Hep	90
Acute hepatitis B	Female	1-5 months	95+ years	Data Rich	-1	3	Education (years per capita)	150
Acute hepatitis B	Female	1-5 months	95+ years	Data Rich	-1	3	LDI (I$ per capita)	--
Acute hepatitis B	Female	1-5 months	95+ years	Global	-1	1	Hepatitis B vaccine coverage, aged through time, COVID-free (proportion)	1000
Acute hepatitis B	Female	1-5 months	95+ years	Global	1	1	Vaccine adjusted HbSAg serporevalence age standardized	1000
Acute hepatitis B	Female	1-5 months	95+ years	Global	1	1	Intravenous drug use (proportion by age)	--
Acute hepatitis B	Female	1-5 months	95+ years	Global	-1	2	Socio-demographic Index	185
Acute hepatitis B	Female	1-5 months	95+ years	Global	-1	2	Healthcare access and quality index	--
Acute hepatitis B	Female	1-5 months	95+ years	Global	1	2	Log-transformed SEV scalar: Hep	506
Acute hepatitis B	Female	1-5 months	95+ years	Global	-1	3	Education (years per capita)	222
Acute hepatitis B	Female	1-5 months	95+ years	Global	-1	3	LDI (I$ per capita)	--

Appendix Table S10: CODEm covariates used, level of covariate, and expected direction of covariate by cause, sex, and age

Cause	Sex	Age Start	Age End	Model Version Type	Direction	Level	Covariate Name	Number of Draws
Acute hepatitis B	Male	1-5 months	95+ years	Data Rich	-1	1	Hepatitis B vaccine coverage, aged through time, COVID-free (proportion)	577
Acute hepatitis B	Male	1-5 months	95+ years	Data Rich	1	1	Vaccine adjusted HbSAg serporevalence age standardized	1000
Acute hepatitis B	Male	1-5 months	95+ years	Data Rich	1	1	Intravenous drug use (proportion by age)	--
Acute hepatitis B	Male	1-5 months	95+ years	Data Rich	-1	2	Healthcare access and quality index	77
Acute hepatitis B	Male	1-5 months	95+ years	Data Rich	-1	2	Socio-demographic Index	169
Acute hepatitis B	Male	1-5 months	95+ years	Data Rich	1	2	Log-transformed SEV scalar: Hep	77
Acute hepatitis B	Male	1-5 months	95+ years	Data Rich	-1	3	Education (years per capita)	160
Acute hepatitis B	Male	1-5 months	95+ years	Data Rich	-1	3	LDI (I$ per capita)	--
Acute hepatitis B	Male	1-5 months	95+ years	Global	-1	1	Hepatitis B vaccine coverage, aged through time, COVID-free (proportion)	449
Acute hepatitis B	Male	1-5 months	95+ years	Global	1	1	Vaccine adjusted HbSAg serporevalence age standardized	551
Acute hepatitis B	Male	1-5 months	95+ years	Global	1	1	Intravenous drug use (proportion by age)	--
Acute hepatitis B	Male	1-5 months	95+ years	Global	-1	2	Socio-demographic Index	88
Acute hepatitis B	Male	1-5 months	95+ years	Global	-1	2	Healthcare access and quality index	279
Acute hepatitis B	Male	1-5 months	95+ years	Global	1	2	Log-transformed SEV scalar: Hep	--
Acute hepatitis B	Male	1-5 months	95+ years	Global	-1	3	Education (years per capita)	400
Acute hepatitis B	Male	1-5 months	95+ years	Global	-1	3	LDI (I$ per capita)	--
Acute hepatitis C	Female	1-5 months	95+ years	Data Rich	1	1	Log-transformed SEV scalar: Hep	624
Acute hepatitis C	Female	1-5 months	95+ years	Data Rich	1	1	Hepatitis C Seroprevalence (anti-HCV) age standardized	1000
Acute hepatitis C	Female	1-5 months	95+ years	Data Rich	-1	2	Healthcare access and quality index	542
Acute hepatitis C	Female	1-5 months	95+ years	Data Rich	-1	2	Socio-demographic Index	542
Acute hepatitis C	Female	1-5 months	95+ years	Data Rich	1	2	Intravenous drug use (proportion by age)	--
Acute hepatitis C	Female	1-5 months	95+ years	Data Rich	-1	3	Education (years per capita)	631
Acute hepatitis C	Female	1-5 months	95+ years	Data Rich	-1	3	LDI (I$ per capita)	--
Acute hepatitis C	Female	1-5 months	95+ years	Global	1	1	Log-transformed SEV scalar: Hep	1000
Acute hepatitis C	Female	1-5 months	95+ years	Global	1	1	Hepatitis C Seroprevalence (anti-HCV) age standardized	--
Acute hepatitis C	Female	1-5 months	95+ years	Global	-1	2	Healthcare access and quality index	593
Acute hepatitis C	Female	1-5 months	95+ years	Global	-1	2	Socio-demographic Index	593
Acute hepatitis C	Female	1-5 months	95+ years	Global	1	2	Intravenous drug use (proportion by age)	--
Acute hepatitis C	Female	1-5 months	95+ years	Global	-1	3	Education (years per capita)	506
Acute hepatitis C	Female	1-5 months	95+ years	Global	-1	3	LDI (I$ per capita)	--
Acute hepatitis C	Male	1-5 months	95+ years	Data Rich	1	1	Hepatitis C Seroprevalence (anti-HCV) age standardized	--
Acute hepatitis C	Male	1-5 months	95+ years	Data Rich	1	1	Intravenous drug use (proportion by age)	--
Acute hepatitis C	Male	1-5 months	95+ years	Data Rich	-1	2	Healthcare access and quality index	726
Acute hepatitis C	Male	1-5 months	95+ years	Data Rich	-1	2	Socio-demographic Index	726
Acute hepatitis C	Male	1-5 months	95+ years	Data Rich	1	2	Log-transformed SEV scalar: Hep	726
Acute hepatitis C	Male	1-5 months	95+ years	Data Rich	-1	3	Education (years per capita)	--
Acute hepatitis C	Male	1-5 months	95+ years	Data Rich	-1	3	LDI (I$ per capita)	--
Acute hepatitis C	Male	1-5 months	95+ years	Global	1	1	Hepatitis C Seroprevalence (anti-HCV) age standardized	1000
Acute hepatitis C	Male	1-5 months	95+ years	Global	1	1	Intravenous drug use (proportion by age)	--
Acute hepatitis C	Male	1-5 months	95+ years	Global	-1	2	Healthcare access and quality index	--
Acute hepatitis C	Male	1-5 months	95+ years	Global	-1	2	Socio-demographic Index	--
Acute hepatitis C	Male	1-5 months	95+ years	Global	1	2	Log-transformed SEV scalar: Hep	--
Acute hepatitis C	Male	1-5 months	95+ years	Global	-1	3	Education (years per capita)	--
Acute hepatitis C	Male	1-5 months	95+ years	Global	-1	3	LDI (I$ per capita)	--
Acute hepatitis E	Female	1-5 months	95+ years	Data Rich	1	1	Proportion of the population living in the classic monsoon region	1000
Acute hepatitis E	Female	1-5 months	95+ years	Data Rich	1	1	Hepatitis E Seroprevalence (anti-HEV) age standardized	--
Acute hepatitis E	Female	1-5 months	95+ years	Data Rich	1	1	Log-transformed SEV scalar: Hep	--
Acute hepatitis E	Female	1-5 months	95+ years	Data Rich	-1	2	Socio-demographic Index	312
Acute hepatitis E	Female	1-5 months	95+ years	Data Rich	-1	2	Healthcare access and quality index	644
Acute hepatitis E	Female	1-5 months	95+ years	Data Rich	1	2	Age- and sex-specific SEV for Unsafe sanitation	362
Acute hepatitis E	Female	1-5 months	95+ years	Data Rich	1	2	Age- and sex-specific SEV for Unsafe water	388
Acute hepatitis E	Female	1-5 months	95+ years	Data Rich	-1	3	Education (years per capita)	--
Acute hepatitis E	Female	1-5 months	95+ years	Data Rich	-1	3	LDI (I$ per capita)	--
Acute hepatitis E	Female	1-5 months	95+ years	Global	1	1	Hepatitis E Seroprevalence (anti-HEV) age standardized	1000
Acute hepatitis E	Female	1-5 months	95+ years	Global	1	1	Log-transformed SEV scalar: Hep	--
Acute hepatitis E	Female	1-5 months	95+ years	Global	-1	2	Healthcare access and quality index	353
Acute hepatitis E	Female	1-5 months	95+ years	Global	-1	2	Socio-demographic Index	--
Acute hepatitis E	Female	1-5 months	95+ years	Global	1	2	Age- and sex-specific SEV for Unsafe water	353
Acute hepatitis E	Female	1-5 months	95+ years	Global	1	2	Proportion of the population living in the classic monsoon region	353
Acute hepatitis E	Female	1-5 months	95+ years	Global	1	2	Age- and sex-specific SEV for Unsafe sanitation	--
Acute hepatitis E	Female	1-5 months	95+ years	Global	-1	3	Education (years per capita)	373
Acute hepatitis E	Female	1-5 months	95+ years	Global	-1	3	LDI (I$ per capita)	--
Acute hepatitis E	Male	1-5 months	95+ years	Data Rich	1	1	Hepatitis E Seroprevalence (anti-HEV) age standardized	1000
Acute hepatitis E	Male	1-5 months	95+ years	Data Rich	1	1	Log-transformed SEV scalar: Hep	--
Acute hepatitis E	Male	1-5 months	95+ years	Data Rich	-1	2	Healthcare access and quality index	--
Acute hepatitis E	Male	1-5 months	95+ years	Data Rich	-1	2	Socio-demographic Index	--
Acute hepatitis E	Male	1-5 months	95+ years	Data Rich	1	2	Age- and sex-specific SEV for Unsafe water	274
Acute hepatitis E	Male	1-5 months	95+ years	Data Rich	1	2	Age- and sex-specific SEV for Unsafe sanitation	397
Acute hepatitis E	Male	1-5 months	95+ years	Data Rich	1	2	Proportion of the population living in the classic monsoon region	--
Acute hepatitis E	Male	1-5 months	95+ years	Data Rich	-1	3	Education (years per capita)	--
Acute hepatitis E	Male	1-5 months	95+ years	Data Rich	-1	3	LDI (I$ per capita)	--
Acute hepatitis E	Male	1-5 months	95+ years	Global	1	1	Hepatitis E Seroprevalence (anti-HEV) age standardized	--
Acute hepatitis E	Male	1-5 months	95+ years	Global	1	1	Log-transformed SEV scalar: Hep	--
Acute hepatitis E	Male	1-5 months	95+ years	Global	-1	2	Socio-demographic Index	269
Acute hepatitis E	Male	1-5 months	95+ years	Global	-1	2	Healthcare access and quality index	294
Acute hepatitis E	Male	1-5 months	95+ years	Global	1	2	Age- and sex-specific SEV for Unsafe water	244
Acute hepatitis E	Male	1-5 months	95+ years	Global	1	2	Age- and sex-specific SEV for Unsafe sanitation	--
Acute hepatitis E	Male	1-5 months	95+ years	Global	1	2	Proportion of the population living in the classic monsoon region	--
Acute hepatitis E	Male	1-5 months	95+ years	Global	-1	3	Education (years per capita)	153
Acute hepatitis E	Male	1-5 months	95+ years	Global	-1	3	LDI (I$ per capita)	--
Other unspecified infectious diseases	Female	0-6 days	95+ years	Data Rich	-1	1	DTP3 Coverage, COVID-free (proportion)	--
Other unspecified infectious diseases	Female	0-6 days	95+ years	Data Rich	-1	2	Improved Water Source (proportion with access)	273
Other unspecified infectious diseases	Female	0-6 days	95+ years	Data Rich	-1	2	Sanitation (proportion with access)	568
Other unspecified infectious diseases	Female	0-6 days	95+ years	Data Rich	-1	2	Healthcare access and quality index	841
Other unspecified infectious diseases	Female	0-6 days	95+ years	Data Rich	-1	3	Socio-demographic Index	159
Other unspecified infectious diseases	Female	0-6 days	95+ years	Data Rich	-1	3	Antenatal Care (1 visit) Coverage (proportion)	367
Other unspecified infectious diseases	Female	0-6 days	95+ years	Global	-1	1	DTP3 Coverage, COVID-free (proportion)	1000
Other unspecified infectious diseases	Female	0-6 days	95+ years	Global	-1	2	Healthcare access and quality index	--
Other unspecified infectious diseases	Female	0-6 days	95+ years	Global	-1	2	Improved Water Source (proportion with access)	--
Other unspecified infectious diseases	Female	0-6 days	95+ years	Global	-1	2	Sanitation (proportion with access)	--
Other unspecified infectious diseases	Female	0-6 days	95+ years	Global	-1	3	Antenatal Care (1 visit) Coverage (proportion)	--
Other unspecified infectious diseases	Female	0-6 days	95+ years	Global	-1	3	Socio-demographic Index	--
Other unspecified infectious diseases	Male	0-6 days	95+ years	Data Rich	-1	1	DTP3 Coverage, COVID-free (proportion)	--
Other unspecified infectious diseases	Male	0-6 days	95+ years	Data Rich	-1	2	Improved Water Source (proportion with access)	640
Other unspecified infectious diseases	Male	0-6 days	95+ years	Data Rich	-1	2	Healthcare access and quality index	--
Other unspecified infectious diseases	Male	0-6 days	95+ years	Data Rich	-1	2	Sanitation (proportion with access)	--
Other unspecified infectious diseases	Male	0-6 days	95+ years	Data Rich	-1	3	Socio-demographic Index	568
Other unspecified infectious diseases	Male	0-6 days	95+ years	Data Rich	-1	3	Antenatal Care (1 visit) Coverage (proportion)	633
Other unspecified infectious diseases	Male	0-6 days	95+ years	Global	-1	1	DTP3 Coverage, COVID-free (proportion)	1000
Other unspecified infectious diseases	Male	0-6 days	95+ years	Global	-1	2	Healthcare access and quality index	--

Appendix Table S10: CODEm covariates used, level of covariate, and expected direction of covariate by cause, sex, and age

Cause	Sex	Age Start	Age End	Model Version Type	Direction	Level	Covariate Name	Number of Draws
Other unspecified infectious diseases	Male	0-6 days	95+ years	Global	-1	2	Improved Water Source (proportion with access)	--
Other unspecified infectious diseases	Male	0-6 days	95+ years	Global	-1	2	Sanitation (proportion with access)	--
Other unspecified infectious diseases	Male	0-6 days	95+ years	Global	-1	3	Antenatal Care (1 visit) Coverage (proportion)	--
Other unspecified infectious diseases	Male	0-6 days	95+ years	Global	-1	3	Socio-demographic Index	--
Neonatal disorders	Female	0-6 days	2-4 years	Data Rich	-1	1	Maternal care and immunization	803
Neonatal disorders	Female	0-6 days	2-4 years	Data Rich	1	1	Log-transformed SEV scalar: Neonatal	736
Neonatal disorders	Female	0-6 days	2-4 years	Data Rich	1	2	Age-standardized SEV for Household air pollution	529
Neonatal disorders	Female	0-6 days	2-4 years	Data Rich	1	2	Age-standardized SEV for Smoking	730
Neonatal disorders	Female	0-6 days	2-4 years	Data Rich	1	2	Age-standardized SEV for Ambient particulate matter	899
Neonatal disorders	Female	0-6 days	2-4 years	Data Rich	1	2	Age-standardized SEV for Low birth weight	--
Neonatal disorders	Female	0-6 days	2-4 years	Data Rich	1	2	Age-standardized SEV for Short gestation	--
Neonatal disorders	Female	0-6 days	2-4 years	Data Rich	-1	3	Antenatal Care (4 visits) Coverage (proportion)	95
Neonatal disorders	Female	0-6 days	2-4 years	Data Rich	-1	3	Antenatal Care (1 visit) Coverage (proportion)	130
Neonatal disorders	Female	0-6 days	2-4 years	Data Rich	-1	3	Healthcare access and quality index	140
Neonatal disorders	Female	0-6 days	2-4 years	Data Rich	-1	3	Proportion of the population with at least 6 years of education, maternal	237
Neonatal disorders	Female	0-6 days	2-4 years	Data Rich	-1	3	Skilled Birth Attendance (proportion)	321
Neonatal disorders	Female	0-6 days	2-4 years	Data Rich	-1	3	Socio-demographic Index	364
Neonatal disorders	Female	0-6 days	2-4 years	Data Rich	-1	3	In-Facility Delivery (proportion)	726
Neonatal disorders	Female	0-6 days	2-4 years	Data Rich	-1	3	Proportion of the population with at least 12 years of education, maternal	920
Neonatal disorders	Female	0-6 days	2-4 years	Data Rich	-1	3	LDI (I$ per capita)	--
Neonatal disorders	Female	0-6 days	2-4 years	Data Rich	1	3	Total Fertility Rate	--
Neonatal disorders	Female	0-6 days	2-4 years	Global	-1	1	Maternal care and immunization	472
Neonatal disorders	Female	0-6 days	2-4 years	Global	1	1	Log-transformed SEV scalar: Neonatal	642
Neonatal disorders	Female	0-6 days	2-4 years	Global	1	2	Age-standardized SEV for Household air pollution	511
Neonatal disorders	Female	0-6 days	2-4 years	Global	1	2	Age-standardized SEV for Smoking	511
Neonatal disorders	Female	0-6 days	2-4 years	Global	1	2	Age-standardized SEV for Ambient particulate matter	--
Neonatal disorders	Female	0-6 days	2-4 years	Global	1	2	Age-standardized SEV for Low birth weight	--
Neonatal disorders	Female	0-6 days	2-4 years	Global	1	2	Age-standardized SEV for Short gestation	--
Neonatal disorders	Female	0-6 days	2-4 years	Global	-1	3	Socio-demographic Index	127
Neonatal disorders	Female	0-6 days	2-4 years	Global	-1	3	Skilled Birth Attendance (proportion)	164
Neonatal disorders	Female	0-6 days	2-4 years	Global	-1	3	Antenatal Care (4 visits) Coverage (proportion)	199
Neonatal disorders	Female	0-6 days	2-4 years	Global	-1	3	Proportion of the population with at least 6 years of education, maternal	313
Neonatal disorders	Female	0-6 days	2-4 years	Global	-1	3	In-Facility Delivery (proportion)	344
Neonatal disorders	Female	0-6 days	2-4 years	Global	-1	3	Antenatal Care (1 visit) Coverage (proportion)	432
Neonatal disorders	Female	0-6 days	2-4 years	Global	-1	3	Proportion of the population with at least 12 years of education, maternal	647
Neonatal disorders	Female	0-6 days	2-4 years	Global	-1	3	Healthcare access and quality index	731
Neonatal disorders	Female	0-6 days	2-4 years	Global	-1	3	LDI (I$ per capita)	--
Neonatal disorders	Female	0-6 days	2-4 years	Global	1	3	Total Fertility Rate	--
Neonatal disorders	Male	0-6 days	2-4 years	Data Rich	-1	1	Maternal care and immunization	472
Neonatal disorders	Male	0-6 days	2-4 years	Data Rich	1	1	Log-transformed SEV scalar: Neonatal	722
Neonatal disorders	Male	0-6 days	2-4 years	Data Rich	1	2	Age-standardized SEV for Smoking	0
Neonatal disorders	Male	0-6 days	2-4 years	Data Rich	1	2	Age-standardized SEV for Ambient particulate matter	332
Neonatal disorders	Male	0-6 days	2-4 years	Data Rich	1	2	Age-standardized SEV for Household air pollution	332
Neonatal disorders	Male	0-6 days	2-4 years	Data Rich	1	2	Age-standardized SEV for Low birth weight	--
Neonatal disorders	Male	0-6 days	2-4 years	Data Rich	1	2	Age-standardized SEV for Short gestation	--
Neonatal disorders	Male	0-6 days	2-4 years	Data Rich	-1	3	Proportion of the population with at least 6 years of education, maternal	40
Neonatal disorders	Male	0-6 days	2-4 years	Data Rich	-1	3	Skilled Birth Attendance (proportion)	96
Neonatal disorders	Male	0-6 days	2-4 years	Data Rich	-1	3	Antenatal Care (1 visit) Coverage (proportion)	123
Neonatal disorders	Male	0-6 days	2-4 years	Data Rich	-1	3	Antenatal Care (4 visits) Coverage (proportion)	164
Neonatal disorders	Male	0-6 days	2-4 years	Data Rich	-1	3	Socio-demographic Index	457
Neonatal disorders	Male	0-6 days	2-4 years	Data Rich	-1	3	Proportion of the population with at least 12 years of education, maternal	492
Neonatal disorders	Male	0-6 days	2-4 years	Data Rich	-1	3	Healthcare access and quality index	599
Neonatal disorders	Male	0-6 days	2-4 years	Data Rich	-1	3	In-Facility Delivery (proportion)	639
Neonatal disorders	Male	0-6 days	2-4 years	Data Rich	-1	3	LDI (I$ per capita)	--
Neonatal disorders	Male	0-6 days	2-4 years	Data Rich	1	3	Total Fertility Rate	--
Neonatal disorders	Male	0-6 days	2-4 years	Global	-1	1	Maternal care and immunization	531
Neonatal disorders	Male	0-6 days	2-4 years	Global	1	1	Log-transformed SEV scalar: Neonatal	745
Neonatal disorders	Male	0-6 days	2-4 years	Global	1	2	Age-standardized SEV for Household air pollution	332
Neonatal disorders	Male	0-6 days	2-4 years	Global	1	2	Age-standardized SEV for Smoking	446
Neonatal disorders	Male	0-6 days	2-4 years	Global	1	2	Age-standardized SEV for Ambient particulate matter	--
Neonatal disorders	Male	0-6 days	2-4 years	Global	1	2	Age-standardized SEV for Low birth weight	--
Neonatal disorders	Male	0-6 days	2-4 years	Global	1	2	Age-standardized SEV for Short gestation	--
Neonatal disorders	Male	0-6 days	2-4 years	Global	-1	3	Proportion of the population with at least 6 years of education, maternal	39
Neonatal disorders	Male	0-6 days	2-4 years	Global	-1	3	Antenatal Care (1 visit) Coverage (proportion)	95
Neonatal disorders	Male	0-6 days	2-4 years	Global	-1	3	Antenatal Care (4 visits) Coverage (proportion)	170
Neonatal disorders	Male	0-6 days	2-4 years	Global	-1	3	Skilled Birth Attendance (proportion)	198
Neonatal disorders	Male	0-6 days	2-4 years	Global	-1	3	In-Facility Delivery (proportion)	328
Neonatal disorders	Male	0-6 days	2-4 years	Global	-1	3	Socio-demographic Index	341
Neonatal disorders	Male	0-6 days	2-4 years	Global	-1	3	Proportion of the population with at least 12 years of education, maternal	525
Neonatal disorders	Male	0-6 days	2-4 years	Global	-1	3	Healthcare access and quality index	619
Neonatal disorders	Male	0-6 days	2-4 years	Global	-1	3	LDI (I$ per capita)	--
Neonatal disorders	Male	0-6 days	2-4 years	Global	1	3	Total Fertility Rate	--
Neonatal preterm birth	Female	0-6 days	2-4 years	Data Rich	-1	1	Maternal care and immunization	1000
Neonatal preterm birth	Female	0-6 days	2-4 years	Data Rich	1	1	Log-transformed SEV scalar: Neonatal	684
Neonatal preterm birth	Female	0-6 days	2-4 years	Data Rich	1	2	Age-standardized SEV for Smoking	161
Neonatal preterm birth	Female	0-6 days	2-4 years	Data Rich	1	2	Age-standardized SEV for Ambient particulate matter	503
Neonatal preterm birth	Female	0-6 days	2-4 years	Data Rich	1	2	Age-standardized SEV for Household air pollution	503
Neonatal preterm birth	Female	0-6 days	2-4 years	Data Rich	1	2	Age-standardized SEV for Low birth weight	--
Neonatal preterm birth	Female	0-6 days	2-4 years	Data Rich	1	2	Age-standardized SEV for Short gestation	--
Neonatal preterm birth	Female	0-6 days	2-4 years	Data Rich	-1	3	Antenatal Care (1 visit) Coverage (proportion)	2
Neonatal preterm birth	Female	0-6 days	2-4 years	Data Rich	-1	3	Skilled Birth Attendance (proportion)	94
Neonatal preterm birth	Female	0-6 days	2-4 years	Data Rich	-1	3	Antenatal Care (4 visits) Coverage (proportion)	101
Neonatal preterm birth	Female	0-6 days	2-4 years	Data Rich	-1	3	Proportion of the population with at least 6 years of education, maternal	153
Neonatal preterm birth	Female	0-6 days	2-4 years	Data Rich	-1	3	Socio-demographic Index	325
Neonatal preterm birth	Female	0-6 days	2-4 years	Data Rich	-1	3	In-Facility Delivery (proportion)	589
Neonatal preterm birth	Female	0-6 days	2-4 years	Data Rich	-1	3	Proportion of the population with at least 12 years of education, maternal	604
Neonatal preterm birth	Female	0-6 days	2-4 years	Data Rich	-1	3	Healthcare access and quality index	726
Neonatal preterm birth	Female	0-6 days	2-4 years	Data Rich	-1	3	LDI (I$ per capita)	--

Appendix Table S10: CODEm covariates used, level of covariate, and expected direction of covariate by cause, sex, and age

Cause	Sex	Age Start	Age End	Model Version Type	Direction	Level	Covariate Name	Number of Draws
Neonatal preterm birth	Female	0-6 days	2-4 years	Data Rich	1	3	Total Fertility Rate	--
Neonatal preterm birth	Female	0-6 days	2-4 years	Global	-1	1	Maternal care and immunization	471
Neonatal preterm birth	Female	0-6 days	2-4 years	Global	1	1	Log-transformed SEV scalar: Neonatal	931
Neonatal preterm birth	Female	0-6 days	2-4 years	Global	1	2	Age-standardized SEV for Ambient particulate matter	367
Neonatal preterm birth	Female	0-6 days	2-4 years	Global	1	2	Age-standardized SEV for Household air pollution	493
Neonatal preterm birth	Female	0-6 days	2-4 years	Global	1	2	Age-standardized SEV for Smoking	493
Neonatal preterm birth	Female	0-6 days	2-4 years	Global	1	2	Age-standardized SEV for Low birth weight	--
Neonatal preterm birth	Female	0-6 days	2-4 years	Global	1	2	Age-standardized SEV for Short gestation	--
Neonatal preterm birth	Female	0-6 days	2-4 years	Global	-1	3	Skilled Birth Attendance (proportion)	5
Neonatal preterm birth	Female	0-6 days	2-4 years	Global	-1	3	In-Facility Delivery (proportion)	17
Neonatal preterm birth	Female	0-6 days	2-4 years	Global	-1	3	Antenatal Care (1 visit) Coverage (proportion)	210
Neonatal preterm birth	Female	0-6 days	2-4 years	Global	-1	3	Antenatal Care (4 visits) Coverage (proportion)	261
Neonatal preterm birth	Female	0-6 days	2-4 years	Global	-1	3	Proportion of the population with at least 6 years of education, maternal	272
Neonatal preterm birth	Female	0-6 days	2-4 years	Global	-1	3	Socio-demographic Index	435
Neonatal preterm birth	Female	0-6 days	2-4 years	Global	-1	3	Proportion of the population with at least 12 years of education, maternal	662
Neonatal preterm birth	Female	0-6 days	2-4 years	Global	-1	3	Healthcare access and quality index	956
Neonatal preterm birth	Female	0-6 days	2-4 years	Global	-1	3	LDI (I$ per capita)	--
Neonatal preterm birth	Female	0-6 days	2-4 years	Global	1	3	Total Fertility Rate	--
Neonatal preterm birth	Male	0-6 days	2-4 years	Data Rich	-1	1	Maternal care and immunization	781
Neonatal preterm birth	Male	0-6 days	2-4 years	Data Rich	1	1	Log-transformed SEV scalar: Neonatal	965
Neonatal preterm birth	Male	0-6 days	2-4 years	Data Rich	1	2	Age-standardized SEV for Smoking	35
Neonatal preterm birth	Male	0-6 days	2-4 years	Data Rich	1	2	Age-standardized SEV for Household air pollution	83
Neonatal preterm birth	Male	0-6 days	2-4 years	Data Rich	1	2	Age-standardized SEV for Ambient particulate matter	572
Neonatal preterm birth	Male	0-6 days	2-4 years	Data Rich	1	2	Age-standardized SEV for Low birth weight	--
Neonatal preterm birth	Male	0-6 days	2-4 years	Data Rich	1	2	Age-standardized SEV for Short gestation	--
Neonatal preterm birth	Male	0-6 days	2-4 years	Data Rich	-1	3	Antenatal Care (4 visits) Coverage (proportion)	123
Neonatal preterm birth	Male	0-6 days	2-4 years	Data Rich	-1	3	Antenatal Care (1 visit) Coverage (proportion)	187
Neonatal preterm birth	Male	0-6 days	2-4 years	Data Rich	-1	3	Proportion of the population with at least 6 years of education, maternal	360
Neonatal preterm birth	Male	0-6 days	2-4 years	Data Rich	-1	3	Socio-demographic Index	442
Neonatal preterm birth	Male	0-6 days	2-4 years	Data Rich	-1	3	Skilled Birth Attendance (proportion)	492
Neonatal preterm birth	Male	0-6 days	2-4 years	Data Rich	-1	3	In-Facility Delivery (proportion)	503
Neonatal preterm birth	Male	0-6 days	2-4 years	Data Rich	-1	3	Proportion of the population with at least 12 years of education, maternal	585
Neonatal preterm birth	Male	0-6 days	2-4 years	Data Rich	-1	3	Healthcare access and quality index	682
Neonatal preterm birth	Male	0-6 days	2-4 years	Data Rich	-1	3	LDI (I$ per capita)	--
Neonatal preterm birth	Male	0-6 days	2-4 years	Data Rich	1	3	Total Fertility Rate	--
Neonatal preterm birth	Male	0-6 days	2-4 years	Global	-1	1	Maternal care and immunization	902
Neonatal preterm birth	Male	0-6 days	2-4 years	Global	1	1	Log-transformed SEV scalar: Neonatal	837
Neonatal preterm birth	Male	0-6 days	2-4 years	Global	1	2	Age-standardized SEV for Ambient particulate matter	735
Neonatal preterm birth	Male	0-6 days	2-4 years	Global	1	2	Age-standardized SEV for Household air pollution	756
Neonatal preterm birth	Male	0-6 days	2-4 years	Global	1	2	Age-standardized SEV for Smoking	756
Neonatal preterm birth	Male	0-6 days	2-4 years	Global	1	2	Age-standardized SEV for Low birth weight	--
Neonatal preterm birth	Male	0-6 days	2-4 years	Global	1	2	Age-standardized SEV for Short gestation	--
Neonatal preterm birth	Male	0-6 days	2-4 years	Global	-1	3	In-Facility Delivery (proportion)	0
Neonatal preterm birth	Male	0-6 days	2-4 years	Global	-1	3	Skilled Birth Attendance (proportion)	9
Neonatal preterm birth	Male	0-6 days	2-4 years	Global	-1	3	Antenatal Care (4 visits) Coverage (proportion)	13
Neonatal preterm birth	Male	0-6 days	2-4 years	Global	-1	3	Antenatal Care (1 visit) Coverage (proportion)	27
Neonatal preterm birth	Male	0-6 days	2-4 years	Global	-1	3	Socio-demographic Index	153
Neonatal preterm birth	Male	0-6 days	2-4 years	Global	-1	3	Proportion of the population with at least 6 years of education, maternal	179
Neonatal preterm birth	Male	0-6 days	2-4 years	Global	-1	3	Proportion of the population with at least 12 years of education, maternal	498
Neonatal preterm birth	Male	0-6 days	2-4 years	Global	-1	3	Healthcare access and quality index	796
Neonatal preterm birth	Male	0-6 days	2-4 years	Global	-1	3	LDI (I$ per capita)	--
Neonatal preterm birth	Male	0-6 days	2-4 years	Global	1	3	Total Fertility Rate	--
Neonatal encephalopathy due to birth asphyxia and trauma	Female	0-6 days	2-4 years	Data Rich	-1	1	Maternal care and immunization	1000
Neonatal encephalopathy due to birth asphyxia and trauma	Female	0-6 days	2-4 years	Data Rich	1	1	Log-transformed SEV scalar: Neonatal	--
Neonatal encephalopathy due to birth asphyxia and trauma	Female	0-6 days	2-4 years	Data Rich	1	2	Age-standardized SEV for Ambient particulate matter	323
Neonatal encephalopathy due to birth asphyxia and trauma	Female	0-6 days	2-4 years	Data Rich	1	2	Age-standardized SEV for Household air pollution	323
Neonatal encephalopathy due to birth asphyxia and trauma	Female	0-6 days	2-4 years	Data Rich	1	2	Age-standardized SEV for Smoking	323
Neonatal encephalopathy due to birth asphyxia and trauma	Female	0-6 days	2-4 years	Data Rich	1	2	Age-standardized SEV for Low birth weight	--
Neonatal encephalopathy due to birth asphyxia and trauma	Female	0-6 days	2-4 years	Data Rich	1	2	Age-standardized SEV for Short gestation	--
Neonatal encephalopathy due to birth asphyxia and trauma	Female	0-6 days	2-4 years	Data Rich	-1	3	In-Facility Delivery (proportion)	66
Neonatal encephalopathy due to birth asphyxia and trauma	Female	0-6 days	2-4 years	Data Rich	-1	3	Antenatal Care (1 visit) Coverage (proportion)	154
Neonatal encephalopathy due to birth asphyxia and trauma	Female	0-6 days	2-4 years	Data Rich	-1	3	Proportion of the population with at least 6 years of education, maternal	227
Neonatal encephalopathy due to birth asphyxia and trauma	Female	0-6 days	2-4 years	Data Rich	-1	3	Antenatal Care (4 visits) Coverage (proportion)	281
Neonatal encephalopathy due to birth asphyxia and trauma	Female	0-6 days	2-4 years	Data Rich	-1	3	Skilled Birth Attendance (proportion)	413
Neonatal encephalopathy due to birth asphyxia and trauma	Female	0-6 days	2-4 years	Data Rich	-1	3	Proportion of the population with at least 12 years of education, maternal	461
Neonatal encephalopathy due to birth asphyxia and trauma	Female	0-6 days	2-4 years	Data Rich	-1	3	Healthcare access and quality index	--
Neonatal encephalopathy due to birth asphyxia and trauma	Female	0-6 days	2-4 years	Data Rich	-1	3	LDI (I$ per capita)	--
Neonatal encephalopathy due to birth asphyxia and trauma	Female	0-6 days	2-4 years	Data Rich	-1	3	Socio-demographic Index	--
Neonatal encephalopathy due to birth asphyxia and trauma	Female	0-6 days	2-4 years	Data Rich	1	3	Total Fertility Rate	--
Neonatal encephalopathy due to birth asphyxia and trauma	Female	0-6 days	2-4 years	Global	-1	1	Maternal care and immunization	1000
Neonatal encephalopathy due to birth asphyxia and trauma	Female	0-6 days	2-4 years	Global	1	1	Log-transformed SEV scalar: Neonatal	--
Neonatal encephalopathy due to birth asphyxia and trauma	Female	0-6 days	2-4 years	Global	1	2	Age-standardized SEV for Household air pollution	176
Neonatal encephalopathy due to birth asphyxia and trauma	Female	0-6 days	2-4 years	Global	1	2	Age-standardized SEV for Smoking	176
Neonatal encephalopathy due to birth asphyxia and trauma	Female	0-6 days	2-4 years	Global	1	2	Age-standardized SEV for Ambient particulate matter	--
Neonatal encephalopathy due to birth asphyxia and trauma	Female	0-6 days	2-4 years	Global	1	2	Age-standardized SEV for Low birth weight	--
Neonatal encephalopathy due to birth asphyxia and trauma	Female	0-6 days	2-4 years	Global	1	2	Age-standardized SEV for Short gestation	--
Neonatal encephalopathy due to birth asphyxia and trauma	Female	0-6 days	2-4 years	Global	-1	3	Proportion of the population with at least 6 years of education, maternal	53
Neonatal encephalopathy due to birth asphyxia and trauma	Female	0-6 days	2-4 years	Global	-1	3	Antenatal Care (4 visits) Coverage (proportion)	174
Neonatal encephalopathy due to birth asphyxia and trauma	Female	0-6 days	2-4 years	Global	-1	3	Healthcare access and quality index	219
Neonatal encephalopathy due to birth asphyxia and trauma	Female	0-6 days	2-4 years	Global	-1	3	Socio-demographic Index	419
Neonatal encephalopathy due to birth asphyxia and trauma	Female	0-6 days	2-4 years	Global	-1	3	In-Facility Delivery (proportion)	505
Neonatal encephalopathy due to birth asphyxia and trauma	Female	0-6 days	2-4 years	Global	-1	3	Proportion of the population with at least 12 years of education, maternal	611
Neonatal encephalopathy due to birth asphyxia and trauma	Female	0-6 days	2-4 years	Global	-1	3	Antenatal Care (1 visit) Coverage (proportion)	--

Appendix Table S10: CODEm covariates used, level of covariate, and expected direction of covariate by cause, sex, and age

Cause	Sex	Age Start	Age End	Model Version Type	Direction	Level	Covariate Name	Number of Draws
Neonatal encephalopathy due to birth asphyxia and trauma	Female	0-6 days	2-4 years	Global	-1	3	LDI (I$ per capita)	--
Neonatal encephalopathy due to birth asphyxia and trauma	Female	0-6 days	2-4 years	Global	-1	3	Skilled Birth Attendance (proportion)	--
Neonatal encephalopathy due to birth asphyxia and trauma	Female	0-6 days	2-4 years	Global	1	3	Total Fertility Rate	--
Neonatal encephalopathy due to birth asphyxia and trauma	Male	0-6 days	2-4 years	Data Rich	-1	1	Maternal care and immunization	885
Neonatal encephalopathy due to birth asphyxia and trauma	Male	0-6 days	2-4 years	Data Rich	1	1	Log-transformed SEV scalar: Neonatal	115
Neonatal encephalopathy due to birth asphyxia and trauma	Male	0-6 days	2-4 years	Data Rich	1	2	Age-standardized SEV for Smoking	156
Neonatal encephalopathy due to birth asphyxia and trauma	Male	0-6 days	2-4 years	Data Rich	1	2	Age-standardized SEV for Ambient particulate matter	160
Neonatal encephalopathy due to birth asphyxia and trauma	Male	0-6 days	2-4 years	Data Rich	1	2	Age-standardized SEV for Household air pollution	211
Neonatal encephalopathy due to birth asphyxia and trauma	Male	0-6 days	2-4 years	Data Rich	1	2	Age-standardized SEV for Low birth weight	--
Neonatal encephalopathy due to birth asphyxia and trauma	Male	0-6 days	2-4 years	Data Rich	1	2	Age-standardized SEV for Short gestation	--
Neonatal encephalopathy due to birth asphyxia and trauma	Male	0-6 days	2-4 years	Data Rich	-1	3	Proportion of the population with at least 6 years of education, maternal	156
Neonatal encephalopathy due to birth asphyxia and trauma	Male	0-6 days	2-4 years	Data Rich	-1	3	Antenatal Care (4 visits) Coverage (proportion)	175
Neonatal encephalopathy due to birth asphyxia and trauma	Male	0-6 days	2-4 years	Data Rich	-1	3	Proportion of the population with at least 12 years of education, maternal	180
Neonatal encephalopathy due to birth asphyxia and trauma	Male	0-6 days	2-4 years	Data Rich	-1	3	Antenatal Care (1 visit) Coverage (proportion)	189
Neonatal encephalopathy due to birth asphyxia and trauma	Male	0-6 days	2-4 years	Data Rich	-1	3	In-Facility Delivery (proportion)	237
Neonatal encephalopathy due to birth asphyxia and trauma	Male	0-6 days	2-4 years	Data Rich	-1	3	Skilled Birth Attendance (proportion)	302
Neonatal encephalopathy due to birth asphyxia and trauma	Male	0-6 days	2-4 years	Data Rich	-1	3	Healthcare access and quality index	--
Neonatal encephalopathy due to birth asphyxia and trauma	Male	0-6 days	2-4 years	Data Rich	-1	3	LDI (I$ per capita)	--
Neonatal encephalopathy due to birth asphyxia and trauma	Male	0-6 days	2-4 years	Data Rich	-1	3	Socio-demographic Index	--
Neonatal encephalopathy due to birth asphyxia and trauma	Male	0-6 days	2-4 years	Data Rich	1	3	Total Fertility Rate	--
Neonatal encephalopathy due to birth asphyxia and trauma	Male	0-6 days	2-4 years	Global	-1	1	Maternal care and immunization	1000
Neonatal encephalopathy due to birth asphyxia and trauma	Male	0-6 days	2-4 years	Global	1	1	Log-transformed SEV scalar: Neonatal	--
Neonatal encephalopathy due to birth asphyxia and trauma	Male	0-6 days	2-4 years	Global	1	2	Age-standardized SEV for Household air pollution	144
Neonatal encephalopathy due to birth asphyxia and trauma	Male	0-6 days	2-4 years	Global	1	2	Age-standardized SEV for Smoking	144
Neonatal encephalopathy due to birth asphyxia and trauma	Male	0-6 days	2-4 years	Global	1	2	Age-standardized SEV for Ambient particulate matter	--
Neonatal encephalopathy due to birth asphyxia and trauma	Male	0-6 days	2-4 years	Global	1	2	Age-standardized SEV for Low birth weight	--
Neonatal encephalopathy due to birth asphyxia and trauma	Male	0-6 days	2-4 years	Global	1	2	Age-standardized SEV for Short gestation	--
Neonatal encephalopathy due to birth asphyxia and trauma	Male	0-6 days	2-4 years	Global	-1	3	Proportion of the population with at least 6 years of education, maternal	18
Neonatal encephalopathy due to birth asphyxia and trauma	Male	0-6 days	2-4 years	Global	-1	3	Healthcare access and quality index	227
Neonatal encephalopathy due to birth asphyxia and trauma	Male	0-6 days	2-4 years	Global	-1	3	In-Facility Delivery (proportion)	275
Neonatal encephalopathy due to birth asphyxia and trauma	Male	0-6 days	2-4 years	Global	-1	3	Socio-demographic Index	371
Neonatal encephalopathy due to birth asphyxia and trauma	Male	0-6 days	2-4 years	Global	-1	3	Antenatal Care (4 visits) Coverage (proportion)	434
Neonatal encephalopathy due to birth asphyxia and trauma	Male	0-6 days	2-4 years	Global	-1	3	Proportion of the population with at least 12 years of education, maternal	446
Neonatal encephalopathy due to birth asphyxia and trauma	Male	0-6 days	2-4 years	Global	-1	3	Antenatal Care (1 visit) Coverage (proportion)	--
Neonatal encephalopathy due to birth asphyxia and trauma	Male	0-6 days	2-4 years	Global	-1	3	LDI (I$ per capita)	--
Neonatal encephalopathy due to birth asphyxia and trauma	Male	0-6 days	2-4 years	Global	-1	3	Skilled Birth Attendance (proportion)	--
Neonatal encephalopathy due to birth asphyxia and trauma	Male	0-6 days	2-4 years	Global	1	3	Total Fertility Rate	--
Neonatal sepsis and other neonatal infections	Female	0-6 days	2-4 years	Data Rich	-1	1	Maternal care and immunization	539
Neonatal sepsis and other neonatal infections	Female	0-6 days	2-4 years	Data Rich	1	1	Log-transformed SEV scalar: Neonatal	1000
Neonatal sepsis and other neonatal infections	Female	0-6 days	2-4 years	Data Rich	1	2	Age-standardized SEV for Ambient particulate matter	180
Neonatal sepsis and other neonatal infections	Female	0-6 days	2-4 years	Data Rich	1	2	Age-standardized SEV for Smoking	180
Neonatal sepsis and other neonatal infections	Female	0-6 days	2-4 years	Data Rich	1	2	Age- and sex-specific SEV for Short gestation	227
Neonatal sepsis and other neonatal infections	Female	0-6 days	2-4 years	Data Rich	1	2	Age- and sex-specific SEV for Low birth weight	391
Neonatal sepsis and other neonatal infections	Female	0-6 days	2-4 years	Data Rich	1	2	Age-standardized SEV for Household air pollution	--
Neonatal sepsis and other neonatal infections	Female	0-6 days	2-4 years	Data Rich	-1	3	Antenatal Care (1 visit) Coverage (proportion)	20
Neonatal sepsis and other neonatal infections	Female	0-6 days	2-4 years	Data Rich	-1	3	Socio-demographic Index	56
Neonatal sepsis and other neonatal infections	Female	0-6 days	2-4 years	Data Rich	-1	3	Antenatal Care (4 visits) Coverage (proportion)	82
Neonatal sepsis and other neonatal infections	Female	0-6 days	2-4 years	Data Rich	-1	3	Skilled Birth Attendance (proportion)	140
Neonatal sepsis and other neonatal infections	Female	0-6 days	2-4 years	Data Rich	-1	3	Proportion of the population with at least 6 years of education, maternal	207
Neonatal sepsis and other neonatal infections	Female	0-6 days	2-4 years	Data Rich	-1	3	In-Facility Delivery (proportion)	319
Neonatal sepsis and other neonatal infections	Female	0-6 days	2-4 years	Data Rich	-1	3	Proportion of the population with at least 12 years of education, maternal	370
Neonatal sepsis and other neonatal infections	Female	0-6 days	2-4 years	Data Rich	-1	3	Healthcare access and quality index	--
Neonatal sepsis and other neonatal infections	Female	0-6 days	2-4 years	Data Rich	-1	3	LDI (I$ per capita)	--
Neonatal sepsis and other neonatal infections	Female	0-6 days	2-4 years	Data Rich	1	3	Total Fertility Rate	--
Neonatal sepsis and other neonatal infections	Female	0-6 days	2-4 years	Global	-1	1	Maternal care and immunization	1000
Neonatal sepsis and other neonatal infections	Female	0-6 days	2-4 years	Global	1	1	Log-transformed SEV scalar: Neonatal	--
Neonatal sepsis and other neonatal infections	Female	0-6 days	2-4 years	Global	1	2	Age-standardized SEV for Ambient particulate matter	409
Neonatal sepsis and other neonatal infections	Female	0-6 days	2-4 years	Global	1	2	Age-standardized SEV for Household air pollution	409
Neonatal sepsis and other neonatal infections	Female	0-6 days	2-4 years	Global	1	2	Age-standardized SEV for Smoking	409
Neonatal sepsis and other neonatal infections	Female	0-6 days	2-4 years	Global	1	2	Age- and sex-specific SEV for Low birth weight	--
Neonatal sepsis and other neonatal infections	Female	0-6 days	2-4 years	Global	1	2	Age- and sex-specific SEV for Short gestation	--
Neonatal sepsis and other neonatal infections	Female	0-6 days	2-4 years	Global	-1	3	Skilled Birth Attendance (proportion)	13
Neonatal sepsis and other neonatal infections	Female	0-6 days	2-4 years	Global	-1	3	In-Facility Delivery (proportion)	66
Neonatal sepsis and other neonatal infections	Female	0-6 days	2-4 years	Global	-1	3	Proportion of the population with at least 12 years of education, maternal	86
Neonatal sepsis and other neonatal infections	Female	0-6 days	2-4 years	Global	-1	3	Proportion of the population with at least 6 years of education, maternal	266
Neonatal sepsis and other neonatal infections	Female	0-6 days	2-4 years	Global	-1	3	Antenatal Care (4 visits) Coverage (proportion)	350
Neonatal sepsis and other neonatal infections	Female	0-6 days	2-4 years	Global	-1	3	Antenatal Care (1 visit) Coverage (proportion)	--
Neonatal sepsis and other neonatal infections	Female	0-6 days	2-4 years	Global	-1	3	Healthcare access and quality index	--
Neonatal sepsis and other neonatal infections	Female	0-6 days	2-4 years	Global	-1	3	LDI (I$ per capita)	--
Neonatal sepsis and other neonatal infections	Female	0-6 days	2-4 years	Global	-1	3	Socio-demographic Index	--
Neonatal sepsis and other neonatal infections	Female	0-6 days	2-4 years	Global	1	3	Total Fertility Rate	--
Neonatal sepsis and other neonatal infections	Male	0-6 days	2-4 years	Data Rich	-1	1	Maternal care and immunization	466
Neonatal sepsis and other neonatal infections	Male	0-6 days	2-4 years	Data Rich	1	1	Log-transformed SEV scalar: Neonatal	708
Neonatal sepsis and other neonatal infections	Male	0-6 days	2-4 years	Data Rich	1	2	Age-standardized SEV for Smoking	618
Neonatal sepsis and other neonatal infections	Male	0-6 days	2-4 years	Data Rich	1	2	Age-standardized SEV for Household air pollution	727
Neonatal sepsis and other neonatal infections	Male	0-6 days	2-4 years	Data Rich	1	2	Age-standardized SEV for Ambient particulate matter	--
Neonatal sepsis and other neonatal infections	Male	0-6 days	2-4 years	Data Rich	1	2	Age-standardized SEV for Low birth weight	--

Appendix Table S10: CODEm covariates used, level of covariate, and expected direction of covariate by cause, sex, and age

Cause	Sex	Age Start	Age End	Model Version Type	Direction	Level	Covariate Name	Number of Draws
Neonatal sepsis and other neonatal infections	Male	0-6 days	2-4 years	Data Rich	1	2	Age-standardized SEV for Short gestation	--
Neonatal sepsis and other neonatal infections	Male	0-6 days	2-4 years	Data Rich	-1	3	Healthcare access and quality index	7
Neonatal sepsis and other neonatal infections	Male	0-6 days	2-4 years	Data Rich	-1	3	Antenatal Care (1 visit) Coverage (proportion)	17
Neonatal sepsis and other neonatal infections	Male	0-6 days	2-4 years	Data Rich	-1	3	Antenatal Care (4 visits) Coverage (proportion)	61
Neonatal sepsis and other neonatal infections	Male	0-6 days	2-4 years	Data Rich	-1	3	Skilled Birth Attendance (proportion)	231
Neonatal sepsis and other neonatal infections	Male	0-6 days	2-4 years	Data Rich	-1	3	Proportion of the population with at least 12 years of education, maternal	258
Neonatal sepsis and other neonatal infections	Male	0-6 days	2-4 years	Data Rich	-1	3	Proportion of the population with at least 6 years of education, maternal	261
Neonatal sepsis and other neonatal infections	Male	0-6 days	2-4 years	Data Rich	-1	3	In-Facility Delivery (proportion)	402
Neonatal sepsis and other neonatal infections	Male	0-6 days	2-4 years	Data Rich	-1	3	LDI (I$ per capita)	--
Neonatal sepsis and other neonatal infections	Male	0-6 days	2-4 years	Data Rich	-1	3	Socio-demographic Index	--
Neonatal sepsis and other neonatal infections	Male	0-6 days	2-4 years	Data Rich	1	3	Total Fertility Rate	--
Neonatal sepsis and other neonatal infections	Male	0-6 days	2-4 years	Global	-1	1	Maternal care and immunization	1000
Neonatal sepsis and other neonatal infections	Male	0-6 days	2-4 years	Global	1	1	Log-transformed SEV scalar: Neonatal	--
Neonatal sepsis and other neonatal infections	Male	0-6 days	2-4 years	Global	1	2	Age-standardized SEV for Ambient particulate matter	188
Neonatal sepsis and other neonatal infections	Male	0-6 days	2-4 years	Global	1	2	Age-standardized SEV for Household air pollution	188
Neonatal sepsis and other neonatal infections	Male	0-6 days	2-4 years	Global	1	2	Age-standardized SEV for Smoking	188
Neonatal sepsis and other neonatal infections	Male	0-6 days	2-4 years	Global	1	2	Age-standardized SEV for Low birth weight	--
Neonatal sepsis and other neonatal infections	Male	0-6 days	2-4 years	Global	1	2	Age-standardized SEV for Short gestation	--
Neonatal sepsis and other neonatal infections	Male	0-6 days	2-4 years	Global	-1	3	Skilled Birth Attendance (proportion)	52
Neonatal sepsis and other neonatal infections	Male	0-6 days	2-4 years	Global	-1	3	Proportion of the population with at least 12 years of education, maternal	61
Neonatal sepsis and other neonatal infections	Male	0-6 days	2-4 years	Global	-1	3	Healthcare access and quality index	188
Neonatal sepsis and other neonatal infections	Male	0-6 days	2-4 years	Global	-1	3	Antenatal Care (4 visits) Coverage (proportion)	199
Neonatal sepsis and other neonatal infections	Male	0-6 days	2-4 years	Global	-1	3	Antenatal Care (1 visit) Coverage (proportion)	297
Neonatal sepsis and other neonatal infections	Male	0-6 days	2-4 years	Global	-1	3	Proportion of the population with at least 6 years of education, maternal	314
Neonatal sepsis and other neonatal infections	Male	0-6 days	2-4 years	Global	-1	3	In-Facility Delivery (proportion)	371
Neonatal sepsis and other neonatal infections	Male	0-6 days	2-4 years	Global	-1	3	LDI (I$ per capita)	--
Neonatal sepsis and other neonatal infections	Male	0-6 days	2-4 years	Global	-1	3	Socio-demographic Index	--
Neonatal sepsis and other neonatal infections	Male	0-6 days	2-4 years	Global	1	3	Total Fertility Rate	--
Hemolytic disease and other neonatal jaundice	Female	0-6 days	2-4 years	Data Rich	-1	1	Maternal care and immunization	912
Hemolytic disease and other neonatal jaundice	Female	0-6 days	2-4 years	Data Rich	1	1	Log-transformed SEV scalar: Neonatal	88
Hemolytic disease and other neonatal jaundice	Female	0-6 days	2-4 years	Data Rich	1	2	Age-standardized SEV for Household air pollution	124
Hemolytic disease and other neonatal jaundice	Female	0-6 days	2-4 years	Data Rich	1	2	Age-standardized SEV for Smoking	124
Hemolytic disease and other neonatal jaundice	Female	0-6 days	2-4 years	Data Rich	1	2	Age-standardized SEV for Ambient particulate matter	148
Hemolytic disease and other neonatal jaundice	Female	0-6 days	2-4 years	Data Rich	1	2	Age-standardized SEV for Low birth weight	--
Hemolytic disease and other neonatal jaundice	Female	0-6 days	2-4 years	Data Rich	1	2	Age-standardized SEV for Short gestation	--
Hemolytic disease and other neonatal jaundice	Female	0-6 days	2-4 years	Data Rich	-1	3	Proportion of the population with at least 6 years of education, maternal	59
Hemolytic disease and other neonatal jaundice	Female	0-6 days	2-4 years	Data Rich	-1	3	Antenatal Care (4 visits) Coverage (proportion)	139
Hemolytic disease and other neonatal jaundice	Female	0-6 days	2-4 years	Data Rich	-1	3	Skilled Birth Attendance (proportion)	206
Hemolytic disease and other neonatal jaundice	Female	0-6 days	2-4 years	Data Rich	-1	3	In-Facility Delivery (proportion)	355
Hemolytic disease and other neonatal jaundice	Female	0-6 days	2-4 years	Data Rich	-1	3	Antenatal Care (1 visit) Coverage (proportion)	364
Hemolytic disease and other neonatal jaundice	Female	0-6 days	2-4 years	Data Rich	-1	3	Proportion of the population with at least 12 years of education, maternal	527
Hemolytic disease and other neonatal jaundice	Female	0-6 days	2-4 years	Data Rich	-1	3	Healthcare access and quality index	591
Hemolytic disease and other neonatal jaundice	Female	0-6 days	2-4 years	Data Rich	-1	3	LDI (I$ per capita)	--
Hemolytic disease and other neonatal jaundice	Female	0-6 days	2-4 years	Data Rich	-1	3	Socio-demographic Index	--
Hemolytic disease and other neonatal jaundice	Female	0-6 days	2-4 years	Data Rich	1	3	Total Fertility Rate	--
Hemolytic disease and other neonatal jaundice	Female	0-6 days	2-4 years	Global	-1	1	Maternal care and immunization	915
Hemolytic disease and other neonatal jaundice	Female	0-6 days	2-4 years	Global	1	1	Log-transformed SEV scalar: Neonatal	85
Hemolytic disease and other neonatal jaundice	Female	0-6 days	2-4 years	Global	1	2	Age-standardized SEV for Ambient particulate matter	185
Hemolytic disease and other neonatal jaundice	Female	0-6 days	2-4 years	Global	1	2	Age-standardized SEV for Smoking	418
Hemolytic disease and other neonatal jaundice	Female	0-6 days	2-4 years	Global	1	2	Age-standardized SEV for Household air pollution	657
Hemolytic disease and other neonatal jaundice	Female	0-6 days	2-4 years	Global	1	2	Age-standardized SEV for Low birth weight	--
Hemolytic disease and other neonatal jaundice	Female	0-6 days	2-4 years	Global	1	2	Age-standardized SEV for Short gestation	--
Hemolytic disease and other neonatal jaundice	Female	0-6 days	2-4 years	Global	-1	3	Skilled Birth Attendance (proportion)	29
Hemolytic disease and other neonatal jaundice	Female	0-6 days	2-4 years	Global	-1	3	Antenatal Care (1 visit) Coverage (proportion)	35
Hemolytic disease and other neonatal jaundice	Female	0-6 days	2-4 years	Global	-1	3	Antenatal Care (4 visits) Coverage (proportion)	48
Hemolytic disease and other neonatal jaundice	Female	0-6 days	2-4 years	Global	-1	3	Socio-demographic Index	126
Hemolytic disease and other neonatal jaundice	Female	0-6 days	2-4 years	Global	-1	3	In-Facility Delivery (proportion)	136
Hemolytic disease and other neonatal jaundice	Female	0-6 days	2-4 years	Global	-1	3	Healthcare access and quality index	146
Hemolytic disease and other neonatal jaundice	Female	0-6 days	2-4 years	Global	-1	3	Proportion of the population with at least 6 years of education, maternal	309
Hemolytic disease and other neonatal jaundice	Female	0-6 days	2-4 years	Global	-1	3	Proportion of the population with at least 12 years of education, maternal	435
Hemolytic disease and other neonatal jaundice	Female	0-6 days	2-4 years	Global	-1	3	LDI (I$ per capita)	--
Hemolytic disease and other neonatal jaundice	Female	0-6 days	2-4 years	Global	1	3	Total Fertility Rate	--
Hemolytic disease and other neonatal jaundice	Male	0-6 days	2-4 years	Data Rich	-1	1	Maternal care and immunization	888
Hemolytic disease and other neonatal jaundice	Male	0-6 days	2-4 years	Data Rich	1	1	Log-transformed SEV scalar: Neonatal	612
Hemolytic disease and other neonatal jaundice	Male	0-6 days	2-4 years	Data Rich	1	2	Age-standardized SEV for Smoking	146
Hemolytic disease and other neonatal jaundice	Male	0-6 days	2-4 years	Data Rich	1	2	Age-standardized SEV for Ambient particulate matter	224
Hemolytic disease and other neonatal jaundice	Male	0-6 days	2-4 years	Data Rich	1	2	Age-standardized SEV for Household air pollution	224
Hemolytic disease and other neonatal jaundice	Male	0-6 days	2-4 years	Data Rich	1	2	Age-standardized SEV for Low birth weight	--
Hemolytic disease and other neonatal jaundice	Male	0-6 days	2-4 years	Data Rich	1	2	Age-standardized SEV for Short gestation	--
Hemolytic disease and other neonatal jaundice	Male	0-6 days	2-4 years	Data Rich	-1	3	Healthcare access and quality index	45
Hemolytic disease and other neonatal jaundice	Male	0-6 days	2-4 years	Data Rich	-1	3	Socio-demographic Index	73
Hemolytic disease and other neonatal jaundice	Male	0-6 days	2-4 years	Data Rich	-1	3	Proportion of the population with at least 6 years of education, maternal	85
Hemolytic disease and other neonatal jaundice	Male	0-6 days	2-4 years	Data Rich	-1	3	Antenatal Care (4 visits) Coverage (proportion)	201
Hemolytic disease and other neonatal jaundice	Male	0-6 days	2-4 years	Data Rich	-1	3	Antenatal Care (1 visit) Coverage (proportion)	391
Hemolytic disease and other neonatal jaundice	Male	0-6 days	2-4 years	Data Rich	-1	3	Skilled Birth Attendance (proportion)	592
Hemolytic disease and other neonatal jaundice	Male	0-6 days	2-4 years	Data Rich	-1	3	In-Facility Delivery (proportion)	611

Appendix Table S10: CODEm covariates used, level of covariate, and expected direction of covariate by cause, sex, and age

Cause	Sex	Age Start	Age End	Model Version Type	Direction	Level	Covariate Name	Number of Draws
Hemolytic disease and other neonatal jaundice	Male	0-6 days	2-4 years	Data Rich	-1	3	Proportion of the population with at least 12 years of education, maternal	930
Hemolytic disease and other neonatal jaundice	Male	0-6 days	2-4 years	Data Rich	-1	3	LDI (I$ per capita)	--
Hemolytic disease and other neonatal jaundice	Male	0-6 days	2-4 years	Data Rich	1	3	Total Fertility Rate	--
Hemolytic disease and other neonatal jaundice	Male	0-6 days	2-4 years	Global	-1	1	Maternal care and immunization	962
Hemolytic disease and other neonatal jaundice	Male	0-6 days	2-4 years	Global	1	1	Log-transformed SEV scalar: Neonatal	217
Hemolytic disease and other neonatal jaundice	Male	0-6 days	2-4 years	Global	1	2	Age-standardized SEV for Smoking	254
Hemolytic disease and other neonatal jaundice	Male	0-6 days	2-4 years	Global	1	2	Age-standardized SEV for Household air pollution	341
Hemolytic disease and other neonatal jaundice	Male	0-6 days	2-4 years	Global	1	2	Age-standardized SEV for Ambient particulate matter	--
Hemolytic disease and other neonatal jaundice	Male	0-6 days	2-4 years	Global	1	2	Age-standardized SEV for Low birth weight	--
Hemolytic disease and other neonatal jaundice	Male	0-6 days	2-4 years	Global	1	2	Age-standardized SEV for Short gestation	--
Hemolytic disease and other neonatal jaundice	Male	0-6 days	2-4 years	Global	-1	3	Antenatal Care (1 visit) Coverage (proportion)	4
Hemolytic disease and other neonatal jaundice	Male	0-6 days	2-4 years	Global	-1	3	Proportion of the population with at least 6 years of education, maternal	14
Hemolytic disease and other neonatal jaundice	Male	0-6 days	2-4 years	Global	-1	3	Skilled Birth Attendance (proportion)	117
Hemolytic disease and other neonatal jaundice	Male	0-6 days	2-4 years	Global	-1	3	Socio-demographic Index	225
Hemolytic disease and other neonatal jaundice	Male	0-6 days	2-4 years	Global	-1	3	In-Facility Delivery (proportion)	423
Hemolytic disease and other neonatal jaundice	Male	0-6 days	2-4 years	Global	-1	3	Antenatal Care (4 visits) Coverage (proportion)	488
Hemolytic disease and other neonatal jaundice	Male	0-6 days	2-4 years	Global	-1	3	Healthcare access and quality index	661
Hemolytic disease and other neonatal jaundice	Male	0-6 days	2-4 years	Global	-1	3	Proportion of the population with at least 12 years of education, maternal	774
Hemolytic disease and other neonatal jaundice	Male	0-6 days	2-4 years	Global	-1	3	LDI (I$ per capita)	--
Hemolytic disease and other neonatal jaundice	Male	0-6 days	2-4 years	Global	1	3	Total Fertility Rate	--
Other neonatal disorders	Female	0-6 days	2-4 years	Data Rich	-1	1	Maternal care and immunization	--
Other neonatal disorders	Female	0-6 days	2-4 years	Data Rich	1	1	Log-transformed SEV scalar: Neonatal	--
Other neonatal disorders	Female	0-6 days	2-4 years	Data Rich	1	2	Age-standardized SEV for Household air pollution	492
Other neonatal disorders	Female	0-6 days	2-4 years	Data Rich	1	2	Age-standardized SEV for Low birth weight	492
Other neonatal disorders	Female	0-6 days	2-4 years	Data Rich	1	2	Age-standardized SEV for Short gestation	492
Other neonatal disorders	Female	0-6 days	2-4 years	Data Rich	1	2	Age-standardized SEV for Ambient particulate matter	--
Other neonatal disorders	Female	0-6 days	2-4 years	Data Rich	1	2	Age-standardized SEV for Smoking	--
Other neonatal disorders	Female	0-6 days	2-4 years	Data Rich	-1	3	Skilled Birth Attendance (proportion)	57
Other neonatal disorders	Female	0-6 days	2-4 years	Data Rich	-1	3	Antenatal Care (4 visits) Coverage (proportion)	69
Other neonatal disorders	Female	0-6 days	2-4 years	Data Rich	-1	3	Socio-demographic Index	99
Other neonatal disorders	Female	0-6 days	2-4 years	Data Rich	-1	3	Healthcare access and quality index	315
Other neonatal disorders	Female	0-6 days	2-4 years	Data Rich	-1	3	Proportion of the population with at least 12 years of education, maternal	329
Other neonatal disorders	Female	0-6 days	2-4 years	Data Rich	-1	3	In-Facility Delivery (proportion)	826
Other neonatal disorders	Female	0-6 days	2-4 years	Data Rich	-1	3	Antenatal Care (1 visit) Coverage (proportion)	--
Other neonatal disorders	Female	0-6 days	2-4 years	Data Rich	-1	3	LDI (I$ per capita)	--
Other neonatal disorders	Female	0-6 days	2-4 years	Data Rich	-1	3	Proportion of the population with at least 6 years of education, maternal	--
Other neonatal disorders	Female	0-6 days	2-4 years	Data Rich	1	3	Total Fertility Rate	--
Other neonatal disorders	Female	0-6 days	2-4 years	Global	-1	1	Maternal care and immunization	--
Other neonatal disorders	Female	0-6 days	2-4 years	Global	1	1	Log-transformed SEV scalar: Neonatal	--
Other neonatal disorders	Female	0-6 days	2-4 years	Global	1	2	Age-standardized SEV for Household air pollution	478
Other neonatal disorders	Female	0-6 days	2-4 years	Global	1	2	Age-standardized SEV for Low birth weight	478
Other neonatal disorders	Female	0-6 days	2-4 years	Global	1	2	Age-standardized SEV for Ambient particulate matter	--
Other neonatal disorders	Female	0-6 days	2-4 years	Global	1	2	Age-standardized SEV for Short gestation	--
Other neonatal disorders	Female	0-6 days	2-4 years	Global	1	2	Age-standardized SEV for Smoking	--
Other neonatal disorders	Female	0-6 days	2-4 years	Global	-1	3	Antenatal Care (1 visit) Coverage (proportion)	47
Other neonatal disorders	Female	0-6 days	2-4 years	Global	-1	3	Proportion of the population with at least 6 years of education, maternal	47
Other neonatal disorders	Female	0-6 days	2-4 years	Global	-1	3	Socio-demographic Index	160
Other neonatal disorders	Female	0-6 days	2-4 years	Global	-1	3	Skilled Birth Attendance (proportion)	178
Other neonatal disorders	Female	0-6 days	2-4 years	Global	-1	3	Proportion of the population with at least 12 years of education, maternal	405
Other neonatal disorders	Female	0-6 days	2-4 years	Global	-1	3	Healthcare access and quality index	515
Other neonatal disorders	Female	0-6 days	2-4 years	Global	-1	3	In-Facility Delivery (proportion)	743
Other neonatal disorders	Female	0-6 days	2-4 years	Global	-1	3	Antenatal Care (4 visits) Coverage (proportion)	870
Other neonatal disorders	Female	0-6 days	2-4 years	Global	-1	3	LDI (I$ per capita)	--
Other neonatal disorders	Female	0-6 days	2-4 years	Global	1	3	Total Fertility Rate	--
Other neonatal disorders	Male	0-6 days	2-4 years	Data Rich	-1	1	Maternal care and immunization	1000
Other neonatal disorders	Male	0-6 days	2-4 years	Data Rich	1	1	Log-transformed SEV scalar: Neonatal	--
Other neonatal disorders	Male	0-6 days	2-4 years	Data Rich	1	2	Age-standardized SEV for Low birth weight	677
Other neonatal disorders	Male	0-6 days	2-4 years	Data Rich	1	2	Age-standardized SEV for Short gestation	677
Other neonatal disorders	Male	0-6 days	2-4 years	Data Rich	1	2	Age-standardized SEV for Ambient particulate matter	--
Other neonatal disorders	Male	0-6 days	2-4 years	Data Rich	1	2	Age-standardized SEV for Household air pollution	--
Other neonatal disorders	Male	0-6 days	2-4 years	Data Rich	1	2	Age-standardized SEV for Smoking	--
Other neonatal disorders	Male	0-6 days	2-4 years	Data Rich	-1	3	Skilled Birth Attendance (proportion)	223
Other neonatal disorders	Male	0-6 days	2-4 years	Data Rich	-1	3	Antenatal Care (1 visit) Coverage (proportion)	--
Other neonatal disorders	Male	0-6 days	2-4 years	Data Rich	-1	3	Antenatal Care (4 visits) Coverage (proportion)	--
Other neonatal disorders	Male	0-6 days	2-4 years	Data Rich	-1	3	Healthcare access and quality index	--
Other neonatal disorders	Male	0-6 days	2-4 years	Data Rich	-1	3	In-Facility Delivery (proportion)	--
Other neonatal disorders	Male	0-6 days	2-4 years	Data Rich	-1	3	LDI (I$ per capita)	--
Other neonatal disorders	Male	0-6 days	2-4 years	Data Rich	-1	3	Proportion of the population with at least 12 years of education, maternal	--
Other neonatal disorders	Male	0-6 days	2-4 years	Data Rich	-1	3	Proportion of the population with at least 6 years of education, maternal	--
Other neonatal disorders	Male	0-6 days	2-4 years	Data Rich	-1	3	Socio-demographic Index	--
Other neonatal disorders	Male	0-6 days	2-4 years	Data Rich	1	3	Total Fertility Rate	--
Other neonatal disorders	Male	0-6 days	2-4 years	Global	-1	1	Maternal care and immunization	1000
Other neonatal disorders	Male	0-6 days	2-4 years	Global	1	1	Log-transformed SEV scalar: Neonatal	--
Other neonatal disorders	Male	0-6 days	2-4 years	Global	1	2	Age-standardized SEV for Low birth weight	299
Other neonatal disorders	Male	0-6 days	2-4 years	Global	1	2	Age-standardized SEV for Short gestation	438
Other neonatal disorders	Male	0-6 days	2-4 years	Global	1	2	Age-standardized SEV for Household air pollution	737
Other neonatal disorders	Male	0-6 days	2-4 years	Global	1	2	Age-standardized SEV for Ambient particulate matter	--
Other neonatal disorders	Male	0-6 days	2-4 years	Global	1	2	Age-standardized SEV for Smoking	--
Other neonatal disorders	Male	0-6 days	2-4 years	Global	-1	3	Proportion of the population with at least 6 years of education, maternal	26
Other neonatal disorders	Male	0-6 days	2-4 years	Global	-1	3	Healthcare access and quality index	48
Other neonatal disorders	Male	0-6 days	2-4 years	Global	-1	3	Skilled Birth Attendance (proportion)	98
Other neonatal disorders	Male	0-6 days	2-4 years	Global	-1	3	Proportion of the population with at least 12 years of education, maternal	158
Other neonatal disorders	Male	0-6 days	2-4 years	Global	-1	3	Antenatal Care (4 visits) Coverage (proportion)	232
Other neonatal disorders	Male	0-6 days	2-4 years	Global	-1	3	Socio-demographic Index	282

Appendix Table S10: CODEm covariates used, level of covariate, and expected direction of covariate by cause, sex, and age

Cause	Sex	Age Start	Age End	Model Version Type	Direction	Level	Covariate Name	Number of Draws
Other neonatal disorders	Male	0-6 days	2-4 years	Global	-1	3	In-Facility Delivery (proportion)	348
Other neonatal disorders	Male	0-6 days	2-4 years	Global	-1	3	Antenatal Care (1 visit) Coverage (proportion)	--
Other neonatal disorders	Male	0-6 days	2-4 years	Global	-1	3	LDI (I$ per capita)	--
Other neonatal disorders	Male	0-6 days	2-4 years	Global	1	3	Total Fertility Rate	--
Nutritional deficiencies	Female	1-5 months	95+ years	Data Rich	-1	1	energy unadjusted(kcal)	56
Nutritional deficiencies	Female	1-5 months	95+ years	Data Rich	1	1	Age-Standardize Prevalence of Severe Anemia	301
Nutritional deficiencies	Female	1-5 months	95+ years	Data Rich	1	1	Age-standardized SEV for Child underweight	305
Nutritional deficiencies	Female	1-5 months	95+ years	Data Rich	1	1	Log-transformed SEV scalar: Diarrhea	409
Nutritional deficiencies	Female	1-5 months	95+ years	Data Rich	1	1	Age-standardized SEV for Child wasting	695
Nutritional deficiencies	Female	1-5 months	95+ years	Data Rich	1	1	Malnutrition Shock mortality rate	--
Nutritional deficiencies	Female	1-5 months	95+ years	Data Rich	-1	2	Maternal care and immunization	0
Nutritional deficiencies	Female	1-5 months	95+ years	Data Rich	-1	2	Healthcare access and quality index	9
Nutritional deficiencies	Female	1-5 months	95+ years	Data Rich	1	2	Rainfall Quintile 2 (proportion)	0
Nutritional deficiencies	Female	1-5 months	95+ years	Data Rich	1	2	Age- and sex-specific SEV for Unsafe water	3
Nutritional deficiencies	Female	1-5 months	95+ years	Data Rich	1	2	Age- and sex-specific SEV for Unsafe sanitation	65
Nutritional deficiencies	Female	1-5 months	95+ years	Data Rich	1	2	Rainfall Quintile 1 (proportion)	111
Nutritional deficiencies	Female	1-5 months	95+ years	Data Rich	1	2	Mortality Rate Due to Death Shocks in Last 10 Years (per 1 person)	--
Nutritional deficiencies	Female	1-5 months	95+ years	Data Rich	-1	3	Education (years per capita)	146
Nutritional deficiencies	Female	1-5 months	95+ years	Data Rich	-1	3	Socio-demographic Index	167
Nutritional deficiencies	Female	1-5 months	95+ years	Data Rich	-1	3	LDI (I$ per capita)	--
Nutritional deficiencies	Female	1-5 months	95+ years	Global	-1	1	energy unadjusted(kcal)	0
Nutritional deficiencies	Female	1-5 months	95+ years	Global	1	1	Age-Standardize Prevalence of Severe Anemia	81
Nutritional deficiencies	Female	1-5 months	95+ years	Global	1	1	Age-standardized SEV for Child wasting	480
Nutritional deficiencies	Female	1-5 months	95+ years	Global	1	1	Log-transformed SEV scalar: Diarrhea	533
Nutritional deficiencies	Female	1-5 months	95+ years	Global	1	1	Age-standardized SEV for Child underweight	1000
Nutritional deficiencies	Female	1-5 months	95+ years	Global	1	1	Malnutrition Shock mortality rate	--
Nutritional deficiencies	Female	1-5 months	95+ years	Global	-1	2	Healthcare access and quality index	0
Nutritional deficiencies	Female	1-5 months	95+ years	Global	-1	2	Maternal care and immunization	0
Nutritional deficiencies	Female	1-5 months	95+ years	Global	1	2	Age- and sex-specific SEV for Unsafe water	0
Nutritional deficiencies	Female	1-5 months	95+ years	Global	1	2	Rainfall Quintile 2 (proportion)	12
Nutritional deficiencies	Female	1-5 months	95+ years	Global	1	2	Rainfall Quintile 1 (proportion)	15
Nutritional deficiencies	Female	1-5 months	95+ years	Global	1	2	Age- and sex-specific SEV for Unsafe sanitation	220
Nutritional deficiencies	Female	1-5 months	95+ years	Global	1	2	Mortality Rate Due to Death Shocks in Last 10 Years (per 1 person)	--
Nutritional deficiencies	Female	1-5 months	95+ years	Global	-1	3	Education (years per capita)	0
Nutritional deficiencies	Female	1-5 months	95+ years	Global	-1	3	Socio-demographic Index	0
Nutritional deficiencies	Female	1-5 months	95+ years	Global	-1	3	LDI (I$ per capita)	--
Nutritional deficiencies	Male	1-5 months	95+ years	Data Rich	-1	1	energy unadjusted(kcal)	956
Nutritional deficiencies	Male	1-5 months	95+ years	Data Rich	1	1	Age-Standardize Prevalence of Severe Anemia	0
Nutritional deficiencies	Male	1-5 months	95+ years	Data Rich	1	1	Age-standardized SEV for Child wasting	1
Nutritional deficiencies	Male	1-5 months	95+ years	Data Rich	1	1	Log-transformed SEV scalar: Diarrhea	197
Nutritional deficiencies	Male	1-5 months	95+ years	Data Rich	1	1	Age-standardized SEV for Child underweight	261
Nutritional deficiencies	Male	1-5 months	95+ years	Data Rich	1	1	Malnutrition Shock mortality rate	--
Nutritional deficiencies	Male	1-5 months	95+ years	Data Rich	-1	2	Maternal care and immunization	0
Nutritional deficiencies	Male	1-5 months	95+ years	Data Rich	-1	2	Healthcare access and quality index	12
Nutritional deficiencies	Male	1-5 months	95+ years	Data Rich	1	2	Rainfall Quintile 1 (proportion)	47
Nutritional deficiencies	Male	1-5 months	95+ years	Data Rich	1	2	Age- and sex-specific SEV for Unsafe sanitation	111
Nutritional deficiencies	Male	1-5 months	95+ years	Data Rich	1	2	Rainfall Quintile 2 (proportion)	123
Nutritional deficiencies	Male	1-5 months	95+ years	Data Rich	1	2	Age- and sex-specific SEV for Unsafe water	138
Nutritional deficiencies	Male	1-5 months	95+ years	Data Rich	1	2	Mortality Rate Due to Death Shocks in Last 10 Years (per 1 person)	--
Nutritional deficiencies	Male	1-5 months	95+ years	Data Rich	-1	3	Education (years per capita)	48
Nutritional deficiencies	Male	1-5 months	95+ years	Data Rich	-1	3	Socio-demographic Index	96
Nutritional deficiencies	Male	1-5 months	95+ years	Data Rich	-1	3	LDI (I$ per capita)	--
Nutritional deficiencies	Male	1-5 months	95+ years	Global	-1	1	energy unadjusted(kcal)	505
Nutritional deficiencies	Male	1-5 months	95+ years	Global	1	1	Age-standardized SEV for Child wasting	628
Nutritional deficiencies	Male	1-5 months	95+ years	Global	1	1	Age-standardized SEV for Child underweight	715
Nutritional deficiencies	Male	1-5 months	95+ years	Global	1	1	Age-Standardize Prevalence of Severe Anemia	750
Nutritional deficiencies	Male	1-5 months	95+ years	Global	1	1	Log-transformed SEV scalar: Diarrhea	938
Nutritional deficiencies	Male	1-5 months	95+ years	Global	1	1	Malnutrition Shock mortality rate	--
Nutritional deficiencies	Male	1-5 months	95+ years	Global	-1	2	Maternal care and immunization	9
Nutritional deficiencies	Male	1-5 months	95+ years	Global	-1	2	Healthcare access and quality index	12
Nutritional deficiencies	Male	1-5 months	95+ years	Global	1	2	Age- and sex-specific SEV for Unsafe water	17
Nutritional deficiencies	Male	1-5 months	95+ years	Global	1	2	Age- and sex-specific SEV for Unsafe sanitation	22
Nutritional deficiencies	Male	1-5 months	95+ years	Global	1	2	Rainfall Quintile 2 (proportion)	46
Nutritional deficiencies	Male	1-5 months	95+ years	Global	1	2	Rainfall Quintile 1 (proportion)	48
Nutritional deficiencies	Male	1-5 months	95+ years	Global	1	2	Mortality Rate Due to Death Shocks in Last 10 Years (per 1 person)	--
Nutritional deficiencies	Male	1-5 months	95+ years	Global	-1	3	Socio-demographic Index	20
Nutritional deficiencies	Male	1-5 months	95+ years	Global	-1	3	Education (years per capita)	92
Nutritional deficiencies	Male	1-5 months	95+ years	Global	-1	3	LDI (I$ per capita)	--
Protein-energy malnutrition	Female	1-5 months	2-4 years	Data Rich	-1	1	energy unadjusted(kcal)	--
Protein-energy malnutrition	Female	1-5 months	2-4 years	Data Rich	1	1	Age-standardized SEV for Child underweight	35
Protein-energy malnutrition	Female	1-5 months	2-4 years	Data Rich	1	1	Age- and sex-specific SEV for Child wasting	290
Protein-energy malnutrition	Female	1-5 months	2-4 years	Data Rich	1	1	Age-Standardize Prevalence of Severe Anemia	656
Protein-energy malnutrition	Female	1-5 months	2-4 years	Data Rich	1	1	Log-transformed SEV scalar: Diarrhea	965
Protein-energy malnutrition	Female	1-5 months	2-4 years	Data Rich	1	1	Malnutrition Shock mortality rate	--
Protein-energy malnutrition	Female	1-5 months	2-4 years	Data Rich	-1	2	Healthcare access and quality index	0
Protein-energy malnutrition	Female	1-5 months	2-4 years	Data Rich	-1	2	Maternal care and immunization	0
Protein-energy malnutrition	Female	1-5 months	2-4 years	Data Rich	1	2	Age- and sex-specific SEV for Unsafe water	1
Protein-energy malnutrition	Female	1-5 months	2-4 years	Data Rich	1	2	Rainfall Quintile 2 (proportion)	10
Protein-energy malnutrition	Female	1-5 months	2-4 years	Data Rich	1	2	Rainfall Quintile 1 (proportion)	11
Protein-energy malnutrition	Female	1-5 months	2-4 years	Data Rich	1	2	Age- and sex-specific SEV for Unsafe sanitation	18
Protein-energy malnutrition	Female	1-5 months	2-4 years	Data Rich	1	2	Mean war mortality rate in the previous ten years	--
Protein-energy malnutrition	Female	1-5 months	2-4 years	Data Rich	-1	3	Education (years per capita)	0
Protein-energy malnutrition	Female	1-5 months	2-4 years	Data Rich	-1	3	Socio-demographic Index	59
Protein-energy malnutrition	Female	1-5 months	2-4 years	Data Rich	-1	3	LDI (I$ per capita)	--
Protein-energy malnutrition	Female	1-5 months	2-4 years	Global	-1	1	energy unadjusted(kcal)	--
Protein-energy malnutrition	Female	1-5 months	2-4 years	Global	1	1	Age-Standardize Prevalence of Severe Anemia	225
Protein-energy malnutrition	Female	1-5 months	2-4 years	Global	1	1	Age- and sex-specific SEV for Child wasting	248
Protein-energy malnutrition	Female	1-5 months	2-4 years	Global	1	1	Age-standardized SEV for Child underweight	867
Protein-energy malnutrition	Female	1-5 months	2-4 years	Global	1	1	Log-transformed SEV scalar: Diarrhea	1000
Protein-energy malnutrition	Female	1-5 months	2-4 years	Global	1	1	Malnutrition Shock mortality rate	--
Protein-energy malnutrition	Female	1-5 months	2-4 years	Global	-1	2	Healthcare access and quality index	0
Protein-energy malnutrition	Female	1-5 months	2-4 years	Global	-1	2	Maternal care and immunization	0
Protein-energy malnutrition	Female	1-5 months	2-4 years	Global	1	2	Age- and sex-specific SEV for Unsafe sanitation	0
Protein-energy malnutrition	Female	1-5 months	2-4 years	Global	1	2	Age- and sex-specific SEV for Unsafe water	0
Protein-energy malnutrition	Female	1-5 months	2-4 years	Global	1	2	Rainfall Quintile 1 (proportion)	0
Protein-energy malnutrition	Female	1-5 months	2-4 years	Global	1	2	Rainfall Quintile 2 (proportion)	5
Protein-energy malnutrition	Female	1-5 months	2-4 years	Global	1	2	Mean war mortality rate in the previous ten years	--
Protein-energy malnutrition	Female	1-5 months	2-4 years	Global	-1	3	Socio-demographic Index	4
Protein-energy malnutrition	Female	1-5 months	2-4 years	Global	-1	3	Education (years per capita)	23
Protein-energy malnutrition	Female	1-5 months	2-4 years	Global	-1	3	LDI (I$ per capita)	--

Appendix Table S10: CODEm covariates used, level of covariate, and expected direction of covariate by cause, sex, and age

Cause	Sex	Age Start	Age End	Model Version Type	Direction	Level	Covariate Name	Number of Draws
Protein-energy malnutrition	Female	5-9 years	95+ years	Data Rich	-1	1	energy unadjusted(kcal)	--
Protein-energy malnutrition	Female	5-9 years	95+ years	Data Rich	1	1	Age-Standardize Prevalence of Severe Anemia	54
Protein-energy malnutrition	Female	5-9 years	95+ years	Data Rich	1	1	Age-standardized SEV for Child underweight	366
Protein-energy malnutrition	Female	5-9 years	95+ years	Data Rich	1	1	Log-transformed SEV scalar: Diarrhea	593
Protein-energy malnutrition	Female	5-9 years	95+ years	Data Rich	1	1	Age- and sex-specific SEV for Child wasting	--
Protein-energy malnutrition	Female	5-9 years	95+ years	Data Rich	1	1	Malnutrition Shock mortality rate	--
Protein-energy malnutrition	Female	5-9 years	95+ years	Data Rich	-1	2	Maternal care and immunization	9
Protein-energy malnutrition	Female	5-9 years	95+ years	Data Rich	-1	2	Healthcare access and quality index	32
Protein-energy malnutrition	Female	5-9 years	95+ years	Data Rich	1	2	Age- and sex-specific SEV for Unsafe sanitation	29
Protein-energy malnutrition	Female	5-9 years	95+ years	Data Rich	1	2	Age- and sex-specific SEV for Unsafe water	43
Protein-energy malnutrition	Female	5-9 years	95+ years	Data Rich	1	2	Rainfall Quintile 2 (proportion)	68
Protein-energy malnutrition	Female	5-9 years	95+ years	Data Rich	1	2	Rainfall Quintile 1 (proportion)	139
Protein-energy malnutrition	Female	5-9 years	95+ years	Data Rich	1	2	Mean war mortality rate in the previous ten years	--
Protein-energy malnutrition	Female	5-9 years	95+ years	Data Rich	-1	3	Socio-demographic Index	47
Protein-energy malnutrition	Female	5-9 years	95+ years	Data Rich	-1	3	Education (years per capita)	56
Protein-energy malnutrition	Female	5-9 years	95+ years	Data Rich	-1	3	LDI (I$ per capita)	--
Protein-energy malnutrition	Female	5-9 years	95+ years	Global	-1	1	energy unadjusted(kcal)	--
Protein-energy malnutrition	Female	5-9 years	95+ years	Global	1	1	Log-transformed SEV scalar: Diarrhea	471
Protein-energy malnutrition	Female	5-9 years	95+ years	Global	1	1	Age-Standardize Prevalence of Severe Anemia	655
Protein-energy malnutrition	Female	5-9 years	95+ years	Global	1	1	Age-standardized SEV for Child underweight	895
Protein-energy malnutrition	Female	5-9 years	95+ years	Global	1	1	Age- and sex-specific SEV for Child wasting	--
Protein-energy malnutrition	Female	5-9 years	95+ years	Global	1	1	Malnutrition Shock mortality rate	--
Protein-energy malnutrition	Female	5-9 years	95+ years	Global	-1	2	Healthcare access and quality index	0
Protein-energy malnutrition	Female	5-9 years	95+ years	Global	-1	2	Maternal care and immunization	0
Protein-energy malnutrition	Female	5-9 years	95+ years	Global	1	2	Age- and sex-specific SEV for Unsafe sanitation	8
Protein-energy malnutrition	Female	5-9 years	95+ years	Global	1	2	Rainfall Quintile 1 (proportion)	66
Protein-energy malnutrition	Female	5-9 years	95+ years	Global	1	2	Rainfall Quintile 2 (proportion)	93
Protein-energy malnutrition	Female	5-9 years	95+ years	Global	1	2	Age- and sex-specific SEV for Unsafe water	144
Protein-energy malnutrition	Female	5-9 years	95+ years	Global	1	2	Mean war mortality rate in the previous ten years	--
Protein-energy malnutrition	Female	5-9 years	95+ years	Global	-1	3	Education (years per capita)	0
Protein-energy malnutrition	Female	5-9 years	95+ years	Global	-1	3	Socio-demographic Index	11
Protein-energy malnutrition	Female	5-9 years	95+ years	Global	-1	3	LDI (I$ per capita)	--
Protein-energy malnutrition	Male	1-5 months	2-4 years	Data Rich	-1	1	energy unadjusted(kcal)	--
Protein-energy malnutrition	Male	1-5 months	2-4 years	Data Rich	1	1	Age-Standardize Prevalence of Severe Anemia	50
Protein-energy malnutrition	Male	1-5 months	2-4 years	Data Rich	1	1	Age-standardized SEV for Child underweight	90
Protein-energy malnutrition	Male	1-5 months	2-4 years	Data Rich	1	1	Age- and sex-specific SEV for Child wasting	453
Protein-energy malnutrition	Male	1-5 months	2-4 years	Data Rich	1	1	Log-transformed SEV scalar: Diarrhea	888
Protein-energy malnutrition	Male	1-5 months	2-4 years	Data Rich	1	1	Malnutrition Shock mortality rate	--
Protein-energy malnutrition	Male	1-5 months	2-4 years	Data Rich	-1	2	Healthcare access and quality index	0
Protein-energy malnutrition	Male	1-5 months	2-4 years	Data Rich	-1	2	Maternal care and immunization	34
Protein-energy malnutrition	Male	1-5 months	2-4 years	Data Rich	1	2	Age- and sex-specific SEV for Unsafe water	14
Protein-energy malnutrition	Male	1-5 months	2-4 years	Data Rich	1	2	Rainfall Quintile 1 (proportion)	19
Protein-energy malnutrition	Male	1-5 months	2-4 years	Data Rich	1	2	Age- and sex-specific SEV for Unsafe sanitation	54
Protein-energy malnutrition	Male	1-5 months	2-4 years	Data Rich	1	2	Rainfall Quintile 2 (proportion)	114
Protein-energy malnutrition	Male	1-5 months	2-4 years	Data Rich	1	2	Mean war mortality rate in the previous ten years	--
Protein-energy malnutrition	Male	1-5 months	2-4 years	Data Rich	-1	3	Education (years per capita)	0
Protein-energy malnutrition	Male	1-5 months	2-4 years	Data Rich	-1	3	Socio-demographic Index	33
Protein-energy malnutrition	Male	1-5 months	2-4 years	Data Rich	-1	3	LDI (I$ per capita)	--
Protein-energy malnutrition	Male	1-5 months	2-4 years	Global	-1	1	energy unadjusted(kcal)	--
Protein-energy malnutrition	Male	1-5 months	2-4 years	Global	1	1	Age-standardized SEV for Child underweight	195
Protein-energy malnutrition	Male	1-5 months	2-4 years	Global	1	1	Age- and sex-specific SEV for Child wasting	319
Protein-energy malnutrition	Male	1-5 months	2-4 years	Global	1	1	Age-Standardize Prevalence of Severe Anemia	598
Protein-energy malnutrition	Male	1-5 months	2-4 years	Global	1	1	Log-transformed SEV scalar: Diarrhea	1000
Protein-energy malnutrition	Male	1-5 months	2-4 years	Global	1	1	Malnutrition Shock mortality rate	--
Protein-energy malnutrition	Male	1-5 months	2-4 years	Global	-1	2	Healthcare access and quality index	0
Protein-energy malnutrition	Male	1-5 months	2-4 years	Global	-1	2	Maternal care and immunization	0
Protein-energy malnutrition	Male	1-5 months	2-4 years	Global	1	2	Age- and sex-specific SEV for Unsafe water	0
Protein-energy malnutrition	Male	1-5 months	2-4 years	Global	1	2	Age- and sex-specific SEV for Unsafe sanitation	6
Protein-energy malnutrition	Male	1-5 months	2-4 years	Global	1	2	Rainfall Quintile 1 (proportion)	19
Protein-energy malnutrition	Male	1-5 months	2-4 years	Global	1	2	Rainfall Quintile 2 (proportion)	53
Protein-energy malnutrition	Male	1-5 months	2-4 years	Global	1	2	Mean war mortality rate in the previous ten years	--
Protein-energy malnutrition	Male	1-5 months	2-4 years	Global	-1	3	Socio-demographic Index	5
Protein-energy malnutrition	Male	1-5 months	2-4 years	Global	-1	3	Education (years per capita)	19
Protein-energy malnutrition	Male	1-5 months	2-4 years	Global	-1	3	LDI (I$ per capita)	--
Protein-energy malnutrition	Male	5-9 years	95+ years	Data Rich	-1	1	energy unadjusted(kcal)	--
Protein-energy malnutrition	Male	5-9 years	95+ years	Data Rich	1	1	Age-Standardize Prevalence of Severe Anemia	61
Protein-energy malnutrition	Male	5-9 years	95+ years	Data Rich	1	1	Age-standardized SEV for Child underweight	369
Protein-energy malnutrition	Male	5-9 years	95+ years	Data Rich	1	1	Log-transformed SEV scalar: Diarrhea	656
Protein-energy malnutrition	Male	5-9 years	95+ years	Data Rich	1	1	Age- and sex-specific SEV for Child wasting	--
Protein-energy malnutrition	Male	5-9 years	95+ years	Data Rich	1	1	Malnutrition Shock mortality rate	--
Protein-energy malnutrition	Male	5-9 years	95+ years	Data Rich	-1	2	Healthcare access and quality index	6
Protein-energy malnutrition	Male	5-9 years	95+ years	Data Rich	-1	2	Maternal care and immunization	31
Protein-energy malnutrition	Male	5-9 years	95+ years	Data Rich	1	2	Age- and sex-specific SEV for Unsafe water	11
Protein-energy malnutrition	Male	5-9 years	95+ years	Data Rich	1	2	Rainfall Quintile 2 (proportion)	124
Protein-energy malnutrition	Male	5-9 years	95+ years	Data Rich	1	2	Rainfall Quintile 1 (proportion)	129
Protein-energy malnutrition	Male	5-9 years	95+ years	Data Rich	1	2	Age- and sex-specific SEV for Unsafe sanitation	194
Protein-energy malnutrition	Male	5-9 years	95+ years	Data Rich	1	2	Mean war mortality rate in the previous ten years	--
Protein-energy malnutrition	Male	5-9 years	95+ years	Data Rich	-1	3	Socio-demographic Index	32
Protein-energy malnutrition	Male	5-9 years	95+ years	Data Rich	-1	3	Education (years per capita)	238
Protein-energy malnutrition	Male	5-9 years	95+ years	Data Rich	-1	3	LDI (I$ per capita)	--
Protein-energy malnutrition	Male	5-9 years	95+ years	Global	-1	1	energy unadjusted(kcal)	--
Protein-energy malnutrition	Male	5-9 years	95+ years	Global	1	1	Log-transformed SEV scalar: Diarrhea	327
Protein-energy malnutrition	Male	5-9 years	95+ years	Global	1	1	Malnutrition Shock mortality rate	354
Protein-energy malnutrition	Male	5-9 years	95+ years	Global	1	1	Age-Standardize Prevalence of Severe Anemia	524
Protein-energy malnutrition	Male	5-9 years	95+ years	Global	1	1	Age-standardized SEV for Child underweight	930
Protein-energy malnutrition	Male	5-9 years	95+ years	Global	1	1	Age- and sex-specific SEV for Child wasting	--
Protein-energy malnutrition	Male	5-9 years	95+ years	Global	-1	2	Healthcare access and quality index	0
Protein-energy malnutrition	Male	5-9 years	95+ years	Global	-1	2	Maternal care and immunization	2
Protein-energy malnutrition	Male	5-9 years	95+ years	Global	1	2	Age- and sex-specific SEV for Unsafe water	10
Protein-energy malnutrition	Male	5-9 years	95+ years	Global	1	2	Age- and sex-specific SEV for Unsafe sanitation	120
Protein-energy malnutrition	Male	5-9 years	95+ years	Global	1	2	Rainfall Quintile 2 (proportion)	162
Protein-energy malnutrition	Male	5-9 years	95+ years	Global	1	2	Rainfall Quintile 1 (proportion)	206
Protein-energy malnutrition	Male	5-9 years	95+ years	Global	1	2	Mean war mortality rate in the previous ten years	--
Protein-energy malnutrition	Male	5-9 years	95+ years	Global	-1	3	Socio-demographic Index	2
Protein-energy malnutrition	Male	5-9 years	95+ years	Global	-1	3	Education (years per capita)	18
Protein-energy malnutrition	Male	5-9 years	95+ years	Global	-1	3	LDI (I$ per capita)	--
Other nutritional deficiencies	Female	1-5 months	95+ years	Data Rich	-1	1	energy unadjusted(kcal)	262
Other nutritional deficiencies	Female	1-5 months	95+ years	Data Rich	1	1	Age-Standardize Prevalence of Severe Anemia	3
Other nutritional deficiencies	Female	1-5 months	95+ years	Data Rich	1	1	Log-transformed SEV scalar: Diarrhea	125

Appendix Table S10: CODEm covariates used, level of covariate, and expected direction of covariate by cause, sex, and age

Cause	Sex	Age Start	Age End	Model Version Type	Direction	Level	Covariate Name	Number of Draws
Other nutritional deficiencies	Female	1-5 months	95+ years	Data Rich	1	1	Age-standardized SEV for Child wasting	735
Other nutritional deficiencies	Female	1-5 months	95+ years	Data Rich	1	1	Age-standardized SEV for Child underweight	899
Other nutritional deficiencies	Female	1-5 months	95+ years	Data Rich	1	1	Malnutrition Shock mortality rate	--
Other nutritional deficiencies	Female	1-5 months	95+ years	Data Rich	-1	2	Maternal care and immunization	6
Other nutritional deficiencies	Female	1-5 months	95+ years	Data Rich	-1	2	Healthcare access and quality index	465
Other nutritional deficiencies	Female	1-5 months	95+ years	Data Rich	1	2	Age- and sex-specific SEV for Unsafe sanitation	0
Other nutritional deficiencies	Female	1-5 months	95+ years	Data Rich	1	2	Rainfall Quintile 2 (proportion)	6
Other nutritional deficiencies	Female	1-5 months	95+ years	Data Rich	1	2	Age- and sex-specific SEV for Unsafe water	7
Other nutritional deficiencies	Female	1-5 months	95+ years	Data Rich	1	2	Mortality Rate Due to Death Shocks in Last 10 Years (per 1 person)	--
Other nutritional deficiencies	Female	1-5 months	95+ years	Data Rich	1	2	Rainfall Quintile 1 (proportion)	--
Other nutritional deficiencies	Female	1-5 months	95+ years	Data Rich	-1	3	Socio-demographic Index	0
Other nutritional deficiencies	Female	1-5 months	95+ years	Data Rich	-1	3	Education (years per capita)	674
Other nutritional deficiencies	Female	1-5 months	95+ years	Data Rich	-1	3	LDI (I$ per capita)	--
Other nutritional deficiencies	Female	1-5 months	95+ years	Global	-1	1	energy unadjusted(kcal)	490
Other nutritional deficiencies	Female	1-5 months	95+ years	Global	1	1	Age-Standardize Prevalence of Severe Anemia	107
Other nutritional deficiencies	Female	1-5 months	95+ years	Global	1	1	Log-transformed SEV scalar: Diarrhea	248
Other nutritional deficiencies	Female	1-5 months	95+ years	Global	1	1	Age-standardized SEV for Child wasting	383
Other nutritional deficiencies	Female	1-5 months	95+ years	Global	1	1	Age-standardized SEV for Child underweight	695
Other nutritional deficiencies	Female	1-5 months	95+ years	Global	1	1	Malnutrition Shock mortality rate	--
Other nutritional deficiencies	Female	1-5 months	95+ years	Global	-1	2	Maternal care and immunization	1
Other nutritional deficiencies	Female	1-5 months	95+ years	Global	-1	2	Healthcare access and quality index	980
Other nutritional deficiencies	Female	1-5 months	95+ years	Global	1	2	Age- and sex-specific SEV for Unsafe sanitation	0
Other nutritional deficiencies	Female	1-5 months	95+ years	Global	1	2	Age- and sex-specific SEV for Unsafe water	0
Other nutritional deficiencies	Female	1-5 months	95+ years	Global	1	2	Rainfall Quintile 2 (proportion)	166
Other nutritional deficiencies	Female	1-5 months	95+ years	Global	1	2	Rainfall Quintile 1 (proportion)	356
Other nutritional deficiencies	Female	1-5 months	95+ years	Global	1	2	Mortality Rate Due to Death Shocks in Last 10 Years (per 1 person)	--
Other nutritional deficiencies	Female	1-5 months	95+ years	Global	-1	3	Socio-demographic Index	30
Other nutritional deficiencies	Female	1-5 months	95+ years	Global	-1	3	Education (years per capita)	194
Other nutritional deficiencies	Female	1-5 months	95+ years	Global	-1	3	LDI (I$ per capita)	--
Other nutritional deficiencies	Male	1-5 months	95+ years	Data Rich	-1	1	energy unadjusted(kcal)	504
Other nutritional deficiencies	Male	1-5 months	95+ years	Data Rich	1	1	Age-Standardize Prevalence of Severe Anemia	0
Other nutritional deficiencies	Male	1-5 months	95+ years	Data Rich	1	1	Log-transformed SEV scalar: Diarrhea	9
Other nutritional deficiencies	Male	1-5 months	95+ years	Data Rich	1	1	Age-standardized SEV for Child underweight	235
Other nutritional deficiencies	Male	1-5 months	95+ years	Data Rich	1	1	Age-standardized SEV for Child wasting	932
Other nutritional deficiencies	Male	1-5 months	95+ years	Data Rich	1	1	Malnutrition Shock mortality rate	--
Other nutritional deficiencies	Male	1-5 months	95+ years	Data Rich	-1	2	Healthcare access and quality index	304
Other nutritional deficiencies	Male	1-5 months	95+ years	Data Rich	-1	2	Maternal care and immunization	315
Other nutritional deficiencies	Male	1-5 months	95+ years	Data Rich	1	2	Age- and sex-specific SEV for Unsafe water	1
Other nutritional deficiencies	Male	1-5 months	95+ years	Data Rich	1	2	Rainfall Quintile 2 (proportion)	5
Other nutritional deficiencies	Male	1-5 months	95+ years	Data Rich	1	2	Age- and sex-specific SEV for Unsafe sanitation	86
Other nutritional deficiencies	Male	1-5 months	95+ years	Data Rich	1	2	Mortality Rate Due to Death Shocks in Last 10 Years (per 1 person)	--
Other nutritional deficiencies	Male	1-5 months	95+ years	Data Rich	1	2	Rainfall Quintile 1 (proportion)	--
Other nutritional deficiencies	Male	1-5 months	95+ years	Data Rich	-1	3	Socio-demographic Index	43
Other nutritional deficiencies	Male	1-5 months	95+ years	Data Rich	-1	3	Education (years per capita)	478
Other nutritional deficiencies	Male	1-5 months	95+ years	Data Rich	-1	3	LDI (I$ per capita)	--
Other nutritional deficiencies	Male	1-5 months	95+ years	Global	-1	1	energy unadjusted(kcal)	--
Other nutritional deficiencies	Male	1-5 months	95+ years	Global	1	1	Log-transformed SEV scalar: Diarrhea	81
Other nutritional deficiencies	Male	1-5 months	95+ years	Global	1	1	Age-standardized SEV for Child wasting	362
Other nutritional deficiencies	Male	1-5 months	95+ years	Global	1	1	Age-standardized SEV for Child underweight	502
Other nutritional deficiencies	Male	1-5 months	95+ years	Global	1	1	Age-Standardize Prevalence of Severe Anemia	517
Other nutritional deficiencies	Male	1-5 months	95+ years	Global	1	1	Malnutrition Shock mortality rate	--
Other nutritional deficiencies	Male	1-5 months	95+ years	Global	-1	2	Maternal care and immunization	0
Other nutritional deficiencies	Male	1-5 months	95+ years	Global	-1	2	Healthcare access and quality index	321
Other nutritional deficiencies	Male	1-5 months	95+ years	Global	1	2	Age- and sex-specific SEV for Unsafe water	0
Other nutritional deficiencies	Male	1-5 months	95+ years	Global	1	2	Rainfall Quintile 1 (proportion)	186
Other nutritional deficiencies	Male	1-5 months	95+ years	Global	1	2	Rainfall Quintile 2 (proportion)	245
Other nutritional deficiencies	Male	1-5 months	95+ years	Global	1	2	Age- and sex-specific SEV for Unsafe sanitation	--
Other nutritional deficiencies	Male	1-5 months	95+ years	Global	1	2	Mortality Rate Due to Death Shocks in Last 10 Years (per 1 person)	--
Other nutritional deficiencies	Male	1-5 months	95+ years	Global	-1	3	Education (years per capita)	501
Other nutritional deficiencies	Male	1-5 months	95+ years	Global	-1	3	Socio-demographic Index	782
Other nutritional deficiencies	Male	1-5 months	95+ years	Global	-1	3	LDI (I$ per capita)	--
Lip and oral cavity cancer	Female	15-19 years	95+ years	Data Rich	1	1	Tobacco (cigarettes per capita)	41
Lip and oral cavity cancer	Female	15-19 years	95+ years	Data Rich	1	1	Cumulative Cigarettes (10 Years)	75
Lip and oral cavity cancer	Female	15-19 years	95+ years	Data Rich	1	1	Cumulative Cigarettes (15 Years)	311
Lip and oral cavity cancer	Female	15-19 years	95+ years	Data Rich	1	1	Cumulative Cigarettes (5 Years)	341
Lip and oral cavity cancer	Female	15-19 years	95+ years	Data Rich	1	1	Cumulative Cigarettes (20 Years)	571
Lip and oral cavity cancer	Female	15-19 years	95+ years	Data Rich	1	1	Liters of alcohol consumed per capita	--
Lip and oral cavity cancer	Female	15-19 years	95+ years	Data Rich	-1	2	Healthcare access and quality index	0
Lip and oral cavity cancer	Female	15-19 years	95+ years	Data Rich	1	2	Age- and sex-specific SEV for High red meat	77
Lip and oral cavity cancer	Female	15-19 years	95+ years	Data Rich	1	2	Age- and sex-specific SEV for Low fruit	260
Lip and oral cavity cancer	Female	15-19 years	95+ years	Data Rich	1	2	Age- and sex-specific SEV for Low vegetables	--
Lip and oral cavity cancer	Female	15-19 years	95+ years	Data Rich	-1	3	Education (years per capita)	4
Lip and oral cavity cancer	Female	15-19 years	95+ years	Data Rich	1	3	LDI (I$ per capita)	--
Lip and oral cavity cancer	Female	15-19 years	95+ years	Data Rich	1	3	Socio-demographic Index	--
Lip and oral cavity cancer	Female	15-19 years	95+ years	Global	1	1	Cumulative Cigarettes (10 Years)	57
Lip and oral cavity cancer	Female	15-19 years	95+ years	Global	1	1	Tobacco (cigarettes per capita)	105
Lip and oral cavity cancer	Female	15-19 years	95+ years	Global	1	1	Cumulative Cigarettes (20 Years)	559
Lip and oral cavity cancer	Female	15-19 years	95+ years	Global	1	1	Liters of alcohol consumed per capita	648
Lip and oral cavity cancer	Female	15-19 years	95+ years	Global	-1	2	Healthcare access and quality index	170
Lip and oral cavity cancer	Female	15-19 years	95+ years	Global	1	2	Age- and sex-specific SEV for Low vegetables	266
Lip and oral cavity cancer	Female	15-19 years	95+ years	Global	1	2	Age- and sex-specific SEV for Low fruit	331
Lip and oral cavity cancer	Female	15-19 years	95+ years	Global	1	2	Age- and sex-specific SEV for High red meat	--
Lip and oral cavity cancer	Female	15-19 years	95+ years	Global	-1	3	Education (years per capita)	199
Lip and oral cavity cancer	Female	15-19 years	95+ years	Global	1	3	Socio-demographic Index	34
Lip and oral cavity cancer	Female	15-19 years	95+ years	Global	1	3	LDI (I$ per capita)	--
Lip and oral cavity cancer	Male	15-19 years	95+ years	Data Rich	1	1	Cumulative Cigarettes (10 Years)	14
Lip and oral cavity cancer	Male	15-19 years	95+ years	Data Rich	1	1	Cumulative Cigarettes (20 Years)	93
Lip and oral cavity cancer	Male	15-19 years	95+ years	Data Rich	1	1	Tobacco (cigarettes per capita)	296
Lip and oral cavity cancer	Male	15-19 years	95+ years	Data Rich	1	1	Liters of alcohol consumed per capita	1000
Lip and oral cavity cancer	Male	15-19 years	95+ years	Data Rich	-1	2	Healthcare access and quality index	1
Lip and oral cavity cancer	Male	15-19 years	95+ years	Data Rich	1	2	Age- and sex-specific SEV for High red meat	14
Lip and oral cavity cancer	Male	15-19 years	95+ years	Data Rich	1	2	Age- and sex-specific SEV for Low fruit	456
Lip and oral cavity cancer	Male	15-19 years	95+ years	Data Rich	1	2	Age- and sex-specific SEV for Low vegetables	516
Lip and oral cavity cancer	Male	15-19 years	95+ years	Data Rich	-1	3	Education (years per capita)	310
Lip and oral cavity cancer	Male	15-19 years	95+ years	Data Rich	1	3	Socio-demographic Index	0
Lip and oral cavity cancer	Male	15-19 years	95+ years	Data Rich	1	3	LDI (I$ per capita)	--
Lip and oral cavity cancer	Male	15-19 years	95+ years	Global	1	1	Tobacco (cigarettes per capita)	100
Lip and oral cavity cancer	Male	15-19 years	95+ years	Global	1	1	Cumulative Cigarettes (20 Years)	142
Lip and oral cavity cancer	Male	15-19 years	95+ years	Global	1	1	Cumulative Cigarettes (10 Years)	176
Lip and oral cavity cancer	Male	15-19 years	95+ years	Global	1	1	Liters of alcohol consumed per capita	988
Lip and oral cavity cancer	Male	15-19 years	95+ years	Global	-1	2	Healthcare access and quality index	523

Appendix Table S10: CODEm covariates used, level of covariate, and expected direction of covariate by cause, sex, and age

Cause	Sex	Age Start	Age End	Model Version Type	Direction	Level	Covariate Name	Number of Draws
Lip and oral cavity cancer	Male	15-19 years	95+ years	Global	1	2	Age- and sex-specific SEV for Low vegetables	114
Lip and oral cavity cancer	Male	15-19 years	95+ years	Global	1	2	Age- and sex-specific SEV for High red meat	199
Lip and oral cavity cancer	Male	15-19 years	95+ years	Global	1	2	Age- and sex-specific SEV for Low fruit	258
Lip and oral cavity cancer	Male	15-19 years	95+ years	Global	-1	3	Education (years per capita)	257
Lip and oral cavity cancer	Male	15-19 years	95+ years	Global	1	3	Socio-demographic Index	138
Lip and oral cavity cancer	Male	15-19 years	95+ years	Global	1	3	LDI (I$ per capita)	--
Nasopharynx cancer	Female	5-9 years	95+ years	Data Rich	1	1	Cumulative Cigarettes (10 Years)	297
Nasopharynx cancer	Female	5-9 years	95+ years	Data Rich	1	1	Cumulative Cigarettes (20 Years)	297
Nasopharynx cancer	Female	5-9 years	95+ years	Data Rich	1	1	Liters of alcohol consumed per capita	703
Nasopharynx cancer	Female	5-9 years	95+ years	Data Rich	1	1	Tobacco (cigarettes per capita)	703
Nasopharynx cancer	Female	5-9 years	95+ years	Data Rich	-1	2	Healthcare access and quality index	--
Nasopharynx cancer	Female	5-9 years	95+ years	Data Rich	1	2	Population Density (over 1000 ppl/sqkm, proportion)	401
Nasopharynx cancer	Female	5-9 years	95+ years	Data Rich	1	2	Age- and sex-specific SEV for Low vegetables	--
Nasopharynx cancer	Female	5-9 years	95+ years	Data Rich	-1	3	Education (years per capita)	225
Nasopharynx cancer	Female	5-9 years	95+ years	Data Rich	-1	3	LDI (I$ per capita)	--
Nasopharynx cancer	Female	5-9 years	95+ years	Data Rich	1	3	Age- and sex-specific SEV for Low fruit	--
Nasopharynx cancer	Female	5-9 years	95+ years	Data Rich	1	3	Socio-demographic Index	--
Nasopharynx cancer	Female	5-9 years	95+ years	Global	1	1	Cumulative Cigarettes (20 Years)	275
Nasopharynx cancer	Female	5-9 years	95+ years	Global	1	1	Tobacco (cigarettes per capita)	292
Nasopharynx cancer	Female	5-9 years	95+ years	Global	1	1	Cumulative Cigarettes (10 Years)	433
Nasopharynx cancer	Female	5-9 years	95+ years	Global	1	1	Liters of alcohol consumed per capita	--
Nasopharynx cancer	Female	5-9 years	95+ years	Global	-1	2	Healthcare access and quality index	219
Nasopharynx cancer	Female	5-9 years	95+ years	Global	1	2	Age- and sex-specific SEV for Low vegetables	262
Nasopharynx cancer	Female	5-9 years	95+ years	Global	1	2	Population Density (over 1000 ppl/sqkm, proportion)	262
Nasopharynx cancer	Female	5-9 years	95+ years	Global	-1	3	Education (years per capita)	406
Nasopharynx cancer	Female	5-9 years	95+ years	Global	-1	3	LDI (I$ per capita)	--
Nasopharynx cancer	Female	5-9 years	95+ years	Global	1	3	Age- and sex-specific SEV for Low fruit	498
Nasopharynx cancer	Female	5-9 years	95+ years	Global	1	3	Socio-demographic Index	--
Nasopharynx cancer	Male	5-9 years	95+ years	Data Rich	1	1	Tobacco (cigarettes per capita)	8
Nasopharynx cancer	Male	5-9 years	95+ years	Data Rich	1	1	Cumulative Cigarettes (20 Years)	283
Nasopharynx cancer	Male	5-9 years	95+ years	Data Rich	1	1	Cumulative Cigarettes (10 Years)	416
Nasopharynx cancer	Male	5-9 years	95+ years	Data Rich	1	1	Liters of alcohol consumed per capita	932
Nasopharynx cancer	Male	5-9 years	95+ years	Data Rich	-1	2	Healthcare access and quality index	0
Nasopharynx cancer	Male	5-9 years	95+ years	Data Rich	1	2	Population Density (over 1000 ppl/sqkm, proportion)	21
Nasopharynx cancer	Male	5-9 years	95+ years	Data Rich	1	2	Age- and sex-specific SEV for Low vegetables	--
Nasopharynx cancer	Male	5-9 years	95+ years	Data Rich	-1	3	Education (years per capita)	300
Nasopharynx cancer	Male	5-9 years	95+ years	Data Rich	-1	3	LDI (I$ per capita)	--
Nasopharynx cancer	Male	5-9 years	95+ years	Data Rich	1	3	Socio-demographic Index	1
Nasopharynx cancer	Male	5-9 years	95+ years	Data Rich	1	3	Age- and sex-specific SEV for Low fruit	487
Nasopharynx cancer	Male	5-9 years	95+ years	Global	1	1	Cumulative Cigarettes (20 Years)	171
Nasopharynx cancer	Male	5-9 years	95+ years	Global	1	1	Cumulative Cigarettes (10 Years)	352
Nasopharynx cancer	Male	5-9 years	95+ years	Global	1	1	Tobacco (cigarettes per capita)	629
Nasopharynx cancer	Male	5-9 years	95+ years	Global	1	1	Liters of alcohol consumed per capita	718
Nasopharynx cancer	Male	5-9 years	95+ years	Global	-1	2	Healthcare access and quality index	872
Nasopharynx cancer	Male	5-9 years	95+ years	Global	1	2	Population Density (over 1000 ppl/sqkm, proportion)	289
Nasopharynx cancer	Male	5-9 years	95+ years	Global	1	2	Age- and sex-specific SEV for Low vegetables	618
Nasopharynx cancer	Male	5-9 years	95+ years	Global	-1	3	Education (years per capita)	91
Nasopharynx cancer	Male	5-9 years	95+ years	Global	-1	3	LDI (I$ per capita)	--
Nasopharynx cancer	Male	5-9 years	95+ years	Global	1	3	Age- and sex-specific SEV for Low fruit	255
Nasopharynx cancer	Male	5-9 years	95+ years	Global	1	3	Socio-demographic Index	465
Other pharynx cancer	Female	20-24 years	95+ years	Data Rich	1	1	Liters of alcohol consumed per capita	431
Other pharynx cancer	Female	20-24 years	95+ years	Data Rich	1	1	Smoking Prevalence	920
Other pharynx cancer	Female	20-24 years	95+ years	Data Rich	-1	2	Healthcare access and quality index	111
Other pharynx cancer	Female	20-24 years	95+ years	Data Rich	1	2	Age- and sex-specific SEV for Low fruit	16
Other pharynx cancer	Female	20-24 years	95+ years	Data Rich	1	2	Age- and sex-specific SEV for Low vegetables	136
Other pharynx cancer	Female	20-24 years	95+ years	Data Rich	1	2	Cumulative Cigarettes (5 Years)	169
Other pharynx cancer	Female	20-24 years	95+ years	Data Rich	1	2	Population Density (over 1000 ppl/sqkm, proportion)	218
Other pharynx cancer	Female	20-24 years	95+ years	Data Rich	1	2	Population Density (under 150 ppl/sqkm, proportion)	--
Other pharynx cancer	Female	20-24 years	95+ years	Data Rich	-1	3	Education (years per capita)	285
Other pharynx cancer	Female	20-24 years	95+ years	Data Rich	1	3	LDI (I$ per capita)	--
Other pharynx cancer	Female	20-24 years	95+ years	Data Rich	1	3	Socio-demographic Index	--
Other pharynx cancer	Female	20-24 years	95+ years	Global	1	1	Smoking Prevalence	1000
Other pharynx cancer	Female	20-24 years	95+ years	Global	1	1	Liters of alcohol consumed per capita	--
Other pharynx cancer	Female	20-24 years	95+ years	Global	-1	2	Healthcare access and quality index	345
Other pharynx cancer	Female	20-24 years	95+ years	Global	1	2	Age- and sex-specific SEV for Low fruit	435
Other pharynx cancer	Female	20-24 years	95+ years	Global	1	2	Age- and sex-specific SEV for Low vegetables	--
Other pharynx cancer	Female	20-24 years	95+ years	Global	1	2	Cumulative Cigarettes (5 Years)	--
Other pharynx cancer	Female	20-24 years	95+ years	Global	1	2	Population Density (over 1000 ppl/sqkm, proportion)	--
Other pharynx cancer	Female	20-24 years	95+ years	Global	1	2	Population Density (under 150 ppl/sqkm, proportion)	--
Other pharynx cancer	Female	20-24 years	95+ years	Global	-1	3	Education (years per capita)	153
Other pharynx cancer	Female	20-24 years	95+ years	Global	1	3	LDI (I$ per capita)	--
Other pharynx cancer	Female	20-24 years	95+ years	Global	1	3	Socio-demographic Index	--
Other pharynx cancer	Male	20-24 years	95+ years	Data Rich	1	1	Liters of alcohol consumed per capita	1000
Other pharynx cancer	Male	20-24 years	95+ years	Data Rich	1	1	Smoking Prevalence	--
Other pharynx cancer	Male	20-24 years	95+ years	Data Rich	-1	2	Healthcare access and quality index	--
Other pharynx cancer	Male	20-24 years	95+ years	Data Rich	1	2	Age- and sex-specific SEV for Low vegetables	176
Other pharynx cancer	Male	20-24 years	95+ years	Data Rich	1	2	Age- and sex-specific SEV for Low fruit	653
Other pharynx cancer	Male	20-24 years	95+ years	Data Rich	1	2	Population Density (under 150 ppl/sqkm, proportion)	653
Other pharynx cancer	Male	20-24 years	95+ years	Data Rich	1	2	Cumulative Cigarettes (5 Years)	--
Other pharynx cancer	Male	20-24 years	95+ years	Data Rich	1	2	Population Density (over 1000 ppl/sqkm, proportion)	--
Other pharynx cancer	Male	20-24 years	95+ years	Data Rich	-1	3	Education (years per capita)	--
Other pharynx cancer	Male	20-24 years	95+ years	Data Rich	1	3	Socio-demographic Index	325
Other pharynx cancer	Male	20-24 years	95+ years	Data Rich	1	3	LDI (I$ per capita)	--
Other pharynx cancer	Male	20-24 years	95+ years	Global	1	1	Smoking Prevalence	489
Other pharynx cancer	Male	20-24 years	95+ years	Global	1	1	Liters of alcohol consumed per capita	1000
Other pharynx cancer	Male	20-24 years	95+ years	Global	-1	2	Healthcare access and quality index	529
Other pharynx cancer	Male	20-24 years	95+ years	Global	1	2	Age- and sex-specific SEV for Low fruit	187
Other pharynx cancer	Male	20-24 years	95+ years	Global	1	2	Population Density (over 1000 ppl/sqkm, proportion)	260
Other pharynx cancer	Male	20-24 years	95+ years	Global	1	2	Age- and sex-specific SEV for Low vegetables	330
Other pharynx cancer	Male	20-24 years	95+ years	Global	1	2	Cumulative Cigarettes (5 Years)	--
Other pharynx cancer	Male	20-24 years	95+ years	Global	1	2	Population Density (under 150 ppl/sqkm, proportion)	--
Other pharynx cancer	Male	20-24 years	95+ years	Global	-1	3	Education (years per capita)	--
Other pharynx cancer	Male	20-24 years	95+ years	Global	1	3	Socio-demographic Index	166
Other pharynx cancer	Male	20-24 years	95+ years	Global	1	3	LDI (I$ per capita)	--
Oesophageal cancer	Female	20-24 years	95+ years	Data Rich	1	1	Liters of alcohol consumed per capita	306
Oesophageal cancer	Female	20-24 years	95+ years	Data Rich	1	1	Smoking Prevalence	867
Oesophageal cancer	Female	20-24 years	95+ years	Data Rich	1	1	Mean BMI	--
Oesophageal cancer	Female	20-24 years	95+ years	Data Rich	-1	2	Healthcare access and quality index	428
Oesophageal cancer	Female	20-24 years	95+ years	Data Rich	1	2	Age- and sex-specific SEV for Low fruit	68
Oesophageal cancer	Female	20-24 years	95+ years	Data Rich	1	2	Age- and sex-specific SEV for Low vegetables	71

Appendix Table S10: CODEm covariates used, level of covariate, and expected direction of covariate by cause, sex, and age

Cause	Sex	Age Start	Age End	Model Version Type	Direction	Level	Covariate Name	Number of Draws
Oesophageal cancer	Female	20-24 years	95+ years	Data Rich	1	2	Tobacco (cigarettes per capita)	200
Oesophageal cancer	Female	20-24 years	95+ years	Data Rich	1	2	Indoor Air Pollution (All Cooking Fuels)	--
Oesophageal cancer	Female	20-24 years	95+ years	Data Rich	-1	3	Improved Water Source (proportion with access)	36
Oesophageal cancer	Female	20-24 years	95+ years	Data Rich	-1	3	Sanitation (proportion with access)	152
Oesophageal cancer	Female	20-24 years	95+ years	Data Rich	-1	3	Education (years per capita)	631
Oesophageal cancer	Female	20-24 years	95+ years	Data Rich	1	3	LDI (I$ per capita)	--
Oesophageal cancer	Female	20-24 years	95+ years	Data Rich	1	3	Socio-demographic Index	--
Oesophageal cancer	Female	20-24 years	95+ years	Global	1	1	Smoking Prevalence	1000
Oesophageal cancer	Female	20-24 years	95+ years	Global	1	1	Liters of alcohol consumed per capita	--
Oesophageal cancer	Female	20-24 years	95+ years	Global	1	1	Mean BMI	--
Oesophageal cancer	Female	20-24 years	95+ years	Global	-1	2	Healthcare access and quality index	--
Oesophageal cancer	Female	20-24 years	95+ years	Global	1	2	Age- and sex-specific SEV for Low fruit	538
Oesophageal cancer	Female	20-24 years	95+ years	Global	1	2	Tobacco (cigarettes per capita)	558
Oesophageal cancer	Female	20-24 years	95+ years	Global	1	2	Indoor Air Pollution (All Cooking Fuels)	635
Oesophageal cancer	Female	20-24 years	95+ years	Global	1	2	Age- and sex-specific SEV for Low vegetables	642
Oesophageal cancer	Female	20-24 years	95+ years	Global	-1	3	Education (years per capita)	313
Oesophageal cancer	Female	20-24 years	95+ years	Global	-1	3	Improved Water Source (proportion with access)	511
Oesophageal cancer	Female	20-24 years	95+ years	Global	-1	3	Sanitation (proportion with access)	528
Oesophageal cancer	Female	20-24 years	95+ years	Global	1	3	LDI (I$ per capita)	--
Oesophageal cancer	Female	20-24 years	95+ years	Global	1	3	Socio-demographic Index	--
Oesophageal cancer	Male	20-24 years	95+ years	Data Rich	1	1	Smoking Prevalence	250
Oesophageal cancer	Male	20-24 years	95+ years	Data Rich	1	1	Liters of alcohol consumed per capita	1000
Oesophageal cancer	Male	20-24 years	95+ years	Data Rich	1	1	Mean BMI	--
Oesophageal cancer	Male	20-24 years	95+ years	Data Rich	-1	2	Healthcare access and quality index	186
Oesophageal cancer	Male	20-24 years	95+ years	Data Rich	1	2	Age- and sex-specific SEV for Low vegetables	186
Oesophageal cancer	Male	20-24 years	95+ years	Data Rich	1	2	Tobacco (cigarettes per capita)	186
Oesophageal cancer	Male	20-24 years	95+ years	Data Rich	1	2	Age- and sex-specific SEV for Low fruit	--
Oesophageal cancer	Male	20-24 years	95+ years	Data Rich	1	2	Indoor Air Pollution (All Cooking Fuels)	--
Oesophageal cancer	Male	20-24 years	95+ years	Data Rich	-1	3	Improved Water Source (proportion with access)	321
Oesophageal cancer	Male	20-24 years	95+ years	Data Rich	-1	3	Education (years per capita)	--
Oesophageal cancer	Male	20-24 years	95+ years	Data Rich	-1	3	Sanitation (proportion with access)	--
Oesophageal cancer	Male	20-24 years	95+ years	Data Rich	1	3	Socio-demographic Index	108
Oesophageal cancer	Male	20-24 years	95+ years	Data Rich	1	3	LDI (I$ per capita)	--
Oesophageal cancer	Male	20-24 years	95+ years	Global	1	1	Mean BMI	164
Oesophageal cancer	Male	20-24 years	95+ years	Global	1	1	Smoking Prevalence	1000
Oesophageal cancer	Male	20-24 years	95+ years	Global	1	1	Liters of alcohol consumed per capita	--
Oesophageal cancer	Male	20-24 years	95+ years	Global	-1	2	Healthcare access and quality index	648
Oesophageal cancer	Male	20-24 years	95+ years	Global	1	2	Indoor Air Pollution (All Cooking Fuels)	211
Oesophageal cancer	Male	20-24 years	95+ years	Global	1	2	Age- and sex-specific SEV for Low vegetables	329
Oesophageal cancer	Male	20-24 years	95+ years	Global	1	2	Age- and sex-specific SEV for Low fruit	594
Oesophageal cancer	Male	20-24 years	95+ years	Global	1	2	Tobacco (cigarettes per capita)	--
Oesophageal cancer	Male	20-24 years	95+ years	Global	-1	3	Sanitation (proportion with access)	128
Oesophageal cancer	Male	20-24 years	95+ years	Global	-1	3	Improved Water Source (proportion with access)	142
Oesophageal cancer	Male	20-24 years	95+ years	Global	-1	3	Education (years per capita)	161
Oesophageal cancer	Male	20-24 years	95+ years	Global	1	3	Socio-demographic Index	144
Oesophageal cancer	Male	20-24 years	95+ years	Global	1	3	LDI (I$ per capita)	--
Stomach cancer	Female	15-19 years	95+ years	Data Rich	1	1	Tobacco (cigarettes per capita)	475
Stomach cancer	Female	15-19 years	95+ years	Data Rich	1	1	Diet high in sodium	1000
Stomach cancer	Female	15-19 years	95+ years	Data Rich	-1	2	Sanitation (proportion with access)	98
Stomach cancer	Female	15-19 years	95+ years	Data Rich	-1	2	Improved Water Source (proportion with access)	117
Stomach cancer	Female	15-19 years	95+ years	Data Rich	-1	2	Healthcare access and quality index	476
Stomach cancer	Female	15-19 years	95+ years	Data Rich	1	2	Age- and sex-specific SEV for Unsafe sanitation	7
Stomach cancer	Female	15-19 years	95+ years	Data Rich	1	2	Age- and sex-specific SEV for Unsafe water	113
Stomach cancer	Female	15-19 years	95+ years	Data Rich	1	2	Cumulative Cigarettes (20 Years)	--
Stomach cancer	Female	15-19 years	95+ years	Data Rich	1	2	Mean BMI	--
Stomach cancer	Female	15-19 years	95+ years	Data Rich	-1	3	Socio-demographic Index	444
Stomach cancer	Female	15-19 years	95+ years	Data Rich	-1	3	Education (years per capita)	648
Stomach cancer	Female	15-19 years	95+ years	Data Rich	1	3	Age- and sex-specific SEV for Low fruit	75
Stomach cancer	Female	15-19 years	95+ years	Data Rich	1	3	Age- and sex-specific SEV for Low vegetables	423
Stomach cancer	Female	15-19 years	95+ years	Data Rich	1	3	LDI (I$ per capita)	--
Stomach cancer	Female	15-19 years	95+ years	Global	1	1	Tobacco (cigarettes per capita)	523
Stomach cancer	Female	15-19 years	95+ years	Global	1	1	Diet high in sodium	842
Stomach cancer	Female	15-19 years	95+ years	Global	-1	2	Sanitation (proportion with access)	201
Stomach cancer	Female	15-19 years	95+ years	Global	-1	2	Improved Water Source (proportion with access)	235
Stomach cancer	Female	15-19 years	95+ years	Global	-1	2	Healthcare access and quality index	357
Stomach cancer	Female	15-19 years	95+ years	Global	1	2	Age- and sex-specific SEV for Unsafe water	43
Stomach cancer	Female	15-19 years	95+ years	Global	1	2	Cumulative Cigarettes (20 Years)	442
Stomach cancer	Female	15-19 years	95+ years	Global	1	2	Age- and sex-specific SEV for Unsafe sanitation	558
Stomach cancer	Female	15-19 years	95+ years	Global	1	2	Mean BMI	--
Stomach cancer	Female	15-19 years	95+ years	Global	-1	3	Socio-demographic Index	216
Stomach cancer	Female	15-19 years	95+ years	Global	-1	3	Education (years per capita)	407
Stomach cancer	Female	15-19 years	95+ years	Global	1	3	Age- and sex-specific SEV for Low vegetables	193
Stomach cancer	Female	15-19 years	95+ years	Global	1	3	Age- and sex-specific SEV for Low fruit	250
Stomach cancer	Female	15-19 years	95+ years	Global	1	3	LDI (I$ per capita)	--
Stomach cancer	Male	15-19 years	95+ years	Data Rich	1	1	Tobacco (cigarettes per capita)	1000
Stomach cancer	Male	15-19 years	95+ years	Data Rich	1	1	Diet high in sodium	--
Stomach cancer	Male	15-19 years	95+ years	Data Rich	-1	2	Sanitation (proportion with access)	52
Stomach cancer	Male	15-19 years	95+ years	Data Rich	-1	2	Improved Water Source (proportion with access)	211
Stomach cancer	Male	15-19 years	95+ years	Data Rich	-1	2	Healthcare access and quality index	631
Stomach cancer	Male	15-19 years	95+ years	Data Rich	1	2	Age- and sex-specific SEV for Unsafe sanitation	211
Stomach cancer	Male	15-19 years	95+ years	Data Rich	1	2	Age- and sex-specific SEV for Unsafe water	211
Stomach cancer	Male	15-19 years	95+ years	Data Rich	1	2	Cumulative Cigarettes (20 Years)	790
Stomach cancer	Male	15-19 years	95+ years	Data Rich	1	2	Mean BMI	--
Stomach cancer	Male	15-19 years	95+ years	Data Rich	-1	3	Socio-demographic Index	273
Stomach cancer	Male	15-19 years	95+ years	Data Rich	-1	3	Education (years per capita)	390
Stomach cancer	Male	15-19 years	95+ years	Data Rich	1	3	Age- and sex-specific SEV for Low fruit	90
Stomach cancer	Male	15-19 years	95+ years	Data Rich	1	3	Age- and sex-specific SEV for Low vegetables	118
Stomach cancer	Male	15-19 years	95+ years	Data Rich	1	3	LDI (I$ per capita)	--
Stomach cancer	Male	15-19 years	95+ years	Global	1	1	Diet high in sodium	431
Stomach cancer	Male	15-19 years	95+ years	Global	1	1	Tobacco (cigarettes per capita)	1000
Stomach cancer	Male	15-19 years	95+ years	Global	-1	2	Improved Water Source (proportion with access)	376
Stomach cancer	Male	15-19 years	95+ years	Global	-1	2	Healthcare access and quality index	392
Stomach cancer	Male	15-19 years	95+ years	Global	-1	2	Sanitation (proportion with access)	396
Stomach cancer	Male	15-19 years	95+ years	Global	1	2	Age- and sex-specific SEV for Unsafe sanitation	376
Stomach cancer	Male	15-19 years	95+ years	Global	1	2	Age- and sex-specific SEV for Unsafe water	412
Stomach cancer	Male	15-19 years	95+ years	Global	1	2	Cumulative Cigarettes (20 Years)	--
Stomach cancer	Male	15-19 years	95+ years	Global	1	2	Mean BMI	--
Stomach cancer	Male	15-19 years	95+ years	Global	-1	3	Education (years per capita)	243
Stomach cancer	Male	15-19 years	95+ years	Global	-1	3	Socio-demographic Index	--
Stomach cancer	Male	15-19 years	95+ years	Global	1	3	Age- and sex-specific SEV for Low vegetables	63
Stomach cancer	Male	15-19 years	95+ years	Global	1	3	Age- and sex-specific SEV for Low fruit	199
Stomach cancer	Male	15-19 years	95+ years	Global	1	3	LDI (I$ per capita)	--

Appendix Table S10: CODEm covariates used, level of covariate, and expected direction of covariate by cause, sex, and age

Cause	Sex	Age Start	Age End	Model Version Type	Direction	Level	Covariate Name	Number of Draws
Colon and rectum cancer	Female	15-19 years	95+ years	Data Rich	-1	1	Total Physical Activity (MET-min/week), Age-specific	--
Colon and rectum cancer	Female	15-19 years	95+ years	Data Rich	1	1	Mean BMI	52
Colon and rectum cancer	Female	15-19 years	95+ years	Data Rich	1	1	Tobacco (cigarettes per capita)	242
Colon and rectum cancer	Female	15-19 years	95+ years	Data Rich	1	1	Age- and sex-specific SEV for High red meat	889
Colon and rectum cancer	Female	15-19 years	95+ years	Data Rich	1	1	Log-transformed SEV scalar: Colorect C	--
Colon and rectum cancer	Female	15-19 years	95+ years	Data Rich	-1	2	pufa adjusted(percent)	--
Colon and rectum cancer	Female	15-19 years	95+ years	Data Rich	1	2	Cumulative Cigarettes (5 Years)	7
Colon and rectum cancer	Female	15-19 years	95+ years	Data Rich	1	2	Diabetes Fasting Plasma Glucose (mmol/L), age-standardized 25+	50
Colon and rectum cancer	Female	15-19 years	95+ years	Data Rich	1	2	Age- and sex-specific SEV for Low fiber	322
Colon and rectum cancer	Female	15-19 years	95+ years	Data Rich	1	2	Age- and sex-specific SEV for Low vegetables	329
Colon and rectum cancer	Female	15-19 years	95+ years	Data Rich	1	2	Liters of alcohol consumed per capita	338
Colon and rectum cancer	Female	15-19 years	95+ years	Data Rich	1	2	Age- and sex-specific SEV for Low calcium	--
Colon and rectum cancer	Female	15-19 years	95+ years	Data Rich	-1	3	Healthcare access and quality index	23
Colon and rectum cancer	Female	15-19 years	95+ years	Data Rich	-1	3	Education (years per capita)	251
Colon and rectum cancer	Female	15-19 years	95+ years	Data Rich	1	3	Socio-demographic Index	123
Colon and rectum cancer	Female	15-19 years	95+ years	Data Rich	1	3	Age- and sex-specific SEV for Low nuts and seeds	251
Colon and rectum cancer	Female	15-19 years	95+ years	Data Rich	1	3	Age- and sex-specific SEV for Low fruit	356
Colon and rectum cancer	Female	15-19 years	95+ years	Data Rich	1	3	Age- and sex-specific SEV for Low milk	419
Colon and rectum cancer	Female	15-19 years	95+ years	Data Rich	1	3	LDI (I$ per capita)	--
Colon and rectum cancer	Female	15-19 years	95+ years	Global	-1	1	Total Physical Activity (MET-min/week), Age-specific	--
Colon and rectum cancer	Female	15-19 years	95+ years	Global	1	1	Age- and sex-specific SEV for High red meat	527
Colon and rectum cancer	Female	15-19 years	95+ years	Global	1	1	Tobacco (cigarettes per capita)	1000
Colon and rectum cancer	Female	15-19 years	95+ years	Global	1	1	Log-transformed SEV scalar: Colorect C	--
Colon and rectum cancer	Female	15-19 years	95+ years	Global	1	1	Mean BMI	--
Colon and rectum cancer	Female	15-19 years	95+ years	Global	-1	2	pufa adjusted(percent)	--
Colon and rectum cancer	Female	15-19 years	95+ years	Global	1	2	Age- and sex-specific SEV for Low vegetables	750
Colon and rectum cancer	Female	15-19 years	95+ years	Global	1	2	Cumulative Cigarettes (5 Years)	750
Colon and rectum cancer	Female	15-19 years	95+ years	Global	1	2	Liters of alcohol consumed per capita	750
Colon and rectum cancer	Female	15-19 years	95+ years	Global	1	2	Age- and sex-specific SEV for Low calcium	--
Colon and rectum cancer	Female	15-19 years	95+ years	Global	1	2	Age- and sex-specific SEV for Low fiber	--
Colon and rectum cancer	Female	15-19 years	95+ years	Global	1	2	Diabetes Fasting Plasma Glucose (mmol/L), age-standardized 25+	--
Colon and rectum cancer	Female	15-19 years	95+ years	Global	-1	3	Healthcare access and quality index	276
Colon and rectum cancer	Female	15-19 years	95+ years	Global	-1	3	Education (years per capita)	344
Colon and rectum cancer	Female	15-19 years	95+ years	Global	1	3	Socio-demographic Index	87
Colon and rectum cancer	Female	15-19 years	95+ years	Global	1	3	Age- and sex-specific SEV for Low fruit	381
Colon and rectum cancer	Female	15-19 years	95+ years	Global	1	3	Age- and sex-specific SEV for Low milk	--
Colon and rectum cancer	Female	15-19 years	95+ years	Global	1	3	Age- and sex-specific SEV for Low nuts and seeds	--
Colon and rectum cancer	Female	15-19 years	95+ years	Global	1	3	LDI (I$ per capita)	--
Colon and rectum cancer	Male	15-19 years	95+ years	Data Rich	-1	1	Total Physical Activity (MET-min/week), Age-specific	--
Colon and rectum cancer	Male	15-19 years	95+ years	Data Rich	1	1	Mean BMI	116
Colon and rectum cancer	Male	15-19 years	95+ years	Data Rich	1	1	Tobacco (cigarettes per capita)	412
Colon and rectum cancer	Male	15-19 years	95+ years	Data Rich	1	1	Age- and sex-specific SEV for High red meat	775
Colon and rectum cancer	Male	15-19 years	95+ years	Data Rich	1	1	Log-transformed SEV scalar: Colorect C	--
Colon and rectum cancer	Male	15-19 years	95+ years	Data Rich	-1	2	pufa adjusted(percent)	--
Colon and rectum cancer	Male	15-19 years	95+ years	Data Rich	1	2	Diabetes Fasting Plasma Glucose (mmol/L), age-standardized 25+	45
Colon and rectum cancer	Male	15-19 years	95+ years	Data Rich	1	2	Cumulative Cigarettes (5 Years)	54
Colon and rectum cancer	Male	15-19 years	95+ years	Data Rich	1	2	Liters of alcohol consumed per capita	60
Colon and rectum cancer	Male	15-19 years	95+ years	Data Rich	1	2	Age- and sex-specific SEV for Low vegetables	256
Colon and rectum cancer	Male	15-19 years	95+ years	Data Rich	1	2	Age- and sex-specific SEV for Low calcium	--
Colon and rectum cancer	Male	15-19 years	95+ years	Data Rich	1	2	Age- and sex-specific SEV for Low fiber	--
Colon and rectum cancer	Male	15-19 years	95+ years	Data Rich	-1	3	Healthcare access and quality index	136
Colon and rectum cancer	Male	15-19 years	95+ years	Data Rich	-1	3	Education (years per capita)	239
Colon and rectum cancer	Male	15-19 years	95+ years	Data Rich	1	3	Age- and sex-specific SEV for Low fruit	61
Colon and rectum cancer	Male	15-19 years	95+ years	Data Rich	1	3	Age- and sex-specific SEV for Low nuts and seeds	97
Colon and rectum cancer	Male	15-19 years	95+ years	Data Rich	1	3	Age- and sex-specific SEV for Low milk	129
Colon and rectum cancer	Male	15-19 years	95+ years	Data Rich	1	3	Socio-demographic Index	435
Colon and rectum cancer	Male	15-19 years	95+ years	Data Rich	1	3	LDI (I$ per capita)	--
Colon and rectum cancer	Male	15-19 years	95+ years	Global	-1	1	Total Physical Activity (MET-min/week), Age-specific	--
Colon and rectum cancer	Male	15-19 years	95+ years	Global	1	1	Mean BMI	148
Colon and rectum cancer	Male	15-19 years	95+ years	Global	1	1	Age- and sex-specific SEV for High red meat	420
Colon and rectum cancer	Male	15-19 years	95+ years	Global	1	1	Tobacco (cigarettes per capita)	1000
Colon and rectum cancer	Male	15-19 years	95+ years	Global	1	1	Log-transformed SEV scalar: Colorect C	--
Colon and rectum cancer	Male	15-19 years	95+ years	Global	-1	2	pufa adjusted(percent)	--
Colon and rectum cancer	Male	15-19 years	95+ years	Global	1	2	Diabetes Fasting Plasma Glucose (mmol/L), age-standardized 25+	96
Colon and rectum cancer	Male	15-19 years	95+ years	Global	1	2	Liters of alcohol consumed per capita	387
Colon and rectum cancer	Male	15-19 years	95+ years	Global	1	2	Age- and sex-specific SEV for Low calcium	--
Colon and rectum cancer	Male	15-19 years	95+ years	Global	1	2	Age- and sex-specific SEV for Low fiber	--
Colon and rectum cancer	Male	15-19 years	95+ years	Global	1	2	Age- and sex-specific SEV for Low vegetables	--
Colon and rectum cancer	Male	15-19 years	95+ years	Global	1	2	Cumulative Cigarettes (20 Years)	--
Colon and rectum cancer	Male	15-19 years	95+ years	Global	-1	3	Healthcare access and quality index	69
Colon and rectum cancer	Male	15-19 years	95+ years	Global	-1	3	Education (years per capita)	--
Colon and rectum cancer	Male	15-19 years	95+ years	Global	1	3	Age- and sex-specific SEV for Low fruit	146
Colon and rectum cancer	Male	15-19 years	95+ years	Global	1	3	Socio-demographic Index	459
Colon and rectum cancer	Male	15-19 years	95+ years	Global	1	3	Age- and sex-specific SEV for Low milk	--
Colon and rectum cancer	Male	15-19 years	95+ years	Global	1	3	Age- and sex-specific SEV for Low nuts and seeds	--
Colon and rectum cancer	Male	15-19 years	95+ years	Global	1	3	LDI (I$ per capita)	--
Liver cancer	Female	0-6 days	95+ years	Data Rich	1	1	Chronic Hepatitis C age standardized	0
Liver cancer	Female	0-6 days	95+ years	Data Rich	1	1	Hepatitis C Seroprevalence (anti-HCV) age standardized	0
Liver cancer	Female	0-6 days	95+ years	Data Rich	1	1	Hepatitis B vaccine coverage (proportion), aged through time, COVID-inclusive	26
Liver cancer	Female	0-6 days	95+ years	Data Rich	1	1	Intravenous drug use (proportion by age)	90
Liver cancer	Female	0-6 days	95+ years	Data Rich	1	1	Alcohol consumption, age standardized, in grams per day	215
Liver cancer	Female	0-6 days	95+ years	Data Rich	1	1	HIV age-standardized prevalence	248
Liver cancer	Female	0-6 days	95+ years	Data Rich	1	1	Vaccine adjusted HbSAg serporevalence age standardized	1000
Liver cancer	Female	0-6 days	95+ years	Data Rich	1	1	Liters of alcohol consumed per capita	--
Liver cancer	Female	0-6 days	95+ years	Data Rich	-1	2	Healthcare access and quality index	0
Liver cancer	Female	0-6 days	95+ years	Data Rich	-1	2	Hepatitis B vaccine coverage, aged through time, COVID-free (proportion)	16
Liver cancer	Female	0-6 days	95+ years	Data Rich	-1	2	Hepatitis B 3-dose coverage, COVID-free (proportion)	--
Liver cancer	Female	0-6 days	95+ years	Data Rich	1	2	Intravenous drug use (age-standardized proportion)	4
Liver cancer	Female	0-6 days	95+ years	Data Rich	1	2	Diabetes Fasting Plasma Glucose (mmol/L), age-standardized 25+	108
Liver cancer	Female	0-6 days	95+ years	Data Rich	1	2	Mean BMI	812
Liver cancer	Female	0-6 days	95+ years	Data Rich	1	2	Cumulative Cigarettes (20 Years)	--
Liver cancer	Female	0-6 days	95+ years	Data Rich	1	2	Tobacco (cigarettes per capita)	--
Liver cancer	Female	0-6 days	95+ years	Data Rich	-1	3	Socio-demographic Index	168

Appendix Table S10: CODEm covariates used, level of covariate, and expected direction of covariate by cause, sex, and age

Cause	Sex	Age Start	Age End	Model Version Type	Direction	Level	Covariate Name	Number of Draws
Liver cancer	Female	0-6 days	95+ years	Data Rich	-1	3	Education (years per capita)	329
Liver cancer	Female	0-6 days	95+ years	Data Rich	-1	3	LDI (I$ per capita)	--
Liver cancer	Female	0-6 days	95+ years	Data Rich	1	3	Age- and sex-specific SEV for High red meat	--
Liver cancer	Female	0-6 days	95+ years	Global	1	1	HIV age-standardized prevalence	229
Liver cancer	Female	0-6 days	95+ years	Global	1	1	Vaccine adjusted HbSAg serporevalence age standardized	860
Liver cancer	Female	0-6 days	95+ years	Global	1	1	Hepatitis C Seroprevalence (anti-HCV) age standardized	1000
Liver cancer	Female	0-6 days	95+ years	Global	1	1	Liters of alcohol consumed per capita	--
Liver cancer	Female	0-6 days	95+ years	Global	-1	2	Hepatitis B vaccine coverage, aged through time, COVID-free (proportion)	105
Liver cancer	Female	0-6 days	95+ years	Global	-1	2	Healthcare access and quality index	432
Liver cancer	Female	0-6 days	95+ years	Global	-1	2	Hepatitis B 3-dose coverage, COVID-free (proportion)	--
Liver cancer	Female	0-6 days	95+ years	Global	1	2	Mean BMI	228
Liver cancer	Female	0-6 days	95+ years	Global	1	2	Intravenous drug use (age-standardized proportion)	489
Liver cancer	Female	0-6 days	95+ years	Global	1	2	Diabetes Fasting Plasma Glucose (mmol/L), age-standardized 25+	555
Liver cancer	Female	0-6 days	95+ years	Global	1	2	Cumulative Cigarettes (20 Years)	--
Liver cancer	Female	0-6 days	95+ years	Global	1	2	Tobacco (cigarettes per capita)	--
Liver cancer	Female	0-6 days	95+ years	Global	-1	3	Education (years per capita)	162
Liver cancer	Female	0-6 days	95+ years	Global	-1	3	Socio-demographic Index	211
Liver cancer	Female	0-6 days	95+ years	Global	-1	3	LDI (I$ per capita)	--
Liver cancer	Female	0-6 days	95+ years	Global	1	3	Age- and sex-specific SEV for High red meat	439
Liver cancer	Male	0-6 days	95+ years	Data Rich	1	1	Hepatitis C Seroprevalence (anti-HCV) age standardized	72
Liver cancer	Male	0-6 days	95+ years	Data Rich	1	1	Vaccine adjusted HbSAg serporevalence age standardized	748
Liver cancer	Male	0-6 days	95+ years	Data Rich	1	1	HIV age-standardized prevalence	817
Liver cancer	Male	0-6 days	95+ years	Data Rich	1	1	Liters of alcohol consumed per capita	--
Liver cancer	Male	0-6 days	95+ years	Data Rich	-1	2	Healthcare access and quality index	--
Liver cancer	Male	0-6 days	95+ years	Data Rich	-1	2	Hepatitis B 3-dose coverage, COVID-free (proportion)	--
Liver cancer	Male	0-6 days	95+ years	Data Rich	-1	2	Hepatitis B vaccine coverage, aged through time, COVID-free (proportion)	--
Liver cancer	Male	0-6 days	95+ years	Data Rich	1	2	Intravenous drug use (age-standardized proportion)	35
Liver cancer	Male	0-6 days	95+ years	Data Rich	1	2	Diabetes Fasting Plasma Glucose (mmol/L), age-standardized 25+	40
Liver cancer	Male	0-6 days	95+ years	Data Rich	1	2	Mean BMI	952
Liver cancer	Male	0-6 days	95+ years	Data Rich	1	2	Cumulative Cigarettes (20 Years)	--
Liver cancer	Male	0-6 days	95+ years	Data Rich	1	2	Tobacco (cigarettes per capita)	--
Liver cancer	Male	0-6 days	95+ years	Data Rich	-1	3	Education (years per capita)	205
Liver cancer	Male	0-6 days	95+ years	Data Rich	-1	3	Socio-demographic Index	351
Liver cancer	Male	0-6 days	95+ years	Data Rich	-1	3	LDI (I$ per capita)	--
Liver cancer	Male	0-6 days	95+ years	Data Rich	1	3	Age- and sex-specific SEV for High red meat	0
Liver cancer	Male	0-6 days	95+ years	Global	1	1	HIV age-standardized prevalence	108
Liver cancer	Male	0-6 days	95+ years	Global	1	1	Hepatitis C Seroprevalence (anti-HCV) age standardized	499
Liver cancer	Male	0-6 days	95+ years	Global	1	1	Vaccine adjusted HbSAg serporevalence age standardized	754
Liver cancer	Male	0-6 days	95+ years	Global	1	1	Liters of alcohol consumed per capita	--
Liver cancer	Male	0-6 days	95+ years	Global	-1	2	Hepatitis B vaccine coverage, aged through time, COVID-free (proportion)	10
Liver cancer	Male	0-6 days	95+ years	Global	-1	2	Healthcare access and quality index	545
Liver cancer	Male	0-6 days	95+ years	Global	-1	2	Hepatitis B 3-dose coverage, COVID-free (proportion)	--
Liver cancer	Male	0-6 days	95+ years	Global	1	2	Mean BMI	460
Liver cancer	Male	0-6 days	95+ years	Global	1	2	Diabetes Fasting Plasma Glucose (mmol/L), age-standardized 25+	672
Liver cancer	Male	0-6 days	95+ years	Global	1	2	Intravenous drug use (age-standardized proportion)	676
Liver cancer	Male	0-6 days	95+ years	Global	1	2	Cumulative Cigarettes (20 Years)	--
Liver cancer	Male	0-6 days	95+ years	Global	1	2	Tobacco (cigarettes per capita)	--
Liver cancer	Male	0-6 days	95+ years	Global	-1	3	Socio-demographic Index	21
Liver cancer	Male	0-6 days	95+ years	Global	-1	3	Education (years per capita)	166
Liver cancer	Male	0-6 days	95+ years	Global	-1	3	LDI (I$ per capita)	--
Liver cancer	Male	0-6 days	95+ years	Global	1	3	Age- and sex-specific SEV for High red meat	201
Gallbladder and biliary tract cancer	Female	20-24 years	95+ years	Data Rich	1	1	Log-transformed SEV scalar: Gallblad C	--
Gallbladder and biliary tract cancer	Female	20-24 years	95+ years	Data Rich	1	1	Mean BMI	--
Gallbladder and biliary tract cancer	Female	20-24 years	95+ years	Data Rich	-1	2	Healthcare access and quality index	777
Gallbladder and biliary tract cancer	Female	20-24 years	95+ years	Data Rich	1	2	Tobacco (cigarettes per capita)	186
Gallbladder and biliary tract cancer	Female	20-24 years	95+ years	Data Rich	1	2	Smoking Prevalence	591
Gallbladder and biliary tract cancer	Female	20-24 years	95+ years	Data Rich	1	2	Liters of alcohol consumed per capita	777
Gallbladder and biliary tract cancer	Female	20-24 years	95+ years	Data Rich	1	2	Age- and sex-specific SEV for Low fruit	--
Gallbladder and biliary tract cancer	Female	20-24 years	95+ years	Data Rich	1	2	Age- and sex-specific SEV for Low vegetables	--
Gallbladder and biliary tract cancer	Female	20-24 years	95+ years	Data Rich	1	2	Cumulative Cigarettes (10 Years)	--
Gallbladder and biliary tract cancer	Female	20-24 years	95+ years	Data Rich	1	2	Cumulative Cigarettes (5 Years)	--
Gallbladder and biliary tract cancer	Female	20-24 years	95+ years	Data Rich	1	2	Diabetes Age-Standardized Prevalence (proportion)	--
Gallbladder and biliary tract cancer	Female	20-24 years	95+ years	Data Rich	-1	3	Socio-demographic Index	223
Gallbladder and biliary tract cancer	Female	20-24 years	95+ years	Data Rich	-1	3	Education (years per capita)	323
Gallbladder and biliary tract cancer	Female	20-24 years	95+ years	Data Rich	1	3	LDI (I$ per capita)	--
Gallbladder and biliary tract cancer	Female	20-24 years	95+ years	Global	1	1	Log-transformed SEV scalar: Gallblad C	--
Gallbladder and biliary tract cancer	Female	20-24 years	95+ years	Global	1	1	Mean BMI	--
Gallbladder and biliary tract cancer	Female	20-24 years	95+ years	Global	-1	2	Healthcare access and quality index	782
Gallbladder and biliary tract cancer	Female	20-24 years	95+ years	Global	1	2	Age- and sex-specific SEV for Low fruit	153
Gallbladder and biliary tract cancer	Female	20-24 years	95+ years	Global	1	2	Smoking Prevalence	380
Gallbladder and biliary tract cancer	Female	20-24 years	95+ years	Global	1	2	Cumulative Cigarettes (5 Years)	439
Gallbladder and biliary tract cancer	Female	20-24 years	95+ years	Global	1	2	Cumulative Cigarettes (10 Years)	496
Gallbladder and biliary tract cancer	Female	20-24 years	95+ years	Global	1	2	Tobacco (cigarettes per capita)	555
Gallbladder and biliary tract cancer	Female	20-24 years	95+ years	Global	1	2	Age- and sex-specific SEV for Low vegetables	--
Gallbladder and biliary tract cancer	Female	20-24 years	95+ years	Global	1	2	Diabetes Age-Standardized Prevalence (proportion)	--
Gallbladder and biliary tract cancer	Female	20-24 years	95+ years	Global	1	2	Liters of alcohol consumed per capita	--
Gallbladder and biliary tract cancer	Female	20-24 years	95+ years	Global	-1	3	Socio-demographic Index	65
Gallbladder and biliary tract cancer	Female	20-24 years	95+ years	Global	-1	3	Education (years per capita)	316
Gallbladder and biliary tract cancer	Female	20-24 years	95+ years	Global	1	3	LDI (I$ per capita)	--
Gallbladder and biliary tract cancer	Male	20-24 years	95+ years	Data Rich	1	1	Log-transformed SEV scalar: Gallblad C	--
Gallbladder and biliary tract cancer	Male	20-24 years	95+ years	Data Rich	1	1	Mean BMI	--
Gallbladder and biliary tract cancer	Male	20-24 years	95+ years	Data Rich	-1	2	Healthcare access and quality index	250
Gallbladder and biliary tract cancer	Male	20-24 years	95+ years	Data Rich	1	2	Cumulative Cigarettes (5 Years)	250
Gallbladder and biliary tract cancer	Male	20-24 years	95+ years	Data Rich	1	2	Age- and sex-specific SEV for Low vegetables	507
Gallbladder and biliary tract cancer	Male	20-24 years	95+ years	Data Rich	1	2	Cumulative Cigarettes (10 Years)	507
Gallbladder and biliary tract cancer	Male	20-24 years	95+ years	Data Rich	1	2	Age- and sex-specific SEV for Low fruit	757
Gallbladder and biliary tract cancer	Male	20-24 years	95+ years	Data Rich	1	2	Liters of alcohol consumed per capita	757
Gallbladder and biliary tract cancer	Male	20-24 years	95+ years	Data Rich	1	2	Smoking Prevalence	757
Gallbladder and biliary tract cancer	Male	20-24 years	95+ years	Data Rich	1	2	Diabetes Age-Standardized Prevalence (proportion)	--
Gallbladder and biliary tract cancer	Male	20-24 years	95+ years	Data Rich	1	2	Tobacco (cigarettes per capita)	--
Gallbladder and biliary tract cancer	Male	20-24 years	95+ years	Data Rich	-1	3	Education (years per capita)	243
Gallbladder and biliary tract cancer	Male	20-24 years	95+ years	Data Rich	-1	3	Socio-demographic Index	537
Gallbladder and biliary tract cancer	Male	20-24 years	95+ years	Data Rich	1	3	LDI (I$ per capita)	--
Gallbladder and biliary tract cancer	Male	20-24 years	95+ years	Global	1	1	Log-transformed SEV scalar: Gallblad C	--
Gallbladder and biliary tract cancer	Male	20-24 years	95+ years	Global	1	1	Mean BMI	--
Gallbladder and biliary tract cancer	Male	20-24 years	95+ years	Global	-1	2	Healthcare access and quality index	--
Gallbladder and biliary tract cancer	Male	20-24 years	95+ years	Global	1	2	Cumulative Cigarettes (10 Years)	160
Gallbladder and biliary tract cancer	Male	20-24 years	95+ years	Global	1	2	Cumulative Cigarettes (5 Years)	620
Gallbladder and biliary tract cancer	Male	20-24 years	95+ years	Global	1	2	Smoking Prevalence	780

Appendix Table S10: CODEm covariates used, level of covariate, and expected direction of covariate by cause, sex, and age								
**Cause**	**Sex**	**Age Start**	**Age End**	**Model Version Type**	**Direction**	**Level**	**Covariate Name**	**Number of Draws**
Gallbladder and biliary tract cancer	Male	20-24 years	95+ years	Global	1	2	Age- and sex-specific SEV for Low fruit	--
Gallbladder and biliary tract cancer	Male	20-24 years	95+ years	Global	1	2	Age- and sex-specific SEV for Low vegetables	--
Gallbladder and biliary tract cancer	Male	20-24 years	95+ years	Global	1	2	Diabetes Age-Standardized Prevalence (proportion)	--
Gallbladder and biliary tract cancer	Male	20-24 years	95+ years	Global	1	2	Liters of alcohol consumed per capita	--
Gallbladder and biliary tract cancer	Male	20-24 years	95+ years	Global	1	2	Tobacco (cigarettes per capita)	--
Gallbladder and biliary tract cancer	Male	20-24 years	95+ years	Global	-1	3	Socio-demographic Index	323
Gallbladder and biliary tract cancer	Male	20-24 years	95+ years	Global	-1	3	Education (years per capita)	344
Gallbladder and biliary tract cancer	Male	20-24 years	95+ years	Global	1	3	LDI (I$ per capita)	--
Pancreatic cancer	Female	15-19 years	95+ years	Data Rich	1	1	Cumulative Cigarettes (10 Years)	350
Pancreatic cancer	Female	15-19 years	95+ years	Data Rich	1	1	Cumulative Cigarettes (20 Years)	849
Pancreatic cancer	Female	15-19 years	95+ years	Data Rich	1	1	Mean BMI	932
Pancreatic cancer	Female	15-19 years	95+ years	Data Rich	1	1	Tobacco (cigarettes per capita)	--
Pancreatic cancer	Female	15-19 years	95+ years	Data Rich	-1	2	Healthcare access and quality index	--
Pancreatic cancer	Female	15-19 years	95+ years	Data Rich	1	2	Age- and sex-specific SEV for High red meat	39
Pancreatic cancer	Female	15-19 years	95+ years	Data Rich	1	2	Diabetes Age-Standardized Prevalence (proportion)	166
Pancreatic cancer	Female	15-19 years	95+ years	Data Rich	1	2	Diabetes Fasting Plasma Glucose (mmol/L), age-standardized 25+	166
Pancreatic cancer	Female	15-19 years	95+ years	Data Rich	1	2	Liters of alcohol consumed per capita	--
Pancreatic cancer	Female	15-19 years	95+ years	Data Rich	1	2	energy unadjusted(kcal)	--
Pancreatic cancer	Female	15-19 years	95+ years	Data Rich	-1	3	Education (years per capita)	--
Pancreatic cancer	Female	15-19 years	95+ years	Data Rich	1	3	Socio-demographic Index	51
Pancreatic cancer	Female	15-19 years	95+ years	Data Rich	1	3	Age- and sex-specific SEV for Low fruit	--
Pancreatic cancer	Female	15-19 years	95+ years	Data Rich	1	3	Age- and sex-specific SEV for Low vegetables	--
Pancreatic cancer	Female	15-19 years	95+ years	Data Rich	1	3	LDI (I$ per capita)	--
Pancreatic cancer	Female	15-19 years	95+ years	Global	1	1	Cumulative Cigarettes (10 Years)	18
Pancreatic cancer	Female	15-19 years	95+ years	Global	1	1	Mean BMI	293
Pancreatic cancer	Female	15-19 years	95+ years	Global	1	1	Tobacco (cigarettes per capita)	330
Pancreatic cancer	Female	15-19 years	95+ years	Global	1	1	Cumulative Cigarettes (20 Years)	742
Pancreatic cancer	Female	15-19 years	95+ years	Global	-1	2	Healthcare access and quality index	--
Pancreatic cancer	Female	15-19 years	95+ years	Global	1	2	Age- and sex-specific SEV for High red meat	4
Pancreatic cancer	Female	15-19 years	95+ years	Global	1	2	Diabetes Fasting Plasma Glucose (mmol/L), age-standardized 25+	12
Pancreatic cancer	Female	15-19 years	95+ years	Global	1	2	Diabetes Age-Standardized Prevalence (proportion)	333
Pancreatic cancer	Female	15-19 years	95+ years	Global	1	2	Liters of alcohol consumed per capita	523
Pancreatic cancer	Female	15-19 years	95+ years	Global	1	2	energy unadjusted(kcal)	550
Pancreatic cancer	Female	15-19 years	95+ years	Global	-1	3	Education (years per capita)	--
Pancreatic cancer	Female	15-19 years	95+ years	Global	1	3	Age- and sex-specific SEV for Low vegetables	180
Pancreatic cancer	Female	15-19 years	95+ years	Global	1	3	Socio-demographic Index	568
Pancreatic cancer	Female	15-19 years	95+ years	Global	1	3	Age- and sex-specific SEV for Low fruit	--
Pancreatic cancer	Female	15-19 years	95+ years	Global	1	3	LDI (I$ per capita)	--
Pancreatic cancer	Male	15-19 years	95+ years	Data Rich	1	1	Tobacco (cigarettes per capita)	77
Pancreatic cancer	Male	15-19 years	95+ years	Data Rich	1	1	Mean BMI	221
Pancreatic cancer	Male	15-19 years	95+ years	Data Rich	1	1	Cumulative Cigarettes (20 Years)	231
Pancreatic cancer	Male	15-19 years	95+ years	Data Rich	1	1	Cumulative Cigarettes (10 Years)	507
Pancreatic cancer	Male	15-19 years	95+ years	Data Rich	-1	2	Healthcare access and quality index	1
Pancreatic cancer	Male	15-19 years	95+ years	Data Rich	1	2	Liters of alcohol consumed per capita	2
Pancreatic cancer	Male	15-19 years	95+ years	Data Rich	1	2	Diabetes Fasting Plasma Glucose (mmol/L), age-standardized 25+	30
Pancreatic cancer	Male	15-19 years	95+ years	Data Rich	1	2	Age- and sex-specific SEV for High red meat	45
Pancreatic cancer	Male	15-19 years	95+ years	Data Rich	1	2	Diabetes Age-Standardized Prevalence (proportion)	125
Pancreatic cancer	Male	15-19 years	95+ years	Data Rich	1	2	energy unadjusted(kcal)	138
Pancreatic cancer	Male	15-19 years	95+ years	Data Rich	-1	3	Education (years per capita)	--
Pancreatic cancer	Male	15-19 years	95+ years	Data Rich	1	3	Age- and sex-specific SEV for Low vegetables	137
Pancreatic cancer	Male	15-19 years	95+ years	Data Rich	1	3	Socio-demographic Index	812
Pancreatic cancer	Male	15-19 years	95+ years	Data Rich	1	3	Age- and sex-specific SEV for Low fruit	--
Pancreatic cancer	Male	15-19 years	95+ years	Data Rich	1	3	LDI (I$ per capita)	--
Pancreatic cancer	Male	15-19 years	95+ years	Global	1	1	Tobacco (cigarettes per capita)	99
Pancreatic cancer	Male	15-19 years	95+ years	Global	1	1	Cumulative Cigarettes (20 Years)	350
Pancreatic cancer	Male	15-19 years	95+ years	Global	1	1	Cumulative Cigarettes (10 Years)	572
Pancreatic cancer	Male	15-19 years	95+ years	Global	1	1	Mean BMI	594
Pancreatic cancer	Male	15-19 years	95+ years	Global	-1	2	Healthcare access and quality index	--
Pancreatic cancer	Male	15-19 years	95+ years	Global	1	2	Diabetes Fasting Plasma Glucose (mmol/L), age-standardized 25+	0
Pancreatic cancer	Male	15-19 years	95+ years	Global	1	2	Age- and sex-specific SEV for High red meat	68
Pancreatic cancer	Male	15-19 years	95+ years	Global	1	2	Diabetes Age-Standardized Prevalence (proportion)	250
Pancreatic cancer	Male	15-19 years	95+ years	Global	1	2	Liters of alcohol consumed per capita	384
Pancreatic cancer	Male	15-19 years	95+ years	Global	1	2	energy unadjusted(kcal)	451
Pancreatic cancer	Male	15-19 years	95+ years	Global	-1	3	Education (years per capita)	--
Pancreatic cancer	Male	15-19 years	95+ years	Global	1	3	Socio-demographic Index	743
Pancreatic cancer	Male	15-19 years	95+ years	Global	1	3	Age- and sex-specific SEV for Low fruit	--
Pancreatic cancer	Male	15-19 years	95+ years	Global	1	3	Age- and sex-specific SEV for Low vegetables	--
Pancreatic cancer	Male	15-19 years	95+ years	Global	1	3	LDI (I$ per capita)	--
Larynx cancer	Female	20-24 years	95+ years	Data Rich	1	1	Liters of alcohol consumed per capita	--
Larynx cancer	Female	20-24 years	95+ years	Data Rich	-1	2	Healthcare access and quality index	--
Larynx cancer	Female	20-24 years	95+ years	Data Rich	1	2	Cumulative Cigarettes (20 Years)	269
Larynx cancer	Female	20-24 years	95+ years	Data Rich	1	2	Age- and sex-specific SEV for Low fruit	511
Larynx cancer	Female	20-24 years	95+ years	Data Rich	1	2	Smoking Prevalence	511
Larynx cancer	Female	20-24 years	95+ years	Data Rich	1	2	Asbestos consumption (metric tons per year per capita)	--
Larynx cancer	Female	20-24 years	95+ years	Data Rich	1	2	Cumulative Cigarettes (10 Years)	--
Larynx cancer	Female	20-24 years	95+ years	Data Rich	1	2	Population Density (over 1000 ppl/sqkm, proportion)	--
Larynx cancer	Female	20-24 years	95+ years	Data Rich	1	3	Age- and sex-specific SEV for Low vegetables	550
Larynx cancer	Female	20-24 years	95+ years	Data Rich	1	3	LDI (I$ per capita)	--
Larynx cancer	Female	20-24 years	95+ years	Data Rich	1	3	Socio-demographic Index	--
Larynx cancer	Female	20-24 years	95+ years	Global	1	1	Liters of alcohol consumed per capita	--
Larynx cancer	Female	20-24 years	95+ years	Global	-1	2	Healthcare access and quality index	867
Larynx cancer	Female	20-24 years	95+ years	Global	1	2	Cumulative Cigarettes (10 Years)	381
Larynx cancer	Female	20-24 years	95+ years	Global	1	2	Smoking Prevalence	381
Larynx cancer	Female	20-24 years	95+ years	Global	1	2	Cumulative Cigarettes (20 Years)	486
Larynx cancer	Female	20-24 years	95+ years	Global	1	2	Age- and sex-specific SEV for Low vegetables	--
Larynx cancer	Female	20-24 years	95+ years	Global	1	2	Asbestos consumption (metric tons per year per capita)	--
Larynx cancer	Female	20-24 years	95+ years	Global	1	2	Population Density (over 1000 ppl/sqkm, proportion)	--
Larynx cancer	Female	20-24 years	95+ years	Global	1	3	Age- and sex-specific SEV for Low fruit	133
Larynx cancer	Female	20-24 years	95+ years	Global	1	3	Socio-demographic Index	365
Larynx cancer	Female	20-24 years	95+ years	Global	1	3	LDI (I$ per capita)	--
Larynx cancer	Male	20-24 years	95+ years	Data Rich	1	1	Liters of alcohol consumed per capita	1000
Larynx cancer	Male	20-24 years	95+ years	Data Rich	-1	2	Healthcare access and quality index	587
Larynx cancer	Male	20-24 years	95+ years	Data Rich	1	2	Age- and sex-specific SEV for Low fruit	169
Larynx cancer	Male	20-24 years	95+ years	Data Rich	1	2	Asbestos consumption (metric tons per year per capita)	587
Larynx cancer	Male	20-24 years	95+ years	Data Rich	1	2	Smoking Prevalence	587
Larynx cancer	Male	20-24 years	95+ years	Data Rich	1	2	Cumulative Cigarettes (10 Years)	--
Larynx cancer	Male	20-24 years	95+ years	Data Rich	1	2	Cumulative Cigarettes (20 Years)	--
Larynx cancer	Male	20-24 years	95+ years	Data Rich	1	2	Population Density (over 1000 ppl/sqkm, proportion)	--
Larynx cancer	Male	20-24 years	95+ years	Data Rich	1	3	Socio-demographic Index	192
Larynx cancer	Male	20-24 years	95+ years	Data Rich	1	3	Age- and sex-specific SEV for Low vegetables	385

Appendix Table S10: CODEm covariates used, level of covariate, and expected direction of covariate by cause, sex, and age

Cause	Sex	Age Start	Age End	Model Version Type	Direction	Level	Covariate Name	Number of Draws
Larynx cancer	Male	20-24 years	95+ years	Data Rich	1	3	LDI (I$ per capita)	--
Larynx cancer	Male	20-24 years	95+ years	Global	1	1	Liters of alcohol consumed per capita	1000
Larynx cancer	Male	20-24 years	95+ years	Global	-1	2	Healthcare access and quality index	--
Larynx cancer	Male	20-24 years	95+ years	Global	1	2	Age- and sex-specific SEV for Low vegetables	398
Larynx cancer	Male	20-24 years	95+ years	Global	1	2	Cumulative Cigarettes (10 Years)	398
Larynx cancer	Male	20-24 years	95+ years	Global	1	2	Cumulative Cigarettes (20 Years)	398
Larynx cancer	Male	20-24 years	95+ years	Global	1	2	Smoking Prevalence	398
Larynx cancer	Male	20-24 years	95+ years	Global	1	2	Asbestos consumption (metric tons per year per capita)	--
Larynx cancer	Male	20-24 years	95+ years	Global	1	2	Population Density (over 1000 ppl/sqkm, proportion)	--
Larynx cancer	Male	20-24 years	95+ years	Global	1	3	Age- and sex-specific SEV for Low fruit	294
Larynx cancer	Male	20-24 years	95+ years	Global	1	3	LDI (I$ per capita)	--
Larynx cancer	Male	20-24 years	95+ years	Global	1	3	Socio-demographic Index	--
Tracheal, bronchus, and lung cancer	Female	15-19 years	95+ years	Data Rich	1	1	Smoking Prevalence	528
Tracheal, bronchus, and lung cancer	Female	15-19 years	95+ years	Data Rich	1	1	Asbestos consumption (metric tons per year per capita)	787
Tracheal, bronchus, and lung cancer	Female	15-19 years	95+ years	Data Rich	-1	2	Healthcare access and quality index	--
Tracheal, bronchus, and lung cancer	Female	15-19 years	95+ years	Data Rich	1	2	Cumulative Cigarettes (10 Years)	7
Tracheal, bronchus, and lung cancer	Female	15-19 years	95+ years	Data Rich	1	2	Outdoor Air Pollution (PM2.5)	27
Tracheal, bronchus, and lung cancer	Female	15-19 years	95+ years	Data Rich	1	2	Residential radon	27
Tracheal, bronchus, and lung cancer	Female	15-19 years	95+ years	Data Rich	1	2	Cumulative Cigarettes (20 Years)	110
Tracheal, bronchus, and lung cancer	Female	15-19 years	95+ years	Data Rich	1	2	Diabetes Fasting Plasma Glucose (mmol/L), age-standardized 25+	172
Tracheal, bronchus, and lung cancer	Female	15-19 years	95+ years	Data Rich	1	2	Indoor Air Pollution (All Cooking Fuels)	--
Tracheal, bronchus, and lung cancer	Female	15-19 years	95+ years	Data Rich	1	2	Secondhand smoke	--
Tracheal, bronchus, and lung cancer	Female	15-19 years	95+ years	Data Rich	-1	3	Education (years per capita)	--
Tracheal, bronchus, and lung cancer	Female	15-19 years	95+ years	Data Rich	1	3	Socio-demographic Index	86
Tracheal, bronchus, and lung cancer	Female	15-19 years	95+ years	Data Rich	1	3	LDI (I$ per capita)	--
Tracheal, bronchus, and lung cancer	Female	15-19 years	95+ years	Global	1	1	Asbestos consumption (metric tons per year per capita)	533
Tracheal, bronchus, and lung cancer	Female	15-19 years	95+ years	Global	1	1	Smoking Prevalence	1000
Tracheal, bronchus, and lung cancer	Female	15-19 years	95+ years	Global	-1	2	Healthcare access and quality index	--
Tracheal, bronchus, and lung cancer	Female	15-19 years	95+ years	Global	1	2	Diabetes Fasting Plasma Glucose (mmol/L), age-standardized 25+	126
Tracheal, bronchus, and lung cancer	Female	15-19 years	95+ years	Global	1	2	Cumulative Cigarettes (10 Years)	205
Tracheal, bronchus, and lung cancer	Female	15-19 years	95+ years	Global	1	2	Cumulative Cigarettes (20 Years)	344
Tracheal, bronchus, and lung cancer	Female	15-19 years	95+ years	Global	1	2	Outdoor Air Pollution (PM2.5)	549
Tracheal, bronchus, and lung cancer	Female	15-19 years	95+ years	Global	1	2	Indoor Air Pollution (All Cooking Fuels)	--
Tracheal, bronchus, and lung cancer	Female	15-19 years	95+ years	Global	1	2	Residential radon	--
Tracheal, bronchus, and lung cancer	Female	15-19 years	95+ years	Global	1	2	Secondhand smoke	--
Tracheal, bronchus, and lung cancer	Female	15-19 years	95+ years	Global	-1	3	Education (years per capita)	--
Tracheal, bronchus, and lung cancer	Female	15-19 years	95+ years	Global	1	3	Socio-demographic Index	474
Tracheal, bronchus, and lung cancer	Female	15-19 years	95+ years	Global	1	3	LDI (I$ per capita)	--
Tracheal, bronchus, and lung cancer	Male	15-19 years	95+ years	Data Rich	1	1	Smoking Prevalence	32
Tracheal, bronchus, and lung cancer	Male	15-19 years	95+ years	Data Rich	1	1	Asbestos consumption (metric tons per year per capita)	968
Tracheal, bronchus, and lung cancer	Male	15-19 years	95+ years	Data Rich	-1	2	Healthcare access and quality index	391
Tracheal, bronchus, and lung cancer	Male	15-19 years	95+ years	Data Rich	1	2	Cumulative Cigarettes (20 Years)	152
Tracheal, bronchus, and lung cancer	Male	15-19 years	95+ years	Data Rich	1	2	Cumulative Cigarettes (10 Years)	294
Tracheal, bronchus, and lung cancer	Male	15-19 years	95+ years	Data Rich	1	2	Secondhand smoke	364
Tracheal, bronchus, and lung cancer	Male	15-19 years	95+ years	Data Rich	1	2	Indoor Air Pollution (All Cooking Fuels)	428
Tracheal, bronchus, and lung cancer	Male	15-19 years	95+ years	Data Rich	1	2	Outdoor Air Pollution (PM2.5)	784
Tracheal, bronchus, and lung cancer	Male	15-19 years	95+ years	Data Rich	1	2	Diabetes Fasting Plasma Glucose (mmol/L), age-standardized 25+	--
Tracheal, bronchus, and lung cancer	Male	15-19 years	95+ years	Data Rich	1	2	Residential radon	--
Tracheal, bronchus, and lung cancer	Male	15-19 years	95+ years	Data Rich	-1	3	Education (years per capita)	398
Tracheal, bronchus, and lung cancer	Male	15-19 years	95+ years	Data Rich	1	3	Socio-demographic Index	114
Tracheal, bronchus, and lung cancer	Male	15-19 years	95+ years	Data Rich	1	3	LDI (I$ per capita)	--
Tracheal, bronchus, and lung cancer	Male	15-19 years	95+ years	Global	1	1	Smoking Prevalence	544
Tracheal, bronchus, and lung cancer	Male	15-19 years	95+ years	Global	1	1	Asbestos consumption (metric tons per year per capita)	1000
Tracheal, bronchus, and lung cancer	Male	15-19 years	95+ years	Global	-1	2	Healthcare access and quality index	746
Tracheal, bronchus, and lung cancer	Male	15-19 years	95+ years	Global	1	2	Cumulative Cigarettes (10 Years)	559
Tracheal, bronchus, and lung cancer	Male	15-19 years	95+ years	Global	1	2	Secondhand smoke	746
Tracheal, bronchus, and lung cancer	Male	15-19 years	95+ years	Global	1	2	Cumulative Cigarettes (20 Years)	896
Tracheal, bronchus, and lung cancer	Male	15-19 years	95+ years	Global	1	2	Outdoor Air Pollution (PM2.5)	896
Tracheal, bronchus, and lung cancer	Male	15-19 years	95+ years	Global	1	2	Diabetes Fasting Plasma Glucose (mmol/L), age-standardized 25+	--
Tracheal, bronchus, and lung cancer	Male	15-19 years	95+ years	Global	1	2	Indoor Air Pollution (All Cooking Fuels)	--
Tracheal, bronchus, and lung cancer	Male	15-19 years	95+ years	Global	1	2	Residential radon	--
Tracheal, bronchus, and lung cancer	Male	15-19 years	95+ years	Global	-1	3	Education (years per capita)	--
Tracheal, bronchus, and lung cancer	Male	15-19 years	95+ years	Global	1	3	Socio-demographic Index	429
Tracheal, bronchus, and lung cancer	Male	15-19 years	95+ years	Global	1	3	LDI (I$ per capita)	--
Malignant skin melanoma	Female	15-19 years	95+ years	Data Rich	1	1	Liters of alcohol consumed per capita	1000
Malignant skin melanoma	Female	15-19 years	95+ years	Data Rich	-1	2	Healthcare access and quality index	--
Malignant skin melanoma	Female	15-19 years	95+ years	Data Rich	-1	2	Latitude 15 to 30 (proportion)	--
Malignant skin melanoma	Female	15-19 years	95+ years	Data Rich	-1	2	Latitude 30 to 45 (proportion)	--
Malignant skin melanoma	Female	15-19 years	95+ years	Data Rich	-1	2	Latitude Over 45 (proportion)	--
Malignant skin melanoma	Female	15-19 years	95+ years	Data Rich	-1	2	Latitude Under 15 (proportion)	--
Malignant skin melanoma	Female	15-19 years	95+ years	Data Rich	-1	3	Education (years per capita)	--
Malignant skin melanoma	Female	15-19 years	95+ years	Data Rich	-1	3	LDI (I$ per capita)	--
Malignant skin melanoma	Female	15-19 years	95+ years	Data Rich	1	3	Socio-demographic Index	568
Malignant skin melanoma	Female	15-19 years	95+ years	Global	1	1	Liters of alcohol consumed per capita	1000
Malignant skin melanoma	Female	15-19 years	95+ years	Global	-1	2	Healthcare access and quality index	--
Malignant skin melanoma	Female	15-19 years	95+ years	Global	-1	2	Latitude 15 to 30 (proportion)	--
Malignant skin melanoma	Female	15-19 years	95+ years	Global	-1	2	Latitude 30 to 45 (proportion)	--
Malignant skin melanoma	Female	15-19 years	95+ years	Global	-1	2	Latitude Over 45 (proportion)	--
Malignant skin melanoma	Female	15-19 years	95+ years	Global	-1	2	Latitude Under 15 (proportion)	--
Malignant skin melanoma	Female	15-19 years	95+ years	Global	-1	3	Education (years per capita)	--
Malignant skin melanoma	Female	15-19 years	95+ years	Global	-1	3	LDI (I$ per capita)	--
Malignant skin melanoma	Female	15-19 years	95+ years	Global	1	3	Socio-demographic Index	--
Malignant skin melanoma	Male	15-19 years	95+ years	Data Rich	1	1	Liters of alcohol consumed per capita	--
Malignant skin melanoma	Male	15-19 years	95+ years	Data Rich	-1	2	Latitude Over 45 (proportion)	208
Malignant skin melanoma	Male	15-19 years	95+ years	Data Rich	-1	2	Latitude Under 15 (proportion)	432
Malignant skin melanoma	Male	15-19 years	95+ years	Data Rich	-1	2	Healthcare access and quality index	--
Malignant skin melanoma	Male	15-19 years	95+ years	Data Rich	-1	2	Latitude 15 to 30 (proportion)	--
Malignant skin melanoma	Male	15-19 years	95+ years	Data Rich	-1	2	Latitude 30 to 45 (proportion)	--
Malignant skin melanoma	Male	15-19 years	95+ years	Data Rich	-1	3	Education (years per capita)	--
Malignant skin melanoma	Male	15-19 years	95+ years	Data Rich	-1	3	LDI (I$ per capita)	--
Malignant skin melanoma	Male	15-19 years	95+ years	Data Rich	1	3	Socio-demographic Index	633
Malignant skin melanoma	Male	15-19 years	95+ years	Global	1	1	Liters of alcohol consumed per capita	--
Malignant skin melanoma	Male	15-19 years	95+ years	Global	-1	2	Healthcare access and quality index	--
Malignant skin melanoma	Male	15-19 years	95+ years	Global	-1	2	Latitude 15 to 30 (proportion)	--
Malignant skin melanoma	Male	15-19 years	95+ years	Global	-1	2	Latitude 30 to 45 (proportion)	--
Malignant skin melanoma	Male	15-19 years	95+ years	Global	-1	2	Latitude Over 45 (proportion)	--
Malignant skin melanoma	Male	15-19 years	95+ years	Global	-1	2	Latitude Under 15 (proportion)	--
Malignant skin melanoma	Male	15-19 years	95+ years	Global	-1	3	Education (years per capita)	--
Malignant skin melanoma	Male	15-19 years	95+ years	Global	-1	3	LDI (I$ per capita)	--
Malignant skin melanoma	Male	15-19 years	95+ years	Global	1	3	Socio-demographic Index	1000
Non-melanoma skin cancer	Female	20-24 years	95+ years	Data Rich	1	1	Smoking Prevalence	130
Non-melanoma skin cancer	Female	20-24 years	95+ years	Data Rich	1	1	Cumulative Cigarettes (15 Years)	340
Non-melanoma skin cancer	Female	20-24 years	95+ years	Data Rich	1	1	Cumulative Cigarettes (5 Years)	530
Non-melanoma skin cancer	Female	20-24 years	95+ years	Data Rich	1	1	Cumulative Cigarettes (10 Years)	--
Non-melanoma skin cancer	Female	20-24 years	95+ years	Data Rich	-1	2	Average latitude	198
Non-melanoma skin cancer	Female	20-24 years	95+ years	Data Rich	-1	2	Healthcare access and quality index	591
Non-melanoma skin cancer	Female	20-24 years	95+ years	Data Rich	-1	3	Education (years per capita)	138
Non-melanoma skin cancer	Female	20-24 years	95+ years	Data Rich	-1	3	LDI (I$ per capita)	--
Non-melanoma skin cancer	Female	20-24 years	95+ years	Data Rich	1	3	Socio-demographic Index	321
Non-melanoma skin cancer	Female	20-24 years	95+ years	Global	1	1	Cumulative Cigarettes (10 Years)	416
Non-melanoma skin cancer	Female	20-24 years	95+ years	Global	1	1	Cumulative Cigarettes (5 Years)	584

Appendix Table S10: CODEm covariates used, level of covariate, and expected direction of covariate by cause, sex, and age

Cause	Sex	Age Start	Age End	Model Version Type	Direction	Level	Covariate Name	Number of Draws
Non-melanoma skin cancer	Female	20-24 years	95+ years	Global	1	1	Cumulative Cigarettes (15 Years)	--
Non-melanoma skin cancer	Female	20-24 years	95+ years	Global	1	1	Smoking Prevalence	--
Non-melanoma skin cancer	Female	20-24 years	95+ years	Global	-1	2	Average latitude	196
Non-melanoma skin cancer	Female	20-24 years	95+ years	Global	-1	2	Healthcare access and quality index	405
Non-melanoma skin cancer	Female	20-24 years	95+ years	Global	-1	3	Education (years per capita)	336
Non-melanoma skin cancer	Female	20-24 years	95+ years	Global	-1	3	LDI (I$ per capita)	--
Non-melanoma skin cancer	Female	20-24 years	95+ years	Global	1	3	Socio-demographic Index	--
Non-melanoma skin cancer	Male	20-24 years	95+ years	Data Rich	1	1	Smoking Prevalence	33
Non-melanoma skin cancer	Male	20-24 years	95+ years	Data Rich	1	1	Cumulative Cigarettes (15 Years)	100
Non-melanoma skin cancer	Male	20-24 years	95+ years	Data Rich	1	1	Cumulative Cigarettes (10 Years)	190
Non-melanoma skin cancer	Male	20-24 years	95+ years	Data Rich	1	1	Cumulative Cigarettes (5 Years)	677
Non-melanoma skin cancer	Male	20-24 years	95+ years	Data Rich	-1	2	Average latitude	388
Non-melanoma skin cancer	Male	20-24 years	95+ years	Data Rich	-1	2	Healthcare access and quality index	--
Non-melanoma skin cancer	Male	20-24 years	95+ years	Data Rich	-1	3	Education (years per capita)	--
Non-melanoma skin cancer	Male	20-24 years	95+ years	Data Rich	-1	3	LDI (I$ per capita)	--
Non-melanoma skin cancer	Male	20-24 years	95+ years	Data Rich	1	3	Socio-demographic Index	580
Non-melanoma skin cancer	Male	20-24 years	95+ years	Global	1	1	Smoking Prevalence	127
Non-melanoma skin cancer	Male	20-24 years	95+ years	Global	1	1	Cumulative Cigarettes (5 Years)	319
Non-melanoma skin cancer	Male	20-24 years	95+ years	Global	1	1	Cumulative Cigarettes (10 Years)	554
Non-melanoma skin cancer	Male	20-24 years	95+ years	Global	1	1	Cumulative Cigarettes (15 Years)	--
Non-melanoma skin cancer	Male	20-24 years	95+ years	Global	-1	2	Healthcare access and quality index	197
Non-melanoma skin cancer	Male	20-24 years	95+ years	Global	-1	2	Average latitude	496
Non-melanoma skin cancer	Male	20-24 years	95+ years	Global	-1	3	Education (years per capita)	--
Non-melanoma skin cancer	Male	20-24 years	95+ years	Global	-1	3	LDI (I$ per capita)	--
Non-melanoma skin cancer	Male	20-24 years	95+ years	Global	1	3	Socio-demographic Index	404
Non-melanoma skin cancer (squamous-cell carcinoma)	Female	20-24 years	95+ years	Data Rich	1	1	Cumulative Cigarettes (10 Years)	255
Non-melanoma skin cancer (squamous-cell carcinoma)	Female	20-24 years	95+ years	Data Rich	1	1	Smoking Prevalence	323
Non-melanoma skin cancer (squamous-cell carcinoma)	Female	20-24 years	95+ years	Data Rich	1	1	Cumulative Cigarettes (15 Years)	422
Non-melanoma skin cancer (squamous-cell carcinoma)	Female	20-24 years	95+ years	Data Rich	1	1	Cumulative Cigarettes (5 Years)	--
Non-melanoma skin cancer (squamous-cell carcinoma)	Female	20-24 years	95+ years	Data Rich	-1	2	Average latitude	156
Non-melanoma skin cancer (squamous-cell carcinoma)	Female	20-24 years	95+ years	Data Rich	-1	2	Healthcare access and quality index	210
Non-melanoma skin cancer (squamous-cell carcinoma)	Female	20-24 years	95+ years	Data Rich	-1	3	LDI (I$ per capita)	83
Non-melanoma skin cancer (squamous-cell carcinoma)	Female	20-24 years	95+ years	Data Rich	-1	3	Education (years per capita)	257
Non-melanoma skin cancer (squamous-cell carcinoma)	Female	20-24 years	95+ years	Data Rich	1	3	Socio-demographic Index	257
Non-melanoma skin cancer (squamous-cell carcinoma)	Female	20-24 years	95+ years	Global	1	1	Cumulative Cigarettes (10 Years)	472
Non-melanoma skin cancer (squamous-cell carcinoma)	Female	20-24 years	95+ years	Global	1	1	Cumulative Cigarettes (15 Years)	528
Non-melanoma skin cancer (squamous-cell carcinoma)	Female	20-24 years	95+ years	Global	1	1	Cumulative Cigarettes (5 Years)	--
Non-melanoma skin cancer (squamous-cell carcinoma)	Female	20-24 years	95+ years	Global	1	1	Smoking Prevalence	--
Non-melanoma skin cancer (squamous-cell carcinoma)	Female	20-24 years	95+ years	Global	-1	2	Average latitude	68
Non-melanoma skin cancer (squamous-cell carcinoma)	Female	20-24 years	95+ years	Global	-1	2	Healthcare access and quality index	634
Non-melanoma skin cancer (squamous-cell carcinoma)	Female	20-24 years	95+ years	Global	-1	3	Education (years per capita)	194
Non-melanoma skin cancer (squamous-cell carcinoma)	Female	20-24 years	95+ years	Global	-1	3	LDI (I$ per capita)	194
Non-melanoma skin cancer (squamous-cell carcinoma)	Female	20-24 years	95+ years	Global	1	3	Socio-demographic Index	249
Non-melanoma skin cancer (squamous-cell carcinoma)	Male	20-24 years	95+ years	Data Rich	1	1	Cumulative Cigarettes (15 Years)	101
Non-melanoma skin cancer (squamous-cell carcinoma)	Male	20-24 years	95+ years	Data Rich	1	1	Cumulative Cigarettes (10 Years)	218
Non-melanoma skin cancer (squamous-cell carcinoma)	Male	20-24 years	95+ years	Data Rich	1	1	Cumulative Cigarettes (5 Years)	316
Non-melanoma skin cancer (squamous-cell carcinoma)	Male	20-24 years	95+ years	Data Rich	1	1	Smoking Prevalence	365
Non-melanoma skin cancer (squamous-cell carcinoma)	Male	20-24 years	95+ years	Data Rich	-1	2	Average latitude	482
Non-melanoma skin cancer (squamous-cell carcinoma)	Male	20-24 years	95+ years	Data Rich	-1	2	Healthcare access and quality index	--
Non-melanoma skin cancer (squamous-cell carcinoma)	Male	20-24 years	95+ years	Data Rich	-1	3	LDI (I$ per capita)	336
Non-melanoma skin cancer (squamous-cell carcinoma)	Male	20-24 years	95+ years	Data Rich	-1	3	Education (years per capita)	--
Non-melanoma skin cancer (squamous-cell carcinoma)	Male	20-24 years	95+ years	Data Rich	1	3	Socio-demographic Index	777
Non-melanoma skin cancer (squamous-cell carcinoma)	Male	20-24 years	95+ years	Global	1	1	Cumulative Cigarettes (15 Years)	221
Non-melanoma skin cancer (squamous-cell carcinoma)	Male	20-24 years	95+ years	Global	1	1	Smoking Prevalence	338
Non-melanoma skin cancer (squamous-cell carcinoma)	Male	20-24 years	95+ years	Global	1	1	Cumulative Cigarettes (5 Years)	441
Non-melanoma skin cancer (squamous-cell carcinoma)	Male	20-24 years	95+ years	Global	1	1	Cumulative Cigarettes (10 Years)	--
Non-melanoma skin cancer (squamous-cell carcinoma)	Male	20-24 years	95+ years	Global	-1	2	Average latitude	385
Non-melanoma skin cancer (squamous-cell carcinoma)	Male	20-24 years	95+ years	Global	-1	2	Healthcare access and quality index	385
Non-melanoma skin cancer (squamous-cell carcinoma)	Male	20-24 years	95+ years	Global	-1	3	LDI (I$ per capita)	224
Non-melanoma skin cancer (squamous-cell carcinoma)	Male	20-24 years	95+ years	Global	-1	3	Education (years per capita)	--
Non-melanoma skin cancer (squamous-cell carcinoma)	Male	20-24 years	95+ years	Global	1	3	Socio-demographic Index	445
Soft tissue and other extraosseous sarcomas	Female	0-6 days	95+ years	Data Rich	1	1	Log-transformed SEV scalar: HIV/AIDS+STIs	--
Soft tissue and other extraosseous sarcomas	Female	0-6 days	95+ years	Data Rich	1	1	Log-transformed age-standardized SEV scalar: HIV/AIDS+STIs	--
Soft tissue and other extraosseous sarcomas	Female	0-6 days	95+ years	Data Rich	-1	2	Socio-demographic Index	671
Soft tissue and other extraosseous sarcomas	Female	0-6 days	95+ years	Data Rich	-1	2	Healthcare access and quality index	--
Soft tissue and other extraosseous sarcomas	Female	0-6 days	95+ years	Data Rich	-1	2	LDI (I$ per capita)	--
Soft tissue and other extraosseous sarcomas	Female	0-6 days	95+ years	Data Rich	-1	2	Universal health coverage	--
Soft tissue and other extraosseous sarcomas	Female	0-6 days	95+ years	Data Rich	-1	3	Education (years per capita)	329
Soft tissue and other extraosseous sarcomas	Female	0-6 days	95+ years	Data Rich	-1	3	Maternal care and immunization	--
Soft tissue and other extraosseous sarcomas	Female	0-6 days	95+ years	Data Rich	1	3	Liters of alcohol consumed per capita	274
Soft tissue and other extraosseous sarcomas	Female	0-6 days	95+ years	Data Rich	1	3	Log-transformed SEV scalar: HIV	--
Soft tissue and other extraosseous sarcomas	Female	0-6 days	95+ years	Data Rich	1	3	Log-transformed age-standardized SEV scalar: HIV	--
Soft tissue and other extraosseous sarcomas	Female	0-6 days	95+ years	Global	-1	2	Universal health coverage	224
Soft tissue and other extraosseous sarcomas	Female	0-6 days	95+ years	Global	-1	2	Healthcare access and quality index	407
Soft tissue and other extraosseous sarcomas	Female	0-6 days	95+ years	Global	-1	2	Socio-demographic Index	442
Soft tissue and other extraosseous sarcomas	Female	0-6 days	95+ years	Global	-1	2	LDI (I$ per capita)	--
Soft tissue and other extraosseous sarcomas	Female	0-6 days	95+ years	Global	-1	3	Maternal care and immunization	188
Soft tissue and other extraosseous sarcomas	Female	0-6 days	95+ years	Global	-1	3	Education (years per capita)	--
Soft tissue and other extraosseous sarcomas	Female	0-6 days	95+ years	Global	1	3	Log-transformed age-standardized SEV scalar: HIV	487
Soft tissue and other extraosseous sarcomas	Female	0-6 days	95+ years	Global	1	3	Liters of alcohol consumed per capita	--
Soft tissue and other extraosseous sarcomas	Female	0-6 days	95+ years	Global	1	3	Log-transformed SEV scalar: HIV	--
Soft tissue and other extraosseous sarcomas	Male	0-6 days	95+ years	Data Rich	-1	2	Healthcare access and quality index	--
Soft tissue and other extraosseous sarcomas	Male	0-6 days	95+ years	Data Rich	-1	2	LDI (I$ per capita)	--
Soft tissue and other extraosseous sarcomas	Male	0-6 days	95+ years	Data Rich	-1	2	Socio-demographic Index	--
Soft tissue and other extraosseous sarcomas	Male	0-6 days	95+ years	Data Rich	-1	2	Universal health coverage	--
Soft tissue and other extraosseous sarcomas	Male	0-6 days	95+ years	Data Rich	-1	3	Education (years per capita)	--
Soft tissue and other extraosseous sarcomas	Male	0-6 days	95+ years	Data Rich	-1	3	Maternal care and immunization	--
Soft tissue and other extraosseous sarcomas	Male	0-6 days	95+ years	Data Rich	1	3	Log-transformed SEV scalar: HIV	454
Soft tissue and other extraosseous sarcomas	Male	0-6 days	95+ years	Data Rich	1	3	Liters of alcohol consumed per capita	--
Soft tissue and other extraosseous sarcomas	Male	0-6 days	95+ years	Data Rich	1	3	Log-transformed age-standardized SEV scalar: HIV	--
Soft tissue and other extraosseous sarcomas	Male	0-6 days	95+ years	Global	-1	2	Healthcare access and quality index	232
Soft tissue and other extraosseous sarcomas	Male	0-6 days	95+ years	Global	-1	2	Universal health coverage	275
Soft tissue and other extraosseous sarcomas	Male	0-6 days	95+ years	Global	-1	2	Socio-demographic Index	493
Soft tissue and other extraosseous sarcomas	Male	0-6 days	95+ years	Global	-1	2	LDI (I$ per capita)	--
Soft tissue and other extraosseous sarcomas	Male	0-6 days	95+ years	Global	-1	3	Maternal care and immunization	381
Soft tissue and other extraosseous sarcomas	Male	0-6 days	95+ years	Global	-1	3	Education (years per capita)	--
Soft tissue and other extraosseous sarcomas	Male	0-6 days	95+ years	Global	1	3	Log-transformed SEV scalar: HIV	72
Soft tissue and other extraosseous sarcomas	Male	0-6 days	95+ years	Global	1	3	Log-transformed age-standardized SEV scalar: HIV	232

Appendix Table S10: CODEm covariates used, level of covariate, and expected direction of covariate by cause, sex, and age

Cause	Sex	Age Start	Age End	Model Version Type	Direction	Level	Covariate Name	Number of Draws
Soft tissue and other extraosseous sarcomas	Male	0-6 days	95+ years	Global	1	3	Liters of alcohol consumed per capita	--
Malignant neoplasm of bone and articular cartilage	Female	12-23 months	95+ years	Data Rich	-1	2	Universal health coverage	97
Malignant neoplasm of bone and articular cartilage	Female	12-23 months	95+ years	Data Rich	-1	2	Healthcare access and quality index	691
Malignant neoplasm of bone and articular cartilage	Female	12-23 months	95+ years	Data Rich	-1	2	Socio-demographic Index	700
Malignant neoplasm of bone and articular cartilage	Female	12-23 months	95+ years	Data Rich	-1	2	LDI (I$ per capita)	--
Malignant neoplasm of bone and articular cartilage	Female	12-23 months	95+ years	Data Rich	-1	3	Maternal care and immunization	332
Malignant neoplasm of bone and articular cartilage	Female	12-23 months	95+ years	Data Rich	-1	3	Health worker density	512
Malignant neoplasm of bone and articular cartilage	Female	12-23 months	95+ years	Data Rich	-1	3	Education (years per capita)	522
Malignant neoplasm of bone and articular cartilage	Female	12-23 months	95+ years	Data Rich	1	3	Age- and sex-specific SEV for Low bone mineral density	112
Malignant neoplasm of bone and articular cartilage	Female	12-23 months	95+ years	Data Rich	1	3	Log-transformed age-standardized SEV scalar: Osteoarth	281
Malignant neoplasm of bone and articular cartilage	Female	12-23 months	95+ years	Data Rich	1	3	Log-transformed SEV scalar: Osteoarth	320
Malignant neoplasm of bone and articular cartilage	Female	12-23 months	95+ years	Data Rich	1	3	Smoking Prevalence	--
Malignant neoplasm of bone and articular cartilage	Female	12-23 months	95+ years	Global	-1	2	Universal health coverage	324
Malignant neoplasm of bone and articular cartilage	Female	12-23 months	95+ years	Global	-1	2	Socio-demographic Index	390
Malignant neoplasm of bone and articular cartilage	Female	12-23 months	95+ years	Global	-1	2	Healthcare access and quality index	547
Malignant neoplasm of bone and articular cartilage	Female	12-23 months	95+ years	Global	-1	2	LDI (I$ per capita)	--
Malignant neoplasm of bone and articular cartilage	Female	12-23 months	95+ years	Global	-1	3	Health worker density	153
Malignant neoplasm of bone and articular cartilage	Female	12-23 months	95+ years	Global	-1	3	Education (years per capita)	--
Malignant neoplasm of bone and articular cartilage	Female	12-23 months	95+ years	Global	-1	3	Maternal care and immunization	--
Malignant neoplasm of bone and articular cartilage	Female	12-23 months	95+ years	Global	1	3	Log-transformed age-standardized SEV scalar: Osteoarth	334
Malignant neoplasm of bone and articular cartilage	Female	12-23 months	95+ years	Global	1	3	Log-transformed SEV scalar: Osteoarth	405
Malignant neoplasm of bone and articular cartilage	Female	12-23 months	95+ years	Global	1	3	Smoking Prevalence	453
Malignant neoplasm of bone and articular cartilage	Female	12-23 months	95+ years	Global	1	3	Age- and sex-specific SEV for Low bone mineral density	--
Malignant neoplasm of bone and articular cartilage	Male	12-23 months	95+ years	Data Rich	-1	2	Universal health coverage	125
Malignant neoplasm of bone and articular cartilage	Male	12-23 months	95+ years	Data Rich	-1	2	Socio-demographic Index	146
Malignant neoplasm of bone and articular cartilage	Male	12-23 months	95+ years	Data Rich	-1	2	Healthcare access and quality index	708
Malignant neoplasm of bone and articular cartilage	Male	12-23 months	95+ years	Data Rich	-1	2	LDI (I$ per capita)	--
Malignant neoplasm of bone and articular cartilage	Male	12-23 months	95+ years	Data Rich	-1	3	Maternal care and immunization	131
Malignant neoplasm of bone and articular cartilage	Male	12-23 months	95+ years	Data Rich	-1	3	Health worker density	155
Malignant neoplasm of bone and articular cartilage	Male	12-23 months	95+ years	Data Rich	-1	3	Education (years per capita)	424
Malignant neoplasm of bone and articular cartilage	Male	12-23 months	95+ years	Data Rich	1	3	Smoking Prevalence	49
Malignant neoplasm of bone and articular cartilage	Male	12-23 months	95+ years	Data Rich	1	3	Log-transformed age-standardized SEV scalar: Osteoarth	328
Malignant neoplasm of bone and articular cartilage	Male	12-23 months	95+ years	Data Rich	1	3	Log-transformed SEV scalar: Osteoarth	410
Malignant neoplasm of bone and articular cartilage	Male	12-23 months	95+ years	Data Rich	1	3	Age- and sex-specific SEV for Low bone mineral density	--
Malignant neoplasm of bone and articular cartilage	Male	12-23 months	95+ years	Global	-1	2	Healthcare access and quality index	249
Malignant neoplasm of bone and articular cartilage	Male	12-23 months	95+ years	Global	-1	2	Socio-demographic Index	288
Malignant neoplasm of bone and articular cartilage	Male	12-23 months	95+ years	Global	-1	2	Universal health coverage	463
Malignant neoplasm of bone and articular cartilage	Male	12-23 months	95+ years	Global	-1	2	LDI (I$ per capita)	--
Malignant neoplasm of bone and articular cartilage	Male	12-23 months	95+ years	Global	-1	3	Health worker density	255
Malignant neoplasm of bone and articular cartilage	Male	12-23 months	95+ years	Global	-1	3	Education (years per capita)	--
Malignant neoplasm of bone and articular cartilage	Male	12-23 months	95+ years	Global	-1	3	Maternal care and immunization	--
Malignant neoplasm of bone and articular cartilage	Male	12-23 months	95+ years	Global	1	3	Log-transformed age-standardized SEV scalar: Osteoarth	317
Malignant neoplasm of bone and articular cartilage	Male	12-23 months	95+ years	Global	1	3	Log-transformed SEV scalar: Osteoarth	572
Malignant neoplasm of bone and articular cartilage	Male	12-23 months	95+ years	Global	1	3	Age- and sex-specific SEV for Low bone mineral density	--
Malignant neoplasm of bone and articular cartilage	Male	12-23 months	95+ years	Global	1	3	Smoking Prevalence	--
Breast cancer	Female	15-19 years	95+ years	Data Rich	1	1	Liters of alcohol consumed per capita	1000
Breast cancer	Female	15-19 years	95+ years	Data Rich	1	1	Mean BMI	--
Breast cancer	Female	15-19 years	95+ years	Data Rich	-1	2	Healthcare access and quality index	344
Breast cancer	Female	15-19 years	95+ years	Data Rich	-1	2	Total Fertility Rate	506
Breast cancer	Female	15-19 years	95+ years	Data Rich	-1	2	Age-Specific Fertility Rate	--
Breast cancer	Female	15-19 years	95+ years	Data Rich	1	2	Age- and sex-specific SEV for Low fruit	162
Breast cancer	Female	15-19 years	95+ years	Data Rich	1	2	Cumulative Cigarettes (10 Years)	195
Breast cancer	Female	15-19 years	95+ years	Data Rich	1	2	Cumulative Cigarettes (20 Years)	195
Breast cancer	Female	15-19 years	95+ years	Data Rich	1	2	Smoking Prevalence	311
Breast cancer	Female	15-19 years	95+ years	Data Rich	1	2	Age- and sex-specific SEV for Low vegetables	--
Breast cancer	Female	15-19 years	95+ years	Data Rich	1	2	Diabetes Fasting Plasma Glucose (mmol/L), age-standardized 25+	--
Breast cancer	Female	15-19 years	95+ years	Data Rich	-1	3	LDI (I$ per capita)	--
Breast cancer	Female	15-19 years	95+ years	Data Rich	1	3	Socio-demographic Index	217
Breast cancer	Female	15-19 years	95+ years	Global	1	1	Liters of alcohol consumed per capita	1000
Breast cancer	Female	15-19 years	95+ years	Global	1	1	Mean BMI	--
Breast cancer	Female	15-19 years	95+ years	Global	-1	2	Healthcare access and quality index	556
Breast cancer	Female	15-19 years	95+ years	Global	-1	2	Total Fertility Rate	896
Breast cancer	Female	15-19 years	95+ years	Global	-1	2	Age-Specific Fertility Rate	--
Breast cancer	Female	15-19 years	95+ years	Global	1	2	Age- and sex-specific SEV for Low fruit	238
Breast cancer	Female	15-19 years	95+ years	Global	1	2	Age- and sex-specific SEV for Low vegetables	255
Breast cancer	Female	15-19 years	95+ years	Global	1	2	Smoking Prevalence	412
Breast cancer	Female	15-19 years	95+ years	Global	1	2	Cumulative Cigarettes (10 Years)	484
Breast cancer	Female	15-19 years	95+ years	Global	1	2	Cumulative Cigarettes (20 Years)	484
Breast cancer	Female	15-19 years	95+ years	Global	1	2	Diabetes Fasting Plasma Glucose (mmol/L), age-standardized 25+	--
Breast cancer	Female	15-19 years	95+ years	Global	-1	3	LDI (I$ per capita)	--
Breast cancer	Female	15-19 years	95+ years	Global	1	3	Socio-demographic Index	363
Breast cancer	Male	15-19 years	95+ years	Data Rich	1	1	Liters of alcohol consumed per capita	1000
Breast cancer	Male	15-19 years	95+ years	Data Rich	1	1	Mean BMI	--
Breast cancer	Male	15-19 years	95+ years	Data Rich	-1	2	Healthcare access and quality index	--
Breast cancer	Male	15-19 years	95+ years	Data Rich	1	2	Age- and sex-specific SEV for Low fruit	--
Breast cancer	Male	15-19 years	95+ years	Data Rich	1	2	Age- and sex-specific SEV for Low vegetables	--
Breast cancer	Male	15-19 years	95+ years	Data Rich	1	2	Cumulative Cigarettes (10 Years)	--
Breast cancer	Male	15-19 years	95+ years	Data Rich	1	2	Cumulative Cigarettes (20 Years)	--
Breast cancer	Male	15-19 years	95+ years	Data Rich	1	2	Diabetes Fasting Plasma Glucose (mmol/L), age-standardized 25+	--
Breast cancer	Male	15-19 years	95+ years	Data Rich	1	2	Smoking Prevalence	--
Breast cancer	Male	15-19 years	95+ years	Data Rich	-1	3	LDI (I$ per capita)	--
Breast cancer	Male	15-19 years	95+ years	Data Rich	1	3	Socio-demographic Index	--
Breast cancer	Male	15-19 years	95+ years	Global	1	1	Liters of alcohol consumed per capita	--
Breast cancer	Male	15-19 years	95+ years	Global	1	1	Mean BMI	--
Breast cancer	Male	15-19 years	95+ years	Global	-1	2	Healthcare access and quality index	1000
Breast cancer	Male	15-19 years	95+ years	Global	1	2	Cumulative Cigarettes (10 Years)	452
Breast cancer	Male	15-19 years	95+ years	Global	1	2	Cumulative Cigarettes (20 Years)	548
Breast cancer	Male	15-19 years	95+ years	Global	1	2	Diabetes Fasting Plasma Glucose (mmol/L), age-standardized 25+	1000
Breast cancer	Male	15-19 years	95+ years	Global	1	2	Age- and sex-specific SEV for Low fruit	--
Breast cancer	Male	15-19 years	95+ years	Global	1	2	Age- and sex-specific SEV for Low vegetables	--
Breast cancer	Male	15-19 years	95+ years	Global	1	2	Smoking Prevalence	--

Appendix Table S10: CODEm covariates used, level of covariate, and expected direction of covariate by cause, sex, and age

Cause	Sex	Age Start	Age End	Model Version Type	Direction	Level	Covariate Name	Number of Draws
Breast cancer	Male	15-19 years	95+ years	Global	-1	3	LDI (I$ per capita)	--
Breast cancer	Male	15-19 years	95+ years	Global	1	3	Socio-demographic Index	407
Cervical cancer	Female	15-19 years	95+ years	Data Rich	1	1	Cumulative Cigarettes (5 Years)	--
Cervical cancer	Female	15-19 years	95+ years	Data Rich	1	1	HIV age-standardized prevalence	--
Cervical cancer	Female	15-19 years	95+ years	Data Rich	1	1	Log-transformed SEV scalar: HIV	--
Cervical cancer	Female	15-19 years	95+ years	Data Rich	1	1	Log-transformed age-standardized SEV scalar: HIV	--
Cervical cancer	Female	15-19 years	95+ years	Data Rich	-1	2	Healthcare access and quality index	497
Cervical cancer	Female	15-19 years	95+ years	Data Rich	1	2	Age- and sex-specific SEV for Low fruit	55
Cervical cancer	Female	15-19 years	95+ years	Data Rich	1	2	Age- and sex-specific SEV for Low vegetables	55
Cervical cancer	Female	15-19 years	95+ years	Data Rich	1	2	Age-Specific Fertility Rate	552
Cervical cancer	Female	15-19 years	95+ years	Data Rich	1	2	Smoking Prevalence	552
Cervical cancer	Female	15-19 years	95+ years	Data Rich	1	2	Total Fertility Rate	552
Cervical cancer	Female	15-19 years	95+ years	Data Rich	-1	3	Socio-demographic Index	166
Cervical cancer	Female	15-19 years	95+ years	Data Rich	-1	3	Education (years per capita)	538
Cervical cancer	Female	15-19 years	95+ years	Data Rich	-1	3	LDI (I$ per capita)	--
Cervical cancer	Female	15-19 years	95+ years	Global	1	1	Cumulative Cigarettes (5 Years)	1000
Cervical cancer	Female	15-19 years	95+ years	Global	1	1	HIV age-standardized prevalence	1000
Cervical cancer	Female	15-19 years	95+ years	Global	1	1	Log-transformed SEV scalar: HIV	--
Cervical cancer	Female	15-19 years	95+ years	Global	1	1	Log-transformed age-standardized SEV scalar: HIV	--
Cervical cancer	Female	15-19 years	95+ years	Global	-1	2	Healthcare access and quality index	--
Cervical cancer	Female	15-19 years	95+ years	Global	1	2	Age- and sex-specific SEV for Low fruit	267
Cervical cancer	Female	15-19 years	95+ years	Global	1	2	Age- and sex-specific SEV for Low vegetables	267
Cervical cancer	Female	15-19 years	95+ years	Global	1	2	Age-Specific Fertility Rate	267
Cervical cancer	Female	15-19 years	95+ years	Global	1	2	Total Fertility Rate	267
Cervical cancer	Female	15-19 years	95+ years	Global	1	2	Smoking Prevalence	--
Cervical cancer	Female	15-19 years	95+ years	Global	-1	3	Education (years per capita)	326
Cervical cancer	Female	15-19 years	95+ years	Global	-1	3	Socio-demographic Index	326
Cervical cancer	Female	15-19 years	95+ years	Global	-1	3	LDI (I$ per capita)	--
Uterine cancer	Female	20-24 years	95+ years	Data Rich	1	1	Mean BMI	1000
Uterine cancer	Female	20-24 years	95+ years	Data Rich	-1	2	Healthcare access and quality index	435
Uterine cancer	Female	20-24 years	95+ years	Data Rich	-1	2	Total Fertility Rate	--
Uterine cancer	Female	20-24 years	95+ years	Data Rich	1	2	Age- and sex-specific SEV for Low fruit	57
Uterine cancer	Female	20-24 years	95+ years	Data Rich	1	2	Cumulative Cigarettes (10 Years)	96
Uterine cancer	Female	20-24 years	95+ years	Data Rich	1	2	Tobacco (cigarettes per capita)	136
Uterine cancer	Female	20-24 years	95+ years	Data Rich	1	2	Cumulative Cigarettes (5 Years)	203
Uterine cancer	Female	20-24 years	95+ years	Data Rich	1	2	Smoking Prevalence	571
Uterine cancer	Female	20-24 years	95+ years	Data Rich	1	2	Age- and sex-specific SEV for Low vegetables	813
Uterine cancer	Female	20-24 years	95+ years	Data Rich	1	2	Diabetes Age-Standardized Prevalence (proportion)	--
Uterine cancer	Female	20-24 years	95+ years	Data Rich	-1	3	Education (years per capita)	395
Uterine cancer	Female	20-24 years	95+ years	Data Rich	1	3	Socio-demographic Index	186
Uterine cancer	Female	20-24 years	95+ years	Data Rich	1	3	LDI (I$ per capita)	--
Uterine cancer	Female	20-24 years	95+ years	Global	1	1	Mean BMI	--
Uterine cancer	Female	20-24 years	95+ years	Global	-1	2	Healthcare access and quality index	496
Uterine cancer	Female	20-24 years	95+ years	Global	-1	2	Total Fertility Rate	--
Uterine cancer	Female	20-24 years	95+ years	Global	1	2	Tobacco (cigarettes per capita)	294
Uterine cancer	Female	20-24 years	95+ years	Global	1	2	Age- and sex-specific SEV for Low fruit	496
Uterine cancer	Female	20-24 years	95+ years	Global	1	2	Age- and sex-specific SEV for Low vegetables	--
Uterine cancer	Female	20-24 years	95+ years	Global	1	2	Cumulative Cigarettes (10 Years)	--
Uterine cancer	Female	20-24 years	95+ years	Global	1	2	Cumulative Cigarettes (5 Years)	--
Uterine cancer	Female	20-24 years	95+ years	Global	1	2	Diabetes Age-Standardized Prevalence (proportion)	--
Uterine cancer	Female	20-24 years	95+ years	Global	1	2	Smoking Prevalence	--
Uterine cancer	Female	20-24 years	95+ years	Global	-1	3	Education (years per capita)	210
Uterine cancer	Female	20-24 years	95+ years	Global	1	3	Socio-demographic Index	229
Uterine cancer	Female	20-24 years	95+ years	Global	1	3	LDI (I$ per capita)	--
Ovarian cancer	Female	15-19 years	95+ years	Data Rich	1	1	Liters of alcohol consumed per capita	1000
Ovarian cancer	Female	15-19 years	95+ years	Data Rich	-1	2	Healthcare access and quality index	186
Ovarian cancer	Female	15-19 years	95+ years	Data Rich	-1	2	Total Fertility Rate	186
Ovarian cancer	Female	15-19 years	95+ years	Data Rich	-1	2	Contraception (Modern) Prevalence (proportion)	--
Ovarian cancer	Female	15-19 years	95+ years	Data Rich	1	2	Asbestos consumption (metric tons per year per capita)	186
Ovarian cancer	Female	15-19 years	95+ years	Data Rich	1	2	Cumulative Cigarettes (10 Years)	186
Ovarian cancer	Female	15-19 years	95+ years	Data Rich	1	2	Cumulative Cigarettes (20 Years)	186
Ovarian cancer	Female	15-19 years	95+ years	Data Rich	1	2	Smoking Prevalence	186
Ovarian cancer	Female	15-19 years	95+ years	Data Rich	1	2	Diabetes Age-Standardized Prevalence (proportion)	--
Ovarian cancer	Female	15-19 years	95+ years	Data Rich	1	2	Mean BMI	--
Ovarian cancer	Female	15-19 years	95+ years	Data Rich	1	2	energy unadjusted(kcal)	--
Ovarian cancer	Female	15-19 years	95+ years	Data Rich	-1	3	Education (years per capita)	268
Ovarian cancer	Female	15-19 years	95+ years	Data Rich	-1	3	LDI (I$ per capita)	--
Ovarian cancer	Female	15-19 years	95+ years	Data Rich	1	3	Age- and sex-specific SEV for Low fruit	--
Ovarian cancer	Female	15-19 years	95+ years	Data Rich	1	3	Age- and sex-specific SEV for Low vegetables	--
Ovarian cancer	Female	15-19 years	95+ years	Data Rich	1	3	Socio-demographic Index	--
Ovarian cancer	Female	15-19 years	95+ years	Global	1	1	Liters of alcohol consumed per capita	1000
Ovarian cancer	Female	15-19 years	95+ years	Global	-1	2	Healthcare access and quality index	679
Ovarian cancer	Female	15-19 years	95+ years	Global	-1	2	Total Fertility Rate	679
Ovarian cancer	Female	15-19 years	95+ years	Global	-1	2	Contraception (Modern) Prevalence (proportion)	--
Ovarian cancer	Female	15-19 years	95+ years	Global	1	2	Smoking Prevalence	141
Ovarian cancer	Female	15-19 years	95+ years	Global	1	2	Cumulative Cigarettes (10 Years)	249
Ovarian cancer	Female	15-19 years	95+ years	Global	1	2	Cumulative Cigarettes (20 Years)	289
Ovarian cancer	Female	15-19 years	95+ years	Global	1	2	Asbestos consumption (metric tons per year per capita)	679
Ovarian cancer	Female	15-19 years	95+ years	Global	1	2	energy unadjusted(kcal)	679
Ovarian cancer	Female	15-19 years	95+ years	Global	1	2	Diabetes Age-Standardized Prevalence (proportion)	--
Ovarian cancer	Female	15-19 years	95+ years	Global	1	2	Mean BMI	--
Ovarian cancer	Female	15-19 years	95+ years	Global	-1	3	Education (years per capita)	--
Ovarian cancer	Female	15-19 years	95+ years	Global	-1	3	LDI (I$ per capita)	--
Ovarian cancer	Female	15-19 years	95+ years	Global	1	3	Age- and sex-specific SEV for Low fruit	141
Ovarian cancer	Female	15-19 years	95+ years	Global	1	3	Socio-demographic Index	284
Ovarian cancer	Female	15-19 years	95+ years	Global	1	3	Age- and sex-specific SEV for Low vegetables	--
Prostate cancer	Male	20-24 years	95+ years	Data Rich	1	1	Log-transformed SEV scalar: Prostate C	1000
Prostate cancer	Male	20-24 years	95+ years	Data Rich	-1	2	Healthcare access and quality index	190
Prostate cancer	Male	20-24 years	95+ years	Data Rich	1	2	Smoking Prevalence	--
Prostate cancer	Male	20-24 years	95+ years	Data Rich	-1	3	Education (years per capita)	308
Prostate cancer	Male	20-24 years	95+ years	Data Rich	-1	3	LDI (I$ per capita)	--
Prostate cancer	Male	20-24 years	95+ years	Data Rich	1	3	Socio-demographic Index	--
Prostate cancer	Male	20-24 years	95+ years	Global	1	1	Log-transformed SEV scalar: Prostate C	--
Prostate cancer	Male	20-24 years	95+ years	Global	-1	2	Healthcare access and quality index	--
Prostate cancer	Male	20-24 years	95+ years	Global	1	2	Smoking Prevalence	726
Prostate cancer	Male	20-24 years	95+ years	Global	-1	3	Education (years per capita)	--
Prostate cancer	Male	20-24 years	95+ years	Global	-1	3	LDI (I$ per capita)	--
Prostate cancer	Male	20-24 years	95+ years	Global	1	3	Socio-demographic Index	--
Testicular cancer	Male	15-19 years	95+ years	Data Rich	-1	2	Healthcare access and quality index	121
Testicular cancer	Male	15-19 years	95+ years	Data Rich	1	2	Age- and sex-specific SEV for Low fruit	121
Testicular cancer	Male	15-19 years	95+ years	Data Rich	1	2	Cumulative Cigarettes (20 Years)	517
Testicular cancer	Male	15-19 years	95+ years	Data Rich	1	2	Cumulative Cigarettes (10 Years)	724
Testicular cancer	Male	15-19 years	95+ years	Data Rich	1	2	Age- and sex-specific SEV for Low vegetables	--
Testicular cancer	Male	15-19 years	95+ years	Data Rich	1	2	Cumulative Cigarettes (15 Years)	--
Testicular cancer	Male	15-19 years	95+ years	Data Rich	1	2	Cumulative Cigarettes (5 Years)	--
Testicular cancer	Male	15-19 years	95+ years	Data Rich	1	2	Smoking Prevalence	--
Testicular cancer	Male	15-19 years	95+ years	Data Rich	1	2	Tobacco (cigarettes per capita)	--

Appendix Table S10: CODEm covariates used, level of covariate, and expected direction of covariate by cause, sex, and age

Cause	Sex	Age Start	Age End	Model Version Type	Direction	Level	Covariate Name	Number of Draws
Testicular cancer	Male	15-19 years	95+ years	Data Rich	-1	3	Education (years per capita)	--
Testicular cancer	Male	15-19 years	95+ years	Data Rich	1	3	Socio-demographic Index	40
Testicular cancer	Male	15-19 years	95+ years	Data Rich	1	3	LDI (I$ per capita)	--
Testicular cancer	Male	15-19 years	95+ years	Global	-1	2	Healthcare access and quality index	939
Testicular cancer	Male	15-19 years	95+ years	Global	1	2	Age- and sex-specific SEV for Low vegetables	5
Testicular cancer	Male	15-19 years	95+ years	Global	1	2	Cumulative Cigarettes (20 Years)	6
Testicular cancer	Male	15-19 years	95+ years	Global	1	2	Smoking Prevalence	54
Testicular cancer	Male	15-19 years	95+ years	Global	1	2	Cumulative Cigarettes (15 Years)	110
Testicular cancer	Male	15-19 years	95+ years	Global	1	2	Cumulative Cigarettes (10 Years)	325
Testicular cancer	Male	15-19 years	95+ years	Global	1	2	Cumulative Cigarettes (5 Years)	442
Testicular cancer	Male	15-19 years	95+ years	Global	1	2	Age- and sex-specific SEV for Low fruit	472
Testicular cancer	Male	15-19 years	95+ years	Global	1	2	Tobacco (cigarettes per capita)	--
Testicular cancer	Male	15-19 years	95+ years	Global	-1	3	Education (years per capita)	3
Testicular cancer	Male	15-19 years	95+ years	Global	1	3	Socio-demographic Index	635
Testicular cancer	Male	15-19 years	95+ years	Global	1	3	LDI (I$ per capita)	--
Kidney cancer	Female	0-6 days	95+ years	Data Rich	1	1	Mean BMI	87
Kidney cancer	Female	0-6 days	95+ years	Data Rich	1	1	Tobacco (cigarettes per capita)	549
Kidney cancer	Female	0-6 days	95+ years	Data Rich	1	1	Cumulative Cigarettes (10 Years)	650
Kidney cancer	Female	0-6 days	95+ years	Data Rich	-1	2	Healthcare access and quality index	117
Kidney cancer	Female	0-6 days	95+ years	Data Rich	1	2	Systolic Blood Pressure (mmHg)	22
Kidney cancer	Female	0-6 days	95+ years	Data Rich	1	2	Diabetes Age-Standardized Prevalence (proportion)	--
Kidney cancer	Female	0-6 days	95+ years	Data Rich	1	2	Liters of alcohol consumed per capita	--
Kidney cancer	Female	0-6 days	95+ years	Data Rich	-1	3	Education (years per capita)	193
Kidney cancer	Female	0-6 days	95+ years	Data Rich	1	3	Socio-demographic Index	176
Kidney cancer	Female	0-6 days	95+ years	Data Rich	1	3	LDI (I$ per capita)	--
Kidney cancer	Female	0-6 days	95+ years	Global	1	1	Mean BMI	414
Kidney cancer	Female	0-6 days	95+ years	Global	1	1	Cumulative Cigarettes (10 Years)	586
Kidney cancer	Female	0-6 days	95+ years	Global	1	1	Tobacco (cigarettes per capita)	1000
Kidney cancer	Female	0-6 days	95+ years	Global	-1	2	Healthcare access and quality index	160
Kidney cancer	Female	0-6 days	95+ years	Global	1	2	Systolic Blood Pressure (mmHg)	137
Kidney cancer	Female	0-6 days	95+ years	Global	1	2	Diabetes Age-Standardized Prevalence (proportion)	--
Kidney cancer	Female	0-6 days	95+ years	Global	1	2	Liters of alcohol consumed per capita	--
Kidney cancer	Female	0-6 days	95+ years	Global	-1	3	Education (years per capita)	--
Kidney cancer	Female	0-6 days	95+ years	Global	1	3	Socio-demographic Index	378
Kidney cancer	Female	0-6 days	95+ years	Global	1	3	LDI (I$ per capita)	--
Kidney cancer	Male	0-6 days	95+ years	Data Rich	1	1	Tobacco (cigarettes per capita)	237
Kidney cancer	Male	0-6 days	95+ years	Data Rich	1	1	Cumulative Cigarettes (10 Years)	325
Kidney cancer	Male	0-6 days	95+ years	Data Rich	1	1	Mean BMI	828
Kidney cancer	Male	0-6 days	95+ years	Data Rich	-1	2	Healthcare access and quality index	189
Kidney cancer	Male	0-6 days	95+ years	Data Rich	1	2	Liters of alcohol consumed per capita	12
Kidney cancer	Male	0-6 days	95+ years	Data Rich	1	2	Diabetes Age-Standardized Prevalence (proportion)	27
Kidney cancer	Male	0-6 days	95+ years	Data Rich	1	2	Systolic Blood Pressure (mmHg)	34
Kidney cancer	Male	0-6 days	95+ years	Data Rich	-1	3	Education (years per capita)	--
Kidney cancer	Male	0-6 days	95+ years	Data Rich	1	3	Socio-demographic Index	209
Kidney cancer	Male	0-6 days	95+ years	Data Rich	1	3	LDI (I$ per capita)	--
Kidney cancer	Male	0-6 days	95+ years	Global	1	1	Tobacco (cigarettes per capita)	1000
Kidney cancer	Male	0-6 days	95+ years	Global	1	1	Cumulative Cigarettes (10 Years)	--
Kidney cancer	Male	0-6 days	95+ years	Global	1	1	Mean BMI	--
Kidney cancer	Male	0-6 days	95+ years	Global	-1	2	Healthcare access and quality index	647
Kidney cancer	Male	0-6 days	95+ years	Global	1	2	Systolic Blood Pressure (mmHg)	647
Kidney cancer	Male	0-6 days	95+ years	Global	1	2	Diabetes Age-Standardized Prevalence (proportion)	--
Kidney cancer	Male	0-6 days	95+ years	Global	1	2	Liters of alcohol consumed per capita	--
Kidney cancer	Male	0-6 days	95+ years	Global	-1	3	Education (years per capita)	--
Kidney cancer	Male	0-6 days	95+ years	Global	1	3	Socio-demographic Index	353
Kidney cancer	Male	0-6 days	95+ years	Global	1	3	LDI (I$ per capita)	--
Bladder cancer	Female	15-19 years	95+ years	Data Rich	-1	1	Healthcare access and quality index	764
Bladder cancer	Female	15-19 years	95+ years	Data Rich	1	1	Cumulative Cigarettes (10 Years)	252
Bladder cancer	Female	15-19 years	95+ years	Data Rich	1	1	Schistosomiasis Prevalence Results	388
Bladder cancer	Female	15-19 years	95+ years	Data Rich	1	1	Socio-demographic Index	835
Bladder cancer	Female	15-19 years	95+ years	Data Rich	1	1	Smoking Prevalence	946
Bladder cancer	Female	15-19 years	95+ years	Data Rich	1	2	Age- and sex-specific SEV for Low vegetables	322
Bladder cancer	Female	15-19 years	95+ years	Data Rich	1	2	Liters of alcohol consumed per capita	345
Bladder cancer	Female	15-19 years	95+ years	Data Rich	1	2	Diabetes Fasting Plasma Glucose (mmol/L), age-standardized 25+	404
Bladder cancer	Female	15-19 years	95+ years	Data Rich	1	3	LDI (I$ per capita)	44
Bladder cancer	Female	15-19 years	95+ years	Data Rich	1	3	Age- and sex-specific SEV for Low fruit	--
Bladder cancer	Female	15-19 years	95+ years	Global	1	1	Schistosomiasis Prevalence Results	750
Bladder cancer	Female	15-19 years	95+ years	Global	1	1	Smoking Prevalence	767
Bladder cancer	Female	15-19 years	95+ years	Global	-1	2	Healthcare access and quality index	408
Bladder cancer	Female	15-19 years	95+ years	Global	1	2	Age- and sex-specific SEV for Low vegetables	471
Bladder cancer	Female	15-19 years	95+ years	Global	1	2	Cumulative Cigarettes (10 Years)	606
Bladder cancer	Female	15-19 years	95+ years	Global	1	2	Liters of alcohol consumed per capita	736
Bladder cancer	Female	15-19 years	95+ years	Global	1	2	Diabetes Fasting Plasma Glucose (mmol/L), age-standardized 25+	--
Bladder cancer	Female	15-19 years	95+ years	Global	1	3	LDI (I$ per capita)	125
Bladder cancer	Female	15-19 years	95+ years	Global	1	3	Age- and sex-specific SEV for Low fruit	176
Bladder cancer	Female	15-19 years	95+ years	Global	1	3	Socio-demographic Index	193
Bladder cancer	Male	15-19 years	95+ years	Data Rich	1	1	Schistosomiasis Prevalence Results	--
Bladder cancer	Male	15-19 years	95+ years	Data Rich	1	1	Smoking Prevalence	--
Bladder cancer	Male	15-19 years	95+ years	Data Rich	-1	2	Healthcare access and quality index	752
Bladder cancer	Male	15-19 years	95+ years	Data Rich	1	2	Age- and sex-specific SEV for Low vegetables	248
Bladder cancer	Male	15-19 years	95+ years	Data Rich	1	2	Cumulative Cigarettes (10 Years)	--
Bladder cancer	Male	15-19 years	95+ years	Data Rich	1	2	Diabetes Fasting Plasma Glucose (mmol/L), age-standardized 25+	--
Bladder cancer	Male	15-19 years	95+ years	Data Rich	1	2	Liters of alcohol consumed per capita	--
Bladder cancer	Male	15-19 years	95+ years	Data Rich	1	3	LDI (I$ per capita)	325
Bladder cancer	Male	15-19 years	95+ years	Data Rich	1	3	Age- and sex-specific SEV for Low fruit	--
Bladder cancer	Male	15-19 years	95+ years	Data Rich	1	3	Socio-demographic Index	--
Bladder cancer	Male	15-19 years	95+ years	Global	1	1	Schistosomiasis Prevalence Results	440
Bladder cancer	Male	15-19 years	95+ years	Global	1	1	Smoking Prevalence	894
Bladder cancer	Male	15-19 years	95+ years	Global	-1	2	Healthcare access and quality index	484
Bladder cancer	Male	15-19 years	95+ years	Global	1	2	Diabetes Fasting Plasma Glucose (mmol/L), age-standardized 25+	283
Bladder cancer	Male	15-19 years	95+ years	Global	1	2	Cumulative Cigarettes (10 Years)	408
Bladder cancer	Male	15-19 years	95+ years	Global	1	2	Age- and sex-specific SEV for Low vegetables	815
Bladder cancer	Male	15-19 years	95+ years	Global	1	2	Liters of alcohol consumed per capita	815
Bladder cancer	Male	15-19 years	95+ years	Global	1	3	Age- and sex-specific SEV for Low fruit	86
Bladder cancer	Male	15-19 years	95+ years	Global	1	3	LDI (I$ per capita)	110
Bladder cancer	Male	15-19 years	95+ years	Global	1	3	Socio-demographic Index	223
Brain and nervous system cancer	Female	0-6 days	95+ years	Data Rich	1	1	Cumulative Cigarettes (10 Years)	248
Brain and nervous system cancer	Female	0-6 days	95+ years	Data Rich	1	1	Smoking Prevalence	752
Brain and nervous system cancer	Female	0-6 days	95+ years	Data Rich	1	1	Liters of alcohol consumed per capita	--
Brain and nervous system cancer	Female	0-6 days	95+ years	Data Rich	-1	2	Healthcare access and quality index	--
Brain and nervous system cancer	Female	0-6 days	95+ years	Data Rich	1	2	Age- and sex-specific SEV for High red meat	427
Brain and nervous system cancer	Female	0-6 days	95+ years	Data Rich	1	2	Low-Density Lipoprotein (mmol/L)	427
Brain and nervous system cancer	Female	0-6 days	95+ years	Data Rich	1	2	Age- and sex-specific SEV for Low fruit	--
Brain and nervous system cancer	Female	0-6 days	95+ years	Data Rich	1	2	Age- and sex-specific SEV for Low vegetables	--
Brain and nervous system cancer	Female	0-6 days	95+ years	Data Rich	1	2	Systolic Blood Pressure (mmHg)	--
Brain and nervous system cancer	Female	0-6 days	95+ years	Data Rich	-1	3	Education (years per capita)	--
Brain and nervous system cancer	Female	0-6 days	95+ years	Data Rich	-1	3	LDI (I$ per capita)	--

Appendix Table S10: CODEm covariates used, level of covariate, and expected direction of covariate by cause, sex, and age

Cause	Sex	Age Start	Age End	Model Version Type	Direction	Level	Covariate Name	Number of Draws
Brain and nervous system cancer	Female	0-6 days	95+ years	Data Rich	1	3	Socio-demographic Index	--
Brain and nervous system cancer	Female	0-6 days	95+ years	Global	1	1	Cumulative Cigarettes (10 Years)	--
Brain and nervous system cancer	Female	0-6 days	95+ years	Global	1	1	Liters of alcohol consumed per capita	--
Brain and nervous system cancer	Female	0-6 days	95+ years	Global	1	1	Smoking Prevalence	--
Brain and nervous system cancer	Female	0-6 days	95+ years	Global	-1	2	Healthcare access and quality index	647
Brain and nervous system cancer	Female	0-6 days	95+ years	Global	1	2	Age- and sex-specific SEV for High red meat	647
Brain and nervous system cancer	Female	0-6 days	95+ years	Global	1	2	Age- and sex-specific SEV for Low fruit	--
Brain and nervous system cancer	Female	0-6 days	95+ years	Global	1	2	Age- and sex-specific SEV for Low vegetables	--
Brain and nervous system cancer	Female	0-6 days	95+ years	Global	1	2	Low-Density Lipoprotein (mmol/L)	--
Brain and nervous system cancer	Female	0-6 days	95+ years	Global	1	2	Systolic Blood Pressure (mmHg)	--
Brain and nervous system cancer	Female	0-6 days	95+ years	Global	-1	3	LDI (I$ per capita)	647
Brain and nervous system cancer	Female	0-6 days	95+ years	Global	-1	3	Education (years per capita)	--
Brain and nervous system cancer	Female	0-6 days	95+ years	Global	1	3	Socio-demographic Index	--
Brain and nervous system cancer	Male	0-6 days	95+ years	Data Rich	1	1	Cumulative Cigarettes (10 Years)	--
Brain and nervous system cancer	Male	0-6 days	95+ years	Data Rich	1	1	Liters of alcohol consumed per capita	--
Brain and nervous system cancer	Male	0-6 days	95+ years	Data Rich	1	1	Smoking Prevalence	--
Brain and nervous system cancer	Male	0-6 days	95+ years	Data Rich	-1	2	Healthcare access and quality index	--
Brain and nervous system cancer	Male	0-6 days	95+ years	Data Rich	1	2	Age- and sex-specific SEV for High red meat	573
Brain and nervous system cancer	Male	0-6 days	95+ years	Data Rich	1	2	Low-Density Lipoprotein (mmol/L)	573
Brain and nervous system cancer	Male	0-6 days	95+ years	Data Rich	1	2	Systolic Blood Pressure (mmHg)	573
Brain and nervous system cancer	Male	0-6 days	95+ years	Data Rich	1	2	Age- and sex-specific SEV for Low fruit	--
Brain and nervous system cancer	Male	0-6 days	95+ years	Data Rich	1	2	Age- and sex-specific SEV for Low vegetables	--
Brain and nervous system cancer	Male	0-6 days	95+ years	Data Rich	-1	3	LDI (I$ per capita)	248
Brain and nervous system cancer	Male	0-6 days	95+ years	Data Rich	-1	3	Education (years per capita)	--
Brain and nervous system cancer	Male	0-6 days	95+ years	Data Rich	1	3	Socio-demographic Index	427
Brain and nervous system cancer	Male	0-6 days	95+ years	Global	1	1	Cumulative Cigarettes (10 Years)	--
Brain and nervous system cancer	Male	0-6 days	95+ years	Global	1	1	Liters of alcohol consumed per capita	--
Brain and nervous system cancer	Male	0-6 days	95+ years	Global	1	1	Smoking Prevalence	--
Brain and nervous system cancer	Male	0-6 days	95+ years	Global	-1	2	Healthcare access and quality index	238
Brain and nervous system cancer	Male	0-6 days	95+ years	Global	1	2	Age- and sex-specific SEV for High red meat	428
Brain and nervous system cancer	Male	0-6 days	95+ years	Global	1	2	Systolic Blood Pressure (mmHg)	428
Brain and nervous system cancer	Male	0-6 days	95+ years	Global	1	2	Age- and sex-specific SEV for Low fruit	--
Brain and nervous system cancer	Male	0-6 days	95+ years	Global	1	2	Age- and sex-specific SEV for Low vegetables	--
Brain and nervous system cancer	Male	0-6 days	95+ years	Global	1	2	Low-Density Lipoprotein (mmol/L)	--
Brain and nervous system cancer	Male	0-6 days	95+ years	Global	-1	3	LDI (I$ per capita)	226
Brain and nervous system cancer	Male	0-6 days	95+ years	Global	-1	3	Education (years per capita)	381
Brain and nervous system cancer	Male	0-6 days	95+ years	Global	1	3	Socio-demographic Index	100
Eye cancer	Female	0-6 days	95+ years	Data Rich	-1	2	Healthcare access and quality index	666
Eye cancer	Female	0-6 days	95+ years	Data Rich	-1	2	Universal health coverage	1000
Eye cancer	Female	0-6 days	95+ years	Data Rich	1	2	Age-standardized melanoma	1000
Eye cancer	Female	0-6 days	95+ years	Data Rich	-1	3	Education (years per capita)	333
Eye cancer	Female	0-6 days	95+ years	Data Rich	-1	3	LDI (I$ per capita)	--
Eye cancer	Female	0-6 days	95+ years	Data Rich	-1	3	Socio-demographic Index	--
Eye cancer	Female	0-6 days	95+ years	Global	-1	2	Healthcare access and quality index	385
Eye cancer	Female	0-6 days	95+ years	Global	-1	2	Universal health coverage	615
Eye cancer	Female	0-6 days	95+ years	Global	1	2	Age-standardized melanoma	218
Eye cancer	Female	0-6 days	95+ years	Global	-1	3	Education (years per capita)	270
Eye cancer	Female	0-6 days	95+ years	Global	-1	3	Socio-demographic Index	304
Eye cancer	Female	0-6 days	95+ years	Global	-1	3	LDI (I$ per capita)	--
Eye cancer	Male	0-6 days	95+ years	Data Rich	-1	2	Education (years per capita)	79
Eye cancer	Male	0-6 days	95+ years	Data Rich	-1	2	Healthcare access and quality index	247
Eye cancer	Male	0-6 days	95+ years	Data Rich	-1	2	Universal health coverage	413
Eye cancer	Male	0-6 days	95+ years	Data Rich	-1	2	Socio-demographic Index	531
Eye cancer	Male	0-6 days	95+ years	Data Rich	-1	2	LDI (I$ per capita)	--
Eye cancer	Male	0-6 days	95+ years	Data Rich	1	2	Age-standardized melanoma	309
Eye cancer	Male	0-6 days	95+ years	Global	-1	2	Education (years per capita)	148
Eye cancer	Male	0-6 days	95+ years	Global	-1	2	Socio-demographic Index	365
Eye cancer	Male	0-6 days	95+ years	Global	-1	2	Healthcare access and quality index	432
Eye cancer	Male	0-6 days	95+ years	Global	-1	2	Universal health coverage	683
Eye cancer	Male	0-6 days	95+ years	Global	-1	2	LDI (I$ per capita)	--
Eye cancer	Male	0-6 days	95+ years	Global	1	2	Age-standardized melanoma	420
Retinoblastoma	Female	0-6 days	5-9 years	Data Rich	-1	2	Healthcare access and quality index	--
Retinoblastoma	Female	0-6 days	5-9 years	Data Rich	-1	2	Universal health coverage	--
Retinoblastoma	Female	0-6 days	5-9 years	Data Rich	-1	3	Education (years per capita)	--
Retinoblastoma	Female	0-6 days	5-9 years	Data Rich	-1	3	LDI (I$ per capita)	--
Retinoblastoma	Female	0-6 days	5-9 years	Data Rich	-1	3	Maternal care and immunization	--
Retinoblastoma	Female	0-6 days	5-9 years	Data Rich	-1	3	Socio-demographic Index	--
Retinoblastoma	Female	0-6 days	5-9 years	Global	-1	2	Universal health coverage	407
Retinoblastoma	Female	0-6 days	5-9 years	Global	-1	2	Healthcare access and quality index	593
Retinoblastoma	Female	0-6 days	5-9 years	Global	-1	3	Socio-demographic Index	506
Retinoblastoma	Female	0-6 days	5-9 years	Global	-1	3	Education (years per capita)	--
Retinoblastoma	Female	0-6 days	5-9 years	Global	-1	3	LDI (I$ per capita)	--
Retinoblastoma	Female	0-6 days	5-9 years	Global	-1	3	Maternal care and immunization	--
Retinoblastoma	Male	0-6 days	5-9 years	Data Rich	-1	2	Healthcare access and quality index	481
Retinoblastoma	Male	0-6 days	5-9 years	Data Rich	-1	2	Universal health coverage	679
Retinoblastoma	Male	0-6 days	5-9 years	Data Rich	-1	3	Socio-demographic Index	169
Retinoblastoma	Male	0-6 days	5-9 years	Data Rich	-1	3	Education (years per capita)	368
Retinoblastoma	Male	0-6 days	5-9 years	Data Rich	-1	3	LDI (I$ per capita)	--
Retinoblastoma	Male	0-6 days	5-9 years	Data Rich	-1	3	Maternal care and immunization	--
Retinoblastoma	Male	0-6 days	5-9 years	Global	-1	2	Healthcare access and quality index	--
Retinoblastoma	Male	0-6 days	5-9 years	Global	-1	2	Universal health coverage	--
Retinoblastoma	Male	0-6 days	5-9 years	Global	-1	3	Education (years per capita)	--
Retinoblastoma	Male	0-6 days	5-9 years	Global	-1	3	LDI (I$ per capita)	--
Retinoblastoma	Male	0-6 days	5-9 years	Global	-1	3	Maternal care and immunization	--
Retinoblastoma	Male	0-6 days	5-9 years	Global	-1	3	Socio-demographic Index	--
Other eye cancers	Female	10-14 years	95+ years	Data Rich	-1	2	Healthcare access and quality index	--
Other eye cancers	Female	10-14 years	95+ years	Data Rich	-1	2	Universal health coverage	--
Other eye cancers	Female	10-14 years	95+ years	Data Rich	1	2	Age-standardized melanoma	--
Other eye cancers	Female	10-14 years	95+ years	Data Rich	-1	3	Education (years per capita)	--
Other eye cancers	Female	10-14 years	95+ years	Data Rich	-1	3	LDI (I$ per capita)	--
Other eye cancers	Female	10-14 years	95+ years	Data Rich	-1	3	Socio-demographic Index	--
Other eye cancers	Female	10-14 years	95+ years	Global	-1	2	Healthcare access and quality index	--
Other eye cancers	Female	10-14 years	95+ years	Global	-1	2	Universal health coverage	--
Other eye cancers	Female	10-14 years	95+ years	Global	1	2	Age-standardized melanoma	--
Other eye cancers	Female	10-14 years	95+ years	Global	-1	3	Education (years per capita)	--
Other eye cancers	Female	10-14 years	95+ years	Global	-1	3	LDI (I$ per capita)	--
Other eye cancers	Female	10-14 years	95+ years	Global	-1	3	Socio-demographic Index	--
Other eye cancers	Male	10-14 years	95+ years	Data Rich	-1	2	Healthcare access and quality index	--
Other eye cancers	Male	10-14 years	95+ years	Data Rich	-1	2	Universal health coverage	--
Other eye cancers	Male	10-14 years	95+ years	Data Rich	1	2	Age-standardized melanoma	--
Other eye cancers	Male	10-14 years	95+ years	Data Rich	-1	3	Education (years per capita)	--
Other eye cancers	Male	10-14 years	95+ years	Data Rich	-1	3	LDI (I$ per capita)	--
Other eye cancers	Male	10-14 years	95+ years	Data Rich	-1	3	Socio-demographic Index	--
Other eye cancers	Male	10-14 years	95+ years	Global	-1	2	Healthcare access and quality index	--
Other eye cancers	Male	10-14 years	95+ years	Global	-1	2	Universal health coverage	--
Other eye cancers	Male	10-14 years	95+ years	Global	1	2	Age-standardized melanoma	--
Other eye cancers	Male	10-14 years	95+ years	Global	-1	3	Education (years per capita)	--
Other eye cancers	Male	10-14 years	95+ years	Global	-1	3	LDI (I$ per capita)	--
Other eye cancers	Male	10-14 years	95+ years	Global	-1	3	Socio-demographic Index	--
Neuroblastoma and other peripheral nervous cell tumours	Female	0-6 days	95+ years	Data Rich	-1	3	Education (years per capita)	208
Neuroblastoma and other peripheral nervous cell tumours	Female	0-6 days	95+ years	Data Rich	-1	3	Healthcare access and quality index	319
Neuroblastoma and other peripheral nervous cell tumours	Female	0-6 days	95+ years	Data Rich	-1	3	Universal health coverage	461
Neuroblastoma and other peripheral nervous cell tumours	Female	0-6 days	95+ years	Data Rich	-1	3	Health worker density	735

Appendix Table S10: CODEm covariates used, level of covariate, and expected direction of covariate by cause, sex, and age

Cause	Sex	Age Start	Age End	Model Version Type	Direction	Level	Covariate Name	Number of Draws
Neuroblastoma and other peripheral nervous cell tumours	Female	0-6 days	95+ years	Data Rich	-1	3	Maternal care and immunization	--
Neuroblastoma and other peripheral nervous cell tumours	Female	0-6 days	95+ years	Data Rich	1	3	Socio-demographic Index	780
Neuroblastoma and other peripheral nervous cell tumours	Female	0-6 days	95+ years	Data Rich	1	3	LDI (I$ per capita)	--
Neuroblastoma and other peripheral nervous cell tumours	Female	0-6 days	95+ years	Data Rich	1	3	Smoking Prevalence	--
Neuroblastoma and other peripheral nervous cell tumours	Female	0-6 days	95+ years	Global	-1	3	Healthcare access and quality index	287
Neuroblastoma and other peripheral nervous cell tumours	Female	0-6 days	95+ years	Global	-1	3	Health worker density	333
Neuroblastoma and other peripheral nervous cell tumours	Female	0-6 days	95+ years	Global	-1	3	Education (years per capita)	--
Neuroblastoma and other peripheral nervous cell tumours	Female	0-6 days	95+ years	Global	-1	3	Maternal care and immunization	--
Neuroblastoma and other peripheral nervous cell tumours	Female	0-6 days	95+ years	Global	-1	3	Universal health coverage	--
Neuroblastoma and other peripheral nervous cell tumours	Female	0-6 days	95+ years	Global	1	3	Smoking Prevalence	623
Neuroblastoma and other peripheral nervous cell tumours	Female	0-6 days	95+ years	Global	1	3	Socio-demographic Index	888
Neuroblastoma and other peripheral nervous cell tumours	Female	0-6 days	95+ years	Global	1	3	LDI (I$ per capita)	--
Neuroblastoma and other peripheral nervous cell tumours	Male	0-6 days	95+ years	Data Rich	-1	3	Universal health coverage	397
Neuroblastoma and other peripheral nervous cell tumours	Male	0-6 days	95+ years	Data Rich	-1	3	Education (years per capita)	432
Neuroblastoma and other peripheral nervous cell tumours	Male	0-6 days	95+ years	Data Rich	-1	3	Healthcare access and quality index	440
Neuroblastoma and other peripheral nervous cell tumours	Male	0-6 days	95+ years	Data Rich	-1	3	Health worker density	905
Neuroblastoma and other peripheral nervous cell tumours	Male	0-6 days	95+ years	Data Rich	-1	3	Maternal care and immunization	--
Neuroblastoma and other peripheral nervous cell tumours	Male	0-6 days	95+ years	Data Rich	1	3	Socio-demographic Index	982
Neuroblastoma and other peripheral nervous cell tumours	Male	0-6 days	95+ years	Data Rich	1	3	LDI (I$ per capita)	--
Neuroblastoma and other peripheral nervous cell tumours	Male	0-6 days	95+ years	Data Rich	1	3	Smoking Prevalence	--
Neuroblastoma and other peripheral nervous cell tumours	Male	0-6 days	95+ years	Global	-1	3	Education (years per capita)	269
Neuroblastoma and other peripheral nervous cell tumours	Male	0-6 days	95+ years	Global	-1	3	Universal health coverage	269
Neuroblastoma and other peripheral nervous cell tumours	Male	0-6 days	95+ years	Global	-1	3	Health worker density	294
Neuroblastoma and other peripheral nervous cell tumours	Male	0-6 days	95+ years	Global	-1	3	Healthcare access and quality index	326
Neuroblastoma and other peripheral nervous cell tumours	Male	0-6 days	95+ years	Global	-1	3	Maternal care and immunization	--
Neuroblastoma and other peripheral nervous cell tumours	Male	0-6 days	95+ years	Global	1	3	Socio-demographic Index	1000
Neuroblastoma and other peripheral nervous cell tumours	Male	0-6 days	95+ years	Global	1	3	LDI (I$ per capita)	--
Neuroblastoma and other peripheral nervous cell tumours	Male	0-6 days	95+ years	Global	1	3	Smoking Prevalence	--
Thyroid cancer	Female	5-9 years	95+ years	Data Rich	1	1	Liters of alcohol consumed per capita	1000
Thyroid cancer	Female	5-9 years	95+ years	Data Rich	-1	2	Healthcare access and quality index	--
Thyroid cancer	Female	5-9 years	95+ years	Data Rich	1	2	Age- and sex-specific SEV for Low vegetables	571
Thyroid cancer	Female	5-9 years	95+ years	Data Rich	1	2	Age- and sex-specific SEV for High red meat	--
Thyroid cancer	Female	5-9 years	95+ years	Data Rich	1	2	Mean BMI	--
Thyroid cancer	Female	5-9 years	95+ years	Data Rich	1	2	Tobacco (cigarettes per capita)	--
Thyroid cancer	Female	5-9 years	95+ years	Data Rich	-1	3	Improved Water Source (proportion with access)	321
Thyroid cancer	Female	5-9 years	95+ years	Data Rich	-1	3	Education (years per capita)	507
Thyroid cancer	Female	5-9 years	95+ years	Data Rich	-1	3	Sanitation (proportion with access)	564
Thyroid cancer	Female	5-9 years	95+ years	Data Rich	1	3	Age- and sex-specific SEV for Low fruit	--
Thyroid cancer	Female	5-9 years	95+ years	Data Rich	1	3	LDI (I$ per capita)	--
Thyroid cancer	Female	5-9 years	95+ years	Data Rich	1	3	Socio-demographic Index	--
Thyroid cancer	Female	5-9 years	95+ years	Global	1	1	Liters of alcohol consumed per capita	--
Thyroid cancer	Female	5-9 years	95+ years	Global	-1	2	Healthcare access and quality index	788
Thyroid cancer	Female	5-9 years	95+ years	Global	1	2	Age- and sex-specific SEV for Low vegetables	149
Thyroid cancer	Female	5-9 years	95+ years	Global	1	2	Tobacco (cigarettes per capita)	149
Thyroid cancer	Female	5-9 years	95+ years	Global	1	2	Age- and sex-specific SEV for High red meat	--
Thyroid cancer	Female	5-9 years	95+ years	Global	1	2	Mean BMI	--
Thyroid cancer	Female	5-9 years	95+ years	Global	-1	3	Sanitation (proportion with access)	76
Thyroid cancer	Female	5-9 years	95+ years	Global	-1	3	Education (years per capita)	182
Thyroid cancer	Female	5-9 years	95+ years	Global	-1	3	Improved Water Source (proportion with access)	854
Thyroid cancer	Female	5-9 years	95+ years	Global	-1	3	Total Physical Activity (MET-min/week), Age-specific	--
Thyroid cancer	Female	5-9 years	95+ years	Global	1	3	Age- and sex-specific SEV for Low fruit	12
Thyroid cancer	Female	5-9 years	95+ years	Global	1	3	Cumulative Cigarettes (10 Years)	113
Thyroid cancer	Female	5-9 years	95+ years	Global	1	3	Cumulative Cigarettes (20 Years)	130
Thyroid cancer	Female	5-9 years	95+ years	Global	1	3	Smoking Prevalence	138
Thyroid cancer	Female	5-9 years	95+ years	Global	1	3	Cumulative Cigarettes (15 Years)	236
Thyroid cancer	Female	5-9 years	95+ years	Global	1	3	Cumulative Cigarettes (5 Years)	328
Thyroid cancer	Female	5-9 years	95+ years	Global	1	3	Diabetes Fasting Plasma Glucose (mmol/L), age-standardized 25+	361
Thyroid cancer	Female	5-9 years	95+ years	Global	1	3	Socio-demographic Index	379
Thyroid cancer	Female	5-9 years	95+ years	Global	1	3	LDI (I$ per capita)	--
Thyroid cancer	Female	5-9 years	95+ years	Global	1	3	Outdoor Air Pollution (PM2.5)	--
Thyroid cancer	Male	5-9 years	95+ years	Data Rich	1	1	Liters of alcohol consumed per capita	1000
Thyroid cancer	Male	5-9 years	95+ years	Data Rich	-1	2	Healthcare access and quality index	--
Thyroid cancer	Male	5-9 years	95+ years	Data Rich	1	2	Age- and sex-specific SEV for Low vegetables	123
Thyroid cancer	Male	5-9 years	95+ years	Data Rich	1	2	Mean BMI	503
Thyroid cancer	Male	5-9 years	95+ years	Data Rich	1	2	Age- and sex-specific SEV for High red meat	--
Thyroid cancer	Male	5-9 years	95+ years	Data Rich	1	2	Tobacco (cigarettes per capita)	--
Thyroid cancer	Male	5-9 years	95+ years	Data Rich	-1	3	Sanitation (proportion with access)	94
Thyroid cancer	Male	5-9 years	95+ years	Data Rich	-1	3	Improved Water Source (proportion with access)	234
Thyroid cancer	Male	5-9 years	95+ years	Data Rich	-1	3	Education (years per capita)	282
Thyroid cancer	Male	5-9 years	95+ years	Data Rich	1	3	Age- and sex-specific SEV for Low fruit	--
Thyroid cancer	Male	5-9 years	95+ years	Data Rich	1	3	LDI (I$ per capita)	--
Thyroid cancer	Male	5-9 years	95+ years	Data Rich	1	3	Socio-demographic Index	--
Thyroid cancer	Male	5-9 years	95+ years	Global	1	1	Liters of alcohol consumed per capita	--
Thyroid cancer	Male	5-9 years	95+ years	Global	-1	2	Healthcare access and quality index	692
Thyroid cancer	Male	5-9 years	95+ years	Global	1	2	Tobacco (cigarettes per capita)	692
Thyroid cancer	Male	5-9 years	95+ years	Global	1	2	Age- and sex-specific SEV for High red meat	--
Thyroid cancer	Male	5-9 years	95+ years	Global	1	2	Age- and sex-specific SEV for Low vegetables	--
Thyroid cancer	Male	5-9 years	95+ years	Global	1	2	Mean BMI	--
Thyroid cancer	Male	5-9 years	95+ years	Global	-1	3	Education (years per capita)	308
Thyroid cancer	Male	5-9 years	95+ years	Global	-1	3	Improved Water Source (proportion with access)	--
Thyroid cancer	Male	5-9 years	95+ years	Global	-1	3	Sanitation (proportion with access)	--
Thyroid cancer	Male	5-9 years	95+ years	Global	1	3	Socio-demographic Index	294
Thyroid cancer	Male	5-9 years	95+ years	Global	1	3	Age- and sex-specific SEV for Low fruit	--
Thyroid cancer	Male	5-9 years	95+ years	Global	1	3	LDI (I$ per capita)	--
Mesothelioma	Female	20-24 years	95+ years	Data Rich	1	1	Smoking Prevalence	344
Mesothelioma	Female	20-24 years	95+ years	Data Rich	1	1	Age- and sex-specific SEV for Occupational asbestos	656
Mesothelioma	Female	20-24 years	95+ years	Data Rich	1	1	Age-standardized SEV for Occupational asbestos	656
Mesothelioma	Female	20-24 years	95+ years	Data Rich	1	1	Asbestos consumption (metric tons per year per capita)	--
Mesothelioma	Female	20-24 years	95+ years	Data Rich	-1	2	Healthcare access and quality index	--
Mesothelioma	Female	20-24 years	95+ years	Data Rich	1	2	Gold production (binary)	311
Mesothelioma	Female	20-24 years	95+ years	Data Rich	1	2	Cumulative Cigarettes (5 Years)	--
Mesothelioma	Female	20-24 years	95+ years	Data Rich	1	2	Indoor Air Pollution (All Cooking Fuels)	--
Mesothelioma	Female	20-24 years	95+ years	Data Rich	1	2	Population Density (over 1000 ppl/sqkm, proportion)	--
Mesothelioma	Female	20-24 years	95+ years	Data Rich	-1	3	Education (years per capita)	--

Appendix Table S10: CODEm covariates used, level of covariate, and expected direction of covariate by cause, sex, and age									
**Cause**	**Sex**	**Age Start**	**Age End**	**Model Version Type**	**Direction**	**Level**	**Covariate Name**	**Number of Draws**	
Mesothelioma	Female	20-24 years	95+ years	Data Rich	-1	3	LDI (I$ per capita)	--	
Mesothelioma	Female	20-24 years	95+ years	Data Rich	1	3	Socio-demographic Index	499	
Mesothelioma	Female	20-24 years	95+ years	Global	1	1	Age- and sex-specific SEV for Occupational asbestos	694	
Mesothelioma	Female	20-24 years	95+ years	Global	1	1	Age-standardized SEV for Occupational asbestos	953	
Mesothelioma	Female	20-24 years	95+ years	Global	1	1	Smoking Prevalence	1000	
Mesothelioma	Female	20-24 years	95+ years	Global	1	1	Asbestos consumption (metric tons per year per capita)	--	
Mesothelioma	Female	20-24 years	95+ years	Global	-1	2	Healthcare access and quality index	--	
Mesothelioma	Female	20-24 years	95+ years	Global	1	2	Population Density (over 1000 ppl/sqkm, proportion)	57	
Mesothelioma	Female	20-24 years	95+ years	Global	1	2	Gold production (binary)	566	
Mesothelioma	Female	20-24 years	95+ years	Global	1	2	Cumulative Cigarettes (5 Years)	--	
Mesothelioma	Female	20-24 years	95+ years	Global	1	2	Indoor Air Pollution (All Cooking Fuels)	--	
Mesothelioma	Female	20-24 years	95+ years	Global	-1	3	Education (years per capita)	--	
Mesothelioma	Female	20-24 years	95+ years	Global	-1	3	LDI (I$ per capita)	--	
Mesothelioma	Female	20-24 years	95+ years	Global	1	3	Socio-demographic Index	443	
Mesothelioma	Male	20-24 years	95+ years	Data Rich	1	1	Smoking Prevalence	301	
Mesothelioma	Male	20-24 years	95+ years	Data Rich	1	1	Asbestos consumption (metric tons per year per capita)	653	
Mesothelioma	Male	20-24 years	95+ years	Data Rich	1	1	Age- and sex-specific SEV for Occupational asbestos	925	
Mesothelioma	Male	20-24 years	95+ years	Data Rich	1	1	Age-standardized SEV for Occupational asbestos	995	
Mesothelioma	Male	20-24 years	95+ years	Data Rich	-1	2	Healthcare access and quality index	--	
Mesothelioma	Male	20-24 years	95+ years	Data Rich	1	2	Gold production (binary)	1	
Mesothelioma	Male	20-24 years	95+ years	Data Rich	1	2	Cumulative Cigarettes (5 Years)	81	
Mesothelioma	Male	20-24 years	95+ years	Data Rich	1	2	Indoor Air Pollution (All Cooking Fuels)	--	
Mesothelioma	Male	20-24 years	95+ years	Data Rich	1	2	Population Density (over 1000 ppl/sqkm, proportion)	--	
Mesothelioma	Male	20-24 years	95+ years	Data Rich	-1	3	Education (years per capita)	--	
Mesothelioma	Male	20-24 years	95+ years	Data Rich	-1	3	LDI (I$ per capita)	--	
Mesothelioma	Male	20-24 years	95+ years	Data Rich	1	3	Socio-demographic Index	465	
Mesothelioma	Male	20-24 years	95+ years	Global	1	1	Smoking Prevalence	368	
Mesothelioma	Male	20-24 years	95+ years	Global	1	1	Asbestos consumption (metric tons per year per capita)	512	
Mesothelioma	Male	20-24 years	95+ years	Global	1	1	Age- and sex-specific SEV for Occupational asbestos	918	
Mesothelioma	Male	20-24 years	95+ years	Global	1	1	Age-standardized SEV for Occupational asbestos	986	
Mesothelioma	Male	20-24 years	95+ years	Global	-1	2	Healthcare access and quality index	109	
Mesothelioma	Male	20-24 years	95+ years	Global	1	2	Indoor Air Pollution (All Cooking Fuels)	99	
Mesothelioma	Male	20-24 years	95+ years	Global	1	2	Gold production (binary)	198	
Mesothelioma	Male	20-24 years	95+ years	Global	1	2	Cumulative Cigarettes (5 Years)	328	
Mesothelioma	Male	20-24 years	95+ years	Global	1	2	Population Density (over 1000 ppl/sqkm, proportion)	--	
Mesothelioma	Male	20-24 years	95+ years	Global	-1	3	Education (years per capita)	--	
Mesothelioma	Male	20-24 years	95+ years	Global	-1	3	LDI (I$ per capita)	--	
Mesothelioma	Male	20-24 years	95+ years	Global	1	3	Socio-demographic Index	142	
Hodgkin lymphoma	Female	2-4 years	95+ years	Data Rich	-1	2	Healthcare access and quality index	1000	
Hodgkin lymphoma	Female	2-4 years	95+ years	Data Rich	-1	3	Education (years per capita)	--	
Hodgkin lymphoma	Female	2-4 years	95+ years	Data Rich	-1	3	LDI (I$ per capita)	--	
Hodgkin lymphoma	Female	2-4 years	95+ years	Data Rich	-1	3	Socio-demographic Index	--	
Hodgkin lymphoma	Female	2-4 years	95+ years	Global	-1	2	Healthcare access and quality index	1000	
Hodgkin lymphoma	Female	2-4 years	95+ years	Global	-1	3	Socio-demographic Index	373	
Hodgkin lymphoma	Female	2-4 years	95+ years	Global	-1	3	Education (years per capita)	--	
Hodgkin lymphoma	Female	2-4 years	95+ years	Global	-1	3	LDI (I$ per capita)	--	
Hodgkin lymphoma	Male	2-4 years	95+ years	Data Rich	-1	2	Healthcare access and quality index	--	
Hodgkin lymphoma	Male	2-4 years	95+ years	Data Rich	-1	3	Socio-demographic Index	1000	
Hodgkin lymphoma	Male	2-4 years	95+ years	Data Rich	-1	3	Education (years per capita)	--	
Hodgkin lymphoma	Male	2-4 years	95+ years	Data Rich	-1	3	LDI (I$ per capita)	--	
Hodgkin lymphoma	Male	2-4 years	95+ years	Global	-1	2	Healthcare access and quality index	1000	
Hodgkin lymphoma	Male	2-4 years	95+ years	Global	-1	3	Education (years per capita)	509	
Hodgkin lymphoma	Male	2-4 years	95+ years	Global	-1	3	LDI (I$ per capita)	--	
Hodgkin lymphoma	Male	2-4 years	95+ years	Global	-1	3	Socio-demographic Index	--	
Non-Hodgkin's lymphoma	Female	12-23 months	95+ years	Data Rich	-1	2	Universal health coverage	0	
Non-Hodgkin's lymphoma	Female	12-23 months	95+ years	Data Rich	-1	2	Healthcare access and quality index	3	
Non-Hodgkin's lymphoma	Female	12-23 months	95+ years	Data Rich	1	2	Cumulative Cigarettes (10 Years)	192	
Non-Hodgkin's lymphoma	Female	12-23 months	95+ years	Data Rich	1	2	Cumulative Cigarettes (20 Years)	213	
Non-Hodgkin's lymphoma	Female	12-23 months	95+ years	Data Rich	1	2	Cumulative Cigarettes (15 Years)	232	
Non-Hodgkin's lymphoma	Female	12-23 months	95+ years	Data Rich	1	2	Cumulative Cigarettes (5 Years)	246	
Non-Hodgkin's lymphoma	Female	12-23 months	95+ years	Data Rich	1	2	Liters of alcohol consumed per capita	471	
Non-Hodgkin's lymphoma	Female	12-23 months	95+ years	Data Rich	1	2	Mean BMI	--	
Non-Hodgkin's lymphoma	Female	12-23 months	95+ years	Data Rich	-1	3	Socio-demographic Index	0	
Non-Hodgkin's lymphoma	Female	12-23 months	95+ years	Data Rich	-1	3	Total Fertility Rate	3	
Non-Hodgkin's lymphoma	Female	12-23 months	95+ years	Data Rich	-1	3	Education (years per capita)	57	
Non-Hodgkin's lymphoma	Female	12-23 months	95+ years	Data Rich	-1	3	LDI (I$ per capita)	--	
Non-Hodgkin's lymphoma	Female	12-23 months	95+ years	Data Rich	1	3	Log-transformed SEV scalar: HIV	0	
Non-Hodgkin's lymphoma	Female	12-23 months	95+ years	Data Rich	1	3	Log-transformed age-standardized SEV scalar: HIV	--	
Non-Hodgkin's lymphoma	Female	12-23 months	95+ years	Global	1	1	Cumulative Cigarettes (15 Years)	106	
Non-Hodgkin's lymphoma	Female	12-23 months	95+ years	Global	1	1	Cumulative Cigarettes (5 Years)	177	
Non-Hodgkin's lymphoma	Female	12-23 months	95+ years	Global	1	1	Hepatitis C Seroprevalence (anti-HCV) age standardized	247	
Non-Hodgkin's lymphoma	Female	12-23 months	95+ years	Global	1	1	Cumulative Cigarettes (10 Years)	436	
Non-Hodgkin's lymphoma	Female	12-23 months	95+ years	Global	1	1	Cumulative Cigarettes (20 Years)	445	
Non-Hodgkin's lymphoma	Female	12-23 months	95+ years	Global	1	1	HIV Prevalence Unadjusted (proportion)	692	
Non-Hodgkin's lymphoma	Female	12-23 months	95+ years	Global	1	1	HIV age-standardized prevalence	--	
Non-Hodgkin's lymphoma	Female	12-23 months	95+ years	Global	1	1	Mean BMI	--	
Non-Hodgkin's lymphoma	Female	12-23 months	95+ years	Global	-1	2	Education (years per capita)	38	
Non-Hodgkin's lymphoma	Female	12-23 months	95+ years	Global	-1	2	Universal health coverage	119	
Non-Hodgkin's lymphoma	Female	12-23 months	95+ years	Global	-1	2	Socio-demographic Index	172	
Non-Hodgkin's lymphoma	Female	12-23 months	95+ years	Global	-1	2	Healthcare access and quality index	225	
Non-Hodgkin's lymphoma	Female	12-23 months	95+ years	Global	-1	2	Total Fertility Rate	313	
Non-Hodgkin's lymphoma	Female	12-23 months	95+ years	Global	-1	2	LDI (I$ per capita)	--	
Non-Hodgkin's lymphoma	Female	12-23 months	95+ years	Global	1	2	Liters of alcohol consumed per capita	--	
Non-Hodgkin's lymphoma	Female	12-23 months	95+ years	Global	1	3	Log-transformed SEV scalar: HIV	--	
Non-Hodgkin's lymphoma	Female	12-23 months	95+ years	Global	1	3	Log-transformed age-standardized SEV scalar: HIV	--	
Non-Hodgkin's lymphoma	Male	12-23 months	95+ years	Data Rich	-1	2	Universal health coverage	102	
Non-Hodgkin's lymphoma	Male	12-23 months	95+ years	Data Rich	-1	2	Healthcare access and quality index	643	
Non-Hodgkin's lymphoma	Male	12-23 months	95+ years	Data Rich	1	2	Cumulative Cigarettes (5 Years)	21	
Non-Hodgkin's lymphoma	Male	12-23 months	95+ years	Data Rich	1	2	Cumulative Cigarettes (20 Years)	117	
Non-Hodgkin's lymphoma	Male	12-23 months	95+ years	Data Rich	1	2	Liters of alcohol consumed per capita	149	
Non-Hodgkin's lymphoma	Male	12-23 months	95+ years	Data Rich	1	2	Cumulative Cigarettes (10 Years)	283	
Non-Hodgkin's lymphoma	Male	12-23 months	95+ years	Data Rich	1	2	Cumulative Cigarettes (15 Years)	--	
Non-Hodgkin's lymphoma	Male	12-23 months	95+ years	Data Rich	1	2	Mean BMI	--	
Non-Hodgkin's lymphoma	Male	12-23 months	95+ years	Data Rich	-1	3	Education (years per capita)	--	
Non-Hodgkin's lymphoma	Male	12-23 months	95+ years	Data Rich	-1	3	LDI (I$ per capita)	--	
Non-Hodgkin's lymphoma	Male	12-23 months	95+ years	Data Rich	-1	3	Socio-demographic Index	--	
Non-Hodgkin's lymphoma	Male	12-23 months	95+ years	Data Rich	1	3	Log-transformed SEV scalar: HIV	--	
Non-Hodgkin's lymphoma	Male	12-23 months	95+ years	Data Rich	1	3	Log-transformed age-standardized SEV scalar: HIV	--	
Non-Hodgkin's lymphoma	Male	12-23 months	95+ years	Global	-1	2	Universal health coverage	150	
Non-Hodgkin's lymphoma	Male	12-23 months	95+ years	Global	-1	2	Healthcare access and quality index	739	
Non-Hodgkin's lymphoma	Male	12-23 months	95+ years	Global	1	2	Liters of alcohol consumed per capita	36	
Non-Hodgkin's lymphoma	Male	12-23 months	95+ years	Global	1	2	Cumulative Cigarettes (10 Years)	66	
Non-Hodgkin's lymphoma	Male	12-23 months	95+ years	Global	1	2	Cumulative Cigarettes (15 Years)	75	
Non-Hodgkin's lymphoma	Male	12-23 months	95+ years	Global	1	2	Cumulative Cigarettes (20 Years)	159	
Non-Hodgkin's lymphoma	Male	12-23 months	95+ years	Global	1	2	Cumulative Cigarettes (5 Years)	280	
Non-Hodgkin's lymphoma	Male	12-23 months	95+ years	Global	1	2	Mean BMI	--	
Non-Hodgkin's lymphoma	Male	12-23 months	95+ years	Global	-1	3	Education (years per capita)	0	
Non-Hodgkin's lymphoma	Male	12-23 months	95+ years	Global	-1	3	Socio-demographic Index	222	
Non-Hodgkin's lymphoma	Male	12-23 months	95+ years	Global	-1	3	LDI (I$ per capita)	--	

Appendix Table S10: CODEm covariates used, level of covariate, and expected direction of covariate by cause, sex, and age

Cause	Sex	Age Start	Age End	Model Version Type	Direction	Level	Covariate Name	Number of Draws
Non-Hodgkin's lymphoma	Male	12-23 months	95+ years	Global	1	3	Log-transformed SEV scalar: HIV	--
Non-Hodgkin's lymphoma	Male	12-23 months	95+ years	Global	1	3	Log-transformed age-standardized SEV scalar: HIV	--
Other non-Hodgkin lymphoma	Female	12-23 months	95+ years	Data Rich	-1	2	Universal health coverage	1
Other non-Hodgkin lymphoma	Female	12-23 months	95+ years	Data Rich	-1	2	Healthcare access and quality index	61
Other non-Hodgkin lymphoma	Female	12-23 months	95+ years	Data Rich	1	2	Cumulative Cigarettes (15 Years)	78
Other non-Hodgkin lymphoma	Female	12-23 months	95+ years	Data Rich	1	2	Cumulative Cigarettes (10 Years)	189
Other non-Hodgkin lymphoma	Female	12-23 months	95+ years	Data Rich	1	2	Cumulative Cigarettes (5 Years)	198
Other non-Hodgkin lymphoma	Female	12-23 months	95+ years	Data Rich	1	2	Cumulative Cigarettes (20 Years)	306
Other non-Hodgkin lymphoma	Female	12-23 months	95+ years	Data Rich	1	2	Liters of alcohol consumed per capita	503
Other non-Hodgkin lymphoma	Female	12-23 months	95+ years	Data Rich	1	2	Mean BMI	--
Other non-Hodgkin lymphoma	Female	12-23 months	95+ years	Data Rich	-1	3	Socio-demographic Index	38
Other non-Hodgkin lymphoma	Female	12-23 months	95+ years	Data Rich	-1	3	Total Fertility Rate	57
Other non-Hodgkin lymphoma	Female	12-23 months	95+ years	Data Rich	-1	3	Education (years per capita)	121
Other non-Hodgkin lymphoma	Female	12-23 months	95+ years	Data Rich	-1	3	LDI (I$ per capita)	--
Other non-Hodgkin lymphoma	Female	12-23 months	95+ years	Data Rich	1	3	Log-transformed SEV scalar: HIV	1
Other non-Hodgkin lymphoma	Female	12-23 months	95+ years	Data Rich	1	3	Log-transformed age-standardized SEV scalar: HIV	--
Other non-Hodgkin lymphoma	Female	12-23 months	95+ years	Global	-1	2	Universal health coverage	1000
Other non-Hodgkin lymphoma	Female	12-23 months	95+ years	Global	-1	2	Healthcare access and quality index	--
Other non-Hodgkin lymphoma	Female	12-23 months	95+ years	Global	1	2	Cumulative Cigarettes (10 Years)	1000
Other non-Hodgkin lymphoma	Female	12-23 months	95+ years	Global	1	2	Liters of alcohol consumed per capita	1000
Other non-Hodgkin lymphoma	Female	12-23 months	95+ years	Global	1	2	Cumulative Cigarettes (15 Years)	--
Other non-Hodgkin lymphoma	Female	12-23 months	95+ years	Global	1	2	Cumulative Cigarettes (20 Years)	--
Other non-Hodgkin lymphoma	Female	12-23 months	95+ years	Global	1	2	Cumulative Cigarettes (5 Years)	--
Other non-Hodgkin lymphoma	Female	12-23 months	95+ years	Global	1	2	Mean BMI	--
Other non-Hodgkin lymphoma	Female	12-23 months	95+ years	Global	-1	3	Education (years per capita)	--
Other non-Hodgkin lymphoma	Female	12-23 months	95+ years	Global	-1	3	LDI (I$ per capita)	--
Other non-Hodgkin lymphoma	Female	12-23 months	95+ years	Global	-1	3	Socio-demographic Index	--
Other non-Hodgkin lymphoma	Female	12-23 months	95+ years	Global	-1	3	Total Fertility Rate	--
Other non-Hodgkin lymphoma	Female	12-23 months	95+ years	Global	1	3	Log-transformed SEV scalar: HIV	--
Other non-Hodgkin lymphoma	Female	12-23 months	95+ years	Global	1	3	Log-transformed age-standardized SEV scalar: HIV	--
Other non-Hodgkin lymphoma	Male	12-23 months	95+ years	Data Rich	-1	2	Universal health coverage	68
Other non-Hodgkin lymphoma	Male	12-23 months	95+ years	Data Rich	-1	2	Healthcare access and quality index	161
Other non-Hodgkin lymphoma	Male	12-23 months	95+ years	Data Rich	1	2	Cumulative Cigarettes (10 Years)	31
Other non-Hodgkin lymphoma	Male	12-23 months	95+ years	Data Rich	1	2	Cumulative Cigarettes (20 Years)	97
Other non-Hodgkin lymphoma	Male	12-23 months	95+ years	Data Rich	1	2	Cumulative Cigarettes (5 Years)	148
Other non-Hodgkin lymphoma	Male	12-23 months	95+ years	Data Rich	1	2	Cumulative Cigarettes (15 Years)	385
Other non-Hodgkin lymphoma	Male	12-23 months	95+ years	Data Rich	1	2	Liters of alcohol consumed per capita	615
Other non-Hodgkin lymphoma	Male	12-23 months	95+ years	Data Rich	1	2	Mean BMI	--
Other non-Hodgkin lymphoma	Male	12-23 months	95+ years	Data Rich	-1	3	Socio-demographic Index	77
Other non-Hodgkin lymphoma	Male	12-23 months	95+ years	Data Rich	-1	3	Education (years per capita)	133
Other non-Hodgkin lymphoma	Male	12-23 months	95+ years	Data Rich	-1	3	LDI (I$ per capita)	--
Other non-Hodgkin lymphoma	Male	12-23 months	95+ years	Data Rich	1	3	Log-transformed SEV scalar: HIV	--
Other non-Hodgkin lymphoma	Male	12-23 months	95+ years	Data Rich	1	3	Log-transformed age-standardized SEV scalar: HIV	--
Other non-Hodgkin lymphoma	Male	12-23 months	95+ years	Global	-1	2	Healthcare access and quality index	117
Other non-Hodgkin lymphoma	Male	12-23 months	95+ years	Global	-1	2	Universal health coverage	351
Other non-Hodgkin lymphoma	Male	12-23 months	95+ years	Global	1	2	Cumulative Cigarettes (10 Years)	69
Other non-Hodgkin lymphoma	Male	12-23 months	95+ years	Global	1	2	Cumulative Cigarettes (5 Years)	122
Other non-Hodgkin lymphoma	Male	12-23 months	95+ years	Global	1	2	Cumulative Cigarettes (15 Years)	381
Other non-Hodgkin lymphoma	Male	12-23 months	95+ years	Global	1	2	Cumulative Cigarettes (20 Years)	428
Other non-Hodgkin lymphoma	Male	12-23 months	95+ years	Global	1	2	Liters of alcohol consumed per capita	510
Other non-Hodgkin lymphoma	Male	12-23 months	95+ years	Global	1	2	Mean BMI	--
Other non-Hodgkin lymphoma	Male	12-23 months	95+ years	Global	-1	3	Education (years per capita)	36
Other non-Hodgkin lymphoma	Male	12-23 months	95+ years	Global	-1	3	Socio-demographic Index	70
Other non-Hodgkin lymphoma	Male	12-23 months	95+ years	Global	-1	3	LDI (I$ per capita)	--
Other non-Hodgkin lymphoma	Male	12-23 months	95+ years	Global	1	3	Log-transformed SEV scalar: HIV	--
Other non-Hodgkin lymphoma	Male	12-23 months	95+ years	Global	1	3	Log-transformed age-standardized SEV scalar: HIV	--
Multiple myeloma	Female	20-24 years	95+ years	Data Rich	1	1	Liters of alcohol consumed per capita	483
Multiple myeloma	Female	20-24 years	95+ years	Data Rich	1	1	Smoking Prevalence	813
Multiple myeloma	Female	20-24 years	95+ years	Data Rich	1	1	Tobacco (cigarettes per capita)	--
Multiple myeloma	Female	20-24 years	95+ years	Data Rich	-1	2	Healthcare access and quality index	--
Multiple myeloma	Female	20-24 years	95+ years	Data Rich	-1	2	Improved Water Source (proportion with access)	--
Multiple myeloma	Female	20-24 years	95+ years	Data Rich	-1	2	Sanitation (proportion with access)	--
Multiple myeloma	Female	20-24 years	95+ years	Data Rich	1	2	Mean BMI	273
Multiple myeloma	Female	20-24 years	95+ years	Data Rich	1	2	Age- and sex-specific SEV for High red meat	313
Multiple myeloma	Female	20-24 years	95+ years	Data Rich	1	2	Age- and sex-specific SEV for Low fruit	--
Multiple myeloma	Female	20-24 years	95+ years	Data Rich	1	2	Age- and sex-specific SEV for Low vegetables	--
Multiple myeloma	Female	20-24 years	95+ years	Data Rich	-1	3	Education (years per capita)	--
Multiple myeloma	Female	20-24 years	95+ years	Data Rich	1	3	Socio-demographic Index	272
Multiple myeloma	Female	20-24 years	95+ years	Data Rich	1	3	LDI (I$ per capita)	--
Multiple myeloma	Female	20-24 years	95+ years	Global	1	1	Liters of alcohol consumed per capita	1000
Multiple myeloma	Female	20-24 years	95+ years	Global	1	1	Smoking Prevalence	--
Multiple myeloma	Female	20-24 years	95+ years	Global	1	1	Tobacco (cigarettes per capita)	--
Multiple myeloma	Female	20-24 years	95+ years	Global	-1	2	Healthcare access and quality index	--
Multiple myeloma	Female	20-24 years	95+ years	Global	-1	2	Improved Water Source (proportion with access)	--
Multiple myeloma	Female	20-24 years	95+ years	Global	-1	2	Sanitation (proportion with access)	--
Multiple myeloma	Female	20-24 years	95+ years	Global	1	2	Age- and sex-specific SEV for High red meat	308
Multiple myeloma	Female	20-24 years	95+ years	Global	1	2	Mean BMI	399
Multiple myeloma	Female	20-24 years	95+ years	Global	1	2	Age- and sex-specific SEV for Low fruit	--
Multiple myeloma	Female	20-24 years	95+ years	Global	1	2	Age- and sex-specific SEV for Low vegetables	--
Multiple myeloma	Female	20-24 years	95+ years	Global	-1	3	Education (years per capita)	--
Multiple myeloma	Female	20-24 years	95+ years	Global	1	3	Socio-demographic Index	381
Multiple myeloma	Female	20-24 years	95+ years	Global	1	3	LDI (I$ per capita)	--
Multiple myeloma	Male	20-24 years	95+ years	Data Rich	1	1	Liters of alcohol consumed per capita	1000
Multiple myeloma	Male	20-24 years	95+ years	Data Rich	1	1	Smoking Prevalence	--
Multiple myeloma	Male	20-24 years	95+ years	Data Rich	1	1	Tobacco (cigarettes per capita)	--
Multiple myeloma	Male	20-24 years	95+ years	Data Rich	-1	2	Healthcare access and quality index	--
Multiple myeloma	Male	20-24 years	95+ years	Data Rich	-1	2	Improved Water Source (proportion with access)	--
Multiple myeloma	Male	20-24 years	95+ years	Data Rich	-1	2	Sanitation (proportion with access)	--
Multiple myeloma	Male	20-24 years	95+ years	Data Rich	1	2	Mean BMI	325
Multiple myeloma	Male	20-24 years	95+ years	Data Rich	1	2	Age- and sex-specific SEV for High red meat	--
Multiple myeloma	Male	20-24 years	95+ years	Data Rich	1	2	Age- and sex-specific SEV for Low fruit	--
Multiple myeloma	Male	20-24 years	95+ years	Data Rich	1	2	Age- and sex-specific SEV for Low vegetables	--
Multiple myeloma	Male	20-24 years	95+ years	Data Rich	-1	3	Education (years per capita)	--
Multiple myeloma	Male	20-24 years	95+ years	Data Rich	1	3	LDI (I$ per capita)	--
Multiple myeloma	Male	20-24 years	95+ years	Data Rich	1	3	Socio-demographic Index	--
Multiple myeloma	Male	20-24 years	95+ years	Global	1	1	Liters of alcohol consumed per capita	1000
Multiple myeloma	Male	20-24 years	95+ years	Global	1	1	Smoking Prevalence	--
Multiple myeloma	Male	20-24 years	95+ years	Global	1	1	Tobacco (cigarettes per capita)	--
Multiple myeloma	Male	20-24 years	95+ years	Global	-1	2	Healthcare access and quality index	--
Multiple myeloma	Male	20-24 years	95+ years	Global	-1	2	Improved Water Source (proportion with access)	--
Multiple myeloma	Male	20-24 years	95+ years	Global	-1	2	Sanitation (proportion with access)	--
Multiple myeloma	Male	20-24 years	95+ years	Global	1	2	Age- and sex-specific SEV for High red meat	546
Multiple myeloma	Male	20-24 years	95+ years	Global	1	2	Mean BMI	546
Multiple myeloma	Male	20-24 years	95+ years	Global	1	2	Age- and sex-specific SEV for Low fruit	--
Multiple myeloma	Male	20-24 years	95+ years	Global	1	2	Age- and sex-specific SEV for Low vegetables	--
Multiple myeloma	Male	20-24 years	95+ years	Global	-1	3	Education (years per capita)	--
Multiple myeloma	Male	20-24 years	95+ years	Global	1	3	LDI (I$ per capita)	--

Appendix Table S10: CODEm covariates used, level of covariate, and expected direction of covariate by cause, sex, and age								
**Cause**	**Sex**	**Age Start**	**Age End**	**Model Version Type**	**Direction**	**Level**	**Covariate Name**	**Number of Draws**
Multiple myeloma	Male	20-24 years	95+ years	Global	1	3	Socio-demographic Index	--
Leukaemia	Female	0-6 days	95+ years	Data Rich	-1	2	Healthcare access and quality index	409
Leukaemia	Female	0-6 days	95+ years	Data Rich	1	2	Cumulative Cigarettes (20 Years)	17
Leukaemia	Female	0-6 days	95+ years	Data Rich	1	2	Liters of alcohol consumed per capita	233
Leukaemia	Female	0-6 days	95+ years	Data Rich	1	2	Cumulative Cigarettes (10 Years)	288
Leukaemia	Female	0-6 days	95+ years	Data Rich	1	2	Tobacco (cigarettes per capita)	922
Leukaemia	Female	0-6 days	95+ years	Data Rich	1	2	Mean BMI	--
Leukaemia	Female	0-6 days	95+ years	Data Rich	-1	3	Education (years per capita)	349
Leukaemia	Female	0-6 days	95+ years	Data Rich	-1	3	Socio-demographic Index	528
Leukaemia	Female	0-6 days	95+ years	Data Rich	1	3	LDI (I$ per capita)	--
Leukaemia	Female	0-6 days	95+ years	Global	-1	2	Healthcare access and quality index	822
Leukaemia	Female	0-6 days	95+ years	Global	1	2	Cumulative Cigarettes (10 Years)	282
Leukaemia	Female	0-6 days	95+ years	Global	1	2	Tobacco (cigarettes per capita)	502
Leukaemia	Female	0-6 days	95+ years	Global	1	2	Cumulative Cigarettes (20 Years)	573
Leukaemia	Female	0-6 days	95+ years	Global	1	2	Liters of alcohol consumed per capita	--
Leukaemia	Female	0-6 days	95+ years	Global	1	2	Mean BMI	--
Leukaemia	Female	0-6 days	95+ years	Global	-1	3	Education (years per capita)	241
Leukaemia	Female	0-6 days	95+ years	Global	-1	3	Socio-demographic Index	285
Leukaemia	Female	0-6 days	95+ years	Global	1	3	LDI (I$ per capita)	--
Leukaemia	Male	0-6 days	95+ years	Data Rich	-1	2	Healthcare access and quality index	777
Leukaemia	Male	0-6 days	95+ years	Data Rich	1	2	Cumulative Cigarettes (10 Years)	134
Leukaemia	Male	0-6 days	95+ years	Data Rich	1	2	Cumulative Cigarettes (20 Years)	138
Leukaemia	Male	0-6 days	95+ years	Data Rich	1	2	Liters of alcohol consumed per capita	260
Leukaemia	Male	0-6 days	95+ years	Data Rich	1	2	Tobacco (cigarettes per capita)	681
Leukaemia	Male	0-6 days	95+ years	Data Rich	1	2	Mean BMI	--
Leukaemia	Male	0-6 days	95+ years	Data Rich	-1	3	Education (years per capita)	144
Leukaemia	Male	0-6 days	95+ years	Data Rich	-1	3	Socio-demographic Index	453
Leukaemia	Male	0-6 days	95+ years	Data Rich	1	3	LDI (I$ per capita)	--
Leukaemia	Male	0-6 days	95+ years	Global	-1	2	Healthcare access and quality index	225
Leukaemia	Male	0-6 days	95+ years	Global	1	2	Cumulative Cigarettes (20 Years)	160
Leukaemia	Male	0-6 days	95+ years	Global	1	2	Cumulative Cigarettes (10 Years)	196
Leukaemia	Male	0-6 days	95+ years	Global	1	2	Tobacco (cigarettes per capita)	644
Leukaemia	Male	0-6 days	95+ years	Global	1	2	Liters of alcohol consumed per capita	--
Leukaemia	Male	0-6 days	95+ years	Global	1	2	Mean BMI	--
Leukaemia	Male	0-6 days	95+ years	Global	-1	3	Socio-demographic Index	156
Leukaemia	Male	0-6 days	95+ years	Global	-1	3	Education (years per capita)	398
Leukaemia	Male	0-6 days	95+ years	Global	1	3	LDI (I$ per capita)	--
Acute lymphoid leukaemia	Female	0-6 days	95+ years	Data Rich	-1	2	Healthcare access and quality index	1000
Acute lymphoid leukaemia	Female	0-6 days	95+ years	Data Rich	1	2	Mean BMI	--
Acute lymphoid leukaemia	Female	0-6 days	95+ years	Data Rich	-1	3	LDI (I$ per capita)	568
Acute lymphoid leukaemia	Female	0-6 days	95+ years	Data Rich	-1	3	Education (years per capita)	--
Acute lymphoid leukaemia	Female	0-6 days	95+ years	Data Rich	1	3	Socio-demographic Index	--
Acute lymphoid leukaemia	Female	0-6 days	95+ years	Global	-1	2	Healthcare access and quality index	1000
Acute lymphoid leukaemia	Female	0-6 days	95+ years	Global	1	2	Mean BMI	--
Acute lymphoid leukaemia	Female	0-6 days	95+ years	Global	-1	3	Education (years per capita)	--
Acute lymphoid leukaemia	Female	0-6 days	95+ years	Global	-1	3	LDI (I$ per capita)	--
Acute lymphoid leukaemia	Female	0-6 days	95+ years	Global	1	3	Socio-demographic Index	--
Acute lymphoid leukaemia	Male	0-6 days	95+ years	Data Rich	-1	2	Healthcare access and quality index	--
Acute lymphoid leukaemia	Male	0-6 days	95+ years	Data Rich	1	2	Mean BMI	--
Acute lymphoid leukaemia	Male	0-6 days	95+ years	Data Rich	-1	3	LDI (I$ per capita)	1000
Acute lymphoid leukaemia	Male	0-6 days	95+ years	Data Rich	-1	3	Education (years per capita)	--
Acute lymphoid leukaemia	Male	0-6 days	95+ years	Data Rich	1	3	Socio-demographic Index	--
Acute lymphoid leukaemia	Male	0-6 days	95+ years	Global	-1	2	Healthcare access and quality index	1000
Acute lymphoid leukaemia	Male	0-6 days	95+ years	Global	1	2	Mean BMI	--
Acute lymphoid leukaemia	Male	0-6 days	95+ years	Global	-1	3	Education (years per capita)	--
Acute lymphoid leukaemia	Male	0-6 days	95+ years	Global	-1	3	LDI (I$ per capita)	--
Acute lymphoid leukaemia	Male	0-6 days	95+ years	Global	1	3	Socio-demographic Index	--
Chronic lymphoid leukaemia	Female	20-24 years	95+ years	Data Rich	-1	2	Healthcare access and quality index	0
Chronic lymphoid leukaemia	Female	20-24 years	95+ years	Data Rich	1	2	Tobacco (cigarettes per capita)	0
Chronic lymphoid leukaemia	Female	20-24 years	95+ years	Data Rich	1	2	Cumulative Cigarettes (10 Years)	15
Chronic lymphoid leukaemia	Female	20-24 years	95+ years	Data Rich	1	2	Cumulative Cigarettes (15 Years)	140
Chronic lymphoid leukaemia	Female	20-24 years	95+ years	Data Rich	1	2	Cumulative Cigarettes (5 Years)	226
Chronic lymphoid leukaemia	Female	20-24 years	95+ years	Data Rich	1	2	Mean BMI	297
Chronic lymphoid leukaemia	Female	20-24 years	95+ years	Data Rich	1	2	Cumulative Cigarettes (20 Years)	310
Chronic lymphoid leukaemia	Female	20-24 years	95+ years	Data Rich	1	2	Liters of alcohol consumed per capita	479
Chronic lymphoid leukaemia	Female	20-24 years	95+ years	Data Rich	1	2	Smoking Prevalence	552
Chronic lymphoid leukaemia	Female	20-24 years	95+ years	Data Rich	-1	3	Education (years per capita)	105
Chronic lymphoid leukaemia	Female	20-24 years	95+ years	Data Rich	-1	3	LDI (I$ per capita)	250
Chronic lymphoid leukaemia	Female	20-24 years	95+ years	Data Rich	1	3	Socio-demographic Index	386
Chronic lymphoid leukaemia	Female	20-24 years	95+ years	Global	-1	2	Healthcare access and quality index	42
Chronic lymphoid leukaemia	Female	20-24 years	95+ years	Global	1	2	Tobacco (cigarettes per capita)	3
Chronic lymphoid leukaemia	Female	20-24 years	95+ years	Global	1	2	Cumulative Cigarettes (20 Years)	96
Chronic lymphoid leukaemia	Female	20-24 years	95+ years	Global	1	2	Smoking Prevalence	141
Chronic lymphoid leukaemia	Female	20-24 years	95+ years	Global	1	2	Cumulative Cigarettes (15 Years)	218
Chronic lymphoid leukaemia	Female	20-24 years	95+ years	Global	1	2	Cumulative Cigarettes (10 Years)	221
Chronic lymphoid leukaemia	Female	20-24 years	95+ years	Global	1	2	Cumulative Cigarettes (5 Years)	221
Chronic lymphoid leukaemia	Female	20-24 years	95+ years	Global	1	2	Mean BMI	405
Chronic lymphoid leukaemia	Female	20-24 years	95+ years	Global	1	2	Liters of alcohol consumed per capita	1000
Chronic lymphoid leukaemia	Female	20-24 years	95+ years	Global	-1	3	Education (years per capita)	162
Chronic lymphoid leukaemia	Female	20-24 years	95+ years	Global	-1	3	LDI (I$ per capita)	231
Chronic lymphoid leukaemia	Female	20-24 years	95+ years	Global	1	3	Socio-demographic Index	27
Chronic lymphoid leukaemia	Male	20-24 years	95+ years	Data Rich	-1	2	Healthcare access and quality index	148
Chronic lymphoid leukaemia	Male	20-24 years	95+ years	Data Rich	1	2	Cumulative Cigarettes (20 Years)	20
Chronic lymphoid leukaemia	Male	20-24 years	95+ years	Data Rich	1	2	Mean BMI	26
Chronic lymphoid leukaemia	Male	20-24 years	95+ years	Data Rich	1	2	Cumulative Cigarettes (10 Years)	85
Chronic lymphoid leukaemia	Male	20-24 years	95+ years	Data Rich	1	2	Cumulative Cigarettes (15 Years)	96
Chronic lymphoid leukaemia	Male	20-24 years	95+ years	Data Rich	1	2	Liters of alcohol consumed per capita	112
Chronic lymphoid leukaemia	Male	20-24 years	95+ years	Data Rich	1	2	Cumulative Cigarettes (5 Years)	185
Chronic lymphoid leukaemia	Male	20-24 years	95+ years	Data Rich	1	2	Tobacco (cigarettes per capita)	366
Chronic lymphoid leukaemia	Male	20-24 years	95+ years	Data Rich	1	2	Smoking Prevalence	465
Chronic lymphoid leukaemia	Male	20-24 years	95+ years	Data Rich	-1	3	LDI (I$ per capita)	20
Chronic lymphoid leukaemia	Male	20-24 years	95+ years	Data Rich	-1	3	Education (years per capita)	--
Chronic lymphoid leukaemia	Male	20-24 years	95+ years	Data Rich	1	3	Socio-demographic Index	783
Chronic lymphoid leukaemia	Male	20-24 years	95+ years	Global	-1	2	Healthcare access and quality index	115
Chronic lymphoid leukaemia	Male	20-24 years	95+ years	Global	1	2	Cumulative Cigarettes (15 Years)	35
Chronic lymphoid leukaemia	Male	20-24 years	95+ years	Global	1	2	Mean BMI	65
Chronic lymphoid leukaemia	Male	20-24 years	95+ years	Global	1	2	Tobacco (cigarettes per capita)	70
Chronic lymphoid leukaemia	Male	20-24 years	95+ years	Global	1	2	Cumulative Cigarettes (10 Years)	97
Chronic lymphoid leukaemia	Male	20-24 years	95+ years	Global	1	2	Smoking Prevalence	122
Chronic lymphoid leukaemia	Male	20-24 years	95+ years	Global	1	2	Cumulative Cigarettes (20 Years)	155
Chronic lymphoid leukaemia	Male	20-24 years	95+ years	Global	1	2	Cumulative Cigarettes (5 Years)	201
Chronic lymphoid leukaemia	Male	20-24 years	95+ years	Global	1	2	Liters of alcohol consumed per capita	385
Chronic lymphoid leukaemia	Male	20-24 years	95+ years	Global	-1	3	LDI (I$ per capita)	107
Chronic lymphoid leukaemia	Male	20-24 years	95+ years	Global	-1	3	Education (years per capita)	138
Chronic lymphoid leukaemia	Male	20-24 years	95+ years	Global	1	3	Socio-demographic Index	6
Acute myeloid leukaemia	Female	0-6 days	95+ years	Data Rich	-1	2	Healthcare access and quality index	--
Acute myeloid leukaemia	Female	0-6 days	95+ years	Data Rich	1	2	Cumulative Cigarettes (20 Years)	325
Acute myeloid leukaemia	Female	0-6 days	95+ years	Data Rich	1	2	Smoking Prevalence	675
Acute myeloid leukaemia	Female	0-6 days	95+ years	Data Rich	1	2	Mean BMI	1000
Acute myeloid leukaemia	Female	0-6 days	95+ years	Data Rich	1	2	Cumulative Cigarettes (10 Years)	--
Acute myeloid leukaemia	Female	0-6 days	95+ years	Data Rich	1	2	Liters of alcohol consumed per capita	--
Acute myeloid leukaemia	Female	0-6 days	95+ years	Data Rich	-1	3	Education (years per capita)	--
Acute myeloid leukaemia	Female	0-6 days	95+ years	Data Rich	1	3	Socio-demographic Index	427
Acute myeloid leukaemia	Female	0-6 days	95+ years	Data Rich	1	3	LDI (I$ per capita)	--
Acute myeloid leukaemia	Female	0-6 days	95+ years	Global	-1	2	Healthcare access and quality index	--
Acute myeloid leukaemia	Female	0-6 days	95+ years	Global	1	2	Smoking Prevalence	222

Appendix Table S10: CODEm covariates used, level of covariate, and expected direction of covariate by cause, sex, and age

Cause	Sex	Age Start	Age End	Model Version Type	Direction	Level	Covariate Name	Number of Draws
Acute myeloid leukaemia	Female	0-6 days	95+ years	Global	1	2	Liters of alcohol consumed per capita	434
Acute myeloid leukaemia	Female	0-6 days	95+ years	Global	1	2	Mean BMI	434
Acute myeloid leukaemia	Female	0-6 days	95+ years	Global	1	2	Cumulative Cigarettes (20 Years)	778
Acute myeloid leukaemia	Female	0-6 days	95+ years	Global	1	2	Cumulative Cigarettes (10 Years)	--
Acute myeloid leukaemia	Female	0-6 days	95+ years	Global	-1	3	Education (years per capita)	--
Acute myeloid leukaemia	Female	0-6 days	95+ years	Global	1	3	LDI (I$ per capita)	--
Acute myeloid leukaemia	Female	0-6 days	95+ years	Global	1	3	Socio-demographic Index	--
Acute myeloid leukaemia	Male	0-6 days	95+ years	Data Rich	-1	2	Healthcare access and quality index	--
Acute myeloid leukaemia	Male	0-6 days	95+ years	Data Rich	1	2	Cumulative Cigarettes (10 Years)	432
Acute myeloid leukaemia	Male	0-6 days	95+ years	Data Rich	1	2	Liters of alcohol consumed per capita	1000
Acute myeloid leukaemia	Male	0-6 days	95+ years	Data Rich	1	2	Mean BMI	1000
Acute myeloid leukaemia	Male	0-6 days	95+ years	Data Rich	1	2	Cumulative Cigarettes (20 Years)	--
Acute myeloid leukaemia	Male	0-6 days	95+ years	Data Rich	1	2	Smoking Prevalence	--
Acute myeloid leukaemia	Male	0-6 days	95+ years	Data Rich	-1	3	Education (years per capita)	--
Acute myeloid leukaemia	Male	0-6 days	95+ years	Data Rich	1	3	LDI (I$ per capita)	--
Acute myeloid leukaemia	Male	0-6 days	95+ years	Data Rich	1	3	Socio-demographic Index	--
Acute myeloid leukaemia	Male	0-6 days	95+ years	Global	-1	2	Healthcare access and quality index	--
Acute myeloid leukaemia	Male	0-6 days	95+ years	Global	1	2	Mean BMI	546
Acute myeloid leukaemia	Male	0-6 days	95+ years	Global	1	2	Liters of alcohol consumed per capita	1000
Acute myeloid leukaemia	Male	0-6 days	95+ years	Global	1	2	Cumulative Cigarettes (10 Years)	--
Acute myeloid leukaemia	Male	0-6 days	95+ years	Global	1	2	Cumulative Cigarettes (20 Years)	--
Acute myeloid leukaemia	Male	0-6 days	95+ years	Global	1	2	Smoking Prevalence	--
Acute myeloid leukaemia	Male	0-6 days	95+ years	Global	-1	3	Education (years per capita)	--
Acute myeloid leukaemia	Male	0-6 days	95+ years	Global	1	3	LDI (I$ per capita)	--
Acute myeloid leukaemia	Male	0-6 days	95+ years	Global	1	3	Socio-demographic Index	--
Chronic myeloid leukaemia	Female	0-6 days	95+ years	Data Rich	-1	2	Healthcare access and quality index	2
Chronic myeloid leukaemia	Female	0-6 days	95+ years	Data Rich	1	2	Cumulative Cigarettes (20 Years)	97
Chronic myeloid leukaemia	Female	0-6 days	95+ years	Data Rich	1	2	Cumulative Cigarettes (5 Years)	102
Chronic myeloid leukaemia	Female	0-6 days	95+ years	Data Rich	1	2	Cumulative Cigarettes (15 Years)	188
Chronic myeloid leukaemia	Female	0-6 days	95+ years	Data Rich	1	2	Smoking Prevalence	260
Chronic myeloid leukaemia	Female	0-6 days	95+ years	Data Rich	1	2	Cumulative Cigarettes (10 Years)	315
Chronic myeloid leukaemia	Female	0-6 days	95+ years	Data Rich	1	2	Liters of alcohol consumed per capita	396
Chronic myeloid leukaemia	Female	0-6 days	95+ years	Data Rich	1	2	Tobacco (cigarettes per capita)	899
Chronic myeloid leukaemia	Female	0-6 days	95+ years	Data Rich	1	2	Mean BMI	--
Chronic myeloid leukaemia	Female	0-6 days	95+ years	Data Rich	-1	3	Socio-demographic Index	27
Chronic myeloid leukaemia	Female	0-6 days	95+ years	Data Rich	-1	3	Education (years per capita)	242
Chronic myeloid leukaemia	Female	0-6 days	95+ years	Data Rich	1	3	LDI (I$ per capita)	--
Chronic myeloid leukaemia	Female	0-6 days	95+ years	Global	-1	2	Healthcare access and quality index	731
Chronic myeloid leukaemia	Female	0-6 days	95+ years	Global	1	2	Cumulative Cigarettes (10 Years)	8
Chronic myeloid leukaemia	Female	0-6 days	95+ years	Global	1	2	Liters of alcohol consumed per capita	183
Chronic myeloid leukaemia	Female	0-6 days	95+ years	Global	1	2	Cumulative Cigarettes (5 Years)	219
Chronic myeloid leukaemia	Female	0-6 days	95+ years	Global	1	2	Cumulative Cigarettes (20 Years)	270
Chronic myeloid leukaemia	Female	0-6 days	95+ years	Global	1	2	Cumulative Cigarettes (15 Years)	422
Chronic myeloid leukaemia	Female	0-6 days	95+ years	Global	1	2	Smoking Prevalence	509
Chronic myeloid leukaemia	Female	0-6 days	95+ years	Global	1	2	Tobacco (cigarettes per capita)	553
Chronic myeloid leukaemia	Female	0-6 days	95+ years	Global	1	2	Mean BMI	--
Chronic myeloid leukaemia	Female	0-6 days	95+ years	Global	-1	3	Education (years per capita)	322
Chronic myeloid leukaemia	Female	0-6 days	95+ years	Global	-1	3	Socio-demographic Index	659
Chronic myeloid leukaemia	Female	0-6 days	95+ years	Global	1	3	LDI (I$ per capita)	--
Chronic myeloid leukaemia	Male	0-6 days	95+ years	Data Rich	-1	2	Healthcare access and quality index	1
Chronic myeloid leukaemia	Male	0-6 days	95+ years	Data Rich	1	2	Cumulative Cigarettes (15 Years)	28
Chronic myeloid leukaemia	Male	0-6 days	95+ years	Data Rich	1	2	Cumulative Cigarettes (5 Years)	46
Chronic myeloid leukaemia	Male	0-6 days	95+ years	Data Rich	1	2	Cumulative Cigarettes (20 Years)	53
Chronic myeloid leukaemia	Male	0-6 days	95+ years	Data Rich	1	2	Cumulative Cigarettes (10 Years)	59
Chronic myeloid leukaemia	Male	0-6 days	95+ years	Data Rich	1	2	Smoking Prevalence	157
Chronic myeloid leukaemia	Male	0-6 days	95+ years	Data Rich	1	2	Liters of alcohol consumed per capita	446
Chronic myeloid leukaemia	Male	0-6 days	95+ years	Data Rich	1	2	Tobacco (cigarettes per capita)	813
Chronic myeloid leukaemia	Male	0-6 days	95+ years	Data Rich	1	2	Mean BMI	--
Chronic myeloid leukaemia	Male	0-6 days	95+ years	Data Rich	-1	3	Socio-demographic Index	61
Chronic myeloid leukaemia	Male	0-6 days	95+ years	Data Rich	-1	3	Education (years per capita)	250
Chronic myeloid leukaemia	Male	0-6 days	95+ years	Data Rich	1	3	LDI (I$ per capita)	--
Chronic myeloid leukaemia	Male	0-6 days	95+ years	Global	-1	2	Healthcare access and quality index	810
Chronic myeloid leukaemia	Male	0-6 days	95+ years	Global	1	2	Tobacco (cigarettes per capita)	26
Chronic myeloid leukaemia	Male	0-6 days	95+ years	Global	1	2	Cumulative Cigarettes (10 Years)	103
Chronic myeloid leukaemia	Male	0-6 days	95+ years	Global	1	2	Cumulative Cigarettes (15 Years)	106
Chronic myeloid leukaemia	Male	0-6 days	95+ years	Global	1	2	Cumulative Cigarettes (20 Years)	116
Chronic myeloid leukaemia	Male	0-6 days	95+ years	Global	1	2	Smoking Prevalence	599
Chronic myeloid leukaemia	Male	0-6 days	95+ years	Global	1	2	Cumulative Cigarettes (5 Years)	609
Chronic myeloid leukaemia	Male	0-6 days	95+ years	Global	1	2	Liters of alcohol consumed per capita	854
Chronic myeloid leukaemia	Male	0-6 days	95+ years	Global	1	2	Mean BMI	--
Chronic myeloid leukaemia	Male	0-6 days	95+ years	Global	-1	3	Socio-demographic Index	264
Chronic myeloid leukaemia	Male	0-6 days	95+ years	Global	-1	3	Education (years per capita)	275
Chronic myeloid leukaemia	Male	0-6 days	95+ years	Global	1	3	LDI (I$ per capita)	--
Other leukaemia	Female	0-6 days	95+ years	Data Rich	1	1	Liters of alcohol consumed per capita	--
Other leukaemia	Female	0-6 days	95+ years	Data Rich	-1	2	Healthcare access and quality index	329
Other leukaemia	Female	0-6 days	95+ years	Data Rich	1	2	Cumulative Cigarettes (20 Years)	274
Other leukaemia	Female	0-6 days	95+ years	Data Rich	1	2	Cumulative Cigarettes (10 Years)	--
Other leukaemia	Female	0-6 days	95+ years	Data Rich	1	2	Mean BMI	--
Other leukaemia	Female	0-6 days	95+ years	Data Rich	1	2	Tobacco (cigarettes per capita)	--
Other leukaemia	Female	0-6 days	95+ years	Data Rich	-1	3	Education (years per capita)	397
Other leukaemia	Female	0-6 days	95+ years	Data Rich	-1	3	Socio-demographic Index	397
Other leukaemia	Female	0-6 days	95+ years	Data Rich	1	3	LDI (I$ per capita)	--
Other leukaemia	Female	0-6 days	95+ years	Global	-1	2	Healthcare access and quality index	--
Other leukaemia	Female	0-6 days	95+ years	Global	1	2	Cumulative Cigarettes (20 Years)	184
Other leukaemia	Female	0-6 days	95+ years	Global	1	2	Mean BMI	303
Other leukaemia	Female	0-6 days	95+ years	Global	1	2	Tobacco (cigarettes per capita)	816
Other leukaemia	Female	0-6 days	95+ years	Global	1	2	Cumulative Cigarettes (10 Years)	--
Other leukaemia	Female	0-6 days	95+ years	Global	1	2	Liters of alcohol consumed per capita	--
Other leukaemia	Female	0-6 days	95+ years	Global	-1	3	Education (years per capita)	170
Other leukaemia	Female	0-6 days	95+ years	Global	-1	3	Socio-demographic Index	452
Other leukaemia	Female	0-6 days	95+ years	Global	1	3	LDI (I$ per capita)	--
Other leukaemia	Male	0-6 days	95+ years	Data Rich	-1	2	Healthcare access and quality index	409
Other leukaemia	Male	0-6 days	95+ years	Data Rich	1	2	Tobacco (cigarettes per capita)	268
Other leukaemia	Male	0-6 days	95+ years	Data Rich	1	2	Cumulative Cigarettes (20 Years)	323
Other leukaemia	Male	0-6 days	95+ years	Data Rich	1	2	Mean BMI	591
Other leukaemia	Male	0-6 days	95+ years	Data Rich	1	2	Cumulative Cigarettes (10 Years)	--
Other leukaemia	Male	0-6 days	95+ years	Data Rich	1	2	Liters of alcohol consumed per capita	--
Other leukaemia	Male	0-6 days	95+ years	Data Rich	-1	3	Education (years per capita)	223
Other leukaemia	Male	0-6 days	95+ years	Data Rich	-1	3	Socio-demographic Index	--
Other leukaemia	Male	0-6 days	95+ years	Data Rich	1	3	LDI (I$ per capita)	223
Other leukaemia	Male	0-6 days	95+ years	Global	-1	2	Healthcare access and quality index	258
Other leukaemia	Male	0-6 days	95+ years	Global	1	2	Cumulative Cigarettes (20 Years)	116
Other leukaemia	Male	0-6 days	95+ years	Global	1	2	Cumulative Cigarettes (10 Years)	216
Other leukaemia	Male	0-6 days	95+ years	Global	1	2	Tobacco (cigarettes per capita)	238
Other leukaemia	Male	0-6 days	95+ years	Global	1	2	Mean BMI	596
Other leukaemia	Male	0-6 days	95+ years	Global	1	2	Liters of alcohol consumed per capita	--
Other leukaemia	Male	0-6 days	95+ years	Global	-1	3	Socio-demographic Index	160
Other leukaemia	Male	0-6 days	95+ years	Global	-1	3	Education (years per capita)	271
Other leukaemia	Male	0-6 days	95+ years	Global	1	3	LDI (I$ per capita)	53
Other malignant cancers	Female	0-6 days	95+ years	Data Rich	1	1	Smoking Prevalence	421
Other malignant cancers	Female	0-6 days	95+ years	Data Rich	1	1	Tobacco (cigarettes per capita)	579
Other malignant cancers	Female	0-6 days	95+ years	Data Rich	-1	2	pufa adjusted(percent)	200
Other malignant cancers	Female	0-6 days	95+ years	Data Rich	-1	2	Healthcare access and quality index	491
Other malignant cancers	Female	0-6 days	95+ years	Data Rich	1	2	Age- and sex-specific SEV for Low fruit	37
Other malignant cancers	Female	0-6 days	95+ years	Data Rich	1	2	Age- and sex-specific SEV for Low nuts and seeds	185

Appendix Table S10: CODEm covariates used, level of covariate, and expected direction of covariate by cause, sex, and age

Cause	Sex	Age Start	Age End	Model Version Type	Direction	Level	Covariate Name	Number of Draws
Other malignant cancers	Female	0-6 days	95+ years	Data Rich	1	2	Age- and sex-specific SEV for Low vegetables	704
Other malignant cancers	Female	0-6 days	95+ years	Data Rich	-1	3	Education (years per capita)	111
Other malignant cancers	Female	0-6 days	95+ years	Data Rich	1	3	Socio-demographic Index	133
Other malignant cancers	Female	0-6 days	95+ years	Data Rich	1	3	LDI (I$ per capita)	--
Other malignant cancers	Female	0-6 days	95+ years	Global	1	1	Smoking Prevalence	547
Other malignant cancers	Female	0-6 days	95+ years	Global	1	1	Tobacco (cigarettes per capita)	619
Other malignant cancers	Female	0-6 days	95+ years	Global	-1	2	pufa adjusted(percent)	318
Other malignant cancers	Female	0-6 days	95+ years	Global	-1	2	Healthcare access and quality index	537
Other malignant cancers	Female	0-6 days	95+ years	Global	1	2	Age- and sex-specific SEV for Low fruit	37
Other malignant cancers	Female	0-6 days	95+ years	Global	1	2	Age- and sex-specific SEV for Low nuts and seeds	65
Other malignant cancers	Female	0-6 days	95+ years	Global	1	2	Age- and sex-specific SEV for Low vegetables	798
Other malignant cancers	Female	0-6 days	95+ years	Global	-1	3	Education (years per capita)	317
Other malignant cancers	Female	0-6 days	95+ years	Global	1	3	Socio-demographic Index	212
Other malignant cancers	Female	0-6 days	95+ years	Global	1	3	LDI (I$ per capita)	--
Other malignant cancers	Male	0-6 days	95+ years	Data Rich	1	1	Smoking Prevalence	428
Other malignant cancers	Male	0-6 days	95+ years	Data Rich	1	1	Tobacco (cigarettes per capita)	572
Other malignant cancers	Male	0-6 days	95+ years	Data Rich	-1	2	pufa adjusted(percent)	120
Other malignant cancers	Male	0-6 days	95+ years	Data Rich	-1	2	Healthcare access and quality index	253
Other malignant cancers	Male	0-6 days	95+ years	Data Rich	1	2	Age- and sex-specific SEV for Low fruit	120
Other malignant cancers	Male	0-6 days	95+ years	Data Rich	1	2	Age- and sex-specific SEV for Low nuts and seeds	174
Other malignant cancers	Male	0-6 days	95+ years	Data Rich	1	2	Age- and sex-specific SEV for Low vegetables	174
Other malignant cancers	Male	0-6 days	95+ years	Data Rich	-1	3	Education (years per capita)	208
Other malignant cancers	Male	0-6 days	95+ years	Data Rich	1	3	LDI (I$ per capita)	--
Other malignant cancers	Male	0-6 days	95+ years	Data Rich	1	3	Socio-demographic Index	--
Other malignant cancers	Male	0-6 days	95+ years	Global	1	1	Smoking Prevalence	328
Other malignant cancers	Male	0-6 days	95+ years	Global	1	1	Tobacco (cigarettes per capita)	672
Other malignant cancers	Male	0-6 days	95+ years	Global	-1	2	pufa adjusted(percent)	92
Other malignant cancers	Male	0-6 days	95+ years	Global	-1	2	Healthcare access and quality index	236
Other malignant cancers	Male	0-6 days	95+ years	Global	1	2	Age- and sex-specific SEV for Low vegetables	92
Other malignant cancers	Male	0-6 days	95+ years	Global	1	2	Age- and sex-specific SEV for Low fruit	133
Other malignant cancers	Male	0-6 days	95+ years	Global	1	2	Age- and sex-specific SEV for Low nuts and seeds	225
Other malignant cancers	Male	0-6 days	95+ years	Global	-1	3	Education (years per capita)	352
Other malignant cancers	Male	0-6 days	95+ years	Global	1	3	LDI (I$ per capita)	--
Other malignant cancers	Male	0-6 days	95+ years	Global	1	3	Socio-demographic Index	--
Other neoplasms	Female	0-6 days	95+ years	Data Rich	1	2	Tobacco (cigarettes per capita)	274
Other neoplasms	Female	0-6 days	95+ years	Data Rich	1	2	Cumulative Cigarettes (10 Years)	353
Other neoplasms	Female	0-6 days	95+ years	Data Rich	1	2	Smoking Prevalence	373
Other neoplasms	Female	0-6 days	95+ years	Data Rich	1	2	Liters of alcohol consumed per capita	--
Other neoplasms	Female	0-6 days	95+ years	Data Rich	-1	3	Maternal care and immunization	100
Other neoplasms	Female	0-6 days	95+ years	Data Rich	-1	3	Education (years per capita)	--
Other neoplasms	Female	0-6 days	95+ years	Data Rich	-1	3	Health worker density	--
Other neoplasms	Female	0-6 days	95+ years	Data Rich	-1	3	Healthcare access and quality index	--
Other neoplasms	Female	0-6 days	95+ years	Data Rich	-1	3	Socio-demographic Index	--
Other neoplasms	Female	0-6 days	95+ years	Data Rich	-1	3	Universal health coverage	--
Other neoplasms	Female	0-6 days	95+ years	Data Rich	1	3	Log-transformed age-standardized SEV scalar: Leukemia	253
Other neoplasms	Female	0-6 days	95+ years	Data Rich	1	3	LDI (I$ per capita)	353
Other neoplasms	Female	0-6 days	95+ years	Data Rich	1	3	Log-transformed SEV scalar: Leukemia	461
Other neoplasms	Female	0-6 days	95+ years	Data Rich	1	3	Mean BMI	561
Other neoplasms	Female	0-6 days	95+ years	Data Rich	1	3	Low-Density Lipoprotein (mmol/L)	--
Other neoplasms	Female	0-6 days	95+ years	Global	-1	3	Maternal care and immunization	5
Other neoplasms	Female	0-6 days	95+ years	Global	-1	3	Healthcare access and quality index	368
Other neoplasms	Female	0-6 days	95+ years	Global	-1	3	Education (years per capita)	--
Other neoplasms	Female	0-6 days	95+ years	Global	-1	3	Socio-demographic Index	--
Other neoplasms	Female	0-6 days	95+ years	Global	-1	3	Universal health coverage	--
Other neoplasms	Female	0-6 days	95+ years	Global	1	3	Low-Density Lipoprotein (mmol/L)	3
Other neoplasms	Female	0-6 days	95+ years	Global	1	3	Smoking Prevalence	100
Other neoplasms	Female	0-6 days	95+ years	Global	1	3	Log-transformed age-standardized SEV scalar: Leukemia	255
Other neoplasms	Female	0-6 days	95+ years	Global	1	3	Cumulative Cigarettes (10 Years)	298
Other neoplasms	Female	0-6 days	95+ years	Global	1	3	Tobacco (cigarettes per capita)	381
Other neoplasms	Female	0-6 days	95+ years	Global	1	3	Log-transformed SEV scalar: Leukemia	870
Other neoplasms	Female	0-6 days	95+ years	Global	1	3	LDI (I$ per capita)	974
Other neoplasms	Female	0-6 days	95+ years	Global	1	3	Liters of alcohol consumed per capita	--
Other neoplasms	Male	0-6 days	95+ years	Data Rich	1	2	Cumulative Cigarettes (10 Years)	--
Other neoplasms	Male	0-6 days	95+ years	Data Rich	1	2	Liters of alcohol consumed per capita	--
Other neoplasms	Male	0-6 days	95+ years	Data Rich	1	2	Smoking Prevalence	--
Other neoplasms	Male	0-6 days	95+ years	Data Rich	1	2	Tobacco (cigarettes per capita)	--
Other neoplasms	Male	0-6 days	95+ years	Data Rich	-1	3	Maternal care and immunization	454
Other neoplasms	Male	0-6 days	95+ years	Data Rich	-1	3	Education (years per capita)	--
Other neoplasms	Male	0-6 days	95+ years	Data Rich	-1	3	Health worker density	--
Other neoplasms	Male	0-6 days	95+ years	Data Rich	-1	3	Healthcare access and quality index	--
Other neoplasms	Male	0-6 days	95+ years	Data Rich	-1	3	Socio-demographic Index	--
Other neoplasms	Male	0-6 days	95+ years	Data Rich	-1	3	Universal health coverage	--
Other neoplasms	Male	0-6 days	95+ years	Data Rich	1	3	Log-transformed age-standardized SEV scalar: Leukemia	546
Other neoplasms	Male	0-6 days	95+ years	Data Rich	1	3	LDI (I$ per capita)	--
Other neoplasms	Male	0-6 days	95+ years	Data Rich	1	3	Log-transformed SEV scalar: Leukemia	--
Other neoplasms	Male	0-6 days	95+ years	Data Rich	1	3	Low-Density Lipoprotein (mmol/L)	--
Other neoplasms	Male	0-6 days	95+ years	Data Rich	1	3	Mean BMI	--
Other neoplasms	Male	0-6 days	95+ years	Global	-1	3	Maternal care and immunization	72
Other neoplasms	Male	0-6 days	95+ years	Global	-1	3	Healthcare access and quality index	132
Other neoplasms	Male	0-6 days	95+ years	Global	-1	3	Universal health coverage	188
Other neoplasms	Male	0-6 days	95+ years	Global	-1	3	Education (years per capita)	--
Other neoplasms	Male	0-6 days	95+ years	Global	-1	3	Socio-demographic Index	--
Other neoplasms	Male	0-6 days	95+ years	Global	1	3	Tobacco (cigarettes per capita)	147
Other neoplasms	Male	0-6 days	95+ years	Global	1	3	Cumulative Cigarettes (10 Years)	163
Other neoplasms	Male	0-6 days	95+ years	Global	1	3	Log-transformed age-standardized SEV scalar: Leukemia	264
Other neoplasms	Male	0-6 days	95+ years	Global	1	3	Smoking Prevalence	379
Other neoplasms	Male	0-6 days	95+ years	Global	1	3	LDI (I$ per capita)	963
Other neoplasms	Male	0-6 days	95+ years	Global	1	3	Liters of alcohol consumed per capita	--
Other neoplasms	Male	0-6 days	95+ years	Global	1	3	Log-transformed SEV scalar: Leukemia	--
Other neoplasms	Male	0-6 days	95+ years	Global	1	3	Low-Density Lipoprotein (mmol/L)	--
Myelodysplastic, myeloproliferative, and other hematopoietic neoplasms	Female	0-6 days	95+ years	Data Rich	-1	2	Healthcare access and quality index	--
Myelodysplastic, myeloproliferative, and other hematopoietic neoplasms	Female	0-6 days	95+ years	Data Rich	1	2	Cumulative Cigarettes (10 Years)	--
Myelodysplastic, myeloproliferative, and other hematopoietic neoplasms	Female	0-6 days	95+ years	Data Rich	1	2	Cumulative Cigarettes (15 Years)	--
Myelodysplastic, myeloproliferative, and other hematopoietic neoplasms	Female	0-6 days	95+ years	Data Rich	1	2	Cumulative Cigarettes (20 Years)	--
Myelodysplastic, myeloproliferative, and other hematopoietic neoplasms	Female	0-6 days	95+ years	Data Rich	1	2	Cumulative Cigarettes (5 Years)	--
Myelodysplastic, myeloproliferative, and other hematopoietic neoplasms	Female	0-6 days	95+ years	Data Rich	1	2	Liters of alcohol consumed per capita	--
Myelodysplastic, myeloproliferative, and other hematopoietic neoplasms	Female	0-6 days	95+ years	Data Rich	1	2	Smoking Prevalence	--
Myelodysplastic, myeloproliferative, and other hematopoietic neoplasms	Female	0-6 days	95+ years	Data Rich	1	2	Tobacco (cigarettes per capita)	--
Myelodysplastic, myeloproliferative, and other hematopoietic neoplasms	Female	0-6 days	95+ years	Data Rich	-1	3	Education (years per capita)	--
Myelodysplastic, myeloproliferative, and other hematopoietic neoplasms	Female	0-6 days	95+ years	Data Rich	1	3	Socio-demographic Index	1000
Myelodysplastic, myeloproliferative, and other hematopoietic neoplasms	Female	0-6 days	95+ years	Data Rich	1	3	LDI (I$ per capita)	--
Myelodysplastic, myeloproliferative, and other hematopoietic neoplasms	Female	0-6 days	95+ years	Global	-1	2	Healthcare access and quality index	--
Myelodysplastic, myeloproliferative, and other hematopoietic neoplasms	Female	0-6 days	95+ years	Global	1	2	Cumulative Cigarettes (5 Years)	50

Appendix Table S10: CODEm covariates used, level of covariate, and expected direction of covariate by cause, sex, and age

Cause	Sex	Age Start	Age End	Model Version Type	Direction	Level	Covariate Name	Number of Draws
Myelodysplastic, myeloproliferative, and other hematopoietic neoplasms	Female	0-6 days	95+ years	Global	1	2	Cumulative Cigarettes (20 Years)	113
Myelodysplastic, myeloproliferative, and other hematopoietic neoplasms	Female	0-6 days	95+ years	Global	1	2	Tobacco (cigarettes per capita)	212
Myelodysplastic, myeloproliferative, and other hematopoietic neoplasms	Female	0-6 days	95+ years	Global	1	2	Liters of alcohol consumed per capita	213
Myelodysplastic, myeloproliferative, and other hematopoietic neoplasms	Female	0-6 days	95+ years	Global	1	2	Cumulative Cigarettes (15 Years)	330
Myelodysplastic, myeloproliferative, and other hematopoietic neoplasms	Female	0-6 days	95+ years	Global	1	2	Cumulative Cigarettes (10 Years)	422
Myelodysplastic, myeloproliferative, and other hematopoietic neoplasms	Female	0-6 days	95+ years	Global	1	2	Smoking Prevalence	--
Myelodysplastic, myeloproliferative, and other hematopoietic neoplasms	Female	0-6 days	95+ years	Global	-1	3	Education (years per capita)	--
Myelodysplastic, myeloproliferative, and other hematopoietic neoplasms	Female	0-6 days	95+ years	Global	1	3	Socio-demographic Index	353
Myelodysplastic, myeloproliferative, and other hematopoietic neoplasms	Female	0-6 days	95+ years	Global	1	3	LDI (I$ per capita)	--
Myelodysplastic, myeloproliferative, and other hematopoietic neoplasms	Male	0-6 days	95+ years	Data Rich	-1	2	Healthcare access and quality index	--
Myelodysplastic, myeloproliferative, and other hematopoietic neoplasms	Male	0-6 days	95+ years	Data Rich	1	2	Cumulative Cigarettes (10 Years)	--
Myelodysplastic, myeloproliferative, and other hematopoietic neoplasms	Male	0-6 days	95+ years	Data Rich	1	2	Cumulative Cigarettes (15 Years)	--
Myelodysplastic, myeloproliferative, and other hematopoietic neoplasms	Male	0-6 days	95+ years	Data Rich	1	2	Cumulative Cigarettes (20 Years)	--
Myelodysplastic, myeloproliferative, and other hematopoietic neoplasms	Male	0-6 days	95+ years	Data Rich	1	2	Cumulative Cigarettes (5 Years)	--
Myelodysplastic, myeloproliferative, and other hematopoietic neoplasms	Male	0-6 days	95+ years	Data Rich	1	2	Liters of alcohol consumed per capita	--
Myelodysplastic, myeloproliferative, and other hematopoietic neoplasms	Male	0-6 days	95+ years	Data Rich	1	2	Smoking Prevalence	--
Myelodysplastic, myeloproliferative, and other hematopoietic neoplasms	Male	0-6 days	95+ years	Data Rich	1	2	Tobacco (cigarettes per capita)	--
Myelodysplastic, myeloproliferative, and other hematopoietic neoplasms	Male	0-6 days	95+ years	Data Rich	-1	3	Education (years per capita)	--
Myelodysplastic, myeloproliferative, and other hematopoietic neoplasms	Male	0-6 days	95+ years	Data Rich	1	3	LDI (I$ per capita)	--
Myelodysplastic, myeloproliferative, and other hematopoietic neoplasms	Male	0-6 days	95+ years	Data Rich	1	3	Socio-demographic Index	--
Myelodysplastic, myeloproliferative, and other hematopoietic neoplasms	Male	0-6 days	95+ years	Global	1	1	Log-transformed age-standardized SEV scalar: Leukemia	1000
Myelodysplastic, myeloproliferative, and other hematopoietic neoplasms	Male	0-6 days	95+ years	Global	1	1	Log-transformed SEV scalar: Leukemia	--
Myelodysplastic, myeloproliferative, and other hematopoietic neoplasms	Male	0-6 days	95+ years	Global	-1	2	Healthcare access and quality index	--
Myelodysplastic, myeloproliferative, and other hematopoietic neoplasms	Male	0-6 days	95+ years	Global	1	2	Cumulative Cigarettes (10 Years)	--
Myelodysplastic, myeloproliferative, and other hematopoietic neoplasms	Male	0-6 days	95+ years	Global	1	2	Cumulative Cigarettes (15 Years)	--
Myelodysplastic, myeloproliferative, and other hematopoietic neoplasms	Male	0-6 days	95+ years	Global	1	2	Cumulative Cigarettes (20 Years)	--
Myelodysplastic, myeloproliferative, and other hematopoietic neoplasms	Male	0-6 days	95+ years	Global	1	2	Cumulative Cigarettes (5 Years)	--
Myelodysplastic, myeloproliferative, and other hematopoietic neoplasms	Male	0-6 days	95+ years	Global	1	2	Liters of alcohol consumed per capita	--
Myelodysplastic, myeloproliferative, and other hematopoietic neoplasms	Male	0-6 days	95+ years	Global	1	2	Smoking Prevalence	--
Myelodysplastic, myeloproliferative, and other hematopoietic neoplasms	Male	0-6 days	95+ years	Global	1	2	Tobacco (cigarettes per capita)	--
Myelodysplastic, myeloproliferative, and other hematopoietic neoplasms	Male	0-6 days	95+ years	Global	-1	3	Education (years per capita)	--
Myelodysplastic, myeloproliferative, and other hematopoietic neoplasms	Male	0-6 days	95+ years	Global	1	3	Socio-demographic Index	398
Myelodysplastic, myeloproliferative, and other hematopoietic neoplasms	Male	0-6 days	95+ years	Global	1	3	LDI (I$ per capita)	--
Non-malignant brain and other central nervous system neoplasms	Female	0-6 days	95+ years	Data Rich	1	1	Cumulative Cigarettes (10 Years)	429
Non-malignant brain and other central nervous system neoplasms	Female	0-6 days	95+ years	Data Rich	1	1	Smoking Prevalence	1000
Non-malignant brain and other central nervous system neoplasms	Female	0-6 days	95+ years	Data Rich	1	1	Liters of alcohol consumed per capita	--
Non-malignant brain and other central nervous system neoplasms	Female	0-6 days	95+ years	Data Rich	-1	2	Healthcare access and quality index	108
Non-malignant brain and other central nervous system neoplasms	Female	0-6 days	95+ years	Data Rich	1	2	Low-Density Lipoprotein (mmol/L)	108
Non-malignant brain and other central nervous system neoplasms	Female	0-6 days	95+ years	Data Rich	1	2	Age- and sex-specific SEV for Low vegetables	294
Non-malignant brain and other central nervous system neoplasms	Female	0-6 days	95+ years	Data Rich	1	2	Age- and sex-specific SEV for High red meat	--
Non-malignant brain and other central nervous system neoplasms	Female	0-6 days	95+ years	Data Rich	1	2	Age- and sex-specific SEV for Low fruit	--
Non-malignant brain and other central nervous system neoplasms	Female	0-6 days	95+ years	Data Rich	1	2	Systolic Blood Pressure (mmHg)	--
Non-malignant brain and other central nervous system neoplasms	Female	0-6 days	95+ years	Data Rich	-1	3	LDI (I$ per capita)	142
Non-malignant brain and other central nervous system neoplasms	Female	0-6 days	95+ years	Data Rich	-1	3	Education (years per capita)	--
Non-malignant brain and other central nervous system neoplasms	Female	0-6 days	95+ years	Data Rich	1	3	Socio-demographic Index	--
Non-malignant brain and other central nervous system neoplasms	Female	0-6 days	95+ years	Global	1	1	Cumulative Cigarettes (10 Years)	225
Non-malignant brain and other central nervous system neoplasms	Female	0-6 days	95+ years	Global	1	1	Smoking Prevalence	1000
Non-malignant brain and other central nervous system neoplasms	Female	0-6 days	95+ years	Global	1	1	Liters of alcohol consumed per capita	--
Non-malignant brain and other central nervous system neoplasms	Female	0-6 days	95+ years	Global	-1	2	Healthcare access and quality index	--
Non-malignant brain and other central nervous system neoplasms	Female	0-6 days	95+ years	Global	1	2	Age- and sex-specific SEV for Low fruit	92
Non-malignant brain and other central nervous system neoplasms	Female	0-6 days	95+ years	Global	1	2	Age- and sex-specific SEV for Low vegetables	261
Non-malignant brain and other central nervous system neoplasms	Female	0-6 days	95+ years	Global	1	2	Low-Density Lipoprotein (mmol/L)	365
Non-malignant brain and other central nervous system neoplasms	Female	0-6 days	95+ years	Global	1	2	Age- and sex-specific SEV for High red meat	--
Non-malignant brain and other central nervous system neoplasms	Female	0-6 days	95+ years	Global	1	2	Systolic Blood Pressure (mmHg)	--
Non-malignant brain and other central nervous system neoplasms	Female	0-6 days	95+ years	Global	-1	3	LDI (I$ per capita)	205
Non-malignant brain and other central nervous system neoplasms	Female	0-6 days	95+ years	Global	-1	3	Education (years per capita)	--
Non-malignant brain and other central nervous system neoplasms	Female	0-6 days	95+ years	Global	1	3	Socio-demographic Index	--
Non-malignant brain and other central nervous system neoplasms	Male	0-6 days	95+ years	Data Rich	1	1	Smoking Prevalence	373
Non-malignant brain and other central nervous system neoplasms	Male	0-6 days	95+ years	Data Rich	1	1	Cumulative Cigarettes (10 Years)	627
Non-malignant brain and other central nervous system neoplasms	Male	0-6 days	95+ years	Data Rich	1	1	Liters of alcohol consumed per capita	--
Non-malignant brain and other central nervous system neoplasms	Male	0-6 days	95+ years	Data Rich	-1	2	Healthcare access and quality index	77
Non-malignant brain and other central nervous system neoplasms	Male	0-6 days	95+ years	Data Rich	1	2	Age- and sex-specific SEV for Low vegetables	183
Non-malignant brain and other central nervous system neoplasms	Male	0-6 days	95+ years	Data Rich	1	2	Systolic Blood Pressure (mmHg)	331
Non-malignant brain and other central nervous system neoplasms	Male	0-6 days	95+ years	Data Rich	1	2	Age- and sex-specific SEV for High red meat	--
Non-malignant brain and other central nervous system neoplasms	Male	0-6 days	95+ years	Data Rich	1	2	Age- and sex-specific SEV for Low fruit	--
Non-malignant brain and other central nervous system neoplasms	Male	0-6 days	95+ years	Data Rich	1	2	Low-Density Lipoprotein (mmol/L)	--
Non-malignant brain and other central nervous system neoplasms	Male	0-6 days	95+ years	Data Rich	-1	3	Education (years per capita)	58
Non-malignant brain and other central nervous system neoplasms	Male	0-6 days	95+ years	Data Rich	-1	3	LDI (I$ per capita)	303
Non-malignant brain and other central nervous system neoplasms	Male	0-6 days	95+ years	Data Rich	1	3	Socio-demographic Index	--
Non-malignant brain and other central nervous system neoplasms	Male	0-6 days	95+ years	Global	1	1	Cumulative Cigarettes (10 Years)	--
Non-malignant brain and other central nervous system neoplasms	Male	0-6 days	95+ years	Global	1	1	Liters of alcohol consumed per capita	--
Non-malignant brain and other central nervous system neoplasms	Male	0-6 days	95+ years	Global	1	1	Smoking Prevalence	--
Non-malignant brain and other central nervous system neoplasms	Male	0-6 days	95+ years	Global	-1	2	Healthcare access and quality index	--
Non-malignant brain and other central nervous system neoplasms	Male	0-6 days	95+ years	Global	1	2	Age- and sex-specific SEV for Low vegetables	546
Non-malignant brain and other central nervous system neoplasms	Male	0-6 days	95+ years	Global	1	2	Age- and sex-specific SEV for High red meat	--
Non-malignant brain and other central nervous system neoplasms	Male	0-6 days	95+ years	Global	1	2	Age- and sex-specific SEV for Low fruit	--
Non-malignant brain and other central nervous system neoplasms	Male	0-6 days	95+ years	Global	1	2	Low-Density Lipoprotein (mmol/L)	--
Non-malignant brain and other central nervous system neoplasms	Male	0-6 days	95+ years	Global	1	2	Systolic Blood Pressure (mmHg)	--
Non-malignant brain and other central nervous system neoplasms	Male	0-6 days	95+ years	Global	-1	3	LDI (I$ per capita)	454
Non-malignant brain and other central nervous system neoplasms	Male	0-6 days	95+ years	Global	-1	3	Education (years per capita)	--
Non-malignant brain and other central nervous system neoplasms	Male	0-6 days	95+ years	Global	1	3	Socio-demographic Index	--

Appendix Table S10: CODEm covariates used, level of covariate, and expected direction of covariate by cause, sex, and age								
**Cause**	**Sex**	**Age Start**	**Age End**	**Model Version Type**	**Direction**	**Level**	**Covariate Name**	**Number of Draws**
Other benign and in situ neoplasms	Female	0-6 days	95+ years	Data Rich	-1	2	Healthcare access and quality index	1000
Other benign and in situ neoplasms	Female	0-6 days	95+ years	Data Rich	1	2	Cumulative Cigarettes (10 Years)	--
Other benign and in situ neoplasms	Female	0-6 days	95+ years	Data Rich	1	2	Smoking Prevalence	--
Other benign and in situ neoplasms	Female	0-6 days	95+ years	Data Rich	1	2	Tobacco (cigarettes per capita)	--
Other benign and in situ neoplasms	Female	0-6 days	95+ years	Data Rich	-1	3	LDI (I$ per capita)	454
Other benign and in situ neoplasms	Female	0-6 days	95+ years	Data Rich	-1	3	Socio-demographic Index	454
Other benign and in situ neoplasms	Female	0-6 days	95+ years	Data Rich	-1	3	Education (years per capita)	--
Other benign and in situ neoplasms	Female	0-6 days	95+ years	Data Rich	1	3	Liters of alcohol consumed per capita	--
Other benign and in situ neoplasms	Female	0-6 days	95+ years	Global	-1	2	Healthcare access and quality index	1000
Other benign and in situ neoplasms	Female	0-6 days	95+ years	Global	1	2	Cumulative Cigarettes (10 Years)	--
Other benign and in situ neoplasms	Female	0-6 days	95+ years	Global	1	2	Smoking Prevalence	--
Other benign and in situ neoplasms	Female	0-6 days	95+ years	Global	1	2	Tobacco (cigarettes per capita)	--
Other benign and in situ neoplasms	Female	0-6 days	95+ years	Global	-1	3	LDI (I$ per capita)	454
Other benign and in situ neoplasms	Female	0-6 days	95+ years	Global	-1	3	Socio-demographic Index	454
Other benign and in situ neoplasms	Female	0-6 days	95+ years	Global	-1	3	Education (years per capita)	--
Other benign and in situ neoplasms	Female	0-6 days	95+ years	Global	1	3	Liters of alcohol consumed per capita	--
Other benign and in situ neoplasms	Male	0-6 days	95+ years	Data Rich	-1	2	Healthcare access and quality index	561
Other benign and in situ neoplasms	Male	0-6 days	95+ years	Data Rich	1	2	Cumulative Cigarettes (10 Years)	174
Other benign and in situ neoplasms	Male	0-6 days	95+ years	Data Rich	1	2	Smoking Prevalence	265
Other benign and in situ neoplasms	Male	0-6 days	95+ years	Data Rich	1	2	Tobacco (cigarettes per capita)	--
Other benign and in situ neoplasms	Male	0-6 days	95+ years	Data Rich	-1	3	LDI (I$ per capita)	100
Other benign and in situ neoplasms	Male	0-6 days	95+ years	Data Rich	-1	3	Education (years per capita)	373
Other benign and in situ neoplasms	Male	0-6 days	95+ years	Data Rich	-1	3	Socio-demographic Index	--
Other benign and in situ neoplasms	Male	0-6 days	95+ years	Data Rich	1	3	Liters of alcohol consumed per capita	--
Other benign and in situ neoplasms	Male	0-6 days	95+ years	Global	-1	2	Healthcare access and quality index	706
Other benign and in situ neoplasms	Male	0-6 days	95+ years	Global	1	2	Cumulative Cigarettes (10 Years)	133
Other benign and in situ neoplasms	Male	0-6 days	95+ years	Global	1	2	Smoking Prevalence	161
Other benign and in situ neoplasms	Male	0-6 days	95+ years	Global	1	2	Tobacco (cigarettes per capita)	--
Other benign and in situ neoplasms	Male	0-6 days	95+ years	Global	-1	3	Education (years per capita)	193
Other benign and in situ neoplasms	Male	0-6 days	95+ years	Global	-1	3	Socio-demographic Index	282
Other benign and in situ neoplasms	Male	0-6 days	95+ years	Global	-1	3	LDI (I$ per capita)	--
Other benign and in situ neoplasms	Male	0-6 days	95+ years	Global	1	3	Liters of alcohol consumed per capita	--
Cardiovascular diseases	Female	0-6 days	95+ years	Data Rich	1	1	Low-Density Lipoprotein (mmol/L)	609
Cardiovascular diseases	Female	0-6 days	95+ years	Data Rich	1	1	Systolic Blood Pressure (mmHg)	718
Cardiovascular diseases	Female	0-6 days	95+ years	Data Rich	1	1	Smoking Prevalence	853
Cardiovascular diseases	Female	0-6 days	95+ years	Data Rich	-1	2	Elevation Over 1500m (proportion)	500
Cardiovascular diseases	Female	0-6 days	95+ years	Data Rich	-1	2	Healthcare access and quality index	819
Cardiovascular diseases	Female	0-6 days	95+ years	Data Rich	1	2	Indoor Air Pollution (All Cooking Fuels)	253
Cardiovascular diseases	Female	0-6 days	95+ years	Data Rich	1	2	Outdoor Air Pollution (PM2.5)	253
Cardiovascular diseases	Female	0-6 days	95+ years	Data Rich	1	2	Diabetes Fasting Plasma Glucose (mmol/L), age-standardized 25+	--
Cardiovascular diseases	Female	0-6 days	95+ years	Data Rich	1	2	Mean BMI	--
Cardiovascular diseases	Female	0-6 days	95+ years	Data Rich	-1	3	Pulses legumes unadjusted(g)	573
Cardiovascular diseases	Female	0-6 days	95+ years	Data Rich	-1	3	LDI (I$ per capita)	591
Cardiovascular diseases	Female	0-6 days	95+ years	Data Rich	1	3	Age- and sex-specific SEV for Low PUFA	69
Cardiovascular diseases	Female	0-6 days	95+ years	Data Rich	1	3	Age- and sex-specific SEV for Low omega-3	116
Cardiovascular diseases	Female	0-6 days	95+ years	Data Rich	1	3	Age- and sex-specific SEV for Low nuts and seeds	134
Cardiovascular diseases	Female	0-6 days	95+ years	Data Rich	1	3	Diet high in trans fatty acids	159
Cardiovascular diseases	Female	0-6 days	95+ years	Data Rich	1	3	Liters of alcohol consumed per capita	417
Cardiovascular diseases	Female	0-6 days	95+ years	Data Rich	1	3	Age- and sex-specific SEV for Low fruit	660
Cardiovascular diseases	Female	0-6 days	95+ years	Data Rich	1	3	Age- and sex-specific SEV for Low vegetables	661
Cardiovascular diseases	Female	0-6 days	95+ years	Global	1	1	Low-Density Lipoprotein (mmol/L)	441
Cardiovascular diseases	Female	0-6 days	95+ years	Global	1	1	Smoking Prevalence	551
Cardiovascular diseases	Female	0-6 days	95+ years	Global	1	1	Systolic Blood Pressure (mmHg)	620
Cardiovascular diseases	Female	0-6 days	95+ years	Global	-1	2	Healthcare access and quality index	853
Cardiovascular diseases	Female	0-6 days	95+ years	Global	-1	2	Elevation Over 1500m (proportion)	--
Cardiovascular diseases	Female	0-6 days	95+ years	Global	1	2	Indoor Air Pollution (All Cooking Fuels)	126
Cardiovascular diseases	Female	0-6 days	95+ years	Global	1	2	Outdoor Air Pollution (PM2.5)	829
Cardiovascular diseases	Female	0-6 days	95+ years	Global	1	2	Diabetes Fasting Plasma Glucose (mmol/L), age-standardized 25+	--
Cardiovascular diseases	Female	0-6 days	95+ years	Global	1	2	Mean BMI	--
Cardiovascular diseases	Female	0-6 days	95+ years	Global	-1	3	Pulses legumes unadjusted(g)	53
Cardiovascular diseases	Female	0-6 days	95+ years	Global	-1	3	LDI (I$ per capita)	638
Cardiovascular diseases	Female	0-6 days	95+ years	Global	1	3	Age- and sex-specific SEV for Low PUFA	2
Cardiovascular diseases	Female	0-6 days	95+ years	Global	1	3	Age- and sex-specific SEV for Low omega-3	15
Cardiovascular diseases	Female	0-6 days	95+ years	Global	1	3	Age- and sex-specific SEV for Low fruit	39
Cardiovascular diseases	Female	0-6 days	95+ years	Global	1	3	Age- and sex-specific SEV for Low nuts and seeds	55
Cardiovascular diseases	Female	0-6 days	95+ years	Global	1	3	Age- and sex-specific SEV for Low vegetables	362
Cardiovascular diseases	Female	0-6 days	95+ years	Global	1	3	Liters of alcohol consumed per capita	571
Cardiovascular diseases	Female	0-6 days	95+ years	Global	1	3	Diet high in trans fatty acids	579
Cardiovascular diseases	Male	0-6 days	95+ years	Data Rich	1	1	Systolic Blood Pressure (mmHg)	513
Cardiovascular diseases	Male	0-6 days	95+ years	Data Rich	1	1	Smoking Prevalence	782
Cardiovascular diseases	Male	0-6 days	95+ years	Data Rich	1	1	Low-Density Lipoprotein (mmol/L)	808
Cardiovascular diseases	Male	0-6 days	95+ years	Data Rich	-1	2	Elevation Over 1500m (proportion)	721
Cardiovascular diseases	Male	0-6 days	95+ years	Data Rich	-1	2	Healthcare access and quality index	956
Cardiovascular diseases	Male	0-6 days	95+ years	Data Rich	1	2	Indoor Air Pollution (All Cooking Fuels)	31
Cardiovascular diseases	Male	0-6 days	95+ years	Data Rich	1	2	Outdoor Air Pollution (PM2.5)	360
Cardiovascular diseases	Male	0-6 days	95+ years	Data Rich	1	2	Diabetes Fasting Plasma Glucose (mmol/L), age-standardized 25+	--
Cardiovascular diseases	Male	0-6 days	95+ years	Data Rich	1	2	Mean BMI	--
Cardiovascular diseases	Male	0-6 days	95+ years	Data Rich	-1	3	LDI (I$ per capita)	639
Cardiovascular diseases	Male	0-6 days	95+ years	Data Rich	-1	3	Pulses legumes unadjusted(g)	798
Cardiovascular diseases	Male	0-6 days	95+ years	Data Rich	1	3	Age- and sex-specific SEV for Low PUFA	3
Cardiovascular diseases	Male	0-6 days	95+ years	Data Rich	1	3	Age- and sex-specific SEV for Low omega-3	4
Cardiovascular diseases	Male	0-6 days	95+ years	Data Rich	1	3	Age- and sex-specific SEV for Low nuts and seeds	85
Cardiovascular diseases	Male	0-6 days	95+ years	Data Rich	1	3	Age- and sex-specific SEV for Low vegetables	433
Cardiovascular diseases	Male	0-6 days	95+ years	Data Rich	1	3	Diet high in trans fatty acids	664
Cardiovascular diseases	Male	0-6 days	95+ years	Data Rich	1	3	Age- and sex-specific SEV for Low fruit	671
Cardiovascular diseases	Male	0-6 days	95+ years	Data Rich	1	3	Liters of alcohol consumed per capita	735
Cardiovascular diseases	Male	0-6 days	95+ years	Global	1	1	Smoking Prevalence	436
Cardiovascular diseases	Male	0-6 days	95+ years	Global	1	1	Systolic Blood Pressure (mmHg)	518
Cardiovascular diseases	Male	0-6 days	95+ years	Global	1	1	Low-Density Lipoprotein (mmol/L)	830
Cardiovascular diseases	Male	0-6 days	95+ years	Global	-1	2	Healthcare access and quality index	756
Cardiovascular diseases	Male	0-6 days	95+ years	Global	-1	2	Elevation Over 1500m (proportion)	--
Cardiovascular diseases	Male	0-6 days	95+ years	Global	1	2	Indoor Air Pollution (All Cooking Fuels)	161
Cardiovascular diseases	Male	0-6 days	95+ years	Global	1	2	Outdoor Air Pollution (PM2.5)	957
Cardiovascular diseases	Male	0-6 days	95+ years	Global	1	2	Diabetes Fasting Plasma Glucose (mmol/L), age-standardized 25+	--
Cardiovascular diseases	Male	0-6 days	95+ years	Global	1	2	Mean BMI	--
Cardiovascular diseases	Male	0-6 days	95+ years	Global	-1	3	Pulses legumes unadjusted(g)	45
Cardiovascular diseases	Male	0-6 days	95+ years	Global	-1	3	LDI (I$ per capita)	574
Cardiovascular diseases	Male	0-6 days	95+ years	Global	1	3	Age- and sex-specific SEV for Low PUFA	0
Cardiovascular diseases	Male	0-6 days	95+ years	Global	1	3	Age- and sex-specific SEV for Low omega-3	0
Cardiovascular diseases	Male	0-6 days	95+ years	Global	1	3	Age- and sex-specific SEV for Low nuts and seeds	85
Cardiovascular diseases	Male	0-6 days	95+ years	Global	1	3	Age- and sex-specific SEV for Low vegetables	101
Cardiovascular diseases	Male	0-6 days	95+ years	Global	1	3	Age- and sex-specific SEV for Low fruit	129
Cardiovascular diseases	Male	0-6 days	95+ years	Global	1	3	Diet high in trans fatty acids	316

Appendix Table S10: CODEm covariates used, level of covariate, and expected direction of covariate by cause, sex, and age

Cause	Sex	Age Start	Age End	Model Version Type	Direction	Level	Covariate Name	Number of Draws
Cardiovascular diseases	Male	0-6 days	95+ years	Global	1	3	Liters of alcohol consumed per capita	451
Rheumatic heart disease	Female	12-23 months	95+ years	Data Rich	-1	1	Sanitation (proportion with access)	209
Rheumatic heart disease	Female	12-23 months	95+ years	Data Rich	-1	1	Improved Water Source (proportion with access)	467
Rheumatic heart disease	Female	12-23 months	95+ years	Data Rich	1	1	Age- and sex-specific SEV for Child underweight	583
Rheumatic heart disease	Female	12-23 months	95+ years	Data Rich	-1	2	Healthcare access and quality index	491
Rheumatic heart disease	Female	12-23 months	95+ years	Data Rich	-1	3	Education (years per capita)	280
Rheumatic heart disease	Female	12-23 months	95+ years	Data Rich	-1	3	LDI (I$ per capita)	--
Rheumatic heart disease	Female	12-23 months	95+ years	Global	-1	1	Sanitation (proportion with access)	368
Rheumatic heart disease	Female	12-23 months	95+ years	Global	-1	1	Improved Water Source (proportion with access)	1000
Rheumatic heart disease	Female	12-23 months	95+ years	Global	1	1	Age- and sex-specific SEV for Child underweight	732
Rheumatic heart disease	Female	12-23 months	95+ years	Global	-1	2	Healthcare access and quality index	--
Rheumatic heart disease	Female	12-23 months	95+ years	Global	-1	3	Education (years per capita)	497
Rheumatic heart disease	Female	12-23 months	95+ years	Global	-1	3	LDI (I$ per capita)	--
Rheumatic heart disease	Female	12-23 months	95+ years	Global	-1	3	Socio-demographic Index	--
Rheumatic heart disease	Male	12-23 months	95+ years	Data Rich	-1	1	Improved Water Source (proportion with access)	632
Rheumatic heart disease	Male	12-23 months	95+ years	Data Rich	-1	1	Sanitation (proportion with access)	763
Rheumatic heart disease	Male	12-23 months	95+ years	Data Rich	1	1	Age- and sex-specific SEV for Child underweight	521
Rheumatic heart disease	Male	12-23 months	95+ years	Data Rich	-1	2	Healthcare access and quality index	--
Rheumatic heart disease	Male	12-23 months	95+ years	Data Rich	-1	3	Education (years per capita)	587
Rheumatic heart disease	Male	12-23 months	95+ years	Data Rich	-1	3	LDI (I$ per capita)	--
Rheumatic heart disease	Male	12-23 months	95+ years	Global	-1	1	Sanitation (proportion with access)	81
Rheumatic heart disease	Male	12-23 months	95+ years	Global	-1	1	Improved Water Source (proportion with access)	233
Rheumatic heart disease	Male	12-23 months	95+ years	Global	1	1	Age- and sex-specific SEV for Child underweight	906
Rheumatic heart disease	Male	12-23 months	95+ years	Global	-1	2	Healthcare access and quality index	599
Rheumatic heart disease	Male	12-23 months	95+ years	Global	-1	3	Socio-demographic Index	288
Rheumatic heart disease	Male	12-23 months	95+ years	Global	-1	3	Education (years per capita)	302
Rheumatic heart disease	Male	12-23 months	95+ years	Global	-1	3	LDI (I$ per capita)	--
Ischaemic heart disease	Female	15-19 years	95+ years	Data Rich	1	1	Systolic Blood Pressure (mmHg)	198
Ischaemic heart disease	Female	15-19 years	95+ years	Data Rich	1	1	Log-transformed SEV scalar: IHD	467
Ischaemic heart disease	Female	15-19 years	95+ years	Data Rich	1	1	Low-Density Lipoprotein (mmol/L)	572
Ischaemic heart disease	Female	15-19 years	95+ years	Data Rich	1	1	Smoking Prevalence	607
Ischaemic heart disease	Female	15-19 years	95+ years	Data Rich	-1	2	Healthcare access and quality index	159
Ischaemic heart disease	Female	15-19 years	95+ years	Data Rich	-1	2	Elevation Over 1500m (proportion)	286
Ischaemic heart disease	Female	15-19 years	95+ years	Data Rich	1	2	Outdoor Air Pollution (PM2.5)	132
Ischaemic heart disease	Female	15-19 years	95+ years	Data Rich	1	2	Indoor Air Pollution (All Cooking Fuels)	334
Ischaemic heart disease	Female	15-19 years	95+ years	Data Rich	1	2	Diabetes Fasting Plasma Glucose (mmol/L), age-standardized 25+	--
Ischaemic heart disease	Female	15-19 years	95+ years	Data Rich	1	2	Mean BMI	--
Ischaemic heart disease	Female	15-19 years	95+ years	Data Rich	-1	3	Pulses legumes unadjusted(g)	8
Ischaemic heart disease	Female	15-19 years	95+ years	Data Rich	-1	3	LDI (I$ per capita)	--
Ischaemic heart disease	Female	15-19 years	95+ years	Data Rich	1	3	Age- and sex-specific SEV for Low PUFA	10
Ischaemic heart disease	Female	15-19 years	95+ years	Data Rich	1	3	Age- and sex-specific SEV for Low omega-3	290
Ischaemic heart disease	Female	15-19 years	95+ years	Data Rich	1	3	Diet high in trans fatty acids	437
Ischaemic heart disease	Female	15-19 years	95+ years	Data Rich	1	3	Liters of alcohol consumed per capita	525
Ischaemic heart disease	Female	15-19 years	95+ years	Data Rich	1	3	Age- and sex-specific SEV for Low fruit	538
Ischaemic heart disease	Female	15-19 years	95+ years	Data Rich	1	3	Age- and sex-specific SEV for Low nuts and seeds	556
Ischaemic heart disease	Female	15-19 years	95+ years	Data Rich	1	3	Age- and sex-specific SEV for Low vegetables	580
Ischaemic heart disease	Female	15-19 years	95+ years	Global	1	1	Systolic Blood Pressure (mmHg)	284
Ischaemic heart disease	Female	15-19 years	95+ years	Global	1	1	Log-transformed SEV scalar: IHD	424
Ischaemic heart disease	Female	15-19 years	95+ years	Global	1	1	Smoking Prevalence	740
Ischaemic heart disease	Female	15-19 years	95+ years	Global	1	1	Low-Density Lipoprotein (mmol/L)	786
Ischaemic heart disease	Female	15-19 years	95+ years	Global	-1	2	Elevation Over 1500m (proportion)	238
Ischaemic heart disease	Female	15-19 years	95+ years	Global	-1	2	Healthcare access and quality index	976
Ischaemic heart disease	Female	15-19 years	95+ years	Global	1	2	Indoor Air Pollution (All Cooking Fuels)	2
Ischaemic heart disease	Female	15-19 years	95+ years	Global	1	2	Outdoor Air Pollution (PM2.5)	717
Ischaemic heart disease	Female	15-19 years	95+ years	Global	1	2	Diabetes Fasting Plasma Glucose (mmol/L), age-standardized 25+	--
Ischaemic heart disease	Female	15-19 years	95+ years	Global	1	2	Mean BMI	--
Ischaemic heart disease	Female	15-19 years	95+ years	Global	-1	3	Pulses legumes unadjusted(g)	0
Ischaemic heart disease	Female	15-19 years	95+ years	Global	-1	3	LDI (I$ per capita)	--
Ischaemic heart disease	Female	15-19 years	95+ years	Global	1	3	Age- and sex-specific SEV for Low omega-3	2
Ischaemic heart disease	Female	15-19 years	95+ years	Global	1	3	Age- and sex-specific SEV for Low vegetables	3
Ischaemic heart disease	Female	15-19 years	95+ years	Global	1	3	Age- and sex-specific SEV for Low fruit	4
Ischaemic heart disease	Female	15-19 years	95+ years	Global	1	3	Age- and sex-specific SEV for Low nuts and seeds	202
Ischaemic heart disease	Female	15-19 years	95+ years	Global	1	3	Age- and sex-specific SEV for Low PUFA	403
Ischaemic heart disease	Female	15-19 years	95+ years	Global	1	3	Diet high in trans fatty acids	436
Ischaemic heart disease	Female	15-19 years	95+ years	Global	1	3	Liters of alcohol consumed per capita	483
Ischaemic heart disease	Male	15-19 years	95+ years	Data Rich	1	1	Systolic Blood Pressure (mmHg)	381
Ischaemic heart disease	Male	15-19 years	95+ years	Data Rich	1	1	Log-transformed SEV scalar: IHD	551
Ischaemic heart disease	Male	15-19 years	95+ years	Data Rich	1	1	Low-Density Lipoprotein (mmol/L)	583
Ischaemic heart disease	Male	15-19 years	95+ years	Data Rich	1	1	Smoking Prevalence	765
Ischaemic heart disease	Male	15-19 years	95+ years	Data Rich	-1	2	Elevation Over 1500m (proportion)	218
Ischaemic heart disease	Male	15-19 years	95+ years	Data Rich	-1	2	Healthcare access and quality index	421
Ischaemic heart disease	Male	15-19 years	95+ years	Data Rich	1	2	Indoor Air Pollution (All Cooking Fuels)	0
Ischaemic heart disease	Male	15-19 years	95+ years	Data Rich	1	2	Outdoor Air Pollution (PM2.5)	28
Ischaemic heart disease	Male	15-19 years	95+ years	Data Rich	1	2	Diabetes Fasting Plasma Glucose (mmol/L), age-standardized 25+	--
Ischaemic heart disease	Male	15-19 years	95+ years	Data Rich	1	2	Mean BMI	--
Ischaemic heart disease	Male	15-19 years	95+ years	Data Rich	-1	3	Pulses legumes unadjusted(g)	256
Ischaemic heart disease	Male	15-19 years	95+ years	Data Rich	-1	3	LDI (I$ per capita)	--
Ischaemic heart disease	Male	15-19 years	95+ years	Data Rich	1	3	Age- and sex-specific SEV for Low omega-3	0
Ischaemic heart disease	Male	15-19 years	95+ years	Data Rich	1	3	Age- and sex-specific SEV for Low vegetables	143
Ischaemic heart disease	Male	15-19 years	95+ years	Data Rich	1	3	Age- and sex-specific SEV for Low PUFA	180
Ischaemic heart disease	Male	15-19 years	95+ years	Data Rich	1	3	Diet high in trans fatty acids	557
Ischaemic heart disease	Male	15-19 years	95+ years	Data Rich	1	3	Age- and sex-specific SEV for Low nuts and seeds	619
Ischaemic heart disease	Male	15-19 years	95+ years	Data Rich	1	3	Age- and sex-specific SEV for Low fruit	637
Ischaemic heart disease	Male	15-19 years	95+ years	Data Rich	1	3	Liters of alcohol consumed per capita	845
Ischaemic heart disease	Male	15-19 years	95+ years	Global	1	1	Systolic Blood Pressure (mmHg)	530
Ischaemic heart disease	Male	15-19 years	95+ years	Global	1	1	Smoking Prevalence	572
Ischaemic heart disease	Male	15-19 years	95+ years	Global	1	1	Log-transformed SEV scalar: IHD	728
Ischaemic heart disease	Male	15-19 years	95+ years	Global	1	1	Low-Density Lipoprotein (mmol/L)	753
Ischaemic heart disease	Male	15-19 years	95+ years	Global	-1	2	Elevation Over 1500m (proportion)	754
Ischaemic heart disease	Male	15-19 years	95+ years	Global	-1	2	Healthcare access and quality index	883
Ischaemic heart disease	Male	15-19 years	95+ years	Global	1	2	Indoor Air Pollution (All Cooking Fuels)	2
Ischaemic heart disease	Male	15-19 years	95+ years	Global	1	2	Outdoor Air Pollution (PM2.5)	740
Ischaemic heart disease	Male	15-19 years	95+ years	Global	1	2	Diabetes Fasting Plasma Glucose (mmol/L), age-standardized 25+	--
Ischaemic heart disease	Male	15-19 years	95+ years	Global	1	2	Mean BMI	--
Ischaemic heart disease	Male	15-19 years	95+ years	Global	-1	3	Pulses legumes unadjusted(g)	0
Ischaemic heart disease	Male	15-19 years	95+ years	Global	-1	3	LDI (I$ per capita)	--
Ischaemic heart disease	Male	15-19 years	95+ years	Global	1	3	Age- and sex-specific SEV for Low omega-3	0
Ischaemic heart disease	Male	15-19 years	95+ years	Global	1	3	Age- and sex-specific SEV for Low fruit	13
Ischaemic heart disease	Male	15-19 years	95+ years	Global	1	3	Age- and sex-specific SEV for Low vegetables	22
Ischaemic heart disease	Male	15-19 years	95+ years	Global	1	3	Age- and sex-specific SEV for Low nuts and seeds	287
Ischaemic heart disease	Male	15-19 years	95+ years	Global	1	3	Age- and sex-specific SEV for Low PUFA	511

Appendix Table S10: CODEm covariates used, level of covariate, and expected direction of covariate by cause, sex, and age								
**Cause**	**Sex**	**Age Start**	**Age End**	**Model Version Type**	**Direction**	**Level**	**Covariate Name**	**Number of Draws**
Ischaemic heart disease	Male	15-19 years	95+ years	Global	1	3	Diet high in trans fatty acids	560
Ischaemic heart disease	Male	15-19 years	95+ years	Global	1	3	Liters of alcohol consumed per capita	632
Stroke	Female	0-6 days	95+ years	Data Rich	1	1	Log-transformed SEV scalar: Stroke	109
Stroke	Female	0-6 days	95+ years	Data Rich	1	1	Low-Density Lipoprotein (mmol/L)	296
Stroke	Female	0-6 days	95+ years	Data Rich	1	1	Smoking Prevalence	628
Stroke	Female	0-6 days	95+ years	Data Rich	1	1	Systolic Blood Pressure (mmHg)	939
Stroke	Female	0-6 days	95+ years	Data Rich	-1	2	Elevation Over 1500m (proportion)	61
Stroke	Female	0-6 days	95+ years	Data Rich	-1	2	Healthcare access and quality index	1000
Stroke	Female	0-6 days	95+ years	Data Rich	1	2	Indoor Air Pollution (All Cooking Fuels)	485
Stroke	Female	0-6 days	95+ years	Data Rich	1	2	Outdoor Air Pollution (PM2.5)	987
Stroke	Female	0-6 days	95+ years	Data Rich	1	2	Diabetes Fasting Plasma Glucose (mmol/L), age-standardized 25+	--
Stroke	Female	0-6 days	95+ years	Data Rich	1	2	Mean BMI	--
Stroke	Female	0-6 days	95+ years	Data Rich	-1	3	Pulses legumes unadjusted(g)	24
Stroke	Female	0-6 days	95+ years	Data Rich	-1	3	LDI (I$ per capita)	--
Stroke	Female	0-6 days	95+ years	Data Rich	1	3	Age- and sex-specific SEV for Low PUFA	0
Stroke	Female	0-6 days	95+ years	Data Rich	1	3	Age- and sex-specific SEV for Low omega-3	0
Stroke	Female	0-6 days	95+ years	Data Rich	1	3	Age- and sex-specific SEV for Low nuts and seeds	106
Stroke	Female	0-6 days	95+ years	Data Rich	1	3	Age- and sex-specific SEV for Low fruit	129
Stroke	Female	0-6 days	95+ years	Data Rich	1	3	Liters of alcohol consumed per capita	192
Stroke	Female	0-6 days	95+ years	Data Rich	1	3	Diet high in trans fatty acids	542
Stroke	Female	0-6 days	95+ years	Data Rich	1	3	Age- and sex-specific SEV for Low vegetables	798
Stroke	Female	0-6 days	95+ years	Global	1	1	Low-Density Lipoprotein (mmol/L)	87
Stroke	Female	0-6 days	95+ years	Global	1	1	Smoking Prevalence	534
Stroke	Female	0-6 days	95+ years	Global	1	1	Log-transformed SEV scalar: Stroke	568
Stroke	Female	0-6 days	95+ years	Global	1	1	Systolic Blood Pressure (mmHg)	610
Stroke	Female	0-6 days	95+ years	Global	-1	2	Healthcare access and quality index	908
Stroke	Female	0-6 days	95+ years	Global	-1	2	Elevation Over 1500m (proportion)	--
Stroke	Female	0-6 days	95+ years	Global	1	2	Indoor Air Pollution (All Cooking Fuels)	107
Stroke	Female	0-6 days	95+ years	Global	1	2	Outdoor Air Pollution (PM2.5)	809
Stroke	Female	0-6 days	95+ years	Global	1	2	Diabetes Fasting Plasma Glucose (mmol/L), age-standardized 25+	--
Stroke	Female	0-6 days	95+ years	Global	1	2	Mean BMI	--
Stroke	Female	0-6 days	95+ years	Global	-1	3	Pulses legumes unadjusted(g)	2
Stroke	Female	0-6 days	95+ years	Global	-1	3	LDI (I$ per capita)	--
Stroke	Female	0-6 days	95+ years	Global	1	3	Age- and sex-specific SEV for Low fruit	0
Stroke	Female	0-6 days	95+ years	Global	1	3	Age- and sex-specific SEV for Low omega-3	0
Stroke	Female	0-6 days	95+ years	Global	1	3	Age- and sex-specific SEV for Low PUFA	2
Stroke	Female	0-6 days	95+ years	Global	1	3	Age- and sex-specific SEV for Low vegetables	15
Stroke	Female	0-6 days	95+ years	Global	1	3	Age- and sex-specific SEV for Low nuts and seeds	62
Stroke	Female	0-6 days	95+ years	Global	1	3	Liters of alcohol consumed per capita	427
Stroke	Female	0-6 days	95+ years	Global	1	3	Diet high in trans fatty acids	527
Stroke	Male	0-6 days	95+ years	Data Rich	1	1	Smoking Prevalence	169
Stroke	Male	0-6 days	95+ years	Data Rich	1	1	Low-Density Lipoprotein (mmol/L)	299
Stroke	Male	0-6 days	95+ years	Data Rich	1	1	Log-transformed SEV scalar: Stroke	374
Stroke	Male	0-6 days	95+ years	Data Rich	1	1	Systolic Blood Pressure (mmHg)	855
Stroke	Male	0-6 days	95+ years	Data Rich	-1	2	Elevation Over 1500m (proportion)	783
Stroke	Male	0-6 days	95+ years	Data Rich	-1	2	Healthcare access and quality index	994
Stroke	Male	0-6 days	95+ years	Data Rich	1	2	Indoor Air Pollution (All Cooking Fuels)	313
Stroke	Male	0-6 days	95+ years	Data Rich	1	2	Outdoor Air Pollution (PM2.5)	750
Stroke	Male	0-6 days	95+ years	Data Rich	1	2	Diabetes Fasting Plasma Glucose (mmol/L), age-standardized 25+	--
Stroke	Male	0-6 days	95+ years	Data Rich	1	2	Mean BMI	--
Stroke	Male	0-6 days	95+ years	Data Rich	-1	3	Pulses legumes unadjusted(g)	112
Stroke	Male	0-6 days	95+ years	Data Rich	-1	3	LDI (I$ per capita)	--
Stroke	Male	0-6 days	95+ years	Data Rich	1	3	Age- and sex-specific SEV for Low PUFA	0
Stroke	Male	0-6 days	95+ years	Data Rich	1	3	Age- and sex-specific SEV for Low omega-3	0
Stroke	Male	0-6 days	95+ years	Data Rich	1	3	Age- and sex-specific SEV for Low nuts and seeds	10
Stroke	Male	0-6 days	95+ years	Data Rich	1	3	Age- and sex-specific SEV for Low fruit	326
Stroke	Male	0-6 days	95+ years	Data Rich	1	3	Age- and sex-specific SEV for Low vegetables	496
Stroke	Male	0-6 days	95+ years	Data Rich	1	3	Liters of alcohol consumed per capita	573
Stroke	Male	0-6 days	95+ years	Data Rich	1	3	Diet high in trans fatty acids	768
Stroke	Male	0-6 days	95+ years	Global	1	1	Low-Density Lipoprotein (mmol/L)	234
Stroke	Male	0-6 days	95+ years	Global	1	1	Log-transformed SEV scalar: Stroke	264
Stroke	Male	0-6 days	95+ years	Global	1	1	Systolic Blood Pressure (mmHg)	308
Stroke	Male	0-6 days	95+ years	Global	1	1	Smoking Prevalence	575
Stroke	Male	0-6 days	95+ years	Global	-1	2	Healthcare access and quality index	977
Stroke	Male	0-6 days	95+ years	Global	-1	2	Elevation Over 1500m (proportion)	--
Stroke	Male	0-6 days	95+ years	Global	1	2	Indoor Air Pollution (All Cooking Fuels)	10
Stroke	Male	0-6 days	95+ years	Global	1	2	Outdoor Air Pollution (PM2.5)	544
Stroke	Male	0-6 days	95+ years	Global	1	2	Diabetes Fasting Plasma Glucose (mmol/L), age-standardized 25+	--
Stroke	Male	0-6 days	95+ years	Global	1	2	Mean BMI	--
Stroke	Male	0-6 days	95+ years	Global	-1	3	Pulses legumes unadjusted(g)	0
Stroke	Male	0-6 days	95+ years	Global	-1	3	LDI (I$ per capita)	--
Stroke	Male	0-6 days	95+ years	Global	1	3	Age- and sex-specific SEV for Low PUFA	0
Stroke	Male	0-6 days	95+ years	Global	1	3	Age- and sex-specific SEV for Low fruit	0
Stroke	Male	0-6 days	95+ years	Global	1	3	Age- and sex-specific SEV for Low omega-3	0
Stroke	Male	0-6 days	95+ years	Global	1	3	Age- and sex-specific SEV for Low vegetables	7
Stroke	Male	0-6 days	95+ years	Global	1	3	Age- and sex-specific SEV for Low nuts and seeds	15
Stroke	Male	0-6 days	95+ years	Global	1	3	Diet high in trans fatty acids	356
Stroke	Male	0-6 days	95+ years	Global	1	3	Liters of alcohol consumed per capita	704
Ischaemic stroke	Female	0-6 days	95+ years	Data Rich	1	1	Systolic Blood Pressure (mmHg)	603
Ischaemic stroke	Female	0-6 days	95+ years	Data Rich	1	1	Log-transformed SEV scalar: Isch Stroke	746
Ischaemic stroke	Female	0-6 days	95+ years	Data Rich	1	1	Low-Density Lipoprotein (mmol/L)	746
Ischaemic stroke	Female	0-6 days	95+ years	Data Rich	1	1	Smoking Prevalence	857
Ischaemic stroke	Female	0-6 days	95+ years	Data Rich	-1	2	Healthcare access and quality index	346
Ischaemic stroke	Female	0-6 days	95+ years	Data Rich	-1	2	Elevation Over 1500m (proportion)	--
Ischaemic stroke	Female	0-6 days	95+ years	Data Rich	1	2	Indoor Air Pollution (All Cooking Fuels)	439
Ischaemic stroke	Female	0-6 days	95+ years	Data Rich	1	2	Diabetes Fasting Plasma Glucose (mmol/L), age-standardized 25+	--
Ischaemic stroke	Female	0-6 days	95+ years	Data Rich	1	2	Mean BMI	--
Ischaemic stroke	Female	0-6 days	95+ years	Data Rich	1	2	Outdoor Air Pollution (PM2.5)	--
Ischaemic stroke	Female	0-6 days	95+ years	Data Rich	-1	3	Pulses legumes unadjusted(g)	44
Ischaemic stroke	Female	0-6 days	95+ years	Data Rich	-1	3	LDI (I$ per capita)	--
Ischaemic stroke	Female	0-6 days	95+ years	Data Rich	1	3	Age- and sex-specific SEV for Low nuts and seeds	42
Ischaemic stroke	Female	0-6 days	95+ years	Data Rich	1	3	Age- and sex-specific SEV for Low omega-3	144
Ischaemic stroke	Female	0-6 days	95+ years	Data Rich	1	3	Age- and sex-specific SEV for Low vegetables	153
Ischaemic stroke	Female	0-6 days	95+ years	Data Rich	1	3	Age- and sex-specific SEV for Low fruit	173
Ischaemic stroke	Female	0-6 days	95+ years	Data Rich	1	3	Diet high in trans fatty acids	268
Ischaemic stroke	Female	0-6 days	95+ years	Data Rich	1	3	Age- and sex-specific SEV for Low PUFA	--
Ischaemic stroke	Female	0-6 days	95+ years	Data Rich	1	3	Liters of alcohol consumed per capita	--
Ischaemic stroke	Female	0-6 days	95+ years	Global	1	1	Low-Density Lipoprotein (mmol/L)	375
Ischaemic stroke	Female	0-6 days	95+ years	Global	1	1	Log-transformed SEV scalar: Isch Stroke	424
Ischaemic stroke	Female	0-6 days	95+ years	Global	1	1	Smoking Prevalence	486

Appendix Table S10: CODEm covariates used, level of covariate, and expected direction of covariate by cause, sex, and age

Cause	Sex	Age Start	Age End	Model Version Type	Direction	Level	Covariate Name	Number of Draws
Ischaemic stroke	Female	0-6 days	95+ years	Global	1	1	Systolic Blood Pressure (mmHg)	644
Ischaemic stroke	Female	0-6 days	95+ years	Global	-1	2	Elevation Over 1500m (proportion)	104
Ischaemic stroke	Female	0-6 days	95+ years	Global	-1	2	Healthcare access and quality index	853
Ischaemic stroke	Female	0-6 days	95+ years	Global	1	2	Indoor Air Pollution (All Cooking Fuels)	59
Ischaemic stroke	Female	0-6 days	95+ years	Global	1	2	Outdoor Air Pollution (PM2.5)	760
Ischaemic stroke	Female	0-6 days	95+ years	Global	1	2	Diabetes Fasting Plasma Glucose (mmol/L), age-standardized 25+	--
Ischaemic stroke	Female	0-6 days	95+ years	Global	1	2	Mean BMI	--
Ischaemic stroke	Female	0-6 days	95+ years	Global	-1	3	Pulses legumes unadjusted(g)	16
Ischaemic stroke	Female	0-6 days	95+ years	Global	-1	3	LDI (I$ per capita)	--
Ischaemic stroke	Female	0-6 days	95+ years	Global	1	3	Age- and sex-specific SEV for Low omega-3	6
Ischaemic stroke	Female	0-6 days	95+ years	Global	1	3	Age- and sex-specific SEV for Low fruit	10
Ischaemic stroke	Female	0-6 days	95+ years	Global	1	3	Age- and sex-specific SEV for Low vegetables	13
Ischaemic stroke	Female	0-6 days	95+ years	Global	1	3	Age- and sex-specific SEV for Low nuts and seeds	18
Ischaemic stroke	Female	0-6 days	95+ years	Global	1	3	Liters of alcohol consumed per capita	219
Ischaemic stroke	Female	0-6 days	95+ years	Global	1	3	Age- and sex-specific SEV for Low PUFA	296
Ischaemic stroke	Female	0-6 days	95+ years	Global	1	3	Diet high in trans fatty acids	338
Ischaemic stroke	Male	0-6 days	95+ years	Data Rich	1	1	Log-transformed SEV scalar: Isch Stroke	227
Ischaemic stroke	Male	0-6 days	95+ years	Data Rich	1	1	Low-Density Lipoprotein (mmol/L)	355
Ischaemic stroke	Male	0-6 days	95+ years	Data Rich	1	1	Smoking Prevalence	454
Ischaemic stroke	Male	0-6 days	95+ years	Data Rich	1	1	Systolic Blood Pressure (mmHg)	653
Ischaemic stroke	Male	0-6 days	95+ years	Data Rich	-1	2	Elevation Over 1500m (proportion)	649
Ischaemic stroke	Male	0-6 days	95+ years	Data Rich	-1	2	Healthcare access and quality index	792
Ischaemic stroke	Male	0-6 days	95+ years	Data Rich	1	2	Outdoor Air Pollution (PM2.5)	488
Ischaemic stroke	Male	0-6 days	95+ years	Data Rich	1	2	Indoor Air Pollution (All Cooking Fuels)	497
Ischaemic stroke	Male	0-6 days	95+ years	Data Rich	1	2	Diabetes Fasting Plasma Glucose (mmol/L), age-standardized 25+	--
Ischaemic stroke	Male	0-6 days	95+ years	Data Rich	1	2	Mean BMI	--
Ischaemic stroke	Male	0-6 days	95+ years	Data Rich	-1	3	Pulses legumes unadjusted(g)	291
Ischaemic stroke	Male	0-6 days	95+ years	Data Rich	-1	3	LDI (I$ per capita)	--
Ischaemic stroke	Male	0-6 days	95+ years	Data Rich	1	3	Age- and sex-specific SEV for Low omega-3	6
Ischaemic stroke	Male	0-6 days	95+ years	Data Rich	1	3	Age- and sex-specific SEV for Low PUFA	12
Ischaemic stroke	Male	0-6 days	95+ years	Data Rich	1	3	Age- and sex-specific SEV for Low nuts and seeds	96
Ischaemic stroke	Male	0-6 days	95+ years	Data Rich	1	3	Diet high in trans fatty acids	198
Ischaemic stroke	Male	0-6 days	95+ years	Data Rich	1	3	Age- and sex-specific SEV for Low vegetables	212
Ischaemic stroke	Male	0-6 days	95+ years	Data Rich	1	3	Liters of alcohol consumed per capita	286
Ischaemic stroke	Male	0-6 days	95+ years	Data Rich	1	3	Age- and sex-specific SEV for Low fruit	323
Ischaemic stroke	Male	0-6 days	95+ years	Global	1	1	Low-Density Lipoprotein (mmol/L)	304
Ischaemic stroke	Male	0-6 days	95+ years	Global	1	1	Log-transformed SEV scalar: Isch Stroke	499
Ischaemic stroke	Male	0-6 days	95+ years	Global	1	1	Systolic Blood Pressure (mmHg)	716
Ischaemic stroke	Male	0-6 days	95+ years	Global	1	1	Smoking Prevalence	766
Ischaemic stroke	Male	0-6 days	95+ years	Global	-1	2	Elevation Over 1500m (proportion)	381
Ischaemic stroke	Male	0-6 days	95+ years	Global	-1	2	Healthcare access and quality index	880
Ischaemic stroke	Male	0-6 days	95+ years	Global	1	2	Indoor Air Pollution (All Cooking Fuels)	54
Ischaemic stroke	Male	0-6 days	95+ years	Global	1	2	Outdoor Air Pollution (PM2.5)	715
Ischaemic stroke	Male	0-6 days	95+ years	Global	1	2	Diabetes Fasting Plasma Glucose (mmol/L), age-standardized 25+	--
Ischaemic stroke	Male	0-6 days	95+ years	Global	1	2	Mean BMI	--
Ischaemic stroke	Male	0-6 days	95+ years	Global	-1	3	Pulses legumes unadjusted(g)	0
Ischaemic stroke	Male	0-6 days	95+ years	Global	-1	3	LDI (I$ per capita)	--
Ischaemic stroke	Male	0-6 days	95+ years	Global	1	3	Age- and sex-specific SEV for Low omega-3	2
Ischaemic stroke	Male	0-6 days	95+ years	Global	1	3	Age- and sex-specific SEV for Low fruit	8
Ischaemic stroke	Male	0-6 days	95+ years	Global	1	3	Age- and sex-specific SEV for Low nuts and seeds	9
Ischaemic stroke	Male	0-6 days	95+ years	Global	1	3	Age- and sex-specific SEV for Low vegetables	9
Ischaemic stroke	Male	0-6 days	95+ years	Global	1	3	Age- and sex-specific SEV for Low PUFA	14
Ischaemic stroke	Male	0-6 days	95+ years	Global	1	3	Liters of alcohol consumed per capita	368
Ischaemic stroke	Male	0-6 days	95+ years	Global	1	3	Diet high in trans fatty acids	389
Intracerebral hemorrhage	Female	0-6 days	95+ years	Data Rich	1	1	Smoking Prevalence	439
Intracerebral hemorrhage	Female	0-6 days	95+ years	Data Rich	1	1	Systolic Blood Pressure (mmHg)	551
Intracerebral hemorrhage	Female	0-6 days	95+ years	Data Rich	1	1	Log-transformed SEV scalar: Intrahem Stroke	694
Intracerebral hemorrhage	Female	0-6 days	95+ years	Data Rich	-1	2	Elevation Over 1500m (proportion)	148
Intracerebral hemorrhage	Female	0-6 days	95+ years	Data Rich	-1	2	Healthcare access and quality index	--
Intracerebral hemorrhage	Female	0-6 days	95+ years	Data Rich	1	2	Outdoor Air Pollution (PM2.5)	453
Intracerebral hemorrhage	Female	0-6 days	95+ years	Data Rich	1	2	Diabetes Fasting Plasma Glucose (mmol/L), age-standardized 25+	--
Intracerebral hemorrhage	Female	0-6 days	95+ years	Data Rich	1	2	Indoor Air Pollution (All Cooking Fuels)	--
Intracerebral hemorrhage	Female	0-6 days	95+ years	Data Rich	1	2	Mean BMI	--
Intracerebral hemorrhage	Female	0-6 days	95+ years	Data Rich	-1	3	Pulses legumes unadjusted(g)	13
Intracerebral hemorrhage	Female	0-6 days	95+ years	Data Rich	-1	3	LDI (I$ per capita)	--
Intracerebral hemorrhage	Female	0-6 days	95+ years	Data Rich	-1	3	Low-Density Lipoprotein (mmol/L)	--
Intracerebral hemorrhage	Female	0-6 days	95+ years	Data Rich	1	3	Diet high in trans fatty acids	28
Intracerebral hemorrhage	Female	0-6 days	95+ years	Data Rich	1	3	Age- and sex-specific SEV for Low fruit	31
Intracerebral hemorrhage	Female	0-6 days	95+ years	Data Rich	1	3	Age- and sex-specific SEV for Low PUFA	83
Intracerebral hemorrhage	Female	0-6 days	95+ years	Data Rich	1	3	Age- and sex-specific SEV for Low nuts and seeds	88
Intracerebral hemorrhage	Female	0-6 days	95+ years	Data Rich	1	3	Age- and sex-specific SEV for Low omega-3	308
Intracerebral hemorrhage	Female	0-6 days	95+ years	Data Rich	1	3	Age- and sex-specific SEV for Low vegetables	393
Intracerebral hemorrhage	Female	0-6 days	95+ years	Data Rich	1	3	Liters of alcohol consumed per capita	--
Intracerebral hemorrhage	Female	0-6 days	95+ years	Global	1	1	Smoking Prevalence	280
Intracerebral hemorrhage	Female	0-6 days	95+ years	Global	1	1	Systolic Blood Pressure (mmHg)	367
Intracerebral hemorrhage	Female	0-6 days	95+ years	Global	1	1	Log-transformed SEV scalar: Intrahem Stroke	923
Intracerebral hemorrhage	Female	0-6 days	95+ years	Global	-1	2	Healthcare access and quality index	715
Intracerebral hemorrhage	Female	0-6 days	95+ years	Global	-1	2	Elevation Over 1500m (proportion)	--
Intracerebral hemorrhage	Female	0-6 days	95+ years	Global	1	2	Outdoor Air Pollution (PM2.5)	553
Intracerebral hemorrhage	Female	0-6 days	95+ years	Global	1	2	Indoor Air Pollution (All Cooking Fuels)	770
Intracerebral hemorrhage	Female	0-6 days	95+ years	Global	1	2	Diabetes Fasting Plasma Glucose (mmol/L), age-standardized 25+	--
Intracerebral hemorrhage	Female	0-6 days	95+ years	Global	1	2	Mean BMI	--
Intracerebral hemorrhage	Female	0-6 days	95+ years	Global	-1	3	Pulses legumes unadjusted(g)	48
Intracerebral hemorrhage	Female	0-6 days	95+ years	Global	-1	3	Low-Density Lipoprotein (mmol/L)	98
Intracerebral hemorrhage	Female	0-6 days	95+ years	Global	-1	3	LDI (I$ per capita)	--
Intracerebral hemorrhage	Female	0-6 days	95+ years	Global	1	3	Age- and sex-specific SEV for Low fruit	7
Intracerebral hemorrhage	Female	0-6 days	95+ years	Global	1	3	Age- and sex-specific SEV for Low omega-3	7
Intracerebral hemorrhage	Female	0-6 days	95+ years	Global	1	3	Age- and sex-specific SEV for Low nuts and seeds	33
Intracerebral hemorrhage	Female	0-6 days	95+ years	Global	1	3	Age- and sex-specific SEV for Low PUFA	44
Intracerebral hemorrhage	Female	0-6 days	95+ years	Global	1	3	Age- and sex-specific SEV for Low vegetables	78
Intracerebral hemorrhage	Female	0-6 days	95+ years	Global	1	3	Diet high in trans fatty acids	437
Intracerebral hemorrhage	Female	0-6 days	95+ years	Global	1	3	Liters of alcohol consumed per capita	--
Intracerebral hemorrhage	Male	0-6 days	95+ years	Data Rich	1	1	Log-transformed SEV scalar: Intrahem Stroke	20
Intracerebral hemorrhage	Male	0-6 days	95+ years	Data Rich	1	1	Smoking Prevalence	618
Intracerebral hemorrhage	Male	0-6 days	95+ years	Data Rich	1	1	Systolic Blood Pressure (mmHg)	1000
Intracerebral hemorrhage	Male	0-6 days	95+ years	Data Rich	-1	2	Elevation Over 1500m (proportion)	85
Intracerebral hemorrhage	Male	0-6 days	95+ years	Data Rich	-1	2	Healthcare access and quality index	182
Intracerebral hemorrhage	Male	0-6 days	95+ years	Data Rich	1	2	Outdoor Air Pollution (PM2.5)	286

Appendix Table S10: CODEm covariates used, level of covariate, and expected direction of covariate by cause, sex, and age

Cause	Sex	Age Start	Age End	Model Version Type	Direction	Level	Covariate Name	Number of Draws
Intracerebral hemorrhage	Male	0-6 days	95+ years	Data Rich	1	2	Indoor Air Pollution (All Cooking Fuels)	520
Intracerebral hemorrhage	Male	0-6 days	95+ years	Data Rich	1	2	Diabetes Fasting Plasma Glucose (mmol/L), age-standardized 25+	--
Intracerebral hemorrhage	Male	0-6 days	95+ years	Data Rich	1	2	Mean BMI	--
Intracerebral hemorrhage	Male	0-6 days	95+ years	Data Rich	-1	3	Low-Density Lipoprotein (mmol/L)	67
Intracerebral hemorrhage	Male	0-6 days	95+ years	Data Rich	-1	3	Pulses legumes unadjusted(g)	214
Intracerebral hemorrhage	Male	0-6 days	95+ years	Data Rich	-1	3	LDI (I$ per capita)	--
Intracerebral hemorrhage	Male	0-6 days	95+ years	Data Rich	1	3	Age- and sex-specific SEV for Low PUFA	80
Intracerebral hemorrhage	Male	0-6 days	95+ years	Data Rich	1	3	Age- and sex-specific SEV for Low fruit	132
Intracerebral hemorrhage	Male	0-6 days	95+ years	Data Rich	1	3	Age- and sex-specific SEV for Low nuts and seeds	142
Intracerebral hemorrhage	Male	0-6 days	95+ years	Data Rich	1	3	Age- and sex-specific SEV for Low omega-3	167
Intracerebral hemorrhage	Male	0-6 days	95+ years	Data Rich	1	3	Diet high in trans fatty acids	413
Intracerebral hemorrhage	Male	0-6 days	95+ years	Data Rich	1	3	Age- and sex-specific SEV for Low vegetables	601
Intracerebral hemorrhage	Male	0-6 days	95+ years	Data Rich	1	3	Liters of alcohol consumed per capita	--
Intracerebral hemorrhage	Male	0-6 days	95+ years	Global	1	1	Systolic Blood Pressure (mmHg)	374
Intracerebral hemorrhage	Male	0-6 days	95+ years	Global	1	1	Log-transformed SEV scalar: Intrahem Stroke	527
Intracerebral hemorrhage	Male	0-6 days	95+ years	Global	1	1	Smoking Prevalence	825
Intracerebral hemorrhage	Male	0-6 days	95+ years	Global	-1	2	Healthcare access and quality index	736
Intracerebral hemorrhage	Male	0-6 days	95+ years	Global	-1	2	Elevation Over 1500m (proportion)	--
Intracerebral hemorrhage	Male	0-6 days	95+ years	Global	1	2	Indoor Air Pollution (All Cooking Fuels)	397
Intracerebral hemorrhage	Male	0-6 days	95+ years	Global	1	2	Outdoor Air Pollution (PM2.5)	691
Intracerebral hemorrhage	Male	0-6 days	95+ years	Global	1	2	Diabetes Fasting Plasma Glucose (mmol/L), age-standardized 25+	--
Intracerebral hemorrhage	Male	0-6 days	95+ years	Global	1	2	Mean BMI	--
Intracerebral hemorrhage	Male	0-6 days	95+ years	Global	-1	3	Pulses legumes unadjusted(g)	7
Intracerebral hemorrhage	Male	0-6 days	95+ years	Global	-1	3	Low-Density Lipoprotein (mmol/L)	264
Intracerebral hemorrhage	Male	0-6 days	95+ years	Global	-1	3	LDI (I$ per capita)	--
Intracerebral hemorrhage	Male	0-6 days	95+ years	Global	1	3	Age- and sex-specific SEV for Low PUFA	100
Intracerebral hemorrhage	Male	0-6 days	95+ years	Global	1	3	Age- and sex-specific SEV for Low nuts and seeds	117
Intracerebral hemorrhage	Male	0-6 days	95+ years	Global	1	3	Age- and sex-specific SEV for Low fruit	136
Intracerebral hemorrhage	Male	0-6 days	95+ years	Global	1	3	Age- and sex-specific SEV for Low omega-3	165
Intracerebral hemorrhage	Male	0-6 days	95+ years	Global	1	3	Age- and sex-specific SEV for Low vegetables	187
Intracerebral hemorrhage	Male	0-6 days	95+ years	Global	1	3	Diet high in trans fatty acids	558
Intracerebral hemorrhage	Male	0-6 days	95+ years	Global	1	3	Liters of alcohol consumed per capita	--
Subarachnoid hemorrhage	Female	0-6 days	95+ years	Data Rich	1	1	Smoking Prevalence	364
Subarachnoid hemorrhage	Female	0-6 days	95+ years	Data Rich	1	1	Systolic Blood Pressure (mmHg)	590
Subarachnoid hemorrhage	Female	0-6 days	95+ years	Data Rich	1	1	Log-transformed SEV scalar: Sub Hem	954
Subarachnoid hemorrhage	Female	0-6 days	95+ years	Data Rich	-1	2	Healthcare access and quality index	216
Subarachnoid hemorrhage	Female	0-6 days	95+ years	Data Rich	-1	3	LDI (I$ per capita)	--
Subarachnoid hemorrhage	Female	0-6 days	95+ years	Data Rich	1	3	Liters of alcohol consumed per capita	--
Subarachnoid hemorrhage	Female	0-6 days	95+ years	Global	1	1	Log-transformed SEV scalar: Sub Hem	293
Subarachnoid hemorrhage	Female	0-6 days	95+ years	Global	1	1	Systolic Blood Pressure (mmHg)	395
Subarachnoid hemorrhage	Female	0-6 days	95+ years	Global	1	1	Smoking Prevalence	733
Subarachnoid hemorrhage	Female	0-6 days	95+ years	Global	-1	2	Healthcare access and quality index	236
Subarachnoid hemorrhage	Female	0-6 days	95+ years	Global	-1	3	LDI (I$ per capita)	--
Subarachnoid hemorrhage	Female	0-6 days	95+ years	Global	1	3	Liters of alcohol consumed per capita	--
Subarachnoid hemorrhage	Male	0-6 days	95+ years	Data Rich	1	1	Log-transformed SEV scalar: Sub Hem	525
Subarachnoid hemorrhage	Male	0-6 days	95+ years	Data Rich	1	1	Systolic Blood Pressure (mmHg)	536
Subarachnoid hemorrhage	Male	0-6 days	95+ years	Data Rich	1	1	Smoking Prevalence	639
Subarachnoid hemorrhage	Male	0-6 days	95+ years	Data Rich	-1	2	Healthcare access and quality index	169
Subarachnoid hemorrhage	Male	0-6 days	95+ years	Data Rich	-1	3	LDI (I$ per capita)	--
Subarachnoid hemorrhage	Male	0-6 days	95+ years	Data Rich	1	3	Liters of alcohol consumed per capita	--
Subarachnoid hemorrhage	Male	0-6 days	95+ years	Global	1	1	Smoking Prevalence	546
Subarachnoid hemorrhage	Male	0-6 days	95+ years	Global	1	1	Log-transformed SEV scalar: Sub Hem	1000
Subarachnoid hemorrhage	Male	0-6 days	95+ years	Global	1	1	Systolic Blood Pressure (mmHg)	--
Subarachnoid hemorrhage	Male	0-6 days	95+ years	Global	-1	2	Healthcare access and quality index	--
Subarachnoid hemorrhage	Male	0-6 days	95+ years	Global	-1	3	LDI (I$ per capita)	--
Subarachnoid hemorrhage	Male	0-6 days	95+ years	Global	1	3	Liters of alcohol consumed per capita	--
Hypertensive heart disease	Female	15-19 years	95+ years	Data Rich	1	1	Systolic Blood Pressure (mmHg)	1000
Hypertensive heart disease	Female	15-19 years	95+ years	Data Rich	-1	2	Healthcare access and quality index	--
Hypertensive heart disease	Female	15-19 years	95+ years	Data Rich	1	2	Smoking Prevalence	601
Hypertensive heart disease	Female	15-19 years	95+ years	Data Rich	1	2	Low-Density Lipoprotein (mmol/L)	--
Hypertensive heart disease	Female	15-19 years	95+ years	Data Rich	1	2	Mean BMI	--
Hypertensive heart disease	Female	15-19 years	95+ years	Data Rich	-1	3	Pulses legumes unadjusted(g)	410
Hypertensive heart disease	Female	15-19 years	95+ years	Data Rich	-1	3	LDI (I$ per capita)	--
Hypertensive heart disease	Female	15-19 years	95+ years	Data Rich	-1	3	Socio-demographic Index	--
Hypertensive heart disease	Female	15-19 years	95+ years	Data Rich	1	3	Age- and sex-specific SEV for Low fruit	55
Hypertensive heart disease	Female	15-19 years	95+ years	Data Rich	1	3	Age- and sex-specific SEV for Low nuts and seeds	121
Hypertensive heart disease	Female	15-19 years	95+ years	Data Rich	1	3	Age- and sex-specific SEV for Low omega-3	368
Hypertensive heart disease	Female	15-19 years	95+ years	Data Rich	1	3	Age- and sex-specific SEV for Low vegetables	680
Hypertensive heart disease	Female	15-19 years	95+ years	Data Rich	1	3	Age- and sex-specific SEV for Low PUFA	--
Hypertensive heart disease	Female	15-19 years	95+ years	Data Rich	1	3	Diet high in trans fatty acids	--
Hypertensive heart disease	Female	15-19 years	95+ years	Data Rich	1	3	Liters of alcohol consumed per capita	--
Hypertensive heart disease	Female	15-19 years	95+ years	Global	1	1	Systolic Blood Pressure (mmHg)	1000
Hypertensive heart disease	Female	15-19 years	95+ years	Global	-1	2	Healthcare access and quality index	--
Hypertensive heart disease	Female	15-19 years	95+ years	Global	1	2	Smoking Prevalence	133
Hypertensive heart disease	Female	15-19 years	95+ years	Global	1	2	Low-Density Lipoprotein (mmol/L)	--
Hypertensive heart disease	Female	15-19 years	95+ years	Global	1	2	Mean BMI	--
Hypertensive heart disease	Female	15-19 years	95+ years	Global	-1	3	LDI (I$ per capita)	--
Hypertensive heart disease	Female	15-19 years	95+ years	Global	-1	3	Pulses legumes unadjusted(g)	--
Hypertensive heart disease	Female	15-19 years	95+ years	Global	-1	3	Socio-demographic Index	--
Hypertensive heart disease	Female	15-19 years	95+ years	Global	1	3	Age- and sex-specific SEV for Low PUFA	161
Hypertensive heart disease	Female	15-19 years	95+ years	Global	1	3	Age- and sex-specific SEV for Low vegetables	204
Hypertensive heart disease	Female	15-19 years	95+ years	Global	1	3	Age- and sex-specific SEV for Low omega-3	282
Hypertensive heart disease	Female	15-19 years	95+ years	Global	1	3	Age- and sex-specific SEV for Low fruit	486
Hypertensive heart disease	Female	15-19 years	95+ years	Global	1	3	Age- and sex-specific SEV for Low nuts and seeds	--
Hypertensive heart disease	Female	15-19 years	95+ years	Global	1	3	Diet high in trans fatty acids	--
Hypertensive heart disease	Female	15-19 years	95+ years	Global	1	3	Liters of alcohol consumed per capita	--
Hypertensive heart disease	Male	15-19 years	95+ years	Data Rich	1	1	Systolic Blood Pressure (mmHg)	--
Hypertensive heart disease	Male	15-19 years	95+ years	Data Rich	-1	2	Healthcare access and quality index	--
Hypertensive heart disease	Male	15-19 years	95+ years	Data Rich	1	2	Low-Density Lipoprotein (mmol/L)	--
Hypertensive heart disease	Male	15-19 years	95+ years	Data Rich	1	2	Mean BMI	--
Hypertensive heart disease	Male	15-19 years	95+ years	Data Rich	1	2	Smoking Prevalence	--
Hypertensive heart disease	Male	15-19 years	95+ years	Data Rich	-1	3	Pulses legumes unadjusted(g)	186
Hypertensive heart disease	Male	15-19 years	95+ years	Data Rich	-1	3	Socio-demographic Index	491
Hypertensive heart disease	Male	15-19 years	95+ years	Data Rich	-1	3	LDI (I$ per capita)	--
Hypertensive heart disease	Male	15-19 years	95+ years	Data Rich	1	3	Age- and sex-specific SEV for Low fruit	186
Hypertensive heart disease	Male	15-19 years	95+ years	Data Rich	1	3	Age- and sex-specific SEV for Low omega-3	546
Hypertensive heart disease	Male	15-19 years	95+ years	Data Rich	1	3	Age- and sex-specific SEV for Low vegetables	591
Hypertensive heart disease	Male	15-19 years	95+ years	Data Rich	1	3	Age- and sex-specific SEV for Low PUFA	--
Hypertensive heart disease	Male	15-19 years	95+ years	Data Rich	1	3	Age- and sex-specific SEV for Low nuts and seeds	--
Hypertensive heart disease	Male	15-19 years	95+ years	Data Rich	1	3	Diet high in trans fatty acids	--

Appendix Table S10: CODEm covariates used, level of covariate, and expected direction of covariate by cause, sex, and age

Cause	Sex	Age Start	Age End	Model Version Type	Direction	Level	Covariate Name	Number of Draws
Hypertensive heart disease	Male	15-19 years	95+ years	Data Rich	1	3	Liters of alcohol consumed per capita	--
Hypertensive heart disease	Male	15-19 years	95+ years	Global	1	1	Systolic Blood Pressure (mmHg)	--
Hypertensive heart disease	Male	15-19 years	95+ years	Global	-1	2	Healthcare access and quality index	706
Hypertensive heart disease	Male	15-19 years	95+ years	Global	1	2	Low-Density Lipoprotein (mmol/L)	--
Hypertensive heart disease	Male	15-19 years	95+ years	Global	1	2	Mean BMI	--
Hypertensive heart disease	Male	15-19 years	95+ years	Global	1	2	Smoking Prevalence	--
Hypertensive heart disease	Male	15-19 years	95+ years	Global	-1	3	Socio-demographic Index	161
Hypertensive heart disease	Male	15-19 years	95+ years	Global	-1	3	LDI (I$ per capita)	--
Hypertensive heart disease	Male	15-19 years	95+ years	Global	-1	3	Pulses legumes unadjusted(g)	--
Hypertensive heart disease	Male	15-19 years	95+ years	Global	1	3	Age- and sex-specific SEV for Low nuts and seeds	133
Hypertensive heart disease	Male	15-19 years	95+ years	Global	1	3	Age- and sex-specific SEV for Low omega-3	244
Hypertensive heart disease	Male	15-19 years	95+ years	Global	1	3	Age- and sex-specific SEV for Low vegetables	552
Hypertensive heart disease	Male	15-19 years	95+ years	Global	1	3	Age- and sex-specific SEV for Low PUFA	--
Hypertensive heart disease	Male	15-19 years	95+ years	Global	1	3	Age- and sex-specific SEV for Low fruit	--
Hypertensive heart disease	Male	15-19 years	95+ years	Global	1	3	Diet high in trans fatty acids	--
Hypertensive heart disease	Male	15-19 years	95+ years	Global	1	3	Liters of alcohol consumed per capita	--
Non-rheumatic valvular heart disease	Female	15-19 years	95+ years	Data Rich	1	1	Systolic Blood Pressure (mmHg)	500
Non-rheumatic valvular heart disease	Female	15-19 years	95+ years	Data Rich	1	1	Smoking Prevalence	1000
Non-rheumatic valvular heart disease	Female	15-19 years	95+ years	Data Rich	-1	2	Healthcare access and quality index	--
Non-rheumatic valvular heart disease	Female	15-19 years	95+ years	Data Rich	1	2	Low-Density Lipoprotein (mmol/L)	--
Non-rheumatic valvular heart disease	Female	15-19 years	95+ years	Data Rich	1	2	Mean BMI	--
Non-rheumatic valvular heart disease	Female	15-19 years	95+ years	Data Rich	-1	3	LDI (I$ per capita)	--
Non-rheumatic valvular heart disease	Female	15-19 years	95+ years	Data Rich	-1	3	Socio-demographic Index	--
Non-rheumatic valvular heart disease	Female	15-19 years	95+ years	Data Rich	1	3	Liters of alcohol consumed per capita	--
Non-rheumatic valvular heart disease	Female	15-19 years	95+ years	Global	1	1	Smoking Prevalence	1000
Non-rheumatic valvular heart disease	Female	15-19 years	95+ years	Global	1	1	Systolic Blood Pressure (mmHg)	1000
Non-rheumatic valvular heart disease	Female	15-19 years	95+ years	Global	-1	2	Healthcare access and quality index	--
Non-rheumatic valvular heart disease	Female	15-19 years	95+ years	Global	1	2	Low-Density Lipoprotein (mmol/L)	--
Non-rheumatic valvular heart disease	Female	15-19 years	95+ years	Global	1	2	Mean BMI	--
Non-rheumatic valvular heart disease	Female	15-19 years	95+ years	Global	-1	3	LDI (I$ per capita)	--
Non-rheumatic valvular heart disease	Female	15-19 years	95+ years	Global	-1	3	Socio-demographic Index	--
Non-rheumatic valvular heart disease	Female	15-19 years	95+ years	Global	1	3	Liters of alcohol consumed per capita	--
Non-rheumatic valvular heart disease	Male	15-19 years	95+ years	Data Rich	1	1	Systolic Blood Pressure (mmHg)	482
Non-rheumatic valvular heart disease	Male	15-19 years	95+ years	Data Rich	1	1	Smoking Prevalence	518
Non-rheumatic valvular heart disease	Male	15-19 years	95+ years	Data Rich	-1	2	Healthcare access and quality index	--
Non-rheumatic valvular heart disease	Male	15-19 years	95+ years	Data Rich	1	2	Mean BMI	253
Non-rheumatic valvular heart disease	Male	15-19 years	95+ years	Data Rich	1	2	Low-Density Lipoprotein (mmol/L)	353
Non-rheumatic valvular heart disease	Male	15-19 years	95+ years	Data Rich	-1	3	Socio-demographic Index	174
Non-rheumatic valvular heart disease	Male	15-19 years	95+ years	Data Rich	-1	3	LDI (I$ per capita)	--
Non-rheumatic valvular heart disease	Male	15-19 years	95+ years	Data Rich	1	3	Liters of alcohol consumed per capita	145
Non-rheumatic valvular heart disease	Male	15-19 years	95+ years	Global	1	1	Smoking Prevalence	556
Non-rheumatic valvular heart disease	Male	15-19 years	95+ years	Global	1	1	Systolic Blood Pressure (mmHg)	1000
Non-rheumatic valvular heart disease	Male	15-19 years	95+ years	Global	-1	2	Healthcare access and quality index	168
Non-rheumatic valvular heart disease	Male	15-19 years	95+ years	Global	1	2	Low-Density Lipoprotein (mmol/L)	168
Non-rheumatic valvular heart disease	Male	15-19 years	95+ years	Global	1	2	Mean BMI	--
Non-rheumatic valvular heart disease	Male	15-19 years	95+ years	Global	-1	3	Socio-demographic Index	288
Non-rheumatic valvular heart disease	Male	15-19 years	95+ years	Global	-1	3	LDI (I$ per capita)	--
Non-rheumatic valvular heart disease	Male	15-19 years	95+ years	Global	1	3	Liters of alcohol consumed per capita	76
Non-rheumatic calcific aortic valve disease	Female	15-19 years	95+ years	Data Rich	1	1	Smoking Prevalence	519
Non-rheumatic calcific aortic valve disease	Female	15-19 years	95+ years	Data Rich	1	1	Systolic Blood Pressure (mmHg)	615
Non-rheumatic calcific aortic valve disease	Female	15-19 years	95+ years	Data Rich	1	1	Mean BMI	720
Non-rheumatic calcific aortic valve disease	Female	15-19 years	95+ years	Data Rich	-1	2	Healthcare access and quality index	--
Non-rheumatic calcific aortic valve disease	Female	15-19 years	95+ years	Data Rich	1	2	Diabetes Fasting Plasma Glucose (mmol/L), age-standardized 25+	111
Non-rheumatic calcific aortic valve disease	Female	15-19 years	95+ years	Data Rich	1	2	Low-Density Lipoprotein (mmol/L)	395
Non-rheumatic calcific aortic valve disease	Female	15-19 years	95+ years	Data Rich	-1	3	LDI (I$ per capita)	--
Non-rheumatic calcific aortic valve disease	Female	15-19 years	95+ years	Data Rich	-1	3	Socio-demographic Index	--
Non-rheumatic calcific aortic valve disease	Female	15-19 years	95+ years	Data Rich	1	3	Liters of alcohol consumed per capita	--
Non-rheumatic calcific aortic valve disease	Female	15-19 years	95+ years	Global	1	1	Systolic Blood Pressure (mmHg)	65
Non-rheumatic calcific aortic valve disease	Female	15-19 years	95+ years	Global	1	1	Smoking Prevalence	575
Non-rheumatic calcific aortic valve disease	Female	15-19 years	95+ years	Global	1	1	Mean BMI	720
Non-rheumatic calcific aortic valve disease	Female	15-19 years	95+ years	Global	-1	2	Healthcare access and quality index	230
Non-rheumatic calcific aortic valve disease	Female	15-19 years	95+ years	Global	1	2	Low-Density Lipoprotein (mmol/L)	180
Non-rheumatic calcific aortic valve disease	Female	15-19 years	95+ years	Global	1	2	Diabetes Fasting Plasma Glucose (mmol/L), age-standardized 25+	--
Non-rheumatic calcific aortic valve disease	Female	15-19 years	95+ years	Global	-1	3	Socio-demographic Index	93
Non-rheumatic calcific aortic valve disease	Female	15-19 years	95+ years	Global	-1	3	LDI (I$ per capita)	--
Non-rheumatic calcific aortic valve disease	Female	15-19 years	95+ years	Global	1	3	Liters of alcohol consumed per capita	--
Non-rheumatic calcific aortic valve disease	Male	15-19 years	95+ years	Data Rich	1	1	Systolic Blood Pressure (mmHg)	524
Non-rheumatic calcific aortic valve disease	Male	15-19 years	95+ years	Data Rich	1	1	Smoking Prevalence	804
Non-rheumatic calcific aortic valve disease	Male	15-19 years	95+ years	Data Rich	1	1	Mean BMI	945
Non-rheumatic calcific aortic valve disease	Male	15-19 years	95+ years	Data Rich	-1	2	Healthcare access and quality index	87
Non-rheumatic calcific aortic valve disease	Male	15-19 years	95+ years	Data Rich	1	2	Diabetes Fasting Plasma Glucose (mmol/L), age-standardized 25+	144
Non-rheumatic calcific aortic valve disease	Male	15-19 years	95+ years	Data Rich	1	2	Low-Density Lipoprotein (mmol/L)	411
Non-rheumatic calcific aortic valve disease	Male	15-19 years	95+ years	Data Rich	-1	3	LDI (I$ per capita)	--
Non-rheumatic calcific aortic valve disease	Male	15-19 years	95+ years	Data Rich	-1	3	Socio-demographic Index	--
Non-rheumatic calcific aortic valve disease	Male	15-19 years	95+ years	Data Rich	1	3	Liters of alcohol consumed per capita	298
Non-rheumatic calcific aortic valve disease	Male	15-19 years	95+ years	Global	1	1	Systolic Blood Pressure (mmHg)	748
Non-rheumatic calcific aortic valve disease	Male	15-19 years	95+ years	Global	1	1	Smoking Prevalence	810
Non-rheumatic calcific aortic valve disease	Male	15-19 years	95+ years	Global	1	1	Mean BMI	--
Non-rheumatic calcific aortic valve disease	Male	15-19 years	95+ years	Global	-1	2	Healthcare access and quality index	161
Non-rheumatic calcific aortic valve disease	Male	15-19 years	95+ years	Global	1	2	Low-Density Lipoprotein (mmol/L)	520
Non-rheumatic calcific aortic valve disease	Male	15-19 years	95+ years	Global	1	2	Diabetes Fasting Plasma Glucose (mmol/L), age-standardized 25+	--
Non-rheumatic calcific aortic valve disease	Male	15-19 years	95+ years	Global	-1	3	LDI (I$ per capita)	--
Non-rheumatic calcific aortic valve disease	Male	15-19 years	95+ years	Global	-1	3	Socio-demographic Index	--
Non-rheumatic calcific aortic valve disease	Male	15-19 years	95+ years	Global	1	3	Liters of alcohol consumed per capita	439
Non-rheumatic degenerative mitral valve disease	Female	15-19 years	95+ years	Data Rich	-1	1	Socio-demographic Index	454
Non-rheumatic degenerative mitral valve disease	Female	15-19 years	95+ years	Data Rich	-1	1	Healthcare access and quality index	546
Non-rheumatic degenerative mitral valve disease	Female	15-19 years	95+ years	Data Rich	-1	1	LDI (I$ per capita)	--
Non-rheumatic degenerative mitral valve disease	Female	15-19 years	95+ years	Global	-1	1	Socio-demographic Index	252
Non-rheumatic degenerative mitral valve disease	Female	15-19 years	95+ years	Global	-1	1	Healthcare access and quality index	563
Non-rheumatic degenerative mitral valve disease	Female	15-19 years	95+ years	Global	-1	1	LDI (I$ per capita)	--
Non-rheumatic degenerative mitral valve disease	Male	15-19 years	95+ years	Data Rich	-1	1	Healthcare access and quality index	323
Non-rheumatic degenerative mitral valve disease	Male	15-19 years	95+ years	Data Rich	-1	1	Socio-demographic Index	409
Non-rheumatic degenerative mitral valve disease	Male	15-19 years	95+ years	Data Rich	-1	1	LDI (I$ per capita)	--
Non-rheumatic degenerative mitral valve disease	Male	15-19 years	95+ years	Global	-1	1	Healthcare access and quality index	1000
Non-rheumatic degenerative mitral valve disease	Male	15-19 years	95+ years	Global	-1	1	LDI (I$ per capita)	--
Non-rheumatic degenerative mitral valve disease	Male	15-19 years	95+ years	Global	-1	1	Socio-demographic Index	--
Other non-rheumatic valve diseases	Female	15-19 years	95+ years	Data Rich	-1	1	Healthcare access and quality index	--
Other non-rheumatic valve diseases	Female	15-19 years	95+ years	Data Rich	1	1	Socio-demographic Index	454
Other non-rheumatic valve diseases	Female	15-19 years	95+ years	Data Rich	1	1	LDI (I$ per capita)	--
Other non-rheumatic valve diseases	Female	15-19 years	95+ years	Global	-1	1	Healthcare access and quality index	--
Other non-rheumatic valve diseases	Female	15-19 years	95+ years	Global	1	1	Socio-demographic Index	509
Other non-rheumatic valve diseases	Female	15-19 years	95+ years	Global	1	1	LDI (I$ per capita)	--
Other non-rheumatic valve diseases	Male	15-19 years	95+ years	Data Rich	-1	1	Healthcare access and quality index	1000
Other non-rheumatic valve diseases	Male	15-19 years	95+ years	Data Rich	1	1	LDI (I$ per capita)	--
Other non-rheumatic valve diseases	Male	15-19 years	95+ years	Data Rich	1	1	Socio-demographic Index	--
Other non-rheumatic valve diseases	Male	15-19 years	95+ years	Global	-1	1	Healthcare access and quality index	1000

Appendix Table S10: CODEm covariates used, level of covariate, and expected direction of covariate by cause, sex, and age								
**Cause**	**Sex**	**Age Start**	**Age End**	**Model Version Type**	**Direction**	**Level**	**Covariate Name**	**Number of Draws**
Other non-rheumatic valve diseases	Male	15-19 years	95+ years	Global	1	1	Socio-demographic Index	454
Other non-rheumatic valve diseases	Male	15-19 years	95+ years	Global	1	1	LDI (I$ per capita)	--
Cardiomyopathy and myocarditis	Female	0-6 days	95+ years	Data Rich	1	1	Log-transformed SEV scalar: CMP	620
Cardiomyopathy and myocarditis	Female	0-6 days	95+ years	Data Rich	1	1	Smoking Prevalence	1000
Cardiomyopathy and myocarditis	Female	0-6 days	95+ years	Data Rich	-1	2	Healthcare access and quality index	160
Cardiomyopathy and myocarditis	Female	0-6 days	95+ years	Data Rich	1	2	Systolic Blood Pressure (mmHg)	160
Cardiomyopathy and myocarditis	Female	0-6 days	95+ years	Data Rich	1	2	Mean BMI	--
Cardiomyopathy and myocarditis	Female	0-6 days	95+ years	Data Rich	-1	3	Socio-demographic Index	244
Cardiomyopathy and myocarditis	Female	0-6 days	95+ years	Data Rich	-1	3	LDI (I$ per capita)	--
Cardiomyopathy and myocarditis	Female	0-6 days	95+ years	Global	1	1	Log-transformed SEV scalar: CMP	682
Cardiomyopathy and myocarditis	Female	0-6 days	95+ years	Global	1	1	Smoking Prevalence	775
Cardiomyopathy and myocarditis	Female	0-6 days	95+ years	Global	-1	2	Healthcare access and quality index	353
Cardiomyopathy and myocarditis	Female	0-6 days	95+ years	Global	1	2	Systolic Blood Pressure (mmHg)	558
Cardiomyopathy and myocarditis	Female	0-6 days	95+ years	Global	1	2	Mean BMI	--
Cardiomyopathy and myocarditis	Female	0-6 days	95+ years	Global	-1	3	LDI (I$ per capita)	--
Cardiomyopathy and myocarditis	Female	0-6 days	95+ years	Global	-1	3	Socio-demographic Index	--
Cardiomyopathy and myocarditis	Male	0-6 days	95+ years	Data Rich	1	1	Log-transformed SEV scalar: CMP	1000
Cardiomyopathy and myocarditis	Male	0-6 days	95+ years	Data Rich	1	1	Smoking Prevalence	--
Cardiomyopathy and myocarditis	Male	0-6 days	95+ years	Data Rich	-1	2	Healthcare access and quality index	223
Cardiomyopathy and myocarditis	Male	0-6 days	95+ years	Data Rich	1	2	Systolic Blood Pressure (mmHg)	223
Cardiomyopathy and myocarditis	Male	0-6 days	95+ years	Data Rich	1	2	Mean BMI	--
Cardiomyopathy and myocarditis	Male	0-6 days	95+ years	Data Rich	-1	3	Socio-demographic Index	186
Cardiomyopathy and myocarditis	Male	0-6 days	95+ years	Data Rich	-1	3	LDI (I$ per capita)	--
Cardiomyopathy and myocarditis	Male	0-6 days	95+ years	Global	1	1	Smoking Prevalence	459
Cardiomyopathy and myocarditis	Male	0-6 days	95+ years	Global	1	1	Log-transformed SEV scalar: CMP	541
Cardiomyopathy and myocarditis	Male	0-6 days	95+ years	Global	-1	2	Healthcare access and quality index	255
Cardiomyopathy and myocarditis	Male	0-6 days	95+ years	Global	1	2	Mean BMI	--
Cardiomyopathy and myocarditis	Male	0-6 days	95+ years	Global	1	2	Systolic Blood Pressure (mmHg)	--
Cardiomyopathy and myocarditis	Male	0-6 days	95+ years	Global	-1	3	Socio-demographic Index	244
Cardiomyopathy and myocarditis	Male	0-6 days	95+ years	Global	-1	3	LDI (I$ per capita)	--
Myocarditis	Female	0-6 days	95+ years	Data Rich	-1	2	Healthcare access and quality index	1000
Myocarditis	Female	0-6 days	95+ years	Data Rich	-1	3	LDI (I$ per capita)	--
Myocarditis	Female	0-6 days	95+ years	Data Rich	-1	3	Socio-demographic Index	--
Myocarditis	Female	0-6 days	95+ years	Global	-1	2	Healthcare access and quality index	1000
Myocarditis	Female	0-6 days	95+ years	Global	-1	3	LDI (I$ per capita)	--
Myocarditis	Female	0-6 days	95+ years	Global	-1	3	Socio-demographic Index	--
Myocarditis	Male	0-6 days	95+ years	Data Rich	-1	2	Healthcare access and quality index	1000
Myocarditis	Male	0-6 days	95+ years	Data Rich	-1	3	Socio-demographic Index	546
Myocarditis	Male	0-6 days	95+ years	Data Rich	-1	3	LDI (I$ per capita)	--
Myocarditis	Male	0-6 days	95+ years	Global	-1	2	Healthcare access and quality index	--
Myocarditis	Male	0-6 days	95+ years	Global	-1	3	Socio-demographic Index	1000
Myocarditis	Male	0-6 days	95+ years	Global	-1	3	LDI (I$ per capita)	--
Alcoholic cardiomyopathy	Female	15-19 years	95+ years	Data Rich	1	1	Log-transformed SEV scalar: CMP	1000
Alcoholic cardiomyopathy	Female	15-19 years	95+ years	Data Rich	1	1	Smoking Prevalence	1000
Alcoholic cardiomyopathy	Female	15-19 years	95+ years	Data Rich	1	1	Age- and sex-specific SEV for Alcohol use	--
Alcoholic cardiomyopathy	Female	15-19 years	95+ years	Data Rich	-1	2	Healthcare access and quality index	--
Alcoholic cardiomyopathy	Female	15-19 years	95+ years	Data Rich	-1	3	LDI (I$ per capita)	--
Alcoholic cardiomyopathy	Female	15-19 years	95+ years	Global	1	1	Smoking Prevalence	293
Alcoholic cardiomyopathy	Female	15-19 years	95+ years	Global	1	1	Log-transformed SEV scalar: CMP	1000
Alcoholic cardiomyopathy	Female	15-19 years	95+ years	Global	1	1	Age- and sex-specific SEV for Alcohol use	--
Alcoholic cardiomyopathy	Female	15-19 years	95+ years	Global	-1	2	Healthcare access and quality index	463
Alcoholic cardiomyopathy	Female	15-19 years	95+ years	Global	-1	3	LDI (I$ per capita)	--
Alcoholic cardiomyopathy	Male	15-19 years	95+ years	Data Rich	1	1	Log-transformed SEV scalar: CMP	1000
Alcoholic cardiomyopathy	Male	15-19 years	95+ years	Data Rich	1	1	Smoking Prevalence	1000
Alcoholic cardiomyopathy	Male	15-19 years	95+ years	Data Rich	1	1	Age- and sex-specific SEV for Alcohol use	--
Alcoholic cardiomyopathy	Male	15-19 years	95+ years	Data Rich	-1	2	Healthcare access and quality index	--
Alcoholic cardiomyopathy	Male	15-19 years	95+ years	Data Rich	-1	3	LDI (I$ per capita)	--
Alcoholic cardiomyopathy	Male	15-19 years	95+ years	Global	1	1	Smoking Prevalence	309
Alcoholic cardiomyopathy	Male	15-19 years	95+ years	Global	1	1	Log-transformed SEV scalar: CMP	1000
Alcoholic cardiomyopathy	Male	15-19 years	95+ years	Global	1	1	Age- and sex-specific SEV for Alcohol use	--
Alcoholic cardiomyopathy	Male	15-19 years	95+ years	Global	-1	2	Healthcare access and quality index	185
Alcoholic cardiomyopathy	Male	15-19 years	95+ years	Global	-1	3	LDI (I$ per capita)	--
Other cardiomyopathy	Female	0-6 days	95+ years	Data Rich	1	1	Systolic Blood Pressure (mmHg)	397
Other cardiomyopathy	Female	0-6 days	95+ years	Data Rich	1	1	Smoking Prevalence	603
Other cardiomyopathy	Female	0-6 days	95+ years	Data Rich	1	1	Log-transformed SEV scalar: CMP	--
Other cardiomyopathy	Female	0-6 days	95+ years	Data Rich	-1	2	Healthcare access and quality index	--
Other cardiomyopathy	Female	0-6 days	95+ years	Data Rich	1	2	Mean BMI	--
Other cardiomyopathy	Female	0-6 days	95+ years	Data Rich	-1	3	Socio-demographic Index	274
Other cardiomyopathy	Female	0-6 days	95+ years	Data Rich	-1	3	LDI (I$ per capita)	--
Other cardiomyopathy	Female	0-6 days	95+ years	Global	1	1	Systolic Blood Pressure (mmHg)	443
Other cardiomyopathy	Female	0-6 days	95+ years	Global	1	1	Smoking Prevalence	557
Other cardiomyopathy	Female	0-6 days	95+ years	Global	1	1	Log-transformed SEV scalar: CMP	--
Other cardiomyopathy	Female	0-6 days	95+ years	Global	-1	2	Healthcare access and quality index	231
Other cardiomyopathy	Female	0-6 days	95+ years	Global	1	2	Mean BMI	--
Other cardiomyopathy	Female	0-6 days	95+ years	Global	-1	3	Socio-demographic Index	354
Other cardiomyopathy	Female	0-6 days	95+ years	Global	-1	3	LDI (I$ per capita)	--
Other cardiomyopathy	Male	0-6 days	95+ years	Data Rich	1	1	Log-transformed SEV scalar: CMP	--
Other cardiomyopathy	Male	0-6 days	95+ years	Data Rich	1	1	Smoking Prevalence	--
Other cardiomyopathy	Male	0-6 days	95+ years	Data Rich	1	1	Systolic Blood Pressure (mmHg)	--
Other cardiomyopathy	Male	0-6 days	95+ years	Data Rich	-1	2	Healthcare access and quality index	--
Other cardiomyopathy	Male	0-6 days	95+ years	Data Rich	1	2	Mean BMI	--
Other cardiomyopathy	Male	0-6 days	95+ years	Data Rich	-1	3	LDI (I$ per capita)	--
Other cardiomyopathy	Male	0-6 days	95+ years	Data Rich	-1	3	Socio-demographic Index	--
Other cardiomyopathy	Male	0-6 days	95+ years	Global	1	1	Systolic Blood Pressure (mmHg)	1000
Other cardiomyopathy	Male	0-6 days	95+ years	Global	1	1	Log-transformed SEV scalar: CMP	--
Other cardiomyopathy	Male	0-6 days	95+ years	Global	1	1	Smoking Prevalence	--
Other cardiomyopathy	Male	0-6 days	95+ years	Global	-1	2	Healthcare access and quality index	--
Other cardiomyopathy	Male	0-6 days	95+ years	Global	1	2	Mean BMI	--
Other cardiomyopathy	Male	0-6 days	95+ years	Global	-1	3	LDI (I$ per capita)	--
Other cardiomyopathy	Male	0-6 days	95+ years	Global	-1	3	Socio-demographic Index	--
Pulmonary Arterial Hypertension	Female	0-6 days	95+ years	Data Rich	-1	1	Socio-demographic Index	1000
Pulmonary Arterial Hypertension	Female	0-6 days	95+ years	Data Rich	1	1	Log-transformed SEV scalar: HIV	454
Pulmonary Arterial Hypertension	Female	0-6 days	95+ years	Data Rich	1	1	Healthcare access and quality index	--
Pulmonary Arterial Hypertension	Female	0-6 days	95+ years	Data Rich	1	1	Schistosomiasis Prevalence Results	--
Pulmonary Arterial Hypertension	Female	0-6 days	95+ years	Global	-1	1	Socio-demographic Index	1000
Pulmonary Arterial Hypertension	Female	0-6 days	95+ years	Global	1	1	Log-transformed SEV scalar: HIV	464
Pulmonary Arterial Hypertension	Female	0-6 days	95+ years	Global	1	1	Healthcare access and quality index	--
Pulmonary Arterial Hypertension	Female	0-6 days	95+ years	Global	1	1	Schistosomiasis Prevalence Results	--
Pulmonary Arterial Hypertension	Male	0-6 days	95+ years	Data Rich	-1	1	Socio-demographic Index	1000
Pulmonary Arterial Hypertension	Male	0-6 days	95+ years	Data Rich	1	1	Healthcare access and quality index	--
Pulmonary Arterial Hypertension	Male	0-6 days	95+ years	Data Rich	1	1	Log-transformed SEV scalar: HIV	--
Pulmonary Arterial Hypertension	Male	0-6 days	95+ years	Data Rich	1	1	Schistosomiasis Prevalence Results	--
Pulmonary Arterial Hypertension	Male	0-6 days	95+ years	Global	-1	1	Socio-demographic Index	1000
Pulmonary Arterial Hypertension	Male	0-6 days	95+ years	Global	1	1	Healthcare access and quality index	--
Pulmonary Arterial Hypertension	Male	0-6 days	95+ years	Global	1	1	Log-transformed SEV scalar: HIV	--
Pulmonary Arterial Hypertension	Male	0-6 days	95+ years	Global	1	1	Schistosomiasis Prevalence Results	--
Atrial fibrillation and flutter	Female	30-34 years	45-49 years	Data Rich	1	1	Smoking Prevalence	--
Atrial fibrillation and flutter	Female	30-34 years	45-49 years	Data Rich	1	1	Systolic Blood Pressure (mmHg)	--
Atrial fibrillation and flutter	Female	30-34 years	45-49 years	Data Rich	1	2	Birth prevalence of CHD	603
Atrial fibrillation and flutter	Female	30-34 years	45-49 years	Data Rich	1	2	Diabetes Fasting Plasma Glucose (mmol/L), age-standardized 25+	--
Atrial fibrillation and flutter	Female	30-34 years	45-49 years	Data Rich	1	2	Live Births 35+ (proportion)	--
Atrial fibrillation and flutter	Female	30-34 years	45-49 years	Data Rich	1	2	Maternal alcohol consumption during pregnancy (proportion)	--
Atrial fibrillation and flutter	Female	30-34 years	45-49 years	Data Rich	-1	3	Healthcare access and quality index	726
Atrial fibrillation and flutter	Female	30-34 years	45-49 years	Data Rich	-1	3	Socio-demographic Index	--
Atrial fibrillation and flutter	Female	30-34 years	45-49 years	Data Rich	1	3	Liters of alcohol consumed per capita	--
Atrial fibrillation and flutter	Female	30-34 years	45-49 years	Global	1	1	Smoking Prevalence	--
Atrial fibrillation and flutter	Female	30-34 years	45-49 years	Global	1	1	Systolic Blood Pressure (mmHg)	--
Atrial fibrillation and flutter	Female	30-34 years	45-49 years	Global	-1	2	Healthcare access and quality index	--

Appendix Table S10: CODEm covariates used, level of covariate, and expected direction of covariate by cause, sex, and age

Cause	Sex	Age Start	Age End	Model Version Type	Direction	Level	Covariate Name	Number of Draws
Atrial fibrillation and flutter	Female	30-34 years	45-49 years	Global	1	2	Live Births 35+ (proportion)	373
Atrial fibrillation and flutter	Female	30-34 years	45-49 years	Global	1	2	Diabetes Fasting Plasma Glucose (mmol/L), age-standardized 25+	627
Atrial fibrillation and flutter	Female	30-34 years	45-49 years	Global	1	2	Birth prevalence of CHD	1000
Atrial fibrillation and flutter	Female	30-34 years	45-49 years	Global	1	2	Maternal alcohol consumption during pregnancy (proportion)	--
Atrial fibrillation and flutter	Female	30-34 years	45-49 years	Global	-1	3	Socio-demographic Index	319
Atrial fibrillation and flutter	Female	30-34 years	45-49 years	Global	1	3	Liters of alcohol consumed per capita	--
Atrial fibrillation and flutter	Female	50-54 years	95+ years	Data Rich	1	1	Log-transformed SEV scalar: A Fib	1000
Atrial fibrillation and flutter	Female	50-54 years	95+ years	Data Rich	1	1	Smoking Prevalence	--
Atrial fibrillation and flutter	Female	50-54 years	95+ years	Data Rich	1	1	Systolic Blood Pressure (mmHg)	--
Atrial fibrillation and flutter	Female	50-54 years	95+ years	Data Rich	-1	2	Healthcare access and quality index	--
Atrial fibrillation and flutter	Female	50-54 years	95+ years	Data Rich	1	2	Diabetes Fasting Plasma Glucose (mmol/L), age-standardized 25+	303
Atrial fibrillation and flutter	Female	50-54 years	95+ years	Data Rich	1	2	Mean BMI	528
Atrial fibrillation and flutter	Female	50-54 years	95+ years	Data Rich	1	2	Low-Density Lipoprotein (mmol/L)	--
Atrial fibrillation and flutter	Female	50-54 years	95+ years	Data Rich	-1	3	Socio-demographic Index	235
Atrial fibrillation and flutter	Female	50-54 years	95+ years	Data Rich	-1	3	LDI (I$ per capita)	--
Atrial fibrillation and flutter	Female	50-54 years	95+ years	Data Rich	-1	3	Pulses legumes unadjusted(g)	--
Atrial fibrillation and flutter	Female	50-54 years	95+ years	Data Rich	1	3	Age- and sex-specific SEV for Low nuts and seeds	133
Atrial fibrillation and flutter	Female	50-54 years	95+ years	Data Rich	1	3	Age- and sex-specific SEV for Low vegetables	284
Atrial fibrillation and flutter	Female	50-54 years	95+ years	Data Rich	1	3	Liters of alcohol consumed per capita	368
Atrial fibrillation and flutter	Female	50-54 years	95+ years	Data Rich	1	3	Age- and sex-specific SEV for Low omega-3	427
Atrial fibrillation and flutter	Female	50-54 years	95+ years	Data Rich	1	3	Age- and sex-specific SEV for Low PUFA	--
Atrial fibrillation and flutter	Female	50-54 years	95+ years	Data Rich	1	3	Age- and sex-specific SEV for Low fruit	--
Atrial fibrillation and flutter	Female	50-54 years	95+ years	Data Rich	1	3	Diet high in trans fatty acids	--
Atrial fibrillation and flutter	Female	50-54 years	95+ years	Global	1	1	Smoking Prevalence	282
Atrial fibrillation and flutter	Female	50-54 years	95+ years	Global	1	1	Log-transformed SEV scalar: A Fib	718
Atrial fibrillation and flutter	Female	50-54 years	95+ years	Global	1	1	Systolic Blood Pressure (mmHg)	--
Atrial fibrillation and flutter	Female	50-54 years	95+ years	Global	-1	2	Healthcare access and quality index	509
Atrial fibrillation and flutter	Female	50-54 years	95+ years	Global	1	2	Mean BMI	509
Atrial fibrillation and flutter	Female	50-54 years	95+ years	Global	1	2	Diabetes Fasting Plasma Glucose (mmol/L), age-standardized 25+	681
Atrial fibrillation and flutter	Female	50-54 years	95+ years	Global	1	2	Low-Density Lipoprotein (mmol/L)	--
Atrial fibrillation and flutter	Female	50-54 years	95+ years	Global	-1	3	Socio-demographic Index	46
Atrial fibrillation and flutter	Female	50-54 years	95+ years	Global	-1	3	LDI (I$ per capita)	--
Atrial fibrillation and flutter	Female	50-54 years	95+ years	Global	-1	3	Pulses legumes unadjusted(g)	--
Atrial fibrillation and flutter	Female	50-54 years	95+ years	Global	1	3	Age- and sex-specific SEV for Low vegetables	255
Atrial fibrillation and flutter	Female	50-54 years	95+ years	Global	1	3	Age- and sex-specific SEV for Low omega-3	352
Atrial fibrillation and flutter	Female	50-54 years	95+ years	Global	1	3	Age- and sex-specific SEV for Low PUFA	--
Atrial fibrillation and flutter	Female	50-54 years	95+ years	Global	1	3	Age- and sex-specific SEV for Low fruit	--
Atrial fibrillation and flutter	Female	50-54 years	95+ years	Global	1	3	Age- and sex-specific SEV for Low nuts and seeds	--
Atrial fibrillation and flutter	Female	50-54 years	95+ years	Global	1	3	Diet high in trans fatty acids	--
Atrial fibrillation and flutter	Female	50-54 years	95+ years	Global	1	3	Liters of alcohol consumed per capita	--
Atrial fibrillation and flutter	Male	30-34 years	45-49 years	Data Rich	1	1	Smoking Prevalence	--
Atrial fibrillation and flutter	Male	30-34 years	45-49 years	Data Rich	1	1	Systolic Blood Pressure (mmHg)	--
Atrial fibrillation and flutter	Male	30-34 years	45-49 years	Data Rich	1	2	Birth prevalence of CHD	494
Atrial fibrillation and flutter	Male	30-34 years	45-49 years	Data Rich	1	2	Live Births 35+ (proportion)	587
Atrial fibrillation and flutter	Male	30-34 years	45-49 years	Data Rich	1	2	Diabetes Fasting Plasma Glucose (mmol/L), age-standardized 25+	711
Atrial fibrillation and flutter	Male	30-34 years	45-49 years	Data Rich	1	2	Maternal alcohol consumption during pregnancy (proportion)	--
Atrial fibrillation and flutter	Male	30-34 years	45-49 years	Data Rich	-1	3	Socio-demographic Index	180
Atrial fibrillation and flutter	Male	30-34 years	45-49 years	Data Rich	-1	3	Healthcare access and quality index	323
Atrial fibrillation and flutter	Male	30-34 years	45-49 years	Data Rich	1	3	Liters of alcohol consumed per capita	--
Atrial fibrillation and flutter	Male	30-34 years	45-49 years	Global	1	1	Smoking Prevalence	--
Atrial fibrillation and flutter	Male	30-34 years	45-49 years	Global	1	1	Systolic Blood Pressure (mmHg)	--
Atrial fibrillation and flutter	Male	30-34 years	45-49 years	Global	-1	2	Healthcare access and quality index	1000
Atrial fibrillation and flutter	Male	30-34 years	45-49 years	Global	1	2	Birth prevalence of CHD	1000
Atrial fibrillation and flutter	Male	30-34 years	45-49 years	Global	1	2	Diabetes Fasting Plasma Glucose (mmol/L), age-standardized 25+	1000
Atrial fibrillation and flutter	Male	30-34 years	45-49 years	Global	1	2	Live Births 35+ (proportion)	1000
Atrial fibrillation and flutter	Male	30-34 years	45-49 years	Global	1	2	Maternal alcohol consumption during pregnancy (proportion)	--
Atrial fibrillation and flutter	Male	30-34 years	45-49 years	Global	-1	3	Socio-demographic Index	--
Atrial fibrillation and flutter	Male	30-34 years	45-49 years	Global	1	3	Liters of alcohol consumed per capita	--
Atrial fibrillation and flutter	Male	50-54 years	95+ years	Data Rich	1	1	Log-transformed SEV scalar: A Fib	1000
Atrial fibrillation and flutter	Male	50-54 years	95+ years	Data Rich	1	1	Smoking Prevalence	--
Atrial fibrillation and flutter	Male	50-54 years	95+ years	Data Rich	1	1	Systolic Blood Pressure (mmHg)	--
Atrial fibrillation and flutter	Male	50-54 years	95+ years	Data Rich	-1	2	Healthcare access and quality index	--
Atrial fibrillation and flutter	Male	50-54 years	95+ years	Data Rich	1	2	Diabetes Fasting Plasma Glucose (mmol/L), age-standardized 25+	831
Atrial fibrillation and flutter	Male	50-54 years	95+ years	Data Rich	1	2	Mean BMI	831
Atrial fibrillation and flutter	Male	50-54 years	95+ years	Data Rich	1	2	Low-Density Lipoprotein (mmol/L)	--
Atrial fibrillation and flutter	Male	50-54 years	95+ years	Data Rich	-1	3	Socio-demographic Index	133
Atrial fibrillation and flutter	Male	50-54 years	95+ years	Data Rich	-1	3	LDI (I$ per capita)	--
Atrial fibrillation and flutter	Male	50-54 years	95+ years	Data Rich	-1	3	Pulses legumes unadjusted(g)	--
Atrial fibrillation and flutter	Male	50-54 years	95+ years	Data Rich	1	3	Age- and sex-specific SEV for Low fruit	160
Atrial fibrillation and flutter	Male	50-54 years	95+ years	Data Rich	1	3	Age- and sex-specific SEV for Low nuts and seeds	192
Atrial fibrillation and flutter	Male	50-54 years	95+ years	Data Rich	1	3	Age- and sex-specific SEV for Low vegetables	321
Atrial fibrillation and flutter	Male	50-54 years	95+ years	Data Rich	1	3	Age- and sex-specific SEV for Low PUFA	--
Atrial fibrillation and flutter	Male	50-54 years	95+ years	Data Rich	1	3	Age- and sex-specific SEV for Low omega-3	--
Atrial fibrillation and flutter	Male	50-54 years	95+ years	Data Rich	1	3	Diet high in trans fatty acids	--
Atrial fibrillation and flutter	Male	50-54 years	95+ years	Data Rich	1	3	Liters of alcohol consumed per capita	--
Atrial fibrillation and flutter	Male	50-54 years	95+ years	Global	1	1	Log-transformed SEV scalar: A Fib	1000
Atrial fibrillation and flutter	Male	50-54 years	95+ years	Global	1	1	Smoking Prevalence	--
Atrial fibrillation and flutter	Male	50-54 years	95+ years	Global	1	1	Systolic Blood Pressure (mmHg)	--
Atrial fibrillation and flutter	Male	50-54 years	95+ years	Global	-1	2	Healthcare access and quality index	--
Atrial fibrillation and flutter	Male	50-54 years	95+ years	Global	1	2	Mean BMI	706
Atrial fibrillation and flutter	Male	50-54 years	95+ years	Global	1	2	Diabetes Fasting Plasma Glucose (mmol/L), age-standardized 25+	--
Atrial fibrillation and flutter	Male	50-54 years	95+ years	Global	1	2	Low-Density Lipoprotein (mmol/L)	--
Atrial fibrillation and flutter	Male	50-54 years	95+ years	Global	-1	3	Pulses legumes unadjusted(g)	204
Atrial fibrillation and flutter	Male	50-54 years	95+ years	Global	-1	3	LDI (I$ per capita)	--
Atrial fibrillation and flutter	Male	50-54 years	95+ years	Global	-1	3	Socio-demographic Index	--
Atrial fibrillation and flutter	Male	50-54 years	95+ years	Global	1	3	Liters of alcohol consumed per capita	133
Atrial fibrillation and flutter	Male	50-54 years	95+ years	Global	1	3	Age- and sex-specific SEV for Low vegetables	244
Atrial fibrillation and flutter	Male	50-54 years	95+ years	Global	1	3	Age- and sex-specific SEV for Low PUFA	--
Atrial fibrillation and flutter	Male	50-54 years	95+ years	Global	1	3	Age- and sex-specific SEV for Low fruit	--
Atrial fibrillation and flutter	Male	50-54 years	95+ years	Global	1	3	Age- and sex-specific SEV for Low nuts and seeds	--
Atrial fibrillation and flutter	Male	50-54 years	95+ years	Global	1	3	Age- and sex-specific SEV for Low omega-3	--
Atrial fibrillation and flutter	Male	50-54 years	95+ years	Global	1	3	Diet high in trans fatty acids	--
Aortic aneurysm	Female	15-19 years	95+ years	Data Rich	1	1	Systolic Blood Pressure (mmHg)	3
Aortic aneurysm	Female	15-19 years	95+ years	Data Rich	1	1	Cumulative Cigarettes (10 Years)	334
Aortic aneurysm	Female	15-19 years	95+ years	Data Rich	1	1	Log-transformed SEV scalar: Aort An	483
Aortic aneurysm	Female	15-19 years	95+ years	Data Rich	1	1	Low-Density Lipoprotein (mmol/L)	785
Aortic aneurysm	Female	15-19 years	95+ years	Data Rich	-1	2	Healthcare access and quality index	14
Aortic aneurysm	Female	15-19 years	95+ years	Data Rich	1	2	Mean BMI	451
Aortic aneurysm	Female	15-19 years	95+ years	Data Rich	-1	3	LDI (I$ per capita)	--
Aortic aneurysm	Female	15-19 years	95+ years	Data Rich	-1	3	Pulses legumes unadjusted(g)	--
Aortic aneurysm	Female	15-19 years	95+ years	Data Rich	-1	3	Socio-demographic Index	--
Aortic aneurysm	Female	15-19 years	95+ years	Data Rich	1	3	Age- and sex-specific SEV for Low fruit	31

Appendix Table S10: CODEm covariates used, level of covariate, and expected direction of covariate by cause, sex, and age

Cause	Sex	Age Start	Age End	Model Version Type	Direction	Level	Covariate Name	Number of Draws
Aortic aneurysm	Female	15-19 years	95+ years	Data Rich	1	3	Liters of alcohol consumed per capita	83
Aortic aneurysm	Female	15-19 years	95+ years	Data Rich	1	3	Age- and sex-specific SEV for Low PUFA	--
Aortic aneurysm	Female	15-19 years	95+ years	Data Rich	1	3	Age- and sex-specific SEV for Low nuts and seeds	--
Aortic aneurysm	Female	15-19 years	95+ years	Data Rich	1	3	Age- and sex-specific SEV for Low omega-3	--
Aortic aneurysm	Female	15-19 years	95+ years	Data Rich	1	3	Age- and sex-specific SEV for Low vegetables	--
Aortic aneurysm	Female	15-19 years	95+ years	Global	1	1	Systolic Blood Pressure (mmHg)	155
Aortic aneurysm	Female	15-19 years	95+ years	Global	1	1	Log-transformed SEV scalar: Aort An	380
Aortic aneurysm	Female	15-19 years	95+ years	Global	1	1	Low-Density Lipoprotein (mmol/L)	503
Aortic aneurysm	Female	15-19 years	95+ years	Global	1	1	Cumulative Cigarettes (10 Years)	746
Aortic aneurysm	Female	15-19 years	95+ years	Global	-1	2	Healthcare access and quality index	15
Aortic aneurysm	Female	15-19 years	95+ years	Global	1	2	Mean BMI	178
Aortic aneurysm	Female	15-19 years	95+ years	Global	-1	3	LDI (I$ per capita)	--
Aortic aneurysm	Female	15-19 years	95+ years	Global	-1	3	Pulses legumes unadjusted(g)	--
Aortic aneurysm	Female	15-19 years	95+ years	Global	-1	3	Socio-demographic Index	--
Aortic aneurysm	Female	15-19 years	95+ years	Global	1	3	Liters of alcohol consumed per capita	622
Aortic aneurysm	Female	15-19 years	95+ years	Global	1	3	Age- and sex-specific SEV for Low PUFA	--
Aortic aneurysm	Female	15-19 years	95+ years	Global	1	3	Age- and sex-specific SEV for Low fruit	--
Aortic aneurysm	Female	15-19 years	95+ years	Global	1	3	Age- and sex-specific SEV for Low nuts and seeds	--
Aortic aneurysm	Female	15-19 years	95+ years	Global	1	3	Age- and sex-specific SEV for Low omega-3	--
Aortic aneurysm	Female	15-19 years	95+ years	Global	1	3	Age- and sex-specific SEV for Low vegetables	--
Aortic aneurysm	Male	15-19 years	95+ years	Data Rich	1	1	Cumulative Cigarettes (10 Years)	137
Aortic aneurysm	Male	15-19 years	95+ years	Data Rich	1	1	Log-transformed SEV scalar: Aort An	220
Aortic aneurysm	Male	15-19 years	95+ years	Data Rich	1	1	Systolic Blood Pressure (mmHg)	454
Aortic aneurysm	Male	15-19 years	95+ years	Data Rich	1	1	Low-Density Lipoprotein (mmol/L)	997
Aortic aneurysm	Male	15-19 years	95+ years	Data Rich	-1	2	Healthcare access and quality index	106
Aortic aneurysm	Male	15-19 years	95+ years	Data Rich	1	2	Mean BMI	146
Aortic aneurysm	Male	15-19 years	95+ years	Data Rich	-1	3	LDI (I$ per capita)	--
Aortic aneurysm	Male	15-19 years	95+ years	Data Rich	-1	3	Pulses legumes unadjusted(g)	--
Aortic aneurysm	Male	15-19 years	95+ years	Data Rich	-1	3	Socio-demographic Index	--
Aortic aneurysm	Male	15-19 years	95+ years	Data Rich	1	3	Age- and sex-specific SEV for Low PUFA	1
Aortic aneurysm	Male	15-19 years	95+ years	Data Rich	1	3	Age- and sex-specific SEV for Low nuts and seeds	2
Aortic aneurysm	Male	15-19 years	95+ years	Data Rich	1	3	Liters of alcohol consumed per capita	205
Aortic aneurysm	Male	15-19 years	95+ years	Data Rich	1	3	Age- and sex-specific SEV for Low fruit	238
Aortic aneurysm	Male	15-19 years	95+ years	Data Rich	1	3	Age- and sex-specific SEV for Low omega-3	--
Aortic aneurysm	Male	15-19 years	95+ years	Data Rich	1	3	Age- and sex-specific SEV for Low vegetables	--
Aortic aneurysm	Male	15-19 years	95+ years	Global	1	1	Cumulative Cigarettes (10 Years)	156
Aortic aneurysm	Male	15-19 years	95+ years	Global	1	1	Systolic Blood Pressure (mmHg)	605
Aortic aneurysm	Male	15-19 years	95+ years	Global	1	1	Low-Density Lipoprotein (mmol/L)	780
Aortic aneurysm	Male	15-19 years	95+ years	Global	1	1	Log-transformed SEV scalar: Aort An	--
Aortic aneurysm	Male	15-19 years	95+ years	Global	-1	2	Healthcare access and quality index	--
Aortic aneurysm	Male	15-19 years	95+ years	Global	1	2	Mean BMI	417
Aortic aneurysm	Male	15-19 years	95+ years	Global	-1	3	LDI (I$ per capita)	--
Aortic aneurysm	Male	15-19 years	95+ years	Global	-1	3	Pulses legumes unadjusted(g)	--
Aortic aneurysm	Male	15-19 years	95+ years	Global	-1	3	Socio-demographic Index	--
Aortic aneurysm	Male	15-19 years	95+ years	Global	1	3	Liters of alcohol consumed per capita	304
Aortic aneurysm	Male	15-19 years	95+ years	Global	1	3	Age- and sex-specific SEV for Low PUFA	--
Aortic aneurysm	Male	15-19 years	95+ years	Global	1	3	Age- and sex-specific SEV for Low fruit	--
Aortic aneurysm	Male	15-19 years	95+ years	Global	1	3	Age- and sex-specific SEV for Low nuts and seeds	--
Aortic aneurysm	Male	15-19 years	95+ years	Global	1	3	Age- and sex-specific SEV for Low omega-3	--
Aortic aneurysm	Male	15-19 years	95+ years	Global	1	3	Age- and sex-specific SEV for Low vegetables	--
Lower extremity peripheral arterial disease	Female	40-44 years	95+ years	Data Rich	1	1	Systolic Blood Pressure (mmHg)	489
Lower extremity peripheral arterial disease	Female	40-44 years	95+ years	Data Rich	1	1	Smoking Prevalence	511
Lower extremity peripheral arterial disease	Female	40-44 years	95+ years	Data Rich	1	1	Log-transformed SEV scalar: PAD	--
Lower extremity peripheral arterial disease	Female	40-44 years	95+ years	Data Rich	1	1	Low-Density Lipoprotein (mmol/L)	--
Lower extremity peripheral arterial disease	Female	40-44 years	95+ years	Data Rich	-1	2	Healthcare access and quality index	132
Lower extremity peripheral arterial disease	Female	40-44 years	95+ years	Data Rich	1	2	Diabetes Fasting Plasma Glucose (mmol/L), age-standardized 25+	402
Lower extremity peripheral arterial disease	Female	40-44 years	95+ years	Data Rich	1	2	Mean BMI	--
Lower extremity peripheral arterial disease	Female	40-44 years	95+ years	Data Rich	-1	3	LDI (I$ per capita)	--
Lower extremity peripheral arterial disease	Female	40-44 years	95+ years	Data Rich	-1	3	Pulses legumes unadjusted(g)	--
Lower extremity peripheral arterial disease	Female	40-44 years	95+ years	Data Rich	-1	3	Socio-demographic Index	--
Lower extremity peripheral arterial disease	Female	40-44 years	95+ years	Data Rich	1	3	Liters of alcohol consumed per capita	164
Lower extremity peripheral arterial disease	Female	40-44 years	95+ years	Data Rich	1	3	Age- and sex-specific SEV for Low vegetables	365
Lower extremity peripheral arterial disease	Female	40-44 years	95+ years	Data Rich	1	3	Age- and sex-specific SEV for Low PUFA	--
Lower extremity peripheral arterial disease	Female	40-44 years	95+ years	Data Rich	1	3	Age- and sex-specific SEV for Low fruit	--
Lower extremity peripheral arterial disease	Female	40-44 years	95+ years	Data Rich	1	3	Age- and sex-specific SEV for Low nuts and seeds	--
Lower extremity peripheral arterial disease	Female	40-44 years	95+ years	Data Rich	1	3	Age- and sex-specific SEV for Low omega-3	--
Lower extremity peripheral arterial disease	Female	40-44 years	95+ years	Global	1	1	Low-Density Lipoprotein (mmol/L)	87
Lower extremity peripheral arterial disease	Female	40-44 years	95+ years	Global	1	1	Log-transformed SEV scalar: PAD	259
Lower extremity peripheral arterial disease	Female	40-44 years	95+ years	Global	1	1	Smoking Prevalence	379
Lower extremity peripheral arterial disease	Female	40-44 years	95+ years	Global	1	1	Systolic Blood Pressure (mmHg)	805
Lower extremity peripheral arterial disease	Female	40-44 years	95+ years	Global	-1	2	Healthcare access and quality index	--
Lower extremity peripheral arterial disease	Female	40-44 years	95+ years	Global	1	2	Diabetes Fasting Plasma Glucose (mmol/L), age-standardized 25+	139
Lower extremity peripheral arterial disease	Female	40-44 years	95+ years	Global	1	2	Mean BMI	404
Lower extremity peripheral arterial disease	Female	40-44 years	95+ years	Global	-1	3	LDI (I$ per capita)	--
Lower extremity peripheral arterial disease	Female	40-44 years	95+ years	Global	-1	3	Pulses legumes unadjusted(g)	--
Lower extremity peripheral arterial disease	Female	40-44 years	95+ years	Global	-1	3	Socio-demographic Index	--
Lower extremity peripheral arterial disease	Female	40-44 years	95+ years	Global	1	3	Age- and sex-specific SEV for Low omega-3	25
Lower extremity peripheral arterial disease	Female	40-44 years	95+ years	Global	1	3	Age- and sex-specific SEV for Low fruit	108
Lower extremity peripheral arterial disease	Female	40-44 years	95+ years	Global	1	3	Liters of alcohol consumed per capita	215
Lower extremity peripheral arterial disease	Female	40-44 years	95+ years	Global	1	3	Age- and sex-specific SEV for Low vegetables	216
Lower extremity peripheral arterial disease	Female	40-44 years	95+ years	Global	1	3	Age- and sex-specific SEV for Low PUFA	--
Lower extremity peripheral arterial disease	Female	40-44 years	95+ years	Global	1	3	Age- and sex-specific SEV for Low nuts and seeds	--
Lower extremity peripheral arterial disease	Male	40-44 years	95+ years	Data Rich	1	1	Systolic Blood Pressure (mmHg)	14
Lower extremity peripheral arterial disease	Male	40-44 years	95+ years	Data Rich	1	1	Low-Density Lipoprotein (mmol/L)	335
Lower extremity peripheral arterial disease	Male	40-44 years	95+ years	Data Rich	1	1	Log-transformed SEV scalar: PAD	565
Lower extremity peripheral arterial disease	Male	40-44 years	95+ years	Data Rich	1	1	Smoking Prevalence	719
Lower extremity peripheral arterial disease	Male	40-44 years	95+ years	Data Rich	-1	2	Healthcare access and quality index	492
Lower extremity peripheral arterial disease	Male	40-44 years	95+ years	Data Rich	1	2	Diabetes Fasting Plasma Glucose (mmol/L), age-standardized 25+	362
Lower extremity peripheral arterial disease	Male	40-44 years	95+ years	Data Rich	1	2	Mean BMI	562
Lower extremity peripheral arterial disease	Male	40-44 years	95+ years	Data Rich	-1	3	LDI (I$ per capita)	--
Lower extremity peripheral arterial disease	Male	40-44 years	95+ years	Data Rich	-1	3	Pulses legumes unadjusted(g)	--
Lower extremity peripheral arterial disease	Male	40-44 years	95+ years	Data Rich	-1	3	Socio-demographic Index	--
Lower extremity peripheral arterial disease	Male	40-44 years	95+ years	Data Rich	1	3	Age- and sex-specific SEV for Low nuts and seeds	47
Lower extremity peripheral arterial disease	Male	40-44 years	95+ years	Data Rich	1	3	Age- and sex-specific SEV for Low fruit	166
Lower extremity peripheral arterial disease	Male	40-44 years	95+ years	Data Rich	1	3	Age- and sex-specific SEV for Low vegetables	380
Lower extremity peripheral arterial disease	Male	40-44 years	95+ years	Data Rich	1	3	Liters of alcohol consumed per capita	557
Lower extremity peripheral arterial disease	Male	40-44 years	95+ years	Data Rich	1	3	Age- and sex-specific SEV for Low PUFA	--
Lower extremity peripheral arterial disease	Male	40-44 years	95+ years	Data Rich	1	3	Age- and sex-specific SEV for Low omega-3	--

Appendix Table S10: CODEm covariates used, level of covariate, and expected direction of covariate by cause, sex, and age

Cause	Sex	Age Start	Age End	Model Version Type	Direction	Level	Covariate Name	Number of Draws
Lower extremity peripheral arterial disease	Male	40-44 years	95+ years	Global	1	1	Low-Density Lipoprotein (mmol/L)	157
Lower extremity peripheral arterial disease	Male	40-44 years	95+ years	Global	1	1	Smoking Prevalence	297
Lower extremity peripheral arterial disease	Male	40-44 years	95+ years	Global	1	1	Systolic Blood Pressure (mmHg)	302
Lower extremity peripheral arterial disease	Male	40-44 years	95+ years	Global	1	1	Log-transformed SEV scalar: PAD	883
Lower extremity peripheral arterial disease	Male	40-44 years	95+ years	Global	-1	2	Healthcare access and quality index	--
Lower extremity peripheral arterial disease	Male	40-44 years	95+ years	Global	1	2	Diabetes Fasting Plasma Glucose (mmol/L), age-standardized 25+	21
Lower extremity peripheral arterial disease	Male	40-44 years	95+ years	Global	1	2	Mean BMI	130
Lower extremity peripheral arterial disease	Male	40-44 years	95+ years	Global	-1	3	LDI (I$ per capita)	--
Lower extremity peripheral arterial disease	Male	40-44 years	95+ years	Global	-1	3	Pulses legumes unadjusted(g)	--
Lower extremity peripheral arterial disease	Male	40-44 years	95+ years	Global	-1	3	Socio-demographic Index	--
Lower extremity peripheral arterial disease	Male	40-44 years	95+ years	Global	1	3	Age- and sex-specific SEV for Low vegetables	4
Lower extremity peripheral arterial disease	Male	40-44 years	95+ years	Global	1	3	Age- and sex-specific SEV for Low fruit	55
Lower extremity peripheral arterial disease	Male	40-44 years	95+ years	Global	1	3	Liters of alcohol consumed per capita	544
Lower extremity peripheral arterial disease	Male	40-44 years	95+ years	Global	1	3	Age- and sex-specific SEV for Low PUFA	--
Lower extremity peripheral arterial disease	Male	40-44 years	95+ years	Global	1	3	Age- and sex-specific SEV for Low nuts and seeds	--
Lower extremity peripheral arterial disease	Male	40-44 years	95+ years	Global	1	3	Age- and sex-specific SEV for Low omega-3	--
Endocarditis	Female	0-6 days	95+ years	Data Rich	-1	1	Improved Water Source (proportion with access)	--
Endocarditis	Female	0-6 days	95+ years	Data Rich	-1	1	Sanitation (proportion with access)	--
Endocarditis	Female	0-6 days	95+ years	Data Rich	1	2	Healthcare access and quality index	671
Endocarditis	Female	0-6 days	95+ years	Data Rich	-1	3	LDI (I$ per capita)	--
Endocarditis	Female	0-6 days	95+ years	Data Rich	1	3	Socio-demographic Index	603
Endocarditis	Female	0-6 days	95+ years	Global	-1	1	Sanitation (proportion with access)	426
Endocarditis	Female	0-6 days	95+ years	Global	-1	1	Improved Water Source (proportion with access)	784
Endocarditis	Female	0-6 days	95+ years	Global	-1	2	Healthcare access and quality index	303
Endocarditis	Female	0-6 days	95+ years	Global	-1	3	Socio-demographic Index	154
Endocarditis	Female	0-6 days	95+ years	Global	-1	3	LDI (I$ per capita)	--
Endocarditis	Male	0-6 days	95+ years	Data Rich	-1	1	Improved Water Source (proportion with access)	--
Endocarditis	Male	0-6 days	95+ years	Data Rich	-1	1	Sanitation (proportion with access)	--
Endocarditis	Male	0-6 days	95+ years	Data Rich	1	2	Healthcare access and quality index	454
Endocarditis	Male	0-6 days	95+ years	Data Rich	-1	3	LDI (I$ per capita)	--
Endocarditis	Male	0-6 days	95+ years	Data Rich	1	3	Socio-demographic Index	546
Endocarditis	Male	0-6 days	95+ years	Global	-1	1	Sanitation (proportion with access)	328
Endocarditis	Male	0-6 days	95+ years	Global	-1	1	Improved Water Source (proportion with access)	672
Endocarditis	Male	0-6 days	95+ years	Global	-1	2	Healthcare access and quality index	274
Endocarditis	Male	0-6 days	95+ years	Global	-1	3	LDI (I$ per capita)	--
Endocarditis	Male	0-6 days	95+ years	Global	-1	3	Socio-demographic Index	--
Other cardiovascular and circulatory diseases	Female	0-6 days	95+ years	Data Rich	1	1	Systolic Blood Pressure (mmHg)	1000
Other cardiovascular and circulatory diseases	Female	0-6 days	95+ years	Data Rich	1	1	Low-Density Lipoprotein (mmol/L)	--
Other cardiovascular and circulatory diseases	Female	0-6 days	95+ years	Data Rich	1	1	Smoking Prevalence	--
Other cardiovascular and circulatory diseases	Female	0-6 days	95+ years	Data Rich	-1	2	Elevation Over 1500m (proportion)	--
Other cardiovascular and circulatory diseases	Female	0-6 days	95+ years	Data Rich	-1	2	Healthcare access and quality index	--
Other cardiovascular and circulatory diseases	Female	0-6 days	95+ years	Data Rich	1	2	Diabetes Fasting Plasma Glucose (mmol/L), age-standardized 25+	475
Other cardiovascular and circulatory diseases	Female	0-6 days	95+ years	Data Rich	1	2	Mean BMI	536
Other cardiovascular and circulatory diseases	Female	0-6 days	95+ years	Data Rich	1	2	Outdoor Air Pollution (PM2.5)	639
Other cardiovascular and circulatory diseases	Female	0-6 days	95+ years	Data Rich	1	2	Indoor Air Pollution (All Cooking Fuels)	--
Other cardiovascular and circulatory diseases	Female	0-6 days	95+ years	Data Rich	-1	3	Socio-demographic Index	548
Other cardiovascular and circulatory diseases	Female	0-6 days	95+ years	Data Rich	-1	3	LDI (I$ per capita)	--
Other cardiovascular and circulatory diseases	Female	0-6 days	95+ years	Data Rich	1	3	Age- and sex-specific SEV for Low fruit	42
Other cardiovascular and circulatory diseases	Female	0-6 days	95+ years	Data Rich	1	3	Age- and sex-specific SEV for Low omega-3	194
Other cardiovascular and circulatory diseases	Female	0-6 days	95+ years	Data Rich	1	3	Age- and sex-specific SEV for Low vegetables	424
Other cardiovascular and circulatory diseases	Female	0-6 days	95+ years	Data Rich	1	3	Liters of alcohol consumed per capita	466
Other cardiovascular and circulatory diseases	Female	0-6 days	95+ years	Data Rich	1	3	Age- and sex-specific SEV for Low legumes	769
Other cardiovascular and circulatory diseases	Female	0-6 days	95+ years	Data Rich	1	3	Age- and sex-specific SEV for Low PUFA	--
Other cardiovascular and circulatory diseases	Female	0-6 days	95+ years	Global	1	1	Systolic Blood Pressure (mmHg)	1000
Other cardiovascular and circulatory diseases	Female	0-6 days	95+ years	Global	1	1	Low-Density Lipoprotein (mmol/L)	--
Other cardiovascular and circulatory diseases	Female	0-6 days	95+ years	Global	1	1	Smoking Prevalence	--
Other cardiovascular and circulatory diseases	Female	0-6 days	95+ years	Global	-1	2	Elevation Over 1500m (proportion)	--
Other cardiovascular and circulatory diseases	Female	0-6 days	95+ years	Global	-1	2	Healthcare access and quality index	--
Other cardiovascular and circulatory diseases	Female	0-6 days	95+ years	Global	1	2	Diabetes Fasting Plasma Glucose (mmol/L), age-standardized 25+	294
Other cardiovascular and circulatory diseases	Female	0-6 days	95+ years	Global	1	2	Indoor Air Pollution (All Cooking Fuels)	294
Other cardiovascular and circulatory diseases	Female	0-6 days	95+ years	Global	1	2	Mean BMI	294
Other cardiovascular and circulatory diseases	Female	0-6 days	95+ years	Global	1	2	Outdoor Air Pollution (PM2.5)	--
Other cardiovascular and circulatory diseases	Female	0-6 days	95+ years	Global	-1	3	Age- and sex-specific SEV for Low nuts and seeds	141
Other cardiovascular and circulatory diseases	Female	0-6 days	95+ years	Global	-1	3	Socio-demographic Index	141
Other cardiovascular and circulatory diseases	Female	0-6 days	95+ years	Global	-1	3	LDI (I$ per capita)	--
Other cardiovascular and circulatory diseases	Female	0-6 days	95+ years	Global	1	3	Age- and sex-specific SEV for Low vegetables	141
Other cardiovascular and circulatory diseases	Female	0-6 days	95+ years	Global	1	3	Liters of alcohol consumed per capita	141
Other cardiovascular and circulatory diseases	Female	0-6 days	95+ years	Global	1	3	Age- and sex-specific SEV for Low fruit	193
Other cardiovascular and circulatory diseases	Female	0-6 days	95+ years	Global	1	3	Age- and sex-specific SEV for Low legumes	423
Other cardiovascular and circulatory diseases	Female	0-6 days	95+ years	Global	1	3	Age- and sex-specific SEV for Low PUFA	--
Other cardiovascular and circulatory diseases	Female	0-6 days	95+ years	Global	1	3	Age- and sex-specific SEV for Low omega-3	--
Other cardiovascular and circulatory diseases	Male	0-6 days	95+ years	Data Rich	1	1	Low-Density Lipoprotein (mmol/L)	--
Other cardiovascular and circulatory diseases	Male	0-6 days	95+ years	Data Rich	1	1	Smoking Prevalence	--
Other cardiovascular and circulatory diseases	Male	0-6 days	95+ years	Data Rich	1	1	Systolic Blood Pressure (mmHg)	--
Other cardiovascular and circulatory diseases	Male	0-6 days	95+ years	Data Rich	-1	2	Healthcare access and quality index	461
Other cardiovascular and circulatory diseases	Male	0-6 days	95+ years	Data Rich	-1	2	Elevation Over 1500m (proportion)	--
Other cardiovascular and circulatory diseases	Male	0-6 days	95+ years	Data Rich	1	2	Outdoor Air Pollution (PM2.5)	220
Other cardiovascular and circulatory diseases	Male	0-6 days	95+ years	Data Rich	1	2	Diabetes Fasting Plasma Glucose (mmol/L), age-standardized 25+	--
Other cardiovascular and circulatory diseases	Male	0-6 days	95+ years	Data Rich	1	2	Indoor Air Pollution (All Cooking Fuels)	--
Other cardiovascular and circulatory diseases	Male	0-6 days	95+ years	Data Rich	1	2	Mean BMI	--
Other cardiovascular and circulatory diseases	Male	0-6 days	95+ years	Data Rich	-1	3	Socio-demographic Index	174
Other cardiovascular and circulatory diseases	Male	0-6 days	95+ years	Data Rich	-1	3	LDI (I$ per capita)	--
Other cardiovascular and circulatory diseases	Male	0-6 days	95+ years	Data Rich	1	3	Age- and sex-specific SEV for Low vegetables	308
Other cardiovascular and circulatory diseases	Male	0-6 days	95+ years	Data Rich	1	3	Age- and sex-specific SEV for Low fruit	419
Other cardiovascular and circulatory diseases	Male	0-6 days	95+ years	Data Rich	1	3	Liters of alcohol consumed per capita	419
Other cardiovascular and circulatory diseases	Male	0-6 days	95+ years	Data Rich	1	3	Age- and sex-specific SEV for Low legumes	627
Other cardiovascular and circulatory diseases	Male	0-6 days	95+ years	Data Rich	1	3	Age- and sex-specific SEV for Low PUFA	--
Other cardiovascular and circulatory diseases	Male	0-6 days	95+ years	Data Rich	1	3	Age- and sex-specific SEV for Low omega-3	--
Other cardiovascular and circulatory diseases	Male	0-6 days	95+ years	Global	1	1	Smoking Prevalence	231

Appendix Table S10: CODEm covariates used, level of covariate, and expected direction of covariate by cause, sex, and age

Cause	Sex	Age Start	Age End	Model Version Type	Direction	Level	Covariate Name	Number of Draws
Other cardiovascular and circulatory diseases	Male	0-6 days	95+ years	Global	1	1	Systolic Blood Pressure (mmHg)	920
Other cardiovascular and circulatory diseases	Male	0-6 days	95+ years	Global	1	1	Low-Density Lipoprotein (mmol/L)	--
Other cardiovascular and circulatory diseases	Male	0-6 days	95+ years	Global	-1	2	Healthcare access and quality index	362
Other cardiovascular and circulatory diseases	Male	0-6 days	95+ years	Global	-1	2	Elevation Over 1500m (proportion)	--
Other cardiovascular and circulatory diseases	Male	0-6 days	95+ years	Global	1	2	Indoor Air Pollution (All Cooking Fuels)	165
Other cardiovascular and circulatory diseases	Male	0-6 days	95+ years	Global	1	2	Diabetes Fasting Plasma Glucose (mmol/L), age-standardized 25+	--
Other cardiovascular and circulatory diseases	Male	0-6 days	95+ years	Global	1	2	Mean BMI	--
Other cardiovascular and circulatory diseases	Male	0-6 days	95+ years	Global	1	2	Outdoor Air Pollution (PM2.5)	--
Other cardiovascular and circulatory diseases	Male	0-6 days	95+ years	Global	-1	3	Socio-demographic Index	243
Other cardiovascular and circulatory diseases	Male	0-6 days	95+ years	Global	-1	3	Age- and sex-specific SEV for Low nuts and seeds	274
Other cardiovascular and circulatory diseases	Male	0-6 days	95+ years	Global	-1	3	LDI (I$ per capita)	--
Other cardiovascular and circulatory diseases	Male	0-6 days	95+ years	Global	1	3	Age- and sex-specific SEV for Low fruit	103
Other cardiovascular and circulatory diseases	Male	0-6 days	95+ years	Global	1	3	Age- and sex-specific SEV for Low legumes	273
Other cardiovascular and circulatory diseases	Male	0-6 days	95+ years	Global	1	3	Age- and sex-specific SEV for Low vegetables	482
Other cardiovascular and circulatory diseases	Male	0-6 days	95+ years	Global	1	3	Age- and sex-specific SEV for Low PUFA	--
Other cardiovascular and circulatory diseases	Male	0-6 days	95+ years	Global	1	3	Age- and sex-specific SEV for Low omega-3	--
Other cardiovascular and circulatory diseases	Male	0-6 days	95+ years	Global	1	3	Liters of alcohol consumed per capita	--
Chronic respiratory diseases	Female	0-6 days	95+ years	Data Rich	1	1	Cumulative Cigarettes (20 Years)	1000
Chronic respiratory diseases	Female	0-6 days	95+ years	Data Rich	1	1	Cumulative Cigarettes (10 Years)	--
Chronic respiratory diseases	Female	0-6 days	95+ years	Data Rich	-1	2	Healthcare access and quality index	233
Chronic respiratory diseases	Female	0-6 days	95+ years	Data Rich	1	2	Outdoor Air Pollution (PM2.5)	301
Chronic respiratory diseases	Female	0-6 days	95+ years	Data Rich	1	2	Smoking Prevalence	301
Chronic respiratory diseases	Female	0-6 days	95+ years	Data Rich	1	2	Elevation Over 1500m (proportion)	534
Chronic respiratory diseases	Female	0-6 days	95+ years	Data Rich	1	2	COVID-19 age-standardized death rate	--
Chronic respiratory diseases	Female	0-6 days	95+ years	Data Rich	-1	3	Socio-demographic Index	426
Chronic respiratory diseases	Female	0-6 days	95+ years	Data Rich	-1	3	Mask use	504
Chronic respiratory diseases	Female	0-6 days	95+ years	Data Rich	1	3	Elevation 500 to 1500m (proportion)	210
Chronic respiratory diseases	Female	0-6 days	95+ years	Data Rich	1	3	Population Density (over 1000 ppl/sqkm, proportion)	--
Chronic respiratory diseases	Female	0-6 days	95+ years	Global	1	1	Cumulative Cigarettes (20 Years)	1000
Chronic respiratory diseases	Female	0-6 days	95+ years	Global	1	1	Cumulative Cigarettes (10 Years)	--
Chronic respiratory diseases	Female	0-6 days	95+ years	Global	-1	2	Healthcare access and quality index	--
Chronic respiratory diseases	Female	0-6 days	95+ years	Global	1	2	Elevation Over 1500m (proportion)	480
Chronic respiratory diseases	Female	0-6 days	95+ years	Global	1	2	Outdoor Air Pollution (PM2.5)	480
Chronic respiratory diseases	Female	0-6 days	95+ years	Global	1	2	Smoking Prevalence	480
Chronic respiratory diseases	Female	0-6 days	95+ years	Global	1	2	COVID-19 age-standardized death rate	--
Chronic respiratory diseases	Female	0-6 days	95+ years	Global	-1	3	Mask use	174
Chronic respiratory diseases	Female	0-6 days	95+ years	Global	-1	3	Socio-demographic Index	--
Chronic respiratory diseases	Female	0-6 days	95+ years	Global	1	3	Elevation 500 to 1500m (proportion)	462
Chronic respiratory diseases	Female	0-6 days	95+ years	Global	1	3	Population Density (over 1000 ppl/sqkm, proportion)	--
Chronic respiratory diseases	Male	0-6 days	95+ years	Data Rich	1	1	Cumulative Cigarettes (10 Years)	114
Chronic respiratory diseases	Male	0-6 days	95+ years	Data Rich	1	1	Cumulative Cigarettes (20 Years)	336
Chronic respiratory diseases	Male	0-6 days	95+ years	Data Rich	1	1	Indoor Air Pollution (All Cooking Fuels)	774
Chronic respiratory diseases	Male	0-6 days	95+ years	Data Rich	-1	2	Healthcare access and quality index	797
Chronic respiratory diseases	Male	0-6 days	95+ years	Data Rich	1	2	Smoking Prevalence	44
Chronic respiratory diseases	Male	0-6 days	95+ years	Data Rich	1	2	Elevation Over 1500m (proportion)	652
Chronic respiratory diseases	Male	0-6 days	95+ years	Data Rich	1	2	Outdoor Air Pollution (PM2.5)	819
Chronic respiratory diseases	Male	0-6 days	95+ years	Data Rich	1	2	COVID-19 age-standardized death rate	--
Chronic respiratory diseases	Male	0-6 days	95+ years	Data Rich	-1	3	Socio-demographic Index	97
Chronic respiratory diseases	Male	0-6 days	95+ years	Data Rich	-1	3	Mask use	177
Chronic respiratory diseases	Male	0-6 days	95+ years	Data Rich	1	3	Elevation 500 to 1500m (proportion)	312
Chronic respiratory diseases	Male	0-6 days	95+ years	Data Rich	1	3	Population Density (over 1000 ppl/sqkm, proportion)	--
Chronic respiratory diseases	Male	0-6 days	95+ years	Global	1	1	Cumulative Cigarettes (20 Years)	159
Chronic respiratory diseases	Male	0-6 days	95+ years	Global	1	1	Smoking Prevalence	204
Chronic respiratory diseases	Male	0-6 days	95+ years	Global	1	1	Cumulative Cigarettes (10 Years)	333
Chronic respiratory diseases	Male	0-6 days	95+ years	Global	1	1	Indoor Air Pollution (All Cooking Fuels)	993
Chronic respiratory diseases	Male	0-6 days	95+ years	Global	-1	2	Healthcare access and quality index	324
Chronic respiratory diseases	Male	0-6 days	95+ years	Global	1	2	Outdoor Air Pollution (PM2.5)	263
Chronic respiratory diseases	Male	0-6 days	95+ years	Global	1	2	Elevation Over 1500m (proportion)	779
Chronic respiratory diseases	Male	0-6 days	95+ years	Global	1	2	COVID-19 age-standardized death rate	--
Chronic respiratory diseases	Male	0-6 days	95+ years	Global	-1	3	Mask use	515
Chronic respiratory diseases	Male	0-6 days	95+ years	Global	-1	3	Socio-demographic Index	543
Chronic respiratory diseases	Male	0-6 days	95+ years	Global	1	3	Elevation 500 to 1500m (proportion)	401
Chronic respiratory diseases	Male	0-6 days	95+ years	Global	1	3	Population Density (over 1000 ppl/sqkm, proportion)	--
Chronic obstructive pulmonary disease	Female	15-19 years	95+ years	Data Rich	1	1	Cumulative Cigarettes (20 Years)	491
Chronic obstructive pulmonary disease	Female	15-19 years	95+ years	Data Rich	1	1	Cumulative Cigarettes (10 Years)	509
Chronic obstructive pulmonary disease	Female	15-19 years	95+ years	Data Rich	1	1	Log-transformed SEV scalar: COPD	--
Chronic obstructive pulmonary disease	Female	15-19 years	95+ years	Data Rich	-1	2	Mask use	421
Chronic obstructive pulmonary disease	Female	15-19 years	95+ years	Data Rich	1	2	Smoking Prevalence	203
Chronic obstructive pulmonary disease	Female	15-19 years	95+ years	Data Rich	1	2	Elevation Over 1500m (proportion)	421
Chronic obstructive pulmonary disease	Female	15-19 years	95+ years	Data Rich	1	2	Indoor Air Pollution (All Cooking Fuels)	421
Chronic obstructive pulmonary disease	Female	15-19 years	95+ years	Data Rich	1	2	COVID-19 age-standardized death rate	--
Chronic obstructive pulmonary disease	Female	15-19 years	95+ years	Data Rich	1	2	Outdoor Air Pollution (PM2.5)	--
Chronic obstructive pulmonary disease	Female	15-19 years	95+ years	Data Rich	-1	3	Socio-demographic Index	51
Chronic obstructive pulmonary disease	Female	15-19 years	95+ years	Data Rich	-1	3	Healthcare access and quality index	726
Chronic obstructive pulmonary disease	Female	15-19 years	95+ years	Global	1	1	Cumulative Cigarettes (20 Years)	68
Chronic obstructive pulmonary disease	Female	15-19 years	95+ years	Global	1	1	Cumulative Cigarettes (10 Years)	75
Chronic obstructive pulmonary disease	Female	15-19 years	95+ years	Global	1	1	Elevation Over 1500m (proportion)	471
Chronic obstructive pulmonary disease	Female	15-19 years	95+ years	Global	1	1	Log-transformed SEV scalar: COPD	755
Chronic obstructive pulmonary disease	Female	15-19 years	95+ years	Global	-1	2	Healthcare access and quality index	455
Chronic obstructive pulmonary disease	Female	15-19 years	95+ years	Global	-1	2	Mask use	473
Chronic obstructive pulmonary disease	Female	15-19 years	95+ years	Global	1	2	Outdoor Air Pollution (PM2.5)	23
Chronic obstructive pulmonary disease	Female	15-19 years	95+ years	Global	1	2	Smoking Prevalence	31
Chronic obstructive pulmonary disease	Female	15-19 years	95+ years	Global	1	2	COVID-19 age-standardized death rate	168
Chronic obstructive pulmonary disease	Female	15-19 years	95+ years	Global	1	2	Indoor Air Pollution (All Cooking Fuels)	542
Chronic obstructive pulmonary disease	Female	15-19 years	95+ years	Global	-1	3	Socio-demographic Index	158
Chronic obstructive pulmonary disease	Male	15-19 years	95+ years	Data Rich	1	1	Cumulative Cigarettes (20 Years)	20
Chronic obstructive pulmonary disease	Male	15-19 years	95+ years	Data Rich	1	1	Log-transformed SEV scalar: COPD	642
Chronic obstructive pulmonary disease	Male	15-19 years	95+ years	Data Rich	1	1	Cumulative Cigarettes (10 Years)	670
Chronic obstructive pulmonary disease	Male	15-19 years	95+ years	Data Rich	-1	2	Healthcare access and quality index	245
Chronic obstructive pulmonary disease	Male	15-19 years	95+ years	Data Rich	-1	2	Mask use	544
Chronic obstructive pulmonary disease	Male	15-19 years	95+ years	Data Rich	1	2	Indoor Air Pollution (All Cooking Fuels)	52
Chronic obstructive pulmonary disease	Male	15-19 years	95+ years	Data Rich	1	2	Elevation Over 1500m (proportion)	256
Chronic obstructive pulmonary disease	Male	15-19 years	95+ years	Data Rich	1	2	Outdoor Air Pollution (PM2.5)	576
Chronic obstructive pulmonary disease	Male	15-19 years	95+ years	Data Rich	1	2	Smoking Prevalence	760
Chronic obstructive pulmonary disease	Male	15-19 years	95+ years	Data Rich	1	2	COVID-19 age-standardized death rate	--
Chronic obstructive pulmonary disease	Male	15-19 years	95+ years	Data Rich	-1	3	Socio-demographic Index	464
Chronic obstructive pulmonary disease	Male	15-19 years	95+ years	Global	1	1	Cumulative Cigarettes (10 Years)	280
Chronic obstructive pulmonary disease	Male	15-19 years	95+ years	Global	1	1	Cumulative Cigarettes (20 Years)	299
Chronic obstructive pulmonary disease	Male	15-19 years	95+ years	Global	1	1	Log-transformed SEV scalar: COPD	421
Chronic obstructive pulmonary disease	Male	15-19 years	95+ years	Global	-1	2	Healthcare access and quality index	372
Chronic obstructive pulmonary disease	Male	15-19 years	95+ years	Global	-1	2	Mask use	494
Chronic obstructive pulmonary disease	Male	15-19 years	95+ years	Global	1	2	Elevation Over 1500m (proportion)	287

Appendix Table S10: CODEm covariates used, level of covariate, and expected direction of covariate by cause, sex, and age

Cause	Sex	Age Start	Age End	Model Version Type	Direction	Level	Covariate Name	Number of Draws
Chronic obstructive pulmonary disease	Male	15-19 years	95+ years	Global	1	2	Indoor Air Pollution (All Cooking Fuels)	494
Chronic obstructive pulmonary disease	Male	15-19 years	95+ years	Global	1	2	Smoking Prevalence	494
Chronic obstructive pulmonary disease	Male	15-19 years	95+ years	Global	1	2	COVID-19 age-standardized death rate	--
Chronic obstructive pulmonary disease	Male	15-19 years	95+ years	Global	1	2	Outdoor Air Pollution (PM2.5)	--
Chronic obstructive pulmonary disease	Male	15-19 years	95+ years	Global	-1	3	Socio-demographic Index	414
Pneumoconiosis	Female	15-19 years	95+ years	Data Rich	1	1	Age-standardized SEV for Occupational asbestos	528
Pneumoconiosis	Female	15-19 years	95+ years	Data Rich	1	1	Log-transformed coal production (per capita)	864
Pneumoconiosis	Female	15-19 years	95+ years	Data Rich	1	1	Age- and sex-specific SEV for Occupational silica	--
Pneumoconiosis	Female	15-19 years	95+ years	Data Rich	1	1	Gold production (kg) per capita, smoothed with 20-year lag	--
Pneumoconiosis	Female	15-19 years	95+ years	Data Rich	1	2	Outdoor Air Pollution (PM2.5)	268
Pneumoconiosis	Female	15-19 years	95+ years	Data Rich	1	2	Asbestos consumption (metric tons per year per capita)	281
Pneumoconiosis	Female	15-19 years	95+ years	Data Rich	1	2	Age- and sex-specific SEV for Occupational beryllium	--
Pneumoconiosis	Female	15-19 years	95+ years	Data Rich	1	2	Cumulative Cigarettes (20 Years)	--
Pneumoconiosis	Female	15-19 years	95+ years	Data Rich	1	2	Indoor Air Pollution (All Cooking Fuels)	--
Pneumoconiosis	Female	15-19 years	95+ years	Data Rich	1	2	Smoking Prevalence	--
Pneumoconiosis	Female	15-19 years	95+ years	Data Rich	-1	3	Socio-demographic Index	101
Pneumoconiosis	Female	15-19 years	95+ years	Data Rich	-1	3	Healthcare access and quality index	118
Pneumoconiosis	Female	15-19 years	95+ years	Global	1	1	Log-transformed coal production (per capita)	1000
Pneumoconiosis	Female	15-19 years	95+ years	Global	1	1	Age- and sex-specific SEV for Occupational silica	--
Pneumoconiosis	Female	15-19 years	95+ years	Global	1	1	Age-standardized SEV for Occupational asbestos	--
Pneumoconiosis	Female	15-19 years	95+ years	Global	1	1	Gold production (kg) per capita, smoothed with 20-year lag	--
Pneumoconiosis	Female	15-19 years	95+ years	Global	1	2	Asbestos consumption (metric tons per year per capita)	593
Pneumoconiosis	Female	15-19 years	95+ years	Global	1	2	Outdoor Air Pollution (PM2.5)	593
Pneumoconiosis	Female	15-19 years	95+ years	Global	1	2	Age- and sex-specific SEV for Occupational beryllium	--
Pneumoconiosis	Female	15-19 years	95+ years	Global	1	2	Cumulative Cigarettes (20 Years)	--
Pneumoconiosis	Female	15-19 years	95+ years	Global	1	2	Indoor Air Pollution (All Cooking Fuels)	--
Pneumoconiosis	Female	15-19 years	95+ years	Global	1	2	Smoking Prevalence	--
Pneumoconiosis	Female	15-19 years	95+ years	Global	-1	3	Healthcare access and quality index	326
Pneumoconiosis	Female	15-19 years	95+ years	Global	-1	3	Socio-demographic Index	548
Pneumoconiosis	Male	15-19 years	95+ years	Data Rich	1	1	Age- and sex-specific SEV for Occupational silica	250
Pneumoconiosis	Male	15-19 years	95+ years	Data Rich	1	1	Log-transformed coal production (per capita)	281
Pneumoconiosis	Male	15-19 years	95+ years	Data Rich	1	1	Age-standardized SEV for Occupational asbestos	804
Pneumoconiosis	Male	15-19 years	95+ years	Data Rich	1	1	Gold production (kg) per capita, smoothed with 20-year lag	--
Pneumoconiosis	Male	15-19 years	95+ years	Data Rich	1	2	Indoor Air Pollution (All Cooking Fuels)	0
Pneumoconiosis	Male	15-19 years	95+ years	Data Rich	1	2	Outdoor Air Pollution (PM2.5)	19
Pneumoconiosis	Male	15-19 years	95+ years	Data Rich	1	2	Cumulative Cigarettes (20 Years)	116
Pneumoconiosis	Male	15-19 years	95+ years	Data Rich	1	2	Asbestos consumption (metric tons per year per capita)	645
Pneumoconiosis	Male	15-19 years	95+ years	Data Rich	1	2	Smoking Prevalence	646
Pneumoconiosis	Male	15-19 years	95+ years	Data Rich	1	2	Age- and sex-specific SEV for Occupational beryllium	--
Pneumoconiosis	Male	15-19 years	95+ years	Data Rich	-1	3	Healthcare access and quality index	473
Pneumoconiosis	Male	15-19 years	95+ years	Data Rich	-1	3	Socio-demographic Index	586
Pneumoconiosis	Male	15-19 years	95+ years	Global	1	1	Age- and sex-specific SEV for Occupational silica	534
Pneumoconiosis	Male	15-19 years	95+ years	Global	1	1	Log-transformed coal production (per capita)	821
Pneumoconiosis	Male	15-19 years	95+ years	Global	1	1	Age-standardized SEV for Occupational asbestos	953
Pneumoconiosis	Male	15-19 years	95+ years	Global	1	1	Gold production (kg) per capita, smoothed with 20-year lag	--
Pneumoconiosis	Male	15-19 years	95+ years	Global	1	2	Indoor Air Pollution (All Cooking Fuels)	56
Pneumoconiosis	Male	15-19 years	95+ years	Global	1	2	Outdoor Air Pollution (PM2.5)	117
Pneumoconiosis	Male	15-19 years	95+ years	Global	1	2	Cumulative Cigarettes (20 Years)	164
Pneumoconiosis	Male	15-19 years	95+ years	Global	1	2	Asbestos consumption (metric tons per year per capita)	264
Pneumoconiosis	Male	15-19 years	95+ years	Global	1	2	Smoking Prevalence	336
Pneumoconiosis	Male	15-19 years	95+ years	Global	1	2	Age- and sex-specific SEV for Occupational beryllium	--
Pneumoconiosis	Male	15-19 years	95+ years	Global	-1	3	Healthcare access and quality index	347
Pneumoconiosis	Male	15-19 years	95+ years	Global	-1	3	Socio-demographic Index	361
Silicosis	Female	15-19 years	95+ years	Data Rich	1	1	Cumulative Cigarettes (20 Years)	362
Silicosis	Female	15-19 years	95+ years	Data Rich	1	1	Age- and sex-specific SEV for Occupational silica	460
Silicosis	Female	15-19 years	95+ years	Data Rich	1	1	Cumulative Cigarettes (10 Years)	508
Silicosis	Female	15-19 years	95+ years	Data Rich	1	1	Gold production (kg) per capita, smoothed with 20-year lag	--
Silicosis	Female	15-19 years	95+ years	Data Rich	-1	2	Healthcare access and quality index	774
Silicosis	Female	15-19 years	95+ years	Data Rich	1	2	Smoking Prevalence	7
Silicosis	Female	15-19 years	95+ years	Data Rich	1	2	Outdoor Air Pollution (PM2.5)	21
Silicosis	Female	15-19 years	95+ years	Data Rich	1	2	Indoor Air Pollution (All Cooking Fuels)	--
Silicosis	Female	15-19 years	95+ years	Data Rich	-1	3	Socio-demographic Index	37
Silicosis	Female	15-19 years	95+ years	Global	1	1	Cumulative Cigarettes (20 Years)	446
Silicosis	Female	15-19 years	95+ years	Global	1	1	Cumulative Cigarettes (10 Years)	554
Silicosis	Female	15-19 years	95+ years	Global	1	1	Age- and sex-specific SEV for Occupational silica	--
Silicosis	Female	15-19 years	95+ years	Global	1	1	Gold production (kg) per capita, smoothed with 20-year lag	--
Silicosis	Female	15-19 years	95+ years	Global	-1	2	Healthcare access and quality index	238
Silicosis	Female	15-19 years	95+ years	Global	1	2	Outdoor Air Pollution (PM2.5)	105
Silicosis	Female	15-19 years	95+ years	Global	1	2	Indoor Air Pollution (All Cooking Fuels)	--
Silicosis	Female	15-19 years	95+ years	Global	1	2	Smoking Prevalence	--
Silicosis	Female	15-19 years	95+ years	Global	-1	3	Socio-demographic Index	463
Silicosis	Male	15-19 years	95+ years	Data Rich	1	1	Age- and sex-specific SEV for Occupational silica	483
Silicosis	Male	15-19 years	95+ years	Data Rich	1	1	Cumulative Cigarettes (10 Years)	796
Silicosis	Male	15-19 years	95+ years	Data Rich	1	1	Cumulative Cigarettes (20 Years)	--
Silicosis	Male	15-19 years	95+ years	Data Rich	1	1	Gold production (kg) per capita, smoothed with 20-year lag	--
Silicosis	Male	15-19 years	95+ years	Data Rich	-1	2	Healthcare access and quality index	174
Silicosis	Male	15-19 years	95+ years	Data Rich	1	2	Indoor Air Pollution (All Cooking Fuels)	22
Silicosis	Male	15-19 years	95+ years	Data Rich	1	2	Smoking Prevalence	88
Silicosis	Male	15-19 years	95+ years	Data Rich	1	2	Outdoor Air Pollution (PM2.5)	308
Silicosis	Male	15-19 years	95+ years	Data Rich	-1	3	Socio-demographic Index	250
Silicosis	Male	15-19 years	95+ years	Global	1	1	Cumulative Cigarettes (20 Years)	219
Silicosis	Male	15-19 years	95+ years	Global	1	1	Age- and sex-specific SEV for Occupational silica	265
Silicosis	Male	15-19 years	95+ years	Global	1	1	Cumulative Cigarettes (10 Years)	707
Silicosis	Male	15-19 years	95+ years	Global	1	1	Gold production (kg) per capita, smoothed with 20-year lag	--
Silicosis	Male	15-19 years	95+ years	Global	-1	2	Healthcare access and quality index	422
Silicosis	Male	15-19 years	95+ years	Global	1	2	Indoor Air Pollution (All Cooking Fuels)	10
Silicosis	Male	15-19 years	95+ years	Global	1	2	Outdoor Air Pollution (PM2.5)	69
Silicosis	Male	15-19 years	95+ years	Global	1	2	Smoking Prevalence	74
Silicosis	Male	15-19 years	95+ years	Global	-1	3	Socio-demographic Index	484
Asbestosis	Female	15-19 years	95+ years	Data Rich	1	1	Age-standardized SEV for Occupational asbestos	573
Asbestosis	Female	15-19 years	95+ years	Data Rich	1	1	Asbestos consumption (metric tons per year per capita)	675
Asbestosis	Female	15-19 years	95+ years	Data Rich	1	1	Cumulative Cigarettes (20 Years)	--
Asbestosis	Female	15-19 years	95+ years	Data Rich	-1	2	Healthcare access and quality index	--
Asbestosis	Female	15-19 years	95+ years	Data Rich	1	2	Cumulative Cigarettes (10 Years)	--
Asbestosis	Female	15-19 years	95+ years	Data Rich	1	2	Cumulative Cigarettes (5 Years)	--
Asbestosis	Female	15-19 years	95+ years	Data Rich	1	2	Indoor Air Pollution (All Cooking Fuels)	--
Asbestosis	Female	15-19 years	95+ years	Data Rich	1	2	Outdoor Air Pollution (PM2.5)	--
Asbestosis	Female	15-19 years	95+ years	Data Rich	1	2	Smoking Prevalence	--
Asbestosis	Female	15-19 years	95+ years	Data Rich	-1	3	Socio-demographic Index	--

Appendix Table S10: CODEm covariates used, level of covariate, and expected direction of covariate by cause, sex, and age

Cause	Sex	Age Start	Age End	Model Version Type	Direction	Level	Covariate Name	Number of Draws
Asbestosis	Female	15-19 years	95+ years	Global	1	1	Asbestos consumption (metric tons per year per capita)	603
Asbestosis	Female	15-19 years	95+ years	Global	1	1	Age-standardized SEV for Occupational asbestos	714
Asbestosis	Female	15-19 years	95+ years	Global	-1	2	Healthcare access and quality index	--
Asbestosis	Female	15-19 years	95+ years	Global	1	2	Outdoor Air Pollution (PM2.5)	386
Asbestosis	Female	15-19 years	95+ years	Global	1	2	Cumulative Cigarettes (10 Years)	--
Asbestosis	Female	15-19 years	95+ years	Global	1	2	Cumulative Cigarettes (20 Years)	--
Asbestosis	Female	15-19 years	95+ years	Global	1	2	Cumulative Cigarettes (5 Years)	--
Asbestosis	Female	15-19 years	95+ years	Global	1	2	Indoor Air Pollution (All Cooking Fuels)	--
Asbestosis	Female	15-19 years	95+ years	Global	1	2	Smoking Prevalence	--
Asbestosis	Female	15-19 years	95+ years	Global	-1	3	Socio-demographic Index	--
Asbestosis	Male	15-19 years	95+ years	Data Rich	1	1	Asbestos consumption (metric tons per year per capita)	568
Asbestosis	Male	15-19 years	95+ years	Data Rich	1	1	Age-standardized SEV for Occupational asbestos	1000
Asbestosis	Male	15-19 years	95+ years	Data Rich	1	1	Cumulative Cigarettes (10 Years)	--
Asbestosis	Male	15-19 years	95+ years	Data Rich	1	1	Cumulative Cigarettes (20 Years)	--
Asbestosis	Male	15-19 years	95+ years	Data Rich	-1	2	Healthcare access and quality index	--
Asbestosis	Male	15-19 years	95+ years	Data Rich	1	2	Indoor Air Pollution (All Cooking Fuels)	--
Asbestosis	Male	15-19 years	95+ years	Data Rich	1	2	Outdoor Air Pollution (PM2.5)	--
Asbestosis	Male	15-19 years	95+ years	Data Rich	1	2	Smoking Prevalence	--
Asbestosis	Male	15-19 years	95+ years	Data Rich	-1	3	Socio-demographic Index	--
Asbestosis	Male	15-19 years	95+ years	Global	1	1	Asbestos consumption (metric tons per year per capita)	353
Asbestosis	Male	15-19 years	95+ years	Global	1	1	Age-standardized SEV for Occupational asbestos	1000
Asbestosis	Male	15-19 years	95+ years	Global	1	1	Cumulative Cigarettes (10 Years)	--
Asbestosis	Male	15-19 years	95+ years	Global	1	1	Cumulative Cigarettes (20 Years)	--
Asbestosis	Male	15-19 years	95+ years	Global	-1	2	Healthcare access and quality index	--
Asbestosis	Male	15-19 years	95+ years	Global	1	2	Outdoor Air Pollution (PM2.5)	294
Asbestosis	Male	15-19 years	95+ years	Global	1	2	Indoor Air Pollution (All Cooking Fuels)	--
Asbestosis	Male	15-19 years	95+ years	Global	1	2	Smoking Prevalence	--
Asbestosis	Male	15-19 years	95+ years	Global	-1	3	Socio-demographic Index	--
Coal workers pneumoconiosis	Female	15-19 years	95+ years	Data Rich	1	1	Cumulative Cigarettes (20 Years)	100
Coal workers pneumoconiosis	Female	15-19 years	95+ years	Data Rich	1	1	Cumulative Cigarettes (10 Years)	302
Coal workers pneumoconiosis	Female	15-19 years	95+ years	Data Rich	1	1	Log-transformed coal production (per capita)	729
Coal workers pneumoconiosis	Female	15-19 years	95+ years	Data Rich	-1	2	Healthcare access and quality index	--
Coal workers pneumoconiosis	Female	15-19 years	95+ years	Data Rich	1	2	Outdoor Air Pollution (PM2.5)	522
Coal workers pneumoconiosis	Female	15-19 years	95+ years	Data Rich	1	2	Indoor Air Pollution (All Cooking Fuels)	--
Coal workers pneumoconiosis	Female	15-19 years	95+ years	Data Rich	1	2	Smoking Prevalence	--
Coal workers pneumoconiosis	Female	15-19 years	95+ years	Data Rich	-1	3	Socio-demographic Index	225
Coal workers pneumoconiosis	Female	15-19 years	95+ years	Global	1	1	Cumulative Cigarettes (10 Years)	174
Coal workers pneumoconiosis	Female	15-19 years	95+ years	Global	1	1	Cumulative Cigarettes (20 Years)	287
Coal workers pneumoconiosis	Female	15-19 years	95+ years	Global	1	1	Log-transformed coal production (per capita)	683
Coal workers pneumoconiosis	Female	15-19 years	95+ years	Global	-1	2	Healthcare access and quality index	207
Coal workers pneumoconiosis	Female	15-19 years	95+ years	Global	1	2	Outdoor Air Pollution (PM2.5)	294
Coal workers pneumoconiosis	Female	15-19 years	95+ years	Global	1	2	Indoor Air Pollution (All Cooking Fuels)	296
Coal workers pneumoconiosis	Female	15-19 years	95+ years	Global	1	2	Smoking Prevalence	--
Coal workers pneumoconiosis	Female	15-19 years	95+ years	Global	-1	3	Socio-demographic Index	165
Coal workers pneumoconiosis	Male	15-19 years	95+ years	Data Rich	1	1	Cumulative Cigarettes (20 Years)	319
Coal workers pneumoconiosis	Male	15-19 years	95+ years	Data Rich	1	1	Cumulative Cigarettes (10 Years)	681
Coal workers pneumoconiosis	Male	15-19 years	95+ years	Data Rich	1	1	Log-transformed coal production (per capita)	--
Coal workers pneumoconiosis	Male	15-19 years	95+ years	Data Rich	-1	2	Healthcare access and quality index	220
Coal workers pneumoconiosis	Male	15-19 years	95+ years	Data Rich	1	2	Outdoor Air Pollution (PM2.5)	220
Coal workers pneumoconiosis	Male	15-19 years	95+ years	Data Rich	1	2	Smoking Prevalence	427
Coal workers pneumoconiosis	Male	15-19 years	95+ years	Data Rich	1	2	Indoor Air Pollution (All Cooking Fuels)	--
Coal workers pneumoconiosis	Male	15-19 years	95+ years	Data Rich	-1	3	Socio-demographic Index	100
Coal workers pneumoconiosis	Male	15-19 years	95+ years	Global	1	1	Cumulative Cigarettes (20 Years)	158
Coal workers pneumoconiosis	Male	15-19 years	95+ years	Global	1	1	Cumulative Cigarettes (10 Years)	600
Coal workers pneumoconiosis	Male	15-19 years	95+ years	Global	1	1	Log-transformed coal production (per capita)	951
Coal workers pneumoconiosis	Male	15-19 years	95+ years	Global	-1	2	Healthcare access and quality index	212
Coal workers pneumoconiosis	Male	15-19 years	95+ years	Global	1	2	Smoking Prevalence	208
Coal workers pneumoconiosis	Male	15-19 years	95+ years	Global	1	2	Indoor Air Pollution (All Cooking Fuels)	227
Coal workers pneumoconiosis	Male	15-19 years	95+ years	Global	1	2	Outdoor Air Pollution (PM2.5)	230
Coal workers pneumoconiosis	Male	15-19 years	95+ years	Global	-1	3	Socio-demographic Index	361
Other pneumoconiosis	Female	15-19 years	95+ years	Data Rich	1	1	Cumulative Cigarettes (20 Years)	1000
Other pneumoconiosis	Female	15-19 years	95+ years	Data Rich	1	1	Age- and sex-specific SEV for Occupational beryllium	--
Other pneumoconiosis	Female	15-19 years	95+ years	Data Rich	1	1	Age- and sex-specific SEV for Occupational silica	--
Other pneumoconiosis	Female	15-19 years	95+ years	Data Rich	1	1	Cumulative Cigarettes (10 Years)	--
Other pneumoconiosis	Female	15-19 years	95+ years	Data Rich	1	1	Gold production (kg) per capita, smoothed with 20-year lag	--
Other pneumoconiosis	Female	15-19 years	95+ years	Data Rich	-1	2	Healthcare access and quality index	--
Other pneumoconiosis	Female	15-19 years	95+ years	Data Rich	1	2	Outdoor Air Pollution (PM2.5)	432
Other pneumoconiosis	Female	15-19 years	95+ years	Data Rich	1	2	Indoor Air Pollution (All Cooking Fuels)	--
Other pneumoconiosis	Female	15-19 years	95+ years	Data Rich	1	2	Proportion of the population working in mining with 20-year lag	--
Other pneumoconiosis	Female	15-19 years	95+ years	Data Rich	1	2	Smoking Prevalence	--
Other pneumoconiosis	Female	15-19 years	95+ years	Data Rich	1	2	Tuberculosis prevalence (age-standardized)	--
Other pneumoconiosis	Female	15-19 years	95+ years	Data Rich	-1	3	Socio-demographic Index	--
Other pneumoconiosis	Female	15-19 years	95+ years	Global	1	1	Cumulative Cigarettes (10 Years)	317
Other pneumoconiosis	Female	15-19 years	95+ years	Global	1	1	Tuberculosis prevalence (age-standardized)	601
Other pneumoconiosis	Female	15-19 years	95+ years	Global	1	1	Cumulative Cigarettes (20 Years)	683
Other pneumoconiosis	Female	15-19 years	95+ years	Global	1	1	Age- and sex-specific SEV for Occupational beryllium	--
Other pneumoconiosis	Female	15-19 years	95+ years	Global	1	1	Age- and sex-specific SEV for Occupational silica	--
Other pneumoconiosis	Female	15-19 years	95+ years	Global	1	1	Gold production (kg) per capita, smoothed with 20-year lag	--
Other pneumoconiosis	Female	15-19 years	95+ years	Global	-1	2	Healthcare access and quality index	68
Other pneumoconiosis	Female	15-19 years	95+ years	Global	1	2	Outdoor Air Pollution (PM2.5)	213
Other pneumoconiosis	Female	15-19 years	95+ years	Global	1	2	Proportion of the population working in mining with 20-year lag	448
Other pneumoconiosis	Female	15-19 years	95+ years	Global	1	2	Indoor Air Pollution (All Cooking Fuels)	--
Other pneumoconiosis	Female	15-19 years	95+ years	Global	1	2	Smoking Prevalence	--
Other pneumoconiosis	Female	15-19 years	95+ years	Global	-1	3	Socio-demographic Index	176
Other pneumoconiosis	Male	15-19 years	95+ years	Data Rich	1	1	Proportion of the population working in mining with 20-year lag	172
Other pneumoconiosis	Male	15-19 years	95+ years	Data Rich	1	1	Age- and sex-specific SEV for Occupational silica	275
Other pneumoconiosis	Male	15-19 years	95+ years	Data Rich	1	1	Cumulative Cigarettes (20 Years)	438
Other pneumoconiosis	Male	15-19 years	95+ years	Data Rich	1	1	Cumulative Cigarettes (10 Years)	522
Other pneumoconiosis	Male	15-19 years	95+ years	Data Rich	1	1	Age- and sex-specific SEV for Occupational beryllium	--
Other pneumoconiosis	Male	15-19 years	95+ years	Data Rich	1	1	Gold production (kg) per capita, smoothed with 20-year lag	--
Other pneumoconiosis	Male	15-19 years	95+ years	Data Rich	-1	2	Healthcare access and quality index	2
Other pneumoconiosis	Male	15-19 years	95+ years	Data Rich	1	2	Indoor Air Pollution (All Cooking Fuels)	16
Other pneumoconiosis	Male	15-19 years	95+ years	Data Rich	1	2	Outdoor Air Pollution (PM2.5)	38
Other pneumoconiosis	Male	15-19 years	95+ years	Data Rich	1	2	Smoking Prevalence	48
Other pneumoconiosis	Male	15-19 years	95+ years	Data Rich	1	2	Tuberculosis prevalence (age-standardized)	232
Other pneumoconiosis	Male	15-19 years	95+ years	Data Rich	-1	3	Socio-demographic Index	41
Other pneumoconiosis	Male	15-19 years	95+ years	Global	1	1	Age- and sex-specific SEV for Occupational silica	133
Other pneumoconiosis	Male	15-19 years	95+ years	Global	1	1	Age- and sex-specific SEV for Occupational beryllium	156
Other pneumoconiosis	Male	15-19 years	95+ years	Global	1	1	Cumulative Cigarettes (20 Years)	525
Other pneumoconiosis	Male	15-19 years	95+ years	Global	1	1	Cumulative Cigarettes (10 Years)	614
Other pneumoconiosis	Male	15-19 years	95+ years	Global	1	1	Tuberculosis prevalence (age-standardized)	706
Other pneumoconiosis	Male	15-19 years	95+ years	Global	1	1	Gold production (kg) per capita, smoothed with 20-year lag	--

Appendix Table S10: CODEm covariates used, level of covariate, and expected direction of covariate by cause, sex, and age

Cause	Sex	Age Start	Age End	Model Version Type	Direction	Level	Covariate Name	Number of Draws
Other pneumoconiosis	Male	15-19 years	95+ years	Global	-1	2	Healthcare access and quality index	170
Other pneumoconiosis	Male	15-19 years	95+ years	Global	1	2	Outdoor Air Pollution (PM2.5)	16
Other pneumoconiosis	Male	15-19 years	95+ years	Global	1	2	Proportion of the population working in mining with 20-year lag	34
Other pneumoconiosis	Male	15-19 years	95+ years	Global	1	2	Indoor Air Pollution (All Cooking Fuels)	103
Other pneumoconiosis	Male	15-19 years	95+ years	Global	1	2	Smoking Prevalence	194
Other pneumoconiosis	Male	15-19 years	95+ years	Global	-1	3	Socio-demographic Index	55
Asthma	Female	12-23 months	95+ years	Data Rich	1	1	Cumulative Cigarettes (10 Years)	--
Asthma	Female	12-23 months	95+ years	Data Rich	1	1	Cumulative Cigarettes (20 Years)	--
Asthma	Female	12-23 months	95+ years	Data Rich	1	1	Log-transformed SEV scalar: Asthma	--
Asthma	Female	12-23 months	95+ years	Data Rich	-1	2	Healthcare access and quality index	360
Asthma	Female	12-23 months	95+ years	Data Rich	-1	2	Proportion of population involved in agricultural activities	--
Asthma	Female	12-23 months	95+ years	Data Rich	1	2	Smoking Prevalence	367
Asthma	Female	12-23 months	95+ years	Data Rich	1	2	Indoor Air Pollution (All Cooking Fuels)	--
Asthma	Female	12-23 months	95+ years	Data Rich	1	2	Outdoor Air Pollution (PM2.5)	--
Asthma	Female	12-23 months	95+ years	Data Rich	-1	3	Socio-demographic Index	481
Asthma	Female	12-23 months	95+ years	Global	1	1	Cumulative Cigarettes (10 Years)	287
Asthma	Female	12-23 months	95+ years	Global	1	1	Cumulative Cigarettes (20 Years)	1000
Asthma	Female	12-23 months	95+ years	Global	1	1	Log-transformed SEV scalar: Asthma	--
Asthma	Female	12-23 months	95+ years	Global	-1	2	Healthcare access and quality index	341
Asthma	Female	12-23 months	95+ years	Global	-1	2	Proportion of population involved in agricultural activities	--
Asthma	Female	12-23 months	95+ years	Global	1	2	Indoor Air Pollution (All Cooking Fuels)	170
Asthma	Female	12-23 months	95+ years	Global	1	2	Outdoor Air Pollution (PM2.5)	341
Asthma	Female	12-23 months	95+ years	Global	1	2	Smoking Prevalence	341
Asthma	Female	12-23 months	95+ years	Global	-1	3	Socio-demographic Index	231
Asthma	Male	12-23 months	95+ years	Data Rich	1	1	Cumulative Cigarettes (10 Years)	1000
Asthma	Male	12-23 months	95+ years	Data Rich	1	1	Cumulative Cigarettes (20 Years)	--
Asthma	Male	12-23 months	95+ years	Data Rich	1	1	Log-transformed SEV scalar: Asthma	--
Asthma	Male	12-23 months	95+ years	Data Rich	-1	2	Healthcare access and quality index	--
Asthma	Male	12-23 months	95+ years	Data Rich	-1	2	Proportion of population involved in agricultural activities	--
Asthma	Male	12-23 months	95+ years	Data Rich	1	2	Indoor Air Pollution (All Cooking Fuels)	568
Asthma	Male	12-23 months	95+ years	Data Rich	1	2	Outdoor Air Pollution (PM2.5)	568
Asthma	Male	12-23 months	95+ years	Data Rich	1	2	Smoking Prevalence	--
Asthma	Male	12-23 months	95+ years	Data Rich	-1	3	Socio-demographic Index	--
Asthma	Male	12-23 months	95+ years	Global	1	1	Cumulative Cigarettes (10 Years)	333
Asthma	Male	12-23 months	95+ years	Global	1	1	Cumulative Cigarettes (20 Years)	667
Asthma	Male	12-23 months	95+ years	Global	1	1	Log-transformed SEV scalar: Asthma	--
Asthma	Male	12-23 months	95+ years	Global	-1	2	Healthcare access and quality index	590
Asthma	Male	12-23 months	95+ years	Global	-1	2	Proportion of population involved in agricultural activities	--
Asthma	Male	12-23 months	95+ years	Global	1	2	Indoor Air Pollution (All Cooking Fuels)	82
Asthma	Male	12-23 months	95+ years	Global	1	2	Smoking Prevalence	126
Asthma	Male	12-23 months	95+ years	Global	1	2	Outdoor Air Pollution (PM2.5)	556
Asthma	Male	12-23 months	95+ years	Global	-1	3	Socio-demographic Index	419
Interstitial lung disease and pulmonary sarcoidosis	Female	12-23 months	95+ years	Data Rich	1	1	Cumulative Cigarettes (10 Years)	--
Interstitial lung disease and pulmonary sarcoidosis	Female	12-23 months	95+ years	Data Rich	1	1	Cumulative Cigarettes (5 Years)	--
Interstitial lung disease and pulmonary sarcoidosis	Female	12-23 months	95+ years	Data Rich	1	1	Smoking Prevalence	--
Interstitial lung disease and pulmonary sarcoidosis	Female	12-23 months	95+ years	Data Rich	-1	2	Healthcare access and quality index	--
Interstitial lung disease and pulmonary sarcoidosis	Female	12-23 months	95+ years	Data Rich	1	2	Elevation Over 1500m (proportion)	432
Interstitial lung disease and pulmonary sarcoidosis	Female	12-23 months	95+ years	Data Rich	1	2	Elevation 500 to 1500m (proportion)	568
Interstitial lung disease and pulmonary sarcoidosis	Female	12-23 months	95+ years	Data Rich	1	2	Indoor Air Pollution (All Cooking Fuels)	--
Interstitial lung disease and pulmonary sarcoidosis	Female	12-23 months	95+ years	Data Rich	1	2	Outdoor Air Pollution (PM2.5)	--
Interstitial lung disease and pulmonary sarcoidosis	Female	12-23 months	95+ years	Data Rich	-1	3	Socio-demographic Index	--
Interstitial lung disease and pulmonary sarcoidosis	Female	12-23 months	95+ years	Global	1	1	Cumulative Cigarettes (10 Years)	--
Interstitial lung disease and pulmonary sarcoidosis	Female	12-23 months	95+ years	Global	1	1	Cumulative Cigarettes (5 Years)	--
Interstitial lung disease and pulmonary sarcoidosis	Female	12-23 months	95+ years	Global	1	1	Indoor Air Pollution (All Cooking Fuels)	--
Interstitial lung disease and pulmonary sarcoidosis	Female	12-23 months	95+ years	Global	1	1	Outdoor Air Pollution (PM2.5)	--
Interstitial lung disease and pulmonary sarcoidosis	Female	12-23 months	95+ years	Global	1	1	Smoking Prevalence	--
Interstitial lung disease and pulmonary sarcoidosis	Female	12-23 months	95+ years	Global	-1	2	Healthcare access and quality index	--
Interstitial lung disease and pulmonary sarcoidosis	Female	12-23 months	95+ years	Global	1	2	Elevation Over 1500m (proportion)	1000
Interstitial lung disease and pulmonary sarcoidosis	Female	12-23 months	95+ years	Global	1	2	Elevation 500 to 1500m (proportion)	--
Interstitial lung disease and pulmonary sarcoidosis	Female	12-23 months	95+ years	Global	-1	3	Socio-demographic Index	--
Interstitial lung disease and pulmonary sarcoidosis	Male	12-23 months	95+ years	Data Rich	1	1	Cumulative Cigarettes (10 Years)	--
Interstitial lung disease and pulmonary sarcoidosis	Male	12-23 months	95+ years	Data Rich	1	1	Cumulative Cigarettes (5 Years)	--
Interstitial lung disease and pulmonary sarcoidosis	Male	12-23 months	95+ years	Data Rich	1	1	Smoking Prevalence	--
Interstitial lung disease and pulmonary sarcoidosis	Male	12-23 months	95+ years	Data Rich	-1	2	Healthcare access and quality index	--
Interstitial lung disease and pulmonary sarcoidosis	Male	12-23 months	95+ years	Data Rich	-1	2	Socio-demographic Index	--
Interstitial lung disease and pulmonary sarcoidosis	Male	12-23 months	95+ years	Data Rich	1	2	Elevation 500 to 1500m (proportion)	1000
Interstitial lung disease and pulmonary sarcoidosis	Male	12-23 months	95+ years	Data Rich	1	2	Elevation Over 1500m (proportion)	--
Interstitial lung disease and pulmonary sarcoidosis	Male	12-23 months	95+ years	Data Rich	1	2	Indoor Air Pollution (All Cooking Fuels)	--
Interstitial lung disease and pulmonary sarcoidosis	Male	12-23 months	95+ years	Data Rich	1	2	Outdoor Air Pollution (PM2.5)	--
Interstitial lung disease and pulmonary sarcoidosis	Male	12-23 months	95+ years	Global	1	1	Cumulative Cigarettes (10 Years)	--
Interstitial lung disease and pulmonary sarcoidosis	Male	12-23 months	95+ years	Global	1	1	Cumulative Cigarettes (20 Years)	--
Interstitial lung disease and pulmonary sarcoidosis	Male	12-23 months	95+ years	Global	1	1	Cumulative Cigarettes (5 Years)	--
Interstitial lung disease and pulmonary sarcoidosis	Male	12-23 months	95+ years	Global	1	1	Smoking Prevalence	--
Interstitial lung disease and pulmonary sarcoidosis	Male	12-23 months	95+ years	Global	-1	2	Healthcare access and quality index	--
Interstitial lung disease and pulmonary sarcoidosis	Male	12-23 months	95+ years	Global	1	2	Elevation 500 to 1500m (proportion)	454
Interstitial lung disease and pulmonary sarcoidosis	Male	12-23 months	95+ years	Global	1	2	Elevation Over 1500m (proportion)	454
Interstitial lung disease and pulmonary sarcoidosis	Male	12-23 months	95+ years	Global	1	2	Outdoor Air Pollution (PM2.5)	546
Interstitial lung disease and pulmonary sarcoidosis	Male	12-23 months	95+ years	Global	1	2	Indoor Air Pollution (All Cooking Fuels)	--
Interstitial lung disease and pulmonary sarcoidosis	Male	12-23 months	95+ years	Global	-1	3	Socio-demographic Index	--
Other chronic respiratory diseases	Female	0-6 days	95+ years	Data Rich	1	1	Indoor Air Pollution (All Cooking Fuels)	360
Other chronic respiratory diseases	Female	0-6 days	95+ years	Data Rich	1	1	Outdoor Air Pollution (PM2.5)	1000
Other chronic respiratory diseases	Female	0-6 days	95+ years	Data Rich	1	1	Cumulative Cigarettes (20 Years)	--
Other chronic respiratory diseases	Female	0-6 days	95+ years	Data Rich	1	1	Cumulative Cigarettes (5 Years)	--
Other chronic respiratory diseases	Female	0-6 days	95+ years	Data Rich	-1	2	Healthcare access and quality index	--
Other chronic respiratory diseases	Female	0-6 days	95+ years	Data Rich	1	2	Mean BMI	208
Other chronic respiratory diseases	Female	0-6 days	95+ years	Data Rich	1	2	Elevation Over 1500m (proportion)	--
Other chronic respiratory diseases	Female	0-6 days	95+ years	Data Rich	1	2	Liters of alcohol consumed per capita	--
Other chronic respiratory diseases	Female	0-6 days	95+ years	Data Rich	1	2	Smoking Prevalence	--
Other chronic respiratory diseases	Female	0-6 days	95+ years	Data Rich	-1	3	Socio-demographic Index	273
Other chronic respiratory diseases	Female	0-6 days	95+ years	Global	1	1	Cumulative Cigarettes (20 Years)	--
Other chronic respiratory diseases	Female	0-6 days	95+ years	Global	1	1	Cumulative Cigarettes (5 Years)	--
Other chronic respiratory diseases	Female	0-6 days	95+ years	Global	1	1	Smoking Prevalence	--
Other chronic respiratory diseases	Female	0-6 days	95+ years	Global	1	2	Elevation Over 1500m (proportion)	274
Other chronic respiratory diseases	Female	0-6 days	95+ years	Global	1	2	Indoor Air Pollution (All Cooking Fuels)	274

Appendix Table S10: CODEm covariates used, level of covariate, and expected direction of covariate by cause, sex, and age								
**Cause**	**Sex**	**Age Start**	**Age End**	**Model Version Type**	**Direction**	**Level**	**Covariate Name**	**Number of Draws**
Other chronic respiratory diseases	Female	0-6 days	95+ years	Global	1	2	Liters of alcohol consumed per capita	--
Other chronic respiratory diseases	Female	0-6 days	95+ years	Global	1	2	Mean BMI	--
Other chronic respiratory diseases	Female	0-6 days	95+ years	Global	1	2	Outdoor Air Pollution (PM2.5)	--
Other chronic respiratory diseases	Female	0-6 days	95+ years	Global	-1	3	Socio-demographic Index	328
Other chronic respiratory diseases	Female	0-6 days	95+ years	Global	-1	3	Healthcare access and quality index	398
Other chronic respiratory diseases	Male	0-6 days	95+ years	Data Rich	1	1	Cumulative Cigarettes (5 Years)	12
Other chronic respiratory diseases	Male	0-6 days	95+ years	Data Rich	1	1	Smoking Prevalence	37
Other chronic respiratory diseases	Male	0-6 days	95+ years	Data Rich	1	1	Indoor Air Pollution (All Cooking Fuels)	446
Other chronic respiratory diseases	Male	0-6 days	95+ years	Data Rich	1	1	Outdoor Air Pollution (PM2.5)	757
Other chronic respiratory diseases	Male	0-6 days	95+ years	Data Rich	1	1	Cumulative Cigarettes (20 Years)	--
Other chronic respiratory diseases	Male	0-6 days	95+ years	Data Rich	-1	2	Healthcare access and quality index	--
Other chronic respiratory diseases	Male	0-6 days	95+ years	Data Rich	1	2	Mean BMI	157
Other chronic respiratory diseases	Male	0-6 days	95+ years	Data Rich	1	2	Elevation Over 1500m (proportion)	393
Other chronic respiratory diseases	Male	0-6 days	95+ years	Data Rich	1	2	Liters of alcohol consumed per capita	--
Other chronic respiratory diseases	Male	0-6 days	95+ years	Data Rich	-1	3	Socio-demographic Index	440
Other chronic respiratory diseases	Male	0-6 days	95+ years	Global	1	1	Cumulative Cigarettes (20 Years)	81
Other chronic respiratory diseases	Male	0-6 days	95+ years	Global	1	1	Smoking Prevalence	97
Other chronic respiratory diseases	Male	0-6 days	95+ years	Global	1	1	Indoor Air Pollution (All Cooking Fuels)	822
Other chronic respiratory diseases	Male	0-6 days	95+ years	Global	1	1	Cumulative Cigarettes (5 Years)	--
Other chronic respiratory diseases	Male	0-6 days	95+ years	Global	1	1	Outdoor Air Pollution (PM2.5)	--
Other chronic respiratory diseases	Male	0-6 days	95+ years	Global	-1	2	Healthcare access and quality index	--
Other chronic respiratory diseases	Male	0-6 days	95+ years	Global	1	2	Elevation Over 1500m (proportion)	267
Other chronic respiratory diseases	Male	0-6 days	95+ years	Global	1	2	Liters of alcohol consumed per capita	268
Other chronic respiratory diseases	Male	0-6 days	95+ years	Global	1	2	Mean BMI	--
Other chronic respiratory diseases	Male	0-6 days	95+ years	Global	-1	3	Socio-demographic Index	555
Digestive diseases	Female	0-6 days	95+ years	Data Rich	-1	1	Sanitation (proportion with access)	989
Digestive diseases	Female	0-6 days	95+ years	Data Rich	1	1	Smoking Prevalence	103
Digestive diseases	Female	0-6 days	95+ years	Data Rich	1	1	Cumulative Cigarettes (10 Years)	145
Digestive diseases	Female	0-6 days	95+ years	Data Rich	1	1	Cumulative Cigarettes (5 Years)	410
Digestive diseases	Female	0-6 days	95+ years	Data Rich	1	1	Liters of alcohol consumed per capita	728
Digestive diseases	Female	0-6 days	95+ years	Data Rich	1	1	Mean BMI	--
Digestive diseases	Female	0-6 days	95+ years	Data Rich	-1	2	Healthcare access and quality index	249
Digestive diseases	Female	0-6 days	95+ years	Data Rich	1	2	Age- and sex-specific SEV for Low fruit	0
Digestive diseases	Female	0-6 days	95+ years	Data Rich	1	2	Age- and sex-specific SEV for Low vegetables	227
Digestive diseases	Female	0-6 days	95+ years	Data Rich	1	2	Age- and sex-specific SEV for High red meat	329
Digestive diseases	Female	0-6 days	95+ years	Data Rich	-1	3	LDI (I$ per capita)	249
Digestive diseases	Female	0-6 days	95+ years	Data Rich	-1	3	Education (years per capita)	501
Digestive diseases	Female	0-6 days	95+ years	Data Rich	-1	3	Socio-demographic Index	--
Digestive diseases	Female	0-6 days	95+ years	Global	-1	1	Sanitation (proportion with access)	988
Digestive diseases	Female	0-6 days	95+ years	Global	1	1	Cumulative Cigarettes (10 Years)	6
Digestive diseases	Female	0-6 days	95+ years	Global	1	1	Cumulative Cigarettes (5 Years)	89
Digestive diseases	Female	0-6 days	95+ years	Global	1	1	Smoking Prevalence	285
Digestive diseases	Female	0-6 days	95+ years	Global	1	1	Liters of alcohol consumed per capita	--
Digestive diseases	Female	0-6 days	95+ years	Global	-1	2	Healthcare access and quality index	429
Digestive diseases	Female	0-6 days	95+ years	Global	1	2	Age- and sex-specific SEV for Low fruit	1
Digestive diseases	Female	0-6 days	95+ years	Global	1	2	Age- and sex-specific SEV for High red meat	199
Digestive diseases	Female	0-6 days	95+ years	Global	1	2	Age- and sex-specific SEV for Low vegetables	307
Digestive diseases	Female	0-6 days	95+ years	Global	1	2	Mean BMI	--
Digestive diseases	Female	0-6 days	95+ years	Global	-1	3	Socio-demographic Index	47
Digestive diseases	Female	0-6 days	95+ years	Global	-1	3	LDI (I$ per capita)	261
Digestive diseases	Female	0-6 days	95+ years	Global	-1	3	Education (years per capita)	703
Digestive diseases	Male	0-6 days	95+ years	Data Rich	-1	1	Sanitation (proportion with access)	250
Digestive diseases	Male	0-6 days	95+ years	Data Rich	1	1	Cumulative Cigarettes (10 Years)	7
Digestive diseases	Male	0-6 days	95+ years	Data Rich	1	1	Smoking Prevalence	157
Digestive diseases	Male	0-6 days	95+ years	Data Rich	1	1	Cumulative Cigarettes (5 Years)	165
Digestive diseases	Male	0-6 days	95+ years	Data Rich	1	1	Liters of alcohol consumed per capita	947
Digestive diseases	Male	0-6 days	95+ years	Data Rich	-1	2	Healthcare access and quality index	566
Digestive diseases	Male	0-6 days	95+ years	Data Rich	1	2	Age- and sex-specific SEV for Low fruit	0
Digestive diseases	Male	0-6 days	95+ years	Data Rich	1	2	Age- and sex-specific SEV for High red meat	88
Digestive diseases	Male	0-6 days	95+ years	Data Rich	1	2	Age- and sex-specific SEV for Low vegetables	93
Digestive diseases	Male	0-6 days	95+ years	Data Rich	1	2	Mean BMI	--
Digestive diseases	Male	0-6 days	95+ years	Data Rich	-1	3	Socio-demographic Index	105
Digestive diseases	Male	0-6 days	95+ years	Data Rich	-1	3	LDI (I$ per capita)	188
Digestive diseases	Male	0-6 days	95+ years	Data Rich	-1	3	Education (years per capita)	675
Digestive diseases	Male	0-6 days	95+ years	Global	-1	1	Sanitation (proportion with access)	927
Digestive diseases	Male	0-6 days	95+ years	Global	1	1	Cumulative Cigarettes (10 Years)	2
Digestive diseases	Male	0-6 days	95+ years	Global	1	1	Cumulative Cigarettes (5 Years)	62
Digestive diseases	Male	0-6 days	95+ years	Global	1	1	Smoking Prevalence	449
Digestive diseases	Male	0-6 days	95+ years	Global	1	1	Liters of alcohol consumed per capita	--
Digestive diseases	Male	0-6 days	95+ years	Global	-1	2	Healthcare access and quality index	249
Digestive diseases	Male	0-6 days	95+ years	Global	1	2	Age- and sex-specific SEV for Low fruit	5
Digestive diseases	Male	0-6 days	95+ years	Global	1	2	Age- and sex-specific SEV for High red meat	101
Digestive diseases	Male	0-6 days	95+ years	Global	1	2	Age- and sex-specific SEV for Low vegetables	331
Digestive diseases	Male	0-6 days	95+ years	Global	1	2	Mean BMI	--
Digestive diseases	Male	0-6 days	95+ years	Global	-1	3	Education (years per capita)	598
Digestive diseases	Male	0-6 days	95+ years	Global	-1	3	LDI (I$ per capita)	609
Digestive diseases	Male	0-6 days	95+ years	Global	-1	3	Socio-demographic Index	--
Cirrhosis and other chronic liver diseases	Female	12-23 months	95+ years	Data Rich	-1	1	Hepatitis B vaccine coverage, aged through time, COVID-free (proportion)	83
Cirrhosis and other chronic liver diseases	Female	12-23 months	95+ years	Data Rich	1	1	Vaccine adjusted HbSAg serporevalence age standardized	571
Cirrhosis and other chronic liver diseases	Female	12-23 months	95+ years	Data Rich	1	1	Liters of alcohol consumed per capita	846
Cirrhosis and other chronic liver diseases	Female	12-23 months	95+ years	Data Rich	1	1	Chronic Hepatitis C age standardized	--
Cirrhosis and other chronic liver diseases	Female	12-23 months	95+ years	Data Rich	-1	2	Healthcare access and quality index	344
Cirrhosis and other chronic liver diseases	Female	12-23 months	95+ years	Data Rich	1	2	Diabetes Age-Standardized Prevalence (proportion)	6
Cirrhosis and other chronic liver diseases	Female	12-23 months	95+ years	Data Rich	1	2	Intravenous drug use (proportion by age)	--
Cirrhosis and other chronic liver diseases	Female	12-23 months	95+ years	Data Rich	1	2	Mean BMI	--
Cirrhosis and other chronic liver diseases	Female	12-23 months	95+ years	Data Rich	-1	3	Socio-demographic Index	54
Cirrhosis and other chronic liver diseases	Female	12-23 months	95+ years	Data Rich	-1	3	Education (years per capita)	211
Cirrhosis and other chronic liver diseases	Female	12-23 months	95+ years	Data Rich	-1	3	LDI (I$ per capita)	--
Cirrhosis and other chronic liver diseases	Female	12-23 months	95+ years	Global	-1	1	Hepatitis B vaccine coverage, aged through time, COVID-free (proportion)	732
Cirrhosis and other chronic liver diseases	Female	12-23 months	95+ years	Global	1	1	Vaccine adjusted HbSAg serporevalence age standardized	268
Cirrhosis and other chronic liver diseases	Female	12-23 months	95+ years	Global	1	1	Chronic Hepatitis C age standardized	--
Cirrhosis and other chronic liver diseases	Female	12-23 months	95+ years	Global	1	1	Liters of alcohol consumed per capita	--
Cirrhosis and other chronic liver diseases	Female	12-23 months	95+ years	Global	-1	2	Healthcare access and quality index	365
Cirrhosis and other chronic liver diseases	Female	12-23 months	95+ years	Global	1	2	Diabetes Age-Standardized Prevalence (proportion)	--
Cirrhosis and other chronic liver diseases	Female	12-23 months	95+ years	Global	1	2	Intravenous drug use (proportion by age)	--
Cirrhosis and other chronic liver diseases	Female	12-23 months	95+ years	Global	1	2	Mean BMI	--
Cirrhosis and other chronic liver diseases	Female	12-23 months	95+ years	Global	-1	3	Education (years per capita)	288
Cirrhosis and other chronic liver diseases	Female	12-23 months	95+ years	Global	-1	3	Socio-demographic Index	306
Cirrhosis and other chronic liver diseases	Female	12-23 months	95+ years	Global	-1	3	LDI (I$ per capita)	--
Cirrhosis and other chronic liver diseases	Male	12-23 months	95+ years	Data Rich	-1	1	Hepatitis B vaccine coverage, aged through time, COVID-free (proportion)	70
Cirrhosis and other chronic liver diseases	Male	12-23 months	95+ years	Data Rich	1	1	Vaccine adjusted HbSAg serporevalence age standardized	665
Cirrhosis and other chronic liver diseases	Male	12-23 months	95+ years	Data Rich	1	1	Liters of alcohol consumed per capita	980

Appendix Table S10: CODEm covariates used, level of covariate, and expected direction of covariate by cause, sex, and age								
**Cause**	**Sex**	**Age Start**	**Age End**	**Model Version Type**	**Direction**	**Level**	**Covariate Name**	**Number of Draws**
Cirrhosis and other chronic liver diseases	Male	12-23 months	95+ years	Data Rich	1	1	Chronic Hepatitis C age standardized	--
Cirrhosis and other chronic liver diseases	Male	12-23 months	95+ years	Data Rich	-1	2	Healthcare access and quality index	476
Cirrhosis and other chronic liver diseases	Male	12-23 months	95+ years	Data Rich	1	2	Intravenous drug use (proportion by age)	366
Cirrhosis and other chronic liver diseases	Male	12-23 months	95+ years	Data Rich	1	2	Diabetes Age-Standardized Prevalence (proportion)	--
Cirrhosis and other chronic liver diseases	Male	12-23 months	95+ years	Data Rich	1	2	Mean BMI	--
Cirrhosis and other chronic liver diseases	Male	12-23 months	95+ years	Data Rich	-1	3	Socio-demographic Index	179
Cirrhosis and other chronic liver diseases	Male	12-23 months	95+ years	Data Rich	-1	3	Education (years per capita)	587
Cirrhosis and other chronic liver diseases	Male	12-23 months	95+ years	Data Rich	-1	3	LDI (I$ per capita)	--
Cirrhosis and other chronic liver diseases	Male	12-23 months	95+ years	Global	-1	1	Hepatitis B vaccine coverage, aged through time, COVID-free (proportion)	600
Cirrhosis and other chronic liver diseases	Male	12-23 months	95+ years	Global	1	1	Vaccine adjusted HbSAg serporevalence age standardized	284
Cirrhosis and other chronic liver diseases	Male	12-23 months	95+ years	Global	1	1	Chronic Hepatitis C age standardized	--
Cirrhosis and other chronic liver diseases	Male	12-23 months	95+ years	Global	1	1	Liters of alcohol consumed per capita	--
Cirrhosis and other chronic liver diseases	Male	12-23 months	95+ years	Global	-1	2	Healthcare access and quality index	221
Cirrhosis and other chronic liver diseases	Male	12-23 months	95+ years	Global	1	2	Intravenous drug use (proportion by age)	235
Cirrhosis and other chronic liver diseases	Male	12-23 months	95+ years	Global	1	2	Diabetes Age-Standardized Prevalence (proportion)	--
Cirrhosis and other chronic liver diseases	Male	12-23 months	95+ years	Global	1	2	Mean BMI	--
Cirrhosis and other chronic liver diseases	Male	12-23 months	95+ years	Global	-1	3	Socio-demographic Index	411
Cirrhosis and other chronic liver diseases	Male	12-23 months	95+ years	Global	-1	3	Education (years per capita)	478
Cirrhosis and other chronic liver diseases	Male	12-23 months	95+ years	Global	-1	3	LDI (I$ per capita)	--
Upper digestive system diseases	Female	6-11 months	95+ years	Data Rich	-1	1	Sanitation (proportion with access)	474
Upper digestive system diseases	Female	6-11 months	95+ years	Data Rich	1	1	Cumulative Cigarettes (5 Years)	139
Upper digestive system diseases	Female	6-11 months	95+ years	Data Rich	1	1	Age- and sex-specific SEV for Unsafe water	173
Upper digestive system diseases	Female	6-11 months	95+ years	Data Rich	1	1	Smoking Prevalence	619
Upper digestive system diseases	Female	6-11 months	95+ years	Data Rich	1	1	Cumulative Cigarettes (10 Years)	759
Upper digestive system diseases	Female	6-11 months	95+ years	Data Rich	-1	2	vegetables unadjusted(g)	806
Upper digestive system diseases	Female	6-11 months	95+ years	Data Rich	-1	2	Healthcare access and quality index	978
Upper digestive system diseases	Female	6-11 months	95+ years	Data Rich	1	2	Liters of alcohol consumed per capita	227
Upper digestive system diseases	Female	6-11 months	95+ years	Data Rich	-1	3	Socio-demographic Index	22
Upper digestive system diseases	Female	6-11 months	95+ years	Data Rich	-1	3	Education (years per capita)	602
Upper digestive system diseases	Female	6-11 months	95+ years	Data Rich	-1	3	LDI (I$ per capita)	--
Upper digestive system diseases	Female	6-11 months	95+ years	Global	-1	1	Sanitation (proportion with access)	109
Upper digestive system diseases	Female	6-11 months	95+ years	Global	1	1	Age- and sex-specific SEV for Unsafe water	13
Upper digestive system diseases	Female	6-11 months	95+ years	Global	1	1	Cumulative Cigarettes (10 Years)	396
Upper digestive system diseases	Female	6-11 months	95+ years	Global	1	1	Cumulative Cigarettes (5 Years)	445
Upper digestive system diseases	Female	6-11 months	95+ years	Global	1	1	Smoking Prevalence	625
Upper digestive system diseases	Female	6-11 months	95+ years	Global	-1	2	vegetables unadjusted(g)	609
Upper digestive system diseases	Female	6-11 months	95+ years	Global	-1	2	Healthcare access and quality index	681
Upper digestive system diseases	Female	6-11 months	95+ years	Global	1	2	Liters of alcohol consumed per capita	74
Upper digestive system diseases	Female	6-11 months	95+ years	Global	-1	3	Socio-demographic Index	263
Upper digestive system diseases	Female	6-11 months	95+ years	Global	-1	3	Education (years per capita)	288
Upper digestive system diseases	Female	6-11 months	95+ years	Global	-1	3	LDI (I$ per capita)	--
Upper digestive system diseases	Male	6-11 months	95+ years	Data Rich	-1	1	Sanitation (proportion with access)	501
Upper digestive system diseases	Male	6-11 months	95+ years	Data Rich	1	1	Age- and sex-specific SEV for Unsafe water	160
Upper digestive system diseases	Male	6-11 months	95+ years	Data Rich	1	1	Cumulative Cigarettes (10 Years)	177
Upper digestive system diseases	Male	6-11 months	95+ years	Data Rich	1	1	Cumulative Cigarettes (5 Years)	382
Upper digestive system diseases	Male	6-11 months	95+ years	Data Rich	1	1	Smoking Prevalence	583
Upper digestive system diseases	Male	6-11 months	95+ years	Data Rich	-1	2	vegetables unadjusted(g)	376
Upper digestive system diseases	Male	6-11 months	95+ years	Data Rich	-1	2	Healthcare access and quality index	697
Upper digestive system diseases	Male	6-11 months	95+ years	Data Rich	1	2	Liters of alcohol consumed per capita	124
Upper digestive system diseases	Male	6-11 months	95+ years	Data Rich	-1	3	Socio-demographic Index	303
Upper digestive system diseases	Male	6-11 months	95+ years	Data Rich	-1	3	Education (years per capita)	678
Upper digestive system diseases	Male	6-11 months	95+ years	Data Rich	-1	3	LDI (I$ per capita)	--
Upper digestive system diseases	Male	6-11 months	95+ years	Global	-1	1	Sanitation (proportion with access)	245
Upper digestive system diseases	Male	6-11 months	95+ years	Global	1	1	Age- and sex-specific SEV for Unsafe water	17
Upper digestive system diseases	Male	6-11 months	95+ years	Global	1	1	Cumulative Cigarettes (10 Years)	320
Upper digestive system diseases	Male	6-11 months	95+ years	Global	1	1	Cumulative Cigarettes (5 Years)	375
Upper digestive system diseases	Male	6-11 months	95+ years	Global	1	1	Smoking Prevalence	788
Upper digestive system diseases	Male	6-11 months	95+ years	Global	-1	2	Healthcare access and quality index	535
Upper digestive system diseases	Male	6-11 months	95+ years	Global	-1	2	vegetables unadjusted(g)	718
Upper digestive system diseases	Male	6-11 months	95+ years	Global	1	2	Liters of alcohol consumed per capita	114
Upper digestive system diseases	Male	6-11 months	95+ years	Global	-1	3	Socio-demographic Index	201
Upper digestive system diseases	Male	6-11 months	95+ years	Global	-1	3	Education (years per capita)	350
Upper digestive system diseases	Male	6-11 months	95+ years	Global	-1	3	LDI (I$ per capita)	--
Peptic ulcer disease	Female	6-11 months	95+ years	Data Rich	-1	1	Sanitation (proportion with access)	223
Peptic ulcer disease	Female	6-11 months	95+ years	Data Rich	1	1	Age- and sex-specific SEV for Unsafe water	10
Peptic ulcer disease	Female	6-11 months	95+ years	Data Rich	1	1	Cumulative Cigarettes (5 Years)	134
Peptic ulcer disease	Female	6-11 months	95+ years	Data Rich	1	1	Cumulative Cigarettes (10 Years)	138
Peptic ulcer disease	Female	6-11 months	95+ years	Data Rich	1	1	Smoking Prevalence	771
Peptic ulcer disease	Female	6-11 months	95+ years	Data Rich	-1	2	vegetables unadjusted(g)	427
Peptic ulcer disease	Female	6-11 months	95+ years	Data Rich	-1	2	Healthcare access and quality index	752
Peptic ulcer disease	Female	6-11 months	95+ years	Data Rich	1	2	Liters of alcohol consumed per capita	472
Peptic ulcer disease	Female	6-11 months	95+ years	Data Rich	-1	3	Socio-demographic Index	205
Peptic ulcer disease	Female	6-11 months	95+ years	Data Rich	-1	3	Education (years per capita)	490
Peptic ulcer disease	Female	6-11 months	95+ years	Data Rich	-1	3	LDI (I$ per capita)	--
Peptic ulcer disease	Female	6-11 months	95+ years	Global	-1	1	Sanitation (proportion with access)	506
Peptic ulcer disease	Female	6-11 months	95+ years	Global	1	1	Age- and sex-specific SEV for Unsafe water	14
Peptic ulcer disease	Female	6-11 months	95+ years	Global	1	1	Cumulative Cigarettes (10 Years)	142
Peptic ulcer disease	Female	6-11 months	95+ years	Global	1	1	Cumulative Cigarettes (5 Years)	444
Peptic ulcer disease	Female	6-11 months	95+ years	Global	1	1	Smoking Prevalence	464
Peptic ulcer disease	Female	6-11 months	95+ years	Global	-1	2	Healthcare access and quality index	681
Peptic ulcer disease	Female	6-11 months	95+ years	Global	-1	2	vegetables unadjusted(g)	748
Peptic ulcer disease	Female	6-11 months	95+ years	Global	1	2	Liters of alcohol consumed per capita	366
Peptic ulcer disease	Female	6-11 months	95+ years	Global	-1	3	Socio-demographic Index	263
Peptic ulcer disease	Female	6-11 months	95+ years	Global	-1	3	Education (years per capita)	333
Peptic ulcer disease	Female	6-11 months	95+ years	Global	-1	3	LDI (I$ per capita)	--
Peptic ulcer disease	Male	6-11 months	95+ years	Data Rich	-1	1	Sanitation (proportion with access)	606
Peptic ulcer disease	Male	6-11 months	95+ years	Data Rich	1	1	Cumulative Cigarettes (10 Years)	4
Peptic ulcer disease	Male	6-11 months	95+ years	Data Rich	1	1	Age- and sex-specific SEV for Unsafe water	219
Peptic ulcer disease	Male	6-11 months	95+ years	Data Rich	1	1	Cumulative Cigarettes (5 Years)	285
Peptic ulcer disease	Male	6-11 months	95+ years	Data Rich	1	1	Smoking Prevalence	561
Peptic ulcer disease	Male	6-11 months	95+ years	Data Rich	-1	2	vegetables unadjusted(g)	101
Peptic ulcer disease	Male	6-11 months	95+ years	Data Rich	-1	2	Healthcare access and quality index	999
Peptic ulcer disease	Male	6-11 months	95+ years	Data Rich	1	2	Liters of alcohol consumed per capita	995
Peptic ulcer disease	Male	6-11 months	95+ years	Data Rich	-1	3	Socio-demographic Index	36
Peptic ulcer disease	Male	6-11 months	95+ years	Data Rich	-1	3	Education (years per capita)	859
Peptic ulcer disease	Male	6-11 months	95+ years	Data Rich	-1	3	LDI (I$ per capita)	--
Peptic ulcer disease	Male	6-11 months	95+ years	Global	-1	1	Sanitation (proportion with access)	340
Peptic ulcer disease	Male	6-11 months	95+ years	Global	1	1	Age- and sex-specific SEV for Unsafe water	27
Peptic ulcer disease	Male	6-11 months	95+ years	Global	1	1	Cumulative Cigarettes (5 Years)	229
Peptic ulcer disease	Male	6-11 months	95+ years	Global	1	1	Cumulative Cigarettes (10 Years)	428
Peptic ulcer disease	Male	6-11 months	95+ years	Global	1	1	Smoking Prevalence	527
Peptic ulcer disease	Male	6-11 months	95+ years	Global	-1	2	vegetables unadjusted(g)	261
Peptic ulcer disease	Male	6-11 months	95+ years	Global	-1	2	Healthcare access and quality index	838
Peptic ulcer disease	Male	6-11 months	95+ years	Global	1	2	Liters of alcohol consumed per capita	167
Peptic ulcer disease	Male	6-11 months	95+ years	Global	-1	3	Socio-demographic Index	324
Peptic ulcer disease	Male	6-11 months	95+ years	Global	-1	3	Education (years per capita)	338
Peptic ulcer disease	Male	6-11 months	95+ years	Global	-1	3	LDI (I$ per capita)	--
Gastritis and duodenitis	Female	6-11 months	95+ years	Data Rich	-1	1	Sanitation (proportion with access)	677
Gastritis and duodenitis	Female	6-11 months	95+ years	Data Rich	1	1	Age- and sex-specific SEV for Unsafe water	323

Appendix Table S10: CODEm covariates used, level of covariate, and expected direction of covariate by cause, sex, and age

Cause	Sex	Age Start	Age End	Model Version Type	Direction	Level	Covariate Name	Number of Draws
Gastritis and duodenitis	Female	6-11 months	95+ years	Data Rich	-1	2	vegetables unadjusted(g)	150
Gastritis and duodenitis	Female	6-11 months	95+ years	Data Rich	-1	2	Healthcare access and quality index	--
Gastritis and duodenitis	Female	6-11 months	95+ years	Data Rich	1	2	Cumulative Cigarettes (5 Years)	149
Gastritis and duodenitis	Female	6-11 months	95+ years	Data Rich	1	2	Smoking Prevalence	450
Gastritis and duodenitis	Female	6-11 months	95+ years	Data Rich	1	2	Cumulative Cigarettes (10 Years)	451
Gastritis and duodenitis	Female	6-11 months	95+ years	Data Rich	1	2	Liters of alcohol consumed per capita	600
Gastritis and duodenitis	Female	6-11 months	95+ years	Data Rich	-1	3	Education (years per capita)	704
Gastritis and duodenitis	Female	6-11 months	95+ years	Data Rich	-1	3	LDI (I$ per capita)	--
Gastritis and duodenitis	Female	6-11 months	95+ years	Data Rich	-1	3	Socio-demographic Index	--
Gastritis and duodenitis	Female	6-11 months	95+ years	Global	-1	1	Sanitation (proportion with access)	306
Gastritis and duodenitis	Female	6-11 months	95+ years	Global	1	1	Age- and sex-specific SEV for Unsafe water	694
Gastritis and duodenitis	Female	6-11 months	95+ years	Global	-1	2	Healthcare access and quality index	362
Gastritis and duodenitis	Female	6-11 months	95+ years	Global	-1	2	vegetables unadjusted(g)	662
Gastritis and duodenitis	Female	6-11 months	95+ years	Global	1	2	Cumulative Cigarettes (10 Years)	99
Gastritis and duodenitis	Female	6-11 months	95+ years	Global	1	2	Smoking Prevalence	99
Gastritis and duodenitis	Female	6-11 months	95+ years	Global	1	2	Liters of alcohol consumed per capita	140
Gastritis and duodenitis	Female	6-11 months	95+ years	Global	1	2	Cumulative Cigarettes (5 Years)	201
Gastritis and duodenitis	Female	6-11 months	95+ years	Global	-1	3	Socio-demographic Index	113
Gastritis and duodenitis	Female	6-11 months	95+ years	Global	-1	3	Education (years per capita)	534
Gastritis and duodenitis	Female	6-11 months	95+ years	Global	-1	3	LDI (I$ per capita)	--
Gastritis and duodenitis	Male	6-11 months	95+ years	Data Rich	-1	1	Sanitation (proportion with access)	1000
Gastritis and duodenitis	Male	6-11 months	95+ years	Data Rich	1	1	Age- and sex-specific SEV for Unsafe water	--
Gastritis and duodenitis	Male	6-11 months	95+ years	Data Rich	-1	2	Healthcare access and quality index	633
Gastritis and duodenitis	Male	6-11 months	95+ years	Data Rich	-1	2	vegetables unadjusted(g)	--
Gastritis and duodenitis	Male	6-11 months	95+ years	Data Rich	1	2	Cumulative Cigarettes (10 Years)	273
Gastritis and duodenitis	Male	6-11 months	95+ years	Data Rich	1	2	Cumulative Cigarettes (5 Years)	360
Gastritis and duodenitis	Male	6-11 months	95+ years	Data Rich	1	2	Liters of alcohol consumed per capita	--
Gastritis and duodenitis	Male	6-11 months	95+ years	Data Rich	1	2	Smoking Prevalence	--
Gastritis and duodenitis	Male	6-11 months	95+ years	Data Rich	-1	3	Education (years per capita)	208
Gastritis and duodenitis	Male	6-11 months	95+ years	Data Rich	-1	3	LDI (I$ per capita)	--
Gastritis and duodenitis	Male	6-11 months	95+ years	Data Rich	-1	3	Socio-demographic Index	--
Gastritis and duodenitis	Male	6-11 months	95+ years	Global	-1	1	Sanitation (proportion with access)	--
Gastritis and duodenitis	Male	6-11 months	95+ years	Global	1	1	Age- and sex-specific SEV for Unsafe water	--
Gastritis and duodenitis	Male	6-11 months	95+ years	Global	-1	2	Healthcare access and quality index	726
Gastritis and duodenitis	Male	6-11 months	95+ years	Global	-1	2	vegetables unadjusted(g)	726
Gastritis and duodenitis	Male	6-11 months	95+ years	Global	1	2	Cumulative Cigarettes (10 Years)	726
Gastritis and duodenitis	Male	6-11 months	95+ years	Global	1	2	Cumulative Cigarettes (5 Years)	--
Gastritis and duodenitis	Male	6-11 months	95+ years	Global	1	2	Liters of alcohol consumed per capita	--
Gastritis and duodenitis	Male	6-11 months	95+ years	Global	1	2	Smoking Prevalence	--
Gastritis and duodenitis	Male	6-11 months	95+ years	Global	-1	3	Socio-demographic Index	274
Gastritis and duodenitis	Male	6-11 months	95+ years	Global	-1	3	Education (years per capita)	627
Gastritis and duodenitis	Male	6-11 months	95+ years	Global	-1	3	LDI (I$ per capita)	--
Appendicitis	Female	12-23 months	95+ years	Data Rich	-1	2	Healthcare access and quality index	564
Appendicitis	Female	12-23 months	95+ years	Data Rich	1	2	Age- and sex-specific SEV for Low fruit	142
Appendicitis	Female	12-23 months	95+ years	Data Rich	1	2	Age- and sex-specific SEV for Low vegetables	436
Appendicitis	Female	12-23 months	95+ years	Data Rich	-1	3	Education (years per capita)	186
Appendicitis	Female	12-23 months	95+ years	Data Rich	-1	3	Socio-demographic Index	321
Appendicitis	Female	12-23 months	95+ years	Data Rich	-1	3	LDI (I$ per capita)	--
Appendicitis	Female	12-23 months	95+ years	Global	-1	2	Healthcare access and quality index	268
Appendicitis	Female	12-23 months	95+ years	Global	1	2	Age- and sex-specific SEV for Low fruit	391
Appendicitis	Female	12-23 months	95+ years	Global	1	2	Age- and sex-specific SEV for Low vegetables	640
Appendicitis	Female	12-23 months	95+ years	Global	-1	3	Education (years per capita)	381
Appendicitis	Female	12-23 months	95+ years	Global	-1	3	LDI (I$ per capita)	--
Appendicitis	Female	12-23 months	95+ years	Global	-1	3	Socio-demographic Index	--
Appendicitis	Male	12-23 months	95+ years	Data Rich	-1	2	Healthcare access and quality index	321
Appendicitis	Male	12-23 months	95+ years	Data Rich	1	2	Age- and sex-specific SEV for Low fruit	250
Appendicitis	Male	12-23 months	95+ years	Data Rich	1	2	Age- and sex-specific SEV for Low vegetables	571
Appendicitis	Male	12-23 months	95+ years	Data Rich	-1	3	Education (years per capita)	243
Appendicitis	Male	12-23 months	95+ years	Data Rich	-1	3	LDI (I$ per capita)	--
Appendicitis	Male	12-23 months	95+ years	Data Rich	-1	3	Socio-demographic Index	--
Appendicitis	Male	12-23 months	95+ years	Global	-1	2	Healthcare access and quality index	--
Appendicitis	Male	12-23 months	95+ years	Global	1	2	Age- and sex-specific SEV for Low fruit	489
Appendicitis	Male	12-23 months	95+ years	Global	1	2	Age- and sex-specific SEV for Low vegetables	1000
Appendicitis	Male	12-23 months	95+ years	Global	-1	3	Education (years per capita)	593
Appendicitis	Male	12-23 months	95+ years	Global	-1	3	LDI (I$ per capita)	--
Appendicitis	Male	12-23 months	95+ years	Global	-1	3	Socio-demographic Index	--
Paralytic ileus and intestinal obstruction	Female	0-6 days	95+ years	Data Rich	-1	2	Healthcare access and quality index	323
Paralytic ileus and intestinal obstruction	Female	0-6 days	95+ years	Data Rich	-1	2	vegetables unadjusted(g)	677
Paralytic ileus and intestinal obstruction	Female	0-6 days	95+ years	Data Rich	-1	2	fruits unadjusted(g)	--
Paralytic ileus and intestinal obstruction	Female	0-6 days	95+ years	Data Rich	-1	3	Education (years per capita)	223
Paralytic ileus and intestinal obstruction	Female	0-6 days	95+ years	Data Rich	-1	3	Socio-demographic Index	268
Paralytic ileus and intestinal obstruction	Female	0-6 days	95+ years	Data Rich	-1	3	LDI (I$ per capita)	--
Paralytic ileus and intestinal obstruction	Female	0-6 days	95+ years	Global	-1	2	vegetables unadjusted(g)	214
Paralytic ileus and intestinal obstruction	Female	0-6 days	95+ years	Global	-1	2	fruits unadjusted(g)	482
Paralytic ileus and intestinal obstruction	Female	0-6 days	95+ years	Global	-1	2	Healthcare access and quality index	662
Paralytic ileus and intestinal obstruction	Female	0-6 days	95+ years	Global	-1	3	Socio-demographic Index	204
Paralytic ileus and intestinal obstruction	Female	0-6 days	95+ years	Global	-1	3	Education (years per capita)	410
Paralytic ileus and intestinal obstruction	Female	0-6 days	95+ years	Global	-1	3	LDI (I$ per capita)	--
Paralytic ileus and intestinal obstruction	Male	0-6 days	95+ years	Data Rich	-1	2	Healthcare access and quality index	210
Paralytic ileus and intestinal obstruction	Male	0-6 days	95+ years	Data Rich	-1	2	fruits unadjusted(g)	418
Paralytic ileus and intestinal obstruction	Male	0-6 days	95+ years	Data Rich	-1	2	vegetables unadjusted(g)	673
Paralytic ileus and intestinal obstruction	Male	0-6 days	95+ years	Data Rich	-1	3	Socio-demographic Index	172
Paralytic ileus and intestinal obstruction	Male	0-6 days	95+ years	Data Rich	-1	3	Education (years per capita)	262
Paralytic ileus and intestinal obstruction	Male	0-6 days	95+ years	Data Rich	-1	3	LDI (I$ per capita)	--
Paralytic ileus and intestinal obstruction	Male	0-6 days	95+ years	Global	-1	2	vegetables unadjusted(g)	371
Paralytic ileus and intestinal obstruction	Male	0-6 days	95+ years	Global	-1	2	fruits unadjusted(g)	529
Paralytic ileus and intestinal obstruction	Male	0-6 days	95+ years	Global	-1	2	Healthcare access and quality index	799
Paralytic ileus and intestinal obstruction	Male	0-6 days	95+ years	Global	-1	3	Socio-demographic Index	134
Paralytic ileus and intestinal obstruction	Male	0-6 days	95+ years	Global	-1	3	Education (years per capita)	494
Paralytic ileus and intestinal obstruction	Male	0-6 days	95+ years	Global	-1	3	LDI (I$ per capita)	--
Inguinal, femoral, and abdominal hernia	Female	0-6 days	95+ years	Data Rich	-1	1	Mean BMI	945
Inguinal, femoral, and abdominal hernia	Female	0-6 days	95+ years	Data Rich	1	1	Cumulative Cigarettes (10 Years)	149
Inguinal, femoral, and abdominal hernia	Female	0-6 days	95+ years	Data Rich	1	1	Cumulative Cigarettes (5 Years)	--
Inguinal, femoral, and abdominal hernia	Female	0-6 days	95+ years	Data Rich	1	1	Smoking Prevalence	--
Inguinal, femoral, and abdominal hernia	Female	0-6 days	95+ years	Data Rich	-1	2	Healthcare access and quality index	656
Inguinal, femoral, and abdominal hernia	Female	0-6 days	95+ years	Data Rich	-1	3	Education (years per capita)	123
Inguinal, femoral, and abdominal hernia	Female	0-6 days	95+ years	Data Rich	-1	3	LDI (I$ per capita)	--
Inguinal, femoral, and abdominal hernia	Female	0-6 days	95+ years	Data Rich	1	3	Socio-demographic Index	282
Inguinal, femoral, and abdominal hernia	Female	0-6 days	95+ years	Global	-1	1	Mean BMI	781
Inguinal, femoral, and abdominal hernia	Female	0-6 days	95+ years	Global	1	1	Smoking Prevalence	54
Inguinal, femoral, and abdominal hernia	Female	0-6 days	95+ years	Global	1	1	Cumulative Cigarettes (10 Years)	71
Inguinal, femoral, and abdominal hernia	Female	0-6 days	95+ years	Global	1	1	Cumulative Cigarettes (5 Years)	94
Inguinal, femoral, and abdominal hernia	Female	0-6 days	95+ years	Global	-1	2	Healthcare access and quality index	496
Inguinal, femoral, and abdominal hernia	Female	0-6 days	95+ years	Global	-1	3	Education (years per capita)	447
Inguinal, femoral, and abdominal hernia	Female	0-6 days	95+ years	Global	-1	3	LDI (I$ per capita)	--
Inguinal, femoral, and abdominal hernia	Female	0-6 days	95+ years	Global	1	3	Socio-demographic Index	--
Inguinal, femoral, and abdominal hernia	Male	0-6 days	95+ years	Data Rich	-1	1	Mean BMI	721
Inguinal, femoral, and abdominal hernia	Male	0-6 days	95+ years	Data Rich	1	1	Cumulative Cigarettes (10 Years)	52
Inguinal, femoral, and abdominal hernia	Male	0-6 days	95+ years	Data Rich	1	1	Smoking Prevalence	121
Inguinal, femoral, and abdominal hernia	Male	0-6 days	95+ years	Data Rich	1	1	Cumulative Cigarettes (5 Years)	158
Inguinal, femoral, and abdominal hernia	Male	0-6 days	95+ years	Data Rich	-1	2	Healthcare access and quality index	476
Inguinal, femoral, and abdominal hernia	Male	0-6 days	95+ years	Data Rich	-1	3	Education (years per capita)	118
Inguinal, femoral, and abdominal hernia	Male	0-6 days	95+ years	Data Rich	-1	3	LDI (I$ per capita)	--
Inguinal, femoral, and abdominal hernia	Male	0-6 days	95+ years	Data Rich	1	3	Socio-demographic Index	--

Appendix Table S10: CODEm covariates used, level of covariate, and expected direction of covariate by cause, sex, and age

Cause	Sex	Age Start	Age End	Model Version Type	Direction	Level	Covariate Name	Number of Draws
Inguinal, femoral, and abdominal hernia	Male	0-6 days	95+ years	Global	-1	1	Mean BMI	667
Inguinal, femoral, and abdominal hernia	Male	0-6 days	95+ years	Global	1	1	Smoking Prevalence	86
Inguinal, femoral, and abdominal hernia	Male	0-6 days	95+ years	Global	1	1	Cumulative Cigarettes (5 Years)	141
Inguinal, femoral, and abdominal hernia	Male	0-6 days	95+ years	Global	1	1	Cumulative Cigarettes (10 Years)	154
Inguinal, femoral, and abdominal hernia	Male	0-6 days	95+ years	Global	-1	2	Healthcare access and quality index	455
Inguinal, femoral, and abdominal hernia	Male	0-6 days	95+ years	Global	-1	3	Education (years per capita)	537
Inguinal, femoral, and abdominal hernia	Male	0-6 days	95+ years	Global	-1	3	LDI (I$ per capita)	--
Inguinal, femoral, and abdominal hernia	Male	0-6 days	95+ years	Global	1	3	Socio-demographic Index	--
Inflammatory bowel disease	Female	2-4 years	95+ years	Data Rich	1	1	Age- and sex-specific SEV for High red meat	--
Inflammatory bowel disease	Female	2-4 years	95+ years	Data Rich	1	1	Age- and sex-specific SEV for Low PUFA	--
Inflammatory bowel disease	Female	2-4 years	95+ years	Data Rich	1	1	Age- and sex-specific SEV for Low fruit	--
Inflammatory bowel disease	Female	2-4 years	95+ years	Data Rich	1	1	Age- and sex-specific SEV for Low vegetables	--
Inflammatory bowel disease	Female	2-4 years	95+ years	Data Rich	-1	2	Healthcare access and quality index	633
Inflammatory bowel disease	Female	2-4 years	95+ years	Data Rich	-1	2	Latitude 15 to 30 (proportion)	--
Inflammatory bowel disease	Female	2-4 years	95+ years	Data Rich	1	2	Latitude 30 to 45 (proportion)	633
Inflammatory bowel disease	Female	2-4 years	95+ years	Data Rich	1	2	Latitude Over 45 (proportion)	--
Inflammatory bowel disease	Female	2-4 years	95+ years	Data Rich	-1	3	Education (years per capita)	--
Inflammatory bowel disease	Female	2-4 years	95+ years	Data Rich	1	3	Socio-demographic Index	208
Inflammatory bowel disease	Female	2-4 years	95+ years	Data Rich	1	3	LDI (I$ per capita)	--
Inflammatory bowel disease	Female	2-4 years	95+ years	Global	1	1	Age- and sex-specific SEV for Low vegetables	1000
Inflammatory bowel disease	Female	2-4 years	95+ years	Global	1	1	Age- and sex-specific SEV for High red meat	--
Inflammatory bowel disease	Female	2-4 years	95+ years	Global	1	1	Age- and sex-specific SEV for Low PUFA	--
Inflammatory bowel disease	Female	2-4 years	95+ years	Global	1	1	Age- and sex-specific SEV for Low fruit	--
Inflammatory bowel disease	Female	2-4 years	95+ years	Global	-1	2	Healthcare access and quality index	593
Inflammatory bowel disease	Female	2-4 years	95+ years	Global	-1	2	Latitude 15 to 30 (proportion)	--
Inflammatory bowel disease	Female	2-4 years	95+ years	Global	1	2	Latitude 30 to 45 (proportion)	--
Inflammatory bowel disease	Female	2-4 years	95+ years	Global	1	2	Latitude Over 45 (proportion)	--
Inflammatory bowel disease	Female	2-4 years	95+ years	Global	-1	3	Education (years per capita)	210
Inflammatory bowel disease	Female	2-4 years	95+ years	Global	1	3	Socio-demographic Index	326
Inflammatory bowel disease	Female	2-4 years	95+ years	Global	1	3	LDI (I$ per capita)	--
Inflammatory bowel disease	Male	2-4 years	95+ years	Data Rich	1	1	Age- and sex-specific SEV for Low vegetables	1000
Inflammatory bowel disease	Male	2-4 years	95+ years	Data Rich	1	1	Age- and sex-specific SEV for High red meat	--
Inflammatory bowel disease	Male	2-4 years	95+ years	Data Rich	1	1	Age- and sex-specific SEV for Low PUFA	--
Inflammatory bowel disease	Male	2-4 years	95+ years	Data Rich	1	1	Age- and sex-specific SEV for Low fruit	--
Inflammatory bowel disease	Male	2-4 years	95+ years	Data Rich	-1	2	Healthcare access and quality index	564
Inflammatory bowel disease	Male	2-4 years	95+ years	Data Rich	-1	2	Latitude 15 to 30 (proportion)	--
Inflammatory bowel disease	Male	2-4 years	95+ years	Data Rich	1	2	Latitude 30 to 45 (proportion)	564
Inflammatory bowel disease	Male	2-4 years	95+ years	Data Rich	1	2	Latitude Over 45 (proportion)	--
Inflammatory bowel disease	Male	2-4 years	95+ years	Data Rich	-1	3	Education (years per capita)	--
Inflammatory bowel disease	Male	2-4 years	95+ years	Data Rich	1	3	Socio-demographic Index	463
Inflammatory bowel disease	Male	2-4 years	95+ years	Data Rich	1	3	LDI (I$ per capita)	--
Inflammatory bowel disease	Male	2-4 years	95+ years	Global	1	1	Age- and sex-specific SEV for Low fruit	153
Inflammatory bowel disease	Male	2-4 years	95+ years	Global	1	1	Age- and sex-specific SEV for Low vegetables	847
Inflammatory bowel disease	Male	2-4 years	95+ years	Global	1	1	Age- and sex-specific SEV for High red meat	--
Inflammatory bowel disease	Male	2-4 years	95+ years	Global	1	1	Age- and sex-specific SEV for Low PUFA	--
Inflammatory bowel disease	Male	2-4 years	95+ years	Global	-1	2	Healthcare access and quality index	443
Inflammatory bowel disease	Male	2-4 years	95+ years	Global	-1	2	Latitude 15 to 30 (proportion)	--
Inflammatory bowel disease	Male	2-4 years	95+ years	Global	1	2	Latitude 30 to 45 (proportion)	--
Inflammatory bowel disease	Male	2-4 years	95+ years	Global	1	2	Latitude Over 45 (proportion)	--
Inflammatory bowel disease	Male	2-4 years	95+ years	Global	-1	3	Education (years per capita)	184
Inflammatory bowel disease	Male	2-4 years	95+ years	Global	1	3	LDI (I$ per capita)	--
Inflammatory bowel disease	Male	2-4 years	95+ years	Global	1	3	Socio-demographic Index	--
Ulcerative colitis	Female	2-4 years	95+ years	Data Rich	1	1	Age- and sex-specific SEV for High red meat	385
Ulcerative colitis	Female	2-4 years	95+ years	Data Rich	1	1	Age- and sex-specific SEV for Low vegetables	881
Ulcerative colitis	Female	2-4 years	95+ years	Data Rich	1	1	Age- and sex-specific SEV for Low PUFA	--
Ulcerative colitis	Female	2-4 years	95+ years	Data Rich	1	1	Age- and sex-specific SEV for Low fruit	--
Ulcerative colitis	Female	2-4 years	95+ years	Data Rich	-1	2	Latitude 15 to 30 (proportion)	127
Ulcerative colitis	Female	2-4 years	95+ years	Data Rich	-1	2	Healthcare access and quality index	--
Ulcerative colitis	Female	2-4 years	95+ years	Data Rich	1	2	Latitude 30 to 45 (proportion)	290
Ulcerative colitis	Female	2-4 years	95+ years	Data Rich	1	2	Latitude Over 45 (proportion)	--
Ulcerative colitis	Female	2-4 years	95+ years	Data Rich	-1	3	Education (years per capita)	--
Ulcerative colitis	Female	2-4 years	95+ years	Data Rich	1	3	Socio-demographic Index	198
Ulcerative colitis	Female	2-4 years	95+ years	Data Rich	1	3	LDI (I$ per capita)	--
Ulcerative colitis	Female	2-4 years	95+ years	Global	1	1	Age- and sex-specific SEV for Low vegetables	1000
Ulcerative colitis	Female	2-4 years	95+ years	Global	1	1	Age- and sex-specific SEV for High red meat	--
Ulcerative colitis	Female	2-4 years	95+ years	Global	1	1	Age- and sex-specific SEV for Low PUFA	--
Ulcerative colitis	Female	2-4 years	95+ years	Global	1	1	Age- and sex-specific SEV for Low fruit	--
Ulcerative colitis	Female	2-4 years	95+ years	Global	-1	2	Healthcare access and quality index	--
Ulcerative colitis	Female	2-4 years	95+ years	Global	-1	2	Latitude 15 to 30 (proportion)	--
Ulcerative colitis	Female	2-4 years	95+ years	Global	1	2	Latitude 30 to 45 (proportion)	--
Ulcerative colitis	Female	2-4 years	95+ years	Global	1	2	Latitude Over 45 (proportion)	--
Ulcerative colitis	Female	2-4 years	95+ years	Global	-1	3	Education (years per capita)	--
Ulcerative colitis	Female	2-4 years	95+ years	Global	1	3	Socio-demographic Index	546
Ulcerative colitis	Female	2-4 years	95+ years	Global	1	3	LDI (I$ per capita)	--
Ulcerative colitis	Male	2-4 years	95+ years	Data Rich	1	1	Age- and sex-specific SEV for High red meat	--
Ulcerative colitis	Male	2-4 years	95+ years	Data Rich	1	1	Age- and sex-specific SEV for Low PUFA	--
Ulcerative colitis	Male	2-4 years	95+ years	Data Rich	1	1	Age- and sex-specific SEV for Low fruit	--
Ulcerative colitis	Male	2-4 years	95+ years	Data Rich	1	1	Age- and sex-specific SEV for Low vegetables	--
Ulcerative colitis	Male	2-4 years	95+ years	Data Rich	-1	2	Latitude 15 to 30 (proportion)	432
Ulcerative colitis	Male	2-4 years	95+ years	Data Rich	-1	2	Healthcare access and quality index	--
Ulcerative colitis	Male	2-4 years	95+ years	Data Rich	1	2	Latitude 30 to 45 (proportion)	432
Ulcerative colitis	Male	2-4 years	95+ years	Data Rich	1	2	Latitude Over 45 (proportion)	--
Ulcerative colitis	Male	2-4 years	95+ years	Data Rich	-1	3	Education (years per capita)	--
Ulcerative colitis	Male	2-4 years	95+ years	Data Rich	1	3	Socio-demographic Index	481
Ulcerative colitis	Male	2-4 years	95+ years	Data Rich	1	3	LDI (I$ per capita)	--
Ulcerative colitis	Male	2-4 years	95+ years	Global	1	1	Age- and sex-specific SEV for Low vegetables	1000
Ulcerative colitis	Male	2-4 years	95+ years	Global	1	1	Age- and sex-specific SEV for High red meat	--
Ulcerative colitis	Male	2-4 years	95+ years	Global	1	1	Age- and sex-specific SEV for Low PUFA	--
Ulcerative colitis	Male	2-4 years	95+ years	Global	1	1	Age- and sex-specific SEV for Low fruit	--
Ulcerative colitis	Male	2-4 years	95+ years	Global	-1	2	Healthcare access and quality index	303
Ulcerative colitis	Male	2-4 years	95+ years	Global	-1	2	Latitude 15 to 30 (proportion)	--
Ulcerative colitis	Male	2-4 years	95+ years	Global	1	2	Latitude 30 to 45 (proportion)	--
Ulcerative colitis	Male	2-4 years	95+ years	Global	1	2	Latitude Over 45 (proportion)	--
Ulcerative colitis	Male	2-4 years	95+ years	Global	-1	3	Education (years per capita)	--
Ulcerative colitis	Male	2-4 years	95+ years	Global	1	3	Socio-demographic Index	394
Ulcerative colitis	Male	2-4 years	95+ years	Global	1	3	LDI (I$ per capita)	--
Crohn's disease	Female	2-4 years	95+ years	Data Rich	1	1	Age- and sex-specific SEV for High red meat	490
Crohn's disease	Female	2-4 years	95+ years	Data Rich	1	1	Age- and sex-specific SEV for Low vegetables	808
Crohn's disease	Female	2-4 years	95+ years	Data Rich	1	1	Age- and sex-specific SEV for Low PUFA	--
Crohn's disease	Female	2-4 years	95+ years	Data Rich	1	1	Age- and sex-specific SEV for Low fruit	--
Crohn's disease	Female	2-4 years	95+ years	Data Rich	-1	2	Healthcare access and quality index	--
Crohn's disease	Female	2-4 years	95+ years	Data Rich	-1	2	Latitude 15 to 30 (proportion)	--
Crohn's disease	Female	2-4 years	95+ years	Data Rich	1	2	Latitude Over 45 (proportion)	226

Appendix Table S10: CODEm covariates used, level of covariate, and expected direction of covariate by cause, sex, and age								
**Cause**	**Sex**	**Age Start**	**Age End**	**Model Version Type**	**Direction**	**Level**	**Covariate Name**	**Number of Draws**
Crohn's disease	Female	2-4 years	95+ years	Data Rich	1	2	Latitude 30 to 45 (proportion)	316
Crohn's disease	Female	2-4 years	95+ years	Data Rich	-1	3	Education (years per capita)	--
Crohn's disease	Female	2-4 years	95+ years	Data Rich	1	3	Socio-demographic Index	577
Crohn's disease	Female	2-4 years	95+ years	Data Rich	1	3	LDI (I$ per capita)	--
Crohn's disease	Female	2-4 years	95+ years	Global	1	1	Age- and sex-specific SEV for Low vegetables	356
Crohn's disease	Female	2-4 years	95+ years	Global	1	1	Age- and sex-specific SEV for High red meat	1000
Crohn's disease	Female	2-4 years	95+ years	Global	1	1	Age- and sex-specific SEV for Low PUFA	--
Crohn's disease	Female	2-4 years	95+ years	Global	1	1	Age- and sex-specific SEV for Low fruit	--
Crohn's disease	Female	2-4 years	95+ years	Global	-1	2	Healthcare access and quality index	--
Crohn's disease	Female	2-4 years	95+ years	Global	-1	2	Latitude 15 to 30 (proportion)	--
Crohn's disease	Female	2-4 years	95+ years	Global	1	2	Latitude 30 to 45 (proportion)	396
Crohn's disease	Female	2-4 years	95+ years	Global	1	2	Latitude Over 45 (proportion)	396
Crohn's disease	Female	2-4 years	95+ years	Global	-1	3	Education (years per capita)	--
Crohn's disease	Female	2-4 years	95+ years	Global	1	3	Socio-demographic Index	428
Crohn's disease	Female	2-4 years	95+ years	Global	1	3	LDI (I$ per capita)	--
Crohn's disease	Male	2-4 years	95+ years	Data Rich	1	1	Age- and sex-specific SEV for Low vegetables	1000
Crohn's disease	Male	2-4 years	95+ years	Data Rich	1	1	Age- and sex-specific SEV for High red meat	--
Crohn's disease	Male	2-4 years	95+ years	Data Rich	1	1	Age- and sex-specific SEV for Low PUFA	--
Crohn's disease	Male	2-4 years	95+ years	Data Rich	1	1	Age- and sex-specific SEV for Low fruit	--
Crohn's disease	Male	2-4 years	95+ years	Data Rich	-1	2	Healthcare access and quality index	--
Crohn's disease	Male	2-4 years	95+ years	Data Rich	-1	2	Latitude 15 to 30 (proportion)	--
Crohn's disease	Male	2-4 years	95+ years	Data Rich	1	2	Latitude 30 to 45 (proportion)	568
Crohn's disease	Male	2-4 years	95+ years	Data Rich	1	2	Latitude Over 45 (proportion)	--
Crohn's disease	Male	2-4 years	95+ years	Data Rich	-1	3	Education (years per capita)	--
Crohn's disease	Male	2-4 years	95+ years	Data Rich	1	3	Socio-demographic Index	633
Crohn's disease	Male	2-4 years	95+ years	Data Rich	1	3	LDI (I$ per capita)	--
Crohn's disease	Male	2-4 years	95+ years	Global	1	1	Age- and sex-specific SEV for High red meat	1000
Crohn's disease	Male	2-4 years	95+ years	Global	1	1	Age- and sex-specific SEV for Low PUFA	--
Crohn's disease	Male	2-4 years	95+ years	Global	1	1	Age- and sex-specific SEV for Low fruit	--
Crohn's disease	Male	2-4 years	95+ years	Global	1	1	Age- and sex-specific SEV for Low vegetables	--
Crohn's disease	Male	2-4 years	95+ years	Global	-1	2	Healthcare access and quality index	--
Crohn's disease	Male	2-4 years	95+ years	Global	-1	2	Latitude 15 to 30 (proportion)	--
Crohn's disease	Male	2-4 years	95+ years	Global	1	2	Latitude 30 to 45 (proportion)	511
Crohn's disease	Male	2-4 years	95+ years	Global	1	2	Latitude Over 45 (proportion)	511
Crohn's disease	Male	2-4 years	95+ years	Global	-1	3	Education (years per capita)	--
Crohn's disease	Male	2-4 years	95+ years	Global	1	3	Socio-demographic Index	352
Crohn's disease	Male	2-4 years	95+ years	Global	1	3	LDI (I$ per capita)	--
Vascular intestinal disorders	Female	2-4 years	95+ years	Data Rich	1	1	Systolic Blood Pressure (mmHg)	753
Vascular intestinal disorders	Female	2-4 years	95+ years	Data Rich	1	1	Low-Density Lipoprotein (mmol/L)	1000
Vascular intestinal disorders	Female	2-4 years	95+ years	Data Rich	1	1	Diabetes Fasting Plasma Glucose (mmol/L), age-standardized 25+	--
Vascular intestinal disorders	Female	2-4 years	95+ years	Data Rich	-1	2	Healthcare access and quality index	591
Vascular intestinal disorders	Female	2-4 years	95+ years	Data Rich	1	2	Mean BMI	145
Vascular intestinal disorders	Female	2-4 years	95+ years	Data Rich	1	2	Smoking Prevalence	591
Vascular intestinal disorders	Female	2-4 years	95+ years	Data Rich	-1	3	Socio-demographic Index	101
Vascular intestinal disorders	Female	2-4 years	95+ years	Data Rich	-1	3	Education (years per capita)	--
Vascular intestinal disorders	Female	2-4 years	95+ years	Data Rich	-1	3	LDI (I$ per capita)	--
Vascular intestinal disorders	Female	2-4 years	95+ years	Data Rich	-1	3	Pulses legumes unadjusted(g)	--
Vascular intestinal disorders	Female	2-4 years	95+ years	Data Rich	1	3	Total Fertility Rate	77
Vascular intestinal disorders	Female	2-4 years	95+ years	Data Rich	1	3	Age- and sex-specific SEV for Low fruit	100
Vascular intestinal disorders	Female	2-4 years	95+ years	Data Rich	1	3	Age- and sex-specific SEV for Low vegetables	340
Vascular intestinal disorders	Female	2-4 years	95+ years	Data Rich	1	3	Age- and sex-specific SEV for Low PUFA	--
Vascular intestinal disorders	Female	2-4 years	95+ years	Data Rich	1	3	Age- and sex-specific SEV for Low nuts and seeds	--
Vascular intestinal disorders	Female	2-4 years	95+ years	Data Rich	1	3	Age- and sex-specific SEV for Low omega-3	--
Vascular intestinal disorders	Female	2-4 years	95+ years	Data Rich	1	3	Liters of alcohol consumed per capita	--
Vascular intestinal disorders	Female	2-4 years	95+ years	Global	1	1	Systolic Blood Pressure (mmHg)	264
Vascular intestinal disorders	Female	2-4 years	95+ years	Global	1	1	Low-Density Lipoprotein (mmol/L)	736
Vascular intestinal disorders	Female	2-4 years	95+ years	Global	1	1	Diabetes Fasting Plasma Glucose (mmol/L), age-standardized 25+	--
Vascular intestinal disorders	Female	2-4 years	95+ years	Global	-1	2	Healthcare access and quality index	130
Vascular intestinal disorders	Female	2-4 years	95+ years	Global	1	2	Smoking Prevalence	496
Vascular intestinal disorders	Female	2-4 years	95+ years	Global	1	2	Mean BMI	--
Vascular intestinal disorders	Female	2-4 years	95+ years	Global	-1	3	Pulses legumes unadjusted(g)	84
Vascular intestinal disorders	Female	2-4 years	95+ years	Global	-1	3	Education (years per capita)	177
Vascular intestinal disorders	Female	2-4 years	95+ years	Global	-1	3	LDI (I$ per capita)	--
Vascular intestinal disorders	Female	2-4 years	95+ years	Global	1	3	Age- and sex-specific SEV for Low vegetables	93
Vascular intestinal disorders	Female	2-4 years	95+ years	Global	1	3	Age- and sex-specific SEV for Low fruit	226
Vascular intestinal disorders	Female	2-4 years	95+ years	Global	1	3	Age- and sex-specific SEV for Low PUFA	--
Vascular intestinal disorders	Female	2-4 years	95+ years	Global	1	3	Age- and sex-specific SEV for Low nuts and seeds	--
Vascular intestinal disorders	Female	2-4 years	95+ years	Global	1	3	Age- and sex-specific SEV for Low omega-3	--
Vascular intestinal disorders	Female	2-4 years	95+ years	Global	1	3	Liters of alcohol consumed per capita	--
Vascular intestinal disorders	Female	2-4 years	95+ years	Global	1	3	Total Fertility Rate	--
Vascular intestinal disorders	Male	2-4 years	95+ years	Data Rich	1	1	Systolic Blood Pressure (mmHg)	369
Vascular intestinal disorders	Male	2-4 years	95+ years	Data Rich	1	1	Low-Density Lipoprotein (mmol/L)	631
Vascular intestinal disorders	Male	2-4 years	95+ years	Data Rich	1	1	Diabetes Fasting Plasma Glucose (mmol/L), age-standardized 25+	--
Vascular intestinal disorders	Male	2-4 years	95+ years	Data Rich	-1	2	Healthcare access and quality index	256
Vascular intestinal disorders	Male	2-4 years	95+ years	Data Rich	1	2	Smoking Prevalence	640
Vascular intestinal disorders	Male	2-4 years	95+ years	Data Rich	1	2	Mean BMI	--
Vascular intestinal disorders	Male	2-4 years	95+ years	Data Rich	-1	3	Education (years per capita)	145
Vascular intestinal disorders	Male	2-4 years	95+ years	Data Rich	-1	3	Socio-demographic Index	190
Vascular intestinal disorders	Male	2-4 years	95+ years	Data Rich	-1	3	LDI (I$ per capita)	--
Vascular intestinal disorders	Male	2-4 years	95+ years	Data Rich	-1	3	Pulses legumes unadjusted(g)	--
Vascular intestinal disorders	Male	2-4 years	95+ years	Data Rich	1	3	Age- and sex-specific SEV for Low omega-3	21
Vascular intestinal disorders	Male	2-4 years	95+ years	Data Rich	1	3	Age- and sex-specific SEV for Low nuts and seeds	64
Vascular intestinal disorders	Male	2-4 years	95+ years	Data Rich	1	3	Total Fertility Rate	65
Vascular intestinal disorders	Male	2-4 years	95+ years	Data Rich	1	3	Age- and sex-specific SEV for Low fruit	159
Vascular intestinal disorders	Male	2-4 years	95+ years	Data Rich	1	3	Age- and sex-specific SEV for Low vegetables	436
Vascular intestinal disorders	Male	2-4 years	95+ years	Data Rich	1	3	Age- and sex-specific SEV for Low PUFA	--
Vascular intestinal disorders	Male	2-4 years	95+ years	Data Rich	1	3	Liters of alcohol consumed per capita	--
Vascular intestinal disorders	Male	2-4 years	95+ years	Global	1	1	Low-Density Lipoprotein (mmol/L)	592
Vascular intestinal disorders	Male	2-4 years	95+ years	Global	1	1	Systolic Blood Pressure (mmHg)	637
Vascular intestinal disorders	Male	2-4 years	95+ years	Global	1	1	Diabetes Fasting Plasma Glucose (mmol/L), age-standardized 25+	--
Vascular intestinal disorders	Male	2-4 years	95+ years	Global	-1	2	Healthcare access and quality index	625
Vascular intestinal disorders	Male	2-4 years	95+ years	Global	1	2	Smoking Prevalence	179
Vascular intestinal disorders	Male	2-4 years	95+ years	Global	1	2	Mean BMI	--
Vascular intestinal disorders	Male	2-4 years	95+ years	Global	-1	3	Education (years per capita)	60
Vascular intestinal disorders	Male	2-4 years	95+ years	Global	-1	3	Pulses legumes unadjusted(g)	309
Vascular intestinal disorders	Male	2-4 years	95+ years	Global	-1	3	LDI (I$ per capita)	--
Vascular intestinal disorders	Male	2-4 years	95+ years	Global	1	3	Total Fertility Rate	15
Vascular intestinal disorders	Male	2-4 years	95+ years	Global	1	3	Age- and sex-specific SEV for Low fruit	91
Vascular intestinal disorders	Male	2-4 years	95+ years	Global	1	3	Age- and sex-specific SEV for Low vegetables	258
Vascular intestinal disorders	Male	2-4 years	95+ years	Global	1	3	Age- and sex-specific SEV for Low PUFA	--
Vascular intestinal disorders	Male	2-4 years	95+ years	Global	1	3	Age- and sex-specific SEV for Low nuts and seeds	--
Vascular intestinal disorders	Male	2-4 years	95+ years	Global	1	3	Age- and sex-specific SEV for Low omega-3	--

Appendix Table S10: CODEm covariates used, level of covariate, and expected direction of covariate by cause, sex, and age

Cause	Sex	Age Start	Age End	Model Version Type	Direction	Level	Covariate Name	Number of Draws
Vascular intestinal disorders	Male	2-4 years	95+ years	Global	1	3	Liters of alcohol consumed per capita	--
Gallbladder and biliary diseases	Female	2-4 years	95+ years	Data Rich	1	1	Age- and sex-specific SEV for Low PUFA	1000
Gallbladder and biliary diseases	Female	2-4 years	95+ years	Data Rich	1	1	Mean BMI	--
Gallbladder and biliary diseases	Female	2-4 years	95+ years	Data Rich	-1	2	Healthcare access and quality index	--
Gallbladder and biliary diseases	Female	2-4 years	95+ years	Data Rich	1	2	Liters of alcohol consumed per capita	568
Gallbladder and biliary diseases	Female	2-4 years	95+ years	Data Rich	1	2	Age- and sex-specific SEV for High red meat	--
Gallbladder and biliary diseases	Female	2-4 years	95+ years	Data Rich	1	2	Population Over 65 (proportion)	--
Gallbladder and biliary diseases	Female	2-4 years	95+ years	Data Rich	-1	3	Education (years per capita)	--
Gallbladder and biliary diseases	Female	2-4 years	95+ years	Data Rich	-1	3	LDI (I$ per capita)	--
Gallbladder and biliary diseases	Female	2-4 years	95+ years	Data Rich	-1	3	Socio-demographic Index	--
Gallbladder and biliary diseases	Female	2-4 years	95+ years	Global	1	1	Age- and sex-specific SEV for Low PUFA	1000
Gallbladder and biliary diseases	Female	2-4 years	95+ years	Global	1	1	Mean BMI	--
Gallbladder and biliary diseases	Female	2-4 years	95+ years	Global	-1	2	Healthcare access and quality index	--
Gallbladder and biliary diseases	Female	2-4 years	95+ years	Global	1	2	Age- and sex-specific SEV for High red meat	--
Gallbladder and biliary diseases	Female	2-4 years	95+ years	Global	1	2	Liters of alcohol consumed per capita	--
Gallbladder and biliary diseases	Female	2-4 years	95+ years	Global	1	2	Population Over 65 (proportion)	--
Gallbladder and biliary diseases	Female	2-4 years	95+ years	Global	-1	3	Education (years per capita)	--
Gallbladder and biliary diseases	Female	2-4 years	95+ years	Global	-1	3	LDI (I$ per capita)	--
Gallbladder and biliary diseases	Female	2-4 years	95+ years	Global	-1	3	Socio-demographic Index	--
Gallbladder and biliary diseases	Male	2-4 years	95+ years	Data Rich	1	1	Age- and sex-specific SEV for Low PUFA	--
Gallbladder and biliary diseases	Male	2-4 years	95+ years	Data Rich	1	1	Mean BMI	--
Gallbladder and biliary diseases	Male	2-4 years	95+ years	Data Rich	-1	2	Healthcare access and quality index	427
Gallbladder and biliary diseases	Male	2-4 years	95+ years	Data Rich	1	2	Liters of alcohol consumed per capita	427
Gallbladder and biliary diseases	Male	2-4 years	95+ years	Data Rich	1	2	Age- and sex-specific SEV for High red meat	--
Gallbladder and biliary diseases	Male	2-4 years	95+ years	Data Rich	1	2	Population Over 65 (proportion)	--
Gallbladder and biliary diseases	Male	2-4 years	95+ years	Data Rich	-1	3	Education (years per capita)	248
Gallbladder and biliary diseases	Male	2-4 years	95+ years	Data Rich	-1	3	Socio-demographic Index	325
Gallbladder and biliary diseases	Male	2-4 years	95+ years	Data Rich	-1	3	LDI (I$ per capita)	--
Gallbladder and biliary diseases	Male	2-4 years	95+ years	Global	1	1	Age- and sex-specific SEV for Low PUFA	1000
Gallbladder and biliary diseases	Male	2-4 years	95+ years	Global	1	1	Mean BMI	--
Gallbladder and biliary diseases	Male	2-4 years	95+ years	Global	-1	2	Healthcare access and quality index	--
Gallbladder and biliary diseases	Male	2-4 years	95+ years	Global	1	2	Age- and sex-specific SEV for High red meat	--
Gallbladder and biliary diseases	Male	2-4 years	95+ years	Global	1	2	Liters of alcohol consumed per capita	--
Gallbladder and biliary diseases	Male	2-4 years	95+ years	Global	1	2	Population Over 65 (proportion)	--
Gallbladder and biliary diseases	Male	2-4 years	95+ years	Global	-1	3	Education (years per capita)	--
Gallbladder and biliary diseases	Male	2-4 years	95+ years	Global	-1	3	LDI (I$ per capita)	--
Gallbladder and biliary diseases	Male	2-4 years	95+ years	Global	-1	3	Socio-demographic Index	--
Pancreatitis	Female	2-4 years	95+ years	Data Rich	1	1	Liters of alcohol consumed per capita	--
Pancreatitis	Female	2-4 years	95+ years	Data Rich	1	1	Log-transformed SEV scalar: Pancreatit	--
Pancreatitis	Female	2-4 years	95+ years	Data Rich	-1	2	Healthcare access and quality index	560
Pancreatitis	Female	2-4 years	95+ years	Data Rich	1	2	Mean BMI	560
Pancreatitis	Female	2-4 years	95+ years	Data Rich	-1	3	Education (years per capita)	440
Pancreatitis	Female	2-4 years	95+ years	Data Rich	-1	3	LDI (I$ per capita)	--
Pancreatitis	Female	2-4 years	95+ years	Data Rich	-1	3	Socio-demographic Index	--
Pancreatitis	Female	2-4 years	95+ years	Global	1	1	Liters of alcohol consumed per capita	--
Pancreatitis	Female	2-4 years	95+ years	Global	1	1	Log-transformed SEV scalar: Pancreatit	--
Pancreatitis	Female	2-4 years	95+ years	Global	-1	2	Healthcare access and quality index	546
Pancreatitis	Female	2-4 years	95+ years	Global	1	2	Mean BMI	--
Pancreatitis	Female	2-4 years	95+ years	Global	-1	3	Education (years per capita)	454
Pancreatitis	Female	2-4 years	95+ years	Global	-1	3	LDI (I$ per capita)	--
Pancreatitis	Female	2-4 years	95+ years	Global	-1	3	Socio-demographic Index	--
Pancreatitis	Male	2-4 years	95+ years	Data Rich	1	1	Liters of alcohol consumed per capita	--
Pancreatitis	Male	2-4 years	95+ years	Data Rich	1	1	Log-transformed SEV scalar: Pancreatit	--
Pancreatitis	Male	2-4 years	95+ years	Data Rich	-1	2	Healthcare access and quality index	--
Pancreatitis	Male	2-4 years	95+ years	Data Rich	1	2	Mean BMI	--
Pancreatitis	Male	2-4 years	95+ years	Data Rich	-1	3	Socio-demographic Index	1000
Pancreatitis	Male	2-4 years	95+ years	Data Rich	-1	3	Education (years per capita)	--
Pancreatitis	Male	2-4 years	95+ years	Data Rich	-1	3	LDI (I$ per capita)	--
Pancreatitis	Male	2-4 years	95+ years	Global	1	1	Liters of alcohol consumed per capita	--
Pancreatitis	Male	2-4 years	95+ years	Global	1	1	Log-transformed SEV scalar: Pancreatit	--
Pancreatitis	Male	2-4 years	95+ years	Global	-1	2	Healthcare access and quality index	546
Pancreatitis	Male	2-4 years	95+ years	Global	1	2	Mean BMI	--
Pancreatitis	Male	2-4 years	95+ years	Global	-1	3	Socio-demographic Index	454
Pancreatitis	Male	2-4 years	95+ years	Global	-1	3	Education (years per capita)	--
Pancreatitis	Male	2-4 years	95+ years	Global	-1	3	LDI (I$ per capita)	--
Diverticular disease of intestines	Female	0-6 days	95+ years	Data Rich	-1	1	Age- and sex-specific SEV for Low fiber	406
Diverticular disease of intestines	Female	0-6 days	95+ years	Data Rich	-1	1	Age- and sex-specific SEV for Low vegetables	846
Diverticular disease of intestines	Female	0-6 days	95+ years	Data Rich	-1	1	Age- and sex-specific SEV for Low fruit	--
Diverticular disease of intestines	Female	0-6 days	95+ years	Data Rich	-1	1	Total Physical Activity (MET-min/week), Age-specific	--
Diverticular disease of intestines	Female	0-6 days	95+ years	Data Rich	1	1	Mean BMI	--
Diverticular disease of intestines	Female	0-6 days	95+ years	Data Rich	-1	2	Healthcare access and quality index	--
Diverticular disease of intestines	Female	0-6 days	95+ years	Data Rich	1	2	Cumulative Cigarettes (5 Years)	107
Diverticular disease of intestines	Female	0-6 days	95+ years	Data Rich	1	2	Liters of alcohol consumed per capita	151
Diverticular disease of intestines	Female	0-6 days	95+ years	Data Rich	1	2	Tobacco (cigarettes per capita)	151
Diverticular disease of intestines	Female	0-6 days	95+ years	Data Rich	1	2	Age- and sex-specific SEV for High red meat	553
Diverticular disease of intestines	Female	0-6 days	95+ years	Data Rich	-1	3	LDI (I$ per capita)	323
Diverticular disease of intestines	Female	0-6 days	95+ years	Data Rich	-1	3	Education (years per capita)	--
Diverticular disease of intestines	Female	0-6 days	95+ years	Data Rich	-1	3	Socio-demographic Index	--
Diverticular disease of intestines	Female	0-6 days	95+ years	Data Rich	1	3	Diabetes Fasting Plasma Glucose (mmol/L), age-standardized 25+	121
Diverticular disease of intestines	Female	0-6 days	95+ years	Global	-1	1	Age- and sex-specific SEV for Low fiber	--
Diverticular disease of intestines	Female	0-6 days	95+ years	Global	-1	1	Age- and sex-specific SEV for Low fruit	--
Diverticular disease of intestines	Female	0-6 days	95+ years	Global	-1	1	Age- and sex-specific SEV for Low vegetables	--
Diverticular disease of intestines	Female	0-6 days	95+ years	Global	-1	1	Total Physical Activity (MET-min/week), Age-specific	--
Diverticular disease of intestines	Female	0-6 days	95+ years	Global	1	1	Mean BMI	--
Diverticular disease of intestines	Female	0-6 days	95+ years	Global	-1	2	Healthcare access and quality index	454
Diverticular disease of intestines	Female	0-6 days	95+ years	Global	1	2	Age- and sex-specific SEV for High red meat	454
Diverticular disease of intestines	Female	0-6 days	95+ years	Global	1	2	Cumulative Cigarettes (5 Years)	--
Diverticular disease of intestines	Female	0-6 days	95+ years	Global	1	2	Liters of alcohol consumed per capita	--
Diverticular disease of intestines	Female	0-6 days	95+ years	Global	1	2	Tobacco (cigarettes per capita)	--
Diverticular disease of intestines	Female	0-6 days	95+ years	Global	-1	3	LDI (I$ per capita)	546
Diverticular disease of intestines	Female	0-6 days	95+ years	Global	-1	3	Education (years per capita)	--
Diverticular disease of intestines	Female	0-6 days	95+ years	Global	-1	3	Socio-demographic Index	--
Diverticular disease of intestines	Female	0-6 days	95+ years	Global	1	3	Diabetes Fasting Plasma Glucose (mmol/L), age-standardized 25+	--
Diverticular disease of intestines	Male	0-6 days	95+ years	Data Rich	-1	1	Age- and sex-specific SEV for Low fiber	346
Diverticular disease of intestines	Male	0-6 days	95+ years	Data Rich	-1	1	Age- and sex-specific SEV for Low fruit	412
Diverticular disease of intestines	Male	0-6 days	95+ years	Data Rich	-1	1	Age- and sex-specific SEV for Low vegetables	588
Diverticular disease of intestines	Male	0-6 days	95+ years	Data Rich	-1	1	Total Physical Activity (MET-min/week), Age-specific	--
Diverticular disease of intestines	Male	0-6 days	95+ years	Data Rich	1	1	Mean BMI	--
Diverticular disease of intestines	Male	0-6 days	95+ years	Data Rich	-1	2	Healthcare access and quality index	--
Diverticular disease of intestines	Male	0-6 days	95+ years	Data Rich	1	2	Tobacco (cigarettes per capita)	19
Diverticular disease of intestines	Male	0-6 days	95+ years	Data Rich	1	2	Liters of alcohol consumed per capita	115

Appendix Table S10: CODEm covariates used, level of covariate, and expected direction of covariate by cause, sex, and age

Cause	Sex	Age Start	Age End	Model Version Type	Direction	Level	Covariate Name	Number of Draws
Diverticular disease of intestines	Male	0-6 days	95+ years	Data Rich	1	2	Cumulative Cigarettes (5 Years)	385
Diverticular disease of intestines	Male	0-6 days	95+ years	Data Rich	1	2	Age- and sex-specific SEV for High red meat	565
Diverticular disease of intestines	Male	0-6 days	95+ years	Data Rich	-1	3	LDI (I$ per capita)	163
Diverticular disease of intestines	Male	0-6 days	95+ years	Data Rich	-1	3	Education (years per capita)	--
Diverticular disease of intestines	Male	0-6 days	95+ years	Data Rich	-1	3	Socio-demographic Index	--
Diverticular disease of intestines	Male	0-6 days	95+ years	Data Rich	1	3	Diabetes Fasting Plasma Glucose (mmol/L), age-standardized 25+	80
Diverticular disease of intestines	Male	0-6 days	95+ years	Global	-1	1	Age- and sex-specific SEV for Low vegetables	1000
Diverticular disease of intestines	Male	0-6 days	95+ years	Global	-1	1	Age- and sex-specific SEV for Low fiber	--
Diverticular disease of intestines	Male	0-6 days	95+ years	Global	-1	1	Age- and sex-specific SEV for Low fruit	--
Diverticular disease of intestines	Male	0-6 days	95+ years	Global	-1	1	Total Physical Activity (MET-min/week), Age-specific	--
Diverticular disease of intestines	Male	0-6 days	95+ years	Global	1	1	Mean BMI	--
Diverticular disease of intestines	Male	0-6 days	95+ years	Global	-1	2	Healthcare access and quality index	--
Diverticular disease of intestines	Male	0-6 days	95+ years	Global	1	2	Tobacco (cigarettes per capita)	373
Diverticular disease of intestines	Male	0-6 days	95+ years	Global	1	2	Age- and sex-specific SEV for High red meat	--
Diverticular disease of intestines	Male	0-6 days	95+ years	Global	1	2	Cumulative Cigarettes (5 Years)	--
Diverticular disease of intestines	Male	0-6 days	95+ years	Global	1	2	Liters of alcohol consumed per capita	--
Diverticular disease of intestines	Male	0-6 days	95+ years	Global	-1	3	Education (years per capita)	274
Diverticular disease of intestines	Male	0-6 days	95+ years	Global	-1	3	LDI (I$ per capita)	274
Diverticular disease of intestines	Male	0-6 days	95+ years	Global	-1	3	Socio-demographic Index	--
Diverticular disease of intestines	Male	0-6 days	95+ years	Global	1	3	Diabetes Fasting Plasma Glucose (mmol/L), age-standardized 25+	--
Other digestive diseases	Female	12-23 months	95+ years	Data Rich	1	1	Cumulative Cigarettes (10 Years)	--
Other digestive diseases	Female	12-23 months	95+ years	Data Rich	1	1	Cumulative Cigarettes (5 Years)	--
Other digestive diseases	Female	12-23 months	95+ years	Data Rich	1	1	Liters of alcohol consumed per capita	--
Other digestive diseases	Female	12-23 months	95+ years	Data Rich	1	1	Smoking Prevalence	--
Other digestive diseases	Female	12-23 months	95+ years	Data Rich	-1	2	Healthcare access and quality index	--
Other digestive diseases	Female	12-23 months	95+ years	Data Rich	-1	2	Improved Water Source (proportion with access)	--
Other digestive diseases	Female	12-23 months	95+ years	Data Rich	-1	2	Sanitation (proportion with access)	--
Other digestive diseases	Female	12-23 months	95+ years	Data Rich	1	2	Age- and sex-specific SEV for High red meat	814
Other digestive diseases	Female	12-23 months	95+ years	Data Rich	1	2	Age- and sex-specific SEV for Low vegetables	814
Other digestive diseases	Female	12-23 months	95+ years	Data Rich	1	2	Diabetes Age-Standardized Prevalence (proportion)	814
Other digestive diseases	Female	12-23 months	95+ years	Data Rich	1	2	Mean BMI	814
Other digestive diseases	Female	12-23 months	95+ years	Data Rich	1	2	Age- and sex-specific SEV for Low PUFA	--
Other digestive diseases	Female	12-23 months	95+ years	Data Rich	1	2	Age- and sex-specific SEV for Low fruit	--
Other digestive diseases	Female	12-23 months	95+ years	Data Rich	-1	3	Education (years per capita)	223
Other digestive diseases	Female	12-23 months	95+ years	Data Rich	-1	3	LDI (I$ per capita)	--
Other digestive diseases	Female	12-23 months	95+ years	Data Rich	1	3	Socio-demographic Index	186
Other digestive diseases	Female	12-23 months	95+ years	Global	1	1	Cumulative Cigarettes (10 Years)	--
Other digestive diseases	Female	12-23 months	95+ years	Global	1	1	Cumulative Cigarettes (5 Years)	--
Other digestive diseases	Female	12-23 months	95+ years	Global	1	1	Liters of alcohol consumed per capita	--
Other digestive diseases	Female	12-23 months	95+ years	Global	1	1	Smoking Prevalence	--
Other digestive diseases	Female	12-23 months	95+ years	Global	-1	2	Improved Water Source (proportion with access)	284
Other digestive diseases	Female	12-23 months	95+ years	Global	-1	2	Sanitation (proportion with access)	309
Other digestive diseases	Female	12-23 months	95+ years	Global	-1	2	Healthcare access and quality index	593
Other digestive diseases	Female	12-23 months	95+ years	Global	1	2	Age- and sex-specific SEV for Low fruit	593
Other digestive diseases	Female	12-23 months	95+ years	Global	1	2	Age- and sex-specific SEV for Low vegetables	593
Other digestive diseases	Female	12-23 months	95+ years	Global	1	2	Diabetes Age-Standardized Prevalence (proportion)	593
Other digestive diseases	Female	12-23 months	95+ years	Global	1	2	Age- and sex-specific SEV for High red meat	--
Other digestive diseases	Female	12-23 months	95+ years	Global	1	2	Age- and sex-specific SEV for Low PUFA	--
Other digestive diseases	Female	12-23 months	95+ years	Global	1	2	Mean BMI	--
Other digestive diseases	Female	12-23 months	95+ years	Global	-1	3	Socio-demographic Index	197
Other digestive diseases	Female	12-23 months	95+ years	Global	-1	3	Education (years per capita)	--
Other digestive diseases	Female	12-23 months	95+ years	Global	-1	3	LDI (I$ per capita)	--
Other digestive diseases	Male	12-23 months	95+ years	Data Rich	1	1	Cumulative Cigarettes (10 Years)	--
Other digestive diseases	Male	12-23 months	95+ years	Data Rich	1	1	Cumulative Cigarettes (5 Years)	--
Other digestive diseases	Male	12-23 months	95+ years	Data Rich	1	1	Liters of alcohol consumed per capita	--
Other digestive diseases	Male	12-23 months	95+ years	Data Rich	1	1	Smoking Prevalence	--
Other digestive diseases	Male	12-23 months	95+ years	Data Rich	-1	2	Healthcare access and quality index	--
Other digestive diseases	Male	12-23 months	95+ years	Data Rich	-1	2	Improved Water Source (proportion with access)	--
Other digestive diseases	Male	12-23 months	95+ years	Data Rich	-1	2	Sanitation (proportion with access)	--
Other digestive diseases	Male	12-23 months	95+ years	Data Rich	1	2	Age- and sex-specific SEV for High red meat	1000
Other digestive diseases	Male	12-23 months	95+ years	Data Rich	1	2	Age- and sex-specific SEV for Low vegetables	1000
Other digestive diseases	Male	12-23 months	95+ years	Data Rich	1	2	Diabetes Age-Standardized Prevalence (proportion)	1000
Other digestive diseases	Male	12-23 months	95+ years	Data Rich	1	2	Mean BMI	1000
Other digestive diseases	Male	12-23 months	95+ years	Data Rich	1	2	Age- and sex-specific SEV for Low PUFA	--
Other digestive diseases	Male	12-23 months	95+ years	Data Rich	1	2	Age- and sex-specific SEV for Low fruit	--
Other digestive diseases	Male	12-23 months	95+ years	Data Rich	-1	3	Education (years per capita)	--
Other digestive diseases	Male	12-23 months	95+ years	Data Rich	-1	3	LDI (I$ per capita)	--
Other digestive diseases	Male	12-23 months	95+ years	Data Rich	-1	3	Socio-demographic Index	--
Other digestive diseases	Male	12-23 months	95+ years	Global	1	1	Cumulative Cigarettes (10 Years)	--
Other digestive diseases	Male	12-23 months	95+ years	Global	1	1	Cumulative Cigarettes (5 Years)	--
Other digestive diseases	Male	12-23 months	95+ years	Global	1	1	Smoking Prevalence	--
Other digestive diseases	Male	12-23 months	95+ years	Global	-1	2	Improved Water Source (proportion with access)	162
Other digestive diseases	Male	12-23 months	95+ years	Global	-1	2	Sanitation (proportion with access)	778
Other digestive diseases	Male	12-23 months	95+ years	Global	-1	2	Healthcare access and quality index	--
Other digestive diseases	Male	12-23 months	95+ years	Global	1	2	Age- and sex-specific SEV for Low fruit	497
Other digestive diseases	Male	12-23 months	95+ years	Global	1	2	Age- and sex-specific SEV for Low vegetables	605
Other digestive diseases	Male	12-23 months	95+ years	Global	1	2	Diabetes Age-Standardized Prevalence (proportion)	940
Other digestive diseases	Male	12-23 months	95+ years	Global	1	2	Age- and sex-specific SEV for High red meat	--
Other digestive diseases	Male	12-23 months	95+ years	Global	1	2	Age- and sex-specific SEV for Low PUFA	--
Other digestive diseases	Male	12-23 months	95+ years	Global	1	2	Mean BMI	--
Other digestive diseases	Male	12-23 months	95+ years	Global	-1	3	Education (years per capita)	72
Other digestive diseases	Male	12-23 months	95+ years	Global	-1	3	Socio-demographic Index	273
Other digestive diseases	Male	12-23 months	95+ years	Global	-1	3	LDI (I$ per capita)	--
Parkinson's disease	Female	20-24 years	95+ years	Data Rich	-1	1	Age- and sex-specific SEV for Low fruit	369
Parkinson's disease	Female	20-24 years	95+ years	Data Rich	-1	1	Cumulative Cigarettes (10 Years)	818
Parkinson's disease	Female	20-24 years	95+ years	Data Rich	-1	2	Healthcare access and quality index	130
Parkinson's disease	Female	20-24 years	95+ years	Data Rich	1	2	Absolute value of average latitude	--
Parkinson's disease	Female	20-24 years	95+ years	Data Rich	1	2	Improved Water Source (proportion with access)	--
Parkinson's disease	Female	20-24 years	95+ years	Data Rich	1	2	Low-Density Lipoprotein (mmol/L)	--
Parkinson's disease	Female	20-24 years	95+ years	Data Rich	1	2	Sanitation (proportion with access)	--
Parkinson's disease	Female	20-24 years	95+ years	Data Rich	-1	3	Education (years per capita)	--
Parkinson's disease	Female	20-24 years	95+ years	Data Rich	1	3	Socio-demographic Index	480
Parkinson's disease	Female	20-24 years	95+ years	Data Rich	1	3	LDI (I$ per capita)	--
Parkinson's disease	Female	20-24 years	95+ years	Global	-1	1	Cumulative Cigarettes (10 Years)	1000
Parkinson's disease	Female	20-24 years	95+ years	Global	-1	1	Age- and sex-specific SEV for Low fruit	--
Parkinson's disease	Female	20-24 years	95+ years	Global	-1	2	Healthcare access and quality index	593
Parkinson's disease	Female	20-24 years	95+ years	Global	1	2	Absolute value of average latitude	--
Parkinson's disease	Female	20-24 years	95+ years	Global	1	2	Improved Water Source (proportion with access)	--
Parkinson's disease	Female	20-24 years	95+ years	Global	1	2	Low-Density Lipoprotein (mmol/L)	--
Parkinson's disease	Female	20-24 years	95+ years	Global	1	2	Sanitation (proportion with access)	--
Parkinson's disease	Female	20-24 years	95+ years	Global	-1	3	Education (years per capita)	210

Appendix Table S10: CODEm covariates used, level of covariate, and expected direction of covariate by cause, sex, and age

Cause	Sex	Age Start	Age End	Model Version Type	Direction	Level	Covariate Name	Number of Draws
Parkinson's disease	Female	20-24 years	95+ years	Global	1	3	LDI (I$ per capita)	--
Parkinson's disease	Female	20-24 years	95+ years	Global	1	3	Socio-demographic Index	--
Parkinson's disease	Male	20-24 years	95+ years	Data Rich	-1	1	Cumulative Cigarettes (10 Years)	307
Parkinson's disease	Male	20-24 years	95+ years	Data Rich	-1	1	Age- and sex-specific SEV for Low fruit	922
Parkinson's disease	Male	20-24 years	95+ years	Data Rich	-1	2	Healthcare access and quality index	366
Parkinson's disease	Male	20-24 years	95+ years	Data Rich	1	2	Sanitation (proportion with access)	128
Parkinson's disease	Male	20-24 years	95+ years	Data Rich	1	2	Improved Water Source (proportion with access)	144
Parkinson's disease	Male	20-24 years	95+ years	Data Rich	1	2	Absolute value of average latitude	--
Parkinson's disease	Male	20-24 years	95+ years	Data Rich	1	2	Low-Density Lipoprotein (mmol/L)	--
Parkinson's disease	Male	20-24 years	95+ years	Data Rich	1	3	Socio-demographic Index	438
Parkinson's disease	Male	20-24 years	95+ years	Global	-1	1	Cumulative Cigarettes (10 Years)	532
Parkinson's disease	Male	20-24 years	95+ years	Global	-1	1	Age- and sex-specific SEV for Low fruit	694
Parkinson's disease	Male	20-24 years	95+ years	Global	-1	2	Healthcare access and quality index	701
Parkinson's disease	Male	20-24 years	95+ years	Global	1	2	Absolute value of average latitude	--
Parkinson's disease	Male	20-24 years	95+ years	Global	1	2	Improved Water Source (proportion with access)	--
Parkinson's disease	Male	20-24 years	95+ years	Global	1	2	Low-Density Lipoprotein (mmol/L)	--
Parkinson's disease	Male	20-24 years	95+ years	Global	1	2	Sanitation (proportion with access)	--
Parkinson's disease	Male	20-24 years	95+ years	Global	1	3	Socio-demographic Index	330
Idiopathic epilepsy	Female	0-6 days	95+ years	Data Rich	1	1	Log-transformed SEV scalar: Idiopathic epilepsy	--
Idiopathic epilepsy	Female	0-6 days	95+ years	Data Rich	1	1	Pigs (per capita)	--
Idiopathic epilepsy	Female	0-6 days	95+ years	Data Rich	1	1	Systolic Blood Pressure (mmHg)	--
Idiopathic epilepsy	Female	0-6 days	95+ years	Data Rich	-1	2	Healthcare access and quality index	432
Idiopathic epilepsy	Female	0-6 days	95+ years	Data Rich	1	2	Mean BMI	432
Idiopathic epilepsy	Female	0-6 days	95+ years	Data Rich	1	2	Low-Density Lipoprotein (mmol/L)	--
Idiopathic epilepsy	Female	0-6 days	95+ years	Data Rich	-1	3	Socio-demographic Index	568
Idiopathic epilepsy	Female	0-6 days	95+ years	Data Rich	-1	3	Education (years per capita)	--
Idiopathic epilepsy	Female	0-6 days	95+ years	Data Rich	-1	3	LDI (I$ per capita)	--
Idiopathic epilepsy	Female	0-6 days	95+ years	Data Rich	1	3	Cumulative Cigarettes (10 Years)	--
Idiopathic epilepsy	Female	0-6 days	95+ years	Data Rich	1	3	Cumulative Cigarettes (5 Years)	--
Idiopathic epilepsy	Female	0-6 days	95+ years	Global	1	1	Pigs (per capita)	1000
Idiopathic epilepsy	Female	0-6 days	95+ years	Global	1	1	Log-transformed SEV scalar: Idiopathic epilepsy	--
Idiopathic epilepsy	Female	0-6 days	95+ years	Global	1	1	Systolic Blood Pressure (mmHg)	--
Idiopathic epilepsy	Female	0-6 days	95+ years	Global	-1	2	Healthcare access and quality index	398
Idiopathic epilepsy	Female	0-6 days	95+ years	Global	1	2	Low-Density Lipoprotein (mmol/L)	--
Idiopathic epilepsy	Female	0-6 days	95+ years	Global	1	2	Mean BMI	--
Idiopathic epilepsy	Female	0-6 days	95+ years	Global	-1	3	Education (years per capita)	328
Idiopathic epilepsy	Female	0-6 days	95+ years	Global	-1	3	LDI (I$ per capita)	--
Idiopathic epilepsy	Female	0-6 days	95+ years	Global	-1	3	Socio-demographic Index	--
Idiopathic epilepsy	Female	0-6 days	95+ years	Global	1	3	Cumulative Cigarettes (10 Years)	--
Idiopathic epilepsy	Female	0-6 days	95+ years	Global	1	3	Cumulative Cigarettes (5 Years)	--
Idiopathic epilepsy	Male	0-6 days	95+ years	Data Rich	1	1	Pigs (per capita)	675
Idiopathic epilepsy	Male	0-6 days	95+ years	Data Rich	1	1	Systolic Blood Pressure (mmHg)	753
Idiopathic epilepsy	Male	0-6 days	95+ years	Data Rich	1	1	Log-transformed SEV scalar: Idiopathic epilepsy	--
Idiopathic epilepsy	Male	0-6 days	95+ years	Data Rich	-1	2	Healthcare access and quality index	--
Idiopathic epilepsy	Male	0-6 days	95+ years	Data Rich	1	2	Low-Density Lipoprotein (mmol/L)	--
Idiopathic epilepsy	Male	0-6 days	95+ years	Data Rich	1	2	Mean BMI	--
Idiopathic epilepsy	Male	0-6 days	95+ years	Data Rich	-1	3	Education (years per capita)	297
Idiopathic epilepsy	Male	0-6 days	95+ years	Data Rich	-1	3	Socio-demographic Index	693
Idiopathic epilepsy	Male	0-6 days	95+ years	Data Rich	-1	3	LDI (I$ per capita)	--
Idiopathic epilepsy	Male	0-6 days	95+ years	Data Rich	1	3	Cumulative Cigarettes (10 Years)	--
Idiopathic epilepsy	Male	0-6 days	95+ years	Data Rich	1	3	Cumulative Cigarettes (5 Years)	--
Idiopathic epilepsy	Male	0-6 days	95+ years	Global	1	1	Pigs (per capita)	1000
Idiopathic epilepsy	Male	0-6 days	95+ years	Global	1	1	Log-transformed SEV scalar: Idiopathic epilepsy	--
Idiopathic epilepsy	Male	0-6 days	95+ years	Global	1	1	Systolic Blood Pressure (mmHg)	--
Idiopathic epilepsy	Male	0-6 days	95+ years	Global	-1	2	Healthcare access and quality index	593
Idiopathic epilepsy	Male	0-6 days	95+ years	Global	1	2	Low-Density Lipoprotein (mmol/L)	--
Idiopathic epilepsy	Male	0-6 days	95+ years	Global	1	2	Mean BMI	--
Idiopathic epilepsy	Male	0-6 days	95+ years	Global	-1	3	Socio-demographic Index	222
Idiopathic epilepsy	Male	0-6 days	95+ years	Global	-1	3	Education (years per capita)	506
Idiopathic epilepsy	Male	0-6 days	95+ years	Global	-1	3	LDI (I$ per capita)	--
Idiopathic epilepsy	Male	0-6 days	95+ years	Global	1	3	Cumulative Cigarettes (10 Years)	--
Idiopathic epilepsy	Male	0-6 days	95+ years	Global	1	3	Cumulative Cigarettes (5 Years)	--
Multiple sclerosis	Female	5-9 years	95+ years	Data Rich	1	1	Absolute value of average latitude	1000
Multiple sclerosis	Female	5-9 years	95+ years	Data Rich	-1	2	Healthcare access and quality index	--
Multiple sclerosis	Female	5-9 years	95+ years	Data Rich	1	2	Low-Density Lipoprotein (mmol/L)	--
Multiple sclerosis	Female	5-9 years	95+ years	Data Rich	-1	3	Education (years per capita)	--
Multiple sclerosis	Female	5-9 years	95+ years	Data Rich	1	3	Prevalence of obesity	568
Multiple sclerosis	Female	5-9 years	95+ years	Data Rich	1	3	Cumulative Cigarettes (10 Years)	--
Multiple sclerosis	Female	5-9 years	95+ years	Data Rich	1	3	Cumulative Cigarettes (5 Years)	--
Multiple sclerosis	Female	5-9 years	95+ years	Data Rich	1	3	Smoking Prevalence	--
Multiple sclerosis	Female	5-9 years	95+ years	Data Rich	1	3	Socio-demographic Index	--
Multiple sclerosis	Female	5-9 years	95+ years	Global	1	1	Absolute value of average latitude	--
Multiple sclerosis	Female	5-9 years	95+ years	Global	-1	2	Healthcare access and quality index	--
Multiple sclerosis	Female	5-9 years	95+ years	Global	1	2	Low-Density Lipoprotein (mmol/L)	--
Multiple sclerosis	Female	5-9 years	95+ years	Global	-1	3	Education (years per capita)	--
Multiple sclerosis	Female	5-9 years	95+ years	Global	1	3	Socio-demographic Index	1000
Multiple sclerosis	Female	5-9 years	95+ years	Global	1	3	Cumulative Cigarettes (10 Years)	--
Multiple sclerosis	Female	5-9 years	95+ years	Global	1	3	Cumulative Cigarettes (5 Years)	--
Multiple sclerosis	Female	5-9 years	95+ years	Global	1	3	Prevalence of obesity	--
Multiple sclerosis	Female	5-9 years	95+ years	Global	1	3	Smoking Prevalence	--
Multiple sclerosis	Male	5-9 years	95+ years	Data Rich	1	1	Absolute value of average latitude	--
Multiple sclerosis	Male	5-9 years	95+ years	Data Rich	-1	2	Healthcare access and quality index	--
Multiple sclerosis	Male	5-9 years	95+ years	Data Rich	1	2	Low-Density Lipoprotein (mmol/L)	--
Multiple sclerosis	Male	5-9 years	95+ years	Data Rich	-1	3	Education (years per capita)	--
Multiple sclerosis	Male	5-9 years	95+ years	Data Rich	-1	3	LDI (I$ per capita)	--
Multiple sclerosis	Male	5-9 years	95+ years	Data Rich	1	3	Socio-demographic Index	1000
Multiple sclerosis	Male	5-9 years	95+ years	Data Rich	1	3	Cumulative Cigarettes (10 Years)	--
Multiple sclerosis	Male	5-9 years	95+ years	Data Rich	1	3	Cumulative Cigarettes (5 Years)	--
Multiple sclerosis	Male	5-9 years	95+ years	Data Rich	1	3	Prevalence of obesity	--
Multiple sclerosis	Male	5-9 years	95+ years	Data Rich	1	3	Smoking Prevalence	--
Multiple sclerosis	Male	5-9 years	95+ years	Global	1	1	Absolute value of average latitude	1000
Multiple sclerosis	Male	5-9 years	95+ years	Global	-1	2	Healthcare access and quality index	--
Multiple sclerosis	Male	5-9 years	95+ years	Global	1	2	Low-Density Lipoprotein (mmol/L)	--
Multiple sclerosis	Male	5-9 years	95+ years	Global	-1	3	Education (years per capita)	--
Multiple sclerosis	Male	5-9 years	95+ years	Global	1	3	Cumulative Cigarettes (10 Years)	159
Multiple sclerosis	Male	5-9 years	95+ years	Global	1	3	Smoking Prevalence	208
Multiple sclerosis	Male	5-9 years	95+ years	Global	1	3	Prevalence of obesity	360
Multiple sclerosis	Male	5-9 years	95+ years	Global	1	3	Cumulative Cigarettes (5 Years)	--
Multiple sclerosis	Male	5-9 years	95+ years	Global	1	3	Socio-demographic Index	--
Motor neuron disease	Female	0-6 days	95+ years	Data Rich	-1	1	Low-Density Lipoprotein (mmol/L)	3
Motor neuron disease	Female	0-6 days	95+ years	Data Rich	-1	1	Age- and sex-specific SEV for Low fruit	50
Motor neuron disease	Female	0-6 days	95+ years	Data Rich	-1	1	Mean BMI	--
Motor neuron disease	Female	0-6 days	95+ years	Data Rich	1	1	Absolute value of average latitude	169
Motor neuron disease	Female	0-6 days	95+ years	Data Rich	1	1	Diabetes Fasting Plasma Glucose (mmol/L), age-standardized 25+	751
Motor neuron disease	Female	0-6 days	95+ years	Data Rich	1	1	Socio-demographic Index	946
Motor neuron disease	Female	0-6 days	95+ years	Data Rich	-1	2	Population-weighted mean temperature	42
Motor neuron disease	Female	0-6 days	95+ years	Data Rich	-1	2	Healthcare access and quality index	--
Motor neuron disease	Female	0-6 days	95+ years	Data Rich	-1	2	Improved Water Source (proportion with access)	--
Motor neuron disease	Female	0-6 days	95+ years	Data Rich	1	2	Sanitation (proportion with access)	696
Motor neuron disease	Female	0-6 days	95+ years	Data Rich	1	3	Education (years per capita)	159
Motor neuron disease	Female	0-6 days	95+ years	Data Rich	1	3	LDI (I$ per capita)	--
Motor neuron disease	Female	0-6 days	95+ years	Global	-1	1	Age- and sex-specific SEV for Low fruit	135
Motor neuron disease	Female	0-6 days	95+ years	Global	-1	1	Low-Density Lipoprotein (mmol/L)	303
Motor neuron disease	Female	0-6 days	95+ years	Global	-1	1	Mean BMI	--
Motor neuron disease	Female	0-6 days	95+ years	Global	1	1	Absolute value of average latitude	506

Appendix Table S10: CODEm covariates used, level of covariate, and expected direction of covariate by cause, sex, and age

Cause	Sex	Age Start	Age End	Model Version Type	Direction	Level	Covariate Name	Number of Draws
Motor neuron disease	Female	0-6 days	95+ years	Global	1	1	Diabetes Fasting Plasma Glucose (mmol/L), age-standardized 25+	586
Motor neuron disease	Female	0-6 days	95+ years	Global	1	1	Socio-demographic Index	740
Motor neuron disease	Female	0-6 days	95+ years	Global	-1	2	Population-weighted mean temperature	197
Motor neuron disease	Female	0-6 days	95+ years	Global	-1	2	Healthcare access and quality index	219
Motor neuron disease	Female	0-6 days	95+ years	Global	-1	2	Improved Water Source (proportion with access)	469
Motor neuron disease	Female	0-6 days	95+ years	Global	1	2	Sanitation (proportion with access)	100
Motor neuron disease	Female	0-6 days	95+ years	Global	1	3	Education (years per capita)	356
Motor neuron disease	Female	0-6 days	95+ years	Global	1	3	LDI (I$ per capita)	--
Motor neuron disease	Male	0-6 days	95+ years	Data Rich	-1	1	Age- and sex-specific SEV for Low fruit	348
Motor neuron disease	Male	0-6 days	95+ years	Data Rich	-1	1	Low-Density Lipoprotein (mmol/L)	--
Motor neuron disease	Male	0-6 days	95+ years	Data Rich	-1	1	Mean BMI	--
Motor neuron disease	Male	0-6 days	95+ years	Data Rich	1	1	Diabetes Fasting Plasma Glucose (mmol/L), age-standardized 25+	287
Motor neuron disease	Male	0-6 days	95+ years	Data Rich	1	1	Socio-demographic Index	433
Motor neuron disease	Male	0-6 days	95+ years	Data Rich	1	1	Absolute value of average latitude	695
Motor neuron disease	Male	0-6 days	95+ years	Data Rich	-1	2	Population-weighted mean temperature	9
Motor neuron disease	Male	0-6 days	95+ years	Data Rich	-1	2	Healthcare access and quality index	--
Motor neuron disease	Male	0-6 days	95+ years	Data Rich	-1	2	Improved Water Source (proportion with access)	--
Motor neuron disease	Male	0-6 days	95+ years	Data Rich	1	2	Sanitation (proportion with access)	248
Motor neuron disease	Male	0-6 days	95+ years	Data Rich	1	3	Education (years per capita)	293
Motor neuron disease	Male	0-6 days	95+ years	Data Rich	1	3	LDI (I$ per capita)	--
Motor neuron disease	Male	0-6 days	95+ years	Global	-1	1	Age- and sex-specific SEV for Low fruit	532
Motor neuron disease	Male	0-6 days	95+ years	Global	-1	1	Low-Density Lipoprotein (mmol/L)	--
Motor neuron disease	Male	0-6 days	95+ years	Global	-1	1	Mean BMI	--
Motor neuron disease	Male	0-6 days	95+ years	Global	1	1	Socio-demographic Index	468
Motor neuron disease	Male	0-6 days	95+ years	Global	1	1	Absolute value of average latitude	532
Motor neuron disease	Male	0-6 days	95+ years	Global	1	1	Diabetes Fasting Plasma Glucose (mmol/L), age-standardized 25+	532
Motor neuron disease	Male	0-6 days	95+ years	Global	-1	2	Improved Water Source (proportion with access)	76
Motor neuron disease	Male	0-6 days	95+ years	Global	-1	2	Healthcare access and quality index	--
Motor neuron disease	Male	0-6 days	95+ years	Global	-1	2	Population-weighted mean temperature	--
Motor neuron disease	Male	0-6 days	95+ years	Global	1	2	Sanitation (proportion with access)	228
Motor neuron disease	Male	0-6 days	95+ years	Global	1	3	Education (years per capita)	152
Motor neuron disease	Male	0-6 days	95+ years	Global	1	3	LDI (I$ per capita)	--
Other neurological disorders	Female	0-6 days	95+ years	Data Rich	1	1	Low-Density Lipoprotein (mmol/L)	1
Other neurological disorders	Female	0-6 days	95+ years	Data Rich	1	1	Age- and sex-specific SEV for High red meat	319
Other neurological disorders	Female	0-6 days	95+ years	Data Rich	1	1	Systolic Blood Pressure (mmHg)	569
Other neurological disorders	Female	0-6 days	95+ years	Data Rich	1	1	Age- and sex-specific SEV for Child underweight	804
Other neurological disorders	Female	0-6 days	95+ years	Data Rich	1	1	Mean BMI	1000
Other neurological disorders	Female	0-6 days	95+ years	Data Rich	1	1	Pigs (per capita)	--
Other neurological disorders	Female	0-6 days	95+ years	Data Rich	-1	2	Age- and sex-specific SEV for Low fruit	514
Other neurological disorders	Female	0-6 days	95+ years	Data Rich	-1	2	Healthcare access and quality index	--
Other neurological disorders	Female	0-6 days	95+ years	Data Rich	1	2	Population Density (over 1000 ppl/sqkm, proportion)	0
Other neurological disorders	Female	0-6 days	95+ years	Data Rich	-1	3	Education (years per capita)	--
Other neurological disorders	Female	0-6 days	95+ years	Data Rich	-1	3	LDI (I$ per capita)	--
Other neurological disorders	Female	0-6 days	95+ years	Data Rich	1	3	Socio-demographic Index	40
Other neurological disorders	Female	0-6 days	95+ years	Data Rich	1	3	Cumulative Cigarettes (10 Years)	114
Other neurological disorders	Female	0-6 days	95+ years	Data Rich	1	3	Smoking Prevalence	176
Other neurological disorders	Female	0-6 days	95+ years	Data Rich	1	3	Cumulative Cigarettes (5 Years)	394
Other neurological disorders	Female	0-6 days	95+ years	Global	1	1	Low-Density Lipoprotein (mmol/L)	114
Other neurological disorders	Female	0-6 days	95+ years	Global	1	1	Age- and sex-specific SEV for High red meat	555
Other neurological disorders	Female	0-6 days	95+ years	Global	1	1	Systolic Blood Pressure (mmHg)	573
Other neurological disorders	Female	0-6 days	95+ years	Global	1	1	Mean BMI	1000
Other neurological disorders	Female	0-6 days	95+ years	Global	1	1	Pigs (per capita)	--
Other neurological disorders	Female	0-6 days	95+ years	Global	-1	2	Age- and sex-specific SEV for Low fruit	281
Other neurological disorders	Female	0-6 days	95+ years	Global	-1	2	Healthcare access and quality index	--
Other neurological disorders	Female	0-6 days	95+ years	Global	1	2	Population Density (over 1000 ppl/sqkm, proportion)	--
Other neurological disorders	Female	0-6 days	95+ years	Global	-1	3	Education (years per capita)	--
Other neurological disorders	Female	0-6 days	95+ years	Global	-1	3	LDI (I$ per capita)	--
Other neurological disorders	Female	0-6 days	95+ years	Global	1	3	Smoking Prevalence	252
Other neurological disorders	Female	0-6 days	95+ years	Global	1	3	Cumulative Cigarettes (10 Years)	286
Other neurological disorders	Female	0-6 days	95+ years	Global	1	3	Cumulative Cigarettes (5 Years)	300
Other neurological disorders	Female	0-6 days	95+ years	Global	1	3	Socio-demographic Index	915
Other neurological disorders	Male	0-6 days	95+ years	Data Rich	1	1	Age- and sex-specific SEV for High red meat	260
Other neurological disorders	Male	0-6 days	95+ years	Data Rich	1	1	Low-Density Lipoprotein (mmol/L)	331
Other neurological disorders	Male	0-6 days	95+ years	Data Rich	1	1	Age- and sex-specific SEV for Child underweight	599
Other neurological disorders	Male	0-6 days	95+ years	Data Rich	1	1	Systolic Blood Pressure (mmHg)	906
Other neurological disorders	Male	0-6 days	95+ years	Data Rich	1	1	Mean BMI	937
Other neurological disorders	Male	0-6 days	95+ years	Data Rich	1	1	Pigs (per capita)	--
Other neurological disorders	Male	0-6 days	95+ years	Data Rich	-1	2	Age- and sex-specific SEV for Low fruit	219
Other neurological disorders	Male	0-6 days	95+ years	Data Rich	-1	2	Healthcare access and quality index	--
Other neurological disorders	Male	0-6 days	95+ years	Data Rich	1	2	Population Density (over 1000 ppl/sqkm, proportion)	277
Other neurological disorders	Male	0-6 days	95+ years	Data Rich	-1	3	Education (years per capita)	--
Other neurological disorders	Male	0-6 days	95+ years	Data Rich	-1	3	LDI (I$ per capita)	--
Other neurological disorders	Male	0-6 days	95+ years	Data Rich	1	3	Socio-demographic Index	120
Other neurological disorders	Male	0-6 days	95+ years	Data Rich	1	3	Cumulative Cigarettes (10 Years)	--
Other neurological disorders	Male	0-6 days	95+ years	Data Rich	1	3	Cumulative Cigarettes (5 Years)	--
Other neurological disorders	Male	0-6 days	95+ years	Data Rich	1	3	Smoking Prevalence	--
Other neurological disorders	Male	0-6 days	95+ years	Global	1	1	Low-Density Lipoprotein (mmol/L)	181
Other neurological disorders	Male	0-6 days	95+ years	Global	1	1	Age- and sex-specific SEV for High red meat	294
Other neurological disorders	Male	0-6 days	95+ years	Global	1	1	Systolic Blood Pressure (mmHg)	362
Other neurological disorders	Male	0-6 days	95+ years	Global	1	1	Age- and sex-specific SEV for Child underweight	483
Other neurological disorders	Male	0-6 days	95+ years	Global	1	1	Mean BMI	1000
Other neurological disorders	Male	0-6 days	95+ years	Global	1	1	Pigs (per capita)	--
Other neurological disorders	Male	0-6 days	95+ years	Global	-1	2	Age- and sex-specific SEV for Low fruit	160
Other neurological disorders	Male	0-6 days	95+ years	Global	-1	2	Healthcare access and quality index	--
Other neurological disorders	Male	0-6 days	95+ years	Global	1	2	Liters of alcohol consumed per capita	--
Other neurological disorders	Male	0-6 days	95+ years	Global	1	2	Population Density (over 1000 ppl/sqkm, proportion)	--
Other neurological disorders	Male	0-6 days	95+ years	Global	-1	3	Education (years per capita)	--
Other neurological disorders	Male	0-6 days	95+ years	Global	-1	3	LDI (I$ per capita)	--
Other neurological disorders	Male	0-6 days	95+ years	Global	1	3	Socio-demographic Index	3
Other neurological disorders	Male	0-6 days	95+ years	Global	1	3	Cumulative Cigarettes (10 Years)	31
Other neurological disorders	Male	0-6 days	95+ years	Global	1	3	Cumulative Cigarettes (5 Years)	207
Other neurological disorders	Male	0-6 days	95+ years	Global	1	3	Smoking Prevalence	--
Anorexia nervosa	Female	5-9 years	45-49 years	Data Rich	-1	1	Age- and sex-specific SEV for Child underweight	--
Anorexia nervosa	Female	5-9 years	45-49 years	Data Rich	1	1	Maternal Education (years per capita)	129
Anorexia nervosa	Female	5-9 years	45-49 years	Data Rich	1	1	Education (years per capita)	--
Anorexia nervosa	Female	5-9 years	45-49 years	Data Rich	1	1	LDI (I$ per capita)	--
Anorexia nervosa	Female	5-9 years	45-49 years	Data Rich	1	1	Sanitation (proportion with access)	--
Anorexia nervosa	Female	5-9 years	45-49 years	Data Rich	-1	2	Healthcare access and quality index	--
Anorexia nervosa	Female	5-9 years	45-49 years	Data Rich	1	3	Socio-demographic Index	--
Anorexia nervosa	Female	5-9 years	45-49 years	Global	-1	1	Age- and sex-specific SEV for Child underweight	--
Anorexia nervosa	Female	5-9 years	45-49 years	Global	1	1	Maternal Education (years per capita)	314
Anorexia nervosa	Female	5-9 years	45-49 years	Global	1	1	Education (years per capita)	--
Anorexia nervosa	Female	5-9 years	45-49 years	Global	1	1	LDI (I$ per capita)	--
Anorexia nervosa	Female	5-9 years	45-49 years	Global	1	1	Sanitation (proportion with access)	--
Anorexia nervosa	Female	5-9 years	45-49 years	Global	-1	2	Healthcare access and quality index	--
Anorexia nervosa	Female	5-9 years	45-49 years	Global	1	3	Socio-demographic Index	252

Appendix Table S10: CODEm covariates used, level of covariate, and expected direction of covariate by cause, sex, and age

Cause	Sex	Age Start	Age End	Model Version Type	Direction	Level	Covariate Name	Number of Draws
Anorexia nervosa	Male	5-9 years	45-49 years	Data Rich	-1	1	Age- and sex-specific SEV for Child underweight	--
Anorexia nervosa	Male	5-9 years	45-49 years	Data Rich	1	1	Education (years per capita)	--
Anorexia nervosa	Male	5-9 years	45-49 years	Data Rich	1	1	LDI (I$ per capita)	--
Anorexia nervosa	Male	5-9 years	45-49 years	Data Rich	1	1	Maternal Education (years per capita)	--
Anorexia nervosa	Male	5-9 years	45-49 years	Data Rich	1	1	Sanitation (proportion with access)	--
Anorexia nervosa	Male	5-9 years	45-49 years	Data Rich	-1	2	Healthcare access and quality index	176
Anorexia nervosa	Male	5-9 years	45-49 years	Data Rich	1	3	Socio-demographic Index	253
Anorexia nervosa	Male	5-9 years	45-49 years	Global	-1	1	Age- and sex-specific SEV for Child underweight	--
Anorexia nervosa	Male	5-9 years	45-49 years	Global	1	1	Education (years per capita)	--
Anorexia nervosa	Male	5-9 years	45-49 years	Global	1	1	LDI (I$ per capita)	--
Anorexia nervosa	Male	5-9 years	45-49 years	Global	1	1	Maternal Education (years per capita)	--
Anorexia nervosa	Male	5-9 years	45-49 years	Global	1	1	Sanitation (proportion with access)	--
Anorexia nervosa	Male	5-9 years	45-49 years	Global	-1	2	Healthcare access and quality index	257
Anorexia nervosa	Male	5-9 years	45-49 years	Global	1	3	Socio-demographic Index	--
Alcohol use disorders	Female	15-19 years	95+ years	Data Rich	1	1	Alcohol consumption, age standardized, in grams per day	199
Alcohol use disorders	Female	15-19 years	95+ years	Data Rich	1	1	Alcohol drinker proportion, age-standardized	962
Alcohol use disorders	Female	15-19 years	95+ years	Data Rich	1	1	Liters of alcohol consumed per capita	--
Alcohol use disorders	Female	15-19 years	95+ years	Data Rich	-1	2	Healthcare access and quality index	993
Alcohol use disorders	Female	15-19 years	95+ years	Data Rich	1	2	Cumulative Cigarettes (10 Years)	--
Alcohol use disorders	Female	15-19 years	95+ years	Data Rich	1	2	Smoking Prevalence	--
Alcohol use disorders	Female	15-19 years	95+ years	Data Rich	-1	3	Education (years per capita)	--
Alcohol use disorders	Female	15-19 years	95+ years	Data Rich	-1	3	LDI (I$ per capita)	--
Alcohol use disorders	Female	15-19 years	95+ years	Data Rich	-1	3	Socio-demographic Index	--
Alcohol use disorders	Female	15-19 years	95+ years	Global	1	1	Alcohol consumption, age standardized, in grams per day	--
Alcohol use disorders	Female	15-19 years	95+ years	Global	1	1	Alcohol drinker proportion, age-standardized	--
Alcohol use disorders	Female	15-19 years	95+ years	Global	1	1	Liters of alcohol consumed per capita	--
Alcohol use disorders	Female	15-19 years	95+ years	Global	-1	2	Healthcare access and quality index	807
Alcohol use disorders	Female	15-19 years	95+ years	Global	1	2	Cumulative Cigarettes (10 Years)	--
Alcohol use disorders	Female	15-19 years	95+ years	Global	1	2	Smoking Prevalence	--
Alcohol use disorders	Female	15-19 years	95+ years	Global	-1	3	Socio-demographic Index	161
Alcohol use disorders	Female	15-19 years	95+ years	Global	-1	3	Education (years per capita)	--
Alcohol use disorders	Female	15-19 years	95+ years	Global	-1	3	LDI (I$ per capita)	--
Alcohol use disorders	Male	15-19 years	95+ years	Data Rich	1	1	Alcohol drinker proportion, age-standardized	193
Alcohol use disorders	Male	15-19 years	95+ years	Data Rich	1	1	Liters of alcohol consumed per capita	968
Alcohol use disorders	Male	15-19 years	95+ years	Data Rich	1	1	Alcohol consumption, age standardized, in grams per day	--
Alcohol use disorders	Male	15-19 years	95+ years	Data Rich	-1	2	Healthcare access and quality index	993
Alcohol use disorders	Male	15-19 years	95+ years	Data Rich	1	2	Cumulative Cigarettes (10 Years)	--
Alcohol use disorders	Male	15-19 years	95+ years	Data Rich	1	2	Smoking Prevalence	--
Alcohol use disorders	Male	15-19 years	95+ years	Data Rich	-1	3	Education (years per capita)	0
Alcohol use disorders	Male	15-19 years	95+ years	Data Rich	-1	3	LDI (I$ per capita)	--
Alcohol use disorders	Male	15-19 years	95+ years	Data Rich	-1	3	Socio-demographic Index	--
Alcohol use disorders	Male	15-19 years	95+ years	Global	1	1	Alcohol drinker proportion, age-standardized	1000
Alcohol use disorders	Male	15-19 years	95+ years	Global	1	1	Alcohol consumption, age standardized, in grams per day	--
Alcohol use disorders	Male	15-19 years	95+ years	Global	1	1	Liters of alcohol consumed per capita	--
Alcohol use disorders	Male	15-19 years	95+ years	Global	-1	2	Healthcare access and quality index	747
Alcohol use disorders	Male	15-19 years	95+ years	Global	1	2	Cumulative Cigarettes (10 Years)	0
Alcohol use disorders	Male	15-19 years	95+ years	Global	1	2	Smoking Prevalence	60
Alcohol use disorders	Male	15-19 years	95+ years	Global	-1	3	Education (years per capita)	190
Alcohol use disorders	Male	15-19 years	95+ years	Global	-1	3	Socio-demographic Index	190
Alcohol use disorders	Male	15-19 years	95+ years	Global	-1	3	LDI (I$ per capita)	--
Drug use disorders	Female	15-19 years	95+ years	Data Rich	1	1	Opioids per million population per day (10 year lag)	542
Drug use disorders	Female	15-19 years	95+ years	Data Rich	1	1	Intravenous drug use (age-standardized proportion)	570
Drug use disorders	Female	15-19 years	95+ years	Data Rich	1	1	Intravenous drug use (proportion by age)	642
Drug use disorders	Female	15-19 years	95+ years	Data Rich	-1	2	Healthcare access and quality index	--
Drug use disorders	Female	15-19 years	95+ years	Data Rich	1	2	Cumulative Cigarettes (10 Years)	--
Drug use disorders	Female	15-19 years	95+ years	Data Rich	1	2	Cumulative Cigarettes (5 Years)	--
Drug use disorders	Female	15-19 years	95+ years	Data Rich	1	2	Smoking Prevalence	--
Drug use disorders	Female	15-19 years	95+ years	Data Rich	1	3	Education (years per capita)	290
Drug use disorders	Female	15-19 years	95+ years	Data Rich	1	3	Socio-demographic Index	537
Drug use disorders	Female	15-19 years	95+ years	Data Rich	1	3	LDI (I$ per capita)	--
Drug use disorders	Female	15-19 years	95+ years	Global	1	1	Intravenous drug use (age-standardized proportion)	802
Drug use disorders	Female	15-19 years	95+ years	Global	1	1	Intravenous drug use (proportion by age)	802
Drug use disorders	Female	15-19 years	95+ years	Global	1	1	Opioids per million population per day (10 year lag)	1000
Drug use disorders	Female	15-19 years	95+ years	Global	-1	2	Healthcare access and quality index	1
Drug use disorders	Female	15-19 years	95+ years	Global	1	2	Cumulative Cigarettes (10 Years)	--
Drug use disorders	Female	15-19 years	95+ years	Global	1	2	Cumulative Cigarettes (5 Years)	--
Drug use disorders	Female	15-19 years	95+ years	Global	1	2	Smoking Prevalence	--
Drug use disorders	Female	15-19 years	95+ years	Global	1	3	Education (years per capita)	6
Drug use disorders	Female	15-19 years	95+ years	Global	1	3	Socio-demographic Index	160
Drug use disorders	Female	15-19 years	95+ years	Global	1	3	LDI (I$ per capita)	--
Drug use disorders	Male	15-19 years	95+ years	Data Rich	1	1	Intravenous drug use (proportion by age)	205
Drug use disorders	Male	15-19 years	95+ years	Data Rich	1	1	Opioids per million population per day (10 year lag)	261
Drug use disorders	Male	15-19 years	95+ years	Data Rich	1	1	Intravenous drug use (age-standardized proportion)	603
Drug use disorders	Male	15-19 years	95+ years	Data Rich	-1	2	Healthcare access and quality index	--
Drug use disorders	Male	15-19 years	95+ years	Data Rich	1	2	Cumulative Cigarettes (10 Years)	--
Drug use disorders	Male	15-19 years	95+ years	Data Rich	1	2	Cumulative Cigarettes (5 Years)	--
Drug use disorders	Male	15-19 years	95+ years	Data Rich	1	2	Smoking Prevalence	--
Drug use disorders	Male	15-19 years	95+ years	Data Rich	1	3	Education (years per capita)	115
Drug use disorders	Male	15-19 years	95+ years	Data Rich	1	3	Socio-demographic Index	885
Drug use disorders	Male	15-19 years	95+ years	Data Rich	1	3	LDI (I$ per capita)	--
Drug use disorders	Male	15-19 years	95+ years	Global	1	1	Intravenous drug use (age-standardized proportion)	2
Drug use disorders	Male	15-19 years	95+ years	Global	1	1	Intravenous drug use (proportion by age)	694
Drug use disorders	Male	15-19 years	95+ years	Global	1	1	Opioids per million population per day (10 year lag)	998
Drug use disorders	Male	15-19 years	95+ years	Global	-1	2	Healthcare access and quality index	0
Drug use disorders	Male	15-19 years	95+ years	Global	1	2	Cumulative Cigarettes (10 Years)	--
Drug use disorders	Male	15-19 years	95+ years	Global	1	2	Cumulative Cigarettes (5 Years)	--
Drug use disorders	Male	15-19 years	95+ years	Global	1	2	Smoking Prevalence	--
Drug use disorders	Male	15-19 years	95+ years	Global	1	3	Education (years per capita)	746
Drug use disorders	Male	15-19 years	95+ years	Global	1	3	Socio-demographic Index	894
Drug use disorders	Male	15-19 years	95+ years	Global	1	3	LDI (I$ per capita)	--
Opioid use disorders	Female	15-19 years	95+ years	Data Rich	1	1	Intravenous drug use (proportion by age)	642
Opioid use disorders	Female	15-19 years	95+ years	Data Rich	1	1	Intravenous drug use (age-standardized proportion)	710
Opioid use disorders	Female	15-19 years	95+ years	Data Rich	1	1	Opioids per million population per day (10 year lag)	915
Opioid use disorders	Female	15-19 years	95+ years	Data Rich	-1	2	Healthcare access and quality index	--
Opioid use disorders	Female	15-19 years	95+ years	Data Rich	1	2	Cumulative Cigarettes (10 Years)	--
Opioid use disorders	Female	15-19 years	95+ years	Data Rich	1	2	Cumulative Cigarettes (5 Years)	--
Opioid use disorders	Female	15-19 years	95+ years	Data Rich	1	2	Smoking Prevalence	--
Opioid use disorders	Female	15-19 years	95+ years	Data Rich	1	3	Socio-demographic Index	67
Opioid use disorders	Female	15-19 years	95+ years	Data Rich	1	3	Education (years per capita)	89
Opioid use disorders	Female	15-19 years	95+ years	Data Rich	1	3	LDI (I$ per capita)	--
Opioid use disorders	Female	15-19 years	95+ years	Global	1	1	Intravenous drug use (age-standardized proportion)	486
Opioid use disorders	Female	15-19 years	95+ years	Global	1	1	Intravenous drug use (proportion by age)	937
Opioid use disorders	Female	15-19 years	95+ years	Global	1	1	Opioids per million population per day (10 year lag)	969
Opioid use disorders	Female	15-19 years	95+ years	Global	-1	2	Healthcare access and quality index	--

Appendix Table S10: CODEm covariates used, level of covariate, and expected direction of covariate by cause, sex, and age

Cause	Sex	Age Start	Age End	Model Version Type	Direction	Level	Covariate Name	Number of Draws
Opioid use disorders	Female	15-19 years	95+ years	Global	1	2	Cumulative Cigarettes (10 Years)	--
Opioid use disorders	Female	15-19 years	95+ years	Global	1	2	Cumulative Cigarettes (5 Years)	--
Opioid use disorders	Female	15-19 years	95+ years	Global	1	2	Smoking Prevalence	--
Opioid use disorders	Female	15-19 years	95+ years	Global	1	3	Socio-demographic Index	75
Opioid use disorders	Female	15-19 years	95+ years	Global	1	3	Education (years per capita)	564
Opioid use disorders	Female	15-19 years	95+ years	Global	1	3	LDI (I$ per capita)	--
Opioid use disorders	Male	15-19 years	95+ years	Data Rich	1	1	Intravenous drug use (proportion by age)	523
Opioid use disorders	Male	15-19 years	95+ years	Data Rich	1	1	Opioids per million population per day (10 year lag)	682
Opioid use disorders	Male	15-19 years	95+ years	Data Rich	1	1	Intravenous drug use (age-standardized proportion)	880
Opioid use disorders	Male	15-19 years	95+ years	Data Rich	1	2	Cumulative Cigarettes (10 Years)	--
Opioid use disorders	Male	15-19 years	95+ years	Data Rich	1	2	Cumulative Cigarettes (5 Years)	--
Opioid use disorders	Male	15-19 years	95+ years	Data Rich	1	2	Smoking Prevalence	--
Opioid use disorders	Male	15-19 years	95+ years	Global	1	1	Intravenous drug use (proportion by age)	166
Opioid use disorders	Male	15-19 years	95+ years	Global	1	1	Intravenous drug use (age-standardized proportion)	962
Opioid use disorders	Male	15-19 years	95+ years	Global	1	1	Opioids per million population per day (10 year lag)	994
Opioid use disorders	Male	15-19 years	95+ years	Global	1	2	Cumulative Cigarettes (10 Years)	--
Opioid use disorders	Male	15-19 years	95+ years	Global	1	2	Cumulative Cigarettes (5 Years)	--
Opioid use disorders	Male	15-19 years	95+ years	Global	1	2	Smoking Prevalence	--
Cocaine use disorders	Female	15-19 years	95+ years	Data Rich	1	1	Intravenous drug use (proportion by age)	299
Cocaine use disorders	Female	15-19 years	95+ years	Data Rich	1	1	Intravenous drug use (age-standardized proportion)	701
Cocaine use disorders	Female	15-19 years	95+ years	Data Rich	1	1	Alcohol drinker proportion, age-standardized	--
Cocaine use disorders	Female	15-19 years	95+ years	Data Rich	1	1	Cumulative Cigarettes (10 Years)	--
Cocaine use disorders	Female	15-19 years	95+ years	Data Rich	1	1	Cumulative Cigarettes (5 Years)	--
Cocaine use disorders	Female	15-19 years	95+ years	Data Rich	1	1	Liters of alcohol consumed per capita	--
Cocaine use disorders	Female	15-19 years	95+ years	Data Rich	1	1	Smoking Prevalence	--
Cocaine use disorders	Female	15-19 years	95+ years	Data Rich	1	1	Tobacco (cigarettes per capita)	--
Cocaine use disorders	Female	15-19 years	95+ years	Data Rich	-1	2	Healthcare access and quality index	--
Cocaine use disorders	Female	15-19 years	95+ years	Data Rich	1	3	Socio-demographic Index	63
Cocaine use disorders	Female	15-19 years	95+ years	Data Rich	1	3	Education (years per capita)	141
Cocaine use disorders	Female	15-19 years	95+ years	Data Rich	1	3	LDI (I$ per capita)	--
Cocaine use disorders	Female	15-19 years	95+ years	Global	1	1	Intravenous drug use (proportion by age)	1000
Cocaine use disorders	Female	15-19 years	95+ years	Global	1	1	Alcohol drinker proportion, age-standardized	--
Cocaine use disorders	Female	15-19 years	95+ years	Global	1	1	Cumulative Cigarettes (10 Years)	--
Cocaine use disorders	Female	15-19 years	95+ years	Global	1	1	Cumulative Cigarettes (5 Years)	--
Cocaine use disorders	Female	15-19 years	95+ years	Global	1	1	Intravenous drug use (age-standardized proportion)	--
Cocaine use disorders	Female	15-19 years	95+ years	Global	1	1	Liters of alcohol consumed per capita	--
Cocaine use disorders	Female	15-19 years	95+ years	Global	1	1	Smoking Prevalence	--
Cocaine use disorders	Female	15-19 years	95+ years	Global	1	1	Tobacco (cigarettes per capita)	--
Cocaine use disorders	Female	15-19 years	95+ years	Global	-1	2	Healthcare access and quality index	801
Cocaine use disorders	Female	15-19 years	95+ years	Global	1	3	Socio-demographic Index	6
Cocaine use disorders	Female	15-19 years	95+ years	Global	1	3	Education (years per capita)	32
Cocaine use disorders	Female	15-19 years	95+ years	Global	1	3	LDI (I$ per capita)	--
Cocaine use disorders	Male	15-19 years	95+ years	Data Rich	1	1	Intravenous drug use (proportion by age)	315
Cocaine use disorders	Male	15-19 years	95+ years	Data Rich	1	1	Liters of alcohol consumed per capita	525
Cocaine use disorders	Male	15-19 years	95+ years	Data Rich	1	1	Intravenous drug use (age-standardized proportion)	880
Cocaine use disorders	Male	15-19 years	95+ years	Data Rich	1	1	Alcohol drinker proportion, age-standardized	--
Cocaine use disorders	Male	15-19 years	95+ years	Data Rich	1	1	Cumulative Cigarettes (10 Years)	--
Cocaine use disorders	Male	15-19 years	95+ years	Data Rich	1	1	Cumulative Cigarettes (5 Years)	--
Cocaine use disorders	Male	15-19 years	95+ years	Data Rich	1	1	Smoking Prevalence	--
Cocaine use disorders	Male	15-19 years	95+ years	Data Rich	1	1	Tobacco (cigarettes per capita)	--
Cocaine use disorders	Male	15-19 years	95+ years	Data Rich	-1	2	Healthcare access and quality index	--
Cocaine use disorders	Male	15-19 years	95+ years	Data Rich	1	3	Socio-demographic Index	108
Cocaine use disorders	Male	15-19 years	95+ years	Data Rich	1	3	Education (years per capita)	--
Cocaine use disorders	Male	15-19 years	95+ years	Data Rich	1	3	LDI (I$ per capita)	--
Cocaine use disorders	Male	15-19 years	95+ years	Global	1	1	Liters of alcohol consumed per capita	141
Cocaine use disorders	Male	15-19 years	95+ years	Global	1	1	Intravenous drug use (age-standardized proportion)	859
Cocaine use disorders	Male	15-19 years	95+ years	Global	1	1	Alcohol drinker proportion, age-standardized	--
Cocaine use disorders	Male	15-19 years	95+ years	Global	1	1	Cumulative Cigarettes (10 Years)	--
Cocaine use disorders	Male	15-19 years	95+ years	Global	1	1	Cumulative Cigarettes (5 Years)	--
Cocaine use disorders	Male	15-19 years	95+ years	Global	1	1	Intravenous drug use (proportion by age)	--
Cocaine use disorders	Male	15-19 years	95+ years	Global	1	1	Smoking Prevalence	--
Cocaine use disorders	Male	15-19 years	95+ years	Global	1	1	Tobacco (cigarettes per capita)	--
Cocaine use disorders	Male	15-19 years	95+ years	Global	-1	2	Healthcare access and quality index	--
Cocaine use disorders	Male	15-19 years	95+ years	Global	1	3	Education (years per capita)	--
Cocaine use disorders	Male	15-19 years	95+ years	Global	1	3	LDI (I$ per capita)	--
Cocaine use disorders	Male	15-19 years	95+ years	Global	1	3	Socio-demographic Index	--
Amphetamine use disorders	Female	15-19 years	95+ years	Data Rich	1	1	Intravenous drug use (proportion by age)	331
Amphetamine use disorders	Female	15-19 years	95+ years	Data Rich	1	1	Intravenous drug use (age-standardized proportion)	669
Amphetamine use disorders	Female	15-19 years	95+ years	Data Rich	1	1	Alcohol drinker proportion, age-standardized	--
Amphetamine use disorders	Female	15-19 years	95+ years	Data Rich	1	1	Cumulative Cigarettes (10 Years)	--
Amphetamine use disorders	Female	15-19 years	95+ years	Data Rich	1	1	Cumulative Cigarettes (5 Years)	--
Amphetamine use disorders	Female	15-19 years	95+ years	Data Rich	1	1	Liters of alcohol consumed per capita	--
Amphetamine use disorders	Female	15-19 years	95+ years	Data Rich	1	1	Smoking Prevalence	--
Amphetamine use disorders	Female	15-19 years	95+ years	Data Rich	1	1	Tobacco (cigarettes per capita)	--
Amphetamine use disorders	Female	15-19 years	95+ years	Data Rich	-1	2	Healthcare access and quality index	--
Amphetamine use disorders	Female	15-19 years	95+ years	Data Rich	1	3	Education (years per capita)	189
Amphetamine use disorders	Female	15-19 years	95+ years	Data Rich	1	3	Socio-demographic Index	189
Amphetamine use disorders	Female	15-19 years	95+ years	Data Rich	1	3	LDI (I$ per capita)	--
Amphetamine use disorders	Female	15-19 years	95+ years	Global	1	1	Intravenous drug use (age-standardized proportion)	1000
Amphetamine use disorders	Female	15-19 years	95+ years	Global	1	1	Alcohol drinker proportion, age-standardized	--
Amphetamine use disorders	Female	15-19 years	95+ years	Global	1	1	Cumulative Cigarettes (10 Years)	--
Amphetamine use disorders	Female	15-19 years	95+ years	Global	1	1	Cumulative Cigarettes (5 Years)	--
Amphetamine use disorders	Female	15-19 years	95+ years	Global	1	1	Intravenous drug use (proportion by age)	--
Amphetamine use disorders	Female	15-19 years	95+ years	Global	1	1	Liters of alcohol consumed per capita	--
Amphetamine use disorders	Female	15-19 years	95+ years	Global	1	1	Smoking Prevalence	--
Amphetamine use disorders	Female	15-19 years	95+ years	Global	1	1	Tobacco (cigarettes per capita)	--
Amphetamine use disorders	Female	15-19 years	95+ years	Global	-1	2	Healthcare access and quality index	--
Amphetamine use disorders	Female	15-19 years	95+ years	Global	1	3	Education (years per capita)	715
Amphetamine use disorders	Female	15-19 years	95+ years	Global	1	3	Socio-demographic Index	715
Amphetamine use disorders	Female	15-19 years	95+ years	Global	1	3	LDI (I$ per capita)	--
Amphetamine use disorders	Male	15-19 years	95+ years	Data Rich	1	1	Liters of alcohol consumed per capita	155
Amphetamine use disorders	Male	15-19 years	95+ years	Data Rich	1	1	Intravenous drug use (proportion by age)	475
Amphetamine use disorders	Male	15-19 years	95+ years	Data Rich	1	1	Intravenous drug use (age-standardized proportion)	557
Amphetamine use disorders	Male	15-19 years	95+ years	Data Rich	1	1	Alcohol drinker proportion, age-standardized	--
Amphetamine use disorders	Male	15-19 years	95+ years	Data Rich	1	1	Cumulative Cigarettes (10 Years)	--
Amphetamine use disorders	Male	15-19 years	95+ years	Data Rich	1	1	Cumulative Cigarettes (5 Years)	--
Amphetamine use disorders	Male	15-19 years	95+ years	Data Rich	1	1	Smoking Prevalence	--
Amphetamine use disorders	Male	15-19 years	95+ years	Data Rich	1	1	Tobacco (cigarettes per capita)	--
Amphetamine use disorders	Male	15-19 years	95+ years	Data Rich	-1	2	Healthcare access and quality index	--
Amphetamine use disorders	Male	15-19 years	95+ years	Data Rich	1	3	Education (years per capita)	380
Amphetamine use disorders	Male	15-19 years	95+ years	Data Rich	1	3	Socio-demographic Index	572
Amphetamine use disorders	Male	15-19 years	95+ years	Data Rich	1	3	LDI (I$ per capita)	--
Amphetamine use disorders	Male	15-19 years	95+ years	Global	1	1	Liters of alcohol consumed per capita	61

Appendix Table S10: CODEm covariates used, level of covariate, and expected direction of covariate by cause, sex, and age

Cause	Sex	Age Start	Age End	Model Version Type	Direction	Level	Covariate Name	Number of Draws
Amphetamine use disorders	Male	15-19 years	95+ years	Global	1	1	Intravenous drug use (proportion by age)	331
Amphetamine use disorders	Male	15-19 years	95+ years	Global	1	1	Intravenous drug use (age-standardized proportion)	875
Amphetamine use disorders	Male	15-19 years	95+ years	Global	1	1	Alcohol drinker proportion, age-standardized	--
Amphetamine use disorders	Male	15-19 years	95+ years	Global	1	1	Cumulative Cigarettes (10 Years)	--
Amphetamine use disorders	Male	15-19 years	95+ years	Global	1	1	Cumulative Cigarettes (5 Years)	--
Amphetamine use disorders	Male	15-19 years	95+ years	Global	1	1	Smoking Prevalence	--
Amphetamine use disorders	Male	15-19 years	95+ years	Global	1	1	Tobacco (cigarettes per capita)	--
Amphetamine use disorders	Male	15-19 years	95+ years	Global	-1	2	Healthcare access and quality index	--
Amphetamine use disorders	Male	15-19 years	95+ years	Global	1	3	Socio-demographic Index	346
Amphetamine use disorders	Male	15-19 years	95+ years	Global	1	3	Education (years per capita)	818
Amphetamine use disorders	Male	15-19 years	95+ years	Global	1	3	LDI (I$ per capita)	--
Other drug use disorders	Female	15-19 years	95+ years	Data Rich	1	1	Alcohol drinker proportion, age-standardized	135
Other drug use disorders	Female	15-19 years	95+ years	Data Rich	1	1	Liters of alcohol consumed per capita	450
Other drug use disorders	Female	15-19 years	95+ years	Data Rich	1	1	Intravenous drug use (age-standardized proportion)	550
Other drug use disorders	Female	15-19 years	95+ years	Data Rich	1	1	Intravenous drug use (proportion by age)	585
Other drug use disorders	Female	15-19 years	95+ years	Data Rich	1	1	Cumulative Cigarettes (10 Years)	--
Other drug use disorders	Female	15-19 years	95+ years	Data Rich	1	1	Cumulative Cigarettes (5 Years)	--
Other drug use disorders	Female	15-19 years	95+ years	Data Rich	1	1	Smoking Prevalence	--
Other drug use disorders	Female	15-19 years	95+ years	Data Rich	1	1	Tobacco (cigarettes per capita)	--
Other drug use disorders	Female	15-19 years	95+ years	Data Rich	-1	2	Healthcare access and quality index	--
Other drug use disorders	Female	15-19 years	95+ years	Data Rich	1	3	Socio-demographic Index	135
Other drug use disorders	Female	15-19 years	95+ years	Data Rich	1	3	Education (years per capita)	315
Other drug use disorders	Female	15-19 years	95+ years	Data Rich	1	3	LDI (I$ per capita)	--
Other drug use disorders	Female	15-19 years	95+ years	Global	1	1	Intravenous drug use (age-standardized proportion)	834
Other drug use disorders	Female	15-19 years	95+ years	Global	1	1	Intravenous drug use (proportion by age)	1000
Other drug use disorders	Female	15-19 years	95+ years	Global	1	1	Liters of alcohol consumed per capita	1000
Other drug use disorders	Female	15-19 years	95+ years	Global	1	1	Alcohol drinker proportion, age-standardized	--
Other drug use disorders	Female	15-19 years	95+ years	Global	1	1	Cumulative Cigarettes (10 Years)	--
Other drug use disorders	Female	15-19 years	95+ years	Global	1	1	Cumulative Cigarettes (5 Years)	--
Other drug use disorders	Female	15-19 years	95+ years	Global	1	1	Smoking Prevalence	--
Other drug use disorders	Female	15-19 years	95+ years	Global	1	1	Tobacco (cigarettes per capita)	--
Other drug use disorders	Female	15-19 years	95+ years	Global	-1	2	Healthcare access and quality index	--
Other drug use disorders	Female	15-19 years	95+ years	Global	1	3	Education (years per capita)	--
Other drug use disorders	Female	15-19 years	95+ years	Global	1	3	LDI (I$ per capita)	--
Other drug use disorders	Female	15-19 years	95+ years	Global	1	3	Socio-demographic Index	--
Other drug use disorders	Male	15-19 years	95+ years	Data Rich	1	1	Liters of alcohol consumed per capita	304
Other drug use disorders	Male	15-19 years	95+ years	Data Rich	1	1	Intravenous drug use (proportion by age)	608
Other drug use disorders	Male	15-19 years	95+ years	Data Rich	1	1	Intravenous drug use (age-standardized proportion)	620
Other drug use disorders	Male	15-19 years	95+ years	Data Rich	1	1	Alcohol drinker proportion, age-standardized	--
Other drug use disorders	Male	15-19 years	95+ years	Data Rich	1	1	Cumulative Cigarettes (10 Years)	--
Other drug use disorders	Male	15-19 years	95+ years	Data Rich	1	1	Cumulative Cigarettes (5 Years)	--
Other drug use disorders	Male	15-19 years	95+ years	Data Rich	1	1	Smoking Prevalence	--
Other drug use disorders	Male	15-19 years	95+ years	Data Rich	1	1	Tobacco (cigarettes per capita)	--
Other drug use disorders	Male	15-19 years	95+ years	Data Rich	-1	2	Healthcare access and quality index	--
Other drug use disorders	Male	15-19 years	95+ years	Data Rich	1	3	Education (years per capita)	228
Other drug use disorders	Male	15-19 years	95+ years	Data Rich	1	3	Socio-demographic Index	228
Other drug use disorders	Male	15-19 years	95+ years	Data Rich	1	3	LDI (I$ per capita)	--
Other drug use disorders	Male	15-19 years	95+ years	Global	1	1	Intravenous drug use (age-standardized proportion)	1000
Other drug use disorders	Male	15-19 years	95+ years	Global	1	1	Alcohol drinker proportion, age-standardized	--
Other drug use disorders	Male	15-19 years	95+ years	Global	1	1	Cumulative Cigarettes (10 Years)	--
Other drug use disorders	Male	15-19 years	95+ years	Global	1	1	Cumulative Cigarettes (5 Years)	--
Other drug use disorders	Male	15-19 years	95+ years	Global	1	1	Intravenous drug use (proportion by age)	--
Other drug use disorders	Male	15-19 years	95+ years	Global	1	1	Liters of alcohol consumed per capita	--
Other drug use disorders	Male	15-19 years	95+ years	Global	1	1	Smoking Prevalence	--
Other drug use disorders	Male	15-19 years	95+ years	Global	1	1	Tobacco (cigarettes per capita)	--
Other drug use disorders	Male	15-19 years	95+ years	Global	-1	2	Healthcare access and quality index	--
Other drug use disorders	Male	15-19 years	95+ years	Global	1	3	Education (years per capita)	779
Other drug use disorders	Male	15-19 years	95+ years	Global	1	3	LDI (I$ per capita)	--
Other drug use disorders	Male	15-19 years	95+ years	Global	1	3	Socio-demographic Index	--
Diabetes mellitus	Female	0-6 days	10-14 years	Data Rich	-1	1	Healthcare access and quality index	1000
Diabetes mellitus	Female	0-6 days	10-14 years	Data Rich	1	2	Absolute value of average latitude	--
Diabetes mellitus	Female	0-6 days	10-14 years	Data Rich	1	2	Live Births 35+ (proportion)	--
Diabetes mellitus	Female	0-6 days	10-14 years	Data Rich	1	2	Live Births 40+ (proportion)	--
Diabetes mellitus	Female	0-6 days	10-14 years	Data Rich	-1	3	Socio-demographic Index	454
Diabetes mellitus	Female	0-6 days	10-14 years	Data Rich	-1	3	Education (years per capita)	--
Diabetes mellitus	Female	0-6 days	10-14 years	Global	-1	1	Healthcare access and quality index	--
Diabetes mellitus	Female	0-6 days	10-14 years	Global	1	2	Absolute value of average latitude	--
Diabetes mellitus	Female	0-6 days	10-14 years	Global	1	2	Live Births 35+ (proportion)	--
Diabetes mellitus	Female	0-6 days	10-14 years	Global	1	2	Live Births 40+ (proportion)	--
Diabetes mellitus	Female	0-6 days	10-14 years	Global	-1	3	Education (years per capita)	454
Diabetes mellitus	Female	0-6 days	10-14 years	Global	-1	3	Socio-demographic Index	546
Diabetes mellitus	Female	15-19 years	95+ years	Data Rich	1	1	Diabetes Age-Standardized Prevalence (proportion)	--
Diabetes mellitus	Female	15-19 years	95+ years	Data Rich	1	1	Diabetes Fasting Plasma Glucose (mmol/L), age-standardized 25+	--
Diabetes mellitus	Female	15-19 years	95+ years	Data Rich	1	1	Mean BMI	--
Diabetes mellitus	Female	15-19 years	95+ years	Data Rich	1	1	Prevalence of obesity	--
Diabetes mellitus	Female	15-19 years	95+ years	Data Rich	-1	2	Age- and sex-specific SEV for Low fruit	--
Diabetes mellitus	Female	15-19 years	95+ years	Data Rich	-1	2	Age- and sex-specific SEV for Low vegetables	--
Diabetes mellitus	Female	15-19 years	95+ years	Data Rich	1	2	Low-Density Lipoprotein (mmol/L)	515
Diabetes mellitus	Female	15-19 years	95+ years	Data Rich	1	2	Age- and sex-specific SEV for Alcohol use	--
Diabetes mellitus	Female	15-19 years	95+ years	Data Rich	1	2	Systolic Blood Pressure (mmHg)	--
Diabetes mellitus	Female	15-19 years	95+ years	Data Rich	1	2	sugar unadjusted(g)	--
Diabetes mellitus	Female	15-19 years	95+ years	Data Rich	-1	3	Education (years per capita)	485
Diabetes mellitus	Female	15-19 years	95+ years	Data Rich	-1	3	Healthcare access and quality index	485
Diabetes mellitus	Female	15-19 years	95+ years	Data Rich	1	3	LDI (I$ per capita)	--
Diabetes mellitus	Female	15-19 years	95+ years	Global	1	1	Diabetes Age-Standardized Prevalence (proportion)	--
Diabetes mellitus	Female	15-19 years	95+ years	Global	1	1	Diabetes Fasting Plasma Glucose (mmol/L), age-standardized 25+	--
Diabetes mellitus	Female	15-19 years	95+ years	Global	1	1	Mean BMI	--
Diabetes mellitus	Female	15-19 years	95+ years	Global	1	1	Prevalence of obesity	--
Diabetes mellitus	Female	15-19 years	95+ years	Global	-1	2	Age- and sex-specific SEV for Low fruit	--
Diabetes mellitus	Female	15-19 years	95+ years	Global	-1	2	Age- and sex-specific SEV for Low vegetables	--
Diabetes mellitus	Female	15-19 years	95+ years	Global	1	2	Age- and sex-specific SEV for Alcohol use	--
Diabetes mellitus	Female	15-19 years	95+ years	Global	1	2	Low-Density Lipoprotein (mmol/L)	--
Diabetes mellitus	Female	15-19 years	95+ years	Global	1	2	Systolic Blood Pressure (mmHg)	--
Diabetes mellitus	Female	15-19 years	95+ years	Global	1	2	sugar unadjusted(g)	--
Diabetes mellitus	Female	15-19 years	95+ years	Global	-1	3	Education (years per capita)	1000
Diabetes mellitus	Female	15-19 years	95+ years	Global	-1	3	Healthcare access and quality index	1000
Diabetes mellitus	Female	15-19 years	95+ years	Global	1	3	LDI (I$ per capita)	--
Diabetes mellitus	Male	0-6 days	10-14 years	Data Rich	-1	1	Healthcare access and quality index	1000
Diabetes mellitus	Male	0-6 days	10-14 years	Data Rich	1	2	Absolute value of average latitude	--
Diabetes mellitus	Male	0-6 days	10-14 years	Data Rich	1	2	Live Births 35+ (proportion)	--
Diabetes mellitus	Male	0-6 days	10-14 years	Data Rich	1	2	Live Births 40+ (proportion)	--
Diabetes mellitus	Male	0-6 days	10-14 years	Data Rich	-1	3	Socio-demographic Index	329
Diabetes mellitus	Male	0-6 days	10-14 years	Data Rich	-1	3	Education (years per capita)	397
Diabetes mellitus	Male	0-6 days	10-14 years	Global	-1	1	Healthcare access and quality index	--
Diabetes mellitus	Male	0-6 days	10-14 years	Global	1	2	Absolute value of average latitude	--
Diabetes mellitus	Male	0-6 days	10-14 years	Global	1	2	Live Births 35+ (proportion)	--
Diabetes mellitus	Male	0-6 days	10-14 years	Global	1	2	Live Births 40+ (proportion)	--

Appendix Table S10: CODEm covariates used, level of covariate, and expected direction of covariate by cause, sex, and age

Cause	Sex	Age Start	Age End	Model Version Type	Direction	Level	Covariate Name	Number of Draws
Diabetes mellitus	Male	0-6 days	10-14 years	Global	-1	3	Education (years per capita)	454
Diabetes mellitus	Male	0-6 days	10-14 years	Global	-1	3	Socio-demographic Index	546
Diabetes mellitus	Male	15-19 years	95+ years	Data Rich	1	1	Diabetes Fasting Plasma Glucose (mmol/L), age-standardized 25+	180
Diabetes mellitus	Male	15-19 years	95+ years	Data Rich	1	1	Prevalence of obesity	240
Diabetes mellitus	Male	15-19 years	95+ years	Data Rich	1	1	Mean BMI	703
Diabetes mellitus	Male	15-19 years	95+ years	Data Rich	1	1	Diabetes Age-Standardized Prevalence (proportion)	786
Diabetes mellitus	Male	15-19 years	95+ years	Data Rich	-1	2	Age- and sex-specific SEV for Low fruit	540
Diabetes mellitus	Male	15-19 years	95+ years	Data Rich	-1	2	Age- and sex-specific SEV for Low vegetables	--
Diabetes mellitus	Male	15-19 years	95+ years	Data Rich	1	2	sugar unadjusted(g)	383
Diabetes mellitus	Male	15-19 years	95+ years	Data Rich	1	2	Low-Density Lipoprotein (mmol/L)	639
Diabetes mellitus	Male	15-19 years	95+ years	Data Rich	1	2	Age- and sex-specific SEV for Alcohol use	--
Diabetes mellitus	Male	15-19 years	95+ years	Data Rich	1	2	Systolic Blood Pressure (mmHg)	--
Diabetes mellitus	Male	15-19 years	95+ years	Data Rich	-1	3	Healthcare access and quality index	257
Diabetes mellitus	Male	15-19 years	95+ years	Data Rich	-1	3	Education (years per capita)	333
Diabetes mellitus	Male	15-19 years	95+ years	Data Rich	1	3	LDI (I$ per capita)	--
Diabetes mellitus	Male	15-19 years	95+ years	Global	1	1	Diabetes Fasting Plasma Glucose (mmol/L), age-standardized 25+	103
Diabetes mellitus	Male	15-19 years	95+ years	Global	1	1	Prevalence of obesity	160
Diabetes mellitus	Male	15-19 years	95+ years	Global	1	1	Diabetes Age-Standardized Prevalence (proportion)	354
Diabetes mellitus	Male	15-19 years	95+ years	Global	1	1	Mean BMI	438
Diabetes mellitus	Male	15-19 years	95+ years	Global	-1	2	Age- and sex-specific SEV for Low fruit	66
Diabetes mellitus	Male	15-19 years	95+ years	Global	-1	2	Age- and sex-specific SEV for Low vegetables	297
Diabetes mellitus	Male	15-19 years	95+ years	Global	1	2	Age- and sex-specific SEV for Alcohol use	--
Diabetes mellitus	Male	15-19 years	95+ years	Global	1	2	Low-Density Lipoprotein (mmol/L)	--
Diabetes mellitus	Male	15-19 years	95+ years	Global	1	2	Systolic Blood Pressure (mmHg)	--
Diabetes mellitus	Male	15-19 years	95+ years	Global	1	2	sugar unadjusted(g)	--
Diabetes mellitus	Male	15-19 years	95+ years	Global	-1	3	Education (years per capita)	226
Diabetes mellitus	Male	15-19 years	95+ years	Global	-1	3	Healthcare access and quality index	608
Diabetes mellitus	Male	15-19 years	95+ years	Global	1	3	LDI (I$ per capita)	--
Diabetes mellitus type 1	Female	0-6 days	95+ years	Data Rich	-1	1	Healthcare access and quality index	1000
Diabetes mellitus type 1	Female	0-6 days	95+ years	Data Rich	1	2	Absolute value of average latitude	--
Diabetes mellitus type 1	Female	0-6 days	95+ years	Data Rich	1	2	Live Births 35+ (proportion)	--
Diabetes mellitus type 1	Female	0-6 days	95+ years	Data Rich	1	2	Live Births 40+ (proportion)	--
Diabetes mellitus type 1	Female	0-6 days	95+ years	Data Rich	-1	3	Socio-demographic Index	274
Diabetes mellitus type 1	Female	0-6 days	95+ years	Data Rich	-1	3	Education (years per capita)	329
Diabetes mellitus type 1	Female	0-6 days	95+ years	Global	-1	1	Healthcare access and quality index	1000
Diabetes mellitus type 1	Female	0-6 days	95+ years	Global	1	2	Absolute value of average latitude	--
Diabetes mellitus type 1	Female	0-6 days	95+ years	Global	1	2	Live Births 35+ (proportion)	--
Diabetes mellitus type 1	Female	0-6 days	95+ years	Global	1	2	Live Births 40+ (proportion)	--
Diabetes mellitus type 1	Female	0-6 days	95+ years	Global	-1	3	Education (years per capita)	--
Diabetes mellitus type 1	Female	0-6 days	95+ years	Global	-1	3	Socio-demographic Index	--
Diabetes mellitus type 1	Male	0-6 days	95+ years	Data Rich	-1	1	Healthcare access and quality index	1000
Diabetes mellitus type 1	Male	0-6 days	95+ years	Data Rich	1	2	Absolute value of average latitude	--
Diabetes mellitus type 1	Male	0-6 days	95+ years	Data Rich	1	2	Live Births 35+ (proportion)	--
Diabetes mellitus type 1	Male	0-6 days	95+ years	Data Rich	1	2	Live Births 40+ (proportion)	--
Diabetes mellitus type 1	Male	0-6 days	95+ years	Data Rich	-1	3	Education (years per capita)	--
Diabetes mellitus type 1	Male	0-6 days	95+ years	Data Rich	-1	3	Socio-demographic Index	--
Diabetes mellitus type 1	Male	0-6 days	95+ years	Global	-1	1	Healthcare access and quality index	1000
Diabetes mellitus type 1	Male	0-6 days	95+ years	Global	1	2	Absolute value of average latitude	--
Diabetes mellitus type 1	Male	0-6 days	95+ years	Global	1	2	Live Births 35+ (proportion)	--
Diabetes mellitus type 1	Male	0-6 days	95+ years	Global	1	2	Live Births 40+ (proportion)	--
Diabetes mellitus type 1	Male	0-6 days	95+ years	Global	-1	3	Education (years per capita)	--
Diabetes mellitus type 1	Male	0-6 days	95+ years	Global	-1	3	Socio-demographic Index	--
Diabetes mellitus type 2	Female	15-19 years	95+ years	Data Rich	1	1	Diabetes Age-Standardized Prevalence (proportion)	--
Diabetes mellitus type 2	Female	15-19 years	95+ years	Data Rich	1	1	Diabetes Fasting Plasma Glucose (mmol/L), age-standardized 25+	--
Diabetes mellitus type 2	Female	15-19 years	95+ years	Data Rich	1	1	Mean BMI	--
Diabetes mellitus type 2	Female	15-19 years	95+ years	Data Rich	1	1	Prevalence of obesity	--
Diabetes mellitus type 2	Female	15-19 years	95+ years	Data Rich	-1	2	Age- and sex-specific SEV for Low fruit	--
Diabetes mellitus type 2	Female	15-19 years	95+ years	Data Rich	-1	2	Age- and sex-specific SEV for Low vegetables	--
Diabetes mellitus type 2	Female	15-19 years	95+ years	Data Rich	1	2	Low-Density Lipoprotein (mmol/L)	454
Diabetes mellitus type 2	Female	15-19 years	95+ years	Data Rich	1	2	Systolic Blood Pressure (mmHg)	454
Diabetes mellitus type 2	Female	15-19 years	95+ years	Data Rich	1	2	Age- and sex-specific SEV for Alcohol use	--
Diabetes mellitus type 2	Female	15-19 years	95+ years	Data Rich	1	2	sugar unadjusted(g)	--
Diabetes mellitus type 2	Female	15-19 years	95+ years	Data Rich	-1	3	Education (years per capita)	546
Diabetes mellitus type 2	Female	15-19 years	95+ years	Data Rich	-1	3	Healthcare access and quality index	546
Diabetes mellitus type 2	Female	15-19 years	95+ years	Data Rich	1	3	LDI (I$ per capita)	--
Diabetes mellitus type 2	Female	15-19 years	95+ years	Global	1	1	Diabetes Age-Standardized Prevalence (proportion)	--
Diabetes mellitus type 2	Female	15-19 years	95+ years	Global	1	1	Diabetes Fasting Plasma Glucose (mmol/L), age-standardized 25+	--
Diabetes mellitus type 2	Female	15-19 years	95+ years	Global	1	1	Mean BMI	--
Diabetes mellitus type 2	Female	15-19 years	95+ years	Global	1	1	Prevalence of obesity	--
Diabetes mellitus type 2	Female	15-19 years	95+ years	Global	-1	2	Age- and sex-specific SEV for Low fruit	--
Diabetes mellitus type 2	Female	15-19 years	95+ years	Global	-1	2	Age- and sex-specific SEV for Low vegetables	--
Diabetes mellitus type 2	Female	15-19 years	95+ years	Global	1	2	Low-Density Lipoprotein (mmol/L)	602
Diabetes mellitus type 2	Female	15-19 years	95+ years	Global	1	2	Age- and sex-specific SEV for Alcohol use	--
Diabetes mellitus type 2	Female	15-19 years	95+ years	Global	1	2	Systolic Blood Pressure (mmHg)	--
Diabetes mellitus type 2	Female	15-19 years	95+ years	Global	1	2	sugar unadjusted(g)	--
Diabetes mellitus type 2	Female	15-19 years	95+ years	Global	-1	3	Healthcare access and quality index	398
Diabetes mellitus type 2	Female	15-19 years	95+ years	Global	-1	3	Education (years per capita)	726
Diabetes mellitus type 2	Female	15-19 years	95+ years	Global	1	3	LDI (I$ per capita)	--
Diabetes mellitus type 2	Male	15-19 years	95+ years	Data Rich	1	1	Diabetes Age-Standardized Prevalence (proportion)	414
Diabetes mellitus type 2	Male	15-19 years	95+ years	Data Rich	1	1	Mean BMI	414
Diabetes mellitus type 2	Male	15-19 years	95+ years	Data Rich	1	1	Diabetes Fasting Plasma Glucose (mmol/L), age-standardized 25+	586
Diabetes mellitus type 2	Male	15-19 years	95+ years	Data Rich	1	1	Prevalence of obesity	586
Diabetes mellitus type 2	Male	15-19 years	95+ years	Data Rich	-1	2	Age- and sex-specific SEV for Low fruit	--
Diabetes mellitus type 2	Male	15-19 years	95+ years	Data Rich	-1	2	Age- and sex-specific SEV for Low vegetables	--
Diabetes mellitus type 2	Male	15-19 years	95+ years	Data Rich	1	2	Age- and sex-specific SEV for Alcohol use	--
Diabetes mellitus type 2	Male	15-19 years	95+ years	Data Rich	1	2	Low-Density Lipoprotein (mmol/L)	--
Diabetes mellitus type 2	Male	15-19 years	95+ years	Data Rich	1	2	Systolic Blood Pressure (mmHg)	--
Diabetes mellitus type 2	Male	15-19 years	95+ years	Data Rich	1	2	sugar unadjusted(g)	--
Diabetes mellitus type 2	Male	15-19 years	95+ years	Data Rich	-1	3	Education (years per capita)	473
Diabetes mellitus type 2	Male	15-19 years	95+ years	Data Rich	-1	3	Healthcare access and quality index	512
Diabetes mellitus type 2	Male	15-19 years	95+ years	Data Rich	1	3	LDI (I$ per capita)	--
Diabetes mellitus type 2	Male	15-19 years	95+ years	Global	1	1	Mean BMI	402
Diabetes mellitus type 2	Male	15-19 years	95+ years	Global	1	1	Diabetes Age-Standardized Prevalence (proportion)	598
Diabetes mellitus type 2	Male	15-19 years	95+ years	Global	1	1	Diabetes Fasting Plasma Glucose (mmol/L), age-standardized 25+	--
Diabetes mellitus type 2	Male	15-19 years	95+ years	Global	1	1	Prevalence of obesity	--
Diabetes mellitus type 2	Male	15-19 years	95+ years	Global	-1	2	Age- and sex-specific SEV for Low fruit	326
Diabetes mellitus type 2	Male	15-19 years	95+ years	Global	-1	2	Age- and sex-specific SEV for Low vegetables	525
Diabetes mellitus type 2	Male	15-19 years	95+ years	Global	1	2	Systolic Blood Pressure (mmHg)	326
Diabetes mellitus type 2	Male	15-19 years	95+ years	Global	1	2	Age- and sex-specific SEV for Alcohol use	--
Diabetes mellitus type 2	Male	15-19 years	95+ years	Global	1	2	Low-Density Lipoprotein (mmol/L)	--
Diabetes mellitus type 2	Male	15-19 years	95+ years	Global	1	2	sugar unadjusted(g)	--
Diabetes mellitus type 2	Male	15-19 years	95+ years	Global	-1	3	Education (years per capita)	489
Diabetes mellitus type 2	Male	15-19 years	95+ years	Global	-1	3	Healthcare access and quality index	675
Diabetes mellitus type 2	Male	15-19 years	95+ years	Global	1	3	LDI (I$ per capita)	--
Chronic kidney disease	Female	0-6 days	95+ years	Data Rich	-1	1	Healthcare access and quality index	--
Chronic kidney disease	Female	0-6 days	95+ years	Data Rich	1	1	Mean BMI	1000

Appendix Table S10: CODEm covariates used, level of covariate, and expected direction of covariate by cause, sex, and age

Cause	Sex	Age Start	Age End	Model Version Type	Direction	Level	Covariate Name	Number of Draws
Chronic kidney disease	Female	0-6 days	95+ years	Data Rich	1	1	Systolic Blood Pressure (mmHg)	--
Chronic kidney disease	Female	0-6 days	95+ years	Data Rich	1	2	Low-Density Lipoprotein (mmol/L)	--
Chronic kidney disease	Female	0-6 days	95+ years	Data Rich	1	2	energy unadjusted(kcal)	--
Chronic kidney disease	Female	0-6 days	95+ years	Data Rich	1	2	red meats unadjusted(g)	--
Chronic kidney disease	Female	0-6 days	95+ years	Data Rich	-1	3	Education (years per capita)	568
Chronic kidney disease	Female	0-6 days	95+ years	Data Rich	-1	3	LDI (I$ per capita)	--
Chronic kidney disease	Female	0-6 days	95+ years	Data Rich	-1	3	Socio-demographic Index	--
Chronic kidney disease	Female	0-6 days	95+ years	Global	-1	1	Healthcare access and quality index	796
Chronic kidney disease	Female	0-6 days	95+ years	Global	1	1	Mean BMI	792
Chronic kidney disease	Female	0-6 days	95+ years	Global	1	1	Systolic Blood Pressure (mmHg)	--
Chronic kidney disease	Female	0-6 days	95+ years	Global	1	2	energy unadjusted(kcal)	92
Chronic kidney disease	Female	0-6 days	95+ years	Global	1	2	Low-Density Lipoprotein (mmol/L)	--
Chronic kidney disease	Female	0-6 days	95+ years	Global	1	2	red meats unadjusted(g)	--
Chronic kidney disease	Female	0-6 days	95+ years	Global	-1	3	Socio-demographic Index	128
Chronic kidney disease	Female	0-6 days	95+ years	Global	-1	3	Education (years per capita)	364
Chronic kidney disease	Female	0-6 days	95+ years	Global	-1	3	LDI (I$ per capita)	--
Chronic kidney disease	Male	0-6 days	95+ years	Data Rich	-1	1	Healthcare access and quality index	633
Chronic kidney disease	Male	0-6 days	95+ years	Data Rich	1	1	Mean BMI	640
Chronic kidney disease	Male	0-6 days	95+ years	Data Rich	1	1	Systolic Blood Pressure (mmHg)	--
Chronic kidney disease	Male	0-6 days	95+ years	Data Rich	1	2	Low-Density Lipoprotein (mmol/L)	--
Chronic kidney disease	Male	0-6 days	95+ years	Data Rich	1	2	energy unadjusted(kcal)	--
Chronic kidney disease	Male	0-6 days	95+ years	Data Rich	1	2	red meats unadjusted(g)	--
Chronic kidney disease	Male	0-6 days	95+ years	Data Rich	-1	3	Education (years per capita)	208
Chronic kidney disease	Male	0-6 days	95+ years	Data Rich	-1	3	LDI (I$ per capita)	--
Chronic kidney disease	Male	0-6 days	95+ years	Data Rich	-1	3	Socio-demographic Index	--
Chronic kidney disease	Male	0-6 days	95+ years	Global	-1	1	Healthcare access and quality index	1000
Chronic kidney disease	Male	0-6 days	95+ years	Global	1	1	Mean BMI	546
Chronic kidney disease	Male	0-6 days	95+ years	Global	1	1	Systolic Blood Pressure (mmHg)	--
Chronic kidney disease	Male	0-6 days	95+ years	Global	1	2	Low-Density Lipoprotein (mmol/L)	--
Chronic kidney disease	Male	0-6 days	95+ years	Global	1	2	energy unadjusted(kcal)	--
Chronic kidney disease	Male	0-6 days	95+ years	Global	1	2	red meats unadjusted(g)	--
Chronic kidney disease	Male	0-6 days	95+ years	Global	-1	3	Education (years per capita)	--
Chronic kidney disease	Male	0-6 days	95+ years	Global	-1	3	LDI (I$ per capita)	--
Chronic kidney disease	Male	0-6 days	95+ years	Global	-1	3	Socio-demographic Index	--
Acute glomerulonephritis	Female	0-6 days	95+ years	Data Rich	-1	2	Sanitation (proportion with access)	86
Acute glomerulonephritis	Female	0-6 days	95+ years	Data Rich	-1	2	Improved Water Source (proportion with access)	399
Acute glomerulonephritis	Female	0-6 days	95+ years	Data Rich	-1	2	Healthcare access and quality index	446
Acute glomerulonephritis	Female	0-6 days	95+ years	Data Rich	1	2	Systolic Blood Pressure (mmHg)	404
Acute glomerulonephritis	Female	0-6 days	95+ years	Data Rich	-1	3	Socio-demographic Index	161
Acute glomerulonephritis	Female	0-6 days	95+ years	Data Rich	-1	3	Education (years per capita)	194
Acute glomerulonephritis	Female	0-6 days	95+ years	Data Rich	-1	3	LDI (I$ per capita)	--
Acute glomerulonephritis	Female	0-6 days	95+ years	Global	-1	2	Sanitation (proportion with access)	192
Acute glomerulonephritis	Female	0-6 days	95+ years	Global	-1	2	Healthcare access and quality index	428
Acute glomerulonephritis	Female	0-6 days	95+ years	Global	-1	2	Improved Water Source (proportion with access)	808
Acute glomerulonephritis	Female	0-6 days	95+ years	Global	1	2	Systolic Blood Pressure (mmHg)	404
Acute glomerulonephritis	Female	0-6 days	95+ years	Global	-1	3	Education (years per capita)	160
Acute glomerulonephritis	Female	0-6 days	95+ years	Global	-1	3	Socio-demographic Index	220
Acute glomerulonephritis	Female	0-6 days	95+ years	Global	-1	3	LDI (I$ per capita)	--
Acute glomerulonephritis	Male	0-6 days	95+ years	Data Rich	-1	2	Improved Water Source (proportion with access)	325
Acute glomerulonephritis	Male	0-6 days	95+ years	Data Rich	-1	2	Healthcare access and quality index	476
Acute glomerulonephritis	Male	0-6 days	95+ years	Data Rich	-1	2	Sanitation (proportion with access)	545
Acute glomerulonephritis	Male	0-6 days	95+ years	Data Rich	1	2	Systolic Blood Pressure (mmHg)	675
Acute glomerulonephritis	Male	0-6 days	95+ years	Data Rich	-1	3	Socio-demographic Index	52
Acute glomerulonephritis	Male	0-6 days	95+ years	Data Rich	-1	3	Education (years per capita)	--
Acute glomerulonephritis	Male	0-6 days	95+ years	Data Rich	-1	3	LDI (I$ per capita)	--
Acute glomerulonephritis	Male	0-6 days	95+ years	Global	-1	2	Improved Water Source (proportion with access)	329
Acute glomerulonephritis	Male	0-6 days	95+ years	Global	-1	2	Healthcare access and quality index	463
Acute glomerulonephritis	Male	0-6 days	95+ years	Global	-1	2	Sanitation (proportion with access)	537
Acute glomerulonephritis	Male	0-6 days	95+ years	Global	1	2	Systolic Blood Pressure (mmHg)	755
Acute glomerulonephritis	Male	0-6 days	95+ years	Global	-1	3	Education (years per capita)	173
Acute glomerulonephritis	Male	0-6 days	95+ years	Global	-1	3	Socio-demographic Index	173
Acute glomerulonephritis	Male	0-6 days	95+ years	Global	-1	3	LDI (I$ per capita)	--
Skin and subcutaneous diseases	Female	0-6 days	95+ years	Data Rich	-1	1	Healthcare access and quality index	564
Skin and subcutaneous diseases	Female	0-6 days	95+ years	Data Rich	-1	1	Improved Water Source (proportion with access)	--
Skin and subcutaneous diseases	Female	0-6 days	95+ years	Data Rich	1	1	Age- and sex-specific SEV for Unsafe sanitation	108
Skin and subcutaneous diseases	Female	0-6 days	95+ years	Data Rich	1	1	Diabetes Fasting Plasma Glucose (mmol/L), by age	429
Skin and subcutaneous diseases	Female	0-6 days	95+ years	Data Rich	1	1	Prevalence of overweight and obesity	1000
Skin and subcutaneous diseases	Female	0-6 days	95+ years	Data Rich	1	2	Cumulative Cigarettes (10 Years)	--
Skin and subcutaneous diseases	Female	0-6 days	95+ years	Data Rich	1	2	Cumulative Cigarettes (5 Years)	--
Skin and subcutaneous diseases	Female	0-6 days	95+ years	Data Rich	1	2	Liters of alcohol consumed per capita	--
Skin and subcutaneous diseases	Female	0-6 days	95+ years	Data Rich	1	2	Smoking Prevalence	--
Skin and subcutaneous diseases	Female	0-6 days	95+ years	Data Rich	-1	3	Socio-demographic Index	186
Skin and subcutaneous diseases	Female	0-6 days	95+ years	Data Rich	-1	3	Education (years per capita)	--
Skin and subcutaneous diseases	Female	0-6 days	95+ years	Data Rich	-1	3	LDI (I$ per capita)	--
Skin and subcutaneous diseases	Female	0-6 days	95+ years	Global	-1	1	Healthcare access and quality index	216
Skin and subcutaneous diseases	Female	0-6 days	95+ years	Global	-1	1	Improved Water Source (proportion with access)	472
Skin and subcutaneous diseases	Female	0-6 days	95+ years	Global	1	1	Age- and sex-specific SEV for Unsafe sanitation	348
Skin and subcutaneous diseases	Female	0-6 days	95+ years	Global	1	1	Diabetes Fasting Plasma Glucose (mmol/L), by age	561
Skin and subcutaneous diseases	Female	0-6 days	95+ years	Global	1	1	Prevalence of overweight and obesity	593
Skin and subcutaneous diseases	Female	0-6 days	95+ years	Global	1	2	Cumulative Cigarettes (10 Years)	--
Skin and subcutaneous diseases	Female	0-6 days	95+ years	Global	1	2	Cumulative Cigarettes (5 Years)	--
Skin and subcutaneous diseases	Female	0-6 days	95+ years	Global	1	2	Liters of alcohol consumed per capita	--
Skin and subcutaneous diseases	Female	0-6 days	95+ years	Global	1	2	Smoking Prevalence	--
Skin and subcutaneous diseases	Female	0-6 days	95+ years	Global	-1	3	Socio-demographic Index	149
Skin and subcutaneous diseases	Female	0-6 days	95+ years	Global	-1	3	Education (years per capita)	196
Skin and subcutaneous diseases	Female	0-6 days	95+ years	Global	-1	3	LDI (I$ per capita)	--
Skin and subcutaneous diseases	Male	0-6 days	95+ years	Data Rich	-1	1	Healthcare access and quality index	568
Skin and subcutaneous diseases	Male	0-6 days	95+ years	Data Rich	-1	1	Improved Water Source (proportion with access)	--
Skin and subcutaneous diseases	Male	0-6 days	95+ years	Data Rich	1	1	Prevalence of overweight and obesity	1000
Skin and subcutaneous diseases	Male	0-6 days	95+ years	Data Rich	1	1	Age- and sex-specific SEV for Unsafe sanitation	--
Skin and subcutaneous diseases	Male	0-6 days	95+ years	Data Rich	1	1	Diabetes Fasting Plasma Glucose (mmol/L), by age	--
Skin and subcutaneous diseases	Male	0-6 days	95+ years	Data Rich	1	2	Cumulative Cigarettes (10 Years)	--
Skin and subcutaneous diseases	Male	0-6 days	95+ years	Data Rich	1	2	Cumulative Cigarettes (5 Years)	--
Skin and subcutaneous diseases	Male	0-6 days	95+ years	Data Rich	1	2	Liters of alcohol consumed per capita	--
Skin and subcutaneous diseases	Male	0-6 days	95+ years	Data Rich	1	2	Smoking Prevalence	--
Skin and subcutaneous diseases	Male	0-6 days	95+ years	Data Rich	-1	3	Education (years per capita)	--
Skin and subcutaneous diseases	Male	0-6 days	95+ years	Data Rich	-1	3	LDI (I$ per capita)	--
Skin and subcutaneous diseases	Male	0-6 days	95+ years	Data Rich	-1	3	Socio-demographic Index	--
Skin and subcutaneous diseases	Male	0-6 days	95+ years	Global	-1	1	Improved Water Source (proportion with access)	341
Skin and subcutaneous diseases	Male	0-6 days	95+ years	Global	-1	1	Healthcare access and quality index	361
Skin and subcutaneous diseases	Male	0-6 days	95+ years	Global	1	1	Age- and sex-specific SEV for Unsafe sanitation	335
Skin and subcutaneous diseases	Male	0-6 days	95+ years	Global	1	1	Diabetes Fasting Plasma Glucose (mmol/L), by age	524
Skin and subcutaneous diseases	Male	0-6 days	95+ years	Global	1	1	Prevalence of overweight and obesity	893
Skin and subcutaneous diseases	Male	0-6 days	95+ years	Global	1	2	Cumulative Cigarettes (10 Years)	--
Skin and subcutaneous diseases	Male	0-6 days	95+ years	Global	1	2	Cumulative Cigarettes (5 Years)	--
Skin and subcutaneous diseases	Male	0-6 days	95+ years	Global	1	2	Liters of alcohol consumed per capita	--
Skin and subcutaneous diseases	Male	0-6 days	95+ years	Global	1	2	Smoking Prevalence	--
Skin and subcutaneous diseases	Male	0-6 days	95+ years	Global	-1	3	Education (years per capita)	32
Skin and subcutaneous diseases	Male	0-6 days	95+ years	Global	-1	3	Socio-demographic Index	98

Appendix Table S10: CODEm covariates used, level of covariate, and expected direction of covariate by cause, sex, and age								
Cause	Sex	Age Start	Age End	Model Version Type	Direction	Level	Covariate Name	Number of Draws
Skin and subcutaneous diseases	Male	0-6 days	95+ years	Global	-1	3	LDI (I$ per capita)	--
Bacterial skin diseases	Female	0-6 days	95+ years	Data Rich	-1	1	Healthcare access and quality index	--
Bacterial skin diseases	Female	0-6 days	95+ years	Data Rich	-1	1	Improved Water Source (proportion with access)	--
Bacterial skin diseases	Female	0-6 days	95+ years	Data Rich	1	1	Age- and sex-specific SEV for Unsafe sanitation	642
Bacterial skin diseases	Female	0-6 days	95+ years	Data Rich	1	1	Diabetes Fasting Plasma Glucose (mmol/L), by age	898
Bacterial skin diseases	Female	0-6 days	95+ years	Data Rich	1	1	Prevalence of overweight and obesity	1000
Bacterial skin diseases	Female	0-6 days	95+ years	Data Rich	1	2	Cumulative Cigarettes (10 Years)	--
Bacterial skin diseases	Female	0-6 days	95+ years	Data Rich	1	2	Cumulative Cigarettes (5 Years)	--
Bacterial skin diseases	Female	0-6 days	95+ years	Data Rich	1	2	Liters of alcohol consumed per capita	--
Bacterial skin diseases	Female	0-6 days	95+ years	Data Rich	1	2	Smoking Prevalence	--
Bacterial skin diseases	Female	0-6 days	95+ years	Data Rich	-1	3	Education (years per capita)	--
Bacterial skin diseases	Female	0-6 days	95+ years	Data Rich	-1	3	LDI (I$ per capita)	--
Bacterial skin diseases	Female	0-6 days	95+ years	Data Rich	-1	3	Socio-demographic Index	--
Bacterial skin diseases	Female	0-6 days	95+ years	Global	-1	1	Healthcare access and quality index	140
Bacterial skin diseases	Female	0-6 days	95+ years	Global	-1	1	Improved Water Source (proportion with access)	476
Bacterial skin diseases	Female	0-6 days	95+ years	Global	1	1	Diabetes Fasting Plasma Glucose (mmol/L), by age	420
Bacterial skin diseases	Female	0-6 days	95+ years	Global	1	1	Age- and sex-specific SEV for Unsafe sanitation	506
Bacterial skin diseases	Female	0-6 days	95+ years	Global	1	1	Prevalence of overweight and obesity	865
Bacterial skin diseases	Female	0-6 days	95+ years	Global	1	2	Cumulative Cigarettes (10 Years)	--
Bacterial skin diseases	Female	0-6 days	95+ years	Global	1	2	Cumulative Cigarettes (5 Years)	--
Bacterial skin diseases	Female	0-6 days	95+ years	Global	1	2	Liters of alcohol consumed per capita	--
Bacterial skin diseases	Female	0-6 days	95+ years	Global	1	2	Smoking Prevalence	--
Bacterial skin diseases	Female	0-6 days	95+ years	Global	-1	3	Socio-demographic Index	21
Bacterial skin diseases	Female	0-6 days	95+ years	Global	-1	3	Education (years per capita)	42
Bacterial skin diseases	Female	0-6 days	95+ years	Global	-1	3	LDI (I$ per capita)	--
Bacterial skin diseases	Male	0-6 days	95+ years	Data Rich	-1	1	Healthcare access and quality index	--
Bacterial skin diseases	Male	0-6 days	95+ years	Data Rich	-1	1	Improved Water Source (proportion with access)	--
Bacterial skin diseases	Male	0-6 days	95+ years	Data Rich	1	1	Diabetes Fasting Plasma Glucose (mmol/L), by age	1000
Bacterial skin diseases	Male	0-6 days	95+ years	Data Rich	1	1	Age- and sex-specific SEV for Unsafe sanitation	--
Bacterial skin diseases	Male	0-6 days	95+ years	Data Rich	1	1	Prevalence of overweight and obesity	--
Bacterial skin diseases	Male	0-6 days	95+ years	Data Rich	1	2	Cumulative Cigarettes (10 Years)	--
Bacterial skin diseases	Male	0-6 days	95+ years	Data Rich	1	2	Cumulative Cigarettes (5 Years)	--
Bacterial skin diseases	Male	0-6 days	95+ years	Data Rich	1	2	Liters of alcohol consumed per capita	--
Bacterial skin diseases	Male	0-6 days	95+ years	Data Rich	1	2	Smoking Prevalence	--
Bacterial skin diseases	Male	0-6 days	95+ years	Data Rich	-1	3	Education (years per capita)	--
Bacterial skin diseases	Male	0-6 days	95+ years	Data Rich	-1	3	LDI (I$ per capita)	--
Bacterial skin diseases	Male	0-6 days	95+ years	Data Rich	-1	3	Socio-demographic Index	--
Bacterial skin diseases	Male	0-6 days	95+ years	Global	-1	1	Healthcare access and quality index	182
Bacterial skin diseases	Male	0-6 days	95+ years	Global	-1	1	Improved Water Source (proportion with access)	576
Bacterial skin diseases	Male	0-6 days	95+ years	Global	1	1	Diabetes Fasting Plasma Glucose (mmol/L), by age	471
Bacterial skin diseases	Male	0-6 days	95+ years	Global	1	1	Age- and sex-specific SEV for Unsafe sanitation	535
Bacterial skin diseases	Male	0-6 days	95+ years	Global	1	1	Prevalence of overweight and obesity	953
Bacterial skin diseases	Male	0-6 days	95+ years	Global	1	2	Cumulative Cigarettes (10 Years)	--
Bacterial skin diseases	Male	0-6 days	95+ years	Global	1	2	Cumulative Cigarettes (5 Years)	--
Bacterial skin diseases	Male	0-6 days	95+ years	Global	1	2	Liters of alcohol consumed per capita	--
Bacterial skin diseases	Male	0-6 days	95+ years	Global	1	2	Smoking Prevalence	--
Bacterial skin diseases	Male	0-6 days	95+ years	Global	-1	3	Socio-demographic Index	15
Bacterial skin diseases	Male	0-6 days	95+ years	Global	-1	3	Education (years per capita)	39
Bacterial skin diseases	Male	0-6 days	95+ years	Global	-1	3	LDI (I$ per capita)	--
Cellulitis	Female	0-6 days	95+ years	Data Rich	-1	1	Healthcare access and quality index	102
Cellulitis	Female	0-6 days	95+ years	Data Rich	1	1	Prevalence of overweight and obesity	358
Cellulitis	Female	0-6 days	95+ years	Data Rich	1	1	Diabetes Fasting Plasma Glucose (mmol/L), by age	1000
Cellulitis	Female	0-6 days	95+ years	Data Rich	-1	2	LDI (I$ per capita)	--
Cellulitis	Female	0-6 days	95+ years	Data Rich	-1	3	Education (years per capita)	--
Cellulitis	Female	0-6 days	95+ years	Global	-1	1	Healthcare access and quality index	152
Cellulitis	Female	0-6 days	95+ years	Global	1	1	Diabetes Fasting Plasma Glucose (mmol/L), by age	496
Cellulitis	Female	0-6 days	95+ years	Global	1	1	Prevalence of overweight and obesity	900
Cellulitis	Female	0-6 days	95+ years	Global	-1	2	LDI (I$ per capita)	--
Cellulitis	Female	0-6 days	95+ years	Global	-1	3	Education (years per capita)	--
Cellulitis	Male	0-6 days	95+ years	Data Rich	-1	1	Healthcare access and quality index	1000
Cellulitis	Male	0-6 days	95+ years	Data Rich	1	1	Prevalence of overweight and obesity	1000
Cellulitis	Male	0-6 days	95+ years	Data Rich	1	1	Diabetes Fasting Plasma Glucose (mmol/L), by age	--
Cellulitis	Male	0-6 days	95+ years	Data Rich	-1	2	LDI (I$ per capita)	--
Cellulitis	Male	0-6 days	95+ years	Data Rich	-1	3	Education (years per capita)	--
Cellulitis	Male	0-6 days	95+ years	Global	-1	1	Healthcare access and quality index	--
Cellulitis	Male	0-6 days	95+ years	Global	1	1	Prevalence of overweight and obesity	546
Cellulitis	Male	0-6 days	95+ years	Global	1	1	Diabetes Fasting Plasma Glucose (mmol/L), by age	1000
Cellulitis	Male	0-6 days	95+ years	Global	-1	2	LDI (I$ per capita)	--
Cellulitis	Male	0-6 days	95+ years	Global	-1	3	Education (years per capita)	--
Pyoderma	Female	0-6 days	95+ years	Data Rich	-1	1	Healthcare access and quality index	--
Pyoderma	Female	0-6 days	95+ years	Data Rich	-1	1	Improved Water Source (proportion with access)	--
Pyoderma	Female	0-6 days	95+ years	Data Rich	1	1	Diabetes Fasting Plasma Glucose (mmol/L), by age	744
Pyoderma	Female	0-6 days	95+ years	Data Rich	1	1	Age- and sex-specific SEV for Unsafe sanitation	898
Pyoderma	Female	0-6 days	95+ years	Data Rich	1	1	Prevalence of overweight and obesity	898
Pyoderma	Female	0-6 days	95+ years	Data Rich	1	2	Cumulative Cigarettes (10 Years)	--
Pyoderma	Female	0-6 days	95+ years	Data Rich	1	2	Cumulative Cigarettes (5 Years)	--
Pyoderma	Female	0-6 days	95+ years	Data Rich	1	2	Liters of alcohol consumed per capita	--
Pyoderma	Female	0-6 days	95+ years	Data Rich	1	2	Smoking Prevalence	--
Pyoderma	Female	0-6 days	95+ years	Data Rich	-1	3	Education (years per capita)	--
Pyoderma	Female	0-6 days	95+ years	Data Rich	-1	3	LDI (I$ per capita)	--
Pyoderma	Female	0-6 days	95+ years	Data Rich	-1	3	Socio-demographic Index	--
Pyoderma	Female	0-6 days	95+ years	Global	-1	1	Healthcare access and quality index	150
Pyoderma	Female	0-6 days	95+ years	Global	-1	1	Improved Water Source (proportion with access)	553
Pyoderma	Female	0-6 days	95+ years	Global	1	1	Age- and sex-specific SEV for Unsafe sanitation	400
Pyoderma	Female	0-6 days	95+ years	Global	1	1	Diabetes Fasting Plasma Glucose (mmol/L), by age	634
Pyoderma	Female	0-6 days	95+ years	Global	1	1	Prevalence of overweight and obesity	962
Pyoderma	Female	0-6 days	95+ years	Global	1	2	Cumulative Cigarettes (10 Years)	--
Pyoderma	Female	0-6 days	95+ years	Global	1	2	Cumulative Cigarettes (5 Years)	--
Pyoderma	Female	0-6 days	95+ years	Global	1	2	Liters of alcohol consumed per capita	--
Pyoderma	Female	0-6 days	95+ years	Global	1	2	Smoking Prevalence	--
Pyoderma	Female	0-6 days	95+ years	Global	-1	3	Socio-demographic Index	99
Pyoderma	Female	0-6 days	95+ years	Global	-1	3	Education (years per capita)	--
Pyoderma	Female	0-6 days	95+ years	Global	-1	3	LDI (I$ per capita)	--
Pyoderma	Male	0-6 days	95+ years	Data Rich	-1	1	Healthcare access and quality index	--
Pyoderma	Male	0-6 days	95+ years	Data Rich	-1	1	Improved Water Source (proportion with access)	--
Pyoderma	Male	0-6 days	95+ years	Data Rich	1	1	Age- and sex-specific SEV for Unsafe sanitation	715
Pyoderma	Male	0-6 days	95+ years	Data Rich	1	1	Prevalence of overweight and obesity	715
Pyoderma	Male	0-6 days	95+ years	Data Rich	1	1	Diabetes Fasting Plasma Glucose (mmol/L), by age	1000
Pyoderma	Male	0-6 days	95+ years	Data Rich	1	2	Cumulative Cigarettes (10 Years)	--
Pyoderma	Male	0-6 days	95+ years	Data Rich	1	2	Cumulative Cigarettes (5 Years)	--

Appendix Table S10: CODEm covariates used, level of covariate, and expected direction of covariate by cause, sex, and age

Cause	Sex	Age Start	Age End	Model Version Type	Direction	Level	Covariate Name	Number of Draws
Pyoderma	Male	0-6 days	95+ years	Data Rich	1	2	Liters of alcohol consumed per capita	--
Pyoderma	Male	0-6 days	95+ years	Data Rich	1	2	Smoking Prevalence	--
Pyoderma	Male	0-6 days	95+ years	Data Rich	-1	3	Education (years per capita)	--
Pyoderma	Male	0-6 days	95+ years	Data Rich	-1	3	LDI (I$ per capita)	--
Pyoderma	Male	0-6 days	95+ years	Data Rich	-1	3	Socio-demographic Index	--
Pyoderma	Male	0-6 days	95+ years	Global	-1	1	Improved Water Source (proportion with access)	139
Pyoderma	Male	0-6 days	95+ years	Global	-1	1	Healthcare access and quality index	396
Pyoderma	Male	0-6 days	95+ years	Global	1	1	Age- and sex-specific SEV for Unsafe sanitation	345
Pyoderma	Male	0-6 days	95+ years	Global	1	1	Diabetes Fasting Plasma Glucose (mmol/L), by age	723
Pyoderma	Male	0-6 days	95+ years	Global	1	1	Prevalence of overweight and obesity	953
Pyoderma	Male	0-6 days	95+ years	Global	1	2	Cumulative Cigarettes (10 Years)	--
Pyoderma	Male	0-6 days	95+ years	Global	1	2	Cumulative Cigarettes (5 Years)	--
Pyoderma	Male	0-6 days	95+ years	Global	1	2	Liters of alcohol consumed per capita	--
Pyoderma	Male	0-6 days	95+ years	Global	1	2	Smoking Prevalence	--
Pyoderma	Male	0-6 days	95+ years	Global	-1	3	Education (years per capita)	--
Pyoderma	Male	0-6 days	95+ years	Global	-1	3	LDI (I$ per capita)	--
Pyoderma	Male	0-6 days	95+ years	Global	-1	3	Socio-demographic Index	--
Decubitus ulcer	Female	12-23 months	95+ years	Data Rich	-1	1	Improved Water Source (proportion with access)	--
Decubitus ulcer	Female	12-23 months	95+ years	Data Rich	1	1	Diabetes Fasting Plasma Glucose (mmol/L), age-standardized 25+	--
Decubitus ulcer	Female	12-23 months	95+ years	Data Rich	1	1	Prevalence of obesity	--
Decubitus ulcer	Female	12-23 months	95+ years	Data Rich	-1	2	Healthcare access and quality index	297
Decubitus ulcer	Female	12-23 months	95+ years	Data Rich	1	2	Cumulative Cigarettes (10 Years)	572
Decubitus ulcer	Female	12-23 months	95+ years	Data Rich	1	2	Cumulative Cigarettes (5 Years)	--
Decubitus ulcer	Female	12-23 months	95+ years	Data Rich	1	2	Liters of alcohol consumed per capita	--
Decubitus ulcer	Female	12-23 months	95+ years	Data Rich	1	2	Smoking Prevalence	--
Decubitus ulcer	Female	12-23 months	95+ years	Data Rich	-1	3	Education (years per capita)	171
Decubitus ulcer	Female	12-23 months	95+ years	Data Rich	-1	3	Socio-demographic Index	356
Decubitus ulcer	Female	12-23 months	95+ years	Data Rich	-1	3	LDI (I$ per capita)	--
Decubitus ulcer	Female	12-23 months	95+ years	Data Rich	1	3	Age- and sex-specific SEV for Unsafe sanitation	--
Decubitus ulcer	Female	12-23 months	95+ years	Global	-1	1	Improved Water Source (proportion with access)	--
Decubitus ulcer	Female	12-23 months	95+ years	Global	1	1	Diabetes Fasting Plasma Glucose (mmol/L), age-standardized 25+	--
Decubitus ulcer	Female	12-23 months	95+ years	Global	1	1	Liters of alcohol consumed per capita	--
Decubitus ulcer	Female	12-23 months	95+ years	Global	1	1	Prevalence of obesity	--
Decubitus ulcer	Female	12-23 months	95+ years	Global	-1	2	Healthcare access and quality index	--
Decubitus ulcer	Female	12-23 months	95+ years	Global	1	2	Cumulative Cigarettes (10 Years)	328
Decubitus ulcer	Female	12-23 months	95+ years	Global	1	2	Cumulative Cigarettes (5 Years)	398
Decubitus ulcer	Female	12-23 months	95+ years	Global	1	2	Smoking Prevalence	--
Decubitus ulcer	Female	12-23 months	95+ years	Global	-1	3	Education (years per capita)	274
Decubitus ulcer	Female	12-23 months	95+ years	Global	-1	3	LDI (I$ per capita)	--
Decubitus ulcer	Female	12-23 months	95+ years	Global	-1	3	Socio-demographic Index	--
Decubitus ulcer	Female	12-23 months	95+ years	Global	1	3	Age- and sex-specific SEV for Unsafe sanitation	--
Decubitus ulcer	Male	12-23 months	95+ years	Data Rich	-1	1	Improved Water Source (proportion with access)	--
Decubitus ulcer	Male	12-23 months	95+ years	Data Rich	1	1	Diabetes Fasting Plasma Glucose (mmol/L), age-standardized 25+	--
Decubitus ulcer	Male	12-23 months	95+ years	Data Rich	1	1	Prevalence of obesity	--
Decubitus ulcer	Male	12-23 months	95+ years	Data Rich	-1	2	Healthcare access and quality index	272
Decubitus ulcer	Male	12-23 months	95+ years	Data Rich	1	2	Cumulative Cigarettes (10 Years)	224
Decubitus ulcer	Male	12-23 months	95+ years	Data Rich	1	2	Smoking Prevalence	362
Decubitus ulcer	Male	12-23 months	95+ years	Data Rich	1	2	Cumulative Cigarettes (5 Years)	--
Decubitus ulcer	Male	12-23 months	95+ years	Data Rich	1	2	Liters of alcohol consumed per capita	--
Decubitus ulcer	Male	12-23 months	95+ years	Data Rich	-1	3	Socio-demographic Index	204
Decubitus ulcer	Male	12-23 months	95+ years	Data Rich	-1	3	Education (years per capita)	374
Decubitus ulcer	Male	12-23 months	95+ years	Data Rich	-1	3	LDI (I$ per capita)	--
Decubitus ulcer	Male	12-23 months	95+ years	Data Rich	1	3	Age- and sex-specific SEV for Unsafe sanitation	--
Decubitus ulcer	Male	12-23 months	95+ years	Global	-1	1	Improved Water Source (proportion with access)	--
Decubitus ulcer	Male	12-23 months	95+ years	Global	1	1	Diabetes Fasting Plasma Glucose (mmol/L), age-standardized 25+	--
Decubitus ulcer	Male	12-23 months	95+ years	Global	1	1	Liters of alcohol consumed per capita	--
Decubitus ulcer	Male	12-23 months	95+ years	Global	1	1	Prevalence of obesity	--
Decubitus ulcer	Male	12-23 months	95+ years	Global	-1	2	Healthcare access and quality index	546
Decubitus ulcer	Male	12-23 months	95+ years	Global	1	2	Smoking Prevalence	454
Decubitus ulcer	Male	12-23 months	95+ years	Global	1	2	Cumulative Cigarettes (10 Years)	546
Decubitus ulcer	Male	12-23 months	95+ years	Global	1	2	Cumulative Cigarettes (5 Years)	--
Decubitus ulcer	Male	12-23 months	95+ years	Global	-1	3	Education (years per capita)	--
Decubitus ulcer	Male	12-23 months	95+ years	Global	-1	3	LDI (I$ per capita)	--
Decubitus ulcer	Male	12-23 months	95+ years	Global	-1	3	Socio-demographic Index	--
Decubitus ulcer	Male	12-23 months	95+ years	Global	1	3	Age- and sex-specific SEV for Unsafe sanitation	--
Other skin and subcutaneous diseases	Female	0-6 days	95+ years	Data Rich	-1	1	Healthcare access and quality index	0
Other skin and subcutaneous diseases	Female	0-6 days	95+ years	Data Rich	-1	1	Improved Water Source (proportion with access)	491
Other skin and subcutaneous diseases	Female	0-6 days	95+ years	Data Rich	1	1	Prevalence of overweight and obesity	0
Other skin and subcutaneous diseases	Female	0-6 days	95+ years	Data Rich	1	1	Age- and sex-specific SEV for Unsafe sanitation	54
Other skin and subcutaneous diseases	Female	0-6 days	95+ years	Data Rich	1	1	Age-standardized SEV for Child underweight	482
Other skin and subcutaneous diseases	Female	0-6 days	95+ years	Data Rich	1	1	Diabetes Fasting Plasma Glucose (mmol/L), by age	779
Other skin and subcutaneous diseases	Female	0-6 days	95+ years	Data Rich	1	2	Cumulative Cigarettes (5 Years)	100
Other skin and subcutaneous diseases	Female	0-6 days	95+ years	Data Rich	1	2	Cumulative Cigarettes (10 Years)	124
Other skin and subcutaneous diseases	Female	0-6 days	95+ years	Data Rich	1	2	Smoking Prevalence	124
Other skin and subcutaneous diseases	Female	0-6 days	95+ years	Data Rich	1	2	Liters of alcohol consumed per capita	--
Other skin and subcutaneous diseases	Female	0-6 days	95+ years	Data Rich	-1	3	Education (years per capita)	966
Other skin and subcutaneous diseases	Female	0-6 days	95+ years	Data Rich	-1	3	LDI (I$ per capita)	--
Other skin and subcutaneous diseases	Female	0-6 days	95+ years	Data Rich	-1	3	Socio-demographic Index	--
Other skin and subcutaneous diseases	Female	0-6 days	95+ years	Global	-1	1	Healthcare access and quality index	1000
Other skin and subcutaneous diseases	Female	0-6 days	95+ years	Global	-1	1	Improved Water Source (proportion with access)	--
Other skin and subcutaneous diseases	Female	0-6 days	95+ years	Global	1	1	Diabetes Fasting Plasma Glucose (mmol/L), by age	697
Other skin and subcutaneous diseases	Female	0-6 days	95+ years	Global	1	1	Prevalence of overweight and obesity	872
Other skin and subcutaneous diseases	Female	0-6 days	95+ years	Global	1	1	Age- and sex-specific SEV for Unsafe sanitation	--
Other skin and subcutaneous diseases	Female	0-6 days	95+ years	Global	1	1	Age-standardized SEV for Child underweight	--
Other skin and subcutaneous diseases	Female	0-6 days	95+ years	Global	1	2	Smoking Prevalence	180
Other skin and subcutaneous diseases	Female	0-6 days	95+ years	Global	1	2	Cumulative Cigarettes (5 Years)	298
Other skin and subcutaneous diseases	Female	0-6 days	95+ years	Global	1	2	Cumulative Cigarettes (10 Years)	305
Other skin and subcutaneous diseases	Female	0-6 days	95+ years	Global	1	2	Liters of alcohol consumed per capita	591
Other skin and subcutaneous diseases	Female	0-6 days	95+ years	Global	-1	3	Education (years per capita)	--
Other skin and subcutaneous diseases	Female	0-6 days	95+ years	Global	-1	3	LDI (I$ per capita)	--
Other skin and subcutaneous diseases	Female	0-6 days	95+ years	Global	-1	3	Socio-demographic Index	--
Other skin and subcutaneous diseases	Male	0-6 days	95+ years	Data Rich	-1	1	Improved Water Source (proportion with access)	202
Other skin and subcutaneous diseases	Male	0-6 days	95+ years	Data Rich	-1	1	Healthcare access and quality index	671
Other skin and subcutaneous diseases	Male	0-6 days	95+ years	Data Rich	1	1	Age-standardized SEV for Child underweight	135
Other skin and subcutaneous diseases	Male	0-6 days	95+ years	Data Rich	1	1	Age- and sex-specific SEV for Unsafe sanitation	326
Other skin and subcutaneous diseases	Male	0-6 days	95+ years	Data Rich	1	1	Diabetes Fasting Plasma Glucose (mmol/L), by age	434
Other skin and subcutaneous diseases	Male	0-6 days	95+ years	Data Rich	1	1	Prevalence of overweight and obesity	533
Other skin and subcutaneous diseases	Male	0-6 days	95+ years	Data Rich	1	2	Cumulative Cigarettes (5 Years)	20
Other skin and subcutaneous diseases	Male	0-6 days	95+ years	Data Rich	1	2	Smoking Prevalence	37
Other skin and subcutaneous diseases	Male	0-6 days	95+ years	Data Rich	1	2	Cumulative Cigarettes (10 Years)	69
Other skin and subcutaneous diseases	Male	0-6 days	95+ years	Data Rich	1	2	Liters of alcohol consumed per capita	157

Appendix Table S10: CODEm covariates used, level of covariate, and expected direction of covariate by cause, sex, and age

Cause	Sex	Age Start	Age End	Model Version Type	Direction	Level	Covariate Name	Number of Draws
Other skin and subcutaneous diseases	Male	0-6 days	95+ years	Data Rich	-1	3	Education (years per capita)	29
Other skin and subcutaneous diseases	Male	0-6 days	95+ years	Data Rich	-1	3	Socio-demographic Index	141
Other skin and subcutaneous diseases	Male	0-6 days	95+ years	Data Rich	-1	3	LDI (I$ per capita)	--
Other skin and subcutaneous diseases	Male	0-6 days	95+ years	Global	-1	1	Healthcare access and quality index	1000
Other skin and subcutaneous diseases	Male	0-6 days	95+ years	Global	-1	1	Improved Water Source (proportion with access)	--
Other skin and subcutaneous diseases	Male	0-6 days	95+ years	Global	1	1	Diabetes Fasting Plasma Glucose (mmol/L), by age	181
Other skin and subcutaneous diseases	Male	0-6 days	95+ years	Global	1	1	Prevalence of overweight and obesity	763
Other skin and subcutaneous diseases	Male	0-6 days	95+ years	Global	1	1	Age- and sex-specific SEV for Unsafe sanitation	--
Other skin and subcutaneous diseases	Male	0-6 days	95+ years	Global	1	1	Age-standardized SEV for Child underweight	--
Other skin and subcutaneous diseases	Male	0-6 days	95+ years	Global	1	2	Liters of alcohol consumed per capita	42
Other skin and subcutaneous diseases	Male	0-6 days	95+ years	Global	1	2	Cumulative Cigarettes (5 Years)	60
Other skin and subcutaneous diseases	Male	0-6 days	95+ years	Global	1	2	Smoking Prevalence	170
Other skin and subcutaneous diseases	Male	0-6 days	95+ years	Global	1	2	Cumulative Cigarettes (10 Years)	287
Other skin and subcutaneous diseases	Male	0-6 days	95+ years	Global	-1	3	Education (years per capita)	--
Other skin and subcutaneous diseases	Male	0-6 days	95+ years	Global	-1	3	LDI (I$ per capita)	--
Other skin and subcutaneous diseases	Male	0-6 days	95+ years	Global	-1	3	Socio-demographic Index	--
Musculoskeletal disorders	Female	1-5 months	95+ years	Data Rich	1	1	Liters of alcohol consumed per capita	--
Musculoskeletal disorders	Female	1-5 months	95+ years	Data Rich	1	1	Mean BMI	--
Musculoskeletal disorders	Female	1-5 months	95+ years	Data Rich	-1	2	Healthcare access and quality index	908
Musculoskeletal disorders	Female	1-5 months	95+ years	Data Rich	1	2	Cumulative Cigarettes (5 Years)	276
Musculoskeletal disorders	Female	1-5 months	95+ years	Data Rich	1	2	Cumulative Cigarettes (10 Years)	606
Musculoskeletal disorders	Female	1-5 months	95+ years	Data Rich	1	2	Smoking Prevalence	641
Musculoskeletal disorders	Female	1-5 months	95+ years	Data Rich	1	2	Low-Density Lipoprotein (mmol/L)	728
Musculoskeletal disorders	Female	1-5 months	95+ years	Data Rich	1	2	Age-standardized bone mineral density among population age 60+ years	841
Musculoskeletal disorders	Female	1-5 months	95+ years	Data Rich	1	2	Education (years per capita)	1000
Musculoskeletal disorders	Female	1-5 months	95+ years	Data Rich	1	2	LDI (I$ per capita)	--
Musculoskeletal disorders	Female	1-5 months	95+ years	Data Rich	1	2	Low bone mineral density	--
Musculoskeletal disorders	Female	1-5 months	95+ years	Data Rich	1	3	Socio-demographic Index	--
Musculoskeletal disorders	Female	1-5 months	95+ years	Global	-1	1	vegetables unadjusted(g)	--
Musculoskeletal disorders	Female	1-5 months	95+ years	Global	1	1	Liters of alcohol consumed per capita	--
Musculoskeletal disorders	Female	1-5 months	95+ years	Global	1	1	Mean BMI	--
Musculoskeletal disorders	Female	1-5 months	95+ years	Global	-1	2	Healthcare access and quality index	326
Musculoskeletal disorders	Female	1-5 months	95+ years	Global	1	2	Age-standardized bone mineral density among population age 60+ years	185
Musculoskeletal disorders	Female	1-5 months	95+ years	Global	1	2	Cumulative Cigarettes (10 Years)	222
Musculoskeletal disorders	Female	1-5 months	95+ years	Global	1	2	Cumulative Cigarettes (5 Years)	267
Musculoskeletal disorders	Female	1-5 months	95+ years	Global	1	2	Low bone mineral density	593
Musculoskeletal disorders	Female	1-5 months	95+ years	Global	1	2	Education (years per capita)	--
Musculoskeletal disorders	Female	1-5 months	95+ years	Global	1	2	LDI (I$ per capita)	--
Musculoskeletal disorders	Female	1-5 months	95+ years	Global	1	2	Low-Density Lipoprotein (mmol/L)	--
Musculoskeletal disorders	Female	1-5 months	95+ years	Global	1	2	Smoking Prevalence	--
Musculoskeletal disorders	Female	1-5 months	95+ years	Global	1	3	Socio-demographic Index	--
Musculoskeletal disorders	Male	1-5 months	95+ years	Data Rich	1	1	Liters of alcohol consumed per capita	--
Musculoskeletal disorders	Male	1-5 months	95+ years	Data Rich	1	1	Mean BMI	--
Musculoskeletal disorders	Male	1-5 months	95+ years	Data Rich	1	1	vegetables unadjusted(g)	--
Musculoskeletal disorders	Male	1-5 months	95+ years	Data Rich	-1	2	Healthcare access and quality index	--
Musculoskeletal disorders	Male	1-5 months	95+ years	Data Rich	1	2	Cumulative Cigarettes (5 Years)	248
Musculoskeletal disorders	Male	1-5 months	95+ years	Data Rich	1	2	Low-Density Lipoprotein (mmol/L)	573
Musculoskeletal disorders	Male	1-5 months	95+ years	Data Rich	1	2	Low bone mineral density	675
Musculoskeletal disorders	Male	1-5 months	95+ years	Data Rich	1	2	Cumulative Cigarettes (10 Years)	752
Musculoskeletal disorders	Male	1-5 months	95+ years	Data Rich	1	2	Age-standardized bone mineral density among population age 60+ years	--
Musculoskeletal disorders	Male	1-5 months	95+ years	Data Rich	1	2	Education (years per capita)	--
Musculoskeletal disorders	Male	1-5 months	95+ years	Data Rich	1	2	LDI (I$ per capita)	--
Musculoskeletal disorders	Male	1-5 months	95+ years	Data Rich	1	2	Smoking Prevalence	--
Musculoskeletal disorders	Male	1-5 months	95+ years	Data Rich	1	3	Socio-demographic Index	--
Musculoskeletal disorders	Male	1-5 months	95+ years	Global	1	1	Liters of alcohol consumed per capita	--
Musculoskeletal disorders	Male	1-5 months	95+ years	Global	1	1	Mean BMI	--
Musculoskeletal disorders	Male	1-5 months	95+ years	Global	1	1	vegetables unadjusted(g)	--
Musculoskeletal disorders	Male	1-5 months	95+ years	Global	-1	2	Healthcare access and quality index	1000
Musculoskeletal disorders	Male	1-5 months	95+ years	Global	1	2	Cumulative Cigarettes (5 Years)	491
Musculoskeletal disorders	Male	1-5 months	95+ years	Global	1	2	Cumulative Cigarettes (10 Years)	509
Musculoskeletal disorders	Male	1-5 months	95+ years	Global	1	2	Education (years per capita)	--
Musculoskeletal disorders	Male	1-5 months	95+ years	Global	1	2	LDI (I$ per capita)	--
Musculoskeletal disorders	Male	1-5 months	95+ years	Global	1	2	Low-Density Lipoprotein (mmol/L)	--
Musculoskeletal disorders	Male	1-5 months	95+ years	Global	1	2	Smoking Prevalence	--
Musculoskeletal disorders	Male	1-5 months	95+ years	Global	1	3	Socio-demographic Index	--
Rheumatoid arthritis	Female	5-9 years	95+ years	Data Rich	-1	1	Healthcare access and quality index	983
Rheumatoid arthritis	Female	5-9 years	95+ years	Data Rich	-1	1	milk unadjusted(g)	--
Rheumatoid arthritis	Female	5-9 years	95+ years	Data Rich	1	1	Smoking Prevalence	59
Rheumatoid arthritis	Female	5-9 years	95+ years	Data Rich	1	1	Cumulative Cigarettes (10 Years)	494
Rheumatoid arthritis	Female	5-9 years	95+ years	Data Rich	1	1	Cumulative Cigarettes (5 Years)	503
Rheumatoid arthritis	Female	5-9 years	95+ years	Data Rich	1	1	Liters of alcohol consumed per capita	--
Rheumatoid arthritis	Female	5-9 years	95+ years	Data Rich	1	2	Mean BMI	727
Rheumatoid arthritis	Female	5-9 years	95+ years	Data Rich	1	2	Low-Density Lipoprotein (mmol/L)	--
Rheumatoid arthritis	Female	5-9 years	95+ years	Data Rich	1	3	Education (years per capita)	263
Rheumatoid arthritis	Female	5-9 years	95+ years	Data Rich	1	3	LDI (I$ per capita)	--
Rheumatoid arthritis	Female	5-9 years	95+ years	Data Rich	1	3	Socio-demographic Index	--
Rheumatoid arthritis	Female	5-9 years	95+ years	Global	-1	1	Healthcare access and quality index	565
Rheumatoid arthritis	Female	5-9 years	95+ years	Global	1	1	Smoking Prevalence	186
Rheumatoid arthritis	Female	5-9 years	95+ years	Global	1	1	Cumulative Cigarettes (5 Years)	249
Rheumatoid arthritis	Female	5-9 years	95+ years	Global	1	1	milk unadjusted(g)	565
Rheumatoid arthritis	Female	5-9 years	95+ years	Global	1	1	Cumulative Cigarettes (10 Years)	--
Rheumatoid arthritis	Female	5-9 years	95+ years	Global	1	1	Liters of alcohol consumed per capita	--
Rheumatoid arthritis	Female	5-9 years	95+ years	Global	1	2	Low-Density Lipoprotein (mmol/L)	--
Rheumatoid arthritis	Female	5-9 years	95+ years	Global	1	2	Mean BMI	--
Rheumatoid arthritis	Female	5-9 years	95+ years	Global	1	3	Education (years per capita)	--
Rheumatoid arthritis	Female	5-9 years	95+ years	Global	1	3	LDI (I$ per capita)	--
Rheumatoid arthritis	Female	5-9 years	95+ years	Global	1	3	Socio-demographic Index	--
Rheumatoid arthritis	Male	5-9 years	95+ years	Data Rich	-1	1	Healthcare access and quality index	1000
Rheumatoid arthritis	Male	5-9 years	95+ years	Data Rich	-1	1	milk unadjusted(g)	--
Rheumatoid arthritis	Male	5-9 years	95+ years	Data Rich	1	1	Cumulative Cigarettes (5 Years)	235
Rheumatoid arthritis	Male	5-9 years	95+ years	Data Rich	1	1	Cumulative Cigarettes (10 Years)	361
Rheumatoid arthritis	Male	5-9 years	95+ years	Data Rich	1	1	Smoking Prevalence	404
Rheumatoid arthritis	Male	5-9 years	95+ years	Data Rich	1	1	Liters of alcohol consumed per capita	--
Rheumatoid arthritis	Male	5-9 years	95+ years	Data Rich	1	2	Low-Density Lipoprotein (mmol/L)	322
Rheumatoid arthritis	Male	5-9 years	95+ years	Data Rich	1	2	Mean BMI	--
Rheumatoid arthritis	Male	5-9 years	95+ years	Data Rich	1	3	Socio-demographic Index	91
Rheumatoid arthritis	Male	5-9 years	95+ years	Data Rich	1	3	Education (years per capita)	146
Rheumatoid arthritis	Male	5-9 years	95+ years	Data Rich	1	3	LDI (I$ per capita)	--
Rheumatoid arthritis	Male	5-9 years	95+ years	Global	-1	1	Healthcare access and quality index	808
Rheumatoid arthritis	Male	5-9 years	95+ years	Global	1	1	milk unadjusted(g)	70
Rheumatoid arthritis	Male	5-9 years	95+ years	Global	1	1	Cumulative Cigarettes (5 Years)	164
Rheumatoid arthritis	Male	5-9 years	95+ years	Global	1	1	Smoking Prevalence	313
Rheumatoid arthritis	Male	5-9 years	95+ years	Global	1	1	Cumulative Cigarettes (10 Years)	446
Rheumatoid arthritis	Male	5-9 years	95+ years	Global	1	1	Liters of alcohol consumed per capita	--
Rheumatoid arthritis	Male	5-9 years	95+ years	Global	1	2	Low-Density Lipoprotein (mmol/L)	525
Rheumatoid arthritis	Male	5-9 years	95+ years	Global	1	2	Mean BMI	--
Rheumatoid arthritis	Male	5-9 years	95+ years	Global	1	3	Socio-demographic Index	147
Rheumatoid arthritis	Male	5-9 years	95+ years	Global	1	3	Education (years per capita)	235
Rheumatoid arthritis	Male	5-9 years	95+ years	Global	1	3	LDI (I$ per capita)	--
Other musculoskeletal disorders	Female	1-5 months	95+ years	Data Rich	1	1	Liters of alcohol consumed per capita	--

Appendix Table S10: CODEm covariates used, level of covariate, and expected direction of covariate by cause, sex, and age

Cause	Sex	Age Start	Age End	Model Version Type	Direction	Level	Covariate Name	Number of Draws
Other musculoskeletal disorders	Female	1-5 months	95+ years	Data Rich	1	1	Mean BMI	--
Other musculoskeletal disorders	Female	1-5 months	95+ years	Data Rich	1	1	vegetables unadjusted(g)	--
Other musculoskeletal disorders	Female	1-5 months	95+ years	Data Rich	-1	2	Healthcare access and quality index	--
Other musculoskeletal disorders	Female	1-5 months	95+ years	Data Rich	1	2	Cumulative Cigarettes (10 Years)	1000
Other musculoskeletal disorders	Female	1-5 months	95+ years	Data Rich	1	2	Cumulative Cigarettes (5 Years)	--
Other musculoskeletal disorders	Female	1-5 months	95+ years	Data Rich	1	2	Education (years per capita)	--
Other musculoskeletal disorders	Female	1-5 months	95+ years	Data Rich	1	2	LDI (I$ per capita)	--
Other musculoskeletal disorders	Female	1-5 months	95+ years	Data Rich	1	2	Low-Density Lipoprotein (mmol/L)	--
Other musculoskeletal disorders	Female	1-5 months	95+ years	Data Rich	1	2	Smoking Prevalence	--
Other musculoskeletal disorders	Female	1-5 months	95+ years	Data Rich	1	3	Socio-demographic Index	--
Other musculoskeletal disorders	Female	1-5 months	95+ years	Global	1	1	Liters of alcohol consumed per capita	--
Other musculoskeletal disorders	Female	1-5 months	95+ years	Global	1	1	Mean BMI	--
Other musculoskeletal disorders	Female	1-5 months	95+ years	Global	1	1	vegetables unadjusted(g)	--
Other musculoskeletal disorders	Female	1-5 months	95+ years	Global	-1	2	Healthcare access and quality index	--
Other musculoskeletal disorders	Female	1-5 months	95+ years	Global	1	2	Low-Density Lipoprotein (mmol/L)	454
Other musculoskeletal disorders	Female	1-5 months	95+ years	Global	1	2	Smoking Prevalence	546
Other musculoskeletal disorders	Female	1-5 months	95+ years	Global	1	2	Cumulative Cigarettes (10 Years)	--
Other musculoskeletal disorders	Female	1-5 months	95+ years	Global	1	2	Cumulative Cigarettes (5 Years)	--
Other musculoskeletal disorders	Female	1-5 months	95+ years	Global	1	2	Education (years per capita)	--
Other musculoskeletal disorders	Female	1-5 months	95+ years	Global	1	2	LDI (I$ per capita)	--
Other musculoskeletal disorders	Female	1-5 months	95+ years	Global	1	3	Socio-demographic Index	--
Other musculoskeletal disorders	Male	1-5 months	95+ years	Data Rich	-1	1	vegetables unadjusted(g)	1000
Other musculoskeletal disorders	Male	1-5 months	95+ years	Data Rich	1	1	Liters of alcohol consumed per capita	--
Other musculoskeletal disorders	Male	1-5 months	95+ years	Data Rich	1	1	Mean BMI	--
Other musculoskeletal disorders	Male	1-5 months	95+ years	Data Rich	-1	2	Healthcare access and quality index	--
Other musculoskeletal disorders	Male	1-5 months	95+ years	Data Rich	1	2	Cumulative Cigarettes (5 Years)	432
Other musculoskeletal disorders	Male	1-5 months	95+ years	Data Rich	1	2	Cumulative Cigarettes (10 Years)	--
Other musculoskeletal disorders	Male	1-5 months	95+ years	Data Rich	1	2	Education (years per capita)	--
Other musculoskeletal disorders	Male	1-5 months	95+ years	Data Rich	1	2	LDI (I$ per capita)	--
Other musculoskeletal disorders	Male	1-5 months	95+ years	Data Rich	1	2	Low-Density Lipoprotein (mmol/L)	--
Other musculoskeletal disorders	Male	1-5 months	95+ years	Data Rich	1	2	Smoking Prevalence	--
Other musculoskeletal disorders	Male	1-5 months	95+ years	Data Rich	1	3	Socio-demographic Index	--
Other musculoskeletal disorders	Male	1-5 months	95+ years	Global	-1	1	vegetables unadjusted(g)	--
Other musculoskeletal disorders	Male	1-5 months	95+ years	Global	1	1	Liters of alcohol consumed per capita	--
Other musculoskeletal disorders	Male	1-5 months	95+ years	Global	1	1	Mean BMI	--
Other musculoskeletal disorders	Male	1-5 months	95+ years	Global	-1	2	Healthcare access and quality index	--
Other musculoskeletal disorders	Male	1-5 months	95+ years	Global	1	2	Cumulative Cigarettes (10 Years)	1000
Other musculoskeletal disorders	Male	1-5 months	95+ years	Global	1	2	Cumulative Cigarettes (5 Years)	--
Other musculoskeletal disorders	Male	1-5 months	95+ years	Global	1	2	Education (years per capita)	--
Other musculoskeletal disorders	Male	1-5 months	95+ years	Global	1	2	LDI (I$ per capita)	--
Other musculoskeletal disorders	Male	1-5 months	95+ years	Global	1	2	Low-Density Lipoprotein (mmol/L)	--
Other musculoskeletal disorders	Male	1-5 months	95+ years	Global	1	2	Smoking Prevalence	--
Other musculoskeletal disorders	Male	1-5 months	95+ years	Global	1	3	Socio-demographic Index	--
Congenital anomalies	Female	0-6 days	65-69 years	Data Rich	-1	1	Folic acid unadjusted (ug)	--
Congenital anomalies	Female	0-6 days	65-69 years	Data Rich	1	1	Birth prevalence of CHD	1000
Congenital anomalies	Female	0-6 days	65-69 years	Data Rich	1	1	Birth prevalence of congenital chromosomal anomalies	--
Congenital anomalies	Female	0-6 days	65-69 years	Data Rich	1	1	Live Births 35+ (proportion)	--
Congenital anomalies	Female	0-6 days	65-69 years	Data Rich	1	1	Maternal alcohol consumption during pregnancy (proportion)	--
Congenital anomalies	Female	0-6 days	65-69 years	Data Rich	-1	2	Antenatal Care (1 visit) Coverage (proportion)	--
Congenital anomalies	Female	0-6 days	65-69 years	Data Rich	-1	2	Antenatal Care (4 visits) Coverage (proportion)	--
Congenital anomalies	Female	0-6 days	65-69 years	Data Rich	-1	2	Composite fortification standard and folic acid inclusion	--
Congenital anomalies	Female	0-6 days	65-69 years	Data Rich	-1	2	Healthcare access and quality index	--
Congenital anomalies	Female	0-6 days	65-69 years	Data Rich	-1	2	In-Facility Delivery (proportion)	--
Congenital anomalies	Female	0-6 days	65-69 years	Data Rich	-1	2	Legality of Abortion	--
Congenital anomalies	Female	0-6 days	65-69 years	Data Rich	1	2	Age-standardized SEV for Smoking	--
Congenital anomalies	Female	0-6 days	65-69 years	Data Rich	-1	3	Maternal Education (years per capita)	--
Congenital anomalies	Female	0-6 days	65-69 years	Data Rich	-1	3	Socio-demographic Index	--
Congenital anomalies	Female	0-6 days	65-69 years	Data Rich	1	3	Age-standardized SEV for Ambient particulate matter	--
Congenital anomalies	Female	0-6 days	65-69 years	Data Rich	1	3	Age-standardized SEV for Household air pollution	--
Congenital anomalies	Female	0-6 days	65-69 years	Data Rich	1	3	Age-standardized SEV for Low fruit	--
Congenital anomalies	Female	0-6 days	65-69 years	Data Rich	1	3	Age-standardized SEV for Low vegetables	--
Congenital anomalies	Female	0-6 days	65-69 years	Data Rich	1	3	Liters of alcohol consumed per capita	--
Congenital anomalies	Female	0-6 days	65-69 years	Global	-1	1	Folic acid unadjusted (ug)	979
Congenital anomalies	Female	0-6 days	65-69 years	Global	1	1	Birth prevalence of CHD	442
Congenital anomalies	Female	0-6 days	65-69 years	Global	1	1	Birth prevalence of congenital chromosomal anomalies	999
Congenital anomalies	Female	0-6 days	65-69 years	Global	1	1	Live Births 35+ (proportion)	--
Congenital anomalies	Female	0-6 days	65-69 years	Global	1	1	Maternal alcohol consumption during pregnancy (proportion)	--
Congenital anomalies	Female	0-6 days	65-69 years	Global	-1	2	Antenatal Care (1 visit) Coverage (proportion)	0
Congenital anomalies	Female	0-6 days	65-69 years	Global	-1	2	Composite fortification standard and folic acid inclusion	200
Congenital anomalies	Female	0-6 days	65-69 years	Global	-1	2	Antenatal Care (4 visits) Coverage (proportion)	254
Congenital anomalies	Female	0-6 days	65-69 years	Global	-1	2	Legality of Abortion	378
Congenital anomalies	Female	0-6 days	65-69 years	Global	-1	2	In-Facility Delivery (proportion)	416
Congenital anomalies	Female	0-6 days	65-69 years	Global	-1	2	Healthcare access and quality index	439
Congenital anomalies	Female	0-6 days	65-69 years	Global	1	2	Age-standardized SEV for Smoking	906
Congenital anomalies	Female	0-6 days	65-69 years	Global	-1	3	Socio-demographic Index	44
Congenital anomalies	Female	0-6 days	65-69 years	Global	-1	3	Maternal Education (years per capita)	285
Congenital anomalies	Female	0-6 days	65-69 years	Global	1	3	Age-standardized SEV for Ambient particulate matter	0
Congenital anomalies	Female	0-6 days	65-69 years	Global	1	3	Age-standardized SEV for Low vegetables	1
Congenital anomalies	Female	0-6 days	65-69 years	Global	1	3	Age-standardized SEV for Household air pollution	32
Congenital anomalies	Female	0-6 days	65-69 years	Global	1	3	Age-standardized SEV for Low fruit	285
Congenital anomalies	Female	0-6 days	65-69 years	Global	1	3	Liters of alcohol consumed per capita	--
Congenital anomalies	Male	0-6 days	65-69 years	Data Rich	-1	1	Folic acid unadjusted (ug)	--
Congenital anomalies	Male	0-6 days	65-69 years	Data Rich	1	1	Birth prevalence of congenital chromosomal anomalies	1000
Congenital anomalies	Male	0-6 days	65-69 years	Data Rich	1	1	Birth prevalence of CHD	--
Congenital anomalies	Male	0-6 days	65-69 years	Data Rich	1	1	Live Births 35+ (proportion)	--
Congenital anomalies	Male	0-6 days	65-69 years	Data Rich	1	1	Maternal alcohol consumption during pregnancy (proportion)	--
Congenital anomalies	Male	0-6 days	65-69 years	Data Rich	-1	2	Antenatal Care (1 visit) Coverage (proportion)	--
Congenital anomalies	Male	0-6 days	65-69 years	Data Rich	-1	2	Antenatal Care (4 visits) Coverage (proportion)	--
Congenital anomalies	Male	0-6 days	65-69 years	Data Rich	-1	2	Composite fortification standard and folic acid inclusion	--
Congenital anomalies	Male	0-6 days	65-69 years	Data Rich	-1	2	Healthcare access and quality index	--
Congenital anomalies	Male	0-6 days	65-69 years	Data Rich	-1	2	In-Facility Delivery (proportion)	--
Congenital anomalies	Male	0-6 days	65-69 years	Data Rich	-1	2	Legality of Abortion	--
Congenital anomalies	Male	0-6 days	65-69 years	Data Rich	1	2	Age-standardized SEV for Smoking	657
Congenital anomalies	Male	0-6 days	65-69 years	Data Rich	-1	3	Maternal Education (years per capita)	329
Congenital anomalies	Male	0-6 days	65-69 years	Data Rich	-1	3	Socio-demographic Index	464
Congenital anomalies	Male	0-6 days	65-69 years	Data Rich	1	3	Age-standardized SEV for Household air pollution	--
Congenital anomalies	Male	0-6 days	65-69 years	Data Rich	1	3	Age-standardized SEV for Low fruit	--
Congenital anomalies	Male	0-6 days	65-69 years	Data Rich	1	3	Age-standardized SEV for Low vegetables	--
Congenital anomalies	Male	0-6 days	65-69 years	Data Rich	1	3	Liters of alcohol consumed per capita	--
Congenital anomalies	Male	0-6 days	65-69 years	Data Rich	1	3	Outdoor Air Pollution (PM2.5)	--
Congenital anomalies	Male	0-6 days	65-69 years	Global	-1	1	Folic acid unadjusted (ug)	208
Congenital anomalies	Male	0-6 days	65-69 years	Global	1	1	Birth prevalence of congenital chromosomal anomalies	489
Congenital anomalies	Male	0-6 days	65-69 years	Global	1	1	Birth prevalence of CHD	1000
Congenital anomalies	Male	0-6 days	65-69 years	Global	1	1	Live Births 35+ (proportion)	--

Appendix Table S10: CODEm covariates used, level of covariate, and expected direction of covariate by cause, sex, and age

Cause	Sex	Age Start	Age End	Model Version Type	Direction	Level	Covariate Name	Number of Draws
Congenital anomalies	Male	0-6 days	65-69 years	Global	1	1	Maternal alcohol consumption during pregnancy (proportion)	--
Congenital anomalies	Male	0-6 days	65-69 years	Global	-1	2	Composite fortification standard and folic acid inclusion	109
Congenital anomalies	Male	0-6 days	65-69 years	Global	-1	2	In-Facility Delivery (proportion)	142
Congenital anomalies	Male	0-6 days	65-69 years	Global	-1	2	Antenatal Care (4 visits) Coverage (proportion)	188
Congenital anomalies	Male	0-6 days	65-69 years	Global	-1	2	Legality of Abortion	237
Congenital anomalies	Male	0-6 days	65-69 years	Global	-1	2	Antenatal Care (1 visit) Coverage (proportion)	--
Congenital anomalies	Male	0-6 days	65-69 years	Global	-1	2	Healthcare access and quality index	--
Congenital anomalies	Male	0-6 days	65-69 years	Global	1	2	Age-standardized SEV for Smoking	484
Congenital anomalies	Male	0-6 days	65-69 years	Global	-1	3	Socio-demographic Index	150
Congenital anomalies	Male	0-6 days	65-69 years	Global	-1	3	Maternal Education (years per capita)	354
Congenital anomalies	Male	0-6 days	65-69 years	Global	1	3	Age-standardized SEV for Low fruit	11
Congenital anomalies	Male	0-6 days	65-69 years	Global	1	3	Age-standardized SEV for Household air pollution	23
Congenital anomalies	Male	0-6 days	65-69 years	Global	1	3	Age-standardized SEV for Low vegetables	153
Congenital anomalies	Male	0-6 days	65-69 years	Global	1	3	Outdoor Air Pollution (PM2.5)	168
Congenital anomalies	Male	0-6 days	65-69 years	Global	1	3	Liters of alcohol consumed per capita	--
Neural tube defects	Female	0-6 days	65-69 years	Data Rich	-1	1	Composite fortification standard and folic acid inclusion	0
Neural tube defects	Female	0-6 days	65-69 years	Data Rich	-1	1	In-Facility Delivery (proportion)	143
Neural tube defects	Female	0-6 days	65-69 years	Data Rich	-1	1	Socio-demographic Index	385
Neural tube defects	Female	0-6 days	65-69 years	Data Rich	-1	1	Folic acid unadjusted (ug)	605
Neural tube defects	Female	0-6 days	65-69 years	Data Rich	-1	2	Legality of Abortion	312
Neural tube defects	Female	0-6 days	65-69 years	Data Rich	-1	2	Healthcare access and quality index	556
Neural tube defects	Female	0-6 days	65-69 years	Data Rich	-1	2	Antenatal Care (1 visit) Coverage (proportion)	--
Neural tube defects	Female	0-6 days	65-69 years	Data Rich	-1	2	Antenatal Care (4 visits) Coverage (proportion)	--
Neural tube defects	Female	0-6 days	65-69 years	Data Rich	1	2	Age-standardized SEV for Smoking	120
Neural tube defects	Female	0-6 days	65-69 years	Data Rich	-1	3	Maternal Education (years per capita)	98
Neural tube defects	Female	0-6 days	65-69 years	Data Rich	1	3	Age-standardized SEV for Household air pollution	1
Neural tube defects	Female	0-6 days	65-69 years	Data Rich	1	3	Age-standardized SEV for Low vegetables	5
Neural tube defects	Female	0-6 days	65-69 years	Data Rich	1	3	Age-standardized SEV for High fasting plasma glucose	205
Neural tube defects	Female	0-6 days	65-69 years	Data Rich	1	3	Liters of alcohol consumed per capita	360
Neural tube defects	Female	0-6 days	65-69 years	Data Rich	1	3	Age-standardized SEV for Low fruit	466
Neural tube defects	Female	0-6 days	65-69 years	Data Rich	1	3	Maternal alcohol consumption during pregnancy (proportion)	--
Neural tube defects	Female	0-6 days	65-69 years	Global	-1	1	Socio-demographic Index	300
Neural tube defects	Female	0-6 days	65-69 years	Global	-1	1	Folic acid unadjusted (ug)	406
Neural tube defects	Female	0-6 days	65-69 years	Global	-1	1	In-Facility Delivery (proportion)	619
Neural tube defects	Female	0-6 days	65-69 years	Global	-1	1	Composite fortification standard and folic acid inclusion	904
Neural tube defects	Female	0-6 days	65-69 years	Global	-1	2	Antenatal Care (1 visit) Coverage (proportion)	0
Neural tube defects	Female	0-6 days	65-69 years	Global	-1	2	Antenatal Care (4 visits) Coverage (proportion)	9
Neural tube defects	Female	0-6 days	65-69 years	Global	-1	2	Legality of Abortion	419
Neural tube defects	Female	0-6 days	65-69 years	Global	-1	2	Healthcare access and quality index	682
Neural tube defects	Female	0-6 days	65-69 years	Global	1	2	Age-standardized SEV for Smoking	267
Neural tube defects	Female	0-6 days	65-69 years	Global	-1	3	Maternal Education (years per capita)	17
Neural tube defects	Female	0-6 days	65-69 years	Global	1	3	Age-standardized SEV for Household air pollution	10
Neural tube defects	Female	0-6 days	65-69 years	Global	1	3	Age-standardized SEV for Low fruit	28
Neural tube defects	Female	0-6 days	65-69 years	Global	1	3	Age-standardized SEV for Low vegetables	81
Neural tube defects	Female	0-6 days	65-69 years	Global	1	3	Age-standardized SEV for High fasting plasma glucose	237
Neural tube defects	Female	0-6 days	65-69 years	Global	1	3	Liters of alcohol consumed per capita	335
Neural tube defects	Female	0-6 days	65-69 years	Global	1	3	Maternal alcohol consumption during pregnancy (proportion)	--
Neural tube defects	Male	0-6 days	65-69 years	Data Rich	-1	1	Socio-demographic Index	462
Neural tube defects	Male	0-6 days	65-69 years	Data Rich	-1	1	Folic acid unadjusted (ug)	818
Neural tube defects	Male	0-6 days	65-69 years	Data Rich	-1	1	Composite fortification standard and folic acid inclusion	--
Neural tube defects	Male	0-6 days	65-69 years	Data Rich	-1	1	In-Facility Delivery (proportion)	--
Neural tube defects	Male	0-6 days	65-69 years	Data Rich	-1	2	Healthcare access and quality index	500
Neural tube defects	Male	0-6 days	65-69 years	Data Rich	-1	2	Legality of Abortion	569
Neural tube defects	Male	0-6 days	65-69 years	Data Rich	-1	2	Antenatal Care (1 visit) Coverage (proportion)	--
Neural tube defects	Male	0-6 days	65-69 years	Data Rich	-1	2	Antenatal Care (4 visits) Coverage (proportion)	--
Neural tube defects	Male	0-6 days	65-69 years	Data Rich	1	2	Age-standardized SEV for Smoking	119
Neural tube defects	Male	0-6 days	65-69 years	Data Rich	-1	3	Maternal Education (years per capita)	34
Neural tube defects	Male	0-6 days	65-69 years	Data Rich	1	3	Age-standardized SEV for Low fruit	129
Neural tube defects	Male	0-6 days	65-69 years	Data Rich	1	3	Liters of alcohol consumed per capita	284
Neural tube defects	Male	0-6 days	65-69 years	Data Rich	1	3	Maternal alcohol consumption during pregnancy (proportion)	357
Neural tube defects	Male	0-6 days	65-69 years	Data Rich	1	3	Age-standardized SEV for High fasting plasma glucose	444
Neural tube defects	Male	0-6 days	65-69 years	Data Rich	1	3	Age-standardized SEV for Household air pollution	--
Neural tube defects	Male	0-6 days	65-69 years	Data Rich	1	3	Age-standardized SEV for Low vegetables	--
Neural tube defects	Male	0-6 days	65-69 years	Global	-1	1	Composite fortification standard and folic acid inclusion	24
Neural tube defects	Male	0-6 days	65-69 years	Global	-1	1	Folic acid unadjusted (ug)	114
Neural tube defects	Male	0-6 days	65-69 years	Global	-1	1	In-Facility Delivery (proportion)	171
Neural tube defects	Male	0-6 days	65-69 years	Global	-1	1	Socio-demographic Index	919
Neural tube defects	Male	0-6 days	65-69 years	Global	-1	2	Antenatal Care (1 visit) Coverage (proportion)	1
Neural tube defects	Male	0-6 days	65-69 years	Global	-1	2	Legality of Abortion	79
Neural tube defects	Male	0-6 days	65-69 years	Global	-1	2	Antenatal Care (4 visits) Coverage (proportion)	248
Neural tube defects	Male	0-6 days	65-69 years	Global	-1	2	Healthcare access and quality index	--
Neural tube defects	Male	0-6 days	65-69 years	Global	1	2	Age-standardized SEV for Smoking	369
Neural tube defects	Male	0-6 days	65-69 years	Global	-1	3	Maternal Education (years per capita)	51
Neural tube defects	Male	0-6 days	65-69 years	Global	1	3	Age-standardized SEV for Low fruit	124
Neural tube defects	Male	0-6 days	65-69 years	Global	1	3	Age-standardized SEV for Household air pollution	292
Neural tube defects	Male	0-6 days	65-69 years	Global	1	3	Liters of alcohol consumed per capita	304
Neural tube defects	Male	0-6 days	65-69 years	Global	1	3	Maternal alcohol consumption during pregnancy (proportion)	308
Neural tube defects	Male	0-6 days	65-69 years	Global	1	3	Age-standardized SEV for Low vegetables	436
Neural tube defects	Male	0-6 days	65-69 years	Global	1	3	Age-standardized SEV for High fasting plasma glucose	527
Congenital heart anomalies	Female	0-6 days	65-69 years	Data Rich	1	1	Birth prevalence of CHD	1000
Congenital heart anomalies	Female	0-6 days	65-69 years	Data Rich	1	1	Maternal alcohol consumption during pregnancy (proportion)	--
Congenital heart anomalies	Female	0-6 days	65-69 years	Data Rich	-1	2	In-Facility Delivery (proportion)	57
Congenital heart anomalies	Female	0-6 days	65-69 years	Data Rich	-1	2	Legality of Abortion	57
Congenital heart anomalies	Female	0-6 days	65-69 years	Data Rich	-1	2	Antenatal Care (1 visit) Coverage (proportion)	--
Congenital heart anomalies	Female	0-6 days	65-69 years	Data Rich	-1	2	Healthcare access and quality index	--
Congenital heart anomalies	Female	0-6 days	65-69 years	Data Rich	-1	2	Socio-demographic Index	--
Congenital heart anomalies	Female	0-6 days	65-69 years	Data Rich	1	2	Age-standardized SEV for High fasting plasma glucose	--
Congenital heart anomalies	Female	0-6 days	65-69 years	Data Rich	1	2	Age-standardized SEV for Smoking	--
Congenital heart anomalies	Female	0-6 days	65-69 years	Data Rich	-1	3	Skilled Birth Attendance (proportion)	111
Congenital heart anomalies	Female	0-6 days	65-69 years	Data Rich	-1	3	Antenatal Care (4 visits) Coverage (proportion)	113
Congenital heart anomalies	Female	0-6 days	65-69 years	Data Rich	-1	3	Maternal Education (years per capita)	345
Congenital heart anomalies	Female	0-6 days	65-69 years	Data Rich	1	3	Liters of alcohol consumed per capita	182
Congenital heart anomalies	Female	0-6 days	65-69 years	Data Rich	1	3	Live Births 35+ (proportion)	--
Congenital heart anomalies	Female	0-6 days	65-69 years	Global	1	1	Birth prevalence of CHD	--
Congenital heart anomalies	Female	0-6 days	65-69 years	Global	1	1	Maternal alcohol consumption during pregnancy (proportion)	--

Appendix Table S10: CODEm covariates used, level of covariate, and expected direction of covariate by cause, sex, and age

Cause	Sex	Age Start	Age End	Model Version Type	Direction	Level	Covariate Name	Number of Draws
Congenital heart anomalies	Female	0-6 days	65-69 years	Global	-1	2	Antenatal Care (1 visit) Coverage (proportion)	1000
Congenital heart anomalies	Female	0-6 days	65-69 years	Global	-1	2	In-Facility Delivery (proportion)	1000
Congenital heart anomalies	Female	0-6 days	65-69 years	Global	-1	2	Healthcare access and quality index	--
Congenital heart anomalies	Female	0-6 days	65-69 years	Global	-1	2	Legality of Abortion	--
Congenital heart anomalies	Female	0-6 days	65-69 years	Global	-1	2	Socio-demographic Index	--
Congenital heart anomalies	Female	0-6 days	65-69 years	Global	1	2	Age-standardized SEV for High fasting plasma glucose	--
Congenital heart anomalies	Female	0-6 days	65-69 years	Global	1	2	Age-standardized SEV for Smoking	--
Congenital heart anomalies	Female	0-6 days	65-69 years	Global	-1	3	Skilled Birth Attendance (proportion)	452
Congenital heart anomalies	Female	0-6 days	65-69 years	Global	-1	3	Antenatal Care (4 visits) Coverage (proportion)	--
Congenital heart anomalies	Female	0-6 days	65-69 years	Global	-1	3	Maternal Education (years per capita)	--
Congenital heart anomalies	Female	0-6 days	65-69 years	Global	1	3	Liters of alcohol consumed per capita	--
Congenital heart anomalies	Female	0-6 days	65-69 years	Global	1	3	Live Births 35+ (proportion)	--
Congenital heart anomalies	Male	0-6 days	65-69 years	Data Rich	1	1	Birth prevalence of CHD	--
Congenital heart anomalies	Male	0-6 days	65-69 years	Data Rich	1	1	Maternal alcohol consumption during pregnancy (proportion)	--
Congenital heart anomalies	Male	0-6 days	65-69 years	Data Rich	-1	2	Antenatal Care (1 visit) Coverage (proportion)	141
Congenital heart anomalies	Male	0-6 days	65-69 years	Data Rich	-1	2	In-Facility Delivery (proportion)	141
Congenital heart anomalies	Male	0-6 days	65-69 years	Data Rich	-1	2	Legality of Abortion	669
Congenital heart anomalies	Male	0-6 days	65-69 years	Data Rich	-1	2	Healthcare access and quality index	--
Congenital heart anomalies	Male	0-6 days	65-69 years	Data Rich	-1	2	Socio-demographic Index	--
Congenital heart anomalies	Male	0-6 days	65-69 years	Data Rich	1	2	Age-standardized SEV for Smoking	528
Congenital heart anomalies	Male	0-6 days	65-69 years	Data Rich	1	2	Age-standardized SEV for High fasting plasma glucose	--
Congenital heart anomalies	Male	0-6 days	65-69 years	Data Rich	-1	3	Antenatal Care (4 visits) Coverage (proportion)	116
Congenital heart anomalies	Male	0-6 days	65-69 years	Data Rich	-1	3	Maternal Education (years per capita)	515
Congenital heart anomalies	Male	0-6 days	65-69 years	Data Rich	-1	3	Skilled Birth Attendance (proportion)	--
Congenital heart anomalies	Male	0-6 days	65-69 years	Data Rich	1	3	Liters of alcohol consumed per capita	364
Congenital heart anomalies	Male	0-6 days	65-69 years	Data Rich	1	3	Live Births 35+ (proportion)	--
Congenital heart anomalies	Male	0-6 days	65-69 years	Global	1	1	Birth prevalence of CHD	1000
Congenital heart anomalies	Male	0-6 days	65-69 years	Global	1	1	Maternal alcohol consumption during pregnancy (proportion)	--
Congenital heart anomalies	Male	0-6 days	65-69 years	Global	-1	2	In-Facility Delivery (proportion)	92
Congenital heart anomalies	Male	0-6 days	65-69 years	Global	-1	2	Legality of Abortion	92
Congenital heart anomalies	Male	0-6 days	65-69 years	Global	-1	2	Antenatal Care (1 visit) Coverage (proportion)	189
Congenital heart anomalies	Male	0-6 days	65-69 years	Global	-1	2	Healthcare access and quality index	--
Congenital heart anomalies	Male	0-6 days	65-69 years	Global	-1	2	Socio-demographic Index	--
Congenital heart anomalies	Male	0-6 days	65-69 years	Global	1	2	Age-standardized SEV for High fasting plasma glucose	--
Congenital heart anomalies	Male	0-6 days	65-69 years	Global	1	2	Age-standardized SEV for Smoking	--
Congenital heart anomalies	Male	0-6 days	65-69 years	Global	-1	3	Antenatal Care (4 visits) Coverage (proportion)	163
Congenital heart anomalies	Male	0-6 days	65-69 years	Global	-1	3	Maternal Education (years per capita)	317
Congenital heart anomalies	Male	0-6 days	65-69 years	Global	-1	3	Skilled Birth Attendance (proportion)	--
Congenital heart anomalies	Male	0-6 days	65-69 years	Global	1	3	Liters of alcohol consumed per capita	--
Congenital heart anomalies	Male	0-6 days	65-69 years	Global	1	3	Live Births 35+ (proportion)	--
Orofacial clefts	Female	0-6 days	2-4 years	Data Rich	-1	1	Composite fortification standard and folic acid inclusion	14
Orofacial clefts	Female	0-6 days	2-4 years	Data Rich	-1	1	Socio-demographic Index	986
Orofacial clefts	Female	0-6 days	2-4 years	Data Rich	-1	2	Legality of Abortion	3
Orofacial clefts	Female	0-6 days	2-4 years	Data Rich	-1	2	Skilled Birth Attendance (proportion)	3
Orofacial clefts	Female	0-6 days	2-4 years	Data Rich	-1	2	Healthcare access and quality index	637
Orofacial clefts	Female	0-6 days	2-4 years	Data Rich	1	2	Age-standardized SEV for Smoking	637
Orofacial clefts	Female	0-6 days	2-4 years	Data Rich	1	2	Age-standardized SEV for High fasting plasma glucose	--
Orofacial clefts	Female	0-6 days	2-4 years	Data Rich	1	2	Maternal alcohol consumption during pregnancy (proportion)	--
Orofacial clefts	Female	0-6 days	2-4 years	Data Rich	-1	3	Antenatal Care (1 visit) Coverage (proportion)	10
Orofacial clefts	Female	0-6 days	2-4 years	Data Rich	-1	3	Antenatal Care (4 visits) Coverage (proportion)	10
Orofacial clefts	Female	0-6 days	2-4 years	Data Rich	-1	3	Maternal Education (years per capita)	346
Orofacial clefts	Female	0-6 days	2-4 years	Data Rich	1	3	Age-standardized SEV for Household air pollution	6
Orofacial clefts	Female	0-6 days	2-4 years	Data Rich	1	3	Age-standardized SEV for Low fruit	177
Orofacial clefts	Female	0-6 days	2-4 years	Data Rich	1	3	Liters of alcohol consumed per capita	538
Orofacial clefts	Female	0-6 days	2-4 years	Data Rich	1	3	Age-standardized SEV for Low vegetables	--
Orofacial clefts	Female	0-6 days	2-4 years	Global	-1	1	Composite fortification standard and folic acid inclusion	550
Orofacial clefts	Female	0-6 days	2-4 years	Global	-1	1	Socio-demographic Index	752
Orofacial clefts	Female	0-6 days	2-4 years	Global	-1	2	Legality of Abortion	10
Orofacial clefts	Female	0-6 days	2-4 years	Global	-1	2	Skilled Birth Attendance (proportion)	10
Orofacial clefts	Female	0-6 days	2-4 years	Global	-1	2	Healthcare access and quality index	742
Orofacial clefts	Female	0-6 days	2-4 years	Global	1	2	Age-standardized SEV for Smoking	818
Orofacial clefts	Female	0-6 days	2-4 years	Global	1	2	Age-standardized SEV for High fasting plasma glucose	--
Orofacial clefts	Female	0-6 days	2-4 years	Global	1	2	Maternal alcohol consumption during pregnancy (proportion)	--
Orofacial clefts	Female	0-6 days	2-4 years	Global	-1	3	Maternal Education (years per capita)	2
Orofacial clefts	Female	0-6 days	2-4 years	Global	-1	3	Antenatal Care (4 visits) Coverage (proportion)	4
Orofacial clefts	Female	0-6 days	2-4 years	Global	-1	3	Antenatal Care (1 visit) Coverage (proportion)	6
Orofacial clefts	Female	0-6 days	2-4 years	Global	1	3	Age-standardized SEV for Household air pollution	6
Orofacial clefts	Female	0-6 days	2-4 years	Global	1	3	Age-standardized SEV for Low fruit	97
Orofacial clefts	Female	0-6 days	2-4 years	Global	1	3	Liters of alcohol consumed per capita	613
Orofacial clefts	Female	0-6 days	2-4 years	Global	1	3	Age-standardized SEV for Low vegetables	--
Orofacial clefts	Male	0-6 days	2-4 years	Data Rich	-1	1	Composite fortification standard and folic acid inclusion	391
Orofacial clefts	Male	0-6 days	2-4 years	Data Rich	-1	1	Socio-demographic Index	609
Orofacial clefts	Male	0-6 days	2-4 years	Data Rich	-1	2	Skilled Birth Attendance (proportion)	347
Orofacial clefts	Male	0-6 days	2-4 years	Data Rich	-1	2	Healthcare access and quality index	568
Orofacial clefts	Male	0-6 days	2-4 years	Data Rich	-1	2	Legality of Abortion	568
Orofacial clefts	Male	0-6 days	2-4 years	Data Rich	1	2	Age-standardized SEV for Smoking	221
Orofacial clefts	Male	0-6 days	2-4 years	Data Rich	1	2	Age-standardized SEV for High fasting plasma glucose	--
Orofacial clefts	Male	0-6 days	2-4 years	Data Rich	1	2	Maternal alcohol consumption during pregnancy (proportion)	--
Orofacial clefts	Male	0-6 days	2-4 years	Data Rich	-1	3	Antenatal Care (1 visit) Coverage (proportion)	31
Orofacial clefts	Male	0-6 days	2-4 years	Data Rich	-1	3	Antenatal Care (4 visits) Coverage (proportion)	31
Orofacial clefts	Male	0-6 days	2-4 years	Data Rich	-1	3	Maternal Education (years per capita)	--
Orofacial clefts	Male	0-6 days	2-4 years	Data Rich	1	3	Age-standardized SEV for Low fruit	15
Orofacial clefts	Male	0-6 days	2-4 years	Data Rich	1	3	Age-standardized SEV for Household air pollution	16
Orofacial clefts	Male	0-6 days	2-4 years	Data Rich	1	3	Liters of alcohol consumed per capita	508
Orofacial clefts	Male	0-6 days	2-4 years	Data Rich	1	3	Age-standardized SEV for Low vegetables	--
Orofacial clefts	Male	0-6 days	2-4 years	Global	-1	1	Composite fortification standard and folic acid inclusion	605
Orofacial clefts	Male	0-6 days	2-4 years	Global	-1	1	Socio-demographic Index	714
Orofacial clefts	Male	0-6 days	2-4 years	Global	-1	2	Skilled Birth Attendance (proportion)	156
Orofacial clefts	Male	0-6 days	2-4 years	Global	-1	2	Healthcare access and quality index	575
Orofacial clefts	Male	0-6 days	2-4 years	Global	-1	2	Legality of Abortion	575
Orofacial clefts	Male	0-6 days	2-4 years	Global	1	2	Age-standardized SEV for Smoking	575
Orofacial clefts	Male	0-6 days	2-4 years	Global	1	2	Age-standardized SEV for High fasting plasma glucose	--
Orofacial clefts	Male	0-6 days	2-4 years	Global	1	2	Maternal alcohol consumption during pregnancy (proportion)	--
Orofacial clefts	Male	0-6 days	2-4 years	Global	-1	3	Antenatal Care (1 visit) Coverage (proportion)	36

Appendix Table S10: CODEm covariates used, level of covariate, and expected direction of covariate by cause, sex, and age

Cause	Sex	Age Start	Age End	Model Version Type	Direction	Level	Covariate Name	Number of Draws
Orofacial clefts	Male	0-6 days	2-4 years	Global	-1	3	Antenatal Care (4 visits) Coverage (proportion)	36
Orofacial clefts	Male	0-6 days	2-4 years	Global	-1	3	Maternal Education (years per capita)	138
Orofacial clefts	Male	0-6 days	2-4 years	Global	1	3	Age-standardized SEV for Household air pollution	36
Orofacial clefts	Male	0-6 days	2-4 years	Global	1	3	Age-standardized SEV for Low fruit	92
Orofacial clefts	Male	0-6 days	2-4 years	Global	1	3	Liters of alcohol consumed per capita	546
Orofacial clefts	Male	0-6 days	2-4 years	Global	1	3	Age-standardized SEV for Low vegetables	--
Down's syndrome	Female	0-6 days	65-69 years	Data Rich	-1	1	Legality of Abortion	63
Down's syndrome	Female	0-6 days	65-69 years	Data Rich	1	1	Live Births 35+ (proportion)	485
Down's syndrome	Female	0-6 days	65-69 years	Data Rich	1	1	Live Births 40+ (proportion)	515
Down's syndrome	Female	0-6 days	65-69 years	Data Rich	1	1	Birth prevalence of congenital chromosomal anomalies	585
Down's syndrome	Female	0-6 days	65-69 years	Data Rich	-1	2	Healthcare access and quality index	--
Down's syndrome	Female	0-6 days	65-69 years	Data Rich	-1	2	In-Facility Delivery (proportion)	--
Down's syndrome	Female	0-6 days	65-69 years	Data Rich	-1	2	Socio-demographic Index	--
Down's syndrome	Female	0-6 days	65-69 years	Data Rich	-1	3	Antenatal Care (1 visit) Coverage (proportion)	--
Down's syndrome	Female	0-6 days	65-69 years	Data Rich	-1	3	Antenatal Care (4 visits) Coverage (proportion)	--
Down's syndrome	Female	0-6 days	65-69 years	Data Rich	-1	3	Maternal Education (years per capita)	--
Down's syndrome	Female	0-6 days	65-69 years	Data Rich	1	3	Age-standardized SEV for Low vegetables	278
Down's syndrome	Female	0-6 days	65-69 years	Data Rich	1	3	Age-standardized SEV for Household air pollution	--
Down's syndrome	Female	0-6 days	65-69 years	Data Rich	1	3	Age-standardized SEV for Smoking	--
Down's syndrome	Female	0-6 days	65-69 years	Data Rich	1	3	Liters of alcohol consumed per capita	--
Down's syndrome	Female	0-6 days	65-69 years	Data Rich	1	3	Maternal alcohol consumption during pregnancy (proportion)	--
Down's syndrome	Female	0-6 days	65-69 years	Global	-1	1	Legality of Abortion	616
Down's syndrome	Female	0-6 days	65-69 years	Global	1	1	Live Births 40+ (proportion)	301
Down's syndrome	Female	0-6 days	65-69 years	Global	1	1	Birth prevalence of congenital chromosomal anomalies	320
Down's syndrome	Female	0-6 days	65-69 years	Global	1	1	Live Births 35+ (proportion)	392
Down's syndrome	Female	0-6 days	65-69 years	Global	-1	2	Healthcare access and quality index	--
Down's syndrome	Female	0-6 days	65-69 years	Global	-1	2	In-Facility Delivery (proportion)	--
Down's syndrome	Female	0-6 days	65-69 years	Global	-1	2	Socio-demographic Index	--
Down's syndrome	Female	0-6 days	65-69 years	Global	-1	3	Antenatal Care (1 visit) Coverage (proportion)	--
Down's syndrome	Female	0-6 days	65-69 years	Global	-1	3	Antenatal Care (4 visits) Coverage (proportion)	--
Down's syndrome	Female	0-6 days	65-69 years	Global	-1	3	Maternal Education (years per capita)	--
Down's syndrome	Female	0-6 days	65-69 years	Global	1	3	Age-standardized SEV for Low vegetables	185
Down's syndrome	Female	0-6 days	65-69 years	Global	1	3	Liters of alcohol consumed per capita	302
Down's syndrome	Female	0-6 days	65-69 years	Global	1	3	Age-standardized SEV for Household air pollution	--
Down's syndrome	Female	0-6 days	65-69 years	Global	1	3	Age-standardized SEV for Smoking	--
Down's syndrome	Female	0-6 days	65-69 years	Global	1	3	Maternal alcohol consumption during pregnancy (proportion)	--
Down's syndrome	Male	0-6 days	65-69 years	Data Rich	-1	1	Legality of Abortion	603
Down's syndrome	Male	0-6 days	65-69 years	Data Rich	1	1	Live Births 40+ (proportion)	433
Down's syndrome	Male	0-6 days	65-69 years	Data Rich	1	1	Live Births 35+ (proportion)	451
Down's syndrome	Male	0-6 days	65-69 years	Data Rich	1	1	Birth prevalence of congenital chromosomal anomalies	461
Down's syndrome	Male	0-6 days	65-69 years	Data Rich	-1	2	Healthcare access and quality index	--
Down's syndrome	Male	0-6 days	65-69 years	Data Rich	-1	2	In-Facility Delivery (proportion)	--
Down's syndrome	Male	0-6 days	65-69 years	Data Rich	-1	2	Socio-demographic Index	--
Down's syndrome	Male	0-6 days	65-69 years	Data Rich	-1	3	Antenatal Care (1 visit) Coverage (proportion)	--
Down's syndrome	Male	0-6 days	65-69 years	Data Rich	-1	3	Antenatal Care (4 visits) Coverage (proportion)	--
Down's syndrome	Male	0-6 days	65-69 years	Data Rich	-1	3	Maternal Education (years per capita)	--
Down's syndrome	Male	0-6 days	65-69 years	Data Rich	1	3	Maternal alcohol consumption during pregnancy (proportion)	60
Down's syndrome	Male	0-6 days	65-69 years	Data Rich	1	3	Age-standardized SEV for Household air pollution	--
Down's syndrome	Male	0-6 days	65-69 years	Data Rich	1	3	Age-standardized SEV for Low vegetables	--
Down's syndrome	Male	0-6 days	65-69 years	Data Rich	1	3	Age-standardized SEV for Smoking	--
Down's syndrome	Male	0-6 days	65-69 years	Data Rich	1	3	Liters of alcohol consumed per capita	--
Down's syndrome	Male	0-6 days	65-69 years	Global	-1	1	Legality of Abortion	396
Down's syndrome	Male	0-6 days	65-69 years	Global	1	1	Live Births 40+ (proportion)	333
Down's syndrome	Male	0-6 days	65-69 years	Global	1	1	Birth prevalence of congenital chromosomal anomalies	433
Down's syndrome	Male	0-6 days	65-69 years	Global	1	1	Live Births 35+ (proportion)	573
Down's syndrome	Male	0-6 days	65-69 years	Global	-1	2	Healthcare access and quality index	--
Down's syndrome	Male	0-6 days	65-69 years	Global	-1	2	In-Facility Delivery (proportion)	--
Down's syndrome	Male	0-6 days	65-69 years	Global	-1	2	Socio-demographic Index	--
Down's syndrome	Male	0-6 days	65-69 years	Global	-1	3	Antenatal Care (1 visit) Coverage (proportion)	--
Down's syndrome	Male	0-6 days	65-69 years	Global	-1	3	Antenatal Care (4 visits) Coverage (proportion)	--
Down's syndrome	Male	0-6 days	65-69 years	Global	-1	3	Maternal Education (years per capita)	--
Down's syndrome	Male	0-6 days	65-69 years	Global	1	3	Liters of alcohol consumed per capita	351
Down's syndrome	Male	0-6 days	65-69 years	Global	1	3	Age-standardized SEV for Household air pollution	--
Down's syndrome	Male	0-6 days	65-69 years	Global	1	3	Age-standardized SEV for Low vegetables	--
Down's syndrome	Male	0-6 days	65-69 years	Global	1	3	Age-standardized SEV for Smoking	--
Down's syndrome	Male	0-6 days	65-69 years	Global	1	3	Maternal alcohol consumption during pregnancy (proportion)	--
Other chromosomal abnormalities	Female	0-6 days	65-69 years	Data Rich	-1	1	Legality of Abortion	98
Other chromosomal abnormalities	Female	0-6 days	65-69 years	Data Rich	1	1	Live Births 35+ (proportion)	1000
Other chromosomal abnormalities	Female	0-6 days	65-69 years	Data Rich	1	1	Live Births 40+ (proportion)	--
Other chromosomal abnormalities	Female	0-6 days	65-69 years	Data Rich	-1	2	Antenatal Care (1 visit) Coverage (proportion)	--
Other chromosomal abnormalities	Female	0-6 days	65-69 years	Data Rich	-1	2	Antenatal Care (4 visits) Coverage (proportion)	--
Other chromosomal abnormalities	Female	0-6 days	65-69 years	Data Rich	-1	2	Healthcare access and quality index	--
Other chromosomal abnormalities	Female	0-6 days	65-69 years	Data Rich	-1	2	In-Facility Delivery (proportion)	--
Other chromosomal abnormalities	Female	0-6 days	65-69 years	Data Rich	-1	2	LDI (I$ per capita)	--
Other chromosomal abnormalities	Female	0-6 days	65-69 years	Data Rich	1	2	Maternal alcohol consumption during pregnancy (proportion)	298
Other chromosomal abnormalities	Female	0-6 days	65-69 years	Data Rich	-1	3	Maternal Education (years per capita)	--
Other chromosomal abnormalities	Female	0-6 days	65-69 years	Data Rich	-1	3	Skilled Birth Attendance (proportion)	--
Other chromosomal abnormalities	Female	0-6 days	65-69 years	Data Rich	-1	3	Socio-demographic Index	--
Other chromosomal abnormalities	Female	0-6 days	65-69 years	Data Rich	1	3	Liters of alcohol consumed per capita	990
Other chromosomal abnormalities	Female	0-6 days	65-69 years	Data Rich	1	3	Age-standardized SEV for Household air pollution	--
Other chromosomal abnormalities	Female	0-6 days	65-69 years	Data Rich	1	3	Age-standardized SEV for Smoking	--
Other chromosomal abnormalities	Female	0-6 days	65-69 years	Global	-1	1	Legality of Abortion	361
Other chromosomal abnormalities	Female	0-6 days	65-69 years	Global	1	1	Live Births 40+ (proportion)	115
Other chromosomal abnormalities	Female	0-6 days	65-69 years	Global	1	1	Live Births 35+ (proportion)	885
Other chromosomal abnormalities	Female	0-6 days	65-69 years	Global	-1	2	Antenatal Care (1 visit) Coverage (proportion)	--
Other chromosomal abnormalities	Female	0-6 days	65-69 years	Global	-1	2	Antenatal Care (4 visits) Coverage (proportion)	--
Other chromosomal abnormalities	Female	0-6 days	65-69 years	Global	-1	2	Healthcare access and quality index	--
Other chromosomal abnormalities	Female	0-6 days	65-69 years	Global	-1	2	In-Facility Delivery (proportion)	--
Other chromosomal abnormalities	Female	0-6 days	65-69 years	Global	-1	2	LDI (I$ per capita)	--
Other chromosomal abnormalities	Female	0-6 days	65-69 years	Global	1	2	Maternal alcohol consumption during pregnancy (proportion)	353
Other chromosomal abnormalities	Female	0-6 days	65-69 years	Global	-1	3	Maternal Education (years per capita)	--
Other chromosomal abnormalities	Female	0-6 days	65-69 years	Global	-1	3	Skilled Birth Attendance (proportion)	--
Other chromosomal abnormalities	Female	0-6 days	65-69 years	Global	-1	3	Socio-demographic Index	--
Other chromosomal abnormalities	Female	0-6 days	65-69 years	Global	1	3	Liters of alcohol consumed per capita	523
Other chromosomal abnormalities	Female	0-6 days	65-69 years	Global	1	3	Age-standardized SEV for Household air pollution	--
Other chromosomal abnormalities	Female	0-6 days	65-69 years	Global	1	3	Age-standardized SEV for Smoking	--

Appendix Table S10: CODEm covariates used, level of covariate, and expected direction of covariate by cause, sex, and age								
**Cause**	**Sex**	**Age Start**	**Age End**	**Model Version Type**	**Direction**	**Level**	**Covariate Name**	**Number of Draws**
Other chromosomal abnormalities	Male	0-6 days	65-69 years	Data Rich	-1	1	Legality of Abortion	699
Other chromosomal abnormalities	Male	0-6 days	65-69 years	Data Rich	1	1	Live Births 40+ (proportion)	35
Other chromosomal abnormalities	Male	0-6 days	65-69 years	Data Rich	1	1	Live Births 35+ (proportion)	965
Other chromosomal abnormalities	Male	0-6 days	65-69 years	Data Rich	-1	2	Antenatal Care (1 visit) Coverage (proportion)	--
Other chromosomal abnormalities	Male	0-6 days	65-69 years	Data Rich	-1	2	Antenatal Care (4 visits) Coverage (proportion)	--
Other chromosomal abnormalities	Male	0-6 days	65-69 years	Data Rich	-1	2	Healthcare access and quality index	--
Other chromosomal abnormalities	Male	0-6 days	65-69 years	Data Rich	-1	2	In-Facility Delivery (proportion)	--
Other chromosomal abnormalities	Male	0-6 days	65-69 years	Data Rich	-1	2	LDI (I$ per capita)	--
Other chromosomal abnormalities	Male	0-6 days	65-69 years	Data Rich	1	2	Maternal alcohol consumption during pregnancy (proportion)	383
Other chromosomal abnormalities	Male	0-6 days	65-69 years	Data Rich	-1	3	Maternal Education (years per capita)	--
Other chromosomal abnormalities	Male	0-6 days	65-69 years	Data Rich	-1	3	Skilled Birth Attendance (proportion)	--
Other chromosomal abnormalities	Male	0-6 days	65-69 years	Data Rich	-1	3	Socio-demographic Index	--
Other chromosomal abnormalities	Male	0-6 days	65-69 years	Data Rich	1	3	Liters of alcohol consumed per capita	103
Other chromosomal abnormalities	Male	0-6 days	65-69 years	Data Rich	1	3	Age-standardized SEV for Household air pollution	--
Other chromosomal abnormalities	Male	0-6 days	65-69 years	Data Rich	1	3	Age-standardized SEV for Smoking	--
Other chromosomal abnormalities	Male	0-6 days	65-69 years	Global	-1	1	Legality of Abortion	--
Other chromosomal abnormalities	Male	0-6 days	65-69 years	Global	1	1	Live Births 40+ (proportion)	3
Other chromosomal abnormalities	Male	0-6 days	65-69 years	Global	1	1	Live Births 35+ (proportion)	997
Other chromosomal abnormalities	Male	0-6 days	65-69 years	Global	-1	2	Antenatal Care (1 visit) Coverage (proportion)	--
Other chromosomal abnormalities	Male	0-6 days	65-69 years	Global	-1	2	Antenatal Care (4 visits) Coverage (proportion)	--
Other chromosomal abnormalities	Male	0-6 days	65-69 years	Global	-1	2	Healthcare access and quality index	--
Other chromosomal abnormalities	Male	0-6 days	65-69 years	Global	-1	2	In-Facility Delivery (proportion)	--
Other chromosomal abnormalities	Male	0-6 days	65-69 years	Global	-1	2	LDI (I$ per capita)	--
Other chromosomal abnormalities	Male	0-6 days	65-69 years	Global	1	2	Maternal alcohol consumption during pregnancy (proportion)	177
Other chromosomal abnormalities	Male	0-6 days	65-69 years	Global	-1	3	Maternal Education (years per capita)	--
Other chromosomal abnormalities	Male	0-6 days	65-69 years	Global	-1	3	Skilled Birth Attendance (proportion)	--
Other chromosomal abnormalities	Male	0-6 days	65-69 years	Global	-1	3	Socio-demographic Index	--
Other chromosomal abnormalities	Male	0-6 days	65-69 years	Global	1	3	Liters of alcohol consumed per capita	698
Other chromosomal abnormalities	Male	0-6 days	65-69 years	Global	1	3	Age-standardized SEV for Household air pollution	--
Other chromosomal abnormalities	Male	0-6 days	65-69 years	Global	1	3	Age-standardized SEV for Smoking	--
Congenital musculoskeletal and limb anomalies	Female	0-6 days	65-69 years	Data Rich	-1	1	Healthcare access and quality index	1000
Congenital musculoskeletal and limb anomalies	Female	0-6 days	65-69 years	Data Rich	-1	1	Legality of Abortion	--
Congenital musculoskeletal and limb anomalies	Female	0-6 days	65-69 years	Data Rich	-1	1	Socio-demographic Index	--
Congenital musculoskeletal and limb anomalies	Female	0-6 days	65-69 years	Data Rich	1	1	Maternal alcohol consumption during pregnancy (proportion)	--
Congenital musculoskeletal and limb anomalies	Female	0-6 days	65-69 years	Data Rich	-1	2	In-Facility Delivery (proportion)	--
Congenital musculoskeletal and limb anomalies	Female	0-6 days	65-69 years	Data Rich	1	2	Age-standardized SEV for High fasting plasma glucose	--
Congenital musculoskeletal and limb anomalies	Female	0-6 days	65-69 years	Data Rich	1	2	Age-standardized SEV for Household air pollution	--
Congenital musculoskeletal and limb anomalies	Female	0-6 days	65-69 years	Data Rich	1	2	Age-standardized SEV for Smoking	--
Congenital musculoskeletal and limb anomalies	Female	0-6 days	65-69 years	Data Rich	-1	3	Antenatal Care (1 visit) Coverage (proportion)	--
Congenital musculoskeletal and limb anomalies	Female	0-6 days	65-69 years	Data Rich	-1	3	Antenatal Care (4 visits) Coverage (proportion)	--
Congenital musculoskeletal and limb anomalies	Female	0-6 days	65-69 years	Data Rich	-1	3	LDI (I$ per capita)	--
Congenital musculoskeletal and limb anomalies	Female	0-6 days	65-69 years	Data Rich	-1	3	Maternal Education (years per capita)	--
Congenital musculoskeletal and limb anomalies	Female	0-6 days	65-69 years	Data Rich	1	3	Age-standardized SEV for Low fruit	--
Congenital musculoskeletal and limb anomalies	Female	0-6 days	65-69 years	Data Rich	1	3	Age-standardized SEV for Low vegetables	--
Congenital musculoskeletal and limb anomalies	Female	0-6 days	65-69 years	Data Rich	1	3	Liters of alcohol consumed per capita	--
Congenital musculoskeletal and limb anomalies	Female	0-6 days	65-69 years	Global	-1	1	Legality of Abortion	229
Congenital musculoskeletal and limb anomalies	Female	0-6 days	65-69 years	Global	-1	1	Healthcare access and quality index	771
Congenital musculoskeletal and limb anomalies	Female	0-6 days	65-69 years	Global	-1	1	Socio-demographic Index	--
Congenital musculoskeletal and limb anomalies	Female	0-6 days	65-69 years	Global	1	1	Maternal alcohol consumption during pregnancy (proportion)	--
Congenital musculoskeletal and limb anomalies	Female	0-6 days	65-69 years	Global	-1	2	In-Facility Delivery (proportion)	307
Congenital musculoskeletal and limb anomalies	Female	0-6 days	65-69 years	Global	1	2	Age-standardized SEV for Household air pollution	106
Congenital musculoskeletal and limb anomalies	Female	0-6 days	65-69 years	Global	1	2	Age-standardized SEV for High fasting plasma glucose	--
Congenital musculoskeletal and limb anomalies	Female	0-6 days	65-69 years	Global	1	2	Age-standardized SEV for Smoking	--
Congenital musculoskeletal and limb anomalies	Female	0-6 days	65-69 years	Global	-1	3	Antenatal Care (1 visit) Coverage (proportion)	--
Congenital musculoskeletal and limb anomalies	Female	0-6 days	65-69 years	Global	-1	3	Antenatal Care (4 visits) Coverage (proportion)	--
Congenital musculoskeletal and limb anomalies	Female	0-6 days	65-69 years	Global	-1	3	LDI (I$ per capita)	--
Congenital musculoskeletal and limb anomalies	Female	0-6 days	65-69 years	Global	-1	3	Maternal Education (years per capita)	--
Congenital musculoskeletal and limb anomalies	Female	0-6 days	65-69 years	Global	1	3	Age-standardized SEV for Low vegetables	134
Congenital musculoskeletal and limb anomalies	Female	0-6 days	65-69 years	Global	1	3	Age-standardized SEV for Low fruit	216
Congenital musculoskeletal and limb anomalies	Female	0-6 days	65-69 years	Global	1	3	Liters of alcohol consumed per capita	--
Congenital musculoskeletal and limb anomalies	Male	0-6 days	65-69 years	Data Rich	-1	1	Legality of Abortion	142
Congenital musculoskeletal and limb anomalies	Male	0-6 days	65-69 years	Data Rich	-1	1	Healthcare access and quality index	858
Congenital musculoskeletal and limb anomalies	Male	0-6 days	65-69 years	Data Rich	-1	1	Socio-demographic Index	--
Congenital musculoskeletal and limb anomalies	Male	0-6 days	65-69 years	Data Rich	1	1	Maternal alcohol consumption during pregnancy (proportion)	--
Congenital musculoskeletal and limb anomalies	Male	0-6 days	65-69 years	Data Rich	-1	2	In-Facility Delivery (proportion)	--
Congenital musculoskeletal and limb anomalies	Male	0-6 days	65-69 years	Data Rich	1	2	Age-standardized SEV for High fasting plasma glucose	325
Congenital musculoskeletal and limb anomalies	Male	0-6 days	65-69 years	Data Rich	1	2	Age-standardized SEV for Household air pollution	--
Congenital musculoskeletal and limb anomalies	Male	0-6 days	65-69 years	Data Rich	1	2	Age-standardized SEV for Smoking	--
Congenital musculoskeletal and limb anomalies	Male	0-6 days	65-69 years	Data Rich	-1	3	Antenatal Care (1 visit) Coverage (proportion)	--
Congenital musculoskeletal and limb anomalies	Male	0-6 days	65-69 years	Data Rich	-1	3	Antenatal Care (4 visits) Coverage (proportion)	--
Congenital musculoskeletal and limb anomalies	Male	0-6 days	65-69 years	Data Rich	-1	3	LDI (I$ per capita)	--
Congenital musculoskeletal and limb anomalies	Male	0-6 days	65-69 years	Data Rich	-1	3	Maternal Education (years per capita)	--
Congenital musculoskeletal and limb anomalies	Male	0-6 days	65-69 years	Data Rich	1	3	Age-standardized SEV for Low vegetables	239
Congenital musculoskeletal and limb anomalies	Male	0-6 days	65-69 years	Data Rich	1	3	Age-standardized SEV for Low fruit	--
Congenital musculoskeletal and limb anomalies	Male	0-6 days	65-69 years	Data Rich	1	3	Liters of alcohol consumed per capita	--
Congenital musculoskeletal and limb anomalies	Male	0-6 days	65-69 years	Global	-1	1	Legality of Abortion	581
Congenital musculoskeletal and limb anomalies	Male	0-6 days	65-69 years	Global	-1	1	Healthcare access and quality index	927
Congenital musculoskeletal and limb anomalies	Male	0-6 days	65-69 years	Global	-1	1	Socio-demographic Index	--
Congenital musculoskeletal and limb anomalies	Male	0-6 days	65-69 years	Global	1	1	Maternal alcohol consumption during pregnancy (proportion)	45
Congenital musculoskeletal and limb anomalies	Male	0-6 days	65-69 years	Global	-1	2	In-Facility Delivery (proportion)	220
Congenital musculoskeletal and limb anomalies	Male	0-6 days	65-69 years	Global	1	2	Age-standardized SEV for Household air pollution	65
Congenital musculoskeletal and limb anomalies	Male	0-6 days	65-69 years	Global	1	2	Age-standardized SEV for High fasting plasma glucose	220
Congenital musculoskeletal and limb anomalies	Male	0-6 days	65-69 years	Global	1	2	Age-standardized SEV for Smoking	--
Congenital musculoskeletal and limb anomalies	Male	0-6 days	65-69 years	Global	-1	3	Antenatal Care (1 visit) Coverage (proportion)	6
Congenital musculoskeletal and limb anomalies	Male	0-6 days	65-69 years	Global	-1	3	Maternal Education (years per capita)	27
Congenital musculoskeletal and limb anomalies	Male	0-6 days	65-69 years	Global	-1	3	Antenatal Care (4 visits) Coverage (proportion)	--

Appendix Table S10: CODEm covariates used, level of covariate, and expected direction of covariate by cause, sex, and age								
**Cause**	**Sex**	**Age Start**	**Age End**	**Model Version Type**	**Direction**	**Level**	**Covariate Name**	**Number of Draws**
Congenital musculoskeletal and limb anomalies	Male	0-6 days	65-69 years	Global	-1	3	LDI (I$ per capita)	--
Congenital musculoskeletal and limb anomalies	Male	0-6 days	65-69 years	Global	1	3	Age-standardized SEV for Low vegetables	7
Congenital musculoskeletal and limb anomalies	Male	0-6 days	65-69 years	Global	1	3	Age-standardized SEV for Low fruit	309
Congenital musculoskeletal and limb anomalies	Male	0-6 days	65-69 years	Global	1	3	Liters of alcohol consumed per capita	--
Urogenital congenital anomalies	Female	0-6 days	65-69 years	Data Rich	1	1	Smoking Prevalence (Reproductive Age Standardized)	321
Urogenital congenital anomalies	Female	0-6 days	65-69 years	Data Rich	1	1	Age-standardized SEV for Smoking	679
Urogenital congenital anomalies	Female	0-6 days	65-69 years	Data Rich	1	1	Maternal alcohol consumption during pregnancy (proportion)	--
Urogenital congenital anomalies	Female	0-6 days	65-69 years	Data Rich	-1	2	Healthcare access and quality index	286
Urogenital congenital anomalies	Female	0-6 days	65-69 years	Data Rich	-1	2	In-Facility Delivery (proportion)	--
Urogenital congenital anomalies	Female	0-6 days	65-69 years	Data Rich	-1	2	Socio-demographic Index	--
Urogenital congenital anomalies	Female	0-6 days	65-69 years	Data Rich	1	2	Age-standardized SEV for Ambient particulate matter	394
Urogenital congenital anomalies	Female	0-6 days	65-69 years	Data Rich	1	2	Age-standardized SEV for Household air pollution	--
Urogenital congenital anomalies	Female	0-6 days	65-69 years	Data Rich	-1	3	Antenatal Care (1 visit) Coverage (proportion)	--
Urogenital congenital anomalies	Female	0-6 days	65-69 years	Data Rich	-1	3	Antenatal Care (4 visits) Coverage (proportion)	--
Urogenital congenital anomalies	Female	0-6 days	65-69 years	Data Rich	-1	3	LDI (I$ per capita)	--
Urogenital congenital anomalies	Female	0-6 days	65-69 years	Data Rich	-1	3	Maternal Education (years per capita)	--
Urogenital congenital anomalies	Female	0-6 days	65-69 years	Data Rich	1	3	Liters of alcohol consumed per capita	--
Urogenital congenital anomalies	Female	0-6 days	65-69 years	Global	1	1	Age-standardized SEV for Smoking	1000
Urogenital congenital anomalies	Female	0-6 days	65-69 years	Global	1	1	Maternal alcohol consumption during pregnancy (proportion)	--
Urogenital congenital anomalies	Female	0-6 days	65-69 years	Global	-1	2	Healthcare access and quality index	--
Urogenital congenital anomalies	Female	0-6 days	65-69 years	Global	-1	2	In-Facility Delivery (proportion)	--
Urogenital congenital anomalies	Female	0-6 days	65-69 years	Global	-1	2	Socio-demographic Index	--
Urogenital congenital anomalies	Female	0-6 days	65-69 years	Global	1	2	Diabetes Age-Standardized Prevalence (proportion)	302
Urogenital congenital anomalies	Female	0-6 days	65-69 years	Global	1	2	Age-standardized SEV for Ambient particulate matter	355
Urogenital congenital anomalies	Female	0-6 days	65-69 years	Global	1	2	Age-standardized SEV for Household air pollution	--
Urogenital congenital anomalies	Female	0-6 days	65-69 years	Global	-1	3	Antenatal Care (1 visit) Coverage (proportion)	--
Urogenital congenital anomalies	Female	0-6 days	65-69 years	Global	-1	3	Antenatal Care (4 visits) Coverage (proportion)	--
Urogenital congenital anomalies	Female	0-6 days	65-69 years	Global	-1	3	LDI (I$ per capita)	--
Urogenital congenital anomalies	Female	0-6 days	65-69 years	Global	-1	3	Maternal Education (years per capita)	--
Urogenital congenital anomalies	Female	0-6 days	65-69 years	Global	1	3	Liters of alcohol consumed per capita	--
Urogenital congenital anomalies	Male	0-6 days	65-69 years	Data Rich	1	1	Age-standardized SEV for Smoking	1000
Urogenital congenital anomalies	Male	0-6 days	65-69 years	Data Rich	1	1	Maternal alcohol consumption during pregnancy (proportion)	--
Urogenital congenital anomalies	Male	0-6 days	65-69 years	Data Rich	-1	2	Healthcare access and quality index	548
Urogenital congenital anomalies	Male	0-6 days	65-69 years	Data Rich	-1	2	In-Facility Delivery (proportion)	--
Urogenital congenital anomalies	Male	0-6 days	65-69 years	Data Rich	-1	2	Socio-demographic Index	--
Urogenital congenital anomalies	Male	0-6 days	65-69 years	Data Rich	1	2	Diabetes Age-Standardized Prevalence (proportion)	548
Urogenital congenital anomalies	Male	0-6 days	65-69 years	Data Rich	1	2	Age-standardized SEV for Ambient particulate matter	--
Urogenital congenital anomalies	Male	0-6 days	65-69 years	Data Rich	1	2	Age-standardized SEV for Household air pollution	--
Urogenital congenital anomalies	Male	0-6 days	65-69 years	Data Rich	-1	3	Antenatal Care (1 visit) Coverage (proportion)	--
Urogenital congenital anomalies	Male	0-6 days	65-69 years	Data Rich	-1	3	Antenatal Care (4 visits) Coverage (proportion)	--
Urogenital congenital anomalies	Male	0-6 days	65-69 years	Data Rich	-1	3	LDI (I$ per capita)	--
Urogenital congenital anomalies	Male	0-6 days	65-69 years	Data Rich	-1	3	Maternal Education (years per capita)	--
Urogenital congenital anomalies	Male	0-6 days	65-69 years	Data Rich	1	3	Liters of alcohol consumed per capita	--
Urogenital congenital anomalies	Male	0-6 days	65-69 years	Global	1	1	Age-standardized SEV for Smoking	--
Urogenital congenital anomalies	Male	0-6 days	65-69 years	Global	1	1	Maternal alcohol consumption during pregnancy (proportion)	--
Urogenital congenital anomalies	Male	0-6 days	65-69 years	Global	-1	2	Healthcare access and quality index	--
Urogenital congenital anomalies	Male	0-6 days	65-69 years	Global	-1	2	In-Facility Delivery (proportion)	--
Urogenital congenital anomalies	Male	0-6 days	65-69 years	Global	-1	2	Socio-demographic Index	--
Urogenital congenital anomalies	Male	0-6 days	65-69 years	Global	1	2	Age-standardized SEV for Ambient particulate matter	481
Urogenital congenital anomalies	Male	0-6 days	65-69 years	Global	1	2	Diabetes Age-Standardized Prevalence (proportion)	481
Urogenital congenital anomalies	Male	0-6 days	65-69 years	Global	1	2	Age-standardized SEV for Household air pollution	--
Urogenital congenital anomalies	Male	0-6 days	65-69 years	Global	-1	3	Antenatal Care (1 visit) Coverage (proportion)	--
Urogenital congenital anomalies	Male	0-6 days	65-69 years	Global	-1	3	Antenatal Care (4 visits) Coverage (proportion)	--
Urogenital congenital anomalies	Male	0-6 days	65-69 years	Global	-1	3	LDI (I$ per capita)	--
Urogenital congenital anomalies	Male	0-6 days	65-69 years	Global	-1	3	Maternal Education (years per capita)	--
Urogenital congenital anomalies	Male	0-6 days	65-69 years	Global	1	3	Liters of alcohol consumed per capita	519
Digestive congenital anomalies	Female	0-6 days	65-69 years	Data Rich	1	1	Age-standardized SEV for Smoking	1000
Digestive congenital anomalies	Female	0-6 days	65-69 years	Data Rich	1	1	Maternal alcohol consumption during pregnancy (proportion)	--
Digestive congenital anomalies	Female	0-6 days	65-69 years	Data Rich	-1	2	Healthcare access and quality index	--
Digestive congenital anomalies	Female	0-6 days	65-69 years	Data Rich	-1	2	In-Facility Delivery (proportion)	--
Digestive congenital anomalies	Female	0-6 days	65-69 years	Data Rich	-1	2	Socio-demographic Index	--
Digestive congenital anomalies	Female	0-6 days	65-69 years	Data Rich	1	2	Age-standardized SEV for High body-mass index	684
Digestive congenital anomalies	Female	0-6 days	65-69 years	Data Rich	1	2	Age-standardized SEV for High fasting plasma glucose	684
Digestive congenital anomalies	Female	0-6 days	65-69 years	Data Rich	1	2	Age-standardized SEV for Household air pollution	--
Digestive congenital anomalies	Female	0-6 days	65-69 years	Data Rich	-1	3	Maternal Education (years per capita)	438
Digestive congenital anomalies	Female	0-6 days	65-69 years	Data Rich	-1	3	Antenatal Care (1 visit) Coverage (proportion)	--
Digestive congenital anomalies	Female	0-6 days	65-69 years	Data Rich	-1	3	Antenatal Care (4 visits) Coverage (proportion)	--
Digestive congenital anomalies	Female	0-6 days	65-69 years	Data Rich	-1	3	LDI (I$ per capita)	--
Digestive congenital anomalies	Female	0-6 days	65-69 years	Data Rich	-1	3	Maternal care and immunization	--
Digestive congenital anomalies	Female	0-6 days	65-69 years	Data Rich	1	3	Liters of alcohol consumed per capita	213
Digestive congenital anomalies	Female	0-6 days	65-69 years	Data Rich	1	3	Age-standardized SEV for Low fruit	651
Digestive congenital anomalies	Female	0-6 days	65-69 years	Data Rich	1	3	Age-standardized SEV for Low vegetables	--
Digestive congenital anomalies	Female	0-6 days	65-69 years	Global	1	1	Age-standardized SEV for Smoking	--
Digestive congenital anomalies	Female	0-6 days	65-69 years	Global	1	1	Maternal alcohol consumption during pregnancy (proportion)	--
Digestive congenital anomalies	Female	0-6 days	65-69 years	Global	-1	2	Healthcare access and quality index	--
Digestive congenital anomalies	Female	0-6 days	65-69 years	Global	-1	2	In-Facility Delivery (proportion)	--
Digestive congenital anomalies	Female	0-6 days	65-69 years	Global	-1	2	Socio-demographic Index	--
Digestive congenital anomalies	Female	0-6 days	65-69 years	Global	1	2	Age-standardized SEV for High fasting plasma glucose	607
Digestive congenital anomalies	Female	0-6 days	65-69 years	Global	1	2	Age-standardized SEV for High body-mass index	--
Digestive congenital anomalies	Female	0-6 days	65-69 years	Global	1	2	Age-standardized SEV for Household air pollution	--
Digestive congenital anomalies	Female	0-6 days	65-69 years	Global	-1	3	Antenatal Care (1 visit) Coverage (proportion)	--
Digestive congenital anomalies	Female	0-6 days	65-69 years	Global	-1	3	Antenatal Care (4 visits) Coverage (proportion)	--
Digestive congenital anomalies	Female	0-6 days	65-69 years	Global	-1	3	LDI (I$ per capita)	--
Digestive congenital anomalies	Female	0-6 days	65-69 years	Global	-1	3	Maternal Education (years per capita)	--
Digestive congenital anomalies	Female	0-6 days	65-69 years	Global	-1	3	Maternal care and immunization	--
Digestive congenital anomalies	Female	0-6 days	65-69 years	Global	1	3	Age-standardized SEV for Low fruit	298
Digestive congenital anomalies	Female	0-6 days	65-69 years	Global	1	3	Liters of alcohol consumed per capita	422
Digestive congenital anomalies	Female	0-6 days	65-69 years	Global	1	3	Age-standardized SEV for Low vegetables	--
Digestive congenital anomalies	Male	0-6 days	65-69 years	Data Rich	1	1	Age-standardized SEV for Smoking	1000
Digestive congenital anomalies	Male	0-6 days	65-69 years	Data Rich	1	1	Maternal alcohol consumption during pregnancy (proportion)	--
Digestive congenital anomalies	Male	0-6 days	65-69 years	Data Rich	-1	2	Healthcare access and quality index	--
Digestive congenital anomalies	Male	0-6 days	65-69 years	Data Rich	-1	2	In-Facility Delivery (proportion)	--
Digestive congenital anomalies	Male	0-6 days	65-69 years	Data Rich	-1	2	Socio-demographic Index	--

Appendix Table S10: CODEm covariates used, level of covariate, and expected direction of covariate by cause, sex, and age

Cause	Sex	Age Start	Age End	Model Version Type	Direction	Level	Covariate Name	Number of Draws
Digestive congenital anomalies	Male	0-6 days	65-69 years	Data Rich	1	2	Age-standardized SEV for High fasting plasma glucose	437
Digestive congenital anomalies	Male	0-6 days	65-69 years	Data Rich	1	2	Age-standardized SEV for High body-mass index	--
Digestive congenital anomalies	Male	0-6 days	65-69 years	Data Rich	1	2	Age-standardized SEV for Household air pollution	--
Digestive congenital anomalies	Male	0-6 days	65-69 years	Data Rich	-1	3	Maternal Education (years per capita)	239
Digestive congenital anomalies	Male	0-6 days	65-69 years	Data Rich	-1	3	Antenatal Care (1 visit) Coverage (proportion)	--
Digestive congenital anomalies	Male	0-6 days	65-69 years	Data Rich	-1	3	Antenatal Care (4 visits) Coverage (proportion)	--
Digestive congenital anomalies	Male	0-6 days	65-69 years	Data Rich	-1	3	LDI (I$ per capita)	--
Digestive congenital anomalies	Male	0-6 days	65-69 years	Data Rich	-1	3	Maternal care and immunization	--
Digestive congenital anomalies	Male	0-6 days	65-69 years	Data Rich	1	3	Liters of alcohol consumed per capita	430
Digestive congenital anomalies	Male	0-6 days	65-69 years	Data Rich	1	3	Age-standardized SEV for Low fruit	--
Digestive congenital anomalies	Male	0-6 days	65-69 years	Data Rich	1	3	Age-standardized SEV for Low vegetables	--
Digestive congenital anomalies	Male	0-6 days	65-69 years	Global	1	1	Age-standardized SEV for Smoking	--
Digestive congenital anomalies	Male	0-6 days	65-69 years	Global	1	1	Maternal alcohol consumption during pregnancy (proportion)	--
Digestive congenital anomalies	Male	0-6 days	65-69 years	Global	-1	2	Healthcare access and quality index	--
Digestive congenital anomalies	Male	0-6 days	65-69 years	Global	-1	2	In-Facility Delivery (proportion)	--
Digestive congenital anomalies	Male	0-6 days	65-69 years	Global	-1	2	Socio-demographic Index	--
Digestive congenital anomalies	Male	0-6 days	65-69 years	Global	1	2	Age-standardized SEV for High body-mass index	--
Digestive congenital anomalies	Male	0-6 days	65-69 years	Global	1	2	Age-standardized SEV for High fasting plasma glucose	--
Digestive congenital anomalies	Male	0-6 days	65-69 years	Global	1	2	Age-standardized SEV for Household air pollution	--
Digestive congenital anomalies	Male	0-6 days	65-69 years	Global	-1	3	Antenatal Care (1 visit) Coverage (proportion)	--
Digestive congenital anomalies	Male	0-6 days	65-69 years	Global	-1	3	Antenatal Care (4 visits) Coverage (proportion)	--
Digestive congenital anomalies	Male	0-6 days	65-69 years	Global	-1	3	LDI (I$ per capita)	--
Digestive congenital anomalies	Male	0-6 days	65-69 years	Global	-1	3	Maternal Education (years per capita)	--
Digestive congenital anomalies	Male	0-6 days	65-69 years	Global	-1	3	Maternal care and immunization	--
Digestive congenital anomalies	Male	0-6 days	65-69 years	Global	1	3	Age-standardized SEV for Low fruit	452
Digestive congenital anomalies	Male	0-6 days	65-69 years	Global	1	3	Age-standardized SEV for Low vegetables	--
Digestive congenital anomalies	Male	0-6 days	65-69 years	Global	1	3	Liters of alcohol consumed per capita	--
Other congenital anomalies	Female	0-6 days	65-69 years	Data Rich	1	1	Live Births 35+ (proportion)	--
Other congenital anomalies	Female	0-6 days	65-69 years	Data Rich	1	1	Maternal alcohol consumption during pregnancy (proportion)	--
Other congenital anomalies	Female	0-6 days	65-69 years	Data Rich	-1	2	Legality of Abortion	601
Other congenital anomalies	Female	0-6 days	65-69 years	Data Rich	-1	2	Healthcare access and quality index	--
Other congenital anomalies	Female	0-6 days	65-69 years	Data Rich	-1	2	In-Facility Delivery (proportion)	--
Other congenital anomalies	Female	0-6 days	65-69 years	Data Rich	1	2	Age-standardized SEV for Smoking	601
Other congenital anomalies	Female	0-6 days	65-69 years	Data Rich	1	2	Age-standardized SEV for Household air pollution	--
Other congenital anomalies	Female	0-6 days	65-69 years	Data Rich	-1	3	Antenatal Care (1 visit) Coverage (proportion)	--
Other congenital anomalies	Female	0-6 days	65-69 years	Data Rich	-1	3	Antenatal Care (4 visits) Coverage (proportion)	--
Other congenital anomalies	Female	0-6 days	65-69 years	Data Rich	-1	3	LDI (I$ per capita)	--
Other congenital anomalies	Female	0-6 days	65-69 years	Data Rich	-1	3	Maternal Education (years per capita)	--
Other congenital anomalies	Female	0-6 days	65-69 years	Data Rich	-1	3	Socio-demographic Index	--
Other congenital anomalies	Female	0-6 days	65-69 years	Data Rich	1	3	Age-standardized SEV for High fasting plasma glucose	308
Other congenital anomalies	Female	0-6 days	65-69 years	Data Rich	1	3	Liters of alcohol consumed per capita	707
Other congenital anomalies	Female	0-6 days	65-69 years	Global	1	1	Live Births 35+ (proportion)	--
Other congenital anomalies	Female	0-6 days	65-69 years	Global	1	1	Maternal alcohol consumption during pregnancy (proportion)	--
Other congenital anomalies	Female	0-6 days	65-69 years	Global	-1	2	In-Facility Delivery (proportion)	257
Other congenital anomalies	Female	0-6 days	65-69 years	Global	-1	2	Healthcare access and quality index	404
Other congenital anomalies	Female	0-6 days	65-69 years	Global	-1	2	Legality of Abortion	661
Other congenital anomalies	Female	0-6 days	65-69 years	Global	1	2	Age-standardized SEV for Smoking	661
Other congenital anomalies	Female	0-6 days	65-69 years	Global	1	2	Age-standardized SEV for Household air pollution	--
Other congenital anomalies	Female	0-6 days	65-69 years	Global	-1	3	Socio-demographic Index	112
Other congenital anomalies	Female	0-6 days	65-69 years	Global	-1	3	Antenatal Care (4 visits) Coverage (proportion)	114
Other congenital anomalies	Female	0-6 days	65-69 years	Global	-1	3	Antenatal Care (1 visit) Coverage (proportion)	--
Other congenital anomalies	Female	0-6 days	65-69 years	Global	-1	3	LDI (I$ per capita)	--
Other congenital anomalies	Female	0-6 days	65-69 years	Global	-1	3	Maternal Education (years per capita)	--
Other congenital anomalies	Female	0-6 days	65-69 years	Global	1	3	Age-standardized SEV for High fasting plasma glucose	364
Other congenital anomalies	Female	0-6 days	65-69 years	Global	1	3	Liters of alcohol consumed per capita	--
Other congenital anomalies	Male	0-6 days	65-69 years	Data Rich	1	1	Live Births 35+ (proportion)	--
Other congenital anomalies	Male	0-6 days	65-69 years	Data Rich	1	1	Maternal alcohol consumption during pregnancy (proportion)	--
Other congenital anomalies	Male	0-6 days	65-69 years	Data Rich	-1	2	Healthcare access and quality index	616
Other congenital anomalies	Male	0-6 days	65-69 years	Data Rich	-1	2	Legality of Abortion	616
Other congenital anomalies	Male	0-6 days	65-69 years	Data Rich	-1	2	In-Facility Delivery (proportion)	--
Other congenital anomalies	Male	0-6 days	65-69 years	Data Rich	1	2	Age-standardized SEV for Household air pollution	--
Other congenital anomalies	Male	0-6 days	65-69 years	Data Rich	1	2	Age-standardized SEV for Smoking	--
Other congenital anomalies	Male	0-6 days	65-69 years	Data Rich	-1	3	Antenatal Care (1 visit) Coverage (proportion)	--
Other congenital anomalies	Male	0-6 days	65-69 years	Data Rich	-1	3	Antenatal Care (4 visits) Coverage (proportion)	--
Other congenital anomalies	Male	0-6 days	65-69 years	Data Rich	-1	3	LDI (I$ per capita)	--
Other congenital anomalies	Male	0-6 days	65-69 years	Data Rich	-1	3	Maternal Education (years per capita)	--
Other congenital anomalies	Male	0-6 days	65-69 years	Data Rich	-1	3	Socio-demographic Index	--
Other congenital anomalies	Male	0-6 days	65-69 years	Data Rich	1	3	Liters of alcohol consumed per capita	227
Other congenital anomalies	Male	0-6 days	65-69 years	Data Rich	1	3	Age-standardized SEV for High fasting plasma glucose	--
Other congenital anomalies	Male	0-6 days	65-69 years	Global	1	1	Live Births 35+ (proportion)	--
Other congenital anomalies	Male	0-6 days	65-69 years	Global	1	1	Maternal alcohol consumption during pregnancy (proportion)	--
Other congenital anomalies	Male	0-6 days	65-69 years	Global	-1	2	In-Facility Delivery (proportion)	182
Other congenital anomalies	Male	0-6 days	65-69 years	Global	-1	2	Healthcare access and quality index	352
Other congenital anomalies	Male	0-6 days	65-69 years	Global	-1	2	Legality of Abortion	787
Other congenital anomalies	Male	0-6 days	65-69 years	Global	1	2	Age-standardized SEV for Household air pollution	253
Other congenital anomalies	Male	0-6 days	65-69 years	Global	1	2	Age-standardized SEV for Smoking	787
Other congenital anomalies	Male	0-6 days	65-69 years	Global	-1	3	Antenatal Care (4 visits) Coverage (proportion)	79
Other congenital anomalies	Male	0-6 days	65-69 years	Global	-1	3	Socio-demographic Index	134
Other congenital anomalies	Male	0-6 days	65-69 years	Global	-1	3	Antenatal Care (1 visit) Coverage (proportion)	--
Other congenital anomalies	Male	0-6 days	65-69 years	Global	-1	3	LDI (I$ per capita)	--
Other congenital anomalies	Male	0-6 days	65-69 years	Global	-1	3	Maternal Education (years per capita)	--
Other congenital anomalies	Male	0-6 days	65-69 years	Global	1	3	Age-standardized SEV for High fasting plasma glucose	235
Other congenital anomalies	Male	0-6 days	65-69 years	Global	1	3	Liters of alcohol consumed per capita	290
Urinary diseases and male infertility	Female	0-6 days	95+ years	Data Rich	-1	2	Healthcare access and quality index	803
Urinary diseases and male infertility	Female	0-6 days	95+ years	Data Rich	1	2	90th percentile climatic temperature in the given country-year.	326
Urinary diseases and male infertility	Female	0-6 days	95+ years	Data Rich	1	2	Mean BMI	826
Urinary diseases and male infertility	Female	0-6 days	95+ years	Data Rich	1	2	Sanitation (proportion with access)	952
Urinary diseases and male infertility	Female	0-6 days	95+ years	Data Rich	-1	3	Education (years per capita)	21
Urinary diseases and male infertility	Female	0-6 days	95+ years	Data Rich	-1	3	Socio-demographic Index	482
Urinary diseases and male infertility	Female	0-6 days	95+ years	Data Rich	-1	3	LDI (I$ per capita)	--
Urinary diseases and male infertility	Female	0-6 days	95+ years	Global	-1	2	Healthcare access and quality index	1000
Urinary diseases and male infertility	Female	0-6 days	95+ years	Global	1	2	Sanitation (proportion with access)	481
Urinary diseases and male infertility	Female	0-6 days	95+ years	Global	1	2	Mean BMI	633
Urinary diseases and male infertility	Female	0-6 days	95+ years	Global	1	2	90th percentile climatic temperature in the given country-year.	--
Urinary diseases and male infertility	Female	0-6 days	95+ years	Global	-1	3	Education (years per capita)	--
Urinary diseases and male infertility	Female	0-6 days	95+ years	Global	-1	3	LDI (I$ per capita)	--

Appendix Table S10: CODEm covariates used, level of covariate, and expected direction of covariate by cause, sex, and age

Cause	Sex	Age Start	Age End	Model Version Type	Direction	Level	Covariate Name	Number of Draws
Urinary diseases and male infertility	Female	0-6 days	95+ years	Global	-1	3	Socio-demographic Index	--
Urinary diseases and male infertility	Male	0-6 days	95+ years	Data Rich	-1	2	Healthcare access and quality index	713
Urinary diseases and male infertility	Male	0-6 days	95+ years	Data Rich	1	2	90th percentile climatic temperature in the given country-year.	115
Urinary diseases and male infertility	Male	0-6 days	95+ years	Data Rich	1	2	Sanitation (proportion with access)	351
Urinary diseases and male infertility	Male	0-6 days	95+ years	Data Rich	1	2	Mean BMI	603
Urinary diseases and male infertility	Male	0-6 days	95+ years	Data Rich	-1	3	Education (years per capita)	29
Urinary diseases and male infertility	Male	0-6 days	95+ years	Data Rich	-1	3	Socio-demographic Index	150
Urinary diseases and male infertility	Male	0-6 days	95+ years	Data Rich	-1	3	LDI (I$ per capita)	--
Urinary diseases and male infertility	Male	0-6 days	95+ years	Global	-1	2	Healthcare access and quality index	1000
Urinary diseases and male infertility	Male	0-6 days	95+ years	Global	1	2	Mean BMI	436
Urinary diseases and male infertility	Male	0-6 days	95+ years	Global	1	2	Sanitation (proportion with access)	493
Urinary diseases and male infertility	Male	0-6 days	95+ years	Global	1	2	90th percentile climatic temperature in the given country-year.	--
Urinary diseases and male infertility	Male	0-6 days	95+ years	Global	-1	3	Education (years per capita)	108
Urinary diseases and male infertility	Male	0-6 days	95+ years	Global	-1	3	LDI (I$ per capita)	--
Urinary diseases and male infertility	Male	0-6 days	95+ years	Global	-1	3	Socio-demographic Index	--
Urinary tract infections and interstitial nephritis	Female	0-6 days	95+ years	Data Rich	1	1	Sanitation (proportion with access)	1000
Urinary tract infections and interstitial nephritis	Female	0-6 days	95+ years	Data Rich	-1	2	Healthcare access and quality index	427
Urinary tract infections and interstitial nephritis	Female	0-6 days	95+ years	Data Rich	-1	2	Education (years per capita)	--
Urinary tract infections and interstitial nephritis	Female	0-6 days	95+ years	Data Rich	-1	2	LDI (I$ per capita)	--
Urinary tract infections and interstitial nephritis	Female	0-6 days	95+ years	Data Rich	-1	3	Socio-demographic Index	325
Urinary tract infections and interstitial nephritis	Female	0-6 days	95+ years	Global	1	1	Sanitation (proportion with access)	--
Urinary tract infections and interstitial nephritis	Female	0-6 days	95+ years	Global	-1	2	Healthcare access and quality index	546
Urinary tract infections and interstitial nephritis	Female	0-6 days	95+ years	Global	-1	2	Education (years per capita)	--
Urinary tract infections and interstitial nephritis	Female	0-6 days	95+ years	Global	-1	2	LDI (I$ per capita)	--
Urinary tract infections and interstitial nephritis	Female	0-6 days	95+ years	Global	-1	3	Socio-demographic Index	--
Urinary tract infections and interstitial nephritis	Male	0-6 days	95+ years	Data Rich	1	1	Sanitation (proportion with access)	1000
Urinary tract infections and interstitial nephritis	Male	0-6 days	95+ years	Data Rich	-1	2	Education (years per capita)	186
Urinary tract infections and interstitial nephritis	Male	0-6 days	95+ years	Data Rich	-1	2	Healthcare access and quality index	321
Urinary tract infections and interstitial nephritis	Male	0-6 days	95+ years	Data Rich	-1	2	LDI (I$ per capita)	--
Urinary tract infections and interstitial nephritis	Male	0-6 days	95+ years	Data Rich	-1	3	Socio-demographic Index	243
Urinary tract infections and interstitial nephritis	Male	0-6 days	95+ years	Global	1	1	Sanitation (proportion with access)	--
Urinary tract infections and interstitial nephritis	Male	0-6 days	95+ years	Global	-1	2	Healthcare access and quality index	326
Urinary tract infections and interstitial nephritis	Male	0-6 days	95+ years	Global	-1	2	Education (years per capita)	489
Urinary tract infections and interstitial nephritis	Male	0-6 days	95+ years	Global	-1	2	LDI (I$ per capita)	--
Urinary tract infections and interstitial nephritis	Male	0-6 days	95+ years	Global	-1	3	Socio-demographic Index	267
Urolithiasis	Female	12-23 months	95+ years	Data Rich	1	1	red meats unadjusted(g)	704
Urolithiasis	Female	12-23 months	95+ years	Data Rich	1	1	90th percentile climatic temperature in the given country-year.	1000
Urolithiasis	Female	12-23 months	95+ years	Data Rich	-1	2	fruits unadjusted(g)	273
Urolithiasis	Female	12-23 months	95+ years	Data Rich	-1	2	Healthcare access and quality index	476
Urolithiasis	Female	12-23 months	95+ years	Data Rich	-1	2	vegetables unadjusted(g)	--
Urolithiasis	Female	12-23 months	95+ years	Data Rich	-1	3	Socio-demographic Index	90
Urolithiasis	Female	12-23 months	95+ years	Data Rich	-1	3	Education (years per capita)	--
Urolithiasis	Female	12-23 months	95+ years	Data Rich	-1	3	LDI (I$ per capita)	--
Urolithiasis	Female	12-23 months	95+ years	Global	1	1	90th percentile climatic temperature in the given country-year.	1000
Urolithiasis	Female	12-23 months	95+ years	Global	1	1	red meats unadjusted(g)	--
Urolithiasis	Female	12-23 months	95+ years	Global	-1	2	Healthcare access and quality index	--
Urolithiasis	Female	12-23 months	95+ years	Global	-1	2	fruits unadjusted(g)	--
Urolithiasis	Female	12-23 months	95+ years	Global	-1	2	vegetables unadjusted(g)	--
Urolithiasis	Female	12-23 months	95+ years	Global	-1	3	Education (years per capita)	--
Urolithiasis	Female	12-23 months	95+ years	Global	-1	3	LDI (I$ per capita)	--
Urolithiasis	Female	12-23 months	95+ years	Global	-1	3	Socio-demographic Index	--
Urolithiasis	Male	12-23 months	95+ years	Data Rich	1	1	90th percentile climatic temperature in the given country-year.	72
Urolithiasis	Male	12-23 months	95+ years	Data Rich	1	1	red meats unadjusted(g)	1000
Urolithiasis	Male	12-23 months	95+ years	Data Rich	-1	2	vegetables unadjusted(g)	282
Urolithiasis	Male	12-23 months	95+ years	Data Rich	-1	2	fruits unadjusted(g)	379
Urolithiasis	Male	12-23 months	95+ years	Data Rich	-1	2	Healthcare access and quality index	--
Urolithiasis	Male	12-23 months	95+ years	Data Rich	-1	3	Education (years per capita)	123
Urolithiasis	Male	12-23 months	95+ years	Data Rich	-1	3	Socio-demographic Index	374
Urolithiasis	Male	12-23 months	95+ years	Data Rich	-1	3	LDI (I$ per capita)	--
Urolithiasis	Male	12-23 months	95+ years	Global	1	1	90th percentile climatic temperature in the given country-year.	--
Urolithiasis	Male	12-23 months	95+ years	Global	1	1	red meats unadjusted(g)	--
Urolithiasis	Male	12-23 months	95+ years	Global	-1	2	Healthcare access and quality index	633
Urolithiasis	Male	12-23 months	95+ years	Global	-1	2	fruits unadjusted(g)	--
Urolithiasis	Male	12-23 months	95+ years	Global	-1	2	vegetables unadjusted(g)	--
Urolithiasis	Male	12-23 months	95+ years	Global	-1	3	Socio-demographic Index	208
Urolithiasis	Male	12-23 months	95+ years	Global	-1	3	Education (years per capita)	--
Urolithiasis	Male	12-23 months	95+ years	Global	-1	3	LDI (I$ per capita)	--
Other urinary diseases	Female	0-6 days	95+ years	Data Rich	1	1	Mean BMI	--
Other urinary diseases	Female	0-6 days	95+ years	Data Rich	-1	2	Education (years per capita)	454
Other urinary diseases	Female	0-6 days	95+ years	Data Rich	-1	2	LDI (I$ per capita)	454
Other urinary diseases	Female	0-6 days	95+ years	Data Rich	-1	2	Healthcare access and quality index	--
Other urinary diseases	Female	0-6 days	95+ years	Data Rich	-1	3	Socio-demographic Index	546
Other urinary diseases	Female	0-6 days	95+ years	Global	1	1	Mean BMI	--
Other urinary diseases	Female	0-6 days	95+ years	Global	-1	2	Healthcare access and quality index	326
Other urinary diseases	Female	0-6 days	95+ years	Global	-1	2	LDI (I$ per capita)	326
Other urinary diseases	Female	0-6 days	95+ years	Global	-1	2	Education (years per capita)	422
Other urinary diseases	Female	0-6 days	95+ years	Global	-1	3	Socio-demographic Index	489
Other urinary diseases	Male	0-6 days	95+ years	Data Rich	1	1	Mean BMI	--
Other urinary diseases	Male	0-6 days	95+ years	Data Rich	-1	2	Healthcare access and quality index	1000
Other urinary diseases	Male	0-6 days	95+ years	Data Rich	-1	2	Education (years per capita)	--
Other urinary diseases	Male	0-6 days	95+ years	Data Rich	-1	2	LDI (I$ per capita)	--
Other urinary diseases	Male	0-6 days	95+ years	Data Rich	-1	3	Socio-demographic Index	--
Other urinary diseases	Male	0-6 days	95+ years	Global	1	1	Mean BMI	--
Other urinary diseases	Male	0-6 days	95+ years	Global	-1	2	Education (years per capita)	546
Other urinary diseases	Male	0-6 days	95+ years	Global	-1	2	Healthcare access and quality index	546
Other urinary diseases	Male	0-6 days	95+ years	Global	-1	2	LDI (I$ per capita)	546
Other urinary diseases	Male	0-6 days	95+ years	Global	-1	3	Socio-demographic Index	454
Gynecological diseases	Female	10-14 years	95+ years	Data Rich	-1	1	Age- and sex-specific SEV for Smoking	--
Gynecological diseases	Female	10-14 years	95+ years	Data Rich	-1	2	Healthcare access and quality index	297
Gynecological diseases	Female	10-14 years	95+ years	Data Rich	-1	2	Skilled Birth Attendance (proportion)	297
Gynecological diseases	Female	10-14 years	95+ years	Data Rich	-1	2	Maternal care and immunization	496
Gynecological diseases	Female	10-14 years	95+ years	Data Rich	1	2	Live Births 35+ (proportion)	--
Gynecological diseases	Female	10-14 years	95+ years	Data Rich	1	2	Total Fertility Rate	--
Gynecological diseases	Female	10-14 years	95+ years	Data Rich	-1	3	Education (years per capita)	207
Gynecological diseases	Female	10-14 years	95+ years	Data Rich	-1	3	Socio-demographic Index	356
Gynecological diseases	Female	10-14 years	95+ years	Data Rich	-1	3	LDI (I$ per capita)	--
Gynecological diseases	Female	10-14 years	95+ years	Global	-1	1	Age- and sex-specific SEV for Smoking	1000
Gynecological diseases	Female	10-14 years	95+ years	Global	-1	2	Healthcare access and quality index	255
Gynecological diseases	Female	10-14 years	95+ years	Global	-1	2	Maternal care and immunization	317
Gynecological diseases	Female	10-14 years	95+ years	Global	-1	2	Skilled Birth Attendance (proportion)	--
Gynecological diseases	Female	10-14 years	95+ years	Global	1	2	Live Births 35+ (proportion)	--
Gynecological diseases	Female	10-14 years	95+ years	Global	1	2	Total Fertility Rate	--

Appendix Table S10: CODEm covariates used, level of covariate, and expected direction of covariate by cause, sex, and age								
**Cause**	**Sex**	**Age Start**	**Age End**	**Model Version Type**	**Direction**	**Level**	**Covariate Name**	**Number of Draws**
Gynecological diseases	Female	10-14 years	95+ years	Global	-1	3	Socio-demographic Index	208
Gynecological diseases	Female	10-14 years	95+ years	Global	-1	3	Education (years per capita)	381
Gynecological diseases	Female	10-14 years	95+ years	Global	-1	3	LDI (I$ per capita)	--
Uterine fibroids	Female	10-14 years	95+ years	Data Rich	-1	1	Age- and sex-specific SEV for Smoking	--
Uterine fibroids	Female	10-14 years	95+ years	Data Rich	-1	2	Skilled Birth Attendance (proportion)	159
Uterine fibroids	Female	10-14 years	95+ years	Data Rich	-1	2	Healthcare access and quality index	360
Uterine fibroids	Female	10-14 years	95+ years	Data Rich	-1	2	Maternal care and immunization	--
Uterine fibroids	Female	10-14 years	95+ years	Data Rich	1	2	Live Births 35+ (proportion)	--
Uterine fibroids	Female	10-14 years	95+ years	Data Rich	1	2	Total Fertility Rate	--
Uterine fibroids	Female	10-14 years	95+ years	Data Rich	-1	3	Education (years per capita)	273
Uterine fibroids	Female	10-14 years	95+ years	Data Rich	-1	3	LDI (I$ per capita)	--
Uterine fibroids	Female	10-14 years	95+ years	Data Rich	-1	3	Socio-demographic Index	--
Uterine fibroids	Female	10-14 years	95+ years	Global	-1	1	Age- and sex-specific SEV for Smoking	--
Uterine fibroids	Female	10-14 years	95+ years	Global	-1	2	Skilled Birth Attendance (proportion)	602
Uterine fibroids	Female	10-14 years	95+ years	Global	-1	2	Healthcare access and quality index	--
Uterine fibroids	Female	10-14 years	95+ years	Global	-1	2	Maternal care and immunization	--
Uterine fibroids	Female	10-14 years	95+ years	Global	1	2	Live Births 35+ (proportion)	--
Uterine fibroids	Female	10-14 years	95+ years	Global	1	2	Total Fertility Rate	--
Uterine fibroids	Female	10-14 years	95+ years	Global	-1	3	Socio-demographic Index	398
Uterine fibroids	Female	10-14 years	95+ years	Global	-1	3	Education (years per capita)	--
Uterine fibroids	Female	10-14 years	95+ years	Global	-1	3	LDI (I$ per capita)	--
Endometriosis	Female	10-14 years	50-54 years	Data Rich	-1	1	Age- and sex-specific SEV for Smoking	--
Endometriosis	Female	10-14 years	50-54 years	Data Rich	-1	2	Healthcare access and quality index	1000
Endometriosis	Female	10-14 years	50-54 years	Data Rich	-1	2	Maternal care and immunization	--
Endometriosis	Female	10-14 years	50-54 years	Data Rich	-1	2	Skilled Birth Attendance (proportion)	--
Endometriosis	Female	10-14 years	50-54 years	Data Rich	1	2	Live Births 35+ (proportion)	1000
Endometriosis	Female	10-14 years	50-54 years	Data Rich	1	2	Total Fertility Rate	--
Endometriosis	Female	10-14 years	50-54 years	Data Rich	-1	3	Education (years per capita)	--
Endometriosis	Female	10-14 years	50-54 years	Data Rich	-1	3	LDI (I$ per capita)	--
Endometriosis	Female	10-14 years	50-54 years	Data Rich	-1	3	Socio-demographic Index	--
Endometriosis	Female	10-14 years	50-54 years	Global	-1	1	Age- and sex-specific SEV for Smoking	1000
Endometriosis	Female	10-14 years	50-54 years	Global	-1	2	Healthcare access and quality index	578
Endometriosis	Female	10-14 years	50-54 years	Global	-1	2	Maternal care and immunization	--
Endometriosis	Female	10-14 years	50-54 years	Global	-1	2	Skilled Birth Attendance (proportion)	--
Endometriosis	Female	10-14 years	50-54 years	Global	1	2	Live Births 35+ (proportion)	--
Endometriosis	Female	10-14 years	50-54 years	Global	1	2	Total Fertility Rate	--
Endometriosis	Female	10-14 years	50-54 years	Global	-1	3	Education (years per capita)	--
Endometriosis	Female	10-14 years	50-54 years	Global	-1	3	LDI (I$ per capita)	--
Endometriosis	Female	10-14 years	50-54 years	Global	-1	3	Socio-demographic Index	--
Genital prolapse	Female	10-14 years	95+ years	Data Rich	-1	1	Age- and sex-specific SEV for Smoking	--
Genital prolapse	Female	10-14 years	95+ years	Data Rich	-1	2	Skilled Birth Attendance (proportion)	432
Genital prolapse	Female	10-14 years	95+ years	Data Rich	-1	2	Healthcare access and quality index	--
Genital prolapse	Female	10-14 years	95+ years	Data Rich	-1	2	Maternal care and immunization	--
Genital prolapse	Female	10-14 years	95+ years	Data Rich	1	2	Total Fertility Rate	432
Genital prolapse	Female	10-14 years	95+ years	Data Rich	1	2	Age-specific cohort cumulative fertility (CCF)	--
Genital prolapse	Female	10-14 years	95+ years	Data Rich	1	2	Live Births 35+ (proportion)	--
Genital prolapse	Female	10-14 years	95+ years	Data Rich	-1	3	Education (years per capita)	568
Genital prolapse	Female	10-14 years	95+ years	Data Rich	-1	3	Socio-demographic Index	568
Genital prolapse	Female	10-14 years	95+ years	Data Rich	-1	3	LDI (I$ per capita)	--
Genital prolapse	Female	10-14 years	95+ years	Global	-1	1	Age- and sex-specific SEV for Smoking	--
Genital prolapse	Female	10-14 years	95+ years	Global	-1	2	Maternal care and immunization	316
Genital prolapse	Female	10-14 years	95+ years	Global	-1	2	Healthcare access and quality index	428
Genital prolapse	Female	10-14 years	95+ years	Global	-1	2	Skilled Birth Attendance (proportion)	428
Genital prolapse	Female	10-14 years	95+ years	Global	1	2	Total Fertility Rate	744
Genital prolapse	Female	10-14 years	95+ years	Global	1	2	Age-specific cohort cumulative fertility (CCF)	--
Genital prolapse	Female	10-14 years	95+ years	Global	1	2	Live Births 35+ (proportion)	--
Genital prolapse	Female	10-14 years	95+ years	Global	-1	3	Socio-demographic Index	160
Genital prolapse	Female	10-14 years	95+ years	Global	-1	3	Education (years per capita)	625
Genital prolapse	Female	10-14 years	95+ years	Global	-1	3	LDI (I$ per capita)	--
Other gynecological diseases	Female	10-14 years	95+ years	Data Rich	-1	1	Age- and sex-specific SEV for Smoking	--
Other gynecological diseases	Female	10-14 years	95+ years	Data Rich	-1	2	Skilled Birth Attendance (proportion)	315
Other gynecological diseases	Female	10-14 years	95+ years	Data Rich	-1	2	Maternal care and immunization	500
Other gynecological diseases	Female	10-14 years	95+ years	Data Rich	-1	2	Healthcare access and quality index	655
Other gynecological diseases	Female	10-14 years	95+ years	Data Rich	1	2	Total Fertility Rate	482
Other gynecological diseases	Female	10-14 years	95+ years	Data Rich	1	2	Live Births 35+ (proportion)	--
Other gynecological diseases	Female	10-14 years	95+ years	Data Rich	-1	3	Socio-demographic Index	46
Other gynecological diseases	Female	10-14 years	95+ years	Data Rich	-1	3	Education (years per capita)	190
Other gynecological diseases	Female	10-14 years	95+ years	Data Rich	-1	3	LDI (I$ per capita)	--
Other gynecological diseases	Female	10-14 years	95+ years	Global	-1	1	Age- and sex-specific SEV for Smoking	--
Other gynecological diseases	Female	10-14 years	95+ years	Global	-1	2	Skilled Birth Attendance (proportion)	189
Other gynecological diseases	Female	10-14 years	95+ years	Global	-1	2	Healthcare access and quality index	385
Other gynecological diseases	Female	10-14 years	95+ years	Global	-1	2	Maternal care and immunization	423
Other gynecological diseases	Female	10-14 years	95+ years	Global	1	2	Total Fertility Rate	466
Other gynecological diseases	Female	10-14 years	95+ years	Global	1	2	Live Births 35+ (proportion)	--
Other gynecological diseases	Female	10-14 years	95+ years	Global	-1	3	Education (years per capita)	313
Other gynecological diseases	Female	10-14 years	95+ years	Global	-1	3	Socio-demographic Index	330
Other gynecological diseases	Female	10-14 years	95+ years	Global	-1	3	LDI (I$ per capita)	--
Hemoglobinopathies and hemolytic anaemias	Female	0-6 days	95+ years	Data Rich	1	1	Probability of Hemoglobin SC disease (SC) and G6PD deficiency at birth	44
Hemoglobinopathies and hemolytic anaemias	Female	0-6 days	95+ years	Data Rich	1	1	Probability of Homozygous sickle cell disease (SS) and G6PD deficiency at birth	126
Hemoglobinopathies and hemolytic anaemias	Female	0-6 days	95+ years	Data Rich	1	1	Probability of Hemoglobin SC disease at birth	214
Hemoglobinopathies and hemolytic anaemias	Female	0-6 days	95+ years	Data Rich	1	1	Hemoglobinopathies Prevalence x Excess Mortality	288
Hemoglobinopathies and hemolytic anaemias	Female	0-6 days	95+ years	Data Rich	1	1	Hemoglobinopathies Prevalence x Excess Mortality (excluding G6PD deficiency)	472
Hemoglobinopathies and hemolytic anaemias	Female	0-6 days	95+ years	Data Rich	1	1	Probability of Homozygous sickle cell disease at birth (SS)	526
Hemoglobinopathies and hemolytic anaemias	Female	0-6 days	95+ years	Data Rich	1	1	Malaria Lysenko PFPR 1 (Holoendemic)	--
Hemoglobinopathies and hemolytic anaemias	Female	0-6 days	95+ years	Data Rich	1	1	Probability of G6PD deficiency at birth	--
Hemoglobinopathies and hemolytic anaemias	Female	0-6 days	95+ years	Data Rich	-1	2	Maternal care and immunization	23
Hemoglobinopathies and hemolytic anaemias	Female	0-6 days	95+ years	Data Rich	-1	2	Healthcare access and quality index	846
Hemoglobinopathies and hemolytic anaemias	Female	0-6 days	95+ years	Data Rich	-1	3	Education (years per capita)	41
Hemoglobinopathies and hemolytic anaemias	Female	0-6 days	95+ years	Data Rich	-1	3	Socio-demographic Index	339
Hemoglobinopathies and hemolytic anaemias	Female	0-6 days	95+ years	Data Rich	-1	3	Latitude Over 45 (proportion)	505
Hemoglobinopathies and hemolytic anaemias	Female	0-6 days	95+ years	Data Rich	-1	3	LDI (I$ per capita)	--
Hemoglobinopathies and hemolytic anaemias	Female	0-6 days	95+ years	Data Rich	-1	3	Latitude 30 to 45 (proportion)	--
Hemoglobinopathies and hemolytic anaemias	Female	0-6 days	95+ years	Data Rich	1	3	Latitude Under 15 (proportion)	532
Hemoglobinopathies and hemolytic anaemias	Female	0-6 days	95+ years	Data Rich	1	3	Latitude 15 to 30 (proportion)	--
Hemoglobinopathies and hemolytic anaemias	Female	0-6 days	95+ years	Global	1	1	Probability of G6PD deficiency at birth	0
Hemoglobinopathies and hemolytic anaemias	Female	0-6 days	95+ years	Global	1	1	Hemoglobinopathies Prevalence x Excess Mortality	5
Hemoglobinopathies and hemolytic anaemias	Female	0-6 days	95+ years	Global	1	1	Hemoglobinopathies Prevalence x Excess Mortality (excluding G6PD deficiency)	6
Hemoglobinopathies and hemolytic anaemias	Female	0-6 days	95+ years	Global	1	1	Probability of Hemoglobin SC disease at birth	31

Appendix Table S10: CODEm covariates used, level of covariate, and expected direction of covariate by cause, sex, and age

Cause	Sex	Age Start	Age End	Model Version Type	Direction	Level	Covariate Name	Number of Draws
Hemoglobinopathies and hemolytic anaemias	Female	0-6 days	95+ years	Global	1	1	Probability of Homozygous sickle cell disease (SS) and G6PD deficiency at birth	121
Hemoglobinopathies and hemolytic anaemias	Female	0-6 days	95+ years	Global	1	1	Malaria Lysenko PFPR 1 (Holoendemic)	128
Hemoglobinopathies and hemolytic anaemias	Female	0-6 days	95+ years	Global	1	1	Probability of Hemoglobin SC disease (SC) and G6PD deficiency at birth	301
Hemoglobinopathies and hemolytic anaemias	Female	0-6 days	95+ years	Global	1	1	Probability of Homozygous sickle cell disease at birth (SS)	798
Hemoglobinopathies and hemolytic anaemias	Female	0-6 days	95+ years	Global	-1	2	Maternal care and immunization	6
Hemoglobinopathies and hemolytic anaemias	Female	0-6 days	95+ years	Global	-1	2	Healthcare access and quality index	985
Hemoglobinopathies and hemolytic anaemias	Female	0-6 days	95+ years	Global	-1	3	Latitude Over 45 (proportion)	3
Hemoglobinopathies and hemolytic anaemias	Female	0-6 days	95+ years	Global	-1	3	Education (years per capita)	6
Hemoglobinopathies and hemolytic anaemias	Female	0-6 days	95+ years	Global	-1	3	Latitude 30 to 45 (proportion)	124
Hemoglobinopathies and hemolytic anaemias	Female	0-6 days	95+ years	Global	-1	3	Socio-demographic Index	371
Hemoglobinopathies and hemolytic anaemias	Female	0-6 days	95+ years	Global	-1	3	LDI (I$ per capita)	--
Hemoglobinopathies and hemolytic anaemias	Female	0-6 days	95+ years	Global	1	3	Latitude Under 15 (proportion)	300
Hemoglobinopathies and hemolytic anaemias	Female	0-6 days	95+ years	Global	1	3	Latitude 15 to 30 (proportion)	--
Hemoglobinopathies and hemolytic anaemias	Male	0-6 days	95+ years	Data Rich	1	1	Probability of Homozygous sickle cell disease at birth (SS)	30
Hemoglobinopathies and hemolytic anaemias	Male	0-6 days	95+ years	Data Rich	1	1	Hemoglobinopathies Prevalence x Excess Mortality	44
Hemoglobinopathies and hemolytic anaemias	Male	0-6 days	95+ years	Data Rich	1	1	Hemoglobinopathies Prevalence x Excess Mortality (excluding G6PD deficiency)	346
Hemoglobinopathies and hemolytic anaemias	Male	0-6 days	95+ years	Data Rich	1	1	Probability of Hemoglobin SC disease at birth	586
Hemoglobinopathies and hemolytic anaemias	Male	0-6 days	95+ years	Data Rich	1	1	Malaria Lysenko PFPR 1 (Holoendemic)	--
Hemoglobinopathies and hemolytic anaemias	Male	0-6 days	95+ years	Data Rich	1	1	Probability of Homozygous sickle cell disease (SS) and G6PD deficiency at birth	--
Hemoglobinopathies and hemolytic anaemias	Male	0-6 days	95+ years	Data Rich	1	1	Probability of G6PD deficiency at birth	--
Hemoglobinopathies and hemolytic anaemias	Male	0-6 days	95+ years	Data Rich	1	1	Probability of Hemoglobin SC disease (SC) and G6PD deficiency at birth	--
Hemoglobinopathies and hemolytic anaemias	Male	0-6 days	95+ years	Data Rich	-1	2	Maternal care and immunization	61
Hemoglobinopathies and hemolytic anaemias	Male	0-6 days	95+ years	Data Rich	-1	2	Healthcare access and quality index	735
Hemoglobinopathies and hemolytic anaemias	Male	0-6 days	95+ years	Data Rich	-1	3	Education (years per capita)	189
Hemoglobinopathies and hemolytic anaemias	Male	0-6 days	95+ years	Data Rich	-1	3	Socio-demographic Index	377
Hemoglobinopathies and hemolytic anaemias	Male	0-6 days	95+ years	Data Rich	-1	3	Latitude Over 45 (proportion)	451
Hemoglobinopathies and hemolytic anaemias	Male	0-6 days	95+ years	Data Rich	-1	3	LDI (I$ per capita)	--
Hemoglobinopathies and hemolytic anaemias	Male	0-6 days	95+ years	Data Rich	-1	3	Latitude 30 to 45 (proportion)	--
Hemoglobinopathies and hemolytic anaemias	Male	0-6 days	95+ years	Data Rich	1	3	Latitude Under 15 (proportion)	343
Hemoglobinopathies and hemolytic anaemias	Male	0-6 days	95+ years	Data Rich	1	3	Latitude 15 to 30 (proportion)	--
Hemoglobinopathies and hemolytic anaemias	Male	0-6 days	95+ years	Global	1	1	Malaria Lysenko PFPR 1 (Holoendemic)	364
Hemoglobinopathies and hemolytic anaemias	Male	0-6 days	95+ years	Global	1	1	Hemoglobinopathies Prevalence x Excess Mortality	366
Hemoglobinopathies and hemolytic anaemias	Male	0-6 days	95+ years	Global	1	1	Probability of Hemoglobin SC disease at birth	490
Hemoglobinopathies and hemolytic anaemias	Male	0-6 days	95+ years	Global	1	1	Hemoglobinopathies Prevalence x Excess Mortality (excluding G6PD deficiency)	508
Hemoglobinopathies and hemolytic anaemias	Male	0-6 days	95+ years	Global	1	1	Probability of Homozygous sickle cell disease at birth (SS)	778
Hemoglobinopathies and hemolytic anaemias	Male	0-6 days	95+ years	Global	1	1	Probability of Homozygous sickle cell disease (SS) and G6PD deficiency at birth	--
Hemoglobinopathies and hemolytic anaemias	Male	0-6 days	95+ years	Global	1	1	Probability of G6PD deficiency at birth	--
Hemoglobinopathies and hemolytic anaemias	Male	0-6 days	95+ years	Global	1	1	Probability of Hemoglobin SC disease (SC) and G6PD deficiency at birth	--
Hemoglobinopathies and hemolytic anaemias	Male	0-6 days	95+ years	Global	-1	2	Maternal care and immunization	12
Hemoglobinopathies and hemolytic anaemias	Male	0-6 days	95+ years	Global	-1	2	Healthcare access and quality index	959
Hemoglobinopathies and hemolytic anaemias	Male	0-6 days	95+ years	Global	-1	3	Latitude 30 to 45 (proportion)	229
Hemoglobinopathies and hemolytic anaemias	Male	0-6 days	95+ years	Global	-1	3	Education (years per capita)	252
Hemoglobinopathies and hemolytic anaemias	Male	0-6 days	95+ years	Global	-1	3	Latitude Over 45 (proportion)	404
Hemoglobinopathies and hemolytic anaemias	Male	0-6 days	95+ years	Global	-1	3	Socio-demographic Index	406
Hemoglobinopathies and hemolytic anaemias	Male	0-6 days	95+ years	Global	-1	3	LDI (I$ per capita)	--
Hemoglobinopathies and hemolytic anaemias	Male	0-6 days	95+ years	Global	1	3	Latitude Under 15 (proportion)	222
Hemoglobinopathies and hemolytic anaemias	Male	0-6 days	95+ years	Global	1	3	Latitude 15 to 30 (proportion)	--
Endocrine, metabolic, blood, and immune disorders	Female	0-6 days	95+ years	Data Rich	1	1	Mean BMI	1000
Endocrine, metabolic, blood, and immune disorders	Female	0-6 days	95+ years	Data Rich	1	1	Liters of alcohol consumed per capita	--
Endocrine, metabolic, blood, and immune disorders	Female	0-6 days	95+ years	Data Rich	1	1	Low-Density Lipoprotein (mmol/L)	--
Endocrine, metabolic, blood, and immune disorders	Female	0-6 days	95+ years	Data Rich	-1	2	Healthcare access and quality index	--
Endocrine, metabolic, blood, and immune disorders	Female	0-6 days	95+ years	Data Rich	-1	3	Socio-demographic Index	248
Endocrine, metabolic, blood, and immune disorders	Female	0-6 days	95+ years	Data Rich	-1	3	Education (years per capita)	427
Endocrine, metabolic, blood, and immune disorders	Female	0-6 days	95+ years	Data Rich	-1	3	LDI (I$ per capita)	--
Endocrine, metabolic, blood, and immune disorders	Female	0-6 days	95+ years	Global	1	1	Mean BMI	--
Endocrine, metabolic, blood, and immune disorders	Female	0-6 days	95+ years	Global	-1	2	Healthcare access and quality index	593
Endocrine, metabolic, blood, and immune disorders	Female	0-6 days	95+ years	Global	1	2	Liters of alcohol consumed per capita	--
Endocrine, metabolic, blood, and immune disorders	Female	0-6 days	95+ years	Global	1	2	Low-Density Lipoprotein (mmol/L)	--
Endocrine, metabolic, blood, and immune disorders	Female	0-6 days	95+ years	Global	-1	3	Education (years per capita)	222
Endocrine, metabolic, blood, and immune disorders	Female	0-6 days	95+ years	Global	-1	3	Socio-demographic Index	222
Endocrine, metabolic, blood, and immune disorders	Female	0-6 days	95+ years	Global	-1	3	LDI (I$ per capita)	--
Endocrine, metabolic, blood, and immune disorders	Male	0-6 days	95+ years	Data Rich	1	1	Mean BMI	--
Endocrine, metabolic, blood, and immune disorders	Male	0-6 days	95+ years	Data Rich	-1	2	Healthcare access and quality index	--
Endocrine, metabolic, blood, and immune disorders	Male	0-6 days	95+ years	Data Rich	1	2	Liters of alcohol consumed per capita	493
Endocrine, metabolic, blood, and immune disorders	Male	0-6 days	95+ years	Data Rich	1	2	Low-Density Lipoprotein (mmol/L)	--
Endocrine, metabolic, blood, and immune disorders	Male	0-6 days	95+ years	Data Rich	-1	3	Socio-demographic Index	108
Endocrine, metabolic, blood, and immune disorders	Male	0-6 days	95+ years	Data Rich	-1	3	Education (years per capita)	142
Endocrine, metabolic, blood, and immune disorders	Male	0-6 days	95+ years	Data Rich	-1	3	LDI (I$ per capita)	--
Endocrine, metabolic, blood, and immune disorders	Male	0-6 days	95+ years	Global	1	1	Mean BMI	--
Endocrine, metabolic, blood, and immune disorders	Male	0-6 days	95+ years	Global	-1	2	Healthcare access and quality index	--
Endocrine, metabolic, blood, and immune disorders	Male	0-6 days	95+ years	Global	1	2	Liters of alcohol consumed per capita	--
Endocrine, metabolic, blood, and immune disorders	Male	0-6 days	95+ years	Global	1	2	Low-Density Lipoprotein (mmol/L)	--
Endocrine, metabolic, blood, and immune disorders	Male	0-6 days	95+ years	Global	-1	3	Socio-demographic Index	353
Endocrine, metabolic, blood, and immune disorders	Male	0-6 days	95+ years	Global	-1	3	Education (years per capita)	373
Endocrine, metabolic, blood, and immune disorders	Male	0-6 days	95+ years	Global	-1	3	LDI (I$ per capita)	--
Thyroid diseases	Female	0-6 days	95+ years	Data Rich	-1	1	Healthcare access and quality index	--
Thyroid diseases	Female	0-6 days	95+ years	Data Rich	1	1	Absolute value of average latitude	--

Appendix Table S10: CODEm covariates used, level of covariate, and expected direction of covariate by cause, sex, and age

Cause	Sex	Age Start	Age End	Model Version Type	Direction	Level	Covariate Name	Number of Draws
Thyroid diseases	Female	0-6 days	95+ years	Data Rich	1	2	Diabetes Age-Standardized Prevalence (proportion)	1000
Thyroid diseases	Female	0-6 days	95+ years	Data Rich	-1	3	Socio-demographic Index	633
Thyroid diseases	Female	0-6 days	95+ years	Data Rich	-1	3	Education (years per capita)	841
Thyroid diseases	Female	0-6 days	95+ years	Data Rich	-1	3	LDI (I$ per capita)	--
Thyroid diseases	Female	0-6 days	95+ years	Data Rich	1	3	Cumulative Cigarettes (5 Years)	360
Thyroid diseases	Female	0-6 days	95+ years	Data Rich	1	3	Smoking Prevalence	481
Thyroid diseases	Female	0-6 days	95+ years	Data Rich	1	3	Mean BMI	841
Thyroid diseases	Female	0-6 days	95+ years	Data Rich	1	3	Cumulative Cigarettes (10 Years)	--
Thyroid diseases	Female	0-6 days	95+ years	Global	-1	1	Healthcare access and quality index	1000
Thyroid diseases	Female	0-6 days	95+ years	Global	1	1	Absolute value of average latitude	--
Thyroid diseases	Female	0-6 days	95+ years	Global	1	2	Diabetes Age-Standardized Prevalence (proportion)	593
Thyroid diseases	Female	0-6 days	95+ years	Global	-1	3	Education (years per capita)	326
Thyroid diseases	Female	0-6 days	95+ years	Global	-1	3	Socio-demographic Index	326
Thyroid diseases	Female	0-6 days	95+ years	Global	-1	3	LDI (I$ per capita)	--
Thyroid diseases	Female	0-6 days	95+ years	Global	1	3	Mean BMI	548
Thyroid diseases	Female	0-6 days	95+ years	Global	1	3	Cumulative Cigarettes (10 Years)	--
Thyroid diseases	Female	0-6 days	95+ years	Global	1	3	Cumulative Cigarettes (5 Years)	--
Thyroid diseases	Female	0-6 days	95+ years	Global	1	3	Smoking Prevalence	--
Thyroid diseases	Male	0-6 days	95+ years	Data Rich	1	1	Diabetes Age-Standardized Prevalence (proportion)	1000
Thyroid diseases	Male	0-6 days	95+ years	Data Rich	1	1	Absolute value of average latitude	--
Thyroid diseases	Male	0-6 days	95+ years	Data Rich	-1	2	Healthcare access and quality index	--
Thyroid diseases	Male	0-6 days	95+ years	Data Rich	-1	3	Socio-demographic Index	360
Thyroid diseases	Male	0-6 days	95+ years	Data Rich	-1	3	Education (years per capita)	432
Thyroid diseases	Male	0-6 days	95+ years	Data Rich	-1	3	LDI (I$ per capita)	--
Thyroid diseases	Male	0-6 days	95+ years	Data Rich	1	3	Cumulative Cigarettes (10 Years)	159
Thyroid diseases	Male	0-6 days	95+ years	Data Rich	1	3	Cumulative Cigarettes (5 Years)	273
Thyroid diseases	Male	0-6 days	95+ years	Data Rich	1	3	Smoking Prevalence	273
Thyroid diseases	Male	0-6 days	95+ years	Data Rich	1	3	Mean BMI	633
Thyroid diseases	Male	0-6 days	95+ years	Global	-1	1	Healthcare access and quality index	1000
Thyroid diseases	Male	0-6 days	95+ years	Global	1	1	Absolute value of average latitude	--
Thyroid diseases	Male	0-6 days	95+ years	Global	1	2	Diabetes Age-Standardized Prevalence (proportion)	--
Thyroid diseases	Male	0-6 days	95+ years	Global	-1	3	Education (years per capita)	--
Thyroid diseases	Male	0-6 days	95+ years	Global	-1	3	LDI (I$ per capita)	--
Thyroid diseases	Male	0-6 days	95+ years	Global	-1	3	Socio-demographic Index	--
Thyroid diseases	Male	0-6 days	95+ years	Global	1	3	Mean BMI	546
Thyroid diseases	Male	0-6 days	95+ years	Global	1	3	Cumulative Cigarettes (10 Years)	--
Thyroid diseases	Male	0-6 days	95+ years	Global	1	3	Cumulative Cigarettes (5 Years)	--
Thyroid diseases	Male	0-6 days	95+ years	Global	1	3	Smoking Prevalence	--
Other endocrine, metabolic, blood, and immune disorders	Female	0-6 days	95+ years	Data Rich	1	1	Mean BMI	--
Other endocrine, metabolic, blood, and immune disorders	Female	0-6 days	95+ years	Data Rich	-1	2	Healthcare access and quality index	427
Other endocrine, metabolic, blood, and immune disorders	Female	0-6 days	95+ years	Data Rich	1	2	Liters of alcohol consumed per capita	427
Other endocrine, metabolic, blood, and immune disorders	Female	0-6 days	95+ years	Data Rich	1	2	Low-Density Lipoprotein (mmol/L)	--
Other endocrine, metabolic, blood, and immune disorders	Female	0-6 days	95+ years	Data Rich	-1	3	Education (years per capita)	--
Other endocrine, metabolic, blood, and immune disorders	Female	0-6 days	95+ years	Data Rich	-1	3	LDI (I$ per capita)	--
Other endocrine, metabolic, blood, and immune disorders	Female	0-6 days	95+ years	Data Rich	-1	3	Socio-demographic Index	--
Other endocrine, metabolic, blood, and immune disorders	Female	0-6 days	95+ years	Global	1	1	Mean BMI	--
Other endocrine, metabolic, blood, and immune disorders	Female	0-6 days	95+ years	Global	-1	2	Healthcare access and quality index	--
Other endocrine, metabolic, blood, and immune disorders	Female	0-6 days	95+ years	Global	1	2	Liters of alcohol consumed per capita	--
Other endocrine, metabolic, blood, and immune disorders	Female	0-6 days	95+ years	Global	1	2	Low-Density Lipoprotein (mmol/L)	--
Other endocrine, metabolic, blood, and immune disorders	Female	0-6 days	95+ years	Global	-1	3	Socio-demographic Index	546
Other endocrine, metabolic, blood, and immune disorders	Female	0-6 days	95+ years	Global	-1	3	Education (years per capita)	--
Other endocrine, metabolic, blood, and immune disorders	Female	0-6 days	95+ years	Global	-1	3	LDI (I$ per capita)	--
Other endocrine, metabolic, blood, and immune disorders	Male	0-6 days	95+ years	Data Rich	1	1	Mean BMI	--
Other endocrine, metabolic, blood, and immune disorders	Male	0-6 days	95+ years	Data Rich	-1	2	Healthcare access and quality index	427
Other endocrine, metabolic, blood, and immune disorders	Male	0-6 days	95+ years	Data Rich	1	2	Liters of alcohol consumed per capita	427
Other endocrine, metabolic, blood, and immune disorders	Male	0-6 days	95+ years	Data Rich	1	2	Low-Density Lipoprotein (mmol/L)	--
Other endocrine, metabolic, blood, and immune disorders	Male	0-6 days	95+ years	Data Rich	-1	3	Socio-demographic Index	325
Other endocrine, metabolic, blood, and immune disorders	Male	0-6 days	95+ years	Data Rich	-1	3	Education (years per capita)	--
Other endocrine, metabolic, blood, and immune disorders	Male	0-6 days	95+ years	Data Rich	-1	3	LDI (I$ per capita)	--
Other endocrine, metabolic, blood, and immune disorders	Male	0-6 days	95+ years	Global	1	1	Mean BMI	--
Other endocrine, metabolic, blood, and immune disorders	Male	0-6 days	95+ years	Global	-1	2	Healthcare access and quality index	398
Other endocrine, metabolic, blood, and immune disorders	Male	0-6 days	95+ years	Global	1	2	Liters of alcohol consumed per capita	--
Other endocrine, metabolic, blood, and immune disorders	Male	0-6 days	95+ years	Global	1	2	Low-Density Lipoprotein (mmol/L)	--
Other endocrine, metabolic, blood, and immune disorders	Male	0-6 days	95+ years	Global	-1	3	Education (years per capita)	294
Other endocrine, metabolic, blood, and immune disorders	Male	0-6 days	95+ years	Global	-1	3	LDI (I$ per capita)	--
Other endocrine, metabolic, blood, and immune disorders	Male	0-6 days	95+ years	Global	-1	3	Socio-demographic Index	--
Sudden infant death syndrome	Female	7-27 days	6-11 months	Data Rich	-1	1	In-Facility Delivery (proportion)	1000
Sudden infant death syndrome	Female	7-27 days	6-11 months	Data Rich	-1	2	Maternal care and immunization	192
Sudden infant death syndrome	Female	7-27 days	6-11 months	Data Rich	-1	2	Skilled Birth Attendance (proportion)	192
Sudden infant death syndrome	Female	7-27 days	6-11 months	Data Rich	-1	2	Healthcare access and quality index	--
Sudden infant death syndrome	Female	7-27 days	6-11 months	Data Rich	-1	3	Education (years per capita)	--
Sudden infant death syndrome	Female	7-27 days	6-11 months	Data Rich	1	3	LDI (I$ per capita)	--
Sudden infant death syndrome	Female	7-27 days	6-11 months	Data Rich	1	3	Socio-demographic Index	--
Sudden infant death syndrome	Female	7-27 days	6-11 months	Data Rich	1	3	Total Fertility Rate	--
Sudden infant death syndrome	Female	7-27 days	6-11 months	Global	-1	1	In-Facility Delivery (proportion)	1000
Sudden infant death syndrome	Female	7-27 days	6-11 months	Global	-1	2	Maternal care and immunization	452
Sudden infant death syndrome	Female	7-27 days	6-11 months	Global	-1	2	Skilled Birth Attendance (proportion)	452
Sudden infant death syndrome	Female	7-27 days	6-11 months	Global	-1	2	Healthcare access and quality index	--
Sudden infant death syndrome	Female	7-27 days	6-11 months	Global	-1	3	Education (years per capita)	--
Sudden infant death syndrome	Female	7-27 days	6-11 months	Global	1	3	LDI (I$ per capita)	--
Sudden infant death syndrome	Female	7-27 days	6-11 months	Global	1	3	Socio-demographic Index	--
Sudden infant death syndrome	Female	7-27 days	6-11 months	Global	1	3	Total Fertility Rate	--
Sudden infant death syndrome	Male	7-27 days	6-11 months	Data Rich	-1	1	In-Facility Delivery (proportion)	1000
Sudden infant death syndrome	Male	7-27 days	6-11 months	Data Rich	-1	2	Maternal care and immunization	166
Sudden infant death syndrome	Male	7-27 days	6-11 months	Data Rich	-1	2	Skilled Birth Attendance (proportion)	166
Sudden infant death syndrome	Male	7-27 days	6-11 months	Data Rich	-1	2	Healthcare access and quality index	--
Sudden infant death syndrome	Male	7-27 days	6-11 months	Data Rich	-1	3	Education (years per capita)	--
Sudden infant death syndrome	Male	7-27 days	6-11 months	Data Rich	1	3	LDI (I$ per capita)	--
Sudden infant death syndrome	Male	7-27 days	6-11 months	Data Rich	1	3	Socio-demographic Index	--
Sudden infant death syndrome	Male	7-27 days	6-11 months	Data Rich	1	3	Total Fertility Rate	--
Sudden infant death syndrome	Male	7-27 days	6-11 months	Global	-1	1	In-Facility Delivery (proportion)	1000
Sudden infant death syndrome	Male	7-27 days	6-11 months	Global	-1	2	Maternal care and immunization	454
Sudden infant death syndrome	Male	7-27 days	6-11 months	Global	-1	2	Skilled Birth Attendance (proportion)	454
Sudden infant death syndrome	Male	7-27 days	6-11 months	Global	-1	2	Healthcare access and quality index	--
Sudden infant death syndrome	Male	7-27 days	6-11 months	Global	-1	3	Education (years per capita)	--
Sudden infant death syndrome	Male	7-27 days	6-11 months	Global	1	3	LDI (I$ per capita)	--
Sudden infant death syndrome	Male	7-27 days	6-11 months	Global	1	3	Socio-demographic Index	--
Sudden infant death syndrome	Male	7-27 days	6-11 months	Global	1	3	Total Fertility Rate	--
Transport injuries	Female	0-6 days	95+ years	Data Rich	1	1	Vehicles - 2 wheels fraction (proportion)	--
Transport injuries	Female	0-6 days	95+ years	Data Rich	1	1	Vehicles - 2+4 wheels (per capita)	--
Transport injuries	Female	0-6 days	95+ years	Data Rich	-1	2	Healthcare access and quality index	834
Transport injuries	Female	0-6 days	95+ years	Data Rich	-1	2	Socio-demographic Index	834
Transport injuries	Female	0-6 days	95+ years	Data Rich	1	2	Population 15 to 30 (proportion)	834

Appendix Table S10: CODEm covariates used, level of covariate, and expected direction of covariate by cause, sex, and age

Cause	Sex	Age Start	Age End	Model Version Type	Direction	Level	Covariate Name	Number of Draws
Transport injuries	Female	0-6 days	95+ years	Data Rich	1	2	Population Density (300-500 ppl/sqkm, proportion)	834
Transport injuries	Female	0-6 days	95+ years	Data Rich	1	2	Liters of alcohol consumed per capita	--
Transport injuries	Female	0-6 days	95+ years	Data Rich	1	2	Population Density (500-1000 ppl/sqkm, proportion)	--
Transport injuries	Female	0-6 days	95+ years	Data Rich	1	2	Population-weighted mean temperature	--
Transport injuries	Female	0-6 days	95+ years	Data Rich	1	3	BAC law professional drivers (quartile)	166
Transport injuries	Female	0-6 days	95+ years	Data Rich	1	3	Rainfall Quintile 5 (proportion)	166
Transport injuries	Female	0-6 days	95+ years	Data Rich	1	3	BAC law general population (quartile)	--
Transport injuries	Female	0-6 days	95+ years	Data Rich	1	3	BAC law youth drivers (quartile)	--
Transport injuries	Female	0-6 days	95+ years	Data Rich	1	3	Speed limit law rural (quartile)	--
Transport injuries	Female	0-6 days	95+ years	Data Rich	1	3	Speed limit law urban (quartile)	--
Transport injuries	Female	0-6 days	95+ years	Global	1	1	Vehicles - 2 wheels fraction (proportion)	1000
Transport injuries	Female	0-6 days	95+ years	Global	1	1	Vehicles - 2+4 wheels (per capita)	--
Transport injuries	Female	0-6 days	95+ years	Global	-1	2	Healthcare access and quality index	385
Transport injuries	Female	0-6 days	95+ years	Global	-1	2	Socio-demographic Index	647
Transport injuries	Female	0-6 days	95+ years	Global	1	2	Population Density (300-500 ppl/sqkm, proportion)	181
Transport injuries	Female	0-6 days	95+ years	Global	1	2	Population Density (500-1000 ppl/sqkm, proportion)	443
Transport injuries	Female	0-6 days	95+ years	Global	1	2	Liters of alcohol consumed per capita	--
Transport injuries	Female	0-6 days	95+ years	Global	1	2	Population 15 to 30 (proportion)	--
Transport injuries	Female	0-6 days	95+ years	Global	1	2	Population-weighted mean temperature	--
Transport injuries	Female	0-6 days	95+ years	Global	1	3	BAC law professional drivers (quartile)	47
Transport injuries	Female	0-6 days	95+ years	Global	1	3	BAC law youth drivers (quartile)	432
Transport injuries	Female	0-6 days	95+ years	Global	1	3	BAC law general population (quartile)	--
Transport injuries	Female	0-6 days	95+ years	Global	1	3	Rainfall Quintile 5 (proportion)	--
Transport injuries	Female	0-6 days	95+ years	Global	1	3	Speed limit law rural (quartile)	--
Transport injuries	Female	0-6 days	95+ years	Global	1	3	Speed limit law urban (quartile)	--
Transport injuries	Male	0-6 days	95+ years	Data Rich	1	1	Vehicles - 2 wheels fraction (proportion)	--
Transport injuries	Male	0-6 days	95+ years	Data Rich	1	1	Vehicles - 2+4 wheels (per capita)	--
Transport injuries	Male	0-6 days	95+ years	Data Rich	-1	2	Healthcare access and quality index	807
Transport injuries	Male	0-6 days	95+ years	Data Rich	-1	2	Socio-demographic Index	--
Transport injuries	Male	0-6 days	95+ years	Data Rich	1	2	Population 15 to 30 (proportion)	807
Transport injuries	Male	0-6 days	95+ years	Data Rich	1	2	Population Density (500-1000 ppl/sqkm, proportion)	807
Transport injuries	Male	0-6 days	95+ years	Data Rich	1	2	Liters of alcohol consumed per capita	--
Transport injuries	Male	0-6 days	95+ years	Data Rich	1	2	Population Density (300-500 ppl/sqkm, proportion)	--
Transport injuries	Male	0-6 days	95+ years	Data Rich	1	2	Population-weighted mean temperature	--
Transport injuries	Male	0-6 days	95+ years	Data Rich	1	3	BAC law youth drivers (quartile)	32
Transport injuries	Male	0-6 days	95+ years	Data Rich	1	3	BAC law professional drivers (quartile)	161
Transport injuries	Male	0-6 days	95+ years	Data Rich	1	3	Rainfall Quintile 5 (proportion)	193
Transport injuries	Male	0-6 days	95+ years	Data Rich	1	3	BAC law general population (quartile)	--
Transport injuries	Male	0-6 days	95+ years	Data Rich	1	3	Speed limit law rural (quartile)	--
Transport injuries	Male	0-6 days	95+ years	Data Rich	1	3	Speed limit law urban (quartile)	--
Transport injuries	Male	0-6 days	95+ years	Global	1	1	Vehicles - 2 wheels fraction (proportion)	1000
Transport injuries	Male	0-6 days	95+ years	Global	1	1	Vehicles - 2+4 wheels (per capita)	--
Transport injuries	Male	0-6 days	95+ years	Global	-1	2	Healthcare access and quality index	588
Transport injuries	Male	0-6 days	95+ years	Global	-1	2	Socio-demographic Index	--
Transport injuries	Male	0-6 days	95+ years	Global	1	2	Population Density (500-1000 ppl/sqkm, proportion)	244
Transport injuries	Male	0-6 days	95+ years	Global	1	2	Population 15 to 30 (proportion)	832
Transport injuries	Male	0-6 days	95+ years	Global	1	2	Population Density (300-500 ppl/sqkm, proportion)	832
Transport injuries	Male	0-6 days	95+ years	Global	1	2	Liters of alcohol consumed per capita	--
Transport injuries	Male	0-6 days	95+ years	Global	1	2	Population-weighted mean temperature	--
Transport injuries	Male	0-6 days	95+ years	Global	1	3	BAC law youth drivers (quartile)	268
Transport injuries	Male	0-6 days	95+ years	Global	1	3	BAC law professional drivers (quartile)	271
Transport injuries	Male	0-6 days	95+ years	Global	1	3	BAC law general population (quartile)	--
Transport injuries	Male	0-6 days	95+ years	Global	1	3	Rainfall Quintile 5 (proportion)	--
Transport injuries	Male	0-6 days	95+ years	Global	1	3	Speed limit law rural (quartile)	--
Transport injuries	Male	0-6 days	95+ years	Global	1	3	Speed limit law urban (quartile)	--
Road injuries	Female	0-6 days	95+ years	Data Rich	1	1	Log-transformed SEV scalar: Road Inj	--
Road injuries	Female	0-6 days	95+ years	Data Rich	1	1	Vehicles - 2 wheels (per capita)	--
Road injuries	Female	0-6 days	95+ years	Data Rich	1	1	Vehicles - 2 wheels fraction (proportion)	--
Road injuries	Female	0-6 days	95+ years	Data Rich	1	1	Vehicles - 2+4 wheels (per capita)	--
Road injuries	Female	0-6 days	95+ years	Data Rich	1	1	Vehicles - 4 wheels (per capita)	--
Road injuries	Female	0-6 days	95+ years	Data Rich	-1	2	Socio-demographic Index	990
Road injuries	Female	0-6 days	95+ years	Data Rich	-1	2	Healthcare access and quality index	--
Road injuries	Female	0-6 days	95+ years	Data Rich	1	2	Population Density (300-500 ppl/sqkm, proportion)	108
Road injuries	Female	0-6 days	95+ years	Data Rich	1	2	Population Density (500-1000 ppl/sqkm, proportion)	902
Road injuries	Female	0-6 days	95+ years	Data Rich	1	2	Population 15 to 30 (proportion)	1000
Road injuries	Female	0-6 days	95+ years	Data Rich	1	2	Liters of alcohol consumed per capita	--
Road injuries	Female	0-6 days	95+ years	Data Rich	1	2	Population-weighted mean temperature	--
Road injuries	Female	0-6 days	95+ years	Data Rich	1	3	BAC law professional drivers (quartile)	8
Road injuries	Female	0-6 days	95+ years	Data Rich	1	3	BAC law youth drivers (quartile)	222
Road injuries	Female	0-6 days	95+ years	Data Rich	1	3	Rainfall Quintile 5 (proportion)	304
Road injuries	Female	0-6 days	95+ years	Data Rich	1	3	BAC law general population (quartile)	--
Road injuries	Female	0-6 days	95+ years	Data Rich	1	3	Speed limit law rural (quartile)	--
Road injuries	Female	0-6 days	95+ years	Data Rich	1	3	Speed limit law urban (quartile)	--
Road injuries	Female	0-6 days	95+ years	Global	1	1	Vehicles - 2 wheels fraction (proportion)	1000
Road injuries	Female	0-6 days	95+ years	Global	1	1	Log-transformed SEV scalar: Road Inj	--
Road injuries	Female	0-6 days	95+ years	Global	1	1	Vehicles - 2 wheels (per capita)	--
Road injuries	Female	0-6 days	95+ years	Global	1	1	Vehicles - 2+4 wheels (per capita)	--
Road injuries	Female	0-6 days	95+ years	Global	1	1	Vehicles - 4 wheels (per capita)	--
Road injuries	Female	0-6 days	95+ years	Global	-1	2	Healthcare access and quality index	868
Road injuries	Female	0-6 days	95+ years	Global	-1	2	Socio-demographic Index	868
Road injuries	Female	0-6 days	95+ years	Global	1	2	Population Density (300-500 ppl/sqkm, proportion)	313
Road injuries	Female	0-6 days	95+ years	Global	1	2	Population Density (500-1000 ppl/sqkm, proportion)	555
Road injuries	Female	0-6 days	95+ years	Global	1	2	Population 15 to 30 (proportion)	868
Road injuries	Female	0-6 days	95+ years	Global	1	2	Liters of alcohol consumed per capita	--
Road injuries	Female	0-6 days	95+ years	Global	1	2	Population-weighted mean temperature	--
Road injuries	Female	0-6 days	95+ years	Global	1	3	Rainfall Quintile 5 (proportion)	150
Road injuries	Female	0-6 days	95+ years	Global	1	3	BAC law professional drivers (quartile)	236
Road injuries	Female	0-6 days	95+ years	Global	1	3	BAC law youth drivers (quartile)	345
Road injuries	Female	0-6 days	95+ years	Global	1	3	BAC law general population (quartile)	--
Road injuries	Female	0-6 days	95+ years	Global	1	3	Speed limit law rural (quartile)	--
Road injuries	Female	0-6 days	95+ years	Global	1	3	Speed limit law urban (quartile)	--
Road injuries	Male	0-6 days	95+ years	Data Rich	1	1	Log-transformed SEV scalar: Road Inj	--
Road injuries	Male	0-6 days	95+ years	Data Rich	1	1	Vehicles - 2 wheels (per capita)	--
Road injuries	Male	0-6 days	95+ years	Data Rich	1	1	Vehicles - 2 wheels fraction (proportion)	--
Road injuries	Male	0-6 days	95+ years	Data Rich	1	1	Vehicles - 2+4 wheels (per capita)	--
Road injuries	Male	0-6 days	95+ years	Data Rich	1	1	Vehicles - 4 wheels (per capita)	--
Road injuries	Male	0-6 days	95+ years	Data Rich	-1	2	Healthcare access and quality index	892
Road injuries	Male	0-6 days	95+ years	Data Rich	-1	2	Socio-demographic Index	892

Appendix Table S10: CODEm covariates used, level of covariate, and expected direction of covariate by cause, sex, and age								
Cause	Sex	Age Start	Age End	Model Version Type	Direction	Level	Covariate Name	Number of Draws
Road injuries	Male	0-6 days	95+ years	Data Rich	1	2	Population-weighted mean temperature	82
Road injuries	Male	0-6 days	95+ years	Data Rich	1	2	Population 15 to 30 (proportion)	892
Road injuries	Male	0-6 days	95+ years	Data Rich	1	2	Population Density (500-1000 ppl/sqkm, proportion)	892
Road injuries	Male	0-6 days	95+ years	Data Rich	1	2	Liters of alcohol consumed per capita	--
Road injuries	Male	0-6 days	95+ years	Data Rich	1	2	Population Density (300-500 ppl/sqkm, proportion)	--
Road injuries	Male	0-6 days	95+ years	Data Rich	1	3	Rainfall Quintile 5 (proportion)	10
Road injuries	Male	0-6 days	95+ years	Data Rich	1	3	BAC law professional drivers (quartile)	24
Road injuries	Male	0-6 days	95+ years	Data Rich	1	3	BAC law youth drivers (quartile)	232
Road injuries	Male	0-6 days	95+ years	Data Rich	1	3	BAC law general population (quartile)	--
Road injuries	Male	0-6 days	95+ years	Data Rich	1	3	Speed limit law rural (quartile)	--
Road injuries	Male	0-6 days	95+ years	Data Rich	1	3	Speed limit law urban (quartile)	--
Road injuries	Male	0-6 days	95+ years	Global	1	1	Vehicles - 2 wheels fraction (proportion)	1000
Road injuries	Male	0-6 days	95+ years	Global	1	1	Log-transformed SEV scalar: Road Inj	--
Road injuries	Male	0-6 days	95+ years	Global	1	1	Vehicles - 2 wheels (per capita)	--
Road injuries	Male	0-6 days	95+ years	Global	1	1	Vehicles - 2+4 wheels (per capita)	--
Road injuries	Male	0-6 days	95+ years	Global	1	1	Vehicles - 4 wheels (per capita)	--
Road injuries	Male	0-6 days	95+ years	Global	-1	2	Healthcare access and quality index	636
Road injuries	Male	0-6 days	95+ years	Global	-1	2	Socio-demographic Index	636
Road injuries	Male	0-6 days	95+ years	Global	1	2	Population 15 to 30 (proportion)	636
Road injuries	Male	0-6 days	95+ years	Global	1	2	Liters of alcohol consumed per capita	--
Road injuries	Male	0-6 days	95+ years	Global	1	2	Population Density (300-500 ppl/sqkm, proportion)	--
Road injuries	Male	0-6 days	95+ years	Global	1	2	Population Density (500-1000 ppl/sqkm, proportion)	--
Road injuries	Male	0-6 days	95+ years	Global	1	2	Population-weighted mean temperature	--
Road injuries	Male	0-6 days	95+ years	Global	1	3	BAC law professional drivers (quartile)	293
Road injuries	Male	0-6 days	95+ years	Global	1	3	BAC law youth drivers (quartile)	308
Road injuries	Male	0-6 days	95+ years	Global	1	3	BAC law general population (quartile)	--
Road injuries	Male	0-6 days	95+ years	Global	1	3	Rainfall Quintile 5 (proportion)	--
Road injuries	Male	0-6 days	95+ years	Global	1	3	Speed limit law rural (quartile)	--
Road injuries	Male	0-6 days	95+ years	Global	1	3	Speed limit law urban (quartile)	--
Pedestrian road injuries	Female	0-6 days	95+ years	Data Rich	1	1	Log-transformed SEV scalar: Pedest	--
Pedestrian road injuries	Female	0-6 days	95+ years	Data Rich	1	1	Vehicles - 2 wheels fraction (proportion)	--
Pedestrian road injuries	Female	0-6 days	95+ years	Data Rich	1	1	Vehicles - 2+4 wheels (per capita)	--
Pedestrian road injuries	Female	0-6 days	95+ years	Data Rich	-1	2	Socio-demographic Index	801
Pedestrian road injuries	Female	0-6 days	95+ years	Data Rich	-1	2	Healthcare access and quality index	--
Pedestrian road injuries	Female	0-6 days	95+ years	Data Rich	1	2	Population Density (500-1000 ppl/sqkm, proportion)	198
Pedestrian road injuries	Female	0-6 days	95+ years	Data Rich	1	2	Population 15 to 30 (proportion)	999
Pedestrian road injuries	Female	0-6 days	95+ years	Data Rich	1	2	Population-weighted mean temperature	999
Pedestrian road injuries	Female	0-6 days	95+ years	Data Rich	1	2	Liters of alcohol consumed per capita	--
Pedestrian road injuries	Female	0-6 days	95+ years	Data Rich	1	2	Population Density (300-500 ppl/sqkm, proportion)	--
Pedestrian road injuries	Female	0-6 days	95+ years	Data Rich	1	3	Rainfall Quintile 5 (proportion)	6
Pedestrian road injuries	Female	0-6 days	95+ years	Data Rich	1	3	BAC law professional drivers (quartile)	7
Pedestrian road injuries	Female	0-6 days	95+ years	Data Rich	1	3	BAC law youth drivers (quartile)	160
Pedestrian road injuries	Female	0-6 days	95+ years	Data Rich	1	3	BAC law general population (quartile)	--
Pedestrian road injuries	Female	0-6 days	95+ years	Data Rich	1	3	Speed limit law rural (quartile)	--
Pedestrian road injuries	Female	0-6 days	95+ years	Data Rich	1	3	Speed limit law urban (quartile)	--
Pedestrian road injuries	Female	0-6 days	95+ years	Global	1	1	Vehicles - 2 wheels fraction (proportion)	1000
Pedestrian road injuries	Female	0-6 days	95+ years	Global	1	1	Log-transformed SEV scalar: Pedest	--
Pedestrian road injuries	Female	0-6 days	95+ years	Global	1	1	Vehicles - 2+4 wheels (per capita)	--
Pedestrian road injuries	Female	0-6 days	95+ years	Global	-1	2	Healthcare access and quality index	--
Pedestrian road injuries	Female	0-6 days	95+ years	Global	-1	2	Socio-demographic Index	--
Pedestrian road injuries	Female	0-6 days	95+ years	Global	1	2	Population 15 to 30 (proportion)	593
Pedestrian road injuries	Female	0-6 days	95+ years	Global	1	2	Population Density (500-1000 ppl/sqkm, proportion)	593
Pedestrian road injuries	Female	0-6 days	95+ years	Global	1	2	Liters of alcohol consumed per capita	--
Pedestrian road injuries	Female	0-6 days	95+ years	Global	1	2	Population Density (300-500 ppl/sqkm, proportion)	--
Pedestrian road injuries	Female	0-6 days	95+ years	Global	1	2	Population-weighted mean temperature	--
Pedestrian road injuries	Female	0-6 days	95+ years	Global	1	3	BAC law youth drivers (quartile)	185
Pedestrian road injuries	Female	0-6 days	95+ years	Global	1	3	Rainfall Quintile 5 (proportion)	452
Pedestrian road injuries	Female	0-6 days	95+ years	Global	1	3	BAC law professional drivers (quartile)	--
Pedestrian road injuries	Female	0-6 days	95+ years	Global	1	3	BAC law general population (quartile)	--
Pedestrian road injuries	Female	0-6 days	95+ years	Global	1	3	Speed limit law rural (quartile)	--
Pedestrian road injuries	Female	0-6 days	95+ years	Global	1	3	Speed limit law urban (quartile)	--
Pedestrian road injuries	Male	0-6 days	95+ years	Data Rich	1	1	Log-transformed SEV scalar: Pedest	--
Pedestrian road injuries	Male	0-6 days	95+ years	Data Rich	1	1	Vehicles - 2 wheels fraction (proportion)	--
Pedestrian road injuries	Male	0-6 days	95+ years	Data Rich	1	1	Vehicles - 2+4 wheels (per capita)	--
Pedestrian road injuries	Male	0-6 days	95+ years	Data Rich	-1	2	Healthcare access and quality index	818
Pedestrian road injuries	Male	0-6 days	95+ years	Data Rich	-1	2	Socio-demographic Index	818
Pedestrian road injuries	Male	0-6 days	95+ years	Data Rich	1	2	Population 15 to 30 (proportion)	818
Pedestrian road injuries	Male	0-6 days	95+ years	Data Rich	1	2	Population Density (300-500 ppl/sqkm, proportion)	818
Pedestrian road injuries	Male	0-6 days	95+ years	Data Rich	1	2	Liters of alcohol consumed per capita	--
Pedestrian road injuries	Male	0-6 days	95+ years	Data Rich	1	2	Population Density (500-1000 ppl/sqkm, proportion)	--
Pedestrian road injuries	Male	0-6 days	95+ years	Data Rich	1	2	Population-weighted mean temperature	--
Pedestrian road injuries	Male	0-6 days	95+ years	Data Rich	1	3	BAC law youth drivers (quartile)	52
Pedestrian road injuries	Male	0-6 days	95+ years	Data Rich	1	3	Rainfall Quintile 5 (proportion)	52
Pedestrian road injuries	Male	0-6 days	95+ years	Data Rich	1	3	BAC law professional drivers (quartile)	130
Pedestrian road injuries	Male	0-6 days	95+ years	Data Rich	1	3	BAC law general population (quartile)	--
Pedestrian road injuries	Male	0-6 days	95+ years	Data Rich	1	3	Speed limit law rural (quartile)	--
Pedestrian road injuries	Male	0-6 days	95+ years	Data Rich	1	3	Speed limit law urban (quartile)	--
Pedestrian road injuries	Male	0-6 days	95+ years	Global	1	1	Log-transformed SEV scalar: Pedest	--
Pedestrian road injuries	Male	0-6 days	95+ years	Global	1	1	Vehicles - 2 wheels fraction (proportion)	--
Pedestrian road injuries	Male	0-6 days	95+ years	Global	1	1	Vehicles - 2+4 wheels (per capita)	--
Pedestrian road injuries	Male	0-6 days	95+ years	Global	-1	2	Socio-demographic Index	780
Pedestrian road injuries	Male	0-6 days	95+ years	Global	-1	2	Healthcare access and quality index	--
Pedestrian road injuries	Male	0-6 days	95+ years	Global	1	2	Population 15 to 30 (proportion)	780
Pedestrian road injuries	Male	0-6 days	95+ years	Global	1	2	Population Density (300-500 ppl/sqkm, proportion)	780
Pedestrian road injuries	Male	0-6 days	95+ years	Global	1	2	Population Density (500-1000 ppl/sqkm, proportion)	780
Pedestrian road injuries	Male	0-6 days	95+ years	Global	1	2	Liters of alcohol consumed per capita	--
Pedestrian road injuries	Male	0-6 days	95+ years	Global	1	2	Population-weighted mean temperature	--
Pedestrian road injuries	Male	0-6 days	95+ years	Global	1	3	BAC law youth drivers (quartile)	220
Pedestrian road injuries	Male	0-6 days	95+ years	Global	1	3	Rainfall Quintile 5 (proportion)	353
Pedestrian road injuries	Male	0-6 days	95+ years	Global	1	3	BAC law professional drivers (quartile)	--
Pedestrian road injuries	Male	0-6 days	95+ years	Global	1	3	BAC law general population (quartile)	--
Pedestrian road injuries	Male	0-6 days	95+ years	Global	1	3	Speed limit law rural (quartile)	--
Pedestrian road injuries	Male	0-6 days	95+ years	Global	1	3	Speed limit law urban (quartile)	--
Cyclist road injuries	Female	12-23 months	90-94 years	Data Rich	1	1	Log-transformed SEV scalar: Cyclist	--
Cyclist road injuries	Female	12-23 months	90-94 years	Data Rich	1	1	Vehicles - 2 wheels fraction (proportion)	--
Cyclist road injuries	Female	12-23 months	90-94 years	Data Rich	1	1	Vehicles - 2+4 wheels (per capita)	--
Cyclist road injuries	Female	12-23 months	90-94 years	Data Rich	-1	2	Healthcare access and quality index	--
Cyclist road injuries	Female	12-23 months	90-94 years	Data Rich	-1	2	Socio-demographic Index	--
Cyclist road injuries	Female	12-23 months	90-94 years	Data Rich	1	2	Population Density (300-500 ppl/sqkm, proportion)	7

Appendix Table S10: CODEm covariates used, level of covariate, and expected direction of covariate by cause, sex, and age								
**Cause**	**Sex**	**Age Start**	**Age End**	**Model Version Type**	**Direction**	**Level**	**Covariate Name**	**Number of Draws**
Cyclist road injuries	Female	12-23 months	90-94 years	Data Rich	1	2	Population Density (500-1000 ppl/sqkm, proportion)	993
Cyclist road injuries	Female	12-23 months	90-94 years	Data Rich	1	2	Liters of alcohol consumed per capita	1000
Cyclist road injuries	Female	12-23 months	90-94 years	Data Rich	1	2	Population 15 to 30 (proportion)	--
Cyclist road injuries	Female	12-23 months	90-94 years	Data Rich	1	2	Population-weighted mean temperature	--
Cyclist road injuries	Female	12-23 months	90-94 years	Data Rich	1	3	BAC law professional drivers (quartile)	161
Cyclist road injuries	Female	12-23 months	90-94 years	Data Rich	1	3	BAC law youth drivers (quartile)	807
Cyclist road injuries	Female	12-23 months	90-94 years	Data Rich	1	3	BAC law general population (quartile)	--
Cyclist road injuries	Female	12-23 months	90-94 years	Data Rich	1	3	Rainfall Quintile 5 (proportion)	--
Cyclist road injuries	Female	12-23 months	90-94 years	Data Rich	1	3	Speed limit law rural (quartile)	--
Cyclist road injuries	Female	12-23 months	90-94 years	Data Rich	1	3	Speed limit law urban (quartile)	--
Cyclist road injuries	Female	12-23 months	90-94 years	Global	1	1	Vehicles - 2+4 wheels (per capita)	748
Cyclist road injuries	Female	12-23 months	90-94 years	Global	1	1	Vehicles - 2 wheels fraction (proportion)	1000
Cyclist road injuries	Female	12-23 months	90-94 years	Global	1	1	Log-transformed SEV scalar: Cyclist	--
Cyclist road injuries	Female	12-23 months	90-94 years	Global	-1	2	Socio-demographic Index	503
Cyclist road injuries	Female	12-23 months	90-94 years	Global	-1	2	Healthcare access and quality index	--
Cyclist road injuries	Female	12-23 months	90-94 years	Global	1	2	Population Density (500-1000 ppl/sqkm, proportion)	236
Cyclist road injuries	Female	12-23 months	90-94 years	Global	1	2	Population 15 to 30 (proportion)	266
Cyclist road injuries	Female	12-23 months	90-94 years	Global	1	2	Population Density (300-500 ppl/sqkm, proportion)	329
Cyclist road injuries	Female	12-23 months	90-94 years	Global	1	2	Population-weighted mean temperature	431
Cyclist road injuries	Female	12-23 months	90-94 years	Global	1	2	Liters of alcohol consumed per capita	--
Cyclist road injuries	Female	12-23 months	90-94 years	Global	1	3	BAC law professional drivers (quartile)	234
Cyclist road injuries	Female	12-23 months	90-94 years	Global	1	3	BAC law youth drivers (quartile)	277
Cyclist road injuries	Female	12-23 months	90-94 years	Global	1	3	BAC law general population (quartile)	--
Cyclist road injuries	Female	12-23 months	90-94 years	Global	1	3	Rainfall Quintile 5 (proportion)	--
Cyclist road injuries	Female	12-23 months	90-94 years	Global	1	3	Speed limit law rural (quartile)	--
Cyclist road injuries	Female	12-23 months	90-94 years	Global	1	3	Speed limit law urban (quartile)	--
Cyclist road injuries	Male	12-23 months	90-94 years	Data Rich	1	1	Vehicles - 2 wheels fraction (proportion)	1000
Cyclist road injuries	Male	12-23 months	90-94 years	Data Rich	1	1	Log-transformed SEV scalar: Cyclist	--
Cyclist road injuries	Male	12-23 months	90-94 years	Data Rich	1	1	Vehicles - 2+4 wheels (per capita)	--
Cyclist road injuries	Male	12-23 months	90-94 years	Data Rich	-1	2	Socio-demographic Index	192
Cyclist road injuries	Male	12-23 months	90-94 years	Data Rich	-1	2	Healthcare access and quality index	801
Cyclist road injuries	Male	12-23 months	90-94 years	Data Rich	1	2	Population 15 to 30 (proportion)	801
Cyclist road injuries	Male	12-23 months	90-94 years	Data Rich	1	2	Liters of alcohol consumed per capita	993
Cyclist road injuries	Male	12-23 months	90-94 years	Data Rich	1	2	Population Density (500-1000 ppl/sqkm, proportion)	993
Cyclist road injuries	Male	12-23 months	90-94 years	Data Rich	1	2	Population-weighted mean temperature	993
Cyclist road injuries	Male	12-23 months	90-94 years	Data Rich	1	2	Population Density (300-500 ppl/sqkm, proportion)	--
Cyclist road injuries	Male	12-23 months	90-94 years	Data Rich	1	3	BAC law youth drivers (quartile)	6
Cyclist road injuries	Male	12-23 months	90-94 years	Data Rich	1	3	BAC law professional drivers (quartile)	32
Cyclist road injuries	Male	12-23 months	90-94 years	Data Rich	1	3	Rainfall Quintile 5 (proportion)	38
Cyclist road injuries	Male	12-23 months	90-94 years	Data Rich	1	3	BAC law general population (quartile)	--
Cyclist road injuries	Male	12-23 months	90-94 years	Data Rich	1	3	Speed limit law rural (quartile)	--
Cyclist road injuries	Male	12-23 months	90-94 years	Data Rich	1	3	Speed limit law urban (quartile)	--
Cyclist road injuries	Male	12-23 months	90-94 years	Global	1	1	Vehicles - 2 wheels fraction (proportion)	320
Cyclist road injuries	Male	12-23 months	90-94 years	Global	1	1	Vehicles - 2+4 wheels (per capita)	680
Cyclist road injuries	Male	12-23 months	90-94 years	Global	1	1	Log-transformed SEV scalar: Cyclist	--
Cyclist road injuries	Male	12-23 months	90-94 years	Global	-1	2	Healthcare access and quality index	423
Cyclist road injuries	Male	12-23 months	90-94 years	Global	-1	2	Socio-demographic Index	744
Cyclist road injuries	Male	12-23 months	90-94 years	Global	1	2	Population Density (500-1000 ppl/sqkm, proportion)	88
Cyclist road injuries	Male	12-23 months	90-94 years	Global	1	2	Population Density (300-500 ppl/sqkm, proportion)	98
Cyclist road injuries	Male	12-23 months	90-94 years	Global	1	2	Population 15 to 30 (proportion)	135
Cyclist road injuries	Male	12-23 months	90-94 years	Global	1	2	Population-weighted mean temperature	321
Cyclist road injuries	Male	12-23 months	90-94 years	Global	1	2	Liters of alcohol consumed per capita	744
Cyclist road injuries	Male	12-23 months	90-94 years	Global	1	3	Rainfall Quintile 5 (proportion)	49
Cyclist road injuries	Male	12-23 months	90-94 years	Global	1	3	BAC law professional drivers (quartile)	156
Cyclist road injuries	Male	12-23 months	90-94 years	Global	1	3	BAC law youth drivers (quartile)	282
Cyclist road injuries	Male	12-23 months	90-94 years	Global	1	3	BAC law general population (quartile)	--
Cyclist road injuries	Male	12-23 months	90-94 years	Global	1	3	Speed limit law rural (quartile)	--
Cyclist road injuries	Male	12-23 months	90-94 years	Global	1	3	Speed limit law urban (quartile)	--
Motorcyclist road injuries	Female	0-6 days	95+ years	Data Rich	1	1	Vehicles - 2 wheels (per capita)	961
Motorcyclist road injuries	Female	0-6 days	95+ years	Data Rich	1	1	Vehicles - 2 wheels fraction (proportion)	1000
Motorcyclist road injuries	Female	0-6 days	95+ years	Data Rich	1	1	Liters of alcohol consumed per capita	--
Motorcyclist road injuries	Female	0-6 days	95+ years	Data Rich	1	1	Log-transformed SEV scalar: Mot Cyc	--
Motorcyclist road injuries	Female	0-6 days	95+ years	Data Rich	-1	2	Socio-demographic Index	39
Motorcyclist road injuries	Female	0-6 days	95+ years	Data Rich	-1	2	Healthcare access and quality index	1000
Motorcyclist road injuries	Female	0-6 days	95+ years	Data Rich	1	2	Population 15 to 30 (proportion)	1000
Motorcyclist road injuries	Female	0-6 days	95+ years	Data Rich	1	2	Population-weighted mean temperature	--
Motorcyclist road injuries	Female	0-6 days	95+ years	Data Rich	1	3	Population Density (300-500 ppl/sqkm, proportion)	1
Motorcyclist road injuries	Female	0-6 days	95+ years	Data Rich	1	3	Population Density (500-1000 ppl/sqkm, proportion)	32
Motorcyclist road injuries	Female	0-6 days	95+ years	Data Rich	1	3	BAC law youth drivers (quartile)	160
Motorcyclist road injuries	Female	0-6 days	95+ years	Data Rich	1	3	BAC law professional drivers (quartile)	--
Motorcyclist road injuries	Female	0-6 days	95+ years	Data Rich	1	3	BAC law general population (quartile)	--
Motorcyclist road injuries	Female	0-6 days	95+ years	Data Rich	1	3	Rainfall Quintile 5 (proportion)	--
Motorcyclist road injuries	Female	0-6 days	95+ years	Data Rich	1	3	Speed limit law rural (quartile)	--
Motorcyclist road injuries	Female	0-6 days	95+ years	Data Rich	1	3	Speed limit law urban (quartile)	--
Motorcyclist road injuries	Female	0-6 days	95+ years	Global	1	1	Vehicles - 2 wheels fraction (proportion)	1000
Motorcyclist road injuries	Female	0-6 days	95+ years	Global	1	1	Liters of alcohol consumed per capita	--
Motorcyclist road injuries	Female	0-6 days	95+ years	Global	1	1	Log-transformed SEV scalar: Mot Cyc	--
Motorcyclist road injuries	Female	0-6 days	95+ years	Global	1	1	Vehicles - 2 wheels (per capita)	--
Motorcyclist road injuries	Female	0-6 days	95+ years	Global	-1	2	Healthcare access and quality index	579
Motorcyclist road injuries	Female	0-6 days	95+ years	Global	-1	2	Socio-demographic Index	--
Motorcyclist road injuries	Female	0-6 days	95+ years	Global	1	2	Population 15 to 30 (proportion)	269
Motorcyclist road injuries	Female	0-6 days	95+ years	Global	1	2	Population-weighted mean temperature	269
Motorcyclist road injuries	Female	0-6 days	95+ years	Global	1	3	BAC law youth drivers (quartile)	292
Motorcyclist road injuries	Female	0-6 days	95+ years	Global	1	3	Population Density (300-500 ppl/sqkm, proportion)	292
Motorcyclist road injuries	Female	0-6 days	95+ years	Global	1	3	Rainfall Quintile 5 (proportion)	387
Motorcyclist road injuries	Female	0-6 days	95+ years	Global	1	3	BAC law professional drivers (quartile)	390
Motorcyclist road injuries	Female	0-6 days	95+ years	Global	1	3	Population Density (500-1000 ppl/sqkm, proportion)	390
Motorcyclist road injuries	Female	0-6 days	95+ years	Global	1	3	BAC law general population (quartile)	--
Motorcyclist road injuries	Female	0-6 days	95+ years	Global	1	3	Speed limit law rural (quartile)	--
Motorcyclist road injuries	Female	0-6 days	95+ years	Global	1	3	Speed limit law urban (quartile)	--
Motorcyclist road injuries	Male	0-6 days	95+ years	Data Rich	1	1	Vehicles - 2 wheels (per capita)	57
Motorcyclist road injuries	Male	0-6 days	95+ years	Data Rich	1	1	Vehicles - 2 wheels fraction (proportion)	943
Motorcyclist road injuries	Male	0-6 days	95+ years	Data Rich	1	1	Liters of alcohol consumed per capita	--
Motorcyclist road injuries	Male	0-6 days	95+ years	Data Rich	1	1	Log-transformed SEV scalar: Mot Cyc	--
Motorcyclist road injuries	Male	0-6 days	95+ years	Data Rich	-1	2	Healthcare access and quality index	998
Motorcyclist road injuries	Male	0-6 days	95+ years	Data Rich	-1	2	Socio-demographic Index	998
Motorcyclist road injuries	Male	0-6 days	95+ years	Data Rich	1	2	Population 15 to 30 (proportion)	998
Motorcyclist road injuries	Male	0-6 days	95+ years	Data Rich	1	2	Population-weighted mean temperature	998

Appendix Table S10: CODEm covariates used, level of covariate, and expected direction of covariate by cause, sex, and age									
**Cause**	**Sex**	**Age Start**	**Age End**	**Model Version Type**	**Direction**	**Level**	**Covariate Name**	**Number of Draws**
Motorcyclist road injuries	Male	0-6 days	95+ years	Data Rich	1	3	Speed limit law rural (quartile)	0
Motorcyclist road injuries	Male	0-6 days	95+ years	Data Rich	1	3	Speed limit law urban (quartile)	0
Motorcyclist road injuries	Male	0-6 days	95+ years	Data Rich	1	3	Population Density (500-1000 ppl/sqkm, proportion)	2
Motorcyclist road injuries	Male	0-6 days	95+ years	Data Rich	1	3	Population Density (300-500 ppl/sqkm, proportion)	13
Motorcyclist road injuries	Male	0-6 days	95+ years	Data Rich	1	3	BAC law youth drivers (quartile)	200
Motorcyclist road injuries	Male	0-6 days	95+ years	Data Rich	1	3	BAC law professional drivers (quartile)	--
Motorcyclist road injuries	Male	0-6 days	95+ years	Data Rich	1	3	BAC law general population (quartile)	--
Motorcyclist road injuries	Male	0-6 days	95+ years	Data Rich	1	3	Rainfall Quintile 5 (proportion)	--
Motorcyclist road injuries	Male	0-6 days	95+ years	Global	1	1	Vehicles - 2 wheels fraction (proportion)	1000
Motorcyclist road injuries	Male	0-6 days	95+ years	Global	1	1	Liters of alcohol consumed per capita	--
Motorcyclist road injuries	Male	0-6 days	95+ years	Global	1	1	Log-transformed SEV scalar: Mot Cyc	--
Motorcyclist road injuries	Male	0-6 days	95+ years	Global	1	1	Vehicles - 2 wheels (per capita)	--
Motorcyclist road injuries	Male	0-6 days	95+ years	Global	-1	2	Healthcare access and quality index	385
Motorcyclist road injuries	Male	0-6 days	95+ years	Global	-1	2	Socio-demographic Index	--
Motorcyclist road injuries	Male	0-6 days	95+ years	Global	1	2	Population 15 to 30 (proportion)	416
Motorcyclist road injuries	Male	0-6 days	95+ years	Global	1	2	Population-weighted mean temperature	801
Motorcyclist road injuries	Male	0-6 days	95+ years	Global	1	3	Rainfall Quintile 5 (proportion)	114
Motorcyclist road injuries	Male	0-6 days	95+ years	Global	1	3	BAC law youth drivers (quartile)	190
Motorcyclist road injuries	Male	0-6 days	95+ years	Global	1	3	Population Density (500-1000 ppl/sqkm, proportion)	307
Motorcyclist road injuries	Male	0-6 days	95+ years	Global	1	3	BAC law professional drivers (quartile)	380
Motorcyclist road injuries	Male	0-6 days	95+ years	Global	1	3	Population Density (300-500 ppl/sqkm, proportion)	386
Motorcyclist road injuries	Male	0-6 days	95+ years	Global	1	3	BAC law general population (quartile)	--
Motorcyclist road injuries	Male	0-6 days	95+ years	Global	1	3	Speed limit law rural (quartile)	--
Motorcyclist road injuries	Male	0-6 days	95+ years	Global	1	3	Speed limit law urban (quartile)	--
Motor vehicle road injuries	Female	0-6 days	95+ years	Data Rich	1	1	Vehicles - 4 wheels (per capita)	--
Motor vehicle road injuries	Female	0-6 days	95+ years	Data Rich	-1	2	Healthcare access and quality index	109
Motor vehicle road injuries	Female	0-6 days	95+ years	Data Rich	-1	2	Socio-demographic Index	--
Motor vehicle road injuries	Female	0-6 days	95+ years	Data Rich	1	2	Population Density (500-1000 ppl/sqkm, proportion)	379
Motor vehicle road injuries	Female	0-6 days	95+ years	Data Rich	1	2	Population 15 to 30 (proportion)	488
Motor vehicle road injuries	Female	0-6 days	95+ years	Data Rich	1	2	Population Density (300-500 ppl/sqkm, proportion)	488
Motor vehicle road injuries	Female	0-6 days	95+ years	Data Rich	1	2	Liters of alcohol consumed per capita	--
Motor vehicle road injuries	Female	0-6 days	95+ years	Data Rich	1	2	Population-weighted mean temperature	--
Motor vehicle road injuries	Female	0-6 days	95+ years	Data Rich	1	3	BAC law professional drivers (quartile)	31
Motor vehicle road injuries	Female	0-6 days	95+ years	Data Rich	1	3	BAC law youth drivers (quartile)	701
Motor vehicle road injuries	Female	0-6 days	95+ years	Data Rich	1	3	BAC law general population (quartile)	--
Motor vehicle road injuries	Female	0-6 days	95+ years	Data Rich	1	3	Log-transformed SEV scalar: Mot Veh	--
Motor vehicle road injuries	Female	0-6 days	95+ years	Data Rich	1	3	Rainfall Quintile 5 (proportion)	--
Motor vehicle road injuries	Female	0-6 days	95+ years	Data Rich	1	3	Speed limit law rural (quartile)	--
Motor vehicle road injuries	Female	0-6 days	95+ years	Data Rich	1	3	Speed limit law urban (quartile)	--
Motor vehicle road injuries	Female	0-6 days	95+ years	Global	1	1	Vehicles - 4 wheels (per capita)	--
Motor vehicle road injuries	Female	0-6 days	95+ years	Global	-1	2	Socio-demographic Index	815
Motor vehicle road injuries	Female	0-6 days	95+ years	Global	-1	2	Healthcare access and quality index	--
Motor vehicle road injuries	Female	0-6 days	95+ years	Global	1	2	Population 15 to 30 (proportion)	815
Motor vehicle road injuries	Female	0-6 days	95+ years	Global	1	2	Population Density (300-500 ppl/sqkm, proportion)	815
Motor vehicle road injuries	Female	0-6 days	95+ years	Global	1	2	Population Density (500-1000 ppl/sqkm, proportion)	815
Motor vehicle road injuries	Female	0-6 days	95+ years	Global	1	2	Liters of alcohol consumed per capita	--
Motor vehicle road injuries	Female	0-6 days	95+ years	Global	1	2	Population-weighted mean temperature	--
Motor vehicle road injuries	Female	0-6 days	95+ years	Global	1	3	BAC law professional drivers (quartile)	407
Motor vehicle road injuries	Female	0-6 days	95+ years	Global	1	3	BAC law youth drivers (quartile)	452
Motor vehicle road injuries	Female	0-6 days	95+ years	Global	1	3	BAC law general population (quartile)	--
Motor vehicle road injuries	Female	0-6 days	95+ years	Global	1	3	Log-transformed SEV scalar: Mot Veh	--
Motor vehicle road injuries	Female	0-6 days	95+ years	Global	1	3	Rainfall Quintile 5 (proportion)	--
Motor vehicle road injuries	Female	0-6 days	95+ years	Global	1	3	Speed limit law rural (quartile)	--
Motor vehicle road injuries	Female	0-6 days	95+ years	Global	1	3	Speed limit law urban (quartile)	--
Motor vehicle road injuries	Male	0-6 days	95+ years	Data Rich	1	1	Vehicles - 4 wheels (per capita)	--
Motor vehicle road injuries	Male	0-6 days	95+ years	Data Rich	-1	2	Healthcare access and quality index	500
Motor vehicle road injuries	Male	0-6 days	95+ years	Data Rich	-1	2	Socio-demographic Index	500
Motor vehicle road injuries	Male	0-6 days	95+ years	Data Rich	1	2	Population 15 to 30 (proportion)	500
Motor vehicle road injuries	Male	0-6 days	95+ years	Data Rich	1	2	Population Density (300-500 ppl/sqkm, proportion)	500
Motor vehicle road injuries	Male	0-6 days	95+ years	Data Rich	1	2	Population Density (500-1000 ppl/sqkm, proportion)	500
Motor vehicle road injuries	Male	0-6 days	95+ years	Data Rich	1	2	Liters of alcohol consumed per capita	--
Motor vehicle road injuries	Male	0-6 days	95+ years	Data Rich	1	2	Population-weighted mean temperature	--
Motor vehicle road injuries	Male	0-6 days	95+ years	Data Rich	1	3	BAC law professional drivers (quartile)	250
Motor vehicle road injuries	Male	0-6 days	95+ years	Data Rich	1	3	BAC law youth drivers (quartile)	500
Motor vehicle road injuries	Male	0-6 days	95+ years	Data Rich	1	3	BAC law general population (quartile)	--
Motor vehicle road injuries	Male	0-6 days	95+ years	Data Rich	1	3	Log-transformed SEV scalar: Mot Veh	--
Motor vehicle road injuries	Male	0-6 days	95+ years	Data Rich	1	3	Rainfall Quintile 5 (proportion)	--
Motor vehicle road injuries	Male	0-6 days	95+ years	Data Rich	1	3	Speed limit law rural (quartile)	--
Motor vehicle road injuries	Male	0-6 days	95+ years	Data Rich	1	3	Speed limit law urban (quartile)	--
Motor vehicle road injuries	Male	0-6 days	95+ years	Global	1	1	Vehicles - 4 wheels (per capita)	--
Motor vehicle road injuries	Male	0-6 days	95+ years	Global	-1	2	Healthcare access and quality index	815
Motor vehicle road injuries	Male	0-6 days	95+ years	Global	-1	2	Socio-demographic Index	815
Motor vehicle road injuries	Male	0-6 days	95+ years	Global	1	2	Population 15 to 30 (proportion)	815
Motor vehicle road injuries	Male	0-6 days	95+ years	Global	1	2	Population Density (300-500 ppl/sqkm, proportion)	815
Motor vehicle road injuries	Male	0-6 days	95+ years	Global	1	2	Population Density (500-1000 ppl/sqkm, proportion)	815
Motor vehicle road injuries	Male	0-6 days	95+ years	Global	1	2	Liters of alcohol consumed per capita	--
Motor vehicle road injuries	Male	0-6 days	95+ years	Global	1	2	Population-weighted mean temperature	--
Motor vehicle road injuries	Male	0-6 days	95+ years	Global	1	3	BAC law professional drivers (quartile)	407
Motor vehicle road injuries	Male	0-6 days	95+ years	Global	1	3	BAC law youth drivers (quartile)	452
Motor vehicle road injuries	Male	0-6 days	95+ years	Global	1	3	BAC law general population (quartile)	--
Motor vehicle road injuries	Male	0-6 days	95+ years	Global	1	3	Log-transformed SEV scalar: Mot Veh	--
Motor vehicle road injuries	Male	0-6 days	95+ years	Global	1	3	Rainfall Quintile 5 (proportion)	--
Motor vehicle road injuries	Male	0-6 days	95+ years	Global	1	3	Speed limit law rural (quartile)	--
Motor vehicle road injuries	Male	0-6 days	95+ years	Global	1	3	Speed limit law urban (quartile)	--
Other road injuries	Female	0-6 days	95+ years	Data Rich	1	1	Log-transformed SEV scalar: Oth Road	--
Other road injuries	Female	0-6 days	95+ years	Data Rich	1	1	Vehicles - 2 wheels fraction (proportion)	--
Other road injuries	Female	0-6 days	95+ years	Data Rich	1	1	Vehicles - 2+4 wheels (per capita)	--
Other road injuries	Female	0-6 days	95+ years	Data Rich	-1	2	Healthcare access and quality index	--
Other road injuries	Female	0-6 days	95+ years	Data Rich	-1	2	Socio-demographic Index	--
Other road injuries	Female	0-6 days	95+ years	Data Rich	1	2	Population 15 to 30 (proportion)	1000
Other road injuries	Female	0-6 days	95+ years	Data Rich	1	2	Liters of alcohol consumed per capita	--
Other road injuries	Female	0-6 days	95+ years	Data Rich	1	2	Population-weighted mean temperature	--
Other road injuries	Female	0-6 days	95+ years	Data Rich	1	3	BAC law professional drivers (quartile)	--
Other road injuries	Female	0-6 days	95+ years	Data Rich	1	3	BAC law general population (quartile)	--
Other road injuries	Female	0-6 days	95+ years	Data Rich	1	3	BAC law youth drivers (quartile)	--
Other road injuries	Female	0-6 days	95+ years	Data Rich	1	3	Rainfall Quintile 5 (proportion)	--
Other road injuries	Female	0-6 days	95+ years	Data Rich	1	3	Speed limit law rural (quartile)	--
Other road injuries	Female	0-6 days	95+ years	Data Rich	1	3	Speed limit law urban (quartile)	--

Appendix Table S10: CODEm covariates used, level of covariate, and expected direction of covariate by cause, sex, and age

Cause	Sex	Age Start	Age End	Model Version Type	Direction	Level	Covariate Name	Number of Draws
Other road injuries	Female	0-6 days	95+ years	Global	1	1	Vehicles - 2 wheels fraction (proportion)	1000
Other road injuries	Female	0-6 days	95+ years	Global	1	1	Log-transformed SEV scalar: Oth Road	--
Other road injuries	Female	0-6 days	95+ years	Global	1	1	Vehicles - 2+4 wheels (per capita)	--
Other road injuries	Female	0-6 days	95+ years	Global	-1	2	Socio-demographic Index	282
Other road injuries	Female	0-6 days	95+ years	Global	-1	2	Healthcare access and quality index	424
Other road injuries	Female	0-6 days	95+ years	Global	1	2	Population 15 to 30 (proportion)	706
Other road injuries	Female	0-6 days	95+ years	Global	1	2	Liters of alcohol consumed per capita	--
Other road injuries	Female	0-6 days	95+ years	Global	1	2	Population-weighted mean temperature	--
Other road injuries	Female	0-6 days	95+ years	Global	1	3	BAC law youth drivers (quartile)	346
Other road injuries	Female	0-6 days	95+ years	Global	1	3	BAC law professional drivers (quartile)	--
Other road injuries	Female	0-6 days	95+ years	Global	1	3	BAC law general population (quartile)	--
Other road injuries	Female	0-6 days	95+ years	Global	1	3	Rainfall Quintile 5 (proportion)	--
Other road injuries	Female	0-6 days	95+ years	Global	1	3	Speed limit law rural (quartile)	--
Other road injuries	Female	0-6 days	95+ years	Global	1	3	Speed limit law urban (quartile)	--
Other road injuries	Male	0-6 days	95+ years	Data Rich	1	1	Log-transformed SEV scalar: Oth Road	--
Other road injuries	Male	0-6 days	95+ years	Data Rich	1	1	Vehicles - 2 wheels fraction (proportion)	--
Other road injuries	Male	0-6 days	95+ years	Data Rich	1	1	Vehicles - 2+4 wheels (per capita)	--
Other road injuries	Male	0-6 days	95+ years	Data Rich	-1	2	Healthcare access and quality index	166
Other road injuries	Male	0-6 days	95+ years	Data Rich	-1	2	Socio-demographic Index	834
Other road injuries	Male	0-6 days	95+ years	Data Rich	1	2	Liters of alcohol consumed per capita	--
Other road injuries	Male	0-6 days	95+ years	Data Rich	1	2	Population 15 to 30 (proportion)	--
Other road injuries	Male	0-6 days	95+ years	Data Rich	1	2	Population-weighted mean temperature	--
Other road injuries	Male	0-6 days	95+ years	Data Rich	1	3	BAC law professional drivers (quartile)	--
Other road injuries	Male	0-6 days	95+ years	Data Rich	1	3	BAC law general population (quartile)	--
Other road injuries	Male	0-6 days	95+ years	Data Rich	1	3	BAC law youth drivers (quartile)	--
Other road injuries	Male	0-6 days	95+ years	Data Rich	1	3	Rainfall Quintile 5 (proportion)	--
Other road injuries	Male	0-6 days	95+ years	Data Rich	1	3	Speed limit law rural (quartile)	--
Other road injuries	Male	0-6 days	95+ years	Data Rich	1	3	Speed limit law urban (quartile)	--
Other road injuries	Male	0-6 days	95+ years	Global	1	1	Vehicles - 2 wheels fraction (proportion)	1000
Other road injuries	Male	0-6 days	95+ years	Global	1	1	Log-transformed SEV scalar: Oth Road	--
Other road injuries	Male	0-6 days	95+ years	Global	1	1	Vehicles - 2+4 wheels (per capita)	--
Other road injuries	Male	0-6 days	95+ years	Global	-1	2	Healthcare access and quality index	398
Other road injuries	Male	0-6 days	95+ years	Global	-1	2	Socio-demographic Index	--
Other road injuries	Male	0-6 days	95+ years	Global	1	2	Population 15 to 30 (proportion)	398
Other road injuries	Male	0-6 days	95+ years	Global	1	2	Liters of alcohol consumed per capita	--
Other road injuries	Male	0-6 days	95+ years	Global	1	2	Population-weighted mean temperature	--
Other road injuries	Male	0-6 days	95+ years	Global	1	3	BAC law youth drivers (quartile)	308
Other road injuries	Male	0-6 days	95+ years	Global	1	3	BAC law professional drivers (quartile)	--
Other road injuries	Male	0-6 days	95+ years	Global	1	3	BAC law general population (quartile)	--
Other road injuries	Male	0-6 days	95+ years	Global	1	3	Rainfall Quintile 5 (proportion)	--
Other road injuries	Male	0-6 days	95+ years	Global	1	3	Speed limit law rural (quartile)	--
Other road injuries	Male	0-6 days	95+ years	Global	1	3	Speed limit law urban (quartile)	--
Other transport injuries	Female	0-6 days	95+ years	Data Rich	1	1	Log-transformed SEV scalar: Oth Trans	--
Other transport injuries	Female	0-6 days	95+ years	Data Rich	1	1	Vehicles - 2 wheels fraction (proportion)	--
Other transport injuries	Female	0-6 days	95+ years	Data Rich	1	1	Vehicles - 2+4 wheels (per capita)	--
Other transport injuries	Female	0-6 days	95+ years	Data Rich	-1	2	Healthcare access and quality index	961
Other transport injuries	Female	0-6 days	95+ years	Data Rich	-1	2	Socio-demographic Index	--
Other transport injuries	Female	0-6 days	95+ years	Data Rich	1	2	Population 15 to 30 (proportion)	137
Other transport injuries	Female	0-6 days	95+ years	Data Rich	1	2	Population Density (500-1000 ppl/sqkm, proportion)	902
Other transport injuries	Female	0-6 days	95+ years	Data Rich	1	2	Liters of alcohol consumed per capita	--
Other transport injuries	Female	0-6 days	95+ years	Data Rich	1	2	Population Density (300-500 ppl/sqkm, proportion)	--
Other transport injuries	Female	0-6 days	95+ years	Data Rich	1	2	Population-weighted mean temperature	--
Other transport injuries	Female	0-6 days	95+ years	Data Rich	1	3	BAC law youth drivers (quartile)	246
Other transport injuries	Female	0-6 days	95+ years	Data Rich	1	3	BAC law professional drivers (quartile)	--
Other transport injuries	Female	0-6 days	95+ years	Data Rich	1	3	BAC law general population (quartile)	--
Other transport injuries	Female	0-6 days	95+ years	Data Rich	1	3	Rainfall Quintile 5 (proportion)	--
Other transport injuries	Female	0-6 days	95+ years	Data Rich	1	3	Speed limit law rural (quartile)	--
Other transport injuries	Female	0-6 days	95+ years	Data Rich	1	3	Speed limit law urban (quartile)	--
Other transport injuries	Female	0-6 days	95+ years	Global	1	1	Vehicles - 2 wheels fraction (proportion)	1000
Other transport injuries	Female	0-6 days	95+ years	Global	1	1	Log-transformed SEV scalar: Oth Trans	--
Other transport injuries	Female	0-6 days	95+ years	Global	1	1	Vehicles - 2+4 wheels (per capita)	--
Other transport injuries	Female	0-6 days	95+ years	Global	-1	2	Healthcare access and quality index	593
Other transport injuries	Female	0-6 days	95+ years	Global	-1	2	Socio-demographic Index	593
Other transport injuries	Female	0-6 days	95+ years	Global	1	2	Population 15 to 30 (proportion)	593
Other transport injuries	Female	0-6 days	95+ years	Global	1	2	Population Density (300-500 ppl/sqkm, proportion)	593
Other transport injuries	Female	0-6 days	95+ years	Global	1	2	Liters of alcohol consumed per capita	--
Other transport injuries	Female	0-6 days	95+ years	Global	1	2	Population Density (500-1000 ppl/sqkm, proportion)	--
Other transport injuries	Female	0-6 days	95+ years	Global	1	2	Population-weighted mean temperature	--
Other transport injuries	Female	0-6 days	95+ years	Global	1	3	BAC law youth drivers (quartile)	531
Other transport injuries	Female	0-6 days	95+ years	Global	1	3	BAC law professional drivers (quartile)	--
Other transport injuries	Female	0-6 days	95+ years	Global	1	3	BAC law general population (quartile)	--
Other transport injuries	Female	0-6 days	95+ years	Global	1	3	Rainfall Quintile 5 (proportion)	--
Other transport injuries	Female	0-6 days	95+ years	Global	1	3	Speed limit law rural (quartile)	--
Other transport injuries	Female	0-6 days	95+ years	Global	1	3	Speed limit law urban (quartile)	--
Other transport injuries	Male	0-6 days	95+ years	Data Rich	1	1	Log-transformed SEV scalar: Oth Trans	--
Other transport injuries	Male	0-6 days	95+ years	Data Rich	1	1	Vehicles - 2 wheels fraction (proportion)	--
Other transport injuries	Male	0-6 days	95+ years	Data Rich	1	1	Vehicles - 2+4 wheels (per capita)	--
Other transport injuries	Male	0-6 days	95+ years	Data Rich	-1	2	Healthcare access and quality index	834
Other transport injuries	Male	0-6 days	95+ years	Data Rich	-1	2	Socio-demographic Index	--
Other transport injuries	Male	0-6 days	95+ years	Data Rich	1	2	Population 15 to 30 (proportion)	834
Other transport injuries	Male	0-6 days	95+ years	Data Rich	1	2	Population Density (300-500 ppl/sqkm, proportion)	834
Other transport injuries	Male	0-6 days	95+ years	Data Rich	1	2	Population Density (500-1000 ppl/sqkm, proportion)	834
Other transport injuries	Male	0-6 days	95+ years	Data Rich	1	2	Liters of alcohol consumed per capita	--
Other transport injuries	Male	0-6 days	95+ years	Data Rich	1	2	Population-weighted mean temperature	--
Other transport injuries	Male	0-6 days	95+ years	Data Rich	1	3	BAC law youth drivers (quartile)	166
Other transport injuries	Male	0-6 days	95+ years	Data Rich	1	3	BAC law professional drivers (quartile)	--
Other transport injuries	Male	0-6 days	95+ years	Data Rich	1	3	BAC law general population (quartile)	--
Other transport injuries	Male	0-6 days	95+ years	Data Rich	1	3	Rainfall Quintile 5 (proportion)	--
Other transport injuries	Male	0-6 days	95+ years	Data Rich	1	3	Speed limit law rural (quartile)	--
Other transport injuries	Male	0-6 days	95+ years	Data Rich	1	3	Speed limit law urban (quartile)	--
Other transport injuries	Male	0-6 days	95+ years	Global	1	1	Vehicles - 2 wheels fraction (proportion)	1000
Other transport injuries	Male	0-6 days	95+ years	Global	1	1	Log-transformed SEV scalar: Oth Trans	--
Other transport injuries	Male	0-6 days	95+ years	Global	1	1	Vehicles - 2+4 wheels (per capita)	--
Other transport injuries	Male	0-6 days	95+ years	Global	-1	2	Healthcare access and quality index	815
Other transport injuries	Male	0-6 days	95+ years	Global	-1	2	Socio-demographic Index	--
Other transport injuries	Male	0-6 days	95+ years	Global	1	2	Population 15 to 30 (proportion)	815
Other transport injuries	Male	0-6 days	95+ years	Global	1	2	Population Density (500-1000 ppl/sqkm, proportion)	815
Other transport injuries	Male	0-6 days	95+ years	Global	1	2	Liters of alcohol consumed per capita	--

Appendix Table S10: CODEm covariates used, level of covariate, and expected direction of covariate by cause, sex, and age

Cause	Sex	Age Start	Age End	Model Version Type	Direction	Level	Covariate Name	Number of Draws
Other transport injuries	Male	0-6 days	95+ years	Global	1	2	Population Density (300-500 ppl/sqkm, proportion)	--
Other transport injuries	Male	0-6 days	95+ years	Global	1	2	Population-weighted mean temperature	--
Other transport injuries	Male	0-6 days	95+ years	Global	1	3	BAC law youth drivers (quartile)	252
Other transport injuries	Male	0-6 days	95+ years	Global	1	3	BAC law general population (quartile)	254
Other transport injuries	Male	0-6 days	95+ years	Global	1	3	BAC law professional drivers (quartile)	--
Other transport injuries	Male	0-6 days	95+ years	Global	1	3	Rainfall Quintile 5 (proportion)	--
Other transport injuries	Male	0-6 days	95+ years	Global	1	3	Speed limit law rural (quartile)	--
Other transport injuries	Male	0-6 days	95+ years	Global	1	3	Speed limit law urban (quartile)	--
Falls	Female	0-6 days	95+ years	Data Rich	1	1	Liters of alcohol consumed per capita	--
Falls	Female	0-6 days	95+ years	Data Rich	1	1	Log-transformed SEV scalar: Falls	--
Falls	Female	0-6 days	95+ years	Data Rich	-1	2	Healthcare access and quality index	--
Falls	Female	0-6 days	95+ years	Data Rich	-1	2	Population-weighted mean temperature	--
Falls	Female	0-6 days	95+ years	Data Rich	-1	3	Socio-demographic Index	--
Falls	Female	0-6 days	95+ years	Data Rich	1	3	Elevation Over 1500m (proportion)	--
Falls	Female	0-6 days	95+ years	Global	1	1	Liters of alcohol consumed per capita	--
Falls	Female	0-6 days	95+ years	Global	1	1	Log-transformed SEV scalar: Falls	--
Falls	Female	0-6 days	95+ years	Global	-1	2	Healthcare access and quality index	--
Falls	Female	0-6 days	95+ years	Global	-1	2	Population-weighted mean temperature	--
Falls	Female	0-6 days	95+ years	Global	-1	3	Socio-demographic Index	--
Falls	Female	0-6 days	95+ years	Global	1	3	Elevation Over 1500m (proportion)	--
Falls	Male	0-6 days	95+ years	Data Rich	1	1	Liters of alcohol consumed per capita	1000
Falls	Male	0-6 days	95+ years	Data Rich	1	1	Log-transformed SEV scalar: Falls	--
Falls	Male	0-6 days	95+ years	Data Rich	-1	2	Healthcare access and quality index	962
Falls	Male	0-6 days	95+ years	Data Rich	-1	2	Population-weighted mean temperature	--
Falls	Male	0-6 days	95+ years	Data Rich	-1	3	Socio-demographic Index	32
Falls	Male	0-6 days	95+ years	Data Rich	1	3	Elevation Over 1500m (proportion)	834
Falls	Male	0-6 days	95+ years	Global	-1	1	Socio-demographic Index	1000
Falls	Male	0-6 days	95+ years	Global	1	1	Liters of alcohol consumed per capita	464
Falls	Male	0-6 days	95+ years	Global	1	1	Log-transformed SEV scalar: Falls	--
Falls	Male	0-6 days	95+ years	Global	-1	2	Healthcare access and quality index	--
Falls	Male	0-6 days	95+ years	Global	-1	2	Population-weighted mean temperature	--
Falls	Male	0-6 days	95+ years	Global	1	3	Elevation Over 1500m (proportion)	534
Drowning	Female	0-6 days	95+ years	Data Rich	-1	1	Landlocked Nation (binary)	6
Drowning	Female	0-6 days	95+ years	Data Rich	-1	1	Maternal Education (years per capita)	1000
Drowning	Female	0-6 days	95+ years	Data Rich	1	1	Log-transformed SEV scalar: Drown	0
Drowning	Female	0-6 days	95+ years	Data Rich	1	1	Population-weighted mean temperature	1
Drowning	Female	0-6 days	95+ years	Data Rich	1	1	Coastal Population within 10km (proportion)	191
Drowning	Female	0-6 days	95+ years	Data Rich	-1	2	Rainfall Quintile 1 (proportion)	--
Drowning	Female	0-6 days	95+ years	Data Rich	1	2	Rainfall Quintile 5 (proportion)	0
Drowning	Female	0-6 days	95+ years	Data Rich	1	2	Elevation Under 100m (proportion)	159
Drowning	Female	0-6 days	95+ years	Data Rich	-1	3	Healthcare access and quality index	0
Drowning	Female	0-6 days	95+ years	Data Rich	-1	3	Socio-demographic Index	0
Drowning	Female	0-6 days	95+ years	Global	-1	1	Maternal Education (years per capita)	503
Drowning	Female	0-6 days	95+ years	Global	-1	1	Landlocked Nation (binary)	685
Drowning	Female	0-6 days	95+ years	Global	1	1	Population-weighted mean temperature	191
Drowning	Female	0-6 days	95+ years	Global	1	1	Coastal Population within 10km (proportion)	290
Drowning	Female	0-6 days	95+ years	Global	1	1	Log-transformed SEV scalar: Drown	--
Drowning	Female	0-6 days	95+ years	Global	-1	2	Rainfall Quintile 1 (proportion)	--
Drowning	Female	0-6 days	95+ years	Global	1	2	Elevation Under 100m (proportion)	167
Drowning	Female	0-6 days	95+ years	Global	1	2	Rainfall Quintile 5 (proportion)	350
Drowning	Female	0-6 days	95+ years	Global	-1	3	Socio-demographic Index	367
Drowning	Female	0-6 days	95+ years	Global	-1	3	Healthcare access and quality index	448
Drowning	Male	0-6 days	95+ years	Data Rich	-1	1	Landlocked Nation (binary)	703
Drowning	Male	0-6 days	95+ years	Data Rich	-1	1	Maternal Education (years per capita)	920
Drowning	Male	0-6 days	95+ years	Data Rich	1	1	Population-weighted mean temperature	279
Drowning	Male	0-6 days	95+ years	Data Rich	1	1	Coastal Population within 10km (proportion)	305
Drowning	Male	0-6 days	95+ years	Data Rich	1	1	Log-transformed SEV scalar: Drown	--
Drowning	Male	0-6 days	95+ years	Data Rich	-1	2	Rainfall Quintile 1 (proportion)	271
Drowning	Male	0-6 days	95+ years	Data Rich	1	2	Rainfall Quintile 5 (proportion)	1
Drowning	Male	0-6 days	95+ years	Data Rich	1	2	Elevation Under 100m (proportion)	13
Drowning	Male	0-6 days	95+ years	Data Rich	-1	3	Socio-demographic Index	21
Drowning	Male	0-6 days	95+ years	Data Rich	-1	3	Healthcare access and quality index	772
Drowning	Male	0-6 days	95+ years	Global	-1	1	Landlocked Nation (binary)	679
Drowning	Male	0-6 days	95+ years	Global	-1	1	Maternal Education (years per capita)	917
Drowning	Male	0-6 days	95+ years	Global	1	1	Population-weighted mean temperature	567
Drowning	Male	0-6 days	95+ years	Global	1	1	Coastal Population within 10km (proportion)	917
Drowning	Male	0-6 days	95+ years	Global	1	1	Log-transformed SEV scalar: Drown	--
Drowning	Male	0-6 days	95+ years	Global	-1	2	Rainfall Quintile 1 (proportion)	--
Drowning	Male	0-6 days	95+ years	Global	1	2	Rainfall Quintile 5 (proportion)	490
Drowning	Male	0-6 days	95+ years	Global	1	2	Elevation Under 100m (proportion)	--
Drowning	Male	0-6 days	95+ years	Global	-1	3	Healthcare access and quality index	509
Drowning	Male	0-6 days	95+ years	Global	-1	3	Socio-demographic Index	--
Fire, heat, and hot substances	Female	0-6 days	95+ years	Data Rich	1	1	Indoor Air Pollution (All Cooking Fuels)	--
Fire, heat, and hot substances	Female	0-6 days	95+ years	Data Rich	1	1	Log-transformed SEV scalar: Fire	--
Fire, heat, and hot substances	Female	0-6 days	95+ years	Data Rich	-1	2	Healthcare access and quality index	--
Fire, heat, and hot substances	Female	0-6 days	95+ years	Data Rich	1	2	Liters of alcohol consumed per capita	--
Fire, heat, and hot substances	Female	0-6 days	95+ years	Data Rich	1	2	Population Density (over 1000 ppl/sqkm, proportion)	--
Fire, heat, and hot substances	Female	0-6 days	95+ years	Data Rich	1	2	Population-weighted mean temperature	--
Fire, heat, and hot substances	Female	0-6 days	95+ years	Data Rich	1	2	Tobacco (cigarettes per capita)	--
Fire, heat, and hot substances	Female	0-6 days	95+ years	Data Rich	-1	3	Socio-demographic Index	1000
Fire, heat, and hot substances	Female	0-6 days	95+ years	Global	1	1	Indoor Air Pollution (All Cooking Fuels)	1000
Fire, heat, and hot substances	Female	0-6 days	95+ years	Global	1	1	Log-transformed SEV scalar: Fire	--
Fire, heat, and hot substances	Female	0-6 days	95+ years	Global	-1	2	Healthcare access and quality index	--
Fire, heat, and hot substances	Female	0-6 days	95+ years	Global	1	2	Tobacco (cigarettes per capita)	546
Fire, heat, and hot substances	Female	0-6 days	95+ years	Global	1	2	Liters of alcohol consumed per capita	--
Fire, heat, and hot substances	Female	0-6 days	95+ years	Global	1	2	Population Density (over 1000 ppl/sqkm, proportion)	--
Fire, heat, and hot substances	Female	0-6 days	95+ years	Global	1	2	Population-weighted mean temperature	--
Fire, heat, and hot substances	Female	0-6 days	95+ years	Global	-1	3	Socio-demographic Index	--
Fire, heat, and hot substances	Male	0-6 days	95+ years	Data Rich	1	1	Indoor Air Pollution (All Cooking Fuels)	1000
Fire, heat, and hot substances	Male	0-6 days	95+ years	Data Rich	1	1	Log-transformed SEV scalar: Fire	--
Fire, heat, and hot substances	Male	0-6 days	95+ years	Data Rich	-1	2	Healthcare access and quality index	--
Fire, heat, and hot substances	Male	0-6 days	95+ years	Data Rich	1	2	Liters of alcohol consumed per capita	--
Fire, heat, and hot substances	Male	0-6 days	95+ years	Data Rich	1	2	Population Density (over 1000 ppl/sqkm, proportion)	--
Fire, heat, and hot substances	Male	0-6 days	95+ years	Data Rich	1	2	Population-weighted mean temperature	--
Fire, heat, and hot substances	Male	0-6 days	95+ years	Data Rich	1	2	Tobacco (cigarettes per capita)	--
Fire, heat, and hot substances	Male	0-6 days	95+ years	Data Rich	-1	3	Socio-demographic Index	--
Fire, heat, and hot substances	Male	0-6 days	95+ years	Global	1	1	Indoor Air Pollution (All Cooking Fuels)	1000
Fire, heat, and hot substances	Male	0-6 days	95+ years	Global	1	1	Log-transformed SEV scalar: Fire	--
Fire, heat, and hot substances	Male	0-6 days	95+ years	Global	-1	2	Healthcare access and quality index	--
Fire, heat, and hot substances	Male	0-6 days	95+ years	Global	1	2	Population-weighted mean temperature	185
Fire, heat, and hot substances	Male	0-6 days	95+ years	Global	1	2	Tobacco (cigarettes per capita)	593
Fire, heat, and hot substances	Male	0-6 days	95+ years	Global	1	2	Liters of alcohol consumed per capita	--

Appendix Table S10: CODEm covariates used, level of covariate, and expected direction of covariate by cause, sex, and age

Cause	Sex	Age Start	Age End	Model Version Type	Direction	Level	Covariate Name	Number of Draws
Fire, heat, and hot substances	Male	0-6 days	95+ years	Global	1	2	Population Density (over 1000 ppl/sqkm, proportion)	--
Fire, heat, and hot substances	Male	0-6 days	95+ years	Global	-1	3	Socio-demographic Index	326
Poisonings	Female	0-6 days	95+ years	Data Rich	-1	1	Maternal Education (years per capita)	0
Poisonings	Female	0-6 days	95+ years	Data Rich	-1	1	Population-weighted mean temperature	1000
Poisonings	Female	0-6 days	95+ years	Data Rich	1	1	Proportion of population involved in agricultural activities	993
Poisonings	Female	0-6 days	95+ years	Data Rich	1	1	Log-transformed SEV scalar: Poison	--
Poisonings	Female	0-6 days	95+ years	Data Rich	-1	2	Socio-demographic Index	0
Poisonings	Female	0-6 days	95+ years	Data Rich	-1	2	Healthcare access and quality index	1000
Poisonings	Female	0-6 days	95+ years	Data Rich	1	2	Liters of alcohol consumed per capita	993
Poisonings	Female	0-6 days	95+ years	Data Rich	-1	3	Population Density (over 1000 ppl/sqkm, proportion)	802
Poisonings	Female	0-6 days	95+ years	Data Rich	1	3	Population Density (under 150 ppl/sqkm, proportion)	32
Poisonings	Female	0-6 days	95+ years	Global	-1	1	Maternal Education (years per capita)	403
Poisonings	Female	0-6 days	95+ years	Global	-1	1	Population-weighted mean temperature	597
Poisonings	Female	0-6 days	95+ years	Global	1	1	Proportion of population involved in agricultural activities	934
Poisonings	Female	0-6 days	95+ years	Global	1	1	Log-transformed SEV scalar: Poison	--
Poisonings	Female	0-6 days	95+ years	Global	-1	2	Healthcare access and quality index	362
Poisonings	Female	0-6 days	95+ years	Global	-1	2	Socio-demographic Index	--
Poisonings	Female	0-6 days	95+ years	Global	1	2	Liters of alcohol consumed per capita	189
Poisonings	Female	0-6 days	95+ years	Global	-1	3	Population Density (over 1000 ppl/sqkm, proportion)	197
Poisonings	Female	0-6 days	95+ years	Global	1	3	Population Density (under 150 ppl/sqkm, proportion)	119
Poisonings	Male	0-6 days	95+ years	Data Rich	-1	1	Population-weighted mean temperature	198
Poisonings	Male	0-6 days	95+ years	Data Rich	-1	1	Maternal Education (years per capita)	990
Poisonings	Male	0-6 days	95+ years	Data Rich	1	1	Proportion of population involved in agricultural activities	187
Poisonings	Male	0-6 days	95+ years	Data Rich	1	1	Log-transformed SEV scalar: Poison	--
Poisonings	Male	0-6 days	95+ years	Data Rich	-1	2	Socio-demographic Index	56
Poisonings	Male	0-6 days	95+ years	Data Rich	-1	2	Healthcare access and quality index	943
Poisonings	Male	0-6 days	95+ years	Data Rich	1	2	Liters of alcohol consumed per capita	1
Poisonings	Male	0-6 days	95+ years	Data Rich	-1	3	Population Density (over 1000 ppl/sqkm, proportion)	273
Poisonings	Male	0-6 days	95+ years	Data Rich	1	3	Population Density (under 150 ppl/sqkm, proportion)	125
Poisonings	Male	0-6 days	95+ years	Global	-1	1	Maternal Education (years per capita)	651
Poisonings	Male	0-6 days	95+ years	Global	-1	1	Population-weighted mean temperature	702
Poisonings	Male	0-6 days	95+ years	Global	1	1	Proportion of population involved in agricultural activities	244
Poisonings	Male	0-6 days	95+ years	Global	1	1	Log-transformed SEV scalar: Poison	--
Poisonings	Male	0-6 days	95+ years	Global	-1	2	Healthcare access and quality index	687
Poisonings	Male	0-6 days	95+ years	Global	-1	2	Socio-demographic Index	--
Poisonings	Male	0-6 days	95+ years	Global	1	2	Liters of alcohol consumed per capita	469
Poisonings	Male	0-6 days	95+ years	Global	-1	3	Population Density (over 1000 ppl/sqkm, proportion)	244
Poisonings	Male	0-6 days	95+ years	Global	1	3	Population Density (under 150 ppl/sqkm, proportion)	124
Poisoning by carbon monoxide	Female	0-6 days	95+ years	Data Rich	-1	1	Population-weighted mean temperature	--
Poisoning by carbon monoxide	Female	0-6 days	95+ years	Data Rich	1	1	Log-transformed SEV scalar: Inj Pois CO	--
Poisoning by carbon monoxide	Female	0-6 days	95+ years	Data Rich	-1	2	Maternal Education (years per capita)	--
Poisoning by carbon monoxide	Female	0-6 days	95+ years	Data Rich	1	2	Indoor Air Pollution (All Cooking Fuels)	33
Poisoning by carbon monoxide	Female	0-6 days	95+ years	Data Rich	1	2	Liters of alcohol consumed per capita	33
Poisoning by carbon monoxide	Female	0-6 days	95+ years	Data Rich	-1	3	Socio-demographic Index	160
Poisoning by carbon monoxide	Female	0-6 days	95+ years	Data Rich	-1	3	Healthcare access and quality index	801
Poisoning by carbon monoxide	Female	0-6 days	95+ years	Data Rich	1	3	Proportion of population involved in agricultural activities	38
Poisoning by carbon monoxide	Female	0-6 days	95+ years	Global	-1	1	Population-weighted mean temperature	--
Poisoning by carbon monoxide	Female	0-6 days	95+ years	Global	1	1	Log-transformed SEV scalar: Inj Pois CO	--
Poisoning by carbon monoxide	Female	0-6 days	95+ years	Global	-1	2	Maternal Education (years per capita)	--
Poisoning by carbon monoxide	Female	0-6 days	95+ years	Global	1	2	Indoor Air Pollution (All Cooking Fuels)	407
Poisoning by carbon monoxide	Female	0-6 days	95+ years	Global	1	2	Liters of alcohol consumed per capita	--
Poisoning by carbon monoxide	Female	0-6 days	95+ years	Global	-1	3	Socio-demographic Index	222
Poisoning by carbon monoxide	Female	0-6 days	95+ years	Global	-1	3	Healthcare access and quality index	326
Poisoning by carbon monoxide	Female	0-6 days	95+ years	Global	1	3	Proportion of population involved in agricultural activities	267
Poisoning by carbon monoxide	Male	0-6 days	95+ years	Data Rich	-1	1	Population-weighted mean temperature	1000
Poisoning by carbon monoxide	Male	0-6 days	95+ years	Data Rich	1	1	Log-transformed SEV scalar: Inj Pois CO	--
Poisoning by carbon monoxide	Male	0-6 days	95+ years	Data Rich	-1	2	Maternal Education (years per capita)	160
Poisoning by carbon monoxide	Male	0-6 days	95+ years	Data Rich	1	2	Indoor Air Pollution (All Cooking Fuels)	6
Poisoning by carbon monoxide	Male	0-6 days	95+ years	Data Rich	1	2	Liters of alcohol consumed per capita	--
Poisoning by carbon monoxide	Male	0-6 days	95+ years	Data Rich	-1	3	Socio-demographic Index	802
Poisoning by carbon monoxide	Male	0-6 days	95+ years	Data Rich	-1	3	Healthcare access and quality index	--
Poisoning by carbon monoxide	Male	0-6 days	95+ years	Data Rich	1	3	Proportion of population involved in agricultural activities	--
Poisoning by carbon monoxide	Male	0-6 days	95+ years	Global	-1	1	Population-weighted mean temperature	1000
Poisoning by carbon monoxide	Male	0-6 days	95+ years	Global	1	1	Log-transformed SEV scalar: Inj Pois CO	--
Poisoning by carbon monoxide	Male	0-6 days	95+ years	Global	-1	2	Maternal Education (years per capita)	269
Poisoning by carbon monoxide	Male	0-6 days	95+ years	Global	1	2	Indoor Air Pollution (All Cooking Fuels)	133
Poisoning by carbon monoxide	Male	0-6 days	95+ years	Global	1	2	Liters of alcohol consumed per capita	--
Poisoning by carbon monoxide	Male	0-6 days	95+ years	Global	-1	3	Socio-demographic Index	217
Poisoning by carbon monoxide	Male	0-6 days	95+ years	Global	-1	3	Healthcare access and quality index	--
Poisoning by carbon monoxide	Male	0-6 days	95+ years	Global	1	3	Proportion of population involved in agricultural activities	170
Poisoning by other means	Female	0-6 days	95+ years	Data Rich	-1	1	Maternal Education (years per capita)	1000
Poisoning by other means	Female	0-6 days	95+ years	Data Rich	1	1	Proportion of population involved in agricultural activities	962
Poisoning by other means	Female	0-6 days	95+ years	Data Rich	1	1	Log-transformed SEV scalar: Inj Pois Oth	--
Poisoning by other means	Female	0-6 days	95+ years	Data Rich	-1	2	Population-weighted mean temperature	967
Poisoning by other means	Female	0-6 days	95+ years	Data Rich	1	2	Liters of alcohol consumed per capita	967
Poisoning by other means	Female	0-6 days	95+ years	Data Rich	-1	3	Socio-demographic Index	32
Poisoning by other means	Female	0-6 days	95+ years	Data Rich	-1	3	Healthcare access and quality index	801
Poisoning by other means	Female	0-6 days	95+ years	Global	-1	1	Maternal Education (years per capita)	--
Poisoning by other means	Female	0-6 days	95+ years	Global	1	1	Log-transformed SEV scalar: Inj Pois Oth	--
Poisoning by other means	Female	0-6 days	95+ years	Global	1	1	Proportion of population involved in agricultural activities	--
Poisoning by other means	Female	0-6 days	95+ years	Global	-1	2	Population-weighted mean temperature	602
Poisoning by other means	Female	0-6 days	95+ years	Global	1	2	Liters of alcohol consumed per capita	--
Poisoning by other means	Female	0-6 days	95+ years	Global	-1	3	Healthcare access and quality index	398
Poisoning by other means	Female	0-6 days	95+ years	Global	-1	3	Socio-demographic Index	726
Poisoning by other means	Male	0-6 days	95+ years	Data Rich	-1	1	Maternal Education (years per capita)	166
Poisoning by other means	Male	0-6 days	95+ years	Data Rich	1	1	Proportion of population involved in agricultural activities	834
Poisoning by other means	Male	0-6 days	95+ years	Data Rich	1	1	Log-transformed SEV scalar: Inj Pois Oth	--
Poisoning by other means	Male	0-6 days	95+ years	Data Rich	-1	2	Population-weighted mean temperature	0
Poisoning by other means	Male	0-6 days	95+ years	Data Rich	1	2	Liters of alcohol consumed per capita	962

Appendix Table S10: CODEm covariates used, level of covariate, and expected direction of covariate by cause, sex, and age

Cause	Sex	Age Start	Age End	Model Version Type	Direction	Level	Covariate Name	Number of Draws
Poisoning by other means	Male	0-6 days	95+ years	Data Rich	-1	3	Healthcare access and quality index	1000
Poisoning by other means	Male	0-6 days	95+ years	Data Rich	-1	3	Socio-demographic Index	--
Poisoning by other means	Male	0-6 days	95+ years	Global	-1	1	Maternal Education (years per capita)	614
Poisoning by other means	Male	0-6 days	95+ years	Global	1	1	Proportion of population involved in agricultural activities	386
Poisoning by other means	Male	0-6 days	95+ years	Global	1	1	Log-transformed SEV scalar: Inj Pois Oth	--
Poisoning by other means	Male	0-6 days	95+ years	Global	-1	2	Population-weighted mean temperature	320
Poisoning by other means	Male	0-6 days	95+ years	Global	1	2	Liters of alcohol consumed per capita	401
Poisoning by other means	Male	0-6 days	95+ years	Global	-1	3	Healthcare access and quality index	528
Poisoning by other means	Male	0-6 days	95+ years	Global	-1	3	Socio-demographic Index	--
Exposure to mechanical forces	Female	0-6 days	95+ years	Data Rich	1	1	Population-weighted mean temperature	--
Exposure to mechanical forces	Female	0-6 days	95+ years	Data Rich	-1	2	Healthcare access and quality index	807
Exposure to mechanical forces	Female	0-6 days	95+ years	Data Rich	-1	2	Population Density (over 1000 ppl/sqkm, proportion)	--
Exposure to mechanical forces	Female	0-6 days	95+ years	Data Rich	1	2	Population Density (under 150 ppl/sqkm, proportion)	193
Exposure to mechanical forces	Female	0-6 days	95+ years	Data Rich	-1	3	Socio-demographic Index	161
Exposure to mechanical forces	Female	0-6 days	95+ years	Global	1	1	Population-weighted mean temperature	--
Exposure to mechanical forces	Female	0-6 days	95+ years	Global	-1	2	Healthcare access and quality index	--
Exposure to mechanical forces	Female	0-6 days	95+ years	Global	-1	2	Population Density (over 1000 ppl/sqkm, proportion)	--
Exposure to mechanical forces	Female	0-6 days	95+ years	Global	1	2	Population Density (under 150 ppl/sqkm, proportion)	602
Exposure to mechanical forces	Female	0-6 days	95+ years	Global	-1	3	Socio-demographic Index	726
Exposure to mechanical forces	Male	0-6 days	95+ years	Data Rich	1	1	Population-weighted mean temperature	--
Exposure to mechanical forces	Male	0-6 days	95+ years	Data Rich	-1	2	Healthcare access and quality index	968
Exposure to mechanical forces	Male	0-6 days	95+ years	Data Rich	-1	2	Population Density (over 1000 ppl/sqkm, proportion)	968
Exposure to mechanical forces	Male	0-6 days	95+ years	Data Rich	1	2	Population Density (under 150 ppl/sqkm, proportion)	968
Exposure to mechanical forces	Male	0-6 days	95+ years	Data Rich	-1	3	Socio-demographic Index	193
Exposure to mechanical forces	Male	0-6 days	95+ years	Global	1	1	Population-weighted mean temperature	--
Exposure to mechanical forces	Male	0-6 days	95+ years	Global	-1	2	Healthcare access and quality index	706
Exposure to mechanical forces	Male	0-6 days	95+ years	Global	-1	2	Population Density (under 150 ppl/sqkm, proportion)	--
Exposure to mechanical forces	Male	0-6 days	95+ years	Global	1	2	Population Density (over 1000 ppl/sqkm, proportion)	--
Exposure to mechanical forces	Male	0-6 days	95+ years	Global	-1	3	Socio-demographic Index	647
Unintentional firearm injuries	Female	0-6 days	95+ years	Data Rich	1	1	Population-weighted mean temperature	1000
Unintentional firearm injuries	Female	0-6 days	95+ years	Data Rich	1	1	Log-transformed SEV scalar: Mech Gun	--
Unintentional firearm injuries	Female	0-6 days	95+ years	Data Rich	-1	2	Healthcare access and quality index	106
Unintentional firearm injuries	Female	0-6 days	95+ years	Data Rich	-1	3	Population Density (over 1000 ppl/sqkm, proportion)	24
Unintentional firearm injuries	Female	0-6 days	95+ years	Data Rich	-1	3	Socio-demographic Index	745
Unintentional firearm injuries	Female	0-6 days	95+ years	Data Rich	1	3	Population Density (under 150 ppl/sqkm, proportion)	671
Unintentional firearm injuries	Female	0-6 days	95+ years	Global	1	1	Population-weighted mean temperature	713
Unintentional firearm injuries	Female	0-6 days	95+ years	Global	1	1	Log-transformed SEV scalar: Mech Gun	905
Unintentional firearm injuries	Female	0-6 days	95+ years	Global	-1	2	Healthcare access and quality index	390
Unintentional firearm injuries	Female	0-6 days	95+ years	Global	-1	3	Population Density (over 1000 ppl/sqkm, proportion)	222
Unintentional firearm injuries	Female	0-6 days	95+ years	Global	-1	3	Socio-demographic Index	483
Unintentional firearm injuries	Female	0-6 days	95+ years	Global	1	3	Population Density (under 150 ppl/sqkm, proportion)	239
Unintentional firearm injuries	Male	0-6 days	95+ years	Data Rich	1	1	Population-weighted mean temperature	1000
Unintentional firearm injuries	Male	0-6 days	95+ years	Data Rich	1	1	Log-transformed SEV scalar: Mech Gun	--
Unintentional firearm injuries	Male	0-6 days	95+ years	Data Rich	-1	2	Healthcare access and quality index	--
Unintentional firearm injuries	Male	0-6 days	95+ years	Data Rich	-1	3	Population Density (over 1000 ppl/sqkm, proportion)	807
Unintentional firearm injuries	Male	0-6 days	95+ years	Data Rich	-1	3	Socio-demographic Index	--
Unintentional firearm injuries	Male	0-6 days	95+ years	Data Rich	1	3	Population Density (under 150 ppl/sqkm, proportion)	161
Unintentional firearm injuries	Male	0-6 days	95+ years	Global	1	1	Log-transformed SEV scalar: Mech Gun	--
Unintentional firearm injuries	Male	0-6 days	95+ years	Global	1	1	Population-weighted mean temperature	--
Unintentional firearm injuries	Male	0-6 days	95+ years	Global	-1	2	Healthcare access and quality index	--
Unintentional firearm injuries	Male	0-6 days	95+ years	Global	-1	3	Population Density (over 1000 ppl/sqkm, proportion)	1000
Unintentional firearm injuries	Male	0-6 days	95+ years	Global	-1	3	Socio-demographic Index	1000
Unintentional firearm injuries	Male	0-6 days	95+ years	Global	1	3	Population Density (under 150 ppl/sqkm, proportion)	--
Other exposure to mechanical forces	Female	0-6 days	95+ years	Data Rich	1	1	Log-transformed SEV scalar: Oth Mech	--
Other exposure to mechanical forces	Female	0-6 days	95+ years	Data Rich	1	1	Population-weighted mean temperature	--
Other exposure to mechanical forces	Female	0-6 days	95+ years	Data Rich	-1	2	Healthcare access and quality index	--
Other exposure to mechanical forces	Female	0-6 days	95+ years	Data Rich	-1	2	Population Density (over 1000 ppl/sqkm, proportion)	--
Other exposure to mechanical forces	Female	0-6 days	95+ years	Data Rich	1	2	Population Density (under 150 ppl/sqkm, proportion)	--
Other exposure to mechanical forces	Female	0-6 days	95+ years	Data Rich	-1	3	Socio-demographic Index	1000
Other exposure to mechanical forces	Female	0-6 days	95+ years	Global	1	1	Log-transformed SEV scalar: Oth Mech	--
Other exposure to mechanical forces	Female	0-6 days	95+ years	Global	1	1	Population-weighted mean temperature	--
Other exposure to mechanical forces	Female	0-6 days	95+ years	Global	-1	2	Population Density (over 1000 ppl/sqkm, proportion)	326
Other exposure to mechanical forces	Female	0-6 days	95+ years	Global	-1	2	Healthcare access and quality index	790
Other exposure to mechanical forces	Female	0-6 days	95+ years	Global	1	2	Population Density (under 150 ppl/sqkm, proportion)	464
Other exposure to mechanical forces	Female	0-6 days	95+ years	Global	-1	3	Socio-demographic Index	422
Other exposure to mechanical forces	Male	0-6 days	95+ years	Data Rich	1	1	Log-transformed SEV scalar: Oth Mech	--
Other exposure to mechanical forces	Male	0-6 days	95+ years	Data Rich	1	1	Population-weighted mean temperature	--
Other exposure to mechanical forces	Male	0-6 days	95+ years	Data Rich	-1	2	Population Density (over 1000 ppl/sqkm, proportion)	193
Other exposure to mechanical forces	Male	0-6 days	95+ years	Data Rich	-1	2	Healthcare access and quality index	--
Other exposure to mechanical forces	Male	0-6 days	95+ years	Data Rich	1	2	Population Density (under 150 ppl/sqkm, proportion)	193
Other exposure to mechanical forces	Male	0-6 days	95+ years	Data Rich	-1	3	Socio-demographic Index	968
Other exposure to mechanical forces	Male	0-6 days	95+ years	Global	1	1	Log-transformed SEV scalar: Oth Mech	--
Other exposure to mechanical forces	Male	0-6 days	95+ years	Global	1	1	Population-weighted mean temperature	--
Other exposure to mechanical forces	Male	0-6 days	95+ years	Global	-1	2	Population Density (over 1000 ppl/sqkm, proportion)	778
Other exposure to mechanical forces	Male	0-6 days	95+ years	Global	-1	2	Healthcare access and quality index	815
Other exposure to mechanical forces	Male	0-6 days	95+ years	Global	1	2	Population Density (under 150 ppl/sqkm, proportion)	407
Other exposure to mechanical forces	Male	0-6 days	95+ years	Global	-1	3	Socio-demographic Index	284
Adverse effects of medical treatment	Female	0-6 days	95+ years	Data Rich	-1	1	Healthcare access and quality index	1000
Adverse effects of medical treatment	Female	0-6 days	95+ years	Data Rich	1	2	Population-weighted mean temperature	--
Adverse effects of medical treatment	Female	0-6 days	95+ years	Data Rich	-1	3	Socio-demographic Index	834
Adverse effects of medical treatment	Female	0-6 days	95+ years	Global	-1	1	Healthcare access and quality index	1000
Adverse effects of medical treatment	Female	0-6 days	95+ years	Global	1	2	Population-weighted mean temperature	627
Adverse effects of medical treatment	Female	0-6 days	95+ years	Global	-1	3	Socio-demographic Index	353
Adverse effects of medical treatment	Male	0-6 days	95+ years	Data Rich	-1	1	Healthcare access and quality index	--
Adverse effects of medical treatment	Male	0-6 days	95+ years	Data Rich	1	2	Population-weighted mean temperature	1000
Adverse effects of medical treatment	Male	0-6 days	95+ years	Data Rich	-1	3	Socio-demographic Index	--
Adverse effects of medical treatment	Male	0-6 days	95+ years	Global	1	1	Healthcare access and quality index	--
Adverse effects of medical treatment	Male	0-6 days	95+ years	Global	1	2	Population-weighted mean temperature	--
Adverse effects of medical treatment	Male	0-6 days	95+ years	Global	-1	3	Socio-demographic Index	1000
Animal contact	Female	0-6 days	95+ years	Data Rich	1	1	Liters of alcohol consumed per capita	--
Animal contact	Female	0-6 days	95+ years	Data Rich	1	1	Log-transformed SEV scalar: Animal	--
Animal contact	Female	0-6 days	95+ years	Data Rich	1	1	Population-weighted mean temperature	--
Animal contact	Female	0-6 days	95+ years	Data Rich	-1	2	Healthcare access and quality index	987
Animal contact	Female	0-6 days	95+ years	Data Rich	1	2	Population 15 to 30 (proportion)	2

Appendix Table S10: CODEm covariates used, level of covariate, and expected direction of covariate by cause, sex, and age

Cause	Sex	Age Start	Age End	Model Version Type	Direction	Level	Covariate Name	Number of Draws
Animal contact	Female	0-6 days	95+ years	Data Rich	-1	3	Population Density (over 1000 ppl/sqkm, proportion)	0
Animal contact	Female	0-6 days	95+ years	Data Rich	-1	3	Socio-demographic Index	754
Animal contact	Female	0-6 days	95+ years	Data Rich	-1	3	Elevation Over 1500m (proportion)	--
Animal contact	Female	0-6 days	95+ years	Data Rich	1	3	Population Density (under 150 ppl/sqkm, proportion)	59
Animal contact	Female	0-6 days	95+ years	Data Rich	1	3	Elevation Under 100m (proportion)	--
Animal contact	Female	0-6 days	95+ years	Global	1	1	Population-weighted mean temperature	1000
Animal contact	Female	0-6 days	95+ years	Global	1	1	Liters of alcohol consumed per capita	--
Animal contact	Female	0-6 days	95+ years	Global	1	1	Log-transformed SEV scalar: Animal	--
Animal contact	Female	0-6 days	95+ years	Global	-1	2	Healthcare access and quality index	548
Animal contact	Female	0-6 days	95+ years	Global	1	2	Population 15 to 30 (proportion)	--
Animal contact	Female	0-6 days	95+ years	Global	-1	3	Socio-demographic Index	267
Animal contact	Female	0-6 days	95+ years	Global	-1	3	Population Density (over 1000 ppl/sqkm, proportion)	593
Animal contact	Female	0-6 days	95+ years	Global	-1	3	Elevation Over 1500m (proportion)	--
Animal contact	Female	0-6 days	95+ years	Global	1	3	Population Density (under 150 ppl/sqkm, proportion)	326
Animal contact	Female	0-6 days	95+ years	Global	1	3	Elevation Under 100m (proportion)	--
Animal contact	Male	0-6 days	95+ years	Data Rich	1	1	Liters of alcohol consumed per capita	--
Animal contact	Male	0-6 days	95+ years	Data Rich	1	1	Log-transformed SEV scalar: Animal	--
Animal contact	Male	0-6 days	95+ years	Data Rich	1	1	Population-weighted mean temperature	--
Animal contact	Male	0-6 days	95+ years	Data Rich	-1	2	Healthcare access and quality index	609
Animal contact	Male	0-6 days	95+ years	Data Rich	1	2	Population 15 to 30 (proportion)	149
Animal contact	Male	0-6 days	95+ years	Data Rich	-1	3	Population Density (over 1000 ppl/sqkm, proportion)	38
Animal contact	Male	0-6 days	95+ years	Data Rich	-1	3	Socio-demographic Index	242
Animal contact	Male	0-6 days	95+ years	Data Rich	-1	3	Elevation Over 1500m (proportion)	--
Animal contact	Male	0-6 days	95+ years	Data Rich	1	3	Population Density (under 150 ppl/sqkm, proportion)	338
Animal contact	Male	0-6 days	95+ years	Data Rich	1	3	Elevation Under 100m (proportion)	--
Animal contact	Male	0-6 days	95+ years	Global	1	1	Log-transformed SEV scalar: Animal	294
Animal contact	Male	0-6 days	95+ years	Global	1	1	Population-weighted mean temperature	706
Animal contact	Male	0-6 days	95+ years	Global	1	1	Liters of alcohol consumed per capita	--
Animal contact	Male	0-6 days	95+ years	Global	-1	2	Healthcare access and quality index	409
Animal contact	Male	0-6 days	95+ years	Global	1	2	Population 15 to 30 (proportion)	222
Animal contact	Male	0-6 days	95+ years	Global	-1	3	Socio-demographic Index	273
Animal contact	Male	0-6 days	95+ years	Global	-1	3	Population Density (over 1000 ppl/sqkm, proportion)	288
Animal contact	Male	0-6 days	95+ years	Global	-1	3	Elevation Over 1500m (proportion)	--
Animal contact	Male	0-6 days	95+ years	Global	1	3	Population Density (under 150 ppl/sqkm, proportion)	502
Animal contact	Male	0-6 days	95+ years	Global	1	3	Elevation Under 100m (proportion)	--
Venomous animal contact	Female	0-6 days	95+ years	Data Rich	1	1	Proportion of population vulnerable to venomous snakebites	1000
Venomous animal contact	Female	0-6 days	95+ years	Data Rich	1	1	Log-transformed SEV scalar: Venom	--
Venomous animal contact	Female	0-6 days	95+ years	Data Rich	1	1	Mean number of venomous snake species	--
Venomous animal contact	Female	0-6 days	95+ years	Data Rich	-1	2	Absolute value of average latitude	38
Venomous animal contact	Female	0-6 days	95+ years	Data Rich	-1	2	Healthcare access and quality index	38
Venomous animal contact	Female	0-6 days	95+ years	Data Rich	1	2	Population-weighted mean temperature	261
Venomous animal contact	Female	0-6 days	95+ years	Data Rich	1	2	Liters of alcohol consumed per capita	--
Venomous animal contact	Female	0-6 days	95+ years	Data Rich	1	2	Proportion of population involved in agricultural activities	--
Venomous animal contact	Female	0-6 days	95+ years	Data Rich	1	2	Rainfall Population-Weighted (mm/yr)	--
Venomous animal contact	Female	0-6 days	95+ years	Data Rich	1	2	Sahel Region of Africa (binary)	--
Venomous animal contact	Female	0-6 days	95+ years	Data Rich	-1	3	Elevation Over 1500m (proportion)	605
Venomous animal contact	Female	0-6 days	95+ years	Data Rich	-1	3	Elevation Under 100m (proportion)	701
Venomous animal contact	Female	0-6 days	95+ years	Data Rich	-1	3	Socio-demographic Index	714
Venomous animal contact	Female	0-6 days	95+ years	Data Rich	-1	3	Population Density (over 1000 ppl/sqkm, proportion)	881
Venomous animal contact	Female	0-6 days	95+ years	Data Rich	1	3	Population Density (under 150 ppl/sqkm, proportion)	104
Venomous animal contact	Female	0-6 days	95+ years	Global	1	1	Mean number of venomous snake species	413
Venomous animal contact	Female	0-6 days	95+ years	Global	1	1	Log-transformed SEV scalar: Venom	586
Venomous animal contact	Female	0-6 days	95+ years	Global	1	1	Proportion of population vulnerable to venomous snakebites	1000
Venomous animal contact	Female	0-6 days	95+ years	Global	-1	2	Healthcare access and quality index	690
Venomous animal contact	Female	0-6 days	95+ years	Global	-1	2	Absolute value of average latitude	--
Venomous animal contact	Female	0-6 days	95+ years	Global	1	2	Proportion of population involved in agricultural activities	174
Venomous animal contact	Female	0-6 days	95+ years	Global	1	2	Rainfall Population-Weighted (mm/yr)	241
Venomous animal contact	Female	0-6 days	95+ years	Global	1	2	Population-weighted mean temperature	511
Venomous animal contact	Female	0-6 days	95+ years	Global	1	2	Liters of alcohol consumed per capita	--
Venomous animal contact	Female	0-6 days	95+ years	Global	1	2	Sahel Region of Africa (binary)	--
Venomous animal contact	Female	0-6 days	95+ years	Global	-1	3	Elevation Under 100m (proportion)	168
Venomous animal contact	Female	0-6 days	95+ years	Global	-1	3	Elevation Over 1500m (proportion)	185
Venomous animal contact	Female	0-6 days	95+ years	Global	-1	3	Population Density (over 1000 ppl/sqkm, proportion)	335
Venomous animal contact	Female	0-6 days	95+ years	Global	-1	3	Socio-demographic Index	356
Venomous animal contact	Female	0-6 days	95+ years	Global	1	3	Population Density (under 150 ppl/sqkm, proportion)	281
Venomous animal contact	Male	0-6 days	95+ years	Data Rich	1	1	Mean number of venomous snake species	366
Venomous animal contact	Male	0-6 days	95+ years	Data Rich	1	1	Log-transformed SEV scalar: Venom	1000
Venomous animal contact	Male	0-6 days	95+ years	Data Rich	1	1	Proportion of population vulnerable to venomous snakebites	--
Venomous animal contact	Male	0-6 days	95+ years	Data Rich	-1	2	Healthcare access and quality index	864
Venomous animal contact	Male	0-6 days	95+ years	Data Rich	-1	2	Absolute value of average latitude	--
Venomous animal contact	Male	0-6 days	95+ years	Data Rich	1	2	Rainfall Population-Weighted (mm/yr)	270
Venomous animal contact	Male	0-6 days	95+ years	Data Rich	1	2	Liters of alcohol consumed per capita	--
Venomous animal contact	Male	0-6 days	95+ years	Data Rich	1	2	Population-weighted mean temperature	--
Venomous animal contact	Male	0-6 days	95+ years	Data Rich	1	2	Proportion of population involved in agricultural activities	--
Venomous animal contact	Male	0-6 days	95+ years	Data Rich	1	2	Sahel Region of Africa (binary)	--
Venomous animal contact	Male	0-6 days	95+ years	Data Rich	-1	3	Elevation Over 1500m (proportion)	217
Venomous animal contact	Male	0-6 days	95+ years	Data Rich	-1	3	Elevation Under 100m (proportion)	234
Venomous animal contact	Male	0-6 days	95+ years	Data Rich	-1	3	Population Density (over 1000 ppl/sqkm, proportion)	589
Venomous animal contact	Male	0-6 days	95+ years	Data Rich	-1	3	Socio-demographic Index	--
Venomous animal contact	Male	0-6 days	95+ years	Data Rich	1	3	Population Density (under 150 ppl/sqkm, proportion)	67
Venomous animal contact	Male	0-6 days	95+ years	Global	1	1	Proportion of population vulnerable to venomous snakebites	417
Venomous animal contact	Male	0-6 days	95+ years	Global	1	1	Log-transformed SEV scalar: Venom	518
Venomous animal contact	Male	0-6 days	95+ years	Global	1	1	Mean number of venomous snake species	826
Venomous animal contact	Male	0-6 days	95+ years	Global	-1	2	Absolute value of average latitude	68
Venomous animal contact	Male	0-6 days	95+ years	Global	-1	2	Healthcare access and quality index	521
Venomous animal contact	Male	0-6 days	95+ years	Global	1	2	Population-weighted mean temperature	96
Venomous animal contact	Male	0-6 days	95+ years	Global	1	2	Rainfall Population-Weighted (mm/yr)	199
Venomous animal contact	Male	0-6 days	95+ years	Global	1	2	Proportion of population involved in agricultural activities	447
Venomous animal contact	Male	0-6 days	95+ years	Global	1	2	Liters of alcohol consumed per capita	--
Venomous animal contact	Male	0-6 days	95+ years	Global	-1	3	Elevation Over 1500m (proportion)	155
Venomous animal contact	Male	0-6 days	95+ years	Global	-1	3	Elevation Under 100m (proportion)	308
Venomous animal contact	Male	0-6 days	95+ years	Global	-1	3	Population Density (over 1000 ppl/sqkm, proportion)	361
Venomous animal contact	Male	0-6 days	95+ years	Global	-1	3	Socio-demographic Index	390
Venomous animal contact	Male	0-6 days	95+ years	Global	1	3	Population Density (under 150 ppl/sqkm, proportion)	280
Non-venomous animal contact	Female	0-6 days	95+ years	Data Rich	1	1	Liters of alcohol consumed per capita	--
Non-venomous animal contact	Female	0-6 days	95+ years	Data Rich	1	1	Log-transformed SEV scalar: Non Ven	--
Non-venomous animal contact	Female	0-6 days	95+ years	Data Rich	1	1	Population-weighted mean temperature	--

Appendix Table S10: CODEm covariates used, level of covariate, and expected direction of covariate by cause, sex, and age

Cause	Sex	Age Start	Age End	Model Version Type	Direction	Level	Covariate Name	Number of Draws
Non-venomous animal contact	Female	0-6 days	95+ years	Data Rich	-1	2	Healthcare access and quality index	--
Non-venomous animal contact	Female	0-6 days	95+ years	Data Rich	-1	3	Elevation Over 1500m (proportion)	--
Non-venomous animal contact	Female	0-6 days	95+ years	Data Rich	-1	3	Population Density (over 1000 ppl/sqkm, proportion)	--
Non-venomous animal contact	Female	0-6 days	95+ years	Data Rich	-1	3	Socio-demographic Index	--
Non-venomous animal contact	Female	0-6 days	95+ years	Data Rich	1	3	Population Density (under 150 ppl/sqkm, proportion)	1000
Non-venomous animal contact	Female	0-6 days	95+ years	Data Rich	1	3	Elevation Under 100m (proportion)	--
Non-venomous animal contact	Female	0-6 days	95+ years	Global	1	1	Log-transformed SEV scalar: Non Ven	1000
Non-venomous animal contact	Female	0-6 days	95+ years	Global	1	1	Liters of alcohol consumed per capita	--
Non-venomous animal contact	Female	0-6 days	95+ years	Global	1	1	Population-weighted mean temperature	--
Non-venomous animal contact	Female	0-6 days	95+ years	Global	-1	2	Healthcare access and quality index	481
Non-venomous animal contact	Female	0-6 days	95+ years	Global	-1	3	Population Density (over 1000 ppl/sqkm, proportion)	282
Non-venomous animal contact	Female	0-6 days	95+ years	Global	-1	3	Elevation Over 1500m (proportion)	--
Non-venomous animal contact	Female	0-6 days	95+ years	Global	-1	3	Socio-demographic Index	--
Non-venomous animal contact	Female	0-6 days	95+ years	Global	1	3	Population Density (under 150 ppl/sqkm, proportion)	566
Non-venomous animal contact	Female	0-6 days	95+ years	Global	1	3	Elevation Under 100m (proportion)	--
Non-venomous animal contact	Male	0-6 days	95+ years	Data Rich	1	1	Liters of alcohol consumed per capita	--
Non-venomous animal contact	Male	0-6 days	95+ years	Data Rich	1	1	Log-transformed SEV scalar: Non Ven	--
Non-venomous animal contact	Male	0-6 days	95+ years	Data Rich	1	1	Population-weighted mean temperature	--
Non-venomous animal contact	Male	0-6 days	95+ years	Data Rich	-1	2	Healthcare access and quality index	819
Non-venomous animal contact	Male	0-6 days	95+ years	Data Rich	-1	3	Population Density (over 1000 ppl/sqkm, proportion)	181
Non-venomous animal contact	Male	0-6 days	95+ years	Data Rich	-1	3	Elevation Over 1500m (proportion)	--
Non-venomous animal contact	Male	0-6 days	95+ years	Data Rich	-1	3	Socio-demographic Index	--
Non-venomous animal contact	Male	0-6 days	95+ years	Data Rich	1	3	Elevation Under 100m (proportion)	--
Non-venomous animal contact	Male	0-6 days	95+ years	Data Rich	1	3	Population Density (under 150 ppl/sqkm, proportion)	--
Non-venomous animal contact	Male	0-6 days	95+ years	Global	-1	1	Population Density (over 1000 ppl/sqkm, proportion)	767
Non-venomous animal contact	Male	0-6 days	95+ years	Global	-1	1	Elevation Over 1500m (proportion)	--
Non-venomous animal contact	Male	0-6 days	95+ years	Global	1	1	Population-weighted mean temperature	131
Non-venomous animal contact	Male	0-6 days	95+ years	Global	1	1	Log-transformed SEV scalar: Non Ven	528
Non-venomous animal contact	Male	0-6 days	95+ years	Global	1	1	Population Density (under 150 ppl/sqkm, proportion)	636
Non-venomous animal contact	Male	0-6 days	95+ years	Global	1	1	Elevation Under 100m (proportion)	--
Non-venomous animal contact	Male	0-6 days	95+ years	Global	1	1	Liters of alcohol consumed per capita	--
Non-venomous animal contact	Male	0-6 days	95+ years	Global	-1	3	Healthcare access and quality index	267
Non-venomous animal contact	Male	0-6 days	95+ years	Global	-1	3	Socio-demographic Index	561
Foreign body	Female	0-6 days	95+ years	Data Rich	1	1	Indoor Air Pollution (All Cooking Fuels)	--
Foreign body	Female	0-6 days	95+ years	Data Rich	1	1	Liters of alcohol consumed per capita	--
Foreign body	Female	0-6 days	95+ years	Data Rich	1	1	Population Over 65 (proportion)	--
Foreign body	Female	0-6 days	95+ years	Data Rich	1	1	Population-weighted mean temperature	--
Foreign body	Female	0-6 days	95+ years	Data Rich	-1	2	Healthcare access and quality index	1000
Foreign body	Female	0-6 days	95+ years	Data Rich	-1	3	Socio-demographic Index	--
Foreign body	Female	0-6 days	95+ years	Global	1	1	Population-weighted mean temperature	454
Foreign body	Female	0-6 days	95+ years	Global	1	1	Indoor Air Pollution (All Cooking Fuels)	1000
Foreign body	Female	0-6 days	95+ years	Global	1	1	Liters of alcohol consumed per capita	--
Foreign body	Female	0-6 days	95+ years	Global	1	1	Population Over 65 (proportion)	--
Foreign body	Female	0-6 days	95+ years	Global	-1	2	Healthcare access and quality index	--
Foreign body	Female	0-6 days	95+ years	Global	-1	3	Socio-demographic Index	--
Foreign body	Male	0-6 days	95+ years	Data Rich	1	1	Indoor Air Pollution (All Cooking Fuels)	--
Foreign body	Male	0-6 days	95+ years	Data Rich	1	1	Liters of alcohol consumed per capita	--
Foreign body	Male	0-6 days	95+ years	Data Rich	1	1	Population Over 65 (proportion)	--
Foreign body	Male	0-6 days	95+ years	Data Rich	1	1	Population-weighted mean temperature	--
Foreign body	Male	0-6 days	95+ years	Data Rich	-1	2	Healthcare access and quality index	1000
Foreign body	Male	0-6 days	95+ years	Data Rich	-1	3	Socio-demographic Index	--
Foreign body	Male	0-6 days	95+ years	Global	1	1	Indoor Air Pollution (All Cooking Fuels)	1000
Foreign body	Male	0-6 days	95+ years	Global	1	1	Liters of alcohol consumed per capita	--
Foreign body	Male	0-6 days	95+ years	Global	1	1	Population Over 65 (proportion)	--
Foreign body	Male	0-6 days	95+ years	Global	1	1	Population-weighted mean temperature	--
Foreign body	Male	0-6 days	95+ years	Global	-1	2	Healthcare access and quality index	--
Foreign body	Male	0-6 days	95+ years	Global	-1	3	Socio-demographic Index	546
Pulmonary aspiration and foreign body in airway	Female	0-6 days	95+ years	Data Rich	1	1	Liters of alcohol consumed per capita	1000
Pulmonary aspiration and foreign body in airway	Female	0-6 days	95+ years	Data Rich	1	1	Log-transformed SEV scalar: F Body Asp	--
Pulmonary aspiration and foreign body in airway	Female	0-6 days	95+ years	Data Rich	1	1	Population-weighted mean temperature	--
Pulmonary aspiration and foreign body in airway	Female	0-6 days	95+ years	Data Rich	-1	2	Healthcare access and quality index	166
Pulmonary aspiration and foreign body in airway	Female	0-6 days	95+ years	Data Rich	1	2	Mean BMI	166
Pulmonary aspiration and foreign body in airway	Female	0-6 days	95+ years	Data Rich	-1	3	Socio-demographic Index	--
Pulmonary aspiration and foreign body in airway	Female	0-6 days	95+ years	Global	1	1	Liters of alcohol consumed per capita	--
Pulmonary aspiration and foreign body in airway	Female	0-6 days	95+ years	Global	1	1	Log-transformed SEV scalar: F Body Asp	--
Pulmonary aspiration and foreign body in airway	Female	0-6 days	95+ years	Global	1	1	Population-weighted mean temperature	--
Pulmonary aspiration and foreign body in airway	Female	0-6 days	95+ years	Global	-1	2	Healthcare access and quality index	1000
Pulmonary aspiration and foreign body in airway	Female	0-6 days	95+ years	Global	1	2	Mean BMI	1000
Pulmonary aspiration and foreign body in airway	Female	0-6 days	95+ years	Global	1	2	Alcohol binge drinker proportion, age-standardized	--
Pulmonary aspiration and foreign body in airway	Female	0-6 days	95+ years	Global	-1	3	Socio-demographic Index	--
Pulmonary aspiration and foreign body in airway	Male	0-6 days	95+ years	Data Rich	1	1	Liters of alcohol consumed per capita	1000
Pulmonary aspiration and foreign body in airway	Male	0-6 days	95+ years	Data Rich	1	1	Log-transformed SEV scalar: F Body Asp	--
Pulmonary aspiration and foreign body in airway	Male	0-6 days	95+ years	Data Rich	1	1	Population-weighted mean temperature	--
Pulmonary aspiration and foreign body in airway	Male	0-6 days	95+ years	Data Rich	-1	2	Healthcare access and quality index	--
Pulmonary aspiration and foreign body in airway	Male	0-6 days	95+ years	Data Rich	1	2	Mean BMI	839
Pulmonary aspiration and foreign body in airway	Male	0-6 days	95+ years	Data Rich	-1	3	Socio-demographic Index	32
Pulmonary aspiration and foreign body in airway	Male	0-6 days	95+ years	Global	1	1	Liters of alcohol consumed per capita	--
Pulmonary aspiration and foreign body in airway	Male	0-6 days	95+ years	Global	1	1	Log-transformed SEV scalar: F Body Asp	--
Pulmonary aspiration and foreign body in airway	Male	0-6 days	95+ years	Global	1	1	Population-weighted mean temperature	--
Pulmonary aspiration and foreign body in airway	Male	0-6 days	95+ years	Global	-1	2	Healthcare access and quality index	491
Pulmonary aspiration and foreign body in airway	Male	0-6 days	95+ years	Global	1	2	Mean BMI	--
Pulmonary aspiration and foreign body in airway	Male	0-6 days	95+ years	Global	-1	3	Socio-demographic Index	509
Foreign body in other body part	Female	0-6 days	95+ years	Data Rich	1	1	Population-weighted mean temperature	1000
Foreign body in other body part	Female	0-6 days	95+ years	Data Rich	1	1	Liters of alcohol consumed per capita	--
Foreign body in other body part	Female	0-6 days	95+ years	Data Rich	1	1	Log-transformed SEV scalar: Oth F Body	--
Foreign body in other body part	Female	0-6 days	95+ years	Data Rich	-1	2	Healthcare access and quality index	161
Foreign body in other body part	Female	0-6 days	95+ years	Data Rich	-1	3	Socio-demographic Index	807
Foreign body in other body part	Female	0-6 days	95+ years	Global	1	1	Population-weighted mean temperature	326
Foreign body in other body part	Female	0-6 days	95+ years	Global	1	1	Log-transformed SEV scalar: Oth F Body	1000
Foreign body in other body part	Female	0-6 days	95+ years	Global	1	1	Liters of alcohol consumed per capita	--
Foreign body in other body part	Female	0-6 days	95+ years	Global	-1	2	Healthcare access and quality index	269

Appendix Table S10: CODEm covariates used, level of covariate, and expected direction of covariate by cause, sex, and age

Cause	Sex	Age Start	Age End	Model Version Type	Direction	Level	Covariate Name	Number of Draws
Foreign body in other body part	Female	0-6 days	95+ years	Global	-1	3	Socio-demographic Index	437
Foreign body in other body part	Male	0-6 days	95+ years	Data Rich	1	1	Liters of alcohol consumed per capita	1000
Foreign body in other body part	Male	0-6 days	95+ years	Data Rich	1	1	Population-weighted mean temperature	1000
Foreign body in other body part	Male	0-6 days	95+ years	Data Rich	1	1	Log-transformed SEV scalar: Oth F Body	--
Foreign body in other body part	Male	0-6 days	95+ years	Data Rich	-1	2	Healthcare access and quality index	--
Foreign body in other body part	Male	0-6 days	95+ years	Data Rich	-1	3	Socio-demographic Index	--
Foreign body in other body part	Male	0-6 days	95+ years	Global	1	1	Liters of alcohol consumed per capita	--
Foreign body in other body part	Male	0-6 days	95+ years	Global	1	1	Log-transformed SEV scalar: Oth F Body	--
Foreign body in other body part	Male	0-6 days	95+ years	Global	1	1	Population-weighted mean temperature	--
Foreign body in other body part	Male	0-6 days	95+ years	Global	-1	2	Healthcare access and quality index	681
Foreign body in other body part	Male	0-6 days	95+ years	Global	-1	3	Socio-demographic Index	672
Electrocution	Female	0-6 days	95+ years	Data Rich	1	1	Log-transformed SEV scalar: Oth Unint	--
Electrocution	Female	0-6 days	95+ years	Data Rich	1	1	Vehicles - 2 wheels (per capita)	--
Electrocution	Female	0-6 days	95+ years	Data Rich	1	1	Vehicles - 4 wheels (per capita)	--
Electrocution	Female	0-6 days	95+ years	Data Rich	-1	2	Healthcare access and quality index	--
Electrocution	Female	0-6 days	95+ years	Data Rich	-1	3	Socio-demographic Index	546
Electrocution	Female	0-6 days	95+ years	Data Rich	-1	3	Population Density (over 1000 ppl/sqkm, proportion)	--
Electrocution	Female	0-6 days	95+ years	Data Rich	1	3	Population Density (under 150 ppl/sqkm, proportion)	454
Electrocution	Female	0-6 days	95+ years	Global	1	1	Log-transformed SEV scalar: Oth Unint	--
Electrocution	Female	0-6 days	95+ years	Global	1	1	Vehicles - 2 wheels (per capita)	--
Electrocution	Female	0-6 days	95+ years	Global	1	1	Vehicles - 4 wheels (per capita)	--
Electrocution	Female	0-6 days	95+ years	Global	-1	2	Healthcare access and quality index	--
Electrocution	Female	0-6 days	95+ years	Global	-1	3	Population Density (over 1000 ppl/sqkm, proportion)	454
Electrocution	Female	0-6 days	95+ years	Global	-1	3	Socio-demographic Index	546
Electrocution	Female	0-6 days	95+ years	Global	1	3	Population Density (under 150 ppl/sqkm, proportion)	--
Electrocution	Male	0-6 days	95+ years	Data Rich	1	1	Log-transformed SEV scalar: Oth Unint	--
Electrocution	Male	0-6 days	95+ years	Data Rich	-1	2	Healthcare access and quality index	--
Electrocution	Male	0-6 days	95+ years	Data Rich	-1	3	Socio-demographic Index	546
Electrocution	Male	0-6 days	95+ years	Data Rich	-1	3	Population Density (over 1000 ppl/sqkm, proportion)	--
Electrocution	Male	0-6 days	95+ years	Data Rich	1	3	Population Density (under 150 ppl/sqkm, proportion)	454
Electrocution	Male	0-6 days	95+ years	Global	1	1	Log-transformed SEV scalar: Oth Unint	--
Electrocution	Male	0-6 days	95+ years	Global	-1	2	Healthcare access and quality index	726
Electrocution	Male	0-6 days	95+ years	Global	-1	3	Population Density (over 1000 ppl/sqkm, proportion)	328
Electrocution	Male	0-6 days	95+ years	Global	-1	3	Socio-demographic Index	--
Electrocution	Male	0-6 days	95+ years	Global	1	3	Population Density (under 150 ppl/sqkm, proportion)	274
Environmental heat and cold exposure	Female	0-6 days	95+ years	Data Rich	-1	1	Healthcare access and quality index	1000
Environmental heat and cold exposure	Female	0-6 days	95+ years	Data Rich	-1	2	Sanitation (proportion with access)	--
Environmental heat and cold exposure	Female	0-6 days	95+ years	Data Rich	1	2	Liters of alcohol consumed per capita	--
Environmental heat and cold exposure	Female	0-6 days	95+ years	Data Rich	-1	3	Education (years per capita)	834
Environmental heat and cold exposure	Female	0-6 days	95+ years	Data Rich	-1	3	90th percentile climatic temperature in the given country-year.	--
Environmental heat and cold exposure	Female	0-6 days	95+ years	Data Rich	-1	3	LDI (I$ per capita)	--
Environmental heat and cold exposure	Female	0-6 days	95+ years	Data Rich	-1	3	Population Density (150-300 ppl/sqkm, proportion)	--
Environmental heat and cold exposure	Female	0-6 days	95+ years	Data Rich	-1	3	Socio-demographic Index	--
Environmental heat and cold exposure	Female	0-6 days	95+ years	Data Rich	1	3	Elevation Over 1500m (proportion)	834
Environmental heat and cold exposure	Female	0-6 days	95+ years	Data Rich	1	3	Population-weighted mean temperature	--
Environmental heat and cold exposure	Female	0-6 days	95+ years	Data Rich	1	3	Rainfall (Quintiles 4-5)	--
Environmental heat and cold exposure	Female	0-6 days	95+ years	Global	1	1	Liters of alcohol consumed per capita	--
Environmental heat and cold exposure	Female	0-6 days	95+ years	Global	-1	2	Healthcare access and quality index	551
Environmental heat and cold exposure	Female	0-6 days	95+ years	Global	-1	3	LDI (I$ per capita)	173
Environmental heat and cold exposure	Female	0-6 days	95+ years	Global	-1	3	Socio-demographic Index	229
Environmental heat and cold exposure	Female	0-6 days	95+ years	Global	-1	3	Education (years per capita)	338
Environmental heat and cold exposure	Female	0-6 days	95+ years	Global	-1	3	Sanitation (proportion with access)	740
Environmental heat and cold exposure	Female	0-6 days	95+ years	Global	-1	3	90th percentile climatic temperature in the given country-year.	--
Environmental heat and cold exposure	Female	0-6 days	95+ years	Global	-1	3	Population Density (150-300 ppl/sqkm, proportion)	--
Environmental heat and cold exposure	Female	0-6 days	95+ years	Global	1	3	Elevation Over 1500m (proportion)	100
Environmental heat and cold exposure	Female	0-6 days	95+ years	Global	1	3	Population-weighted mean temperature	--
Environmental heat and cold exposure	Female	0-6 days	95+ years	Global	1	3	Rainfall (Quintiles 4-5)	--
Environmental heat and cold exposure	Male	0-6 days	95+ years	Data Rich	1	1	Liters of alcohol consumed per capita	--
Environmental heat and cold exposure	Male	0-6 days	95+ years	Data Rich	-1	2	90th percentile climatic temperature in the given country-year.	--
Environmental heat and cold exposure	Male	0-6 days	95+ years	Data Rich	-1	2	Healthcare access and quality index	--
Environmental heat and cold exposure	Male	0-6 days	95+ years	Data Rich	-1	3	Socio-demographic Index	1000
Environmental heat and cold exposure	Male	0-6 days	95+ years	Data Rich	-1	3	Population Density (150-300 ppl/sqkm, proportion)	--
Environmental heat and cold exposure	Male	0-6 days	95+ years	Data Rich	-1	3	Sanitation (proportion with access)	--
Environmental heat and cold exposure	Male	0-6 days	95+ years	Data Rich	1	3	Elevation Over 1500m (proportion)	--
Environmental heat and cold exposure	Male	0-6 days	95+ years	Data Rich	1	3	Population-weighted mean temperature	--
Environmental heat and cold exposure	Male	0-6 days	95+ years	Data Rich	1	3	Rainfall (Quintiles 4-5)	--
Environmental heat and cold exposure	Male	0-6 days	95+ years	Global	1	1	Liters of alcohol consumed per capita	--
Environmental heat and cold exposure	Male	0-6 days	95+ years	Global	-1	2	90th percentile climatic temperature in the given country-year.	--
Environmental heat and cold exposure	Male	0-6 days	95+ years	Global	-1	2	Healthcare access and quality index	--
Environmental heat and cold exposure	Male	0-6 days	95+ years	Global	1	2	Population-weighted mean temperature	--
Environmental heat and cold exposure	Male	0-6 days	95+ years	Global	-1	3	Sanitation (proportion with access)	1000
Environmental heat and cold exposure	Male	0-6 days	95+ years	Global	-1	3	Socio-demographic Index	1000
Environmental heat and cold exposure	Male	0-6 days	95+ years	Global	-1	3	Population Density (150-300 ppl/sqkm, proportion)	--
Environmental heat and cold exposure	Male	0-6 days	95+ years	Global	1	3	Elevation Over 1500m (proportion)	--
Environmental heat and cold exposure	Male	0-6 days	95+ years	Global	1	3	Rainfall (Quintiles 4-5)	--
Other unintentional injuries	Female	0-6 days	95+ years	Data Rich	1	1	Liters of alcohol consumed per capita	--
Other unintentional injuries	Female	0-6 days	95+ years	Data Rich	1	1	Log-transformed SEV scalar: Oth Unint	--
Other unintentional injuries	Female	0-6 days	95+ years	Data Rich	1	1	Population-weighted mean temperature	--
Other unintentional injuries	Female	0-6 days	95+ years	Data Rich	1	1	Vehicles - 2 wheels (per capita)	--
Other unintentional injuries	Female	0-6 days	95+ years	Data Rich	1	1	Vehicles - 4 wheels (per capita)	--
Other unintentional injuries	Female	0-6 days	95+ years	Data Rich	-1	2	Healthcare access and quality index	--
Other unintentional injuries	Female	0-6 days	95+ years	Data Rich	-1	3	Socio-demographic Index	834
Other unintentional injuries	Female	0-6 days	95+ years	Data Rich	-1	3	Population Density (over 1000 ppl/sqkm, proportion)	--
Other unintentional injuries	Female	0-6 days	95+ years	Data Rich	1	3	Population Density (under 150 ppl/sqkm, proportion)	166
Other unintentional injuries	Female	0-6 days	95+ years	Global	1	1	Liters of alcohol consumed per capita	--
Other unintentional injuries	Female	0-6 days	95+ years	Global	1	1	Log-transformed SEV scalar: Oth Unint	--
Other unintentional injuries	Female	0-6 days	95+ years	Global	1	1	Population-weighted mean temperature	--
Other unintentional injuries	Female	0-6 days	95+ years	Global	1	1	Vehicles - 2 wheels (per capita)	--
Other unintentional injuries	Female	0-6 days	95+ years	Global	1	1	Vehicles - 4 wheels (per capita)	--
Other unintentional injuries	Female	0-6 days	95+ years	Global	-1	2	Healthcare access and quality index	746
Other unintentional injuries	Female	0-6 days	95+ years	Global	-1	3	Population Density (over 1000 ppl/sqkm, proportion)	430
Other unintentional injuries	Female	0-6 days	95+ years	Global	-1	3	Socio-demographic Index	712
Other unintentional injuries	Female	0-6 days	95+ years	Global	1	3	Population Density (under 150 ppl/sqkm, proportion)	366
Other unintentional injuries	Male	0-6 days	95+ years	Data Rich	1	1	Liters of alcohol consumed per capita	1000
Other unintentional injuries	Male	0-6 days	95+ years	Data Rich	1	1	Log-transformed SEV scalar: Oth Unint	--

Appendix Table S10: CODEm covariates used, level of covariate, and expected direction of covariate by cause, sex, and age

Cause	Sex	Age Start	Age End	Model Version Type	Direction	Level	Covariate Name	Number of Draws
Other unintentional injuries	Male	0-6 days	95+ years	Data Rich	1	1	Population-weighted mean temperature	--
Other unintentional injuries	Male	0-6 days	95+ years	Data Rich	1	1	Vehicles - 2 wheels (per capita)	--
Other unintentional injuries	Male	0-6 days	95+ years	Data Rich	1	1	Vehicles - 4 wheels (per capita)	--
Other unintentional injuries	Male	0-6 days	95+ years	Data Rich	-1	2	Healthcare access and quality index	--
Other unintentional injuries	Male	0-6 days	95+ years	Data Rich	-1	3	Population Density (over 1000 ppl/sqkm, proportion)	600
Other unintentional injuries	Male	0-6 days	95+ years	Data Rich	-1	3	Socio-demographic Index	--
Other unintentional injuries	Male	0-6 days	95+ years	Data Rich	1	3	Population Density (under 150 ppl/sqkm, proportion)	--
Other unintentional injuries	Male	0-6 days	95+ years	Global	1	1	Liters of alcohol consumed per capita	--
Other unintentional injuries	Male	0-6 days	95+ years	Global	1	1	Log-transformed SEV scalar: Oth Unint	--
Other unintentional injuries	Male	0-6 days	95+ years	Global	1	1	Population-weighted mean temperature	--
Other unintentional injuries	Male	0-6 days	95+ years	Global	1	1	Vehicles - 2 wheels (per capita)	--
Other unintentional injuries	Male	0-6 days	95+ years	Global	1	1	Vehicles - 4 wheels (per capita)	--
Other unintentional injuries	Male	0-6 days	95+ years	Global	-1	2	Healthcare access and quality index	542
Other unintentional injuries	Male	0-6 days	95+ years	Global	-1	3	Population Density (over 1000 ppl/sqkm, proportion)	537
Other unintentional injuries	Male	0-6 days	95+ years	Global	-1	3	Socio-demographic Index	634
Other unintentional injuries	Male	0-6 days	95+ years	Global	1	3	Population Density (under 150 ppl/sqkm, proportion)	265
Self-harm	Female	10-14 years	95+ years	Data Rich	1	1	Log-transformed SEV scalar: Self Harm	573
Self-harm	Female	10-14 years	95+ years	Data Rich	1	1	Major depressive disorder	797
Self-harm	Female	10-14 years	95+ years	Data Rich	1	1	Liters of alcohol consumed per capita	--
Self-harm	Female	10-14 years	95+ years	Data Rich	1	1	Population-weighted mean temperature	--
Self-harm	Female	10-14 years	95+ years	Data Rich	-1	2	Population Density (over 1000 ppl/sqkm, proportion)	270
Self-harm	Female	10-14 years	95+ years	Data Rich	-1	2	Healthcare access and quality index	461
Self-harm	Female	10-14 years	95+ years	Data Rich	-1	2	Population Density (300-500 ppl/sqkm, proportion)	--
Self-harm	Female	10-14 years	95+ years	Data Rich	-1	2	Population Density (500-1000 ppl/sqkm, proportion)	--
Self-harm	Female	10-14 years	95+ years	Data Rich	1	2	Population Density (150-300 ppl/sqkm, proportion)	331
Self-harm	Female	10-14 years	95+ years	Data Rich	1	2	Population Density (under 150 ppl/sqkm, proportion)	331
Self-harm	Female	10-14 years	95+ years	Data Rich	-1	3	Socio-demographic Index	245
Self-harm	Female	10-14 years	95+ years	Data Rich	-1	3	Education (years per capita)	262
Self-harm	Female	10-14 years	95+ years	Data Rich	-1	3	LDI (I$ per capita)	--
Self-harm	Female	10-14 years	95+ years	Global	1	1	Population-weighted mean temperature	174
Self-harm	Female	10-14 years	95+ years	Global	1	1	Log-transformed SEV scalar: Self Harm	420
Self-harm	Female	10-14 years	95+ years	Global	1	1	Major depressive disorder	722
Self-harm	Female	10-14 years	95+ years	Global	1	1	Liters of alcohol consumed per capita	--
Self-harm	Female	10-14 years	95+ years	Global	-1	2	Population Density (over 1000 ppl/sqkm, proportion)	272
Self-harm	Female	10-14 years	95+ years	Global	-1	2	Healthcare access and quality index	--
Self-harm	Female	10-14 years	95+ years	Global	-1	2	Population Density (300-500 ppl/sqkm, proportion)	--
Self-harm	Female	10-14 years	95+ years	Global	-1	2	Population Density (500-1000 ppl/sqkm, proportion)	--
Self-harm	Female	10-14 years	95+ years	Global	1	2	Population Density (under 150 ppl/sqkm, proportion)	54
Self-harm	Female	10-14 years	95+ years	Global	1	2	Population Density (150-300 ppl/sqkm, proportion)	161
Self-harm	Female	10-14 years	95+ years	Global	-1	3	Socio-demographic Index	225
Self-harm	Female	10-14 years	95+ years	Global	-1	3	Education (years per capita)	650
Self-harm	Female	10-14 years	95+ years	Global	-1	3	LDI (I$ per capita)	--
Self-harm	Male	10-14 years	95+ years	Data Rich	1	1	Major depressive disorder	1000
Self-harm	Male	10-14 years	95+ years	Data Rich	1	1	Liters of alcohol consumed per capita	--
Self-harm	Male	10-14 years	95+ years	Data Rich	1	1	Log-transformed SEV scalar: Self Harm	--
Self-harm	Male	10-14 years	95+ years	Data Rich	1	1	Population-weighted mean temperature	--
Self-harm	Male	10-14 years	95+ years	Data Rich	-1	2	Population Density (over 1000 ppl/sqkm, proportion)	269
Self-harm	Male	10-14 years	95+ years	Data Rich	-1	2	Healthcare access and quality index	--
Self-harm	Male	10-14 years	95+ years	Data Rich	-1	2	Population Density (300-500 ppl/sqkm, proportion)	--
Self-harm	Male	10-14 years	95+ years	Data Rich	-1	2	Population Density (500-1000 ppl/sqkm, proportion)	--
Self-harm	Male	10-14 years	95+ years	Data Rich	1	2	Population Density (150-300 ppl/sqkm, proportion)	327
Self-harm	Male	10-14 years	95+ years	Data Rich	1	2	Population Density (under 150 ppl/sqkm, proportion)	327
Self-harm	Male	10-14 years	95+ years	Data Rich	-1	3	Socio-demographic Index	112
Self-harm	Male	10-14 years	95+ years	Data Rich	-1	3	Education (years per capita)	117
Self-harm	Male	10-14 years	95+ years	Data Rich	-1	3	LDI (I$ per capita)	--
Self-harm	Male	10-14 years	95+ years	Global	1	1	Major depressive disorder	1000
Self-harm	Male	10-14 years	95+ years	Global	1	1	Liters of alcohol consumed per capita	--
Self-harm	Male	10-14 years	95+ years	Global	1	1	Log-transformed SEV scalar: Self Harm	--
Self-harm	Male	10-14 years	95+ years	Global	1	1	Population-weighted mean temperature	--
Self-harm	Male	10-14 years	95+ years	Global	-1	2	Healthcare access and quality index	473
Self-harm	Male	10-14 years	95+ years	Global	-1	2	Population Density (over 1000 ppl/sqkm, proportion)	496
Self-harm	Male	10-14 years	95+ years	Global	-1	2	Population Density (300-500 ppl/sqkm, proportion)	--
Self-harm	Male	10-14 years	95+ years	Global	-1	2	Population Density (500-1000 ppl/sqkm, proportion)	--
Self-harm	Male	10-14 years	95+ years	Global	1	2	Population Density (150-300 ppl/sqkm, proportion)	429
Self-harm	Male	10-14 years	95+ years	Global	1	2	Population Density (under 150 ppl/sqkm, proportion)	--
Self-harm	Male	10-14 years	95+ years	Global	-1	3	Socio-demographic Index	44
Self-harm	Male	10-14 years	95+ years	Global	-1	3	Education (years per capita)	64
Self-harm	Male	10-14 years	95+ years	Global	-1	3	LDI (I$ per capita)	--
Self-harm by hanging, strangulation, and suffocation	Female	10-14 years	95+ years	Data Rich	1	1	Liters of alcohol consumed per capita	1000
Self-harm by hanging, strangulation, and suffocation	Female	10-14 years	95+ years	Data Rich	1	1	Major depressive disorder	--
Self-harm by hanging, strangulation, and suffocation	Female	10-14 years	95+ years	Data Rich	1	1	Population-weighted mean temperature	--
Self-harm by hanging, strangulation, and suffocation	Female	10-14 years	95+ years	Data Rich	-1	2	Population Density (500-1000 ppl/sqkm, proportion)	546
Self-harm by hanging, strangulation, and suffocation	Female	10-14 years	95+ years	Data Rich	-1	2	Healthcare access and quality index	--
Self-harm by hanging, strangulation, and suffocation	Female	10-14 years	95+ years	Data Rich	-1	2	Population Density (300-500 ppl/sqkm, proportion)	--
Self-harm by hanging, strangulation, and suffocation	Female	10-14 years	95+ years	Data Rich	-1	2	Population Density (over 1000 ppl/sqkm, proportion)	--
Self-harm by hanging, strangulation, and suffocation	Female	10-14 years	95+ years	Data Rich	1	2	Population Density (150-300 ppl/sqkm, proportion)	--
Self-harm by hanging, strangulation, and suffocation	Female	10-14 years	95+ years	Data Rich	1	2	Population Density (under 150 ppl/sqkm, proportion)	--
Self-harm by hanging, strangulation, and suffocation	Female	10-14 years	95+ years	Data Rich	-1	3	Education (years per capita)	--
Self-harm by hanging, strangulation, and suffocation	Female	10-14 years	95+ years	Data Rich	-1	3	LDI (I$ per capita)	--
Self-harm by hanging, strangulation, and suffocation	Female	10-14 years	95+ years	Data Rich	-1	3	Socio-demographic Index	--
Self-harm by hanging, strangulation, and suffocation	Female	10-14 years	95+ years	Global	1	1	Major depressive disorder	1000
Self-harm by hanging, strangulation, and suffocation	Female	10-14 years	95+ years	Global	1	1	Liters of alcohol consumed per capita	--
Self-harm by hanging, strangulation, and suffocation	Female	10-14 years	95+ years	Global	1	1	Population-weighted mean temperature	--
Self-harm by hanging, strangulation, and suffocation	Female	10-14 years	95+ years	Global	-1	2	Population Density (500-1000 ppl/sqkm, proportion)	647
Self-harm by hanging, strangulation, and suffocation	Female	10-14 years	95+ years	Global	-1	2	Healthcare access and quality index	--
Self-harm by hanging, strangulation, and suffocation	Female	10-14 years	95+ years	Global	-1	2	Population Density (300-500 ppl/sqkm, proportion)	--
Self-harm by hanging, strangulation, and suffocation	Female	10-14 years	95+ years	Global	-1	2	Population Density (over 1000 ppl/sqkm, proportion)	--
Self-harm by hanging, strangulation, and suffocation	Female	10-14 years	95+ years	Global	1	2	Population Density (150-300 ppl/sqkm, proportion)	--
Self-harm by hanging, strangulation, and suffocation	Female	10-14 years	95+ years	Global	1	2	Population Density (under 150 ppl/sqkm, proportion)	--
Self-harm by hanging, strangulation, and suffocation	Female	10-14 years	95+ years	Global	-1	3	Education (years per capita)	274

Appendix Table S10: CODEm covariates used, level of covariate, and expected direction of covariate by cause, sex, and age

Cause	Sex	Age Start	Age End	Model Version Type	Direction	Level	Covariate Name	Number of Draws
Self-harm by hanging, strangulation, and suffocation	Female	10-14 years	95+ years	Global	-1	3	LDI (I$ per capita)	--
Self-harm by hanging, strangulation, and suffocation	Female	10-14 years	95+ years	Global	-1	3	Socio-demographic Index	--
Self-harm by hanging, strangulation, and suffocation	Male	10-14 years	95+ years	Data Rich	1	1	Liters of alcohol consumed per capita	409
Self-harm by hanging, strangulation, and suffocation	Male	10-14 years	95+ years	Data Rich	1	1	Major depressive disorder	591
Self-harm by hanging, strangulation, and suffocation	Male	10-14 years	95+ years	Data Rich	1	1	Population-weighted mean temperature	--
Self-harm by hanging, strangulation, and suffocation	Male	10-14 years	95+ years	Data Rich	-1	2	Population Density (300-500 ppl/sqkm, proportion)	491
Self-harm by hanging, strangulation, and suffocation	Male	10-14 years	95+ years	Data Rich	-1	2	Population Density (500-1000 ppl/sqkm, proportion)	491
Self-harm by hanging, strangulation, and suffocation	Male	10-14 years	95+ years	Data Rich	-1	2	Healthcare access and quality index	--
Self-harm by hanging, strangulation, and suffocation	Male	10-14 years	95+ years	Data Rich	-1	2	Population Density (over 1000 ppl/sqkm, proportion)	--
Self-harm by hanging, strangulation, and suffocation	Male	10-14 years	95+ years	Data Rich	1	2	Population Density (150-300 ppl/sqkm, proportion)	--
Self-harm by hanging, strangulation, and suffocation	Male	10-14 years	95+ years	Data Rich	1	2	Population Density (under 150 ppl/sqkm, proportion)	--
Self-harm by hanging, strangulation, and suffocation	Male	10-14 years	95+ years	Data Rich	-1	3	Education (years per capita)	--
Self-harm by hanging, strangulation, and suffocation	Male	10-14 years	95+ years	Data Rich	-1	3	LDI (I$ per capita)	--
Self-harm by hanging, strangulation, and suffocation	Male	10-14 years	95+ years	Data Rich	-1	3	Socio-demographic Index	--
Self-harm by hanging, strangulation, and suffocation	Male	10-14 years	95+ years	Global	1	1	Liters of alcohol consumed per capita	--
Self-harm by hanging, strangulation, and suffocation	Male	10-14 years	95+ years	Global	1	1	Major depressive disorder	--
Self-harm by hanging, strangulation, and suffocation	Male	10-14 years	95+ years	Global	1	1	Population-weighted mean temperature	--
Self-harm by hanging, strangulation, and suffocation	Male	10-14 years	95+ years	Global	-1	2	Healthcare access and quality index	--
Self-harm by hanging, strangulation, and suffocation	Male	10-14 years	95+ years	Global	-1	2	Population Density (300-500 ppl/sqkm, proportion)	--
Self-harm by hanging, strangulation, and suffocation	Male	10-14 years	95+ years	Global	-1	2	Population Density (500-1000 ppl/sqkm, proportion)	--
Self-harm by hanging, strangulation, and suffocation	Male	10-14 years	95+ years	Global	-1	2	Population Density (over 1000 ppl/sqkm, proportion)	--
Self-harm by hanging, strangulation, and suffocation	Male	10-14 years	95+ years	Global	1	2	Population Density (under 150 ppl/sqkm, proportion)	726
Self-harm by hanging, strangulation, and suffocation	Male	10-14 years	95+ years	Global	1	2	Population Density (150-300 ppl/sqkm, proportion)	--
Self-harm by hanging, strangulation, and suffocation	Male	10-14 years	95+ years	Global	-1	3	Education (years per capita)	602
Self-harm by hanging, strangulation, and suffocation	Male	10-14 years	95+ years	Global	-1	3	LDI (I$ per capita)	--
Self-harm by hanging, strangulation, and suffocation	Male	10-14 years	95+ years	Global	-1	3	Socio-demographic Index	--
Self-harm by fire, heat, and hot substances	Female	10-14 years	95+ years	Data Rich	1	1	Major depressive disorder	328
Self-harm by fire, heat, and hot substances	Female	10-14 years	95+ years	Data Rich	1	1	Liters of alcohol consumed per capita	867
Self-harm by fire, heat, and hot substances	Female	10-14 years	95+ years	Data Rich	1	1	Population-weighted mean temperature	--
Self-harm by fire, heat, and hot substances	Female	10-14 years	95+ years	Data Rich	-1	2	Healthcare access and quality index	629
Self-harm by fire, heat, and hot substances	Female	10-14 years	95+ years	Data Rich	-1	2	Population Density (300-500 ppl/sqkm, proportion)	--
Self-harm by fire, heat, and hot substances	Female	10-14 years	95+ years	Data Rich	-1	2	Population Density (500-1000 ppl/sqkm, proportion)	--
Self-harm by fire, heat, and hot substances	Female	10-14 years	95+ years	Data Rich	-1	2	Population Density (over 1000 ppl/sqkm, proportion)	--
Self-harm by fire, heat, and hot substances	Female	10-14 years	95+ years	Data Rich	1	2	Population Density (150-300 ppl/sqkm, proportion)	568
Self-harm by fire, heat, and hot substances	Female	10-14 years	95+ years	Data Rich	1	2	Population Density (under 150 ppl/sqkm, proportion)	--
Self-harm by fire, heat, and hot substances	Female	10-14 years	95+ years	Data Rich	-1	3	Socio-demographic Index	560
Self-harm by fire, heat, and hot substances	Female	10-14 years	95+ years	Data Rich	-1	3	Education (years per capita)	626
Self-harm by fire, heat, and hot substances	Female	10-14 years	95+ years	Data Rich	-1	3	LDI (I$ per capita)	--
Self-harm by fire, heat, and hot substances	Female	10-14 years	95+ years	Global	1	1	Liters of alcohol consumed per capita	--
Self-harm by fire, heat, and hot substances	Female	10-14 years	95+ years	Global	1	1	Major depressive disorder	--
Self-harm by fire, heat, and hot substances	Female	10-14 years	95+ years	Global	1	1	Population-weighted mean temperature	--
Self-harm by fire, heat, and hot substances	Female	10-14 years	95+ years	Global	-1	2	Healthcare access and quality index	--
Self-harm by fire, heat, and hot substances	Female	10-14 years	95+ years	Global	-1	2	Population Density (300-500 ppl/sqkm, proportion)	--
Self-harm by fire, heat, and hot substances	Female	10-14 years	95+ years	Global	-1	2	Population Density (500-1000 ppl/sqkm, proportion)	--
Self-harm by fire, heat, and hot substances	Female	10-14 years	95+ years	Global	-1	2	Population Density (over 1000 ppl/sqkm, proportion)	--
Self-harm by fire, heat, and hot substances	Female	10-14 years	95+ years	Global	1	2	Population Density (150-300 ppl/sqkm, proportion)	--
Self-harm by fire, heat, and hot substances	Female	10-14 years	95+ years	Global	1	2	Population Density (under 150 ppl/sqkm, proportion)	--
Self-harm by fire, heat, and hot substances	Female	10-14 years	95+ years	Global	-1	3	Education (years per capita)	1000
Self-harm by fire, heat, and hot substances	Female	10-14 years	95+ years	Global	-1	3	LDI (I$ per capita)	--
Self-harm by fire, heat, and hot substances	Female	10-14 years	95+ years	Global	-1	3	Socio-demographic Index	--
Self-harm by fire, heat, and hot substances	Male	10-14 years	95+ years	Data Rich	1	1	Liters of alcohol consumed per capita	--
Self-harm by fire, heat, and hot substances	Male	10-14 years	95+ years	Data Rich	1	1	Major depressive disorder	--
Self-harm by fire, heat, and hot substances	Male	10-14 years	95+ years	Data Rich	1	1	Population-weighted mean temperature	--
Self-harm by fire, heat, and hot substances	Male	10-14 years	95+ years	Data Rich	-1	2	Population Density (500-1000 ppl/sqkm, proportion)	1000
Self-harm by fire, heat, and hot substances	Male	10-14 years	95+ years	Data Rich	-1	2	Healthcare access and quality index	--
Self-harm by fire, heat, and hot substances	Male	10-14 years	95+ years	Data Rich	-1	2	Population Density (300-500 ppl/sqkm, proportion)	--
Self-harm by fire, heat, and hot substances	Male	10-14 years	95+ years	Data Rich	-1	2	Population Density (over 1000 ppl/sqkm, proportion)	--
Self-harm by fire, heat, and hot substances	Male	10-14 years	95+ years	Data Rich	1	2	Population Density (150-300 ppl/sqkm, proportion)	--
Self-harm by fire, heat, and hot substances	Male	10-14 years	95+ years	Data Rich	1	2	Population Density (under 150 ppl/sqkm, proportion)	--
Self-harm by fire, heat, and hot substances	Male	10-14 years	95+ years	Data Rich	-1	3	Education (years per capita)	--
Self-harm by fire, heat, and hot substances	Male	10-14 years	95+ years	Data Rich	-1	3	LDI (I$ per capita)	--
Self-harm by fire, heat, and hot substances	Male	10-14 years	95+ years	Data Rich	-1	3	Socio-demographic Index	--
Self-harm by fire, heat, and hot substances	Male	10-14 years	95+ years	Global	1	1	Liters of alcohol consumed per capita	--
Self-harm by fire, heat, and hot substances	Male	10-14 years	95+ years	Global	1	1	Major depressive disorder	--
Self-harm by fire, heat, and hot substances	Male	10-14 years	95+ years	Global	1	1	Population-weighted mean temperature	--
Self-harm by fire, heat, and hot substances	Male	10-14 years	95+ years	Global	-1	2	Healthcare access and quality index	1000
Self-harm by fire, heat, and hot substances	Male	10-14 years	95+ years	Global	-1	2	Population Density (300-500 ppl/sqkm, proportion)	--
Self-harm by fire, heat, and hot substances	Male	10-14 years	95+ years	Global	-1	2	Population Density (500-1000 ppl/sqkm, proportion)	--
Self-harm by fire, heat, and hot substances	Male	10-14 years	95+ years	Global	-1	2	Population Density (over 1000 ppl/sqkm, proportion)	--
Self-harm by fire, heat, and hot substances	Male	10-14 years	95+ years	Global	1	2	Population Density (150-300 ppl/sqkm, proportion)	--
Self-harm by fire, heat, and hot substances	Male	10-14 years	95+ years	Global	1	2	Population Density (under 150 ppl/sqkm, proportion)	--
Self-harm by fire, heat, and hot substances	Male	10-14 years	95+ years	Global	-1	3	Education (years per capita)	--
Self-harm by fire, heat, and hot substances	Male	10-14 years	95+ years	Global	-1	3	LDI (I$ per capita)	--
Self-harm by fire, heat, and hot substances	Male	10-14 years	95+ years	Global	-1	3	Socio-demographic Index	--
Self-harm by firearm	Female	10-14 years	95+ years	Data Rich	1	1	Major depressive disorder	1000
Self-harm by firearm	Female	10-14 years	95+ years	Data Rich	1	1	Liters of alcohol consumed per capita	--
Self-harm by firearm	Female	10-14 years	95+ years	Data Rich	1	1	Log-transformed SEV scalar: Self Harm	--
Self-harm by firearm	Female	10-14 years	95+ years	Data Rich	1	1	Population-weighted mean temperature	--
Self-harm by firearm	Female	10-14 years	95+ years	Data Rich	-1	2	Healthcare access and quality index	527
Self-harm by firearm	Female	10-14 years	95+ years	Data Rich	-1	2	Population Density (300-500 ppl/sqkm, proportion)	--
Self-harm by firearm	Female	10-14 years	95+ years	Data Rich	-1	2	Population Density (500-1000 ppl/sqkm, proportion)	--
Self-harm by firearm	Female	10-14 years	95+ years	Data Rich	-1	2	Population Density (over 1000 ppl/sqkm, proportion)	--
Self-harm by firearm	Female	10-14 years	95+ years	Data Rich	1	2	Population Density (150-300 ppl/sqkm, proportion)	--
Self-harm by firearm	Female	10-14 years	95+ years	Data Rich	1	2	Population Density (under 150 ppl/sqkm, proportion)	--
Self-harm by firearm	Female	10-14 years	95+ years	Data Rich	-1	3	Socio-demographic Index	120
Self-harm by firearm	Female	10-14 years	95+ years	Data Rich	-1	3	Education (years per capita)	398
Self-harm by firearm	Female	10-14 years	95+ years	Data Rich	-1	3	LDI (I$ per capita)	--
Self-harm by firearm	Female	10-14 years	95+ years	Global	1	1	Liters of alcohol consumed per capita	--
Self-harm by firearm	Female	10-14 years	95+ years	Global	1	1	Log-transformed SEV scalar: Self Harm	--

Appendix Table S10: CODEm covariates used, level of covariate, and expected direction of covariate by cause, sex, and age								
**Cause**	**Sex**	**Age Start**	**Age End**	**Model Version Type**	**Direction**	**Level**	**Covariate Name**	**Number of Draws**
Self-harm by firearm	Female	10-14 years	95+ years	Global	1	1	Major depressive disorder	--
Self-harm by firearm	Female	10-14 years	95+ years	Global	1	1	Population-weighted mean temperature	--
Self-harm by firearm	Female	10-14 years	95+ years	Global	-1	2	Healthcare access and quality index	511
Self-harm by firearm	Female	10-14 years	95+ years	Global	-1	2	Population Density (over 1000 ppl/sqkm, proportion)	511
Self-harm by firearm	Female	10-14 years	95+ years	Global	-1	2	Population Density (300-500 ppl/sqkm, proportion)	--
Self-harm by firearm	Female	10-14 years	95+ years	Global	-1	2	Population Density (500-1000 ppl/sqkm, proportion)	--
Self-harm by firearm	Female	10-14 years	95+ years	Global	1	2	Population Density (150-300 ppl/sqkm, proportion)	100
Self-harm by firearm	Female	10-14 years	95+ years	Global	1	2	Population Density (under 150 ppl/sqkm, proportion)	--
Self-harm by firearm	Female	10-14 years	95+ years	Global	-1	3	Education (years per capita)	160
Self-harm by firearm	Female	10-14 years	95+ years	Global	-1	3	Socio-demographic Index	362
Self-harm by firearm	Female	10-14 years	95+ years	Global	-1	3	LDI (I$ per capita)	--
Self-harm by firearm	Male	10-14 years	95+ years	Data Rich	1	1	Liters of alcohol consumed per capita	--
Self-harm by firearm	Male	10-14 years	95+ years	Data Rich	1	1	Log-transformed SEV scalar: Self Harm	--
Self-harm by firearm	Male	10-14 years	95+ years	Data Rich	1	1	Major depressive disorder	--
Self-harm by firearm	Male	10-14 years	95+ years	Data Rich	1	1	Population-weighted mean temperature	--
Self-harm by firearm	Male	10-14 years	95+ years	Data Rich	-1	2	Healthcare access and quality index	--
Self-harm by firearm	Male	10-14 years	95+ years	Data Rich	-1	2	Population Density (300-500 ppl/sqkm, proportion)	--
Self-harm by firearm	Male	10-14 years	95+ years	Data Rich	-1	2	Population Density (500-1000 ppl/sqkm, proportion)	--
Self-harm by firearm	Male	10-14 years	95+ years	Data Rich	-1	2	Population Density (over 1000 ppl/sqkm, proportion)	--
Self-harm by firearm	Male	10-14 years	95+ years	Data Rich	1	2	Population Density (150-300 ppl/sqkm, proportion)	327
Self-harm by firearm	Male	10-14 years	95+ years	Data Rich	1	2	Population Density (under 150 ppl/sqkm, proportion)	444
Self-harm by firearm	Male	10-14 years	95+ years	Data Rich	-1	3	Education (years per capita)	212
Self-harm by firearm	Male	10-14 years	95+ years	Data Rich	-1	3	Socio-demographic Index	434
Self-harm by firearm	Male	10-14 years	95+ years	Data Rich	-1	3	LDI (I$ per capita)	--
Self-harm by firearm	Male	10-14 years	95+ years	Global	1	1	Major depressive disorder	1000
Self-harm by firearm	Male	10-14 years	95+ years	Global	1	1	Liters of alcohol consumed per capita	--
Self-harm by firearm	Male	10-14 years	95+ years	Global	1	1	Log-transformed SEV scalar: Self Harm	--
Self-harm by firearm	Male	10-14 years	95+ years	Global	1	1	Population-weighted mean temperature	--
Self-harm by firearm	Male	10-14 years	95+ years	Global	-1	2	Population Density (over 1000 ppl/sqkm, proportion)	636
Self-harm by firearm	Male	10-14 years	95+ years	Global	-1	2	Healthcare access and quality index	--
Self-harm by firearm	Male	10-14 years	95+ years	Global	-1	2	Population Density (300-500 ppl/sqkm, proportion)	--
Self-harm by firearm	Male	10-14 years	95+ years	Global	-1	2	Population Density (500-1000 ppl/sqkm, proportion)	--
Self-harm by firearm	Male	10-14 years	95+ years	Global	1	2	Population Density (150-300 ppl/sqkm, proportion)	636
Self-harm by firearm	Male	10-14 years	95+ years	Global	1	2	Population Density (under 150 ppl/sqkm, proportion)	--
Self-harm by firearm	Male	10-14 years	95+ years	Global	-1	3	Education (years per capita)	193
Self-harm by firearm	Male	10-14 years	95+ years	Global	-1	3	Socio-demographic Index	194
Self-harm by firearm	Male	10-14 years	95+ years	Global	-1	3	LDI (I$ per capita)	--
Self-harm by poisoning pesticides	Female	10-14 years	95+ years	Data Rich	1	1	Log-transformed SEV scalar: Self Harm	460
Self-harm by poisoning pesticides	Female	10-14 years	95+ years	Data Rich	1	1	Major depressive disorder	970
Self-harm by poisoning pesticides	Female	10-14 years	95+ years	Data Rich	1	1	Liters of alcohol consumed per capita	--
Self-harm by poisoning pesticides	Female	10-14 years	95+ years	Data Rich	1	1	Population-weighted mean temperature	--
Self-harm by poisoning pesticides	Female	10-14 years	95+ years	Data Rich	-1	2	Population Density (over 1000 ppl/sqkm, proportion)	73
Self-harm by poisoning pesticides	Female	10-14 years	95+ years	Data Rich	-1	2	Healthcare access and quality index	124
Self-harm by poisoning pesticides	Female	10-14 years	95+ years	Data Rich	-1	2	Population Density (500-1000 ppl/sqkm, proportion)	334
Self-harm by poisoning pesticides	Female	10-14 years	95+ years	Data Rich	-1	2	Population Density (300-500 ppl/sqkm, proportion)	--
Self-harm by poisoning pesticides	Female	10-14 years	95+ years	Data Rich	1	2	Population Density (150-300 ppl/sqkm, proportion)	14
Self-harm by poisoning pesticides	Female	10-14 years	95+ years	Data Rich	1	2	Population Density (under 150 ppl/sqkm, proportion)	373
Self-harm by poisoning pesticides	Female	10-14 years	95+ years	Data Rich	-1	3	Socio-demographic Index	35
Self-harm by poisoning pesticides	Female	10-14 years	95+ years	Data Rich	-1	3	Education (years per capita)	114
Self-harm by poisoning pesticides	Female	10-14 years	95+ years	Data Rich	-1	3	LDI (I$ per capita)	--
Self-harm by poisoning pesticides	Female	10-14 years	95+ years	Global	1	1	Log-transformed SEV scalar: Self Harm	1000
Self-harm by poisoning pesticides	Female	10-14 years	95+ years	Global	1	1	Liters of alcohol consumed per capita	--
Self-harm by poisoning pesticides	Female	10-14 years	95+ years	Global	1	1	Major depressive disorder	--
Self-harm by poisoning pesticides	Female	10-14 years	95+ years	Global	1	1	Population-weighted mean temperature	--
Self-harm by poisoning pesticides	Female	10-14 years	95+ years	Global	-1	2	Population Density (over 1000 ppl/sqkm, proportion)	604
Self-harm by poisoning pesticides	Female	10-14 years	95+ years	Global	-1	2	Healthcare access and quality index	--
Self-harm by poisoning pesticides	Female	10-14 years	95+ years	Global	-1	2	Population Density (300-500 ppl/sqkm, proportion)	--
Self-harm by poisoning pesticides	Female	10-14 years	95+ years	Global	-1	2	Population Density (500-1000 ppl/sqkm, proportion)	--
Self-harm by poisoning pesticides	Female	10-14 years	95+ years	Global	1	2	Population Density (150-300 ppl/sqkm, proportion)	--
Self-harm by poisoning pesticides	Female	10-14 years	95+ years	Global	1	2	Population Density (under 150 ppl/sqkm, proportion)	--
Self-harm by poisoning pesticides	Female	10-14 years	95+ years	Global	-1	3	Socio-demographic Index	334
Self-harm by poisoning pesticides	Female	10-14 years	95+ years	Global	-1	3	Education (years per capita)	362
Self-harm by poisoning pesticides	Female	10-14 years	95+ years	Global	-1	3	LDI (I$ per capita)	--
Self-harm by poisoning pesticides	Male	10-14 years	95+ years	Data Rich	1	1	Major depressive disorder	114
Self-harm by poisoning pesticides	Male	10-14 years	95+ years	Data Rich	1	1	Liters of alcohol consumed per capita	886
Self-harm by poisoning pesticides	Male	10-14 years	95+ years	Data Rich	1	1	Log-transformed SEV scalar: Self Harm	--
Self-harm by poisoning pesticides	Male	10-14 years	95+ years	Data Rich	1	1	Population-weighted mean temperature	--
Self-harm by poisoning pesticides	Male	10-14 years	95+ years	Data Rich	-1	2	Population Density (over 1000 ppl/sqkm, proportion)	166
Self-harm by poisoning pesticides	Male	10-14 years	95+ years	Data Rich	-1	2	Healthcare access and quality index	--
Self-harm by poisoning pesticides	Male	10-14 years	95+ years	Data Rich	-1	2	Population Density (300-500 ppl/sqkm, proportion)	--
Self-harm by poisoning pesticides	Male	10-14 years	95+ years	Data Rich	-1	2	Population Density (500-1000 ppl/sqkm, proportion)	--
Self-harm by poisoning pesticides	Male	10-14 years	95+ years	Data Rich	1	2	Population Density (under 150 ppl/sqkm, proportion)	276
Self-harm by poisoning pesticides	Male	10-14 years	95+ years	Data Rich	1	2	Population Density (150-300 ppl/sqkm, proportion)	344
Self-harm by poisoning pesticides	Male	10-14 years	95+ years	Data Rich	-1	3	Socio-demographic Index	186
Self-harm by poisoning pesticides	Male	10-14 years	95+ years	Data Rich	-1	3	Education (years per capita)	281
Self-harm by poisoning pesticides	Male	10-14 years	95+ years	Data Rich	-1	3	LDI (I$ per capita)	--
Self-harm by poisoning pesticides	Male	10-14 years	95+ years	Global	1	1	Major depressive disorder	1000
Self-harm by poisoning pesticides	Male	10-14 years	95+ years	Global	1	1	Liters of alcohol consumed per capita	--
Self-harm by poisoning pesticides	Male	10-14 years	95+ years	Global	1	1	Log-transformed SEV scalar: Self Harm	--
Self-harm by poisoning pesticides	Male	10-14 years	95+ years	Global	1	1	Population-weighted mean temperature	--
Self-harm by poisoning pesticides	Male	10-14 years	95+ years	Global	-1	2	Healthcare access and quality index	437
Self-harm by poisoning pesticides	Male	10-14 years	95+ years	Global	-1	2	Population Density (300-500 ppl/sqkm, proportion)	--
Self-harm by poisoning pesticides	Male	10-14 years	95+ years	Global	-1	2	Population Density (500-1000 ppl/sqkm, proportion)	--
Self-harm by poisoning pesticides	Male	10-14 years	95+ years	Global	-1	2	Population Density (over 1000 ppl/sqkm, proportion)	--
Self-harm by poisoning pesticides	Male	10-14 years	95+ years	Global	1	2	Population Density (150-300 ppl/sqkm, proportion)	--
Self-harm by poisoning pesticides	Male	10-14 years	95+ years	Global	1	2	Population Density (under 150 ppl/sqkm, proportion)	--
Self-harm by poisoning pesticides	Male	10-14 years	95+ years	Global	-1	3	Education (years per capita)	161
Self-harm by poisoning pesticides	Male	10-14 years	95+ years	Global	-1	3	LDI (I$ per capita)	--
Self-harm by poisoning pesticides	Male	10-14 years	95+ years	Global	-1	3	Socio-demographic Index	--
Self-harm by non pesticide substance and gas	Female	10-14 years	95+ years	Data Rich	1	1	Major depressive disorder	1000
Self-harm by non pesticide substance and gas	Female	10-14 years	95+ years	Data Rich	1	1	Liters of alcohol consumed per capita	--
Self-harm by non pesticide substance and gas	Female	10-14 years	95+ years	Data Rich	1	1	Log-transformed SEV scalar: Self Harm	--

Appendix Table S10: CODEm covariates used, level of covariate, and expected direction of covariate by cause, sex, and age

Cause	Sex	Age Start	Age End	Model Version Type	Direction	Level	Covariate Name	Number of Draws
Self-harm by non pesticide substance and gas	Female	10-14 years	95+ years	Data Rich	1	1	Population-weighted mean temperature	--
Self-harm by non pesticide substance and gas	Female	10-14 years	95+ years	Data Rich	-1	2	Healthcare access and quality index	--
Self-harm by non pesticide substance and gas	Female	10-14 years	95+ years	Data Rich	-1	2	Population Density (300-500 ppl/sqkm, proportion)	--
Self-harm by non pesticide substance and gas	Female	10-14 years	95+ years	Data Rich	-1	2	Population Density (500-1000 ppl/sqkm, proportion)	--
Self-harm by non pesticide substance and gas	Female	10-14 years	95+ years	Data Rich	-1	2	Population Density (over 1000 ppl/sqkm, proportion)	--
Self-harm by non pesticide substance and gas	Female	10-14 years	95+ years	Data Rich	1	2	Population Density (150-300 ppl/sqkm, proportion)	--
Self-harm by non pesticide substance and gas	Female	10-14 years	95+ years	Data Rich	1	2	Population Density (under 150 ppl/sqkm, proportion)	--
Self-harm by non pesticide substance and gas	Female	10-14 years	95+ years	Data Rich	-1	3	Education (years per capita)	--
Self-harm by non pesticide substance and gas	Female	10-14 years	95+ years	Data Rich	-1	3	LDI (I$ per capita)	--
Self-harm by non pesticide substance and gas	Female	10-14 years	95+ years	Data Rich	-1	3	Socio-demographic Index	--
Self-harm by non pesticide substance and gas	Female	10-14 years	95+ years	Global	1	1	Liters of alcohol consumed per capita	--
Self-harm by non pesticide substance and gas	Female	10-14 years	95+ years	Global	1	1	Log-transformed SEV scalar: Self Harm	--
Self-harm by non pesticide substance and gas	Female	10-14 years	95+ years	Global	1	1	Major depressive disorder	--
Self-harm by non pesticide substance and gas	Female	10-14 years	95+ years	Global	1	1	Population-weighted mean temperature	--
Self-harm by non pesticide substance and gas	Female	10-14 years	95+ years	Global	-1	2	Healthcare access and quality index	--
Self-harm by non pesticide substance and gas	Female	10-14 years	95+ years	Global	-1	2	Population Density (300-500 ppl/sqkm, proportion)	--
Self-harm by non pesticide substance and gas	Female	10-14 years	95+ years	Global	-1	2	Population Density (500-1000 ppl/sqkm, proportion)	--
Self-harm by non pesticide substance and gas	Female	10-14 years	95+ years	Global	-1	2	Population Density (over 1000 ppl/sqkm, proportion)	--
Self-harm by non pesticide substance and gas	Female	10-14 years	95+ years	Global	1	2	Population Density (150-300 ppl/sqkm, proportion)	1000
Self-harm by non pesticide substance and gas	Female	10-14 years	95+ years	Global	1	2	Population Density (under 150 ppl/sqkm, proportion)	--
Self-harm by non pesticide substance and gas	Female	10-14 years	95+ years	Global	-1	3	Education (years per capita)	--
Self-harm by non pesticide substance and gas	Female	10-14 years	95+ years	Global	-1	3	LDI (I$ per capita)	--
Self-harm by non pesticide substance and gas	Female	10-14 years	95+ years	Global	-1	3	Socio-demographic Index	--
Self-harm by non pesticide substance and gas	Male	10-14 years	95+ years	Data Rich	1	1	Major depressive disorder	1000
Self-harm by non pesticide substance and gas	Male	10-14 years	95+ years	Data Rich	1	1	Liters of alcohol consumed per capita	--
Self-harm by non pesticide substance and gas	Male	10-14 years	95+ years	Data Rich	1	1	Log-transformed SEV scalar: Self Harm	--
Self-harm by non pesticide substance and gas	Male	10-14 years	95+ years	Data Rich	1	1	Population-weighted mean temperature	--
Self-harm by non pesticide substance and gas	Male	10-14 years	95+ years	Data Rich	-1	2	Healthcare access and quality index	--
Self-harm by non pesticide substance and gas	Male	10-14 years	95+ years	Data Rich	-1	2	Population Density (300-500 ppl/sqkm, proportion)	--
Self-harm by non pesticide substance and gas	Male	10-14 years	95+ years	Data Rich	-1	2	Population Density (500-1000 ppl/sqkm, proportion)	--
Self-harm by non pesticide substance and gas	Male	10-14 years	95+ years	Data Rich	-1	2	Population Density (over 1000 ppl/sqkm, proportion)	--
Self-harm by non pesticide substance and gas	Male	10-14 years	95+ years	Data Rich	1	2	Population Density (150-300 ppl/sqkm, proportion)	--
Self-harm by non pesticide substance and gas	Male	10-14 years	95+ years	Data Rich	1	2	Population Density (under 150 ppl/sqkm, proportion)	--
Self-harm by non pesticide substance and gas	Male	10-14 years	95+ years	Data Rich	-1	3	Education (years per capita)	--
Self-harm by non pesticide substance and gas	Male	10-14 years	95+ years	Data Rich	-1	3	LDI (I$ per capita)	--
Self-harm by non pesticide substance and gas	Male	10-14 years	95+ years	Data Rich	-1	3	Socio-demographic Index	--
Self-harm by non pesticide substance and gas	Male	10-14 years	95+ years	Global	1	1	Major depressive disorder	1000
Self-harm by non pesticide substance and gas	Male	10-14 years	95+ years	Global	1	1	Liters of alcohol consumed per capita	--
Self-harm by non pesticide substance and gas	Male	10-14 years	95+ years	Global	1	1	Log-transformed SEV scalar: Self Harm	--
Self-harm by non pesticide substance and gas	Male	10-14 years	95+ years	Global	1	1	Population-weighted mean temperature	--
Self-harm by non pesticide substance and gas	Male	10-14 years	95+ years	Global	-1	2	Healthcare access and quality index	--
Self-harm by non pesticide substance and gas	Male	10-14 years	95+ years	Global	-1	2	Population Density (300-500 ppl/sqkm, proportion)	--
Self-harm by non pesticide substance and gas	Male	10-14 years	95+ years	Global	-1	2	Population Density (500-1000 ppl/sqkm, proportion)	--
Self-harm by non pesticide substance and gas	Male	10-14 years	95+ years	Global	-1	2	Population Density (over 1000 ppl/sqkm, proportion)	--
Self-harm by non pesticide substance and gas	Male	10-14 years	95+ years	Global	1	2	Population Density (150-300 ppl/sqkm, proportion)	491
Self-harm by non pesticide substance and gas	Male	10-14 years	95+ years	Global	1	2	Population Density (under 150 ppl/sqkm, proportion)	--
Self-harm by non pesticide substance and gas	Male	10-14 years	95+ years	Global	-1	3	Education (years per capita)	--
Self-harm by non pesticide substance and gas	Male	10-14 years	95+ years	Global	-1	3	LDI (I$ per capita)	--
Self-harm by non pesticide substance and gas	Male	10-14 years	95+ years	Global	-1	3	Socio-demographic Index	--
Self-harm by other specified means	Female	10-14 years	95+ years	Data Rich	1	1	Major depressive disorder	529
Self-harm by other specified means	Female	10-14 years	95+ years	Data Rich	1	1	Liters of alcohol consumed per capita	1000
Self-harm by other specified means	Female	10-14 years	95+ years	Data Rich	1	1	Log-transformed SEV scalar: Self Harm	--
Self-harm by other specified means	Female	10-14 years	95+ years	Data Rich	1	1	Population-weighted mean temperature	--
Self-harm by other specified means	Female	10-14 years	95+ years	Data Rich	-1	2	Population Density (500-1000 ppl/sqkm, proportion)	57
Self-harm by other specified means	Female	10-14 years	95+ years	Data Rich	-1	2	Population Density (300-500 ppl/sqkm, proportion)	188
Self-harm by other specified means	Female	10-14 years	95+ years	Data Rich	-1	2	Healthcare access and quality index	382
Self-harm by other specified means	Female	10-14 years	95+ years	Data Rich	-1	2	Population Density (over 1000 ppl/sqkm, proportion)	--
Self-harm by other specified means	Female	10-14 years	95+ years	Data Rich	1	2	Population Density (150-300 ppl/sqkm, proportion)	--
Self-harm by other specified means	Female	10-14 years	95+ years	Data Rich	1	2	Population Density (under 150 ppl/sqkm, proportion)	--
Self-harm by other specified means	Female	10-14 years	95+ years	Data Rich	-1	3	Socio-demographic Index	242
Self-harm by other specified means	Female	10-14 years	95+ years	Data Rich	-1	3	Education (years per capita)	472
Self-harm by other specified means	Female	10-14 years	95+ years	Global	1	1	Liters of alcohol consumed per capita	861
Self-harm by other specified means	Female	10-14 years	95+ years	Global	1	1	Major depressive disorder	1000
Self-harm by other specified means	Female	10-14 years	95+ years	Global	1	1	Log-transformed SEV scalar: Self Harm	--
Self-harm by other specified means	Female	10-14 years	95+ years	Global	1	1	Population-weighted mean temperature	--
Self-harm by other specified means	Female	10-14 years	95+ years	Global	-1	2	Population Density (500-1000 ppl/sqkm, proportion)	372
Self-harm by other specified means	Female	10-14 years	95+ years	Global	-1	2	Population Density (300-500 ppl/sqkm, proportion)	523
Self-harm by other specified means	Female	10-14 years	95+ years	Global	-1	2	Healthcare access and quality index	635
Self-harm by other specified means	Female	10-14 years	95+ years	Global	-1	2	Population Density (over 1000 ppl/sqkm, proportion)	--
Self-harm by other specified means	Female	10-14 years	95+ years	Global	1	2	Population Density (150-300 ppl/sqkm, proportion)	--
Self-harm by other specified means	Female	10-14 years	95+ years	Global	1	2	Population Density (under 150 ppl/sqkm, proportion)	--
Self-harm by other specified means	Female	10-14 years	95+ years	Global	-1	3	Socio-demographic Index	251
Self-harm by other specified means	Female	10-14 years	95+ years	Global	-1	3	Education (years per capita)	404
Self-harm by other specified means	Female	10-14 years	95+ years	Global	-1	3	LDI (I$ per capita)	--
Self-harm by other specified means	Male	10-14 years	95+ years	Data Rich	1	1	Liters of alcohol consumed per capita	294
Self-harm by other specified means	Male	10-14 years	95+ years	Data Rich	1	1	Log-transformed SEV scalar: Self Harm	475
Self-harm by other specified means	Male	10-14 years	95+ years	Data Rich	1	1	Major depressive disorder	771
Self-harm by other specified means	Male	10-14 years	95+ years	Data Rich	1	1	Population-weighted mean temperature	--
Self-harm by other specified means	Male	10-14 years	95+ years	Data Rich	-1	2	Population Density (300-500 ppl/sqkm, proportion)	120
Self-harm by other specified means	Male	10-14 years	95+ years	Data Rich	-1	2	Population Density (500-1000 ppl/sqkm, proportion)	120
Self-harm by other specified means	Male	10-14 years	95+ years	Data Rich	-1	2	Healthcare access and quality index	354
Self-harm by other specified means	Male	10-14 years	95+ years	Data Rich	-1	2	Population Density (over 1000 ppl/sqkm, proportion)	--
Self-harm by other specified means	Male	10-14 years	95+ years	Data Rich	1	2	Population Density (150-300 ppl/sqkm, proportion)	--
Self-harm by other specified means	Male	10-14 years	95+ years	Data Rich	1	2	Population Density (under 150 ppl/sqkm, proportion)	--
Self-harm by other specified means	Male	10-14 years	95+ years	Data Rich	-1	3	Socio-demographic Index	381

Appendix Table S10: CODEm covariates used, level of covariate, and expected direction of covariate by cause, sex, and age

Cause	Sex	Age Start	Age End	Model Version Type	Direction	Level	Covariate Name	Number of Draws
Self-harm by other specified means	Male	10-14 years	95+ years	Data Rich	-1	3	Education (years per capita)	433
Self-harm by other specified means	Male	10-14 years	95+ years	Global	1	1	Liters of alcohol consumed per capita	282
Self-harm by other specified means	Male	10-14 years	95+ years	Global	1	1	Major depressive disorder	440
Self-harm by other specified means	Male	10-14 years	95+ years	Global	1	1	Log-transformed SEV scalar: Self Harm	718
Self-harm by other specified means	Male	10-14 years	95+ years	Global	1	1	Population-weighted mean temperature	--
Self-harm by other specified means	Male	10-14 years	95+ years	Global	-1	2	Population Density (500-1000 ppl/sqkm, proportion)	207
Self-harm by other specified means	Male	10-14 years	95+ years	Global	-1	2	Population Density (300-500 ppl/sqkm, proportion)	425
Self-harm by other specified means	Male	10-14 years	95+ years	Global	-1	2	Healthcare access and quality index	759
Self-harm by other specified means	Male	10-14 years	95+ years	Global	-1	2	Population Density (over 1000 ppl/sqkm, proportion)	--
Self-harm by other specified means	Male	10-14 years	95+ years	Global	1	2	Population Density (150-300 ppl/sqkm, proportion)	--
Self-harm by other specified means	Male	10-14 years	95+ years	Global	1	2	Population Density (under 150 ppl/sqkm, proportion)	--
Self-harm by other specified means	Male	10-14 years	95+ years	Global	-1	3	Socio-demographic Index	164
Self-harm by other specified means	Male	10-14 years	95+ years	Global	-1	3	Education (years per capita)	386
Self-harm by other specified means	Male	10-14 years	95+ years	Global	-1	3	LDI (I$ per capita)	--
Interpersonal violence	Female	0-6 days	95+ years	Data Rich	1	1	Population-weighted mean temperature	224
Interpersonal violence	Female	0-6 days	95+ years	Data Rich	1	1	Population 15 to 30 males (proportion)	298
Interpersonal violence	Female	0-6 days	95+ years	Data Rich	1	1	Education Relative Inequality (Gini)	350
Interpersonal violence	Female	0-6 days	95+ years	Data Rich	1	1	Log-transformed SEV scalar: Violence	669
Interpersonal violence	Female	0-6 days	95+ years	Data Rich	1	1	Liters of alcohol consumed per capita	--
Interpersonal violence	Female	0-6 days	95+ years	Data Rich	-1	2	Healthcare access and quality index	237
Interpersonal violence	Female	0-6 days	95+ years	Data Rich	1	2	Population Density (over 1000 ppl/sqkm, proportion)	--
Interpersonal violence	Female	0-6 days	95+ years	Data Rich	-1	3	Education (years per capita)	72
Interpersonal violence	Female	0-6 days	95+ years	Data Rich	-1	3	Socio-demographic Index	236
Interpersonal violence	Female	0-6 days	95+ years	Data Rich	-1	3	LDI (I$ per capita)	--
Interpersonal violence	Female	0-6 days	95+ years	Global	1	1	Log-transformed SEV scalar: Violence	169
Interpersonal violence	Female	0-6 days	95+ years	Global	1	1	Education Relative Inequality (Gini)	227
Interpersonal violence	Female	0-6 days	95+ years	Global	1	1	Population-weighted mean temperature	387
Interpersonal violence	Female	0-6 days	95+ years	Global	1	1	Population 15 to 30 males (proportion)	567
Interpersonal violence	Female	0-6 days	95+ years	Global	1	1	Liters of alcohol consumed per capita	--
Interpersonal violence	Female	0-6 days	95+ years	Global	-1	2	Healthcare access and quality index	613
Interpersonal violence	Female	0-6 days	95+ years	Global	1	2	Population Density (over 1000 ppl/sqkm, proportion)	--
Interpersonal violence	Female	0-6 days	95+ years	Global	-1	3	Socio-demographic Index	55
Interpersonal violence	Female	0-6 days	95+ years	Global	-1	3	Education (years per capita)	346
Interpersonal violence	Female	0-6 days	95+ years	Global	-1	3	LDI (I$ per capita)	--
Interpersonal violence	Male	0-6 days	95+ years	Data Rich	1	1	Liters of alcohol consumed per capita	69
Interpersonal violence	Male	0-6 days	95+ years	Data Rich	1	1	Education Relative Inequality (Gini)	164
Interpersonal violence	Male	0-6 days	95+ years	Data Rich	1	1	Population 15 to 30 males (proportion)	355
Interpersonal violence	Male	0-6 days	95+ years	Data Rich	1	1	Population-weighted mean temperature	674
Interpersonal violence	Male	0-6 days	95+ years	Data Rich	1	1	Log-transformed SEV scalar: Violence	--
Interpersonal violence	Male	0-6 days	95+ years	Data Rich	-1	2	Healthcare access and quality index	262
Interpersonal violence	Male	0-6 days	95+ years	Data Rich	1	2	Population Density (over 1000 ppl/sqkm, proportion)	378
Interpersonal violence	Male	0-6 days	95+ years	Data Rich	-1	3	Education (years per capita)	27
Interpersonal violence	Male	0-6 days	95+ years	Data Rich	-1	3	Socio-demographic Index	238
Interpersonal violence	Male	0-6 days	95+ years	Data Rich	-1	3	LDI (I$ per capita)	--
Interpersonal violence	Male	0-6 days	95+ years	Global	1	1	Population 15 to 30 males (proportion)	396
Interpersonal violence	Male	0-6 days	95+ years	Global	1	1	Education Relative Inequality (Gini)	516
Interpersonal violence	Male	0-6 days	95+ years	Global	1	1	Population-weighted mean temperature	635
Interpersonal violence	Male	0-6 days	95+ years	Global	1	1	Liters of alcohol consumed per capita	--
Interpersonal violence	Male	0-6 days	95+ years	Global	1	1	Log-transformed SEV scalar: Violence	--
Interpersonal violence	Male	0-6 days	95+ years	Global	-1	2	Healthcare access and quality index	286
Interpersonal violence	Male	0-6 days	95+ years	Global	1	2	Population Density (over 1000 ppl/sqkm, proportion)	--
Interpersonal violence	Male	0-6 days	95+ years	Global	-1	3	Education (years per capita)	98
Interpersonal violence	Male	0-6 days	95+ years	Global	-1	3	Socio-demographic Index	111
Interpersonal violence	Male	0-6 days	95+ years	Global	-1	3	LDI (I$ per capita)	--
Assault by firearm	Female	0-6 days	95+ years	Data Rich	1	1	Population-weighted mean temperature	0
Assault by firearm	Female	0-6 days	95+ years	Data Rich	1	1	Education Relative Inequality (Gini)	7
Assault by firearm	Female	0-6 days	95+ years	Data Rich	1	1	Log-transformed SEV scalar: Viol Gun	193
Assault by firearm	Female	0-6 days	95+ years	Data Rich	1	1	Population 15 to 30 males (proportion)	1000
Assault by firearm	Female	0-6 days	95+ years	Data Rich	1	1	Liters of alcohol consumed per capita	--
Assault by firearm	Female	0-6 days	95+ years	Data Rich	-1	2	Healthcare access and quality index	0
Assault by firearm	Female	0-6 days	95+ years	Data Rich	1	2	Population Density (over 1000 ppl/sqkm, proportion)	--
Assault by firearm	Female	0-6 days	95+ years	Data Rich	-1	3	Socio-demographic Index	32
Assault by firearm	Female	0-6 days	95+ years	Data Rich	-1	3	Education (years per capita)	801
Assault by firearm	Female	0-6 days	95+ years	Data Rich	-1	3	LDI (I$ per capita)	--
Assault by firearm	Female	0-6 days	95+ years	Global	1	1	Education Relative Inequality (Gini)	55
Assault by firearm	Female	0-6 days	95+ years	Global	1	1	Population-weighted mean temperature	505
Assault by firearm	Female	0-6 days	95+ years	Global	1	1	Population 15 to 30 males (proportion)	546
Assault by firearm	Female	0-6 days	95+ years	Global	1	1	Log-transformed SEV scalar: Viol Gun	693
Assault by firearm	Female	0-6 days	95+ years	Global	1	1	Liters of alcohol consumed per capita	--
Assault by firearm	Female	0-6 days	95+ years	Global	-1	2	Healthcare access and quality index	539
Assault by firearm	Female	0-6 days	95+ years	Global	1	2	Population Density (over 1000 ppl/sqkm, proportion)	--
Assault by firearm	Female	0-6 days	95+ years	Global	-1	3	Education (years per capita)	56
Assault by firearm	Female	0-6 days	95+ years	Global	-1	3	Socio-demographic Index	222
Assault by firearm	Female	0-6 days	95+ years	Global	-1	3	LDI (I$ per capita)	--
Assault by firearm	Male	0-6 days	95+ years	Data Rich	1	1	Liters of alcohol consumed per capita	0
Assault by firearm	Male	0-6 days	95+ years	Data Rich	1	1	Log-transformed SEV scalar: Viol Gun	0
Assault by firearm	Male	0-6 days	95+ years	Data Rich	1	1	Education Relative Inequality (Gini)	32
Assault by firearm	Male	0-6 days	95+ years	Data Rich	1	1	Population-weighted mean temperature	166
Assault by firearm	Male	0-6 days	95+ years	Data Rich	1	1	Population 15 to 30 males (proportion)	1000
Assault by firearm	Male	0-6 days	95+ years	Data Rich	-1	2	Healthcare access and quality index	--
Assault by firearm	Male	0-6 days	95+ years	Data Rich	1	2	Population Density (over 1000 ppl/sqkm, proportion)	0
Assault by firearm	Male	0-6 days	95+ years	Data Rich	-1	3	Socio-demographic Index	6
Assault by firearm	Male	0-6 days	95+ years	Data Rich	-1	3	Education (years per capita)	961
Assault by firearm	Male	0-6 days	95+ years	Data Rich	-1	3	LDI (I$ per capita)	--
Assault by firearm	Male	0-6 days	95+ years	Global	1	1	Population-weighted mean temperature	193
Assault by firearm	Male	0-6 days	95+ years	Global	1	1	Log-transformed SEV scalar: Viol Gun	359
Assault by firearm	Male	0-6 days	95+ years	Global	1	1	Education Relative Inequality (Gini)	441
Assault by firearm	Male	0-6 days	95+ years	Global	1	1	Population 15 to 30 males (proportion)	870
Assault by firearm	Male	0-6 days	95+ years	Global	1	1	Liters of alcohol consumed per capita	--
Assault by firearm	Male	0-6 days	95+ years	Global	-1	2	Healthcare access and quality index	320
Assault by firearm	Male	0-6 days	95+ years	Global	1	2	Population Density (over 1000 ppl/sqkm, proportion)	44
Assault by firearm	Male	0-6 days	95+ years	Global	-1	3	Socio-demographic Index	107
Assault by firearm	Male	0-6 days	95+ years	Global	-1	3	Education (years per capita)	211
Assault by firearm	Male	0-6 days	95+ years	Global	-1	3	LDI (I$ per capita)	--
Assault by sharp object	Female	0-6 days	95+ years	Data Rich	1	1	Population-weighted mean temperature	187
Assault by sharp object	Female	0-6 days	95+ years	Data Rich	1	1	Liters of alcohol consumed per capita	303

Appendix Table S10: CODEm covariates used, level of covariate, and expected direction of covariate by cause, sex, and age

Cause	Sex	Age Start	Age End	Model Version Type	Direction	Level	Covariate Name	Number of Draws
Assault by sharp object	Female	0-6 days	95+ years	Data Rich	1	1	Population 15 to 30 males (proportion)	317
Assault by sharp object	Female	0-6 days	95+ years	Data Rich	1	1	Log-transformed SEV scalar: Viol Knife	419
Assault by sharp object	Female	0-6 days	95+ years	Data Rich	1	1	Education Relative Inequality (Gini)	572
Assault by sharp object	Female	0-6 days	95+ years	Data Rich	-1	2	Healthcare access and quality index	39
Assault by sharp object	Female	0-6 days	95+ years	Data Rich	1	2	Population Density (over 1000 ppl/sqkm, proportion)	59
Assault by sharp object	Female	0-6 days	95+ years	Data Rich	-1	3	Socio-demographic Index	118
Assault by sharp object	Female	0-6 days	95+ years	Data Rich	-1	3	Education (years per capita)	166
Assault by sharp object	Female	0-6 days	95+ years	Data Rich	-1	3	LDI (I$ per capita)	--
Assault by sharp object	Female	0-6 days	95+ years	Global	1	1	Population 15 to 30 males (proportion)	171
Assault by sharp object	Female	0-6 days	95+ years	Global	1	1	Education Relative Inequality (Gini)	242
Assault by sharp object	Female	0-6 days	95+ years	Global	1	1	Log-transformed SEV scalar: Viol Knife	667
Assault by sharp object	Female	0-6 days	95+ years	Global	1	1	Population-weighted mean temperature	794
Assault by sharp object	Female	0-6 days	95+ years	Global	1	1	Liters of alcohol consumed per capita	--
Assault by sharp object	Female	0-6 days	95+ years	Global	-1	2	Healthcare access and quality index	494
Assault by sharp object	Female	0-6 days	95+ years	Global	1	2	Population Density (over 1000 ppl/sqkm, proportion)	--
Assault by sharp object	Female	0-6 days	95+ years	Global	-1	3	Education (years per capita)	94
Assault by sharp object	Female	0-6 days	95+ years	Global	-1	3	Socio-demographic Index	230
Assault by sharp object	Female	0-6 days	95+ years	Global	-1	3	LDI (I$ per capita)	--
Assault by sharp object	Male	0-6 days	95+ years	Data Rich	1	1	Liters of alcohol consumed per capita	49
Assault by sharp object	Male	0-6 days	95+ years	Data Rich	1	1	Education Relative Inequality (Gini)	182
Assault by sharp object	Male	0-6 days	95+ years	Data Rich	1	1	Log-transformed SEV scalar: Viol Knife	445
Assault by sharp object	Male	0-6 days	95+ years	Data Rich	1	1	Population-weighted mean temperature	548
Assault by sharp object	Male	0-6 days	95+ years	Data Rich	1	1	Population 15 to 30 males (proportion)	644
Assault by sharp object	Male	0-6 days	95+ years	Data Rich	-1	2	Healthcare access and quality index	265
Assault by sharp object	Male	0-6 days	95+ years	Data Rich	1	2	Population Density (over 1000 ppl/sqkm, proportion)	49
Assault by sharp object	Male	0-6 days	95+ years	Data Rich	-1	3	Education (years per capita)	110
Assault by sharp object	Male	0-6 days	95+ years	Data Rich	-1	3	Socio-demographic Index	363
Assault by sharp object	Male	0-6 days	95+ years	Data Rich	-1	3	LDI (I$ per capita)	--
Assault by sharp object	Male	0-6 days	95+ years	Global	1	1	Education Relative Inequality (Gini)	95
Assault by sharp object	Male	0-6 days	95+ years	Global	1	1	Population-weighted mean temperature	388
Assault by sharp object	Male	0-6 days	95+ years	Global	1	1	Log-transformed SEV scalar: Viol Knife	472
Assault by sharp object	Male	0-6 days	95+ years	Global	1	1	Population 15 to 30 males (proportion)	962
Assault by sharp object	Male	0-6 days	95+ years	Global	1	1	Liters of alcohol consumed per capita	--
Assault by sharp object	Male	0-6 days	95+ years	Global	-1	2	Healthcare access and quality index	635
Assault by sharp object	Male	0-6 days	95+ years	Global	1	2	Population Density (over 1000 ppl/sqkm, proportion)	--
Assault by sharp object	Male	0-6 days	95+ years	Global	-1	3	Education (years per capita)	157
Assault by sharp object	Male	0-6 days	95+ years	Global	-1	3	Socio-demographic Index	270
Assault by sharp object	Male	0-6 days	95+ years	Global	-1	3	LDI (I$ per capita)	--
Assault by other means	Female	0-6 days	95+ years	Data Rich	1	1	Population 15 to 30 males (proportion)	250
Assault by other means	Female	0-6 days	95+ years	Data Rich	1	1	Education Relative Inequality (Gini)	427
Assault by other means	Female	0-6 days	95+ years	Data Rich	1	1	Population-weighted mean temperature	568
Assault by other means	Female	0-6 days	95+ years	Data Rich	1	1	Log-transformed SEV scalar: Oth Viol	685
Assault by other means	Female	0-6 days	95+ years	Data Rich	1	1	Liters of alcohol consumed per capita	--
Assault by other means	Female	0-6 days	95+ years	Data Rich	-1	2	Healthcare access and quality index	--
Assault by other means	Female	0-6 days	95+ years	Data Rich	1	2	Population Density (over 1000 ppl/sqkm, proportion)	26
Assault by other means	Female	0-6 days	95+ years	Data Rich	-1	3	Education (years per capita)	389
Assault by other means	Female	0-6 days	95+ years	Data Rich	-1	3	Socio-demographic Index	505
Assault by other means	Female	0-6 days	95+ years	Data Rich	-1	3	LDI (I$ per capita)	--
Assault by other means	Female	0-6 days	95+ years	Global	1	1	Education Relative Inequality (Gini)	41
Assault by other means	Female	0-6 days	95+ years	Global	1	1	Population-weighted mean temperature	273
Assault by other means	Female	0-6 days	95+ years	Global	1	1	Log-transformed SEV scalar: Oth Viol	559
Assault by other means	Female	0-6 days	95+ years	Global	1	1	Population 15 to 30 males (proportion)	847
Assault by other means	Female	0-6 days	95+ years	Global	1	1	Liters of alcohol consumed per capita	--
Assault by other means	Female	0-6 days	95+ years	Global	-1	2	Healthcare access and quality index	900
Assault by other means	Female	0-6 days	95+ years	Global	1	2	Population Density (over 1000 ppl/sqkm, proportion)	--
Assault by other means	Female	0-6 days	95+ years	Global	-1	3	Education (years per capita)	170
Assault by other means	Female	0-6 days	95+ years	Global	-1	3	Socio-demographic Index	206
Assault by other means	Female	0-6 days	95+ years	Global	-1	3	LDI (I$ per capita)	--
Assault by other means	Male	0-6 days	95+ years	Data Rich	1	1	Population 15 to 30 males (proportion)	340
Assault by other means	Male	0-6 days	95+ years	Data Rich	1	1	Education Relative Inequality (Gini)	607
Assault by other means	Male	0-6 days	95+ years	Data Rich	1	1	Population-weighted mean temperature	663
Assault by other means	Male	0-6 days	95+ years	Data Rich	1	1	Liters of alcohol consumed per capita	--
Assault by other means	Male	0-6 days	95+ years	Data Rich	1	1	Log-transformed SEV scalar: Oth Viol	--
Assault by other means	Male	0-6 days	95+ years	Data Rich	-1	2	Healthcare access and quality index	66
Assault by other means	Male	0-6 days	95+ years	Data Rich	1	2	Population Density (over 1000 ppl/sqkm, proportion)	171
Assault by other means	Male	0-6 days	95+ years	Data Rich	-1	3	Education (years per capita)	121
Assault by other means	Male	0-6 days	95+ years	Data Rich	-1	3	Socio-demographic Index	217
Assault by other means	Male	0-6 days	95+ years	Data Rich	-1	3	LDI (I$ per capita)	--
Assault by other means	Male	0-6 days	95+ years	Global	1	1	Education Relative Inequality (Gini)	145
Assault by other means	Male	0-6 days	95+ years	Global	1	1	Population 15 to 30 males (proportion)	461
Assault by other means	Male	0-6 days	95+ years	Global	1	1	Population-weighted mean temperature	922
Assault by other means	Male	0-6 days	95+ years	Global	1	1	Liters of alcohol consumed per capita	--
Assault by other means	Male	0-6 days	95+ years	Global	1	1	Log-transformed SEV scalar: Oth Viol	--
Assault by other means	Male	0-6 days	95+ years	Global	-1	2	Healthcare access and quality index	634
Assault by other means	Male	0-6 days	95+ years	Global	1	2	Population Density (over 1000 ppl/sqkm, proportion)	--
Assault by other means	Male	0-6 days	95+ years	Global	-1	3	Socio-demographic Index	280
Assault by other means	Male	0-6 days	95+ years	Global	-1	3	Education (years per capita)	285
Assault by other means	Male	0-6 days	95+ years	Global	-1	3	LDI (I$ per capita)	--
Executions and police conflict	Female	1-5 months	95+ years	Data Rich	1	1	Population-weighted mean temperature	1000
Executions and police conflict	Female	1-5 months	95+ years	Data Rich	1	1	Education Relative Inequality (Gini)	--
Executions and police conflict	Female	1-5 months	95+ years	Data Rich	1	1	Liters of alcohol consumed per capita	--
Executions and police conflict	Female	1-5 months	95+ years	Data Rich	1	1	Population 15 to 30 males (proportion)	--
Executions and police conflict	Female	1-5 months	95+ years	Data Rich	-1	2	Healthcare access and quality index	839
Executions and police conflict	Female	1-5 months	95+ years	Data Rich	-1	2	Socio-demographic Index	--
Executions and police conflict	Female	1-5 months	95+ years	Data Rich	1	2	Population Density (over 1000 ppl/sqkm, proportion)	--
Executions and police conflict	Female	1-5 months	95+ years	Data Rich	-1	3	LDI (I$ per capita)	--
Executions and police conflict	Female	1-5 months	95+ years	Data Rich	1	3	Education (years per capita)	32
Executions and police conflict	Female	1-5 months	95+ years	Global	1	1	Population-weighted mean temperature	1000
Executions and police conflict	Female	1-5 months	95+ years	Global	1	1	Liters of alcohol consumed per capita	--
Executions and police conflict	Female	1-5 months	95+ years	Global	1	1	Population 15 to 30 males (proportion)	--
Executions and police conflict	Female	1-5 months	95+ years	Global	-1	2	Socio-demographic Index	222
Executions and police conflict	Female	1-5 months	95+ years	Global	-1	2	Healthcare access and quality index	593
Executions and police conflict	Female	1-5 months	95+ years	Global	1	2	Population Density (over 1000 ppl/sqkm, proportion)	--
Executions and police conflict	Female	1-5 months	95+ years	Global	-1	3	LDI (I$ per capita)	--
Executions and police conflict	Female	1-5 months	95+ years	Global	1	3	Education (years per capita)	284
Executions and police conflict	Male	1-5 months	95+ years	Data Rich	1	1	Population-weighted mean temperature	1000
Executions and police conflict	Male	1-5 months	95+ years	Data Rich	1	1	Education Relative Inequality (Gini)	--

Appendix Table S10: CODEm covariates used, level of covariate, and expected direction of covariate by cause, sex, and age								
**Cause**	**Sex**	**Age Start**	**Age End**	**Model Version Type**	**Direction**	**Level**	**Covariate Name**	**Number of Draws**
Executions and police conflict	Male	1-5 months	95+ years	Data Rich	1	1	Liters of alcohol consumed per capita	--
Executions and police conflict	Male	1-5 months	95+ years	Data Rich	1	1	Population 15 to 30 males (proportion)	--
Executions and police conflict	Male	1-5 months	95+ years	Data Rich	-1	2	Healthcare access and quality index	962
Executions and police conflict	Male	1-5 months	95+ years	Data Rich	-1	2	Socio-demographic Index	--
Executions and police conflict	Male	1-5 months	95+ years	Data Rich	1	2	Population Density (over 1000 ppl/sqkm, proportion)	962
Executions and police conflict	Male	1-5 months	95+ years	Data Rich	-1	3	LDI (I$ per capita)	--
Executions and police conflict	Male	1-5 months	95+ years	Data Rich	1	3	Education (years per capita)	166
Executions and police conflict	Male	1-5 months	95+ years	Global	1	1	Population-weighted mean temperature	308
Executions and police conflict	Male	1-5 months	95+ years	Global	1	1	Population 15 to 30 males (proportion)	1000
Executions and police conflict	Male	1-5 months	95+ years	Global	1	1	Liters of alcohol consumed per capita	--
Executions and police conflict	Male	1-5 months	95+ years	Global	-1	2	Healthcare access and quality index	--
Executions and police conflict	Male	1-5 months	95+ years	Global	-1	2	Socio-demographic Index	--
Executions and police conflict	Male	1-5 months	95+ years	Global	1	2	Population Density (over 1000 ppl/sqkm, proportion)	398
Executions and police conflict	Male	1-5 months	95+ years	Global	-1	3	LDI (I$ per capita)	--
Executions and police conflict	Male	1-5 months	95+ years	Global	1	3	Education (years per capita)	--

Appendix Table S11: Comparison of GBD 2021 and GBD 2023 covariates and level of covariates used in cause of death modeling									
Cause	Model Version Type	Sex	Covariate	Level 1 GBD 2021	Level 2 GBD 2021	Level 3 GBD 2021	Level 1 GBD 2023	Level 2 GBD 2023	Level 3 GBD 2023
Sexually transmitted infections excluding HIV	Data Rich	Female	LDI (I$ per capita)			X			X
Sexually transmitted infections excluding HIV	Global	Female	LDI (I$ per capita)			X			X
Sexually transmitted infections excluding HIV	Data Rich	Female	Antenatal Care (1 visit) Coverage (proportion)			X			X
Sexually transmitted infections excluding HIV	Global	Female	Antenatal Care (1 visit) Coverage (proportion)			X			X
Sexually transmitted infections excluding HIV	Data Rich	Female	Antenatal Care (4 visits) Coverage (proportion)			X			X
Sexually transmitted infections excluding HIV	Global	Female	Antenatal Care (4 visits) Coverage (proportion)			X			X
Sexually transmitted infections excluding HIV	Data Rich	Female	Maternal care and immunization		X			X	
Sexually transmitted infections excluding HIV	Global	Female	Maternal care and immunization		X			X	
Sexually transmitted infections excluding HIV	Data Rich	Female	Education (years per capita)		X			X	
Sexually transmitted infections excluding HIV	Global	Female	Education (years per capita)		X			X	
Sexually transmitted infections excluding HIV	Data Rich	Female	Healthcare access and quality index		X			X	
Sexually transmitted infections excluding HIV	Global	Female	Healthcare access and quality index		X			X	
Sexually transmitted infections excluding HIV	Data Rich	Female	Legality of Abortion		X			X	
Sexually transmitted infections excluding HIV	Global	Female	Legality of Abortion		X			X	
Sexually transmitted infections excluding HIV	Data Rich	Female	Age-Specific Fertility Rate		X			X	
Sexually transmitted infections excluding HIV	Global	Female	Age-Specific Fertility Rate		X			X	
Sexually transmitted infections excluding HIV	Data Rich	Female	Total Fertility Rate		X			X	
Sexually transmitted infections excluding HIV	Global	Female	Total Fertility Rate		X			X	
Sexually transmitted infections excluding HIV	Data Rich	Female	Syphilis prevalence (proportion)	X			X		
Sexually transmitted infections excluding HIV	Global	Female	Syphilis prevalence (proportion)	X			X		
Sexually transmitted infections excluding HIV	Data Rich	Male	LDI (I$ per capita)			X			X
Sexually transmitted infections excluding HIV	Global	Male	LDI (I$ per capita)			X			X
Sexually transmitted infections excluding HIV	Data Rich	Male	Antenatal Care (1 visit) Coverage (proportion)			X			X
Sexually transmitted infections excluding HIV	Global	Male	Antenatal Care (1 visit) Coverage (proportion)			X			X
Sexually transmitted infections excluding HIV	Data Rich	Male	Antenatal Care (4 visits) Coverage (proportion)			X			X
Sexually transmitted infections excluding HIV	Global	Male	Antenatal Care (4 visits) Coverage (proportion)			X			X
Sexually transmitted infections excluding HIV	Data Rich	Male	Maternal care and immunization		X			X	
Sexually transmitted infections excluding HIV	Global	Male	Maternal care and immunization		X			X	
Sexually transmitted infections excluding HIV	Data Rich	Male	Education (years per capita)		X			X	
Sexually transmitted infections excluding HIV	Global	Male	Education (years per capita)		X			X	
Sexually transmitted infections excluding HIV	Data Rich	Male	Healthcare access and quality index		X			X	
Sexually transmitted infections excluding HIV	Global	Male	Healthcare access and quality index		X			X	
Sexually transmitted infections excluding HIV	Data Rich	Male	Legality of Abortion		X			X	
Sexually transmitted infections excluding HIV	Global	Male	Legality of Abortion		X			X	
Sexually transmitted infections excluding HIV	Data Rich	Male	Age-Specific Fertility Rate		X			X	
Sexually transmitted infections excluding HIV	Global	Male	Age-Specific Fertility Rate		X			X	
Sexually transmitted infections excluding HIV	Data Rich	Male	Total Fertility Rate		X			X	
Sexually transmitted infections excluding HIV	Global	Male	Total Fertility Rate		X			X	
Sexually transmitted infections excluding HIV	Data Rich	Male	Syphilis prevalence (proportion)	X			X		
Sexually transmitted infections excluding HIV	Global	Male	Syphilis prevalence (proportion)	X			X		
Lower respiratory infections	Data Rich	Female	Education (years per capita)			X			X
Lower respiratory infections	Global	Female	Education (years per capita)			X			X
Lower respiratory infections	Data Rich	Female	LDI (I$ per capita)			X			X
Lower respiratory infections	Global	Female	LDI (I$ per capita)			X			X
Lower respiratory infections	Global	Female	Socio-demographic Index			X			X
Lower respiratory infections	Data Rich	Female	Socio-demographic Index			X			X
Lower respiratory infections	Global	Female	Maternal Education (years per capita)			X			X
Lower respiratory infections	Data Rich	Female	Maternal Education (years per capita)			X			X
Lower respiratory infections	Data Rich	Female	Age- and sex-specific SEV for Unsafe sanitation			X			X
Lower respiratory infections	Global	Female	Age- and sex-specific SEV for Unsafe sanitation			X			X
Lower respiratory infections	Global	Female	Age- and sex-specific SEV for Unsafe sanitation			X			X
Lower respiratory infections	Data Rich	Female	Age- and sex-specific SEV for Unsafe sanitation			X			X
Lower respiratory infections	Global	Female	Population Density (over 1000 ppl/sqkm, proportion)			X			X
Lower respiratory infections	Data Rich	Female	Population Density (over 1000 ppl/sqkm, proportion)			X			X
Lower respiratory infections	Data Rich	Female	Socio-demographic Index			X			X
Lower respiratory infections	Global	Female	Socio-demographic Index			X			X
Lower respiratory infections	Data Rich	Female	Healthcare access and quality index		X			X	
Lower respiratory infections	Global	Female	Healthcare access and quality index		X			X	
Lower respiratory infections	Global	Female	Healthcare access and quality index		X			X	
Lower respiratory infections	Data Rich	Female	Healthcare access and quality index		X			X	
Lower respiratory infections	Global	Female	Outdoor Air Pollution (PM2.5)		X			X	
Lower respiratory infections	Data Rich	Female	Outdoor Air Pollution (PM2.5)		X			X	
Lower respiratory infections	Global	Female	Secondhand smoke		X			X	
Lower respiratory infections	Data Rich	Female	Secondhand smoke		X			X	
Lower respiratory infections	Data Rich	Female	No access to handwashing facility		X			X	
Lower respiratory infections	Global	Female	No access to handwashing facility		X			X	
Lower respiratory infections	Global	Female	Zinc deficiency		X			X	
Lower respiratory infections	Data Rich	Female	Zinc deficiency		X			X	
Lower respiratory infections	Data Rich	Female	Age-standardized proportion adult underweight		X			X	
Lower respiratory infections	Global	Female	Age-standardized proportion adult underweight		X			X	
Lower respiratory infections	Global	Female	Age-standardized SEV for Handwashing		X			X	
Lower respiratory infections	Data Rich	Female	Age-standardized SEV for Handwashing		X			X	
Lower respiratory infections	Global	Female	Age- and sex-specific SEV for Ambient particulate matter		X			X	
Lower respiratory infections	Data Rich	Female	Age- and sex-specific SEV for Ambient particulate matter		X			X	
Lower respiratory infections	Global	Female	Age- and sex-specific SEV for Household air pollution		X			X	
Lower respiratory infections	Data Rich	Female	Age- and sex-specific SEV for Household air pollution		X			X	
Lower respiratory infections	Global	Female	Age- and sex-specific SEV for Child underweight	X			X		
Lower respiratory infections	Data Rich	Female	Age- and sex-specific SEV for Child underweight	X			X		
Lower respiratory infections	Data Rich	Female	Smoking Prevalence	X			X		
Lower respiratory infections	Global	Female	Smoking Prevalence	X			X		
Lower respiratory infections	Global	Female	Age- and sex-specific SEV for Child wasting	X			X		
Lower respiratory infections	Data Rich	Female	Age- and sex-specific SEV for Child wasting	X			X		
Lower respiratory infections	Data Rich	Female	Indoor Air Pollution (All Cooking Fuels)	X			X		
Lower respiratory infections	Global	Female	Indoor Air Pollution (All Cooking Fuels)	X			X		
Lower respiratory infections	Global	Female	Indoor Air Pollution (All Cooking Fuels)	X			X		
Lower respiratory infections	Data Rich	Female	Indoor Air Pollution (All Cooking Fuels)	X			X		
Lower respiratory infections	Data Rich	Female	Outdoor Air Pollution (PM2.5)	X			X		
Lower respiratory infections	Global	Female	Outdoor Air Pollution (PM2.5)	X			X		
Lower respiratory infections	Data Rich	Female	Secondhand smoke	X			X		
Lower respiratory infections	Global	Female	Secondhand smoke	X			X		
Lower respiratory infections	Data Rich	Female	Log-transformed SEV scalar: LRI	X			X		
Lower respiratory infections	Global	Female	Log-transformed SEV scalar: LRI	X			X		
Lower respiratory infections	Global	Female	Log-transformed SEV scalar: LRI	X			X		
Lower respiratory infections	Data Rich	Female	Log-transformed SEV scalar: LRI	X			X		
Lower respiratory infections	Global	Female	Antibiotics for LRI	X			X		
Lower respiratory infections	Data Rich	Female	Antibiotics for LRI	X			X		
Lower respiratory infections	Global	Female	Age- and sex-specific SEV for Child stunting	X			X		
Lower respiratory infections	Data Rich	Female	Age- and sex-specific SEV for Child stunting	X			X		
Lower respiratory infections	Data Rich	Female	PCV3 lagged five year coverage, COVID-inclusive (proportion)					X	
Lower respiratory infections	Global	Female	PCV3 lagged five year coverage, COVID-inclusive (proportion)					X	
Lower respiratory infections	Data Rich	Female	DTP3 lagged five year coverage, COVID-inclusive (proportion)					X	

Appendix Table S11: Comparison of GBD 2021 and GBD 2023 covariates and level of covariates used in cause of death modeling

Cause	Model Version Type	Sex	Covariate	Level 1 GBD 2021	Level 2 GBD 2021	Level 3 GBD 2021	Level 1 GBD 2023	Level 2 GBD 2023	Level 3 GBD 2023
Lower respiratory infections	Global	Female	DTP3 lagged five year coverage, COVID-inclusive (proportion)					X	
Lower respiratory infections	Global	Female	DTP3 lagged five year coverage, COVID-inclusive (proportion)					X	
Lower respiratory infections	Data Rich	Female	DTP3 lagged five year coverage, COVID-inclusive (proportion)					X	
Lower respiratory infections	Data Rich	Female	Mask use					X	
Lower respiratory infections	Global	Female	Mask use					X	
Lower respiratory infections	Global	Female	Mask use					X	
Lower respiratory infections	Data Rich	Female	Mask use					X	
Lower respiratory infections	Global	Female	PCV3 lagged five year coverage, COVID-inclusive (proportion)				X		
Lower respiratory infections	Data Rich	Female	PCV3 lagged five year coverage, COVID-inclusive (proportion)				X		
Lower respiratory infections	Global	Female	Hib3 lagged five year coverage, COVID-inclusive (proportion)				X		
Lower respiratory infections	Data Rich	Female	Hib3 lagged five year coverage, COVID-inclusive (proportion)				X		
Lower respiratory infections	Data Rich	Male	Education (years per capita)			X			X
Lower respiratory infections	Global	Male	Education (years per capita)			X			X
Lower respiratory infections	Data Rich	Male	LDI (I$ per capita)			X			X
Lower respiratory infections	Global	Male	LDI (I$ per capita)			X			X
Lower respiratory infections	Global	Male	Socio-demographic Index			X			X
Lower respiratory infections	Data Rich	Male	Socio-demographic Index			X			X
Lower respiratory infections	Global	Male	Maternal Education (years per capita)			X			X
Lower respiratory infections	Data Rich	Male	Maternal Education (years per capita)			X			X
Lower respiratory infections	Data Rich	Male	Age- and sex-specific SEV for Unsafe sanitation			X			X
Lower respiratory infections	Global	Male	Age- and sex-specific SEV for Unsafe sanitation			X			X
Lower respiratory infections	Global	Male	Age- and sex-specific SEV for Unsafe sanitation			X			X
Lower respiratory infections	Data Rich	Male	Age- and sex-specific SEV for Unsafe sanitation			X			X
Lower respiratory infections	Global	Male	Population Density (over 1000 ppl/sqkm, proportion)			X			X
Lower respiratory infections	Data Rich	Male	Population Density (over 1000 ppl/sqkm, proportion)			X			X
Lower respiratory infections	Data Rich	Male	Socio-demographic Index			X			X
Lower respiratory infections	Global	Male	Socio-demographic Index			X			X
Lower respiratory infections	Data Rich	Male	Healthcare access and quality index		X			X	
Lower respiratory infections	Global	Male	Healthcare access and quality index		X			X	
Lower respiratory infections	Global	Male	Healthcare access and quality index		X			X	
Lower respiratory infections	Data Rich	Male	Healthcare access and quality index		X			X	
Lower respiratory infections	Global	Male	Outdoor Air Pollution (PM2.5)		X			X	
Lower respiratory infections	Data Rich	Male	Outdoor Air Pollution (PM2.5)		X			X	
Lower respiratory infections	Global	Male	Secondhand smoke		X			X	
Lower respiratory infections	Data Rich	Male	Secondhand smoke		X			X	
Lower respiratory infections	Data Rich	Male	No access to handwashing facility		X			X	
Lower respiratory infections	Global	Male	No access to handwashing facility		X			X	
Lower respiratory infections	Global	Male	Zinc deficiency		X			X	
Lower respiratory infections	Data Rich	Male	Zinc deficiency		X			X	
Lower respiratory infections	Data Rich	Male	Age-standardized proportion adult underweight		X			X	
Lower respiratory infections	Global	Male	Age-standardized proportion adult underweight		X			X	
Lower respiratory infections	Global	Male	Age-standardized SEV for Handwashing		X			X	
Lower respiratory infections	Data Rich	Male	Age-standardized SEV for Handwashing		X			X	
Lower respiratory infections	Global	Male	Age- and sex-specific SEV for Ambient particulate matter		X			X	
Lower respiratory infections	Data Rich	Male	Age- and sex-specific SEV for Ambient particulate matter		X			X	
Lower respiratory infections	Global	Male	Age- and sex-specific SEV for Household air pollution		X			X	
Lower respiratory infections	Data Rich	Male	Age- and sex-specific SEV for Household air pollution		X			X	
Lower respiratory infections	Global	Male	Age- and sex-specific SEV for Child underweight	X			X		
Lower respiratory infections	Data Rich	Male	Age- and sex-specific SEV for Child underweight	X			X		
Lower respiratory infections	Data Rich	Male	Smoking Prevalence	X			X		
Lower respiratory infections	Global	Male	Smoking Prevalence	X			X		
Lower respiratory infections	Global	Male	Age- and sex-specific SEV for Child wasting	X			X		
Lower respiratory infections	Data Rich	Male	Age- and sex-specific SEV for Child wasting	X			X		
Lower respiratory infections	Data Rich	Male	Indoor Air Pollution (All Cooking Fuels)	X			X		
Lower respiratory infections	Global	Male	Indoor Air Pollution (All Cooking Fuels)	X			X		
Lower respiratory infections	Global	Male	Indoor Air Pollution (All Cooking Fuels)	X			X		
Lower respiratory infections	Data Rich	Male	Indoor Air Pollution (All Cooking Fuels)	X			X		
Lower respiratory infections	Data Rich	Male	Outdoor Air Pollution (PM2.5)	X			X		
Lower respiratory infections	Global	Male	Outdoor Air Pollution (PM2.5)	X			X		
Lower respiratory infections	Data Rich	Male	Secondhand smoke	X			X		
Lower respiratory infections	Global	Male	Secondhand smoke	X			X		
Lower respiratory infections	Data Rich	Male	Log-transformed SEV scalar: LRI	X			X		
Lower respiratory infections	Global	Male	Log-transformed SEV scalar: LRI	X			X		
Lower respiratory infections	Global	Male	Log-transformed SEV scalar: LRI	X			X		
Lower respiratory infections	Data Rich	Male	Log-transformed SEV scalar: LRI	X			X		
Lower respiratory infections	Global	Male	Antibiotics for LRI	X			X		
Lower respiratory infections	Data Rich	Male	Antibiotics for LRI	X			X		
Lower respiratory infections	Global	Male	Age- and sex-specific SEV for Child stunting	X			X		
Lower respiratory infections	Data Rich	Male	Age- and sex-specific SEV for Child stunting	X			X		
Lower respiratory infections	Data Rich	Male	PCV3 lagged five year coverage, COVID-inclusive (proportion)					X	
Lower respiratory infections	Global	Male	PCV3 lagged five year coverage, COVID-inclusive (proportion)					X	
Lower respiratory infections	Data Rich	Male	DTP3 lagged five year coverage, COVID-inclusive (proportion)					X	
Lower respiratory infections	Global	Male	DTP3 lagged five year coverage, COVID-inclusive (proportion)					X	
Lower respiratory infections	Global	Male	DTP3 lagged five year coverage, COVID-inclusive (proportion)					X	
Lower respiratory infections	Data Rich	Male	DTP3 lagged five year coverage, COVID-inclusive (proportion)					X	
Lower respiratory infections	Data Rich	Male	Mask use					X	
Lower respiratory infections	Global	Male	Mask use					X	
Lower respiratory infections	Global	Male	Mask use					X	
Lower respiratory infections	Data Rich	Male	Mask use					X	
Lower respiratory infections	Global	Male	PCV3 lagged five year coverage, COVID-inclusive (proportion)				X		
Lower respiratory infections	Data Rich	Male	PCV3 lagged five year coverage, COVID-inclusive (proportion)				X		
Lower respiratory infections	Global	Male	Hib3 lagged five year coverage, COVID-inclusive (proportion)				X		
Lower respiratory infections	Data Rich	Male	Hib3 lagged five year coverage, COVID-inclusive (proportion)				X		
Upper respiratory infections	Data Rich	Female	Education (years per capita)			X			X
Upper respiratory infections	Global	Female	Education (years per capita)			X			X
Upper respiratory infections	Data Rich	Female	LDI (I$ per capita)			X			X
Upper respiratory infections	Global	Female	LDI (I$ per capita)			X			X
Upper respiratory infections	Data Rich	Female	Socio-demographic Index			X			X
Upper respiratory infections	Global	Female	Socio-demographic Index			X			X
Upper respiratory infections	Data Rich	Female	Healthcare access and quality index		X			X	
Upper respiratory infections	Global	Female	Healthcare access and quality index		X			X	
Upper respiratory infections	Data Rich	Female	Indoor Air Pollution (All Cooking Fuels)		X			X	
Upper respiratory infections	Global	Female	Indoor Air Pollution (All Cooking Fuels)		X			X	
Upper respiratory infections	Data Rich	Female	Outdoor Air Pollution (PM2.5)		X			X	
Upper respiratory infections	Global	Female	Outdoor Air Pollution (PM2.5)		X			X	
Upper respiratory infections	Data Rich	Female	Smoking Prevalence	X			X		
Upper respiratory infections	Global	Female	Smoking Prevalence	X			X		
Upper respiratory infections	Data Rich	Male	Education (years per capita)			X			X
Upper respiratory infections	Global	Male	Education (years per capita)			X			X
Upper respiratory infections	Data Rich	Male	LDI (I$ per capita)			X			X
Upper respiratory infections	Global	Male	LDI (I$ per capita)			X			X
Upper respiratory infections	Data Rich	Male	Socio-demographic Index			X			X
Upper respiratory infections	Global	Male	Socio-demographic Index			X			X
Upper respiratory infections	Data Rich	Male	Healthcare access and quality index		X			X	
Upper respiratory infections	Global	Male	Healthcare access and quality index		X			X	
Upper respiratory infections	Data Rich	Male	Indoor Air Pollution (All Cooking Fuels)		X			X	
Upper respiratory infections	Global	Male	Indoor Air Pollution (All Cooking Fuels)		X			X	
Upper respiratory infections	Data Rich	Male	Outdoor Air Pollution (PM2.5)		X			X	
Upper respiratory infections	Global	Male	Outdoor Air Pollution (PM2.5)		X			X	
Upper respiratory infections	Data Rich	Male	Smoking Prevalence	X			X		
Upper respiratory infections	Global	Male	Smoking Prevalence	X			X		
Otitis media	Data Rich	Female	Education (years per capita)			X			X

Appendix Table S11: Comparison of GBD 2021 and GBD 2023 covariates and level of covariates used in cause of death modeling

Cause	Model Version Type	Sex	Covariate	Level 1 GBD 2021	Level 2 GBD 2021	Level 3 GBD 2021	Level 1 GBD 2023	Level 2 GBD 2023	Level 3 GBD 2023
Otitis media	Global	Female	Education (years per capita)			X			X
Otitis media	Data Rich	Female	LDI (I$ per capita)			X			X
Otitis media	Global	Female	LDI (I$ per capita)			X			X
Otitis media	Data Rich	Female	Socio-demographic Index			X			X
Otitis media	Global	Female	Socio-demographic Index			X			X
Otitis media	Data Rich	Female	Healthcare access and quality index		X			X	
Otitis media	Global	Female	Healthcare access and quality index		X			X	
Otitis media	Data Rich	Female	Indoor Air Pollution (All Cooking Fuels)		X			X	
Otitis media	Global	Female	Indoor Air Pollution (All Cooking Fuels)		X			X	
Otitis media	Data Rich	Female	Outdoor Air Pollution (PM2.5)		X			X	
Otitis media	Global	Female	Outdoor Air Pollution (PM2.5)		X			X	
Otitis media	Data Rich	Female	Smoking Prevalence	X			X		
Otitis media	Global	Female	Smoking Prevalence	X			X		
Otitis media	Data Rich	Female	Log-transformed SEV scalar: Otitis	X			X		
Otitis media	Global	Female	Log-transformed SEV scalar: Otitis	X			X		
Otitis media	Data Rich	Male	Education (years per capita)			X			X
Otitis media	Global	Male	Education (years per capita)			X			X
Otitis media	Data Rich	Male	LDI (I$ per capita)			X			X
Otitis media	Global	Male	LDI (I$ per capita)			X			X
Otitis media	Data Rich	Male	Socio-demographic Index			X			X
Otitis media	Global	Male	Socio-demographic Index			X			X
Otitis media	Data Rich	Male	Healthcare access and quality index		X			X	
Otitis media	Global	Male	Healthcare access and quality index		X			X	
Otitis media	Data Rich	Male	Indoor Air Pollution (All Cooking Fuels)		X			X	
Otitis media	Global	Male	Indoor Air Pollution (All Cooking Fuels)		X			X	
Otitis media	Data Rich	Male	Outdoor Air Pollution (PM2.5)		X			X	
Otitis media	Global	Male	Outdoor Air Pollution (PM2.5)		X			X	
Otitis media	Data Rich	Male	Smoking Prevalence	X			X		
Otitis media	Global	Male	Smoking Prevalence	X			X		
Otitis media	Data Rich	Male	Log-transformed SEV scalar: Otitis	X			X		
Otitis media	Global	Male	Log-transformed SEV scalar: Otitis	X			X		
Diarrhoeal diseases	Global	Female	Education (years per capita)			X			X
Diarrhoeal diseases	Data Rich	Female	Education (years per capita)			X			X
Diarrhoeal diseases	Global	Female	LDI (I$ per capita)			X			X
Diarrhoeal diseases	Data Rich	Female	LDI (I$ per capita)			X			X
Diarrhoeal diseases	Global	Female	LDI (I$ per capita)			X			X
Diarrhoeal diseases	Data Rich	Female	LDI (I$ per capita)			X			X
Diarrhoeal diseases	Global	Female	Socio-demographic Index			X			X
Diarrhoeal diseases	Data Rich	Female	Socio-demographic Index			X			X
Diarrhoeal diseases	Global	Female	Socio-demographic Index			X			X
Diarrhoeal diseases	Data Rich	Female	Socio-demographic Index			X			X
Diarrhoeal diseases	Global	Female	Maternal Education (years per capita)			X			X
Diarrhoeal diseases	Data Rich	Female	Maternal Education (years per capita)			X			X
Diarrhoeal diseases	Global	Female	No access to handwashing facility			X			X
Diarrhoeal diseases	Data Rich	Female	No access to handwashing facility			X			X
Diarrhoeal diseases	Global	Female	No access to handwashing facility			X			X
Diarrhoeal diseases	Data Rich	Female	No access to handwashing facility			X			X
Diarrhoeal diseases	Global	Female	Age-standardized proportion adult underweight			X			X
Diarrhoeal diseases	Data Rich	Female	Age-standardized proportion adult underweight			X			X
Diarrhoeal diseases	Global	Female	Healthcare access and quality index		X			X	
Diarrhoeal diseases	Data Rich	Female	Healthcare access and quality index		X			X	
Diarrhoeal diseases	Global	Female	Healthcare access and quality index		X			X	
Diarrhoeal diseases	Data Rich	Female	Healthcare access and quality index		X			X	
Diarrhoeal diseases	Global	Female	ORS (oral rehydration)		X			X	
Diarrhoeal diseases	Data Rich	Female	ORS (oral rehydration)		X			X	
Diarrhoeal diseases	Global	Female	Zinc deficiency		X			X	
Diarrhoeal diseases	Data Rich	Female	Zinc deficiency		X			X	
Diarrhoeal diseases	Global	Female	Zinc treatment for diarrhea		X			X	
Diarrhoeal diseases	Data Rich	Female	Zinc treatment for diarrhea		X			X	
Diarrhoeal diseases	Global	Female	Sanitation (proportion with access)	X			X		
Diarrhoeal diseases	Data Rich	Female	Sanitation (proportion with access)	X			X		
Diarrhoeal diseases	Global	Female	Sanitation (proportion with access)	X			X		
Diarrhoeal diseases	Data Rich	Female	Sanitation (proportion with access)	X			X		
Diarrhoeal diseases	Global	Female	Improved Water Source (proportion with access)	X			X		
Diarrhoeal diseases	Data Rich	Female	Improved Water Source (proportion with access)	X			X		
Diarrhoeal diseases	Global	Female	Improved Water Source (proportion with access)	X			X		
Diarrhoeal diseases	Data Rich	Female	Improved Water Source (proportion with access)	X			X		
Diarrhoeal diseases	Global	Female	ORS (oral rehydration)	X			X		
Diarrhoeal diseases	Data Rich	Female	ORS (oral rehydration)	X			X		
Diarrhoeal diseases	Global	Female	RotaC lagged five year coverage, COVID-inclusive (proportion)					X	
Diarrhoeal diseases	Data Rich	Female	RotaC lagged five year coverage, COVID-inclusive (proportion)					X	
Diarrhoeal diseases	Global	Female	RotaC lagged five year coverage, COVID-inclusive (proportion)				X		
Diarrhoeal diseases	Data Rich	Female	RotaC lagged five year coverage, COVID-inclusive (proportion)				X		
Diarrhoeal diseases	Global	Male	Education (years per capita)			X			X
Diarrhoeal diseases	Data Rich	Male	Education (years per capita)			X			X
Diarrhoeal diseases	Global	Male	LDI (I$ per capita)			X			X
Diarrhoeal diseases	Data Rich	Male	LDI (I$ per capita)			X			X
Diarrhoeal diseases	Global	Male	LDI (I$ per capita)			X			X
Diarrhoeal diseases	Data Rich	Male	LDI (I$ per capita)			X			X
Diarrhoeal diseases	Global	Male	Socio-demographic Index			X			X
Diarrhoeal diseases	Data Rich	Male	Socio-demographic Index			X			X
Diarrhoeal diseases	Global	Male	Socio-demographic Index			X			X
Diarrhoeal diseases	Data Rich	Male	Socio-demographic Index			X			X
Diarrhoeal diseases	Global	Male	Maternal Education (years per capita)			X			X
Diarrhoeal diseases	Data Rich	Male	Maternal Education (years per capita)			X			X
Diarrhoeal diseases	Global	Male	No access to handwashing facility			X			X
Diarrhoeal diseases	Data Rich	Male	No access to handwashing facility			X			X
Diarrhoeal diseases	Global	Male	No access to handwashing facility			X			X
Diarrhoeal diseases	Data Rich	Male	No access to handwashing facility			X			X
Diarrhoeal diseases	Global	Male	Age-standardized proportion adult underweight			X			X
Diarrhoeal diseases	Data Rich	Male	Age-standardized proportion adult underweight			X			X
Diarrhoeal diseases	Global	Male	Healthcare access and quality index		X			X	
Diarrhoeal diseases	Data Rich	Male	Healthcare access and quality index		X			X	
Diarrhoeal diseases	Global	Male	Healthcare access and quality index		X			X	
Diarrhoeal diseases	Data Rich	Male	Healthcare access and quality index		X			X	
Diarrhoeal diseases	Global	Male	ORS (oral rehydration)		X			X	
Diarrhoeal diseases	Data Rich	Male	ORS (oral rehydration)		X			X	
Diarrhoeal diseases	Global	Male	Zinc deficiency		X			X	
Diarrhoeal diseases	Data Rich	Male	Zinc deficiency		X			X	
Diarrhoeal diseases	Global	Male	Zinc treatment for diarrhea		X			X	
Diarrhoeal diseases	Data Rich	Male	Zinc treatment for diarrhea		X			X	
Diarrhoeal diseases	Global	Male	Sanitation (proportion with access)	X			X		
Diarrhoeal diseases	Data Rich	Male	Sanitation (proportion with access)	X			X		
Diarrhoeal diseases	Global	Male	Sanitation (proportion with access)	X			X		
Diarrhoeal diseases	Data Rich	Male	Sanitation (proportion with access)	X			X		
Diarrhoeal diseases	Global	Male	Improved Water Source (proportion with access)	X			X		
Diarrhoeal diseases	Data Rich	Male	Improved Water Source (proportion with access)	X			X		
Diarrhoeal diseases	Global	Male	Improved Water Source (proportion with access)	X			X		
Diarrhoeal diseases	Data Rich	Male	Improved Water Source (proportion with access)	X			X		
Diarrhoeal diseases	Global	Male	ORS (oral rehydration)	X			X		
Diarrhoeal diseases	Data Rich	Male	ORS (oral rehydration)	X			X		
Diarrhoeal diseases	Global	Male	RotaC lagged five year coverage, COVID-inclusive (proportion)					X	
Diarrhoeal diseases	Data Rich	Male	RotaC lagged five year coverage, COVID-inclusive (proportion)					X	
Diarrhoeal diseases	Global	Male	RotaC lagged five year coverage, COVID-inclusive (proportion)				X		
Diarrhoeal diseases	Data Rich	Male	RotaC lagged five year coverage, COVID-inclusive (proportion)				X		
Rabies	Data Rich	Female	Population Density (500-1000 ppl/sqkm, proportion)			X			X
Rabies	Global	Female	Population Density (500-1000 ppl/sqkm, proportion)			X			X
Rabies	Data Rich	Female	Population Density (under 150 ppl/sqkm, proportion)			X			X
Rabies	Global	Female	Population Density (under 150 ppl/sqkm, proportion)			X			X
Rabies	Data Rich	Female	Maternal care and immunization		X			X	

Appendix Table S11: Comparison of GBD 2021 and GBD 2023 covariates and level of covariates used in cause of death modeling

Cause	Model Version Type	Sex	Covariate	Level 1 GBD 2021	Level 2 GBD 2021	Level 3 GBD 2021	Level 1 GBD 2023	Level 2 GBD 2023	Level 3 GBD 2023
Rabies	Data Rich	Female	Healthcare access and quality index		X			X	
Rabies	Global	Female	Healthcare access and quality index		X			X	
Rabies	Data Rich	Female	Skilled Birth Attendance (proportion)		X			X	
Rabies	Global	Female	Skilled Birth Attendance (proportion)		X			X	
Rabies	Data Rich	Female	Socio-demographic Index	X			X		
Rabies	Global	Female	Socio-demographic Index	X			X		
Rabies	Data Rich	Female	Antenatal Care (4 visits) Coverage (proportion)	X			X		
Rabies	Global	Female	Antenatal Care (4 visits) Coverage (proportion)	X			X		
Rabies	Data Rich	Female	In-Facility Delivery (proportion)	X			X		
Rabies	Global	Female	In-Facility Delivery (proportion)	X			X		
Rabies	Data Rich	Male	Population Density (500-1000 ppl/sqkm, proportion)			X			X
Rabies	Global	Male	Population Density (500-1000 ppl/sqkm, proportion)			X			X
Rabies	Data Rich	Male	Population Density (under 150 ppl/sqkm, proportion)			X			X
Rabies	Global	Male	Population Density (under 150 ppl/sqkm, proportion)			X			X
Rabies	Data Rich	Male	Maternal care and immunization		X			X	
Rabies	Data Rich	Male	Healthcare access and quality index		X			X	
Rabies	Global	Male	Healthcare access and quality index		X			X	
Rabies	Data Rich	Male	Skilled Birth Attendance (proportion)		X			X	
Rabies	Global	Male	Skilled Birth Attendance (proportion)		X			X	
Rabies	Data Rich	Male	Socio-demographic Index	X			X		
Rabies	Global	Male	Socio-demographic Index	X			X		
Rabies	Data Rich	Male	Antenatal Care (4 visits) Coverage (proportion)	X			X		
Rabies	Global	Male	Antenatal Care (4 visits) Coverage (proportion)	X			X		
Rabies	Data Rich	Male	In-Facility Delivery (proportion)	X			X		
Rabies	Global	Male	In-Facility Delivery (proportion)	X			X		
Other neglected tropical diseases	Data Rich	Female	Education (years per capita)			X			X
Other neglected tropical diseases	Global	Female	Education (years per capita)			X			X
Other neglected tropical diseases	Data Rich	Female	LDI (I$ per capita)			X			X
Other neglected tropical diseases	Global	Female	LDI (I$ per capita)			X			X
Other neglected tropical diseases	Data Rich	Female	Socio-demographic Index			X			X
Other neglected tropical diseases	Global	Female	Socio-demographic Index			X			X
Other neglected tropical diseases	Data Rich	Female	Sanitation (proportion with access)		X			X	
Other neglected tropical diseases	Global	Female	Sanitation (proportion with access)		X			X	
Other neglected tropical diseases	Data Rich	Female	Rainfall Quintile 5 (proportion)		X			X	
Other neglected tropical diseases	Global	Female	Rainfall Quintile 5 (proportion)		X			X	
Other neglected tropical diseases	Data Rich	Female	Healthcare access and quality index	X			X		
Other neglected tropical diseases	Global	Female	Healthcare access and quality index	X			X		
Other neglected tropical diseases	Data Rich	Female	Latitude Under 15 (proportion)	X			X		
Other neglected tropical diseases	Global	Female	Latitude Under 15 (proportion)	X			X		
Other neglected tropical diseases	Data Rich	Male	Education (years per capita)			X			X
Other neglected tropical diseases	Global	Male	Education (years per capita)			X			X
Other neglected tropical diseases	Data Rich	Male	LDI (I$ per capita)			X			X
Other neglected tropical diseases	Global	Male	LDI (I$ per capita)			X			X
Other neglected tropical diseases	Data Rich	Male	Socio-demographic Index			X			X
Other neglected tropical diseases	Global	Male	Socio-demographic Index			X			X
Other neglected tropical diseases	Data Rich	Male	Sanitation (proportion with access)		X			X	
Other neglected tropical diseases	Global	Male	Sanitation (proportion with access)		X			X	
Other neglected tropical diseases	Data Rich	Male	Rainfall Quintile 5 (proportion)		X			X	
Other neglected tropical diseases	Global	Male	Rainfall Quintile 5 (proportion)		X			X	
Other neglected tropical diseases	Data Rich	Male	Healthcare access and quality index	X			X		
Other neglected tropical diseases	Global	Male	Healthcare access and quality index	X			X		
Other neglected tropical diseases	Data Rich	Male	Latitude Under 15 (proportion)	X			X		
Other neglected tropical diseases	Global	Male	Latitude Under 15 (proportion)	X			X		
Meningitis	Global	Female	LDI (I$ per capita)			X			X
Meningitis	Data Rich	Female	LDI (I$ per capita)			X			X
Meningitis	Data Rich	Female	LDI (I$ per capita)			X			X
Meningitis	Global	Female	LDI (I$ per capita)			X			X
Meningitis	Global	Female	Socio-demographic Index			X			X
Meningitis	Data Rich	Female	Socio-demographic Index			X			X
Meningitis	Data Rich	Female	Socio-demographic Index			X			X
Meningitis	Global	Female	Socio-demographic Index			X			X
Meningitis	Global	Female	Sanitation (proportion with access)			X			X
Meningitis	Data Rich	Female	Sanitation (proportion with access)			X			X
Meningitis	Data Rich	Female	Sanitation (proportion with access)			X			X
Meningitis	Global	Female	Sanitation (proportion with access)			X			X
Meningitis	Global	Female	Maternal Education (years per capita)			X			X
Meningitis	Data Rich	Female	Maternal Education (years per capita)			X			X
Meningitis	Data Rich	Female	Maternal Education (years per capita)			X			X
Meningitis	Global	Female	Maternal Education (years per capita)			X			X
Meningitis	Global	Female	Maternal care and immunization		X			X	
Meningitis	Data Rich	Female	Maternal care and immunization		X			X	
Meningitis	Data Rich	Female	Maternal care and immunization		X			X	
Meningitis	Global	Female	Maternal care and immunization		X			X	
Meningitis	Global	Female	Healthcare access and quality index		X			X	
Meningitis	Data Rich	Female	Healthcare access and quality index		X			X	
Meningitis	Data Rich	Female	Healthcare access and quality index		X			X	
Meningitis	Global	Female	Healthcare access and quality index		X			X	
Meningitis	Global	Female	Improved Water Source (proportion with access)		X			X	
Meningitis	Data Rich	Female	Improved Water Source (proportion with access)		X			X	
Meningitis	Data Rich	Female	Improved Water Source (proportion with access)		X			X	
Meningitis	Global	Female	Improved Water Source (proportion with access)		X			X	
Meningitis	Global	Female	Age- and sex-specific SEV for Child underweight		X			X	
Meningitis	Data Rich	Female	Age- and sex-specific SEV for Child underweight		X			X	
Meningitis	Global	Female	meningitis belt (proportion)	X			X		
Meningitis	Data Rich	Female	meningitis belt (proportion)	X			X		
Meningitis	Data Rich	Female	meningitis belt (proportion)	X			X		
Meningitis	Global	Female	meningitis belt (proportion)	X			X		
Meningitis	Global	Female	Proportion of total population covered by menafrivac initiative (meningitis meningococcal type A vaccine)	X			X		
Meningitis	Data Rich	Female	Proportion of total population covered by menafrivac initiative (meningitis meningococcal type A vaccine)	X			X		
Meningitis	Data Rich	Female	Proportion of total population covered by menafrivac initiative (meningitis meningococcal type A vaccine)	X			X		
Meningitis	Global	Female	Proportion of total population covered by menafrivac initiative (meningitis meningococcal type A vaccine)	X			X		
Meningitis	Global	Female	Hib3 transformed: population-level coverage, including indirect effects (proportion)	X			X		
Meningitis	Data Rich	Female	Hib3 transformed: population-level coverage, including indirect effects (proportion)	X			X		
Meningitis	Data Rich	Female	Hib3 transformed: population-level coverage, including indirect effects (proportion)	X			X		
Meningitis	Global	Female	Hib3 transformed: population-level coverage, including indirect effects (proportion)	X			X		
Meningitis	Global	Female	PCV3 lagged five year coverage, COVID-inclusive (proportion)				X		
Meningitis	Data Rich	Female	PCV3 lagged five year coverage, COVID-inclusive (proportion)				X		
Meningitis	Data Rich	Female	PCV3 lagged five year coverage, COVID-inclusive (proportion)				X		
Meningitis	Global	Female	PCV3 lagged five year coverage, COVID-inclusive (proportion)				X		
Meningitis	Global	Male	LDI (I$ per capita)			X			X
Meningitis	Data Rich	Male	LDI (I$ per capita)			X			X
Meningitis	Data Rich	Male	LDI (I$ per capita)			X			X
Meningitis	Global	Male	LDI (I$ per capita)			X			X
Meningitis	Global	Male	Socio-demographic Index			X			X
Meningitis	Data Rich	Male	Socio-demographic Index			X			X
Meningitis	Data Rich	Male	Socio-demographic Index			X			X
Meningitis	Global	Male	Socio-demographic Index			X			X
Meningitis	Global	Male	Sanitation (proportion with access)			X			X
Meningitis	Data Rich	Male	Sanitation (proportion with access)			X			X
Meningitis	Data Rich	Male	Sanitation (proportion with access)			X			X

Appendix Table S11: Comparison of GBD 2021 and GBD 2023 covariates and level of covariates used in cause of death modeling

Cause	Model Version Type	Sex	Covariate	Level 1 GBD 2021	Level 2 GBD 2021	Level 3 GBD 2021	Level 1 GBD 2023	Level 2 GBD 2023	Level 3 GBD 2023
Meningitis	Global	Male	Sanitation (proportion with access)			X			X
Meningitis	Global	Male	Maternal Education (years per capita)			X			X
Meningitis	Data Rich	Male	Maternal Education (years per capita)			X			X
Meningitis	Data Rich	Male	Maternal Education (years per capita)			X			X
Meningitis	Global	Male	Maternal Education (years per capita)			X			X
Meningitis	Global	Male	Maternal care and immunization		X			X	
Meningitis	Data Rich	Male	Maternal care and immunization		X			X	
Meningitis	Data Rich	Male	Maternal care and immunization		X			X	
Meningitis	Global	Male	Maternal care and immunization		X			X	
Meningitis	Global	Male	Healthcare access and quality index		X			X	
Meningitis	Data Rich	Male	Healthcare access and quality index		X			X	
Meningitis	Data Rich	Male	Healthcare access and quality index		X			X	
Meningitis	Global	Male	Healthcare access and quality index		X			X	
Meningitis	Global	Male	Improved Water Source (proportion with access)		X			X	
Meningitis	Data Rich	Male	Improved Water Source (proportion with access)		X			X	
Meningitis	Data Rich	Male	Improved Water Source (proportion with access)		X			X	
Meningitis	Global	Male	Improved Water Source (proportion with access)		X			X	
Meningitis	Global	Male	Age- and sex-specific SEV for Child underweight		X			X	
Meningitis	Data Rich	Male	Age- and sex-specific SEV for Child underweight		X			X	
Meningitis	Global	Male	meningitis belt (proportion)	X			X		
Meningitis	Data Rich	Male	meningitis belt (proportion)	X			X		
Meningitis	Data Rich	Male	meningitis belt (proportion)	X			X		
Meningitis	Global	Male	meningitis belt (proportion)	X			X		
Meningitis	Global	Male	Proportion of total population covered by menafrivac initiative (meningitis meningococcal type A vaccine)	X			X		
Meningitis	Data Rich	Male	Proportion of total population covered by menafrivac initiative (meningitis meningococcal type A vaccine)	X			X		
Meningitis	Data Rich	Male	Proportion of total population covered by menafrivac initiative (meningitis meningococcal type A vaccine)	X			X		
Meningitis	Global	Male	Proportion of total population covered by menafrivac initiative (meningitis meningococcal type A vaccine)	X			X		
Meningitis	Global	Male	Hib3 transformed: population-level coverage, including indirect effects (proportion)	X			X		
Meningitis	Data Rich	Male	Hib3 transformed: population-level coverage, including indirect effects (proportion)	X			X		
Meningitis	Data Rich	Male	Hib3 transformed: population-level coverage, including indirect effects (proportion)	X			X		
Meningitis	Global	Male	Hib3 transformed: population-level coverage, including indirect effects (proportion)	X			X		
Meningitis	Global	Male	PCV3 lagged five year coverage, COVID-inclusive (proportion)				X		
Meningitis	Data Rich	Male	PCV3 lagged five year coverage, COVID-inclusive (proportion)				X		
Meningitis	Data Rich	Male	PCV3 lagged five year coverage, COVID-inclusive (proportion)				X		
Meningitis	Global	Male	PCV3 lagged five year coverage, COVID-inclusive (proportion)				X		
Encephalitis	Data Rich	Female	Socio-demographic Index			X			X
Encephalitis	Global	Female	Socio-demographic Index			X			X
Encephalitis	Data Rich	Female	DTP3 Coverage, COVID-free (proportion)			X			X
Encephalitis	Global	Female	DTP3 Coverage, COVID-free (proportion)			X			X
Encephalitis	Data Rich	Female	In-Facility Delivery (proportion)			X			X
Encephalitis	Global	Female	In-Facility Delivery (proportion)			X			X
Encephalitis	Data Rich	Female	Sanitation (proportion with access)			X			X
Encephalitis	Global	Female	Sanitation (proportion with access)			X			X
Encephalitis	Data Rich	Female	Improved Water Source (proportion with access)			X			X
Encephalitis	Global	Female	Improved Water Source (proportion with access)			X			X
Encephalitis	Data Rich	Female	Maternal Education (years per capita)			X			X
Encephalitis	Global	Female	Maternal Education (years per capita)			X			X
Encephalitis	Data Rich	Female	Maternal care and immunization		X			X	
Encephalitis	Global	Female	Maternal care and immunization		X			X	
Encephalitis	Data Rich	Female	LDI (I$ per capita)		X			X	
Encephalitis	Global	Female	LDI (I$ per capita)		X			X	
Encephalitis	Data Rich	Female	Healthcare access and quality index		X			X	
Encephalitis	Global	Female	Healthcare access and quality index		X			X	
Encephalitis	Data Rich	Female	Japanese encelphalitis endemic area (binary)	X			X		
Encephalitis	Global	Female	Japanese encelphalitis endemic area (binary)	X			X		
Encephalitis	Data Rich	Female	Age- and sex-specific SEV for Child underweight	X			X		
Encephalitis	Global	Female	Age- and sex-specific SEV for Child underweight	X			X		
Encephalitis	Data Rich	Male	Socio-demographic Index			X			X
Encephalitis	Global	Male	Socio-demographic Index			X			X
Encephalitis	Data Rich	Male	DTP3 Coverage, COVID-free (proportion)			X			X
Encephalitis	Global	Male	DTP3 Coverage, COVID-free (proportion)			X			X
Encephalitis	Data Rich	Male	In-Facility Delivery (proportion)			X			X
Encephalitis	Global	Male	In-Facility Delivery (proportion)			X			X
Encephalitis	Data Rich	Male	Sanitation (proportion with access)			X			X
Encephalitis	Global	Male	Sanitation (proportion with access)			X			X
Encephalitis	Data Rich	Male	Improved Water Source (proportion with access)			X			X
Encephalitis	Global	Male	Improved Water Source (proportion with access)			X			X
Encephalitis	Data Rich	Male	Maternal Education (years per capita)			X			X
Encephalitis	Global	Male	Maternal Education (years per capita)			X			X
Encephalitis	Data Rich	Male	Maternal care and immunization		X			X	
Encephalitis	Global	Male	Maternal care and immunization		X			X	
Encephalitis	Data Rich	Male	LDI (I$ per capita)		X			X	
Encephalitis	Global	Male	LDI (I$ per capita)		X			X	
Encephalitis	Data Rich	Male	Healthcare access and quality index		X			X	
Encephalitis	Global	Male	Healthcare access and quality index		X			X	
Encephalitis	Data Rich	Male	Japanese encelphalitis endemic area (binary)	X			X		
Encephalitis	Global	Male	Japanese encelphalitis endemic area (binary)	X			X		
Encephalitis	Data Rich	Male	Age- and sex-specific SEV for Child underweight	X			X		
Encephalitis	Global	Male	Age- and sex-specific SEV for Child underweight	X			X		
Tetanus	Data Rich	Female	Education (years per capita)			X			X
Tetanus	Global	Female	Education (years per capita)			X			X
Tetanus	Data Rich	Female	Education (years per capita)			X			X
Tetanus	Global	Female	Education (years per capita)			X			X
Tetanus	Data Rich	Female	LDI (I$ per capita)			X			X
Tetanus	Global	Female	LDI (I$ per capita)			X			X
Tetanus	Data Rich	Female	LDI (I$ per capita)			X			X
Tetanus	Global	Female	LDI (I$ per capita)			X			X
Tetanus	Data Rich	Female	Socio-demographic Index			X			X
Tetanus	Global	Female	Socio-demographic Index			X			X
Tetanus	Data Rich	Female	Socio-demographic Index			X			X
Tetanus	Global	Female	Socio-demographic Index			X			X
Tetanus	Data Rich	Female	Sanitation (proportion with access)			X			X
Tetanus	Global	Female	Sanitation (proportion with access)			X			X
Tetanus	Data Rich	Female	Healthcare access and quality index		X			X	
Tetanus	Global	Female	Healthcare access and quality index		X			X	
Tetanus	Data Rich	Female	Healthcare access and quality index		X			X	
Tetanus	Global	Female	Healthcare access and quality index		X			X	
Tetanus	Data Rich	Female	Skilled Birth Attendance (proportion)		X			X	
Tetanus	Global	Female	Skilled Birth Attendance (proportion)		X			X	
Tetanus	Data Rich	Female	In-Facility Delivery (proportion)		X			X	
Tetanus	Global	Female	In-Facility Delivery (proportion)		X			X	
Tetanus	Data Rich	Female	tetanus toxoid maternal protection at birth	X			X		
Tetanus	Global	Female	tetanus toxoid maternal protection at birth	X			X		

Appendix Table S11: Comparison of GBD 2021 and GBD 2023 covariates and level of covariates used in cause of death modeling

Cause	Model Version Type	Sex	Covariate	Level 1 GBD 2021	Level 2 GBD 2021	Level 3 GBD 2021	Level 1 GBD 2023	Level 2 GBD 2023	Level 3 GBD 2023
Tetanus	Data Rich	Female	DTP3 lagged five year coverage, COVID-inclusive (proportion)				X		
Tetanus	Global	Female	DTP3 lagged five year coverage, COVID-inclusive (proportion)				X		
Tetanus	Data Rich	Female	DTP3 lagged five year coverage, COVID-inclusive (proportion)				X		
Tetanus	Global	Female	DTP3 lagged five year coverage, COVID-inclusive (proportion)				X		
Tetanus	Data Rich	Male	Education (years per capita)			X			X
Tetanus	Global	Male	Education (years per capita)			X			X
Tetanus	Data Rich	Male	Education (years per capita)			X			X
Tetanus	Global	Male	Education (years per capita)			X			X
Tetanus	Data Rich	Male	LDI (I$ per capita)			X			X
Tetanus	Global	Male	LDI (I$ per capita)			X			X
Tetanus	Data Rich	Male	LDI (I$ per capita)			X			X
Tetanus	Global	Male	LDI (I$ per capita)			X			X
Tetanus	Data Rich	Male	Socio-demographic Index			X			X
Tetanus	Global	Male	Socio-demographic Index			X			X
Tetanus	Data Rich	Male	Socio-demographic Index			X			X
Tetanus	Global	Male	Socio-demographic Index			X			X
Tetanus	Data Rich	Male	Sanitation (proportion with access)			X			X
Tetanus	Global	Male	Sanitation (proportion with access)			X			X
Tetanus	Data Rich	Male	Healthcare access and quality index		X			X	
Tetanus	Global	Male	Healthcare access and quality index		X			X	
Tetanus	Data Rich	Male	Healthcare access and quality index		X			X	
Tetanus	Global	Male	Healthcare access and quality index		X			X	
Tetanus	Data Rich	Male	Skilled Birth Attendance (proportion)		X			X	
Tetanus	Global	Male	Skilled Birth Attendance (proportion)		X			X	
Tetanus	Data Rich	Male	In-Facility Delivery (proportion)		X			X	
Tetanus	Global	Male	In-Facility Delivery (proportion)		X			X	
Tetanus	Data Rich	Male	tetanus toxoid maternal protection at birth	X			X		
Tetanus	Global	Male	tetanus toxoid maternal protection at birth	X			X		
Tetanus	Data Rich	Male	DTP3 lagged five year coverage, COVID-inclusive (proportion)				X		
Tetanus	Global	Male	DTP3 lagged five year coverage, COVID-inclusive (proportion)				X		
Tetanus	Data Rich	Male	DTP3 lagged five year coverage, COVID-inclusive (proportion)				X		
Tetanus	Global	Male	DTP3 lagged five year coverage, COVID-inclusive (proportion)				X		
Acute hepatitis	Global	Female	Education (years per capita)			X			X
Acute hepatitis	Data Rich	Female	Education (years per capita)			X			X
Acute hepatitis	Global	Female	LDI (I$ per capita)			X			X
Acute hepatitis	Data Rich	Female	LDI (I$ per capita)			X			X
Acute hepatitis	Global	Female	Socio-demographic Index		X			X	
Acute hepatitis	Data Rich	Female	Socio-demographic Index		X			X	
Acute hepatitis	Global	Female	Healthcare access and quality index		X			X	
Acute hepatitis	Data Rich	Female	Healthcare access and quality index		X			X	
Acute hepatitis	Global	Female	Age- and sex-specific SEV for Unsafe water		X			X	
Acute hepatitis	Data Rich	Female	Age- and sex-specific SEV for Unsafe water		X			X	
Acute hepatitis	Global	Female	Age- and sex-specific SEV for Unsafe sanitation		X			X	
Acute hepatitis	Data Rich	Female	Age- and sex-specific SEV for Unsafe sanitation		X			X	
Acute hepatitis	Global	Female	Hepatitis B vaccine coverage, aged through time, COVID-free (proportion)		X			X	
Acute hepatitis	Data Rich	Female	Hepatitis B vaccine coverage, aged through time, COVID-free (proportion)		X			X	
Acute hepatitis	Global	Female	Intravenous drug use (proportion by age)		X			X	
Acute hepatitis	Data Rich	Female	Intravenous drug use (proportion by age)		X			X	
Acute hepatitis	Global	Female	Hepatitis C Seroprevalence (anti-HCV) age standardized	X			X		
Acute hepatitis	Data Rich	Female	Hepatitis C Seroprevalence (anti-HCV) age standardized	X			X		
Acute hepatitis	Global	Female	Vaccine adjusted HbSAg serporevalence age standardized	X			X		
Acute hepatitis	Data Rich	Female	Vaccine adjusted HbSAg serporevalence age standardized	X			X		
Acute hepatitis	Global	Female	Log-transformed SEV scalar: Hep	X			X		
Acute hepatitis	Data Rich	Female	Log-transformed SEV scalar: Hep	X			X		
Acute hepatitis	Global	Female	Hepatitis A Seroprevalence (anti-HAV) age standardized	X			X		
Acute hepatitis	Data Rich	Female	Hepatitis A Seroprevalence (anti-HAV) age standardized	X			X		
Acute hepatitis	Global	Female	Hepatitis E Seroprevalence (anti-HEV) age standardized	X			X		
Acute hepatitis	Data Rich	Female	Hepatitis E Seroprevalence (anti-HEV) age standardized	X			X		
Acute hepatitis	Global	Male	Education (years per capita)			X			X
Acute hepatitis	Data Rich	Male	Education (years per capita)			X			X
Acute hepatitis	Global	Male	LDI (I$ per capita)			X			X
Acute hepatitis	Data Rich	Male	LDI (I$ per capita)			X			X
Acute hepatitis	Global	Male	Socio-demographic Index		X			X	
Acute hepatitis	Data Rich	Male	Socio-demographic Index		X			X	
Acute hepatitis	Global	Male	Healthcare access and quality index		X			X	
Acute hepatitis	Data Rich	Male	Healthcare access and quality index		X			X	
Acute hepatitis	Global	Male	Age- and sex-specific SEV for Unsafe water		X			X	
Acute hepatitis	Data Rich	Male	Age- and sex-specific SEV for Unsafe water		X			X	
Acute hepatitis	Global	Male	Age- and sex-specific SEV for Unsafe sanitation		X			X	
Acute hepatitis	Data Rich	Male	Age- and sex-specific SEV for Unsafe sanitation		X			X	
Acute hepatitis	Global	Male	Hepatitis B vaccine coverage, aged through time, COVID-free (proportion)		X			X	
Acute hepatitis	Data Rich	Male	Hepatitis B vaccine coverage, aged through time, COVID-free (proportion)		X			X	
Acute hepatitis	Global	Male	Intravenous drug use (proportion by age)		X			X	
Acute hepatitis	Data Rich	Male	Intravenous drug use (proportion by age)		X			X	
Acute hepatitis	Global	Male	Hepatitis C Seroprevalence (anti-HCV) age standardized	X			X		
Acute hepatitis	Data Rich	Male	Hepatitis C Seroprevalence (anti-HCV) age standardized	X			X		
Acute hepatitis	Global	Male	Vaccine adjusted HbSAg serporevalence age standardized	X			X		
Acute hepatitis	Data Rich	Male	Vaccine adjusted HbSAg serporevalence age standardized	X			X		
Acute hepatitis	Global	Male	Log-transformed SEV scalar: Hep	X			X		
Acute hepatitis	Data Rich	Male	Log-transformed SEV scalar: Hep	X			X		
Acute hepatitis	Global	Male	Hepatitis A Seroprevalence (anti-HAV) age standardized	X			X		
Acute hepatitis	Data Rich	Male	Hepatitis A Seroprevalence (anti-HAV) age standardized	X			X		
Acute hepatitis	Global	Male	Hepatitis E Seroprevalence (anti-HEV) age standardized	X			X		
Acute hepatitis	Data Rich	Male	Hepatitis E Seroprevalence (anti-HEV) age standardized	X			X		
Acute hepatitis A	Data Rich	Female	Education (years per capita)			X			X
Acute hepatitis A	Global	Female	Education (years per capita)			X			X
Acute hepatitis A	Data Rich	Female	LDI (I$ per capita)			X			X
Acute hepatitis A	Global	Female	LDI (I$ per capita)			X			X
Acute hepatitis A	Data Rich	Female	Socio-demographic Index		X			X	
Acute hepatitis A	Global	Female	Socio-demographic Index		X			X	
Acute hepatitis A	Data Rich	Female	Healthcare access and quality index		X			X	
Acute hepatitis A	Global	Female	Healthcare access and quality index		X			X	
Acute hepatitis A	Data Rich	Female	Age- and sex-specific SEV for Unsafe water		X			X	
Acute hepatitis A	Global	Female	Age- and sex-specific SEV for Unsafe water		X			X	
Acute hepatitis A	Data Rich	Female	Age- and sex-specific SEV for Unsafe sanitation		X			X	
Acute hepatitis A	Global	Female	Age- and sex-specific SEV for Unsafe sanitation		X			X	
Acute hepatitis A	Data Rich	Female	Log-transformed SEV scalar: Hep	X			X		
Acute hepatitis A	Global	Female	Log-transformed SEV scalar: Hep	X			X		
Acute hepatitis A	Data Rich	Female	Hepatitis A Seroprevalence (anti-HAV) age standardized	X			X		
Acute hepatitis A	Global	Female	Hepatitis A Seroprevalence (anti-HAV) age standardized	X			X		
Acute hepatitis A	Data Rich	Male	Education (years per capita)			X			X
Acute hepatitis A	Global	Male	Education (years per capita)			X			X
Acute hepatitis A	Data Rich	Male	LDI (I$ per capita)			X			X
Acute hepatitis A	Global	Male	LDI (I$ per capita)			X			X
Acute hepatitis A	Data Rich	Male	Socio-demographic Index		X			X	
Acute hepatitis A	Global	Male	Socio-demographic Index		X			X	
Acute hepatitis A	Data Rich	Male	Healthcare access and quality index		X			X	
Acute hepatitis A	Global	Male	Healthcare access and quality index		X			X	

Appendix Table S11: Comparison of GBD 2021 and GBD 2023 covariates and level of covariates used in cause of death modeling

Cause	Model Version Type	Sex	Covariate	Level 1 GBD 2021	Level 2 GBD 2021	Level 3 GBD 2021	Level 1 GBD 2023	Level 2 GBD 2023	Level 3 GBD 2023
Acute hepatitis A	Data Rich	Male	Age- and sex-specific SEV for Unsafe water		X			X	
Acute hepatitis A	Global	Male	Age- and sex-specific SEV for Unsafe water		X			X	
Acute hepatitis A	Data Rich	Male	Age- and sex-specific SEV for Unsafe sanitation		X			X	
Acute hepatitis A	Global	Male	Age- and sex-specific SEV for Unsafe sanitation		X			X	
Acute hepatitis A	Data Rich	Male	Log-transformed SEV scalar: Hep	X			X		
Acute hepatitis A	Global	Male	Log-transformed SEV scalar: Hep	X			X		
Acute hepatitis A	Data Rich	Male	Hepatitis A Seroprevalence (anti-HAV) age standardized	X			X		
Acute hepatitis A	Global	Male	Hepatitis A Seroprevalence (anti-HAV) age standardized	X			X		
Acute hepatitis B	Data Rich	Female	Education (years per capita)			X			X
Acute hepatitis B	Global	Female	Education (years per capita)			X			X
Acute hepatitis B	Data Rich	Female	LDI (I$ per capita)			X			X
Acute hepatitis B	Global	Female	LDI (I$ per capita)			X			X
Acute hepatitis B	Data Rich	Female	Socio-demographic Index		X			X	
Acute hepatitis B	Global	Female	Socio-demographic Index		X			X	
Acute hepatitis B	Data Rich	Female	Healthcare access and quality index		X			X	
Acute hepatitis B	Global	Female	Healthcare access and quality index		X			X	
Acute hepatitis B	Data Rich	Female	Log-transformed SEV scalar: Hep		X			X	
Acute hepatitis B	Global	Female	Log-transformed SEV scalar: Hep		X			X	
Acute hepatitis B	Data Rich	Female	Hepatitis B vaccine coverage, aged through time, COVID-free (proportion)	X			X		
Acute hepatitis B	Global	Female	Hepatitis B vaccine coverage, aged through time, COVID-free (proportion)	X			X		
Acute hepatitis B	Data Rich	Female	Vaccine adjusted HbSAg serporevalence age standardized	X			X		
Acute hepatitis B	Global	Female	Vaccine adjusted HbSAg serporevalence age standardized	X			X		
Acute hepatitis B	Data Rich	Female	Intravenous drug use (proportion by age)	X			X		
Acute hepatitis B	Global	Female	Intravenous drug use (proportion by age)	X			X		
Acute hepatitis B	Data Rich	Male	Education (years per capita)			X			X
Acute hepatitis B	Global	Male	Education (years per capita)			X			X
Acute hepatitis B	Data Rich	Male	LDI (I$ per capita)			X			X
Acute hepatitis B	Global	Male	LDI (I$ per capita)			X			X
Acute hepatitis B	Data Rich	Male	Socio-demographic Index		X			X	
Acute hepatitis B	Global	Male	Socio-demographic Index		X			X	
Acute hepatitis B	Data Rich	Male	Healthcare access and quality index		X			X	
Acute hepatitis B	Global	Male	Healthcare access and quality index		X			X	
Acute hepatitis B	Data Rich	Male	Log-transformed SEV scalar: Hep		X			X	
Acute hepatitis B	Global	Male	Log-transformed SEV scalar: Hep		X			X	
Acute hepatitis B	Data Rich	Male	Hepatitis B vaccine coverage, aged through time, COVID-free (proportion)	X			X		
Acute hepatitis B	Global	Male	Hepatitis B vaccine coverage, aged through time, COVID-free (proportion)	X			X		
Acute hepatitis B	Data Rich	Male	Vaccine adjusted HbSAg serporevalence age standardized	X			X		
Acute hepatitis B	Global	Male	Vaccine adjusted HbSAg serporevalence age standardized	X			X		
Acute hepatitis B	Data Rich	Male	Intravenous drug use (proportion by age)	X			X		
Acute hepatitis B	Global	Male	Intravenous drug use (proportion by age)	X			X		
Acute hepatitis C	Data Rich	Female	Education (years per capita)			X			X
Acute hepatitis C	Global	Female	Education (years per capita)			X			X
Acute hepatitis C	Data Rich	Female	LDI (I$ per capita)			X			X
Acute hepatitis C	Global	Female	LDI (I$ per capita)			X			X
Acute hepatitis C	Data Rich	Female	Socio-demographic Index		X			X	
Acute hepatitis C	Global	Female	Socio-demographic Index		X			X	
Acute hepatitis C	Data Rich	Female	Healthcare access and quality index		X			X	
Acute hepatitis C	Global	Female	Healthcare access and quality index		X			X	
Acute hepatitis C	Data Rich	Female	Intravenous drug use (proportion by age)		X			X	
Acute hepatitis C	Global	Female	Intravenous drug use (proportion by age)		X			X	
Acute hepatitis C	Data Rich	Female	Hepatitis C Seroprevalence (anti-HCV) age standardized	X			X		
Acute hepatitis C	Global	Female	Hepatitis C Seroprevalence (anti-HCV) age standardized	X			X		
Acute hepatitis C	Data Rich	Female	Log-transformed SEV scalar: Hep	X			X		
Acute hepatitis C	Global	Female	Log-transformed SEV scalar: Hep	X			X		
Acute hepatitis C	Data Rich	Male	Education (years per capita)			X			X
Acute hepatitis C	Global	Male	Education (years per capita)			X			X
Acute hepatitis C	Data Rich	Male	LDI (I$ per capita)			X			X
Acute hepatitis C	Global	Male	LDI (I$ per capita)			X			X
Acute hepatitis C	Data Rich	Male	Socio-demographic Index		X			X	
Acute hepatitis C	Global	Male	Socio-demographic Index		X			X	
Acute hepatitis C	Data Rich	Male	Healthcare access and quality index		X			X	
Acute hepatitis C	Global	Male	Healthcare access and quality index		X			X	
Acute hepatitis C	Data Rich	Male	Log-transformed SEV scalar: Hep		X			X	
Acute hepatitis C	Global	Male	Log-transformed SEV scalar: Hep		X			X	
Acute hepatitis C	Data Rich	Male	Hepatitis C Seroprevalence (anti-HCV) age standardized	X			X		
Acute hepatitis C	Global	Male	Hepatitis C Seroprevalence (anti-HCV) age standardized	X			X		
Acute hepatitis C	Data Rich	Male	Intravenous drug use (proportion by age)	X			X		
Acute hepatitis C	Global	Male	Intravenous drug use (proportion by age)	X			X		
Acute hepatitis E	Data Rich	Female	Education (years per capita)			X			X
Acute hepatitis E	Global	Female	Education (years per capita)			X			X
Acute hepatitis E	Data Rich	Female	LDI (I$ per capita)			X			X
Acute hepatitis E	Global	Female	LDI (I$ per capita)			X			X
Acute hepatitis E	Data Rich	Female	Socio-demographic Index		X			X	
Acute hepatitis E	Global	Female	Socio-demographic Index		X			X	
Acute hepatitis E	Data Rich	Female	Healthcare access and quality index		X			X	
Acute hepatitis E	Global	Female	Healthcare access and quality index		X			X	
Acute hepatitis E	Data Rich	Female	Age- and sex-specific SEV for Unsafe water		X			X	
Acute hepatitis E	Global	Female	Age- and sex-specific SEV for Unsafe water		X			X	
Acute hepatitis E	Data Rich	Female	Age- and sex-specific SEV for Unsafe sanitation		X			X	
Acute hepatitis E	Global	Female	Age- and sex-specific SEV for Unsafe sanitation		X			X	
Acute hepatitis E	Global	Female	Proportion of the population living in the classic monsoon region		X			X	
Acute hepatitis E	Data Rich	Female	Log-transformed SEV scalar: Hep	X			X		
Acute hepatitis E	Global	Female	Log-transformed SEV scalar: Hep	X			X		
Acute hepatitis E	Data Rich	Female	Hepatitis E Seroprevalence (anti-HEV) age standardized	X			X		
Acute hepatitis E	Global	Female	Hepatitis E Seroprevalence (anti-HEV) age standardized	X			X		
Acute hepatitis E	Data Rich	Female	Proportion of the population living in the classic monsoon region	X			X		
Acute hepatitis E	Data Rich	Male	Education (years per capita)			X			X
Acute hepatitis E	Global	Male	Education (years per capita)			X			X
Acute hepatitis E	Data Rich	Male	LDI (I$ per capita)			X			X
Acute hepatitis E	Global	Male	LDI (I$ per capita)			X			X
Acute hepatitis E	Data Rich	Male	Socio-demographic Index		X			X	
Acute hepatitis E	Global	Male	Socio-demographic Index		X			X	
Acute hepatitis E	Data Rich	Male	Healthcare access and quality index		X			X	
Acute hepatitis E	Global	Male	Healthcare access and quality index		X			X	
Acute hepatitis E	Data Rich	Male	Age- and sex-specific SEV for Unsafe water		X			X	
Acute hepatitis E	Global	Male	Age- and sex-specific SEV for Unsafe water		X			X	
Acute hepatitis E	Data Rich	Male	Age- and sex-specific SEV for Unsafe sanitation		X			X	
Acute hepatitis E	Global	Male	Age- and sex-specific SEV for Unsafe sanitation		X			X	
Acute hepatitis E	Data Rich	Male	Proportion of the population living in the classic monsoon region		X			X	
Acute hepatitis E	Global	Male	Proportion of the population living in the classic monsoon region		X			X	
Acute hepatitis E	Data Rich	Male	Log-transformed SEV scalar: Hep	X			X		
Acute hepatitis E	Global	Male	Log-transformed SEV scalar: Hep	X			X		
Acute hepatitis E	Data Rich	Male	Hepatitis E Seroprevalence (anti-HEV) age standardized	X			X		
Acute hepatitis E	Global	Male	Hepatitis E Seroprevalence (anti-HEV) age standardized	X			X		
Other unspecified infectious diseases	Data Rich	Female	Socio-demographic Index			X			X
Other unspecified infectious diseases	Global	Female	Socio-demographic Index			X			X
Other unspecified infectious diseases	Data Rich	Female	Antenatal Care (1 visit) Coverage (proportion)			X			X
Other unspecified infectious diseases	Global	Female	Antenatal Care (1 visit) Coverage (proportion)			X			X
Other unspecified infectious diseases	Data Rich	Female	Healthcare access and quality index		X			X	
Other unspecified infectious diseases	Global	Female	Healthcare access and quality index		X			X	
Other unspecified infectious diseases	Data Rich	Female	Sanitation (proportion with access)		X			X	

Appendix Table S11: Comparison of GBD 2021 and GBD 2023 covariates and level of covariates used in cause of death modeling									
Cause	Model Version Type	Sex	Covariate	Level 1 GBD 2021	Level 2 GBD 2021	Level 3 GBD 2021	Level 1 GBD 2023	Level 2 GBD 2023	Level 3 GBD 2023
Other unspecified infectious diseases	Global	Female	Sanitation (proportion with access)		X			X	
Other unspecified infectious diseases	Data Rich	Female	Improved Water Source (proportion with access)		X			X	
Other unspecified infectious diseases	Global	Female	Improved Water Source (proportion with access)		X			X	
Other unspecified infectious diseases	Data Rich	Female	DTP3 Coverage, COVID-free (proportion)	X			X		
Other unspecified infectious diseases	Global	Female	DTP3 Coverage, COVID-free (proportion)	X			X		
Other unspecified infectious diseases	Data Rich	Male	Socio-demographic Index			X			X
Other unspecified infectious diseases	Global	Male	Socio-demographic Index			X			X
Other unspecified infectious diseases	Data Rich	Male	Antenatal Care (1 visit) Coverage (proportion)			X			X
Other unspecified infectious diseases	Global	Male	Antenatal Care (1 visit) Coverage (proportion)			X			X
Other unspecified infectious diseases	Data Rich	Male	Healthcare access and quality index		X			X	
Other unspecified infectious diseases	Global	Male	Healthcare access and quality index		X			X	
Other unspecified infectious diseases	Data Rich	Male	Sanitation (proportion with access)		X			X	
Other unspecified infectious diseases	Global	Male	Sanitation (proportion with access)		X			X	
Other unspecified infectious diseases	Data Rich	Male	Improved Water Source (proportion with access)		X			X	
Other unspecified infectious diseases	Global	Male	Improved Water Source (proportion with access)		X			X	
Other unspecified infectious diseases	Data Rich	Male	DTP3 Coverage, COVID-free (proportion)	X			X		
Other unspecified infectious diseases	Global	Male	DTP3 Coverage, COVID-free (proportion)	X			X		
Neonatal disorders	Global	Female	LDI (I$ per capita)			X			X
Neonatal disorders	Data Rich	Female	LDI (I$ per capita)			X			X
Neonatal disorders	Global	Female	Skilled Birth Attendance (proportion)			X			X
Neonatal disorders	Data Rich	Female	Skilled Birth Attendance (proportion)			X			X
Neonatal disorders	Global	Female	Antenatal Care (1 visit) Coverage (proportion)			X			X
Neonatal disorders	Data Rich	Female	Antenatal Care (1 visit) Coverage (proportion)			X			X
Neonatal disorders	Global	Female	Antenatal Care (4 visits) Coverage (proportion)			X			X
Neonatal disorders	Data Rich	Female	Antenatal Care (4 visits) Coverage (proportion)			X			X
Neonatal disorders	Global	Female	Total Fertility Rate			X			X
Neonatal disorders	Data Rich	Female	Total Fertility Rate			X			X
Neonatal disorders	Global	Female	In-Facility Delivery (proportion)			X			X
Neonatal disorders	Data Rich	Female	In-Facility Delivery (proportion)			X			X
Neonatal disorders	Global	Female	Socio-demographic Index		X				X
Neonatal disorders	Data Rich	Female	Socio-demographic Index		X				X
Neonatal disorders	Global	Female	Healthcare access and quality index		X				X
Neonatal disorders	Data Rich	Female	Healthcare access and quality index		X				X
Neonatal disorders	Global	Female	Proportion of the population with at least 6 years of education, maternal		X				X
Neonatal disorders	Data Rich	Female	Proportion of the population with at least 6 years of education, maternal		X				X
Neonatal disorders	Global	Female	Proportion of the population with at least 12 years of education, maternal		X				X
Neonatal disorders	Data Rich	Female	Proportion of the population with at least 12 years of education, maternal		X				X
Neonatal disorders	Global	Female	Age-standardized SEV for Ambient particulate matter	X				X	
Neonatal disorders	Data Rich	Female	Age-standardized SEV for Ambient particulate matter	X				X	
Neonatal disorders	Global	Female	Age-standardized SEV for Household air pollution	X				X	
Neonatal disorders	Data Rich	Female	Age-standardized SEV for Household air pollution	X				X	
Neonatal disorders	Global	Female	Age-standardized SEV for Short gestation	X				X	
Neonatal disorders	Data Rich	Female	Age-standardized SEV for Short gestation	X				X	
Neonatal disorders	Global	Female	Age-standardized SEV for Low birth weight	X				X	
Neonatal disorders	Data Rich	Female	Age-standardized SEV for Low birth weight	X				X	
Neonatal disorders	Global	Female	Age-standardized SEV for Smoking	X				X	
Neonatal disorders	Data Rich	Female	Age-standardized SEV for Smoking	X				X	
Neonatal disorders	Global	Female	Maternal care and immunization	X			X		
Neonatal disorders	Data Rich	Female	Maternal care and immunization	X			X		
Neonatal disorders	Global	Female	Log-transformed SEV scalar: Neonatal				X		
Neonatal disorders	Data Rich	Female	Log-transformed SEV scalar: Neonatal				X		
Neonatal disorders	Global	Male	LDI (I$ per capita)			X			X
Neonatal disorders	Data Rich	Male	LDI (I$ per capita)			X			X
Neonatal disorders	Global	Male	Skilled Birth Attendance (proportion)			X			X
Neonatal disorders	Data Rich	Male	Skilled Birth Attendance (proportion)			X			X
Neonatal disorders	Global	Male	Antenatal Care (1 visit) Coverage (proportion)			X			X
Neonatal disorders	Data Rich	Male	Antenatal Care (1 visit) Coverage (proportion)			X			X
Neonatal disorders	Global	Male	Antenatal Care (4 visits) Coverage (proportion)			X			X
Neonatal disorders	Data Rich	Male	Antenatal Care (4 visits) Coverage (proportion)			X			X
Neonatal disorders	Global	Male	Total Fertility Rate			X			X
Neonatal disorders	Data Rich	Male	Total Fertility Rate			X			X
Neonatal disorders	Global	Male	In-Facility Delivery (proportion)			X			X
Neonatal disorders	Data Rich	Male	In-Facility Delivery (proportion)			X			X
Neonatal disorders	Global	Male	Socio-demographic Index		X				X
Neonatal disorders	Data Rich	Male	Socio-demographic Index		X				X
Neonatal disorders	Global	Male	Healthcare access and quality index		X				X
Neonatal disorders	Data Rich	Male	Healthcare access and quality index		X				X
Neonatal disorders	Global	Male	Proportion of the population with at least 6 years of education, maternal		X				X
Neonatal disorders	Data Rich	Male	Proportion of the population with at least 6 years of education, maternal		X				X
Neonatal disorders	Global	Male	Proportion of the population with at least 12 years of education, maternal		X				X
Neonatal disorders	Data Rich	Male	Proportion of the population with at least 12 years of education, maternal		X				X
Neonatal disorders	Global	Male	Age-standardized SEV for Ambient particulate matter	X				X	
Neonatal disorders	Data Rich	Male	Age-standardized SEV for Ambient particulate matter	X				X	
Neonatal disorders	Global	Male	Age-standardized SEV for Household air pollution	X				X	
Neonatal disorders	Data Rich	Male	Age-standardized SEV for Household air pollution	X				X	
Neonatal disorders	Global	Male	Age-standardized SEV for Short gestation	X				X	
Neonatal disorders	Data Rich	Male	Age-standardized SEV for Short gestation	X				X	
Neonatal disorders	Global	Male	Age-standardized SEV for Low birth weight	X				X	
Neonatal disorders	Data Rich	Male	Age-standardized SEV for Low birth weight	X				X	
Neonatal disorders	Global	Male	Age-standardized SEV for Smoking	X				X	
Neonatal disorders	Data Rich	Male	Age-standardized SEV for Smoking	X				X	
Neonatal disorders	Global	Male	Maternal care and immunization	X			X		
Neonatal disorders	Data Rich	Male	Maternal care and immunization	X			X		
Neonatal disorders	Global	Male	Log-transformed SEV scalar: Neonatal				X		
Neonatal disorders	Data Rich	Male	Log-transformed SEV scalar: Neonatal				X		
Neonatal preterm birth	Global	Female	LDI (I$ per capita)			X			X
Neonatal preterm birth	Data Rich	Female	LDI (I$ per capita)			X			X
Neonatal preterm birth	Global	Female	Skilled Birth Attendance (proportion)			X			X
Neonatal preterm birth	Data Rich	Female	Skilled Birth Attendance (proportion)			X			X
Neonatal preterm birth	Global	Female	Antenatal Care (1 visit) Coverage (proportion)			X			X
Neonatal preterm birth	Data Rich	Female	Antenatal Care (1 visit) Coverage (proportion)			X			X
Neonatal preterm birth	Global	Female	Antenatal Care (4 visits) Coverage (proportion)			X			X
Neonatal preterm birth	Data Rich	Female	Antenatal Care (4 visits) Coverage (proportion)			X			X
Neonatal preterm birth	Global	Female	Total Fertility Rate			X			X
Neonatal preterm birth	Data Rich	Female	Total Fertility Rate			X			X
Neonatal preterm birth	Global	Female	In-Facility Delivery (proportion)			X			X
Neonatal preterm birth	Data Rich	Female	In-Facility Delivery (proportion)			X			X
Neonatal preterm birth	Global	Female	Socio-demographic Index		X				X
Neonatal preterm birth	Data Rich	Female	Socio-demographic Index		X				X
Neonatal preterm birth	Global	Female	Healthcare access and quality index		X				X
Neonatal preterm birth	Data Rich	Female	Healthcare access and quality index		X				X

Appendix Table S11: Comparison of GBD 2021 and GBD 2023 covariates and level of covariates used in cause of death modeling									
Cause	Model Version Type	Sex	Covariate	Level 1 GBD 2021	Level 2 GBD 2021	Level 3 GBD 2021	Level 1 GBD 2023	Level 2 GBD 2023	Level 3 GBD 2023
Neonatal preterm birth	Global	Female	Proportion of the population with at least 6 years of education, maternal		X				X
Neonatal preterm birth	Data Rich	Female	Proportion of the population with at least 6 years of education, maternal		X				X
Neonatal preterm birth	Global	Female	Proportion of the population with at least 12 years of education, maternal		X				X
Neonatal preterm birth	Data Rich	Female	Proportion of the population with at least 12 years of education, maternal		X				X
Neonatal preterm birth	Global	Female	Age-standardized SEV for Ambient particulate matter	X				X	
Neonatal preterm birth	Data Rich	Female	Age-standardized SEV for Ambient particulate matter	X				X	
Neonatal preterm birth	Global	Female	Age-standardized SEV for Household air pollution	X				X	
Neonatal preterm birth	Data Rich	Female	Age-standardized SEV for Household air pollution	X				X	
Neonatal preterm birth	Global	Female	Age-standardized SEV for Short gestation	X				X	
Neonatal preterm birth	Data Rich	Female	Age-standardized SEV for Short gestation	X				X	
Neonatal preterm birth	Global	Female	Age-standardized SEV for Low birth weight	X				X	
Neonatal preterm birth	Data Rich	Female	Age-standardized SEV for Low birth weight	X				X	
Neonatal preterm birth	Global	Female	Age-standardized SEV for Smoking	X				X	
Neonatal preterm birth	Data Rich	Female	Age-standardized SEV for Smoking	X				X	
Neonatal preterm birth	Global	Female	Maternal care and immunization	X			X		
Neonatal preterm birth	Data Rich	Female	Maternal care and immunization	X			X		
Neonatal preterm birth	Global	Female	Log-transformed SEV scalar: Neonatal				X		
Neonatal preterm birth	Data Rich	Female	Log-transformed SEV scalar: Neonatal				X		
Neonatal preterm birth	Data Rich	Male	LDI (I$ per capita)			X			X
Neonatal preterm birth	Global	Male	LDI (I$ per capita)			X			X
Neonatal preterm birth	Data Rich	Male	Skilled Birth Attendance (proportion)			X			X
Neonatal preterm birth	Global	Male	Skilled Birth Attendance (proportion)			X			X
Neonatal preterm birth	Data Rich	Male	Antenatal Care (1 visit) Coverage (proportion)			X			X
Neonatal preterm birth	Global	Male	Antenatal Care (1 visit) Coverage (proportion)			X			X
Neonatal preterm birth	Data Rich	Male	Antenatal Care (4 visits) Coverage (proportion)			X			X
Neonatal preterm birth	Global	Male	Antenatal Care (4 visits) Coverage (proportion)			X			X
Neonatal preterm birth	Data Rich	Male	Total Fertility Rate			X			X
Neonatal preterm birth	Global	Male	Total Fertility Rate			X			X
Neonatal preterm birth	Data Rich	Male	In-Facility Delivery (proportion)			X			X
Neonatal preterm birth	Global	Male	In-Facility Delivery (proportion)			X			X
Neonatal preterm birth	Data Rich	Male	Socio-demographic Index		X				X
Neonatal preterm birth	Global	Male	Socio-demographic Index		X				X
Neonatal preterm birth	Data Rich	Male	Healthcare access and quality index		X				X
Neonatal preterm birth	Global	Male	Healthcare access and quality index		X				X
Neonatal preterm birth	Data Rich	Male	Proportion of the population with at least 6 years of education, maternal		X				X
Neonatal preterm birth	Global	Male	Proportion of the population with at least 6 years of education, maternal		X				X
Neonatal preterm birth	Data Rich	Male	Proportion of the population with at least 12 years of education, maternal		X				X
Neonatal preterm birth	Global	Male	Proportion of the population with at least 12 years of education, maternal		X				X
Neonatal preterm birth	Data Rich	Male	Age-standardized SEV for Ambient particulate matter	X				X	
Neonatal preterm birth	Global	Male	Age-standardized SEV for Ambient particulate matter	X				X	
Neonatal preterm birth	Data Rich	Male	Age-standardized SEV for Household air pollution	X				X	
Neonatal preterm birth	Global	Male	Age-standardized SEV for Household air pollution	X				X	
Neonatal preterm birth	Data Rich	Male	Age-standardized SEV for Short gestation	X				X	
Neonatal preterm birth	Global	Male	Age-standardized SEV for Short gestation	X				X	
Neonatal preterm birth	Data Rich	Male	Age-standardized SEV for Low birth weight	X				X	
Neonatal preterm birth	Global	Male	Age-standardized SEV for Low birth weight	X				X	
Neonatal preterm birth	Data Rich	Male	Age-standardized SEV for Smoking	X				X	
Neonatal preterm birth	Global	Male	Age-standardized SEV for Smoking	X				X	
Neonatal preterm birth	Data Rich	Male	Maternal care and immunization	X			X		
Neonatal preterm birth	Global	Male	Maternal care and immunization	X			X		
Neonatal preterm birth	Data Rich	Male	Log-transformed SEV scalar: Neonatal				X		
Neonatal preterm birth	Global	Male	Log-transformed SEV scalar: Neonatal				X		
Neonatal encephalopathy due to birth asphyxia and trauma	Global	Female	LDI (I$ per capita)			X			X
Neonatal encephalopathy due to birth asphyxia and trauma	Data Rich	Female	LDI (I$ per capita)			X			X
Neonatal encephalopathy due to birth asphyxia and trauma	Global	Female	Skilled Birth Attendance (proportion)			X			X
Neonatal encephalopathy due to birth asphyxia and trauma	Data Rich	Female	Skilled Birth Attendance (proportion)			X			X
Neonatal encephalopathy due to birth asphyxia and trauma	Data Rich	Female	Antenatal Care (1 visit) Coverage (proportion)			X			X
Neonatal encephalopathy due to birth asphyxia and trauma	Global	Female	Antenatal Care (4 visits) Coverage (proportion)			X			X
Neonatal encephalopathy due to birth asphyxia and trauma	Data Rich	Female	Antenatal Care (4 visits) Coverage (proportion)			X			X
Neonatal encephalopathy due to birth asphyxia and trauma	Global	Female	Total Fertility Rate			X			X
Neonatal encephalopathy due to birth asphyxia and trauma	Data Rich	Female	Total Fertility Rate			X			X
Neonatal encephalopathy due to birth asphyxia and trauma	Global	Female	In-Facility Delivery (proportion)			X			X
Neonatal encephalopathy due to birth asphyxia and trauma	Data Rich	Female	In-Facility Delivery (proportion)			X			X
Neonatal encephalopathy due to birth asphyxia and trauma	Global	Female	Socio-demographic Index		X				X
Neonatal encephalopathy due to birth asphyxia and trauma	Data Rich	Female	Socio-demographic Index		X				X
Neonatal encephalopathy due to birth asphyxia and trauma	Global	Female	Healthcare access and quality index		X				X
Neonatal encephalopathy due to birth asphyxia and trauma	Data Rich	Female	Healthcare access and quality index		X				X
Neonatal encephalopathy due to birth asphyxia and trauma	Global	Female	Proportion of the population with at least 6 years of education, maternal		X				X
Neonatal encephalopathy due to birth asphyxia and trauma	Data Rich	Female	Proportion of the population with at least 6 years of education, maternal		X				X
Neonatal encephalopathy due to birth asphyxia and trauma	Global	Female	Proportion of the population with at least 12 years of education, maternal		X				X
Neonatal encephalopathy due to birth asphyxia and trauma	Data Rich	Female	Proportion of the population with at least 12 years of education, maternal		X				X
Neonatal encephalopathy due to birth asphyxia and trauma	Global	Female	Antenatal Care (1 visit) Coverage (proportion)	X					X
Neonatal encephalopathy due to birth asphyxia and trauma	Global	Female	Age-standardized SEV for Ambient particulate matter	X				X	
Neonatal encephalopathy due to birth asphyxia and trauma	Data Rich	Female	Age-standardized SEV for Ambient particulate matter	X				X	
Neonatal encephalopathy due to birth asphyxia and trauma	Global	Female	Age-standardized SEV for Household air pollution	X				X	
Neonatal encephalopathy due to birth asphyxia and trauma	Data Rich	Female	Age-standardized SEV for Household air pollution	X				X	
Neonatal encephalopathy due to birth asphyxia and trauma	Global	Female	Age-standardized SEV for Short gestation	X				X	
Neonatal encephalopathy due to birth asphyxia and trauma	Data Rich	Female	Age-standardized SEV for Short gestation	X				X	
Neonatal encephalopathy due to birth asphyxia and trauma	Global	Female	Age-standardized SEV for Low birth weight	X				X	
Neonatal encephalopathy due to birth asphyxia and trauma	Data Rich	Female	Age-standardized SEV for Low birth weight	X				X	
Neonatal encephalopathy due to birth asphyxia and trauma	Global	Female	Age-standardized SEV for Smoking	X				X	
Neonatal encephalopathy due to birth asphyxia and trauma	Data Rich	Female	Age-standardized SEV for Smoking	X				X	
Neonatal encephalopathy due to birth asphyxia and trauma	Global	Female	Maternal care and immunization	X			X		
Neonatal encephalopathy due to birth asphyxia and trauma	Data Rich	Female	Maternal care and immunization	X			X		
Neonatal encephalopathy due to birth asphyxia and trauma	Global	Female	Log-transformed SEV scalar: Neonatal				X		
Neonatal encephalopathy due to birth asphyxia and trauma	Data Rich	Female	Log-transformed SEV scalar: Neonatal				X		
Neonatal encephalopathy due to birth asphyxia and trauma	Data Rich	Male	LDI (I$ per capita)			X			X
Neonatal encephalopathy due to birth asphyxia and trauma	Global	Male	LDI (I$ per capita)			X			X
Neonatal encephalopathy due to birth asphyxia and trauma	Data Rich	Male	Skilled Birth Attendance (proportion)			X			X
Neonatal encephalopathy due to birth asphyxia and trauma	Global	Male	Skilled Birth Attendance (proportion)			X			X

Appendix Table S11: Comparison of GBD 2021 and GBD 2023 covariates and level of covariates used in cause of death modeling

Cause	Model Version Type	Sex	Covariate	Level 1 GBD 2021	Level 2 GBD 2021	Level 3 GBD 2021	Level 1 GBD 2023	Level 2 GBD 2023	Level 3 GBD 2023
Neonatal encephalopathy due to birth asphyxia and trauma	Data Rich	Male	Antenatal Care (1 visit) Coverage (proportion)			X			X
Neonatal encephalopathy due to birth asphyxia and trauma	Data Rich	Male	Antenatal Care (4 visits) Coverage (proportion)			X			X
Neonatal encephalopathy due to birth asphyxia and trauma	Global	Male	Antenatal Care (4 visits) Coverage (proportion)			X			X
Neonatal encephalopathy due to birth asphyxia and trauma	Data Rich	Male	Total Fertility Rate			X			X
Neonatal encephalopathy due to birth asphyxia and trauma	Global	Male	Total Fertility Rate			X			X
Neonatal encephalopathy due to birth asphyxia and trauma	Data Rich	Male	In-Facility Delivery (proportion)			X			X
Neonatal encephalopathy due to birth asphyxia and trauma	Global	Male	In-Facility Delivery (proportion)			X			X
Neonatal encephalopathy due to birth asphyxia and trauma	Data Rich	Male	Socio-demographic Index		X				X
Neonatal encephalopathy due to birth asphyxia and trauma	Global	Male	Socio-demographic Index		X				X
Neonatal encephalopathy due to birth asphyxia and trauma	Data Rich	Male	Healthcare access and quality index		X				X
Neonatal encephalopathy due to birth asphyxia and trauma	Global	Male	Healthcare access and quality index		X				X
Neonatal encephalopathy due to birth asphyxia and trauma	Data Rich	Male	Proportion of the population with at least 6 years of education, maternal		X				X
Neonatal encephalopathy due to birth asphyxia and trauma	Global	Male	Proportion of the population with at least 6 years of education, maternal		X				X
Neonatal encephalopathy due to birth asphyxia and trauma	Data Rich	Male	Proportion of the population with at least 12 years of education, maternal		X				X
Neonatal encephalopathy due to birth asphyxia and trauma	Global	Male	Proportion of the population with at least 12 years of education, maternal		X				X
Neonatal encephalopathy due to birth asphyxia and trauma	Global	Male	Antenatal Care (1 visit) Coverage (proportion)	X					X
Neonatal encephalopathy due to birth asphyxia and trauma	Data Rich	Male	Age-standardized SEV for Ambient particulate matter	X				X	
Neonatal encephalopathy due to birth asphyxia and trauma	Global	Male	Age-standardized SEV for Ambient particulate matter	X				X	
Neonatal encephalopathy due to birth asphyxia and trauma	Data Rich	Male	Age-standardized SEV for Household air pollution	X				X	
Neonatal encephalopathy due to birth asphyxia and trauma	Global	Male	Age-standardized SEV for Household air pollution	X				X	
Neonatal encephalopathy due to birth asphyxia and trauma	Data Rich	Male	Age-standardized SEV for Short gestation	X				X	
Neonatal encephalopathy due to birth asphyxia and trauma	Global	Male	Age-standardized SEV for Short gestation	X				X	
Neonatal encephalopathy due to birth asphyxia and trauma	Data Rich	Male	Age-standardized SEV for Low birth weight	X				X	
Neonatal encephalopathy due to birth asphyxia and trauma	Global	Male	Age-standardized SEV for Low birth weight	X				X	
Neonatal encephalopathy due to birth asphyxia and trauma	Data Rich	Male	Age-standardized SEV for Smoking	X				X	
Neonatal encephalopathy due to birth asphyxia and trauma	Global	Male	Age-standardized SEV for Smoking	X				X	
Neonatal encephalopathy due to birth asphyxia and trauma	Data Rich	Male	Maternal care and immunization	X			X		
Neonatal encephalopathy due to birth asphyxia and trauma	Global	Male	Maternal care and immunization	X			X		
Neonatal encephalopathy due to birth asphyxia and trauma	Data Rich	Male	Log-transformed SEV scalar: Neonatal				X		
Neonatal encephalopathy due to birth asphyxia and trauma	Global	Male	Log-transformed SEV scalar: Neonatal				X		
Neonatal sepsis and other neonatal infections	Data Rich	Female	LDI (I$ per capita)			X			X
Neonatal sepsis and other neonatal infections	Global	Female	LDI (I$ per capita)			X			X
Neonatal sepsis and other neonatal infections	Data Rich	Female	Skilled Birth Attendance (proportion)			X			X
Neonatal sepsis and other neonatal infections	Global	Female	Skilled Birth Attendance (proportion)			X			X
Neonatal sepsis and other neonatal infections	Data Rich	Female	Antenatal Care (1 visit) Coverage (proportion)			X			X
Neonatal sepsis and other neonatal infections	Global	Female	Antenatal Care (1 visit) Coverage (proportion)			X			X
Neonatal sepsis and other neonatal infections	Data Rich	Female	Antenatal Care (4 visits) Coverage (proportion)			X			X
Neonatal sepsis and other neonatal infections	Global	Female	Antenatal Care (4 visits) Coverage (proportion)			X			X
Neonatal sepsis and other neonatal infections	Data Rich	Female	Total Fertility Rate			X			X
Neonatal sepsis and other neonatal infections	Global	Female	Total Fertility Rate			X			X
Neonatal sepsis and other neonatal infections	Data Rich	Female	In-Facility Delivery (proportion)			X			X
Neonatal sepsis and other neonatal infections	Global	Female	In-Facility Delivery (proportion)			X			X
Neonatal sepsis and other neonatal infections	Data Rich	Female	Socio-demographic Index		X				X
Neonatal sepsis and other neonatal infections	Global	Female	Socio-demographic Index		X				X
Neonatal sepsis and other neonatal infections	Data Rich	Female	Healthcare access and quality index		X				X
Neonatal sepsis and other neonatal infections	Global	Female	Healthcare access and quality index		X				X
Neonatal sepsis and other neonatal infections	Data Rich	Female	Proportion of the population with at least 6 years of education, maternal		X				X
Neonatal sepsis and other neonatal infections	Global	Female	Proportion of the population with at least 6 years of education, maternal		X				X
Neonatal sepsis and other neonatal infections	Data Rich	Female	Proportion of the population with at least 12 years of education, maternal		X				X
Neonatal sepsis and other neonatal infections	Global	Female	Proportion of the population with at least 12 years of education, maternal		X				X
Neonatal sepsis and other neonatal infections	Data Rich	Female	Age-standardized SEV for Ambient particulate matter	X				X	
Neonatal sepsis and other neonatal infections	Global	Female	Age-standardized SEV for Ambient particulate matter	X				X	
Neonatal sepsis and other neonatal infections	Data Rich	Female	Age-standardized SEV for Household air pollution	X				X	
Neonatal sepsis and other neonatal infections	Global	Female	Age-standardized SEV for Household air pollution	X				X	
Neonatal sepsis and other neonatal infections	Data Rich	Female	Age-standardized SEV for Smoking	X				X	
Neonatal sepsis and other neonatal infections	Global	Female	Age-standardized SEV for Smoking	X				X	
Neonatal sepsis and other neonatal infections	Data Rich	Female	Age- and sex-specific SEV for Short gestation	X				X	
Neonatal sepsis and other neonatal infections	Global	Female	Age- and sex-specific SEV for Short gestation	X				X	
Neonatal sepsis and other neonatal infections	Data Rich	Female	Age- and sex-specific SEV for Low birth weight	X				X	
Neonatal sepsis and other neonatal infections	Global	Female	Age- and sex-specific SEV for Low birth weight	X				X	
Neonatal sepsis and other neonatal infections	Data Rich	Female	Maternal care and immunization	X			X		
Neonatal sepsis and other neonatal infections	Global	Female	Maternal care and immunization	X			X		
Neonatal sepsis and other neonatal infections	Data Rich	Female	Log-transformed SEV scalar: Neonatal				X		
Neonatal sepsis and other neonatal infections	Global	Female	Log-transformed SEV scalar: Neonatal				X		
Neonatal sepsis and other neonatal infections	Data Rich	Male	LDI (I$ per capita)			X			X
Neonatal sepsis and other neonatal infections	Global	Male	LDI (I$ per capita)			X			X
Neonatal sepsis and other neonatal infections	Data Rich	Male	Skilled Birth Attendance (proportion)			X			X
Neonatal sepsis and other neonatal infections	Global	Male	Skilled Birth Attendance (proportion)			X			X
Neonatal sepsis and other neonatal infections	Data Rich	Male	Antenatal Care (1 visit) Coverage (proportion)			X			X
Neonatal sepsis and other neonatal infections	Global	Male	Antenatal Care (1 visit) Coverage (proportion)			X			X
Neonatal sepsis and other neonatal infections	Data Rich	Male	Antenatal Care (4 visits) Coverage (proportion)			X			X
Neonatal sepsis and other neonatal infections	Global	Male	Antenatal Care (4 visits) Coverage (proportion)			X			X
Neonatal sepsis and other neonatal infections	Data Rich	Male	Total Fertility Rate			X			X
Neonatal sepsis and other neonatal infections	Global	Male	Total Fertility Rate			X			X
Neonatal sepsis and other neonatal infections	Data Rich	Male	In-Facility Delivery (proportion)			X			X
Neonatal sepsis and other neonatal infections	Global	Male	In-Facility Delivery (proportion)			X			X
Neonatal sepsis and other neonatal infections	Data Rich	Male	Socio-demographic Index		X				X
Neonatal sepsis and other neonatal infections	Global	Male	Socio-demographic Index		X				X
Neonatal sepsis and other neonatal infections	Data Rich	Male	Healthcare access and quality index		X				X
Neonatal sepsis and other neonatal infections	Global	Male	Healthcare access and quality index		X				X
Neonatal sepsis and other neonatal infections	Data Rich	Male	Proportion of the population with at least 6 years of education, maternal		X				X
Neonatal sepsis and other neonatal infections	Global	Male	Proportion of the population with at least 6 years of education, maternal		X				X
Neonatal sepsis and other neonatal infections	Data Rich	Male	Proportion of the population with at least 12 years of education, maternal		X				X
Neonatal sepsis and other neonatal infections	Global	Male	Proportion of the population with at least 12 years of education, maternal		X				X

Appendix Table S11: Comparison of GBD 2021 and GBD 2023 covariates and level of covariates used in cause of death modeling

Cause	Model Version Type	Sex	Covariate	Level 1 GBD 2021	Level 2 GBD 2021	Level 3 GBD 2021	Level 1 GBD 2023	Level 2 GBD 2023	Level 3 GBD 2023
Neonatal sepsis and other neonatal infections	Data Rich	Male	Age-standardized SEV for Ambient particulate matter	X				X	
Neonatal sepsis and other neonatal infections	Global	Male	Age-standardized SEV for Ambient particulate matter	X				X	
Neonatal sepsis and other neonatal infections	Data Rich	Male	Age-standardized SEV for Household air pollution	X				X	
Neonatal sepsis and other neonatal infections	Global	Male	Age-standardized SEV for Household air pollution	X				X	
Neonatal sepsis and other neonatal infections	Data Rich	Male	Age-standardized SEV for Short gestation	X				X	
Neonatal sepsis and other neonatal infections	Global	Male	Age-standardized SEV for Short gestation	X				X	
Neonatal sepsis and other neonatal infections	Data Rich	Male	Age-standardized SEV for Low birth weight	X				X	
Neonatal sepsis and other neonatal infections	Global	Male	Age-standardized SEV for Low birth weight	X				X	
Neonatal sepsis and other neonatal infections	Data Rich	Male	Age-standardized SEV for Smoking	X				X	
Neonatal sepsis and other neonatal infections	Global	Male	Age-standardized SEV for Smoking	X				X	
Neonatal sepsis and other neonatal infections	Data Rich	Male	Maternal care and immunization	X			X		
Neonatal sepsis and other neonatal infections	Global	Male	Maternal care and immunization	X			X		
Neonatal sepsis and other neonatal infections	Data Rich	Male	Log-transformed SEV scalar: Neonatal				X		
Neonatal sepsis and other neonatal infections	Global	Male	Log-transformed SEV scalar: Neonatal				X		
Hemolytic disease and other neonatal jaundice	Data Rich	Female	LDI (I$ per capita)			X			X
Hemolytic disease and other neonatal jaundice	Global	Female	LDI (I$ per capita)			X			X
Hemolytic disease and other neonatal jaundice	Data Rich	Female	Skilled Birth Attendance (proportion)			X			X
Hemolytic disease and other neonatal jaundice	Global	Female	Skilled Birth Attendance (proportion)			X			X
Hemolytic disease and other neonatal jaundice	Data Rich	Female	Antenatal Care (1 visit) Coverage (proportion)			X			X
Hemolytic disease and other neonatal jaundice	Global	Female	Antenatal Care (1 visit) Coverage (proportion)			X			X
Hemolytic disease and other neonatal jaundice	Data Rich	Female	Antenatal Care (4 visits) Coverage (proportion)			X			X
Hemolytic disease and other neonatal jaundice	Global	Female	Antenatal Care (4 visits) Coverage (proportion)			X			X
Hemolytic disease and other neonatal jaundice	Data Rich	Female	Total Fertility Rate			X			X
Hemolytic disease and other neonatal jaundice	Global	Female	Total Fertility Rate			X			X
Hemolytic disease and other neonatal jaundice	Data Rich	Female	In-Facility Delivery (proportion)			X			X
Hemolytic disease and other neonatal jaundice	Global	Female	In-Facility Delivery (proportion)			X			X
Hemolytic disease and other neonatal jaundice	Data Rich	Female	Socio-demographic Index		X				X
Hemolytic disease and other neonatal jaundice	Global	Female	Socio-demographic Index		X				X
Hemolytic disease and other neonatal jaundice	Data Rich	Female	Healthcare access and quality index		X				X
Hemolytic disease and other neonatal jaundice	Global	Female	Healthcare access and quality index		X				X
Hemolytic disease and other neonatal jaundice	Data Rich	Female	Proportion of the population with at least 6 years of education, maternal		X				X
Hemolytic disease and other neonatal jaundice	Global	Female	Proportion of the population with at least 6 years of education, maternal		X				X
Hemolytic disease and other neonatal jaundice	Data Rich	Female	Proportion of the population with at least 12 years of education, maternal		X				X
Hemolytic disease and other neonatal jaundice	Global	Female	Proportion of the population with at least 12 years of education, maternal		X				X
Hemolytic disease and other neonatal jaundice	Data Rich	Female	Age-standardized SEV for Ambient particulate matter	X				X	
Hemolytic disease and other neonatal jaundice	Global	Female	Age-standardized SEV for Ambient particulate matter	X				X	
Hemolytic disease and other neonatal jaundice	Data Rich	Female	Age-standardized SEV for Household air pollution	X				X	
Hemolytic disease and other neonatal jaundice	Global	Female	Age-standardized SEV for Household air pollution	X				X	
Hemolytic disease and other neonatal jaundice	Data Rich	Female	Age-standardized SEV for Short gestation	X				X	
Hemolytic disease and other neonatal jaundice	Global	Female	Age-standardized SEV for Short gestation	X				X	
Hemolytic disease and other neonatal jaundice	Data Rich	Female	Age-standardized SEV for Low birth weight	X				X	
Hemolytic disease and other neonatal jaundice	Global	Female	Age-standardized SEV for Low birth weight	X				X	
Hemolytic disease and other neonatal jaundice	Data Rich	Female	Age-standardized SEV for Smoking	X				X	
Hemolytic disease and other neonatal jaundice	Global	Female	Age-standardized SEV for Smoking	X				X	
Hemolytic disease and other neonatal jaundice	Data Rich	Female	Maternal care and immunization	X			X		
Hemolytic disease and other neonatal jaundice	Global	Female	Maternal care and immunization	X			X		
Hemolytic disease and other neonatal jaundice	Data Rich	Female	Log-transformed SEV scalar: Neonatal				X		
Hemolytic disease and other neonatal jaundice	Global	Female	Log-transformed SEV scalar: Neonatal				X		
Hemolytic disease and other neonatal jaundice	Data Rich	Male	LDI (I$ per capita)			X			X
Hemolytic disease and other neonatal jaundice	Global	Male	LDI (I$ per capita)			X			X
Hemolytic disease and other neonatal jaundice	Data Rich	Male	Skilled Birth Attendance (proportion)			X			X
Hemolytic disease and other neonatal jaundice	Global	Male	Skilled Birth Attendance (proportion)			X			X
Hemolytic disease and other neonatal jaundice	Data Rich	Male	Antenatal Care (1 visit) Coverage (proportion)			X			X
Hemolytic disease and other neonatal jaundice	Global	Male	Antenatal Care (1 visit) Coverage (proportion)			X			X
Hemolytic disease and other neonatal jaundice	Data Rich	Male	Antenatal Care (4 visits) Coverage (proportion)			X			X
Hemolytic disease and other neonatal jaundice	Global	Male	Antenatal Care (4 visits) Coverage (proportion)			X			X
Hemolytic disease and other neonatal jaundice	Data Rich	Male	Total Fertility Rate			X			X
Hemolytic disease and other neonatal jaundice	Global	Male	Total Fertility Rate			X			X
Hemolytic disease and other neonatal jaundice	Data Rich	Male	In-Facility Delivery (proportion)			X			X
Hemolytic disease and other neonatal jaundice	Global	Male	In-Facility Delivery (proportion)			X			X
Hemolytic disease and other neonatal jaundice	Data Rich	Male	Socio-demographic Index		X				X
Hemolytic disease and other neonatal jaundice	Global	Male	Socio-demographic Index		X				X
Hemolytic disease and other neonatal jaundice	Data Rich	Male	Healthcare access and quality index		X				X
Hemolytic disease and other neonatal jaundice	Global	Male	Healthcare access and quality index		X				X
Hemolytic disease and other neonatal jaundice	Data Rich	Male	Proportion of the population with at least 6 years of education, maternal		X				X
Hemolytic disease and other neonatal jaundice	Global	Male	Proportion of the population with at least 6 years of education, maternal		X				X
Hemolytic disease and other neonatal jaundice	Data Rich	Male	Proportion of the population with at least 12 years of education, maternal		X				X
Hemolytic disease and other neonatal jaundice	Global	Male	Proportion of the population with at least 12 years of education, maternal		X				X
Hemolytic disease and other neonatal jaundice	Data Rich	Male	Age-standardized SEV for Ambient particulate matter	X				X	
Hemolytic disease and other neonatal jaundice	Global	Male	Age-standardized SEV for Ambient particulate matter	X				X	
Hemolytic disease and other neonatal jaundice	Data Rich	Male	Age-standardized SEV for Household air pollution	X				X	
Hemolytic disease and other neonatal jaundice	Global	Male	Age-standardized SEV for Household air pollution	X				X	
Hemolytic disease and other neonatal jaundice	Data Rich	Male	Age-standardized SEV for Short gestation	X				X	
Hemolytic disease and other neonatal jaundice	Global	Male	Age-standardized SEV for Short gestation	X				X	
Hemolytic disease and other neonatal jaundice	Data Rich	Male	Age-standardized SEV for Low birth weight	X				X	
Hemolytic disease and other neonatal jaundice	Global	Male	Age-standardized SEV for Low birth weight	X				X	
Hemolytic disease and other neonatal jaundice	Data Rich	Male	Age-standardized SEV for Smoking	X				X	
Hemolytic disease and other neonatal jaundice	Global	Male	Age-standardized SEV for Smoking	X				X	
Hemolytic disease and other neonatal jaundice	Data Rich	Male	Maternal care and immunization	X			X		
Hemolytic disease and other neonatal jaundice	Global	Male	Maternal care and immunization	X			X		
Hemolytic disease and other neonatal jaundice	Data Rich	Male	Log-transformed SEV scalar: Neonatal				X		
Hemolytic disease and other neonatal jaundice	Global	Male	Log-transformed SEV scalar: Neonatal				X		
Other neonatal disorders	Data Rich	Female	LDI (I$ per capita)			X			X
Other neonatal disorders	Global	Female	LDI (I$ per capita)			X			X
Other neonatal disorders	Data Rich	Female	Skilled Birth Attendance (proportion)			X			X
Other neonatal disorders	Global	Female	Skilled Birth Attendance (proportion)			X			X
Other neonatal disorders	Data Rich	Female	Antenatal Care (1 visit) Coverage (proportion)			X			X

Appendix Table S11: Comparison of GBD 2021 and GBD 2023 covariates and level of covariates used in cause of death modeling									
**Cause**	**Model Version Type**	**Sex**	**Covariate**	**Level 1 GBD 2021**	**Level 2 GBD 2021**	**Level 3 GBD 2021**	**Level 1 GBD 2023**	**Level 2 GBD 2023**	**Level 3 GBD 2023**
Other neonatal disorders	Global	Female	Antenatal Care (1 visit) Coverage (proportion)			X			X
Other neonatal disorders	Data Rich	Female	Antenatal Care (4 visits) Coverage (proportion)			X			X
Other neonatal disorders	Global	Female	Antenatal Care (4 visits) Coverage (proportion)			X			X
Other neonatal disorders	Data Rich	Female	Total Fertility Rate			X			X
Other neonatal disorders	Global	Female	Total Fertility Rate			X			X
Other neonatal disorders	Data Rich	Female	In-Facility Delivery (proportion)			X			X
Other neonatal disorders	Global	Female	In-Facility Delivery (proportion)			X			X
Other neonatal disorders	Data Rich	Female	Socio-demographic Index		X				X
Other neonatal disorders	Global	Female	Socio-demographic Index		X				X
Other neonatal disorders	Data Rich	Female	Healthcare access and quality index		X				X
Other neonatal disorders	Global	Female	Healthcare access and quality index		X				X
Other neonatal disorders	Data Rich	Female	Proportion of the population with at least 6 years of education, maternal		X				X
Other neonatal disorders	Global	Female	Proportion of the population with at least 6 years of education, maternal		X				X
Other neonatal disorders	Data Rich	Female	Proportion of the population with at least 12 years of education, maternal		X				X
Other neonatal disorders	Global	Female	Proportion of the population with at least 12 years of education, maternal		X				X
Other neonatal disorders	Data Rich	Female	Age-standardized SEV for Ambient particulate matter	X				X	
Other neonatal disorders	Global	Female	Age-standardized SEV for Ambient particulate matter	X				X	
Other neonatal disorders	Data Rich	Female	Age-standardized SEV for Household air pollution	X				X	
Other neonatal disorders	Global	Female	Age-standardized SEV for Household air pollution	X				X	
Other neonatal disorders	Data Rich	Female	Age-standardized SEV for Short gestation	X				X	
Other neonatal disorders	Global	Female	Age-standardized SEV for Short gestation	X				X	
Other neonatal disorders	Data Rich	Female	Age-standardized SEV for Low birth weight	X				X	
Other neonatal disorders	Global	Female	Age-standardized SEV for Low birth weight	X				X	
Other neonatal disorders	Data Rich	Female	Age-standardized SEV for Smoking	X				X	
Other neonatal disorders	Global	Female	Age-standardized SEV for Smoking	X				X	
Other neonatal disorders	Data Rich	Female	Maternal care and immunization	X			X		
Other neonatal disorders	Global	Female	Maternal care and immunization	X			X		
Other neonatal disorders	Data Rich	Female	Log-transformed SEV scalar: Neonatal				X		
Other neonatal disorders	Global	Female	Log-transformed SEV scalar: Neonatal				X		
Other neonatal disorders	Data Rich	Male	LDI (I$ per capita)			X			X
Other neonatal disorders	Global	Male	LDI (I$ per capita)			X			X
Other neonatal disorders	Data Rich	Male	Skilled Birth Attendance (proportion)			X			X
Other neonatal disorders	Global	Male	Skilled Birth Attendance (proportion)			X			X
Other neonatal disorders	Data Rich	Male	Antenatal Care (1 visit) Coverage (proportion)			X			X
Other neonatal disorders	Global	Male	Antenatal Care (1 visit) Coverage (proportion)			X			X
Other neonatal disorders	Data Rich	Male	Antenatal Care (4 visits) Coverage (proportion)			X			X
Other neonatal disorders	Global	Male	Antenatal Care (4 visits) Coverage (proportion)			X			X
Other neonatal disorders	Data Rich	Male	Total Fertility Rate			X			X
Other neonatal disorders	Global	Male	Total Fertility Rate			X			X
Other neonatal disorders	Data Rich	Male	In-Facility Delivery (proportion)			X			X
Other neonatal disorders	Global	Male	In-Facility Delivery (proportion)			X			X
Other neonatal disorders	Data Rich	Male	Socio-demographic Index		X				X
Other neonatal disorders	Global	Male	Socio-demographic Index		X				X
Other neonatal disorders	Data Rich	Male	Healthcare access and quality index		X				X
Other neonatal disorders	Global	Male	Healthcare access and quality index		X				X
Other neonatal disorders	Data Rich	Male	Proportion of the population with at least 6 years of education, maternal		X				X
Other neonatal disorders	Global	Male	Proportion of the population with at least 6 years of education, maternal		X				X
Other neonatal disorders	Data Rich	Male	Proportion of the population with at least 12 years of education, maternal		X				X
Other neonatal disorders	Global	Male	Proportion of the population with at least 12 years of education, maternal		X				X
Other neonatal disorders	Data Rich	Male	Age-standardized SEV for Ambient particulate matter	X				X	
Other neonatal disorders	Global	Male	Age-standardized SEV for Ambient particulate matter	X				X	
Other neonatal disorders	Data Rich	Male	Age-standardized SEV for Household air pollution	X				X	
Other neonatal disorders	Global	Male	Age-standardized SEV for Household air pollution	X				X	
Other neonatal disorders	Data Rich	Male	Age-standardized SEV for Short gestation	X				X	
Other neonatal disorders	Global	Male	Age-standardized SEV for Short gestation	X				X	
Other neonatal disorders	Data Rich	Male	Age-standardized SEV for Low birth weight	X				X	
Other neonatal disorders	Global	Male	Age-standardized SEV for Low birth weight	X				X	
Other neonatal disorders	Data Rich	Male	Age-standardized SEV for Smoking	X				X	
Other neonatal disorders	Global	Male	Age-standardized SEV for Smoking	X				X	
Other neonatal disorders	Data Rich	Male	Maternal care and immunization	X			X		
Other neonatal disorders	Global	Male	Maternal care and immunization	X			X		
Other neonatal disorders	Data Rich	Male	Log-transformed SEV scalar: Neonatal				X		
Other neonatal disorders	Global	Male	Log-transformed SEV scalar: Neonatal				X		
Nutritional deficiencies	Data Rich	Female	Education (years per capita)			X			X
Nutritional deficiencies	Global	Female	Education (years per capita)			X			X
Nutritional deficiencies	Data Rich	Female	LDI (I$ per capita)			X			X
Nutritional deficiencies	Global	Female	LDI (I$ per capita)			X			X
Nutritional deficiencies	Data Rich	Female	Socio-demographic Index			X			X
Nutritional deficiencies	Global	Female	Socio-demographic Index		X				X
Nutritional deficiencies	Data Rich	Female	Maternal care and immunization		X			X	
Nutritional deficiencies	Global	Female	Maternal care and immunization		X			X	
Nutritional deficiencies	Data Rich	Female	Healthcare access and quality index		X			X	
Nutritional deficiencies	Global	Female	Healthcare access and quality index		X			X	
Nutritional deficiencies	Data Rich	Female	Rainfall Quintile 1 (proportion)		X			X	
Nutritional deficiencies	Global	Female	Rainfall Quintile 1 (proportion)		X			X	
Nutritional deficiencies	Data Rich	Female	Rainfall Quintile 2 (proportion)		X			X	
Nutritional deficiencies	Global	Female	Rainfall Quintile 2 (proportion)		X			X	
Nutritional deficiencies	Global	Female	Age- and sex-specific SEV for Unsafe water		X			X	
Nutritional deficiencies	Global	Female	Age- and sex-specific SEV for Unsafe sanitation		X			X	
Nutritional deficiencies	Data Rich	Female	Mortality Rate Due to Death Shocks in Last 10 Years (per 1 person)		X			X	
Nutritional deficiencies	Global	Female	Mortality Rate Due to Death Shocks in Last 10 Years (per 1 person)		X			X	
Nutritional deficiencies	Data Rich	Female	Age-Standardize Prevalence of Severe Anemia	X			X		
Nutritional deficiencies	Global	Female	Age-Standardize Prevalence of Severe Anemia	X			X		
Nutritional deficiencies	Data Rich	Female	Log-transformed SEV scalar: Diarrhea	X			X		
Nutritional deficiencies	Global	Female	Log-transformed SEV scalar: Diarrhea	X			X		
Nutritional deficiencies	Data Rich	Female	energy unadjusted(kcal)	X			X		
Nutritional deficiencies	Global	Female	energy unadjusted(kcal)	X			X		
Nutritional deficiencies	Data Rich	Female	Age-standardized SEV for Child underweight	X			X		
Nutritional deficiencies	Global	Female	Age-standardized SEV for Child underweight	X			X		
Nutritional deficiencies	Data Rich	Female	Age-standardized SEV for Child wasting	X			X		
Nutritional deficiencies	Global	Female	Age-standardized SEV for Child wasting	X			X		
Nutritional deficiencies	Data Rich	Female	Age- and sex-specific SEV for Unsafe water					X	
Nutritional deficiencies	Data Rich	Female	Age- and sex-specific SEV for Unsafe sanitation					X	
Nutritional deficiencies	Data Rich	Female	Malnutrition Shock mortality rate				X		
Nutritional deficiencies	Global	Female	Malnutrition Shock mortality rate				X		
Nutritional deficiencies	Global	Male	Education (years per capita)			X			X
Nutritional deficiencies	Data Rich	Male	Education (years per capita)			X			X
Nutritional deficiencies	Global	Male	LDI (I$ per capita)			X			X
Nutritional deficiencies	Data Rich	Male	LDI (I$ per capita)			X			X
Nutritional deficiencies	Data Rich	Male	Socio-demographic Index			X			X
Nutritional deficiencies	Global	Male	Socio-demographic Index		X				X
Nutritional deficiencies	Global	Male	Maternal care and immunization		X			X	
Nutritional deficiencies	Data Rich	Male	Maternal care and immunization		X			X	
Nutritional deficiencies	Global	Male	Healthcare access and quality index		X			X	

Appendix Table S11: Comparison of GBD 2021 and GBD 2023 covariates and level of covariates used in cause of death modeling									
Cause	Model Version Type	Sex	Covariate	Level 1 GBD 2021	Level 2 GBD 2021	Level 3 GBD 2021	Level 1 GBD 2023	Level 2 GBD 2023	Level 3 GBD 2023
Nutritional deficiencies	Data Rich	Male	Healthcare access and quality index		X			X	
Nutritional deficiencies	Global	Male	Rainfall Quintile 1 (proportion)		X			X	
Nutritional deficiencies	Data Rich	Male	Rainfall Quintile 1 (proportion)		X			X	
Nutritional deficiencies	Global	Male	Rainfall Quintile 2 (proportion)		X			X	
Nutritional deficiencies	Data Rich	Male	Rainfall Quintile 2 (proportion)		X			X	
Nutritional deficiencies	Global	Male	Age- and sex-specific SEV for Unsafe water		X			X	
Nutritional deficiencies	Global	Male	Age- and sex-specific SEV for Unsafe sanitation		X			X	
Nutritional deficiencies	Global	Male	Mortality Rate Due to Death Shocks in Last 10 Years (per 1 person)		X			X	
Nutritional deficiencies	Data Rich	Male	Mortality Rate Due to Death Shocks in Last 10 Years (per 1 person)		X			X	
Nutritional deficiencies	Global	Male	Age-Standardize Prevalence of Severe Anemia	X			X		
Nutritional deficiencies	Data Rich	Male	Age-Standardize Prevalence of Severe Anemia	X			X		
Nutritional deficiencies	Global	Male	Log-transformed SEV scalar: Diarrhea	X			X		
Nutritional deficiencies	Data Rich	Male	Log-transformed SEV scalar: Diarrhea	X			X		
Nutritional deficiencies	Global	Male	energy unadjusted(kcal)	X			X		
Nutritional deficiencies	Data Rich	Male	energy unadjusted(kcal)	X			X		
Nutritional deficiencies	Global	Male	Age-standardized SEV for Child underweight	X			X		
Nutritional deficiencies	Data Rich	Male	Age-standardized SEV for Child underweight	X			X		
Nutritional deficiencies	Global	Male	Age-standardized SEV for Child wasting	X			X		
Nutritional deficiencies	Data Rich	Male	Age-standardized SEV for Child wasting	X			X		
Nutritional deficiencies	Data Rich	Male	Age- and sex-specific SEV for Unsafe water					X	
Nutritional deficiencies	Data Rich	Male	Age- and sex-specific SEV for Unsafe sanitation					X	
Nutritional deficiencies	Global	Male	Malnutrition Shock mortality rate				X		
Nutritional deficiencies	Data Rich	Male	Malnutrition Shock mortality rate				X		
Protein-energy malnutrition	Data Rich	Female	Education (years per capita)			X			X
Protein-energy malnutrition	Global	Female	Education (years per capita)			X			X
Protein-energy malnutrition	Data Rich	Female	Education (years per capita)			X			X
Protein-energy malnutrition	Global	Female	Education (years per capita)			X			X
Protein-energy malnutrition	Data Rich	Female	LDI (I$ per capita)			X			X
Protein-energy malnutrition	Global	Female	LDI (I$ per capita)			X			X
Protein-energy malnutrition	Data Rich	Female	LDI (I$ per capita)			X			X
Protein-energy malnutrition	Global	Female	LDI (I$ per capita)			X			X
Protein-energy malnutrition	Data Rich	Female	Socio-demographic Index			X			X
Protein-energy malnutrition	Data Rich	Female	Socio-demographic Index		X				X
Protein-energy malnutrition	Global	Female	Socio-demographic Index		X				X
Protein-energy malnutrition	Global	Female	Socio-demographic Index		X				X
Protein-energy malnutrition	Data Rich	Female	Maternal care and immunization		X			X	
Protein-energy malnutrition	Global	Female	Maternal care and immunization		X			X	
Protein-energy malnutrition	Data Rich	Female	Maternal care and immunization		X			X	
Protein-energy malnutrition	Global	Female	Maternal care and immunization		X			X	
Protein-energy malnutrition	Data Rich	Female	Healthcare access and quality index		X			X	
Protein-energy malnutrition	Global	Female	Healthcare access and quality index		X			X	
Protein-energy malnutrition	Data Rich	Female	Healthcare access and quality index		X			X	
Protein-energy malnutrition	Global	Female	Healthcare access and quality index		X			X	
Protein-energy malnutrition	Data Rich	Female	Rainfall Quintile 1 (proportion)		X			X	
Protein-energy malnutrition	Global	Female	Rainfall Quintile 1 (proportion)		X			X	
Protein-energy malnutrition	Data Rich	Female	Rainfall Quintile 1 (proportion)		X			X	
Protein-energy malnutrition	Global	Female	Rainfall Quintile 1 (proportion)		X			X	
Protein-energy malnutrition	Data Rich	Female	Rainfall Quintile 2 (proportion)		X			X	
Protein-energy malnutrition	Global	Female	Rainfall Quintile 2 (proportion)		X			X	
Protein-energy malnutrition	Data Rich	Female	Rainfall Quintile 2 (proportion)		X			X	
Protein-energy malnutrition	Global	Female	Rainfall Quintile 2 (proportion)		X			X	
Protein-energy malnutrition	Data Rich	Female	Age- and sex-specific SEV for Unsafe water		X			X	
Protein-energy malnutrition	Global	Female	Age- and sex-specific SEV for Unsafe water		X			X	
Protein-energy malnutrition	Data Rich	Female	Age- and sex-specific SEV for Unsafe water		X			X	
Protein-energy malnutrition	Global	Female	Age- and sex-specific SEV for Unsafe water		X			X	
Protein-energy malnutrition	Data Rich	Female	Age- and sex-specific SEV for Unsafe sanitation		X			X	
Protein-energy malnutrition	Global	Female	Age- and sex-specific SEV for Unsafe sanitation		X			X	
Protein-energy malnutrition	Data Rich	Female	Age- and sex-specific SEV for Unsafe sanitation		X			X	
Protein-energy malnutrition	Global	Female	Age- and sex-specific SEV for Unsafe sanitation		X			X	
Protein-energy malnutrition	Data Rich	Female	Age-Standardize Prevalence of Severe Anemia	X			X		
Protein-energy malnutrition	Global	Female	Age-Standardize Prevalence of Severe Anemia	X			X		
Protein-energy malnutrition	Data Rich	Female	Age-Standardize Prevalence of Severe Anemia	X			X		
Protein-energy malnutrition	Global	Female	Age-Standardize Prevalence of Severe Anemia	X			X		
Protein-energy malnutrition	Data Rich	Female	Log-transformed SEV scalar: Diarrhea	X			X		
Protein-energy malnutrition	Global	Female	Log-transformed SEV scalar: Diarrhea	X			X		
Protein-energy malnutrition	Data Rich	Female	Log-transformed SEV scalar: Diarrhea	X			X		
Protein-energy malnutrition	Global	Female	Log-transformed SEV scalar: Diarrhea	X			X		
Protein-energy malnutrition	Data Rich	Female	energy unadjusted(kcal)	X			X		
Protein-energy malnutrition	Global	Female	energy unadjusted(kcal)	X			X		
Protein-energy malnutrition	Data Rich	Female	energy unadjusted(kcal)	X			X		
Protein-energy malnutrition	Global	Female	energy unadjusted(kcal)	X			X		
Protein-energy malnutrition	Data Rich	Female	Malnutrition Shock mortality rate	X			X		
Protein-energy malnutrition	Data Rich	Female	Age- and sex-specific SEV for Child wasting	X			X		
Protein-energy malnutrition	Global	Female	Age- and sex-specific SEV for Child wasting	X			X		
Protein-energy malnutrition	Data Rich	Female	Mean war mortality rate in the previous ten years					X	
Protein-energy malnutrition	Global	Female	Mean war mortality rate in the previous ten years					X	
Protein-energy malnutrition	Data Rich	Female	Mean war mortality rate in the previous ten years					X	
Protein-energy malnutrition	Global	Female	Mean war mortality rate in the previous ten years					X	
Protein-energy malnutrition	Data Rich	Female	Malnutrition Shock mortality rate				X		
Protein-energy malnutrition	Global	Female	Malnutrition Shock mortality rate				X		
Protein-energy malnutrition	Global	Female	Malnutrition Shock mortality rate				X		
Protein-energy malnutrition	Data Rich	Female	Age-standardized SEV for Child underweight				X		
Protein-energy malnutrition	Global	Female	Age-standardized SEV for Child underweight				X		
Protein-energy malnutrition	Data Rich	Female	Age-standardized SEV for Child underweight				X		
Protein-energy malnutrition	Global	Female	Age-standardized SEV for Child underweight				X		
Protein-energy malnutrition	Data Rich	Female	Age- and sex-specific SEV for Child wasting				X		
Protein-energy malnutrition	Global	Female	Age- and sex-specific SEV for Child wasting				X		
Protein-energy malnutrition	Data Rich	Male	Education (years per capita)			X			X
Protein-energy malnutrition	Global	Male	Education (years per capita)			X			X
Protein-energy malnutrition	Data Rich	Male	Education (years per capita)			X			X
Protein-energy malnutrition	Global	Male	Education (years per capita)			X			X
Protein-energy malnutrition	Data Rich	Male	LDI (I$ per capita)			X			X
Protein-energy malnutrition	Global	Male	LDI (I$ per capita)			X			X
Protein-energy malnutrition	Data Rich	Male	LDI (I$ per capita)			X			X
Protein-energy malnutrition	Global	Male	LDI (I$ per capita)			X			X
Protein-energy malnutrition	Data Rich	Male	Socio-demographic Index			X			X
Protein-energy malnutrition	Global	Male	Socio-demographic Index		X				X
Protein-energy malnutrition	Data Rich	Male	Socio-demographic Index		X				X
Protein-energy malnutrition	Global	Male	Socio-demographic Index		X				X
Protein-energy malnutrition	Data Rich	Male	Maternal care and immunization		X			X	
Protein-energy malnutrition	Global	Male	Maternal care and immunization		X			X	
Protein-energy malnutrition	Data Rich	Male	Maternal care and immunization		X			X	
Protein-energy malnutrition	Global	Male	Maternal care and immunization		X			X	
Protein-energy malnutrition	Data Rich	Male	Healthcare access and quality index		X			X	
Protein-energy malnutrition	Global	Male	Healthcare access and quality index		X			X	
Protein-energy malnutrition	Data Rich	Male	Healthcare access and quality index		X			X	
Protein-energy malnutrition	Global	Male	Healthcare access and quality index		X			X	
Protein-energy malnutrition	Data Rich	Male	Rainfall Quintile 1 (proportion)		X			X	
Protein-energy malnutrition	Global	Male	Rainfall Quintile 1 (proportion)		X			X	
Protein-energy malnutrition	Data Rich	Male	Rainfall Quintile 1 (proportion)		X			X	
Protein-energy malnutrition	Global	Male	Rainfall Quintile 1 (proportion)		X			X	
Protein-energy malnutrition	Data Rich	Male	Rainfall Quintile 2 (proportion)		X			X	
Protein-energy malnutrition	Global	Male	Rainfall Quintile 2 (proportion)		X			X	
Protein-energy malnutrition	Data Rich	Male	Rainfall Quintile 2 (proportion)		X			X	
Protein-energy malnutrition	Global	Male	Rainfall Quintile 2 (proportion)		X			X	

Appendix Table S11: Comparison of GBD 2021 and GBD 2023 covariates and level of covariates used in cause of death modeling

Cause	Model Version Type	Sex	Covariate	Level 1 GBD 2021	Level 2 GBD 2021	Level 3 GBD 2021	Level 1 GBD 2023	Level 2 GBD 2023	Level 3 GBD 2023
Protein-energy malnutrition	Data Rich	Male	Age- and sex-specific SEV for Unsafe water		X			X	
Protein-energy malnutrition	Global	Male	Age- and sex-specific SEV for Unsafe water		X			X	
Protein-energy malnutrition	Data Rich	Male	Age- and sex-specific SEV for Unsafe water		X			X	
Protein-energy malnutrition	Global	Male	Age- and sex-specific SEV for Unsafe water		X			X	
Protein-energy malnutrition	Data Rich	Male	Age- and sex-specific SEV for Unsafe sanitation		X			X	
Protein-energy malnutrition	Global	Male	Age- and sex-specific SEV for Unsafe sanitation		X			X	
Protein-energy malnutrition	Data Rich	Male	Age- and sex-specific SEV for Unsafe sanitation		X			X	
Protein-energy malnutrition	Global	Male	Age- and sex-specific SEV for Unsafe sanitation		X			X	
Protein-energy malnutrition	Data Rich	Male	Age-Standardize Prevalence of Severe Anemia	X			X		
Protein-energy malnutrition	Global	Male	Age-Standardize Prevalence of Severe Anemia	X			X		
Protein-energy malnutrition	Data Rich	Male	Age-Standardize Prevalence of Severe Anemia	X			X		
Protein-energy malnutrition	Global	Male	Age-Standardize Prevalence of Severe Anemia	X			X		
Protein-energy malnutrition	Data Rich	Male	Log-transformed SEV scalar: Diarrhea	X			X		
Protein-energy malnutrition	Global	Male	Log-transformed SEV scalar: Diarrhea	X			X		
Protein-energy malnutrition	Data Rich	Male	Log-transformed SEV scalar: Diarrhea	X			X		
Protein-energy malnutrition	Global	Male	Log-transformed SEV scalar: Diarrhea	X			X		
Protein-energy malnutrition	Data Rich	Male	energy unadjusted(kcal)	X			X		
Protein-energy malnutrition	Global	Male	energy unadjusted(kcal)	X			X		
Protein-energy malnutrition	Data Rich	Male	energy unadjusted(kcal)	X			X		
Protein-energy malnutrition	Global	Male	energy unadjusted(kcal)	X			X		
Protein-energy malnutrition	Data Rich	Male	Malnutrition Shock mortality rate	X			X		
Protein-energy malnutrition	Data Rich	Male	Age- and sex-specific SEV for Child wasting	X			X		
Protein-energy malnutrition	Global	Male	Age- and sex-specific SEV for Child wasting	X			X		
Protein-energy malnutrition	Data Rich	Male	Mean war mortality rate in the previous ten years					X	
Protein-energy malnutrition	Global	Male	Mean war mortality rate in the previous ten years					X	
Protein-energy malnutrition	Data Rich	Male	Mean war mortality rate in the previous ten years					X	
Protein-energy malnutrition	Global	Male	Mean war mortality rate in the previous ten years					X	
Protein-energy malnutrition	Global	Male	Malnutrition Shock mortality rate				X		
Protein-energy malnutrition	Data Rich	Male	Malnutrition Shock mortality rate				X		
Protein-energy malnutrition	Global	Male	Malnutrition Shock mortality rate				X		
Protein-energy malnutrition	Data Rich	Male	Age-standardized SEV for Child underweight				X		
Protein-energy malnutrition	Global	Male	Age-standardized SEV for Child underweight				X		
Protein-energy malnutrition	Data Rich	Male	Age-standardized SEV for Child underweight				X		
Protein-energy malnutrition	Global	Male	Age-standardized SEV for Child underweight				X		
Protein-energy malnutrition	Data Rich	Male	Age- and sex-specific SEV for Child wasting				X		
Protein-energy malnutrition	Global	Male	Age- and sex-specific SEV for Child wasting				X		
Other nutritional deficiencies	Global	Female	Education (years per capita)			X			X
Other nutritional deficiencies	Data Rich	Female	Education (years per capita)			X			X
Other nutritional deficiencies	Global	Female	LDI (I$ per capita)			X			X
Other nutritional deficiencies	Data Rich	Female	LDI (I$ per capita)			X			X
Other nutritional deficiencies	Global	Female	Socio-demographic Index		X				X
Other nutritional deficiencies	Data Rich	Female	Socio-demographic Index		X				X
Other nutritional deficiencies	Global	Female	Maternal care and immunization		X			X	
Other nutritional deficiencies	Data Rich	Female	Maternal care and immunization		X			X	
Other nutritional deficiencies	Global	Female	Healthcare access and quality index		X			X	
Other nutritional deficiencies	Data Rich	Female	Healthcare access and quality index		X			X	
Other nutritional deficiencies	Global	Female	Rainfall Quintile 1 (proportion)		X			X	
Other nutritional deficiencies	Data Rich	Female	Rainfall Quintile 1 (proportion)		X			X	
Other nutritional deficiencies	Global	Female	Rainfall Quintile 2 (proportion)		X			X	
Other nutritional deficiencies	Data Rich	Female	Rainfall Quintile 2 (proportion)		X			X	
Other nutritional deficiencies	Global	Female	Age- and sex-specific SEV for Unsafe water		X			X	
Other nutritional deficiencies	Data Rich	Female	Age- and sex-specific SEV for Unsafe water		X			X	
Other nutritional deficiencies	Global	Female	Age- and sex-specific SEV for Unsafe sanitation		X			X	
Other nutritional deficiencies	Data Rich	Female	Age- and sex-specific SEV for Unsafe sanitation		X			X	
Other nutritional deficiencies	Global	Female	Mortality Rate Due to Death Shocks in Last 10 Years (per 1 person)		X			X	
Other nutritional deficiencies	Data Rich	Female	Mortality Rate Due to Death Shocks in Last 10 Years (per 1 person)		X			X	
Other nutritional deficiencies	Data Rich	Female	energy unadjusted(kcal)		X		X		
Other nutritional deficiencies	Global	Female	Age-Standardize Prevalence of Severe Anemia	X			X		
Other nutritional deficiencies	Data Rich	Female	Age-Standardize Prevalence of Severe Anemia	X			X		
Other nutritional deficiencies	Global	Female	Log-transformed SEV scalar: Diarrhea	X			X		
Other nutritional deficiencies	Data Rich	Female	Log-transformed SEV scalar: Diarrhea	X			X		
Other nutritional deficiencies	Global	Female	energy unadjusted(kcal)	X			X		
Other nutritional deficiencies	Global	Female	Age-standardized SEV for Child underweight	X			X		
Other nutritional deficiencies	Data Rich	Female	Age-standardized SEV for Child underweight	X			X		
Other nutritional deficiencies	Global	Female	Malnutrition Shock mortality rate				X		
Other nutritional deficiencies	Data Rich	Female	Malnutrition Shock mortality rate				X		
Other nutritional deficiencies	Global	Female	Age-standardized SEV for Child wasting				X		
Other nutritional deficiencies	Data Rich	Female	Age-standardized SEV for Child wasting				X		
Other nutritional deficiencies	Global	Male	Education (years per capita)			X			X
Other nutritional deficiencies	Data Rich	Male	Education (years per capita)			X			X
Other nutritional deficiencies	Global	Male	LDI (I$ per capita)			X			X
Other nutritional deficiencies	Data Rich	Male	LDI (I$ per capita)			X			X
Other nutritional deficiencies	Global	Male	Socio-demographic Index		X				X
Other nutritional deficiencies	Data Rich	Male	Socio-demographic Index		X				X
Other nutritional deficiencies	Global	Male	Maternal care and immunization		X			X	
Other nutritional deficiencies	Data Rich	Male	Maternal care and immunization		X			X	
Other nutritional deficiencies	Global	Male	Healthcare access and quality index		X			X	
Other nutritional deficiencies	Data Rich	Male	Healthcare access and quality index		X			X	
Other nutritional deficiencies	Global	Male	Rainfall Quintile 1 (proportion)		X			X	
Other nutritional deficiencies	Data Rich	Male	Rainfall Quintile 1 (proportion)		X			X	
Other nutritional deficiencies	Global	Male	Rainfall Quintile 2 (proportion)		X			X	
Other nutritional deficiencies	Data Rich	Male	Rainfall Quintile 2 (proportion)		X			X	
Other nutritional deficiencies	Global	Male	Age- and sex-specific SEV for Unsafe water		X			X	
Other nutritional deficiencies	Data Rich	Male	Age- and sex-specific SEV for Unsafe water		X			X	
Other nutritional deficiencies	Global	Male	Age- and sex-specific SEV for Unsafe sanitation		X			X	
Other nutritional deficiencies	Data Rich	Male	Age- and sex-specific SEV for Unsafe sanitation		X			X	
Other nutritional deficiencies	Global	Male	Mortality Rate Due to Death Shocks in Last 10 Years (per 1 person)		X			X	
Other nutritional deficiencies	Data Rich	Male	Mortality Rate Due to Death Shocks in Last 10 Years (per 1 person)		X			X	
Other nutritional deficiencies	Data Rich	Male	energy unadjusted(kcal)		X		X		
Other nutritional deficiencies	Global	Male	Age-Standardize Prevalence of Severe Anemia	X			X		
Other nutritional deficiencies	Data Rich	Male	Age-Standardize Prevalence of Severe Anemia	X			X		
Other nutritional deficiencies	Global	Male	Log-transformed SEV scalar: Diarrhea	X			X		
Other nutritional deficiencies	Data Rich	Male	Log-transformed SEV scalar: Diarrhea	X			X		
Other nutritional deficiencies	Global	Male	energy unadjusted(kcal)	X			X		
Other nutritional deficiencies	Global	Male	Age-standardized SEV for Child underweight	X			X		
Other nutritional deficiencies	Data Rich	Male	Age-standardized SEV for Child underweight	X			X		
Other nutritional deficiencies	Global	Male	Malnutrition Shock mortality rate				X		
Other nutritional deficiencies	Data Rich	Male	Malnutrition Shock mortality rate				X		
Other nutritional deficiencies	Global	Male	Age-standardized SEV for Child wasting				X		
Other nutritional deficiencies	Data Rich	Male	Age-standardized SEV for Child wasting				X		
Lip and oral cavity cancer	Global	Female	Education (years per capita)			X			X
Lip and oral cavity cancer	Data Rich	Female	Education (years per capita)			X			X
Lip and oral cavity cancer	Global	Female	LDI (I$ per capita)			X			X
Lip and oral cavity cancer	Data Rich	Female	LDI (I$ per capita)			X			X
Lip and oral cavity cancer	Global	Female	Socio-demographic Index			X			X
Lip and oral cavity cancer	Data Rich	Female	Socio-demographic Index			X			X
Lip and oral cavity cancer	Global	Female	Healthcare access and quality index		X			X	
Lip and oral cavity cancer	Data Rich	Female	Healthcare access and quality index		X			X	

Appendix Table S11: Comparison of GBD 2021 and GBD 2023 covariates and level of covariates used in cause of death modeling

Cause	Model Version Type	Sex	Covariate	Level 1 GBD 2021	Level 2 GBD 2021	Level 3 GBD 2021	Level 1 GBD 2023	Level 2 GBD 2023	Level 3 GBD 2023
Lip and oral cavity cancer	Global	Female	Age- and sex-specific SEV for Low fruit		X			X	
Lip and oral cavity cancer	Data Rich	Female	Age- and sex-specific SEV for Low fruit		X			X	
Lip and oral cavity cancer	Global	Female	Age- and sex-specific SEV for Low vegetables		X			X	
Lip and oral cavity cancer	Data Rich	Female	Age- and sex-specific SEV for Low vegetables		X			X	
Lip and oral cavity cancer	Global	Female	Age- and sex-specific SEV for High red meat		X			X	
Lip and oral cavity cancer	Data Rich	Female	Age- and sex-specific SEV for High red meat		X			X	
Lip and oral cavity cancer	Global	Female	Tobacco (cigarettes per capita)	X			X		
Lip and oral cavity cancer	Data Rich	Female	Tobacco (cigarettes per capita)	X			X		
Lip and oral cavity cancer	Global	Female	Cumulative Cigarettes (10 Years)	X			X		
Lip and oral cavity cancer	Data Rich	Female	Cumulative Cigarettes (10 Years)	X			X		
Lip and oral cavity cancer	Global	Female	Liters of alcohol consumed per capita	X			X		
Lip and oral cavity cancer	Data Rich	Female	Liters of alcohol consumed per capita	X			X		
Lip and oral cavity cancer	Global	Female	Cumulative Cigarettes (20 Years)	X			X		
Lip and oral cavity cancer	Data Rich	Female	Cumulative Cigarettes (20 Years)	X			X		
Lip and oral cavity cancer	Data Rich	Female	Cumulative Cigarettes (5 Years)				X		
Lip and oral cavity cancer	Data Rich	Female	Cumulative Cigarettes (15 Years)				X		
Lip and oral cavity cancer	Global	Male	Education (years per capita)			X			X
Lip and oral cavity cancer	Data Rich	Male	Education (years per capita)			X			X
Lip and oral cavity cancer	Global	Male	LDI (I$ per capita)			X			X
Lip and oral cavity cancer	Data Rich	Male	LDI (I$ per capita)			X			X
Lip and oral cavity cancer	Global	Male	Socio-demographic Index			X			X
Lip and oral cavity cancer	Data Rich	Male	Socio-demographic Index			X			X
Lip and oral cavity cancer	Global	Male	Healthcare access and quality index		X			X	
Lip and oral cavity cancer	Data Rich	Male	Healthcare access and quality index		X			X	
Lip and oral cavity cancer	Global	Male	Age- and sex-specific SEV for Low fruit		X			X	
Lip and oral cavity cancer	Data Rich	Male	Age- and sex-specific SEV for Low fruit		X			X	
Lip and oral cavity cancer	Global	Male	Age- and sex-specific SEV for Low vegetables		X			X	
Lip and oral cavity cancer	Data Rich	Male	Age- and sex-specific SEV for Low vegetables		X			X	
Lip and oral cavity cancer	Global	Male	Age- and sex-specific SEV for High red meat		X			X	
Lip and oral cavity cancer	Data Rich	Male	Age- and sex-specific SEV for High red meat		X			X	
Lip and oral cavity cancer	Global	Male	Tobacco (cigarettes per capita)	X			X		
Lip and oral cavity cancer	Data Rich	Male	Tobacco (cigarettes per capita)	X			X		
Lip and oral cavity cancer	Global	Male	Cumulative Cigarettes (10 Years)	X			X		
Lip and oral cavity cancer	Data Rich	Male	Cumulative Cigarettes (10 Years)	X			X		
Lip and oral cavity cancer	Global	Male	Liters of alcohol consumed per capita	X			X		
Lip and oral cavity cancer	Data Rich	Male	Liters of alcohol consumed per capita	X			X		
Lip and oral cavity cancer	Global	Male	Cumulative Cigarettes (20 Years)	X			X		
Lip and oral cavity cancer	Data Rich	Male	Cumulative Cigarettes (20 Years)	X			X		
Nasopharynx cancer	Global	Female	Education (years per capita)			X			X
Nasopharynx cancer	Data Rich	Female	Education (years per capita)			X			X
Nasopharynx cancer	Global	Female	LDI (I$ per capita)			X			X
Nasopharynx cancer	Data Rich	Female	LDI (I$ per capita)			X			X
Nasopharynx cancer	Global	Female	Socio-demographic Index			X			X
Nasopharynx cancer	Data Rich	Female	Socio-demographic Index			X			X
Nasopharynx cancer	Global	Female	Age- and sex-specific SEV for Low fruit			X			X
Nasopharynx cancer	Data Rich	Female	Age- and sex-specific SEV for Low fruit			X			X
Nasopharynx cancer	Global	Female	Healthcare access and quality index		X			X	
Nasopharynx cancer	Data Rich	Female	Healthcare access and quality index		X			X	
Nasopharynx cancer	Global	Female	Age- and sex-specific SEV for Low vegetables		X			X	
Nasopharynx cancer	Data Rich	Female	Age- and sex-specific SEV for Low vegetables		X			X	
Nasopharynx cancer	Global	Female	Population Density (over 1000 ppl/sqkm, proportion)		X			X	
Nasopharynx cancer	Data Rich	Female	Population Density (over 1000 ppl/sqkm, proportion)		X			X	
Nasopharynx cancer	Global	Female	Tobacco (cigarettes per capita)	X			X		
Nasopharynx cancer	Data Rich	Female	Tobacco (cigarettes per capita)	X			X		
Nasopharynx cancer	Global	Female	Cumulative Cigarettes (10 Years)	X			X		
Nasopharynx cancer	Data Rich	Female	Cumulative Cigarettes (10 Years)	X			X		
Nasopharynx cancer	Global	Female	Liters of alcohol consumed per capita	X			X		
Nasopharynx cancer	Data Rich	Female	Liters of alcohol consumed per capita	X			X		
Nasopharynx cancer	Global	Female	Cumulative Cigarettes (20 Years)	X			X		
Nasopharynx cancer	Data Rich	Female	Cumulative Cigarettes (20 Years)	X			X		
Nasopharynx cancer	Global	Male	Education (years per capita)			X			X
Nasopharynx cancer	Data Rich	Male	Education (years per capita)			X			X
Nasopharynx cancer	Global	Male	LDI (I$ per capita)			X			X
Nasopharynx cancer	Data Rich	Male	LDI (I$ per capita)			X			X
Nasopharynx cancer	Global	Male	Socio-demographic Index			X			X
Nasopharynx cancer	Data Rich	Male	Socio-demographic Index			X			X
Nasopharynx cancer	Global	Male	Age- and sex-specific SEV for Low fruit			X			X
Nasopharynx cancer	Data Rich	Male	Age- and sex-specific SEV for Low fruit			X			X
Nasopharynx cancer	Global	Male	Healthcare access and quality index		X			X	
Nasopharynx cancer	Data Rich	Male	Healthcare access and quality index		X			X	
Nasopharynx cancer	Global	Male	Age- and sex-specific SEV for Low vegetables		X			X	
Nasopharynx cancer	Data Rich	Male	Age- and sex-specific SEV for Low vegetables		X			X	
Nasopharynx cancer	Global	Male	Population Density (over 1000 ppl/sqkm, proportion)		X			X	
Nasopharynx cancer	Data Rich	Male	Population Density (over 1000 ppl/sqkm, proportion)		X			X	
Nasopharynx cancer	Global	Male	Tobacco (cigarettes per capita)	X			X		
Nasopharynx cancer	Data Rich	Male	Tobacco (cigarettes per capita)	X			X		
Nasopharynx cancer	Global	Male	Cumulative Cigarettes (10 Years)	X			X		
Nasopharynx cancer	Data Rich	Male	Cumulative Cigarettes (10 Years)	X			X		
Nasopharynx cancer	Global	Male	Liters of alcohol consumed per capita	X			X		
Nasopharynx cancer	Data Rich	Male	Liters of alcohol consumed per capita	X			X		
Nasopharynx cancer	Global	Male	Cumulative Cigarettes (20 Years)	X			X		
Nasopharynx cancer	Data Rich	Male	Cumulative Cigarettes (20 Years)	X			X		
Other pharynx cancer	Global	Female	Education (years per capita)			X			X
Other pharynx cancer	Data Rich	Female	Education (years per capita)			X			X
Other pharynx cancer	Global	Female	LDI (I$ per capita)			X			X
Other pharynx cancer	Data Rich	Female	LDI (I$ per capita)			X			X
Other pharynx cancer	Global	Female	Socio-demographic Index			X			X
Other pharynx cancer	Data Rich	Female	Socio-demographic Index			X			X
Other pharynx cancer	Global	Female	Healthcare access and quality index		X			X	
Other pharynx cancer	Data Rich	Female	Healthcare access and quality index		X			X	
Other pharynx cancer	Global	Female	Population Density (under 150 ppl/sqkm, proportion)		X			X	
Other pharynx cancer	Data Rich	Female	Population Density (under 150 ppl/sqkm, proportion)		X			X	
Other pharynx cancer	Global	Female	Age- and sex-specific SEV for Low fruit		X			X	
Other pharynx cancer	Data Rich	Female	Age- and sex-specific SEV for Low fruit		X			X	
Other pharynx cancer	Global	Female	Age- and sex-specific SEV for Low vegetables		X			X	
Other pharynx cancer	Data Rich	Female	Age- and sex-specific SEV for Low vegetables		X			X	
Other pharynx cancer	Global	Female	Cumulative Cigarettes (5 Years)		X			X	
Other pharynx cancer	Data Rich	Female	Cumulative Cigarettes (5 Years)		X			X	
Other pharynx cancer	Global	Female	Population Density (over 1000 ppl/sqkm, proportion)		X			X	
Other pharynx cancer	Data Rich	Female	Population Density (over 1000 ppl/sqkm, proportion)		X			X	
Other pharynx cancer	Global	Female	Smoking Prevalence	X			X		
Other pharynx cancer	Data Rich	Female	Smoking Prevalence	X			X		
Other pharynx cancer	Global	Female	Liters of alcohol consumed per capita	X			X		
Other pharynx cancer	Data Rich	Female	Liters of alcohol consumed per capita	X			X		
Other pharynx cancer	Global	Male	Education (years per capita)			X			X
Other pharynx cancer	Data Rich	Male	Education (years per capita)			X			X
Other pharynx cancer	Global	Male	LDI (I$ per capita)			X			X
Other pharynx cancer	Data Rich	Male	LDI (I$ per capita)			X			X
Other pharynx cancer	Global	Male	Socio-demographic Index			X			X
Other pharynx cancer	Data Rich	Male	Socio-demographic Index			X			X
Other pharynx cancer	Global	Male	Healthcare access and quality index		X			X	
Other pharynx cancer	Data Rich	Male	Healthcare access and quality index		X			X	
Other pharynx cancer	Global	Male	Population Density (under 150 ppl/sqkm, proportion)		X			X	
Other pharynx cancer	Data Rich	Male	Population Density (under 150 ppl/sqkm, proportion)		X			X	

Appendix Table S11: Comparison of GBD 2021 and GBD 2023 covariates and level of covariates used in cause of death modeling									
**Cause**	**Model Version Type**	**Sex**	**Covariate**	**Level 1 GBD 2021**	**Level 2 GBD 2021**	**Level 3 GBD 2021**	**Level 1 GBD 2023**	**Level 2 GBD 2023**	**Level 3 GBD 2023**
Other pharynx cancer	Global	Male	Age- and sex-specific SEV for Low fruit		X			X	
Other pharynx cancer	Data Rich	Male	Age- and sex-specific SEV for Low fruit		X			X	
Other pharynx cancer	Global	Male	Age- and sex-specific SEV for Low vegetables		X			X	
Other pharynx cancer	Data Rich	Male	Age- and sex-specific SEV for Low vegetables		X			X	
Other pharynx cancer	Global	Male	Cumulative Cigarettes (5 Years)		X			X	
Other pharynx cancer	Data Rich	Male	Cumulative Cigarettes (5 Years)		X			X	
Other pharynx cancer	Global	Male	Population Density (over 1000 ppl/sqkm, proportion)		X			X	
Other pharynx cancer	Data Rich	Male	Population Density (over 1000 ppl/sqkm, proportion)		X			X	
Other pharynx cancer	Global	Male	Smoking Prevalence	X			X		
Other pharynx cancer	Data Rich	Male	Smoking Prevalence	X			X		
Other pharynx cancer	Global	Male	Liters of alcohol consumed per capita	X			X		
Other pharynx cancer	Data Rich	Male	Liters of alcohol consumed per capita	X			X		
Oesophageal cancer	Data Rich	Female	Education (years per capita)			X			X
Oesophageal cancer	Global	Female	Education (years per capita)			X			X
Oesophageal cancer	Data Rich	Female	LDI (I$ per capita)			X			X
Oesophageal cancer	Global	Female	LDI (I$ per capita)			X			X
Oesophageal cancer	Data Rich	Female	Socio-demographic Index			X			X
Oesophageal cancer	Global	Female	Socio-demographic Index			X			X
Oesophageal cancer	Data Rich	Female	Sanitation (proportion with access)			X			X
Oesophageal cancer	Global	Female	Sanitation (proportion with access)			X			X
Oesophageal cancer	Data Rich	Female	Improved Water Source (proportion with access)			X			X
Oesophageal cancer	Global	Female	Improved Water Source (proportion with access)			X			X
Oesophageal cancer	Data Rich	Female	Tobacco (cigarettes per capita)		X			X	
Oesophageal cancer	Global	Female	Tobacco (cigarettes per capita)		X			X	
Oesophageal cancer	Data Rich	Female	Healthcare access and quality index		X			X	
Oesophageal cancer	Global	Female	Healthcare access and quality index		X			X	
Oesophageal cancer	Data Rich	Female	Indoor Air Pollution (All Cooking Fuels)		X			X	
Oesophageal cancer	Global	Female	Indoor Air Pollution (All Cooking Fuels)		X			X	
Oesophageal cancer	Data Rich	Female	Age- and sex-specific SEV for Low fruit		X			X	
Oesophageal cancer	Global	Female	Age- and sex-specific SEV for Low fruit		X			X	
Oesophageal cancer	Data Rich	Female	Age- and sex-specific SEV for Low vegetables		X			X	
Oesophageal cancer	Global	Female	Age- and sex-specific SEV for Low vegetables		X			X	
Oesophageal cancer	Data Rich	Female	Smoking Prevalence	X			X		
Oesophageal cancer	Global	Female	Smoking Prevalence	X			X		
Oesophageal cancer	Data Rich	Female	Liters of alcohol consumed per capita	X			X		
Oesophageal cancer	Global	Female	Liters of alcohol consumed per capita	X			X		
Oesophageal cancer	Data Rich	Female	Mean BMI	X			X		
Oesophageal cancer	Global	Female	Mean BMI	X			X		
Oesophageal cancer	Data Rich	Male	Education (years per capita)			X			X
Oesophageal cancer	Global	Male	Education (years per capita)			X			X
Oesophageal cancer	Data Rich	Male	LDI (I$ per capita)			X			X
Oesophageal cancer	Global	Male	LDI (I$ per capita)			X			X
Oesophageal cancer	Data Rich	Male	Socio-demographic Index			X			X
Oesophageal cancer	Global	Male	Socio-demographic Index			X			X
Oesophageal cancer	Data Rich	Male	Sanitation (proportion with access)			X			X
Oesophageal cancer	Global	Male	Sanitation (proportion with access)			X			X
Oesophageal cancer	Data Rich	Male	Improved Water Source (proportion with access)			X			X
Oesophageal cancer	Global	Male	Improved Water Source (proportion with access)			X			X
Oesophageal cancer	Data Rich	Male	Tobacco (cigarettes per capita)		X			X	
Oesophageal cancer	Global	Male	Tobacco (cigarettes per capita)		X			X	
Oesophageal cancer	Data Rich	Male	Healthcare access and quality index		X			X	
Oesophageal cancer	Global	Male	Healthcare access and quality index		X			X	
Oesophageal cancer	Data Rich	Male	Indoor Air Pollution (All Cooking Fuels)		X			X	
Oesophageal cancer	Global	Male	Indoor Air Pollution (All Cooking Fuels)		X			X	
Oesophageal cancer	Data Rich	Male	Age- and sex-specific SEV for Low fruit		X			X	
Oesophageal cancer	Global	Male	Age- and sex-specific SEV for Low fruit		X			X	
Oesophageal cancer	Data Rich	Male	Age- and sex-specific SEV for Low vegetables		X			X	
Oesophageal cancer	Global	Male	Age- and sex-specific SEV for Low vegetables		X			X	
Oesophageal cancer	Data Rich	Male	Smoking Prevalence	X			X		
Oesophageal cancer	Global	Male	Smoking Prevalence	X			X		
Oesophageal cancer	Data Rich	Male	Liters of alcohol consumed per capita	X			X		
Oesophageal cancer	Global	Male	Liters of alcohol consumed per capita	X			X		
Oesophageal cancer	Data Rich	Male	Mean BMI	X			X		
Oesophageal cancer	Global	Male	Mean BMI	X			X		
Stomach cancer	Global	Female	Education (years per capita)			X			X
Stomach cancer	Data Rich	Female	Education (years per capita)			X			X
Stomach cancer	Global	Female	LDI (I$ per capita)			X			X
Stomach cancer	Data Rich	Female	LDI (I$ per capita)			X			X
Stomach cancer	Global	Female	Socio-demographic Index			X			X
Stomach cancer	Data Rich	Female	Socio-demographic Index			X			X
Stomach cancer	Global	Female	Age- and sex-specific SEV for Low fruit			X			X
Stomach cancer	Data Rich	Female	Age- and sex-specific SEV for Low fruit			X			X
Stomach cancer	Global	Female	Age- and sex-specific SEV for Low vegetables			X			X
Stomach cancer	Data Rich	Female	Age- and sex-specific SEV for Low vegetables			X			X
Stomach cancer	Global	Female	Healthcare access and quality index		X			X	
Stomach cancer	Data Rich	Female	Healthcare access and quality index		X			X	
Stomach cancer	Global	Female	Mean BMI		X			X	
Stomach cancer	Data Rich	Female	Mean BMI		X			X	
Stomach cancer	Global	Female	Sanitation (proportion with access)		X			X	
Stomach cancer	Data Rich	Female	Sanitation (proportion with access)		X			X	
Stomach cancer	Global	Female	Improved Water Source (proportion with access)		X			X	
Stomach cancer	Data Rich	Female	Improved Water Source (proportion with access)		X			X	
Stomach cancer	Global	Female	Age- and sex-specific SEV for Unsafe water		X			X	
Stomach cancer	Data Rich	Female	Age- and sex-specific SEV for Unsafe water		X			X	
Stomach cancer	Global	Female	Age- and sex-specific SEV for Unsafe sanitation		X			X	
Stomach cancer	Data Rich	Female	Age- and sex-specific SEV for Unsafe sanitation		X			X	
Stomach cancer	Global	Female	Cumulative Cigarettes (20 Years)		X			X	
Stomach cancer	Data Rich	Female	Cumulative Cigarettes (20 Years)		X			X	
Stomach cancer	Global	Female	Tobacco (cigarettes per capita)	X			X		
Stomach cancer	Data Rich	Female	Tobacco (cigarettes per capita)	X			X		
Stomach cancer	Global	Female	Diet high in sodium	X			X		
Stomach cancer	Data Rich	Female	Diet high in sodium	X			X		
Stomach cancer	Global	Male	Education (years per capita)			X			X
Stomach cancer	Data Rich	Male	Education (years per capita)			X			X
Stomach cancer	Global	Male	LDI (I$ per capita)			X			X
Stomach cancer	Data Rich	Male	LDI (I$ per capita)			X			X
Stomach cancer	Global	Male	Socio-demographic Index			X			X
Stomach cancer	Data Rich	Male	Socio-demographic Index			X			X
Stomach cancer	Global	Male	Age- and sex-specific SEV for Low fruit			X			X
Stomach cancer	Data Rich	Male	Age- and sex-specific SEV for Low fruit			X			X
Stomach cancer	Global	Male	Age- and sex-specific SEV for Low vegetables			X			X
Stomach cancer	Data Rich	Male	Age- and sex-specific SEV for Low vegetables			X			X
Stomach cancer	Global	Male	Healthcare access and quality index		X			X	
Stomach cancer	Data Rich	Male	Healthcare access and quality index		X			X	
Stomach cancer	Global	Male	Mean BMI		X			X	
Stomach cancer	Data Rich	Male	Mean BMI		X			X	
Stomach cancer	Global	Male	Sanitation (proportion with access)		X			X	
Stomach cancer	Data Rich	Male	Sanitation (proportion with access)		X			X	
Stomach cancer	Global	Male	Improved Water Source (proportion with access)		X			X	
Stomach cancer	Data Rich	Male	Improved Water Source (proportion with access)		X			X	
Stomach cancer	Global	Male	Age- and sex-specific SEV for Unsafe water		X			X	
Stomach cancer	Data Rich	Male	Age- and sex-specific SEV for Unsafe water		X			X	

Appendix Table S11: Comparison of GBD 2021 and GBD 2023 covariates and level of covariates used in cause of death modeling

Cause	Model Version Type	Sex	Covariate	Level 1 GBD 2021	Level 2 GBD 2021	Level 3 GBD 2021	Level 1 GBD 2023	Level 2 GBD 2023	Level 3 GBD 2023
Stomach cancer	Global	Male	Age- and sex-specific SEV for Unsafe sanitation		X			X	
Stomach cancer	Data Rich	Male	Age- and sex-specific SEV for Unsafe sanitation		X			X	
Stomach cancer	Global	Male	Cumulative Cigarettes (20 Years)		X			X	
Stomach cancer	Data Rich	Male	Cumulative Cigarettes (20 Years)		X			X	
Stomach cancer	Global	Male	Tobacco (cigarettes per capita)	X			X		
Stomach cancer	Data Rich	Male	Tobacco (cigarettes per capita)	X			X		
Stomach cancer	Global	Male	Diet high in sodium	X			X		
Stomach cancer	Data Rich	Male	Diet high in sodium	X			X		
Colon and rectum cancer	Data Rich	Female	Education (years per capita)			X			X
Colon and rectum cancer	Global	Female	Education (years per capita)			X			X
Colon and rectum cancer	Data Rich	Female	LDI (I$ per capita)			X			X
Colon and rectum cancer	Global	Female	LDI (I$ per capita)			X			X
Colon and rectum cancer	Data Rich	Female	Socio-demographic Index			X			X
Colon and rectum cancer	Global	Female	Socio-demographic Index			X			X
Colon and rectum cancer	Data Rich	Female	Healthcare access and quality index			X			X
Colon and rectum cancer	Global	Female	Healthcare access and quality index			X			X
Colon and rectum cancer	Data Rich	Female	Age- and sex-specific SEV for Low fruit			X			X
Colon and rectum cancer	Global	Female	Age- and sex-specific SEV for Low fruit			X			X
Colon and rectum cancer	Data Rich	Female	Age- and sex-specific SEV for Low nuts and seeds			X			X
Colon and rectum cancer	Global	Female	Age- and sex-specific SEV for Low nuts and seeds			X			X
Colon and rectum cancer	Data Rich	Female	Age- and sex-specific SEV for Low milk			X			X
Colon and rectum cancer	Global	Female	Age- and sex-specific SEV for Low milk			X			X
Colon and rectum cancer	Data Rich	Female	Liters of alcohol consumed per capita		X			X	
Colon and rectum cancer	Global	Female	Liters of alcohol consumed per capita		X			X	
Colon and rectum cancer	Data Rich	Female	Diabetes Fasting Plasma Glucose (mmol/L), age-standardized 25+		X			X	
Colon and rectum cancer	Global	Female	Diabetes Fasting Plasma Glucose (mmol/L), age-standardized 25+		X			X	
Colon and rectum cancer	Data Rich	Female	Age- and sex-specific SEV for Low vegetables		X			X	
Colon and rectum cancer	Global	Female	Age- and sex-specific SEV for Low vegetables		X			X	
Colon and rectum cancer	Data Rich	Female	Cumulative Cigarettes (5 Years)		X			X	
Colon and rectum cancer	Global	Female	Cumulative Cigarettes (5 Years)		X			X	
Colon and rectum cancer	Data Rich	Female	Age- and sex-specific SEV for Low fiber		X			X	
Colon and rectum cancer	Global	Female	Age- and sex-specific SEV for Low fiber		X			X	
Colon and rectum cancer	Data Rich	Female	pufa adjusted(percent)		X			X	
Colon and rectum cancer	Global	Female	pufa adjusted(percent)		X			X	
Colon and rectum cancer	Data Rich	Female	Age- and sex-specific SEV for Low calcium		X			X	
Colon and rectum cancer	Global	Female	Age- and sex-specific SEV for Low calcium		X			X	
Colon and rectum cancer	Data Rich	Female	Tobacco (cigarettes per capita)	X			X		
Colon and rectum cancer	Global	Female	Tobacco (cigarettes per capita)	X			X		
Colon and rectum cancer	Data Rich	Female	Mean BMI	X			X		
Colon and rectum cancer	Global	Female	Mean BMI	X			X		
Colon and rectum cancer	Data Rich	Female	Total Physical Activity (MET-min/week), Age-specific	X			X		
Colon and rectum cancer	Global	Female	Total Physical Activity (MET-min/week), Age-specific	X			X		
Colon and rectum cancer	Data Rich	Female	Age- and sex-specific SEV for High red meat	X			X		
Colon and rectum cancer	Global	Female	Age- and sex-specific SEV for High red meat	X			X		
Colon and rectum cancer	Data Rich	Female	Log-transformed SEV scalar: Colorect C	X			X		
Colon and rectum cancer	Global	Female	Log-transformed SEV scalar: Colorect C	X			X		
Colon and rectum cancer	Data Rich	Male	Education (years per capita)			X			X
Colon and rectum cancer	Global	Male	Education (years per capita)			X			X
Colon and rectum cancer	Data Rich	Male	LDI (I$ per capita)			X			X
Colon and rectum cancer	Global	Male	LDI (I$ per capita)			X			X
Colon and rectum cancer	Data Rich	Male	Socio-demographic Index			X			X
Colon and rectum cancer	Global	Male	Socio-demographic Index			X			X
Colon and rectum cancer	Data Rich	Male	Healthcare access and quality index			X			X
Colon and rectum cancer	Global	Male	Healthcare access and quality index			X			X
Colon and rectum cancer	Data Rich	Male	Age- and sex-specific SEV for Low fruit			X			X
Colon and rectum cancer	Global	Male	Age- and sex-specific SEV for Low fruit			X			X
Colon and rectum cancer	Data Rich	Male	Age- and sex-specific SEV for Low nuts and seeds			X			X
Colon and rectum cancer	Global	Male	Age- and sex-specific SEV for Low nuts and seeds			X			X
Colon and rectum cancer	Data Rich	Male	Age- and sex-specific SEV for Low milk			X			X
Colon and rectum cancer	Global	Male	Age- and sex-specific SEV for Low milk			X			X
Colon and rectum cancer	Data Rich	Male	Liters of alcohol consumed per capita		X			X	
Colon and rectum cancer	Global	Male	Liters of alcohol consumed per capita		X			X	
Colon and rectum cancer	Data Rich	Male	Diabetes Fasting Plasma Glucose (mmol/L), age-standardized 25+		X			X	
Colon and rectum cancer	Global	Male	Diabetes Fasting Plasma Glucose (mmol/L), age-standardized 25+		X			X	
Colon and rectum cancer	Data Rich	Male	Age- and sex-specific SEV for Low vegetables		X			X	
Colon and rectum cancer	Global	Male	Age- and sex-specific SEV for Low vegetables		X			X	
Colon and rectum cancer	Data Rich	Male	Cumulative Cigarettes (5 Years)		X			X	
Colon and rectum cancer	Data Rich	Male	Age- and sex-specific SEV for Low fiber		X			X	
Colon and rectum cancer	Global	Male	Age- and sex-specific SEV for Low fiber		X			X	
Colon and rectum cancer	Data Rich	Male	pufa adjusted(percent)		X			X	
Colon and rectum cancer	Global	Male	pufa adjusted(percent)		X			X	
Colon and rectum cancer	Data Rich	Male	Age- and sex-specific SEV for Low calcium		X			X	
Colon and rectum cancer	Global	Male	Age- and sex-specific SEV for Low calcium		X			X	
Colon and rectum cancer	Global	Male	Cumulative Cigarettes (20 Years)		X			X	
Colon and rectum cancer	Data Rich	Male	Tobacco (cigarettes per capita)	X			X		
Colon and rectum cancer	Global	Male	Tobacco (cigarettes per capita)	X			X		
Colon and rectum cancer	Data Rich	Male	Mean BMI	X			X		
Colon and rectum cancer	Global	Male	Mean BMI	X			X		
Colon and rectum cancer	Data Rich	Male	Total Physical Activity (MET-min/week), Age-specific	X			X		
Colon and rectum cancer	Global	Male	Total Physical Activity (MET-min/week), Age-specific	X			X		
Colon and rectum cancer	Data Rich	Male	Age- and sex-specific SEV for High red meat	X			X		
Colon and rectum cancer	Global	Male	Age- and sex-specific SEV for High red meat	X			X		
Colon and rectum cancer	Data Rich	Male	Log-transformed SEV scalar: Colorect C	X			X		
Colon and rectum cancer	Global	Male	Log-transformed SEV scalar: Colorect C	X			X		
Liver cancer	Global	Female	Education (years per capita)			X			X
Liver cancer	Data Rich	Female	Education (years per capita)			X			X
Liver cancer	Global	Female	LDI (I$ per capita)			X			X
Liver cancer	Data Rich	Female	LDI (I$ per capita)			X			X
Liver cancer	Global	Female	Socio-demographic Index			X			X
Liver cancer	Data Rich	Female	Socio-demographic Index			X			X
Liver cancer	Global	Female	Age- and sex-specific SEV for High red meat			X			X
Liver cancer	Data Rich	Female	Age- and sex-specific SEV for High red meat			X			X
Liver cancer	Global	Female	Tobacco (cigarettes per capita)		X			X	
Liver cancer	Data Rich	Female	Tobacco (cigarettes per capita)		X			X	
Liver cancer	Global	Female	Healthcare access and quality index		X			X	
Liver cancer	Data Rich	Female	Healthcare access and quality index		X			X	
Liver cancer	Global	Female	Mean BMI		X			X	
Liver cancer	Data Rich	Female	Mean BMI		X			X	
Liver cancer	Global	Female	Diabetes Fasting Plasma Glucose (mmol/L), age-standardized 25+		X			X	
Liver cancer	Data Rich	Female	Diabetes Fasting Plasma Glucose (mmol/L), age-standardized 25+		X			X	
Liver cancer	Global	Female	Cumulative Cigarettes (20 Years)		X			X	
Liver cancer	Data Rich	Female	Cumulative Cigarettes (20 Years)		X			X	
Liver cancer	Global	Female	Hepatitis B 3-dose coverage, COVID-free (proportion)		X			X	
Liver cancer	Data Rich	Female	Hepatitis B 3-dose coverage, COVID-free (proportion)		X			X	
Liver cancer	Global	Female	Intravenous drug use (age-standardized proportion)		X			X	
Liver cancer	Data Rich	Female	Intravenous drug use (age-standardized proportion)		X			X	
Liver cancer	Global	Female	Hepatitis B vaccine coverage, aged through time, COVID-free (proportion)		X			X	

Appendix Table S11: Comparison of GBD 2021 and GBD 2023 covariates and level of covariates used in cause of death modeling

Cause	Model Version Type	Sex	Covariate	Level 1 GBD 2021	Level 2 GBD 2021	Level 3 GBD 2021	Level 1 GBD 2023	Level 2 GBD 2023	Level 3 GBD 2023
Liver cancer	Data Rich	Female	Hepatitis B vaccine coverage, aged through time, COVID-free (proportion)		X			X	
Liver cancer	Global	Female	Liters of alcohol consumed per capita	X			X		
Liver cancer	Data Rich	Female	Liters of alcohol consumed per capita	X			X		
Liver cancer	Global	Female	HIV age-standardized prevalence	X			X		
Liver cancer	Data Rich	Female	HIV age-standardized prevalence	X			X		
Liver cancer	Global	Female	Hepatitis C Seroprevalence (anti-HCV) age standardized	X			X		
Liver cancer	Data Rich	Female	Hepatitis C Seroprevalence (anti-HCV) age standardized	X			X		
Liver cancer	Global	Female	Vaccine adjusted HbSAg serporevalence age standardized	X			X		
Liver cancer	Data Rich	Female	Vaccine adjusted HbSAg serporevalence age standardized	X			X		
Liver cancer	Data Rich	Female	Intravenous drug use (proportion by age)				X		
Liver cancer	Data Rich	Female	Alcohol consumption, age standardized, in grams per day				X		
Liver cancer	Data Rich	Female	Chronic Hepatitis C age standardized				X		
Liver cancer	Data Rich	Female	Hepatitis B vaccine coverage (proportion), aged through time, COVID-inclusive				X		
Liver cancer	Global	Male	Education (years per capita)			X			X
Liver cancer	Data Rich	Male	Education (years per capita)			X			X
Liver cancer	Global	Male	LDI (I$ per capita)			X			X
Liver cancer	Data Rich	Male	LDI (I$ per capita)			X			X
Liver cancer	Global	Male	Socio-demographic Index			X			X
Liver cancer	Data Rich	Male	Socio-demographic Index			X			X
Liver cancer	Global	Male	Age- and sex-specific SEV for High red meat			X			X
Liver cancer	Data Rich	Male	Age- and sex-specific SEV for High red meat			X			X
Liver cancer	Global	Male	Tobacco (cigarettes per capita)		X			X	
Liver cancer	Data Rich	Male	Tobacco (cigarettes per capita)		X			X	
Liver cancer	Global	Male	Healthcare access and quality index		X			X	
Liver cancer	Data Rich	Male	Healthcare access and quality index		X			X	
Liver cancer	Global	Male	Mean BMI		X			X	
Liver cancer	Data Rich	Male	Mean BMI		X			X	
Liver cancer	Global	Male	Diabetes Fasting Plasma Glucose (mmol/L), age-standardized 25+		X			X	
Liver cancer	Data Rich	Male	Diabetes Fasting Plasma Glucose (mmol/L), age-standardized 25+		X			X	
Liver cancer	Global	Male	Cumulative Cigarettes (20 Years)		X			X	
Liver cancer	Data Rich	Male	Cumulative Cigarettes (20 Years)		X			X	
Liver cancer	Global	Male	Hepatitis B 3-dose coverage, COVID-free (proportion)		X			X	
Liver cancer	Data Rich	Male	Hepatitis B 3-dose coverage, COVID-free (proportion)		X			X	
Liver cancer	Global	Male	Intravenous drug use (age-standardized proportion)		X			X	
Liver cancer	Data Rich	Male	Intravenous drug use (age-standardized proportion)		X			X	
Liver cancer	Global	Male	Hepatitis B vaccine coverage, aged through time, COVID-free (proportion)		X			X	
Liver cancer	Data Rich	Male	Hepatitis B vaccine coverage, aged through time, COVID-free (proportion)		X			X	
Liver cancer	Global	Male	Liters of alcohol consumed per capita	X			X		
Liver cancer	Data Rich	Male	Liters of alcohol consumed per capita	X			X		
Liver cancer	Global	Male	HIV age-standardized prevalence	X			X		
Liver cancer	Data Rich	Male	HIV age-standardized prevalence	X			X		
Liver cancer	Global	Male	Hepatitis C Seroprevalence (anti-HCV) age standardized	X			X		
Liver cancer	Data Rich	Male	Hepatitis C Seroprevalence (anti-HCV) age standardized	X			X		
Liver cancer	Global	Male	Vaccine adjusted HbSAg serporevalence age standardized	X			X		
Liver cancer	Data Rich	Male	Vaccine adjusted HbSAg serporevalence age standardized	X			X		
Gallbladder and biliary tract cancer	Global	Female	Education (years per capita)			X			X
Gallbladder and biliary tract cancer	Data Rich	Female	Education (years per capita)			X			X
Gallbladder and biliary tract cancer	Global	Female	LDI (I$ per capita)			X			X
Gallbladder and biliary tract cancer	Data Rich	Female	LDI (I$ per capita)			X			X
Gallbladder and biliary tract cancer	Global	Female	Socio-demographic Index			X			X
Gallbladder and biliary tract cancer	Data Rich	Female	Socio-demographic Index			X			X
Gallbladder and biliary tract cancer	Global	Female	Tobacco (cigarettes per capita)		X			X	
Gallbladder and biliary tract cancer	Data Rich	Female	Tobacco (cigarettes per capita)		X			X	
Gallbladder and biliary tract cancer	Global	Female	Cumulative Cigarettes (10 Years)		X			X	
Gallbladder and biliary tract cancer	Data Rich	Female	Cumulative Cigarettes (10 Years)		X			X	
Gallbladder and biliary tract cancer	Global	Female	Smoking Prevalence		X			X	
Gallbladder and biliary tract cancer	Data Rich	Female	Smoking Prevalence		X			X	
Gallbladder and biliary tract cancer	Global	Female	Healthcare access and quality index		X			X	
Gallbladder and biliary tract cancer	Data Rich	Female	Healthcare access and quality index		X			X	
Gallbladder and biliary tract cancer	Global	Female	Liters of alcohol consumed per capita		X			X	
Gallbladder and biliary tract cancer	Data Rich	Female	Liters of alcohol consumed per capita		X			X	
Gallbladder and biliary tract cancer	Global	Female	Age- and sex-specific SEV for Low fruit		X			X	
Gallbladder and biliary tract cancer	Data Rich	Female	Age- and sex-specific SEV for Low fruit		X			X	
Gallbladder and biliary tract cancer	Global	Female	Age- and sex-specific SEV for Low vegetables		X			X	
Gallbladder and biliary tract cancer	Data Rich	Female	Age- and sex-specific SEV for Low vegetables		X			X	
Gallbladder and biliary tract cancer	Global	Female	Cumulative Cigarettes (5 Years)		X			X	
Gallbladder and biliary tract cancer	Data Rich	Female	Cumulative Cigarettes (5 Years)		X			X	
Gallbladder and biliary tract cancer	Global	Female	Diabetes Age-Standardized Prevalence (proportion)		X			X	
Gallbladder and biliary tract cancer	Data Rich	Female	Diabetes Age-Standardized Prevalence (proportion)		X			X	
Gallbladder and biliary tract cancer	Global	Female	Mean BMI	X			X		
Gallbladder and biliary tract cancer	Data Rich	Female	Mean BMI	X			X		
Gallbladder and biliary tract cancer	Global	Female	Log-transformed SEV scalar: Gallblad C	X			X		
Gallbladder and biliary tract cancer	Data Rich	Female	Log-transformed SEV scalar: Gallblad C	X			X		
Gallbladder and biliary tract cancer	Global	Male	Education (years per capita)			X			X
Gallbladder and biliary tract cancer	Data Rich	Male	Education (years per capita)			X			X
Gallbladder and biliary tract cancer	Global	Male	LDI (I$ per capita)			X			X
Gallbladder and biliary tract cancer	Data Rich	Male	LDI (I$ per capita)			X			X
Gallbladder and biliary tract cancer	Global	Male	Socio-demographic Index			X			X
Gallbladder and biliary tract cancer	Data Rich	Male	Socio-demographic Index			X			X
Gallbladder and biliary tract cancer	Global	Male	Tobacco (cigarettes per capita)		X			X	
Gallbladder and biliary tract cancer	Data Rich	Male	Tobacco (cigarettes per capita)		X			X	
Gallbladder and biliary tract cancer	Global	Male	Cumulative Cigarettes (10 Years)		X			X	
Gallbladder and biliary tract cancer	Data Rich	Male	Cumulative Cigarettes (10 Years)		X			X	
Gallbladder and biliary tract cancer	Global	Male	Smoking Prevalence		X			X	
Gallbladder and biliary tract cancer	Data Rich	Male	Smoking Prevalence		X			X	
Gallbladder and biliary tract cancer	Global	Male	Healthcare access and quality index		X			X	
Gallbladder and biliary tract cancer	Data Rich	Male	Healthcare access and quality index		X			X	
Gallbladder and biliary tract cancer	Global	Male	Liters of alcohol consumed per capita		X			X	
Gallbladder and biliary tract cancer	Data Rich	Male	Liters of alcohol consumed per capita		X			X	
Gallbladder and biliary tract cancer	Global	Male	Age- and sex-specific SEV for Low fruit		X			X	
Gallbladder and biliary tract cancer	Data Rich	Male	Age- and sex-specific SEV for Low fruit		X			X	
Gallbladder and biliary tract cancer	Global	Male	Age- and sex-specific SEV for Low vegetables		X			X	
Gallbladder and biliary tract cancer	Data Rich	Male	Age- and sex-specific SEV for Low vegetables		X			X	
Gallbladder and biliary tract cancer	Global	Male	Cumulative Cigarettes (5 Years)		X			X	
Gallbladder and biliary tract cancer	Data Rich	Male	Cumulative Cigarettes (5 Years)		X			X	
Gallbladder and biliary tract cancer	Global	Male	Diabetes Age-Standardized Prevalence (proportion)		X			X	
Gallbladder and biliary tract cancer	Data Rich	Male	Diabetes Age-Standardized Prevalence (proportion)		X			X	
Gallbladder and biliary tract cancer	Global	Male	Mean BMI	X			X		
Gallbladder and biliary tract cancer	Data Rich	Male	Mean BMI	X			X		
Gallbladder and biliary tract cancer	Global	Male	Log-transformed SEV scalar: Gallblad C	X			X		
Gallbladder and biliary tract cancer	Data Rich	Male	Log-transformed SEV scalar: Gallblad C	X			X		
Pancreatic cancer	Global	Female	Education (years per capita)			X			X
Pancreatic cancer	Data Rich	Female	Education (years per capita)			X			X
Pancreatic cancer	Global	Female	LDI (I$ per capita)			X			X
Pancreatic cancer	Data Rich	Female	LDI (I$ per capita)			X			X
Pancreatic cancer	Global	Female	Socio-demographic Index			X			X
Pancreatic cancer	Data Rich	Female	Socio-demographic Index			X			X
Pancreatic cancer	Global	Female	Age- and sex-specific SEV for Low fruit			X			X
Pancreatic cancer	Data Rich	Female	Age- and sex-specific SEV for Low fruit			X			X

Appendix Table S11: Comparison of GBD 2021 and GBD 2023 covariates and level of covariates used in cause of death modeling

Cause	Model Version Type	Sex	Covariate	Level 1 GBD 2021	Level 2 GBD 2021	Level 3 GBD 2021	Level 1 GBD 2023	Level 2 GBD 2023	Level 3 GBD 2023
Pancreatic cancer	Global	Female	Age- and sex-specific SEV for Low vegetables			X			X
Pancreatic cancer	Data Rich	Female	Age- and sex-specific SEV for Low vegetables			X			X
Pancreatic cancer	Global	Female	Healthcare access and quality index		X			X	
Pancreatic cancer	Data Rich	Female	Healthcare access and quality index		X			X	
Pancreatic cancer	Global	Female	Liters of alcohol consumed per capita		X			X	
Pancreatic cancer	Data Rich	Female	Liters of alcohol consumed per capita		X			X	
Pancreatic cancer	Global	Female	energy unadjusted(kcal)		X			X	
Pancreatic cancer	Data Rich	Female	energy unadjusted(kcal)		X			X	
Pancreatic cancer	Global	Female	Diabetes Fasting Plasma Glucose (mmol/L), age-standardized 25+		X			X	
Pancreatic cancer	Data Rich	Female	Diabetes Fasting Plasma Glucose (mmol/L), age-standardized 25+		X			X	
Pancreatic cancer	Global	Female	Age- and sex-specific SEV for High red meat		X			X	
Pancreatic cancer	Data Rich	Female	Age- and sex-specific SEV for High red meat		X			X	
Pancreatic cancer	Global	Female	Diabetes Age-Standardized Prevalence (proportion)		X			X	
Pancreatic cancer	Data Rich	Female	Diabetes Age-Standardized Prevalence (proportion)		X			X	
Pancreatic cancer	Global	Female	Tobacco (cigarettes per capita)	X			X		
Pancreatic cancer	Data Rich	Female	Tobacco (cigarettes per capita)	X			X		
Pancreatic cancer	Global	Female	Cumulative Cigarettes (10 Years)	X			X		
Pancreatic cancer	Data Rich	Female	Cumulative Cigarettes (10 Years)	X			X		
Pancreatic cancer	Global	Female	Mean BMI	X			X		
Pancreatic cancer	Data Rich	Female	Mean BMI	X			X		
Pancreatic cancer	Global	Female	Cumulative Cigarettes (20 Years)	X			X		
Pancreatic cancer	Data Rich	Female	Cumulative Cigarettes (20 Years)	X			X		
Pancreatic cancer	Global	Male	Education (years per capita)			X			X
Pancreatic cancer	Data Rich	Male	Education (years per capita)			X			X
Pancreatic cancer	Global	Male	LDI (I$ per capita)			X			X
Pancreatic cancer	Data Rich	Male	LDI (I$ per capita)			X			X
Pancreatic cancer	Global	Male	Socio-demographic Index			X			X
Pancreatic cancer	Data Rich	Male	Socio-demographic Index			X			X
Pancreatic cancer	Global	Male	Age- and sex-specific SEV for Low fruit			X			X
Pancreatic cancer	Data Rich	Male	Age- and sex-specific SEV for Low fruit			X			X
Pancreatic cancer	Global	Male	Age- and sex-specific SEV for Low vegetables			X			X
Pancreatic cancer	Data Rich	Male	Age- and sex-specific SEV for Low vegetables			X			X
Pancreatic cancer	Global	Male	Healthcare access and quality index		X			X	
Pancreatic cancer	Data Rich	Male	Healthcare access and quality index		X			X	
Pancreatic cancer	Global	Male	Liters of alcohol consumed per capita		X			X	
Pancreatic cancer	Data Rich	Male	Liters of alcohol consumed per capita		X			X	
Pancreatic cancer	Global	Male	energy unadjusted(kcal)		X			X	
Pancreatic cancer	Data Rich	Male	energy unadjusted(kcal)		X			X	
Pancreatic cancer	Global	Male	Diabetes Fasting Plasma Glucose (mmol/L), age-standardized 25+		X			X	
Pancreatic cancer	Data Rich	Male	Diabetes Fasting Plasma Glucose (mmol/L), age-standardized 25+		X			X	
Pancreatic cancer	Global	Male	Age- and sex-specific SEV for High red meat		X			X	
Pancreatic cancer	Data Rich	Male	Age- and sex-specific SEV for High red meat		X			X	
Pancreatic cancer	Global	Male	Diabetes Age-Standardized Prevalence (proportion)		X			X	
Pancreatic cancer	Data Rich	Male	Diabetes Age-Standardized Prevalence (proportion)		X			X	
Pancreatic cancer	Global	Male	Tobacco (cigarettes per capita)	X			X		
Pancreatic cancer	Data Rich	Male	Tobacco (cigarettes per capita)	X			X		
Pancreatic cancer	Global	Male	Cumulative Cigarettes (10 Years)	X			X		
Pancreatic cancer	Data Rich	Male	Cumulative Cigarettes (10 Years)	X			X		
Pancreatic cancer	Global	Male	Mean BMI	X			X		
Pancreatic cancer	Data Rich	Male	Mean BMI	X			X		
Pancreatic cancer	Global	Male	Cumulative Cigarettes (20 Years)	X			X		
Pancreatic cancer	Data Rich	Male	Cumulative Cigarettes (20 Years)	X			X		
Larynx cancer	Data Rich	Female	LDI (I$ per capita)			X			X
Larynx cancer	Global	Female	LDI (I$ per capita)			X			X
Larynx cancer	Data Rich	Female	Socio-demographic Index			X			X
Larynx cancer	Global	Female	Socio-demographic Index			X			X
Larynx cancer	Global	Female	Age- and sex-specific SEV for Low fruit			X			X
Larynx cancer	Data Rich	Female	Age- and sex-specific SEV for Low vegetables			X			X
Larynx cancer	Data Rich	Female	Cumulative Cigarettes (10 Years)		X			X	
Larynx cancer	Global	Female	Cumulative Cigarettes (10 Years)		X			X	
Larynx cancer	Data Rich	Female	Smoking Prevalence		X			X	
Larynx cancer	Global	Female	Smoking Prevalence		X			X	
Larynx cancer	Data Rich	Female	Healthcare access and quality index		X			X	
Larynx cancer	Global	Female	Healthcare access and quality index		X			X	
Larynx cancer	Data Rich	Female	Age- and sex-specific SEV for Low fruit		X			X	
Larynx cancer	Global	Female	Age- and sex-specific SEV for Low vegetables		X			X	
Larynx cancer	Data Rich	Female	Cumulative Cigarettes (20 Years)		X			X	
Larynx cancer	Global	Female	Cumulative Cigarettes (20 Years)		X			X	
Larynx cancer	Data Rich	Female	Asbestos consumption (metric tons per year per capita)		X			X	
Larynx cancer	Global	Female	Asbestos consumption (metric tons per year per capita)		X			X	
Larynx cancer	Data Rich	Female	Population Density (over 1000 ppl/sqkm, proportion)		X			X	
Larynx cancer	Global	Female	Population Density (over 1000 ppl/sqkm, proportion)		X			X	
Larynx cancer	Data Rich	Female	Liters of alcohol consumed per capita	X			X		
Larynx cancer	Global	Female	Liters of alcohol consumed per capita	X			X		
Larynx cancer	Data Rich	Male	LDI (I$ per capita)			X			X
Larynx cancer	Global	Male	LDI (I$ per capita)			X			X
Larynx cancer	Data Rich	Male	Socio-demographic Index			X			X
Larynx cancer	Global	Male	Socio-demographic Index			X			X
Larynx cancer	Global	Male	Age- and sex-specific SEV for Low fruit			X			X
Larynx cancer	Data Rich	Male	Age- and sex-specific SEV for Low vegetables			X			X
Larynx cancer	Data Rich	Male	Cumulative Cigarettes (10 Years)		X			X	
Larynx cancer	Global	Male	Cumulative Cigarettes (10 Years)		X			X	
Larynx cancer	Data Rich	Male	Smoking Prevalence		X			X	
Larynx cancer	Global	Male	Smoking Prevalence		X			X	
Larynx cancer	Data Rich	Male	Healthcare access and quality index		X			X	
Larynx cancer	Global	Male	Healthcare access and quality index		X			X	
Larynx cancer	Data Rich	Male	Age- and sex-specific SEV for Low fruit		X			X	
Larynx cancer	Global	Male	Age- and sex-specific SEV for Low vegetables		X			X	
Larynx cancer	Data Rich	Male	Cumulative Cigarettes (20 Years)		X			X	
Larynx cancer	Global	Male	Cumulative Cigarettes (20 Years)		X			X	
Larynx cancer	Data Rich	Male	Asbestos consumption (metric tons per year per capita)		X			X	
Larynx cancer	Global	Male	Asbestos consumption (metric tons per year per capita)		X			X	
Larynx cancer	Data Rich	Male	Population Density (over 1000 ppl/sqkm, proportion)		X			X	
Larynx cancer	Global	Male	Population Density (over 1000 ppl/sqkm, proportion)		X			X	
Larynx cancer	Data Rich	Male	Liters of alcohol consumed per capita	X			X		
Larynx cancer	Global	Male	Liters of alcohol consumed per capita	X			X		
Tracheal, bronchus, and lung cancer	Global	Female	Education (years per capita)			X			X
Tracheal, bronchus, and lung cancer	Data Rich	Female	Education (years per capita)			X			X
Tracheal, bronchus, and lung cancer	Global	Female	LDI (I$ per capita)			X			X
Tracheal, bronchus, and lung cancer	Data Rich	Female	LDI (I$ per capita)			X			X
Tracheal, bronchus, and lung cancer	Global	Female	Socio-demographic Index			X			X
Tracheal, bronchus, and lung cancer	Data Rich	Female	Socio-demographic Index			X			X
Tracheal, bronchus, and lung cancer	Global	Female	Cumulative Cigarettes (10 Years)		X			X	
Tracheal, bronchus, and lung cancer	Data Rich	Female	Cumulative Cigarettes (10 Years)		X			X	
Tracheal, bronchus, and lung cancer	Global	Female	Healthcare access and quality index		X			X	
Tracheal, bronchus, and lung cancer	Data Rich	Female	Healthcare access and quality index		X			X	
Tracheal, bronchus, and lung cancer	Global	Female	Diabetes Fasting Plasma Glucose (mmol/L), age-standardized 25+		X			X	
Tracheal, bronchus, and lung cancer	Data Rich	Female	Diabetes Fasting Plasma Glucose (mmol/L), age-standardized 25+		X			X	
Tracheal, bronchus, and lung cancer	Global	Female	Indoor Air Pollution (All Cooking Fuels)		X			X	
Tracheal, bronchus, and lung cancer	Data Rich	Female	Indoor Air Pollution (All Cooking Fuels)		X			X	
Tracheal, bronchus, and lung cancer	Global	Female	Outdoor Air Pollution (PM2.5)		X			X	
Tracheal, bronchus, and lung cancer	Data Rich	Female	Outdoor Air Pollution (PM2.5)		X			X	
Tracheal, bronchus, and lung cancer	Global	Female	Cumulative Cigarettes (20 Years)		X			X	

Appendix Table S11: Comparison of GBD 2021 and GBD 2023 covariates and level of covariates used in cause of death modeling

Cause	Model Version Type	Sex	Covariate	Level 1 GBD 2021	Level 2 GBD 2021	Level 3 GBD 2021	Level 1 GBD 2023	Level 2 GBD 2023	Level 3 GBD 2023
Tracheal, bronchus, and lung cancer	Data Rich	Female	Cumulative Cigarettes (20 Years)		X			X	
Tracheal, bronchus, and lung cancer	Global	Female	Residential radon		X			X	
Tracheal, bronchus, and lung cancer	Data Rich	Female	Residential radon		X			X	
Tracheal, bronchus, and lung cancer	Global	Female	Secondhand smoke		X			X	
Tracheal, bronchus, and lung cancer	Data Rich	Female	Secondhand smoke		X			X	
Tracheal, bronchus, and lung cancer	Global	Female	Smoking Prevalence	X			X		
Tracheal, bronchus, and lung cancer	Data Rich	Female	Smoking Prevalence	X			X		
Tracheal, bronchus, and lung cancer	Global	Female	Asbestos consumption (metric tons per year per capita)	X			X		
Tracheal, bronchus, and lung cancer	Data Rich	Female	Asbestos consumption (metric tons per year per capita)	X			X		
Tracheal, bronchus, and lung cancer	Global	Male	Education (years per capita)			X			X
Tracheal, bronchus, and lung cancer	Data Rich	Male	Education (years per capita)			X			X
Tracheal, bronchus, and lung cancer	Global	Male	LDI (I$ per capita)			X			X
Tracheal, bronchus, and lung cancer	Data Rich	Male	LDI (I$ per capita)			X			X
Tracheal, bronchus, and lung cancer	Global	Male	Socio-demographic Index			X			X
Tracheal, bronchus, and lung cancer	Data Rich	Male	Socio-demographic Index			X			X
Tracheal, bronchus, and lung cancer	Global	Male	Cumulative Cigarettes (10 Years)		X			X	
Tracheal, bronchus, and lung cancer	Data Rich	Male	Cumulative Cigarettes (10 Years)		X			X	
Tracheal, bronchus, and lung cancer	Global	Male	Healthcare access and quality index		X			X	
Tracheal, bronchus, and lung cancer	Data Rich	Male	Healthcare access and quality index		X			X	
Tracheal, bronchus, and lung cancer	Global	Male	Diabetes Fasting Plasma Glucose (mmol/L), age-standardized 25+		X			X	
Tracheal, bronchus, and lung cancer	Data Rich	Male	Diabetes Fasting Plasma Glucose (mmol/L), age-standardized 25+		X			X	
Tracheal, bronchus, and lung cancer	Global	Male	Indoor Air Pollution (All Cooking Fuels)		X			X	
Tracheal, bronchus, and lung cancer	Data Rich	Male	Indoor Air Pollution (All Cooking Fuels)		X			X	
Tracheal, bronchus, and lung cancer	Global	Male	Outdoor Air Pollution (PM2.5)		X			X	
Tracheal, bronchus, and lung cancer	Data Rich	Male	Outdoor Air Pollution (PM2.5)		X			X	
Tracheal, bronchus, and lung cancer	Global	Male	Cumulative Cigarettes (20 Years)		X			X	
Tracheal, bronchus, and lung cancer	Data Rich	Male	Cumulative Cigarettes (20 Years)		X			X	
Tracheal, bronchus, and lung cancer	Global	Male	Residential radon		X			X	
Tracheal, bronchus, and lung cancer	Data Rich	Male	Residential radon		X			X	
Tracheal, bronchus, and lung cancer	Global	Male	Secondhand smoke		X			X	
Tracheal, bronchus, and lung cancer	Data Rich	Male	Secondhand smoke		X			X	
Tracheal, bronchus, and lung cancer	Global	Male	Smoking Prevalence	X			X		
Tracheal, bronchus, and lung cancer	Data Rich	Male	Smoking Prevalence	X			X		
Tracheal, bronchus, and lung cancer	Global	Male	Asbestos consumption (metric tons per year per capita)	X			X		
Tracheal, bronchus, and lung cancer	Data Rich	Male	Asbestos consumption (metric tons per year per capita)	X			X		
Malignant skin melanoma	Data Rich	Female	Education (years per capita)			X			X
Malignant skin melanoma	Global	Female	Education (years per capita)			X			X
Malignant skin melanoma	Data Rich	Female	LDI (I$ per capita)			X			X
Malignant skin melanoma	Global	Female	LDI (I$ per capita)			X			X
Malignant skin melanoma	Data Rich	Female	Socio-demographic Index			X			X
Malignant skin melanoma	Global	Female	Socio-demographic Index			X			X
Malignant skin melanoma	Data Rich	Female	Healthcare access and quality index		X			X	
Malignant skin melanoma	Global	Female	Healthcare access and quality index		X			X	
Malignant skin melanoma	Data Rich	Female	Latitude Under 15 (proportion)		X			X	
Malignant skin melanoma	Global	Female	Latitude Under 15 (proportion)		X			X	
Malignant skin melanoma	Data Rich	Female	Latitude 15 to 30 (proportion)		X			X	
Malignant skin melanoma	Global	Female	Latitude 15 to 30 (proportion)		X			X	
Malignant skin melanoma	Data Rich	Female	Latitude 30 to 45 (proportion)		X			X	
Malignant skin melanoma	Global	Female	Latitude 30 to 45 (proportion)		X			X	
Malignant skin melanoma	Data Rich	Female	Latitude Over 45 (proportion)		X			X	
Malignant skin melanoma	Global	Female	Latitude Over 45 (proportion)		X			X	
Malignant skin melanoma	Data Rich	Female	Liters of alcohol consumed per capita	X			X		
Malignant skin melanoma	Global	Female	Liters of alcohol consumed per capita	X			X		
Malignant skin melanoma	Data Rich	Male	Education (years per capita)			X			X
Malignant skin melanoma	Global	Male	Education (years per capita)			X			X
Malignant skin melanoma	Data Rich	Male	LDI (I$ per capita)			X			X
Malignant skin melanoma	Global	Male	LDI (I$ per capita)			X			X
Malignant skin melanoma	Data Rich	Male	Socio-demographic Index			X			X
Malignant skin melanoma	Global	Male	Socio-demographic Index			X			X
Malignant skin melanoma	Data Rich	Male	Healthcare access and quality index		X			X	
Malignant skin melanoma	Global	Male	Healthcare access and quality index		X			X	
Malignant skin melanoma	Data Rich	Male	Latitude Under 15 (proportion)		X			X	
Malignant skin melanoma	Global	Male	Latitude Under 15 (proportion)		X			X	
Malignant skin melanoma	Data Rich	Male	Latitude 15 to 30 (proportion)		X			X	
Malignant skin melanoma	Global	Male	Latitude 15 to 30 (proportion)		X			X	
Malignant skin melanoma	Data Rich	Male	Latitude 30 to 45 (proportion)		X			X	
Malignant skin melanoma	Global	Male	Latitude 30 to 45 (proportion)		X			X	
Malignant skin melanoma	Data Rich	Male	Latitude Over 45 (proportion)		X			X	
Malignant skin melanoma	Global	Male	Latitude Over 45 (proportion)		X			X	
Malignant skin melanoma	Data Rich	Male	Liters of alcohol consumed per capita	X			X		
Malignant skin melanoma	Global	Male	Liters of alcohol consumed per capita	X			X		
Non-melanoma skin cancer	Data Rich	Female	Education (years per capita)			X			X
Non-melanoma skin cancer	Global	Female	Education (years per capita)			X			X
Non-melanoma skin cancer	Data Rich	Female	LDI (I$ per capita)			X			X
Non-melanoma skin cancer	Global	Female	LDI (I$ per capita)			X			X
Non-melanoma skin cancer	Data Rich	Female	Socio-demographic Index			X			X
Non-melanoma skin cancer	Global	Female	Socio-demographic Index			X			X
Non-melanoma skin cancer	Data Rich	Female	Healthcare access and quality index		X			X	
Non-melanoma skin cancer	Global	Female	Healthcare access and quality index		X			X	
Non-melanoma skin cancer	Data Rich	Female	Average latitude		X			X	
Non-melanoma skin cancer	Global	Female	Average latitude		X			X	
Non-melanoma skin cancer	Data Rich	Female	Cumulative Cigarettes (10 Years)	X			X		
Non-melanoma skin cancer	Global	Female	Cumulative Cigarettes (10 Years)	X			X		
Non-melanoma skin cancer	Data Rich	Female	Smoking Prevalence	X			X		
Non-melanoma skin cancer	Global	Female	Smoking Prevalence	X			X		
Non-melanoma skin cancer	Data Rich	Female	Cumulative Cigarettes (5 Years)	X			X		
Non-melanoma skin cancer	Global	Female	Cumulative Cigarettes (5 Years)	X			X		
Non-melanoma skin cancer	Data Rich	Female	Cumulative Cigarettes (15 Years)	X			X		
Non-melanoma skin cancer	Global	Female	Cumulative Cigarettes (15 Years)	X			X		
Non-melanoma skin cancer	Data Rich	Male	Education (years per capita)			X			X
Non-melanoma skin cancer	Global	Male	Education (years per capita)			X			X
Non-melanoma skin cancer	Data Rich	Male	LDI (I$ per capita)			X			X
Non-melanoma skin cancer	Global	Male	LDI (I$ per capita)			X			X
Non-melanoma skin cancer	Data Rich	Male	Socio-demographic Index			X			X
Non-melanoma skin cancer	Global	Male	Socio-demographic Index			X			X
Non-melanoma skin cancer	Data Rich	Male	Healthcare access and quality index		X			X	
Non-melanoma skin cancer	Global	Male	Healthcare access and quality index		X			X	
Non-melanoma skin cancer	Data Rich	Male	Average latitude		X			X	
Non-melanoma skin cancer	Global	Male	Average latitude		X			X	
Non-melanoma skin cancer	Data Rich	Male	Cumulative Cigarettes (10 Years)	X			X		
Non-melanoma skin cancer	Global	Male	Cumulative Cigarettes (10 Years)	X			X		
Non-melanoma skin cancer	Data Rich	Male	Smoking Prevalence	X			X		
Non-melanoma skin cancer	Global	Male	Smoking Prevalence	X			X		
Non-melanoma skin cancer	Data Rich	Male	Cumulative Cigarettes (5 Years)	X			X		
Non-melanoma skin cancer	Global	Male	Cumulative Cigarettes (5 Years)	X			X		
Non-melanoma skin cancer	Data Rich	Male	Cumulative Cigarettes (15 Years)	X			X		
Non-melanoma skin cancer	Global	Male	Cumulative Cigarettes (15 Years)	X			X		
Non-melanoma skin cancer (squamous-cell carcinoma)	Data Rich	Female	Education (years per capita)			X			X
Non-melanoma skin cancer (squamous-cell carcinoma)	Global	Female	Education (years per capita)			X			X
Non-melanoma skin cancer (squamous-cell carcinoma)	Data Rich	Female	LDI (I$ per capita)			X			X
Non-melanoma skin cancer (squamous-cell carcinoma)	Global	Female	LDI (I$ per capita)			X			X
Non-melanoma skin cancer (squamous-cell carcinoma)	Data Rich	Female	Socio-demographic Index			X			X
Non-melanoma skin cancer (squamous-cell carcinoma)	Global	Female	Socio-demographic Index			X			X
Non-melanoma skin cancer (squamous-cell carcinoma)	Data Rich	Female	Healthcare access and quality index		X			X	
Non-melanoma skin cancer (squamous-cell carcinoma)	Global	Female	Healthcare access and quality index		X			X	
Non-melanoma skin cancer (squamous-cell carcinoma)	Data Rich	Female	Average latitude		X			X	
Non-melanoma skin cancer (squamous-cell carcinoma)	Global	Female	Average latitude		X			X	
Non-melanoma skin cancer (squamous-cell carcinoma)	Data Rich	Female	Cumulative Cigarettes (10 Years)	X			X		
Non-melanoma skin cancer (squamous-cell carcinoma)	Global	Female	Cumulative Cigarettes (10 Years)	X			X		
Non-melanoma skin cancer (squamous-cell carcinoma)	Data Rich	Female	Smoking Prevalence	X			X		
Non-melanoma skin cancer (squamous-cell carcinoma)	Global	Female	Smoking Prevalence	X			X		
Non-melanoma skin cancer (squamous-cell carcinoma)	Data Rich	Female	Cumulative Cigarettes (5 Years)	X			X		

Appendix Table S11: Comparison of GBD 2021 and GBD 2023 covariates and level of covariates used in cause of death modeling									
**Cause**	**Model Version Type**	**Sex**	**Covariate**	**Level 1 GBD 2021**	**Level 2 GBD 2021**	**Level 3 GBD 2021**	**Level 1 GBD 2023**	**Level 2 GBD 2023**	**Level 3 GBD 2023**
Non-melanoma skin cancer (squamous-cell carcinoma)	Global	Female	Cumulative Cigarettes (5 Years)	X			X		
Non-melanoma skin cancer (squamous-cell carcinoma)	Data Rich	Female	Cumulative Cigarettes (15 Years)	X			X		
Non-melanoma skin cancer (squamous-cell carcinoma)	Global	Female	Cumulative Cigarettes (15 Years)	X			X		
Non-melanoma skin cancer (squamous-cell carcinoma)	Data Rich	Male	Education (years per capita)			X			X
Non-melanoma skin cancer (squamous-cell carcinoma)	Global	Male	Education (years per capita)			X			X
Non-melanoma skin cancer (squamous-cell carcinoma)	Data Rich	Male	LDI (I$ per capita)			X			X
Non-melanoma skin cancer (squamous-cell carcinoma)	Global	Male	LDI (I$ per capita)			X			X
Non-melanoma skin cancer (squamous-cell carcinoma)	Data Rich	Male	Socio-demographic Index			X			X
Non-melanoma skin cancer (squamous-cell carcinoma)	Global	Male	Socio-demographic Index			X			X
Non-melanoma skin cancer (squamous-cell carcinoma)	Data Rich	Male	Healthcare access and quality index		X			X	
Non-melanoma skin cancer (squamous-cell carcinoma)	Global	Male	Healthcare access and quality index		X			X	
Non-melanoma skin cancer (squamous-cell carcinoma)	Data Rich	Male	Average latitude		X			X	
Non-melanoma skin cancer (squamous-cell carcinoma)	Global	Male	Average latitude		X			X	
Non-melanoma skin cancer (squamous-cell carcinoma)	Data Rich	Male	Cumulative Cigarettes (10 Years)	X			X		
Non-melanoma skin cancer (squamous-cell carcinoma)	Global	Male	Cumulative Cigarettes (10 Years)	X			X		
Non-melanoma skin cancer (squamous-cell carcinoma)	Data Rich	Male	Smoking Prevalence	X			X		
Non-melanoma skin cancer (squamous-cell carcinoma)	Global	Male	Smoking Prevalence	X			X		
Non-melanoma skin cancer (squamous-cell carcinoma)	Data Rich	Male	Cumulative Cigarettes (5 Years)	X			X		
Non-melanoma skin cancer (squamous-cell carcinoma)	Global	Male	Cumulative Cigarettes (5 Years)	X			X		
Non-melanoma skin cancer (squamous-cell carcinoma)	Data Rich	Male	Cumulative Cigarettes (15 Years)	X			X		
Non-melanoma skin cancer (squamous-cell carcinoma)	Global	Male	Cumulative Cigarettes (15 Years)	X			X		
Soft tissue and other extraosseous sarcomas	Data Rich	Female	Maternal care and immunization			X			X
Soft tissue and other extraosseous sarcomas	Global	Female	Maternal care and immunization			X			X
Soft tissue and other extraosseous sarcomas	Data Rich	Female	Education (years per capita)			X			X
Soft tissue and other extraosseous sarcomas	Global	Female	Education (years per capita)			X			X
Soft tissue and other extraosseous sarcomas	Data Rich	Female	Liters of alcohol consumed per capita			X			X
Soft tissue and other extraosseous sarcomas	Global	Female	Liters of alcohol consumed per capita			X			X
Soft tissue and other extraosseous sarcomas	Data Rich	Female	Log-transformed SEV scalar: HIV			X			X
Soft tissue and other extraosseous sarcomas	Global	Female	Log-transformed SEV scalar: HIV			X			X
Soft tissue and other extraosseous sarcomas	Data Rich	Female	Log-transformed age-standardized SEV scalar: HIV			X			X
Soft tissue and other extraosseous sarcomas	Global	Female	Log-transformed age-standardized SEV scalar: HIV			X			X
Soft tissue and other extraosseous sarcomas	Data Rich	Female	LDI (I$ per capita)		X			X	
Soft tissue and other extraosseous sarcomas	Global	Female	LDI (I$ per capita)		X			X	
Soft tissue and other extraosseous sarcomas	Data Rich	Female	Socio-demographic Index		X			X	
Soft tissue and other extraosseous sarcomas	Global	Female	Socio-demographic Index		X			X	
Soft tissue and other extraosseous sarcomas	Data Rich	Female	Universal health coverage		X			X	
Soft tissue and other extraosseous sarcomas	Global	Female	Universal health coverage		X			X	
Soft tissue and other extraosseous sarcomas	Data Rich	Female	Healthcare access and quality index		X			X	
Soft tissue and other extraosseous sarcomas	Global	Female	Healthcare access and quality index		X			X	
Soft tissue and other extraosseous sarcomas	Data Rich	Female	Log-transformed SEV scalar: HIV/AIDS+STIs				X		
Soft tissue and other extraosseous sarcomas	Data Rich	Female	Log-transformed age-standardized SEV scalar: HIV/AIDS+STIs				X		
Soft tissue and other extraosseous sarcomas	Data Rich	Male	Maternal care and immunization			X			X
Soft tissue and other extraosseous sarcomas	Global	Male	Maternal care and immunization			X			X
Soft tissue and other extraosseous sarcomas	Data Rich	Male	Education (years per capita)			X			X
Soft tissue and other extraosseous sarcomas	Global	Male	Education (years per capita)			X			X
Soft tissue and other extraosseous sarcomas	Data Rich	Male	Liters of alcohol consumed per capita			X			X
Soft tissue and other extraosseous sarcomas	Global	Male	Liters of alcohol consumed per capita			X			X
Soft tissue and other extraosseous sarcomas	Data Rich	Male	Log-transformed SEV scalar: HIV			X			X
Soft tissue and other extraosseous sarcomas	Global	Male	Log-transformed SEV scalar: HIV			X			X
Soft tissue and other extraosseous sarcomas	Data Rich	Male	Log-transformed age-standardized SEV scalar: HIV			X			X
Soft tissue and other extraosseous sarcomas	Global	Male	Log-transformed age-standardized SEV scalar: HIV			X			X
Soft tissue and other extraosseous sarcomas	Data Rich	Male	LDI (I$ per capita)		X			X	
Soft tissue and other extraosseous sarcomas	Global	Male	LDI (I$ per capita)		X			X	
Soft tissue and other extraosseous sarcomas	Data Rich	Male	Socio-demographic Index		X			X	
Soft tissue and other extraosseous sarcomas	Global	Male	Socio-demographic Index		X			X	
Soft tissue and other extraosseous sarcomas	Data Rich	Male	Universal health coverage		X			X	
Soft tissue and other extraosseous sarcomas	Global	Male	Universal health coverage		X			X	
Soft tissue and other extraosseous sarcomas	Data Rich	Male	Healthcare access and quality index		X			X	
Soft tissue and other extraosseous sarcomas	Global	Male	Healthcare access and quality index		X			X	
Malignant neoplasm of bone and articular cartilage	Data Rich	Female	Maternal care and immunization			X			X
Malignant neoplasm of bone and articular cartilage	Global	Female	Maternal care and immunization			X			X
Malignant neoplasm of bone and articular cartilage	Data Rich	Female	Education (years per capita)			X			X
Malignant neoplasm of bone and articular cartilage	Global	Female	Education (years per capita)			X			X
Malignant neoplasm of bone and articular cartilage	Data Rich	Female	Smoking Prevalence			X			X
Malignant neoplasm of bone and articular cartilage	Global	Female	Smoking Prevalence			X			X
Malignant neoplasm of bone and articular cartilage	Data Rich	Female	Health worker density			X			X
Malignant neoplasm of bone and articular cartilage	Global	Female	Health worker density			X			X
Malignant neoplasm of bone and articular cartilage	Data Rich	Female	Log-transformed SEV scalar: Osteoarth			X			X
Malignant neoplasm of bone and articular cartilage	Global	Female	Log-transformed SEV scalar: Osteoarth			X			X
Malignant neoplasm of bone and articular cartilage	Data Rich	Female	Log-transformed age-standardized SEV scalar: Osteoarth			X			X
Malignant neoplasm of bone and articular cartilage	Global	Female	Log-transformed age-standardized SEV scalar: Osteoarth			X			X
Malignant neoplasm of bone and articular cartilage	Data Rich	Female	Age- and sex-specific SEV for Low bone mineral density			X			X
Malignant neoplasm of bone and articular cartilage	Global	Female	Age- and sex-specific SEV for Low bone mineral density			X			X
Malignant neoplasm of bone and articular cartilage	Data Rich	Female	LDI (I$ per capita)		X			X	
Malignant neoplasm of bone and articular cartilage	Global	Female	LDI (I$ per capita)		X			X	
Malignant neoplasm of bone and articular cartilage	Data Rich	Female	Socio-demographic Index		X			X	
Malignant neoplasm of bone and articular cartilage	Global	Female	Socio-demographic Index		X			X	
Malignant neoplasm of bone and articular cartilage	Data Rich	Female	Universal health coverage		X			X	
Malignant neoplasm of bone and articular cartilage	Global	Female	Universal health coverage		X			X	
Malignant neoplasm of bone and articular cartilage	Data Rich	Female	Healthcare access and quality index		X			X	
Malignant neoplasm of bone and articular cartilage	Global	Female	Healthcare access and quality index		X			X	
Malignant neoplasm of bone and articular cartilage	Data Rich	Male	Maternal care and immunization			X			X
Malignant neoplasm of bone and articular cartilage	Global	Male	Maternal care and immunization			X			X
Malignant neoplasm of bone and articular cartilage	Data Rich	Male	Education (years per capita)			X			X
Malignant neoplasm of bone and articular cartilage	Global	Male	Education (years per capita)			X			X
Malignant neoplasm of bone and articular cartilage	Data Rich	Male	Smoking Prevalence			X			X
Malignant neoplasm of bone and articular cartilage	Global	Male	Smoking Prevalence			X			X
Malignant neoplasm of bone and articular cartilage	Data Rich	Male	Health worker density			X			X
Malignant neoplasm of bone and articular cartilage	Global	Male	Health worker density			X			X
Malignant neoplasm of bone and articular cartilage	Data Rich	Male	Log-transformed SEV scalar: Osteoarth			X			X
Malignant neoplasm of bone and articular cartilage	Global	Male	Log-transformed SEV scalar: Osteoarth			X			X
Malignant neoplasm of bone and articular cartilage	Data Rich	Male	Log-transformed age-standardized SEV scalar: Osteoarth			X			X
Malignant neoplasm of bone and articular cartilage	Global	Male	Log-transformed age-standardized SEV scalar: Osteoarth			X			X
Malignant neoplasm of bone and articular cartilage	Data Rich	Male	Age- and sex-specific SEV for Low bone mineral density			X			X
Malignant neoplasm of bone and articular cartilage	Global	Male	Age- and sex-specific SEV for Low bone mineral density			X			X
Malignant neoplasm of bone and articular cartilage	Data Rich	Male	LDI (I$ per capita)		X			X	
Malignant neoplasm of bone and articular cartilage	Global	Male	LDI (I$ per capita)		X			X	
Malignant neoplasm of bone and articular cartilage	Data Rich	Male	Socio-demographic Index		X			X	
Malignant neoplasm of bone and articular cartilage	Global	Male	Socio-demographic Index		X			X	

Appendix Table S11: Comparison of GBD 2021 and GBD 2023 covariates and level of covariates used in cause of death modeling

Cause	Model Version Type	Sex	Covariate	Level 1 GBD 2021	Level 2 GBD 2021	Level 3 GBD 2021	Level 1 GBD 2023	Level 2 GBD 2023	Level 3 GBD 2023
Malignant neoplasm of bone and articular cartilage	Data Rich	Male	Universal health coverage		X			X	
Malignant neoplasm of bone and articular cartilage	Global	Male	Universal health coverage		X			X	
Malignant neoplasm of bone and articular cartilage	Data Rich	Male	Healthcare access and quality index		X			X	
Malignant neoplasm of bone and articular cartilage	Global	Male	Healthcare access and quality index		X			X	
Breast cancer	Global	Female	LDI (I$ per capita)			X			X
Breast cancer	Data Rich	Female	LDI (I$ per capita)			X			X
Breast cancer	Global	Female	Socio-demographic Index			X			X
Breast cancer	Data Rich	Female	Socio-demographic Index			X			X
Breast cancer	Global	Female	Cumulative Cigarettes (10 Years)		X			X	
Breast cancer	Data Rich	Female	Cumulative Cigarettes (10 Years)		X			X	
Breast cancer	Global	Female	Smoking Prevalence		X			X	
Breast cancer	Data Rich	Female	Smoking Prevalence		X			X	
Breast cancer	Global	Female	Healthcare access and quality index		X			X	
Breast cancer	Data Rich	Female	Healthcare access and quality index		X			X	
Breast cancer	Global	Female	Diabetes Fasting Plasma Glucose (mmol/L), age-standardized 25+		X			X	
Breast cancer	Data Rich	Female	Diabetes Fasting Plasma Glucose (mmol/L), age-standardized 25+		X			X	
Breast cancer	Global	Female	Age- and sex-specific SEV for Low fruit		X			X	
Breast cancer	Data Rich	Female	Age- and sex-specific SEV for Low fruit		X			X	
Breast cancer	Global	Female	Age- and sex-specific SEV for Low vegetables		X			X	
Breast cancer	Data Rich	Female	Age- and sex-specific SEV for Low vegetables		X			X	
Breast cancer	Global	Female	Cumulative Cigarettes (20 Years)		X			X	
Breast cancer	Data Rich	Female	Cumulative Cigarettes (20 Years)		X			X	
Breast cancer	Global	Female	Age-Specific Fertility Rate		X			X	
Breast cancer	Data Rich	Female	Age-Specific Fertility Rate		X			X	
Breast cancer	Global	Female	Total Fertility Rate		X			X	
Breast cancer	Data Rich	Female	Total Fertility Rate		X			X	
Breast cancer	Global	Female	Liters of alcohol consumed per capita	X			X		
Breast cancer	Data Rich	Female	Liters of alcohol consumed per capita	X			X		
Breast cancer	Global	Female	Mean BMI	X			X		
Breast cancer	Data Rich	Female	Mean BMI	X			X		
Breast cancer	Data Rich	Male	LDI (I$ per capita)			X			X
Breast cancer	Global	Male	LDI (I$ per capita)			X			X
Breast cancer	Data Rich	Male	Socio-demographic Index			X			X
Breast cancer	Global	Male	Socio-demographic Index			X			X
Breast cancer	Data Rich	Male	Cumulative Cigarettes (10 Years)		X			X	
Breast cancer	Global	Male	Cumulative Cigarettes (10 Years)		X			X	
Breast cancer	Data Rich	Male	Smoking Prevalence		X			X	
Breast cancer	Global	Male	Smoking Prevalence		X			X	
Breast cancer	Data Rich	Male	Healthcare access and quality index		X			X	
Breast cancer	Global	Male	Healthcare access and quality index		X			X	
Breast cancer	Data Rich	Male	Diabetes Fasting Plasma Glucose (mmol/L), age-standardized 25+		X			X	
Breast cancer	Global	Male	Diabetes Fasting Plasma Glucose (mmol/L), age-standardized 25+		X			X	
Breast cancer	Data Rich	Male	Age- and sex-specific SEV for Low fruit		X			X	
Breast cancer	Global	Male	Age- and sex-specific SEV for Low fruit		X			X	
Breast cancer	Data Rich	Male	Age- and sex-specific SEV for Low vegetables		X			X	
Breast cancer	Global	Male	Age- and sex-specific SEV for Low vegetables		X			X	
Breast cancer	Data Rich	Male	Cumulative Cigarettes (20 Years)		X			X	
Breast cancer	Global	Male	Cumulative Cigarettes (20 Years)		X			X	
Breast cancer	Data Rich	Male	Liters of alcohol consumed per capita	X			X		
Breast cancer	Global	Male	Liters of alcohol consumed per capita	X			X		
Breast cancer	Data Rich	Male	Mean BMI	X			X		
Breast cancer	Global	Male	Mean BMI	X			X		
Cervical cancer	Data Rich	Female	Education (years per capita)			X			X
Cervical cancer	Global	Female	Education (years per capita)			X			X
Cervical cancer	Data Rich	Female	LDI (I$ per capita)			X			X
Cervical cancer	Global	Female	LDI (I$ per capita)			X			X
Cervical cancer	Data Rich	Female	Socio-demographic Index			X			X
Cervical cancer	Global	Female	Socio-demographic Index			X			X
Cervical cancer	Data Rich	Female	Smoking Prevalence		X			X	
Cervical cancer	Global	Female	Smoking Prevalence		X			X	
Cervical cancer	Data Rich	Female	Healthcare access and quality index		X			X	
Cervical cancer	Global	Female	Healthcare access and quality index		X			X	
Cervical cancer	Data Rich	Female	Age- and sex-specific SEV for Low fruit		X			X	
Cervical cancer	Global	Female	Age- and sex-specific SEV for Low fruit		X			X	
Cervical cancer	Data Rich	Female	Age- and sex-specific SEV for Low vegetables		X			X	
Cervical cancer	Global	Female	Age- and sex-specific SEV for Low vegetables		X			X	
Cervical cancer	Data Rich	Female	Age-Specific Fertility Rate		X			X	
Cervical cancer	Global	Female	Age-Specific Fertility Rate		X			X	
Cervical cancer	Data Rich	Female	Total Fertility Rate		X			X	
Cervical cancer	Global	Female	Total Fertility Rate		X			X	
Cervical cancer	Data Rich	Female	Log-transformed SEV scalar: HIV	X			X		
Cervical cancer	Global	Female	Log-transformed SEV scalar: HIV	X			X		
Cervical cancer	Data Rich	Female	Log-transformed age-standardized SEV scalar: HIV	X			X		
Cervical cancer	Global	Female	Log-transformed age-standardized SEV scalar: HIV	X			X		
Cervical cancer	Data Rich	Female	Cumulative Cigarettes (5 Years)	X			X		
Cervical cancer	Global	Female	Cumulative Cigarettes (5 Years)	X			X		
Cervical cancer	Data Rich	Female	HIV age-standardized prevalence	X			X		
Cervical cancer	Global	Female	HIV age-standardized prevalence	X			X		
Uterine cancer	Data Rich	Female	Education (years per capita)			X			X
Uterine cancer	Global	Female	Education (years per capita)			X			X
Uterine cancer	Data Rich	Female	LDI (I$ per capita)			X			X
Uterine cancer	Global	Female	LDI (I$ per capita)			X			X
Uterine cancer	Data Rich	Female	Socio-demographic Index			X			X
Uterine cancer	Global	Female	Socio-demographic Index			X			X
Uterine cancer	Data Rich	Female	Tobacco (cigarettes per capita)		X			X	
Uterine cancer	Global	Female	Tobacco (cigarettes per capita)		X			X	
Uterine cancer	Data Rich	Female	Cumulative Cigarettes (10 Years)		X			X	
Uterine cancer	Global	Female	Cumulative Cigarettes (10 Years)		X			X	
Uterine cancer	Data Rich	Female	Smoking Prevalence		X			X	
Uterine cancer	Global	Female	Smoking Prevalence		X			X	
Uterine cancer	Data Rich	Female	Healthcare access and quality index		X			X	
Uterine cancer	Global	Female	Healthcare access and quality index		X			X	
Uterine cancer	Data Rich	Female	Age- and sex-specific SEV for Low fruit		X			X	
Uterine cancer	Global	Female	Age- and sex-specific SEV for Low fruit		X			X	
Uterine cancer	Data Rich	Female	Age- and sex-specific SEV for Low vegetables		X			X	
Uterine cancer	Global	Female	Age- and sex-specific SEV for Low vegetables		X			X	
Uterine cancer	Data Rich	Female	Cumulative Cigarettes (5 Years)		X			X	
Uterine cancer	Global	Female	Cumulative Cigarettes (5 Years)		X			X	
Uterine cancer	Data Rich	Female	Total Fertility Rate		X			X	
Uterine cancer	Global	Female	Total Fertility Rate		X			X	
Uterine cancer	Data Rich	Female	Diabetes Age-Standardized Prevalence (proportion)		X			X	
Uterine cancer	Global	Female	Diabetes Age-Standardized Prevalence (proportion)		X			X	
Uterine cancer	Data Rich	Female	Mean BMI	X			X		
Uterine cancer	Global	Female	Mean BMI	X			X		
Ovarian cancer	Data Rich	Female	Education (years per capita)			X			X
Ovarian cancer	Global	Female	Education (years per capita)			X			X
Ovarian cancer	Data Rich	Female	LDI (I$ per capita)			X			X
Ovarian cancer	Global	Female	LDI (I$ per capita)			X			X
Ovarian cancer	Data Rich	Female	Socio-demographic Index			X			X
Ovarian cancer	Global	Female	Socio-demographic Index			X			X
Ovarian cancer	Data Rich	Female	Age- and sex-specific SEV for Low fruit			X			X
Ovarian cancer	Global	Female	Age- and sex-specific SEV for Low fruit			X			X
Ovarian cancer	Data Rich	Female	Age- and sex-specific SEV for Low vegetables			X			X
Ovarian cancer	Global	Female	Age- and sex-specific SEV for Low vegetables			X			X
Ovarian cancer	Data Rich	Female	Cumulative Cigarettes (10 Years)		X			X	
Ovarian cancer	Global	Female	Cumulative Cigarettes (10 Years)		X			X	
Ovarian cancer	Data Rich	Female	Smoking Prevalence		X			X	
Ovarian cancer	Global	Female	Smoking Prevalence		X			X	

Appendix Table S11: Comparison of GBD 2021 and GBD 2023 covariates and level of covariates used in cause of death modeling									
Cause	Model Version Type	Sex	Covariate	Level 1 GBD 2021	Level 2 GBD 2021	Level 3 GBD 2021	Level 1 GBD 2023	Level 2 GBD 2023	Level 3 GBD 2023
Ovarian cancer	Data Rich	Female	Healthcare access and quality index		X			X	
Ovarian cancer	Global	Female	Healthcare access and quality index		X			X	
Ovarian cancer	Data Rich	Female	Mean BMI		X			X	
Ovarian cancer	Global	Female	Mean BMI		X			X	
Ovarian cancer	Data Rich	Female	energy unadjusted(kcal)		X			X	
Ovarian cancer	Global	Female	energy unadjusted(kcal)		X			X	
Ovarian cancer	Data Rich	Female	Cumulative Cigarettes (20 Years)		X			X	
Ovarian cancer	Global	Female	Cumulative Cigarettes (20 Years)		X			X	
Ovarian cancer	Data Rich	Female	Asbestos consumption (metric tons per year per capita)		X			X	
Ovarian cancer	Global	Female	Asbestos consumption (metric tons per year per capita)		X			X	
Ovarian cancer	Data Rich	Female	Total Fertility Rate		X			X	
Ovarian cancer	Global	Female	Total Fertility Rate		X			X	
Ovarian cancer	Data Rich	Female	Diabetes Age-Standardized Prevalence (proportion)		X			X	
Ovarian cancer	Global	Female	Diabetes Age-Standardized Prevalence (proportion)		X			X	
Ovarian cancer	Data Rich	Female	Contraception (Modern) Prevalence (proportion)		X			X	
Ovarian cancer	Global	Female	Contraception (Modern) Prevalence (proportion)		X			X	
Ovarian cancer	Data Rich	Female	Liters of alcohol consumed per capita	X			X		
Ovarian cancer	Global	Female	Liters of alcohol consumed per capita	X			X		
Prostate cancer	Data Rich	Male	Education (years per capita)			X			X
Prostate cancer	Global	Male	Education (years per capita)			X			X
Prostate cancer	Data Rich	Male	LDI (I$ per capita)			X			X
Prostate cancer	Global	Male	LDI (I$ per capita)			X			X
Prostate cancer	Data Rich	Male	Socio-demographic Index			X			X
Prostate cancer	Global	Male	Socio-demographic Index			X			X
Prostate cancer	Data Rich	Male	Smoking Prevalence		X			X	
Prostate cancer	Global	Male	Smoking Prevalence		X			X	
Prostate cancer	Data Rich	Male	Healthcare access and quality index		X			X	
Prostate cancer	Global	Male	Healthcare access and quality index		X			X	
Prostate cancer	Data Rich	Male	Log-transformed SEV scalar: Prostate C	X			X		
Prostate cancer	Global	Male	Log-transformed SEV scalar: Prostate C	X			X		
Testicular cancer	Global	Male	Education (years per capita)			X			X
Testicular cancer	Data Rich	Male	Education (years per capita)			X			X
Testicular cancer	Global	Male	LDI (I$ per capita)			X			X
Testicular cancer	Data Rich	Male	LDI (I$ per capita)			X			X
Testicular cancer	Global	Male	Socio-demographic Index			X			X
Testicular cancer	Data Rich	Male	Socio-demographic Index			X			X
Testicular cancer	Global	Male	Tobacco (cigarettes per capita)		X			X	
Testicular cancer	Data Rich	Male	Tobacco (cigarettes per capita)		X			X	
Testicular cancer	Global	Male	Cumulative Cigarettes (10 Years)		X			X	
Testicular cancer	Data Rich	Male	Cumulative Cigarettes (10 Years)		X			X	
Testicular cancer	Global	Male	Smoking Prevalence		X			X	
Testicular cancer	Data Rich	Male	Smoking Prevalence		X			X	
Testicular cancer	Global	Male	Healthcare access and quality index		X			X	
Testicular cancer	Data Rich	Male	Healthcare access and quality index		X			X	
Testicular cancer	Global	Male	Age- and sex-specific SEV for Low fruit		X			X	
Testicular cancer	Data Rich	Male	Age- and sex-specific SEV for Low fruit		X			X	
Testicular cancer	Global	Male	Age- and sex-specific SEV for Low vegetables		X			X	
Testicular cancer	Data Rich	Male	Age- and sex-specific SEV for Low vegetables		X			X	
Testicular cancer	Global	Male	Cumulative Cigarettes (5 Years)		X			X	
Testicular cancer	Data Rich	Male	Cumulative Cigarettes (5 Years)		X			X	
Testicular cancer	Global	Male	Cumulative Cigarettes (20 Years)		X			X	
Testicular cancer	Data Rich	Male	Cumulative Cigarettes (20 Years)		X			X	
Testicular cancer	Global	Male	Cumulative Cigarettes (15 Years)		X			X	
Testicular cancer	Data Rich	Male	Cumulative Cigarettes (15 Years)		X			X	
Kidney cancer	Data Rich	Female	Education (years per capita)			X			X
Kidney cancer	Global	Female	Education (years per capita)			X			X
Kidney cancer	Data Rich	Female	LDI (I$ per capita)			X			X
Kidney cancer	Global	Female	LDI (I$ per capita)			X			X
Kidney cancer	Data Rich	Female	Socio-demographic Index			X			X
Kidney cancer	Global	Female	Socio-demographic Index			X			X
Kidney cancer	Data Rich	Female	Healthcare access and quality index		X			X	
Kidney cancer	Global	Female	Healthcare access and quality index		X			X	
Kidney cancer	Data Rich	Female	Liters of alcohol consumed per capita		X			X	
Kidney cancer	Global	Female	Liters of alcohol consumed per capita		X			X	
Kidney cancer	Data Rich	Female	Systolic Blood Pressure (mmHg)		X			X	
Kidney cancer	Global	Female	Systolic Blood Pressure (mmHg)		X			X	
Kidney cancer	Data Rich	Female	Diabetes Age-Standardized Prevalence (proportion)		X			X	
Kidney cancer	Global	Female	Diabetes Age-Standardized Prevalence (proportion)		X			X	
Kidney cancer	Data Rich	Female	Tobacco (cigarettes per capita)	X			X		
Kidney cancer	Global	Female	Tobacco (cigarettes per capita)	X			X		
Kidney cancer	Data Rich	Female	Cumulative Cigarettes (10 Years)	X			X		
Kidney cancer	Global	Female	Cumulative Cigarettes (10 Years)	X			X		
Kidney cancer	Data Rich	Female	Mean BMI	X			X		
Kidney cancer	Global	Female	Mean BMI	X			X		
Kidney cancer	Data Rich	Male	Education (years per capita)			X			X
Kidney cancer	Global	Male	Education (years per capita)			X			X
Kidney cancer	Data Rich	Male	LDI (I$ per capita)			X			X
Kidney cancer	Global	Male	LDI (I$ per capita)			X			X
Kidney cancer	Data Rich	Male	Socio-demographic Index			X			X
Kidney cancer	Global	Male	Socio-demographic Index			X			X
Kidney cancer	Data Rich	Male	Healthcare access and quality index		X			X	
Kidney cancer	Global	Male	Healthcare access and quality index		X			X	
Kidney cancer	Data Rich	Male	Liters of alcohol consumed per capita		X			X	
Kidney cancer	Global	Male	Liters of alcohol consumed per capita		X			X	
Kidney cancer	Data Rich	Male	Systolic Blood Pressure (mmHg)		X			X	
Kidney cancer	Global	Male	Systolic Blood Pressure (mmHg)		X			X	
Kidney cancer	Data Rich	Male	Diabetes Age-Standardized Prevalence (proportion)		X			X	
Kidney cancer	Global	Male	Diabetes Age-Standardized Prevalence (proportion)		X			X	
Kidney cancer	Data Rich	Male	Tobacco (cigarettes per capita)	X			X		
Kidney cancer	Global	Male	Tobacco (cigarettes per capita)	X			X		
Kidney cancer	Data Rich	Male	Cumulative Cigarettes (10 Years)	X			X		
Kidney cancer	Global	Male	Cumulative Cigarettes (10 Years)	X			X		
Kidney cancer	Data Rich	Male	Mean BMI	X			X		
Kidney cancer	Global	Male	Mean BMI	X			X		
Bladder cancer	Global	Female	LDI (I$ per capita)			X			X
Bladder cancer	Data Rich	Female	LDI (I$ per capita)			X			X
Bladder cancer	Global	Female	Socio-demographic Index			X			X
Bladder cancer	Global	Female	Age- and sex-specific SEV for Low fruit			X			X
Bladder cancer	Data Rich	Female	Age- and sex-specific SEV for Low fruit			X			X
Bladder cancer	Global	Female	Cumulative Cigarettes (10 Years)		X			X	
Bladder cancer	Global	Female	Healthcare access and quality index		X			X	
Bladder cancer	Global	Female	Liters of alcohol consumed per capita		X			X	
Bladder cancer	Data Rich	Female	Liters of alcohol consumed per capita		X			X	
Bladder cancer	Global	Female	Diabetes Fasting Plasma Glucose (mmol/L), age-standardized 25+		X			X	
Bladder cancer	Data Rich	Female	Diabetes Fasting Plasma Glucose (mmol/L), age-standardized 25+		X			X	
Bladder cancer	Global	Female	Age- and sex-specific SEV for Low vegetables		X			X	
Bladder cancer	Data Rich	Female	Age- and sex-specific SEV for Low vegetables		X			X	
Bladder cancer	Data Rich	Female	Socio-demographic Index			X	X		
Bladder cancer	Data Rich	Female	Cumulative Cigarettes (10 Years)		X		X		
Bladder cancer	Data Rich	Female	Healthcare access and quality index		X		X		
Bladder cancer	Global	Female	Smoking Prevalence	X			X		
Bladder cancer	Data Rich	Female	Smoking Prevalence	X			X		
Bladder cancer	Global	Female	Schistosomiasis Prevalence Results	X			X		
Bladder cancer	Data Rich	Female	Schistosomiasis Prevalence Results	X			X		
Bladder cancer	Global	Male	LDI (I$ per capita)			X			X
Bladder cancer	Data Rich	Male	LDI (I$ per capita)			X			X
Bladder cancer	Global	Male	Socio-demographic Index			X			X
Bladder cancer	Data Rich	Male	Socio-demographic Index			X			X
Bladder cancer	Global	Male	Age- and sex-specific SEV for Low fruit			X			X
Bladder cancer	Data Rich	Male	Age- and sex-specific SEV for Low fruit			X			X

Appendix Table S11: Comparison of GBD 2021 and GBD 2023 covariates and level of covariates used in cause of death modeling									
Cause	Model Version Type	Sex	Covariate	Level 1 GBD 2021	Level 2 GBD 2021	Level 3 GBD 2021	Level 1 GBD 2023	Level 2 GBD 2023	Level 3 GBD 2023
Bladder cancer	Global	Male	Cumulative Cigarettes (10 Years)		X			X	
Bladder cancer	Data Rich	Male	Cumulative Cigarettes (10 Years)		X			X	
Bladder cancer	Global	Male	Healthcare access and quality index		X			X	
Bladder cancer	Data Rich	Male	Healthcare access and quality index		X			X	
Bladder cancer	Global	Male	Liters of alcohol consumed per capita		X			X	
Bladder cancer	Data Rich	Male	Liters of alcohol consumed per capita		X			X	
Bladder cancer	Global	Male	Diabetes Fasting Plasma Glucose (mmol/L), age-standardized 25+		X			X	
Bladder cancer	Data Rich	Male	Diabetes Fasting Plasma Glucose (mmol/L), age-standardized 25+		X			X	
Bladder cancer	Global	Male	Age- and sex-specific SEV for Low vegetables		X			X	
Bladder cancer	Data Rich	Male	Age- and sex-specific SEV for Low vegetables		X			X	
Bladder cancer	Global	Male	Smoking Prevalence	X			X		
Bladder cancer	Data Rich	Male	Smoking Prevalence	X			X		
Bladder cancer	Global	Male	Schistosomiasis Prevalence Results	X			X		
Bladder cancer	Data Rich	Male	Schistosomiasis Prevalence Results	X			X		
Brain and nervous system cancer	Data Rich	Female	Education (years per capita)			X			X
Brain and nervous system cancer	Global	Female	Education (years per capita)			X			X
Brain and nervous system cancer	Data Rich	Female	LDI (I$ per capita)			X			X
Brain and nervous system cancer	Global	Female	LDI (I$ per capita)			X			X
Brain and nervous system cancer	Data Rich	Female	Socio-demographic Index			X			X
Brain and nervous system cancer	Global	Female	Socio-demographic Index			X			X
Brain and nervous system cancer	Data Rich	Female	Low-Density Lipoprotein (mmol/L)		X			X	
Brain and nervous system cancer	Global	Female	Low-Density Lipoprotein (mmol/L)		X			X	
Brain and nervous system cancer	Data Rich	Female	Healthcare access and quality index		X			X	
Brain and nervous system cancer	Global	Female	Healthcare access and quality index		X			X	
Brain and nervous system cancer	Data Rich	Female	Systolic Blood Pressure (mmHg)		X			X	
Brain and nervous system cancer	Global	Female	Systolic Blood Pressure (mmHg)		X			X	
Brain and nervous system cancer	Data Rich	Female	Age- and sex-specific SEV for Low fruit		X			X	
Brain and nervous system cancer	Global	Female	Age- and sex-specific SEV for Low fruit		X			X	
Brain and nervous system cancer	Data Rich	Female	Age- and sex-specific SEV for Low vegetables		X			X	
Brain and nervous system cancer	Global	Female	Age- and sex-specific SEV for Low vegetables		X			X	
Brain and nervous system cancer	Data Rich	Female	Age- and sex-specific SEV for High red meat		X			X	
Brain and nervous system cancer	Global	Female	Age- and sex-specific SEV for High red meat		X			X	
Brain and nervous system cancer	Data Rich	Female	Cumulative Cigarettes (10 Years)	X			X		
Brain and nervous system cancer	Global	Female	Cumulative Cigarettes (10 Years)	X			X		
Brain and nervous system cancer	Data Rich	Female	Smoking Prevalence	X			X		
Brain and nervous system cancer	Global	Female	Smoking Prevalence	X			X		
Brain and nervous system cancer	Data Rich	Female	Liters of alcohol consumed per capita	X			X		
Brain and nervous system cancer	Global	Female	Liters of alcohol consumed per capita	X			X		
Brain and nervous system cancer	Data Rich	Male	Education (years per capita)			X			X
Brain and nervous system cancer	Global	Male	Education (years per capita)			X			X
Brain and nervous system cancer	Data Rich	Male	LDI (I$ per capita)			X			X
Brain and nervous system cancer	Global	Male	LDI (I$ per capita)			X			X
Brain and nervous system cancer	Data Rich	Male	Socio-demographic Index			X			X
Brain and nervous system cancer	Global	Male	Socio-demographic Index			X			X
Brain and nervous system cancer	Data Rich	Male	Low-Density Lipoprotein (mmol/L)		X			X	
Brain and nervous system cancer	Global	Male	Low-Density Lipoprotein (mmol/L)		X			X	
Brain and nervous system cancer	Data Rich	Male	Healthcare access and quality index		X			X	
Brain and nervous system cancer	Global	Male	Healthcare access and quality index		X			X	
Brain and nervous system cancer	Data Rich	Male	Systolic Blood Pressure (mmHg)		X			X	
Brain and nervous system cancer	Global	Male	Systolic Blood Pressure (mmHg)		X			X	
Brain and nervous system cancer	Data Rich	Male	Age- and sex-specific SEV for Low fruit		X			X	
Brain and nervous system cancer	Global	Male	Age- and sex-specific SEV for Low fruit		X			X	
Brain and nervous system cancer	Data Rich	Male	Age- and sex-specific SEV for Low vegetables		X			X	
Brain and nervous system cancer	Global	Male	Age- and sex-specific SEV for Low vegetables		X			X	
Brain and nervous system cancer	Data Rich	Male	Age- and sex-specific SEV for High red meat		X			X	
Brain and nervous system cancer	Global	Male	Age- and sex-specific SEV for High red meat		X			X	
Brain and nervous system cancer	Data Rich	Male	Cumulative Cigarettes (10 Years)	X			X		
Brain and nervous system cancer	Global	Male	Cumulative Cigarettes (10 Years)	X			X		
Brain and nervous system cancer	Data Rich	Male	Smoking Prevalence	X			X		
Brain and nervous system cancer	Global	Male	Smoking Prevalence	X			X		
Brain and nervous system cancer	Data Rich	Male	Liters of alcohol consumed per capita	X			X		
Brain and nervous system cancer	Global	Male	Liters of alcohol consumed per capita	X			X		
Eye cancer	Global	Female	Education (years per capita)			X			X
Eye cancer	Data Rich	Female	Education (years per capita)			X			X
Eye cancer	Global	Female	LDI (I$ per capita)			X			X
Eye cancer	Data Rich	Female	LDI (I$ per capita)			X			X
Eye cancer	Global	Female	Socio-demographic Index			X			X
Eye cancer	Data Rich	Female	Socio-demographic Index			X			X
Eye cancer	Global	Female	Universal health coverage		X			X	
Eye cancer	Data Rich	Female	Universal health coverage		X			X	
Eye cancer	Global	Female	Healthcare access and quality index		X			X	
Eye cancer	Data Rich	Female	Healthcare access and quality index		X			X	
Eye cancer	Global	Female	Age-standardized melanoma		X			X	
Eye cancer	Data Rich	Female	Age-standardized melanoma		X			X	
Eye cancer	Global	Male	Education (years per capita)		X			X	
Eye cancer	Data Rich	Male	Education (years per capita)		X			X	
Eye cancer	Global	Male	LDI (I$ per capita)		X			X	
Eye cancer	Data Rich	Male	LDI (I$ per capita)		X			X	
Eye cancer	Global	Male	Socio-demographic Index		X			X	
Eye cancer	Data Rich	Male	Socio-demographic Index		X			X	
Eye cancer	Global	Male	Universal health coverage		X			X	
Eye cancer	Data Rich	Male	Universal health coverage		X			X	
Eye cancer	Global	Male	Healthcare access and quality index		X			X	
Eye cancer	Data Rich	Male	Healthcare access and quality index		X			X	
Eye cancer	Global	Male	Age-standardized melanoma		X			X	
Eye cancer	Data Rich	Male	Age-standardized melanoma		X			X	
Retinoblastoma	Global	Female	Education (years per capita)			X			X
Retinoblastoma	Data Rich	Female	Education (years per capita)			X			X
Retinoblastoma	Global	Female	LDI (I$ per capita)			X			X
Retinoblastoma	Data Rich	Female	LDI (I$ per capita)			X			X
Retinoblastoma	Global	Female	Socio-demographic Index			X			X
Retinoblastoma	Data Rich	Female	Socio-demographic Index			X			X
Retinoblastoma	Global	Female	Maternal care and immunization			X			X
Retinoblastoma	Data Rich	Female	Maternal care and immunization			X			X
Retinoblastoma	Global	Female	Universal health coverage		X			X	
Retinoblastoma	Data Rich	Female	Universal health coverage		X			X	
Retinoblastoma	Global	Female	Healthcare access and quality index		X			X	
Retinoblastoma	Data Rich	Female	Healthcare access and quality index		X			X	
Retinoblastoma	Global	Male	Education (years per capita)			X			X
Retinoblastoma	Data Rich	Male	Education (years per capita)			X			X
Retinoblastoma	Global	Male	LDI (I$ per capita)			X			X
Retinoblastoma	Data Rich	Male	LDI (I$ per capita)			X			X
Retinoblastoma	Global	Male	Socio-demographic Index			X			X
Retinoblastoma	Data Rich	Male	Socio-demographic Index			X			X
Retinoblastoma	Global	Male	Maternal care and immunization			X			X
Retinoblastoma	Data Rich	Male	Maternal care and immunization			X			X
Retinoblastoma	Global	Male	Universal health coverage		X			X	
Retinoblastoma	Data Rich	Male	Universal health coverage		X			X	
Retinoblastoma	Global	Male	Healthcare access and quality index		X			X	
Retinoblastoma	Data Rich	Male	Healthcare access and quality index		X			X	
Other eye cancers	Global	Female	Education (years per capita)			X			X
Other eye cancers	Data Rich	Female	Education (years per capita)			X			X
Other eye cancers	Global	Female	LDI (I$ per capita)			X			X
Other eye cancers	Data Rich	Female	LDI (I$ per capita)			X			X
Other eye cancers	Global	Female	Socio-demographic Index			X			X
Other eye cancers	Data Rich	Female	Socio-demographic Index			X			X
Other eye cancers	Global	Female	Universal health coverage		X			X	
Other eye cancers	Data Rich	Female	Universal health coverage		X			X	
Other eye cancers	Global	Female	Healthcare access and quality index		X			X	
Other eye cancers	Data Rich	Female	Healthcare access and quality index		X			X	
Other eye cancers	Global	Female	Age-standardized melanoma		X			X	
Other eye cancers	Data Rich	Female	Age-standardized melanoma		X			X	
Other eye cancers	Global	Male	Education (years per capita)			X			X
Other eye cancers	Data Rich	Male	Education (years per capita)			X			X
Other eye cancers	Global	Male	LDI (I$ per capita)			X			X

Appendix Table S11: Comparison of GBD 2021 and GBD 2023 covariates and level of covariates used in cause of death modeling									
**Cause**	**Model Version Type**	**Sex**	**Covariate**	**Level 1 GBD 2021**	**Level 2 GBD 2021**	**Level 3 GBD 2021**	**Level 1 GBD 2023**	**Level 2 GBD 2023**	**Level 3 GBD 2023**
Other eye cancers	Data Rich	Male	LDI (I$ per capita)			X			X
Other eye cancers	Global	Male	Socio-demographic Index			X			X
Other eye cancers	Data Rich	Male	Socio-demographic Index			X			X
Other eye cancers	Global	Male	Universal health coverage		X			X	
Other eye cancers	Data Rich	Male	Universal health coverage		X			X	
Other eye cancers	Global	Male	Healthcare access and quality index		X			X	
Other eye cancers	Data Rich	Male	Healthcare access and quality index		X			X	
Other eye cancers	Global	Male	Age-standardized melanoma		X			X	
Other eye cancers	Data Rich	Male	Age-standardized melanoma		X			X	
Neuroblastoma and other peripheral nervous cell tumours	Data Rich	Female	Maternal care and immunization			X			X
Neuroblastoma and other peripheral nervous cell tumours	Global	Female	Maternal care and immunization			X			X
Neuroblastoma and other peripheral nervous cell tumours	Data Rich	Female	Education (years per capita)			X			X
Neuroblastoma and other peripheral nervous cell tumours	Global	Female	Education (years per capita)			X			X
Neuroblastoma and other peripheral nervous cell tumours	Data Rich	Female	LDI (I$ per capita)			X			X
Neuroblastoma and other peripheral nervous cell tumours	Global	Female	LDI (I$ per capita)			X			X
Neuroblastoma and other peripheral nervous cell tumours	Data Rich	Female	Socio-demographic Index			X			X
Neuroblastoma and other peripheral nervous cell tumours	Global	Female	Socio-demographic Index			X			X
Neuroblastoma and other peripheral nervous cell tumours	Data Rich	Female	Universal health coverage			X			X
Neuroblastoma and other peripheral nervous cell tumours	Global	Female	Universal health coverage			X			X
Neuroblastoma and other peripheral nervous cell tumours	Data Rich	Female	Healthcare access and quality index			X			X
Neuroblastoma and other peripheral nervous cell tumours	Global	Female	Healthcare access and quality index			X			X
Neuroblastoma and other peripheral nervous cell tumours	Data Rich	Female	Health worker density			X			X
Neuroblastoma and other peripheral nervous cell tumours	Global	Female	Health worker density			X			X
Neuroblastoma and other peripheral nervous cell tumours	Data Rich	Female	Smoking Prevalence			X			X
Neuroblastoma and other peripheral nervous cell tumours	Global	Female	Smoking Prevalence			X			X
Neuroblastoma and other peripheral nervous cell tumours	Data Rich	Male	Maternal care and immunization			X			X
Neuroblastoma and other peripheral nervous cell tumours	Global	Male	Maternal care and immunization			X			X
Neuroblastoma and other peripheral nervous cell tumours	Data Rich	Male	Education (years per capita)			X			X
Neuroblastoma and other peripheral nervous cell tumours	Global	Male	Education (years per capita)			X			X
Neuroblastoma and other peripheral nervous cell tumours	Data Rich	Male	LDI (I$ per capita)			X			X
Neuroblastoma and other peripheral nervous cell tumours	Global	Male	LDI (I$ per capita)			X			X
Neuroblastoma and other peripheral nervous cell tumours	Data Rich	Male	Socio-demographic Index			X			X
Neuroblastoma and other peripheral nervous cell tumours	Global	Male	Socio-demographic Index			X			X
Neuroblastoma and other peripheral nervous cell tumours	Data Rich	Male	Universal health coverage			X			X
Neuroblastoma and other peripheral nervous cell tumours	Global	Male	Universal health coverage			X			X
Neuroblastoma and other peripheral nervous cell tumours	Data Rich	Male	Healthcare access and quality index			X			X
Neuroblastoma and other peripheral nervous cell tumours	Global	Male	Healthcare access and quality index			X			X
Neuroblastoma and other peripheral nervous cell tumours	Data Rich	Male	Health worker density			X			X
Neuroblastoma and other peripheral nervous cell tumours	Global	Male	Health worker density			X			X
Neuroblastoma and other peripheral nervous cell tumours	Data Rich	Male	Smoking Prevalence			X			X
Neuroblastoma and other peripheral nervous cell tumours	Global	Male	Smoking Prevalence			X			X
Thyroid cancer	Global	Female	Education (years per capita)			X			X
Thyroid cancer	Data Rich	Female	Education (years per capita)			X			X
Thyroid cancer	Global	Female	LDI (I$ per capita)			X			X
Thyroid cancer	Data Rich	Female	LDI (I$ per capita)			X			X
Thyroid cancer	Global	Female	Socio-demographic Index			X			X
Thyroid cancer	Data Rich	Female	Socio-demographic Index			X			X
Thyroid cancer	Global	Female	Sanitation (proportion with access)			X			X
Thyroid cancer	Data Rich	Female	Sanitation (proportion with access)			X			X
Thyroid cancer	Global	Female	Improved Water Source (proportion with access)			X			X
Thyroid cancer	Data Rich	Female	Improved Water Source (proportion with access)			X			X
Thyroid cancer	Global	Female	Age- and sex-specific SEV for Low fruit			X			X
Thyroid cancer	Data Rich	Female	Age- and sex-specific SEV for Low fruit			X			X
Thyroid cancer	Global	Female	Tobacco (cigarettes per capita)		X			X	
Thyroid cancer	Data Rich	Female	Tobacco (cigarettes per capita)		X			X	
Thyroid cancer	Global	Female	Healthcare access and quality index		X			X	
Thyroid cancer	Data Rich	Female	Healthcare access and quality index		X			X	
Thyroid cancer	Global	Female	Mean BMI		X			X	
Thyroid cancer	Data Rich	Female	Mean BMI		X			X	
Thyroid cancer	Global	Female	Age- and sex-specific SEV for Low vegetables		X			X	
Thyroid cancer	Data Rich	Female	Age- and sex-specific SEV for Low vegetables		X			X	
Thyroid cancer	Global	Female	Age- and sex-specific SEV for High red meat		X			X	
Thyroid cancer	Data Rich	Female	Age- and sex-specific SEV for High red meat		X			X	
Thyroid cancer	Global	Female	Liters of alcohol consumed per capita	X			X		
Thyroid cancer	Data Rich	Female	Liters of alcohol consumed per capita	X			X		
Thyroid cancer	Global	Female	Cumulative Cigarettes (10 Years)						X
Thyroid cancer	Global	Female	Smoking Prevalence						X
Thyroid cancer	Global	Female	Diabetes Fasting Plasma Glucose (mmol/L), age-standardized 25+						X
Thyroid cancer	Global	Female	Outdoor Air Pollution (PM2.5)						X
Thyroid cancer	Global	Female	Cumulative Cigarettes (5 Years)						X
Thyroid cancer	Global	Female	Total Physical Activity (MET-min/week), Age-specific						X
Thyroid cancer	Global	Female	Cumulative Cigarettes (20 Years)						X
Thyroid cancer	Global	Female	Cumulative Cigarettes (15 Years)						X
Thyroid cancer	Global	Male	Education (years per capita)			X			X
Thyroid cancer	Data Rich	Male	Education (years per capita)			X			X
Thyroid cancer	Global	Male	LDI (I$ per capita)			X			X
Thyroid cancer	Data Rich	Male	LDI (I$ per capita)			X			X
Thyroid cancer	Global	Male	Socio-demographic Index			X			X
Thyroid cancer	Data Rich	Male	Socio-demographic Index			X			X
Thyroid cancer	Global	Male	Sanitation (proportion with access)			X			X
Thyroid cancer	Data Rich	Male	Sanitation (proportion with access)			X			X
Thyroid cancer	Global	Male	Improved Water Source (proportion with access)			X			X
Thyroid cancer	Data Rich	Male	Improved Water Source (proportion with access)			X			X
Thyroid cancer	Global	Male	Age- and sex-specific SEV for Low fruit			X			X
Thyroid cancer	Data Rich	Male	Age- and sex-specific SEV for Low fruit			X			X
Thyroid cancer	Global	Male	Tobacco (cigarettes per capita)		X			X	
Thyroid cancer	Data Rich	Male	Tobacco (cigarettes per capita)		X			X	
Thyroid cancer	Global	Male	Healthcare access and quality index		X			X	
Thyroid cancer	Data Rich	Male	Healthcare access and quality index		X			X	
Thyroid cancer	Global	Male	Mean BMI		X			X	
Thyroid cancer	Data Rich	Male	Mean BMI		X			X	
Thyroid cancer	Global	Male	Age- and sex-specific SEV for Low vegetables		X			X	
Thyroid cancer	Data Rich	Male	Age- and sex-specific SEV for Low vegetables		X			X	
Thyroid cancer	Global	Male	Age- and sex-specific SEV for High red meat		X			X	
Thyroid cancer	Data Rich	Male	Age- and sex-specific SEV for High red meat		X			X	
Thyroid cancer	Global	Male	Liters of alcohol consumed per capita	X			X		
Thyroid cancer	Data Rich	Male	Liters of alcohol consumed per capita	X			X		
Mesothelioma	Data Rich	Female	Education (years per capita)			X			X
Mesothelioma	Global	Female	Education (years per capita)			X			X
Mesothelioma	Data Rich	Female	LDI (I$ per capita)			X			X
Mesothelioma	Global	Female	LDI (I$ per capita)			X			X
Mesothelioma	Data Rich	Female	Socio-demographic Index			X			X
Mesothelioma	Global	Female	Socio-demographic Index			X			X
Mesothelioma	Data Rich	Female	Healthcare access and quality index		X			X	
Mesothelioma	Global	Female	Healthcare access and quality index		X			X	
Mesothelioma	Data Rich	Female	Indoor Air Pollution (All Cooking Fuels)		X			X	
Mesothelioma	Global	Female	Indoor Air Pollution (All Cooking Fuels)		X			X	
Mesothelioma	Data Rich	Female	Cumulative Cigarettes (5 Years)		X			X	

Appendix Table S11: Comparison of GBD 2021 and GBD 2023 covariates and level of covariates used in cause of death modeling

Cause	Model Version Type	Sex	Covariate	Level 1 GBD 2021	Level 2 GBD 2021	Level 3 GBD 2021	Level 1 GBD 2023	Level 2 GBD 2023	Level 3 GBD 2023
Mesothelioma	Global	Female	Cumulative Cigarettes (5 Years)		X			X	
Mesothelioma	Data Rich	Female	Population Density (over 1000 ppl/sqkm, proportion)		X			X	
Mesothelioma	Global	Female	Population Density (over 1000 ppl/sqkm, proportion)		X			X	
Mesothelioma	Data Rich	Female	Gold production (binary)		X			X	
Mesothelioma	Global	Female	Gold production (binary)		X			X	
Mesothelioma	Data Rich	Female	Smoking Prevalence	X			X		
Mesothelioma	Global	Female	Smoking Prevalence	X			X		
Mesothelioma	Data Rich	Female	Asbestos consumption (metric tons per year per capita)	X			X		
Mesothelioma	Global	Female	Asbestos consumption (metric tons per year per capita)	X			X		
Mesothelioma	Data Rich	Female	Age- and sex-specific SEV for Occupational asbestos	X			X		
Mesothelioma	Global	Female	Age- and sex-specific SEV for Occupational asbestos	X			X		
Mesothelioma	Data Rich	Female	Age-standardized SEV for Occupational asbestos	X			X		
Mesothelioma	Global	Female	Age-standardized SEV for Occupational asbestos	X			X		
Mesothelioma	Global	Male	Education (years per capita)			X			X
Mesothelioma	Data Rich	Male	Education (years per capita)			X			X
Mesothelioma	Global	Male	LDI (I$ per capita)			X			X
Mesothelioma	Data Rich	Male	LDI (I$ per capita)			X			X
Mesothelioma	Global	Male	Socio-demographic Index			X			X
Mesothelioma	Data Rich	Male	Socio-demographic Index			X			X
Mesothelioma	Global	Male	Healthcare access and quality index		X			X	
Mesothelioma	Data Rich	Male	Healthcare access and quality index		X			X	
Mesothelioma	Global	Male	Indoor Air Pollution (All Cooking Fuels)		X			X	
Mesothelioma	Data Rich	Male	Indoor Air Pollution (All Cooking Fuels)		X			X	
Mesothelioma	Global	Male	Cumulative Cigarettes (5 Years)		X			X	
Mesothelioma	Data Rich	Male	Cumulative Cigarettes (5 Years)		X			X	
Mesothelioma	Global	Male	Population Density (over 1000 ppl/sqkm, proportion)		X			X	
Mesothelioma	Data Rich	Male	Population Density (over 1000 ppl/sqkm, proportion)		X			X	
Mesothelioma	Global	Male	Gold production (binary)		X			X	
Mesothelioma	Data Rich	Male	Gold production (binary)		X			X	
Mesothelioma	Global	Male	Smoking Prevalence	X			X		
Mesothelioma	Data Rich	Male	Smoking Prevalence	X			X		
Mesothelioma	Global	Male	Asbestos consumption (metric tons per year per capita)	X			X		
Mesothelioma	Data Rich	Male	Asbestos consumption (metric tons per year per capita)	X			X		
Mesothelioma	Global	Male	Age- and sex-specific SEV for Occupational asbestos	X			X		
Mesothelioma	Data Rich	Male	Age- and sex-specific SEV for Occupational asbestos	X			X		
Mesothelioma	Global	Male	Age-standardized SEV for Occupational asbestos	X			X		
Mesothelioma	Data Rich	Male	Age-standardized SEV for Occupational asbestos	X			X		
Hodgkin lymphoma	Data Rich	Female	Education (years per capita)			X			X
Hodgkin lymphoma	Global	Female	Education (years per capita)			X			X
Hodgkin lymphoma	Data Rich	Female	LDI (I$ per capita)			X			X
Hodgkin lymphoma	Global	Female	LDI (I$ per capita)			X			X
Hodgkin lymphoma	Data Rich	Female	Socio-demographic Index			X			X
Hodgkin lymphoma	Global	Female	Socio-demographic Index			X			X
Hodgkin lymphoma	Data Rich	Female	Healthcare access and quality index		X			X	
Hodgkin lymphoma	Global	Female	Healthcare access and quality index		X			X	
Hodgkin lymphoma	Data Rich	Male	Education (years per capita)			X			X
Hodgkin lymphoma	Global	Male	Education (years per capita)			X			X
Hodgkin lymphoma	Data Rich	Male	LDI (I$ per capita)			X			X
Hodgkin lymphoma	Global	Male	LDI (I$ per capita)			X			X
Hodgkin lymphoma	Data Rich	Male	Socio-demographic Index			X			X
Hodgkin lymphoma	Global	Male	Socio-demographic Index			X			X
Hodgkin lymphoma	Data Rich	Male	Healthcare access and quality index		X			X	
Hodgkin lymphoma	Global	Male	Healthcare access and quality index		X			X	
Non-Hodgkin's lymphoma	Data Rich	Female	Education (years per capita)			X			X
Non-Hodgkin's lymphoma	Data Rich	Female	LDI (I$ per capita)			X			X
Non-Hodgkin's lymphoma	Data Rich	Female	Socio-demographic Index			X			X
Non-Hodgkin's lymphoma	Data Rich	Female	Total Fertility Rate			X			X
Non-Hodgkin's lymphoma	Data Rich	Female	Log-transformed SEV scalar: HIV			X			X
Non-Hodgkin's lymphoma	Global	Female	Log-transformed SEV scalar: HIV			X			X
Non-Hodgkin's lymphoma	Data Rich	Female	Log-transformed age-standardized SEV scalar: HIV			X			X
Non-Hodgkin's lymphoma	Global	Female	Log-transformed age-standardized SEV scalar: HIV			X			X
Non-Hodgkin's lymphoma	Global	Female	Education (years per capita)			X		X	
Non-Hodgkin's lymphoma	Global	Female	LDI (I$ per capita)			X		X	
Non-Hodgkin's lymphoma	Global	Female	Socio-demographic Index			X		X	
Non-Hodgkin's lymphoma	Global	Female	Total Fertility Rate			X		X	
Non-Hodgkin's lymphoma	Data Rich	Female	Cumulative Cigarettes (10 Years)		X			X	
Non-Hodgkin's lymphoma	Data Rich	Female	Universal health coverage		X			X	
Non-Hodgkin's lymphoma	Global	Female	Universal health coverage		X			X	
Non-Hodgkin's lymphoma	Data Rich	Female	Healthcare access and quality index		X			X	
Non-Hodgkin's lymphoma	Global	Female	Healthcare access and quality index		X			X	
Non-Hodgkin's lymphoma	Data Rich	Female	Mean BMI		X			X	
Non-Hodgkin's lymphoma	Data Rich	Female	Liters of alcohol consumed per capita		X			X	
Non-Hodgkin's lymphoma	Global	Female	Liters of alcohol consumed per capita		X			X	
Non-Hodgkin's lymphoma	Data Rich	Female	Cumulative Cigarettes (5 Years)		X			X	
Non-Hodgkin's lymphoma	Data Rich	Female	Cumulative Cigarettes (20 Years)		X			X	
Non-Hodgkin's lymphoma	Data Rich	Female	Cumulative Cigarettes (15 Years)		X			X	
Non-Hodgkin's lymphoma	Global	Female	Cumulative Cigarettes (10 Years)		X		X		
Non-Hodgkin's lymphoma	Global	Female	Mean BMI		X		X		
Non-Hodgkin's lymphoma	Global	Female	Cumulative Cigarettes (5 Years)		X		X		
Non-Hodgkin's lymphoma	Global	Female	Cumulative Cigarettes (20 Years)		X		X		
Non-Hodgkin's lymphoma	Global	Female	Cumulative Cigarettes (15 Years)		X		X		
Non-Hodgkin's lymphoma	Global	Female	HIV age-standardized prevalence				X		
Non-Hodgkin's lymphoma	Global	Female	Hepatitis C Seroprevalence (anti-HCV) age standardized				X		
Non-Hodgkin's lymphoma	Global	Female	HIV Prevalence Unadjusted (proportion)				X		
Non-Hodgkin's lymphoma	Data Rich	Male	Education (years per capita)			X			X
Non-Hodgkin's lymphoma	Global	Male	Education (years per capita)			X			X
Non-Hodgkin's lymphoma	Data Rich	Male	LDI (I$ per capita)			X			X
Non-Hodgkin's lymphoma	Global	Male	LDI (I$ per capita)			X			X
Non-Hodgkin's lymphoma	Data Rich	Male	Socio-demographic Index			X			X
Non-Hodgkin's lymphoma	Global	Male	Socio-demographic Index			X			X
Non-Hodgkin's lymphoma	Data Rich	Male	Log-transformed SEV scalar: HIV			X			X
Non-Hodgkin's lymphoma	Global	Male	Log-transformed SEV scalar: HIV			X			X
Non-Hodgkin's lymphoma	Data Rich	Male	Log-transformed age-standardized SEV scalar: HIV			X			X
Non-Hodgkin's lymphoma	Global	Male	Log-transformed age-standardized SEV scalar: HIV			X			X
Non-Hodgkin's lymphoma	Data Rich	Male	Cumulative Cigarettes (10 Years)		X			X	
Non-Hodgkin's lymphoma	Global	Male	Cumulative Cigarettes (10 Years)		X			X	
Non-Hodgkin's lymphoma	Data Rich	Male	Universal health coverage		X			X	
Non-Hodgkin's lymphoma	Global	Male	Universal health coverage		X			X	
Non-Hodgkin's lymphoma	Data Rich	Male	Healthcare access and quality index		X			X	
Non-Hodgkin's lymphoma	Global	Male	Healthcare access and quality index		X			X	
Non-Hodgkin's lymphoma	Data Rich	Male	Mean BMI		X			X	
Non-Hodgkin's lymphoma	Global	Male	Mean BMI		X			X	
Non-Hodgkin's lymphoma	Data Rich	Male	Liters of alcohol consumed per capita		X			X	
Non-Hodgkin's lymphoma	Global	Male	Liters of alcohol consumed per capita		X			X	
Non-Hodgkin's lymphoma	Data Rich	Male	Cumulative Cigarettes (5 Years)		X			X	
Non-Hodgkin's lymphoma	Global	Male	Cumulative Cigarettes (5 Years)		X			X	
Non-Hodgkin's lymphoma	Data Rich	Male	Cumulative Cigarettes (20 Years)		X			X	
Non-Hodgkin's lymphoma	Global	Male	Cumulative Cigarettes (20 Years)		X			X	
Non-Hodgkin's lymphoma	Data Rich	Male	Cumulative Cigarettes (15 Years)		X			X	
Non-Hodgkin's lymphoma	Global	Male	Cumulative Cigarettes (15 Years)		X			X	
Other non-Hodgkin lymphoma	Data Rich	Female	Education (years per capita)			X			X
Other non-Hodgkin lymphoma	Global	Female	Education (years per capita)			X			X
Other non-Hodgkin lymphoma	Data Rich	Female	LDI (I$ per capita)			X			X
Other non-Hodgkin lymphoma	Global	Female	LDI (I$ per capita)			X			X
Other non-Hodgkin lymphoma	Data Rich	Female	Socio-demographic Index			X			X
Other non-Hodgkin lymphoma	Global	Female	Socio-demographic Index			X			X
Other non-Hodgkin lymphoma	Data Rich	Female	Total Fertility Rate			X			X
Other non-Hodgkin lymphoma	Global	Female	Total Fertility Rate			X			X
Other non-Hodgkin lymphoma	Data Rich	Female	Log-transformed SEV scalar: HIV			X			X
Other non-Hodgkin lymphoma	Global	Female	Log-transformed SEV scalar: HIV			X			X
Other non-Hodgkin lymphoma	Data Rich	Female	Log-transformed age-standardized SEV scalar: HIV			X			X

Appendix Table S11: Comparison of GBD 2021 and GBD 2023 covariates and level of covariates used in cause of death modeling

Cause	Model Version Type	Sex	Covariate	Level 1 GBD 2021	Level 2 GBD 2021	Level 3 GBD 2021	Level 1 GBD 2023	Level 2 GBD 2023	Level 3 GBD 2023
Other non-Hodgkin lymphoma	Global	Female	Log-transformed age-standardized SEV scalar: HIV			X			X
Other non-Hodgkin lymphoma	Data Rich	Female	Cumulative Cigarettes (10 Years)		X			X	
Other non-Hodgkin lymphoma	Global	Female	Cumulative Cigarettes (10 Years)		X			X	
Other non-Hodgkin lymphoma	Data Rich	Female	Universal health coverage		X			X	
Other non-Hodgkin lymphoma	Global	Female	Universal health coverage		X			X	
Other non-Hodgkin lymphoma	Data Rich	Female	Healthcare access and quality index		X			X	
Other non-Hodgkin lymphoma	Global	Female	Healthcare access and quality index		X			X	
Other non-Hodgkin lymphoma	Data Rich	Female	Mean BMI		X			X	
Other non-Hodgkin lymphoma	Global	Female	Mean BMI		X			X	
Other non-Hodgkin lymphoma	Data Rich	Female	Liters of alcohol consumed per capita		X			X	
Other non-Hodgkin lymphoma	Global	Female	Liters of alcohol consumed per capita		X			X	
Other non-Hodgkin lymphoma	Data Rich	Female	Cumulative Cigarettes (5 Years)		X			X	
Other non-Hodgkin lymphoma	Global	Female	Cumulative Cigarettes (5 Years)		X			X	
Other non-Hodgkin lymphoma	Data Rich	Female	Cumulative Cigarettes (20 Years)		X			X	
Other non-Hodgkin lymphoma	Global	Female	Cumulative Cigarettes (20 Years)		X			X	
Other non-Hodgkin lymphoma	Data Rich	Female	Cumulative Cigarettes (15 Years)		X			X	
Other non-Hodgkin lymphoma	Global	Female	Cumulative Cigarettes (15 Years)		X			X	
Other non-Hodgkin lymphoma	Data Rich	Male	Education (years per capita)			X			X
Other non-Hodgkin lymphoma	Global	Male	Education (years per capita)			X			X
Other non-Hodgkin lymphoma	Data Rich	Male	LDI (I$ per capita)			X			X
Other non-Hodgkin lymphoma	Global	Male	LDI (I$ per capita)			X			X
Other non-Hodgkin lymphoma	Data Rich	Male	Socio-demographic Index			X			X
Other non-Hodgkin lymphoma	Global	Male	Socio-demographic Index			X			X
Other non-Hodgkin lymphoma	Data Rich	Male	Log-transformed SEV scalar: HIV			X			X
Other non-Hodgkin lymphoma	Global	Male	Log-transformed SEV scalar: HIV			X			X
Other non-Hodgkin lymphoma	Data Rich	Male	Log-transformed age-standardized SEV scalar: HIV			X			X
Other non-Hodgkin lymphoma	Global	Male	Log-transformed age-standardized SEV scalar: HIV			X			X
Other non-Hodgkin lymphoma	Data Rich	Male	Cumulative Cigarettes (10 Years)		X			X	
Other non-Hodgkin lymphoma	Global	Male	Cumulative Cigarettes (10 Years)		X			X	
Other non-Hodgkin lymphoma	Data Rich	Male	Universal health coverage		X			X	
Other non-Hodgkin lymphoma	Global	Male	Universal health coverage		X			X	
Other non-Hodgkin lymphoma	Data Rich	Male	Healthcare access and quality index		X			X	
Other non-Hodgkin lymphoma	Global	Male	Healthcare access and quality index		X			X	
Other non-Hodgkin lymphoma	Data Rich	Male	Mean BMI		X			X	
Other non-Hodgkin lymphoma	Global	Male	Mean BMI		X			X	
Other non-Hodgkin lymphoma	Data Rich	Male	Liters of alcohol consumed per capita		X			X	
Other non-Hodgkin lymphoma	Global	Male	Liters of alcohol consumed per capita		X			X	
Other non-Hodgkin lymphoma	Data Rich	Male	Cumulative Cigarettes (5 Years)		X			X	
Other non-Hodgkin lymphoma	Global	Male	Cumulative Cigarettes (5 Years)		X			X	
Other non-Hodgkin lymphoma	Data Rich	Male	Cumulative Cigarettes (20 Years)		X			X	
Other non-Hodgkin lymphoma	Global	Male	Cumulative Cigarettes (20 Years)		X			X	
Other non-Hodgkin lymphoma	Data Rich	Male	Cumulative Cigarettes (15 Years)		X			X	
Other non-Hodgkin lymphoma	Global	Male	Cumulative Cigarettes (15 Years)		X			X	
Multiple myeloma	Global	Female	Education (years per capita)			X			X
Multiple myeloma	Data Rich	Female	Education (years per capita)			X			X
Multiple myeloma	Global	Female	LDI (I$ per capita)			X			X
Multiple myeloma	Data Rich	Female	LDI (I$ per capita)			X			X
Multiple myeloma	Global	Female	Socio-demographic Index			X			X
Multiple myeloma	Data Rich	Female	Socio-demographic Index			X			X
Multiple myeloma	Global	Female	Healthcare access and quality index		X			X	
Multiple myeloma	Data Rich	Female	Healthcare access and quality index		X			X	
Multiple myeloma	Global	Female	Mean BMI		X			X	
Multiple myeloma	Data Rich	Female	Mean BMI		X			X	
Multiple myeloma	Global	Female	Sanitation (proportion with access)		X			X	
Multiple myeloma	Data Rich	Female	Sanitation (proportion with access)		X			X	
Multiple myeloma	Global	Female	Improved Water Source (proportion with access)		X			X	
Multiple myeloma	Data Rich	Female	Improved Water Source (proportion with access)		X			X	
Multiple myeloma	Global	Female	Age- and sex-specific SEV for Low fruit		X			X	
Multiple myeloma	Data Rich	Female	Age- and sex-specific SEV for Low fruit		X			X	
Multiple myeloma	Global	Female	Age- and sex-specific SEV for Low vegetables		X			X	
Multiple myeloma	Data Rich	Female	Age- and sex-specific SEV for Low vegetables		X			X	
Multiple myeloma	Global	Female	Age- and sex-specific SEV for High red meat		X			X	
Multiple myeloma	Data Rich	Female	Age- and sex-specific SEV for High red meat		X			X	
Multiple myeloma	Global	Female	Tobacco (cigarettes per capita)	X			X		
Multiple myeloma	Data Rich	Female	Tobacco (cigarettes per capita)	X			X		
Multiple myeloma	Global	Female	Smoking Prevalence	X			X		
Multiple myeloma	Data Rich	Female	Smoking Prevalence	X			X		
Multiple myeloma	Global	Female	Liters of alcohol consumed per capita	X			X		
Multiple myeloma	Data Rich	Female	Liters of alcohol consumed per capita	X			X		
Multiple myeloma	Global	Male	Education (years per capita)			X			X
Multiple myeloma	Data Rich	Male	Education (years per capita)			X			X
Multiple myeloma	Global	Male	LDI (I$ per capita)			X			X
Multiple myeloma	Data Rich	Male	LDI (I$ per capita)			X			X
Multiple myeloma	Global	Male	Socio-demographic Index			X			X
Multiple myeloma	Data Rich	Male	Socio-demographic Index			X			X
Multiple myeloma	Global	Male	Healthcare access and quality index		X			X	
Multiple myeloma	Data Rich	Male	Healthcare access and quality index		X			X	
Multiple myeloma	Global	Male	Mean BMI		X			X	
Multiple myeloma	Data Rich	Male	Mean BMI		X			X	
Multiple myeloma	Global	Male	Sanitation (proportion with access)		X			X	
Multiple myeloma	Data Rich	Male	Sanitation (proportion with access)		X			X	
Multiple myeloma	Global	Male	Improved Water Source (proportion with access)		X			X	
Multiple myeloma	Data Rich	Male	Improved Water Source (proportion with access)		X			X	
Multiple myeloma	Global	Male	Age- and sex-specific SEV for Low fruit		X			X	
Multiple myeloma	Data Rich	Male	Age- and sex-specific SEV for Low fruit		X			X	
Multiple myeloma	Global	Male	Age- and sex-specific SEV for Low vegetables		X			X	
Multiple myeloma	Data Rich	Male	Age- and sex-specific SEV for Low vegetables		X			X	
Multiple myeloma	Global	Male	Age- and sex-specific SEV for High red meat		X			X	
Multiple myeloma	Data Rich	Male	Age- and sex-specific SEV for High red meat		X			X	
Multiple myeloma	Global	Male	Tobacco (cigarettes per capita)	X			X		
Multiple myeloma	Data Rich	Male	Tobacco (cigarettes per capita)	X			X		
Multiple myeloma	Global	Male	Smoking Prevalence	X			X		
Multiple myeloma	Data Rich	Male	Smoking Prevalence	X			X		
Multiple myeloma	Global	Male	Liters of alcohol consumed per capita	X			X		
Multiple myeloma	Data Rich	Male	Liters of alcohol consumed per capita	X			X		
Leukaemia	Data Rich	Female	Education (years per capita)			X			X
Leukaemia	Global	Female	Education (years per capita)			X			X
Leukaemia	Data Rich	Female	LDI (I$ per capita)			X			X
Leukaemia	Global	Female	LDI (I$ per capita)			X			X
Leukaemia	Data Rich	Female	Socio-demographic Index			X			X
Leukaemia	Global	Female	Socio-demographic Index			X			X
Leukaemia	Data Rich	Female	Tobacco (cigarettes per capita)		X			X	
Leukaemia	Global	Female	Tobacco (cigarettes per capita)		X			X	
Leukaemia	Data Rich	Female	Cumulative Cigarettes (10 Years)		X			X	
Leukaemia	Global	Female	Cumulative Cigarettes (10 Years)		X			X	
Leukaemia	Data Rich	Female	Healthcare access and quality index		X			X	
Leukaemia	Global	Female	Healthcare access and quality index		X			X	
Leukaemia	Data Rich	Female	Mean BMI		X			X	
Leukaemia	Global	Female	Mean BMI		X			X	
Leukaemia	Data Rich	Female	Liters of alcohol consumed per capita		X			X	
Leukaemia	Global	Female	Liters of alcohol consumed per capita		X			X	
Leukaemia	Data Rich	Female	Cumulative Cigarettes (20 Years)		X			X	
Leukaemia	Global	Female	Cumulative Cigarettes (20 Years)		X			X	
Leukaemia	Data Rich	Male	Education (years per capita)			X			X
Leukaemia	Global	Male	Education (years per capita)			X			X
Leukaemia	Data Rich	Male	LDI (I$ per capita)			X			X
Leukaemia	Global	Male	LDI (I$ per capita)			X			X
Leukaemia	Data Rich	Male	Socio-demographic Index			X			X
Leukaemia	Global	Male	Socio-demographic Index			X			X
Leukaemia	Data Rich	Male	Tobacco (cigarettes per capita)		X			X	
Leukaemia	Global	Male	Tobacco (cigarettes per capita)		X			X	

Appendix Table S11: Comparison of GBD 2021 and GBD 2023 covariates and level of covariates used in cause of death modeling

Cause	Model Version Type	Sex	Covariate	Level 1 GBD 2021	Level 2 GBD 2021	Level 3 GBD 2021	Level 1 GBD 2023	Level 2 GBD 2023	Level 3 GBD 2023
Leukaemia	Data Rich	Male	Cumulative Cigarettes (10 Years)		X			X	
Leukaemia	Global	Male	Cumulative Cigarettes (10 Years)		X			X	
Leukaemia	Data Rich	Male	Healthcare access and quality index		X			X	
Leukaemia	Global	Male	Healthcare access and quality index		X			X	
Leukaemia	Data Rich	Male	Mean BMI		X			X	
Leukaemia	Global	Male	Mean BMI		X			X	
Leukaemia	Data Rich	Male	Liters of alcohol consumed per capita		X			X	
Leukaemia	Global	Male	Liters of alcohol consumed per capita		X			X	
Leukaemia	Data Rich	Male	Cumulative Cigarettes (20 Years)		X			X	
Leukaemia	Global	Male	Cumulative Cigarettes (20 Years)		X			X	
Acute lymphoid leukaemia	Data Rich	Female	Education (years per capita)			X			X
Acute lymphoid leukaemia	Global	Female	Education (years per capita)			X			X
Acute lymphoid leukaemia	Data Rich	Female	LDI (I$ per capita)			X			X
Acute lymphoid leukaemia	Global	Female	LDI (I$ per capita)			X			X
Acute lymphoid leukaemia	Data Rich	Female	Socio-demographic Index			X			X
Acute lymphoid leukaemia	Global	Female	Socio-demographic Index			X			X
Acute lymphoid leukaemia	Data Rich	Female	Healthcare access and quality index		X			X	
Acute lymphoid leukaemia	Global	Female	Healthcare access and quality index		X			X	
Acute lymphoid leukaemia	Data Rich	Female	Mean BMI		X			X	
Acute lymphoid leukaemia	Global	Female	Mean BMI		X			X	
Acute lymphoid leukaemia	Data Rich	Male	Education (years per capita)			X			X
Acute lymphoid leukaemia	Global	Male	Education (years per capita)			X			X
Acute lymphoid leukaemia	Data Rich	Male	LDI (I$ per capita)			X			X
Acute lymphoid leukaemia	Global	Male	LDI (I$ per capita)			X			X
Acute lymphoid leukaemia	Data Rich	Male	Socio-demographic Index			X			X
Acute lymphoid leukaemia	Global	Male	Socio-demographic Index			X			X
Acute lymphoid leukaemia	Data Rich	Male	Healthcare access and quality index		X			X	
Acute lymphoid leukaemia	Global	Male	Healthcare access and quality index		X			X	
Acute lymphoid leukaemia	Data Rich	Male	Mean BMI		X			X	
Acute lymphoid leukaemia	Global	Male	Mean BMI		X			X	
Chronic lymphoid leukaemia	Data Rich	Female	Education (years per capita)			X			X
Chronic lymphoid leukaemia	Global	Female	Education (years per capita)			X			X
Chronic lymphoid leukaemia	Data Rich	Female	LDI (I$ per capita)			X			X
Chronic lymphoid leukaemia	Global	Female	LDI (I$ per capita)			X			X
Chronic lymphoid leukaemia	Data Rich	Female	Socio-demographic Index			X			X
Chronic lymphoid leukaemia	Global	Female	Socio-demographic Index			X			X
Chronic lymphoid leukaemia	Data Rich	Female	Tobacco (cigarettes per capita)		X			X	
Chronic lymphoid leukaemia	Global	Female	Tobacco (cigarettes per capita)		X			X	
Chronic lymphoid leukaemia	Data Rich	Female	Cumulative Cigarettes (10 Years)		X			X	
Chronic lymphoid leukaemia	Global	Female	Cumulative Cigarettes (10 Years)		X			X	
Chronic lymphoid leukaemia	Data Rich	Female	Healthcare access and quality index		X			X	
Chronic lymphoid leukaemia	Global	Female	Healthcare access and quality index		X			X	
Chronic lymphoid leukaemia	Data Rich	Female	Mean BMI		X			X	
Chronic lymphoid leukaemia	Global	Female	Mean BMI		X			X	
Chronic lymphoid leukaemia	Data Rich	Female	Liters of alcohol consumed per capita		X			X	
Chronic lymphoid leukaemia	Global	Female	Liters of alcohol consumed per capita		X			X	
Chronic lymphoid leukaemia	Data Rich	Female	Smoking Prevalence		X			X	
Chronic lymphoid leukaemia	Global	Female	Smoking Prevalence		X			X	
Chronic lymphoid leukaemia	Data Rich	Female	Cumulative Cigarettes (5 Years)		X			X	
Chronic lymphoid leukaemia	Global	Female	Cumulative Cigarettes (5 Years)		X			X	
Chronic lymphoid leukaemia	Data Rich	Female	Cumulative Cigarettes (20 Years)		X			X	
Chronic lymphoid leukaemia	Global	Female	Cumulative Cigarettes (20 Years)		X			X	
Chronic lymphoid leukaemia	Data Rich	Female	Cumulative Cigarettes (15 Years)		X			X	
Chronic lymphoid leukaemia	Global	Female	Cumulative Cigarettes (15 Years)		X			X	
Chronic lymphoid leukaemia	Data Rich	Male	Education (years per capita)			X			X
Chronic lymphoid leukaemia	Global	Male	Education (years per capita)			X			X
Chronic lymphoid leukaemia	Data Rich	Male	LDI (I$ per capita)			X			X
Chronic lymphoid leukaemia	Global	Male	LDI (I$ per capita)			X			X
Chronic lymphoid leukaemia	Data Rich	Male	Socio-demographic Index			X			X
Chronic lymphoid leukaemia	Global	Male	Socio-demographic Index			X			X
Chronic lymphoid leukaemia	Data Rich	Male	Tobacco (cigarettes per capita)		X			X	
Chronic lymphoid leukaemia	Global	Male	Tobacco (cigarettes per capita)		X			X	
Chronic lymphoid leukaemia	Data Rich	Male	Cumulative Cigarettes (10 Years)		X			X	
Chronic lymphoid leukaemia	Global	Male	Cumulative Cigarettes (10 Years)		X			X	
Chronic lymphoid leukaemia	Data Rich	Male	Healthcare access and quality index		X			X	
Chronic lymphoid leukaemia	Global	Male	Healthcare access and quality index		X			X	
Chronic lymphoid leukaemia	Data Rich	Male	Mean BMI		X			X	
Chronic lymphoid leukaemia	Global	Male	Mean BMI		X			X	
Chronic lymphoid leukaemia	Data Rich	Male	Liters of alcohol consumed per capita		X			X	
Chronic lymphoid leukaemia	Global	Male	Liters of alcohol consumed per capita		X			X	
Chronic lymphoid leukaemia	Data Rich	Male	Smoking Prevalence		X			X	
Chronic lymphoid leukaemia	Global	Male	Smoking Prevalence		X			X	
Chronic lymphoid leukaemia	Data Rich	Male	Cumulative Cigarettes (5 Years)		X			X	
Chronic lymphoid leukaemia	Global	Male	Cumulative Cigarettes (5 Years)		X			X	
Chronic lymphoid leukaemia	Data Rich	Male	Cumulative Cigarettes (20 Years)		X			X	
Chronic lymphoid leukaemia	Global	Male	Cumulative Cigarettes (20 Years)		X			X	
Chronic lymphoid leukaemia	Data Rich	Male	Cumulative Cigarettes (15 Years)		X			X	
Chronic lymphoid leukaemia	Global	Male	Cumulative Cigarettes (15 Years)		X			X	
Acute myeloid leukaemia	Data Rich	Female	Education (years per capita)			X			X
Acute myeloid leukaemia	Global	Female	Education (years per capita)			X			X
Acute myeloid leukaemia	Data Rich	Female	LDI (I$ per capita)			X			X
Acute myeloid leukaemia	Global	Female	LDI (I$ per capita)			X			X
Acute myeloid leukaemia	Data Rich	Female	Socio-demographic Index			X			X
Acute myeloid leukaemia	Global	Female	Socio-demographic Index			X			X
Acute myeloid leukaemia	Data Rich	Female	Cumulative Cigarettes (10 Years)		X			X	
Acute myeloid leukaemia	Global	Female	Cumulative Cigarettes (10 Years)		X			X	
Acute myeloid leukaemia	Data Rich	Female	Smoking Prevalence		X			X	
Acute myeloid leukaemia	Global	Female	Smoking Prevalence		X			X	
Acute myeloid leukaemia	Data Rich	Female	Healthcare access and quality index		X			X	
Acute myeloid leukaemia	Global	Female	Healthcare access and quality index		X			X	
Acute myeloid leukaemia	Data Rich	Female	Mean BMI		X			X	
Acute myeloid leukaemia	Global	Female	Mean BMI		X			X	
Acute myeloid leukaemia	Data Rich	Female	Liters of alcohol consumed per capita		X			X	
Acute myeloid leukaemia	Global	Female	Liters of alcohol consumed per capita		X			X	
Acute myeloid leukaemia	Data Rich	Female	Cumulative Cigarettes (20 Years)		X			X	
Acute myeloid leukaemia	Global	Female	Cumulative Cigarettes (20 Years)		X			X	
Acute myeloid leukaemia	Data Rich	Male	Education (years per capita)			X			X
Acute myeloid leukaemia	Global	Male	Education (years per capita)			X			X
Acute myeloid leukaemia	Data Rich	Male	LDI (I$ per capita)			X			X
Acute myeloid leukaemia	Global	Male	LDI (I$ per capita)			X			X
Acute myeloid leukaemia	Data Rich	Male	Socio-demographic Index			X			X
Acute myeloid leukaemia	Global	Male	Socio-demographic Index			X			X
Acute myeloid leukaemia	Data Rich	Male	Cumulative Cigarettes (10 Years)		X			X	
Acute myeloid leukaemia	Global	Male	Cumulative Cigarettes (10 Years)		X			X	
Acute myeloid leukaemia	Data Rich	Male	Smoking Prevalence		X			X	
Acute myeloid leukaemia	Global	Male	Smoking Prevalence		X			X	
Acute myeloid leukaemia	Data Rich	Male	Healthcare access and quality index		X			X	
Acute myeloid leukaemia	Global	Male	Healthcare access and quality index		X			X	
Acute myeloid leukaemia	Data Rich	Male	Mean BMI		X			X	
Acute myeloid leukaemia	Global	Male	Mean BMI		X			X	
Acute myeloid leukaemia	Data Rich	Male	Liters of alcohol consumed per capita		X			X	
Acute myeloid leukaemia	Global	Male	Liters of alcohol consumed per capita		X			X	
Acute myeloid leukaemia	Data Rich	Male	Cumulative Cigarettes (20 Years)		X			X	
Acute myeloid leukaemia	Global	Male	Cumulative Cigarettes (20 Years)		X			X	
Chronic myeloid leukaemia	Data Rich	Female	Education (years per capita)			X			X
Chronic myeloid leukaemia	Global	Female	Education (years per capita)			X			X
Chronic myeloid leukaemia	Data Rich	Female	LDI (I$ per capita)			X			X
Chronic myeloid leukaemia	Global	Female	LDI (I$ per capita)			X			X
Chronic myeloid leukaemia	Data Rich	Female	Socio-demographic Index			X			X
Chronic myeloid leukaemia	Global	Female	Socio-demographic Index			X			X
Chronic myeloid leukaemia	Data Rich	Female	Tobacco (cigarettes per capita)		X			X	
Chronic myeloid leukaemia	Global	Female	Tobacco (cigarettes per capita)		X			X	
Chronic myeloid leukaemia	Data Rich	Female	Cumulative Cigarettes (10 Years)		X			X	
Chronic myeloid leukaemia	Global	Female	Cumulative Cigarettes (10 Years)		X			X	
Chronic myeloid leukaemia	Data Rich	Female	Smoking Prevalence		X			X	
Chronic myeloid leukaemia	Global	Female	Smoking Prevalence		X			X	
Chronic myeloid leukaemia	Data Rich	Female	Healthcare access and quality index		X			X	
Chronic myeloid leukaemia	Global	Female	Healthcare access and quality index		X			X	
Chronic myeloid leukaemia	Data Rich	Female	Mean BMI		X			X	
Chronic myeloid leukaemia	Global	Female	Mean BMI		X			X	
Chronic myeloid leukaemia	Data Rich	Female	Liters of alcohol consumed per capita		X			X	

Appendix Table S11: Comparison of GBD 2021 and GBD 2023 covariates and level of covariates used in cause of death modeling

Cause	Model Version Type	Sex	Covariate	Level 1 GBD 2021	Level 2 GBD 2021	Level 3 GBD 2021	Level 1 GBD 2023	Level 2 GBD 2023	Level 3 GBD 2023
Chronic myeloid leukaemia	Global	Female	Liters of alcohol consumed per capita		X			X	
Chronic myeloid leukaemia	Data Rich	Female	Cumulative Cigarettes (5 Years)		X			X	
Chronic myeloid leukaemia	Global	Female	Cumulative Cigarettes (5 Years)		X			X	
Chronic myeloid leukaemia	Data Rich	Female	Cumulative Cigarettes (20 Years)		X			X	
Chronic myeloid leukaemia	Global	Female	Cumulative Cigarettes (20 Years)		X			X	
Chronic myeloid leukaemia	Data Rich	Female	Cumulative Cigarettes (15 Years)		X			X	
Chronic myeloid leukaemia	Global	Female	Cumulative Cigarettes (15 Years)		X			X	
Chronic myeloid leukaemia	Data Rich	Male	Education (years per capita)			X			X
Chronic myeloid leukaemia	Global	Male	Education (years per capita)			X			X
Chronic myeloid leukaemia	Data Rich	Male	LDI (I$ per capita)			X			X
Chronic myeloid leukaemia	Global	Male	LDI (I$ per capita)			X			X
Chronic myeloid leukaemia	Data Rich	Male	Socio-demographic Index			X			X
Chronic myeloid leukaemia	Global	Male	Socio-demographic Index			X			X
Chronic myeloid leukaemia	Data Rich	Male	Tobacco (cigarettes per capita)		X			X	
Chronic myeloid leukaemia	Global	Male	Tobacco (cigarettes per capita)		X			X	
Chronic myeloid leukaemia	Data Rich	Male	Cumulative Cigarettes (10 Years)		X			X	
Chronic myeloid leukaemia	Global	Male	Cumulative Cigarettes (10 Years)		X			X	
Chronic myeloid leukaemia	Data Rich	Male	Smoking Prevalence		X			X	
Chronic myeloid leukaemia	Global	Male	Smoking Prevalence		X			X	
Chronic myeloid leukaemia	Data Rich	Male	Healthcare access and quality index		X			X	
Chronic myeloid leukaemia	Global	Male	Healthcare access and quality index		X			X	
Chronic myeloid leukaemia	Data Rich	Male	Mean BMI		X			X	
Chronic myeloid leukaemia	Global	Male	Mean BMI		X			X	
Chronic myeloid leukaemia	Data Rich	Male	Liters of alcohol consumed per capita		X			X	
Chronic myeloid leukaemia	Global	Male	Liters of alcohol consumed per capita		X			X	
Chronic myeloid leukaemia	Data Rich	Male	Cumulative Cigarettes (5 Years)		X			X	
Chronic myeloid leukaemia	Global	Male	Cumulative Cigarettes (5 Years)		X			X	
Chronic myeloid leukaemia	Data Rich	Male	Cumulative Cigarettes (20 Years)		X			X	
Chronic myeloid leukaemia	Global	Male	Cumulative Cigarettes (20 Years)		X			X	
Chronic myeloid leukaemia	Data Rich	Male	Cumulative Cigarettes (15 Years)		X			X	
Chronic myeloid leukaemia	Global	Male	Cumulative Cigarettes (15 Years)		X			X	
Other leukaemia	Data Rich	Female	Education (years per capita)			X			X
Other leukaemia	Global	Female	Education (years per capita)			X			X
Other leukaemia	Data Rich	Female	LDI (I$ per capita)			X			X
Other leukaemia	Global	Female	LDI (I$ per capita)			X			X
Other leukaemia	Data Rich	Female	Socio-demographic Index			X			X
Other leukaemia	Global	Female	Socio-demographic Index			X			X
Other leukaemia	Data Rich	Female	Tobacco (cigarettes per capita)		X			X	
Other leukaemia	Global	Female	Tobacco (cigarettes per capita)		X			X	
Other leukaemia	Data Rich	Female	Cumulative Cigarettes (10 Years)		X			X	
Other leukaemia	Global	Female	Cumulative Cigarettes (10 Years)		X			X	
Other leukaemia	Data Rich	Female	Healthcare access and quality index		X			X	
Other leukaemia	Global	Female	Healthcare access and quality index		X			X	
Other leukaemia	Data Rich	Female	Mean BMI		X			X	
Other leukaemia	Global	Female	Mean BMI		X			X	
Other leukaemia	Global	Female	Liters of alcohol consumed per capita		X			X	
Other leukaemia	Data Rich	Female	Cumulative Cigarettes (20 Years)		X			X	
Other leukaemia	Global	Female	Cumulative Cigarettes (20 Years)		X			X	
Other leukaemia	Data Rich	Female	Liters of alcohol consumed per capita	X			X		
Other leukaemia	Data Rich	Male	Education (years per capita)			X			X
Other leukaemia	Global	Male	Education (years per capita)			X			X
Other leukaemia	Data Rich	Male	LDI (I$ per capita)			X			X
Other leukaemia	Global	Male	LDI (I$ per capita)			X			X
Other leukaemia	Data Rich	Male	Socio-demographic Index			X			X
Other leukaemia	Global	Male	Socio-demographic Index			X			X
Other leukaemia	Data Rich	Male	Tobacco (cigarettes per capita)		X			X	
Other leukaemia	Global	Male	Tobacco (cigarettes per capita)		X			X	
Other leukaemia	Data Rich	Male	Cumulative Cigarettes (10 Years)		X			X	
Other leukaemia	Global	Male	Cumulative Cigarettes (10 Years)		X			X	
Other leukaemia	Data Rich	Male	Healthcare access and quality index		X			X	
Other leukaemia	Global	Male	Healthcare access and quality index		X			X	
Other leukaemia	Data Rich	Male	Mean BMI		X			X	
Other leukaemia	Global	Male	Mean BMI		X			X	
Other leukaemia	Data Rich	Male	Liters of alcohol consumed per capita		X			X	
Other leukaemia	Global	Male	Liters of alcohol consumed per capita		X			X	
Other leukaemia	Data Rich	Male	Cumulative Cigarettes (20 Years)		X			X	
Other leukaemia	Global	Male	Cumulative Cigarettes (20 Years)		X			X	
Other malignant cancers	Data Rich	Female	Education (years per capita)			X			X
Other malignant cancers	Global	Female	Education (years per capita)			X			X
Other malignant cancers	Data Rich	Female	LDI (I$ per capita)			X			X
Other malignant cancers	Global	Female	LDI (I$ per capita)			X			X
Other malignant cancers	Data Rich	Female	Socio-demographic Index			X			X
Other malignant cancers	Global	Female	Socio-demographic Index			X			X
Other malignant cancers	Data Rich	Female	Healthcare access and quality index		X			X	
Other malignant cancers	Global	Female	Healthcare access and quality index		X			X	
Other malignant cancers	Data Rich	Female	Age- and sex-specific SEV for Low fruit		X			X	
Other malignant cancers	Global	Female	Age- and sex-specific SEV for Low fruit		X			X	
Other malignant cancers	Data Rich	Female	Age- and sex-specific SEV for Low vegetables		X			X	
Other malignant cancers	Global	Female	Age- and sex-specific SEV for Low vegetables		X			X	
Other malignant cancers	Data Rich	Female	Age- and sex-specific SEV for Low nuts and seeds		X			X	
Other malignant cancers	Global	Female	Age- and sex-specific SEV for Low nuts and seeds		X			X	
Other malignant cancers	Data Rich	Female	pufa adjusted(percent)		X			X	
Other malignant cancers	Global	Female	pufa adjusted(percent)		X			X	
Other malignant cancers	Data Rich	Female	Tobacco (cigarettes per capita)	X			X		
Other malignant cancers	Global	Female	Tobacco (cigarettes per capita)	X			X		
Other malignant cancers	Data Rich	Female	Smoking Prevalence	X			X		
Other malignant cancers	Global	Female	Smoking Prevalence	X			X		
Other malignant cancers	Data Rich	Male	Education (years per capita)			X			X
Other malignant cancers	Global	Male	Education (years per capita)			X			X
Other malignant cancers	Data Rich	Male	LDI (I$ per capita)			X			X
Other malignant cancers	Global	Male	LDI (I$ per capita)			X			X
Other malignant cancers	Data Rich	Male	Socio-demographic Index			X			X
Other malignant cancers	Global	Male	Socio-demographic Index			X			X
Other malignant cancers	Data Rich	Male	Healthcare access and quality index		X			X	
Other malignant cancers	Global	Male	Healthcare access and quality index		X			X	
Other malignant cancers	Data Rich	Male	Age- and sex-specific SEV for Low fruit		X			X	
Other malignant cancers	Global	Male	Age- and sex-specific SEV for Low fruit		X			X	
Other malignant cancers	Data Rich	Male	Age- and sex-specific SEV for Low vegetables		X			X	
Other malignant cancers	Global	Male	Age- and sex-specific SEV for Low vegetables		X			X	
Other malignant cancers	Data Rich	Male	Age- and sex-specific SEV for Low nuts and seeds		X			X	
Other malignant cancers	Global	Male	Age- and sex-specific SEV for Low nuts and seeds		X			X	
Other malignant cancers	Data Rich	Male	pufa adjusted(percent)		X			X	
Other malignant cancers	Global	Male	pufa adjusted(percent)		X			X	
Other malignant cancers	Data Rich	Male	Tobacco (cigarettes per capita)	X			X		
Other malignant cancers	Global	Male	Tobacco (cigarettes per capita)	X			X		
Other malignant cancers	Data Rich	Male	Smoking Prevalence	X			X		
Other malignant cancers	Global	Male	Smoking Prevalence	X			X		
Other neoplasms	Global	Female	Maternal care and immunization			X			X
Other neoplasms	Data Rich	Female	Maternal care and immunization			X			X
Other neoplasms	Global	Female	Tobacco (cigarettes per capita)			X			X
Other neoplasms	Global	Female	Cumulative Cigarettes (10 Years)			X			X
Other neoplasms	Global	Female	Education (years per capita)			X			X
Other neoplasms	Data Rich	Female	Education (years per capita)			X			X
Other neoplasms	Global	Female	LDI (I$ per capita)			X			X
Other neoplasms	Data Rich	Female	LDI (I$ per capita)			X			X
Other neoplasms	Global	Female	Low-Density Lipoprotein (mmol/L)			X			X
Other neoplasms	Data Rich	Female	Low-Density Lipoprotein (mmol/L)			X			X
Other neoplasms	Global	Female	Smoking Prevalence			X			X
Other neoplasms	Global	Female	Log-transformed SEV scalar: Leukemia			X			X
Other neoplasms	Data Rich	Female	Log-transformed SEV scalar: Leukemia			X			X
Other neoplasms	Global	Female	Log-transformed age-standardized SEV scalar: Leukemia			X			X
Other neoplasms	Data Rich	Female	Log-transformed age-standardized SEV scalar: Leukemia			X			X
Other neoplasms	Global	Female	Socio-demographic Index			X			X
Other neoplasms	Data Rich	Female	Socio-demographic Index			X			X
Other neoplasms	Global	Female	Universal health coverage			X			X

Appendix Table S11: Comparison of GBD 2021 and GBD 2023 covariates and level of covariates used in cause of death modeling

Cause	Model Version Type	Sex	Covariate	Level 1 GBD 2021	Level 2 GBD 2021	Level 3 GBD 2021	Level 1 GBD 2023	Level 2 GBD 2023	Level 3 GBD 2023
Other neoplasms	Data Rich	Female	Universal health coverage			X			X
Other neoplasms	Global	Female	Healthcare access and quality index			X			X
Other neoplasms	Data Rich	Female	Healthcare access and quality index			X			X
Other neoplasms	Global	Female	Liters of alcohol consumed per capita			X			X
Other neoplasms	Data Rich	Female	Mean BMI			X			X
Other neoplasms	Data Rich	Female	Health worker density			X			X
Other neoplasms	Data Rich	Female	Tobacco (cigarettes per capita)		X			X	
Other neoplasms	Data Rich	Female	Cumulative Cigarettes (10 Years)		X			X	
Other neoplasms	Data Rich	Female	Smoking Prevalence		X			X	
Other neoplasms	Data Rich	Female	Liters of alcohol consumed per capita		X			X	
Other neoplasms	Global	Male	Maternal care and immunization			X			X
Other neoplasms	Data Rich	Male	Maternal care and immunization			X			X
Other neoplasms	Global	Male	Tobacco (cigarettes per capita)			X			X
Other neoplasms	Global	Male	Cumulative Cigarettes (10 Years)			X			X
Other neoplasms	Global	Male	Education (years per capita)			X			X
Other neoplasms	Data Rich	Male	Education (years per capita)			X			X
Other neoplasms	Global	Male	LDI (I$ per capita)			X			X
Other neoplasms	Data Rich	Male	LDI (I$ per capita)			X			X
Other neoplasms	Global	Male	Low-Density Lipoprotein (mmol/L)			X			X
Other neoplasms	Data Rich	Male	Low-Density Lipoprotein (mmol/L)			X			X
Other neoplasms	Global	Male	Smoking Prevalence			X			X
Other neoplasms	Global	Male	Log-transformed SEV scalar: Leukemia			X			X
Other neoplasms	Data Rich	Male	Log-transformed SEV scalar: Leukemia			X			X
Other neoplasms	Global	Male	Log-transformed age-standardized SEV scalar: Leukemia			X			X
Other neoplasms	Data Rich	Male	Log-transformed age-standardized SEV scalar: Leukemia			X			X
Other neoplasms	Global	Male	Socio-demographic Index			X			X
Other neoplasms	Data Rich	Male	Socio-demographic Index			X			X
Other neoplasms	Global	Male	Universal health coverage			X			X
Other neoplasms	Data Rich	Male	Universal health coverage			X			X
Other neoplasms	Global	Male	Healthcare access and quality index			X			X
Other neoplasms	Data Rich	Male	Healthcare access and quality index			X			X
Other neoplasms	Global	Male	Liters of alcohol consumed per capita			X			X
Other neoplasms	Data Rich	Male	Mean BMI			X			X
Other neoplasms	Data Rich	Male	Health worker density			X			X
Other neoplasms	Data Rich	Male	Tobacco (cigarettes per capita)		X			X	
Other neoplasms	Data Rich	Male	Cumulative Cigarettes (10 Years)		X			X	
Other neoplasms	Data Rich	Male	Smoking Prevalence		X			X	
Other neoplasms	Data Rich	Male	Liters of alcohol consumed per capita		X			X	
Myelodysplastic, myeloproliferative, and other hematopoietic neoplasms	Data Rich	Female	Education (years per capita)			X			X
Myelodysplastic, myeloproliferative, and other hematopoietic neoplasms	Global	Female	Education (years per capita)			X			X
Myelodysplastic, myeloproliferative, and other hematopoietic neoplasms	Data Rich	Female	LDI (I$ per capita)			X			X
Myelodysplastic, myeloproliferative, and other hematopoietic neoplasms	Global	Female	LDI (I$ per capita)			X			X
Myelodysplastic, myeloproliferative, and other hematopoietic neoplasms	Data Rich	Female	Socio-demographic Index			X			X
Myelodysplastic, myeloproliferative, and other hematopoietic neoplasms	Global	Female	Socio-demographic Index			X			X
Myelodysplastic, myeloproliferative, and other hematopoietic neoplasms	Data Rich	Female	Tobacco (cigarettes per capita)		X			X	
Myelodysplastic, myeloproliferative, and other hematopoietic neoplasms	Global	Female	Tobacco (cigarettes per capita)		X			X	
Myelodysplastic, myeloproliferative, and other hematopoietic neoplasms	Data Rich	Female	Cumulative Cigarettes (10 Years)		X			X	
Myelodysplastic, myeloproliferative, and other hematopoietic neoplasms	Global	Female	Cumulative Cigarettes (10 Years)		X			X	
Myelodysplastic, myeloproliferative, and other hematopoietic neoplasms	Data Rich	Female	Healthcare access and quality index		X			X	
Myelodysplastic, myeloproliferative, and other hematopoietic neoplasms	Global	Female	Healthcare access and quality index		X			X	
Myelodysplastic, myeloproliferative, and other hematopoietic neoplasms	Data Rich	Female	Liters of alcohol consumed per capita		X			X	
Myelodysplastic, myeloproliferative, and other hematopoietic neoplasms	Global	Female	Liters of alcohol consumed per capita		X			X	
Myelodysplastic, myeloproliferative, and other hematopoietic neoplasms	Data Rich	Female	Smoking Prevalence		X			X	
Myelodysplastic, myeloproliferative, and other hematopoietic neoplasms	Global	Female	Smoking Prevalence		X			X	
Myelodysplastic, myeloproliferative, and other hematopoietic neoplasms	Data Rich	Female	Cumulative Cigarettes (5 Years)		X			X	
Myelodysplastic, myeloproliferative, and other hematopoietic neoplasms	Global	Female	Cumulative Cigarettes (5 Years)		X			X	
Myelodysplastic, myeloproliferative, and other hematopoietic neoplasms	Data Rich	Female	Cumulative Cigarettes (20 Years)		X			X	
Myelodysplastic, myeloproliferative, and other hematopoietic neoplasms	Global	Female	Cumulative Cigarettes (20 Years)		X			X	
Myelodysplastic, myeloproliferative, and other hematopoietic neoplasms	Data Rich	Female	Cumulative Cigarettes (15 Years)		X			X	
Myelodysplastic, myeloproliferative, and other hematopoietic neoplasms	Global	Female	Cumulative Cigarettes (15 Years)		X			X	
Myelodysplastic, myeloproliferative, and other hematopoietic neoplasms	Data Rich	Male	Education (years per capita)			X			X
Myelodysplastic, myeloproliferative, and other hematopoietic neoplasms	Global	Male	Education (years per capita)			X			X
Myelodysplastic, myeloproliferative, and other hematopoietic neoplasms	Data Rich	Male	LDI (I$ per capita)			X			X
Myelodysplastic, myeloproliferative, and other hematopoietic neoplasms	Global	Male	LDI (I$ per capita)			X			X
Myelodysplastic, myeloproliferative, and other hematopoietic neoplasms	Data Rich	Male	Socio-demographic Index			X			X
Myelodysplastic, myeloproliferative, and other hematopoietic neoplasms	Global	Male	Socio-demographic Index			X			X
Myelodysplastic, myeloproliferative, and other hematopoietic neoplasms	Data Rich	Male	Tobacco (cigarettes per capita)		X			X	
Myelodysplastic, myeloproliferative, and other hematopoietic neoplasms	Global	Male	Tobacco (cigarettes per capita)		X			X	
Myelodysplastic, myeloproliferative, and other hematopoietic neoplasms	Data Rich	Male	Cumulative Cigarettes (10 Years)		X			X	
Myelodysplastic, myeloproliferative, and other hematopoietic neoplasms	Global	Male	Cumulative Cigarettes (10 Years)		X			X	
Myelodysplastic, myeloproliferative, and other hematopoietic neoplasms	Data Rich	Male	Healthcare access and quality index		X			X	
Myelodysplastic, myeloproliferative, and other hematopoietic neoplasms	Global	Male	Healthcare access and quality index		X			X	
Myelodysplastic, myeloproliferative, and other hematopoietic neoplasms	Data Rich	Male	Liters of alcohol consumed per capita		X			X	
Myelodysplastic, myeloproliferative, and other hematopoietic neoplasms	Global	Male	Liters of alcohol consumed per capita		X			X	
Myelodysplastic, myeloproliferative, and other hematopoietic neoplasms	Data Rich	Male	Smoking Prevalence		X			X	
Myelodysplastic, myeloproliferative, and other hematopoietic neoplasms	Global	Male	Smoking Prevalence		X			X	
Myelodysplastic, myeloproliferative, and other hematopoietic neoplasms	Data Rich	Male	Cumulative Cigarettes (5 Years)		X			X	
Myelodysplastic, myeloproliferative, and other hematopoietic neoplasms	Global	Male	Cumulative Cigarettes (5 Years)		X			X	
Myelodysplastic, myeloproliferative, and other hematopoietic neoplasms	Data Rich	Male	Cumulative Cigarettes (20 Years)		X			X	
Myelodysplastic, myeloproliferative, and other hematopoietic neoplasms	Global	Male	Cumulative Cigarettes (20 Years)		X			X	
Myelodysplastic, myeloproliferative, and other hematopoietic neoplasms	Data Rich	Male	Cumulative Cigarettes (15 Years)		X			X	
Myelodysplastic, myeloproliferative, and other hematopoietic neoplasms	Global	Male	Cumulative Cigarettes (15 Years)		X			X	
Myelodysplastic, myeloproliferative, and other hematopoietic neoplasms	Global	Male	Log-transformed SEV scalar: Leukemia	X			X		
Myelodysplastic, myeloproliferative, and other hematopoietic neoplasms	Global	Male	Log-transformed age-standardized SEV scalar: Leukemia	X			X		
Non-malignant brain and other central nervous system neoplasms	Global	Female	Education (years per capita)						X
Non-malignant brain and other central nervous system neoplasms	Data Rich	Female	Education (years per capita)						X
Non-malignant brain and other central nervous system neoplasms	Global	Female	LDI (I$ per capita)						X
Non-malignant brain and other central nervous system neoplasms	Data Rich	Female	LDI (I$ per capita)						X
Non-malignant brain and other central nervous system neoplasms	Global	Female	Socio-demographic Index						X
Non-malignant brain and other central nervous system neoplasms	Data Rich	Female	Socio-demographic Index						X
Non-malignant brain and other central nervous system neoplasms	Global	Female	Low-Density Lipoprotein (mmol/L)					X	
Non-malignant brain and other central nervous system neoplasms	Data Rich	Female	Low-Density Lipoprotein (mmol/L)					X	
Non-malignant brain and other central nervous system neoplasms	Global	Female	Healthcare access and quality index					X	
Non-malignant brain and other central nervous system neoplasms	Data Rich	Female	Healthcare access and quality index					X	
Non-malignant brain and other central nervous system neoplasms	Global	Female	Systolic Blood Pressure (mmHg)					X	
Non-malignant brain and other central nervous system neoplasms	Data Rich	Female	Systolic Blood Pressure (mmHg)					X	
Non-malignant brain and other central nervous system neoplasms	Global	Female	Age- and sex-specific SEV for Low fruit					X	
Non-malignant brain and other central nervous system neoplasms	Data Rich	Female	Age- and sex-specific SEV for Low fruit					X	
Non-malignant brain and other central nervous system neoplasms	Global	Female	Age- and sex-specific SEV for Low vegetables					X	
Non-malignant brain and other central nervous system neoplasms	Data Rich	Female	Age- and sex-specific SEV for Low vegetables					X	

Appendix Table S11: Comparison of GBD 2021 and GBD 2023 covariates and level of covariates used in cause of death modeling

Cause	Model Version Type	Sex	Covariate	Level 1 GBD 2021	Level 2 GBD 2021	Level 3 GBD 2021	Level 1 GBD 2023	Level 2 GBD 2023	Level 3 GBD 2023
Non-malignant brain and other central nervous system neoplasms	Global	Female	Age- and sex-specific SEV for High red meat					X	
Non-malignant brain and other central nervous system neoplasms	Data Rich	Female	Age- and sex-specific SEV for High red meat					X	
Non-malignant brain and other central nervous system neoplasms	Global	Female	Cumulative Cigarettes (10 Years)				X		
Non-malignant brain and other central nervous system neoplasms	Data Rich	Female	Cumulative Cigarettes (10 Years)				X		
Non-malignant brain and other central nervous system neoplasms	Global	Female	Smoking Prevalence				X		
Non-malignant brain and other central nervous system neoplasms	Data Rich	Female	Smoking Prevalence				X		
Non-malignant brain and other central nervous system neoplasms	Global	Female	Liters of alcohol consumed per capita				X		
Non-malignant brain and other central nervous system neoplasms	Data Rich	Female	Liters of alcohol consumed per capita				X		
Non-malignant brain and other central nervous system neoplasms	Global	Male	Education (years per capita)						X
Non-malignant brain and other central nervous system neoplasms	Data Rich	Male	Education (years per capita)						X
Non-malignant brain and other central nervous system neoplasms	Global	Male	LDI (I$ per capita)						X
Non-malignant brain and other central nervous system neoplasms	Data Rich	Male	LDI (I$ per capita)						X
Non-malignant brain and other central nervous system neoplasms	Global	Male	Socio-demographic Index						X
Non-malignant brain and other central nervous system neoplasms	Data Rich	Male	Socio-demographic Index						X
Non-malignant brain and other central nervous system neoplasms	Global	Male	Low-Density Lipoprotein (mmol/L)					X	
Non-malignant brain and other central nervous system neoplasms	Data Rich	Male	Low-Density Lipoprotein (mmol/L)					X	
Non-malignant brain and other central nervous system neoplasms	Global	Male	Healthcare access and quality index					X	
Non-malignant brain and other central nervous system neoplasms	Data Rich	Male	Healthcare access and quality index					X	
Non-malignant brain and other central nervous system neoplasms	Global	Male	Systolic Blood Pressure (mmHg)					X	
Non-malignant brain and other central nervous system neoplasms	Data Rich	Male	Systolic Blood Pressure (mmHg)					X	
Non-malignant brain and other central nervous system neoplasms	Global	Male	Age- and sex-specific SEV for Low fruit					X	
Non-malignant brain and other central nervous system neoplasms	Data Rich	Male	Age- and sex-specific SEV for Low fruit					X	
Non-malignant brain and other central nervous system neoplasms	Global	Male	Age- and sex-specific SEV for Low vegetables					X	
Non-malignant brain and other central nervous system neoplasms	Data Rich	Male	Age- and sex-specific SEV for Low vegetables					X	
Non-malignant brain and other central nervous system neoplasms	Global	Male	Age- and sex-specific SEV for High red meat					X	
Non-malignant brain and other central nervous system neoplasms	Data Rich	Male	Age- and sex-specific SEV for High red meat					X	
Non-malignant brain and other central nervous system neoplasms	Global	Male	Cumulative Cigarettes (10 Years)				X		
Non-malignant brain and other central nervous system neoplasms	Data Rich	Male	Cumulative Cigarettes (10 Years)				X		
Non-malignant brain and other central nervous system neoplasms	Global	Male	Smoking Prevalence				X		
Non-malignant brain and other central nervous system neoplasms	Data Rich	Male	Smoking Prevalence				X		
Non-malignant brain and other central nervous system neoplasms	Global	Male	Liters of alcohol consumed per capita				X		
Non-malignant brain and other central nervous system neoplasms	Data Rich	Male	Liters of alcohol consumed per capita				X		
Other benign and in situ neoplasms	Data Rich	Female	Education (years per capita)						X
Other benign and in situ neoplasms	Global	Female	Education (years per capita)						X
Other benign and in situ neoplasms	Data Rich	Female	LDI (I$ per capita)						X
Other benign and in situ neoplasms	Global	Female	LDI (I$ per capita)						X
Other benign and in situ neoplasms	Data Rich	Female	Socio-demographic Index						X
Other benign and in situ neoplasms	Global	Female	Socio-demographic Index						X
Other benign and in situ neoplasms	Data Rich	Female	Liters of alcohol consumed per capita						X
Other benign and in situ neoplasms	Global	Female	Liters of alcohol consumed per capita						X
Other benign and in situ neoplasms	Data Rich	Female	Tobacco (cigarettes per capita)					X	
Other benign and in situ neoplasms	Global	Female	Tobacco (cigarettes per capita)					X	
Other benign and in situ neoplasms	Data Rich	Female	Cumulative Cigarettes (10 Years)					X	
Other benign and in situ neoplasms	Global	Female	Cumulative Cigarettes (10 Years)					X	
Other benign and in situ neoplasms	Data Rich	Female	Smoking Prevalence					X	
Other benign and in situ neoplasms	Global	Female	Smoking Prevalence					X	
Other benign and in situ neoplasms	Data Rich	Female	Healthcare access and quality index					X	
Other benign and in situ neoplasms	Global	Female	Healthcare access and quality index					X	
Other benign and in situ neoplasms	Data Rich	Male	Education (years per capita)						X
Other benign and in situ neoplasms	Global	Male	Education (years per capita)						X
Other benign and in situ neoplasms	Data Rich	Male	LDI (I$ per capita)						X
Other benign and in situ neoplasms	Global	Male	LDI (I$ per capita)						X
Other benign and in situ neoplasms	Data Rich	Male	Socio-demographic Index						X
Other benign and in situ neoplasms	Global	Male	Socio-demographic Index						X
Other benign and in situ neoplasms	Data Rich	Male	Liters of alcohol consumed per capita						X
Other benign and in situ neoplasms	Global	Male	Liters of alcohol consumed per capita						X
Other benign and in situ neoplasms	Data Rich	Male	Tobacco (cigarettes per capita)					X	
Other benign and in situ neoplasms	Global	Male	Tobacco (cigarettes per capita)					X	
Other benign and in situ neoplasms	Data Rich	Male	Cumulative Cigarettes (10 Years)					X	
Other benign and in situ neoplasms	Global	Male	Cumulative Cigarettes (10 Years)					X	
Other benign and in situ neoplasms	Data Rich	Male	Smoking Prevalence					X	
Other benign and in situ neoplasms	Global	Male	Smoking Prevalence					X	
Other benign and in situ neoplasms	Data Rich	Male	Healthcare access and quality index					X	
Other benign and in situ neoplasms	Global	Male	Healthcare access and quality index					X	
Cardiovascular diseases	Data Rich	Female	LDI (I$ per capita)			X			X
Cardiovascular diseases	Global	Female	LDI (I$ per capita)			X			X
Cardiovascular diseases	Data Rich	Female	Liters of alcohol consumed per capita			X			X
Cardiovascular diseases	Global	Female	Liters of alcohol consumed per capita			X			X
Cardiovascular diseases	Data Rich	Female	Pulses legumes unadjusted(g)			X			X
Cardiovascular diseases	Global	Female	Pulses legumes unadjusted(g)			X			X
Cardiovascular diseases	Data Rich	Female	Diet high in trans fatty acids			X			X
Cardiovascular diseases	Global	Female	Diet high in trans fatty acids			X			X
Cardiovascular diseases	Data Rich	Female	Age- and sex-specific SEV for Low fruit			X			X
Cardiovascular diseases	Global	Female	Age- and sex-specific SEV for Low fruit			X			X
Cardiovascular diseases	Data Rich	Female	Age- and sex-specific SEV for Low vegetables			X			X
Cardiovascular diseases	Global	Female	Age- and sex-specific SEV for Low vegetables			X			X
Cardiovascular diseases	Data Rich	Female	Age- and sex-specific SEV for Low nuts and seeds			X			X
Cardiovascular diseases	Global	Female	Age- and sex-specific SEV for Low nuts and seeds			X			X
Cardiovascular diseases	Data Rich	Female	Age- and sex-specific SEV for Low omega-3			X			X
Cardiovascular diseases	Global	Female	Age- and sex-specific SEV for Low omega-3			X			X
Cardiovascular diseases	Data Rich	Female	Age- and sex-specific SEV for Low PUFA			X			X
Cardiovascular diseases	Global	Female	Age- and sex-specific SEV for Low PUFA			X			X
Cardiovascular diseases	Data Rich	Female	Healthcare access and quality index		X			X	
Cardiovascular diseases	Global	Female	Healthcare access and quality index		X			X	
Cardiovascular diseases	Data Rich	Female	Mean BMI		X			X	
Cardiovascular diseases	Global	Female	Mean BMI		X			X	
Cardiovascular diseases	Data Rich	Female	Diabetes Fasting Plasma Glucose (mmol/L), age-standardized 25+		X			X	
Cardiovascular diseases	Global	Female	Diabetes Fasting Plasma Glucose (mmol/L), age-standardized 25+		X			X	
Cardiovascular diseases	Data Rich	Female	Indoor Air Pollution (All Cooking Fuels)		X			X	
Cardiovascular diseases	Global	Female	Indoor Air Pollution (All Cooking Fuels)		X			X	
Cardiovascular diseases	Data Rich	Female	Outdoor Air Pollution (PM2.5)		X			X	
Cardiovascular diseases	Global	Female	Outdoor Air Pollution (PM2.5)		X			X	
Cardiovascular diseases	Data Rich	Female	Elevation Over 1500m (proportion)		X			X	
Cardiovascular diseases	Global	Female	Elevation Over 1500m (proportion)		X			X	
Cardiovascular diseases	Data Rich	Female	Low-Density Lipoprotein (mmol/L)	X			X		
Cardiovascular diseases	Global	Female	Low-Density Lipoprotein (mmol/L)	X			X		
Cardiovascular diseases	Data Rich	Female	Smoking Prevalence	X			X		
Cardiovascular diseases	Global	Female	Smoking Prevalence	X			X		
Cardiovascular diseases	Data Rich	Female	Systolic Blood Pressure (mmHg)	X			X		
Cardiovascular diseases	Global	Female	Systolic Blood Pressure (mmHg)	X			X		
Cardiovascular diseases	Global	Male	LDI (I$ per capita)			X			X
Cardiovascular diseases	Data Rich	Male	LDI (I$ per capita)			X			X
Cardiovascular diseases	Global	Male	Liters of alcohol consumed per capita			X			X
Cardiovascular diseases	Data Rich	Male	Liters of alcohol consumed per capita			X			X
Cardiovascular diseases	Global	Male	Pulses legumes unadjusted(g)			X			X
Cardiovascular diseases	Data Rich	Male	Pulses legumes unadjusted(g)			X			X
Cardiovascular diseases	Global	Male	Diet high in trans fatty acids			X			X
Cardiovascular diseases	Data Rich	Male	Diet high in trans fatty acids			X			X

Appendix Table S11: Comparison of GBD 2021 and GBD 2023 covariates and level of covariates used in cause of death modeling

Cause	Model Version Type	Sex	Covariate	Level 1 GBD 2021	Level 2 GBD 2021	Level 3 GBD 2021	Level 1 GBD 2023	Level 2 GBD 2023	Level 3 GBD 2023
Cardiovascular diseases	Global	Male	Age- and sex-specific SEV for Low fruit			X			X
Cardiovascular diseases	Data Rich	Male	Age- and sex-specific SEV for Low fruit			X			X
Cardiovascular diseases	Global	Male	Age- and sex-specific SEV for Low vegetables			X			X
Cardiovascular diseases	Data Rich	Male	Age- and sex-specific SEV for Low vegetables			X			X
Cardiovascular diseases	Global	Male	Age- and sex-specific SEV for Low nuts and seeds			X			X
Cardiovascular diseases	Data Rich	Male	Age- and sex-specific SEV for Low nuts and seeds			X			X
Cardiovascular diseases	Global	Male	Age- and sex-specific SEV for Low omega-3			X			X
Cardiovascular diseases	Data Rich	Male	Age- and sex-specific SEV for Low omega-3			X			X
Cardiovascular diseases	Global	Male	Age- and sex-specific SEV for Low PUFA			X			X
Cardiovascular diseases	Data Rich	Male	Age- and sex-specific SEV for Low PUFA			X			X
Cardiovascular diseases	Global	Male	Healthcare access and quality index		X			X	
Cardiovascular diseases	Data Rich	Male	Healthcare access and quality index		X			X	
Cardiovascular diseases	Global	Male	Mean BMI		X			X	
Cardiovascular diseases	Data Rich	Male	Mean BMI		X			X	
Cardiovascular diseases	Global	Male	Diabetes Fasting Plasma Glucose (mmol/L), age-standardized 25+		X			X	
Cardiovascular diseases	Data Rich	Male	Diabetes Fasting Plasma Glucose (mmol/L), age-standardized 25+		X			X	
Cardiovascular diseases	Global	Male	Indoor Air Pollution (All Cooking Fuels)		X			X	
Cardiovascular diseases	Data Rich	Male	Indoor Air Pollution (All Cooking Fuels)		X			X	
Cardiovascular diseases	Global	Male	Outdoor Air Pollution (PM2.5)		X			X	
Cardiovascular diseases	Data Rich	Male	Outdoor Air Pollution (PM2.5)		X			X	
Cardiovascular diseases	Global	Male	Elevation Over 1500m (proportion)		X			X	
Cardiovascular diseases	Data Rich	Male	Elevation Over 1500m (proportion)		X			X	
Cardiovascular diseases	Global	Male	Low-Density Lipoprotein (mmol/L)	X			X		
Cardiovascular diseases	Data Rich	Male	Low-Density Lipoprotein (mmol/L)	X			X		
Cardiovascular diseases	Global	Male	Smoking Prevalence	X			X		
Cardiovascular diseases	Data Rich	Male	Smoking Prevalence	X			X		
Cardiovascular diseases	Global	Male	Systolic Blood Pressure (mmHg)	X			X		
Cardiovascular diseases	Data Rich	Male	Systolic Blood Pressure (mmHg)	X			X		
Rheumatic heart disease	Data Rich	Female	Education (years per capita)			X			X
Rheumatic heart disease	Global	Female	Education (years per capita)			X			X
Rheumatic heart disease	Data Rich	Female	LDI (I$ per capita)			X			X
Rheumatic heart disease	Global	Female	LDI (I$ per capita)			X			X
Rheumatic heart disease	Data Rich	Female	Healthcare access and quality index		X			X	
Rheumatic heart disease	Global	Female	Healthcare access and quality index		X			X	
Rheumatic heart disease	Data Rich	Female	Sanitation (proportion with access)	X			X		
Rheumatic heart disease	Global	Female	Sanitation (proportion with access)	X			X		
Rheumatic heart disease	Data Rich	Female	Improved Water Source (proportion with access)	X			X		
Rheumatic heart disease	Global	Female	Improved Water Source (proportion with access)	X			X		
Rheumatic heart disease	Data Rich	Female	Age- and sex-specific SEV for Child underweight	X			X		
Rheumatic heart disease	Global	Female	Age- and sex-specific SEV for Child underweight	X			X		
Rheumatic heart disease	Global	Female	Socio-demographic Index						X
Rheumatic heart disease	Data Rich	Male	Education (years per capita)			X			X
Rheumatic heart disease	Global	Male	Education (years per capita)			X			X
Rheumatic heart disease	Data Rich	Male	LDI (I$ per capita)			X			X
Rheumatic heart disease	Global	Male	LDI (I$ per capita)			X			X
Rheumatic heart disease	Global	Male	Socio-demographic Index			X			X
Rheumatic heart disease	Data Rich	Male	Healthcare access and quality index		X			X	
Rheumatic heart disease	Global	Male	Healthcare access and quality index		X			X	
Rheumatic heart disease	Data Rich	Male	Sanitation (proportion with access)	X			X		
Rheumatic heart disease	Global	Male	Sanitation (proportion with access)	X			X		
Rheumatic heart disease	Data Rich	Male	Improved Water Source (proportion with access)	X			X		
Rheumatic heart disease	Global	Male	Improved Water Source (proportion with access)	X			X		
Rheumatic heart disease	Data Rich	Male	Age- and sex-specific SEV for Child underweight	X			X		
Rheumatic heart disease	Global	Male	Age- and sex-specific SEV for Child underweight	X			X		
Ischaemic heart disease	Data Rich	Female	LDI (I$ per capita)			X			X
Ischaemic heart disease	Global	Female	LDI (I$ per capita)			X			X
Ischaemic heart disease	Data Rich	Female	Liters of alcohol consumed per capita			X			X
Ischaemic heart disease	Global	Female	Liters of alcohol consumed per capita			X			X
Ischaemic heart disease	Data Rich	Female	Pulses legumes unadjusted(g)			X			X
Ischaemic heart disease	Global	Female	Pulses legumes unadjusted(g)			X			X
Ischaemic heart disease	Data Rich	Female	Diet high in trans fatty acids			X			X
Ischaemic heart disease	Global	Female	Diet high in trans fatty acids			X			X
Ischaemic heart disease	Data Rich	Female	Age- and sex-specific SEV for Low fruit			X			X
Ischaemic heart disease	Global	Female	Age- and sex-specific SEV for Low fruit			X			X
Ischaemic heart disease	Data Rich	Female	Age- and sex-specific SEV for Low vegetables			X			X
Ischaemic heart disease	Global	Female	Age- and sex-specific SEV for Low vegetables			X			X
Ischaemic heart disease	Data Rich	Female	Age- and sex-specific SEV for Low nuts and seeds			X			X
Ischaemic heart disease	Global	Female	Age- and sex-specific SEV for Low nuts and seeds			X			X
Ischaemic heart disease	Data Rich	Female	Age- and sex-specific SEV for Low omega-3			X			X
Ischaemic heart disease	Global	Female	Age- and sex-specific SEV for Low omega-3			X			X
Ischaemic heart disease	Data Rich	Female	Age- and sex-specific SEV for Low PUFA			X			X
Ischaemic heart disease	Global	Female	Age- and sex-specific SEV for Low PUFA			X			X
Ischaemic heart disease	Data Rich	Female	Healthcare access and quality index		X			X	
Ischaemic heart disease	Global	Female	Healthcare access and quality index		X			X	
Ischaemic heart disease	Data Rich	Female	Diabetes Fasting Plasma Glucose (mmol/L), age-standardized 25+		X			X	
Ischaemic heart disease	Global	Female	Diabetes Fasting Plasma Glucose (mmol/L), age-standardized 25+		X			X	
Ischaemic heart disease	Data Rich	Female	Mean BMI		X			X	
Ischaemic heart disease	Global	Female	Mean BMI		X			X	
Ischaemic heart disease	Data Rich	Female	Indoor Air Pollution (All Cooking Fuels)		X			X	
Ischaemic heart disease	Global	Female	Indoor Air Pollution (All Cooking Fuels)		X			X	
Ischaemic heart disease	Data Rich	Female	Outdoor Air Pollution (PM2.5)		X			X	
Ischaemic heart disease	Global	Female	Outdoor Air Pollution (PM2.5)		X			X	
Ischaemic heart disease	Data Rich	Female	Elevation Over 1500m (proportion)		X			X	
Ischaemic heart disease	Global	Female	Elevation Over 1500m (proportion)		X			X	
Ischaemic heart disease	Data Rich	Female	Low-Density Lipoprotein (mmol/L)	X			X		
Ischaemic heart disease	Global	Female	Low-Density Lipoprotein (mmol/L)	X			X		
Ischaemic heart disease	Data Rich	Female	Smoking Prevalence	X			X		
Ischaemic heart disease	Global	Female	Smoking Prevalence	X			X		
Ischaemic heart disease	Data Rich	Female	Systolic Blood Pressure (mmHg)	X			X		
Ischaemic heart disease	Global	Female	Systolic Blood Pressure (mmHg)	X			X		
Ischaemic heart disease	Data Rich	Female	Log-transformed SEV scalar: IHD	X			X		
Ischaemic heart disease	Global	Female	Log-transformed SEV scalar: IHD	X			X		
Ischaemic heart disease	Data Rich	Male	LDI (I$ per capita)			X			X
Ischaemic heart disease	Global	Male	LDI (I$ per capita)			X			X
Ischaemic heart disease	Data Rich	Male	Liters of alcohol consumed per capita			X			X
Ischaemic heart disease	Global	Male	Liters of alcohol consumed per capita			X			X
Ischaemic heart disease	Data Rich	Male	Pulses legumes unadjusted(g)			X			X
Ischaemic heart disease	Global	Male	Pulses legumes unadjusted(g)			X			X
Ischaemic heart disease	Data Rich	Male	Diet high in trans fatty acids			X			X
Ischaemic heart disease	Global	Male	Diet high in trans fatty acids			X			X
Ischaemic heart disease	Data Rich	Male	Age- and sex-specific SEV for Low fruit			X			X
Ischaemic heart disease	Global	Male	Age- and sex-specific SEV for Low fruit			X			X
Ischaemic heart disease	Data Rich	Male	Age- and sex-specific SEV for Low vegetables			X			X
Ischaemic heart disease	Global	Male	Age- and sex-specific SEV for Low vegetables			X			X
Ischaemic heart disease	Data Rich	Male	Age- and sex-specific SEV for Low nuts and seeds			X			X
Ischaemic heart disease	Global	Male	Age- and sex-specific SEV for Low nuts and seeds			X			X
Ischaemic heart disease	Data Rich	Male	Age- and sex-specific SEV for Low omega-3			X			X
Ischaemic heart disease	Global	Male	Age- and sex-specific SEV for Low omega-3			X			X
Ischaemic heart disease	Data Rich	Male	Age- and sex-specific SEV for Low PUFA			X			X
Ischaemic heart disease	Global	Male	Age- and sex-specific SEV for Low PUFA			X			X
Ischaemic heart disease	Data Rich	Male	Healthcare access and quality index		X			X	

Appendix Table S11: Comparison of GBD 2021 and GBD 2023 covariates and level of covariates used in cause of death modeling									
Cause	Model Version Type	Sex	Covariate	Level 1 GBD 2021	Level 2 GBD 2021	Level 3 GBD 2021	Level 1 GBD 2023	Level 2 GBD 2023	Level 3 GBD 2023
Ischaemic heart disease	Global	Male	Healthcare access and quality index		X			X	
Ischaemic heart disease	Data Rich	Male	Diabetes Fasting Plasma Glucose (mmol/L), age-standardized 25+		X			X	
Ischaemic heart disease	Global	Male	Diabetes Fasting Plasma Glucose (mmol/L), age-standardized 25+		X			X	
Ischaemic heart disease	Data Rich	Male	Mean BMI		X			X	
Ischaemic heart disease	Global	Male	Mean BMI		X			X	
Ischaemic heart disease	Data Rich	Male	Indoor Air Pollution (All Cooking Fuels)		X			X	
Ischaemic heart disease	Global	Male	Indoor Air Pollution (All Cooking Fuels)		X			X	
Ischaemic heart disease	Data Rich	Male	Outdoor Air Pollution (PM2.5)		X			X	
Ischaemic heart disease	Global	Male	Outdoor Air Pollution (PM2.5)		X			X	
Ischaemic heart disease	Data Rich	Male	Elevation Over 1500m (proportion)		X			X	
Ischaemic heart disease	Global	Male	Elevation Over 1500m (proportion)		X			X	
Ischaemic heart disease	Data Rich	Male	Low-Density Lipoprotein (mmol/L)	X			X		
Ischaemic heart disease	Global	Male	Low-Density Lipoprotein (mmol/L)	X			X		
Ischaemic heart disease	Data Rich	Male	Smoking Prevalence	X			X		
Ischaemic heart disease	Global	Male	Smoking Prevalence	X			X		
Ischaemic heart disease	Data Rich	Male	Systolic Blood Pressure (mmHg)	X			X		
Ischaemic heart disease	Global	Male	Systolic Blood Pressure (mmHg)	X			X		
Ischaemic heart disease	Data Rich	Male	Log-transformed SEV scalar: IHD	X			X		
Ischaemic heart disease	Global	Male	Log-transformed SEV scalar: IHD	X			X		
Stroke	Data Rich	Female	LDI (I$ per capita)			X			X
Stroke	Global	Female	LDI (I$ per capita)			X			X
Stroke	Data Rich	Female	Liters of alcohol consumed per capita			X			X
Stroke	Global	Female	Liters of alcohol consumed per capita			X			X
Stroke	Data Rich	Female	Pulses legumes unadjusted(g)			X			X
Stroke	Global	Female	Pulses legumes unadjusted(g)			X			X
Stroke	Data Rich	Female	Diet high in trans fatty acids			X			X
Stroke	Global	Female	Diet high in trans fatty acids			X			X
Stroke	Data Rich	Female	Age- and sex-specific SEV for Low fruit			X			X
Stroke	Global	Female	Age- and sex-specific SEV for Low fruit			X			X
Stroke	Data Rich	Female	Age- and sex-specific SEV for Low vegetables			X			X
Stroke	Global	Female	Age- and sex-specific SEV for Low vegetables			X			X
Stroke	Data Rich	Female	Age- and sex-specific SEV for Low nuts and seeds			X			X
Stroke	Global	Female	Age- and sex-specific SEV for Low nuts and seeds			X			X
Stroke	Data Rich	Female	Age- and sex-specific SEV for Low omega-3			X			X
Stroke	Global	Female	Age- and sex-specific SEV for Low omega-3			X			X
Stroke	Data Rich	Female	Age- and sex-specific SEV for Low PUFA			X			X
Stroke	Global	Female	Age- and sex-specific SEV for Low PUFA			X			X
Stroke	Data Rich	Female	Healthcare access and quality index		X			X	
Stroke	Global	Female	Healthcare access and quality index		X			X	
Stroke	Data Rich	Female	Diabetes Fasting Plasma Glucose (mmol/L), age-standardized 25+		X			X	
Stroke	Global	Female	Diabetes Fasting Plasma Glucose (mmol/L), age-standardized 25+		X			X	
Stroke	Data Rich	Female	Mean BMI		X			X	
Stroke	Global	Female	Mean BMI		X			X	
Stroke	Data Rich	Female	Indoor Air Pollution (All Cooking Fuels)		X			X	
Stroke	Global	Female	Indoor Air Pollution (All Cooking Fuels)		X			X	
Stroke	Data Rich	Female	Outdoor Air Pollution (PM2.5)		X			X	
Stroke	Global	Female	Outdoor Air Pollution (PM2.5)		X			X	
Stroke	Data Rich	Female	Elevation Over 1500m (proportion)		X			X	
Stroke	Global	Female	Elevation Over 1500m (proportion)		X			X	
Stroke	Data Rich	Female	Low-Density Lipoprotein (mmol/L)	X			X		
Stroke	Global	Female	Low-Density Lipoprotein (mmol/L)	X			X		
Stroke	Data Rich	Female	Smoking Prevalence	X			X		
Stroke	Global	Female	Smoking Prevalence	X			X		
Stroke	Data Rich	Female	Systolic Blood Pressure (mmHg)	X			X		
Stroke	Global	Female	Systolic Blood Pressure (mmHg)	X			X		
Stroke	Data Rich	Female	Log-transformed SEV scalar: Stroke	X			X		
Stroke	Global	Female	Log-transformed SEV scalar: Stroke	X			X		
Stroke	Global	Male	LDI (I$ per capita)			X			X
Stroke	Data Rich	Male	LDI (I$ per capita)			X			X
Stroke	Global	Male	Liters of alcohol consumed per capita			X			X
Stroke	Data Rich	Male	Liters of alcohol consumed per capita			X			X
Stroke	Global	Male	Pulses legumes unadjusted(g)			X			X
Stroke	Data Rich	Male	Pulses legumes unadjusted(g)			X			X
Stroke	Global	Male	Diet high in trans fatty acids			X			X
Stroke	Data Rich	Male	Diet high in trans fatty acids			X			X
Stroke	Global	Male	Age- and sex-specific SEV for Low fruit			X			X
Stroke	Data Rich	Male	Age- and sex-specific SEV for Low fruit			X			X
Stroke	Global	Male	Age- and sex-specific SEV for Low vegetables			X			X
Stroke	Data Rich	Male	Age- and sex-specific SEV for Low vegetables			X			X
Stroke	Global	Male	Age- and sex-specific SEV for Low nuts and seeds			X			X
Stroke	Data Rich	Male	Age- and sex-specific SEV for Low nuts and seeds			X			X
Stroke	Global	Male	Age- and sex-specific SEV for Low omega-3			X			X
Stroke	Data Rich	Male	Age- and sex-specific SEV for Low omega-3			X			X
Stroke	Global	Male	Age- and sex-specific SEV for Low PUFA			X			X
Stroke	Data Rich	Male	Age- and sex-specific SEV for Low PUFA			X			X
Stroke	Global	Male	Healthcare access and quality index		X			X	
Stroke	Data Rich	Male	Healthcare access and quality index		X			X	
Stroke	Global	Male	Diabetes Fasting Plasma Glucose (mmol/L), age-standardized 25+		X			X	
Stroke	Data Rich	Male	Diabetes Fasting Plasma Glucose (mmol/L), age-standardized 25+		X			X	
Stroke	Global	Male	Mean BMI		X			X	
Stroke	Data Rich	Male	Mean BMI		X			X	
Stroke	Global	Male	Indoor Air Pollution (All Cooking Fuels)		X			X	
Stroke	Data Rich	Male	Indoor Air Pollution (All Cooking Fuels)		X			X	
Stroke	Global	Male	Outdoor Air Pollution (PM2.5)		X			X	
Stroke	Data Rich	Male	Outdoor Air Pollution (PM2.5)		X			X	
Stroke	Global	Male	Elevation Over 1500m (proportion)		X			X	
Stroke	Data Rich	Male	Elevation Over 1500m (proportion)		X			X	
Stroke	Global	Male	Low-Density Lipoprotein (mmol/L)	X			X		
Stroke	Data Rich	Male	Low-Density Lipoprotein (mmol/L)	X			X		
Stroke	Global	Male	Smoking Prevalence	X			X		
Stroke	Data Rich	Male	Smoking Prevalence	X			X		
Stroke	Global	Male	Systolic Blood Pressure (mmHg)	X			X		
Stroke	Data Rich	Male	Systolic Blood Pressure (mmHg)	X			X		
Stroke	Global	Male	Log-transformed SEV scalar: Stroke	X			X		
Stroke	Data Rich	Male	Log-transformed SEV scalar: Stroke	X			X		
Ischaemic stroke	Data Rich	Female	LDI (I$ per capita)			X			X
Ischaemic stroke	Global	Female	LDI (I$ per capita)			X			X
Ischaemic stroke	Data Rich	Female	Liters of alcohol consumed per capita			X			X
Ischaemic stroke	Global	Female	Liters of alcohol consumed per capita			X			X
Ischaemic stroke	Data Rich	Female	Pulses legumes unadjusted(g)			X			X
Ischaemic stroke	Global	Female	Pulses legumes unadjusted(g)			X			X
Ischaemic stroke	Data Rich	Female	Diet high in trans fatty acids			X			X
Ischaemic stroke	Global	Female	Diet high in trans fatty acids			X			X
Ischaemic stroke	Data Rich	Female	Age- and sex-specific SEV for Low fruit			X			X
Ischaemic stroke	Global	Female	Age- and sex-specific SEV for Low fruit			X			X
Ischaemic stroke	Data Rich	Female	Age- and sex-specific SEV for Low vegetables			X			X
Ischaemic stroke	Global	Female	Age- and sex-specific SEV for Low vegetables			X			X
Ischaemic stroke	Data Rich	Female	Age- and sex-specific SEV for Low nuts and seeds			X			X
Ischaemic stroke	Global	Female	Age- and sex-specific SEV for Low nuts and seeds			X			X
Ischaemic stroke	Data Rich	Female	Age- and sex-specific SEV for Low omega-3			X			X
Ischaemic stroke	Global	Female	Age- and sex-specific SEV for Low omega-3			X			X
Ischaemic stroke	Data Rich	Female	Age- and sex-specific SEV for Low PUFA			X			X

Appendix Table S11: Comparison of GBD 2021 and GBD 2023 covariates and level of covariates used in cause of death modeling

Cause	Model Version Type	Sex	Covariate	Level 1 GBD 2021	Level 2 GBD 2021	Level 3 GBD 2021	Level 1 GBD 2023	Level 2 GBD 2023	Level 3 GBD 2023
Ischaemic stroke	Global	Female	Age- and sex-specific SEV for Low PUFA			X			X
Ischaemic stroke	Data Rich	Female	Healthcare access and quality index		X			X	
Ischaemic stroke	Global	Female	Healthcare access and quality index		X			X	
Ischaemic stroke	Data Rich	Female	Diabetes Fasting Plasma Glucose (mmol/L), age-standardized 25+		X			X	
Ischaemic stroke	Global	Female	Diabetes Fasting Plasma Glucose (mmol/L), age-standardized 25+		X			X	
Ischaemic stroke	Data Rich	Female	Mean BMI		X			X	
Ischaemic stroke	Global	Female	Mean BMI		X			X	
Ischaemic stroke	Data Rich	Female	Indoor Air Pollution (All Cooking Fuels)		X			X	
Ischaemic stroke	Global	Female	Indoor Air Pollution (All Cooking Fuels)		X			X	
Ischaemic stroke	Data Rich	Female	Outdoor Air Pollution (PM2.5)		X			X	
Ischaemic stroke	Global	Female	Outdoor Air Pollution (PM2.5)		X			X	
Ischaemic stroke	Data Rich	Female	Elevation Over 1500m (proportion)		X			X	
Ischaemic stroke	Global	Female	Elevation Over 1500m (proportion)		X			X	
Ischaemic stroke	Data Rich	Female	Low-Density Lipoprotein (mmol/L)	X			X		
Ischaemic stroke	Global	Female	Low-Density Lipoprotein (mmol/L)	X			X		
Ischaemic stroke	Data Rich	Female	Smoking Prevalence	X			X		
Ischaemic stroke	Global	Female	Smoking Prevalence	X			X		
Ischaemic stroke	Data Rich	Female	Systolic Blood Pressure (mmHg)	X			X		
Ischaemic stroke	Global	Female	Systolic Blood Pressure (mmHg)	X			X		
Ischaemic stroke	Data Rich	Female	Log-transformed SEV scalar: Isch Stroke	X			X		
Ischaemic stroke	Global	Female	Log-transformed SEV scalar: Isch Stroke	X			X		
Ischaemic stroke	Global	Male	LDI (I$ per capita)			X			X
Ischaemic stroke	Data Rich	Male	LDI (I$ per capita)			X			X
Ischaemic stroke	Global	Male	Liters of alcohol consumed per capita			X			X
Ischaemic stroke	Data Rich	Male	Liters of alcohol consumed per capita			X			X
Ischaemic stroke	Global	Male	Pulses legumes unadjusted(g)			X			X
Ischaemic stroke	Data Rich	Male	Pulses legumes unadjusted(g)			X			X
Ischaemic stroke	Global	Male	Diet high in trans fatty acids			X			X
Ischaemic stroke	Data Rich	Male	Diet high in trans fatty acids			X			X
Ischaemic stroke	Global	Male	Age- and sex-specific SEV for Low fruit			X			X
Ischaemic stroke	Data Rich	Male	Age- and sex-specific SEV for Low fruit			X			X
Ischaemic stroke	Global	Male	Age- and sex-specific SEV for Low vegetables			X			X
Ischaemic stroke	Data Rich	Male	Age- and sex-specific SEV for Low vegetables			X			X
Ischaemic stroke	Global	Male	Age- and sex-specific SEV for Low nuts and seeds			X			X
Ischaemic stroke	Data Rich	Male	Age- and sex-specific SEV for Low nuts and seeds			X			X
Ischaemic stroke	Global	Male	Age- and sex-specific SEV for Low omega-3			X			X
Ischaemic stroke	Data Rich	Male	Age- and sex-specific SEV for Low omega-3			X			X
Ischaemic stroke	Global	Male	Age- and sex-specific SEV for Low PUFA			X			X
Ischaemic stroke	Data Rich	Male	Age- and sex-specific SEV for Low PUFA			X			X
Ischaemic stroke	Global	Male	Healthcare access and quality index		X			X	
Ischaemic stroke	Data Rich	Male	Healthcare access and quality index		X			X	
Ischaemic stroke	Global	Male	Diabetes Fasting Plasma Glucose (mmol/L), age-standardized 25+		X			X	
Ischaemic stroke	Data Rich	Male	Diabetes Fasting Plasma Glucose (mmol/L), age-standardized 25+		X			X	
Ischaemic stroke	Global	Male	Mean BMI		X			X	
Ischaemic stroke	Data Rich	Male	Mean BMI		X			X	
Ischaemic stroke	Global	Male	Indoor Air Pollution (All Cooking Fuels)		X			X	
Ischaemic stroke	Data Rich	Male	Indoor Air Pollution (All Cooking Fuels)		X			X	
Ischaemic stroke	Global	Male	Outdoor Air Pollution (PM2.5)		X			X	
Ischaemic stroke	Data Rich	Male	Outdoor Air Pollution (PM2.5)		X			X	
Ischaemic stroke	Global	Male	Elevation Over 1500m (proportion)		X			X	
Ischaemic stroke	Data Rich	Male	Elevation Over 1500m (proportion)		X			X	
Ischaemic stroke	Global	Male	Low-Density Lipoprotein (mmol/L)	X			X		
Ischaemic stroke	Data Rich	Male	Low-Density Lipoprotein (mmol/L)	X			X		
Ischaemic stroke	Global	Male	Smoking Prevalence	X			X		
Ischaemic stroke	Data Rich	Male	Smoking Prevalence	X			X		
Ischaemic stroke	Global	Male	Systolic Blood Pressure (mmHg)	X			X		
Ischaemic stroke	Data Rich	Male	Systolic Blood Pressure (mmHg)	X			X		
Ischaemic stroke	Global	Male	Log-transformed SEV scalar: Isch Stroke	X			X		
Ischaemic stroke	Data Rich	Male	Log-transformed SEV scalar: Isch Stroke	X			X		
Intracerebral hemorrhage	Data Rich	Female	LDI (I$ per capita)			X			X
Intracerebral hemorrhage	Global	Female	LDI (I$ per capita)			X			X
Intracerebral hemorrhage	Data Rich	Female	Low-Density Lipoprotein (mmol/L)			X			X
Intracerebral hemorrhage	Global	Female	Low-Density Lipoprotein (mmol/L)			X			X
Intracerebral hemorrhage	Data Rich	Female	Liters of alcohol consumed per capita			X			X
Intracerebral hemorrhage	Global	Female	Liters of alcohol consumed per capita			X			X
Intracerebral hemorrhage	Data Rich	Female	Pulses legumes unadjusted(g)			X			X
Intracerebral hemorrhage	Global	Female	Pulses legumes unadjusted(g)			X			X
Intracerebral hemorrhage	Data Rich	Female	Diet high in trans fatty acids			X			X
Intracerebral hemorrhage	Global	Female	Diet high in trans fatty acids			X			X
Intracerebral hemorrhage	Data Rich	Female	Age- and sex-specific SEV for Low fruit			X			X
Intracerebral hemorrhage	Global	Female	Age- and sex-specific SEV for Low fruit			X			X
Intracerebral hemorrhage	Global	Female	Age- and sex-specific SEV for Low vegetables			X			X
Intracerebral hemorrhage	Data Rich	Female	Age- and sex-specific SEV for Low nuts and seeds			X			X
Intracerebral hemorrhage	Global	Female	Age- and sex-specific SEV for Low nuts and seeds			X			X
Intracerebral hemorrhage	Data Rich	Female	Age- and sex-specific SEV for Low omega-3			X			X
Intracerebral hemorrhage	Global	Female	Age- and sex-specific SEV for Low omega-3			X			X
Intracerebral hemorrhage	Data Rich	Female	Age- and sex-specific SEV for Low PUFA			X			X
Intracerebral hemorrhage	Global	Female	Age- and sex-specific SEV for Low PUFA			X			X
Intracerebral hemorrhage	Data Rich	Female	Healthcare access and quality index		X			X	
Intracerebral hemorrhage	Global	Female	Healthcare access and quality index		X			X	
Intracerebral hemorrhage	Data Rich	Female	Diabetes Fasting Plasma Glucose (mmol/L), age-standardized 25+		X			X	
Intracerebral hemorrhage	Global	Female	Diabetes Fasting Plasma Glucose (mmol/L), age-standardized 25+		X			X	
Intracerebral hemorrhage	Data Rich	Female	Mean BMI		X			X	
Intracerebral hemorrhage	Global	Female	Mean BMI		X			X	
Intracerebral hemorrhage	Data Rich	Female	Indoor Air Pollution (All Cooking Fuels)		X			X	
Intracerebral hemorrhage	Global	Female	Indoor Air Pollution (All Cooking Fuels)		X			X	
Intracerebral hemorrhage	Data Rich	Female	Outdoor Air Pollution (PM2.5)		X			X	
Intracerebral hemorrhage	Global	Female	Outdoor Air Pollution (PM2.5)		X			X	
Intracerebral hemorrhage	Data Rich	Female	Elevation Over 1500m (proportion)		X			X	
Intracerebral hemorrhage	Global	Female	Elevation Over 1500m (proportion)		X			X	
Intracerebral hemorrhage	Data Rich	Female	Smoking Prevalence	X			X		
Intracerebral hemorrhage	Global	Female	Smoking Prevalence	X			X		
Intracerebral hemorrhage	Data Rich	Female	Systolic Blood Pressure (mmHg)	X			X		
Intracerebral hemorrhage	Global	Female	Systolic Blood Pressure (mmHg)	X			X		
Intracerebral hemorrhage	Data Rich	Female	Log-transformed SEV scalar: Intrahem Stroke	X			X		
Intracerebral hemorrhage	Global	Female	Log-transformed SEV scalar: Intrahem Stroke	X			X		
Intracerebral hemorrhage	Data Rich	Female	Age- and sex-specific SEV for Low vegetables						X
Intracerebral hemorrhage	Data Rich	Male	LDI (I$ per capita)			X			X
Intracerebral hemorrhage	Global	Male	LDI (I$ per capita)			X			X
Intracerebral hemorrhage	Data Rich	Male	Low-Density Lipoprotein (mmol/L)			X			X
Intracerebral hemorrhage	Global	Male	Low-Density Lipoprotein (mmol/L)			X			X
Intracerebral hemorrhage	Data Rich	Male	Liters of alcohol consumed per capita			X			X
Intracerebral hemorrhage	Global	Male	Liters of alcohol consumed per capita			X			X
Intracerebral hemorrhage	Data Rich	Male	Pulses legumes unadjusted(g)			X			X
Intracerebral hemorrhage	Global	Male	Pulses legumes unadjusted(g)			X			X
Intracerebral hemorrhage	Data Rich	Male	Diet high in trans fatty acids			X			X
Intracerebral hemorrhage	Global	Male	Diet high in trans fatty acids			X			X
Intracerebral hemorrhage	Data Rich	Male	Age- and sex-specific SEV for Low fruit			X			X
Intracerebral hemorrhage	Global	Male	Age- and sex-specific SEV for Low fruit			X			X
Intracerebral hemorrhage	Data Rich	Male	Age- and sex-specific SEV for Low vegetables			X			X
Intracerebral hemorrhage	Global	Male	Age- and sex-specific SEV for Low vegetables			X			X

Appendix Table S11: Comparison of GBD 2021 and GBD 2023 covariates and level of covariates used in cause of death modeling

Cause	Model Version Type	Sex	Covariate	Level 1 GBD 2021	Level 2 GBD 2021	Level 3 GBD 2021	Level 1 GBD 2023	Level 2 GBD 2023	Level 3 GBD 2023
Intracerebral hemorrhage	Data Rich	Male	Age- and sex-specific SEV for Low nuts and seeds			X			X
Intracerebral hemorrhage	Global	Male	Age- and sex-specific SEV for Low nuts and seeds			X			X
Intracerebral hemorrhage	Data Rich	Male	Age- and sex-specific SEV for Low omega 3			X			X
Intracerebral hemorrhage	Global	Male	Age- and sex-specific SEV for Low omega 3			X			X
Intracerebral hemorrhage	Data Rich	Male	Age- and sex-specific SEV for Low PUFA			X			X
Intracerebral hemorrhage	Global	Male	Age- and sex-specific SEV for Low PUFA			X			X
Intracerebral hemorrhage	Data Rich	Male	Healthcare access and quality index		X			X	
Intracerebral hemorrhage	Global	Male	Healthcare access and quality index		X			X	
Intracerebral hemorrhage	Data Rich	Male	Diabetes Fasting Plasma Glucose (mmol/L), age-standardized 25+		X			X	
Intracerebral hemorrhage	Global	Male	Diabetes Fasting Plasma Glucose (mmol/L), age-standardized 25+		X			X	
Intracerebral hemorrhage	Data Rich	Male	Mean BMI		X			X	
Intracerebral hemorrhage	Global	Male	Mean BMI		X			X	
Intracerebral hemorrhage	Data Rich	Male	Indoor Air Pollution (All Cooking Fuels)		X			X	
Intracerebral hemorrhage	Global	Male	Indoor Air Pollution (All Cooking Fuels)		X			X	
Intracerebral hemorrhage	Data Rich	Male	Outdoor Air Pollution (PM2.5)		X			X	
Intracerebral hemorrhage	Global	Male	Outdoor Air Pollution (PM2.5)		X			X	
Intracerebral hemorrhage	Data Rich	Male	Elevation Over 1500m (proportion)		X			X	
Intracerebral hemorrhage	Global	Male	Elevation Over 1500m (proportion)		X			X	
Intracerebral hemorrhage	Data Rich	Male	Smoking Prevalence	X			X		
Intracerebral hemorrhage	Global	Male	Smoking Prevalence	X			X		
Intracerebral hemorrhage	Data Rich	Male	Systolic Blood Pressure (mmHg)	X			X		
Intracerebral hemorrhage	Global	Male	Systolic Blood Pressure (mmHg)	X			X		
Intracerebral hemorrhage	Data Rich	Male	Log-transformed SEV scalar: Intrahem Stroke	X			X		
Intracerebral hemorrhage	Global	Male	Log-transformed SEV scalar: Intrahem Stroke	X			X		
Subarachnoid hemorrhage	Data Rich	Female	LDI (I$ per capita)			X			X
Subarachnoid hemorrhage	Global	Female	LDI (I$ per capita)			X			X
Subarachnoid hemorrhage	Data Rich	Female	Liters of alcohol consumed per capita			X			X
Subarachnoid hemorrhage	Global	Female	Liters of alcohol consumed per capita			X			X
Subarachnoid hemorrhage	Data Rich	Female	Healthcare access and quality index		X			X	
Subarachnoid hemorrhage	Global	Female	Healthcare access and quality index		X			X	
Subarachnoid hemorrhage	Data Rich	Female	Smoking Prevalence	X			X		
Subarachnoid hemorrhage	Global	Female	Smoking Prevalence	X			X		
Subarachnoid hemorrhage	Data Rich	Female	Systolic Blood Pressure (mmHg)	X			X		
Subarachnoid hemorrhage	Global	Female	Systolic Blood Pressure (mmHg)	X			X		
Subarachnoid hemorrhage	Data Rich	Female	Log-transformed SEV scalar: Sub Hem	X			X		
Subarachnoid hemorrhage	Global	Female	Log-transformed SEV scalar: Sub Hem	X			X		
Subarachnoid hemorrhage	Data Rich	Male	LDI (I$ per capita)			X			X
Subarachnoid hemorrhage	Global	Male	LDI (I$ per capita)			X			X
Subarachnoid hemorrhage	Data Rich	Male	Liters of alcohol consumed per capita			X			X
Subarachnoid hemorrhage	Global	Male	Liters of alcohol consumed per capita			X			X
Subarachnoid hemorrhage	Data Rich	Male	Healthcare access and quality index		X			X	
Subarachnoid hemorrhage	Global	Male	Healthcare access and quality index		X			X	
Subarachnoid hemorrhage	Data Rich	Male	Smoking Prevalence	X			X		
Subarachnoid hemorrhage	Global	Male	Smoking Prevalence	X			X		
Subarachnoid hemorrhage	Data Rich	Male	Systolic Blood Pressure (mmHg)	X			X		
Subarachnoid hemorrhage	Global	Male	Systolic Blood Pressure (mmHg)	X			X		
Subarachnoid hemorrhage	Data Rich	Male	Log-transformed SEV scalar: Sub Hem	X			X		
Subarachnoid hemorrhage	Global	Male	Log-transformed SEV scalar: Sub Hem	X			X		
Hypertensive heart disease	Global	Female	LDI (I$ per capita)			X			X
Hypertensive heart disease	Data Rich	Female	LDI (I$ per capita)			X			X
Hypertensive heart disease	Global	Female	Socio-demographic Index			X			X
Hypertensive heart disease	Data Rich	Female	Socio-demographic Index			X			X
Hypertensive heart disease	Global	Female	Liters of alcohol consumed per capita			X			X
Hypertensive heart disease	Data Rich	Female	Liters of alcohol consumed per capita			X			X
Hypertensive heart disease	Global	Female	Pulses legumes unadjusted(g)			X			X
Hypertensive heart disease	Data Rich	Female	Pulses legumes unadjusted(g)			X			X
Hypertensive heart disease	Global	Female	Diet high in trans fatty acids			X			X
Hypertensive heart disease	Data Rich	Female	Diet high in trans fatty acids			X			X
Hypertensive heart disease	Global	Female	Age- and sex-specific SEV for Low fruit			X			X
Hypertensive heart disease	Data Rich	Female	Age- and sex-specific SEV for Low fruit			X			X
Hypertensive heart disease	Global	Female	Age- and sex-specific SEV for Low vegetables			X			X
Hypertensive heart disease	Data Rich	Female	Age- and sex-specific SEV for Low vegetables			X			X
Hypertensive heart disease	Global	Female	Age- and sex-specific SEV for Low nuts and seeds			X			X
Hypertensive heart disease	Data Rich	Female	Age- and sex-specific SEV for Low nuts and seeds			X			X
Hypertensive heart disease	Global	Female	Age- and sex-specific SEV for Low omega 3			X			X
Hypertensive heart disease	Data Rich	Female	Age- and sex-specific SEV for Low omega 3			X			X
Hypertensive heart disease	Global	Female	Age- and sex-specific SEV for Low PUFA			X			X
Hypertensive heart disease	Data Rich	Female	Age- and sex-specific SEV for Low PUFA			X			X
Hypertensive heart disease	Global	Female	Low-Density Lipoprotein (mmol/L)		X			X	
Hypertensive heart disease	Data Rich	Female	Low-Density Lipoprotein (mmol/L)		X			X	
Hypertensive heart disease	Global	Female	Smoking Prevalence		X			X	
Hypertensive heart disease	Data Rich	Female	Smoking Prevalence		X			X	
Hypertensive heart disease	Global	Female	Healthcare access and quality index		X			X	
Hypertensive heart disease	Data Rich	Female	Healthcare access and quality index		X			X	
Hypertensive heart disease	Global	Female	Mean BMI		X			X	
Hypertensive heart disease	Data Rich	Female	Mean BMI		X			X	
Hypertensive heart disease	Global	Female	Systolic Blood Pressure (mmHg)	X			X		
Hypertensive heart disease	Data Rich	Female	Systolic Blood Pressure (mmHg)	X			X		
Hypertensive heart disease	Global	Male	LDI (I$ per capita)			X			X
Hypertensive heart disease	Data Rich	Male	LDI (I$ per capita)			X			X
Hypertensive heart disease	Global	Male	Socio-demographic Index			X			X
Hypertensive heart disease	Data Rich	Male	Socio-demographic Index			X			X
Hypertensive heart disease	Global	Male	Liters of alcohol consumed per capita			X			X
Hypertensive heart disease	Data Rich	Male	Liters of alcohol consumed per capita			X			X
Hypertensive heart disease	Global	Male	Pulses legumes unadjusted(g)			X			X
Hypertensive heart disease	Data Rich	Male	Pulses legumes unadjusted(g)			X			X
Hypertensive heart disease	Global	Male	Diet high in trans fatty acids			X			X
Hypertensive heart disease	Data Rich	Male	Diet high in trans fatty acids			X			X
Hypertensive heart disease	Global	Male	Age- and sex-specific SEV for Low fruit			X			X
Hypertensive heart disease	Data Rich	Male	Age- and sex-specific SEV for Low fruit			X			X
Hypertensive heart disease	Global	Male	Age- and sex-specific SEV for Low vegetables			X			X
Hypertensive heart disease	Data Rich	Male	Age- and sex-specific SEV for Low vegetables			X			X
Hypertensive heart disease	Global	Male	Age- and sex-specific SEV for Low nuts and seeds			X			X
Hypertensive heart disease	Data Rich	Male	Age- and sex-specific SEV for Low nuts and seeds			X			X
Hypertensive heart disease	Global	Male	Age- and sex-specific SEV for Low omega 3			X			X
Hypertensive heart disease	Data Rich	Male	Age- and sex-specific SEV for Low omega 3			X			X
Hypertensive heart disease	Global	Male	Age- and sex-specific SEV for Low PUFA			X			X
Hypertensive heart disease	Data Rich	Male	Age- and sex-specific SEV for Low PUFA			X			X
Hypertensive heart disease	Global	Male	Low-Density Lipoprotein (mmol/L)		X			X	
Hypertensive heart disease	Data Rich	Male	Low-Density Lipoprotein (mmol/L)		X			X	
Hypertensive heart disease	Global	Male	Smoking Prevalence		X			X	
Hypertensive heart disease	Data Rich	Male	Smoking Prevalence		X			X	
Hypertensive heart disease	Global	Male	Healthcare access and quality index		X			X	
Hypertensive heart disease	Data Rich	Male	Healthcare access and quality index		X			X	
Hypertensive heart disease	Global	Male	Mean BMI		X			X	
Hypertensive heart disease	Data Rich	Male	Mean BMI		X			X	
Hypertensive heart disease	Global	Male	Systolic Blood Pressure (mmHg)	X			X		
Hypertensive heart disease	Data Rich	Male	Systolic Blood Pressure (mmHg)	X			X		
Non-rheumatic valvular heart disease	Data Rich	Female	LDI (I$ per capita)			X			X
Non-rheumatic valvular heart disease	Global	Female	LDI (I$ per capita)			X			X
Non-rheumatic valvular heart disease	Data Rich	Female	Socio-demographic Index			X			X
Non-rheumatic valvular heart disease	Global	Female	Socio-demographic Index			X			X
Non-rheumatic valvular heart disease	Data Rich	Female	Liters of alcohol consumed per capita			X			X

Appendix Table S11: Comparison of GBD 2021 and GBD 2023 covariates and level of covariates used in cause of death modeling

Cause	Model Version Type	Sex	Covariate	Level 1 GBD 2021	Level 2 GBD 2021	Level 3 GBD 2021	Level 1 GBD 2023	Level 2 GBD 2023	Level 3 GBD 2023
Non-rheumatic valvular heart disease	Global	Female	Liters of alcohol consumed per capita			X			X
Non-rheumatic valvular heart disease	Data Rich	Female	Low-Density Lipoprotein (mmol/L)		X			X	
Non-rheumatic valvular heart disease	Global	Female	Low-Density Lipoprotein (mmol/L)		X			X	
Non-rheumatic valvular heart disease	Data Rich	Female	Healthcare access and quality index		X			X	
Non-rheumatic valvular heart disease	Global	Female	Healthcare access and quality index		X			X	
Non-rheumatic valvular heart disease	Data Rich	Female	Mean BMI		X			X	
Non-rheumatic valvular heart disease	Global	Female	Mean BMI		X			X	
Non-rheumatic valvular heart disease	Data Rich	Female	Smoking Prevalence	X			X		
Non-rheumatic valvular heart disease	Global	Female	Smoking Prevalence	X			X		
Non-rheumatic valvular heart disease	Data Rich	Female	Systolic Blood Pressure (mmHg)	X			X		
Non-rheumatic valvular heart disease	Global	Female	Systolic Blood Pressure (mmHg)	X			X		
Non-rheumatic valvular heart disease	Data Rich	Male	LDI (I$ per capita)			X			X
Non-rheumatic valvular heart disease	Global	Male	LDI (I$ per capita)			X			X
Non-rheumatic valvular heart disease	Data Rich	Male	Socio-demographic Index			X			X
Non-rheumatic valvular heart disease	Global	Male	Socio-demographic Index			X			X
Non-rheumatic valvular heart disease	Data Rich	Male	Liters of alcohol consumed per capita			X			X
Non-rheumatic valvular heart disease	Global	Male	Liters of alcohol consumed per capita			X			X
Non-rheumatic valvular heart disease	Data Rich	Male	Low-Density Lipoprotein (mmol/L)		X			X	
Non-rheumatic valvular heart disease	Global	Male	Low-Density Lipoprotein (mmol/L)		X			X	
Non-rheumatic valvular heart disease	Data Rich	Male	Healthcare access and quality index		X			X	
Non-rheumatic valvular heart disease	Global	Male	Healthcare access and quality index		X			X	
Non-rheumatic valvular heart disease	Data Rich	Male	Mean BMI		X			X	
Non-rheumatic valvular heart disease	Global	Male	Mean BMI		X			X	
Non-rheumatic valvular heart disease	Data Rich	Male	Smoking Prevalence	X			X		
Non-rheumatic valvular heart disease	Global	Male	Smoking Prevalence	X			X		
Non-rheumatic valvular heart disease	Data Rich	Male	Systolic Blood Pressure (mmHg)	X			X		
Non-rheumatic valvular heart disease	Global	Male	Systolic Blood Pressure (mmHg)	X			X		
Non-rheumatic calcific aortic valve disease	Global	Female	LDI (I$ per capita)			X			X
Non-rheumatic calcific aortic valve disease	Data Rich	Female	LDI (I$ per capita)			X			X
Non-rheumatic calcific aortic valve disease	Global	Female	Socio-demographic Index			X			X
Non-rheumatic calcific aortic valve disease	Data Rich	Female	Socio-demographic Index			X			X
Non-rheumatic calcific aortic valve disease	Global	Female	Liters of alcohol consumed per capita			X			X
Non-rheumatic calcific aortic valve disease	Data Rich	Female	Liters of alcohol consumed per capita			X			X
Non-rheumatic calcific aortic valve disease	Global	Female	Low-Density Lipoprotein (mmol/L)		X			X	
Non-rheumatic calcific aortic valve disease	Data Rich	Female	Low-Density Lipoprotein (mmol/L)		X			X	
Non-rheumatic calcific aortic valve disease	Global	Female	Healthcare access and quality index		X			X	
Non-rheumatic calcific aortic valve disease	Data Rich	Female	Healthcare access and quality index		X			X	
Non-rheumatic calcific aortic valve disease	Global	Female	Diabetes Fasting Plasma Glucose (mmol/L), age-standardized 25+		X			X	
Non-rheumatic calcific aortic valve disease	Data Rich	Female	Diabetes Fasting Plasma Glucose (mmol/L), age-standardized 25+		X			X	
Non-rheumatic calcific aortic valve disease	Global	Female	Smoking Prevalence	X			X		
Non-rheumatic calcific aortic valve disease	Data Rich	Female	Smoking Prevalence	X			X		
Non-rheumatic calcific aortic valve disease	Global	Female	Mean BMI	X			X		
Non-rheumatic calcific aortic valve disease	Data Rich	Female	Mean BMI	X			X		
Non-rheumatic calcific aortic valve disease	Global	Female	Systolic Blood Pressure (mmHg)	X			X		
Non-rheumatic calcific aortic valve disease	Data Rich	Female	Systolic Blood Pressure (mmHg)	X			X		
Non-rheumatic calcific aortic valve disease	Global	Male	LDI (I$ per capita)			X			X
Non-rheumatic calcific aortic valve disease	Data Rich	Male	LDI (I$ per capita)			X			X
Non-rheumatic calcific aortic valve disease	Global	Male	Socio-demographic Index			X			X
Non-rheumatic calcific aortic valve disease	Data Rich	Male	Socio-demographic Index			X			X
Non-rheumatic calcific aortic valve disease	Global	Male	Liters of alcohol consumed per capita			X			X
Non-rheumatic calcific aortic valve disease	Data Rich	Male	Liters of alcohol consumed per capita			X			X
Non-rheumatic calcific aortic valve disease	Global	Male	Low-Density Lipoprotein (mmol/L)		X			X	
Non-rheumatic calcific aortic valve disease	Data Rich	Male	Low-Density Lipoprotein (mmol/L)		X			X	
Non-rheumatic calcific aortic valve disease	Global	Male	Healthcare access and quality index		X			X	
Non-rheumatic calcific aortic valve disease	Data Rich	Male	Healthcare access and quality index		X			X	
Non-rheumatic calcific aortic valve disease	Global	Male	Diabetes Fasting Plasma Glucose (mmol/L), age-standardized 25+		X			X	
Non-rheumatic calcific aortic valve disease	Data Rich	Male	Diabetes Fasting Plasma Glucose (mmol/L), age-standardized 25+		X			X	
Non-rheumatic calcific aortic valve disease	Global	Male	Smoking Prevalence	X			X		
Non-rheumatic calcific aortic valve disease	Data Rich	Male	Smoking Prevalence	X			X		
Non-rheumatic calcific aortic valve disease	Global	Male	Mean BMI	X			X		
Non-rheumatic calcific aortic valve disease	Data Rich	Male	Mean BMI	X			X		
Non-rheumatic calcific aortic valve disease	Global	Male	Systolic Blood Pressure (mmHg)	X			X		
Non-rheumatic calcific aortic valve disease	Data Rich	Male	Systolic Blood Pressure (mmHg)	X			X		
Non-rheumatic degenerative mitral valve disease	Global	Female	LDI (I$ per capita)	X			X		
Non-rheumatic degenerative mitral valve disease	Data Rich	Female	LDI (I$ per capita)	X			X		
Non-rheumatic degenerative mitral valve disease	Global	Female	Socio-demographic Index	X			X		
Non-rheumatic degenerative mitral valve disease	Data Rich	Female	Socio-demographic Index	X			X		
Non-rheumatic degenerative mitral valve disease	Global	Female	Healthcare access and quality index	X			X		
Non-rheumatic degenerative mitral valve disease	Data Rich	Female	Healthcare access and quality index	X			X		
Non-rheumatic degenerative mitral valve disease	Global	Male	LDI (I$ per capita)	X			X		
Non-rheumatic degenerative mitral valve disease	Data Rich	Male	LDI (I$ per capita)	X			X		
Non-rheumatic degenerative mitral valve disease	Global	Male	Socio-demographic Index	X			X		
Non-rheumatic degenerative mitral valve disease	Data Rich	Male	Socio-demographic Index	X			X		
Non-rheumatic degenerative mitral valve disease	Global	Male	Healthcare access and quality index	X			X		
Non-rheumatic degenerative mitral valve disease	Data Rich	Male	Healthcare access and quality index	X			X		
Other non-rheumatic valve diseases	Global	Female	Socio-demographic Index		X		X		
Other non-rheumatic valve diseases	Data Rich	Female	Socio-demographic Index		X		X		
Other non-rheumatic valve diseases	Global	Female	Healthcare access and quality index	X			X		
Other non-rheumatic valve diseases	Data Rich	Female	Healthcare access and quality index	X			X		
Other non-rheumatic valve diseases	Global	Female	LDI (I$ per capita)				X		
Other non-rheumatic valve diseases	Data Rich	Female	LDI (I$ per capita)				X		
Other non-rheumatic valve diseases	Global	Male	Socio-demographic Index		X		X		
Other non-rheumatic valve diseases	Data Rich	Male	Socio-demographic Index		X		X		
Other non-rheumatic valve diseases	Global	Male	Healthcare access and quality index	X			X		
Other non-rheumatic valve diseases	Data Rich	Male	Healthcare access and quality index	X			X		
Other non-rheumatic valve diseases	Global	Male	LDI (I$ per capita)				X		
Other non-rheumatic valve diseases	Data Rich	Male	LDI (I$ per capita)				X		
Cardiomyopathy and myocarditis	Data Rich	Female	LDI (I$ per capita)			X			X
Cardiomyopathy and myocarditis	Global	Female	LDI (I$ per capita)			X			X
Cardiomyopathy and myocarditis	Data Rich	Female	Socio-demographic Index			X			X
Cardiomyopathy and myocarditis	Global	Female	Socio-demographic Index			X			X
Cardiomyopathy and myocarditis	Data Rich	Female	Healthcare access and quality index		X			X	
Cardiomyopathy and myocarditis	Global	Female	Healthcare access and quality index		X			X	
Cardiomyopathy and myocarditis	Data Rich	Female	Mean BMI		X			X	
Cardiomyopathy and myocarditis	Global	Female	Mean BMI		X			X	
Cardiomyopathy and myocarditis	Data Rich	Female	Systolic Blood Pressure (mmHg)		X			X	
Cardiomyopathy and myocarditis	Global	Female	Systolic Blood Pressure (mmHg)		X			X	
Cardiomyopathy and myocarditis	Data Rich	Female	Smoking Prevalence	X			X		
Cardiomyopathy and myocarditis	Global	Female	Smoking Prevalence	X			X		
Cardiomyopathy and myocarditis	Data Rich	Female	Log-transformed SEV scalar: CMP	X			X		
Cardiomyopathy and myocarditis	Global	Female	Log-transformed SEV scalar: CMP	X			X		
Cardiomyopathy and myocarditis	Data Rich	Male	LDI (I$ per capita)			X			X
Cardiomyopathy and myocarditis	Global	Male	LDI (I$ per capita)			X			X
Cardiomyopathy and myocarditis	Data Rich	Male	Socio-demographic Index			X			X
Cardiomyopathy and myocarditis	Global	Male	Socio-demographic Index			X			X
Cardiomyopathy and myocarditis	Data Rich	Male	Healthcare access and quality index		X			X	
Cardiomyopathy and myocarditis	Global	Male	Healthcare access and quality index		X			X	
Cardiomyopathy and myocarditis	Data Rich	Male	Mean BMI		X			X	
Cardiomyopathy and myocarditis	Global	Male	Mean BMI		X			X	
Cardiomyopathy and myocarditis	Data Rich	Male	Systolic Blood Pressure (mmHg)		X			X	
Cardiomyopathy and myocarditis	Global	Male	Systolic Blood Pressure (mmHg)		X			X	
Cardiomyopathy and myocarditis	Data Rich	Male	Smoking Prevalence	X			X		
Cardiomyopathy and myocarditis	Global	Male	Smoking Prevalence	X			X		
Cardiomyopathy and myocarditis	Data Rich	Male	Log-transformed SEV scalar: CMP	X			X		
Cardiomyopathy and myocarditis	Global	Male	Log-transformed SEV scalar: CMP	X			X		
Myocarditis	Global	Female	LDI (I$ per capita)			X			X
Myocarditis	Data Rich	Female	LDI (I$ per capita)			X			X
Myocarditis	Global	Female	Socio-demographic Index			X			X
Myocarditis	Data Rich	Female	Socio-demographic Index			X			X
Myocarditis	Global	Female	Healthcare access and quality index		X			X	
Myocarditis	Data Rich	Female	Healthcare access and quality index		X			X	
Myocarditis	Global	Male	LDI (I$ per capita)			X			X
Myocarditis	Data Rich	Male	LDI (I$ per capita)			X			X
Myocarditis	Global	Male	Socio-demographic Index			X			X
Myocarditis	Data Rich	Male	Socio-demographic Index			X			X

Appendix Table S11: Comparison of GBD 2021 and GBD 2023 covariates and level of covariates used in cause of death modeling

Cause	Model Version Type	Sex	Covariate	Level 1 GBD 2021	Level 2 GBD 2021	Level 3 GBD 2021	Level 1 GBD 2023	Level 2 GBD 2023	Level 3 GBD 2023
Myocarditis	Global	Male	Healthcare access and quality index		X			X	
Myocarditis	Data Rich	Male	Healthcare access and quality index		X			X	
Alcoholic cardiomyopathy	Global	Female	LDI (I$ per capita)			X			X
Alcoholic cardiomyopathy	Data Rich	Female	LDI (I$ per capita)			X			X
Alcoholic cardiomyopathy	Global	Female	Healthcare access and quality index		X			X	
Alcoholic cardiomyopathy	Data Rich	Female	Healthcare access and quality index		X			X	
Alcoholic cardiomyopathy	Global	Female	Smoking Prevalence	X			X		
Alcoholic cardiomyopathy	Data Rich	Female	Smoking Prevalence	X			X		
Alcoholic cardiomyopathy	Global	Female	Age- and sex-specific SEV for Alcohol use	X			X		
Alcoholic cardiomyopathy	Data Rich	Female	Age- and sex-specific SEV for Alcohol use	X			X		
Alcoholic cardiomyopathy	Global	Female	Log-transformed SEV scalar: CMP	X			X		
Alcoholic cardiomyopathy	Data Rich	Female	Log-transformed SEV scalar: CMP	X			X		
Alcoholic cardiomyopathy	Data Rich	Male	LDI (I$ per capita)			X			X
Alcoholic cardiomyopathy	Global	Male	LDI (I$ per capita)			X			X
Alcoholic cardiomyopathy	Data Rich	Male	Healthcare access and quality index		X			X	
Alcoholic cardiomyopathy	Global	Male	Healthcare access and quality index		X			X	
Alcoholic cardiomyopathy	Data Rich	Male	Smoking Prevalence	X			X		
Alcoholic cardiomyopathy	Global	Male	Smoking Prevalence	X			X		
Alcoholic cardiomyopathy	Data Rich	Male	Age- and sex-specific SEV for Alcohol use	X			X		
Alcoholic cardiomyopathy	Global	Male	Age- and sex-specific SEV for Alcohol use	X			X		
Alcoholic cardiomyopathy	Data Rich	Male	Log-transformed SEV scalar: CMP	X			X		
Alcoholic cardiomyopathy	Global	Male	Log-transformed SEV scalar: CMP	X			X		
Other cardiomyopathy	Data Rich	Female	LDI (I$ per capita)			X			X
Other cardiomyopathy	Global	Female	LDI (I$ per capita)			X			X
Other cardiomyopathy	Data Rich	Female	Socio-demographic Index			X			X
Other cardiomyopathy	Global	Female	Socio-demographic Index			X			X
Other cardiomyopathy	Data Rich	Female	Healthcare access and quality index		X			X	
Other cardiomyopathy	Global	Female	Healthcare access and quality index		X			X	
Other cardiomyopathy	Data Rich	Female	Mean BMI		X			X	
Other cardiomyopathy	Global	Female	Mean BMI		X			X	
Other cardiomyopathy	Data Rich	Female	Smoking Prevalence	X			X		
Other cardiomyopathy	Global	Female	Smoking Prevalence	X			X		
Other cardiomyopathy	Data Rich	Female	Systolic Blood Pressure (mmHg)	X			X		
Other cardiomyopathy	Global	Female	Systolic Blood Pressure (mmHg)	X			X		
Other cardiomyopathy	Data Rich	Female	Log-transformed SEV scalar: CMP	X			X		
Other cardiomyopathy	Global	Female	Log-transformed SEV scalar: CMP	X			X		
Other cardiomyopathy	Data Rich	Male	LDI (I$ per capita)			X			X
Other cardiomyopathy	Global	Male	LDI (I$ per capita)			X			X
Other cardiomyopathy	Data Rich	Male	Socio-demographic Index			X			X
Other cardiomyopathy	Global	Male	Socio-demographic Index			X			X
Other cardiomyopathy	Data Rich	Male	Healthcare access and quality index		X			X	
Other cardiomyopathy	Global	Male	Healthcare access and quality index		X			X	
Other cardiomyopathy	Data Rich	Male	Mean BMI		X			X	
Other cardiomyopathy	Global	Male	Mean BMI		X			X	
Other cardiomyopathy	Data Rich	Male	Smoking Prevalence	X			X		
Other cardiomyopathy	Global	Male	Smoking Prevalence	X			X		
Other cardiomyopathy	Data Rich	Male	Systolic Blood Pressure (mmHg)	X			X		
Other cardiomyopathy	Global	Male	Systolic Blood Pressure (mmHg)	X			X		
Other cardiomyopathy	Data Rich	Male	Log-transformed SEV scalar: CMP	X			X		
Other cardiomyopathy	Global	Male	Log-transformed SEV scalar: CMP	X			X		
Pulmonary Arterial Hypertension	Global	Female	Socio-demographic Index	X			X		
Pulmonary Arterial Hypertension	Data Rich	Female	Socio-demographic Index	X			X		
Pulmonary Arterial Hypertension	Global	Female	Healthcare access and quality index	X			X		
Pulmonary Arterial Hypertension	Data Rich	Female	Healthcare access and quality index	X			X		
Pulmonary Arterial Hypertension	Global	Female	Log-transformed SEV scalar: HIV	X			X		
Pulmonary Arterial Hypertension	Data Rich	Female	Log-transformed SEV scalar: HIV	X			X		
Pulmonary Arterial Hypertension	Global	Female	Schistosomiasis Prevalence Results	X			X		
Pulmonary Arterial Hypertension	Data Rich	Female	Schistosomiasis Prevalence Results	X			X		
Pulmonary Arterial Hypertension	Global	Male	Socio-demographic Index	X			X		
Pulmonary Arterial Hypertension	Data Rich	Male	Socio-demographic Index	X			X		
Pulmonary Arterial Hypertension	Global	Male	Healthcare access and quality index	X			X		
Pulmonary Arterial Hypertension	Data Rich	Male	Healthcare access and quality index	X			X		
Pulmonary Arterial Hypertension	Global	Male	Log-transformed SEV scalar: HIV	X			X		
Pulmonary Arterial Hypertension	Data Rich	Male	Log-transformed SEV scalar: HIV	X			X		
Pulmonary Arterial Hypertension	Global	Male	Schistosomiasis Prevalence Results	X			X		
Pulmonary Arterial Hypertension	Data Rich	Male	Schistosomiasis Prevalence Results	X			X		
Atrial fibrillation and flutter	Global	Female	LDI (I$ per capita)			X			X
Atrial fibrillation and flutter	Data Rich	Female	LDI (I$ per capita)			X			X
Atrial fibrillation and flutter	Global	Female	Socio-demographic Index			X			X
Atrial fibrillation and flutter	Data Rich	Female	Socio-demographic Index			X			X
Atrial fibrillation and flutter	Global	Female	Liters of alcohol consumed per capita			X			X
Atrial fibrillation and flutter	Data Rich	Female	Liters of alcohol consumed per capita			X			X
Atrial fibrillation and flutter	Global	Female	Pulses legumes unadjusted(g)			X			X
Atrial fibrillation and flutter	Data Rich	Female	Pulses legumes unadjusted(g)			X			X
Atrial fibrillation and flutter	Global	Female	Diet high in trans fatty acids			X			X
Atrial fibrillation and flutter	Data Rich	Female	Diet high in trans fatty acids			X			X
Atrial fibrillation and flutter	Global	Female	Age- and sex-specific SEV for Low fruit			X			X
Atrial fibrillation and flutter	Data Rich	Female	Age- and sex-specific SEV for Low fruit			X			X
Atrial fibrillation and flutter	Global	Female	Age- and sex-specific SEV for Low vegetables			X			X
Atrial fibrillation and flutter	Data Rich	Female	Age- and sex-specific SEV for Low vegetables			X			X
Atrial fibrillation and flutter	Global	Female	Age- and sex-specific SEV for Low nuts and seeds			X			X
Atrial fibrillation and flutter	Data Rich	Female	Age- and sex-specific SEV for Low nuts and seeds			X			X
Atrial fibrillation and flutter	Global	Female	Age- and sex-specific SEV for Low omega-3			X			X
Atrial fibrillation and flutter	Data Rich	Female	Age- and sex-specific SEV for Low omega-3			X			X
Atrial fibrillation and flutter	Global	Female	Age- and sex-specific SEV for Low PUFA			X			X
Atrial fibrillation and flutter	Data Rich	Female	Age- and sex-specific SEV for Low PUFA			X			X
Atrial fibrillation and flutter	Global	Female	Low-Density Lipoprotein (mmol/L)		X			X	
Atrial fibrillation and flutter	Data Rich	Female	Low-Density Lipoprotein (mmol/L)		X			X	
Atrial fibrillation and flutter	Global	Female	Healthcare access and quality index		X			X	
Atrial fibrillation and flutter	Data Rich	Female	Healthcare access and quality index		X			X	
Atrial fibrillation and flutter	Global	Female	Mean BMI		X			X	
Atrial fibrillation and flutter	Data Rich	Female	Mean BMI		X			X	
Atrial fibrillation and flutter	Global	Female	Diabetes Fasting Plasma Glucose (mmol/L), age-standardized 25+		X			X	
Atrial fibrillation and flutter	Data Rich	Female	Diabetes Fasting Plasma Glucose (mmol/L), age-standardized 25+		X			X	
Atrial fibrillation and flutter	Global	Female	Smoking Prevalence	X			X		
Atrial fibrillation and flutter	Data Rich	Female	Smoking Prevalence	X			X		
Atrial fibrillation and flutter	Global	Female	Systolic Blood Pressure (mmHg)	X			X		
Atrial fibrillation and flutter	Data Rich	Female	Systolic Blood Pressure (mmHg)	X			X		
Atrial fibrillation and flutter	Global	Female	Log-transformed SEV scalar: A Fib	X			X		
Atrial fibrillation and flutter	Data Rich	Female	Log-transformed SEV scalar: A Fib	X			X		
Atrial fibrillation and flutter	Global	Female	Socio-demographic Index						X
Atrial fibrillation and flutter	Data Rich	Female	Socio-demographic Index						X
Atrial fibrillation and flutter	Data Rich	Female	Healthcare access and quality index						X
Atrial fibrillation and flutter	Global	Female	Liters of alcohol consumed per capita						X
Atrial fibrillation and flutter	Data Rich	Female	Liters of alcohol consumed per capita						X
Atrial fibrillation and flutter	Global	Female	Healthcare access and quality index					X	
Atrial fibrillation and flutter	Global	Female	Live Births 35+ (proportion)					X	
Atrial fibrillation and flutter	Data Rich	Female	Live Births 35+ (proportion)					X	
Atrial fibrillation and flutter	Global	Female	Maternal alcohol consumption during pregnancy (proportion)					X	
Atrial fibrillation and flutter	Data Rich	Female	Maternal alcohol consumption during pregnancy (proportion)					X	
Atrial fibrillation and flutter	Global	Female	Diabetes Fasting Plasma Glucose (mmol/L), age-standardized 25+					X	
Atrial fibrillation and flutter	Data Rich	Female	Diabetes Fasting Plasma Glucose (mmol/L), age-standardized 25+					X	
Atrial fibrillation and flutter	Global	Female	Birth prevalence of CHD					X	
Atrial fibrillation and flutter	Data Rich	Female	Birth prevalence of CHD					X	
Atrial fibrillation and flutter	Global	Female	Smoking Prevalence				X		
Atrial fibrillation and flutter	Data Rich	Female	Smoking Prevalence				X		
Atrial fibrillation and flutter	Global	Female	Systolic Blood Pressure (mmHg)				X		
Atrial fibrillation and flutter	Data Rich	Female	Systolic Blood Pressure (mmHg)				X		
Atrial fibrillation and flutter	Global	Male	LDI (I$ per capita)			X			X
Atrial fibrillation and flutter	Data Rich	Male	LDI (I$ per capita)			X			X
Atrial fibrillation and flutter	Global	Male	Socio-demographic Index			X			X
Atrial fibrillation and flutter	Data Rich	Male	Socio-demographic Index			X			X
Atrial fibrillation and flutter	Global	Male	Liters of alcohol consumed per capita			X			X

Appendix Table S11: Comparison of GBD 2021 and GBD 2023 covariates and level of covariates used in cause of death modeling									
**Cause**	**Model Version Type**	**Sex**	**Covariate**	**Level 1 GBD 2021**	**Level 2 GBD 2021**	**Level 3 GBD 2021**	**Level 1 GBD 2023**	**Level 2 GBD 2023**	**Level 3 GBD 2023**
Atrial fibrillation and flutter	Data Rich	Male	Liters of alcohol consumed per capita			X			X
Atrial fibrillation and flutter	Global	Male	Pulses legumes unadjusted(g)			X			X
Atrial fibrillation and flutter	Data Rich	Male	Pulses legumes unadjusted(g)			X			X
Atrial fibrillation and flutter	Global	Male	Diet high in trans fatty acids			X			X
Atrial fibrillation and flutter	Data Rich	Male	Diet high in trans fatty acids			X			X
Atrial fibrillation and flutter	Global	Male	Age- and sex-specific SEV for Low fruit			X			X
Atrial fibrillation and flutter	Data Rich	Male	Age- and sex-specific SEV for Low fruit			X			X
Atrial fibrillation and flutter	Global	Male	Age- and sex-specific SEV for Low vegetables			X			X
Atrial fibrillation and flutter	Data Rich	Male	Age- and sex-specific SEV for Low vegetables			X			X
Atrial fibrillation and flutter	Global	Male	Age- and sex-specific SEV for Low nuts and seeds			X			X
Atrial fibrillation and flutter	Data Rich	Male	Age- and sex-specific SEV for Low nuts and seeds			X			X
Atrial fibrillation and flutter	Global	Male	Age- and sex-specific SEV for Low omega-3			X			X
Atrial fibrillation and flutter	Data Rich	Male	Age- and sex-specific SEV for Low omega-3			X			X
Atrial fibrillation and flutter	Global	Male	Age- and sex-specific SEV for Low PUFA			X			X
Atrial fibrillation and flutter	Data Rich	Male	Age- and sex-specific SEV for Low PUFA			X			X
Atrial fibrillation and flutter	Global	Male	Low-Density Lipoprotein (mmol/L)		X			X	
Atrial fibrillation and flutter	Data Rich	Male	Low-Density Lipoprotein (mmol/L)		X			X	
Atrial fibrillation and flutter	Global	Male	Healthcare access and quality index		X			X	
Atrial fibrillation and flutter	Data Rich	Male	Healthcare access and quality index		X			X	
Atrial fibrillation and flutter	Global	Male	Mean BMI		X			X	
Atrial fibrillation and flutter	Data Rich	Male	Mean BMI		X			X	
Atrial fibrillation and flutter	Global	Male	Diabetes Fasting Plasma Glucose (mmol/L), age-standardized 25+		X			X	
Atrial fibrillation and flutter	Data Rich	Male	Diabetes Fasting Plasma Glucose (mmol/L), age-standardized 25+		X			X	
Atrial fibrillation and flutter	Global	Male	Smoking Prevalence	X			X		
Atrial fibrillation and flutter	Data Rich	Male	Smoking Prevalence	X			X		
Atrial fibrillation and flutter	Global	Male	Systolic Blood Pressure (mmHg)	X			X		
Atrial fibrillation and flutter	Data Rich	Male	Systolic Blood Pressure (mmHg)	X			X		
Atrial fibrillation and flutter	Global	Male	Log-transformed SEV scalar: A Fib	X			X		
Atrial fibrillation and flutter	Data Rich	Male	Log-transformed SEV scalar: A Fib	X			X		
Atrial fibrillation and flutter	Global	Male	Socio-demographic Index						X
Atrial fibrillation and flutter	Data Rich	Male	Socio-demographic Index						X
Atrial fibrillation and flutter	Data Rich	Male	Healthcare access and quality index						X
Atrial fibrillation and flutter	Global	Male	Liters of alcohol consumed per capita						X
Atrial fibrillation and flutter	Data Rich	Male	Liters of alcohol consumed per capita						X
Atrial fibrillation and flutter	Global	Male	Healthcare access and quality index					X	
Atrial fibrillation and flutter	Global	Male	Live Births 35+ (proportion)					X	
Atrial fibrillation and flutter	Data Rich	Male	Live Births 35+ (proportion)					X	
Atrial fibrillation and flutter	Global	Male	Maternal alcohol consumption during pregnancy (proportion)					X	
Atrial fibrillation and flutter	Data Rich	Male	Maternal alcohol consumption during pregnancy (proportion)					X	
Atrial fibrillation and flutter	Global	Male	Diabetes Fasting Plasma Glucose (mmol/L), age-standardized 25+					X	
Atrial fibrillation and flutter	Data Rich	Male	Diabetes Fasting Plasma Glucose (mmol/L), age-standardized 25+					X	
Atrial fibrillation and flutter	Global	Male	Birth prevalence of CHD					X	
Atrial fibrillation and flutter	Data Rich	Male	Birth prevalence of CHD					X	
Atrial fibrillation and flutter	Global	Male	Smoking Prevalence				X		
Atrial fibrillation and flutter	Data Rich	Male	Smoking Prevalence				X		
Atrial fibrillation and flutter	Global	Male	Systolic Blood Pressure (mmHg)				X		
Atrial fibrillation and flutter	Data Rich	Male	Systolic Blood Pressure (mmHg)				X		
Aortic aneurysm	Data Rich	Female	LDI (I$ per capita)			X			X
Aortic aneurysm	Global	Female	LDI (I$ per capita)			X			X
Aortic aneurysm	Data Rich	Female	Socio-demographic Index			X			X
Aortic aneurysm	Global	Female	Socio-demographic Index			X			X
Aortic aneurysm	Data Rich	Female	Liters of alcohol consumed per capita			X			X
Aortic aneurysm	Global	Female	Liters of alcohol consumed per capita			X			X
Aortic aneurysm	Data Rich	Female	Pulses legumes unadjusted(g)			X			X
Aortic aneurysm	Global	Female	Pulses legumes unadjusted(g)			X			X
Aortic aneurysm	Data Rich	Female	Age- and sex-specific SEV for Low fruit			X			X
Aortic aneurysm	Global	Female	Age- and sex-specific SEV for Low fruit			X			X
Aortic aneurysm	Data Rich	Female	Age- and sex-specific SEV for Low vegetables			X			X
Aortic aneurysm	Global	Female	Age- and sex-specific SEV for Low vegetables			X			X
Aortic aneurysm	Data Rich	Female	Age- and sex-specific SEV for Low nuts and seeds			X			X
Aortic aneurysm	Global	Female	Age- and sex-specific SEV for Low nuts and seeds			X			X
Aortic aneurysm	Data Rich	Female	Age- and sex-specific SEV for Low omega-3			X			X
Aortic aneurysm	Global	Female	Age- and sex-specific SEV for Low omega-3			X			X
Aortic aneurysm	Data Rich	Female	Age- and sex-specific SEV for Low PUFA			X			X
Aortic aneurysm	Global	Female	Age- and sex-specific SEV for Low PUFA			X			X
Aortic aneurysm	Data Rich	Female	Healthcare access and quality index		X			X	
Aortic aneurysm	Global	Female	Healthcare access and quality index		X			X	
Aortic aneurysm	Data Rich	Female	Mean BMI		X			X	
Aortic aneurysm	Global	Female	Mean BMI		X			X	
Aortic aneurysm	Data Rich	Female	Cumulative Cigarettes (10 Years)	X			X		
Aortic aneurysm	Global	Female	Cumulative Cigarettes (10 Years)	X			X		
Aortic aneurysm	Data Rich	Female	Low-Density Lipoprotein (mmol/L)	X			X		
Aortic aneurysm	Global	Female	Low-Density Lipoprotein (mmol/L)	X			X		
Aortic aneurysm	Data Rich	Female	Systolic Blood Pressure (mmHg)	X			X		
Aortic aneurysm	Global	Female	Systolic Blood Pressure (mmHg)	X			X		
Aortic aneurysm	Data Rich	Female	Log-transformed SEV scalar: Aort An	X			X		
Aortic aneurysm	Global	Female	Log-transformed SEV scalar: Aort An	X			X		
Aortic aneurysm	Data Rich	Male	LDI (I$ per capita)			X			X
Aortic aneurysm	Global	Male	LDI (I$ per capita)			X			X
Aortic aneurysm	Data Rich	Male	Socio-demographic Index			X			X
Aortic aneurysm	Global	Male	Socio-demographic Index			X			X
Aortic aneurysm	Data Rich	Male	Liters of alcohol consumed per capita			X			X
Aortic aneurysm	Global	Male	Liters of alcohol consumed per capita			X			X
Aortic aneurysm	Data Rich	Male	Pulses legumes unadjusted(g)			X			X
Aortic aneurysm	Global	Male	Pulses legumes unadjusted(g)			X			X
Aortic aneurysm	Data Rich	Male	Age- and sex-specific SEV for Low fruit			X			X
Aortic aneurysm	Global	Male	Age- and sex-specific SEV for Low fruit			X			X
Aortic aneurysm	Data Rich	Male	Age- and sex-specific SEV for Low vegetables			X			X
Aortic aneurysm	Global	Male	Age- and sex-specific SEV for Low vegetables			X			X
Aortic aneurysm	Data Rich	Male	Age- and sex-specific SEV for Low nuts and seeds			X			X
Aortic aneurysm	Global	Male	Age- and sex-specific SEV for Low nuts and seeds			X			X
Aortic aneurysm	Data Rich	Male	Age- and sex-specific SEV for Low omega-3			X			X
Aortic aneurysm	Global	Male	Age- and sex-specific SEV for Low omega-3			X			X
Aortic aneurysm	Data Rich	Male	Age- and sex-specific SEV for Low PUFA			X			X
Aortic aneurysm	Global	Male	Age- and sex-specific SEV for Low PUFA			X			X
Aortic aneurysm	Data Rich	Male	Healthcare access and quality index		X			X	
Aortic aneurysm	Global	Male	Healthcare access and quality index		X			X	
Aortic aneurysm	Data Rich	Male	Mean BMI		X			X	
Aortic aneurysm	Global	Male	Mean BMI		X			X	
Aortic aneurysm	Data Rich	Male	Cumulative Cigarettes (10 Years)	X			X		
Aortic aneurysm	Global	Male	Cumulative Cigarettes (10 Years)	X			X		
Aortic aneurysm	Data Rich	Male	Low-Density Lipoprotein (mmol/L)	X			X		
Aortic aneurysm	Global	Male	Low-Density Lipoprotein (mmol/L)	X			X		
Aortic aneurysm	Data Rich	Male	Systolic Blood Pressure (mmHg)	X			X		
Aortic aneurysm	Global	Male	Systolic Blood Pressure (mmHg)	X			X		
Aortic aneurysm	Data Rich	Male	Log-transformed SEV scalar: Aort An	X			X		
Aortic aneurysm	Global	Male	Log-transformed SEV scalar: Aort An	X			X		
Lower extremity peripheral arterial disease	Global	Female	LDI (I$ per capita)			X			X
Lower extremity peripheral arterial disease	Data Rich	Female	LDI (I$ per capita)			X			X
Lower extremity peripheral arterial disease	Global	Female	Socio-demographic Index			X			X
Lower extremity peripheral arterial disease	Data Rich	Female	Socio-demographic Index			X			X
Lower extremity peripheral arterial disease	Global	Female	Liters of alcohol consumed per capita			X			X

Appendix Table S11: Comparison of GBD 2021 and GBD 2023 covariates and level of covariates used in cause of death modeling

Cause	Model Version Type	Sex	Covariate	Level 1 GBD 2021	Level 2 GBD 2021	Level 3 GBD 2021	Level 1 GBD 2023	Level 2 GBD 2023	Level 3 GBD 2023
Lower extremity peripheral arterial disease	Data Rich	Female	Liters of alcohol consumed per capita			X			X
Lower extremity peripheral arterial disease	Global	Female	Pulses legumes unadjusted(g)			X			X
Lower extremity peripheral arterial disease	Data Rich	Female	Pulses legumes unadjusted(g)			X			X
Lower extremity peripheral arterial disease	Global	Female	Age- and sex-specific SEV for Low fruit			X			X
Lower extremity peripheral arterial disease	Data Rich	Female	Age- and sex-specific SEV for Low fruit			X			X
Lower extremity peripheral arterial disease	Global	Female	Age- and sex-specific SEV for Low vegetables			X			X
Lower extremity peripheral arterial disease	Data Rich	Female	Age- and sex-specific SEV for Low vegetables			X			X
Lower extremity peripheral arterial disease	Global	Female	Age- and sex-specific SEV for Low nuts and seeds			X			X
Lower extremity peripheral arterial disease	Data Rich	Female	Age- and sex-specific SEV for Low nuts and seeds			X			X
Lower extremity peripheral arterial disease	Global	Female	Age- and sex-specific SEV for Low omega-3			X			X
Lower extremity peripheral arterial disease	Data Rich	Female	Age- and sex-specific SEV for Low omega-3			X			X
Lower extremity peripheral arterial disease	Global	Female	Age- and sex-specific SEV for Low PUFA			X			X
Lower extremity peripheral arterial disease	Data Rich	Female	Age- and sex-specific SEV for Low PUFA			X			X
Lower extremity peripheral arterial disease	Global	Female	Healthcare access and quality index		X			X	
Lower extremity peripheral arterial disease	Data Rich	Female	Healthcare access and quality index		X			X	
Lower extremity peripheral arterial disease	Global	Female	Mean BMI		X			X	
Lower extremity peripheral arterial disease	Data Rich	Female	Mean BMI		X			X	
Lower extremity peripheral arterial disease	Global	Female	Diabetes Fasting Plasma Glucose (mmol/L), age-standardized 25+		X			X	
Lower extremity peripheral arterial disease	Data Rich	Female	Diabetes Fasting Plasma Glucose (mmol/L), age-standardized 25+		X			X	
Lower extremity peripheral arterial disease	Global	Female	Low-Density Lipoprotein (mmol/L)	X			X		
Lower extremity peripheral arterial disease	Data Rich	Female	Low-Density Lipoprotein (mmol/L)	X			X		
Lower extremity peripheral arterial disease	Global	Female	Smoking Prevalence	X			X		
Lower extremity peripheral arterial disease	Data Rich	Female	Smoking Prevalence	X			X		
Lower extremity peripheral arterial disease	Global	Female	Systolic Blood Pressure (mmHg)	X			X		
Lower extremity peripheral arterial disease	Data Rich	Female	Systolic Blood Pressure (mmHg)	X			X		
Lower extremity peripheral arterial disease	Global	Female	Log-transformed SEV scalar: PAD	X			X		
Lower extremity peripheral arterial disease	Data Rich	Female	Log-transformed SEV scalar: PAD	X			X		
Lower extremity peripheral arterial disease	Global	Male	LDI (I$ per capita)			X			X
Lower extremity peripheral arterial disease	Data Rich	Male	LDI (I$ per capita)			X			X
Lower extremity peripheral arterial disease	Global	Male	Socio-demographic Index			X			X
Lower extremity peripheral arterial disease	Data Rich	Male	Socio-demographic Index			X			X
Lower extremity peripheral arterial disease	Global	Male	Liters of alcohol consumed per capita			X			X
Lower extremity peripheral arterial disease	Data Rich	Male	Liters of alcohol consumed per capita			X			X
Lower extremity peripheral arterial disease	Global	Male	Pulses legumes unadjusted(g)			X			X
Lower extremity peripheral arterial disease	Data Rich	Male	Pulses legumes unadjusted(g)			X			X
Lower extremity peripheral arterial disease	Global	Male	Age- and sex-specific SEV for Low fruit			X			X
Lower extremity peripheral arterial disease	Data Rich	Male	Age- and sex-specific SEV for Low fruit			X			X
Lower extremity peripheral arterial disease	Global	Male	Age- and sex-specific SEV for Low vegetables			X			X
Lower extremity peripheral arterial disease	Data Rich	Male	Age- and sex-specific SEV for Low vegetables			X			X
Lower extremity peripheral arterial disease	Global	Male	Age- and sex-specific SEV for Low nuts and seeds			X			X
Lower extremity peripheral arterial disease	Data Rich	Male	Age- and sex-specific SEV for Low nuts and seeds			X			X
Lower extremity peripheral arterial disease	Global	Male	Age- and sex-specific SEV for Low omega-3			X			X
Lower extremity peripheral arterial disease	Data Rich	Male	Age- and sex-specific SEV for Low omega-3			X			X
Lower extremity peripheral arterial disease	Global	Male	Age- and sex-specific SEV for Low PUFA			X			X
Lower extremity peripheral arterial disease	Data Rich	Male	Age- and sex-specific SEV for Low PUFA			X			X
Lower extremity peripheral arterial disease	Global	Male	Healthcare access and quality index		X			X	
Lower extremity peripheral arterial disease	Data Rich	Male	Healthcare access and quality index		X			X	
Lower extremity peripheral arterial disease	Global	Male	Mean BMI		X			X	
Lower extremity peripheral arterial disease	Data Rich	Male	Mean BMI		X			X	
Lower extremity peripheral arterial disease	Global	Male	Diabetes Fasting Plasma Glucose (mmol/L), age-standardized 25+		X			X	
Lower extremity peripheral arterial disease	Data Rich	Male	Diabetes Fasting Plasma Glucose (mmol/L), age-standardized 25+		X			X	
Lower extremity peripheral arterial disease	Global	Male	Low-Density Lipoprotein (mmol/L)	X			X		
Lower extremity peripheral arterial disease	Data Rich	Male	Low-Density Lipoprotein (mmol/L)	X			X		
Lower extremity peripheral arterial disease	Global	Male	Smoking Prevalence	X			X		
Lower extremity peripheral arterial disease	Data Rich	Male	Smoking Prevalence	X			X		
Lower extremity peripheral arterial disease	Global	Male	Systolic Blood Pressure (mmHg)	X			X		
Lower extremity peripheral arterial disease	Data Rich	Male	Systolic Blood Pressure (mmHg)	X			X		
Lower extremity peripheral arterial disease	Global	Male	Log-transformed SEV scalar: PAD	X			X		
Lower extremity peripheral arterial disease	Data Rich	Male	Log-transformed SEV scalar: PAD	X			X		
Endocarditis	Data Rich	Female	LDI (I$ per capita)			X			X
Endocarditis	Global	Female	LDI (I$ per capita)			X			X
Endocarditis	Data Rich	Female	Socio-demographic Index			X			X
Endocarditis	Global	Female	Socio-demographic Index			X			X
Endocarditis	Data Rich	Female	Healthcare access and quality index		X			X	
Endocarditis	Global	Female	Healthcare access and quality index		X			X	
Endocarditis	Data Rich	Female	Sanitation (proportion with access)	X			X		
Endocarditis	Global	Female	Sanitation (proportion with access)	X			X		
Endocarditis	Data Rich	Female	Improved Water Source (proportion with access)	X			X		
Endocarditis	Global	Female	Improved Water Source (proportion with access)	X			X		
Endocarditis	Global	Male	LDI (I$ per capita)			X			X
Endocarditis	Data Rich	Male	LDI (I$ per capita)			X			X
Endocarditis	Global	Male	Socio-demographic Index			X			X
Endocarditis	Data Rich	Male	Socio-demographic Index			X			X
Endocarditis	Global	Male	Healthcare access and quality index		X			X	
Endocarditis	Data Rich	Male	Healthcare access and quality index		X			X	
Endocarditis	Global	Male	Sanitation (proportion with access)	X			X		
Endocarditis	Data Rich	Male	Sanitation (proportion with access)	X			X		
Endocarditis	Global	Male	Improved Water Source (proportion with access)	X			X		
Endocarditis	Data Rich	Male	Improved Water Source (proportion with access)	X			X		
Other cardiovascular and circulatory diseases	Data Rich	Female	LDI (I$ per capita)			X			X
Other cardiovascular and circulatory diseases	Global	Female	LDI (I$ per capita)			X			X
Other cardiovascular and circulatory diseases	Data Rich	Female	Socio-demographic Index			X			X
Other cardiovascular and circulatory diseases	Global	Female	Socio-demographic Index			X			X
Other cardiovascular and circulatory diseases	Data Rich	Female	Liters of alcohol consumed per capita			X			X
Other cardiovascular and circulatory diseases	Global	Female	Liters of alcohol consumed per capita			X			X
Other cardiovascular and circulatory diseases	Data Rich	Female	Age- and sex-specific SEV for Low fruit			X			X
Other cardiovascular and circulatory diseases	Global	Female	Age- and sex-specific SEV for Low fruit			X			X
Other cardiovascular and circulatory diseases	Data Rich	Female	Age- and sex-specific SEV for Low vegetables			X			X
Other cardiovascular and circulatory diseases	Global	Female	Age- and sex-specific SEV for Low vegetables			X			X
Other cardiovascular and circulatory diseases	Global	Female	Age- and sex-specific SEV for Low nuts and seeds			X			X
Other cardiovascular and circulatory diseases	Data Rich	Female	Age- and sex-specific SEV for Low omega-3			X			X
Other cardiovascular and circulatory diseases	Global	Female	Age- and sex-specific SEV for Low omega-3			X			X
Other cardiovascular and circulatory diseases	Data Rich	Female	Age- and sex-specific SEV for Low PUFA			X			X
Other cardiovascular and circulatory diseases	Global	Female	Age- and sex-specific SEV for Low PUFA			X			X
Other cardiovascular and circulatory diseases	Data Rich	Female	Age- and sex-specific SEV for Low legumes			X			X
Other cardiovascular and circulatory diseases	Global	Female	Age- and sex-specific SEV for Low legumes			X			X
Other cardiovascular and circulatory diseases	Data Rich	Female	Healthcare access and quality index		X			X	
Other cardiovascular and circulatory diseases	Global	Female	Healthcare access and quality index		X			X	
Other cardiovascular and circulatory diseases	Global	Female	Mean BMI		X			X	
Other cardiovascular and circulatory diseases	Data Rich	Female	Diabetes Fasting Plasma Glucose (mmol/L), age-standardized 25+		X			X	
Other cardiovascular and circulatory diseases	Global	Female	Diabetes Fasting Plasma Glucose (mmol/L), age-standardized 25+		X			X	
Other cardiovascular and circulatory diseases	Data Rich	Female	Mean BMI		X			X	
Other cardiovascular and circulatory diseases	Data Rich	Female	Indoor Air Pollution (All Cooking Fuels)		X			X	
Other cardiovascular and circulatory diseases	Global	Female	Indoor Air Pollution (All Cooking Fuels)		X			X	
Other cardiovascular and circulatory diseases	Data Rich	Female	Outdoor Air Pollution (PM2.5)		X			X	
Other cardiovascular and circulatory diseases	Global	Female	Outdoor Air Pollution (PM2.5)		X			X	

Appendix Table S11: Comparison of GBD 2021 and GBD 2023 covariates and level of covariates used in cause of death modeling									
Cause	Model Version Type	Sex	Covariate	Level 1 GBD 2021	Level 2 GBD 2021	Level 3 GBD 2021	Level 1 GBD 2023	Level 2 GBD 2023	Level 3 GBD 2023
Other cardiovascular and circulatory diseases	Data Rich	Female	Elevation Over 1500m (proportion)		X			X	
Other cardiovascular and circulatory diseases	Global	Female	Elevation Over 1500m (proportion)		X			X	
Other cardiovascular and circulatory diseases	Data Rich	Female	Low-Density Lipoprotein (mmol/L)	X			X		
Other cardiovascular and circulatory diseases	Global	Female	Low-Density Lipoprotein (mmol/L)	X			X		
Other cardiovascular and circulatory diseases	Data Rich	Female	Smoking Prevalence	X			X		
Other cardiovascular and circulatory diseases	Global	Female	Smoking Prevalence	X			X		
Other cardiovascular and circulatory diseases	Data Rich	Female	Systolic Blood Pressure (mmHg)	X			X		
Other cardiovascular and circulatory diseases	Global	Female	Systolic Blood Pressure (mmHg)	X			X		
Other cardiovascular and circulatory diseases	Data Rich	Male	LDI (I$ per capita)			X			X
Other cardiovascular and circulatory diseases	Global	Male	LDI (I$ per capita)			X			X
Other cardiovascular and circulatory diseases	Data Rich	Male	Socio-demographic Index			X			X
Other cardiovascular and circulatory diseases	Global	Male	Socio-demographic Index			X			X
Other cardiovascular and circulatory diseases	Data Rich	Male	Liters of alcohol consumed per capita			X			X
Other cardiovascular and circulatory diseases	Global	Male	Liters of alcohol consumed per capita			X			X
Other cardiovascular and circulatory diseases	Data Rich	Male	Age- and sex-specific SEV for Low fruit			X			X
Other cardiovascular and circulatory diseases	Global	Male	Age- and sex-specific SEV for Low fruit			X			X
Other cardiovascular and circulatory diseases	Data Rich	Male	Age- and sex-specific SEV for Low vegetables			X			X
Other cardiovascular and circulatory diseases	Global	Male	Age- and sex-specific SEV for Low vegetables			X			X
Other cardiovascular and circulatory diseases	Global	Male	Age- and sex-specific SEV for Low nuts and seeds			X			X
Other cardiovascular and circulatory diseases	Data Rich	Male	Age- and sex-specific SEV for Low omega-3			X			X
Other cardiovascular and circulatory diseases	Global	Male	Age- and sex-specific SEV for Low omega-3			X			X
Other cardiovascular and circulatory diseases	Data Rich	Male	Age- and sex-specific SEV for Low PUFA			X			X
Other cardiovascular and circulatory diseases	Global	Male	Age- and sex-specific SEV for Low PUFA			X			X
Other cardiovascular and circulatory diseases	Data Rich	Male	Age- and sex-specific SEV for Low legumes			X			X
Other cardiovascular and circulatory diseases	Global	Male	Age- and sex-specific SEV for Low legumes			X			X
Other cardiovascular and circulatory diseases	Data Rich	Male	Healthcare access and quality index		X			X	
Other cardiovascular and circulatory diseases	Global	Male	Healthcare access and quality index		X			X	
Other cardiovascular and circulatory diseases	Global	Male	Mean BMI		X			X	
Other cardiovascular and circulatory diseases	Data Rich	Male	Diabetes Fasting Plasma Glucose (mmol/L), age-standardized 25+		X			X	
Other cardiovascular and circulatory diseases	Global	Male	Diabetes Fasting Plasma Glucose (mmol/L), age-standardized 25+		X			X	
Other cardiovascular and circulatory diseases	Data Rich	Male	Mean BMI		X			X	
Other cardiovascular and circulatory diseases	Data Rich	Male	Indoor Air Pollution (All Cooking Fuels)		X			X	
Other cardiovascular and circulatory diseases	Global	Male	Indoor Air Pollution (All Cooking Fuels)		X			X	
Other cardiovascular and circulatory diseases	Data Rich	Male	Outdoor Air Pollution (PM2.5)		X			X	
Other cardiovascular and circulatory diseases	Global	Male	Outdoor Air Pollution (PM2.5)		X			X	
Other cardiovascular and circulatory diseases	Data Rich	Male	Elevation Over 1500m (proportion)		X			X	
Other cardiovascular and circulatory diseases	Global	Male	Elevation Over 1500m (proportion)		X			X	
Other cardiovascular and circulatory diseases	Data Rich	Male	Low-Density Lipoprotein (mmol/L)	X			X		
Other cardiovascular and circulatory diseases	Global	Male	Low-Density Lipoprotein (mmol/L)	X			X		
Other cardiovascular and circulatory diseases	Data Rich	Male	Smoking Prevalence	X			X		
Other cardiovascular and circulatory diseases	Global	Male	Smoking Prevalence	X			X		
Other cardiovascular and circulatory diseases	Data Rich	Male	Systolic Blood Pressure (mmHg)	X			X		
Other cardiovascular and circulatory diseases	Global	Male	Systolic Blood Pressure (mmHg)	X			X		
Chronic respiratory diseases	Data Rich	Female	Socio-demographic Index			X			X
Chronic respiratory diseases	Global	Female	Socio-demographic Index			X			X
Chronic respiratory diseases	Data Rich	Female	Population Density (over 1000 ppl/sqkm, proportion)			X			X
Chronic respiratory diseases	Global	Female	Population Density (over 1000 ppl/sqkm, proportion)			X			X
Chronic respiratory diseases	Data Rich	Female	Elevation 500 to 1500m (proportion)			X			X
Chronic respiratory diseases	Global	Female	Elevation 500 to 1500m (proportion)			X			X
Chronic respiratory diseases	Data Rich	Female	Healthcare access and quality index		X			X	
Chronic respiratory diseases	Data Rich	Female	Smoking Prevalence		X			X	
Chronic respiratory diseases	Global	Female	Smoking Prevalence		X			X	
Chronic respiratory diseases	Data Rich	Female	Outdoor Air Pollution (PM2.5)		X			X	
Chronic respiratory diseases	Global	Female	Outdoor Air Pollution (PM2.5)		X			X	
Chronic respiratory diseases	Data Rich	Female	Elevation Over 1500m (proportion)		X			X	
Chronic respiratory diseases	Global	Female	Elevation Over 1500m (proportion)		X			X	
Chronic respiratory diseases	Global	Female	Healthcare access and quality index	X				X	
Chronic respiratory diseases	Global	Female	Cumulative Cigarettes (10 Years)	X			X		
Chronic respiratory diseases	Data Rich	Female	Cumulative Cigarettes (10 Years)	X			X		
Chronic respiratory diseases	Data Rich	Female	Mask use						X
Chronic respiratory diseases	Global	Female	Mask use						X
Chronic respiratory diseases	Data Rich	Female	COVID-19 age-standardized death rate					X	
Chronic respiratory diseases	Global	Female	COVID-19 age-standardized death rate					X	
Chronic respiratory diseases	Global	Female	Cumulative Cigarettes (20 Years)				X		
Chronic respiratory diseases	Data Rich	Female	Cumulative Cigarettes (20 Years)				X		
Chronic respiratory diseases	Data Rich	Male	Socio-demographic Index			X			X
Chronic respiratory diseases	Global	Male	Socio-demographic Index			X			X
Chronic respiratory diseases	Data Rich	Male	Population Density (over 1000 ppl/sqkm, proportion)			X			X
Chronic respiratory diseases	Global	Male	Population Density (over 1000 ppl/sqkm, proportion)			X			X
Chronic respiratory diseases	Data Rich	Male	Elevation 500 to 1500m (proportion)			X			X
Chronic respiratory diseases	Global	Male	Elevation 500 to 1500m (proportion)			X			X
Chronic respiratory diseases	Data Rich	Male	Healthcare access and quality index		X			X	
Chronic respiratory diseases	Global	Male	Healthcare access and quality index		X			X	
Chronic respiratory diseases	Data Rich	Male	Smoking Prevalence		X			X	
Chronic respiratory diseases	Data Rich	Male	Outdoor Air Pollution (PM2.5)		X			X	
Chronic respiratory diseases	Global	Male	Outdoor Air Pollution (PM2.5)		X			X	
Chronic respiratory diseases	Data Rich	Male	Elevation Over 1500m (proportion)		X			X	
Chronic respiratory diseases	Global	Male	Elevation Over 1500m (proportion)		X			X	
Chronic respiratory diseases	Data Rich	Male	Indoor Air Pollution (All Cooking Fuels)		X		X		
Chronic respiratory diseases	Global	Male	Smoking Prevalence	X			X		
Chronic respiratory diseases	Global	Male	Indoor Air Pollution (All Cooking Fuels)	X			X		
Chronic respiratory diseases	Data Rich	Male	Cumulative Cigarettes (10 Years)	X			X		
Chronic respiratory diseases	Global	Male	Cumulative Cigarettes (10 Years)	X			X		
Chronic respiratory diseases	Data Rich	Male	Mask use						X
Chronic respiratory diseases	Global	Male	Mask use						X
Chronic respiratory diseases	Data Rich	Male	COVID-19 age-standardized death rate					X	
Chronic respiratory diseases	Global	Male	COVID-19 age-standardized death rate					X	
Chronic respiratory diseases	Data Rich	Male	Cumulative Cigarettes (20 Years)				X		
Chronic respiratory diseases	Global	Male	Cumulative Cigarettes (20 Years)				X		
Chronic obstructive pulmonary disease	Global	Female	Socio-demographic Index			X			X
Chronic obstructive pulmonary disease	Data Rich	Female	Socio-demographic Index			X			X
Chronic obstructive pulmonary disease	Global	Female	Smoking Prevalence		X			X	
Chronic obstructive pulmonary disease	Data Rich	Female	Smoking Prevalence		X			X	
Chronic obstructive pulmonary disease	Global	Female	Indoor Air Pollution (All Cooking Fuels)		X			X	
Chronic obstructive pulmonary disease	Data Rich	Female	Indoor Air Pollution (All Cooking Fuels)		X			X	
Chronic obstructive pulmonary disease	Data Rich	Female	Healthcare access and quality index	X					X
Chronic obstructive pulmonary disease	Global	Female	Healthcare access and quality index	X				X	
Chronic obstructive pulmonary disease	Global	Female	Outdoor Air Pollution (PM2.5)	X				X	
Chronic obstructive pulmonary disease	Data Rich	Female	Outdoor Air Pollution (PM2.5)	X				X	
Chronic obstructive pulmonary disease	Data Rich	Female	Elevation Over 1500m (proportion)	X				X	
Chronic obstructive pulmonary disease	Global	Female	Elevation Over 1500m (proportion)	X			X		
Chronic obstructive pulmonary disease	Global	Female	Cumulative Cigarettes (10 Years)	X			X		
Chronic obstructive pulmonary disease	Data Rich	Female	Cumulative Cigarettes (10 Years)	X			X		
Chronic obstructive pulmonary disease	Global	Female	Log-transformed SEV scalar: COPD	X			X		
Chronic obstructive pulmonary disease	Data Rich	Female	Log-transformed SEV scalar: COPD	X			X		
Chronic obstructive pulmonary disease	Global	Female	Mask use					X	

Appendix Table S11: Comparison of GBD 2021 and GBD 2023 covariates and level of covariates used in cause of death modeling

Cause	Model Version Type	Sex	Covariate	Level 1 GBD 2021	Level 2 GBD 2021	Level 3 GBD 2021	Level 1 GBD 2023	Level 2 GBD 2023	Level 3 GBD 2023
Chronic obstructive pulmonary disease	Data Rich	Female	Mask use					X	
Chronic obstructive pulmonary disease	Global	Female	COVID-19 age-standardized death rate					X	
Chronic obstructive pulmonary disease	Data Rich	Female	COVID-19 age-standardized death rate					X	
Chronic obstructive pulmonary disease	Global	Female	Cumulative Cigarettes (20 Years)				X		
Chronic obstructive pulmonary disease	Data Rich	Female	Cumulative Cigarettes (20 Years)				X		
Chronic obstructive pulmonary disease	Data Rich	Male	Socio-demographic Index			X			X
Chronic obstructive pulmonary disease	Global	Male	Socio-demographic Index			X			X
Chronic obstructive pulmonary disease	Data Rich	Male	Healthcare access and quality index		X			X	
Chronic obstructive pulmonary disease	Global	Male	Healthcare access and quality index		X			X	
Chronic obstructive pulmonary disease	Data Rich	Male	Smoking Prevalence		X			X	
Chronic obstructive pulmonary disease	Global	Male	Smoking Prevalence		X			X	
Chronic obstructive pulmonary disease	Data Rich	Male	Indoor Air Pollution (All Cooking Fuels)		X			X	
Chronic obstructive pulmonary disease	Global	Male	Indoor Air Pollution (All Cooking Fuels)		X			X	
Chronic obstructive pulmonary disease	Data Rich	Male	Outdoor Air Pollution (PM2.5)	X				X	
Chronic obstructive pulmonary disease	Global	Male	Outdoor Air Pollution (PM2.5)	X				X	
Chronic obstructive pulmonary disease	Data Rich	Male	Elevation Over 1500m (proportion)	X				X	
Chronic obstructive pulmonary disease	Global	Male	Elevation Over 1500m (proportion)	X				X	
Chronic obstructive pulmonary disease	Data Rich	Male	Cumulative Cigarettes (10 Years)	X			X		
Chronic obstructive pulmonary disease	Global	Male	Cumulative Cigarettes (10 Years)	X			X		
Chronic obstructive pulmonary disease	Data Rich	Male	Cumulative Cigarettes (20 Years)	X			X		
Chronic obstructive pulmonary disease	Global	Male	Cumulative Cigarettes (20 Years)	X			X		
Chronic obstructive pulmonary disease	Data Rich	Male	Log-transformed SEV scalar: COPD	X			X		
Chronic obstructive pulmonary disease	Global	Male	Log-transformed SEV scalar: COPD	X			X		
Chronic obstructive pulmonary disease	Data Rich	Male	Mask use					X	
Chronic obstructive pulmonary disease	Global	Male	Mask use					X	
Chronic obstructive pulmonary disease	Data Rich	Male	COVID-19 age-standardized death rate					X	
Chronic obstructive pulmonary disease	Global	Male	COVID-19 age-standardized death rate					X	
Pneumoconiosis	Global	Female	Socio-demographic Index			X			X
Pneumoconiosis	Data Rich	Female	Socio-demographic Index			X			X
Pneumoconiosis	Global	Female	Healthcare access and quality index		X				X
Pneumoconiosis	Data Rich	Female	Healthcare access and quality index		X				X
Pneumoconiosis	Global	Female	Smoking Prevalence		X			X	
Pneumoconiosis	Data Rich	Female	Smoking Prevalence		X			X	
Pneumoconiosis	Global	Female	Indoor Air Pollution (All Cooking Fuels)		X			X	
Pneumoconiosis	Data Rich	Female	Indoor Air Pollution (All Cooking Fuels)		X			X	
Pneumoconiosis	Global	Female	Asbestos consumption (metric tons per year per capita)	X				X	
Pneumoconiosis	Data Rich	Female	Asbestos consumption (metric tons per year per capita)	X				X	
Pneumoconiosis	Data Rich	Female	Age- and sex-specific SEV for Occupational beryllium	X				X	
Pneumoconiosis	Global	Female	Log-transformed coal production (per capita)	X			X		
Pneumoconiosis	Data Rich	Female	Log-transformed coal production (per capita)	X			X		
Pneumoconiosis	Data Rich	Female	Age- and sex-specific SEV for Occupational silica	X			X		
Pneumoconiosis	Global	Female	Gold production (kg) per capita, smoothed with 20-year lag	X			X		
Pneumoconiosis	Data Rich	Female	Gold production (kg) per capita, smoothed with 20-year lag	X			X		
Pneumoconiosis	Global	Female	Outdoor Air Pollution (PM2.5)					X	
Pneumoconiosis	Data Rich	Female	Outdoor Air Pollution (PM2.5)					X	
Pneumoconiosis	Global	Female	Cumulative Cigarettes (20 Years)					X	
Pneumoconiosis	Data Rich	Female	Cumulative Cigarettes (20 Years)					X	
Pneumoconiosis	Global	Female	Age- and sex-specific SEV for Occupational beryllium					X	
Pneumoconiosis	Global	Female	Age-standardized SEV for Occupational asbestos				X		
Pneumoconiosis	Data Rich	Female	Age-standardized SEV for Occupational asbestos				X		
Pneumoconiosis	Global	Female	Age- and sex-specific SEV for Occupational silica				X		
Pneumoconiosis	Global	Male	Socio-demographic Index			X			X
Pneumoconiosis	Data Rich	Male	Socio-demographic Index			X			X
Pneumoconiosis	Global	Male	Healthcare access and quality index		X				X
Pneumoconiosis	Data Rich	Male	Healthcare access and quality index		X				X
Pneumoconiosis	Global	Male	Smoking Prevalence		X			X	
Pneumoconiosis	Data Rich	Male	Smoking Prevalence		X			X	
Pneumoconiosis	Global	Male	Indoor Air Pollution (All Cooking Fuels)		X			X	
Pneumoconiosis	Data Rich	Male	Indoor Air Pollution (All Cooking Fuels)		X			X	
Pneumoconiosis	Global	Male	Asbestos consumption (metric tons per year per capita)	X				X	
Pneumoconiosis	Data Rich	Male	Asbestos consumption (metric tons per year per capita)	X				X	
Pneumoconiosis	Data Rich	Male	Age- and sex-specific SEV for Occupational beryllium	X				X	
Pneumoconiosis	Global	Male	Log-transformed coal production (per capita)	X			X		
Pneumoconiosis	Data Rich	Male	Log-transformed coal production (per capita)	X			X		
Pneumoconiosis	Data Rich	Male	Age- and sex-specific SEV for Occupational silica	X			X		
Pneumoconiosis	Global	Male	Gold production (kg) per capita, smoothed with 20-year lag	X			X		
Pneumoconiosis	Data Rich	Male	Gold production (kg) per capita, smoothed with 20-year lag	X			X		
Pneumoconiosis	Global	Male	Outdoor Air Pollution (PM2.5)					X	
Pneumoconiosis	Data Rich	Male	Outdoor Air Pollution (PM2.5)					X	
Pneumoconiosis	Global	Male	Cumulative Cigarettes (20 Years)					X	
Pneumoconiosis	Data Rich	Male	Cumulative Cigarettes (20 Years)					X	
Pneumoconiosis	Global	Male	Age- and sex-specific SEV for Occupational beryllium					X	
Pneumoconiosis	Global	Male	Age-standardized SEV for Occupational asbestos				X		
Pneumoconiosis	Data Rich	Male	Age-standardized SEV for Occupational asbestos				X		
Pneumoconiosis	Global	Male	Age- and sex-specific SEV for Occupational silica				X		
Silicosis	Data Rich	Female	Socio-demographic Index			X			X
Silicosis	Global	Female	Socio-demographic Index			X			X
Silicosis	Data Rich	Female	Healthcare access and quality index		X			X	
Silicosis	Global	Female	Healthcare access and quality index		X			X	
Silicosis	Data Rich	Female	Smoking Prevalence		X			X	
Silicosis	Global	Female	Smoking Prevalence		X			X	
Silicosis	Data Rich	Female	Indoor Air Pollution (All Cooking Fuels)		X			X	
Silicosis	Global	Female	Indoor Air Pollution (All Cooking Fuels)		X			X	
Silicosis	Data Rich	Female	Age- and sex-specific SEV for Occupational silica	X			X		
Silicosis	Data Rich	Female	Gold production (kg) per capita, smoothed with 20-year lag	X			X		
Silicosis	Global	Female	Gold production (kg) per capita, smoothed with 20-year lag	X			X		
Silicosis	Data Rich	Female	Outdoor Air Pollution (PM2.5)					X	
Silicosis	Global	Female	Outdoor Air Pollution (PM2.5)					X	
Silicosis	Data Rich	Female	Cumulative Cigarettes (10 Years)				X		
Silicosis	Global	Female	Cumulative Cigarettes (10 Years)				X		
Silicosis	Data Rich	Female	Cumulative Cigarettes (20 Years)				X		
Silicosis	Global	Female	Cumulative Cigarettes (20 Years)				X		
Silicosis	Global	Female	Age- and sex-specific SEV for Occupational silica				X		
Silicosis	Data Rich	Male	Socio-demographic Index			X			X
Silicosis	Global	Male	Socio-demographic Index			X			X
Silicosis	Data Rich	Male	Healthcare access and quality index		X			X	
Silicosis	Global	Male	Healthcare access and quality index		X			X	
Silicosis	Data Rich	Male	Smoking Prevalence		X			X	
Silicosis	Global	Male	Smoking Prevalence		X			X	
Silicosis	Data Rich	Male	Indoor Air Pollution (All Cooking Fuels)		X			X	
Silicosis	Global	Male	Indoor Air Pollution (All Cooking Fuels)		X			X	
Silicosis	Data Rich	Male	Age- and sex-specific SEV for Occupational silica	X			X		
Silicosis	Data Rich	Male	Gold production (kg) per capita, smoothed with 20-year lag	X			X		
Silicosis	Global	Male	Gold production (kg) per capita, smoothed with 20-year lag	X			X		
Silicosis	Data Rich	Male	Outdoor Air Pollution (PM2.5)					X	
Silicosis	Global	Male	Outdoor Air Pollution (PM2.5)					X	
Silicosis	Data Rich	Male	Cumulative Cigarettes (10 Years)				X		
Silicosis	Global	Male	Cumulative Cigarettes (10 Years)				X		
Silicosis	Data Rich	Male	Cumulative Cigarettes (20 Years)				X		
Silicosis	Global	Male	Cumulative Cigarettes (20 Years)				X		
Silicosis	Global	Male	Age- and sex-specific SEV for Occupational silica				X		
Asbestosis	Data Rich	Female	Socio-demographic Index			X			X
Asbestosis	Global	Female	Socio-demographic Index			X			X

Appendix Table S11: Comparison of GBD 2021 and GBD 2023 covariates and level of covariates used in cause of death modeling

Cause	Model Version Type	Sex	Covariate	Level 1 GBD 2021	Level 2 GBD 2021	Level 3 GBD 2021	Level 1 GBD 2023	Level 2 GBD 2023	Level 3 GBD 2023
Asbestosis	Data Rich	Female	Cumulative Cigarettes (10 Years)		X			X	
Asbestosis	Global	Female	Cumulative Cigarettes (10 Years)		X			X	
Asbestosis	Data Rich	Female	Healthcare access and quality index		X			X	
Asbestosis	Global	Female	Healthcare access and quality index		X			X	
Asbestosis	Data Rich	Female	Smoking Prevalence		X			X	
Asbestosis	Global	Female	Smoking Prevalence		X			X	
Asbestosis	Data Rich	Female	Indoor Air Pollution (All Cooking Fuels)		X			X	
Asbestosis	Global	Female	Indoor Air Pollution (All Cooking Fuels)		X			X	
Asbestosis	Data Rich	Female	Cumulative Cigarettes (5 Years)		X			X	
Asbestosis	Global	Female	Cumulative Cigarettes (5 Years)		X			X	
Asbestosis	Data Rich	Female	Asbestos consumption (metric tons per year per capita)	X			X		
Asbestosis	Global	Female	Asbestos consumption (metric tons per year per capita)	X			X		
Asbestosis	Data Rich	Female	Outdoor Air Pollution (PM2.5)					X	
Asbestosis	Global	Female	Outdoor Air Pollution (PM2.5)					X	
Asbestosis	Global	Female	Cumulative Cigarettes (20 Years)					X	
Asbestosis	Data Rich	Female	Cumulative Cigarettes (20 Years)				X		
Asbestosis	Data Rich	Female	Age-standardized SEV for Occupational asbestos				X		
Asbestosis	Global	Female	Age-standardized SEV for Occupational asbestos				X		
Asbestosis	Data Rich	Male	Socio-demographic Index			X			X
Asbestosis	Global	Male	Socio-demographic Index			X			X
Asbestosis	Data Rich	Male	Healthcare access and quality index		X			X	
Asbestosis	Global	Male	Healthcare access and quality index		X			X	
Asbestosis	Global	Male	Smoking Prevalence		X			X	
Asbestosis	Data Rich	Male	Indoor Air Pollution (All Cooking Fuels)		X			X	
Asbestosis	Global	Male	Indoor Air Pollution (All Cooking Fuels)		X			X	
Asbestosis	Data Rich	Male	Smoking Prevalence	X				X	
Asbestosis	Data Rich	Male	Asbestos consumption (metric tons per year per capita)	X			X		
Asbestosis	Global	Male	Asbestos consumption (metric tons per year per capita)	X			X		
Asbestosis	Data Rich	Male	Outdoor Air Pollution (PM2.5)					X	
Asbestosis	Global	Male	Outdoor Air Pollution (PM2.5)					X	
Asbestosis	Data Rich	Male	Cumulative Cigarettes (10 Years)				X		
Asbestosis	Global	Male	Cumulative Cigarettes (10 Years)				X		
Asbestosis	Data Rich	Male	Cumulative Cigarettes (20 Years)				X		
Asbestosis	Global	Male	Cumulative Cigarettes (20 Years)				X		
Asbestosis	Data Rich	Male	Age-standardized SEV for Occupational asbestos				X		
Asbestosis	Global	Male	Age-standardized SEV for Occupational asbestos				X		
Coal workers pneumoconiosis	Global	Female	Socio-demographic Index			X			X
Coal workers pneumoconiosis	Data Rich	Female	Socio-demographic Index			X			X
Coal workers pneumoconiosis	Global	Female	Healthcare access and quality index		X			X	
Coal workers pneumoconiosis	Data Rich	Female	Healthcare access and quality index		X			X	
Coal workers pneumoconiosis	Global	Female	Smoking Prevalence		X			X	
Coal workers pneumoconiosis	Data Rich	Female	Smoking Prevalence		X			X	
Coal workers pneumoconiosis	Global	Female	Indoor Air Pollution (All Cooking Fuels)		X			X	
Coal workers pneumoconiosis	Data Rich	Female	Indoor Air Pollution (All Cooking Fuels)		X			X	
Coal workers pneumoconiosis	Global	Female	Log-transformed coal production (per capita)	X			X		
Coal workers pneumoconiosis	Data Rich	Female	Log-transformed coal production (per capita)	X			X		
Coal workers pneumoconiosis	Global	Female	Outdoor Air Pollution (PM2.5)					X	
Coal workers pneumoconiosis	Data Rich	Female	Outdoor Air Pollution (PM2.5)					X	
Coal workers pneumoconiosis	Global	Female	Cumulative Cigarettes (10 Years)				X		
Coal workers pneumoconiosis	Data Rich	Female	Cumulative Cigarettes (10 Years)				X		
Coal workers pneumoconiosis	Global	Female	Cumulative Cigarettes (20 Years)				X		
Coal workers pneumoconiosis	Data Rich	Female	Cumulative Cigarettes (20 Years)				X		
Coal workers pneumoconiosis	Global	Male	Socio-demographic Index			X			X
Coal workers pneumoconiosis	Data Rich	Male	Socio-demographic Index			X			X
Coal workers pneumoconiosis	Global	Male	Healthcare access and quality index		X			X	
Coal workers pneumoconiosis	Data Rich	Male	Healthcare access and quality index		X			X	
Coal workers pneumoconiosis	Global	Male	Smoking Prevalence		X			X	
Coal workers pneumoconiosis	Data Rich	Male	Smoking Prevalence		X			X	
Coal workers pneumoconiosis	Global	Male	Indoor Air Pollution (All Cooking Fuels)		X			X	
Coal workers pneumoconiosis	Data Rich	Male	Indoor Air Pollution (All Cooking Fuels)		X			X	
Coal workers pneumoconiosis	Global	Male	Log-transformed coal production (per capita)	X			X		
Coal workers pneumoconiosis	Data Rich	Male	Log-transformed coal production (per capita)	X			X		
Coal workers pneumoconiosis	Global	Male	Outdoor Air Pollution (PM2.5)					X	
Coal workers pneumoconiosis	Data Rich	Male	Outdoor Air Pollution (PM2.5)					X	
Coal workers pneumoconiosis	Global	Male	Cumulative Cigarettes (10 Years)				X		
Coal workers pneumoconiosis	Data Rich	Male	Cumulative Cigarettes (10 Years)				X		
Coal workers pneumoconiosis	Global	Male	Cumulative Cigarettes (20 Years)				X		
Coal workers pneumoconiosis	Data Rich	Male	Cumulative Cigarettes (20 Years)				X		
Other pneumoconiosis	Data Rich	Female	Socio-demographic Index			X			X
Other pneumoconiosis	Global	Female	Socio-demographic Index			X			X
Other pneumoconiosis	Data Rich	Female	Healthcare access and quality index		X			X	
Other pneumoconiosis	Global	Female	Healthcare access and quality index		X			X	
Other pneumoconiosis	Data Rich	Female	Smoking Prevalence		X			X	
Other pneumoconiosis	Global	Female	Smoking Prevalence		X			X	
Other pneumoconiosis	Data Rich	Female	Indoor Air Pollution (All Cooking Fuels)		X			X	
Other pneumoconiosis	Global	Female	Indoor Air Pollution (All Cooking Fuels)		X			X	
Other pneumoconiosis	Data Rich	Female	Tuberculosis prevalence (age-standardized)	X				X	
Other pneumoconiosis	Data Rich	Female	Age- and sex-specific SEV for Occupational beryllium	X			X		
Other pneumoconiosis	Global	Female	Tuberculosis prevalence (age-standardized)	X			X		
Other pneumoconiosis	Data Rich	Female	Outdoor Air Pollution (PM2.5)					X	
Other pneumoconiosis	Global	Female	Outdoor Air Pollution (PM2.5)					X	
Other pneumoconiosis	Data Rich	Female	Proportion of the population working in mining with 20-year lag					X	
Other pneumoconiosis	Global	Female	Proportion of the population working in mining with 20-year lag					X	
Other pneumoconiosis	Data Rich	Female	Cumulative Cigarettes (10 Years)				X		
Other pneumoconiosis	Global	Female	Cumulative Cigarettes (10 Years)				X		
Other pneumoconiosis	Data Rich	Female	Cumulative Cigarettes (20 Years)				X		
Other pneumoconiosis	Global	Female	Cumulative Cigarettes (20 Years)				X		
Other pneumoconiosis	Global	Female	Age- and sex-specific SEV for Occupational beryllium				X		
Other pneumoconiosis	Data Rich	Female	Age- and sex-specific SEV for Occupational silica				X		
Other pneumoconiosis	Global	Female	Age- and sex-specific SEV for Occupational silica				X		
Other pneumoconiosis	Data Rich	Female	Gold production (kg) per capita, smoothed with 20-year lag				X		
Other pneumoconiosis	Global	Female	Gold production (kg) per capita, smoothed with 20-year lag				X		
Other pneumoconiosis	Global	Male	Socio-demographic Index			X			X
Other pneumoconiosis	Data Rich	Male	Socio-demographic Index			X			X
Other pneumoconiosis	Global	Male	Healthcare access and quality index		X			X	
Other pneumoconiosis	Data Rich	Male	Healthcare access and quality index		X			X	
Other pneumoconiosis	Global	Male	Smoking Prevalence		X			X	
Other pneumoconiosis	Data Rich	Male	Smoking Prevalence		X			X	
Other pneumoconiosis	Global	Male	Indoor Air Pollution (All Cooking Fuels)		X			X	
Other pneumoconiosis	Data Rich	Male	Indoor Air Pollution (All Cooking Fuels)		X			X	
Other pneumoconiosis	Data Rich	Male	Tuberculosis prevalence (age-standardized)	X				X	
Other pneumoconiosis	Data Rich	Male	Age- and sex-specific SEV for Occupational beryllium	X			X		
Other pneumoconiosis	Global	Male	Tuberculosis prevalence (age-standardized)	X			X		
Other pneumoconiosis	Global	Male	Outdoor Air Pollution (PM2.5)					X	
Other pneumoconiosis	Data Rich	Male	Outdoor Air Pollution (PM2.5)					X	
Other pneumoconiosis	Global	Male	Proportion of the population working in mining with 20-year lag					X	
Other pneumoconiosis	Global	Male	Cumulative Cigarettes (10 Years)				X		
Other pneumoconiosis	Data Rich	Male	Cumulative Cigarettes (10 Years)				X		
Other pneumoconiosis	Global	Male	Cumulative Cigarettes (20 Years)				X		
Other pneumoconiosis	Data Rich	Male	Cumulative Cigarettes (20 Years)				X		
Other pneumoconiosis	Global	Male	Age- and sex-specific SEV for Occupational beryllium				X		
Other pneumoconiosis	Global	Male	Age- and sex-specific SEV for Occupational silica				X		
Other pneumoconiosis	Data Rich	Male	Age- and sex-specific SEV for Occupational silica				X		
Other pneumoconiosis	Global	Male	Gold production (kg) per capita, smoothed with 20-year lag				X		

Appendix Table S11: Comparison of GBD 2021 and GBD 2023 covariates and level of covariates used in cause of death modeling									
Cause	Model Version Type	Sex	Covariate	Level 1 GBD 2021	Level 2 GBD 2021	Level 3 GBD 2021	Level 1 GBD 2023	Level 2 GBD 2023	Level 3 GBD 2023
Other pneumoconiosis	Data Rich	Male	Gold production (kg) per capita, smoothed with 20-year lag				X		
Other pneumoconiosis	Data Rich	Male	Proportion of the population working in mining with 20-year lag				X		
Asthma	Global	Female	Socio-demographic Index			X			X
Asthma	Data Rich	Female	Socio-demographic Index			X			X
Asthma	Global	Female	Smoking Prevalence		X			X	
Asthma	Data Rich	Female	Smoking Prevalence		X			X	
Asthma	Global	Female	Indoor Air Pollution (All Cooking Fuels)		X			X	
Asthma	Data Rich	Female	Indoor Air Pollution (All Cooking Fuels)		X			X	
Asthma	Global	Female	Outdoor Air Pollution (PM2.5)		X			X	
Asthma	Data Rich	Female	Outdoor Air Pollution (PM2.5)		X			X	
Asthma	Global	Female	Healthcare access and quality index	X				X	
Asthma	Data Rich	Female	Healthcare access and quality index	X				X	
Asthma	Global	Female	Cumulative Cigarettes (10 Years)	X			X		
Asthma	Data Rich	Female	Cumulative Cigarettes (10 Years)	X			X		
Asthma	Global	Female	Log-transformed SEV scalar: Asthma	X			X		
Asthma	Data Rich	Female	Log-transformed SEV scalar: Asthma	X			X		
Asthma	Global	Female	Proportion of population involved in agricultural activities					X	
Asthma	Data Rich	Female	Proportion of population involved in agricultural activities					X	
Asthma	Global	Female	Cumulative Cigarettes (20 Years)				X		
Asthma	Data Rich	Female	Cumulative Cigarettes (20 Years)				X		
Asthma	Global	Male	Socio-demographic Index			X			X
Asthma	Data Rich	Male	Socio-demographic Index			X			X
Asthma	Global	Male	Smoking Prevalence		X			X	
Asthma	Data Rich	Male	Smoking Prevalence		X			X	
Asthma	Global	Male	Indoor Air Pollution (All Cooking Fuels)		X			X	
Asthma	Data Rich	Male	Indoor Air Pollution (All Cooking Fuels)		X			X	
Asthma	Global	Male	Outdoor Air Pollution (PM2.5)		X			X	
Asthma	Data Rich	Male	Outdoor Air Pollution (PM2.5)		X			X	
Asthma	Global	Male	Healthcare access and quality index	X				X	
Asthma	Data Rich	Male	Healthcare access and quality index	X				X	
Asthma	Global	Male	Cumulative Cigarettes (10 Years)	X			X		
Asthma	Data Rich	Male	Cumulative Cigarettes (10 Years)	X			X		
Asthma	Global	Male	Log-transformed SEV scalar: Asthma	X			X		
Asthma	Data Rich	Male	Log-transformed SEV scalar: Asthma	X			X		
Asthma	Global	Male	Proportion of population involved in agricultural activities					X	
Asthma	Data Rich	Male	Proportion of population involved in agricultural activities					X	
Asthma	Global	Male	Cumulative Cigarettes (20 Years)				X		
Asthma	Data Rich	Male	Cumulative Cigarettes (20 Years)				X		
Interstitial lung disease and pulmonary sarcoidosis	Global	Female	Socio-demographic Index			X			X
Interstitial lung disease and pulmonary sarcoidosis	Data Rich	Female	Socio-demographic Index			X			X
Interstitial lung disease and pulmonary sarcoidosis	Global	Female	Healthcare access and quality index		X			X	
Interstitial lung disease and pulmonary sarcoidosis	Data Rich	Female	Healthcare access and quality index		X			X	
Interstitial lung disease and pulmonary sarcoidosis	Data Rich	Female	Indoor Air Pollution (All Cooking Fuels)		X			X	
Interstitial lung disease and pulmonary sarcoidosis	Data Rich	Female	Outdoor Air Pollution (PM2.5)		X			X	
Interstitial lung disease and pulmonary sarcoidosis	Global	Female	Elevation Over 1500m (proportion)		X			X	
Interstitial lung disease and pulmonary sarcoidosis	Data Rich	Female	Elevation Over 1500m (proportion)		X			X	
Interstitial lung disease and pulmonary sarcoidosis	Global	Female	Elevation 500 to 1500m (proportion)		X			X	
Interstitial lung disease and pulmonary sarcoidosis	Data Rich	Female	Elevation 500 to 1500m (proportion)		X			X	
Interstitial lung disease and pulmonary sarcoidosis	Global	Female	Cumulative Cigarettes (10 Years)	X			X		
Interstitial lung disease and pulmonary sarcoidosis	Data Rich	Female	Cumulative Cigarettes (10 Years)	X			X		
Interstitial lung disease and pulmonary sarcoidosis	Global	Female	Smoking Prevalence	X			X		
Interstitial lung disease and pulmonary sarcoidosis	Data Rich	Female	Smoking Prevalence	X			X		
Interstitial lung disease and pulmonary sarcoidosis	Global	Female	Indoor Air Pollution (All Cooking Fuels)	X			X		
Interstitial lung disease and pulmonary sarcoidosis	Global	Female	Outdoor Air Pollution (PM2.5)	X			X		
Interstitial lung disease and pulmonary sarcoidosis	Global	Female	Cumulative Cigarettes (5 Years)	X			X		
Interstitial lung disease and pulmonary sarcoidosis	Data Rich	Female	Cumulative Cigarettes (5 Years)	X			X		
Interstitial lung disease and pulmonary sarcoidosis	Global	Male	Socio-demographic Index			X			X
Interstitial lung disease and pulmonary sarcoidosis	Data Rich	Male	Socio-demographic Index			X		X	
Interstitial lung disease and pulmonary sarcoidosis	Global	Male	Healthcare access and quality index		X			X	
Interstitial lung disease and pulmonary sarcoidosis	Data Rich	Male	Healthcare access and quality index		X			X	
Interstitial lung disease and pulmonary sarcoidosis	Global	Male	Indoor Air Pollution (All Cooking Fuels)		X			X	
Interstitial lung disease and pulmonary sarcoidosis	Data Rich	Male	Indoor Air Pollution (All Cooking Fuels)		X			X	
Interstitial lung disease and pulmonary sarcoidosis	Global	Male	Outdoor Air Pollution (PM2.5)		X			X	
Interstitial lung disease and pulmonary sarcoidosis	Data Rich	Male	Outdoor Air Pollution (PM2.5)		X			X	
Interstitial lung disease and pulmonary sarcoidosis	Global	Male	Elevation Over 1500m (proportion)		X			X	
Interstitial lung disease and pulmonary sarcoidosis	Data Rich	Male	Elevation Over 1500m (proportion)		X			X	
Interstitial lung disease and pulmonary sarcoidosis	Global	Male	Elevation 500 to 1500m (proportion)		X			X	
Interstitial lung disease and pulmonary sarcoidosis	Data Rich	Male	Elevation 500 to 1500m (proportion)		X			X	
Interstitial lung disease and pulmonary sarcoidosis	Global	Male	Cumulative Cigarettes (10 Years)	X			X		
Interstitial lung disease and pulmonary sarcoidosis	Data Rich	Male	Cumulative Cigarettes (10 Years)	X			X		
Interstitial lung disease and pulmonary sarcoidosis	Global	Male	Smoking Prevalence	X			X		
Interstitial lung disease and pulmonary sarcoidosis	Data Rich	Male	Smoking Prevalence	X			X		
Interstitial lung disease and pulmonary sarcoidosis	Global	Male	Cumulative Cigarettes (5 Years)	X			X		
Interstitial lung disease and pulmonary sarcoidosis	Data Rich	Male	Cumulative Cigarettes (5 Years)	X			X		
Interstitial lung disease and pulmonary sarcoidosis	Global	Male	Cumulative Cigarettes (20 Years)	X			X		
Other chronic respiratory diseases	Global	Female	Socio-demographic Index			X			X
Other chronic respiratory diseases	Data Rich	Female	Socio-demographic Index			X			X
Other chronic respiratory diseases	Global	Female	Healthcare access and quality index		X				X
Other chronic respiratory diseases	Data Rich	Female	Healthcare access and quality index		X			X	
Other chronic respiratory diseases	Global	Female	Elevation Over 1500m (proportion)		X			X	
Other chronic respiratory diseases	Data Rich	Female	Elevation Over 1500m (proportion)		X			X	
Other chronic respiratory diseases	Data Rich	Female	Smoking Prevalence	X				X	
Other chronic respiratory diseases	Global	Female	Indoor Air Pollution (All Cooking Fuels)	X				X	
Other chronic respiratory diseases	Global	Female	Outdoor Air Pollution (PM2.5)	X				X	
Other chronic respiratory diseases	Global	Female	Smoking Prevalence	X			X		
Other chronic respiratory diseases	Data Rich	Female	Indoor Air Pollution (All Cooking Fuels)	X			X		
Other chronic respiratory diseases	Data Rich	Female	Outdoor Air Pollution (PM2.5)	X			X		
Other chronic respiratory diseases	Global	Female	Cumulative Cigarettes (5 Years)	X			X		
Other chronic respiratory diseases	Data Rich	Female	Cumulative Cigarettes (5 Years)	X			X		
Other chronic respiratory diseases	Global	Female	Mean BMI					X	
Other chronic respiratory diseases	Data Rich	Female	Mean BMI					X	
Other chronic respiratory diseases	Global	Female	Liters of alcohol consumed per capita					X	
Other chronic respiratory diseases	Data Rich	Female	Liters of alcohol consumed per capita					X	
Other chronic respiratory diseases	Global	Female	Cumulative Cigarettes (20 Years)				X		
Other chronic respiratory diseases	Data Rich	Female	Cumulative Cigarettes (20 Years)				X		
Other chronic respiratory diseases	Global	Male	Socio-demographic Index			X			X
Other chronic respiratory diseases	Data Rich	Male	Socio-demographic Index			X			X
Other chronic respiratory diseases	Global	Male	Healthcare access and quality index		X			X	
Other chronic respiratory diseases	Data Rich	Male	Healthcare access and quality index		X			X	
Other chronic respiratory diseases	Global	Male	Elevation Over 1500m (proportion)		X			X	
Other chronic respiratory diseases	Data Rich	Male	Elevation Over 1500m (proportion)		X			X	
Other chronic respiratory diseases	Global	Male	Smoking Prevalence	X			X		
Other chronic respiratory diseases	Data Rich	Male	Smoking Prevalence	X			X		
Other chronic respiratory diseases	Global	Male	Indoor Air Pollution (All Cooking Fuels)	X			X		
Other chronic respiratory diseases	Data Rich	Male	Indoor Air Pollution (All Cooking Fuels)	X			X		
Other chronic respiratory diseases	Global	Male	Outdoor Air Pollution (PM2.5)	X			X		
Other chronic respiratory diseases	Data Rich	Male	Outdoor Air Pollution (PM2.5)	X			X		

Appendix Table S11: Comparison of GBD 2021 and GBD 2023 covariates and level of covariates used in cause of death modeling									
Cause	Model Version Type	Sex	Covariate	Level 1 GBD 2021	Level 2 GBD 2021	Level 3 GBD 2021	Level 1 GBD 2023	Level 2 GBD 2023	Level 3 GBD 2023
Other chronic respiratory diseases	Global	Male	Cumulative Cigarettes (5 Years)	X			X		
Other chronic respiratory diseases	Data Rich	Male	Cumulative Cigarettes (5 Years)	X			X		
Other chronic respiratory diseases	Global	Male	Mean BMI					X	
Other chronic respiratory diseases	Data Rich	Male	Mean BMI					X	
Other chronic respiratory diseases	Global	Male	Liters of alcohol consumed per capita					X	
Other chronic respiratory diseases	Data Rich	Male	Liters of alcohol consumed per capita					X	
Other chronic respiratory diseases	Global	Male	Cumulative Cigarettes (20 Years)				X		
Other chronic respiratory diseases	Data Rich	Male	Cumulative Cigarettes (20 Years)				X		
Digestive diseases	Data Rich	Female	Education (years per capita)			X			X
Digestive diseases	Global	Female	Education (years per capita)			X			X
Digestive diseases	Data Rich	Female	LDI (I$ per capita)			X			X
Digestive diseases	Global	Female	LDI (I$ per capita)			X			X
Digestive diseases	Data Rich	Female	Socio-demographic Index			X			X
Digestive diseases	Global	Female	Socio-demographic Index			X			X
Digestive diseases	Data Rich	Female	Healthcare access and quality index		X			X	
Digestive diseases	Global	Female	Healthcare access and quality index		X			X	
Digestive diseases	Global	Female	Mean BMI		X			X	
Digestive diseases	Data Rich	Female	Age- and sex-specific SEV for Low fruit		X			X	
Digestive diseases	Global	Female	Age- and sex-specific SEV for Low fruit		X			X	
Digestive diseases	Data Rich	Female	Age- and sex-specific SEV for Low vegetables		X			X	
Digestive diseases	Global	Female	Age- and sex-specific SEV for Low vegetables		X			X	
Digestive diseases	Data Rich	Female	Age- and sex-specific SEV for High red meat		X			X	
Digestive diseases	Global	Female	Age- and sex-specific SEV for High red meat		X			X	
Digestive diseases	Data Rich	Female	Cumulative Cigarettes (10 Years)	X			X		
Digestive diseases	Data Rich	Female	Smoking Prevalence	X			X		
Digestive diseases	Data Rich	Female	Liters of alcohol consumed per capita	X			X		
Digestive diseases	Data Rich	Female	Sanitation (proportion with access)	X			X		
Digestive diseases	Global	Female	Sanitation (proportion with access)	X			X		
Digestive diseases	Global	Female	Liters of alcohol consumed per capita	X			X		
Digestive diseases	Global	Female	Smoking Prevalence	X			X		
Digestive diseases	Data Rich	Female	Mean BMI	X			X		
Digestive diseases	Data Rich	Female	Cumulative Cigarettes (5 Years)	X			X		
Digestive diseases	Global	Female	Cumulative Cigarettes (10 Years)	X			X		
Digestive diseases	Global	Female	Cumulative Cigarettes (5 Years)	X			X		
Digestive diseases	Data Rich	Male	Education (years per capita)			X			X
Digestive diseases	Global	Male	Education (years per capita)			X			X
Digestive diseases	Data Rich	Male	LDI (I$ per capita)			X			X
Digestive diseases	Global	Male	LDI (I$ per capita)			X			X
Digestive diseases	Data Rich	Male	Socio-demographic Index			X			X
Digestive diseases	Global	Male	Socio-demographic Index			X			X
Digestive diseases	Data Rich	Male	Healthcare access and quality index		X			X	
Digestive diseases	Global	Male	Healthcare access and quality index		X			X	
Digestive diseases	Global	Male	Mean BMI		X			X	
Digestive diseases	Data Rich	Male	Mean BMI		X			X	
Digestive diseases	Data Rich	Male	Age- and sex-specific SEV for Low fruit		X			X	
Digestive diseases	Global	Male	Age- and sex-specific SEV for Low fruit		X			X	
Digestive diseases	Data Rich	Male	Age- and sex-specific SEV for Low vegetables		X			X	
Digestive diseases	Global	Male	Age- and sex-specific SEV for Low vegetables		X			X	
Digestive diseases	Data Rich	Male	Age- and sex-specific SEV for High red meat		X			X	
Digestive diseases	Global	Male	Age- and sex-specific SEV for High red meat		X			X	
Digestive diseases	Data Rich	Male	Cumulative Cigarettes (10 Years)	X			X		
Digestive diseases	Data Rich	Male	Smoking Prevalence	X			X		
Digestive diseases	Data Rich	Male	Liters of alcohol consumed per capita	X			X		
Digestive diseases	Data Rich	Male	Sanitation (proportion with access)	X			X		
Digestive diseases	Global	Male	Sanitation (proportion with access)	X			X		
Digestive diseases	Global	Male	Liters of alcohol consumed per capita	X			X		
Digestive diseases	Global	Male	Smoking Prevalence	X			X		
Digestive diseases	Data Rich	Male	Cumulative Cigarettes (5 Years)	X			X		
Digestive diseases	Global	Male	Cumulative Cigarettes (10 Years)	X			X		
Digestive diseases	Global	Male	Cumulative Cigarettes (5 Years)	X			X		
Cirrhosis and other chronic liver diseases	Data Rich	Female	Education (years per capita)			X			X
Cirrhosis and other chronic liver diseases	Global	Female	Education (years per capita)			X			X
Cirrhosis and other chronic liver diseases	Data Rich	Female	LDI (I$ per capita)			X			X
Cirrhosis and other chronic liver diseases	Global	Female	LDI (I$ per capita)			X			X
Cirrhosis and other chronic liver diseases	Data Rich	Female	Socio-demographic Index			X			X
Cirrhosis and other chronic liver diseases	Global	Female	Socio-demographic Index			X			X
Cirrhosis and other chronic liver diseases	Data Rich	Female	Healthcare access and quality index		X			X	
Cirrhosis and other chronic liver diseases	Global	Female	Healthcare access and quality index		X			X	
Cirrhosis and other chronic liver diseases	Global	Female	Mean BMI		X			X	
Cirrhosis and other chronic liver diseases	Data Rich	Female	Mean BMI		X			X	
Cirrhosis and other chronic liver diseases	Data Rich	Female	Intravenous drug use (proportion by age)		X			X	
Cirrhosis and other chronic liver diseases	Global	Female	Intravenous drug use (proportion by age)		X			X	
Cirrhosis and other chronic liver diseases	Data Rich	Female	Diabetes Age-Standardized Prevalence (proportion)		X			X	
Cirrhosis and other chronic liver diseases	Global	Female	Diabetes Age-Standardized Prevalence (proportion)		X			X	
Cirrhosis and other chronic liver diseases	Data Rich	Female	Liters of alcohol consumed per capita	X			X		
Cirrhosis and other chronic liver diseases	Global	Female	Liters of alcohol consumed per capita	X			X		
Cirrhosis and other chronic liver diseases	Data Rich	Female	Hepatitis B vaccine coverage, aged through time, COVID-free (proportion)	X			X		
Cirrhosis and other chronic liver diseases	Global	Female	Hepatitis B vaccine coverage, aged through time, COVID-free (proportion)	X			X		
Cirrhosis and other chronic liver diseases	Data Rich	Female	Vaccine adjusted HbSAg serporevalence age standardized	X			X		
Cirrhosis and other chronic liver diseases	Global	Female	Vaccine adjusted HbSAg serporevalence age standardized	X			X		
Cirrhosis and other chronic liver diseases	Data Rich	Female	Chronic Hepatitis C age standardized	X			X		
Cirrhosis and other chronic liver diseases	Global	Female	Chronic Hepatitis C age standardized	X			X		
Cirrhosis and other chronic liver diseases	Data Rich	Male	Education (years per capita)			X			X
Cirrhosis and other chronic liver diseases	Global	Male	Education (years per capita)			X			X
Cirrhosis and other chronic liver diseases	Data Rich	Male	LDI (I$ per capita)			X			X
Cirrhosis and other chronic liver diseases	Global	Male	LDI (I$ per capita)			X			X
Cirrhosis and other chronic liver diseases	Data Rich	Male	Socio-demographic Index			X			X
Cirrhosis and other chronic liver diseases	Global	Male	Socio-demographic Index			X			X
Cirrhosis and other chronic liver diseases	Data Rich	Male	Healthcare access and quality index		X			X	
Cirrhosis and other chronic liver diseases	Global	Male	Healthcare access and quality index		X			X	
Cirrhosis and other chronic liver diseases	Global	Male	Mean BMI		X			X	
Cirrhosis and other chronic liver diseases	Data Rich	Male	Mean BMI		X			X	
Cirrhosis and other chronic liver diseases	Data Rich	Male	Intravenous drug use (proportion by age)		X			X	
Cirrhosis and other chronic liver diseases	Global	Male	Intravenous drug use (proportion by age)		X			X	
Cirrhosis and other chronic liver diseases	Data Rich	Male	Diabetes Age-Standardized Prevalence (proportion)		X			X	
Cirrhosis and other chronic liver diseases	Global	Male	Diabetes Age-Standardized Prevalence (proportion)		X			X	
Cirrhosis and other chronic liver diseases	Data Rich	Male	Liters of alcohol consumed per capita	X			X		
Cirrhosis and other chronic liver diseases	Global	Male	Liters of alcohol consumed per capita	X			X		
Cirrhosis and other chronic liver diseases	Data Rich	Male	Hepatitis B vaccine coverage, aged through time, COVID-free (proportion)	X			X		
Cirrhosis and other chronic liver diseases	Global	Male	Hepatitis B vaccine coverage, aged through time, COVID-free (proportion)	X			X		
Cirrhosis and other chronic liver diseases	Data Rich	Male	Vaccine adjusted HbSAg serporevalence age standardized	X			X		
Cirrhosis and other chronic liver diseases	Global	Male	Vaccine adjusted HbSAg serporevalence age standardized	X			X		
Cirrhosis and other chronic liver diseases	Data Rich	Male	Chronic Hepatitis C age standardized	X			X		
Cirrhosis and other chronic liver diseases	Global	Male	Chronic Hepatitis C age standardized	X			X		
Upper digestive system diseases	Data Rich	Female	Education (years per capita)			X			X
Upper digestive system diseases	Global	Female	Education (years per capita)			X			X
Upper digestive system diseases	Data Rich	Female	LDI (I$ per capita)			X			X
Upper digestive system diseases	Global	Female	LDI (I$ per capita)			X			X
Upper digestive system diseases	Data Rich	Female	Socio-demographic Index			X			X
Upper digestive system diseases	Global	Female	Socio-demographic Index			X			X
Upper digestive system diseases	Data Rich	Female	Healthcare access and quality index		X			X	
Upper digestive system diseases	Global	Female	Healthcare access and quality index		X			X	
Upper digestive system diseases	Data Rich	Female	Liters of alcohol consumed per capita		X			X	

Appendix Table S11: Comparison of GBD 2021 and GBD 2023 covariates and level of covariates used in cause of death modeling

Cause	Model Version Type	Sex	Covariate	Level 1 GBD 2021	Level 2 GBD 2021	Level 3 GBD 2021	Level 1 GBD 2023	Level 2 GBD 2023	Level 3 GBD 2023
Upper digestive system diseases	Global	Female	Liters of alcohol consumed per capita		X			X	
Upper digestive system diseases	Data Rich	Female	vegetables unadjusted(g)		X			X	
Upper digestive system diseases	Global	Female	vegetables unadjusted(g)		X			X	
Upper digestive system diseases	Data Rich	Female	Sanitation (proportion with access)	X			X		
Upper digestive system diseases	Global	Female	Sanitation (proportion with access)	X			X		
Upper digestive system diseases	Data Rich	Female	Smoking Prevalence	X			X		
Upper digestive system diseases	Global	Female	Smoking Prevalence	X			X		
Upper digestive system diseases	Data Rich	Female	Age- and sex-specific SEV for Unsafe water	X			X		
Upper digestive system diseases	Global	Female	Age- and sex-specific SEV for Unsafe water	X			X		
Upper digestive system diseases	Data Rich	Female	Cumulative Cigarettes (10 Years)	X			X		
Upper digestive system diseases	Global	Female	Cumulative Cigarettes (10 Years)	X			X		
Upper digestive system diseases	Data Rich	Female	Cumulative Cigarettes (5 Years)	X			X		
Upper digestive system diseases	Global	Female	Cumulative Cigarettes (5 Years)	X			X		
Upper digestive system diseases	Data Rich	Male	Education (years per capita)			X			X
Upper digestive system diseases	Global	Male	Education (years per capita)			X			X
Upper digestive system diseases	Data Rich	Male	LDI (I$ per capita)			X			X
Upper digestive system diseases	Global	Male	LDI (I$ per capita)			X			X
Upper digestive system diseases	Data Rich	Male	Socio-demographic Index			X			X
Upper digestive system diseases	Global	Male	Socio-demographic Index			X			X
Upper digestive system diseases	Data Rich	Male	Healthcare access and quality index		X			X	
Upper digestive system diseases	Global	Male	Healthcare access and quality index		X			X	
Upper digestive system diseases	Data Rich	Male	Liters of alcohol consumed per capita		X			X	
Upper digestive system diseases	Global	Male	Liters of alcohol consumed per capita		X			X	
Upper digestive system diseases	Data Rich	Male	vegetables unadjusted(g)		X			X	
Upper digestive system diseases	Global	Male	vegetables unadjusted(g)		X			X	
Upper digestive system diseases	Data Rich	Male	Sanitation (proportion with access)	X			X		
Upper digestive system diseases	Global	Male	Sanitation (proportion with access)	X			X		
Upper digestive system diseases	Data Rich	Male	Smoking Prevalence	X			X		
Upper digestive system diseases	Global	Male	Smoking Prevalence	X			X		
Upper digestive system diseases	Data Rich	Male	Age- and sex-specific SEV for Unsafe water	X			X		
Upper digestive system diseases	Global	Male	Age- and sex-specific SEV for Unsafe water	X			X		
Upper digestive system diseases	Data Rich	Male	Cumulative Cigarettes (10 Years)	X			X		
Upper digestive system diseases	Global	Male	Cumulative Cigarettes (10 Years)	X			X		
Upper digestive system diseases	Data Rich	Male	Cumulative Cigarettes (5 Years)	X			X		
Upper digestive system diseases	Global	Male	Cumulative Cigarettes (5 Years)	X			X		
Peptic ulcer disease	Data Rich	Female	Education (years per capita)			X			X
Peptic ulcer disease	Global	Female	Education (years per capita)			X			X
Peptic ulcer disease	Data Rich	Female	LDI (I$ per capita)			X			X
Peptic ulcer disease	Global	Female	LDI (I$ per capita)			X			X
Peptic ulcer disease	Data Rich	Female	Socio-demographic Index			X			X
Peptic ulcer disease	Global	Female	Socio-demographic Index			X			X
Peptic ulcer disease	Data Rich	Female	Healthcare access and quality index		X			X	
Peptic ulcer disease	Global	Female	Healthcare access and quality index		X			X	
Peptic ulcer disease	Data Rich	Female	Liters of alcohol consumed per capita		X			X	
Peptic ulcer disease	Global	Female	Liters of alcohol consumed per capita		X			X	
Peptic ulcer disease	Data Rich	Female	vegetables unadjusted(g)		X			X	
Peptic ulcer disease	Global	Female	vegetables unadjusted(g)		X			X	
Peptic ulcer disease	Data Rich	Female	Cumulative Cigarettes (10 Years)	X			X		
Peptic ulcer disease	Data Rich	Female	Smoking Prevalence	X			X		
Peptic ulcer disease	Data Rich	Female	Sanitation (proportion with access)	X			X		
Peptic ulcer disease	Global	Female	Sanitation (proportion with access)	X			X		
Peptic ulcer disease	Global	Female	Smoking Prevalence	X			X		
Peptic ulcer disease	Data Rich	Female	Age- and sex-specific SEV for Unsafe water	X			X		
Peptic ulcer disease	Global	Female	Age- and sex-specific SEV for Unsafe water	X			X		
Peptic ulcer disease	Data Rich	Female	Cumulative Cigarettes (5 Years)	X			X		
Peptic ulcer disease	Global	Female	Cumulative Cigarettes (10 Years)	X			X		
Peptic ulcer disease	Global	Female	Cumulative Cigarettes (5 Years)	X			X		
Peptic ulcer disease	Data Rich	Male	Education (years per capita)			X			X
Peptic ulcer disease	Global	Male	Education (years per capita)			X			X
Peptic ulcer disease	Data Rich	Male	LDI (I$ per capita)			X			X
Peptic ulcer disease	Global	Male	LDI (I$ per capita)			X			X
Peptic ulcer disease	Data Rich	Male	Socio-demographic Index			X			X
Peptic ulcer disease	Global	Male	Socio-demographic Index			X			X
Peptic ulcer disease	Data Rich	Male	Healthcare access and quality index		X			X	
Peptic ulcer disease	Global	Male	Healthcare access and quality index		X			X	
Peptic ulcer disease	Data Rich	Male	Liters of alcohol consumed per capita		X			X	
Peptic ulcer disease	Global	Male	Liters of alcohol consumed per capita		X			X	
Peptic ulcer disease	Data Rich	Male	vegetables unadjusted(g)		X			X	
Peptic ulcer disease	Global	Male	vegetables unadjusted(g)		X			X	
Peptic ulcer disease	Data Rich	Male	Cumulative Cigarettes (10 Years)	X			X		
Peptic ulcer disease	Data Rich	Male	Smoking Prevalence	X			X		
Peptic ulcer disease	Data Rich	Male	Sanitation (proportion with access)	X			X		
Peptic ulcer disease	Global	Male	Sanitation (proportion with access)	X			X		
Peptic ulcer disease	Global	Male	Smoking Prevalence	X			X		
Peptic ulcer disease	Data Rich	Male	Age- and sex-specific SEV for Unsafe water	X			X		
Peptic ulcer disease	Global	Male	Age- and sex-specific SEV for Unsafe water	X			X		
Peptic ulcer disease	Data Rich	Male	Cumulative Cigarettes (5 Years)	X			X		
Peptic ulcer disease	Global	Male	Cumulative Cigarettes (10 Years)	X			X		
Peptic ulcer disease	Global	Male	Cumulative Cigarettes (5 Years)	X			X		
Gastritis and duodenitis	Data Rich	Female	Education (years per capita)			X			X
Gastritis and duodenitis	Global	Female	Education (years per capita)			X			X
Gastritis and duodenitis	Data Rich	Female	LDI (I$ per capita)			X			X
Gastritis and duodenitis	Global	Female	LDI (I$ per capita)			X			X
Gastritis and duodenitis	Data Rich	Female	Socio-demographic Index			X			X
Gastritis and duodenitis	Global	Female	Socio-demographic Index			X			X
Gastritis and duodenitis	Data Rich	Female	Cumulative Cigarettes (10 Years)		X			X	
Gastritis and duodenitis	Data Rich	Female	Smoking Prevalence		X			X	
Gastritis and duodenitis	Data Rich	Female	Healthcare access and quality index		X			X	
Gastritis and duodenitis	Global	Female	Healthcare access and quality index		X			X	
Gastritis and duodenitis	Data Rich	Female	Liters of alcohol consumed per capita		X			X	
Gastritis and duodenitis	Global	Female	Liters of alcohol consumed per capita		X			X	
Gastritis and duodenitis	Global	Female	Smoking Prevalence		X			X	
Gastritis and duodenitis	Data Rich	Female	Cumulative Cigarettes (5 Years)		X			X	
Gastritis and duodenitis	Data Rich	Female	vegetables unadjusted(g)		X			X	
Gastritis and duodenitis	Global	Female	vegetables unadjusted(g)		X			X	
Gastritis and duodenitis	Global	Female	Cumulative Cigarettes (10 Years)		X			X	
Gastritis and duodenitis	Global	Female	Cumulative Cigarettes (5 Years)		X			X	
Gastritis and duodenitis	Data Rich	Female	Sanitation (proportion with access)	X			X		
Gastritis and duodenitis	Global	Female	Sanitation (proportion with access)	X			X		
Gastritis and duodenitis	Data Rich	Female	Age- and sex-specific SEV for Unsafe water	X			X		
Gastritis and duodenitis	Global	Female	Age- and sex-specific SEV for Unsafe water	X			X		
Gastritis and duodenitis	Data Rich	Male	Education (years per capita)			X			X
Gastritis and duodenitis	Global	Male	Education (years per capita)			X			X
Gastritis and duodenitis	Data Rich	Male	LDI (I$ per capita)			X			X
Gastritis and duodenitis	Global	Male	LDI (I$ per capita)			X			X
Gastritis and duodenitis	Data Rich	Male	Socio-demographic Index			X			X
Gastritis and duodenitis	Global	Male	Socio-demographic Index			X			X
Gastritis and duodenitis	Data Rich	Male	Cumulative Cigarettes (10 Years)		X			X	
Gastritis and duodenitis	Data Rich	Male	Smoking Prevalence		X			X	
Gastritis and duodenitis	Data Rich	Male	Healthcare access and quality index		X			X	
Gastritis and duodenitis	Global	Male	Healthcare access and quality index		X			X	
Gastritis and duodenitis	Data Rich	Male	Liters of alcohol consumed per capita		X			X	
Gastritis and duodenitis	Global	Male	Liters of alcohol consumed per capita		X			X	
Gastritis and duodenitis	Global	Male	Smoking Prevalence		X			X	
Gastritis and duodenitis	Data Rich	Male	Cumulative Cigarettes (5 Years)		X			X	
Gastritis and duodenitis	Data Rich	Male	vegetables unadjusted(g)		X			X	
Gastritis and duodenitis	Global	Male	vegetables unadjusted(g)		X			X	
Gastritis and duodenitis	Global	Male	Cumulative Cigarettes (10 Years)		X			X	
Gastritis and duodenitis	Global	Male	Cumulative Cigarettes (5 Years)		X			X	
Gastritis and duodenitis	Data Rich	Male	Sanitation (proportion with access)	X			X		
Gastritis and duodenitis	Global	Male	Sanitation (proportion with access)	X			X		
Gastritis and duodenitis	Data Rich	Male	Age- and sex-specific SEV for Unsafe water	X			X		
Gastritis and duodenitis	Global	Male	Age- and sex-specific SEV for Unsafe water	X			X		
Appendicitis	Data Rich	Female	Education (years per capita)			X			X
Appendicitis	Global	Female	Education (years per capita)			X			X

Appendix Table S11: Comparison of GBD 2021 and GBD 2023 covariates and level of covariates used in cause of death modeling

Cause	Model Version Type	Sex	Covariate	Level 1 GBD 2021	Level 2 GBD 2021	Level 3 GBD 2021	Level 1 GBD 2023	Level 2 GBD 2023	Level 3 GBD 2023
Appendicitis	Data Rich	Female	LDI (I$ per capita)			X			X
Appendicitis	Global	Female	LDI (I$ per capita)			X			X
Appendicitis	Data Rich	Female	Socio-demographic Index			X			X
Appendicitis	Global	Female	Socio-demographic Index			X			X
Appendicitis	Data Rich	Female	Healthcare access and quality index		X			X	
Appendicitis	Global	Female	Healthcare access and quality index		X			X	
Appendicitis	Data Rich	Female	Age- and sex-specific SEV for Low fruit		X			X	
Appendicitis	Global	Female	Age- and sex-specific SEV for Low fruit		X			X	
Appendicitis	Data Rich	Female	Age- and sex-specific SEV for Low vegetables		X			X	
Appendicitis	Global	Female	Age- and sex-specific SEV for Low vegetables		X			X	
Appendicitis	Data Rich	Male	Education (years per capita)			X			X
Appendicitis	Global	Male	Education (years per capita)			X			X
Appendicitis	Data Rich	Male	LDI (I$ per capita)			X			X
Appendicitis	Global	Male	LDI (I$ per capita)			X			X
Appendicitis	Data Rich	Male	Socio-demographic Index			X			X
Appendicitis	Global	Male	Socio-demographic Index			X			X
Appendicitis	Data Rich	Male	Healthcare access and quality index		X			X	
Appendicitis	Global	Male	Healthcare access and quality index		X			X	
Appendicitis	Data Rich	Male	Age- and sex-specific SEV for Low fruit		X			X	
Appendicitis	Global	Male	Age- and sex-specific SEV for Low fruit		X			X	
Appendicitis	Data Rich	Male	Age- and sex-specific SEV for Low vegetables		X			X	
Appendicitis	Global	Male	Age- and sex-specific SEV for Low vegetables		X			X	
Paralytic ileus and intestinal obstruction	Data Rich	Female	Education (years per capita)			X			X
Paralytic ileus and intestinal obstruction	Global	Female	Education (years per capita)			X			X
Paralytic ileus and intestinal obstruction	Data Rich	Female	LDI (I$ per capita)			X			X
Paralytic ileus and intestinal obstruction	Global	Female	LDI (I$ per capita)			X			X
Paralytic ileus and intestinal obstruction	Data Rich	Female	Socio-demographic Index			X			X
Paralytic ileus and intestinal obstruction	Global	Female	Socio-demographic Index			X			X
Paralytic ileus and intestinal obstruction	Data Rich	Female	Healthcare access and quality index		X			X	
Paralytic ileus and intestinal obstruction	Global	Female	Healthcare access and quality index		X			X	
Paralytic ileus and intestinal obstruction	Data Rich	Female	vegetables unadjusted(g)		X			X	
Paralytic ileus and intestinal obstruction	Global	Female	vegetables unadjusted(g)		X			X	
Paralytic ileus and intestinal obstruction	Data Rich	Female	fruits unadjusted(g)		X			X	
Paralytic ileus and intestinal obstruction	Global	Female	fruits unadjusted(g)		X			X	
Paralytic ileus and intestinal obstruction	Data Rich	Male	Education (years per capita)			X			X
Paralytic ileus and intestinal obstruction	Global	Male	Education (years per capita)			X			X
Paralytic ileus and intestinal obstruction	Data Rich	Male	LDI (I$ per capita)			X			X
Paralytic ileus and intestinal obstruction	Global	Male	LDI (I$ per capita)			X			X
Paralytic ileus and intestinal obstruction	Data Rich	Male	Socio-demographic Index			X			X
Paralytic ileus and intestinal obstruction	Global	Male	Socio-demographic Index			X			X
Paralytic ileus and intestinal obstruction	Data Rich	Male	Healthcare access and quality index		X			X	
Paralytic ileus and intestinal obstruction	Global	Male	Healthcare access and quality index		X			X	
Paralytic ileus and intestinal obstruction	Data Rich	Male	vegetables unadjusted(g)		X			X	
Paralytic ileus and intestinal obstruction	Global	Male	vegetables unadjusted(g)		X			X	
Paralytic ileus and intestinal obstruction	Data Rich	Male	fruits unadjusted(g)		X			X	
Paralytic ileus and intestinal obstruction	Global	Male	fruits unadjusted(g)		X			X	
Inguinal, femoral, and abdominal hernia	Data Rich	Female	Education (years per capita)			X			X
Inguinal, femoral, and abdominal hernia	Global	Female	Education (years per capita)			X			X
Inguinal, femoral, and abdominal hernia	Data Rich	Female	LDI (I$ per capita)			X			X
Inguinal, femoral, and abdominal hernia	Global	Female	LDI (I$ per capita)			X			X
Inguinal, femoral, and abdominal hernia	Data Rich	Female	Socio-demographic Index			X			X
Inguinal, femoral, and abdominal hernia	Global	Female	Socio-demographic Index			X			X
Inguinal, femoral, and abdominal hernia	Data Rich	Female	Healthcare access and quality index		X			X	
Inguinal, femoral, and abdominal hernia	Global	Female	Healthcare access and quality index		X			X	
Inguinal, femoral, and abdominal hernia	Data Rich	Female	Mean BMI	X			X		
Inguinal, femoral, and abdominal hernia	Global	Female	Mean BMI	X			X		
Inguinal, femoral, and abdominal hernia	Data Rich	Female	Smoking Prevalence	X			X		
Inguinal, femoral, and abdominal hernia	Global	Female	Smoking Prevalence	X			X		
Inguinal, femoral, and abdominal hernia	Data Rich	Female	Cumulative Cigarettes (10 Years)	X			X		
Inguinal, femoral, and abdominal hernia	Global	Female	Cumulative Cigarettes (10 Years)	X			X		
Inguinal, femoral, and abdominal hernia	Data Rich	Female	Cumulative Cigarettes (5 Years)	X			X		
Inguinal, femoral, and abdominal hernia	Global	Female	Cumulative Cigarettes (5 Years)	X			X		
Inguinal, femoral, and abdominal hernia	Data Rich	Male	Education (years per capita)			X			X
Inguinal, femoral, and abdominal hernia	Global	Male	Education (years per capita)			X			X
Inguinal, femoral, and abdominal hernia	Data Rich	Male	LDI (I$ per capita)			X			X
Inguinal, femoral, and abdominal hernia	Global	Male	LDI (I$ per capita)			X			X
Inguinal, femoral, and abdominal hernia	Data Rich	Male	Socio-demographic Index			X			X
Inguinal, femoral, and abdominal hernia	Global	Male	Socio-demographic Index			X			X
Inguinal, femoral, and abdominal hernia	Data Rich	Male	Healthcare access and quality index		X			X	
Inguinal, femoral, and abdominal hernia	Global	Male	Healthcare access and quality index		X			X	
Inguinal, femoral, and abdominal hernia	Data Rich	Male	Mean BMI	X			X		
Inguinal, femoral, and abdominal hernia	Global	Male	Mean BMI	X			X		
Inguinal, femoral, and abdominal hernia	Data Rich	Male	Smoking Prevalence	X			X		
Inguinal, femoral, and abdominal hernia	Global	Male	Smoking Prevalence	X			X		
Inguinal, femoral, and abdominal hernia	Data Rich	Male	Cumulative Cigarettes (10 Years)	X			X		
Inguinal, femoral, and abdominal hernia	Global	Male	Cumulative Cigarettes (10 Years)	X			X		
Inguinal, femoral, and abdominal hernia	Data Rich	Male	Cumulative Cigarettes (5 Years)	X			X		
Inguinal, femoral, and abdominal hernia	Global	Male	Cumulative Cigarettes (5 Years)	X			X		
Inflammatory bowel disease	Data Rich	Female	Education (years per capita)			X			X
Inflammatory bowel disease	Global	Female	Education (years per capita)			X			X
Inflammatory bowel disease	Data Rich	Female	LDI (I$ per capita)			X			X
Inflammatory bowel disease	Global	Female	LDI (I$ per capita)			X			X
Inflammatory bowel disease	Data Rich	Female	Socio-demographic Index			X			X
Inflammatory bowel disease	Global	Female	Socio-demographic Index			X			X
Inflammatory bowel disease	Data Rich	Female	Healthcare access and quality index		X			X	
Inflammatory bowel disease	Global	Female	Healthcare access and quality index		X			X	
Inflammatory bowel disease	Data Rich	Female	Latitude 15 to 30 (proportion)		X			X	
Inflammatory bowel disease	Global	Female	Latitude 15 to 30 (proportion)		X			X	
Inflammatory bowel disease	Data Rich	Female	Latitude 30 to 45 (proportion)		X			X	
Inflammatory bowel disease	Global	Female	Latitude 30 to 45 (proportion)		X			X	
Inflammatory bowel disease	Data Rich	Female	Latitude Over 45 (proportion)		X			X	
Inflammatory bowel disease	Global	Female	Latitude Over 45 (proportion)		X			X	
Inflammatory bowel disease	Data Rich	Female	Age- and sex-specific SEV for Low fruit	X			X		
Inflammatory bowel disease	Global	Female	Age- and sex-specific SEV for Low fruit	X			X		
Inflammatory bowel disease	Data Rich	Female	Age- and sex-specific SEV for Low vegetables	X			X		
Inflammatory bowel disease	Global	Female	Age- and sex-specific SEV for Low vegetables	X			X		
Inflammatory bowel disease	Data Rich	Female	Age- and sex-specific SEV for Low PUFA	X			X		
Inflammatory bowel disease	Global	Female	Age- and sex-specific SEV for Low PUFA	X			X		
Inflammatory bowel disease	Data Rich	Female	Age- and sex-specific SEV for High red meat	X			X		
Inflammatory bowel disease	Global	Female	Age- and sex-specific SEV for High red meat	X			X		
Inflammatory bowel disease	Data Rich	Male	Education (years per capita)			X			X
Inflammatory bowel disease	Global	Male	Education (years per capita)			X			X
Inflammatory bowel disease	Data Rich	Male	LDI (I$ per capita)			X			X
Inflammatory bowel disease	Global	Male	LDI (I$ per capita)			X			X
Inflammatory bowel disease	Data Rich	Male	Socio-demographic Index			X			X
Inflammatory bowel disease	Global	Male	Socio-demographic Index			X			X
Inflammatory bowel disease	Data Rich	Male	Healthcare access and quality index		X			X	
Inflammatory bowel disease	Global	Male	Healthcare access and quality index		X			X	
Inflammatory bowel disease	Data Rich	Male	Latitude 15 to 30 (proportion)		X			X	
Inflammatory bowel disease	Global	Male	Latitude 15 to 30 (proportion)		X			X	
Inflammatory bowel disease	Data Rich	Male	Latitude 30 to 45 (proportion)		X			X	
Inflammatory bowel disease	Global	Male	Latitude 30 to 45 (proportion)		X			X	
Inflammatory bowel disease	Data Rich	Male	Latitude Over 45 (proportion)		X			X	
Inflammatory bowel disease	Global	Male	Latitude Over 45 (proportion)		X			X	
Inflammatory bowel disease	Data Rich	Male	Age- and sex-specific SEV for Low fruit	X			X		
Inflammatory bowel disease	Global	Male	Age- and sex-specific SEV for Low fruit	X			X		
Inflammatory bowel disease	Data Rich	Male	Age- and sex-specific SEV for Low vegetables	X			X		
Inflammatory bowel disease	Global	Male	Age- and sex-specific SEV for Low vegetables	X			X		
Inflammatory bowel disease	Data Rich	Male	Age- and sex-specific SEV for Low PUFA	X			X		
Inflammatory bowel disease	Global	Male	Age- and sex-specific SEV for Low PUFA	X			X		
Inflammatory bowel disease	Data Rich	Male	Age- and sex-specific SEV for High red meat	X			X		
Inflammatory bowel disease	Global	Male	Age- and sex-specific SEV for High red meat	X			X		
Ulcerative colitis	Data Rich	Female	Education (years per capita)						X
Ulcerative colitis	Global	Female	Education (years per capita)						X

Appendix Table S11: Comparison of GBD 2021 and GBD 2023 covariates and level of covariates used in cause of death modeling									
**Cause**	**Model Version Type**	**Sex**	**Covariate**	**Level 1 GBD 2021**	**Level 2 GBD 2021**	**Level 3 GBD 2021**	**Level 1 GBD 2023**	**Level 2 GBD 2023**	**Level 3 GBD 2023**
Ulcerative colitis	Data Rich	Female	LDI (I$ per capita)						X
Ulcerative colitis	Global	Female	LDI (I$ per capita)						X
Ulcerative colitis	Data Rich	Female	Socio-demographic Index						X
Ulcerative colitis	Global	Female	Socio-demographic Index						X
Ulcerative colitis	Data Rich	Female	Healthcare access and quality index					X	
Ulcerative colitis	Global	Female	Healthcare access and quality index					X	
Ulcerative colitis	Data Rich	Female	Latitude 15 to 30 (proportion)					X	
Ulcerative colitis	Global	Female	Latitude 15 to 30 (proportion)					X	
Ulcerative colitis	Data Rich	Female	Latitude 30 to 45 (proportion)					X	
Ulcerative colitis	Global	Female	Latitude 30 to 45 (proportion)					X	
Ulcerative colitis	Data Rich	Female	Latitude Over 45 (proportion)					X	
Ulcerative colitis	Global	Female	Latitude Over 45 (proportion)					X	
Ulcerative colitis	Data Rich	Female	Age- and sex-specific SEV for Low fruit				X		
Ulcerative colitis	Global	Female	Age- and sex-specific SEV for Low fruit				X		
Ulcerative colitis	Data Rich	Female	Age- and sex-specific SEV for Low vegetables				X		
Ulcerative colitis	Global	Female	Age- and sex-specific SEV for Low vegetables				X		
Ulcerative colitis	Data Rich	Female	Age- and sex-specific SEV for Low PUFA				X		
Ulcerative colitis	Global	Female	Age- and sex-specific SEV for Low PUFA				X		
Ulcerative colitis	Data Rich	Female	Age- and sex-specific SEV for High red meat				X		
Ulcerative colitis	Global	Female	Age- and sex-specific SEV for High red meat				X		
Ulcerative colitis	Data Rich	Male	Education (years per capita)						X
Ulcerative colitis	Global	Male	Education (years per capita)						X
Ulcerative colitis	Data Rich	Male	LDI (I$ per capita)						X
Ulcerative colitis	Global	Male	LDI (I$ per capita)						X
Ulcerative colitis	Data Rich	Male	Socio-demographic Index						X
Ulcerative colitis	Global	Male	Socio-demographic Index						X
Ulcerative colitis	Data Rich	Male	Healthcare access and quality index					X	
Ulcerative colitis	Global	Male	Healthcare access and quality index					X	
Ulcerative colitis	Data Rich	Male	Latitude 15 to 30 (proportion)					X	
Ulcerative colitis	Global	Male	Latitude 15 to 30 (proportion)					X	
Ulcerative colitis	Data Rich	Male	Latitude 30 to 45 (proportion)					X	
Ulcerative colitis	Global	Male	Latitude 30 to 45 (proportion)					X	
Ulcerative colitis	Data Rich	Male	Latitude Over 45 (proportion)					X	
Ulcerative colitis	Global	Male	Latitude Over 45 (proportion)					X	
Ulcerative colitis	Data Rich	Male	Age- and sex-specific SEV for Low fruit				X		
Ulcerative colitis	Global	Male	Age- and sex-specific SEV for Low fruit				X		
Ulcerative colitis	Data Rich	Male	Age- and sex-specific SEV for Low vegetables				X		
Ulcerative colitis	Global	Male	Age- and sex-specific SEV for Low vegetables				X		
Ulcerative colitis	Data Rich	Male	Age- and sex-specific SEV for Low PUFA				X		
Ulcerative colitis	Global	Male	Age- and sex-specific SEV for Low PUFA				X		
Ulcerative colitis	Data Rich	Male	Age- and sex-specific SEV for High red meat				X		
Ulcerative colitis	Global	Male	Age- and sex-specific SEV for High red meat				X		
Crohn's disease	Data Rich	Female	Education (years per capita)						X
Crohn's disease	Global	Female	Education (years per capita)						X
Crohn's disease	Data Rich	Female	LDI (I$ per capita)						X
Crohn's disease	Global	Female	LDI (I$ per capita)						X
Crohn's disease	Data Rich	Female	Socio-demographic Index						X
Crohn's disease	Global	Female	Socio-demographic Index						X
Crohn's disease	Data Rich	Female	Healthcare access and quality index					X	
Crohn's disease	Global	Female	Healthcare access and quality index					X	
Crohn's disease	Data Rich	Female	Latitude 15 to 30 (proportion)					X	
Crohn's disease	Global	Female	Latitude 15 to 30 (proportion)					X	
Crohn's disease	Data Rich	Female	Latitude 30 to 45 (proportion)					X	
Crohn's disease	Global	Female	Latitude 30 to 45 (proportion)					X	
Crohn's disease	Data Rich	Female	Latitude Over 45 (proportion)					X	
Crohn's disease	Global	Female	Latitude Over 45 (proportion)					X	
Crohn's disease	Data Rich	Female	Age- and sex-specific SEV for Low fruit				X		
Crohn's disease	Global	Female	Age- and sex-specific SEV for Low fruit				X		
Crohn's disease	Data Rich	Female	Age- and sex-specific SEV for Low vegetables				X		
Crohn's disease	Global	Female	Age- and sex-specific SEV for Low vegetables				X		
Crohn's disease	Data Rich	Female	Age- and sex-specific SEV for Low PUFA				X		
Crohn's disease	Global	Female	Age- and sex-specific SEV for Low PUFA				X		
Crohn's disease	Data Rich	Female	Age- and sex-specific SEV for High red meat				X		
Crohn's disease	Global	Female	Age- and sex-specific SEV for High red meat				X		
Crohn's disease	Data Rich	Male	Education (years per capita)						X
Crohn's disease	Global	Male	Education (years per capita)						X
Crohn's disease	Data Rich	Male	LDI (I$ per capita)						X
Crohn's disease	Global	Male	LDI (I$ per capita)						X
Crohn's disease	Data Rich	Male	Socio-demographic Index						X
Crohn's disease	Global	Male	Socio-demographic Index						X
Crohn's disease	Data Rich	Male	Healthcare access and quality index					X	
Crohn's disease	Global	Male	Healthcare access and quality index					X	
Crohn's disease	Data Rich	Male	Latitude 15 to 30 (proportion)					X	
Crohn's disease	Global	Male	Latitude 15 to 30 (proportion)					X	
Crohn's disease	Data Rich	Male	Latitude 30 to 45 (proportion)					X	
Crohn's disease	Global	Male	Latitude 30 to 45 (proportion)					X	
Crohn's disease	Data Rich	Male	Latitude Over 45 (proportion)					X	
Crohn's disease	Global	Male	Latitude Over 45 (proportion)					X	
Crohn's disease	Data Rich	Male	Age- and sex-specific SEV for Low fruit				X		
Crohn's disease	Global	Male	Age- and sex-specific SEV for Low fruit				X		
Crohn's disease	Data Rich	Male	Age- and sex-specific SEV for Low vegetables				X		
Crohn's disease	Global	Male	Age- and sex-specific SEV for Low vegetables				X		
Crohn's disease	Data Rich	Male	Age- and sex-specific SEV for Low PUFA				X		
Crohn's disease	Global	Male	Age- and sex-specific SEV for Low PUFA				X		
Crohn's disease	Data Rich	Male	Age- and sex-specific SEV for High red meat				X		
Crohn's disease	Global	Male	Age- and sex-specific SEV for High red meat				X		
Vascular intestinal disorders	Data Rich	Female	Education (years per capita)			X			X
Vascular intestinal disorders	Global	Female	Education (years per capita)			X			X
Vascular intestinal disorders	Data Rich	Female	LDI (I$ per capita)			X			X
Vascular intestinal disorders	Global	Female	LDI (I$ per capita)			X			X
Vascular intestinal disorders	Data Rich	Female	Socio-demographic Index			X			X
Vascular intestinal disorders	Data Rich	Female	Liters of alcohol consumed per capita			X			X
Vascular intestinal disorders	Global	Female	Liters of alcohol consumed per capita			X			X
Vascular intestinal disorders	Data Rich	Female	Pulses legumes unadjusted(g)			X			X
Vascular intestinal disorders	Global	Female	Pulses legumes unadjusted(g)			X			X
Vascular intestinal disorders	Data Rich	Female	Age- and sex-specific SEV for Low fruit			X			X
Vascular intestinal disorders	Global	Female	Age- and sex-specific SEV for Low fruit			X			X
Vascular intestinal disorders	Data Rich	Female	Age- and sex-specific SEV for Low vegetables			X			X
Vascular intestinal disorders	Global	Female	Age- and sex-specific SEV for Low vegetables			X			X
Vascular intestinal disorders	Data Rich	Female	Age- and sex-specific SEV for Low nuts and seeds			X			X
Vascular intestinal disorders	Global	Female	Age- and sex-specific SEV for Low nuts and seeds			X			X
Vascular intestinal disorders	Data Rich	Female	Age- and sex-specific SEV for Low omega-3			X			X
Vascular intestinal disorders	Global	Female	Age- and sex-specific SEV for Low omega-3			X			X
Vascular intestinal disorders	Data Rich	Female	Age- and sex-specific SEV for Low PUFA			X			X
Vascular intestinal disorders	Global	Female	Age- and sex-specific SEV for Low PUFA			X			X
Vascular intestinal disorders	Data Rich	Female	Total Fertility Rate			X			X
Vascular intestinal disorders	Global	Female	Total Fertility Rate			X			X
Vascular intestinal disorders	Data Rich	Female	Smoking Prevalence		X			X	
Vascular intestinal disorders	Data Rich	Female	Healthcare access and quality index		X			X	
Vascular intestinal disorders	Global	Female	Healthcare access and quality index		X			X	
Vascular intestinal disorders	Global	Female	Mean BMI		X			X	
Vascular intestinal disorders	Global	Female	Smoking Prevalence		X			X	
Vascular intestinal disorders	Data Rich	Female	Mean BMI		X			X	
Vascular intestinal disorders	Data Rich	Female	Low-Density Lipoprotein (mmol/L)	X			X		

Appendix Table S11: Comparison of GBD 2021 and GBD 2023 covariates and level of covariates used in cause of death modeling									
**Cause**	**Model Version Type**	**Sex**	**Covariate**	**Level 1 GBD 2021**	**Level 2 GBD 2021**	**Level 3 GBD 2021**	**Level 1 GBD 2023**	**Level 2 GBD 2023**	**Level 3 GBD 2023**
Vascular intestinal disorders	Global	Female	Low-Density Lipoprotein (mmol/L)	X			X		
Vascular intestinal disorders	Data Rich	Female	Diabetes Fasting Plasma Glucose (mmol/L), age-standardized 25+	X			X		
Vascular intestinal disorders	Global	Female	Diabetes Fasting Plasma Glucose (mmol/L), age-standardized 25+	X			X		
Vascular intestinal disorders	Data Rich	Female	Systolic Blood Pressure (mmHg)	X			X		
Vascular intestinal disorders	Global	Female	Systolic Blood Pressure (mmHg)	X			X		
Vascular intestinal disorders	Data Rich	Male	Education (years per capita)			X			X
Vascular intestinal disorders	Global	Male	Education (years per capita)			X			X
Vascular intestinal disorders	Data Rich	Male	LDI (I$ per capita)			X			X
Vascular intestinal disorders	Global	Male	LDI (I$ per capita)			X			X
Vascular intestinal disorders	Data Rich	Male	Socio-demographic Index			X			X
Vascular intestinal disorders	Data Rich	Male	Liters of alcohol consumed per capita			X			X
Vascular intestinal disorders	Global	Male	Liters of alcohol consumed per capita			X			X
Vascular intestinal disorders	Data Rich	Male	Pulses legumes unadjusted(g)			X			X
Vascular intestinal disorders	Global	Male	Pulses legumes unadjusted(g)			X			X
Vascular intestinal disorders	Data Rich	Male	Age- and sex-specific SEV for Low fruit			X			X
Vascular intestinal disorders	Global	Male	Age- and sex-specific SEV for Low fruit			X			X
Vascular intestinal disorders	Data Rich	Male	Age- and sex-specific SEV for Low vegetables			X			X
Vascular intestinal disorders	Global	Male	Age- and sex-specific SEV for Low vegetables			X			X
Vascular intestinal disorders	Data Rich	Male	Age- and sex-specific SEV for Low nuts and seeds			X			X
Vascular intestinal disorders	Global	Male	Age- and sex-specific SEV for Low nuts and seeds			X			X
Vascular intestinal disorders	Data Rich	Male	Age- and sex-specific SEV for Low omega-3			X			X
Vascular intestinal disorders	Global	Male	Age- and sex-specific SEV for Low omega-3			X			X
Vascular intestinal disorders	Data Rich	Male	Age- and sex-specific SEV for Low PUFA			X			X
Vascular intestinal disorders	Global	Male	Age- and sex-specific SEV for Low PUFA			X			X
Vascular intestinal disorders	Data Rich	Male	Total Fertility Rate			X			X
Vascular intestinal disorders	Global	Male	Total Fertility Rate			X			X
Vascular intestinal disorders	Data Rich	Male	Smoking Prevalence		X			X	
Vascular intestinal disorders	Data Rich	Male	Healthcare access and quality index		X			X	
Vascular intestinal disorders	Global	Male	Healthcare access and quality index		X			X	
Vascular intestinal disorders	Global	Male	Mean BMI		X			X	
Vascular intestinal disorders	Global	Male	Smoking Prevalence		X			X	
Vascular intestinal disorders	Data Rich	Male	Mean BMI		X			X	
Vascular intestinal disorders	Data Rich	Male	Low-Density Lipoprotein (mmol/L)	X			X		
Vascular intestinal disorders	Global	Male	Low-Density Lipoprotein (mmol/L)	X			X		
Vascular intestinal disorders	Data Rich	Male	Diabetes Fasting Plasma Glucose (mmol/L), age-standardized 25+	X			X		
Vascular intestinal disorders	Global	Male	Diabetes Fasting Plasma Glucose (mmol/L), age-standardized 25+	X			X		
Vascular intestinal disorders	Data Rich	Male	Systolic Blood Pressure (mmHg)	X			X		
Vascular intestinal disorders	Global	Male	Systolic Blood Pressure (mmHg)	X			X		
Gallbladder and biliary diseases	Data Rich	Female	Education (years per capita)			X			X
Gallbladder and biliary diseases	Global	Female	Education (years per capita)			X			X
Gallbladder and biliary diseases	Data Rich	Female	LDI (I$ per capita)			X			X
Gallbladder and biliary diseases	Global	Female	LDI (I$ per capita)			X			X
Gallbladder and biliary diseases	Data Rich	Female	Socio-demographic Index			X			X
Gallbladder and biliary diseases	Global	Female	Socio-demographic Index			X			X
Gallbladder and biliary diseases	Data Rich	Female	Healthcare access and quality index		X			X	
Gallbladder and biliary diseases	Global	Female	Healthcare access and quality index		X			X	
Gallbladder and biliary diseases	Data Rich	Female	Liters of alcohol consumed per capita		X			X	
Gallbladder and biliary diseases	Global	Female	Liters of alcohol consumed per capita		X			X	
Gallbladder and biliary diseases	Data Rich	Female	Age- and sex-specific SEV for High red meat		X			X	
Gallbladder and biliary diseases	Global	Female	Age- and sex-specific SEV for High red meat		X			X	
Gallbladder and biliary diseases	Data Rich	Female	Population Over 65 (proportion)		X			X	
Gallbladder and biliary diseases	Global	Female	Population Over 65 (proportion)		X			X	
Gallbladder and biliary diseases	Global	Female	Mean BMI	X			X		
Gallbladder and biliary diseases	Data Rich	Female	Mean BMI	X			X		
Gallbladder and biliary diseases	Data Rich	Female	Age- and sex-specific SEV for Low PUFA	X			X		
Gallbladder and biliary diseases	Global	Female	Age- and sex-specific SEV for Low PUFA	X			X		
Gallbladder and biliary diseases	Data Rich	Male	Education (years per capita)			X			X
Gallbladder and biliary diseases	Global	Male	Education (years per capita)			X			X
Gallbladder and biliary diseases	Data Rich	Male	LDI (I$ per capita)			X			X
Gallbladder and biliary diseases	Global	Male	LDI (I$ per capita)			X			X
Gallbladder and biliary diseases	Data Rich	Male	Socio-demographic Index			X			X
Gallbladder and biliary diseases	Global	Male	Socio-demographic Index			X			X
Gallbladder and biliary diseases	Data Rich	Male	Healthcare access and quality index		X			X	
Gallbladder and biliary diseases	Global	Male	Healthcare access and quality index		X			X	
Gallbladder and biliary diseases	Data Rich	Male	Liters of alcohol consumed per capita		X			X	
Gallbladder and biliary diseases	Global	Male	Liters of alcohol consumed per capita		X			X	
Gallbladder and biliary diseases	Data Rich	Male	Age- and sex-specific SEV for High red meat		X			X	
Gallbladder and biliary diseases	Global	Male	Age- and sex-specific SEV for High red meat		X			X	
Gallbladder and biliary diseases	Data Rich	Male	Population Over 65 (proportion)		X			X	
Gallbladder and biliary diseases	Global	Male	Population Over 65 (proportion)		X			X	
Gallbladder and biliary diseases	Global	Male	Mean BMI	X			X		
Gallbladder and biliary diseases	Data Rich	Male	Mean BMI	X			X		
Gallbladder and biliary diseases	Data Rich	Male	Age- and sex-specific SEV for Low PUFA	X			X		
Gallbladder and biliary diseases	Global	Male	Age- and sex-specific SEV for Low PUFA	X			X		
Pancreatitis	Data Rich	Female	Education (years per capita)			X			X
Pancreatitis	Global	Female	Education (years per capita)			X			X
Pancreatitis	Data Rich	Female	LDI (I$ per capita)			X			X
Pancreatitis	Global	Female	LDI (I$ per capita)			X			X
Pancreatitis	Data Rich	Female	Socio-demographic Index			X			X
Pancreatitis	Global	Female	Socio-demographic Index			X			X
Pancreatitis	Data Rich	Female	Healthcare access and quality index		X			X	
Pancreatitis	Global	Female	Healthcare access and quality index		X			X	
Pancreatitis	Global	Female	Mean BMI		X			X	
Pancreatitis	Data Rich	Female	Mean BMI		X			X	
Pancreatitis	Data Rich	Female	Liters of alcohol consumed per capita	X			X		
Pancreatitis	Global	Female	Liters of alcohol consumed per capita	X			X		
Pancreatitis	Data Rich	Female	Log-transformed SEV scalar: Pancreatit	X			X		
Pancreatitis	Global	Female	Log-transformed SEV scalar: Pancreatit	X			X		
Pancreatitis	Data Rich	Male	Education (years per capita)			X			X
Pancreatitis	Global	Male	Education (years per capita)			X			X
Pancreatitis	Data Rich	Male	LDI (I$ per capita)			X			X
Pancreatitis	Global	Male	LDI (I$ per capita)			X			X
Pancreatitis	Data Rich	Male	Socio-demographic Index			X			X
Pancreatitis	Global	Male	Socio-demographic Index			X			X
Pancreatitis	Data Rich	Male	Healthcare access and quality index		X			X	
Pancreatitis	Global	Male	Healthcare access and quality index		X			X	
Pancreatitis	Global	Male	Mean BMI		X			X	
Pancreatitis	Data Rich	Male	Mean BMI		X			X	
Pancreatitis	Data Rich	Male	Liters of alcohol consumed per capita	X			X		
Pancreatitis	Global	Male	Liters of alcohol consumed per capita	X			X		
Pancreatitis	Data Rich	Male	Log-transformed SEV scalar: Pancreatit	X			X		
Pancreatitis	Global	Male	Log-transformed SEV scalar: Pancreatit	X			X		
Diverticular disease of intestines	Data Rich	Female	Education (years per capita)						X
Diverticular disease of intestines	Global	Female	Education (years per capita)						X
Diverticular disease of intestines	Data Rich	Female	LDI (I$ per capita)						X
Diverticular disease of intestines	Global	Female	LDI (I$ per capita)						X
Diverticular disease of intestines	Data Rich	Female	Socio-demographic Index						X
Diverticular disease of intestines	Global	Female	Socio-demographic Index						X
Diverticular disease of intestines	Data Rich	Female	Diabetes Fasting Plasma Glucose (mmol/L), age-standardized 25+						X
Diverticular disease of intestines	Global	Female	Diabetes Fasting Plasma Glucose (mmol/L), age-standardized 25+						X
Diverticular disease of intestines	Data Rich	Female	Tobacco (cigarettes per capita)					X	
Diverticular disease of intestines	Global	Female	Tobacco (cigarettes per capita)					X	
Diverticular disease of intestines	Data Rich	Female	Healthcare access and quality index					X	
Diverticular disease of intestines	Global	Female	Healthcare access and quality index					X	
Diverticular disease of intestines	Data Rich	Female	Liters of alcohol consumed per capita					X	
Diverticular disease of intestines	Global	Female	Liters of alcohol consumed per capita					X	

Appendix Table S11: Comparison of GBD 2021 and GBD 2023 covariates and level of covariates used in cause of death modeling

Cause	Model Version Type	Sex	Covariate	Level 1 GBD 2021	Level 2 GBD 2021	Level 3 GBD 2021	Level 1 GBD 2023	Level 2 GBD 2023	Level 3 GBD 2023
Diverticular disease of intestines	Data Rich	Female	Cumulative Cigarettes (5 Years)					X	
Diverticular disease of intestines	Global	Female	Cumulative Cigarettes (5 Years)					X	
Diverticular disease of intestines	Data Rich	Female	Age- and sex-specific SEV for High red meat					X	
Diverticular disease of intestines	Global	Female	Age- and sex-specific SEV for High red meat					X	
Diverticular disease of intestines	Data Rich	Female	Mean BMI				X		
Diverticular disease of intestines	Global	Female	Mean BMI				X		
Diverticular disease of intestines	Data Rich	Female	Age- and sex-specific SEV for Low fruit				X		
Diverticular disease of intestines	Global	Female	Age- and sex-specific SEV for Low fruit				X		
Diverticular disease of intestines	Data Rich	Female	Age- and sex-specific SEV for Low vegetables				X		
Diverticular disease of intestines	Global	Female	Age- and sex-specific SEV for Low vegetables				X		
Diverticular disease of intestines	Data Rich	Female	Total Physical Activity (MET-min/week), Age-specific				X		
Diverticular disease of intestines	Global	Female	Total Physical Activity (MET-min/week), Age-specific				X		
Diverticular disease of intestines	Data Rich	Female	Age- and sex-specific SEV for Low fiber				X		
Diverticular disease of intestines	Global	Female	Age- and sex-specific SEV for Low fiber				X		
Diverticular disease of intestines	Data Rich	Male	Education (years per capita)						X
Diverticular disease of intestines	Global	Male	Education (years per capita)						X
Diverticular disease of intestines	Data Rich	Male	LDI (I$ per capita)						X
Diverticular disease of intestines	Global	Male	LDI (I$ per capita)						X
Diverticular disease of intestines	Data Rich	Male	Socio-demographic Index						X
Diverticular disease of intestines	Global	Male	Socio-demographic Index						X
Diverticular disease of intestines	Data Rich	Male	Diabetes Fasting Plasma Glucose (mmol/L), age-standardized 25+						X
Diverticular disease of intestines	Global	Male	Diabetes Fasting Plasma Glucose (mmol/L), age-standardized 25+						X
Diverticular disease of intestines	Data Rich	Male	Tobacco (cigarettes per capita)					X	
Diverticular disease of intestines	Global	Male	Tobacco (cigarettes per capita)					X	
Diverticular disease of intestines	Data Rich	Male	Healthcare access and quality index					X	
Diverticular disease of intestines	Global	Male	Healthcare access and quality index					X	
Diverticular disease of intestines	Data Rich	Male	Liters of alcohol consumed per capita					X	
Diverticular disease of intestines	Global	Male	Liters of alcohol consumed per capita					X	
Diverticular disease of intestines	Data Rich	Male	Cumulative Cigarettes (5 Years)					X	
Diverticular disease of intestines	Global	Male	Cumulative Cigarettes (5 Years)					X	
Diverticular disease of intestines	Data Rich	Male	Age- and sex-specific SEV for High red meat					X	
Diverticular disease of intestines	Global	Male	Age- and sex-specific SEV for High red meat					X	
Diverticular disease of intestines	Data Rich	Male	Mean BMI				X		
Diverticular disease of intestines	Global	Male	Mean BMI				X		
Diverticular disease of intestines	Data Rich	Male	Age- and sex-specific SEV for Low fruit				X		
Diverticular disease of intestines	Global	Male	Age- and sex-specific SEV for Low fruit				X		
Diverticular disease of intestines	Data Rich	Male	Age- and sex-specific SEV for Low vegetables				X		
Diverticular disease of intestines	Global	Male	Age- and sex-specific SEV for Low vegetables				X		
Diverticular disease of intestines	Data Rich	Male	Total Physical Activity (MET-min/week), Age-specific				X		
Diverticular disease of intestines	Global	Male	Total Physical Activity (MET-min/week), Age-specific				X		
Diverticular disease of intestines	Data Rich	Male	Age- and sex-specific SEV for Low fiber				X		
Diverticular disease of intestines	Global	Male	Age- and sex-specific SEV for Low fiber				X		
Other digestive diseases	Data Rich	Female	Education (years per capita)			X			X
Other digestive diseases	Global	Female	Education (years per capita)			X			X
Other digestive diseases	Data Rich	Female	LDI (I$ per capita)			X			X
Other digestive diseases	Global	Female	LDI (I$ per capita)			X			X
Other digestive diseases	Data Rich	Female	Socio-demographic Index			X			X
Other digestive diseases	Global	Female	Socio-demographic Index			X			X
Other digestive diseases	Data Rich	Female	Healthcare access and quality index		X			X	
Other digestive diseases	Global	Female	Healthcare access and quality index		X			X	
Other digestive diseases	Global	Female	Mean BMI		X			X	
Other digestive diseases	Data Rich	Female	Sanitation (proportion with access)		X			X	
Other digestive diseases	Global	Female	Sanitation (proportion with access)		X			X	
Other digestive diseases	Data Rich	Female	Improved Water Source (proportion with access)		X			X	
Other digestive diseases	Global	Female	Improved Water Source (proportion with access)		X			X	
Other digestive diseases	Data Rich	Female	Mean BMI		X			X	
Other digestive diseases	Data Rich	Female	Age- and sex-specific SEV for Low fruit		X			X	
Other digestive diseases	Global	Female	Age- and sex-specific SEV for Low fruit		X			X	
Other digestive diseases	Data Rich	Female	Age- and sex-specific SEV for Low vegetables		X			X	
Other digestive diseases	Global	Female	Age- and sex-specific SEV for Low vegetables		X			X	
Other digestive diseases	Data Rich	Female	Age- and sex-specific SEV for Low PUFA		X			X	
Other digestive diseases	Global	Female	Age- and sex-specific SEV for Low PUFA		X			X	
Other digestive diseases	Data Rich	Female	Age- and sex-specific SEV for High red meat		X			X	
Other digestive diseases	Global	Female	Age- and sex-specific SEV for High red meat		X			X	
Other digestive diseases	Data Rich	Female	Diabetes Age-Standardized Prevalence (proportion)		X			X	
Other digestive diseases	Global	Female	Diabetes Age-Standardized Prevalence (proportion)		X			X	
Other digestive diseases	Data Rich	Female	Cumulative Cigarettes (10 Years)	X			X		
Other digestive diseases	Data Rich	Female	Smoking Prevalence	X			X		
Other digestive diseases	Data Rich	Female	Liters of alcohol consumed per capita	X			X		
Other digestive diseases	Global	Female	Liters of alcohol consumed per capita	X			X		
Other digestive diseases	Global	Female	Smoking Prevalence	X			X		
Other digestive diseases	Data Rich	Female	Cumulative Cigarettes (5 Years)	X			X		
Other digestive diseases	Global	Female	Cumulative Cigarettes (10 Years)	X			X		
Other digestive diseases	Global	Female	Cumulative Cigarettes (5 Years)	X			X		
Other digestive diseases	Data Rich	Male	Education (years per capita)			X			X
Other digestive diseases	Global	Male	Education (years per capita)			X			X
Other digestive diseases	Data Rich	Male	LDI (I$ per capita)			X			X
Other digestive diseases	Global	Male	LDI (I$ per capita)			X			X
Other digestive diseases	Data Rich	Male	Socio-demographic Index			X			X
Other digestive diseases	Global	Male	Socio-demographic Index			X			X
Other digestive diseases	Data Rich	Male	Healthcare access and quality index		X			X	
Other digestive diseases	Global	Male	Healthcare access and quality index		X			X	
Other digestive diseases	Global	Male	Mean BMI		X			X	
Other digestive diseases	Data Rich	Male	Sanitation (proportion with access)		X			X	
Other digestive diseases	Global	Male	Sanitation (proportion with access)		X			X	
Other digestive diseases	Data Rich	Male	Improved Water Source (proportion with access)		X			X	
Other digestive diseases	Global	Male	Improved Water Source (proportion with access)		X			X	
Other digestive diseases	Data Rich	Male	Mean BMI		X			X	
Other digestive diseases	Data Rich	Male	Age- and sex-specific SEV for Low fruit		X			X	
Other digestive diseases	Global	Male	Age- and sex-specific SEV for Low fruit		X			X	
Other digestive diseases	Data Rich	Male	Age- and sex-specific SEV for Low vegetables		X			X	
Other digestive diseases	Global	Male	Age- and sex-specific SEV for Low vegetables		X			X	
Other digestive diseases	Data Rich	Male	Age- and sex-specific SEV for Low PUFA		X			X	
Other digestive diseases	Global	Male	Age- and sex-specific SEV for Low PUFA		X			X	
Other digestive diseases	Data Rich	Male	Age- and sex-specific SEV for High red meat		X			X	
Other digestive diseases	Global	Male	Age- and sex-specific SEV for High red meat		X			X	
Other digestive diseases	Data Rich	Male	Diabetes Age-Standardized Prevalence (proportion)		X			X	
Other digestive diseases	Global	Male	Diabetes Age-Standardized Prevalence (proportion)		X			X	
Other digestive diseases	Data Rich	Male	Cumulative Cigarettes (10 Years)	X			X		
Other digestive diseases	Data Rich	Male	Smoking Prevalence	X			X		
Other digestive diseases	Data Rich	Male	Liters of alcohol consumed per capita	X			X		
Other digestive diseases	Global	Male	Smoking Prevalence	X			X		
Other digestive diseases	Data Rich	Male	Cumulative Cigarettes (5 Years)	X			X		
Other digestive diseases	Global	Male	Cumulative Cigarettes (10 Years)	X			X		
Other digestive diseases	Global	Male	Cumulative Cigarettes (5 Years)	X			X		
Parkinson's disease	Data Rich	Female	Education (years per capita)			X			X
Parkinson's disease	Global	Female	Education (years per capita)			X			X
Parkinson's disease	Data Rich	Female	LDI (I$ per capita)			X			X
Parkinson's disease	Global	Female	LDI (I$ per capita)			X			X
Parkinson's disease	Data Rich	Female	Socio-demographic Index			X			X

Appendix Table S11: Comparison of GBD 2021 and GBD 2023 covariates and level of covariates used in cause of death modeling									
**Cause**	**Model Version Type**	**Sex**	**Covariate**	**Level 1 GBD 2021**	**Level 2 GBD 2021**	**Level 3 GBD 2021**	**Level 1 GBD 2023**	**Level 2 GBD 2023**	**Level 3 GBD 2023**
Parkinson's disease	Global	Female	Socio-demographic Index			X			X
Parkinson's disease	Data Rich	Female	Low-Density Lipoprotein (mmol/L)		X			X	
Parkinson's disease	Global	Female	Low-Density Lipoprotein (mmol/L)		X			X	
Parkinson's disease	Data Rich	Female	Healthcare access and quality index		X			X	
Parkinson's disease	Global	Female	Healthcare access and quality index		X			X	
Parkinson's disease	Data Rich	Female	Sanitation (proportion with access)		X			X	
Parkinson's disease	Global	Female	Sanitation (proportion with access)		X			X	
Parkinson's disease	Data Rich	Female	Improved Water Source (proportion with access)		X			X	
Parkinson's disease	Global	Female	Improved Water Source (proportion with access)		X			X	
Parkinson's disease	Data Rich	Female	Absolute value of average latitude		X			X	
Parkinson's disease	Global	Female	Absolute value of average latitude		X			X	
Parkinson's disease	Data Rich	Female	Cumulative Cigarettes (10 Years)	X			X		
Parkinson's disease	Global	Female	Cumulative Cigarettes (10 Years)	X			X		
Parkinson's disease	Data Rich	Female	Age- and sex-specific SEV for Low fruit	X			X		
Parkinson's disease	Global	Female	Age- and sex-specific SEV for Low fruit	X			X		
Parkinson's disease	Data Rich	Male	Socio-demographic Index			X			X
Parkinson's disease	Global	Male	Socio-demographic Index			X			X
Parkinson's disease	Data Rich	Male	Low-Density Lipoprotein (mmol/L)		X			X	
Parkinson's disease	Global	Male	Low-Density Lipoprotein (mmol/L)		X			X	
Parkinson's disease	Data Rich	Male	Healthcare access and quality index		X			X	
Parkinson's disease	Global	Male	Healthcare access and quality index		X			X	
Parkinson's disease	Data Rich	Male	Sanitation (proportion with access)		X			X	
Parkinson's disease	Global	Male	Sanitation (proportion with access)		X			X	
Parkinson's disease	Data Rich	Male	Improved Water Source (proportion with access)		X			X	
Parkinson's disease	Global	Male	Improved Water Source (proportion with access)		X			X	
Parkinson's disease	Data Rich	Male	Absolute value of average latitude		X			X	
Parkinson's disease	Global	Male	Absolute value of average latitude		X			X	
Parkinson's disease	Data Rich	Male	Cumulative Cigarettes (10 Years)	X			X		
Parkinson's disease	Global	Male	Cumulative Cigarettes (10 Years)	X			X		
Parkinson's disease	Data Rich	Male	Age- and sex-specific SEV for Low fruit	X			X		
Parkinson's disease	Global	Male	Age- and sex-specific SEV for Low fruit	X			X		
Idiopathic epilepsy	Global	Female	Cumulative Cigarettes (10 Years)			X			X
Idiopathic epilepsy	Data Rich	Female	Cumulative Cigarettes (10 Years)			X			X
Idiopathic epilepsy	Global	Female	Education (years per capita)			X			X
Idiopathic epilepsy	Data Rich	Female	Education (years per capita)			X			X
Idiopathic epilepsy	Global	Female	LDI (I$ per capita)			X			X
Idiopathic epilepsy	Data Rich	Female	LDI (I$ per capita)			X			X
Idiopathic epilepsy	Global	Female	Socio-demographic Index			X			X
Idiopathic epilepsy	Data Rich	Female	Socio-demographic Index			X			X
Idiopathic epilepsy	Global	Female	Cumulative Cigarettes (5 Years)			X			X
Idiopathic epilepsy	Data Rich	Female	Cumulative Cigarettes (5 Years)			X			X
Idiopathic epilepsy	Global	Female	Low-Density Lipoprotein (mmol/L)		X			X	
Idiopathic epilepsy	Data Rich	Female	Low-Density Lipoprotein (mmol/L)		X			X	
Idiopathic epilepsy	Global	Female	Healthcare access and quality index		X			X	
Idiopathic epilepsy	Data Rich	Female	Healthcare access and quality index		X			X	
Idiopathic epilepsy	Global	Female	Mean BMI		X			X	
Idiopathic epilepsy	Data Rich	Female	Mean BMI		X			X	
Idiopathic epilepsy	Global	Female	Systolic Blood Pressure (mmHg)	X			X		
Idiopathic epilepsy	Data Rich	Female	Systolic Blood Pressure (mmHg)	X			X		
Idiopathic epilepsy	Global	Female	Pigs (per capita)	X			X		
Idiopathic epilepsy	Data Rich	Female	Pigs (per capita)	X			X		
Idiopathic epilepsy	Global	Female	Log-transformed SEV scalar: Idiopathic epilepsy	X			X		
Idiopathic epilepsy	Data Rich	Female	Log-transformed SEV scalar: Idiopathic epilepsy	X			X		
Idiopathic epilepsy	Global	Male	Cumulative Cigarettes (10 Years)			X			X
Idiopathic epilepsy	Data Rich	Male	Cumulative Cigarettes (10 Years)			X			X
Idiopathic epilepsy	Global	Male	Education (years per capita)			X			X
Idiopathic epilepsy	Data Rich	Male	Education (years per capita)			X			X
Idiopathic epilepsy	Global	Male	LDI (I$ per capita)			X			X
Idiopathic epilepsy	Data Rich	Male	LDI (I$ per capita)			X			X
Idiopathic epilepsy	Global	Male	Socio-demographic Index			X			X
Idiopathic epilepsy	Data Rich	Male	Socio-demographic Index			X			X
Idiopathic epilepsy	Global	Male	Cumulative Cigarettes (5 Years)			X			X
Idiopathic epilepsy	Data Rich	Male	Cumulative Cigarettes (5 Years)			X			X
Idiopathic epilepsy	Global	Male	Low-Density Lipoprotein (mmol/L)		X			X	
Idiopathic epilepsy	Data Rich	Male	Low-Density Lipoprotein (mmol/L)		X			X	
Idiopathic epilepsy	Global	Male	Healthcare access and quality index		X			X	
Idiopathic epilepsy	Data Rich	Male	Healthcare access and quality index		X			X	
Idiopathic epilepsy	Global	Male	Mean BMI		X			X	
Idiopathic epilepsy	Data Rich	Male	Mean BMI		X			X	
Idiopathic epilepsy	Global	Male	Systolic Blood Pressure (mmHg)	X			X		
Idiopathic epilepsy	Data Rich	Male	Systolic Blood Pressure (mmHg)	X			X		
Idiopathic epilepsy	Global	Male	Pigs (per capita)	X			X		
Idiopathic epilepsy	Data Rich	Male	Pigs (per capita)	X			X		
Idiopathic epilepsy	Global	Male	Log-transformed SEV scalar: Idiopathic epilepsy	X			X		
Idiopathic epilepsy	Data Rich	Male	Log-transformed SEV scalar: Idiopathic epilepsy	X			X		
Multiple sclerosis	Global	Female	Cumulative Cigarettes (10 Years)			X			X
Multiple sclerosis	Data Rich	Female	Cumulative Cigarettes (10 Years)			X			X
Multiple sclerosis	Global	Female	Education (years per capita)			X			X
Multiple sclerosis	Data Rich	Female	Education (years per capita)			X			X
Multiple sclerosis	Global	Female	Smoking Prevalence			X			X
Multiple sclerosis	Data Rich	Female	Smoking Prevalence			X			X
Multiple sclerosis	Global	Female	Socio-demographic Index			X			X
Multiple sclerosis	Data Rich	Female	Socio-demographic Index			X			X
Multiple sclerosis	Global	Female	Cumulative Cigarettes (5 Years)			X			X
Multiple sclerosis	Data Rich	Female	Cumulative Cigarettes (5 Years)			X			X
Multiple sclerosis	Global	Female	Low-Density Lipoprotein (mmol/L)		X			X	
Multiple sclerosis	Data Rich	Female	Low-Density Lipoprotein (mmol/L)		X			X	
Multiple sclerosis	Global	Female	Healthcare access and quality index		X			X	
Multiple sclerosis	Data Rich	Female	Healthcare access and quality index		X			X	
Multiple sclerosis	Global	Female	Absolute value of average latitude	X			X		
Multiple sclerosis	Data Rich	Female	Absolute value of average latitude	X			X		
Multiple sclerosis	Global	Female	Prevalence of obesity						X
Multiple sclerosis	Data Rich	Female	Prevalence of obesity						X
Multiple sclerosis	Data Rich	Male	Cumulative Cigarettes (10 Years)			X			X
Multiple sclerosis	Global	Male	Cumulative Cigarettes (10 Years)			X			X
Multiple sclerosis	Data Rich	Male	Education (years per capita)			X			X
Multiple sclerosis	Global	Male	Education (years per capita)			X			X
Multiple sclerosis	Data Rich	Male	LDI (I$ per capita)			X			X
Multiple sclerosis	Data Rich	Male	Smoking Prevalence			X			X
Multiple sclerosis	Global	Male	Smoking Prevalence			X			X
Multiple sclerosis	Data Rich	Male	Socio-demographic Index			X			X
Multiple sclerosis	Global	Male	Socio-demographic Index			X			X
Multiple sclerosis	Data Rich	Male	Cumulative Cigarettes (5 Years)			X			X
Multiple sclerosis	Global	Male	Cumulative Cigarettes (5 Years)			X			X
Multiple sclerosis	Data Rich	Male	Low-Density Lipoprotein (mmol/L)		X			X	
Multiple sclerosis	Global	Male	Low-Density Lipoprotein (mmol/L)		X			X	
Multiple sclerosis	Data Rich	Male	Healthcare access and quality index		X			X	
Multiple sclerosis	Global	Male	Healthcare access and quality index		X			X	
Multiple sclerosis	Data Rich	Male	Absolute value of average latitude	X			X		
Multiple sclerosis	Global	Male	Absolute value of average latitude	X			X		
Multiple sclerosis	Data Rich	Male	Prevalence of obesity						X
Multiple sclerosis	Global	Male	Prevalence of obesity						X
Motor neuron disease	Data Rich	Female	Education (years per capita)			X			X
Motor neuron disease	Global	Female	Education (years per capita)			X			X
Motor neuron disease	Data Rich	Female	LDI (I$ per capita)			X			X
Motor neuron disease	Global	Female	LDI (I$ per capita)			X			X
Motor neuron disease	Data Rich	Female	Healthcare access and quality index		X			X	
Motor neuron disease	Global	Female	Healthcare access and quality index		X			X	
Motor neuron disease	Data Rich	Female	Sanitation (proportion with access)		X			X	
Motor neuron disease	Global	Female	Sanitation (proportion with access)		X			X	
Motor neuron disease	Data Rich	Female	Improved Water Source (proportion with access)		X			X	
Motor neuron disease	Global	Female	Improved Water Source (proportion with access)		X			X	
Motor neuron disease	Data Rich	Female	Population-weighted mean temperature		X			X	
Motor neuron disease	Global	Female	Population-weighted mean temperature		X			X	
Motor neuron disease	Data Rich	Female	Low-Density Lipoprotein (mmol/L)	X			X		
Motor neuron disease	Global	Female	Low-Density Lipoprotein (mmol/L)	X			X		
Motor neuron disease	Data Rich	Female	Socio-demographic Index	X			X		
Motor neuron disease	Global	Female	Socio-demographic Index	X			X		

Appendix Table S11: Comparison of GBD 2021 and GBD 2023 covariates and level of covariates used in cause of death modeling

Cause	Model Version Type	Sex	Covariate	Level 1 GBD 2021	Level 2 GBD 2021	Level 3 GBD 2021	Level 1 GBD 2023	Level 2 GBD 2023	Level 3 GBD 2023
Motor neuron disease	Data Rich	Female	Diabetes Fasting Plasma Glucose (mmol/L), age-standardized 25+	X			X		
Motor neuron disease	Global	Female	Diabetes Fasting Plasma Glucose (mmol/L), age-standardized 25+	X			X		
Motor neuron disease	Data Rich	Female	Mean BMI	X			X		
Motor neuron disease	Global	Female	Mean BMI	X			X		
Motor neuron disease	Data Rich	Female	Age- and sex-specific SEV for Low fruit	X			X		
Motor neuron disease	Global	Female	Age- and sex-specific SEV for Low fruit	X			X		
Motor neuron disease	Data Rich	Female	Absolute value of average latitude	X			X		
Motor neuron disease	Global	Female	Absolute value of average latitude	X			X		
Motor neuron disease	Data Rich	Male	Education (years per capita)			X			X
Motor neuron disease	Global	Male	Education (years per capita)			X			X
Motor neuron disease	Data Rich	Male	LDI (I$ per capita)			X			X
Motor neuron disease	Global	Male	LDI (I$ per capita)			X			X
Motor neuron disease	Data Rich	Male	Healthcare access and quality index		X			X	
Motor neuron disease	Global	Male	Healthcare access and quality index		X			X	
Motor neuron disease	Data Rich	Male	Sanitation (proportion with access)		X			X	
Motor neuron disease	Global	Male	Sanitation (proportion with access)		X			X	
Motor neuron disease	Data Rich	Male	Improved Water Source (proportion with access)		X			X	
Motor neuron disease	Global	Male	Improved Water Source (proportion with access)		X			X	
Motor neuron disease	Data Rich	Male	Population-weighted mean temperature		X			X	
Motor neuron disease	Global	Male	Population-weighted mean temperature		X			X	
Motor neuron disease	Data Rich	Male	Low-Density Lipoprotein (mmol/L)	X			X		
Motor neuron disease	Global	Male	Low-Density Lipoprotein (mmol/L)	X			X		
Motor neuron disease	Data Rich	Male	Socio-demographic Index	X			X		
Motor neuron disease	Global	Male	Socio-demographic Index	X			X		
Motor neuron disease	Data Rich	Male	Diabetes Fasting Plasma Glucose (mmol/L), age-standardized 25+	X			X		
Motor neuron disease	Global	Male	Diabetes Fasting Plasma Glucose (mmol/L), age-standardized 25+	X			X		
Motor neuron disease	Data Rich	Male	Mean BMI	X			X		
Motor neuron disease	Global	Male	Mean BMI	X			X		
Motor neuron disease	Data Rich	Male	Age- and sex-specific SEV for Low fruit	X			X		
Motor neuron disease	Global	Male	Age- and sex-specific SEV for Low fruit	X			X		
Motor neuron disease	Data Rich	Male	Absolute value of average latitude	X			X		
Motor neuron disease	Global	Male	Absolute value of average latitude	X			X		
Other neurological disorders	Global	Female	Cumulative Cigarettes (10 Years)			X			X
Other neurological disorders	Data Rich	Female	Cumulative Cigarettes (10 Years)			X			X
Other neurological disorders	Global	Female	Education (years per capita)			X			X
Other neurological disorders	Data Rich	Female	Education (years per capita)			X			X
Other neurological disorders	Global	Female	LDI (I$ per capita)			X			X
Other neurological disorders	Data Rich	Female	LDI (I$ per capita)			X			X
Other neurological disorders	Global	Female	Smoking Prevalence			X			X
Other neurological disorders	Data Rich	Female	Smoking Prevalence			X			X
Other neurological disorders	Global	Female	Socio-demographic Index			X			X
Other neurological disorders	Data Rich	Female	Socio-demographic Index			X			X
Other neurological disorders	Global	Female	Cumulative Cigarettes (5 Years)			X			X
Other neurological disorders	Data Rich	Female	Cumulative Cigarettes (5 Years)			X			X
Other neurological disorders	Global	Female	Healthcare access and quality index		X			X	
Other neurological disorders	Data Rich	Female	Healthcare access and quality index		X			X	
Other neurological disorders	Global	Female	Age- and sex-specific SEV for Low fruit		X			X	
Other neurological disorders	Data Rich	Female	Age- and sex-specific SEV for Low fruit		X			X	
Other neurological disorders	Global	Female	Population Density (over 1000 ppl/sqkm, proportion)		X			X	
Other neurological disorders	Data Rich	Female	Population Density (over 1000 ppl/sqkm, proportion)		X			X	
Other neurological disorders	Global	Female	Low-Density Lipoprotein (mmol/L)	X			X		
Other neurological disorders	Data Rich	Female	Low-Density Lipoprotein (mmol/L)	X			X		
Other neurological disorders	Global	Female	Mean BMI	X			X		
Other neurological disorders	Data Rich	Female	Mean BMI	X			X		
Other neurological disorders	Data Rich	Female	Age- and sex-specific SEV for Child underweight	X			X		
Other neurological disorders	Global	Female	Systolic Blood Pressure (mmHg)	X			X		
Other neurological disorders	Data Rich	Female	Systolic Blood Pressure (mmHg)	X			X		
Other neurological disorders	Global	Female	Age- and sex-specific SEV for High red meat	X			X		
Other neurological disorders	Data Rich	Female	Age- and sex-specific SEV for High red meat	X			X		
Other neurological disorders	Global	Female	Pigs (per capita)	X			X		
Other neurological disorders	Data Rich	Female	Pigs (per capita)	X			X		
Other neurological disorders	Global	Male	Cumulative Cigarettes (10 Years)			X			X
Other neurological disorders	Data Rich	Male	Cumulative Cigarettes (10 Years)			X			X
Other neurological disorders	Global	Male	Education (years per capita)			X			X
Other neurological disorders	Data Rich	Male	Education (years per capita)			X			X
Other neurological disorders	Global	Male	LDI (I$ per capita)			X			X
Other neurological disorders	Data Rich	Male	LDI (I$ per capita)			X			X
Other neurological disorders	Global	Male	Smoking Prevalence			X			X
Other neurological disorders	Data Rich	Male	Smoking Prevalence			X			X
Other neurological disorders	Global	Male	Socio-demographic Index			X			X
Other neurological disorders	Data Rich	Male	Socio-demographic Index			X			X
Other neurological disorders	Global	Male	Cumulative Cigarettes (5 Years)			X			X
Other neurological disorders	Data Rich	Male	Cumulative Cigarettes (5 Years)			X			X
Other neurological disorders	Global	Male	Healthcare access and quality index		X			X	
Other neurological disorders	Data Rich	Male	Healthcare access and quality index		X			X	
Other neurological disorders	Global	Male	Liters of alcohol consumed per capita		X			X	
Other neurological disorders	Global	Male	Age- and sex-specific SEV for Low fruit		X			X	
Other neurological disorders	Data Rich	Male	Age- and sex-specific SEV for Low fruit		X			X	
Other neurological disorders	Global	Male	Population Density (over 1000 ppl/sqkm, proportion)		X			X	
Other neurological disorders	Data Rich	Male	Population Density (over 1000 ppl/sqkm, proportion)		X			X	
Other neurological disorders	Global	Male	Low-Density Lipoprotein (mmol/L)	X			X		
Other neurological disorders	Data Rich	Male	Low-Density Lipoprotein (mmol/L)	X			X		
Other neurological disorders	Global	Male	Mean BMI	X			X		
Other neurological disorders	Data Rich	Male	Mean BMI	X			X		
Other neurological disorders	Global	Male	Age- and sex-specific SEV for Child underweight	X			X		
Other neurological disorders	Data Rich	Male	Age- and sex-specific SEV for Child underweight	X			X		
Other neurological disorders	Global	Male	Systolic Blood Pressure (mmHg)	X			X		
Other neurological disorders	Data Rich	Male	Systolic Blood Pressure (mmHg)	X			X		
Other neurological disorders	Global	Male	Age- and sex-specific SEV for High red meat	X			X		
Other neurological disorders	Data Rich	Male	Age- and sex-specific SEV for High red meat	X			X		
Other neurological disorders	Global	Male	Pigs (per capita)	X			X		
Other neurological disorders	Data Rich	Male	Pigs (per capita)	X			X		
Anorexia nervosa	Global	Female	Socio-demographic Index			X			X
Anorexia nervosa	Data Rich	Female	Socio-demographic Index			X			X
Anorexia nervosa	Global	Female	Healthcare access and quality index		X			X	
Anorexia nervosa	Data Rich	Female	Healthcare access and quality index		X			X	
Anorexia nervosa	Global	Female	Education (years per capita)	X			X		
Anorexia nervosa	Data Rich	Female	Education (years per capita)	X			X		
Anorexia nervosa	Global	Female	LDI (I$ per capita)	X			X		
Anorexia nervosa	Data Rich	Female	LDI (I$ per capita)	X			X		
Anorexia nervosa	Global	Female	Sanitation (proportion with access)	X			X		
Anorexia nervosa	Data Rich	Female	Sanitation (proportion with access)	X			X		
Anorexia nervosa	Global	Female	Maternal Education (years per capita)	X			X		
Anorexia nervosa	Data Rich	Female	Maternal Education (years per capita)	X			X		
Anorexia nervosa	Global	Female	Age- and sex-specific SEV for Child underweight	X			X		
Anorexia nervosa	Data Rich	Female	Age- and sex-specific SEV for Child underweight	X			X		
Anorexia nervosa	Global	Male	Socio-demographic Index			X			X
Anorexia nervosa	Data Rich	Male	Socio-demographic Index			X			X
Anorexia nervosa	Global	Male	Healthcare access and quality index		X			X	
Anorexia nervosa	Data Rich	Male	Healthcare access and quality index		X			X	
Anorexia nervosa	Global	Male	Education (years per capita)	X			X		
Anorexia nervosa	Data Rich	Male	Education (years per capita)	X			X		
Anorexia nervosa	Global	Male	LDI (I$ per capita)	X			X		
Anorexia nervosa	Data Rich	Male	LDI (I$ per capita)	X			X		
Anorexia nervosa	Global	Male	Sanitation (proportion with access)	X			X		
Anorexia nervosa	Data Rich	Male	Sanitation (proportion with access)	X			X		
Anorexia nervosa	Global	Male	Maternal Education (years per capita)	X			X		
Anorexia nervosa	Data Rich	Male	Maternal Education (years per capita)	X			X		

Appendix Table S11: Comparison of GBD 2021 and GBD 2023 covariates and level of covariates used in cause of death modeling

Cause	Model Version Type	Sex	Covariate	Level 1 GBD 2021	Level 2 GBD 2021	Level 3 GBD 2021	Level 1 GBD 2023	Level 2 GBD 2023	Level 3 GBD 2023
Anorexia nervosa	Global	Male	Age- and sex-specific SEV for Child underweight	X			X		
Anorexia nervosa	Data Rich	Male	Age- and sex-specific SEV for Child underweight	X			X		
Alcohol use disorders	Data Rich	Female	Education (years per capita)			X			X
Alcohol use disorders	Global	Female	Education (years per capita)			X			X
Alcohol use disorders	Data Rich	Female	LDI (I$ per capita)			X			X
Alcohol use disorders	Global	Female	LDI (I$ per capita)			X			X
Alcohol use disorders	Data Rich	Female	Socio-demographic Index			X			X
Alcohol use disorders	Global	Female	Socio-demographic Index			X			X
Alcohol use disorders	Data Rich	Female	Cumulative Cigarettes (10 Years)		X			X	
Alcohol use disorders	Global	Female	Cumulative Cigarettes (10 Years)		X			X	
Alcohol use disorders	Data Rich	Female	Smoking Prevalence		X			X	
Alcohol use disorders	Global	Female	Smoking Prevalence		X			X	
Alcohol use disorders	Data Rich	Female	Healthcare access and quality index		X			X	
Alcohol use disorders	Global	Female	Healthcare access and quality index		X			X	
Alcohol use disorders	Data Rich	Female	Liters of alcohol consumed per capita	X			X		
Alcohol use disorders	Global	Female	Liters of alcohol consumed per capita	X			X		
Alcohol use disorders	Data Rich	Female	Alcohol consumption, age standardized, in grams per day	X			X		
Alcohol use disorders	Global	Female	Alcohol consumption, age standardized, in grams per day	X			X		
Alcohol use disorders	Data Rich	Female	Alcohol drinker proportion, age-standardized	X			X		
Alcohol use disorders	Global	Female	Alcohol drinker proportion, age-standardized	X			X		
Alcohol use disorders	Data Rich	Male	Education (years per capita)			X			X
Alcohol use disorders	Global	Male	Education (years per capita)			X			X
Alcohol use disorders	Data Rich	Male	LDI (I$ per capita)			X			X
Alcohol use disorders	Global	Male	LDI (I$ per capita)			X			X
Alcohol use disorders	Data Rich	Male	Socio-demographic Index			X			X
Alcohol use disorders	Global	Male	Socio-demographic Index			X			X
Alcohol use disorders	Data Rich	Male	Cumulative Cigarettes (10 Years)		X			X	
Alcohol use disorders	Global	Male	Cumulative Cigarettes (10 Years)		X			X	
Alcohol use disorders	Data Rich	Male	Smoking Prevalence		X			X	
Alcohol use disorders	Global	Male	Smoking Prevalence		X			X	
Alcohol use disorders	Data Rich	Male	Healthcare access and quality index		X			X	
Alcohol use disorders	Global	Male	Healthcare access and quality index		X			X	
Alcohol use disorders	Data Rich	Male	Liters of alcohol consumed per capita	X			X		
Alcohol use disorders	Global	Male	Liters of alcohol consumed per capita	X			X		
Alcohol use disorders	Data Rich	Male	Alcohol consumption, age standardized, in grams per day	X			X		
Alcohol use disorders	Global	Male	Alcohol consumption, age standardized, in grams per day	X			X		
Alcohol use disorders	Data Rich	Male	Alcohol drinker proportion, age-standardized	X			X		
Alcohol use disorders	Global	Male	Alcohol drinker proportion, age-standardized	X			X		
Drug use disorders	Data Rich	Female	Education (years per capita)			X			X
Drug use disorders	Global	Female	Education (years per capita)			X			X
Drug use disorders	Data Rich	Female	LDI (I$ per capita)			X			X
Drug use disorders	Global	Female	LDI (I$ per capita)			X			X
Drug use disorders	Data Rich	Female	Socio-demographic Index			X			X
Drug use disorders	Global	Female	Socio-demographic Index			X			X
Drug use disorders	Data Rich	Female	Cumulative Cigarettes (10 Years)		X			X	
Drug use disorders	Global	Female	Cumulative Cigarettes (10 Years)		X			X	
Drug use disorders	Data Rich	Female	Smoking Prevalence		X			X	
Drug use disorders	Global	Female	Smoking Prevalence		X			X	
Drug use disorders	Data Rich	Female	Healthcare access and quality index		X			X	
Drug use disorders	Global	Female	Healthcare access and quality index		X			X	
Drug use disorders	Data Rich	Female	Cumulative Cigarettes (5 Years)		X			X	
Drug use disorders	Global	Female	Cumulative Cigarettes (5 Years)		X			X	
Drug use disorders	Data Rich	Female	Intravenous drug use (age-standardized proportion)	X			X		
Drug use disorders	Global	Female	Intravenous drug use (age-standardized proportion)	X			X		
Drug use disorders	Data Rich	Female	Intravenous drug use (proportion by age)	X			X		
Drug use disorders	Global	Female	Intravenous drug use (proportion by age)	X			X		
Drug use disorders	Data Rich	Female	Opioids per million population per day (10 year lag)	X			X		
Drug use disorders	Global	Female	Opioids per million population per day (10 year lag)	X			X		
Drug use disorders	Data Rich	Male	Education (years per capita)			X			X
Drug use disorders	Global	Male	Education (years per capita)			X			X
Drug use disorders	Data Rich	Male	LDI (I$ per capita)			X			X
Drug use disorders	Global	Male	LDI (I$ per capita)			X			X
Drug use disorders	Data Rich	Male	Socio-demographic Index			X			X
Drug use disorders	Global	Male	Socio-demographic Index			X			X
Drug use disorders	Data Rich	Male	Cumulative Cigarettes (10 Years)		X			X	
Drug use disorders	Global	Male	Cumulative Cigarettes (10 Years)		X			X	
Drug use disorders	Data Rich	Male	Smoking Prevalence		X			X	
Drug use disorders	Global	Male	Smoking Prevalence		X			X	
Drug use disorders	Data Rich	Male	Healthcare access and quality index		X			X	
Drug use disorders	Global	Male	Healthcare access and quality index		X			X	
Drug use disorders	Data Rich	Male	Cumulative Cigarettes (5 Years)		X			X	
Drug use disorders	Global	Male	Cumulative Cigarettes (5 Years)		X			X	
Drug use disorders	Data Rich	Male	Intravenous drug use (age-standardized proportion)	X			X		
Drug use disorders	Global	Male	Intravenous drug use (age-standardized proportion)	X			X		
Drug use disorders	Data Rich	Male	Intravenous drug use (proportion by age)	X			X		
Drug use disorders	Global	Male	Intravenous drug use (proportion by age)	X			X		
Drug use disorders	Data Rich	Male	Opioids per million population per day (10 year lag)	X			X		
Drug use disorders	Global	Male	Opioids per million population per day (10 year lag)	X			X		
Opioid use disorders	Data Rich	Female	Education (years per capita)			X			X
Opioid use disorders	Global	Female	Education (years per capita)			X			X
Opioid use disorders	Data Rich	Female	LDI (I$ per capita)			X			X
Opioid use disorders	Global	Female	LDI (I$ per capita)			X			X
Opioid use disorders	Data Rich	Female	Socio-demographic Index			X			X
Opioid use disorders	Global	Female	Socio-demographic Index			X			X
Opioid use disorders	Data Rich	Female	Cumulative Cigarettes (10 Years)		X			X	
Opioid use disorders	Global	Female	Cumulative Cigarettes (10 Years)		X			X	
Opioid use disorders	Data Rich	Female	Smoking Prevalence		X			X	
Opioid use disorders	Global	Female	Smoking Prevalence		X			X	
Opioid use disorders	Data Rich	Female	Healthcare access and quality index		X			X	
Opioid use disorders	Global	Female	Healthcare access and quality index		X			X	
Opioid use disorders	Data Rich	Female	Cumulative Cigarettes (5 Years)		X			X	
Opioid use disorders	Global	Female	Cumulative Cigarettes (5 Years)		X			X	
Opioid use disorders	Data Rich	Female	Intravenous drug use (age-standardized proportion)	X			X		
Opioid use disorders	Global	Female	Intravenous drug use (age-standardized proportion)	X			X		
Opioid use disorders	Data Rich	Female	Intravenous drug use (proportion by age)	X			X		
Opioid use disorders	Global	Female	Intravenous drug use (proportion by age)	X			X		
Opioid use disorders	Data Rich	Female	Opioids per million population per day (10 year lag)	X			X		
Opioid use disorders	Global	Female	Opioids per million population per day (10 year lag)	X			X		
Opioid use disorders	Data Rich	Male	Cumulative Cigarettes (10 Years)		X			X	
Opioid use disorders	Global	Male	Cumulative Cigarettes (10 Years)		X			X	
Opioid use disorders	Data Rich	Male	Smoking Prevalence		X			X	
Opioid use disorders	Global	Male	Smoking Prevalence		X			X	
Opioid use disorders	Data Rich	Male	Cumulative Cigarettes (5 Years)		X			X	
Opioid use disorders	Global	Male	Cumulative Cigarettes (5 Years)		X			X	
Opioid use disorders	Data Rich	Male	Intravenous drug use (age-standardized proportion)	X			X		
Opioid use disorders	Global	Male	Intravenous drug use (age-standardized proportion)	X			X		
Opioid use disorders	Data Rich	Male	Intravenous drug use (proportion by age)	X			X		
Opioid use disorders	Global	Male	Intravenous drug use (proportion by age)	X			X		
Opioid use disorders	Data Rich	Male	Opioids per million population per day (10 year lag)	X			X		
Opioid use disorders	Global	Male	Opioids per million population per day (10 year lag)	X			X		
Cocaine use disorders	Data Rich	Female	Education (years per capita)			X			X
Cocaine use disorders	Global	Female	Education (years per capita)			X			X
Cocaine use disorders	Data Rich	Female	LDI (I$ per capita)			X			X
Cocaine use disorders	Global	Female	LDI (I$ per capita)			X			X
Cocaine use disorders	Data Rich	Female	Socio-demographic Index			X			X
Cocaine use disorders	Global	Female	Socio-demographic Index			X			X
Cocaine use disorders	Data Rich	Female	Healthcare access and quality index		X			X	

Appendix Table S11: Comparison of GBD 2021 and GBD 2023 covariates and level of covariates used in cause of death modeling									
Cause	Model Version Type	Sex	Covariate	Level 1 GBD 2021	Level 2 GBD 2021	Level 3 GBD 2021	Level 1 GBD 2023	Level 2 GBD 2023	Level 3 GBD 2023
Cocaine use disorders	Global	Female	Healthcare access and quality index		X			X	
Cocaine use disorders	Data Rich	Female	Tobacco (cigarettes per capita)	X			X		
Cocaine use disorders	Global	Female	Tobacco (cigarettes per capita)	X			X		
Cocaine use disorders	Data Rich	Female	Cumulative Cigarettes (10 Years)	X			X		
Cocaine use disorders	Global	Female	Cumulative Cigarettes (10 Years)	X			X		
Cocaine use disorders	Data Rich	Female	Smoking Prevalence	X			X		
Cocaine use disorders	Global	Female	Smoking Prevalence	X			X		
Cocaine use disorders	Data Rich	Female	Liters of alcohol consumed per capita	X			X		
Cocaine use disorders	Global	Female	Liters of alcohol consumed per capita	X			X		
Cocaine use disorders	Data Rich	Female	Cumulative Cigarettes (5 Years)	X			X		
Cocaine use disorders	Global	Female	Cumulative Cigarettes (5 Years)	X			X		
Cocaine use disorders	Data Rich	Female	Intravenous drug use (age-standardized proportion)	X			X		
Cocaine use disorders	Global	Female	Intravenous drug use (age-standardized proportion)	X			X		
Cocaine use disorders	Data Rich	Female	Intravenous drug use (proportion by age)	X			X		
Cocaine use disorders	Global	Female	Intravenous drug use (proportion by age)	X			X		
Cocaine use disorders	Data Rich	Female	Alcohol drinker proportion, age-standardized	X			X		
Cocaine use disorders	Global	Female	Alcohol drinker proportion, age-standardized	X			X		
Cocaine use disorders	Data Rich	Male	Education (years per capita)			X			X
Cocaine use disorders	Global	Male	Education (years per capita)			X			X
Cocaine use disorders	Data Rich	Male	LDI (I$ per capita)			X			X
Cocaine use disorders	Global	Male	LDI (I$ per capita)			X			X
Cocaine use disorders	Data Rich	Male	Socio-demographic Index			X			X
Cocaine use disorders	Global	Male	Socio-demographic Index			X			X
Cocaine use disorders	Data Rich	Male	Healthcare access and quality index		X			X	
Cocaine use disorders	Global	Male	Healthcare access and quality index		X			X	
Cocaine use disorders	Data Rich	Male	Tobacco (cigarettes per capita)	X			X		
Cocaine use disorders	Global	Male	Tobacco (cigarettes per capita)	X			X		
Cocaine use disorders	Data Rich	Male	Cumulative Cigarettes (10 Years)	X			X		
Cocaine use disorders	Global	Male	Cumulative Cigarettes (10 Years)	X			X		
Cocaine use disorders	Data Rich	Male	Smoking Prevalence	X			X		
Cocaine use disorders	Global	Male	Smoking Prevalence	X			X		
Cocaine use disorders	Data Rich	Male	Liters of alcohol consumed per capita	X			X		
Cocaine use disorders	Global	Male	Liters of alcohol consumed per capita	X			X		
Cocaine use disorders	Data Rich	Male	Cumulative Cigarettes (5 Years)	X			X		
Cocaine use disorders	Global	Male	Cumulative Cigarettes (5 Years)	X			X		
Cocaine use disorders	Data Rich	Male	Intravenous drug use (age-standardized proportion)	X			X		
Cocaine use disorders	Global	Male	Intravenous drug use (age-standardized proportion)	X			X		
Cocaine use disorders	Data Rich	Male	Intravenous drug use (proportion by age)	X			X		
Cocaine use disorders	Global	Male	Intravenous drug use (proportion by age)	X			X		
Cocaine use disorders	Data Rich	Male	Alcohol drinker proportion, age-standardized	X			X		
Cocaine use disorders	Global	Male	Alcohol drinker proportion, age-standardized	X			X		
Amphetamine use disorders	Global	Female	Education (years per capita)			X			X
Amphetamine use disorders	Data Rich	Female	Education (years per capita)			X			X
Amphetamine use disorders	Global	Female	LDI (I$ per capita)			X			X
Amphetamine use disorders	Data Rich	Female	LDI (I$ per capita)			X			X
Amphetamine use disorders	Global	Female	Socio-demographic Index			X			X
Amphetamine use disorders	Data Rich	Female	Socio-demographic Index			X			X
Amphetamine use disorders	Global	Female	Healthcare access and quality index		X			X	
Amphetamine use disorders	Data Rich	Female	Healthcare access and quality index		X			X	
Amphetamine use disorders	Global	Female	Tobacco (cigarettes per capita)	X			X		
Amphetamine use disorders	Data Rich	Female	Tobacco (cigarettes per capita)	X			X		
Amphetamine use disorders	Global	Female	Cumulative Cigarettes (10 Years)	X			X		
Amphetamine use disorders	Data Rich	Female	Cumulative Cigarettes (10 Years)	X			X		
Amphetamine use disorders	Global	Female	Smoking Prevalence	X			X		
Amphetamine use disorders	Data Rich	Female	Smoking Prevalence	X			X		
Amphetamine use disorders	Global	Female	Liters of alcohol consumed per capita	X			X		
Amphetamine use disorders	Data Rich	Female	Liters of alcohol consumed per capita	X			X		
Amphetamine use disorders	Global	Female	Cumulative Cigarettes (5 Years)	X			X		
Amphetamine use disorders	Data Rich	Female	Cumulative Cigarettes (5 Years)	X			X		
Amphetamine use disorders	Global	Female	Intravenous drug use (age-standardized proportion)	X			X		
Amphetamine use disorders	Data Rich	Female	Intravenous drug use (age-standardized proportion)	X			X		
Amphetamine use disorders	Global	Female	Intravenous drug use (proportion by age)	X			X		
Amphetamine use disorders	Data Rich	Female	Intravenous drug use (proportion by age)	X			X		
Amphetamine use disorders	Global	Female	Alcohol drinker proportion, age-standardized	X			X		
Amphetamine use disorders	Data Rich	Female	Alcohol drinker proportion, age-standardized	X			X		
Amphetamine use disorders	Global	Male	Education (years per capita)			X			X
Amphetamine use disorders	Data Rich	Male	Education (years per capita)			X			X
Amphetamine use disorders	Global	Male	LDI (I$ per capita)			X			X
Amphetamine use disorders	Data Rich	Male	LDI (I$ per capita)			X			X
Amphetamine use disorders	Global	Male	Socio-demographic Index			X			X
Amphetamine use disorders	Data Rich	Male	Socio-demographic Index			X			X
Amphetamine use disorders	Global	Male	Healthcare access and quality index		X			X	
Amphetamine use disorders	Data Rich	Male	Healthcare access and quality index		X			X	
Amphetamine use disorders	Global	Male	Tobacco (cigarettes per capita)	X			X		
Amphetamine use disorders	Data Rich	Male	Tobacco (cigarettes per capita)	X			X		
Amphetamine use disorders	Global	Male	Cumulative Cigarettes (10 Years)	X			X		
Amphetamine use disorders	Data Rich	Male	Cumulative Cigarettes (10 Years)	X			X		
Amphetamine use disorders	Global	Male	Smoking Prevalence	X			X		
Amphetamine use disorders	Data Rich	Male	Smoking Prevalence	X			X		
Amphetamine use disorders	Global	Male	Liters of alcohol consumed per capita	X			X		
Amphetamine use disorders	Data Rich	Male	Liters of alcohol consumed per capita	X			X		
Amphetamine use disorders	Global	Male	Cumulative Cigarettes (5 Years)	X			X		
Amphetamine use disorders	Data Rich	Male	Cumulative Cigarettes (5 Years)	X			X		
Amphetamine use disorders	Global	Male	Intravenous drug use (age-standardized proportion)	X			X		
Amphetamine use disorders	Data Rich	Male	Intravenous drug use (age-standardized proportion)	X			X		
Amphetamine use disorders	Global	Male	Intravenous drug use (proportion by age)	X			X		
Amphetamine use disorders	Data Rich	Male	Intravenous drug use (proportion by age)	X			X		
Amphetamine use disorders	Global	Male	Alcohol drinker proportion, age-standardized	X			X		
Amphetamine use disorders	Data Rich	Male	Alcohol drinker proportion, age-standardized	X			X		
Other drug use disorders	Data Rich	Female	Education (years per capita)			X			X
Other drug use disorders	Global	Female	Education (years per capita)			X			X
Other drug use disorders	Data Rich	Female	LDI (I$ per capita)			X			X
Other drug use disorders	Global	Female	LDI (I$ per capita)			X			X
Other drug use disorders	Data Rich	Female	Socio-demographic Index			X			X
Other drug use disorders	Global	Female	Socio-demographic Index			X			X
Other drug use disorders	Data Rich	Female	Healthcare access and quality index		X			X	
Other drug use disorders	Global	Female	Healthcare access and quality index		X			X	
Other drug use disorders	Data Rich	Female	Tobacco (cigarettes per capita)	X			X		
Other drug use disorders	Global	Female	Tobacco (cigarettes per capita)	X			X		
Other drug use disorders	Data Rich	Female	Cumulative Cigarettes (10 Years)	X			X		
Other drug use disorders	Global	Female	Cumulative Cigarettes (10 Years)	X			X		
Other drug use disorders	Data Rich	Female	Smoking Prevalence	X			X		
Other drug use disorders	Global	Female	Smoking Prevalence	X			X		
Other drug use disorders	Data Rich	Female	Liters of alcohol consumed per capita	X			X		
Other drug use disorders	Global	Female	Liters of alcohol consumed per capita	X			X		
Other drug use disorders	Data Rich	Female	Cumulative Cigarettes (5 Years)	X			X		
Other drug use disorders	Global	Female	Cumulative Cigarettes (5 Years)	X			X		
Other drug use disorders	Data Rich	Female	Intravenous drug use (age-standardized proportion)	X			X		
Other drug use disorders	Global	Female	Intravenous drug use (age-standardized proportion)	X			X		
Other drug use disorders	Data Rich	Female	Intravenous drug use (proportion by age)	X			X		
Other drug use disorders	Global	Female	Intravenous drug use (proportion by age)	X			X		
Other drug use disorders	Data Rich	Female	Alcohol drinker proportion, age-standardized	X			X		
Other drug use disorders	Global	Female	Alcohol drinker proportion, age-standardized	X			X		
Other drug use disorders	Data Rich	Male	Education (years per capita)			X			X
Other drug use disorders	Global	Male	Education (years per capita)			X			X
Other drug use disorders	Data Rich	Male	LDI (I$ per capita)			X			X

Appendix Table S11: Comparison of GBD 2021 and GBD 2023 covariates and level of covariates used in cause of death modeling									
Cause	Model Version Type	Sex	Covariate	Level 1 GBD 2021	Level 2 GBD 2021	Level 3 GBD 2021	Level 1 GBD 2023	Level 2 GBD 2023	Level 3 GBD 2023
Other drug use disorders	Global	Male	LDI (I$ per capita)			X			X
Other drug use disorders	Data Rich	Male	Socio-demographic Index			X			X
Other drug use disorders	Global	Male	Socio-demographic Index			X			X
Other drug use disorders	Data Rich	Male	Healthcare access and quality index		X			X	
Other drug use disorders	Global	Male	Healthcare access and quality index		X			X	
Other drug use disorders	Data Rich	Male	Tobacco (cigarettes per capita)	X			X		
Other drug use disorders	Global	Male	Tobacco (cigarettes per capita)	X			X		
Other drug use disorders	Data Rich	Male	Cumulative Cigarettes (10 Years)	X			X		
Other drug use disorders	Global	Male	Cumulative Cigarettes (10 Years)	X			X		
Other drug use disorders	Data Rich	Male	Smoking Prevalence	X			X		
Other drug use disorders	Global	Male	Smoking Prevalence	X			X		
Other drug use disorders	Data Rich	Male	Liters of alcohol consumed per capita	X			X		
Other drug use disorders	Global	Male	Liters of alcohol consumed per capita	X			X		
Other drug use disorders	Data Rich	Male	Cumulative Cigarettes (5 Years)	X			X		
Other drug use disorders	Global	Male	Cumulative Cigarettes (5 Years)	X			X		
Other drug use disorders	Data Rich	Male	Intravenous drug use (age-standardized proportion)	X			X		
Other drug use disorders	Global	Male	Intravenous drug use (age-standardized proportion)	X			X		
Other drug use disorders	Data Rich	Male	Intravenous drug use (proportion by age)	X			X		
Other drug use disorders	Global	Male	Intravenous drug use (proportion by age)	X			X		
Other drug use disorders	Data Rich	Male	Alcohol drinker proportion, age-standardized	X			X		
Other drug use disorders	Global	Male	Alcohol drinker proportion, age-standardized	X			X		
Diabetes mellitus	Data Rich	Female	Education (years per capita)			X			X
Diabetes mellitus	Global	Female	Education (years per capita)			X			X
Diabetes mellitus	Data Rich	Female	Education (years per capita)			X			X
Diabetes mellitus	Global	Female	Education (years per capita)			X			X
Diabetes mellitus	Data Rich	Female	LDI (I$ per capita)			X			X
Diabetes mellitus	Global	Female	LDI (I$ per capita)			X			X
Diabetes mellitus	Data Rich	Female	Socio-demographic Index			X			X
Diabetes mellitus	Global	Female	Socio-demographic Index			X			X
Diabetes mellitus	Data Rich	Female	Healthcare access and quality index			X			X
Diabetes mellitus	Global	Female	Healthcare access and quality index			X			X
Diabetes mellitus	Data Rich	Female	Low-Density Lipoprotein (mmol/L)		X			X	
Diabetes mellitus	Global	Female	Low-Density Lipoprotein (mmol/L)		X			X	
Diabetes mellitus	Global	Female	Live Births 35+ (proportion)		X			X	
Diabetes mellitus	Data Rich	Female	Systolic Blood Pressure (mmHg)		X			X	
Diabetes mellitus	Global	Female	Systolic Blood Pressure (mmHg)		X			X	
Diabetes mellitus	Data Rich	Female	Age- and sex-specific SEV for Low fruit		X			X	
Diabetes mellitus	Global	Female	Age- and sex-specific SEV for Low fruit		X			X	
Diabetes mellitus	Data Rich	Female	Age- and sex-specific SEV for Low vegetables		X			X	
Diabetes mellitus	Global	Female	Age- and sex-specific SEV for Low vegetables		X			X	
Diabetes mellitus	Data Rich	Female	Absolute value of average latitude		X			X	
Diabetes mellitus	Global	Female	Absolute value of average latitude		X			X	
Diabetes mellitus	Data Rich	Female	sugar unadjusted(g)		X			X	
Diabetes mellitus	Global	Female	sugar unadjusted(g)		X			X	
Diabetes mellitus	Data Rich	Female	Age- and sex-specific SEV for Alcohol use		X			X	
Diabetes mellitus	Global	Female	Age- and sex-specific SEV for Alcohol use		X			X	
Diabetes mellitus	Global	Female	Live Births 40+ (proportion)		X			X	
Diabetes mellitus	Data Rich	Female	Live Births 35+ (proportion)		X			X	
Diabetes mellitus	Data Rich	Female	Live Births 40+ (proportion)		X			X	
Diabetes mellitus	Data Rich	Female	Healthcare access and quality index	X			X		
Diabetes mellitus	Global	Female	Healthcare access and quality index	X			X		
Diabetes mellitus	Data Rich	Female	Mean BMI	X			X		
Diabetes mellitus	Global	Female	Mean BMI	X			X		
Diabetes mellitus	Data Rich	Female	Diabetes Fasting Plasma Glucose (mmol/L), age-standardized 25+	X			X		
Diabetes mellitus	Global	Female	Diabetes Fasting Plasma Glucose (mmol/L), age-standardized 25+	X			X		
Diabetes mellitus	Data Rich	Female	Prevalence of obesity	X			X		
Diabetes mellitus	Global	Female	Prevalence of obesity	X			X		
Diabetes mellitus	Data Rich	Female	Diabetes Age-Standardized Prevalence (proportion)	X			X		
Diabetes mellitus	Global	Female	Diabetes Age-Standardized Prevalence (proportion)	X			X		
Diabetes mellitus	Data Rich	Male	Education (years per capita)			X			X
Diabetes mellitus	Global	Male	Education (years per capita)			X			X
Diabetes mellitus	Data Rich	Male	Education (years per capita)			X			X
Diabetes mellitus	Global	Male	Education (years per capita)			X			X
Diabetes mellitus	Data Rich	Male	LDI (I$ per capita)			X			X
Diabetes mellitus	Global	Male	LDI (I$ per capita)			X			X
Diabetes mellitus	Data Rich	Male	Socio-demographic Index			X			X
Diabetes mellitus	Global	Male	Socio-demographic Index			X			X
Diabetes mellitus	Data Rich	Male	Healthcare access and quality index			X			X
Diabetes mellitus	Global	Male	Healthcare access and quality index			X			X
Diabetes mellitus	Data Rich	Male	Low-Density Lipoprotein (mmol/L)		X			X	
Diabetes mellitus	Global	Male	Low-Density Lipoprotein (mmol/L)		X			X	
Diabetes mellitus	Global	Male	Live Births 35+ (proportion)		X			X	
Diabetes mellitus	Data Rich	Male	Systolic Blood Pressure (mmHg)		X			X	
Diabetes mellitus	Global	Male	Systolic Blood Pressure (mmHg)		X			X	
Diabetes mellitus	Data Rich	Male	Age- and sex-specific SEV for Low fruit		X			X	
Diabetes mellitus	Global	Male	Age- and sex-specific SEV for Low fruit		X			X	
Diabetes mellitus	Data Rich	Male	Age- and sex-specific SEV for Low vegetables		X			X	
Diabetes mellitus	Global	Male	Age- and sex-specific SEV for Low vegetables		X			X	
Diabetes mellitus	Data Rich	Male	Absolute value of average latitude		X			X	
Diabetes mellitus	Global	Male	Absolute value of average latitude		X			X	
Diabetes mellitus	Data Rich	Male	sugar unadjusted(g)		X			X	
Diabetes mellitus	Global	Male	sugar unadjusted(g)		X			X	
Diabetes mellitus	Data Rich	Male	Age- and sex-specific SEV for Alcohol use		X			X	
Diabetes mellitus	Global	Male	Age- and sex-specific SEV for Alcohol use		X			X	
Diabetes mellitus	Global	Male	Live Births 40+ (proportion)		X			X	
Diabetes mellitus	Data Rich	Male	Live Births 35+ (proportion)		X			X	
Diabetes mellitus	Data Rich	Male	Live Births 40+ (proportion)		X			X	
Diabetes mellitus	Data Rich	Male	Healthcare access and quality index	X			X		
Diabetes mellitus	Global	Male	Healthcare access and quality index	X			X		
Diabetes mellitus	Data Rich	Male	Mean BMI	X			X		
Diabetes mellitus	Global	Male	Mean BMI	X			X		
Diabetes mellitus	Data Rich	Male	Diabetes Fasting Plasma Glucose (mmol/L), age-standardized 25+	X			X		
Diabetes mellitus	Global	Male	Diabetes Fasting Plasma Glucose (mmol/L), age-standardized 25+	X			X		
Diabetes mellitus	Data Rich	Male	Prevalence of obesity	X			X		
Diabetes mellitus	Global	Male	Prevalence of obesity	X			X		
Diabetes mellitus	Data Rich	Male	Diabetes Age-Standardized Prevalence (proportion)	X			X		
Diabetes mellitus	Global	Male	Diabetes Age-Standardized Prevalence (proportion)	X			X		
Diabetes mellitus type 1	Data Rich	Female	Education (years per capita)			X			X
Diabetes mellitus type 1	Global	Female	Education (years per capita)			X			X
Diabetes mellitus type 1	Data Rich	Female	Socio-demographic Index			X			X
Diabetes mellitus type 1	Global	Female	Socio-demographic Index			X			X
Diabetes mellitus type 1	Global	Female	Live Births 35+ (proportion)		X			X	
Diabetes mellitus type 1	Data Rich	Female	Absolute value of average latitude		X			X	
Diabetes mellitus type 1	Global	Female	Absolute value of average latitude		X			X	
Diabetes mellitus type 1	Global	Female	Live Births 40+ (proportion)		X			X	
Diabetes mellitus type 1	Data Rich	Female	Live Births 35+ (proportion)		X			X	
Diabetes mellitus type 1	Data Rich	Female	Live Births 40+ (proportion)		X			X	
Diabetes mellitus type 1	Data Rich	Female	Healthcare access and quality index	X			X		
Diabetes mellitus type 1	Global	Female	Healthcare access and quality index	X			X		
Diabetes mellitus type 1	Data Rich	Male	Education (years per capita)			X			X
Diabetes mellitus type 1	Global	Male	Education (years per capita)			X			X
Diabetes mellitus type 1	Data Rich	Male	Socio-demographic Index			X			X
Diabetes mellitus type 1	Global	Male	Socio-demographic Index			X			X
Diabetes mellitus type 1	Global	Male	Live Births 35+ (proportion)		X			X	
Diabetes mellitus type 1	Data Rich	Male	Absolute value of average latitude		X			X	
Diabetes mellitus type 1	Global	Male	Absolute value of average latitude		X			X	
Diabetes mellitus type 1	Global	Male	Live Births 40+ (proportion)		X			X	
Diabetes mellitus type 1	Data Rich	Male	Live Births 35+ (proportion)		X			X	
Diabetes mellitus type 1	Data Rich	Male	Live Births 40+ (proportion)		X			X	
Diabetes mellitus type 1	Data Rich	Male	Healthcare access and quality index	X			X		
Diabetes mellitus type 1	Global	Male	Healthcare access and quality index	X			X		
Diabetes mellitus type 2	Data Rich	Female	Education (years per capita)			X			X

Appendix Table S11: Comparison of GBD 2021 and GBD 2023 covariates and level of covariates used in cause of death modeling

Cause	Model Version Type	Sex	Covariate	Level 1 GBD 2021	Level 2 GBD 2021	Level 3 GBD 2021	Level 1 GBD 2023	Level 2 GBD 2023	Level 3 GBD 2023
Diabetes mellitus type 2	Global	Female	Education (years per capita)			X			X
Diabetes mellitus type 2	Data Rich	Female	LDI (I$ per capita)			X			X
Diabetes mellitus type 2	Global	Female	LDI (I$ per capita)			X			X
Diabetes mellitus type 2	Data Rich	Female	Healthcare access and quality index			X			X
Diabetes mellitus type 2	Global	Female	Healthcare access and quality index			X			X
Diabetes mellitus type 2	Data Rich	Female	Low-Density Lipoprotein (mmol/L)		X			X	
Diabetes mellitus type 2	Global	Female	Low-Density Lipoprotein (mmol/L)		X			X	
Diabetes mellitus type 2	Data Rich	Female	Systolic Blood Pressure (mmHg)		X			X	
Diabetes mellitus type 2	Global	Female	Systolic Blood Pressure (mmHg)		X			X	
Diabetes mellitus type 2	Data Rich	Female	Age- and sex-specific SEV for Low fruit		X			X	
Diabetes mellitus type 2	Global	Female	Age- and sex-specific SEV for Low fruit		X			X	
Diabetes mellitus type 2	Data Rich	Female	Age- and sex-specific SEV for Low vegetables		X			X	
Diabetes mellitus type 2	Global	Female	Age- and sex-specific SEV for Low vegetables		X			X	
Diabetes mellitus type 2	Data Rich	Female	sugar unadjusted(g)		X			X	
Diabetes mellitus type 2	Global	Female	sugar unadjusted(g)		X			X	
Diabetes mellitus type 2	Data Rich	Female	Age- and sex-specific SEV for Alcohol use		X			X	
Diabetes mellitus type 2	Global	Female	Age- and sex-specific SEV for Alcohol use		X			X	
Diabetes mellitus type 2	Data Rich	Female	Mean BMI	X			X		
Diabetes mellitus type 2	Global	Female	Mean BMI	X			X		
Diabetes mellitus type 2	Data Rich	Female	Diabetes Fasting Plasma Glucose (mmol/L), age-standardized 25+	X			X		
Diabetes mellitus type 2	Global	Female	Diabetes Fasting Plasma Glucose (mmol/L), age-standardized 25+	X			X		
Diabetes mellitus type 2	Data Rich	Female	Prevalence of obesity	X			X		
Diabetes mellitus type 2	Global	Female	Prevalence of obesity	X			X		
Diabetes mellitus type 2	Data Rich	Female	Diabetes Age-Standardized Prevalence (proportion)	X			X		
Diabetes mellitus type 2	Global	Female	Diabetes Age-Standardized Prevalence (proportion)	X			X		
Diabetes mellitus type 2	Data Rich	Male	Education (years per capita)			X			X
Diabetes mellitus type 2	Global	Male	Education (years per capita)			X			X
Diabetes mellitus type 2	Data Rich	Male	LDI (I$ per capita)			X			X
Diabetes mellitus type 2	Global	Male	LDI (I$ per capita)			X			X
Diabetes mellitus type 2	Data Rich	Male	Healthcare access and quality index			X			X
Diabetes mellitus type 2	Global	Male	Healthcare access and quality index			X			X
Diabetes mellitus type 2	Data Rich	Male	Low-Density Lipoprotein (mmol/L)		X			X	
Diabetes mellitus type 2	Global	Male	Low-Density Lipoprotein (mmol/L)		X			X	
Diabetes mellitus type 2	Data Rich	Male	Systolic Blood Pressure (mmHg)		X			X	
Diabetes mellitus type 2	Global	Male	Systolic Blood Pressure (mmHg)		X			X	
Diabetes mellitus type 2	Data Rich	Male	Age- and sex-specific SEV for Low fruit		X			X	
Diabetes mellitus type 2	Global	Male	Age- and sex-specific SEV for Low fruit		X			X	
Diabetes mellitus type 2	Data Rich	Male	Age- and sex-specific SEV for Low vegetables		X			X	
Diabetes mellitus type 2	Global	Male	Age- and sex-specific SEV for Low vegetables		X			X	
Diabetes mellitus type 2	Data Rich	Male	sugar unadjusted(g)		X			X	
Diabetes mellitus type 2	Global	Male	sugar unadjusted(g)		X			X	
Diabetes mellitus type 2	Data Rich	Male	Age- and sex-specific SEV for Alcohol use		X			X	
Diabetes mellitus type 2	Global	Male	Age- and sex-specific SEV for Alcohol use		X			X	
Diabetes mellitus type 2	Data Rich	Male	Mean BMI	X			X		
Diabetes mellitus type 2	Global	Male	Mean BMI	X			X		
Diabetes mellitus type 2	Data Rich	Male	Diabetes Fasting Plasma Glucose (mmol/L), age-standardized 25+	X			X		
Diabetes mellitus type 2	Global	Male	Diabetes Fasting Plasma Glucose (mmol/L), age-standardized 25+	X			X		
Diabetes mellitus type 2	Data Rich	Male	Prevalence of obesity	X			X		
Diabetes mellitus type 2	Global	Male	Prevalence of obesity	X			X		
Diabetes mellitus type 2	Data Rich	Male	Diabetes Age-Standardized Prevalence (proportion)	X			X		
Diabetes mellitus type 2	Global	Male	Diabetes Age-Standardized Prevalence (proportion)	X			X		
Chronic kidney disease	Global	Female	Education (years per capita)			X			X
Chronic kidney disease	Data Rich	Female	Education (years per capita)			X			X
Chronic kidney disease	Global	Female	LDI (I$ per capita)			X			X
Chronic kidney disease	Data Rich	Female	LDI (I$ per capita)			X			X
Chronic kidney disease	Global	Female	Socio-demographic Index			X			X
Chronic kidney disease	Data Rich	Female	Socio-demographic Index			X			X
Chronic kidney disease	Global	Female	Low-Density Lipoprotein (mmol/L)		X			X	
Chronic kidney disease	Data Rich	Female	Low-Density Lipoprotein (mmol/L)		X			X	
Chronic kidney disease	Global	Female	energy unadjusted(kcal)		X			X	
Chronic kidney disease	Data Rich	Female	energy unadjusted(kcal)		X			X	
Chronic kidney disease	Global	Female	red meats unadjusted(g)		X			X	
Chronic kidney disease	Data Rich	Female	red meats unadjusted(g)		X			X	
Chronic kidney disease	Global	Female	Healthcare access and quality index	X			X		
Chronic kidney disease	Data Rich	Female	Healthcare access and quality index	X			X		
Chronic kidney disease	Global	Female	Mean BMI	X			X		
Chronic kidney disease	Data Rich	Female	Mean BMI	X			X		
Chronic kidney disease	Global	Female	Systolic Blood Pressure (mmHg)	X			X		
Chronic kidney disease	Data Rich	Female	Systolic Blood Pressure (mmHg)	X			X		
Chronic kidney disease	Global	Male	Education (years per capita)			X			X
Chronic kidney disease	Data Rich	Male	Education (years per capita)			X			X
Chronic kidney disease	Global	Male	LDI (I$ per capita)			X			X
Chronic kidney disease	Data Rich	Male	LDI (I$ per capita)			X			X
Chronic kidney disease	Global	Male	Socio-demographic Index			X			X
Chronic kidney disease	Data Rich	Male	Socio-demographic Index			X			X
Chronic kidney disease	Global	Male	Low-Density Lipoprotein (mmol/L)		X			X	
Chronic kidney disease	Data Rich	Male	Low-Density Lipoprotein (mmol/L)		X			X	
Chronic kidney disease	Global	Male	energy unadjusted(kcal)		X			X	
Chronic kidney disease	Data Rich	Male	energy unadjusted(kcal)		X			X	
Chronic kidney disease	Global	Male	red meats unadjusted(g)		X			X	
Chronic kidney disease	Data Rich	Male	red meats unadjusted(g)		X			X	
Chronic kidney disease	Global	Male	Healthcare access and quality index	X			X		
Chronic kidney disease	Data Rich	Male	Healthcare access and quality index	X			X		
Chronic kidney disease	Global	Male	Mean BMI	X			X		
Chronic kidney disease	Data Rich	Male	Mean BMI	X			X		
Chronic kidney disease	Global	Male	Systolic Blood Pressure (mmHg)	X			X		
Chronic kidney disease	Data Rich	Male	Systolic Blood Pressure (mmHg)	X			X		
Acute glomerulonephritis	Data Rich	Female	Education (years per capita)			X			X
Acute glomerulonephritis	Global	Female	Education (years per capita)			X			X
Acute glomerulonephritis	Data Rich	Female	LDI (I$ per capita)			X			X
Acute glomerulonephritis	Global	Female	LDI (I$ per capita)			X			X
Acute glomerulonephritis	Data Rich	Female	Socio-demographic Index			X			X
Acute glomerulonephritis	Global	Female	Socio-demographic Index			X			X
Acute glomerulonephritis	Data Rich	Female	Healthcare access and quality index		X			X	
Acute glomerulonephritis	Global	Female	Healthcare access and quality index		X			X	
Acute glomerulonephritis	Data Rich	Female	Sanitation (proportion with access)		X			X	
Acute glomerulonephritis	Global	Female	Sanitation (proportion with access)		X			X	
Acute glomerulonephritis	Data Rich	Female	Improved Water Source (proportion with access)		X			X	
Acute glomerulonephritis	Global	Female	Improved Water Source (proportion with access)		X			X	
Acute glomerulonephritis	Data Rich	Female	Systolic Blood Pressure (mmHg)		X			X	
Acute glomerulonephritis	Global	Female	Systolic Blood Pressure (mmHg)		X			X	
Acute glomerulonephritis	Data Rich	Male	Education (years per capita)			X			X
Acute glomerulonephritis	Global	Male	Education (years per capita)			X			X
Acute glomerulonephritis	Data Rich	Male	LDI (I$ per capita)			X			X
Acute glomerulonephritis	Global	Male	LDI (I$ per capita)			X			X
Acute glomerulonephritis	Data Rich	Male	Socio-demographic Index			X			X
Acute glomerulonephritis	Global	Male	Socio-demographic Index			X			X
Acute glomerulonephritis	Data Rich	Male	Healthcare access and quality index		X			X	
Acute glomerulonephritis	Global	Male	Healthcare access and quality index		X			X	
Acute glomerulonephritis	Data Rich	Male	Sanitation (proportion with access)		X			X	
Acute glomerulonephritis	Global	Male	Sanitation (proportion with access)		X			X	
Acute glomerulonephritis	Data Rich	Male	Improved Water Source (proportion with access)		X			X	
Acute glomerulonephritis	Global	Male	Improved Water Source (proportion with access)		X			X	
Acute glomerulonephritis	Data Rich	Male	Systolic Blood Pressure (mmHg)		X			X	
Acute glomerulonephritis	Global	Male	Systolic Blood Pressure (mmHg)		X			X	
Skin and subcutaneous diseases	Data Rich	Female	Education (years per capita)			X			X
Skin and subcutaneous diseases	Global	Female	Education (years per capita)			X			X
Skin and subcutaneous diseases	Data Rich	Female	LDI (I$ per capita)			X			X
Skin and subcutaneous diseases	Global	Female	LDI (I$ per capita)			X			X
Skin and subcutaneous diseases	Data Rich	Female	Socio-demographic Index			X			X
Skin and subcutaneous diseases	Global	Female	Socio-demographic Index			X			X
Skin and subcutaneous diseases	Data Rich	Female	Cumulative Cigarettes (10 Years)		X			X	
Skin and subcutaneous diseases	Global	Female	Cumulative Cigarettes (10 Years)		X			X	
Skin and subcutaneous diseases	Data Rich	Female	Smoking Prevalence		X			X	

Appendix Table S11: Comparison of GBD 2021 and GBD 2023 covariates and level of covariates used in cause of death modeling

Cause	Model Version Type	Sex	Covariate	Level 1 GBD 2021	Level 2 GBD 2021	Level 3 GBD 2021	Level 1 GBD 2023	Level 2 GBD 2023	Level 3 GBD 2023
Skin and subcutaneous diseases	Global	Female	Smoking Prevalence		X			X	
Skin and subcutaneous diseases	Data Rich	Female	Liters of alcohol consumed per capita		X			X	
Skin and subcutaneous diseases	Global	Female	Liters of alcohol consumed per capita		X			X	
Skin and subcutaneous diseases	Data Rich	Female	Cumulative Cigarettes (5 Years)		X			X	
Skin and subcutaneous diseases	Global	Female	Cumulative Cigarettes (5 Years)		X			X	
Skin and subcutaneous diseases	Data Rich	Female	Healthcare access and quality index	X			X		
Skin and subcutaneous diseases	Global	Female	Healthcare access and quality index	X			X		
Skin and subcutaneous diseases	Data Rich	Female	Improved Water Source (proportion with access)	X			X		
Skin and subcutaneous diseases	Global	Female	Improved Water Source (proportion with access)	X			X		
Skin and subcutaneous diseases	Data Rich	Female	Age- and sex-specific SEV for Unsafe sanitation	X			X		
Skin and subcutaneous diseases	Global	Female	Age- and sex-specific SEV for Unsafe sanitation	X			X		
Skin and subcutaneous diseases	Data Rich	Female	Prevalence of overweight and obesity	X			X		
Skin and subcutaneous diseases	Global	Female	Prevalence of overweight and obesity	X			X		
Skin and subcutaneous diseases	Data Rich	Female	Diabetes Fasting Plasma Glucose (mmol/L), by age	X			X		
Skin and subcutaneous diseases	Global	Female	Diabetes Fasting Plasma Glucose (mmol/L), by age	X			X		
Skin and subcutaneous diseases	Data Rich	Male	Education (years per capita)			X			X
Skin and subcutaneous diseases	Global	Male	Education (years per capita)			X			X
Skin and subcutaneous diseases	Data Rich	Male	LDI (I$ per capita)			X			X
Skin and subcutaneous diseases	Global	Male	LDI (I$ per capita)			X			X
Skin and subcutaneous diseases	Data Rich	Male	Socio-demographic Index			X			X
Skin and subcutaneous diseases	Global	Male	Socio-demographic Index			X			X
Skin and subcutaneous diseases	Data Rich	Male	Cumulative Cigarettes (10 Years)		X			X	
Skin and subcutaneous diseases	Global	Male	Cumulative Cigarettes (10 Years)		X			X	
Skin and subcutaneous diseases	Data Rich	Male	Smoking Prevalence		X			X	
Skin and subcutaneous diseases	Global	Male	Smoking Prevalence		X			X	
Skin and subcutaneous diseases	Data Rich	Male	Liters of alcohol consumed per capita		X			X	
Skin and subcutaneous diseases	Global	Male	Liters of alcohol consumed per capita		X			X	
Skin and subcutaneous diseases	Data Rich	Male	Cumulative Cigarettes (5 Years)		X			X	
Skin and subcutaneous diseases	Global	Male	Cumulative Cigarettes (5 Years)		X			X	
Skin and subcutaneous diseases	Data Rich	Male	Healthcare access and quality index	X			X		
Skin and subcutaneous diseases	Global	Male	Healthcare access and quality index	X			X		
Skin and subcutaneous diseases	Data Rich	Male	Improved Water Source (proportion with access)	X			X		
Skin and subcutaneous diseases	Global	Male	Improved Water Source (proportion with access)	X			X		
Skin and subcutaneous diseases	Data Rich	Male	Age- and sex-specific SEV for Unsafe sanitation	X			X		
Skin and subcutaneous diseases	Global	Male	Age- and sex-specific SEV for Unsafe sanitation	X			X		
Skin and subcutaneous diseases	Data Rich	Male	Prevalence of overweight and obesity	X			X		
Skin and subcutaneous diseases	Global	Male	Prevalence of overweight and obesity	X			X		
Skin and subcutaneous diseases	Data Rich	Male	Diabetes Fasting Plasma Glucose (mmol/L), by age	X			X		
Skin and subcutaneous diseases	Global	Male	Diabetes Fasting Plasma Glucose (mmol/L), by age	X			X		
Bacterial skin diseases	Data Rich	Female	Education (years per capita)			X			X
Bacterial skin diseases	Global	Female	Education (years per capita)			X			X
Bacterial skin diseases	Data Rich	Female	LDI (I$ per capita)			X			X
Bacterial skin diseases	Global	Female	LDI (I$ per capita)			X			X
Bacterial skin diseases	Data Rich	Female	Socio-demographic Index			X			X
Bacterial skin diseases	Global	Female	Socio-demographic Index			X			X
Bacterial skin diseases	Data Rich	Female	Cumulative Cigarettes (10 Years)		X			X	
Bacterial skin diseases	Global	Female	Cumulative Cigarettes (10 Years)		X			X	
Bacterial skin diseases	Data Rich	Female	Smoking Prevalence		X			X	
Bacterial skin diseases	Global	Female	Smoking Prevalence		X			X	
Bacterial skin diseases	Data Rich	Female	Liters of alcohol consumed per capita		X			X	
Bacterial skin diseases	Global	Female	Liters of alcohol consumed per capita		X			X	
Bacterial skin diseases	Data Rich	Female	Cumulative Cigarettes (5 Years)		X			X	
Bacterial skin diseases	Global	Female	Cumulative Cigarettes (5 Years)		X			X	
Bacterial skin diseases	Data Rich	Female	Healthcare access and quality index	X			X		
Bacterial skin diseases	Global	Female	Healthcare access and quality index	X			X		
Bacterial skin diseases	Data Rich	Female	Improved Water Source (proportion with access)	X			X		
Bacterial skin diseases	Global	Female	Improved Water Source (proportion with access)	X			X		
Bacterial skin diseases	Data Rich	Female	Age- and sex-specific SEV for Unsafe sanitation	X			X		
Bacterial skin diseases	Global	Female	Age- and sex-specific SEV for Unsafe sanitation	X			X		
Bacterial skin diseases	Data Rich	Female	Prevalence of overweight and obesity	X			X		
Bacterial skin diseases	Global	Female	Prevalence of overweight and obesity	X			X		
Bacterial skin diseases	Data Rich	Female	Diabetes Fasting Plasma Glucose (mmol/L), by age	X			X		
Bacterial skin diseases	Global	Female	Diabetes Fasting Plasma Glucose (mmol/L), by age	X			X		
Bacterial skin diseases	Data Rich	Male	Education (years per capita)			X			X
Bacterial skin diseases	Global	Male	Education (years per capita)			X			X
Bacterial skin diseases	Data Rich	Male	LDI (I$ per capita)			X			X
Bacterial skin diseases	Global	Male	LDI (I$ per capita)			X			X
Bacterial skin diseases	Data Rich	Male	Socio-demographic Index			X			X
Bacterial skin diseases	Global	Male	Socio-demographic Index			X			X
Bacterial skin diseases	Data Rich	Male	Cumulative Cigarettes (10 Years)		X			X	
Bacterial skin diseases	Global	Male	Cumulative Cigarettes (10 Years)		X			X	
Bacterial skin diseases	Data Rich	Male	Smoking Prevalence		X			X	
Bacterial skin diseases	Global	Male	Smoking Prevalence		X			X	
Bacterial skin diseases	Data Rich	Male	Liters of alcohol consumed per capita		X			X	
Bacterial skin diseases	Global	Male	Liters of alcohol consumed per capita		X			X	
Bacterial skin diseases	Data Rich	Male	Cumulative Cigarettes (5 Years)		X			X	
Bacterial skin diseases	Global	Male	Cumulative Cigarettes (5 Years)		X			X	
Bacterial skin diseases	Data Rich	Male	Healthcare access and quality index	X			X		
Bacterial skin diseases	Global	Male	Healthcare access and quality index	X			X		
Bacterial skin diseases	Data Rich	Male	Improved Water Source (proportion with access)	X			X		
Bacterial skin diseases	Global	Male	Improved Water Source (proportion with access)	X			X		
Bacterial skin diseases	Data Rich	Male	Age- and sex-specific SEV for Unsafe sanitation	X			X		
Bacterial skin diseases	Global	Male	Age- and sex-specific SEV for Unsafe sanitation	X			X		
Bacterial skin diseases	Data Rich	Male	Prevalence of overweight and obesity	X			X		
Bacterial skin diseases	Global	Male	Prevalence of overweight and obesity	X			X		
Bacterial skin diseases	Data Rich	Male	Diabetes Fasting Plasma Glucose (mmol/L), by age	X			X		
Bacterial skin diseases	Global	Male	Diabetes Fasting Plasma Glucose (mmol/L), by age	X			X		
Cellulitis	Global	Female	Education (years per capita)			X			X
Cellulitis	Data Rich	Female	Education (years per capita)			X			X
Cellulitis	Global	Female	LDI (I$ per capita)		X			X	
Cellulitis	Data Rich	Female	LDI (I$ per capita)		X			X	
Cellulitis	Global	Female	Healthcare access and quality index	X			X		
Cellulitis	Data Rich	Female	Healthcare access and quality index	X			X		
Cellulitis	Global	Female	Prevalence of overweight and obesity	X			X		
Cellulitis	Data Rich	Female	Prevalence of overweight and obesity	X			X		
Cellulitis	Global	Female	Diabetes Fasting Plasma Glucose (mmol/L), by age	X			X		
Cellulitis	Data Rich	Female	Diabetes Fasting Plasma Glucose (mmol/L), by age	X			X		
Cellulitis	Global	Male	Education (years per capita)			X			X
Cellulitis	Data Rich	Male	Education (years per capita)			X			X
Cellulitis	Global	Male	LDI (I$ per capita)		X			X	
Cellulitis	Data Rich	Male	LDI (I$ per capita)		X			X	
Cellulitis	Global	Male	Healthcare access and quality index	X			X		
Cellulitis	Data Rich	Male	Healthcare access and quality index	X			X		
Cellulitis	Global	Male	Prevalence of overweight and obesity	X			X		
Cellulitis	Data Rich	Male	Prevalence of overweight and obesity	X			X		
Cellulitis	Global	Male	Diabetes Fasting Plasma Glucose (mmol/L), by age	X			X		
Cellulitis	Data Rich	Male	Diabetes Fasting Plasma Glucose (mmol/L), by age	X			X		
Pyoderma	Global	Female	Education (years per capita)			X			X
Pyoderma	Data Rich	Female	Education (years per capita)			X			X
Pyoderma	Global	Female	LDI (I$ per capita)			X			X
Pyoderma	Data Rich	Female	LDI (I$ per capita)			X			X
Pyoderma	Global	Female	Socio-demographic Index			X			X

Appendix Table S11: Comparison of GBD 2021 and GBD 2023 covariates and level of covariates used in cause of death modeling

Cause	Model Version Type	Sex	Covariate	Level 1 GBD 2021	Level 2 GBD 2021	Level 3 GBD 2021	Level 1 GBD 2023	Level 2 GBD 2023	Level 3 GBD 2023
Pyoderma	Data Rich	Female	Socio-demographic Index			X			X
Pyoderma	Global	Female	Cumulative Cigarettes (10 Years)		X			X	
Pyoderma	Data Rich	Female	Cumulative Cigarettes (10 Years)		X			X	
Pyoderma	Global	Female	Smoking Prevalence		X			X	
Pyoderma	Data Rich	Female	Smoking Prevalence		X			X	
Pyoderma	Global	Female	Liters of alcohol consumed per capita		X			X	
Pyoderma	Data Rich	Female	Liters of alcohol consumed per capita		X			X	
Pyoderma	Global	Female	Cumulative Cigarettes (5 Years)		X			X	
Pyoderma	Data Rich	Female	Cumulative Cigarettes (5 Years)		X			X	
Pyoderma	Global	Female	Healthcare access and quality index	X			X		
Pyoderma	Data Rich	Female	Healthcare access and quality index	X			X		
Pyoderma	Global	Female	Improved Water Source (proportion with access)	X			X		
Pyoderma	Data Rich	Female	Improved Water Source (proportion with access)	X			X		
Pyoderma	Global	Female	Age- and sex-specific SEV for Unsafe sanitation	X			X		
Pyoderma	Data Rich	Female	Age- and sex-specific SEV for Unsafe sanitation	X			X		
Pyoderma	Global	Female	Prevalence of overweight and obesity	X			X		
Pyoderma	Data Rich	Female	Prevalence of overweight and obesity	X			X		
Pyoderma	Global	Female	Diabetes Fasting Plasma Glucose (mmol/L), by age	X			X		
Pyoderma	Data Rich	Female	Diabetes Fasting Plasma Glucose (mmol/L), by age	X			X		
Pyoderma	Global	Male	Education (years per capita)			X			X
Pyoderma	Data Rich	Male	Education (years per capita)			X			X
Pyoderma	Global	Male	LDI (I$ per capita)			X			X
Pyoderma	Data Rich	Male	LDI (I$ per capita)			X			X
Pyoderma	Global	Male	Socio-demographic Index			X			X
Pyoderma	Data Rich	Male	Socio-demographic Index			X			X
Pyoderma	Global	Male	Cumulative Cigarettes (10 Years)		X			X	
Pyoderma	Data Rich	Male	Cumulative Cigarettes (10 Years)		X			X	
Pyoderma	Global	Male	Smoking Prevalence		X			X	
Pyoderma	Data Rich	Male	Smoking Prevalence		X			X	
Pyoderma	Global	Male	Liters of alcohol consumed per capita		X			X	
Pyoderma	Data Rich	Male	Liters of alcohol consumed per capita		X			X	
Pyoderma	Global	Male	Cumulative Cigarettes (5 Years)		X			X	
Pyoderma	Data Rich	Male	Cumulative Cigarettes (5 Years)		X			X	
Pyoderma	Global	Male	Healthcare access and quality index	X			X		
Pyoderma	Data Rich	Male	Healthcare access and quality index	X			X		
Pyoderma	Global	Male	Improved Water Source (proportion with access)	X			X		
Pyoderma	Data Rich	Male	Improved Water Source (proportion with access)	X			X		
Pyoderma	Global	Male	Age- and sex-specific SEV for Unsafe sanitation	X			X		
Pyoderma	Data Rich	Male	Age- and sex-specific SEV for Unsafe sanitation	X			X		
Pyoderma	Global	Male	Prevalence of overweight and obesity	X			X		
Pyoderma	Data Rich	Male	Prevalence of overweight and obesity	X			X		
Pyoderma	Global	Male	Diabetes Fasting Plasma Glucose (mmol/L), by age	X			X		
Pyoderma	Data Rich	Male	Diabetes Fasting Plasma Glucose (mmol/L), by age	X			X		
Decubitus ulcer	Global	Female	Education (years per capita)			X			X
Decubitus ulcer	Data Rich	Female	Education (years per capita)			X			X
Decubitus ulcer	Global	Female	LDI (I$ per capita)			X			X
Decubitus ulcer	Data Rich	Female	LDI (I$ per capita)			X			X
Decubitus ulcer	Global	Female	Socio-demographic Index			X			X
Decubitus ulcer	Data Rich	Female	Socio-demographic Index			X			X
Decubitus ulcer	Global	Female	Age- and sex-specific SEV for Unsafe sanitation			X			X
Decubitus ulcer	Data Rich	Female	Age- and sex-specific SEV for Unsafe sanitation			X			X
Decubitus ulcer	Global	Female	Cumulative Cigarettes (10 Years)		X			X	
Decubitus ulcer	Data Rich	Female	Cumulative Cigarettes (10 Years)		X			X	
Decubitus ulcer	Global	Female	Smoking Prevalence		X			X	
Decubitus ulcer	Data Rich	Female	Smoking Prevalence		X			X	
Decubitus ulcer	Global	Female	Healthcare access and quality index		X			X	
Decubitus ulcer	Data Rich	Female	Healthcare access and quality index		X			X	
Decubitus ulcer	Data Rich	Female	Liters of alcohol consumed per capita		X			X	
Decubitus ulcer	Global	Female	Cumulative Cigarettes (5 Years)		X			X	
Decubitus ulcer	Data Rich	Female	Cumulative Cigarettes (5 Years)		X			X	
Decubitus ulcer	Global	Female	Liters of alcohol consumed per capita	X			X		
Decubitus ulcer	Global	Female	Improved Water Source (proportion with access)	X			X		
Decubitus ulcer	Data Rich	Female	Improved Water Source (proportion with access)	X			X		
Decubitus ulcer	Global	Female	Diabetes Fasting Plasma Glucose (mmol/L), age-standardized 25+	X			X		
Decubitus ulcer	Data Rich	Female	Diabetes Fasting Plasma Glucose (mmol/L), age-standardized 25+	X			X		
Decubitus ulcer	Global	Female	Prevalence of obesity	X			X		
Decubitus ulcer	Data Rich	Female	Prevalence of obesity	X			X		
Decubitus ulcer	Global	Male	Education (years per capita)			X			X
Decubitus ulcer	Data Rich	Male	Education (years per capita)			X			X
Decubitus ulcer	Global	Male	LDI (I$ per capita)			X			X
Decubitus ulcer	Data Rich	Male	LDI (I$ per capita)			X			X
Decubitus ulcer	Global	Male	Socio-demographic Index			X			X
Decubitus ulcer	Data Rich	Male	Socio-demographic Index			X			X
Decubitus ulcer	Global	Male	Age- and sex-specific SEV for Unsafe sanitation			X			X
Decubitus ulcer	Data Rich	Male	Age- and sex-specific SEV for Unsafe sanitation			X			X
Decubitus ulcer	Global	Male	Cumulative Cigarettes (10 Years)		X			X	
Decubitus ulcer	Data Rich	Male	Cumulative Cigarettes (10 Years)		X			X	
Decubitus ulcer	Global	Male	Smoking Prevalence		X			X	
Decubitus ulcer	Data Rich	Male	Smoking Prevalence		X			X	
Decubitus ulcer	Global	Male	Healthcare access and quality index		X			X	
Decubitus ulcer	Data Rich	Male	Healthcare access and quality index		X			X	
Decubitus ulcer	Data Rich	Male	Liters of alcohol consumed per capita		X			X	
Decubitus ulcer	Global	Male	Cumulative Cigarettes (5 Years)		X			X	
Decubitus ulcer	Data Rich	Male	Cumulative Cigarettes (5 Years)		X			X	
Decubitus ulcer	Global	Male	Liters of alcohol consumed per capita	X			X		
Decubitus ulcer	Global	Male	Improved Water Source (proportion with access)	X			X		
Decubitus ulcer	Data Rich	Male	Improved Water Source (proportion with access)	X			X		
Decubitus ulcer	Global	Male	Diabetes Fasting Plasma Glucose (mmol/L), age-standardized 25+	X			X		
Decubitus ulcer	Data Rich	Male	Diabetes Fasting Plasma Glucose (mmol/L), age-standardized 25+	X			X		
Decubitus ulcer	Global	Male	Prevalence of obesity	X			X		
Decubitus ulcer	Data Rich	Male	Prevalence of obesity	X			X		
Other skin and subcutaneous diseases	Data Rich	Female	Education (years per capita)			X			X
Other skin and subcutaneous diseases	Global	Female	Education (years per capita)			X			X
Other skin and subcutaneous diseases	Data Rich	Female	LDI (I$ per capita)			X			X
Other skin and subcutaneous diseases	Global	Female	LDI (I$ per capita)			X			X
Other skin and subcutaneous diseases	Data Rich	Female	Socio-demographic Index			X			X
Other skin and subcutaneous diseases	Global	Female	Socio-demographic Index			X			X
Other skin and subcutaneous diseases	Data Rich	Female	Cumulative Cigarettes (10 Years)		X			X	
Other skin and subcutaneous diseases	Global	Female	Cumulative Cigarettes (10 Years)		X			X	
Other skin and subcutaneous diseases	Data Rich	Female	Smoking Prevalence		X			X	
Other skin and subcutaneous diseases	Global	Female	Smoking Prevalence		X			X	
Other skin and subcutaneous diseases	Data Rich	Female	Liters of alcohol consumed per capita		X			X	
Other skin and subcutaneous diseases	Global	Female	Liters of alcohol consumed per capita		X			X	
Other skin and subcutaneous diseases	Data Rich	Female	Cumulative Cigarettes (5 Years)		X			X	
Other skin and subcutaneous diseases	Global	Female	Cumulative Cigarettes (5 Years)		X			X	
Other skin and subcutaneous diseases	Data Rich	Female	Healthcare access and quality index	X			X		
Other skin and subcutaneous diseases	Global	Female	Healthcare access and quality index	X			X		
Other skin and subcutaneous diseases	Data Rich	Female	Improved Water Source (proportion with access)	X			X		
Other skin and subcutaneous diseases	Global	Female	Improved Water Source (proportion with access)	X			X		
Other skin and subcutaneous diseases	Data Rich	Female	Age- and sex-specific SEV for Unsafe sanitation	X			X		
Other skin and subcutaneous diseases	Global	Female	Age- and sex-specific SEV for Unsafe sanitation	X			X		
Other skin and subcutaneous diseases	Data Rich	Female	Age-standardized SEV for Child underweight	X			X		
Other skin and subcutaneous diseases	Global	Female	Age-standardized SEV for Child underweight	X			X		

Appendix Table S11: Comparison of GBD 2021 and GBD 2023 covariates and level of covariates used in cause of death modeling

Cause	Model Version Type	Sex	Covariate	Level 1 GBD 2021	Level 2 GBD 2021	Level 3 GBD 2021	Level 1 GBD 2023	Level 2 GBD 2023	Level 3 GBD 2023
Other skin and subcutaneous diseases	Data Rich	Female	Prevalence of overweight and obesity	X			X		
Other skin and subcutaneous diseases	Global	Female	Prevalence of overweight and obesity	X			X		
Other skin and subcutaneous diseases	Data Rich	Female	Diabetes Fasting Plasma Glucose (mmol/L), by age	X			X		
Other skin and subcutaneous diseases	Global	Female	Diabetes Fasting Plasma Glucose (mmol/L), by age	X			X		
Other skin and subcutaneous diseases	Data Rich	Male	Education (years per capita)			X			X
Other skin and subcutaneous diseases	Global	Male	Education (years per capita)			X			X
Other skin and subcutaneous diseases	Data Rich	Male	LDI (I$ per capita)			X			X
Other skin and subcutaneous diseases	Global	Male	LDI (I$ per capita)			X			X
Other skin and subcutaneous diseases	Data Rich	Male	Socio-demographic Index			X			X
Other skin and subcutaneous diseases	Global	Male	Socio-demographic Index			X			X
Other skin and subcutaneous diseases	Data Rich	Male	Cumulative Cigarettes (10 Years)		X			X	
Other skin and subcutaneous diseases	Global	Male	Cumulative Cigarettes (10 Years)		X			X	
Other skin and subcutaneous diseases	Data Rich	Male	Smoking Prevalence		X			X	
Other skin and subcutaneous diseases	Global	Male	Smoking Prevalence		X			X	
Other skin and subcutaneous diseases	Data Rich	Male	Liters of alcohol consumed per capita		X			X	
Other skin and subcutaneous diseases	Global	Male	Liters of alcohol consumed per capita		X			X	
Other skin and subcutaneous diseases	Data Rich	Male	Cumulative Cigarettes (5 Years)		X			X	
Other skin and subcutaneous diseases	Global	Male	Cumulative Cigarettes (5 Years)		X			X	
Other skin and subcutaneous diseases	Data Rich	Male	Healthcare access and quality index	X			X		
Other skin and subcutaneous diseases	Global	Male	Healthcare access and quality index	X			X		
Other skin and subcutaneous diseases	Data Rich	Male	Improved Water Source (proportion with access)	X			X		
Other skin and subcutaneous diseases	Global	Male	Improved Water Source (proportion with access)	X			X		
Other skin and subcutaneous diseases	Data Rich	Male	Age- and sex-specific SEV for Unsafe sanitation	X			X		
Other skin and subcutaneous diseases	Global	Male	Age- and sex-specific SEV for Unsafe sanitation	X			X		
Other skin and subcutaneous diseases	Data Rich	Male	Age-standardized SEV for Child underweight	X			X		
Other skin and subcutaneous diseases	Global	Male	Age-standardized SEV for Child underweight	X			X		
Other skin and subcutaneous diseases	Data Rich	Male	Prevalence of overweight and obesity	X			X		
Other skin and subcutaneous diseases	Global	Male	Prevalence of overweight and obesity	X			X		
Other skin and subcutaneous diseases	Data Rich	Male	Diabetes Fasting Plasma Glucose (mmol/L), by age	X			X		
Other skin and subcutaneous diseases	Global	Male	Diabetes Fasting Plasma Glucose (mmol/L), by age	X			X		
Musculoskeletal disorders	Global	Female	Socio-demographic Index			X			X
Musculoskeletal disorders	Data Rich	Female	Socio-demographic Index			X			X
Musculoskeletal disorders	Global	Female	Cumulative Cigarettes (10 Years)		X			X	
Musculoskeletal disorders	Data Rich	Female	Cumulative Cigarettes (10 Years)		X			X	
Musculoskeletal disorders	Global	Female	Education (years per capita)		X			X	
Musculoskeletal disorders	Data Rich	Female	Education (years per capita)		X			X	
Musculoskeletal disorders	Global	Female	LDI (I$ per capita)		X			X	
Musculoskeletal disorders	Data Rich	Female	LDI (I$ per capita)		X			X	
Musculoskeletal disorders	Global	Female	Low-Density Lipoprotein (mmol/L)		X			X	
Musculoskeletal disorders	Data Rich	Female	Low-Density Lipoprotein (mmol/L)		X			X	
Musculoskeletal disorders	Global	Female	Smoking Prevalence		X			X	
Musculoskeletal disorders	Data Rich	Female	Smoking Prevalence		X			X	
Musculoskeletal disorders	Global	Female	Healthcare access and quality index		X			X	
Musculoskeletal disorders	Data Rich	Female	Healthcare access and quality index		X			X	
Musculoskeletal disorders	Global	Female	Cumulative Cigarettes (5 Years)		X			X	
Musculoskeletal disorders	Data Rich	Female	Cumulative Cigarettes (5 Years)		X			X	
Musculoskeletal disorders	Global	Female	Low bone mineral density		X			X	
Musculoskeletal disorders	Data Rich	Female	Low bone mineral density		X			X	
Musculoskeletal disorders	Global	Female	Age-standardized bone mineral density among population age 60+ years		X			X	
Musculoskeletal disorders	Data Rich	Female	Age-standardized bone mineral density among population age 60+ years		X			X	
Musculoskeletal disorders	Global	Female	Mean BMI	X			X		
Musculoskeletal disorders	Data Rich	Female	Mean BMI	X			X		
Musculoskeletal disorders	Global	Female	Liters of alcohol consumed per capita	X			X		
Musculoskeletal disorders	Data Rich	Female	Liters of alcohol consumed per capita	X			X		
Musculoskeletal disorders	Global	Female	vegetables unadjusted(g)	X			X		
Musculoskeletal disorders	Global	Male	Socio-demographic Index			X			X
Musculoskeletal disorders	Data Rich	Male	Socio-demographic Index			X			X
Musculoskeletal disorders	Global	Male	Cumulative Cigarettes (10 Years)		X			X	
Musculoskeletal disorders	Data Rich	Male	Cumulative Cigarettes (10 Years)		X			X	
Musculoskeletal disorders	Global	Male	Education (years per capita)		X			X	
Musculoskeletal disorders	Data Rich	Male	Education (years per capita)		X			X	
Musculoskeletal disorders	Global	Male	LDI (I$ per capita)		X			X	
Musculoskeletal disorders	Data Rich	Male	LDI (I$ per capita)		X			X	
Musculoskeletal disorders	Global	Male	Low-Density Lipoprotein (mmol/L)		X			X	
Musculoskeletal disorders	Data Rich	Male	Low-Density Lipoprotein (mmol/L)		X			X	
Musculoskeletal disorders	Global	Male	Smoking Prevalence		X			X	
Musculoskeletal disorders	Data Rich	Male	Smoking Prevalence		X			X	
Musculoskeletal disorders	Global	Male	Healthcare access and quality index		X			X	
Musculoskeletal disorders	Data Rich	Male	Healthcare access and quality index		X			X	
Musculoskeletal disorders	Global	Male	Cumulative Cigarettes (5 Years)		X			X	
Musculoskeletal disorders	Data Rich	Male	Cumulative Cigarettes (5 Years)		X			X	
Musculoskeletal disorders	Data Rich	Male	Low bone mineral density		X			X	
Musculoskeletal disorders	Data Rich	Male	Age-standardized bone mineral density among population age 60+ years		X			X	
Musculoskeletal disorders	Global	Male	Liters of alcohol consumed per capita	X			X		
Musculoskeletal disorders	Data Rich	Male	Mean BMI	X			X		
Musculoskeletal disorders	Data Rich	Male	Liters of alcohol consumed per capita	X			X		
Musculoskeletal disorders	Global	Male	Mean BMI	X			X		
Musculoskeletal disorders	Global	Male	vegetables unadjusted(g)	X			X		
Musculoskeletal disorders	Data Rich	Male	vegetables unadjusted(g)	X			X		
Rheumatoid arthritis	Global	Female	Education (years per capita)			X			X
Rheumatoid arthritis	Data Rich	Female	Education (years per capita)			X			X
Rheumatoid arthritis	Global	Female	LDI (I$ per capita)			X			X
Rheumatoid arthritis	Data Rich	Female	LDI (I$ per capita)			X			X
Rheumatoid arthritis	Global	Female	Socio-demographic Index			X			X
Rheumatoid arthritis	Data Rich	Female	Socio-demographic Index			X			X
Rheumatoid arthritis	Global	Female	Low-Density Lipoprotein (mmol/L)		X			X	
Rheumatoid arthritis	Data Rich	Female	Low-Density Lipoprotein (mmol/L)		X			X	
Rheumatoid arthritis	Global	Female	Mean BMI		X			X	
Rheumatoid arthritis	Data Rich	Female	Mean BMI		X			X	
Rheumatoid arthritis	Global	Female	Cumulative Cigarettes (10 Years)	X			X		
Rheumatoid arthritis	Data Rich	Female	Cumulative Cigarettes (10 Years)	X			X		
Rheumatoid arthritis	Global	Female	Smoking Prevalence	X			X		
Rheumatoid arthritis	Data Rich	Female	Smoking Prevalence	X			X		
Rheumatoid arthritis	Global	Female	Healthcare access and quality index	X			X		
Rheumatoid arthritis	Data Rich	Female	Healthcare access and quality index	X			X		
Rheumatoid arthritis	Global	Female	Liters of alcohol consumed per capita	X			X		
Rheumatoid arthritis	Data Rich	Female	Liters of alcohol consumed per capita	X			X		
Rheumatoid arthritis	Global	Female	Cumulative Cigarettes (5 Years)	X			X		
Rheumatoid arthritis	Data Rich	Female	Cumulative Cigarettes (5 Years)	X			X		
Rheumatoid arthritis	Global	Female	milk unadjusted(g)	X			X		
Rheumatoid arthritis	Data Rich	Female	milk unadjusted(g)	X			X		
Rheumatoid arthritis	Global	Male	Education (years per capita)			X			X
Rheumatoid arthritis	Data Rich	Male	Education (years per capita)			X			X
Rheumatoid arthritis	Global	Male	LDI (I$ per capita)			X			X
Rheumatoid arthritis	Data Rich	Male	LDI (I$ per capita)			X			X
Rheumatoid arthritis	Global	Male	Socio-demographic Index			X			X
Rheumatoid arthritis	Data Rich	Male	Socio-demographic Index			X			X
Rheumatoid arthritis	Global	Male	Low-Density Lipoprotein (mmol/L)		X			X	
Rheumatoid arthritis	Data Rich	Male	Low-Density Lipoprotein (mmol/L)		X			X	
Rheumatoid arthritis	Global	Male	Mean BMI		X			X	
Rheumatoid arthritis	Data Rich	Male	Mean BMI		X			X	
Rheumatoid arthritis	Global	Male	Cumulative Cigarettes (10 Years)	X			X		
Rheumatoid arthritis	Data Rich	Male	Cumulative Cigarettes (10 Years)	X			X		
Rheumatoid arthritis	Global	Male	Smoking Prevalence	X			X		
Rheumatoid arthritis	Data Rich	Male	Smoking Prevalence	X			X		
Rheumatoid arthritis	Global	Male	Healthcare access and quality index	X			X		
Rheumatoid arthritis	Data Rich	Male	Healthcare access and quality index	X			X		
Rheumatoid arthritis	Global	Male	Liters of alcohol consumed per capita	X			X		
Rheumatoid arthritis	Data Rich	Male	Liters of alcohol consumed per capita	X			X		
Rheumatoid arthritis	Global	Male	Cumulative Cigarettes (5 Years)	X			X		

Appendix Table S11: Comparison of GBD 2021 and GBD 2023 covariates and level of covariates used in cause of death modeling

Cause	Model Version Type	Sex	Covariate	Level 1 GBD 2021	Level 2 GBD 2021	Level 3 GBD 2021	Level 1 GBD 2023	Level 2 GBD 2023	Level 3 GBD 2023
Rheumatoid arthritis	Data Rich	Male	Cumulative Cigarettes (5 Years)	X			X		
Rheumatoid arthritis	Global	Male	milk unadjusted(g)	X			X		
Rheumatoid arthritis	Data Rich	Male	milk unadjusted(g)	X			X		
Other musculoskeletal disorders	Data Rich	Female	Socio-demographic Index			X			X
Other musculoskeletal disorders	Global	Female	Socio-demographic Index			X			X
Other musculoskeletal disorders	Data Rich	Female	Cumulative Cigarettes (10 Years)		X			X	
Other musculoskeletal disorders	Global	Female	Cumulative Cigarettes (10 Years)		X			X	
Other musculoskeletal disorders	Data Rich	Female	Education (years per capita)		X			X	
Other musculoskeletal disorders	Global	Female	Education (years per capita)		X			X	
Other musculoskeletal disorders	Data Rich	Female	LDI (I$ per capita)		X			X	
Other musculoskeletal disorders	Global	Female	LDI (I$ per capita)		X			X	
Other musculoskeletal disorders	Data Rich	Female	Low-Density Lipoprotein (mmol/L)		X			X	
Other musculoskeletal disorders	Global	Female	Low-Density Lipoprotein (mmol/L)		X			X	
Other musculoskeletal disorders	Data Rich	Female	Smoking Prevalence		X			X	
Other musculoskeletal disorders	Global	Female	Smoking Prevalence		X			X	
Other musculoskeletal disorders	Data Rich	Female	Healthcare access and quality index		X			X	
Other musculoskeletal disorders	Global	Female	Healthcare access and quality index		X			X	
Other musculoskeletal disorders	Data Rich	Female	Cumulative Cigarettes (5 Years)		X			X	
Other musculoskeletal disorders	Global	Female	Cumulative Cigarettes (5 Years)		X			X	
Other musculoskeletal disorders	Data Rich	Female	Mean BMI	X			X		
Other musculoskeletal disorders	Global	Female	Mean BMI	X			X		
Other musculoskeletal disorders	Data Rich	Female	Liters of alcohol consumed per capita	X			X		
Other musculoskeletal disorders	Global	Female	Liters of alcohol consumed per capita	X			X		
Other musculoskeletal disorders	Data Rich	Female	vegetables unadjusted(g)	X			X		
Other musculoskeletal disorders	Global	Female	vegetables unadjusted(g)	X			X		
Other musculoskeletal disorders	Data Rich	Male	Socio-demographic Index			X			X
Other musculoskeletal disorders	Global	Male	Socio-demographic Index			X			X
Other musculoskeletal disorders	Data Rich	Male	Cumulative Cigarettes (10 Years)		X			X	
Other musculoskeletal disorders	Global	Male	Cumulative Cigarettes (10 Years)		X			X	
Other musculoskeletal disorders	Data Rich	Male	Education (years per capita)		X			X	
Other musculoskeletal disorders	Global	Male	Education (years per capita)		X			X	
Other musculoskeletal disorders	Data Rich	Male	LDI (I$ per capita)		X			X	
Other musculoskeletal disorders	Global	Male	LDI (I$ per capita)		X			X	
Other musculoskeletal disorders	Data Rich	Male	Low-Density Lipoprotein (mmol/L)		X			X	
Other musculoskeletal disorders	Global	Male	Low-Density Lipoprotein (mmol/L)		X			X	
Other musculoskeletal disorders	Data Rich	Male	Smoking Prevalence		X			X	
Other musculoskeletal disorders	Global	Male	Smoking Prevalence		X			X	
Other musculoskeletal disorders	Data Rich	Male	Healthcare access and quality index		X			X	
Other musculoskeletal disorders	Global	Male	Healthcare access and quality index		X			X	
Other musculoskeletal disorders	Data Rich	Male	Cumulative Cigarettes (5 Years)		X			X	
Other musculoskeletal disorders	Global	Male	Cumulative Cigarettes (5 Years)		X			X	
Other musculoskeletal disorders	Data Rich	Male	Mean BMI	X			X		
Other musculoskeletal disorders	Global	Male	Mean BMI	X			X		
Other musculoskeletal disorders	Data Rich	Male	Liters of alcohol consumed per capita	X			X		
Other musculoskeletal disorders	Global	Male	Liters of alcohol consumed per capita	X			X		
Other musculoskeletal disorders	Data Rich	Male	vegetables unadjusted(g)	X			X		
Other musculoskeletal disorders	Global	Male	vegetables unadjusted(g)	X			X		
Congenital anomalies	Data Rich	Female	Socio-demographic Index			X			X
Congenital anomalies	Global	Female	Socio-demographic Index			X			X
Congenital anomalies	Data Rich	Female	Maternal Education (years per capita)			X			X
Congenital anomalies	Global	Female	Maternal Education (years per capita)			X			X
Congenital anomalies	Data Rich	Female	Age-standardized SEV for Ambient particulate matter			X			X
Congenital anomalies	Global	Female	Age-standardized SEV for Ambient particulate matter			X			X
Congenital anomalies	Data Rich	Female	Age-standardized SEV for Household air pollution			X			X
Congenital anomalies	Global	Female	Age-standardized SEV for Household air pollution			X			X
Congenital anomalies	Data Rich	Female	Age-standardized SEV for Low fruit			X			X
Congenital anomalies	Global	Female	Age-standardized SEV for Low fruit			X			X
Congenital anomalies	Data Rich	Female	Age-standardized SEV for Low vegetables			X			X
Congenital anomalies	Global	Female	Age-standardized SEV for Low vegetables			X			X
Congenital anomalies	Data Rich	Female	Liters of alcohol consumed per capita			X			X
Congenital anomalies	Global	Female	Liters of alcohol consumed per capita			X			X
Congenital anomalies	Data Rich	Female	Healthcare access and quality index		X			X	
Congenital anomalies	Global	Female	Healthcare access and quality index		X			X	
Congenital anomalies	Data Rich	Female	Legality of Abortion		X			X	
Congenital anomalies	Global	Female	Legality of Abortion		X			X	
Congenital anomalies	Data Rich	Female	Antenatal Care (1 visit) Coverage (proportion)		X			X	
Congenital anomalies	Global	Female	Antenatal Care (1 visit) Coverage (proportion)		X			X	
Congenital anomalies	Data Rich	Female	Antenatal Care (4 visits) Coverage (proportion)		X			X	
Congenital anomalies	Global	Female	Antenatal Care (4 visits) Coverage (proportion)		X			X	
Congenital anomalies	Data Rich	Female	In-Facility Delivery (proportion)		X			X	
Congenital anomalies	Global	Female	In-Facility Delivery (proportion)		X			X	
Congenital anomalies	Data Rich	Female	Age-standardized SEV for Smoking		X			X	
Congenital anomalies	Global	Female	Age-standardized SEV for Smoking		X			X	
Congenital anomalies	Data Rich	Female	Composite fortification standard and folic acid inclusion		X			X	
Congenital anomalies	Global	Female	Composite fortification standard and folic acid inclusion		X			X	
Congenital anomalies	Data Rich	Female	Maternal alcohol consumption during pregnancy (proportion)	X			X		
Congenital anomalies	Global	Female	Maternal alcohol consumption during pregnancy (proportion)	X			X		
Congenital anomalies	Data Rich	Female	Live Births 35+ (proportion)	X			X		
Congenital anomalies	Global	Female	Live Births 35+ (proportion)	X			X		
Congenital anomalies	Data Rich	Female	Birth prevalence of CHD	X			X		
Congenital anomalies	Global	Female	Birth prevalence of CHD	X			X		
Congenital anomalies	Data Rich	Female	Birth prevalence of congenital chromosomal anomalies	X			X		
Congenital anomalies	Global	Female	Birth prevalence of congenital chromosomal anomalies	X			X		
Congenital anomalies	Data Rich	Female	Folic acid unadjusted (ug)	X			X		
Congenital anomalies	Global	Female	Folic acid unadjusted (ug)	X			X		
Congenital anomalies	Data Rich	Male	Socio-demographic Index			X			X
Congenital anomalies	Global	Male	Socio-demographic Index			X			X
Congenital anomalies	Data Rich	Male	Maternal Education (years per capita)			X			X
Congenital anomalies	Global	Male	Maternal Education (years per capita)			X			X
Congenital anomalies	Data Rich	Male	Age-standardized SEV for Household air pollution			X			X
Congenital anomalies	Global	Male	Age-standardized SEV for Household air pollution			X			X
Congenital anomalies	Data Rich	Male	Age-standardized SEV for Low fruit			X			X
Congenital anomalies	Global	Male	Age-standardized SEV for Low fruit			X			X
Congenital anomalies	Data Rich	Male	Age-standardized SEV for Low vegetables			X			X
Congenital anomalies	Global	Male	Age-standardized SEV for Low vegetables			X			X
Congenital anomalies	Data Rich	Male	Liters of alcohol consumed per capita			X			X
Congenital anomalies	Global	Male	Liters of alcohol consumed per capita			X			X
Congenital anomalies	Global	Male	Outdoor Air Pollution (PM2.5)			X			X
Congenital anomalies	Data Rich	Male	Healthcare access and quality index		X			X	
Congenital anomalies	Global	Male	Healthcare access and quality index		X			X	
Congenital anomalies	Data Rich	Male	Legality of Abortion		X			X	
Congenital anomalies	Global	Male	Legality of Abortion		X			X	
Congenital anomalies	Data Rich	Male	Antenatal Care (1 visit) Coverage (proportion)		X			X	
Congenital anomalies	Global	Male	Antenatal Care (1 visit) Coverage (proportion)		X			X	
Congenital anomalies	Data Rich	Male	Antenatal Care (4 visits) Coverage (proportion)		X			X	
Congenital anomalies	Global	Male	Antenatal Care (4 visits) Coverage (proportion)		X			X	
Congenital anomalies	Data Rich	Male	In-Facility Delivery (proportion)		X			X	
Congenital anomalies	Global	Male	In-Facility Delivery (proportion)		X			X	
Congenital anomalies	Data Rich	Male	Age-standardized SEV for Smoking		X			X	
Congenital anomalies	Global	Male	Age-standardized SEV for Smoking		X			X	
Congenital anomalies	Data Rich	Male	Composite fortification standard and folic acid inclusion		X			X	
Congenital anomalies	Global	Male	Composite fortification standard and folic acid inclusion		X			X	
Congenital anomalies	Data Rich	Male	Maternal alcohol consumption during pregnancy (proportion)	X			X		
Congenital anomalies	Global	Male	Maternal alcohol consumption during pregnancy (proportion)	X			X		
Congenital anomalies	Data Rich	Male	Live Births 35+ (proportion)	X			X		
Congenital anomalies	Global	Male	Live Births 35+ (proportion)	X			X		

Appendix Table S11: Comparison of GBD 2021 and GBD 2023 covariates and level of covariates used in cause of death modeling

Cause	Model Version Type	Sex	Covariate	Level 1 GBD 2021	Level 2 GBD 2021	Level 3 GBD 2021	Level 1 GBD 2023	Level 2 GBD 2023	Level 3 GBD 2023
Congenital anomalies	Data Rich	Male	Birth prevalence of CHD	X			X		
Congenital anomalies	Global	Male	Birth prevalence of CHD	X			X		
Congenital anomalies	Data Rich	Male	Birth prevalence of congenital chromosomal anomalies	X			X		
Congenital anomalies	Global	Male	Birth prevalence of congenital chromosomal anomalies	X			X		
Congenital anomalies	Data Rich	Male	Folic acid unadjusted (ug)	X			X		
Congenital anomalies	Global	Male	Folic acid unadjusted (ug)	X			X		
Congenital anomalies	Data Rich	Male	Outdoor Air Pollution (PM2.5)						X
Neural tube defects	Data Rich	Female	Maternal Education (years per capita)			X			X
Neural tube defects	Global	Female	Maternal Education (years per capita)			X			X
Neural tube defects	Data Rich	Female	Age-standardized SEV for Household air pollution			X			X
Neural tube defects	Global	Female	Age-standardized SEV for Household air pollution			X			X
Neural tube defects	Data Rich	Female	Maternal alcohol consumption during pregnancy (proportion)			X			X
Neural tube defects	Global	Female	Maternal alcohol consumption during pregnancy (proportion)			X			X
Neural tube defects	Data Rich	Female	Age-standardized SEV for Low fruit			X			X
Neural tube defects	Global	Female	Age-standardized SEV for Low fruit			X			X
Neural tube defects	Data Rich	Female	Age-standardized SEV for Low vegetables			X			X
Neural tube defects	Global	Female	Age-standardized SEV for Low vegetables			X			X
Neural tube defects	Data Rich	Female	Age-standardized SEV for High fasting plasma glucose			X			X
Neural tube defects	Global	Female	Age-standardized SEV for High fasting plasma glucose			X			X
Neural tube defects	Data Rich	Female	Liters of alcohol consumed per capita			X			X
Neural tube defects	Global	Female	Liters of alcohol consumed per capita			X			X
Neural tube defects	Data Rich	Female	Healthcare access and quality index		X			X	
Neural tube defects	Global	Female	Healthcare access and quality index		X			X	
Neural tube defects	Data Rich	Female	Legality of Abortion		X			X	
Neural tube defects	Global	Female	Legality of Abortion		X			X	
Neural tube defects	Data Rich	Female	Antenatal Care (1 visit) Coverage (proportion)		X			X	
Neural tube defects	Global	Female	Antenatal Care (1 visit) Coverage (proportion)		X			X	
Neural tube defects	Data Rich	Female	Antenatal Care (4 visits) Coverage (proportion)		X			X	
Neural tube defects	Global	Female	Antenatal Care (4 visits) Coverage (proportion)		X			X	
Neural tube defects	Data Rich	Female	Age-standardized SEV for Smoking		X			X	
Neural tube defects	Global	Female	Age-standardized SEV for Smoking		X			X	
Neural tube defects	Data Rich	Female	Socio-demographic Index	X			X		
Neural tube defects	Global	Female	Socio-demographic Index	X			X		
Neural tube defects	Data Rich	Female	In-Facility Delivery (proportion)	X			X		
Neural tube defects	Global	Female	In-Facility Delivery (proportion)	X			X		
Neural tube defects	Data Rich	Female	Folic acid unadjusted (ug)	X			X		
Neural tube defects	Global	Female	Folic acid unadjusted (ug)	X			X		
Neural tube defects	Data Rich	Female	Composite fortification standard and folic acid inclusion	X			X		
Neural tube defects	Global	Female	Composite fortification standard and folic acid inclusion	X			X		
Neural tube defects	Data Rich	Male	Maternal Education (years per capita)			X			X
Neural tube defects	Global	Male	Maternal Education (years per capita)			X			X
Neural tube defects	Data Rich	Male	Age-standardized SEV for Household air pollution			X			X
Neural tube defects	Global	Male	Age-standardized SEV for Household air pollution			X			X
Neural tube defects	Data Rich	Male	Maternal alcohol consumption during pregnancy (proportion)			X			X
Neural tube defects	Global	Male	Maternal alcohol consumption during pregnancy (proportion)			X			X
Neural tube defects	Data Rich	Male	Age-standardized SEV for Low fruit			X			X
Neural tube defects	Global	Male	Age-standardized SEV for Low fruit			X			X
Neural tube defects	Data Rich	Male	Age-standardized SEV for Low vegetables			X			X
Neural tube defects	Global	Male	Age-standardized SEV for Low vegetables			X			X
Neural tube defects	Data Rich	Male	Age-standardized SEV for High fasting plasma glucose			X			X
Neural tube defects	Global	Male	Age-standardized SEV for High fasting plasma glucose			X			X
Neural tube defects	Data Rich	Male	Liters of alcohol consumed per capita			X			X
Neural tube defects	Global	Male	Liters of alcohol consumed per capita			X			X
Neural tube defects	Data Rich	Male	Healthcare access and quality index		X			X	
Neural tube defects	Global	Male	Healthcare access and quality index		X			X	
Neural tube defects	Data Rich	Male	Legality of Abortion		X			X	
Neural tube defects	Global	Male	Legality of Abortion		X			X	
Neural tube defects	Data Rich	Male	Antenatal Care (1 visit) Coverage (proportion)		X			X	
Neural tube defects	Global	Male	Antenatal Care (1 visit) Coverage (proportion)		X			X	
Neural tube defects	Data Rich	Male	Antenatal Care (4 visits) Coverage (proportion)		X			X	
Neural tube defects	Global	Male	Antenatal Care (4 visits) Coverage (proportion)		X			X	
Neural tube defects	Data Rich	Male	Age-standardized SEV for Smoking		X			X	
Neural tube defects	Global	Male	Age-standardized SEV for Smoking		X			X	
Neural tube defects	Data Rich	Male	Socio-demographic Index	X			X		
Neural tube defects	Global	Male	Socio-demographic Index	X			X		
Neural tube defects	Data Rich	Male	In-Facility Delivery (proportion)	X			X		
Neural tube defects	Global	Male	In-Facility Delivery (proportion)	X			X		
Neural tube defects	Data Rich	Male	Folic acid unadjusted (ug)	X			X		
Neural tube defects	Global	Male	Folic acid unadjusted (ug)	X			X		
Neural tube defects	Data Rich	Male	Composite fortification standard and folic acid inclusion	X			X		
Neural tube defects	Global	Male	Composite fortification standard and folic acid inclusion	X			X		
Congenital heart anomalies	Data Rich	Female	Skilled Birth Attendance (proportion)			X			X
Congenital heart anomalies	Global	Female	Skilled Birth Attendance (proportion)			X			X
Congenital heart anomalies	Data Rich	Female	Antenatal Care (4 visits) Coverage (proportion)			X			X
Congenital heart anomalies	Global	Female	Antenatal Care (4 visits) Coverage (proportion)			X			X
Congenital heart anomalies	Data Rich	Female	Maternal Education (years per capita)			X			X
Congenital heart anomalies	Global	Female	Maternal Education (years per capita)			X			X
Congenital heart anomalies	Data Rich	Female	Liters of alcohol consumed per capita			X			X
Congenital heart anomalies	Global	Female	Liters of alcohol consumed per capita			X			X
Congenital heart anomalies	Data Rich	Female	Live Births 35+ (proportion)			X			X
Congenital heart anomalies	Global	Female	Live Births 35+ (proportion)			X			X
Congenital heart anomalies	Data Rich	Female	Socio-demographic Index		X			X	
Congenital heart anomalies	Global	Female	Socio-demographic Index		X			X	
Congenital heart anomalies	Data Rich	Female	Healthcare access and quality index		X			X	
Congenital heart anomalies	Global	Female	Healthcare access and quality index		X			X	
Congenital heart anomalies	Data Rich	Female	Legality of Abortion		X			X	
Congenital heart anomalies	Global	Female	Legality of Abortion		X			X	
Congenital heart anomalies	Data Rich	Female	Antenatal Care (1 visit) Coverage (proportion)		X			X	
Congenital heart anomalies	Global	Female	Antenatal Care (1 visit) Coverage (proportion)		X			X	
Congenital heart anomalies	Data Rich	Female	In-Facility Delivery (proportion)		X			X	
Congenital heart anomalies	Global	Female	In-Facility Delivery (proportion)		X			X	
Congenital heart anomalies	Data Rich	Female	Age-standardized SEV for Smoking		X			X	
Congenital heart anomalies	Global	Female	Age-standardized SEV for Smoking		X			X	
Congenital heart anomalies	Data Rich	Female	Age-standardized SEV for High fasting plasma glucose		X			X	
Congenital heart anomalies	Global	Female	Age-standardized SEV for High fasting plasma glucose		X			X	
Congenital heart anomalies	Data Rich	Female	Maternal alcohol consumption during pregnancy (proportion)	X			X		
Congenital heart anomalies	Global	Female	Maternal alcohol consumption during pregnancy (proportion)	X			X		
Congenital heart anomalies	Data Rich	Female	Birth prevalence of CHD	X			X		
Congenital heart anomalies	Global	Female	Birth prevalence of CHD	X			X		
Congenital heart anomalies	Data Rich	Male	Skilled Birth Attendance (proportion)			X			X
Congenital heart anomalies	Global	Male	Skilled Birth Attendance (proportion)			X			X
Congenital heart anomalies	Data Rich	Male	Antenatal Care (4 visits) Coverage (proportion)			X			X
Congenital heart anomalies	Global	Male	Antenatal Care (4 visits) Coverage (proportion)			X			X
Congenital heart anomalies	Data Rich	Male	Maternal Education (years per capita)			X			X
Congenital heart anomalies	Global	Male	Maternal Education (years per capita)			X			X
Congenital heart anomalies	Data Rich	Male	Liters of alcohol consumed per capita			X			X

Appendix Table S11: Comparison of GBD 2021 and GBD 2023 covariates and level of covariates used in cause of death modeling

Cause	Model Version Type	Sex	Covariate	Level 1 GBD 2021	Level 2 GBD 2021	Level 3 GBD 2021	Level 1 GBD 2023	Level 2 GBD 2023	Level 3 GBD 2023
Congenital heart anomalies	Global	Male	Liters of alcohol consumed per capita			X			X
Congenital heart anomalies	Data Rich	Male	Live Births 35+ (proportion)			X			X
Congenital heart anomalies	Global	Male	Live Births 35+ (proportion)			X			X
Congenital heart anomalies	Data Rich	Male	Socio-demographic Index		X			X	
Congenital heart anomalies	Global	Male	Socio-demographic Index		X			X	
Congenital heart anomalies	Data Rich	Male	Healthcare access and quality index		X			X	
Congenital heart anomalies	Global	Male	Healthcare access and quality index		X			X	
Congenital heart anomalies	Data Rich	Male	Legality of Abortion		X			X	
Congenital heart anomalies	Global	Male	Legality of Abortion		X			X	
Congenital heart anomalies	Data Rich	Male	Antenatal Care (1 visit) Coverage (proportion)		X			X	
Congenital heart anomalies	Global	Male	Antenatal Care (1 visit) Coverage (proportion)		X			X	
Congenital heart anomalies	Data Rich	Male	In-Facility Delivery (proportion)		X			X	
Congenital heart anomalies	Global	Male	In-Facility Delivery (proportion)		X			X	
Congenital heart anomalies	Data Rich	Male	Age-standardized SEV for Smoking		X			X	
Congenital heart anomalies	Global	Male	Age-standardized SEV for Smoking		X			X	
Congenital heart anomalies	Data Rich	Male	Age-standardized SEV for High fasting plasma glucose		X			X	
Congenital heart anomalies	Global	Male	Age-standardized SEV for High fasting plasma glucose		X			X	
Congenital heart anomalies	Data Rich	Male	Maternal alcohol consumption during pregnancy (proportion)	X			X		
Congenital heart anomalies	Global	Male	Maternal alcohol consumption during pregnancy (proportion)	X			X		
Congenital heart anomalies	Data Rich	Male	Birth prevalence of CHD	X			X		
Congenital heart anomalies	Global	Male	Birth prevalence of CHD	X			X		
Orofacial clefts	Data Rich	Female	Antenatal Care (1 visit) Coverage (proportion)			X			X
Orofacial clefts	Global	Female	Antenatal Care (1 visit) Coverage (proportion)			X			X
Orofacial clefts	Data Rich	Female	Antenatal Care (4 visits) Coverage (proportion)			X			X
Orofacial clefts	Global	Female	Antenatal Care (4 visits) Coverage (proportion)			X			X
Orofacial clefts	Data Rich	Female	Maternal Education (years per capita)			X			X
Orofacial clefts	Global	Female	Maternal Education (years per capita)			X			X
Orofacial clefts	Global	Female	Age-standardized SEV for Household air pollution			X			X
Orofacial clefts	Data Rich	Female	Age-standardized SEV for Low fruit			X			X
Orofacial clefts	Global	Female	Age-standardized SEV for Low fruit			X			X
Orofacial clefts	Data Rich	Female	Age-standardized SEV for Low vegetables			X			X
Orofacial clefts	Global	Female	Age-standardized SEV for Low vegetables			X			X
Orofacial clefts	Data Rich	Female	Liters of alcohol consumed per capita			X			X
Orofacial clefts	Global	Female	Liters of alcohol consumed per capita			X			X
Orofacial clefts	Data Rich	Female	Healthcare access and quality index		X			X	
Orofacial clefts	Global	Female	Healthcare access and quality index		X			X	
Orofacial clefts	Data Rich	Female	Skilled Birth Attendance (proportion)		X			X	
Orofacial clefts	Global	Female	Skilled Birth Attendance (proportion)		X			X	
Orofacial clefts	Data Rich	Female	Legality of Abortion		X			X	
Orofacial clefts	Global	Female	Legality of Abortion		X			X	
Orofacial clefts	Data Rich	Female	Age-standardized SEV for Smoking		X			X	
Orofacial clefts	Global	Female	Age-standardized SEV for Smoking		X			X	
Orofacial clefts	Data Rich	Female	Maternal alcohol consumption during pregnancy (proportion)		X			X	
Orofacial clefts	Global	Female	Maternal alcohol consumption during pregnancy (proportion)		X			X	
Orofacial clefts	Data Rich	Female	Age-standardized SEV for High fasting plasma glucose		X			X	
Orofacial clefts	Global	Female	Age-standardized SEV for High fasting plasma glucose		X			X	
Orofacial clefts	Data Rich	Female	Socio-demographic Index	X			X		
Orofacial clefts	Global	Female	Socio-demographic Index	X			X		
Orofacial clefts	Data Rich	Female	Composite fortification standard and folic acid inclusion	X			X		
Orofacial clefts	Global	Female	Composite fortification standard and folic acid inclusion	X			X		
Orofacial clefts	Data Rich	Female	Age-standardized SEV for Household air pollution						X
Orofacial clefts	Data Rich	Male	Antenatal Care (1 visit) Coverage (proportion)			X			X
Orofacial clefts	Global	Male	Antenatal Care (1 visit) Coverage (proportion)			X			X
Orofacial clefts	Data Rich	Male	Antenatal Care (4 visits) Coverage (proportion)			X			X
Orofacial clefts	Global	Male	Antenatal Care (4 visits) Coverage (proportion)			X			X
Orofacial clefts	Data Rich	Male	Maternal Education (years per capita)			X			X
Orofacial clefts	Global	Male	Maternal Education (years per capita)			X			X
Orofacial clefts	Global	Male	Age-standardized SEV for Household air pollution			X			X
Orofacial clefts	Data Rich	Male	Age-standardized SEV for Low fruit			X			X
Orofacial clefts	Global	Male	Age-standardized SEV for Low fruit			X			X
Orofacial clefts	Data Rich	Male	Age-standardized SEV for Low vegetables			X			X
Orofacial clefts	Global	Male	Age-standardized SEV for Low vegetables			X			X
Orofacial clefts	Data Rich	Male	Liters of alcohol consumed per capita			X			X
Orofacial clefts	Global	Male	Liters of alcohol consumed per capita			X			X
Orofacial clefts	Data Rich	Male	Healthcare access and quality index		X			X	
Orofacial clefts	Global	Male	Healthcare access and quality index		X			X	
Orofacial clefts	Data Rich	Male	Skilled Birth Attendance (proportion)		X			X	
Orofacial clefts	Global	Male	Skilled Birth Attendance (proportion)		X			X	
Orofacial clefts	Data Rich	Male	Legality of Abortion		X			X	
Orofacial clefts	Global	Male	Legality of Abortion		X			X	
Orofacial clefts	Data Rich	Male	Age-standardized SEV for Smoking		X			X	
Orofacial clefts	Global	Male	Age-standardized SEV for Smoking		X			X	
Orofacial clefts	Data Rich	Male	Maternal alcohol consumption during pregnancy (proportion)		X			X	
Orofacial clefts	Global	Male	Maternal alcohol consumption during pregnancy (proportion)		X			X	
Orofacial clefts	Data Rich	Male	Age-standardized SEV for High fasting plasma glucose		X			X	
Orofacial clefts	Global	Male	Age-standardized SEV for High fasting plasma glucose		X			X	
Orofacial clefts	Data Rich	Male	Socio-demographic Index	X			X		
Orofacial clefts	Global	Male	Socio-demographic Index	X			X		
Orofacial clefts	Data Rich	Male	Composite fortification standard and folic acid inclusion	X			X		
Orofacial clefts	Global	Male	Composite fortification standard and folic acid inclusion	X			X		
Orofacial clefts	Data Rich	Male	Age-standardized SEV for Household air pollution						X
Down's syndrome	Data Rich	Female	Antenatal Care (1 visit) Coverage (proportion)			X			X
Down's syndrome	Global	Female	Antenatal Care (1 visit) Coverage (proportion)			X			X
Down's syndrome	Data Rich	Female	Antenatal Care (4 visits) Coverage (proportion)			X			X
Down's syndrome	Global	Female	Antenatal Care (4 visits) Coverage (proportion)			X			X
Down's syndrome	Data Rich	Female	Maternal Education (years per capita)			X			X
Down's syndrome	Global	Female	Maternal Education (years per capita)			X			X
Down's syndrome	Data Rich	Female	Age-standardized SEV for Household air pollution			X			X
Down's syndrome	Global	Female	Age-standardized SEV for Household air pollution			X			X
Down's syndrome	Data Rich	Female	Age-standardized SEV for Smoking			X			X
Down's syndrome	Global	Female	Age-standardized SEV for Smoking			X			X
Down's syndrome	Data Rich	Female	Maternal alcohol consumption during pregnancy (proportion)			X			X
Down's syndrome	Global	Female	Maternal alcohol consumption during pregnancy (proportion)			X			X
Down's syndrome	Data Rich	Female	Age-standardized SEV for Low vegetables			X			X
Down's syndrome	Global	Female	Age-standardized SEV for Low vegetables			X			X
Down's syndrome	Data Rich	Female	Liters of alcohol consumed per capita			X			X
Down's syndrome	Global	Female	Liters of alcohol consumed per capita			X			X
Down's syndrome	Data Rich	Female	Socio-demographic Index		X			X	
Down's syndrome	Global	Female	Socio-demographic Index		X			X	
Down's syndrome	Data Rich	Female	Healthcare access and quality index		X			X	
Down's syndrome	Global	Female	Healthcare access and quality index		X			X	
Down's syndrome	Data Rich	Female	In-Facility Delivery (proportion)		X			X	
Down's syndrome	Global	Female	In-Facility Delivery (proportion)		X			X	
Down's syndrome	Data Rich	Female	Legality of Abortion	X			X		
Down's syndrome	Global	Female	Legality of Abortion	X			X		

Appendix Table S11: Comparison of GBD 2021 and GBD 2023 covariates and level of covariates used in cause of death modeling

Cause	Model Version Type	Sex	Covariate	Level 1 GBD 2021	Level 2 GBD 2021	Level 3 GBD 2021	Level 1 GBD 2023	Level 2 GBD 2023	Level 3 GBD 2023
Down's syndrome	Data Rich	Female	Live Births 35+ (proportion)	X			X		
Down's syndrome	Global	Female	Live Births 35+ (proportion)	X			X		
Down's syndrome	Data Rich	Female	Live Births 40+ (proportion)	X			X		
Down's syndrome	Global	Female	Live Births 40+ (proportion)	X			X		
Down's syndrome	Data Rich	Female	Birth prevalence of congenital chromosomal anomalies	X			X		
Down's syndrome	Global	Female	Birth prevalence of congenital chromosomal anomalies	X			X		
Down's syndrome	Data Rich	Male	Antenatal Care (1 visit) Coverage (proportion)			X			X
Down's syndrome	Global	Male	Antenatal Care (1 visit) Coverage (proportion)			X			X
Down's syndrome	Data Rich	Male	Antenatal Care (4 visits) Coverage (proportion)			X			X
Down's syndrome	Global	Male	Antenatal Care (4 visits) Coverage (proportion)			X			X
Down's syndrome	Data Rich	Male	Maternal Education (years per capita)			X			X
Down's syndrome	Global	Male	Maternal Education (years per capita)			X			X
Down's syndrome	Data Rich	Male	Age-standardized SEV for Household air pollution			X			X
Down's syndrome	Global	Male	Age-standardized SEV for Household air pollution			X			X
Down's syndrome	Data Rich	Male	Age-standardized SEV for Smoking			X			X
Down's syndrome	Global	Male	Age-standardized SEV for Smoking			X			X
Down's syndrome	Data Rich	Male	Maternal alcohol consumption during pregnancy (proportion)			X			X
Down's syndrome	Global	Male	Maternal alcohol consumption during pregnancy (proportion)			X			X
Down's syndrome	Data Rich	Male	Age-standardized SEV for Low vegetables			X			X
Down's syndrome	Global	Male	Age-standardized SEV for Low vegetables			X			X
Down's syndrome	Data Rich	Male	Liters of alcohol consumed per capita			X			X
Down's syndrome	Global	Male	Liters of alcohol consumed per capita			X			X
Down's syndrome	Data Rich	Male	Socio-demographic Index		X			X	
Down's syndrome	Global	Male	Socio-demographic Index		X			X	
Down's syndrome	Data Rich	Male	Healthcare access and quality index		X			X	
Down's syndrome	Global	Male	Healthcare access and quality index		X			X	
Down's syndrome	Data Rich	Male	In-Facility Delivery (proportion)		X			X	
Down's syndrome	Global	Male	In-Facility Delivery (proportion)		X			X	
Down's syndrome	Data Rich	Male	Legality of Abortion	X			X		
Down's syndrome	Global	Male	Legality of Abortion	X			X		
Down's syndrome	Data Rich	Male	Live Births 35+ (proportion)	X			X		
Down's syndrome	Global	Male	Live Births 35+ (proportion)	X			X		
Down's syndrome	Data Rich	Male	Live Births 40+ (proportion)	X			X		
Down's syndrome	Global	Male	Live Births 40+ (proportion)	X			X		
Down's syndrome	Data Rich	Male	Birth prevalence of congenital chromosomal anomalies	X			X		
Down's syndrome	Global	Male	Birth prevalence of congenital chromosomal anomalies	X			X		
Other chromosomal abnormalities	Data Rich	Female	Socio-demographic Index			X			X
Other chromosomal abnormalities	Global	Female	Socio-demographic Index			X			X
Other chromosomal abnormalities	Data Rich	Female	Skilled Birth Attendance (proportion)			X			X
Other chromosomal abnormalities	Global	Female	Skilled Birth Attendance (proportion)			X			X
Other chromosomal abnormalities	Data Rich	Female	Maternal Education (years per capita)			X			X
Other chromosomal abnormalities	Global	Female	Maternal Education (years per capita)			X			X
Other chromosomal abnormalities	Data Rich	Female	Age-standardized SEV for Household air pollution			X			X
Other chromosomal abnormalities	Global	Female	Age-standardized SEV for Household air pollution			X			X
Other chromosomal abnormalities	Data Rich	Female	Age-standardized SEV for Smoking			X			X
Other chromosomal abnormalities	Global	Female	Age-standardized SEV for Smoking			X			X
Other chromosomal abnormalities	Data Rich	Female	Liters of alcohol consumed per capita			X			X
Other chromosomal abnormalities	Global	Female	Liters of alcohol consumed per capita			X			X
Other chromosomal abnormalities	Data Rich	Female	LDI (I$ per capita)		X			X	
Other chromosomal abnormalities	Global	Female	LDI (I$ per capita)		X			X	
Other chromosomal abnormalities	Data Rich	Female	Healthcare access and quality index		X			X	
Other chromosomal abnormalities	Global	Female	Healthcare access and quality index		X			X	
Other chromosomal abnormalities	Data Rich	Female	Antenatal Care (1 visit) Coverage (proportion)		X			X	
Other chromosomal abnormalities	Global	Female	Antenatal Care (1 visit) Coverage (proportion)		X			X	
Other chromosomal abnormalities	Data Rich	Female	Antenatal Care (4 visits) Coverage (proportion)		X			X	
Other chromosomal abnormalities	Global	Female	Antenatal Care (4 visits) Coverage (proportion)		X			X	
Other chromosomal abnormalities	Data Rich	Female	In-Facility Delivery (proportion)		X			X	
Other chromosomal abnormalities	Global	Female	In-Facility Delivery (proportion)		X			X	
Other chromosomal abnormalities	Data Rich	Female	Maternal alcohol consumption during pregnancy (proportion)		X			X	
Other chromosomal abnormalities	Global	Female	Maternal alcohol consumption during pregnancy (proportion)		X			X	
Other chromosomal abnormalities	Data Rich	Female	Legality of Abortion	X			X		
Other chromosomal abnormalities	Global	Female	Legality of Abortion	X			X		
Other chromosomal abnormalities	Data Rich	Female	Live Births 35+ (proportion)	X			X		
Other chromosomal abnormalities	Global	Female	Live Births 35+ (proportion)	X			X		
Other chromosomal abnormalities	Data Rich	Female	Live Births 40+ (proportion)	X			X		
Other chromosomal abnormalities	Global	Female	Live Births 40+ (proportion)	X			X		
Other chromosomal abnormalities	Data Rich	Male	Socio-demographic Index			X			X
Other chromosomal abnormalities	Global	Male	Socio-demographic Index			X			X
Other chromosomal abnormalities	Data Rich	Male	Skilled Birth Attendance (proportion)			X			X
Other chromosomal abnormalities	Global	Male	Skilled Birth Attendance (proportion)			X			X
Other chromosomal abnormalities	Data Rich	Male	Maternal Education (years per capita)			X			X
Other chromosomal abnormalities	Global	Male	Maternal Education (years per capita)			X			X
Other chromosomal abnormalities	Data Rich	Male	Age-standardized SEV for Household air pollution			X			X
Other chromosomal abnormalities	Global	Male	Age-standardized SEV for Household air pollution			X			X
Other chromosomal abnormalities	Data Rich	Male	Age-standardized SEV for Smoking			X			X
Other chromosomal abnormalities	Global	Male	Age-standardized SEV for Smoking			X			X
Other chromosomal abnormalities	Data Rich	Male	Liters of alcohol consumed per capita			X			X
Other chromosomal abnormalities	Global	Male	Liters of alcohol consumed per capita			X			X
Other chromosomal abnormalities	Data Rich	Male	LDI (I$ per capita)		X			X	
Other chromosomal abnormalities	Global	Male	LDI (I$ per capita)		X			X	
Other chromosomal abnormalities	Data Rich	Male	Healthcare access and quality index		X			X	
Other chromosomal abnormalities	Global	Male	Healthcare access and quality index		X			X	
Other chromosomal abnormalities	Data Rich	Male	Antenatal Care (1 visit) Coverage (proportion)		X			X	
Other chromosomal abnormalities	Global	Male	Antenatal Care (1 visit) Coverage (proportion)		X			X	
Other chromosomal abnormalities	Data Rich	Male	Antenatal Care (4 visits) Coverage (proportion)		X			X	
Other chromosomal abnormalities	Global	Male	Antenatal Care (4 visits) Coverage (proportion)		X			X	
Other chromosomal abnormalities	Data Rich	Male	In-Facility Delivery (proportion)		X			X	
Other chromosomal abnormalities	Global	Male	In-Facility Delivery (proportion)		X			X	
Other chromosomal abnormalities	Data Rich	Male	Maternal alcohol consumption during pregnancy (proportion)		X			X	
Other chromosomal abnormalities	Global	Male	Maternal alcohol consumption during pregnancy (proportion)		X			X	
Other chromosomal abnormalities	Data Rich	Male	Legality of Abortion	X			X		
Other chromosomal abnormalities	Global	Male	Legality of Abortion	X			X		
Other chromosomal abnormalities	Data Rich	Male	Live Births 35+ (proportion)	X			X		
Other chromosomal abnormalities	Global	Male	Live Births 35+ (proportion)	X			X		
Other chromosomal abnormalities	Data Rich	Male	Live Births 40+ (proportion)	X			X		
Other chromosomal abnormalities	Global	Male	Live Births 40+ (proportion)	X			X		
Congenital musculoskeletal and limb anomalies	Data Rich	Female	LDI (I$ per capita)			X			X
Congenital musculoskeletal and limb anomalies	Global	Female	LDI (I$ per capita)			X			X
Congenital musculoskeletal and limb anomalies	Data Rich	Female	Antenatal Care (1 visit) Coverage (proportion)			X			X
Congenital musculoskeletal and limb anomalies	Global	Female	Antenatal Care (1 visit) Coverage (proportion)			X			X
Congenital musculoskeletal and limb anomalies	Data Rich	Female	Antenatal Care (4 visits) Coverage (proportion)			X			X
Congenital musculoskeletal and limb anomalies	Global	Female	Antenatal Care (4 visits) Coverage (proportion)			X			X
Congenital musculoskeletal and limb anomalies	Data Rich	Female	Maternal Education (years per capita)			X			X
Congenital musculoskeletal and limb anomalies	Global	Female	Maternal Education (years per capita)			X			X
Congenital musculoskeletal and limb anomalies	Data Rich	Female	Age-standardized SEV for Low fruit			X			X
Congenital musculoskeletal and limb anomalies	Global	Female	Age-standardized SEV for Low fruit			X			X
Congenital musculoskeletal and limb anomalies	Data Rich	Female	Age-standardized SEV for Low vegetables			X			X

Appendix Table S11: Comparison of GBD 2021 and GBD 2023 covariates and level of covariates used in cause of death modeling

Cause	Model Version Type	Sex	Covariate	Level 1 GBD 2021	Level 2 GBD 2021	Level 3 GBD 2021	Level 1 GBD 2023	Level 2 GBD 2023	Level 3 GBD 2023
Congenital musculoskeletal and limb anomalies	Global	Female	Age-standardized SEV for Low vegetables			X			X
Congenital musculoskeletal and limb anomalies	Data Rich	Female	Liters of alcohol consumed per capita			X			X
Congenital musculoskeletal and limb anomalies	Global	Female	Liters of alcohol consumed per capita			X			X
Congenital musculoskeletal and limb anomalies	Data Rich	Female	In-Facility Delivery (proportion)		X			X	
Congenital musculoskeletal and limb anomalies	Global	Female	In-Facility Delivery (proportion)		X			X	
Congenital musculoskeletal and limb anomalies	Data Rich	Female	Age-standardized SEV for Household air pollution		X			X	
Congenital musculoskeletal and limb anomalies	Global	Female	Age-standardized SEV for Household air pollution		X			X	
Congenital musculoskeletal and limb anomalies	Data Rich	Female	Age-standardized SEV for Smoking		X			X	
Congenital musculoskeletal and limb anomalies	Global	Female	Age-standardized SEV for Smoking		X			X	
Congenital musculoskeletal and limb anomalies	Data Rich	Female	Age-standardized SEV for High fasting plasma glucose		X			X	
Congenital musculoskeletal and limb anomalies	Global	Female	Age-standardized SEV for High fasting plasma glucose		X			X	
Congenital musculoskeletal and limb anomalies	Data Rich	Female	Socio-demographic Index		X		X		
Congenital musculoskeletal and limb anomalies	Global	Female	Socio-demographic Index		X		X		
Congenital musculoskeletal and limb anomalies	Data Rich	Female	Healthcare access and quality index		X		X		
Congenital musculoskeletal and limb anomalies	Global	Female	Healthcare access and quality index		X		X		
Congenital musculoskeletal and limb anomalies	Data Rich	Female	Legality of Abortion	X			X		
Congenital musculoskeletal and limb anomalies	Global	Female	Legality of Abortion	X			X		
Congenital musculoskeletal and limb anomalies	Data Rich	Female	Maternal alcohol consumption during pregnancy (proportion)	X			X		
Congenital musculoskeletal and limb anomalies	Global	Female	Maternal alcohol consumption during pregnancy (proportion)	X			X		
Congenital musculoskeletal and limb anomalies	Data Rich	Male	LDI (I$ per capita)			X			X
Congenital musculoskeletal and limb anomalies	Global	Male	LDI (I$ per capita)			X			X
Congenital musculoskeletal and limb anomalies	Data Rich	Male	Antenatal Care (1 visit) Coverage (proportion)			X			X
Congenital musculoskeletal and limb anomalies	Global	Male	Antenatal Care (1 visit) Coverage (proportion)			X			X
Congenital musculoskeletal and limb anomalies	Data Rich	Male	Antenatal Care (4 visits) Coverage (proportion)			X			X
Congenital musculoskeletal and limb anomalies	Global	Male	Antenatal Care (4 visits) Coverage (proportion)			X			X
Congenital musculoskeletal and limb anomalies	Data Rich	Male	Maternal Education (years per capita)			X			X
Congenital musculoskeletal and limb anomalies	Global	Male	Maternal Education (years per capita)			X			X
Congenital musculoskeletal and limb anomalies	Data Rich	Male	Age-standardized SEV for Low fruit			X			X
Congenital musculoskeletal and limb anomalies	Global	Male	Age-standardized SEV for Low fruit			X			X
Congenital musculoskeletal and limb anomalies	Data Rich	Male	Age-standardized SEV for Low vegetables			X			X
Congenital musculoskeletal and limb anomalies	Global	Male	Age-standardized SEV for Low vegetables			X			X
Congenital musculoskeletal and limb anomalies	Data Rich	Male	Liters of alcohol consumed per capita			X			X
Congenital musculoskeletal and limb anomalies	Global	Male	Liters of alcohol consumed per capita			X			X
Congenital musculoskeletal and limb anomalies	Data Rich	Male	In-Facility Delivery (proportion)		X			X	
Congenital musculoskeletal and limb anomalies	Global	Male	In-Facility Delivery (proportion)		X			X	
Congenital musculoskeletal and limb anomalies	Data Rich	Male	Age-standardized SEV for Household air pollution		X			X	
Congenital musculoskeletal and limb anomalies	Global	Male	Age-standardized SEV for Household air pollution		X			X	
Congenital musculoskeletal and limb anomalies	Data Rich	Male	Age-standardized SEV for Smoking		X			X	
Congenital musculoskeletal and limb anomalies	Global	Male	Age-standardized SEV for Smoking		X			X	
Congenital musculoskeletal and limb anomalies	Data Rich	Male	Age-standardized SEV for High fasting plasma glucose		X			X	
Congenital musculoskeletal and limb anomalies	Global	Male	Age-standardized SEV for High fasting plasma glucose		X			X	
Congenital musculoskeletal and limb anomalies	Data Rich	Male	Socio-demographic Index		X		X		
Congenital musculoskeletal and limb anomalies	Global	Male	Socio-demographic Index		X		X		
Congenital musculoskeletal and limb anomalies	Data Rich	Male	Healthcare access and quality index		X		X		
Congenital musculoskeletal and limb anomalies	Global	Male	Healthcare access and quality index		X		X		
Congenital musculoskeletal and limb anomalies	Data Rich	Male	Legality of Abortion	X			X		
Congenital musculoskeletal and limb anomalies	Global	Male	Legality of Abortion	X			X		
Congenital musculoskeletal and limb anomalies	Data Rich	Male	Maternal alcohol consumption during pregnancy (proportion)	X			X		
Congenital musculoskeletal and limb anomalies	Global	Male	Maternal alcohol consumption during pregnancy (proportion)	X			X		
Urogenital congenital anomalies	Data Rich	Female	LDI (I$ per capita)			X			X
Urogenital congenital anomalies	Global	Female	LDI (I$ per capita)			X			X
Urogenital congenital anomalies	Data Rich	Female	Antenatal Care (1 visit) Coverage (proportion)			X			X
Urogenital congenital anomalies	Global	Female	Antenatal Care (1 visit) Coverage (proportion)			X			X
Urogenital congenital anomalies	Data Rich	Female	Antenatal Care (4 visits) Coverage (proportion)			X			X
Urogenital congenital anomalies	Global	Female	Antenatal Care (4 visits) Coverage (proportion)			X			X
Urogenital congenital anomalies	Data Rich	Female	Maternal Education (years per capita)			X			X
Urogenital congenital anomalies	Global	Female	Maternal Education (years per capita)			X			X
Urogenital congenital anomalies	Data Rich	Female	Liters of alcohol consumed per capita			X			X
Urogenital congenital anomalies	Global	Female	Liters of alcohol consumed per capita			X			X
Urogenital congenital anomalies	Data Rich	Female	Socio-demographic Index		X			X	
Urogenital congenital anomalies	Global	Female	Socio-demographic Index		X			X	
Urogenital congenital anomalies	Data Rich	Female	Healthcare access and quality index		X			X	
Urogenital congenital anomalies	Global	Female	Healthcare access and quality index		X			X	
Urogenital congenital anomalies	Data Rich	Female	In-Facility Delivery (proportion)		X			X	
Urogenital congenital anomalies	Global	Female	In-Facility Delivery (proportion)		X			X	
Urogenital congenital anomalies	Data Rich	Female	Age-standardized SEV for Ambient particulate matter		X			X	
Urogenital congenital anomalies	Global	Female	Age-standardized SEV for Ambient particulate matter		X			X	
Urogenital congenital anomalies	Data Rich	Female	Age-standardized SEV for Household air pollution		X			X	
Urogenital congenital anomalies	Global	Female	Age-standardized SEV for Household air pollution		X			X	
Urogenital congenital anomalies	Global	Female	Diabetes Age-Standardized Prevalence (proportion)		X			X	
Urogenital congenital anomalies	Data Rich	Female	Age-standardized SEV for Smoking	X			X		
Urogenital congenital anomalies	Global	Female	Age-standardized SEV for Smoking	X			X		
Urogenital congenital anomalies	Data Rich	Female	Maternal alcohol consumption during pregnancy (proportion)	X			X		
Urogenital congenital anomalies	Global	Female	Maternal alcohol consumption during pregnancy (proportion)	X			X		
Urogenital congenital anomalies	Data Rich	Female	Smoking Prevalence (Reproductive Age Standardized)	X			X		
Urogenital congenital anomalies	Data Rich	Male	LDI (I$ per capita)			X			X
Urogenital congenital anomalies	Global	Male	LDI (I$ per capita)			X			X
Urogenital congenital anomalies	Data Rich	Male	Antenatal Care (1 visit) Coverage (proportion)			X			X
Urogenital congenital anomalies	Global	Male	Antenatal Care (1 visit) Coverage (proportion)			X			X
Urogenital congenital anomalies	Data Rich	Male	Antenatal Care (4 visits) Coverage (proportion)			X			X
Urogenital congenital anomalies	Global	Male	Antenatal Care (4 visits) Coverage (proportion)			X			X
Urogenital congenital anomalies	Data Rich	Male	Maternal Education (years per capita)			X			X
Urogenital congenital anomalies	Global	Male	Maternal Education (years per capita)			X			X
Urogenital congenital anomalies	Data Rich	Male	Liters of alcohol consumed per capita			X			X
Urogenital congenital anomalies	Global	Male	Liters of alcohol consumed per capita			X			X
Urogenital congenital anomalies	Data Rich	Male	Socio-demographic Index		X			X	
Urogenital congenital anomalies	Global	Male	Socio-demographic Index		X			X	
Urogenital congenital anomalies	Data Rich	Male	Healthcare access and quality index		X			X	
Urogenital congenital anomalies	Global	Male	Healthcare access and quality index		X			X	
Urogenital congenital anomalies	Data Rich	Male	In-Facility Delivery (proportion)		X			X	
Urogenital congenital anomalies	Global	Male	In-Facility Delivery (proportion)		X			X	
Urogenital congenital anomalies	Data Rich	Male	Age-standardized SEV for Ambient particulate matter		X			X	
Urogenital congenital anomalies	Global	Male	Age-standardized SEV for Ambient particulate matter		X			X	
Urogenital congenital anomalies	Data Rich	Male	Age-standardized SEV for Household air pollution		X			X	
Urogenital congenital anomalies	Global	Male	Age-standardized SEV for Household air pollution		X			X	
Urogenital congenital anomalies	Data Rich	Male	Diabetes Age-Standardized Prevalence (proportion)		X			X	

Appendix Table S11: Comparison of GBD 2021 and GBD 2023 covariates and level of covariates used in cause of death modeling									
Cause	Model Version Type	Sex	Covariate	Level 1 GBD 2021	Level 2 GBD 2021	Level 3 GBD 2021	Level 1 GBD 2023	Level 2 GBD 2023	Level 3 GBD 2023
Urogenital congenital anomalies	Global	Male	Diabetes Age-Standardized Prevalence (proportion)		X			X	
Urogenital congenital anomalies	Data Rich	Male	Age-standardized SEV for Smoking	X			X		
Urogenital congenital anomalies	Global	Male	Age-standardized SEV for Smoking	X			X		
Urogenital congenital anomalies	Data Rich	Male	Maternal alcohol consumption during pregnancy (proportion)	X			X		
Urogenital congenital anomalies	Global	Male	Maternal alcohol consumption during pregnancy (proportion)	X			X		
Digestive congenital anomalies	Data Rich	Female	Maternal care and immunization			X			X
Digestive congenital anomalies	Global	Female	Maternal care and immunization			X			X
Digestive congenital anomalies	Data Rich	Female	LDI (I$ per capita)			X			X
Digestive congenital anomalies	Global	Female	LDI (I$ per capita)			X			X
Digestive congenital anomalies	Data Rich	Female	Antenatal Care (1 visit) Coverage (proportion)			X			X
Digestive congenital anomalies	Global	Female	Antenatal Care (1 visit) Coverage (proportion)			X			X
Digestive congenital anomalies	Data Rich	Female	Antenatal Care (4 visits) Coverage (proportion)			X			X
Digestive congenital anomalies	Global	Female	Antenatal Care (4 visits) Coverage (proportion)			X			X
Digestive congenital anomalies	Data Rich	Female	Maternal Education (years per capita)			X			X
Digestive congenital anomalies	Global	Female	Maternal Education (years per capita)			X			X
Digestive congenital anomalies	Data Rich	Female	Age-standardized SEV for Low fruit			X			X
Digestive congenital anomalies	Global	Female	Age-standardized SEV for Low fruit			X			X
Digestive congenital anomalies	Data Rich	Female	Age-standardized SEV for Low vegetables			X			X
Digestive congenital anomalies	Global	Female	Age-standardized SEV for Low vegetables			X			X
Digestive congenital anomalies	Data Rich	Female	Liters of alcohol consumed per capita			X			X
Digestive congenital anomalies	Global	Female	Liters of alcohol consumed per capita			X			X
Digestive congenital anomalies	Data Rich	Female	Socio-demographic Index		X			X	
Digestive congenital anomalies	Global	Female	Socio-demographic Index		X			X	
Digestive congenital anomalies	Data Rich	Female	Healthcare access and quality index		X			X	
Digestive congenital anomalies	Global	Female	Healthcare access and quality index		X			X	
Digestive congenital anomalies	Data Rich	Female	In-Facility Delivery (proportion)		X			X	
Digestive congenital anomalies	Global	Female	In-Facility Delivery (proportion)		X			X	
Digestive congenital anomalies	Data Rich	Female	Age-standardized SEV for Household air pollution		X			X	
Digestive congenital anomalies	Global	Female	Age-standardized SEV for Household air pollution		X			X	
Digestive congenital anomalies	Data Rich	Female	Age-standardized SEV for High fasting plasma glucose		X			X	
Digestive congenital anomalies	Global	Female	Age-standardized SEV for High fasting plasma glucose		X			X	
Digestive congenital anomalies	Data Rich	Female	Age-standardized SEV for High body-mass index		X			X	
Digestive congenital anomalies	Global	Female	Age-standardized SEV for High body-mass index		X			X	
Digestive congenital anomalies	Data Rich	Female	Age-standardized SEV for Smoking	X			X		
Digestive congenital anomalies	Global	Female	Age-standardized SEV for Smoking	X			X		
Digestive congenital anomalies	Data Rich	Female	Maternal alcohol consumption during pregnancy (proportion)	X			X		
Digestive congenital anomalies	Global	Female	Maternal alcohol consumption during pregnancy (proportion)	X			X		
Digestive congenital anomalies	Data Rich	Male	Maternal care and immunization			X			X
Digestive congenital anomalies	Global	Male	Maternal care and immunization			X			X
Digestive congenital anomalies	Data Rich	Male	LDI (I$ per capita)			X			X
Digestive congenital anomalies	Global	Male	LDI (I$ per capita)			X			X
Digestive congenital anomalies	Data Rich	Male	Antenatal Care (1 visit) Coverage (proportion)			X			X
Digestive congenital anomalies	Global	Male	Antenatal Care (1 visit) Coverage (proportion)			X			X
Digestive congenital anomalies	Data Rich	Male	Antenatal Care (4 visits) Coverage (proportion)			X			X
Digestive congenital anomalies	Global	Male	Antenatal Care (4 visits) Coverage (proportion)			X			X
Digestive congenital anomalies	Data Rich	Male	Maternal Education (years per capita)			X			X
Digestive congenital anomalies	Global	Male	Maternal Education (years per capita)			X			X
Digestive congenital anomalies	Data Rich	Male	Age-standardized SEV for Low fruit			X			X
Digestive congenital anomalies	Global	Male	Age-standardized SEV for Low fruit			X			X
Digestive congenital anomalies	Data Rich	Male	Age-standardized SEV for Low vegetables			X			X
Digestive congenital anomalies	Global	Male	Age-standardized SEV for Low vegetables			X			X
Digestive congenital anomalies	Data Rich	Male	Liters of alcohol consumed per capita			X			X
Digestive congenital anomalies	Global	Male	Liters of alcohol consumed per capita			X			X
Digestive congenital anomalies	Data Rich	Male	Socio-demographic Index		X			X	
Digestive congenital anomalies	Global	Male	Socio-demographic Index		X			X	
Digestive congenital anomalies	Data Rich	Male	Healthcare access and quality index		X			X	
Digestive congenital anomalies	Global	Male	Healthcare access and quality index		X			X	
Digestive congenital anomalies	Data Rich	Male	In-Facility Delivery (proportion)		X			X	
Digestive congenital anomalies	Global	Male	In-Facility Delivery (proportion)		X			X	
Digestive congenital anomalies	Data Rich	Male	Age-standardized SEV for Household air pollution		X			X	
Digestive congenital anomalies	Global	Male	Age-standardized SEV for Household air pollution		X			X	
Digestive congenital anomalies	Data Rich	Male	Age-standardized SEV for High fasting plasma glucose		X			X	
Digestive congenital anomalies	Global	Male	Age-standardized SEV for High fasting plasma glucose		X			X	
Digestive congenital anomalies	Data Rich	Male	Age-standardized SEV for High body-mass index		X			X	
Digestive congenital anomalies	Global	Male	Age-standardized SEV for High body-mass index		X			X	
Digestive congenital anomalies	Data Rich	Male	Age-standardized SEV for Smoking	X			X		
Digestive congenital anomalies	Global	Male	Age-standardized SEV for Smoking	X			X		
Digestive congenital anomalies	Data Rich	Male	Maternal alcohol consumption during pregnancy (proportion)	X			X		
Digestive congenital anomalies	Global	Male	Maternal alcohol consumption during pregnancy (proportion)	X			X		
Other congenital anomalies	Data Rich	Female	LDI (I$ per capita)			X			X
Other congenital anomalies	Global	Female	LDI (I$ per capita)			X			X
Other congenital anomalies	Data Rich	Female	Socio-demographic Index			X			X
Other congenital anomalies	Global	Female	Socio-demographic Index			X			X
Other congenital anomalies	Data Rich	Female	Antenatal Care (1 visit) Coverage (proportion)			X			X
Other congenital anomalies	Global	Female	Antenatal Care (1 visit) Coverage (proportion)			X			X
Other congenital anomalies	Data Rich	Female	Antenatal Care (4 visits) Coverage (proportion)			X			X
Other congenital anomalies	Global	Female	Antenatal Care (4 visits) Coverage (proportion)			X			X
Other congenital anomalies	Data Rich	Female	Maternal Education (years per capita)			X			X
Other congenital anomalies	Global	Female	Maternal Education (years per capita)			X			X
Other congenital anomalies	Data Rich	Female	Age-standardized SEV for High fasting plasma glucose			X			X
Other congenital anomalies	Global	Female	Age-standardized SEV for High fasting plasma glucose			X			X
Other congenital anomalies	Data Rich	Female	Liters of alcohol consumed per capita			X			X
Other congenital anomalies	Global	Female	Liters of alcohol consumed per capita			X			X
Other congenital anomalies	Data Rich	Female	Healthcare access and quality index		X			X	
Other congenital anomalies	Global	Female	Healthcare access and quality index		X			X	
Other congenital anomalies	Data Rich	Female	Legality of Abortion		X			X	
Other congenital anomalies	Global	Female	Legality of Abortion		X			X	
Other congenital anomalies	Data Rich	Female	In-Facility Delivery (proportion)		X			X	
Other congenital anomalies	Global	Female	In-Facility Delivery (proportion)		X			X	
Other congenital anomalies	Data Rich	Female	Age-standardized SEV for Household air pollution		X			X	
Other congenital anomalies	Global	Female	Age-standardized SEV for Household air pollution		X			X	
Other congenital anomalies	Data Rich	Female	Age-standardized SEV for Smoking		X			X	
Other congenital anomalies	Global	Female	Age-standardized SEV for Smoking		X			X	
Other congenital anomalies	Data Rich	Female	Maternal alcohol consumption during pregnancy (proportion)	X			X		
Other congenital anomalies	Global	Female	Maternal alcohol consumption during pregnancy (proportion)	X			X		
Other congenital anomalies	Data Rich	Female	Live Births 35+ (proportion)	X			X		
Other congenital anomalies	Global	Female	Live Births 35+ (proportion)	X			X		
Other congenital anomalies	Data Rich	Male	LDI (I$ per capita)			X			X
Other congenital anomalies	Global	Male	LDI (I$ per capita)			X			X
Other congenital anomalies	Data Rich	Male	Socio-demographic Index			X			X
Other congenital anomalies	Global	Male	Socio-demographic Index			X			X
Other congenital anomalies	Data Rich	Male	Antenatal Care (1 visit) Coverage (proportion)			X			X
Other congenital anomalies	Global	Male	Antenatal Care (1 visit) Coverage (proportion)			X			X
Other congenital anomalies	Data Rich	Male	Antenatal Care (4 visits) Coverage (proportion)			X			X
Other congenital anomalies	Global	Male	Antenatal Care (4 visits) Coverage (proportion)			X			X
Other congenital anomalies	Data Rich	Male	Maternal Education (years per capita)			X			X

Appendix Table S11: Comparison of GBD 2021 and GBD 2023 covariates and level of covariates used in cause of death modeling

Cause	Model Version Type	Sex	Covariate	Level 1 GBD 2021	Level 2 GBD 2021	Level 3 GBD 2021	Level 1 GBD 2023	Level 2 GBD 2023	Level 3 GBD 2023
Other congenital anomalies	Global	Male	Maternal Education (years per capita)			X			X
Other congenital anomalies	Data Rich	Male	Age-standardized SEV for High fasting plasma glucose			X			X
Other congenital anomalies	Global	Male	Age-standardized SEV for High fasting plasma glucose			X			X
Other congenital anomalies	Data Rich	Male	Liters of alcohol consumed per capita			X			X
Other congenital anomalies	Global	Male	Liters of alcohol consumed per capita			X			X
Other congenital anomalies	Data Rich	Male	Healthcare access and quality index		X			X	
Other congenital anomalies	Global	Male	Healthcare access and quality index		X			X	
Other congenital anomalies	Data Rich	Male	Legality of Abortion		X			X	
Other congenital anomalies	Global	Male	Legality of Abortion		X			X	
Other congenital anomalies	Data Rich	Male	In-Facility Delivery (proportion)		X			X	
Other congenital anomalies	Global	Male	In-Facility Delivery (proportion)		X			X	
Other congenital anomalies	Data Rich	Male	Age-standardized SEV for Household air pollution		X			X	
Other congenital anomalies	Global	Male	Age-standardized SEV for Household air pollution		X			X	
Other congenital anomalies	Data Rich	Male	Age-standardized SEV for Smoking		X			X	
Other congenital anomalies	Global	Male	Age-standardized SEV for Smoking		X			X	
Other congenital anomalies	Data Rich	Male	Maternal alcohol consumption during pregnancy (proportion)	X			X		
Other congenital anomalies	Global	Male	Maternal alcohol consumption during pregnancy (proportion)	X			X		
Other congenital anomalies	Data Rich	Male	Live Births 35+ (proportion)	X			X		
Other congenital anomalies	Global	Male	Live Births 35+ (proportion)	X			X		
Urinary diseases and male infertility	Data Rich	Female	Education (years per capita)			X			X
Urinary diseases and male infertility	Global	Female	Education (years per capita)			X			X
Urinary diseases and male infertility	Data Rich	Female	LDI (I$ per capita)			X			X
Urinary diseases and male infertility	Global	Female	LDI (I$ per capita)			X			X
Urinary diseases and male infertility	Data Rich	Female	Socio-demographic Index			X			X
Urinary diseases and male infertility	Global	Female	Socio-demographic Index			X			X
Urinary diseases and male infertility	Data Rich	Female	Healthcare access and quality index		X			X	
Urinary diseases and male infertility	Global	Female	Healthcare access and quality index		X			X	
Urinary diseases and male infertility	Data Rich	Female	Mean BMI		X			X	
Urinary diseases and male infertility	Global	Female	Mean BMI		X			X	
Urinary diseases and male infertility	Data Rich	Female	Sanitation (proportion with access)		X			X	
Urinary diseases and male infertility	Global	Female	Sanitation (proportion with access)		X			X	
Urinary diseases and male infertility	Data Rich	Female	90th percentile climatic temperature in the given country-year.		X			X	
Urinary diseases and male infertility	Global	Female	90th percentile climatic temperature in the given country-year.		X			X	
Urinary diseases and male infertility	Data Rich	Male	Education (years per capita)			X			X
Urinary diseases and male infertility	Global	Male	Education (years per capita)			X			X
Urinary diseases and male infertility	Data Rich	Male	LDI (I$ per capita)			X			X
Urinary diseases and male infertility	Global	Male	LDI (I$ per capita)			X			X
Urinary diseases and male infertility	Data Rich	Male	Socio-demographic Index			X			X
Urinary diseases and male infertility	Global	Male	Socio-demographic Index			X			X
Urinary diseases and male infertility	Data Rich	Male	Healthcare access and quality index		X			X	
Urinary diseases and male infertility	Global	Male	Healthcare access and quality index		X			X	
Urinary diseases and male infertility	Data Rich	Male	Mean BMI		X			X	
Urinary diseases and male infertility	Global	Male	Mean BMI		X			X	
Urinary diseases and male infertility	Data Rich	Male	Sanitation (proportion with access)		X			X	
Urinary diseases and male infertility	Global	Male	Sanitation (proportion with access)		X			X	
Urinary diseases and male infertility	Data Rich	Male	90th percentile climatic temperature in the given country-year.		X			X	
Urinary diseases and male infertility	Global	Male	90th percentile climatic temperature in the given country-year.		X			X	
Urinary tract infections and interstitial nephritis	Global	Female	Socio-demographic Index			X			X
Urinary tract infections and interstitial nephritis	Data Rich	Female	Socio-demographic Index			X			X
Urinary tract infections and interstitial nephritis	Global	Female	Education (years per capita)		X			X	
Urinary tract infections and interstitial nephritis	Data Rich	Female	Education (years per capita)		X			X	
Urinary tract infections and interstitial nephritis	Global	Female	LDI (I$ per capita)		X			X	
Urinary tract infections and interstitial nephritis	Data Rich	Female	LDI (I$ per capita)		X			X	
Urinary tract infections and interstitial nephritis	Global	Female	Healthcare access and quality index		X			X	
Urinary tract infections and interstitial nephritis	Data Rich	Female	Healthcare access and quality index		X			X	
Urinary tract infections and interstitial nephritis	Global	Female	Sanitation (proportion with access)	X			X		
Urinary tract infections and interstitial nephritis	Data Rich	Female	Sanitation (proportion with access)	X			X		
Urinary tract infections and interstitial nephritis	Global	Male	Socio-demographic Index			X			X
Urinary tract infections and interstitial nephritis	Data Rich	Male	Socio-demographic Index			X			X
Urinary tract infections and interstitial nephritis	Global	Male	Education (years per capita)		X			X	
Urinary tract infections and interstitial nephritis	Data Rich	Male	Education (years per capita)		X			X	
Urinary tract infections and interstitial nephritis	Global	Male	LDI (I$ per capita)		X			X	
Urinary tract infections and interstitial nephritis	Data Rich	Male	LDI (I$ per capita)		X			X	
Urinary tract infections and interstitial nephritis	Global	Male	Healthcare access and quality index		X			X	
Urinary tract infections and interstitial nephritis	Data Rich	Male	Healthcare access and quality index		X			X	
Urinary tract infections and interstitial nephritis	Global	Male	Sanitation (proportion with access)	X			X		
Urinary tract infections and interstitial nephritis	Data Rich	Male	Sanitation (proportion with access)	X			X		
Urolithiasis	Global	Female	Education (years per capita)			X			X
Urolithiasis	Data Rich	Female	Education (years per capita)			X			X
Urolithiasis	Global	Female	LDI (I$ per capita)			X			X
Urolithiasis	Data Rich	Female	LDI (I$ per capita)			X			X
Urolithiasis	Global	Female	Socio-demographic Index			X			X
Urolithiasis	Data Rich	Female	Socio-demographic Index			X			X
Urolithiasis	Global	Female	Healthcare access and quality index		X			X	
Urolithiasis	Data Rich	Female	Healthcare access and quality index		X			X	
Urolithiasis	Global	Female	vegetables unadjusted(g)		X			X	
Urolithiasis	Data Rich	Female	vegetables unadjusted(g)		X			X	
Urolithiasis	Global	Female	fruits unadjusted(g)		X			X	
Urolithiasis	Data Rich	Female	fruits unadjusted(g)		X			X	
Urolithiasis	Global	Female	red meats unadjusted(g)	X			X		
Urolithiasis	Data Rich	Female	red meats unadjusted(g)	X			X		
Urolithiasis	Global	Female	90th percentile climatic temperature in the given country-year.	X			X		
Urolithiasis	Data Rich	Female	90th percentile climatic temperature in the given country-year.	X			X		
Urolithiasis	Data Rich	Male	Education (years per capita)			X			X
Urolithiasis	Global	Male	Education (years per capita)			X			X
Urolithiasis	Data Rich	Male	LDI (I$ per capita)			X			X
Urolithiasis	Global	Male	LDI (I$ per capita)			X			X
Urolithiasis	Data Rich	Male	Socio-demographic Index			X			X
Urolithiasis	Global	Male	Socio-demographic Index			X			X
Urolithiasis	Data Rich	Male	Healthcare access and quality index		X			X	
Urolithiasis	Global	Male	Healthcare access and quality index		X			X	
Urolithiasis	Data Rich	Male	vegetables unadjusted(g)		X			X	
Urolithiasis	Data Rich	Male	fruits unadjusted(g)		X			X	
Urolithiasis	Data Rich	Male	red meats unadjusted(g)	X			X		
Urolithiasis	Data Rich	Male	90th percentile climatic temperature in the given country-year.	X			X		
Urolithiasis	Global	Male	90th percentile climatic temperature in the given country-year.	X			X		
Urolithiasis	Global	Male	vegetables unadjusted(g)					X	
Urolithiasis	Global	Male	fruits unadjusted(g)					X	
Urolithiasis	Global	Male	red meats unadjusted(g)				X		
Other urinary diseases	Global	Female	Socio-demographic Index			X			X
Other urinary diseases	Data Rich	Female	Socio-demographic Index			X			X
Other urinary diseases	Global	Female	Education (years per capita)		X			X	
Other urinary diseases	Data Rich	Female	Education (years per capita)		X			X	
Other urinary diseases	Global	Female	LDI (I$ per capita)		X			X	
Other urinary diseases	Data Rich	Female	LDI (I$ per capita)		X			X	
Other urinary diseases	Global	Female	Healthcare access and quality index		X			X	
Other urinary diseases	Data Rich	Female	Healthcare access and quality index		X			X	
Other urinary diseases	Global	Female	Mean BMI	X			X		
Other urinary diseases	Data Rich	Female	Mean BMI	X			X		
Other urinary diseases	Global	Male	Socio-demographic Index			X			X
Other urinary diseases	Data Rich	Male	Socio-demographic Index			X			X
Other urinary diseases	Global	Male	Education (years per capita)		X			X	

Appendix Table S11: Comparison of GBD 2021 and GBD 2023 covariates and level of covariates used in cause of death modeling

Cause	Model Version Type	Sex	Covariate	Level 1 GBD 2021	Level 2 GBD 2021	Level 3 GBD 2021	Level 1 GBD 2023	Level 2 GBD 2023	Level 3 GBD 2023
Other urinary diseases	Data Rich	Male	Education (years per capita)		X			X	
Other urinary diseases	Global	Male	LDI (I$ per capita)		X			X	
Other urinary diseases	Data Rich	Male	LDI (I$ per capita)		X			X	
Other urinary diseases	Global	Male	Healthcare access and quality index		X			X	
Other urinary diseases	Data Rich	Male	Healthcare access and quality index		X			X	
Other urinary diseases	Global	Male	Mean BMI	X			X		
Other urinary diseases	Data Rich	Male	Mean BMI	X			X		
Gynecological diseases	Global	Female	Education (years per capita)			X			X
Gynecological diseases	Data Rich	Female	Education (years per capita)			X			X
Gynecological diseases	Global	Female	LDI (I$ per capita)			X			X
Gynecological diseases	Data Rich	Female	LDI (I$ per capita)			X			X
Gynecological diseases	Global	Female	Socio-demographic Index			X			X
Gynecological diseases	Data Rich	Female	Socio-demographic Index			X			X
Gynecological diseases	Global	Female	Maternal care and immunization		X			X	
Gynecological diseases	Data Rich	Female	Maternal care and immunization		X			X	
Gynecological diseases	Global	Female	Healthcare access and quality index		X			X	
Gynecological diseases	Data Rich	Female	Healthcare access and quality index		X			X	
Gynecological diseases	Global	Female	Live Births 35+ (proportion)		X			X	
Gynecological diseases	Data Rich	Female	Live Births 35+ (proportion)		X			X	
Gynecological diseases	Global	Female	Skilled Birth Attendance (proportion)		X			X	
Gynecological diseases	Data Rich	Female	Skilled Birth Attendance (proportion)		X			X	
Gynecological diseases	Global	Female	Total Fertility Rate		X			X	
Gynecological diseases	Data Rich	Female	Total Fertility Rate		X			X	
Gynecological diseases	Global	Female	Age- and sex-specific SEV for Smoking	X			X		
Gynecological diseases	Data Rich	Female	Age- and sex-specific SEV for Smoking	X			X		
Uterine fibroids	Data Rich	Female	Education (years per capita)			X			X
Uterine fibroids	Global	Female	Education (years per capita)			X			X
Uterine fibroids	Data Rich	Female	LDI (I$ per capita)			X			X
Uterine fibroids	Global	Female	LDI (I$ per capita)			X			X
Uterine fibroids	Data Rich	Female	Socio-demographic Index			X			X
Uterine fibroids	Global	Female	Socio-demographic Index			X			X
Uterine fibroids	Data Rich	Female	Maternal care and immunization		X			X	
Uterine fibroids	Global	Female	Maternal care and immunization		X			X	
Uterine fibroids	Data Rich	Female	Healthcare access and quality index		X			X	
Uterine fibroids	Global	Female	Healthcare access and quality index		X			X	
Uterine fibroids	Data Rich	Female	Skilled Birth Attendance (proportion)		X			X	
Uterine fibroids	Global	Female	Skilled Birth Attendance (proportion)		X			X	
Uterine fibroids	Data Rich	Female	Total Fertility Rate		X			X	
Uterine fibroids	Global	Female	Total Fertility Rate		X			X	
Uterine fibroids	Data Rich	Female	Live Births 35+ (proportion)		X			X	
Uterine fibroids	Global	Female	Live Births 35+ (proportion)		X			X	
Uterine fibroids	Data Rich	Female	Age- and sex-specific SEV for Smoking	X			X		
Uterine fibroids	Global	Female	Age- and sex-specific SEV for Smoking	X			X		
Endometriosis	Global	Female	Education (years per capita)			X			X
Endometriosis	Data Rich	Female	Education (years per capita)			X			X
Endometriosis	Global	Female	LDI (I$ per capita)			X			X
Endometriosis	Data Rich	Female	LDI (I$ per capita)			X			X
Endometriosis	Global	Female	Socio-demographic Index			X			X
Endometriosis	Data Rich	Female	Socio-demographic Index			X			X
Endometriosis	Global	Female	Maternal care and immunization		X			X	
Endometriosis	Data Rich	Female	Maternal care and immunization		X			X	
Endometriosis	Global	Female	Healthcare access and quality index		X			X	
Endometriosis	Data Rich	Female	Healthcare access and quality index		X			X	
Endometriosis	Global	Female	Live Births 35+ (proportion)		X			X	
Endometriosis	Data Rich	Female	Live Births 35+ (proportion)		X			X	
Endometriosis	Global	Female	Skilled Birth Attendance (proportion)		X			X	
Endometriosis	Data Rich	Female	Skilled Birth Attendance (proportion)		X			X	
Endometriosis	Global	Female	Total Fertility Rate		X			X	
Endometriosis	Data Rich	Female	Total Fertility Rate		X			X	
Endometriosis	Global	Female	Age- and sex-specific SEV for Smoking	X			X		
Endometriosis	Data Rich	Female	Age- and sex-specific SEV for Smoking	X			X		
Genital prolapse	Data Rich	Female	Education (years per capita)			X			X
Genital prolapse	Global	Female	Education (years per capita)			X			X
Genital prolapse	Data Rich	Female	LDI (I$ per capita)			X			X
Genital prolapse	Global	Female	LDI (I$ per capita)			X			X
Genital prolapse	Data Rich	Female	Socio-demographic Index			X			X
Genital prolapse	Global	Female	Socio-demographic Index			X			X
Genital prolapse	Data Rich	Female	Maternal care and immunization		X			X	
Genital prolapse	Global	Female	Maternal care and immunization		X			X	
Genital prolapse	Data Rich	Female	Healthcare access and quality index		X			X	
Genital prolapse	Global	Female	Healthcare access and quality index		X			X	
Genital prolapse	Data Rich	Female	Skilled Birth Attendance (proportion)		X			X	
Genital prolapse	Global	Female	Skilled Birth Attendance (proportion)		X			X	
Genital prolapse	Data Rich	Female	Total Fertility Rate		X			X	
Genital prolapse	Global	Female	Total Fertility Rate		X			X	
Genital prolapse	Data Rich	Female	Live Births 35+ (proportion)		X			X	
Genital prolapse	Global	Female	Live Births 35+ (proportion)		X			X	
Genital prolapse	Data Rich	Female	Age- and sex-specific SEV for Smoking	X			X		
Genital prolapse	Global	Female	Age- and sex-specific SEV for Smoking	X			X		
Genital prolapse	Data Rich	Female	Age-specific cohort cumulative fertility (CCF)					X	
Genital prolapse	Global	Female	Age-specific cohort cumulative fertility (CCF)					X	
Other gynecological diseases	Data Rich	Female	Education (years per capita)			X			X
Other gynecological diseases	Global	Female	Education (years per capita)			X			X
Other gynecological diseases	Data Rich	Female	LDI (I$ per capita)			X			X
Other gynecological diseases	Global	Female	LDI (I$ per capita)			X			X
Other gynecological diseases	Data Rich	Female	Socio-demographic Index			X			X
Other gynecological diseases	Global	Female	Socio-demographic Index			X			X
Other gynecological diseases	Data Rich	Female	Maternal care and immunization		X			X	
Other gynecological diseases	Global	Female	Maternal care and immunization		X			X	
Other gynecological diseases	Data Rich	Female	Healthcare access and quality index		X			X	
Other gynecological diseases	Global	Female	Healthcare access and quality index		X			X	
Other gynecological diseases	Data Rich	Female	Skilled Birth Attendance (proportion)		X			X	
Other gynecological diseases	Global	Female	Skilled Birth Attendance (proportion)		X			X	
Other gynecological diseases	Data Rich	Female	Total Fertility Rate		X			X	
Other gynecological diseases	Global	Female	Total Fertility Rate		X			X	
Other gynecological diseases	Data Rich	Female	Live Births 35+ (proportion)		X			X	
Other gynecological diseases	Global	Female	Live Births 35+ (proportion)		X			X	
Other gynecological diseases	Data Rich	Female	Age- and sex-specific SEV for Smoking	X			X		
Other gynecological diseases	Global	Female	Age- and sex-specific SEV for Smoking	X			X		
Hemoglobinopathies and hemolytic anaemias	Global	Female	Education (years per capita)			X			X
Hemoglobinopathies and hemolytic anaemias	Data Rich	Female	Education (years per capita)			X			X
Hemoglobinopathies and hemolytic anaemias	Global	Female	LDI (I$ per capita)			X			X
Hemoglobinopathies and hemolytic anaemias	Data Rich	Female	LDI (I$ per capita)			X			X
Hemoglobinopathies and hemolytic anaemias	Global	Female	Socio-demographic Index			X			X
Hemoglobinopathies and hemolytic anaemias	Data Rich	Female	Socio-demographic Index			X			X
Hemoglobinopathies and hemolytic anaemias	Global	Female	Latitude Under 15 (proportion)			X			X
Hemoglobinopathies and hemolytic anaemias	Data Rich	Female	Latitude Under 15 (proportion)			X			X
Hemoglobinopathies and hemolytic anaemias	Global	Female	Latitude 15 to 30 (proportion)			X			X
Hemoglobinopathies and hemolytic anaemias	Data Rich	Female	Latitude 15 to 30 (proportion)			X			X
Hemoglobinopathies and hemolytic anaemias	Global	Female	Latitude 30 to 45 (proportion)			X			X
Hemoglobinopathies and hemolytic anaemias	Data Rich	Female	Latitude 30 to 45 (proportion)			X			X
Hemoglobinopathies and hemolytic anaemias	Global	Female	Latitude Over 45 (proportion)			X			X
Hemoglobinopathies and hemolytic anaemias	Data Rich	Female	Latitude Over 45 (proportion)			X			X
Hemoglobinopathies and hemolytic anaemias	Global	Female	Maternal care and immunization		X			X	
Hemoglobinopathies and hemolytic anaemias	Data Rich	Female	Maternal care and immunization		X			X	
Hemoglobinopathies and hemolytic anaemias	Global	Female	Healthcare access and quality index		X			X	

Appendix Table S11: Comparison of GBD 2021 and GBD 2023 covariates and level of covariates used in cause of death modeling

Cause	Model Version Type	Sex	Covariate	Level 1 GBD 2021	Level 2 GBD 2021	Level 3 GBD 2021	Level 1 GBD 2023	Level 2 GBD 2023	Level 3 GBD 2023
Hemoglobinopathies and hemolytic anaemias	Data Rich	Female	Healthcare access and quality index		X			X	
Hemoglobinopathies and hemolytic anaemias	Global	Female	Malaria Lysenko PFPR 1 (Holoendemic)	X			X		
Hemoglobinopathies and hemolytic anaemias	Data Rich	Female	Malaria Lysenko PFPR 1 (Holoendemic)	X			X		
Hemoglobinopathies and hemolytic anaemias	Data Rich	Female	Hemoglobinopathies Prevalence x Excess Mortality	X			X		
Hemoglobinopathies and hemolytic anaemias	Data Rich	Female	Hemoglobinopathies Prevalence x Excess Mortality (excluding G6PD deficiency)	X			X		
Hemoglobinopathies and hemolytic anaemias	Global	Female	Hemoglobinopathies Prevalence x Excess Mortality				X		
Hemoglobinopathies and hemolytic anaemias	Global	Female	Hemoglobinopathies Prevalence x Excess Mortality (excluding G6PD deficiency)				X		
Hemoglobinopathies and hemolytic anaemias	Global	Female	Probability of Homozygous sickle cell disease at birth (SS)				X		
Hemoglobinopathies and hemolytic anaemias	Data Rich	Female	Probability of Homozygous sickle cell disease at birth (SS)				X		
Hemoglobinopathies and hemolytic anaemias	Global	Female	Probability of Hemoglobin SC disease at birth				X		
Hemoglobinopathies and hemolytic anaemias	Data Rich	Female	Probability of Hemoglobin SC disease at birth				X		
Hemoglobinopathies and hemolytic anaemias	Global	Female	Probability of Homozygous sickle cell disease (SS) and G6PD deficiency at birth				X		
Hemoglobinopathies and hemolytic anaemias	Data Rich	Female	Probability of Homozygous sickle cell disease (SS) and G6PD deficiency at birth				X		
Hemoglobinopathies and hemolytic anaemias	Global	Female	Probability of Hemoglobin SC disease (SC) and G6PD deficiency at birth				X		
Hemoglobinopathies and hemolytic anaemias	Data Rich	Female	Probability of Hemoglobin SC disease (SC) and G6PD deficiency at birth				X		
Hemoglobinopathies and hemolytic anaemias	Global	Female	Probability of G6PD deficiency at birth				X		
Hemoglobinopathies and hemolytic anaemias	Data Rich	Female	Probability of G6PD deficiency at birth				X		
Hemoglobinopathies and hemolytic anaemias	Data Rich	Male	Education (years per capita)			X			X
Hemoglobinopathies and hemolytic anaemias	Global	Male	Education (years per capita)			X			X
Hemoglobinopathies and hemolytic anaemias	Data Rich	Male	LDI (I$ per capita)			X			X
Hemoglobinopathies and hemolytic anaemias	Global	Male	LDI (I$ per capita)			X			X
Hemoglobinopathies and hemolytic anaemias	Data Rich	Male	Socio-demographic Index			X			X
Hemoglobinopathies and hemolytic anaemias	Global	Male	Socio-demographic Index			X			X
Hemoglobinopathies and hemolytic anaemias	Data Rich	Male	Latitude Under 15 (proportion)			X			X
Hemoglobinopathies and hemolytic anaemias	Global	Male	Latitude Under 15 (proportion)			X			X
Hemoglobinopathies and hemolytic anaemias	Data Rich	Male	Latitude 15 to 30 (proportion)			X			X
Hemoglobinopathies and hemolytic anaemias	Global	Male	Latitude 15 to 30 (proportion)			X			X
Hemoglobinopathies and hemolytic anaemias	Data Rich	Male	Latitude 30 to 45 (proportion)			X			X
Hemoglobinopathies and hemolytic anaemias	Global	Male	Latitude 30 to 45 (proportion)			X			X
Hemoglobinopathies and hemolytic anaemias	Data Rich	Male	Latitude Over 45 (proportion)			X			X
Hemoglobinopathies and hemolytic anaemias	Global	Male	Latitude Over 45 (proportion)			X			X
Hemoglobinopathies and hemolytic anaemias	Data Rich	Male	Maternal care and immunization		X			X	
Hemoglobinopathies and hemolytic anaemias	Global	Male	Maternal care and immunization		X			X	
Hemoglobinopathies and hemolytic anaemias	Data Rich	Male	Healthcare access and quality index		X			X	
Hemoglobinopathies and hemolytic anaemias	Global	Male	Healthcare access and quality index		X			X	
Hemoglobinopathies and hemolytic anaemias	Data Rich	Male	Malaria Lysenko PFPR 1 (Holoendemic)	X			X		
Hemoglobinopathies and hemolytic anaemias	Global	Male	Malaria Lysenko PFPR 1 (Holoendemic)	X			X		
Hemoglobinopathies and hemolytic anaemias	Data Rich	Male	Hemoglobinopathies Prevalence x Excess Mortality	X			X		
Hemoglobinopathies and hemolytic anaemias	Data Rich	Male	Hemoglobinopathies Prevalence x Excess Mortality (excluding G6PD deficiency)	X			X		
Hemoglobinopathies and hemolytic anaemias	Global	Male	Hemoglobinopathies Prevalence x Excess Mortality				X		
Hemoglobinopathies and hemolytic anaemias	Global	Male	Hemoglobinopathies Prevalence x Excess Mortality (excluding G6PD deficiency)				X		
Hemoglobinopathies and hemolytic anaemias	Data Rich	Male	Probability of Homozygous sickle cell disease at birth (SS)				X		
Hemoglobinopathies and hemolytic anaemias	Global	Male	Probability of Homozygous sickle cell disease at birth (SS)				X		
Hemoglobinopathies and hemolytic anaemias	Data Rich	Male	Probability of Hemoglobin SC disease at birth				X		
Hemoglobinopathies and hemolytic anaemias	Global	Male	Probability of Hemoglobin SC disease at birth				X		
Hemoglobinopathies and hemolytic anaemias	Data Rich	Male	Probability of Homozygous sickle cell disease (SS) and G6PD deficiency at birth				X		
Hemoglobinopathies and hemolytic anaemias	Global	Male	Probability of Homozygous sickle cell disease (SS) and G6PD deficiency at birth				X		
Hemoglobinopathies and hemolytic anaemias	Data Rich	Male	Probability of Hemoglobin SC disease (SC) and G6PD deficiency at birth				X		
Hemoglobinopathies and hemolytic anaemias	Global	Male	Probability of Hemoglobin SC disease (SC) and G6PD deficiency at birth				X		
Hemoglobinopathies and hemolytic anaemias	Data Rich	Male	Probability of G6PD deficiency at birth				X		
Hemoglobinopathies and hemolytic anaemias	Global	Male	Probability of G6PD deficiency at birth				X		
Endocrine, metabolic, blood, and immune disorders	Global	Female	Education (years per capita)			X			X
Endocrine, metabolic, blood, and immune disorders	Data Rich	Female	Education (years per capita)			X			X
Endocrine, metabolic, blood, and immune disorders	Global	Female	LDI (I$ per capita)			X			X
Endocrine, metabolic, blood, and immune disorders	Data Rich	Female	LDI (I$ per capita)			X			X
Endocrine, metabolic, blood, and immune disorders	Global	Female	Socio-demographic Index			X			X
Endocrine, metabolic, blood, and immune disorders	Data Rich	Female	Socio-demographic Index			X			X
Endocrine, metabolic, blood, and immune disorders	Global	Female	Low-Density Lipoprotein (mmol/L)		X			X	
Endocrine, metabolic, blood, and immune disorders	Global	Female	Healthcare access and quality index		X			X	
Endocrine, metabolic, blood, and immune disorders	Data Rich	Female	Healthcare access and quality index		X			X	
Endocrine, metabolic, blood, and immune disorders	Global	Female	Liters of alcohol consumed per capita		X			X	
Endocrine, metabolic, blood, and immune disorders	Data Rich	Female	Low-Density Lipoprotein (mmol/L)		X		X		
Endocrine, metabolic, blood, and immune disorders	Data Rich	Female	Liters of alcohol consumed per capita		X		X		
Endocrine, metabolic, blood, and immune disorders	Global	Female	Mean BMI	X			X		
Endocrine, metabolic, blood, and immune disorders	Data Rich	Female	Mean BMI	X			X		
Endocrine, metabolic, blood, and immune disorders	Global	Male	Education (years per capita)			X			X
Endocrine, metabolic, blood, and immune disorders	Data Rich	Male	Education (years per capita)			X			X
Endocrine, metabolic, blood, and immune disorders	Global	Male	LDI (I$ per capita)			X			X
Endocrine, metabolic, blood, and immune disorders	Data Rich	Male	LDI (I$ per capita)			X			X
Endocrine, metabolic, blood, and immune disorders	Global	Male	Socio-demographic Index			X			X
Endocrine, metabolic, blood, and immune disorders	Data Rich	Male	Socio-demographic Index			X			X
Endocrine, metabolic, blood, and immune disorders	Global	Male	Low-Density Lipoprotein (mmol/L)		X			X	
Endocrine, metabolic, blood, and immune disorders	Data Rich	Male	Low-Density Lipoprotein (mmol/L)		X			X	
Endocrine, metabolic, blood, and immune disorders	Global	Male	Healthcare access and quality index		X			X	
Endocrine, metabolic, blood, and immune disorders	Data Rich	Male	Healthcare access and quality index		X			X	
Endocrine, metabolic, blood, and immune disorders	Global	Male	Liters of alcohol consumed per capita		X			X	
Endocrine, metabolic, blood, and immune disorders	Data Rich	Male	Liters of alcohol consumed per capita		X			X	
Endocrine, metabolic, blood, and immune disorders	Global	Male	Mean BMI	X			X		
Endocrine, metabolic, blood, and immune disorders	Data Rich	Male	Mean BMI	X			X		
Thyroid diseases	Data Rich	Female	Cumulative Cigarettes (10 Years)						X
Thyroid diseases	Global	Female	Cumulative Cigarettes (10 Years)						X
Thyroid diseases	Data Rich	Female	Education (years per capita)						X
Thyroid diseases	Global	Female	Education (years per capita)						X
Thyroid diseases	Data Rich	Female	LDI (I$ per capita)						X
Thyroid diseases	Global	Female	LDI (I$ per capita)						X
Thyroid diseases	Data Rich	Female	Smoking Prevalence						X
Thyroid diseases	Global	Female	Smoking Prevalence						X

Appendix Table S11: Comparison of GBD 2021 and GBD 2023 covariates and level of covariates used in cause of death modeling

Cause	Model Version Type	Sex	Covariate	Level 1 GBD 2021	Level 2 GBD 2021	Level 3 GBD 2021	Level 1 GBD 2023	Level 2 GBD 2023	Level 3 GBD 2023
Thyroid diseases	Data Rich	Female	Socio-demographic Index						X
Thyroid diseases	Global	Female	Socio-demographic Index						X
Thyroid diseases	Data Rich	Female	Mean BMI						X
Thyroid diseases	Global	Female	Mean BMI						X
Thyroid diseases	Data Rich	Female	Cumulative Cigarettes (5 Years)						X
Thyroid diseases	Global	Female	Cumulative Cigarettes (5 Years)						X
Thyroid diseases	Data Rich	Female	Diabetes Age-Standardized Prevalence (proportion)					X	
Thyroid diseases	Global	Female	Diabetes Age-Standardized Prevalence (proportion)					X	
Thyroid diseases	Data Rich	Female	Healthcare access and quality index				X		
Thyroid diseases	Global	Female	Healthcare access and quality index				X		
Thyroid diseases	Data Rich	Female	Absolute value of average latitude				X		
Thyroid diseases	Global	Female	Absolute value of average latitude				X		
Thyroid diseases	Data Rich	Male	Cumulative Cigarettes (10 Years)						X
Thyroid diseases	Global	Male	Cumulative Cigarettes (10 Years)						X
Thyroid diseases	Data Rich	Male	Education (years per capita)						X
Thyroid diseases	Global	Male	Education (years per capita)						X
Thyroid diseases	Data Rich	Male	LDI (I$ per capita)						X
Thyroid diseases	Global	Male	LDI (I$ per capita)						X
Thyroid diseases	Data Rich	Male	Smoking Prevalence						X
Thyroid diseases	Global	Male	Smoking Prevalence						X
Thyroid diseases	Data Rich	Male	Socio-demographic Index						X
Thyroid diseases	Global	Male	Socio-demographic Index						X
Thyroid diseases	Data Rich	Male	Mean BMI						X
Thyroid diseases	Global	Male	Mean BMI						X
Thyroid diseases	Data Rich	Male	Cumulative Cigarettes (5 Years)						X
Thyroid diseases	Global	Male	Cumulative Cigarettes (5 Years)						X
Thyroid diseases	Data Rich	Male	Healthcare access and quality index					X	
Thyroid diseases	Global	Male	Diabetes Age-Standardized Prevalence (proportion)					X	
Thyroid diseases	Global	Male	Healthcare access and quality index				X		
Thyroid diseases	Data Rich	Male	Absolute value of average latitude				X		
Thyroid diseases	Global	Male	Absolute value of average latitude				X		
Thyroid diseases	Data Rich	Male	Diabetes Age-Standardized Prevalence (proportion)				X		
Other endocrine, metabolic, blood, and immune disorders	Data Rich	Female	Education (years per capita)						X
Other endocrine, metabolic, blood, and immune disorders	Global	Female	Education (years per capita)						X
Other endocrine, metabolic, blood, and immune disorders	Data Rich	Female	LDI (I$ per capita)						X
Other endocrine, metabolic, blood, and immune disorders	Global	Female	LDI (I$ per capita)						X
Other endocrine, metabolic, blood, and immune disorders	Data Rich	Female	Socio-demographic Index						X
Other endocrine, metabolic, blood, and immune disorders	Global	Female	Socio-demographic Index						X
Other endocrine, metabolic, blood, and immune disorders	Data Rich	Female	Low-Density Lipoprotein (mmol/L)					X	
Other endocrine, metabolic, blood, and immune disorders	Global	Female	Low-Density Lipoprotein (mmol/L)					X	
Other endocrine, metabolic, blood, and immune disorders	Data Rich	Female	Healthcare access and quality index					X	
Other endocrine, metabolic, blood, and immune disorders	Global	Female	Healthcare access and quality index					X	
Other endocrine, metabolic, blood, and immune disorders	Data Rich	Female	Liters of alcohol consumed per capita					X	
Other endocrine, metabolic, blood, and immune disorders	Global	Female	Liters of alcohol consumed per capita					X	
Other endocrine, metabolic, blood, and immune disorders	Data Rich	Female	Mean BMI				X		
Other endocrine, metabolic, blood, and immune disorders	Global	Female	Mean BMI				X		
Other endocrine, metabolic, blood, and immune disorders	Data Rich	Male	Education (years per capita)						X
Other endocrine, metabolic, blood, and immune disorders	Global	Male	Education (years per capita)						X
Other endocrine, metabolic, blood, and immune disorders	Data Rich	Male	LDI (I$ per capita)						X
Other endocrine, metabolic, blood, and immune disorders	Global	Male	LDI (I$ per capita)						X
Other endocrine, metabolic, blood, and immune disorders	Data Rich	Male	Socio-demographic Index						X
Other endocrine, metabolic, blood, and immune disorders	Global	Male	Socio-demographic Index						X
Other endocrine, metabolic, blood, and immune disorders	Data Rich	Male	Low-Density Lipoprotein (mmol/L)					X	
Other endocrine, metabolic, blood, and immune disorders	Global	Male	Low-Density Lipoprotein (mmol/L)					X	
Other endocrine, metabolic, blood, and immune disorders	Data Rich	Male	Healthcare access and quality index					X	
Other endocrine, metabolic, blood, and immune disorders	Global	Male	Healthcare access and quality index					X	
Other endocrine, metabolic, blood, and immune disorders	Data Rich	Male	Liters of alcohol consumed per capita					X	
Other endocrine, metabolic, blood, and immune disorders	Global	Male	Liters of alcohol consumed per capita					X	
Other endocrine, metabolic, blood, and immune disorders	Data Rich	Male	Mean BMI				X		
Other endocrine, metabolic, blood, and immune disorders	Global	Male	Mean BMI				X		
Sudden infant death syndrome	Data Rich	Female	Education (years per capita)			X			X
Sudden infant death syndrome	Global	Female	Education (years per capita)			X			X
Sudden infant death syndrome	Data Rich	Female	LDI (I$ per capita)			X			X
Sudden infant death syndrome	Global	Female	LDI (I$ per capita)			X			X
Sudden infant death syndrome	Data Rich	Female	Socio-demographic Index			X			X
Sudden infant death syndrome	Global	Female	Socio-demographic Index			X			X
Sudden infant death syndrome	Data Rich	Female	Total Fertility Rate			X			X
Sudden infant death syndrome	Global	Female	Total Fertility Rate			X			X
Sudden infant death syndrome	Data Rich	Female	Maternal care and immunization		X			X	
Sudden infant death syndrome	Global	Female	Maternal care and immunization		X			X	
Sudden infant death syndrome	Data Rich	Female	Healthcare access and quality index		X			X	
Sudden infant death syndrome	Global	Female	Healthcare access and quality index		X			X	
Sudden infant death syndrome	Data Rich	Female	Skilled Birth Attendance (proportion)		X			X	
Sudden infant death syndrome	Global	Female	Skilled Birth Attendance (proportion)		X			X	
Sudden infant death syndrome	Data Rich	Female	In-Facility Delivery (proportion)	X			X		
Sudden infant death syndrome	Global	Female	In-Facility Delivery (proportion)	X			X		
Sudden infant death syndrome	Data Rich	Male	Education (years per capita)			X			X
Sudden infant death syndrome	Global	Male	Education (years per capita)			X			X
Sudden infant death syndrome	Data Rich	Male	LDI (I$ per capita)			X			X
Sudden infant death syndrome	Global	Male	LDI (I$ per capita)			X			X
Sudden infant death syndrome	Data Rich	Male	Socio-demographic Index			X			X
Sudden infant death syndrome	Global	Male	Socio-demographic Index			X			X
Sudden infant death syndrome	Data Rich	Male	Total Fertility Rate			X			X
Sudden infant death syndrome	Global	Male	Total Fertility Rate			X			X
Sudden infant death syndrome	Data Rich	Male	Maternal care and immunization		X			X	
Sudden infant death syndrome	Global	Male	Maternal care and immunization		X			X	
Sudden infant death syndrome	Data Rich	Male	Healthcare access and quality index		X			X	
Sudden infant death syndrome	Global	Male	Healthcare access and quality index		X			X	
Sudden infant death syndrome	Data Rich	Male	Skilled Birth Attendance (proportion)		X			X	
Sudden infant death syndrome	Global	Male	Skilled Birth Attendance (proportion)		X			X	
Sudden infant death syndrome	Data Rich	Male	In-Facility Delivery (proportion)	X			X		
Sudden infant death syndrome	Global	Male	In-Facility Delivery (proportion)	X			X		
Transport injuries	Data Rich	Female	Rainfall Quintile 5 (proportion)			X			X
Transport injuries	Global	Female	Rainfall Quintile 5 (proportion)			X			X
Transport injuries	Data Rich	Female	Socio-demographic Index		X			X	
Transport injuries	Global	Female	Socio-demographic Index		X			X	
Transport injuries	Data Rich	Female	Healthcare access and quality index		X			X	
Transport injuries	Global	Female	Healthcare access and quality index		X			X	
Transport injuries	Data Rich	Female	Population Density (500-1000 ppl/sqkm, proportion)		X			X	
Transport injuries	Global	Female	Population Density (500-1000 ppl/sqkm, proportion)		X			X	
Transport injuries	Data Rich	Female	Population-weighted mean temperature		X			X	
Transport injuries	Global	Female	Population-weighted mean temperature		X			X	
Transport injuries	Data Rich	Female	Population Density (300-500 ppl/sqkm, proportion)		X			X	
Transport injuries	Global	Female	Population Density (300-500 ppl/sqkm, proportion)		X			X	
Transport injuries	Data Rich	Female	Population 15 to 30 (proportion)		X			X	
Transport injuries	Global	Female	Population 15 to 30 (proportion)		X			X	
Transport injuries	Data Rich	Female	BAC law general population (quartile)	X					X
Transport injuries	Global	Female	BAC law general population (quartile)	X					X
Transport injuries	Data Rich	Female	BAC law youth drivers (quartile)	X					X
Transport injuries	Global	Female	BAC law youth drivers (quartile)	X					X
Transport injuries	Data Rich	Female	BAC law professional drivers (quartile)	X					X
Transport injuries	Global	Female	BAC law professional drivers (quartile)	X					X
Transport injuries	Data Rich	Female	Speed limit law urban (quartile)	X					X

Appendix Table S11: Comparison of GBD 2021 and GBD 2023 covariates and level of covariates used in cause of death modeling

Cause	Model Version Type	Sex	Covariate	Level 1 GBD 2021	Level 2 GBD 2021	Level 3 GBD 2021	Level 1 GBD 2023	Level 2 GBD 2023	Level 3 GBD 2023
Transport injuries	Global	Female	Speed limit law urban (quartile)	X					X
Transport injuries	Data Rich	Female	Speed limit law rural (quartile)	X					X
Transport injuries	Global	Female	Speed limit law rural (quartile)	X					X
Transport injuries	Data Rich	Female	Liters of alcohol consumed per capita	X				X	
Transport injuries	Global	Female	Liters of alcohol consumed per capita	X				X	
Transport injuries	Data Rich	Female	Vehicles - 2+4 wheels (per capita)	X			X		
Transport injuries	Global	Female	Vehicles - 2+4 wheels (per capita)	X			X		
Transport injuries	Data Rich	Female	Vehicles - 2 wheels fraction (proportion)	X			X		
Transport injuries	Global	Female	Vehicles - 2 wheels fraction (proportion)	X			X		
Transport injuries	Data Rich	Male	Rainfall Quintile 5 (proportion)			X			X
Transport injuries	Global	Male	Rainfall Quintile 5 (proportion)			X			X
Transport injuries	Data Rich	Male	Socio-demographic Index		X			X	
Transport injuries	Global	Male	Socio-demographic Index		X			X	
Transport injuries	Data Rich	Male	Healthcare access and quality index		X			X	
Transport injuries	Global	Male	Healthcare access and quality index		X			X	
Transport injuries	Data Rich	Male	Population Density (500-1000 ppl/sqkm, proportion)		X			X	
Transport injuries	Global	Male	Population Density (500-1000 ppl/sqkm, proportion)		X			X	
Transport injuries	Data Rich	Male	Population-weighted mean temperature		X			X	
Transport injuries	Global	Male	Population-weighted mean temperature		X			X	
Transport injuries	Data Rich	Male	Population Density (300-500 ppl/sqkm, proportion)		X			X	
Transport injuries	Global	Male	Population Density (300-500 ppl/sqkm, proportion)		X			X	
Transport injuries	Data Rich	Male	Population 15 to 30 (proportion)		X			X	
Transport injuries	Global	Male	Population 15 to 30 (proportion)		X			X	
Transport injuries	Data Rich	Male	BAC law general population (quartile)	X					X
Transport injuries	Global	Male	BAC law general population (quartile)	X					X
Transport injuries	Data Rich	Male	BAC law youth drivers (quartile)	X					X
Transport injuries	Global	Male	BAC law youth drivers (quartile)	X					X
Transport injuries	Data Rich	Male	BAC law professional drivers (quartile)	X					X
Transport injuries	Global	Male	BAC law professional drivers (quartile)	X					X
Transport injuries	Data Rich	Male	Speed limit law urban (quartile)	X					X
Transport injuries	Global	Male	Speed limit law urban (quartile)	X					X
Transport injuries	Data Rich	Male	Speed limit law rural (quartile)	X					X
Transport injuries	Global	Male	Speed limit law rural (quartile)	X					X
Transport injuries	Data Rich	Male	Liters of alcohol consumed per capita	X				X	
Transport injuries	Global	Male	Liters of alcohol consumed per capita	X				X	
Transport injuries	Data Rich	Male	Vehicles - 2+4 wheels (per capita)	X			X		
Transport injuries	Global	Male	Vehicles - 2+4 wheels (per capita)	X			X		
Transport injuries	Data Rich	Male	Vehicles - 2 wheels fraction (proportion)	X			X		
Transport injuries	Global	Male	Vehicles - 2 wheels fraction (proportion)	X			X		
Road injuries	Data Rich	Female	Rainfall Quintile 5 (proportion)			X			X
Road injuries	Global	Female	Rainfall Quintile 5 (proportion)			X			X
Road injuries	Global	Female	Socio-demographic Index			X		X	
Road injuries	Data Rich	Female	Socio-demographic Index		X			X	
Road injuries	Data Rich	Female	Healthcare access and quality index		X			X	
Road injuries	Global	Female	Healthcare access and quality index		X			X	
Road injuries	Data Rich	Female	Population Density (500-1000 ppl/sqkm, proportion)		X			X	
Road injuries	Global	Female	Population Density (500-1000 ppl/sqkm, proportion)		X			X	
Road injuries	Data Rich	Female	Population-weighted mean temperature		X			X	
Road injuries	Global	Female	Population-weighted mean temperature		X			X	
Road injuries	Data Rich	Female	Population Density (300-500 ppl/sqkm, proportion)		X			X	
Road injuries	Global	Female	Population Density (300-500 ppl/sqkm, proportion)		X			X	
Road injuries	Data Rich	Female	Population 15 to 30 (proportion)		X			X	
Road injuries	Global	Female	Population 15 to 30 (proportion)		X			X	
Road injuries	Data Rich	Female	BAC law general population (quartile)	X					X
Road injuries	Global	Female	BAC law general population (quartile)	X					X
Road injuries	Data Rich	Female	BAC law youth drivers (quartile)	X					X
Road injuries	Global	Female	BAC law youth drivers (quartile)	X					X
Road injuries	Data Rich	Female	BAC law professional drivers (quartile)	X					X
Road injuries	Global	Female	BAC law professional drivers (quartile)	X					X
Road injuries	Data Rich	Female	Speed limit law urban (quartile)	X					X
Road injuries	Global	Female	Speed limit law urban (quartile)	X					X
Road injuries	Data Rich	Female	Speed limit law rural (quartile)	X					X
Road injuries	Global	Female	Speed limit law rural (quartile)	X					X
Road injuries	Data Rich	Female	Liters of alcohol consumed per capita	X				X	
Road injuries	Global	Female	Liters of alcohol consumed per capita	X				X	
Road injuries	Data Rich	Female	Vehicles - 2+4 wheels (per capita)	X			X		
Road injuries	Global	Female	Vehicles - 2+4 wheels (per capita)	X			X		
Road injuries	Data Rich	Female	Vehicles - 2 wheels fraction (proportion)	X			X		
Road injuries	Global	Female	Vehicles - 2 wheels fraction (proportion)	X			X		
Road injuries	Data Rich	Female	Vehicles - 2 wheels (per capita)	X			X		
Road injuries	Global	Female	Vehicles - 2 wheels (per capita)	X			X		
Road injuries	Data Rich	Female	Vehicles - 4 wheels (per capita)	X			X		
Road injuries	Global	Female	Vehicles - 4 wheels (per capita)	X			X		
Road injuries	Data Rich	Female	Log-transformed SEV scalar: Road Inj	X			X		
Road injuries	Global	Female	Log-transformed SEV scalar: Road Inj	X			X		
Road injuries	Data Rich	Male	Rainfall Quintile 5 (proportion)			X			X
Road injuries	Global	Male	Rainfall Quintile 5 (proportion)			X			X
Road injuries	Global	Male	Socio-demographic Index			X		X	
Road injuries	Global	Male	BAC law general population (quartile)		X				X
Road injuries	Global	Male	BAC law youth drivers (quartile)		X				X
Road injuries	Global	Male	BAC law professional drivers (quartile)		X				X
Road injuries	Global	Male	Speed limit law urban (quartile)		X				X
Road injuries	Global	Male	Speed limit law rural (quartile)		X				X
Road injuries	Data Rich	Male	Socio-demographic Index		X			X	
Road injuries	Data Rich	Male	Healthcare access and quality index		X			X	
Road injuries	Global	Male	Healthcare access and quality index		X			X	
Road injuries	Data Rich	Male	Population Density (500-1000 ppl/sqkm, proportion)		X			X	
Road injuries	Global	Male	Population Density (500-1000 ppl/sqkm, proportion)		X			X	
Road injuries	Data Rich	Male	Population-weighted mean temperature		X			X	
Road injuries	Global	Male	Population-weighted mean temperature		X			X	
Road injuries	Data Rich	Male	Population Density (300-500 ppl/sqkm, proportion)		X			X	
Road injuries	Global	Male	Population Density (300-500 ppl/sqkm, proportion)		X			X	
Road injuries	Data Rich	Male	Population 15 to 30 (proportion)		X			X	
Road injuries	Global	Male	Population 15 to 30 (proportion)		X			X	
Road injuries	Data Rich	Male	BAC law general population (quartile)	X					X
Road injuries	Data Rich	Male	BAC law youth drivers (quartile)	X					X
Road injuries	Data Rich	Male	BAC law professional drivers (quartile)	X					X
Road injuries	Data Rich	Male	Speed limit law urban (quartile)	X					X
Road injuries	Data Rich	Male	Speed limit law rural (quartile)	X					X
Road injuries	Data Rich	Male	Liters of alcohol consumed per capita	X				X	
Road injuries	Global	Male	Liters of alcohol consumed per capita	X				X	
Road injuries	Data Rich	Male	Vehicles - 2+4 wheels (per capita)	X			X		
Road injuries	Global	Male	Vehicles - 2+4 wheels (per capita)	X			X		
Road injuries	Data Rich	Male	Vehicles - 2 wheels fraction (proportion)	X			X		
Road injuries	Global	Male	Vehicles - 2 wheels fraction (proportion)	X			X		
Road injuries	Data Rich	Male	Vehicles - 2 wheels (per capita)	X			X		
Road injuries	Global	Male	Vehicles - 2 wheels (per capita)	X			X		
Road injuries	Data Rich	Male	Vehicles - 4 wheels (per capita)	X			X		
Road injuries	Global	Male	Vehicles - 4 wheels (per capita)	X			X		
Road injuries	Data Rich	Male	Log-transformed SEV scalar: Road Inj	X			X		
Road injuries	Global	Male	Log-transformed SEV scalar: Road Inj	X			X		

Appendix Table S11: Comparison of GBD 2021 and GBD 2023 covariates and level of covariates used in cause of death modeling

Cause	Model Version Type	Sex	Covariate	Level 1 GBD 2021	Level 2 GBD 2021	Level 3 GBD 2021	Level 1 GBD 2023	Level 2 GBD 2023	Level 3 GBD 2023
Pedestrian road injuries	Data Rich	Female	Rainfall Quintile 5 (proportion)			X			X
Pedestrian road injuries	Global	Female	Rainfall Quintile 5 (proportion)			X			X
Pedestrian road injuries	Data Rich	Female	Socio-demographic Index		X			X	
Pedestrian road injuries	Global	Female	Socio-demographic Index		X			X	
Pedestrian road injuries	Data Rich	Female	Healthcare access and quality index		X			X	
Pedestrian road injuries	Global	Female	Healthcare access and quality index		X			X	
Pedestrian road injuries	Data Rich	Female	Population Density (500-1000 ppl/sqkm, proportion)		X			X	
Pedestrian road injuries	Global	Female	Population Density (500-1000 ppl/sqkm, proportion)		X			X	
Pedestrian road injuries	Data Rich	Female	Population-weighted mean temperature		X			X	
Pedestrian road injuries	Global	Female	Population-weighted mean temperature		X			X	
Pedestrian road injuries	Data Rich	Female	Population Density (300-500 ppl/sqkm, proportion)		X			X	
Pedestrian road injuries	Global	Female	Population Density (300-500 ppl/sqkm, proportion)		X			X	
Pedestrian road injuries	Data Rich	Female	Population 15 to 30 (proportion)		X			X	
Pedestrian road injuries	Global	Female	Population 15 to 30 (proportion)		X			X	
Pedestrian road injuries	Data Rich	Female	BAC law general population (quartile)	X					X
Pedestrian road injuries	Global	Female	BAC law general population (quartile)	X					X
Pedestrian road injuries	Data Rich	Female	BAC law youth drivers (quartile)	X					X
Pedestrian road injuries	Global	Female	BAC law youth drivers (quartile)	X					X
Pedestrian road injuries	Data Rich	Female	BAC law professional drivers (quartile)	X					X
Pedestrian road injuries	Global	Female	BAC law professional drivers (quartile)	X					X
Pedestrian road injuries	Data Rich	Female	Speed limit law urban (quartile)	X					X
Pedestrian road injuries	Global	Female	Speed limit law urban (quartile)	X					X
Pedestrian road injuries	Data Rich	Female	Speed limit law rural (quartile)	X					X
Pedestrian road injuries	Global	Female	Speed limit law rural (quartile)	X					X
Pedestrian road injuries	Data Rich	Female	Liters of alcohol consumed per capita	X				X	
Pedestrian road injuries	Global	Female	Liters of alcohol consumed per capita	X				X	
Pedestrian road injuries	Data Rich	Female	Vehicles - 2+4 wheels (per capita)	X			X		
Pedestrian road injuries	Global	Female	Vehicles - 2+4 wheels (per capita)	X			X		
Pedestrian road injuries	Data Rich	Female	Vehicles - 2 wheels fraction (proportion)	X			X		
Pedestrian road injuries	Global	Female	Vehicles - 2 wheels fraction (proportion)	X			X		
Pedestrian road injuries	Data Rich	Female	Log-transformed SEV scalar: Pedest	X			X		
Pedestrian road injuries	Global	Female	Log-transformed SEV scalar: Pedest	X			X		
Pedestrian road injuries	Data Rich	Male	Rainfall Quintile 5 (proportion)			X			X
Pedestrian road injuries	Global	Male	Rainfall Quintile 5 (proportion)			X			X
Pedestrian road injuries	Data Rich	Male	Socio-demographic Index		X			X	
Pedestrian road injuries	Global	Male	Socio-demographic Index		X			X	
Pedestrian road injuries	Data Rich	Male	Healthcare access and quality index		X			X	
Pedestrian road injuries	Global	Male	Healthcare access and quality index		X			X	
Pedestrian road injuries	Data Rich	Male	Population Density (500-1000 ppl/sqkm, proportion)		X			X	
Pedestrian road injuries	Global	Male	Population Density (500-1000 ppl/sqkm, proportion)		X			X	
Pedestrian road injuries	Global	Male	Population-weighted mean temperature		X			X	
Pedestrian road injuries	Data Rich	Male	Population Density (300-500 ppl/sqkm, proportion)		X			X	
Pedestrian road injuries	Global	Male	Population Density (300-500 ppl/sqkm, proportion)		X			X	
Pedestrian road injuries	Data Rich	Male	Population 15 to 30 (proportion)		X			X	
Pedestrian road injuries	Global	Male	Population 15 to 30 (proportion)		X			X	
Pedestrian road injuries	Data Rich	Male	BAC law general population (quartile)	X					X
Pedestrian road injuries	Global	Male	BAC law general population (quartile)	X					X
Pedestrian road injuries	Data Rich	Male	BAC law youth drivers (quartile)	X					X
Pedestrian road injuries	Global	Male	BAC law youth drivers (quartile)	X					X
Pedestrian road injuries	Data Rich	Male	BAC law professional drivers (quartile)	X					X
Pedestrian road injuries	Global	Male	BAC law professional drivers (quartile)	X					X
Pedestrian road injuries	Data Rich	Male	Speed limit law urban (quartile)	X					X
Pedestrian road injuries	Global	Male	Speed limit law urban (quartile)	X					X
Pedestrian road injuries	Data Rich	Male	Speed limit law rural (quartile)	X					X
Pedestrian road injuries	Global	Male	Speed limit law rural (quartile)	X					X
Pedestrian road injuries	Data Rich	Male	Liters of alcohol consumed per capita	X				X	
Pedestrian road injuries	Global	Male	Liters of alcohol consumed per capita	X				X	
Pedestrian road injuries	Data Rich	Male	Population-weighted mean temperature	X				X	
Pedestrian road injuries	Data Rich	Male	Vehicles - 2+4 wheels (per capita)	X			X		
Pedestrian road injuries	Global	Male	Vehicles - 2+4 wheels (per capita)	X			X		
Pedestrian road injuries	Data Rich	Male	Vehicles - 2 wheels fraction (proportion)	X			X		
Pedestrian road injuries	Global	Male	Vehicles - 2 wheels fraction (proportion)	X			X		
Pedestrian road injuries	Data Rich	Male	Log-transformed SEV scalar: Pedest	X			X		
Pedestrian road injuries	Global	Male	Log-transformed SEV scalar: Pedest	X			X		
Cyclist road injuries	Data Rich	Female	Rainfall Quintile 5 (proportion)			X			X
Cyclist road injuries	Global	Female	Rainfall Quintile 5 (proportion)			X			X
Cyclist road injuries	Data Rich	Female	Socio-demographic Index		X			X	
Cyclist road injuries	Global	Female	Socio-demographic Index		X			X	
Cyclist road injuries	Data Rich	Female	Healthcare access and quality index		X			X	
Cyclist road injuries	Global	Female	Healthcare access and quality index		X			X	
Cyclist road injuries	Data Rich	Female	Population Density (500-1000 ppl/sqkm, proportion)		X			X	
Cyclist road injuries	Global	Female	Population Density (500-1000 ppl/sqkm, proportion)		X			X	
Cyclist road injuries	Data Rich	Female	Population-weighted mean temperature		X			X	
Cyclist road injuries	Global	Female	Population-weighted mean temperature		X			X	
Cyclist road injuries	Data Rich	Female	Population Density (300-500 ppl/sqkm, proportion)		X			X	
Cyclist road injuries	Global	Female	Population Density (300-500 ppl/sqkm, proportion)		X			X	
Cyclist road injuries	Data Rich	Female	Population 15 to 30 (proportion)		X			X	
Cyclist road injuries	Global	Female	Population 15 to 30 (proportion)		X			X	
Cyclist road injuries	Data Rich	Female	BAC law general population (quartile)	X					X
Cyclist road injuries	Global	Female	BAC law general population (quartile)	X					X
Cyclist road injuries	Data Rich	Female	BAC law youth drivers (quartile)	X					X
Cyclist road injuries	Global	Female	BAC law youth drivers (quartile)	X					X
Cyclist road injuries	Data Rich	Female	BAC law professional drivers (quartile)	X					X
Cyclist road injuries	Global	Female	BAC law professional drivers (quartile)	X					X
Cyclist road injuries	Data Rich	Female	Speed limit law urban (quartile)	X					X
Cyclist road injuries	Global	Female	Speed limit law urban (quartile)	X					X
Cyclist road injuries	Data Rich	Female	Speed limit law rural (quartile)	X					X
Cyclist road injuries	Global	Female	Speed limit law rural (quartile)	X					X
Cyclist road injuries	Data Rich	Female	Liters of alcohol consumed per capita	X				X	
Cyclist road injuries	Global	Female	Liters of alcohol consumed per capita	X				X	
Cyclist road injuries	Data Rich	Female	Vehicles - 2+4 wheels (per capita)	X			X		
Cyclist road injuries	Global	Female	Vehicles - 2+4 wheels (per capita)	X			X		
Cyclist road injuries	Data Rich	Female	Vehicles - 2 wheels fraction (proportion)	X			X		
Cyclist road injuries	Global	Female	Vehicles - 2 wheels fraction (proportion)	X			X		
Cyclist road injuries	Data Rich	Female	Log-transformed SEV scalar: Cyclist	X			X		
Cyclist road injuries	Global	Female	Log-transformed SEV scalar: Cyclist	X			X		
Cyclist road injuries	Data Rich	Male	Rainfall Quintile 5 (proportion)			X			X
Cyclist road injuries	Global	Male	Rainfall Quintile 5 (proportion)			X			X
Cyclist road injuries	Data Rich	Male	Socio-demographic Index		X			X	
Cyclist road injuries	Global	Male	Socio-demographic Index		X			X	
Cyclist road injuries	Data Rich	Male	Healthcare access and quality index		X			X	
Cyclist road injuries	Global	Male	Healthcare access and quality index		X			X	
Cyclist road injuries	Data Rich	Male	Population Density (500-1000 ppl/sqkm, proportion)		X			X	
Cyclist road injuries	Global	Male	Population Density (500-1000 ppl/sqkm, proportion)		X			X	
Cyclist road injuries	Data Rich	Male	Population-weighted mean temperature		X			X	
Cyclist road injuries	Global	Male	Population-weighted mean temperature		X			X	
Cyclist road injuries	Data Rich	Male	Population Density (300-500 ppl/sqkm, proportion)		X			X	
Cyclist road injuries	Global	Male	Population Density (300-500 ppl/sqkm, proportion)		X			X	
Cyclist road injuries	Data Rich	Male	Population 15 to 30 (proportion)		X			X	
Cyclist road injuries	Global	Male	Population 15 to 30 (proportion)		X			X	
Cyclist road injuries	Data Rich	Male	BAC law general population (quartile)	X					X

Appendix Table S11: Comparison of GBD 2021 and GBD 2023 covariates and level of covariates used in cause of death modeling									
**Cause**	**Model Version Type**	**Sex**	**Covariate**	**Level 1 GBD 2021**	**Level 2 GBD 2021**	**Level 3 GBD 2021**	**Level 1 GBD 2023**	**Level 2 GBD 2023**	**Level 3 GBD 2023**
Cyclist road injuries	Global	Male	BAC law general population (quartile)	X					X
Cyclist road injuries	Data Rich	Male	BAC law youth drivers (quartile)	X					X
Cyclist road injuries	Global	Male	BAC law youth drivers (quartile)	X					X
Cyclist road injuries	Data Rich	Male	BAC law professional drivers (quartile)	X					X
Cyclist road injuries	Global	Male	BAC law professional drivers (quartile)	X					X
Cyclist road injuries	Data Rich	Male	Speed limit law urban (quartile)	X					X
Cyclist road injuries	Global	Male	Speed limit law urban (quartile)	X					X
Cyclist road injuries	Data Rich	Male	Speed limit law rural (quartile)	X					X
Cyclist road injuries	Global	Male	Speed limit law rural (quartile)	X					X
Cyclist road injuries	Data Rich	Male	Liters of alcohol consumed per capita	X				X	
Cyclist road injuries	Global	Male	Liters of alcohol consumed per capita	X				X	
Cyclist road injuries	Data Rich	Male	Vehicles - 2+4 wheels (per capita)	X			X		
Cyclist road injuries	Global	Male	Vehicles - 2+4 wheels (per capita)	X			X		
Cyclist road injuries	Data Rich	Male	Vehicles - 2 wheels fraction (proportion)	X			X		
Cyclist road injuries	Global	Male	Vehicles - 2 wheels fraction (proportion)	X			X		
Cyclist road injuries	Data Rich	Male	Log-transformed SEV scalar: Cyclist	X			X		
Cyclist road injuries	Global	Male	Log-transformed SEV scalar: Cyclist	X			X		
Motorcyclist road injuries	Data Rich	Female	Rainfall Quintile 5 (proportion)			X			X
Motorcyclist road injuries	Global	Female	Rainfall Quintile 5 (proportion)			X			X
Motorcyclist road injuries	Data Rich	Female	Population Density (500-1000 ppl/sqkm, proportion)		X				X
Motorcyclist road injuries	Global	Female	Population Density (500-1000 ppl/sqkm, proportion)		X				X
Motorcyclist road injuries	Data Rich	Female	Population Density (300-500 ppl/sqkm, proportion)		X				X
Motorcyclist road injuries	Global	Female	Population Density (300-500 ppl/sqkm, proportion)		X				X
Motorcyclist road injuries	Data Rich	Female	Socio-demographic Index		X			X	
Motorcyclist road injuries	Global	Female	Socio-demographic Index		X			X	
Motorcyclist road injuries	Data Rich	Female	Healthcare access and quality index		X			X	
Motorcyclist road injuries	Global	Female	Healthcare access and quality index		X			X	
Motorcyclist road injuries	Data Rich	Female	Population-weighted mean temperature		X			X	
Motorcyclist road injuries	Global	Female	Population-weighted mean temperature		X			X	
Motorcyclist road injuries	Data Rich	Female	Population 15 to 30 (proportion)		X			X	
Motorcyclist road injuries	Global	Female	Population 15 to 30 (proportion)		X			X	
Motorcyclist road injuries	Data Rich	Female	BAC law general population (quartile)	X					X
Motorcyclist road injuries	Global	Female	BAC law general population (quartile)	X					X
Motorcyclist road injuries	Data Rich	Female	BAC law youth drivers (quartile)	X					X
Motorcyclist road injuries	Global	Female	BAC law youth drivers (quartile)	X					X
Motorcyclist road injuries	Data Rich	Female	BAC law professional drivers (quartile)	X					X
Motorcyclist road injuries	Global	Female	BAC law professional drivers (quartile)	X					X
Motorcyclist road injuries	Data Rich	Female	Speed limit law urban (quartile)	X					X
Motorcyclist road injuries	Global	Female	Speed limit law urban (quartile)	X					X
Motorcyclist road injuries	Data Rich	Female	Speed limit law rural (quartile)	X					X
Motorcyclist road injuries	Global	Female	Speed limit law rural (quartile)	X					X
Motorcyclist road injuries	Data Rich	Female	Liters of alcohol consumed per capita	X			X		
Motorcyclist road injuries	Global	Female	Liters of alcohol consumed per capita	X			X		
Motorcyclist road injuries	Data Rich	Female	Vehicles - 2 wheels fraction (proportion)	X			X		
Motorcyclist road injuries	Global	Female	Vehicles - 2 wheels fraction (proportion)	X			X		
Motorcyclist road injuries	Data Rich	Female	Log-transformed SEV scalar: Mot Cyc	X			X		
Motorcyclist road injuries	Global	Female	Log-transformed SEV scalar: Mot Cyc	X			X		
Motorcyclist road injuries	Data Rich	Female	Vehicles - 2 wheels (per capita)				X		
Motorcyclist road injuries	Global	Female	Vehicles - 2 wheels (per capita)				X		
Motorcyclist road injuries	Data Rich	Male	Rainfall Quintile 5 (proportion)			X			X
Motorcyclist road injuries	Global	Male	Rainfall Quintile 5 (proportion)			X			X
Motorcyclist road injuries	Data Rich	Male	Population Density (500-1000 ppl/sqkm, proportion)		X				X
Motorcyclist road injuries	Global	Male	Population Density (500-1000 ppl/sqkm, proportion)		X				X
Motorcyclist road injuries	Data Rich	Male	Population Density (300-500 ppl/sqkm, proportion)		X				X
Motorcyclist road injuries	Global	Male	Population Density (300-500 ppl/sqkm, proportion)		X				X
Motorcyclist road injuries	Data Rich	Male	Socio-demographic Index		X			X	
Motorcyclist road injuries	Global	Male	Socio-demographic Index		X			X	
Motorcyclist road injuries	Data Rich	Male	Healthcare access and quality index		X			X	
Motorcyclist road injuries	Global	Male	Healthcare access and quality index		X			X	
Motorcyclist road injuries	Data Rich	Male	Population-weighted mean temperature		X			X	
Motorcyclist road injuries	Global	Male	Population-weighted mean temperature		X			X	
Motorcyclist road injuries	Data Rich	Male	Population 15 to 30 (proportion)		X			X	
Motorcyclist road injuries	Global	Male	Population 15 to 30 (proportion)		X			X	
Motorcyclist road injuries	Data Rich	Male	BAC law general population (quartile)	X					X
Motorcyclist road injuries	Global	Male	BAC law general population (quartile)	X					X
Motorcyclist road injuries	Data Rich	Male	BAC law youth drivers (quartile)	X					X
Motorcyclist road injuries	Global	Male	BAC law youth drivers (quartile)	X					X
Motorcyclist road injuries	Data Rich	Male	BAC law professional drivers (quartile)	X					X
Motorcyclist road injuries	Global	Male	BAC law professional drivers (quartile)	X					X
Motorcyclist road injuries	Data Rich	Male	Speed limit law urban (quartile)	X					X
Motorcyclist road injuries	Global	Male	Speed limit law urban (quartile)	X					X
Motorcyclist road injuries	Data Rich	Male	Speed limit law rural (quartile)	X					X
Motorcyclist road injuries	Global	Male	Speed limit law rural (quartile)	X					X
Motorcyclist road injuries	Data Rich	Male	Liters of alcohol consumed per capita	X			X		
Motorcyclist road injuries	Global	Male	Liters of alcohol consumed per capita	X			X		
Motorcyclist road injuries	Data Rich	Male	Vehicles - 2 wheels fraction (proportion)	X			X		
Motorcyclist road injuries	Global	Male	Vehicles - 2 wheels fraction (proportion)	X			X		
Motorcyclist road injuries	Data Rich	Male	Log-transformed SEV scalar: Mot Cyc	X			X		
Motorcyclist road injuries	Global	Male	Log-transformed SEV scalar: Mot Cyc	X			X		
Motorcyclist road injuries	Data Rich	Male	Vehicles - 2 wheels (per capita)				X		
Motorcyclist road injuries	Global	Male	Vehicles - 2 wheels (per capita)				X		
Motor vehicle road injuries	Data Rich	Female	Rainfall Quintile 5 (proportion)			X			X
Motor vehicle road injuries	Global	Female	Rainfall Quintile 5 (proportion)			X			X
Motor vehicle road injuries	Data Rich	Female	Socio-demographic Index		X			X	
Motor vehicle road injuries	Global	Female	Socio-demographic Index		X			X	
Motor vehicle road injuries	Data Rich	Female	Healthcare access and quality index		X			X	
Motor vehicle road injuries	Global	Female	Healthcare access and quality index		X			X	
Motor vehicle road injuries	Data Rich	Female	Population Density (500-1000 ppl/sqkm, proportion)		X			X	
Motor vehicle road injuries	Global	Female	Population Density (500-1000 ppl/sqkm, proportion)		X			X	
Motor vehicle road injuries	Data Rich	Female	Population-weighted mean temperature		X			X	
Motor vehicle road injuries	Global	Female	Population-weighted mean temperature		X			X	
Motor vehicle road injuries	Data Rich	Female	Population Density (300-500 ppl/sqkm, proportion)		X			X	
Motor vehicle road injuries	Global	Female	Population Density (300-500 ppl/sqkm, proportion)		X			X	
Motor vehicle road injuries	Data Rich	Female	Population 15 to 30 (proportion)		X			X	
Motor vehicle road injuries	Global	Female	Population 15 to 30 (proportion)		X			X	
Motor vehicle road injuries	Data Rich	Female	BAC law general population (quartile)	X					X
Motor vehicle road injuries	Global	Female	BAC law general population (quartile)	X					X
Motor vehicle road injuries	Data Rich	Female	BAC law youth drivers (quartile)	X					X
Motor vehicle road injuries	Global	Female	BAC law youth drivers (quartile)	X					X
Motor vehicle road injuries	Data Rich	Female	BAC law professional drivers (quartile)	X					X
Motor vehicle road injuries	Global	Female	BAC law professional drivers (quartile)	X					X
Motor vehicle road injuries	Data Rich	Female	Speed limit law urban (quartile)	X					X
Motor vehicle road injuries	Global	Female	Speed limit law urban (quartile)	X					X
Motor vehicle road injuries	Data Rich	Female	Speed limit law rural (quartile)	X					X
Motor vehicle road injuries	Global	Female	Speed limit law rural (quartile)	X					X
Motor vehicle road injuries	Data Rich	Female	Log-transformed SEV scalar: Mot Veh	X					X
Motor vehicle road injuries	Global	Female	Log-transformed SEV scalar: Mot Veh	X					X
Motor vehicle road injuries	Data Rich	Female	Liters of alcohol consumed per capita	X				X	
Motor vehicle road injuries	Global	Female	Liters of alcohol consumed per capita	X				X	

Appendix Table S11: Comparison of GBD 2021 and GBD 2023 covariates and level of covariates used in cause of death modeling									
**Cause**	**Model Version Type**	**Sex**	**Covariate**	**Level 1 GBD 2021**	**Level 2 GBD 2021**	**Level 3 GBD 2021**	**Level 1 GBD 2023**	**Level 2 GBD 2023**	**Level 3 GBD 2023**
Motor vehicle road injuries	Data Rich	Female	Vehicles - 4 wheels (per capita)	X			X		
Motor vehicle road injuries	Global	Female	Vehicles - 4 wheels (per capita)	X			X		
Motor vehicle road injuries	Data Rich	Male	Rainfall Quintile 5 (proportion)			X			X
Motor vehicle road injuries	Global	Male	Rainfall Quintile 5 (proportion)			X			X
Motor vehicle road injuries	Data Rich	Male	Socio-demographic Index		X			X	
Motor vehicle road injuries	Global	Male	Socio-demographic Index		X			X	
Motor vehicle road injuries	Data Rich	Male	Healthcare access and quality index		X			X	
Motor vehicle road injuries	Global	Male	Healthcare access and quality index		X			X	
Motor vehicle road injuries	Data Rich	Male	Population Density (500-1000 ppl/sqkm, proportion)		X			X	
Motor vehicle road injuries	Global	Male	Population Density (500-1000 ppl/sqkm, proportion)		X			X	
Motor vehicle road injuries	Data Rich	Male	Population-weighted mean temperature		X			X	
Motor vehicle road injuries	Global	Male	Population-weighted mean temperature		X			X	
Motor vehicle road injuries	Data Rich	Male	Population Density (300-500 ppl/sqkm, proportion)		X			X	
Motor vehicle road injuries	Global	Male	Population Density (300-500 ppl/sqkm, proportion)		X			X	
Motor vehicle road injuries	Data Rich	Male	Population 15 to 30 (proportion)		X			X	
Motor vehicle road injuries	Global	Male	Population 15 to 30 (proportion)		X			X	
Motor vehicle road injuries	Data Rich	Male	BAC law general population (quartile)	X					X
Motor vehicle road injuries	Global	Male	BAC law general population (quartile)	X					X
Motor vehicle road injuries	Data Rich	Male	BAC law youth drivers (quartile)	X					X
Motor vehicle road injuries	Global	Male	BAC law youth drivers (quartile)	X					X
Motor vehicle road injuries	Data Rich	Male	BAC law professional drivers (quartile)	X					X
Motor vehicle road injuries	Global	Male	BAC law professional drivers (quartile)	X					X
Motor vehicle road injuries	Data Rich	Male	Speed limit law urban (quartile)	X					X
Motor vehicle road injuries	Global	Male	Speed limit law urban (quartile)	X					X
Motor vehicle road injuries	Data Rich	Male	Speed limit law rural (quartile)	X					X
Motor vehicle road injuries	Global	Male	Speed limit law rural (quartile)	X					X
Motor vehicle road injuries	Data Rich	Male	Log-transformed SEV scalar: Mot Veh	X					X
Motor vehicle road injuries	Global	Male	Log-transformed SEV scalar: Mot Veh	X					X
Motor vehicle road injuries	Data Rich	Male	Liters of alcohol consumed per capita	X				X	
Motor vehicle road injuries	Global	Male	Liters of alcohol consumed per capita	X				X	
Motor vehicle road injuries	Data Rich	Male	Vehicles - 4 wheels (per capita)	X			X		
Motor vehicle road injuries	Global	Male	Vehicles - 4 wheels (per capita)	X			X		
Other road injuries	Data Rich	Female	Rainfall Quintile 5 (proportion)			X			X
Other road injuries	Global	Female	Rainfall Quintile 5 (proportion)			X			X
Other road injuries	Data Rich	Female	Socio-demographic Index			X		X	
Other road injuries	Global	Female	Socio-demographic Index			X		X	
Other road injuries	Data Rich	Female	Healthcare access and quality index		X			X	
Other road injuries	Global	Female	Healthcare access and quality index		X			X	
Other road injuries	Data Rich	Female	Population-weighted mean temperature		X			X	
Other road injuries	Global	Female	Population-weighted mean temperature		X			X	
Other road injuries	Data Rich	Female	Population 15 to 30 (proportion)		X			X	
Other road injuries	Global	Female	Population 15 to 30 (proportion)		X			X	
Other road injuries	Data Rich	Female	BAC law general population (quartile)	X					X
Other road injuries	Global	Female	BAC law general population (quartile)	X					X
Other road injuries	Data Rich	Female	BAC law youth drivers (quartile)	X					X
Other road injuries	Global	Female	BAC law youth drivers (quartile)	X					X
Other road injuries	Data Rich	Female	BAC law professional drivers (quartile)	X					X
Other road injuries	Global	Female	BAC law professional drivers (quartile)	X					X
Other road injuries	Data Rich	Female	Speed limit law urban (quartile)	X					X
Other road injuries	Global	Female	Speed limit law urban (quartile)	X					X
Other road injuries	Data Rich	Female	Speed limit law rural (quartile)	X					X
Other road injuries	Global	Female	Speed limit law rural (quartile)	X					X
Other road injuries	Data Rich	Female	Liters of alcohol consumed per capita	X				X	
Other road injuries	Global	Female	Liters of alcohol consumed per capita	X				X	
Other road injuries	Data Rich	Female	Vehicles - 2+4 wheels (per capita)	X			X		
Other road injuries	Global	Female	Vehicles - 2+4 wheels (per capita)	X			X		
Other road injuries	Data Rich	Female	Vehicles - 2 wheels fraction (proportion)	X			X		
Other road injuries	Global	Female	Vehicles - 2 wheels fraction (proportion)	X			X		
Other road injuries	Data Rich	Female	Log-transformed SEV scalar: Oth Road	X			X		
Other road injuries	Global	Female	Log-transformed SEV scalar: Oth Road	X			X		
Other road injuries	Data Rich	Male	Rainfall Quintile 5 (proportion)			X			X
Other road injuries	Global	Male	Rainfall Quintile 5 (proportion)			X			X
Other road injuries	Data Rich	Male	Socio-demographic Index			X		X	
Other road injuries	Global	Male	Socio-demographic Index		X			X	
Other road injuries	Data Rich	Male	Healthcare access and quality index		X			X	
Other road injuries	Global	Male	Healthcare access and quality index		X			X	
Other road injuries	Data Rich	Male	Population-weighted mean temperature		X			X	
Other road injuries	Global	Male	Population-weighted mean temperature		X			X	
Other road injuries	Data Rich	Male	Population 15 to 30 (proportion)		X			X	
Other road injuries	Global	Male	Population 15 to 30 (proportion)		X			X	
Other road injuries	Data Rich	Male	BAC law general population (quartile)	X					X
Other road injuries	Global	Male	BAC law general population (quartile)	X					X
Other road injuries	Data Rich	Male	BAC law youth drivers (quartile)	X					X
Other road injuries	Global	Male	BAC law youth drivers (quartile)	X					X
Other road injuries	Data Rich	Male	BAC law professional drivers (quartile)	X					X
Other road injuries	Global	Male	BAC law professional drivers (quartile)	X					X
Other road injuries	Data Rich	Male	Speed limit law urban (quartile)	X					X
Other road injuries	Global	Male	Speed limit law urban (quartile)	X					X
Other road injuries	Data Rich	Male	Speed limit law rural (quartile)	X					X
Other road injuries	Global	Male	Speed limit law rural (quartile)	X					X
Other road injuries	Data Rich	Male	Liters of alcohol consumed per capita	X				X	
Other road injuries	Global	Male	Liters of alcohol consumed per capita	X				X	
Other road injuries	Data Rich	Male	Vehicles - 2+4 wheels (per capita)	X			X		
Other road injuries	Global	Male	Vehicles - 2+4 wheels (per capita)	X			X		
Other road injuries	Data Rich	Male	Vehicles - 2 wheels fraction (proportion)	X			X		
Other road injuries	Global	Male	Vehicles - 2 wheels fraction (proportion)	X			X		
Other road injuries	Data Rich	Male	Log-transformed SEV scalar: Oth Road	X			X		
Other road injuries	Global	Male	Log-transformed SEV scalar: Oth Road	X			X		
Other transport injuries	Global	Female	Rainfall Quintile 5 (proportion)			X			X
Other transport injuries	Data Rich	Female	Rainfall Quintile 5 (proportion)			X			X
Other transport injuries	Global	Female	Socio-demographic Index		X			X	
Other transport injuries	Data Rich	Female	Socio-demographic Index		X			X	
Other transport injuries	Global	Female	Healthcare access and quality index		X			X	
Other transport injuries	Data Rich	Female	Healthcare access and quality index		X			X	
Other transport injuries	Global	Female	Population Density (500-1000 ppl/sqkm, proportion)		X			X	
Other transport injuries	Data Rich	Female	Population Density (500-1000 ppl/sqkm, proportion)		X			X	
Other transport injuries	Global	Female	Population-weighted mean temperature		X			X	
Other transport injuries	Data Rich	Female	Population-weighted mean temperature		X			X	
Other transport injuries	Global	Female	Population Density (300-500 ppl/sqkm, proportion)		X			X	
Other transport injuries	Data Rich	Female	Population Density (300-500 ppl/sqkm, proportion)		X			X	
Other transport injuries	Global	Female	Population 15 to 30 (proportion)		X			X	
Other transport injuries	Data Rich	Female	Population 15 to 30 (proportion)		X			X	
Other transport injuries	Global	Female	BAC law general population (quartile)	X					X
Other transport injuries	Data Rich	Female	BAC law general population (quartile)	X					X
Other transport injuries	Global	Female	BAC law youth drivers (quartile)	X					X
Other transport injuries	Data Rich	Female	BAC law youth drivers (quartile)	X					X
Other transport injuries	Global	Female	BAC law professional drivers (quartile)	X					X
Other transport injuries	Data Rich	Female	BAC law professional drivers (quartile)	X					X
Other transport injuries	Global	Female	Speed limit law urban (quartile)	X					X
Other transport injuries	Data Rich	Female	Speed limit law urban (quartile)	X					X
Other transport injuries	Global	Female	Speed limit law rural (quartile)	X					X
Other transport injuries	Data Rich	Female	Speed limit law rural (quartile)	X					X

Appendix Table S11: Comparison of GBD 2021 and GBD 2023 covariates and level of covariates used in cause of death modeling

Cause	Model Version Type	Sex	Covariate	Level 1 GBD 2021	Level 2 GBD 2021	Level 3 GBD 2021	Level 1 GBD 2023	Level 2 GBD 2023	Level 3 GBD 2023
Other transport injuries	Global	Female	Liters of alcohol consumed per capita	X				X	
Other transport injuries	Data Rich	Female	Liters of alcohol consumed per capita	X				X	
Other transport injuries	Global	Female	Vehicles - 2+4 wheels (per capita)	X			X		
Other transport injuries	Data Rich	Female	Vehicles - 2+4 wheels (per capita)	X			X		
Other transport injuries	Global	Female	Vehicles - 2 wheels fraction (proportion)	X			X		
Other transport injuries	Data Rich	Female	Vehicles - 2 wheels fraction (proportion)	X			X		
Other transport injuries	Global	Female	Log-transformed SEV scalar: Oth Trans	X			X		
Other transport injuries	Data Rich	Female	Log-transformed SEV scalar: Oth Trans	X			X		
Other transport injuries	Global	Male	Rainfall Quintile 5 (proportion)			X			X
Other transport injuries	Data Rich	Male	Rainfall Quintile 5 (proportion)			X			X
Other transport injuries	Global	Male	Socio-demographic Index		X			X	
Other transport injuries	Data Rich	Male	Socio-demographic Index		X			X	
Other transport injuries	Global	Male	Healthcare access and quality index		X			X	
Other transport injuries	Data Rich	Male	Healthcare access and quality index		X			X	
Other transport injuries	Global	Male	Population Density (500-1000 ppl/sqkm, proportion)		X			X	
Other transport injuries	Data Rich	Male	Population Density (500-1000 ppl/sqkm, proportion)		X			X	
Other transport injuries	Global	Male	Population-weighted mean temperature		X			X	
Other transport injuries	Data Rich	Male	Population-weighted mean temperature		X			X	
Other transport injuries	Global	Male	Population Density (300-500 ppl/sqkm, proportion)		X			X	
Other transport injuries	Data Rich	Male	Population Density (300-500 ppl/sqkm, proportion)		X			X	
Other transport injuries	Global	Male	Population 15 to 30 (proportion)		X			X	
Other transport injuries	Data Rich	Male	Population 15 to 30 (proportion)		X			X	
Other transport injuries	Global	Male	BAC law general population (quartile)	X					X
Other transport injuries	Data Rich	Male	BAC law general population (quartile)	X					X
Other transport injuries	Global	Male	BAC law youth drivers (quartile)	X					X
Other transport injuries	Data Rich	Male	BAC law youth drivers (quartile)	X					X
Other transport injuries	Global	Male	BAC law professional drivers (quartile)	X					X
Other transport injuries	Data Rich	Male	BAC law professional drivers (quartile)	X					X
Other transport injuries	Global	Male	Speed limit law urban (quartile)	X					X
Other transport injuries	Data Rich	Male	Speed limit law urban (quartile)	X					X
Other transport injuries	Global	Male	Speed limit law rural (quartile)	X					X
Other transport injuries	Data Rich	Male	Speed limit law rural (quartile)	X					X
Other transport injuries	Global	Male	Liters of alcohol consumed per capita	X				X	
Other transport injuries	Data Rich	Male	Liters of alcohol consumed per capita	X				X	
Other transport injuries	Global	Male	Vehicles - 2+4 wheels (per capita)	X			X		
Other transport injuries	Data Rich	Male	Vehicles - 2+4 wheels (per capita)	X			X		
Other transport injuries	Global	Male	Vehicles - 2 wheels fraction (proportion)	X			X		
Other transport injuries	Data Rich	Male	Vehicles - 2 wheels fraction (proportion)	X			X		
Other transport injuries	Global	Male	Log-transformed SEV scalar: Oth Trans	X			X		
Other transport injuries	Data Rich	Male	Log-transformed SEV scalar: Oth Trans	X			X		
Falls	Data Rich	Female	Socio-demographic Index			X			X
Falls	Global	Female	Socio-demographic Index			X			X
Falls	Data Rich	Female	Elevation Over 1500m (proportion)			X			X
Falls	Global	Female	Elevation Over 1500m (proportion)			X			X
Falls	Data Rich	Female	Healthcare access and quality index		X			X	
Falls	Global	Female	Healthcare access and quality index		X			X	
Falls	Data Rich	Female	Population-weighted mean temperature		X			X	
Falls	Global	Female	Population-weighted mean temperature		X			X	
Falls	Data Rich	Female	Liters of alcohol consumed per capita	X			X		
Falls	Global	Female	Liters of alcohol consumed per capita	X			X		
Falls	Data Rich	Female	Log-transformed SEV scalar: Falls	X			X		
Falls	Global	Female	Log-transformed SEV scalar: Falls	X			X		
Falls	Data Rich	Male	Socio-demographic Index			X			X
Falls	Global	Male	Elevation Over 1500m (proportion)			X			X
Falls	Data Rich	Male	Elevation Over 1500m (proportion)			X			X
Falls	Global	Male	Healthcare access and quality index		X			X	
Falls	Data Rich	Male	Healthcare access and quality index		X			X	
Falls	Global	Male	Population-weighted mean temperature		X			X	
Falls	Data Rich	Male	Population-weighted mean temperature		X			X	
Falls	Global	Male	Socio-demographic Index			X	X		
Falls	Global	Male	Liters of alcohol consumed per capita	X			X		
Falls	Data Rich	Male	Liters of alcohol consumed per capita	X			X		
Falls	Global	Male	Log-transformed SEV scalar: Falls	X			X		
Falls	Data Rich	Male	Log-transformed SEV scalar: Falls	X			X		
Drowning	Global	Female	Socio-demographic Index			X			X
Drowning	Data Rich	Female	Socio-demographic Index			X			X
Drowning	Global	Female	Elevation Under 100m (proportion)		X			X	
Drowning	Data Rich	Female	Elevation Under 100m (proportion)		X			X	
Drowning	Global	Female	Rainfall Quintile 1 (proportion)	X				X	
Drowning	Data Rich	Female	Rainfall Quintile 1 (proportion)	X				X	
Drowning	Global	Female	Rainfall Quintile 5 (proportion)	X				X	
Drowning	Data Rich	Female	Rainfall Quintile 5 (proportion)	X				X	
Drowning	Global	Female	Population-weighted mean temperature	X			X		
Drowning	Data Rich	Female	Population-weighted mean temperature	X			X		
Drowning	Global	Female	Coastal Population within 10km (proportion)	X			X		
Drowning	Data Rich	Female	Coastal Population within 10km (proportion)	X			X		
Drowning	Global	Female	Landlocked Nation (binary)	X			X		
Drowning	Data Rich	Female	Landlocked Nation (binary)	X			X		
Drowning	Global	Female	Log-transformed SEV scalar: Drown	X			X		
Drowning	Data Rich	Female	Log-transformed SEV scalar: Drown	X			X		
Drowning	Global	Female	Healthcare access and quality index						X
Drowning	Data Rich	Female	Healthcare access and quality index						X
Drowning	Global	Female	Maternal Education (years per capita)				X		
Drowning	Data Rich	Female	Maternal Education (years per capita)				X		
Drowning	Global	Male	Socio-demographic Index			X			X
Drowning	Data Rich	Male	Socio-demographic Index			X			X
Drowning	Global	Male	Elevation Under 100m (proportion)		X			X	
Drowning	Data Rich	Male	Elevation Under 100m (proportion)		X			X	
Drowning	Global	Male	Rainfall Quintile 1 (proportion)	X				X	
Drowning	Data Rich	Male	Rainfall Quintile 1 (proportion)	X				X	
Drowning	Global	Male	Rainfall Quintile 5 (proportion)	X				X	
Drowning	Data Rich	Male	Rainfall Quintile 5 (proportion)	X				X	
Drowning	Global	Male	Population-weighted mean temperature	X			X		
Drowning	Data Rich	Male	Population-weighted mean temperature	X			X		
Drowning	Global	Male	Coastal Population within 10km (proportion)	X			X		
Drowning	Data Rich	Male	Coastal Population within 10km (proportion)	X			X		
Drowning	Global	Male	Landlocked Nation (binary)	X			X		
Drowning	Data Rich	Male	Landlocked Nation (binary)	X			X		
Drowning	Global	Male	Log-transformed SEV scalar: Drown	X			X		
Drowning	Data Rich	Male	Log-transformed SEV scalar: Drown	X			X		
Drowning	Global	Male	Healthcare access and quality index						X
Drowning	Data Rich	Male	Healthcare access and quality index						X
Drowning	Global	Male	Maternal Education (years per capita)				X		
Drowning	Data Rich	Male	Maternal Education (years per capita)				X		
Fire, heat, and hot substances	Data Rich	Female	Socio-demographic Index			X			X
Fire, heat, and hot substances	Global	Female	Socio-demographic Index			X			X
Fire, heat, and hot substances	Data Rich	Female	Tobacco (cigarettes per capita)		X			X	
Fire, heat, and hot substances	Global	Female	Tobacco (cigarettes per capita)		X			X	
Fire, heat, and hot substances	Data Rich	Female	Healthcare access and quality index		X			X	
Fire, heat, and hot substances	Global	Female	Healthcare access and quality index		X			X	
Fire, heat, and hot substances	Data Rich	Female	Population Density (over 1000 ppl/sqkm, proportion)		X			X	
Fire, heat, and hot substances	Global	Female	Population Density (over 1000 ppl/sqkm, proportion)		X			X	
Fire, heat, and hot substances	Data Rich	Female	Indoor Air Pollution (All Cooking Fuels)		X		X		

Appendix Table S11: Comparison of GBD 2021 and GBD 2023 covariates and level of covariates used in cause of death modeling									
**Cause**	**Model Version Type**	**Sex**	**Covariate**	**Level 1 GBD 2021**	**Level 2 GBD 2021**	**Level 3 GBD 2021**	**Level 1 GBD 2023**	**Level 2 GBD 2023**	**Level 3 GBD 2023**
Fire, heat, and hot substances	Global	Female	Indoor Air Pollution (All Cooking Fuels)		X		X		
Fire, heat, and hot substances	Data Rich	Female	Population-weighted mean temperature	X				X	
Fire, heat, and hot substances	Global	Female	Population-weighted mean temperature	X				X	
Fire, heat, and hot substances	Data Rich	Female	Log-transformed SEV scalar: Fire	X			X		
Fire, heat, and hot substances	Global	Female	Log-transformed SEV scalar: Fire	X			X		
Fire, heat, and hot substances	Data Rich	Female	Liters of alcohol consumed per capita					X	
Fire, heat, and hot substances	Global	Female	Liters of alcohol consumed per capita					X	
Fire, heat, and hot substances	Data Rich	Male	Socio-demographic Index			X			X
Fire, heat, and hot substances	Global	Male	Socio-demographic Index			X			X
Fire, heat, and hot substances	Data Rich	Male	Tobacco (cigarettes per capita)		X			X	
Fire, heat, and hot substances	Global	Male	Tobacco (cigarettes per capita)		X			X	
Fire, heat, and hot substances	Data Rich	Male	Healthcare access and quality index		X			X	
Fire, heat, and hot substances	Global	Male	Healthcare access and quality index		X			X	
Fire, heat, and hot substances	Data Rich	Male	Population Density (over 1000 ppl/sqkm, proportion)		X			X	
Fire, heat, and hot substances	Global	Male	Population Density (over 1000 ppl/sqkm, proportion)		X			X	
Fire, heat, and hot substances	Data Rich	Male	Indoor Air Pollution (All Cooking Fuels)		X		X		
Fire, heat, and hot substances	Global	Male	Indoor Air Pollution (All Cooking Fuels)		X		X		
Fire, heat, and hot substances	Data Rich	Male	Population-weighted mean temperature	X				X	
Fire, heat, and hot substances	Global	Male	Population-weighted mean temperature	X				X	
Fire, heat, and hot substances	Data Rich	Male	Log-transformed SEV scalar: Fire	X			X		
Fire, heat, and hot substances	Global	Male	Log-transformed SEV scalar: Fire	X			X		
Fire, heat, and hot substances	Data Rich	Male	Liters of alcohol consumed per capita					X	
Fire, heat, and hot substances	Global	Male	Liters of alcohol consumed per capita					X	
Poisonings	Data Rich	Female	Socio-demographic Index			X		X	
Poisonings	Global	Female	Socio-demographic Index			X		X	
Poisonings	Data Rich	Female	Population Density (under 150 ppl/sqkm, proportion)		X				X
Poisonings	Global	Female	Population Density (under 150 ppl/sqkm, proportion)		X				X
Poisonings	Data Rich	Female	Population Density (over 1000 ppl/sqkm, proportion)		X				X
Poisonings	Global	Female	Population Density (over 1000 ppl/sqkm, proportion)		X				X
Poisonings	Data Rich	Female	Healthcare access and quality index		X			X	
Poisonings	Global	Female	Healthcare access and quality index		X			X	
Poisonings	Data Rich	Female	Liters of alcohol consumed per capita	X				X	
Poisonings	Global	Female	Liters of alcohol consumed per capita	X				X	
Poisonings	Data Rich	Female	Population-weighted mean temperature	X			X		
Poisonings	Global	Female	Population-weighted mean temperature	X			X		
Poisonings	Data Rich	Female	Log-transformed SEV scalar: Poison	X			X		
Poisonings	Global	Female	Log-transformed SEV scalar: Poison	X			X		
Poisonings	Data Rich	Female	Maternal Education (years per capita)				X		
Poisonings	Global	Female	Maternal Education (years per capita)				X		
Poisonings	Data Rich	Female	Proportion of population involved in agricultural activities				X		
Poisonings	Global	Female	Proportion of population involved in agricultural activities				X		
Poisonings	Data Rich	Male	Socio-demographic Index			X		X	
Poisonings	Global	Male	Socio-demographic Index			X		X	
Poisonings	Data Rich	Male	Population Density (under 150 ppl/sqkm, proportion)		X				X
Poisonings	Global	Male	Population Density (under 150 ppl/sqkm, proportion)		X				X
Poisonings	Data Rich	Male	Population Density (over 1000 ppl/sqkm, proportion)		X				X
Poisonings	Global	Male	Population Density (over 1000 ppl/sqkm, proportion)		X				X
Poisonings	Data Rich	Male	Healthcare access and quality index		X			X	
Poisonings	Global	Male	Healthcare access and quality index		X			X	
Poisonings	Data Rich	Male	Liters of alcohol consumed per capita	X				X	
Poisonings	Global	Male	Liters of alcohol consumed per capita	X				X	
Poisonings	Data Rich	Male	Population-weighted mean temperature	X			X		
Poisonings	Global	Male	Population-weighted mean temperature	X			X		
Poisonings	Data Rich	Male	Log-transformed SEV scalar: Poison	X			X		
Poisonings	Global	Male	Log-transformed SEV scalar: Poison	X			X		
Poisonings	Data Rich	Male	Maternal Education (years per capita)				X		
Poisonings	Global	Male	Maternal Education (years per capita)				X		
Poisonings	Data Rich	Male	Proportion of population involved in agricultural activities				X		
Poisonings	Global	Male	Proportion of population involved in agricultural activities				X		
Poisoning by carbon monoxide	Global	Female	Socio-demographic Index			X			X
Poisoning by carbon monoxide	Data Rich	Female	Socio-demographic Index			X			X
Poisoning by carbon monoxide	Global	Female	Healthcare access and quality index			X			X
Poisoning by carbon monoxide	Data Rich	Female	Healthcare access and quality index			X			X
Poisoning by carbon monoxide	Global	Female	Population-weighted mean temperature		X		X		
Poisoning by carbon monoxide	Data Rich	Female	Population-weighted mean temperature		X		X		
Poisoning by carbon monoxide	Global	Female	Liters of alcohol consumed per capita	X				X	
Poisoning by carbon monoxide	Data Rich	Female	Liters of alcohol consumed per capita	X				X	
Poisoning by carbon monoxide	Global	Female	Log-transformed SEV scalar: Inj Pois CO	X			X		
Poisoning by carbon monoxide	Data Rich	Female	Log-transformed SEV scalar: Inj Pois CO	X			X		
Poisoning by carbon monoxide	Global	Female	Proportion of population involved in agricultural activities						X
Poisoning by carbon monoxide	Data Rich	Female	Proportion of population involved in agricultural activities						X
Poisoning by carbon monoxide	Global	Female	Maternal Education (years per capita)					X	
Poisoning by carbon monoxide	Data Rich	Female	Maternal Education (years per capita)					X	
Poisoning by carbon monoxide	Global	Female	Indoor Air Pollution (All Cooking Fuels)					X	
Poisoning by carbon monoxide	Data Rich	Female	Indoor Air Pollution (All Cooking Fuels)					X	
Poisoning by carbon monoxide	Data Rich	Male	Socio-demographic Index			X			X
Poisoning by carbon monoxide	Global	Male	Socio-demographic Index			X			X
Poisoning by carbon monoxide	Data Rich	Male	Healthcare access and quality index			X			X
Poisoning by carbon monoxide	Global	Male	Healthcare access and quality index			X			X
Poisoning by carbon monoxide	Data Rich	Male	Population-weighted mean temperature		X		X		
Poisoning by carbon monoxide	Global	Male	Population-weighted mean temperature		X		X		
Poisoning by carbon monoxide	Data Rich	Male	Liters of alcohol consumed per capita	X				X	
Poisoning by carbon monoxide	Global	Male	Liters of alcohol consumed per capita	X				X	
Poisoning by carbon monoxide	Data Rich	Male	Log-transformed SEV scalar: Inj Pois CO	X			X		
Poisoning by carbon monoxide	Global	Male	Log-transformed SEV scalar: Inj Pois CO	X			X		
Poisoning by carbon monoxide	Data Rich	Male	Proportion of population involved in agricultural activities						X
Poisoning by carbon monoxide	Global	Male	Proportion of population involved in agricultural activities						X
Poisoning by carbon monoxide	Data Rich	Male	Maternal Education (years per capita)					X	
Poisoning by carbon monoxide	Global	Male	Maternal Education (years per capita)					X	
Poisoning by carbon monoxide	Data Rich	Male	Indoor Air Pollution (All Cooking Fuels)					X	
Poisoning by carbon monoxide	Global	Male	Indoor Air Pollution (All Cooking Fuels)					X	
Poisoning by other means	Global	Female	Socio-demographic Index			X			X
Poisoning by other means	Data Rich	Female	Socio-demographic Index			X			X
Poisoning by other means	Global	Female	Healthcare access and quality index			X			X
Poisoning by other means	Data Rich	Female	Healthcare access and quality index			X			X
Poisoning by other means	Global	Female	Liters of alcohol consumed per capita	X				X	
Poisoning by other means	Data Rich	Female	Liters of alcohol consumed per capita	X				X	
Poisoning by other means	Global	Female	Population-weighted mean temperature	X				X	
Poisoning by other means	Data Rich	Female	Population-weighted mean temperature	X				X	
Poisoning by other means	Global	Female	Log-transformed SEV scalar: Inj Pois Oth	X			X		
Poisoning by other means	Data Rich	Female	Log-transformed SEV scalar: Inj Pois Oth	X			X		
Poisoning by other means	Global	Female	Maternal Education (years per capita)				X		

Appendix Table S11: Comparison of GBD 2021 and GBD 2023 covariates and level of covariates used in cause of death modeling

Cause	Model Version Type	Sex	Covariate	Level 1 GBD 2021	Level 2 GBD 2021	Level 3 GBD 2021	Level 1 GBD 2023	Level 2 GBD 2023	Level 3 GBD 2023
Poisoning by other means	Data Rich	Female	Maternal Education (years per capita)				X		
Poisoning by other means	Global	Female	Proportion of population involved in agricultural activities				X		
Poisoning by other means	Data Rich	Female	Proportion of population involved in agricultural activities				X		
Poisoning by other means	Global	Male	Socio-demographic Index			X			X
Poisoning by other means	Data Rich	Male	Socio-demographic Index			X			X
Poisoning by other means	Global	Male	Healthcare access and quality index			X			X
Poisoning by other means	Data Rich	Male	Healthcare access and quality index			X			X
Poisoning by other means	Global	Male	Liters of alcohol consumed per capita	X				X	
Poisoning by other means	Data Rich	Male	Liters of alcohol consumed per capita	X				X	
Poisoning by other means	Global	Male	Population-weighted mean temperature	X				X	
Poisoning by other means	Data Rich	Male	Population-weighted mean temperature	X				X	
Poisoning by other means	Global	Male	Log-transformed SEV scalar: Inj Pois Oth	X			X		
Poisoning by other means	Data Rich	Male	Log-transformed SEV scalar: Inj Pois Oth	X			X		
Poisoning by other means	Global	Male	Maternal Education (years per capita)				X		
Poisoning by other means	Data Rich	Male	Maternal Education (years per capita)				X		
Poisoning by other means	Global	Male	Proportion of population involved in agricultural activities				X		
Poisoning by other means	Data Rich	Male	Proportion of population involved in agricultural activities				X		
Exposure to mechanical forces	Data Rich	Female	Socio-demographic Index			X			X
Exposure to mechanical forces	Global	Female	Socio-demographic Index			X			X
Exposure to mechanical forces	Data Rich	Female	Healthcare access and quality index		X			X	
Exposure to mechanical forces	Global	Female	Healthcare access and quality index		X			X	
Exposure to mechanical forces	Data Rich	Female	Population Density (under 150 ppl/sqkm, proportion)		X			X	
Exposure to mechanical forces	Global	Female	Population Density (under 150 ppl/sqkm, proportion)		X			X	
Exposure to mechanical forces	Data Rich	Female	Population Density (over 1000 ppl/sqkm, proportion)		X			X	
Exposure to mechanical forces	Global	Female	Population Density (over 1000 ppl/sqkm, proportion)		X			X	
Exposure to mechanical forces	Data Rich	Female	Population-weighted mean temperature	X			X		
Exposure to mechanical forces	Global	Female	Population-weighted mean temperature	X			X		
Exposure to mechanical forces	Data Rich	Male	Socio-demographic Index			X			X
Exposure to mechanical forces	Global	Male	Socio-demographic Index			X			X
Exposure to mechanical forces	Data Rich	Male	Healthcare access and quality index		X			X	
Exposure to mechanical forces	Global	Male	Healthcare access and quality index		X			X	
Exposure to mechanical forces	Data Rich	Male	Population Density (under 150 ppl/sqkm, proportion)		X			X	
Exposure to mechanical forces	Global	Male	Population Density (under 150 ppl/sqkm, proportion)		X			X	
Exposure to mechanical forces	Data Rich	Male	Population Density (over 1000 ppl/sqkm, proportion)		X			X	
Exposure to mechanical forces	Global	Male	Population Density (over 1000 ppl/sqkm, proportion)		X			X	
Exposure to mechanical forces	Data Rich	Male	Population-weighted mean temperature	X			X		
Exposure to mechanical forces	Global	Male	Population-weighted mean temperature	X			X		
Unintentional firearm injuries	Data Rich	Female	Socio-demographic Index			X			X
Unintentional firearm injuries	Global	Female	Socio-demographic Index			X			X
Unintentional firearm injuries	Data Rich	Female	Population Density (under 150 ppl/sqkm, proportion)			X			X
Unintentional firearm injuries	Global	Female	Population Density (under 150 ppl/sqkm, proportion)			X			X
Unintentional firearm injuries	Data Rich	Female	Population Density (over 1000 ppl/sqkm, proportion)			X			X
Unintentional firearm injuries	Global	Female	Population Density (over 1000 ppl/sqkm, proportion)			X			X
Unintentional firearm injuries	Data Rich	Female	Healthcare access and quality index		X			X	
Unintentional firearm injuries	Global	Female	Healthcare access and quality index		X			X	
Unintentional firearm injuries	Data Rich	Female	Population-weighted mean temperature	X			X		
Unintentional firearm injuries	Global	Female	Population-weighted mean temperature	X			X		
Unintentional firearm injuries	Data Rich	Female	Log-transformed SEV scalar: Mech Gun	X			X		
Unintentional firearm injuries	Global	Female	Log-transformed SEV scalar: Mech Gun	X			X		
Unintentional firearm injuries	Data Rich	Male	Socio-demographic Index			X			X
Unintentional firearm injuries	Global	Male	Socio-demographic Index			X			X
Unintentional firearm injuries	Data Rich	Male	Population Density (under 150 ppl/sqkm, proportion)			X			X
Unintentional firearm injuries	Global	Male	Population Density (under 150 ppl/sqkm, proportion)			X			X
Unintentional firearm injuries	Data Rich	Male	Population Density (over 1000 ppl/sqkm, proportion)			X			X
Unintentional firearm injuries	Global	Male	Population Density (over 1000 ppl/sqkm, proportion)			X			X
Unintentional firearm injuries	Data Rich	Male	Healthcare access and quality index		X			X	
Unintentional firearm injuries	Global	Male	Healthcare access and quality index		X			X	
Unintentional firearm injuries	Data Rich	Male	Population-weighted mean temperature	X			X		
Unintentional firearm injuries	Global	Male	Population-weighted mean temperature	X			X		
Unintentional firearm injuries	Data Rich	Male	Log-transformed SEV scalar: Mech Gun	X			X		
Unintentional firearm injuries	Global	Male	Log-transformed SEV scalar: Mech Gun	X			X		
Other exposure to mechanical forces	Data Rich	Female	Socio-demographic Index			X			X
Other exposure to mechanical forces	Global	Female	Socio-demographic Index			X			X
Other exposure to mechanical forces	Data Rich	Female	Healthcare access and quality index		X			X	
Other exposure to mechanical forces	Global	Female	Healthcare access and quality index		X			X	
Other exposure to mechanical forces	Data Rich	Female	Population Density (under 150 ppl/sqkm, proportion)		X			X	
Other exposure to mechanical forces	Global	Female	Population Density (under 150 ppl/sqkm, proportion)		X			X	
Other exposure to mechanical forces	Data Rich	Female	Population Density (over 1000 ppl/sqkm, proportion)		X			X	
Other exposure to mechanical forces	Global	Female	Population Density (over 1000 ppl/sqkm, proportion)		X			X	
Other exposure to mechanical forces	Data Rich	Female	Population-weighted mean temperature	X			X		
Other exposure to mechanical forces	Global	Female	Population-weighted mean temperature	X			X		
Other exposure to mechanical forces	Data Rich	Female	Log-transformed SEV scalar: Oth Mech	X			X		
Other exposure to mechanical forces	Global	Female	Log-transformed SEV scalar: Oth Mech	X			X		
Other exposure to mechanical forces	Data Rich	Male	Socio-demographic Index			X			X
Other exposure to mechanical forces	Global	Male	Socio-demographic Index			X			X
Other exposure to mechanical forces	Data Rich	Male	Healthcare access and quality index		X			X	
Other exposure to mechanical forces	Global	Male	Healthcare access and quality index		X			X	
Other exposure to mechanical forces	Data Rich	Male	Population Density (under 150 ppl/sqkm, proportion)		X			X	
Other exposure to mechanical forces	Global	Male	Population Density (under 150 ppl/sqkm, proportion)		X			X	
Other exposure to mechanical forces	Data Rich	Male	Population Density (over 1000 ppl/sqkm, proportion)		X			X	
Other exposure to mechanical forces	Global	Male	Population Density (over 1000 ppl/sqkm, proportion)		X			X	
Other exposure to mechanical forces	Data Rich	Male	Population-weighted mean temperature	X			X		
Other exposure to mechanical forces	Global	Male	Population-weighted mean temperature	X			X		
Other exposure to mechanical forces	Data Rich	Male	Log-transformed SEV scalar: Oth Mech	X			X		
Other exposure to mechanical forces	Global	Male	Log-transformed SEV scalar: Oth Mech	X			X		
Adverse effects of medical treatment	Global	Female	Socio-demographic Index			X			X
Adverse effects of medical treatment	Data Rich	Female	Socio-demographic Index			X			X
Adverse effects of medical treatment	Global	Female	Healthcare access and quality index		X		X		
Adverse effects of medical treatment	Data Rich	Female	Healthcare access and quality index		X		X		
Adverse effects of medical treatment	Global	Female	Population-weighted mean temperature	X				X	
Adverse effects of medical treatment	Data Rich	Female	Population-weighted mean temperature	X				X	
Adverse effects of medical treatment	Global	Male	Socio-demographic Index			X			X
Adverse effects of medical treatment	Data Rich	Male	Socio-demographic Index			X			X
Adverse effects of medical treatment	Global	Male	Healthcare access and quality index		X		X		
Adverse effects of medical treatment	Data Rich	Male	Healthcare access and quality index		X		X		
Adverse effects of medical treatment	Global	Male	Population-weighted mean temperature	X				X	
Adverse effects of medical treatment	Data Rich	Male	Population-weighted mean temperature	X				X	
Animal contact	Data Rich	Female	Socio-demographic Index			X			X
Animal contact	Global	Female	Socio-demographic Index			X			X
Animal contact	Data Rich	Female	Population Density (under 150 ppl/sqkm, proportion)			X			X
Animal contact	Global	Female	Population Density (under 150 ppl/sqkm, proportion)			X			X
Animal contact	Data Rich	Female	Elevation Over 1500m (proportion)			X			X
Animal contact	Global	Female	Elevation Over 1500m (proportion)			X			X
Animal contact	Data Rich	Female	Population Density (over 1000 ppl/sqkm, proportion)			X			X

Appendix Table S11: Comparison of GBD 2021 and GBD 2023 covariates and level of covariates used in cause of death modeling

Cause	Model Version Type	Sex	Covariate	Level 1 GBD 2021	Level 2 GBD 2021	Level 3 GBD 2021	Level 1 GBD 2023	Level 2 GBD 2023	Level 3 GBD 2023
Animal contact	Global	Female	Population Density (over 1000 ppl/sqkm, proportion)			X			X
Animal contact	Data Rich	Female	Elevation Under 100m (proportion)			X			X
Animal contact	Global	Female	Elevation Under 100m (proportion)			X			X
Animal contact	Data Rich	Female	Healthcare access and quality index		X			X	
Animal contact	Global	Female	Healthcare access and quality index		X			X	
Animal contact	Data Rich	Female	Population 15 to 30 (proportion)		X			X	
Animal contact	Global	Female	Population 15 to 30 (proportion)		X			X	
Animal contact	Data Rich	Female	Liters of alcohol consumed per capita	X			X		
Animal contact	Global	Female	Liters of alcohol consumed per capita	X			X		
Animal contact	Data Rich	Female	Population-weighted mean temperature	X			X		
Animal contact	Global	Female	Population-weighted mean temperature	X			X		
Animal contact	Data Rich	Female	Log-transformed SEV scalar: Animal	X			X		
Animal contact	Global	Female	Log-transformed SEV scalar: Animal	X			X		
Animal contact	Data Rich	Male	Socio-demographic Index			X			X
Animal contact	Global	Male	Socio-demographic Index			X			X
Animal contact	Data Rich	Male	Population Density (under 150 ppl/sqkm, proportion)			X			X
Animal contact	Global	Male	Population Density (under 150 ppl/sqkm, proportion)			X			X
Animal contact	Data Rich	Male	Elevation Over 1500m (proportion)			X			X
Animal contact	Global	Male	Elevation Over 1500m (proportion)			X			X
Animal contact	Data Rich	Male	Population Density (over 1000 ppl/sqkm, proportion)			X			X
Animal contact	Global	Male	Population Density (over 1000 ppl/sqkm, proportion)			X			X
Animal contact	Data Rich	Male	Elevation Under 100m (proportion)			X			X
Animal contact	Global	Male	Elevation Under 100m (proportion)			X			X
Animal contact	Data Rich	Male	Healthcare access and quality index		X			X	
Animal contact	Global	Male	Healthcare access and quality index		X			X	
Animal contact	Data Rich	Male	Population 15 to 30 (proportion)		X			X	
Animal contact	Global	Male	Population 15 to 30 (proportion)		X			X	
Animal contact	Data Rich	Male	Liters of alcohol consumed per capita	X			X		
Animal contact	Global	Male	Liters of alcohol consumed per capita	X			X		
Animal contact	Data Rich	Male	Population-weighted mean temperature	X			X		
Animal contact	Global	Male	Population-weighted mean temperature	X			X		
Animal contact	Data Rich	Male	Log-transformed SEV scalar: Animal	X			X		
Animal contact	Global	Male	Log-transformed SEV scalar: Animal	X			X		
Venomous animal contact	Global	Female	Socio-demographic Index			X			X
Venomous animal contact	Data Rich	Female	Socio-demographic Index			X			X
Venomous animal contact	Global	Female	Population Density (under 150 ppl/sqkm, proportion)			X			X
Venomous animal contact	Data Rich	Female	Population Density (under 150 ppl/sqkm, proportion)			X			X
Venomous animal contact	Global	Female	Elevation Over 1500m (proportion)			X			X
Venomous animal contact	Data Rich	Female	Elevation Over 1500m (proportion)			X			X
Venomous animal contact	Global	Female	Population Density (over 1000 ppl/sqkm, proportion)			X			X
Venomous animal contact	Data Rich	Female	Population Density (over 1000 ppl/sqkm, proportion)			X			X
Venomous animal contact	Global	Female	Elevation Under 100m (proportion)			X			X
Venomous animal contact	Data Rich	Female	Elevation Under 100m (proportion)			X			X
Venomous animal contact	Global	Female	Healthcare access and quality index		X			X	
Venomous animal contact	Data Rich	Female	Healthcare access and quality index		X			X	
Venomous animal contact	Global	Female	Liters of alcohol consumed per capita		X			X	
Venomous animal contact	Data Rich	Female	Liters of alcohol consumed per capita		X			X	
Venomous animal contact	Global	Female	Absolute value of average latitude		X			X	
Venomous animal contact	Data Rich	Female	Absolute value of average latitude		X			X	
Venomous animal contact	Global	Female	Population-weighted mean temperature		X			X	
Venomous animal contact	Data Rich	Female	Population-weighted mean temperature		X			X	
Venomous animal contact	Global	Female	Proportion of population involved in agricultural activities		X			X	
Venomous animal contact	Data Rich	Female	Proportion of population involved in agricultural activities		X			X	
Venomous animal contact	Global	Female	Rainfall Population-Weighted (mm/yr)		X			X	
Venomous animal contact	Data Rich	Female	Rainfall Population-Weighted (mm/yr)		X			X	
Venomous animal contact	Global	Female	Sahel Region of Africa (binary)		X			X	
Venomous animal contact	Data Rich	Female	Sahel Region of Africa (binary)		X			X	
Venomous animal contact	Global	Female	Log-transformed SEV scalar: Venom	X			X		
Venomous animal contact	Data Rich	Female	Log-transformed SEV scalar: Venom	X			X		
Venomous animal contact	Global	Female	Proportion of population vulnerable to venomous snakebites	X			X		
Venomous animal contact	Data Rich	Female	Proportion of population vulnerable to venomous snakebites	X			X		
Venomous animal contact	Global	Female	Mean number of venomous snake species	X			X		
Venomous animal contact	Data Rich	Female	Mean number of venomous snake species	X			X		
Venomous animal contact	Global	Male	Socio-demographic Index			X			X
Venomous animal contact	Data Rich	Male	Socio-demographic Index			X			X
Venomous animal contact	Global	Male	Population Density (under 150 ppl/sqkm, proportion)			X			X
Venomous animal contact	Data Rich	Male	Population Density (under 150 ppl/sqkm, proportion)			X			X
Venomous animal contact	Global	Male	Elevation Over 1500m (proportion)			X			X
Venomous animal contact	Data Rich	Male	Elevation Over 1500m (proportion)			X			X
Venomous animal contact	Global	Male	Population Density (over 1000 ppl/sqkm, proportion)			X			X
Venomous animal contact	Data Rich	Male	Population Density (over 1000 ppl/sqkm, proportion)			X			X
Venomous animal contact	Global	Male	Elevation Under 100m (proportion)			X			X
Venomous animal contact	Data Rich	Male	Elevation Under 100m (proportion)			X			X
Venomous animal contact	Global	Male	Healthcare access and quality index		X			X	
Venomous animal contact	Data Rich	Male	Healthcare access and quality index		X			X	
Venomous animal contact	Global	Male	Liters of alcohol consumed per capita		X			X	
Venomous animal contact	Data Rich	Male	Liters of alcohol consumed per capita		X			X	
Venomous animal contact	Global	Male	Absolute value of average latitude		X			X	
Venomous animal contact	Data Rich	Male	Absolute value of average latitude		X			X	
Venomous animal contact	Global	Male	Population-weighted mean temperature		X			X	
Venomous animal contact	Data Rich	Male	Population-weighted mean temperature		X			X	
Venomous animal contact	Global	Male	Proportion of population involved in agricultural activities		X			X	
Venomous animal contact	Data Rich	Male	Proportion of population involved in agricultural activities		X			X	
Venomous animal contact	Global	Male	Rainfall Population-Weighted (mm/yr)		X			X	
Venomous animal contact	Data Rich	Male	Rainfall Population-Weighted (mm/yr)		X			X	
Venomous animal contact	Data Rich	Male	Sahel Region of Africa (binary)		X			X	
Venomous animal contact	Global	Male	Log-transformed SEV scalar: Venom	X			X		
Venomous animal contact	Data Rich	Male	Log-transformed SEV scalar: Venom	X			X		
Venomous animal contact	Global	Male	Proportion of population vulnerable to venomous snakebites	X			X		
Venomous animal contact	Data Rich	Male	Proportion of population vulnerable to venomous snakebites	X			X		
Venomous animal contact	Global	Male	Mean number of venomous snake species	X			X		
Venomous animal contact	Data Rich	Male	Mean number of venomous snake species	X			X		
Non-venomous animal contact	Data Rich	Female	Socio-demographic Index			X			X
Non-venomous animal contact	Global	Female	Socio-demographic Index			X			X
Non-venomous animal contact	Data Rich	Female	Population Density (under 150 ppl/sqkm, proportion)			X			X
Non-venomous animal contact	Global	Female	Population Density (under 150 ppl/sqkm, proportion)			X			X
Non-venomous animal contact	Data Rich	Female	Elevation Over 1500m (proportion)			X			X
Non-venomous animal contact	Global	Female	Elevation Over 1500m (proportion)			X			X
Non-venomous animal contact	Data Rich	Female	Population Density (over 1000 ppl/sqkm, proportion)			X			X
Non-venomous animal contact	Global	Female	Population Density (over 1000 ppl/sqkm, proportion)			X			X
Non-venomous animal contact	Data Rich	Female	Elevation Under 100m (proportion)			X			X
Non-venomous animal contact	Global	Female	Elevation Under 100m (proportion)			X			X
Non-venomous animal contact	Data Rich	Female	Healthcare access and quality index		X			X	
Non-venomous animal contact	Global	Female	Healthcare access and quality index		X			X	
Non-venomous animal contact	Data Rich	Female	Liters of alcohol consumed per capita	X			X		
Non-venomous animal contact	Global	Female	Liters of alcohol consumed per capita	X			X		
Non-venomous animal contact	Data Rich	Female	Population-weighted mean temperature	X			X		
Non-venomous animal contact	Global	Female	Population-weighted mean temperature	X			X		
Non-venomous animal contact	Data Rich	Female	Log-transformed SEV scalar: Non Ven	X			X		

Appendix Table S11: Comparison of GBD 2021 and GBD 2023 covariates and level of covariates used in cause of death modeling

Cause	Model Version Type	Sex	Covariate	Level 1 GBD 2021	Level 2 GBD 2021	Level 3 GBD 2021	Level 1 GBD 2023	Level 2 GBD 2023	Level 3 GBD 2023
Non-venomous animal contact	Global	Female	Log-transformed SEV scalar: Non Ven	X			X		
Non-venomous animal contact	Data Rich	Male	Socio-demographic Index			X			X
Non-venomous animal contact	Global	Male	Socio-demographic Index			X			X
Non-venomous animal contact	Global	Male	Healthcare access and quality index			X			X
Non-venomous animal contact	Data Rich	Male	Population Density (under 150 ppl/sqkm, proportion)			X			X
Non-venomous animal contact	Data Rich	Male	Elevation Over 1500m (proportion)			X			X
Non-venomous animal contact	Data Rich	Male	Population Density (over 1000 ppl/sqkm, proportion)			X			X
Non-venomous animal contact	Data Rich	Male	Elevation Under 100m (proportion)			X			X
Non-venomous animal contact	Data Rich	Male	Healthcare access and quality index		X			X	
Non-venomous animal contact	Data Rich	Male	Liters of alcohol consumed per capita	X			X		
Non-venomous animal contact	Global	Male	Liters of alcohol consumed per capita	X			X		
Non-venomous animal contact	Global	Male	Population Density (under 150 ppl/sqkm, proportion)	X			X		
Non-venomous animal contact	Global	Male	Elevation Over 1500m (proportion)	X			X		
Non-venomous animal contact	Data Rich	Male	Population-weighted mean temperature	X			X		
Non-venomous animal contact	Global	Male	Population-weighted mean temperature	X			X		
Non-venomous animal contact	Global	Male	Population Density (over 1000 ppl/sqkm, proportion)	X			X		
Non-venomous animal contact	Global	Male	Elevation Under 100m (proportion)	X			X		
Non-venomous animal contact	Data Rich	Male	Log-transformed SEV scalar: Non Ven	X			X		
Non-venomous animal contact	Global	Male	Log-transformed SEV scalar: Non Ven	X			X		
Foreign body	Data Rich	Female	Socio-demographic Index			X			X
Foreign body	Global	Female	Socio-demographic Index			X			X
Foreign body	Data Rich	Female	Healthcare access and quality index		X			X	
Foreign body	Global	Female	Healthcare access and quality index		X			X	
Foreign body	Data Rich	Female	Liters of alcohol consumed per capita	X			X		
Foreign body	Global	Female	Liters of alcohol consumed per capita	X			X		
Foreign body	Data Rich	Female	Indoor Air Pollution (All Cooking Fuels)	X			X		
Foreign body	Global	Female	Indoor Air Pollution (All Cooking Fuels)	X			X		
Foreign body	Data Rich	Female	Population-weighted mean temperature	X			X		
Foreign body	Global	Female	Population-weighted mean temperature	X			X		
Foreign body	Data Rich	Female	Population Over 65 (proportion)	X			X		
Foreign body	Global	Female	Population Over 65 (proportion)	X			X		
Foreign body	Data Rich	Male	Socio-demographic Index			X			X
Foreign body	Global	Male	Socio-demographic Index			X			X
Foreign body	Data Rich	Male	Healthcare access and quality index		X			X	
Foreign body	Global	Male	Healthcare access and quality index		X			X	
Foreign body	Data Rich	Male	Liters of alcohol consumed per capita	X			X		
Foreign body	Global	Male	Liters of alcohol consumed per capita	X			X		
Foreign body	Data Rich	Male	Indoor Air Pollution (All Cooking Fuels)	X			X		
Foreign body	Global	Male	Indoor Air Pollution (All Cooking Fuels)	X			X		
Foreign body	Data Rich	Male	Population-weighted mean temperature	X			X		
Foreign body	Global	Male	Population-weighted mean temperature	X			X		
Foreign body	Data Rich	Male	Population Over 65 (proportion)	X			X		
Foreign body	Global	Male	Population Over 65 (proportion)	X			X		
Pulmonary aspiration and foreign body in airway	Data Rich	Female	Socio-demographic Index			X			X
Pulmonary aspiration and foreign body in airway	Global	Female	Socio-demographic Index			X			X
Pulmonary aspiration and foreign body in airway	Data Rich	Female	Healthcare access and quality index		X			X	
Pulmonary aspiration and foreign body in airway	Global	Female	Healthcare access and quality index		X			X	
Pulmonary aspiration and foreign body in airway	Data Rich	Female	Mean BMI		X			X	
Pulmonary aspiration and foreign body in airway	Global	Female	Mean BMI		X			X	
Pulmonary aspiration and foreign body in airway	Global	Female	Alcohol binge drinker proportion, age-standardized		X			X	
Pulmonary aspiration and foreign body in airway	Data Rich	Female	Liters of alcohol consumed per capita	X			X		
Pulmonary aspiration and foreign body in airway	Global	Female	Liters of alcohol consumed per capita	X			X		
Pulmonary aspiration and foreign body in airway	Data Rich	Female	Population-weighted mean temperature	X			X		
Pulmonary aspiration and foreign body in airway	Global	Female	Population-weighted mean temperature	X			X		
Pulmonary aspiration and foreign body in airway	Data Rich	Female	Log-transformed SEV scalar: F Body Asp	X			X		
Pulmonary aspiration and foreign body in airway	Global	Female	Log-transformed SEV scalar: F Body Asp	X			X		
Pulmonary aspiration and foreign body in airway	Data Rich	Male	Socio-demographic Index			X			X
Pulmonary aspiration and foreign body in airway	Global	Male	Socio-demographic Index			X			X
Pulmonary aspiration and foreign body in airway	Data Rich	Male	Healthcare access and quality index		X			X	
Pulmonary aspiration and foreign body in airway	Global	Male	Healthcare access and quality index		X			X	
Pulmonary aspiration and foreign body in airway	Data Rich	Male	Mean BMI		X			X	
Pulmonary aspiration and foreign body in airway	Global	Male	Mean BMI		X			X	
Pulmonary aspiration and foreign body in airway	Data Rich	Male	Liters of alcohol consumed per capita	X			X		
Pulmonary aspiration and foreign body in airway	Global	Male	Liters of alcohol consumed per capita	X			X		
Pulmonary aspiration and foreign body in airway	Data Rich	Male	Population-weighted mean temperature	X			X		
Pulmonary aspiration and foreign body in airway	Global	Male	Population-weighted mean temperature	X			X		
Pulmonary aspiration and foreign body in airway	Data Rich	Male	Log-transformed SEV scalar: F Body Asp	X			X		
Pulmonary aspiration and foreign body in airway	Global	Male	Log-transformed SEV scalar: F Body Asp	X			X		
Foreign body in other body part	Data Rich	Female	Socio-demographic Index			X			X
Foreign body in other body part	Global	Female	Socio-demographic Index			X			X
Foreign body in other body part	Data Rich	Female	Healthcare access and quality index		X			X	
Foreign body in other body part	Global	Female	Healthcare access and quality index		X			X	
Foreign body in other body part	Data Rich	Female	Liters of alcohol consumed per capita	X			X		
Foreign body in other body part	Global	Female	Liters of alcohol consumed per capita	X			X		
Foreign body in other body part	Data Rich	Female	Population-weighted mean temperature	X			X		
Foreign body in other body part	Global	Female	Population-weighted mean temperature	X			X		
Foreign body in other body part	Data Rich	Female	Log-transformed SEV scalar: Oth F Body	X			X		
Foreign body in other body part	Global	Female	Log-transformed SEV scalar: Oth F Body	X			X		
Foreign body in other body part	Data Rich	Male	Socio-demographic Index			X			X
Foreign body in other body part	Global	Male	Socio-demographic Index			X			X
Foreign body in other body part	Data Rich	Male	Healthcare access and quality index		X			X	
Foreign body in other body part	Global	Male	Healthcare access and quality index		X			X	
Foreign body in other body part	Data Rich	Male	Liters of alcohol consumed per capita	X			X		
Foreign body in other body part	Global	Male	Liters of alcohol consumed per capita	X			X		
Foreign body in other body part	Data Rich	Male	Population-weighted mean temperature	X			X		
Foreign body in other body part	Global	Male	Population-weighted mean temperature	X			X		
Foreign body in other body part	Data Rich	Male	Log-transformed SEV scalar: Oth F Body	X			X		
Foreign body in other body part	Global	Male	Log-transformed SEV scalar: Oth F Body	X			X		
Electrocution	Data Rich	Female	Socio-demographic Index						X
Electrocution	Global	Female	Socio-demographic Index						X
Electrocution	Data Rich	Female	Population Density (under 150 ppl/sqkm, proportion)						X
Electrocution	Global	Female	Population Density (under 150 ppl/sqkm, proportion)						X
Electrocution	Data Rich	Female	Population Density (over 1000 ppl/sqkm, proportion)						X
Electrocution	Global	Female	Population Density (over 1000 ppl/sqkm, proportion)						X
Electrocution	Data Rich	Female	Healthcare access and quality index					X	
Electrocution	Global	Female	Healthcare access and quality index					X	
Electrocution	Data Rich	Female	Vehicles - 2 wheels (per capita)				X		
Electrocution	Global	Female	Vehicles - 2 wheels (per capita)				X		
Electrocution	Data Rich	Female	Vehicles - 4 wheels (per capita)				X		
Electrocution	Global	Female	Vehicles - 4 wheels (per capita)				X		
Electrocution	Data Rich	Female	Log-transformed SEV scalar: Oth Unint				X		
Electrocution	Global	Female	Log-transformed SEV scalar: Oth Unint				X		
Electrocution	Data Rich	Male	Socio-demographic Index						X

Appendix Table S11: Comparison of GBD 2021 and GBD 2023 covariates and level of covariates used in cause of death modeling

Cause	Model Version Type	Sex	Covariate	Level 1 GBD 2021	Level 2 GBD 2021	Level 3 GBD 2021	Level 1 GBD 2023	Level 2 GBD 2023	Level 3 GBD 2023
Electrocution	Global	Male	Socio-demographic Index						X
Electrocution	Data Rich	Male	Population Density (under 150 ppl/sqkm, proportion)						X
Electrocution	Global	Male	Population Density (under 150 ppl/sqkm, proportion)						X
Electrocution	Data Rich	Male	Population Density (over 1000 ppl/sqkm, proportion)						X
Electrocution	Global	Male	Population Density (over 1000 ppl/sqkm, proportion)						X
Electrocution	Data Rich	Male	Healthcare access and quality index					X	
Electrocution	Global	Male	Healthcare access and quality index					X	
Electrocution	Data Rich	Male	Log-transformed SEV scalar: Oth Unint				X		
Electrocution	Global	Male	Log-transformed SEV scalar: Oth Unint				X		
Environmental heat and cold exposure	Global	Female	Education (years per capita)			X			X
Environmental heat and cold exposure	Data Rich	Female	Education (years per capita)			X			X
Environmental heat and cold exposure	Global	Female	LDI (I$ per capita)			X			X
Environmental heat and cold exposure	Data Rich	Female	LDI (I$ per capita)			X			X
Environmental heat and cold exposure	Global	Female	Socio-demographic Index			X			X
Environmental heat and cold exposure	Data Rich	Female	Socio-demographic Index			X			X
Environmental heat and cold exposure	Global	Female	Sanitation (proportion with access)			X			X
Environmental heat and cold exposure	Global	Female	Elevation Over 1500m (proportion)			X			X
Environmental heat and cold exposure	Data Rich	Female	Elevation Over 1500m (proportion)			X			X
Environmental heat and cold exposure	Global	Female	Population-weighted mean temperature			X			X
Environmental heat and cold exposure	Data Rich	Female	Population-weighted mean temperature			X			X
Environmental heat and cold exposure	Global	Female	Population Density (150-300 ppl/sqkm, proportion)			X			X
Environmental heat and cold exposure	Data Rich	Female	Population Density (150-300 ppl/sqkm, proportion)			X			X
Environmental heat and cold exposure	Global	Female	Rainfall (Quintiles 4-5)			X			X
Environmental heat and cold exposure	Data Rich	Female	Rainfall (Quintiles 4-5)			X			X
Environmental heat and cold exposure	Global	Female	90th percentile climatic temperature in the given country-year.			X			X
Environmental heat and cold exposure	Data Rich	Female	90th percentile climatic temperature in the given country-year.			X			X
Environmental heat and cold exposure	Data Rich	Female	Sanitation (proportion with access)			X		X	
Environmental heat and cold exposure	Global	Female	Healthcare access and quality index		X			X	
Environmental heat and cold exposure	Data Rich	Female	Healthcare access and quality index		X		X		
Environmental heat and cold exposure	Data Rich	Female	Liters of alcohol consumed per capita					X	
Environmental heat and cold exposure	Global	Female	Liters of alcohol consumed per capita				X		
Environmental heat and cold exposure	Data Rich	Male	Socio-demographic Index			X			X
Environmental heat and cold exposure	Global	Male	Socio-demographic Index			X			X
Environmental heat and cold exposure	Data Rich	Male	Sanitation (proportion with access)			X			X
Environmental heat and cold exposure	Global	Male	Sanitation (proportion with access)			X			X
Environmental heat and cold exposure	Data Rich	Male	Elevation Over 1500m (proportion)			X			X
Environmental heat and cold exposure	Global	Male	Elevation Over 1500m (proportion)			X			X
Environmental heat and cold exposure	Data Rich	Male	Population-weighted mean temperature			X			X
Environmental heat and cold exposure	Data Rich	Male	Population Density (150-300 ppl/sqkm, proportion)			X			X
Environmental heat and cold exposure	Global	Male	Population Density (150-300 ppl/sqkm, proportion)			X			X
Environmental heat and cold exposure	Data Rich	Male	Rainfall (Quintiles 4-5)			X			X
Environmental heat and cold exposure	Global	Male	Rainfall (Quintiles 4-5)			X			X
Environmental heat and cold exposure	Global	Male	Population-weighted mean temperature			X		X	
Environmental heat and cold exposure	Data Rich	Male	90th percentile climatic temperature in the given country-year.			X		X	
Environmental heat and cold exposure	Global	Male	90th percentile climatic temperature in the given country-year.			X		X	
Environmental heat and cold exposure	Data Rich	Male	Healthcare access and quality index		X			X	
Environmental heat and cold exposure	Global	Male	Healthcare access and quality index		X			X	
Environmental heat and cold exposure	Data Rich	Male	Liters of alcohol consumed per capita				X		
Environmental heat and cold exposure	Global	Male	Liters of alcohol consumed per capita				X		
Other unintentional injuries	Data Rich	Female	Socio-demographic Index			X			X
Other unintentional injuries	Global	Female	Socio-demographic Index			X			X
Other unintentional injuries	Data Rich	Female	Population Density (under 150 ppl/sqkm, proportion)			X			X
Other unintentional injuries	Global	Female	Population Density (under 150 ppl/sqkm, proportion)			X			X
Other unintentional injuries	Data Rich	Female	Population Density (over 1000 ppl/sqkm, proportion)			X			X
Other unintentional injuries	Global	Female	Population Density (over 1000 ppl/sqkm, proportion)			X			X
Other unintentional injuries	Data Rich	Female	Healthcare access and quality index		X			X	
Other unintentional injuries	Global	Female	Healthcare access and quality index		X			X	
Other unintentional injuries	Data Rich	Female	Liters of alcohol consumed per capita	X			X		
Other unintentional injuries	Global	Female	Liters of alcohol consumed per capita	X			X		
Other unintentional injuries	Data Rich	Female	Population-weighted mean temperature	X			X		
Other unintentional injuries	Global	Female	Population-weighted mean temperature	X			X		
Other unintentional injuries	Data Rich	Female	Vehicles - 2 wheels (per capita)	X			X		
Other unintentional injuries	Global	Female	Vehicles - 2 wheels (per capita)	X			X		
Other unintentional injuries	Data Rich	Female	Vehicles - 4 wheels (per capita)	X			X		
Other unintentional injuries	Global	Female	Vehicles - 4 wheels (per capita)	X			X		
Other unintentional injuries	Data Rich	Female	Log-transformed SEV scalar: Oth Unint	X			X		
Other unintentional injuries	Global	Female	Log-transformed SEV scalar: Oth Unint	X			X		
Other unintentional injuries	Data Rich	Male	Socio-demographic Index			X			X
Other unintentional injuries	Global	Male	Socio-demographic Index			X			X
Other unintentional injuries	Data Rich	Male	Population Density (under 150 ppl/sqkm, proportion)			X			X
Other unintentional injuries	Global	Male	Population Density (under 150 ppl/sqkm, proportion)			X			X
Other unintentional injuries	Data Rich	Male	Population Density (over 1000 ppl/sqkm, proportion)			X			X
Other unintentional injuries	Global	Male	Population Density (over 1000 ppl/sqkm, proportion)			X			X
Other unintentional injuries	Data Rich	Male	Healthcare access and quality index		X			X	
Other unintentional injuries	Global	Male	Healthcare access and quality index		X			X	
Other unintentional injuries	Data Rich	Male	Liters of alcohol consumed per capita	X			X		
Other unintentional injuries	Global	Male	Liters of alcohol consumed per capita	X			X		
Other unintentional injuries	Data Rich	Male	Population-weighted mean temperature	X			X		
Other unintentional injuries	Global	Male	Population-weighted mean temperature	X			X		
Other unintentional injuries	Data Rich	Male	Vehicles - 2 wheels (per capita)	X			X		
Other unintentional injuries	Global	Male	Vehicles - 2 wheels (per capita)	X			X		
Other unintentional injuries	Data Rich	Male	Vehicles - 4 wheels (per capita)	X			X		
Other unintentional injuries	Global	Male	Vehicles - 4 wheels (per capita)	X			X		
Other unintentional injuries	Data Rich	Male	Log-transformed SEV scalar: Oth Unint	X			X		
Other unintentional injuries	Global	Male	Log-transformed SEV scalar: Oth Unint	X			X		
Self-harm	Global	Female	Education (years per capita)			X			X
Self-harm	Data Rich	Female	Education (years per capita)			X			X
Self-harm	Global	Female	LDI (I$ per capita)			X			X
Self-harm	Data Rich	Female	LDI (I$ per capita)			X			X
Self-harm	Global	Female	Socio-demographic Index			X			X
Self-harm	Data Rich	Female	Socio-demographic Index			X			X
Self-harm	Global	Female	Healthcare access and quality index		X			X	
Self-harm	Data Rich	Female	Healthcare access and quality index		X			X	
Self-harm	Global	Female	Population Density (500-1000 ppl/sqkm, proportion)		X			X	
Self-harm	Data Rich	Female	Population Density (500-1000 ppl/sqkm, proportion)		X			X	
Self-harm	Global	Female	Population Density (under 150 ppl/sqkm, proportion)		X			X	
Self-harm	Data Rich	Female	Population Density (under 150 ppl/sqkm, proportion)		X			X	
Self-harm	Global	Female	Population Density (over 1000 ppl/sqkm, proportion)		X			X	
Self-harm	Data Rich	Female	Population Density (over 1000 ppl/sqkm, proportion)		X			X	
Self-harm	Global	Female	Population Density (150-300 ppl/sqkm, proportion)		X			X	
Self-harm	Data Rich	Female	Population Density (150-300 ppl/sqkm, proportion)		X			X	
Self-harm	Global	Female	Population Density (300-500 ppl/sqkm, proportion)		X			X	
Self-harm	Data Rich	Female	Population Density (300-500 ppl/sqkm, proportion)		X			X	
Self-harm	Global	Female	Liters of alcohol consumed per capita	X			X		
Self-harm	Data Rich	Female	Liters of alcohol consumed per capita	X			X		

Appendix Table S11: Comparison of GBD 2021 and GBD 2023 covariates and level of covariates used in cause of death modeling

Cause	Model Version Type	Sex	Covariate	Level 1 GBD 2021	Level 2 GBD 2021	Level 3 GBD 2021	Level 1 GBD 2023	Level 2 GBD 2023	Level 3 GBD 2023
Self-harm	Global	Female	Population-weighted mean temperature	X			X		
Self-harm	Data Rich	Female	Population-weighted mean temperature	X			X		
Self-harm	Global	Female	Log-transformed SEV scalar: Self Harm	X			X		
Self-harm	Data Rich	Female	Log-transformed SEV scalar: Self Harm	X			X		
Self-harm	Global	Female	Major depressive disorder	X			X		
Self-harm	Data Rich	Female	Major depressive disorder	X			X		
Self-harm	Global	Male	Education (years per capita)			X			X
Self-harm	Data Rich	Male	Education (years per capita)			X			X
Self-harm	Global	Male	LDI (I$ per capita)			X			X
Self-harm	Data Rich	Male	LDI (I$ per capita)			X			X
Self-harm	Global	Male	Socio-demographic Index			X			X
Self-harm	Data Rich	Male	Socio-demographic Index			X			X
Self-harm	Global	Male	Healthcare access and quality index		X			X	
Self-harm	Data Rich	Male	Healthcare access and quality index		X			X	
Self-harm	Global	Male	Population Density (500-1000 ppl/sqkm, proportion)		X			X	
Self-harm	Data Rich	Male	Population Density (500-1000 ppl/sqkm, proportion)		X			X	
Self-harm	Global	Male	Population Density (under 150 ppl/sqkm, proportion)		X			X	
Self-harm	Data Rich	Male	Population Density (under 150 ppl/sqkm, proportion)		X			X	
Self-harm	Global	Male	Population Density (over 1000 ppl/sqkm, proportion)		X			X	
Self-harm	Data Rich	Male	Population Density (over 1000 ppl/sqkm, proportion)		X			X	
Self-harm	Global	Male	Population Density (150-300 ppl/sqkm, proportion)		X			X	
Self-harm	Data Rich	Male	Population Density (150-300 ppl/sqkm, proportion)		X			X	
Self-harm	Global	Male	Population Density (300-500 ppl/sqkm, proportion)		X			X	
Self-harm	Data Rich	Male	Population Density (300-500 ppl/sqkm, proportion)		X			X	
Self-harm	Global	Male	Liters of alcohol consumed per capita	X			X		
Self-harm	Data Rich	Male	Liters of alcohol consumed per capita	X			X		
Self-harm	Global	Male	Population-weighted mean temperature	X			X		
Self-harm	Data Rich	Male	Population-weighted mean temperature	X			X		
Self-harm	Global	Male	Major depressive disorder	X			X		
Self-harm	Data Rich	Male	Major depressive disorder	X			X		
Self-harm	Global	Male	Log-transformed SEV scalar: Self Harm				X		
Self-harm	Data Rich	Male	Log-transformed SEV scalar: Self Harm				X		
Self-harm by hanging, strangulation, and suffocation	Global	Female	Education (years per capita)						X
Self-harm by hanging, strangulation, and suffocation	Data Rich	Female	Education (years per capita)						X
Self-harm by hanging, strangulation, and suffocation	Global	Female	LDI (I$ per capita)						X
Self-harm by hanging, strangulation, and suffocation	Data Rich	Female	LDI (I$ per capita)						X
Self-harm by hanging, strangulation, and suffocation	Global	Female	Socio-demographic Index						X
Self-harm by hanging, strangulation, and suffocation	Data Rich	Female	Socio-demographic Index						X
Self-harm by hanging, strangulation, and suffocation	Global	Female	Healthcare access and quality index					X	
Self-harm by hanging, strangulation, and suffocation	Data Rich	Female	Healthcare access and quality index					X	
Self-harm by hanging, strangulation, and suffocation	Global	Female	Population Density (500-1000 ppl/sqkm, proportion)					X	
Self-harm by hanging, strangulation, and suffocation	Data Rich	Female	Population Density (500-1000 ppl/sqkm, proportion)					X	
Self-harm by hanging, strangulation, and suffocation	Global	Female	Population Density (under 150 ppl/sqkm, proportion)					X	
Self-harm by hanging, strangulation, and suffocation	Data Rich	Female	Population Density (under 150 ppl/sqkm, proportion)					X	
Self-harm by hanging, strangulation, and suffocation	Global	Female	Population Density (over 1000 ppl/sqkm, proportion)					X	
Self-harm by hanging, strangulation, and suffocation	Data Rich	Female	Population Density (over 1000 ppl/sqkm, proportion)					X	
Self-harm by hanging, strangulation, and suffocation	Global	Female	Population Density (150-300 ppl/sqkm, proportion)					X	
Self-harm by hanging, strangulation, and suffocation	Data Rich	Female	Population Density (150-300 ppl/sqkm, proportion)					X	
Self-harm by hanging, strangulation, and suffocation	Global	Female	Population Density (300-500 ppl/sqkm, proportion)					X	
Self-harm by hanging, strangulation, and suffocation	Data Rich	Female	Population Density (300-500 ppl/sqkm, proportion)					X	
Self-harm by hanging, strangulation, and suffocation	Global	Female	Liters of alcohol consumed per capita				X		
Self-harm by hanging, strangulation, and suffocation	Data Rich	Female	Liters of alcohol consumed per capita				X		
Self-harm by hanging, strangulation, and suffocation	Global	Female	Population-weighted mean temperature				X		
Self-harm by hanging, strangulation, and suffocation	Data Rich	Female	Population-weighted mean temperature				X		
Self-harm by hanging, strangulation, and suffocation	Global	Female	Major depressive disorder				X		
Self-harm by hanging, strangulation, and suffocation	Data Rich	Female	Major depressive disorder				X		
Self-harm by hanging, strangulation, and suffocation	Global	Male	Education (years per capita)						X
Self-harm by hanging, strangulation, and suffocation	Data Rich	Male	Education (years per capita)						X
Self-harm by hanging, strangulation, and suffocation	Global	Male	LDI (I$ per capita)						X
Self-harm by hanging, strangulation, and suffocation	Data Rich	Male	LDI (I$ per capita)						X
Self-harm by hanging, strangulation, and suffocation	Global	Male	Socio-demographic Index						X
Self-harm by hanging, strangulation, and suffocation	Data Rich	Male	Socio-demographic Index						X
Self-harm by hanging, strangulation, and suffocation	Global	Male	Healthcare access and quality index					X	
Self-harm by hanging, strangulation, and suffocation	Data Rich	Male	Healthcare access and quality index					X	
Self-harm by hanging, strangulation, and suffocation	Global	Male	Population Density (500-1000 ppl/sqkm, proportion)					X	
Self-harm by hanging, strangulation, and suffocation	Data Rich	Male	Population Density (500-1000 ppl/sqkm, proportion)					X	
Self-harm by hanging, strangulation, and suffocation	Global	Male	Population Density (under 150 ppl/sqkm, proportion)					X	
Self-harm by hanging, strangulation, and suffocation	Data Rich	Male	Population Density (under 150 ppl/sqkm, proportion)					X	
Self-harm by hanging, strangulation, and suffocation	Global	Male	Population Density (over 1000 ppl/sqkm, proportion)					X	
Self-harm by hanging, strangulation, and suffocation	Data Rich	Male	Population Density (over 1000 ppl/sqkm, proportion)					X	
Self-harm by hanging, strangulation, and suffocation	Global	Male	Population Density (150-300 ppl/sqkm, proportion)					X	
Self-harm by hanging, strangulation, and suffocation	Data Rich	Male	Population Density (150-300 ppl/sqkm, proportion)					X	
Self-harm by hanging, strangulation, and suffocation	Global	Male	Population Density (300-500 ppl/sqkm, proportion)					X	
Self-harm by hanging, strangulation, and suffocation	Data Rich	Male	Population Density (300-500 ppl/sqkm, proportion)					X	
Self-harm by hanging, strangulation, and suffocation	Global	Male	Liters of alcohol consumed per capita				X		
Self-harm by hanging, strangulation, and suffocation	Data Rich	Male	Liters of alcohol consumed per capita				X		
Self-harm by hanging, strangulation, and suffocation	Global	Male	Population-weighted mean temperature				X		
Self-harm by hanging, strangulation, and suffocation	Data Rich	Male	Population-weighted mean temperature				X		
Self-harm by hanging, strangulation, and suffocation	Global	Male	Major depressive disorder				X		
Self-harm by hanging, strangulation, and suffocation	Data Rich	Male	Major depressive disorder				X		
Self-harm by fire, heat, and hot substances	Global	Female	Education (years per capita)						X
Self-harm by fire, heat, and hot substances	Data Rich	Female	Education (years per capita)						X
Self-harm by fire, heat, and hot substances	Global	Female	LDI (I$ per capita)						X
Self-harm by fire, heat, and hot substances	Data Rich	Female	LDI (I$ per capita)						X
Self-harm by fire, heat, and hot substances	Global	Female	Socio-demographic Index						X
Self-harm by fire, heat, and hot substances	Data Rich	Female	Socio-demographic Index						X
Self-harm by fire, heat, and hot substances	Global	Female	Healthcare access and quality index					X	
Self-harm by fire, heat, and hot substances	Data Rich	Female	Healthcare access and quality index					X	
Self-harm by fire, heat, and hot substances	Global	Female	Population Density (500-1000 ppl/sqkm, proportion)					X	
Self-harm by fire, heat, and hot substances	Data Rich	Female	Population Density (500-1000 ppl/sqkm, proportion)					X	
Self-harm by fire, heat, and hot substances	Global	Female	Population Density (under 150 ppl/sqkm, proportion)					X	
Self-harm by fire, heat, and hot substances	Data Rich	Female	Population Density (under 150 ppl/sqkm, proportion)					X	
Self-harm by fire, heat, and hot substances	Global	Female	Population Density (over 1000 ppl/sqkm, proportion)					X	
Self-harm by fire, heat, and hot substances	Data Rich	Female	Population Density (over 1000 ppl/sqkm, proportion)					X	
Self-harm by fire, heat, and hot substances	Global	Female	Population Density (150-300 ppl/sqkm, proportion)					X	
Self-harm by fire, heat, and hot substances	Data Rich	Female	Population Density (150-300 ppl/sqkm, proportion)					X	
Self-harm by fire, heat, and hot substances	Global	Female	Population Density (300-500 ppl/sqkm, proportion)					X	

Appendix Table S11: Comparison of GBD 2021 and GBD 2023 covariates and level of covariates used in cause of death modeling									
Cause	Model Version Type	Sex	Covariate	Level 1 GBD 2021	Level 2 GBD 2021	Level 3 GBD 2021	Level 1 GBD 2023	Level 2 GBD 2023	Level 3 GBD 2023
Self-harm by fire, heat, and hot substances	Data Rich	Female	Population Density (300-500 ppl/sqkm, proportion)					X	
Self-harm by fire, heat, and hot substances	Global	Female	Liters of alcohol consumed per capita				X		
Self-harm by fire, heat, and hot substances	Data Rich	Female	Liters of alcohol consumed per capita				X		
Self-harm by fire, heat, and hot substances	Global	Female	Population-weighted mean temperature				X		
Self-harm by fire, heat, and hot substances	Data Rich	Female	Population-weighted mean temperature				X		
Self-harm by fire, heat, and hot substances	Global	Female	Major depressive disorder				X		
Self-harm by fire, heat, and hot substances	Data Rich	Female	Major depressive disorder				X		
Self-harm by fire, heat, and hot substances	Global	Male	Education (years per capita)						X
Self-harm by fire, heat, and hot substances	Data Rich	Male	Education (years per capita)						X
Self-harm by fire, heat, and hot substances	Global	Male	LDI (I$ per capita)						X
Self-harm by fire, heat, and hot substances	Data Rich	Male	LDI (I$ per capita)						X
Self-harm by fire, heat, and hot substances	Global	Male	Socio-demographic Index						X
Self-harm by fire, heat, and hot substances	Data Rich	Male	Socio-demographic Index						X
Self-harm by fire, heat, and hot substances	Global	Male	Healthcare access and quality index					X	
Self-harm by fire, heat, and hot substances	Data Rich	Male	Healthcare access and quality index					X	
Self-harm by fire, heat, and hot substances	Global	Male	Population Density (500-1000 ppl/sqkm, proportion)					X	
Self-harm by fire, heat, and hot substances	Data Rich	Male	Population Density (500-1000 ppl/sqkm, proportion)					X	
Self-harm by fire, heat, and hot substances	Global	Male	Population Density (under 150 ppl/sqkm, proportion)					X	
Self-harm by fire, heat, and hot substances	Data Rich	Male	Population Density (under 150 ppl/sqkm, proportion)					X	
Self-harm by fire, heat, and hot substances	Global	Male	Population Density (over 1000 ppl/sqkm, proportion)					X	
Self-harm by fire, heat, and hot substances	Data Rich	Male	Population Density (over 1000 ppl/sqkm, proportion)					X	
Self-harm by fire, heat, and hot substances	Global	Male	Population Density (150-300 ppl/sqkm, proportion)					X	
Self-harm by fire, heat, and hot substances	Data Rich	Male	Population Density (150-300 ppl/sqkm, proportion)					X	
Self-harm by fire, heat, and hot substances	Global	Male	Population Density (300-500 ppl/sqkm, proportion)					X	
Self-harm by fire, heat, and hot substances	Data Rich	Male	Population Density (300-500 ppl/sqkm, proportion)					X	
Self-harm by fire, heat, and hot substances	Global	Male	Liters of alcohol consumed per capita				X		
Self-harm by fire, heat, and hot substances	Data Rich	Male	Liters of alcohol consumed per capita				X		
Self-harm by fire, heat, and hot substances	Global	Male	Population-weighted mean temperature				X		
Self-harm by fire, heat, and hot substances	Data Rich	Male	Population-weighted mean temperature				X		
Self-harm by fire, heat, and hot substances	Global	Male	Major depressive disorder				X		
Self-harm by fire, heat, and hot substances	Data Rich	Male	Major depressive disorder				X		
Self-harm by firearm	Global	Female	Education (years per capita)			X			X
Self-harm by firearm	Data Rich	Female	Education (years per capita)			X			X
Self-harm by firearm	Global	Female	LDI (I$ per capita)			X			X
Self-harm by firearm	Data Rich	Female	LDI (I$ per capita)			X			X
Self-harm by firearm	Global	Female	Socio-demographic Index			X			X
Self-harm by firearm	Data Rich	Female	Socio-demographic Index			X			X
Self-harm by firearm	Global	Female	Healthcare access and quality index		X			X	
Self-harm by firearm	Data Rich	Female	Healthcare access and quality index		X			X	
Self-harm by firearm	Global	Female	Population Density (500-1000 ppl/sqkm, proportion)		X			X	
Self-harm by firearm	Data Rich	Female	Population Density (500-1000 ppl/sqkm, proportion)		X			X	
Self-harm by firearm	Global	Female	Population Density (under 150 ppl/sqkm, proportion)		X			X	
Self-harm by firearm	Data Rich	Female	Population Density (under 150 ppl/sqkm, proportion)		X			X	
Self-harm by firearm	Global	Female	Population Density (over 1000 ppl/sqkm, proportion)		X			X	
Self-harm by firearm	Data Rich	Female	Population Density (over 1000 ppl/sqkm, proportion)		X			X	
Self-harm by firearm	Global	Female	Population Density (150-300 ppl/sqkm, proportion)		X			X	
Self-harm by firearm	Data Rich	Female	Population Density (150-300 ppl/sqkm, proportion)		X			X	
Self-harm by firearm	Global	Female	Population Density (300-500 ppl/sqkm, proportion)		X			X	
Self-harm by firearm	Data Rich	Female	Population Density (300-500 ppl/sqkm, proportion)		X			X	
Self-harm by firearm	Global	Female	Liters of alcohol consumed per capita	X			X		
Self-harm by firearm	Data Rich	Female	Liters of alcohol consumed per capita	X			X		
Self-harm by firearm	Global	Female	Population-weighted mean temperature	X			X		
Self-harm by firearm	Data Rich	Female	Population-weighted mean temperature	X			X		
Self-harm by firearm	Global	Female	Log-transformed SEV scalar: Self Harm	X			X		
Self-harm by firearm	Data Rich	Female	Log-transformed SEV scalar: Self Harm	X			X		
Self-harm by firearm	Global	Female	Major depressive disorder	X			X		
Self-harm by firearm	Data Rich	Female	Major depressive disorder	X			X		
Self-harm by firearm	Global	Male	Education (years per capita)			X			X
Self-harm by firearm	Data Rich	Male	Education (years per capita)			X			X
Self-harm by firearm	Global	Male	LDI (I$ per capita)			X			X
Self-harm by firearm	Data Rich	Male	LDI (I$ per capita)			X			X
Self-harm by firearm	Global	Male	Socio-demographic Index			X			X
Self-harm by firearm	Data Rich	Male	Socio-demographic Index			X			X
Self-harm by firearm	Global	Male	Healthcare access and quality index		X			X	
Self-harm by firearm	Data Rich	Male	Healthcare access and quality index		X			X	
Self-harm by firearm	Global	Male	Population Density (500-1000 ppl/sqkm, proportion)		X			X	
Self-harm by firearm	Data Rich	Male	Population Density (500-1000 ppl/sqkm, proportion)		X			X	
Self-harm by firearm	Global	Male	Population Density (under 150 ppl/sqkm, proportion)		X			X	
Self-harm by firearm	Data Rich	Male	Population Density (under 150 ppl/sqkm, proportion)		X			X	
Self-harm by firearm	Global	Male	Population Density (over 1000 ppl/sqkm, proportion)		X			X	
Self-harm by firearm	Data Rich	Male	Population Density (over 1000 ppl/sqkm, proportion)		X			X	
Self-harm by firearm	Global	Male	Population Density (150-300 ppl/sqkm, proportion)		X			X	
Self-harm by firearm	Data Rich	Male	Population Density (150-300 ppl/sqkm, proportion)		X			X	
Self-harm by firearm	Global	Male	Population Density (300-500 ppl/sqkm, proportion)		X			X	
Self-harm by firearm	Data Rich	Male	Population Density (300-500 ppl/sqkm, proportion)		X			X	
Self-harm by firearm	Global	Male	Liters of alcohol consumed per capita	X			X		
Self-harm by firearm	Data Rich	Male	Liters of alcohol consumed per capita	X			X		
Self-harm by firearm	Global	Male	Population-weighted mean temperature	X			X		
Self-harm by firearm	Data Rich	Male	Population-weighted mean temperature	X			X		
Self-harm by firearm	Global	Male	Log-transformed SEV scalar: Self Harm	X			X		
Self-harm by firearm	Data Rich	Male	Log-transformed SEV scalar: Self Harm	X			X		
Self-harm by firearm	Global	Male	Major depressive disorder	X			X		
Self-harm by firearm	Data Rich	Male	Major depressive disorder	X			X		
Self-harm by poisoning pesticides	Global	Female	Education (years per capita)						X
Self-harm by poisoning pesticides	Data Rich	Female	Education (years per capita)						X
Self-harm by poisoning pesticides	Global	Female	LDI (I$ per capita)						X
Self-harm by poisoning pesticides	Data Rich	Female	LDI (I$ per capita)						X
Self-harm by poisoning pesticides	Global	Female	Socio-demographic Index						X
Self-harm by poisoning pesticides	Data Rich	Female	Socio-demographic Index						X
Self-harm by poisoning pesticides	Global	Female	Healthcare access and quality index					X	
Self-harm by poisoning pesticides	Data Rich	Female	Healthcare access and quality index					X	
Self-harm by poisoning pesticides	Global	Female	Population Density (500-1000 ppl/sqkm, proportion)					X	
Self-harm by poisoning pesticides	Data Rich	Female	Population Density (500-1000 ppl/sqkm, proportion)					X	
Self-harm by poisoning pesticides	Global	Female	Population Density (under 150 ppl/sqkm, proportion)					X	
Self-harm by poisoning pesticides	Data Rich	Female	Population Density (under 150 ppl/sqkm, proportion)					X	
Self-harm by poisoning pesticides	Global	Female	Population Density (over 1000 ppl/sqkm, proportion)					X	
Self-harm by poisoning pesticides	Data Rich	Female	Population Density (over 1000 ppl/sqkm, proportion)					X	
Self-harm by poisoning pesticides	Global	Female	Population Density (150-300 ppl/sqkm, proportion)					X	
Self-harm by poisoning pesticides	Data Rich	Female	Population Density (150-300 ppl/sqkm, proportion)					X	
Self-harm by poisoning pesticides	Global	Female	Population Density (300-500 ppl/sqkm, proportion)					X	
Self-harm by poisoning pesticides	Data Rich	Female	Population Density (300-500 ppl/sqkm, proportion)					X	
Self-harm by poisoning pesticides	Global	Female	Liters of alcohol consumed per capita				X		
Self-harm by poisoning pesticides	Data Rich	Female	Liters of alcohol consumed per capita				X		
Self-harm by poisoning pesticides	Global	Female	Population-weighted mean temperature				X		

Appendix Table S11: Comparison of GBD 2021 and GBD 2023 covariates and level of covariates used in cause of death modeling									
**Cause**	**Model Version Type**	**Sex**	**Covariate**	**Level 1 GBD 2021**	**Level 2 GBD 2021**	**Level 3 GBD 2021**	**Level 1 GBD 2023**	**Level 2 GBD 2023**	**Level 3 GBD 2023**
Self-harm by poisoning pesticides	Data Rich	Female	Population-weighted mean temperature				X		
Self-harm by poisoning pesticides	Global	Female	Log-transformed SEV scalar: Self Harm				X		
Self-harm by poisoning pesticides	Data Rich	Female	Log-transformed SEV scalar: Self Harm				X		
Self-harm by poisoning pesticides	Global	Female	Major depressive disorder				X		
Self-harm by poisoning pesticides	Data Rich	Female	Major depressive disorder				X		
Self-harm by poisoning pesticides	Global	Male	Education (years per capita)						X
Self-harm by poisoning pesticides	Data Rich	Male	Education (years per capita)						X
Self-harm by poisoning pesticides	Global	Male	LDI (I$ per capita)						X
Self-harm by poisoning pesticides	Data Rich	Male	LDI (I$ per capita)						X
Self-harm by poisoning pesticides	Global	Male	Socio-demographic Index						X
Self-harm by poisoning pesticides	Data Rich	Male	Socio-demographic Index						X
Self-harm by poisoning pesticides	Global	Male	Healthcare access and quality index					X	
Self-harm by poisoning pesticides	Data Rich	Male	Healthcare access and quality index					X	
Self-harm by poisoning pesticides	Global	Male	Population Density (500-1000 ppl/sqkm, proportion)					X	
Self-harm by poisoning pesticides	Data Rich	Male	Population Density (500-1000 ppl/sqkm, proportion)					X	
Self-harm by poisoning pesticides	Global	Male	Population Density (under 150 ppl/sqkm, proportion)					X	
Self-harm by poisoning pesticides	Data Rich	Male	Population Density (under 150 ppl/sqkm, proportion)					X	
Self-harm by poisoning pesticides	Global	Male	Population Density (over 1000 ppl/sqkm, proportion)					X	
Self-harm by poisoning pesticides	Data Rich	Male	Population Density (over 1000 ppl/sqkm, proportion)					X	
Self-harm by poisoning pesticides	Global	Male	Population Density (150-300 ppl/sqkm, proportion)					X	
Self-harm by poisoning pesticides	Data Rich	Male	Population Density (150-300 ppl/sqkm, proportion)					X	
Self-harm by poisoning pesticides	Global	Male	Population Density (300-500 ppl/sqkm, proportion)					X	
Self-harm by poisoning pesticides	Data Rich	Male	Population Density (300-500 ppl/sqkm, proportion)					X	
Self-harm by poisoning pesticides	Global	Male	Liters of alcohol consumed per capita				X		
Self-harm by poisoning pesticides	Data Rich	Male	Liters of alcohol consumed per capita				X		
Self-harm by poisoning pesticides	Global	Male	Population-weighted mean temperature				X		
Self-harm by poisoning pesticides	Data Rich	Male	Population-weighted mean temperature				X		
Self-harm by poisoning pesticides	Global	Male	Log-transformed SEV scalar: Self Harm				X		
Self-harm by poisoning pesticides	Data Rich	Male	Log-transformed SEV scalar: Self Harm				X		
Self-harm by poisoning pesticides	Global	Male	Major depressive disorder				X		
Self-harm by poisoning pesticides	Data Rich	Male	Major depressive disorder				X		
Self-harm by non pesticide substance and gas	Global	Female	Education (years per capita)						X
Self-harm by non pesticide substance and gas	Data Rich	Female	Education (years per capita)						X
Self-harm by non pesticide substance and gas	Global	Female	LDI (I$ per capita)						X
Self-harm by non pesticide substance and gas	Data Rich	Female	LDI (I$ per capita)						X
Self-harm by non pesticide substance and gas	Global	Female	Socio-demographic Index						X
Self-harm by non pesticide substance and gas	Data Rich	Female	Socio-demographic Index						X
Self-harm by non pesticide substance and gas	Global	Female	Healthcare access and quality index					X	
Self-harm by non pesticide substance and gas	Data Rich	Female	Healthcare access and quality index					X	
Self-harm by non pesticide substance and gas	Global	Female	Population Density (500-1000 ppl/sqkm, proportion)					X	
Self-harm by non pesticide substance and gas	Data Rich	Female	Population Density (500-1000 ppl/sqkm, proportion)					X	
Self-harm by non pesticide substance and gas	Global	Female	Population Density (under 150 ppl/sqkm, proportion)					X	
Self-harm by non pesticide substance and gas	Data Rich	Female	Population Density (under 150 ppl/sqkm, proportion)					X	
Self-harm by non pesticide substance and gas	Global	Female	Population Density (over 1000 ppl/sqkm, proportion)					X	
Self-harm by non pesticide substance and gas	Data Rich	Female	Population Density (over 1000 ppl/sqkm, proportion)					X	
Self-harm by non pesticide substance and gas	Global	Female	Population Density (150-300 ppl/sqkm, proportion)					X	
Self-harm by non pesticide substance and gas	Data Rich	Female	Population Density (150-300 ppl/sqkm, proportion)					X	
Self-harm by non pesticide substance and gas	Global	Female	Population Density (300-500 ppl/sqkm, proportion)					X	
Self-harm by non pesticide substance and gas	Data Rich	Female	Population Density (300-500 ppl/sqkm, proportion)					X	
Self-harm by non pesticide substance and gas	Global	Female	Liters of alcohol consumed per capita				X		
Self-harm by non pesticide substance and gas	Data Rich	Female	Liters of alcohol consumed per capita				X		
Self-harm by non pesticide substance and gas	Global	Female	Population-weighted mean temperature				X		
Self-harm by non pesticide substance and gas	Data Rich	Female	Population-weighted mean temperature				X		
Self-harm by non pesticide substance and gas	Global	Female	Log-transformed SEV scalar: Self Harm				X		
Self-harm by non pesticide substance and gas	Data Rich	Female	Log-transformed SEV scalar: Self Harm				X		
Self-harm by non pesticide substance and gas	Global	Female	Major depressive disorder				X		
Self-harm by non pesticide substance and gas	Data Rich	Female	Major depressive disorder				X		
Self-harm by non pesticide substance and gas	Global	Male	Education (years per capita)						X
Self-harm by non pesticide substance and gas	Data Rich	Male	Education (years per capita)						X
Self-harm by non pesticide substance and gas	Global	Male	LDI (I$ per capita)						X
Self-harm by non pesticide substance and gas	Data Rich	Male	LDI (I$ per capita)						X
Self-harm by non pesticide substance and gas	Global	Male	Socio-demographic Index						X
Self-harm by non pesticide substance and gas	Data Rich	Male	Socio-demographic Index						X
Self-harm by non pesticide substance and gas	Global	Male	Healthcare access and quality index					X	
Self-harm by non pesticide substance and gas	Data Rich	Male	Healthcare access and quality index					X	
Self-harm by non pesticide substance and gas	Global	Male	Population Density (500-1000 ppl/sqkm, proportion)					X	
Self-harm by non pesticide substance and gas	Data Rich	Male	Population Density (500-1000 ppl/sqkm, proportion)					X	
Self-harm by non pesticide substance and gas	Global	Male	Population Density (under 150 ppl/sqkm, proportion)					X	
Self-harm by non pesticide substance and gas	Data Rich	Male	Population Density (under 150 ppl/sqkm, proportion)					X	
Self-harm by non pesticide substance and gas	Global	Male	Population Density (over 1000 ppl/sqkm, proportion)					X	
Self-harm by non pesticide substance and gas	Data Rich	Male	Population Density (over 1000 ppl/sqkm, proportion)					X	
Self-harm by non pesticide substance and gas	Global	Male	Population Density (150-300 ppl/sqkm, proportion)					X	
Self-harm by non pesticide substance and gas	Data Rich	Male	Population Density (150-300 ppl/sqkm, proportion)					X	
Self-harm by non pesticide substance and gas	Global	Male	Population Density (300-500 ppl/sqkm, proportion)					X	
Self-harm by non pesticide substance and gas	Data Rich	Male	Population Density (300-500 ppl/sqkm, proportion)					X	
Self-harm by non pesticide substance and gas	Global	Male	Liters of alcohol consumed per capita				X		
Self-harm by non pesticide substance and gas	Data Rich	Male	Liters of alcohol consumed per capita				X		
Self-harm by non pesticide substance and gas	Global	Male	Population-weighted mean temperature				X		
Self-harm by non pesticide substance and gas	Data Rich	Male	Population-weighted mean temperature				X		
Self-harm by non pesticide substance and gas	Global	Male	Log-transformed SEV scalar: Self Harm				X		
Self-harm by non pesticide substance and gas	Data Rich	Male	Log-transformed SEV scalar: Self Harm				X		
Self-harm by non pesticide substance and gas	Global	Male	Major depressive disorder				X		
Self-harm by non pesticide substance and gas	Data Rich	Male	Major depressive disorder				X		
Self-harm by other specified means	Global	Female	Education (years per capita)			X			X
Self-harm by other specified means	Data Rich	Female	Education (years per capita)			X			X
Self-harm by other specified means	Global	Female	LDI (I$ per capita)			X			X
Self-harm by other specified means	Global	Female	Socio-demographic Index			X			X
Self-harm by other specified means	Data Rich	Female	Socio-demographic Index			X			X
Self-harm by other specified means	Global	Female	Healthcare access and quality index		X			X	
Self-harm by other specified means	Data Rich	Female	Healthcare access and quality index		X			X	
Self-harm by other specified means	Global	Female	Population Density (500-1000 ppl/sqkm, proportion)		X			X	
Self-harm by other specified means	Data Rich	Female	Population Density (500-1000 ppl/sqkm, proportion)		X			X	
Self-harm by other specified means	Global	Female	Population Density (under 150 ppl/sqkm, proportion)		X			X	
Self-harm by other specified means	Data Rich	Female	Population Density (under 150 ppl/sqkm, proportion)		X			X	
Self-harm by other specified means	Global	Female	Population Density (over 1000 ppl/sqkm, proportion)		X			X	
Self-harm by other specified means	Data Rich	Female	Population Density (over 1000 ppl/sqkm, proportion)		X			X	

Appendix Table S11: Comparison of GBD 2021 and GBD 2023 covariates and level of covariates used in cause of death modeling									
**Cause**	**Model Version Type**	**Sex**	**Covariate**	**Level 1 GBD 2021**	**Level 2 GBD 2021**	**Level 3 GBD 2021**	**Level 1 GBD 2023**	**Level 2 GBD 2023**	**Level 3 GBD 2023**
Self-harm by other specified means	Global	Female	Population Density (150-300 ppl/sqkm, proportion)		X			X	
Self-harm by other specified means	Data Rich	Female	Population Density (150-300 ppl/sqkm, proportion)		X			X	
Self-harm by other specified means	Global	Female	Population Density (300-500 ppl/sqkm, proportion)		X			X	
Self-harm by other specified means	Data Rich	Female	Population Density (300-500 ppl/sqkm, proportion)		X			X	
Self-harm by other specified means	Global	Female	Liters of alcohol consumed per capita	X			X		
Self-harm by other specified means	Data Rich	Female	Liters of alcohol consumed per capita	X			X		
Self-harm by other specified means	Global	Female	Population-weighted mean temperature	X			X		
Self-harm by other specified means	Data Rich	Female	Population-weighted mean temperature	X			X		
Self-harm by other specified means	Global	Female	Log-transformed SEV scalar: Self Harm	X			X		
Self-harm by other specified means	Data Rich	Female	Log-transformed SEV scalar: Self Harm	X			X		
Self-harm by other specified means	Global	Female	Major depressive disorder	X			X		
Self-harm by other specified means	Data Rich	Female	Major depressive disorder	X			X		
Self-harm by other specified means	Global	Male	Education (years per capita)			X			X
Self-harm by other specified means	Data Rich	Male	Education (years per capita)			X			X
Self-harm by other specified means	Global	Male	LDI (I$ per capita)			X			X
Self-harm by other specified means	Global	Male	Socio-demographic Index			X			X
Self-harm by other specified means	Data Rich	Male	Socio-demographic Index			X			X
Self-harm by other specified means	Global	Male	Healthcare access and quality index		X			X	
Self-harm by other specified means	Data Rich	Male	Healthcare access and quality index		X			X	
Self-harm by other specified means	Global	Male	Population Density (500-1000 ppl/sqkm, proportion)		X			X	
Self-harm by other specified means	Data Rich	Male	Population Density (500-1000 ppl/sqkm, proportion)		X			X	
Self-harm by other specified means	Global	Male	Population Density (under 150 ppl/sqkm, proportion)		X			X	
Self-harm by other specified means	Data Rich	Male	Population Density (under 150 ppl/sqkm, proportion)		X			X	
Self-harm by other specified means	Global	Male	Population Density (over 1000 ppl/sqkm, proportion)		X			X	
Self-harm by other specified means	Data Rich	Male	Population Density (over 1000 ppl/sqkm, proportion)		X			X	
Self-harm by other specified means	Global	Male	Population Density (150-300 ppl/sqkm, proportion)		X			X	
Self-harm by other specified means	Data Rich	Male	Population Density (150-300 ppl/sqkm, proportion)		X			X	
Self-harm by other specified means	Global	Male	Population Density (300-500 ppl/sqkm, proportion)		X			X	
Self-harm by other specified means	Data Rich	Male	Population Density (300-500 ppl/sqkm, proportion)		X			X	
Self-harm by other specified means	Global	Male	Liters of alcohol consumed per capita	X			X		
Self-harm by other specified means	Data Rich	Male	Liters of alcohol consumed per capita	X			X		
Self-harm by other specified means	Global	Male	Population-weighted mean temperature	X			X		
Self-harm by other specified means	Data Rich	Male	Population-weighted mean temperature	X			X		
Self-harm by other specified means	Global	Male	Log-transformed SEV scalar: Self Harm	X			X		
Self-harm by other specified means	Data Rich	Male	Log-transformed SEV scalar: Self Harm	X			X		
Self-harm by other specified means	Global	Male	Major depressive disorder	X			X		
Self-harm by other specified means	Data Rich	Male	Major depressive disorder	X			X		
Interpersonal violence	Global	Female	Education (years per capita)			X			X
Interpersonal violence	Data Rich	Female	Education (years per capita)			X			X
Interpersonal violence	Global	Female	LDI (I$ per capita)			X			X
Interpersonal violence	Data Rich	Female	LDI (I$ per capita)			X			X
Interpersonal violence	Global	Female	Socio-demographic Index			X			X
Interpersonal violence	Data Rich	Female	Socio-demographic Index			X			X
Interpersonal violence	Global	Female	Healthcare access and quality index		X			X	
Interpersonal violence	Data Rich	Female	Healthcare access and quality index		X			X	
Interpersonal violence	Global	Female	Population Density (over 1000 ppl/sqkm, proportion)		X			X	
Interpersonal violence	Data Rich	Female	Population Density (over 1000 ppl/sqkm, proportion)		X			X	
Interpersonal violence	Global	Female	Liters of alcohol consumed per capita	X			X		
Interpersonal violence	Data Rich	Female	Liters of alcohol consumed per capita	X			X		
Interpersonal violence	Global	Female	Population-weighted mean temperature	X			X		
Interpersonal violence	Data Rich	Female	Population-weighted mean temperature	X			X		
Interpersonal violence	Global	Female	Education Relative Inequality (Gini)	X			X		
Interpersonal violence	Data Rich	Female	Education Relative Inequality (Gini)	X			X		
Interpersonal violence	Global	Female	Population 15 to 30 males (proportion)	X			X		
Interpersonal violence	Data Rich	Female	Population 15 to 30 males (proportion)	X			X		
Interpersonal violence	Global	Female	Log-transformed SEV scalar: Violence	X			X		
Interpersonal violence	Data Rich	Female	Log-transformed SEV scalar: Violence	X			X		
Interpersonal violence	Global	Male	Education (years per capita)			X			X
Interpersonal violence	Data Rich	Male	Education (years per capita)			X			X
Interpersonal violence	Global	Male	LDI (I$ per capita)			X			X
Interpersonal violence	Data Rich	Male	LDI (I$ per capita)			X			X
Interpersonal violence	Global	Male	Socio-demographic Index			X			X
Interpersonal violence	Data Rich	Male	Socio-demographic Index			X			X
Interpersonal violence	Global	Male	Healthcare access and quality index		X			X	
Interpersonal violence	Data Rich	Male	Healthcare access and quality index		X			X	
Interpersonal violence	Global	Male	Population Density (over 1000 ppl/sqkm, proportion)		X			X	
Interpersonal violence	Data Rich	Male	Population Density (over 1000 ppl/sqkm, proportion)		X			X	
Interpersonal violence	Global	Male	Liters of alcohol consumed per capita	X			X		
Interpersonal violence	Data Rich	Male	Liters of alcohol consumed per capita	X			X		
Interpersonal violence	Global	Male	Population-weighted mean temperature	X			X		
Interpersonal violence	Data Rich	Male	Population-weighted mean temperature	X			X		
Interpersonal violence	Global	Male	Education Relative Inequality (Gini)	X			X		
Interpersonal violence	Data Rich	Male	Education Relative Inequality (Gini)	X			X		
Interpersonal violence	Global	Male	Population 15 to 30 males (proportion)	X			X		
Interpersonal violence	Data Rich	Male	Population 15 to 30 males (proportion)	X			X		
Interpersonal violence	Global	Male	Log-transformed SEV scalar: Violence	X			X		
Interpersonal violence	Data Rich	Male	Log-transformed SEV scalar: Violence	X			X		
Assault by firearm	Global	Female	Education (years per capita)			X			X
Assault by firearm	Data Rich	Female	Education (years per capita)			X			X
Assault by firearm	Global	Female	LDI (I$ per capita)			X			X
Assault by firearm	Data Rich	Female	LDI (I$ per capita)			X			X
Assault by firearm	Global	Female	Socio-demographic Index			X			X
Assault by firearm	Data Rich	Female	Socio-demographic Index			X			X
Assault by firearm	Global	Female	Healthcare access and quality index		X			X	
Assault by firearm	Data Rich	Female	Healthcare access and quality index		X			X	
Assault by firearm	Global	Female	Population Density (over 1000 ppl/sqkm, proportion)		X			X	
Assault by firearm	Data Rich	Female	Population Density (over 1000 ppl/sqkm, proportion)		X			X	
Assault by firearm	Global	Female	Liters of alcohol consumed per capita	X			X		
Assault by firearm	Data Rich	Female	Liters of alcohol consumed per capita	X			X		
Assault by firearm	Global	Female	Population-weighted mean temperature	X			X		
Assault by firearm	Data Rich	Female	Population-weighted mean temperature	X			X		
Assault by firearm	Global	Female	Education Relative Inequality (Gini)	X			X		
Assault by firearm	Data Rich	Female	Education Relative Inequality (Gini)	X			X		
Assault by firearm	Global	Female	Population 15 to 30 males (proportion)	X			X		
Assault by firearm	Data Rich	Female	Population 15 to 30 males (proportion)	X			X		
Assault by firearm	Global	Female	Log-transformed SEV scalar: Viol Gun	X			X		
Assault by firearm	Data Rich	Female	Log-transformed SEV scalar: Viol Gun	X			X		
Assault by firearm	Global	Male	Education (years per capita)			X			X
Assault by firearm	Data Rich	Male	Education (years per capita)			X			X
Assault by firearm	Global	Male	LDI (I$ per capita)			X			X
Assault by firearm	Data Rich	Male	LDI (I$ per capita)			X			X
Assault by firearm	Global	Male	Socio-demographic Index			X			X
Assault by firearm	Data Rich	Male	Socio-demographic Index			X			X
Assault by firearm	Global	Male	Healthcare access and quality index		X			X	
Assault by firearm	Data Rich	Male	Healthcare access and quality index		X			X	
Assault by firearm	Global	Male	Population Density (over 1000 ppl/sqkm, proportion)		X			X	
Assault by firearm	Data Rich	Male	Population Density (over 1000 ppl/sqkm, proportion)		X			X	

Appendix Table S11: Comparison of GBD 2021 and GBD 2023 covariates and level of covariates used in cause of death modeling

Cause	Model Version Type	Sex	Covariate	Level 1 GBD 2021	Level 2 GBD 2021	Level 3 GBD 2021	Level 1 GBD 2023	Level 2 GBD 2023	Level 3 GBD 2023
Assault by firearm	Global	Male	Liters of alcohol consumed per capita	X			X		
Assault by firearm	Data Rich	Male	Liters of alcohol consumed per capita	X			X		
Assault by firearm	Global	Male	Population-weighted mean temperature	X			X		
Assault by firearm	Data Rich	Male	Population-weighted mean temperature	X			X		
Assault by firearm	Global	Male	Education Relative Inequality (Gini)	X			X		
Assault by firearm	Data Rich	Male	Education Relative Inequality (Gini)	X			X		
Assault by firearm	Global	Male	Population 15 to 30 males (proportion)	X			X		
Assault by firearm	Data Rich	Male	Population 15 to 30 males (proportion)	X			X		
Assault by firearm	Global	Male	Log-transformed SEV scalar: Viol Gun	X			X		
Assault by firearm	Data Rich	Male	Log-transformed SEV scalar: Viol Gun	X			X		
Assault by sharp object	Global	Female	Education (years per capita)			X			X
Assault by sharp object	Data Rich	Female	Education (years per capita)			X			X
Assault by sharp object	Global	Female	LDI (I$ per capita)			X			X
Assault by sharp object	Data Rich	Female	LDI (I$ per capita)			X			X
Assault by sharp object	Global	Female	Socio-demographic Index			X			X
Assault by sharp object	Data Rich	Female	Socio-demographic Index			X			X
Assault by sharp object	Global	Female	Healthcare access and quality index		X			X	
Assault by sharp object	Data Rich	Female	Healthcare access and quality index		X			X	
Assault by sharp object	Global	Female	Population Density (over 1000 ppl/sqkm, proportion)		X			X	
Assault by sharp object	Data Rich	Female	Population Density (over 1000 ppl/sqkm, proportion)		X			X	
Assault by sharp object	Data Rich	Female	Population-weighted mean temperature		X		X		
Assault by sharp object	Global	Female	Liters of alcohol consumed per capita	X			X		
Assault by sharp object	Data Rich	Female	Liters of alcohol consumed per capita	X			X		
Assault by sharp object	Global	Female	Population-weighted mean temperature	X			X		
Assault by sharp object	Global	Female	Education Relative Inequality (Gini)	X			X		
Assault by sharp object	Data Rich	Female	Education Relative Inequality (Gini)	X			X		
Assault by sharp object	Global	Female	Population 15 to 30 males (proportion)	X			X		
Assault by sharp object	Data Rich	Female	Population 15 to 30 males (proportion)	X			X		
Assault by sharp object	Global	Female	Log-transformed SEV scalar: Viol Knife	X			X		
Assault by sharp object	Data Rich	Female	Log-transformed SEV scalar: Viol Knife	X			X		
Assault by sharp object	Global	Male	Education (years per capita)			X			X
Assault by sharp object	Data Rich	Male	Education (years per capita)			X			X
Assault by sharp object	Global	Male	LDI (I$ per capita)			X			X
Assault by sharp object	Data Rich	Male	LDI (I$ per capita)			X			X
Assault by sharp object	Global	Male	Socio-demographic Index			X			X
Assault by sharp object	Data Rich	Male	Socio-demographic Index			X			X
Assault by sharp object	Global	Male	Healthcare access and quality index		X			X	
Assault by sharp object	Data Rich	Male	Healthcare access and quality index		X			X	
Assault by sharp object	Global	Male	Population Density (over 1000 ppl/sqkm, proportion)		X			X	
Assault by sharp object	Data Rich	Male	Population Density (over 1000 ppl/sqkm, proportion)		X			X	
Assault by sharp object	Global	Male	Liters of alcohol consumed per capita	X			X		
Assault by sharp object	Data Rich	Male	Liters of alcohol consumed per capita	X			X		
Assault by sharp object	Global	Male	Population-weighted mean temperature	X			X		
Assault by sharp object	Data Rich	Male	Population-weighted mean temperature	X			X		
Assault by sharp object	Global	Male	Education Relative Inequality (Gini)	X			X		
Assault by sharp object	Data Rich	Male	Education Relative Inequality (Gini)	X			X		
Assault by sharp object	Global	Male	Population 15 to 30 males (proportion)	X			X		
Assault by sharp object	Data Rich	Male	Population 15 to 30 males (proportion)	X			X		
Assault by sharp object	Global	Male	Log-transformed SEV scalar: Viol Knife	X			X		
Assault by sharp object	Data Rich	Male	Log-transformed SEV scalar: Viol Knife	X			X		
Assault by other means	Global	Female	Education (years per capita)			X			X
Assault by other means	Data Rich	Female	Education (years per capita)			X			X
Assault by other means	Global	Female	LDI (I$ per capita)			X			X
Assault by other means	Data Rich	Female	LDI (I$ per capita)			X			X
Assault by other means	Global	Female	Socio-demographic Index			X			X
Assault by other means	Data Rich	Female	Socio-demographic Index			X			X
Assault by other means	Global	Female	Healthcare access and quality index		X			X	
Assault by other means	Data Rich	Female	Healthcare access and quality index		X			X	
Assault by other means	Global	Female	Population Density (over 1000 ppl/sqkm, proportion)		X			X	
Assault by other means	Data Rich	Female	Population Density (over 1000 ppl/sqkm, proportion)		X			X	
Assault by other means	Global	Female	Liters of alcohol consumed per capita	X			X		
Assault by other means	Data Rich	Female	Liters of alcohol consumed per capita	X			X		
Assault by other means	Global	Female	Population-weighted mean temperature	X			X		
Assault by other means	Data Rich	Female	Population-weighted mean temperature	X			X		
Assault by other means	Global	Female	Education Relative Inequality (Gini)	X			X		
Assault by other means	Data Rich	Female	Education Relative Inequality (Gini)	X			X		
Assault by other means	Global	Female	Population 15 to 30 males (proportion)	X			X		
Assault by other means	Data Rich	Female	Population 15 to 30 males (proportion)	X			X		
Assault by other means	Global	Female	Log-transformed SEV scalar: Oth Viol	X			X		
Assault by other means	Data Rich	Female	Log-transformed SEV scalar: Oth Viol	X			X		
Assault by other means	Global	Male	Education (years per capita)			X			X
Assault by other means	Data Rich	Male	Education (years per capita)			X			X
Assault by other means	Global	Male	LDI (I$ per capita)			X			X
Assault by other means	Data Rich	Male	LDI (I$ per capita)			X			X
Assault by other means	Global	Male	Socio-demographic Index			X			X
Assault by other means	Data Rich	Male	Socio-demographic Index			X			X
Assault by other means	Global	Male	Healthcare access and quality index		X			X	
Assault by other means	Data Rich	Male	Healthcare access and quality index		X			X	
Assault by other means	Global	Male	Population Density (over 1000 ppl/sqkm, proportion)		X			X	
Assault by other means	Data Rich	Male	Population Density (over 1000 ppl/sqkm, proportion)		X			X	
Assault by other means	Global	Male	Liters of alcohol consumed per capita	X			X		
Assault by other means	Data Rich	Male	Liters of alcohol consumed per capita	X			X		
Assault by other means	Global	Male	Population-weighted mean temperature	X			X		
Assault by other means	Data Rich	Male	Population-weighted mean temperature	X			X		
Assault by other means	Global	Male	Education Relative Inequality (Gini)	X			X		
Assault by other means	Data Rich	Male	Education Relative Inequality (Gini)	X			X		
Assault by other means	Global	Male	Population 15 to 30 males (proportion)	X			X		
Assault by other means	Data Rich	Male	Population 15 to 30 males (proportion)	X			X		
Assault by other means	Global	Male	Log-transformed SEV scalar: Oth Viol	X			X		
Assault by other means	Data Rich	Male	Log-transformed SEV scalar: Oth Viol	X			X		
Executions and police conflict	Data Rich	Female	Education (years per capita)			X			X
Executions and police conflict	Global	Female	Education (years per capita)			X			X
Executions and police conflict	Data Rich	Female	LDI (I$ per capita)			X			X
Executions and police conflict	Global	Female	LDI (I$ per capita)			X			X
Executions and police conflict	Data Rich	Female	Socio-demographic Index		X			X	
Executions and police conflict	Global	Female	Socio-demographic Index		X			X	
Executions and police conflict	Data Rich	Female	Healthcare access and quality index		X			X	
Executions and police conflict	Global	Female	Healthcare access and quality index		X			X	
Executions and police conflict	Data Rich	Female	Population Density (over 1000 ppl/sqkm, proportion)		X			X	
Executions and police conflict	Global	Female	Population Density (over 1000 ppl/sqkm, proportion)		X			X	
Executions and police conflict	Data Rich	Female	Liters of alcohol consumed per capita	X			X		
Executions and police conflict	Global	Female	Liters of alcohol consumed per capita	X			X		
Executions and police conflict	Data Rich	Female	Population-weighted mean temperature	X			X		
Executions and police conflict	Global	Female	Population-weighted mean temperature	X			X		
Executions and police conflict	Data Rich	Female	Population 15 to 30 males (proportion)	X			X		
Executions and police conflict	Global	Female	Population 15 to 30 males (proportion)	X			X		
Executions and police conflict	Data Rich	Female	Education Relative Inequality (Gini)				X		
Executions and police conflict	Data Rich	Male	Education (years per capita)			X			X

Appendix Table S11: Comparison of GBD 2021 and GBD 2023 covariates and level of covariates used in cause of death modeling

Cause	Model Version Type	Sex	Covariate	Level 1 GBD 2021	Level 2 GBD 2021	Level 3 GBD 2021	Level 1 GBD 2023	Level 2 GBD 2023	Level 3 GBD 2023
Executions and police conflict	Global	Male	Education (years per capita)			X			X
Executions and police conflict	Data Rich	Male	LDI (I$ per capita)			X			X
Executions and police conflict	Global	Male	LDI (I$ per capita)			X			X
Executions and police conflict	Data Rich	Male	Socio-demographic Index		X			X	
Executions and police conflict	Global	Male	Socio-demographic Index		X			X	
Executions and police conflict	Data Rich	Male	Healthcare access and quality index		X			X	
Executions and police conflict	Global	Male	Healthcare access and quality index		X			X	
Executions and police conflict	Data Rich	Male	Population Density (over 1000 ppl/sqkm, proportion)		X			X	
Executions and police conflict	Global	Male	Population Density (over 1000 ppl/sqkm, proportion)		X			X	
Executions and police conflict	Data Rich	Male	Liters of alcohol consumed per capita	X			X		
Executions and police conflict	Global	Male	Liters of alcohol consumed per capita	X			X		
Executions and police conflict	Data Rich	Male	Population-weighted mean temperature	X			X		
Executions and police conflict	Global	Male	Population-weighted mean temperature	X			X		
Executions and police conflict	Data Rich	Male	Education Relative Inequality (Gini)	X			X		
Executions and police conflict	Data Rich	Male	Population 15 to 30 males (proportion)	X			X		
Executions and police conflict	Global	Male	Population 15 to 30 males (proportion)	X			X		

Appendix Table S12: CODEm predictive validity results by cause, model type, sex, and age

Cause	Sex	Age Start	Age End	RMSE In-Sample	RMSE Out-of-Sample	Trend In-Sample	Trend Out-of-Sample	Coverage In-Sample	Coverage Out-of-Sample
Sexually transmitted infections excluding HIV [Global]	Female	10-14 years	95+ years	0.656193	0.809108	0.497032	0.459767	0.988532	0.980201
Sexually transmitted infections excluding HIV [Global]	Male	10-14 years	95+ years	0.785884	1.17277	0.564526	0.580205	0.979214	0.951923
Sexually transmitted infections excluding HIV [Data Rich]	Female	10-14 years	95+ years	0.291383	0.460498	0.241984	0.319254	0.997293	0.995991
Sexually transmitted infections excluding HIV [Data Rich]	Male	10-14 years	95+ years	0.51074	0.758491	0.428188	0.441666	0.986042	0.980766
Lower respiratory infections [Global]	Female	0-6 days	2-4 years	0.233615	0.410132	0.163863	0.157338	0.999588	0.991
Lower respiratory infections [Global]	Female	5-9 years	95+ years	0.232484	0.438612	0.145361	0.148725	0.999452	0.98752
Lower respiratory infections [Global]	Male	0-6 days	2-4 years	0.234147	0.418379	0.161189	0.164058	0.999598	0.989243
Lower respiratory infections [Global]	Male	5-9 years	95+ years	0.233115	0.443208	0.145151	0.150649	0.99948	0.984789
Lower respiratory infections [Data Rich]	Female	0-6 days	2-4 years	0.116347	0.250854	0.0805327	0.128474	0.999931	0.998742
Lower respiratory infections [Data Rich]	Female	5-9 years	95+ years	0.112413	0.246439	0.0892367	0.151542	0.999985	0.999469
Lower respiratory infections [Data Rich]	Male	0-6 days	2-4 years	0.116562	0.257758	0.078238	0.130335	0.999931	0.998336
Lower respiratory infections [Data Rich]	Male	5-9 years	95+ years	0.109462	0.25036	0.0871617	0.151685	0.999989	0.999359
Upper respiratory infections [Global]	Female	0-6 days	95+ years	0.671489	1.07545	0.399768	0.371265	0.986384	0.964899
Upper respiratory infections [Global]	Male	0-6 days	95+ years	0.687869	1.0658	0.416021	0.37651	0.98485	0.967287
Upper respiratory infections [Data Rich]	Female	0-6 days	95+ years	0.378621	0.768492	0.319194	0.294961	0.994688	0.990046
Upper respiratory infections [Data Rich]	Male	0-6 days	95+ years	0.393763	0.685699	0.329711	0.304825	0.993714	0.988401
Otitis media [Global]	Female	0-6 days	95+ years	1.17983	2.05742	0.83154	0.872917	0.97755	0.907118
Otitis media [Global]	Male	0-6 days	95+ years	1.13694	1.92042	0.777746	0.809641	0.983978	0.928017
Otitis media [Data Rich]	Female	0-6 days	95+ years	1.51875	1.88024	0.835899	0.806037	0.963242	0.921427
Otitis media [Data Rich]	Male	0-6 days	95+ years	1.08838	1.7966	0.699652	0.663806	0.986738	0.956617
Diarrhoeal diseases [Global]	Female	0-6 days	2-4 years	0.34138	0.659816	0.263855	0.254193	0.999675	0.987695
Diarrhoeal diseases [Global]	Female	5-9 years	95+ years	0.324743	0.72746	0.169213	0.186151	0.999822	0.981285
Diarrhoeal diseases [Global]	Male	0-6 days	2-4 years	0.341231	0.654386	0.258008	0.232381	0.999677	0.988805
Diarrhoeal diseases [Global]	Male	5-9 years	95+ years	0.400056	0.682089	0.175141	0.185215	0.999742	0.98905
Diarrhoeal diseases [Data Rich]	Female	0-6 days	2-4 years	0.153148	0.357618	0.108438	0.1966	0.999977	0.998576
Diarrhoeal diseases [Data Rich]	Female	5-9 years	95+ years	0.138649	0.35525	0.105967	0.208657	0.99979	0.999367
Diarrhoeal diseases [Data Rich]	Male	0-6 days	2-4 years	0.154778	0.351462	0.107517	0.189332	0.999988	0.998601
Diarrhoeal diseases [Data Rich]	Male	5-9 years	95+ years	0.142556	0.356262	0.106598	0.203149	0.999856	0.999255
Rabies [Global]	Female	1-5 months	95+ years	0.877899	1.71692	0.651517	0.673566	0.987197	0.920887
Rabies [Global]	Male	1-5 months	95+ years	0.851084	1.67638	0.635405	0.666947	0.988237	0.927016
Rabies [Data Rich]	Female	1-5 months	95+ years	0.490973	1.23518	0.41425	0.598694	0.994585	0.970566
Rabies [Data Rich]	Male	1-5 months	95+ years	0.496794	1.20628	0.423741	0.580184	0.994421	0.965643
Other neglected tropical diseases [Global]	Female	0-6 days	95+ years	0.634451	1.23675	0.369542	0.385344	0.992235	0.955152
Other neglected tropical diseases [Global]	Male	0-6 days	95+ years	0.670769	1.26991	0.390423	0.417708	0.991684	0.955189
Other neglected tropical diseases [Data Rich]	Female	0-6 days	95+ years	0.390208	0.974522	0.293966	0.362882	0.995135	0.990822
Other neglected tropical diseases [Data Rich]	Male	0-6 days	95+ years	0.410567	0.929415	0.324192	0.3675	0.994335	0.988977
Meningitis [Global]	Female	0-6 days	2-4 years	0.360024	0.542253	0.291136	0.234406	0.998904	0.992782
Meningitis [Global]	Female	5-9 years	95+ years	0.288588	0.486932	0.175037	0.17055	0.998582	0.993047
Meningitis [Global]	Male	0-6 days	2-4 years	0.332194	0.498987	0.218564	0.215065	0.999459	0.993575
Meningitis [Global]	Male	5-9 years	95+ years	0.293684	0.489886	0.178075	0.178732	0.998519	0.991529
Meningitis [Data Rich]	Female	0-6 days	2-4 years	0.115614	0.253616	0.0879465	0.134965	1	0.999919
Meningitis [Data Rich]	Female	5-9 years	95+ years	0.120141	0.23484	0.0959599	0.144646	0.999996	0.999928
Meningitis [Data Rich]	Male	0-6 days	2-4 years	0.119591	0.263032	0.0892996	0.141371	1	0.999824
Meningitis [Data Rich]	Male	5-9 years	95+ years	0.11736	0.236204	0.094676	0.148196	1	0.99995
Encephalitis [Global]	Female	0-6 days	95+ years	0.551448	0.867131	0.349558	0.359812	0.991992	0.976272
Encephalitis [Global]	Male	0-6 days	95+ years	0.54284	0.858671	0.336539	0.348374	0.993202	0.974877
Encephalitis [Data Rich]	Female	0-6 days	95+ years	0.337691	0.628884	0.271296	0.406223	0.995697	0.990547
Encephalitis [Data Rich]	Male	0-6 days	95+ years	0.312862	0.584503	0.253733	0.362519	0.99645	0.992089
Tetanus [Global]	Female	0-6 days	6-11 months	0.991314	1.59182	0.940984	0.965323	0.966589	0.923302
Tetanus [Global]	Female	12-23 months	95+ years	0.963657	1.63435	0.740955	0.737947	0.978182	0.925362
Tetanus [Global]	Male	0-6 days	6-11 months	1.01363	1.60312	1.01083	0.94876	0.968859	0.933409
Tetanus [Global]	Male	12-23 months	95+ years	1.03959	1.59325	0.752738	0.755385	0.985144	0.948254
Tetanus [Data Rich]	Female	0-6 days	6-11 months	0.726095	0.953281	0.628962	0.625414	0.974713	0.955838
Tetanus [Data Rich]	Female	12-23 months	95+ years	0.857646	1.17115	0.714277	0.750531	0.976478	0.958253
Tetanus [Data Rich]	Male	0-6 days	6-11 months	0.690504	0.997115	0.595826	0.697551	0.981126	0.963202
Tetanus [Data Rich]	Male	12-23 months	95+ years	0.76984	1.06336	0.655565	0.727594	0.980782	0.963618
Acute hepatitis [Global]	Female	1-5 months	95+ years	0.452106	0.86831	0.316547	0.372903	0.996922	0.977628
Acute hepatitis [Global]	Male	1-5 months	95+ years	0.430375	0.83165	0.309655	0.343834	0.996978	0.97892
Acute hepatitis [Data Rich]	Female	1-5 months	95+ years	0.368381	0.714928	0.299785	0.447748	0.997736	0.994194
Acute hepatitis [Data Rich]	Male	1-5 months	95+ years	0.361241	0.722885	0.299609	0.465396	0.997282	0.99299
Acute hepatitis A [Global]	Female	1-5 months	95+ years	0.622157	1.08525	0.444563	0.467445	0.995189	0.967261
Acute hepatitis A [Global]	Male	1-5 months	95+ years	0.569364	1.10337	0.411324	0.447106	0.99465	0.951228
Acute hepatitis A [Data Rich]	Female	1-5 months	95+ years	0.507791	0.911477	0.429953	0.478122	0.995971	0.985642
Acute hepatitis A [Data Rich]	Male	1-5 months	95+ years	0.47509	0.915935	0.393764	0.474967	0.996596	0.989667
Acute hepatitis B [Global]	Female	1-5 months	95+ years	0.643464	1.0965	0.374325	0.416877	0.995804	0.961177
Acute hepatitis B [Global]	Male	1-5 months	95+ years	0.618613	1.11619	0.401431	0.460224	0.990872	0.958543
Acute hepatitis B [Data Rich]	Female	1-5 months	95+ years	0.416732	1.39049	0.334635	0.49157	0.997052	0.985353
Acute hepatitis B [Data Rich]	Male	1-5 months	95+ years	0.448548	1.4768	0.368968	0.562944	0.992853	0.98091
Acute hepatitis C [Global]	Female	1-5 months	95+ years	0.547906	0.912273	0.292825	0.291032	0.999024	0.97231
Acute hepatitis C [Global]	Male	1-5 months	95+ years	0.596788	0.920805	0.281798	0.281396	0.999093	0.975715
Acute hepatitis C [Data Rich]	Female	1-5 months	95+ years	0.21496	0.859219	0.185189	0.3555	0.999769	0.997397
Acute hepatitis C [Data Rich]	Male	1-5 months	95+ years	0.199705	0.76241	0.168408	0.302001	0.999838	0.997826
Acute hepatitis E [Global]	Female	1-5 months	95+ years	0.661785	0.979577	0.472986	0.464048	0.998336	0.979519
Acute hepatitis E [Global]	Male	1-5 months	95+ years	0.621936	0.970571	0.446014	0.455316	0.999167	0.98127
Acute hepatitis E [Data Rich]	Female	1-5 months	95+ years	0.51243	0.874647	0.449105	0.417784	0.998259	0.991991
Acute hepatitis E [Data Rich]	Male	1-5 months	95+ years	0.498643	0.736088	0.431229	0.391823	0.99926	0.995682
Other unspecified infectious diseases [Global]	Female	0-6 days	95+ years	0.684576	0.874845	0.567012	0.516806	0.990838	0.975261
Other unspecified infectious diseases [Global]	Male	0-6 days	95+ years	0.693107	0.935933	0.556124	0.504452	0.992268	0.974619
Other unspecified infectious diseases [Data Rich]	Female	0-6 days	95+ years	0.132513	0.399018	0.0979193	0.193888	0.999986	0.999762
Other unspecified infectious diseases [Data Rich]	Male	0-6 days	95+ years	0.129715	0.387444	0.0967775	0.186534	0.999975	0.999745
Neonatal disorders [Global]	Female	0-6 days	2-4 years	0.195098	0.338827	0.112676	0.112341	0.999863	0.995518
Neonatal disorders [Global]	Male	0-6 days	2-4 years	0.19394	0.335071	0.113415	0.11135	0.999907	0.99483
Neonatal disorders [Data Rich]	Female	0-6 days	2-4 years	0.103164	0.227834	0.0626507	0.101597	0.999965	0.998884
Neonatal disorders [Data Rich]	Male	0-6 days	2-4 years	0.101125	0.224825	0.0631115	0.102811	0.999953	0.998246
Neonatal preterm birth [Global]	Female	0-6 days	2-4 years	0.208734	0.360115	0.129296	0.127482	0.999612	0.994728
Neonatal preterm birth [Global]	Male	0-6 days	2-4 years	0.212717	0.363952	0.132084	0.127026	0.999614	0.994397
Neonatal preterm birth [Data Rich]	Female	0-6 days	2-4 years	0.109882	0.275531	0.0709711	0.104938	0.999975	0.999405
Neonatal preterm birth [Data Rich]	Male	0-6 days	2-4 years	0.105534	0.2762	0.0679435	0.103919	0.999987	0.999303
Neonatal encephalopathy due to birth asphyxia and trauma [Global]	Female	0-6 days	2-4 years	0.239326	0.420416	0.136995	0.133638	0.999794	0.997332
Neonatal encephalopathy due to birth asphyxia and trauma [Global]	Male	0-6 days	2-4 years	0.251384	0.416742	0.139009	0.13533	0.999813	0.99675
Neonatal encephalopathy due to birth asphyxia and trauma [Data Rich]	Female	0-6 days	2-4 years	0.115169	0.345189	0.0794303	0.121186	0.999975	0.999477
Neonatal encephalopathy due to birth asphyxia and trauma [Data Rich]	Male	0-6 days	2-4 years	0.11628	0.355465	0.0807479	0.118541	0.999987	0.999329
Neonatal sepsis and other neonatal infections [Global]	Female	0-6 days	2-4 years	0.371021	0.569636	0.269437	0.264423	0.997164	0.988856
Neonatal sepsis and other neonatal infections [Global]	Male	0-6 days	2-4 years	0.334999	0.517017	0.235504	0.220275	0.998725	0.994882
Neonatal sepsis and other neonatal infections [Data Rich]	Female	0-6 days	2-4 years	0.193114	0.476232	0.159278	0.232194	0.99881	0.995525
Neonatal sepsis and other neonatal infections [Data Rich]	Male	0-6 days	2-4 years	0.178908	0.457518	0.142727	0.181759	0.999124	0.997634
Hemolytic disease and other neonatal jaundice [Global]	Female	0-6 days	2-4 years	0.770139	1.10212	0.603421	0.629243	0.979326	0.958346
Hemolytic disease and other neonatal jaundice [Global]	Male	0-6 days	2-4 years	0.649986	1.00453	0.519796	0.519323	0.979595	0.961905
Hemolytic disease and other neonatal jaundice [Data Rich]	Female	0-6 days	2-4 years	0.706884	1.02774	0.609192	0.549338	0.977249	0.960759
Hemolytic disease and other neonatal jaundice [Data Rich]	Male	0-6 days	2-4 years	0.603264	0.826034	0.52576	0.490227	0.979861	0.969261
Other neonatal disorders [Global]	Female	0-6 days	2-4 years	0.282672	0.52265	0.183069	0.180691	0.999323	0.993949
Other neonatal disorders [Global]	Male	0-6 days	2-4 years	0.295806	0.528364	0.179018	0.180793	0.999307	0.993495
Other neonatal disorders [Data Rich]	Female	0-6 days	2-4 years	0.135007	0.423207	0.0971879	0.191404	0.999912	0.999184
Other neonatal disorders [Data Rich]	Male	0-6 days	2-4 years	0.14256	0.449706	0.0990861	0.186442	0.9997	0.998703
Nutritional deficiencies [Global]	Female	1-5 months	95+ years	0.143412	0.568101	0.0977531	0.137119	0.999409	0.975161
Nutritional deficiencies [Global]	Male	1-5 months	95+ years	0.244261	0.581667	0.151418	0.155978	0.999677	0.985668
Nutritional deficiencies [Data Rich]	Female	1-5 months	95+ years	0.119478	0.304206	0.0861618	0.169986	0.999982	0.999558
Nutritional deficiencies [Data Rich]	Male	1-5 months	95+ years	0.162263	0.308235	0.118181	0.164953	0.999939	0.999767
Protein-energy malnutrition [Global]	Female	1-5 months	2-4 years	0.29952	0.729098	0.182271	0.204416	0.999417	0.97183
Protein-energy malnutrition [Global]	Female	5-9 years	95+ years	0.335778	0.772153	0.196236	0.211156	0.998324	0.968494
Protein-energy malnutrition [Global]	Male	1-5 months	2-4 years	0.282667	0.664048	0.171537	0.187156	0.999379	0.979483
Protein-energy malnutrition [Global]	Male	5-9 years	95+ years	0.319234	0.708499	0.191031	0.203854	0.998721	0.975938
Protein-energy malnutrition [Data Rich]	Female	1-5 months	2-4 years	0.141542	0.505449	0.100608	0.221284	0.999982	0.999264
Protein-energy malnutrition [Data Rich]	Female	5-9 years	95+ years	0.141895	0.531936	0.105578	0.231671	0.99997	0.999467
Protein-energy malnutrition [Data Rich]	Male	1-5 months	2-4 years	0.131381	0.515996	0.0929953	0.194783	1	0.999322
Protein-energy malnutrition [Data Rich]	Male	5-9 years	95+ years	0.130776	0.492399	0.102362	0.211307	0.999985	0.999704
Other nutritional deficiencies [Global]	Female	1-5 months	95+ years	0.376967	0.758253	0.256799	0.263819	0.99667	0.984211
Other nutritional deficiencies [Global]	Male	1-5 months	95+ years	0.362415	0.751041	0.248031	0.259008	0.996801	0.986619
Other nutritional deficiencies [Data Rich]	Female	1-5 months	95+ years	0.259211	0.45779	0.193897	0.204734	0.998541	0.996718
Other nutritional deficiencies [Data Rich]	Male	1-5 months	95+ years	0.257864	0.434464	0.19676	0.221155	0.998304	0.996904
Lip and oral cavity cancer [Global]	Female	15-19 years	95+ years	0.210086	0.346367	0.14674	0.145013	0.999295	0.996952
Lip and oral cavity cancer [Global]	Male	15-19 years	95+ years	0.191948	0.356422	0.141826	0.145084	0.999685	0.992354
Lip and oral cavity cancer [Data Rich]	Female	15-19 years	95+ years	0.113933	0.227005	0.0900668	0.155626	0.999972	0.999788
Lip and oral cavity cancer [Data Rich]	Male	15-19 years	95+ years	0.106154	0.220991	0.0844764	0.148703	0.999992	0.999813
Nasopharynx cancer [Global]	Female	5-9 years	95+ years	0.256854	0.470129	0.177206	0.186116	0.998741	0.994403

Appendix Table S12: CODEm predictive validity results by cause, model type, sex, and age									
**Cause**	**Sex**	**Age Start**	**Age End**	**RMSE In-Sample**	**RMSE Out-of-Sample**	**Trend In-Sample**	**Trend Out-of-Sample**	**Coverage In-Sample**	**Coverage Out-of-Sample**
Nasopharynx cancer [Global]	Male	5-9 years	95+ years	0.210411	0.456501	0.141814	0.150265	0.999728	0.989936
Nasopharynx cancer [Data Rich]	Female	5-9 years	95+ years	0.194994	0.283884	0.150123	0.198937	0.999551	0.998684
Nasopharynx cancer [Data Rich]	Male	5-9 years	95+ years	0.150494	0.245569	0.108633	0.141666	0.999974	0.999866
Other pharynx cancer [Global]	Female	20-24 years	95+ years	0.296219	0.461091	0.174507	0.17781	0.998817	0.994397
Other pharynx cancer [Global]	Male	20-24 years	95+ years	0.244116	0.46772	0.165441	0.174073	0.999216	0.989089
Other pharynx cancer [Data Rich]	Female	20-24 years	95+ years	0.14218	0.278281	0.0926644	0.167379	0.999987	0.998751
Other pharynx cancer [Data Rich]	Male	20-24 years	95+ years	0.116233	0.261713	0.0866386	0.166137	0.999991	0.999631
Oesophageal cancer [Global]	Female	20-24 years	95+ years	0.209556	0.462769	0.142525	0.143535	0.999681	0.983586
Oesophageal cancer [Global]	Male	20-24 years	95+ years	0.189482	0.411375	0.134752	0.138508	0.999669	0.979144
Oesophageal cancer [Data Rich]	Female	20-24 years	95+ years	0.10557	0.204588	0.0841862	0.138567	0.999966	0.999698
Oesophageal cancer [Data Rich]	Male	20-24 years	95+ years	0.104488	0.206529	0.083425	0.146638	0.999983	0.999418
Stomach cancer [Global]	Female	15-19 years	95+ years	0.178159	0.341786	0.130111	0.135141	0.999676	0.98848
Stomach cancer [Global]	Male	15-19 years	95+ years	0.189562	0.358729	0.128869	0.132722	0.999613	0.981992
Stomach cancer [Data Rich]	Female	15-19 years	95+ years	0.105351	0.195219	0.0852236	0.13104	0.999964	0.999066
Stomach cancer [Data Rich]	Male	15-19 years	95+ years	0.103341	0.194053	0.0830231	0.129781	0.999968	0.999217
Colon and rectum cancer [Global]	Female	15-19 years	95+ years	0.183472	0.295703	0.128929	0.130385	0.999705	0.993043
Colon and rectum cancer [Global]	Male	15-19 years	95+ years	0.181979	0.304793	0.132008	0.137589	0.999618	0.990014
Colon and rectum cancer [Data Rich]	Female	15-19 years	95+ years	0.101624	0.194717	0.0818527	0.138301	0.999936	0.999178
Colon and rectum cancer [Data Rich]	Male	15-19 years	95+ years	0.102382	0.196563	0.0829476	0.142803	0.999952	0.998698
Liver cancer [Global]	Female	0-6 days	95+ years	0.249693	0.475806	0.176764	0.184676	0.999175	0.991433
Liver cancer [Global]	Male	0-6 days	95+ years	0.245112	0.484242	0.17504	0.182245	0.998455	0.983559
Liver cancer [Data Rich]	Female	0-6 days	95+ years	0.141716	0.327648	0.101018	0.191755	0.999728	0.999328
Liver cancer [Data Rich]	Male	0-6 days	95+ years	0.144966	0.319652	0.0996465	0.176498	0.999749	0.998785
Gallbladder and biliary tract cancer [Global]	Female	20-24 years	95+ years	0.220773	0.398287	0.143021	0.147336	0.999706	0.988298
Gallbladder and biliary tract cancer [Global]	Male	20-24 years	95+ years	0.237787	0.389343	0.152392	0.155421	0.99962	0.99213
Gallbladder and biliary tract cancer [Data Rich]	Female	20-24 years	95+ years	0.107607	0.235082	0.0852739	0.142841	0.999943	0.999498
Gallbladder and biliary tract cancer [Data Rich]	Male	20-24 years	95+ years	0.112383	0.255644	0.0900915	0.160773	0.999969	0.999665
Pancreatic cancer [Global]	Female	15-19 years	95+ years	0.190464	0.333813	0.140568	0.147986	0.999488	0.994875
Pancreatic cancer [Global]	Male	15-19 years	95+ years	0.184295	0.316478	0.136218	0.142463	0.999558	0.993319
Pancreatic cancer [Data Rich]	Female	15-19 years	95+ years	0.110693	0.225541	0.0872269	0.151479	0.999938	0.99918
Pancreatic cancer [Data Rich]	Male	15-19 years	95+ years	0.109028	0.22851	0.0860364	0.153908	0.999942	0.998993
Larynx cancer [Global]	Female	20-24 years	95+ years	0.279739	0.485046	0.166005	0.166224	0.999511	0.993116
Larynx cancer [Global]	Male	20-24 years	95+ years	0.264148	0.431025	0.141242	0.141539	0.999835	0.986493
Larynx cancer [Data Rich]	Female	20-24 years	95+ years	0.124742	0.248042	0.0945579	0.172496	0.999996	0.999721
Larynx cancer [Data Rich]	Male	20-24 years	95+ years	0.112131	0.215508	0.0866632	0.142168	0.999974	0.999464
Tracheal, bronchus, and lung cancer [Global]	Female	15-19 years	95+ years	0.186812	0.363213	0.129272	0.136561	0.999743	0.978948
Tracheal, bronchus, and lung cancer [Global]	Male	15-19 years	95+ years	0.1932	0.363302	0.124202	0.132593	0.999498	0.966618
Tracheal, bronchus, and lung cancer [Data Rich]	Female	15-19 years	95+ years	0.108383	0.215081	0.0858141	0.15653	0.999972	0.998889
Tracheal, bronchus, and lung cancer [Data Rich]	Male	15-19 years	95+ years	0.100398	0.195328	0.0798169	0.132604	0.999912	0.997113
Malignant skin melanoma [Global]	Female	15-19 years	95+ years	0.307316	0.450724	0.184846	0.197173	0.998573	0.995326
Malignant skin melanoma [Global]	Male	15-19 years	95+ years	0.299861	0.463229	0.17215	0.185186	0.999322	0.993398
Malignant skin melanoma [Data Rich]	Female	15-19 years	95+ years	0.119241	0.243161	0.0975857	0.172314	1	0.999533
Malignant skin melanoma [Data Rich]	Male	15-19 years	95+ years	0.122556	0.26762	0.0996514	0.186017	1	0.999687
Non-melanoma skin cancer [Global]	Female	20-24 years	95+ years	0.292715	0.50194	0.194731	0.195459	0.998695	0.988794
Non-melanoma skin cancer [Global]	Male	20-24 years	95+ years	0.262794	0.478371	0.178925	0.182617	0.999292	0.990931
Non-melanoma skin cancer [Data Rich]	Female	20-24 years	95+ years	0.133455	0.318463	0.105535	0.200934	0.999942	0.99936
Non-melanoma skin cancer [Data Rich]	Male	20-24 years	95+ years	0.127866	0.330376	0.102425	0.193983	0.999973	0.999629
Non-melanoma skin cancer (squamous-cell carcinoma) [Global]	Female	20-24 years	95+ years	0.288753	0.486236	0.194914	0.204409	0.998596	0.991904
Non-melanoma skin cancer (squamous-cell carcinoma) [Global]	Male	20-24 years	95+ years	0.264009	0.461975	0.180063	0.196769	0.999326	0.992361
Non-melanoma skin cancer (squamous-cell carcinoma) [Data Rich]	Female	20-24 years	95+ years	0.133631	0.318362	0.105597	0.187114	0.999951	0.999329
Non-melanoma skin cancer (squamous-cell carcinoma) [Data Rich]	Male	20-24 years	95+ years	0.128296	0.344166	0.102555	0.18795	0.999978	0.999651
Soft tissue and other extraosseous sarcomas [Global]	Female	0-6 days	95+ years	0.277257	0.437728	0.16764	0.155394	0.99876	0.996011
Soft tissue and other extraosseous sarcomas [Global]	Male	0-6 days	95+ years	0.281554	0.435444	0.157705	0.15508	0.998658	0.995772
Soft tissue and other extraosseous sarcomas [Data Rich]	Female	0-6 days	95+ years	0.126818	0.332669	0.099169	0.166035	0.999936	0.999405
Soft tissue and other extraosseous sarcomas [Data Rich]	Male	0-6 days	95+ years	0.127657	0.344045	0.095736	0.163252	0.999874	0.999586
Malignant neoplasm of bone and articular cartilage [Global]	Female	12-23 months	95+ years	0.364173	0.531808	0.261305	0.278359	0.996778	0.991905
Malignant neoplasm of bone and articular cartilage [Global]	Male	12-23 months	95+ years	0.370542	0.542159	0.249696	0.263489	0.996815	0.991717
Malignant neoplasm of bone and articular cartilage [Data Rich]	Female	12-23 months	95+ years	0.159217	0.311106	0.112063	0.180995	0.999306	0.998923
Malignant neoplasm of bone and articular cartilage [Data Rich]	Male	12-23 months	95+ years	0.138312	0.308907	0.107055	0.17528	0.999676	0.999534
Breast cancer [Global]	Female	15-19 years	95+ years	0.193996	0.311076	0.131387	0.136746	0.999565	0.98968
Breast cancer [Global]	Male	15-19 years	95+ years	0.368098	0.576575	0.225141	0.241821	0.9984	0.992913
Breast cancer [Data Rich]	Female	15-19 years	95+ years	0.103919	0.200364	0.0838307	0.145563	0.9999	0.998111
Breast cancer [Data Rich]	Male	15-19 years	95+ years	0.170411	0.413502	0.11837	0.220053	0.999643	0.999032
Cervical cancer [Global]	Female	15-19 years	95+ years	0.226963	0.361748	0.13564	0.134617	0.99984	0.994699
Cervical cancer [Data Rich]	Female	15-19 years	95+ years	0.108432	0.207446	0.0851864	0.140129	0.999928	0.99972
Uterine cancer [Global]	Female	20-24 years	95+ years	0.237595	0.361737	0.139934	0.141324	0.999857	0.997966
Uterine cancer [Data Rich]	Female	20-24 years	95+ years	0.102469	0.212839	0.0804228	0.135373	0.999987	0.999833
Ovarian cancer [Global]	Female	15-19 years	95+ years	0.207552	0.33052	0.143844	0.154997	0.999641	0.992475
Ovarian cancer [Data Rich]	Female	15-19 years	95+ years	0.109948	0.23179	0.0896165	0.156975	0.999946	0.999143
Prostate cancer [Global]	Male	20-24 years	95+ years	0.23886	0.36605	0.1396	0.141806	0.999619	0.989633
Prostate cancer [Data Rich]	Male	20-24 years	95+ years	0.106149	0.2233	0.083378	0.148713	0.999953	0.998903
Testicular cancer [Global]	Male	15-19 years	95+ years	0.244456	0.441864	0.181654	0.176253	0.999152	0.995152
Testicular cancer [Data Rich]	Male	15-19 years	95+ years	0.128047	0.286225	0.105879	0.168685	0.999988	0.999574
Kidney cancer [Global]	Female	0-6 days	95+ years	0.265878	0.416286	0.163403	0.165861	0.999463	0.99517
Kidney cancer [Global]	Male	0-6 days	95+ years	0.264911	0.391427	0.141427	0.142303	0.999607	0.995613
Kidney cancer [Data Rich]	Female	0-6 days	95+ years	0.116366	0.274597	0.0883282	0.154888	0.99998	0.999506
Kidney cancer [Data Rich]	Male	0-6 days	95+ years	0.114482	0.253644	0.0836427	0.145175	0.99996	0.999473
Bladder cancer [Global]	Female	15-19 years	95+ years	0.204972	0.364546	0.143286	0.144742	0.999563	0.996185
Bladder cancer [Global]	Male	15-19 years	95+ years	0.190812	0.337974	0.138913	0.144894	0.999718	0.993357
Bladder cancer [Data Rich]	Female	15-19 years	95+ years	0.110214	0.22443	0.0865417	0.154471	0.999996	0.999533
Bladder cancer [Data Rich]	Male	15-19 years	95+ years	0.109379	0.220478	0.0873041	0.150612	0.999963	0.999124
Brain and nervous system cancer [Global]	Female	0-6 days	95+ years	0.248084	0.389178	0.13502	0.143105	0.999748	0.995475
Brain and nervous system cancer [Global]	Male	0-6 days	95+ years	0.231154	0.368562	0.135731	0.144909	0.999528	0.995385
Brain and nervous system cancer [Data Rich]	Female	0-6 days	95+ years	0.106741	0.242175	0.0815269	0.138448	0.999988	0.999702
Brain and nervous system cancer [Data Rich]	Male	0-6 days	95+ years	0.106245	0.249195	0.082003	0.142122	0.999989	0.999537
Eye cancer [Global]	Female	0-6 days	95+ years	0.723331	0.847318	0.558073	0.514045	0.987521	0.976409
Eye cancer [Global]	Male	0-6 days	95+ years	0.775126	0.892215	0.596002	0.575336	0.982967	0.972589
Eye cancer [Data Rich]	Female	0-6 days	95+ years	0.4754	0.563803	0.388674	0.380285	0.995092	0.991359
Eye cancer [Data Rich]	Male	0-6 days	95+ years	0.45936	0.583017	0.399533	0.432774	0.992234	0.987468
Retinoblastoma [Global]	Female	0-6 days	5-9 years	1.23294	1.60622	0.980918	0.978727	0.971146	0.939139
Retinoblastoma [Global]	Male	0-6 days	5-9 years	1.23463	1.5429	0.957585	0.912189	0.971115	0.939697
Retinoblastoma [Data Rich]	Female	0-6 days	5-9 years	1.05348	1.35174	0.927582	1.00311	0.965148	0.939515
Retinoblastoma [Data Rich]	Male	0-6 days	5-9 years	1.02627	1.2721	0.896494	0.928034	0.969049	0.946114
Other eye cancers [Global]	Female	10-14 years	95+ years	0.797316	0.857998	0.587898	0.523737	0.984359	0.971545
Other eye cancers [Global]	Male	10-14 years	95+ years	0.79974	0.880333	0.614344	0.550849	0.982181	0.971497
Other eye cancers [Data Rich]	Female	10-14 years	95+ years	0.444872	0.482741	0.364439	0.308936	0.996765	0.994444
Other eye cancers [Data Rich]	Male	10-14 years	95+ years	0.385665	0.486305	0.326024	0.333868	0.99829	0.996412
Neuroblastoma and other peripheral nervous cell tumours [Global]	Female	0-6 days	95+ years	0.330838	0.578397	0.20126	0.219564	0.998899	0.994188
Neuroblastoma and other peripheral nervous cell tumours [Global]	Male	0-6 days	95+ years	0.353595	0.614512	0.191045	0.208292	0.999274	0.99211
Neuroblastoma and other peripheral nervous cell tumours [Data Rich]	Female	0-6 days	95+ years	0.199297	0.456795	0.15989	0.162958	0.999591	0.999101
Neuroblastoma and other peripheral nervous cell tumours [Data Rich]	Male	0-6 days	95+ years	0.203702	0.492419	0.154872	0.193364	0.999812	0.998918
Thyroid cancer [Global]	Female	5-9 years	95+ years	0.213996	0.355537	0.151245	0.152611	0.999503	0.998121
Thyroid cancer [Global]	Male	5-9 years	95+ years	0.27603	0.389446	0.160503	0.167478	0.99939	0.99805
Thyroid cancer [Data Rich]	Female	5-9 years	95+ years	0.114199	0.238048	0.0864821	0.147647	0.999996	0.999823
Thyroid cancer [Data Rich]	Male	5-9 years	95+ years	0.115504	0.253887	0.0881059	0.157519	0.999996	0.999843
Mesothelioma [Global]	Female	20-24 years	95+ years	0.269168	0.486952	0.187308	0.203266	0.998642	0.994483
Mesothelioma [Global]	Male	20-24 years	95+ years	0.232232	0.476443	0.167614	0.185103	0.999238	0.996228
Mesothelioma [Data Rich]	Female	20-24 years	95+ years	0.132178	0.333323	0.0991841	0.202799	0.999791	0.999321
Mesothelioma [Data Rich]	Male	20-24 years	95+ years	0.107512	0.245653	0.0818553	0.158153	0.999801	0.999638
Hodgkin lymphoma [Global]	Female	2-4 years	95+ years	0.345588	0.546777	0.192572	0.199598	0.999259	0.992104
Hodgkin lymphoma [Global]	Male	2-4 years	95+ years	0.378141	0.564351	0.182844	0.190858	0.999548	0.992683
Hodgkin lymphoma [Data Rich]	Female	2-4 years	95+ years	0.127473	0.287692	0.0972144	0.16281	0.999982	0.999638
Hodgkin lymphoma [Data Rich]	Male	2-4 years	95+ years	0.127331	0.302892	0.0944461	0.147418	0.999993	0.999553
Non-Hodgkin's lymphoma [Global]	Female	12-23 months	95+ years	0.191784	0.352306	0.132526	0.135686	0.99992	0.997294
Non-Hodgkin's lymphoma [Global]	Male	12-23 months	95+ years	0.190949	0.346145	0.13461	0.137606	0.999918	0.997036
Non-Hodgkin's lymphoma [Data Rich]	Female	12-23 months	95+ years	0.104703	0.229364	0.0811724	0.145022	0.999993	0.999928
Non-Hodgkin's lymphoma [Data Rich]	Male	12-23 months	95+ years	0.105394	0.234761	0.0829517	0.146667	1	0.99997
Other non-Hodgkin lymphoma [Global]	Female	12-23 months	95+ years	0.259966	0.36631	0.130917	0.135611	0.999867	0.998544
Other non-Hodgkin lymphoma [Global]	Male	12-23 months	95+ years	0.190702	0.354253	0.131009	0.134746	0.99981	0.997599
Other non-Hodgkin lymphoma [Data Rich]	Female	12-23 months	95+ years	0.110091	0.330631	0.0840411	0.140046	0.999826	0.999794
Other non-Hodgkin lymphoma [Data Rich]	Male	12-23 months	95+ years	0.10902	0.334036	0.0842989	0.138756	0.999783	0.999675
Multiple myeloma [Global]	Female	20-24 years	95+ years	0.333348	0.495018	0.226211	0.256963	0.995565	0.99166
Multiple myeloma [Global]	Male	20-24 years	95+ years	0.343418	0.469189	0.213918	0.238332	0.995776	0.989778
Multiple myeloma [Data Rich]	Female	20-24 years	95+ years	0.112628	0.237025	0.0868636	0.147318	0.999751	0.999446

Appendix Table S12: CODEm predictive validity results by cause, model type, sex, and age

Cause	Sex	Age Start	Age End	RMSE In-Sample	RMSE Out-of-Sample	Trend In-Sample	Trend Out-of-Sample	Coverage In-Sample	Coverage Out-of-Sample
Multiple myeloma [Data Rich]	Male	20-24 years	95+ years	0.114058	0.250626	0.0887323	0.154023	0.999706	0.999392
Leukaemia [Global]	Female	0-6 days	95+ years	0.200317	0.323824	0.167592	0.174761	0.999778	0.995637
Leukaemia [Global]	Male	0-6 days	95+ years	0.203844	0.326354	0.137403	0.142295	0.999801	0.995198
Leukaemia [Data Rich]	Female	0-6 days	95+ years	0.10521	0.2109	0.0813327	0.132873	0.999947	0.999622
Leukaemia [Data Rich]	Male	0-6 days	95+ years	0.106613	0.215902	0.0821763	0.136548	0.999964	0.999561
Acute lymphoid leukaemia [Global]	Female	0-6 days	95+ years	0.790922	0.95575	0.482954	0.483399	0.993917	0.988924
Acute lymphoid leukaemia [Global]	Male	0-6 days	95+ years	0.719389	0.870941	0.446229	0.440699	0.994479	0.989887
Acute lymphoid leukaemia [Data Rich]	Female	0-6 days	95+ years	0.180594	0.479686	0.134838	0.211869	0.999234	0.998594
Acute lymphoid leukaemia [Data Rich]	Male	0-6 days	95+ years	0.135481	0.463638	0.105516	0.189389	0.999659	0.999032
Chronic lymphoid leukaemia [Global]	Female	20-24 years	95+ years	0.618671	1.10608	0.399243	0.371074	0.991595	0.962327
Chronic lymphoid leukaemia [Global]	Male	20-24 years	95+ years	0.590201	0.827262	0.377039	0.356447	0.993887	0.972932
Chronic lymphoid leukaemia [Data Rich]	Female	20-24 years	95+ years	0.164737	0.400663	0.101168	0.176263	0.999079	0.995816
Chronic lymphoid leukaemia [Data Rich]	Male	20-24 years	95+ years	0.120403	0.337014	0.0901838	0.162402	0.999828	0.999031
Acute myeloid leukaemia [Global]	Female	0-6 days	95+ years	0.773685	0.880577	0.505279	0.496642	0.993722	0.98859
Acute myeloid leukaemia [Global]	Male	0-6 days	95+ years	0.766198	0.961836	0.495325	0.517377	0.993618	0.9887
Acute myeloid leukaemia [Data Rich]	Female	0-6 days	95+ years	0.163374	0.490194	0.137626	0.219027	0.999106	0.998357
Acute myeloid leukaemia [Data Rich]	Male	0-6 days	95+ years	0.169828	0.452943	0.135193	0.236643	0.9988	0.997516
Chronic myeloid leukaemia [Global]	Female	0-6 days	95+ years	0.668584	1.10266	0.443652	0.454454	0.992983	0.971523
Chronic myeloid leukaemia [Global]	Male	0-6 days	95+ years	0.656453	0.931691	0.430871	0.397322	0.992627	0.972491
Chronic myeloid leukaemia [Data Rich]	Female	0-6 days	95+ years	0.267416	0.638104	0.178137	0.179673	0.998803	0.992418
Chronic myeloid leukaemia [Data Rich]	Male	0-6 days	95+ years	0.215304	0.541348	0.138551	0.151804	0.999558	0.995823
Other leukaemia [Global]	Female	0-6 days	95+ years	0.751294	1.11775	0.378459	0.343876	0.994916	0.973148
Other leukaemia [Global]	Male	0-6 days	95+ years	0.606472	0.97883	0.372567	0.376176	0.99365	0.965175
Other leukaemia [Data Rich]	Female	0-6 days	95+ years	0.300667	0.783131	0.168188	0.206487	0.998025	0.986487
Other leukaemia [Data Rich]	Male	0-6 days	95+ years	0.256995	0.673536	0.175553	0.194724	0.997837	0.99166
Other malignant cancers [Global]	Female	0-6 days	95+ years	0.203559	0.3459	0.142419	0.144959	0.999627	0.99615
Other malignant cancers [Global]	Male	0-6 days	95+ years	0.231306	0.369	0.148585	0.149545	0.999738	0.995449
Other malignant cancers [Data Rich]	Female	0-6 days	95+ years	0.110662	0.227933	0.0875512	0.1346	0.999901	0.999567
Other malignant cancers [Data Rich]	Male	0-6 days	95+ years	0.107332	0.246646	0.0827862	0.128328	0.999963	0.999736
Other neoplasms [Global]	Female	0-6 days	95+ years	0.268577	0.616986	0.160495	0.172151	0.999509	0.985031
Other neoplasms [Global]	Male	0-6 days	95+ years	0.308879	0.611955	0.194242	0.179756	0.999312	0.985134
Other neoplasms [Data Rich]	Female	0-6 days	95+ years	0.168986	0.658211	0.104485	0.196913	0.999842	0.997208
Other neoplasms [Data Rich]	Male	0-6 days	95+ years	0.166039	0.697391	0.119075	0.208542	0.999716	0.99747
Myelodysplastic, myeloproliferative, and other hematopoietic neoplasms [Global]	Female	0-6 days	95+ years	0.423138	0.75679	0.264428	0.228657	0.995662	0.98214
Myelodysplastic, myeloproliferative, and other hematopoietic neoplasms [Global]	Male	0-6 days	95+ years	0.496742	0.784421	0.229034	0.215236	0.997628	0.984049
Myelodysplastic, myeloproliferative, and other hematopoietic neoplasms [Data Rich]	Female	0-6 days	95+ years	0.253049	0.778061	0.17928	0.269979	0.998065	0.995865
Myelodysplastic, myeloproliferative, and other hematopoietic neoplasms [Data Rich]	Male	0-6 days	95+ years	0.220486	0.755094	0.154055	0.287115	0.999126	0.997587
Non-malignant brain and other central nervous system neoplasms [Global]	Female	0-6 days	95+ years	0.334277	0.592428	0.185597	0.172956	0.999447	0.991985
Non-malignant brain and other central nervous system neoplasms [Global]	Male	0-6 days	95+ years	0.425236	0.659532	0.210582	0.177385	0.99927	0.992262
Non-malignant brain and other central nervous system neoplasms [Data Rich]	Female	0-6 days	95+ years	0.164758	0.595202	0.109918	0.182086	0.999912	0.997127
Non-malignant brain and other central nervous system neoplasms [Data Rich]	Male	0-6 days	95+ years	0.164946	0.639765	0.12464	0.194726	0.999557	0.997655
Other benign and in situ neoplasms [Global]	Female	0-6 days	95+ years	0.500266	1.10295	0.163746	0.169571	0.999915	0.951758
Other benign and in situ neoplasms [Global]	Male	0-6 days	95+ years	0.405486	1.09224	0.158754	0.165725	0.999936	0.946737
Other benign and in situ neoplasms [Data Rich]	Female	0-6 days	95+ years	0.177001	0.930205	0.136013	0.166695	1	0.961521
Other benign and in situ neoplasms [Data Rich]	Male	0-6 days	95+ years	0.17347	0.930644	0.132924	0.161528	1	0.951625
Cardiovascular diseases [Global]	Female	0-6 days	95+ years	0.180238	0.323161	0.115044	0.119724	0.999747	0.981198
Cardiovascular diseases [Global]	Male	0-6 days	95+ years	0.167366	0.330122	0.112826	0.117502	0.999414	0.970273
Cardiovascular diseases [Data Rich]	Female	0-6 days	95+ years	0.102407	0.212339	0.0743535	0.110078	0.999922	0.998007
Cardiovascular diseases [Data Rich]	Male	0-6 days	95+ years	0.0993994	0.215349	0.0723873	0.108324	0.999916	0.997029
Rheumatic heart disease [Global]	Female	12-23 months	95+ years	0.262575	0.543282	0.140707	0.142711	0.999927	0.981808
Rheumatic heart disease [Global]	Male	12-23 months	95+ years	0.247768	0.498456	0.149253	0.153429	0.999919	0.992216
Rheumatic heart disease [Data Rich]	Female	12-23 months	95+ years	0.12373	0.316889	0.0892242	0.137228	0.999983	0.999565
Rheumatic heart disease [Data Rich]	Male	12-23 months	95+ years	0.134464	0.363619	0.0947014	0.142184	0.999997	0.999756
Ischaemic heart disease [Global]	Female	15-19 years	95+ years	0.159664	0.337213	0.110257	0.114066	0.999349	0.960133
Ischaemic heart disease [Global]	Male	15-19 years	95+ years	0.150452	0.338593	0.10544	0.111525	0.999197	0.953887
Ischaemic heart disease [Data Rich]	Female	15-19 years	95+ years	0.0908847	0.188306	0.0709932	0.111808	0.999697	0.996076
Ischaemic heart disease [Data Rich]	Male	15-19 years	95+ years	0.0858	0.182141	0.0672042	0.11041	0.999855	0.994522
Stroke [Global]	Female	0-6 days	95+ years	0.191326	0.356249	0.120996	0.123446	0.999819	0.989395
Stroke [Global]	Male	0-6 days	95+ years	0.185151	0.371293	0.12305	0.127363	0.999786	0.986147
Stroke [Data Rich]	Female	0-6 days	95+ years	0.114988	0.231386	0.0809617	0.119296	0.999961	0.999312
Stroke [Data Rich]	Male	0-6 days	95+ years	0.113449	0.233345	0.0805856	0.120347	0.999978	0.999096
Ischaemic stroke [Global]	Female	0-6 days	95+ years	0.197421	0.404394	0.116036	0.119531	0.999772	0.98623
Ischaemic stroke [Global]	Male	0-6 days	95+ years	0.1952	0.405425	0.11775	0.121413	0.999839	0.98931
Ischaemic stroke [Data Rich]	Female	0-6 days	95+ years	0.115006	0.362714	0.077898	0.116048	0.999967	0.999721
Ischaemic stroke [Data Rich]	Male	0-6 days	95+ years	0.117312	0.294794	0.0786796	0.114295	0.999958	0.999688
Intracerebral hemorrhage [Global]	Female	0-6 days	95+ years	0.209415	0.390799	0.123642	0.12651	0.999802	0.990459
Intracerebral hemorrhage [Global]	Male	0-6 days	95+ years	0.187521	0.381094	0.120749	0.125077	0.99986	0.991595
Intracerebral hemorrhage [Data Rich]	Female	0-6 days	95+ years	0.13085	0.394598	0.0837499	0.126963	0.999973	0.999347
Intracerebral hemorrhage [Data Rich]	Male	0-6 days	95+ years	0.117135	0.355109	0.0806851	0.125503	0.999982	0.99952
Subarachnoid hemorrhage [Global]	Female	0-6 days	95+ years	0.262403	0.439536	0.137945	0.144046	0.999588	0.993984
Subarachnoid hemorrhage [Global]	Male	0-6 days	95+ years	0.337463	0.499432	0.145838	0.150278	0.999355	0.989124
Subarachnoid hemorrhage [Data Rich]	Female	0-6 days	95+ years	0.116434	0.305762	0.0798217	0.12479	0.999946	0.999436
Subarachnoid hemorrhage [Data Rich]	Male	0-6 days	95+ years	0.116683	0.304448	0.0799153	0.12877	0.999988	0.999718
Hypertensive heart disease [Global]	Female	15-19 years	95+ years	0.300703	0.603522	0.148397	0.151608	0.999591	0.954347
Hypertensive heart disease [Global]	Male	15-19 years	95+ years	0.294913	0.557782	0.159361	0.159162	0.999309	0.963667
Hypertensive heart disease [Data Rich]	Female	15-19 years	95+ years	0.108437	0.319329	0.0803372	0.137691	0.999917	0.998823
Hypertensive heart disease [Data Rich]	Male	15-19 years	95+ years	0.11035	0.336791	0.084314	0.145911	0.999934	0.999048
Non-rheumatic valvular heart disease [Global]	Female	15-19 years	95+ years	0.395216	0.597992	0.167533	0.165993	0.999833	0.989354
Non-rheumatic valvular heart disease [Global]	Male	15-19 years	95+ years	0.282669	0.532835	0.150446	0.153088	0.999953	0.989528
Non-rheumatic valvular heart disease [Data Rich]	Female	15-19 years	95+ years	0.110106	0.409144	0.0837385	0.167268	0.999979	0.999717
Non-rheumatic valvular heart disease [Data Rich]	Male	15-19 years	95+ years	0.108316	0.370475	0.0834114	0.156111	0.999983	0.999715
Non-rheumatic calcific aortic valve disease [Global]	Female	15-19 years	95+ years	0.309699	0.606714	0.152225	0.154856	0.999894	0.985234
Non-rheumatic calcific aortic valve disease [Global]	Male	15-19 years	95+ years	0.260928	0.587453	0.146586	0.150613	0.999891	0.981279
Non-rheumatic calcific aortic valve disease [Data Rich]	Female	15-19 years	95+ years	0.116174	0.399383	0.0852308	0.165337	0.999974	0.999832
Non-rheumatic calcific aortic valve disease [Data Rich]	Male	15-19 years	95+ years	0.10626	0.369904	0.0811288	0.149916	1	0.999782
Non-rheumatic degenerative mitral valve disease [Global]	Female	15-19 years	95+ years	0.428395	0.721961	0.200943	0.185689	0.998916	0.984955
Non-rheumatic degenerative mitral valve disease [Global]	Male	15-19 years	95+ years	0.431641	0.71135	0.190032	0.177666	0.999309	0.983984
Non-rheumatic degenerative mitral valve disease [Data Rich]	Female	15-19 years	95+ years	0.129963	0.421892	0.0936347	0.149782	0.999876	0.999358
Non-rheumatic degenerative mitral valve disease [Data Rich]	Male	15-19 years	95+ years	0.124157	0.365254	0.095771	0.147946	0.999824	0.999383
Other non-rheumatic valve diseases [Global]	Female	15-19 years	95+ years	0.740536	1.20264	0.436839	0.468359	0.991152	0.952107
Other non-rheumatic valve diseases [Global]	Male	15-19 years	95+ years	0.870033	1.27413	0.557822	0.553781	0.985839	0.944673
Other non-rheumatic valve diseases [Data Rich]	Female	15-19 years	95+ years	0.644572	0.864626	0.424896	0.445813	0.99219	0.990524
Other non-rheumatic valve diseases [Data Rich]	Male	15-19 years	95+ years	0.766077	0.908851	0.5123	0.449805	0.989381	0.986087
Cardiomyopathy and myocarditis [Global]	Female	0-6 days	95+ years	0.291986	0.611088	0.177513	0.181104	0.999413	0.979326
Cardiomyopathy and myocarditis [Global]	Male	0-6 days	95+ years	0.312287	0.556327	0.164364	0.17132	0.999572	0.977073
Cardiomyopathy and myocarditis [Data Rich]	Female	0-6 days	95+ years	0.136848	0.399086	0.103149	0.169709	0.999916	0.999428
Cardiomyopathy and myocarditis [Data Rich]	Male	0-6 days	95+ years	0.129865	0.381287	0.0963557	0.167795	0.999879	0.999277
Myocarditis [Global]	Female	0-6 days	95+ years	0.464861	0.745034	0.222554	0.223013	0.998609	0.988435
Myocarditis [Global]	Male	0-6 days	95+ years	0.442796	0.70391	0.223248	0.238514	0.998498	0.988146
Myocarditis [Data Rich]	Female	0-6 days	95+ years	0.214678	0.42706	0.169897	0.192653	0.9997	0.9993
Myocarditis [Data Rich]	Male	0-6 days	95+ years	0.226555	0.458839	0.180797	0.226372	0.99945	0.998886
Alcoholic cardiomyopathy [Global]	Female	15-19 years	95+ years	0.491294	0.8649	0.315985	0.308034	0.997179	0.978605
Alcoholic cardiomyopathy [Global]	Male	15-19 years	95+ years	0.443383	0.750922	0.275283	0.279018	0.998015	0.986382
Alcoholic cardiomyopathy [Data Rich]	Female	15-19 years	95+ years	0.265629	0.630322	0.212004	0.203391	0.999228	0.997417
Alcoholic cardiomyopathy [Data Rich]	Male	15-19 years	95+ years	0.227754	0.499659	0.18194	0.178003	0.998281	0.996312
Other cardiomyopathy [Global]	Female	0-6 days	95+ years	0.354449	0.654449	0.16954	0.172451	0.999828	0.987027
Other cardiomyopathy [Global]	Male	0-6 days	95+ years	0.408503	0.638175	0.167163	0.16873	0.999717	0.979924
Other cardiomyopathy [Data Rich]	Female	0-6 days	95+ years	0.14016	0.421004	0.102882	0.175313	0.999977	0.99971
Other cardiomyopathy [Data Rich]	Male	0-6 days	95+ years	0.136138	0.390274	0.101391	0.17475	0.999936	0.999572
Pulmonary Arterial Hypertension [Global]	Female	0-6 days	95+ years	0.425229	0.710568	0.198231	0.201394	0.999887	0.988048
Pulmonary Arterial Hypertension [Global]	Male	0-6 days	95+ years	0.545986	0.807203	0.227955	0.224746	0.999325	0.983684
Pulmonary Arterial Hypertension [Data Rich]	Female	0-6 days	95+ years	0.205102	0.53378	0.151494	0.196387	0.999979	0.996075
Pulmonary Arterial Hypertension [Data Rich]	Male	0-6 days	95+ years	0.22056	0.560585	0.170231	0.216526	0.999732	0.992343
Atrial fibrillation and flutter [Global]	Female	30-34 years	45-49 years	0.258974	0.34861	0.0950642	0.0969282	0.999411	0.999047
Atrial fibrillation and flutter [Global]	Female	50-54 years	95+ years	0.107877	0.212623	0.0553543	0.0579471	0.999839	0.991089
Atrial fibrillation and flutter [Global]	Male	30-34 years	45-49 years	0.206455	0.303344	0.0945114	0.0959834	0.999985	0.999764
Atrial fibrillation and flutter [Global]	Male	50-54 years	95+ years	0.125791	0.224899	0.0577231	0.0597734	0.99987	0.993212
Atrial fibrillation and flutter [Data Rich]	Female	30-34 years	45-49 years	0.118933	0.271498	0.0579319	0.104979	1	0.99973
Atrial fibrillation and flutter [Data Rich]	Female	50-54 years	95+ years	0.0514482	0.1732	0.0355933	0.0594508	0.999961	0.9992
Atrial fibrillation and flutter [Data Rich]	Male	30-34 years	45-49 years	0.0783098	0.225216	0.0599751	0.0935804	1	1
Atrial fibrillation and flutter [Data Rich]	Male	50-54 years	95+ years	0.0499111	0.169913	0.0341722	0.0601391	0.999937	0.999474

Appendix Table S12: CODEm predictive validity results by cause, model type, sex, and age

Cause	Sex	Age Start	Age End	RMSE In-Sample	RMSE Out-of-Sample	Trend In-Sample	Trend Out-of-Sample	Coverage In-Sample	Coverage Out-of-Sample
Aortic aneurysm [Global]	Female	15-19 years	95+ years	0.335751	0.582754	0.200618	0.208601	0.996617	0.983313
Aortic aneurysm [Global]	Male	15-19 years	95+ years	0.275681	0.517969	0.168655	0.17385	0.997717	0.980694
Aortic aneurysm [Data Rich]	Female	15-19 years	95+ years	0.119468	0.316593	0.0925277	0.160746	0.999949	0.999511
Aortic aneurysm [Data Rich]	Male	15-19 years	95+ years	0.113532	0.30501	0.0854634	0.143587	0.999957	0.999237
Lower extremity peripheral arterial disease [Global]	Female	40-44 years	95+ years	0.356337	0.655357	0.241886	0.187009	0.997451	0.976968
Lower extremity peripheral arterial disease [Global]	Male	40-44 years	95+ years	0.302276	0.605198	0.207828	0.168188	0.998485	0.977402
Lower extremity peripheral arterial disease [Data Rich]	Female	40-44 years	95+ years	0.157962	0.514993	0.125794	0.220124	0.999198	0.990031
Lower extremity peripheral arterial disease [Data Rich]	Male	40-44 years	95+ years	0.13509	0.386304	0.109424	0.166668	0.999621	0.989848
Endocarditis [Global]	Female	0-6 days	95+ years	0.275991	0.568785	0.157123	0.164521	0.999864	0.992495
Endocarditis [Global]	Male	0-6 days	95+ years	0.372191	0.598146	0.175321	0.181354	0.999406	0.989053
Endocarditis [Data Rich]	Female	0-6 days	95+ years	0.125998	0.443043	0.0843415	0.158357	0.999973	0.999691
Endocarditis [Data Rich]	Male	0-6 days	95+ years	0.125923	0.439535	0.0856673	0.159026	0.999979	0.999596
Other cardiovascular and circulatory diseases [Global]	Female	0-6 days	95+ years	0.289261	0.491074	0.154648	0.164327	0.999477	0.990209
Other cardiovascular and circulatory diseases [Global]	Male	0-6 days	95+ years	0.25486	0.470095	0.157783	0.167692	0.999125	0.991111
Other cardiovascular and circulatory diseases [Data Rich]	Female	0-6 days	95+ years	0.121666	0.282232	0.087311	0.140327	0.999948	0.999638
Other cardiovascular and circulatory diseases [Data Rich]	Male	0-6 days	95+ years	0.121393	0.273531	0.0894945	0.143836	0.999983	0.999714
Chronic respiratory diseases [Global]	Female	0-6 days	95+ years	0.459078	0.925239	0.129913	0.137743	0.999795	0.964685
Chronic respiratory diseases [Global]	Male	0-6 days	95+ years	0.269227	0.689497	0.125924	0.129193	0.999578	0.970731
Chronic respiratory diseases [Data Rich]	Female	0-6 days	95+ years	0.165591	1.50536	0.0872492	0.167052	0.999766	0.995558
Chronic respiratory diseases [Data Rich]	Male	0-6 days	95+ years	0.143119	1.10114	0.085733	0.139541	0.999921	0.997669
Chronic obstructive pulmonary disease [Global]	Female	15-19 years	95+ years	0.182307	0.458441	0.11955	0.127105	0.999854	0.973449
Chronic obstructive pulmonary disease [Global]	Male	15-19 years	95+ years	0.183882	0.390476	0.117594	0.122755	0.999897	0.976877
Chronic obstructive pulmonary disease [Data Rich]	Female	15-19 years	95+ years	0.107887	0.315242	0.0814969	0.138403	0.999974	0.999315
Chronic obstructive pulmonary disease [Data Rich]	Male	15-19 years	95+ years	0.0998803	0.25528	0.0791427	0.127545	0.999987	0.998933
Pneumoconiosis [Global]	Female	15-19 years	95+ years	0.693659	1.18829	0.395561	0.39955	0.993657	0.962307
Pneumoconiosis [Global]	Male	15-19 years	95+ years	0.465515	1.12121	0.311027	0.301865	0.993547	0.93645
Pneumoconiosis [Data Rich]	Female	15-19 years	95+ years	0.428502	0.83775	0.340841	0.404982	0.994226	0.989155
Pneumoconiosis [Data Rich]	Male	15-19 years	95+ years	0.297892	0.563624	0.229754	0.226029	0.998019	0.996672
Silicosis [Global]	Female	15-19 years	95+ years	0.76343	1.29784	0.491849	0.515087	0.993884	0.946096
Silicosis [Global]	Male	15-19 years	95+ years	0.608144	1.45175	0.414446	0.442323	0.989444	0.906015
Silicosis [Data Rich]	Female	15-19 years	95+ years	0.538735	0.997229	0.436936	0.513255	0.995102	0.980451
Silicosis [Data Rich]	Male	15-19 years	95+ years	0.428334	0.952844	0.334834	0.311222	0.992893	0.987293
Asbestosis [Global]	Female	15-19 years	95+ years	0.741263	1.31859	0.464813	0.484891	0.994022	0.947343
Asbestosis [Global]	Male	15-19 years	95+ years	0.700269	1.15844	0.390688	0.411215	0.991567	0.962236
Asbestosis [Data Rich]	Female	15-19 years	95+ years	0.509153	1.12928	0.415608	0.526556	0.995118	0.980251
Asbestosis [Data Rich]	Male	15-19 years	95+ years	0.351423	0.809124	0.282835	0.320328	0.996248	0.993301
Coal workers pneumoconiosis [Global]	Female	15-19 years	95+ years	0.788974	1.38079	0.59488	0.605848	0.990996	0.957358
Coal workers pneumoconiosis [Global]	Male	15-19 years	95+ years	0.668815	1.43581	0.490589	0.4687	0.988006	0.912785
Coal workers pneumoconiosis [Data Rich]	Female	15-19 years	95+ years	0.623022	1.1641	0.535155	0.61863	0.988733	0.961375
Coal workers pneumoconiosis [Data Rich]	Male	15-19 years	95+ years	0.529871	1.04487	0.431939	0.36102	0.990274	0.979399
Other pneumoconiosis [Global]	Female	15-19 years	95+ years	0.835114	1.49574	0.581599	0.572615	0.986829	0.904819
Other pneumoconiosis [Global]	Male	15-19 years	95+ years	0.705492	1.46428	0.530512	0.525868	0.982321	0.90462
Other pneumoconiosis [Data Rich]	Female	15-19 years	95+ years	0.636343	1.20409	0.540863	0.564622	0.98441	0.971057
Other pneumoconiosis [Data Rich]	Male	15-19 years	95+ years	0.569427	1.01917	0.476243	0.438343	0.986773	0.9765
Asthma [Global]	Female	12-23 months	95+ years	0.325729	0.556889	0.145652	0.145004	0.99997	0.99074
Asthma [Global]	Male	12-23 months	95+ years	0.299419	0.588009	0.166526	0.161791	0.999821	0.982995
Asthma [Data Rich]	Female	12-23 months	95+ years	0.138114	0.375171	0.0990127	0.139201	0.999993	0.998907
Asthma [Data Rich]	Male	12-23 months	95+ years	0.150552	0.478138	0.118445	0.145128	0.999941	0.999467
Interstitial lung disease and pulmonary sarcoidosis [Global]	Female	12-23 months	95+ years	0.476689	0.708895	0.205029	0.204351	0.999353	0.984791
Interstitial lung disease and pulmonary sarcoidosis [Global]	Male	12-23 months	95+ years	0.518596	0.766438	0.209757	0.210986	0.999085	0.983142
Interstitial lung disease and pulmonary sarcoidosis [Data Rich]	Female	12-23 months	95+ years	0.199144	0.543953	0.125579	0.18958	0.998464	0.995433
Interstitial lung disease and pulmonary sarcoidosis [Data Rich]	Male	12-23 months	95+ years	0.216566	0.57779	0.134163	0.195614	0.999289	0.992484
Other chronic respiratory diseases [Global]	Female	0-6 days	95+ years	0.466892	0.691585	0.263796	0.273153	0.997485	0.983585
Other chronic respiratory diseases [Global]	Male	0-6 days	95+ years	0.381559	0.688333	0.232612	0.240444	0.999511	0.980671
Other chronic respiratory diseases [Data Rich]	Female	0-6 days	95+ years	0.252848	0.663096	0.201184	0.228931	0.999606	0.997551
Other chronic respiratory diseases [Data Rich]	Male	0-6 days	95+ years	0.25279	0.714515	0.200925	0.224674	0.999556	0.997452
Digestive diseases [Global]	Female	0-6 days	95+ years	0.175812	0.379814	0.117886	0.123728	0.999803	0.972757
Digestive diseases [Global]	Male	0-6 days	95+ years	0.178452	0.377951	0.117143	0.122587	0.999598	0.968651
Digestive diseases [Data Rich]	Female	0-6 days	95+ years	0.110944	0.24861	0.0807823	0.131994	0.999997	0.998994
Digestive diseases [Data Rich]	Male	0-6 days	95+ years	0.109031	0.243026	0.0793063	0.129926	0.999986	0.998349
Cirrhosis and other chronic liver diseases [Global]	Female	12-23 months	95+ years	0.206235	0.430264	0.130083	0.138347	0.999828	0.981308
Cirrhosis and other chronic liver diseases [Global]	Male	12-23 months	95+ years	0.205726	0.43935	0.127232	0.13664	0.99965	0.967294
Cirrhosis and other chronic liver diseases [Data Rich]	Female	12-23 months	95+ years	0.116974	0.241709	0.0875642	0.149462	0.999993	0.999101
Cirrhosis and other chronic liver diseases [Data Rich]	Male	12-23 months	95+ years	0.114154	0.240556	0.0848653	0.146111	0.99998	0.998267
Upper digestive system diseases [Global]	Female	6-11 months	95+ years	0.224269	0.483025	0.13963	0.134126	0.999165	0.988975
Upper digestive system diseases [Global]	Male	6-11 months	95+ years	0.218956	0.453074	0.135516	0.136277	0.999139	0.991111
Upper digestive system diseases [Data Rich]	Female	6-11 months	95+ years	0.117686	0.23417	0.087384	0.130814	0.999984	0.999567
Upper digestive system diseases [Data Rich]	Male	6-11 months	95+ years	0.117989	0.230249	0.0878268	0.130733	0.999994	0.99938
Peptic ulcer disease [Global]	Female	6-11 months	95+ years	0.207339	0.48341	0.134514	0.133698	0.999817	0.98733
Peptic ulcer disease [Global]	Male	6-11 months	95+ years	0.204762	0.428176	0.134118	0.135658	0.999869	0.988941
Peptic ulcer disease [Data Rich]	Female	6-11 months	95+ years	0.127292	0.33792	0.0906563	0.132253	0.999974	0.999366
Peptic ulcer disease [Data Rich]	Male	6-11 months	95+ years	0.131071	0.317509	0.0903171	0.132823	0.999987	0.999135
Gastritis and duodenitis [Global]	Female	6-11 months	95+ years	0.341816	0.763546	0.192802	0.191903	0.998379	0.977861
Gastritis and duodenitis [Global]	Male	6-11 months	95+ years	0.473306	0.743196	0.223996	0.212505	0.997219	0.97996
Gastritis and duodenitis [Data Rich]	Female	6-11 months	95+ years	0.211649	0.600842	0.162411	0.180032	0.999568	0.999082
Gastritis and duodenitis [Data Rich]	Male	6-11 months	95+ years	0.214718	0.577693	0.167064	0.200613	0.999391	0.998782
Appendicitis [Global]	Female	12-23 months	95+ years	0.324864	0.551467	0.176462	0.181617	0.999418	0.993616
Appendicitis [Global]	Male	12-23 months	95+ years	0.337731	0.550223	0.167533	0.171643	0.999773	0.994431
Appendicitis [Data Rich]	Female	12-23 months	95+ years	0.202096	0.340601	0.154501	0.194645	0.999773	0.999349
Appendicitis [Data Rich]	Male	12-23 months	95+ years	0.191344	0.320445	0.147115	0.177394	0.999967	0.999728
Paralytic ileus and intestinal obstruction [Global]	Female	0-6 days	95+ years	0.248088	0.399764	0.152731	0.152148	0.999606	0.995426
Paralytic ileus and intestinal obstruction [Global]	Male	0-6 days	95+ years	0.243771	0.403805	0.153139	0.153414	0.999742	0.995902
Paralytic ileus and intestinal obstruction [Data Rich]	Female	0-6 days	95+ years	0.119783	0.269127	0.0858754	0.142575	0.999994	0.999774
Paralytic ileus and intestinal obstruction [Data Rich]	Male	0-6 days	95+ years	0.121362	0.282541	0.0868526	0.146364	0.999994	0.999896
Inguinal, femoral, and abdominal hernia [Global]	Female	0-6 days	95+ years	0.388225	0.585965	0.254444	0.242578	0.996125	0.992344
Inguinal, femoral, and abdominal hernia [Global]	Male	0-6 days	95+ years	0.355562	0.592057	0.218907	0.211646	0.997484	0.992902
Inguinal, femoral, and abdominal hernia [Data Rich]	Female	0-6 days	95+ years	0.128383	0.348547	0.0966554	0.137179	0.999769	0.999086
Inguinal, femoral, and abdominal hernia [Data Rich]	Male	0-6 days	95+ years	0.149016	0.382448	0.0917143	0.142634	0.999761	0.998177
Inflammatory bowel disease [Global]	Female	2-4 years	95+ years	0.393622	0.602457	0.214235	0.19264	0.998618	0.99132
Inflammatory bowel disease [Global]	Male	2-4 years	95+ years	0.339385	0.538939	0.188217	0.189757	0.999443	0.993578
Inflammatory bowel disease [Data Rich]	Female	2-4 years	95+ years	0.15318	0.56051	0.12292	0.206585	0.999689	0.997924
Inflammatory bowel disease [Data Rich]	Male	2-4 years	95+ years	0.144429	0.469151	0.112051	0.196473	0.999969	0.999409
Ulcerative colitis [Global]	Female	2-4 years	95+ years	0.481392	0.730503	0.23493	0.232551	0.998294	0.98561
Ulcerative colitis [Global]	Male	2-4 years	95+ years	0.375541	0.632385	0.208891	0.218162	0.999352	0.987946
Ulcerative colitis [Data Rich]	Female	2-4 years	95+ years	0.237272	0.781046	0.185065	0.241689	0.999308	0.997035
Ulcerative colitis [Data Rich]	Male	2-4 years	95+ years	0.211452	0.612131	0.163878	0.223102	0.99966	0.998807
Crohn's disease [Global]	Female	2-4 years	95+ years	0.347948	0.571218	0.219439	0.220202	0.998965	0.991622
Crohn's disease [Global]	Male	2-4 years	95+ years	0.337697	0.555969	0.217663	0.228207	0.999037	0.993852
Crohn's disease [Data Rich]	Female	2-4 years	95+ years	0.225217	0.434951	0.179632	0.224464	0.999574	0.998788
Crohn's disease [Data Rich]	Male	2-4 years	95+ years	0.229791	0.412028	0.189241	0.233865	0.999375	0.998562
Vascular intestinal disorders [Global]	Female	2-4 years	95+ years	0.275089	0.516076	0.178048	0.175033	0.998507	0.987916
Vascular intestinal disorders [Global]	Male	2-4 years	95+ years	0.31266	0.580126	0.202976	0.174567	0.99841	0.986856
Vascular intestinal disorders [Data Rich]	Female	2-4 years	95+ years	0.135886	0.36446	0.0967244	0.138669	0.99972	0.99873
Vascular intestinal disorders [Data Rich]	Male	2-4 years	95+ years	0.150722	0.361675	0.107512	0.141633	0.999567	0.998571
Gallbladder and biliary diseases [Global]	Female	2-4 years	95+ years	0.398843	0.595872	0.156579	0.168316	0.999109	0.988583
Gallbladder and biliary diseases [Global]	Male	2-4 years	95+ years	0.385259	0.560716	0.161853	0.173314	0.999111	0.991505
Gallbladder and biliary diseases [Data Rich]	Female	2-4 years	95+ years	0.14602	0.321331	0.0863571	0.150539	0.999983	0.99857
Gallbladder and biliary diseases [Data Rich]	Male	2-4 years	95+ years	0.134645	0.27857	0.0876235	0.153925	0.999993	0.999303
Pancreatitis [Global]	Female	2-4 years	95+ years	0.347845	0.478909	0.160619	0.155257	0.998003	0.993378
Pancreatitis [Global]	Male	2-4 years	95+ years	0.334049	0.487509	0.155083	0.155919	0.998341	0.986456
Pancreatitis [Data Rich]	Female	2-4 years	95+ years	0.131071	0.300711	0.0905128	0.150396	0.999986	0.999387
Pancreatitis [Data Rich]	Male	2-4 years	95+ years	0.119031	0.301175	0.0892974	0.152906	0.999996	0.999579
Diverticular disease of intestines [Global]	Female	0-6 days	95+ years	0.552276	0.82307	0.255032	0.238162	0.997156	0.983205
Diverticular disease of intestines [Global]	Male	0-6 days	95+ years	0.506796	0.817999	0.253328	0.22695	0.996869	0.982447
Diverticular disease of intestines [Data Rich]	Female	0-6 days	95+ years	0.207258	0.513386	0.126469	0.191187	0.999395	0.995504
Diverticular disease of intestines [Data Rich]	Male	0-6 days	95+ years	0.194563	0.528701	0.1277	0.213195	0.999384	0.996348
Other digestive diseases [Global]	Female	12-23 months	95+ years	0.336769	0.530464	0.170801	0.173126	0.999135	0.994576
Other digestive diseases [Global]	Male	12-23 months	95+ years	0.363779	0.548697	0.203685	0.190788	0.996839	0.99331
Other digestive diseases [Data Rich]	Female	12-23 months	95+ years	0.112027	0.263801	0.0813794	0.149638	0.99999	0.999894
Other digestive diseases [Data Rich]	Male	12-23 months	95+ years	0.112731	0.269735	0.0810097	0.149803	0.999993	0.999898

Appendix Table S12: CODEm predictive validity results by cause, model type, sex, and age

Cause	Sex	Age Start	Age End	RMSE In-Sample	RMSE Out-of-Sample	Trend In-Sample	Trend Out-of-Sample	Coverage In-Sample	Coverage Out-of-Sample
Parkinson's disease [Global]	Female	20-24 years	95+ years	0.336124	0.440515	0.065456	0.0690184	0.999148	0.995138
Parkinson's disease [Global]	Male	20-24 years	95+ years	0.161786	0.272702	0.0634304	0.0676284	0.999859	0.998891
Parkinson's disease [Data Rich]	Female	20-24 years	95+ years	0.148023	0.486412	0.0425607	0.0973389	0.999486	0.99545
Parkinson's disease [Data Rich]	Male	20-24 years	95+ years	0.0698796	0.215676	0.0390493	0.0764525	0.999991	0.999695
Idiopathic epilepsy [Global]	Female	0-6 days	95+ years	0.36664	0.574585	0.162247	0.160214	0.999316	0.991072
Idiopathic epilepsy [Global]	Male	0-6 days	95+ years	0.323834	0.520317	0.152911	0.153524	0.999613	0.993379
Idiopathic epilepsy [Data Rich]	Female	0-6 days	95+ years	0.137565	0.297109	0.0985703	0.162455	0.999969	0.999559
Idiopathic epilepsy [Data Rich]	Male	0-6 days	95+ years	0.124964	0.274067	0.0921156	0.159961	0.999983	0.999746
Multiple sclerosis [Global]	Female	5-9 years	95+ years	0.30245	0.835425	0.23024	0.232308	0.998248	0.976556
Multiple sclerosis [Global]	Male	5-9 years	95+ years	0.403346	1.08041	0.361207	0.371475	0.995389	0.961121
Multiple sclerosis [Data Rich]	Female	5-9 years	95+ years	0.250878	0.472634	0.17192	0.22274	0.998952	0.996709
Multiple sclerosis [Data Rich]	Male	5-9 years	95+ years	0.253757	0.422502	0.180198	0.201873	0.999048	0.996093
Motor neuron disease [Global]	Female	0-6 days	95+ years	0.506764	1.00399	0.286995	0.287679	0.9914	0.961038
Motor neuron disease [Global]	Male	0-6 days	95+ years	0.77127	1.03882	0.326563	0.313142	0.987969	0.958513
Motor neuron disease [Data Rich]	Female	0-6 days	95+ years	0.185706	0.904322	0.136977	0.268158	0.999326	0.996888
Motor neuron disease [Data Rich]	Male	0-6 days	95+ years	0.177886	0.830229	0.136662	0.262624	0.999365	0.997496
Other neurological disorders [Global]	Female	0-6 days	95+ years	0.422285	0.864155	0.281278	0.290894	0.995531	0.972548
Other neurological disorders [Global]	Male	0-6 days	95+ years	0.415888	0.881554	0.275068	0.275744	0.993667	0.970749
Other neurological disorders [Data Rich]	Female	0-6 days	95+ years	0.19528	0.475748	0.159928	0.217471	0.999202	0.997578
Other neurological disorders [Data Rich]	Male	0-6 days	95+ years	0.171603	0.437571	0.13853	0.190609	0.999394	0.998588
Anorexia nervosa [Global]	Female	5-9 years	45-49 years	1.16032	1.80992	0.913663	0.913241	0.952929	0.892903
Anorexia nervosa [Global]	Male	5-9 years	45-49 years	1.60557	2.60339	1.2143	1.30403	0.902626	0.719648
Anorexia nervosa [Data Rich]	Female	5-9 years	45-49 years	0.96914	1.85495	0.766026	0.879194	0.972947	0.943903
Anorexia nervosa [Data Rich]	Male	5-9 years	45-49 years	1.42349	2.18897	1.14446	1.33697	0.912565	0.865643
Alcohol use disorders [Global]	Female	15-19 years	95+ years	0.291943	0.722869	0.194246	0.203452	0.998438	0.96766
Alcohol use disorders [Global]	Male	15-19 years	95+ years	0.276918	0.632858	0.172428	0.180462	0.997714	0.959381
Alcohol use disorders [Data Rich]	Female	15-19 years	95+ years	0.12564	0.305589	0.0970361	0.195944	0.999963	0.998941
Alcohol use disorders [Data Rich]	Male	15-19 years	95+ years	0.116129	0.287146	0.0898095	0.175247	0.999902	0.996704
Drug use disorders [Global]	Female	15-19 years	95+ years	0.373512	0.827114	0.252399	0.244622	0.997428	0.977545
Drug use disorders [Global]	Male	15-19 years	95+ years	0.349286	0.766418	0.244741	0.236368	0.998346	0.977471
Drug use disorders [Data Rich]	Female	15-19 years	95+ years	0.249981	0.430579	0.192004	0.276829	0.999163	0.998896
Drug use disorders [Data Rich]	Male	15-19 years	95+ years	0.240868	0.414881	0.180855	0.249543	0.999653	0.999433
Opioid use disorders [Global]	Female	15-19 years	95+ years	0.367842	0.783303	0.265094	0.264709	0.997365	0.980166
Opioid use disorders [Global]	Male	15-19 years	95+ years	0.317839	0.704793	0.212267	0.205557	0.998927	0.982225
Opioid use disorders [Data Rich]	Female	15-19 years	95+ years	0.284116	0.576968	0.232212	0.300447	0.998353	0.996855
Opioid use disorders [Data Rich]	Male	15-19 years	95+ years	0.241416	0.57594	0.172791	0.240042	0.999499	0.999092
Cocaine use disorders [Global]	Female	15-19 years	95+ years	0.38801	0.92926	0.282046	0.288196	0.99709	0.978862
Cocaine use disorders [Global]	Male	15-19 years	95+ years	0.355713	0.819067	0.231844	0.235031	0.998768	0.981341
Cocaine use disorders [Data Rich]	Female	15-19 years	95+ years	0.265671	0.616287	0.205937	0.231361	0.998383	0.997833
Cocaine use disorders [Data Rich]	Male	15-19 years	95+ years	0.221563	0.558428	0.167601	0.210487	0.999474	0.998823
Amphetamine use disorders [Global]	Female	15-19 years	95+ years	0.424944	0.859659	0.269825	0.254938	0.997575	0.986816
Amphetamine use disorders [Global]	Male	15-19 years	95+ years	0.41228	0.858621	0.273814	0.277114	0.998137	0.986358
Amphetamine use disorders [Data Rich]	Female	15-19 years	95+ years	0.248397	0.584286	0.19502	0.214167	0.998515	0.996963
Amphetamine use disorders [Data Rich]	Male	15-19 years	95+ years	0.280614	0.614564	0.213907	0.266574	0.999128	0.995863
Other drug use disorders [Global]	Female	15-19 years	95+ years	0.320524	0.796904	0.210489	0.186777	0.997844	0.98525
Other drug use disorders [Global]	Male	15-19 years	95+ years	0.305141	0.652252	0.195332	0.185863	0.999221	0.989427
Other drug use disorders [Data Rich]	Female	15-19 years	95+ years	0.21583	0.500533	0.16917	0.220069	0.999048	0.998504
Other drug use disorders [Data Rich]	Male	15-19 years	95+ years	0.198947	0.425261	0.149019	0.19937	0.999653	0.99926
Diabetes mellitus [Global]	Female	0-6 days	10-14 years	0.336956	0.485995	0.133293	0.132991	0.999606	0.996748
Diabetes mellitus [Global]	Female	15-19 years	95+ years	0.323747	0.479332	0.143979	0.14538	0.999663	0.976718
Diabetes mellitus [Global]	Male	0-6 days	10-14 years	0.314441	0.440337	0.127475	0.124065	0.999698	0.997308
Diabetes mellitus [Global]	Male	15-19 years	95+ years	0.216633	0.411779	0.141678	0.147663	0.999667	0.976005
Diabetes mellitus [Data Rich]	Female	0-6 days	10-14 years	0.117499	0.264322	0.0741968	0.111845	0.999977	0.999898
Diabetes mellitus [Data Rich]	Female	15-19 years	95+ years	0.108457	0.259045	0.0818865	0.135207	0.999927	0.998646
Diabetes mellitus [Data Rich]	Male	0-6 days	10-14 years	0.109755	0.245523	0.0710048	0.111174	0.999977	0.999961
Diabetes mellitus [Data Rich]	Male	15-19 years	95+ years	0.112371	0.25657	0.0845434	0.146366	0.999939	0.999038
Diabetes mellitus type 1 [Global]	Female	0-6 days	95+ years	0.302474	0.462303	0.120066	0.117189	0.999875	0.99373
Diabetes mellitus type 1 [Global]	Male	0-6 days	95+ years	0.288054	0.438513	0.114424	0.11411	0.999924	0.994349
Diabetes mellitus type 1 [Data Rich]	Female	0-6 days	95+ years	0.127234	0.377045	0.0874527	0.0928372	0.999974	0.999913
Diabetes mellitus type 1 [Data Rich]	Male	0-6 days	95+ years	0.120278	0.347893	0.0819257	0.0967196	0.999981	0.999961
Diabetes mellitus type 2 [Global]	Female	15-19 years	95+ years	0.291511	0.489686	0.135137	0.13556	0.999881	0.980142
Diabetes mellitus type 2 [Global]	Male	15-19 years	95+ years	0.237885	0.432959	0.134696	0.139396	0.999898	0.982304
Diabetes mellitus type 2 [Data Rich]	Female	15-19 years	95+ years	0.107972	0.359012	0.077069	0.121227	0.999939	0.999411
Diabetes mellitus type 2 [Data Rich]	Male	15-19 years	95+ years	0.110785	0.341373	0.0783486	0.126668	0.999917	0.999523
Chronic kidney disease [Global]	Female	0-6 days	95+ years	0.2445	0.43507	0.122475	0.128048	0.999746	0.991349
Chronic kidney disease [Global]	Male	0-6 days	95+ years	0.281105	0.435558	0.123566	0.127642	0.99978	0.989772
Chronic kidney disease [Data Rich]	Female	0-6 days	95+ years	0.108979	0.269621	0.0740394	0.134948	0.999989	0.999739
Chronic kidney disease [Data Rich]	Male	0-6 days	95+ years	0.109142	0.265333	0.0749941	0.135324	0.999997	0.999798
Acute glomerulonephritis [Global]	Female	0-6 days	95+ years	0.676486	1.20345	0.419946	0.426062	0.988518	0.963313
Acute glomerulonephritis [Global]	Male	0-6 days	95+ years	0.750007	1.23427	0.472523	0.48624	0.986398	0.958356
Acute glomerulonephritis [Data Rich]	Female	0-6 days	95+ years	0.425058	0.900739	0.347001	0.334792	0.993088	0.98871
Acute glomerulonephritis [Data Rich]	Male	0-6 days	95+ years	0.492389	0.918624	0.411006	0.36955	0.988951	0.983125
Skin and subcutaneous diseases [Global]	Female	0-6 days	95+ years	0.2822	0.590266	0.14123	0.149098	0.999289	0.990542
Skin and subcutaneous diseases [Global]	Male	0-6 days	95+ years	0.277962	0.562822	0.142573	0.152929	0.999211	0.989957
Skin and subcutaneous diseases [Data Rich]	Female	0-6 days	95+ years	0.142556	0.351414	0.0858691	0.157685	0.999977	0.999456
Skin and subcutaneous diseases [Data Rich]	Male	0-6 days	95+ years	0.1382	0.385551	0.0887602	0.169352	0.999994	0.999772
Bacterial skin diseases [Global]	Female	0-6 days	95+ years	0.213771	0.697354	0.163715	0.191975	0.999709	0.99864
Bacterial skin diseases [Global]	Male	0-6 days	95+ years	0.218452	0.677068	0.170291	0.200814	0.99984	0.998198
Bacterial skin diseases [Data Rich]	Female	0-6 days	95+ years	0.185396	0.503633	0.111085	0.219245	0.999965	0.999155
Bacterial skin diseases [Data Rich]	Male	0-6 days	95+ years	0.181584	0.52297	0.115082	0.249672	0.999983	0.999637
Cellulitis [Global]	Female	0-6 days	95+ years	0.43367	0.896057	0.217854	0.233644	0.997973	0.976229
Cellulitis [Global]	Male	0-6 days	95+ years	0.489707	0.84195	0.240315	0.266045	0.998204	0.978943
Cellulitis [Data Rich]	Female	0-6 days	95+ years	0.260087	0.913281	0.195584	0.264822	0.998858	0.992647
Cellulitis [Data Rich]	Male	0-6 days	95+ years	0.278376	0.921147	0.220692	0.311866	0.998553	0.991607
Pyoderma [Global]	Female	0-6 days	95+ years	0.33768	0.767591	0.199094	0.220515	0.999041	0.986577
Pyoderma [Global]	Male	0-6 days	95+ years	0.364807	0.759753	0.201861	0.222033	0.999445	0.989673
Pyoderma [Data Rich]	Female	0-6 days	95+ years	0.229727	0.729147	0.172659	0.263966	0.999712	0.997788
Pyoderma [Data Rich]	Male	0-6 days	95+ years	0.227364	0.703402	0.170177	0.258982	0.999764	0.998578
Decubitus ulcer [Global]	Female	12-23 months	95+ years	0.572074	1.05499	0.216974	0.237025	0.996574	0.964345
Decubitus ulcer [Global]	Male	12-23 months	95+ years	0.638953	1.06088	0.29171	0.294397	0.994272	0.965915
Decubitus ulcer [Data Rich]	Female	12-23 months	95+ years	0.209025	0.667855	0.136876	0.160487	0.999789	0.999194
Decubitus ulcer [Data Rich]	Male	12-23 months	95+ years	0.266966	0.699288	0.183409	0.200076	0.998804	0.997369
Other skin and subcutaneous diseases [Global]	Female	0-6 days	95+ years	0.378978	0.795025	0.248524	0.255569	0.996289	0.969703
Other skin and subcutaneous diseases [Global]	Male	0-6 days	95+ years	0.492763	0.932184	0.365881	0.382325	0.992134	0.972993
Other skin and subcutaneous diseases [Data Rich]	Female	0-6 days	95+ years	0.32112	0.576479	0.225998	0.303593	0.997364	0.99439
Other skin and subcutaneous diseases [Data Rich]	Male	0-6 days	95+ years	0.437629	0.710759	0.356191	0.419517	0.993029	0.99012
Musculoskeletal disorders [Global]	Female	1-5 months	95+ years	0.318416	0.552592	0.144287	0.15289	0.999448	0.987356
Musculoskeletal disorders [Global]	Male	1-5 months	95+ years	0.356752	0.546849	0.185938	0.17459	0.998552	0.990659
Musculoskeletal disorders [Data Rich]	Female	1-5 months	95+ years	0.127595	0.345517	0.0865004	0.150202	0.999953	0.998933
Musculoskeletal disorders [Data Rich]	Male	1-5 months	95+ years	0.125807	0.33451	0.098175	0.156151	0.999919	0.999174
Rheumatoid arthritis [Global]	Female	5-9 years	95+ years	0.36665	0.635369	0.203419	0.15575	0.997935	0.98708
Rheumatoid arthritis [Global]	Male	5-9 years	95+ years	0.322496	0.67269	0.200865	0.191884	0.997968	0.984849
Rheumatoid arthritis [Data Rich]	Female	5-9 years	95+ years	0.128783	0.374248	0.0980662	0.144086	0.999702	0.999188
Rheumatoid arthritis [Data Rich]	Male	5-9 years	95+ years	0.158415	0.377011	0.0969496	0.152199	0.999911	0.997727
Other musculoskeletal disorders [Global]	Female	1-5 months	95+ years	0.332099	0.485427	0.145929	0.152376	0.999845	0.994522
Other musculoskeletal disorders [Global]	Male	1-5 months	95+ years	0.36007	0.486306	0.186981	0.174723	0.998201	0.995412
Other musculoskeletal disorders [Data Rich]	Female	1-5 months	95+ years	0.128978	0.33416	0.0869992	0.146977	0.999926	0.999077
Other musculoskeletal disorders [Data Rich]	Male	1-5 months	95+ years	0.129143	0.319996	0.0981162	0.157832	0.999847	0.999237
Congenital anomalies [Global]	Female	0-6 days	65-69 years	0.181117	0.328688	0.129807	0.130471	0.999775	0.994502
Congenital anomalies [Global]	Male	0-6 days	65-69 years	0.181936	0.353757	0.130301	0.131251	0.999824	0.992579
Congenital anomalies [Data Rich]	Female	0-6 days	65-69 years	0.237161	0.287295	0.114655	0.125946	0.999952	0.999819
Congenital anomalies [Data Rich]	Male	0-6 days	65-69 years	0.174885	0.24592	0.115632	0.127465	0.999963	0.999861
Neural tube defects [Global]	Female	0-6 days	65-69 years	0.32581	0.577433	0.220538	0.229039	0.99778	0.989997
Neural tube defects [Global]	Male	0-6 days	65-69 years	0.336709	0.580428	0.240767	0.231698	0.996981	0.990598
Neural tube defects [Data Rich]	Female	0-6 days	65-69 years	0.254126	0.543138	0.193167	0.23402	0.998592	0.9964
Neural tube defects [Data Rich]	Male	0-6 days	65-69 years	0.284292	0.505733	0.218133	0.204565	0.997632	0.994507
Congenital heart anomalies [Global]	Female	0-6 days	65-69 years	0.269634	0.430645	0.142105	0.142786	0.999869	0.995364
Congenital heart anomalies [Global]	Male	0-6 days	65-69 years	0.214881	0.391985	0.139268	0.138201	0.999873	0.994721
Congenital heart anomalies [Data Rich]	Female	0-6 days	65-69 years	0.126433	0.368231	0.0902497	0.126659	1	0.999511
Congenital heart anomalies [Data Rich]	Male	0-6 days	65-69 years	0.125317	0.376746	0.0905492	0.126152	0.999996	0.9991
Orofacial clefts [Global]	Female	0-6 days	2-4 years	1.35524	1.86235	1.11693	1.09412	0.924152	0.855024
Orofacial clefts [Global]	Male	0-6 days	2-4 years	1.26076	1.72228	0.918447	0.922144	0.96992	0.914955
Orofacial clefts [Data Rich]	Female	0-6 days	2-4 years	1.50289	1.95558	1.24697	1.08651	0.919483	0.889525
Orofacial clefts [Data Rich]	Male	0-6 days	2-4 years	1.29957	1.84963	1.01369	0.921472	0.954499	0.911846
Down's syndrome [Global]	Female	0-6 days	65-69 years	0.347032	0.733785	0.176044	0.185009	0.999791	0.984407
Down's syndrome [Global]	Male	0-6 days	65-69 years	0.288681	0.763773	0.185489	0.202422	0.999449	0.978067
Down's syndrome [Data Rich]	Female	0-6 days	65-69 years	0.628221	0.900383	0.174142	0.20564	0.999669	0.993284
Down's syndrome [Data Rich]	Male	0-6 days	65-69 years	0.620534	1.03256	0.183406	0.24248	0.999461	0.993377
Other chromosomal abnormalities [Global]	Female	0-6 days	65-69 years	0.257856	0.610815	0.167419	0.174414	0.999536	0.99073
Other chromosomal abnormalities [Global]	Male	0-6 days	65-69 years	0.253745	0.606704	0.170338	0.176832	0.9995	0.992085
Other chromosomal abnormalities [Data Rich]	Female	0-6 days	65-69 years	0.185151	0.579492	0.143715	0.185379	0.999788	0.999
Other chromosomal abnormalities [Data Rich]	Male	0-6 days	65-69 years	0.190108	0.640431	0.149157	0.194551	0.999759	0.998344
Congenital musculoskeletal and limb anomalies [Global]	Female	0-6 days	65-69 years	0.438001	0.627486	0.316932	0.318512	0.994466	0.991291

Appendix Table S12: CODEm predictive validity results by cause, model type, sex, and age

Cause	Sex	Age Start	Age End	RMSE In-Sample	RMSE Out-of-Sample	Trend In-Sample	Trend Out-of-Sample	Coverage In-Sample	Coverage Out-of-Sample
Congenital musculoskeletal and limb anomalies [Global]	Male	0-6 days	65-69 years	0.324508	0.55343	0.275144	0.28064	0.996421	0.992193
Congenital musculoskeletal and limb anomalies [Data Rich]	Female	0-6 days	65-69 years	0.513149	0.613621	0.32068	0.317938	0.994405	0.993276
Congenital musculoskeletal and limb anomalies [Data Rich]	Male	0-6 days	65-69 years	0.342342	0.517762	0.279874	0.326967	0.99684	0.99589
Urogenital congenital anomalies [Global]	Female	0-6 days	65-69 years	0.705294	0.953964	0.604785	0.607688	0.992277	0.985463
Urogenital congenital anomalies [Global]	Male	0-6 days	65-69 years	0.649112	0.86238	0.491694	0.480488	0.993033	0.983928
Urogenital congenital anomalies [Data Rich]	Female	0-6 days	65-69 years	0.379935	0.535112	0.286458	0.330601	0.996351	0.994367
Urogenital congenital anomalies [Data Rich]	Male	0-6 days	65-69 years	0.315603	0.429576	0.159171	0.194493	0.999854	0.999057
Digestive congenital anomalies [Global]	Female	0-6 days	65-69 years	0.307726	0.492068	0.168542	0.15386	0.998953	0.994828
Digestive congenital anomalies [Global]	Male	0-6 days	65-69 years	0.350741	0.496946	0.182996	0.177869	0.998621	0.996312
Digestive congenital anomalies [Data Rich]	Female	0-6 days	65-69 years	0.296011	0.498344	0.165426	0.147832	0.999264	0.998665
Digestive congenital anomalies [Data Rich]	Male	0-6 days	65-69 years	0.29401	0.46856	0.179753	0.165909	0.998594	0.998233
Other congenital anomalies [Global]	Female	0-6 days	65-69 years	0.315673	0.509471	0.195765	0.186155	0.997826	0.990328
Other congenital anomalies [Global]	Male	0-6 days	65-69 years	0.437794	0.541773	0.201089	0.194709	0.997891	0.997147
Other congenital anomalies [Data Rich]	Female	0-6 days	65-69 years	0.177172	0.343425	0.102378	0.139943	1	0.999679
Other congenital anomalies [Data Rich]	Male	0-6 days	65-69 years	0.155734	0.439524	0.0988187	0.155805	1	0.999554
Urinary diseases and male infertility [Global]	Female	0-6 days	95+ years	0.148533	0.503216	0.09708	0.136913	0.999766	0.974949
Urinary diseases and male infertility [Global]	Male	0-6 days	95+ years	0.151521	0.497385	0.103973	0.146673	0.999688	0.975606
Urinary diseases and male infertility [Data Rich]	Female	0-6 days	95+ years	0.120756	0.284725	0.0841456	0.155945	0.999995	0.99964
Urinary diseases and male infertility [Data Rich]	Male	0-6 days	95+ years	0.117628	0.284085	0.0874886	0.164303	0.999992	0.999688
Urinary tract infections and interstitial nephritis [Global]	Female	0-6 days	95+ years	0.377649	0.567842	0.17039	0.171643	0.999717	0.987039
Urinary tract infections and interstitial nephritis [Global]	Male	0-6 days	95+ years	0.374358	0.586691	0.181117	0.180884	0.999685	0.987752
Urinary tract infections and interstitial nephritis [Data Rich]	Female	0-6 days	95+ years	0.122222	0.364136	0.0851774	0.160467	0.999977	0.999419
Urinary tract infections and interstitial nephritis [Data Rich]	Male	0-6 days	95+ years	0.122378	0.36862	0.0890275	0.163026	0.999969	0.999612
Urolithiasis [Global]	Female	12-23 months	95+ years	0.301531	0.715176	0.217377	0.227092	0.997411	0.97657
Urolithiasis [Global]	Male	12-23 months	95+ years	0.323668	0.673573	0.237561	0.233382	0.997064	0.981482
Urolithiasis [Data Rich]	Female	12-23 months	95+ years	0.21334	0.444996	0.167434	0.179067	0.99907	0.99837
Urolithiasis [Data Rich]	Male	12-23 months	95+ years	0.221626	0.509376	0.178738	0.192921	0.998799	0.998103
Other urinary diseases [Global]	Female	0-6 days	95+ years	0.643385	0.91777	0.389697	0.380977	0.986243	0.966533
Other urinary diseases [Global]	Male	0-6 days	95+ years	0.53326	0.717408	0.293138	0.293374	0.994449	0.983031
Other urinary diseases [Data Rich]	Female	0-6 days	95+ years	0.33234	0.656193	0.262659	0.223849	0.996739	0.991838
Other urinary diseases [Data Rich]	Male	0-6 days	95+ years	0.237911	0.560927	0.186353	0.205329	0.998911	0.997646
Gynecological diseases [Global]	Female	10-14 years	95+ years	0.449259	0.733086	0.28348	0.274774	0.99559	0.981042
Gynecological diseases [Data Rich]	Female	10-14 years	95+ years	0.280482	0.434025	0.232954	0.228559	0.996723	0.99483
Uterine fibroids [Global]	Female	10-14 years	95+ years	0.634681	1.03276	0.397573	0.397414	0.990127	0.964757
Uterine fibroids [Data Rich]	Female	10-14 years	95+ years	0.444055	0.889255	0.374285	0.376378	0.990094	0.976473
Endometriosis [Global]	Female	10-14 years	50-54 years	1.11254	1.64543	0.774573	0.785032	0.988342	0.944957
Endometriosis [Data Rich]	Female	10-14 years	50-54 years	1.05589	1.37849	0.735714	0.790961	0.98994	0.97588
Genital prolapse [Global]	Female	10-14 years	95+ years	0.866384	1.27616	0.694744	0.701487	0.973466	0.936434
Genital prolapse [Data Rich]	Female	10-14 years	95+ years	0.70058	1.29786	0.609303	0.603125	0.970375	0.942479
Other gynecological diseases [Global]	Female	10-14 years	95+ years	0.446377	0.747803	0.322045	0.322766	0.99481	0.981896
Other gynecological diseases [Data Rich]	Female	10-14 years	95+ years	0.349634	0.775122	0.300475	0.297704	0.996632	0.989675
Hemoglobinopathies and hemolytic anaemias [Global]	Female	0-6 days	95+ years	0.237496	0.434923	0.15414	0.145884	0.999244	0.996613
Hemoglobinopathies and hemolytic anaemias [Global]	Male	0-6 days	95+ years	0.243741	0.421801	0.169257	0.151757	0.999487	0.996049
Hemoglobinopathies and hemolytic anaemias [Data Rich]	Female	0-6 days	95+ years	0.133114	0.283642	0.094015	0.14294	0.999914	0.999129
Hemoglobinopathies and hemolytic anaemias [Data Rich]	Male	0-6 days	95+ years	0.136212	0.291852	0.0999297	0.141137	0.999906	0.999409
Endocrine, metabolic, blood, and immune disorders [Global]	Female	0-6 days	95+ years	0.308996	0.479764	0.161918	0.165717	0.999193	0.992942
Endocrine, metabolic, blood, and immune disorders [Global]	Male	0-6 days	95+ years	0.325239	0.493293	0.170015	0.177415	0.999404	0.993136
Endocrine, metabolic, blood, and immune disorders [Data Rich]	Female	0-6 days	95+ years	0.119203	0.264077	0.0907067	0.163842	0.999986	0.999677
Endocrine, metabolic, blood, and immune disorders [Data Rich]	Male	0-6 days	95+ years	0.122094	0.266596	0.0936323	0.1748	0.999986	0.999629
Thyroid diseases [Global]	Female	0-6 days	95+ years	0.326837	0.56387	0.160561	0.168754	0.999766	0.995787
Thyroid diseases [Global]	Male	0-6 days	95+ years	0.372418	0.592704	0.192273	0.197344	0.998923	0.995293
Thyroid diseases [Data Rich]	Female	0-6 days	95+ years	0.190772	0.552998	0.138448	0.180601	0.999888	0.999375
Thyroid diseases [Data Rich]	Male	0-6 days	95+ years	0.20849	0.538238	0.161082	0.204229	0.999742	0.999097
Other endocrine, metabolic, blood, and immune disorders [Global]	Female	0-6 days	95+ years	0.303836	0.485672	0.160649	0.164764	0.999771	0.994928
Other endocrine, metabolic, blood, and immune disorders [Global]	Male	0-6 days	95+ years	0.289624	0.460358	0.164799	0.168642	0.999684	0.995079
Other endocrine, metabolic, blood, and immune disorders [Data Rich]	Female	0-6 days	95+ years	0.124988	0.415123	0.0948558	0.16326	0.999956	0.999111
Other endocrine, metabolic, blood, and immune disorders [Data Rich]	Male	0-6 days	95+ years	0.126363	0.404397	0.0971403	0.172264	0.999984	0.999284
Sudden infant death syndrome [Global]	Female	7-27 days	6-11 months	0.641271	1.12489	0.424635	0.43409	0.995522	0.964758
Sudden infant death syndrome [Global]	Male	7-27 days	6-11 months	0.647122	0.973387	0.391055	0.428022	0.995456	0.961554
Sudden infant death syndrome [Data Rich]	Female	7-27 days	6-11 months	0.452905	0.931847	0.376001	0.492747	0.994436	0.967433
Sudden infant death syndrome [Data Rich]	Male	7-27 days	6-11 months	0.420494	0.938402	0.34171	0.473838	0.995348	0.966949
Transport injuries [Global]	Female	0-6 days	95+ years	0.235298	0.426439	0.139517	0.142317	0.999855	0.990745
Transport injuries [Global]	Male	0-6 days	95+ years	0.229418	0.398713	0.130569	0.134006	0.999922	0.989082
Transport injuries [Data Rich]	Female	0-6 days	95+ years	0.119737	0.250372	0.0907146	0.141654	0.999983	0.999612
Transport injuries [Data Rich]	Male	0-6 days	95+ years	0.114875	0.246349	0.0870857	0.134887	0.999994	0.998939
Road injuries [Global]	Female	0-6 days	95+ years	0.292874	0.469658	0.16749	0.172375	0.998109	0.989178
Road injuries [Global]	Male	0-6 days	95+ years	0.292916	0.476067	0.157198	0.158177	0.99799	0.982899
Road injuries [Data Rich]	Female	0-6 days	95+ years	0.121538	0.251857	0.092047	0.147546	0.999986	0.999564
Road injuries [Data Rich]	Male	0-6 days	95+ years	0.116576	0.245743	0.088486	0.140148	0.999925	0.998933
Pedestrian road injuries [Global]	Female	0-6 days	95+ years	0.395723	0.612135	0.198974	0.197602	0.996784	0.984902
Pedestrian road injuries [Global]	Male	0-6 days	95+ years	0.403112	0.565	0.196518	0.193611	0.995855	0.986423
Pedestrian road injuries [Data Rich]	Female	0-6 days	95+ years	0.132188	0.385926	0.0980307	0.144671	0.999997	0.99974
Pedestrian road injuries [Data Rich]	Male	0-6 days	95+ years	0.127669	0.388413	0.0955579	0.139181	0.999994	0.999689
Cyclist road injuries [Global]	Female	12-23 months	90-94 years	0.636854	0.912291	0.339832	0.326033	0.98785	0.97809
Cyclist road injuries [Global]	Male	12-23 months	90-94 years	0.411304	0.71965	0.230434	0.225371	0.990511	0.976016
Cyclist road injuries [Data Rich]	Female	12-23 months	90-94 years	0.232229	0.585353	0.185726	0.200651	0.999475	0.998516
Cyclist road injuries [Data Rich]	Male	12-23 months	90-94 years	0.177527	0.510752	0.13563	0.174219	0.999888	0.999512
Motorcyclist road injuries [Global]	Female	0-6 days	95+ years	0.348152	0.682563	0.214156	0.21867	0.997505	0.986364
Motorcyclist road injuries [Global]	Male	0-6 days	95+ years	0.319367	0.603546	0.170761	0.174284	0.997507	0.983618
Motorcyclist road injuries [Data Rich]	Female	0-6 days	95+ years	0.159633	0.642891	0.118935	0.186461	0.99988	0.998367
Motorcyclist road injuries [Data Rich]	Male	0-6 days	95+ years	0.123797	0.525935	0.0859238	0.144054	0.99999	0.999139
Motor vehicle road injuries [Global]	Female	0-6 days	95+ years	0.342504	0.525052	0.175154	0.174201	0.997613	0.986307
Motor vehicle road injuries [Global]	Male	0-6 days	95+ years	0.322475	0.506517	0.163675	0.168872	0.997996	0.985442
Motor vehicle road injuries [Data Rich]	Female	0-6 days	95+ years	0.121832	0.374067	0.0913558	0.143883	0.999969	0.999522
Motor vehicle road injuries [Data Rich]	Male	0-6 days	95+ years	0.120142	0.365322	0.0899626	0.139435	0.999957	0.9993
Other road injuries [Global]	Female	0-6 days	95+ years	0.655574	0.977085	0.331792	0.330548	0.988488	0.974811
Other road injuries [Global]	Male	0-6 days	95+ years	0.702219	0.971506	0.32058	0.286447	0.987158	0.966686
Other road injuries [Data Rich]	Female	0-6 days	95+ years	0.25885	0.962753	0.198308	0.286644	0.999206	0.986747
Other road injuries [Data Rich]	Male	0-6 days	95+ years	0.238375	0.911313	0.185544	0.265383	0.999656	0.990699
Other transport injuries [Global]	Female	0-6 days	95+ years	0.42791	0.673588	0.232683	0.23991	0.997804	0.987831
Other transport injuries [Global]	Male	0-6 days	95+ years	0.374183	0.631598	0.182625	0.187325	0.998235	0.986825
Other transport injuries [Data Rich]	Female	0-6 days	95+ years	0.155525	0.446649	0.12195	0.233847	0.999931	0.999526
Other transport injuries [Data Rich]	Male	0-6 days	95+ years	0.127135	0.423531	0.0911825	0.163113	0.999922	0.99959
Falls [Global]	Female	0-6 days	95+ years	0.313619	0.478236	0.170214	0.172682	0.999469	0.991084
Falls [Global]	Male	0-6 days	95+ years	0.277372	0.429826	0.151277	0.154145	0.999664	0.992115
Falls [Data Rich]	Female	0-6 days	95+ years	0.116103	0.244149	0.0832381	0.146579	0.999994	0.999926
Falls [Data Rich]	Male	0-6 days	95+ years	0.112962	0.233309	0.0825095	0.14369	0.999997	0.999931
Drowning [Global]	Female	0-6 days	95+ years	0.259383	0.499972	0.194688	0.194333	0.99936	0.992894
Drowning [Global]	Male	0-6 days	95+ years	0.260534	0.475517	0.193989	0.186648	0.999644	0.990468
Drowning [Data Rich]	Female	0-6 days	95+ years	0.132935	0.265208	0.100312	0.16322	0.999959	0.999611
Drowning [Data Rich]	Male	0-6 days	95+ years	0.125148	0.249454	0.0947644	0.152219	0.999989	0.999209
Fire, heat, and hot substances [Global]	Female	0-6 days	95+ years	0.376018	0.574564	0.192452	0.186409	0.998459	0.991454
Fire, heat, and hot substances [Global]	Male	0-6 days	95+ years	0.340337	0.519348	0.190486	0.175471	0.99898	0.991255
Fire, heat, and hot substances [Data Rich]	Female	0-6 days	95+ years	0.119022	0.241583	0.0940013	0.145728	0.999961	0.999504
Fire, heat, and hot substances [Data Rich]	Male	0-6 days	95+ years	0.120151	0.258383	0.0901024	0.151988	0.999986	0.999702
Poisonings [Global]	Female	0-6 days	95+ years	0.409661	0.691751	0.273099	0.272686	0.998536	0.989854
Poisonings [Global]	Male	0-6 days	95+ years	0.336318	0.631267	0.268624	0.253516	0.998832	0.986977
Poisonings [Data Rich]	Female	0-6 days	95+ years	0.149786	0.356361	0.116908	0.220857	0.999913	0.999458
Poisonings [Data Rich]	Male	0-6 days	95+ years	0.140766	0.315704	0.108057	0.176056	0.999978	0.999638
Poisoning by carbon monoxide [Global]	Female	0-6 days	95+ years	0.503845	0.842061	0.292924	0.289514	0.998417	0.987821
Poisoning by carbon monoxide [Global]	Male	0-6 days	95+ years	0.433111	0.776935	0.250359	0.265962	0.997987	0.984808
Poisoning by carbon monoxide [Data Rich]	Female	0-6 days	95+ years	0.292121	0.495068	0.239012	0.360976	0.997523	0.995093
Poisoning by carbon monoxide [Data Rich]	Male	0-6 days	95+ years	0.253284	0.383451	0.206101	0.261468	0.998975	0.997233
Poisoning by other means [Global]	Female	0-6 days	95+ years	0.510276	0.899091	0.23362	0.243234	0.997472	0.977128
Poisoning by other means [Global]	Male	0-6 days	95+ years	0.388055	0.932025	0.214473	0.223229	0.99908	0.975106
Poisoning by other means [Data Rich]	Female	0-6 days	95+ years	0.230459	0.623467	0.1802	0.225979	0.999215	0.997948
Poisoning by other means [Data Rich]	Male	0-6 days	95+ years	0.228753	0.787408	0.174618	0.192238	0.999789	0.998496
Exposure to mechanical forces [Global]	Female	0-6 days	95+ years	0.474013	0.645619	0.393517	0.365754	0.998918	0.99471
Exposure to mechanical forces [Global]	Male	0-6 days	95+ years	0.433172	0.613832	0.361685	0.36844	0.999264	0.991368
Exposure to mechanical forces [Data Rich]	Female	0-6 days	95+ years	0.146036	0.334422	0.0993845	0.177838	0.999947	0.99923
Exposure to mechanical forces [Data Rich]	Male	0-6 days	95+ years	0.126708	0.293926	0.0926558	0.144109	0.999936	0.99966
Unintentional firearm injuries [Global]	Female	0-6 days	95+ years	0.55602	1.02201	0.35769	0.377433	0.996347	0.970106
Unintentional firearm injuries [Global]	Male	0-6 days	95+ years	0.663012	0.912099	0.309012	0.296158	0.996839	0.982274
Unintentional firearm injuries [Data Rich]	Female	0-6 days	95+ years	0.314077	0.779494	0.250203	0.374058	0.997854	0.99511

Appendix Table S12: CODEm predictive validity results by cause, model type, sex, and age									
**Cause**	**Sex**	**Age Start**	**Age End**	**RMSE In-Sample**	**RMSE Out-of-Sample**	**Trend In-Sample**	**Trend Out-of-Sample**	**Coverage In-Sample**	**Coverage Out-of-Sample**
Unintentional firearm injuries [Data Rich]	Male	0-6 days	95+ years	0.279354	0.898149	0.219857	0.23098	0.99811	0.993482
Other exposure to mechanical forces [Global]	Female	0-6 days	95+ years	0.479435	0.659311	0.416865	0.385703	0.999055	0.99423
Other exposure to mechanical forces [Global]	Male	0-6 days	95+ years	0.440064	0.631201	0.38098	0.362004	0.999244	0.993586
Other exposure to mechanical forces [Data Rich]	Female	0-6 days	95+ years	0.151693	0.492871	0.105135	0.178586	0.999931	0.998961
Other exposure to mechanical forces [Data Rich]	Male	0-6 days	95+ years	0.129178	0.41595	0.0951125	0.136947	0.999929	0.999599
Adverse effects of medical treatment [Global]	Female	0-6 days	95+ years	0.311932	0.498744	0.151423	0.155972	0.999877	0.992956
Adverse effects of medical treatment [Global]	Male	0-6 days	95+ years	0.360377	0.550493	0.163553	0.171062	0.999843	0.991986
Adverse effects of medical treatment [Data Rich]	Female	0-6 days	95+ years	0.125268	0.298223	0.087968	0.159757	0.999997	0.999776
Adverse effects of medical treatment [Data Rich]	Male	0-6 days	95+ years	0.127817	0.324138	0.0951615	0.171135	0.999989	0.99985
Animal contact [Global]	Female	0-6 days	95+ years	0.464954	0.80436	0.251301	0.274848	0.997661	0.98263
Animal contact [Global]	Male	0-6 days	95+ years	0.342572	0.695805	0.213253	0.222121	0.998408	0.985867
Animal contact [Data Rich]	Female	0-6 days	95+ years	0.279328	0.510587	0.222335	0.33349	0.998212	0.996467
Animal contact [Data Rich]	Male	0-6 days	95+ years	0.225041	0.511196	0.177708	0.271512	0.999665	0.998888
Venomous animal contact [Global]	Female	0-6 days	95+ years	0.473958	0.893171	0.35343	0.402825	0.993791	0.968085
Venomous animal contact [Global]	Male	0-6 days	95+ years	0.437366	0.772283	0.340661	0.350454	0.993028	0.975247
Venomous animal contact [Data Rich]	Female	0-6 days	95+ years	0.409665	0.975816	0.344476	0.539713	0.995775	0.989277
Venomous animal contact [Data Rich]	Male	0-6 days	95+ years	0.379412	0.819555	0.332867	0.484934	0.993725	0.989176
Non-venomous animal contact [Global]	Female	0-6 days	95+ years	0.574455	0.973718	0.3375	0.373837	0.994354	0.975721
Non-venomous animal contact [Global]	Male	0-6 days	95+ years	0.41895	0.849662	0.274742	0.301688	0.995719	0.982503
Non-venomous animal contact [Data Rich]	Female	0-6 days	95+ years	0.362094	0.790618	0.301248	0.450478	0.997072	0.991796
Non-venomous animal contact [Data Rich]	Male	0-6 days	95+ years	0.281218	0.693494	0.225321	0.300975	0.998458	0.996342
Foreign body [Global]	Female	0-6 days	95+ years	0.361935	0.554883	0.160141	0.164424	0.999334	0.992112
Foreign body [Global]	Male	0-6 days	95+ years	0.359891	0.516281	0.169025	0.167325	0.99929	0.991268
Foreign body [Data Rich]	Female	0-6 days	95+ years	0.117322	0.296742	0.0855468	0.157552	0.999992	0.999667
Foreign body [Data Rich]	Male	0-6 days	95+ years	0.11747	0.283949	0.0855747	0.149901	0.999989	0.999529
Pulmonary aspiration and foreign body in airway [Global]	Female	0-6 days	95+ years	0.347311	0.523319	0.159341	0.163007	0.999578	0.994938
Pulmonary aspiration and foreign body in airway [Global]	Male	0-6 days	95+ years	0.335918	0.510375	0.163415	0.16937	0.99962	0.991012
Pulmonary aspiration and foreign body in airway [Data Rich]	Female	0-6 days	95+ years	0.119781	0.429413	0.0882854	0.152363	0.999994	0.999443
Pulmonary aspiration and foreign body in airway [Data Rich]	Male	0-6 days	95+ years	0.119036	0.394185	0.0871947	0.147106	0.999988	0.999086
Foreign body in other body part [Global]	Female	0-6 days	95+ years	0.574335	0.901967	0.342585	0.358674	0.994392	0.973884
Foreign body in other body part [Global]	Male	0-6 days	95+ years	0.612252	0.902807	0.337087	0.345611	0.992269	0.976228
Foreign body in other body part [Data Rich]	Female	0-6 days	95+ years	0.334039	0.697939	0.269308	0.391354	0.997208	0.99288
Foreign body in other body part [Data Rich]	Male	0-6 days	95+ years	0.336083	0.765148	0.268261	0.379675	0.997266	0.994072
Electrocution [Global]	Female	0-6 days	95+ years	0.936925	1.30691	0.622169	0.657314	0.974006	0.929839
Electrocution [Global]	Male	0-6 days	95+ years	0.545755	0.814738	0.330918	0.332541	0.992772	0.981456
Electrocution [Data Rich]	Female	0-6 days	95+ years	0.722786	1.27953	0.615951	0.818191	0.966748	0.943737
Electrocution [Data Rich]	Male	0-6 days	95+ years	0.383042	0.687687	0.31615	0.24787	0.992606	0.988691
Environmental heat and cold exposure [Global]	Female	0-6 days	95+ years	0.476325	0.807326	0.279851	0.287093	0.996716	0.983217
Environmental heat and cold exposure [Global]	Male	0-6 days	95+ years	0.52309	0.759882	0.255301	0.253758	0.996764	0.986716
Environmental heat and cold exposure [Data Rich]	Female	0-6 days	95+ years	0.161258	0.464593	0.130714	0.258846	0.999848	0.998741
Environmental heat and cold exposure [Data Rich]	Male	0-6 days	95+ years	0.140613	0.356739	0.111564	0.216244	0.999941	0.999265
Other unintentional injuries [Global]	Female	0-6 days	95+ years	0.465094	0.72589	0.221784	0.227944	0.997976	0.987478
Other unintentional injuries [Global]	Male	0-6 days	95+ years	0.425805	0.651921	0.198434	0.208115	0.998063	0.98748
Other unintentional injuries [Data Rich]	Female	0-6 days	95+ years	0.221422	0.447277	0.164151	0.239778	0.999697	0.998726
Other unintentional injuries [Data Rich]	Male	0-6 days	95+ years	0.198817	0.480071	0.137888	0.239894	0.999703	0.999057
Self-harm [Global]	Female	10-14 years	95+ years	0.204366	0.4362	0.140815	0.141482	0.999772	0.985014
Self-harm [Global]	Male	10-14 years	95+ years	0.212787	0.427163	0.136614	0.141785	0.999865	0.981424
Self-harm [Data Rich]	Female	10-14 years	95+ years	0.109429	0.233155	0.0886376	0.162096	0.999996	0.999809
Self-harm [Data Rich]	Male	10-14 years	95+ years	0.107239	0.241225	0.0857989	0.169892	0.999988	0.999388
Self-harm by hanging, strangulation, and suffocation [Global]	Female	10-14 years	95+ years	0.358992	0.638733	0.174262	0.187247	0.999655	0.986233
Self-harm by hanging, strangulation, and suffocation [Global]	Male	10-14 years	95+ years	0.325835	0.602094	0.165586	0.179023	0.999325	0.973859
Self-harm by hanging, strangulation, and suffocation [Data Rich]	Female	10-14 years	95+ years	0.141762	0.53605	0.109863	0.222132	0.999883	0.998794
Self-harm by hanging, strangulation, and suffocation [Data Rich]	Male	10-14 years	95+ years	0.129901	0.483883	0.10418	0.218185	0.999935	0.997274
Self-harm by fire, heat, and hot substances [Global]	Female	10-14 years	95+ years	0.749636	1.11072	0.401248	0.442119	0.990015	0.95666
Self-harm by fire, heat, and hot substances [Global]	Male	10-14 years	95+ years	0.787632	1.20352	0.429988	0.462495	0.989258	0.955617
Self-harm by fire, heat, and hot substances [Data Rich]	Female	10-14 years	95+ years	0.24923	0.855238	0.216918	0.484138	0.997848	0.989578
Self-harm by fire, heat, and hot substances [Data Rich]	Male	10-14 years	95+ years	0.216109	0.717923	0.181093	0.345713	0.999165	0.991725
Self-harm by firearm [Global]	Female	10-14 years	95+ years	0.455734	0.791808	0.299779	0.278807	0.997925	0.98446
Self-harm by firearm [Global]	Male	10-14 years	95+ years	0.323106	0.639379	0.191557	0.181445	0.99935	0.978554
Self-harm by firearm [Data Rich]	Female	10-14 years	95+ years	0.190245	0.591928	0.157044	0.266671	0.999665	0.99786
Self-harm by firearm [Data Rich]	Male	10-14 years	95+ years	0.116768	0.342691	0.0949339	0.149669	0.999978	0.998619
Self-harm by poisoning pesticides [Global]	Female	10-14 years	95+ years	0.536526	1.03989	0.313247	0.31556	0.994433	0.96514
Self-harm by poisoning pesticides [Global]	Male	10-14 years	95+ years	0.553286	0.98385	0.295089	0.296666	0.994527	0.967182
Self-harm by poisoning pesticides [Data Rich]	Female	10-14 years	95+ years	0.209162	0.878593	0.176674	0.319873	0.99939	0.99533
Self-harm by poisoning pesticides [Data Rich]	Male	10-14 years	95+ years	0.200713	0.816144	0.163463	0.333888	0.999238	0.99553
Self-harm by non pesticide substance and gas [Global]	Female	10-14 years	95+ years	0.865997	1.31134	0.403953	0.406296	0.984811	0.957144
Self-harm by non pesticide substance and gas [Global]	Male	10-14 years	95+ years	0.82794	1.3077	0.399482	0.390697	0.986002	0.958221
Self-harm by non pesticide substance and gas [Data Rich]	Female	10-14 years	95+ years	0.194646	0.794532	0.165225	0.336535	0.998351	0.996347
Self-harm by non pesticide substance and gas [Data Rich]	Male	10-14 years	95+ years	0.206893	0.734301	0.175853	0.306357	0.997996	0.995626
Self-harm by other specified means [Global]	Female	10-14 years	95+ years	0.268047	0.499471	0.180472	0.165238	0.999137	0.988462
Self-harm by other specified means [Global]	Male	10-14 years	95+ years	0.251508	0.503021	0.168398	0.177952	0.999691	0.992159
Self-harm by other specified means [Data Rich]	Female	10-14 years	95+ years	0.117739	0.385367	0.0946461	0.160741	0.999926	0.999153
Self-harm by other specified means [Data Rich]	Male	10-14 years	95+ years	0.115159	0.383581	0.0888719	0.173959	0.999996	0.999709
Interpersonal violence [Global]	Female	0-6 days	95+ years	0.279631	0.537986	0.193765	0.208677	0.998807	0.990759
Interpersonal violence [Global]	Male	0-6 days	95+ years	0.276846	0.5555	0.189587	0.203277	0.998602	0.980422
Interpersonal violence [Data Rich]	Female	0-6 days	95+ years	0.132146	0.260231	0.103154	0.165524	0.999835	0.999314
Interpersonal violence [Data Rich]	Male	0-6 days	95+ years	0.130889	0.28068	0.0982588	0.162885	0.999793	0.999172
Assault by firearm [Global]	Female	0-6 days	95+ years	0.417145	0.755233	0.306306	0.286854	0.99522	0.983876
Assault by firearm [Global]	Male	0-6 days	95+ years	0.317255	0.688476	0.21535	0.222958	0.998104	0.969494
Assault by firearm [Data Rich]	Female	0-6 days	95+ years	0.231289	0.584801	0.191919	0.29791	0.998894	0.993057
Assault by firearm [Data Rich]	Male	0-6 days	95+ years	0.160107	0.536989	0.125044	0.208704	0.999783	0.994107
Assault by sharp object [Global]	Female	0-6 days	95+ years	0.322614	0.599918	0.20537	0.219596	0.99799	0.99238
Assault by sharp object [Global]	Male	0-6 days	95+ years	0.282958	0.542371	0.171619	0.164613	0.999023	0.991958
Assault by sharp object [Data Rich]	Female	0-6 days	95+ years	0.153824	0.474968	0.126851	0.246918	0.999762	0.998473
Assault by sharp object [Data Rich]	Male	0-6 days	95+ years	0.144428	0.418305	0.102576	0.153187	0.999925	0.999226
Assault by other means [Global]	Female	0-6 days	95+ years	0.259795	0.4606	0.166325	0.17019	0.999095	0.994605
Assault by other means [Global]	Male	0-6 days	95+ years	0.252991	0.461335	0.151369	0.153646	0.999439	0.995145
Assault by other means [Data Rich]	Female	0-6 days	95+ years	0.133521	0.355965	0.101122	0.183754	0.99991	0.999504
Assault by other means [Data Rich]	Male	0-6 days	95+ years	0.122012	0.403969	0.0866973	0.14055	0.999959	0.999134
Executions and police conflict [Global]	Female	1-5 months	95+ years	0.786819	1.34685	0.503319	0.539826	0.995553	0.952517
Executions and police conflict [Global]	Male	1-5 months	95+ years	1.0658	1.67999	0.743432	0.767557	0.989276	0.919447
Executions and police conflict [Data Rich]	Female	1-5 months	95+ years	0.557483	1.01932	0.48746	0.49996	0.994169	0.979119
Executions and police conflict [Data Rich]	Male	1-5 months	95+ years	0.85544	1.22892	0.777574	0.701133	0.984338	0.95534

Appendix Table S13. GATHER checklist of information that should be included in reports of global health estimates, with description of compliance and location of information for "Global burden of 292 causes of death in 204 countries and territories and 661 subnational locations, 1990–2023: a systematic analysis for the Global Burden of Disease Study 2023".

#	GATHER checklist item	Description of compliance	Reference
**Objectives and funding**			
1	Define the indicators, populations, and time periods for which estimates were made.	Narrative provided in paper and methods appendix describing indicators, definitions, and populations	Manuscript (Methods) and Appendix 1 section 2.
2	List the funding sources for the work.	Funding sources listed in paper	Manuscript (Funding) and Appendix 1 section 2.6.
**Data Inputs**			
For all data inputs from multiple sources that are synthesized as part of the study:			
3	Describe how the data were identified and how the data were accessed.	Narrative description of data seeking methods provided	Manuscript (Methods) and Appendix 1 section 3
4	Specify the inclusion and exclusion criteria. Identify all ad-hoc exclusions.	Narrative about inclusion and exclusion criteria by data type provided; ad-hoc exclusions in cause-specific write ups	Appendix 1 section 3.1.
5	Provide information on all included data sources and their main characteristics. For each data source used, report reference information or contact name/institution, population represented, data collection method, year(s) of data collection, sex and age range, diagnostic criteria or measurement method, and sample size, as relevant.	An interactive, online data source tool that provides metadata for data sources by component, geography, cause, risk, or impairment has been developed	For detailed information on data sources and estimates, please visit the Global Health Data Exchange GBD 2023 website at https://ghdx.healthdata.org/gbd-2023/sources
6	Identify and describe any categories of input data that have potentially important biases (e.g., based on characteristics listed in item 5).	Summary of known biases by cause included in methods appendix	Appendix 1 section 3 and in each cause methods write up, found in section 9.
For data inputs that contribute to the analysis but were not synthesized as part of the study:			
7	Describe and give sources for any other data inputs.	Included in online data source tool	Other data inputs are accessible on the Global Health Data Exchange<br><br>https://ghdx.healthdata.org/gbd-2023/sources
For all data inputs:			

8	Provide all data inputs in a file format from which data can be efficiently extracted (e.g., a spreadsheet as opposed to a PDF), including all relevant meta-data listed in item 5. For any data inputs that cannot be shared due to ethical or legal reasons, such as third-party ownership, provide a contact name or the name of the institution that retains the right to the data.	Downloads of input data are available through an online data repository; input data not available in this form will be made available upon request.	Data inputs are accessible on the Global Health Data Exchange<br><br>https://ghdx.healthdata.org/gbd-2023/sources
**Data analysis**			
9	Provide a conceptual overview of the data analysis method. A diagram may be helpful.	Flow diagrams of the overall methodological processes, as well as cause-specific modeling processes, have been provided	Manuscript (Methods) and Appendix 1 figure S1.
10	Provide a detailed description of all steps of the analysis, including mathematical formulae. This description should cover, as relevant, data cleaning, data pre-processing, data adjustments and weighting of data sources, and mathematical or statistical model(s).	Flow diagrams and corresponding methodological write-ups for each cause, as well as the databases and modeling processes, have been provided	Manuscript (Methods) and Appendix 1, sections 2, 6, and 9).
11	Describe how candidate models were evaluated and how the final model(s) were selected.	Provided in the methodological write-ups	Appendix 1 section 4.
12	Provide the results of an evaluation of model performance, if done, as well as the results of any relevant sensitivity analysis.	Provided in the methodological write-ups	Appendix 1 section 4 and figure S7.
13	Describe methods for calculating uncertainty of the estimates. State which sources of uncertainty were, and were not, accounted for in the uncertainty analysis.	Provided in the methodological write-ups	Manuscript (Methods) Appendix 1 section 4.
14	State how analytic or statistical source code used to generate estimates can be accessed.	Access statement provided	Code is provided at https://ghdx.healthdata.org/gbd-2023/code
**Results and Discussion**			

15	Provide published estimates in a file format from which data can be efficiently extracted.	GBD 2023 results are available through online data visualization tools, the Global Health Data Exchange, and the online data query tool	Manuscript, supplementary results, and online data tools (data visualization tools, data query tools, and the Global Health Data Exchange);<br><br>https://ghdx.healthdata.org/record/ihme-data/gbd-2023-cause-specific-mortality-1990-2023
16	Report a quantitative measure of the uncertainty of the estimates (e.g. uncertainty intervals).	Uncertainty intervals are provided with all results	Uncertinty is available at:<br><br>https://ghdx.healthdata.org/record/ihme-data/gbd-2023-cause-specific-mortality-1990-2023
17	Interpret results in light of existing evidence. If updating a previous set of estimates, describe the reasons for changes in estimates.	Discussion of methodological changes between GBD rounds provided in the narrative of the manuscript and methods appendix	Manuscript (Research in Context, Methods and Discussion) and Appendix 1 cause write-ups section 9.
18	Discuss limitations of the estimates. Include a discussion of any modeling assumptions or data limitations that affect interpretation of the estimates.	Discussion of limitations provided in the narrative of the manuscript, as well as in the methodological write-ups in the methods appendix	Manuscript (Limitations) and Appendix 1 sections 3 and 4.

Section 9: CoD cause-specific modelling descriptions

Acute endocarditis

Flowchart

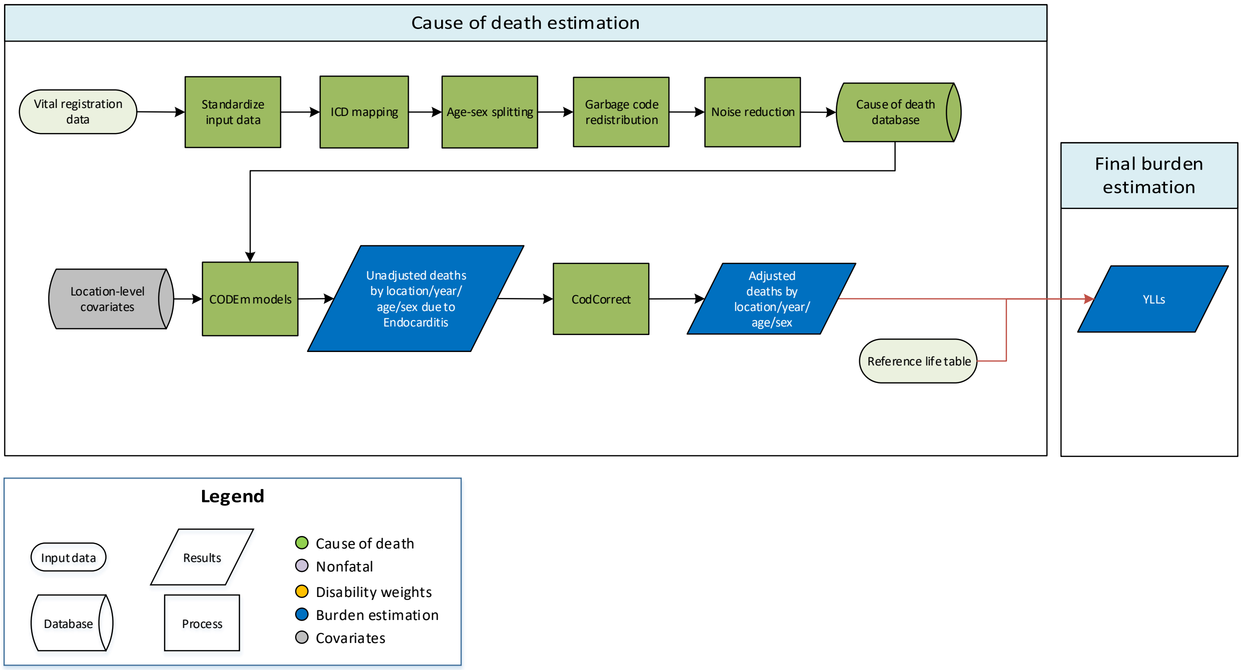

Input data and methodological summary for endocarditis

Input data

Vital registration data were used to model endocarditis. We outliered data in Mozambique as these were non-representative for sub-Saharan Africa and were causing regional estimates to be implausibly low. In addition, data from Cabo Verde, Ghana, and Palau were also outliered due to poor data quality leading to implausible results. We also outliered ICD-8 data that were discontinuous from the rest of the data series and created an implausible time trend.

Crosswalk by code system

Most data sources used for cause of death estimation are ICD-coded. The ICD periodically releases new versions, which can introduce an implausible disjoint in the time series that is indicative of a change in the coding practice, rather than a true change in the epidemiological trend of the disease. In GBD 2023, certain causes and countries using the ICD-9 code systems were adjusted to address the disjoint in the time trend and number of deaths between the older ICD-9 code systems and the more recent ICD-10 code systems. Endocarditis was corrected in Bulgaria (1990–2004), Canada (1986–1999), Spain (1980–1998), France (1980–1999), the USA (1980–1998), Hungary (1980–1995), Croatia (1985–1994), the UK (1980–1999), Greece (1987–2008), Italy (1980–2002), Mexico (1980–1997), and Mauritius (1980–2004) to account for this code system disjoint. This was done using a meta-regression tool described as a crosswalk, by taking systematically biased datapoints and estimating their unbiased value. Using this modelling tool, correction factors were created based on the more recent and more reliable ICD-10-

coded data. The crosswalk creates matched pairs between the last three years of data using the ICD-9 code system and the first three years of data using the ICD-10 code system. The correction is applied at the child cause level for all Level 2 causes, and after the correction is applied the individual child causes are raked to the parent cause to maintain a consistent number of deaths across the cause hierarchy.

Modelling strategy

We used a standard CODEm approach to model deaths from endocarditis. The model uses an empirical Bayesian noise reduction algorithm that uses grouped data by region and data type as a prior to better reflect regional patterns. For GBD 2023, the direction of the covariates for access to sanitation and improved water access were flipped from 1 to –1, and Healthcare Access Quality Index and Socio-demographic Index from –1 to 1. The covariates selected for inclusion in the CODEm ensemble modelling process are listed in Table 3. Aside from this change, there have been no substantive changes to the modelling strategy since GBD 2021.

Table 3. Covariates used in endocarditis mortality modelling

Level	Covariate	Direction
1	Sanitation (proportion with access)	–1
	Improved water (proportion)	–1
2	Healthcare Access and Quality Index	1
3	Socio-demographic Index	1
	Log-transformed lag distributed income per capital (I$)	–1

Acute glomerulonephritis

Flowchart

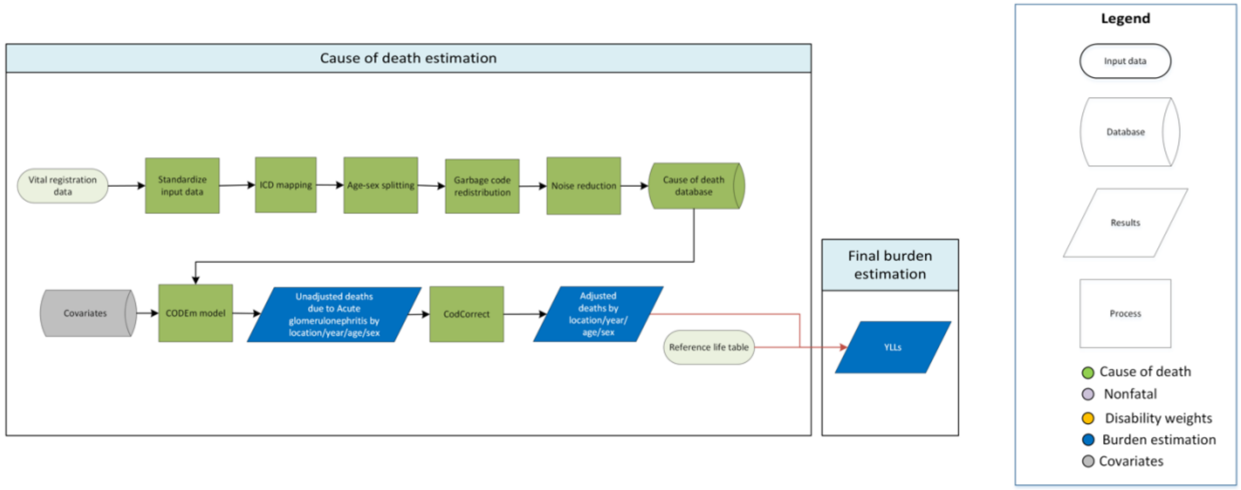

Input data and methodological summary for acute glomerulonephritis

Input data

Data used to estimate mortality due to acute glomerulonephritis consisted of vital registration (VR) data from the cause of death (CoD) database. A list of ICD codes that were mapped to this cause, a list of vital registration sources included in the CoD database and their quality as indicated by star-ratings, and the steps used to process these data for modelling can be found in the section of this appendix on the causes of death database and the tables and figures it refers to. As described there in greater detail, CoD data processing was broadly similar to previous rounds of GBD, but two novel data processing steps were employed in preparing data for acute glomerulonephritis that were not needed prior to GBD 2023: crosswalk by code system and COVID-19 corrections. The former of these is particularly relevant to acute glomerulonephritis. In brief, most data sources used for CoD estimation are ICD-coded. The ICD periodically releases new versions, which can introduce an implausible disjoint in the time series that is indicative of a change in the coding practice, rather than a true change in the epidemiological trend of the disease. In GBD 2023, certain causes and countries using the ICD-9 code systems were adjusted to address the disjoint in the time trend and number of deaths between the older ICD-9 code systems and the more recent ICD-10 code systems. Acute glomerulonephritis was corrected in New Zealand (1980–1999), Japan (1980–1994), Greece (1980–2013), and Mauritius (1980–2004) to account for this code system disjoint. This was done using a meta-regression tool described as a crosswalk, by taking systematically biased datapoints and estimating their unbiased value. Using this modelling tool, correction factors were created based on the more recent and more reliable ICD-10-coded data. The crosswalk creates matched pairs between the last three years of data using the ICD-9 code system and the first three years of data using the ICD-10 code system. The correction is applied at the child cause

level for all Level 2 causes, and after the correction is applied, the individual child causes are raked to the parent cause to maintain a consistent number of deaths across the cause hierarchy.

Data exclusions

After all data processing steps were complete, datapoints for all locations and years were systematically reviewed to identify outliers or other data limitations not fully overcome by CoD data processing methods. For acute glomerulonephritis, the following were identified and excluded prior to modelling.

In some Russian subnationals and the country of Tajikistan, a limitation in the noise-reduction method resulted in data that were implausibly high compared to national and regional averages, respectively. Kiribati data were excluded due to limited coverage of the population resulting in zero estimated deaths. Palau and Tonga data were excluded because having only one year of data from a small population, even after region-level noise reduction, made it difficult to distinguish true extreme values from sampling error that was insufficiently addressed with current noise-reduction algorithms. In Iran and its subnationals as well as Oman, data were excluded where discontinuities between different coding systems resulted in implausible time trends. Ghana and Zimbabwe data were excluded for being implausibly low outliers compared to other data in the region. Finally, for India and its subnationals, data from India Medical Certification of Cause of Death State-Level Tabulations were excluded because of lack of representation among rural areas and bias towards representation of only hospital deaths.

Modelling strategy

The estimation strategy used for fatal acute glomerulonephritis in GBD 2023 was largely similar to that used in GBD 2021. A standard CODEm model was used (see the section of this appendix on CODEm for details). Models were generated separately for males in data-rich countries, males in all countries (referred to as a global model), females in data-rich countries, and females in all countries (global model). Fatal estimation for this cause was carried out for ages 0 days to 95+ years. Covariates offered to the CODEm algorithm for selection based on out-of-sample performance are listed with their permitted directions of association in the table below.

Table 1. Covariates offered for selection in acute glomerulonephritis mortality modelling

Level	Covariate	Direction
2	Mean systolic blood pressure (mmHg)	+
	Sanitation (proportion with access)	-
	Improved water sources (proportion with access)	-
	Healthcare Access and Quality Index	-
3	Socio-demographic Index	-
	Education (years per capita)	-
	Log lag distributed income (I$ per capita)	-

In the ensemble models selected by the CODEm algorithm based on out-of-sample predictive validity, all covariates were selected for female global, male global, and female data-rich models. All but education (years per capita) were chosen for the male data-rich model.

We hybridised the results of global and data-rich models to acquire unadjusted results, which we adjusted using CoDCorrect and compared to the reference life table to calculate final YLLs.

ACUTE HEPATITIS

Flowchart

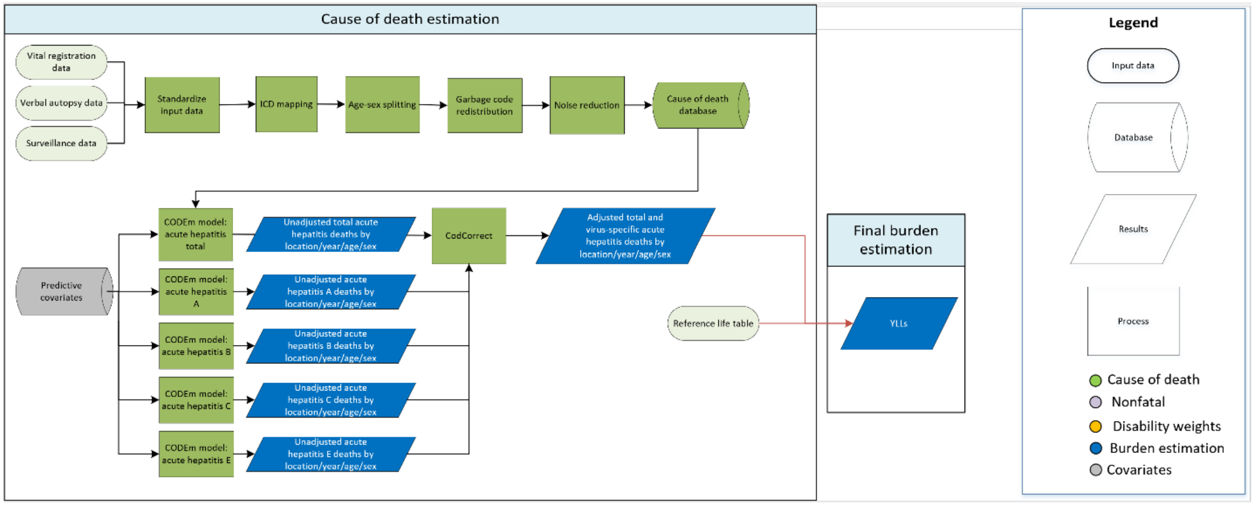

Input Data and Methodological Summary for Acute Hepatitis

In the GBD methodology, 'acute hepatitis' refers to acute viral hepatitis caused by hepatitis A, B, C, or E viruses. Mortality estimates for total acute hepatitis and for each specific virus type are modeled using the standard Causes of Death Ensemble (CODEm) modelling approach.

Input data

Data used to estimate the mortality envelope of acute hepatitis consisted of vital registration (VR) and verbal autopsy (VA) from the CoD database. In contrast, only VR data were utilized for specific virus types. A list of VR and VA sources included in the CoD database, their quality as indicated by star-ratings, and the steps used to process these data for modelling can be found in the section of this appendix on the CoD database, and the tables and figures it refers to.

The table below (Table 1) shows the ICD codes mapped directly to acute hepatitides. All viral hepatitis deaths were included in the database for total acute hepatitis; those that specified virus type (A, B, or E) were mapped directly to separate databases by viral type. Additionally, acute delta infections of hepatitis B carrier deaths were mapped to acute hepatitis B.

Table 1: List of International Classification of Disease (ICD) codes for acute hepatitis

Cause	ICD system	Codes
Acute hepatitis A	10	B15,B15.0,B15.9
Acute hepatitis A	9	070.0,070.1
Acute hepatitis B	10	B16,B16.0,B16.1,B16.2,B17.0,B19.1,B19.10,B19.11,P35.3
Acute hepatitis B	9	070.2,070.20,070.21,070.42,070.52
Acute hepatitis E	10	B17.2
Acute hepatitis E	9	070.43,070.53

Deaths directly attributable to acute hepatitis C virus infection are rare, and hepatology experts in and out of the GBD Collaborator network have asserted that most deaths recorded as due to acute hepatitis C as the underlying cause of death are likely misclassified and instead represent decompensated cirrhosis or other chronic hepatitis C deaths. Consequently, deaths assigned codes for acute hepatitis C (ICD9: 070.41 and 070.51 and ICD10: B17.1) as underlying cause of death are treated as garbage codes and redistributed to other underlying causes of death in COD data processing. The mortality input data for acute hepatitis C modelling were themselves derived exclusively from deaths redistributed from garbage codes.

No substantive changes in redistribution of garbage codes impacting acute hepatitis were made since GBD 2019. We continued to redistribute unspecified hepatitis deaths based on a review of multiple cause of death data in GBD 2019, in which we found that when unspecified hepatitis ICD codes are assigned as underlying cause of death, ICD codes reflecting the presence of chronic liver disease were frequently found in the cause of death chain. Consequently, unspecified hepatitis deaths were redistributed mostly to cirrhosis and other chronic liver diseases, and a small proportion were redistributed to acute hepatitis A, B, C and E according to their observed proportions in the directly mapped data (for cirrhosis, A, B and E) and in the proportion of 2% (for C).

COD data processing was broadly similar to previous rounds of GBD, but one novel data processing step was employed in preparing data for acute hepatitis that was not needed prior to GBD 2023 - COVID corrections – which are described elsewhere in this appendix.

Data exclusions

After all data processing steps were complete, data-points for all locations and years were systematically reviewed to identify outliers or other data limitations not fully overcome by COD data processing methods. For the acute hepatitis envelope, the following were identified and excluded prior to modelling:

Super-region	Countries	Years and types excluded
Central Europe, Eastern Europe and Central Asia	No exclusions	
High-income	Greenland	All years, vital registration

Southeast Asia, East Asia and Oceania	No exclusions	
Latin America & Caribbean	Guyana<br>Ceara, Brazil	1991 and 1992, vital registration for Guyana<br>1993 verbal autopsy (sole year) for Ceara, Brazil
North Africa & Middle East	Morocco	1988, verbal autopsy (sole year)
Sub-Saharan Africa	No exclusions	
South Asia	Nepal<br>Pakistan<br>India	2007, verbal autopsy (sole year)<br>2006, verbal autopsy (sole year)<br>Department of Economics and Statistics' Medically Certified Causes of Death records in India excluded due to inclusion of hospital deaths in urban areas only (India Sample Registration System is preferred source)

Modelling strategy

The mortality estimation strategy used for fatal acute hepatitis and its specific virus types are largely similar to the methods used in GBD 2021. A standard CODEm model was used to model deaths due to acute hepatitis (see the section of this appendix on CODEm for details). We also modeled acute hepatitis A, B, C, and E in separate CODEm models. Models were generated separately for males in data-rich countries, males in all countries (referred to as a global model), females in data-rich countries, and females in all countries (global model). Fatal estimation for this cause was restricted to ages 1 month – 95+ years. We hybridised the results of global and data-rich models to acquire unadjusted results. The following tables report the covariates presented for possible selection in each model.

Table 2: Covariates used in parent acute hepatitis mortality modelling

Level	Covariate	Direction
1	SEV scalar age-standardised hepatitis	+
	Vaccine-adjusted HBsAg seroprevalence, age-standardised	+
	Seroprevalence (anti-HCV), age-standardised	+
	Seroprevalence (anti-HAV), age-standardised	+
	Seroprevalence (anti-HEV), age-standardised	+
2	Healthcare Access and Quality Index	-
	SEV unsafe sanitation	+
	SEV unsafe water	+
	Socio-demographic Index	-
	Hep B vaccine coverage proportion, aged through time	-
	Injection drug use proportion by age	+
3	Education (years per capita)	-
	Lag distributed income (LDI) (ln transformation)	-

Table 3: Covariates used in acute hepatitis A mortality modelling

Level	Covariate	Direction
1	SEV scalar (hepatitis)	+
	Seroprevalence (anti-HAV), age-standardised	+
2	Healthcare Access and Quality Index	-
	SEV unsafe sanitation	+
	SEV unsafe water	+
	Socio-demographic Index	-
3	Education (years per capita)	-
	Lag distributed income (LDI) (ln transformation)	-

Table 4: Covariates used in acute hepatitis B mortality modelling

Level	Covariate	Direction
1	Vaccine adjusted HBsAg seroprevalence, age-standardised	+

	Injection drug use proportion by age	+
	Hep B vaccine coverage proportion, aged through time	-
2	Healthcare Access and Quality Index	-
	Socio-demographic Index	-
	SEV scalar (hepatitis)	+
3	Education (years per capita)	-
	Lag distributed income (LDI) (ln transformation)	-

Table 5: Covariates used in acute hepatitis C mortality modelling

Level	Covariate	Direction
1	Seroprevalence (anti-HCV), age-standardised	+
	Injection drug use proportion by age	+
2	Healthcare Access and Quality Index	-
	Socio-demographic Index	-
	SEV scalar (hepatitis)	+
3	Education (years per capita)	-
	Lag distributed income (LDI) (ln transformation)	-

Table 6: Covariates used in acute hepatitis E mortality modelling

Level	Covariate	Direction
1	SEV scalar (hepatitis)	+
	Seroprevalence (anti-HEV), age-standardised	+
2	Healthcare Access and Quality Index	-
	SEV unsafe sanitation	+
	SEV unsafe water	+
	Socio-demographic Index	-
	Proportion of the population living in the classic monsoon region	+
3	Education (years per capita)	-

	Lag distributed income (LDI) (ln transformation)	-

The unadjusted results of virus-specific deaths were rescaled to fit within the envelope defined by the total acute hepatitis CODEm model through the CoDCorrect process. Acute hepatitis, overall, was rescaled up the GBD cause hierarchy so that all cause-specific deaths added up to all-cause mortality for each year, age, sex and location. Adjusted deaths were compared to the reference life table to calculate final YLLs.

Alcohol use disorders

Flowchart

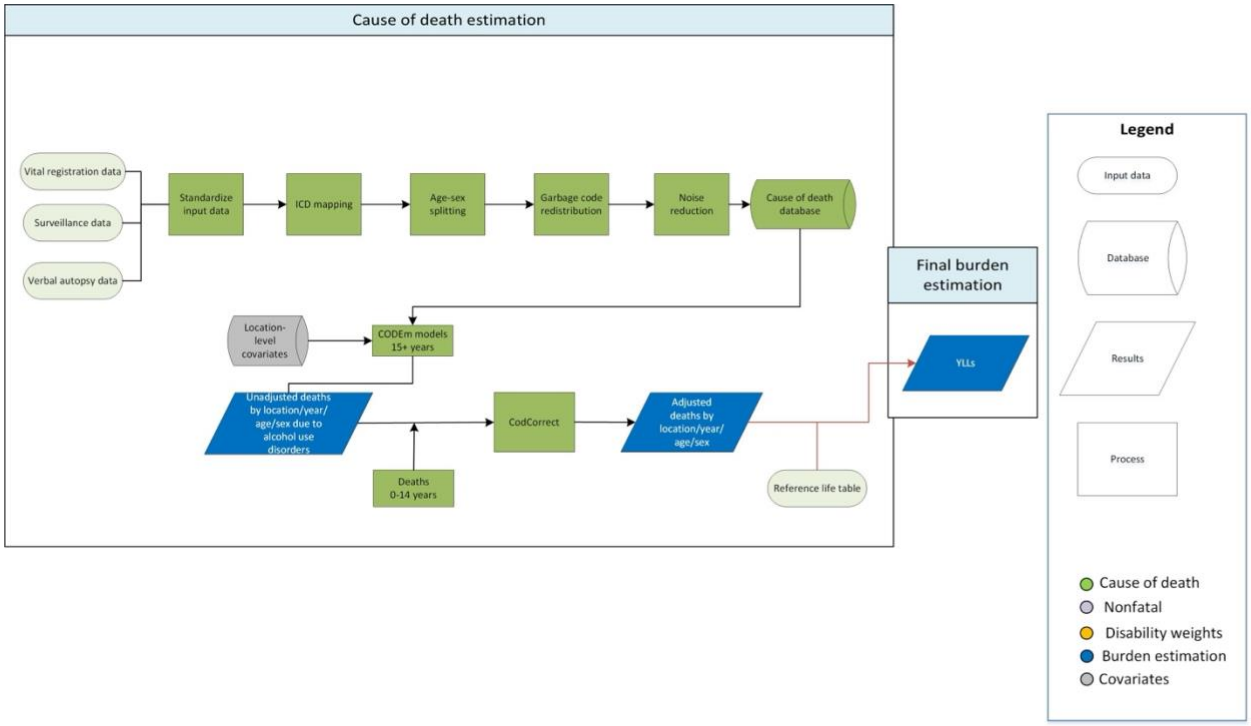

Input data and methodological summary for alcohol use disorders

Input data

All data for alcohol use disorders mortality were from vital registration, China surveillance, and verbal autopsy. Datapoints from countries with sparse and heterogeneous data were excluded if they led to implausible fluctuations in death counts and regional patterns. This exclusion was confirmed with in-country collaborators and subject matter experts. For instance, data from India's Medical Certification of Cause of Death for alcohol use disorders were omitted due to unrealistically low estimates. Additionally, in this round, data coded under outdated ICD schemes, such as ICD-9 and ICD-8, which resulted in erratic trends in mortality, were omitted. Conversely, data coded under more recent schemes like ICD-10, which provided more consistent trends, were retained. An example of this approach is the exclusion of all ICD-9 BTL data for select countries in eastern Europe and central Latin America. In the GBD 2023, data were derived from a comprehensive set of ICD-10 codes, specifically E24.4, F10, G31.2, G62.1, G72.1, P04.3, Q86.0, R78.0, X45, X65, and Y15. Additionally, we expanded our data to include codes Y90 and Y91 this round.

Modelling strategy

In the GBD 2023 round, the modelling strategy for alcohol use disorders remained largely unchanged. Alcohol use disorders have a unique modelling strategy introduced in GBD 2021. Deaths are primarily modelled in older age groups (15+) using the standard Cause of Death Ensemble modelling (CODEm) approach (see Appendix 1, Section 4). However, occasional deaths in younger age groups (0–14) are also recorded in the raw cause of death (CoD) data. For example, while alcohol use disorders are modelled in CODEm for ages 15+, rare deaths in younger age groups are identified from reported death data (mostly vital registrations) in locations where such reports are available. These rare deaths are not modelled but are directly added to the unadjusted death estimates after the CODEm process.

The combined unadjusted death estimates from CODEm models for ages 15+ and the rare deaths data for ages 0–14 for alcohol use disorders then undergo CoDCorrect adjustment. During CoDCorrect, unadjusted estimates from this cause are summed and fit to the distribution of deaths within the overall substance use disorders cause hierarchy. This process ensures consistency and alignment within the substance use disorders hierarchy, as detailed in Appendix 1, Section 4.

Model covariate inclusion was based on empirical evidence and expert feedback, which resulted in a set of model covariates that reflected alcohol consumption, smoking, education, health system access, domestic income, and Socio-demographic Index (SDI).

Key change from GBD 2021:
In the GBD 2023 round, the alcohol binge drinking covariate was excluded from the model because it had not been updated since GBD 2019, and there are no plans to incorporate new data for this variable in the foreseeable future.

Table 1: Covariates used in alcohol use disorders mortality model

Level	Covariate	Direction
1	Alcohol consumption (litres per capita)	+
	Alcohol consumption, age-standardised, in grams per day	+
	Alcohol drinker proportion, age-standardised	+
2	Cumulative cigarettes (10 years)	+
	Smoking prevalence	+
	Healthcare Access and Quality Index	-
3	Log LDI (I$ per capita)	-
	Education (years per capita)	-
	Socio-demographic Index	-

Alcoholic cardiomyopathy

Flowchart

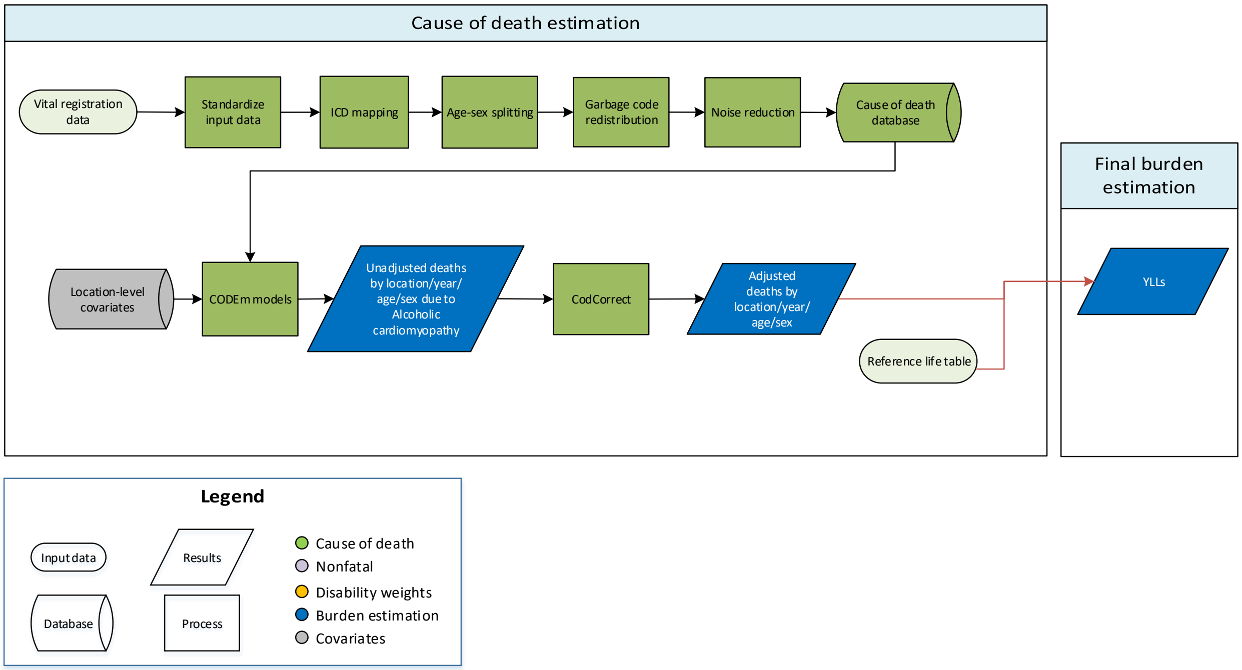

Input data and methodological summary for alcoholic cardiomyopathy

Input data

Vital registration data were used to model deaths due to alcoholic cardiomyopathy. We outliered all datapoints in Bosnia and Herzegovina, Montenegro, North Macedonia, and Egypt due to implausibly high cause fractions likely caused by regional patterns in the use of ICD codes for cardiomyopathies. We also outliered subsets of data in Slovenia and Greece due to implausible patterns. Additionally, we outliered tabulated ICD-10 datapoints in locations such as Kazakhstan where unreliable estimates caused an abrupt inconsistency with detailed ICD-10 data.

Crosswalk by code system

Most data sources used for cause of death estimation are ICD-coded. The ICD periodically releases new versions, which can introduce an implausible disjoint in the time series that is indicative of a change in the coding practice, rather than a true change in the epidemiological trend of the disease. In GBD 2023, certain causes and countries using the ICD-9 code systems were adjusted to address the disjoint in the time trend and number of deaths between the older ICD-9 code systems and the more recent ICD-10 code systems. Alcoholic cardiomyopathy was corrected in Bulgaria (1990–2004), Canada (1986–1999), Spain (1980–1998), France (1980–1999), the USA (1980–1998), Hungary (1980–1995), Croatia (1985–1994), the UK (1980–1999), Greece (1987–2008), Italy (1980–2002), Mexico (1980–1997), and Mauritius

(1980–2004) to account for this code system disjoint. This was done using a meta-regression tool described as a crosswalk, by taking systematically biased datapoints and estimating their unbiased value. Using this modelling tool, correction factors were created based on the more recent and more reliable ICD-10 coded data. The crosswalk creates matched pairs between the last three years of data using the ICD-9 code system and the first three years of data using the ICD-10 code system. The correction is applied at the child cause level for all Level 2 causes, and after the correction is applied the individual child causes are raked to the parent cause to maintain a consistent number of deaths across the cause hierarchy.

Modelling strategy

We used a standard CODEm approach to model deaths from alcoholic cardiomyopathy. The model uses an empirical Bayesian noise reduction algorithm that uses grouped data by region and data type as a prior to better reflect regional patterns. The covariates selected for inclusion in the CODEm modelling process can be found in Table 2. There have been no substantive changes to the modelling strategy since GBD 2021.

Table 2. Covariates used in alcoholic cardiomyopathy mortality modelling

Level	Covariate	Direction
1	Log transformed summary exposure value, cardiomyopathy (CMP)	1
	Smoking prevalence	1
	Summary exposure value, alcohol consumption	1
2	Healthcare Access and Quality Index	-1
3	Log-transformed lag distributed income per capita (I$)	-1

Alzheimer's disease and other dementias

Flowchart

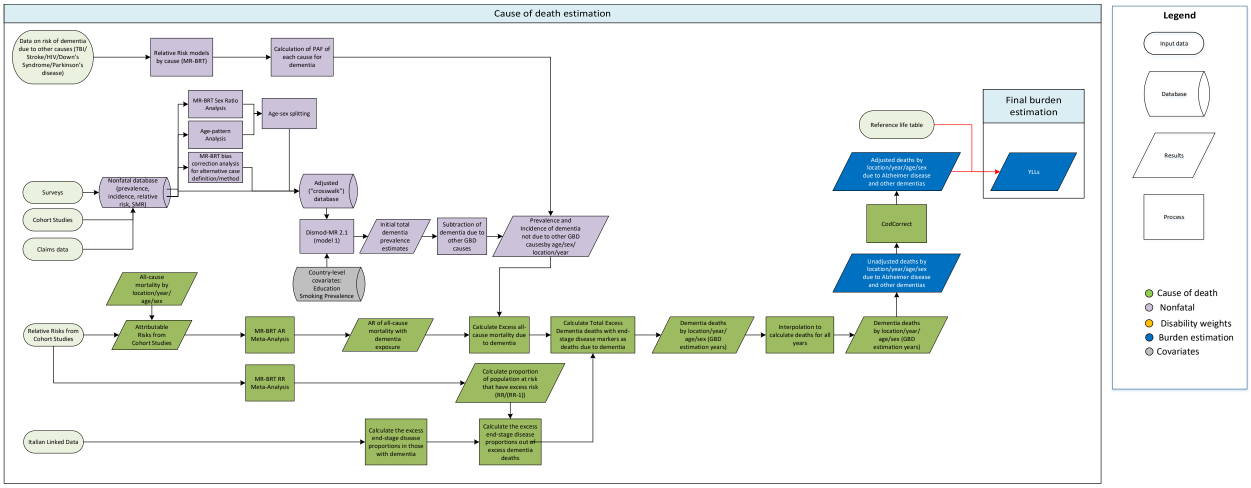

Input data

Since GBD 2019, fatal modelling removed reliance on vital registration data (described in more detail in the "Modelling strategy" section). Instead, two source types were extracted:

(1) Literature on the relative risk of all-cause mortality given the exposure of dementia. Relative risk sources were identified through a systematic review using PubMed with the following search terms:

> "((Excess mortality[Title/Abstract] OR Standardized mortality[Title/Abstract] OR survival[Title/Abstract] OR (relative risk[Title/Abstract] AND (mortality[Title/Abstract] OR death[Title/Abstract]))) AND (dementia[Title/Abstract] OR alzheimer's disease[Title/Abstract] OR "dementia"[MeSH Terms] OR "alzheimer disease"[MeSH Terms]) AND ("1980/01/01"[PDAT] : "2018/08/14"[PDAT]) NOT (animals[MeSH] NOT humans[MeSH])),"

This yielded 4470 total hits, of which 34 studies were marked for extraction (Table 1). Overall, the data were heterogeneous and varied in the exposure category measured (all dementia, Alzheimer's disease, cognitive impairment) and in the different factors controlled for in analyses.

(2) Linked vital registration and hospitalisation data. We used mortality records linked to inpatient records, covering all deaths from 2003 to 2017 in the Emilia-Romagna region of Italy.

Table 1: Results of systematic review on all-cause excess mortality with dementia

N		60
Region name (%)	East Asia	4 (6.7)
	Eastern sub-Saharan Africa	2 (3.3)
	High income Asia Pacific	4 (6.7)
	High income North America	22 (36.7)
	North Africa and Middle East	1 (1.7)
	Tropical Latin America	1 (1.7)
	Western Europe	26 (43.3)
Exposure (%)	Alzheimer's disease	11 (18.3)
	Cognitive impairment	10 (16.7)
	Other dementia	35 (58.3)
	Vascular dementia	4 (6.7)
Conducted in clinical setting (%)	Clinical setting	10 (16.7)
	Population representative	50 (83.3)
Controlled for education (%)	Controlled	32 (53.3)
	No control	28 (46.7)
Controlled for basic CVD info (%)	Controlled	33 (55.0)
	No control	27 (45.0)
Extensive CVD control (%)	Controlled	15 (25.0)
	No control	45 (75.0)
Controlled for smoking and alcohol (%)	Controlled	11 (18.3)
	No control	49 (81.7)
Controlled for factors in causal pathway (%)	Controlled	13 (21.7)
	No control	47 (78.3)

Modelling strategy

Overview

Dementia mortality rates have increased more than five-fold since 1980 in high-quality vital registration systems such as in the USA and Scandinavia. We have not seen an equivalent increase in prevalence and incidence data sources. If at all, there has been a modest decline in incidence and prevalence of dementia in studies in the UK and the USA.[1,2] Also, the greater than 20-fold variation in mortality rates of dementia between countries is much greater than the four-fold difference in prevalence and incidence between countries. As it is unlikely that case fatality from dementia has dramatically increased

[1] Akushevich I, Kravchenko J, Ukraintseva S, Arbeev K, Yashin AI. Time trends of incidence of age-associated diseases in the US elderly population: Medicare-based analysis. *Age and ageing*. 2013 Jul 1;42(4):494-500.

[2] Matthews FE, Arthur A, Barnes LE, Bond J, Jagger C, Robinson L, Brayne C, Medical Research Council Cognitive Function and Ageing Collaboration. A two-decade comparison of prevalence of dementia in individuals aged 65 years and older from three geographical areas of England: results of the Cognitive Function and Ageing Study I and II. *The Lancet*. 2013 Nov 1;382(9902):1405-12.

over the time period and that it would differ by a very large margin between countries, the hypothesis is that certifying and coding practices have changed over time and at a different pace between countries. To avoid spurious large trends over time in the fatal component of the burden of dementia, we decided in GBD 2013 to make dementia mortality rates consistent with the most recent rates relative to prevalence of countries that are most likely to certify or code dementia as an underlying cause of death. This approach was applied again for GBD 2017 with some modifications. For GBD 2019 onward, the fatal modelling process was redesigned to avoid the need for using estimates only from the highest dementia mortality locations. This was accomplished with an attributable risk model based on a systematic review of cohort studies and relative risk data, and end-stage disease proportions from linked hospital and death records. The modelling process is described below.

Modelling steps

Relative risk data

First, using relative risk data extracted from studies identified by systematic review, we calculated attributable risk using the GBD estimate of all-cause mortality rate for a given study location and time, using the following formula:

$$Attributable\ Risk = (Relative\ Risk - 1) * All\text{-}Cause\ Mortality$$

We then conducted a meta-regression analysis using MR-BRT (see appendix section 4) on the attributable risk data, using covariates for age, sex, exposure category (all dementia, Alzheimer's disease, cognitive impairment), whether the study was conducted in a clinical sample, and categories indicating different types of variables that were controlled for in the component studies (educational attainment, cardiovascular disease comorbidities, smoking and alcohol consumption, and daily activities or residence in a nursing home). Relative risks were estimated using a second Bayesian bias-reduction meta-regression model using the same studies identified through systematic review. Regression results for relative risk and attributable risk analyses are displayed below.

Figure 1. Meta-regression results for relative risks and attributable risk of dementia mortality

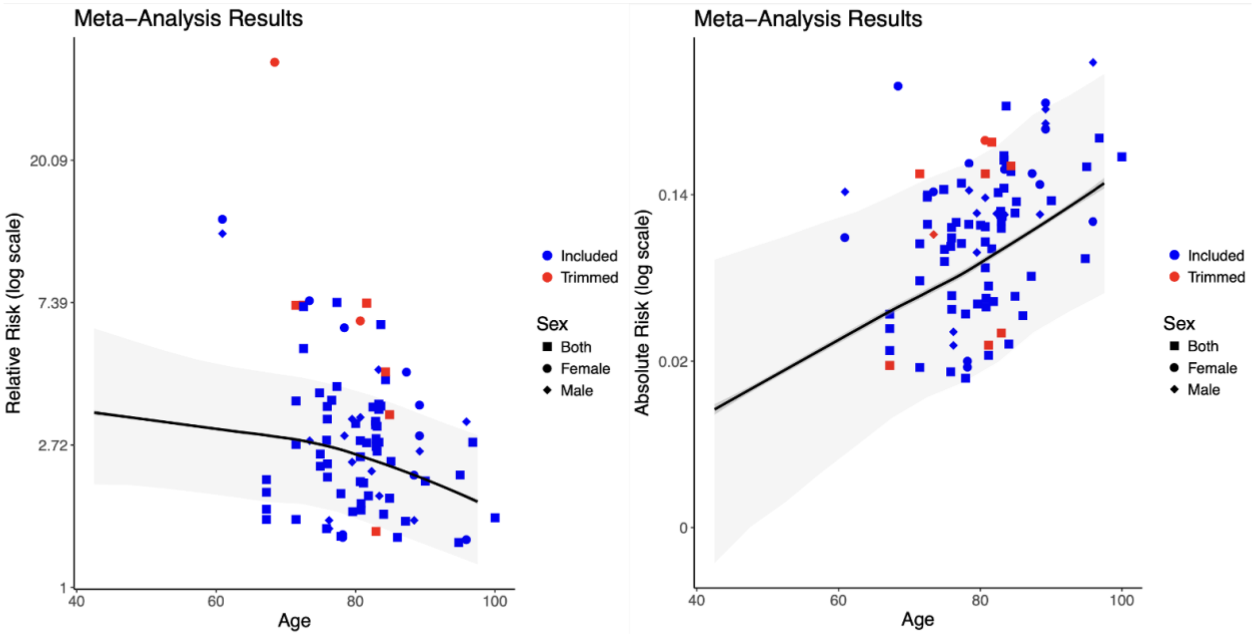

Meta-regression results were used to calculate the total number of excess deaths due to dementia as the product of our prevalence estimates (post-adjustment for dementia caused by other GBD diseases) and our estimates of attributable risk. See the non-fatal write-up on dementia for details on prevalence calculations.

Linked data

The excess deaths calculated through the multiplication of attributable risk and prevalence represent the total number of excess deaths due to having dementia, which likely includes deaths due to other conditions, such as cardiovascular diseases, that are more common in those with dementia as compared to the general population due to common underlying risk factors such as high blood pressure, smoking, and lower educational attainment. To subset this total number of excess dementia deaths to calculate the number of deaths that were caused by dementia, we completed an analysis of linked clinical and mortality data. We used mortality records linked to inpatient records, covering all deaths from 2003 to 2017 in the Emilia-Romagna region of Italy. Using these data, we looked for markers of severe, end-stage disease in the clinical records up to one year before death.

To select these markers, for each ICD code that appeared in the data we calculated the difference in the proportion of individuals who died with dementia and had a record of each code in the year before death and the proportion of individuals who died without dementia and had a record of the same code in the year before death. We reviewed the 150 codes with the highest difference and selected codes that indicated end-stage disease, excluding codes for conditions such as cardiovascular disease. Codes for decubitus ulcer, malnutrition, sepsis, pneumonia, urinary tract infections, falling from bed, senility, dehydration, sodium imbalance, muscular wasting, bronchitis, dysphagia, hip fracture, and bedridden status were used as indicators of severe disease.

To determine the proportion of excess deaths that were caused by dementia, we calculated the proportion of dementia deaths that had clinical markers of end-stage disease in the year before death, above and beyond the occurrence of end-stage disease markers in those who died without dementia

(Figure 2). The subtraction of the proportions with end-stage disease markers in those without dementia from the proportions in those with dementia represents the proportion of individuals who are assumed to have died with severe, end-stage dementia out of total deaths in those with dementia.

Figure 2. Percentage of individuals who died with and without dementia with end-stage conditions in the last year before death

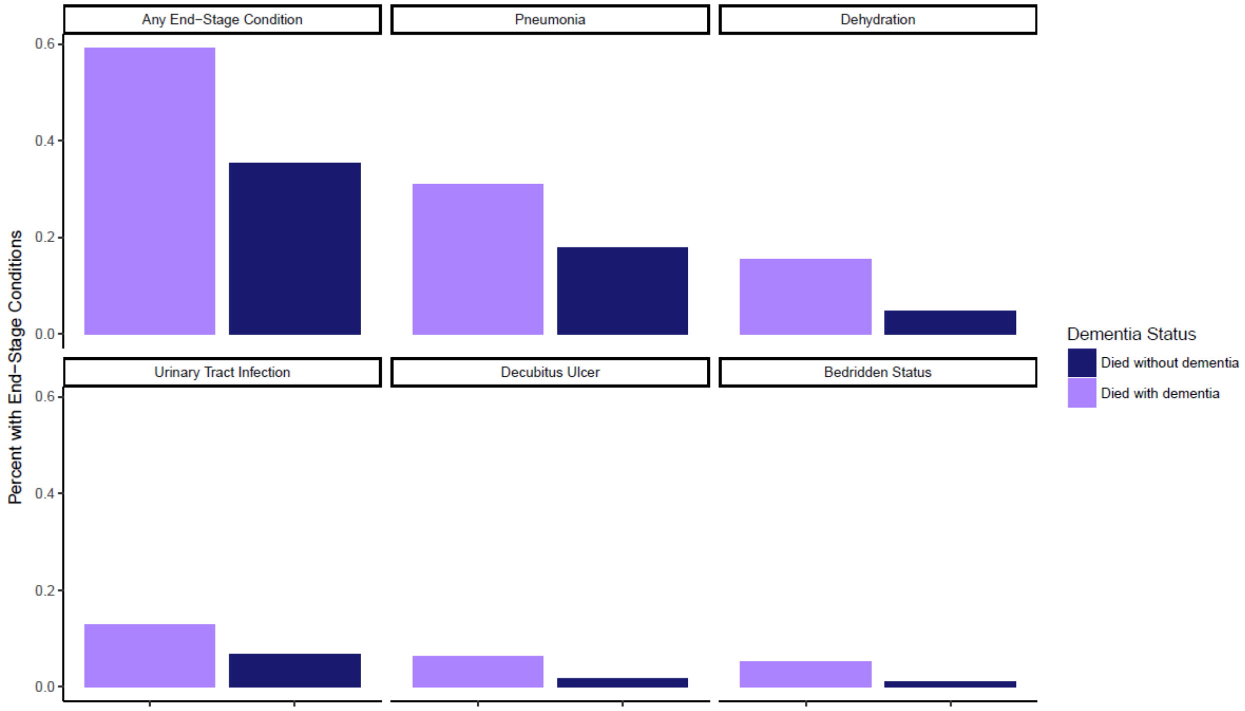

Calculation of deaths due to dementia

To apply these estimates to the total excess deaths we then adjusted these proportions to calculate the proportion of individuals who died with severe, end-stage dementia out of excess dementia deaths using the formula:

$$\frac{Died\ with\ Severe\ Disease}{Excess\ Dementia\ Deaths} = \frac{Died\ with\ Severe\ Disease}{Total\ Dementia\ Deaths} * \frac{Relative\ Risk}{Relative\ Risk - 1}$$

We then calculated the number of deaths due to dementia as the product of total excess dementia deaths and the proportion of those who died with severe disease out of excess dementia deaths. These final estimates of deaths due to dementia were then used to adjust data on causes of death from all other causes in vital registration systems.

Interpolation for all years

Finally, we used log-linear interpolation to interpolate these results (limited to 1990, 1995, 2000, 2005, 2010, 2015, 2017, 2019, 2020, 2021, 2022, and 2023) to create estimates for the entire time series from 1980 to 2023. Socio-demographic Index was used as a covariate to extrapolate back to the year 1980.

Amphetamine use disorder

Flowchart

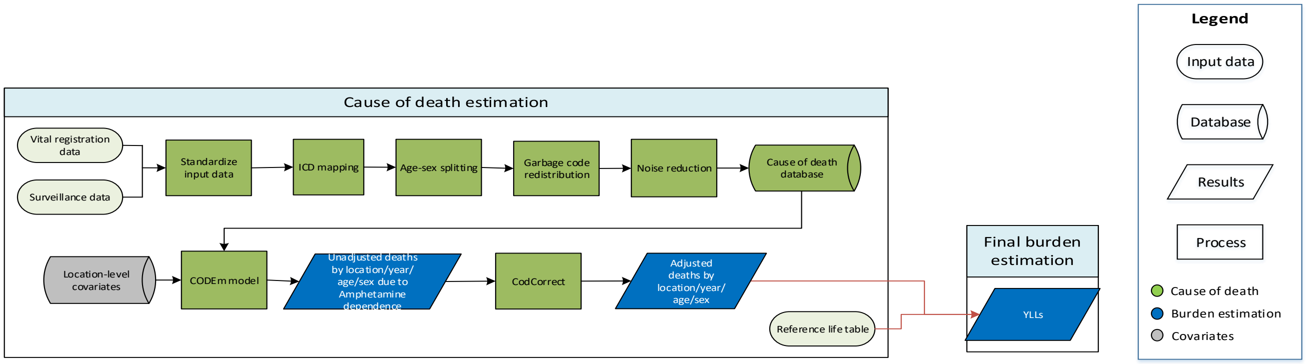

Input data and methodological summary for amphetamine use disorders

Input data

Data used to estimate amphetamine use disorders mortality were from vital registration and surveillance sources from the cause of death (CoD) database. Data from countries with sparse yet heterogeneous data were excluded as the data exaggerated fluctuations in deaths and gave implausible regional patterns, according to in-country and subject matter experts. Excluded data were typically from lower-income countries. A full description of changes to coding and redistribution are described in the write-up focusing on drug use disorders.

Modelling strategy

In the GBD 2023 round, the modelling strategy for amphetamine use disorders remained largely unchanged. The standard Cause of Death Ensemble modelling (CODEm) approach was used to estimate deaths due to amphetamine use disorders (for details, see appendix 1, section 4). Deaths are modelled in age groups 15 years and older, assuming that drug-related deaths from intentional use in younger age groups are extremely rare and there are not sufficient data at the population level to make estimates.

Model covariate inclusion was based on empirical evidence and expert feedback, which resulted in a set of model covariates that reflected alcohol consumption, smoking, education, health system access, domestic income, and Socio-demographic Index (SDI) (Table 1).

Table 1: Covariate used in the amphetamine use disorders modelling

**Level**	**Covariate**	**Direction**
1	Alcohol (litres per capita)	+
	Current drinking prevalence	+
	Intravenous drug use, age-standardised	+

	Intravenous drug use, age-specific	+
	Cumulative cigarettes (10 years)	+
	Cumulative cigarettes (5 years)	+
	Cigarettes per capita	+
	Smoking prevalence	+
2	Healthcare Access and Quality Index	-
3	log LDI (I$ per capita)	+
	Education (years per capita)	+
	Socio-demographic Index	+

Anorexia nervosa

Flowchart

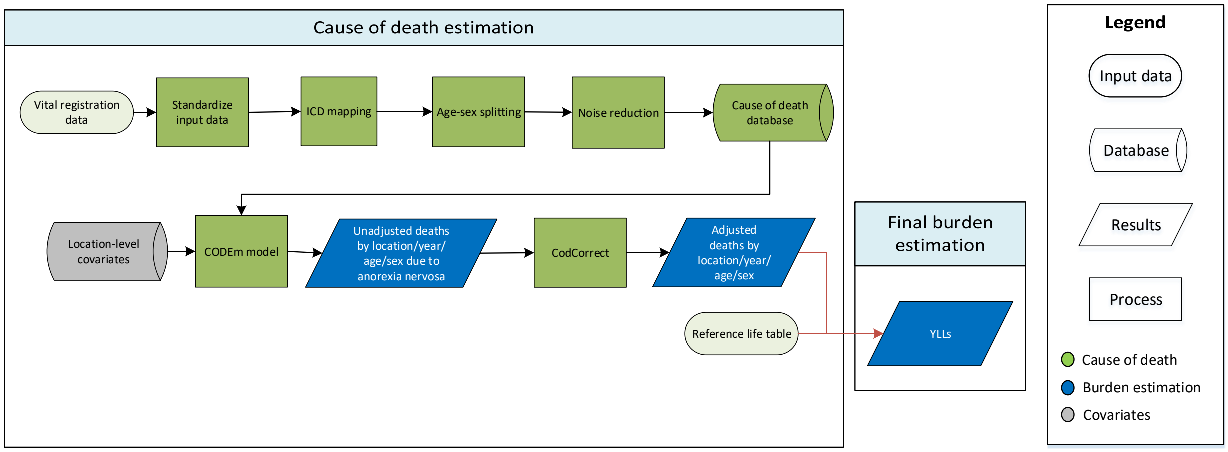

Input data and methodological summary for anorexia nervosa

Data used to estimate anorexia nervosa mortality included centrally prepped vital registration data from the cause of death (CoD) database.

Modelling strategy

We have made no substantive changes in the modelling strategy from GBD 2021.

Anorexia nervosa was modelled using the standard CODEm approach and came under the eating disorders parent model. Age was restricted to deaths occurring between 5 and 49 years based on expert advice and patterns of prevalence seen in the non-fatal model. Several covariates were applied to this model and are listed in the table below, along with the direction in which they were applied.

Level	Covariate	Direction
1	Education (years per capita)	+
	Log LDI (I$ per capita)	+
	Age- and sex-specific SEV for child underweight	-
	Sanitation (proportion with access)	+
	Maternal education (years per capita)	+
2	Healthcare Access and Quality Index	-
3	Socio-demographic Index	+

In GBD 2013, anorexia nervosa deaths were extrapolated from the eating disorders model, which was modelled through a negative binomial approach. This approach was changed in GBD 2015, with anorexia nervosa being modelled as a standard CODEm model, as no additional benefit was seen from using the custom modelling approach. GBD 2023 continued to utilise the same approach.

Deaths from garbage codes were not redistributed to anorexia nervosa because deaths due to dehydration in low- and middle-income countries would likely bias the age, sex, and geographical distribution of deaths observed. For example, testing of this process showed that while only a relatively small proportion of dehydration garbage code deaths were redistributed to anorexia nervosa, this added a comparatively large number of deaths to the model, particularly in regions with higher rates of infectious diseases. The redistributed deaths were also applied equally between males and females despite the prevalence of anorexia nervosa being known to be up to ten times higher in females.

Aortic aneurysm

Flowchart

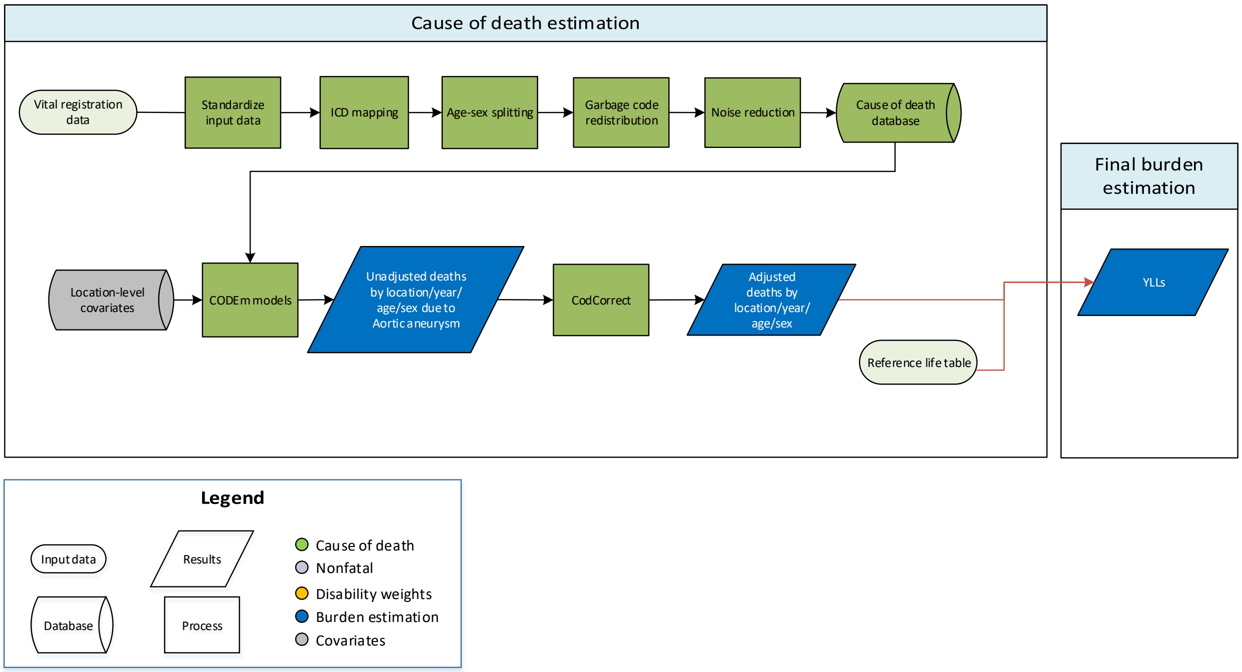

Input data and methodological summary for aortic aneurism

Input data

Vital registration data were used to model cause-specific mortality for aortic aneurysm. We outliered data from Ethiopia and Russia subnationals as they were improbable values in comparison with other data in the location or with the rest of their regions. We also outliered ICD-8 data that were discontinuous with the rest of the time series and created implausible time trends. In addition, we outliered a subset of vital registration datapoints in Latin America due to implausibly high values at the oldest age groups that resulted in inconsistencies in time trends. Subsets of data in Guyana were outliered due to implausibly low values compared with other data in the country.

Crosswalk by code system

Most data sources used for cause of death estimation are ICD-coded. The ICD periodically releases new versions, which can introduce an implausible disjoint in the time series that is indicative of a change in the coding practice, rather than a true change in the epidemiological trend of the disease. In GBD 2023, certain causes and countries using the ICD-9 code systems were adjusted to address the disjoint in the time trend and number of deaths between the older ICD-9 code systems and the more recent ICD-10 code systems. Aortic aneurism was corrected in Bulgaria (1990–2004), Canada (1986–1999), Spain (1980–1998), France (1980–1999), the USA (1980–1998), Hungary (1980–1995), Croatia (1985–1994),

the UK (1980–1999), Greece (1987–2008), Italy (1980–2002), Mexico (1980–1997), and Mauritius (1980–2004) to account for this code system disjoint. This was done using a meta-regression tool described as a crosswalk, by taking systematically biased datapoints and estimating their unbiased value. Using this modelling tool, correction factors were created based on the more recent and more reliable ICD-10-coded data. The crosswalk creates matched pairs between the last three years of data using the ICD-9 code system and the first three years of data using the ICD-10 code system. The correction is applied at the child cause level for all Level 2 causes, and after the correction is applied the individual child causes are raked to the parent cause to maintain a consistent number of deaths across the cause hierarchy.

Modelling strategy

We used a standard CODEm approach to model deaths from aortic aneurysm. The model uses an empirical Bayesian noise reduction algorithm that uses grouped data by region and data type as prior to better reflect regional patterns. The covariates selected for inclusion in the CODEm modelling process can be found in Table 1. For GBD 2023, the direction of the covariate Socio-demographic Index was flipped from 1 to –1. Aside from this change, there have been no substantive changes to the modelling strategy since GBD 2021.

Table 1. Covariates used in aortic aneurysm mortality modelling

Level	Covariate	Direction
1	Summary exposure variable, aortic aneurysm	1
	Mean low-density lipoprotein (mmol/L)	1
	Cumulative cigarettes (10 yrs)	1
	Mean systolic blood pressure (mmHg)	1
2	Mean body-mass index (kg/m^2)	1
	Healthcare Access and Quality Index	–1
3	Lag-distributed income per capita (I$)	–1
	Socio-demographic Index	–1
	Summary exposure value, diet low in omega-3	1
	Summary exposure value, diet low in fruits	1
	Summary exposure value, diet low in vegetables	1
	Summary exposure value, diet low in nuts and seeds	1
	Pulses/legumes (g/capita, unadjusted)	–1
	Summary exposure value, diet low in polyunsaturated fatty acids PUFA	1
	Alcohol (litres per capita)	1

Appendicitis

Flowchart

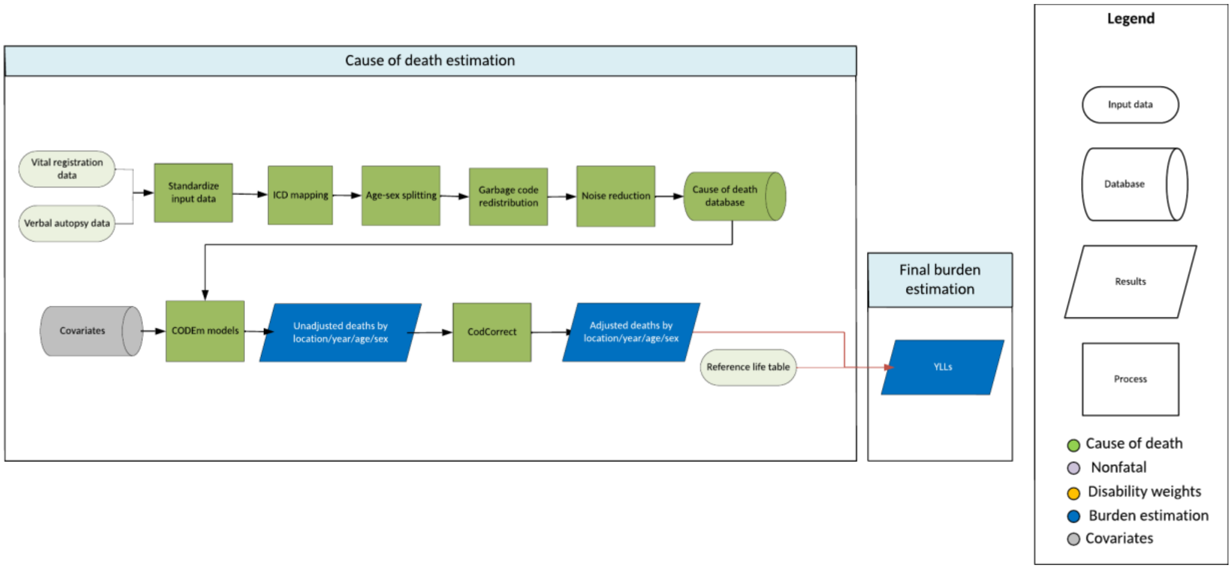

Input data and methodological summary for appendicitis

Appendicitis is a Level 3 cause in the GBD hierarchy.

Input data

Data used to estimate mortality of appendicitis consisted of vital registration (VR) and verbal autopsy data (VA) from the cause of death (CoD) database. A list of ICD codes that were mapped to this cause, a list of vital registration sources included in the CoD database and their quality as indicated by star-ratings, and the steps used to process these data for modelling can be found in the section of this appendix on the causes of death database and the tables and figures it refers to. CoD data processing was broadly similar to previous rounds of GBD, but one novel data processing step was employed in preparing data for appendicitis that was not needed prior to GBD 2023—COVID-19 corrections—which are described elsewhere in this appendix.

After all data processing steps were complete, datapoints for all locations and years were systematically reviewed to identify outliers or other data limitations not fully overcome by CoD data processing methods, and selected sources were excluded from analysis.

Modelling strategy

The estimation strategy used to estimate mortality due to appendicitis was not substantively changed between GBD 2021 and GBD 2023. A standard CODEm model was used to model deaths due to appendicitis (see appendix section on CODEm method for details). Models were generated separately for males in data-rich countries, males in all countries (referred to as a global model), females in data-rich countries, and females in all countries (global model). Fatal estimation for this cause was restricted to ages 1–95+ years.

The following table presents a full list of covariates presented to the CODEm algorithm for selection in models of appendicitis mortality.

Table 1. Covariates used in appendicitis mortality modelling

Level	Covariate	Direction
2	Age-sex-specific scaled exposure variable for low fruit consumption	+
	Age-sex-specific scaled exposure variable for low vegetable consumption	+
	Healthcare Access and Quality Index	-
3	Socio-demographic Index	-
	Education (years per capita)	-
	Log LDI ($I per capita)	-

We hybridised separate global and data-rich models to acquire unadjusted results, which we adjusted using CoDCorrect. Adjustment in CoDCorrect included rescaling unadjusted death estimates for appendicitis alongside other Level 3 digestive disease death estimates so that they summed to total Level 2 digestive disease deaths, which were then rescaled along with other causes in the hierarchy to ultimately sum to all-cause counts of death. Adjusted (post-CoDCorrect) deaths were then compared to the reference life table to calculate final YLLs due to appendicitis.

Ascariasis

Flowchart

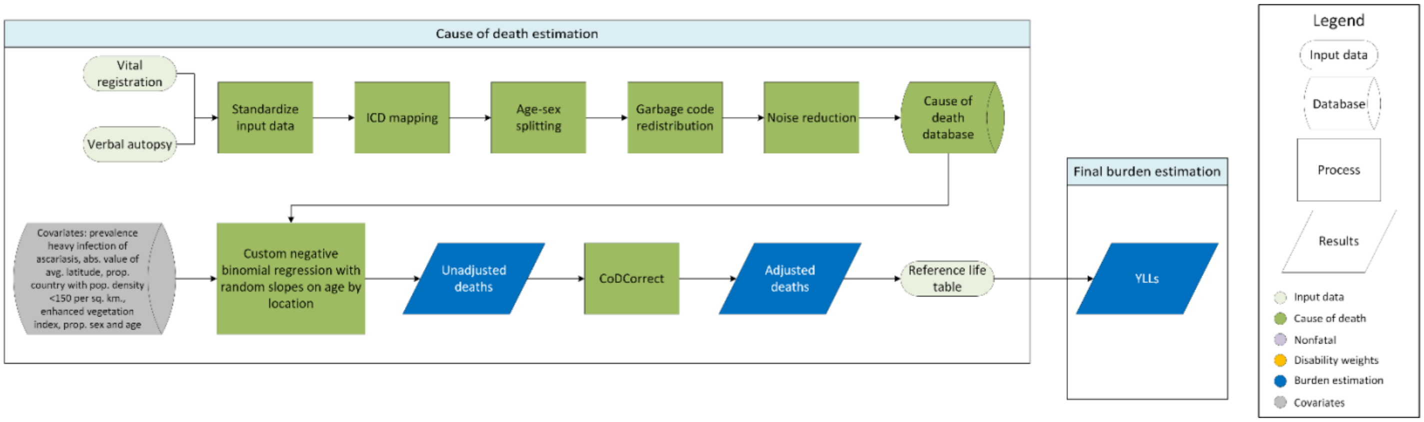

Input data and methodological summary for ascariasis

Input data

To estimate mortality due to ascariasis, country-year-age-sex-specific verbal autopsy and vital registration data were used. Covariates used included prevalence of heavy infection of ascariasis, the absolute value of average latitude, the proportion of the country with population density under 150 people per square kilometer, enhanced vegetation index, sex, and age.

Geographical restrictions

We conducted a literature review (last updated for GBD 2017) to determine the geographical extent of the disease and classify locations based on whether the disease is absent or present in each year. Locations that were geographically restricted in any given year did not have estimates made for them. Of note, we did not attempt a complete systematic review, since a single high-quality source could offer sufficient evidence of presence. Evidence of absence or presence was not available for every location for each year. Assumptions made for missing years took into consideration the epidemiological characteristics of the disease.

If evidence indicated disease presence for two non-consecutive years, we assumed presence for all years between the two. If evidence indicated disease absence for two non-consecutive years, we assumed absence for all years between the two. If evidence indicated a change in status (ie, from absent to present, or present to absent) between two non-consecutive years, then we conducted targeted searches to ascertain the relevant year of introduction or elimination for that location. In the cases where presence or absence information was missing for the start or end years of our study interval without evidence of any introduction or elimination events within the interval, we applied the status of the first and last presence/absence observations, respectively, to all years between the interval bound and the observation year. Table 1 shows the search strings and associated yield for each of the databases queried.

Table 1. Geographical restriction search strings

Database	Search string	Yield
PubMed	(Ascariasis[Title/Abstract] OR Ascaris[Title/Abstract] OR "A. lumbricoides"[Title/Abstract] OR Ascaris[MeSH] OR Trichuris[Title/Abstract] OR Trichuriasis[Title/Abstract] OR "Whip Worm"[Title/Abstract] OR "T. trichura"[Title/Abstract] OR Trichuris[MeSH] OR Hookworm[Title/Abstract] OR "A. duodenale"[Title/Abstract] OR "Ancylostoma duodenale"[Title/Abstract] OR ancylostomiasis[Title/Abstract] OR "N. americanus"[Title/Abstract] OR "Necator americanus"[Title/Abstract] OR necatoriasis[Title/Abstract] OR Ancylostoma [MeSH] OR Necator[MeSH]) AND (prevalence[Title/Abstract] OR incidence[Title/Abstract] OR epidemiology[Title/Abstract] OR surveillance[Title/Abstract]) NOT(Animals[MeSH] NOT Humans[MeSH])	2376
Web of Science	(Ascariasis OR Ascaris OR A. lumbricoides OR Trichuris OR Trichuriasis OR Whip Worm OR T. trichura OR Hookworm OR A. duodenale OR Ancylostoma duodenale OR anclyostomiasis OR N. americanus OR Necator americanus OR necatoriasis) AND TOPIC:(prevalence OR incidence OR epidemiology OR surveillance) NOTTOPIC: ((Animals NOT Humans))<br>Timespan: 1980-2016. Indexes: SCI-EXPANDED, SSCI, A&HCI, ESCI.	2266
SCOPUS	TITLE-ABS_KEY (ascariasis OR ascaris OR a. lumbricoides OR trichuris OR trichuriasis OR whip worm OR t. trichura OR hookworm OR a. duodenale OR ancylostoma duodenale OR anclyostomiasis OR n. americanus OR necator americanus OR necatoriasis) AND PUBYEAR>1979	29

These papers were used to classify location-years for all locations and years present in the literature. Additionally, systematic literature reviews, meta-analyses, national health statistics publications, and collaborator input were used to classify location-years not present in the literature review wherever possible.

Modelling strategy

A negative binomial model was used to estimate deaths from ascariasis with random intercepts for locations and random slopes for age groups by location. A multivariate normal distribution using the mean and variance-covariance matrix from the model was used to generate 1000 draws of deaths due to ascariasis. The final model was selected based on how well the estimated number fit the input data and how plausible the predicted distribution of disease was over time and with age.

Changes from GBD 2021 to GBD 2023

There were no substantive changes to the modelling strategy for GBD 2023.

Asthma

Flowchart

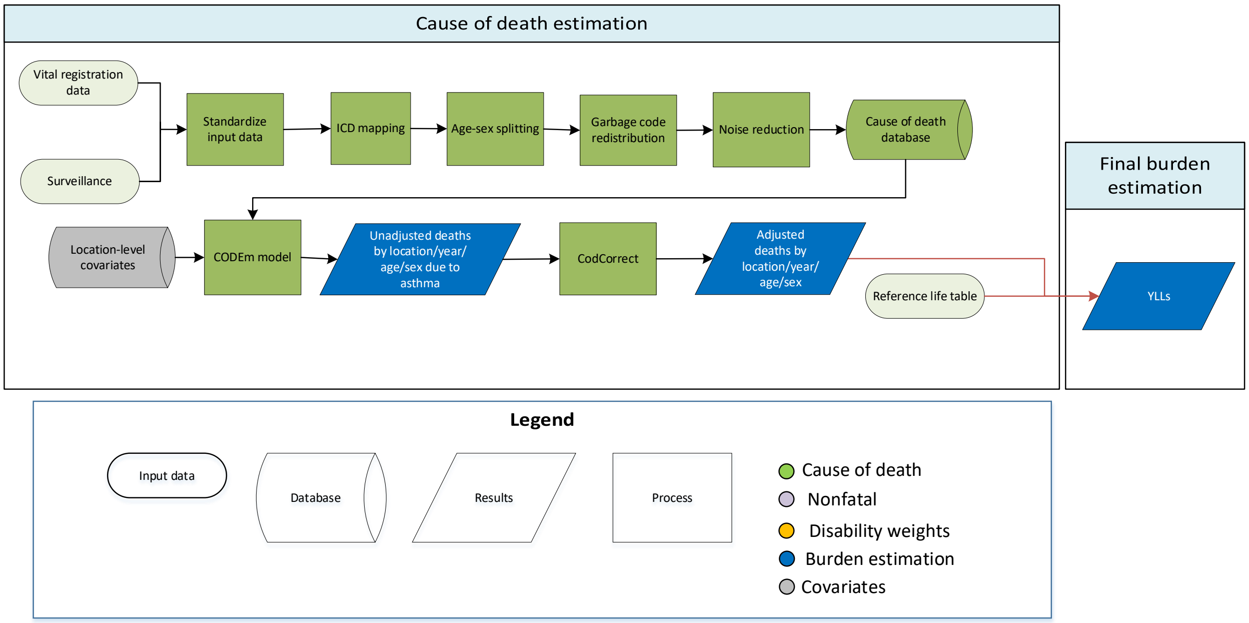

Input data and methodological summary for asthma

Input data

Data used to estimate asthma mortality included vital registration and surveillance data from the cause of death (COD) database. Verbal autopsy data were not included and were instead mapped to an overall chronic respiratory model. Our outlier criteria excluded datapoints that (1) were implausibly high or low relative to global or regional patterns, (2) substantially conflicted with established age or temporal patterns, or (3) significantly conflicted with other data sources conducted from the same locations or locations with similar characteristics (ie, Socio-demographic Index).

Modelling strategy

There were no substantive changes to the modelling approach this round. The standard Cause of Death Ensemble modelling (CODEm) approach was used to estimate deaths due to asthma. Separate models were conducted for male and female mortality, and the age range for both models was 1 to 95+ years.

The following table lists the covariates included in the model. This requires that the covariate selected for the model must have the directional relationship with chronic respiratory deaths. For GBD 2023, COVID-19-related covariates were tested but ultimately dropped from a final model because of inconclusive evidence about the association between COVID-19 and increased asthma mortality. Redundant covariates were dropped to improve model fitting in CODEm. Covariate directions were selected based on the strength of the evidence.

Table 1. Covariates used in asthma mortality modelling

Level	Covariate	Direction
1	Cumulative cigarettes (10 years)	+
	Cumulative cigarettes (20 years)	+
	Log-transformed SEV scalar: Asthma	+
2	Indoor air pollution (all cooking fuels)	+
	Outdoor air pollution (PM 2.5)	+
	Smoking prevalence	+
	Healthcare Access and Quality Index	-
3	Socio-demographic Index	-

Atrial fibrillation and flutter

Flowchart

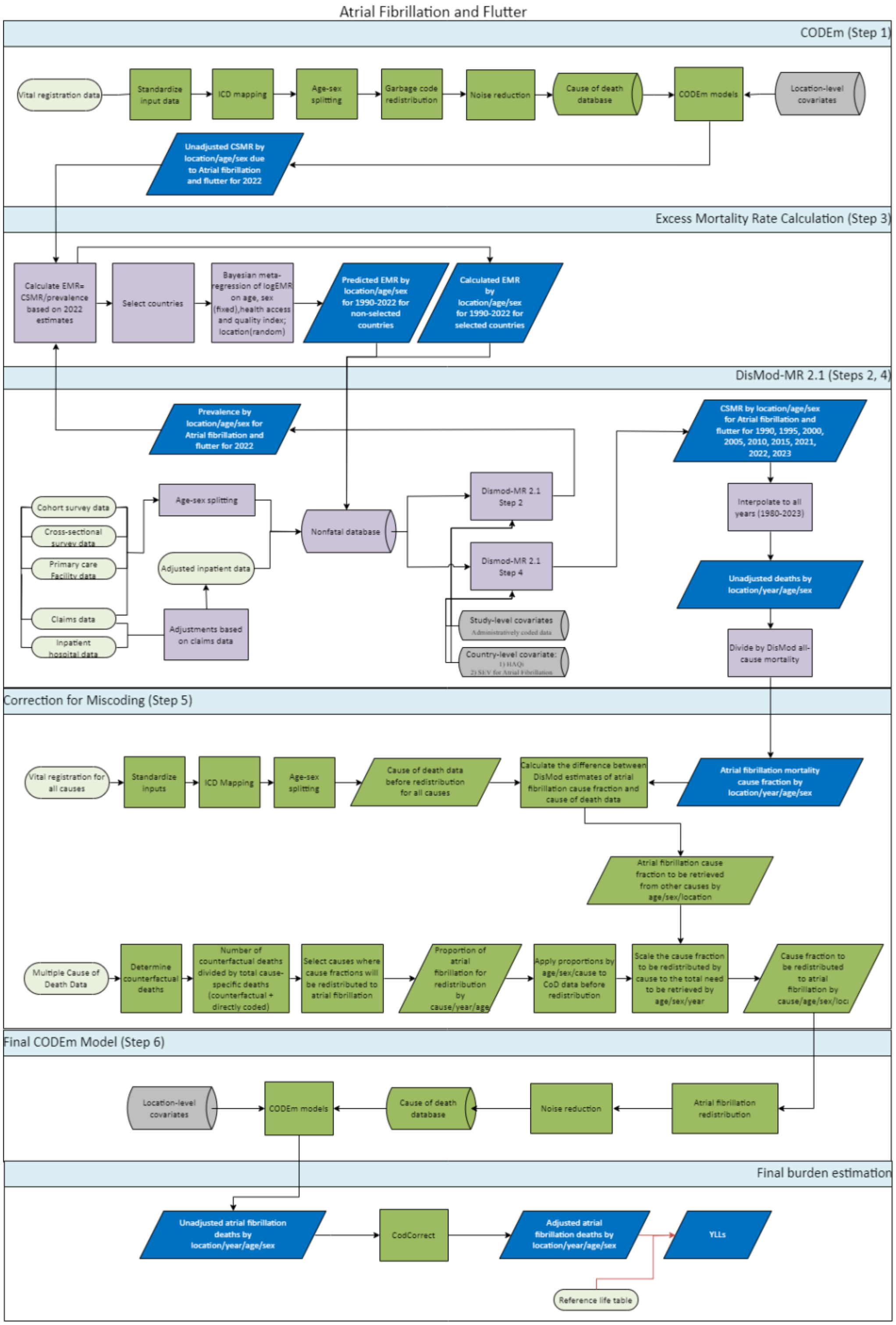

Input data and methodological summary for atrial fibrillation and flutter

Input data

Vital registration (VR) data: We outliered all ICD-8 and ICD-9 datapoints that were discontinuous from other data in the time series and created an unlikely time trend; ICD-9 data in Bulgaria and Poland were correspondingly outliered. We also outliered ICD-10 datapoints in American Samoa, Kyrgyzstan, Uzbekistan, Bahrain, Qatar, and Syria that were implausibly high compared to other locations in the region. Additionally, we outliered data from 2020 in Peru that were implausibly high compared to the rest of the time series for all ages.

Most data sources used for cause of death estimation are ICD-coded. The ICD periodically releases new versions, which can introduce an implausible disjoint in the time series that is indicative of a change in the coding practice, rather than a true change in the epidemiological trend of the disease. In GBD 2023, certain causes and countries using the ICD-9 code systems were adjusted to address the disjoint in the time trend and number of deaths between the older ICD-9 code systems and the more recent ICD-10 code systems. Atrial fibrillation and flutter was corrected in Bulgaria (1990–2004), Canada (1986–1999), Spain (1980–1998), France (1980–1999), the USA (1980–1998), Hungary (1980–1995), Croatia (1985–1994), the UK (1980–1999), Greece (1987–2008), Italy (1980–2002), Mexico (1980–2002), and Mauritius (1980–2004) to account for this code system disjoint. This was done using a meta-regression tool described as a crosswalk, by taking systematically biased datapoints and estimating their unbiased value. Using this modelling tool, correction factors were created based on the more recent and more reliable ICD-10 coded data. The crosswalk creates matched pairs between the last three years of data using the ICD-9 code system and the first three years of data using the ICD-10 code system. The correction is applied at the child cause level for all Level 2 causes, and after the correction is applied the individual child causes are raked to the parent cause to maintain a consistent number of deaths across the cause hierarchy.

Modelling strategy

In order to address large increases in deaths reports as due to atrial fibrillation, which we believe represent changes in coding practices and not a true rise in these deaths,[1] we used an integrated approach that combined DisMod-MR 2.1 and CODEm models to estimate deaths from atrial fibrillation and flutter. This approach allowed us to adjust estimates to more accurately reflect the number of deaths for which atrial fibrillation was the true underlying cause of death. The overarching strategy was designed to better inform estimated atrial fibrillation death estimates from vital registration with everything we know about atrial fibrillation prevalence, incidence, and survival from all other population-based sources.

The modelling steps are illustrated in the above flowchart. Covariates included in the DisMod-MR 2.1 and CODEm models can be found in the table below. In Step 1, we estimated deaths for atrial fibrillation using a standard CODEm approach with unadjusted vital registration data. In Step 2, we estimated prevalence rates in DisMod-MR 2.1 using data from published reports of cross-sectional surveys, cohort studies, and primary care facility data. In part, the stability of prevalence over time is what has informed our prior that atrial fibrillation deaths have not increased rapidly as suggested in the raw vital registration data. We also used claims data covering inpatient and outpatient visits for the USA along with inpatient hospital data from other locations. Inpatient hospital data were adjusted using age- and sex-specific information for: 1) readmission within one year; 2) primary diagnosis code to secondary

codes; and 3) the ratio of inpatient to outpatient visits. We set priors of no remission, excess mortality, or incidence prior to age 30.

In Step 3, we calculated the excess mortality rate (EMR) for 2022 (defined as the cause-specific mortality rate (CSMR) estimated from CODEm divided by the prevalence per capita estimated from DisMod-MR 2.1). We then selected 21 countries based on four conditions: 1) ranking of 4 or 5 stars on the system for assessing the quality of VR data; 2) prevalence data available from the literature were included in the DisMod-MR 2.1 model in Step 2; 3) prevalence per capita ≥0.005; and 4) CSMR ≥0.00002. Using information from these countries as input data, we ran a Bayesian meta-regression (MR-BRT) of logEMR on sex, age, Healthcare Access and Quality Index, and location. Sex, age, and Healthcare Access and Quality Index were treated as fixed effects for the regression, while location was considered a random effect. We then predicted age- and sex-specific EMR using the results of this regression for all non-selected countries. Countries included in the regression were assigned their directly calculated values. These EMR datapoints were assigned to the time period 1990–2022 and uploaded into the non-fatal database in order to be used in modelling.

In Step 4, we reran DisMod-MR 2.1 including the EMR estimated in Step 3 as input data using the same priors as in Step 2 to obtain CSMR estimates from DisMod-MR 2.1 that are consistent with the available data for incidence and prevalence.

In Step 5, the DisMod-MR 2.1 CSMR estimates were used to determine the correction needed for the VR data in the misdiagnosis correction step of the cause of death data processing. A full description of this process can be found in Section 2.6. Briefly, as DisMod-MR 2.1 only generates estimates for nine years (1990, 1995, 2000, 2005, 2010, 2015, 2021, 2022, 2023), the period 1990–2023 was interpolated using a log-linear approach. Estimates for 1980–1989 were generated via regression on the entire time series, using Socio-demographic Index as a predictor. Next, the difference between the cause fraction estimated by DisMod-MR 2.1 and the cause fraction in the VR data was calculated. This yielded the cause fraction that would need to be retrieved from other causes. After this process of reassigning deaths, the cause fraction data are processed through the standard redistribution and noise reduction processes.

In Step 6, these adjusted cause fraction data are then used as inputs for a final CODEm model using the covariates listed in the table below. For GBD 2023, we ran CODEm for two distinct age-stratified models to capture the different relationships of covariates according to age. Global and data-rich CODEm models were run separately for age groups 30–49 and 50+ using differing covariates. Covariates chosen for the 30–49 CODEm model were those related to developing atrial fibrillation and flutter in younger ages, including alcohol use and congenital heart disease.[2] The covariates can be found in table 1a and 1b below. Since GBD 2021, CODEm has also switched from using an ensemble of rate and cause-fraction models to only rate models. The results from the CODEm model are processed through CoDCorrect; these post-CoDCorrected results are the final estimates for cause-specific mortality for atrial fibrillation and flutter.

Table 1a. Covariates used in atrial fibrillation and flutter mortality modelling ages 30–49

Level	Covariate	Direction
1	Smoking prevalence	1
	Systolic blood pressure (mmHg)	1
2	Fasting plasma glucose	1
	Percentage of births in over 35-year-olds	1
	Alcohol consumption during pregnancy	1
	Birth prevalence of congenital heart disease	1
3	Socio-demographic Index	–1
	Alcohol (litres per capita)	1
	Healthcare Access and Quality Index	–1

Table 1b. Covariates used in atrial fibrillation and flutter mortality modelling ages 50+

Level	Covariate	Direction
1	Summary exposure variable, atrial fibrillation	1
	Smoking prevalence	1
	Systolic blood pressure (mmHg)	1
2	Mean BMI	1
	Fasting plasma glucose	1
	Healthcare Access and Quality Index	–1
	Cholesterol (total, mean per capita)	1
3	Log-transformed lag distributed income per capita (I$)	–1
	Socio-demographic Index	–1
	Summary exposure value, omega-3	1
	Summary exposure value, fruits	1
	Summary exposure value, vegetables	1
	Summary exposure value, nuts and seeds	1
	Pulses/legumes (kcal/capita, unadjusted)	–1
	Summary exposure value, PUFA	1
	Alcohol (litres per capita)	1
	Trans fatty acid	1

DisMod-MR 2.1 Covariates – Step 2

Covariate	Parameter	Beta	Exponentiated beta
SEV scalar: Atrial fibrillation	Prevalence	0.91 (0.86 to 0.95)	2.48 (2.36 to 2.60)
Healthcare Access and Quality Index	Excess mortality rate	–0.12 (–0.13 to –0.11)	0.89 (0.87 to 0.90)

DisMod-MR 2.1 Covariates – Step 4

Covariate	Parameter	Beta	Exponentiated beta
SEV scalar: Atrial fibrillation	Prevalence	0.77 (0.75 to 0.80)	2.16 (2.12 to 2.21)
Healthcare Access and Quality Index	Excess mortality rate	–0.005 (–0.0051 to –0.0049)	1.00 (0.99 to 1.00)

References

1 Duncan ME, Pitcher A, Goldacre MJ. Atrial fibrillation as a cause of death increased steeply in England between 1995 and 2010. *Europace*. 2014 Jun;16(6):797-802. doi: 10.1093/europace/eut388. Epub 2013 Dec 30. PMID: 24381330.

2 Aggarwal N, Selvendran S, Raphael CE, Vassiliou V. Atrial Fibrillation in the Young: A Neurologist's Nightmare. *Neurol Res Int*. 2015;2015:374352. doi: 10.1155/2015/374352. Epub 2015 Apr 2. PMID: 25922764; PMCID: PMC4398952.

Bacterial skin diseases

Flowchart

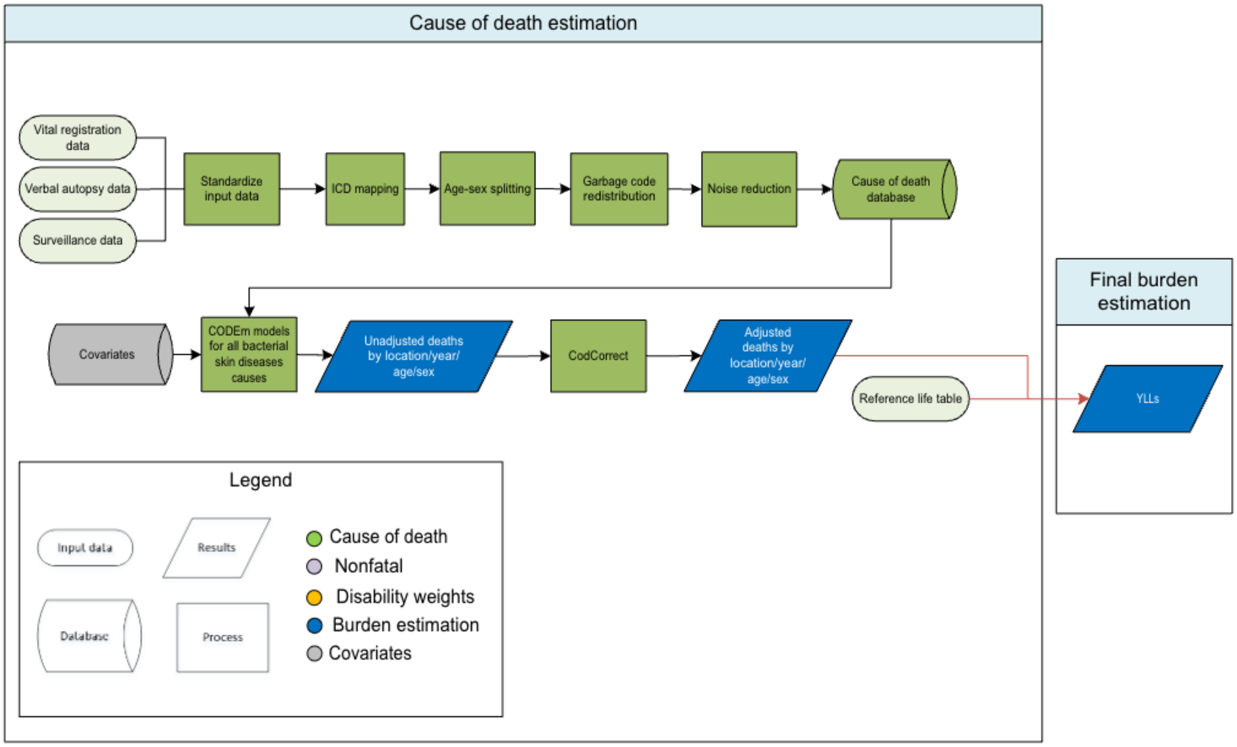

Input data and methodological summary for bacterial skin diseases

Input data

Data sources used to estimate mortality due to bacterial skin diseases consisted of vital registration, verbal autopsy, and surveillance data from the cause of death (CoD) database. Outlier criteria excluded datapoints that were implausibly under/over-reporting relative to global or regional patterns and data from datasets with small populations.

Modelling strategy

The bacterial skin diseases model is estimated by CODEm for the parent cause as well as a CODEm model for each of the child causes: pyoderma and cellulitis. For all these models, standard CODEm parameters were used along with the CoD database and location-level covariates as inputs. During CoDCorrect, all the child causes get squeezed to the parent cause.

Compared to GBD 2021, there are several updates that were made to the bacterial skin diseases estimation strategy. First, we have received new data for bacterial skin diseases for multiple location-years. There were 131 data sources added to the cause of death database, which led to improved estimates, particularly for recent years. Key location-years that impacted the bacterial skin disease estimates were from Bangladesh, the Philippines, India, Malaysia, Pakistan, the USA, China, and Brazil.

Additionally, in GBD 2023, CODEm began using an all-cause mortality envelope that is inclusive of HIV, whereas in previous rounds, the all-cause mortality envelope was HIV-free. This can lead to changes in cause fractions and rates when compared to previous GBD rounds.

Table 1. Covariates used in bacterial skin diseases mortality modelling

Level	Covariate	Direction
1	Summary exposure value (SEV) scalar for unsafe sanitation	+
	Prevalence of overweight and obesity	+
	Healthcare Access and Quality Index	-
	Diabetes fasting plasma glucose (mmol/L), by age	+
	Improved water source (proportion with access)	-
2	Alcohol (litres per capita)	+
	Cumulative cigarettes (5 years)	+
	Cumulative cigarettes (10 years)	+
	Smoking prevalence	+
3	Education (years per capita)	-
	Lag-distributed income (per capita)	-
	Socio-demographic Index	-

Table 2. Covariates used in pyoderma mortality modelling

Level	Covariate	Direction
1	Improved water source (proportion with access)	-
	Prevalence of overweight and obesity	+
	Healthcare Access and Quality Index	-
	Diabetes fasting plasma glucose (mmol/L), by age	+
	Unsafe sanitation (summary exposure value)	+
2	Alcohol (litres per capita)	+
	Cumulative cigarettes (10 years)	+
	Cumulative cigarettes (5 years)	+
	Smoking prevalence	+
3	Lag-distributed income (per capita)	-
	Education (years per capita)	-
	Socio-demographic Index	-

Table 3. Covariates used in cellulitis mortality modelling

Level	Covariate	Direction
1	Healthcare Access and Quality Index	-
	Diabetes fasting plasma glucose (mmol/L), by age	+
	Prevalence of overweight and obesity	+
2	Lag-distributed income (per capita)	-
3	Education (years per capita)	-

Cancers

Flowchart

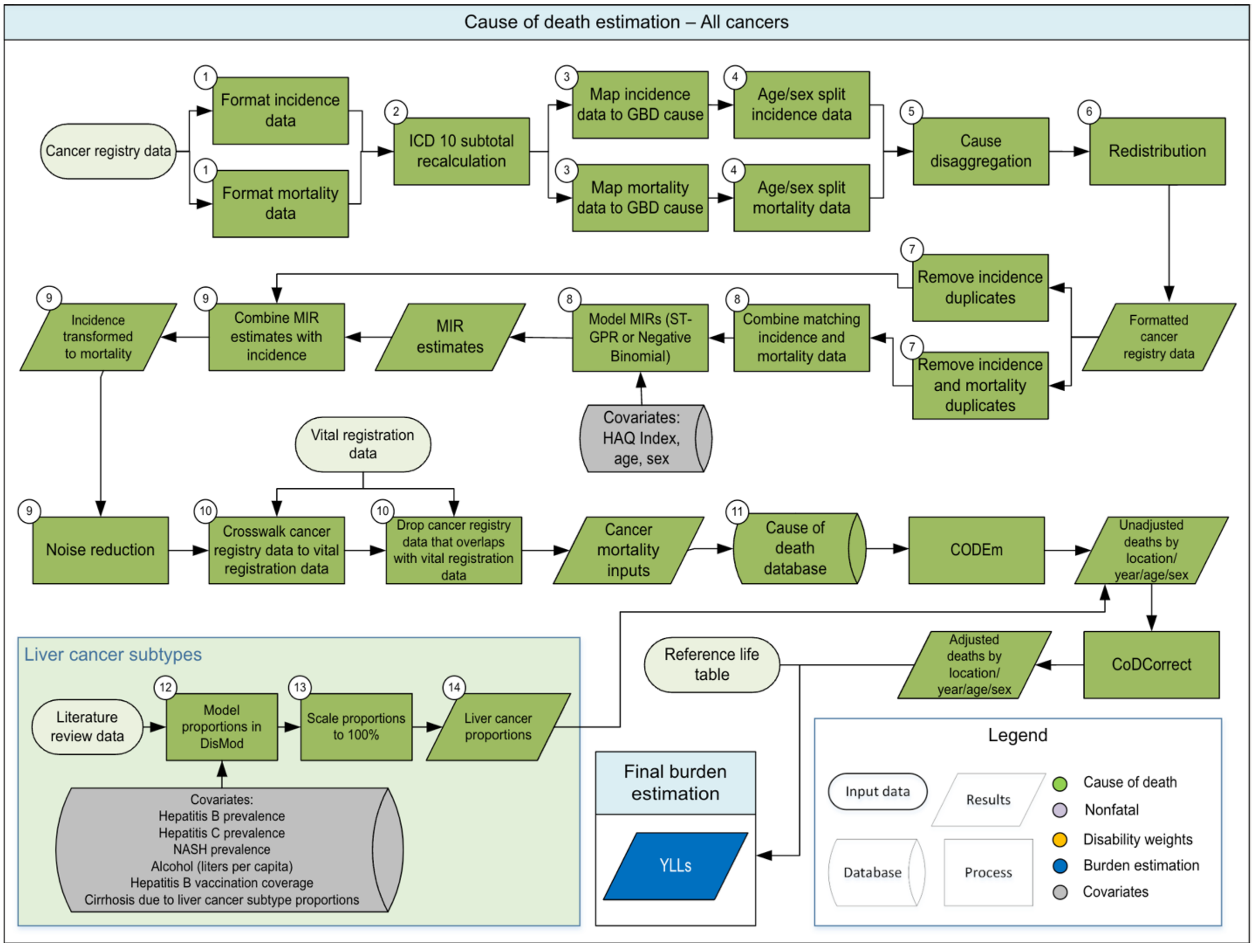

Figure 1: Flowchart of cause of death estimation for all cancers

Abbreviations: CODEm, cause of death ensemble model; DisMod-MR, disease model – Bayesian meta-regression; GBD, Global Burden of Disease Study; HAQ Index, Healthcare Access and Quality Index; ICD, International classification of diseases; MIR, mortality-to-incidence ratio; NASH, non-alcoholic steatohepatitis; ST-GPR, spaciotemporal Gaussian process regression; YLLs, years of life lost

Input data and methodological summary for all cancers except for non-melanoma skin cancer

Input data

The GBD cause of death (CoD) database contains cancer mortality data originating from multiple sources, including vital registration (VR), verbal autopsy (VA), and cancer registry (CR) data. The cancer registry mortality estimates that are uploaded into the CoD database stem from cancer registry incidence data that have been transformed to mortality estimates through the use of mortality-to-incidence ratios (MIRs).

Data-seeking processes

Cancer mortality data in the cause of death database other than cancer registry data

Sources for cancer mortality data other than cancer registry data are described in the CoD section of the Capstone Appendix.

Cancer registry data

We used data from GBD 2021 and added cancer registry data from Benin, China, Congo, Côte d'Ivoire, Eswatini, Ghana, Kenya, Lithuania, Mali, Mozambique, Niger, Nigeria, North Macedonia, Spain, Tanzania, Türkiye, Uganda, and Zambia.

Inclusion and exclusion criteria

Only population-based cancer registries were included, with inclusion criteria that they included all cancers (ie, were not specialty registries for a subset of cancer types), reported data for all age groups (with the exception of paediatric cancer registries), and reported data for both sexes. Pathology-based and hospital-based cancer registries were excluded. Redundant cancer registry data were excluded from either the final incidence data input or the MIR model input if a more detailed source (eg, providing more detailed age or diagnostic groups) was available for the same population. Preference was given to registries with national coverage over those with subnational coverage, except those from countries where the GBD study provides subnational estimates. Data were excluded if the coverage population was unknown, except for in high Socio-demographic Index (SDI) quintile locations with full geographical coverage where the GBD estimated population could be substituted.

Bias of categories of input data

Cancer registry data can be biased in multiple ways. A high proportion of ill-defined cancer cases in the cancer registry data requires redistribution of these cases to other cancers, which introduces a potential for bias. Changes between coding systems can lead to artificial differences in disease estimates; however, we adjust for this bias by mapping the different coding systems to GBD cancer causes. Incomplete overlap of different coding systems and data sources can also lead to artificial shifts in the estimates over time, which we lessen through temporal smoothing and crosswalk adjustments. Underreporting of cancers that require advanced diagnostic techniques (eg, leukaemia, brain, pancreatic, and liver cancer) can be an issue in cancer registries from lower-income countries. On the other hand, misclassification of metastatic sites as primary cancer can lead to overestimation of cancer sites that are common sites for metastases (eg, brain or liver cancer). Since many cancer registries are located in urban areas, the representativeness of the registry for the general non-urban population can also be problematic. The accuracy of mortality data reported by a subset of cancer registries usually depends on the quality of the vital registration system. If the vital registration system is incomplete or of poor quality, the mortality-to-incidence ratio can be biased to lower ratios.

Data for liver cancer aetiology splits

Additional data are utilised to proportionally split the age ≥10 years liver cancer total (which excludes hepatoblastoma) into the five aetiology groups included in the GBD: 1) liver cancer due to hepatitis B, 2) liver cancer due to hepatitis C, 3) liver cancer due to alcohol, 4) liver cancer due to non-alcoholic steatohepatitis (NASH), and 5) liver cancer due to other causes. To find data to inform these

proportions, a systematic literature search was performed in PubMed on 8/13/2015 using the following search string:

> “("liver neoplasms"[All Fields] OR "HCC"[All Fields] OR "liver cancer"[All Fields] OR "Carcinoma, Hepatocellular"[Mesh]) AND (("hepatitis B"[All Fields] OR "Hepatitis B"[Mesh] OR "Hepatitis B virus"[Mesh] OR "Hepatitis B Antibodies"[Mesh] OR "Hepatitis B Antigens"[Mesh]) OR ("hepatitis C"[All Fields] OR "Hepatitis C"[Mesh] OR "hepatitis C antibodies"[MESH] OR "Hepatitis C Antigens"[Mesh] OR "Hepacivirus"[Mesh]) OR ("alcohol"[All Fields] OR "Alcohol Drinking"[Mesh] OR "Alcohol-Related Disorders"[Mesh] OR "Alcoholism"[Mesh] OR "Alcohol-Induced Disorders"[Mesh])) NOT (animals[MeSH] NOT humans[MeSH])”.

Studies were included if they provided proportion data that were representative for the location covered. Several studies not initially found through this search were added because they were included in the meta-analysis by de Martel and colleagues.[1] We also included the study by Hong and colleagues after the authors provided us with additional data on the overlap in aetiologies.[2]

For each study, the proportions of liver cancer due to each of the five specific aetiologies were calculated. Cases were considered to be due to NASH when the manuscript explicitly listed the aetiology to be NASH or non-alcoholic fatty liver disease (NAFLD). Cases where the aetiology was listed as “cryptogenic”, “idiopathic”, or “unknown” were assessed as either implicitly NASH (included within the “NASH” category when alcohol, hepatitis B, and hepatitis C were reported), or of ambiguous aetiology (included within the “other causes” category when any of alcohol, hepatitis B, or hepatitis C could not be ruled out). These implicit NASH proportions were adjusted to the explicit NASH proportions using the GBD meta-regression—Bayesian, regularised, trimmed (MR-BRT) method, which is detailed elsewhere in the GBD summary papers. In manuscripts where the aetiology for a case was not known but major categories could not be ruled out (for example, if the study tested for hepatitis B and C but did not assess alcohol use), only the explicitly defined proportions were included (in this example, including proportions for hepatitis B and C, and excluding any remainder). Any remaining named aetiologies were included under a combined “other” group (for example, haemochromatosis, autoimmune hepatitis, Wilson’s disease, etc.). If multiple aetiologies were reported for any cases, these were assigned proportionally to the individual aetiologies reported in the study. Sex-combined sources were split into sex-specific proportions using the GBD MR-BRT method detailed elsewhere the GBD summary papers.

Modelling strategy

Steps of analysis and data transformation processes

Cancer registry data go through multiple processing steps before entering the CoD database.

1. Format incidence and mortality data. First, the original data are transformed into standardised files, which includes standardisation of format, categorisation, and registry names. See #1 in the flowchart.

2. Subtotal recalculation. Some cancer registries report individual codes as well as aggregated totals. An example of this would be where the registry data report C18, C19, and C20 individually, and also the aggregated group of C18–C20 (colon and rectum cancer). The data processing step “subtotal recalculation” verifies these totals and subtracts the values of any individual codes from the aggregates so that no cases and deaths are counted more than once between specific codes and aggregated groupings. See #2 in the flowchart.

3. Map data to GBD causes. Cancer registry incidence data and cancer registry mortality data are mapped to GBD causes. A different map is used for incidence and for mortality data because of different assumptions around misclassification of ICD 'D' prefix codes. For incidence data, these D codes are assumed to be correctly assigned to incident cases and are not mapped to a GBD cause because they are not malignant cancers. In contrast, for mortality data these D codes do get mapped to the GBD cause, because it is assumed that a death that was assigned a 'D' code was misclassified and should have been assigned the relevant 'C' code instead. The maps also differ because of the assumption that there are no deaths for certain cancers. One example is basal cell carcinoma of the skin. In the cancer registry incidence data, basal cell carcinoma is mapped to non-melanoma skin cancer (basal cell carcinoma). However, if basal cell carcinoma is recorded in the cancer registry mortality data, the deaths are instead mapped to non-melanoma skin cancer (squamous cell carcinoma) under the assumption that they were actually misclassified squamous cell skin cancers. Another example is benign or in situ neoplasms. Because cancer registries do not collect non-malignant neoplasms in a standardised way, any benign or in situ neoplasms reported in a cancer registry incidence dataset are excluded. The same neoplasms reported in a cancer registry mortality dataset are instead mapped to the respective invasive cancer. For example, cases of 'ductal carcinoma in situ' in a cancer registry incidence dataset are excluded, while deaths from 'ductal carcinoma in situ' in a cancer registry mortality dataset are mapped to breast cancer. The exception is for codes for benign tumours of the brain, which are mapped to 'other benign and in situ neoplasms' rather than brain cancer, as these non-invasive tumors can still cause death through intracranial pressure. See #3 in the flowchart.

4. Age/sex splitting. Cancer registry data are standardised to the GBD age groups. For each cancer, the minimum age group estimated was determined as the youngest age group where SEER reported at least 50 cases over the period 1990 to 2015.[3] Reference global age-specific incidence rates were generated using select comprehensive cancer registry datasets, such as from SEER,[4] NORDCAN,[5] CI5,[6–16] and IICC.[17] Reference age-specific mortality rates were generated using age weights from CoD VR data, as described elsewhere in the GBD summary papers. For incidence or mortality datasets that require age splitting, global age-specific proportions are then generated by applying the age-specific rates to the overall registry population to produce the expected number of cases (or deaths for a mortality dataset) for that registry by age. The expected number of cases (or deaths) for each sex, age, and cancer were normalised to 1, creating final, age-specific proportions. These proportions were then applied to the total number of cases (or deaths) by sex and cancer to get the GBD age group-specific number of cases (or deaths) related to that dataset. See #4 in the flowchart.

In the rare case that the cancer registry only contains data for both sexes combined, the age-specific cases or deaths are split and reassigned to separate sexes using the same weights that are used for the age splitting process. Starting from the expected number of deaths, global proportions are generated by sex for each age. For example, if for ages 15–19 years old there are 6 expected deaths for males and 4 expected deaths for females from cause of death data, then 60% of the combined-sex deaths for ages 15–19 years would be assigned to males and the remaining 40% would be assigned to females.

5. Cause disaggregation. Data for cause entries that are aggregates of GBD causes were redistributed across those GBD causes. Examples of these aggregated causes include some cancer registries reporting ICD-10 codes C00–C14 together as 'lip, oral cavity, and pharyngeal cancer'. These groups are broken down into sub-causes that can be individually mapped to single GBD causes. In this example, the more specific ICD-10 codes within C00–C14 are 'lip and oral cavity cancer' (C00–C08), 'nasopharyngeal cancer'

(C11), 'cancer of other parts of the pharynx' (C09–C10, C12–C13), and 'malignant neoplasm of other and ill-defined sites in the lip, oral cavity, and pharynx' (C14). To redistribute the data, weights were created using the same "rate-applied-to-population" method employed in age/sex splitting (see step four above). For the undefined code (C14 in the example) an "average all cancer" weight was used, calculated on the high-quality cancer registry data from SEER,[3] NORDCAN,[5] and CI5[6–16] by dividing the sum of the cases across these registries by the combined population across these registries. Then, proportions were generated by sub-cause for each aggregate cause as in the sex splitting example above (see step 4). The total number of cases from the aggregated group (C00–C14) was recalculated for each subgroup and the undefined code (C14). C14 was then redistributed as a "garbage code" in step six. For some exceptions, C44 (non-melanoma skin cancer), C46 (Kaposi sarcoma), and C74 (malignant neoplasm of adrenal gland), fixed proportions were used to redistribute into GBD causes. Non-melanoma skin cancer processing is described below under the section specific to these cancers. C46 entries were proportionally redistributed to either HIV or to the GBD cause 'soft tissue and other extraosseous sarcomas', with the redistribution proportion varying by GBD region (ranging from 7.7% to HIV in the south Asia region to 93% HIV in the southern sub-Saharan Africa region); this proportion was an update from GBD 2021 where the proportion was based on age, and led to fewer C46 deaths being redistributed to HIV than in previous GBD rounds. C74 entries were redistributed to 'neuroblastoma and other peripheral nervous cell tumours' and 'other malignant neoplasms', with percentages varying by age (younger age groups have a greater proportion redistributed to 'neuroblastoma and other peripheral nervous cell tumours'). See #5 in the flowchart.

6. Redistribution. Unspecified ICD codes ("garbage codes") such as "ill-defined cancer site" (for example, C76 or C80) are redistributed across relevant causes estimated within the GBD hierarchy. Redistribution of cancer registry incidence and mortality data mirrored the redistribution process and maps used in the cause of death database, as detailed elsewhere in the GBD summary papers. See #6 in the flowchart.

7. Removal of duplicates. Duplicate or redundant data sources were removed from the processed cancer registry datasets. Duplicate sources were present if, for example, a cancer registry was part of the CI5[6–16] database but we also had data from that registry directly. Redundancies occurred and were removed as described in the "Inclusion and exclusion criteria" section above, where more detailed data were available, or when national registry data could replace regionally representative data. From here, two parallel selection processes were run: one to generate input data for the mortality-to-incidence ratio (MIR) models, and one to generate incidence for final mortality estimation. When creating the final incidence input, higher priority was given to registry data from the most standardised source, whereas for the MIR model input, only sources that reported both incidence and mortality were used. See #7 in the flowchart.

8. Combine matching incidence and mortality data and model MIRs. The processed incidence and mortality data from cancer registries were matched by cancer, age, sex, year, and location to generate MIRs. These MIRs were used as input for further modelling, using one of two approaches, depending on the cancer. See #8 in the flowchart

As in previous GBD cycles, MIRs for most cancers were estimated with a three-step modelling approach using the general GBD spatiotemporal Gaussian process regression (ST-GPR) method. The first step used a linear model for logit-transformed MIR as the outcome, with covariates for sex, categorical age group

(number of categories depending on cancer type), and Healthcare Access and Quality (HAQ) Index as a covariates.[18] For the MIRs, this initial linear prior used the following general form:

$$\text{logit}\left(MIR_{c,a,s,t}\right) = \alpha + \beta_1(HAQIndex)_{c,t} + \beta_2\mathrm{I}_s + \sum_{a}^{A}\beta_a\mathrm{I}_a + \epsilon$$

MIR: mortality-to-incidence ratio
c: country (or subnational for subnationally modelled locations), a: age group, t: time (years); s: sex
HAQIndex: Healthcare Access and Quality Index
I: indicator variable
ϵ: error term

Results from the final linear model were used as input for spatiotemporal smoothing and a Gaussian process regression. The ST-GPR model has three main hyperparameters that control for smoothing across time, age, and geography.[19] These hyperparameter values were iteratively evaluated for balancing model performance with local data availability in GBD 2019, and were not changed for GBD 2023. The time adjustment parameter lambda (λ) aims to borrow strength from neighbouring time points (ie, the value in this year is highly correlated with the value in the previous year but less so further back in time) and was set to 0.05. The age adjustment parameter omega (ω) borrows strength from data in neighbouring age groups and was set to 0.5. The space adjustment parameter zeta (ξ) aims to borrow strength across the hierarchy of geographical locations and was set to 0.01. For the remaining parameters in the Gaussian process regression, we set amplitude to 1 (influences fluctuation from the mean function) and set the scale value to 10 (influences the time distance over which points are correlated). Additional details on ST-GPR are described elsewhere in the GBD summary papers. These models were used to obtain MIR estimates for all combinations of GBD cause, age group, sex, year, and location. Datapoints were outliered manually if they clearly influenced the model in an unrealistic way. For example, a datapoint was marked as an outlier if it created a single year, single age group spike in model predictions that was inconsistent with the trend suggested by surrounding datapoints.

As in GBD 2021, a subset of cancers used a different approach than ST-GPR to model MIRs because of differences in data availability, the age distribution of cases and deaths, the reliability of the modelled estimates, and the GBD cause hierarchy. For retinoblastoma and hepatoblastoma, we modelled MIRs using a negative binomial regression approach for GBD 2023. The negative binomial approach was used for these two cancers causes because it allows modelling of count data with overdispersion (meaning the mean and variance are allowed to differ in the underlying distribution), which was determined to be needed due to the relatively rare deaths for these cancer causes. MIRs were estimated for each age-sex-year-location using a negative binomial regression run in R (version 4.4.0) using glm.nb from the MASS package. We used categorical age and HAQ Index as covariates and offset by the logarithm of cases. For five additional cancers that had been modelled using negative binomial models in GBD 2021, in GBD 2023 these were able to be modelled using the ST-GPR methods described above due to greater data availability in this round.

New for GBD2023, we processed incidence and mortality data from paediatric-specific cancer registries independent of the all-ages cancer registries and included them in our MIR models, allowing for more accurate paediatric estimates. For all causes that existed in GBD 2021, data-cleaning steps for MIR estimation were similar as for GBD 2021. For each cancer, MIRs from locations in HAQ Index quintiles 1–

4 were dropped if they were below the median of MIRs from locations in HAQ Index quintile 5. We also dropped MIRs from locations in HAQ Index quintiles 1–4 if the MIRs were above an outlier threshold calculated as the third quartile + 1.5 * IQR (inter-quartile range). We dropped all MIR data that were based on fewer than 15 incident cases to avoid excessive variation in the ratio due to small numbers. An exception to this threshold was made for mesothelioma, acute myeloid leukaemia and, new this round, acute lymphoid leukaemia, where instead we dropped MIRs that were based on fewer than ten cases because of lower data availability for these three cancers. For the lower end of the age spectrum where cancers are generally rarer, we previously aggregated incidence and mortality to the youngest five-year age bin where SEER[3] reported at least 50 cases from 1990 to 2015, to avoid unstable MIR predictions in young age groups because of too few cases or deaths. For GBD 2023, instead of using the SEER-based age threshold, we allowed age groups with greater than ten observations and more than one unique location to retain their age-specific data and be modelled independently. However, below this new threshold, the MIR estimates were then copied down to all younger GBD age groups estimated for that cancer.

For the nine new cancer causes first estimated in GBD 2021, additional data processing steps were used to help stabilise the input data and MIR estimates. First, for retinoblastoma and hepatoblastoma, data were aggregated across sexes and across bins of ten calendar years. Data were then only excluded if there were less than 0.01 cases since such small values were a result of redistribution and were leading to implausibly high MIRs. As cancer registry mortality data were limited for retinoblastoma, we supplemented with mortality data from vital registration systems. For this cause, cancer registry incidence was matched with vital registration mortality by age-sex-year-location. These cancer registry–vital registration matched inputs were processed the same as the standard matched inputs.

Since MIRs can be above 1, especially in older age groups and for cancers with low cure rates, we used the 95th percentile (by age group) of the cleaned dataset (detailed above) to cap the MIR input data. These "upper cap" values were used to allow MIRs over 1 in some age groups but to constrain the MIRs to a maximum level. The addition of new data for GBD 2023 led to slightly different upper caps compared to GBD 2021 (see upper cap values for GBD 2023 below). For GBD 2023, we no longer set a minimum upper cap of 1 (regardless of the 95th percentile) for paediatric age groups (under 20 years).

**Age group (years)**	**0–4**	**5–9**	**10–14**	**15–19**	**20–24**	**25–29**	**30–34**	**35–39**	**40–44**	**45–49**
**Upper cap**	0.678	0.750	0.793	0.961	0.954	0.798	0.752	0.812	0.859	0.866

**Age group (years)**	**50–54**	**55–59**	**60–64**	**65–69**	**70–74**	**75–79**	**80–84**	**85–89**	**90–94**	**95+**
**Upper cap**	0.893	0.925	0.973	1.02	1.06	1.16	1.28	1.41	1.61	1.84

Any MIR values over this upper cap were winsorised to the cap value. To run the logit model in ST-GPR, the input data were first divided by the upper caps to get proportional data ranging from 0 to 1. Model predictions from ST-GPR were then rescaled back to MIRs by multiplying the scaled predictions by the

upper caps. To constrain the MIRs at the lower end, we used the fifth percentile of the cancer and age-specific cleaned MIR input data to winsorise all model predictions below this lower cap.

9. Generate mortality estimates from incidence and MIRs. Final estimated MIRs were matched with the cleaned cancer registry incidence dataset finalised in the seventh step (#7 in the flowchart) to generate mortality estimates:

$$MIR_{estimates} * \ incidence_{registry} = mortality_{CR\ inputs}$$

These mortality estimates were then smoothed by a Bayesian noise-reduction algorithm to deal with zero counts; this is also applied to the CoD data inputs (VR and VA data), as detailed elsewhere in the GBD summary papers. See #9 in the flowchart.

10. Compositional bias and crosswalk approach. Compositional bias can occur when data from multiple reporting systems are used across time, for example cancer registry (CR) and vital registration (VR) data. When time trends switch between reporting systems, a disjoint can occur that is an artifact of underlying differences between the reporting systems (eg, how cancer data are collected, reported, coded, etc.) rather than actual changes in cancer mortality that occurred over time. In GBD 2023, we newly applied a crosswalk using RegMod (more information here: https://github.com/ihmeuw-msca/regmod) that takes systematically biased datapoints and estimates their unbiased value. This crosswalk adjustment was applied to the CR data, leveraging the gold-standard VR data to provide more appropriate time trends.

To correct for compositional bias due to systematic differences between CR and VR data, this crosswalk approach was applied to all causes and locations with CR data. The crosswalk method applied depended on the number of years of overlapping CR and VR data for a given location. For those locations with greater than two years of overlap, a spline method was used; for those locations with less than two years of overlap, a monotonic method was used; for those locations that only had CR data (ie, no VR data) or the model did not converge, the pre-crosswalked CR data was retained. Pre-crosswalked CR data was also retained in the locations where the only VR data available was from 2019 or later, due to concerns that effects of the COVID-19 pandemic made the VR data less reliable to use for crosswalk purposes.

Compositional bias can also occur when there are multiple data sources for the same point in time. Once crosswalking was complete, CR data that had a direct overlap in the VR timeseries was dropped. This process substantially decreased the amount of CR data that directly contributed to mortality estimates, which is reflected in the lower source counts compared to previous rounds. The CR data remaining after this exclusion thus reflects CR contributions to mortality beyond the data from VR systems. See #10 in the flowchart.

11. Upload CR data to CoD database. These data were then uploaded into the CoD database as CR data. See #11 in the flowchart.

Cancer-specific mortality modelling then followed the general CODEm process using the totality of VA, VR, and CR data inputs.

Liver cancer aetiology split models

The proportion data from the liver cancer systematic literature review (see section above "Data for liver cancer etiology splits") were used as input for five separate DisMod-MR 2.1 models to determine the proportion of liver cancers due to the five aetiologies for all locations, sexes, years, and GBD age groups (see #12 in the flowchart). For liver cancer due to alcohol, a prior value of 0% was set for ages 0 to 5 years. For liver cancer due to hepatitis B and hepatitis C, a prior value of 0% was set between ages 0 and 0.01 years. The covariates used differed by aetiology model and direction. The liver cancer due to alcohol model included positive covariates (with the beta prior minimum set at zero) for the litres of alcohol consumed per capita and the age-standardised proportion of alcohol drinkers. The liver cancer due to hepatitis B model included a positive covariate for age-standardised vaccine-adjusted HBsAg seroprevalence and a negative covariate for ten-year lagged hepatitis B three-dose vaccine coverage. The liver cancer due to hepatitis C model included a positive covariate for age-standardised chronic hepatitis C. The liver cancer due to NASH model included positive covariates for mean body-mass index, the age-standardised prevalence of obesity, and the prevalence of NASH and non-alcoholic fatty liver disease.

Since the five aetiology proportion models were run independently of each other, the final proportion estimates were scaled to sum to 100% within each age, sex, year, and location, by dividing each proportion by the sum of the five (see #13 in the flowchart). For the liver cancer aetiology mortality estimates, we multiplied the parent cause "liver cancer" deaths (excluding deaths in ages less than 10, which are assigned to hepatoblastoma, see below) by the corresponding scaled proportions (see #14 in the flowchart). Single cause estimates were later adjusted to fit into the separately modelled all-cause mortality in the CoDCorrect process. CoDCorrect also combines these five subtypes with the hepatoblastoma estimates (see below) to create a new total liver cancer estimate across all ages.

Hepatoblastoma estimation

For GBD 2023, all mortality estimates under age 10 from the liver cancer parent model were assigned to hepatoblastoma. No other aetiology was assigned to these under-10 hepatoblastoma estimates. While hepatoblastoma is not the only type of liver cancer that children under 10 can die from, the GBD currently only estimates Level 4 mortality from liver cancer under 10 years in the cause hepatoblastoma. Independent modelling using observed hepatoblastoma data and consideration of other liver cancer types and aetiologies under 10 years of age are anticipated in the future, depending on data availability.

Interpretation of results

Cancer mortality estimates for GBD 2023 can differ from the GBD 2021 results for multiple reasons. New and updated cancer mortality data were added from vital registration system data, verbal autopsy studies, and cancer registry incidence data, MIRs were informed by a substantial amount of new paediatric cancer registry data and modelling updates, and we introduced a new method of crosswalking CR data and dropping CR mortality input data that overlapped with VR data. For GBD 2023 there are large relative increases in the mortality estimates for 'Other neoplasms' and the underlying cause 'Other benign and in situ neoplasms'; these increases are due to fixing an error during the CoDCorrect process that was excluding deaths in the underlying cause 'Other benign and in situ neoplasms' in previous cycles, and the inclusion of codes for benign tumours of the brain that were inadvertently dropped in previous rounds, as these non-invasive tumours can still cause death through intracranial pressure.

The other group producing global and country-level cancer mortality estimates is the International Agency for Research on Cancer (IARC) with their GLOBOCAN.[20] Substantially different methods between the GBD study and GLOBOCAN can lead to differences in results. For GLOBOCAN, estimates are produced separately at the national level, using several different regression or imputation models differentially by country depending on the data available.[21] For the GBD, cancer estimation occurs globally across all locations following a consistent, well-documented ensemble modelling approach that includes relevant covariate data, which allows for cross-validation of models as well as determination of uncertainty. Another major difference is the ability in the GBD study to adjust single cause estimates to the all-cause mortality envelope, which is determined independently. This allows correction for the underdiagnosis of cancer in countries with inadequate diagnostic resources. Redistribution of a fraction of undefined causes of death to certain cancers is another methodological advantage of the GBD study as compared to GLOBOCAN, and estimates for cancer mortality can therefore differ substantially in countries with a large proportion of undefined causes of death in their vital registration data or a large proportion of undefined cancer cases in their cancer registry data. There are also differences in the inclusion and categorisation of cancer types reported; for instance, basal cell carcinoma cases are included in both GBD and GLOBOCAN mortality estimates in addition to GBD total cancer incidence estimates, but excluded in GLOBOCAN incidence estimates; and a handful of cancers are individually reported in GBD which are not reported separately in GLOBOCAN (eg, malignant neoplasm of bone and articular cartilage) or are reported in GLOBOCAN (eg, penis cancer), which in the GBD are included within the "other malignant neoplasms" cause.[20]

Limitations

There are certain limitations to consider when interpreting the GBD mortality cancer estimates. First, even though every effort is made to include the most recently available data for each country, data-seeking resources are not limitless, and new data cannot always be accessed as soon as they are made available. It is therefore possible that the GBD study does not include all available data sources for cancer incidence or cancer mortality. Second, different redistribution methods can potentially change the cancer estimates substantially if the data sources used for the estimated location contain a large number of undefined causes; however, neglecting to account for these undefined deaths would likely introduce an even greater bias in the disease estimates. Third, using mortality-to-incidence ratios to transform cancer registry incidence data to mortality estimates requires accurate MIRs. For GBD 2023, we have made further refinements to the estimation of MIRs, but the method remains sensitive to under-diagnosis of cancer cases or under-ascertainment of cancer deaths. However, given that the majority of data used for cancer mortality estimation come from vital registration data and not cancer registry data, this is not a major limitation.

Input data and methodological summary for non-melanoma skin cancer (squamous cell carcinoma)

In the GBD framework, non-melanoma skin cancer (NMSC) estimates include both squamous cell carcinoma (both incidence and mortality) and basal cell carcinoma (incidence only). This section describes the methods for squamous cell carcinoma estimation, while a description of methods for basal cell carcinoma estimates can be found elsewhere in the GBD summary papers.

Input data

Data-seeking processes

Since squamous cell carcinomas are usually not recorded by cancer registries, only vital registration system data were used as input for the squamous cell carcinoma mortality modelling.

Inclusion and exclusion criteria

Inclusion and exclusion criteria followed the same methods as for the vital registration data sources, as described elsewhere in the GBD summary papers.

Bias of categories of input data

The potential biases of the input data are the same as for other cancers (see above).

Modelling strategy

Overall methodological process

Vital registration system data were used as input to model deaths due to squamous cell skin cancer in CODEm.

Steps of analysis and data transformation processes

Since mortality estimates for non-melanoma skin cancer are produced for squamous cell carcinoma under the assumption that basal cell carcinoma causes no deaths, all mortality reported as ICD-10 code "C44" or ICD-9 code "173" were mapped to the GBD cause "squamous cell carcinoma".

Model performance and sensitivity

The modelling strategy, performance and sensitivity, and uncertainty estimation for non-melanoma skin cancer (squamous cell carcinoma) followed the same general CODEm process as other cancer causes, as described in the GBD summary papers.

Interpretation of results

Although the data availability for non-melanoma skin cancer is a challenge, it is a common incident cancer and thus has been included in the GBD framework since GBD 2016. Non-melanoma skin cancer (NMSC) incidence and mortality estimates are not widely available from other sources. GLOBOCAN,[20,21] for example, reported cases and deaths due to non-melanoma skin cancer for the first time in their 2018 release; these GLOBOCAN NMSC incident case estimates exclude basal cell carcinoma, while GLOBOCAN NMSC death estimates include all types of NMSC.

Limitations

Cancer registry data for non-melanoma skin cancer incidence must be interpreted with caution due to a substantial amount of under-reporting, and because of common rules that only the first non-melanoma

skin cancer has to be registered. Many cancer registries therefore do not report non-melanoma skin cancers at all. Information regarding whether or not cancer registries capture non-melanoma skin cancer is not consistently available. Therefore, no cancer registry data were used to estimate deaths due to squamous cell carcinoma of the skin. For vital registration data, we make the assumption that there are no deaths due to basal cell non-melanoma skin cancer, and therefore all deaths attributed to basal cell carcinoma were included instead as squamous cell carcinoma.

Covariates by cancer cause

Acute lymphoid leukaemia

Level	Covariate	Direction
2	*Mean BMI*	+
	Healthcare Access and Quality Index	–
3	*Education (years per capita)*	–
	LDI (I$ per capita)	–
	Socio-demographic Index	+

Acute myeloid leukaemia

Level	Covariate	Direction
2	*Litres of alcohol consumed per capita*	+
	Mean BMI	+
	Cumulative cigarettes (10 years)	+
	Cumulative cigarettes (20 years)	+
	Smoking prevalence	+
	Healthcare Access and Quality Index	–
3	*Education (years per capita)*	–
	LDI (I$ per capita)	+
	Socio-demographic Index	+

Bladder cancer

Level	Covariate	Direction
1	*Schistosomiasis prevalence (proportion)*	+
	Smoking prevalence	+
2	*Litres of alcohol consumed per capita*	+
	Diabetes fasting plasma glucose (mmol/L), age-standardised 25+	+
	Age- and sex-specific SEV for low vegetables	+
	Healthcare Access and Quality Index	–
	Cumulative cigarettes (10 years)	+
3	*Age- and sex-specific SEV for low fruits*	+
	LDI (I$ per capita)	+
	Socio-demographic Index	+

Brain and central nervous system cancer

Level	Covariate	Direction
1	*Litres of alcohol consumed per capita*	+
	Cumulative cigarettes (10 years)	+
	Smoking prevalence	+
2	*Low-density lipoprotein (mmol/L)*	+
	Systolic blood pressure (mmHg)	+
	Age- and sex-specific SEV for high red meat	+
	Age- and sex-specific SEV for low vegetables	+
	Age- and sex-specific SEV for low fruit	+
	Healthcare Access and Quality Index	–
3	*Education (years per capita)*	–
	LDI (I$ per capita)	–
	Socio-demographic Index	+

Breast cancer

Level	Covariate	Direction
1	*Litres of alcohol consumed per capita*	+
	Mean BMI	+
2	*Age-specific fertility rate*	–
	Total fertility rate	–
	Age- and sex-specific SEV for low fruit	+
	Age- and sex-specific SEV for low vegetables	+
	Cumulative cigarettes (10 years)	+
	Cumulative cigarettes (20 years)	+
	Smoking prevalence	+
	Diabetes fasting plasma glucose (mmol/L), age-standardised 25+	+
	Healthcare Access and Quality Index	–
3	*LDI (I$ per capita)*	–
	Socio-demographic Index	+

Burkitt lymphoma

Level	Covariate	Direction
1	*Malaria incidence adjusted for antimalarial coverage and drug effectiveness*	+
	Malaria incidence map	+
	Log-transformed SEV scalar: HIV	+
	Log-transformed age-standardised SEV scalar: HIV	+
2	*Universal health coverage*	–
	Healthcare Access and Quality Index	–
3	*Maternal care and immunisation*	–
	Education (years per capita)	–
	LDI (I$ per capita)	–
	Socio-demographic Index	–

Cervical cancer

Level	Covariate	Direction
1	*Cumulative cigarettes (5 years)*	+
	Log-transformed age-standardised SEV scalar: HIV	+
	HIV age-standardised prevalence	+
	Log-transformed SEV scalar: HIV	+
2	*Age-specific fertility rate*	+
	Total fertility rate	+
	Smoking prevalence	+
	Age- and sex-specific SEV for low fruit	+
	Age- and sex-specific SEV for low vegetables	+
	Healthcare Access and Quality Index	–
3	*Education (years per capita)*	–
	LDI (I$ per capita)	–
	Socio-demographic Index	–

Chronic lymphoid leukaemia

Level	Covariate	Direction
2	*Litres of alcohol consumed per capita*	+
	Mean BMI	+
	Cumulative cigarettes (5 years)	+
	Cumulative cigarettes (10 years)	+
	Cumulative cigarettes (15 years)	+
	Cumulative cigarettes (20 years)	+
	Tobacco (cigarettes per capita)	+
	Smoking prevalence	+
	Healthcare Access and Quality Index	–
3	*Education (years per capita)*	–
	LDI (I$ per capita)	–
	Socio-demographic Index	+

Chronic myeloid leukaemia

Level	Covariate	Direction
2	*Litres of alcohol consumed per capita*	+
	Mean BMI	+
	Cumulative cigarettes (5 years)	+
	Cumulative cigarettes (10 years)	+
	Cumulative cigarettes (15 years)	+
	Cumulative cigarettes (20 years)	+
	Tobacco (cigarettes per capita)	+
	Smoking prevalence	+
	Healthcare Access and Quality Index	–
3	*Education (years per capita)*	–
	LDI (I$ per capita)	+
	Socio-demographic Index	–

Colon and rectum cancer

Level	Covariate	Direction
1	Mean BMI	+
	Tobacco (cigarettes per capita)	+
	Total physical activity (MET-min/week), age-specific	–
	Log-transformed SEV scalar: Colorectal cancer	+
	Age- and sex-specific SEV for high red meat	+
2	Litres of alcohol consumed per capita	+
	PUFA adjusted (percent)	–
	Age- and sex-specific SEV for low vegetables	+
	Age- and sex-specific SEV for low fibre	+
	Age- and sex-specific SEV for low calcium	+
	Cumulative cigarettes (20 years)	+
	Diabetes fasting plasma glucose (mmol/L), age-standardised 25+	+
3	Education (years per capita)	–
	Age- and sex-specific SEV for low milk	+
	Age- and sex-specific SEV for low fruit	+
	Age- and sex-specific SEV for low nuts and seeds	+
	Healthcare Access and Quality Index	–
	LDI (I$ per capita)	+
	Socio-demographic Index	+

Eye cancer

Level	Covariate	Direction
2	Universal health coverage	–
	Age-standardised melanoma	+
	Healthcare Access and Quality Index	–
	Index LDI (I$ per capita)	–
	Socio-demographic Index	–
	Education (years per capita)	–

Gallbladder and biliary tract cancer

Level	Covariate	Direction
1	Log-transformed SEV scalar: Gallbladder cancer	+
	Mean BMI	+
2	Litres of alcohol consumed per capita	+
	Cumulative cigarettes (5 years)	+
	Cumulative cigarettes (10 years)	+
	Smoking prevalence	+
	Tobacco (cigarettes per capita)	+
	Age- and sex-specific SEV for low fruit	+
	Age- and sex-specific SEV for low vegetables	+
	Diabetes age-standardised prevalence (proportion)	+
	Healthcare Access and Quality Index	–
3	Education (years per capita)	–
	LDI (I$ per capita)	+
	Socio-demographic Index	-

Hepatoblastoma (liver cancer parent)

Level	Covariate	Direction
1	*Litres of alcohol consumed per capita*	+
	HIV age-standardised prevalence	+
	Vaccine-adjusted HBsAg seroprevalence age-standardised	+
	Hepatitis C seroprevalence (anti-HCV) age-standardised	+
2	*Hepatitis B 3-dose coverage (proportion)*	–
	Hepatitis B vaccine coverage (proportion), aged through time	–
	Intravenous drug use (age-standardised proportion)	+
	Cumulative cigarettes (20 years)	+
	Mean BMI	+
	Tobacco (cigarettes per capita)	+
	Healthcare Access and Quality Index	–
	Diabetes fasting plasma glucose (mmol/L), age-standardised 25+	+
3	*Education (years per capita)*	–
	Age- and sex-specific SEV for high red meat	+
	LDI (I$ per capita)	–
	Socio-demographic Index	–

Hodgkin lymphoma

Level	Covariate	Direction
2	*Healthcare Access and Quality Index*	–
3	*Education (years per capita)*	–
	LDI (I$ per capita)	–
	Socio-demographic Index	–

Kidney cancer

Level	Covariate	Direction
1	*Tobacco (cigarettes per capita)*	+
	Cumulative cigarettes (10 years)	+
	Mean BMI	+
2	*Litres of alcohol consumed per capita*	+
	Diabetes age-standardised prevalence (proportion)	+
	Systolic blood pressure (mmHg)	+
	Healthcare Access and Quality Index	–
3	*Education (years per capita)*	–
	LDI (I$ per capita)	+
	Socio-demographic Index	+

Larynx cancer

Level	Covariate	Direction
1	Litres of alcohol consumed per capita	+
2	Smoking prevalence	+
	Asbestos consumption (metric tons per year per capita)	+
	Age- and sex-specific SEV for low vegetables	+
	Cumulative cigarettes (10 years)	+
	Cumulative cigarettes (20 years)	+
	Population density (over 1000 ppl/sqkm, proportion)	+
	Healthcare Access and Quality Index	–
3	Age- and sex-specific SEV for low fruit	+
	LDI (I$ per capita)	+
	Socio-demographic Index	+

Leukaemia

Level	Covariate	Direction
2	Litres of alcohol consumed per capita	+
	Mean BMI	+
	Cumulative cigarettes (10 years)	+
	Cumulative cigarettes (20 years)	+
	Tobacco (cigarettes per capita)	+
	Healthcare Access and Quality Index	–
3	Education (years per capita)	–
	LDI (I$ per capita)	+
	Socio-demographic Index	–

Lip and oral cavity cancer

Level	Covariate	Direction
1	Litres of alcohol consumed per capita	+
	Cumulative cigarettes (10 years)	+
	Cumulative cigarettes (20 years)	+
	Tobacco (cigarettes per capita)	+
2	Age- and sex-specific SEV for high red meat	+
	Age- and sex-specific SEV for low vegetables	+
	Age- and sex-specific SEV for low fruit	+
	Healthcare Access and Quality Index	–
3	Education (years per capita)	–
	LDI (I$ per capita)	+
	Socio-demographic Index	+

Liver cancer (Level 3 parent model)

Level	Covariate	Direction
1	*Litres of alcohol consumed per capita*	+
	HIV age-standardised prevalence	+
	Vaccine-adjusted HBsAg seroprevalence age-standardised	+
	Hepatitis C seroprevalence (anti-HCV) age-standardised	+
2	*Hepatitis B 3-dose coverage (proportion)*	–
	Hepatitis B vaccine coverage (proportion), aged through time	–
	Intravenous drug use (age-standardised proportion)	+
	Cumulative cigarettes (20 years)	+
	Mean BMI	+
	Tobacco (cigarettes per capita)	+
	Healthcare Access and Quality Index	–
	Diabetes fasting plasma glucose (mmol/L), age-standardised 25+	+
3	*Education (years per capita)*	–
	Age- and sex-specific SEV for high red meat	+
	LDI (I$ per capita)	–
	Socio-demographic Index	–

Malignant neoplasm of bone and articular cartilage

Level	Covariate	Direction
2	*Universal health coverage*	–
	Socio-demographic Index	–
	LDI (I$ per capita)	–
	Healthcare Access and Quality Index	–
3	*Smoking prevalence*	+
	Health worker density	–
	Education (years per capita)	–
	Age- and sex-specific SEV for low bone mineral density	+
	Maternal care and immunisation	–
	Log-transformed SEV scalar: Osteoarthritis	+
	Log-transformed age-standardised SEV scalar: Osteoarthritis	+

Malignant skin melanoma

Level	Covariate	Direction
1	*Litres of alcohol consumed per capita*	+
2	*Latitude under 15 (proportion)*	–
	Latitude 15 to 30 (proportion)	–
	Latitude 30 to 45 (proportion)	–
	Latitude over 45 (proportion)	–
	Healthcare Access and Quality Index	–
3	*Education (years per capita)*	–
	LDI (I$ per capita)	–
	Socio-demographic Index	+

Mesothelioma

Level	Covariate	Direction
1	Asbestos consumption (metric tons per year per capita)	+
	Age- and sex-specific SEV for Occupational asbestos	+
	Age-standardised SEV for occupational asbestos	+
	Smoking prevalence	+
2	Gold production (binary)	+
	Indoor air pollution (all cooking fuels)	+
	Population density (over 1000 ppl/sqkm, proportion)	+
	Healthcare Access and Quality Index	–
	Cumulative cigarettes (5 years)	+
3	Education (years per capita)	–
	LDI (I$ per capita)	–
	Socio-demographic Index	+

Multiple myeloma

Level	Covariate	Direction
1	Litres of alcohol consumed per capita	+
	Smoking prevalence	+
	Tobacco (cigarettes per capita)	+
2	Age- and sex-specific SEV for low vegetables	+
	Age- and sex-specific SEV for low fruits	+
	Age- and sex-specific SEV for high red meat	+
	Mean BMI	+
	Sanitation (proportion with access)	–
	Improved water source (proportion with access)	–
	Healthcare Access and Quality Index	–
3	Education (years per capita)	–
	LDI (I$ per capita)	+
	Socio-demographic Index	+

Myelodysplastic, myeloproliferative, and other haemopoietic neoplasms

Level	Covariate	Direction
1	Log-transformed age-standardised SEV scalar: Leukaemia	+
	Log-transformed SEV scalar: Leukaemia	+
2	Litres of alcohol consumed per capita	+
	Cumulative cigarettes (5 years)	+
	Cumulative cigarettes (10 years)	+
	Cumulative cigarettes (15 years)	+
	Cumulative cigarettes (20 years)	+
	Smoking prevalence	+
	Tobacco (cigarettes per capita)	+
	Healthcare Access and Quality Index	–
3	Education (years per capita)	–
	LDI (I$ per capita)	+
	Socio-demographic Index	+

Nasopharynx cancer

Level	Covariate	Direction
1	Litres of alcohol consumed per capita	+
	Cumulative cigarettes (10 years)	+
	Cumulative cigarettes (20 years)	+
	Tobacco (cigarettes per capita)	+
2	Age- and sex-specific SEV for low vegetables	+
	Population density (over 1000 ppl/sqkm, proportion)	+
	Healthcare Access and Quality Index	–
3	Education (years per capita)	–
	Age- and sex-specific SEV for low fruit	+
	LDI (I$ per capita)	–
	Socio-demographic Index	+

Neuroblastoma and other peripheral nervous cell tumours

Level	Covariate	Direction
3	Smoking prevalence	+
	Health worker density	–
	Healthcare Access and Quality Index	–
	Education (years per capita)	–
	LDI (I$ per capita)	+
	Socio-demographic Index	+
	Maternal care and immunisation	–
	Universal health coverage	–

Non-Hodgkin lymphoma

Level	Covariate	Direction
2	Cumulative cigarettes (5 years)	+
	Cumulative cigarettes (10 years)	+
	Cumulative cigarettes (15 years)	+
	Cumulative cigarettes (20 years)	+
	Litres of alcohol consumed per capita	+
	Mean BMI	+
	Universal health coverage	–
	Healthcare Access and Quality Index	–
3	Log-transformed SEV scalar: HIV	+
	Log-transformed age-standardised SEV scalar: HIV	+
	Total fertility rate	–
	Education (years per capita)	–
	LDI (I$ per capita)	–
	Socio-demographic Index	–

Non-melanoma skin cancer & squamous cell carcinoma

Level	*Covariate*	*Direction*
1	*Cumulative cigarettes (5 years)*	+
	Cumulative cigarettes (10 years)	+
	Cumulative cigarettes (15 years)	+
	Smoking prevalence	+
2	*Average latitude*	−
	Healthcare Access and Quality Index	−
3	*Education (years per capita)*	−
	LDI (I$ per capita)	−
	Socio-demographic Index	+

Oesophageal cancer

Level	*Covariate*	*Direction*
1	*Litres of alcohol consumed per capita*	+
	Mean BMI	+
	Smoking prevalence	+
2	*Indoor air pollution (all cooking fuels)*	+
	Tobacco (cigarettes per capita)	+
	Age- and sex-specific SEV for low vegetables	+
	Age- and sex-specific SEV for low fruit	+
	Healthcare Access and Quality Index	−
3	*Education (years per capita)*	−
	Sanitation (proportion with access)	−
	Improved water source (proportion with access)	−
	LDI (I$ per capita)	+
	Socio-demographic Index	+

Other benign and in situ neoplasms

Level	*Covariate*	*Direction*
2	*Tobacco (cigarettes per capita)*	+
	Cumulative cigarettes (10 years)	+
	Smoking prevalence	+
	Healthcare Access and Quality Index	−
3	*LDI (I$ per capita)*	−
	Socio-demographic Index	−
	Litres of alcohol consumed per capita	+
	Education (years per capita)	−

Other eye cancers

Level	Covariate	Direction
2	*Universal health coverage*	–
	Age-standardised melanoma	+
	Healthcare Access and Quality Index	–
3	*Index LDI (I$ per capita)*	–
	Socio-demographic Index	–
	Education (years per capita)	–

Other leukaemia

Level	Covariate	Direction
2	*Litres of alcohol consumed per capita*	+
	Mean BMI	+
	Cumulative cigarettes (10 years)	+
	Cumulative cigarettes (20 years)	+
	Tobacco (cigarettes per capita)	+
	Healthcare Access and Quality Index	–
3	*Education (years per capita)*	–
	LDI (I$ per capita)	+
	Socio-demographic Index	–

Other malignant neoplasms

Level	Covariate	Direction
1	*Smoking prevalence*	+
	Tobacco (cigarettes per capita)	+
2	*Age- and sex-specific SEV for low vegetables*	+
	Age- and sex-specific SEV for low fruits	+
	Age- and sex-specific SEV for low nuts and seeds	+
	PUFA adjusted (percent)	–
	Healthcare Access and Quality Index	–
3	*Education (years per capita)*	–
	LDI (I$ per capita)	+
	Socio-demographic Index	+

Other neoplasms

Level	Covariate	Direction
3	*Healthcare Access and Quality Index*	–
	Education (years per capita)	–
	LDI (I$ per capita)	+
	Socio-demographic Index	–
	Tobacco (cigarettes per capita)	+
	Cumulative cigarettes (10 years)	+
	Cholesterol (total, mean per capita)	+
	Smoking prevalence	+
	Maternal care and immunisation	–
	Log-transformed SEV scalar: Leukaemia	+
	Log-transformed age-standardised SEV scalar: Leukaemia	+
	Universal health coverage	–
	Litres of alcohol consumed per capita	+

Other non-Hodgkin lymphoma

Level	Covariate	Direction
2	*Cumulative cigarettes (5 years)*	+
	Cumulative cigarettes (10 years)	+
	Cumulative cigarettes (15 years)	+
	Cumulative cigarettes (20 years)	+
	Litres of alcohol consumed per capita	+
	Mean BMI	+
	Universal health coverage	–
	Healthcare Access and Quality Index	–
3	*Log-transformed SEV scalar: HIV*	+
	Log-transformed age-standardised SEV scalar: HIV	+
	Total fertility rate	-
	Education (years per capita)	–
	LDI (I$ per capita)	–
	Socio-demographic Index	–

Other pharynx cancer

Level	Covariate	Direction
1	*Litres of alcohol consumed per capita*	+
	Smoking prevalence	+
2	*Cumulative cigarettes (5 years)*	+
	Age- and sex-specific SEV for low fruit	+
	Age- and sex-specific SEV for low vegetables	+
	Population density (over 1000 ppl/sqkm, proportion)	+
	Population density (under 150 ppl/sqkm, proportion)	+
	Healthcare Access and Quality Index	–
3	*Education (years per capita)*	–
	LDI (I$ per capita)	+
	Socio-demographic Index	+

Ovarian cancer

Level	Covariate	Direction
1	*Litres of alcohol consumed per capita*	+
2	*Asbestos consumption (metric tons per year per capita)*	+
	Smoking prevalence	+
	Total fertility rate	–
	Energy unadjusted (kcal)	+
	Mean BMI	+
	Contraception (modern) prevalence (proportion)	–
	Diabetes age-standardised prevalence (proportion)	+
	Cumulative cigarettes (10 years)	+
	Cumulative cigarettes (20 years)	+
	Healthcare Access and Quality Index	–
3	*Education (years per capita)*	–
	Age- and sex-specific SEV for low fruits	+
	Age- and sex-specific SEV for low vegetables	+
	LDI (I$ per capita)	–
	Socio-demographic Index	+

Pancreatic cancer

Level	Covariate	Direction
1	*Cumulative cigarettes (10 years)*	+
	Cumulative cigarettes (20 years)	+
	Tobacco (cigarettes per capita)	+
	Mean BMI	+
2	*Age- and sex-specific SEV for high red meat*	+
	Litres of alcohol consumed per capita	+
	Energy unadjusted (kcal)	+
	Diabetes fasting plasma glucose (mmol/L), age-standardised 25+	+
	Diabetes age-standardised prevalence (proportion)	+
	Healthcare Access and Quality Index	–
3	*Education (years per capita)*	–
	Age- and sex-specific SEV for low fruit	+
	LDI (I$ per capita)	+
	Socio-demographic Index	+
	Age- and sex-specific SEV for low vegetables	+

Prostate cancer

Level	Covariate	Direction
1	*SEV scalar: Prostate cancer*	+
2	*Smoking prevalence*	+
	Healthcare Access and Quality Index	–
3	*Education (years per capita)*	–
	LDI (I$ per capita)	–
	Socio-demographic Index	+

Retinoblastoma

Level	Covariate	Direction
2	*Universal health coverage*	–
	Healthcare Access and Quality Index	–
3	*LDI (I$ per capita)*	–
	Maternal care and immunisation	–
	Socio-demographic Index	–
	Education (years per capita)	–

Soft tissue and other extraosseous sarcomas

Level	Covariate	Direction
2	*Healthcare Access and Quality Index*	–
	LDI (I$ per capita)	–
	Socio-demographic Index	–
	Universal health coverage	–
3	*Education (years per capita)*	–
	Maternal care and immunisation	–
	Log-transformed SEV scalar: HIV	+
	Log-transformed age-standardised SEV scalar: HIV	+
	Litres of alcohol consumed per capita	+

Stomach cancer

Level	Covariate	Direction
1	*Diet high in sodium*	+
	Tobacco (cigarettes per capita)	+
2	*Cumulative cigarettes (20 years)*	+
	Age- and sex-specific SEV for unsafe water	+
	Age- and sex-specific SEV for unsafe sanitation	+
	Mean BMI	+
	Sanitation (proportion with access)	–
	Improved water source (proportion with access)	–
	Healthcare Access and Quality Index	–
3	*Education (years per capita)*	–
	Age- and sex-specific SEV for low fruit	+
	Age- and sex-specific SEV for low vegetables	+
	LDI (I$ per capita)	+
	Socio-demographic Index	–

Testicular cancer

Level	Covariate	Direction
2	*Cumulative cigarettes (5 years)*	+
	Cumulative cigarettes (10 years)	+
	Cumulative cigarettes (15 years)	+
	Cumulative cigarettes (20 years)	+
	Tobacco (cigarettes per capita)	+
	Smoking prevalence	+
	Age- and sex-specific SEV for low fruits	+
	Age- and sex-specific SEV for low vegetables	+
	Healthcare Access and Quality Index	–
3	*Education (years per capita)*	–
	LDI (I$ per capita)	+
	Socio-demographic Index	+

Thyroid cancer

Level	Covariate	Direction
1	*Litres of alcohol consumed per capita*	+
2	*Age- and sex-specific SEV for low vegetables*	+
	Age- and sex-specific SEV for high red meat	+
	Tobacco (cigarettes per capita)	+
	Mean BMI	+
	Healthcare Access and Quality Index	–
3	*Education (years per capita)*	–
	Sanitation (proportion with access)	–
	Improved water source (proportion with access)	–
	Age- and sex-specific SEV for low fruits	+
	LDI (I$ per capita)	+
	Socio-demographic Index	+

Tracheal, bronchus, and lung cancer

Level	Covariate	Direction
1	*Asbestos consumption (metric tons per year per capita)*	+
	Smoking prevalence	+
2	*Indoor air pollution (all cooking fuels)*	+
	Second-hand smoke	+
	Cumulative cigarettes (10 years)	+
	Cumulative cigarettes (20 years)	+
	Outdoor air pollution ($PM_{2.5}$)	+
	Residential radon	+
	Diabetes fasting plasma glucose (mmol/L), age-standardised 25+	+
	Healthcare Access and Quality Index	–
3	*Education (years per capita)*	–
	LDI (I$ per capita)	+
	Socio-demographic Index	+

Uterine cancer

Level	Covariate	Direction
1	*Mean BMI*	+
2	*Cumulative cigarettes (5 years)*	+
	Cumulative cigarettes (10 years)	+
	Smoking prevalence	+
	Tobacco (cigarettes per capita)	+
	Diabetes age-standardised prevalence (proportion)	+
	Total fertility rate	−
	Age- and sex-specific SEV for low fruit	+
	Age- and sex-specific SEV for low vegetables	+
	Healthcare Access and Quality Index	−
3	*Education (years per capita)*	−
	LDI (I$ per capita)	+
	Socio-demographic Index	+

References

1 de Martel C, Maucort-Boulch D, Plummer M, Franceschi S. World-wide relative contribution of hepatitis B and C viruses in hepatocellular carcinoma. *Hepatology* 2015; **62**: 1190–200.

2 Hong TP, Gow P, Fink M, *et al.* Novel population-based study finding higher than reported hepatocellular carcinoma incidence suggests an updated approach is needed. *Hepatology* 2016; **63**: 1205–12.

3 Surveillance, Epidemiology, and End Results (SEER) Program (www.seer.cancer.gov) SEER*Stat Database: Incidence - SEER 18 Regs Research Data + Hurricane Katrina Impacted Louisiana Cases, Nov 2017 Sub (1973-2015 varying) - Linked To County Attributes - Total U.S., 1969-2016 Counties, National Cancer Institute, DCCPS, Surveillance Research Program, released April 2018, based on the November 2017 submission.

4 Surveillance, Epidemiology, and End Results (SEER) Program (www.Seer.Cancer.Gov) SEER*Stat Database: Incidence - SEER 18.

5 Engholm G, Ferlay J, Christensen N, *et al.* NORDCAN--a Nordic tool for cancer information, planning, quality control and research. *Acta Oncol* 2010; **49**: 725–36.

6 R D, P P, JAH W. Cancer Incidence in Five Continents Volume I. https://publications.iarc.fr/Non-Series-Publications/Other-Non-Series-Publications/Cancer-Incidence-In-Five-Continents-Volume-I-1966 (accessed Feb 24, 2021).

7 Doll R, Muir C, Waterhouse J. Cancer Incidence in Five Continents: Volume II–1970. Springer Science & Business Media, 2012.

8 Waterhouse J, Muir C, Correa P, Powell J. Cancer Incidence in Five Continents III. Lyon: IARC; 1976.

9 Waterhouse J, Muir C, Shanmugaratnam K, Powell J. Cancer Incidence in Five Continents IV. Lyon: IARC; 1982.

10 Muir C, Mack T, Powell J, Whelan S. Cancer Incidence in Five Continents V. Lyon: IARC; 1987.

11 Parkin D, Muir C, Whelan S, Gao Y, Ferlay J, Powell J. Cancer Incidence in Five Continents VI. Lyon: IARC; 1992.

12 Parkin D, Whelan S, Ferlay J, Raymond L, Young J. Cancer Incidence in Five Continents VII. Lyon: IARC; 1997.

13 Parkin D, Whelan S, Ferlay J, Teppo L, Thomas D. Cancer Incidence in Five Continents VIII. Lyon: IARC; 2002.

14 Curado M, Edwards B, Shin H, et al. Cancer Incidence in Five Continents IX. Lyon: IARC; 2007. http://www.iarc.fr/en/publ.

15 Forman D, Bray F, Brewster D, et al. Cancer Incidence in Five Continents X. http://ci5.iarc.fr. Published 2013.

16 Bray F, Colombet M, Mery L, Piñeros M, Znaor A, Zanetti R and Ferlay J, editors (2017). Cancer Incidence in Five Contine.

17 Steliarova-Foucher E, Colombet M, Ries LAG, *et al.* International incidence of childhood cancer, 2001–10: a population-based registry study. *The Lancet Oncology* 2017; **18**: 719–31.

18 Barber RM, Fullman N, Sorensen RJD, *et al.* Healthcare Access and Quality Index based on mortality from causes amenable to personal health care in 195 countries and territories, 1990–2015: a novel analysis from the Global Burden of Disease Study 2015. *The Lancet* 2017; **390**: 231–66.

19 Murray CJL, Aravkin AY, Zheng P, *et al.* Global burden of 87 risk factors in 204 countries and territories, 1990–2019: a systematic analysis for the Global Burden of Disease Study 2019. *The Lancet* 2020; **396**: 1223–49.

20 Sung H, Ferlay J, Siegel RL, *et al.* Global Cancer Statistics 2020: GLOBOCAN Estimates of Incidence and Mortality Worldwide for 36 Cancers in 185 Countries. *CA: A Cancer Journal for Clinicians* 2021; **71**: 209–49.

21 Ferlay J, Colombet M, Soerjomataram I, *et al.* Estimating the global cancer incidence and mortality in 2018: GLOBOCAN sources and methods. *International Journal of Cancer* 2019; **144**: 1941–53.

Cardiomyopathy and myocarditis

Flowchart

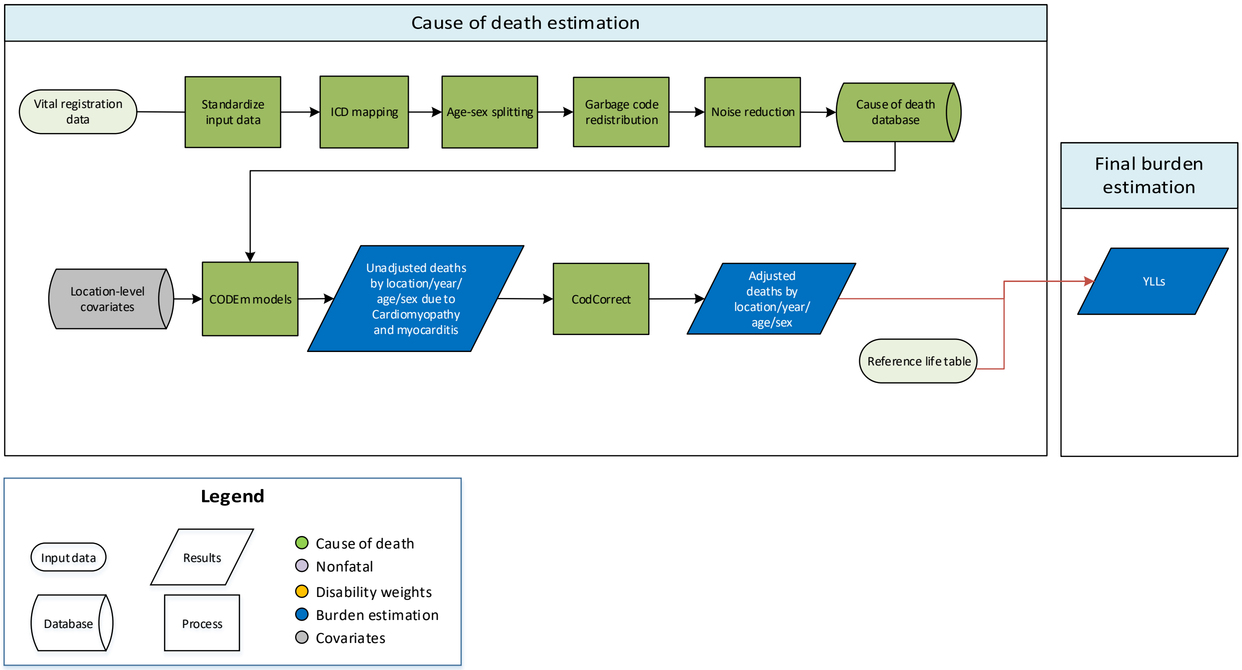

Input data and methodological summary for cardiomyopathy and myocarditis

Input data

Vital registration data were used to model deaths due to cardiomyopathy and myocarditis. We outliered all datapoints in Bosnia and Herzegovina, Montenegro, North Macedonia, Serbia, and Egypt due to implausible values likely caused by local coding practices. We also outliered ICD-8 and ICD-9-BTL datapoints in countries where they were discontinuous with other data in the time series or were implausibly high or low. In addition, we outliered ICD-10 tabulated data in Kazakhstan which were implausibly high compared to other data in the country.

Modelling strategy

We used a standard CODEm approach to model deaths from cardiomyopathy and myocarditis. The model uses an empirical Bayesian noise reduction algorithm that uses grouped data by region and data type as a prior to better reflect regional patterns. The covariates selected for inclusion in the CODEm modelling process can be found in Table 1. There have been no substantive changes to the modelling strategy since GBD 2021.

Table 1. Covariates used in cardiomyopathy and myocarditis mortality modelling

Level	Covariate	Direction

1	Log-transformed summary exposure variable, cardiomyopathy (CMP)	1
	Smoking prevalence	1
2	Mean body mass index (kg/m^2)	1
	Healthcare Access and Quality Index	–1
	Mean systolic blood pressure (mmHg)	1
3	Log transformed lag distributed income per capita (I$)	–1
	Socio-demographic Index	–1

Cardiovascular diseases

Flowchart

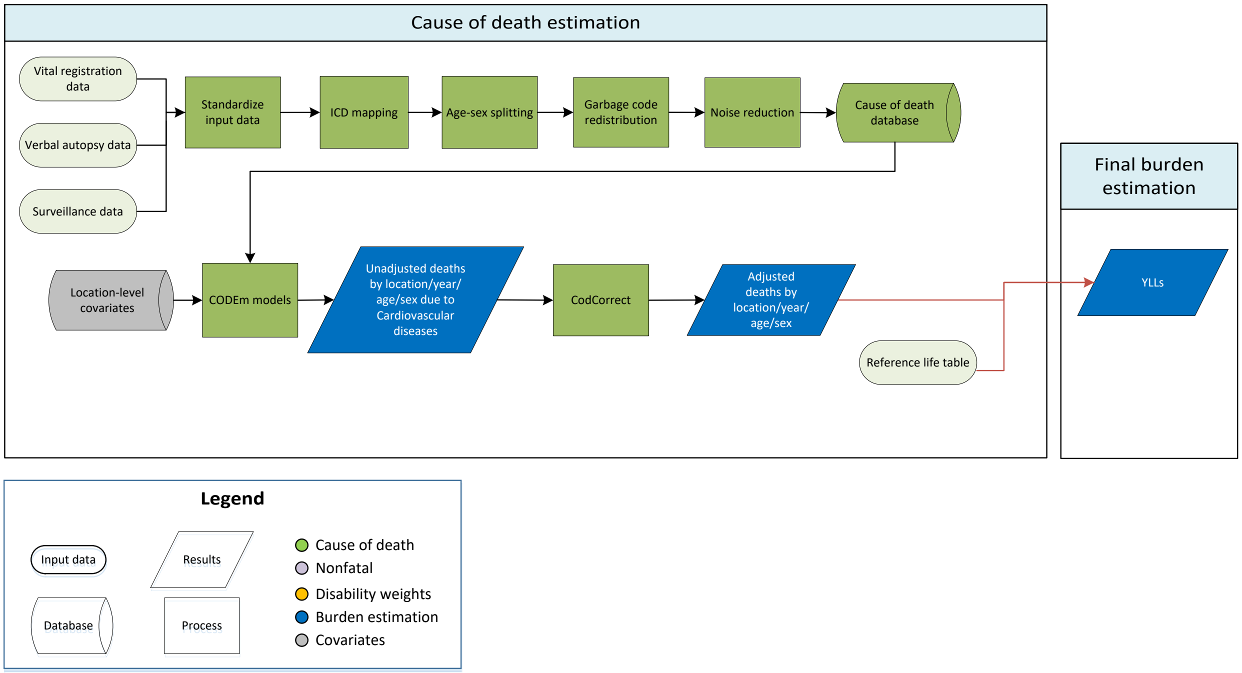

Input data and methodological summary for cardiovascular diseases

Input data

Vital registration and verbal autopsy data were used to model the parent cardiovascular envelope. For GBD 2023, all verbal autopsy data sources included in the cardiovascular envelope were systematically reviewed. In order to maximise the reliability of the included data, verbal autopsy studies that did not meet the World Health Organization standards were excluded.[1] In addition, non-representative subnational verbal autopsies from a number of Indian states and verbal autopsy data in Nepal and Papua New Guinea that were implausible in terms of time and age trends were outliered. Verbal autopsy data were systematically outliered among under-15 age groups. Data for children under 5 from the Child Health and Mortality Prevention Surveillance Network Program across countries[2] (ie, Mozambique, South Africa, Somalia, and Sierra Leone) were systematically excluded from the analyses of cardiovascular disease causes of death due to implausible values. Data sources that were implausibly low in all age groups as well as ICD-8 and ICD-9-BTL datapoints that were inconsistent with the rest of the data and created implausible time trends were also outliered. In addition, implausibly high ICD-10 data from the 150 England Upper Tier Local Authorities 2014–2018 were outliered. Additionally, datapoints from Venezuela, Ecuador, United Arab Emirates, Zambia, Montenegro, and Ethiopia subnationals were outliered due to inconsistencies with the rest of the region.

Crosswalk by code system

Most data sources used for cause of death estimation are ICD-coded. The ICD periodically releases new versions, which can introduce an implausible disjoint in the time series that is indicative of a change in the coding practice, rather than a true change in the epidemiological trend of the disease. In GBD 2023, certain causes and countries using the ICD-9 code systems were adjusted to address the disjoint in the time trend and number of deaths between the older ICD-9 code systems and the more recent ICD-10 code systems. Cardiovascular disease was corrected in Bulgaria (1990–2004), Canada (1986–1999), Spain (1980–1998), France (1980–1999), the USA (1980–1998), Hungary (1980–1995), Croatia (1985–1994), the UK (1980–1999), Greece (1987–2008), Italy (1980–2002), Mexico (1980–1997), and Mauritius (1980–2004) to account for this code system disjoint. This was done using a meta-regression tool described as a crosswalk, by taking systematically biased datapoints and estimating their unbiased value. Using this modelling tool, correction factors were created based on the more recent and more reliable ICD-10-coded data. The crosswalk creates matched pairs between the last three years of data using the ICD-9 code system and the first three years of data using the ICD-10 code system. The correction is applied at the child cause level for all Level 2 causes, and after the correction is applied the individual child causes are raked to the parent cause to maintain a consistent number of deaths across the cause hierarchy.

Modelling strategy

We used a standard CODEm approach to model deaths from cardiovascular diseases. The model uses an empirical Bayesian noise reduction algorithm that uses grouped data by region and data type as prior to better reflect regional patterns. The covariates included in the ensemble modelling process are listed in Table 1. There are no substantive changes from the approach used in GBD 2021.

Table 1. Covariates used in cardiovascular diseases mortality modelling

Level	Covariate	Direction
1	Mean low-density lipoprotein (mmol/L)	1
	Smoking prevalence	1
	Mean systolic blood pressure (mmHg)	1
2	Mean body-mass index (kg/m^2)	1
	Elevation over 1500 m (proportion)	-1
	Mean fasting plasma glucose (mmol/L)	1
	Outdoor air pollution ($PM_{2.5}$)	1
	Indoor air pollution (all cooking fuels)	1
	Healthcare Access and Quality Index	-1
3	Lag-distributed income per capita (I$)	-1
	Summary exposure value, diet low in omega-3 fatty acids	1
	Summary exposure value, diet low in fruits	1
	Summary exposure value, diet low in vegetables	1
	Summary exposure value, diet low in nuts and seeds	1
	Pulses/legumes (g per capita, unadjusted)	-1
	Summary exposure value, diet low in polyunsaturated fatty acids (PUFA)	1
	Alcohol (litres per capita)	1
	Diet high in trans fatty acids	1

References

1 WHO | Methodological trends in studies based on verbal autopsies before and after published guidelines. https://www.who.int/bulletin/volumes/87/9/07-049288/en/ (accessed April 22, 2021).

2 Centers for Disease Control and Prevention (CDC), Child Health and Mortality Prevention Surveillance (CHAMPS) Network, Deloitte Consulting LLP, Emory Global Health Institute (EGHI), International Association of National Public Health Institutes (IANPHI), Task Force for Global Health, Emory University. Child Health and Mortality Prevention Surveillance (CHAMPS) Network Program.

Chagas disease

Flowchart

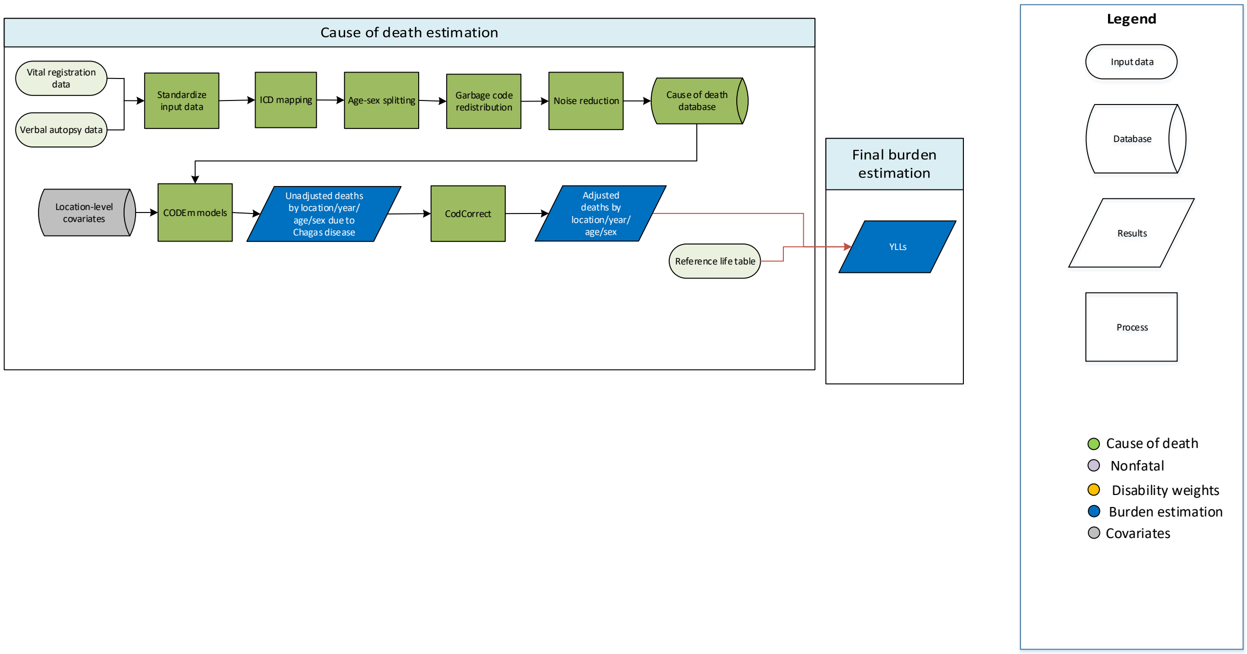

Input data and methodological summary for Chagas disease

Input data

We modelled Chagas mortality using all available data in the cause of death (CoD) database. Datapoints were outliered if they reported an improbable number of Chagas deaths (eg, zero Chagas deaths in a hyper-endemic country) or if their inclusion in the model yielded distorted trends.

Modelling strategy

We modelled Chagas mortality using a CODEm model of all Chagas-endemic countries of Latin America using all data in the COD database. Estimates of Chagas mortality in endemic countries were drawn from the CODEm model. Estimates of mortality in countries without known endemic transmission were added as imported cases if reported through vital registration systems.

The CODEm models included two covariates, as detailed in Table 1.

Table 1. Covariates. Summary of covariates used in Chagas disease mortality modelling

Level	Covariate	Direction
2	Healthcare Access and Quality Index	-
	Socio-demographic Index	-

Changes from GBD 2021 to GBD 2023

There were no substantive changes to the modelling strategy for GBD 2023.

Chronic kidney disease

Flowchart

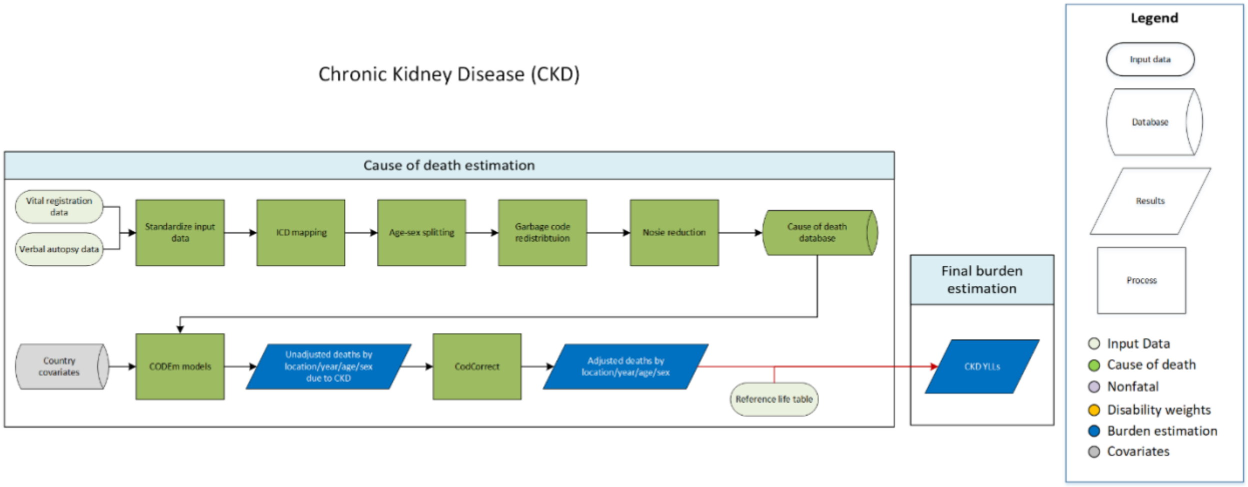

Input data and methodological summary for chronic kidney disease

Input data

Vital registration and verbal autopsy data were used to model mortality due to chronic kidney disease. Data were standardised and mapped according to the GBD causes of death ICD mapping method. These data were then age-sex split, and appropriate redistribution of garbage code data was performed. Datapoints that violated well-established age or time trends or that resulted in extremely high or low cause fractions were marked as outliers and excluded.

In GBD 2023, deaths due to congenital kidney anomalies, such as cystic kidney disease, were no longer considered deaths due to CKD. These deaths (ICD-10 codes Q61 and Q62) are now captured under the urogenital congenital anomalies cause.

Modelling strategy

We have made no substantive changes in the modelling strategy for fatal chronic kidney disease for GBD 2023. A standard Cause of Death Ensemble model (CODEm) with location-level covariates was used to model deaths due to chronic kidney disease. Additional information on methods can be found in appendix 1, section 4.

The full list of covariates used in the GBD 2023 model is displayed below.

Table 1. Covariates used in chronic kidney disease mortality modelling

Level	Covariate	Direction
1	Mean systolic blood pressure (mmHg)	+
	Mean BMI	+
	Healthcare Access and Quality Index	–
2	Mean cholesterol	+

	Total calories available per capita per day	+
	Red meat unadjusted (kcal per capita)	+
3	Socio-demographic Index	–
	Education (years per capita)	–
	LDI (I$ per capita)	–

Chronic kidney disease subtypes

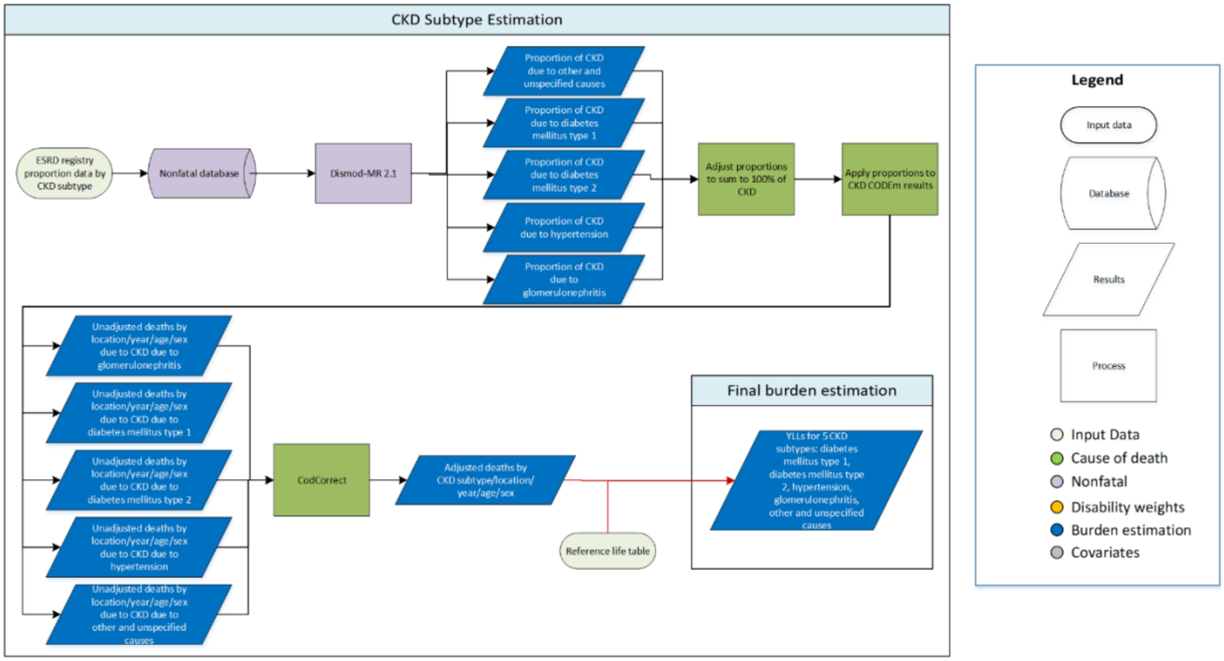

Input data and methodological summary for chronic kidney disease subtypes

Input data

We estimated deaths due to five subtypes of chronic kidney disease: diabetes mellitus (DM) type 1, diabetes mellitus (DM) type 2, hypertension, glomerulonephritis, and other and unspecified causes. Data from end-stage renal disease registries were used to estimate the proportion of CKD mortality attributable to each CKD subtype. Vital registration (VR) data were excluded from subtype-specific estimates, as aetiology coding in VR sources was considered to be of highly variable quality between countries.

Modelling strategy

We utilised data primarily from end-stage kidney disease registries that included CKD aetiologies to model CKD-death aetiology proportions. Each aetiology was modelled with the disease model—Bayesian meta-regression (DisMod-MR 2.1, for methods description see appendix 1, section 2) tool to obtain estimates of each by location, year, age, and sex. Data for CKD due to overall DM were more widely available than data by type of DM. To make use of all available data, we modelled the proportion of CKD due to overall DM with age-standardised diabetes prevalence as a county-level covariate. The

proportions of CKD due to DM type 1 and DM type 2 were then scaled to sum to the proportion of overall DM by location, year, age, and sex. Mean systolic blood pressure (mmHg) was used as a country-level covariate in the CKD due to hypertension model.

Then the results from all five aetiology-specific models were adjusted proportionally so that estimates across the aetiologies sum to 100%. These adjusted proportions were applied to the overall CKD CODEm model to obtain estimates of CKD mortality due to each aetiology by each location, year, age, and sex combination.

Table 2. Summary of covariates used in the CKD aetiology DisMod-MR meta-regression models

Model	Covariate	Type	Parameter	Exponentiated beta (95% uncertainty interval)
CKD due to diabetes mellitus proportion	Age-standardised diabetes prevalence	Country-level	Proportion	2.71 (2.69–2.72)
CKD due to hypertension proportion	Mean systolic blood pressure	Country-level	Proportion	1.00 (1.00–1.01)

Chronic obstructive pulmonary disease

Flowchart

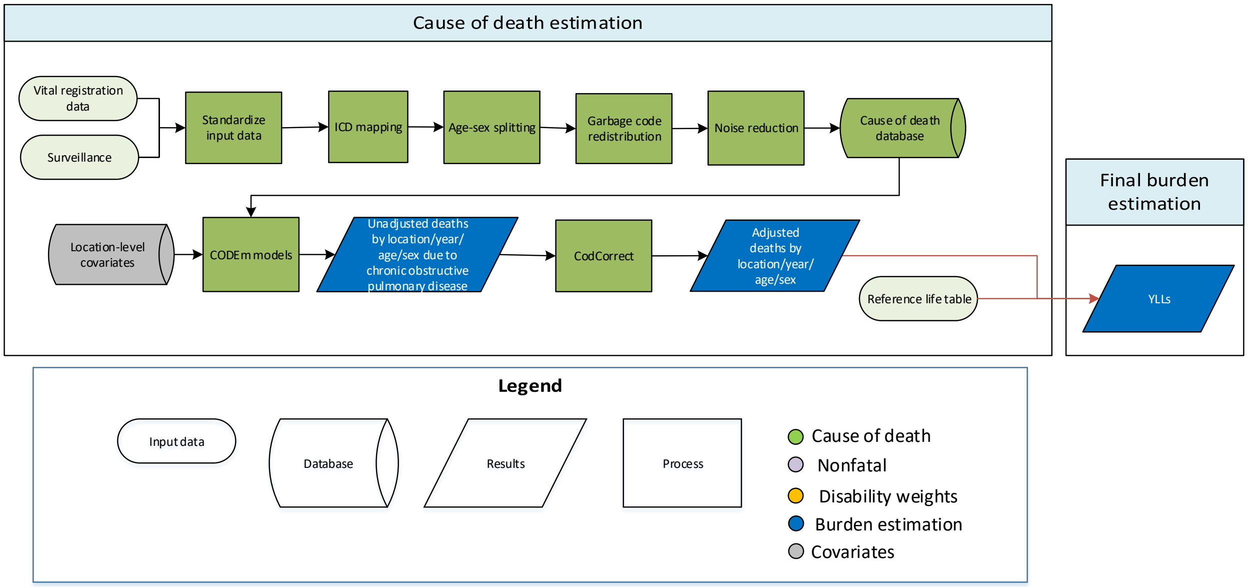

Input data and methodological summary for chronic obstructive pulmonary disease

Input data

Data used to estimate chronic obstructive pulmonary disease (COPD) mortality included vital registration and surveillance data from the cause of death (CoD) database. Verbal autopsy data were not included and were instead mapped to an overall chronic respiratory disease model. Our outlier criteria excluded datapoints that (1) were implausibly high or low, (2) substantially conflicted with established age or temporal patterns, or (3) substantially conflicted with other data sources conducted from the same locations or locations with similar characteristics (ie, Socio-demographic Index).

Modelling strategy

There were no substantive changes to the modelling approach this round. The standard Cause of Death Ensemble modelling (CODEm) approach was used to estimate deaths due to COPD (see appendix 1, section 4 for additional details). Individual models were run for male and female mortality, and the age range for both models was 15 to 95+ years. The earliest age we model COPD is 15, as the disease typically takes years or decades to develop and is unlikely to cause severe outcomes, such as death, in younger age groups.

The following table lists the covariates included in the model. For GBD 2023, updates to covariate selection included incorporating covariates related to COVID-19 mortality and mask wearing to account for the association between COVID-19 infection and COPD mortality. Covariate directions were selected based on the strength of the evidence.

Table 1. Covariates used in COPD mortality modelling

Level	Covariate	Direction
1	Cumulative cigarettes (10 years)	+
	Cumulative cigarettes (20 years)	+
	Log-transformed SEV scalar: COPD	+
2	Indoor air pollution (all cooking fuels)	+
	Outdoor air pollution (PM 2.5)	+
	Smoking prevalence	+
	Healthcare Access and Quality Index	+
	Mask use	-
	COVID-19 age-standardised death rate	+
	Proportion population over 1500 m elevation	+
3	Socio-demographic Index	-

Chronic respiratory disease

Flowchart

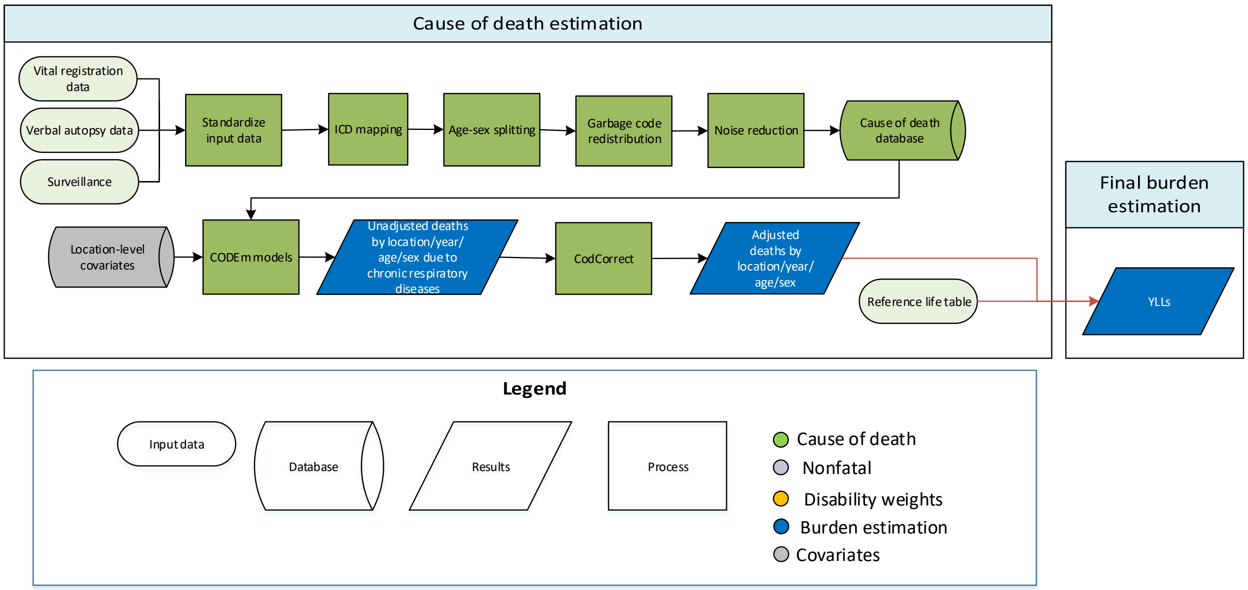

Input data and methodological summary for chronic respiratory disease

Input data

Sources used to estimate chronic respiratory disease mortality included vital registration and verbal autopsy data, and surveillance data from China. Our outlier criteria excluded datapoints that (1) were implausibly high or low, (2) substantially conflicted with established age or temporal patterns, or (3) significantly conflicted with other data sources conducted from the same locations or locations with similar characteristics (ie, Socio-demographic Index).

Modelling strategy

There were no substantive changes to the modelling approach this round. The standard Cause of Death Ensemble modelling (CODEm) approach was used to estimate deaths due to chronic respiratory diseases (see appendix 1, section 4 for additional details). Separate models were conducted for male and female mortality, and the age range for both models was 0 to 95+ years.

Chronic respiratory diseases served as an envelope to the following causes:

- chronic obstructive pulmonary disease
- pneumoconiosis (silicosis, asbestosis, coal worker’s pneumoconiosis, other pneumoconiosis)
- asthma
- interstitial lung disease and pulmonary sarcoidosis

- other chronic respiratory diseases

The unadjusted death estimates for the individual chronic respiratory disease causes are summed and fit to the distribution of deaths estimated for the mortality envelope during the CoDCorrect adjustment process. This results in deaths recorded using non-specific coding systems, such as verbal autopsy, being included in the parent model and redistributed to the child models proportionately. This approach assumes that deaths reported in non-specific data sources have the same underlying distribution of specific causes as deaths reported in more specific data sources.

The following table lists the covariates included in the model. This requires that the covariate selected for the model must have the directional relationship with chronic respiratory deaths. For GBD 2023, additional COVID-19-related covariates (COVID-19 age-standardised death rate, mask use) were added to account for the association between chronic respiratory disease mortality and COVID-19. Covariate directions were selected based on the strength of evidence from scientific literature.

Table 1. Covariates used in chronic respiratory disease mortality modelling

Level	Covariate	Direction
1	Cumulative cigarettes (10 years)	+
	Cumulative cigarettes (20 years)	+
	Indoor air pollution (all cooking fuels)	+
2	Outdoor air pollution (PM 2.5)	+
	Smoking prevalence	+
	Proportion of population at elevation over 1500 m	+
	COVID-19 age-standardised death rate	+
	Healthcare Access and Quality Index	-
	Population density (over 1000 ppl/km^2, proportion)	+
3	Mask use	-
	Socio-demographic Index	-

Cirrhosis and other chronic liver diseases

Flowchart

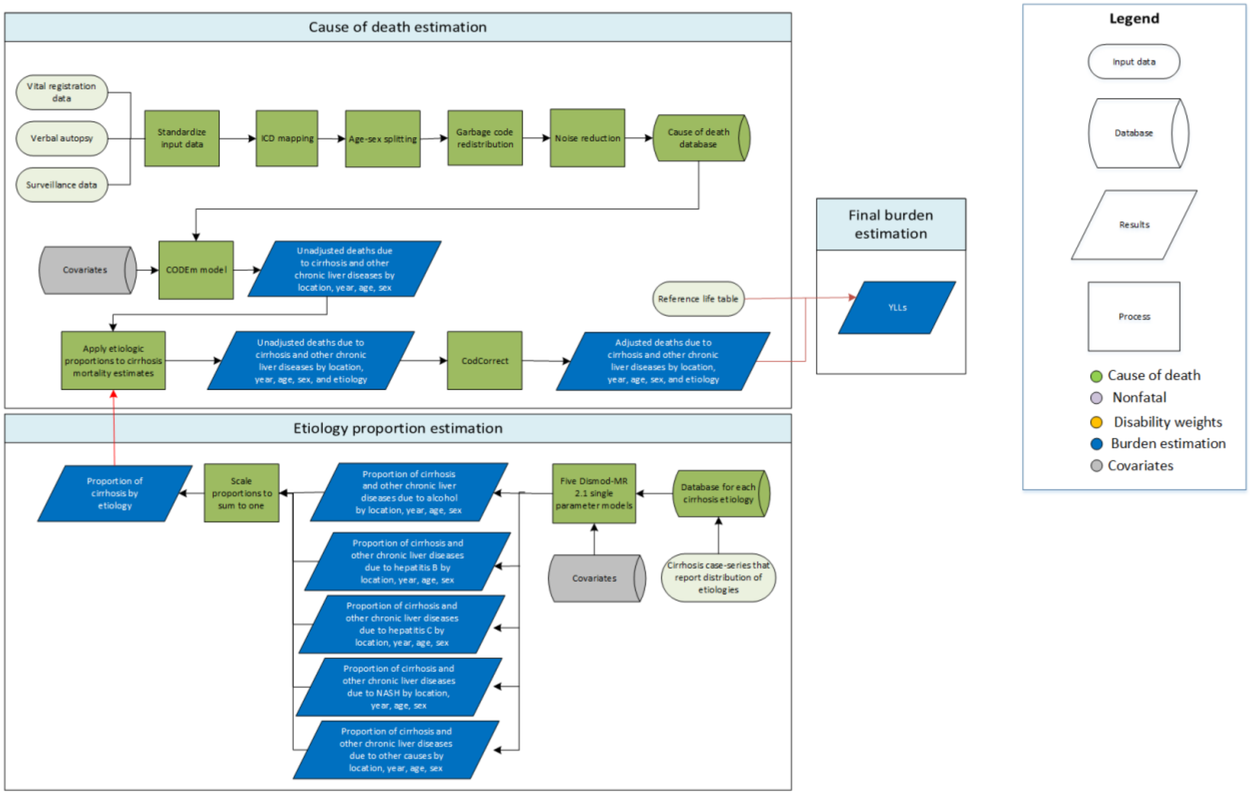

Input data and methodological summary for cirrhosis

In GBD, this Level 3 cause of death encompasses deaths due to cirrhosis and other chronic liver diseases (referred to as "cirrhosis" thereafter) caused by five aetiologies: alcohol use, chronic hepatitis B, chronic hepatitis C, non-alcoholic steatohepatitis (NASH, now known as metabolic dysfunction-associated steatohepatitis or MASH), and other causes such as Wilson's disease, hemochromatosis, and autoimmune disease. Total mortality due to this cause is modelled using causes of death (CoD) data in a standard Cause of Death Ensemble model (CODEm). The mortality due to the five aetiologies mentioned above are estimated by splitting the total by proportions of cirrhosis cases attributable to alcohol, chronic hepatitis B, chronic hepatitis C, non-alcoholic steatohepatitis, and other causes, which are estimated separately (see below for details).

Input data

Data used to estimate the mortality of total cirrhosis and other chronic liver diseases consisted of vital registration (VR), verbal autopsy (VA), and DHIS surveys from the CoD database. A list of ICD codes that were mapped to this cause, a list of sources included in the CoD database and the data quality as indicated by star-ratings for VR sources, and the steps used to process these data for modelling can be found in the section of this appendix on the CoD database and the tables and figures it refers to. In brief, processing of data for cirrhosis mortality estimation has been broadly similar in GBD 2019, 2021, and 2023, and it should be noted that a large fraction of deaths coded as "unspecified hepatitis" in vital

registration data are redistributed to cirrhosis. This decision was informed by a review of multiple cause of death data, which revealed that many deaths coded with "unspecified hepatitis" had cirrhosis in the cause of death chain. As a result, deaths coded as "unspecified hepatitis" are proportionally redistributed to cirrhosis and other valid GBD causes based on the observed frequency of underlying causes in directly mapped data sources.

One novel data processing step was employed in GBD 2023 in preparing data for cirrhosis that was not needed prior to GBD 2023—COVID-19 corrections—which are described elsewhere in this appendix.

Data exclusions

After all data processing steps were complete, datapoints for all locations and years were systematically reviewed to identify outliers or other data limitations not fully overcome by CoD data processing methods. For total cirrhosis and other chronic liver diseases, the following were identified and excluded prior to modelling:

Super-region	Countries	Years and types excluded
Central Europe, eastern Europe, and central Asia	No exclusions	
High income	No exclusions	
Southeast Asia, east Asia, and Oceania	Cambodia<br>Myanmar<br>Kiribati	2009, verbal autopsy (sole year)<br>2007, 2016, 2017, verbal autopsy (all years)<br>All years, vital registration
Latin America and Caribbean	No exclusions	
North Africa and Middle East	Afghanistan<br>Türkiye (2002 data)	2015, verbal autopsy (sole year)<br>2006, verbal autopsy (vital registration is preferred source)
Sub-Saharan Africa	Democratic Republic of Congo<br>Somalia<br><br>Kenya<br>Ethiopia<br>South Africa	1986, verbal autopsy (sole year)<br>2017, verbal autopsy (sole year)<br><br>2019, Child Health and Mortality Prevention Surveillance (verbal autopsy and vital registration are preferred sources)
South Asia	Nepal<br><br>India	2007, verbal autopsy (sole year)<br><br><br>Department of Economics and Statistics' Medically Certified Causes of Death records in India excluded due to inclusion of hospital deaths in urban areas only (India Sample Registration System is preferred source)

Data that were modelled to inform aetiological proportions of the total cirrhosis envelope came from a total of 105 case-series studies that report the proportions of cirrhosis cases attributable to alcohol, chronic hepatitis B, chronic hepatitis C, NASH (now known as MASH), and other causes. These studies were identified from a series of systematic literature reviews and contributions to the Global Health Data Exchange (GHDx) from network collaborators. The latest systematic review was undertaken for GBD 2021 using the following search string:

(((((((hepatitis b[Title/Abstract] OR "hepatitis b antibod*"[Title/Abstract] OR "hepatitis b antigens"[Title/Abstract] OR hbsag[Title/Abstract])) OR (hepatitis c[Title/Abstract] OR "hepatitis c antibod*"[Title/Abstract] OR "hepatitis c antigens"[Title/Abstract] OR "anti-hcv"[Title/Abstract] OR HCV-RNA[Title/Abstract]))))) AND (alcohol* OR "alcoholic disorders" OR cirrhosis))) AND (NAFLD OR "non-alcoholic fatty liver disease" OR NAFL)

Review and extraction of sources identified by this search string were batched to allow for serial data updates even prior to completion of the systematic review. See the non-fatal methods appendix on cirrhosis estimation for additional details on the case-series data identified and how they are processed. This is the PRISMA diagram of the portion of the systematic literature review completed and added in GBD 2021:

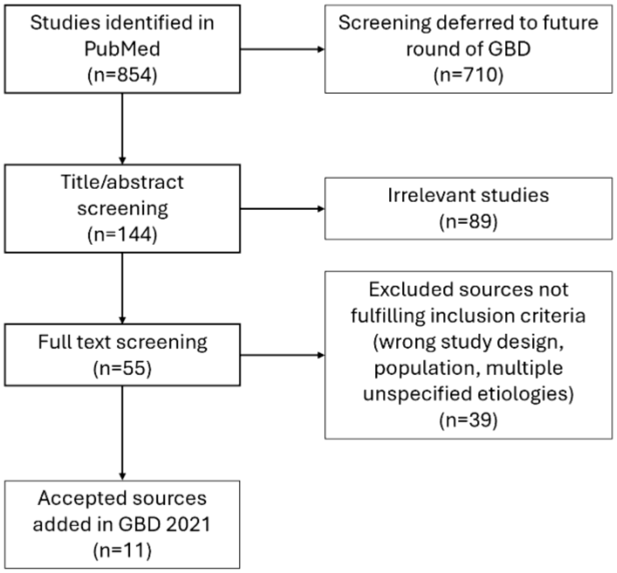

Modelling strategy

We modelled total cirrhosis and other chronic liver disease mortality using a standard CODEm approach. Further details on this method can be found in the appendix section on cause of death modelling methods. Fatal estimation for this cause was restricted to ages 1–95+ years. We hybridised the results of global and data-rich models to acquire unadjusted results. Predictive covariates entered for selection in this CODEm model are shown in Table 1. No substantive changes were made in GBD 2023 from the modelling strategy used in GBD 2021.

Table 1: Covariates used in CODem model for cirrhosis and other chronic liver diseases (total, without regard to aetiology)

Level	Covariate	Direction
1	Litres of alcohol consumed per capita	+
	Vaccine-adjusted HbSAg seroprevalence, age-standardised	+
	Chronic hepatitis C, age-standardised	+
	Hepatitis B vaccine coverage proportion, aged through time	-
2	Mean BMI	+
	Healthcare Access and Quality Index	-
	Diabetes prevalence, age-standardised	+
	Intravenous drug use (proportion by age)	+
3	Education (years per capita)	-
	Lag-distributed income (LDI) (ln transformation)	-
	Socio-demographic index	-

Proportions of cirrhosis due to alcohol, hepatitis B, hepatitis C, NASH (now known as MASH), and other causes were modeled using DisMod-MR 2.1. Proportions from the five aetiology models were then rescaled to sum to one (at the draw level) and used to split the total cirrhosis mortality estimates from CODEm. The summary of DisMod model covariates is listed in Tables 2-6. See the non-fatal methods appendix on cirrhosis estimation for additional details on modelling of these aetiological proportions.

Table 2: Covariates used in the proportion of cirrhosis due to hepatitis B DisMod-MR meta-regression model

Covariate	Exponentiated beta (95% uncertainty interval)
Vaccine adjusted HBsAg seroprevalence age standardised	1.64 (1.06–2.57)
Proportion of liver cancer due to hepatitis B, age-standardised	1.28 (1.02–1.72)
Hepatitis B vaccine coverage (proportion), aged through time	0.55 (0.38–0.88)
Proportion of cirrhosis due to alcohol	0.46 (0.37–0.65)
Proportion of cirrhosis due to hepatitis C	0.65 (0.45–0.93)
Proportion of cirrhosis due to other causes	0.68 (0.47–0.94)
Proportion of cirrhosis due to NASH	0.59 (0.39–0.94)

Table 3: Covariates used in the proportion of cirrhosis due to hepatitis C DisMod-MR meta-regression model

Covariate	Exponentiated beta (95% uncertainty interval)
Chronic hepatitis C, age-standardised	1.79 (1.10–2.63)
Proportion of liver cancer due to hepatitis C, age-standardised	1.86 (1.19–2.63)

Proportion of cirrhosis due to alcohol	0.41 (0.37–0.50)
Proportion of cirrhosis due to hepatitis B	0.53 (0.38–0.80)
Proportion of cirrhosis due to other causes	0.94 (0.82–1.00)
Proportion of cirrhosis due to NASH	0.63 (0.41–0.94)

Table 4: Covariates used in the proportion of cirrhosis due to alcohol DisMod-MR meta-regression model

Covariate	Exponentiated beta (95% uncertainty interval)
Litres of alcohol consumed per capita	1.01 (1.00–1.03)
Alcohol drinker proportion, age-standardised	1.58 (1.14–2.19)
Proportion of liver cancer due to alcohol, age-standardised	1.39 (1.02–2.17)

Table 5: Covariates used in the proportion of cirrhosis due to other causes DisMod-MR meta-regression model

Covariate	Exponentiated beta (95% uncertainty interval)
Proportion of liver cancer due to other causes, age-standardised	1.91 (1.22–2.64)

Table 6: Covariates used in the proportion of cirrhosis due to NASH DisMod-MR meta-regression model

Covariate	Exponentiated beta (95% uncertainty interval)
Mean BMI	1.27 (1.06–1.54)
Prevalence of obesity	2.19 (1.06–5.45)
NAFLD/NASH prevalence	3.22 (1.27–6.78)
Proportion of liver cancer due to NASH, age-standardised	2.30 (1.07–5.75)

The unadjusted results of cause-specific death estimation were then rescaled up the GBD cause hierarchy so that all cause-specific deaths added up to all-cause mortality for each year, age, sex, and location. Adjusted deaths were compared to the reference life table to calculate final YLLs.

Cocaine use disorder

Flowchart

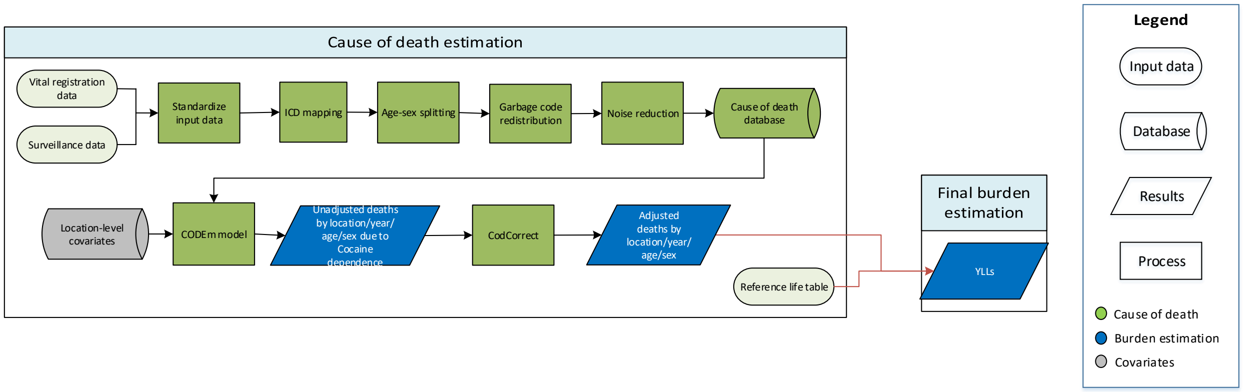

Input data and methodological summary for cocaine use disorders

Input data

Data used to estimate cocaine use disorders mortality were from vital registration and surveillance sources from the cause of death (CoD) database. Data from countries with sparse yet heterogeneous data were excluded as the data exaggerated fluctuations in deaths and gave implausible regional patterns, according to in-country and subject matter experts. Excluded data were typically from low- and middle-income countries. A full description of changes to coding and redistribution is described in the write-up on drug use disorders.

Modelling strategy

In the GBD 2023 round, the modelling strategy for cocaine use disorders remained largely unchanged.

The standard Cause of Death Ensemble modelling (CODEm) approach was used to estimate deaths due to cocaine use disorders (see, appendix 1, section 4). Deaths are modelled in age groups 15 years and older, assuming that cocaine use disorders-related deaths from intentional use in younger age groups are extremely rare and there are not sufficient data at the population level to make estimates. Model covariate inclusion was based on empirical evidence and expert feedback, which resulted in a set of model covariates that reflected alcohol consumption, smoking, education, health system access, income per capita, and Socio-demographic Index (SDI) (Table 1).

Table 1: Covariates used in the cocaine use disorders mortality modelling

Level	Covariate	Direction
1	Alcohol (litres per capita)	+
	Current drinking prevalence	+

	Intravenous drug use, age-standardised	+
	Intravenous drug use, age-specific	+
	Cumulative cigarettes (10 years)	+
	Cumulative cigarettes (5 years)	+
	Cigarettes per capita	+
	Smoking prevalence	+
2	Healthcare Access and Quality Index	-
3	Log LDI (I$ per capita)	+
	Education (years per capita)	+
	Socio-demographic Index	+

Congenital birth defects: neural tube defects, congenital heart anomalies, orofacial clefts, Down syndrome, Turner syndrome, Klinefelter syndrome, other chromosomal disorders, congenital musculoskeletal anomalies, urogenital congenital anomalies, digestive congenital anomalies, and other congenital birth defects

Flowchart

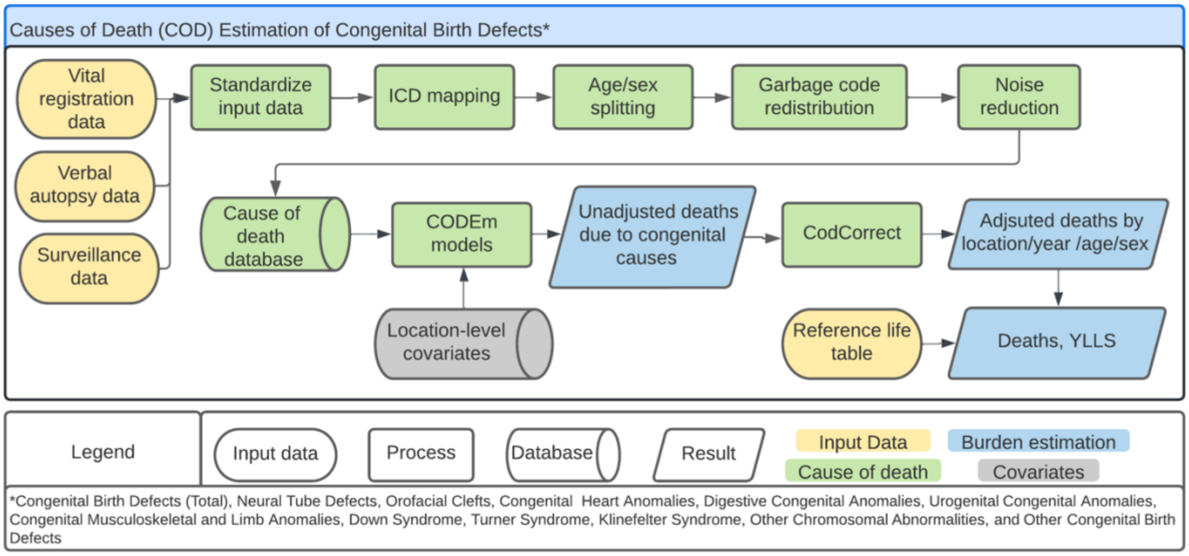

Input data

For GBD 2023, input data for estimating mortality due to congenital anomalies were centrally extracted, processed, and stored in the cause of death (CoD) database. Vital registration (VR) was the dominant data type, followed by verbal autopsy (VA) and surveillance. Those CoD data sources that specified the sub-cause of birth defect were included in estimation of both the parent congenital anomalies model as well as in subtype-specific models.

For GBD 2023, the majority of VA data were outliered in those over 5 years old as the age patterns were unreliable and led to poor model performance in the under-5 age groups. We also excluded some data sources from the parent model where only a subset of sub-causes were specified (eg, congenital heart disease, neural tube defects, and other congenital anomalies) and the sum of the sub-causes clearly represented systematic under-reporting of one of the sub-causes. Systematic under-reporting was suspected when sex- and age-specific rates were more than an order of magnitude lower than neighbouring or comparable locations. Data sources for those locations were still included by default for sub-cause-specific models because under-reporting of the total was not assumed to necessarily be associated with under-reporting of all of the component conditions.

Modelling strategy

All types of congenital anomalies were estimated using Cause of Death Ensemble modelling (CODEm) for GBD 2023, as was done for previous iterations of the GBD study. Specific causes included neural tube defects, congenital heart anomalies, orofacial clefts, Down syndrome, other chromosomal anomalies, congenital musculoskeletal anomalies, urogenital congenital anomalies, digestive congenital anomalies, and other congenital birth defects. We assumed no mortality from either Klinefelter syndrome or Turner syndrome, for which we model non-fatal outcomes only. For GBD 2023, we modelled congenital anomalies as a cause of death for ages 0–69 years only, assuming that all mortality from congenital conditions occurs before age 70 years of age.

Table 1: Covariates tested for CODEm model of overall congenital birth defects

Covariate	Transformation	Level	Direction
Maternal alcohol consumption during pregnancy (proportion)	None	1	+
In-facility delivery (proportion)	None	1	-
Livebirths 35+ (proportion)	None	1	+
Folic acid unadjusted (µg)	None	1	-
Folic acid fortification index	None	1	-
Birth prevalence of congenital heart disease	None	1	+
Birth prevalence of chromosomal anomalies	None	1	+
Legality of abortion	None	2	-
Antenatal care (1 visit) coverage (proportion)	None	2	-
Age-standardised summary exposure value (SEV) of smoking	None	2	+
Antenatal care (4 visits) coverage (proportion)	None	2	-
Healthcare Access and Quality Index	None	2	-
Maternal education (years per capita)	None	3	-
Alcohol (litres per capita)	None	3	+
Age-standardised SEV of low fruits	None	3	+
Outdoor air pollution (PM2.5)	None	3	+
Age-standardised SEV of household air pollution	None	3	+
Socio-demographic Index	None	3	-
Age-standardised SEV of low vegetables	None	3	+

Table 2: Covariates tested for CODEm model of neural tube defects

Covariate	Transformation	Level	Direction
In-facility delivery (proportion)	None	1	-
Folic acid unadjusted (µg)	None	1	-
Folic acid fortification index	None	1	-
Healthcare Access and Quality Index	None	2	-
Antenatal care (1 visit) coverage (proportion)	None	2	-
Antenatal care (4 visits) coverage (proportion)	None	2	-
Age-standardised SEV of smoking	None	2	+
Age-standardised SEV of low fruits	None	3	+
Age-standardised SEV of low vegetables	None	3	+
Maternal education (years per capita)	None	3	-
Socio-demographic Index	None	3	-
Legality of abortion	None	2	-
Maternal alcohol consumption during pregnancy (proportion)	None	3	+
Age-standardised SEV of household air pollution	None	3	+

Age-standardised SEV of fasting plasma glucose	None	3	+
Litres of alcohol consumed per capita	None	3	+

Table 3: Covariates selected for CODEm model of congenital heart anomalies

Covariate	Transformation	Level	Direction
Maternal alcohol consumption during pregnancy (proportion)	None	1	+
Birth prevalence of congenital heart disease	None	1	+
Socio-demographic Index	Log	2	-
Age-standardised SEV of smoking	None	2	+
Age-standardised SEV of diabetes	None	2	+
Healthcare Access and Quality Index	None	2	-
Legality of abortion	None	2	-
Antenatal care (1 visit) coverage (proportion)	None	2	-
In-facility delivery (proportion)	None	2	-
Maternal education (years per capita)	None	3	-
Alcohol (litres per capita)	None	3	+
Antenatal care (4 visits) coverage (proportion)	None	3	-
Skilled birth attendance (proportion)	None	3	-
Livebirths 35+ (proportion)	None	3	+

Table 4: Covariates selected for CODEm model of cleft lip and cleft palate

Covariate	Transformation	Level	Direction
Socio-demographic Index	None	1	-
Folic acid fortification index	None	1	-
Age-standardised SEV of diabetes	None	2	+
Maternal alcohol consumption during pregnancy (proportion)	None	2	+
Healthcare Access and Quality Index	None	2	-
Legality of abortion	None	2	-
Skilled birth attendance (proportion)	None	2	-
Age-standardised SEV of smoking	None	2	+
Age-standardised SEV of household air pollution	None	3	+
Age-standardised SEV of low vegetables	None	3	+
Alcohol (litres per capita)	None	3	+
Antenatal care (4 visits) coverage (proportion)	None	3	-
Maternal education (years per capita)	None	3	-
Age-standardised SEV of low fruits	None	3	+
Antenatal care (1 visit) coverage (proportion)	None	3	-

Table 5: Covariates selected for CODEm model of Down syndrome

Covariate	Transformation	Level	Direction
Livebirths 35+ (proportion)	None	1	+
Legality of abortion	None	1	-
Livebirths 40+ (proportion)	None	1	+
Birth prevalence of chromosomal anomalies	None	1	+
Socio-demographic Index	None	2	-
In-facility delivery (proportion)	None	2	-
Healthcare Access and Quality Index	None	2	-
Maternal alcohol consumption during pregnancy (proportion)	None	3	+
Antenatal care (1 visit) coverage (proportion)	None	3	-

Maternal education (years per capita)	None	3	-
Age-standardised SEV of household air pollution	None	3	+
Antenatal care (4 visits) coverage (proportion)	None	3	-
Age-standardised SEV of low vegetables	None	3	-
Age-standardised SEV of smoking	None	3	+
Litres of alcohol consumed per capita	None	3	+

Table 6: Covariates selected for CODEm model of other chromosomal abnormalities

Covariate	Transformation	Level	Direction
Livebirths 35+ (proportion)	None	1	+
Livebirths 40+ (proportion)	None	1	+
Legality of abortion	None	1	-
Lag-distributed income (LDI) (I$ per capita)	Log	2	-
Healthcare Access and Quality Index	None	2	-
Antenatal care (4 visits) coverage (proportion)	None	2	-
Antenatal care (1 visit) coverage (proportion)	None	2	-
In-facility delivery (proportion)	None	2	-
Maternal alcohol consumption during pregnancy (proportion)	None	2	+
Socio-demographic Index	None	3	-
Alcohol (litres per capita)	None	3	+
Age-standardised SEV of smoking	None	3	+
Age-standardised SEV of household air pollution	None	3	+
Maternal education (years per capita)	None	3	-
Skilled birth attendance (proportion)	None	3	-

Table 7: Covariates selected for CODEm model of congenital musculoskeletal and limb anomalies

Covariate	Transformation	Level	Direction
Maternal alcohol consumption during pregnancy (proportion)	None	1	+
Legality of abortion	None	1	-
Socio-demographic Index	None	1	-
Healthcare Access and Quality Index	None	1	-
In-facility delivery (proportion)	None	2	-
Ag-standardised SEV of diabetes	None	2	+
Age-standardised SEV of household air pollution	None	2	+
Age-standardised SEV of smoking	None	2	+
Antenatal care (4 visits) coverage (proportion)	None	3	-
Alcohol (litres per capita)	None	3	+
Age-standardised SEV of low fruits	None	3	+
Age-standardised SEV of low vegetables	None	3	+
Maternal education (years per capita)	None	3	-
Antenatal care (1 visit) coverage (proportion)	None	3	-
LDI per capita	Log	3	-

Table 8: Covariates selected for CODEm model of urogenital congenital anomalies

Covariate	Transformation	Level	Direction
Age-standardised SEV of smoking	None	1	+
Maternal alcohol consumption during pregnancy (proportion)	None	1	+
Healthcare Access and Quality Index	None	2	-
Diabetes age-standardised prevalence (proportion)	None	2	+

Socio-demographic Index	None	2	-
Age-standardised SEV of outdoor air pollution	None	2	+
In-facility delivery (proportion)	None	2	-
Age-standardised SEV of household air pollution	None	2	+
Antenatal care (1 visit) coverage (proportion)	None	3	-
Alcohol (litres per capita)	None	3	+
Maternal education (years per capita)	None	3	-
LDI (I$ per capita)	Log	3	-
Antenatal care (4 visits) coverage (proportion)	None	3	-

Table 9: Covariates selected for CODEm model of digestive congenital anomalies

Covariate	Transformation	Level	Direction
Maternal alcohol consumption during pregnancy (proportion)	None	1	+
Age-standardised SEV of smoking	None	1	+
Age-standardised SEV of household air pollution	None	2	+
Age-standardised SEV for high fasting plasma glucose	None	2	+
Socio-demographic Index	None	2	-
Age-standardised SEV for high body-mass index	None	2	+
In-facility delivery (proportion)	None	2	-
Healthcare Access and Quality Index	None	2	-
Alcohol (litres per capita)	None	3	+
Maternal education (years per capita)	None	3	-
Age-standardised SEV of low vegetables	None	3	+
Antenatal care (1 visit) coverage (proportion)	None	3	-
Antenatal care (4 visits) coverage (proportion)	None	3	-
Age-standardised SEV of low fruits	None	3	+
LDI (I$ per capita)	Log	3	-
Maternal care and immunisation	None	3	-

Table 10: Covariates selected for CODEm model of other congenital birth defects

Covariate	Transformation	Level	Direction
Maternal alcohol consumption during pregnancy (proportion)	None	1	+
Livebirths 35+ (proportion)	None	1	+
Age-standardised SEV for smoking	None	2	+
Maternal education (years per capita)	None	2	-
Legality of abortion	None	2	-
In-facility delivery (proportion)	None	2	-
Age-standardised SEV of household air pollution	None	2	+
Healthcare Access and Quality Index	None	2	-
Antenatal care (1 visit) coverage (proportion)	None	3	-
Age-standardised SEV for high fasting plasma glucose	None	3	+
LDI (I$ per capita)	Log	3	-
Socio-demographic Index	None	3	-
Antenatal care (4 visits) coverage (proportion)	None	3	-
Alcohol (litres per capita)	None	3	+

Cystic echinococcosis

Flowchart

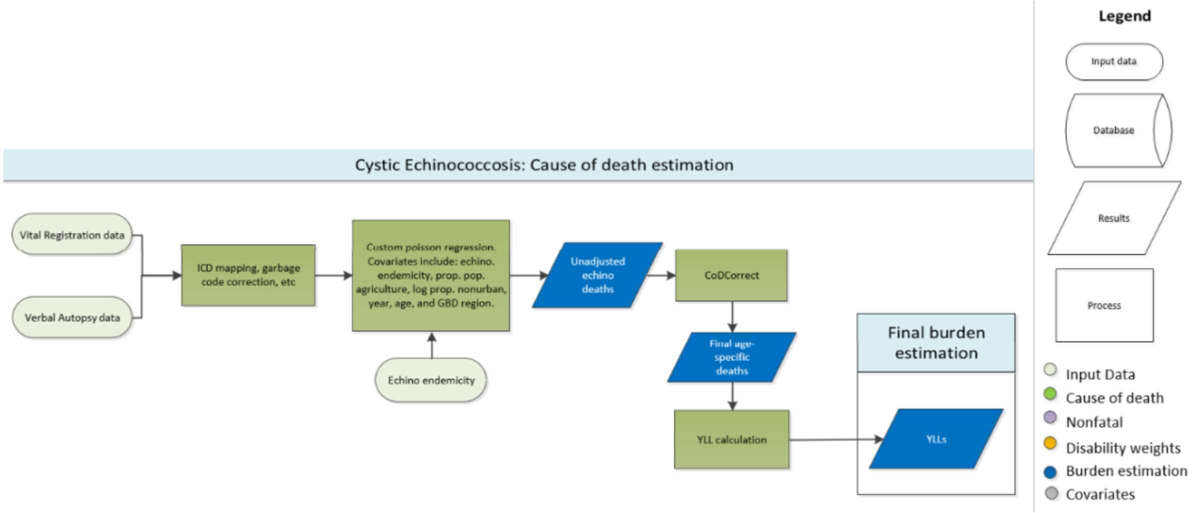

Input data and methodological summary for cystic echinococcosis

Input data

Mortality due to cystic echinococcosis (CE) was modelled using vital registration data and covariates. The Mortality and Cause of Death team provided country-year-age-sex-specific vital registration. The ICD codes mapped to cystic echinococcosis are as presented in Table 1.

Table 1: ICD-9 codes mapped to CE

ICD Code	ICD Name
122	Echinococcosis
122.0	*Echinococcus granulosus* infection of liver
122.1	*Echinococcus granulosus* infection of lung
122.2	*Echinococcus granulosus* infection of thyroid
122.3	*Echinococcus granulosus* infection, other
122.4	*Echinococcus granulosus* infection, unspecified
122.8	Echinococcosis unspecified, of liver
122.9	Echinococcosis other and unspecified

Table 2: ICD-10 codes mapped to CE

ICD Code	ICD Name
B67.0	*Echinococcus granulosus* infection of liver
B67.1	*Echinococcus granulosus* infection of lung
B67.2	*Echinococcus granulosus* infection of bone
B67.3	*Echinococcus granulosus* infection, other and multiple sites
B67.31	*Echinococcus granulosus* infection, thyroid gland
B67.32	*Echinococcus granulosus* infection, multiple sites
B67.39	*Echinococcus granulosus* infection, other sites
B67.4	*Echinococcus granulosus* infection, unspecified
B67.8	Echinococcosis, unspecified, of liver
B67.9	Echinococcosis, other and unspecified
B67.90	Echinococcosis, unspecified
B67.99	Other echinococcosis

As vital registration data are not available for all locations, ages, and years, we modelled cause-specific mortality as a function of several predictive covariates.

We created a categorical cystic echinococcosis endemicity covariate based on expert opinion and an endemicity map published by WHO.[1] We assigned GBD locations to one of four categories: probable absence, rare and/or sporadic transmission, suspected and/or confirmed transmission, and high endemic areas.

We based further selection of covariates on a meta-analysis of potential risk factors associated with cystic echinococcosis.[2] According to the meta-analysis, statistically significant potential risk factors include living in rural endemic areas, slaughtering, feeding dogs with viscera, and low income. Hence, we also included two other covariates: the proportion of the population participating in agricultural activities and the log of proportion non-urban.

Geographical restrictions

Although a full literature review was last conducted in GBD 2015, in GBD 2019 we reviewed all references pertaining to CE in "Global Distribution of Alveolar and Cystic Echinococcosis" by Deplazes and colleagues[3] and supplemented with targeted searches to classify location-years in PubMed and the GHDx. Locations that were geographically restricted in any given year did not have estimates made for them but could have imported cases attributed to them at a later stage. Evidence of absence or presence was not available for every location for each year, and so assumptions were made for missing years by taking into consideration the epidemiological characteristics of the disease. If evidence indicated disease presence at a given point in time, we assumed presence for all years. If evidence indicated disease absence, we assumed absence for all years. If evidence indicated a change in status (ie, from absent to present, or present to absent) between two non-consecutive years, then we conducted targeted searches to ascertain the relevant year of introduction or elimination for that location. In the cases where presence or absence information was missing from the start or end years of our study interval (1990–2015) without evidence of any introduction or elimination events within the

interval, we applied the status of the first and last presence/absence observations, respectively, to all years between the interval bound and the observation year.

Modelling strategy

We implemented a Poisson regression model to estimate deaths due to cystic echinococcosis. The Poisson regression was selected due to its suitability for modelling count data that are not over-dispersed. Covariates for the model, including echinococcosis endemicity, log of lag-distributed income per capita, proportion of the population participating in agricultural activities, and education (years per capita), were incorporated into the model to influence the global trend due to paucity of data. Random effects were used on location with random slopes on age by location. A multivariate normal distribution using the mean and variance-covariance matrix from the model was used to generate 1000 draws of deaths due to cystic echinococcosis. The final model was selected based on how well the estimated numbers fit the input data and how plausible the predicted distribution of disease was over time and with age.

Changes from GBD 2021 to GBD 2023

There were no substantive changes to the modelling strategy for GBD 2023.

References

1. World Health Organization (2010). Global Echinococcosis Granulosus and Cystic Echinococcosis (hydatidosis) Worldwide 2009.

2. Possenti A, Manzano-Román R, Sanchez-Ovejero C, Boufana B, La Torre G, Siles-Lucas M, Casulli A. Potential risk factors associated with human cystic echinococcosis: systematic review and meta-analysis. *PLoS Neglected Tropical Diseases*. 2016;10(11):e0005114. doi: 10.1371/journal.pntd.0005114.

3. Deplazes P, Rinaldi L, Rojas CA, Torgerson PR, Harandi MF, Romig T, Antolova D, Schurer JM, Lahmar S, Cringoli GJ, Magambo J. Global distribution of alveolar and cystic echinococcosis. Advances in Parasitology. 2017 Jan 1;95:315-493.

Cysticercosis

Flowchart

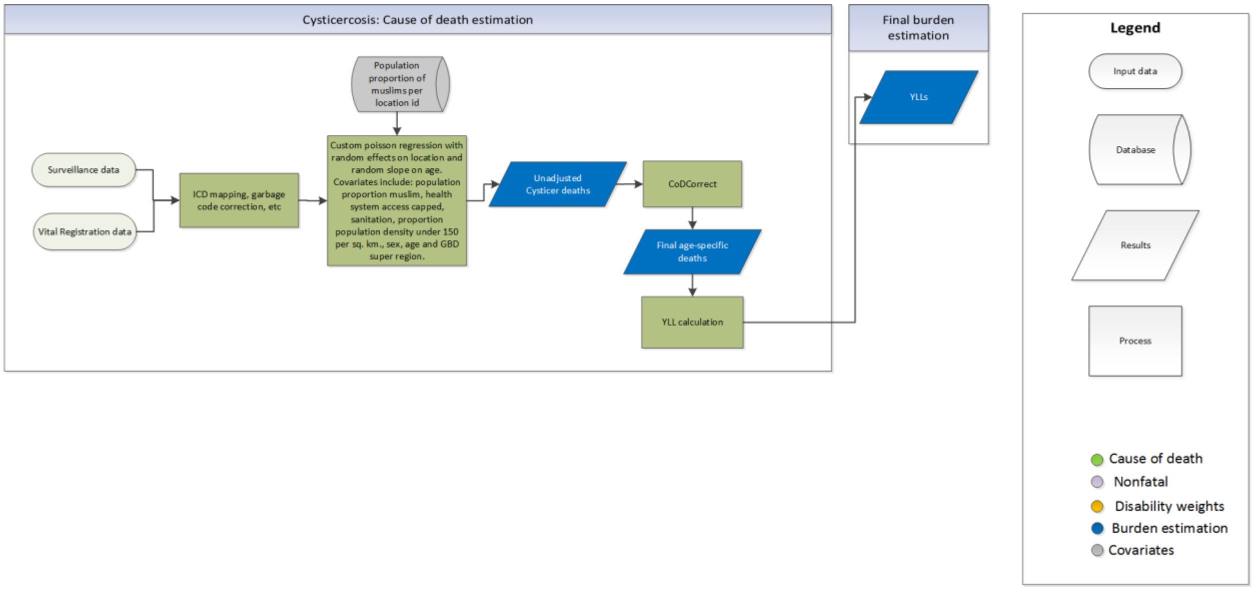

Input data and methodological summary for cysticercosis

Input data

Mortality data for fatal cases of cysticercosis relied on vital registration and surveillance data from endemic countries. In addition, we used data from the Pew Research Center on the proportion of the population that is Muslim by country. The primary covariates adjusted for in the fatal model were proportion of the population that is Muslim; a composite indicator of maternal care and immunisation coverage (combining estimates of antenatal care, in-facility delivery skilled birth attendance, and vaccine [DTP3 and MCV1] coverage); proportion of the population with access to sanitation; proportion of the country with population density under 150 people per square kilometer; sex; age; and GBD super-region.

Geographical restrictions

We conducted a literature review to determine the geographical extent of the disease and classify locations based on whether the disease is absent or present in each year. Locations that were geographically restricted in any given year did not have estimates made for them but could have imported cases attributed to them at a later stage. Of note, we did not attempt a complete systematic review of geographical restrictions, since a single high-quality source could offer sufficient evidence of its presence. Evidence of absence or presence was not available for every location for each year, so assumptions were made for missing years by taking into consideration the epidemiological characteristics of the disease. If evidence indicated disease presence for two non-consecutive years, we assumed presence for all years between the two. If evidence indicated disease absence for two non-consecutive years, we assumed absence for all years between the two. If evidence indicated a change in

status (ie, from absent to present or present to absent) between two non-consecutive years, we conducted targeted searches to ascertain the relevant year of introduction or elimination for that location. In the cases where presence or absence information was missing for the start or end years of the study interval (1990–2023) and without evidence of any introduction or elimination events within the interval, we applied the status of the first and last presence and absence observations, respectively, to all years between the interval bound and the observation year. For cysticercosis, we performed targeted searches to classify location-years in PubMed and Google Scholar. The map was populated by 21 peer-reviewed articles, meta-analyses, and WHO reports.

Modelling strategy

Globally, deaths attributed to cysticercosis are relatively low. Therefore, a Poisson model was used to model cysticercosis deaths due to its suitability for count data. This model choice was validated by tests for overdispersion. Random intercepts were used on location with random slopes on age by location. A multivariate normal distribution using the mean and variance-covariance matrix from the model was used to generate 1000 draws of deaths due to cysticercosis.

Since the Pew Research Center has data on proportion of Muslims only by country, we applied the national proportions to subnational locations. We acknowledge that this solution does not account for sometimes large, expected differences in proportions of Muslims within a country and that the proportion of the population who identify as Muslim is an incomplete proxy for cysticercosis exposure. We continue to investigate data sources that could allow for the creation of more granular and more proximate covariates to represent cysticercosis risk. In addition to the proportion of Muslims, we used maternal care and immunisation, proportion with access to sanitation (in logit space), and proportion of the country with population density under 150 people per square kilometer as covariates.

Changes from GBD 2021 to GBD 2023

There were no substantive changes to the modelling strategy for GBD 2023.

Decubitus ulcer

Flowchart

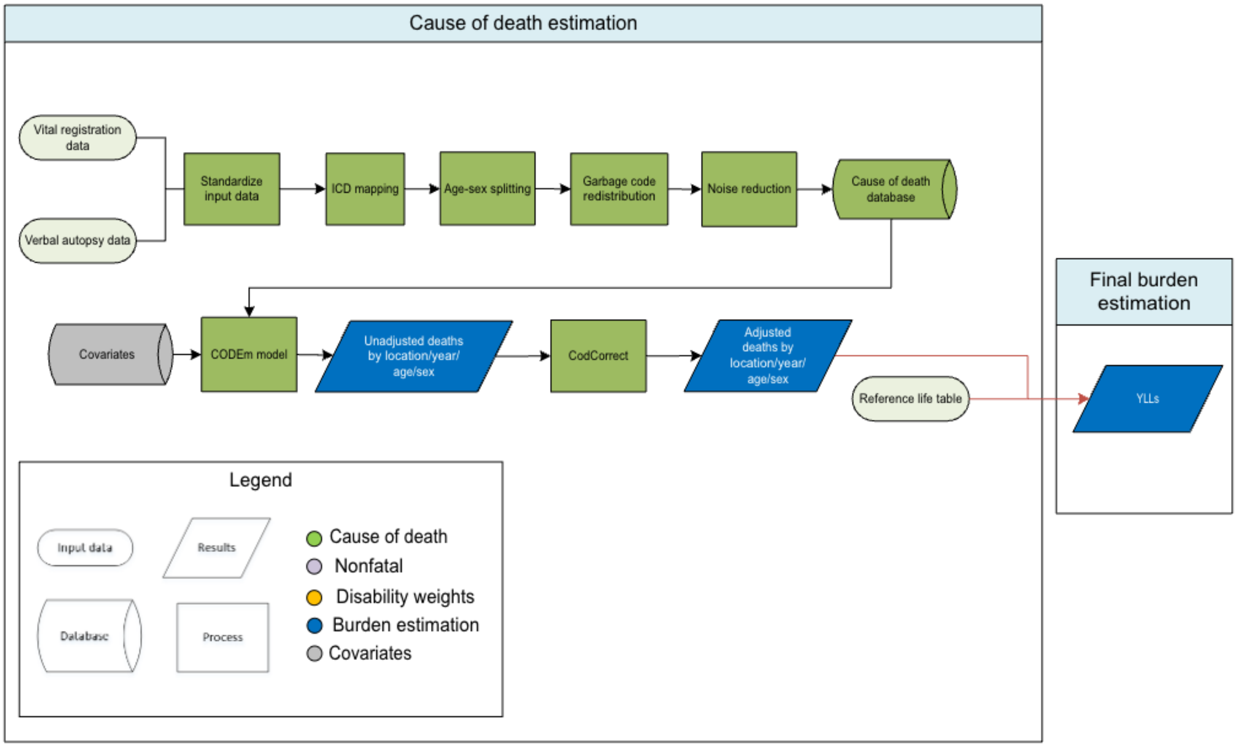

Input data and methodological summary for decubitus ulcer

Input data

Data used to estimate mortality due to decubitus ulcer consisted of vital registration and verbal autopsy data from the cause of death (CoD) database. Outlier criteria excluded datapoints that were implausibly under/over-reporting relative to global or regional patterns and data from datasets with small populations.

Modelling strategy

The decubitus ulcer model is estimated by CODEm model with standard CODEm parameters along with the CoD database and location-level covariates as inputs. We hybridised separate global and data-rich models to acquire unadjusted results, which we finalised and adjusted using CoDCorrect to reach final years of life lost (YLLs) due to decubitus ulcer.

Compared to GBD 2021, there are several updates that were made to the decubitus ulcer estimation strategy. First, we have received new data for decubitus ulcer for multiple location-years. There were 102 data sources added to the cause of death database, which led to improved estimates, particularly for recent years. Key location-years that impacted the decubitus ulcer estimates were from Bangladesh, Indonesia, the Philippines, Malaysia, Ethiopia, the USA, Brazil, Saudi Arabia, China, and India.

Additionally, in GBD 2023, CODEm began using an all-cause mortality envelope that is inclusive of HIV, whereas in previous rounds, the all-cause mortality envelope was HIV-free. This can lead to changes in cause fractions and rates when compared to previous GBD rounds.

Table 1. Covariates used in decubitus ulcer mortality modelling

Level	Covariate	Direction
1	Alcohol (litres per capita)	+
	Prevalence of overweight and obesity	+
	Diabetes fasting plasma glucose (mmol/L), by age	+
	Improved water source (proportion with access)	-
2	Healthcare Access and Quality Index	-
	Cumulative cigarettes (5 years)	+
	Cumulative cigarettes (10 years)	+
	Smoking prevalence	+
3	Education (years per capita)	-
	Summary exposure variable (SEV) scalar for unsafe sanitation	+
	Socio-demographic Index	-
	Lag-distributed income (per capita)	-

Dengue

Flowchart

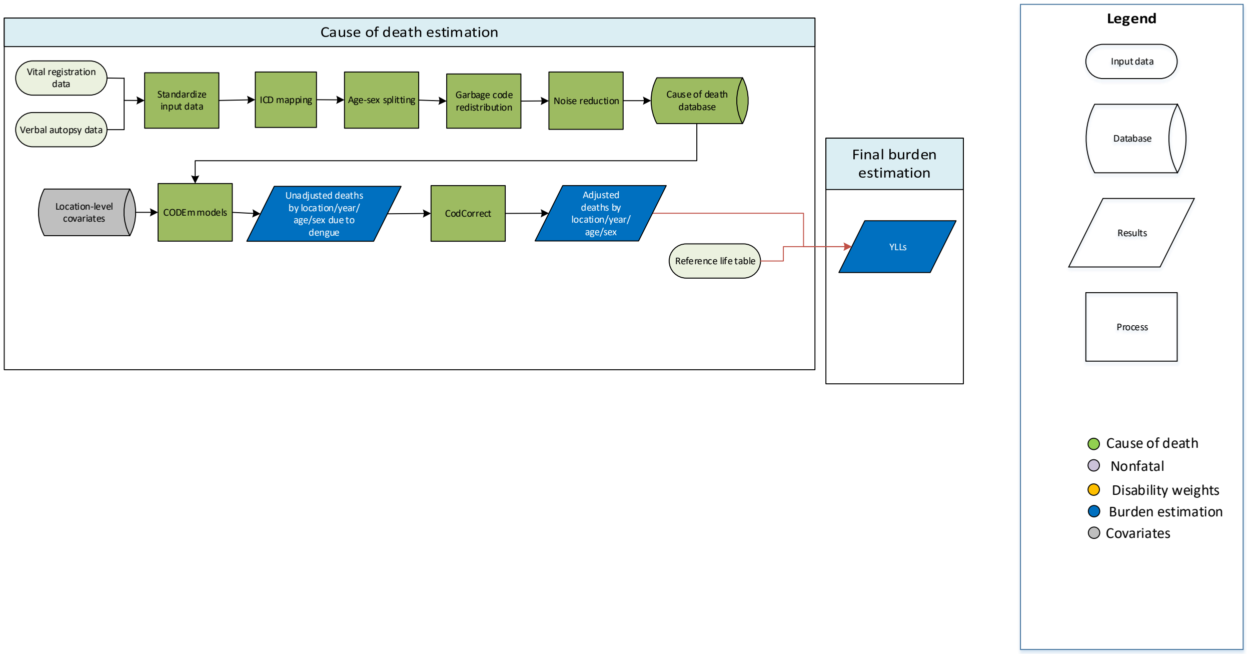

Input data and methodological summary for dengue

Input data

We modelled dengue mortality using all available data in the cause of death database. Datapoints were outliered if they reported an improbably low number of dengue deaths (eg, zero dengue deaths in a hyper-endemic country) or an improbably high number of dengue deaths.

Geographical restrictions

We last updated the geographical restrictions for GBD 2019. The geographical restrictions determine whether a location is considered non-endemic (and, therefore, will have estimates based on the imported case model) in a given year. We derived our geographical restrictions for 2010 from Brady and colleagues.[1] We have also refreshed our literature review to determine locations and years in which dengue was introduced or eliminated, to allow for time-varying geographical restrictions.

Modelling strategy

We modelled dengue mortality using a three-model hybrid approach: 1) a CODEm model of all dengue-endemic locations using all data in the CoD database for these locations; 2) a shocks model to account for outbreaks; and 3) estimates of mortality from imported cases in non-endemic, data-rich countries. Where dengue deaths were reported in non-endemic data-rich countries, we produced non-zero

estimates by drawing from a beta distribution based on number of reported deaths and the underlying sample size.

We use country-level covariates to inform our model. The *Level* is the associated strength of relationship between the covariate and LRI mortality, ranked from 1 (proximally related) to 3 (distally related). The direction is the forced direction of the association between the covariate and dengue mortality (Table 1).

Table 1. CODEm model covariates and directions

Level	Covariate	Direction
1	Population density (over 1000 ppl/km^2, proportion)	+
	Population weighted probability of dengue transmission	+
2	Health system access	-
	Latitude under 15 (proportion)	+
	Elevation under 100 m (proportion)	+
	Rainfall quintile 4 (proportion)	+
	Rainfall quintile 5 (proportion)	+
	Dengue outbreaks (binary)	+
3	Education (years per capita)	-
	LDI (1$ per capita)	-

Changes from GBD 2021 to GBD 2023

There have been no substantive changes to the modelling strategy for GBD 2023.

References

1. Brady OJ, Gething PW, Bhatt S, Messina JP, Brownstein JS, Hoen AG, et al. Refining the Global Spatial Limits of Dengue Virus Transmission by Evidence-Based Consensus. *PLoS Negl Trop Dis*. 2012 Aug 7;6(8):e1760.

Diabetes mellitus

Diabetes mellitus mortality was estimated for overall diabetes mellitus, diabetes mellitus type 1, and diabetes mellitus type 2 in GBD 2023.

Overall diabetes mellitus

Flowchart

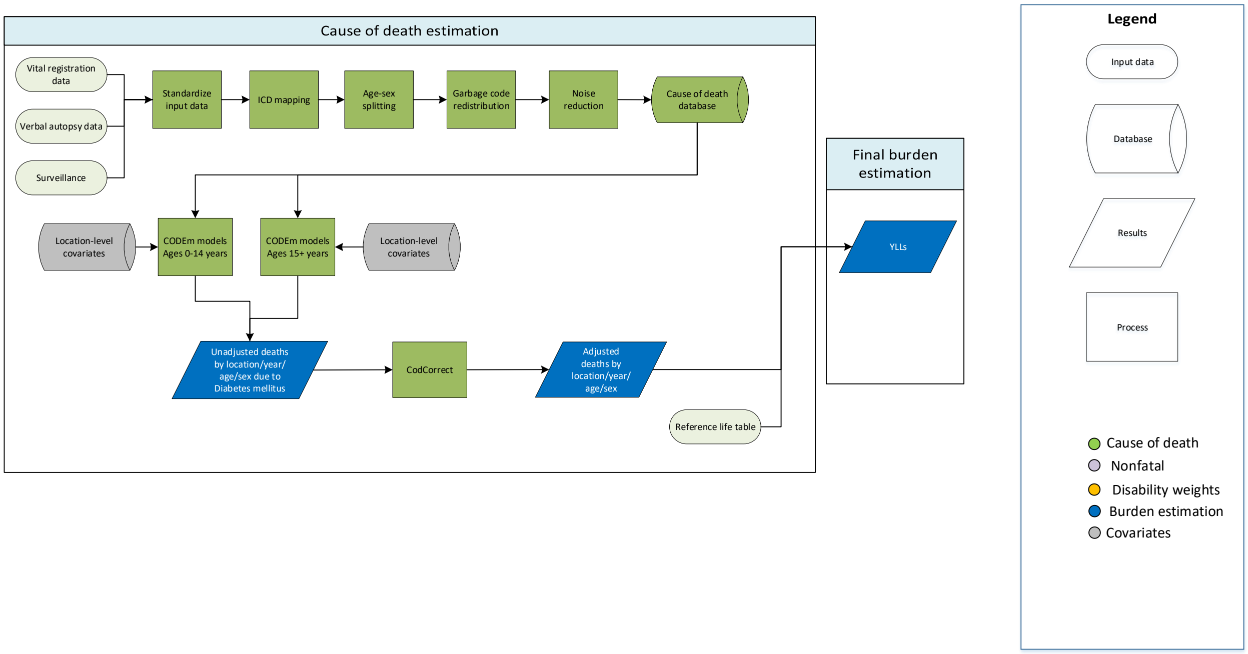

Input data and methodological summary for diabetes mellitus

Input data

Overall diabetes mellitus mortality was estimated using deaths directly attributed to diabetes mellitus. We used verbal autopsy and vital registration data as inputs into the model.

Verbal autopsy data: We outliered datapoints from sources where there were zero deaths estimated in an age group as this was not realistic for deaths due to diabetes and we determined that these data sources were unreliable.

Vital registration data: We systematically outliered all data from the India Medical Certification of Cause of Death report since the source of the data was unreliable according to expert opinion; ICD-9-BTL datapoints that were inconsistent with the rest of the data series and created unlikely time trends; Ethiopia District Health Informatic Software 2 Cause of Death data as unexpectedly low compared to Ethiopia verbal autopsy data in the same years; Kazakhstan data from the WHO mortality database for years 2013 and onward given unrealistic increases in mortality according to expert opinion.

Crosswalk by code system

Most data sources used for cause of death estimation are ICD-coded. The ICD periodically releases new versions, which can introduce an implausible disjoint in the time series that is indicative of a change in the coding practice, rather than a true change in the epidemiological trend of the disease. In GBD 2023, certain causes and countries using the ICD-9 code systems were adjusted to address the disjoint in the time trend and number of deaths between the older ICD-9 code systems and the more recent ICD-10 code systems. Overall diabetes was corrected in four locations (years): New Zealand (1980–1999), Japan (1980-1994), Greece (1980-2013) and Mauritius (1980-2004) to account for this code system disjoint. This was done using a meta-regression tool described as a crosswalk, by taking systematically biased data points and estimating their unbiased value. Using this modeling tool, correction factors were created based on the more recent and more reliable ICD-10 coded data. The crosswalk creates matched pairs between the last three years of data using the ICD-9 code system and the first three years of data using the ICD-10 code system. The correction is applied at the overall diabetes level, and after the correction is applied, estimates from type 1 diabetes and type 2 diabetes are raked to total diabetes to maintain a consistent number of deaths across the cause hierarchy.

Modelling strategy

The Cause of Death Ensemble model (CODEm)[1] was used for deaths due to diabetes estimation. Additional information on CODEm methods can be found in appendix 1, section 3 of the reference article.

In the overall diabetes mellitus model, we used two models to estimate overall diabetes deaths with different age restrictions. This is because deaths in younger age groups are almost exclusively due to type 1 diabetes, while deaths in older ages are primarily due to type 2 diabetes. This allowed us to select predictive covariates that are specific to the pathophysiology of diabetes type 1 and type 2. We set the younger age model from 0 to 14 years and the older age model from 15 to 95+ years. We determined the age threshold based on evidence of the onset of diabetes type 2 occurring at younger ages.

Covariate selection

The following table lists the covariates included in the model. This requires that the covariate selected for the model must have the directional relationship with diabetes mellitus deaths. For GBD 2023, no significant updates were made for diabetes mellitus covariate selection. Covariate directions were selected based on the strength of the evidence of association with diabetes mortality. For the under-15 age model, covariates were selected based on the existing literature. However, genetic and autoimmune factors were not included, given the lack of existing covariates within the GBD covariate database. We also included HAQ Index, given its immediate link to insulin access and survival for type 1 diabetes.

Table 1. Covariates used in diabetes mellitus mortality modelling

Model	Level	Covariate	Direction
0–14 years	1	Healthcare Access and Quality (HAQ) Index	-
	2	Latitude	+
	2	Percentage of births occurring in women >35 years old	+
	2	Percentage of births occurring in women >40 years old	+

	3	Socio-demographic Index	-
	3	Education years per capita	-
15-+ years	1	Age-standardised mean fasting plasma glucose (mmol/L)	+
	1	Age-standardised prevalence of diabetes	+
	1	Mean BMI	+
	1	Prevalence of obesity	+
	2	Mean cholesterol	+
	2	Mean systolic blood pressure	+
	2	Age- and sex-specific summary exposure variable for low fruit	-
	2	Unadjusted grams of sugar	+
	2	Age- and sex-specific summary exposure variable for low vegetables	-
	2	Age- and sex-specific summary exposure variable for alcohol use	+
	3	Healthcare Access and Quality Index	-
	3	Education years per capita	-
	3	Lag-distributed income per capita	+

Diabetes mellitus type 1 and type 2

Flowchart

Diabetes mellitus type 1

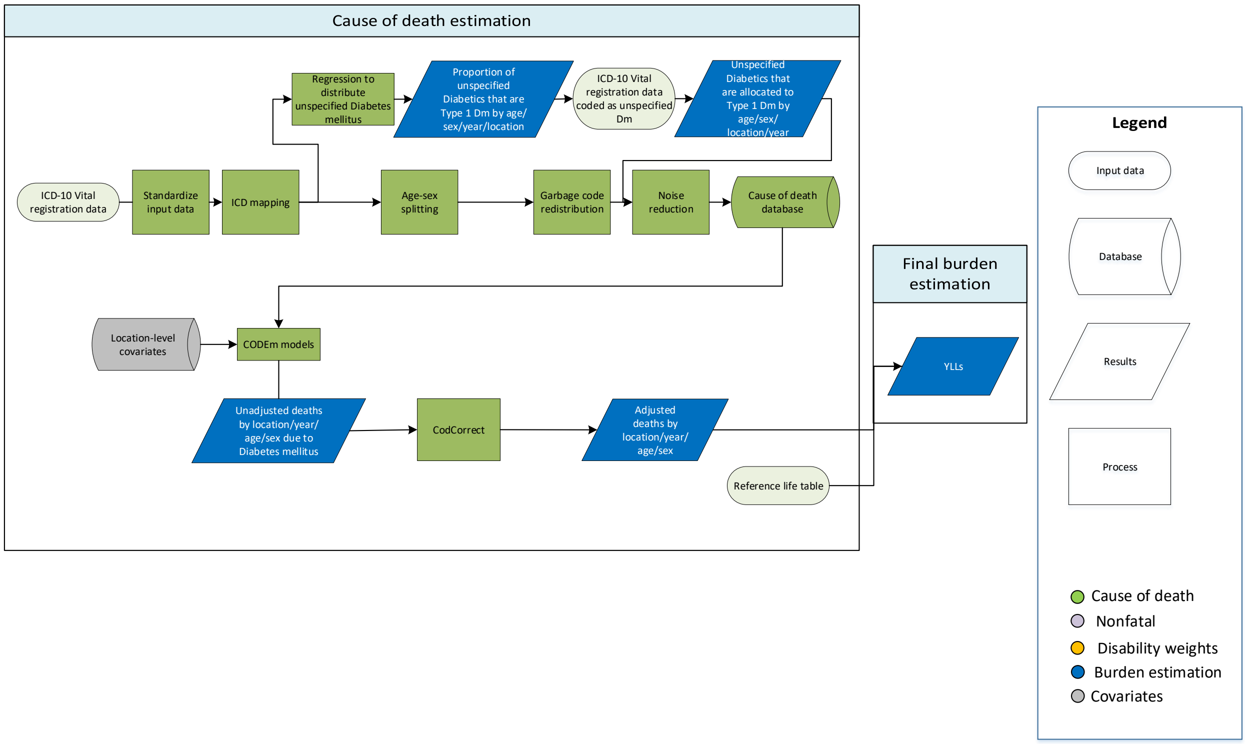

Diabetes mellitus type 2

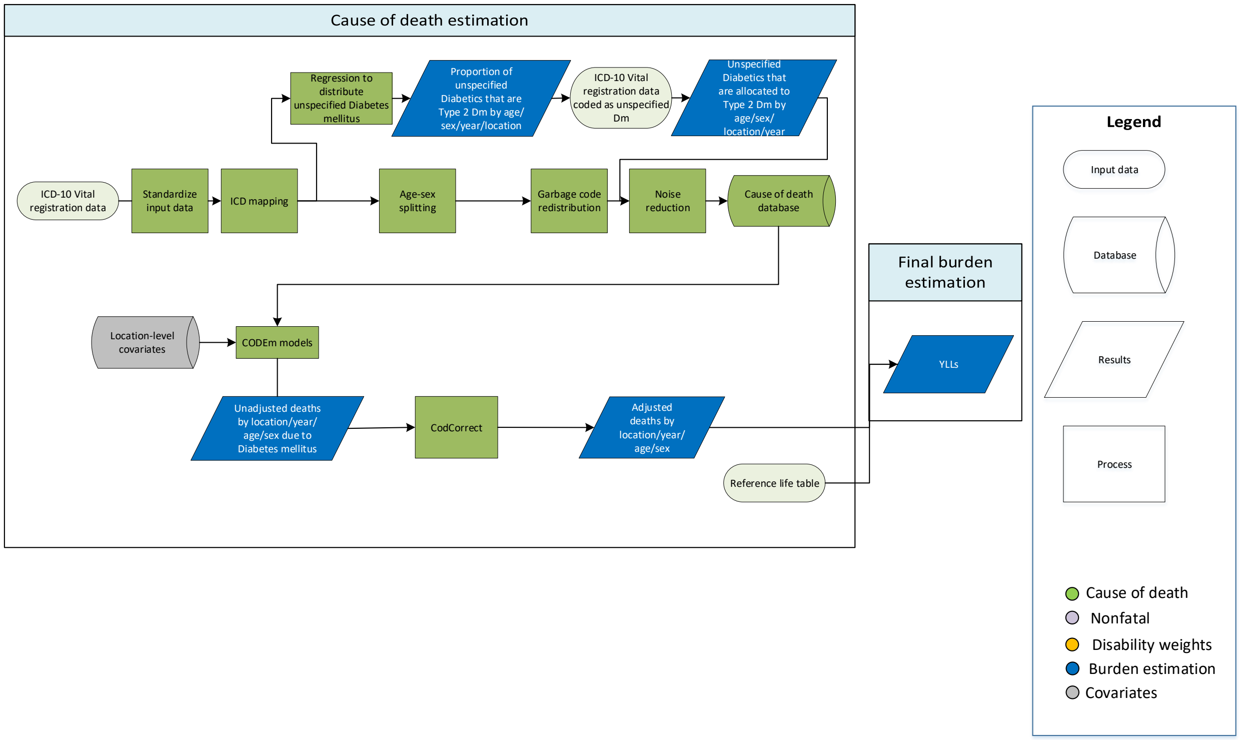

Input data and methodological summary for type 1 and type 2 diabetes mellitus

Input data

Type-specific diabetes mellitus mortality was estimated using deaths from vital registration sources in ICD-10 codes only. Diabetes type-specific information was not available in ICD-9 codes or deaths determined by verbal autopsy.

There were two unique data manipulation steps that occurred to prepare the data as part of the modelling process.

1. We assumed that all deaths <15 years were due to type 1 regardless of the ICD-10 code assigned to the death. We imposed 100% attribution of diabetes mellitus deaths in <15 years to type 1 diabetes mellitus.
2. ICD-10 diabetes data were reported as type 1, type 2, or unspecified. We assumed that all type 1 deaths in persons >45 years were unspecified regardless of the ICD-10 code assigned to the death because we found an unreasonably high proportion of deaths due to diabetes were assigned to type 1 diabetes. We developed a regression to estimate the fraction of unspecified diabetes mellitus that was type 1 and type 2. We only used data from 703 country-years to inform the regression. The selected country-years had more than 50% of the deaths coded to type-specific deaths AND at least 70% of type-specific deaths in people >50 years were coded to type 2. Since there was a separate regression to estimate the proportion of type 1 diabetes mellitus and type 2 diabetes mellitus, we scaled the predicted proportions to 1. These scaled proportions were then applied to the number of deaths coded to unspecified diabetes in each location, year, sex where ICD-10 data were reported.

<u>Regression equations</u>:

Type 1:

$$logit\left(\frac{number\ type\ 1\ DM}{number\ total\ DM}\right) \sim logit\left(\frac{number\ unspecified\ DM}{number\ total\ DM}\right) + \beta_1 age\ group + \beta_2 age\text{-}st\ prev\ obesity * age\ group + age\text{-}st\ prev\ obesity$$

Type 2:

$$logit\left(\frac{number\ type\ 2\ DM}{number\ total\ DM}\right) \sim logit\left(\frac{number\ unspecified\ DM}{number\ total\ DM}\right) + \beta_1 age\ group + \beta_2 age\text{-}st\ prev\ obesity * age\ group + age\text{-}st\ prev\ obesity$$

Modelling strategy

CODEm was used for deaths due to diabetes mellitus type-specific estimation.

Deaths in younger age groups are almost exclusively due to type 1 diabetes, while deaths in older ages are primarily due to type 2 diabetes. To account for this age pattern, we set the age range of the diabetes type 1 model to 0–95+ years and the age range of the diabetes type 2 model to 15–95+ years. We used the same covariates in the diabetes type 1 model and diabetes type 2 model as the 0–14 year and 15–95+ year in the overall diabetes models, respectively.

Covariate selection

The following are the covariates included in the model. We selected the same covariates for the type 1 diabetes model as the 0–14-year diabetes parent model and the type 2 diabetes model as the 15–95+ year diabetes parent model. For GBD 2023, no significant updates were made for the type-specific diabetes covariate selection. Covariate directions were selected based on the strength of association with diabetes mortality. Type 1 model covariates were selected based on the existing literature. However, genetic and autoimmune factors were not included, given the lack of existing covariates within the GBD covariate database. We also included HAQ Index, given its immediate link to insulin access and survival for type 1 diabetes.

Table 2. Covariates used in diabetes mellitus type 1 and type 2 mortality modelling

Model	Level	Covariate	Direction
Type 1	1	Healthcare Access and Quality Index	-
	2	Latitude	+
	2	Percentage of births occurring in women >35 years old	+
	2	Percentage of births occurring in women >40 years old	+
	3	Socio-demographic Index	-
	3	Education years per capita	-
Type 2	1	Age-standardised mean fasting plasma glucose (mmol/L)	+

	1	Age-standardised prevalence of diabetes	+
	1	Mean BMI	+
	1	Prevalence of obesity	+
	2	Mean cholesterol	+
	2	Mean systolic blood pressure	+
	2	Age- and sex-specific summary exposure variable for low fruit	-
	2	Unadjusted grams of sugar	+
	2	Age- and sex-specific summary exposure variable for low vegetables	-
	2	Age- and sex-specific summary exposure variable for alcohol use	+
	3	Healthcare Access and Quality Index	-
	3	Education years per capita	-
	3	Lag-distributed income per capita	+

Diarrhoeal diseases

Flowchart

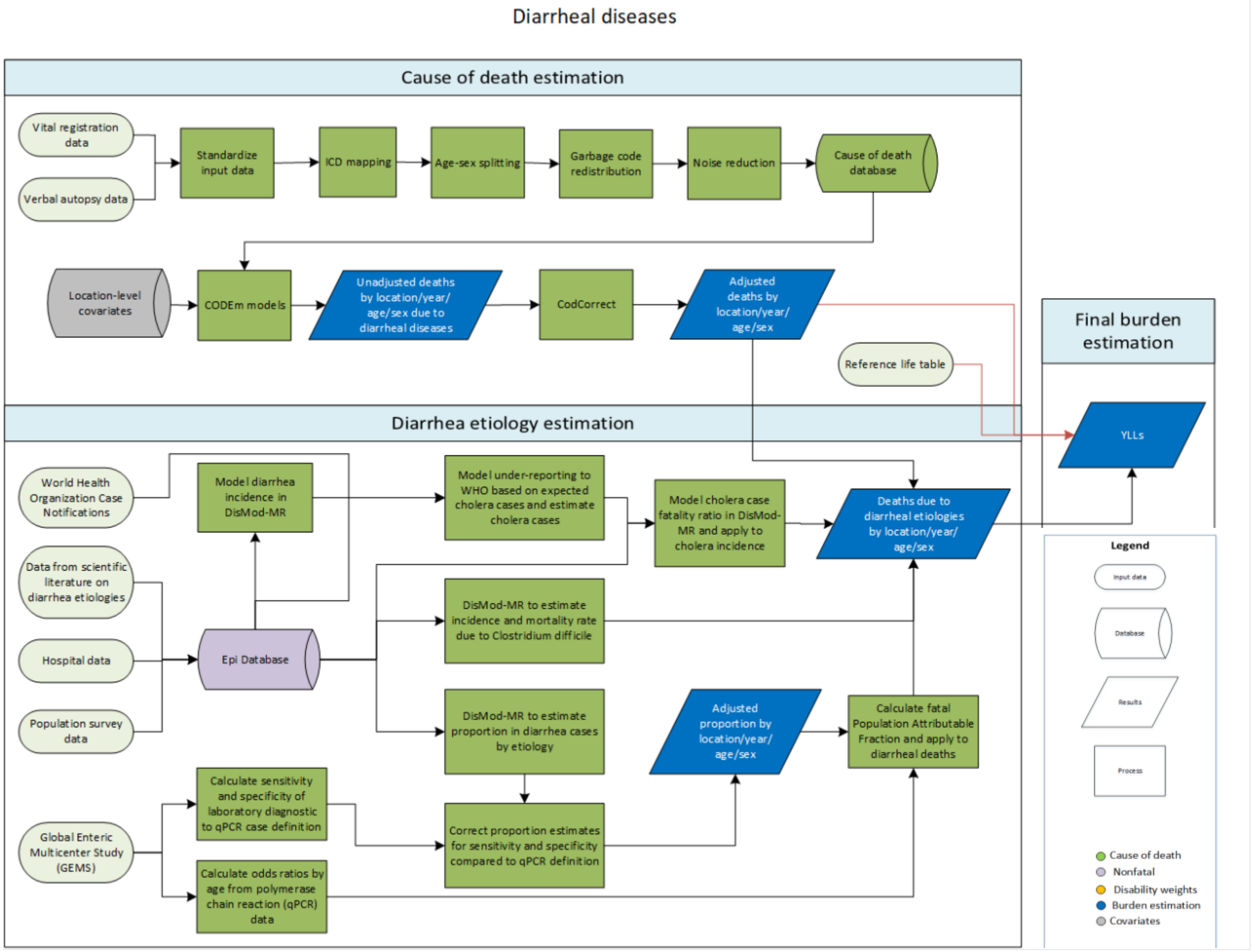

Diarrhoeal diseases are a cause of death in GBD. We also estimated the attributable deaths from 15 diarrhoeal aetiologies using an independent modelling strategy. These pathways are shown in the flowchart above and will be described in this report.

Input data and methodological summary for diarrhoeal diseases

Input data

Cause of death. We used all available data from vital registration systems, surveillance systems, and verbal autopsy. Datapoints that violated well-established age or time trends were determined to be outliers. We also excluded early neonatal mortality data in the Philippines (1994–1998), India Civil Registration System data, and medically certified cause of death (MCCD) data in all states (1986–2013).

Aetiologies. The second type of data describe diarrhoea aetiologies. There are 15 aetiologies in GBD 2023 for diarrhoea: adenovirus, *Aeromonas, Campylobacter, Vibrio cholerae, Clostridium difficile, Cryptosporidium*, *Entamoeba histolytica,* enteropathogenic *E coli* (EPEC), enterotoxigenic *E coli* (ETEC),

norovirus, rotavirus, non-typhoidal *Salmonella, Shigella,* astrovirus, and sapovirus. We extracted data on all aetiologies from scientific literature that reported the proportion of diarrhoea cases that tested positive for each pathogen. We completed a systematic literature review covering the period December 2020 to January 2024 for diarrhoea prevalence, incidence, and diarrhoea aetiologies. Inclusion criteria included diarrhoea as the case definition, studies with a sample size of at least 100, and studies with at least one year of follow-up. We excluded studies that reported on diarrhoeal outbreaks exclusively and we also excluded studies that reported acute gastroenteritis with or without diarrhoea.

We searched articles using a PubMed search term that combined non-specific and aetiology-specific diarrhoea using the following search string:

(diarrhoea*[title/abstract] OR "Diarrhea"[Mesh] OR diarrhea*[title/abstract]) AND (2020/12/01:2024/01/09[PDat]) AND (inciden*[title/abstract] OR incidence[Mesh] OR prevalent*[title/abstract] OR cases[title/abstract] OR prevalence[Mesh] OR epidemiology[title/abstract] OR epidemiology[Mesh] OR salmonella[title/abstract] OR salmonella[Mesh] OR aeromona*[title/abstract] OR aeromonas[Mesh] OR shigell*[title/abstract] OR shigella[Mesh] OR enteropathogenic[title/abstract] OR enterotoxigenic[title/abstract] OR "Enterotoxigenic Escherichia coli"[Mesh] OR "Enteropathogenic Escherichia coli"[Mesh] OR *coli[title/abstract] OR campylobacter[title/abstract] OR campylobacter[Mesh] OR amoebiasis[title/abstract] OR "Amebiasis"[Mesh] OR entamoeb*[title/abstract] OR cryptosporid*[title/abstract] OR "Cryptosporidiosis"[Mesh] OR rotavir*[title/abstract] OR rotavirus[Mesh] OR norovirus[title/abstract] norovirus[Mesh] OR adenovirus[title/abstract] OR "Adenovirus Infections, Human"[Mesh] OR clostridium*[title/abstract] OR Clostridioides difficile[title/abstract] OR "Clostridioides difficile"[Mesh] OR c. diff*[title/abstract] OR “astrovir*”[Mesh] OR “astrovir*”*[title/abstract] OR “sapovir*”[Mesh] OR “sapovir*”*[title/abstract] OR etiolog*[title/abstract] OR aetiolog*[title/abstract] OR pathogen*[title/abstract]) NOT (appendicitis[title/abstract] OR esophag*[title/abstract] OR gastritis[title/abstract] OR liver[title/abstract] OR case report[title/abstract] OR case-report[title/abstract] OR "Case Reports" [Publication Type] OR therapy[title] OR Crohn[title/abstract] OR "inflammatory bowel"[title/abstract] OR irritable[title/abstract] OR travel*[title] OR Outbreak[title] OR Review[ptyp]) NOT (animals[MeSH] NOT humans[MeSH])

We identified 2, 504 studies, of which 88 met our inclusion criteria. We extracted data for location, sex, year, and age.

Figure 1. Diarrhoeal disease systematic review PRISMA diagram

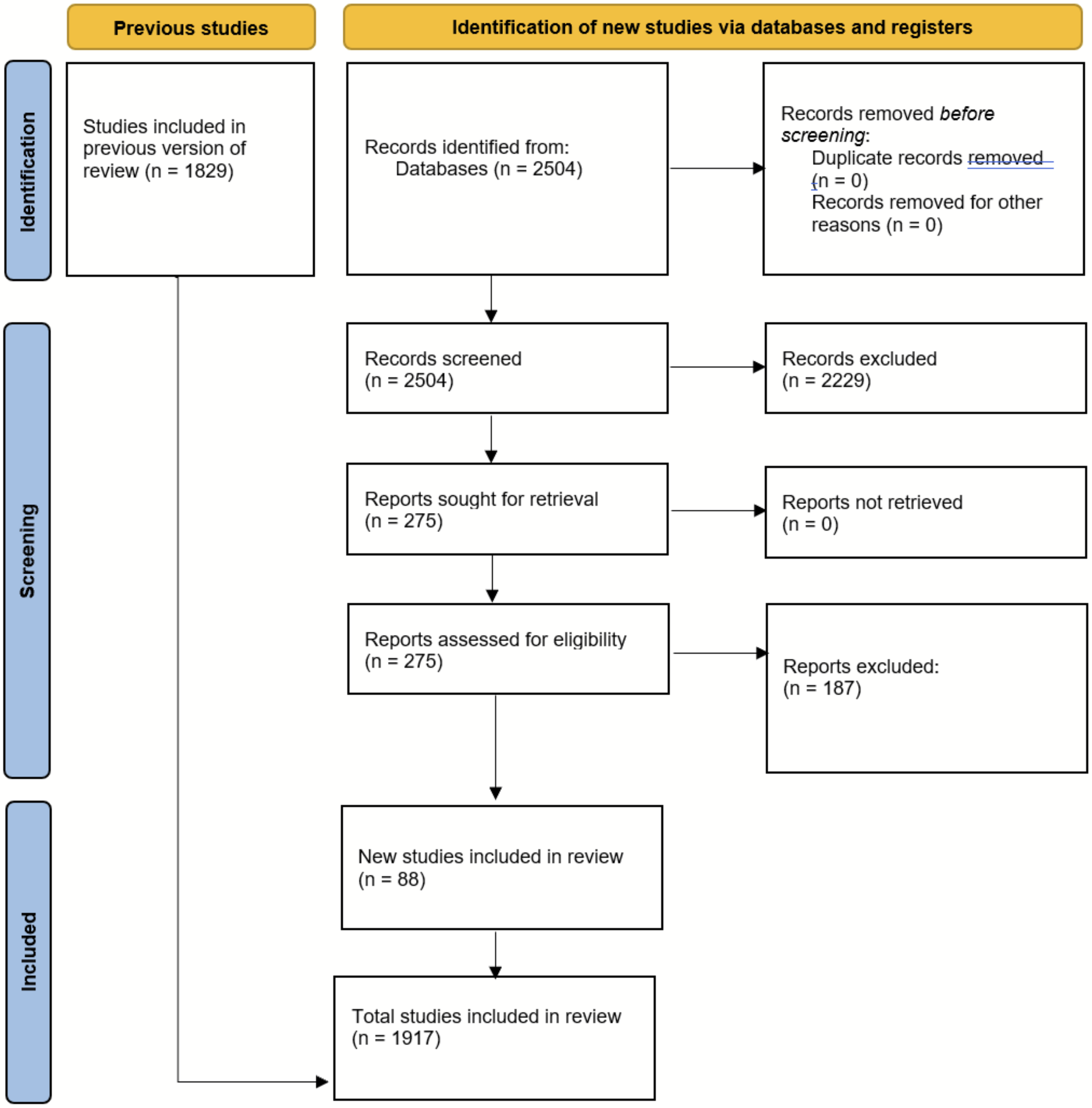

To update our cholera proportion estimates, we used an additional PubMed search string spanning from January 2019 to December 2023:

(cholera*[title/abstract] OR cholera[Mesh]) AND (diarrhea*[title/abstract] OR diarrhoea*[title/abstract] OR diarrhea[Mesh]) AND (epidemiolog*[title/abstract] OR epidemiology[Mesh] OR inciden*[title/abstract] OR incidence[Mesh] OR prevalent*[title/abstract] OR prevalence[Mesh] OR death*[title/abstract] OR mortality[title/abstract] OR mortality[Mesh] OR fatal*[title/abstract] OR case*[title/abstract] OR proportion*[title/abstract]) NOT ("case report"[title/abstract] OR "case reports"[Publication Type] OR review[Publication Type]) AND (("2019/01/01"[PDat] : "2023/12/21"[PDat])) NOT (animals[MeSH] NOT humans[MeSH])

We identified 244 studies, of which seven met our inclusion criteria.

Figure 2. Cholera proportion systematic review PRISMA diagram

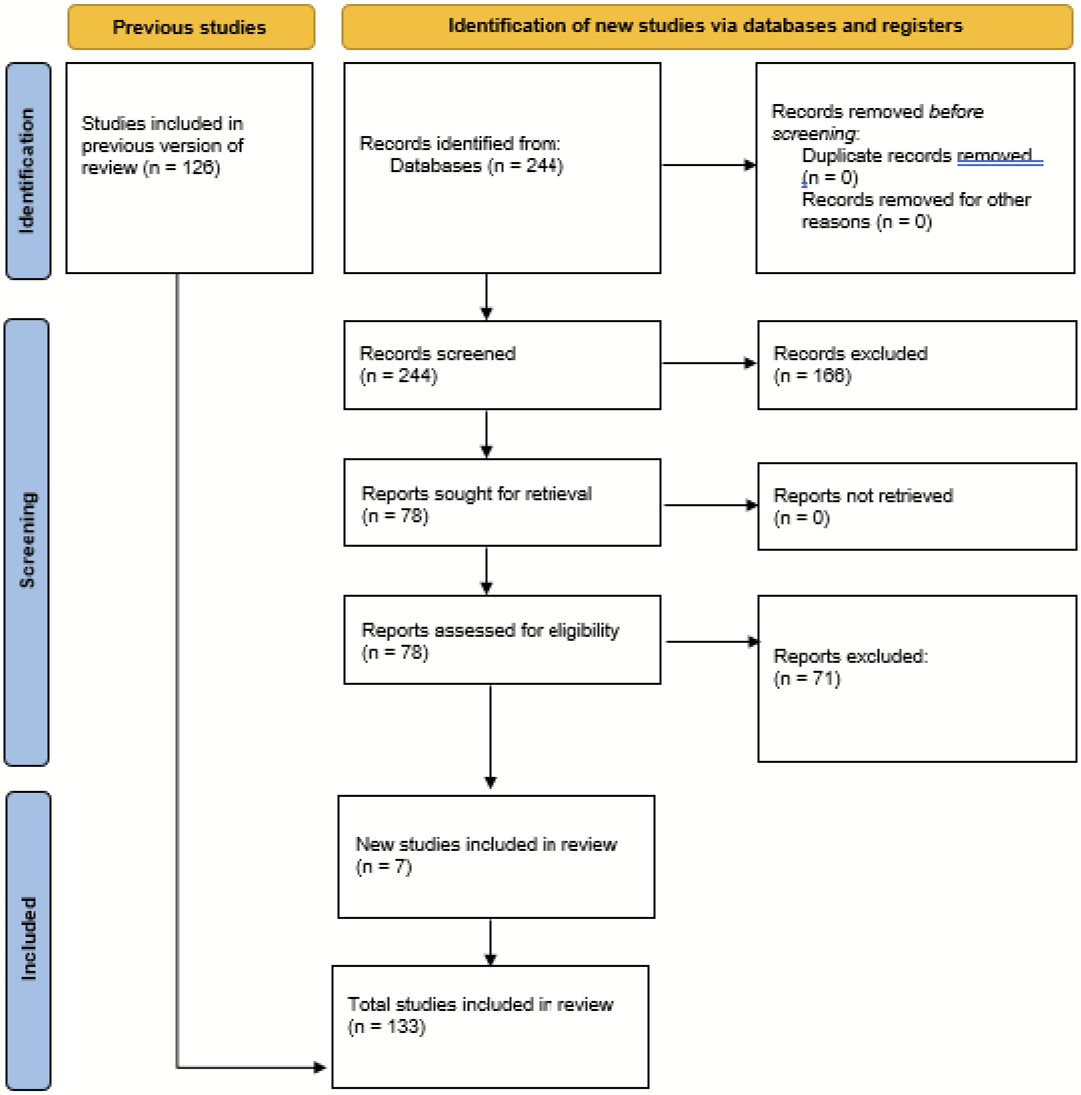

We used the Global Enteric Multicenter Study (GEMS), a seven-site, case-control study of moderate-to-severe diarrhoea in children under 5 years,[1] and the MAL-ED study,[2] a multi-site birth cohort, to calculate odds ratios for the diarrhoeal pathogens. We analysed raw data for a systematic reanalysis, representative of the distribution of cases and controls by age and site that were tested for the presence of pathogen using quantitative polymerase chain reaction (qPCR).[3]

Data that did not use qPCR for detection were adjusted for sensitivity and specificity prior to modelling in order to standardise data regardless of detection method. Adjusting these data prior to modelling allowed us to adjust only data that did not use qPCR, as well as better control for values at extreme bounds, and capture uncertainty in modelling.

Case-fatality rate (CFR) data for *Clostridium difficile* was collected from ICD-coded hospital records from Austria, Brazil, Canada, Italy, Mexico, New Zealand, and the USA. ICD codes A04.7 (ICD-10) and 008.45 (ICD-9) were used to identify intestinal infections with *Clostridium difficile*. Supplemental data for Romania collected as part of the International Nosocomial Infection Control Consortium (DOI: 10.1016/j.ajic.2016.01.005) were also included. We standardised age and sex across all datasets to the following most-detailed groups using the GBD causes of death age-sex splitting algorithm for age: 0–6, 7–27, and 28–364 days, and 1–4, 5–9, 10–14, 15–19, 20–24, 25–29, 30–34, 35–39, 40–44, 45–49, 50–54, 55–59, 60–64, 65–69, 70–74, 75–79, 80–84, 85–89, 90–94, 95+ years; and sex: male and female. This algorithm is based on the assumption that age-sex pattern of the death or case rate for a given infectious syndrome or pathogen is inherent to the pathology of the disease and is therefore constant across location and year. Crude case-fatality rates were then estimated for the input data for each GBD age group and sex.

CFR data for cholera were extracted from an updated systematic review with sources identified using the following PubMed search string capturing sources from January 2016 to October 2024. We identified 354 sources, of which 39 met our inclusion criteria. Additionally, two surveillance data series were updated for India and Malawi.

(Cholera*[TiAb] OR cholera [MeSH] OR “vibrio cholerae” [MeSH]) AND ("case fatality"[TiAb] OR “mortality”[TiAb] OR “cfr”[TiAb]) NOT (animals[MESH] NOT humans[MESH]) ("2016/01/01"[PDAT] : "2024/10/10"[PDAT])

Figure 3. Cholera CFR systematic review PRISMA diagram

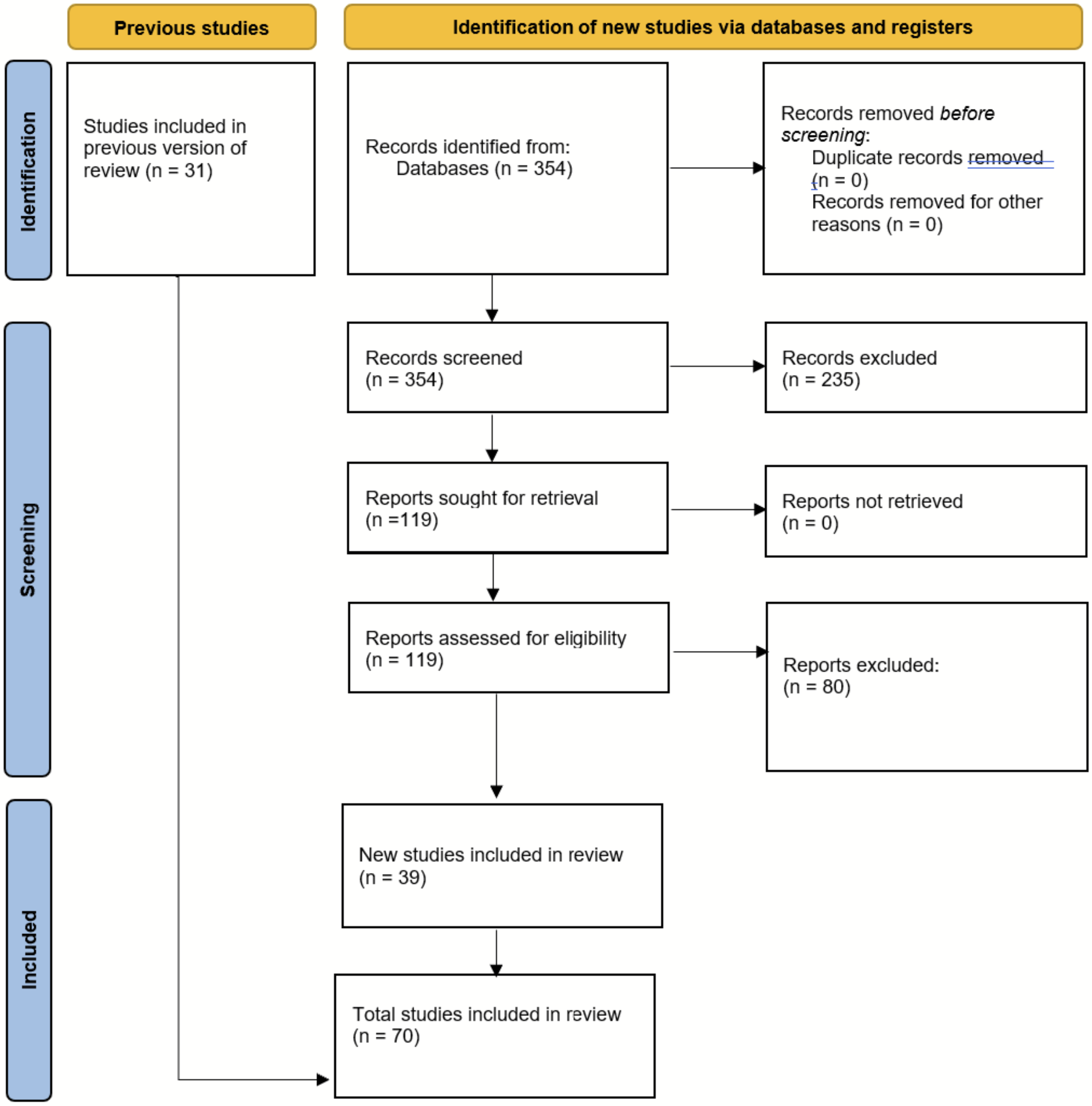

We also conducted an update to our rotavirus vaccine efficacy data with a PubMed search string beginning in December 2020 and ending in October 2024.

(((rotavirus[title/abstract] AND vaccine[title/abstract] AND (efficacy[title/abstract] OR effectiveness[title/abstract]) AND (2020/12/31[PDAT] : 2024/10/22[PDAT])))) NOT Review[Publication Type] NOT (animals[MeSH] NOT humans[MeSH])

We identified 185 studies from this search string along with 108 additional studies identified by reviewing meta-analyses captured by the search string. Of these 293 sources, 14 met our inclusion criteria. Adding on to the inclusion criteria of our larger diarrhoea systematic review mentioned previously, data were included if they pertained to under-5 children. From these sources, we extracted rotavirus vaccine efficacy relevant to rotavirus cases by location, under-5 age groups, year, sex, vaccine type, and number of doses.

Figure 4. Rotavirus vaccine efficacy systematic review PRISMA diagram

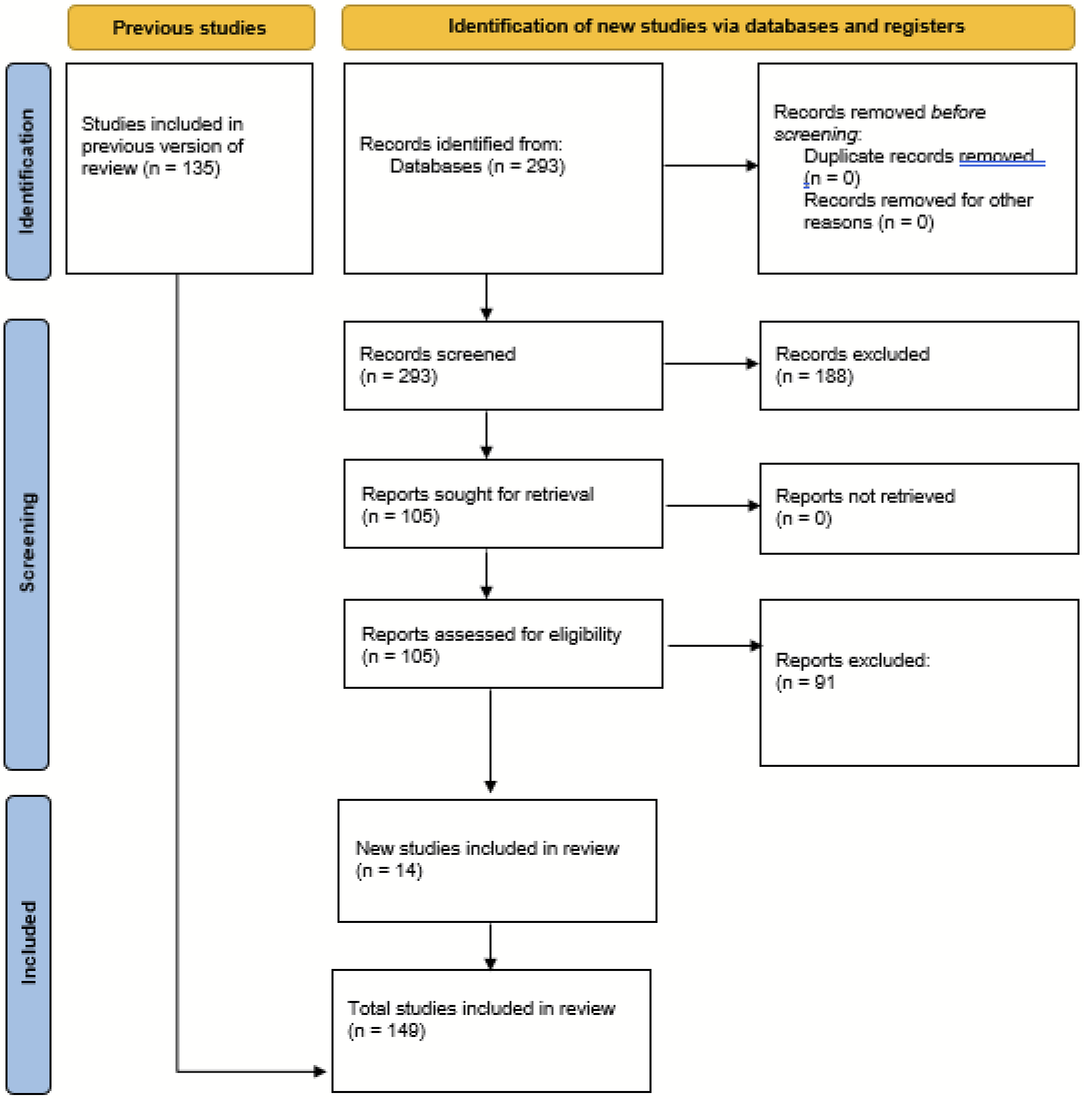

Modelling strategy

Cause of death. Diarrhoeal disease mortality was estimated in the Cause of Death Ensemble modelling platform (CODEm). We estimated diarrhoea mortality separately for males and females and for children under 5 years and older than 5 years. We used country-level covariates to inform our CODEm models (Table 1).

In past GBD cycles, estimates of rotavirus vaccine coverage among infants in the modelled year were used as the primary covariate for this linear regression. In GBD 2021, we began using a lagged mean of rotavirus vaccine coverage calculated over a rolling, five-year interval in order to capture population-level vaccine-derived immunity among under-5-year-olds, including coverage both in the current year and in recent years.

Table 1. Covariates used in diarrhoea mortality modelling. Table 1A shows the covariates used in the 0–4 years model, and Table 2B shows the covariates used in the 5–95+ years model. The *Level* represents the

strength of the association between the covariate and diarrhoea mortality from 1 (proximally related) to 3 (distally related). The *Direction* indicates the positive or negative association between the covariate and diarrhoea mortality.

Table 1A. Covariates used in the 0–4 years model

Level	Covariate	Direction
1	Oral rehydration solution treatment	-
	Rotavirus vaccine coverage (proportion)	-
	Sanitation (proportion with access)	-
	Improved water source (proportion with access)	-
2	Zinc deficiency	+
	Zinc treatment for diarrhoea	-
	Healthcare Access and Quality Index	-
3	Maternal education years	-
	Socio-demographic Index (SDI)	-
	Lag-distributed income (LDI) per capita	-
	No access to handwashing facility	+

Table 1B. Covariates used in the 5–95+ years model

Level	Covariate	Direction
1	Sanitation (proportion with access)	-
	Improved water source (proportion with access)	-
2	Healthcare Access and Quality Index	-
	Rotavirus vaccine coverage (proportion)	-
	Oral rehydration solution treatment	-
3	Education years per capita	-
	Lag-distributed income (LDI) per capita	-
	Adult underweight	+
	Socio-demographic Index (SDI)	-
	No access to handwashing facility	+

Aetiologies. We estimated diarrhoeal disease aetiologies independently from overall diarrhoea mortality using a counterfactual strategy for enteric adenovirus, *Aeromonas*, *Entamoeba histolytica* (amoebiasis), *Campylobacter*, *Cryptosporidium*, EPEC, (ETEC), norovirus, non-typhoidal *Salmonella* infections, rotavirus, *Shigella,* astrovirus, and sapovirus. *Vibrio cholerae* and *C difficile* were modelled separately.

Diarrhoeal aetiologies are attributed to diarrhoeal deaths using a counterfactual approach. We calculated a population attributable fraction (PAF) from the proportion of severe diarrhoea cases that are positive for each aetiology. The PAF represents the relative reduction in diarrhoea mortality if there was no exposure to a given aetiology. As diarrhoea can be caused by multiple pathogens and the pathogens may co-infect, PAFs can overlap and are not scaled to sum to 100%. We calculated the PAF from the proportion of severe diarrhoea cases that are positive for each aetiology. We assumed that hospitalised diarrhoea cases are a proxy of severe and fatal cases. We used the following formula to estimate PAF:[4]

$$PAF = Proportion * (1 - \frac{1}{OR})$$

Where *Proportion* is the proportion of diarrhoea cases positive for an aetiology and *OR* is the odds ratio of diarrhoea given the presence of the pathogen.

We dichotomised the continuous qPCR test result using the value of the cycle threshold (Ct) that most accurately discriminated between cases and controls. The Ct values range from 0 to 35 cycles, representing the relative concentration of the target gene in the stool sample. A low value indicates a higher concentration of the pathogen, while a value of 35 indicates the absence of the target in the sample. We used the lower Ct value when we had multiple Ct values for the cutpoint. The case definition for each pathogen is a Ct value that is below the established cutoff point.

We used a mixed effects conditional logistic regression model to calculate the odds ratio for ages under 1 year and 1–4 years old for each of our pathogens. The stool samples from cases and controls in GEMS were used exclusively to calculate these odds ratios as we assumed that the association between pathogens and moderate-to-severe diarrhoea is a proxy for fatal outcomes. The odds ratio for ages 1–4 years was applied to all GBD age groups over 5 years. There were three pathogen-age odds ratios that were not statistically significant: *Aeromonas* and amoebiasis in under 1 year and *Campylobacter* in 1–4 years. The mean value of the odds ratio was above 1 in all three cases, so we transformed the odds ratios for these three exceptions only in log space such that exponentiated values could not be below 1. The transformation was:

$$Odds\ ratio = exp(log(OR) – 1)) + 1$$

We modelled the proportion data using the Bayesian meta-regression tool DisMod-MR to estimate the proportion of positive diarrhoea cases for each separate aetiology by location/year/age/sex and to adjust for the covariates. We used the estimated sensitivity and specificity of the original laboratory diagnostic test results from the pooled GEMS and MAL-ED qPCR stool samples compared to the qPCR test result to adjust our proportion before we modelled the proportions:[5]

$$Proportion_{True} = \frac{(Proportion_{Observed} + Specificity - 1)}{(Sensitivity + Specificity - 1)}$$

We used this correction to account for the fact that the proportions we used are based on a new test that is not consistent with the laboratory-based case definition (qPCR versus GEMS conventional laboratory testing for pathogens).[6] Because differences in the type of PCR used in the original (non-reference qPCR diagnostic) between GEMS and MAL-ED in detecting norovirus, we combined the sensitivity and specificity results for norovirus such that 50% of the draws were coming from GEMS test results exclusively and 50% of the draws were coming from MAL-ED test results exclusively. Additionally, because the original laboratory diagnostic technique used for *Campylobacter* in MAL-ED was one not commonly used, we only used GEMS to determine the sensitivity and specificity of bacterial culture compared to qPCR in detecting *Campylobacter.*[7]

To be consistent with the odds ratios that we obtained, we adjusted our proportion estimates of any EPEC to typical EPEC only. This adjustment was informed by all available data that reported both atypical and typical EPEC. We applied the same approach to differentiate between heat-stable toxin- (ST) and heat-labile toxin-producing (LT) ETEC. For the first time, GBD 2019 split these serotypes so that estimates from GBD 2019 onward represent the diarrhoeal disease burden attributable to ST-ETEC. This was based on work showing that ST-ETEC was much more pathogenic than LT-ETEC. As some of our proportion data were extracted for any ETEC, we determined a proportion of all ETEC that produced ST from all available data reporting both ETEC and ST-ETEC and applied that ratio to data representing all ETEC so that they represented ST-ETEC only.

For *Vibrio cholerae* (cholera), we used the literature review to estimate the expected number of cholera cases for each country-year using the incidence of diarrhoea (estimated using DisMod-MR) and the proportion of diarrhoea cases that are positive for cholera. We assigned cholera PAF using odds ratios from the qPCR results to estimate a number of cholera-attributable cases. We compared this expected number of cholera cases to the number reported to the World Health Organization at the country-year level.[8] We modelled the under-reporting fraction to correct the cholera case notification data for all countries using health system access and the diarrhoea SEV scalar to predict total cholera cases. We used the age-specific proportion of positive cholera samples in DisMod-MR and our incidence estimates to predict the number of cholera cases for each age/sex/year/location. Finally, we modelled the case-fatality ratio of cholera using DisMod-MR and estimated the number of cholera deaths.

For *C difficile*, we modelled incidence and mortality in DisMod-MR for each age, sex, year, location. DisMod-MR is a Bayesian meta-regression tool that uses spatiotemporal information as priors to estimate prevalence, incidence, remission, and mortality for *C difficile* infection. DisMod-MR uses a compartmental model to relate prevalence, incidence, remission, and mortality. We set remission in our model to 1 month.

C difficile CFRs were calculated using MR-BRT, a meta-analytic mixed effects structure. The main model can be specified as follows:

$$logit(y_i) = X_i\beta + u_i 1 + \epsilon_i, \qquad \epsilon_i \sim N(0, \Sigma_i), \qquad u_i \sim N(0, \gamma)$$

where

- y_i contains CFRs for data source i
- Design matrix X_i contains as columns the following covariates:
  - HAQ Index
  - dummy-coded indicator for age group
    - neonatal–5 years, 5–50 years, 50–70 years, and 70 years and older
  - dummy-coded ICU indicator for data source
    - 1 if data source only compiles information on ICU patients, 0 if a mix between ICU/non-ICU patients
  - dummy-coded indicator for pathogen
- β are fixed effect multipliers
- ϵ_i are observation error terms with known variances
- u_i are data source-specific random intercepts with unknown covariance γ

We also implemented a prior on γ, the data source random effect. Many input data sources cover only a single country, leading to low variability in HAQ Index within each data source. Such collinearity adversely influenced the accuracy of the estimated effect of HAQ Index, which was instrumental in extrapolating trends from the input data to global results. To emphasise the contribution of HAQ Index over data source in the modelled estimates, we implemented a strong Gaussian prior (mean 0, standard error 0.001) on γ. Predictions for *Clostridium difficile* CFRs were generated for each country and age group as a function of each country's HAQ Index, assuming mixed ICU/non-ICU patients. *C difficile* excess mortality rate was calculated using CFR with the following, with duration assumed to be 1.0 month (0.3–1.7):

$$EMR = -\ln(1 - CFR)/duration$$

For rotavirus, we made a change to the process of estimating attributable fraction to explicitly account for rotavirus vaccine efficacy beginning in GBD 2019. The impact of the rotavirus vaccine is dependent on modelled vaccine coverage for a location-year and on the rotavirus vaccine efficacy (VE). There are numerous studies that demonstrate a difference in VE by location.[9] We determined that SDI was the best predictor of rotavirus VE, and we used a meta-regression with this covariate to predict the rotavirus VE by location where the VE was higher in areas with larger SDI values and followed a logit-linear distribution.

Starting from GBD 2019, we explicitly incorporated the results from our analysis of VE to produce more robust estimates of the proportion of diarrhoea that has rotavirus over time and space. We assumed that the impact of the vaccine can be represented as one minus the product of the estimated vaccine coverage and VE.

$$Vaccine\ impact = 1 - vaccine\ coverage * vaccine\ efficacy$$

Both of these values vary in time and space but not by age. To avoid discontinuities in our model, we adjusted the input proportion data to remove the impact of the rotavirus vaccine by dividing the observed proportion by the vaccine impact.

$$Rotavirus\ proportion_{Adjusted} = \frac{Rotavirus\ proportion}{1 - Cov_{RotaV} * VE_{Modeled}}$$

The result is the modelled proportion of diarrhoea positive for rotavirus in the absence of the vaccine. This modelled value is then multiplied by the impact of the rotavirus vaccine to determine the estimated proportion of diarrhoea positive for rotavirus in the presence of the vaccine. Our modified attributable fraction is then:

$$DisModPAF = Modelled\ Proportion\ (from\ DisMod)\ * \left(1 - \frac{1}{OR}\right)$$

The last step is to account for the expected impact of the rotavirus vaccine. We do this using the equation below:

$$PAF_{Rota} = DisModPAF * \frac{(1 - Cov_{RotaV} * VE_{Modeled})}{(1 - DisModPAF * Cov_{RotaV} * VE_{Modeled})}$$

where the final attributable fraction for rotavirus is the product of the PAF estimated in DisMod-MR and the expected reduction in that PAF given modelled vaccine coverage and modelled VE by location-year, and this value is only applied to children 28 days to 5 years old. The product of the rotavirus attributable fraction and the number of deaths or cases of diarrhoea is the number of deaths and cases caused by rotavirus.

References

1 Kotloff KL, Nataro JP, Blackwelder WC, *et al.* Burden and aetiology of diarrhoeal disease in infants and young children in developing countries (the Global Enteric Multicenter Study, GEMS): a prospective, case-control study. *Lancet* 2013; **382**: 209–22.

2 Platts-Mills JA, Liu J, Rogawski ET, *et al.* Use of quantitative molecular diagnostic methods to assess the aetiology, burden, and clinical characteristics of diarrhoea in children in low-resource settings: a reanalysis of the MAL-ED cohort study. *Lancet Glob Health* 2018; **6**: e1309–18.

3 Liu J, Gratz J, Amour C, *et al.* A laboratory-developed TaqMan Array Card for simultaneous detection of 19 enteropathogens. *J Clin Microbiol* 2013; **51**: 472–80.

4 Miettinen OS. Proportion of disease caused or prevented by a given exposure, trait or intervention. *Am J Epidemiol* 1974; **99**: 325–32.

5 Reiczigel J, Földi J, Ozsvári L. Exact confidence limits for prevalence of a disease with an imperfect diagnostic test. *Epidemiol Infect* 2010; **138**: 1674–8.

6 Platts-Mills JA, Operario DJ, Houpt ER. Molecular diagnosis of diarrhea: current status and future potential. *Curr Infect Dis Rep* 2012; **14**: 41–6.

7 Platts-Mills JA, Liu J, Gratz J, *et al.* Detection of Campylobacter in stool and determination of significance by culture, enzyme immunoassay, and PCR in developing countries. *J Clin Microbiol* 2014; **52**: 1074–80.

8 World Health Organization. Global Health Observatory data repository: Cholera. 2016. http://apps.who.int/gho/data/node.main.174?lang=en (accessed Aug 25, 2016).

9 Lamberti LM, Ashraf S, Walker CLF, Black RE. A Systematic Review of the Effect of Rotavirus Vaccination on Diarrhea Outcomes Among Children Younger Than 5 Years. *Pediatr Infect Dis J* 2016; **35**: 992–8.

Digestive diseases

Flowchart

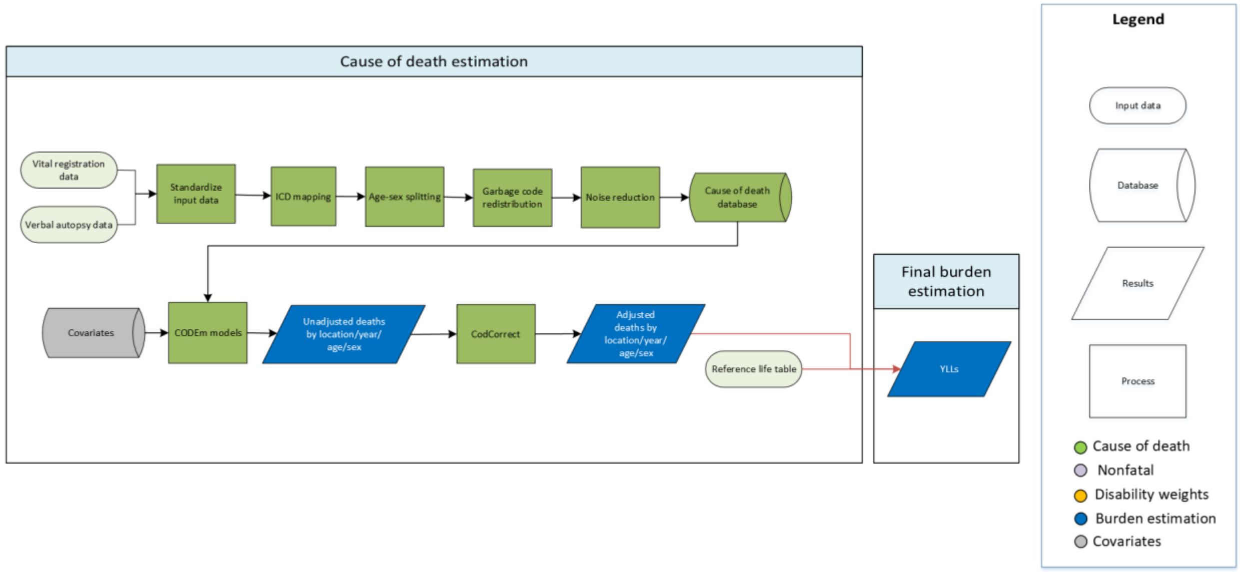

Input data and methodological summary for digestive diseases

Digestive diseases comprise a Level 2 cause in the GBD cause hierarchy.

Input data

Data used to estimate mortality of digestive diseases consisted of vital registration (VR) and verbal autopsy data (VA) from the cause of death (CoD) database. The data in digestive diseases consisted of aggregated data from all specific Level 3 digestive disease causes (ie, cirrhosis and other chronic liver diseases; upper digestive system diseases; appendicitis; vascular intestinal disorders; paralytic ileus and intestinal obstruction; inguinal, femoral, and abdominal hernias; inflammatory bowel disease; gallbladder and biliary diseases; pancreatitis) as well as unique datapoints from deaths reported with a set of non-specific digestive disease codes (ie, other digestive diseases). A list of ICD codes that were mapped to this cause, a list of vital registration sources included in the CoD database and their quality as indicated by star-ratings, and the steps used to process CoD data prior to modelling can be found in the section of this appendix on the causes of death database. CoD data processing was broadly similar to previous rounds of GBD, but one novel data processing step was employed in preparing data for digestive diseases that was not needed prior to GBD 2023—COVID-19 corrections—which are described in elsewhere in this appendix.

After all data processing steps were complete, datapoints for all locations and years were systematically reviewed to identify outliers or other data limitations not fully overcome by CoD data processing methods. The following data were excluded based on this review:

Super-region	Countries	Years and types excluded
Central Europe, eastern Europe, and central Asia	Kazakhstan	2013, 2014, 2015, 2017, vital registration

High income	No exclusions	
Latin America and Caribbean	Honduras	1988, 1990, vital registration
North Africa and Middle East	Palestine<br>Saudi Arabia<br>Türkiye	Prior to 2010, vital registration<br>1999, 2001, 2007, vital registration<br>2002, verbal autopsy (vital registration is preferred source)
South Asia	Nepal<br>India	2007, verbal autopsy (sole year)<br>Department of Economics and Statistics' Medically Certified Causes of Death records in India excluded due to inclusion of hospital deaths in urban areas only (India Sample Registration System is preferred source)
Sub-Saharan Africa	Cabo Verde<br>Ghana	1980, ICD-8A-coded vital registration<br>2007, vital registration
Southeast Asia, east Asia, and Oceania	Indonesia<br>Kiribati<br>Palau	Single years and age-groups of verbal autopsy data for some subnational locations<br>All years, vital registration<br>2013, vital registration (sole year)

Modelling strategy

For modelling fatal burden of digestive diseases, no substantive changes were made in GBD 2023 from the modelling strategy used in GBD 2021. A standard CODEm model was used to model deaths due to digestive diseases (see appendix section on CODEm method for details). Models were generated separately for males in data-rich countries, males in all countries (referred to as a global model), females in data-rich countries, and females in all countries (global model). We hybridised separate global and data-rich models to acquire unadjusted results. The following table has the full list of covariates presented to the CODEm algorithm for possible inclusion in the digestive disease mortality model.

Table 1. Covariates used in digestive diseases mortality modelling

Level	Covariate	Direction
1	Sanitation (proportion with access)	-
	Cumulative cigarettes (10 years)	+
	Cumulative cigarettes (5 years)	+
	Smoking prevalence	+
	Alcohol (litres per capita)	+
2	Mean BMI	+
	Age-sex-specific scaled exposure variable for low fruit consumption	+
	Age-sex-specific scaled exposure variable for low vegetable consumption	+

	Age-sex-specific scaled exposure variable for high red meat consumption	+
	Healthcare Access and Quality Index	-
3	Socio-demographic Index	-
	Education (year per capita)	-
	Log LDI ($I per capita)	-

Adjustment in CoDCorrect included rescaling unadjusted death estimates for all Level 3 digestive disease causes (cirrhosis and other chronic liver diseases; upper digestive system diseases; appendicitis; vascular intestinal disorders; paralytic ileus and intestinal obstruction; inguinal, femoral, and abdominal hernias; inflammatory bowel disease; gallbladder and biliary diseases; pancreatitis; other digestive diseases) to sum to overall digestive disease deaths, which were then rescaled alongside other causes of death to sum to all-cause counts of death. Adjusted age-sex-specific results were compared to reference life tables to calculate YLLs.

Diphtheria

Flowchart

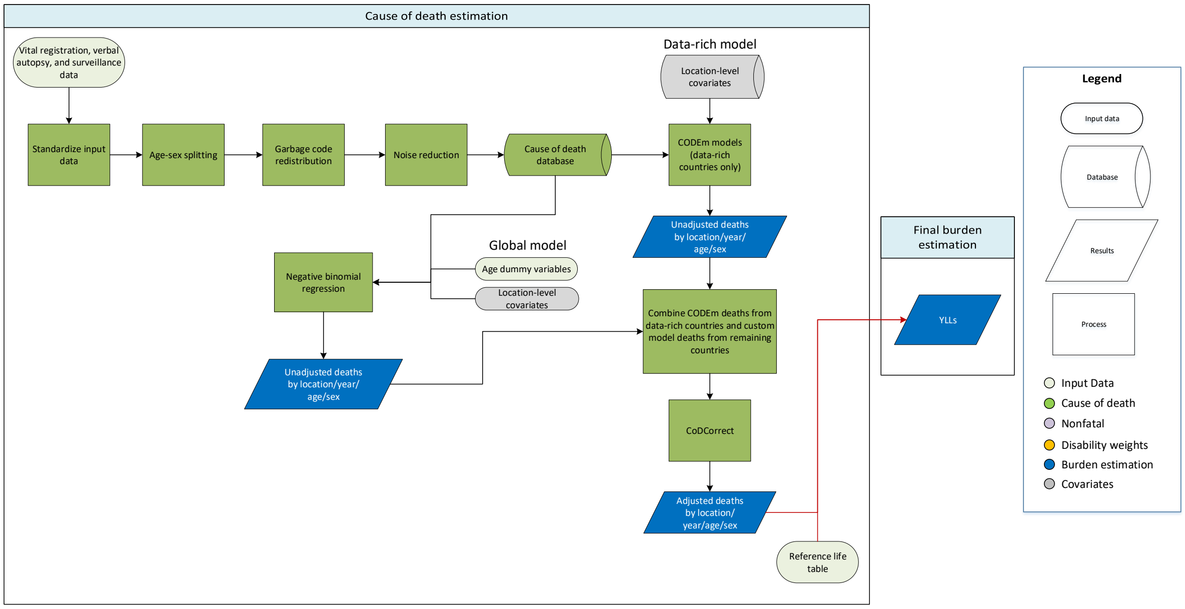

Input data and methodological summary for diphtheria

Input data

Diphtheria cause of death (CoD) data for GBD 2023 included vital registration, verbal autopsy, and surveillance sources from locations where data were available. We excluded COD data if they were highly incongruent with other available data from the same location or locations with similar sociodemographic characteristics.

Modelling strategy

We used two distinct methods to estimate diphtheria mortality for different countries based on the quality of the available cause-of-death data. We used a Cause of Death Ensemble modelling strategy (CODEm) for countries with well-defined cause of death registration systems. For the remaining countries, we used a custom negative binomial regression model. Each approach is further described in detail below. For all locations, we produced estimates for post-neonatal to 59 years of age.

Data-rich countries

For GBD 2023, our CODEm modelling strategy in data-rich settings included spatiotemporal Gaussian process regression (ST-GPR) and linear mixed effects models in the ensemble. We used the covariates outlined in Table 1 to inform CODEm predictions. Average COVID-inclusive diphtheria-pertussis-tetanus third dose (DTP3) lagged coverage over the previous five years was used as a covariate, accounting for

COVID effects directly within the model. In comparison, in GBD 2021, COVID-free vaccine coverage estimates were used in the model with post-hoc adjustments made to account for COVID impacts.

Table 1. Covariates. Summary of covariates used in the data-rich diphtheria cause of death model

Level	Covariate	Direction
1	Average COVID-inclusive diphtheria-tetanus-pertussis third-dose vaccination coverage (DTP3) over the past five years	-
	Healthcare Access and Quality (HAQ) Index	-
	Age- and sex-specific SEV for child wasting	+
3	Lag-distributed income (LDI)	-
	Socio-demographic Index (SDI)	-
	Mean years of education per capita	-

Custom count model

Our custom mortality model for non-data-rich locations also used CoD data as available by location. We excluded data with extremely high cause fractions (ie, greater than the 99.9th percentile of all diphtheria cause fractions). We used a negative binomial model with a log link to model diphtheria cause fractions representing the number of deaths due to diphtheria as a proportion of the all-cause mortality envelope. The five-year rolling COVID-inclusive DTP3 vaccine coverage and Healthcare Access and Quality (HAQ) Index were used as covariates along with dummy variables for each GBD age group. Due to the significant impact of COVID-19 on disease-specific cause fractions within the mortality envelope, we excluded post-2019 data from the model. We used the following model formula:

$$Y_{ij} = \beta_0 + \beta_1\, DTP3_{ij} + \beta_2\, HAQ_{ij} + \beta_a\, age_a + e_{ij}$$

where Y_{ij} is the log-transformed cause fraction (counts of deaths with an offset of the total number of deaths); β_0 is the fixed-effect intercept; β_1 is the fixed-effects slope on vaccine coverage; β_2 is the fixed-effects slope on Healthcare Access and Quality; β_a is the fixed-effects slope on age_a, the dummy variable for each GBD age group in the estimation; e_{ij} is the residual; i is the year; and j is the location.

Uncertainty was estimated by taking 1000 samples from the posterior distribution based on the variance-covariance matrix, and a random sample of the dispersion parameter from a gamma distribution. Results were summarised as the mean of all samples (draws) and an associated 95% uncertainty interval (the 2.5th and 97.5th percentile of all draws).

Changes from GBD 2021 to GBD 2023

The major substantive change to our modelling strategy for GBD 2023 was the handling of COVID impacts on diphtheria mortality. In GBD 2023, we used a five-year lagged COVID-inclusive DTP3 mean vaccine coverage covariate to account for COVID effects directly within the model. In non-data-rich models, we excluded post-2019 data due to the significant impact of COVID-19 on disease-specific cause fractions within the mortality envelope.

Diverticular disease

Flowchart

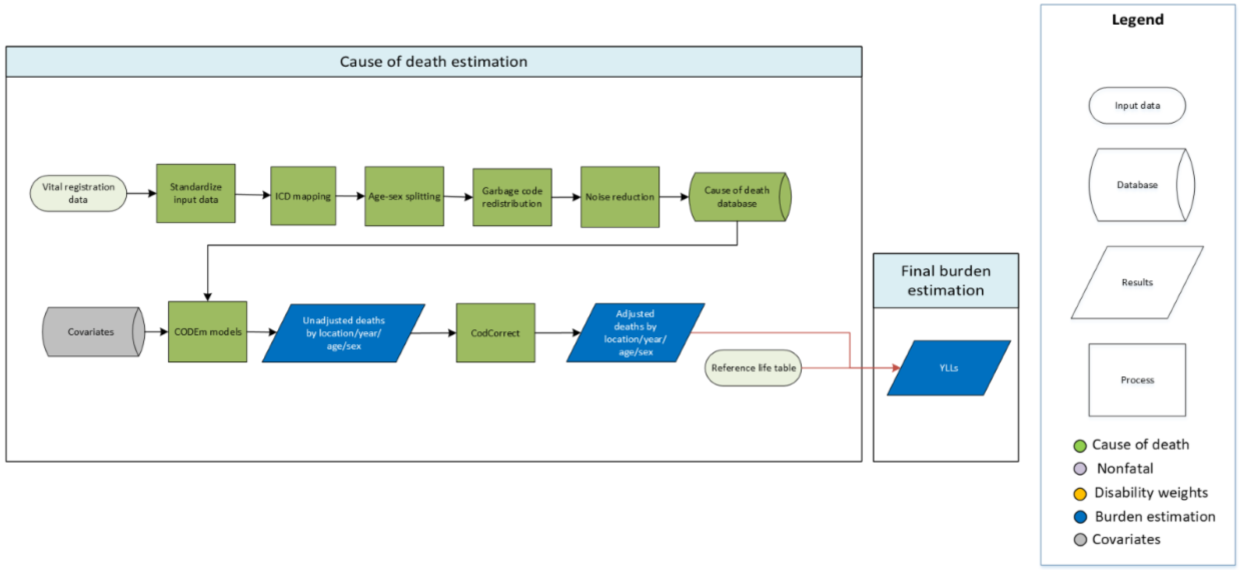

Input data and methodological summary for diverticular disease

Diverticular disease comprises a Level 3 cause in the GBD hierarchy.

Input data

Data used to estimate mortality due to diverticular disease consisted of vital registration (VR) from the cause of death (CoD) database. A list of ICD codes that were mapped to this cause, a list of vital registration sources included in the CoD database and their quality as indicated by star-ratings, and the steps used to process these data for modelling (including corrections for misclassification related to the COVID-19 pandemic) can be found in the section of this appendix on the CoD database and the tables and figures it refers to. Prior to GBD 2023, ICD codes related to diverticular disease were mapped to the cause “other digestive diseases”.

After all data processing steps were complete, datapoints for all locations and years were systematically reviewed to identify outliers or other data limitations not fully overcome by CoD data processing methods, and selected sources were excluded.

Modelling strategy

Diverticular disease fatal burden was estimated as part of GBD for the first time in GBD 2023. A standard CODEm model was used to model deaths due to diverticular disease (see appendix section on CODEm method for details). Models were generated separately for males in data-rich countries, males in all countries (referred to as a global model), females in data-rich countries, and females in all countries (global model). Fatal estimation for this cause was restricted to ages 0 days to 95+ years.

The following table has the full list of covariates presented to the CODEm algorithm for selection in models of diverticular disease mortality.

Table 1. Covariates used in diverticular disease mortality modelling

Level	Covariate	Direction
1	BMI (mean)	+
	Total physical activity (MET-min/week), age-specific	-
	Age- and sex-specific SEV for low fruit	-
	Age- and sex-specific SEV for low vegetables	-
	Age- and sex-specific SEV for low fibre	-
2	Tobacco (cigarettes per capita)	+
	Cumulative cigarettes (5 years)	+
	Healthcare Access and Quality Index	-
	Age- and sex-specific SEV for high red meat	+
	Litres of alcohol consumed per capita	+
3	Diabetes fasting plasma glucose (mmol/L), age-standardised 25+	+
	Socio-demographic Index	-
	Education (years per capita)	-
	Log LDI ($I per capita)	-

We hybridised separate global and data-rich models to acquire unadjusted results, which we adjusted using CoDCorrect. Adjustment in CoDCorrect included rescaling unadjusted diverticular disease death estimates alongside other Level 3 digestive disease deaths to sum to overall digestive disease deaths, which were then rescaled along other causes to sum up the hierarchy to all-cause counts of death. Adjusted deaths were compared to the reference life table to calculate final YLLs due to diverticular disease.

Drug use disorders

Flowchart

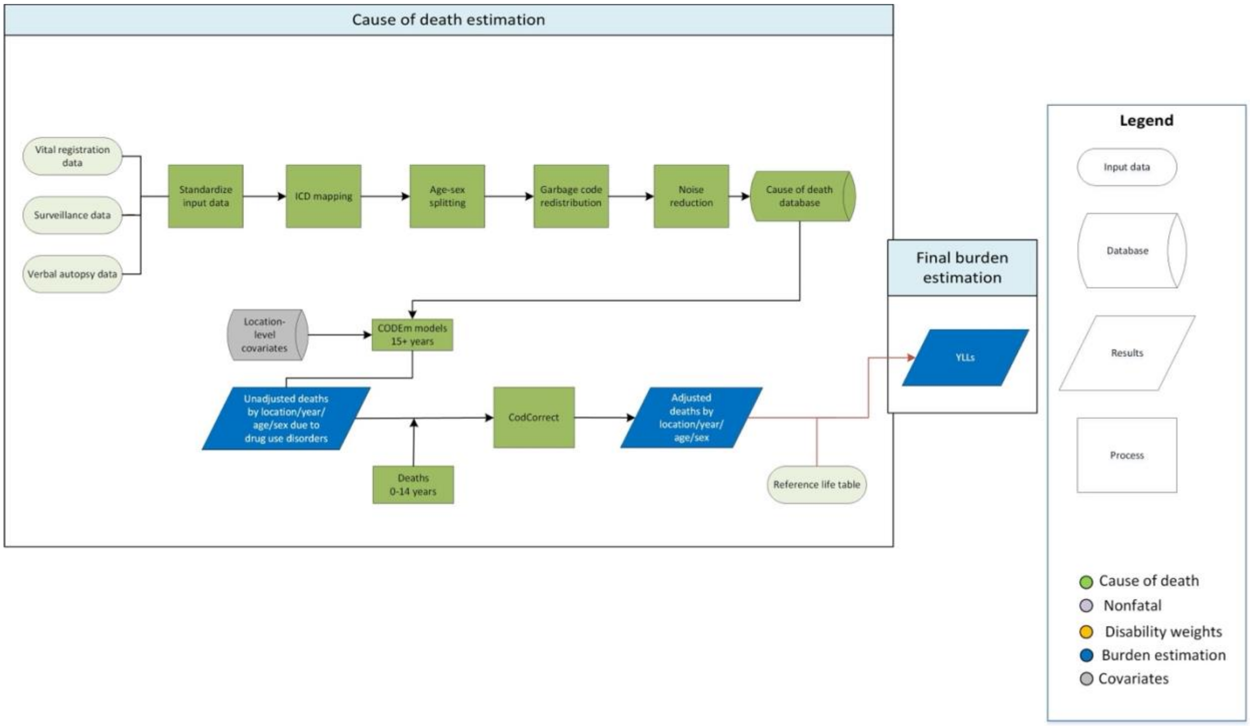

Input data and methodological summary for drug use disorders

Input data

All data were from vital registration, verbal autopsy, and surveillance sources. Data from countries with sparse yet heterogeneous data were excluded as the data exaggerated fluctuations in deaths and gave implausible regional patterns determined by in-country experts and subject experts. Excluded data were primarily from low- and middle-income countries.

A persistent challenge in modelling drug use disorders is addressing ICD codes classified as "garbage codes." Among the most relevant are codes for accidental poisonings (X40–X44 and X49). Over 90% of these cases involve exposure to narcotics, psychodysleptics, or other drugs, with more than 97% occurring in individuals aged 15–65 years. These deaths are often the result of deliberate ingestion leading to unintentional poisoning rather than accidental poisoning.

Using multiple cause-of-death (CoD) data from countries such as the USA, Mexico, Brazil, Taiwan*, Italy, Colombia, Australia, and various European nations, deaths coded under X40–X44 were identified and reassigned to the specific drug use disorder categories outlined in Table 1 (additional details in Appendix 1, Section 4). Table 1 lists the ICD-10 codes corresponding to these drug use disorders.

The redistribution process, detailed in Table 2, follows an algorithm that accounts for the relative fatality risk of different substances. For instance, opioids, being highly fatal in combination with other drugs, are assigned a proportionate share of deaths. If 40% of deaths involve opioids as a contributing cause, 40% of those deaths are redistributed to opioid-related categories.

In the final categorisation, deaths involving substances like cannabis and psychoactive drugs were grouped under "other drug use disorders" due to their comparatively lower fatality risks when evaluated alongside substances like opioids, cocaine, and methamphetamine.

Table 1. ICD-10 codes for substances or drugs used to assign deaths coded to an underlying cause of unintentional poisoning by using multiple CoD data

**Category**	**ICD-10 Codes**
**Accidental poisoning codes**	All X40, X41, X42, X43, X44 codes
**Opioid codes**	T40.0, T40.1, T40.2, T40.3, T40.4, T40.6, F11.0, F11.1, F11.2, F11.3, F11.4, F11.5, F11.6, F11.7, F11.8, F11.9
**Amphetamine codes**	T43.6, F15.0, F15.1, F15.2, F15.3, F15.4, F15.5, F15.6, F15.7, F15.8, F15.9
**Cocaine codes**	T40.5, F14.0, F14.1, F14.2, F14.3, F14.4, F14.5, F14.6, F14.7, F14.8, F14.9
**Psychoactive and psychedelic codes**	T40.8, T40.9, F16.0, F16.1, F16.2, F16.3, F16.4, F16.5, F16.6, F16.7, F16.8, F16.9
**Alcohol codes**	T51.0, T51.1, T51.2, T51.3, T51.8, T51.9, F10.0, F10.1, F10.2, F10.3, F10.4, F10.5, F10.6, F10.7, F10.8, F10.9
**Cannabis codes**	T40.7, F12.0, F12.1, F12.2, F12.3, F12.4, F12.5, F12.6, F12.7, F12.8, F12.9

Table 2. Algorithm for the selection and assignment of a substance or drug use cause of death for deaths coded to an underlying cause of unintentional poisoning using multiple cause of death data

Selection algorithm						
	Opioids	Cannabis	Cocaine	Amphetamine	Alcohol	Psychoactive and psychedelic
Opioids	Opioids	Opioids	Opioids	Opioids	Opioids	Opioids
Cannabis	Opioids	Cannabis	Cocaine	Amphetamine	Alcohol	Psychoactive and psychedelic
Cocaine	Opioids	Cocaine	Cocaine	Amphetamine	Cocaine +alcohol	Cocaine
Amphetamine	Opioids	Amphetamine	Amphetamine +cocaine	Amphetamine	Amphetamine+ alcohol	Amphetamine
Alcohol	Opioids	Alcohol	Cocaine +alcohol	Amphetamine + alcohol	Alcohol	Psychoactive and psychedelic
Psychoactive and psychedelic	Opioids	Psychoactive and psychedelic	Cocaine	Amphetamine	Psychoactive and psychedelic	Psychoactive and psychedelic

Modelling strategy

In the GBD 2023 round, the modelling strategy for drug use disorders remained largely unchanged. The standard Cause of Death Ensemble modelling (CODEm) approach (detailed in appendix 1 section 4) was used to estimate deaths due to drug use disorders.

Drug use disorders served as a "parent" to the following causes:

- Amphetamine use disorders
- Opioid use disorders
- Cocaine use disorders
- Other drug use disorders
    - While the CODEm modelling tool was used to predict estimates of drug use deaths in persons >15 years, we also recognised that deaths in children <15 years can occur as a result of exposure to drugs in the maternal womb. This cause attempts to capture these deaths. However, due to the rarity of these events and lack of data, our approach is to directly add deaths recorded in vital registration (ICD-10: Q86.0; P04.3 for alcohol-related deaths, P96.1 for opioid-related deaths, and P044 for newborn affected by maternal use of drugs or addiction) for ages younger than 15 as a post-processing step, rather than being modelled.

Covariates were selected based on their directional relationship with drug use deaths (Table 3). Level 1 covariates, with the strongest associations, include:

- Intravenous drug use prevalence
- Opioid consumption per million inhabitants (measured as daily doses per million inhabitants, derived from International Narcotics Control Board data, INCB).

Covariate directions were determined by evidence strength.

Table 3. Covariates used in drug use disorders mortality modelling

Level	Covariate	Direction
1	Intravenous drug use, age-standardised	+
	Intravenous drug use, age-specific	+
	Opioid standard doses per million per day (10-year lag)	+
2	Cumulative cigarettes (10 years)	+
	Cumulative cigarettes (5 years)	+
	Smoking prevalence	+
	Healthcare Access and Quality Index	-
3	Log LDI (I$ per capita)	+
	Education (years per capita)	+
	Socio-demographic Index	+

References

1. Johnson, S.C., Cunningham, M., Dippenaar, I.N. *et al.* Public health utility of cause of death data: applying empirical algorithms to improve data quality. *BMC Med Inform Decis Mak* **21,** 175 (2021). https://doi.org/10.1186/s12911-021-01501-1

Ebola virus disease

Flowchart

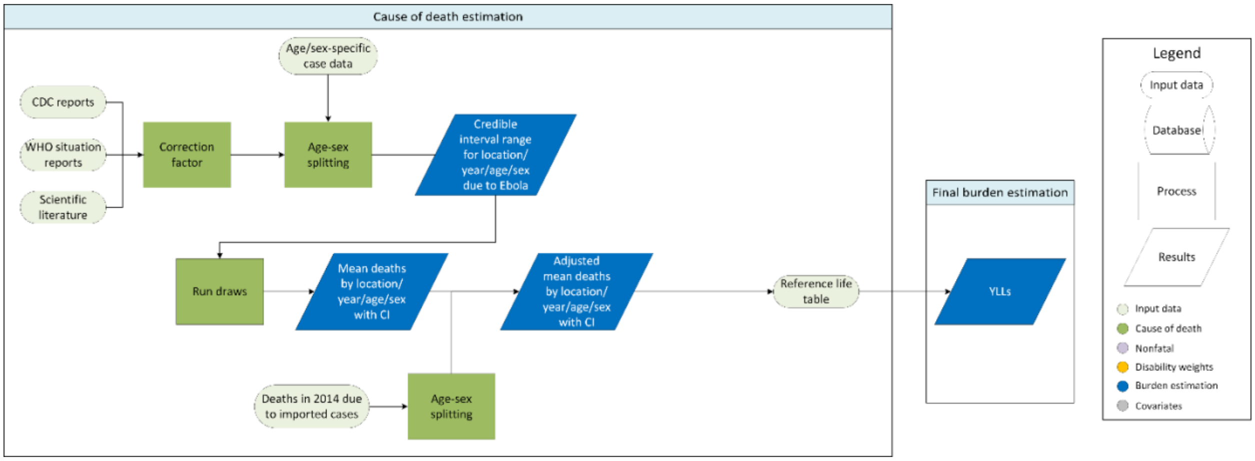

Input data and methodological summary for Ebola virus disease

Input data

The input data for deaths due to Ebola virus disease (EVD) came in three forms: (i) total case count tallies provided by the Centers for Disease Control (CDC), (ii) total case count tallies for the Democratic Republic of the Congo (DRC) outbreak from 2018 to 2020 provided by the World Health Organization (WHO), and (iii) literature searches for reported deaths due to EVD not captured by the aforementioned outbreaks. This is further explained below:

i. *CDC estimates for the West African outbreak, 2014–2016*

The final tallies, as reported by the CDC, were used for each of the three worst-affected countries (Liberia, Guinea, and Sierra Leone). Death data from Guinea ranged from February 18, 2014, to September 27, 2015, with data from Liberia ranging from March 20, 2014, to May 4, 2015, and data from Sierra Leone ranging from May 21, 2015, to September 28, 2015. In order to capture the small number of fatalities outside of these core three countries, WHO situation reports supplemented the CDC estimates. Fatalities were reported in the USA (specifically Texas), Mali, and Nigeria.[1] All deaths in the USA, Mali, and Nigeria occurred in 2014. Additional age- and sex-specific information could only be obtained for the deaths that occurred in the USA.

CDC estimates for the Guinea and DRC outbreaks, 2021

The final tallies, as reported by the CDC, were used for each country. Death data from Guinea ranged from February 14, 2021, to June 19, 2021, and data from DRC for two outbreaks, ranging from February 7, 2021, to May 3, 2021, and October 8, 2021, to December 16, 2021.[8] All deaths occurred in 2021.

ii. *WHO estimates for DRC, 2018–2020*

Final case counts in the DRC, as reported by WHO External Situation reports, were used. This included the outbreak in the Equateur Province[2] in 2018, and the outbreak from 2018 to 2020 in Ituri, North Kivu, and South Kivu Provinces.[3,4,5] The latter outbreak included one case reported for Uganda.[6] This DRC data were further supplemented by CDC case counts of the Equateur Province outbreak[7] from June 1, 2020, to November 18, 2020.

WHO estimates for DRC and Uganda, 2022

Final death counts in DRC and Uganda, as reported by WHO weekly bulletins, were used. This included the outbreaks in Equateur Province[9] from April 23, 2022, to July 3, 2022, and North Kivu Province[10] from August 21, 2022, to September 27, 2022, in DRC, and Mubende District[11] from September 20, 2022, to December 3, 2022, in Uganda.

iii. *Literature searches for reported deaths due to Ebola outside of the above outbreaks*

Using a previous review of historical outbreaks,[12,13] original articles describing the progression of historical outbreaks were consulted, and this work was supplemented by additional searches. This resulted in datasets describing each outbreak with variable degrees of detail – some fully describing the age- and sex-specific breakdown of all deaths (eg, Rosello and colleagues[14]) and others simply providing the final total. Only confirmed or probable deaths were included; suspected EVD deaths were omitted. Outbreaks that spanned multiple years, in the absence of sufficient data providing an accurate breakdown, were apportioned between the years by evenly assigning a uniform number of deaths to each month of the outbreak's duration.

Table 1. Tabulation of death metadata

Outbreak	Number of deaths	Sex metadata	Age metadata	Year metadata
Côte d'Ivoire 1994	No deaths	N/A	N/A	N/A
Gabon 1994/1995	Georges 1999	Imputed	Imputed	Georges 1999
Democratic Republic of the Congo 1995	Rosello 2015	Rosello 2015	Rosello 2015 [94.5% coverage]	Rosello 2015
Gabon 1996	Milleliri 2004	Imputed	Imputed	Milleliri 2004
Gabon 1996/1997	Milleliri 2004	Imputed	Imputed	Imputed
Uganda 2000/2001	Okware 2002	Imputed	Imputed	Imputed
Congo 2002/2003	Kuhn 2008	Imputed	Imputed	Imputed
Congo 2003	Boumandouki 2005	Imputed	Imputed	Boumandouki 2005
South Sudan 2004	WHO 2004	WHO 2004	WHO 2004 [42.86% coverage]	WHO 2004
Congo 2005	Nkoghe 2011	Nkoghe 2011	Nkoghe 2011	Nkoghe 2011
Democratic Republic of the Congo 2007	Rosello 2015	Rosello 2015	Rosello 2015	Rosello 2015
Uganda 2007	Wamala 2010	Wamala 2010	Imputed	Wamala 2010

Democratic Republic of the Congo 2008	Rosello 2015	Rosello 2015	Rosello 2015	Rosello 2015
Uganda 2011	Shoemaker 2012	Shoemaker 2012	Shoemaker 2012	Shoemaker 2012
Democratic Republic of the Congo 2012	Rosello 2015	Rosello 2015	Rosello 2015	Rosello 2015
Uganda 2012	Albarino 2013	Imputed	Imputed	Albarino 2013
Uganda 2012/2013	Albarino 2013	Imputed	Imputed	Imputed
West Africa 2013/2015	WHO/CDC	Imputed	Imputed	WHO/CDC
Democratic Republic of the Congo 2014	Rosello 2015	Rosello 2015	Rosello 2015	Rosello 2015
Democratic Republic of the Congo, Equateur province 2018	WHO 2018	Imputed	Imputed	WHO 2018
Democratic Republic of the Congo, North Kivu 2018/2019/2020	WHO 2018, WHO 2019, WHO 2020	Imputed	Imputed	WHO 2018, WHO 2019, WHO 2020
Democratic Republic of the Congo, Equateur province 2020	CDC 2020	Imputed	Imputed	CDC 2020
Guinea, N'Zérékoré prefecture 2021	CDC 2021	Imputed	Imputed	CDC 2021
Democratic Republic of the Congo, North Kivu province 2021	CDC 2021	Imputed	Imputed	CDC 2021
Democratic Republic of the Congo, Equateur province 2022	WHO 2022	Imputed	Imputed	WHO 2022
Democratic Republic of the Congo, North Kivu province 2022	WHO 2022	Imputed	Imputed	WHO 2022
Uganda, Mubende district 2022	WHO 2022	Imputed	Imputed	WHO 2022

Modelling strategy

Data on deaths resulting from imported cases from 2014 were used as specific count data, as it was assumed to be an accurate representation of the cases and outbreaks in these countries, all of which were on high alert for importation of cases.[15,16]

The other input data were processed prior to inclusion in GBD to account for any potential under-reporting of deaths. A meta-analysis of existing under-reporting studies from the literature was performed, using a random effects model with a DerSimonian-Laird estimator. A variety of sources were included, capturing several different estimation processes, all identified by literature review. Figure 1 below shows the different effect sizes of the various studies,[17-22],as well as the resulting GBD 2016 correction factor, with the GBD 2015 correction factor for reference. The correction factor ranged from 1.4580 to 2.5475, with a mean of 2.0027. For GBD 2023, the GBD 2016 factor was used.

Figure 1. Effect sizes of sources for under-reporting

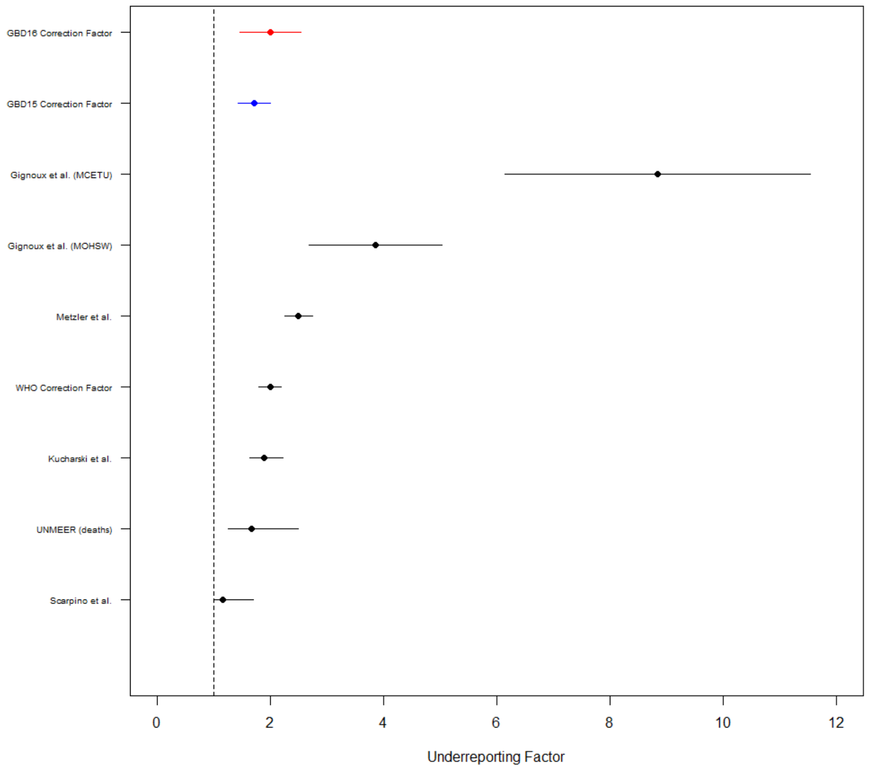

In order to capture this potential variation, all input data were multiplied by the lower and upper limit of this estimated correction factor; these numbers then provided the lower and upper bounds from which draw values were taken. For outbreaks where no data were supplied for age and/or sex, the pattern observed in the age- and sex-specific case data was used to apportion these total values.

1000 draws were taken from a normal distribution fitted between these lower and upper bound values, which generated mean estimates stratified by age, sex, location, and year along with credible intervals

for these numbers. These estimates were then adjusted by including the count data for imported cases from 2014.

Data on Ebola outbreaks prior to 2014 are sparse, and as a result many values derived from the West African outbreak were assumed to be valid for historical outbreaks as well. This may mask significant differences that exist between these outbreaks, some of which were caused by different species of Ebola virus. To minimise this problem, we chose to implement a data-driven approach – for those outbreaks where sufficiently detailed historical data could be obtained, these were used in preference to any assumed age/sex breakdown.

Changes from GBD 2021 to GBD 2023

There have been no substantive changes to the modelling strategy for GBD 2023.

References

1 World Health Organization. Ebola Situation Reports. 2016. Interview (accessed March 14, 2016).

2 World Health Organization (WHO). WHO Ebola Situation Report 2018 - Number 17. 2018.

3 World Health Organization (WHO). WHO Ebola Situation Report 2019 - Number 69. 2019.

4 World Health Organization (WHO). WHO Ebola Situation Report 2019 - Number 73. 2019.

5 World Health Organization (WHO). WHO Ebola Situation Report 2019 - Number 98. 2020.

6 World Health Organization (WHO). WHO Ebola Situation Report 2019 - Number 45. 2019.

7 Centers for Disease Control and Prevention (CDC). Ebola (Ebola Virus Disease). 2020 Democratic Republic of the Congo, Equateur Province. 2020. https://www-cdc-gov.offcampus.lib.washington.edu/vhf/ebola/outbreaks/drc/2020-june.html (accessed January 11, 2021).

8 Centers for Disease Control and Prevention (CDC). Ebola (Ebola Virus Disease). 2021 Democratic Republic of the Congo, North Kivu Province & Guinea, N'Zérékoré prefecture. 2021. https://www.cdc.gov/vhf/ebola/history/chronology.html (accessed July 27, 2022).

9 World Health Organization (WHO). WHO Outbreak News Bulletin. 2022 Democratic Republic of the Congo, Equateur Province. 2022. https://www.who.int/emergencies/disease-outbreak-news/item/2022-DON398 (accessed July 4, 2022).

10 World Health Organization (WHO). WHO Weekly Bulletin on Outbreaks and other Emergencies 2022 - Number 40. 2022.

11 World Health Organization (WHO). WHO Weekly Bulletin on Outbreaks and other Emergencies 2023 - Number 1. 2023.

12 Pigott DM, Golding N, Mylne A, *et al.* Mapping the zoonotic niche of Ebola virus disease in Africa. *Elife* 2014; **3**: e04395.

13 Mylne A, Brady OJ, Huang Z, *et al.* A comprehensive database of the geographic spread of past human Ebola outbreaks. *Sci Data* 2014; **1**: 140042.

14 Rosello A, Mossoko M, Flasche S, *et al.* Ebola virus disease in the Democratic Republic of the Congo, 1976-2014. *Elife* 2015; **4**. DOI:10.7554/eLife.09015.

15 Fasina FO, Shittu A, Lazarus D, *et al.* Transmission dynamics and control of Ebola virus disease outbreak in Nigeria, July to September 2014. *Euro Surveill* 2014; **19**: 20920.

16 Althaus CL, Low N, Musa EO, Shuaib F, Gsteiger S. Ebola virus disease outbreak in Nigeria: Transmission dynamics and rapid control. *Epidemics* 2015; **11**: 80–4.

17 Gignoux E, Idowu R, Bawo L, *et al.* Use of Capture-Recapture to Estimate Underreporting of Ebola Virus Disease, Montserrado County, Liberia. *Emerg Infect Dis* 2015; **21**: 2265–7.

18 Meltzer MI, Atkins CY, Santibanez S, *et al.* Estimating the future number of cases in the Ebola epidemic--Liberia and Sierra Leone, 2014-2015. *MMWR Suppl* 2014; **63**: 1–14.

19 Scarpino S V, Iamarino A, Wells C, *et al.* Epidemiological and viral genomic sequence analysis of the 2014 ebola outbreak reveals clustered transmission. *Clin Infect Dis* 2015; **60**: 1079–82.

20 Kucharski AJ, Camacho A, Flasche S, Glover RE, Edmunds WJ, Funk S. Measuring the impact of Ebola control measures in Sierra Leone. *Proc Natl Acad Sci U S A* 2015; **112**: 14366–71.

21 UNMEER. Sierra Leone: Ebola emergency Weekly Situation Report No. 7. 2014 https://www.humanitarianresponse.info/system/files/documents/files/UNMEER_NERC_SitRep_07Dec.pdf.

22 Enserink M. How many Ebola cases are there really? | Science | AAAS. 2014. http://www.sciencemag.org/news/2014/10/how-many-ebola-cases-are-there-really (accessed Jan 28, 2017).

Encephalitis

Flowchart

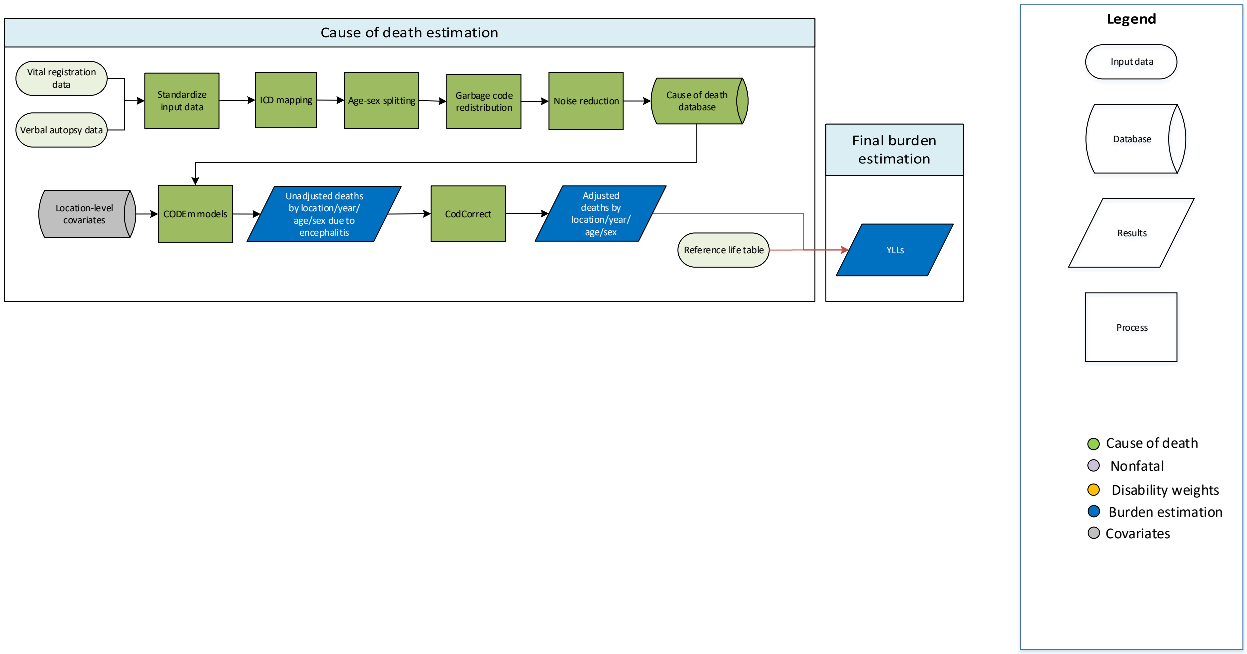

Input data and methodological summary for encephalitis

Input data

For GBD 2023, vital registration and verbal autopsy data were used to model this cause. We outliered data in instances where garbage code redistribution and noise reduction, in combination with small sample sizes, resulted in unreasonable cause fractions when compared to regional, super-regional, and global rates, and data that violated well-established time or age trends. Outliering methods were consistent across both vital registration and verbal autopsy data.

Modelling strategy

We modelled deaths due to encephalitis with a standard CODEm model using the cause of death database and location-level covariates as inputs. We hybridised separate global and data-rich models to acquire unadjusted results, which were adjusted using CoDCorrect to estimate final years of life lost due to encephalitis.

There were no substantive changes from GBD 2021 in terms of modelling strategy. A full list of covariate inputs in the published model can be found in Table 1. Covariates were weighted and selected based on the ensemble modelling process.

Table 1. Covariates used in encephalitis mortality modelling

Level	Covariate	Direction
1	Japanese encephalitis endemic area binary	+
	Age- and sex-specific summary exposure value (SEV) for child underweight	+
2	Log-transformed lag distributed income	-
	Healthcare Access and Quality Index	-
	Maternal care and immunisation	-
3	Squared proportion of in-facility deliveries	-
	Socio-demographic Index	-
	Logit-transformed sanitation (proportion with access)	-
	Logit-transformed water (proportion with access)	-
	DTP3 coverage	-
	Maternal education (years per capita)	-

Endocrine, metabolic, blood, and immune disorders

Flowchart

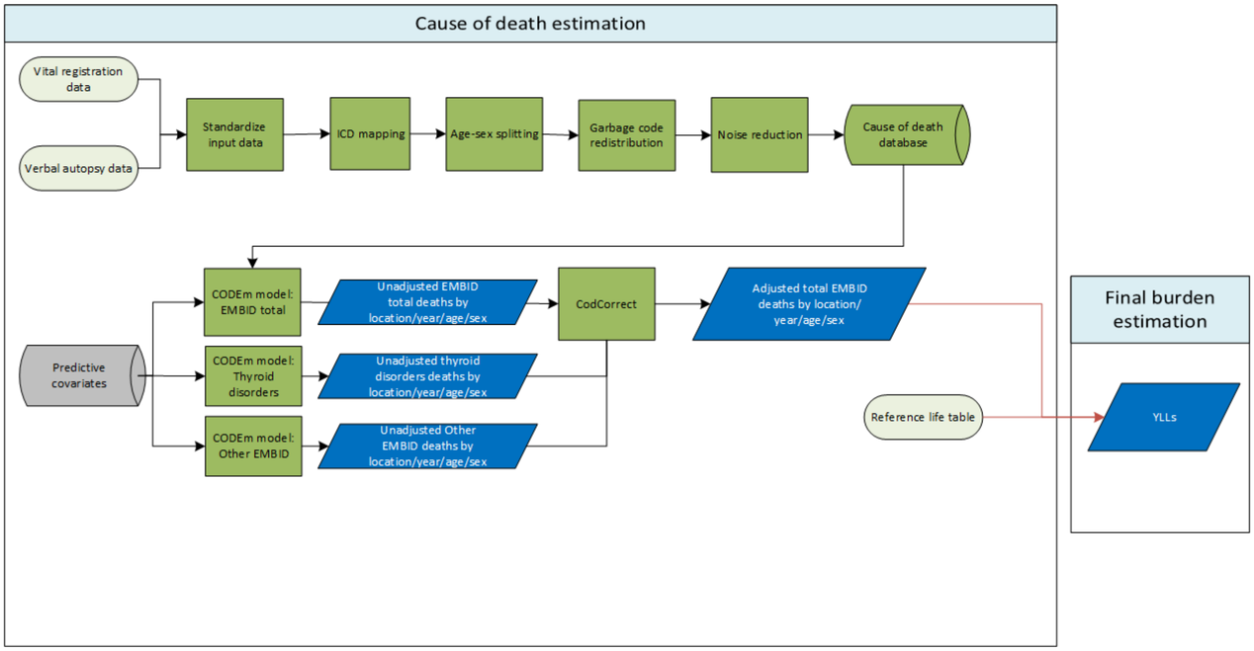

Input data and methodological summary for endocrine, metabolic, blood, and immune disorders

Endocrine, metabolic, blood, and immune disorders (EMBID) is a Level 3 cause of death in the GBD hierarchy and comprises a residual group of conditions that do not map to other causes within the diabetes, urogenital, blood, and endocrine disease hierarchy in GBD. This residual group consists of thyroid disorders, rare metabolic and immune disorders, and blood disorders not resulting in anaemia. From the ICD chapter on endocrine, metabolic, and immune disorders (the E chapter), GBD's definition of EMBID excludes the codes for nutritional deficiencies, diabetes, and anaemia, which are modelled as separate causes; as well as those for obesity and hypercholesterolemia, which are modelled as risk factors, not diseases.

Starting in GBD 2021, we began estimating deaths due to thyroid disorders separately from other conditions within EMBID, named "Other endocrine, metabolic, blood, and immune disorders" (other EMBID), as two Level 4 causes of EMBID (Level 3) in the GBD cause list. All deaths related to thyroid disorders and other EMBID were included in the database for EMBID. Separate statistical models were fit to estimate mortality for total EMBID, thyroid disorders, and other EMBID, and they were subsequently combined during CoDCorrect, all described below.

Input data

Data used to estimate mortality due to EMBID (Level 3) consisted of vital registration (VR) and verbal autopsy (VA) from the CoD database. In contrast, only VR data were used for two Level 4 causes of EMBID: thyroid disorders and other EMBID. A list of VR and VA sources included in the CoD database, an

assessment of their quality as indicated by star-ratings, and the steps used to process these data for modelling can be found in the section of this appendix on the CoD database and the tables and figures it refers to. CoD data processing was broadly similar to previous rounds of GBD, but one novel data processing step was employed in preparing data for EMBID that was not needed prior to GBD 2023–COVID-19 corrections–which are described in the aforementioned appendix section.

Data exclusions

After all data processing steps were complete, datapoints for all locations and years were systematically reviewed to identify outliers or other data limitations not fully overcome by CoD data processing methods. For EMBID, the following were identified and excluded prior to modelling:

Super-region	Countries	Years and types excluded
Central Europe, eastern Europe, and central Asia	No exclusions	
High income	Austria	ICD-9-coded vital registration data prior to 2002
Southeast Asia, east Asia, and Oceania	Viet Nam	2008, verbal autopsy (sole year)
Latin America and Caribbean	No exclusions	
North Africa and Middle East	Afghanistan<br>Türkiye	2008, verbal autopsy (sole year)<br>2006, verbal autopsy (vital registration is preferred source)
Sub-Saharan Africa	Mali	2020 Child Health and Mortality Prevention Surveillance data
South Asia	Pakistan<br>Bangladesh<br><br>India	2006, verbal autopsy (sole year)<br>All years, verbal autopsy<br><br>Department of Economics and Statistics' Medically Certified Causes of Death records in India excluded due to inclusion of hospital deaths in urban areas only (India Sample Registration System is preferred source)

Modelling strategy

The mortality estimation strategy used for EMBID and its Level 4 causes, thyroid disorders and other EMBID, are largely similar to the methods used in GBD 2021, using standard CODEm models. (See the section of this appendix on CODEm for details of this method.) Separately for EMBID, thyroid disorders, and other EMBID, models were generated for males in data-rich countries, males in all countries (referred to as a global model), females in data-rich countries, and females in all countries (global model). Fatal estimation for this cause was restricted to ages 0 days to 95+ years. The following tables report the covariates tested for possible selection in models for each cause.

Table 1. Covariates used in endocrine, blood, metabolic, and immune disorders CODEm modelling

Level	Covariate	Direction
1	Mean BMI	+
2	Mean cholesterol	+
	Alcohol (litres per capita)	+
	Healthcare Access and Quality Index	-
3	Socio-demographic Index	-
	Education (year per capita)	-
	Log LDI ($I per capita)	-

Table 2. Covariates used in thyroid disorders CODEm modelling

Level	Covariate	Direction
1	Absolute value of average latitude	+
	Healthcare Access and Quality Index	-
2	Diabetes age-standardised prevalence (proportion)	+
3	Cumulative cigarettes (10 years)	+
	Cumulative cigarettes (5 years)	+
	Smoking prevalence	+
	Mean BMI	+
	Socio-demographic Index	-
	Education (year per capita)	-
	Log LDI ($I per capita)	-

Table 3. Covariates used in other endocrine, blood, metabolic, and immune disorders CODEm modelling

Level	Covariate	Direction
1	Mean BMI	+
2	Mean cholesterol	+
	Alcohol (litres per capita)	+
	Healthcare Access and Quality Index	-
3	Socio-demographic Index	-
	Education (year per capita)	-
	Log LDI ($I per capita)	-

We hybridised the results of global and data-rich models to acquire unadjusted results. The unadjusted (CODEm) results of thyroid disorders and other EMBID were rescaled to fit within the total defined by the EMBID results, which were likewise scaled alongside other Level 3 non-communicable disease causes of death, and so on through the GBD cause hierarchy in the CoDCorrect process, such that ultimately all causes totaled all-cause mortality for every year, age, sex, and location combination. Adjusted results were compared to the reference life table to calculate YLLs for each cause.

Fatal discontinuities

Flowchart

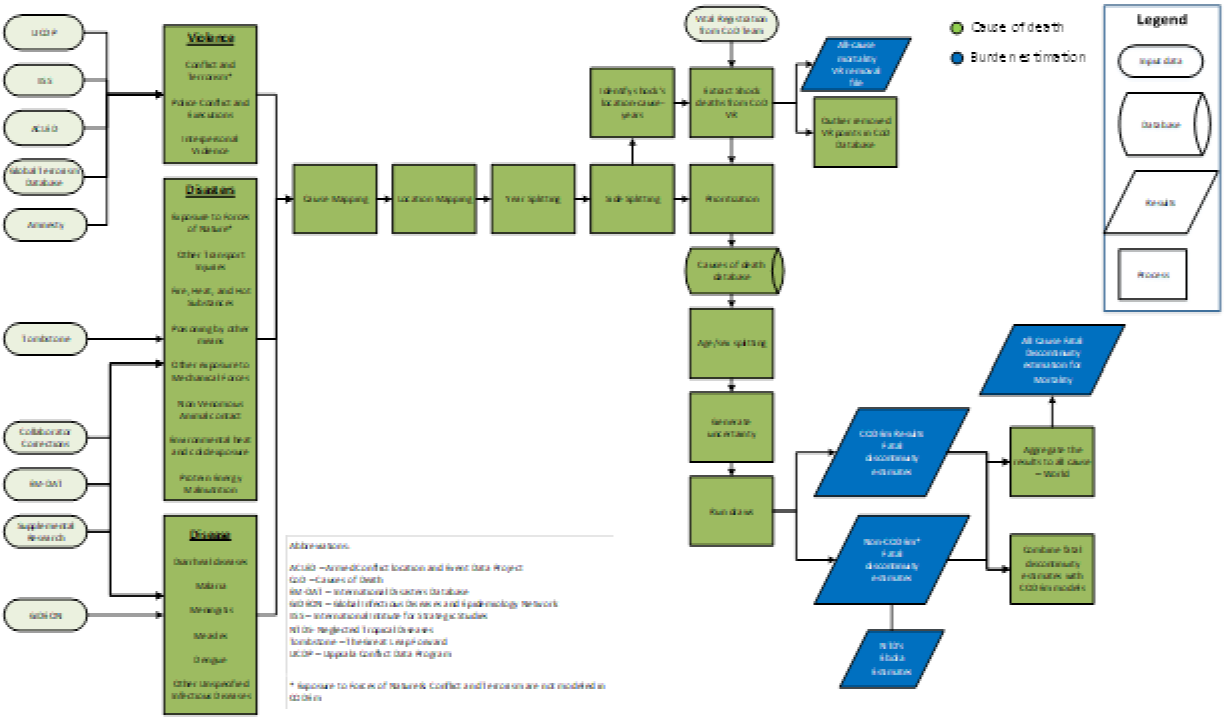

Input data and methodological summary for fatal discontinuities

Fatal discontinuities are defined as events that are stochastic in nature and cannot be modelled because they do not have a predictable time trend. Some causes have both fatal discontinuities, as well as a continuous background mortality that has a smooth time trend and can be modelled; these include police violence and executions; interpersonal violence; other transport injuries; fire, heat, and hot substances; poisoning by other means; other exposure to mechanical forces; non-venomous animal contact; environmental heat and cold exposure; protein-energy malnutrition; diarrhoeal disease; malaria; meningitis; encephalitis; diphtheria; measles; dengue; and other unspecified infectious disease. Causes without a continuous background mortality that are exclusively estimated using the fatal discontinuity method are conflict and terrorism, and exposure to forces of nature. Any other causes are not captured in fatal discontinuities.

Input data

Overall

Input data for fatal discontinuities are compiled from a range of sources, including country vital registration (VR) data; international databases that capture several cause-specific fatal discontinuities;

and supplemental data in the presence of known issues with data quality or representativeness, or time lags in reporting. Below more detail is provided on the different input data sources by sub-causes of fatal discontinuities.

Only discontinuity (non-CODEm)

For causes that are not modelled in CODEm, all of the deaths captured in VR are considered to be fatal discontinuities. Deaths that are extracted from cause-specific VR are then subtracted from the all-cause VR data used in the all-cause mortality estimation process.

Conflict and terrorism

In GBD 2023, war is defined as "*a state of armed conflict between states, governments, societies, and paramilitary groups. It is generally characterised by extreme violence, aggression, destruction, and mortality and the use of regular or irregular military forces".*[14] Terrorism is defined as *"the unlawful use or threatened use of force or violence against individuals or property in an attempt to coerce or intimidate governments or societies to achieve political, religious, or ideological objectives"*.[13] Data for conflict and terrorism came from the Uppsala Conflict Data Program (UCDP), International Institute for Strategic Studies (IISS), Armed Conflict Location & Event Data Project (ACLED), Global Terrorism Database (GTD), and vital registration (VR) and other supplemental data sources. Causes were assigned for each event using the source's cause coding and any description from the notes available.

Data source name	Date accessed	Years of data downloaded	Type of data included
**Uppsala Conflict Data Program**[31]			
Georeferenced Event Dataset, Version 19.1	3/5/2024	1989-2022	UCDP battles, non-state, and one-sided conflict deaths with the most disaggregated location information available
PRIO Battles Deaths Dataset, Version 3.1	3/5/2024	1946-2021	Armed conflict (civil wars, etc.)
**International Institute for Strategic Studies**			
Armed Conflict Dataset	11/17/2016	1997-2016	Insurgency, Inter-state, Intra-state conflict deaths
**Robert S. Strauss Center for International Security and Law**			
Armed Conflict Location and Event Dataset (ACLED)	3/3/2024	1997-2023	Actions of opposition groups, governments, and militias in selected locations in Africa, Asia, and the Middle East specifying the exact location and date of battle events, transfers of military control, headquarter establishment, civilian violence, and rioting
**University of Maryland, Global Terrorism Database**			
Global Terrorism Database (GTD)	9/12/2023	1970-2020	Attacks aimed at attaining political, economic, religious, or social goal, includes evidence of intention to coerce, action was outside precepts of International Humanitarian Law.
**University of Chicago, Chicago Project on Security and Threats**			
Suicide Attack Database (CPOST SAD)	11/26/2017	1982-2016	Attacks in which an attacker kills him/herself in a deliberate attempt to kill others, includes only attacks perpetrated by non-state actors

Amnesty International			
Amnesty	9/12/2023	1991-2022	Police conflict and executions

Four major conflicts were identified that were not represented in these databases: 1997 civil conflict in Albania;[4] 1971 genocide in Bangladesh;[5] 1972 genocide in Burundi;[6] and 1993 genocide in Burundi.[6] In these cases, literature sources were used to account for these fatal discontinuities. Deaths of migrants who die en route are split into their respective countries of origin using patterns found from the Missing Migrants Project.[11]

Exposure to forces of nature

In GBD 2023, exposure to forces of nature is defined as *"A force which is beyond human control".*[12] The Centre for Research on the Epidemiology of Disasters' International Disaster Database (EM-DAT)[7] served as the primary non-VR source of fatal discontinuities due to exposure to forces of nature (ie, natural disasters, lightning, earthquake, volcanic eruption, avalanche, storms, and floods). Data from EM-DAT were last accessed February 28, 2024. Supplemental online research was conducted for events where EM-DAT and VR were not up to date.

Partial discontinuity (CODEm)

For causes modelled in CODEm that have fatal discontinuities hiding in the time trend, a process was established to avoid duplication of fatal discontinuity deaths in CODEm and the fatal discontinuity estimates. First, location-cause-years were identified through outside non-VR sources. If these location-cause-years also had VR death estimates that were greater than the average of the immediate surrounding years, the difference between the identified year and the average of the surrounding years was included in the relevant cause for the fatal discontinuities database. The extracted deaths for all fatal discontinuity causes from VR are then subtracted from the all-cause VR data used in the all-cause mortality estimation process.

Police conflict and executions

In GBD 2023, police conflict and executions is defined as "*The lawful use or threatened use of force or violence against individual or group of people or property in an attempt to achieve political or socioeconomic objectives for a state".*[15] Data for police conflict and executions mainly came from Amnesty International, but other sources such as UCDP, ACLED, and VR that reported deaths due to legal intervention were also cause-mapped to police conflict and executions.

Homicide

In GBD 2023, homicide is defined as "*The use of violence against an individual or group of people without the motivation of political, religious, or ideological objectives.*"[15] Data for homicide come from VR, IISS, GED, ACLED, and other supplements. Events are mapped to homicide where the notes found in the raw data indicate gang violence. Deaths from IISS, GED, and ACLED were then split among three homicide sub-types; physical violence by firearms, physical violence by sharp object, and physical violence by other means, based on the rates calculated from VR by country if available, and by region if country VR was unavailable.

Protein-energy malnutrition (PEM)

Protein-energy malnutrition is defined as *"a lack of dietary protein and/or energy"*[16] and covers famines as well as severe droughts. The primary source for PEM, other than VR, is EM-DAT. Supplemental online research was conducted for events where EM-DAT and VR were not up to date. The Tombstone report was used to estimate deaths attributed to the famine during the Great Leap Forward in China in the 1960s.[8]

Other injury causes

Other injury causes include other transport injuries (eg, plane, train, and boat accidents); poisonings; fire, heat, and hot substances; and other exposure to mechanical forces (eg, building collapse). The primary data source other than VR for these events is EM-DAT. Supplemental online research was conducted for events where EM-DAT and VR were not up to date.

Meningococcal meningitis and other diseases

In GBD 2023, fatal discontinuities due to a subset of infectious diseases were estimated, including meningococcal meningitis (or meningococcal infection), and diarrhoeal disease caused by cholera, dengue, and malaria. These infectious diseases were first included on the fatal discontinuity cause list for GBD 2016 because (1) their current modelling strategies with the Cause of Death Ensemble model (CODEm) do not optimally capture the potentially highly variable – or epidemic – mortality levels and trends characteristic of these two causes; and (2) they can contribute to significant total fatalities in a given location-year. Other infectious diseases for which the latter is true – high death rates in the presence of an outbreak or epidemic – are currently modelled with alternative cause of death methods (eg, natural history models for measles and yellow fever), which allow for greater variation year-over-year if or when outbreaks occur. The Global Infectious Diseases and Epidemiology Network (GIDEON) and EM-DAT served as the primary data sources for collating cholera and meningococcal meningitis or meningococcal infection death reports.[9,10] For any year that cholera or meningococcal meningitis deaths were recorded in a country or territory covered by the GBD, reported deaths were directly extracted from 1950 to 2020. If GIDEON or EMDAT had reporting gaps in cholera or meningococcal meningitis deaths, and the World Health Organization (WHO) reports had coverage for those years, the WHO reports were used. For the Yemen cholera outbreak in 2016 and 2017, estimates from local collaborators were used in the absence of other data sources.

Location mapping

Every event in the fatal discontinuities database was mapped to a GBD location using a four-step process that includes the following steps in succession: manual mapping, string matching, GPS overlay, and geocoding. If an event was manually mapped, the location was assigned without the use of any other map types. In manual mapping, events are manually assigned to locations by matching the location provided in the raw data to a GBD location. During string matching, an event's location strings are directly compared to the GBD ASCII location names. During GPS overlay, events that have GPS coordinates provided are overlaid onto a map of GBD locations. If the event is placed over a GBD most-detailed location, the event is assigned to that location. During geocoding, the event's location string is entered into Open Street Maps, which returns GPS coordinates. These coordinates are processed using GPS overlay to return GBD locations. This hierarchy provides results where the results of manual mappings are considered the most reliable, followed successively by string matching, GPS coordinates, and then geocoding.

Side splitting

Many fatal discontinuities, such as war, have deaths that are reported across multiple locations. In these instances, deaths are split across the population from both locations, unless estimates by side are provided by the source itself or if weights are provided by another source. If the resulting locations are at the most detailed level according to GBD, no further splitting is needed. If a location is not most detailed, the deaths are distributed among the child locations by population.

Prioritisation

Choosing between multiple sources for same event (prioritisation)
Where multiple sources reported shock deaths for the same location-year-cause, a cause-specific prioritisation scheme was followed that reflected the available detail in the cause-specific datasets. For example, the Georeferenced Event Dataset from UCDP was prioritised above all other non-VR sources because it included detail on how deaths were distributed between multiple actors and locations in each conflict event. In most cases, VR from 4- or 5-star locations was used where available. In some cases, VR from 4- or 5-star locations was not chosen if there were well-known data quality issues or discrepancies in the cause of death data reporting related to a particular event (eg, supplemental death data for Louisiana were used for Hurricane Katrina because of established data reporting issues).

Assigning uncertainty and generating draws

Uncertainty intervals for deaths due to conflict and terrorism were generated using UCDP high and low death estimates, except in the case of Iraq for 2003–2016. During this time period, deaths due to conflict and terrorism in Iraq were estimated using a combination of supplemental sources. The source

found with the lowest number of deaths, Iraq Body Count (IBC),[2] was used as the lower bound of the uncertainty interval from 2003 to 2016. Estimates from the Iraq Mortality Study (IMS) by Hagopian and colleagues[3] from 2003 to 2006, the deadliest years of the war, were used to scale deaths to generate the upper uncertainty interval limits using the following formula:

$$deaths_{GBD\,2017,\ high} = deaths_{IBC} \cdot \overline{\left[\frac{deaths_{IMS}}{deaths_{IBC}}\right]}_{2003-2006}$$

GBD 2023 used the average ratio between IMS and IBC reported deaths between 2003 and 2006, multiplied by the number of deaths reported by the IBC. This high estimate was carried forward through 2017 under the assumption that the Iraq Body Count similarly undercounts the number of deaths due to the ongoing civil war in Iraq. The final, best estimate for conflict and terrorism deaths in Iraq from 2003 to 2016 is the midpoint of the high and low estimates given above.

In cases where low and high estimates were not included in the available data, the regional average uncertainty interval was applied to the available death estimate across all fatal discontinuity causes.

A log-normal distribution was assumed, using mean death rates and standard error based on high and low estimates. In the case that standard error was less than 10e-8, the draws were set equal to the mean rate. 1000 draws were sampled from this log-normal distribution. These 1000 draws were then converted back to count space and used for final calculations of means and uncertainty intervals.

Modelling strategy

In GBD 2023, no new methods were introduced to the estimation of fatal discontinuities. Instead, resources were used to find reliable information for the recent conflicts as well as reviewing country profiles. One event that was notably challenging to find an estimate for was the war in Tigray, Ethiopia, from 2020 to 2023. This conflict one of the largest estimate disparities between sources that we have observed. ACLED provides ~25 thousand deaths over the time period while UCDP provides ~321 thousand deaths. The number cited by UCDP is the number that is generally tied to the conflict online. However, we are not comfortable enough with the underlying sources to use the estimate in GBD until more evidence is gathered. UCDP reports ~321 thousand deaths for this conflict, but at least 235 thousand of those deaths come from underlying sources that cite an alleged comment(s) by Field Marshal Berhanu. For this event we are using ACLED's estimate of roughly ~25 thousand. ACLED is constructing their estimate by counting the confirmed casualties from the conflict. Over time their estimate will increase.

References

1 UCDP/PRIO Armed Conflict Dataset Codebook. Uppsala Conflict Data Program (UCDP); Centre for the Study of Civil Wars, International Peace Research Institute, Oslo (PRIO), 2013.
2 Iraq Body Count. https://www.iraqbodycount.org/database/ (accessed May 8, 2017).

3 Hagopian A, Flaxman AD, Takaro TK, *et al.* Mortality in Iraq Associated with the 2003–2011 War and Occupation: Findings from a National Cluster Sample Survey by the University Collaborative Iraq Mortality Study. *PLOS Medicine* 2013; **10**: e1001533.
4 Jarvis C. The Rise and Fall of Albania's Pyramid Schemes. *F&D* 2000; **37**. http://www.imf.org/external/pubs/ft/fandd/2000/03/jarvis.htm.
5 Obermeyer Z, Murray CJL, Gakidou E. Fifty years of violent war deaths from Vietnam to Bosnia: analysis of data from the world health survey programme. *BMJ* 2008; **336**: 1482–6.
6 Milton L. Rwanda, 1994: International incompetence produces genocide. 1994 https://ezproxy.uwc.edu/login?url=http://search.proquest.com/docview/234405747?accountid=42411.
7 Centre for Research on the Epidemiology of Disasters (CRED). EM-DAT: The OFDA/CRED International Disaster Database. Brussels, Belgium: Catholic University of Leuven
8 Jisheng Y, Friedman E, Guo J, Mosher S. *Tombstone: The Great Chinese Famine, 1958-1962*. New York: Farrar, Straus and Giroux (Macmillan), 2012.
9 Inc GI, Berger DS. Cholera: Global Status: 2017 edition. GIDEON Informatics Inc, 2017.
10 Inc GI, Berger DS. Bacterial Meningitis: Global Status: 2017 edition. GIDEON Informatics Inc, 2017.
11 Missing Migrants Project. 2021 [cited 2021May19]. Available from: https://missingmigrants.iom.int/
12 Act of God [Internet]. Wikipedia. Wikimedia Foundation; 2021 [cited 2021May19]. Available from: https://en.wikipedia.org/wiki/Act_of_God
13 Nato. NATO's military concept for defence against terrorism [Internet]. NATO. [cited 2021May19]. Available from: https://www.nato.int/cps/en/natohq/topics_69482.htm
14 projects Cto W. War [Internet]. Wikiquote. Wikimedia Foundation, Inc.; 2021 [cited 2021May19]. Available from: https://en.wikiquote.org/wiki/War
15 Alex Schmid, *Terrorism - The Definitional Problem*, 36 Case W. Res. J. Int'l L. 375 (2004) Available at: https://scholarlycommons.law.case.edu/jil/vol36/iss2/8 –
17 Protein-Energy Malnutrition Definition. Merck Manual Professional Edition; 2007 [cited 2021May19]. Available from: http://healthinsight.org/Internal/assets/Nursing%20Home/PRU%20-%20Protein%20Energy%20and%20Malnutrition.pdf

Gallbladder and biliary diseases

Flowchart

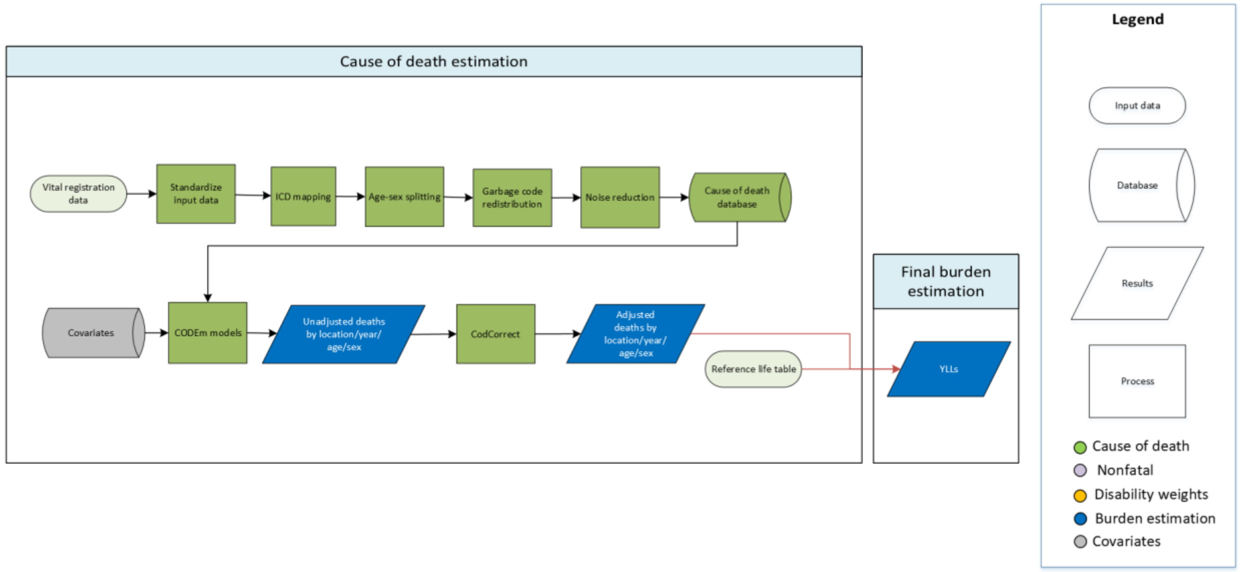

Input data and methodological summary for gallbladder and biliary diseases

Gallbladder and biliary diseases comprise a Level 3 cause in the GBD hierarchy.

Input data

Data used to estimate mortality of gallbladder and biliary diseases consisted of vital registration (VR) from the cause of death (CoD) database. A list of ICD codes that were mapped to this cause, a list of vital registration sources included in the CoD database and their quality as indicated by star-ratings, and the steps used to process these data for modelling, can be found in the section of this appendix on the causes of death database and the tables and figures it refers to. COD data processing was broadly similar to previous rounds of GBD, but one novel data processing step was employed in preparing data for gallbladder and biliary diseases that was not needed prior to GBD 2023—COVID-19 corrections—which are described elsewhere in this appendix.

After all data processing steps were complete, datapoints for all locations and years were systematically reviewed to identify outliers or other data limitations not fully overcome by CoD data processing methods, and selected sources were excluded from analysis.

Modelling strategy

The estimation strategy used to estimate mortality due to gallbladder and biliary diseases was not substantively changed between GBD 2021 and GBD 2023. A standard CODEm model was used to model deaths due to gallbladder and biliary diseases (see appendix section on CODEm method for details). Models were generated separately for males in data-rich countries, males in all countries (referred to as a global model), females in data-rich countries, and females in all countries (global model). Fatal estimation for this cause was restricted to ages 2 years to 95+ years.

The following table has the full list of covariates presented to the CODEm algorithm for selection in models of gallbladder and biliary diseases mortality.

Table 1. Covariates used in gallbladder and biliary diseases mortality modelling

Level	Covariate	Direction
1	Age-sex-specific scaled exposure variable for low polyunsaturated fatty acids	+
	BMI (mean)	+
2	Alcohol (litres per capita)	+
	Healthcare Access and Quality Index	-
	Age-sex-specific scaled exposure variable for high red meat consumption	+
	Population over 65 (proportion)	+
3	Socio-demographic Index	-
	Education (years per capita)	-
	Log LDI ($I per capita)	-

We hybridised separate global and data-rich models to acquire unadjusted results, which we adjusted using CoDCorrect. Adjustment in CoDCorrect included rescaling unadjusted gallbladder and biliary diseases death estimates alongside other Level 3 digestive disease deaths to sum to overall digestive disease deaths, which were then scaled alongside other causes to sum up the hierarchy to all-cause counts of death. Adjusted deaths were compared to the reference life table to calculate final YLLs due to gallbladder and biliary disease.

Gastritis and duodenitis

Flowchart

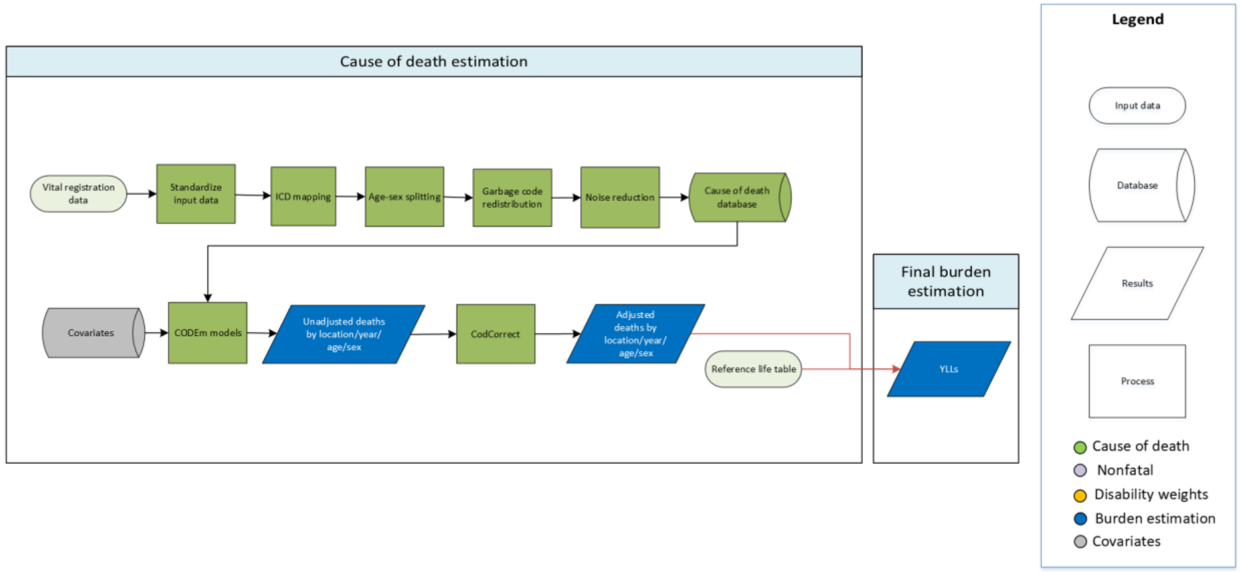

Input data and methodological summary for gastritis and duodenitis

Gastritis and duodenitis comprise a Level 4 cause in the GBD cause hierarchy.

Input data

Data used to estimate mortality of gastritis and duodenitis consisted of vital registration (VR) from the cause of death (CoD) database. A list of ICD codes that were mapped to this cause, a list of vital registration sources included in the CoD database and their quality as indicated by star-ratings, and the steps used to process these data for modelling can be found in the section of this appendix on the causes of death database.

After all data processing steps were complete, datapoints for all locations and years were systematically reviewed to identify outliers or other data limitations not fully overcome by CoD data processing methods, and select sources were excluded based on this review.

Modelling strategy

For modelling fatal burden of gastritis and duodenitis, no substantive changes were made in GBD 2023 from the modelling strategy used in GBD 2021. A standard CODEm model was used to model deaths due to gastritis and duodenitis (see appendix section on CODEm method for details). Models were generated separately for males in data-rich countries, males in all countries (referred to as a global model), females in data-rich countries, and females in all countries (global model). Fatal estimation for this cause was restricted to ages 0.5 years to 95+ years. We hybridised separate global and data-rich models to acquire unadjusted results. The following table has the full list of covariates presented to the CODEm algorithm for possible inclusion in the gastritis and duodenitis mortality model.

Table 1. Covariates used in gastritis and duodenitis mortality modelling

Level	Covariate	Direction
1	Sanitation, proportion with access	-
	Scaled exposure variable for unsafe water source	+
2	Smoking prevalence	+
	Cumulative cigarettes (10 years)	+
	Cumulative cigarettes (5 years)	+
	Litres of alcohol consumed per capita	+
	Vegetables (grams, unadjusted)	-
	Healthcare Access and Quality Index	-
3	Lag-distributed income (per capita)	-
	Education (years per capita)	-
	Socio-demographic Index	-

Unadjusted estimates of gastritis and duodenitis deaths were rescaled alongside peptic ulcer disease to sum to all upper digestive disease deaths, as part of the CoDCorrect process. These were then rescaled alongside deaths due to other causes to sum to all-cause deaths. Adjusted deaths were then compared to reference life tables to calculate YLLs.

Gynaecological diseases

Flowchart

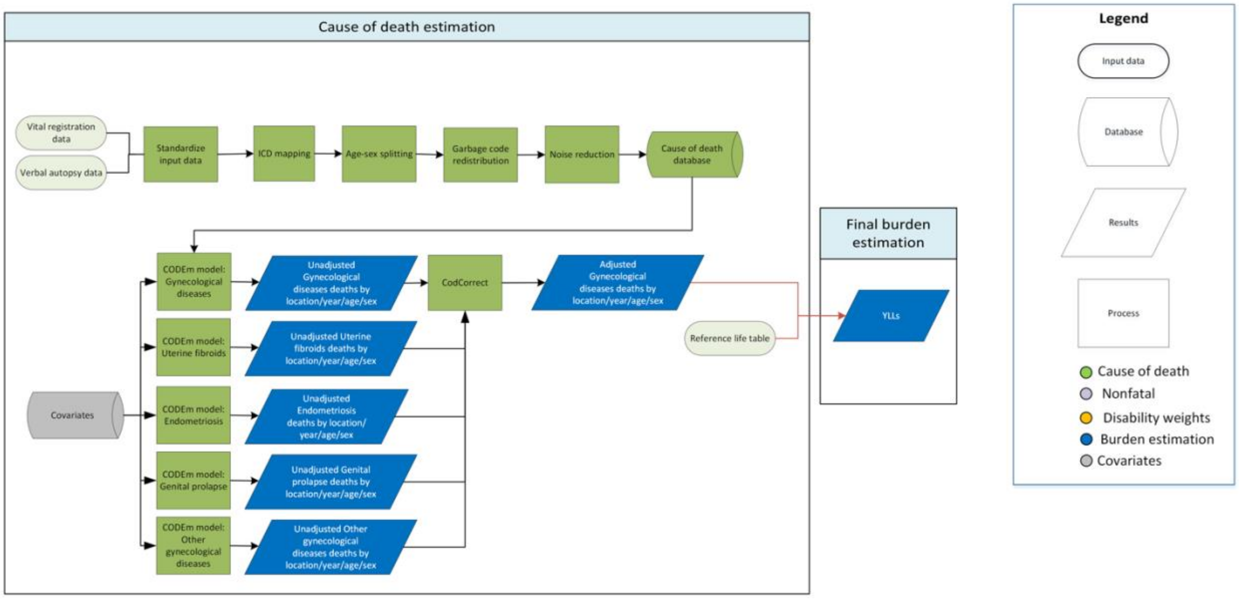

Input data and methodological summary for gynaecological diseases

Gynaecological diseases comprise a Level 3 cause of death in the GBD hierarchy, with uterine fibroids, endometriosis, genital prolapse, and other gynaecological diseases as Level 4 causes of death. (Other Level 4 causes contribute to gynaecological disease health loss, but they are not considered causes of death.)

Input data

For GBD 2023, vital registration (VR) and verbal autopsy (VA) data were used to estimate deaths for gynaecological diseases in aggregate. Among its Level 4 sub-causes of death, uterine fibroids utilised VR and VA data, while endometriosis, genital prolapse, and other gynaecological disorders utilised only VR data. Other gynaecological disorders include inflammatory disease of cervix uteri, diseases of Bartholin's gland, other inflammation of vagina and vulva, vulvovaginal ulceration and inflammation, and non-inflammatory disorders of ovary, fallopian tube, and broad ligament. As in previous GBD cycles, we reassigned deaths due to leiomyomas and other benign uterine tumors to uterine fibroids, and we assumed no deaths from premenstrual syndrome, primary infertility, and polycystic ovarian syndrome, which we model as non-fatal outcomes only. A list of ICD codes that were mapped to the gynaecological causes of death, a list of vital registration sources included in the CoD database and their quality as indicated by star-ratings, and the steps used to process these data for modelling can be found in the section of this appendix on the causes of death database and the tables and figures it refers to. CoD data processing was broadly similar to previous rounds of GBD, but one novel data processing step was employed in preparing data for gynaecological diseases that was not needed prior to GBD 2023—COVID-19 corrections—which are described in the aforementioned appendix section.

Data exclusions

After all data processing steps were complete, datapoints for all locations and years were systematically reviewed to identify outliers or other data limitations not fully overcome by CoD data processing methods. For gynaecological diseases, the following were identified and excluded prior to modeling: We excluded ICD-9 datapoints from former Soviet Union nations such as Armenia, Azerbaijan, Belarus, Estonia, Georgia, Kazakhstan, Kyrgyzstan, Latvia, Lithuania, the Republic of Moldova, Tajikistan, Turkmenistan, Ukraine and its subnationals, and Uzbekistan due to the fact that USSR ICD-9 tabulations did not record any deaths due to gynaecological causes for any location or year, raising the question whether these were true measurements of zero or if this method of coding and tabulation did not capture gynaecological disease deaths. Likewise, ICD-8 tabulations did not record any deaths due to gynaecological causes for any location or year and were excluded for the same reason; impacted locations included: Norway, Denmark, Finland, Sweden, Cabo Verde, and Mauritius.

Some early years of data in China reported zero deaths due to gynecological diseases for any age group and were excluded. In Viet Nam, we excluded one year of VA data that was several orders of magnitude higher than any other data in the region. The earliest year of data in Zimbabwe was excluded for being three times as high as any other data in the region.

We excluded all datapoints in Indonesian subnationals that only had one year of vital registration data in that subnational location. This was because single years of data tended to produce implausible discontinuities in spatial and temporal trends, it was not possible to distinguish true variation from insufficient noise reduction, and stabler trends were achieved by borrowing strength from neighbouring locations with multiple years of data and predictive covariates. This included single years of data in Bangka-Belitung Islands, Banten, Jambi, Central Kalimantan, Riau Islands, Lampung, Papua, Central Sulawesi, and Yogyakarta. We also excluded single years of data in Macao Special Administrative Region of China, Palau, Burkina Faso, and São Tomé.

For India and its subnationals, data from India Medical Certification of Cause of Death State-Level Tabulations were excluded in favor of the India Sample Registration System data, because the latter source covered both urban and rural locations, and used a sample that was more representative of both in-hospital and out-of-hospital deaths. Kiribati data were excluded due to inadequate population coverage. In some Ethiopian subnationals, District Health Information Software 2 (DHIS2) data were excluded in favor of Ethiopia’s VA data to avoid compositional bias and maintain the source believed to have more correct assignment of underlying cause of death, since the VA data were more similar to all other VA and VR data in the region. In Iran and its subnationals, data were excluded where data discontinuities between different coding systems resulted in implausible time trends.

Northern Mariana Islands, Greenland, Andorra, Monaco, and San Marino data were excluded where existing noise-reduction algorithms were insufficient to overcome the large amount of noise in measuring this very rare cause of death in very small populations.

Decisions to exclude CoD data sources above were made via assessment of the Level 3 gynaecological diseases dataset and were carried down to Level 4 causes below it in the hierarchy.

Modelling strategy

For gynaecological diseases in aggregate and for each sub-cause, we completed data-rich (DR) and global models for females aged 10 years and older in the Cause of Death Ensemble modelling (CODEm)

tool. More information about this tool can be found in the "Cause of death modelling methods: CODEm" section of this appendix. Models for each of the gynaecological diseases included the nine covariates in the table below for possible selection in their final models. No substantive changes were made in GBD 2023 from the modelling strategy used in GBD 2021.

Table 1. Covariates offered for selection in gynaecological diseases mortality modelling

Level	Covariate	Direction
1	Age- and sex-specific SEV for smoking	-
2	Proportion of livebirths by mothers aged 35 years and older	+
	Skilled birth attendance proportion	-
	Total fertility rate (TFR)	+
	Maternal care and immunisation	-
	Healthcare Access and Quality (HAQ) Index	-
3	Log lag-distributed income (LDI) ($I per capita)	-
	Education (years per capita)	-
	Socio-demographic Index (SDI)	-

In the ensemble models selected by the CODEm algorithm based on out-of-sample predictive validity, SEV for smoking, HAQ Index, maternal care and immunisation, education, LDI, and SDI were chosen for the female global model. Skilled birth attendance, HAQ Index, maternal care and immunisation, education, LDI, and SDI were chosen for the female data-rich model.

Input data and methodological summary for uterine fibroids

Uterine fibroids comprise a Level 4 cause of death in the GBD hierarchy.

Flowchart

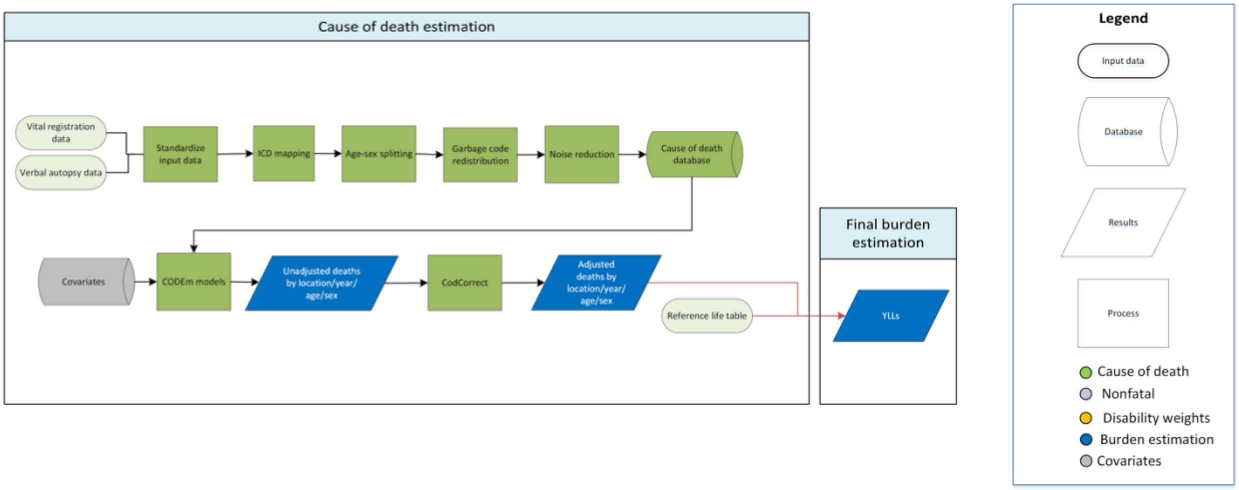

Input data

The same data and exclusion strategies were applied to uterine fibroids as for its parent cause, gynaecological diseases, described above. In addition, we excluded data for this cause in Oceania and the Caribbean, where existing noise-reduction algorithms were insufficient to overcome the large amount of noise in measuring this very rare cause of death in very small populations. Burkina Faso and São Tomé and Príncipe had no data for this cause. Belarus and Syria only had one year of data instead of multiple as in the parent, so these single years of data were excluded under the same principle of having only one year of data from a small population, even after region-level noise reduction, made it difficult to distinguish true extreme values from sampling error that was insufficiently addressed with current noise-reduction algorithms.

Modelling strategy

The same modelling strategy was utilised for uterine fibroids as for its parent cause, gynaecological diseases, described above. No substantive changes were made in GBD 2023 from the modelling strategy used in GBD 2021.

See Table 1 for covariates offered for selection in uterine fibroids mortality modelling.

In the ensemble models selected by the CODEm algorithm based on out-of-sample predictive validity, skilled birth attendance, LDI, and SDI were chosen for the female global model. Skilled birth attendance, HAQ Index, education, and LDI were chosen for the female data-rich model.

Adjustment in CoDCorrect included fitting unadjusted death estimates for all other specific and non-specific gynaecological diseases to overall gynaecological diseases deaths, which were then adjusted with all other causes to sum to all-cause counts of death.

Input data and methodological summary for endometriosis

Endometriosis is a Level 4 cause of death in the GBD hierarchy.

Flowchart

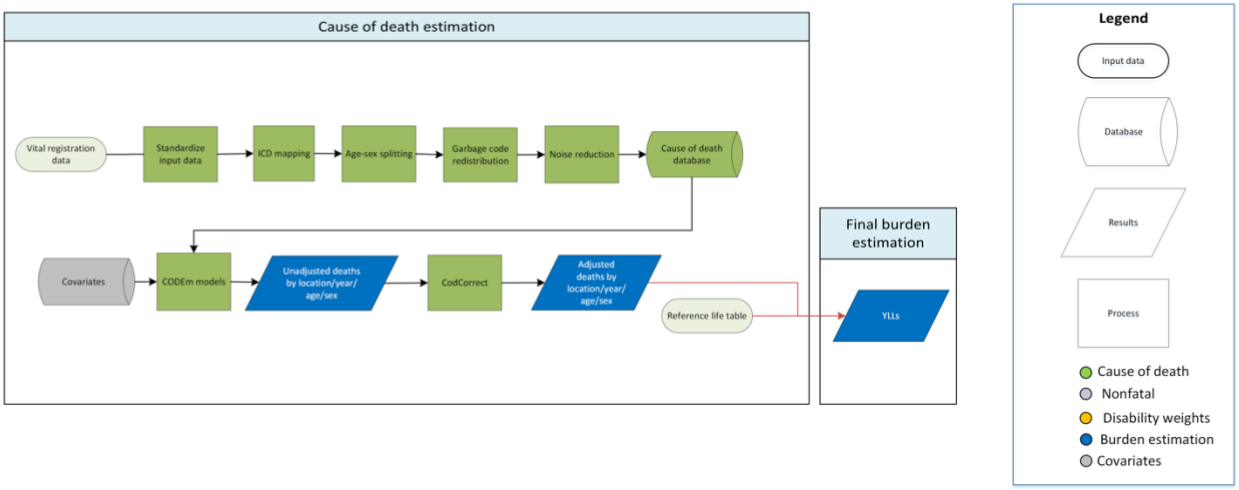

Input data

The same data and exclusion strategies were applied to uterine fibroids as for its parent cause, gynaecological diseases, described above. In addition, we excluded data for this cause in Oceania and the Caribbean, where existing noise-reduction algorithms were insufficient to overcome the large amount of noise in measuring this very rare cause of death in very small populations. Ethiopia, Burkina Faso, and São Tomé and Príncipe had no data for this cause. Belarus and Syria only had one year of data instead of multiple as in the parent, so these single years of data were excluded under the same principle of having only one year of data from a small population, even after region-level noise reduction, made it difficult to distinguish true extreme values from sampling error that was insufficiently addressed with current noise reduction algorithms. Zimbabwe data were excluded for being two orders of magnitude greater than all other data in the region.

Modelling strategy

The same modelling strategy was utilised for endometriosis as for its parent cause, gynaecological diseases, described above. No substantive changes were made in GBD 2023 from the modelling strategy used in GBD 2021. Fatal estimation for this cause was restricted to ages 10–54 years.

See Table 1 for covariates offered for selection in endometriosis mortality modelling.

In the ensemble models selected by the CODEm algorithm based on out-of-sample predictive validity, SEV for smoking and HAQ Index were chosen for the female global model. Proportion of livebirths by mothers aged 35 years and older and HAQ Index were chosen for the female data-rich model.

Adjustment in CoDCorrect included fitting unadjusted death estimates for all other specific and non-specific gynaecological diseases to overall gynaecological diseases deaths, which were then adjusted with all other causes to sum to all-cause counts of death.

Input data and methodological summary for genital prolapse

Genital prolapse is a Level 4 cause of death in the GBD hierarchy.

Flowchart

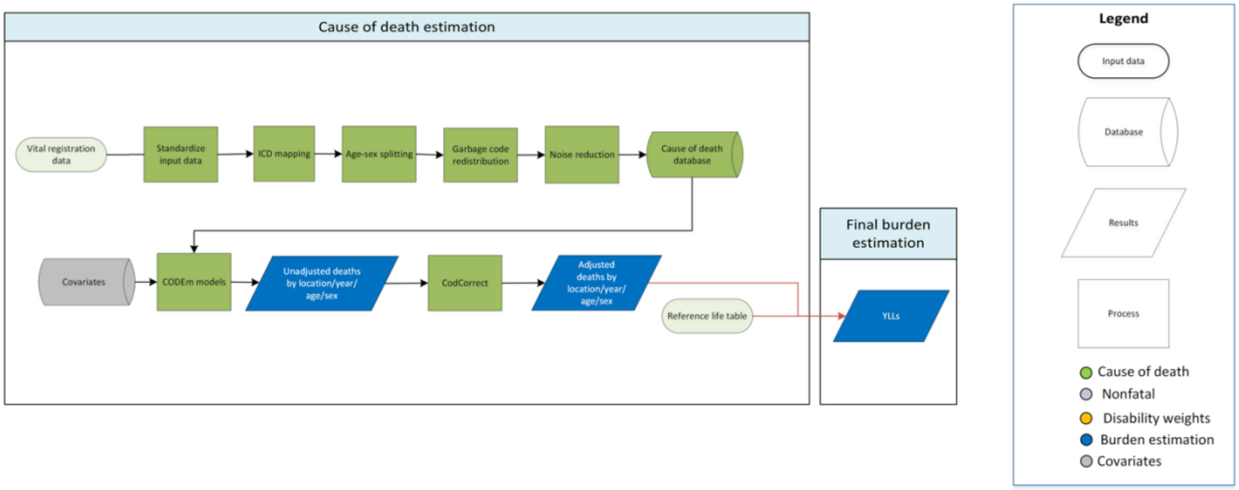

Input data

The same data and exclusion strategies were applied to genital prolapse as for its parent cause, gynaecological diseases, described above. In addition, we excluded data for this cause in Oceania and the Caribbean, where existing noise-reduction algorithms were insufficient to overcome the large amount of noise in measuring this very rare cause of death in very small populations. Indonesia, Viet Nam, India, Ethiopia, Burkina Faso, and São Tomé and Príncipe had no data for this cause. Belarus and Syria only had one year of data instead of multiple as in the parent, so these single years of data were excluded under the same principle of having only one year of data from a small population, even after region-level noise reduction, made it difficult to distinguish true extreme values from sampling error that was insufficiently addressed with current noise-reduction algorithms. Zimbabwe data were excluded for being two orders of magnitude greater than all other data in the region.

Modeling strategy

The same modeling strategy was utilised for genital prolapse as for its parent cause, gynaecological diseases, described above. No substantive changes were made in GBD 2023 from the modelling strategy used in GBD 2021. Fatal estimation for this cause was restricted to ages 10–95+ years.

Table 2. Covariates offered for selection in genital prolapse mortality modelling

Level	Covariate	Direction
1	Age- and sex-specific SEV for smoking	-
	Age-specific cohort cumulative fertility (CCF)	+
	Proportion of livebirths by mothers aged 35 years and older	+
2	Skilled birth attendance proportion	-
	Total fertility rate (TFR)	+
	Maternal care and immunisation	-
	Healthcare Access and Quality (HAQ) Index	-
3	Log lag-distributed income (LDI) ($I per capita)	-

Education (years per capita)	-
Socio-demographic Index (SDI)	-

In the ensemble models selected by the CODEm algorithm based on out-of-sample predictive validity, skilled birth attendance, TFR, maternal care and immunisation, HAQ Index, LDI, education, and SDI were chosen for the female global model. Skilled birth attendance, TFR, education, and SDI were chosen for the female data-rich model.

Adjustment in CoDCorrect included fitting unadjusted death estimates for all other specific and non-specific gynaecological diseases to overall gynaecological diseases deaths, which were then adjusted with all other causes to sum to all-cause counts of death.

Input data and methodological summary for other gynaecological diseases

Other gynaecological diseases comprise a Level 4 cause of death in the GBD hierarchy.

Flowchart

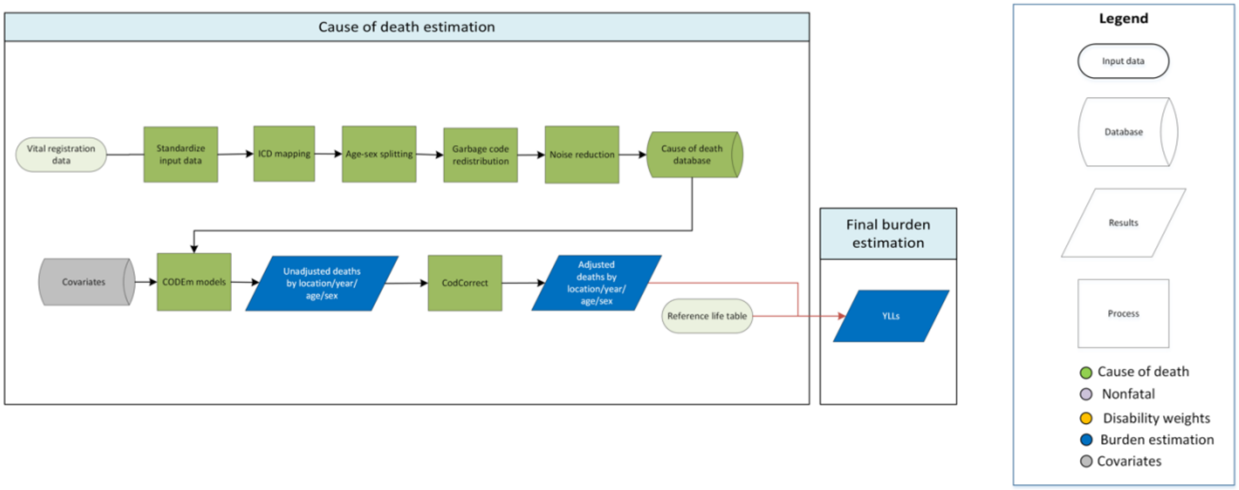

Input data

The same data and outliering strategies were applied to this child cause. In addition, we excluded data for this cause in Oceania and the Caribbean, as current noise-reduction algorithms, even applied at the regional level, are insufficient in these countries due to the combination of rare causes and small population sizes. Indonesia, Viet Nam, India, Burkina Faso, and São Tomé and Príncipe had no data for this cause.

Modelling strategy

The same modelling strategy was utilised for other gynaecological diseases as for its parent cause, gynaecological diseases, described above. No substantive changes were made in GBD 2023 from the modelling strategy used in GBD 2021. Fatal estimation for this cause was restricted to ages 10–95+ years.

See Table 1 for covariates offered for selection in other gynaecological diseases mortality modelling.

In the ensemble models selected by the CODEm algorithm based on out-of-sample predictive validity, skilled birth attendance, TFR, maternal care and immunisation, HAQ Index, LDI, education, and SDI were chosen for the female global and female data-rich models.

Adjustment in CoDCorrect included fitting unadjusted death estimates for all other specific and non-specific gynaecological diseases to overall gynaecological diseases deaths, which were then adjusted with all other causes to sum to all-cause counts of death.

Haemoglobinopathies and haemolytic anaemias

This summary covers the following sub-causes: sickle cell disorders, thalassaemias, glucose-6-phosphate dehydrogenase (G6PD) deficiency, and other haemoglobinopathies and haemolytic anaemias

Fatal models

Flowchart

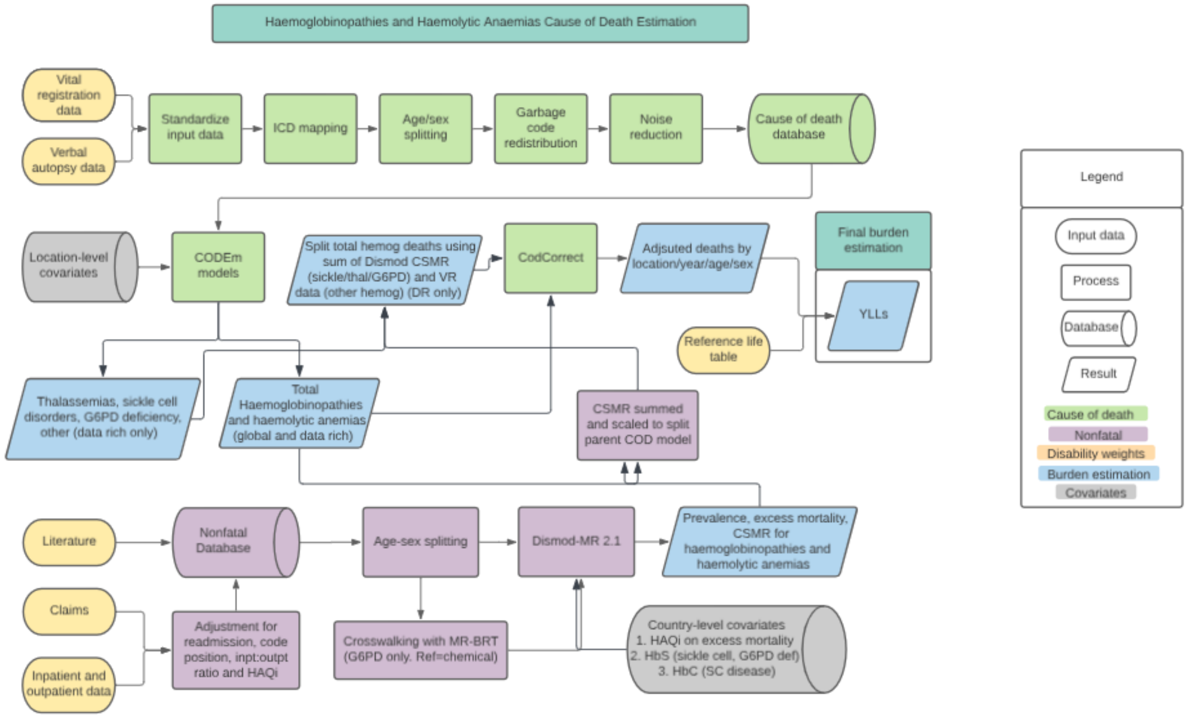

Input data and methodological summary for haemoglobinopathies and haemolytic anaemias fatal models

Input data

Input data to CODEm models were centrally processed along with all other specific causes of death and stored in the cause of death (CoD) database. Data processing steps are described elsewhere. It should be noted that updates to garbage code redistribution algorithms in GBD 2023 had substantial impact on the CODEm input data in some location-year-age-sex combinations. Outliers were identified as those data where age patterns or temporal patterns were inconsistent with neighbouring age groups or

locations, or where sparse data were predicting implausible overall temporal or age patterns for a given location.

Modelling strategy

For GBD 2023, our approach was as follows, which is the same as was used in GBD 2021 and combines several approaches to inform final cause-specific mortality results:

Total haemoglobinopathies and sub-causes in data-rich locations

First, we used Cause of Death Ensemble modelling (CODEm) models across all age groups and years to inform estimates for the parent GBD cause of haemoglobinopathies and haemolytic anaemias as well as for each of the sub-causes in data-rich locations. CODEm models were run separately for males and females; one model was run for all locations (global) and a separate one for all "data-rich" locations using standard GBD 2023 settings for each. Covariates used in each of the CODEm models, along with their level and direction, are shown in the table below. Other haemoglobinopathies and haemolytic anaemias has several covariates unique to it, reflecting the risk factors for aplastic anaemias that constitute a large proportion of this cause category.

Table 1. Covariates used in haemoglobinopathies and haemolytic anaemias (cause 613) CODEm models (data-rich and global models)

Level	Covariate	Direction
1	Haemoglobinopathies prevalence * excess mortality	+
	Sickle cell and thalassaemias prevalence * excess mortality	+
	Probability of homozygous sickle cell disease at birth (SS)	+
	Probability of haemoglobin SC disease at birth	+
	Probability of homozygous sickle cell disease (SS) and G6PD deficiency at birth	+
	Probability of haemoglobin SC disease (SC) and G6PD deficiency at birth	+
	G6PD deficiency from MAP	+
	Malaria Lysenko PFPR 1 (holoendemic)	+
2	Maternal care and immunisation (MCI)	-
	Healthcare Access and Quality Index	-
3	Socio-demographic Index	-
	Lag-distributed income (LN-transformed)	-
	Population proportion (0-15 latitude)	+
	Population proportion (15-30 latitude)	+
	Population proportion (30-45 latitude)	-
	Population proportion (45+ latitude)	-
	Education (years per capita)	-

**Level refers to a categorical assessment of the strength of mechanistic relationship between the covariate and mortality (1 = more likely; 3 = less likely); direction refers to the direction of the relationship (1 = positive correlation; –1 = negative correlation).*

Table 2. Covariates used in thalassaemias (cause 614) CODEm models (data-rich)

Level	Covariate	Direction
1	Haemoglobinopathies prevalence * excess mortality	+
	Sickle cell and thalassaemias prevalence * excess mortality	+
	Malaria Lysenko PFPR 1 (holoendemic)	+
2	Healthcare Access and Quality Index	-
	Population proportion (0-15 latitude)	+
	Population proportion (15-30 latitude)	+
	Population proportion (30-45 latitude)	-
	Population proportion (45+ latitude)	-
3	Socio-demographic Index	-
	Education (proportion with 6+ years schooling)	-
	Education (proportion with 12+ years schooling)	-

**Level refers to a categorical assessment of the strength of mechanistic relationship between the covariate and mortality (1 = more likely; 3 = less likely); direction refers to the direction of the relationship (1 = positive correlation; –1 = negative correlation).*

Table 3. Covariates used in sickle cell disorders (cause 615) CODEm models (data-rich)

Level	Covariate	Direction
1	Haemoglobinopathies prevalence * excess mortality	+
	Sickle cell and thalassaemias prevalence * excess mortality	+
	Probability of homozygous sickle cell disease at birth (SS)	+
	Probability of haemoglobin SC disease at birth	+
	Probability of homozygous sickle cell disease (SS) and G6PD deficiency at birth	+
	Probability of haemoglobin SC disease (SC) and G6PD deficiency at birth	+
	Malaria Lysenko PFPR 1 (Holoendemic)	+
2	Maternal care and immunisation (MCI)	-
	Healthcare Access and Quality Index	-
3	Socio-demographic Index	-
	Lag-distributed income (LN-transformed)	-
	Population proportion (0-15 latitude)	+
	Population proportion (15-30 latitude)	+
	Population proportion (30-45 latitude)	-
	Population proportion (45+ latitude)	-
	Education (years per capita)	-
	Education (proportion with 6+ years schooling)	-
	Education (proportion with 12+ years schooling)	-

**Level refers to a categorical assessment of the strength of mechanistic relationship between the covariate and mortality (1 = more likely; 3 = less likely); direction refers to the direction of the relationship (1 = positive correlation; –1 = negative correlation).*

Table 4. Covariates used in G6PD (cause 616) CODEm models (data-rich and global models)

Level	Covariate	Direction
1	Probability of haemoglobin S trait and G6PD deficiency at birth	+
	Probability of haemoglobin C trait and G6PD deficiency at birth	+
	Haemoglobinopathies prevalence * excess mortality	+
	Sickle cell and thalassaemias prevalence * excess mortality	+
	Probability of homozygous sickle cell disease (SS) and G6PD deficiency at birth	+
	Probability of haemoglobin SC disease (SC) and G6PD deficiency at birth	+
	G6PD deficiency from MAP	+
	Malaria Lysenko PFPR 1 (Holoendemic)	+
2	Healthcare Access and Quality Index	-
	Population proportion (0-15 latitude)	+
	Population proportion (15-30 latitude)	+
	Population proportion (30-45 latitude)	-
	Population proportion (45+ latitude)	-
3	Socio-demographic Index	-
	Education (proportion with 6+ years schooling)	-
	Education (proportion with 12+ years schooling)	-

**Level refers to a categorical assessment of the strength of mechanistic relationship between the covariate and mortality (1 = more likely; 3 = less likely); direction refers to the direction of the relationship (1 = positive correlation; –1 = negative correlation).*

Table 5. Covariates used in other haemoglobinopathies and haemolytic anaemias (cause 618) CODEm models (data-rich)

Level	Covariate	Direction
1	HIV prevalence unadjusted (proportion)	+
	Log-transformed SEV scalar: leukaemia	+
	Age- and sex-specific SEV for unsafe water	+
	Age- and sex-specific SEV for unsafe sanitation	+
2	Healthcare Access and Quality Index	-
	Socio-demographic Index	-
	Age-standardized SEV for alcohol use	+
	Age- and sex-specific SEV for high body-mass index	+
	Haemoglobinopathies prevalence * excess mortality	+
	Sickle cell and thalassaemias prevalence * excess mortality	+
3	Education (years per capita)	-
	Education (proportion with 6+ years schooling)	-
	Education (proportion with 12+ years schooling)	-
	Temperature_90_perc	+

**Level refers to a categorical assessment of the strength of mechanistic relationship between the covariate and mortality (1 = more likely; 3 = less likely); direction refers to the direction of the relationship (1 = positive correlation; –1 = negative correlation).*

Sub-causes in non-data-rich locations

Second, in non-data-rich locations, estimates for "other haemoglobinopathies" (input 1) were informed by vital registration (VR) cause of death data from data-rich locations in the CoD database. The need to use this approach reflects an almost complete absence of non-VR data for this heterogeneous cause grouping. We used VR data to calculate a year-pooled, data-rich average cause-specific mortality rate (CSMR) for each age group, sex, and ten-year-range from 1980 to 2024. To smooth out jagged time trends, data were grouped into ten-year spans, with recent years set up with custom spans (2014–2024) to accommodate data availability.

Third, in non-data-rich locations, estimates for thalassaemias, sickle cell disorders, and G6PD deficiency used were replaced with scaled cause-specific mortality rate (CSMR) estimates from DisMod-MR 2.1 models. DisMod-MR 2.1 uses a compartment model to produce CSMR estimates that are internally consistent with the CODEm results for all haemoglobinopathies combined. DisMod-MR 2.1 produces cause-specific mortality estimates that are internally consistent with data and expert priors for incidence (= 0 after birth), prevalence, remission (= 0), and excess mortality (informed by cohort survival studies).

There were three distinct DisMod-MR 2.1 models for thalassaemias (beta-thalassaemia major, haemoglobin E/beta-thalassaemia, and haemoglobin H disease), three distinct models for sickle cell disorders (homozygous sickle cell and severe sickle cell/beta-thalassaemia, haemoglobin SC disease, and mild sickle cell/beta-thalassaemia) and a single DisMod MR 2.1 model for G6PD deficiency. We derived CSMR results from DisMod-MR 2.1, summed to match the GBD cause level, then interpolated between estimation years to obtain a set of location-age-sex-specific estimates for all estimation years (1980–2023) (input 2). After having a complete set of pooled VR (other haemoglobinopathies) and DisMod-MR 2.1-derived (thalassaemias, sickle cell disorders, G6PD deficiency) results, we summed them and calculated the proportion of total haemoglobinopathies cause-specific mortality due to each sub-cause. Finally, we applied this proportion to CSMR estimates from the CODEm total haemoglobinopathies model (input 3), and merged these with the data-rich CODEm results above, to create a set of scaled cause-specific mortality results for each sub-cause. (See below for more details on the contents of these models, which is reproduced from the GBD 2023 Diseases and Injuries Supplement).

Limitations and discussion

The primary limitation of our estimation is data availability, especially in the locations thought to have the highest burden. We elected a hybrid approach of CODEm, VR data pooling, and DisMod-MR 2.1 to improve the quality of estimates in data-poor locations, but even frequency and survival data are relatively sparse in many locations, leading to relatively large uncertainty. Another major limitation of assessing cause-specific mortality for haemoglobinopathies is that the proximal cause of death in many with haemoglobinopathies is infections such as malaria, lower respiratory infections, and diarrhoea, or due to cardiovascular diseases such as ischaemic heart disease or stroke, and are associated with increased risk of death during pregnancy. In locations with poor diagnostic capabilities and high

infectious burden, it is thus very plausible that mortality due to haemoglobinopathies may be even higher. This disconnect between a full assessment of excess mortality (that includes all of the proximal causes) and the central CoD data sources and processing (which only includes "underlying" cause) limits the utility of CoD data for informing non-fatal models directly. Future work is aimed to address these disconnects and limitations.

Non-fatal models

As described above, estimates of mortality due to haemoglobinopathies and haemolytic anemias are informed by the non-fatal estimation process for these causes – namely, DisMod-MR 2.1 models that are used to produce mortality estimates in non-data-rich locations. These methods are described in the GBD 2023 Diseases and Injuries Supplement. Below, for convenience, we have reproduced the description of the non-fatal modelling process for haemoglobinopathies and haemolytic anemias from that Supplement.

This document describes the non-fatal disease burden modelling process for GBD 2023 for each of sickle cell disorders, thalassaemias, glucose-6-phosphate dehydrogenase (G6PD) deficiency, sickle cell trait, thalassaemia trait, hemizygous G6PD deficiency, and other haemoglobinopathies and haemolytic anaemias.

Flowchart

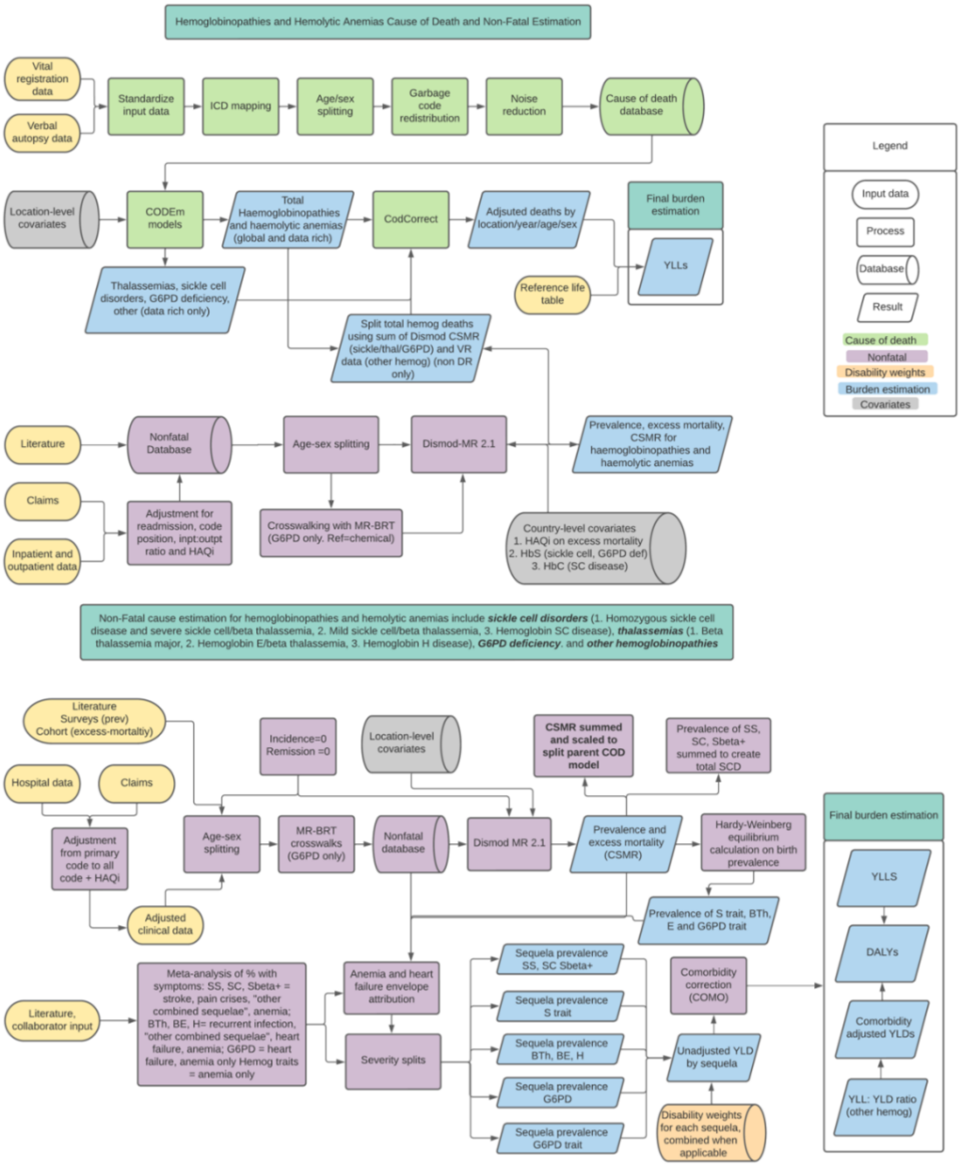

Input data and methodological summary for haemoglobinopathies and haemolytic anaemias

Case definition

Haemoglobinopathies and haemolytic anaemias span four GBD causes: thalassaemias, sickle cell disorders, G6PD deficiency, and other haemoglobinopathies and haemolytic anaemias. Case definitions for each of the types of thalassaemias and sickle cell were based on genotype. G6PD deficiency is an X-linked recessive genetic disease, and our reference definition was based on quantitative decline in G6PD activity during reagent (ie, chemical) testing; genotype or other testing was an acceptable alternate definition and adjusted as described below. Sickle cell trait, thalassaemia trait, and hemizygous G6PD deficiency were all similarly defined by genotype. They were estimated from the component disease models' estimates of birth prevalence assuming Hardy-Weinberg equilibrium. YLDs due to other haemoglobinopathies and haemolytic anaemias were estimated assuming the YLD-to-YLL ratio for each

age, sex, location, and year was similar to that of the aggregate of sickle cell, thalassaemias, and G6PD deficiency. Most conditions in this group are aplastic anaemias.

Several unique combinations of genetic mutations lead to distinct phenotypes with different natural history, which has led us to estimate several distinct subtypes of thalassaemias and sickle cell disorders. The three thalassaemia models included 1) beta-thalassaemia major, 2) haemoglobin E/beta-thalassaemia, and 3) haemoglobin H disease (genotype = - - / - alpha). Sickle cell models included 1) homozygous sickle cell and severe sickle cell/beta-thalassaemia where the latter genotype had either a severe version of the sickle gene (assumed to always be the case if unspecified and west of the Arabian peninsula) or a nonsense (as opposed to reduced activity) mutation at the other beta haemoglobin gene locus; 2) haemoglobin sickle cell disease; and 3) "mild" sickle cell-beta-thalassaemia. G6PD deficiency was estimated in a single model.

Input data

Three sources of data were used for DisMod-MR 2.1 models: literature (generally from community prevalence surveys, birth screening, and cohort studies), claims data, and ICD-9 and ICD-10 hospital discharge data that were adjusted for ICD code position, readmission, inpatient-to-outpatient ratio, and location-specific Healthcare Access and Quality (HAQ) Index. Of note, there were no hospital data available for haemoglobin E/beta-thalassaemia, haemoglobin H disease, or G6PD deficiency. Our last comprehensive literature review was completed in GBD 2017, where we identified data on prevalence, excess mortality rate, or with-condition mortality rate. Age-specific survival probabilities from cohort studies were converted to corresponding with-condition mortality rates.

The systematic literature review used the following search strings in PubMed:

> (G6PD[Title/Abstract] OR G6PD deficiency[Title/Abstract] OR glucose-6 phosphate dehydrogenase[Title/Abstract] OR glucose-6-phosphate dehydrogenase deficiency[Title/Abstract] AND (survival[Title/Abstract] OR mortality[Title/Abstract] OR prevalence[Title/Abstract] OR incidence[Title/Abstract]) AND (2013/01/01[PDat] : 2016/12/31[PDat])) AND "humans"[MeSH Terms]
>
> (sickle cell[Title/Abstract] AND (mortality[Title/Abstract] OR survival[Title/Abstract] OR prevalence[Title/Abstract] OR incidence[Title/Abstract]) AND (2013/04/01[PDat] : 2016/12/31[PDat])) AND "humans"[MeSH Terms]
>
> (thalassemias [Title/Abstract] AND (prevalence[Title/Abstract] OR incidence[Title/Abstract] OR survival[Title/Abstract] OR mortality[Title/Abstract]) AND (2013/01/01[PDat] : 2016/12/31[PDat])) AND "humans"[MeSH Terms]

The search was completed on July 5, 2016, and supplemented similar searches that were completed for GBD 2010 and GBD 2013. The G6PD deficiency search yielded 120 results, of which 57 were selected for full text review and 32 were extracted. The sickle cell search yielded 488 results, of which 49 were selected for full text review and 22 were extracted. The thalassaemias search yielded 27 results, ten had full text review, and four were extracted.

We extracted prevalence data from population-level and community surveys as well as with-condition mortality and excess mortality data from cohort studies. Age-specific survival proportions were

converted to with-condition mortality rates as needed. We also included data from hospital and claims data for a subset of haemoglobinopathy models, including beta-thalassaemia major, haemoglobin E/beta-thalassaemia, homozygous sickle cell and severe sickle cell/beta-thalassaemia, haemoglobin SC disease, and mild sickle cell/beta-thalassaemia.

Processing of clinical administrative data (ie, hospital and claims) was based on ICD-9 and ICD-10 codes as listed in Table 1. The extraction and processing of hospital and claims data is described separately.

Figure 1. PRISMA diagram of GBD 2016 literature review

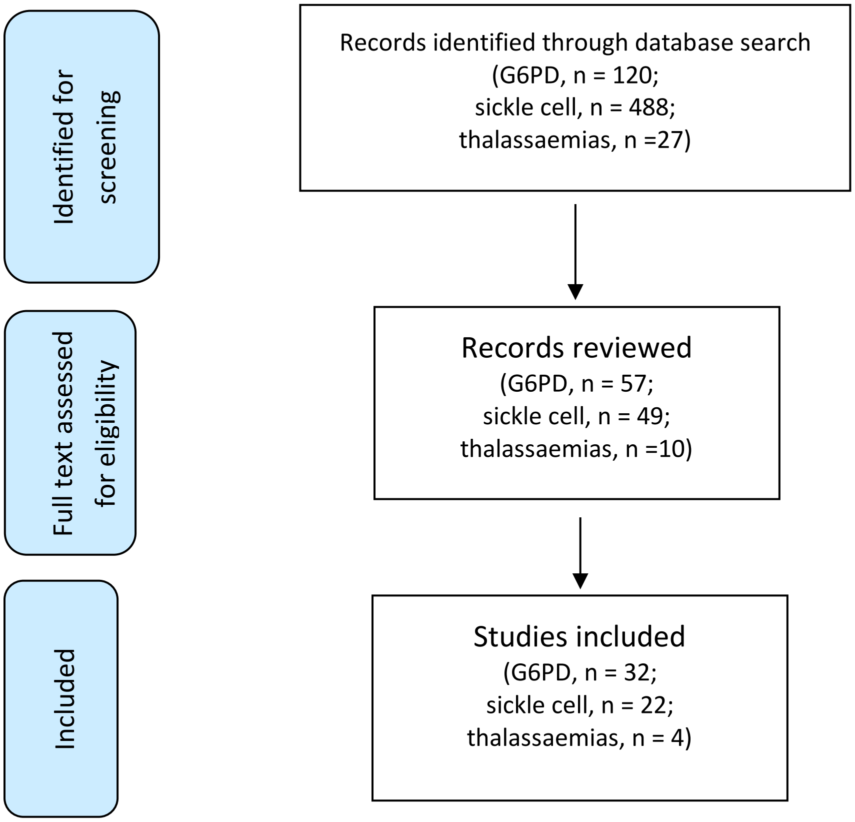

Table 1. Data inputs for modelling prevalence of haemoglobinopathies and haemolytic anaemias

**Condition**	**New sources**	**Total sources**	**Countries with data**
Haemoglobinopathies and haemolytic anaemias (all measures)	94	790	138
Prevalence	66	752	138
Excess mortality rate	20	25	13
With-condition mortality rate	11	16	11
Other	0	9	6

Condition	New sources	Total sources	Countries with data
Thalassaemias (all measures)	69	354	94
Prevalence	63	348	94
Excess mortality rate	5	6	6
With-condition mortality rate	1	1	1
Sickle cell disorders (all measures)	88	556	117
Prevalence	66	533	117
Excess mortality rate	15	19	9
With-condition mortality rate	10	15	10
G6PD deficiency (all measures)	0	185	69
Prevalence	0	176	68
Other	0	9	6

Table 2. International classification of diseases codes for haemoglobinopathies and haemolytic anaemias in GBD 2023 cause of death analysis

Condition	ICD-10 code	ICD-9 code
Thalassaemias	D56	282.4
Sickle cell disorders	D57	282.5–282.6
G6PD deficiency	D55	282.2–282.3
Other haemoglobinopathies and haemolytic anaemias	D58–D64.8	282.0–282.1, 282.7–285.8

Data processing

Data processing strategies did not change from GBD 2021 such that we conducted age-sex splitting and crosswalking in the same methods detailed as follows:

The first step of the process was age-sex splitting. For any datum that did not entirely fit within a GBD age group or was for both sexes combined, the observation was split to be multiple age-sex-specific datapoints based on the age and sex pattern predicted by GBD 2019 DisMod-MR 2.1 models. For thalassaemias and sickle cell disorders, this was the only processing completed.

For G6PD deficiency, we crosswalked all data to the reference definition of chemical test. We began by evaluating the number of observations of each alternate definition that matched with a corresponding observation from the reference definition. A match was considered "within" study if it was from the same data source and an exact match for age, sex, location, and year. A match was considered "between" study if it was from the same GBD location, GBD age group, and sex, and the midpoint of the study was within five years of the midpoint of the reference definition observation. Because the prevalence of G6PD deficiency itself can vary between studies, and the difference between reagent and chemical testing is expected to be a largely constant phenomenon, we restricted the crosswalk only to

be based on within-study matches. There were no matches for diagnostics that were not based on either genetic or reagent testing. All of these data were therefore dropped from the model. The total number of datapoints and matches is shown in the table below.

Table 3. Datapoints and matches between alternate and reference definitions

	Reference (cv_dx_chemical)	Alternate #1 (cv_dx_genetic)	Alternate #2 (cv_dx_other)
Number of datapoints	6370	2578	9
Within-study matches to reference	--	397	0

The ratio of prevalence from alternate:reference was calculated, log-transformed, the standard error of the ratio calculated using the delta method, and all were analysed using meta-regression—Bayesian, regularised, trimmed (MR-BRT), a meta-regression tool developed for GBD 2019. We tested the relationships as a function of sex, age, and the variability as a function of location (grouped into super-regions). Only sex remained a significant predictor, so it was the only additional factor included in the final crosswalk model. We trimmed 10% of the data from the MR-BRT model. Our covariate betas for each of the included covariates in the model are summarised in the table below.

Table 4. MR-BRT crosswalk adjustment factors for haemoglobinopathies and haemolytic anaemias

<table>
<tr><th>Data input</th><th>Reference or alternative case definition</th><th>Gamma</th><th>Beta coefficient, log (95% CI)</th><th>Adjustment factor*</th></tr>
<tr><td>Chemical test</td><td>Reference</td><td rowspan="3">0.06</td><td>---</td><td>---</td></tr>
<tr><td>Genetic test</td><td>Alternative</td><td>0.291 (–0.175 to 0.755)</td><td>1.33 (0.84–2.13)</td></tr>
<tr><td>Sex</td><td>Alternative</td><td>–0.027</td><td></td></tr>
</table>

**MR-BRT crosswalk adjustments can be interpreted as the factor the alternative case definition is adjusted by to reflect what it would have been had it been measured using the reference case definition. If the log beta coefficient is negative, then the alternative is adjusted up to the reference. If the log beta coefficient is positive, then the alternative is adjusted down to the reference. The adjustment factor column is the exponentiated beta coefficient and can be interpreted as the relative rate between the two case definitions.*

Modelling strategy

The three sickle cell prevalence sub-causes were summed to create a fourth, total sickle cell disorders model. Covariates for the prevalence of haemoglobin S (HbS) and haemoglobin C (HbC) to the sickle cell and G6PD deficiency models bS and HbC rasters were summarised into GBD geographies from Malaria Atlas Project publications on them and assumed to be invariant over time and age. We estimated the non-fatal burden of haemoglobinopathies in four parts.

DisMod-MR 2.1 modelling of disease

First, we used the datasets described above to estimate prevalence for each age-sex-location-year using DisMod-MR 2.1. Natural-log-transformed lag-distributed income per capita was used as a covariate on excess mortality for most models. HbS and HbC allele frequency covariates were used as covariates on prevalence for each of the subtypes of sickle cell disorders and for G6PD deficiency, where the effect

size and predictive power were expectedly much smaller. HAQ Index was also used as a covariate for excess mortality rate in the homozygous sickle cell and severe sickle cell/beta-thalassaemia model. A full table of all the location-level covariates and their effect sizes is shown below.

Final models were selected on a combination of qualitative and quantitative goodness of fit to input data, plausibility of geographical and temporal trends, consistency of age pattern, and, when available, comparison with other published studies on haemoglobinopathy epidemiology. Directionality, magnitude, and plausibility of study-level and country-level covariates was also considered in the process of model development. Of note, due to the nature of statistical modelling, final results do not always cover the values reported in input data.

Table 5. Covariate, parameter, beta, and exponentiated beta values for each model

Model	Covariate	Parameter	Beta	Exponentiated beta
Beta-thalassaemia major	UHC	EMR	−0.026 (−0.047 to −0.0037)	0.97 (0.95–1.00)
Haemoglobin E/beta-thalassaemia	UHC	EMR	−0.025 (−0.05 to −0.0024)	0.98 (0.95–1.00)
Haemoglobin E/beta-thalassaemia	Year	Prev	0.020 (0.018 to 0.020)	1.02 (1.02 to 1.02)
Haemoglobin H disease	Year	Prev	−0.018 (−0.019 to −0.017)	0.98 (0.98 to 0.98)
Homozygous sickle cell and severe sickle cell/beta-thal	(HbS)^2	Prev	49.94 (49.90 to 50.00)	5.02e+21 (4.69e+21 to 5.18e+21)
Homozygous sickle cell and severe sickle cell/beta-thal	UHC	EMR	−0.028 (−0.048 to −0.0036)	0.97 (0.95 to 1.00)
Haemoglobin SC disease	HbS	Prev	19.99 (19.98 to 20.00)	4.82e+8 (4.76e+8 to 4.85e+8)
Haemoglobin SC disease	HbC	Prev	10.00 (9.99 to 10.00)	2.19e+4 (2.18e+4 to 2.20e+4)
Haemoglobin SC disease	UHC	EMR	−0.024 (−0.046 to −0.0038)	0.98 (0.96 to 1.00)
Mild sickle cell/beta-thalassaemia	HbS	Prev	19.99 (19.97 to 20.00)	4.80e+8 (4.71e+8 to 4.85e+8)
Mild sickle cell/beta-thalassaemia	UHC	EMR	−0.025 (−0.048 to −0.0029)	0.98 (0.95 to 1.00)
G6PD deficiency	|Latitude|	Prev	−0.003 (−0.0045 to −0.0016)	1.00 (1.00 to 1.00)
G6PD deficiency	HbC	Prev	0.068 (0.0031 to 0.17)	1.07 (1.00 to 1.19)

G6PD deficiency	HbS	Prev	0.12<br>(0.0043 to 0.40)	1.13<br>(1.00 to 1.50)

Abbreviations: UHC=universal health coverage. EMR=excess mortality rate. Prev=prevalence. HbS=haemoglobin S trait prevalence. HbC=haemoglobin C trait prevalence.

Hardy-Weinberg equilibrium to estimate carrier prevalence

Second, we calculated prevalence of haemoglobinopathy traits (sickle cell trait, haemoglobin E trait, haemoglobin beta trait, hemizygous G6PD) by back-calculating from birth prevalence estimates from corresponding DisMod-MR 2.1 models, assuming Hardy-Weinberg equilibrium and no excess mortality. Because G6PD deficiency is an X-linked disease, hemizygous G6PD can only occur in females.

Severity distributions and sequelae of disease

With the exception of anaemia, only homozygous individuals were considered to experience disability. Estimated sequelae of thalassaemias included anaemia (described separately), heart failure (described separately), and periodic severe infection. Another series of common, but not universal, sequelae also occur in those with thalassaemias, including splenomegaly, skeletal deformity, delayed growth/puberty, diabetes, hypothyroidism, and leg ulcers. Given sparse data on the occurrence of these sequelae, they were approximated with a health state named “other combined sequelae of thalassaemia,” for which we used the disability weight (DW) corresponding to a health state of “generic uncomplicated disease, anxiety about diagnosis and daily medication” which, of note, was also used to approximate the disability for those with cancer in remission. For sickle cell disorders, we similarly estimated YLDs for anaemia (described separately), stroke, and pain crises separately and approximated the myriad additional complications of sickle cell disease with the health state “other combined sequelae of sickle cell disease.” The only sequelae estimated for G6PD deficiency were anaemia (described separately) and heart failure (described separately). Notably, however, G6PD deficiency is considered to be asymptomatic for a vast majority of those with the condition, with only a very small subset of around 1 in 1 million having chronic haemolysis (Class I disease) and approximately 1% having periodic haemolytic episodes (Class II disease) with exposure to environmental, pharmaceutical, or food products. Females heterozygous for G6PD deficiency exhibit chimerism, as one X chromosome becomes dominant in each of the red blood cells, so we estimated half as many heterozygous females will be symptomatic as homozygous females. Table 6 has all the disabling health states that were included in calculation of YLDs for haemoglobinopathies and haemolytic anaemias.

Anaemia causal attribution

The age- and sex-specific anaemia prevalence for each of the haemoglobinopathies, as well as the estimates of anaemia due to carrier/trait state, were analysed as part of overall anaemia causal attribution for GBD 2023. The details of the anaemia analysis are described separately in the “Anaemia Impairment” section. Briefly, after estimating total anaemia, a series of counterfactual distributions are generated based on the prevalence of each anaemia-causing condition and the quantitative effect that the condition has on haemoglobin concentration in the blood, a so-called “haemoglobin shift” that was derived by meta-analysing cohort studies, observational studies, or trials comparing the haemotological status of those with as compared to without the disease. Due to limited data on haemoglobin shift, all were assumed to be invariant over age, sex, location, and year.

YLL:YLD ratio for other haemoglobinopathies and haemolytic anaemias

Finally, we calculated the YLD-to-YLL ratio for all haemoglobinopathies and then applied it to YLLs estimated for other haemoglobinopathies and haemolytic anaemias in our cause-specific mortality analysis. Quantitative crosswalk results for each model are shown below.

Table 6. Health states for haemoglobinopathies and haemolytic anaemias

Severity level	Lay description	DW (95% CI)	Cause
Mild anaemia	Feels slightly tired and weak at times, but this does not interfere with normal daily activities.	0.004 (0.001–0.008)	All
Moderate anaemia	Feels moderate fatigue, weakness, and shortness of breath after exercise, making daily activities more difficult.	0.052 (0.034–0.076)	All
Severe anaemia	Feels very weak, tired, and short of breath, and has problems with activities that require physical effort or deep concentration.	0.149 (0.101–0.209)	All
Severe abdominopelvic problem (proxy for vaso-occlusive crisis)	Has severe pain in the belly and feels nauseous. The person is anxious and unable to carry out daily activities.	0.324 (0.22–0.442)	Sickle cell disorders
Stroke, long-term consequences, moderate plus cognition problems	Has some difficulty in moving around, in using the hands for lifting and holding things, dressing and grooming, and in speaking. The person is often forgetful and confused.	0.316 (0.206–0.437)	Sickle cell disorders
Combined sequelae of disease (approximation of all other sequelae)	Has a chronic disease that requires medication every day and causes some worry but minimal interference with daily activities.	0.012 (0.006-0.023)	Sickle cell disorders
Controlled, medically managed heart failure	has a chronic disease that requires medication every day and causes some worry but minimal interference with daily activities.	0.049 (0.031-0.072)	Thalassaemias
Mild heart failure	Is short of breath and easily tires with moderate physical activity, such as walking uphill or more than a quarter-mile on level ground. The person feels comfortable at rest or during activities requiring less effort.	0.041 (0.026–0.062)	Thalassaemias
Moderate heart failure	Is short of breath and easily tires with minimal physical activity, such as walking only a short distance. The person feels comfortable at rest but avoids moderate activity.	0.072 (0.047–0.103)	Thalassaemias
Severe heart failure	Is short of breath and feels tired when at rest. The person avoids any physical activity for fear of worsening the breathing problems.	0.179 (0.122–0.251)	Thalassaemias
Severe infection	Has a high fever and pain, and feels very weak, which causes great difficulty with daily activities.	0.133 (0.088–0.19)	Thalassaemias

HIV/AIDS—multidrug-resistant tuberculosis without extensive drug resistance, HIV/AIDS—extensively drug-resistant tuberculosis, and HIV/AIDS—drug-susceptible tuberculosis

Flowchart

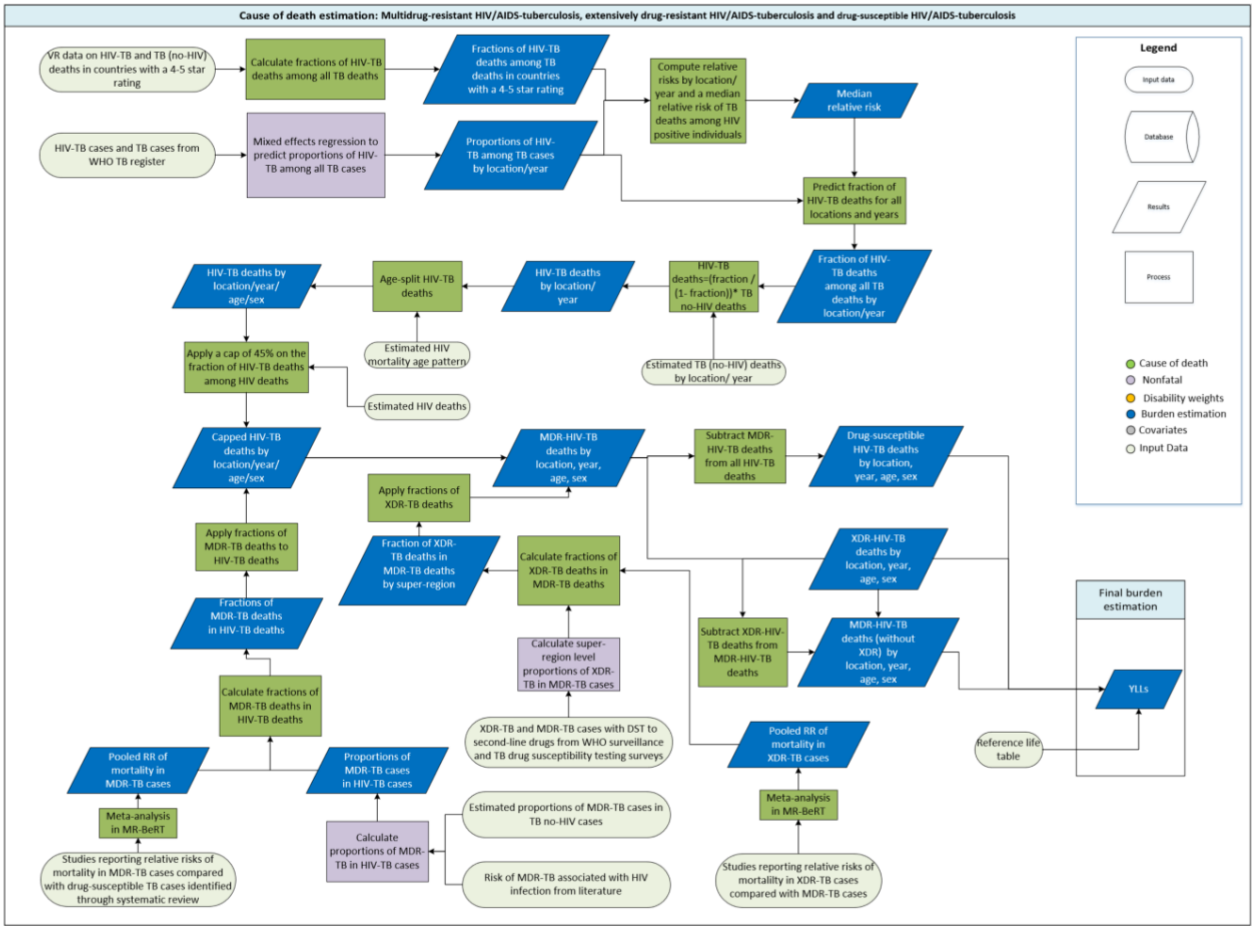

Input data and methodological summary for HIV/AIDS—multidrug-resistant tuberculosis without extensive drug resistance, HIV/AIDS—extensively drug-resistant tuberculosis, and HIV/AIDS—drug-susceptible tuberculosis

Input data

Input data for HIV/AIDS-tuberculosis (HIV-TB) mortality estimation include (i) vital registration data from countries with a four- or five-star rating where cause of death data for directly coded HIV-TB and tuberculosis (TB) were available, and (ii) the number of TB cases (new and re-treatment) recorded as HIV-positive and the number of TB cases (new and re-treatment) with an HIV test result recorded in the TB register from the World Health Organization (WHO). We excluded data from countries with ten HIV-

TB deaths or less. We also excluded data that were largely conflicting with the majority of data for other years from the same country.

Input data for estimation of multidrug-resistant and extensively drug-resistant HIV-TB include: (i) the number of drug-resistant cases by type (multidrug-resistant tuberculosis [MDR-TB], extensively drug-resistant tuberculosis [XDR-TB], all TB cases with a drug sensitivity testing [DST] result for isoniazid and rifampicin, and MDR-TB cases with DST for second-line drugs) from routine surveillance and surveys reported to WHO. Additional input data include relative risks of mortality in MDR-TB cases compared with drug-susceptible TB cases, and relative risks of mortality in XDR-TB cases compared with MDR-TB cases reported by studies identified through our systematic review, and the risk of MDR-TB associated with HIV infection from the literature.[1] Our systematic review was not updated for GBD 2023.

Modelling strategy

To determine TB deaths in HIV-positive individuals, we first computed the fraction of HIV-TB deaths among all TB deaths using vital registration data from countries with a four- or five-star rating. We also calculated the proportion of TB cases that are HIV-positive (ie, number of TB cases recorded as HIV-positive/number of TB cases with an HIV test result recorded in the WHO TB register). We used these proportions as input data for a mixed effects regression to predict the proportions of HIV-TB cases among all TB cases for all locations and years using an adult HIV death rate covariate. We estimated the fraction of HIV-TB deaths among all TB deaths in each location and year $(D_{c,y})$, defined by

$$D_{c,y} = \frac{P_{c,y}RR}{P_{c,y}RR + 1 - P_{c,y}}$$

where $P_{c,y}$ is the proportion of HIV-TB cases among all TB cases and *RR* is the relative risk of TB deaths in HIV positive individuals, defined by:

$$RR = \frac{D_{c,y}P_{c,y} - D_{c,y}}{D_{c,y}P_{c,y} - P_{c,y}}$$

We took the median relative risk (RR) from each calculation. We then applied the median RR and the predicted proportions of HIV-TB cases among all TB cases to get the fractions of HIV-TB deaths among all TB deaths for all locations and years. Location-year-specific HIV-TB deaths were then calculated using the following equation:

$$Deaths_{HIV-TB} = \frac{D_{c,y}}{1 - D_{c,y}} Deaths_{TB}$$

where $Deaths_{TB}$ is location-year-specific deaths from the CODEm TB no-HIV model. Finally, we applied the age-sex pattern of the HIV mortality estimates to these HIV-TB deaths to generate location-year-age-sex-specific HIV-TB deaths. As the HIV-TB deaths were estimated based on the fraction of HIV-TB deaths among all TB deaths, the total number of HIV-TB deaths could exceed the total number of HIV deaths in

some locations. To avoid this, we applied a cap of 45% on the fraction of HIV-TB deaths among HIV deaths, based on a review by Cox and colleagues, 2010,[2] and a systematic review and meta-analysis by Ford and colleagues, 2016.[3]

To split HIV-TB into HIV-MDR-TB and HIV-drug-susceptible-TB, we first calculated the proportion of HIV-MDR-TB among all HIV-TB cases ($P_{MDR-HIVc,y,a,s}$) for each location, year, age, and sex using the following formula:

$$P_{MDR-HIVc,y,a,s} = P_{MDRnoHIVc,y,a,s} RR_{HIV}$$

where $P_{MDRnoHIVc,y,a,s}$ is the estimated proportion of MDR-TB among HIV-negative TB cases for each location, year, age, and sex (see MDR-TB modelling strategy for the detail) and RR_{HIV} is the relative risk of MDR-TB associated with HIV infection.

We then computed the fraction of HIV-MDR-TB deaths among all HIV-TB deaths ($D_{MDR-HIVc,y,a,s}$) using the following formula:

$$D_{MDR-HIVc,y,a,s} = \frac{P_{MDR-HIVc,y,a,s} RR_{MDR}}{P_{MDR-HIVc,y,a,s} RR_{MDR} + 1 - P_{MDR-HIVc,y,a,s}}$$

where RR_{MDR} is the pooled relative risk of mortality in MDR-TB cases compared with drug-susceptible TB cases. In GBD 2020, the pooled relative risk was derived from a meta-analysis in the meta-regression with Bayesian priors, regularisation, and trimming (MR-BRT) model. After derivation of the pooled relative risk, we then applied the predicted HIV-MDR-TB death fractions to all HIV-TB death estimates to generate HIV-MDR-TB deaths by location, year, age, and sex. Next, we subtracted HIV-MDR-TB deaths from all HIV-TB deaths at the 1000-draw level to generate drug-susceptible HIV-TB deaths by location, year, age, and sex.

To separate out HIV-XDR-TB from HIV-MDR-TB, we aggregated the XDR-TB cases and MDR-TB cases (with DST for second-line drugs) up to the super-region level and calculated the super-region-level proportions of XDR-TB among MDR-TB cases. Next, we computed the super-region-specific fraction of XDR-TB deaths among all MDR-TB deaths (D_{XDRsr}) using the following formula:

$$D_{XDRsr} = \frac{P_{XDRsr} RR_{XDR}}{P_{XDRsr} RR_{XDR} + 1 - P_{XDRsr}}$$

where P_{XDRsr} is the proportion of XDR-TB among MDR-TB cases for each super-region, and RR_{XDR} is the pooled relative risk of mortality in XDR-TB cases compared with MDR-TB cases. Similar to the pooled relative risk for MDR-TB, the derivation of the pooled relative risk of mortality in XDR-TB was computed with a meta-analysis in the MR-BRT model for GBD 2020. The fractions were then applied to MDR-TB deaths in corresponding countries within the super-regions to produce XDR-TB deaths by location, age, and sex for the most recent year of estimation. We linearly extrapolated XDR-TB mortality rates back,

assuming the mortality rates were zero in 1992, one year before 1993 when XDR-TB was first recorded in USA surveillance data.[4] Finally, we subtracted HIV-XDR-TB deaths from HIV-MDR-TB deaths to generate HIV-MDR-TB (without extensive drug resistance) deaths by location, year, age, and sex.

References

1. Mesfin YM, Hailemariam D, Biadglign S, Kibret KT. Association between HIV/AIDS and multi-drug resistance tuberculosis: a systematic review and meta-analysis. *PLoS One*. 2014;9(1):e82235.
2. Cox JA, Lukande RL, Lucas S, Nelson AM, Van Marck E, Colebunders R. Autopsy causes of death in HIV-positive individuals in sub-Saharan Africa and correlation with clinical diagnoses. *AIDS Rev* 2010; **12**: 183–94.
3. Ford N, Matteelli A, Shubber Z, *et al.* TB as a cause of hospitalization and in-hospital mortality among people living with HIV worldwide: a systematic review and meta-analysis. *J Int AIDS Soc* 2016; **19**: 20714.
4. Centers for Disease Control and Prevention (CDC). Extensively Drug-Resistant Tuberculosis --- United States, 1993—2006. MMWR. 2007; 56(11);250-253

HIV/AIDS

Flowchart

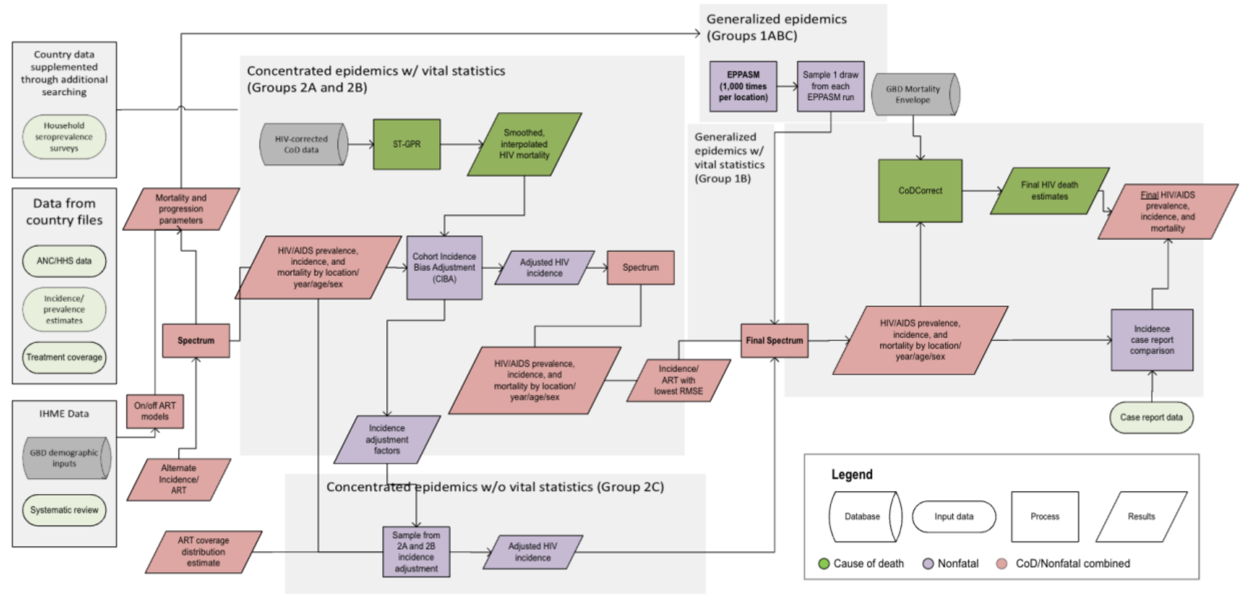

Input data and methodological summary for HIV/AIDS

Input data

Prevalence data

Household seroprevalence surveys
Geographically representative HIV seroprevalence survey results were used as inputs to the model for countries with generalised HIV epidemics where available. From these surveys, we used age- and sex-specific prevalence data.

GBD demographic inputs

Location-specific population, births, fertility, migration, and HIV-free survival rates from GBD 2021 were used as inputs in modelling all locations.

Data from countries

The files compiled by UNAIDS for their HIV/AIDS estimation process were our main source of data for producing estimates of HIV burden. The files are often built by within-country experts with the support of UNAIDS, which publishes estimates annually on behalf of countries and only shares their files when permission is granted. The files contain the HIV-specific information which is needed to run the Spectrum,[4] and Estimation and Projection Package-Age Sex (EPP-ASM)[5] models. Spectrum and EPP-ASM require the following input data: AIDS mortality among people living with HIV with and without ART, CD4 progression among people living with HIV not on ART, ART coverage among adults and children, cotrimoxazole coverage among children, coverage of breastfeeding among women living with HIV, prevention of mother-to-child transmission (PMTCT) coverage, and CD4 thresholds for treatment eligibility. EPP-ASM additionally uses HIV prevalence data from surveillance sites and representative surveys. Antenatal care clinic (ANC) and treatment coverage data from UNAIDS were used in modelling Group 1 countries. We extracted all of these data from the proprietary format used by UNAIDS.

The EPP-ASM and Spectrum models used for GBD estimation vary slightly from those used by UNAIDS, with details on this variation included below. On top of the differences in model structure, we integrate our estimates of input model parameters, including new transition parameters and demographic rates. The differences between our estimates and UNAIDS' estimates reflect differences in model structure, model parameters, and the location-specific data used to calibrate our models.

Vital registration data

We used all available sources of cause of death data and sample registration data from the GBD Causes of Death database after garbage code redistribution and HIV/AIDS mis-coding correction in Group 2 countries and India.[3,7] There are two different cause of death data sources for HIV/AIDS in China: the Disease Surveillance Points (DSP) system and the Notifiable Infectious Disease Reporting (NIDR) system. Both systems are administered by the Chinese Center for Disease Control and Prevention, but the reported number of deaths due to HIV is significantly lower in DSP. Therefore, we have used the provincial-level ratio of deaths due to HIV/AIDS from NIDR to those from DSP, choosing the larger ratio between years 2013 and 2014, and scaled the reported deaths in the DSP system, which is in turn used in the spatiotemporal Gaussian process regression (ST-GPR).

On-ART mortality literature data

GBD 2023 used the same set of literature data extracted for GBD 2021.[1]

To be included, studies must include only HIV-positive people over the age of 15 who receive antiretroviral therapy (ART) but who were ART-naïve prior to the study. In addition, studies must report either a duration-specific (time since initiation of ART) mortality proportion or a hazard ratio across age or sex, and must not include children.

For duration-specific survival data, studies must report uncertainty on mortality estimates or provide stratum-specific sample sizes and must include duration-specific data to allow for calculation of 0–6, 7–12, or 13–24-month conditional mortality. In addition, studies must either report separate mortality and loss-to-follow-up (LTFU) curves, be corrected for LTFU using vital registration data or double sampling, or be conducted in a high-income setting. Finally, studies must report the percentage of participants who are male and the median age of participants.

Hazard ratio data for ages or sexes can only be used if the hazard ratios are controlled for other variables of interest (age, sex, and CD4 category). In GBD 2021, we included 61 studies, 13 of which were new this cycle. Of these studies, we added ten to inform the estimation age-sex hazard ratios, and three studies informed LFTU curves.

Off-ART mortality and CD4 progression literature data

In GBD 2013, we systematically reviewed the literature on mortality without ART to characterise uncertainty in the progression and death rates. We searched terms related to pre-ART or ART-naïve survival since seroconversion.[8] After screening, we identified 13 cohort studies that included the cohorts used by UNAIDS, from which we extracted survival at each one-year point after infection. Screening for additional, recently published studies in GBD 2015, GBD 2016, and GBD 2017 identified no new cohort studies for inclusion in this analysis. We did not search for new studies in GBD 2019 or GBD 2021.

Modelling strategy

Case definition

Infection with the human immunodeficiency virus (HIV) causes influenza-like symptoms during the acute period following infection and can lead to acquired immunodeficiency syndrome (AIDS) if untreated. HIV attacks the immune system of its host, leaving infected individuals more susceptible to opportunistic infections like tuberculosis. Although there are two different subtypes of HIV, HIV-1 and HIV-2, no distinction is made in our estimation process or presentation of results. For HIV, ICD-10 codes are B20-B24, C46-C469, D84.9; ICD-9 codes are 042-044, 112-118 (after 1980), 130 (after 1980), 136.3-136.8 (after 1980), 176.0-176.9 (after 1980), 279 (after 1980); and ICD-9 BTL codes are B184-B185.

Country groupings

Countries were divided into groups: Groups 1A, 1B, and 2A, 2B, and 2C. Group 1 includes countries with HIV prevalence data from antenatal care clinics or nationally or subnationally representative population-based seroprevalence surveys. Group 1A included countries with a peak of at least 0.5% prevalence, and Group 1B includes countries with a peak of at least 0.25% prevalence and vital registration completeness less than 65%.

The remaining countries made up Group 2, which are further subdivided in Group 2A, 2B, and 2C based on availability of vital registration data. Group 2A consisted of countries with high-quality vital registration data; Group 2B consisted of countries with available cause of death data that is not high-quality; and Group 2C countries were those without any vital registration data. Quality was measured based on a star rating system as described elsewhere.[3]

Both groups of countries relied on the same approach to modelling on- and off-antiretroviral therapy (ART) mortality, as described below.

Results were aggregated by super-region as defined by the Global Burden of Disease study. These super-regions are depicted in figure S2.

Figure S1. HIV-specific country groupings based on data availability

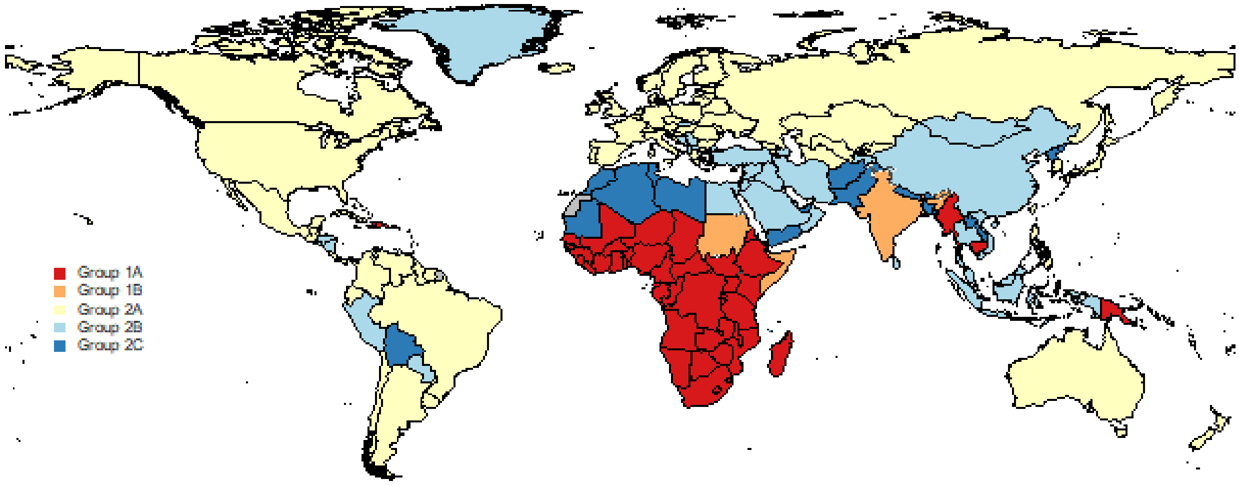

Figure S2. Global Burden of Disease 2021 super-regions

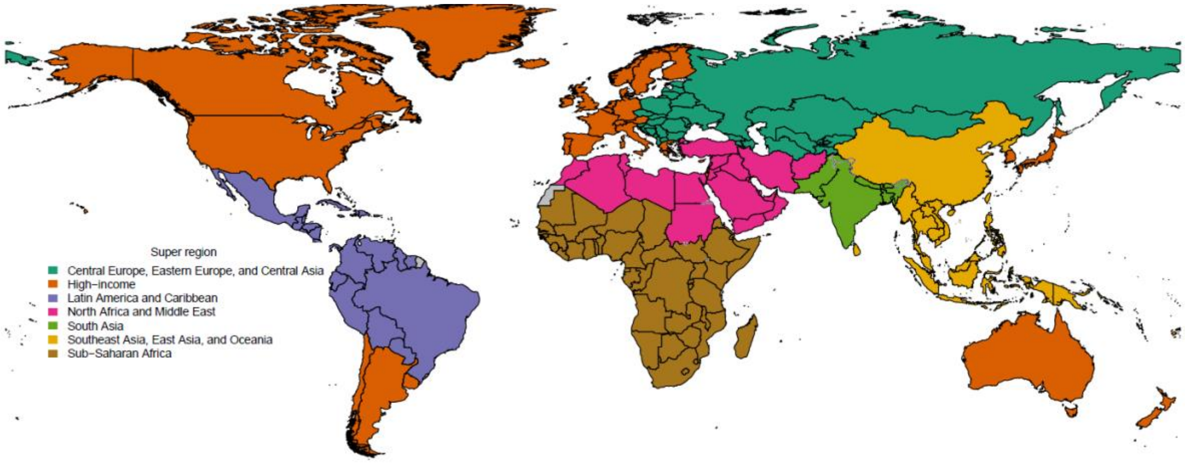

On-ART, off-ART mortality and CD4 progression parameters

On-ART mortality

We employ the meta-regression tool meta-regression with Bayesian priors, regularisation, and Trimming (MR-BRT), developed for the Global Burden of Disease (GBD) study 2019 for modelling. This tool combines the Bayesian framework with mixed-effects models to incorporate prior knowledge in the form of regularisation or constraints in the optimisation. This approach addresses some of the limitations of traditional meta-analyses by exploring between-study heterogeneity; accounting for small numbers of studies; allowing for nonlinear measurements, priors, constraints, and observation measurement errors. Specifically, we modelled the non-linear relationship between baseline CD4 and mortality, weighted each observation by the inverse of its reported variance, captured between-study heterogeneity through a random effects model, and applied a monotonic decreasing prior on baseline CD4 levels.

The modelling has two stages. In the first stage, CD4 is modelled as a continuous variable. The outcome (logit of conditional probability of mortality after subtracting the background mortality) is exclusively regressed against the spline of the measures of CD4 to ensure the non-linear dynamics are captured by aggregating data from all studies. In this model, a spline degree of 2 is employed with a total of 4 knots, and 5% of the datapoints are trimmed. In the second stage, the other covariates are integrated into a mixed-effects model along with the fitted values from the first stage: age (15–24, 25–34, 35–44, 44–54, 55–100), sex (male, female), time since initiation of ART (0–6, 7–12, 13–24 months). The modelling is implemented in three regions, and we model each region separately: sub-Saharan Africa, high-income, other countries.

We corrected reported probabilities of death for loss to follow-up using an approach developed by Verguet and colleagues.[2] Verguet and colleagues used tracing and follow-up studies to empirically estimate the relationship between death in LTFU and the rate of LTFU.

To create estimates of sex-specific hazard ratios, we use the *metan* function in Stata to create estimates of relative risks separately by region, using female age groups as the reference group within each age strata. The age and sex hazard ratios were applied to the study-level mortality rates, accounting for the distribution of ages and sexes in the mortality data. We then subtracted HIV-free mortality from the model life table process to calculate study-level age-sex HIV-specific mortality.

We then used MR-BRT to synthesise the age-sex-split study-level data into estimates of conditional probability of death over initial CD4 count. We replaced our on-ART mortality rates with those estimated off treatment if they were higher. Prior to analysing the data, we apply a logit transformation of the outcome variable, which is the conditional probability of mortality after individuals initiate treatment with ART. This transformation, along with the corresponding variance transformation, enables the data to be analysed using a powerful Gaussian mixed effects model (MR-BRT), with random (Gaussian) effects by study. The random errors in our model are assumed to follow a normal distribution. The link function used in our meta-regression model is the logit function. The random-effect term in our model accounts for between-study variability. This term is assumed to follow a normal distribution, capturing the heterogeneity across different studies included in the meta-analysis.

We estimate mortality for each region in its own DisMod model based on data from the IeDEA cohort collaboration[3] and include a covariate for year as mortality among the LFTU has been found to decline in recent years.[4] Finally, in cases where on-ART rates were higher, we replaced our estimated on-ART mortality rates by rates off ART to account for progression to lower CD4 categories. This ensured individuals would not experience higher mortality when they entered treatment in Spectrum or EPP-ASM.

Off-ART mortality and CD4 progression

Following UNAIDS assumptions, off-ART mortality and CD4 progression is modelled as shown in the figure below.

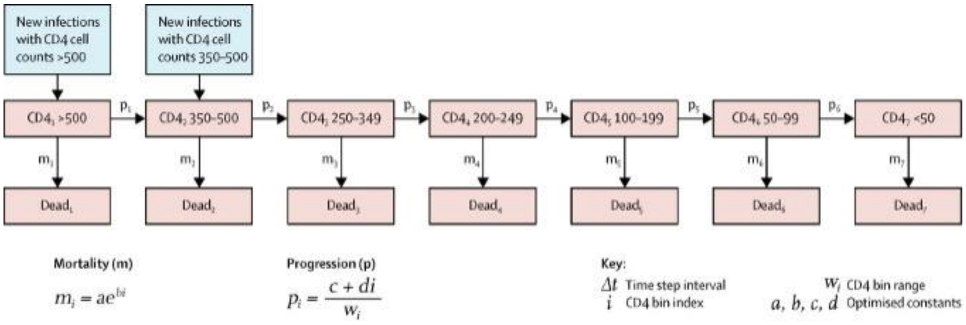

The death and progression rates between CD4 categories vary by age according to four age groups: 15–24 years, 25–34 years, 35–44 years, and 45 years or older. We modelled the logit of the conditional probability of death between years in these studies using the following formula:

$$logit(m_{ijk}) = \beta_0 + \sum_{i=1}^{4} \beta_{1i} a_i + \sum_{j=1}^{12} \beta_{2j} t_j + u_k + \epsilon_{ijk}$$

In the formula, *m* is conditional probability of death from year t_j to t_{j+1}, a_i is an indicator variable for age group at seroconversion (15–24 years, 25–34 years, 35–44 years, and 45 years or older), t_j is an indicator variable of year since seroconversion, and u_k is a study-level random effect. The categories of the variable t_j are one-year intervals up to 12 years since seroconversion, after which data availability was sparse. The baseline level was the lowest category for each indicator, so 15–24 years for age at seroconversion and 0 years for years since seroconversion. The model assumed a multivariate normal distribution for the study-level random effects, u_k, and the error term, ε, after the logit transformation of the conditional probabilities of death.

By sampling the multivariate normal distribution represented by the fitted mean and variance-covariance matrix, we generated 1000 survival curves for each age group that capture the systematic variation in survival across the available studies. For each of the 1000 survival curves, we used a framework modelled after the UNAIDS optimisation framework in which we find a set of progression and death rates that minimises the sum of the squared errors for the fit to the survival curve.[5,6]

Disease model

We used two different components to derive year-, age-, and sex-specific estimates of HIV incidence, prevalence, and mortality depending on locations' availability of data and extent of HIV burden, as described below:

1. EPP-ASM was used to estimate incidence, prevalence, and mortality that are consistent with serosurveillance data from antenatal care clinics and/or prevalence surveys.
2. Spectrum is a compartmental HIV progression model used to generate age-sex-specific incidence, prevalence, and death rates from input incidence and prevalence curves and assumptions about intervention scale-up and local variation in epidemiology. This model was used in conjunction with EPP-ASM for India and for all Group 2 countries.

Group 1: EPP–ASM

53 countries – as well as subnational locations in India, Kenya, Ethiopia, Nigeria, and South Africa – were included in Group 1 with available ANC data and/or at least one geographically representative HIV

seroprevalence survey. For all these locations we used EPP-ASM, which was updated to incorporate the new ANC bias adjustment.

In EPP-ASM,[7] the transmission rate, r(t), is a simple transmission model applied at each time step (1/10 of a year) to the population. 'r' represents the number of new cases expected to emanate from a single case. Over 3000 iterations, a new r(t) is drawn, the full epidemic is determined and compared to the observed prevalence data to determine its likelihood. Beyond the end of the data, a prior distribution on r(t) helps to determine how we should expect the epidemic to behave. This assumption was different in EPP-ASM versus EPP. In EPP-ASM in most countries, we extended a random walk into the future based on the 'r-hybrid' r(t). The r-hybrid assumes a logistic decay until the year 2003, a linear interpolation until year 2008, and a random walk form after this.

For GBD 2023, we continued to use our modified version of EPP-ASM both to improve the fit to data and to generate paediatric estimates. We built a paediatric module in EPP-ASM that mirrored early updates to the paediatric module in Spectrum.[8] This child module included CD4 progression and CD4-specific mortality rates taken from a model fit to survival data from IeDEA and child initiation of ART based on ART distribution data from IeDEA. Perinatal and breastfeeding transmission was calculated as a function of prevalence among pregnant women and PMTCT programme data. We were thus able to utilise EPP-ASM to produce HIV incidence, prevalence, and mortality estimates for all ages. Additionally, we improved fit to prevalence data through allowing flexibility in the age distribution of incidence over time. We parameterised the ratio of incidence among ages 15–24:25+ as a constant before year 2000 and a linear regression thereafter. This allowed for the shifts in the age distribution of incidence observed over the course of the HIV epidemic to be reflected in our results. Finally, we utilised GBD demographic inputs and substituted in our own assumptions about HIV progression rates and on/off-ART mortality.

To incorporate uncertainty in our demographic and progression parameters, we ran EPP-ASM with separate draws of CD4 progression, on- and off-ART mortality rates, fertility, and HIV-free mortality. This process produced 1000 posterior distributions for each of the locations that make up Group 1. For every location in the group, we sampled one draw from each of the sets of EPP-ASM results to create a final distribution. By sampling one draw from each set, we ensured that the distribution of mortality parameters dictating the relationship between incidence and prevalence aligned with those used in the GBD demographics estimates.

We also continued to use the approach implemented in GBD 2019[9] to address selection bias resulting from temporal and geographical variation in ANC reporting. The ANC data which EPP-ASM uses cannot be assumed as representative of HIV prevalence in the full population. This is especially the case when there are minimal or no nationally representative prevalence surveys to anchor estimates, as in the early epidemic.[10]

EPP-ASM has embedded approaches to adjust for the bias associated with using prevalence among ANC-site-attending pregnant women to estimate prevalence among the both-sexes population. For the bias between pregnant women and the national both-sexes population, it makes assumptions around the difference in total fertility rate among HIV-positive and HIV-negative women, and the difference in prevalence between men and women. For the bias associated with the data coming from ANC sites, the specification of the likelihood of observed ANC data includes random intercepts for each clinic. The random intercepts allow each site's baseline prevalence to vary randomly around the overall mean prevalence. In other words, factors that could drive differences between sites' HIV prevalence levels are "adjusted" for.

However, the embedded approach does not explicitly account for the fact that the location of the clinic in space may also drive its HIV prevalence level. For example, we might expect rural sites to be more correlated than urban sites. Thus, to further adjust for this bias, we used an offset term that represents the difference in the prevalence among the national, both-sexes population and the prevalence among the female, pregnant population associated with an ANC site location. The offset term was derived for each location as the difference between the adjusted prevalence in a given site-year and the adjusted national prevalence in that year. These estimates are adjusted for covariates that are thought to influence prevalence, for example, access to health-care facilities, malaria incidence, and male circumcision.

Thus, our final strategy for estimating the likelihood of the observed ANC data was:

$$W_{st} = \varphi^{-1}(\rho_t) + \vartheta_{st} + u_s + e_{st}$$

$$e_{st} \sim N(0, \sigma_{st}^2)$$

$$u_s \sim N(0, \sigma_s^2)$$

Where:
W_{st} = the probit transformed prevalence at site *s* and time *t*
ρ_t= The national prevalence adjusted to represent prevalence among pregnant women from the model simulation
ϑ_{st} = The offset term representing the difference between the adjusted prevalence in a given site-year and the adjusted national prevalence in that year
φ^{-1} = probit transformation
e_{st} = Site-specific error term
u_s = Site-specific intercept

Group 2: Spectrum

For GBD 2013, we created an exact replica of Spectrum in Python. This enabled us to run thousands of iterations of the model at once on our computing cluster and allowed for more flexible input data structures. Additionally, we scaled all input values by a uniformly sampled factor between 0.9 and 1.1 to generate estimates with realistic ranges of uncertainty. For example, if treatment retention rates across CD4 categories were 0.906, 0.759, 0.787, 0.795, 0.785, 0.756, 0.813, and 0.700, we multiplied each number by an array of equivalent size that contained factors ranging from 0.9 to 1.1. At each draw, the array would contain different, randomly selected factors in the same range. Further, we previously improved our sex-specific modelling strategy in Spectrum by sex-splitting incidence based on a model fit to the sex ratio of prevalence observed in countries with representative surveys and updated the Spectrum paediatric module to reflect changes made by UNAIDS.[13] Our child module was revised to include CD4 progression and CD4-specific mortality rates taken from a model fit to survival data from IeDEA. Finally, we updated child initiation of ART to include data on ART distribution from IeDEA. These changes were retained in GBD 2021.

ART coverage distribution
Spectrum determines the number of people initiating ART treatment across each CD4 category based on eligibility criteria, and the number of expected deaths and untreated people. In other words, groups with a large proportion of people living with HIV and high numbers of expected deaths initiated the most individuals into treatment.

We improved the basis for this distribution using survey microdata and country-level wealth information. Three relevant surveys were available: Uganda AIS 2011 and Kenya AIS 2007 and 2012. These surveys conducted CD4 count measurements and include a question regarding the amount of time that an individual receiving ART had been enrolled in treatment. Survey data provide cross-sectional CD4 count information; however, the Spectrum modelling framework tracks individuals by categorical CD4 count at the initiation of treatment. To crosswalk the cross-sectional survey data into estimates of CD4 count at treatment initiation, we built a model using relevant cohort data which tracked changes in CD4 count after initiation of treatment to translate an individual's current CD4 count and duration on treatment into CD4 count at initiation of treatment. The functional form for changes in CD4 count as a function of duration on treatment was a natural spline on duration with knots at 3, 12, 24, and 36 months, and an interaction between initial CD4 count and duration.

After crosswalking, we predicted the probability of being on treatment as a function of individual income (measured through an asset-based index), stratified by CD4 count, age, and sex. The results of this prediction were translated into country-specific age-sex-year-CD4 count probabilities of coverage using a conversion factor between individual income and lag-distributed GDP per capita. We used stochastic frontier analysis to constrain the maximum possible coverage for a given degree of income and CD4 count.

Predicted probabilities of coverage were input to Spectrum to inform the distribution, and not the overall level, of ART treatment by CD4 count. Within Spectrum, the probabilities of coverage are converted to counts of expected individuals on treatment in each CD4 count group. These are scaled to the distribution across CD4 count groups to match the input data on the number of people on ART coming from UNAIDS country files. In cases where the predicted number of individuals initiating treatment exceeds the total number of untreated individuals in a CD4 count group, we reallocate treatment evenly to other CD4 count groups.

Group 2: Countries without survey data and vital registration data

33 countries had neither geographically representative seroprevalence surveys nor reliable vital registration systems, these make up group 2C locations. To produce estimates of HIV burden in these countries, we used Spectrum to produce estimates of burden. As above, the estimates of incidence, prevalence, and mortality were incorporated into the rest of the machinery via the reckoning process.

Spatiotemporal Gaussian process regression (ST-GPR)

Countries with vital registration data (Groups 2A and 2B)

Vital registration is one of the highest-quality sources of data on HIV burden in many countries, so generating estimates that are consistent with these data with necessary adjustment to account for any potential under-reporting is critical. We identified 121 countries – as well as 760 subnational locations from China, Japan, Indonesia, India, Mexico, Sweden, the Philippines, Poland, Italy, the UK, Ukraine, Russia, New Zealand, Iran, Norway, and the USA – with usable points of vital registration data, verbal autopsy (VA) data, or sample registration system (SRS) data. In India, Vietnam, and Indonesia, we used SRS and VA data, respectively, as input mortality for CIBA. For India, we extracted the resulting age-sex distribution of incidence but scaled the level to match the adult incidence rate estimated from EPP for each state.

We estimated full time series for HIV deaths using ST-GPR fit to available cause of death data. We analysed mortality trends using ST-GPR starting in 1981, the year that HIV was first identified in the

USA.[11] For ST-GPR, we adjusted the lambda (time weight) and GPR scale according to the completeness of vital registration data, with 4- and 5-star quality vital registration using parameters designed to follow the data more closely. We produced separate splines by country/age group, up to the peak year of death rate. We then ran a linear regression with fixed effects on region, age, and sex. Following this, we ran space-time residual smoothing, in which time, age, and space weights are used to inform smoothing of the residuals between datapoints and the linear regression estimate. From this process, we generated space-time estimates with the applied weights, along with the median absolute deviation (MAD) of the space-time estimates from the data. The MAD was calculated at various levels of the geographical hierarchy (eg, subnational and national), and was added into the data variance term. The data variance and space-time estimates were then analysed using Gaussian process regression to return a final estimate of mortality along with uncertainty. ST-GPR deaths were used as final deaths in group 2A and group 2B.

Although Spectrum produces HIV mortality estimates that are within the realm of possibility in most countries using the incidence curves provided in the UNAIDS country files, it is a deterministic model that has not yet been integrated into an optimisable framework. Therefore, in order to "fit" it to vital registration data, we need to adjust input incidence. In contrast to GBD 2019 and previous cycles, in addition to adjusting input incidence, we determined the most plausible best treatment input based on fit to vital registration as well.

Additional adjustments

Additional adjustments enabled us to use case surveillance data and HIV mortality estimated as part of the GBD all-cause mortality life table process. In countries and territories with high-quality case notification data, we scaled incidence results to align with case reports after accounting for an assumed average of five years' lag to diagnosis. In previous GBD cycles, HIV mortality went through a "reckoning process" that was intended to be a method of reconciling separate estimates of HIV mortality (and its resulting effect on estimates of HIV-free and all-cause mortality) in Group 1 countries by averaging estimates of HIV mortality from the model life table process and EPP-ASM. Additional details on the reckoning can be found elsewhere.[12] In GBD 2023, this processed was removed and HIV deaths instead went through the standard GBD 2023 CoDCorrect process.[13]

Changes for GBD 2023

In GBD 2023, we ran three individual MR-BRT models to produce on-ART mortality rates for input in our disease models. This represented a shift away from 90 different models used in GBD 2021. The new MR-BRT modelling approach for on-ART mortality is implemented in three regions, which are modelled separately as sub-Saharan Africa, high-income, and other countries.

For GBD 2023, ST-GPR was run using a generalised set of hyperparameters, rather than the country-specific hyperparameters in previous cycles. This was done to ensure a consistent method for estimating mortality for countries with vital registration data. These generalised hyperparameters result in trends that better incorporate regional estimates with country-specific mortality data to give country-level estimates with greater spatial smoothing than GBD 2021 and previous rounds.

In previous GBD cycles, HIV incidence for Group 1 was "reconciled" by averaging estimates of HIV mortality from the model life table process and EPP-ASM.[1] In GBD 2023, we no longer averaged the two sets of estimates, and instead HIV mortality estimates were processed in the standard GBD CoDCorrection process, which is described in further detail in the CoDCorrect section of this appendix.

References

1 Carter A, Zhang M, Tram KH, *et al.* Global, regional, and national burden of HIV/AIDS, 1990–2021, and forecasts to 2050, for 204 countries and territories: the Global Burden of Disease Study 2021. *The Lancet HIV* 2024; **11**: e807–22.

2 Verguet S, Lim SS, Murray CJL, Gakidou E, Salomon JA. Incorporating Loss to Follow-up in Estimates of Survival Among HIV-Infected Individuals in Sub-Saharan Africa Enrolled in Antiretroviral Therapy Programs. *Journal of Infectious Diseases* 2013; **207**: 72–9.

3 Tarwater PM, Margolick JB, Jin J, *et al.* Increase and plateau of CD4 T-cell counts in the 3 1/2 years after initiation of potent antiretroviral therapy. *Journal of Acquired Immune Deficiency Syndromes* 2001; **27**: 168–75.

4 Zhou J, Kumarasamy N, Ditangco R, *et al.* The TREAT Asia HIV Observational Database. *J Acquir Immune Defic Syndr* 2005; **38**: 174–9.

5 Ghys PD, Żaba B, Prins M. Survival and mortality of people infected with HIV in low and middle income countries: results from the extended ALPHA network. *AIDS* 2007; **21**: S1–4.

6 Hallett TB, Zaba B, Todd J, *et al.* Estimating Incidence from Prevalence in Generalised HIV Epidemics: Methods and Validation. *PLoS Med* 2008; **5**: e80.

7 Eaton JW, Brown T, Puckett R, *et al.* The Estimation and Projection Package Age-Sex Model and the r-hybrid model: new tools for estimating HIV incidence trends in sub-Saharan Africa. *AIDS* 2019; **33**: S235–44.

8 Stover J, Glaubius R, Mofenson L, *et al.* Updates to the Spectrum/AIM model for estimating key HIV indicators at national and subnational levels. *AIDS* 2019; **33**: S227–34.

9 Jahagirdar D, Walters MK, Novotney A, *et al.* Global, regional, and national sex-specific burden and control of the HIV epidemic, 1990–2019, for 204 countries and territories: the Global Burden of Diseases Study 2019. *The Lancet HIV* 2021; **8**: e633–51.

10 Ng M, Gakidou E, Murray CJ, Lim SS. A comparison of missing data procedures for addressing selection bias in HIV sentinel surveillance data. *Popul Health Metr* 2013; **11**: 12.

11 Pneumocystis Pneumonia --- Los Angeles. https://www.cdc.gov/mmwr/preview/mmwrhtml/june_5.htm (accessed Feb 12, 2024).

12 Vos T, Lim SS, Abbafati C, *et al.* Global burden of 369 diseases and injuries in 204 countries and territories, 1990–2019: a systematic analysis for the Global Burden of Disease Study 2019. *The Lancet* 2020; **396**: 1204–22.

13 GBD 2021 Causes of Death Collaborators. Global burden of 288 causes of death and life expectancy decomposition in 204 countries and territories and 811 subnational locations, 1990-2021: a systematic analysis for the Global Burden of Disease Study 2021. *Lancet* 2024; **403**: 2100–32.

Human African trypanosomiasis (HAT)

Flowchart

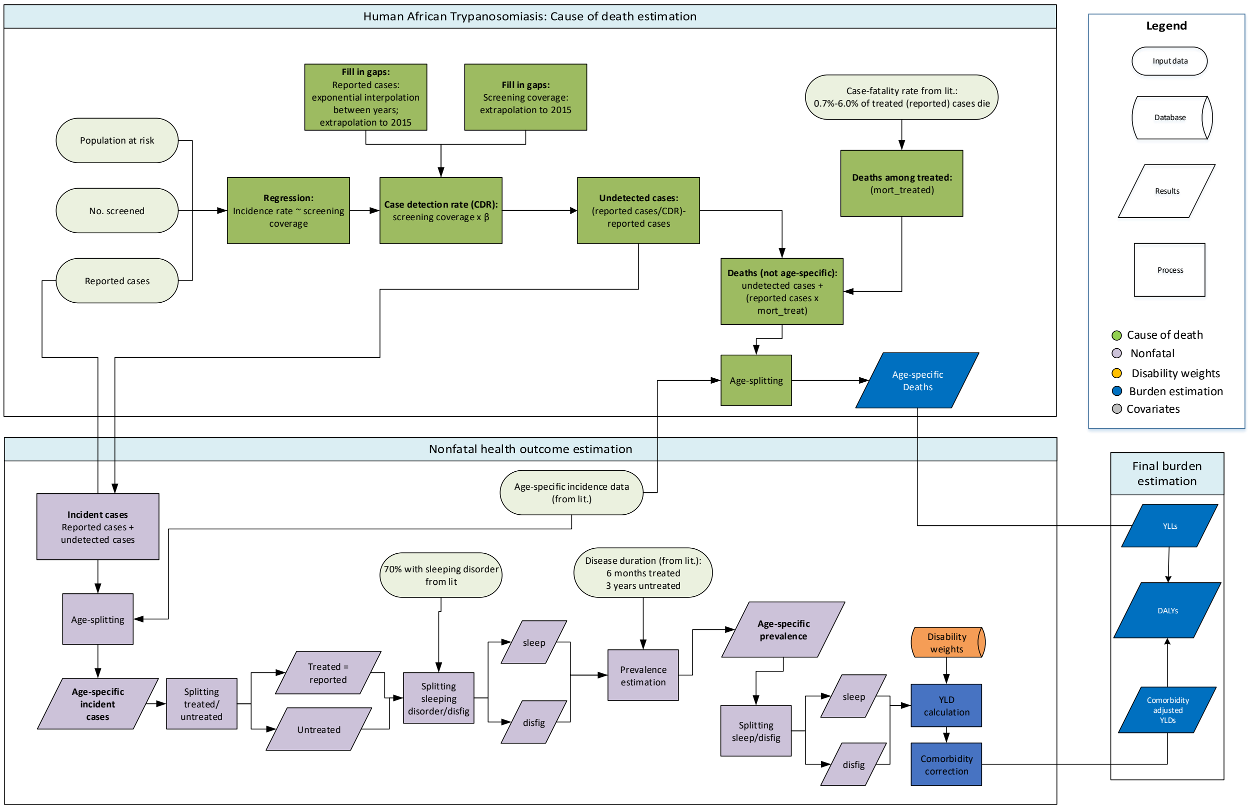

Input data and methodological summary for human African trypanosomiasis (HAT)

Human African trypanosomiasis (HAT), also known as sleeping sickness, is a vector-borne disease that is transmitted by the bite of the tsetse fly. It is caused by the parasite *Trypanosoma brucei* with two subspecies, namely *T.b. rhodesience* and *T.b. gambiense*. Cases are diagnosed through laboratory methods which rest on finding the parasite in body fluid or tissue by microscopy. In highly endemic or epidemic areas where the likelihood of false positives in serological tests is deemed lower, a seropositive individual is considered affected even in the absence of parasitological confirmation. The ICD-10 codes for HAT are B56.0, B56.1, and B56.9.

Input data

Data sources for GBD 2023 included the following:

1. Annual case totals 1980–2022: National-level annual case totals from 1990 to 2022 were obtained from WHO's publicly available dataset.[1]

Subnational data:

Kenya: Deaths due to HAT were attributed to Busia County. Identification of subnational locations for Kenyan case data were obtained via studies published in the peer-reviewed literature[2] and review of maps published via the WHO HAT Atlas.[3]

2. Age/sex data: Data on the age and sex distribution of HAT were last updated for GBD 2017. Cases were extracted from the peer-reviewed literature via a systematic review of sources identified in PubMed using the following search string:

   ((African trypanosomiasis[Title/Abstract] AND (incidence[Title/Abstract] OR burden[Title/Abstract] OR prevalence[Title/Abstract] OR community[Title/Abstract])) AND (“1990”[Date – Publication] : “2017”[Date – Publication]))

   This yielded 219 studies, of which only three met the inclusion criteria and were extracted. The inclusion criteria were:
   1. Studies representative of the national population
   2. Population-based studies
   3. Studies with primary data on incidence
   4. Studies of human African trypanosomiasis (excluded studies on animal African trypanosomiasis)

3. Population at risk estimates 1980–2015: population at risk estimates from GBD 2010 ArcGIS analysis using geocoded case notifications for 2000 to 2009[4] and population Count Grid estimates from Gridded Population of the World 3.[5]

4. Screening coverage: Data on active versus passive screening coverage were obtained from a Weekly Epidemiological Report[6] identifying the population screened from 1997 to 2004 at the national level.

5. Geographical restrictions: Data file of all GBD locations, defining location as either endemic or non-endemic for HAT. Estimates are not produced for non-endemic countries, nor are they generated for countries with a history of HAT transmission but no data reported by WHO from 1990–2018.

Modelling strategy

Geographical restrictions

For countries historically considered endemic for HAT, but which have no reported case data or estimate of the population at risk, estimates are not produced. These countries include Botswana, Ethiopia, Guinea-Bissau, and Rwanda.

Among countries where population at risk data are available, if no cases were reported to WHO, we assume the incidence of HAT is zero for those years and generate model estimates accordingly.

Modelling steps

The cause of death model for HAT was implemented as follows:

1. The incidence of reported HAT cases among the population at risk was calculated as the total number of reported cases divided by the population at risk estimates generated by the GBD working group for the period 1980–2015. Population at risk estimates for 2016–2023 were generated by assuming an annual 2% rate of population growth.

2. To estimate the number of cases that were likely undetected by country and year, a multilevel mixed-effects linear regression of log-transformed incidence rate (ratio of reported HAT cases to population at risk) on log-transformed screening coverage[6] (ratio of number screened for HAT to population at risk), with country random effects, was performed. Gaps were then filled using interpolation between years and extrapolation from 2019 to 2023 for reported cases. This model generates a beta-coefficient which is used to estimate the case detection rate (see step 4).

   For countries with particularly sparse data on screening coverage, we used alternative approaches to avoid excessive extrapolation. Among countries with data reported only for years 1997–2004, the proportion of the at-risk population screened from 1997 was used retrospectively for the period 1980–1996, and the screening coverage from 2004 was carried forward from 2005 to 2023. For countries with no screening data reported, we used the mean screening coverage for the region.

3. To construct an estimate of total deaths, we first assume that all detected cases receive treatment, and that mortality among the treated occurs for a small proportion of cases. Deaths among detected cases are estimated by generating 1000 draws of mortality among treated cases, assuming that between 0.7% and 6.0% of all reported (and therefore assumed to have received treatment) cases die.[7-9]

4. We then assume that all undetected cases experience mortality. This is estimated via generation of 1000 draws of the case detection rate (CDR), given the expected screening coverage from the regression (in step 2). Undetected deaths were then estimated as the difference between the ratio of reported cases to CDR and reported cases (reported cases/CDR – reported cases).

5. Estimates of death were obtained by adding the deaths among treated cases to the total number of undetected cases. Without information on sex-specific incidence or deaths, death rates between both sexes were equal.

6. Finally, an age pattern was applied to the mortality estimates using the incidence studies from Sudan,[10] DRC,[11] and Uganda.[12] The age pattern in GBD 2023 employed a cubic spline to account for the higher risk of infection among working-age adults.

Changes from GBD 2021 to GBD 2023

There have been no substantive changes to the modelling strategy for GBD 2023.

References

1. The Global Health Observatory, WHO. “Human African Trypanosomiasis (Sleeping Sickness).” https://www.who.int/data/gho/data/themes/topics/human-african-trypanosomiasis.
2. Rutto JJ, Osano O, Thuranira EG, Kurgat RK, Odenyo VA. Socio-economic and cultural determinants of human african trypanosomiasis at the Kenya - Uganda transboundary. *PLoS Negl Trop Dis* 2013; **7**(4): e2186.
3. Mapping the distribution of human African trypanosomiasis, WHO. “The Atlas of Human African Trypanosomiasis (2000-2018): Kenya.” http://quarry.essi.upc.edu:8080/who/Distribution/HAT_Kenya_2000-2018.jpg.
4. Simarro PP, Cecchi G, Paone M, et al. The Atlas of human African trypanosomiasis: a contribution to global mapping of neglected tropical diseases. *Int J Health Geogr* 2010; **9**: 57.
5. Earth Science Data Systems, NASA. “Gridded Population of the World, Version 3 (GPWv3): Population Density Grid | NASA Earthdata.” Earth Science Data Systems, NASA. https://www.earthdata.nasa.gov/data/catalog/sedac-ciesin-sedac-gpwv3-popdens-3.00.
6. World Health Organization. Human African trypanosomiasis (sleeping sickness): epidemiological update. *Weekly epidemiological record* 2006; **February 24**(8): 69-80.
7. Kato CD, Nanteza A, Mugasa C, Edyelu A, Matovu E, Alibu VP. Clinical profiles, disease outcome and co-morbidities among T. b. rhodesiense sleeping sickness patients in Uganda. *PLoS One* 2015; **10**(2): e0118370.
8. Balasegaram M, Harris S, Checchi F, Hamel C, Karunakara U. Treatment outcomes and risk factors for relapse in patients with early-stage human African trypanosomiasis (HAT) in the Republic of the Congo. *Bull World Health Organ* 2006; **84**(10): 777-82.
9. Odiit M, Kansiime F, Enyaru JC. Duration of symptoms and case fatality of sleeping sickness caused by Trypanosoma brucei rhodesiense in Tororo, Uganda. *East Afr Med J* 1997; **74**(12): 792-5.
10. Moore A, Richer M, Enrile M, Losio E, Roberts J, Levy D. Resurgence of sleeping sickness in Tambura County, Sudan. *Am J Trop Med Hyg* 1999; **61**(2): 315-8.
11. Lutumba P, Makieya E, Shaw A, Meheus F, Boelaert M. Human African trypanosomiasis in a rural community, Democratic Republic of Congo. *Emerg Infect Dis* 2007; **13**(2): 248-54.
12. Fevre EM, Odiit M, Coleman PG, Woolhouse ME, Welburn SC. Estimating the burden of rhodesiense sleeping sickness during an outbreak in Serere, eastern Uganda. *BMC Public Health* 2008; **8**: 96.

Hypertensive heart disease

Flowchart

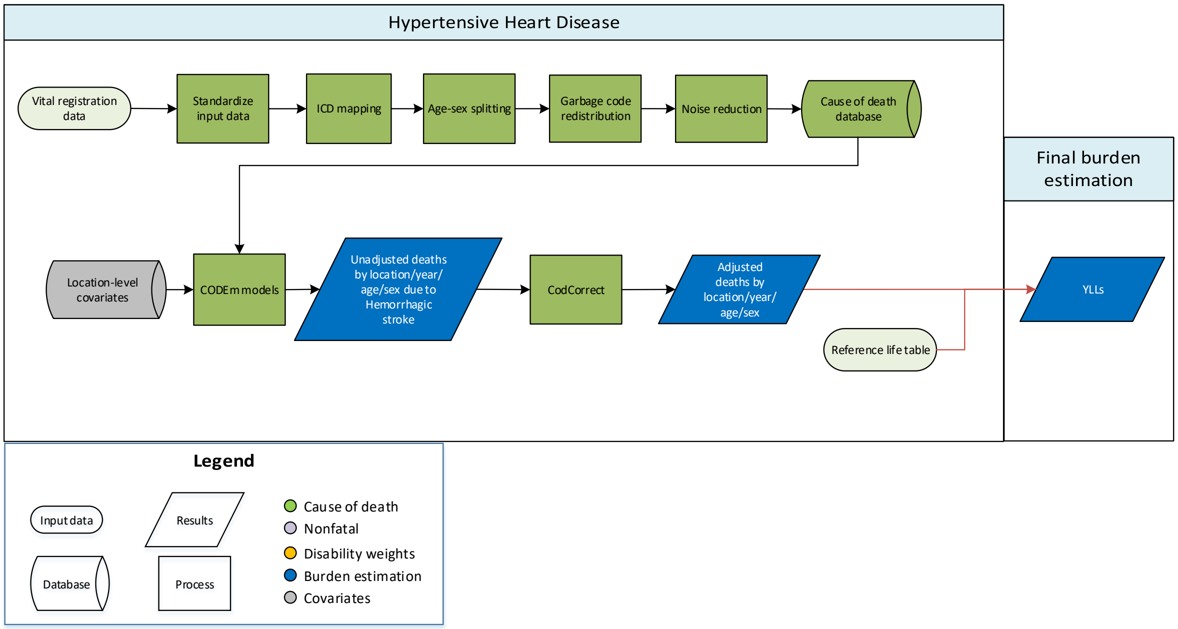

Input data and methodological summary for hypertensive heart disease

Input data

Vital registration data were used to model cause-specific mortality for hypertensive heart disease. ICD-8 datapoints from Norway and Sweden which caused implausible time trends were outliered. We outliered ICD-9-BTL datapoints, which were inconsistent with the rest of the data and created implausible time trends. ICD-9 and ICD-10 datapoints in Turkmenistan and Uzbekistan were outliered due to being implausibly high compared to other data in the region. In addition, we outliered vital registration data from Grenada in 2017, Saudi Arabia, Ukraine, and Armenia in 2018, Croatia, Russia, Mexico, Ethiopia subnationals, and Germany in 2019–2021 were outliered for being implausible values across all age groups.

Most data sources used for cause of death estimation are ICD-coded. The ICD periodically releases new versions, which can introduce an implausible disjoint in the time series that is indicative of a change in the coding practice, rather than a true change in the epidemiological trend of the disease. In GBD 2023, certain causes and countries using the ICD-9 code systems were adjusted to address the disjoint in the time trend and number of deaths between the older ICD-9 code systems and the more recent ICD-10 code systems. Hypertensive heart disease was corrected in Bulgaria (1990–2004), Canada (1986–1999), Spain (1980–1998), France (1980–1999), the USA (1980–1998), Hungary (1980–1995), Croatia (1985–1994), the UK (1980–1999), Greece (1987–2008), Italy (1980–2002), Mexico (1980–2002), and Mauritius (1980–2004) to account for this code system disjoint. This was done using a meta-regression tool described as a crosswalk, by taking systematically biased datapoints and estimating their unbiased

value. Using this modelling tool, correction factors were created based on the more recent and more reliable ICD-10 coded data. The crosswalk creates matched pairs between the last three years of data using the ICD-9 code system and the first three years of data using the ICD-10 code system. The correction is applied at the child cause level for all Level 2 causes, and after the correction is applied the individual child causes are raked to the parent cause to maintain a consistent number of deaths across the cause hierarchy.

Modelling strategy

We used a standard CODEm approach to model deaths from hypertensive heart disease. Since GBD 2021, CODEm has switched from using an ensemble of rate and cause-fraction models to only rate models. We also changed the direction of the Socio-demographic Index covariate from positive to negative with consistency with other similar covariates. Otherwise there were no substantial changes in CODEm modelling since GBD 2021. The covariates included in the ensemble modelling process are listed in the table below.

Table 1. Covariates used in hypertensive heart disease mortality modelling

Level	Covariate	Direction
1	Systolic blood pressure (mmHg)	1
2	Cholesterol (total, mean per capita)	1
	Smoking prevalence	1
	Mean BMI	1
	Healthcare Access and Quality Index	–1
3	Lag-distributed income per capita (I$)	–1
	Socio-demographic Index	–1
	Alcohol (litres per capita)	1
	Summary exposure value, omega-3	1
	Summary exposure value, fruits	1
	Summary exposure value, nuts and seeds	1
	Summary exposure value, PUFA	1
	Summary exposure value, vegetables	1
	Pulses/legumes (kcal per capita, unadjusted)	–1
	Trans fatty acid (percent)	1

Idiopathic epilepsy

Flowchart

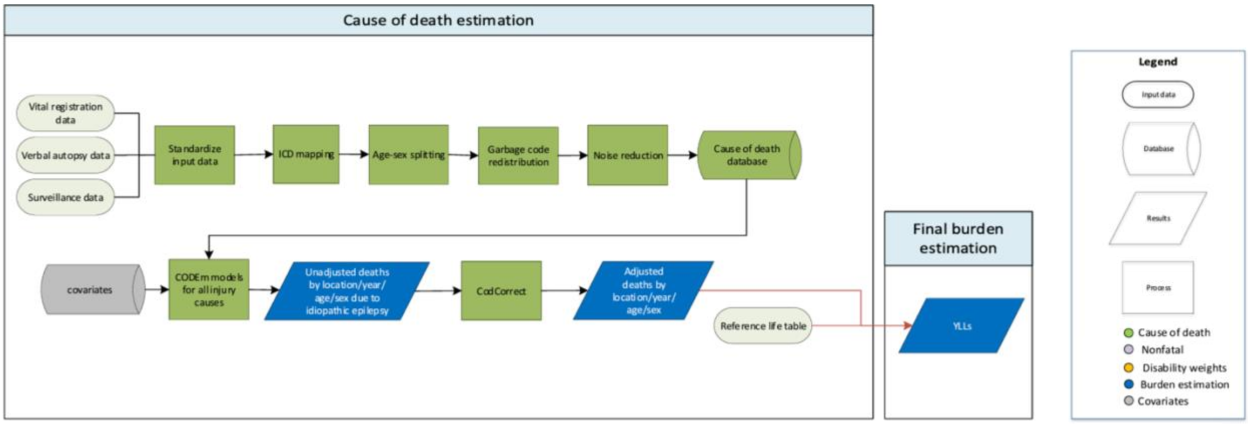

Input data and methodological summary for idiopathic epilepsy

Input data

Data used to estimate epilepsy mortality included vital registration (VR), verbal autopsy, and China mortality surveillance data from the cause of death (CoD) database. Our outlier criteria were to exclude datapoints that were (1) implausibly high or low relative to global or regional patterns, (2) substantially conflicted with established age or temporal patterns, or (3) substantially conflicted with other data sources based in the same locations or locations with similar characteristics (ie, Socio-demographic Index). In GBD 2023, it was determined that data sources from India which capture only a subset of deaths in the country generally reported lower death counts compared to more comprehensive sources. This discrepancy created a misleading impression that epilepsy mortality rates are increasing over time. To address this, idiopathic epilepsy estimates for India were informed exclusively by sample registration system (SRS) data, which provide broader and more representative coverage.

Modelling strategy

The standard Cause of Death Ensemble modelling (CODEm) approach was used to estimate deaths due to idiopathic epilepsy. Separate models were conducted for male and female mortality, and the age range for both models was 38 days to 95+ years. Unadjusted death estimates were adjusted using CoDCorrect to produce final estimates of deaths and years of life lost (YLLs).[1] See appendix 1 section 4 of the reference article for further information.

The following table lists the covariates included in the model. For GBD 2023, no significant updates were made for idiopathic epilepsy covariate selection. Covariate directions were selected based on the strength of the evidence.

Table 1. Covariates used in idiopathic epilepsy mortality modelling

Level	Covariate	Direction
1	Pigs (per capita)	+
	SEV scalar: epilepsy	+
	Mean systolic blood pressure (mmHg)	+
2	Healthcare Access and Quality Index	-
	Mean body-mass index	+
	Mean serum total cholesterol (mmol/L)	+
3	Cumulative cigarettes (10 years)	+
	Cumulative cigarettes (5 years)	+
	Education (years per capita)	-
	Log LDI (per capita)	-

Inflammatory bowel disease

Flowchart

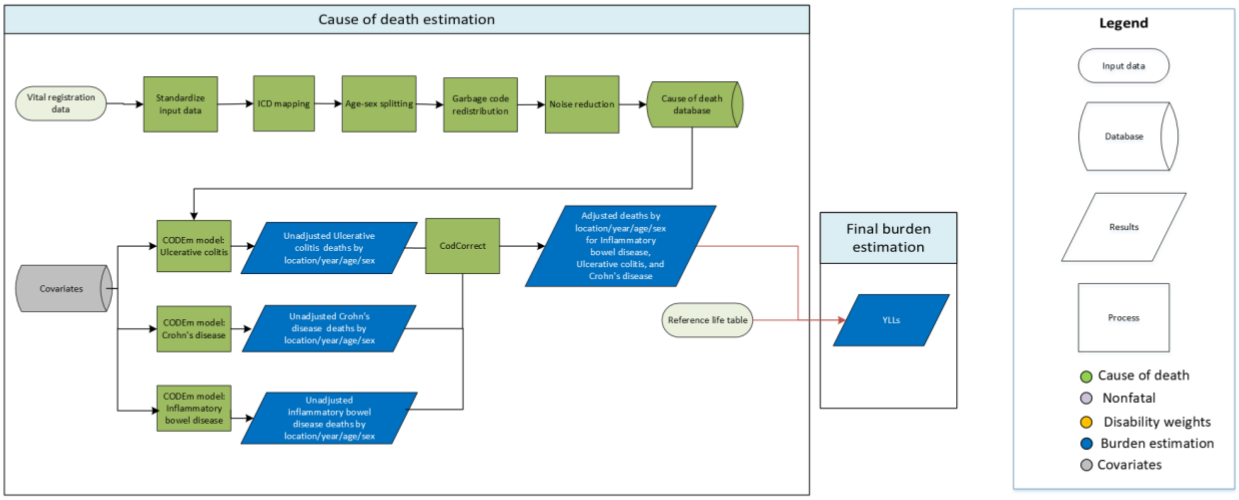

Input data and methodological summary for inflammatory bowel disease

Inflammatory bowel disease (IBD) is a Level 3 cause in the GBD hierarchy and has for many rounds of GBD estimation. Starting in GBD 2021, we began estimating deaths due to ulcerative colitis (UC) and Crohn's disease (CD) specifically, as two Level 4 child causes of IBD in the GBD cause hierarchy. All deaths related to UC and CD were included in the database for IBD. Separate statistical models were fit to estimate mortality for IBD, UC, and CD, and the results were harmonised in the CoDCorrect process, described below.

Input data

Data used to estimate IBD mortality consisted of vital registration (VR) from the cause of death (CoD) database. A list of ICD codes that were mapped to this cause, a list of vital registration sources included in the CoD database and their quality as indicated by star-ratings, and the steps used to process these data for modelling can be found in the section of this appendix on the CoD database and the tables and figures it refers to. CoD data processing was broadly similar to previous rounds of GBD, but one novel data processing step was employed in preparing data for inflammatory bowel disease that was not needed prior to GBD 2023 – COVID corrections – which are described elsewhere in this appendix.

Data exclusions

After all data processing steps were complete, datapoints for all locations and years were systematically reviewed to identify outliers or other data limitations not fully overcome by COD data processing methods, and selected sources were excluded from analysis. For IBD, the following were identified and excluded prior to modelling:

Super-region	Countries with outliers	Years and types excluded

Central Europe, eastern Europe, and central Asia	Armenia<br>Belarus<br>Mongolia<br>Tajikistan	2007, vital registration<br>2018, vital registration (sole year)<br><br>All years, vital registration<br>2016 and 2017, vital registration (all years)
High income	Australia<br>Canada<br>Northern Island<br>Scotland	2013, vital registration<br>2011, vital registration<br>2011, 2012, 2013, vital registration<br>2011-2014, vital registration
Latin America and Caribbean	Antigua and Barbuda<br>Dominica<br>Grenada<br>Saint Lucia	1996, vital registration<br>2010, vital registration<br>2015, 2017, vital registration<br>1996, 2015, vital registration
North Africa and Middle East	Bahrain<br>Lebanon<br>Oman<br>Palestine<br>Qatar	All years, vital registration<br>2017, vital registration<br>All years, vital registration<br>Prior to 2018, vital registration<br>Prior to 2019, vital registration
South Asia	No exclusions	
Sub-Saharan Africa	No exclusions	
Southeast Asia, east Asia, and Oceania	Kiribati	All years, vital registration

Modelling strategy

The strategy used to estimate mortality due to IBD has not substantively changed between GBD 2021 and GBD 2023. Standard CODEm models were used to separately model deaths due to IBD, UC, and CD (see appendix section on CODEm method for details). For each cause, models were generated separately for males in data-rich countries, males in all countries (referred to as a global model), females in data-rich countries, and females in all countries (global model). Fatal estimation for these causes was restricted to ages 2 years to 95+ years. The following table has the full list of covariates presented to the CODEm algorithm for selection in models of IBD, UC, and CD; the presented covariates were the same for all three of these GBD causes, but selection based on out-of-sample performance was carried out by the CODEm algorithm separately for each cause.

Table 1. Covariates used in inflammatory bowel disease mortality modelling (IBD, UC, and CD)

Level	Covariate	Direction
1	Age-sex-specific scaled exposure variable for low polyunsaturated fatty acids consumption	+
	Age-sex-specific scaled exposure variable for low fruit consumption	+
	Age-sex-specific scaled exposure variable for low vegetable consumption	+

	Age-sex-specific scaled exposure variable for high red meat consumption	+
2	Healthcare Access and Quality Index	-
	Latitude 15 to 30 (proportion)	-
	Latitude 30 to 45 (proportion)	+
	Latitude 45 plus (proportion)	+
3	Socio-demographic Index	+
	Education (years per capita)	-
	Log LDI ($I per capita)	+

We hybridised separate global and data-rich models to acquire unadjusted results, which we adjusted using CoDCorrect. Adjustment in CoDCorrect included rescaling unadjusted death estimates for Level 4 child causes of IBD (ie, UC and CD) to overall inflammatory bowel disease deaths, which were then rescaled alongside other causes to sum up the hierarchy to all-cause counts of death. Adjusted deaths were compared to the reference life table to calculate final YLLs due to inflammatory bowel disease.

Inguinal, femoral, and abdominal hernia

Flowchart

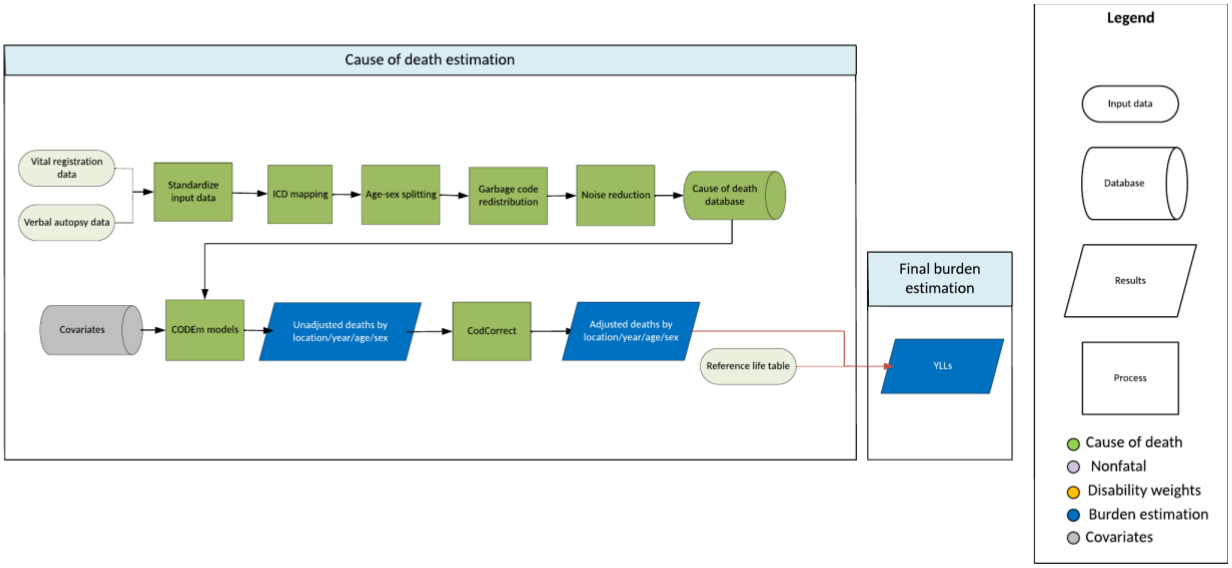

Input data and methodological summary for inguinal, femoral, and abdominal hernia

Inguinal, femoral, and abdominal hernia comprise a Level 3 cause in the GBD hierarchy.

Input data

Data used to estimate mortality of inguinal, femoral, and abdominal hernia consisted of vital registration (VR) and verbal autopsy data (VA) from the cause of death (CoD) database. A list of ICD codes that were mapped to this cause, a list of vital registration sources included in the CoD database and their quality as indicated by star-ratings, and the steps used to process these data for modelling can be found in the section of this appendix on the CoD database and the tables and figures it refers to. CoD data processing was broadly similar to previous rounds of GBD, but one novel data processing step was employed in preparing data for inguinal, femoral, and abdominal hernia that was not needed prior to GBD 2023—COVID-19 corrections—which are described elsewhere in this appendix.

After all data processing steps were complete, datapoints for all locations and years were systematically reviewed to identify outliers or other data limitations not fully overcome by CoD data processing methods, and selected sources were excluded from analysis.

Modelling strategy

The strategy used to estimate mortality due to inguinal, femoral, and abdominal hernia was not substantively changed between GBD 2021 and GBD 2023. A standard CODEm model was used to model deaths due to inguinal, femoral, and abdominal hernia (see appendix section on CODEm method for details). Models were generated separately for males in data-rich countries, males in all countries (referred to as a global model), females in data-rich countries, and females in all countries (global

model). Fatal estimation for this cause was restricted to ages 0 days to 95+ years. The following table has the full list of covariates presented to the CODEm algorithm for selection in models of inguinal, femoral, and abdominal hernia mortality.

Table 1. Covariates used in inguinal, femoral, and abdominal hernia mortality modelling

Level	Covariate	Direction
1	BMI (mean)	-
	Cumulative cigarettes (10 years)	+
	Cumulative cigarettes (5 years)	+
	Smoking prevalence	+
2	Healthcare Access and Quality Index	-
3	Socio-demographic Index	+
	Education (years per capita)	-
	Log LDI ($I per capita)	-

We hybridised separate global and data-rich models to acquire unadjusted results, which we adjusted using CoDCorrect. Adjustment in CoDCorrect included rescaling unadjusted inguinal, femoral, and abdominal hernia death estimates alongside other Level 3 digestive disease deaths to sum to overall digestive disease deaths, which were then rescaled along with other causes to sum up the hierarchy to all-cause counts of death. Adjusted deaths were compared to the reference life table to calculate final YLLs due to inguinal, femoral, and abdominal hernia.

Injuries

Flowchart

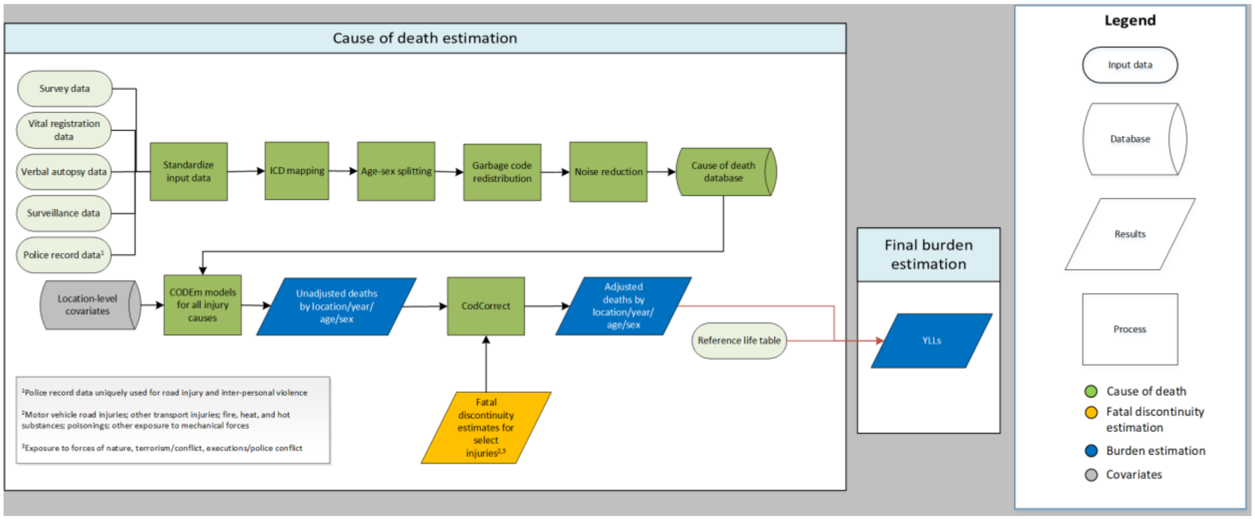

Input Data and Methodological Summary for Injuries

Injuries are a Level 1 condition within the GBD hierarchy. We estimated mortality for 37 causes of injury, which are grouped into three categories: transport injuries, unintentional injuries, and self-harm and interpersonal violence. The table below lists each of the injuries for which we make estimates.

Table 1: 3 injuries here for which GBD makes fatal estimates

**Transport injuries**	**Unintentional injuries**	**Self-harm and interpersonal violence**
Transport injuries	Falls	Self-harm
Road injuries	Drowning	Self-harm by firearm
Pedestrian road injuries	Fire, heat, and hot substances	Self-harm by other specified means
Cyclist road injuries	Poisonings	Interpersonal violence
Motorcyclist road injuries	Poisoning by carbon monoxide	Physical violence by firearm
Motor vehicle road injuries	Poisoning by other means	Physical violence by sharp object
Other road injuries	Exposure to mechanical forces	Physical violence by other means
Other transport injuries	Unintentional firearm injuries	Conflict and terrorism
	Other exposure to mechanical forces	Executions and police conflict
	Adverse effects of medical treatment	
	Animal contact	
	Venomous animal contact	
	Non-venomous animal contact	
	Foreign body	

	Pulmonary aspiration and foreign body in airway	
	Foreign body in other body part	
	Environmental exposure to heat and cold	
	Exposure to forces of nature	
	Other unintentional injuries	
	Electrocution	

Input data

We estimated injury mortality using data from vital registration, verbal autopsy, mortality surveillance, censuses, surveys, and police record data. Police and crime reports were data sources uniquely used for the estimation of deaths from transport and road traffic injuries, and interpersonal violence. The police data were collected from published studies, national agencies, and institutional surveys such as the United Nations Crime Trends Survey and the WHO Global Status Report on Road Safety Survey. Police records were not used for countries with vital registration data unless the recorded number of road injury and interpersonal violence deaths from police records exceeded deaths in the vital registration data.

Infrequently, datapoints were marked as outliers. Outlier criteria excluded datapoints that (1) were implausibly high or low relative to global or regional patterns based on subject-matter or in-country experts, (2) substantially conflicted with established age or temporal patterns, or (3) significantly conflicted with higher-quality data sources conducted from the same locations or locations with similar characteristics (ie, Socio-demographic Index).

Modelling strategy

Overview

In GBD 2023, the standard CODEm modelling approach was applied to estimate deaths due to all causes of injury, excluding fatal discontinuities (see "Fatal discontinuities" section below, and appendix 1 section 4 for more about the CoD modelling approach). Refer to the table at the end of this section for a complete list of the cause-of-injury categories, modelling strategies, and covariate changes from GBD 2021.

GBD injury codes and categories

The International Classification of Diseases (ICD) was used to classify injuries. In GBD, injury incidence and death are defined as ICD-9 codes E000–E999 and ICD-10 chapters V to Y. There is one exception: deaths and cases of alcohol poisoning and drug overdoses are classified under drug and alcohol use disorders and modelled outside of the injury framework. Injury causes were organised into 29 mutually exclusive and collectively exhaustive external cause-of-injury categories.

Preparation of data

The preparation of cause-of-death data includes age-sex splitting, smoothing, and outlier detection. These steps are described in detail by Naghavi and colleagues and Lozano and colleagues.[1,2,3] We also found that some deaths are assigned an ICD code that links to a condition that does not lead to death (eg, senility), are overly vague and could be attributed to more than one cause of death (ie,"Exposure to unspecified factor" [X59 in ICD-10 and E887 in ICD-9]), are undetermined intent codes (Y10-Y34 in ICD-

10 and E980-E988 in ICD-9), are intermediate causes of death (ie, septicemia or peritonitis) or are an ill-defined and unknown cause of mortality (R99). We consider these codes as "garbage codes" and redistribute them based on methods described in GBD 2019.[4]

In countries with non-detail ICD code data, cause-of-injury categories were proportionally split into sublevel cause-of-injury categories. The sublevel cause-of-injury causes were created in the CoDCorrect process. For GBD iterations of 2015, 2016, 2017, 2019, 2021, and 2023, the proportions were based on post-mortem investigation of injury deaths as described in the paper by Matzopoulos and colleagues 2015.[5]

We added police data for road injuries and interpersonal violence in countries with sparse or absent cause of death data even though we know from countries with near-complete vital registration data that police records tend to underestimate the true level of deaths. However, we applied police data estimates in instances where reported deaths were higher than vital registration numbers. In these select instances, we were advised by in-country experts that the police data were more complete than vital registration data.

Due to nationwide protests by the Black Lives Matter movement in the USA in 2020 and increased national attention on the epidemic of police violence against Black Americans, input data on police conflict and executions for the USA were reviewed for completeness. We determined that the USA National Vital Statistics System (NVSS) systematically under-reports deaths due to police violence by about 50% every year. In order to quantify this bias, we ran a network meta-regression on NVSS data with direct comparisons by state and year to Mapping Police Violence (MPV),[7] an alternate open-source database that catalogs deaths due to police violence, and indirect comparisons to an additional source, Fatal Encounters (FE).[8] We believe that they more accurately capture deaths due to police violence because of they use open-source methodologies to identify police violence deaths, rather than relying on death certificates.[9,10,11] The regression included a fixed effect on state to capture different under-reporting rates across states but assumed that under-reporting rates are constant across age, sex, and year. Additionally, since MPV does not attempt to capture police killed by civilians and neither MPV nor FE attempts to capture executions, death counts from the FBI's Law Enforcement Officers Killed and Assaulted database and the Death Penalty Information Center (DPIC) were added to these data sources in order to conform them to the GBD definition of executions and police conflict.[12,13] During data processing, we added police conflict deaths in USA NVSS by pulling deaths from a selection of redistribution packages and GBD causes that are likely to contain the miscoded deaths. We took away deaths from interpersonal violence and related garbage packages first, then from GBD causes falls and exposure to mechanical forces and garbage packages exposure to unspecified factor (X59) and ill-defined. These groups of causes were selected based on literature review that found homicide as the main source of miscoded police conflict deaths.[9,14,15] Record linkage between NVSS and open-source databases has shown that interpersonal violence is the most common underlying cause of death listed on death certificates for mis-assigned police violence deaths.[6]

Injuries estimated as fatal discontinuities

Fatal discontinuity events, defined as spikes in a time series of deaths that are unexpected and introduce a "shock" of deaths that is not predictable through modelling, were estimated for ten injury causes that are also modelled in CODEm. These causes included "Other transport injuries", "Fire, heat,

and hot substances”, “Poisoning by other means”, “Other exposure to mechanical forces”, “Non-venomous animal contact”, “Environmental heat and cold exposure”, “Physical violence by firearm”, “Physical violence by sharp object”, “Physical violence by other means”, and “Executions and police conflict”. Final fatal discontinuity estimations for these causes were merged with CODEm results post-CoDCorrect to produce final cause of death results. For these causes, vital registration data are prioritised. Only when there are known inconsistencies in vital registration death estimates are “shock” deaths merged with CODEm results.

“Exposure to forces of nature,” and “Conflict and terrorism” are injuries that were modelled outside of the CODEm process only using fatal discontinuity estimation. Details on the fatal discontinuity estimation process can be found in its own section of this appendix.

Covariates

The following covariates were tested for each injury cause of death model. For GBD 2023, all covariates were re-evaluated to include additional known covariates that are associated with increased mortality for each injury cause. Education and lag-distributed income per capita (LDI) were systematically dropped from each cause because Healthcare Access and Quality Index and Socio-demographic Index covariates capture similar constructs.

TRANSPORT INJURIES

Table 2: Covariate changes from GBD 2021 to GBD 2023

ID	Cause	Modelling strategy	Covariate changes from GBD 2021
1	Transport injuries	CODEm	Dropped: Education, LDI
1.1	Road injuries	CODEm	Dropped: Education, LDI
1.1.1	Pedestrian road injuries	CODEm	Dropped: Education, LDI
1.1.2	Cyclist road injuries	CODEm	Dropped: Education, LDI
1.1.3	Motorcyclist road injuries	CODEm	Dropped: Education, LDI<br>Added: Vehicles – 2 wheels, per capita
1.1.4	Motor vehicle road injuries	CODEm	Dropped: Education, LDI
1.1.5	Other road injuries	CODEm	Dropped: Education, LDI
1.2	Other transport injuries	CODEm and fatal discontinuity estimation	Dropped: Education, LDI

Table 2.1: Transport injuries covariate levels and directions

Covariate	Transport injuries		Road injuries		Pedestrian road injuries		Cyclist road injuries		Motorcyclist road injuries		Motor vehicle road injuries		Other road injuries		Other transport injuries	
	Level	Direction	Level	Direction	Level	Direction	Level	Direction	Level	Direction	Level	Direction	Level	Direction	Level	Direction

BAC law professional drivers (quartile)	1	1	3	1	3	1	3	1	3	1	3	1	3	1	3	1
BAC law general population (quartile)	1	1	3	1	3	1	3	1	3	1	3	1	3	1	3	1
BAC law youth drivers (quartile)	1	1	3	1	3	1	3	1	3	1	3	1	3	1	3	1
Litres of alcohol consumed per capita	1	1	2	1	2	1	2	1	1	1	2	1	2	1	2	1
Speed limit law rural (quartile)	1	1	3	1	3	1	3	1	3	1	3	1	3	1	3	1
Speed limit law urban (quartile)	1	1	3	1	3	1	3	1	3	1	3	1	3	1	3	1
Vehicles - 2 wheels (per capita)	NA	NA	1	1	NA	NA	NA	NA	1	1	NA	NA	NA	NA	NA	NA
Vehicles - 2 wheels fraction (proportion)	1	1	1	1	1	1	1	1	1	1	NA	NA	1	1	1	1
Vehicles - 2+4 wheels (per capita)	1	1	1	1	1	1	1	1	NA	NA	NA	NA	1	1	1	1
Vehicles - 4 wheels (per capita)	NA	NA	1	1	NA	NA	NA	NA	NA	NA	1	1	NA	NA	NA	NA
Healthcare Access and Quality Index	2	-1	2	-1	2	-1	2	-1	2	-1	2	-1	2	-1	2	-1
Population 15 to 30 (proportion)	2	1	2	1	2	1	2	1	2	1	2	1	2	1	2	1
Population density (300-500 ppl/sqkm, proportion)	2	1	2	1	2	1	2	1	2	1	2	1	NA	NA	2	1
Population density (500-1000 ppl/sqkm, proportion)	2	1	2	1	2	1	2	1	2	1	2	1	NA	NA	2	1
Population-weighted mean temperature	2	1	2	1	2	1	2	1	2	1	2	1	2	1	2	1
Socio-demographic Index	2	-1	2	-1	2	-1	2	-1	2	-1	2	-1	2	-1	2	-1
Rainfall quintile 5 (proportion)	3	1	3	1	3	1	3	1	3	1	3	1	3	1	3	1
Log-transformed SEV scalar: Road Inj	NA	NA	1	1	NA	NA	NA	NA	NA	NA	NA	NA	NA	NA	NA	NA
Log-transformed SEV scalar: Pedest	NA	NA	NA	NA	1	1	NA	NA	NA	NA	NA	NA	NA	NA	NA	NA
Log-transformed SEV scalar: Cyclist	NA	NA	NA	NA	NA	NA	1	1	NA	NA	NA	NA	NA	NA	NA	NA
Log-transformed SEV scalar: Mot Cyc	NA	NA	NA	NA	NA	NA	NA	NA	1	1	NA	NA	NA	NA	NA	NA
Log-transformed SEV scalar: Mot Veh	NA	NA	NA	NA	NA	NA	NA	NA	NA	NA	1	1	NA	NA	NA	NA

Log-transformed SEV scalar: Oth Road	NA	NA	NA	NA	NA	NA	NA	NA	NA	NA	NA	NA	1	1	NA	NA
Log-transformed SEV scalar: Oth Trans	NA	NA	NA	NA	NA	NA	NA	NA	NA	NA	NA	NA	NA	NA	1	1

Covariate level is grouped by strong biological link (1), strong evidence of a relationship (2), or weak relationship (3). The direction indicates whether the covariate and cause of death change in the same direction (1) or opposite (-1).

Figure 2a: Transport injuries covariate influence plot

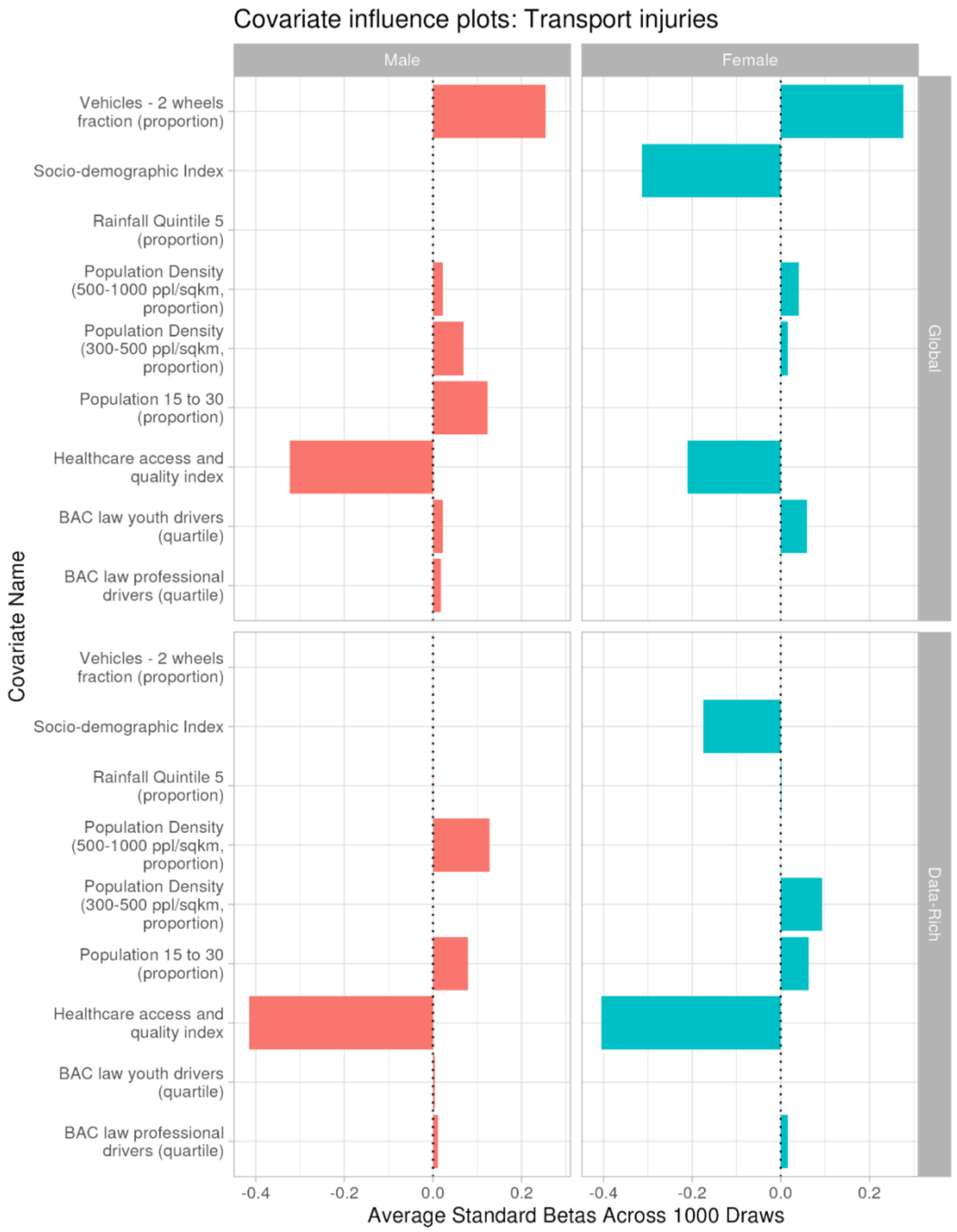

Figure 2b: Road injuries covariate influence plot

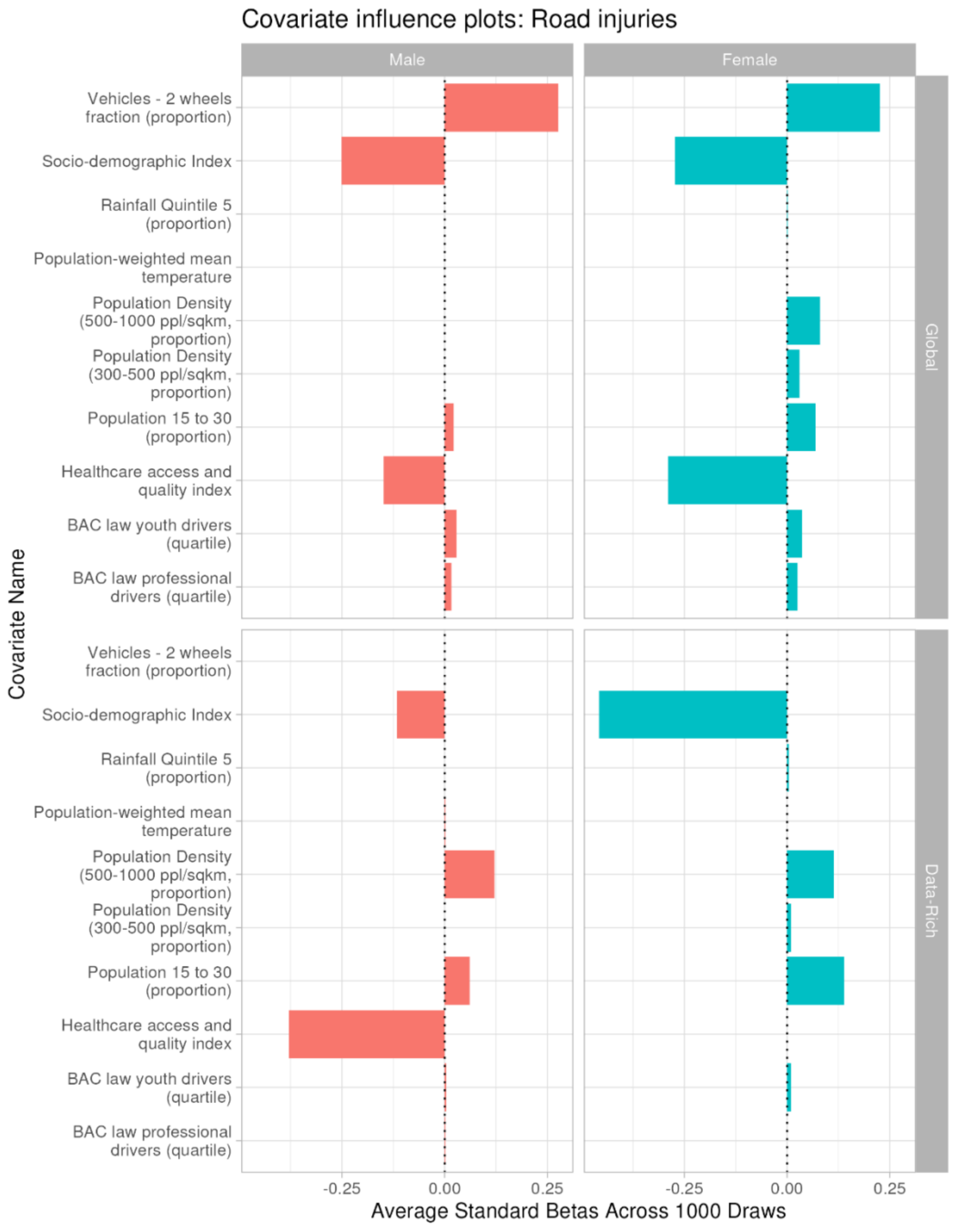
Covariate influence plots: Road injuries
Male
Female
Global
Data-Rich
Covariate Name
Vehicles - 2 wheels fraction (proportion)
Socio-demographic Index
Rainfall Quintile 5 (proportion)
Population-weighted mean temperature
Population Density (500-1000 ppl/sqkm, proportion)
Population Density (300-500 ppl/sqkm, proportion)
Population 15 to 30 (proportion)
Healthcare access and quality index
BAC law youth drivers (quartile)
BAC law professional drivers (quartile)
-0.25
0.00
0.25
Average Standard Betas Across 1000 Draws

UNINTENTIONAL INJURIES

Table 3: Covariate changes from GBD 2021 to GBD 2023

ID	Cause	Modelling strategy	Covariate changes from GBD 2021
2	Unintentional injuries	Not modelled at parent cause level	
2.1	Falls	CODEm	Dropped: Education, LDI
2.2	Drowning	CODEm	Added: Maternal education<br>Dropped: LDI, education
2.3	Fire, heat, and hot substances	CODEm and fatal discontinuity estimation	Dropped: Education, LDI<br>Added: Litres of alcohol consumed per capita
2.4	Poisonings	CODEm	Added: Maternal education, Proportion of population involved in agricultural activities<br>Dropped: Education, LDI
2.4.1	Poisoning by carbon monoxide	CODEm	Added: Maternal education, Proportion of population involved in agricultural activities, Indoor Air Pollution (All Cooking Fuels)<br>Dropped: Education, LDI
2.4.2	Poisoning by other means	CODEm and fatal discontinuity estimation	Added: Maternal education, Proportion of population involved in agricultural activities<br>Dropped: Education, LDI
2.5	Exposure to mechanical forces	CODEm	Dropped: Education, LDI
2.5.1	Unintentional firearm injuries	CODEm	Dropped: Education, LDI, Population-weighted mean temperature
2.5.2	Other exposure to mechanical forces	CODEm and fatal discontinuity estimation	Dropped: Education, LDI
2.6	Adverse effects of medical treatment	CODEm	Dropped: Education, LDI
2.7	Animal contact	CODEm	Dropped: Education, LDI
2.7.1	Venomous animal contact	CODEm	Dropped: Education, LDI
2.7.2	Non-venomous animal contact	CODEm and fatal discontinuity estimation	Dropped: Education, LDI
2.8	Foreign body	CODEm	Dropped: Education, LDI

			Added: Foreign body SEV scalar
2.8.1	Pulmonary aspiration and foreign body in airway	CODEm	Dropped: Education, LDI
2.8.2	Foreign body in other body part	CODEm	Dropped: Education, LDI
2.9	Environmental exposure to heat and cold	CODEm and fatal discontinuity estimation	Dropped: LDI and education only for male models, elevation 500 m – 1500 m for both<br>Added: Litres of alcohol consumed per capita
2.10	Exposure to forces of nature	Fatal discontinuity estimation	
2.11	Other unintentional injuries	CODEm and fatal discontinuity estimation	Dropped: Education, LDI
2.12	Electrocution	CODEm	Added: Population density (under 150 ppl/sqkm, proportion), population density (over 1000 ppl/sqkm, proportion), unintentional injuries SEV scalar, Socio-demographic Index, Healthcare Access and Quality Index

Table 3.1: Unintentional injuries covariate levels and directions

Covariate	Falls		Drowning		Fire, heat, and hot substances	
	Level	Direction	Level	Direction	Level	Direction
Litres of alcohol consumed per capita	1	1	NA	NA	2	1
Log-transformed SEV scalar: Falls	1	1	NA	NA	NA	NA
Healthcare Access and Quality Index	2	-1	3	-1	2	-1
Population-weighted mean temperature	2	-1	1	1	2	1
Elevation over 1500 m (proportion)	3	1	NA	NA	NA	NA
Socio-demographic Index	3	-1	3	-1	3	-1
Coastal population within 10 km (proportion)	NA	NA	1	1	NA	NA
Landlocked nation (binary)	NA	NA	1	-1	NA	NA

Log-transformed SEV scalar: Drown	NA	NA	1	1	NA	NA
Rainfall quintile 1 (proportion)	NA	NA	2	-1	NA	NA
Rainfall quintile 5 (proportion)	NA	NA	2	1	NA	NA
Elevation under 100 m (proportion)	NA	NA	2	1	NA	NA
Log-transformed SEV scalar: Fire	NA	NA	NA	NA	1	1
Indoor air pollution (all cooking fuels)	NA	NA	NA	NA	1	1
Population density (over 1000 ppl/sqkm, proportion)	NA	NA	NA	NA	2	1
Tobacco (cigarettes per capita)	NA	NA	NA	NA	2	1

Covariate level is grouped by strong biological link (1), strong evidence of a relationship (2), or weak relationship (3). The direction indicates whether the covariate and cause of death change in the same direction (1) or opposite (-1).

Table 3.2: Poisonings covariate levels and directions

Covariate	Poisonings		Poisonings by carbon monoxide		Poisoning by other means	
	Level	Direction	Level	Direction	Level	Direction
Maternal education (years per capita)	1	-1	1	-1	1	-1
Litres of alcohol consumed per capita	2	1	2	1	2	1
Healthcare Access and Quality Index	2	-1	3	-1	3	-1
Population-weighted mean temperature	1	-1	1	-1	2	-1
Socio-demographic Index	2	-1	3	-1	3	-1
Population density (over 1000 ppl/sqkm, proportion)	3	-1	NA	NA	NA	NA
Population density (under 150 ppl/sqkm, proportion)	3	1	NA	NA	NA	NA
Proportion of population involved in agricultural activities	1	1	3	1	1	1
Log-transformed SEV scalar: Poison	1	1	NA	NA	NA	NA

Log-transformed SEV scalar: Inj pois CO	NA	NA	1	1	NA	NA
Log-transformed SEV scalar: Inj pois Oth	NA	NA	NA	NA	1	1

Covariate level is grouped by strong biological link (1), strong evidence of a relationship (2), or weak relationship (3). The direction indicates whether the covariate and cause of death change in the same direction (1) or opposite (-1).

Table 3.3: Exposure to mechanical forces covariate levels and directions

Covariate	Exposure to mechanical forces		Unintentional firearm injuries		Other exposure to mechanical forces	
	Level	Direction	Level	Direction	Level	Direction
Education (years per capita)	NA	NA	3	-1	NA	NA
Healthcare Access and Quality Index	2	-1	2	-1	2	-1
Population-weighted mean temperature	1	1	1	1	1	1
LDI (I$ per capita)	NA	NA	3	-1	NA	NA
Socio-demographic Index	3	-1	3	-1	3	-1
Population density (over 1000 ppl/sqkm, proportion)	2	1	3	-1	2	-1
Population density (under 150 ppl/sqkm, proportion)	2	-1	2	-1	2	1
Log-transformed SEV scalar: Mech gun	NA	NA	1	1	NA	NA
Log-transformed SEV scalar: Oth mech	NA.	NA	NA	NA	1	1

Covariate level is grouped by strong biological link (1), strong evidence of a relationship (2), or weak relationship (3). The direction indicates whether the covariate and cause of death change in the same direction (1) or opposite (-1).

Table 3.4: Animal contact covariate levels and directions

Covariate	Animal contact		Venomous animal contact		Non-venomous animal contact	
	Level	Direction	Level	Direction	Level	Direction
Litres of alcohol consumed per capita	1	1	NA	NA	1	1
Healthcare Access and Quality Index	2	-1	2	1	2[a]	-1
Population-weighted mean temperature	1	1	2	1	1	1
Elevation over 1500 m (proportion)	3	-1	3	-1	3[b]	-1

LDI (I$ per capita)	NA	NA	NA	NA	NA	NA
Socio-demographic Index	3	-1	3	-1	3	-1
Elevation under 100 m (proportion)	3	1	3	-1	3[b]	1
Population density (over 1000 ppl/sqkm, proportion)	3	-1	3	-1	3[b]	-1
Population density (under 150 ppl/sqkm, proportion)	3	1	3	1	3[b]	1
Log-transformed SEV scalar: Animal	1	1	NA	NA	NA	NA
Population 15 to 30 (proportion)	2	1	NA	NA	NA	NA
Log-transformed SEV scalar: Venom	NA	NA	1	1	NA	NA
Mean number of venomous snake species	NA	NA	1	1	NA	NA
Proportion of population vulnerable to snake species	NA	NA	1	1	NA	NA
Absolute value of average latitude	NA	NA	2	-1	NA	NA
Rainfall population-weighted (mm/yr)	NA	NA	2	1	NA	NA
Proportion of population involved in agricultural activities	NA	NA	2	1	NA	NA
Sahel region of Africa (binary)	NA	NA	2[d]	1	NA	NA
Urbanicity	NA	NA	2	-1	NA	NA
Log-transformed SEV scalar: Non ven	NA	NA	NA	NA	1	1

a: Used at Level 3 in male global model
b: Used at Level 1 in male global model, Level 3 for the other three models
c: Not used in male global model
Covariate level is grouped by strong biological link (1), strong evidence of a relationship (2), or weak relationship (3). The direction indicates whether the covariate and cause of death change in the same direction (1) or opposite (-1).

Table 3.5: Foreign body covariate levels and directions

Covariate	Foreign body		Pulmonary aspiration and foreign body in airway		Foreign body in other body part	
	Level	Directions	Level	Directions	Level	Directions
Education (years per capita)	NA	NA	NA	NA	NA	NA

Litres of alcohol consumed per capita	1	1	1	1	1	1
Healthcare Access and Quality Index	2	-1	2	-1	2	-1
Population-weighted mean temperature	1	1	1	1	1	1
LDI (I$ per capita)	NA	NA	NA	NA	NA	NA
Socio-demographic Index	3	-1	3	-1	3	-1
Indoor air pollution (all cooking fuels)	1	1	NA	NA	NA	NA
Population over 65 (proportion)	1	1	NA	NA	NA	NA
Log-transformed SEV scalar: Foreign body asp	NA	NA	1	1	NA	NA
Alcohol binge drinker proportion, age-standardised	NA	NA	2[k]	1	NA	NA
Mean BMI	NA	NA	2	1	NA	NA
Log-transformed SEV scalar: Other foreign body	NA	NA	NA	NA	1	1
Log-transformed SEV scalar: Foreign body	1	1	NA	NA	NA	NA

k: Only used in the female global model
Covariate level is grouped by strong biological link (1), strong evidence of a relationship (2), or weak relationship (3). The direction indicates whether the covariate and cause of death change in the same direction (1) or opposite (-1).

Table 3.6: Other unintentional injuries covariates level and directions

Covariate	Adverse effects of medical treatment		Other unintentional injuries		Environmental exposure to heat and cold		Electrocution	
	Level	Direction	Level	Direction	Level	Direction	Level	Direction
Education (years per capita)	NA	NA	NA	NA	3	-1	NA	NA
Healthcare Access and Quality Index	1	-1	2	-1	2	-1	2	-1
Population-weighted mean temperature	2	1	1	1	3	1	NA	NA
LDI (I$ per capita)	NA	NA	NA	NA	3	-1	NA	NA
Socio-demographic Index	3	-1	3	-1	3	-1	3	-1

Population density (over 1000 ppl/sqkm proportion)	NA	NA	3	-1	NA	NA	3	-1
Population density (under 150 ppl/sqkm proportion)	NA	NA	3	1	NA	NA	3	1
Population density (150-300 ppl/sqkm proportion)	NA	NA	NA	NA	3	-1	NA	NA
Log-transformed SEV scalar: Oth unintent	NA	NA	3	1	NA	NA	1	1
Vehicles – 2 wheels (per capita)	NA	NA	1	1	NA	NA	NA	NA
Vehicles – 4 wheels (per capita)	NA	NA	1	1	NA	NA	NA	NA
Elevation over 1500 m (proportion)	NA	NA	1	1	3	1	NA	NA
90th percentile climatic temperature in the given county-year	NA	NA	NA	NA	2	-1	NA	NA
Rainfall (quintiles 4-5)	NA	NA	NA	NA	3	1	NA	NA
Sanitation (proportion with access)	NA	NA	NA	NA	3	-1	NA	NA
Litres of alcohol consumed per capita	NA	NA	1	1	1	1	NA	NA

Covariate level is grouped by strong biological link (1), strong evidence of a relationship (2), or weak relationship (3). The direction indicates whether the covariate and cause of death change in the same direction (1) or opposite (-1).

Figure 3a: Drowning covariate influence plot

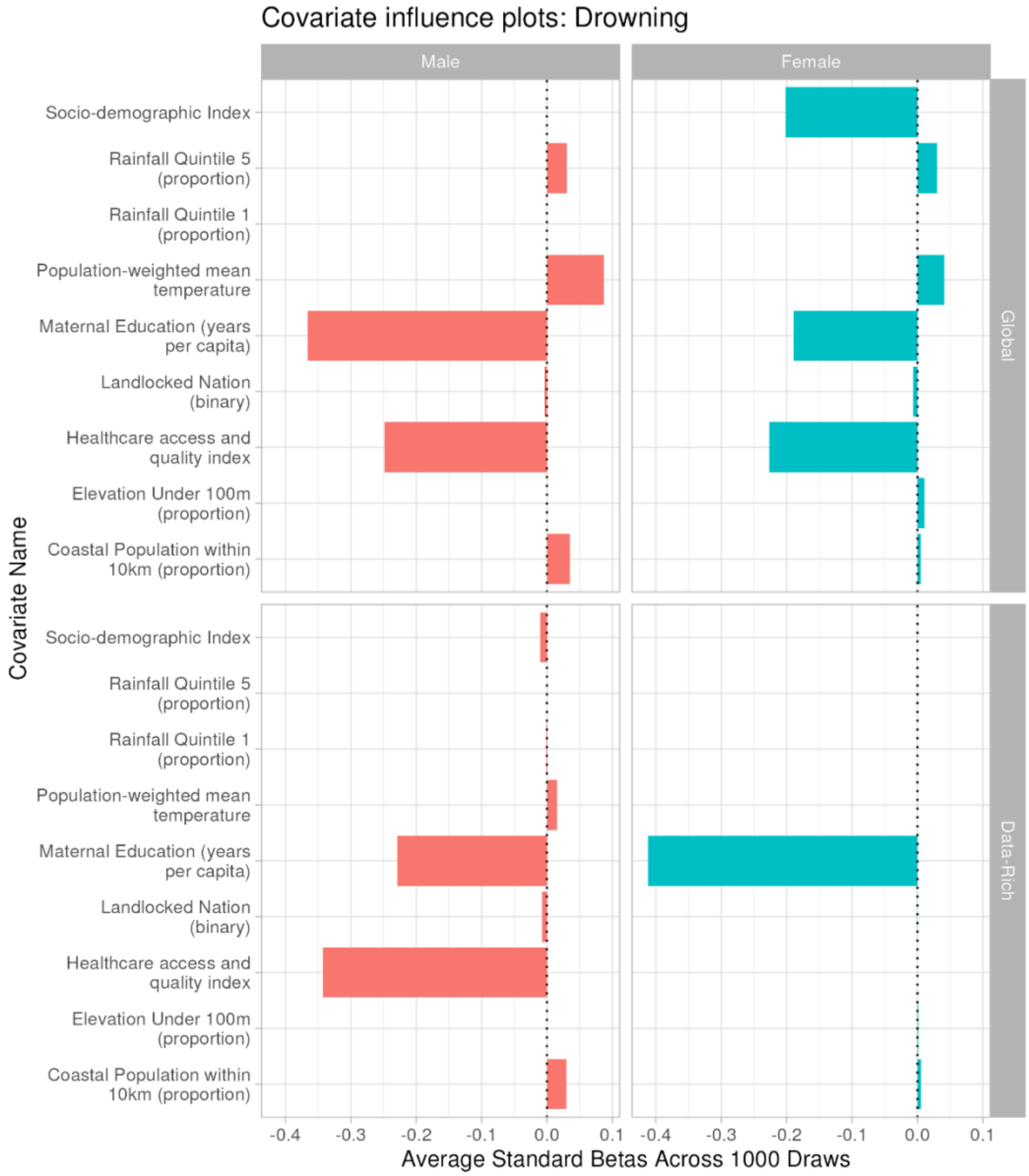

Figure 3b: Falls covariate influence plot

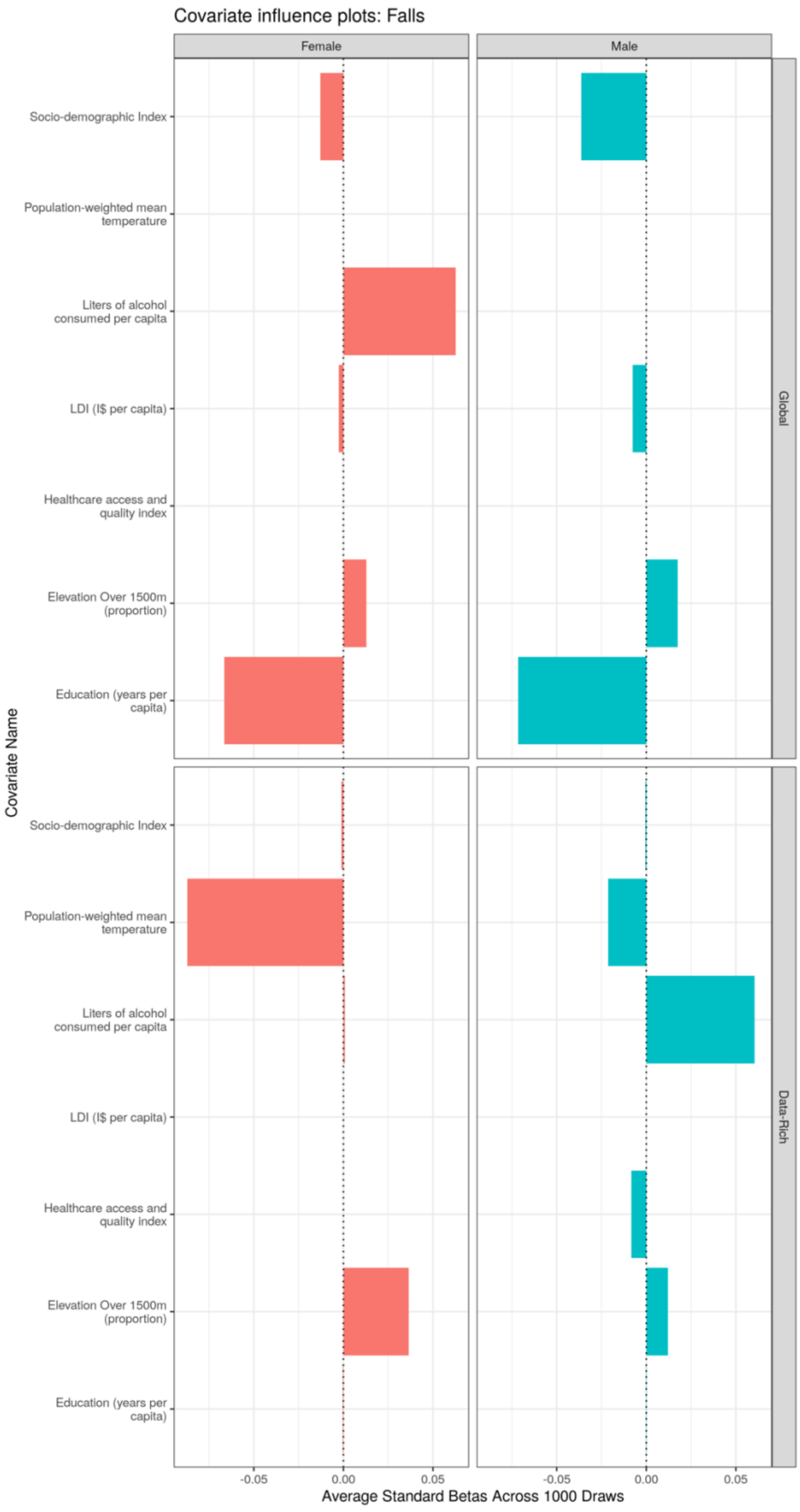

Figure 3c: Fire, heat, and hot substances covariate influence plot

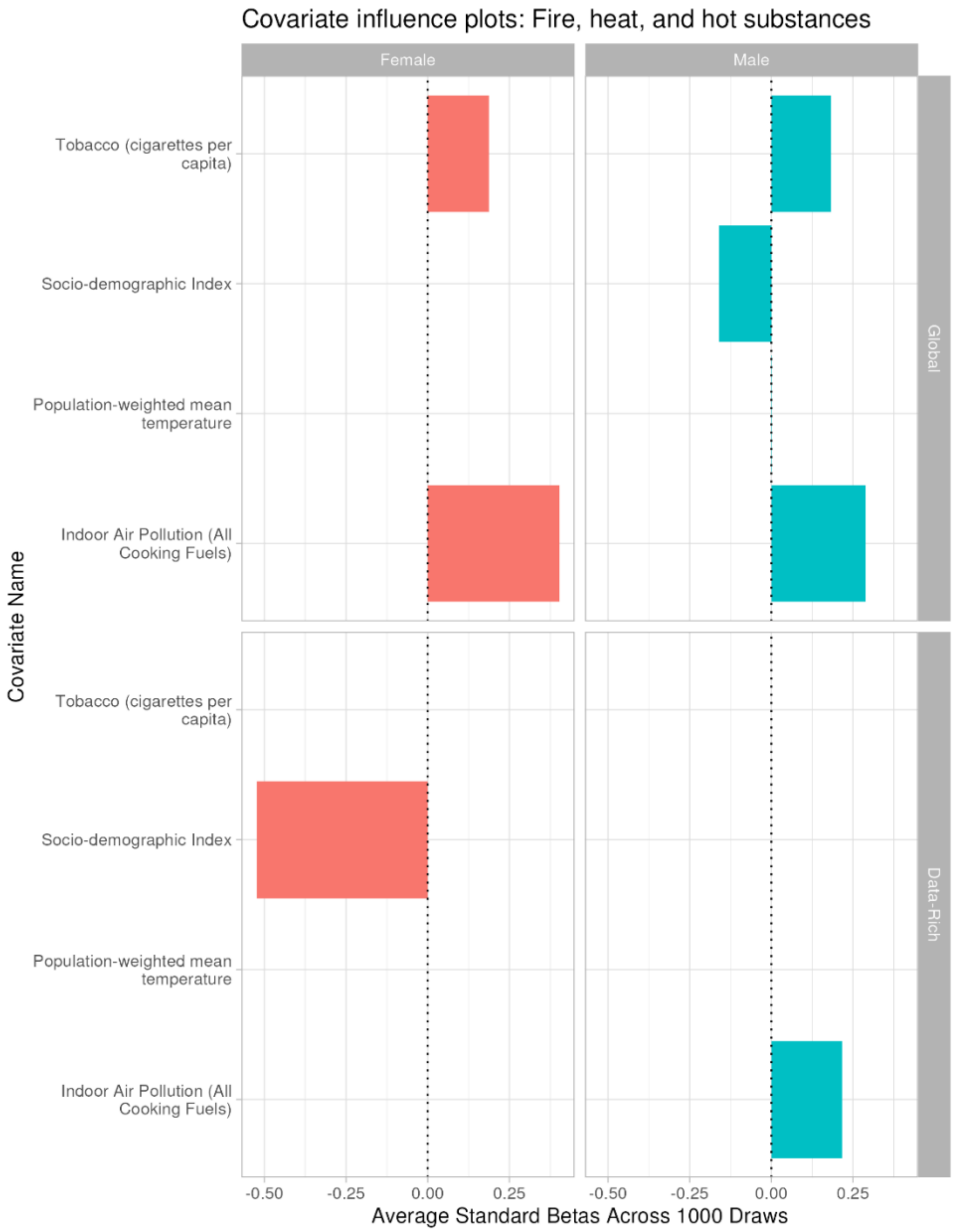

Figure 3d: Poisonings covariate influence plot

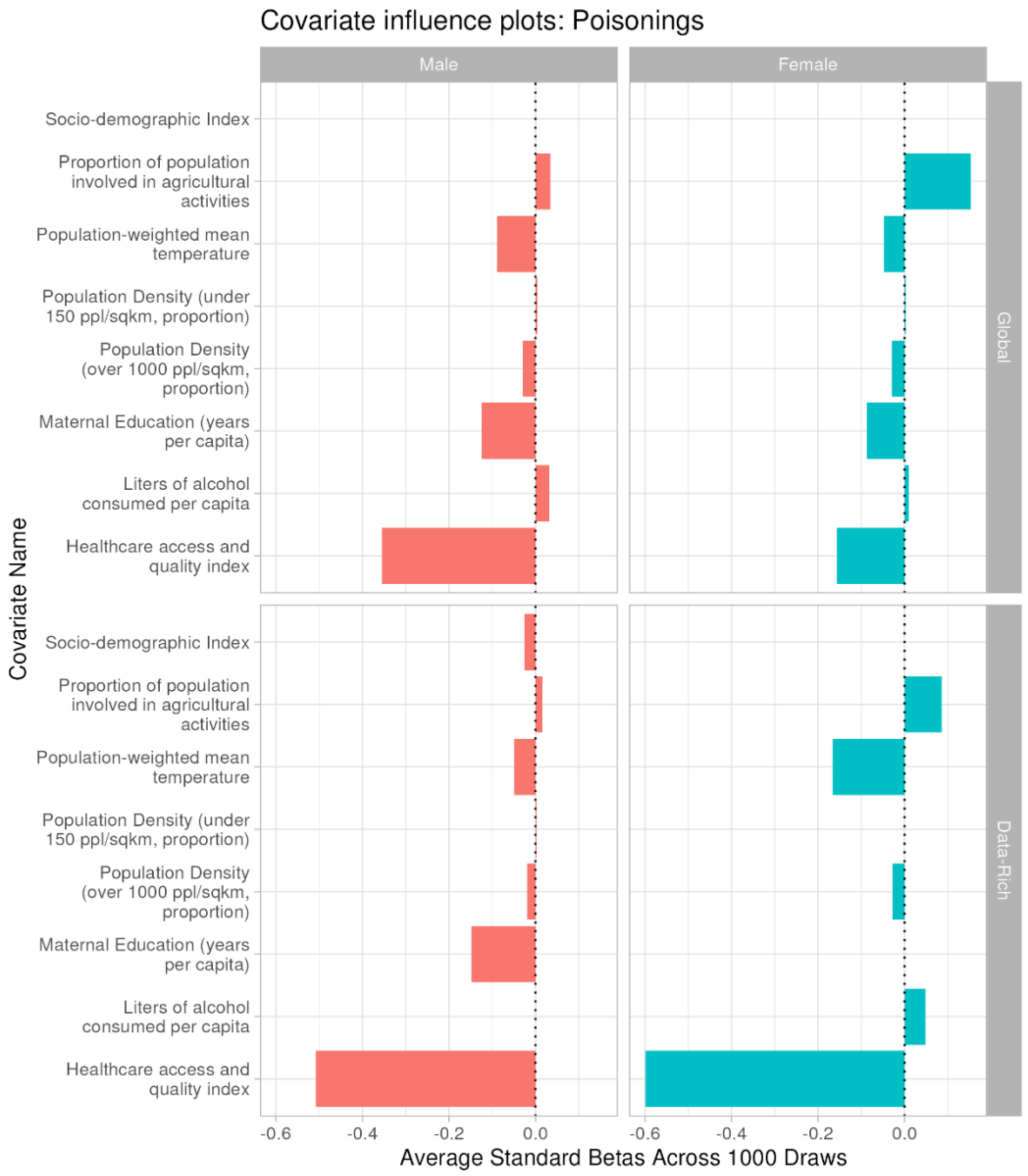

Figure 3e: Exposure to mechanical forces covariate influence plot

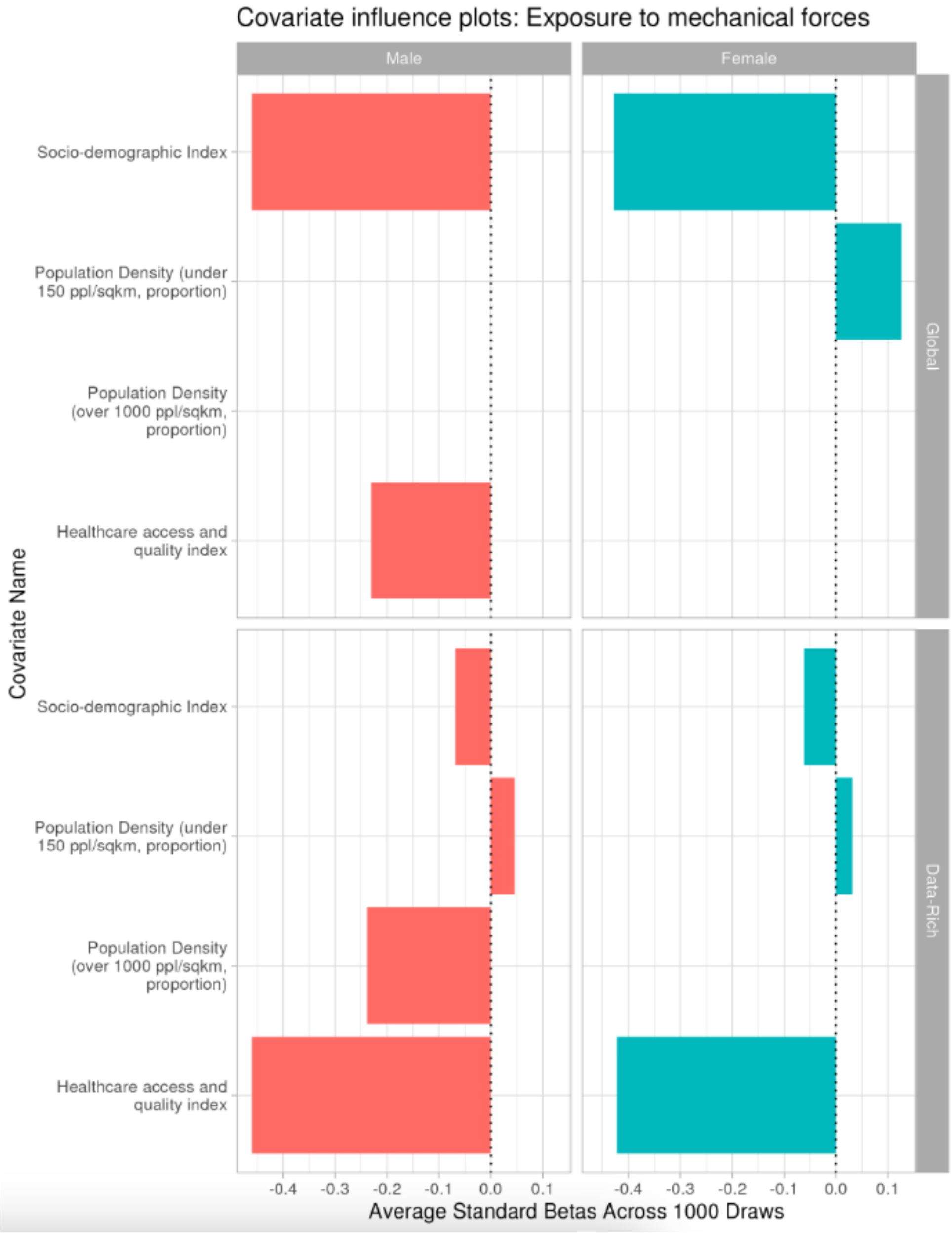

Figure 3f: Animal contact covariate influence plot

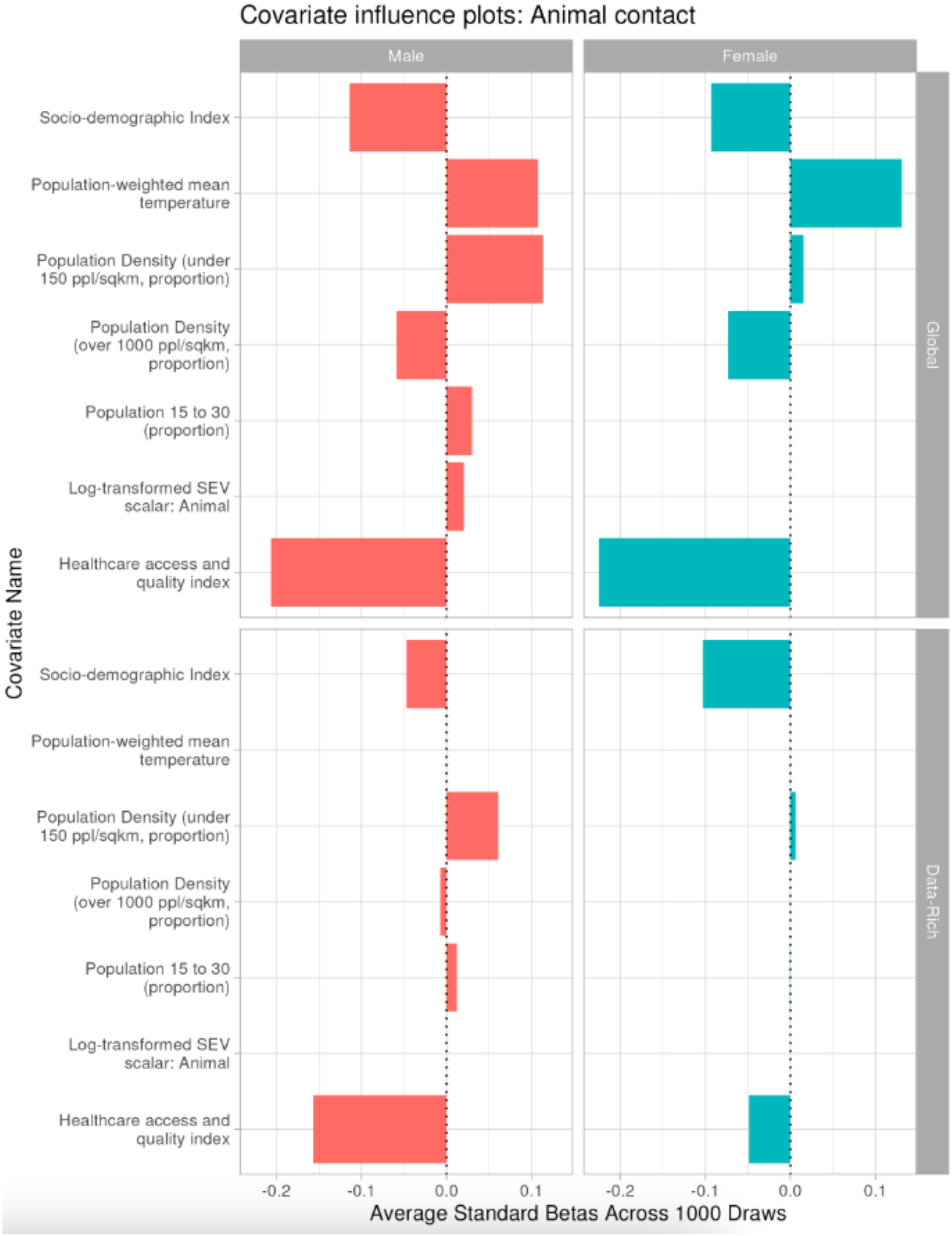

Figure 3g: Foreign body covariate influence plot

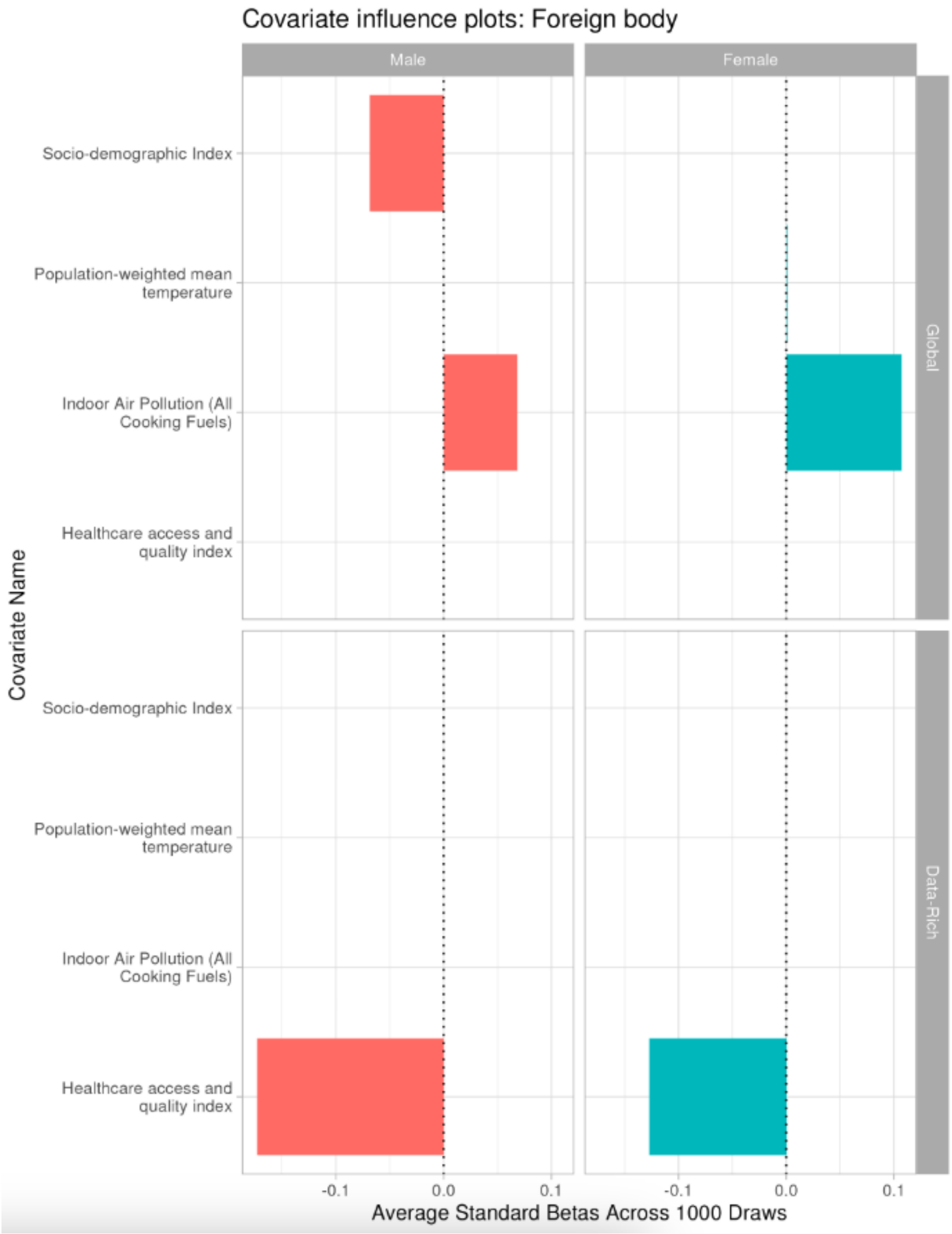

Figure 3h: Other unintentional injuries covariate influence plot

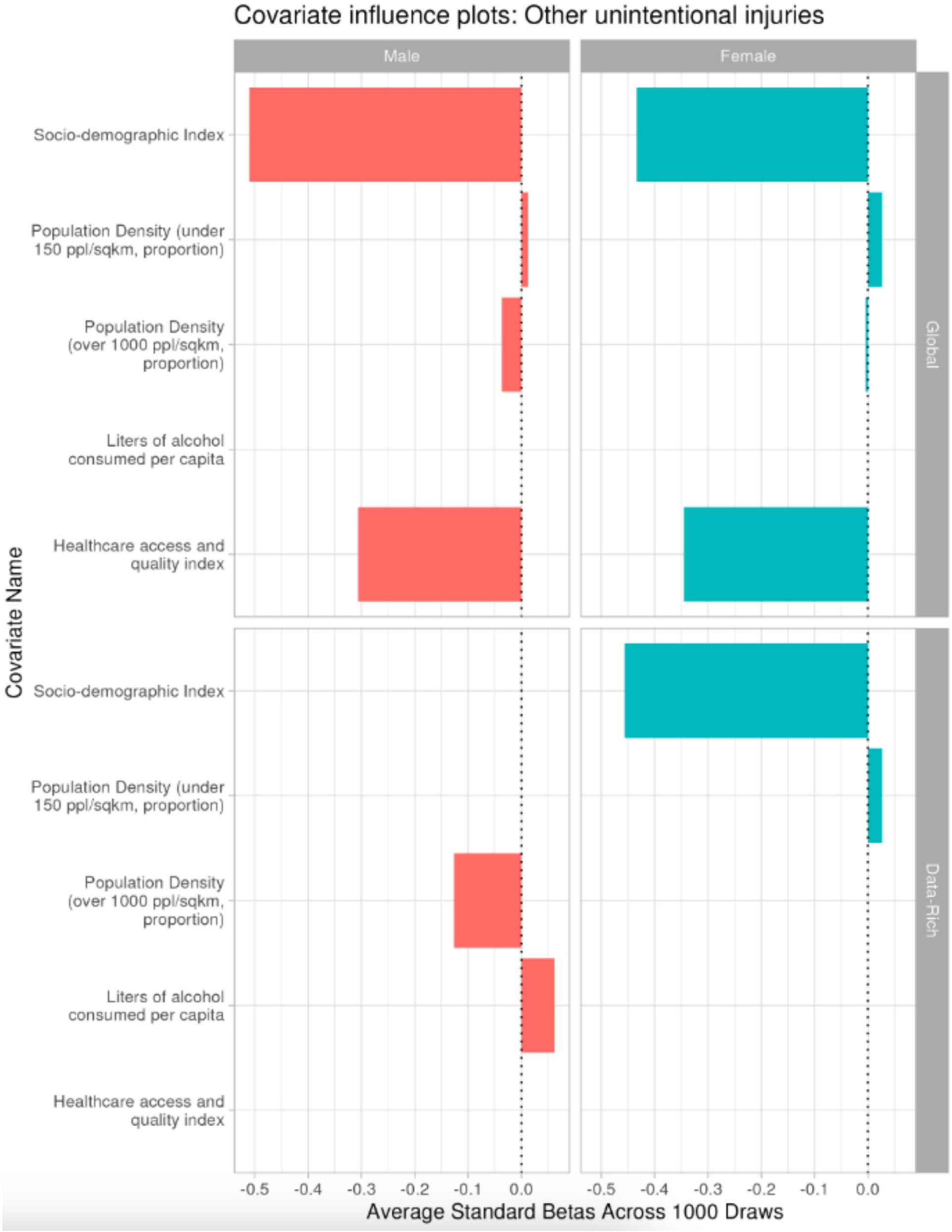

Figure 3i: Adverse effects of medical treatment covariate influence plot

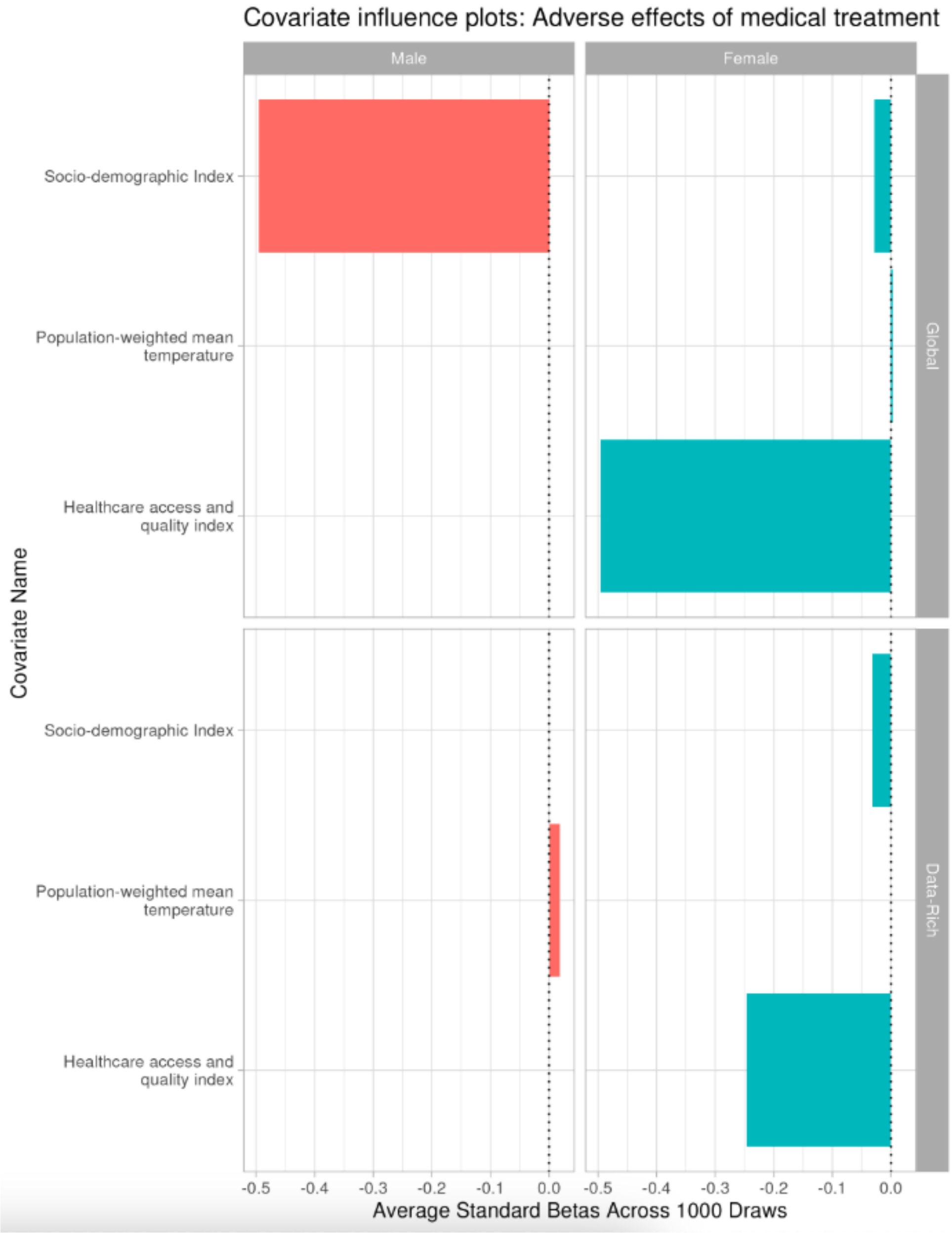

Figure 3j: Environmental heat and cold exposure

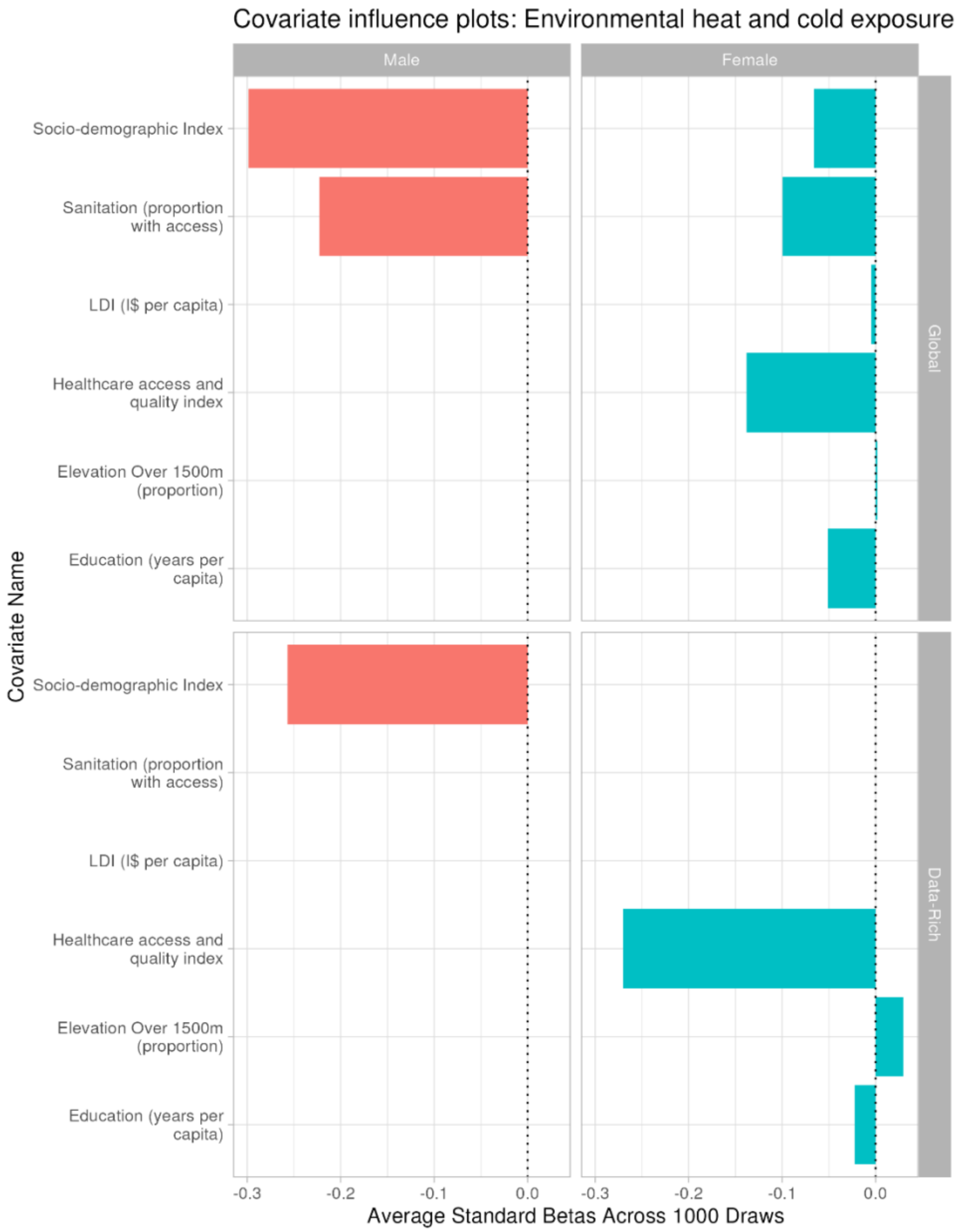

Figure 3k: Electrocution

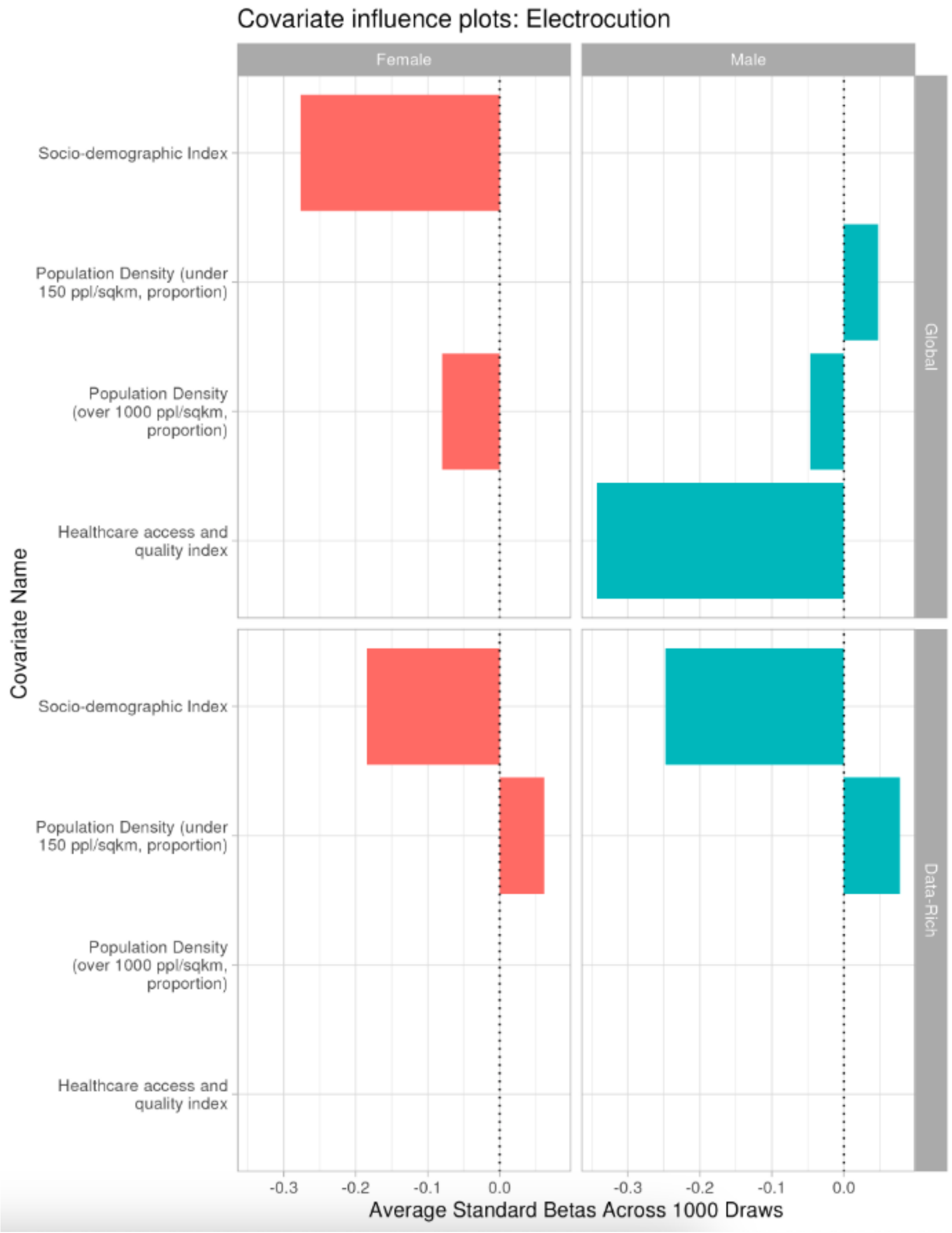

SELF-HARM AND INTERPERSONAL VIOLENCE

Table 4: Covariate changes from GBD 2021 to GBD 2023

ID	Cause	Modelling strategy	Covariate changes from GBD 2021
3	Self-harm and interpersonal violence	Not modelled at parent cause level	

3.1	Self-harm	CODEm	Dropped: 12-month non-partner sexual violence, Proportion of the population that is Muslim
3.1.3	Self-harm by firearm	CODEm	Dropped: 12-month non-partner sexual violence
3.1.6	Self-harm by other specified means	CODEm	Dropped: 12-month non-partner sexual violence
3.2	Interpersonal violence	CODEm	
3.2.1	Physical violence by firearm	CODEm and fatal discontinuity estimation	
3.2.2	Physical violence by sharp object	CODEm and fatal discontinuity estimation	
3.2.3	Physical violence by other means	CODEm and fatal discontinuity estimation	
3.3	Conflict and terrorism	Fatal discontinuity estimation	
3.4	Executions and police conflict	CODEm and fatal discontinuity estimation	

Table 4.1. Self-harm covariate levels and directions

Covariate	Self-harm		Self-harm by firearm		Self-harm by other specified means	
	Level	Direction	Level	Direction	Level	Direction
Litres of alcohol consumed per capita	1	1	1	1	1	1
Log-transformed SEV scalar: Self-harm	1	1	1	1	1	1

Major depressive disorder	1	1	1	1	1	1
Population-weighted mean temperature	1	1	1	1	1	1
Healthcare Access and Quality Index	2	-1	2	-1	2	-1
Population density (150-300 ppl/sqkm, proportion)	2	1	2	1	2	1
Population density (300-500 ppl/sqkm, proportion)	2	-1	2	-1	2	-1
Population density (500-1000 ppl/sqkm, proportion)	2	-1	2	-1	2	-1
Population density (over 1000 ppl/sqkm, proportion)	2	-1	2	-1	2	-1
Population density (under 150 ppl/sqkm, proportion)	2	1	2	1	2	1
Education (years per capita)	3	-1	3	-1	3	-1
LDI (I$ per capita)	3	-1	3	-1	3	-1
Socio-demographic Index	3	-1	3	-1	3	-1

Covariate level is grouped by strong biological link (1), strong evidence of a relationship (2), or weak relationship (3). The direction indicates whether the covariate and cause of death change in the same direction (1) or opposite (-1).

Table 4.2: Interpersonal violence covariate level and directions

Covariate	Interpersonal violence		Execution and police conflict	
	Level	Direction	Level	Direction
Litres of alcohol consumed per capita	1	1	1	1
Log-transformed SEV scalar: Interpersonal Violence	1	1	NA	NA
Healthcare Access and Quality Index	2	1	2	-1
Population density (150-300 ppl/sqkm, proportion)	NA	NA	NA	NA
Population density (over 1000 ppl/sqkm, proportion)	2	1	2	1
Population density (under 150 ppl/sqkm, proportion)	NA	NA	NA	NA
Education (years per capita)	3	-1	3	1
LDI (I$ per capita)	3	-1	3	-1
Socio-demographic Index	3	-1	2	-1
Education relative inequality (Gini)	1	1	NA	NA
Log-transformed SEV scalar: Violence	NA	NA	NA	NA
Population 15 to 30 males (proportion)	1	1	1	1
Log-transformed SEV scalar: Viol gun	1	1	NA	NA
Population-weighted mean temperature	1	1	1	1

Covariate level is grouped by strong biological link (1), strong evidence of a relationship (2), or weak relationship (3). The direction indicates whether the covariate and cause of death change in the same direction (1) or opposite (-1).

Table 4.3: Physical violence covariate level and directions

Covariate	Physical violence by firearm		Physical violence by sharp object		Physical violence by other means	
	Level	Direction	Level	Direction	Level	Direction
Litres of alcohol consumed per capita	1	1	1	1	1	1
Population-weighted mean temperature	1	1	1	1	1	1
Healthcare Access and Quality Index	2	-1	2	-1	2	-1

Population density (over 1000 ppl/sqkm, proportion)	2	1	2	1	2	1
Education (years per capita)	3	-1	3	-1	3	-1
LDI (I$ per capita)	3	-1	3	-1	3	-1
Socio-demographic Index	3	-1	3	-1	3	-1
Education relative inequality (Gini)	1	1	1	1	1	1
Population 15 to 30 males (proportion)	1	1	1	1	1	1
Log-transformed SEV scalar: Viol gun	1	1	NA	NA	NA	NA
Log-transformed SEV scalar: Viol knife	NA	NA	1	1	NA	NA
Log-transformed SEV scalar: Oth viol	NA	NA	NA	NA	1	1

Covariate level is grouped by strong biological link (1), strong evidence of a relationship (2), or weak relationship (3). The direction indicates whether the covariate and cause of death change in the same direction (1) or opposite (-1).

Figure 4a: Self-harm covariate influence plot

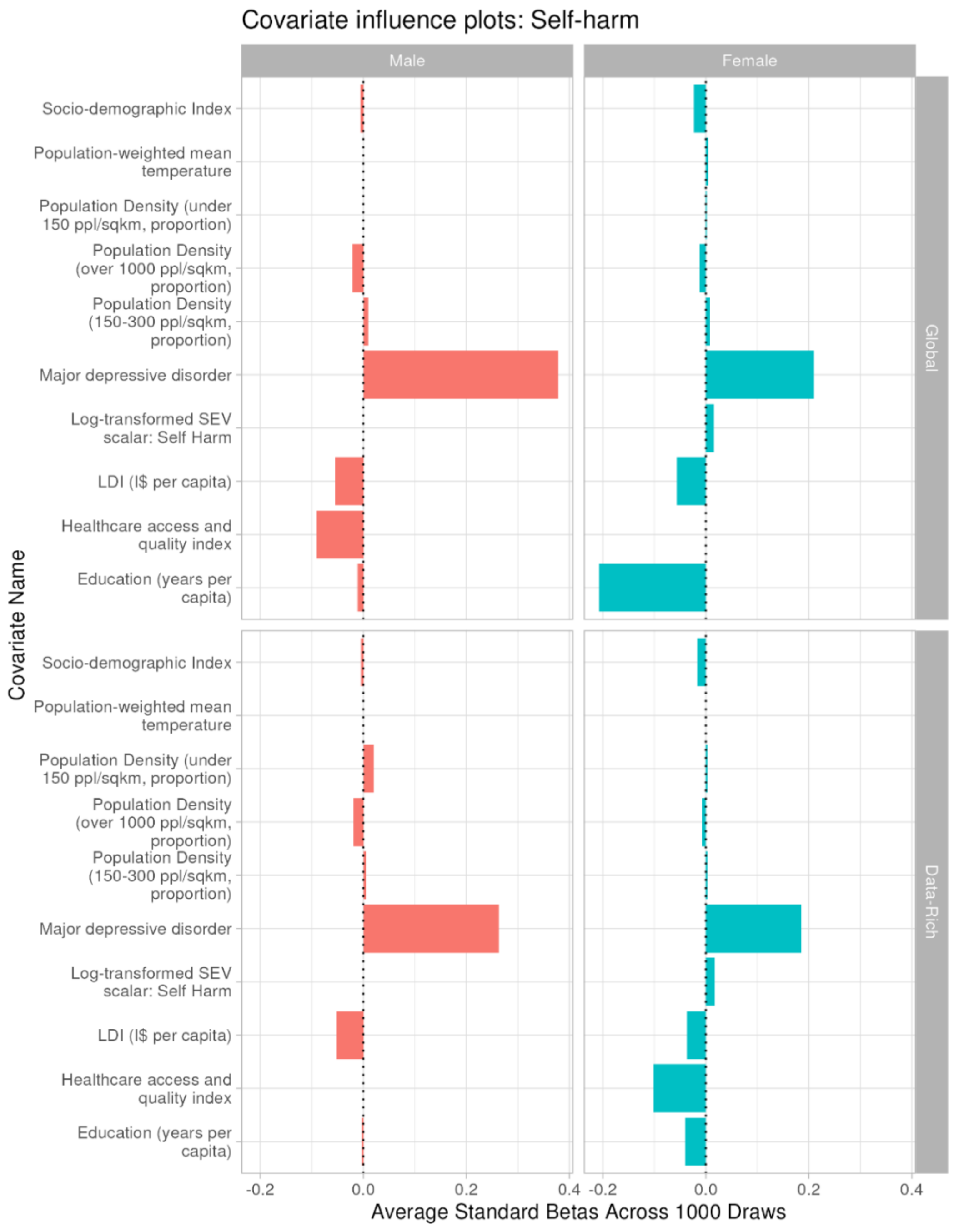

Figure 4b: Interpersonal violence covariate influence plot

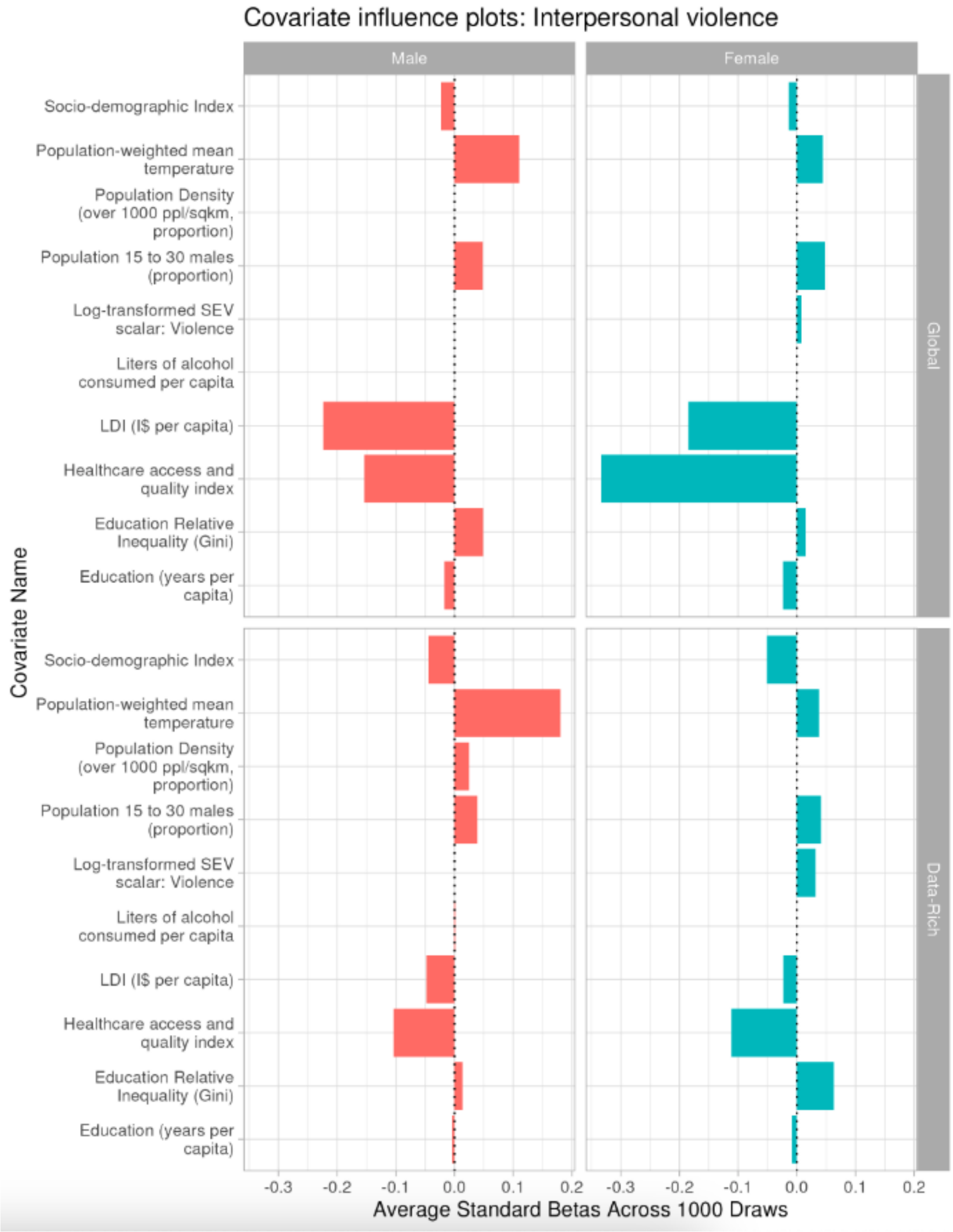

Figure 4d: Police conflict and executions covariate influence plot

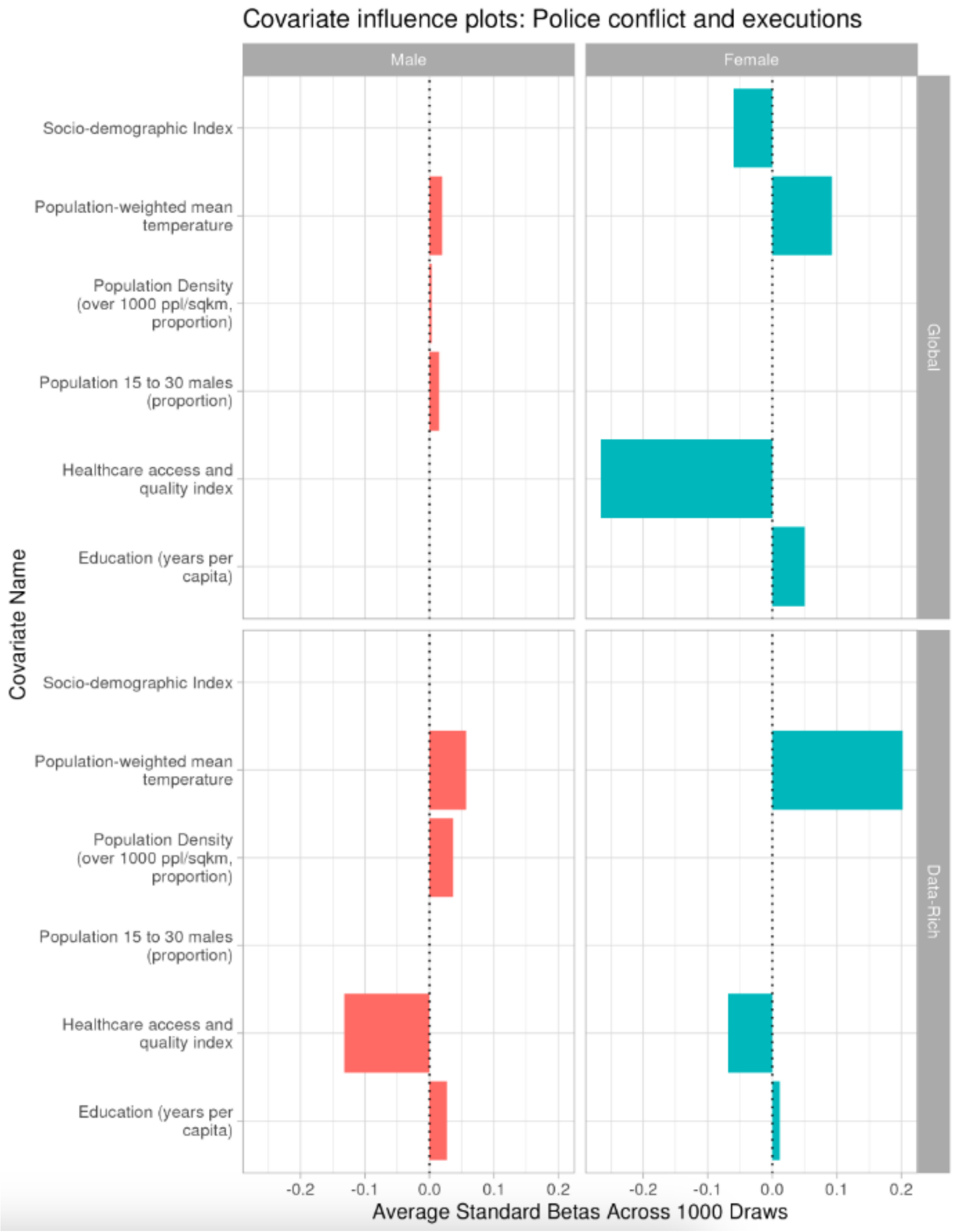

References

1 Lozano R, Naghavi M, Foreman K, *et al.* Global and regional mortality from 235 causes of death for 20 age groups in 1990 and 2010: a systematic analysis for the Global Burden of Disease Study 2010. *The Lancet* 2012; **380**: 2095–128.

2 Global, regional, and national age–sex specific all-cause and cause-specific mortality for 240 causes of death, 1990–2013: a systematic analysis for the Global Burden of Disease Study 2013. *The Lancet* 2015; **385**: 117–71.

3 Global, regional, and national life expectancy, all-cause mortality, and cause-specific mortality for 249 causes of death, 1980-2015: a systematic analysis for the Global Burden of Disease Study 2015. *The Lancet* 2016; **388**: 1459-1544.

4 Johnson, S.C., Cunningham, M., Dippenaar, I.N. *et al.* Public health utility of cause of death data: applying empirical algorithms to improve data quality. *BMC Med Inform Decis Mak* **21**, 175 (2021). https://doi.org/10.1186/s12911-021-01501-1

5 Matzopoulos R, Prinsloo M, Wyk VP, Gwebushe N, Mathews S, et al. Injury-related mortality in South Africa: a retrospective descriptive study of postmortem investigations. *Bull World Health Organ* 2015; **93**: 303–13.

6 Feldman JM, Gruskin S, Coull BA, Krieger N (2017) Quantifying underreporting of law-enforcement-related deaths in United States vital statistics and news-media-based data sources: A capture–recapture analysis. *PLOS Medicine* 14(10): e1002399.

7. Samuel S, DeRay M. Mapping Police Violence (United States). 2021.

8. Burghart B. Fatal Encounters Database (United States). 2021.

9. Banks D, Ruddle P, Kennedy E, Planty M/ Arrest-Related Deaths Program Redesign Study. *Bureau of Justice Statistics* 2016. No. NCJ 250112.

10. Feldman JM, Gruskin S, Coull BA, Krieger N. Killed by Police: Validity of Media-Based Data and Misclassification of Death Certificates in Massachusetts, 2004–2016. *American Journal of Public Health* 2017. 107, no. 10: pp. 1624-1626.

11. Ozkan T, Worrall JL, Zettler H. Validating media-driven and crowdsourced police shooting data: a research note. *Journal of Crime and Justice* 2018. 41:3, 334-345.

12. United States Department of Justice, Federal Bureau of Investigation. Uniform Crime Reporting Program Data: Law Enforcement Officers Killed and Assaulted (LEOKA). 2019.

13. Death Penalty Information Center (DPIC). United States Death Penalty Execution Database. Washington, DC, United States of America: Death Penalty Information Center (DPIC). 2020.

14. Loftin C, Wiersema B, McDowall D, Dobrin A. Underreporting of Justifiable Homicides Committed by Police Officers in the United States, 1976–1998. *American Journal of Public Health* 2003. 93, no. 7: pp. 1117-1121.

15. Loftin C, McDowall D, Xie M. Underreporting of Homicides by Police Officers in the United States, 1976–2013. *Homicide Studies* 2017. 21(2):159-174.

Interstitial lung disease and pulmonary sarcoidosis

Flowchart

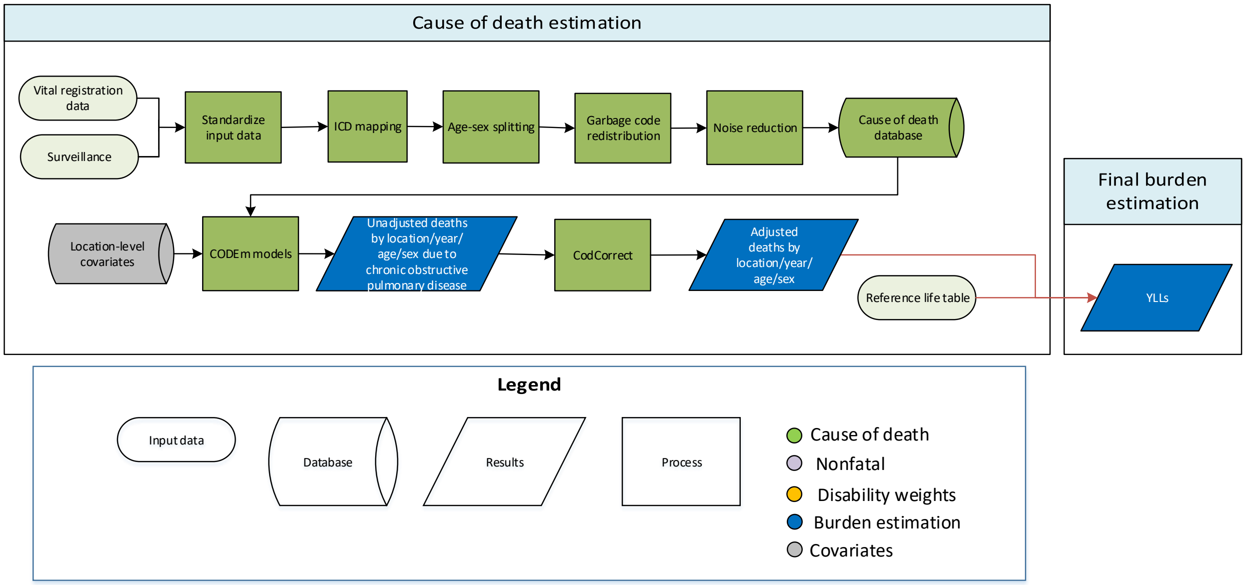

Input data and methodological summary for interstitial lung disease (ILD) and pulmonary sarcoidosis

Input data

Data used to estimate interstitial lung disease and pulmonary sarcoidosis mortality included vital registration and surveillance data from the cause of death (CoD) database. Verbal autopsy data were not included and were instead mapped to an overall chronic respiratory disease model. Our outlier criteria excluded datapoints that (1) were implausibly high or low, (2) substantially conflicted with established age or temporal patterns, or (3) substantially conflicted with other data sources conducted from the same locations or locations with similar characteristics (ie, Socio-demographic Index).

Modelling strategy

There were no substantive changes to the modelling approach this round. The standard Cause of Death Ensemble modelling (CODEm) approach (detailed in appendix 1 section 4) was used to estimate deaths due to interstitial lung disease and pulmonary sarcoidosis. Separate models were conducted for male and female mortality, and the age range for both models was 2 to 95+ years. For GBD 2023, we removed the occupational professionals covariates due to a lack of predictive quality on ILD mortality.

The following table lists the covariates included in the model. This requires that the covariate selected for the model must have a directional relationship with ILD. For GBD 2023, we added additional cumulative cigarette consumption covariates at 10 and 20 years to better capture the increased

likelihood of mortality due to higher quantities and duration of cigarette consumption. Covariate directions were selected based on the strength of the evidence from scientific literature.

Table 1. Covariates used in interstitial lung disease and pulmonary sarcoidosis mortality modelling

Level	Covariate	Direction
1	Cumulative cigarettes (5 years)	+
	Cumulative cigarettes (10 years)	+
	Cumulative cigarettes (20 years)	+
	Smoking prevalence	+
2	Indoor air pollution (all cooking fuels)	+
	Outdoor air pollution (PM 2.5)	+
	Proportion population between 500 m and 1500 m elevation	+
	Healthcare Access and Quality Index	-
	Proportion population over 1500 m elevation	+
3	Socio-demographic Index	-

Intracerebral haemorrhage

Flowchart

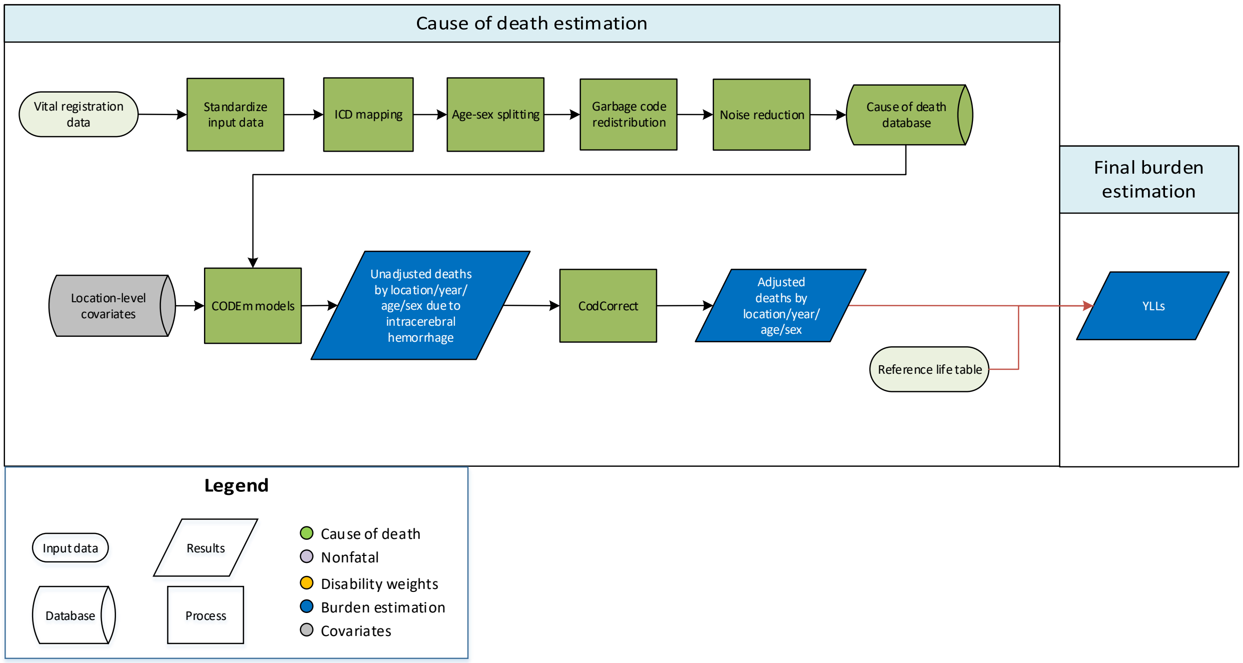

Input data and methodological summary for intracerebral haemorrhage

Input data

Vital registration data were used to model intracerebral haemorrhage. We outliered ICD-8 datapoints which were inconsistent with the rest of the data and created implausible time trends. In addition, we outliered vital registration datapoints in certain Latin American countries due to implausibly high values at the oldest age groups resulting in inconsistencies in time trends. ICD-9 data in Poland pre-1995 were outliered for inconsistency with the remainder of the time series, including ICD-10 data. We also outliered implausibly high ICD-10 vital registration data in Montenegro, Ghana, Cabo Verde, and Palestine. In addition, we outliered ICD-10 datapoints from sources in Oman, Iran, and some subnationals of Ethiopia which were implausibly low in all age groups.

Most data sources used for cause of death estimation are ICD-coded. The ICD periodically releases new versions, which can introduce an implausible disjoint in the time series that is indicative of a change in the coding practice, rather than a true change in the epidemiological trend of the disease. In GBD 2023, certain causes and countries using the ICD-9 code systems were adjusted to address the disjoint in the time trend and number of deaths between the older ICD-9 code systems and the more recent ICD-10 code systems. Intracerebral haemorrhage was corrected in Bulgaria (1990–2004), Canada (1986–1999), Spain (1980–1998), France (1980–1999), the USA (1980–1998), Hungary (1980–1995), Croatia (1985–1994), the UK (1980–1999), Greece (1987–2008), Italy (1980–2002), Mexico (1980–2002), and Mauritius (1980–2004) to account for this code system disjoint. This was done using a meta-regression tool described as a crosswalk, by taking systematically biased datapoints and estimating their unbiased

value. Using this modeling tool, correction factors were created based on the more recent and more reliable ICD-10 coded data. The crosswalk creates matched pairs between the last three years of data using the ICD-9 code system and the first three years of data using the ICD-10 code system. The correction is applied at the child cause level for all Level 2 causes, and after the correction is applied the individual child causes are raked to the parent cause to maintain a consistent number of deaths across the cause hierarchy.

Modelling strategy

We used a standard CODEm approach to model deaths from intracerebral haemorrhage. Since GBD 2021, CODEm has switched from using an ensemble of rate and cause-fraction models to only rate models. Otherwise, there were no substantial changes in CODEm modelling since GBD 2021. The covariates included in the ensemble modelling process are listed in the table below.

Table: Selected covariates for CODEm models, intracerebral haemorrhage

Level	Covariate	Direction
1	Summary exposure variable, intracerebral haemorrhage	1
	Smoking prevalence	1
	Systolic blood pressure (mmHg)	1
2	Mean BMI	1
	Elevation over 1,500 m (proportion)	–1
	Fasting plasma glucose	1
	Outdoor pollution ($PM_{2.5}$)	1
	Indoor air pollution	1
	Healthcare Access and Quality Index	–1
3	Log-transformed lag-distributed income per capita (I$)	–1
	Summary exposure value, omega-3	1
	Summary exposure value, fruits	1
	Summary exposure value, vegetables	1
	Summary exposure value, nuts and seeds	1
	Pulses/legumes (kcal per capita, unadjusted)	–1
	Summary exposure value, PUFA adjusted (percent)	1
	Alcohol (litres per capita)	1
	LDL cholesterol (mean per capita)	–1
	Trans fatty acid	1

Invasive non-typhoidal *Salmonella* (iNTS)

Flowchart

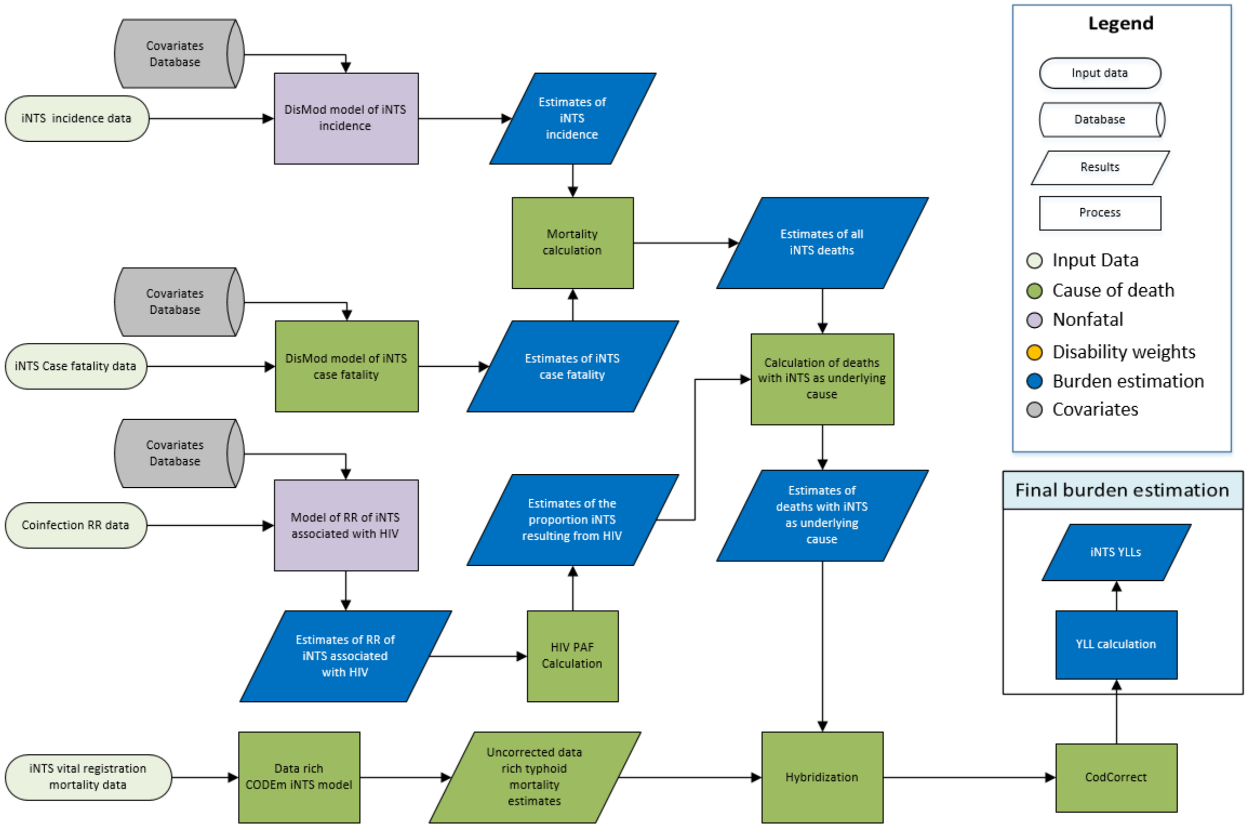

Input data and methodological summary for iNTS

Input data

Our CODEm model used all available data in the cause of death database from data-rich countries. No data were outliered for this cause. Incidence estimates for the natural history model are modelled using an incidence dataset based principally on prospective cohort studies and facility-based surveillance. Similarly, data on case fatality and co-infection come from prospective cohort studies and facility-based surveillance. We conducted a systematic review to update literature-based incidence and case fatality data, adding 23 datapoints from one case fatality and three incidence data sources (Figure 1).

Modelling strategy

We model iNTS deaths using a hybrid modelling strategy with two components: 1) for data-rich locations we estimate iNTS mortality using a CODEm model of CoD data; and 2) in all other locations (ie, not data-rich) we use a natural history model in which we derive deaths as the product of cases and case fatality.

The CODEm model included three covariates:

Level	Covariate	Direction
1	SEV unsafe water	+
	Malaria incidence adjusted for antimalarial coverage and drug effectiveness	+
	HIV mortality rate	+

For the natural history model, we estimate iNTS deaths as the product of cases and case fatality. Incidence was modelled with DisMod-MR 2.1, using the HIV mortality rate, malaria incidence adjusted for antimalarial coverage and drug effectiveness, and the summary exposure value (SEV) – unsafe water, as covariates. We estimated the relative risk of iNTS comparing people with HIV to those without using a negative binomial model with log-age and log of the summary exposure value (SEV) for water as predictors. We used the resulting relative risk estimates and HIV prevalence estimates to calculate the proportion of iNTS that was attributable to HIV in each location, year, age, and sex. Using these proportions, we divided iNTS cases into those that were attributable to HIV and those that were not. We modelled case fatality by age and Socio-demographic Index (SDI) separately for those with and without HIV using a generalised additive model, parameterising age with P-splines, and estimated mortality as the product of incidence and case fatality. Where iNTS occurs among those with HIV, we assume that iNTS is an opportunistic infection and that HIV is therefore the underlying cause of death. We therefore estimate deaths with iNTS as the underlying cause as total iNTS deaths times the proportion of cases not attributable to HIV.

We have made no substantive changes to our modelling strategy between GBD 2021 and 2023.

Figure 1: PRISMA flow diagram

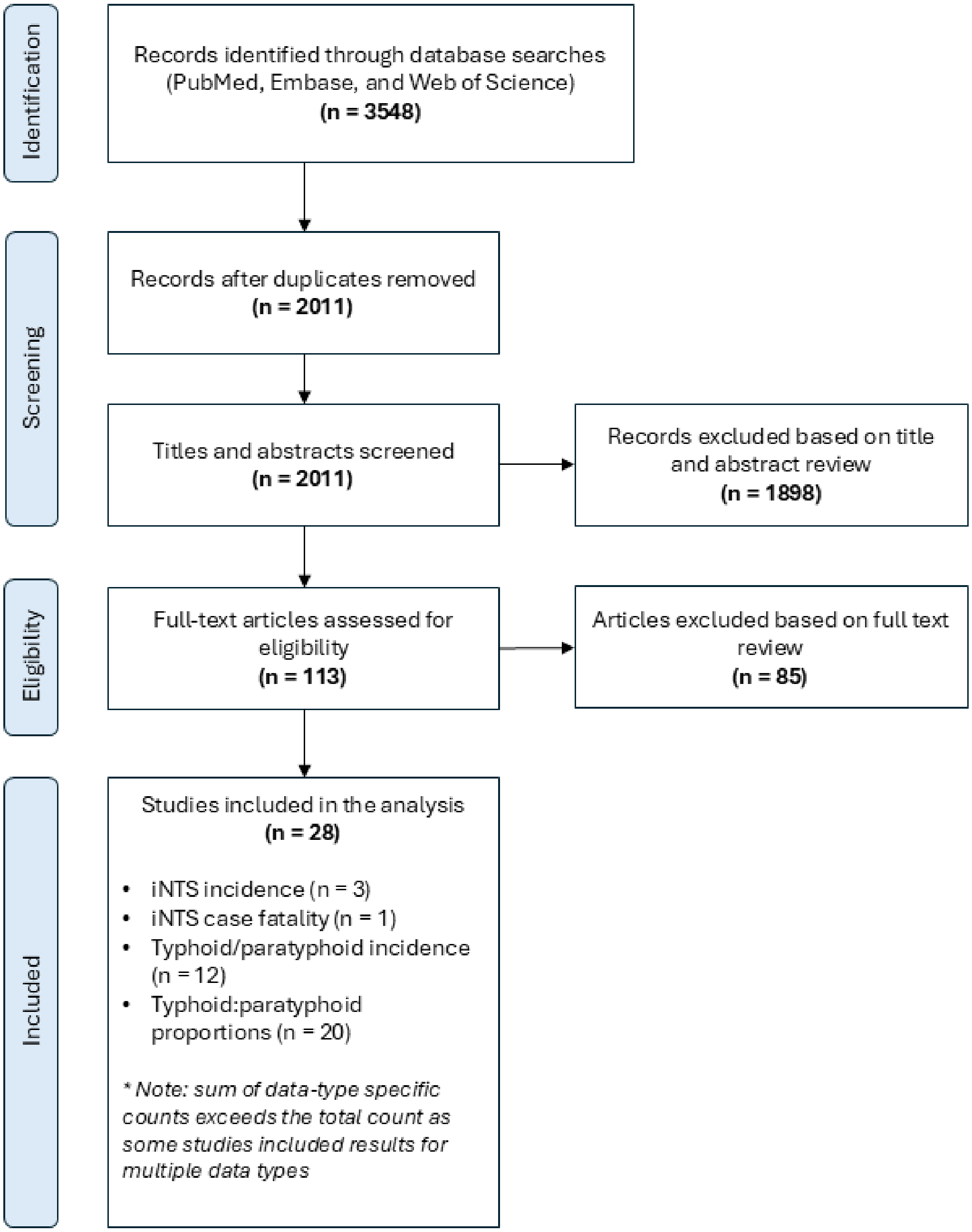

Ischaemic heart disease

Flowchart

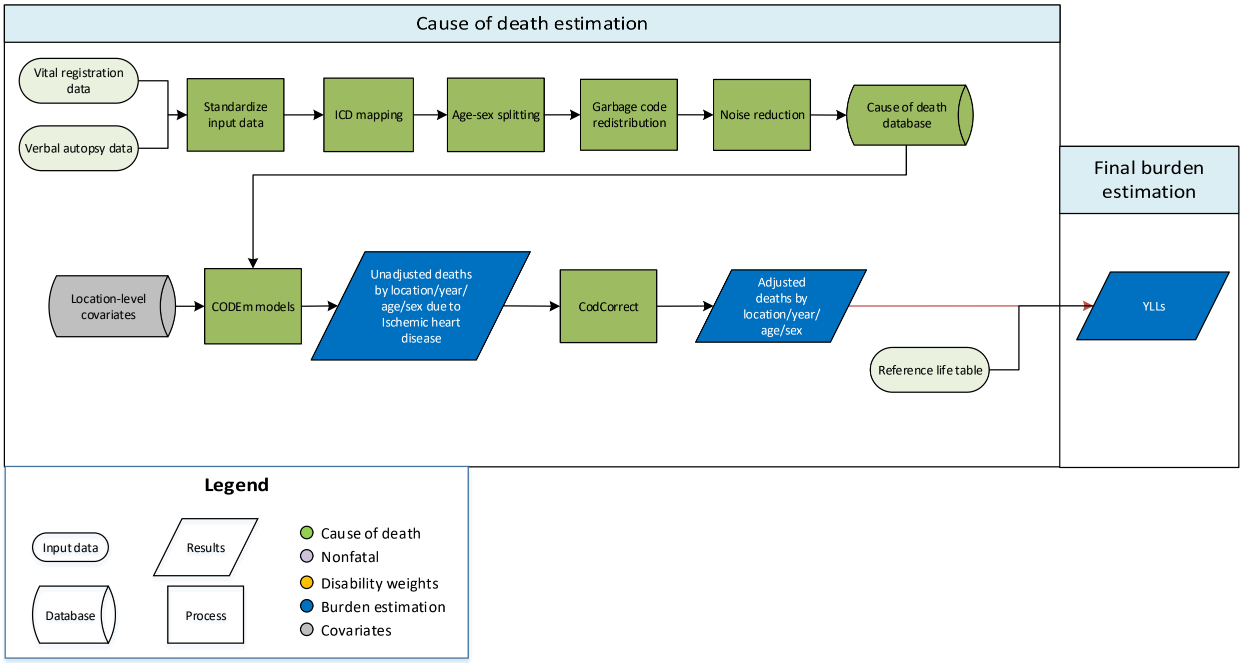

Input data and methodological summary for ischaemic heart disease

Input data

Vital registration and verbal autopsy data were used to model ischaemic heart disease.

To maximise the reliability of data included in the model, verbal autopsy studies that did not meet the World Health Organization standards were excluded.[1] Verbal autopsy studies which included only populations under 30 years were also excluded. In addition, we outliered non-representative subnational verbal autopsies from several Indian states and verbal autopsy data in Nepal, Sri Lanka, Bangladesh, and Papua New Guinea that were implausible in terms of time and age trends. We also outliered verbal autopsy data in countries and subnational locations where high-quality vital registration data were available.

After evaluating the available vital registration data, we outliered ICD-8 and ICD-9-BTL datapoints from Germany and Mauritius which were inconsistent with the rest of the data and created implausible time trends. We also outliered vital registration data in several Indian states identified by experts as poor-quality. We outliered recent vital registration data in and Croatia in 2019 and 2020 that were implausibly different from the preceding vital registration time series. We outliered ICD-10 data in England from 2014 to 2018 in ages 25–34 due to an implausible increase in these years.

Most data sources used for cause of death estimation are ICD-coded. The ICD periodically releases new versions, which can introduce an implausible disjoint in the time series that is indicative of a change in the coding practice, rather than a true change in the epidemiological trend of the disease. In GBD 2023, certain causes and countries using the ICD-9 code systems were adjusted to address the disjoint in the time trend and number of deaths between the older ICD-9 code systems and the more recent ICD-10 code systems. Ischaemic heart disease was corrected in Bulgaria (1990–2004), Canada (1986–1999), Spain (1980–1998), France (1980–1999), the USA (1980–1998), Hungary (1980–1995), Croatia (1985–1994), the UK (1980–1999), Greece (1987–2008), Italy (1980–2002), Mexico (1980–2002), and Mauritius (1980–2004) to account for this code system disjoint. This was done using a meta-regression tool described as a crosswalk, by taking systematically biased datapoints and estimating their unbiased value. Using this modelling tool, correction factors were created based on the more recent and more reliable ICD-10 coded data. The crosswalk creates matched pairs between the last three years of data using the ICD-9 code system and the first three years of data using the ICD-10 code system. The correction is applied at the child cause level for all Level 2 causes, and after the correction is applied the individual child causes are raked to the parent cause to maintain a consistent number of deaths across the cause hierarchy.

Modelling strategy

We used a standard CODEm approach to model deaths from ischaemic heart disease. Since GBD 2021, CODEm has switched from using an ensemble of rate and cause-fraction models to only rate models. Otherwise, there were no substantial changes in CODEm modelling since GBD 2021.The covariates included in the ensemble modelling process are listed in the table below. No changes to the covariates were made for GBD 2023.

Table 1: Covariates used in cardiovascular diseases mortality modelling

Level	Covariate	Direction
1	Summary exposure value, IHD	1
	Cholesterol (total, mean per capita)	1
	Smoking prevalence	1
	Systolic blood pressure (mmHg)	1
2	Mean BMI	1
	Elevation over 1500 m (proportion)	–1
	Fasting plasma glucose	1
	Outdoor pollution ($PM_{2.5}$)	1
	Indoor air pollution	1
	Healthcare Access and Quality Index	–1
3	Lag-distributed income per capita (I$)	–1
	Summary exposure value, omega-3	1
	Summary exposure value, fruits	1
	Summary exposure value, vegetables	1
	Summary exposure value, nuts and seeds	1
	Pulses/legumes (kcal per capita, unadjusted)	–1

Summary exposure value, PUFA (percent, adjusted)	1
Alcohol (litres per capita)	1
Trans fatty acid	1

References

1 WHO | Methodological trends in studies based on verbal autopsies before and after published guidelines. https://www.who.int/bulletin/volumes/87/9/07-049288/en/ (accessed April 22, 2021).

Ischaemic stroke

Flowchart

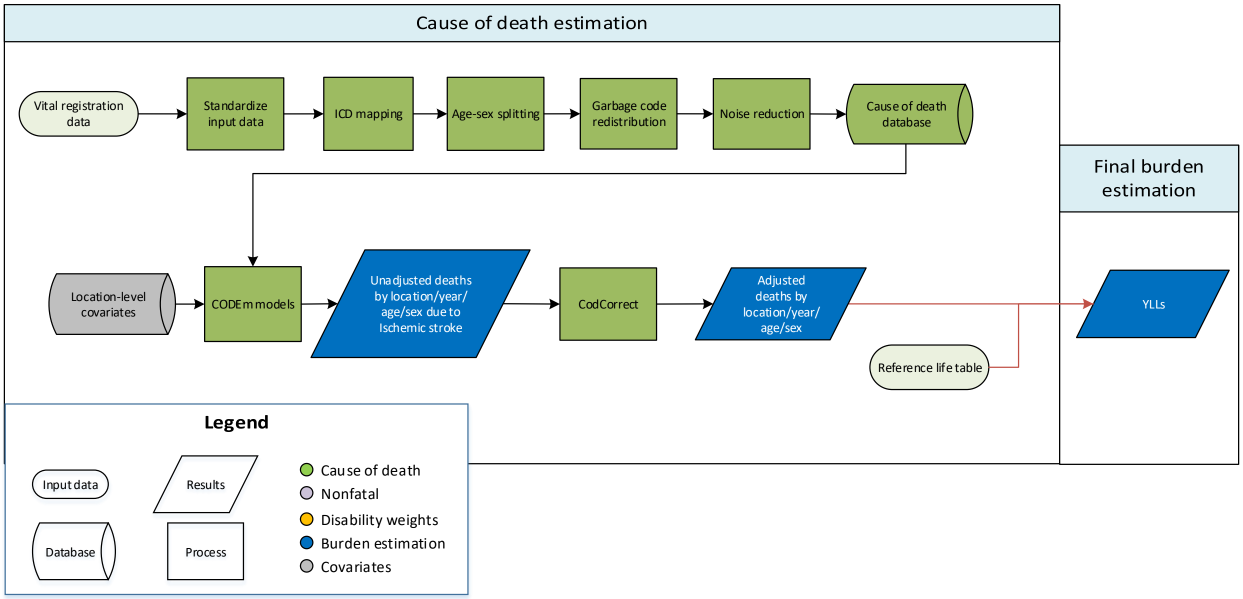

Input data and methodological summary for ischaemic stroke

Input data

Vital registration data were used to model deaths from ischaemic stroke. We outliered ICD-8 datapoints which were inconsistent with the rest of the data and created implausible time trends. We outliered ICD-9 datapoints in Poland and Saint Vincent and the Grenadines that were inconsistent with the rest of the time series of ICD-10 vital registration data. We also outliered data in 2020 and 2021 in the Chechen Republic that were implausibly low compared to the previous time series of data. In addition, we outliered ICD-10 datapoints from sources in Ethiopian subnational locations, Cabo Verde, Montenegro, Iran, Oman, Saudi Arabia, and Egypt which were implausibly low in all age groups and caused regional estimates to be too low.

Most data sources used for cause of death estimation are ICD-coded. The ICD periodically releases new versions, which can introduce an implausible disjoint in the time series that is indicative of a change in the coding practice, rather than a true change in the epidemiological trend of the disease. In GBD 2023, certain causes and countries using the ICD-9 code systems were adjusted to address the disjoint in the time trend and number of deaths between the older ICD-9 code systems and the more recent ICD-10 code systems. Ischaemic stroke was corrected in Bulgaria (1990–2004), Canada (1986–1999), Spain (1980–1998), France (1980–1999), the USA (1980–1998), Hungary (1980–1995), Croatia (1985–1994), the UK (1980–1999), Greece (1987–2008), Italy (1980–2002), Mexico (1980–2002), and Mauritius (1980–2004) to account for this code system disjoint. This was done using a meta-regression tool described as a crosswalk, by taking systematically biased datapoints and estimating their unbiased value. Using this modelling tool, correction factors were created based on the more recent and more reliable ICD-10-coded data. The crosswalk creates matched pairs between the last three years of data using the ICD-9

code system and the first three years of data using the ICD-10 code system. The correction is applied at the child cause level for all Level 2 causes, and after the correction is applied the individual child causes are raked to the parent cause to maintain a consistent number of deaths across the cause hierarchy.

Modelling strategy

We used a standard CODEm approach to model deaths from ischaemic stroke. Since GBD 2021, CODEm has switched from using an ensemble of rate and cause-fraction models to only rate models. Otherwise, there were no substantial changes in CODEm modelling since GBD 2021.

The covariates included in the ensemble modelling process are listed in the table below.

Table 1. Covariates used in ischaemic stroke mortality modelling

Level	Covariate	Direction
1	Summary exposure variable, ischaemic stroke	1
	LDL cholesterol (mean per capita)	1
	Smoking prevalence	1
	Systolic blood pressure (mmHg)	1
2	Mean BMI	1
	Elevation over 1,500 m (proportion)	–1
	Fasting plasma glucose	1
	Outdoor pollution ($PM_{2.5}$)	1
	Indoor air pollution	1
	Healthcare Access and Quality Index	–1
3	Log-transformed lag distributed income per capita (I$)	–1
	Summary exposure value, omega-3	1
	Summary exposure value, fruits	1
	Summary exposure value, vegetables	1
	Summary exposure value, nuts and seeds	1
	Pulses/legumes (kcal per capita, unadjusted)	–1
	Summary exposure value, PUFA adjusted (percent)	1
	Alcohol (litres per capita)	1
	Trans fatty acid	1

Lower extremity peripheral artery disease

Flowchart

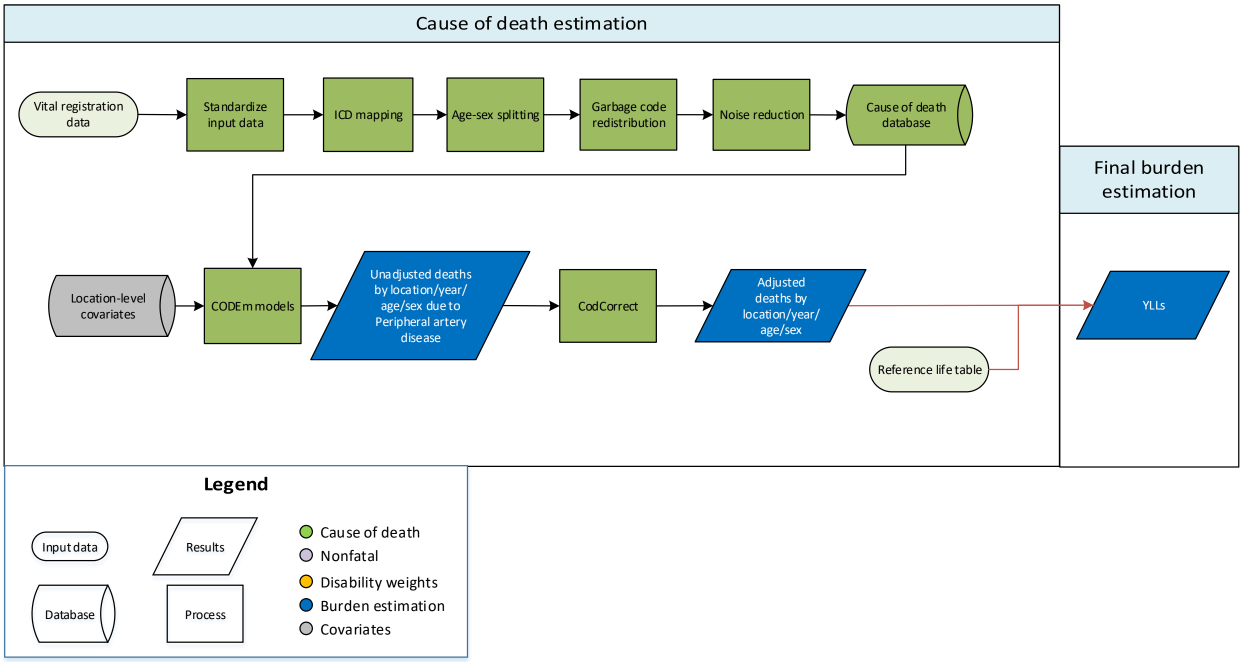

Input data and methodological summary for lower extremity peripheral artery disease

Vital registration data were used to model lower extremity peripheral artery disease. We outliered all datapoints with less than one death in Egypt per expert review. ICD-8 datapoints from Norway and Sweden with implausible values and discontinuous with the rest of the time series were outliered. ICD-9 data in Bulgaria, Czechia, North Macedonia, Poland, Estonia, Latvia, Lithuania, the Republic of Moldova, and England were outliered due to inconsistency with corresponding ICD-10 data later in the time series. Implausibly low ICD-10 datapoints were outliered in Finland, American Samoa, Guam, Kiribati, and Palau. All ICD data in Kazakhstan were outliered due to implausibly high values compared to the rest of the region.

Most data sources used for cause of death estimation are ICD-coded. The ICD periodically releases new versions, which can introduce an implausible disjoint in the time series that is indicative of a change in the coding practice, rather than a true change in the epidemiological trend of the disease. In GBD 2023, certain causes and countries using the ICD-9 code systems were adjusted to address the disjoint in the time trend and number of deaths between the older ICD-9 code systems and the more recent ICD-10 code systems. Lower extremity peripheral arterial disease was corrected in Bulgaria (1990–2004), Canada (1986–1999), Spain (1980–1998), France (1980–1999), the USA (1980–1998), Hungary (1980–1995), Croatia (1985–1994), the UK (1980–1999), Greece (1987–2008), Italy (1980–2002), Mexico (1980–2002), and Mauritius (1980–2004) to account for this code system disjoint. This was done using a meta-regression tool described as a crosswalk, by taking systematically biased datapoints and estimating

their unbiased value. Using this modelling tool, correction factors were created based on the more recent and more reliable ICD-10-coded data. The crosswalk creates matched pairs between the last three years of data using the ICD-9 code system and the first three years of data using the ICD-10 code system. The correction is applied at the child cause level for all Level 2 causes, and after the correction is applied the individual child causes are raked to the parent cause to maintain a consistent number of deaths across the cause hierarchy.

Modelling strategy

We used a standard CODEm approach to model deaths from peripheral artery disease. Since GBD 2021, CODEm has switched from using an ensemble of rate and cause-fraction models to only rate models. We also changed the direction of the Socio-demographic Index and Healthcare Access and Quality Index covariates from positive to negative. Otherwise, there were no substantial changes in CODEm modelling since GBD 2021.

The covariates included in the ensemble modelling process are listed in the table below.

Table 1. Covariates used in peripheral artery disease mortality modelling

Level	Covariate	Direction
1	Summary exposure variable, PAD	1
	Systolic blood pressure (mmHg)	1
	Cholesterol (total, mean per capita)	1
	Smoking prevalence	1
2	Mean body mass index (kg/m^2)	–1
	Healthcare Access and Quality Index	–1
	High fasting plasma glucose (mmol/L)	–1
3	Lag0distributed income per capita (I$)	–1
	Socio-demographic Index	–1
	Summary exposure value, omega-3	1
	Summary exposure value, fruits	1
	Summary exposure value, vegetables	1
	Summary exposure value, nuts and seeds	1
	Pulses/legumes (kcal/capita, unadjusted)	–1
	Summary exposure value, polyunsaturated fatty acids	1
	Alcohol (litres per capita)	1

Lower respiratory infections (LRI)

Flowchart

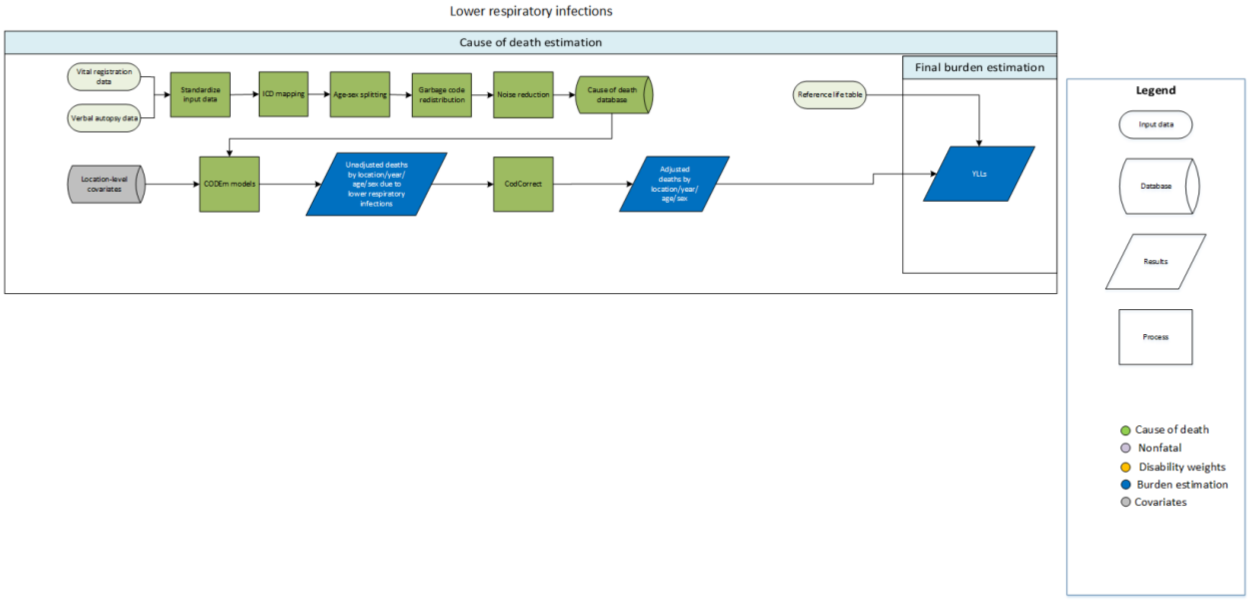

Input data and methodological summary for lower respiratory infections

Input data

Lower respiratory infection (LRI) mortality was estimated in CODEm. We estimated LRI mortality separately for males and females and for children under 5 years and 5 years and older. We used all available data from vital registration systems, surveillance systems, and verbal autopsy. We checked for and excluded outliers from our data by country or region.

Modelling strategy

LRI fatal modelling occurs using CODEm. Because of starkly different patterns, LRI CODEm models include under 5 years and 5–95+ years. Like all models of mortality in GBD, LRI mortality models are single-cause, requiring in effect that the sum of all mortality models must be equal to the all-cause mortality envelope. We correct LRI mortality estimates, and other causes of mortality, by rescaling them according to the uncertainty around the cause-specific mortality rate. This process is called CoDCorrect and is essential to ensure internal consistency among causes of death.

We use a lagged mean of PCV3, Hib3, and DTP3 vaccine coverage calculated over a rolling, five-year interval to capture population-level vaccine-derived immunity among under-5-year-olds, including coverage both in the current year and in recent years.

Table 1A. Covariates used in under 5 years model

Level	Covariate	Direction

**1**	Childhood stunting summary exposure value (SEV)	+
	Childhood underweight SEV	+
	Childhood wasting SEV	+
	Indoor air pollution	+
	LRI SEV	+
	Antibiotics for LRI	-
	Hib3 vaccine coverage proportion, lagged	-
	PCV3 vaccine coverage proportion, lagged	-
**2**	Secondhand smoking prevalence	+
	Zinc deficiency	+
	DTP3 vaccine coverage proportion, lagged	-
	Healthcare Access and Quality Index	-
	Ambient particulate matter SEV	+
	Household air pollution SEV	+
	Outdoor air pollution ($PM_{2.5}$)	+
	Handwashing SEV	+
**3**	Sanitation SEV	+
	Population density >1000/km^2	+
	Maternal education	-
	Socio-demographic Index	-

Note on Table 1A: The *Level* is the associated strength of relationship between the covariate and LRI mortality, ranked from 1 (proximally related) to 3 (distally related). *Direction* is the direction of the association between the covariate and LRI mortality.

Table 1B. Covariates used in 5–95+ years model

Level	Covariate	Direction
**1**	Indoor air pollution	+
	LRI SEV	+
	Outdoor air pollution ($PM_{2.5}$)	+
	Secondhand smoking prevalence	+
	Smoking prevalence	+
**2**	DTP3 vaccine coverage proportion, lagged	-
	Adult underweight	+
	Healthcare Access and Quality Index	-
	PCV3 vaccine coverage proportion, lagged	-
	Handwashing access	+
**3**	Education years per capita	-
	Lag distributed income per capita	-
	Socio-demographic Index	-
	Sanitation SEV	+

Note on Table 1B: The *Level* is the associated strength of relationship between the covariate and LRI mortality, ranked from 1 (proximally related) to 3 (distally related). *Direction* is the direction of the association between the covariate and LRI mortality.

Aetiology estimation

LRI aetiologies are now produced by IHME's Antimicrobial Resistance Collaborators. Further reading on the data and methods used to estimate these proportions were published in September 2024.[1]

References

1. Naghavi M, Vollset SE, Ikuta KS, Swetschinski LR, Gray AP, Wool EE, Aguilar GR, Mestrovic T, Smith G, Han C, Hsu RL. Global burden of bacterial antimicrobial resistance 1990–2021: a systematic analysis with forecasts to 2050. The Lancet. 2024 Sep 28;404(10459):1199-226.

Malaria

Flowchart

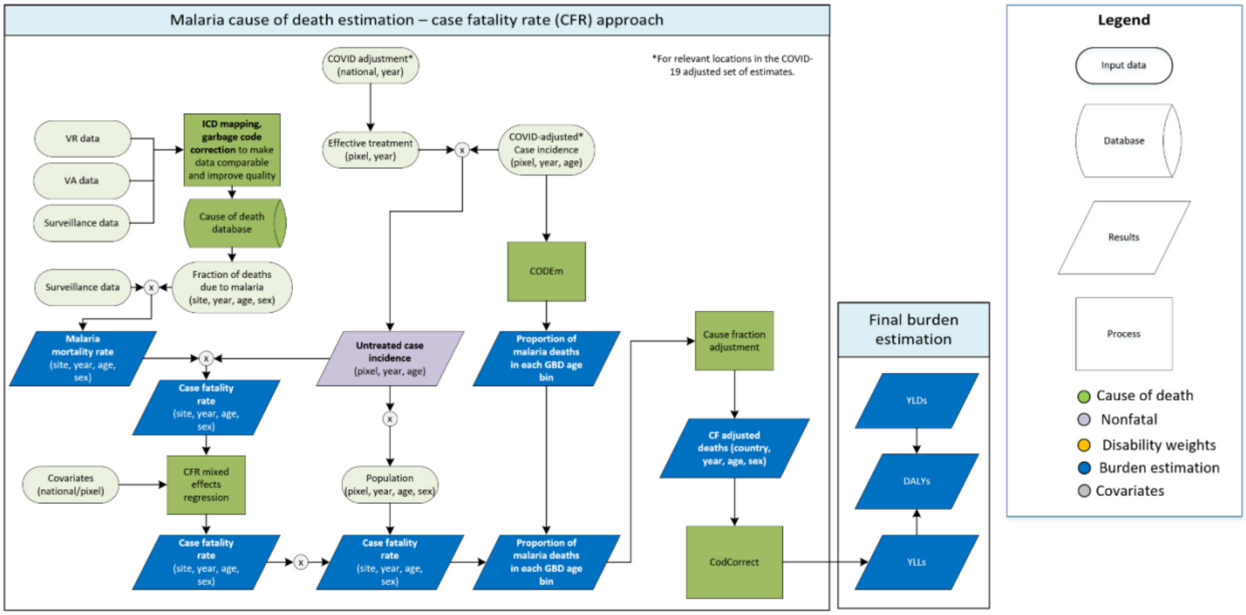

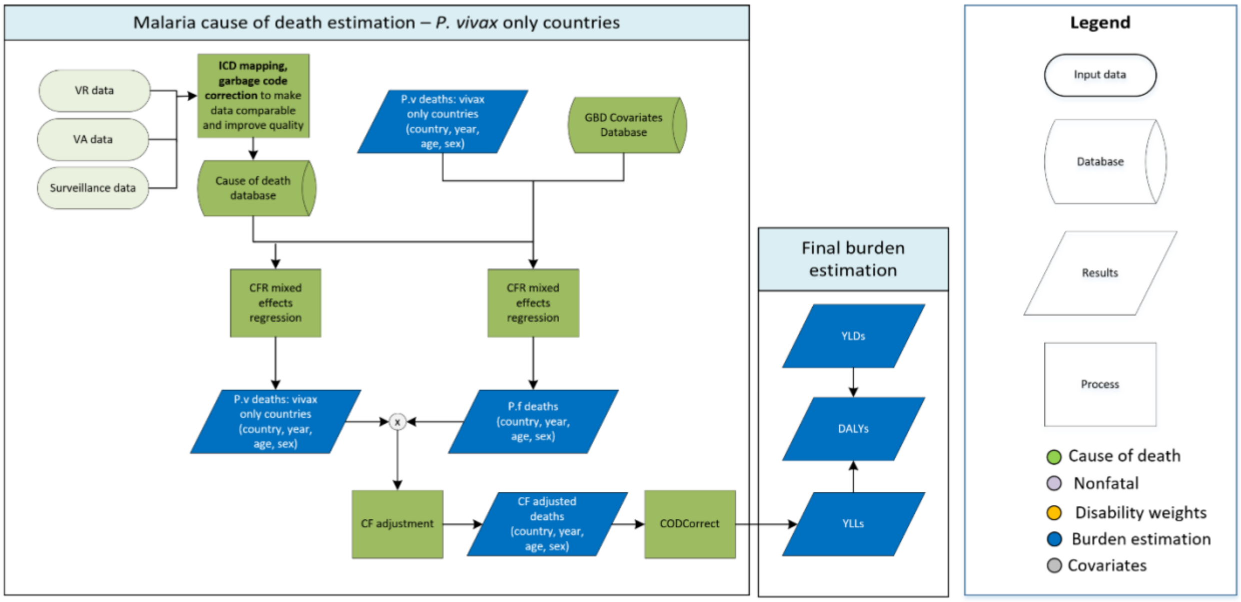

Input data and methodological summary for malaria

Variability in the presence of *Plasmodium falciparum* necessitated distinct approaches for estimating malaria mortality. In countries with *Plasmodium falciparum*, which is responsible for the vast majority of malaria deaths and for which there is cause of death evidence available, this species was considered the only cause of malaria deaths. For these countries, we applied a model that generates a geographically heterogeneous case-fatality rate (CFR) grid, which we then intersect with untreated incidence to determine the number of deaths. Pixel-level death totals are then aggregated to the admin level to produce the GBD estimates. In countries where the only species present was *Plasmodium vivax*, a very simplistic model was used instead to attribute some nominal number of deaths to malaria.

Input data

The cause of death (CoD) data included vital registration, verbal autopsy, and surveillance data from the GBD database. For the CFR model, we only used CoD data (mostly verbal autopsy) for which we have been able to successfully geo-reference the site (ie, find associated geographical coordinates). Systematic literature reviews for malaria were not conducted. Our outlier criteria excluded datapoints that (i) were implausibly high or low relative to global or regional patterns, (ii) substantially conflicted with established age or temporal patterns, or (iii) significantly conflicted with other data sources conducted from the same locations or locations with similar characteristics (ie, local Socio-demographic Index).

A COVID-19 adjustment was applied to the years 2020–2022 utilizing PULSE surveys, from WHO, conducted by country government officials on health-care service disruption.[1-4] This adjustment was applied to antimalarial effective treatment rates, which had a double effect on mortality estimation, by not only impacting prevalence results, and subsequently incidence, but also the proportion of untreated cases, and therefore the uCFR among all malaria cases. Please see Dzianach and colleagues (2023)[5] for further details on how the adjustments were derived.

Currently these adjustments have only been applied to 26 countries located in Africa (see full list below) due to the lack of a complementary approach to introducing the adjustments for countries located outside of Africa. For countries in which a national survey was conducted after the onset of the pandemic, the new empirical data on treatment seeking for fever superseded the need to model these disruptions, and no adjustment was applied for the study-years. At the time our estimates were generated, these were the best data available to help account for the impact of COVID on malaria. As new data become available ,we will continue to refine our adjustment approach.

COVID-19 adjustments were applied to the following countries: Angola, Burundi, Benin, Central African Republic, Côte d'Ivoire, Cameroon, Democratic Republic of the Congo, Congo, Gabon, Ghana, Guinea-Bissau, Equatorial Guinea, Kenya (and subnationals), Liberia, Mozambique, Malawi, Nigeria (and subnationals), Rwanda, Sudan, Sierra Leone, Somalia, South Sudan, Chad, Togo, Uganda, and Zambia.

Modelling strategy

For most GBD causes, epidemiological measures may be used as covariates in a traditional CODEm approach, if at all. To estimate the fatal burden of *P. falciparum* malaria in Africa, we used

epidemiological measures in our estimation process directly. The Malaria Atlas Project (MAP) at the Telethon Kids Institute has generated updated spatiotemporal "cubes" estimating clinical incidence (rates and case counts) for each 5x5-km pixel, by year, from 1980 to 2023. MAP has also generated an equivalent spatiotemporal prediction of effective treatment with an antimalarial drug (combining treatment seeking, the fraction of malaria cases receiving different classes of antimalarial, and the estimated country-year-specific efficacy of each antimalarial class though time). This estimated effective treatment rate was combined with the incidence rate cube to derive a third cube estimating the incidence of untreated cases at the pixel level.

For each site-year for which CoD malaria cause fraction (CF) data were available, we (i) estimated a site-year-specific malaria mortality rate as the product of malaria CF and all-cause mortality rate (with the latter drawn from national-level values); (ii) divided the malaria mortality rate by the site-year-specific estimate of untreated malaria incidence rate (drawn from the MAP cube) to estimate a site-year-specific case-fatality rate (CFR) among untreated malaria cases. These derived site-year-specific CFR values were then used in a geostatistical model to estimate pixel-year CFR for each 5x5-km grid cell. The response variable for this model was logit all-ages CFR (for untreated cases), and Gaussian likelihood was used. The model included a separate intercept for each IHME super-region. Similarly, each continent was given its own smooth temporal effect (random walk of order 2). There was no global intercept or global temporal term as some continents had many datapoints, while Africa, in particular, had very few. The fixed effect covariates used were travel time to cities, proportion of adults, proportion of infants, log country-year all-cause mortality, and sickle cell anaemia rate (proportion of heterozygotes). Finally, a sample location random effect was included (and not used in prediction) to account for sampling biases between sites.

Pixel-year predictions of CFR were then multiplied by the untreated incidence rate rasters from the MAP cube to yield pixel-year mortality rate estimates, which were then multiplied by pixel-year population to derive pixel-year malaria death counts. Pixel-level results were then aggregated to yield the GBD national and subnational death estimates. By applying this logic over a set of raster realisations, we created a distribution of results from which we obtained measures of uncertainty.

To age-split the deaths, we relied on the age-specific death ratios that emerged from a separate CODEm modelling strategy. This strategy was carried out in eight parts: males <5 years, males ≥5 years, females <5 years, and females ≥5 years for countries inside and outside of Africa. The resulting predicted age patterns were used to distribute the country-year mortality estimates proportionally into the 23 GBD age bins. The covariates used in CODEm were as follows:

Table 1: Covariates used in malaria mortality modelling

Level	Covariate	Direction
1	Pf-only incidence	1
1	Effective antimalarial treatment	–1

For countries where the exclusive strain of malaria was *P. vivax*, deaths were estimated using a zero-inflated negative binomial mixed model where the outcome is study deaths. The model included as fixed effects the logarithm of mortality rate, age, and sex. Locations were included as random effects.

The results from the *P. falciparum* and *P. vivax* models were collated to produce overall malaria mortality.

Changes from GBD 2021 to GBD 2023

The *Plasmodium knowlesi* inclusion to the non-fatal estimates for Malaysia also impacted death results as, for lack of applicable information published on deaths related to this parasite, we used the same untreated case-fatality rate as use used for *P. falciparum*. A minor modelling correction also relates to our derivation of deaths in countries with only *P. vivax* malaria, which we model for countries without *P. falciparum* incidence. In rare cases, our previous approach allowed the uCFR to increase in countries that switched from having both species to only having *P. vivax.* Given this parasite is far less likely to lead to death, we capped the uCFR to never exceed the lowest value calculated in years with both species present.

The second substantive change to the modelling strategy for GBD 2023 was the application of a CF adjustment. To avoid implausibly high CFs in the malaria fatal results, a sex- and age-specific CF adjustment was applied prior to CoDCorrect. First, we estimated the sex-specific age pattern of malaria mortality. Using MR-BRT (meta-regression-Bayesian, regularised, trimmed) we fit a model with a spline on age to sex- and age-specific maximum malaria mortality CFs, including only malaria mortality data from locations in sub-Saharan Africa with a sample size greater than 20 (see Figures 1 and 2). For the under-5 age groups, the data were selected from multiple locations, while the 5 and older age groups only used mortality data from Mozambique due to it consistently having the highest CFs. The adjustment was applied to location-year-sex-age groups where the CF exceeded that of the sex- and age-specific MR-BRT results.

Figure 1: Malaria MR-BRT model with spline on age for females fit to age-specific maximum malaria mortality CF data

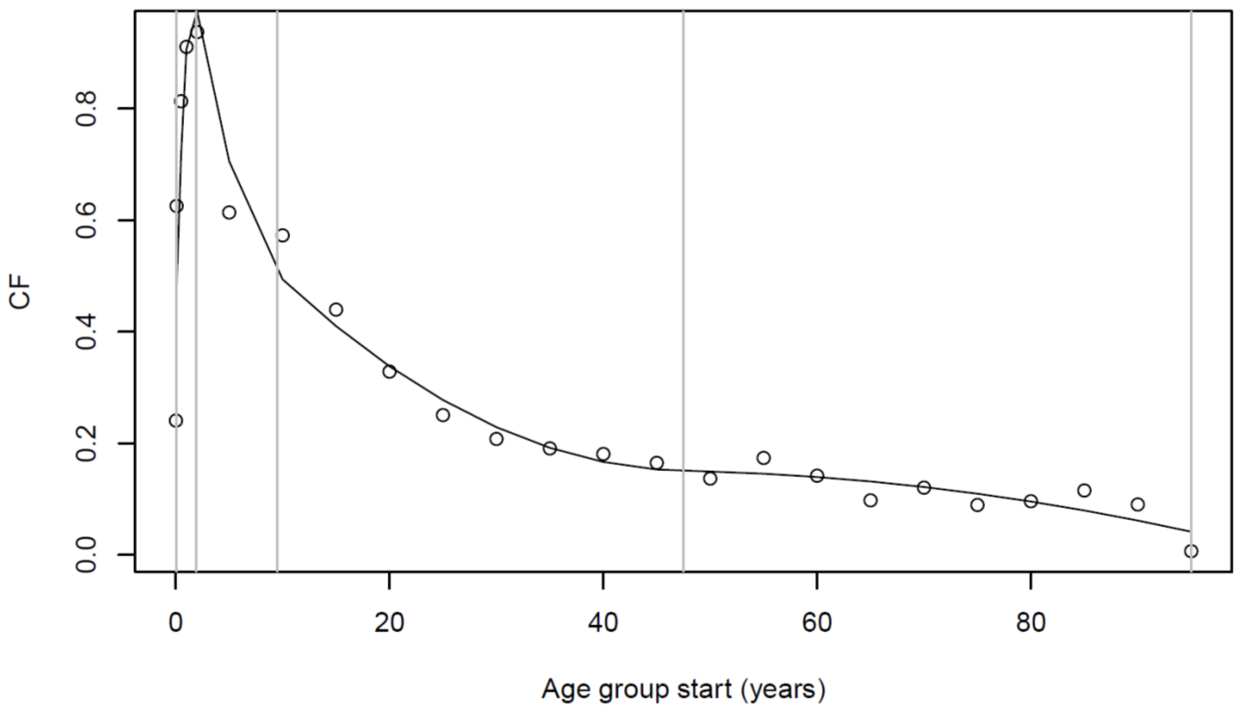

Figure 2: Malaria MR-BRT model with spline on age for males fit to age-specific maximum malaria mortality CF data

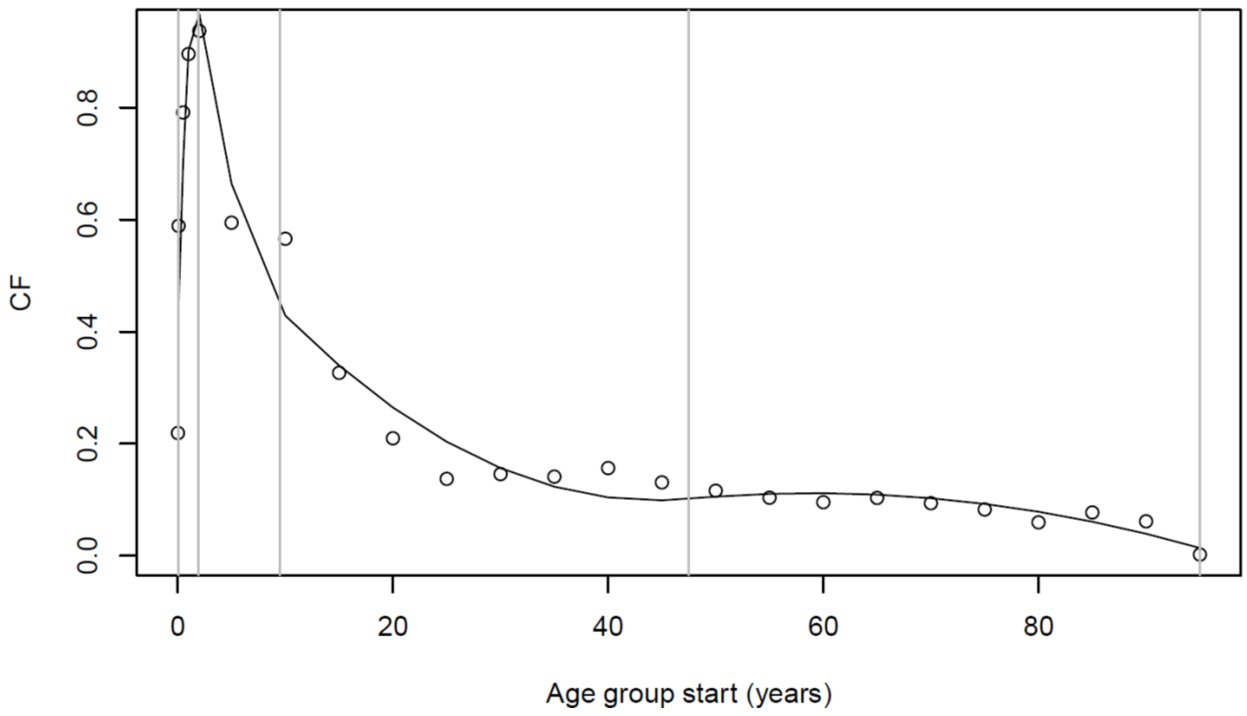

References

1. Pulse survey on continuity of essential health services during the COVID-19 pandemic: interim report, 27 August 2020. Geneva: World Health Organization; 2020 (https://www.who.int/publications/i/item/WHO-2019-nCoV-EHS_continuity-survey-2020.1).
2. Second round of the national pulse survey on continuity of essential health services during the COVID-19 pandemic: January−March 2021. Geneva: World Health Organization; 2021 (https://apps.who.int/iris/bitstream/handle/10665/340937/WHO-2019-nCoV-EHScontinuity-survey-2021.1-eng.pdf?sequence=1&isAllowed=y).
3. Third round of the global pulse survey on continuity of essential health services during the COVID-19 pandemic. Geneva: World Health Organization; 2022 (https://www.who.int/publications/i/item/WHO-2019-nCoV-EHS_continuity-survey-2022.1).
4. Fourth round of the global pulse survey on continuity of essential health services during the COVID-19 pandemic. Geneva: World Health Organization; 2023 (https://www.who.int/publications/i/item/WHO-2019-nCoV-EHS_continuity-survey-2023.1)
5. Dzianach, P.A.; Rumisha, S.F.; Lubinda, J.; Saddler, A.; Van den Berg, M.; Gelaw, Y.A., Harris, J.R.; Vargas-Ruiz, C.A.; Cameron, E.; Gething, P.W.; Weiss, D.J. Evaluating COVID-19 related disruptions to healthcare accessibility in 2020-2021 and its potential effects on malaria burden in Sub-Saharan Africa. *Trop. Med. Infect. Dis.* **2023**, *7*, x. https://doi.org/10.3390/xxxxx

Maternal disorders

Flowchart

Figure 1: Analytical flowchart for the estimation of maternal mortality for GBD 2023

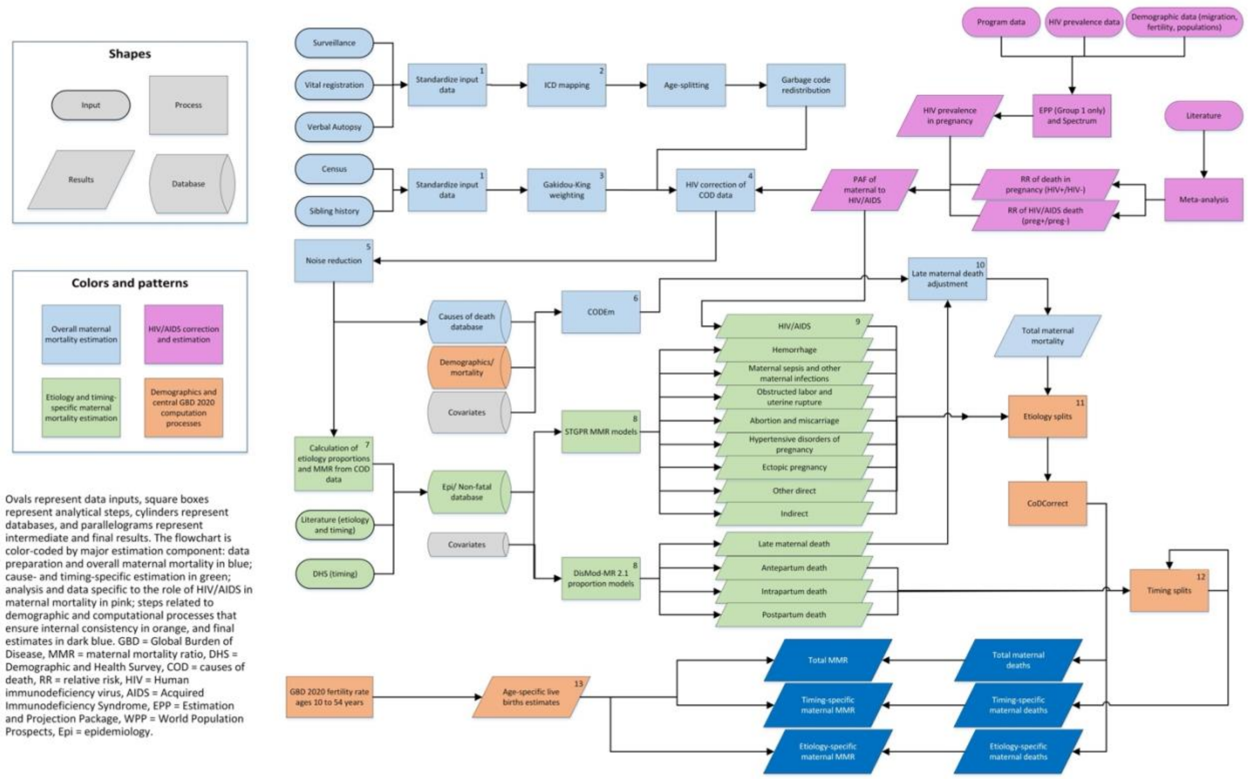

Input data and methodological summary for maternal disorders

Maternal disorders is a Level 3 cause of death in the GBD cause hierarchy and comprises ten Level 4 subcauses: maternal hemorrhage, maternal sepsis and other maternal infections, maternal hypertensive disorders, maternal obstructed labour and uterine rupture, maternal abortion and miscarriage, ectopic pregnancy, other (direct) maternal deaths, maternal deaths aggravated by HIV/AIDS (aka indirect maternal deaths due to the exacerbating effects of pregnancy on HIV/AIDS), indirect maternal deaths (excluding those due to HIV/AIDS), and late maternal deaths. Maternal disorders mortality captures comprehensive maternal deaths as defined by the World Health Organization.

Input data

Models of maternal mortality used centrally prepped data stored in the cause of death (CoD) database, including data sources that comprehensively assign cause of death to an entire sample of deaths and are used for other cause-specific mortality estimates in GBD (ie, vital registration, verbal autopsy), as well as sources that specifically measure maternal or pregnancy-associated deaths (ie, maternal mortality surveillance systems or confidential enquiries, other targeted surveys, censuses, sibling histories from large household surveys). A list of ICD codes that were mapped to maternal disorders, a list of vital registration sources included in the CoD database and their quality as indicated by star-ratings, and the steps used to process these diverse data types data prior to modelling can be found in the section of this appendix on the causes of death database and the tables and figures it refers to.

In addition to the CoD database, maternal mortality models also used data sources and reports identified through a systematic literature search, which is updated iteratively. Our systematic literature review for maternal disorders includes overall maternal mortality, cause-specific maternal mortality, incidence of pregnancy complications by type, relative risk of mortality in pregnancy in HIV-positive versus HIV-negative women, and relative risk of mortality in HIV-positive women who are pregnant versus non-pregnant. We updated this search on February 2, 2022, using the following search string:

(((("Postpartum Hemorrhage" OR "Uterine Hemorrhage") OR (maternal[Title/Abstract] OR pregnan*[Title/Abstract] OR mothers) AND (haemorrhag*[Title/Abstract] OR hemorrhag*[Title/Abstract]) NOT "case report"[All fields]) OR (("induced abortion" OR "Therapeutic abortion" OR "legal Abortion" OR "medical abortion" OR "miscarriage" OR "Abortion, Induced"[Mesh] OR "Abortion, Therapeutic"[Mesh] OR "Abortion, Legal"[Mesh] OR "ectopic Pregnancy") NOT ("case report"[Title/Abstract] OR "birth defect"[Title/Abstract] OR congenital[Title/Abstract])) OR ("obstructed labour" OR "obstructed labor" OR "labour dystocia" OR "labor dystocia" OR dystocia OR "cephalopelvic disproportion" OR "cephalo-pelvic disproportion") OR (("obstetric fistula" OR "vesicovaginal fistula") OR "rectovaginal fistula") OR (("Puerperal Infection"[Mesh] OR "Puerperal Infection" OR ((maternal[Title/Abstract] OR pregnan*[Title/Abstract]) AND (Sepsis OR infection[Title/Abstract]))) NOT "case report") OR ((pre-eclampsia[Title/Abstract] OR preeclampsia[Title/Abstract] OR eclampsia[Title/Abstract] OR Pre-Eclampsia[Mesh] OR Eclampsia[Mesh] OR "Hypertension, Pregnancy-Induced"[Mesh] OR "pregnancy induced hypertension"[Title/Abstract] OR "gestational hypertension"[Title/Abstract] OR "Hypertensive disorders of pregnancy"[Title/Abstract]) NOT ("case report" OR "kidney donor"[Title/Abstract] OR "kidney donors"[Title/Abstract] OR polymorphism*[Title/Abstract] OR endotheli*[Title/Abstract]))) OR ((("maternal mortality"[Title/Abstract] OR "maternal death"[Title/Abstract] OR "maternal deaths"[Title/Abstract] OR "MM"[Title/Abstract] OR "confidential enquiry"[Title/Abstract] OR "confidential inquiry"[Title/Abstract] OR ((obstetric[Title/Abstract] OR pregnan*[Title/Abstract]) AND (etiology[Title/Abstract] OR cause[Title/Abstract] OR pattern[Title/Abstract]) AND (death[Title/Abstract] OR mortality[Title/Abstract]))) NOT (fetal[Title/Abstract] OR newborn*[Title/Abstract] OR neonatal[Title/Abstract] OR "case report" [Title/Abstract] OR "case study" [Title/Abstract] OR pathogenesis[Title/Abstract] OR thromboprophylaxis[Title/Abstract])) OR ((("maternal mortality"[Title/Abstract] OR "maternal death"[Title/Abstract] OR "maternal deaths"[Title/Abstract] OR "MMR"[Title/Abstract]) AND ("Afghanistan"[Title/Abstract] OR "Albania"[Title/Abstract] OR "Algeria"[Title/Abstract] OR "Andorra"[Title/Abstract] OR "Angola"[Title/Abstract] OR "Antigua and Barbuda"[Title/Abstract] OR "Argentina"[Title/Abstract] OR "Armenia"[Title/Abstract] OR "Azerbaijan"[Title/Abstract] OR "Bahrain"[Title/Abstract] OR "Bangladesh"[Title/Abstract] OR "Barbados"[Title/Abstract] OR "Belarus"[Title/Abstract] OR "Belize"[Title/Abstract] OR "Benin"[Title/Abstract] OR "Bhutan"[Title/Abstract] OR "Bolivia"[Title/Abstract] OR "Bosnia and Herzegovina"[Title/Abstract] OR "Botswana"[Title/Abstract] OR "Brazil"[Title/Abstract] OR "Brunei"[Title/Abstract] OR "Bulgaria"[Title/Abstract] OR "Burkina Faso"[Title/Abstract] OR "Burundi"[Title/Abstract] OR "Cambodia"[Title/Abstract] OR "Cameroon"[Title/Abstract] OR "Cape Verde"[Title/Abstract] OR "Central African Republic"[Title/Abstract] OR "Chad"[Title/Abstract] OR "China"[Title/Abstract] OR "Colombia"[Title/Abstract] OR "Comoros"[Title/Abstract] OR "Congo"[Title/Abstract] OR "Costa Rica"[Title/Abstract] OR "Croatia"[Title/Abstract] OR "Cuba"[Title/Abstract] OR "Cyprus"[Title/Abstract] OR "Côte d'Ivoire"[Title/Abstract] OR "Democratic Republic of the Congo"[Title/Abstract] OR "Djibouti"[Title/Abstract] OR "Dominica"[Title/Abstract] OR "Dominican Republic"[Title/Abstract] OR "Ecuador"[Title/Abstract] OR "Egypt"[Title/Abstract] OR "El Salvador"[Title/Abstract] OR "Equatorial Guinea"[Title/Abstract] OR "Eritrea"[Title/Abstract] OR "Ethiopia"[Title/Abstract] OR "Federated States of Micronesia"[Title/Abstract] OR "Fiji"[Title/Abstract] OR "Gabon"[Title/Abstract] OR "Georgia"[Title/Abstract] OR "Ghana"[Title/Abstract] OR "Grenada"[Title/Abstract] OR "Guatemala"[Title/Abstract] OR "Guinea"[Title/Abstract] OR "Guinea-Bissau"[Title/Abstract] OR "Guyana"[Title/Abstract] OR "Haiti"[Title/Abstract] OR "Honduras"[Title/Abstract] OR "India"[Title/Abstract] OR "Indonesia"[Title/Abstract] OR "Iran"[Title/Abstract] OR "Iraq"[Title/Abstract] OR "Jamaica"[Title/Abstract] OR "Jordan"[Title/Abstract] OR "Kazakhstan"[Title/Abstract] OR "Kenya"[Title/Abstract] OR "Kiribati"[Title/Abstract] OR "Kuwait"[Title/Abstract] OR "Kyrgyzstan"[Title/Abstract] OR "Laos"[Title/Abstract] OR "Latvia"[Title/Abstract] OR "Lebanon"[Title/Abstract] OR "Lesotho"[Title/Abstract] OR "Liberia"[Title/Abstract] OR "Libya"[Title/Abstract] OR "Lithuania"[Title/Abstract] OR "Macedonia"[Title/Abstract] OR "Madagascar"[Title/Abstract] OR "Malawi"[Title/Abstract] OR "Malaysia"[Title/Abstract] OR "Maldives"[Title/Abstract] OR "Mali"[Title/Abstract] OR "Malta"[Title/Abstract] OR "Marshall Islands"[Title/Abstract] OR "Mauritania"[Title/Abstract] OR "Mauritius"[Title/Abstract] OR "Moldova"[Title/Abstract] OR "Mongolia"[Title/Abstract] OR "Montenegro"[Title/Abstract] OR "Morocco"[Title/Abstract] OR "Mozambique"[Title/Abstract] OR "Myanmar"[Title/Abstract] OR "Namibia"[Title/Abstract] OR "Nepal"[Title/Abstract] OR "Nicaragua"[Title/Abstract] OR "Niger"[Title/Abstract] OR "Nigeria"[Title/Abstract] OR "North Korea"[Title/Abstract] OR "Oman"[Title/Abstract] OR "Pakistan"[Title/Abstract] OR "Palestine"[Title/Abstract] OR "Panama"[Title/Abstract] OR "Papua New Guinea"[Title/Abstract] OR "Paraguay"[Title/Abstract] OR "Peru"[Title/Abstract] OR "Philippines"[Title/Abstract] OR "Qatar"[Title/Abstract] OR "Romania"[Title/Abstract] OR "Russia"[Title/Abstract] OR "Rwanda"[Title/Abstract] OR "Saint Lucia"[Title/Abstract] OR "Saint Vincent and the Grenadines"[Title/Abstract] OR "Samoa"[Title/Abstract] OR "Saudi Arabia"[Title/Abstract] OR "Senegal"[Title/Abstract] OR "Serbia"[Title/Abstract] OR "Seychelles"[Title/Abstract] OR "Sierra Leone"[Title/Abstract] OR "Singapore"[Title/Abstract] OR "Solomon Islands"[Title/Abstract] OR "Somalia"[Title/Abstract] OR "South Africa"[Title/Abstract] OR "South Sudan"[Title/Abstract] OR "Sri Lanka"[Title/Abstract] OR "Sudan"[Title/Abstract] OR "Suriname"[Title/Abstract] OR "Swaziland"[Title/Abstract] OR "Syria"[Title/Abstract] OR "São Tomé and Principe"[Title/Abstract] OR "Taiwan"[Title/Abstract] OR "Tajikistan"[Title/Abstract] OR "Tanzania"[Title/Abstract] OR "Thailand"[Title/Abstract] OR "The Bahamas"[Title/Abstract] OR "The Gambia"[Title/Abstract] OR "Timor-Leste"[Title/Abstract] OR "Togo"[Title/Abstract] OR "Tonga"[Title/Abstract] OR "Trinidad and Tobago"[Title/Abstract] OR "Tunisia"[Title/Abstract] OR "Turkmenistan"[Title/Abstract] OR "Uganda"[Title/Abstract] OR "Ukraine"[Title/Abstract] OR "United Arab Emirates"[Title/Abstract] OR "Uruguay"[Title/Abstract] OR "Uzbekistan"[Title/Abstract] OR "Vanuatu"[Title/Abstract] OR "Venezuela"[Title/Abstract] OR "Vietnam"[Title/Abstract] OR "Yemen"[Title/Abstract] OR "Zambia"[Title/Abstract] OR "Zimbabwe"[Title/Abstract])) NOT ("demographic and health survey"[Title/Abstract] OR "demographic and health surveys "[Title/Abstract] OR DHS[Title/Abstract] OR "reproductive health survey"[Title/Abstract] OR "reproductive health surveys"[Title/Abstract] OR RHS[Title/Abstract])) OR ((HIV[Title/Abstract] OR "Acquired Immunodeficiency Syndrome"[Title/Abstract] OR AIDS[Title/Abstract]) AND (pregnan*[Title/Abstract] OR "postpartum"[Title/Abstract] OR "post partum"[Title/Abstract]) AND ("mortality"[Title/Abstract] OR "death"[Title/Abstract]) NOT "case report")) AND (2019/05/10[PDat] : 3000[PDat]) NOT (animals[MeSH] NOT humans[MeSH]))

For GBD 2023, a total of 10 435 new, deduplicated published reports were identified and had their title and abstract reviewed. Of these, 499 sources were selected for full text review, and 163 met criteria for inclusion for one or more indicator of maternal disorder burden. We did not identify any new sources for the subcause of maternal deaths aggravated by HIV.

COD data processing was broadly similar to previous rounds of GBD, but one novel data processing step was employed in preparing data for maternal disorders that was not needed prior to GBD 2023—COVID-19 corrections—which are described elsewhere in this appendix.

Figure 2: PRISMA 2020 flow diagram

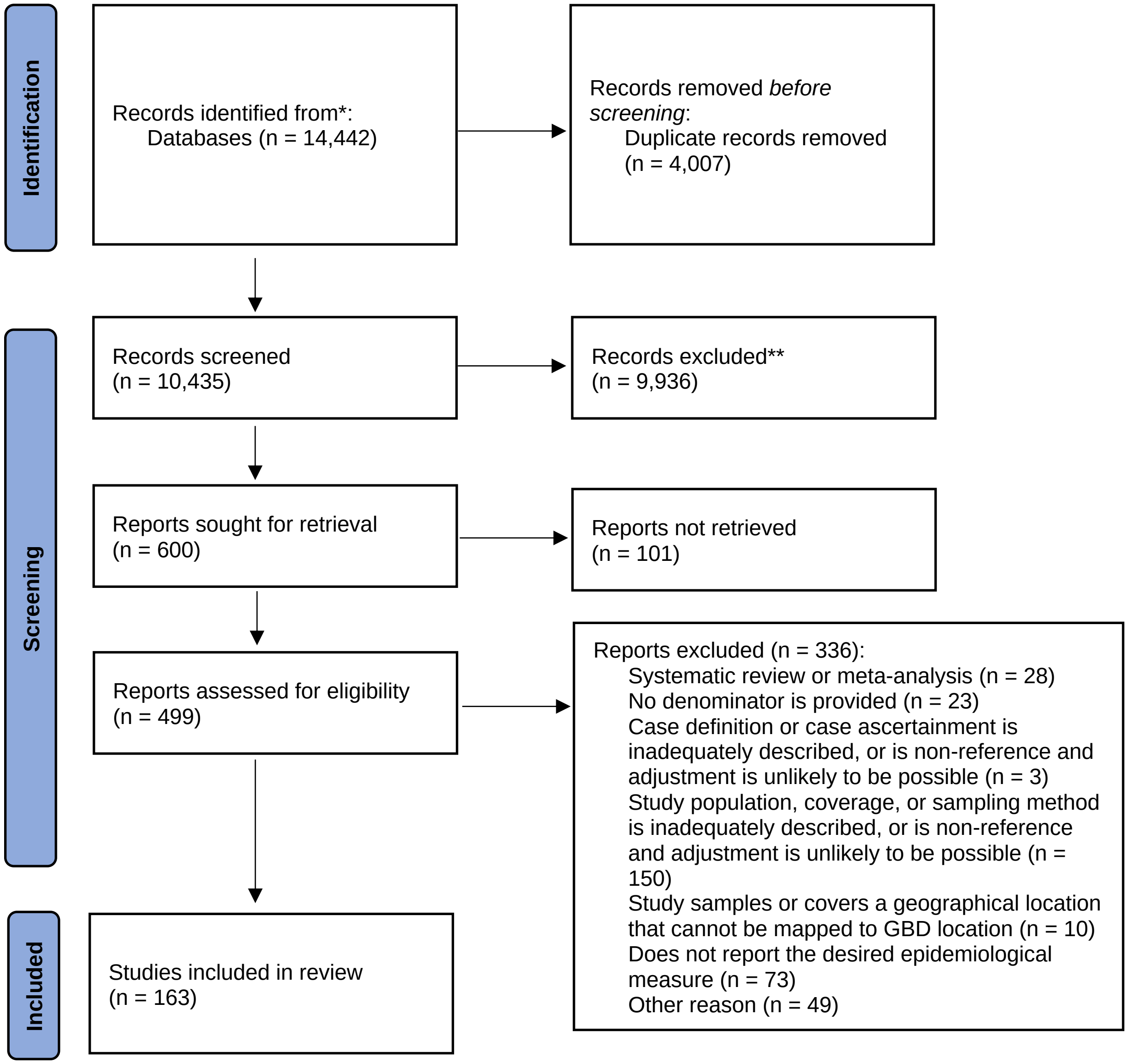

When available, we extracted overall maternal mortality data as a cause fraction, or the number of maternal deaths divided by all deaths of women of reproductive age, for modelling using Cause of Death Ensemble modeling (CODEm). For subcause-specific maternal mortality, we preferentially extracted subcause-specific maternal mortality ratios (MMR, or subcause-specific deaths per 100,000 livebirths) for modelling using spatiotemporal Gaussian process regression (ST-GPR). For sources that reported other metrics (eg, overall maternal mortality as MMR, subcause-specific maternal mortality as cause fraction, or either outcome as a population rate or count), we extracted the best available metric and

converted to the preferred metric using GBD all-cause mortality, population, and age-specific fertility estimates. Estimation of these demographic quantities is described in capstone publications for each round of GBD.

For sources that reported pregnancy-associated deaths, we first removed all HIV deaths during data processing. To determine the number of non-incidental maternal deaths due to HIV, we combined estimated HIV prevalence in pregnancy with relative risk (RR) of mortality during pregnancy for HIV-positive women compared to HIV-negative women to calculate a population attributable fraction that was then divided between incidental and maternal deaths based on RR of death in HIV-positive women who are pregnant versus not pregnant. Maternal (non-incidental) HIV deaths were then added to HIV-free estimates for all sources. These processes are described in more detail in the appendix section on HIV/AIDS estimation.

For late maternal deaths, we used unprocessed CoD data from the subset of locations where ICD codes for late maternal deaths appear in vital registration (VR) data; we assumed that any location that has never reported a late maternal death in its VR system does not capture any late maternal deaths. These CoD data were supplemented with late maternal death data identified during our systematic review, described above. We extracted these data as the proportion of total maternal deaths that occurred 43 to 365 days after the end of pregnancy.

After all data processing steps were complete, datapoints for all locations and years were systematically reviewed to identify outliers or other data limitations not fully overcome by CoD data processing methods. For maternal disorders, we systematically identified and excluded select location-years prior to modelling as follows:

- In the Central Europe, eastern Europe, and central Asia super-region, we excluded survey and census data in favor of vital registration data for Mongolia and Belarus. We also excluded data for Romania prior to 1990 for systematically high and implausible estimates in the ICD-9-BTL coding system.
- In the high-income super-region, we excluded select years of data for Andorra, Monaco, and San Marino for low coverage. We also excluded surveillance data for several years in Denmark and Ireland due to the compositional bias introduced into the time series when combined with vital registration data.
- In the Latin America and the Caribbean super-region, we excluded vital registration data in favor of surveillance and/or sibling history data for Peru, El Salvador, and Guatemala. For Colombia, we excluded data for years using ICD-9 codes due to a time-series disconnect between ICD-9 and ICD-10. We also excluded data prior to 1992 for Grenada, Saint Lucia, and Suriname due to a known coding consistency issue in ICD-9-BTL data.
- In the north Africa and the Middle East super-region, we excluded selected years of data for Saudi Arabia due to low data completeness and a high percentage of garbage-coded data. We also excluded survey and census data for Iraq due to low coverage, and we excluded vital registration in favor of verbal autopsy and survey/census data for Egypt.
- In the south Asia super-region, we excluded Medical Certification of Cause of Death (MCCD) data for India because this source only covers hospital deaths in urban areas. (India Sample Registration System is the preferred source.)
- In the southeast Asia, east Asia, and Oceania super-region, we excluded surveillance data in the Philippines in favor of vital registration and sibling history data. In Sri Lanka, we excluded vital

registration data in favor of survey and census data. In Viet Nam, we excluded select years for known low data coverage. In China, we excluded vital registration – sample and vital registration data in favor of surveillance in most provinces; however, in some provinces where surveillance sources had known issues systematically biasing estimates, we excluded surveillance data in favor of vital registration – sample and vital registration sources.

- In sub-Saharan Africa, we excluded select years due to a recall bias issue in sibling history data for multiple countries: Congo, Gabon, Comoros, Ethiopia, Eswatini, Lesotho, Côte d'Ivoire, the Gambia, Ghana, Liberia, Mauritania, Nigeria, São Tomé and Príncipe, and Sierra Leone. In Madagascar, Zimbabwe, and Ghana, we also excluded vital registration data in favor of sibling history data.
- We excluded literature sources for Tanzania, Nigeria, Senegal, Sierra Leone, Mali, Honduras, Morocco, Lebanon, Bangladesh, Syria, and Algeria that were inadvertently processed as cause fractions that excluded HIV deaths in the denominator.

We made other ad hoc data exclusions for specific datapoints where age patterns or temporal patterns were inconsistent, implausible, or reflected known issues with specific years or age groups (eg, unreliable data for terminal age groups).

Modelling strategy

Overall maternal mortality

Overall maternal mortality was estimated using CODEm. Fatal estimation for maternal mortality was restricted to ages 10–54 years. Two separate models were run, one of which was for data-rich countries, and one of which was for all countries (which we refer to as the global model). Covariates tested for inclusion in the data-rich and global models and their level and directionality are show in the table below:

Table 1: Covariates tested for inclusion in CODEm models of overall maternal mortality

Level	Covariate	Direction
Level 1	Age-specific fertility rate<br>Total fertility rate (log-transformed)<br>Maternal education (years per capita)<br>In-facility delivery (proportion)<br>Skilled birth attendance (proportion)<br>Neonatal mortality ratio (log-transformed)<br>Age-specific HIV mortality in females 10–54 (log-transformed)<br>COVID-19 age-standardised death rate *(data-rich model only)*	+<br>+<br>–<br>–<br>–<br>+<br>+
Level 2	Antenatal care 1-visit coverage (proportion)	-
	Antenatal care 4-visits coverage (proportion)	-
	Age-standardised wasting (weight-for-height) summary exposure value (SEV)	+
	Age-standardised stunting (height-for-age) SEV	+
	Healthcare Access and Quality Index	-
	Age- and sex-specific SEV for high body-mass index (BMI)	+
	Age- and sex-specific SEV for high blood pressure (SBP)	+
	Underweight women of reproductive age	+

Level 3	Socio-demographic Index<br>Mortality shock (cumulative rate in last 10 years)<br>LDI (log-transformed)	-<br>+<br>-

In the ensemble models selected by the CODEm algorithm based on out-of-sample predictive validity, 16 covariates were selected the final global CODEm model; the two covariates that were not selected were age- and sex-specific SEV for high blood pressure (SBP) and mortality shock (cumulative rate in last ten years). For the final data-rich CODEm model, 14 covariates were selected. Those covariates not selected were age- and sex-specific SEV for high blood pressure (SBP), morality shock (cumulative rate in last ten years), underweight women of reproductive age, Socio-demographic Index, and log-transformed LDI.

Subcause-specific maternal mortality

We used spatiotemporal Gaussian process regression (ST-GPR) to estimate MMR for each of eight maternal subcauses (the Level 4 GBD causes: maternal hemorrhage, maternal sepsis and other maternal infections, maternal hypertensive disorders, maternal obstructed labour and uterine rupture, maternal abortion and miscarriage, ectopic pregnancy, other (direct) maternal deaths, indirect maternal deaths [excluding those due to HIV/AIDS]). This modelling strategy required data to be in standard, five-year GBD age groups; for all input data that were not extracted in standard GBD age groups, we applied the global age pattern of available CoD data for that respective subcause. ST-GPR also requires variance for each datapoint, so we ran a LOESS regression on the input data by year and used the variance of the residuals as variance for that datapoint.

Stage 1 predictions for ST-GPR were generated using an ensemble of regressions where weighting of each component model was based on out-of-sample coverage prediction performance. This approach allowed us to test inclusion of multiple covariates and to specify the direction of relationships between covariates and the subcause outcome of interest. Covariates were specific for each subcause model, as shown in the table below:

Table 2: Covariates used in generation of ensemble stage 1 predictions of subcause-specific maternal mortality ST-GPR models

Maternal subcause	Country-level covariates	Direction
Maternal haemorrhage	In-facility delivery (proportion)<br>Skilled birth attendance (proportion)<br>Age- and sex-specific SEV for unsafe sanitation<br>Neonatal mortality ratio (log-transformed)<br>Maternal education<br>Healthcare Access and Quality Index	-<br>-<br>+<br>+<br>-<br>-
Maternal hypertensive disorders	Age- and sex-specific SEV for high fasting plasma glucose (FPG)<br>Age- and sex-specific SEV for high blood pressure (SBP)<br>Neonatal mortality ratio (log-transformed)<br>Antenatal care 1-visit coverage (proportion)<br>Antenatal care 4-visits coverage (proportion)<br>Healthcare Access and Quality Index	+<br>+<br>+<br>-<br>-<br>-
Obstructed labour and uterine rupture	In-facility delivery (proportion)<br>Skilled birth attendance (proportion)	-<br>-

	Underweight women of reproductive age<br>Neonatal mortality ratio (log-transformed)<br>Age-standardised wasting (weight-for-height) SEV<br>Age-standardised stunting (height-for-age) SEV	+<br>+<br>-<br>+
Abortion and miscarriage	Abortion legality<br>Antenatal care 1-visit coverage (proportion)<br>Antenatal care 4-visits coverage (proportion)<br>Maternal education<br>Healthcare Access and Quality Index	-<br>-<br>-<br>-<br>-
Ectopic pregnancy	Abortion legality<br>Pelvic inflammatory disease age-standardised prevalence<br>Antenatal care 1-visit coverage (proportion)<br>Antenatal care 4-visits coverage (proportion)<br>Maternal education<br>Healthcare Access and Quality Index	-<br>+<br>-<br>-<br>-<br>-
Maternal sepsis and other maternal infections	In-facility delivery (proportion)<br>Skilled birth attendance (proportion)<br>Age- and sex-specific SEV for unsafe sanitation<br>Age- and sex-specific SEV for high fasting plasma glucose (FPG)<br>Antenatal care 1-visit coverage (proportion)<br>Antenatal care 4-visits coverage (proportion)<br>LDI (log-transformed)<br>Healthcare Access and Quality Index	-<br>-<br>+<br>+<br>-<br>-<br>-<br>-
Other maternal deaths	In-facility delivery (proportion)<br>Skilled birth attendance (proportion)<br>Antenatal care 1-visit coverage (proportion)<br>Antenatal care 4-visits coverage (proportion)<br>LDI (log-transformed)<br>Maternal education<br>Healthcare Access and Quality Index	-<br>-<br>-<br>-<br>-<br>-<br>-
Indirect maternal deaths	In-facility delivery (proportion)<br>Skilled birth attendance (proportion)<br>Antenatal care 1-visit coverage (proportion)<br>Antenatal care 4-visits coverage (proportion)<br>LDI (log-transformed)<br>Maternal education<br>Healthcare Access and Quality Index	-<br>-<br>-<br>-<br>-<br>-<br>-

Maternal deaths aggravated by HIV/AIDS (aka indirect maternal deaths due to the exacerbating effects of pregnancy on HIV/AIDS)

Deaths estimated as maternal (non-incidental) HIV deaths (ie, those appended to HIV-free estimates, as described in the section above for overall maternal mortality) are directly used as estimates for this subcause of maternal deaths.

Late maternal deaths

We ran a single-parameter model in DisMod-MR 2.1 to estimate the proportion of all maternal deaths that were late. We used the late proportion data described previously and HAQ Index as a country-level covariate.

Rescaling of estimates

We used results of the late proportion model to expand the total estimate of maternal deaths upward to account for presumed missing late maternal deaths in one of two situations: in locations with vital registration systems reporting late maternal deaths, we late-corrected years prior to the year these deaths were first reported; in locations where late maternal deaths data are not reported, we late-corrected all location-years. Subcause-specific estimates were derived by scaling the results from the ST-GPR subcause-specific models in relation to each other; these subcause-specific proportions were then multiplied by the estimated total maternal deaths (excluding late maternal deaths and indirect maternal deaths due to HIV) for that age group, location, and year. Maternal mortality estimates were further scaled in CoDCorrect, which rescales causes of death in GBD to one another and within a common set of demographics including population size, fertility, and all-cause mortality.

Cause-specific limitations

Pandemic effects on maternal mortality are captured in the data-rich CODEm model, reflecting availability of pandemic-year data and inclusion of a COVID mortality-related covariate; for non-data-rich locations, however, empirical pandemic-year data are sparse, and including this covariate resulted in implausibly high estimates for pandemic years. We plan to explore additional methods of capturing pandemic-year trends in locations with no or minimal pandemic year data in future rounds of GBD, either through CODEm or through custom analyses.

In GBD 2023, all-cause mortality estimates are notably different from GBD 2021, especially in some locations with a high burden of maternal mortality. This change, which reflects an improvement to demographic estimation methodology, has resulted in higher estimates of MMR across the time series globally and in several countries compared to previous iterations of GBD.

Measles

Flowchart

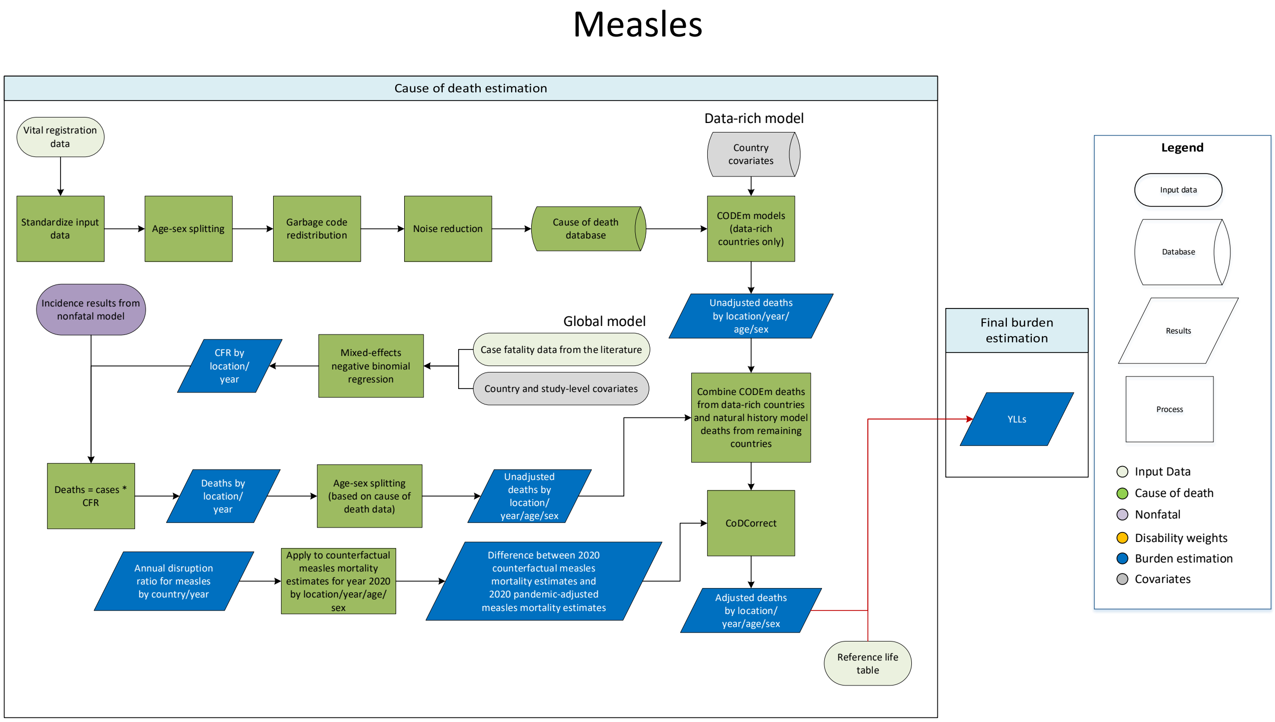

Input data and methodological summary for measles

Input data

Measles cause of death (CoD) data for GBD 2023 included vital registration, verbal autopsy, and surveillance sources from all locations where data were available. We included data from 22 new sources from a systematic review of scientific literature on measles case-fatality ratios, bringing our total sources to 255 (see Figure 1).

Modelling strategy overview

The GBD 2023 measles mortality estimates were generated in one of two ways depending on the quality of available cause of death data. For countries with well-defined cause of death registration systems, we used a Cause of Death Ensemble model (CODEm). For the remaining countries, we used estimates of incident measles cases from our non-fatal models and multiplied those by case-fatality rate estimates. For all countries, we produced measles mortality estimates for all age groups between 6 months and 64 years.

Data-rich countries

For data-rich countries modelled in CODEm, we used the covariates listed in Table 1 to inform predictions. New in GBD 2023, we included a number of covariates to adjust for the impact of the

COVID-19 pandemic on measles mortality. These covariates included a COVID-inclusive moving average of first dose of measles-containing vaccine (MCV1) coverage over the past five years, per capita COVID-infection, and masking outside the home.

Table 1. Covariates. Summary of covariates used in the data-rich measles cause of death model

Level	Covariate	Direction
1	Average COVID-inclusive measles-containing vaccination dose one (MCV1) coverage over the past five years	-
	Per capita COVID infection	+/-
	Proportion of population reporting always wearing a mask when leaving home	-
2	Healthcare Access and Quality (HAQ) Index	-
3	Socio-demographic Index (SDI)	-
	Mean years of education per capita	-

Natural history model

A natural history model was used to estimate measles mortality in non-data-rich locations where mortality data are sparse. To derive mortality, measles incidence estimates were multiplied by measles case-fatality ratio (CFR) estimates. As described in the non-fatal measles modelling methods appendix, case notifications informing the measles non-fatal model came from the World Health Organization (WHO) Joint Reporting Form (JRF) and national surveillance systems where subnational data were available. Log-transformed measles incidence was modelled using a mixed effects linear regression model with vaccination coverage (rolling means of COVID-inclusive MCV1 and MCV2 over the preceding five years), five-year lagged supplemental immunisation activities (SIA) coverage and masking outside the home as covariates. New in GBD 2023, we included a masking covariate to account for the impact of COVID-19 mitigation strategies on the incidence of measles. This approach differs from the COVID-19 adjustment used in GBD 2021, wherein COVID-free counterfactual estimates of measles incidence were modelled, followed by a post-hoc adjustment for the impact of the pandemic.

The measles CFR data were obtained through systematic reviews of the literature last updated for GBD 2023. This search was conducted in PubMed using the following search string: *((((measles[MeSH Terms] OR measles) AND (mortality[MeSH Terms] OR mortality OR "case fatality rate" OR "case fatality ratio" OR "case fatality"))) AND ("2016"[Date - Publication] : "2019"[Date - Publication]))*.

With the available measles CFR input data, we estimated a location- and year-specific case fatality ratio using a negative binomial model. We included the following covariates in the model: Socio-demographic Index (SDI) as a country-level covariate, hospitalised versus outpatient, outbreak versus endemic, and rural or urban/mixed as study-level covariates. In GBD 2023, we removed the location-specific random effect that had previously been included in the model to avoid undue influence of a limited number of studies from a geographical region. In GBD 2021, population-level data from non-data-rich locations were excluded from the model. In GBD 2023, all population-level data were included. The model was formulated as follows:

$$Y_{ij} = \beta_0 + \beta_1 SDI_{ij} + \beta_2 hospital_{ij} + \beta_3 outbreak_{ij} + \beta_4 rural_{ij} + e_{ij}$$

where Y_{ij} is the number of deaths (using measles cases as the offset term); β_0 is the fixed-effect intercept; β_1, β_2, β_3, and β_4 are the fixed-effects slopes on the Socio-demographic Index (SDI) and hospital, outbreak, and rurality study-level covariates; e_{ij} is the residual; i is the year; and j is the location. Studies were weighted by sample size (number of measles cases) when fitting the CFR model. When predicting CFR from this model for each location and year, covariates were set to reflect non-hospital, non-outbreak, and urban (non-rural) settings. Uncertainty was estimated by taking 1000 samples from predictions calculated from the variance-covariance matrix.

Measles deaths were calculated as:

$$deaths = incidence * CFR$$

This calculation was replicated for each of the 1000 samples of the posterior distribution of both the incidence and CFR models to produce estimates of measles deaths for each location and year. These all-age, all-sex estimates were then split using an age-sex distribution pattern of measles mortality extracted from the cause of death database. All estimates were then summarised as the mean of the samples (draws) along with a 95% uncertainty interval (the 2.5^{th} and 97.5^{th} percentile of all draws).

Changes from GBD 2021 to GBD 2023

The major substantive change in our modelling strategy for GBD 2023 was in the methods used for estimating COVID impact on measles mortality and our approach to CFR modelling. In GBD 2023, we incorporated COVID-inclusive vaccine coverage and mask use as covariates directly in our models, whereas in GBD 2021, the impact of COVID-19 was handled as a post-hoc adjustment. In our GBD 2023 CFR model, we included population-level data from non-data-rich locations and removed the location-specific random effect.

Figure 1: PRISMA 2020 flow diagram for measles CFR

From: Page MJ, McKenzie JE, Bossuyt PM, et al. The PRISMA 2020 statement: an updated guideline for reporting systematic reviews. *BMJ*. 2021;372:n71. Published 2021 Mar 29. doi:10.1136/bmj.n71

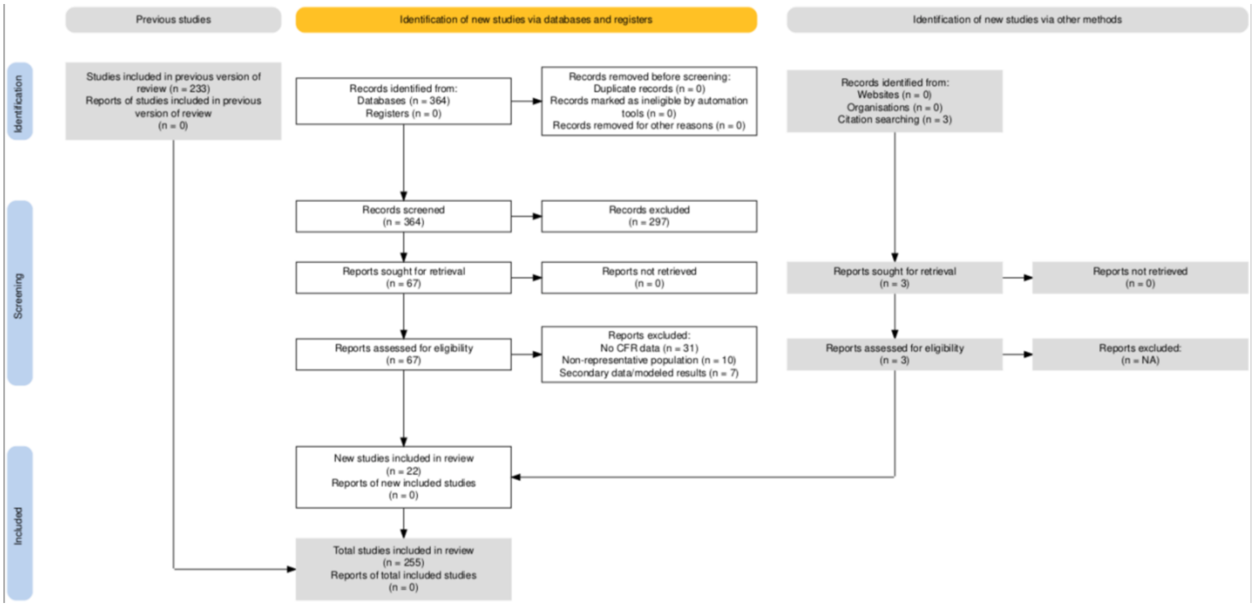

Meningitis

Flowchart

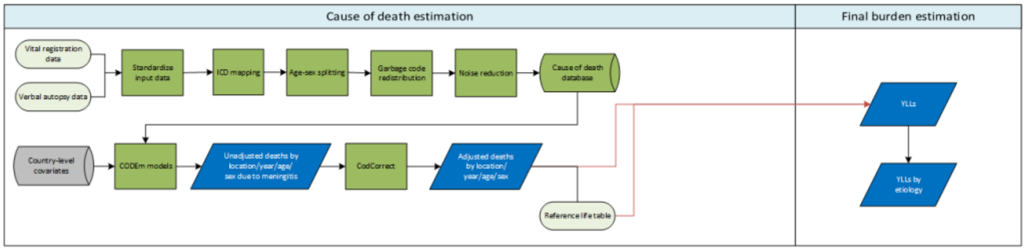

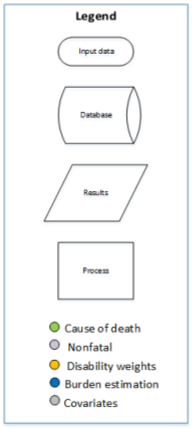

Input data and methodological summary for meningitis

Input data

Input data for the overall meningitis model came from the cause of death database, which includes vital registration (VR) and verbal autopsy (VA) data. We outliered data in instances where garbage code redistribution and noise reduction, in combination with small sample sizes, resulted in unreasonable cause fractions when compared to regional, super-regional, and global rates, and data that violated well-established time or age trends. Outliering methods were consistent across both VR and VA data.

Modelling strategy

We modelled deaths due to all meningitis with two CODEm models, separately for each sex and two age categories – under 5 and 5 years and older. The mortality trends differ substantially between children and adults, and a significant number of data sources only have data for children under 5. The two models used the same covariates (with the exception of the covariate for underweight, which is age-specific in the under-5 model and age-standardised in the 5+ model) and otherwise standard CODEm parameters. The final sex-specific models for deaths due to all meningitis were a hybridised model of separate global and data-rich models for males and females.

In past GBD cycles, estimates of PCV3 vaccine coverage among infants in the modelled year were used as the primary covariate for this linear regression. Starting in GBD 2021, we used a lagged mean of PCV3 vaccine coverage calculated over a rolling, five-year interval in order to capture population-level vaccine-derived immunity among under-5-year-olds, including coverage both in the current year and in recent years. For Hib3 vaccine coverage, we use a version of the lagged Hib3 covariate with an additional transformation applied to account for indirect effects from herd immunity. The Hib vaccine is

one of the most effective vaccines in use, with 95% to 100% efficacy.[1] This results in a highly pronounced herd immunity effect, meaning that the reduction in invasive Hib disease seen in the population is greater than what would be expected with direct coverage alone. This effect was quantified by Majumder and colleagues, who performed a meta-analysis to determine an indirect effect multiplier for vaccine coverage.[2] We used the equations from the Wolfson method for expected protection from invasive Hib provided in this meta-analysis to transform the Hib3 covariate.

Table 1. Covariates used in meningitis mortality modelling (0–4 years, 5–95+ years)

Level	Covariate	Direction
1	Meningitis belt (proportion of population in belt)	+
	MenAfriVac coverage	-
	Hib3 vaccine coverage proportion, indirect	-
	PCV3 vaccine coverage proportion, lagged, COVID-inclusive	-
2	Age- and sex-specific summary exposure value (SEV) for child underweight	+
	Logit-transformed water (proportion with access)	-
	Maternal care and immunisation	-
	Healthcare Access and Quality (HAQ) Index	-
3	Log-transformed lag-distributed income	-
	Sanitation (proportion with access)	-
	Maternal education (years per capita)	-
	Socio-demographic Index	-

Aetiology estimation

Meningitis aetiologies are now produced by IHME's Antimicrobial Resistance Collaborators. Further reading on the data and methods used to estimate these proportions were published in September 2024.[1]

References

1. Naghavi M, Vollset SE, Ikuta KS, Swetschinski LR, Gray AP, Wool EE, Aguilar GR, Mestrovic T, Smith G, Han C, Hsu RL. Global burden of bacterial antimicrobial resistance 1990–2021: a systematic analysis with forecasts to 2050. The Lancet. 2024 Sep 28;404(10459):1199-226.

---

[1] Hamborsky J, Kroger A, editors. Epidemiology and prevention of vaccine-preventable diseases, E-Book: The Pink Book. Public Health Foundation; 2015 Oct 19. Chapter 8: *Haemophilus influenzae*.

[2] Majumder A. *Quantifying the Indirect Effects of* Haemophilus Influenzae *type b Vaccination in Children Under 5 Years-Old* (Doctoral dissertation, Johns Hopkins University).

[3] Naghavi M, Vollset SE, Ikuta KS, Swetschinski LR, Gray AP, Wool EE, et al. Global burden of bacterial antimicrobial resistance 1990–2021: a systematic analysis with forecasts to 2050. The Lancet. 2024 Sep 28;404(10459):1199–226.

Motor neuron disease

Flowchart

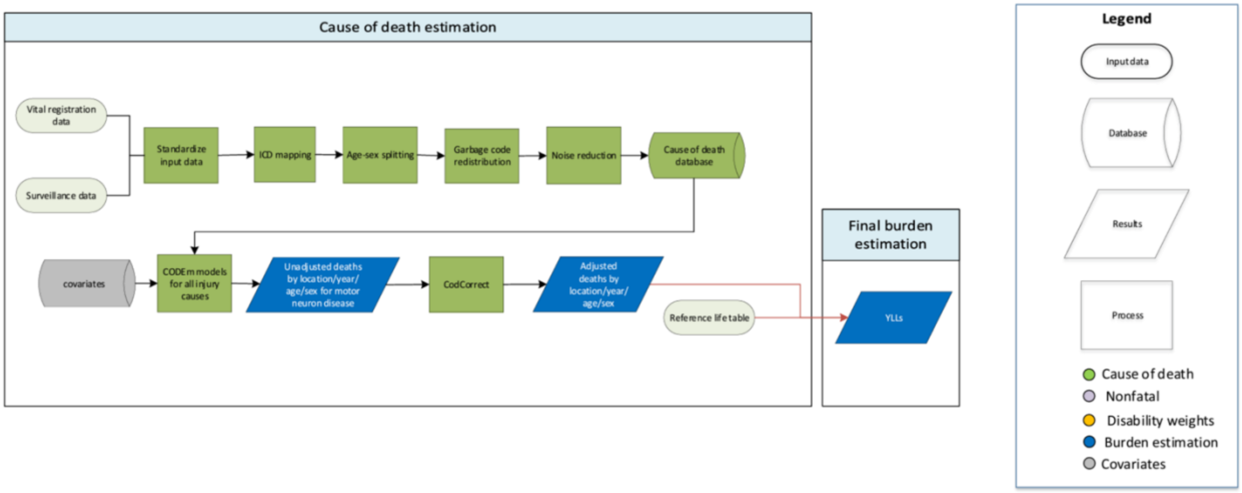

Input data and methodological summary for motor neuron disease

Input data

Data used to estimate motor neuron disease mortality included vital registration and surveillance data. Our outlier criteria were to exclude datapoints that (1) were implausibly high or low, (2) substantially conflicted with established age or temporal patterns, or (3) substantially conflicted with other data sources from the same locations or locations with similar characteristics (ie, Socio-demographic Index).

Modelling strategy

The standard Cause of Death Ensemble modelling (CODEm) approach was used to estimate deaths due to motor neuron disease. Separate models were conducted for male and female mortality, and the age range for both models was 0 days to 95+ years. Unadjusted death estimates were adjusted using CoDCorrect to produce final estimates of deaths and years of life lost (YLLs). See appendix 1 section 4 of the reference article for further information.

The following table lists the covariates included in the model. CODEm requires that the covariate selected for the model must have the directional relationship with motor neuron disease deaths. For GBD 2023, no significant updates were made for motor neuron disease covariate selection. Covariate directions were selected based on the strength of the evidence.

Table 1. Covariates used in motor neuron disease mortality modelling

Level	Covariate	Direction
1	Mean total body-mass index (kg/m^2)	-
	Mean serum total cholesterol (mmol/L)	-
	Absolute value of average latitude	+
	Mean diabetes fasting plasma glucose (mmol/L)	+
	Fruit consumption (grams per day, adjusted)	-
	Socio-demographic Index	+
	Healthcare Access and Quality Index	-
2	Population-weighted mean temperature	-
	Sanitation (proportion with access)	+
	Improved water source (proportion with access)	-
3	Education (years per capita)	+
	Log-transformed LDI (per capita)	+

Multidrug-resistant tuberculosis, extensively drug-resistant tuberculosis, and drug-susceptible tuberculosis

Flowchart

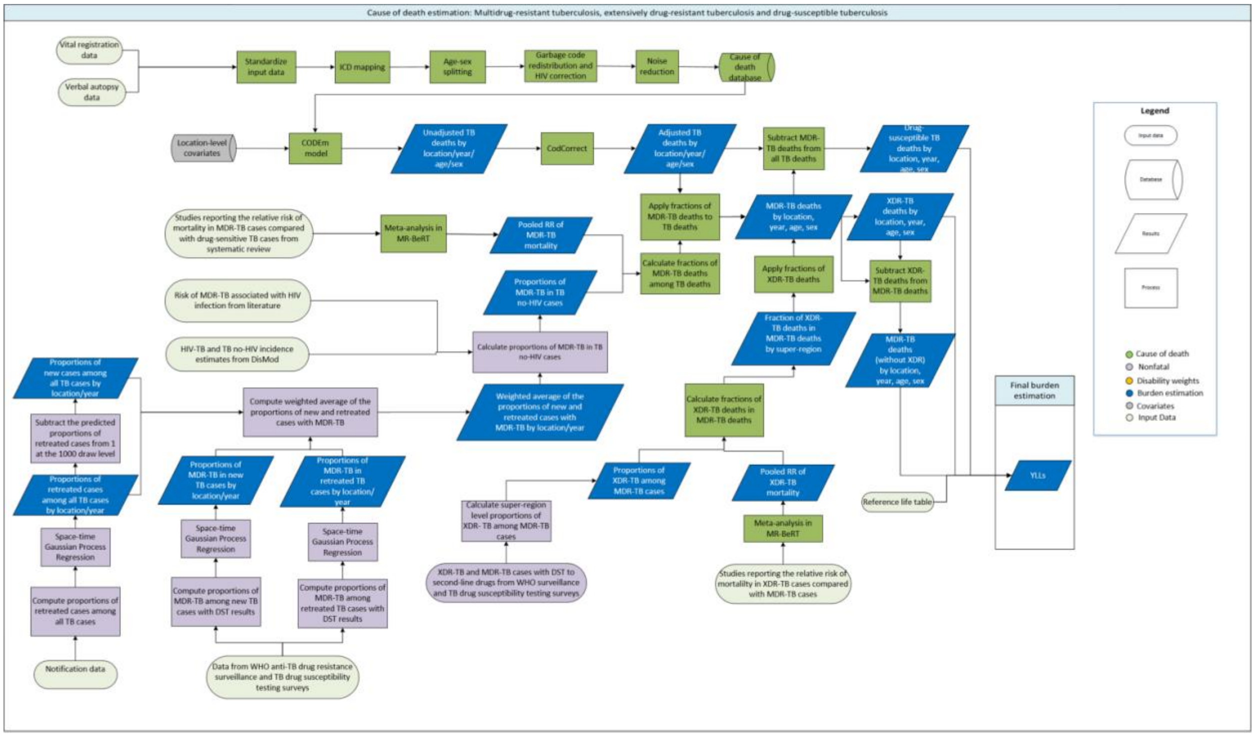

Input data and methodological summary for multidrug-resistant tuberculosis, extensively drug-resistant tuberculosis, and drug-susceptible tuberculosis

Input data

Input data include: (i) the number of drug-resistant cases by type (multidrug-resistant tuberculosis [MDR-TB], extensively drug-resistant tuberculosis [XDR-TB], all TB cases with a drug-susceptible testing [DST] result for isoniazid and rifampicin, and MDR-TB cases with DST for second-line drugs) from routine surveillance and surveys reported to the World Health Organization, (ii) data from studies (identified through our systematic review) reporting on the relative risk of death in MDR-TB cases compared with non-MDR TB (drug-susceptible TB) cases, and the relative risk of death in XDR-TB cases compared with MDR-TB cases, and (iii) the risk of MDR-TB associated with HIV infection from the literature.[1] The systematic review was not updated in GBD 2023 and leveraged data inputs from the systematic review conducted in GBD 2021.

Modelling strategy

We ran spatiotemporal Gaussian process regressions to predict the proportions of new TB cases with MDR-TB, proportions of retreated TB cases with MDR-TB, and proportions of retreated cases among all TB cases for all locations and years. We also calculated the proportions of new TB cases among all TB cases. We then computed the weighted average of the proportions of new and retreated cases with MDR-TB at the 1000-draw level. We then used the weighted average proportions of MDR-TB, along with the HIV-TB and TB no-HIV incidence estimates (from our modelling of non-fatal TB), and the relative risk of MDR-TB associated with HIV infection from the literature[1] to compute the proportions of MDR-TB cases among HIV-negative TB cases $\left(P_{MDRnoHIVc,y,a,s}\right)$ by location, year, age, and sex using the following formula:

$$P_{MDRnoHIVc,y,a,s} = \frac{MDR_{c,y}}{\left(1 + \left(RR_{HIV}\frac{HIVTB_{c,y,a,s}}{TBnoHIV_{c,y,a,s}}\right)\right) TBnoHIV_{c,y,a,s}}$$

where $MDR_{c,y}$ is the number of all MDR-TB cases among HIV-positive and HIV-negative individuals by location and year, RR_{HIV} is the relative risk of MDR-TB associated with HIV infection, $HIVTB_{c,y,a,s}$ is the number of HIV-TB incident cases by location, year, age, and sex, and $TBnoHIV_{c,y,a,s}$ is the number of TB no-HIV incident cases by location, year, age, and sex.

We then computed the fraction of MDR-TB deaths among all HIV-negative TB deaths ($D_{MDRnoHIVc,y,a,s}$) using the following formula:

$$D_{MDRnoHIVc,y,a,s} = \frac{P_{MDRnoHIVc,y,a,s}RR_{MDR}}{P_{MDRnoHIVc,y,a,s}RR_{MDR} + 1 - P_{MDRnoHIVc,y,a,s}}$$

where RR_{MDR} is the relative risk of death in MDR-TB cases compared with drug-susceptible TB cases. In GBD 2021, the pooled relative risk was derived from a meta-analysis in the meta-regression with Bayesian priors, regularisation, and trimming (MR-BRT) model. After derivation of the pooled relative risk, we then applied the predicted HIV-MDR-TB death fractions to all HIV-TB death estimates to generate HIV-MDR-TB deaths by location, year, age, and sex. Next, we subtracted MDR-TB deaths from all TB deaths to generate drug-susceptible TB deaths by location, year, age, and sex.

To separate out XDR-TB from MDR-TB, we aggregated the XDR-TB cases and MDR-TB cases (with DST for second-line drugs) up to the super-region level and calculated the super-region-level proportions of XDR-TB among MDR-TB cases. Next, we computed the super-region-specific fractions of XDR-TB deaths among all MDR-TB deaths (D_{XDRsr}) using the following formula:

$$D_{XDRsr} = \frac{P_{XDRsr}RR_{XDR}}{P_{XDRsr}RR_{XDR} + 1 - P_{XDRsr}}$$

where P_{XDRsr} is the proportion of XDR-TB among MDR-TB cases for each super-region, and RR_{XDR} is the pooled relative risk of mortality in XDR-TB cases compared with MDR-TB cases. Similar to the pooled

relative risk for MDR-TB, the derivation of the pooled relative risk of mortality in XDR-TB was computed with a meta-analysis in the MR-BRT model for GBD 2021. These fractions were then applied to MDR-TB deaths in corresponding countries within the super-regions to produce XDR-TB deaths by location, age, and sex for the most recent year of estimation. We linearly extrapolated XDR-TB mortality rates back, assuming the mortality rates were zero in 1992, one year before 1993 when XDR-TB was first recorded in USA surveillance data.[2] Finally, we subtracted XDR-TB deaths from MDR-TB deaths to generate MDR-TB (without extensive drug resistance) deaths by location, year, age, and sex.

References

1. Mesfin YM, Hailemariam D, Biadglign S, Kibret KT. Association between HIV/AIDS and multi-drug resistance tuberculosis: a systematic review and meta-analysis. *PLoS One*. 2014;9(1):e82235.
2. Centers for Disease Control and Prevention (CDC). Extensively Drug-Resistant Tuberculosis --- United States, 1993—2006. MMWR. 2007; 56(11);250-253

Multiple sclerosis

Flowchart

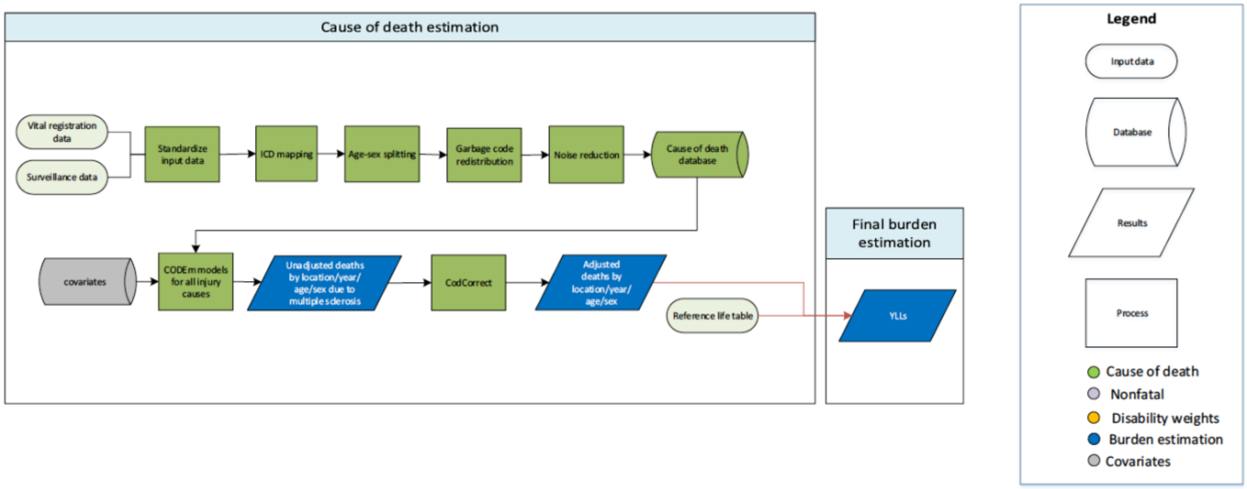

Input data and methodological summary for multiple sclerosis

Input data

Data used to estimate multiple sclerosis mortality included vital registration and surveillance data from the cause of death (CoD) database. Our outlier criteria were to exclude datapoints that (1) were implausibly high or low, (2) substantially conflicted with established age or temporal patterns, or (3) substantially conflicted with other data sources conducted from the same locations or locations with similar characteristics (ie, Socio-demographic Index). In particular, where data processing could not resolve discrepancies between different coding systems for the same location over time, one system was selected as more reliable and the other was excluded. In GBD 2023, this affected Kazakhstan, where ICD- 9-BTL-tabulated vital registration data were available for 1991–2003 and ICD-10-coded vital registration were available for 2013 onward. The raw ICD-10 data for 2013 onward were five-fold higher than the raw ICD-9-BTL (1992–2003), causing an implausible time pattern for multiple sclerosis. As such, the ICD-10-coded data were excluded. For China, two primary issues were observed in the data processing for MS. First, most MS deaths in China occurred in ages over 70, which appears unrealistic when compared to age patterns observed in data-rich countries like the USA. Second, the reported ICD-10-coded data from China contained implausibly low estimates for MS. To fix the issues in age pattern, MS deaths in China for those aged 70 and over were excluded from data processing. To address the remaining low ICD-10-coded data for those under 70, the ICD-9-coded data were strengthened during the noise reduction step, allowing

the ICD-10-coded data to instead take on the more reasonable pattern observed in the ICD-9-coded years.

Modelling strategy

The standard Cause of Death Ensemble modelling (CODEm) approach was used to estimate deaths due to multiple sclerosis. Separate models were conducted for male and female mortality, and the age range for both models was 5 to 95+ years. Unadjusted death estimates were adjusted using CoDCorrect to produce final estimates of deaths and years of life lost (YLLs). See appendix 1 section 4 of the reference article for further information.

The following table lists the covariates included in the model. CODEm requires that the covariate selected for the model must have a directional relationship with multiple sclerosis deaths. For GBD 2023, obesity prevalence was newly included in the multiple sclerosis model. Covariate directions were selected based on the strength of the evidence.

Table 1. Covariates used in multiple sclerosis mortality modelling

Level	Covariate	Direction
1	Absolute value of average latitude	+
2	Mean serum total cholesterol (mmol/L)	+
	Healthcare Access and Quality Index	-
3	Cumulative cigarettes (10 years)	+
	Cumulative cigarettes (5 years)	+
	Education (years per capita)	-
	Log-transformed LDI (per capita)	-
	Smoking prevalence	+
	Socio-demographic Index	+
	Obesity prevalence	+

Musculoskeletal disorders

Flowchart

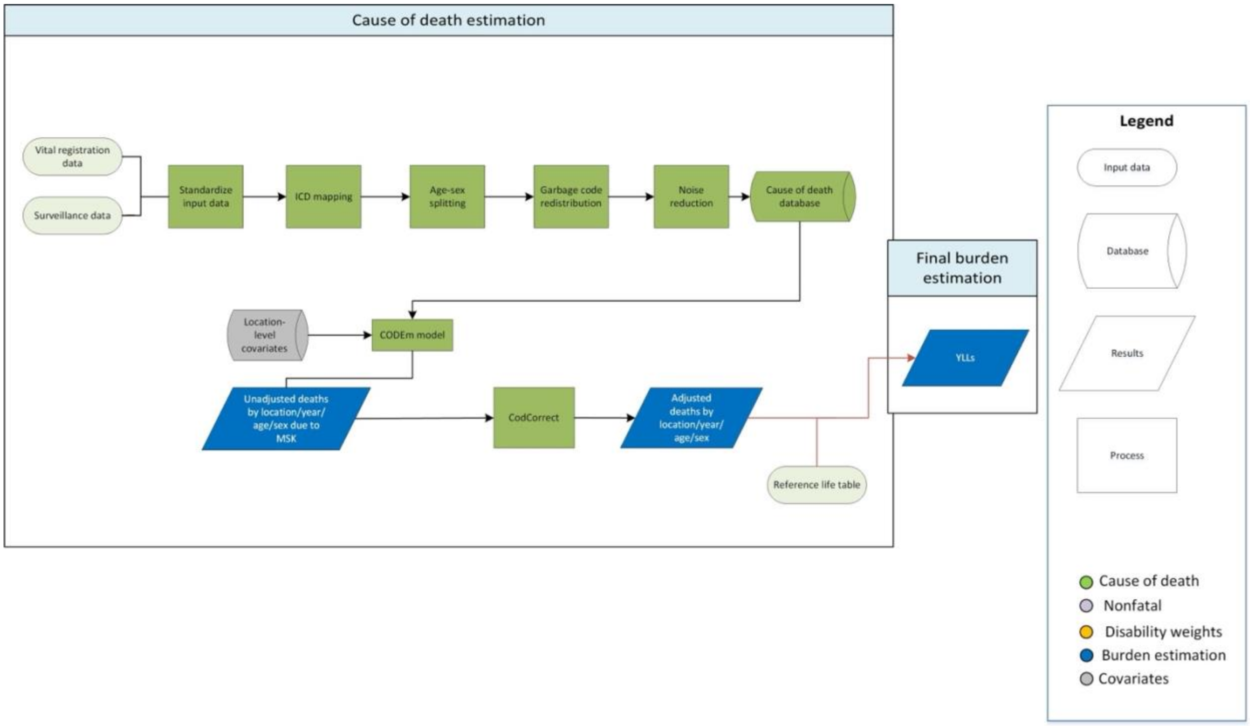

Input data and methodological summary for musculoskeletal disorders

Input data

Data used to estimate mortality from musculoskeletal disorders (MSKs) included vital registration (VR) and China disease surveillance data from the cause of death (CoD) database. Our outlier criteria excluded datapoints that (1) were implausibly high or low relative to global or regional patterns according to subject matter experts, (2) substantially conflicted with established age or temporal patterns, or (3) significantly conflicted with other data sources based from the same locations or locations with similar characteristics (ie, Socio-demographic Index), and (4) from verbal autopsy (VA) sources due to the inability of verbal autopsy to accurately capture most musculoskeletal conditions.

In GBD 2023, certain data were excluded based on quality concerns and input from in-country and subject matter experts:

- **Sparse and heterogeneous data**: Data from countries with sparse but heterogeneous data were excluded because they exaggerated death fluctuations and produced implausible regional trends. For example, verbal autopsy (VA) data from Sierra Leone were excluded for this reason.

- **Discontinuity in time trends**: Data were excluded when changes in coding systems caused time trend discontinuities, leading to implausibly high or low estimates. For instance, ICD-10 data from Kyrgyzstan before 2010 were excluded.

- **Voronezh Oblast data**: All vital registration (VR) data from Voronezh Oblast were excluded due to data quality issues, including implausibly high death estimates caused by coding practices for rare diseases in a low-population region.

- **Bangladesh data**: Data from Bangladesh were outliered because verbal autopsy methods in this location unreliably capture most musculoskeletal-related deaths.

- **India subnational data**: Data from Bihar and Uttar Pradesh subnationals were excluded due to implausibly high death estimates, as determined by subject matter experts.

Modelling strategy

In the GBD 2023 round, the modelling strategy for MSKs remained largely unchanged. The standard CODEm (Cause of Death Ensemble model) modelling approach was applied to estimate deaths due to musculoskeletal disorders (see appendix 1, section 4). The CODEm model for musculoskeletal disorders is limited by a lack of strong predictive covariates. Many are selected as a proxy for Socio-demographic Index (SDI), as many musculoskeletal disorders are autoimmune conditions, which tend to have increasing prevalence with SDI. The following table lists the covariates included in the model. Covariate directions were selected based on the strength of the evidence. This requires that the covariate selected for the model must have a directional relationship with musculoskeletal disorder deaths.

Table 1. Covariates used in musculoskeletal disorder mortality modelling

Level	Covariate	Direction
1	Mean BMI	+
	Vegetables (g), unadjusted	-
	Alcohol consumption (litres per capita)	+
2	Cumulative cigarettes (10 years)	+
	Cumulative cigarettes (5 years)	+
	Education (years per capita)	+
	Log-transformed LDI: lag-distributed income ($ per capita)	+
	Mean cholesterol	+
	Smoking prevalence	+
	Healthcare Access and Quality Index	-
	Age-standardised low bone mineral density	+
	Low bone mineral density	+
	Age-standardised bone mineral density among population age 60+ years	+
3	SDI: Socio-demographic Index	+

Myocarditis

Flowchart

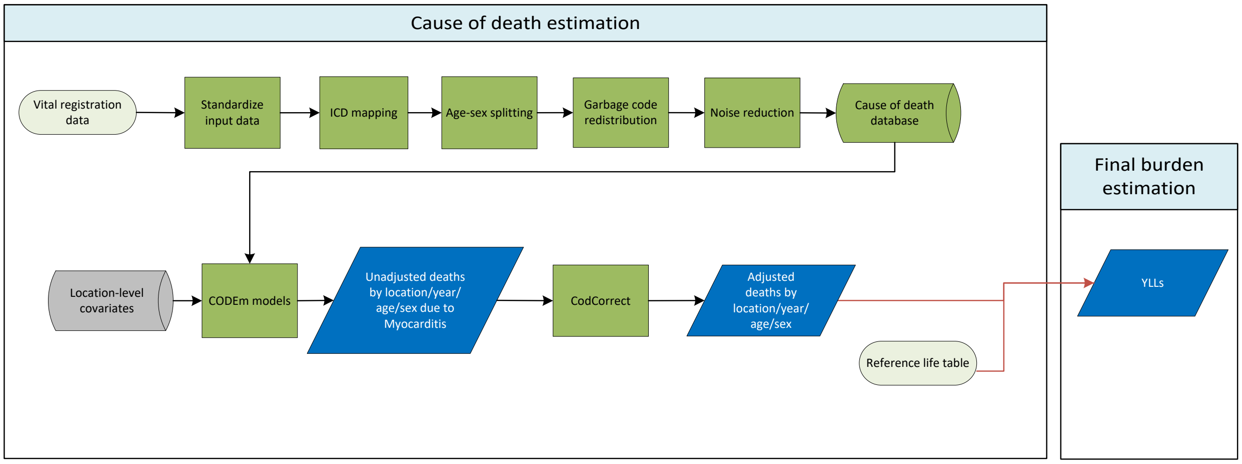

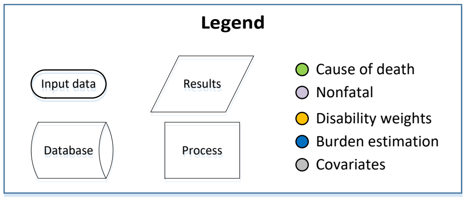

Input data and methodological summary for myocarditis

Input data

Vital registration data were used to model deaths due to myocarditis. We outliered all ICD-10 data in Egypt due to an implausibly high cause fraction compared to other locations in the region. ICD-10 tabulated data in Kazakhstan were outliered due to being much higher compared to the ICD-10 data in the country. Subsets of data from Bosnia and Herzegovina, Russia, Guyana, Cook Islands, Lebanon, Romania, Republic of Crimea, Sri Lanka, and western sub-Saharan Africa (Cabo Verde and Ghana) were also outliered due to implausibly high values and time trends.

Crosswalk by code system

Most data sources used for cause of death estimation are ICD-coded. The ICD periodically releases new versions, which can introduce an implausible disjoint in the time series that is indicative of a change in the coding practice, rather than a true change in the epidemiological trend of the disease. In GBD 2023, certain causes and countries using the ICD-9 code systems were adjusted to address the disjoint in the time trend and number of deaths between the older ICD-9 code systems and the more recent ICD-10 code systems. Myocarditis was corrected in Bulgaria (1990–2004), Canada (1986–1999), Spain (1980–1998), France (1980–1999), the USA (1980–1998), Hungary (1980–1995), Croatia (1985–1994), the UK (1980–1999), Greece (1987–2008), Italy (1980–2002), Mexico (1980–1997), and Mauritius (1980–2004) to account for this code system disjoint. This was done using a meta-regression tool described as a crosswalk, by taking systematically biased datapoints and estimating their unbiased value. Using this modeling tool, correction factors were created based on the more recent and more reliable ICD-10-

coded data. The crosswalk creates matched pairs between the last three years of data using the ICD-9 code system and the first three years of data using the ICD-10 code system. The correction is applied at the child cause level for all Level 2 causes, and after the correction is applied the individual child causes are raked to the parent cause to maintain a consistent number of deaths across the cause hierarchy.

Modelling strategy

We used a standard CODEm approach to model deaths from myocarditis. The model uses an empirical Bayesian noise reduction algorithm that uses grouped data by region and data type as a prior to better reflect regional patterns. For GBD 2023, we returned the direction of all variables from 1 to –1. The covariates selected for evaluation in the CODEm ensemble modelling process can be found in Table 3. Aside from this change, there have been no substantive changes to the modelling strategy since GBD 2021.

Table 3. Covariates used in myocarditis mortality modelling

Level	Covariate	Direction
2	Healthcare Access and Quality Index	–1
	Log-transformed lag-distributed income per capital (I$)	–1
3	Socio-demographic index	–1

Neonatal disorders

Flowchart

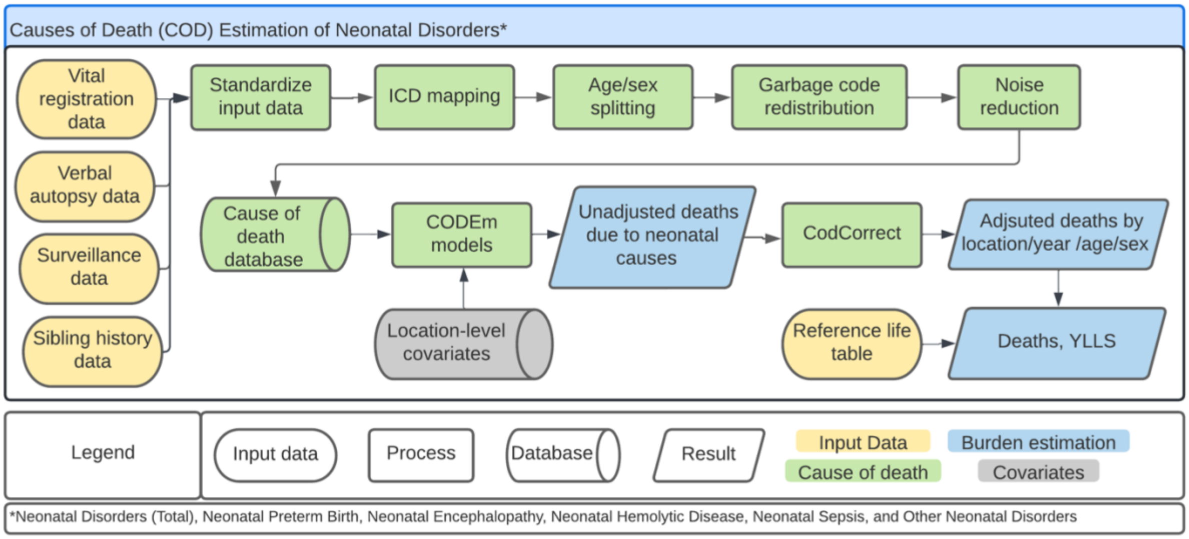

Input data and methodological summary for neonatal disorders

Mortality for five causes is modelled within “neonatal disorders”: neonatal preterm birth complications, neonatal encephalopathy due to birth asphyxia and trauma, neonatal sepsis and other neonatal infections, haemolytic disease and other neonatal jaundice, and other neonatal disorders. An overall neonatal disorders “parent” envelope is also estimated, to which all neonatal causes are squeezed.

Input data

Vital registration and surveillance were the majority of data sources used to estimate number of deaths from each condition. Only deaths among males and females under age 5 were modelled, in four separate age groups: early neonatal period, late neonatal period, post-neonatal period, and 1-4 years. Datapoints were selected as outliers if they were implausibly high or low, or significantly conflicted with established age or temporal patterns.

As of GBD 2019, neonatal disorders began using data from the Child Health and Mortality Prevention Surveillance (CHAMPS) Program in Bangladesh, Kenya, Mozambique, South Africa, and Mali, which provides minimally invasive tissue sampling (MITS) data on under-5 causes of death. For each subsequent GBD study, more MITS data were added to the models.

Though verbal autopsy data were available, validation studies suggest that verbal autopsy methods tend to be less accurate for cause of death ascertainment in the neonatal age groups.[1-4] Thus, the majority of verbal autopsy data were excluded. Verbal autopsy data were used, however, in the Indian states, in which they were the only data source.

Modelling strategy

The standard CODEm modelling approach was used to model each of the neonatal conditions. All neonatal causes used the following pool of covariates in covariate selection:

Table 1. Covariates used in neonatal disorders mortality modelling

Level	Covariate	Direction
1	Maternal care and immunisation	-
	All risk factors SEV scalar for neonatal disorders	+
2	Age-standardised SEV for household air pollution	+
	Age-standardised SEV for short gestation	+
	Age-standardised SEV for low birthweight	+
	Age-standardised SEV for smoking	+
	Age-standardised SEV for ambient particulate matter	+
3	Antenatal care (1 visit) coverage (proportion) Livebirths 35+ (proportion)	-
	Antenatal care (4 visits) coverage (proportion)	-
	In-facility delivery (proportion)	-
	Lag-distributed income per capita (I$ per capita)	-
	Skilled birth attendance (proportion)	-
	Total fertility rate	+
	Socio-demographic Index	-
	Healthcare Access and Quality Index	-
	Proportion of the population with at least 6 years of education, maternal	-
	Proportion of the population with at least 12 years of education, maternal	-

References

1. Anker M, Black RE, Coldham C, *et al.* A Standard Verbal Autopsy Method for Investigating Causes of Death in Infants and Children. Geneva, Switzerland: World Health Organization Department of Communicable Disease Surveillance and Response; The Johns Hopkins School of Hygiene and Public Health; The London School of Hygiene and Tropical Medicine, 1999.

2. Kalter HD, Gray RH, Black RE, Gultiano SA. Validation of postmortem interviews to ascertain selected causes of death in children. *Int J Epidemiol* 1990; **19**: 380–6.

3. Quigley MA, Armstrong Schellenberg JR, Snow RW. Algorithms for verbal autopsies: a validation study in Kenyan children. *Bull World Health Organ* 1996; **74**: 147–54.

4. Snow RW, Armstrong JR, Forster D, *et al.* Childhood deaths in Africa: uses and limitations of verbal autopsies. *The Lancet* 1992; **340**: 351–5.

Non-rheumatic valvular heart disease

Non-rheumatic calcific aortic valve disease
Non-rheumatic degenerative mitral valve disease
Other non-rheumatic valvular heart diseases

Flowchart

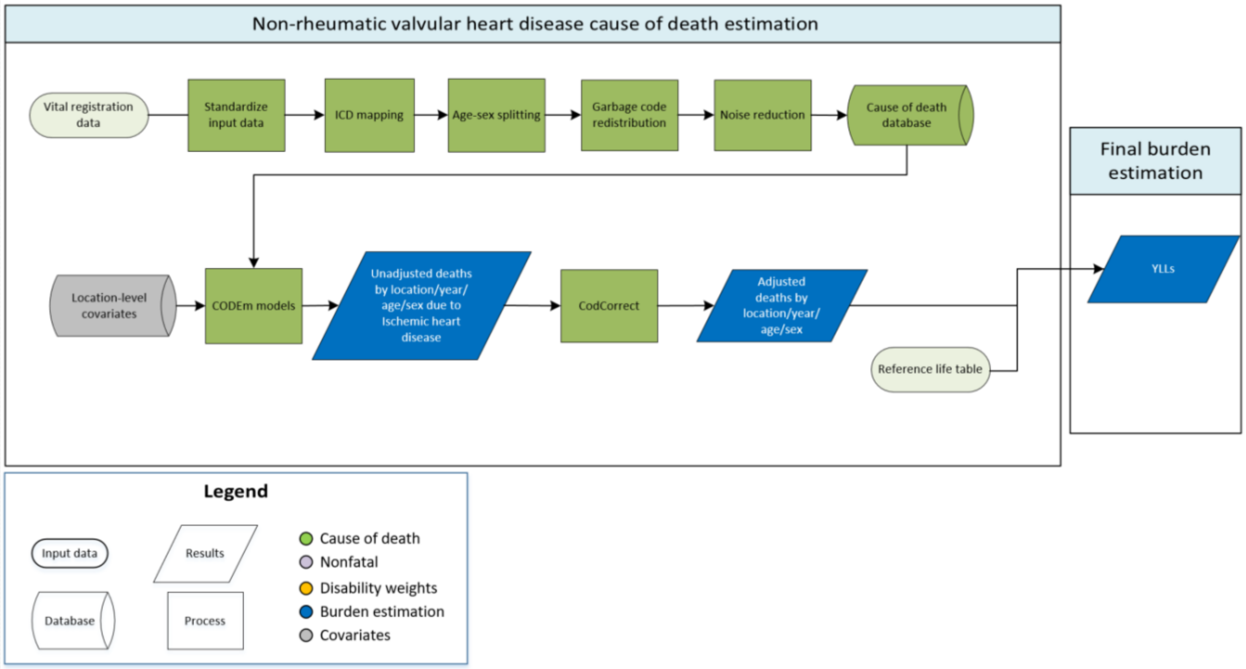

Input data and methodological summary for non-rheumatic valvular heart disease

Input data

Vital registration data were used to model the Level 3 parent cause of non-rheumatic valvular heart disease, and the Level 4 child causes of non-rheumatic calcific valve disease, non-rheumatic degenerative mitral valve disease, and other non-rheumatic valvular diseases. We outliered datapoints in specific locations that created implausible temporal or geographical patterns for each cause.

Non-rheumatic valvular heart disease

For non-rheumatic valvular heart disease, these datapoints included ICD-9- and ICD-10-coded data in several locations in Oceania including Kiribati, Saint Vincent and the Grenadines, American Samoa, Guam, Fiji, Federated States of Micronesia, and Northern Mariana Islands. We also outliered ICD-10-coded datapoints in Iran, as well as sampled vital registration data for all locations in China.

Non-rheumatic calcific aortic valve disease

For non-rheumatic calcific aortic valve disease, these datapoints included ICD-9- and ICD-10-coded data in several locations in Oceania including Kiribati, American Samoa, Guam, Fiji, and Northern Mariana Islands. We also outliered ICD-9-coded datapoints for the years 1980 to 1989 for all locations within Poland.

Non-rheumatic degenerative mitral valve disease

For non-rheumatic degenerative mitral valve disease, these datapoints included ICD9-coded data in Belgium, and Ecuador, ICD9- and ICD10-coded data in France, and ICD10-coded data in Malaysia.

Other non-rheumatic valvular heart diseases

For other non-rheumatic valvular heart disease, these datapoints included ICD-9-coded data in several locations, including Scotland and Northern Ireland for the years 1980 to 1991. We also outliered ICD-9- and ICD-10-coded data in several locations in Oceania, including Kiribati, Fiji, American Samoa, and Guam.

Crosswalk by code system

Most data sources used for cause of death estimation are ICD-coded. The ICD periodically releases new versions, which can introduce an implausible disjoint in the time series that is indicative of a change in the coding practice, rather than a true change in the epidemiological trend of the disease. In GBD 2023, certain causes and countries using the ICD-9 code systems were adjusted to address the disjoint in the time trend and number of deaths between the older ICD-9 code systems and the more recent ICD-10 code systems. Non-rheumatic calcific aortic valve disease, non-rheumatic valvular mitral disease, and other non-rheumatic valvular heart diseases were corrected in Bulgaria, Canada, Spain, France, the USA, Hungary, Croatia, the UK, Greece, Italy, Mexico, and Mauritius from 1980 to 2008 to account for code system disjoints. This was done using a meta-regression tool described as a crosswalk, by taking systematically biased datapoints and estimating their unbiased value. Using this modelling tool, correction factors were created based on the more recent and more reliable ICD-10-coded data. The crosswalk creates matched pairs between the last three years of data using the ICD-9 code system and the first three years of data using the ICD-10 code system. The correction is applied at the child cause level for all Level 2 causes, and after the correction is applied the individual child causes are raked to the parent cause to maintain a consistent number of deaths across the cause hierarchy.

Modelling strategy

We used a standard CODEm approach to model deaths from non-rheumatic valvular heart disease, non-rheumatic calcific valve disease, non-rheumatic degenerative mitral valve disease, and other non-rheumatic valvular diseases. Apart from the overall updates to data processing for ICD code system described above and the correction for misclassified COVID deaths described elsewhere in this appendix, there have been no updates to the modelling strategy since GBD 2021.

Table 1. Covariates used in non-rheumatic valvular heart disease mortality modelling

Level	Covariate	Direction
1	Smoking prevalence	1
	Systolic blood pressure (mmHg)	1
2	LDL-cholesterol (mmol/L)	1
	Mean BMI (kg/m^2)	1
	Healthcare Access and Quality Index	–1
3	Lag-distributed income per capita (I$)	–1
	Socio-demographic Index	–1
	Alcohol (litres per capita)	1

Table 2. Covariates used in non-rheumatic calcific aortic valve disease mortality modelling

Level	Covariate	Direction
1	Smoking prevalence	1
	Mean BMI (kg/m^2)	1
	Systolic blood pressure (mmHg)	1
2	LDL cholesterol (mmol/L)	1
	Healthcare Access and Quality Index	–1
	Fasting plasma glucose (mmol/L)	1
3	Lag-distributed income per capita (I$)	–1
	Socio-demographic Index	–1
	Alcohol (litres per capita)	1

Table 3. Covariates used in non-rheumatic degenerative mitral valve disease mortality modelling

Level	Covariate	Direction
1	Healthcare Access and Quality Index	–1
	Lag-distributed income per capita (I$)	–1
	Socio-demographic Index	–1

Table 4. Covariates used in other non-rheumatic valvular heart diseases mortality modelling

Level	Covariate	Direction
1	Summary exposure value, non-rheumatic valve disease	1
	Healthcare Access and Quality Index	–1
2	Socio-demographic Index	1

Nutritional deficiencies: *Parent nutritional deficiencies, protein-energy malnutrition, and other nutritional deficiencies*

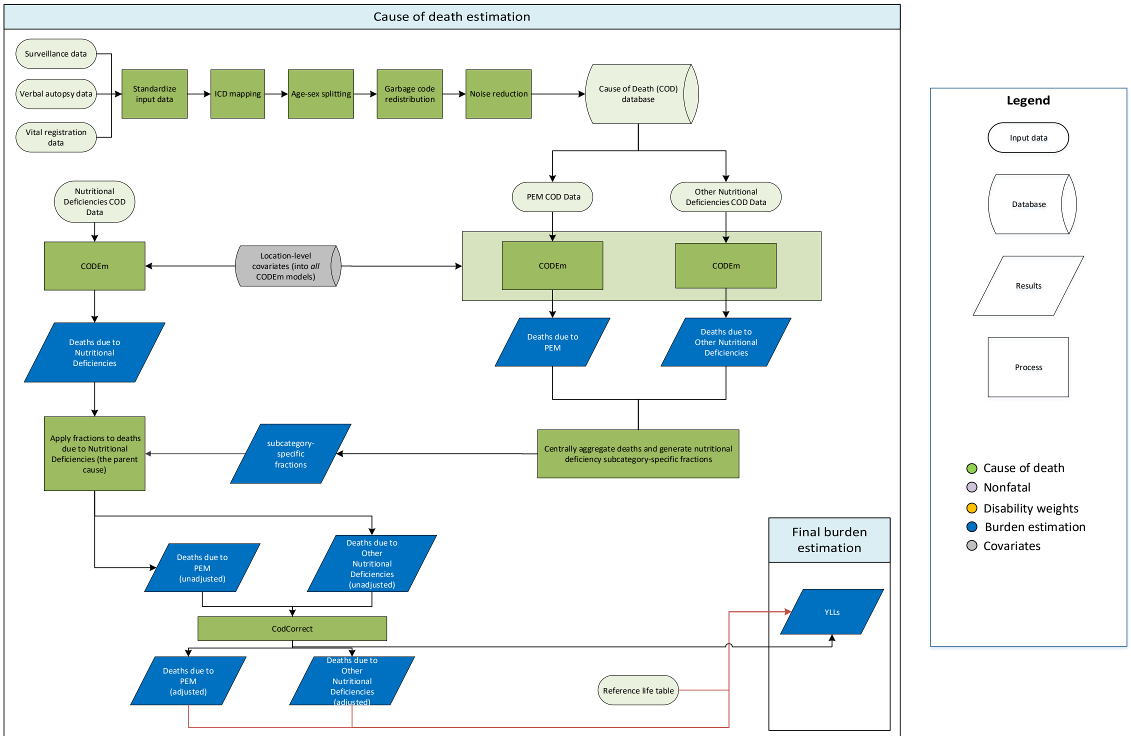

Input data and methodological summary for nutritional deficiencies

Input data

Vital registration (VR), verbal autopsy (VA), and surveillance data were used to model deaths due to nutritional deficiencies. In GBD 2023, data from District Health Information system 2 (DHIS2) are included. We outliered data that were largely conflicting with the majority of data from other studies conducted either in the same countries or different countries (with similar socio-demographic characteristics) in the same region. ICD codes, which can be interpreted as case definitions, for each of the nutritional deficiencies are listed in Table 1.

Table 1. ICD-10 codes included in the nutritional deficiency models

GBD cause	ICD-10 code
Protein-energy malnutrition	E40-E46.9 (Kwashiorkor, nutritional marasmus, marasmic kwashiorkor, unspecified severe protein-calorie malnutrition, moderate protein-calorie malnutrition, mild protein-calorie malnutrition, retarded development following protein-calorie malnutrition, unspecified protein-calorie malnutrition)
Other nutritional deficiencies	D51-D52.0 (vitamin B12 deficiency anaemia and folate deficiency anaemia)

Other nutritional deficiencies	D52.8-D53.9 (other nutritional anaemias)
Other nutritional deficiencies	D64.3 (other sideroblastic anaemias)
Other nutritional deficiencies	E51-E61.9 (thiamine, niacin, other B group vitamins, ascorbic acid, vitamin D, other vitamin, dietary calcium, dietary selenium, dietary zinc, and other nutrient element deficiencies)
Other nutritional deficiencies	E63-E64.0 (other nutritional deficiencies and sequelae of protein-calorie malnutrition)
Other nutritional deficiencies	E64.2-E64.9 (sequelae of vitamin C deficiency, rickets, other nutritional deficiencies, and unspecified nutritional deficiencies)
Other nutritional deficiencies	M12.1-M12.19 (Kashin-Beck disease)
Garbage code	D50, D50.0 and D50.9 (unspecified anaemia)

Modelling strategy

Data and data processing methods were updated centrally by the cause of death team for GBD 2023. We made updates to the covariates used and the levels of covariates in each of the nutritional deficiencies cause of death (CoD) models, as described in Table 2. In GBD 2023, we ensured consistency in the covariates used across the nutritional deficiencies CoD models. Apart from these updates, we did not make any substantive modelling strategy change from GBD 2021.

We used a standard Cause of Death Ensemble model (CODEm) approach to model deaths from nutritional deficiencies. We estimated mortality for the nutritional deficiencies in two steps. First, a nutritional deficiencies CoD model was run to generate mortality estimates for total nutritional deficiencies. Then, we ran individual CoD models for each sub-category of nutritional deficiencies: protein-energy malnutrition and other nutritional deficiencies, separately. Protein-energy malnutrition was modelled separately for age groups under 5 and 5 and older to accurately capture the data trends and patterns. Estimates from the two nutritional sub-categories were then scaled at the 1000-draw level in CoDCorrect to match that for total nutritional deficiencies.

The CoD model covariates (including level and direction) used for each of the models are listed in the table 2.

Table 2. Covariates used in nutritional deficiencies CoD models

**Nutritional deficiencies (overall)**		
**Level**	**Covariate**	**Direction**
1	Age-standardised prevalence of severe anaemia	+
	Age-standardised summary exposure value (SEV) for child underweight	+
	SEV for child wasting	+
	Malnutrition shock mortality rate	+
	SEV_scalar_diarrhoea	+
	Total kcal per person per day availability	-
2	Population living in the first world quintile (least) of annual rainfall	+

	Population living in the second world quintile (second least) of annual rainfall	+
	Unsafe sanitation SEV	+
	Unsafe water SEV	+
	Mortality rate due to war shocks	+
	Healthcare Access and Quality Index	-
	Maternal care and immunisation	-
3	Education (years per capita)	-
	Lag-distributed income per capita	-
	Socio-demographic Index	-
**Protein-energy malnutrition**		
**Level**	**Covariate**	**Direction**
1	Age-standardised prevalence of severe anaemia	+
	Age-standardised SEV for child underweight	+
	SEV for child wasting	+
	Malnutrition shock mortality rate	+
	SEV_scalar_diarrhoea	+
	Total kcal per person per day availability	-
2	Population living in the first world quintile (least) of annual rainfall	+
	Population living in the second world quintile (second least) of annual rainfall	+
	Unsafe sanitation SEV	+
	Unsafe water SEV	+
	Mortality rate due to war shocks	+
	Healthcare Access and Quality Index	-
	Maternal care and immunisation	-
3	Education (years per capita)	-
	Lag-distributed income per capita	-
	Socio-demographic Index	-
**Other nutritional deficiencies**		
**Level**	**Covariate**	**Direction**
1	Age-standardised prevalence of severe anaemia	+
	Age-standardised SEV for child underweight	+
	SEV for child wasting	+
	Malnutrition shock mortality rate	+
	SEV_scalar_diarrhoea	+
	Total kcal per person per day availability	-
2	Population living in the first world quintile (least) of annual rainfall	+
	Population living in the second world quintile (second least) of annual rainfall	+
	Unsafe sanitation SEV	+
	Unsafe water SEV	+
	Mortality rate due to war shocks	+
	Healthcare Access and Quality Index	-
	Maternal care and immunisation	-
3	Education (years per capita)	-
	Lag-distributed income per capita	-
	Socio-demographic Index	-

Nutritional deficiencies: *Parent nutritional deficiencies, protein-energy malnutrition, and other nutritional deficiencies*

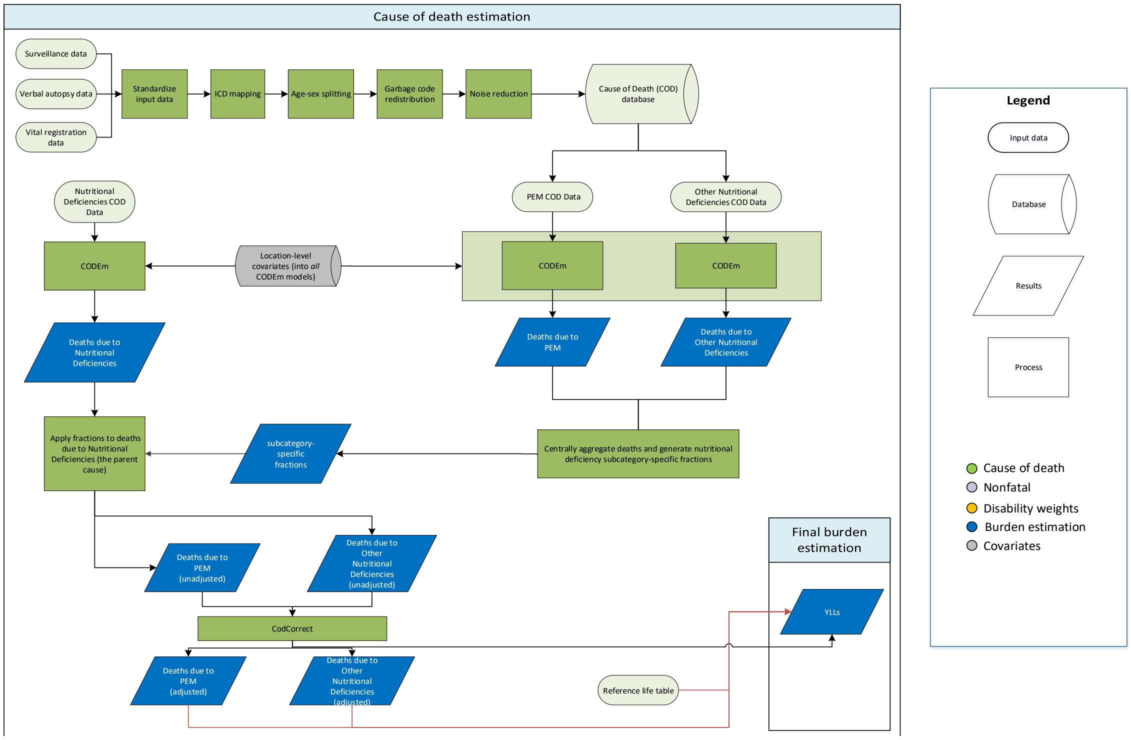

Input data and methodological summary for nutritional deficiencies

Input data

Vital registration (VR), verbal autopsy (VA), and surveillance data were used to model deaths due to nutritional deficiencies. In GBD 2023, data from District Health Information system 2 (DHIS2) are included. We outliered data that were largely conflicting with the majority of data from other studies conducted either in the same countries or different countries (with similar socio-demographic characteristics) in the same region. ICD codes, which can be interpreted as case definitions, for each of the nutritional deficiencies are listed in Table 1 below.

Table 1. ICD-10 codes included in the nutritional deficiency models

GBD cause	ICD-10 code
Protein-energy malnutrition	E40-E46.9 (Kwashiorkor, Nutritional marasmus, Marasmic kwashiorkor, Unspecified severe protein-calorie malnutrition, Moderate protein-calorie malnutrition, Mild protein-calorie malnutrition, Retarded development following protein-calorie malnutrition, Unspecified protein-calorie malnutrition)
Other nutritional deficiencies	D51-D52.0 (vitamin B12 deficiency anaemia and folate deficiency anaemia)

Other nutritional deficiencies	D52.8-D53.9 (other nutritional anaemias)
Other nutritional deficiencies	D64.3 (other sideroblastic anaemias)
Other nutritional deficiencies	E51-E61.9 (thiamine, niacin, other B group vitamins, ascorbic acid, vitamin D, other vitamin, dietary calcium, dietary selenium, dietary zinc, and other nutrient element deficiencies)
Other nutritional deficiencies	E63-E64.0 (other nutritional deficiencies and sequelae of protein-calorie malnutrition)
Other nutritional deficiencies	E64.2-E64.9 (sequelae of vitamin C deficiency, rickets, other nutritional deficiencies, and unspecified nutritional deficiencies)
Other nutritional deficiencies	M12.1-M12.19 (Kashin-Beck disease)
Garbage code	D50, D50.0 and D50.9 (unspecified anaemia)

Modelling strategy

Data and data processing methods were updated centrally by the cause of death team for GBD 2023. We made updates to the covariates used and the levels of covariates in each of the nutritional deficiencies Cause of Death (CoD) models, as described in Table 2. In GBD 2023, we ensured consistency in the covariates used across the Nutritional Deficiencies CoD models. Apart from these updates, we did not make any substantive modelling strategy change from GBD 2021.

We used a standard Cause of Death Ensemble model (CODEm) approach to model deaths from nutritional deficiencies. We estimated mortality for the nutritional deficiencies in two steps. First, a nutritional deficiencies CoD model was run to generate mortality estimates for total nutritional deficiencies. Then, we run individual CoD models for each sub-category of nutritional deficiencies: protein-energy malnutrition and other nutritional deficiencies, separately. Protein-energy malnutrition was modelled separately for age groups under 5 and 5 and older to accurately capture the data trends and patterns. Estimates from the two nutritional sub-categories were then scaled at the 1000-draw level in CoDCorrect to match that for total nutritional deficiencies.

The CoD model covariates (including level and direction) used for each of the models are listed in table 2.

Table 2. Covariates used in nutritional deficiencies CoD models

Nutritional deficiencies (overall)		
**Level**	**Covariate**	**Direction**
1	Age-standardised prevalence of severe anaemia	+
	Age-standardised summary exposure value (SEV) for child underweight	+
	SEV for child wasting	+
	Malnutrition shock mortality rate	+
	SEV_scalar_diarrhoea	+
	Total kcal per person per day availability	-
2	Population living in the first world quintile (least) of annual rainfall	+

	Population living in the second world quintile (second least) of annual rainfall	+
	Unsafe sanitation SEV	+
	Unsafe water SEV	+
	Mortality rate due to war shocks	+
	Healthcare Access and Quality Index	-
	Maternal care and immunisation	-
3	Education (years per capita)	-
	Lag-distributed income per capita	-
	Socio-demographic Index	-
**Protein-energy malnutrition**		
**Level**	**Covariate**	**Direction**
1	Age-standardised prevalence of severe anaemia	+
	Age-standardised SEV for child underweight	+
	SEV for child wasting	+
	Malnutrition shock mortality rate	+
	SEV_scalar_diarrhoea	+
	Total kcal per person per day availability	-
2	Population living in the first world quintile (least) of annual rainfall	+
	Population living in the second world quintile (second least) of annual rainfall	+
	Unsafe sanitation SEV	+
	Unsafe water SEV	+
	Mortality rate due to war shocks	+
	Healthcare Access and Quality Index	-
	Maternal care and immunisation	-
3	Education (years per capita)	-
	Lag-distributed income per capita	-
	Socio-demographic Index	-
**Other nutritional deficiencies**		
**Level**	**Covariate**	**Direction**
1	Age-standardised prevalence of severe anaemia	+
	Age-standardised SEV for child underweight	+
	SEV for child wasting	+
	Malnutrition shock mortality rate	+
	SEV_scalar_diarrhoea	+
	Total kcal per person per day availability	-
2	Population living in the first world quintile (least) of annual rainfall	+
	Population living in the second world quintile (second least) of annual rainfall	+
	Unsafe sanitation SEV	+
	Unsafe water SEV	+
	Mortality rate due to war shocks	+
	Healthcare Access and Quality Index	-
	Maternal care and immunisation	-
3	Education (years per capita)	-
	Lag-distributed income per capita	-
	Socio-demographic Index	-

Opioid use disorders

Flowchart

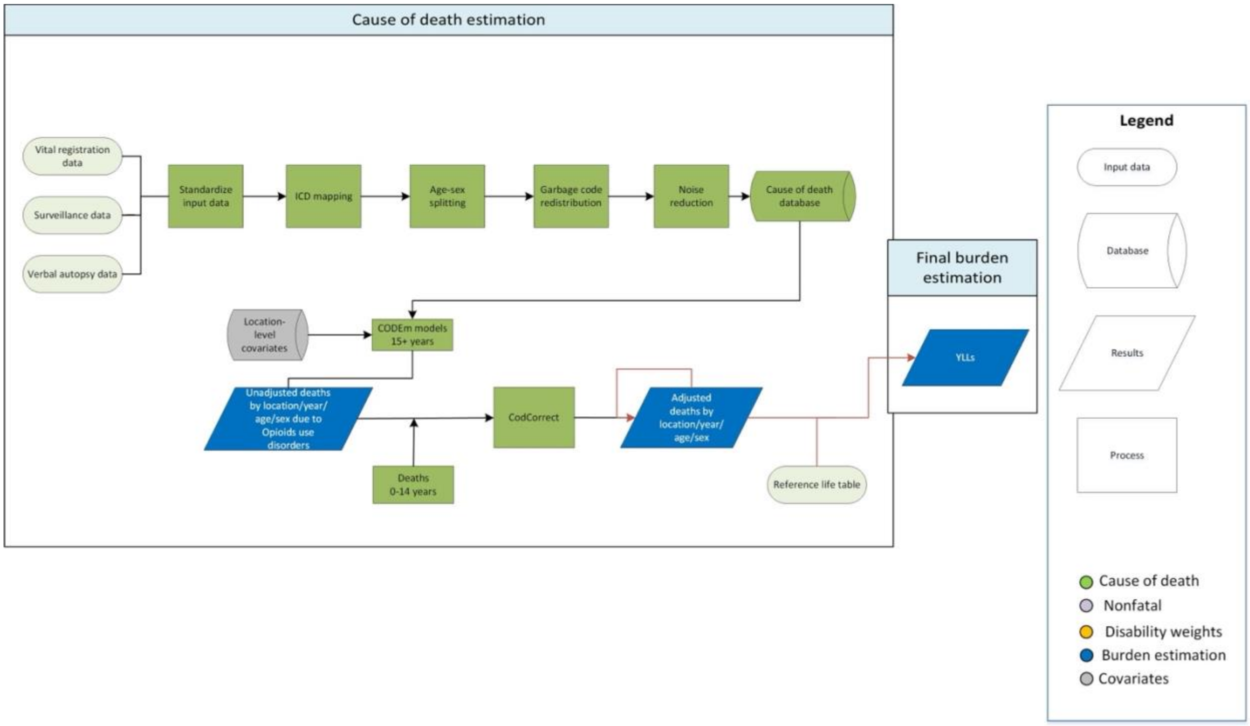

Input data and methodological summary for opioid use disorders

Input data

All input data were from vital registration and surveillance sources. Data from countries with sparse yet heterogeneous data were excluded as the data exaggerated fluctuations in deaths and gave implausible regional patterns, according to in-country and subject matter experts. Excluded data were typically from low- and middle-income countries. The locations for which there were the most data included North America, Australia, western Europe, and parts of Latin America.

Modelling strategy

In the GBD 2023 round, the modelling strategy for opioid use disorders remained largely unchanged, continuing the unique approach introduced in GBD 2021. Due to differing age patterns, deaths from opioid use disorders are primarily modelled in older age groups (15+) using the standard Cause of Death Ensemble modelling (CODEm) approach. However, occasional deaths in younger age groups (0–14) are still recorded in the raw cause of death (CoD) data. For example, while opioid use disorders are modelled in CODEm for ages 15+, rare deaths in younger age groups are identified from reported raw death data in locations where such reports are available. These rare deaths are not modelled but are directly added to the unadjusted death estimates after the CODEm process.

The combined unadjusted death estimates from the CODEm models (ages 15+) and the rare deaths data (ages 0–14) for opioid use disorders then undergo CoDCorrect adjustment. During this process,

unadjusted estimates from opioid use disorders and other “child” causes, including amphetamine, cocaine, and other drug use disorders, are summed and fit to the overall distribution of deaths within the drug use disorders “parent” model. The rare deaths in younger ages are also incorporated into the parent model after its separate CODEm run and before its own CoDCorrect adjustment. The parent model subsequently informs the results for the child models, ensuring that deaths are redistributed appropriately, collectively sum to the parent, and are available across the entire age spectrum for locations where these rare events were reported.

When data sources, such as verbal autopsy reports, lack sufficient detail to attribute deaths to specific child causes, the deaths are included in the parent model. The parent model then guides the distribution of deaths among the child models, ensuring consistency, accuracy, and alignment with the total number of deaths captured in the drug use disorders hierarchy.

Several covariates are particularly important for the opioid use disorder models to be able to capture the rapid increases in opioid use disorder deaths recently observed in the United States. These include intravenous drug use prevalence from the model used to estimate exposure for the drug use as a risk analyses, and opioid consumption per million inhabitants per day. The latter covariate was derived from data from the International Narcotics Control Board (INCB), which measures *“defined daily doses for statistical purposes” (S-DDD),* which translates all different opioids of different types and dosages into comparable units to quantify consumption in different countries.

Table 1: Covariates used in the opioids use disorders mortality modelling

Level	Covariate	Direction
1	Intravenous drug use, age-standardised	+
	Intravenous drug use, age-specific	+
	Opioid standard doses per million per day (10-year lag)	+
2	Cumulative cigarettes (10 years)	+
	Cumulative cigarettes (5 years)	+
	Opium cultivation bin	+
	Smoking prevalence	+
	Healthcare Access and Quality Index	-

Other cardiomyopathy

Flowchart

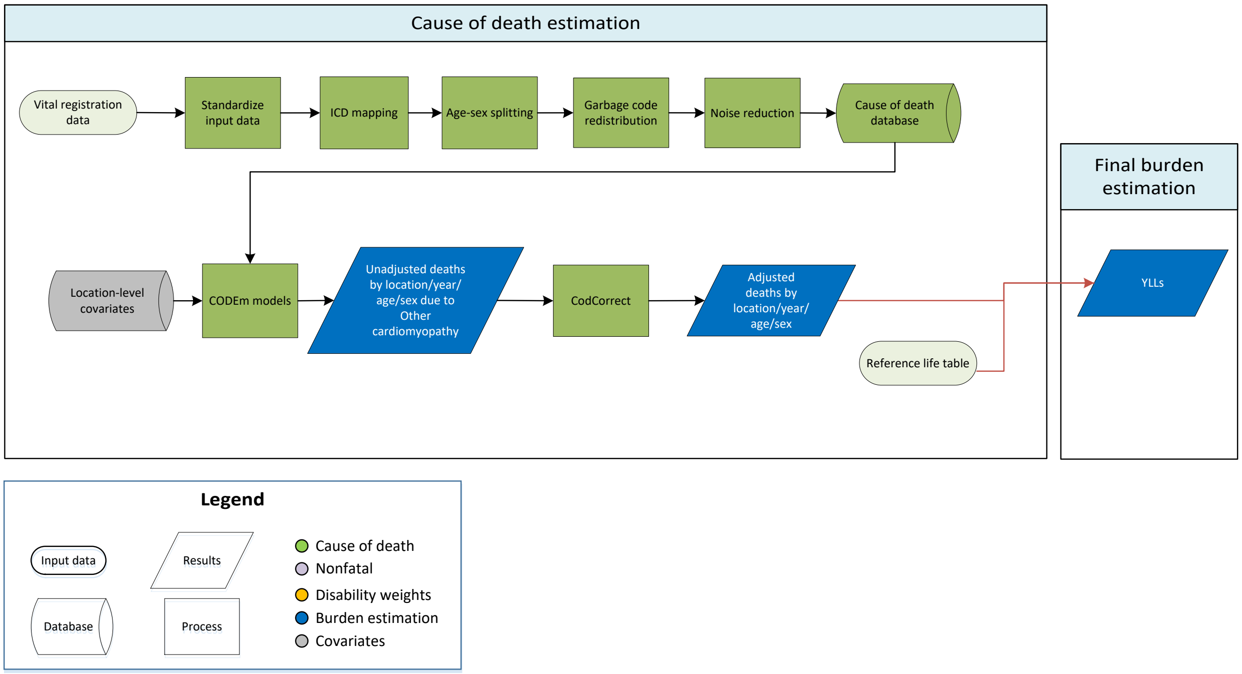

Input data and methodological summary for other cardiomyopathy

Input data

Vital registration data were used to model deaths due to other cardiomyopathy. We outliered all datapoints in Bosnia and Herzegovina, Montenegro, North Macedonia, and Serbia due to implausibly high values, which we attributed to variation in local coding practices after review with experts. In addition, we outliered ICD-10 and ICD-10 tabulated data in Kazakhstan that were much higher than the most recent ICD-10 data.

Crosswalk by code system

Most data sources used for cause of death estimation are ICD-coded. The ICD periodically releases new versions, which can introduce an implausible disjoint in the time series that is indicative of a change in the coding practice, rather than a true change in the epidemiological trend of the disease. In GBD 2023, certain causes and countries using the ICD-9 code systems were adjusted to address the disjoint in the time trend and number of deaths between the older ICD-9 code systems and the more recent ICD-10 code systems. Other cardiomyopathy was corrected in Bulgaria (1990–2004), Canada (1986–1999), Spain (1980–1998), France (1980–1999), the USA (1980–1998), Hungary (1980–1995), Croatia (1985–1994), the UK (1980–1999), Greece (1987–2008), Italy (1980–2002), Mexico (1980–1997), and Mauritius (1980–2004) to account for this code system disjoint. This was done using a meta-regression tool described as a crosswalk, by taking systematically biased datapoints and estimating their unbiased value. Using this modelling tool, correction factors were created based on the more recent and more reliable ICD-10-

coded data. The crosswalk creates matched pairs between the last three years of data using the ICD-9 code system and the first three years of data using the ICD-10 code system. The correction is applied at the child cause level for all Level 2 causes, and after the correction is applied the individual child causes are raked to the parent cause to maintain a consistent number of deaths across the cause hierarchy.

Modelling strategy

We used a standard CODEm approach to model deaths from other cardiomyopathy. The model uses an empirical Bayesian noise reduction algorithm that uses grouped data by region and data type as a prior to better reflect regional patterns. The covariates selected for inclusion in the CODEm modelling process can be found in Table 2. For GBD 2023, the direction of the covariate Socio-demographic Index was flipped from 1 to –1. Aside from this change, there have been no substantive changes to the modelling strategy since GBD 2021.

Table 1. Covariates used in other cardiomyopathy mortality modelling

Level	Covariate	Direction
1	Summary exposure variable, cardiomyopathy (CMP)	1
	Mean systolic blood pressure (mmHg)	1
	Smoking prevalence	1
2	Mean body-mass index (kg/m^2)	1
	Healthcare Access and Quality Index	–1
3	Log-transformed lag-distributed income per capita (I$)	–1
	Socio-demographic Index	–1

Other cardiovascular and circulatory diseases

Flowchart

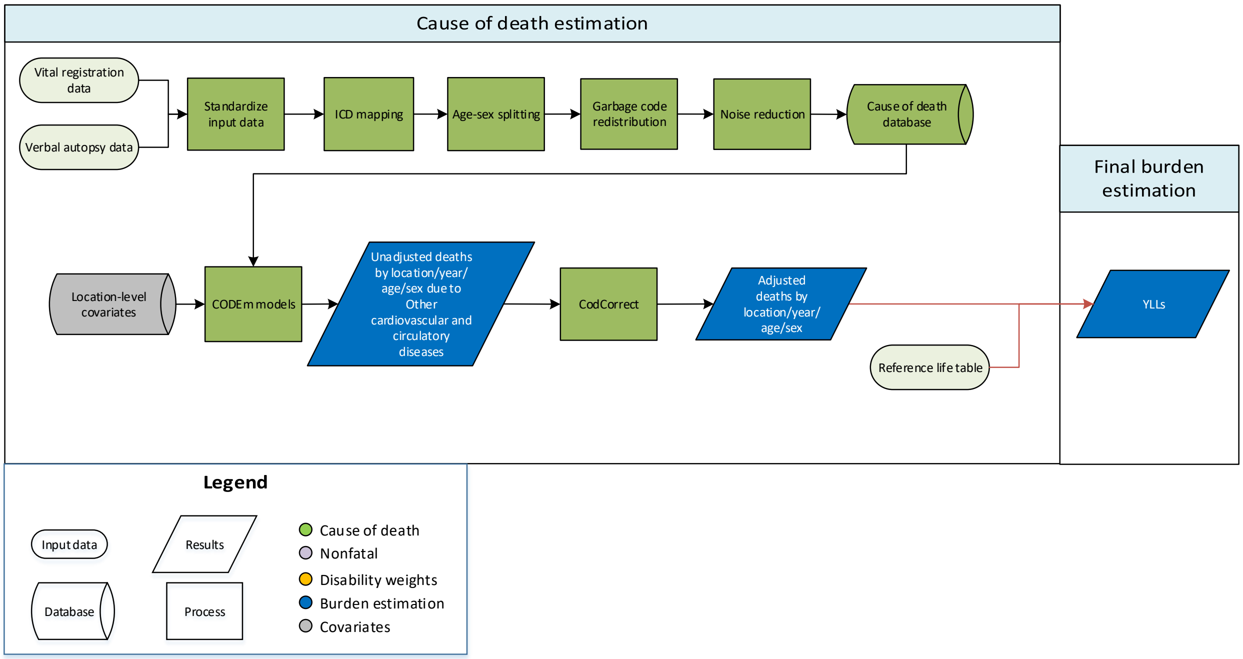

Input data and methodological summary for other cardiovascular and circulatory diseases

Input data

Vital registration and verbal autopsy data were used to model other cardiovascular and circulatory diseases. In 2020, ICD codes for pulmonary arterial hypertension (ICD-10 code I27.0 and ICD-9 code 416) were removed from this cause, as pulmonary arterial hypertension is modelled separately. We outliered ICD-8 and ICD-9-BTL datapoints that were inconsistent with the rest of the data and created implausible time trends. We also outliered ICD-8 datapoints which were not nationally representative. In addition, we outliered ICD-10 datapoints from Oman and the United Arab Emirates in all age groups that were causing the regional estimates to be improbably high. We also outliered ICD-10 datapoints from sources in some subnationals of Ethiopia which were implausibly low in all age groups

Most data sources used for cause of death estimation are ICD-coded. The ICD periodically releases new versions, which can introduce an implausible disjoint in the time series that is indicative of a change in the coding practice, rather than a true change in the epidemiological trend of the disease. In GBD 2023, certain causes and countries using the ICD-9 code systems were adjusted to address the disjoint in the time trend and number of deaths between the older ICD-9 code systems and the more recent ICD-10 code systems. Other cardiovascular and circulatory diseases was corrected in Bulgaria (1990–2004), Canada (1986–1999), Spain (1980–1998), France (1980–1999), the USA (1980–1998), Hungary (1980–1995), Croatia (1985–1994), the UK (1980–1999), Greece (1987–2008), Italy (1980–2002), Mexico (1980–2002), and Mauritius (1980–2004) to account for this code system disjoint. This was done using a

meta-regression tool described as a crosswalk, by taking systematically biased datapoints and estimating their unbiased value. Using this modelling tool, correction factors were created based on the more recent and more reliable ICD-10-coded data. The crosswalk creates matched pairs between the last three years of data using the ICD-9 code system and the first three years of data using the ICD-10 code system. The correction is applied at the child cause level for all Level 2 causes, and after the correction is applied the individual child causes are raked to the parent cause to maintain a consistent number of deaths across the cause hierarchy.

Modelling strategy

We used a standard CODEm approach to model deaths from other circulatory and cardiovascular diseases. Covariates selected for inclusion in the ensemble model are listed in the table below. Since GBD 2021, CODEm has switched from using an ensemble of rate and cause-fraction models to only rate models. For GBD 2023, we also added Socio-demographic Index as a covariate in CODEm for consistency with other cardiovascular causes. No other significant changes were made from GBD 2021.

Table 1. Covariates used in other cardiovascular diseases mortality modelling

Level	Covariate	Direction
1	Summary exposure value (SEV) other CVD	1
1	LDL cholesterol (mean per capita)	1
1	Smoking prevalence	1
1	Systolic blood pressure (mmHg)	1
2	Mean body-mass index (kg/m^2)	1
2	Elevation over 1,500m (proportion)	–1
2	Fasting plasma glucose	1
2	Outdoor pollution ($PM_{2.5}$)	1
2	Indoor air pollution	1
2	Healthcare Access and Quality Index	–1
3	Log-transformed lag-distributed income per capita (I$)	–1
3	Socio-demographic Index	–1
3	Summary exposure value, omega-3	1
3	Summary exposure value, fruits	1
3	Summary exposure value, vegetables	1
3	Summary exposure value, nuts and seeds	–1
3	Summary exposure value, low legumes	1
3	Summary exposure value, PUFA adjusted (percent)	1
3	Alcohol (litres per capita)	1

Other chronic respiratory diseases

Flowchart

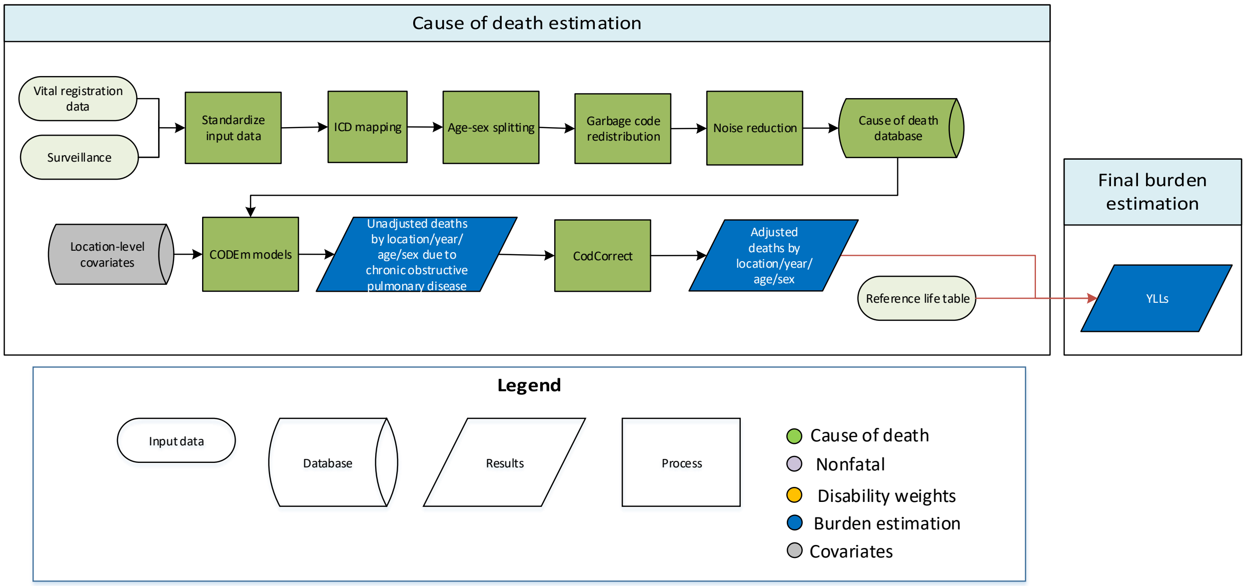

Input data and methodological summary for other chronic respiratory diseases

Input data

Other chronic respiratory diseases is a residual cause group that encompasses many distinct respiratory conditions, included in Table 2 and 3 are the ICD-9 and ICD-10 codes that make up the cause. Data used to estimate other chronic respiratory disease mortality included vital registration and surveillance data from the cause of death (CoD) database. Verbal autopsy data were not included and were instead mapped to an overall chronic respiratory disease model. Our outlier criteria excluded datapoints where there were known ICD coding issues leading to implausible under- or overestimations. An example of an outlier data source is Iran Vital Registration 2001–2012, where there were near-zero deaths coded to this cause group in certain subnational locations.

Modelling strategy

There were no substantive changes to the modelling approach this round. The standard Cause of Death Ensemble modelling (CODEm) approach was used to estimate deaths due to other chronic respiratory diseases (see appendix 1, section 4 for additional details). Separate models were conducted for male and female mortality, and the age range for both models was 0 to 95+ years.

The following table lists the covariates included in the model. This requires that the covariate selected for the model must have a directional relationship with other chronic respiratory diseases. For GBD 2023, new covariates were added as options for potential covariate inclusion related to sleep apnea (litres of alcohol consumed per capita, and mean BMI), the condition that makes up the largest

percentage of deaths in this residual cause group. Covariates selected in the final global and data-rich models for males and females include cumulative cigarettes (5 years), cumulative cigarettes (20 years), indoor air pollution (all cooking fuels), outdoor air pollution ($PM_{2.5}$), mean BMI, litres of alcohol consumed per capita, Healthcare Access and Quality Index, proportion of population over 1500 m in elevation, and Socio-demographic Index.

Table 1. Covariates used in other chronic respiratory diseases mortality modelling

Level	Covariate	Direction
1	Cumulative cigarettes (5 years)	+
	Cumulative cigarettes (10 years)	+
	Cumulative cigarettes (20 years)	+
	Indoor air pollution (all cooking fuels)	+
	Outdoor air pollution (PM 2.5)	+
2	Mean BMI	+
	Litres of alcohol consumed per capita	+
	Healthcare Access and Quality Index	-
	Proportion population over 1500 m elevation	+
3	Socio-demographic Index	-

Table 2. ICD-9 codes for other chronic respiratory diseases

Code system	ICD code	Code name
ICD9_detail	327.2	Organic sleep apnea
ICD9_detail	327.2	Organic sleep disorders unspecified
ICD9_detail	327.21	Primary central sleep apnea
ICD9_detail	327.22	High altitude periodic breathing
ICD9_detail	327.23	Obstructive sleep apnea (adult)(pediatric)
ICD9_detail	327.24	Idiopathic sleep related non-obstructive alveolar hypoventilation
ICD9_detail	327.25	Congenital central alveolar hypoventilation syndrome
ICD9_detail	327.26	Sleep related hypoventilation/hypoxemia in conditions classifiable elsewhere
ICD9_detail	327.27	Central sleep apnea in conditions classified elsewhere
ICD9_detail	327.29	Other organic sleep apnea
ICD9_detail	327.3	Circadian rhythm sleep disorder

ICD9_detail	327.3	Organic sleep disorders unspecified
ICD9_detail	327.31	Organic sleep disorders delayed sleep phase type
ICD9_detail	327.32	Organic sleep disorders advanced sleep phase type
ICD9_detail	327.33	Organic sleep disorders irregular sleep-wake type
ICD9_detail	327.34	Organic sleep disorders free-running type
ICD9_detail	327.35	Organic sleep disorders jet lag type
ICD9_detail	327.36	Organic sleep disorders shift work type
ICD9_detail	327.37	Organic sleep disorders in conditions classified elsewhere
ICD9_detail	327.39	Other circadian rhythm sleep disorder
ICD9_detail	327.4	Organic parasomnia
ICD9_detail	327.4	Organic sleep disorders unspecified
ICD9_detail	327.41	Confusional arousals
ICD9_detail	327.42	REM sleep behavior disorder
ICD9_detail	327.43	Recurrent isolated sleep paralysis
ICD9_detail	327.44	Parasomnia in conditions classified elsewhere
ICD9_detail	327.49	Other organic parasomnia
ICD9_detail	327.5	Organic sleep related movement disorders
ICD9_detail	327.51	Periodic limb movement disorder
ICD9_detail	327.52	Sleep related leg cramps
ICD9_detail	327.53	Sleep related bruxism
ICD9_detail	327.59	Other organic sleep related movement disorders
ICD9_detail	327.8	Other organic sleep disorders
ICD9_detail	780.57	Unspecified sleep apnea
ICD9_detail	786.03	Apnea
ICD9_detail	470	Deviated nasal septum
ICD9_detail	478.3	Paralysis of vocal cords or larynx
ICD9_detail	478.3	Other diseases of upper respiratory tract unspecified
ICD9_detail	478.31	Unilateral paralysis of vocal cords or larynx, partial
ICD9_detail	478.32	Unilateral paralysis of vocal cords or larynx, complete
ICD9_detail	478.33	Bilateral paralysis of vocal cords or larynx, partial
ICD9_detail	478.34	Bilateral paralysis of vocal cords or larynx, complete
ICD9_detail	478.4	Polyp of vocal cord or larynx
ICD9_detail	478.5	Other diseases of vocal cords
ICD9_detail	478.6	Edema of larynx
ICD9_detail	478.7	Other diseases of larynx not elsewhere classified
ICD9_detail	478.7	Unspecified disease of larynx
ICD9_detail	478.71	Cellulitis and perichondritis of larynx
ICD9_detail	478.74	Stenosis of larynx
ICD9_detail	478.75	Laryngeal spasm
ICD9_detail	478.79	Other diseases of larynx, not elsewhere classified
ICD9_detail	478.8	Upper respiratory tract hypersensitivity reaction, site unspecified
ICD9_detail	478.9	Other and unspecified diseases of upper respiratory tract
ICD9_detail	479	Other and unspecified diseases of upper respiratory tract

ICD9_detail	495	Extrinsic allergic alveolitis
ICD9_detail	495	Farmers' lung
ICD9_detail	495.1	Bagassosis
ICD9_detail	495.2	Bird-fanciers' lung
ICD9_detail	495.3	Suberosis
ICD9_detail	495.4	Malt workers' lung
ICD9_detail	495.5	Mushroom workers' lung
ICD9_detail	495.6	Maple bark-strippers' lung
ICD9_detail	495.7	"Ventilation" pneumonitis
ICD9_detail	495.8	Other specified allergic alveolitis and pneumonitis
ICD9_detail	495.9	Unspecified allergic alveolitis and pneumonitis
ICD9_detail	506	Respiratory conditions due to chemical fumes and vapors
ICD9_detail	506	Bronchitis and pneumonitis due to fumes and vapors
ICD9_detail	506.1	Acute pulmonary edema due to fumes and vapors
ICD9_detail	506.2	Upper respiratory inflammation due to fumes and vapors
ICD9_detail	506.3	Other acute and subacute respiratory conditions due to fumes and vapors
ICD9_detail	506.4	Chronic respiratory conditions due to fumes and vapors
ICD9_detail	506.9	Unspecified respiratory conditions due to fumes and vapors
ICD9_detail	508	Respiratory conditions due to other and unspecified external agents
ICD9_detail	508	Acute pulmonary manifestations due to radiation
ICD9_detail	508.1	Chronic and other pulmonary manifestations due to radiation
ICD9_detail	508.2	Respiratory conditions due to smoke inhalation
ICD9_detail	508.8	Respiratory conditions due to other specified external agents
ICD9_detail	508.9	Respiratory conditions due to unspecified external agent
ICD9_detail	509	There is not this code in ICD-9 site, but we have in this data
ICD9_detail	517	Lung involvement in conditions classified elsewhere
ICD9_detail	517.1	Rheumatic pneumonia
ICD9_detail	517.2	Lung involvement in systemic sclerosis
ICD9_detail	517.3	Acute chest syndrome
ICD9_detail	517.8	Lung involvement in other diseases classified elsewhere
ICD9_detail	518.6	Allergic bronchopulmonary aspergillosis
ICD9_detail	518.9	
ICD9_detail	519.1	Other diseases of trachea and bronchus not elsewhere classified
ICD9_detail	519.11	Acute bronchospasm
ICD9_detail	519.19	Other diseases of trachea and bronchus
ICD9_detail	519.2	Mediastinitis
ICD9_detail	519.3	Other diseases of mediastinum, not elsewhere classified
ICD9_detail	519.4	Disorders of diaphragm

Table 3. ICD-10 codes for other chronic respiratory diseases

Code system	ICD code	Code name
ICD10	G47.3	Sleep apnea
ICD10	G47.30	Sleep apnea, unspecified
ICD10	G47.31	Primary central sleep apnea
ICD10	G47.32	High altitude periodic breathing
ICD10	G47.33	Obstructive sleep apnea (adult) (pediatric)
ICD10	G47.34	Idiopathic sleep related nonobstructive alveolar hypoventilation
ICD10	G47.35	Congenital central alveolar hypoventilation syndrome
ICD10	G47.36	Sleep related hypoventilation in conditions classified elsewhere
ICD10	G47.37	Central sleep apnea in conditions classified elsewhere
ICD10	G47.39	Other sleep apnea

ICD10	J34.1	Cyst and mucocele of nose and nasal sinus
ICD10	J34.2	Deviated nasal septum
ICD10	J34.3	Hypertrophy of nasal turbinates
ICD10	J34.8	Other specified disorders of nose and nasal sinuses
ICD10	J34.81	Nasal mucositis (ulcerative)
ICD10	J34.89	Other specified disorders of nose and nasal sinuses
ICD10	J34.9	Unspecified disorder of nose and nasal sinuses
ICD10	J38	Diseases of vocal cords and larynx, not elsewhere classified
ICD10	J38.0	Paralysis of vocal cords and larynx
ICD10	J38.00	Paralysis of vocal cords and larynx, unspecified
ICD10	J38.01	Paralysis of vocal cords and larynx, unilateral
ICD10	J38.02	Paralysis of vocal cords and larynx, bilateral
ICD10	J38.1	Polyp of vocal cord and larynx
ICD10	J38.2	Nodules of vocal cords
ICD10	J38.3	Other diseases of vocal cords
ICD10	J38.4	Edema of larynx
ICD10	J38.5	Laryngeal spasm
ICD10	J38.6	Stenosis of larynx
ICD10	J38.7	Other diseases of larynx
ICD10	J39	Other diseases of upper respiratory tract
ICD10	J39.2	Other diseases of pharynx
ICD10	J39.3	Upper respiratory tract hypersensitivity reaction, site unspecified
ICD10	J39.8	Other specified diseases of upper respiratory tract
ICD10	J39.9	Disease of upper respiratory tract, unspecified
ICD10	J66	Airway disease due to specific organic dust
ICD10	J66.0	Byssinosis
ICD10	J66.1	Flax-dressers' disease
ICD10	J66.2	Cannabinosis
ICD10	J66.8	Airway disease due to other specific organic dusts
ICD10	J67	Hypersensitivity pneumonitis due to organic dust
ICD10	J67.0	Farmer's lung
ICD10	J67.1	Bagassosis
ICD10	J67.2	Bird fancier's lung
ICD10	J67.3	Suberosis
ICD10	J67.4	Maltworker's lung
ICD10	J67.5	Mushroom-worker's lung
ICD10	J67.6	Maple-bark-stripper's lung
ICD10	J67.7	Air conditioner and humidifier lung
ICD10	J67.8	Hypersensitivity pneumonitis due to other organic dusts
ICD10	J67.9	Hypersensitivity pneumonitis due to unspecified organic dust
ICD10	J68	Respiratory conditions due to inhalation of chemicals, gases, fumes and vapors
ICD10	J68.0	Bronchitis and pneumonitis due to chemicals, gases, fumes and vapors

ICD10	J68.1	Pulmonary edema due to chemicals, gases, fumes and vapors
ICD10	J68.2	Upper respiratory inflammation due to chemicals, gases, fumes and vapors, not elsewhere classified
ICD10	J68.3	Other acute and subacute respiratory conditions due to chemicals, gases, fumes and vapors
ICD10	J68.4	Chronic respiratory conditions due to chemicals, gases, fumes and vapors
ICD10	J68.8	Other respiratory conditions due to chemicals, gases, fumes and vapors
ICD10	J68.9	Unspecified respiratory condition due to chemicals, gases, fumes and vapors
ICD10	J70	Respiratory conditions due to other external agents
ICD10	J70.8	Respiratory conditions due to other specified external agents
ICD10	J70.9	Respiratory conditions due to unspecified external agent
ICD10	J82	Pulmonary eosinophilia, not elsewhere classified
ICD10	J91	Pleural effusion in conditions classified elsewhere
ICD10	J91.8	Pleural effusion in other conditions classified elsewhere
ICD10	J92	Pleural plaque
ICD10	J92.9	Pleural plaque without asbestos

Other digestive diseases

Flowchart

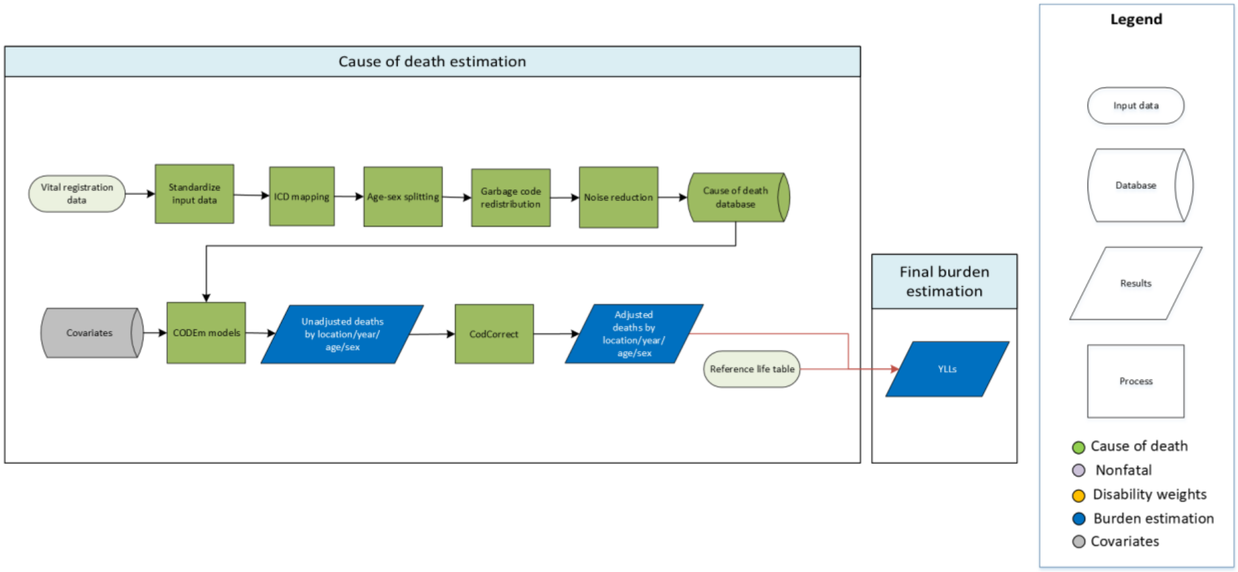

Input data and methodological summary for other digestive diseases

Other digestive diseases comprise a Level 3 cause in the GBD hierarchy.

Input data

Data used to estimate mortality of other digestive diseases consisted of vital registration (VR) from the cause of death (CoD) database. The data in other digestive diseases consist of unique datapoints from deaths reported with a set of non-specific digestive disease codes (see appendix section on ICD mapping for details). The ICD codes that map to this cause were substantially revised between GBD 2021 and GBD 2023, insofar as a new cause was introduced to the GBD cause list: diverticular disease of the intestines. All codes for this new cause were previously mapped to other digestive diseases and now map to the new cause, leading to lower values for data processed for other digestive diseases across all CoD sources. After mapping, subsequent data processing steps, such as garbage code redistribution and noise reduction, were broadly similar to previous rounds of GBD. One novel data processing step was employed in preparing data for other digestive diseases that was not needed prior to GBD 2023—COVID-19 corrections—which are described elsewhere in this appendix.

After all data processing steps were complete, datapoints for all locations and years were systematically reviewed to identify outliers or other data limitations not fully overcome by CoD data processing methods, and selected sources were excluded from analysis.

Modelling strategy

After re-mapping ICD-coded COD data, as described above, the estimation strategy used to estimate mortality due to other digestive diseases was not substantively changed between GBD 2021 and GBD 2023. A standard CODEm model was used to model deaths due to other digestive diseases (see appendix section on CODEm method for details). Models were generated separately for males in data-

rich countries, males in all countries (referred to as a global model), females in data-rich countries, and females in all countries (global model). Fatal estimation for this cause was restricted to ages 1 year to 95+ years.

The following table has the full list of covariates presented to the CODEm algorithm for selection in models of other digestive diseases mortality.

Table 1. Covariates used in other digestive diseases mortality modelling

Level	Covariate	Direction
1	Cumulative cigarettes (10 years)	+
	Cumulative cigarettes (5 years)	+
	Smoking prevalence	+
	Alcohol (litres per capita)	+
2	Diabetes age-standardised prevalence (proportion)	+
	BMI (mean)	+
	Sanitation (proportion with access)	-
	Improved water source (proportion with access)	-
	Age-sex-specific scaled exposure variable for low polyunsaturated fatty acids consumption	+
	Age-sex-specific scaled exposure variable for low fruit consumption	+
	Age-sex-specific scaled exposure variable for low vegetable consumption	+
	Age-sex-specific scaled exposure variable for high red meat consumption	+
	Healthcare Access and Quality Index	-
3	Socio-demographic Index	-
	Education (years per capita)	-
	Log LDI ($I per capita)	-

We hybridised separate global and data-rich models to acquire unadjusted results, which we adjusted using CoDCorrect. Adjustment in CoDCorrect included rescaling unadjusted other digestive disease death estimates alongside other Level 3 digestive disease deaths to sum to overall digestive disease deaths (a Level 2 cause), which were then rescaled along other causes to sum up the hierarchy to all-cause counts of death. Adjusted deaths were compared to the reference life table to calculate final YLLs due to other digestive diseases.

Other drug use disorders

Flowchart

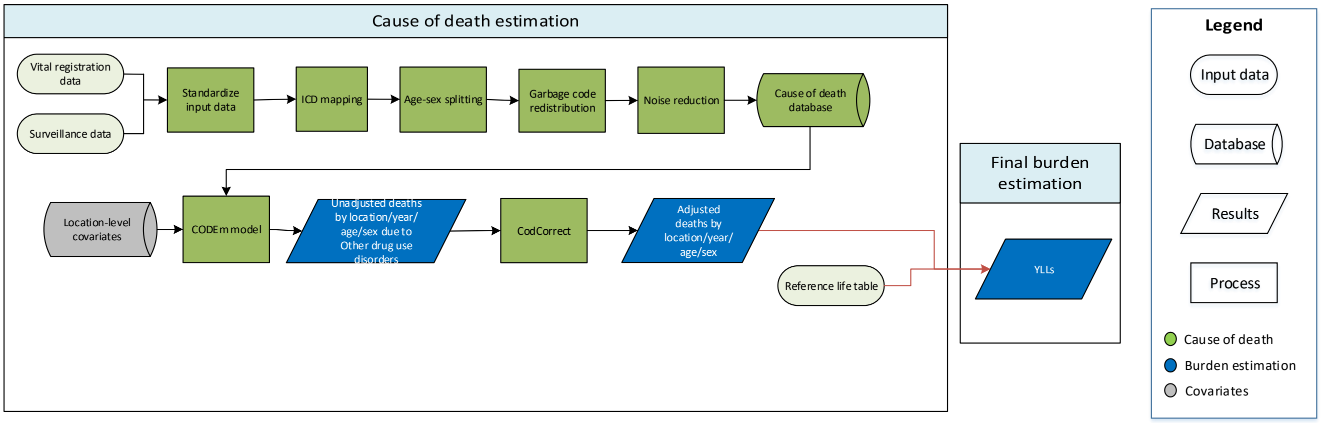

Input data and methodological summary for other drug use disorders

Input data

All data were from vital registration and surveillance sources. Data from countries with sparse yet heterogeneous data were excluded as the data exaggerated fluctuations in deaths and gave implausible regional patterns. Excluded data were typically from lower-income countries. A full description of changes to coding and redistribution approaches is described in the write-up on drug use disorders and in appendix 1 section 4.

Modelling strategy

In the GBD 2023 round, the modelling strategy for other drug use disorders remained largely unchanged. The standard Cause of Death Ensemble modelling (CODEm) approach (detailed in Appendix 1, Section 4) was used to estimate deaths from other drug use disorders. Deaths are primarily modelled in age groups 15 years and older, assuming that drug-related deaths in younger age groups are unlikely to be due to intentional drug use. Deaths due to unintentional drug use are captured in the unintentional poisoning injury cause. This model encompasses deaths from all illicit drug use except opioids, cocaine, and amphetamines. Other drug use disorders are classified as a “child” cause within the broader “parent” model for drug use disorders.

Covariates included in the model were selected based on empirical evidence and expert input of the relationship to the cause (development or survival), resulting in a set of variables representing alcohol consumption, smoking, education, health care access, domestic income, and the Socio-demographic Index (SDI), as detailed in Table 1.

Table 1: Covariates used in the other drug use disorders mortality modelling

Level	Covariate	Direction
1	Alcohol (litres per capita)	+
	Current drinking prevalence	+
	Intravenous drug use, age-standardised	+
	Intravenous drug use, age-specific	+
	Cumulative cigarettes (10 years)	+
	Cumulative cigarettes (5 years)	+
	Cigarettes per capita	+
	Smoking prevalence	+
2	Healthcare Access and Quality Index	-
3	log LDI (I$ per capita)	+
	education (years per capita)	+
	Socio-demographic Index	+

Other intestinal infectious diseases

Flowchart

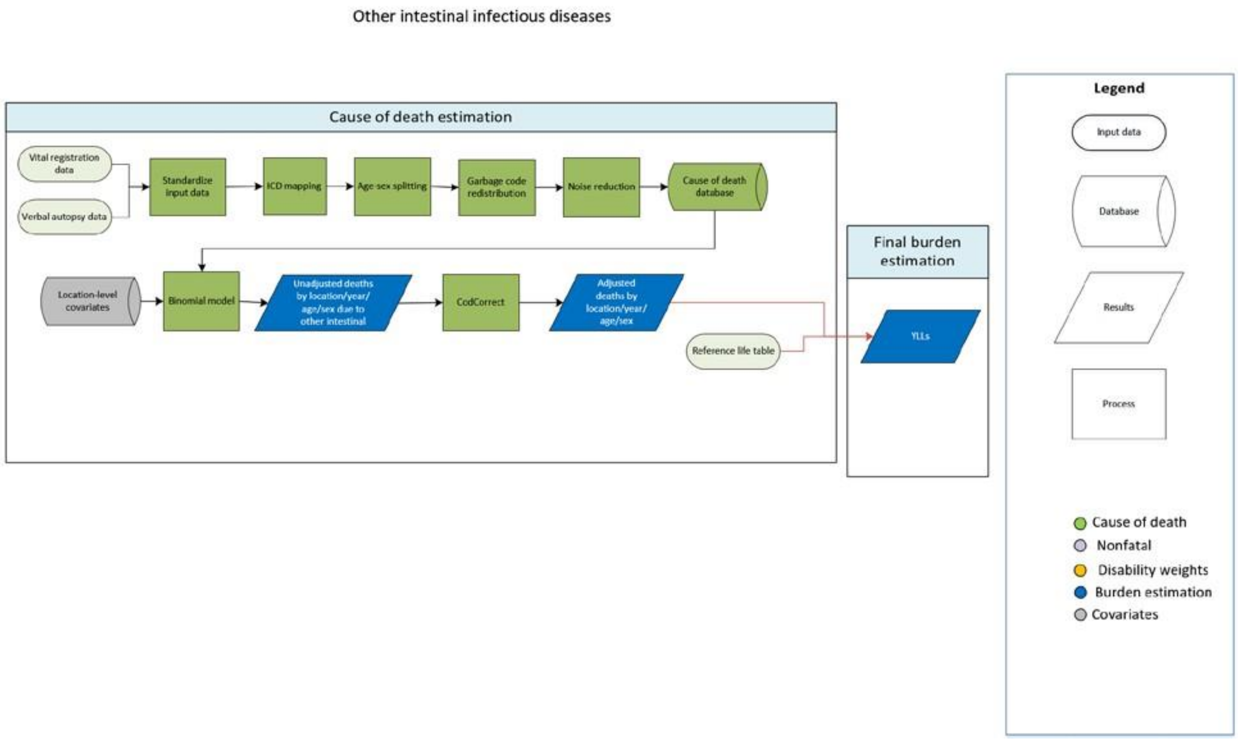

Input data and methodological summary for other intestinal infectious diseases

Input data

We modelled other intestinal infectious disease mortality using all available data in the cause of death (CoD) database. Datapoints were outliered if they reported an improbable number of deaths or if their inclusion in the model yielded distorted trends. In some cases, multiple data sources for the same location differed dramatically in both their quality and reported other intestinal infectious disease mortality (eg, a verbal autopsy and vital registration source). In these cases, the lower-quality data source was outliered.

Modelling strategy

We modelled other intestinal infectious disease mortality using a custom binomial model of all data in the CoD database. The custom model was used because of very small death counts. We used the number of cause-specific deaths as the outcome, with the all-cause mortality envelope as the exposure term. We included the square root of Socio-demographic Index, age group, and sex as covariates, and included a random effect on region.

We have made no substantive changes to the modelling strategy in GBD 2023.

Other intestinal infectious diseases

Flowchart

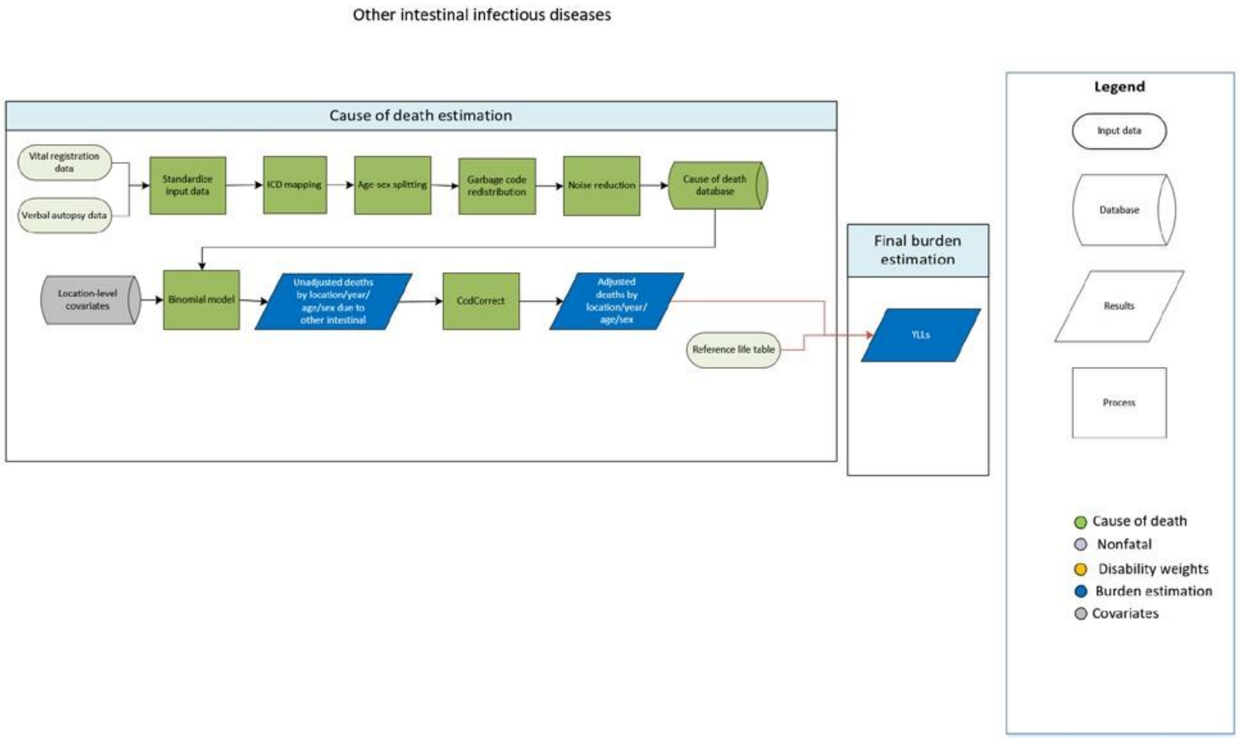

Input data and methodological summary for other intestinal infectious diseases

Input data

We modelled other intestinal infectious disease mortality using all available data in the cause of death (CoD) database. Datapoints were outliered if they reported an improbable number of deaths or if their inclusion in the model yielded distorted trends. In some cases, multiple data sources for the same location differed dramatically in both their quality and reported other intestinal infectious disease mortality (eg, a verbal autopsy and vital registration source). In these cases, the lower-quality data source was outliered.

Modelling strategy

We modelled other intestinal infectious disease mortality using a custom binomial model of all data in the CoD database. The custom model was used because of very small death counts. We used the number of cause-specific deaths as the outcome, with the all-cause mortality envelope as the exposure term. We included the square root of Socio-demographic Index, age group, and sex as covariates, and included a random effect on region.

We have made no substantive changes to the modelling strategy in GBD 2023.

Other musculoskeletal disorders

Flowchart

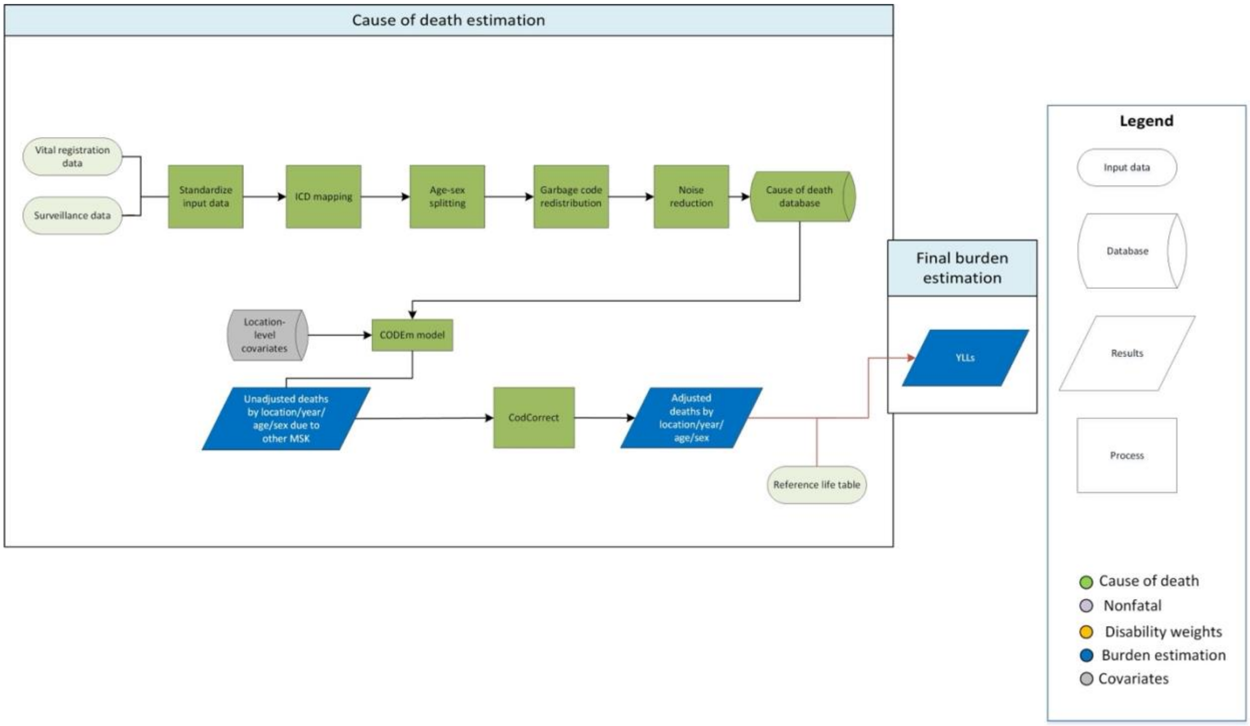

Input data and methodological summary for other musculoskeletal disorders

Input data

Data used to estimate mortality for other musculoskeletal disorders (MSK) included vital registration records and China Disease Surveillance data from the cause of death database. Datapoints were excluded based on the following criteria:

1. **Implausible values**: Datapoints that were implausibly high or low compared to global or regional patterns, as assessed by subject matter experts.
2. **Conflicts with established patterns**: Data that substantially conflicted with known age-specific or temporal trends.
3. **Conflicts with comparable locations**: Data that significantly conflicted with other sources from the same location or from locations with similar characteristics (eg, Socio-demographic Index).
4. **Verbal autopsy limitations**: Data from verbal autopsy sources were excluded due to their inability to accurately capture most musculoskeletal conditions.

For example, ICD-9 data from the US NVSS Mortality data in New Mexico were excluded because the transition to ICD-10 introduced time trend discontinuities and resulted in implausibly low death estimates.

Globally, 60% of all ICD-10-coded deaths for other MSK were coded to autoimmune disorders (like systemic lupus erythematosus and systemic sclerosis), 21% to osteoporosis, 7% to pyogenic arthritis, and 4% to spinal deformities.

Modelling strategy

In the GBD 2023 round, the modelling strategy for other MSKs remained largely unchanged.
The standard CODEm (Cause of Death Ensemble model) modelling approach was applied to estimate deaths due to other musculoskeletal disorders, and general methods are described elsewhere (appendix 1 section 4). We applied the same covariates used in GBD 2021. The CODEm model for other musculoskeletal disorders is limited by a lack of strong predictive covariates. The following table lists the covariates included in the model. Covariate levels were selected based on the strength of the evidence, and the direction was selected based on the expected direction of the relationship.

Table 1. Covariates used in other MSK mortality modelling

Level	Covariate	Direction
1	Mean BMI	+
	Vegetables (g), unadjusted	-
	Alcohol consumption (litres per capita)	+
2	Cumulative cigarettes (10 years)	+
	Cumulative cigarettes (5 years)	+
	Education (years per capita)	+
	Log-transformed LDI: lag-distributed income ($ per capita)	+
	Mean cholesterol[1]	+
	Smoking prevalence	+
	Healthcare Access and Quality Index	-
3	SDI: Socio-demographic Index	+

[1] This covariate is quantified based on LDL cholesterol.

Other neglected tropical diseases (NTDs)

Flowchart

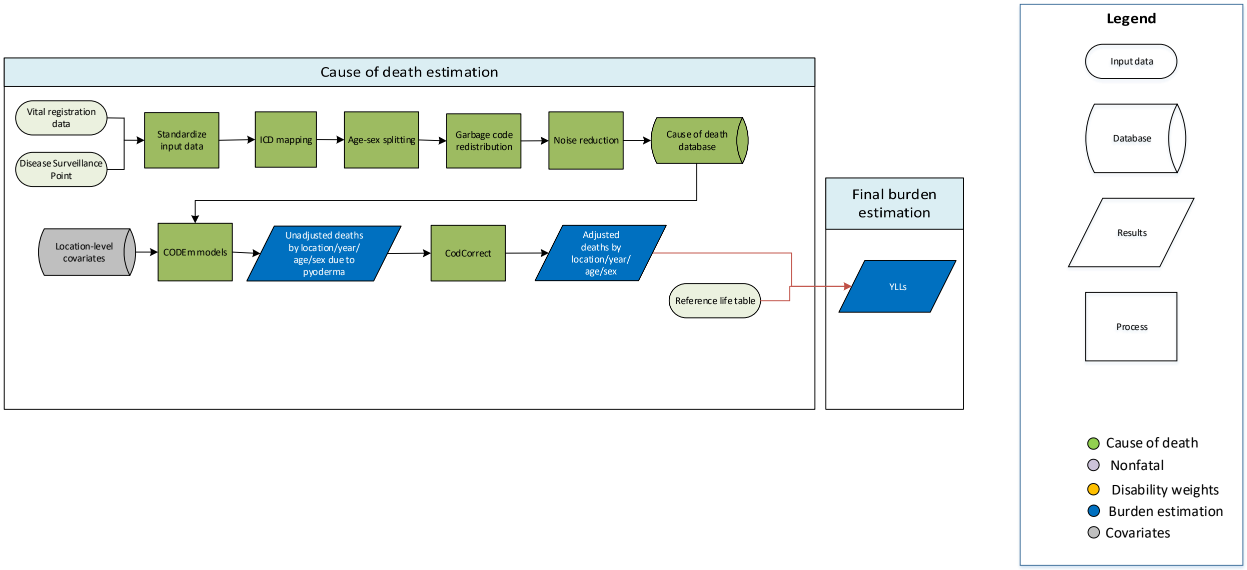

Input data and methodological summary for other neglected tropical diseases (NTDs)

There are many diverse types of neglected tropical diseases, which are encompassed by the following ICD-10 codes:

A68 Relapsing fevers

A68.0 Louse-borne relapsing fever

A68.1 Tick-borne relapsing fever

A68.9 Relapsing fever, unspecified

A69.5 There is not this code in ICD-10 site, but we have this in mortality data

A69.8 Other specified spirochetal infections

A69.9 Spirochetal infection, unspecified

A75 Typhus fever

A75.0 Epidemic louse-borne typhus fever due to *Rickettsia prowazekii*

A75.1 Recrudescent typhus [Brill's disease]

A75.2 Typhus fever due to *Rickettsia typhi*

A75.3 Typhus fever due to *Rickettsia tsutsugamushi*

A75.9 Typhus fever, unspecified

A77 Spotted fever [tick-borne rickettsioses]

A77.0 Spotted fever due to *Rickettsia rickettsii*

A77.1 Spotted fever due to *Rickettsia conorii*

A77.2 Spotted fever due to *Rickettsia siberica*

A77.3 Spotted fever due to *Rickettsia australis*

A77.8 Other spotted fevers

A77.9 Spotted fever, unspecified

A78 Q fever

A79 Other rickettsioses

A79.0 Trench fever

A79.1 Rickettsial pox due to *Rickettsia akari*

A79.8 Other specified rickettsioses

A79.81 Rickettsiosis due to *Ehrlichia sennetsu*

A79.89 Other specified rickettsioses

A79.9 Rickettsiosis, unspecified

A92 Other mosquito-borne viral fevers

A92.0 Chikungunya virus disease

A92.1 O'nyong-nyong fever

A92.2 Venezuelan equine fever

A92.3 West Nile virus infection

A92.30 West Nile virus infection, unspecified

A92.31 West Nile virus infection with encephalitis

A92.32 West Nile virus infection with other neurologic manifestation

A92.39 West Nile virus infection with other complications

A92.4 Rift Valley fever

A92.8 Other specified mosquito-borne viral fevers

A92.9 Mosquito-borne viral fever, unspecified

A93	Other arthropod-borne viral fevers, not elsewhere classified
A93.0	Oropouche virus disease
A93.1	Sandfly fever
A93.2	Colorado tick fever
A93.8	Other specified arthropod-borne viral fevers
A94	Unspecified arthropod-borne viral fever
A94.0	Unspecified arthropod-borne viral fever
A96	Arenaviral haemorrhagic fever
A96.0	Junin haemorrhagic fever
A96.1	Machupo haemorrhagic fever
A96.2	Lassa fever
A96.8	Other arenaviral haemorrhagic fevers
A96.9	Arenaviral haemorrhagic fever, unspecified
A98	Other viral haemorrhagic fevers, not elsewhere classified
A98.0	Crimean-Congo haemorrhagic fever
A98.1	Omsk haemorrhagic fever
A98.2	Kyasanur Forest disease
A98.3	Marburg virus disease
A98.5	Haemorrhagic fever with renal syndrome
A98.8	Other specified viral haemorrhagic fevers
B33.1	Ross River disease
B60	Other protozoal diseases, not elsewhere classified
B60.0	Babesiosis
B60.1	Acanthamebiasis
B60.10	Acanthamebiasis, unspecified
B60.11	Meningoencephalitis due to *Acanthamoeba* (*culbertsoni*)
B60.12	Conjunctivitis due to *Acanthamoeba*
B60.13	Keratoconjunctivitis due to *Acanthamoeba*
B60.19	Other acanthamebic disease

B60.2 Naegleriasis

B60.8 Other specified protozoal diseases

B67.5 *Echinococcus multilocularis* infection of liver

B67.6 *Echinococcus multilocularis* infection, other and multiple sites

B67.61 *Echinococcus multilocularis* infection, multiple sites

B67.69 *Echinococcus multilocularis* infection, other sites

B67.7 *Echinococcus multilocularis* infection, unspecified

B70 Diphyllobothriasis and sparganosis

B70.0 Diphyllobothriasis

B70.1 Sparganosis

B71 Other cestode infections

B71.0 Hymenolepiasis

B71.1 Dipylidiasis

B71.8 Other specified cestode infections

B71.9 Cestode infection, unspecified

B74.3 Loiasis

B74.4 Mansonelliasis

B74.8 Other filariases

B74.9 Filariasis, unspecified

B75 Trichinellosis

B78 Strongyloidiasis

B78.0 Intestinal strongyloidiasis

B78.1 Cutaneous strongyloidiasis

B78.7 Disseminated strongyloidiasis

B78.9 Strongyloidiasis, unspecified

B83 Other helminthiases

B83.0 Visceral larva migrans

B83.1 Gnathostomiasis

B83.2 Angiostrongyliasis due to *Parastrongylus cantonensis*

B83.3 Syngamiasis

B83.4 Internal hirudiniasis

B83.8 Other specified helminthiases

Input data

We modelled other neglected tropical disease mortality using all available data in the cause of death database. Datapoints were outliered if they reported an improbable number of deaths or if their inclusion in the model yielded distorted trends.

Modelling strategy

We modelled other neglected tropical disease mortality using a two-model hybrid approach: 1) a global Cause of Death Ensemble modelling (CODEm) model of all locations, using all data in the cause of death (COD) database; and 2) a CODEm model restricted to data-rich countries.

Level	Covariate	Direction
1	Healthcare Access and Quality Index	−
	Proportion of the population living between 0 and 15 degrees latitude	+
2	Proportion of the population living in the fifth quintile of rainfall	+
	Sanitation	−
3	Education (years per capita)	−
	Lag-distributed income (per capita)	−
	Socio-demographic Index	−

Changes from GBD 2021 to GBD 2023

There were no substantive changes to the modelling strategy for GBD 2023.

Other neurological disorders

Flowchart

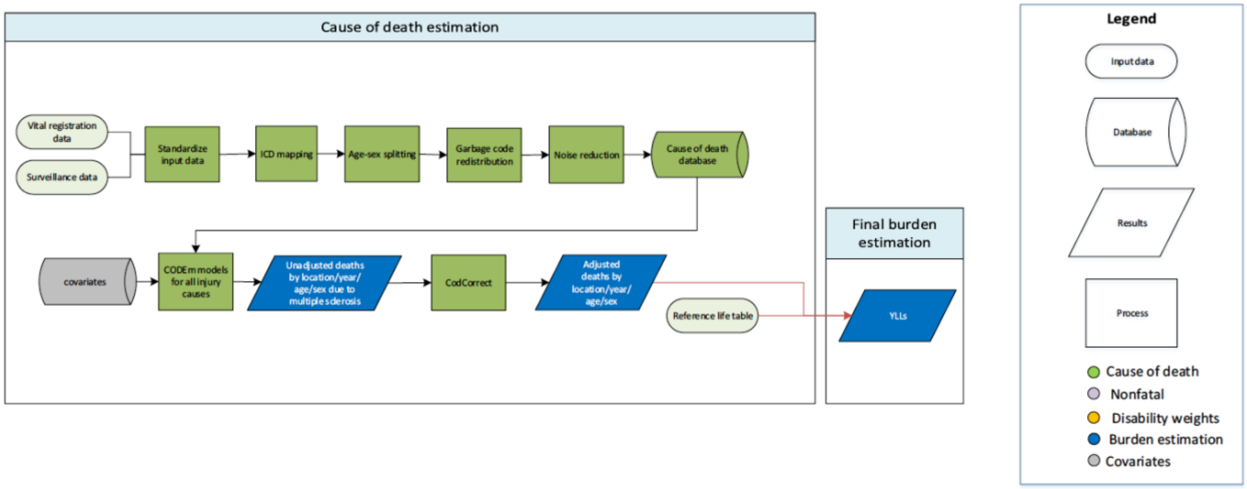

Input data and methodological summary for other neurological disorders

Input data

Data used to estimate other neurological disorders included vital registration and surveillance data. Our outlier criteria were to exclude datapoints that (1) were implausibly high or low, (2) substantially conflicted with established age or temporal patterns, or (3) significantly conflicted with other data sources conducted from the same locations or locations with similar characteristics (ie, Socio-demographic Index). In particular,

- Data excluded as outliers in GBD 2021 continued to be excluded in GBD 2023.
- ICD-10 data were available for Kazakhstan for 2013 onward but were marked as outliers as the raw data were ten-fold greater than the previously modelled mean.
- Similarly, Brunei data from 2011 to 2018 were marked as outliers because they were more than three-fold higher than the median for countries in the high-income Asia Pacific region.

Table 1. ICD-9-CM and ICD-10-CM codes included in other neurological disorders category

Condition type	ICD codes	Conditions covered
Neurodegenerative disorders	**ICD-9**: 330, 330.1, 330.2, 330.3, 330.4, 330.8, 330.9, 331.7<br>**ICD-10**: F02.2, G13, G13.1, G13.2, G13.8, G23, G23.0, G23.1, G23.2, G23.5, G23.8, G23.9	Cerebral degenerations usually manifest in childhood; Cerebral lipidoses; Cerebral degeneration in generalized lipidoses; Cerebral degeneration of childhood in other diseases classified elsewhere; Other specified cerebral degenerations in childhood; Unspecified cerebral degeneration in childhood; Cerebral degeneration in diseases classified elsewhere; Dementia in other diseases classified elsewhere; Systemic atrophies

		primarily affecting central nervous system in diseases classified elsewhere; Other systemic atrophy primarily affecting central nervous system in neoplastic disease; Systemic atrophy primarily affecting the central nervous system in myxedema; Systemic atrophy primarily affecting central nervous system in other diseases classified elsewhere; Other degenerative diseases of basal ganglia; Hallervorden-Spatz disease; Progressive supranuclear ophthalmoplegia [Steele-Richardson-Olszewski]; Striatonigral degeneration; Other specified degenerative diseases of basal ganglia; Degenerative disease of basal ganglia, unspecified
Movement disorders	**ICD-9**: 333, 333.1, 333.2, 333.3, 333.5, 333.6, 333.7, 333.71, 333.72, 333.79, 333.8, 333.81, 333.83, 333.84, 333.85, 333.89, 333.9, 333.91, 333.92, 333.93, 333.94, 333.99<br>**ICD-10**: G24, G24.1, G24.2, G24.3, G24.4, G24.5, G24.8, G24.9, G25, G25.0, G25.2, G25.3, G25.5, G25.8, G25.81, G25.82, G25.83, G25.89, G25.9, G26, G26.0	Other extrapyramidal disease and abnormal movement disorders; Essential and other specified forms of tremor; Myoclonus; Tics of organic origin; Other choreas; Genetic torsion dystonia; Acquired torsion dystonia; Athetoid cerebral palsy; Acute dystonia due to drugs; Other acquired torsion dystonia; Fragments of torsion dystonia; Blepharospasm; Orofacial dyskinesia; Spasmodic torticollis; Organic writers' cramp; Subacute dyskinesia due to drugs; Other fragments of torsion dystonia; Unspecified extrapyramidal disease and abnormal movement disorder; Stiff-man syndrome; Neuroleptic malignant syndrome; Benign shuddering attacks; Restless legs syndrome (RLS); Idiopathic nonfamilial dystonia
Spinocerebellar disorders	**ICD-9**: 334, 334.1, 334.2, 334.3, 334.4, 334.8, 334.9<br>**ICD-10**: G11, G11.0, G11.1, G11.2, G11.3, G11.4, G11.8, G11.9	Spinocerebellar disease; Hereditary spastic paraplegia; Primary cerebellar degeneration; Other cerebellar ataxia; Cerebellar ataxia in diseases classified elsewhere; Other spinocerebellar diseases; Spinocerebellar disease unspecified; Hereditary ataxia; Congenital nonprogressive ataxia; Early-onset cerebellar ataxia; Late-onset cerebellar ataxia; Cerebellar ataxia with defective DNA repair; Hereditary spastic paraplegia; Other hereditary ataxias; Hereditary ataxia, unspecified
Neuromuscular disorders	**ICD-9**: 358, 358.01, 358.2, 358.3, 358.31, 358.39, 358.8, 358.9, 359, 359.3, 359.4, 359.5, 359.6, 359.7, 359.71, 359.79, 359.8, 359.81, 359.89, 359.9	Myoneural disorders; Myoneural disorders without (acute) exacerbation; Myoneural disorders with (acute) exacerbation; Toxic myoneural disorders; Lambert-Eaton syndrome; Lambert-Eaton syndrome in neoplastic disease; Lambert-Eaton syndrome in other diseases classified elsewhere; Other specified myoneural disorders; Myoneural disorders unspecified; Muscular dystrophies and other

	**ICD-10:** G13.0, G70.8, G70.80, G70.81, G70.89, G70.9, G73, G73.0, G73.1, G73.2, G73.3, G73.4, G73.5, G73.6, G73.7	myopathies; Periodic paralysis; Toxic myopathy; Myopathy in endocrine diseases classified elsewhere; Symptomatic inflammatory myopathy in diseases classified elsewhere; Inflammatory and immune myopathies, NEC; Inclusion body myositis; Other inflammatory and immune myopathies, NEC; Other myopathies; Critical illness myopathy; Other myopathies; Myopathy, unspecified; Paraneoplastic neuromyopathy and neuropathy; Disorders of myoneural junction and muscle in diseases classified elsewhere; Myasthenic syndromes in other diseases classified elsewhere; Myopathy in infectious and parasitic diseases classified elsewhere
Peripheral neuropathies	**ICD-9**: 356, 356.1, 356.2, 356.3, 356.4, 356.8, 356.9, 357, 357.1, 357.3, 357.4, 357.7<br>**ICD-10:** G61, G61.0, G61.1, G61.8, G61.81, G61.89, G61.9	Hereditary and idiopathic peripheral neuropathy; Peroneal muscular atrophy; Hereditary sensory neuropathy; Refsum's disease; Idiopathic progressive polyneuropathy; Other specified idiopathic peripheral neuropathy; Unspecified hereditary and idiopathic peripheral neuropathy; Acute infective polyneuritis; Polyneuropathy in collagen vascular disease; Polyneuropathy in malignant disease; Polyneuropathy in other diseases classified elsewhere; Polyneuropathy due to other toxic agents
Spinal cord disorders	**ICD-9**: 336, 336.1, 336.2, 336.3, 336.8, 336.9<br>**ICD-10:** G95, G95.0, G95.1, G95.11, G95.19, G95.2, G95.29, G95.3, G95.6, G95.8, G95.81, G95.89, G95.9	Other diseases of spinal cord; Syringomyelia and syringobulbia; Vascular myelopathies; Subacute combined degeneration of spinal cord in diseases classified elsewhere; Myelopathy in other diseases classified elsewhere; Other myelopathy; Unspecified disease of spinal cord; Acute infarction of spinal cord (embolic) (nonembolic); Other vascular myelopathies; Other and unspecified cord compression; Unspecified cord compression; Other cord compression; Conus medullaris syndrome
Demyelinating disorders	**ICD-9**: 341, 341.1, 341.2, 341.21, 341.22, 341.8, 341.9<br>**ICD-10:** G36, G36.0, G36.1, G36.8, G36.9, G37, G37.0, G37.1, G37.2, G37.3, G37.4, G37.5, G37.8, G37.9	Other demyelinating diseases of central nervous system; Neuromyelitis optica; Schilder's disease; Acute (transverse) myelitis; Other demyelinating diseases of central nervous system NOS; Other demyelinating diseases of central nervous system in conditions classified elsewhere; Idiopathic transverse myelitis; Other demyelinating diseases of central nervous system; Demyelinating disease of central nervous system, unspecified; Acute and subacute hemorrhagic leukoencephalitis [Hurst]; Other specified acute disseminated demyelination; Acute disseminated demyelination, unspecified;

		Diffuse sclerosis of central nervous system; Central demyelination of corpus callosum; Central pontine myelinolysis; Acute transverse myelitis in demyelinating disease of central nervous system; Subacute necrotizing myelitis of central nervous system; Concentric sclerosis [Balo] of central nervous system; Other specified demyelinating diseases of central nervous system; Demyelinating disease of central nervous system, unspecified
Autonomic nervous system disorders	**ICD-9**: 337, 337.01, 337.09, 337.1, 337.2, 337.21, 337.22, 337.29, 337.3, 337.9<br>**ICD-10**: G90, G90.0, G90.01, G90.09, G90.1, G90.2, G90.3, G90.4, G90.5, G90.50, G90.51, G90.52, G90.59, G90.8, G90.9	Disorders of the autonomic nervous system; Carotid sinus syndrome; Other idiopathic peripheral autonomic neuropathy; Peripheral autonomic neuropathy in disorders classified elsewhere; Reflex sympathetic dystrophy; Disorders of the autonomic nervous system unspecified; Disorders of the autonomic nervous system of the upper limb; Disorders of the autonomic nervous system of the lower limb; Disorders of the autonomic nervous system of other specified site; Autonomic dysreflexia; Unspecified disorder of autonomic nervous system; Idiopathic peripheral autonomic neuropathy; Familial dysautonomia [Riley-Day]; Horner's syndrome; Multi-system degeneration of the autonomic nervous system; Complex regional pain syndrome I (CRPS I); Complex regional pain syndrome I, unspecified; Complex regional pain syndrome I of upper limb; Complex regional pain syndrome I of right upper limb; Complex regional pain syndrome I of left upper limb; Complex regional pain syndrome I of upper limb, bilateral; Complex regional pain syndrome I of unspecified upper limb; Complex regional pain syndrome I of lower limb; Complex regional pain syndrome I of right lower limb; Complex regional pain syndrome I of left lower limb; Complex regional pain syndrome I of lower limb, bilateral; Complex regional pain syndrome I of unspecified lower limb; Complex regional pain syndrome I of other specified site
Other disorders of nervous system	**ICD-9**: 349, 349.2, 349.3, 349.39, 349.8, 349.9	Other and unspecified disorders of the nervous system; Disorders of meninges, not elsewhere classified; Dural tear; Other dural tear; Other specified disorders of nervous system; Other nerve root and plexus disorders; Unspecified nerve root and plexus disorder

Modelling strategy

The standard Cause of Death Ensemble modelling (CODEm) approach (as described in the reference appendix 1 section 4) was used to estimate deaths due to other neurological conditions. Separate models were conducted for male and female mortality, and the age range for both models was 28 days to 95+ years. CoDCorrect was used to produce final estimates of number of deaths and years of life lost (YLLs). CODEm requires that the covariate selected for the model must have the directional relationship with other neurological disorders deaths. For GBD 2023, no significant updates were made for other neurological disorders covariate selection. Covariate directions were selected based on the strength of the evidence.

Table 2. Covariates used in other neurological disorders mortality modelling

Level	Covariate	Direction
1	Mean total body-mass index	+
	Mean serum total cholesterol (mmol/L)	+
	Mean systolic blood pressure (mmHg)	+
	Pigs per capita	+
	Age-standardised SEV for underweight children	+
	Red meat consumption, adjusted	+
2	Population density over 1000 per square kilometer pct	+
	Healthcare Access and Quality Index	-
	Fruit consumption (grams per day, adjusted)	-
3	Cumulative cigarettes (10 years)	+
	Cumulative cigarettes (5 years)	+
	Education (years per capita)	-
	Log-transformed LDI (per capita)	-
	Smoking prevalence	+
	Socio-demographic Index	+

Other skin and subcutaneous diseases

Flowchart

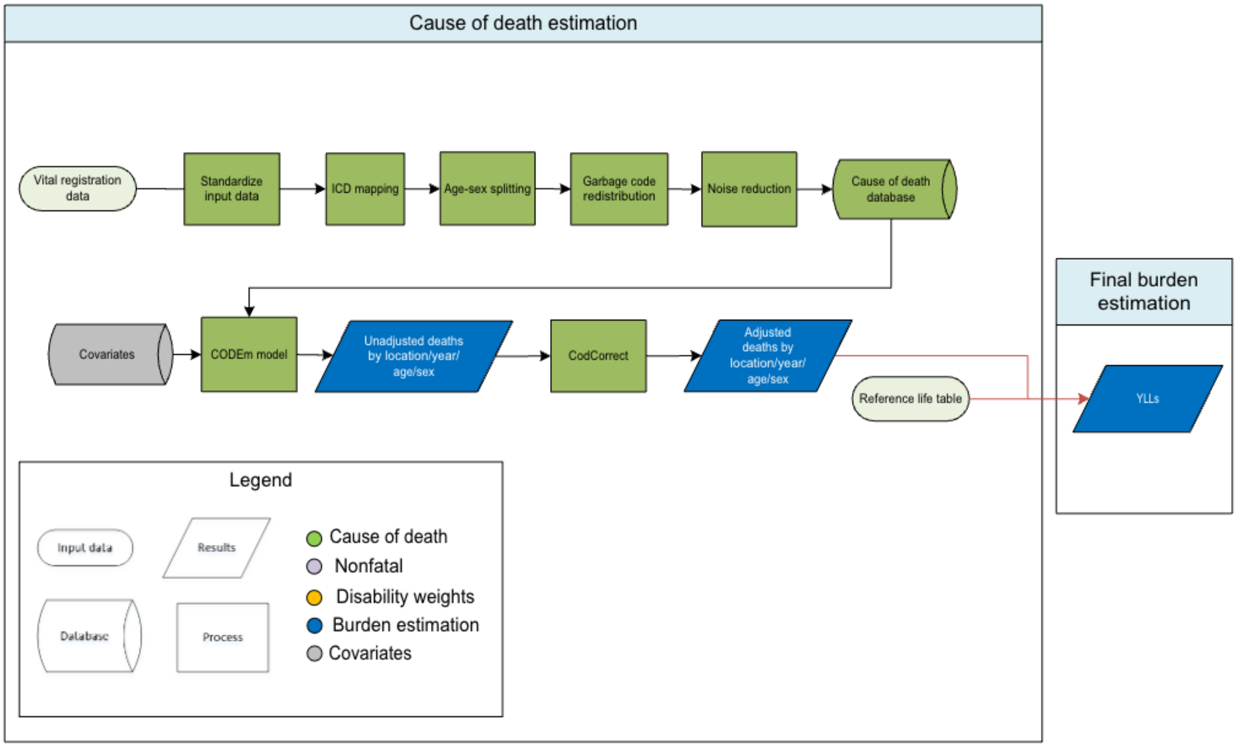

Input data and methodological summary for other skin and subcutaneous diseases

Input data

Data used to estimate mortality due to other skin and subcutaneous diseases consisted of vital registration and verbal autopsy data from the cause of death (CoD) database. Outlier criteria excluded datapoints that were implausibly under/over-reporting relative to global or regional patterns and data from datasets with small populations.

Modelling strategy

The other skin and subcutaneous diseases model is estimated by CODEm model with standard CODEm parameters along with the CoD database and location-level covariates as inputs. We hybridised separate global and data-rich models to acquire unadjusted results, which we finalised and adjusted using CoDCorrect to reach final years of life lost (YLLs) due to other skin and subcutaneous diseases.

Compared to GBD 2021, there are several updates that were made to the model estimation strategy. First, we have received new data for other skin and subcutaneous diseases for multiple location-years. There were 101 data sources added to the cause of death database, which led to improved estimates, particularly for recent years. Key location-years that impacted our estimates were from Bangladesh, Indonesia, Ethiopia, Nigeria, Brazil, China, and India.

Additionally, in GBD 2023, CODEm began using an all-cause mortality envelope that is inclusive of HIV, whereas in previous rounds, the all-cause mortality envelope was HIV-free. This can lead to changes in cause fractions and rates when compared to previous GBD rounds.

Table 1. Covariates used in other skin and subcutaneous diseases mortality modelling

Level	Covariate	Direction
1	Age-standardised summary exposure value (SEV) for child underweight	
	Improved water source (proportion with access)	
	SEV scalar for unsafe sanitation	
	Diabetes fasting plasma glucose (mmol/L), by age	
	Healthcare Access and Quality Index	
	Prevalence of overweight and obesity	
2	Smoking prevalence	
	Alcohol (litres per capita)	
	Cumulative cigarettes (5 years)	
	Cumulative cigarettes (10 years)	
3	Education (years per capita)	
	Lag-distributed income (per capita)	
	Socio-demographic Index	

Other unspecified infectious diseases

Flowchart

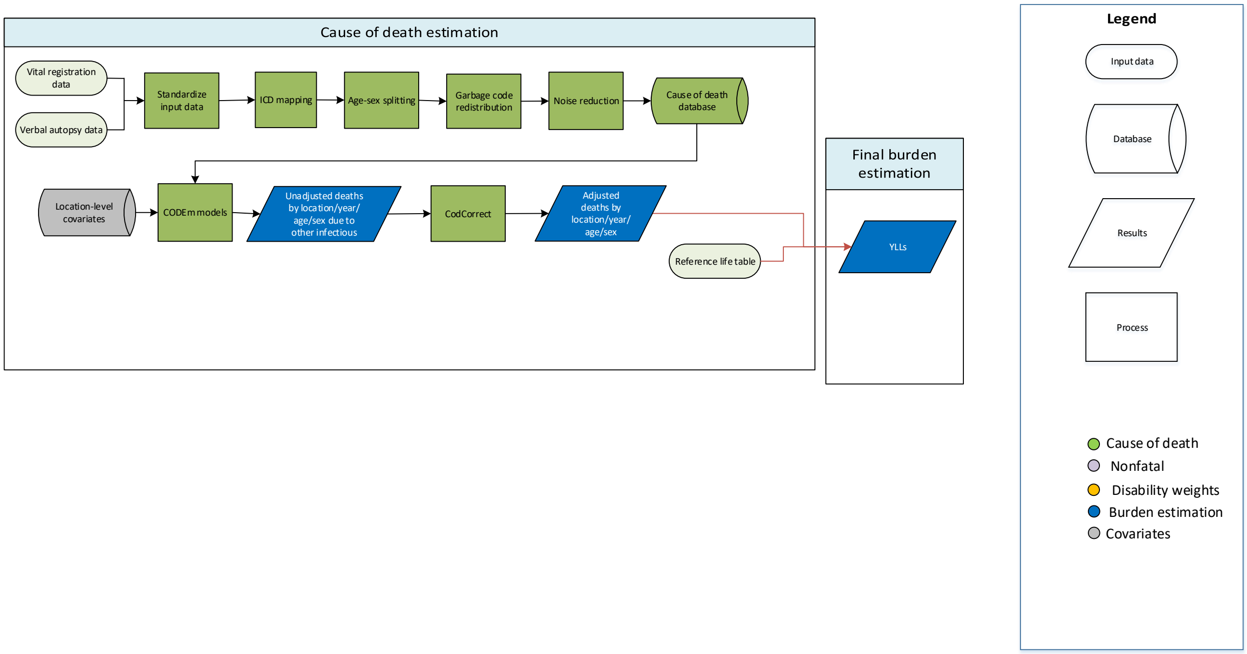

Input data and methodological summary for other unspecified infectious diseases

Input data

We modelled other infectious disease mortality using all available data in the cause of death database. Datapoints were outliered if they reported an improbable number of deaths or if their inclusion in the model yielded distorted trends.

Modelling strategy

We modelled other unspecified infectious disease mortality using a two-model hybrid approach: 1) a global CODEm model of all locations, using all data in the CoD database; and 2) a CODEm model restricted to data-rich countries. We have made no substantive changes to the modelling strategy since GBD 2017.

Table 1. Covariates used in other unspecified infectious diseases mortality modelling

Level	Covariate name	Level	Direction
1	DPT3 coverage	1	-
2	Healthcare Access and Quality Index	2	-
	Sanitation proportion	2	-
	Improved water source proportion	2	-
3	Socio-demographic Index	3	-
	Antenatal care coverage proportion	3	-

Otitis media

Flowchart

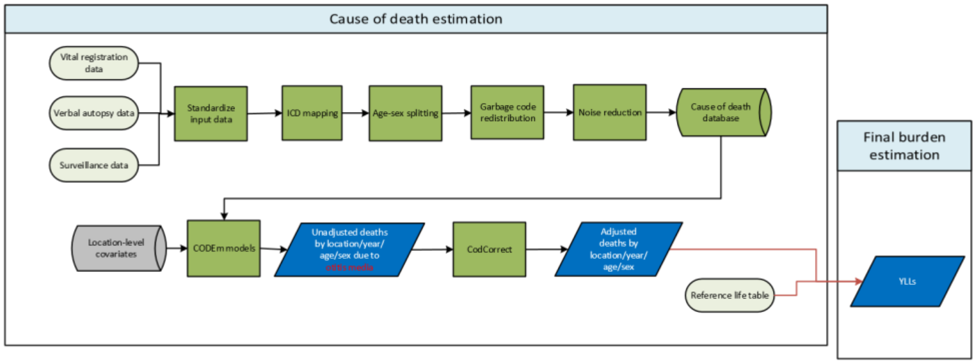

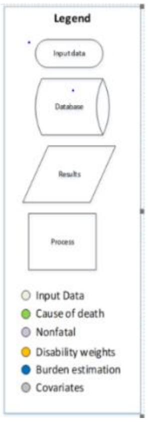

Input data and methodological summary for otitis media

Input data

Vital registration, verbal autopsy, and surveillance data were used. Outliers were identified by systematic examination of datapoints. Datapoints that violated well-established age or time trends were inconsistent with other country- or region-specific points, or that resulted in extremely high or low mortality rates were determined to be outliers.

Modelling strategy

A general CODEm modelling strategy was used. There were no substantive changes from GBD 2021 in terms of modelling strategy. The covariates used are displayed in Table 1.

Table 1. Covariates used in otitis media mortality modelling

Level	Covariate	Direction
1	Otitis summary exposure value (SEV)	+
	Smoking prevalence	+
2	Indoor pollution	+
	Healthcare Access and Quality Index	-
	Outdoor pollution ($PM_{2.5}$)	+
3	Socio-demographic Index (SDI)	-
	Log-transformed lag-distributed income	-
	Education (years per capita)	-

Pancreatitis

Flowchart

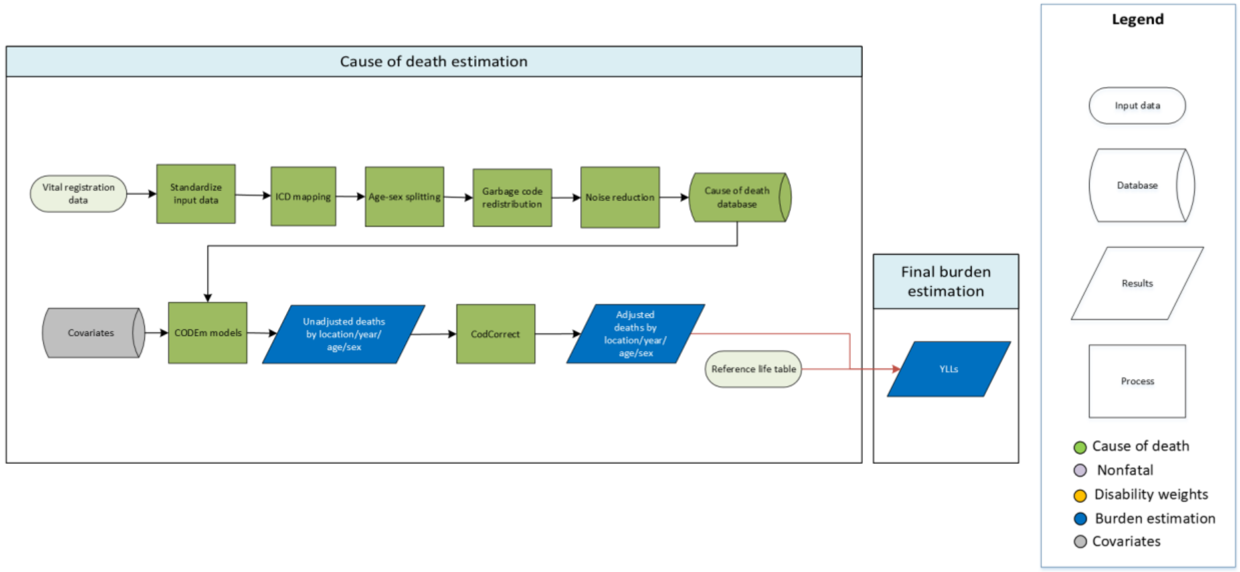

Input data and methodological summary for pancreatitis

Pancreatitis comprise a Level 3 cause in the GBD hierarchy.

Input data

Data used to estimate mortality of pancreatitis consisted of vital registration (VR) from the cause of death (CoD) database. A list of ICD codes that were mapped to this cause, a list of vital registration sources included in the CoD database and their quality as indicated by star-ratings, and the steps used to process these data for modelling can be found in the section of this appendix on the causes of death database and the tables and figures it refers to. COD data processing was broadly similar to previous rounds of GBD, but one novel data processing step was employed in preparing data for pancreatitis that was not needed prior to GBD 2023—COVID-19 corrections—which are described elsewhere in this appendix. After all data processing steps were complete, datapoints for all locations and years were systematically reviewed to identify outliers or other data limitations not fully overcome by CoD data processing methods, and selected sources were excluded from analysis.

Modelling strategy

The strategy used to estimate mortality due to pancreatitis was not substantively changed between GBD 2021 and GBD 2023. A standard CODEm model was used to model deaths due to pancreatitis (see appendix section on CODEm method for details). Models were generated separately for males in data-rich countries, males in all countries (referred to as a global model), females in data-rich countries, and females in all countries (global model). Fatal estimation for this cause was restricted to ages 2–95+ years.

The following table has the full list of covariates presented to the CODEm algorithm for selection in models of pancreatitis mortality.

Table 1. Covariates used in pancreatitis mortality modelling

Level	Covariate	Direction
1	Log-transformed scaled exposure variable for pancreatitis	+
	Alcohol (litres per capita)	+
2	Healthcare Access and Quality Index	-
	BMI (mean)	+
3	Socio-demographic Index	-
	Education (years per capita)	-
	Log LDI ($I per capita)	-

We hybridised separate global and data-rich models to acquire unadjusted results, which we adjusted using CoDCorrect. Adjustment in CoDCorrect included rescaling unadjusted pancreatitis death estimates alongside other Level 3 digestive disease deaths to sum to overall digestive disease deaths, which were then rescaled along other causes to sum up the hierarchy to all-cause counts of death. Adjusted deaths were compared to the reference life table to calculate final YLLs due to pancreatitis.

Paralytic ileus and intestinal obstruction

Flowchart

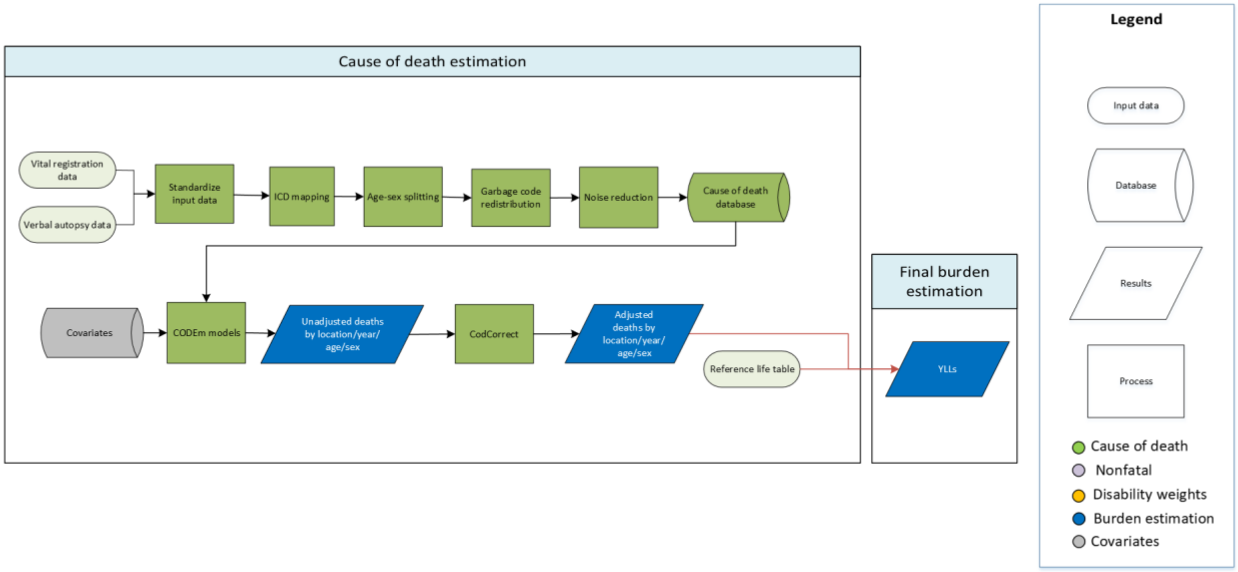

Input data and methodological summary for paralytic ileus and intestinal obstruction

Paralytic ileus and intestinal obstruction comprise a Level 3 cause in the GBD cause hierarchy.

Input data

Data used to estimate mortality due to paralytic ileus and intestinal obstruction consisted of vital registration (VR) and verbal autopsy data (VA) from the cause of death (CoD) database. A list of ICD codes that were mapped to this cause, a list of vital registration sources included in the CoD database and their quality as indicated by star-ratings, and the steps used to process these data for modelling can be found in the section of this appendix on the causes of death database and the tables and figures it refers to. CoD data processing was broadly similar to previous rounds of GBD, but one novel data processing step was employed in preparing data for paralytic ileus and intestinal obstruction that was not needed prior to GBD 2023—COVID-19 corrections—which are described elsewhere in this appendix.

After all data processing steps were complete, datapoints for all locations and years were systematically reviewed to identify outliers or other data limitations not fully overcome by CoD data processing methods, and selected sources were excluded from analysis.

Modelling strategy

The modelling strategy used to estimate mortality due to fatal paralytic ileus and intestinal obstruction was not substantively changed between GBD 2021 and GBD 2023. A standard CODEm model was used to model deaths due to paralytic ileus and intestinal obstruction (see appendix section on CODEm method for details). Models were generated separately for males in data-rich countries, males in all countries (referred to as a global model), females in data-rich countries, and females in all countries (global model). Fatal estimation for this cause was restricted to ages 0 days to 95+ years. The following

table has the full list of covariates presented to the CODEm algorithm for potential inclusion in the models for paralytic ileus and intestinal obstruction.

Table 1. Covariates used in paralytic ileus and intestinal obstruction mortality modelling

Level	Covariate	Direction
2	Fruit consumption (unadjusted, kcal per capita)	-
	Vegetable consumption (unadjusted, kcal per capita)	-
	Healthcare Access and Quality Index	-
3	Socio-demographic Index	-
	Education (years per capita)	-
	Log LDI ($I per capita)	-

We hybridised separate global and data-rich models to acquire unadjusted results, which we adjusted using CoDCorrect. Adjustment in CoDCorrect included rescaling unadjusted death estimates for paralytic ileus and intestinal obstruction alongside other Level 3 digestive diseases to sum to overall digestive disease deaths, which were then rescaled along with other causes up the cause hierarchy to sum to all-cause counts of death. Adjusted deaths were compared to the reference life table to calculate final YLLs due to paralytic ileus and intestinal obstruction.

Paratyphoid fever

Flowchart

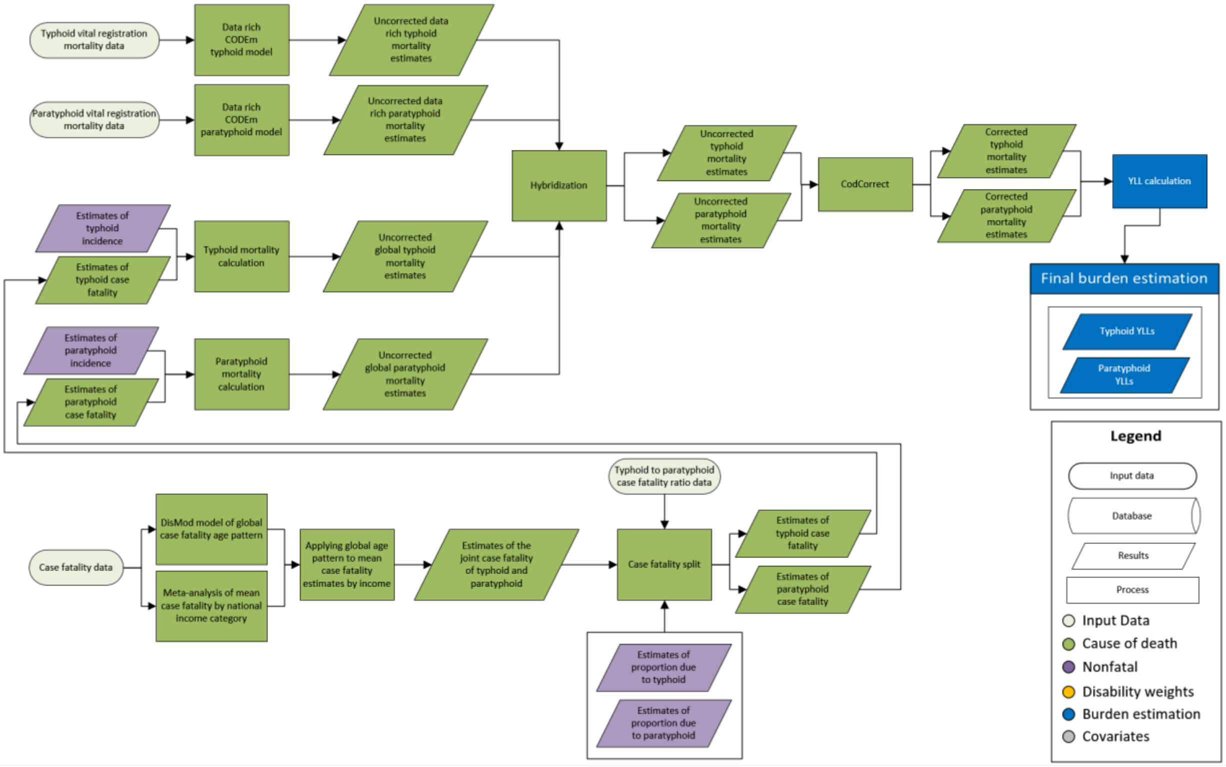

Input data and methodological summary for paratyphoid fever

Input data

Our CODEm model used all available data in the cause of death database from data-rich countries. No data were outliered for this cause. For the natural history model, our incidence dataset included a combination of data from prospective cohort studies and national surveillance systems. Similarly, data on proportions due to typhoid and paratyphoid included a combination of prospective cohort studies and national surveillance systems, and facility- and lab-based studies. Case fatality data were from national surveillance systems and hospital databases.

Modelling strategy

We model paratyphoid deaths using a hybrid modelling strategy with two components: 1) for data-rich locations, we estimate paratyphoid mortality using a CODEm model of CoD data; and 2) in all other locations (ie, not data-rich), we use a natural history model in which we derive deaths as the product of cases and case fatality.

The CODEm model included six covariates:

Level	Covariate	Direction
1	Sanitation (proportion with access)	-
	Improved water source (proportion of the population with access)	-
	Proportion of the population living in the Indian Ocean monsoon belt	+
	SEV unsafe water	+
	SEV unsafe sanitation	+
2	Healthcare Access and Quality Index	-

For the natural history model, we first model total incidence of typhoid and paratyphoid combined. Second, we model the proportion of this total due to typhoid and the proportion due to paratyphoid. Third, we estimate case fatality by age and national income category for typhoid and paratyphoid combined. Fourth, we use data on the relative fatality of typhoid and paratyphoid to split the joint case fatality estimates into typhoid- and paratyphoid-specific case fatality estimates. Finally, we estimate cause-specific mortality rates as the product of incidence and case fatality.

Before modelling incidence, we applied four adjustments to the incidence data: 1) diagnostic sensitivity adjustment, 2) passive surveillance adjustment, 3) typhoid-only adjustment, and 4) age/sex splits. Incidence data were inflated to account for poor diagnostic sensitivity, based on an internal meta-analysis of the sensitivity of blood culture, the most common diagnostic used for typhoid: we estimate a sensitivity of 60.3% (50.3–68.8). We performed a crosswalk adjustment for incomplete case capture data from passive versus active surveillance, with active surveillance as the reference, using a MR-BRT model and adjusted the data before modelling. Where incidence data were from studies that only tested for typhoid fever, we used estimates from our aetiological proportion models to adjust these typhoid-only sources and calculated an adjusted joint incidence (ie, including both typhoid and paratyphoid cases) by dividing the typhoid-only incidence by the estimated proportion due to typhoid. We performed this calculation using posterior simulation with 1000 draws to propagate uncertainty from both the incidence data and the proportion estimate. Finally, where incidence data were reported for both sexes combined or for age categories spanning more than 25 years, we produced datapoints that were age- and sex-specific based on a MR-BRT model of sex ratios and a DisMod model of age patterns.

Total incidence was modelled using DisMod-MR, using the summary exposure values (SEV) for unsafe sanitation and the proportion of the population living in the Indian Ocean monsoon belt as covariates. Similarly, we used a DisMod model to estimate aetiological proportions, using a single model of the proportion of enteric fever due to *Salmonella* Partyphi. We model the proportion due to paratyphoid instead of the proportion due to typhoid as we've found that this approach results in better a better model fit for sub-Saharan Africa, where the proportion of enteric fever due to *Salmonella* Typhi approaches 1.0, as DisMod performs better with proportions that are near-zero, than with proportions that are near-one.

Case fatality data were too limited to allow for a complete DisMod model, or to allow for varying estimates by time and space. We had sufficient data, however, to estimate case fatality by age and by three categories of national income. We used DisMod to extract a global age pattern in case fatality, and

meta-regression to estimate the mean case fatality by income category. Finally, we estimated the relative risk of death from typhoid relative to paratyphoid based on data from Chinese surveillance and used that relative risk to estimate case fatality separately for typhoid and paratyphoid, by age and income.

Finally, we estimated paratyphoid mortality as the product of total incidence, the proportion of the total due to paratyphoid, and case fatality for paratyphoid. We propagated uncertainty through every step of the modelling process by pulling 1000 draws from the distribution of each model component (eg, incidence, proportion due to paratyphoid, overall case fatality, case fatality age pattern, relative fatalness of typhoid versus paratyphoid), and performing all calculations at the draw level.

Changes from GBD 2021 to GBD 2023

We updated our systematic review for GBD 2023 (Figure 1) and updated the code pipeline to improve robustness and efficiency but made no substantive changes to our modelling strategy for typhoid and paratyphoid between GBD 2021 and GBD 2023.

Figure 1: PRISMA flow diagram

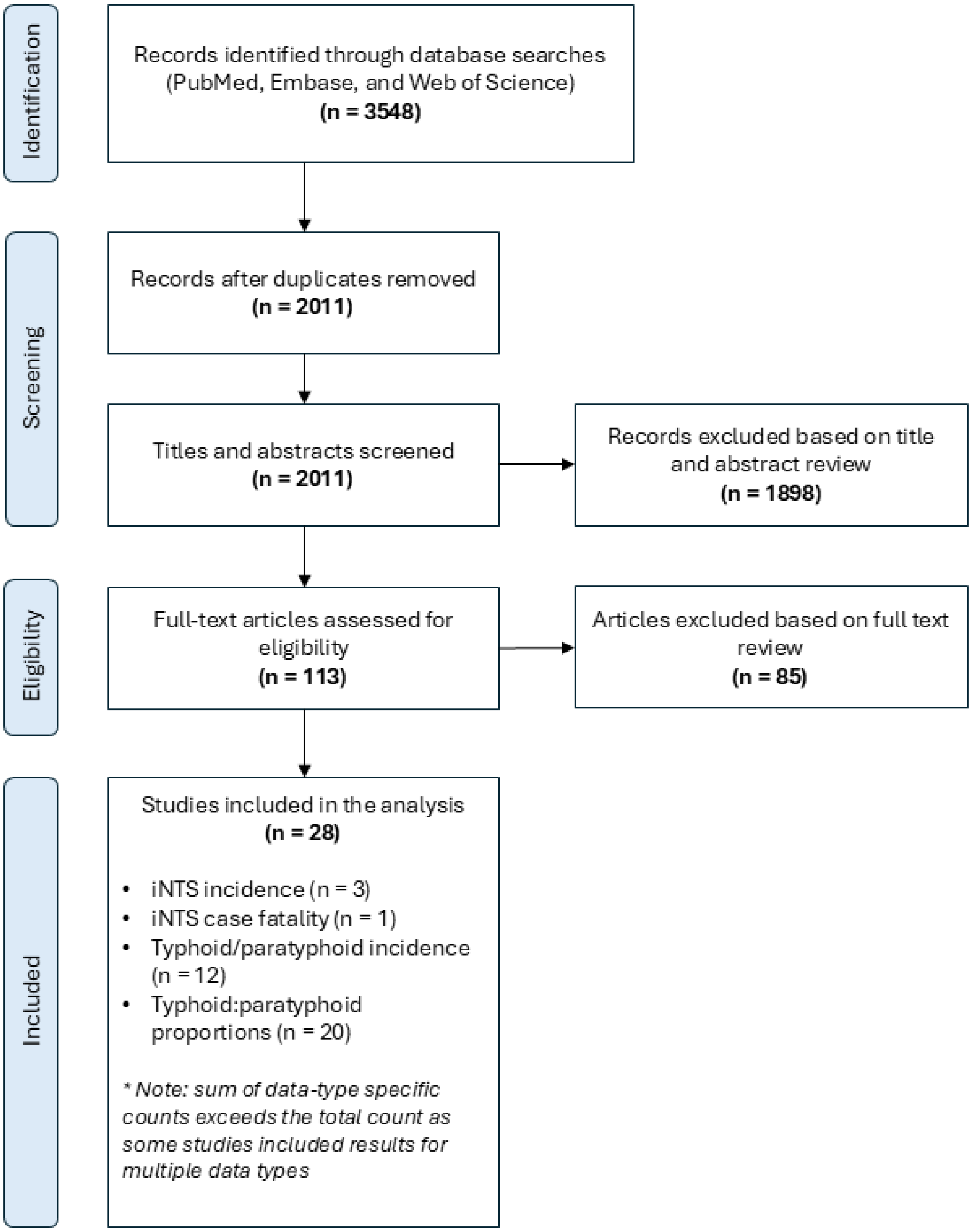

Parkinson’s disease

Flowchart

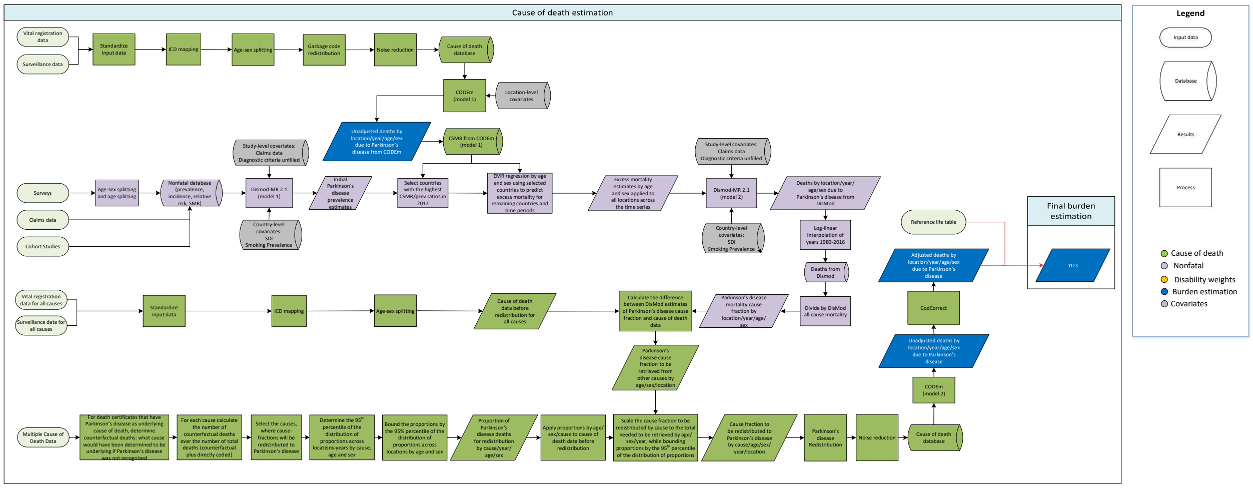

Input data

In GBD 2023, data used to estimate deaths due to Parkinson’s disease included mortality data from vital registration (VR) systems and non-fatal and mortality data from surveys and insurance claims sources, as described below.

For incidence and prevalence, a systematic review was conducted from September 2015 to August 2017 for GBD 2019 with the following search terms to capture studies for Parkinson’s disease: (Parkinson disease[Title/Abstract] OR Parkinson's disease[Title/Abstract]) AND (epidemiology[Title/Abstract] OR prevalence[Title/Abstract] OR incidence[Title/Abstract]) AND ("2015/09/31"[PDAT] : "2017/08/23"[PDAT]). Inclusion criteria comprised studies that reported prevalence, incidence, remission rate, excess mortality rate, relative risk of mortality, standardised mortality ratio, or with-condition mortality rate. Studies with no clearly defined sample or that drew from specific clinic/patient organisations were excluded. We also added USA claims data for 2011 and 2012–2015. For GBD 2023, no further prevalence or incidence data were added to inform fatal modelling. While we collect incidence data, we do not use these data to inform our estimates of Parkinson’s disease because it is difficult to determine the onset of Parkinson’s disease (versus parkinsonism); therefore, we preferentially use prevalence data.

Modelling strategy

Overview

Parkinson’s disease mortality rates have more than doubled since 1980 in high-quality VR systems such as in the USA, Canada, Australia, France, Germany, the UK, and Finland, while other European countries like the Netherlands, Sweden, and Norway have not seen such increases over time. We have not seen an

equivalent increase in prevalence and incidence data sources. Additionally, the greater than 15-fold variation in mortality rates of Parkinson's disease between countries is much greater the three-fold difference in prevalence and incidence between high-income countries. As it is unlikely that case fatality from Parkinson's disease has dramatically increased over the time period and that it would differ by a very large margin between countries, the hypothesis is that certifying and coding practices have changed over time and at a different pace between countries. For GBD 2016 onward, we decided to employ a modelling strategy that avoids spurious large trends over time in the fatal component of the burden of Parkinson's disease by making Parkinson's mortality rates consistent with the rates observed in estimation years, relative to prevalence in countries that are most likely to certify or code Parkinson's disease as an underlying cause of death.

Modelling steps

Fatal modelling for Parkinson's disease is described in the following steps. The Multiple Cause of Death (MCOD) Parkinson's disease inputs were identical to those used in the GBD 2019 capstone publication.[1]

First, we ran a Cause of Death Ensemble (CODEm) model (see appendix 1 section 4) for Parkinson's disease and extracted the mortality rates by age, sex, and geography. The covariates used in this intermediary model are displayed in the table that follows this paragraph; some covariates have a direction of zero because this model was run in GBD 2017. The final Parkinson's model for GBD 2023 has a negative or positive direction specified for all covariates (see Table 4).

Table 1. Covariates used in Parkinson's mortality modelling

Level	Covariate	Direction
1	Cumulative cigarette consumption (10 years)	-
2	Absolute latitude	+
	Cholesterol (total, mean per capita)	+
	Sanitation (proportion with access)	0
	Improved water source (proportion with access)	0
	Fruit consumption adjusted (g)	-
	Healthcare Access and Quality Index	-
3	Education (years per capita)	-
	Socio-demographic Index	+
	Lag-distributed income	0

Second, we ran a DisMod-MR 2.1 model (see appendix 1 section 4) with all data on incidence, prevalence, and mortality risk (relative risk, standardised mortality rate, or with-condition mortality rates) and a setting of zero remission and extracted prevalence by age, sex, and geography. Studies where the case definition of two of the four cardinal symptoms of Parkinson's disease (tremors/trembling, bradykinesia, stiffness of limbs and torso, posture instability) were not filled were adjusted using MR-BRT (see appendix 1 section 1), a meta-regression framework, to studies using the reference case definition. No random effects were used in the model in order to prevent spurious inflation of regional differences due to differences in measurement and measurement error.

Third, we selected the seven countries (France, England, the USA, the Netherlands, Finland, Scotland, and Wales) with the highest cause-specific mortality rate (from step 1) to prevalence (from step 2) ratio in 2017, which also had an age-standardised prevalence rate greater than 0.0005, and a population greater than 1 million.

Fourth, we used a linear effects regression with dummies on age group and sex to predict excess mortality (EMR) (ie, the ratio of cause-specific mortality rate and prevalence) by age and sex, the results of which are found in the Tables 2 and 3.

Table 2: Fixed effect coefficients of EMR regression. Outcome: ln(EMR)

Independent variables	Coef	Std. error	P value	95% confidence interval	
Male	0.288	0.036	0.000	0.218	0.358
Age 40–59	-3.25	0.076	0.000	-3.399	-3.101
Age 60–64	-2.557	0.076	0.000	-2.706	-2.407
Age 65–69	-2.021	0.076	0.000	-2.17	-1.871
Age 70–74	-1.42	0.076	0.000	-1.57	-1.271
Age 75–80	-0.898	0.076	0.000	-1.047	-0.749
Age 80–84	-0.502	0.076	0.000	-0.651	-0.352
Age 85–89	-0.248	0.076	0.001	-0.397	-0.099
Age 90–94	-0.047	0.076	0.537	-0.196	0.102
Constant	-2.357	0.057	0.000	-2.469	-2.246

Table 3: Predicted EMR values by age and sex (95% CI)

	Male	Female
Age 40–59	0.005 (0.004–0.005)	0.004 (0.003–0.004)
Age 60–64	0.01 (0.009–0.011)	0.007 (0.007–0.008)
Age 65–69	0.017 (0.015–0.019)	0.013 (0.011–0.014)
Age 70–74	0.031 (0.027–0.034)	0.023 (0.02–0.025)
Age 75–80	0.051 (0.046–0.057)	0.039 (0.035–0.043)
Age 80–84	0.076 (0.068–0.085)	0.058 (0.052–0.064)
Age 85–89	0.099 (0.089–0.111)	0.074 (0.066–0.083)
Age 90–94	0.12 (0.108–0.135)	0.09 (0.081–0.1)
Age 95+	0.126 (0.113–0.142)	0.095 (0.085–0.106)

Fifth, these estimates were added to a second DisMod-MR 2.1 model. For the countries included in the regression, we allowed them to retain their original EMR values when the age-standardised EMR for a country was higher than the age-standardised EMR prediction generated from the regression. These countries retained their age- and sex-specific ratios and entered those also as pertaining to the full estimation period. Smoking prevalence was used as a country-level covariate. We excluded data for standardised mortality ratio, with-condition mortality rate, and relative risk as we wanted to estimate cause-specific mortality rates that were consistent with the level of excess mortality from the seven chosen countries.

Sixth, we took the predictions of cause-specific mortality by age, sex, geography, and year that DisMod-MR 2.1 calculated as being consistent with the data on incidence, prevalence, and the priors on excess mortality from step five. Because DisMod-MR 2.1 produces estimates in five-year intervals only, we

expanded the time series by log-linear interpolation; values for 1980–1990 were generated using a regression on the entire time series with Socio-demographic Index included as a predictor. We divided this cause-specific mortality by the all-cause mortality used in DisMod-MR 2.1 to calculate the Parkinson's disease cause-fraction based on prevalence data and the excess mortality derived from countries most likely to code to Parkinson's disease as a cause of death.

Seventh, we calculated the difference between this cause-fraction derived from DisMod and the cause-fraction derived from the cause of death data prep process before redistribution in order to get the amount of cause-fraction that needed to be retrieved from other causes through the Parkinson's disease redistribution process.

Eighth, to calculate where these Parkinson's disease deaths should be retrieved from, we analysed MCOD data. We only used data from the USA and asserted that the data from 2010 to 2015 are the reference data because during this period the increases in coding to Parkinson's disease as a cause of death levelled off.

Ninth, for deaths where Parkinson's disease is the underlying cause of death in the years 2010–2015, we calculated what the underlying cause of death would have been in the counterfactual scenario in which Parkinson's disease had not been recognised. To calculate this counterfactual, we examined the causes listed in part one of the chain of the death certificate. For each death certificate chain, we looked across the entire dataset from 1980 to 2015 and determined what the distribution of underlying causes of death was in individuals with that particular death certificate chain. Then, we assigned the counterfactual deaths proportionally to the causes that are listed as underlying in these death certificates. If, over the time period, there were fewer than 1000 death certificates that had exactly the same death certificate chain, then we included all death certificate chains that had those same causes, but which could additionally include other causes in the chain as well. To assign counterfactual deaths for these chains, we further subsetted the data to death certificate chains where any of the causes in the original death certificate chain were listed as underlying, determined the distribution of underlying causes of death among just this subset, and then assigned counterfactual deaths proportionally in the same manner.

Tenth, once we determined the counterfactual causes of death stemming from all Parkinson's disease deaths from 2010 to 2015, we calculated the proportion of deaths by cause that should be Parkinson's disease deaths according to the reference data by taking the counterfactual deaths for each cause and dividing by the sum of the counterfactual deaths for that cause plus the directly coded deaths for that cause.

Eleventh, we applied the proportions to cause of death data in cause-fraction space and scaled the cause fractions to the total mortality cause-fraction to be retrieved based on the DisMod-MR 2.1 model. We set caps on the percentage of deaths that were moved by age, sex, and cause. The caps were determined by finding the 95th percentile of the percentages of deaths moved in each age-sex-cause category across all high-quality data source locations. The causes of death data were then processed using general redistribution strategies and noise reduction described.

Finally, the data derived from this process were used in a final CODEm model, adjusting for covariates. For GBD 2023, the covariates and their specified directionality and level are shown below in Table 4.

These results were then adjusted through CoDCorrect (see appendix 1 section 4) and become the final cause of death estimates for Parkinson's disease.

Table 4. Covariates tested for GBD 2023

Level	Covariate	Direction
1	Cumulative cigarette consumption (10 years)	-
	Diet low in fruits, adjusted (g)	-
2	Absolute latitude	+
	Low density lipoprotein (mmol/L)	+
	Sanitation (proportion with access)	+
	Improved water source (proportion with access)	+
	Healthcare Access and Quality Index	-
3	Socio-demographic Index	+

Figure 1 shows the influence of each selected covariate on the four CODEm models (female global, female data-rich, male global, and male data-rich). A positive standardised beta (to the right) means that the covariate was associated with increased death. A negative standardised beta (to the left) means the covariate was associated with decreased death.

Figure 1. Covariate influence plots for Parkinson's disease

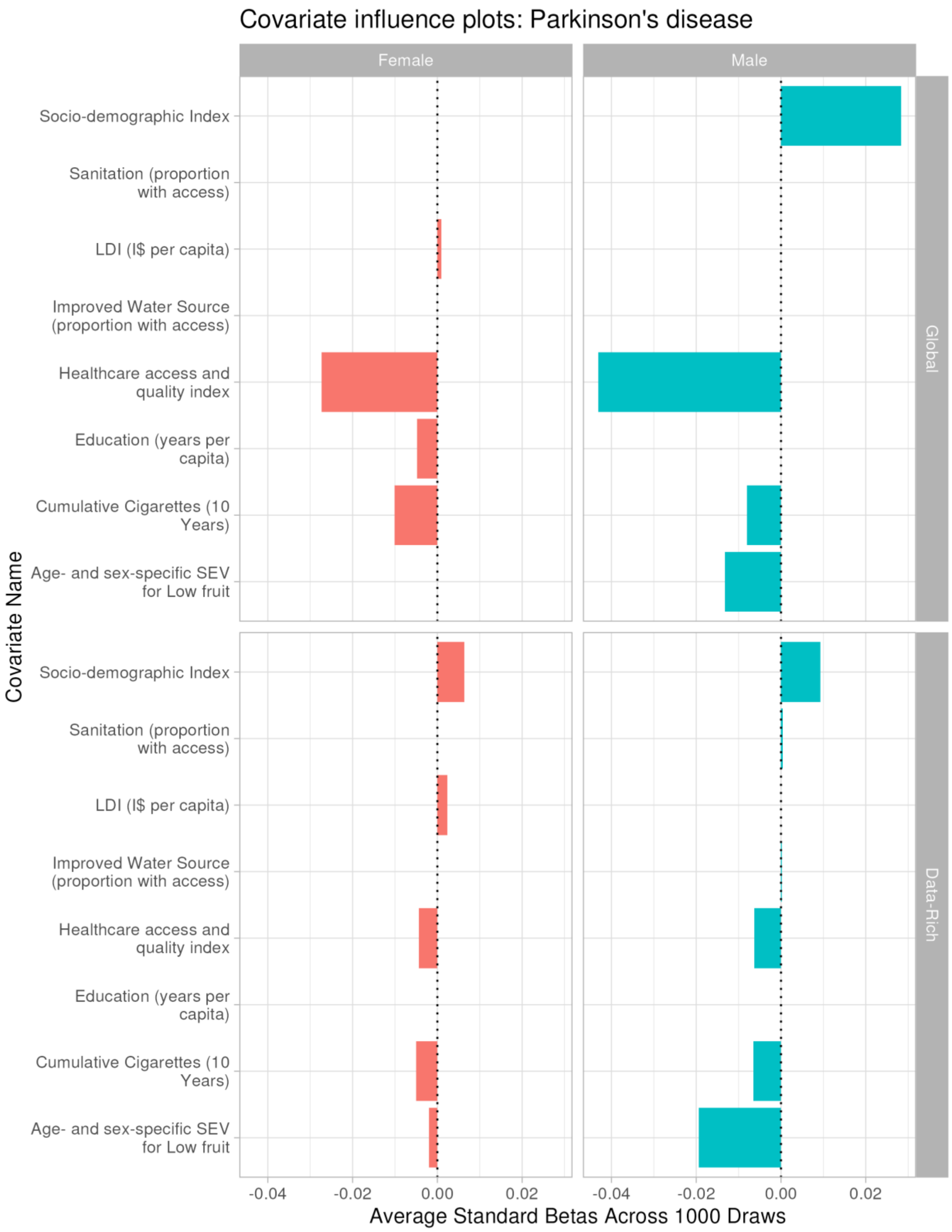
Covariate influence plots: Parkinson's disease
Female
Male
Global
Data-Rich
Covariate Name
Socio-demographic Index
Sanitation (proportion with access)
LDI (I$ per capita)
Improved Water Source (proportion with access)
Healthcare access and quality index
Education (years per capita)
Cumulative Cigarettes (10 Years)
Age- and sex-specific SEV for Low fruit
-0.04
-0.02
0.00
0.02
Average Standard Betas Across 1000 Draws

References

[1]Vos T, Lim SS, Abbafati C, et al. Global burden of 369 diseases and injuries in 204 countries and territories, 1990–2019: a systematic analysis for the Global Burden of Disease Study 2019. *The Lancet* 2020; 396: 1204–22. doi: https://doi.org/10.1016/S0140-6736(20)30925-9

Peptic ulcer disease

Flowchart

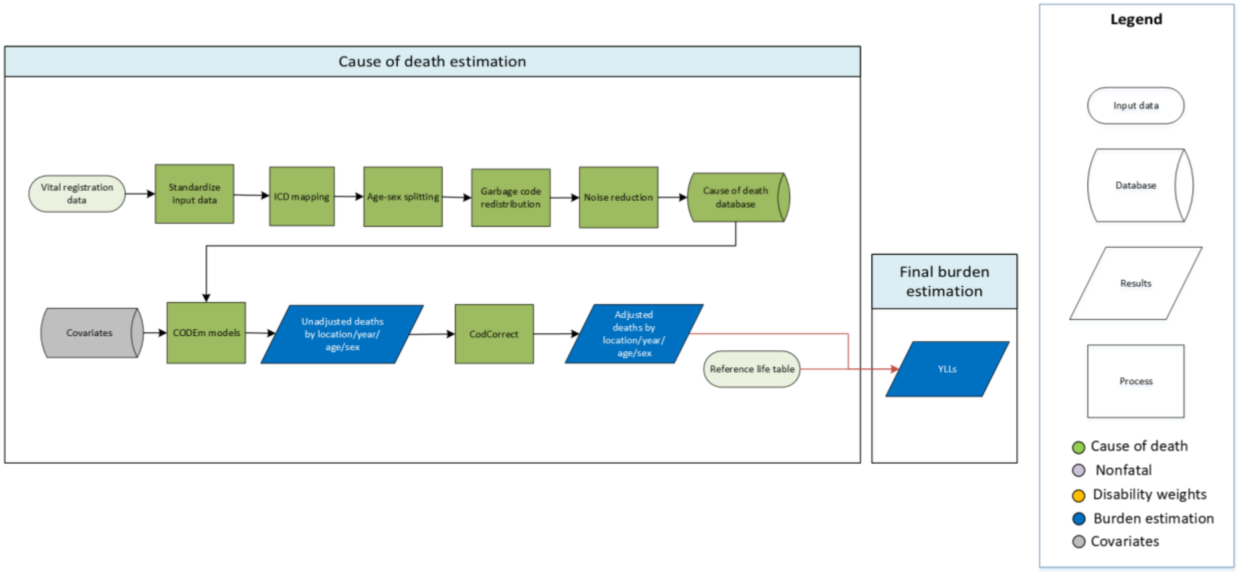

Input data and methodological summary for peptic ulcer disease

Peptic ulcer disease is a Level 4 cause in the GBD cause hierarchy.

Input data

Data used to estimate mortality of peptic ulcer disease consisted of vital registration (VR) from the cause of death (CoD) database. A list of ICD codes that were mapped to this cause, a list of vital registration sources included in the CoD database and their quality as indicated by star-ratings, and the steps used to process these data for modelling can be found in the section of this appendix on the causes of death database and the tables and figures it refers to. CoD data processing was broadly similar to previous rounds of GBD, but one novel data processing step was employed in preparing data for peptic ulcer disease that was not needed prior to GBD 2023—COVID-19 corrections—which are described elsewhere in this appendix.

After all data processing steps were complete, datapoints for all locations and years were systematically reviewed to identify outliers or other data limitations not fully overcome by CoD data processing, and data were excluded as indicated by that review.

Modelling strategy

No substantive changes were made to the strategy for fatal peptic ulcer disease burden in GBD 2023 relative to GBD 2021. A standard CODEm model was used to model deaths due to peptic ulcer disease (see appendix section on CODEm method for details). Models were generated separately for males in data-rich countries, males in all countries (referred to as a global model), females in data-rich countries, and females in all countries (global model). Fatal estimation for this cause was restricted to ages 0.5 years to 95+ years. We hybridised separate global and data-rich models to acquire unadjusted results.

The following table has the full list of covariates presented to the CODEm algorithm for possible inclusion in the peptic ulcer disease mortality model.

Table 1. Covariates used in peptic ulcer disease mortality modelling

Level	Covariate	Direction
1	Sanitation, proportion with access	-
	Scaled exposure variable for unsafe water source	+
	Smoking prevalence	+
	Cumulative cigarettes (10 years)	+
	Cumulative cigarettes (5 years)	+
2	Litres of alcohol consumed per capita	+
	Vegetables (grams, unadjusted)	-
	Healthcare Access and Quality Index	-
3	Lag distributed income (per capita)	-
	Education (years per capita)	-
	Socio-demographic Index	-

Unadjusted peptic ulcer disease deaths were then rescaled alongside gastritis and duodenitis deaths to sum to upper digestive disease deaths, as part of the CoDCorrect process, which rescales all cause-specific deaths to sum to all-cause counts of death. Rescaled, age-sex-specific peptic ulcer disease deaths were compared to the reference life table to calculate final YLLs due to peptic ulcer disease.

Pertussis (whooping cough)

Flowchart

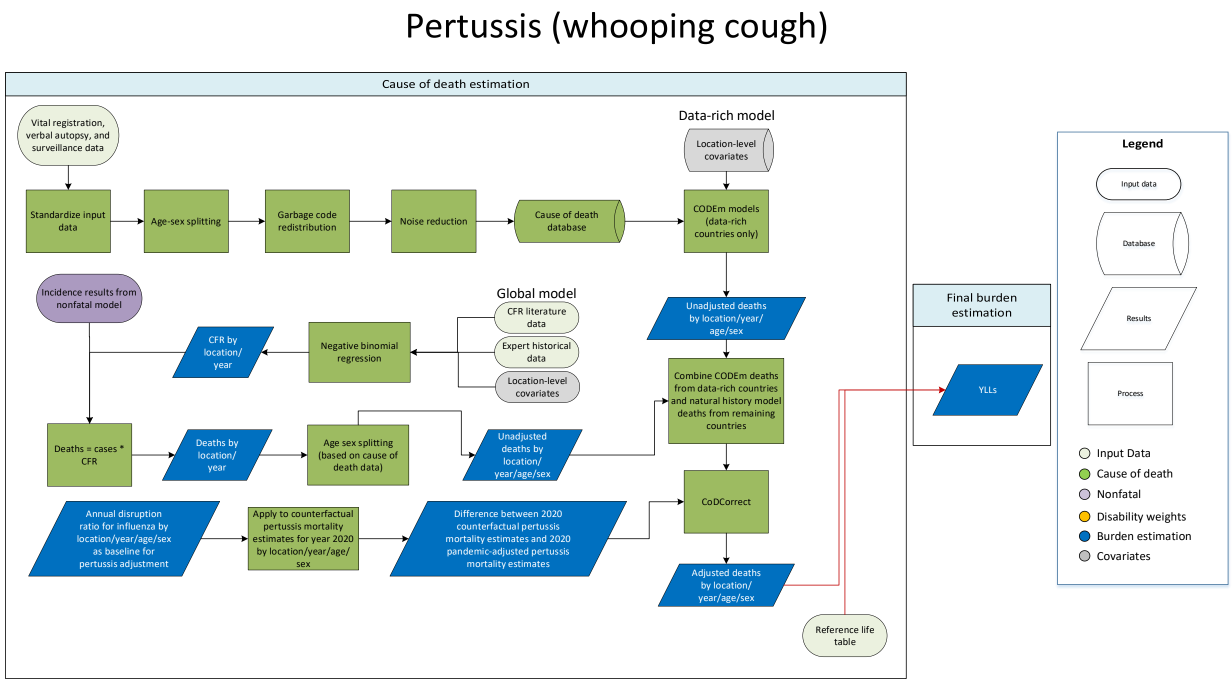

Input data and methodological summary for pertussis

Input data

Pertussis cause of death (COD) data for GBD 2023 included vital registration, verbal autopsy, and surveillance sources from all locations where data were available. For our case-fatality ratio model, we used data from the same systematic review of the scientific literature used in GBD 2021.

Modelling strategy

We used two strategies to develop GBD 2023 pertussis mortality estimates, depending on the quality of the available cause-of-death data. For countries with well-defined cause-of-death data registration systems, we used a Cause of Death Ensemble model (CODEm). For the remaining countries, we multiplied our non-fatal pertussis incidence estimates by our case-fatality ratio estimates. For all countries, we produced estimates for all age groups between post-neonatal and 59 years.

Data-rich countries

For data-rich countries modelled in CODEm, we used the covariates listed in Table 1 to inform predictions. As in the previous GBD cycle, we only included spatiotemporal Gaussian process regression (ST-GPR) models in the final CODEm ensemble. In GBD 2023, we estimated the impact of the COVID-19 pandemic on pertussis mortality by incorporating COVID-specific covariates in the model. These covariates included COVID-inclusive third dose diphtheria-tetanus-pertussis vaccine (DTP3) coverage

lagged over five years, per capita COVID-infection, and masking outside the home. In GBD 2021, we made post-hoc adjustments to pertussis mortality estimates to account for the impact of COVID-19.

Table 1. Covariates. Summary of covariates used in the data-rich pertussis cause of death model

Level	Covariate	Direction
1	Average COVID-inclusive diphtheria-tetanus-pertussis third dose (DTP3) vaccination coverage over the past five years	-
	Per capita COVID infection	-
	Proportion of population reporting always wearing a mask when leaving home	-
	Age- and sex-specific SEV for child underweight	+
	Healthcare Access and Quality (HAQ) Index	-
3	Lag-distributed income (LDI)	-
	Socio-demographic Index (SDI)	-
	Mean years of education per capita	-

Natural history model

The pertussis natural history model uses GBD incidence estimates and pertussis case-fatality ratio (CFR) estimates to calculate pertussis mortality in non-data-rich locations. As described in the non-fatal pertussis modelling appendix, case notifications informing the pertussis incidence model come from the World Health Organization (WHO) Joint Reporting Form (JRF) and are supplemented by historical pertussis case and vaccination surveillance data from the UK. Pertussis log-transformed incidence is estimated using a mixed effects linear regression model with DTP3 vaccination coverage and masking outside the home as covariates. New in GBD 2023, we included the masking covariate to account for the impact of COVID-19 mitigation strategies on the incidence of pertussis. This approach differs from the COVID-19 adjustment used in GBD 2021, wherein COVID-free counterfactual estimates of pertussis incidence were modelled, followed by a post-hoc adjustment for the impact of the pandemic.

The pertussis CFR data were obtained through a systematic review of the literature last updated for GBD 2021. To estimate CFR for all location-years, we fit a negative binomial model to the available pertussis CFR data, using the Healthcare Access and Quality (HAQ) Index as a covariate:

$$Y_{ij} = \beta_0 + \beta_1 HAQ_{ij} + e_{ij}$$

Pertussis deaths were then calculated as:

$$deaths = incidence * CFR$$

This calculation was replicated at the draw level 1000 times to produce uncertainty estimates. These draw-level estimates were sex- and age-split using the global sex and age distribution patterns found in the cause of death data. We present the data as the mean of samples from the posterior distribution (draws) and a 95% uncertainty interval (the 2.5th and 97.5th percentile of all draws).

Changes from GBD 2021 to GBD 2023

The major substantive change in our modelling strategy for GBD 2023 is in the handling of COVID-19 impact on pertussis mortality. In GBD 2023, we used a five-year lagged COVID-inclusive DTP3 mean vaccine coverage covariate and a mask use covariate to account for COVID effects directly within the model.

Pertussis (whooping cough)

Flowchart

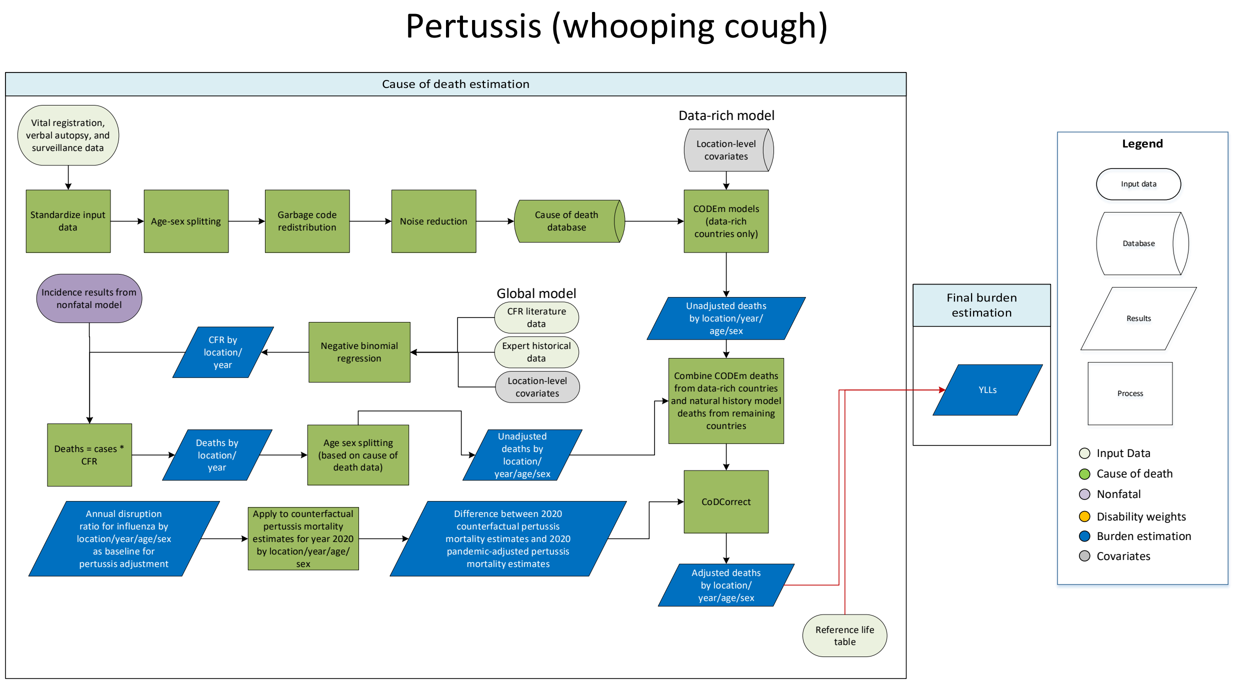

Input data and methodological summary for pertussis

Input data

Pertussis cause of death (COD) data for GBD 2023 included vital registration, verbal autopsy, and surveillance sources from all locations where data were available. For our case-fatality ratio model, we used data from the same systematic review of the scientific literature used in GBD 2021.

Modelling strategy

We used two strategies to develop GBD 2023 pertussis mortality estimates, depending on the quality of the available cause-of-death data. For countries with well-defined cause-of-death data registration systems, we used a Cause of Death Ensemble model (CODEm). For the remaining countries, we multiplied our non-fatal pertussis incidence estimates by our case-fatality ratio estimates. For all countries, we produced estimates for all age groups between post-neonatal and 59 years.

Data-rich countries

For data-rich countries modelled in CODEm, we used the covariates listed in Table 1 to inform predictions. As in the previous GBD cycle, we only included spatiotemporal Gaussian process regression (ST-GPR) models in the final CODEm ensemble. In GBD 2023, we estimated the impact of the COVID-19 pandemic on pertussis mortality by incorporating COVID-specific covariates in the model. These covariates included COVID-inclusive third dose diphtheria-tetanus-pertussis vaccine (DTP3) coverage

lagged over five years, per capita COVID-infection, and masking outside the home. In GBD 2021, we made post-hoc adjustments to pertussis mortality estimates to account for the impact of COVID-19.

Table 1. Covariates. Summary of covariates used in the data-rich pertussis cause of death model

Level	Covariate	Direction
1	Average COVID-inclusive diphtheria-tetanus-pertussis third dose (DTP3) vaccination coverage over the past five years	-
	Per capita COVID infection	-
	Proportion of population reporting always wearing a mask when leaving home	-
	Age- and sex-specific SEV for child underweight	+
	Healthcare Access and Quality (HAQ) Index	-
3	Lag-distributed income (LDI)	-
	Socio-demographic Index (SDI)	-
	Mean years of education per capita	-

Natural history model

The pertussis natural history model uses GBD incidence estimates and pertussis case-fatality ratio (CFR) estimates to calculate pertussis mortality in non-data-rich locations. As described in the non-fatal pertussis modelling appendix, case notifications informing the pertussis incidence model come from the World Health Organization (WHO) Joint Reporting Form (JRF) and are supplemented by historical pertussis case and vaccination surveillance data from the UK. Pertussis log-transformed incidence is estimated using a mixed effects linear regression model with DTP3 vaccination coverage and masking outside the home as covariates. New in GBD 2023, we included the masking covariate to account for the impact of COVID-19 mitigation strategies on the incidence of pertussis. This approach differs from the COVID-19 adjustment used in GBD 2021, wherein COVID-free counterfactual estimates of pertussis incidence were modelled, followed by a post-hoc adjustment for the impact of the pandemic.

The pertussis CFR data were obtained through a systematic review of the literature last updated for GBD 2021. To estimate CFR for all location-years, we fit a negative binomial model to the available pertussis CFR data, using the Healthcare Access and Quality (HAQ) Index as a covariate:

$$Y_{ij} = \beta_0 + \beta_1 HAQ_{ij} + e_{ij}$$

Pertussis deaths were then calculated as:

$$deaths = incidence * CFR$$

This calculation was replicated at the draw level 1000 times to produce uncertainty estimates. These draw-level estimates were sex- and age-split using the global sex and age distribution patterns found in the cause of death data. We present the data as the mean of samples from the posterior distribution (draws) and a 95% uncertainty interval (the 2.5th and 97.5th percentile of all draws).

Changes from GBD 2021 to GBD 2023

The major substantive change in our modelling strategy for GBD 2023 is in the handling of COVID-19 impact on pertussis mortality. In GBD 2023, we used a five-year lagged COVID-inclusive DTP3 mean vaccine coverage covariate and a mask use covariate to account for COVID effects directly within the model.

Pneumoconiosis

Flowchart

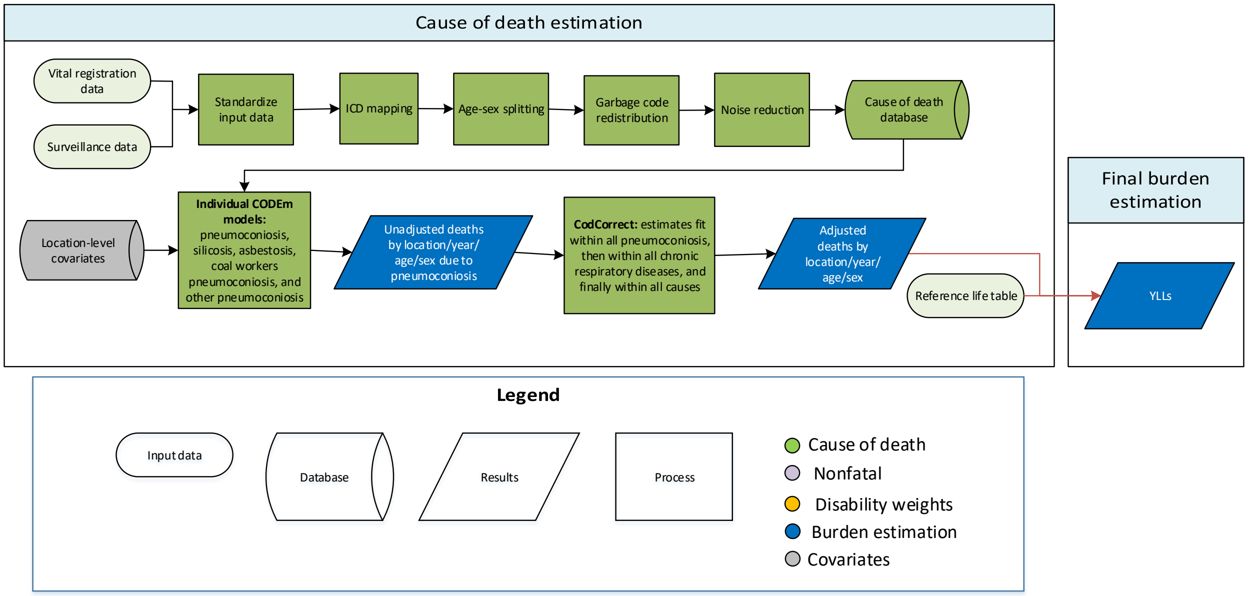

Input data and methodological summary for pneumoconiosis

Input data

Data used to estimate pneumoconiosis mortality included vital registration and China mortality surveillance data from the cause of death (CoD) database. Our outlier criteria excluded datapoints that (1) were implausibly high or low based on previous data or expert collaborator knowledge, (2) substantially conflicted with established age or temporal patterns, or (3) substantially conflicted with data from the same locations or locations with similar characteristics (ie, Socio-demographic Index).

Modelling strategy

The standard Cause of Death Ensemble modelling (CODEm) approach was used to estimate deaths due to pneumoconiosis (see appendix 1, section 4 for more details). Separate models were conducted for male and female mortality, and the age range for both models was 15 to 95+ years. We ran separate models for total pneumoconiosis, silicosis, asbestosis, coal worker's pneumoconiosis, and other pneumoconiosis. Parent pneumoconiosis and the four subtypes have an age restriction at 15 years because pneumoconiosis only occurs from occupational exposure to mineral dusts and generally takes years from the onset of exposure to develop.

The total pneumoconiosis model serves as an envelope model for silicosis, asbestosis, coal worker's pneumoconiosis, and other pneumoconiosis models. The mortality estimates from pneumoconiosis disease models were then fit into the chronic respiratory envelope, which is the parent cause for pneumoconiosis disease. Finally, the chronic respiratory disease envelope is fit into the all-cause mortality envelope.

The following tables list the covariates included in each of the child models. The direction of the expected relationship between the covariate and disease is also provided. Covariate levels were selected based on the strength of the evidence.

Table 1a. Covariates used in parent pneumoconiosis mortality modelling

Level	Covariate	Direction
1	Log-transformed coal production (per capita)	+
	Age-standardised SEV for occupational asbestos	+
	Age- and sex-specific SEV for occupational silica	+
	Gold production (km) per capita, smoothed with 20-year lag	+
2	Age- and sex-specific SEV for occupational beryllium	+
	Cumulative cigarettes (20 years)	+
	Smoking prevalence	+
	Asbestos consumption (metric tons per year per capita)	+
	Indoor air pollution (all cooking fuels)	+
	Outdoor air pollution (PM 2.5)	+
3	Socio-demographic Index	-
	Healthcare Access and Quality Index	-

Table 1b. Covariates used in silicosis mortality modelling

Level	Covariate	Direction
1	Cumulative cigarettes (10 years)	+
	Cumulative cigarettes (20 years)	+
	Age- and sex-specific SEV for occupational silica	+
	Gold production (km) per capita, smoothed with 20-year lag	+
2	Indoor air pollution (all cooking fuels)	+
	Outdoor air pollution (PM 2.5)	+
	Smoking prevalence	+
	Healthcare Access and Quality Index	-
3	Socio-demographic Index	-

Table 1c. Covariates used in asbestosis mortality modelling

Level	Covariate	Direction
1	Cumulative cigarettes (5 years)[a]	+
	Cumulative cigarettes (10 years)	+
	Cumulative cigarettes (20 years)	+
	Age-standardised SEV for occupational asbestos	+
	Asbestos consumption (metric tons per year per capita)	+

2	Smoking prevalence	+
	Indoor air pollution (all cooking fuels)	+
	Outdoor air pollution (PM 2.5)	+
	Healthcare Access and Quality Index	-
3	Socio-demographic Index	-

Table 1d. Covariates used in coal worker's pneumoconiosis mortality modelling

Level	Covariate	Direction
1	Cumulative cigarettes (10 years)	+
	Cumulative cigarettes (20 years)	+
	Log-transformed coal production (per capita)	+
2	Outdoor air pollution (PM 2.5)	+
	Indoor air pollution (all cooking fuels)	+
	Smoking prevalence	+
	Healthcare Access and Quality Index	-
3	Socio-demographic Index	-

Table 1e. Covariates used in other pneumoconiosis mortality modelling

Level	Covariate	Direction
1	Cumulative cigarettes (10 years)	+
	Cumulative cigarettes (20 years)	+
	Age and sex-specific SEV for occupational beryllium	+
	Age- and sex-specific SEV for occupational silica	+
	Proportion of population working in mining (20-year lag)	+
	Gold production (km) per capita, smoothed with 20-year lag	+
2	Tuberculosis prevalence	+
	Outdoor air pollution (PM 2.5)	+
	Indoor air pollution (all cooking fuels)	+
	Smoking prevalence	+
	Healthcare Access and Quality Index	-
3	Socio-demographic Index	-

[a]only in the female global and data-rich models

Pulmonary arterial hypertension

Flowchart

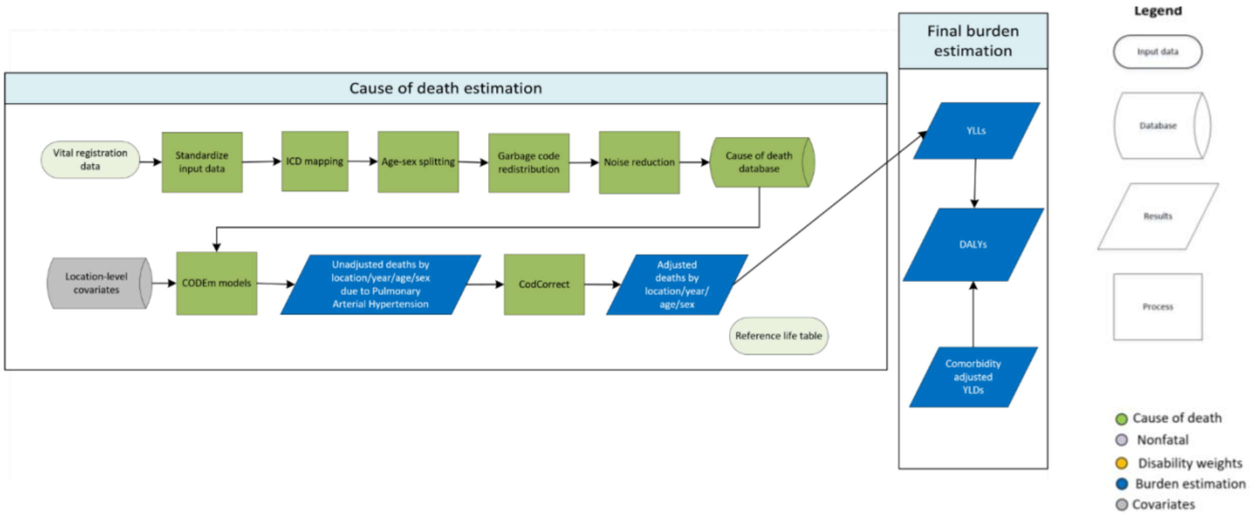

Input data and methodological summary for pulmonary arterial hypertension

Input data

Vital registration data were used to model pulmonary arterial hypertension (PAH). ICD codes 416 and I27.0 were used. Clinical guidelines, available ICD codes, and ICD coding practices have changed over time for PAH; after review of the literature and raw data, these codes were chosen as they capture PAH and not pulmonary hypertension groups 2–5. Specifically, we excluded the code I27.2, which may include other pulmonary diseases in addition to PAH. We outliered ICD-8, ICD-9-BTL, and ICD-10 tabulated datapoints, which we believe do not have sufficient detail to distinguish PAH from other pulmonary diseases. In countries where the introduction of I27.0 caused a drastic change in PAH mortality estimates, either in level or temporal trend, we excluded data before the introduction of I27.0. Figure 1 illustrates this change in the USA; data before 2003 were excluded, and I27.0 alone represents PAH. In addition, we outliered ICD-10 datapoints from sources in some subnationals of Ethiopia which were implausibly low in all age groups. We also outliered ICD-10 in all subnationals of Japan from in years 2017–2020 due to an implausible disjoint from the previous years in the time-series. We also outliered a number of ICD-10 datapoints in locations in Oceania (Palau, American Samoa, Guam, Kiribati, Northern Mariana Islands, Cabo Verde) that were implausibly low and were setting a low mortality estimate for the region.

Figure 1: Rate of PAH, as estimated by three ICD codes, in the USA, Belgium, and France

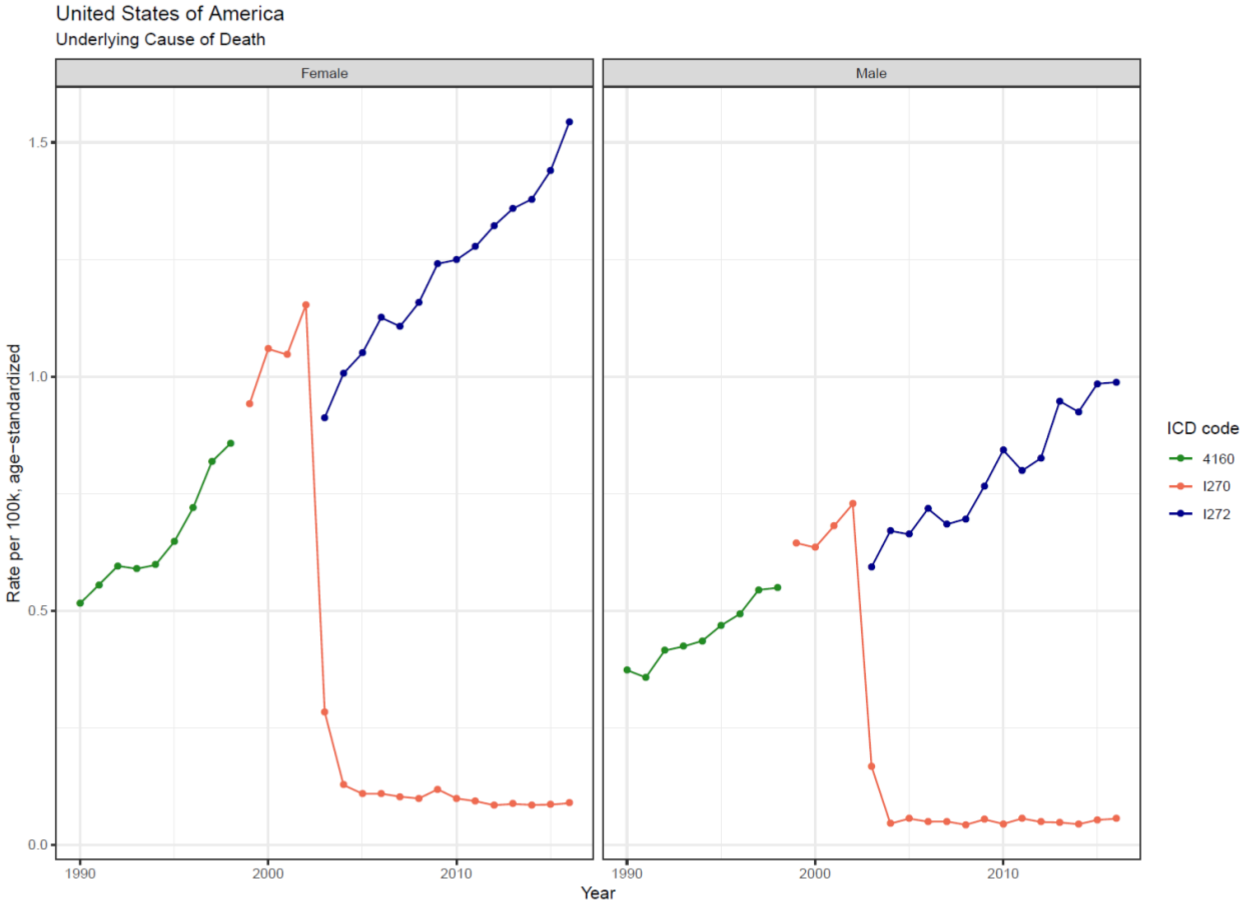

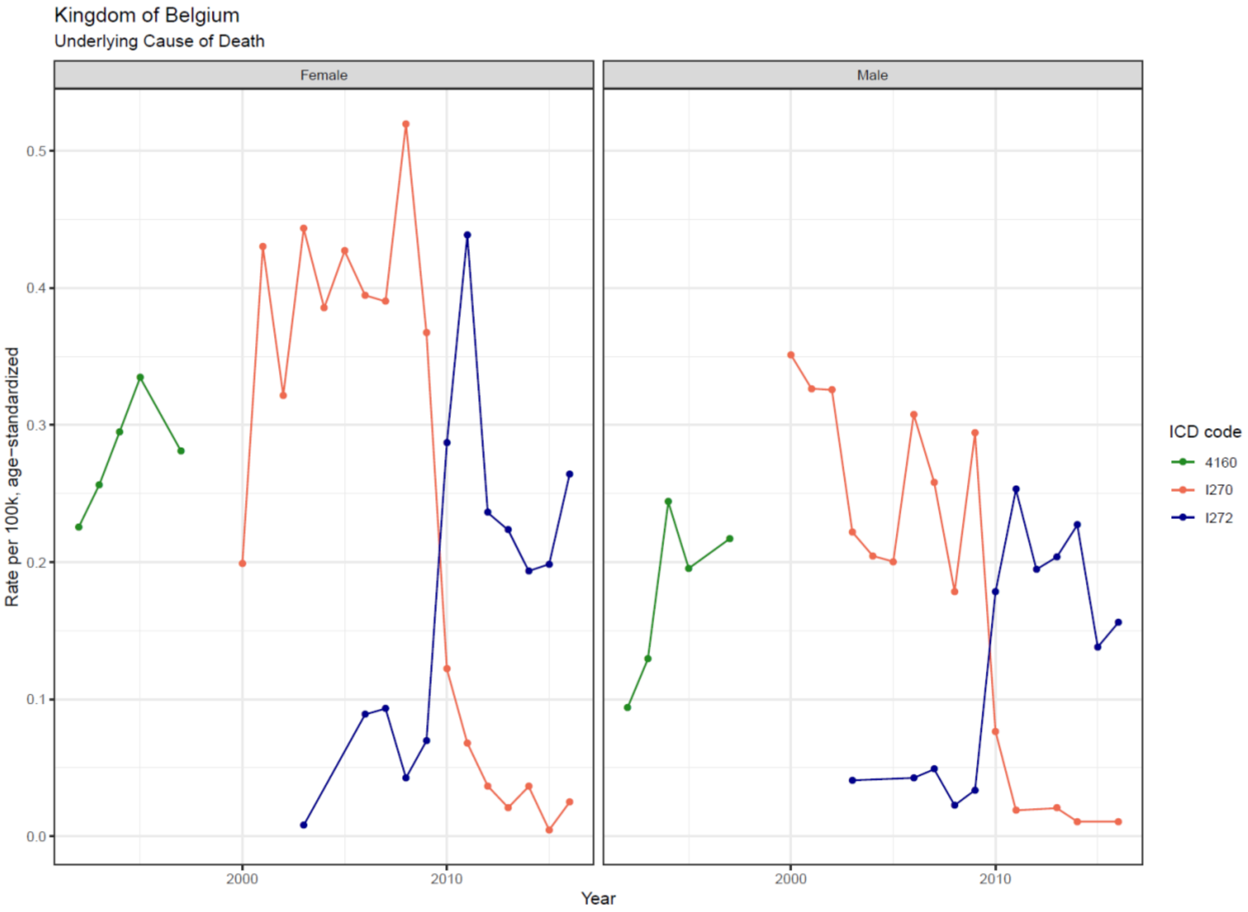
Kingdom of Belgium
Underlying Cause of Death
Female
Male
Rate per 100k, age-standardized
0.0
0.1
0.2
0.3
0.4
0.5
2000
2010
Year
ICD code
4160
I270
I272

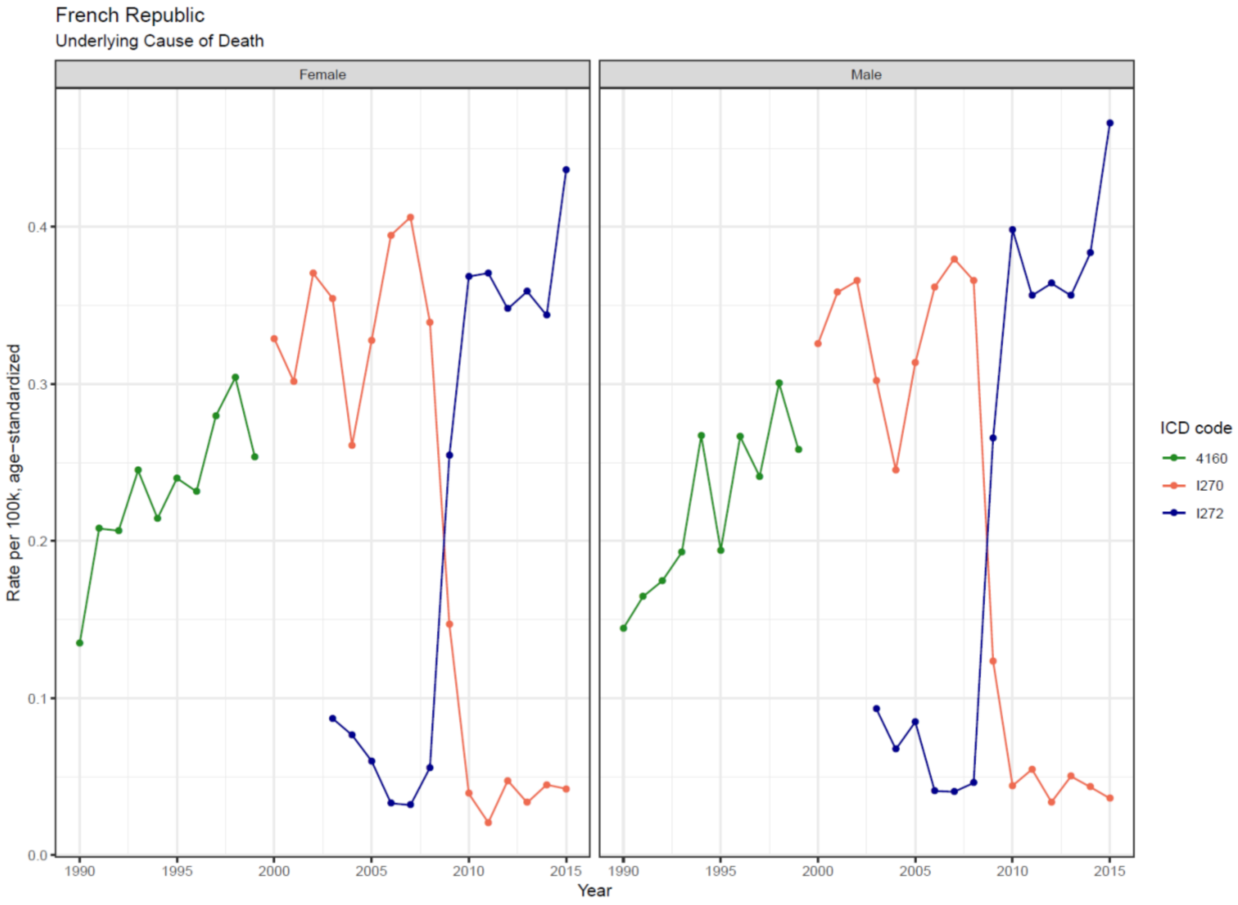

Most data sources used for cause of death estimation are ICD-coded. The ICD periodically releases new versions, which can introduce an implausible disjoint in the time series that is indicative of a change in the coding practice, rather than a true change in the epidemiological trend of the disease. In GBD 2023, certain causes and countries using the ICD-9 code systems were adjusted to address the disjoint in the time trend and number of deaths between the older ICD-9 code systems and the more recent ICD-10 code systems. Pulmonary arterial hypertension was corrected in Bulgaria (1990–2004), Canada (1986–1999), Spain (1980–1998), France (1980–1999), the USA (1980–1998), Hungary (1980–1995), Croatia (1985–1994), the UK (1980–1999), Greece (1987–2008), Italy (1980–2002), Mexico (1980–2002), and Mauritius (1980–2004) to account for this code system disjoint. This was done using a meta-regression tool described as a crosswalk, by taking systematically biased datapoints and estimating their unbiased value. Using this modelling tool, correction factors were created based on the more recent and more reliable ICD-10-coded data. The crosswalk creates matched pairs between the last three years of data using the ICD-9 code system and the first three years of data using the ICD-10 code system. The correction is applied at the child cause level for all Level 2 causes, and after the correction is applied the individual child causes are raked to the parent cause to maintain a consistent number of deaths across the cause hierarchy.

Modelling strategy

We used a standard CODEm approach to model deaths from pulmonary arterial hypertension. The covariates used, along with their transformations, importance levels, and imposed directions, are reported by cause in the tables below. Schistosomiasis and HIV were chosen as covariates because these diseases are underlying causes of PAH, and they are major drivers of PAH mortality in some locations. SDI and HAQ Index were assigned a negative direction to reflect how treatment, screening, and medication can lower mortality of PAH in locations with high SDI or HAQ Index values. Since GBD 2021, CODEm has switched from using an ensemble of rate and cause-fraction models to only rate models. We also added an additional covariate for lag-distributed income. Otherwise, there were no substantial changes in CODEm modelling since GBD 2021.

The covariates included in the ensemble modelling process are listed in the table below.

Table 1. Covariates used in pulmonary arterial hypertension mortality modelling

Level	Covariate	Direction
1	Prevalence of schistosomiasis	1
	Summary exposure value (SEV), HIV	1
	Socio-demographic Index	–1
	Healthcare Access and Quality Index	–1
	Lag-distributed income per capita (LDI)	–1

Rabies

Flowchart

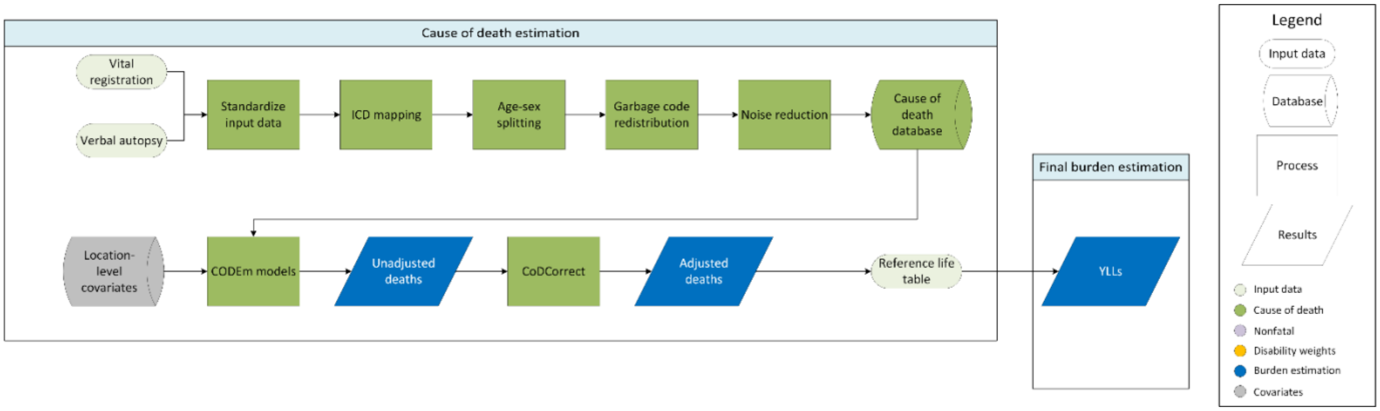

Input data and methodological summary for rabies

Input data

We modelled rabies mortality using all available data in the cause of death database. Datapoints were outliered if they reported an improbable number of rabies deaths (eg, zero rabies deaths in a hyper-endemic country) or if their inclusion in the model yielded distorted trends. In some cases, multiple data sources for the same location differed dramatically both in their quality and reported rabies mortality. In these cases, the lower-quality data source was outliered.

Modelling strategy

We modelled rabies mortality using a two-model hybrid approach: 1) a global (GLB) Cause of Death Ensemble modelling (CODEm) model of all locations, using all data in the cause of death (CoD) database; and 2) a CODEm model restricted to data-rich (DR) countries. The CODEm models included eight covariates:

Table 1. Covariates used in rabies mortality modelling

Level	Covariate	Direction	GLB or DR
1	Antenatal care coverage (4 visits)	-	GLB, DR
	Socio-demographic Index	-	GLB, DR
	In-facility delivery coverage	-	GLB, DR
2	Healthcare Access and Quality Index	-	GLB, DR
	Skilled birth attendance coverage	-	GLB, DR
	Maternal care and immunisation	-	DR
3	Population density, 500–1000 per km^2	+	GLB, DR
	Population density, <150 per km^2	+	GLB, DR

Changes from GBD 2021 to GBD 2023

There have been no substantive changes to the modelling strategy for GBD 2023.

Rheumatic heart disease

Flowchart

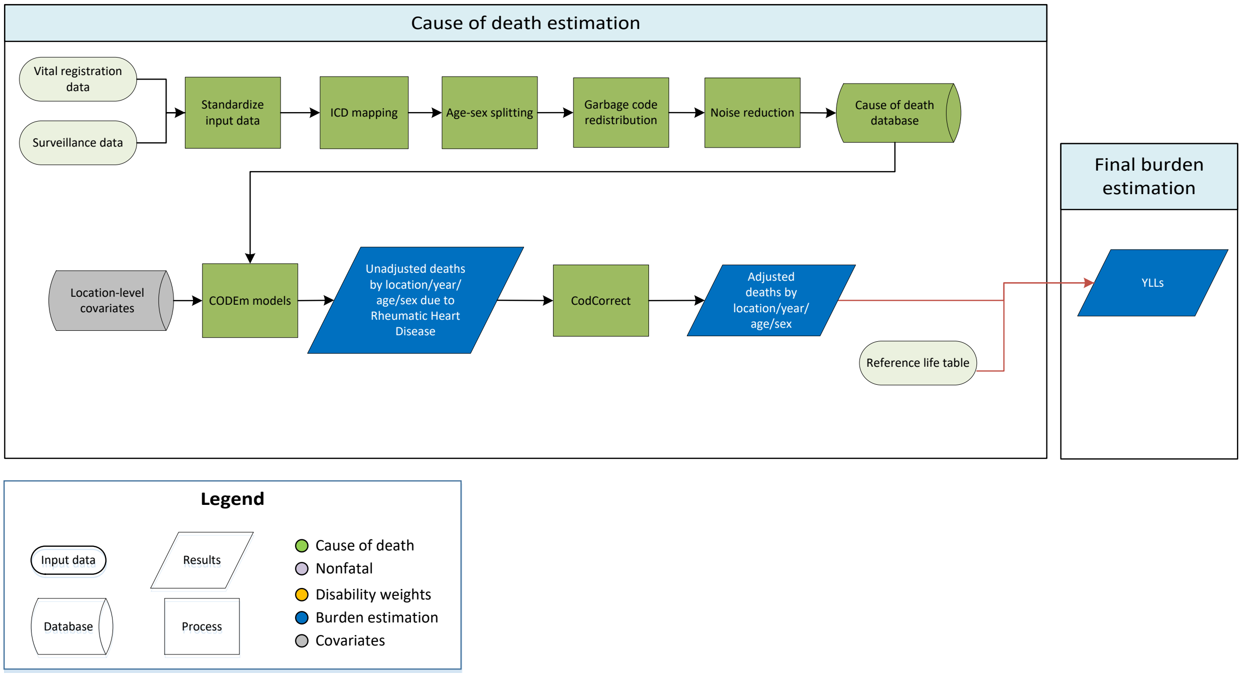

Input data and methodological summary for rheumatic heart disease

Input data

Vital registration data were used to model rheumatic heart disease. We outliered datapoints in specific locations that created implausible temporal or geographical patterns. These included ICD-8-coded data in Denmark, Finland, and Mauritius, ICD-9-coded data in Mongolia, Belgium, Honduras, Egypt, Russia, and Seychelles, and ICD-10-coded data in Belarus and Oman.

Crosswalk by code system

Most data sources used for cause of death estimation are ICD-coded. The ICD periodically releases new versions, which can introduce an implausible disjoint in the time series that is indicative of a change in the coding practice, rather than a true change in the epidemiological trend of the disease. In GBD 2023, certain causes and countries using the ICD-9 code systems were adjusted to address the disjoint in the time trend and number of deaths between the older ICD-9 code systems and the more recent ICD-10 code systems. Rheumatic heart disease was corrected in Bulgaria, Canada, Spain, France, the USA, Hungary, Croatia, the UK, Greece, Italy, Mexico, and Mauritius between 1980 to 2008 to account for this code system disjoint. This was done using a meta-regression tool described as a crosswalk, by taking systematically biased datapoints and estimating their unbiased value. Using this modelling tool, correction factors were created based on the more recent and more reliable ICD-10-coded data. The crosswalk creates matched pairs between the last three years of data using the ICD-9 code system and

the first three years of data using the ICD-10 code system. The correction is applied at the child cause level for all Level 2 causes, and after the correction is applied the individual child causes are raked to the parent cause to maintain a consistent number of deaths across the cause hierarchy.

Modelling strategy

We used a standard CODEm approach to model deaths from rheumatic heart disease. Apart from the overall updates to data processing for ICD code system described above and the correction for misclassified COVID deaths described elsewhere in this appendix, there have been no updates to the modelling strategy since GBD 2021.

Table 1: Covariates used in rheumatic heart disease mortality modelling

Level	Covariate	Direction
1	Improved water (proportion with access)	–1
1	Age- and sex-specific SEV scalar for child underweight	1
1	Sanitation (proportion with access)	–1
2	Healthcare Access and Quality Index	–1
3	Lag-distributed income per capita (I$)	–1
3	Socio-demographic Index	–1
3	Education (years per capita)	–1

Rheumatoid arthritis

Flowchart

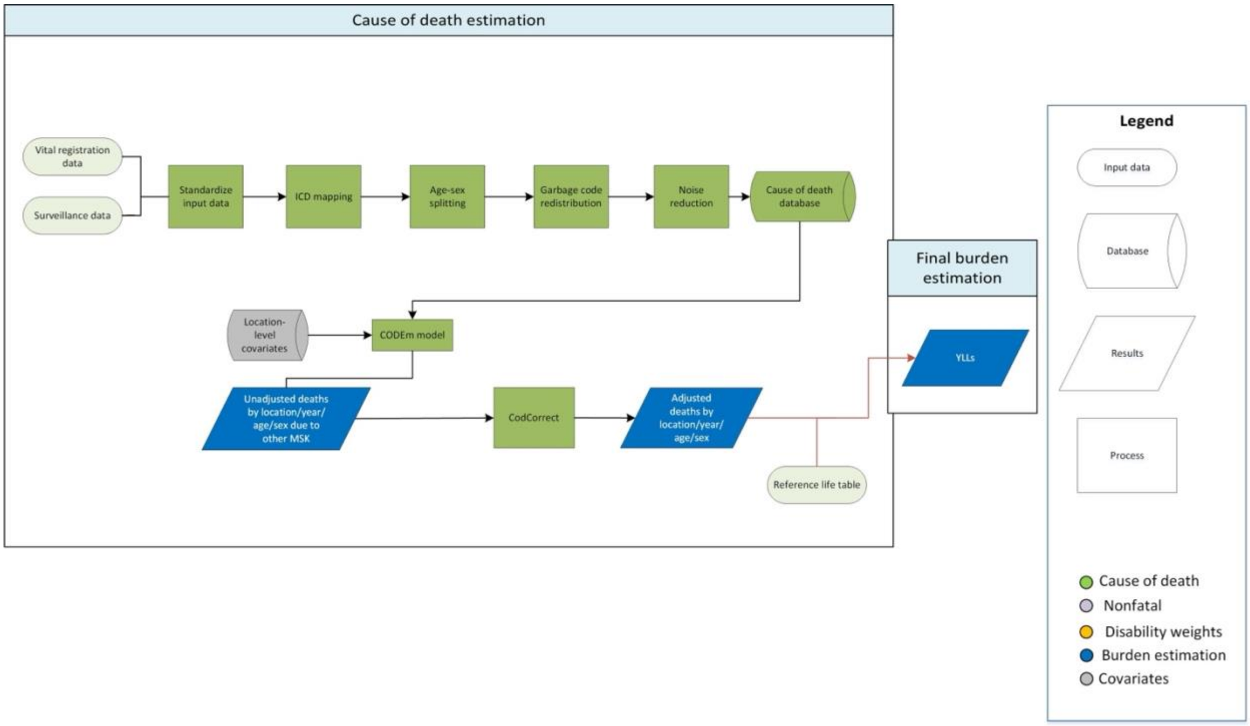

Input data and methodological summary for rheumatoid arthritis

Input data

Data used to estimate rheumatoid arthritis mortality included vital registration records and China Disease Surveillance data from the cause of death database. Datapoints were excluded (outliered) if they met any of the following criteria:

1. **Implausible values**: Datapoints were excluded if they were implausibly high or low compared to global or regional patterns, as assessed by subject matter experts.
2. **Conflict with established patterns**: Data that substantially conflicted with known age-specific or temporal trends were excluded.
3. **Conflict with comparable locations**: Datapoints were excluded if they significantly conflicted with other sources from the same location or from locations with similar characteristics (eg, Socio-demographic Index).
4. **Verbal autopsy limitations**: Data from verbal autopsy sources were excluded due to their inability to accurately capture most musculoskeletal conditions.

Specific exclusions included:

- All vital registration (VR) data from **Voronezh Oblast, Kazakhstan, and Ghana** due to data quality concerns, which led to implausibly high death estimates caused by coding practices, as identified by subject matter experts.
- Russian mortality ICD-9 data from the **Chukotka Autonomous Area** were excluded because changes in coding systems introduced discontinuities in time trends.

Modelling strategy

The standard CODEm (Cause of Death Ensemble model) modelling approach was applied to estimate deaths due to rheumatoid arthritis. We applied the same covariates used in GBD 2021. General methods are described in appendix 1 section 4. The following table lists the covariates included in the model. The direction corresponds to the expected relationship between the covariate and disease. Covariate level was selected based on the strength of the evidence.

Table 1. Covariates used in rheumatoid arthritis mortality modelling

Level	Covariate	Direction
1	Cumulative cigarettes (10 years)	+
	Cumulative cigarettes (5 years)	+
	Smoking prevalence	+
	Milk (g), unadjusted	
	Healthcare Access and Quality Index	-
	Alcohol consumption (litres per capita)	+
2	Mean BMI	+
	Mean cholesterol[1]	+
3	Education (years per capita)	+
	Log-transformed LDI: lag-distributed income ($ per capita)	+
	SDI: Socio-demographic Index	-

[1] This covariate refers to LDL cholesterol.

Schistosomiasis

Flowchart

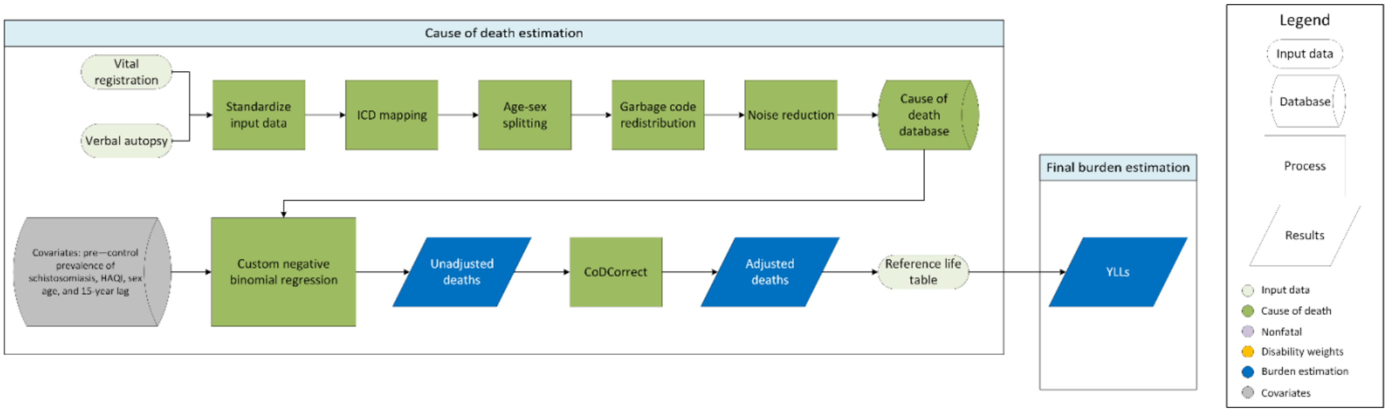

Input data and methodological summary for schistosomiasis

Input data

To estimate mortality due to schistosomiasis, data on deaths and prevalence of infection were used. We used the prevalence data prepared for GBD 2023. Further information on prevalence data is available in the non-fatal write-up for this cause. In addition, country-year-age-sex-specific verbal autopsy and vital registration data were used in the mortality model.

Geographical restrictions

We conducted a literature review (last updated for GBD 2017) to determine the geographical extent of the disease and classify locations based on whether the disease is absent or present in each year. Locations that were geographically restricted in any given year did not have estimates made for them but could have imported cases attributed to them at a later stage. Evidence of absence or presence was not available for every location for each year, and so assumptions were made for missing years by taking into consideration the epidemiological characteristics of the disease. If evidence indicated disease presence for two non-consecutive years, we assumed presence for all years between the two. If evidence indicated disease absence for two non-consecutive years, we assumed absence for all years between the two. If evidence indicated a change in status (ie, from absent to present, or present to absent) between two non-consecutive years, then we conducted targeted searches to ascertain the relevant year of introduction or elimination for that location. In the cases where presence or absence information was missing for the start or end years of our study interval without evidence of any introduction or elimination events within the interval, we applied the status of the first and last presence/absence observations respectively to all years between the interval bound and the observation year. For schistosomiasis, we used a combination of Chitsulo and colleagues' *The global status of schistosomiasis and its control* (1) and WHO's *Preventive chemotherapy in human helminthiasis* (2) report as a baseline. Where country-level endemicity statuses conflicted between the two sources, we searched PubMed and Google Scholar for country- and subnational-specific endemicity status. Our search yielded 22 sources that were used to develop our annual geographical restriction map for schistosomiasis.

Modelling strategy

To estimate deaths due to schistosomiasis, a negative binomial regression model of country-year-age-sex-specific deaths on natural log-transformed age-standardised schistosomiasis infection prevalence with a 15-year lag was used. The negative binomial regression was selected due to its suitability for modelling count data. In addition, there are relatively low numbers of deaths attributable to schistosomiasis. Indicator variables for endemic Brazil subnationals and South Africa subnationals were used to allow the model to follow data in those areas. A multivariate normal distribution using the mean and variance-covariance matrix from the model was used to generate 1000 draws of deaths due to schistosomiasis.

Models were evaluated by assessing the AIC and plotting the predicted deaths against time, age, and sex. In addition, the Cause of Death visualisation tool was used to evaluate time trends across locations, age, and sex. A map of the global distribution of schistosomiasis across age groups was also used to assess the changes in death rates over time. The final model was selected based on how well the estimated numbers fit the input data and how plausible the predicted distribution of disease was over time and with age.

Changes from GBD 2021 to GBD 2023

There have been no substantive changes to the modelling strategy for GBD 2023.

References

1. Chitsulo, L., Engels, D., Montresor, A., & Savioli, L. (2000). The global status of schistosomiasis and its control. Acta Tropica, 77(1), 41-51. doi:10.1016/s0001-706x(00)00122-4

2. World Health Organization (2006). Preventive chemotherapy in human helminthiasis: coordinated use of anthelminthic drugs in control interventions: a manual for health professionals and programme managers.

Sexually transmitted infections excluding HIV

Mortality due to sexually transmitted infections excluding HIV in adults (adult STIs) and mortality due to congenital syphilis are estimated separately and aggregated to calculate total fatal burden for sexually transmitted infections excluding HIV.

Input data and methodological summary for sexually transmitted infections excluding HIV – adults

Flowchart

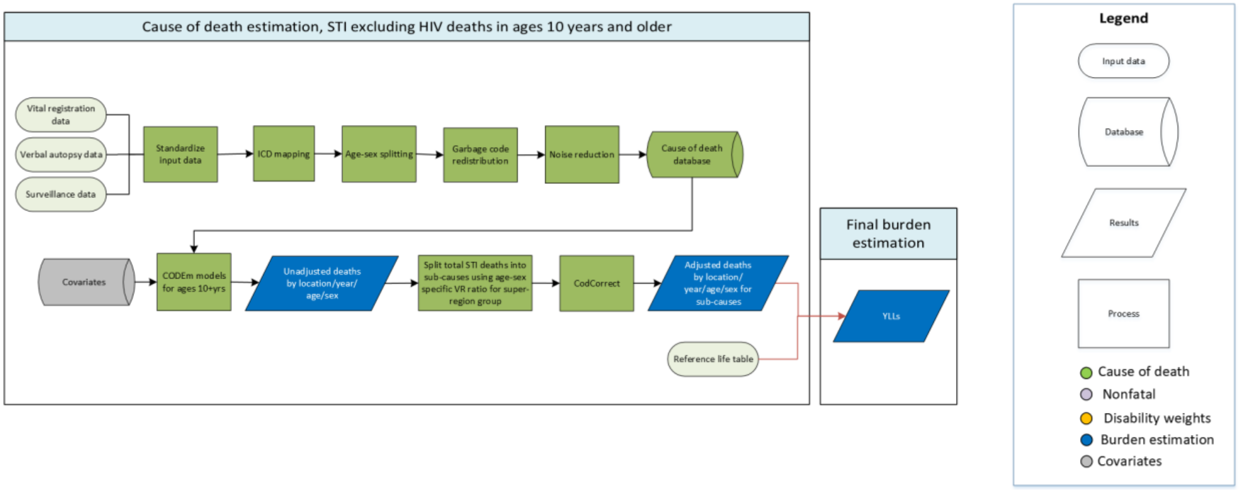

Input data

Total adult deaths due to STI excluding HIV were modelled in aggregate for males and females 10 years and older using centrally processed vital registration (VR), verbal autopsy (VA), and surveillance data from the cause of death (CoD) database. These data included deaths from all geographies and coding systems for syphilis, chlamydial infection, gonococcal infection, and other STIs excluding HIV. A list of ICD codes that were mapped to this cause, a list of vital registration sources included in the CoD database and their quality as indicated by star-ratings, and the steps used to process these data for modelling can be found in the section of this appendix on the causes of death database and the tables and figures it refers to. CoD data processing was broadly similar to previous rounds of GBD, but one novel data processing step was employed in preparing data for STIs excluding HIV that was not needed prior to GBD 2023—COVID-19 corrections—which are described in the aforementioned appendix section.

Data exclusions: After all data processing steps were complete, datapoints for all locations and years were systematically reviewed to identify outliers or other data limitations not fully overcome by CoD data processing methods. For adult STIs, the following were identified and excluded prior to modelling.

VR data from ICD-8A coding were excluded in Denmark, Finland, Switzerland, Mauritius, and Cabo Verde because this coding system did not accurately capture STI deaths. Data in both North Macedonia and the Cook Islands reported zero deaths due to STIs excluding HIV for all years and both sexes, suggesting that this is not an underlying cause of death that certifiers use in these countries. For Ukraine and its

subnationals, data were excluded where discontinuities between different coding systems resulted in implausible time trends. Data in Greenland were excluded where existing noise-reduction algorithms were insufficient to overcome the large amount of noise in measuring this very rare cause of death in very small populations. Cambodia, Viet Nam, Pakistan and its subnationals, Nepal, São Tomé and Príncipe, and Türkiye were excluded because having only one year of data from a small population and/or a small sample size from the source, even after region-level noise reduction, made it difficult to distinguish true extreme values from sampling error that was insufficiently addressed with current noise-reduction algorithms. Kiribati data were excluded due to inadequate population coverage. For India and its subnationals, data from India Medical Certification of Cause of Death State-Level Tabulations were excluded in favor of the India Sample Registration System data, because the latter source covered both urban and rural locations and used a sample that was more representative of both in-hospital and out-of-hospital deaths. In Ethiopia and some of its subnationals, District Health Information Software 2 (DHIS2) data were excluded in favor of Ethiopia's VA data to avoid compositional bias and maintain the source believed to have more correct assignment of underlying cause of death, since the VA data were more similar to all other VA and VR data in the region. Zimbabwe data were excluded for being at least six times higher than other data in its region.

Modelling strategy

Sexually transmitted infections excluding HIV – total

No substantive changes were made to the strategy used to estimate adult mortality due to STIs excluding HIV in GBD 2023 compared to the strategy used in GBD 2021. A standard CODEm model was used. Models were generated separately for males in data-rich countries, males in all countries (referred to as a global model), females in data-rich countries, and females in all countries (global model). Fatal estimation for this cause was restricted to ages 10–95+ years.

Table 1: Covariates offered for selection in adult STI mortality modelling

Level	Covariate	Direction
1	Syphilis prevalence	+
2	Abortion legality	-
	Age-specific fertility rate	+
	Education (years per capita)	-
	Total fertility rate	+
	Maternal care and immunisation (MCI)	-
	Healthcare Access and Quality (HAQ) Index	-
3	Antenatal care (ANC) coverage, 1+ visits	-
	Antenatal care (ANC) coverage, 4+ visits	-
	Log lag-distributed income (LDI) ($I per capita)	-

In the ensemble models selected by the CODEm algorithm based on out-of-sample predictive validity, all covariates except syphilis prevalence were selected for the female global model. Syphilis prevalence, MCI, legality of abortion, HAQ Index, ANC coverage 1+ visits, ANC coverage 4+ visits, and LDI per capita were chosen for the male global model. All covariates were chosen for the female data-rich model, and

all covariates except age-specific fertility rate were chosen for the male data-rich model. We hybridised separate global and data-rich models to acquire unadjusted results, which we adjusted using CoDCorrect and compared to the reference life table to calculate final YLLs due to adult STIs.

Sub-causes (chlamydial infection, gonococcal infection, syphilis, other STIs)

To produce estimates of deaths specifically due to syphilis, chlamydial infection, gonococcal infection, and other STIs, estimates from the total model were divided according to proportions that were estimated from all available cause-specific VR data from the CoD database. Trichomoniasis and HSV-2 were assumed not to cause mortality. Chlamydial infection was assumed to not to cause death in males. Therefore, for males, the STI CODEm model was split into deaths due to syphilis, gonococcal infection, or other STI. For females, the STI CODEm model was split into deaths due to syphilis, chlamydial infection, gonococcal infection, or other STI.

In GBD 2017, cause-specific VR data were summed by age group and sex, then scaled to the total STI death model in order to calculate proportions for each specific infection. These proportions were then applied to all locations. Beginning in GBD 2019, to account for geographical variation in proportions, cause-specific VR data were summed by age group, sex, and super-region, then scaled to the total. Unfortunately, the CoD database had very sparse data on STI causes of death in sub-Saharan Africa and north Africa and the Middle East, which resulted in implausible proportions estimated for these super-regions. As a result, the decision was made to calculate cause-specific proportions by age, sex, and two super-region groups.

Table 2: Super-region groups for STI sub-cause proportions

Super-region group	Super-regions included
ALMA	• *Southeast Asia, east Asia, and Oceania*<br>• *Latin America and the Caribbean*<br>• *North Africa and the Middle East*<br>• *South Asia*<br>• *Sub-Saharan Africa*
CECAH	• *Central Europe, eastern Europe, and central Asia*<br>• *High income*

The proportional splits for males and females have subsequently been updated using data from the GBD 2023 CoD database. The results for each of these two super-region groups are shown in the below figures.

Figure 1: Adult STI proportional splits from VR data

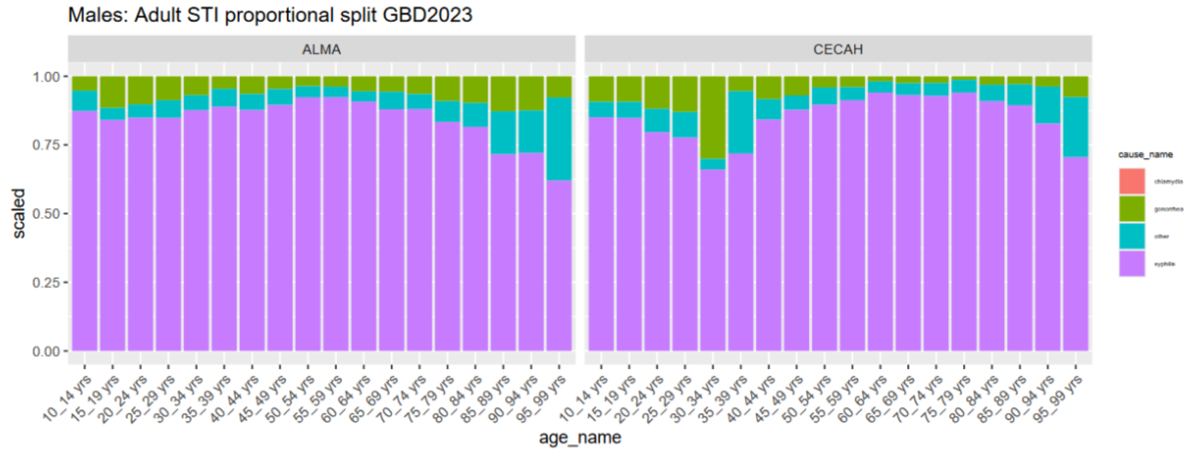
Males: Adult STI proportional split GBD2023
ALMA
CECAH
scaled
1.00
0.75
0.50
0.25
0.00
10_14 yrs
15_19 yrs
20_24 yrs
25_29 yrs
30_34 yrs
35_39 yrs
40_44 yrs
45_49 yrs
50_54 yrs
55_59 yrs
60_64 yrs
65_69 yrs
70_74 yrs
75_79 yrs
80_84 yrs
85_89 yrs
90_94 yrs
95_99 yrs
age_name
cause_name
chlamydia
gonorrhea
other
syphilis

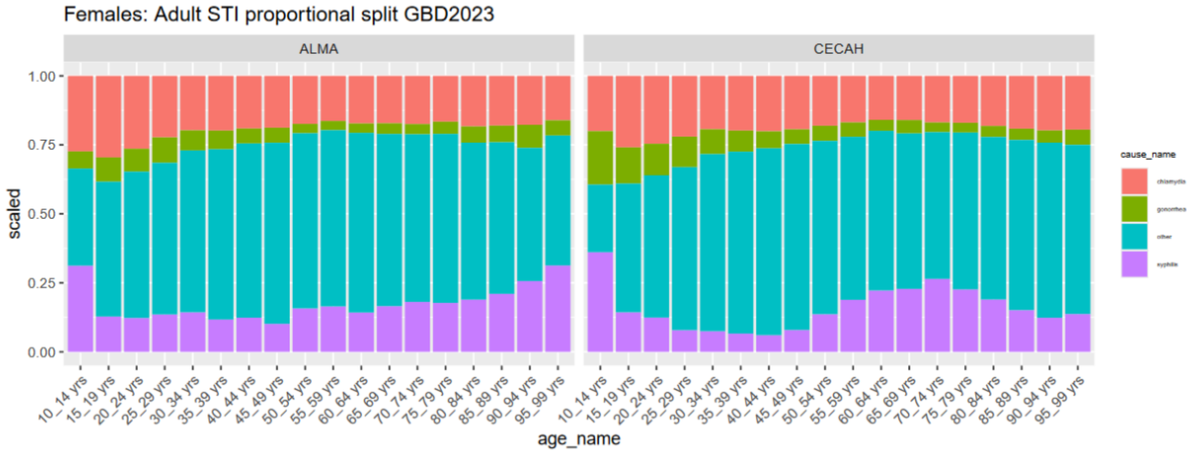
Females: Adult STI proportional split GBD2023
ALMA
CECAH
scaled
1.00
0.75
0.50
0.25
0.00
10_14 yrs
15_19 yrs
20_24 yrs
25_29 yrs
30_34 yrs
35_39 yrs
40_44 yrs
45_49 yrs
50_54 yrs
55_59 yrs
60_64 yrs
65_69 yrs
70_74 yrs
75_79 yrs
80_84 yrs
85_89 yrs
90_94 yrs
95_99 yrs
age_name
cause_name
chlamydia
gonorrhea
other
syphilis

Input data and methodological summary for congenital syphilis

Congenital syphilis arises from the transmission of syphilis from mother to child, in the womb or during childbirth. We model deaths due to congenital syphilis for males and females aged 0 to 9 years. Of all STIs excluding HIV, only syphilis is regarded as causing deaths in children under 10 years. In GBD 2017, congenital syphilis deaths were estimated in all locations with a natural history model. However, after GBD 2017, we found that our natural history model exceeded the number of deaths recorded by countries with high-quality vital registration (VR) and a record of investment into the eradication of congenital syphilis. To produce more plausible estimates based on data considered to be highly complete and reliable, we decided that congenital syphilis deaths in data-rich countries would be estimated in a CODEm model. We continue to use the natural history model to produce estimates for countries with no or lesser-quality VR (data-sparse). Outputs for data-sparse countries produced in the natural history model are combined with outputs for data-rich countries produced in the CODEm model, then passed on to the CoDCorrect process as a hybrid model and included in final GBD estimates of mortality due to congenital syphilis. Please see the "cause of death modelling methods: central computation" section of the appendix for more information. In the sections below, the input data and the modelling strategy for each method are described.

Flowchart

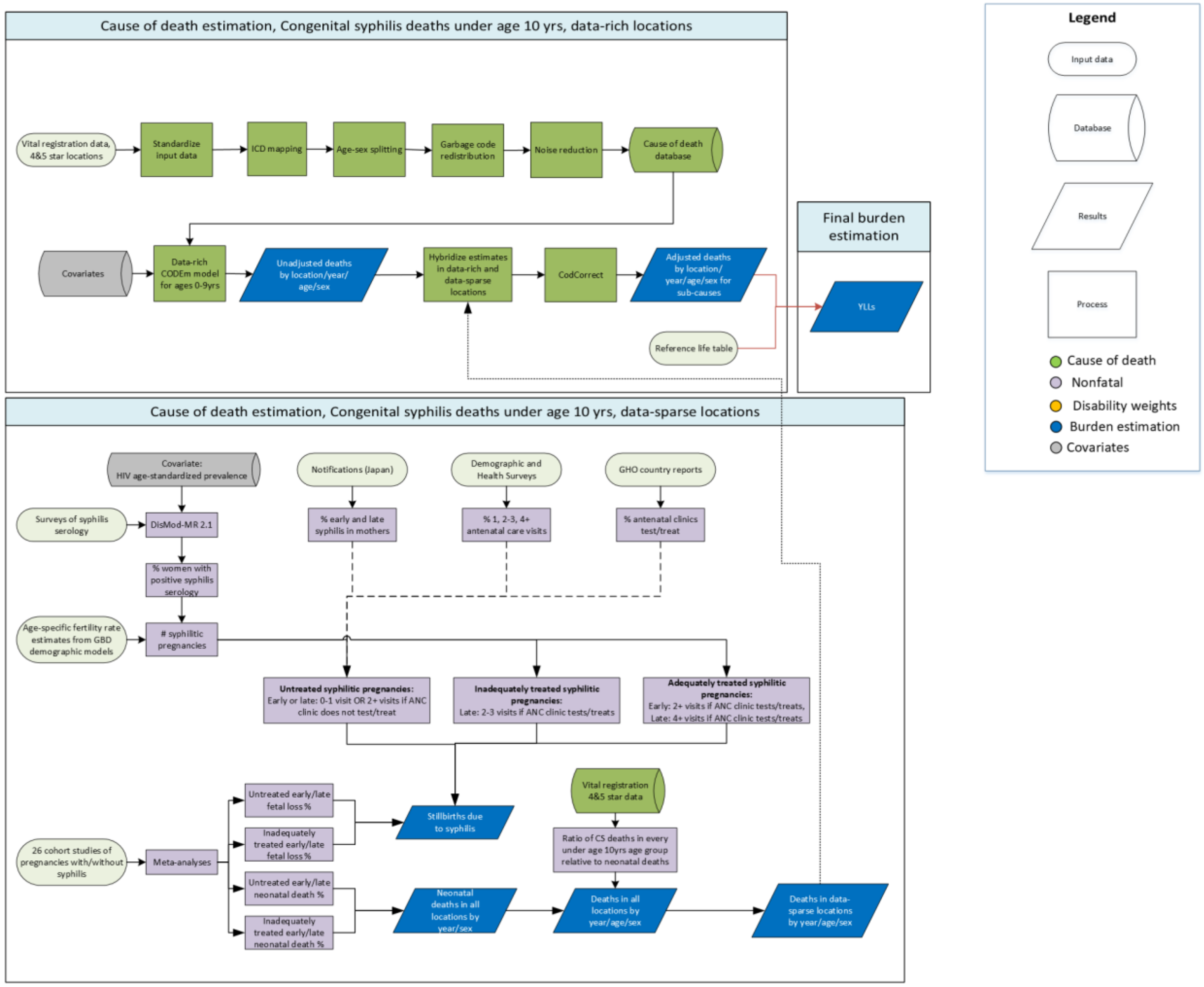

Input data

CODEm

Deaths due to congenital syphilis (CS) in data-rich countries were modelled using centrally processed vital registration data from the cause of death (CoD) database. A list of ICD codes that were mapped to this cause, a list of vital registration sources included in the CoD database and their quality as indicated by star-ratings, and the steps used to process these data for modelling can be found in the section of this appendix on the causes of death database and the tables and figures it refers to. CoD data processing was broadly similar to previous rounds of GBD, but one novel data processing step was employed in preparing data for congenital syphilis that was not needed prior to GBD 2023—COVID corrections—which are described in the aforementioned appendix section.

Data exclusions: After all data processing steps were complete, datapoints for all locations and years were systematically reviewed to identify outliers or other data limitations not fully overcome by CoD data processing methods. The same data and exclusion strategies were applied to congenital syphilis as for its parent cause, STIs excluding HIV, as described above, with the following differences: Cambodia, Viet Nam, Nepal, and Pakistan had no data for this cause. Azerbaijan, Belarus, Syria, and Kenya only had one year of data instead of multiple as in the parent, so these single years of data were excluded under the same principle of having only one year of data from a small population, even after region-level noise reduction, made it difficult to distinguish true extreme values from sampling error that was insufficiently addressed with current noise-reduction algorithms.

Natural history

Five different inputs were used to model the natural history of congenital syphilis. Inputs were drawn from both data-rich and data-sparse locations, and the model produced outputs for all location-years. Outputs for data-sparse location-years were passed on to the hybrid model that went into CoDCorrect and subsequently included in final GBD estimates of mortality due to congenital syphilis.

Our first inputs were estimates of positive syphilis serology in women of reproductive age from our DisMod model of syphilis seroprevalence. A detailed description of these estimates can be found in the non-fatal methods appendix for sexually transmitted infections (STIs) under the syphilis subheading. Our second inputs were age-specific fertility rates (ASFRs) estimated in the GBD 2023 demographic analyses. Please see the GBD 2023 demographics capstone paper for methodological information about the ASFRs. Third, we used GBD estimates of the number of antenatal care (ANC) visits per pregnant woman. Fourth, we used surveillance data that included disease stage at time of diagnosis by age and gender, which we use to determine the relative proportions of early versus late symptomatic syphilis among pregnant women.[1,2] Fifth, we used data from the Global Health Observatory on the proportion of women who access ANC services who are tested for syphilis and the proportion of positive ANC attendees who receive treatment for syphilis.[3] Sixth, we used cohort studies on the risk of fetal loss and neonatal death in women who have syphilis infection during pregnancy.

In GBD 2017, 11 studies on the risk of fetal loss and neonatal death in syphilitic women were collected through recommendations from our GBD Collaborator Network. In GBD 2019, we conducted a systematic review of congenital syphilis. The search string below was run on April 4, 2019, through PubMed. It returned 1675 articles. After title/abstract review, 442 articles remained for full-text screening. Of these, 165 were deemed eligible for data extraction. These sources included information on the following: syphilis during pregnancy, stillbirth, spontaneous abortion, preterm birth, low

birthweight, neonatal death, transmission of CS, and sequelae of CS. 15 of the articles on stillbirth, spontaneous abortion, and neonatal death were combined with the 11 studies from GBD 2017 and included in a meta-analysis of excess neonatal death and fetal loss. Of the remaining 150 articles, sources on the vertical transmission of CS or the sequela of CS were utilised in the non-fatal estimation of congenital syphilis. Sources on syphilis during pregnancy were utilised in the non-fatal estimation of adult syphilis seroprevalence. Please see the sexually transmitted infections (STIs) section of the non-fatal appendix for further information on methodology. Articles on preterm birth and low birthweight were not currently used in CS estimation but were catalogued in IHME's database, the Global Health Data Exchange (GHDx).

(syphilis[tiab] OR "treponema pallidum"[tiab]) AND ((pregnan[tiab] OR fetal[tiab] OR foetal[tiab] or fetus*[tiab] OR foetus*[tiab] OR neonat*[tiab] OR infan*[tiab] OR newborn*[tiab] OR congenital[tiab]) OR ((vertical*[tiab] OR maternal[tiab] OR mother[tiab] OR fetomaternal[tiab]) AND transmi*[tiab])) AND (outcomes[tiab] OR sequela*[tiab] OR manifestation*[tiab] OR morbidity*[tiab] OR diagnos*[tiab] OR hutchinson*[tiab])*

Modelling strategy

CODEm

A standard CODEm model was used to model deaths due to congenital syphilis in data-rich countries. Models were generated separately for males in data-rich countries and females in data-rich countries. Fatal estimation for this cause was restricted to ages 0–9 years.

Table 3: Covariates offered for selection in congenital syphilis data-rich mortality modelling

Level	Covariate	Direction
1	Syphilis prevalence	+
	Antenatal care (ANC) coverage, 1+ visits	-
	Antenatal care (ANC) coverage, 4+ visits	-
	Maternal care and immunisation (MCI)	-
2	Legality of abortion	-
	Age-specific fertility rate	+
	Total fertility rate	+
3	Education (years per capita)	-
	Healthcare Access and Quality (HAQ) Index	-
	Log lag-distributed income (LDI) ($I per capita)	-

In the ensemble models selected by the CODEm algorithm based on out-of-sample predictive validity, syphilis prevalence, MCI, legality of abortion, education, HAQ Index, and LDI per capita were chosen for both the female data-rich and the male data-rich models.

Natural history

Our natural history model for congenital syphilis mortality began with the estimation of pregnancies that are at risk (ie, that are exposed to gestational syphilis infection). To calculate this, we multiplied the prevalence of positive syphilis serology in women of childbearing age by age-specific fertility rates.

Next, we incorporated five separate measures that allowed us to estimate the number of stillbirths and neonatal deaths resulting from pregnancies exposed to gestational syphilis. These were: 1) the proportion of women accessing antenatal (ANC) clinics who are tested for syphilis, and, among those who test positive, the proportion of women who are treated for syphilis; 2) the proportion of women in each location-year who attend ANC clinics 0–1, 2–3, or 4+ times per pregnancy, as a proxy for adequacy of treatment; 3) the proportion of infected mothers who are either in the early or in the late symptomatic stage of syphilis, which informs treatment needs; 4) excess risk of stillbirth and neonatal death in pregnancies exposed to gestational syphilis infection by treatment status and disease stage; and 5) ratios of syphilis death for every age group up to 10 years of age, relative to neonatal deaths.

1) ANC testing and treatment data were obtained from 132 countries via the Global Health Observatory. The first of these measures was the proportion of ANC attendees who are tested for syphilis at their first visit. The second was the proportion of infected women who receive treatment if they test positive for syphilis. These data were entered into a ST-GPR model to estimate these measures for all year-age-location combinations with Socio-demographic Index (SDI) as a covariate.

2) The distribution of the number of skilled antenatal care visits during pregnancy was produced by internal GBD analyses of maternal care. Data from geographically representative household surveys that include self-reported care received during pregnancy, specifically number of ANC visits, were used to model three levels of ANC care received: 1 visit, 2+ visits, and 4+ visits. ANC coverage was extracted as number of visits per most recent livebirth, or, if number was not available, an indicator if whether any care was received.[4]

3) Detailed notification data from Japan on the stage of syphilis infection in pregnant women diagnosed during antenatal screening were used to determine the proportion of women with early or late syphilis by age group.

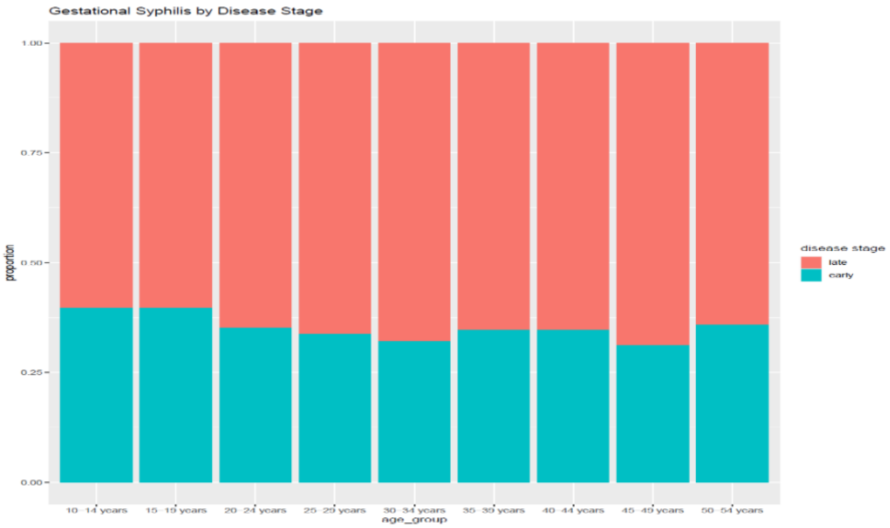

4) The excess risk of stillbirth and neonatal death in pregnancies exposed versus unexposed to gestational syphilis infection was estimated in a meta-analysis described below.

5) 4- and 5-star vital registration data on deaths from congenital syphilis for males and females in every age group up to 10 years (early neonatal, late neonatal, 1–5 months, 6–11 months, 12–23 months, 2–4 years, 5–9 years) was used to calculate a ratio of deaths for every age group relative to neonatal deaths.

Measures 1–4 were used to estimate total fetal loss and neonatal death from congenital syphilis. The fifth measure allowed us to disaggregate neonatal deaths into the early and late neonatal groups, and to estimate the number of deaths in infants in the post-neonatal stage up until age 9 years.

For measure 4, the excess risk of fetal loss and neonatal death for mothers with gestational syphilis infection relative to mothers without gestational syphilis infection was estimated using a meta-analysis of 26 cohort studies. Risks were calculated specified by treatment status of the mother. The year of study, and hence, baseline rates of fetal loss and neonatal death, varied across the cohort studies included in the meta-analysis, but these differences were accounted for by calculating excess risk as the rate in pregnancies exposed to gestational syphilis infection minus the rate observed in pregnancies unexposed to gestational syphilis infection from the same study. For the subset of studies that only reported rates of fetal loss and neonatal death in pregnancies exposed to gestational syphilis infection, excess risk rates were calculated by subtracting off the year-age-location-specific all-cause stillbirth and all-cause neonatal mortality rates estimated by the GBD demographics team. Please see the GBD 2023 demographics capstone paper for methodological information on these rates. Forest plots of the excess risk data from cohort studies are below. Values of mortality from women of unknown treatment status were excluded from the analysis.

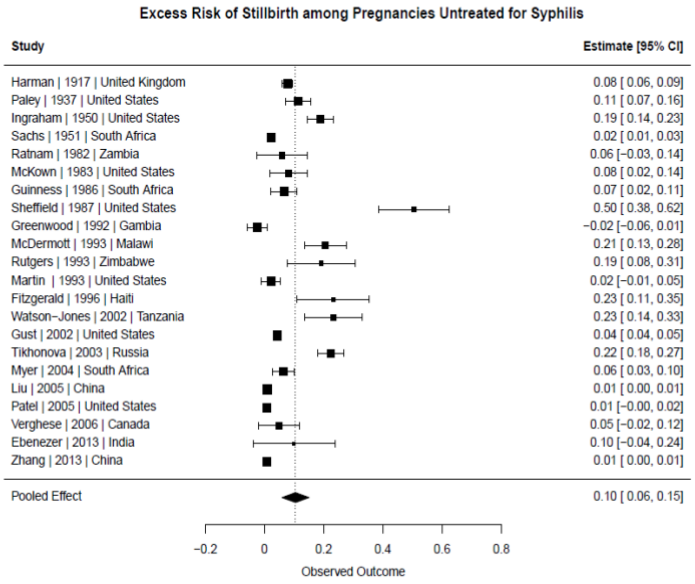

Excess Risk of Neonatal Death among Pregnancies Untreated for Syphilis

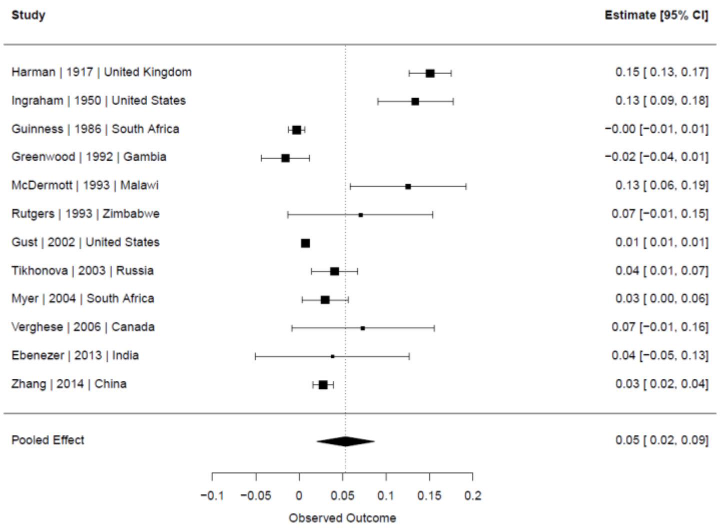

Excess Risk of Stillbirth among Pregnancies Inadequately Treated for Syphilis

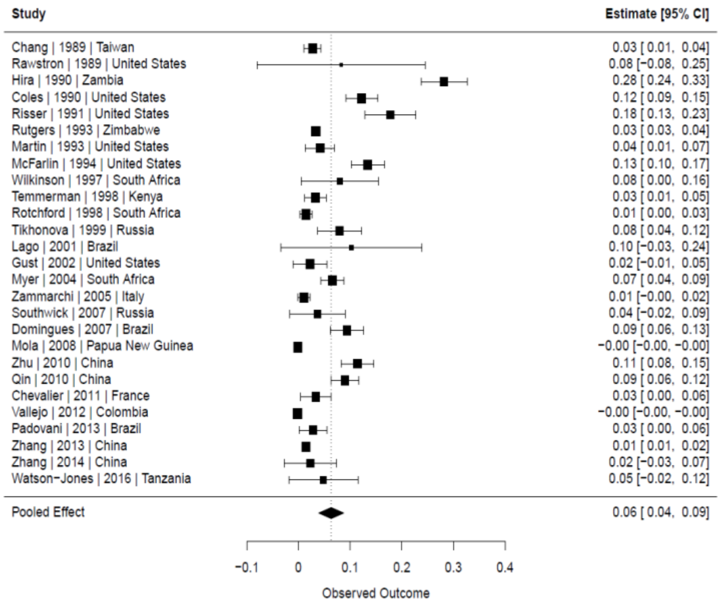

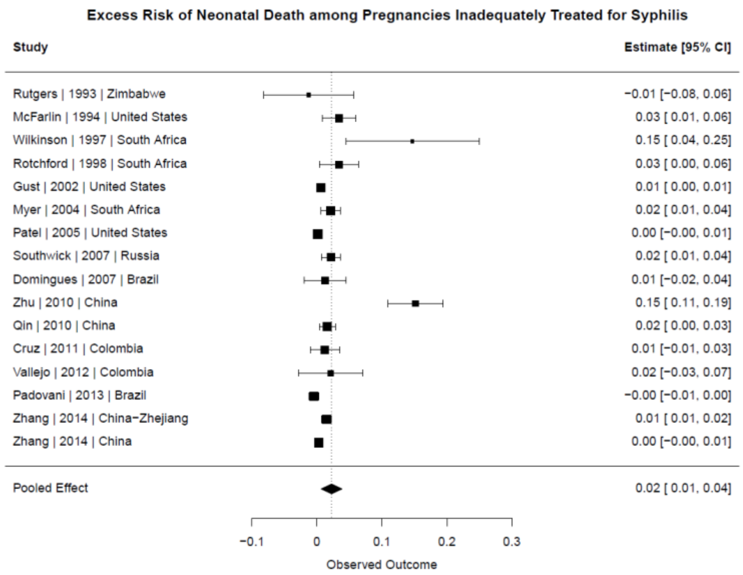

No excess mortality or fetal loss was assumed for adequately treated cases of maternal syphilis. To confirm this assumption, a comparison of the neonatal mortality rates between adequately treated women and uninfected women showed a smaller proportion of babies from adequately treated women died than babies from uninfected women.

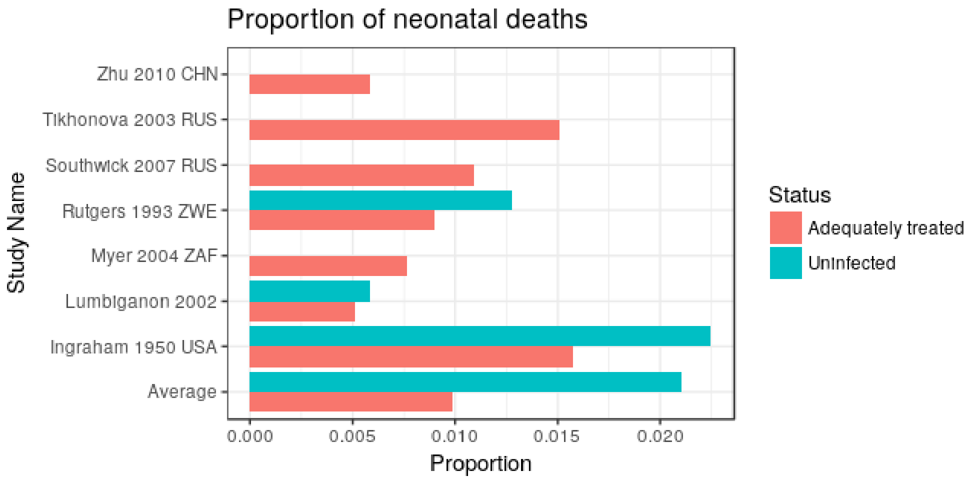

To combine these measures and obtain the final death counts:

We multiplied the number of pregnancies exposed to gestational syphilis infection by the proportions of mothers attending antenatal clinics at least one, two to three, or four times during pregnancy, the probability of attending a clinic that tests and treats, and the proportions of early and late syphilis in pregnant women. This gave us the number of pregnancies of untreated status, inadequately treated status, or adequately treated status. These three groups are estimated because treatment status impacts the risk of fetal loss and neonatal death. The recommendation throughout literature was that individuals with early syphilis infection require one dose of penicillin to be adequately treated, while those with late syphilis infection are recommended three doses of penicillin for adequate treatment. We assumed that women need to attend an ANC clinic at least two times – once to undergo syphilis testing,

and a second time to receive test results and begin treatment. Thus, for those with early infection, 0–1 ANC visits indicated untreated status, and two or more visits indicated adequately treated status. For those with late infection, 0–1 visits indicated untreated status, 2–3 visits indicated inadequately treated status, and 4+ visits indicated adequately treatment status.

After the number of pregnancies in each treatment group was calculated, we multiplied the untreated and the inadequately treated categories by the excess risk of fetal loss specific to each treatment category. This produced the number of excess stillbirths in pregnancies of each treatment category. We subtracted the number of excess stillbirths in each treatment category from the pregnancies of each respective treatment category to get the number of livebirths born from pregnancies of each treatment category. We then multiplied the livebirths born from untreated and inadequately treated pregnancies by the excess risk of neonatal death specific to each treatment category. This produced the number of neonatal deaths for each treatment category. The numbers of neonatal deaths were then summed for all treatment categories to determine the total number of neonatal deaths due to congenital syphilis.

Finally, we distributed neonatal deaths across the early and late neonatal age groups and estimated the number of deaths for the 1–5 months, 6–11 months, 12–23 months, 2–4 years, and 5–9 years age groups. In GBD 2017, ratios for each age group relative to neonatal deaths were calculated using vital registration (VR) data from all location-years. However, this produced implausible differences between males and females in the estimated ratios. To solve this, starting in GBD 2019, only 4- and 5-star VR data were used to calculate ratios of deaths for every age group relative to neonatal deaths. A further explanation of the star rating system can be found in the "GBD 2023 Causes of Death database: Causes of death data star rating calculation" section of the appendix. We multiplied the ratios calculated from high-quality VR data by our estimated number of neonatal deaths.

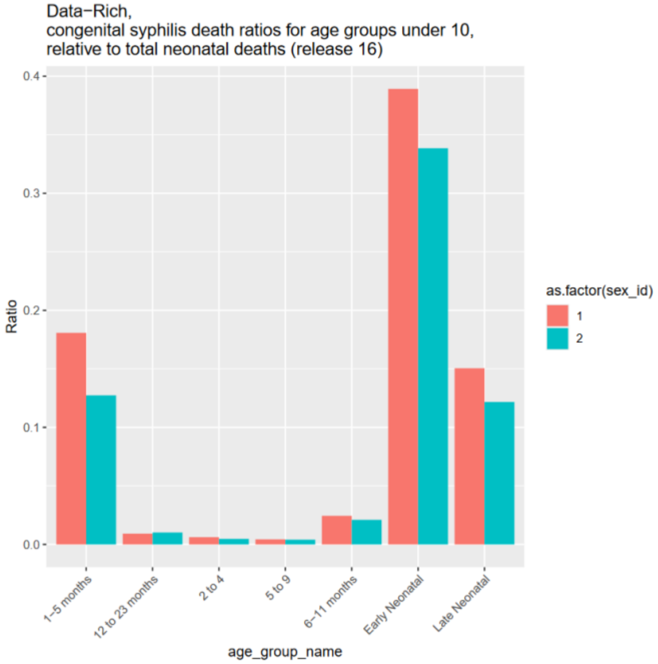

Subsequently, the sex- and age-specific congenital syphilis deaths estimated in the natural history model for data-sparse location-years were hybridised with the deaths estimated in the CODEm model for data-

rich locations, and the hybrid model results were uploaded to the causes of death database and entered into the CoDCorrect process.

References

1 National Institute of Infectious Diseases (Japan). Sexually transmitted diseases in Japan as of 2007. 2008 https://idsc.niid.go.jp/iasr/29/343/tpc343.html.

2 National Institute of Infectious Diseases (Japan). Japan Syphilis 2008-2014. 2015 https://www.niid.go.jp/niid/images/idsc/iasr/36/420e.pdf.

3 World Health Organization. Global Health Observatory data repository - Syphilis in pregnancy - Data by country. WHO. https://apps.who.int/gho/data/node.main.A1358STI?lang=en (accessed Oct 21, 2024).

4 Gardner WM. Global, Regional, and National Estimates of Complete Coverage of Maternal Obstetric Care Interventions, 1990-2019. *Diss* 2020; published online Aug 14. http://hdl.handle.net/1773/45723 (accessed Oct 21, 2024).

Skin and subcutaneous diseases

Flowchart

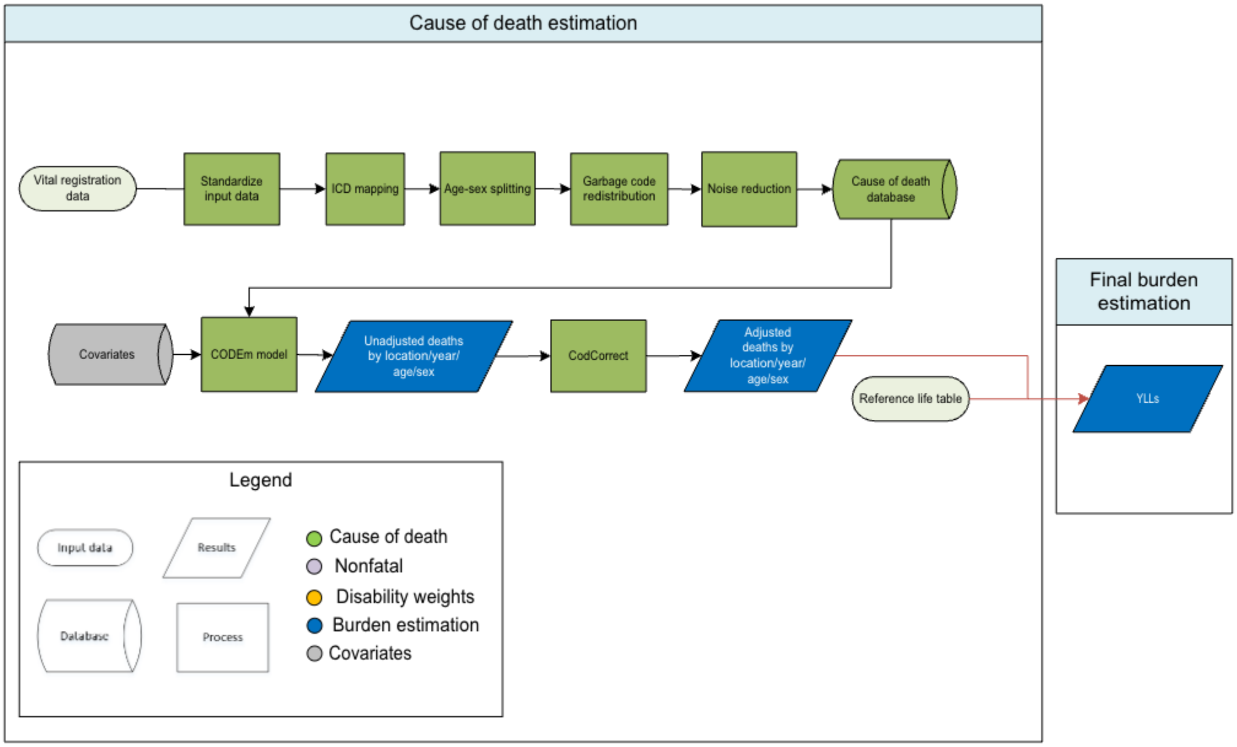

Input data and methodological summary for skin and subcutaneous diseases

Input data

Data used to estimate mortality due to skin and subcutaneous diseases consisted of vital registration and verbal autopsy data from the cause of death (CoD) database. Outlier criteria excluded datapoints that were implausibly under/over-reporting relative to global or regional patterns and data from datasets with small populations. The data in skin and subcutaneous diseases consist of aggregated data from all other specific skin diseases (cellulitis, pyoderma, decubitus ulcer) as well as unique datapoints from unspecified codes of skin and subcutaneous disease.

Modelling strategy

The skin and subcutaneous diseases model is estimated by CODEm model with standard CODEm parameters along with the CoD database and location-level covariates as inputs. We hybridised separate global and data-rich models to acquire unadjusted results, which we finalised and adjusted using CoDCorrect to reach final years of life lost (YLLs) due to skin and subcutaneous diseases.

Compared to GBD 2021, there are several updates that were made to the model estimation strategy. First, we have received new data for skin and subcutaneous diseases for multiple location-years. 101 data sources were added to the cause of death database, which led to improved estimates, particularly

for recent years. Key location-years that impacted our estimates were from Bangladesh, Indonesia, Ethiopia, Nigeria, Brazil, China, and India. Additionally, in GBD 2023, CODEm began using an all-cause mortality envelope that is inclusive of HIV, whereas in previous rounds, the all-cause mortality envelope was HIV-free. This can lead to changes in cause fractions and rates when compared to previous GBD rounds.

Table 1. Covariates used in skin and subcutaneous diseases mortality modelling

Level	Covariate	Direction
1	LDI (per capita)	-
	Improved water source (proportion with access)	-
	SEV scalar for unsafe sanitation	+
	Diabetes fasting plasma glucose (mmol/L), by age	+
	Healthcare Access and Quality Index	-
	Prevalence of overweight and obesity	+
3	Education (years per capita)	-
	Socio-demographic Index	-

Stroke

Flowchart

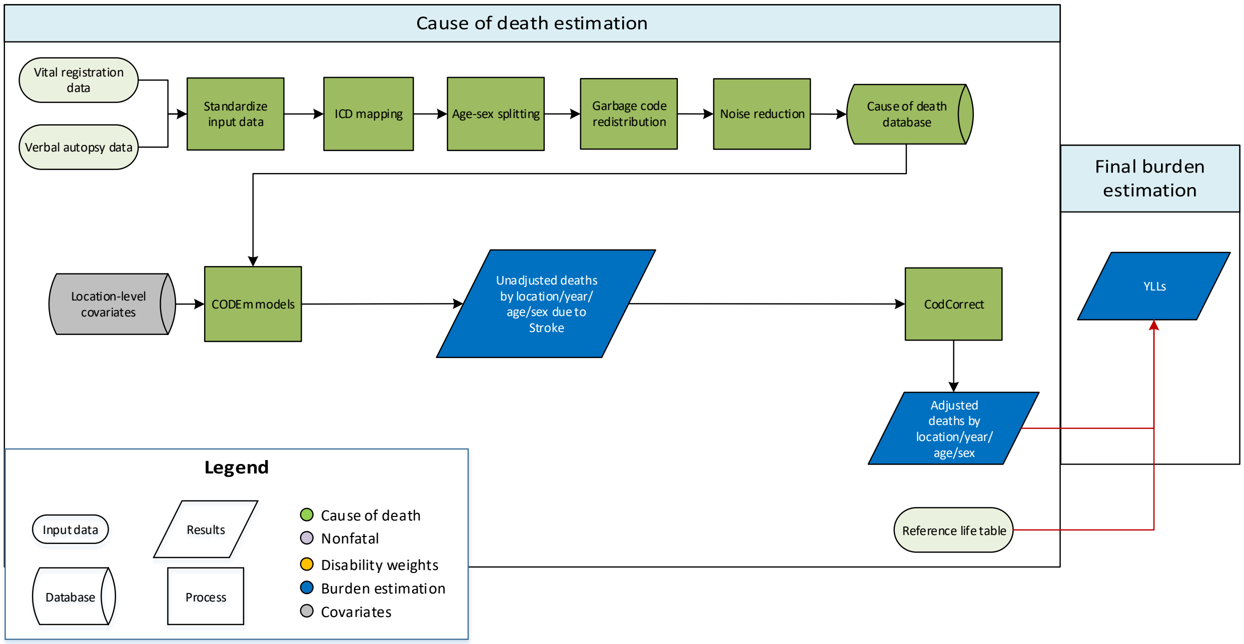

Input data and methodological summary for stroke

Input data

Vital registration and verbal autopsy data were used to model stroke. To maximise the reliability of data included in the model, verbal autopsy studies that did not meet World Health Organization standards were excluded. Verbal autopsy studies which included only populations under 30 years were also excluded. In addition, we outliered non-representative subnational verbal autopsies from several Indian states and verbal autopsy data in Nepal, Sri Lanka, Bangladesh, and Papua New Guinea that were implausible in terms of time and age trends. We also outliered verbal autopsy data in countries and subnational locations where high-quality vital registration data were available.

After evaluating the available vital registration data, we outliered ICD-8 data for stroke. We additionally outliered ICD-9 data in Poland, Russia, Bulgaria, and Egypt for inconsistency in the time series with ICD-10 data. We also outliered ICD-10 data in Sri Lanka, Azerbaijan, Grenada, Egypt, and Saudi Arabia, and Cabo Verde.

Most data sources used for cause of death estimation are ICD-coded. The ICD periodically releases new versions, which can introduce an implausible disjoint in the time series that is indicative of a change in the coding practice, rather than a true change in the epidemiological trend of the disease. In GBD 2023, certain causes and countries using the ICD-9 code systems were adjusted to address the disjoint in the time trend and number of deaths between the older ICD-9 code systems and the more recent ICD-10 code systems. Stroke was corrected in Bulgaria (1990–2004), Canada (1986–1999), Spain (1980–1998), France (1980–1999), the USA (1980–1998), Hungary (1980–1995), Croatia (1985–1994), the UK (1980–

1999), Greece (1987–2008), Italy (1980–2002), Mexico (1980–2002), and Mauritius (1980–2004) to account for this code system disjoint. This was done using a meta-regression tool described as a crosswalk, by taking systematically biased datapoints and estimating their unbiased value. Using this modelling tool, correction factors were created based on the more recent and more reliable ICD-10-coded data. The crosswalk creates matched pairs between the last three years of data using the ICD-9 code system and the first three years of data using the ICD-10 code system. The correction is applied at the child cause level for all Level 2 causes, and after the correction is applied the individual child causes are raked to the parent cause to maintain a consistent number of deaths across the cause hierarchy.

Modelling strategy

We used a standard CODEm approach to model deaths from stroke. Since GBD 2021, CODEm has switched from using an ensemble of rate and cause-fraction models to only rate models. Otherwise, there were no substantial changes in CODEm modelling since GBD 2021.

The covariates included in the ensemble modelling process are listed in the table below.

Table 1. Covariates used in stroke mortality modelling

Level	Covariate	Direction
1	Summary exposure variable, stroke	1
	LDL cholesterol (mean per capita)	1
	Smoking prevalence	1
	Systolic blood pressure (mmHg)	1
2	Mean body-mass index (kg/m^2)	1
	Elevation over 1,500 m (proportion)	–1
	Fasting plasma glucose	1
	Outdoor pollution ($PM_{2.5}$)	1
	Indoor air pollution	1
	Healthcare Access and Quality Index	–1
3	Log-transformed lag-distributed income per capita (I$)	–1
	Summary exposure value, omega-3	1
	Summary exposure value, fruits	1
	Summary exposure value, vegetables	1
	Summary exposure value, nuts and seeds	1
	Pulses/legumes (kcal per capita, unadjusted)	–1
	Summary exposure value, PUFA, adjusted (percent)	1
	Alcohol (litres per capita)	1
	Trans fatty acid	1

Subarachnoid haemorrhage

Flowchart

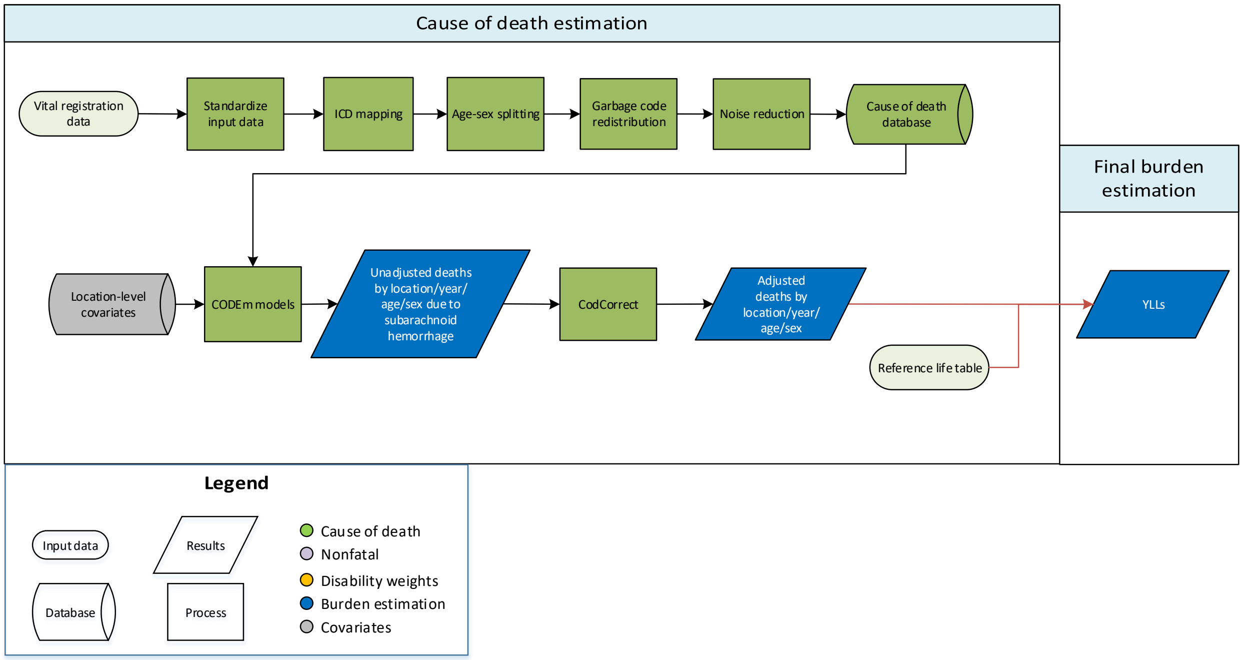

Input data and methodological summary for subarachnoid haemorrhage

Input data

Vital registration data were used to model subarachnoid haemorrhage. We outliered ICD-8 datapoints which were inconsistent with the rest of the data and created implausible time trends. In addition, we outliered vital registration data in Tibet, Ghana, and Cabo Verde that were implausibly high for all years and age groups. ICD-9 data in Poland, Dominican Republic, Saint Vincent and the Grenadines, and Greece were outliered due to inconsistency with corresponding ICD-10 data. We also outliered vital registration data in Egypt, Oman, Montenegro, Tajikistan, and Palestine that were implausibly high. In addition, we outliered ICD-10 datapoints from sources in Iran, Georgia, and subnationals of Ethiopia which were implausibly low in all age groups.

Most data sources used for cause of death estimation are ICD-coded. The ICD periodically releases new versions, which can introduce an implausible disjoint in the time series that is indicative of a change in the coding practice, rather than a true change in the epidemiological trend of the disease. In GBD 2023, certain causes and countries using the ICD-9 code systems were adjusted to address the disjoint in the time trend and number of deaths between the older ICD-9 code systems and the more recent ICD-10 code systems. Subarachnoid haemorrhage was corrected in Bulgaria (1990–2004), Canada (1986–1999), Spain (1980–1998), France (1980–1999), the USA (1980–1998), Hungary (1980–1995), Croatia (1985–1994), the UK (1980–1999), Greece (1987–2008), Italy (1980–2002), Mexico (1980–2002), and Mauritius (1980–2004) to account for this code system disjoint. This was done using a meta-regression tool described as a crosswalk, by taking systematically biased datapoints and estimating their unbiased

value. Using this modelling tool, correction factors were created based on the more recent and more reliable ICD-10-coded data. The crosswalk creates matched pairs between the last three years of data using the ICD-9 code system and the first three years of data using the ICD-10 code system. The correction is applied at the child cause level for all Level 2 causes, and after the correction is applied the individual child causes are raked to the parent cause to maintain a consistent number of deaths across the cause hierarchy.

Modelling strategy

We used a standard CODEm approach to model deaths from subarachnoid haemorrhage. Since GBD 2021, CODEm has switched from using an ensemble of rate and cause-fraction models to only rate models. Otherwise, there were no substantial changes in CODEm modelling since GBD 2021.

The covariates included in the ensemble modelling process are listed in the table below.

Table 1. Covariates used in subarachnoid haemorrhage mortality modelling

Level	Covariate	Direction
1	Summary exposure variable, subarachnoid haemorrhage	1
	Smoking prevalence	1
	Systolic blood pressure (mmHg)	1
2	Healthcare Access and Quality Index	–1
3	Log-transformed lag-distributed income per capita (I$)	–1
	Alcohol (litres per capita)	1

Sudden infant death syndrome

Flowchart

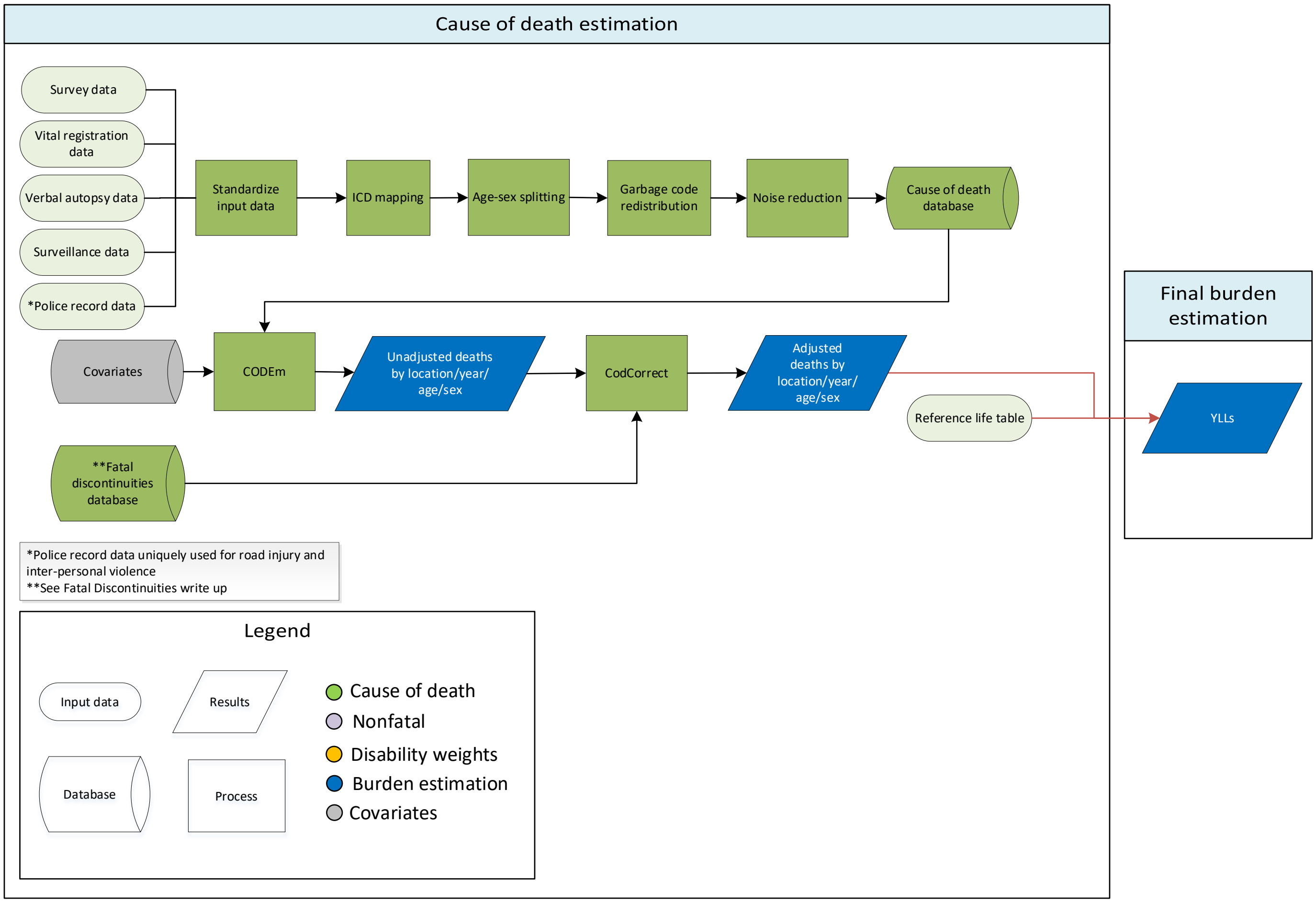

Input data and methodological summary for sudden infant death syndrome

Input data

Vital registration (VR) data from the cause of death (CoD) database was used for the estimation of sudden infant death syndrome (SIDS). Data that presented implausible deviations from established time trends were outliered and not included in the modelling process described below.

Lastly, for SIDS specifically, only VR data from countries with high-quality vital registration systems were included. Only high-quality VR systems are used due to this cause's high probability for miscoding with other neonatal disorders in lower-quality collection systems.

Modelling strategy

SIDS is estimated by the Cause of Death Ensemble model (CODEm). SIDS models use standard CODEm parameters, alongside data from the CoD database and location-level covariates as inputs.

There were no major adjustments to the modelling process for SIDS between GBD 2021 and GBD 2023. There were, however, an additional 312 country-years of new VR data added in GBD 2023, contributing to the available SIDS input data.

The primary challenge in the estimation of SIDS is the difficulty in diagnosing, or potentially miscoding of this cause in lower-quality cause of death data collection systems, as mentioned above. For this reason, the only CoD data available to inform CODEm come from high-quality VR systems. This means for countries without high-quality collection systems, SIDS estimates are informed entirely from covariates and neighbouring regional trends where high-quality data are available.

Table 1. Covariates used in sudden infant death syndrome mortality modelling

Level	Covariate	Direction
1	In-facility delivery (proportion)	-
2	Skilled birth attendant (proportion)	-
	Maternal care and immunisation	-
	Healthcare Access and Quality Index	-
3	Education (years per capita)	-
	Lag-distributed income (per capita)	+
	Total fertility rate	+
	Socio-demographic Index	+

Tetanus

Flowchart

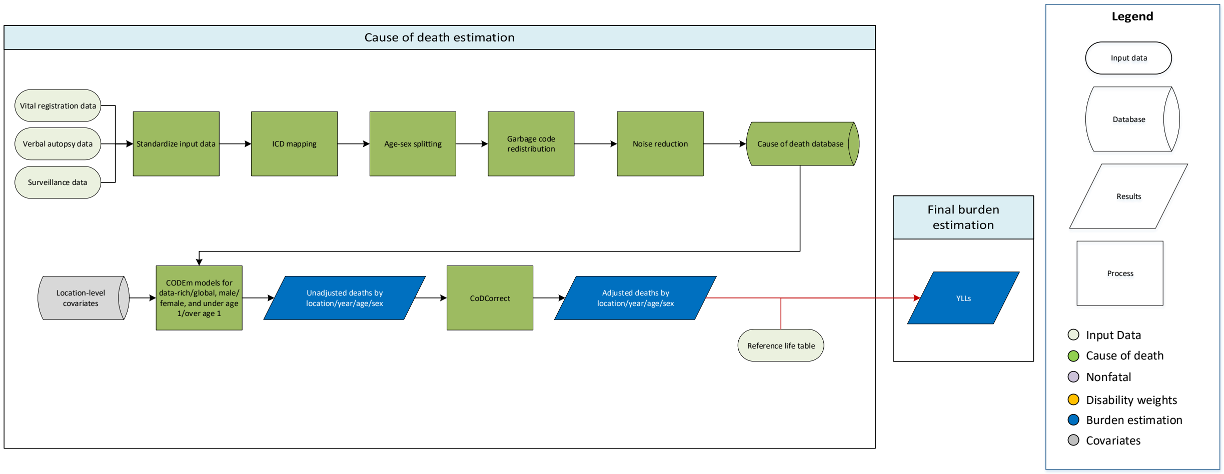

Input data and methodological summary for tetanus

Input data

Tetanus cause of death (COD) data for GBD 2023 included vital registration, verbal autopsy, and surveillance sources from all locations as available. We excluded prepared CoD data if they were highly incongruent with other available data from the same location or locations of similar sociodemographic characteristics.

Modelling strategy

We used a Cause of Death Ensemble modelling (CODEm) approach to compute age-, sex-, location-, and year-specific estimates. In GBD 2023, both count and rate models were included in the final ensemble. Separate, sex-specific models were run for neonatal tetanus (under-1-year age groups) and all other tetanus (1 year to 95+ age groups). We also stratified models by vital registration data quality, running both "data-rich" and global models for each age- and sex-specific group. Following model completion, the data-rich and global model outputs were combined to produce a single set of estimates for all locations by sex and age (under-1 and over-1 age groups).

Table 1a lists the covariates used in the data-rich and global under-1 models, and table 1b the covariates in the over-1 model. To account for the impact of COVID-19 on tetanus vaccination coverage, we used the COVID-inclusive average lagged third dose diphtheria-tetanus-pertussis vaccine (DTP3) coverage over the past five years as a covariate.

Table 1a. Covariates. Summary of covariates used in the under-1 tetanus cause of death model

Level	Covariate	Direction

1	Average COVID-inclusive diphtheria-tetanus-pertussis third dose (DTP3) vaccination coverage over the past five years	-
	Tetanus toxoid coverage	-
2	In-facility deliveries (proportion)	-
	Skilled birth attendance (proportion)	-
	Healthcare Access and Quality (HAQ) Index	-
3	Lag-distributed income (LDI)	-
	Socio-demographic Index (SDI)	-
	Mean years of education per capita	-

Table 1b. Covariates. Summary of covariates used in the over-1 tetanus cause of death model

Level	Covariate	Direction
1	Average COVID-inclusive diphtheria-tetanus-pertussis third dose (DTP3) vaccination coverage over the past five years	-
2	Healthcare Access and Quality (HAQ) Index	-
3	Sanitation access (proportion)	-
	Lag-distributed income (LDI)	-
	Socio-demographic Index (SDI)	-
	Mean years of education per capita	-

Changes from GBD 2021 to GBD 2023

There were no substantive changes to the modelling strategy for GBD 2023.

Tuberculosis

Flowchart

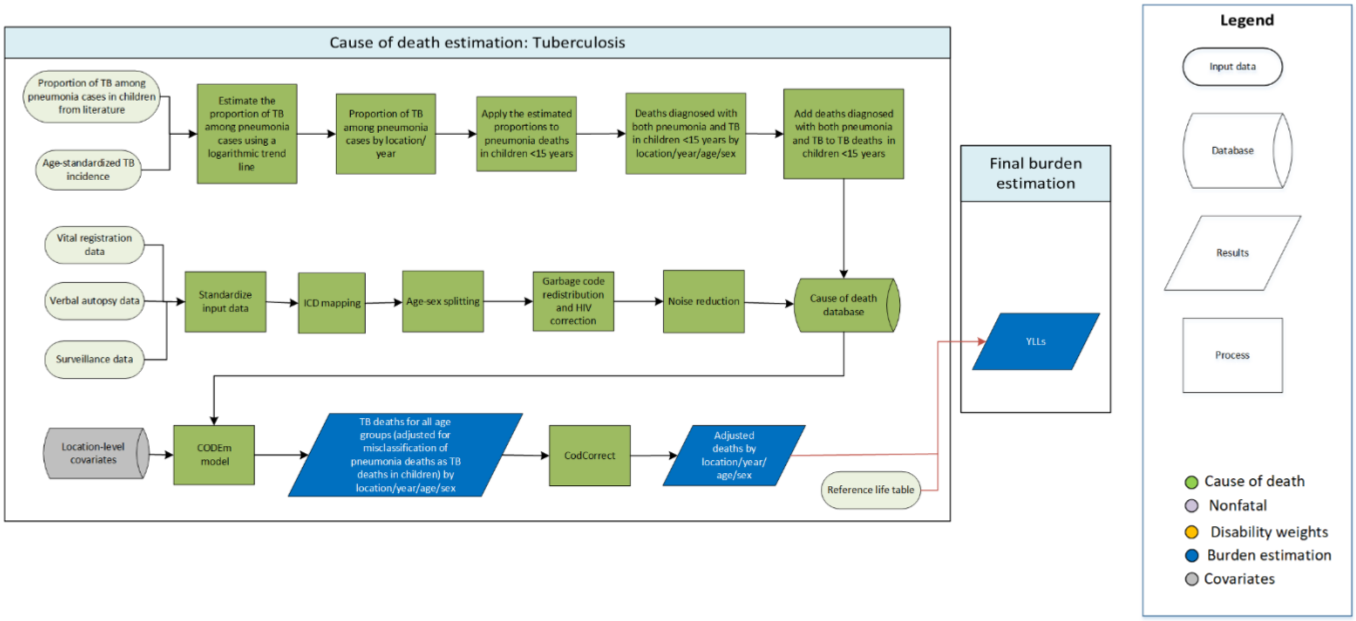

Input data and methodological summary for tuberculosis

Input data

Input data for modelling tuberculosis (TB) mortality among HIV-negative individuals include vital registration, verbal autopsy, and surveillance data. Vital registration data were adjusted for garbage coding (including ill-defined codes and the use of intermediate causes) following GBD algorithms and misclassified HIV deaths (ie, HIV deaths being assigned to other underlying causes of death such as tuberculosis or diarrhoea because of stigma or misdiagnosis).

Verbal autopsy data in countries with age-standardised HIV prevalence greater than 5% were removed because of a high probability of misclassification, as verbal autopsy studies have poor validity in distinguishing HIV deaths from HIV-TB deaths.

Modelling strategy

A general CODEm modelling strategy was used. We continued to use the TB strain prevalence-weighted transmission risk and cigarettes per capita covariate that were introduced in GBD 2017. Other location-level covariates included in the CODEm model were the same as in previous GBD cycles: adult underweight proportion, alcohol (litres per capita), diabetes (fasting plasma glucose mmol/L), education (years per capita), Healthcare Access and Quality Index, lag-distributed income, indoor air pollution, outdoor air pollution, population density, prevalence of active tuberculosis, prevalence of latent tuberculosis infection, smoking prevalence, Socio-demographic Index, and a summary exposure variable reflecting the average exposure to all of the risk factors.

Covariate table

	Covariate	Direction
Level 1	TB prevalence<br>Latent TB infection prevalence<br>SEV scalar<br>Litres of alcohol consumed per capita<br>Smoking prevalence<br>Cigarettes per capita<br>Fasting plasma glucose	+<br>+<br>+<br>+<br>+<br>+<br>+
Level 2	HAQ Index<br>Adult underweight proportion<br>Indoor air pollution<br>Outdoor air pollution<br>Population density	-<br>+<br>+<br>+<br>+
Level 3	Log LDI<br>Education (years per capita)<br>Socio-demographic Index (SDI)	-<br>-<br>-

Correcting for a potential misclassification of tuberculosis deaths as pneumonia deaths in children

Since GBD 2017, we have addressed the potential for misclassification of TB deaths as pneumonia deaths among children in locations with high TB burden. First, we estimated the proportion of tuberculosis among pneumonia cases as a function of age-standardised TB incidence using data from eight clinical studies[2,3,4,5,6,7,8,9] reporting the proportion of pneumonia cases that had tuberculosis (or the data to calculate them) and the age-standardised TB incidence estimates. We used a logarithmic trend line to fit these data. We applied the estimated proportions to pneumonia deaths reported in data among children younger than 15 years to compute the number of deaths diagnosed with both pneumonia and TB, which were then added to child TB data. Following this correction in our input data, the CODEm model was run to provide location-year-age-sex specific estimates. This is a departure from GBD 2017, where the estimated proportions were applied after CODEm. Finally, the CODEm estimates were adjusted using CoDCorrect, which ensures that the number of deaths from each cause add up to all-cause mortality deaths for a given year.

References

1. Graham SM, Sismanidis C, Menzies HJ, Marais BJ, Detjen AK, Black RE. Importance of tuberculosis control to address child survival. *Lancet* 2014; **383**(9928): 1605-7.
2. Adegbola RA, Falade AG, Sam BE, et al. The etiology of pneumonia in malnourished and well-nourished Gambian children. *Pediatr Infect Dis J* 1994; 13: 975–82.

3. Chisti MJ, Graham SM, Duke T, et al. A prospective study of the prevalence of tuberculosis and bacteraemia in Bangladeshi children with severe malnutrition and pneumonia including an evaluation of Xpert MTB/RIF assay. *PloS One* 2014; 9: e93776.
4. Madhi SA, Petersen K, Madhi A, Khoosal M, Klugman KP. Increased disease burden and antibiotic resistance of bacteria causing severe community-acquired lower respiratory tract infections in human immunodeficiency virus type 1-infected children. *Clin Infect Dis* 2000; 31: 170–76.
5. McNally LM, Jeena PM, Gajee K, et al. Effect of age, polymicrobial disease, and maternal HIV status on treatment response and cause of severe pneumonia in South African children: a prospective descriptive study. *Lancet* 2007; 369: 1440–51.
6. Moore DP, Klugman KP, Madhi SA. Role of Streptococcus pneumoniae in hospitalisation for acute community-acquired pneumonia associated with culture-confirmed Mycobacterium tuberculosis in children: a pneumococcal conjugate vaccine probe study. *Pediatr Infect Dis J* 2010; 29: 1099–104.
7. Nantongo JM, Wobudeya E, Mupere E, et al. High incidence of pulmonary tuberculosis in children admitted with severe pneumonia in Uganda. *BMC Pediatr* 2013; 13: 16.
8. Zar HJ, Hanslo D, Tannenbaum E, et al. Aetiology and outcome of pneumonia in human immunodeficiency virus-infected children hospitalized in South Africa. *Acta Paediatr* 2001; 90: 119–25.
9. Moore DP, Higdon MM, Hammitt LL, Prosperi C, DeLuca AN, Da Silva P, Baillie VL, Adrian PV, Mudau A, Deloria Knoll M, Feikin DR. The incremental value of repeated induced sputum and gastric aspirate samples for the diagnosis of pulmonary tuberculosis in young children with acute community-acquired pneumonia. *Clinical Infectious Diseases*. 2017 May 27;64(suppl_3):S309-16.

TB strain prevalence-weighted transmission risk covariate

In GBD 2017, we incorporated a TB covariate that incorporated data on the global distribution of TB strains and the relative risk of transmission associated with those strains. We continued the use of this covariate in GBD 2019. For this covariate, we defined TB strains according to the seven phylogenetic lineages of the *Mycobacterium tuberculosis* complex (MTBC) identified by S. Gagneaux and colleagues.[1] We determined the global distribution of these strains using a systematic review of human TB molecular epidemiology studies from 1990 to 2017 in PubMed and Scopus, as described in greater detail elsewhere.[2] All studies that used population-based sampling methods or collected isolates from all culture-positive TB cases in a given location and time period were included. All genotypes that could be converted to phylogenetic lineages were extracted, including genotypes determined by spoligotyping, MIRU-VNTR typing, and PCR or whole-genome sequencing. Studies of sub-populations, such as prison populations or drug-resistant cases only, were excluded. In total, 206 studies representing 85 countries and over 200,000 bacterial isolates were included. In GBD 2019, the systematic review was updated, which yielded an additional 18 studies published between 2017 and 2019, but we did not update it in GBD 2023 due to lack of bandwidth. A map of these strains highlighted the widespread global distribution of Euro-American Lineage 4 strains and East Asian Lineage 2 strains, and the geographical restriction of Lineage 5 and 6 strains to West Africa. Thirty of these studies also reported transmission chains associated with bacterial genotypes, as defined by genetic clustering.[3]

We used spatiotemporal Gaussian process regression (ST-GPR) to model the distribution of each strain in each GBD location across all ages and sexes, as described in greater detail elsewhere.[4] The covariates tested in each model included HIV age-standardised prevalence, population density, and a custom-made human movement covariate. The human movement covariate took into account (1) immigration and emigration patterns[5] and (2) airplane passenger flow[6] to and from each country. In the ST-GPR models we assumed strong correlation and smoothing over both space and time. We then used a random-effects meta-analysis to determine the relative risk (RR) of transmission associated with each strain, as defined by genetic clustering. We used the most widespread strains, Euro-American Lineage 4 strains, as the reference group. We found that East Asian Lineage 2 strains were associated with increased risk of transmission overall (relative risk [95% CI] = 1.24 [1.07, 1.45]), while West African Lineage 5 and 6 strains were associated with reduced transmission (relative risk [95% CI] = 0.61 [0.43, 0.86]). We used the following formula to calculate a TB strain prevalence-weighted risk of transmission based on these estimates:

$$\sum_{i=1}^{n} Pr_i RR_i$$ i=TB strain; Pr=proportion; RR=relative risk

References

1. Comas I, Coscolla M, Luo T, *et al.* Out-of-Africa migration and Neolithic coexpansion of *Mycobacterium tuberculosis* with modern humans. *Nat Genet* 2013; **45**: 1176–82.
2. Wiens KE, Woyczynski LP, Ledesma JR, *et al*. Global variation in bacterial strains that cause tuberculosis disease: a systematic review and meta-analysis. *BMC Medicine 2018*; 16:196.
3. Dheda K, Gumbo T, Maartens G, *et al.* The epidemiology, pathogenesis, transmission, diagnosis, and management of multidrug-resistant, extensively drug-resistant, and incurable tuberculosis. *Lancet Respir Med* 2017; **5**: 291–360.
4. Manuscript in preparation.
5. United Nations Population Division. United Nations Trends in International Migrant Stock: The 2015 Revision. New York City, United States: United Nations Population Division, 2015.
6. Huang Z, Wu X, Garcia AJ, *et al*. An open-access modeled passenger flow matrix for the global air network in 2010. *PLoS ONE* **8(5)**: e64317.

Typhoid fever

Flowchart

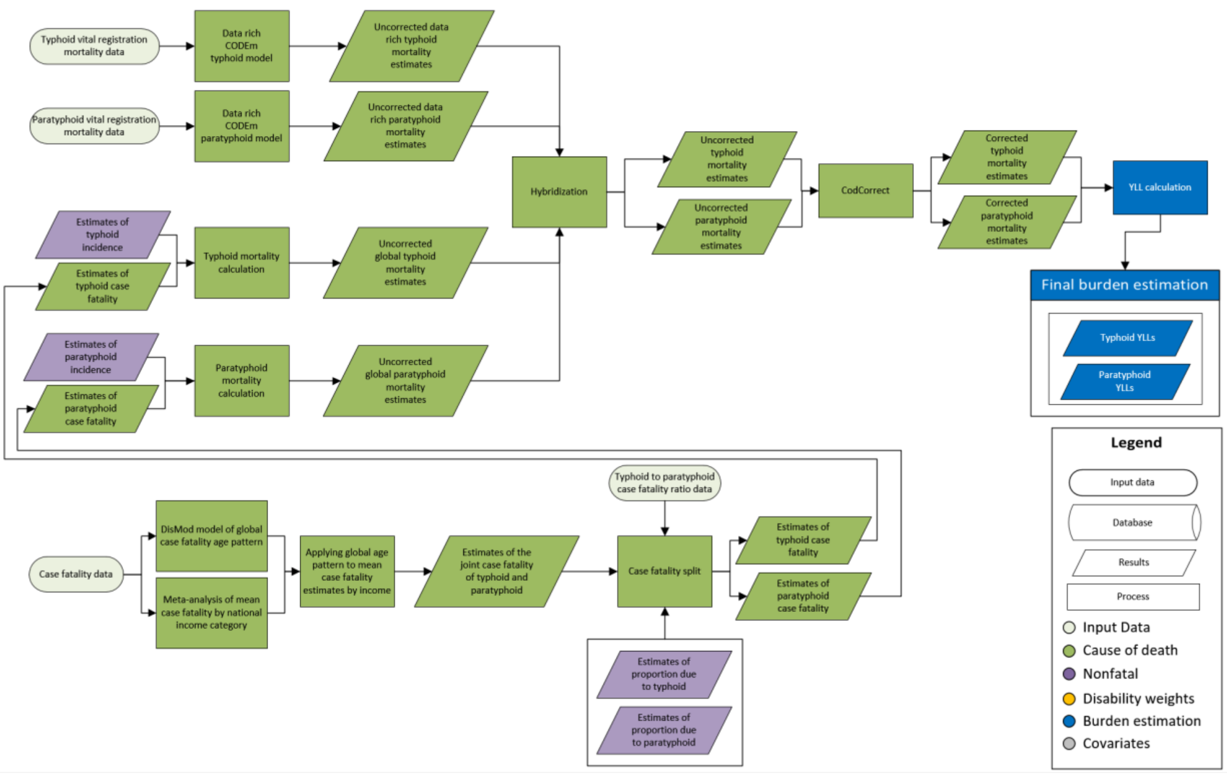

Input data and methodological summary for typhoid fever

Input data

Our CODEm model used all available data in the cause of death database from data-rich countries. No data were outliered for this cause. For the natural history model, our incidence dataset included a combination of data from prospective cohort studies and national surveillance systems. Similarly, data on proportions due to typhoid and paratyphoid included a combination of prospective cohort studies and national surveillance systems, and facility- and lab-based studies. Case fatality data were from national surveillance systems and hospital databases.

Modelling strategy

We model typhoid deaths using a hybrid modelling strategy with two components: 1) for data-rich locations we estimate typhoid mortality using a CODEm model of CoD data; and 2) in all other locations (ie, not data-rich) we use a natural history model in which we derive deaths as the product of cases and case fatality.

The CODEm model included six covariates:

Level	Covariate	Direction
1	Sanitation (proportion with access)	-
	Improved water source (proportion of the population with access)	-
	Proportion of the population living in the Indian Ocean monsoon belt	+
	SEV unsafe water	+
	SEV unsafe sanitation	+
2	Healthcare Access and Quality Index	-

For the natural history model, we first model total incidence of typhoid and paratyphoid combined. Second, we model the proportion of this total due to typhoid and the proportion due to paratyphoid. Third, we estimate case fatality by age and national income category for typhoid and paratyphoid combined. Fourth, we use data on the relative fatality of typhoid and paratyphoid to split the joint case fatality estimates into typhoid- and paratyphoid-specific case fatality estimates. Finally, we estimate cause-specific mortality rates as the product of incidence and case fatality.

Before modelling incidence, we applied four adjustments to the incidence data: 1) diagnostic sensitivity adjustment, 2) passive surveillance adjustment, 3) typhoid-only adjustment, and 4) age/sex splits. Incidence data were inflated to account for poor diagnostic sensitivity, based on an internal meta-analysis of the sensitivity of blood culture, the most common diagnostic used for typhoid: we estimate a sensitivity of 60.3% (50.3–68.8). We performed a crosswalk adjustment for incomplete case capture data from passive versus active surveillance, with active surveillance as the reference, using a MR-BRT model and adjusted the data before modelling. Where incidence data were from studies that only tested for typhoid fever, we used estimates from our aetiological proportion models to adjust these typhoid-only sources and calculated an adjusted joint incidence (ie, including both typhoid and paratyphoid cases) by dividing the typhoid-only incidence by the estimated proportion due to typhoid. We performed this calculation using posterior simulation with 1000 draws to propagate uncertainty from both the incidence data and the proportion estimate. Finally, where incidence data were reported for both sexes combined or for age categories spanning more than 25 years, we produced datapoints that were age- and sex-specific based on a MR-BRT model of sex ratios and a DisMod model of age patterns.

Total incidence was modelled using DisMod-MR, using the summary exposure values (SEV) for unsafe sanitation and the proportion of the population living in the Indian Ocean monsoon belt as covariates. Similarly, we used a DisMod model to estimate aetiological proportions, using a single model of the proportion of enteric fever due to *Salmonella* Paratyphi. We model the proportion due to paratyphoid instead of the proportion due to typhoid as we've found that this approach results in better a better model fit for sub-Saharan Africa, where the proportion of enteric fever due to *Salmonella* Typhi approaches 1.0, as DisMod performs better with proportions that are near-zero, than with proportions that are near-one.

Case fatality data were too limited to allow for a complete DisMod model or to allow for varying estimates by time and space. We had sufficient data, however, to estimate case fatality by age and by three categories of national income. We used DisMod to extract a global age pattern in case fatality, and meta-regression to estimate the mean case fatality by income category. Finally, we estimated the

relative risk of death from typhoid relative to paratyphoid based on data from Chinese surveillance and used that relative risk to estimate case fatality separately for typhoid and paratyphoid, by age and income.

Finally, we estimated typhoid mortality as the product of total incidence, the proportion of the total due to typhoid, and case fatality for typhoid. We propagated uncertainty through every step of the modelling process by pulling 1000 draws from the distribution of each model component (eg, incidence, proportion due to typhoid, overall case fatality, case fatality age pattern, relative fatalness of typhoid versus paratyphoid), and performing all calculations at the draw level.

Changes from GBD 2021 to GBD 2023

We updated our systematic review for GBD 2023 (Figure 1) and updated the code pipeline to improve robustness and efficiency but made no substantive changes to our modelling strategy for typhoid and paratyphoid between GBD 2021 and GBD 2023.

Figure 1: PRISMA flow diagram

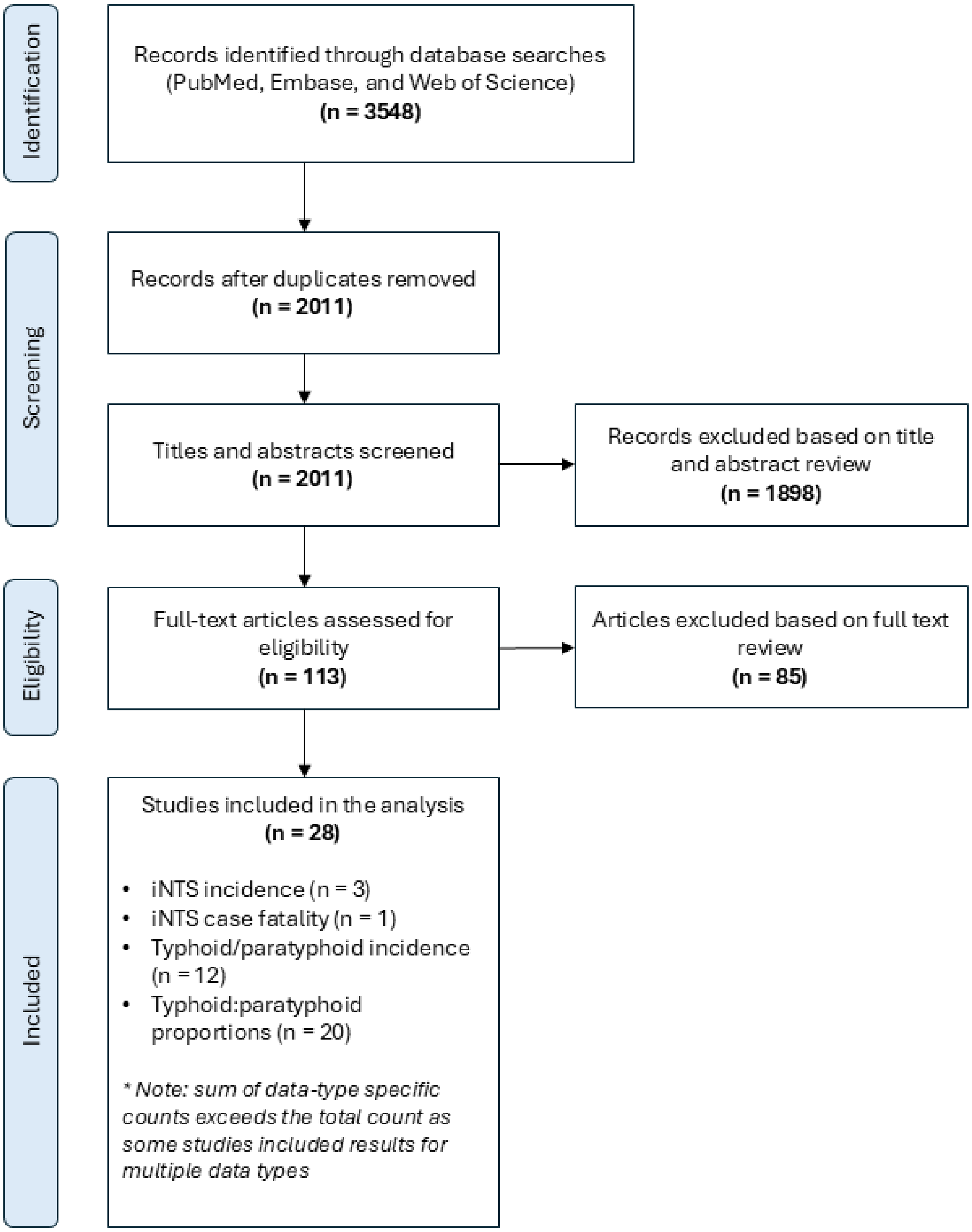

Upper digestive diseases

Flowchart

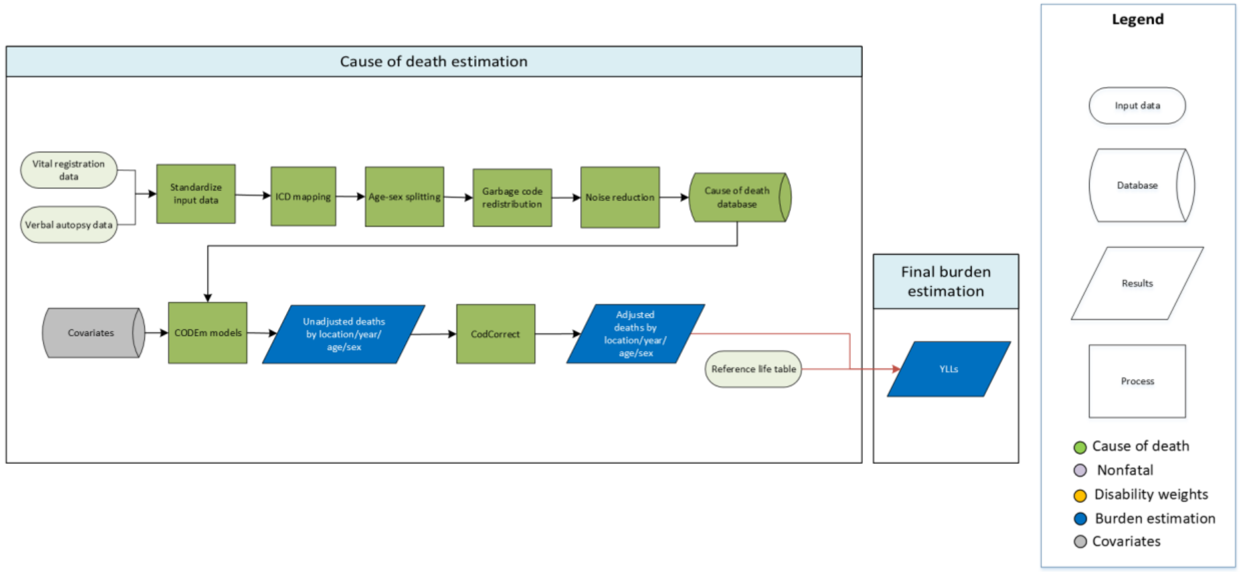

Input data and methodological summary for upper digestive diseases

Upper digestive diseases comprise a Level 3 cause in the GBD cause hierarchy.

Input data

Data used to estimate mortality of upper digestive diseases consisted of vital registration (VR) and verbal autopsy data (VA) from the cause of death (CoD) database. Upper digestive disease data aggregate deaths due to peptic ulcer disease and gastritis and duodenitis, which are also modelled separately. For sources of data that were considered too low-quality to definitively assign peptic ulcer or gastritis deaths to one of these two causes, data were included only in the upper digestive disease dataset. A list of ICD codes that were mapped to this cause, a list of vital registration sources included in the CoD database and their quality as indicated by star-ratings, and the steps used to process CoD data prior to modelling can be found in the section of this appendix on the causes of death database. CoD data processing was broadly similar to previous rounds of GBD, but one novel data processing step was employed in preparing data for upper digestive diseases that was not needed prior to GBD 2023—COVID corrections—which are described elsewhere in this appendix.

Data exclusions

After all data processing steps were complete, datapoints for all locations and years were systematically reviewed to identify outliers or other data limitations not fully overcome by CoD data processing methods, and select sources were excluded based on this review. For upper digestive diseases, the following were identified and excluded prior to modelling:

Super-region	Countries	Years and sources excluded

Central Europe, eastern Europe, and central Asia	No exclusions	
High income	No exclusions	
Latin America and Caribbean	No exclusions	
North Africa and Middle East	No exclusions	
South Asia	India	Department of Economics and Statistics' Medically Certified Causes of Death records in India excluded due to inclusion of hospital deaths in urban areas only. (India Sample Registration System is the preferred source.)
Sub-Saharan Africa	No exclusions	
Southeast Asia, east Asia, and Oceania	Indonesia<br>Cook Islands<br>Kiribati<br>Riau Islands<br>Viet Nam	Selected years and age groups in some subnational locations, verbal autopsy<br>All years, vital registration<br>All years, vital registration<br>2013, verbal autopsy (sole year)<br>2008, verbal autopsy (sole year)

Modelling strategy

The modelling strategy used to estimate fatal burden due to upper digestive diseases was largely similar in GBD 2023 compared to GBD 2021. A standard CODEm model was used to model deaths due to upper digestive diseases (see appendix section on CODEm method for details). Models were generated separately for males in data-rich countries, males in all countries (referred to as a global model), females in data-rich countries, and females in all countries (global model). Fatal estimation for this cause was restricted to ages 0.5 years to 95+ years. We hybridised separate global and data-rich models to acquire unadjusted results. The following table has the full list of covariates presented to the CODEm algorithm for possible inclusion in the upper digestive disease mortality model.

Table 1. Covariates used in upper digestive diseases mortality modelling

Level	Covariate	Direction
1	Sanitation, proportion with access	-
	Scaled exposure variable for unsafe water source	+
	Smoking prevalence	+
	Cumulative cigarettes (10 years)	+
	Cumulative cigarettes (5 years)	+
2	Litres of alcohol consumed per capita	+
	Vegetables (grams, unadjusted)	-
	Healthcare Access and Quality Index	-
3	Lag-distributed income (per capita)	-
	Education (years per capita)	-
	Socio-demographic Index	-

Adjustment in CoDCorrect included rescaling estimates for peptic ulcer disease and gastritis and duodenitis to all upper digestive disease deaths, then adjustment of upper digestive disease deaths with deaths due to other causes to sum to all-cause counts of death. Adjusted age-sex-specific results were compared to reference life tables to calculate YLLs.

Upper respiratory infections

Flowchart

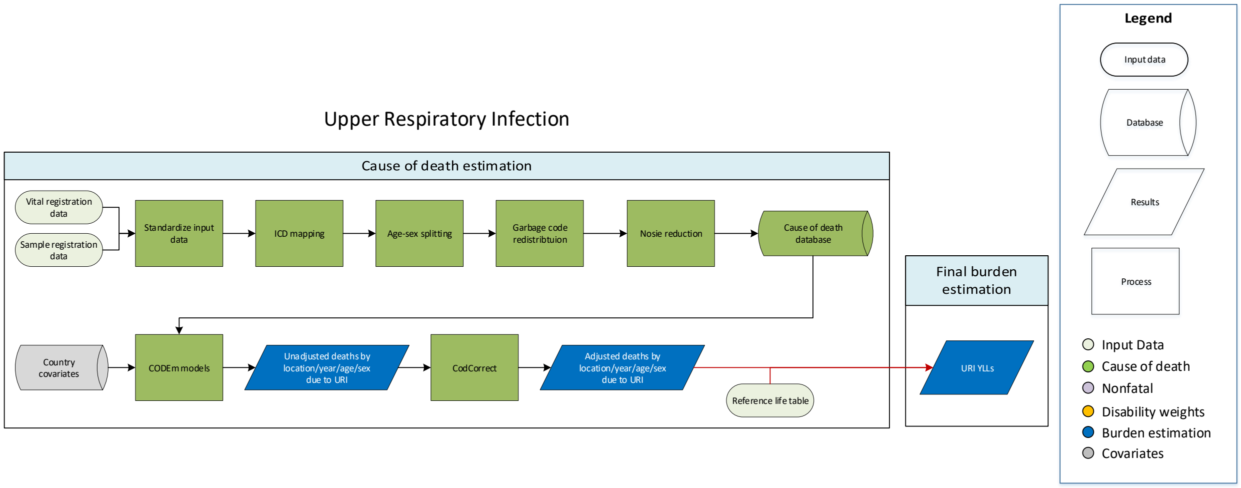

Input data and methodological summary for upper respiratory infections

Input data

Vital registration and surveillance data from the cause of death (CoD) database were used. Outliers were identified by systematic examination of datapoints. Datapoints that violated well-established age or time trends, were inconsistent with other country- or region-specific points, or that resulted in extremely high or low mortality rates were determined to be outliers.

Modelling strategy

A generic CODEm approach was used to estimate mortality due to upper respiratory infections (URI) in GBD 2023. In GBD 2016, mortality from URI was modelled using a negative binomial regression. It was determined that a negative binomial regression was an appropriate approach for estimating URI because of the small number of deaths due to URI in the CoD database. However, due to changes in how we redistribute cause of death codes, more deaths were attributed to URI in the CoD database, and thus it was determined that a generic CODEm approach was feasible for estimating URI mortality in GBD 2017. The covariates used are displayed below. We have made no substantive changes to the modelling strategy in 2023.

Table 1. Covariates used in upper respiratory infections mortality modelling

Level	Covariate	Direction
1	Smoking prevalence	+
2	Indoor pollution	+
	Outdoor pollution ($PM_{2.5}$)	+
	Healthcare Access and Quality Index	-
3	Socio-demographic Index	-
	Lag-distributed income	-

	Education (years per capita)	-

Urinary diseases and male infertility

Flowchart

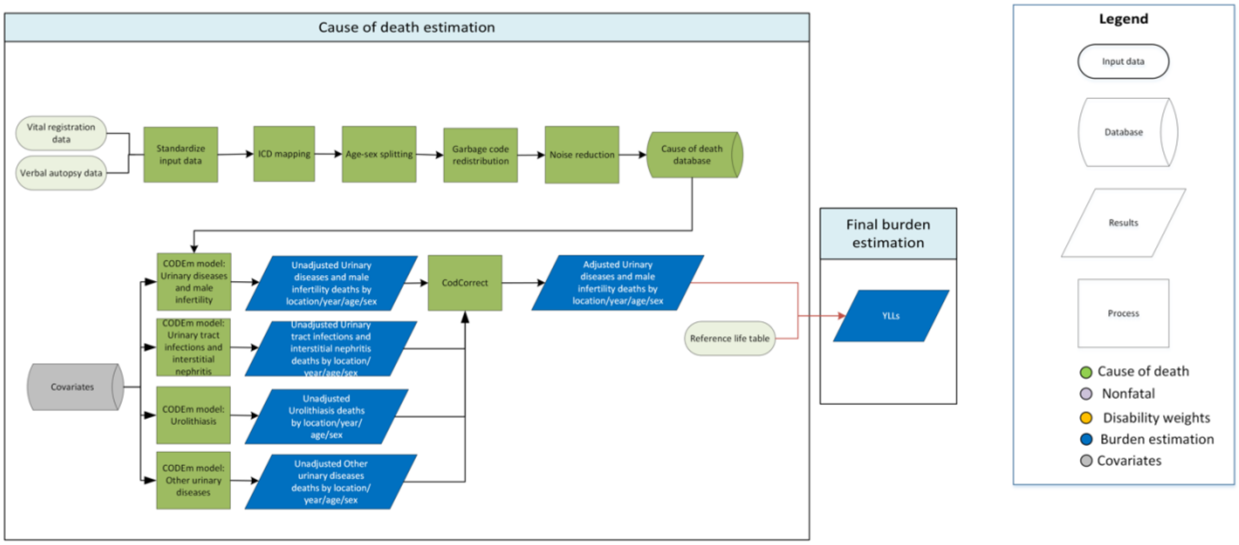

Input data and methodological summary for urinary diseases and male infertility

Urinary diseases and male infertility comprise a Level 3 cause in the GBD hierarchy, with five Level 4 causes, three of which contribute to mortality: urinary tract infection and interstitial nephritis, urolithiasis, and other urinary diseases. (Other Level 4 causes that contribute to urinary diseases and male infertility only contribute nonfatal burden: benign prostatic hypertrophy and male infertility.)

Input data

Data used to estimate mortality of urinary diseases and male infertility consisted of vital registration data (VR) and verbal autopsy (VA) data from the cause of death (CoD) database. The data in urinary diseases (Level 3) consist of aggregated data from its Level 4 fatal child causes. Of the child causes, urinary tract infection and interstitial nephritis as well as urolithiasis both utilised VR and VA data, while other urinary diseases only utilised VR data. A list of ICD codes that were mapped to these causes, a list of vital registration sources included in the CoD database and their quality as indicated by star-ratings, and the steps used to process these data for modelling can be found in the section of this appendix on the CoD database and the tables and figures it refers to. CoD data processing was broadly similar to previous rounds of GBD 2023, but one novel data processing step was employed in preparing data for urinary diseases and male infertility that was not needed prior to GBD 2023—COVID-19 corrections—which are described in the aforementioned appendix section.

Data exclusions: After all CoD data processing steps were complete, datapoints for all locations and years were systematically reviewed to identify outliers or other data limitations not fully overcome by CoD data processing methods. For urinary diseases and male infertility, the following were identified and excluded prior to modelling:

In Iran and its subnationals, older data were excluded where discontinuities between different coding systems resulted in implausible time trends. Kiribati data were excluded due to inadequate population

coverage. In Kazakhstan, ICD-10 data were excluded due to an issue with death assignment in that code system and country. In some Ethiopian subnationals, District Health Information Software 2 (DHIS2) data were excluded in favor of Ethiopia's VA data to avoid compositional bias and maintain the source believed to have more correct assignment of underlying cause of death, since the VA data were more similar to all other VA and VR data in the region. For India and its subnationals, data from India Medical Certification of Cause of Death State-Level Tabulations were excluded in favor of the India Sample Registration System data, because the latter source covered both urban and rural locations and used a sample that was more representative of both in-hospital and out-of-hospital deaths. In Pakistani subnationals, Nepal, Burkina Faso, Malawi, São Tomé, Indonesian subnationals, Palau, Tonga, and Viet Nam, data were excluded because having only one year of data from a small population, even after region-level noise reduction, made it difficult to distinguish true extreme values from sampling error that was insufficiently addressed with current noise-reduction algorithms.

Modelling strategy

The estimation strategy used for fatal urinary diseases and male infertility is largely similar to methods used in GBD 2021; no substantive changes to modelling were made for GBD 2023. A standard CODEm model was used (see appendix section on CODEm method for details). Models were generated separately for males in data-rich countries, males in all countries (referred to as a global model), females in data-rich countries, and females in all countries (global model). Fatal estimation for this cause was restricted to ages 0 days to 95+ years. We hybridised separate global and data-rich models to acquire unadjusted results. The following table shows the covariates offered to the CODEm model for testing and selection for all models.

Table 1. Covariates offered for selection in urinary diseases and male infertility mortality modelling

Level	Covariate	Direction
2	Temperature (90th percentile)	+
	Sanitation (proportion with access)	+
	Mean BMI	+
	Healthcare Access and Quality (HAQ) Index	-
3	Socio-demographic Index (SDI)	-
	Education (years per capita)	-
	Log lag-distributed income (LDI) ($I per capita)	-

In the ensemble models selected by the CODEm algorithm based on out-of-sample predictive validity, HAQ Index, mean BMI, and sanitation were chosen for the female global model. Education, HAQ Index, mean BMI, and sanitation were chosen for the male global model. All covariates were chosen for the female data-rich and male data-rich models.

We rescaled deaths due to urinary diseases from CODEm alongside other Level 3 causes of death, according to the GBD cause hierarchy, using CoDCorrect to ensure ultimately all cause-specific deaths summed to all-cause mortality for every year, age, sex and location. Adjusted urinary disease deaths were compared to the reference life table to calculate final YLLs due to urinary diseases and male infertility.

Input data and methodological summary for urinary tract infections and interstitial nephritis

Urinary tract infections and interstitial nephritis comprise a Level 4 cause in the GBD hierarchy.

Flowchart

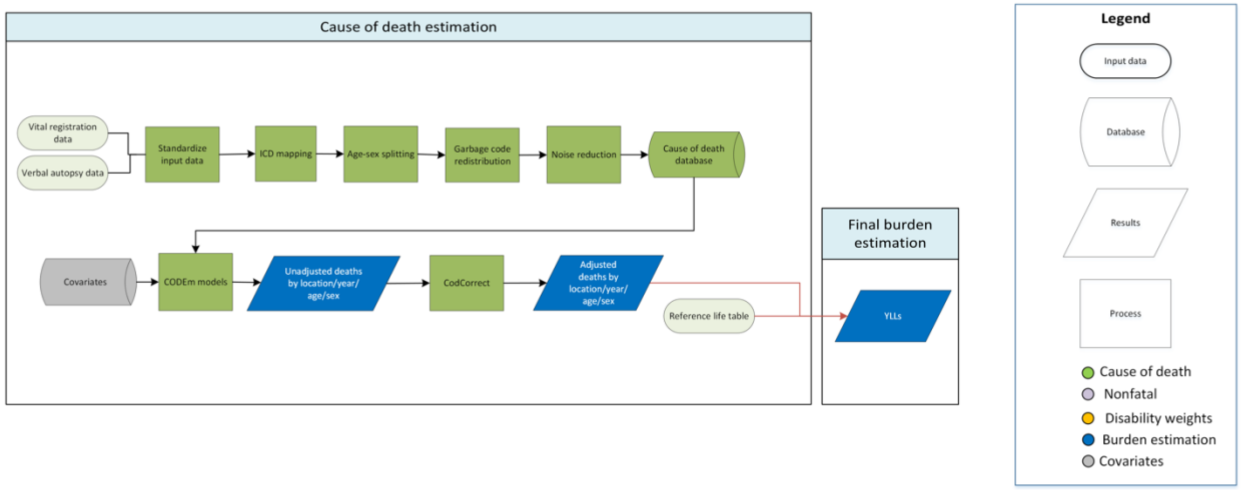

Input data

The same data and exclusion strategies were applied to urinary tract infections and interstitial nephritis as for its parent cause, urinary diseases and male infertility, described above, with the following differences: Tonga and São Tomé and Príncipe had no data for this cause. Kenya and Tanzania only had one year of VA data instead of multiple as in the parent, so these single years of data were excluded under the same principle of having only one year of data from a small population, even after region-level noise reduction, made it difficult to distinguish true extreme values from sampling error that was insufficiently addressed with current noise-reduction algorithms.

Modelling strategy

The same modelling strategy was utilised for urinary tract infections and interstitial nephritis as for its parent cause, urinary diseases and male infertility, described above. No substantive changes were made in GBD 2023 from the modelling strategy used in GBD 2021. Fatal estimation for this cause was restricted to ages 0 days to 95+ years.

Table 2. Covariates offered for selection in urinary tract infections and interstitial nephritis mortality modelling

Level	Covariate	Direction
1	Sanitation (proportion with access)	+
	Education (years per capita)	+
2	Log lag-distributed income (LDI) ($I per capita)	-
	Healthcare Access and Quality (HAQ) Index	-
3	Socio-demographic Index (SDI)	-

In the ensemble models selected by the CODEm algorithm based on out-of-sample predictive validity, LDI per capita and HAQ Index were chosen for the female global model. LDI per capita, education, HAQ

Index, and SDI were chosen for the male global model. Sanitation, HAQ Index, and SDI were chosen for the female data-rich model, and all covariates were chosen for the male data-rich model.

Adjustment in CoDCorrect included fitting unadjusted death estimates for urinary tract infection and interstitial nephritis, urolithiasis, and other urinary diseases to overall urinary disease deaths, which were then adjusted with all other causes to sum to all-cause counts of death.

Input data and methodological summary for urolithiasis

Urolithiasis is a Level 4 cause in the GBD hierarchy.

Flowchart

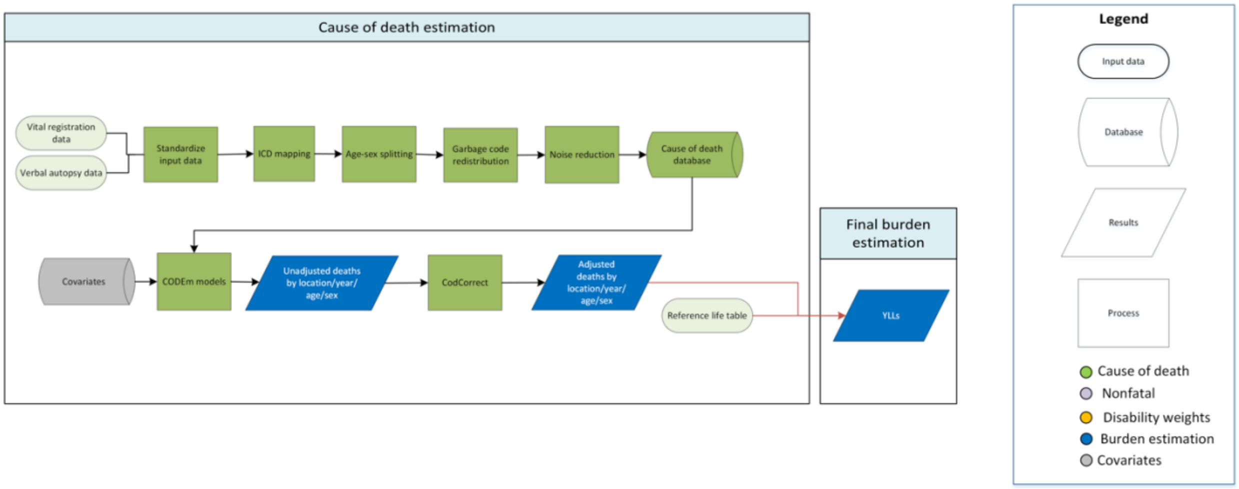

Input data

The same data and exclusion strategies were applied to urolithiasis as for its parent cause, urinary diseases and male infertility, described above, with the following differences: Tonga, Burkina Faso, and São Tomé and Príncipe had no data for this cause. Azerbaijan, Belarus, Syria, Kenya, and Zambia only had one year of data instead of multiple as in the parent, so these single years of data were excluded under the same principle of having only one year of data from a small population, even after region-level noise reduction, made it difficult to distinguish true extreme values from sampling error that was insufficiently addressed with current noise-reduction algorithms.

Modelling strategy

The same modelling strategy was utilised for urolithiasis as for its parent cause, urinary diseases and male infertility, with the exception of age restrictions. Fatal estimation for this cause was restricted to ages 12 months to 95+ years. No substantive changes were made in GBD 2023 from the modelling strategy used in GBD 2021.

Table 3. Covariates offered for selection in urolithiasis mortality modelling

Level	Covariate	Direction
1	Temperature (90th percentile)	+
	Red meat consumption (unadjusted, kcal per capita)	+

	Fruit consumption (unadjusted, kcal per capita)	-
2	Vegetable consumption (unadjusted, kcal per capita)	-
	Healthcare Access and Quality Index	-
	Socio-demographic Index (SDI)	-
3	Education (years per capita)	-
	Log lag distributed income (LDI) ($I per capita)	-

In the ensemble models selected by the CODEm algorithm based on out-of-sample predictive validity, temperature was chosen for the female global model. LDI per capita, HAQ Index, and SDI were chosen for the male global model. Temperature, red meat consumption, fruit consumption, HAQ Index, LDI per capita, and SDI were chosen for the female data-rich model. Temperature, red meat consumption, fruit consumption, vegetable consumption, SDI, and education were chosen for the male data-rich model.

Adjustment in CoDCorrect included fitting unadjusted death estimates for urolithiasis alongside urinary tract infection and interstitial nephritis and other urinary diseases to sum to overall urinary disease deaths, which were then adjusted with all other causes to sum to all-cause counts of death.

Input data and methodological summary for other urinary diseases

Other urinary diseases comprise a Level 4 cause in the GBD hierarchy.

Flowchart

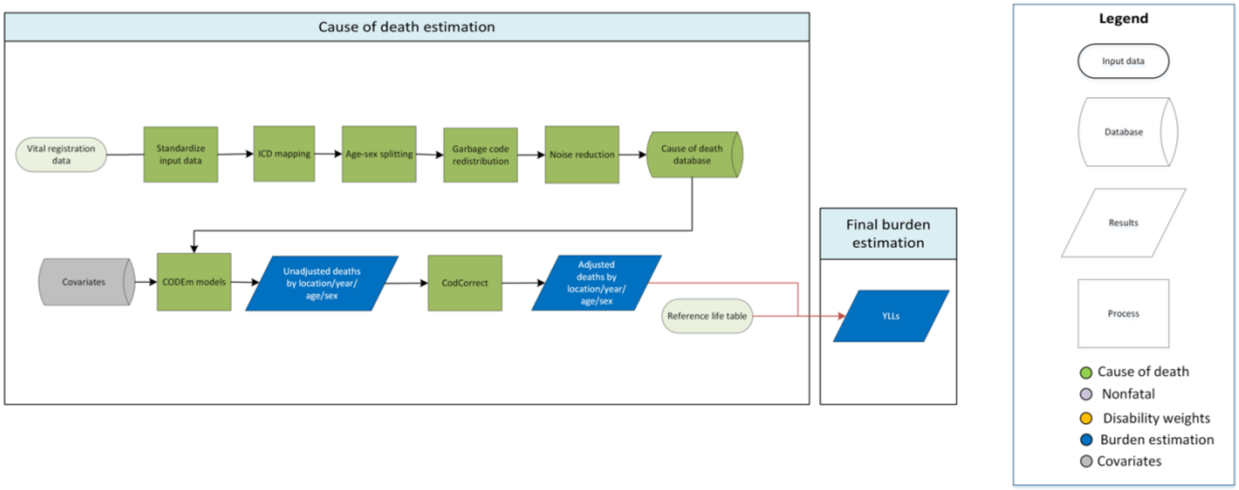

Input data

The same data and exclusion strategies were applied to other urinary diseases as for its parent cause, urinary diseases and male infertility, described above, with the following differences: Viet Nam, Tonga, Pakistan, Nepal, Malawi, Burkina Faso, and São Tomé and Príncipe had no data for this cause. Azerbaijan, Belarus, and Syria only had one year of data instead of multiple as in the parent, so these single years of data were excluded under the same principle of having only one year of data from a small population, even after region-level noise reduction, made it difficult to distinguish true extreme values from sampling error that was insufficiently addressed with current noise-reduction algorithms.

Modelling strategy

The same modelling strategy was utilised for other urinary diseases as for its parent cause, urinary diseases and male infertility, described above. No substantive changes were made in GBD 2023 from the modelling strategy used in GBD 2021. Fatal estimation for this cause was restricted to ages 0 days to 95+ years.

Table 4. Covariates offered for selection in other urinary diseases mortality modelling

Level	Covariate	Direction
1	Mean BMI	+
2	Education (years per capita)	-
	Log lag-distributed income (LDI) ($I per capita)	-
	Healthcare Access and Quality (HAQ) Index	-
3	Socio-demographic Index (SDI)	-

In the ensemble models selected by the CODEm algorithm based on out-of-sample predictive validity, education, LDI, HAQ Index, and SDI were chosen for the female global and male global models. Education, LDI, and SDI were chosen for the female data-rich model, and HAQ Index was chosen for the male data-rich model.

Adjustment in CoDCorrect included fitting unadjusted death estimates for other urinary diseases alongside urinary tract infections and interstitial nephritis and urolithiasis to sum to overall urinary disease deaths, which were then adjusted with all other causes to sum to all-cause counts of death.

Varicella and herpes zoster

Flowchart

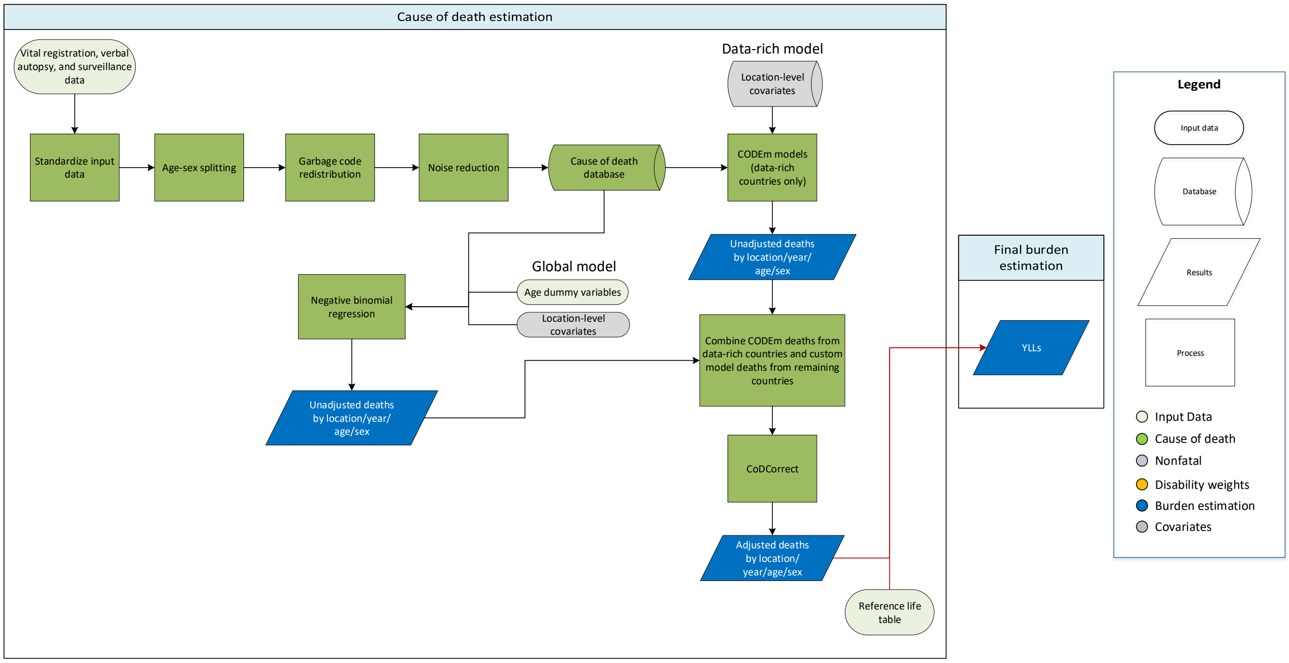

Input data and methodological summary for varicella and herpes zoster

Input data

Varicella and herpes zoster cause of death (CoD) data for GBD 2023 included vital registration, verbal autopsy, and surveillance sources from all locations as available. Cause of death data used in modelling include both primary varicella and herpes zoster–related diagnostic codes. We excluded CoD data if they were highly incongruent with other available data from the same location or locations of similar sociodemographic characteristics.

Modelling strategy

The modelling strategy for varicella and herpes zoster did not change significantly since GBD 2021. We used two distinct methods to estimate varicella and herpes zoster mortality based on the quality of cause of death registration systems in each country. For countries with well-defined cause of death registration systems we used a rate-based Cause of Death Ensemble modelling strategy (CODEm). For the remaining countries, we used a count-based negative binomial regression model. Each approach is described in further detail below.

Data-rich countries

For data-rich countries, the covariates listed in Table 1 were used to inform CODEm predictions.

Table 1. Covariates. Summary of covariates used in the data-rich varicella and herpes zoster cause of death model

Level	Covariate	Direction
1	Healthcare Access and Quality (HAQ) Index	-
	Age- and sex-specific SEV for child underweight	+
	Age- and sex-specific SEV for child wasting	+
3	Lag-distributed income (LDI)	-
	Mean years of education per capita	-
	Sanitation access (proportion)	-
	Population density over 1000 people per square kilometer (proportion)	+
	Socio-demographic Index (SDI)	-

Custom count model

Our custom counts mortality model for non-data-rich locations fits a negative binomial regression to available varicella and herpes zoster CoD data. We modelled counts of deaths using the Healthcare Access and Quality (HAQ) Index and age dummy variables with location-, year-, age-, and sex-specific population as the offset:

$$Y_{ij} = \beta_0 + \beta_1\, HAQ_{ij} + age_{a\,ij} + e_{ij}$$

where Y_{ij} is the log-transformed number of varicella and herpes zoster deaths offset by population size; β_0 is the fixed-effect intercept; β_1 is the fixed-effects slope on location- and year-specific HAQ_{ij}; $age_{a\,ij}$ is a dummy variable for each GBD age group in the estimation; e_{ij} is the residual; *i* is the year; and *j* is the location. We excluded studies that report a varicella and herpes zoster–specific cause fraction (the fraction of deaths due to varicella and herpes zoster) higher than the 99th percentile of all reported cause fractions. Uncertainty was estimated by taking 1000 samples from the variance-covariance matrix and a random sample of the dispersion parameter from a gamma distribution.

Changes from GBD 2021 to GBD 2023

There were no substantive changes to the modelling strategy for GBD 2023.

Vascular intestinal disorders

Flowchart

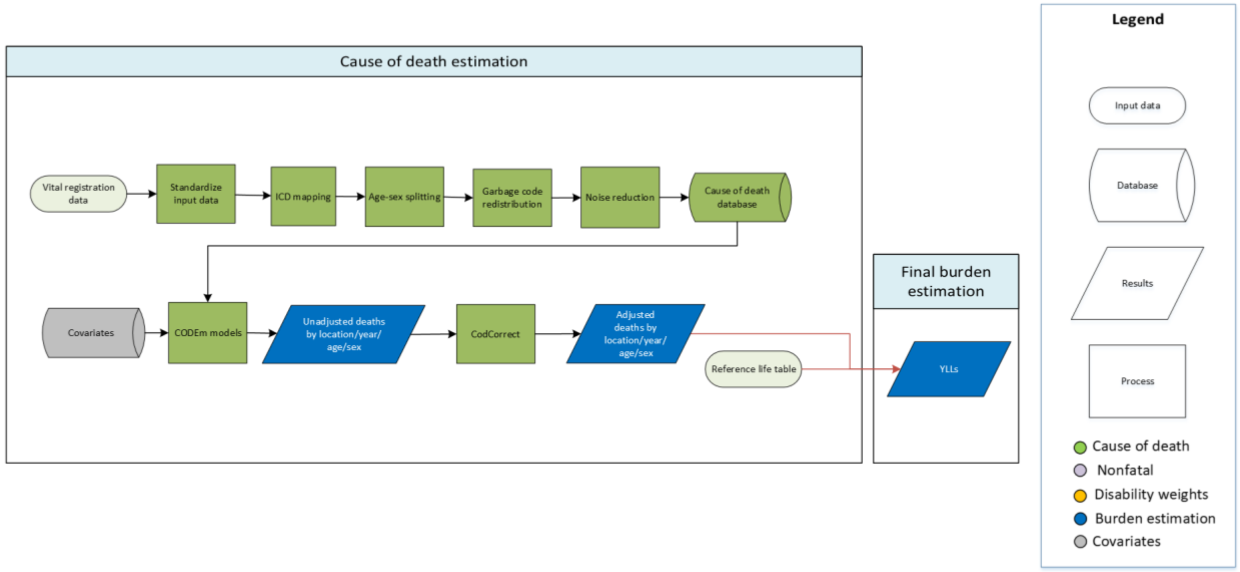

Input data and methodological summary for vascular intestinal disorders

Vascular intestinal disorders comprise a Level 3 cause in the GBD hierarchy.

Input data

Data used to estimate mortality of vascular intestinal disorders consisted of vital registration (VR) from the cause of death (CoD) database. A list of ICD codes that were mapped to this cause, a list of vital registration sources included in the CoD database and their quality as indicated by star-ratings, and the steps used to process these data for modelling can be found in the section of this appendix on the CoD database and the tables and figures it refers to. CoD data processing was broadly similar to previous rounds of GBD, but one novel data processing step was employed in preparing data for vascular intestinal disorders—COVID-19 corrections—which are described elsewhere in this appendix.

After all data processing steps were complete, datapoints for all locations and years were systematically reviewed to identify outliers or other data limitations not fully overcome by CoD data processing methods, and selected sources were excluded from analysis.

Modelling strategy

The strategy used to estimate mortality due to vascular intestinal disorders was not substantively changed between GBD 2021 and GBD 2023. A standard CODEm model was used to model deaths due to vascular intestinal disorders (see appendix section on CODEm method for details). Models were generated separately for males in data-rich countries, males in all countries (referred to as a global model), females in data-rich countries, and females in all countries (global model). Fatal estimation for this cause was restricted to ages 2–95+ years.

The following table has the full list of covariates presented to the CODEm algorithm for selection in models of vascular intestinal disorders mortality.

Table 1. Covariates used in vascular intestinal disorders mortality modelling

Level	Covariate	Direction
1	Fasting plasma glucose	+
	Cholesterol (total, mean per capita)	+
	Systolic blood pressures (mmHg)	+
2	BMI (mean)	+
	Smoking prevalence	+
	Healthcare Access and Quality Index	-
3	Socio-demographic Index	-
	Education (years per capita)	-
	Log LDI ($I per capita)	-
	Pulses/legumes (kcal/capita, adjusted)	-
	Age-sex-specific scaled exposure variable for low fruit consumption	+
	Age-sex-specific scaled exposure variable for low vegetable consumption	+
	Age-sex-specific scaled exposure variable for high red meat consumption	+
	Age-sex-specific scaled exposure variable for low fish consumption	+
	Age-sex-specific scaled exposure variable for low nut consumption	+
	Consumption of high trans-unsaturated fatty acids	+
	Alcohol (litres per capita)	+

We hybridised separate global and data-rich models to acquire unadjusted results, which we adjusted using CoDCorrect. Adjustment in CoDCorrect included rescaling unadjusted vascular intestinal disorder death estimates alongside other Level 3 digestive disease deaths to sum to overall digestive disease deaths, which were then rescaled alongside other causes to sum up the hierarchy to all-cause counts of death. Adjusted deaths were compared to the reference life table to calculate final YLLs due to vascular intestinal disorders.

Visceral leishmaniasis

Flowchart

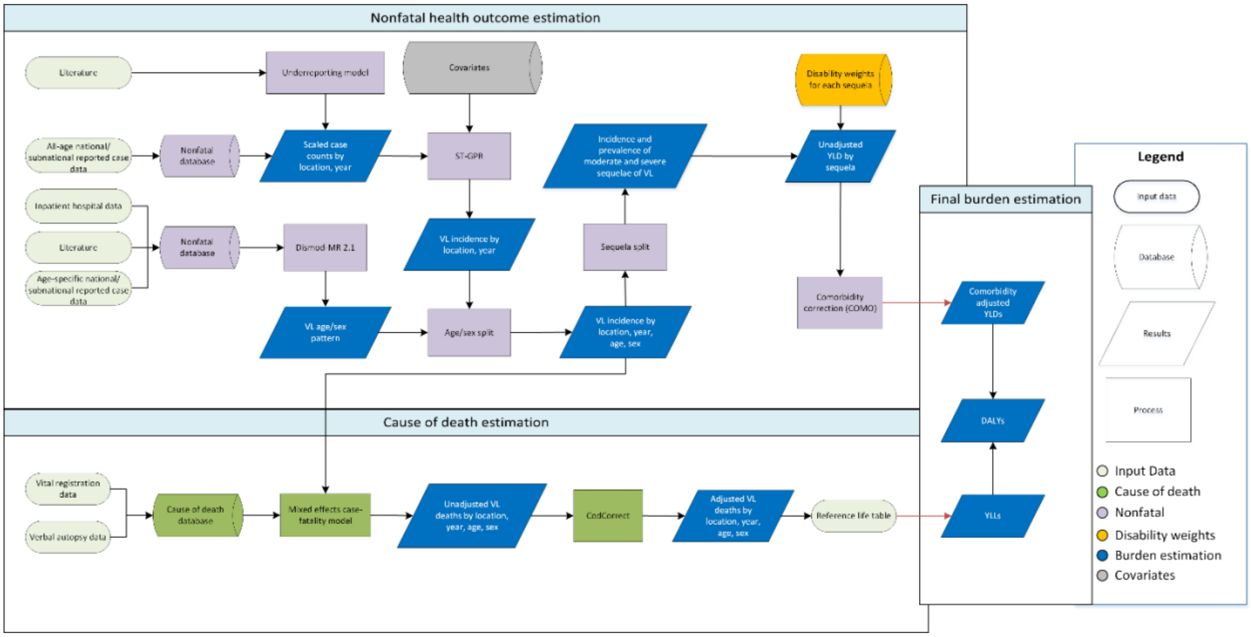

Input data and methodological summary for visceral leishmaniasis

Input data

Deaths were extracted from a variety of sources, ranging from vital registration (VR) records to verbal autopsy (VA) assessments. Deaths assigned to visceral leishmaniasis (VL) were processed following central cause of death processing, outlined elsewhere in this methods appendix.

Modelling strategy

The fatal estimation process for visceral leishmaniasis builds from incident case notification data, described in more detail in another section of this appendix. Briefly, incident case data that are representative of the GBD geographical location are adjusted for under-reporting. The upscaled all-age, both-sex case counts are modelled using spatiotemporal Gaussian process regression (ST-GPR) to impute for missing location-year combinations as well as to account for further biases and inaccuracies in reporting. Datasets that disaggregate VL cases by age and sex are modelled using DisMod-MR to produce a global age-sex split, which is applied to the all-age, both-sex envelope estimates resulting from ST-GPR. The mean incidence estimates are compared with estimated death counts to generate a case-fatality rate model that is subsequently used to estimate deaths for each age, sex, location, and year.

Deaths were modelled using a mixed effect model parameterising case-fatality rate, with data derived from taking attributed-death data and dividing it by the mean predicted incident cases.

$$Logit(Case\ Fatality\ Rate) \sim Age + Sex + (Age|Super\ Region/Region)$$

Only data from countries defined as present or protocol present were used, as these represent locations that are generalisable to all endemic regions for VL. The deaths in non-endemic countries, while not used in the case-fatality rate model, are subsequently added back into the death envelope as-is by central computation. For African and European countries as well as South Sudan from 1990–1994, we assumed custom case-fatality rates. These assumptions were more consistent with external literature of visceral leishmaniasis case-fatality rates. For African case-fatality rates, 1000 draws were taken from a uniform distribution between 0.10 and 0.30 (Alvar and colleagues 2012, Martins-Melo and colleagues 2014).[1,2] For European case-fatality rates, including endemic Italian subnationals, we drew 1000 draws from a uniform distribution between 0.06 and 0.10,[2] and we assumed a 0.69 case-fatality rate for South Sudan between the years of 1990 and 1994, based on data reported during the VL epidemic from the late 1980s to 1994.[3]

Case-fatality rate estimates had high uncertainty in some geographies. In general, female mean case-fatality rates were higher than male case-fatality rates. Typically, an all-age estimate of 10% case-fatality rate is discussed when looking at visceral leishmaniasis.[1] Final fatal estimates of VL were calculated as the product of incident case estimates and case fatality.

Limitations

Known limitations for the VL model will be the focus in future GBD rounds and engagement with collaborators. Given the focus on location-representative estimates, the existing model is based upon national case counts. This excludes a large resource of published literature and grey literature focused on site-specific surveillance or surveys. In the future, there is a need to identify an independent resource to aid in quantifying the population at risk.

Age-sex patterns are highly reflective of the countries from which data are obtained. Importantly, there is a large skew in information coming from Brazil. This information has potential biases due to the nature of the data inputs (notification and hospital data) and the corresponding age-sex variation in health-seeking behaviours which may not be generalisable to other settings.

Changes from GBD 2021 to GBD 2023

There have been no substantive changes to the modelling strategy for GBD 2023.

References

1. Alvar J, Vélez ID, Bern C, *et al.* Leishmaniasis Worldwide and Global Estimates of Its Incidence. *PLoS One* 2012; **7**. DOI:10.1371/journal.pone.0035671.

2. Martins-Melo FR, Lima M da S, Ramos AN, Alencar CH, Heukelbach J. Mortality and case fatality due to visceral leishmaniasis in Brazil: a nationwide analysis of epidemiology, trends and spatial patterns. *PLoS One* 2014; **9**: e93770.

3. Seaman J, Mercer AJ, Sondorp E. The epidemic of visceral leishmaniasis in western Upper Nile, southern Sudan: course and impact from 1984 to 1994. *Int J Epidemiol* 1996; **25**: 862–71.

Yellow fever

Flowchart

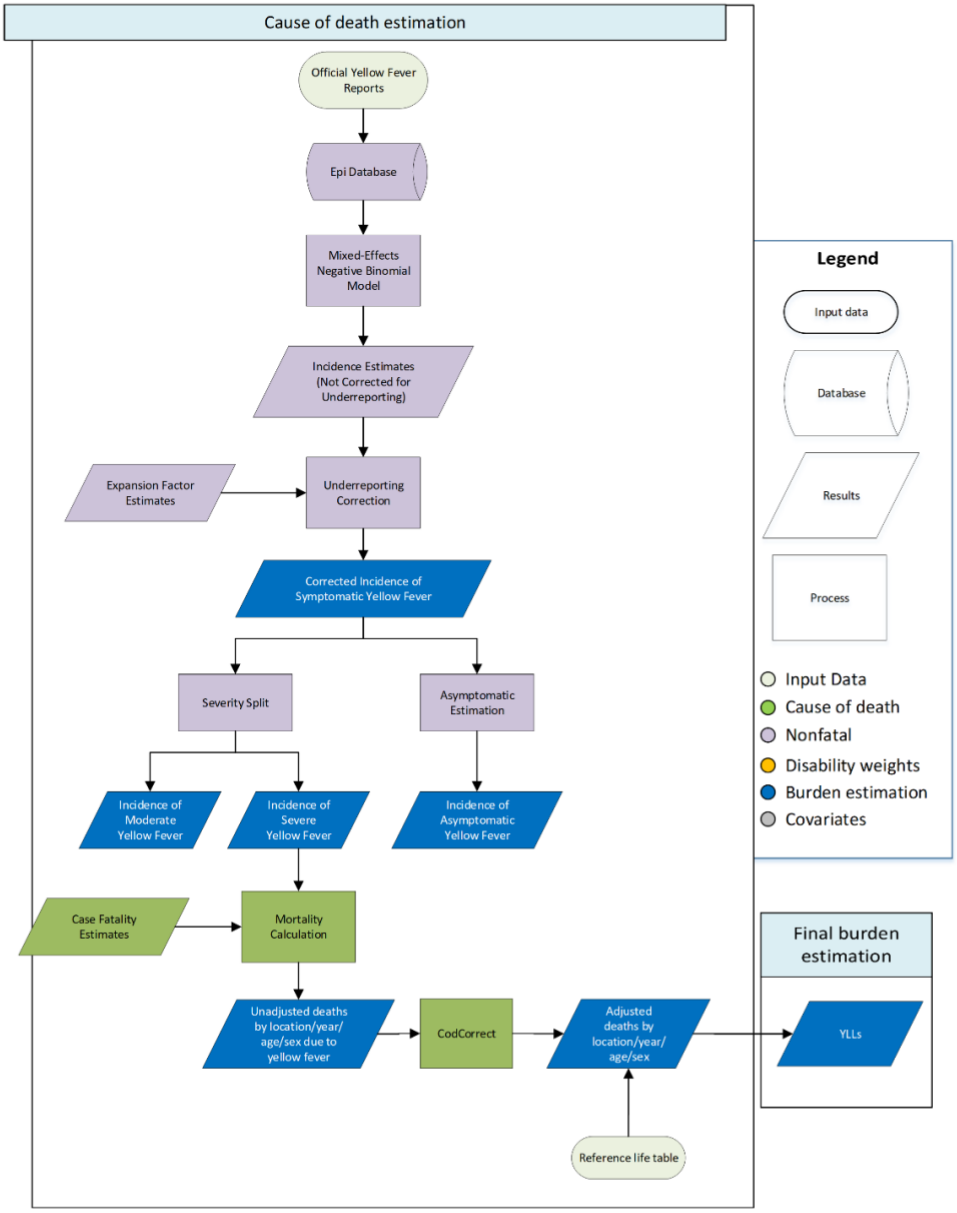

Input data and methodological summary for yellow fever

Input data

Case data come from official case reports filed with the World Health Organization. Data on case fatality come from published studies of yellow fever fatality. Data on deaths in non-endemic countries are restricted to only vital registration data.

Modelling strategy

We model yellow fever deaths using a hybrid approach. For countries in which yellow fever is endemic, we use a natural history approach in which we estimate deaths as the product of severe incident cases and case fatality. For non-endemic countries, we allow for deaths among imported cases where we have vital registration data indicating yellow fever deaths. That is, we assume no yellow fever deaths in non-endemic countries; however, where yellow fever deaths are reported in vital registration data, we accept those as true imported yellow fever deaths.

We model reported cases using a mixed-effects negative binomial model, described in detail elsewhere in this appendix. Based on published estimates, we assume that 27% of symptomatic cases will be severe.[1] We performed a meta-analysis of case fatality using data from published studies of yellow fever fatality (see Table 1 below). Studies tend to report deaths among those with severe infection (eg, hospitalised cases), rather than among all cases. We assume that no deaths occur with asymptomatic infection or among those with only moderate symptoms. With that, we estimate deaths as the product of severe cases and case fatality.

Table 1. Metadata for case-fatality assumptions

Each record lists a citation, GBD location of relevance, year, and output values used in modelling.			
Citation	GBD location	Time period	Case fatality rate<br>(# deaths/# reported cases)
Reiter *et al.* First recorded outbreak of yellow fever in Kenya, 1992–1993[2]	Kenya	1992–1993	5/26
Soghaier *et al*. Yellow Fever outbreak in Darfur, Sudan in October 2012[3]	Sudan	2012	5/7
Thonnon *et al.* Re-emergence of yellow fever in Senegal in 1995[4]	Senegal	1995	15/79
Tuboi *et al.* Clinical and epidemiological characteristics of yellow fever in Brazil: analysis of reported cases 1998–2002[5]	Brazil	1998–2002	11/251
Vasconcelos *et al.* Epidemic of jungle yellow fever in Brazil, 2000: implications of climatic alterations in disease spread[6]	Brazil	2000	39/77
Vasconcelos *et al.* An epidemic of sylvatic yellow fever in the southeast region of Maranhao State, Brazil, 1993–1994: epidemiologic and entomologic findings[7]	Brazil	1993–1994	13/74

Wamala *et al.* Epidemiological and laboratory characterization of a yellow fever outbreak in northern Uganda[8]	Uganda	2010–2011	7/13

We accept deaths reported in vital registration data as true imported deaths. We adjust total death estimates to account for the high case burden observed in the 2017–2018 outbreak in Brazil. We used reported deaths from Brazilian vital registration data from 2017 to derive an age and sex distribution of these deaths, and simulated uncertainty for case totals from a Poisson distribution to inflate modelled death estimates to account for this outbreak.

Changes from GBD 2021 to GBD 2023

There have been no substantive changes to the modelling strategy for GBD 2023.

References

1. Johansson MA, Vasconcelos PFC, Staples JE. The whole iceberg: estimating the incidence of yellow fever virus infection from the number of severe cases. *Trans R Soc Trop Med Hyg* 2014; 108: 482–7.

2. Reiter P, Cordellier R, Ouma JO, *et al.* First recorded outbreak of yellow fever in Kenya, 1992-1993. II. Entomologic investigations. *Am J Trop Med Hyg* 1998; **59**: 650–6.

3. Soghaier MA, Hagar A, Abbas MA, Elmangory MM, Eltahir KM, Sall AA. Yellow Fever outbreak in Darfur, Sudan in October 2012; the initial outbreak investigation report. *J Infect Public Health* 2013; **6**: 370–6.

4. Thonnon J, Fontenille D, Tall A, *et al.* Re-emergence of yellow fever in Senegal in 1995. *Am J Trop Med Hyg* 1998; **59**: 108–14.

5. Tuboi SH, Costa ZGA, da Costa Vasconcelos PF, Hatch D. Clinical and epidemiological characteristics of yellow fever in Brazil: analysis of reported cases 1998-2002. *Trans R Soc Trop Med Hyg* 2007; **101**: 169–75.

6. Vasconcelos PF, Costa ZG, Travassos Da Rosa ES, *et al.* Epidemic of jungle yellow fever in Brazil, 2000: implications of climatic alterations in disease spread. *J Med Virol* 2001; **65**: 598–604.

7. Vasconcelos PF, Rodrigues SG, Degallier N, *et al.* An epidemic of sylvatic yellow fever in the southeast region of Maranhao State, Brazil, 1993-1994: epidemiologic and entomologic findings. *Am J Trop Med Hyg* 1997; **57**: 132–7.

8. Wamala JF, Malimbo M, Okot CL, *et al.* Epidemiological and laboratory characterization of a yellow fever outbreak in northern Uganda, October 2010-January 2011. *Int J Infect Dis* 2012; **16**: e536-542.

Zika virus disease

Flowchart

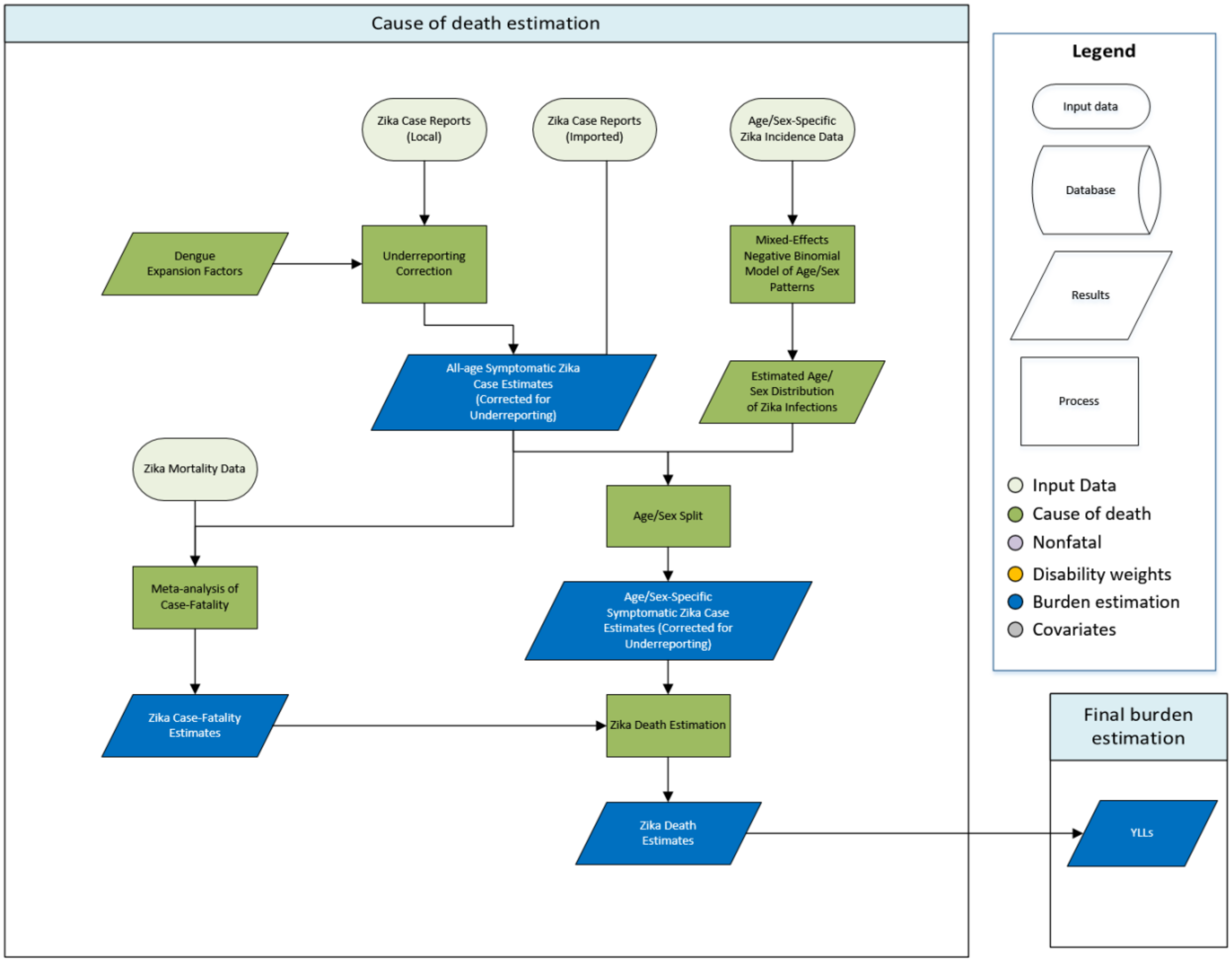

Input data

The mortality data used for estimating deaths attributable to Zika virus are from official reports, primarily from the Pan American Health Organization (PAHO), in which deaths attributed to Zika virus infection were reported for the period 2015–2018. Overall, a total of 22 deaths were reported in Brazil, Suriname, and Puerto Rico during this period. Of these cases, the majority were among adult males. Incidence data used in the model are from the Zika symptomatic incidence case estimates for GBD 2023.

Modelling strategy

We modelled Zika deaths using a global case-fatality rate (CFR) model. The numerator was the all-age and all-sex death totals reported in the period from all endemic locations. The denominator was the all-age and all-sex symptomatic Zika incidence case counts from 2015 to 2017 (years for which reported deaths were available) from all endemic locations. We used a binomial distribution to generate 1000

draws of a global, all-age, all-sex CFR. Then, we multiplied the CFR draws by draws of age-specific and sex-specific symptomatic Zika incidence case counts, by location and year, to generate draws of fatal case estimates, by age, sex, location, and year. For locations and years where the reported number of deaths was higher than the estimated value from this approach, we used a location-specific and year-specific CFR, rather than the global CFR. For these location-years, reported deaths came from either the Pan American Health Organization or Brazil's Ministry of Health, and incidence rate came from the non-fatal Zika model, which is described elsewhere.

Limitations

We did not apply any adjustments for the COVID-19 pandemic to Zika virus disease due to a lack of available data quantifying the impacts of the pandemic on NTD epidemiology.

Changes from GBD 2021 to GBD 2023

There have been no substantive changes to the modelling strategy for GBD 2023.

*UN Convention lists Taiwan as a Province of China
