# Supplementary material for: Global burden of 292 causes of death in 204 countries and territories and 660 subnational locations, 1990–2023: a systematic analysis for the Global Burden of Disease Study 2023
Source: Lancet. 2025 Oct 18;406(10513):1811–72. doi: 10.1016/S0140-6736(25)01917-8 (PMC12535838; doi:10.1016/S0140-6736(25)01917-8)

Appendix 2: supplementary results to “Global burden of 292 causes of death in 204 countries and territories and 660 subnational locations, 1990–2023: a systematic analysis for the Global Burden of Disease Study 2023”

This supplement provides additional figures containing more detailed results for “Global burden of 292 causes of death in 204 countries and territories and 660 subnational locations, 1990–2023: a systematic analysis for the Global Burden of Disease Study 2023”

All supplementary tables can be accessed here https://ghdx.healthdata.org/record/ihme-data/gbd-2023-cause-specific-mortality-1990-2023

List of supplementary results tables and figures

Tables

Table S1. Total number of site years by cause and source type for GBD2023.

Table S2. Reported number of Covid deaths before and after Covid misclassification correction.

Table S3. Top causes containing misclassified COVID-19 (reported) by location, 2020-2022.

Table S4. Global, all age, both sex YLLs for preventable diseases from 1990 to 2023.

Table S5. All age, both sex deaths and mortality rates due to COVID-19 from 2020-2023.

Table S6. Probability of death before age 70, and mean age of death for the top 50 causes, all locations.

Table S7. Age-Standardised mortality rate per 100,000 and all-age deaths by location and cause (both sexes).

Table S8. Age-standardised mortality rate per 100,000 and all-age deaths by location and cause (males).

Table S9. Age-standardised mortality rate per 100,00 and all-age deaths by location and cause (females).

Table S10. Age-standardised rate of YLLs per 100,000 and all-age YLLs by location and cause (both-sex).

Table S11. All cause probability of death before age 70 by location and sex.

Table S12. Global, top 100 causes by age-standardised death rate per 100,000: 1990, 2019, 2021, 2023, and the percent change between years.

Table S13. Global, top 100 causes by age-standardised YLLs per 100,000: 1990, 2019, 2021, 2023, and the percent change between years.

Table S14. Mean age of death correlation with SDI by cause-sex.

Table S15. Mean age of death by cause and location (both).

Table S16. Mean age of death by cause and location (females).

Table S17. Mean age of death by cause and location (males).

Table S18. Percent of global conflict and terror deaths by region-sex from 1995 to 2023.

Table S19. Percent change in age specific mortality rates between 2000 and 2023 for all causes by region.

Figures

Figure S1. Percent change of mortality rate and age-standardised mortality rate from previous year. 3

Figure S2. Leading Level 3 causes of global age-standardised YLL rate per 100,000 for 1990, 2019, 2021, and 2023 for males and females combined. 4

Figure S3. Global, all age, both sex COVID-19 deaths by year and age....5

Figure S4. Comparison of mean age of death for chronic kidney disease, stroke, tracheal, bronchus, and lung cancer, and tuberculosis between 4 GBD regions, both sexes. 6

Figure S5. Cardiovascular disease age-standardised death rates, age-standardised rate of YLLs, and 70q0 by super-region....10

Figure S6. Level 1 causes: Net percent change in probability of death before 70 between 2000 and 2023 12

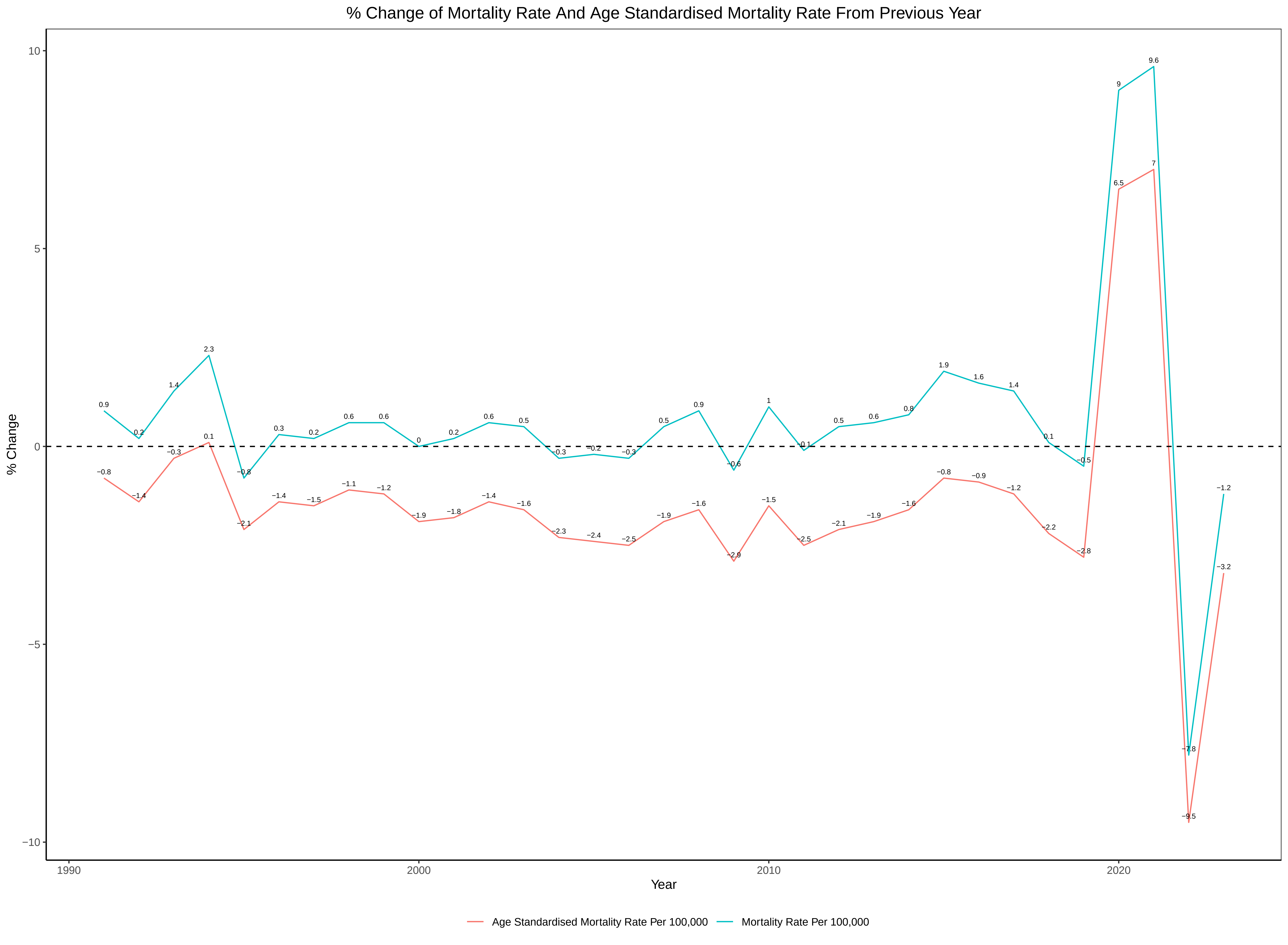

% Change of Mortality Rate And Age Standardised Mortality Rate From Previous Year
% Change
Year
1990
2000
2010
2020
10
5
0
−5
−10
Age Standardised Mortality Rate Per 100,000
Mortality Rate Per 100,000

Leading Level 3 causes of global age-standardised YLL rate per 100,000 for 1990, 2019, 2021, and 2023 for males and females combined

Leading causes 1990	Age-standardized rate of YLLs (per 100,000) 1990
1 Neonatal disorders	4487.8 (4212.3 to 4761.6)
2 Diarrheal diseases	3668.6 (2957.7 to 4542.2)
3 Lower respiratory infect	3458.1 (3002.3 to 3957.1)
4 Stroke	3150.6 (2789.8 to 3463.4)
5 Ischemic heart disease	3131.9 (2892.4 to 3381.5)
6 Tuberculosis	1610.4 (1232.2 to 2088.5)
7 Measles	1566.4 (675.6 to 2630.3)
8 Road injuries	1225.7 (971.1 to 1458.1)
9 Malaria	1041.9 (524.4 to 1864.6)
10 Congenital defects	1011.4 (819.9 to 1252.9)
11 COPD	979.5 (765.2 to 1131.2)
12 Cirrhosis liver	769.7 (655.0 to 889.3)
13 Lung cancer	690.1 (649.0 to 764.6)
14 Self-harm	626.2 (534.7 to 705.9)
15 Protein-energy malnutrition	623.7 (489.1 to 811.5)
16 Drowning	612.9 (499.0 to 746.4)
17 Meningitis	599.3 (451.8 to 752.2)
18 Stomach cancer	586.1 (509.5 to 657.8)
19 Maternal disorders	456.3 (399.9 to 532.4)
20 Typhoid & paratyph	430.4 (222.2 to 707.7)
21 Diabetes	414.3 (358.4 to 477.6)
22 Chronic kidney disease	413.6 (351.4 to 472.9)
23 Interpersonal violence	407.2 (335.8 to 471.2)
25 Hypertensive heart disease	399.4 (292.9 to 494.1)
28 HIV/AIDS	375.6 (339.8 to 419.2)
29 Colorectal cancer	353.4 (330.4 to 375.5)
31 Alzheimer's disease	303.5 (74.9 to 779.8)

Leading causes 2019	Age-standardized rate of YLLs (per 100,000) 2019
1 Neonatal disorders	2487.9 (2351.4 to 2618.7)
2 Ischemic heart disease	2030.4 (1885.6 to 2176.7)
3 Stroke	1636.2 (1506.9 to 1799.7)
4 Lower respiratory infect	1503.4 (1323.5 to 1709.6)
5 Diarrheal diseases	852.2 (656.1 to 1079.1)
6 Road injuries	807.9 (638.2 to 940.1)
7 Congenital defects	714.9 (575.5 to 884.4)
8 Malaria	680.6 (298.4 to 1180.3)
9 COPD	670.6 (585.4 to 791.2)
10 HIV/AIDS	572.3 (517.2 to 645.1)
11 Tuberculosis	538.0 (436.9 to 691.5)
12 Cirrhosis liver	504.7 (447.5 to 568.6)
13 Lung cancer	494.2 (453.2 to 533.5)
14 Diabetes	488.7 (426.0 to 551.4)
15 Self-harm	412.8 (364.4 to 467.2)
16 Chronic kidney disease	391.2 (346.0 to 440.3)
17 Alzheimer's disease	311.1 (77.8 to 768.4)
18 Interpersonal violence	306.0 (275.2 to 348.8)
19 Hypertensive heart disease	294.8 (231.6 to 354.1)
20 Colorectal cancer	266.9 (247.6 to 288.7)
21 Meningitis	259.7 (200.8 to 333.5)
22 Stomach cancer	245.9 (220.4 to 285.1)
24 Drowning	241.4 (194.1 to 300.9)
26 Maternal disorders	200.9 (175.1 to 229.2)
27 Protein-energy malnutrition	176.7 (137.0 to 225.6)
29 Measles	160.4 (69.8 to 285.3)
39 Typhoid & paratyph	102.7 (53.9 to 166.6)

Leading causes 2021	Age-standardized rate of YLLs (per 100,000) 2021
1 COVID-19	2983.1 (2884.7 to 3069.0)
2 Neonatal disorders	2450.7 (2298.0 to 2596.9)
3 Ischemic heart disease	2087.7 (1927.1 to 2232.0)
4 Stroke	1630.9 (1486.1 to 1793.5)
5 Lower respiratory infect	1167.2 (1017.0 to 1354.5)
6 Road injuries	836.1 (649.5 to 981.7)
7 Diarrheal diseases	790.5 (596.8 to 1026.3)
8 Congenital defects	763.6 (597.5 to 935.3)
9 Malaria	759.8 (320.7 to 1341.3)
10 COPD	594.7 (500.7 to 706.0)
11 HIV/AIDS	521.7 (460.4 to 595.0)
12 Diabetes	517.8 (451.6 to 591.6)
13 Lung cancer	499.9 (456.6 to 543.4)
14 Cirrhosis liver	494.2 (429.9 to 555.8)
15 Tuberculosis	483.3 (394.6 to 615.8)
16 Self-harm	418.9 (364.9 to 466.3)
17 Chronic kidney disease	405.3 (354.4 to 457.6)
18 Interpersonal violence	316.6 (280.1 to 363.4)
19 Alzheimer's disease	313.5 (78.1 to 779.4)
20 Hypertensive heart disease	305.2 (240.2 to 374.8)
21 Colorectal cancer	272.5 (252.7 to 296.8)

Leading causes 2023	Age-standardized rate of YLLs (per 100,000) 2023
1 Neonatal disorders	2398.9 (2219.7 to 2575.4)
2 Ischemic heart disease	2020.0 (1852.7 to 2212.8)
3 Stroke	1552.5 (1381.1 to 1713.2)
4 Lower respiratory infect	1391.9 (1215.8 to 1601.7)
5 Road injuries	826.9 (636.8 to 987.2)
6 Congenital defects	753.7 (586.8 to 956.6)
7 Malaria	732.1 (280.5 to 1358.1)
8 Diarrheal diseases	706.0 (531.1 to 931.4)
9 COPD	659.7 (563.7 to 788.5)
10 Diabetes	506.2 (426.9 to 586.5)
11 Lung cancer	499.5 (453.8 to 544.3)
12 HIV/AIDS	497.8 (439.2 to 568.8)
13 Cirrhosis liver	473.2 (413.0 to 535.6)
14 Tuberculosis	455.2 (349.3 to 570.1)
15 Self-harm	413.9 (358.2 to 465.7)
16 Chronic kidney disease	409.4 (351.1 to 466.8)
17 Alzheimer's disease	315.8 (78.8 to 771.3)
18 Hypertensive heart disease	303.6 (241.5 to 380.2)
19 Interpersonal violence	302.4 (264.5 to 358.0)
20 Colorectal cancer	275.5 (250.7 to 300.3)
25 COVID-19	233.3 (219.0 to 244.0)

Legend:
Communicable, maternal, neonatal and nutritional
Non-communicable
Injuries

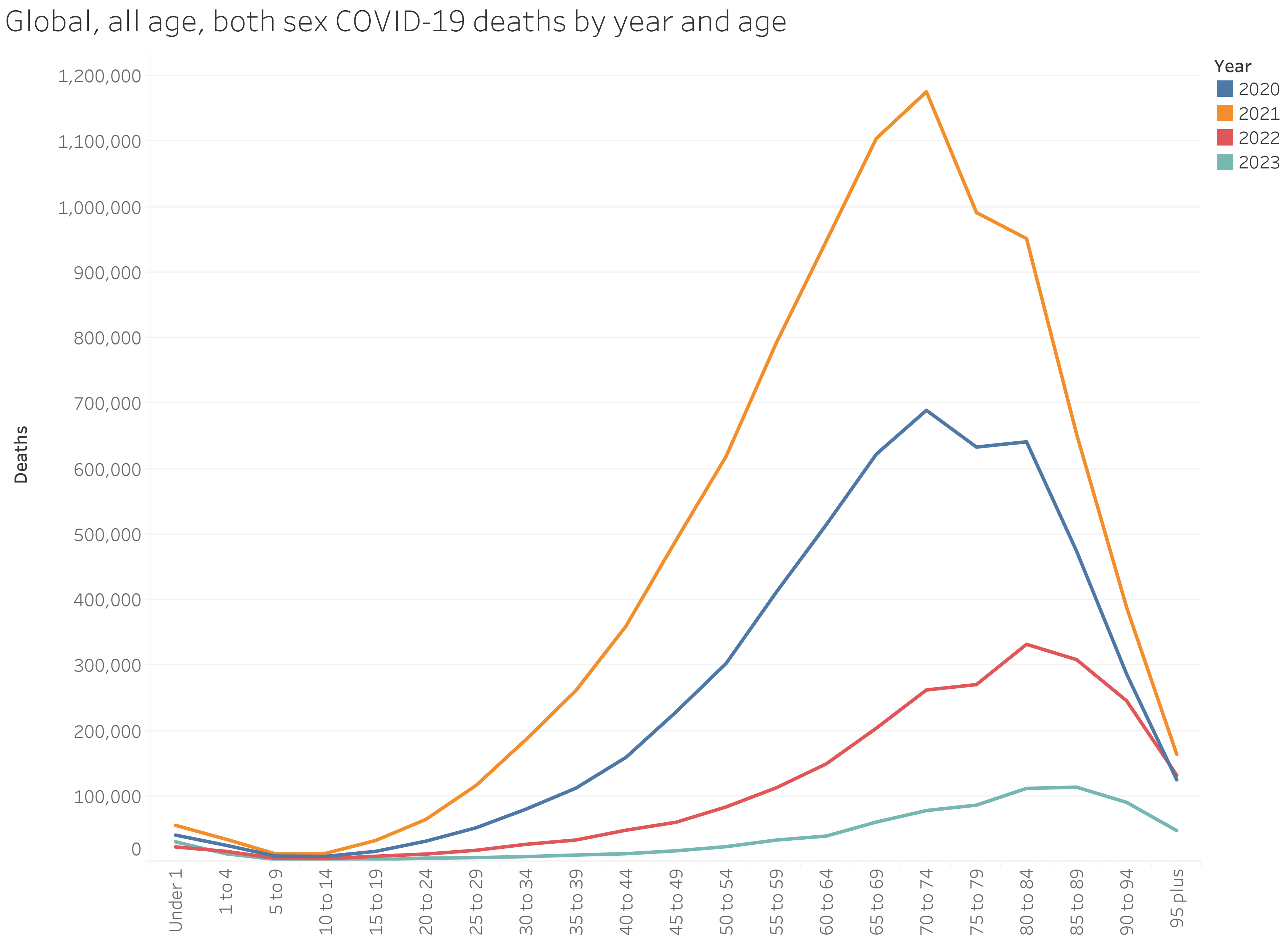
Global, all age, both sex COVID-19 deaths by year and age
Year
2020
2021
2022
2023
Deaths
1,200,000
1,100,000
1,000,000
900,000
800,000
700,000
600,000
500,000
400,000
300,000
200,000
100,000
0
Under 1
1 to 4
5 to 9
10 to 14
15 to 19
20 to 24
25 to 29
30 to 34
35 to 39
40 to 44
45 to 49
50 to 54
55 to 59
60 to 64
65 to 69
70 to 74
75 to 79
80 to 84
85 to 89
90 to 94
95 plus

Figure S4: Comparison of mean age of death for chronic kidney disease, stroke, tracheal, bronchus, and lung cancer, and tuberculosis between 4 GBD regions, both sexes

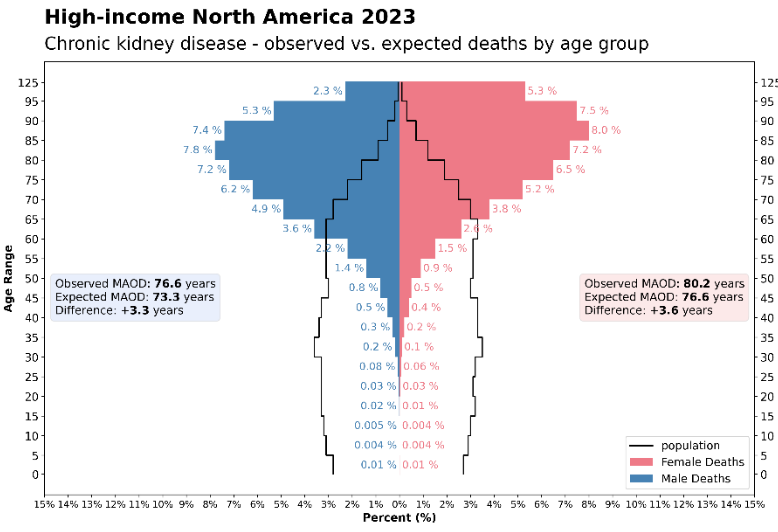

The expected mean age of death is the result of calculating the mean age of death after applying the global mortality rate on a country's population by age and sex for a given cause

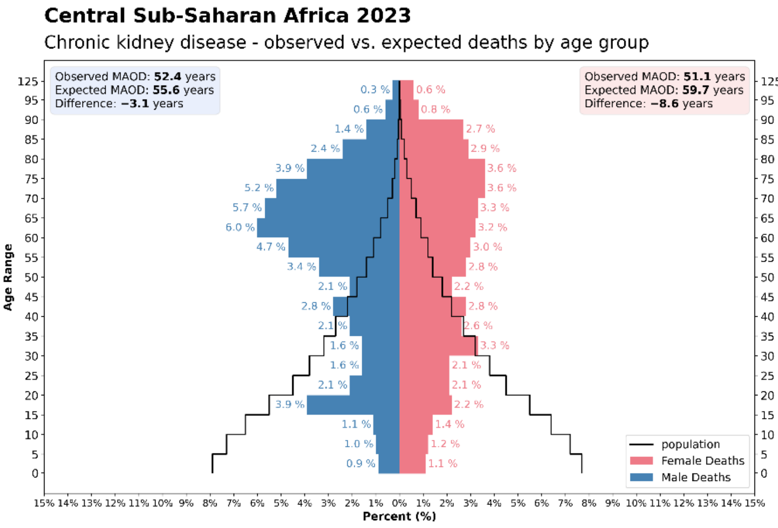

The expected mean age of death is the result of calculating the mean age of death after applying the global mortality rate on a country's population by age and sex for a given cause

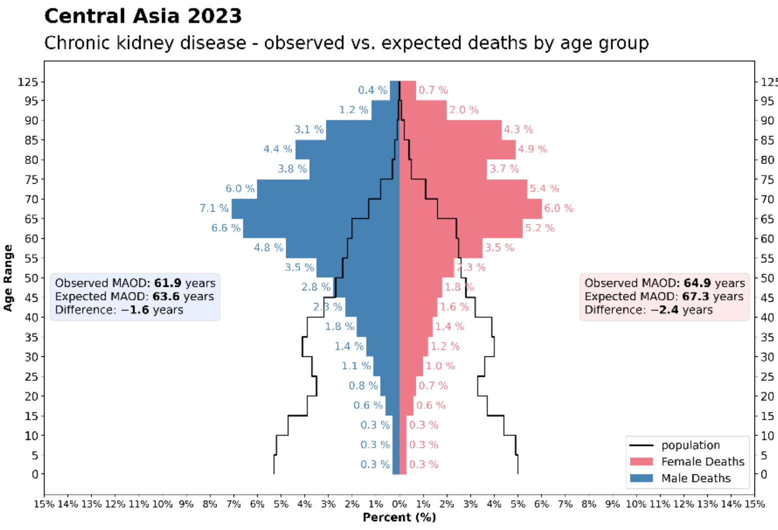

The expected mean age of death is the result of calculating the mean age of death after applying the global mortality rate on a country's population by age and sex for a given cause

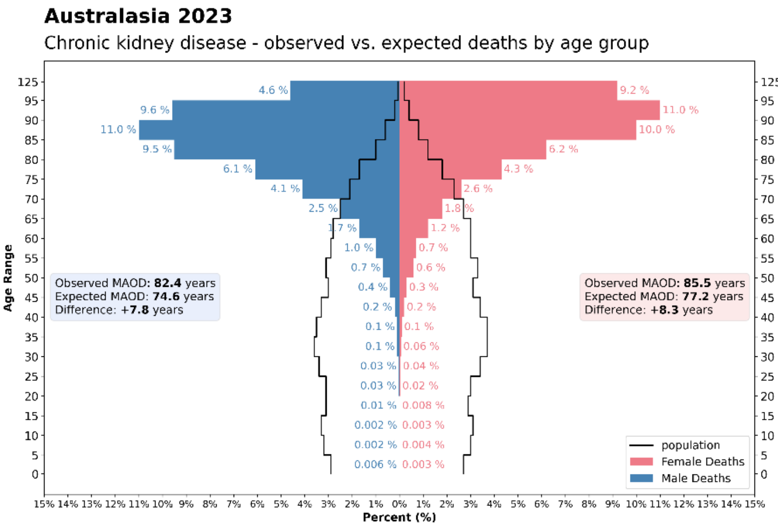

The expected mean age of death is the result of calculating the mean age of death after applying the global mortality rate on a country's population by age and sex for a given cause

Western Sub-Saharan Africa 2023

Stroke - observed vs. expected deaths by age group

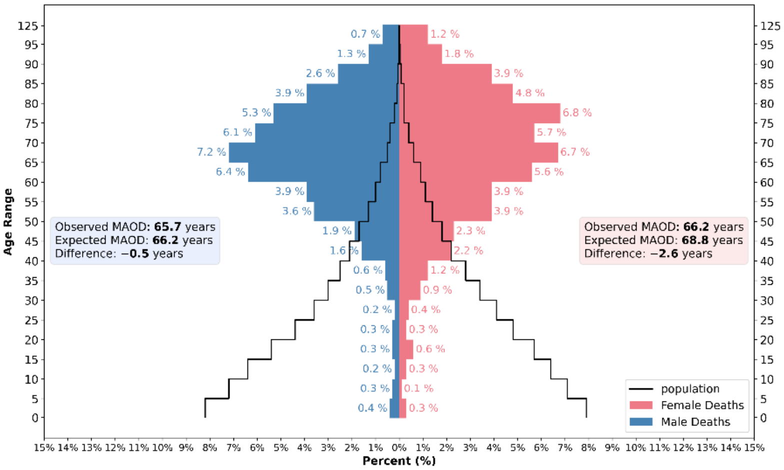

The expected mean age of death is the result of calculating the mean age of death after applying the global mortality rate on a country's population by age and sex for a given cause

Western Europe 2023

Stroke - observed vs. expected deaths by age group

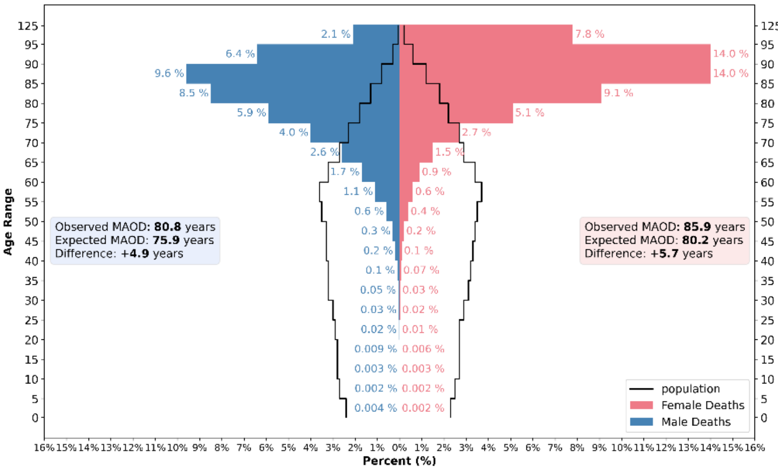

The expected mean age of death is the result of calculating the mean age of death after applying the global mortality rate on a country's population by age and sex for a given cause

North Africa and Middle East 2023

Stroke - observed vs. expected deaths by age group

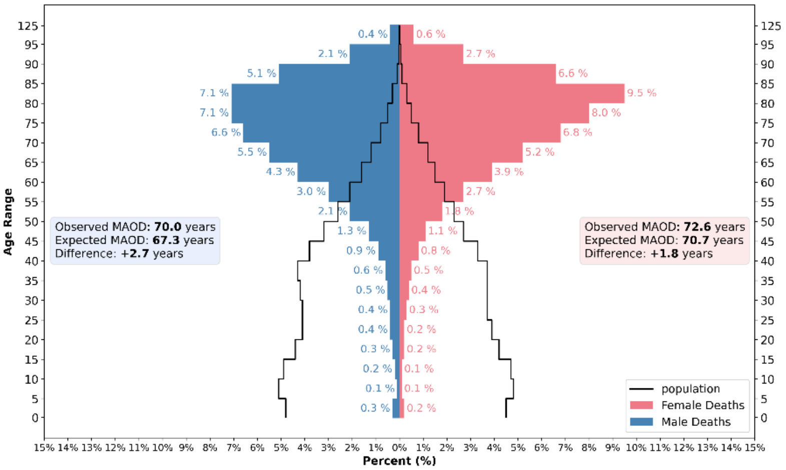

The expected mean age of death is the result of calculating the mean age of death after applying the global mortality rate on a country's population by age and sex for a given cause

Eastern Europe 2023

Stroke - observed vs. expected deaths by age group

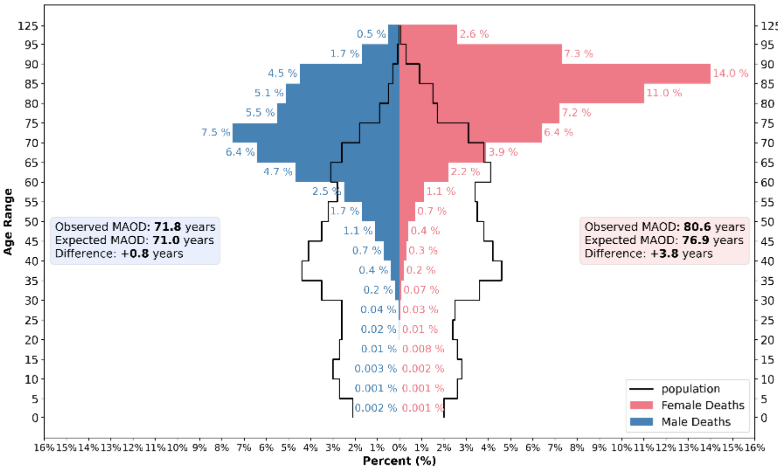

The expected mean age of death is the result of calculating the mean age of death after applying the global mortality rate on a country's population by age and sex for a given cause

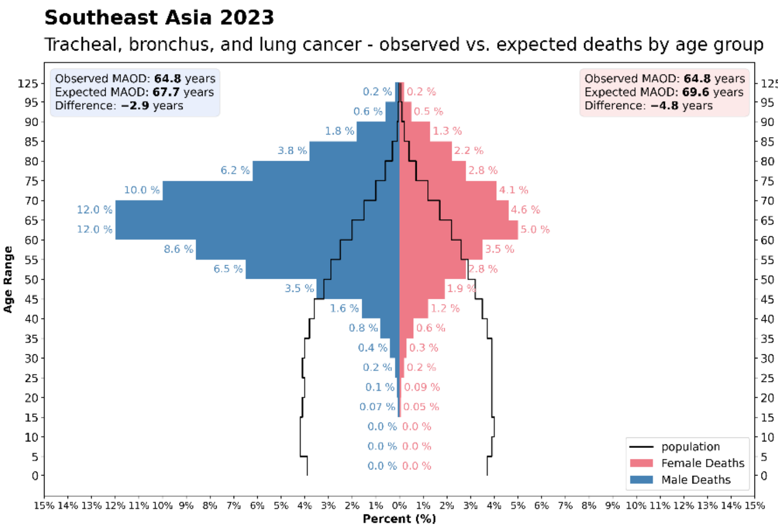

The expected mean age of death is the result of calculating the mean age of death after applying the global mortality rate on a country's population by age and sex for a given cause

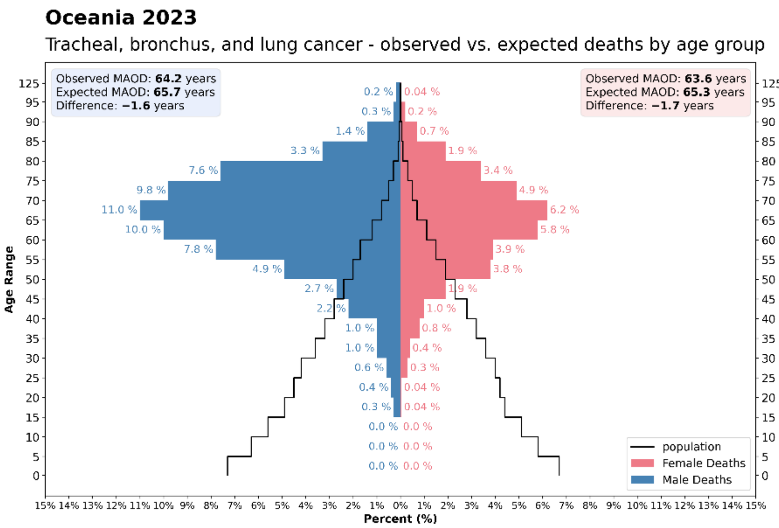

The expected mean age of death is the result of calculating the mean age of death after applying the global mortality rate on a country's population by age and sex for a given cause

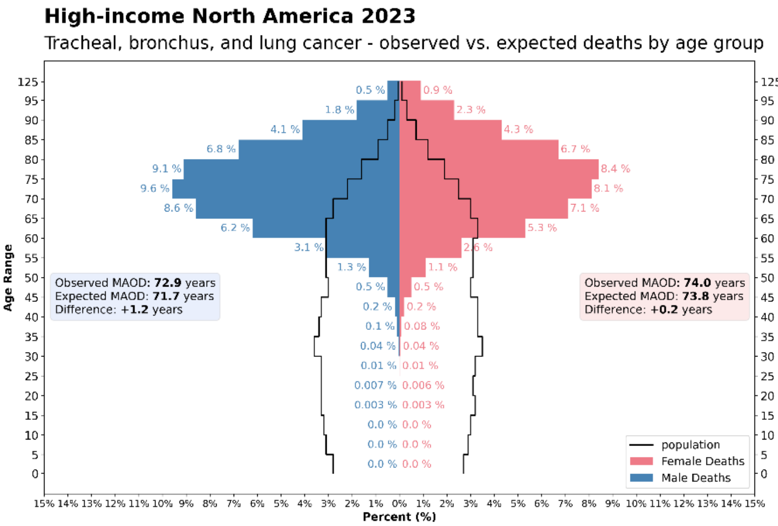

The expected mean age of death is the result of calculating the mean age of death after applying the global mortality rate on a country's population by age and sex for a given cause

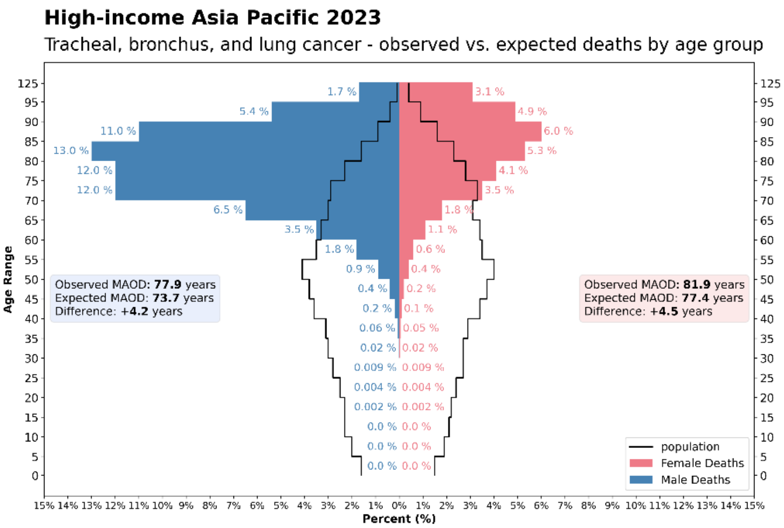

The expected mean age of death is the result of calculating the mean age of death after applying the global mortality rate on a country's population by age and sex for a given cause

Western Sub-Saharan Africa 2023

Tuberculosis - observed vs. expected deaths by age group

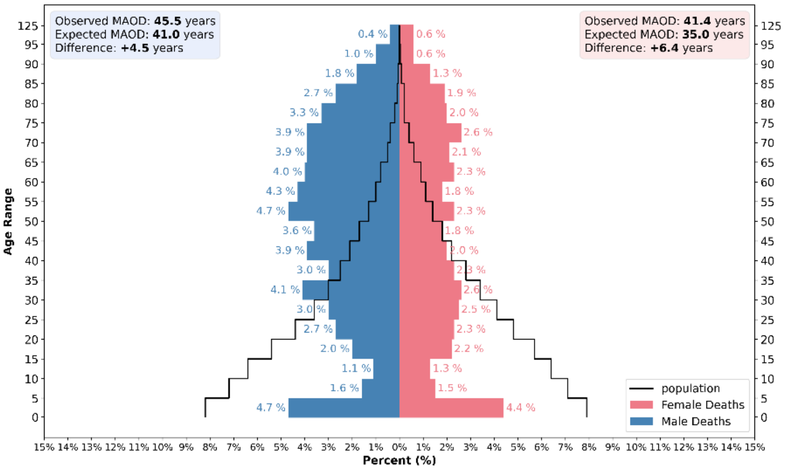

The expected mean age of death is the result of calculating the mean age of death after applying the global mortality rate on a country's population by age and sex for a given cause

North Africa and Middle East 2023

Tuberculosis - observed vs. expected deaths by age group

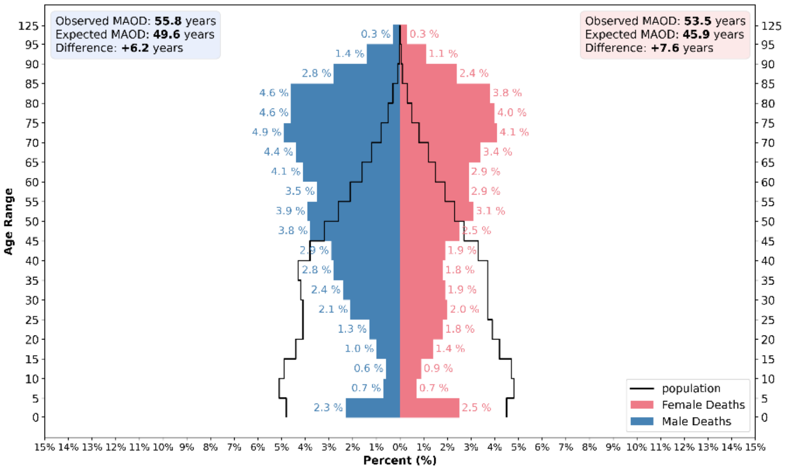

The expected mean age of death is the result of calculating the mean age of death after applying the global mortality rate on a country's population by age and sex for a given cause

High-income North America 2023

Tuberculosis - observed vs. expected deaths by age group

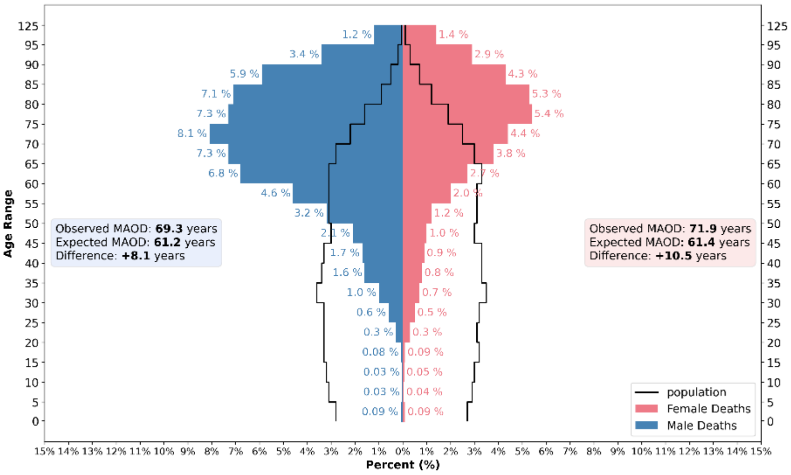

The expected mean age of death is the result of calculating the mean age of death after applying the global mortality rate on a country's population by age and sex for a given cause

High-income Asia Pacific 2023

Tuberculosis - observed vs. expected deaths by age group

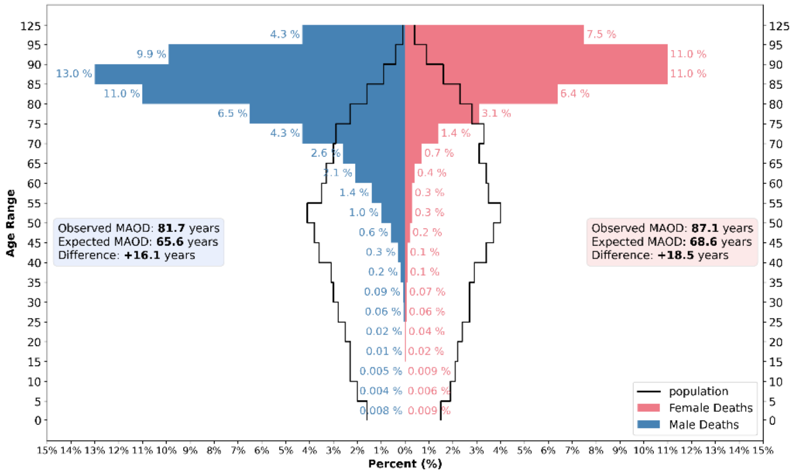

The expected mean age of death is the result of calculating the mean age of death after applying the global mortality rate on a country's population by age and sex for a given cause

Cardiovascular disease - Age standardised death rates, Age standardised rate of YLLs, and 70q0 by super region

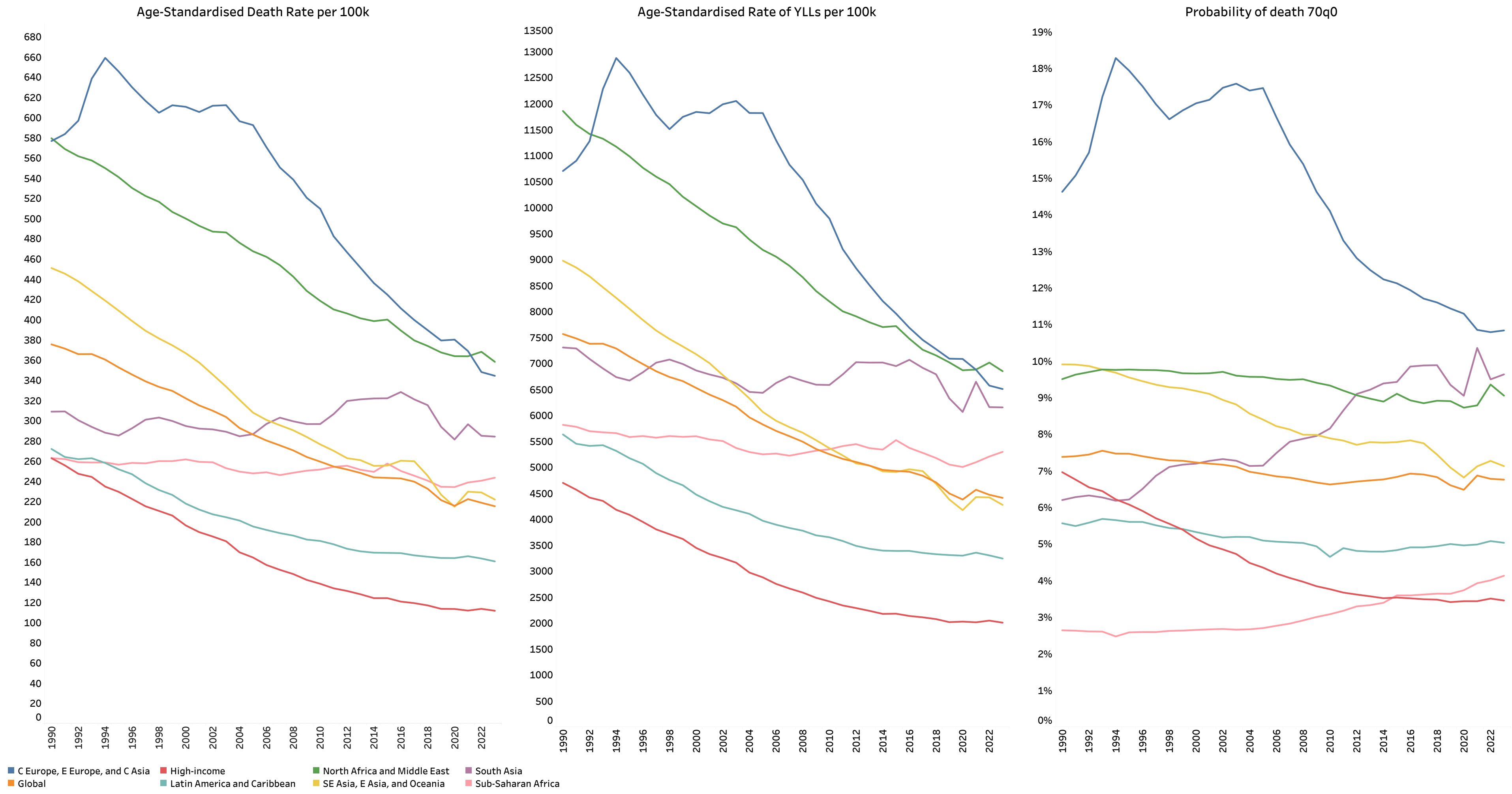

Neoplasms - Age standardised death rates, Age standardised rate of YLLs, and 70q0 by super region

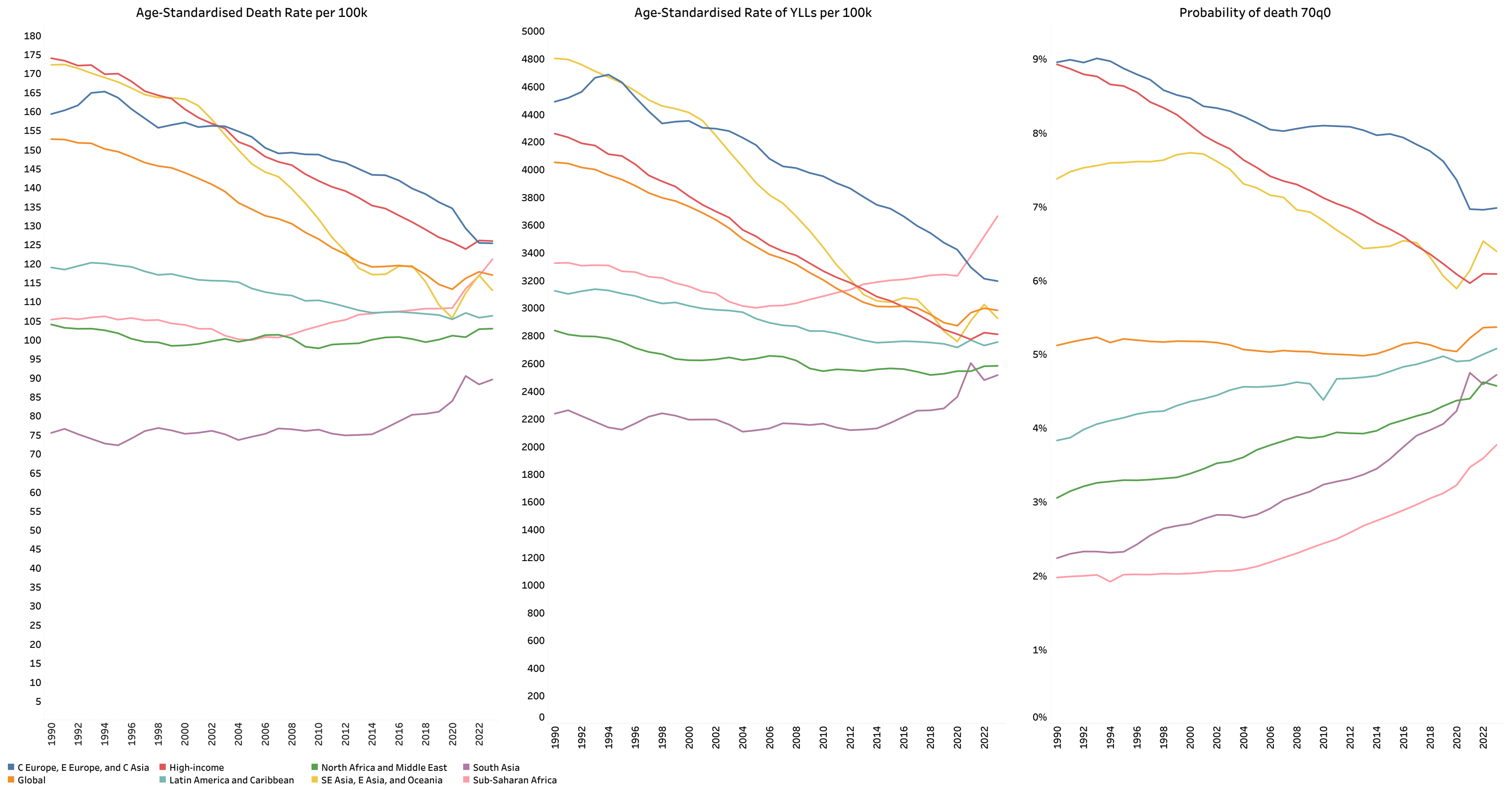

Figure S6. Level 1 Causes - Net percent change in probability of death before 70 between 2000 and 2023

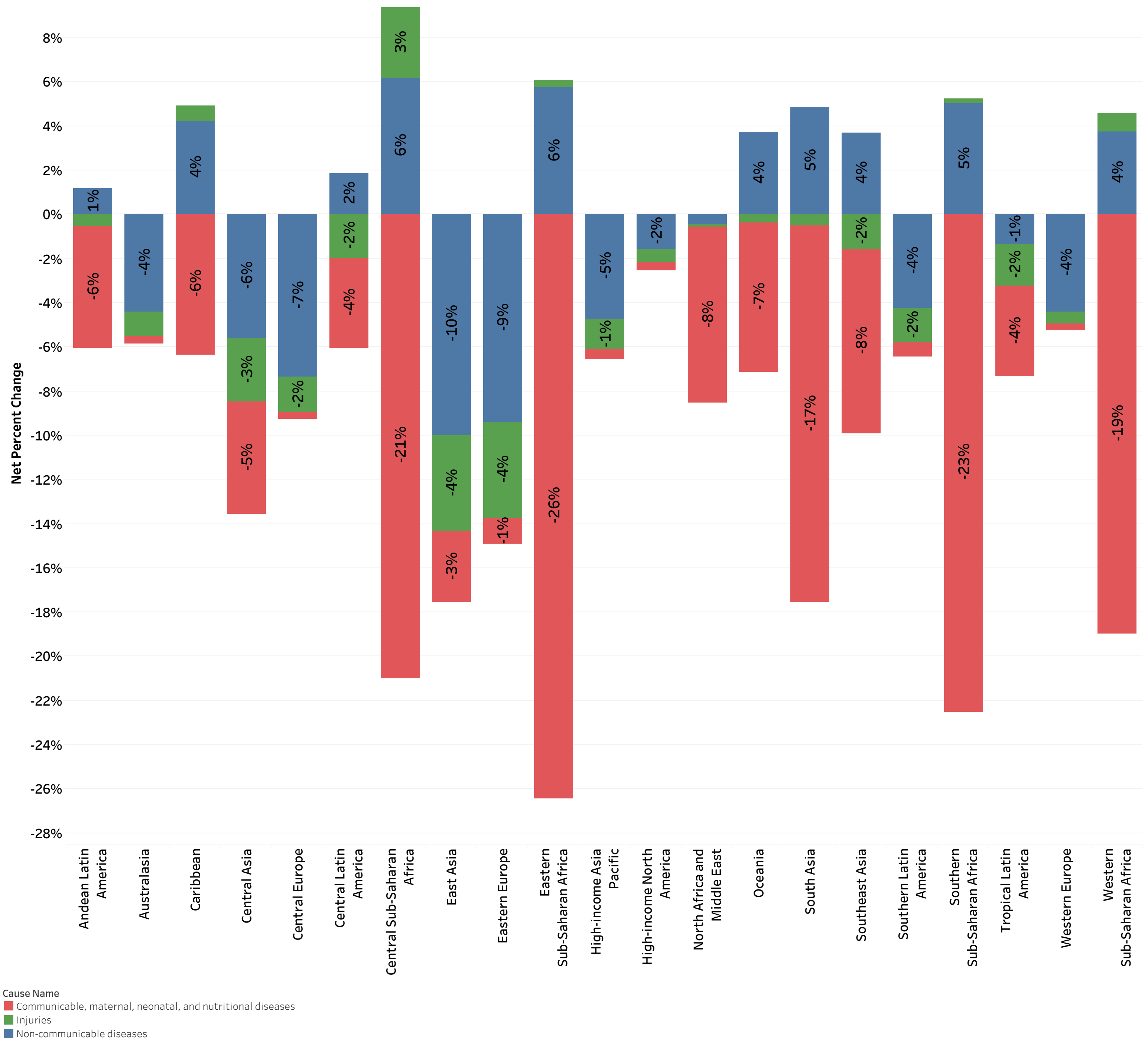

Supplement: Supplementary appendix 2 [file mmc2.pdf]
