# Supplementary material for: Global burden of 292 causes of death in 204 countries and territories and 660 subnational locations, 1990–2023: a systematic analysis for the Global Burden of Disease Study 2023
Source: Lancet. 2025 Oct 18;406(10513):1811–72. doi: 10.1016/S0140-6736(25)01917-8 (PMC12535838; doi:10.1016/S0140-6736(25)01917-8)
Supplement: Supplementary appendix 3 [file mmc3.pdf]

OneMod Writeup

1. Introduction

OneMod is an orchestration tool that allows fitting stagewise statistical models with multiple components. In principle, the user can fit any set of stages, including MSCA tools as well as custom stages. Here, we describe key tools that are frequently used in multiple projects.

Core OneMod components include Rover, which is used for variable selection, SpXMod, which implements parametric fitting with covariates, intercepts, trends, and splines, and KReg, which implements nonparametric fitting using Kernel methods. OneMod allows the user to link stages with these components (as well other custom components) using an over-arching likelihood, with priors and offsets used to pass information between stages and tools. The binomial likelihood (a particular example of general linear models [7]) is used frequently, and we focus on it here.

We first describe the Rover variable selection strategy. Then we describe stagewise modeling within binomial models. Finally we describe SpXMod, and KReg as core parametric and non-parametric modeling components, respectively.

2. Variable Selection using Rover

Variable selection is a key problem in statistical modeling. In particular, given a number of candidate covariates, we want to choose parsimonious models with good predictive performance. This strategy splits the division of responsibility between experts, who choose important covariates that *may* inform prediction, and the model, which tests and selects a set based on a quality metric.

The approach we use is similar to Bayesian Model Averaging [8]. Given n covariates, we could in principle consider 2^n models obtained from the power set. Then we could use a quality metric to judge the performance of each model, and aggregate predictions based on this metric. The BMA approach imposes priors on the space of possible models, with uniform a simple choice, and then uses the posterior marginal likelihood of the covariate choice, marginalizing out all choices of coefficients, as the quality metric. Since our emphasis is selection rather than inference, we use out-of-sample model prediction as our metric.

Beyond choice of metric, the most important feature of the Rover approach is the strategy for traversing the model space. The exhaustive strategy fails as the number of candidate covariates exceeds 10 or so, since this requires fitting over 1000 models, and the model number doubles with each additional covariate. As a result, we build the set of models over which to aggregate sequentially, using forward and backward strategies.

Forward Model.

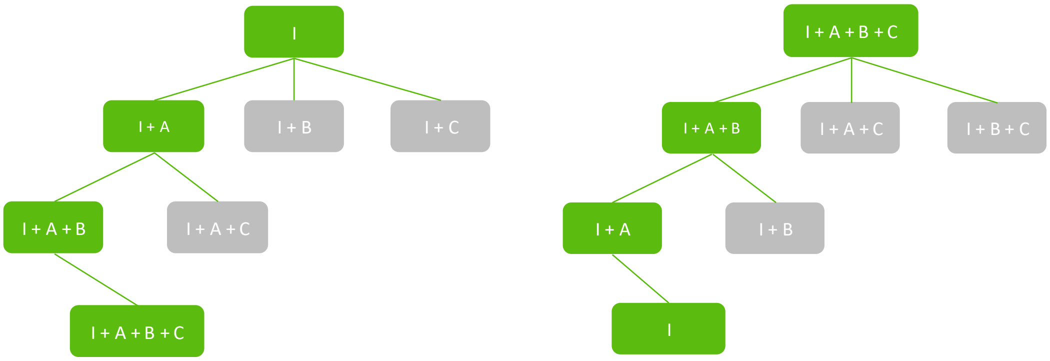

Figure 1. Rover Forward (left) and Backward (Right) Exploration Strategies. Each strategy yields a set of considered models, along with their estimated multipliers, uncertainty, and out of sample performance, which can be used to aggregate results and select covariates.

- Starting with the intercept-only model, we consider all n 1-covariate models, and choose the k best based on out of sample performance across a given set of holdouts.
- For each of the k best models (and corresponding k covariates), we consider all possible 2-covariate models that include the chosen covariate and one other covariate, and choose the k best child models based on out of sample performance across a set of holdouts.
- We continue until we arrive at the saturated model with n covariates, and include all models considered in the final set over which we aggregate. We evaluate every considered model on the full set of data, and come up with aggregate predictions, weighing the multipliers and uncertainty of each model on all data using out of sample performance metric for that model across holdout sets.

Backward Model.

- Starting with the full model (n covariates), we consider all n $n-1$-covariate models, and choose the k best based on out of sample performance across a given set of holdouts.
- For each of the k best models (and corresponding k covariates), we consider all possible $n-2$-covariate models that exclude the covariate excluded from the previous step, and one other other covariate, and choose the k best child models based on out of sample performance across a set of holdouts.
- We continue until we arrive at the intercept-only model, and include all models considered in the final set over which we aggregate. We evaluate every considered model on the full set of data, and come up with aggregate predictions, weighing the multipliers and uncertainty of each model on all data using out of sample performance metric for that model across holdout sets.

The strategies are not mutually exclusive, and can be used in combination to obtain a larger set of models. After obtaining all candidate models using either or both of the strategies, we compute aggregated estimates of the covariate multipliers and uncertainty by combining the model estimates weighted by their out of sample performance.

Further processing may include selection of covariates whose multipliers were significantly different from zero based on the aggregated results, or selection of covariate multipliers that were significantly different from

zero in multiple analyses (such as across age groups). The pictorial representation of the forward and backward strategies with $k = 1$ for three candidate covariates is shown in Figure 1.

3. Stagewise Binomial Modeling using Offsets

Given an observation p_i, associated weight N_i, and feature vector $a_i \in \mathbb{R}^n$, the contribution to the binomial negative log-likelihood is given by

$$\ell(y_i; p_i, N_i) = -N_i \left[y_i \log(p_i) - (1 - y_i) \log(1 - p_i) \right]. \tag{1}$$

The generalized modeling framework supposes a shared linear predictor between all the p_i, with

$$p_i(\theta) = \text{expit}(a_i^T \theta) = \frac{1}{1 + \exp(-a_i^T \theta)}.$$

An **offset** is a constant term within the linear predictor. This simple idea enables stagewise model fitting. For example, suppose we partition the parameter θ into

$$\theta = \begin{bmatrix} \theta_1 \\ \theta_2 \end{bmatrix}.$$

where θ_1 and θ_2 are two blocks of covariates. It may be desirable to first fit a model using θ_1. For example, θ_1 may include global information that can be borrowed across location, such as covariates, while θ_2 may include location -specific information, such as intercepts and trends.

The predictor for the first stage can now be written as

$$p_i(\theta_1) = \text{expit}(a_{i,1}^T \theta_1).$$

We can then fit

$$\hat{\theta}_1 = \arg\min_{\theta_1} \sum_i \ell(y_i; p_i(\theta_1), N_i).$$

Having obtained $\hat{\theta}_1$, we can lock the vector to the estimate, and for the second stage, consider

$$p_i(\theta_2 | \hat{\theta}_1) = \text{expit}(a_{i,1}^T \hat{\theta}_1 + a_{i,2}^T \theta_2) \tag{2}$$

where the term $a_{i,1}^T \hat{\theta}_1$ is the offset, and we now fit the remaining components of the model by solving

$$\hat{\theta}_2 = \arg\min_{\theta_2} \sum_i \ell(y_i; p_i(\theta_2 | \hat{\theta}_1), N_i)$$

The stagewise approach is a key technique for modeling outcomes informed by differential levels of sparsity, and offers great flexibility in combining results from different model types, such as parametric and nonparametric pieces as described below.

4. SpXMod: Functional Specification of Complex GLM Models

The SpXMod tool allows specification of complex regularized generalized linear models (GLMs), allowing the user to provide dimensions of variation for intercepts, coefficients, and splines [3].

4.1. Group-specific coefficients and similarity priors

SpXMod provides a flexible modeling interface to allow group-specific effects of covariates, as well as similarity priors that can smooth these same effects across the dimension of interest.

For example, consider the dimension of **age** in the model, parametrized by age group $j = 1, \dots\dots, M$. Consider now that the effective parameters of interest, θ_j, may be age-specific, and that data $y_{i,j}$ and features $a_{i,j}$ are age-specific as well. Then we can specify a model that allows age-specific effects but imposes a relational prior:

$$\min_{\theta_1,\dots,\theta_M} \sum_{i,j} \ell(y_{i,j}; p_{i,j}, N_{i,j}) + \frac{1}{2}\theta^T C \theta.$$

where

$$p_{i,j} = \text{expit}(a_{i,j}^T \theta_{i,j})$$

and C is a matrix that enforces a similarity prior on the θ parameters. For example, suppose we assume that adjacent age-groups should not vary significantly. This corresponds to a regularization term of the form

$$\gamma \sum_j \|\theta_j - \theta_{j-1}\|^2$$

and so we would have

$$C = \gamma \begin{bmatrix} -I & I & 0 & \dots & 0 \\ 0 & -I & I & 0 & \dots \\ \ddots & \ddots & \ddots & \ddots & \\ 0 & 0 & \dots & -I & I \end{bmatrix}. \tag{3}$$

While we used age in the construction, coefficients can vary across any relevant dimension or dimension combination. Key examples we come back to later on include location, where intercepts are location-specific, and location-age, where intercepts are specific to both location and age. The smoothing priors become ever more important in constraining the problems as there is more freedom for coefficients and intercepts to vary across multiple dimensions. To make this point more clear, one can view imposing a large penalty, for example, a large γ in (3), as a way to impose coefficients and intercepts that are constant across relevant dimensions such as age. Thus the framework can interpolate between more common assumptions where all data are used to inform a single intercept and feature-specific multipliers and, on the other end, complete flexibility where the model not borrow strength across groups in fitting group-specific intercepts and covariate multipliers.

4.2. Spline Specification

The coefficients θ_j may be understood as covariate multipliers for specific covariates. However, they may also correspond to splines, as detailed in this section. Taken together, the two sections show that SpXMod can specify correlated splines that vary across dimensions of interest.

For general background on splines and spline regression see [3] and [5].

For a spline with polynomial degree p and k knots placed at locations along the domain x, $t_0, ..., t_k$, we need $p+k$ basis elements to construct the spline basis, denoted s_j^p. These basis elements can be constructed recursively.

The standard B-spline basis is generated recursively: By default we assume $c_0^i = \emptyset$, $c_{i+k+1}^i = \emptyset$ and $s_0^i(t) = 0$, $s_{i+k+1}^i(t) = 0$, for all $i \geq 0$.

- $i = 0$,

$$c_j^0 = [t_{j-1}, t_j), \quad s_j^0(t) = \delta_{c_j^0}(t), \quad j = 1, \ldots, k$$

- $i > 0$,

$$c_j^i = c_{j-1}^{i-1} \cup c_j^{i-1}, \quad s_j^i(t) = s_{j-1}^{i-1}(t) l(t; c_{j-1}^{i-1}) + s_j^{i-1}(t) r(t; c_j^{i-1}), \quad j = 1, \ldots, i+k$$

where

$$l(t;c) = \begin{cases} (t - \inf c)/(\sup c - \inf c), & c \neq \emptyset \\ 0, & c = \emptyset \end{cases},$$

$$r(t;c) = \begin{cases} (t - \sup c)/(\inf c - \sup c), & c \neq \emptyset \\ 0, & c = \emptyset \end{cases}.$$

as illustrated in Figure 2.

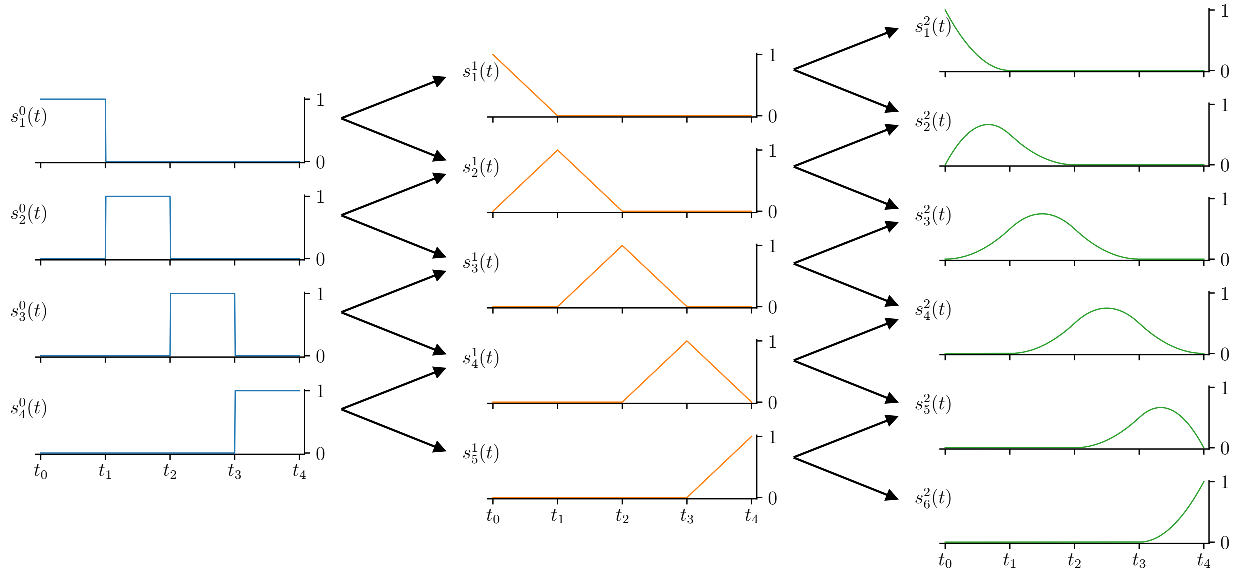

Figure 2. Generation of bspline basis elements (orders 0, 1, 2).

With knowledge of the degree and knots placement, we can construct a *design matrix* X based on an input vector x, where the jth column of the design matrix is given by the expression

$$X_{\cdot,j} = \begin{bmatrix} s_j^p(t_0) \\ \vdots \\ s_j^p(t_k) \end{bmatrix}. \tag{4}$$

Intercepts and trends can be seen as specific basis functions, and play a key role in the demographics analysis.

5. KReg: Kronecker-Factored Multivariate Kernel Regression

Kernel regression [6] allows us to fit observations directly, balancing available observations with similarity across dimensions. We solve

$$\hat{f} = \arg\min_f \sum_{i=1}^{n} \ell(f_i|y_i, N_i) + \frac{\lambda}{2} f^T P f$$

where f is the entire vector of linear predictions of interest and $P = K^{-1}$, with K the Kernel matrix that encodes the covariance structure through similarities across dimensions, such as age, time, and location.

This model corresponds to computing the MAP estimator of f given a Gaussian process prior

$$f \sim GP(0, \frac{1}{\lambda} K),$$

with fixed covariance K, and where all available information is communicated to the model through the kernel matrix K. In the following subsections, we discuss several useful kernels, and show how to efficiently model the action of the kernels across dimensions in higher-dimensional analysis.

5.1. Specific Kernels

KREg makes use of several standard kernels, an can incorporate user defined kernels. In the demographics analysis, we rely on different versions fo the Matern kernel for age and time dimensions. For comparison, we also specify the Radial Basis Function (RBF) [2] kernel familiar to many readers; see e.g. [10] for an accessible overview.

For all kernels, define

$$d = \|\mathbf{x} - \mathbf{y}\|_2 = \sqrt{\sum_{i=1}^{n} (x_i - y_i)^2}. \tag{5}$$

Radial Basis Function (RBF) Kernel.

$$k(\mathbf{x}, \mathbf{y}) = \exp\left(-\frac{d^2}{2\gamma^2}\right), \tag{6}$$

with d defined in (5). This kernel is widely used to model very smooth functions, and the underlying assumption that the unknown function is infinitely differentiable is not always desirable, and can induce oscillations when fitting it to data arising from more realistic processes.

Matern Kernel. The Matern $3/2$ kernel function is defined as:

$$k(\mathbf{x}, \mathbf{y}) = \left(1 + \frac{\sqrt{3}\, d}{\rho}\right) \exp\left(-\frac{\sqrt{3}\, d}{\rho}\right), \tag{7}$$

with d defined in (5). In dimension 1, this models functions that are one time differentiable and can help avoid overshoot/oscillations. The Matern kernel is frequently used in model-based geostatistics for this reason [4].

Linear Kernel. The linear kernel is parametrized by location and scale, and is defined by

$$k(x,y) = (x-a)^T(y-a)/b^2 \tag{8}$$

5.2. Efficient Kernel Regression Through Kronecker product

The vector f is inherently high-dimensional, as it includes all estimates of interest. For example, in the demographics case, the dimension would be

$$D = 2 \times 70 \times 20 \times 905 = 2,534,000,$$

where we consider 2 sexes, 70 years, 20 age groups, and 905 locations. Thus a naive application of Kernel regression with coupling across all dimensions would require computing and inverting a $2.5M \times 2.5M$ dense matrix at each iteration, which is not feasible. However, by imposing a product structure on our choice of covariance kernel and assuming fixed grids for each dimension, the resulting kernel matrix is available in Kronecker factored form. This greatly reduces memory and computational cost and makes the fully coupled dense problem computationally tractable.

Kronecker Product. The Kronecker product is an operation on two matrices A and B, resulting in a larger block matrix. If A is an $m \times n$ matrix and B is a $p \times q$ matrix, their Kronecker product $A \otimes B$ is an $(mp) \times (nq)$ matrix defined as:

$$A \otimes B = \begin{bmatrix} a_{11}B & a_{12}B & \cdots & a_{1n}B \\ a_{21}B & a_{22}B & \cdots & a_{2n}B \\ \vdots & \vdots & \ddots & \vdots \\ a_{m1}B & a_{m2}B & \cdots & a_{mn}B \end{bmatrix}$$

Each element a_{ij} of matrix A is multiplied by the entire matrix B, resulting in a block matrix.

While we handle arbitrarily many blocks of variables, we detail the case of two blocks clarity. The Kronecker structure for Kernels [1] arises from the following assumptions:

1. x can be partitioned into two groups of variables, $x = (a, b)$, so that $x_i = (a_i, b_i)$.
2. Assume observations lie on a uniform grid in terms of a_i, b_i, so that there are grids of values $(a_k)_{k=1}^{n_a}$, $(b_j)_{j=1}^{n_b}$, and $x_{kn_a+j} = (a_k, b_j)$ for $k = 1, \ldots, n_a$, $j = 1, \ldots, n_b$.
3. The covariance function $k(x_i, x_j)$ factorizes into $k(x_i, x_j) = k((a_i, b_i), (a_j, b_j)) = k_a(a_i, a_j)k_b(b_i, b_j)$.

Using this factorization, we can write:

$$K = K_a \otimes K_b, \quad K_a = (k_a(a_i, a_j))_{i,j=1}^{n_a}, \quad K_b = (k_b(b_i, b_j))_{i,j=1}^{n_b}$$

By properties of the Kronecker product,

$$P = P_a \otimes P_b, \quad P_a = K_a^{-1}, \quad P_b = K_b^{-1}$$

The optimization problem then becomes:

$$f^* = \arg\min_f \left(\sum_{i=1}^n \ell_i(f_i) + \frac{\lambda}{2} f^\top (P_a \otimes P_b) f \right)$$

The Kronecker factorization simplifies both direct and indirect approaches to the optimization problem.

Decoupling. In practice, we often assume further decoupling across sexes and locations which instead leads to 1810 separate estimation problems of size 1400. This allows us to adapt hyperparameters to different locations as needed for a more desirable model fit. Indeed, conditioning on other features and the coupling induced by SpXMod, the additional variation that we want to fit using KReg doesn't appear to benefit much from coupling across locations.

5.3. KReg with Offsets

KReg can communicate with previous model stages (such as SpXMod) using offsets. For each reported observation y_i, we let g_i represent the predicted value from any previous steps.

The offset Kernel regression model is given by

$$\hat{f} = \arg\min_f \sum_{i=1}^n \ell(f_i + g_i | y_i, N_i) + \frac{\lambda}{2} f^T P f. \tag{9}$$

Specifically, for any elements of f with observations, we shift the prediction f_i from the kernel regression in the likelihood by the offset g_i predicted from the previous model step.

In the case a grid point i is unobserved, the model effectively sets $N_i = 0$, weighting the negative log likelihood by zero there.

In the limiting case of no observations, then f will revert to zero, and our prediction reverts to g.

5.4. Uncertainty Calibration

The model (9) provides classic asymptotic uncertainty for the estimates f_i:

$$f_i \sim N(\hat{f}_i, \nabla^2 \ell|_{\hat{f}_i} + \lambda P)^{-1}. \tag{10}$$

However, from the form of the variance, it is clear that as we strengthen the prior kernel smoothing matrix P, we decrease the posterior variance as well.

To mitigate the effect of the prior, we use a calibration strategy for uncertainty analogous to the one frequently employed in weather prediction [9].

Once we obtain model-based asymptotic uncertainty for each f_i, we pick a granularity (either location or region) and scale each set of uncertainties so that residuals corresponding to observations has variance 1.

Specifically, the variance of the prediction intervals for the Kernel regression model is given by

$$V_p = V(f_i) + \frac{1}{\hat{p}(1-\hat{p})w}$$

where the second term corresponds to the reported variance from a binomial model. We pick α so that the observed residuals, when scaled by $\sqrt{\alpha V_p}$, have variance 1.

References

[1] A. Airola and T. Pahikkala. Fast kronecker product kernel methods via generalized vec trick. *IEEE transactions on neural networks and learning systems*, 29(8):3374–3387, 2017.

[2] M. D. Buhmann. Radial basis functions. *Acta numerica*, 9:1–38, 2000.

[3] C. De Boor, C. De Boor, E.-U. Mathématicien, C. De Boor, and C. De Boor. *A practical guide to splines*, volume 27. springer-verlag New York, 1978.

[4] P. J. Diggle, J. A. Tawn, and R. A. Moyeed. Model-based geostatistics. *Journal of the Royal Statistical Society Series C: Applied Statistics*, 47(3):299–350, 1998.

[5] J. H. Friedman. Multivariate adaptive regression splines. *The annals of statistics*, pages 1–67, 1991.

[6] T. Hofmann, B. Schölkopf, and A. J. Smola. Kernel methods in machine learning. 2008.

[7] J. A. Nelder and R. W. Wedderburn. Generalized linear models. *Journal of the Royal Statistical Society Series A: Statistics in Society*, 135(3):370–384, 1972.

[8] A. E. Raftery, D. Madigan, and J. A. Hoeting. Bayesian model averaging for linear regression models. *Journal of the American Statistical Association*, 92(437):179–191, 1997.

[9] J. Slingo and T. Palmer. Uncertainty in weather and climate prediction. *Philosophical Transactions of the Royal Society A: Mathematical, Physical and Engineering Sciences*, 369(1956):4751–4767, 2011.

[10] C. K. Williams and C. E. Rasmussen. *Gaussian processes for machine learning*, volume 2. MIT press Cambridge, MA, 2006.
