# Supplementary material for: Global burden of 292 causes of death in 204 countries and territories and 660 subnational locations, 1990–2023: a systematic analysis for the Global Burden of Disease Study 2023
Source: Lancet. 2025 Oct 18;406(10513):1811–72. doi: 10.1016/S0140-6736(25)01917-8 (PMC12535838; doi:10.1016/S0140-6736(25)01917-8)
Supplement: Supplementary appendix 4 [file mmc4.pdf]

Appendix 4: Authorship appendix to "Global burden of 292 causes of death in 204 countries and territories and 660 subnational locations, 1990–2023: a systematic analysis for the Global Burden of Disease Study 2023"

This appendix provides further authorship detail for "Global burden of 292 causes of death in 204 countries and territories and 660 subnational locations, 1990–2023: a systematic analysis for the Global Burden of Disease Study 2023"

Table of Contents

GBD 2023 Causes of Death Collaborators **2**

Affiliations **13**

Authors' Contributions **67**

- Managing the overall research enterprise 67
- Writing the first draft of the manuscript 67
- Primary responsibility for applying analytical methods to produce estimates 67
- Primary responsibility for seeking, cataloguing, extracting, or cleaning data; designing or coding figures and tables 67
- Providing data or critical feedback on data sources 67
- Developing methods or computational machinery 72
- Providing critical feedback on methods or results 73
- Drafting the work or revising it critically for important intellectual content 82
- Managing the estimation or publications process 90

GBD 2023 Causes of Death Collaborators

Mohsen Naghavi,* Hmwe Hmwe Kyu,* Bhoomadevi A, Mohammad Amin Aalipour, Hasan Aalruz, Hazim S Ababneh, Bedru J Abafita, Ukachukwu O Abaraogu, Cristiana Abbafati, Madineh Abbasi, Faezeh Abbaspour, Hedayat Abbastabar, Abdallah H A Abd Al Magied, Samar Abd ElHafeez, Ashraf Nabiel Abdalla, Mohammed Altigani Abdalla, Emad M Abdallah, Barkhad Aden Abdeeq, Nadin M I Abdel Razeq, Ahmed Abdelrahman Abdelgalil, Reda Abdel-Hameed, Michael Abdelmasseh, Mahmoud Abdelnabi, Wael M Abdel-Rahman, Arman Abdous, Mostafa M Abdrabou, Jeza Muhamad Abdul Aziz, Deldar Morad Abdulah, Auwal Abdullahi, Toufik Abdul-Rahman, Habtamu Abebe Getahun, Aidin Abedi, Armita Abedi, Parisa Abedi, Asrat Agalu Abejew, Roberto Ariel Abeldaño Zuñiga, Shehab Uddin Al Abid, Syed Hani Abidi, Alemwork Abie, Olugbenga Olusola Abiodun, Richard Gyan Aboagye, Shady Abohashem, Hassan Abolhassani, Ulric Sena Abonie, Nagah M Abourashed, Mohamed Abouzid, Dmitry Abramov, Lucas Guimarães Abreu, Dariush Abtahi, Rana Kamal Abu Farha, Fuad Hamdi A Abuadas, Aminu Kende Abubakar, Nermeen Abu-Elala, Eman Abu-Gharbieh, Sawsan Abuhammad, Ahmad Y Abuhelwa, Hana J Abukhadijah, Niveen ME Abu-Rmeileh, Salahdein Aburuz, Dina Abushanab, Manfred Mario Kokou Accrombessi, Anirudh Balakrishna Acharya, Apurba Acharya, Ousman Adal, Lisa C Adams, Abdu A Adamu, Isaac Yeboah Addo, Oluwafemi Atanda Adeagbo, Tajudeen Adesanmi Adebisi, Isaac Akinkunmi Adedeji, Kamoru Ademola Adedokun, Oluwatobi E Adegbile, Nurudeen A Adegoke, Olumide Thomas Adeleke, Bulcha Guye Adema, Bashir Aden, Isaac Ayodeji Adesina, Miracle Ayomikun Adesina, Juliana Bunmi Adetunji, Habeeb Omoponle Adewuyi, Temitayo Esther Adeyeoluwa, Mache Tsadik Adhana, Ripon Kumar Adhikary, Usha Adiga, Tanin Adl Parvar, Mohd Adnan, Qorinah Estiningtyas Sakilah Adnani, Prince Owusu Adoma, Leticia Akua Adzigbli, David Adzrago, Giuseppina Affinito, Ahmed M Afifi, Clifford Afoakwah, Aanuoluwapo Adeyimika Afolabi, Rotimi Felix Afolabi, Vlad-Adrian Afrăsânie, Saira Afzal, Gizachew Beykaso Agafari, Suneth Buddhika Agampodi, Thilini Chanchala Agampodi, Navidha Aggarwal, Mahdi Aghaalikhani, Sepehr Aghajanian, Seyed Mohammad Kazem Aghamir, Feleke Doyore Agide, César Agostinis Sobrinho, Anurag Agrawal, Williams Agyemang-Duah, Mahsa Ahadi, Bright Opoku Ahinkorah, Aqeel Ahmad, Danish Ahmad, Faisal Ahmad, Ijaz Ahmad, Khabir Ahmad, Khurshid Ahmad, Sajjad Ahmad, Tauseef Ahmad, Waqas Ahmad, Negar Sadat Ahmadi, Ali Ahmed, Ayman Ahmed, Gasha Salih Ahmed, Haroon Ahmed, Junaid Ahmed, Luai A Ahmed, MD Faisal Ahmed, Mehrunnisha Sharif Ahmed, Meqdad Saleh Ahmed, Muktar Beshir Ahmed, Mushood Ahmed, Shabbir Ahmed, Sindew Mahmud Ahmed, Syed Anees Ahmed, Gulzhanat Aimagambetova, Marjan Ajami, Budi Aji, Hossein Akbarialiabad, Saeid Akbarifard, Oluwasefunmi Akeju, Roland Eghoghosoa Akhigbe, Ruslan Akhmedullin, Olufemi Ambrose Akinkuotu, Mohammed Ahmed Akkaif, Wole Akosile, Ashley E Akrami, Ralph Kwame Akyea, Alaa Al Amiry, Salah Al Awaidy, Syed Mahfuz Al Hasan, Ammar Al Homsi, Mohammad Khaled Al Nawayseh, Omar Al Omari, Zain Al Ta'ani, Yazan Al Thaher, Omar Ali Mohammed Al Zaabi, Mohammad Ahmmad Mahmoud Al Zoubi, Mousa Ali Al-Abbadi, Tariq A Alalwan, Ziyad Al-Aly, Khurshid Alam, Manjurul Alam, Mohammad Khursheed Alam, Mostafa Alam, Rasmieh Mustafa Al-Amer, Abebaw Alamrew, Amani Alansari, Turki M Alanzi, Fahmi Y Al-Ashwal, Mohammed Albashtawy, Wafa A Aldhaleei, Mohammed S Aldossary, Robert W Aldridge, Shereen M Aleidi, Bezawit Abeje Alemayehu, Fentahun Alemnew, Melaku Birhanu Alemu, Kefyalew Addis Alene, Ayman Al-Eyadhy, Ali M Alfalki, Fahad D Algahtani, Abdelazeem M Algammal, Khairat Al-Habbal, Nma Bida Alhaji, Samar Al-Hajj, Fadwa Naji Alhalaiqa, Mohammed Khaled Al-Hanawi, Khalid A Alhasan, Ashraf Alhumaidi, Fahad A Alhumaydhi, Amjad Ali, Haroon Muhammad Ali, Irfan Ali, Liaqat Ali, Maratab Ali, Mohammad Daud Ali, Mohammed Usman Ali, Rafat Ali, Shahid Ali, Syed Shujait Ali, Waad Ali, Gianfranco Alicandro, Montaha Al-Iede, Sheikh Mohammad Alif, Hamid Alinejad Rokny, Morteza Alipour, Samah W Al-Jabi, Mohamad Aljofan, Moath Saleh Aljohani,

Syed Mohamed Aljunid, Ahmad Alkhatib, Mustafa Alkhawam, Peter Allebeck, Khaled S Allemailem, Mohammed Z Allouh, Wesam Taher Almagharbeh, Sabah Al-Marwani, Nihad A Almasri, Joseph Uy Almazan, Hesham M Al-Mekhlafi, Omar Almidani, Amr Almobayed, Khaldoon Aied Alnawafleh, Hasan Yaser Alniss, Margret Beaula Alocious Sukumar, Mahmoud A Alomari, Mohammad R Alosta, Jaber S Alqahtani, Saleh A Alqahtani, Mohammad R Alqudimat, Ahmad Rajeh Al-Qudimat, Intima Alrimawi, Sahel Majed Alrousan, Salman Khalifah Al-Sabah, Mohammed A Alsabri, Zaid Altaany, Awais Altaf, Alaa B Al-Tammemi, Jaffar A Al-Tawfiq, Malik A Althobiani, Khalid A Altirkawi, Javier Alvarez-Galvez, Nelson Alvis-Guzman, Nelson J Alvis-Zakzuk, Hassan Alwafi, Mohammad Al-Wardat, Yaser Mohammed Al-Worafi, Hany Aly, Mohammad Sharif Ibrahim Alyahya, Hosam Alzahrani, Karem H Alzoubi, Adel Sharaf Al-Zubairi, Ekiyor Joseph Amafah, Joy Amafah, Reza Amani-Beni, Faten Amer, Bardia Amidi, Amr Amin, Tarek Tawfik Amin, Alireza Amindarolzarbi, Saeed Amini, Ehsan Amini-Salehi, Majid Aminzare, Sohrab Amiri, Joanne O Amlag, Dickson A Amugsi, Ganiyu Adeniyi Amusa, Filippos Anagnostakis, Roshan A Ananda, Nazanin Anaraki, Robert Ancuceanu, Deanna Anderlini, David B Anderson, Nguyen Hoang Anh, Abdul-Azeez Adeyemi Anjorin, Samuel Egyakwa Ankomah, Kabilan Annadurai, Sumbul Ansari, Alireza Ansari-Moghaddam, Catherine M Antony, Ernoiz Antriyandarti, Boluwatife Stephen Anuoluwa, Iyadunni Adesola Anuoluwa, Saeid Anvari, Saleha Anwar, Sumadi Lukman Anwar, Razique Anwer, Shahnawaz Anwer, Anayochukwu Edward Anyasodor, Francis Appiah, Juan Pablo Arab, Hossein Arabi, Jalal Arabloo, Mosab Arafat, Daniel T Araki, Aleksandr Y Aravkin, Demelash Areda, Getnet Mesfin Aregu, Jorge Arias de la Torre, Ghazal Arjmand, Benedetta Armocida, Johan Ärnlöv, Jesu Arockiaraj, Mahwish Arooj, Anton A Artamonov, Ashokan Arumugam, Deepavalli Arumuganainar, Umesh Raj Aryal, Nurila Aryntayeva, Mahsa Asadi Anar, Muhammad Asaduzzaman, Syed Mohammed Basheeruddin Asdaq, Mulusew Andualem A Asemahagn, Mulu Tiruneh Asemu, Saeed Asgary, Mohammad Asghari-Jafarabadi, Syed Amir Ashraf, Tahira Ashraf, Mitra Ashrafi, Milad Ashrafizadeh, Bernard Kwadwo Yeboah Asiamah-Asare, Saeed Aslani, Yuni Asri, Batyrbek Assembekov, Seyyed Shamsadin Athari, Alok Atreya, Julie Alaere Atta, Zeenah A Atwan, Khursheed Aurangzeb, Marcel Ausloos, Abolfazl Avan, Núbia Carelli Pereira Avelar, Sana Javaid Awan, Adedapo Wasiu Awotidebe, Lemessa Assefa A Ayana, Haleh Ayatollahi, Yusuf Oloruntoyin Ayipo, Seyed Mohammad Ayyoubzadeh, Sina Azadnajafabad, Arian Azadnia, James Mba Azam, Alireza Azarboo, Zelalem Nigussie Azene, Gulrez Shah Azhar, Amirali Azimi, Farya Azimi, Mohd Yusmaidie Aziz, Sadat Abdulla Aziz, Amin Azizan, Ahmed Y Azzam, Giridhara Rathnaiah Babu, Youngoh Bae, Arvind Bagga, Nasser Bagheri, Sara Bagheri, Elahe Baghizadeh, Fereshteh Baghizadeh, Sana Baghizadeh, Khlood K Baghlaf, Najmeh Bahmanziari, Ruhai Bai, Mohamed Ibrahem Baklola, Abdulaziz T Bako, Wondu Feyisa Balcha, Maher Balkis, Jose Balmori-de-la-Miyar, Mohammadreza Balooch Hasankhani, Ovidiu Constantin Baltatu, Soham Bandyopadhyay, Palash Chandra Banik, Noel C Barengo, Suzanne Lyn Barker-Collo, Hiba Jawdat Barqawi, Amadou Barrow, Sandra Barteit, Lingkan Barua, MD Abu Bashar, Shahid Bashir, Guido Basile, Rehana Basri, Quique Bassat, Mohammad-Mahdi Bastan, Abdul-Monim Batiha, Kavita Batra, Matteo Bauckneht, Mahdis Bayat, Mohammad Amin Bayat Tork, Thomas Beaney, Neeraj Bedi, Narasimha M Beeraka, Massimiliano Beghi, Jina Behjati, Bezawit K Bekele, Almaz Nibret Belay, Demeke Mesfin Belay, Asnake Gashaw Belayneh, Melesse Belayneh, Abel Cherkos Belete, Gokce Belge Bilgin, Muhammad Bashir Bello, Olorunjuwon Omolaja Bello, Umar Muhammad Bello, Luis Belo, Apostolos Beloukas, Riyad Bendardaf, Isabela M Bensenor, Samiun Nazrin Bente Kamal Tune, Maria Bergami, Alemshet Yirga Berhie, Abiye Assefa Berihun, Amiel Nazer C Bermudez, Robert S Bernstein, Gregory J Bertolacci, Paola Bertuccio, Paulo J G Bettencourt, Ajeet Singh Bhadoria, Akshaya Srikanth Bhagavathula, Neeraj Bhala, Buna Bhandari, Kayleigh Bhangdia, Charmi Bhanushali, Nikha Bhardwaj, Pankaj Bhardwaj, Ashish Bhargava, Sonu Bhaskar, Anup Bhat, Priyadarshini Bhattacharjee,

Shuvarthi Bhattacharjee, Gurjit Kaur Bhatti, Jasvinder Singh Bhatti, Mohiuddin Ahmed Bhuiyan, Zulfiqar A Bhutta, Soumitra S Bhuyan, Haoran Bi, Sibhatu Kassa Biadgilign, Raluca Bievel-Radulescu, Naif Kandash Binsaleh, Catherine Bisignano, Atanu Biswas, Bijit Biswas, Mohammad Shahangir Biswas, Ahmad Naoras Bitar, Molalegne Bitew, Bruno Bizzozero-Peroni, Tone Bjørge, Virginia Bodolica, Eyob Ketema Bogale, Lucimere Bohn, Obasanjo Afolabi Bolarinwa, Paria Bolourinejad, Aime Bonny, Sri Harsha Boppana, Hamed Borhany, Mina Borran, Sudipta Bose, Samuel Adolf Bosoka, Alejandro Botero Carvajal, Soufiane Boufous, Christopher Boxe, Dejana Braithwaite, Luisa C Brant, Michael Brauer, Nicholas J K Breitborde, Susanne Breitner, Hermann Brenner, Edmond D Brewer, Maria L Bringas Vega, Julie Brown, Annie J Browne, Traolach Brugha, Raffaele Bugiardini, Norma B Bulamu, Tsion Samuel Bunare, Danilo Buonsenso, Richard A Burns, Akeem Olayinka Busari, Felix Busch, Yasser Bustanji, Nadeem Shafique Butt, Zahid A Butt, Sanjay C J, Tianji Cai, Rose Cairns, Mehtap Çakmak Barsbay, Daniela Calina, Luis Alberto Cámera, Luciana Aparecida Campos, Ismael Campos-Nonato, Fan Cao, Si Cao, Angelo Capodici, Rosario Cárdenas, Giulia Carreras, Juan Jesus Carrero, Andrea Carugno, Andre F Carvalho, Felix Carvalho, Márcia Carvalho, Ana Paula Carvalho-e-Silva, Joao Mauricio Castaldelli-Maia, Carlos A Castañeda-Orjuela, Giulio Castelpietra, Ferrán Catalá-López, Alberico L Catapano, Maria Sofia Cattaruzza, Luca Cegolon, Francieli Cembranel, Muthia Cenderadewi, Kelly M Cercy, Ester Cerin, Pamela Roxana Chacón-Uscamaita, Chiranjib Chakraborty, Sandip Chakraborty, Joht Singh Chandan, Rama Mohan Chandika, Miyuru Chandradasa, Baskaran Chandrasekaran, Vijay Kumar Chattu, Victoria Chatzimavridou-Grigoriadou, Anis Ahmad Chaudhary, Sirshendu Chaudhuri, Akhilanand Chaurasia, An-Tian Chen, Catherine S Chen, Guangjin Chen, Haiyan Chen, Hana Chen, Haowei Chen, Hui Chen, Rucheng Chen, Shanquan Chen, Simiao Chen, Xiang Chen, Haojin Cheng, Ka Ching Cheung, Nicholas WS Chew, Gerald Chi, Fatemeh Chichagi, Izumi Chihara, Odgerel Chimed-Ochir, Patrick R Ching, Jesus Lorenzo Chirinos-Caceres, Daniel Youngwhan Cho, William C S Cho, Bryan Chong, Yuen Yu Chong, Hou In Chou, Enayet Karim Chowdhury, Sreshtha Chowdhury, Hanne Christensen, Ting-Wu Chuang, Isaac Sunday Chukwu, Erin Chung, Sheng-Chia Chung, Sunghyun Chung, Muhammad Chutiyami, Arrigo Francesco Giuseppe Cicero, Cain C T Clark, Fred Cohen, Alyssa Columbus, Joao Conde, Stephen E Congly, Nathalie Conrad, Leslie Trumbull Cooper, Alexandru Corlateanu, Samuele Cortese, Paolo Angelo Cortesi, Claudia Cosma, Ewerton Cousin, Emma Johnson Cowart, Michael H Criqui, Andrew Crist, Jessica A Cruz, Natalia Cruz-Martins, Xiaolin Cui, Garland T Culbreth, Patricia Cullen, Matthew Cunningham, Nour Dababo, Ali Dabbagh, Omid Dadras, Tukur Dahiru, Xiaochen Dai, Zhaoli Dai, Mayank Dalakoti, Koustuv Dalal, Gloria Dalla Costa, Emanuele D'Amico, Roy Arokiam Daniel, Lucio D'Anna, Pojsakorn Danpanichkul, Samuel E Danso, Samuel Demissie Darcho, Latefa Ali Dardas, Chengetai Dare, Bahar Darouei, Reza Darvishi Cheshmeh Soltani, Sayan Kumar Das, Claudio Alberto Dávila-Cervantes, Nicole Davis Weaver, Dimash Davletov, Kairat Davletov, Fernando Pio De la Hoz, Alejandro de la Torre-Luque, Edward Christopher Dee, Sindhura Deekonda, Amanda Deen, Louisa Degenhardt, Paria Dehesh, Pouria Delbari, Laura Delgado-Ortiz, Mohammad Delsoz, Andreas K Demetriades, Edgar Denova-Gutiérrez, Tadios Niguss Derese, Ismail Dergaa, Kebede Deribe, Hunegnaw Almaw Derseh, Nikolaos Dervenis, Emina Dervišević, Hardik Dineshbhai Desai, Abraham Aregay Desta, Vinoth Gnana Chellaiyan Devanbu, Pradeep Kumar Devarakonda, Syed Masudur Rahman Dewan, Arkadeep Dhali, Kuldeep Dhama, Sreedhar Dharmagadda, Mandira Lamichhane Dhimal, Meghnath Dhimal, Bibha Dhungel, Marcello Di Pumpo, Diana Dias da Silva, Daniel Diaz, Luis Antonio Diaz, Kimia Didehvar, Elangovan Dilipan, Lauren K Dillard, Xueting Ding, Saeid Doaei, Sushil Dohare, Klara Georgieva Dokova, Mario D'Oria, Fariba Dorostkar, E Ray Dorsey, Ojas Prakashbhai Doshi, Leila Doshmangir, Robert Kokou Dowou, Menayit Tamrat Dresse, Tim Robert Driscoll, Ashel Chelsea Dsouza, Jiang Du, John Dube, Judy R Dubno, Emeka W Dumbili, Samuel C Dumith, Bruce B

Duncan, Andre Rodrigues Duraes, Oyewole Christopher Durojaiye, Ashit Kumar Dutta, Siddhartha Dutta, Sulagna Dutta, Osamudiamen Ebohon, Ejemai Eboreime, Lamiaa Labieb Mahmoud Ebraheim, Alireza Ebrahimi, Mohammad Hossein Ebrahimi, Abdelaziz Ed-Dra, David Edvardsson, Ferry Efendi, Behrad Eftekhari, Foolad Eghbali, Ashkan Eighaei Sedeh, Terje Andreas Eikemo, Ebrahim Eini, Michael Ekholuenetale, Temitope Cyrus Ekundayo, Rabie Adel El Arab, Abdelfatteh EL Omri, Maysaa El Sayed Zaki, Mohamed Ahmed Eladl, Reza Elahi, Said El-Ashker, Rana Elbeshbeishy, Noha Mousaad Elemam, Ghada Metwally Tawfik ElGohary, Muhammed Elhadi, Mohamed Elhoumed, Waseem El-Huneidi, Omar Abdelsadek Abdou Elmeligy, Mohamed A Elmonem, Rami Elmorsi, Mohamed Hassan Elnaem, Gihan ELNahas, Mohammed Elshaer, Ibrahim Elsohaby, Abdelgawad Salah Eltahawy, Tadele Emagneneh, Misganu Endriyas, Ryenchindorj Erkhembayar, Christopher Imokhuede Esezobor, Derese Eshetu, Majid Eslami, Narges Eslami, Rafaela Cavalheiro do Espírito Santo, Kara Estep, Oghenowede Eyawo, Ugochukwu Anthony Eze, Elochukwu Ezenwankwo, Heidar Fadavian, Adeniyi Francis Fagbamigbe, Omotayo Francis Fagbule, Ayesha Fahim, Saman Fahimi, Aamir Fahira, Ildar Ravisovich Fakhradiyev, Aliasghar Fakhri-Demeshghieh, Luca Falzone, Qiping Fan, Mohammad Farahmand, Ali Faramarzi, Mohammad Fareed, Zaki Farhana, Liliana Faria, Carla Sofia e Sá Farinha, MoezAlIslam Ezzat Mahmoud Faris, Andre Faro, Syed Muhammad Yousaf Farooq, Hossein Farrokhpour, Fatemeh Farshad, Farima Farsi, Folorunso Oludayo Fasina, Modupe Margaret Fasina, Ali Fatehizadeh, Davood Fathi, Zareen Fatima, Mohammad Fayaz, Pooria Fazeli, Valery L Feigin, Alireza Feizkhah, Gelana Fekadu, Ginenus Fekadu, Ulrich Membe Femoe, Talukdar Raian Ferdous, Seyed-Mohammad Fereshtehnejad, Rodrigo Fernandez-Jimenez, Pietro Ferrara, Alize J Ferrari, Nuno Ferreira, Getahun Fetensa, Bikila Regassa Feyisa, Alexander Finnemore, Claudio Fiorilla, Florian Fischer, Ida Fitriana, Federica Fogacci, Morenike Oluwatoyin Folayan, Artem Alekseevich Fomenkov, Marco Fonzo, Lisa M Force, Daniela Fortuna, Matteo Foschi, Maryam Fotouhi, Kayode Raphael Fowobaje, Richard Charles Franklin, Alberto Freitas, Jinming Fu, Takeshi Fukumoto, Ami Fukunaga, John E Fuller, Sridevi G, Peter Andras Gaal, Muktar A Gadanya, Dominic Dormenyo Gadeka, Lebo Francina Gafane-Matemane, Márió Gajdács, Yaseen Galali, Dinara Galiyeva, Silvano Gallus, Dhanraj Ganapathy, Balasankar Ganesan, Shivaprakash Gangachannaiah, Xiang Gao, Yijie Gao, Bashiru Garba, Miguel Garcia-Argibay, David Garcia-Azorin, William M Gardner, Wendy Paola Gastélum Espinoza, Zisis Gatzioufas, Prem Gautam, Rupesh K Gautam, Bamba Gaye, Hong-Han Ge, Feven Sahle Gebre, Miglas Welay Gebregergis, Mesfin Gebrehiwot, Miesa Gelchu, Stefano Gelibter, Nsikakabasi Samuel George, Lemma Getacher, Genanew K Getahun, Kalab Yigermal Gete, Peter W Gething, Keyghobad Ghadiri, Fataneh Ghadirian, Amir Ghaffari Jolfayi, Arin Ghamkhar, Shakiba Ghasemi Assl, Fariba Ghassemi, Ramy Mohamed Ghazy, Sama Ghoba, Maryam Gholamalizadeh, Zainab Gholami, Nasim Gholizadeh, Zeinab Ghorbani, Elena Ghotbi, Arun Ghuge, Alessandro Gialluisi, Konstantinos Giannakis, Syed Abdullah Gilani, Tiffany K Gill, Bikash Ranjan Giri, Alem Abera Girmay, Alessandro Girombelli, Laszlo Göbölös, Anil Kumar Goel, Archit Goel, Rajesh Kumar Goel, Lay Hoon Goh, Kimiya Gohari, Mahaveer Golechha, Ali Golestani, Davide Golinelli, Melika Golmohammadi, Wenping Gong, Alessandra C Goulart, Ayman Grada, Simon Matthew Graham, Michal Grivna, Shi-Yang Guan, Giovanni Guarducci, Mohammed Ibrahim Mohialdeen Gubari, Mesay Dechasa Gudeta, Avirup Guha, Stefano Guicciardi, Sheffali Gulati, Sasidhar Gunturu, Cui Guo, Xingzhi Guo, Zhaoyu Guo, Zhifeng Guo, Bhawna Gupta, Gaurav Gupta, Lalit Gupta, Rajeev Gupta, Reyna Alma Gutiérrez, Roberth Steven Gutiérrez-Murillo, Jose Guzman-Esquivel, Abrham Tesfaye Habteyes, Awoke Derbie Habteyohannes, Tesfahun Simon Hadaro, Najah R Hadi, Zahra Hadian, Abdul Hafiz, Sarah Hafsia, Faraidoon Haghdoost, Arian Haghtalab, Nguyen Hai Nam, Addisalem Haile, Demewoz Haile, Pritam Halder, Sebastian Haller, Rabih Halwani, Kosar Hikmat Hama Aziz, Islam M Hamad, Randah R Hamadeh, Samer Hamidi, Erin B Hamilton,

Ahmad Hammoud, Chieh Han, Hannah Han, Asif Hanif, Nasrin Hanifi, Graeme J Hankey, Fahad Hanna, Ashanul Haque, Md Nuruzzaman Haque, Obaid I Haque, Arief Hargono, Andy Martahan Andreas Hariandja, Josep Maria Haro, Ashley Ann Harris, Ahmed I Hasaballah, Faizul Hasan, Md Kamrul Hasan, Towhid Hasan, Hamidreza Hasani, Ali Hasanpour- Dehkordi, Mohammad Hashem Hashempur, Nada Tawfig Hashim, Ammarah Hasnain, Abbas M Hassan, Amr Hassan, Ibrahim Nagmeldin Hassan, Ikrama Hassan, Nageeb Hassan, Omed Hassan Ahmed, Yusuf Hassan Wada, Mahgol Sadat Hassan Zadeh Tabatabaei, Soheil Hassanipour, Lasanthi Wathsala Hathagoda, Johannes Haubold, Rasmus J Havmoeller, Simon I Hay, Youssef Hbid, Jiawei He, Jeffrey J Hebert, Golnaz Heidari, Mohammad Heidari, Mojtaba Heydari, Kamal Hezam, Yuta Hiraike, Nobuyuki Horita, Alamgir Hossain, Lubna Hossain, Md Belal Hossain, Md Mahbub Hossain, Md Sabbir Hossain, Mohammad Bellal Hossain, Fatemeh Sadat Hosseini, Mehdi Hosseinzadeh, Mihaela Hostiuc, Sorin Hostiuc, Peter J Hotez, Priya Hotwani, Hanno Hoven, Chengxi Hu, Yifei Hu, Junjie Huang, Weijun Huang, Yefei Huang, Yuting Huang, Zhenyao Huang, Mega Hasanul Huda, Ayesha Humayun, Waqar Husain, Kiavash Hushmandi, Javid Hussain, Nawfal R Hussein, Mohamed Ibrahim Husseiny, Luigi Francesco Iannone, Segun Emmanuel Ibitoye, Khalid S Ibrahim, Ramzi Ibrahim, Reem Ibrahim, Umar Idris Ibrahim, Anel Ibrayeva, Fidelia Ida, Kevin S Ikuta, Olayinka Stephen Ilesanmi, Irena M Ilic, Milena D Ilic, Muhammad Hamza Ilyas, Mohammad Tarique Imam, Masoud Imani, Lucius Chidiebere Imoh, Arit Inok, Meesha Iqbal, Mujahid Iqbal, Lalu Muhammad Irham, Mustafa Alhaji Isa, Benni Iskandar, Teresa R Iskander, Md Rabiul Islam, Md Shahinul Islam, Md Shariful Islam, Sheikh Mohammed Shariful Islam, Farhad Islami, Faisal Ismail, Nahlah Elkudssiah Ismail, Yerlan Ismoldayev, Gaetano Isola, Masao Iwagami, Ihoghosa Osamuyi Iyamu, Vinothini J, Jalil Jaafari, Louis Jacob, Kathryn H Jacobsen, Ali Jadidi, Farhad Jadidi-Niaragh, Mohammadsadegh Jafari, Morteza Jafarinia, Abdollah Jafarzadeh, Shabbar Jaffar, Haitham Jahrami, Ammar Abdulrahman Jairoun, Vikash Jaiswal, Sanobar Jaka, Mihajlo Jakovljevic, Reza Jalilzadeh Yengejeh, Mohamed Jalloh, Armaan Jamal, Qazi Mohammad Sajid Jamal, Jazlan Jamaluddin, Jerin James, Hasan Jamil, Safayet Jamil, Roland Dominic G Jamora, Masoud Jamshidi, Shaghayegh JamshidiRastabi, Rajiv Janardhanan, Chinmay T Jani, Esmaeil Jarrahi, Tahereh Javaheri, Syed Sarmad Javaid, Anita Javanmardi, Javad Javidnia, Talha Jawaid, Qassim Jawell Odah Abed, Sathish Kumar Jayapal, Shubha Jayaram, Ruwan Duminda Jayasinghe, Yovanthi Anurangi Jayasinghe, Sun Ha Jee, Jayakumar Jeganathan, Diptismita Jena, Seogsong Jeong, Bijay Mukesh Jeswani, Vivekanand Jha, John S Ji, Min Jiang, Wenyi Jin, Nabi Jomehzadeh, Jost B Jonas, Tamas Joo, Abu Jor, Abel Joseph, Nitin Joseph, Meha Joshi, George Joy, Jacek Jerzy Jozwiak, Mikk Jürisson, Vaishali K, Billingsley Kaambwa, Ali Kabir, Zubair Kabir, Rajendra Kadel, Dler H Hussein Kadir, Ashish Kumar Kakkar, Pradnya Vishal Kakodkar, Rizwan Kalani, Khalil Kalavani, Feroze Kaliyadan, Sanjay Kalra, Md Moustafa Kamal, Mehnaz Kamal, Sivesh Kathir Kamarajah, Rajesh Kamath, Saltanat Kamenova, Arun Kamireddy, Ramat T Kamorudeen, Devanish Narasimhasanth Kamtam, Naser Kamyari, Oleksandr Kamyshnyi, Mona Kanaan, Saddam Fuad Kanaan, Jiseung Kang, Kehinde Kazeem Kanmodi, Suthanthira Kannan S, Rami S Kantar, Debasish Kar, Sujita Kumar Kar, Paschalis Karakasis, Jafar Karami, Reema A Karasneh, Mohammad Amin Karimi, Salah Eddin Karimi, Arman Karimi Behnagh, Mohmed Isaqali Karobari, Tomasz M Karpiński, Adarsh Katamreddy, Joonas H Kauppila, Kanica Kaushal, Foad Kazemi, Nastaran Kazemi Rad, Sina Kazemian, Hafte Kahsay Kebede, Yabets Tesfaye Kebede, Tibebeselassie S Keflie, Swetha N Kempegowda, Salima Kerai, Jessica A Kerr, Vikash Ranjan Keshri, Kamyab Keshtkar, Emmanuelle Kesse-Guyot, Reza Khademi, Yousef Saleh Khader, Sidra Khalid, Hazim O Khalifa, Anas Husam Khalifeh, Anees Ahmed Khalil, Anita Khalili, Pantea Khalili, Alireza Khalilian, Ghazaleh Khalili-Tanha, Mohamed Khalis, Faham Khamesipour, Ajmal Khan, Fayaz Khan, Gulfaraz Khan, Iman Waheed Khan, Maseer Khan, Md Abdullah Saeed Khan, Mohammad Jobair Khan, Muhammad Hamza Khan, Muhammad Mueed Khan,

Muhammad Umair Khan, Muhammad Umer Khan, Salman Ali Khan, Serab Khan, Sumaiya Khan, Ubaid Khan, Yusuf Saleem Khan, Zahid Khan, Vishnu Khanal, Shaghayegh Khanmohammadi, Sameer Uttamaro Khasbage, Zenith Khashim, Khaled Khatab, Haitham Khatatbeh, Moawiah Mohammad Khatatbeh, Mahalaqua Nazli Khatib, Kavin Khatri, Hamid Reza Khayat Kashani, Khalid A Kheirallah, Sunil Kumar Khokhar, Najmaddin Salih Husen Khoshnaw, Atulya Aman Khosla, Ardeshir Khosravi, Farbod Khosravi, Sepehr Khosravi, Mahmood Khosrowjerdi, P Ratan Khuman, Zemene Demelash Kifle, Hye Jun Kim, Jinho Kim, Kwanghyun Kim, Min Seo Kim, Yun Jin Kim, Ruth W Kimokoti, Tadele Kinati, Yohannes Kinfu, Sanjay Kini B, Mary Kirk, Adnan Kisa, Sezer Kisa, Katarzyna Kissimova-Skarbek, Tegene Atamenta Kitaw, Mika Kivimäki, Abdul Basith KM, Shivakumar KM, Ann Kristin Skrindo Knudsen, Nazarii Kobyliak, Jonathan M Kocarnik, Sonali Kochhar, Prakash Babu Kodali, Michail Kokkorakis, Ali-Asghar Kolahi, Diana Gladys Kolieghu Tcheumeni, Kairi Kolves, Joyce Komesuor, Farzad Kompani, Aida Kondybayeva, Isaac Koomson, Gerbrand Koren, Tapos Kormoker, Vladimir Andreevich Korshunov, Oleksii Korzh, Soewarta Kosen, Karel Kostev, Parvaiz A Koul, Irene Akwo Kretchy, James-Paul Kretchy, Kewal Krishan, Chong-Han Kua, Ananya Kuanar, Barthelemy Kuate Defo, Mohammed Kuddus, Ilari Kuitunen, Mukhtar Kulimbet, Shweta Kulshreshtha, Dewesh Kumar, Dhasarathi Kumar, Jogender Kumar, Kamal Kumar, Mukesh Kumar, Nitesh Kumar, Nithin Kumar, Tarun Kumar, Tushar Kumar, Vijay Kumar, Vikash Kumar, Subramanian Kumaran, Jibin Kunjavara, Setor K Kunutsor, Almagul Kurmanova, Om P Kurmi, Maria Dyah Kurniasari, Krishna Prasad Kurpad, Asep Kusnali, Christina Yeni Kustanti, Dian Kusuma, Tezer Kutluk, Assylkhan Kuttybayev, Evans F Kyei, Grace Kwakyewaa Kyei, Frank Kyei-Arthur, Ville Kytö, Pallavi L C, Adriano La Vecchia, Carlo La Vecchia, Alessio Lachi, Muhammad Awwal Ladan, Abraham K Lagat, Chandrakant Lahariya, Daphne Teck Ching Lai, Balzhan Lakanova, Anita Lakhani, Tea Lallukka, Judit Lám, Iván Landires, Berthold Langguth, Ariane Laplante-Lévesque, Laura Lara-Castor, Savita Lasrado, Kamaluddin Latief, Areeba Latif, Mahrukh Latif, Jerrald Lau, Paolo Lauriola, Aliyu Lawan, Teniola Lawanson, Harriet L S Lawford, Eilean Rathinasamy Lazarus, Dai Quang Le, Duc Tin Le, Thao Thi Thu Le, Caterina Ledda, Ivan Lee, Paul H Lee, Seung Won Lee, Yo Han Lee, James Leigh, Vasileios Leivaditis, Matthew J Lennon, Matilde Leonardi, Elvynna Leong, Negin Letafatkar, Chengfeng Li, Hui Li, Jiaying Li, Jie Li, Ming-Chieh Li, Si Li, Wei Li, Weilong Li, Zhaolong Adrian Li, Zhengrui Li, Yanxue Lian, Chen Liao, Stephen S Lim, Jialing Lin, Queran Lin, Shuzhi Lin, Daniel Lindholm, Christine Linehan, Yuewei Ling, Shai Linn, Haipeng Liu, Jue Liu, Xianliang Liu, Xiaofeng Liu, Xuefeng Liu, Zhe Liu, Zhenyu Liu, Erand Llanaj, Michael J Loftus, Valerie Lohner, José Francisco López-Gil, Platon D Lopukhov, Stefan Lorkowski, Rafael Lozano, Shanjie Luan, Jailos Lubinda, Taraneh Lucas, Giancarlo Lucchetti, Alessandra Lugo, Raimundas Lunevicius, Huaxia Luo, Lisha Luo, Susu Luo, Lei Lv, Miltiadis D Lytras, Ellina Lytvyak, Kevin Sheng-Kai Ma, Zheng Feei Ma, Raymond Saa-Eru Maalman, Kelsey Lynn Maass, Mahmoud Mabrok, Nikolaos Machairas, Monika Machoy, Seyed Ataollah Madinezad, Aurea Marilia Madureira-Carvalho, Pasquale Maffia, Sasikumar Mahalingam, Samatar Abshir Mahamed, Nozad Hussein Mahmood, Shakeel Ahmed Ibne Mahmood, Alireza Mahmoudi, My Tra Mai, Hao Mai Xuan, Rituparna Maiti, Marek Majdan, Abdelrahman M Makram, Omar M Makram, Mohammad-Reza Malekpour, Reza Malekzadeh, Hardeep Singh Malhotra, Ahmad Azam Malik, Farihah Malik, Deborah Carvalho Malta, Mustapha Mangdow, Jyothsna Manikkath, Yosef Manla, Fahmida Mannan, Farheen Mansoor, Marjan Mansourian, Mohammad Ali Mansournia, Lorenzo Giovanni Mantovani, Changkun Mao, Tahir Maqbool, Bishnu P Marasini, Hamid Reza Marateb, Joemer C Maravilla, Adilson Marques, Bernardo Alfonso Martinez-Guerra, Ramon Martinez-Piedra, Daniela Martini, Santi Martini, Francisco Rogerlândio Martins-Melo, Miquel Martorell, Winfried März, Roy Rillera Marzo, Sammer Marzouk, Sugeng Mashudi, Stefano Masi, Yasith Mathangasinghe, Stephanie Mathieson, Alexander G Mathioudakis, Medha Mathur, Neeta Mathur, Rita Mattiello, Richard James

Maude, Pallab K Maulik, Miranda L May, Mahsa Mayeli, Mohsen Mazidi, Antonio Mazzotti, Ikechukwu Innocent Mbachu, Martin McKee, Michael A McPhail, Steven M McPhail, Rishi P Mediratta, Jitendra Meena, Medhin Mehari, Riffat Mehboob, Ravi Mehrotra, Vini Mehta, Tesfahun Mekene Meto, Hadush Negash Meles, Addisu Melese, Satish Melwani, Aishe Memetova, Walter Mendoza, Godfred Antony Menezes, Ritesh G Menezes, Emiru Ayalew Mengistie, George A Mensah, Sultan Ayoub Meo, Michelangelo Mercogliano, Atte Meretoja, Tuomo J Meretoja, Tomislav Mestrovic, Chamila Dinushi Kukulege Mettananda, Sachith Mettananda, Mohamed M M Metwally, Adquate Mhlanga, Tomasz Miazgowski, Irmina Maria Michalek, Andrea Michelerio, Hiwot Soboksa Mideksa, Kebadnew Mulatu Mihretie, Ted R Miller, Giuseppe Minervini, Wai-kit Ming, GK Mini, Mojgan Mirghafourvand, Andreea Mirica, Alireza Mirkheshti, Seyed Ali Mirshahvalad, Mizan Kiros Mirutse, Maryam Mirzaei, Archana Mishra, Ashim Mishra, Vinaytosh Mishra, Philip B Mitchell, Sayan Mitra, Chaitanya Mittal, Mohammadreza Mobayen, Madeline E Moberg, Shivani Modi, Ahmed Ismail Mohamed, Heba M Mohamed, Jama Mohamed, Mona Gamal Mohamed, Nouh Saad Mohamed, Khabab Abbasher Hussien Mohamed Ahmed, Taj Mohammad, Abdolreza Mohammadi, Mohammad Reza Mohammadi, Abdollah Mohammadian-Hafshejani, Ibrahim Mohammadzadeh, Abdulwase Mohammed, Ammas Siraj Mohammed, Hussen Mohammed, Omer Mohammed, Shafiu Mohammed, Suleiman Mohammed, Yahaya Mohammed, Mohammad Mohseni, Tsz-ngai Mok, Amin Mokari-Yamchi, Ali H Mokdad, Sabrina Molinaro, Amirabbas Mollaei, Shaher Momani, Lorenzo Monasta, Amirabbas Monazzami, Himel Mondal, Marco Montalti, Yousef Moradi, Mohammad Moradi-Joo, Maziar Moradi-Lakeh, Paula Moraga, Lidia Morawska, Rafael Silveira Moreira, Mahmoud M Morsy, Reza Mosaddeghi Heris, Jonathan F Mosser, Elias Mossialos, Maha Motavvef, Vincent Mougin, Asma Mousavi, Seyede Zohre Mousavi, Amin Mousavi Khaneghah, Seyed Mohamad Sadegh Mousavi Kiasary, Amanda Movo, Hagar Lotfy Mowafy, Kimia Mozahheb Yousefi, Matías Mrejen, Rabia Mubarak, Sumaira Mubarik, Steward Mudenda, Faraz Mughal, Syed Aun Muhammad, Muhammad Solihuddin Muhtar, Oscar J Mujica, Sukhes Mukherjee, Sumoni Mukherjee, Amartya Mukhopadhyay, M A Muktadir, Sileshi Mulatu, Francesk Mulita, Chalie Mulugeta, Damaris Felistus Mulwa, Javier Muñoz Laguna, Anjana Munshi, Efren Murillo-Zamora, Ali Mushtaq, Mubarak Taiwo Mustapha, Sathish Muthu, Saravanan Muthupandian, Claude Mambo Muvunyi, Woojae Myung, Amin Nabavi, Fatemehzahra Naddafi, Ayoub Nafei, Ahamarshan Jayaraman Nagarajan, Ganesh R Naik, Gurudatta Naik, Firzan Nainu, Sanjeev Nair, Hastyar Hama Rashid Najmuldeen, Noureddin Nakhostin Ansari, Gopal Nambi, Ni Gusti Ayu Nanditha, Vinay Nangia, Jobert Richie Nansseu, Ibrahim A Naqid, Aparna Ichalangod Narayana, Shumaila Nargus, Delaram Narimani Davani, Yvonne Nartey, Bruno Ramos Nascimento, Gustavo G Nascimento, Abdallah Y Naser, Abdulqadir J Nashwan, Hamide Nasiri, Mahmoud Nassar, Zuhair S Natto, Javaid Nauman, Samidi Nirasha Kumari Navaratna, Biswa Prakash Nayak, Shalini Ganesh Nayak, Vinod C Nayak, Shumaila Naz, Athare Nazri-Panjaki, G Takop Nchanji, Sabina Onyinye Nduaguba, Amanuel Tebabal Nega, Meti T Negassa, Chernet Tafere Negesse, Ionut Negoi, Ruxandra Irina Negoi, Alina Gabriela Negru, Chakib Nejjari, Samata Nepal, Olivia D Nesbit, Henok Biresaw Netsere, Marie Ng, Georges Nguefack-Tsague, Josephine W Ngunjiri, Cuong Tat Nguyen, Dang Nguyen, Huong-Dung Thi Nguyen, Nghia Phu Nguyen, The Phuong Nguyen, Van Thanh Nguyen, Ambe Marius Ngwa, Robina Khan Niazi, Luciano Nieddu, Yeshambel T Nigatu, Ali Nikoobar, Vikram Niranjan, Abebe Melis Nisro, Chukwudi A Nnaji, Shuhei Nomura, Syed Toukir Ahmed Noor, Mohammadamin Noorafrooz, Mamoona Noreen, Masoud Noroozi, Jean Jacques Noubiap, Mehran Nouri, Taylor Noyes, Valentine C Nriagu, Chisom Adaobi Nri-Ezedi, Jean Claude Nshimiyimana, Fred Nugen, Mengistu H Nunemo, Nurfatimah Nurfatimah, Dieta Nurrika, Sylvester Dodzi Nyadanu, Felix Kwasi Nyande, Bogdan Oancea, Ramez M Odat, Fabio Massimo Oddi, Ismail A Odetokun, Oluwakemi Ololade Odukoya, Joseph

Kojo Oduro, Michael Safo Oduro, Oluwafunmilayo Tosin Ogundeko-Olugbami, Abiola Ogunkoya, Oluwafunmbi Ebenezer Ogunmiluyi, In-Hwan Oh, Sarah Oh, Hassan Okati-Aliabad, Sylvester Reuben Okeke, Deborah Oluwatosin Okeke-Obayemi, Akinkunmi Paul Okekunle, Olalekan John Okesanya, Osaretin Christabel Okonji, Bolanle Adeyemi Ola, Oluwaseyi Isaiah Olabisi, Oladotun Victor Olalusi, Matthew Idowu Olatubi, Arão Belitardo Oliveira, Gláucia Maria Moraes Oliveira, Abdulhakeem Abayomi Olorukooba, Oluseye Olalekan Oludoye, Ronald Olum, Bolajoko Olubukunola Olusanya, Jacob Olusegun Olusanya, Oluwafemi G Oluwole, Folorunsho Bright Omage, Goran Latif Omer, Abidemi E Omonisi, Kanyin Liane Ong, Sandersan Onie, Obinna E Onwujekwe, Oluwaseyi Aina Gbolade Opesemowo, John Nelson Opio, Marcel Opitz, Aksoltan Shyhdurdyevna Oradova, Michal Ordak, Verner N Orish, Raffaele Ornello, Atakan Orscelik, Alberto Ortiz, Esteban Ortiz-Prado, Augustus Osborne, Samuel M Ostroff, John W Ostrominski, Uchechukwu Levi Osuagwu, Olayinka Osuolale, Elham H Othman, Adrian Otoiu, Abdu Oumer, Jerry John Ouner, Amel Ouyahia, Mayowa O Owolabi, Irene Amoakoh Owusu, Oladayo Ayobami Oyebanji, Kolapo Oyebola, Tope Oyelade, Kehinde Adewole Oyeniran, Oyetunde T Oyeyemi, Ilker Ozsahin, Mahesh P A, Kevin Pacheco-Barrios, Alicia Padron-Monedero, Jagadish Rao Padubidri, Dimpal Manilal Paija, Anton Pak, Yeganeh Pakbaz, Pramod Kumar Pal, Tamás Palicz, Raffaele Palladino, Tejasri Paluvai, Feng Pan, Sujogya Kumar Panda, Songhomitra Panda-Jonas, Deepshikha Pande Katare, Seithikurippu R Pandi-Perumal, Victoria Pando-Robles, Apurvakumar Pandya, Helena Ullyartha Pangaribuan, Georgios D Panos, Leonidas D Panos, Ioannis Pantazopoulos, Anca Pantea Stoian, Giovanni Paolino, Mario Virgilio Papa, Ilias Papadimopoulos, Paraskevi Papadopoulou, Peyvand Parhizkar Roudsari, Romil R Parikh, Chulwoo Park, Seoyeon Park, Arpit Parmar, Roberto Passera, Jay Patel, Mitesh Patel, Neel Navinkumar Patel, Sangram Kishor Patel, Satyananda Patel, Bharat Smita Umakant Patil, Shankargouda Patil, Dimitrios Patoulias, Apurba Patra, Venkata Suresh Patthipati, Shrikant Pawar, Shubhadarshini Pawar, Hamidreza Pazoki Toroudi, Neil Pearce, Amy E Peden, Paolo Pedersini, Jarmila Pekarcikova, Louise Penberthy, Veincent Christian Filipino Pepito, Emmanuel K Peprah, Prince Peprah, João Perdigão, Gavin Pereira, Gladymar Perez Chacon, Arokiasamy Perianayagam, Norberto Perico, Simone Perna, Konrad Pesudovs, Pavlo Petakh, Ionela-Roxana Petcu, Olumuyiwa James Peter, Fanny Emily Petermann-Rocha, William A Petri, Hoang Nhat Pham, Hoang Tran Pham, Tung Thanh Pham, Anil K Philip, Michael R Phillips, Zahra Zahid Piracha, Edoardo Pirera, Moein Piroozkhkah, Saeed Pirouzpanah, Enrico Pisoni, Evgenii Plotnikov, Indrashis Podder, Dimitri Poddighe, Roman V Polibin, Ramesh Poluru, Arjun Pon Avudaiappan, Ville T Ponkilainen, Ion Popa, Djordje S Popovic, Thantrira Porntaveetus, Sajjad Pourasghary, Reza Pourbabaki, Farzad Pourghazi, Naeimeh Pourtaheri, Sergio I Prada, Jalandhar Pradhan, Rifky Octavia Pradipta, Akila Prashant, Elton Junio Sady Prates, Natalie Pritchett, Harsh Priya, Nicola Riccardo Pugliese, Jagadeesh Puvvula, Nameer Hashim Qasim, Ibrahim Qattea, Xiang Qi, Zhipeng Qi, Yanan Qiao, Zahiruddin Syed Quazi, Navid Rabiee, Reza Rabiei, Basuki Rachmat, Raghu Anekal Radhakrishnan, Venkatraman Radhakrishnan, Maja R Radojčić, Negar Radpour, Hadi Raeisi Shahraki, Lida Rafati, Ibrar Rafique, Pracheth Raghuveer, Fakher Rahim, Hawbash Mohammed-Amin Rahim, Sajjad Rahimi, Vafa Rahimi-Movaghar, Fryad Majeed Rahman, Mahbubur Rahman, Md Mosfequr Rahman, Mohammad Hifz Ur Rahman, Mohammad Meshbahur Rahman, Mosiur Rahman, Amir Masoud Rahmani, Saeed Rahmani, Masoud Rahmati, Ghasem Rahmatpour Rokni, Hakim Rahmoune, Diego Raimondo, Ivano Raimondo, Sunil Kumar Raina, Jeffrey Pradeep Raj, Adarsh Raja, Sathish Rajaa, Erta Rajabi, Gunaseelan Rajendran, Judah Rajendran, Vinoth Rajendran, Shaman Rajindrajith, Pushp Lata Rajpoot, Prashant Rajput, Mahmoud Mohammed Ramadan, Majed Ramadan, Kadar Ramadhan, Chitra Ramasamy, Shakthi Kumaran Ramasamy, Zahra Ramezani, Marzieh Ramezani Farani, Robinson Ramírez-Vélez, Juwel Rana, Kirtan Rana, Shailendra Singh Rana, Chhabi Lal Ranabhat, Nemanja Rancic, Smitha

Rani, Fatemeh - Ranjbar Noei, Chythra R Rao, Kumuda Rao, Mithun Rao, Davide Rasella, Sina Rashedi, Vahid Rashedi, Mamunur Rashid, Mohammad-Mahdi Rashidi, Mohammad Aziz Rasouli, Ashkan Rasouli-Saravani, Azad Rasul, Devarajan Rathish, Abdur Rauf, Santosh Kumar Rauniyar, Ilari Rautalin, Ramin Ravangard, David Laith Rawaf, Lal Rawal, Reza Rawassizadeh, Bahman Razi, C Mahony Reategui-Rivera, Elrashdy Redwan, Aqeeb Ur Rehman, Faizan Ur Rehman, Wajiha Rehman, Lennart Reifels, Rainer Reile, Giuseppe Remuzzi, Bhageerathy Reshmi, Stefano Restaino, Luis Felipe Reyes, Mina Rezaei, Nazila Rezaei, Nima Rezaei, Mohsen Rezaeian, Donya Rezazadeh Eidgahi, Taeho Gregory Rhee, Yohanes Andy Rias, Antonio Luiz P Ribeiro, Tércia Moreira Ribeiro da Silva, Jennifer Rickard, Moattar Raza Rizvi, Hannah Elizabeth Robinson-Oden, Hermano Alexandre Lima Rocha, João Rocha Rocha-Gomes, Mónica Rodrigues, Thales Philipe Rodrigues da Silva, Jefferson Antonio Buendia Rodriguez, Leonardo Roever, Peter Rohloff, Iftitakhur Rohmah, Susanne Röhr, David Rojas-Rueda, Megan L Rolfzen, Debby Syahru Romadlon, Michele Romoli, Luca Ronfani, Kevin T Root, Emily Rosenblad, Amirhossein Roshanshad, Morteza Rostamian, Gregory A Roth, Kunle Rotimi, Himanshu Sekhar Rout, Hanieh Rouzbahani, Reza Rouzbahani, Jemma V Rowlands, Adrija Roy, Bedanta Roy, Priyanka Roy, Sharmistha Roy, Shubhanjali Roy, Simanta Roy, Parameswari Royapuram Parthasarathy, Enrico Rubagotti, Susan Fred Rumisha, Michele Russo, Godfrey Mutashambara Rwegerera, Aly M A Saad, Michela Sabbatucci, Maha Mohamed Saber-Ayad, Siamak Sabour, Perminder S Sachdev, Seyed Kiarash Sadat Rafiei, Basema Ahmad Saddik, Bashdar Abuzed Sadee, Tarannom Sadegh, Ehsan Sadeghi, Erfan Sadeghi, Fatemeh Sadeghi-Ghyassi, Mohd Saeed, Umar Saeed, Maryam Saeedi, Mahdi Safdarian, Sare Safi, Sher Zaman Safi, Rajesh Sagar, Mastooreh Sagharichi, Amene Saghazadeh, Dominic Sagoe, Indranil Saha, Nondo Saha, Fatemeh Saheb Sharif-Askari, Narjes Saheb Sharif-Askari, Amirhossein Sahebkar, Kirti Sundar Sahu, Zahra Saif, S Mohammad Sajadi, Md Refat Uz Zaman Sajib, Mirza Rizwan Sajid, Dorsa Salabat, Payman Salamati, Luciane B Salaroli, Mohamed A Saleh, Leili Salehi, Mahdi Salehi, Marwa Rashad Salem, Mohammed Z Y Salem, Aanuoluwa James Salemcity, Dauda Salihu, Sohrab Salimi, Malik Sallam, Hossein Samadi Kafil, Jayami Eshana Samaranayake, Saad Samargandy, Waqas Sami, Yoseph Leonardo Samodra, Abdallah M Samy, Sandeep G Sangle, Elaheh Sanjari, Sathish Sankar, Francesco Sanmarchi, Francesca Sanna, Damian F Santomauro, Itamar S Santos, Lucas H C C Santos, Milena M Santric-Milicevic, Adekunle Sanyaolu, Bruno Piassi Sao Jose, Krishna Prasad Sapkota, Sivan Yegnanarayana Iyer Saraswathy, Yaser Sarikhani, Hemen Sarma, Mohammad Sarmadi, Gargi Sachin Sarode, Sachin C Sarode, Benn Sartorius, Arash Sarveazad, Michele Sassano, Mukesh Kumar Sathya Narayanan, Maheswar Satpathy, Reza Sattarpour, Davide Sattin, Mehrdad Savabi Far, Monika Sawhney, Sangeeta Gopal Saxena, Ganesh Kumar Saya, Abu Sayeed, Christophe Schinckus, Jurgen Carlo Schmidt, Maria Inês Schmidt, Rachel D Schneider, Art Schuermans, Austin E Schumacher, Aletta Elisabeth Schutte, Ghil Schwarz, David C Schwebel, Falk Schwendicke, Sneha Annie Sebastian, Amin Sedigh, Soraya Seedat, Mario Šekerija, Muthamizh Selvamani, Vimalraj Selvaraj, Yuliya Semenova, Mohammad H Semreen, Fikadu Waltengus Sendeku, Pallav Sengupta, Yigit Can Senol, Subramanian Senthilkumaran, Sadaf G Sepanlou, Edson Serván-Mori, Yashendra Sethi, Seyed Mohammad Seyed Alshohadaei, Allen Seylani, Abubakar Sha'aban, Mahan Shafie, Arezoo Shafieioun, Shazlin Shaharudin, Muhammad Shahbaz, Samiah Shahid, Syed Ahsan Shahid, Endrit Shahini, Fatemeh Shahrahmani, Hamid R Shahsavari, Moyad Jamal Shahwan, Masood Ali Shaikh, Alireza Shakeri, Ali Shakerimoghaddam, Ali S Shalash, Muhammad Aaqib Shamim, Farzane Shams, Mehran Shams-Beyranvand, Anas Shamsi, Alfiya Shamsutdinova, Dan Shan, Shan Shan, Mohd Shanawaz, Amin Sharifan, Javad Sharifi Rad, Avimanu Sharma, Bhoopesh Kumar Sharma, Bunty Sharma, Gaurav Sharma, Kamal Sharma, Kamlesh Sharma, Manoj Sharma, Ravi Kumar Sharma, Ujjawal Sharma, Vishal Sharma, Shamee Shastry, Maryam Shayan, Babangida Shehu Bappah, Fateme Sheida, Ali Sheidaei, Ali

Sheikhy, Rekha Raghuveer Shenoy, Samendra P Sherchan, B Suresh Kumar Shetty, Shiran Shetty, Fanchao Shi, Fang Shi, Amir Shiani, Belayneh Fentahun Shibesh, Kenji Shibuya, Desalegn Shiferaw, Tariku Shimels, Md Monir Hossain Shimul, Min-Jeong Shin, Rahman Shiri, Reza Shirkoohi, Aminu Shittu, Abdul-karim Olayinka Shitu, Ivy Shiue, Velizar Shivarov, Nathan A Shlobin, Ambreen Shoaib, Shayan Shojaei, Sina Shool, Seyed Afshin Shorofi, Sunil Shrestha, Suleiman Adeiza Shuaibu, Kerem Shuval, Zahra Siavashpour, Nicole Remaliah Samantha Sibuyi, Emmanuel Edwar Siddig, Ahmed Kamal Siddiqi, Diego Augusto Santos Silva, João Pedro Silva, Luís Manuel Lopes Rodrigues Silva, Padam Prasad Simkhada, Biagio Simonetti, Abhinav Singh, Amit Singh, Balbir Bagicha Singh, Baljinder Singh, Bhim Pratap Singh, Harmanjit Singh, Harpreet Singh, Jasvinder A Singh, Jawahar Singh, Kalpana Singh, Mayank Singh, Narinder Pal Singh, Paramdeep Singh, Poornima Suryanath Singh, Puneetpal Singh, Rakesh K Singh, Samer Singh, Satwinder Singh, Surendra Singh, Surjit Singh, Mukesh Kumar Sinha, Robert Sinto, Sarah Brooke Sirota, Dagne Feleke Siyoum, Natia Skhvitaridze, Anna Aleksandrovna Skryabina, David A Sleet, Mahdieh SobhZahedi, Marzieh Soheili, MdSalman Sohel, Somaye Sohrabi, Shipra Solanki, Lencho Kajela Solbana, Solikhah Solikhah, Sameh S M Soliman, Weiyi Song, Aayushi Sood, Prashant Sood, Soroush Soraneh, Reed J D Sorensen, Joan B Soriano, Fernando Sousa, Marco Aurelio Sousa, Ireneous N Soyiri, Ceren Soylu, Michael Spartalis, Chandrashekhar T Sreeramareddy, Suresh Kumar Srinivasamurthy, Shyamkumar Sriram, Prateek Srivastav, Devin Bailey Srivastava, Lauryn K Stafford, Jeffrey D Stanaway, Muhammad Haroon Stanikzai, Nadine Steckling-Muschack, Dan J Stein, Caitlyn Steiner, Jaimie D Steinmetz, Paschalis Steiropoulos, Blossom Christa Maree Stephan, Aleksandar Stevanović, Leo Stockfelt, Sebastian Straube, Jacob L Stubbs, Peter Stubbs, Omer Subasi, Narayan Subedi, Alisha Suhag, Hasnat Sujon, Thitiporn Sukaew, Surajo Kamilu Sulaiman, Auwal Garba Suleiman, Muritala Suleiman Odidi, Muhammad Suleman, Mark J M Sullman, Anusha Sultan Meo, Haitong Zhe Sun, Jing Sun, Mao-ling Sun, Xiaodong Sun, Xiaohui Sun, Zhong Sun, Zhuanlan Sun, Suraj Sundaragiri, Thanigaivel Sundaram, Johan Sundström, David Sunkersing, Sumam Sunny, Vinay Suresh, Hani Susianti, Chandan Kumar Swain, Vivianne M Swart, Dayinta Annisa Syaiful, Tasmin L Symons, Lukasz Szarpak, Mindy D Szeto, Sree Sudha T Y, Payam Tabaee Damavandi, Rafael Tabarés-Seisdedos, Fatemeh Sadat Tabatabaei, Seyed Shahaboddin Tabatabaei, Seyyed Mohammad Tabatabaei, Seyed-Amir Tabatabaeizadeh, Shima Tabatabai, Celine Tabche, Mohammad Tabish, Takahiro Tabuchi, Getu Ferenji Tadesse, Farzad Taghizadeh-Hesary, Zanan Mohammed-Ameen Taha, Jabeen Taiba, Shima Tajabadi, Iman M Talaat, Mircea Tampa, Jacques Lukenze Tamuzi, Ker-Kan Tan, Mohammad Tanashat, Haosu Tang, Mohsan Tanveer, Abiyu Abadi Tareke, Sarvenaz Taridashti, Ingan Ukur Tarigan, Mengistie Kassahun Tariku, Saba Tariq, Aigul Yelgondiyevna Tazhiyeva, Tarilate Temedie-Asogwa, Mohamad-Hani Temsah, Masayuki Teramoto, Azimeraw Arega Tesfu, Nahom Worku Teshager, Gizachew A Tessema, Jay Tewari, Alireza Teymouri, Chandan Kumar Thakur, Kavumpurathu Raman Thankappan, Rekha Thapar, Ismaeel Tharwat, Samar Tharwat, Rasiah Thayakaran, Muthu Thiruvengadam, Manuel Sebastian Thomas, Wei Tian, Jansje Henny Vera Ticoalu, Madi Tleshev, Sojit Tomo, Marcello Tonelli, Roman Topor-Madry, Mathilde Touvier, Marcos Roberto Tovani-Palone, Khaled Trabelsi, Quynh Thuy Huong Tran, Tam Quoc Minh Tran, Thang Huu Tran, Nguyen Tran Minh Duc, Domenico Trico, Indang Trihandini, Manjari Tripathi, Tulika Tripathi, Samuel Joseph Tromans, Quynh Xuan Nguyen Truong, Thien Tan Tri Tai Truyen, Gary Tse, Vasilis-Spyridon Tseriotis, Evangelia Eirini Tsermpini, Lorainne Tudor Car, Munkhtuya Tumurkhuu, Zhouting Tuo, Biruk Shalmeno Tusa, Sok Cin Tye, Stefanos Tyrovolas, Aniefiok John Udoakang, Atta Ullah, Himayat Ullah, Saeed Ullah, Muhammad Umair, Hauwa Onozasi Umar, Lawan Umar, Muhammad Umar,[‡] Muhammad Umar,[§] Shehu Salihu Umar, Eduardo A Undurraga, Bhaskaran Unnikrishnan, Dinesh Upadhya, Era Upadhyay, Dipan Uppal, Daniele Urso, Jibrin Sammani Usman, Kelechi Julian Uzor, Hande

Uzunçıbuk, Pratyusha Vadagam, Asokan Govindaraj Vaithinathan, Pascual R Valdez, Mario Valenti, Zahir Vally, Jef Van den Eynde, Javad Varasteh, Joe Varghese, Pavani Varma, Tommi Juhani Vasankari, Sampara Vasishta, Srivatsa Surya Vasudevan, Alireza Vaysi, Siavash Vaziri, Narayanaswamy Venketasubramanian, Madhur Verma, Megan Verma, Poonam Verma, Massimiliano Veroux, Georgios-Ioannis Verras, Simone Vidale, Mathavaswami Vijayageetha, Simone Villa, Jorge Hugo Villafañe, Leonardo Villani, David Villarreal-Zegarra, Francesco S Violante, Senthil Visaga Ambi, Luciano Magalhães Vitorino, Vasily Vlassov, Stein Emil Vollset, Avina Vongpradith, Theo Vos, Mehdi Vosoughi, Elpida Vounzoulaki, Linh Vu, Isidora S Vujcic, Krishna Dhavan Vyas, Henok Toga Wada, Yasir Waheed, Mohd Wahid, Mugi Wahidin, Mandaras Tariku Walde, Megha Walia, Jin-Yi Wan, Arvinder Wander, Fang Wang, Fulin Wang, Junshi Wang, Liang Wang, Qingzhi Wang, Ruixuan Wang, Shu Wang, Wanzhou Wang, Xing Wang, Xuequan Wang, Yan Wang, Yanzhong Wang, Yichen Wang, Youxin Wang, Yuan-Pang Wang, Zhihua Wang, Tanveer A Wani, Mary Njeri Wanjau, Ahmed Bilal Waqar, Muhammad Waqas, John W Ward, Paul Ward, Toyiba Hiyaru Wassie, Stefanie Watson, Ishanka Weerasekara, Fei-Long Wei, Xueying Wei, Robert G Weintraub, Daniel J Weiss, Eli J Weiss, Katherine M Wells, Andrea Werdecker, Ronny Westerman, Taweewat Wiangkham, Yohanes Cakrapradipta Wibowo, Dakshitha Praneeth Wickramasinghe, Nuwan Darshana Wickramasinghe, Samuel Wiebe, Angga Wilandika, Peter Willeit, Shadrach Wilson, Andrew Awuah Wireko, Charles Shey Wiysonge, Abay Tadesse Woday, Bogdan Wojtyniak, Nathnael Abera Woldehana, Dawit Habte Woldeyes, Axel Walter Wolf, Tewodros Eshete Wonde, Yen Jun Wong, Daniel Tarekegn Worede, Abdulhalik Workicho, Minichil Chanie Worku, Ai-Min Wu, Chenkai Wu, Felicia Wu, James Fan Wu, Jinyi Wu, Peng Wu, Zenghong Wu, Yihun Miskir Wubie, Ratna Dwi Wulandari, Zhijia Xia, Guangqin Xiao, Lishun Xiao, Na Xiao, Wanqing Xie, Site Xu, Suowen Xu, Xiaoyue Xu, Yvonne Yiru Xu, Mukesh Kumar Yadav, Vikas Yadav, Mahnaz Yadollahi, Saba Yahoo (Syed), Galal Yahya, Kazumasa Yamagishi, Guangcan Yan, Haibo Yang, Yuichiro Yano, Haiqiang Yao, Laiang Yao, Amir Yarahmadi, Habib Yaribeygi, Haya Yasin, Mohamed A Yassin, Yuichi Yasufuku, Sanni Yaya, Pengpeng Ye, Meghdad Yeganeh, Ali Cem Yekdeş, Mohammad Hossein YektaKooshali, Kuanysh A Yergaliyev, Subah Abderehim Yesuf, Saber Yezli, Siyan Yi, Dehui Yin, Paul Yip, Malede Berihun Yismaw, Yazachew Engida Yismaw, Dong Keon Yon, Naohiro Yonemoto, Seok-Jun Yoon, Mustafa Z Younis, Saideh Yousefi, Abdilahi Yousuf, Chuanhua Yu, Yong Yu, Hui Yuan, Faith H Yuh, Ghazala Yunus, Umar Yunusa, Siddhesh Zadey, Vesna Zadnik, Mubashir Zafar, Manijeh Zaghampour, Emilia Zainal Abidin, Fathiah Zakham, Nazar Zaki, Giulia Zamagni, Nelson Zamora, Hussaini Zandam, Aurora Zanghì, Heather J Zar, Kourosh Zarea, Mohammed Zawiah, Mohammed G M Zeariya, Abay Mulu Zenebe, Sebastian Zensen, Nejimu Biza Zepro, Eyael M Zeru, Tiansong Zhan, Yongle Zhan, Beijian Zhang, Casper J P Zhang, Haijun Zhang, Julio Min Fei Zhang, Kexin Zhang, Liqun Zhang, Meixin Zhang, Xiaoyi Zhang, Xiu-Hang Zhang, Yunquan Zhang, Zhiqiang Zhang, Sholpan Bolatovna Zhangelova, Hanqing Zhao, Jianhui Zhao, Jiefeng Zhao, Yang Zhao, Zhongyi Zhao, Anthony Zhong, Claire Chenwen Zhong, Jiayan Zhou, Juexiao Zhou, Bin Zhu, Abzal Zhumagaliuly, Magdalena Zielińska, Ghazal Zoghi, Mohamed Ali Zoromba, Zhiyong Zou, Rafat Mohammad Zrieq, Liesl J Zuhlke, Lilik Zuhriyah, Alimuddin Zumla, Ahed H Zyoud, Sa'ed H Zyoud, Shaher H Zyoud, Eve E Wool,[†] and Christopher J L Murray.[†]

*joint first authors

†joint senior authors

Affiliations

Institute for Health Metrics and Evaluation (Prof M Naghavi PhD, H H Kyu PhD, R W Aldridge PhD, J O Amlag MPH, C M Antony MA, D T Araki MPH, A Y Aravkin PhD, G J Bertolacci MS, K Bhangdia MS, C Bisignano MPH, Prof M Brauer DSc, E D Brewer, K M Cercy BS, C S Chen BA, I Chihara PhD, E Chung MD, E Cousin PhD, E J Cowart BS, A Crist BSc, J A Cruz BS, G T Culbreth PhD, M Cunningham MSc, X Dai PhD, N Davis Weaver MPH, A Deen MPH, Prof L Degenhardt PhD, K Estep MPA, Prof V L Feigin PhD, A J Ferrari PhD, L M Force MD, J E Fuller MLIS, W M Gardner MPH, S Ghoba MS, D Haile PhD, E B Hamilton MPH, C Han BA, H Han MSc, A A Harris MPH, Prof S I Hay FMedSci, J He MSc, K S Ikuta MD, M Kirk MPH, J M Kocarnik PhD, L Lara-Castor PhD, Prof S S Lim PhD, Prof R Lozano MD, K L Maass PhD, M L May MPH, T Mestrovic PhD, M E Moberg MS, Prof A H Mokdad PhD, J F Mosser MD, V Mougin BA, A Movo MPH, N Nanditha PhD, O D Nesbit MA, M Ng PhD, T Noyes MPH, K L Ong PhD, S M Ostroff PhD, L Penberthy MS, N Pritchett DrPH, H E Robinson-Oden MLIS, E Rosenblad MPH, G A Roth MD, D F Santomauro PhD, R D Schneider MPPM, A E Schumacher PhD, S B Sirota MA, R J D Sorensen PhD, L K Stafford MS, J D Stanaway PhD, C Steiner MPH, J D Steinmetz PhD, V M Swart MPH, Me Verma MSc, Prof S E Vollset DrPH, A Vongpradith BA, Prof T Vos PhD, S Watson MS, E J Weiss BS, K M Wells BA, S Wilson BS, Y Xu MPH, F H Yuh MPA, M Zhang MS, E E Wool MPH, Prof C J L Murray DPhil), Department of Health Metrics Sciences, School of Medicine (Prof M Naghavi PhD, H H Kyu PhD, R W Aldridge PhD, A Y Aravkin PhD, E Cousin PhD, X Dai PhD, L M Force MD, Prof S I Hay FMedSci, L Lara-Castor PhD, Prof S S Lim PhD, Prof R Lozano MD, Prof A H Mokdad PhD, G A Roth MD, B Sartorius PhD, J D Stanaway PhD, Prof S E Vollset DrPH, Prof T Vos PhD, Prof C J L Murray DPhil), Department of Applied Mathematics (A Y Aravkin PhD), Department of Pediatrics (E Chung MD), Department of Neurology (R Kalani MD), Department of Radiology (F Khosravi MD), Cardiothoracic Imaging Section (F Khosravi MD), Department of Global Health (S Kochhar MD, R J D Sorensen PhD), Henry M Jackson School of International Studies (S M Ostroff PhD), Division of Cardiology (G A Roth MD), University of Washington, Seattle, WA, USA; Amity Institute of Public Health (Prof B A PhD), Amity University, Uttar Pradesh, India; Department of Anesthesiology (Prof D Abtahi MD, Prof A Mirkheshti MD, S Salimi MD, S Seyed Alshohadaei MD, A Shakeri MD), National Nutrition and Food Technology Research Institute (M Ajami PhD, Z Hadian PhD), Department of Oral and Maxillofacial Surgery (Mo Alam MSc), School of Medicine (G Arjmand MD, M Asadi Anar MD, J Behjati MD, M Golmohammadi MD, S Madinezad MD, S Sadat Rafiei MD), Research Institute of Dental Sciences (Prof S Asgary MSc), School of Medical Education and Learning Technologies (S Bagheri PhD, S Sohrabi PhD), Shahid Rajii Hospital (E Baghizadeh MD), Anesthesiology Research Center (F Baghizadeh MD), Cancer Research Center (M Bayat MD, M Gholamalizadeh PhD), Internal Medicine Department (H Borhany MD), Department of Anesthesia, Critical Care and Pain Medicine (Prof A Dabbagh MD), Department of Community Nutrition (S Doaei PhD), School of Nursing and Midwifery (F Ghadirian PhD), Faculty of Medicine (A Ghaffari Jolfayi MD, M Sagharichi Dipl), Obstetrics and Gynecology Department (E Ghotbi MD), Department of General Medicine (M Karimi MD), Department of Neurosurgery (H Khayat Kashani MD), Social Determinants of Health Research Center (Prof A Kolahi MD, A Nikoobar BSc, M Rashidi MD), Injury Prevention and Safety Promotion Research Center (T Lucas PhD), Skull Base Research Center (I Mohammadzadeh MD), Department of Audiology, School of Rehabilitation (S Mousavi PhD), Department of Health Information Technology and Management (R Rabiei PhD), Department of Nephrology and Urology (N Radpour MD), Department of Immunology (A Rasouli-Saravani PhD), Department of Epidemiology (Prof S Sabour PhD), School of Public Health (S Sadat Rafiei MD), Ophthalmic Research Center (S Safi PhD), Ophthalmic Research Center (ORC) (M Shayan MD), Radio-Oncology Department of Shohadaye Tajrish Hospital (Z Siavashpour PhD), Neurology

Department (S Tabatabaei MD), Department of Medical Education (S Tabatabai PhD), Shahid Beheshti University of Medical Sciences, Tehran, Iran (M Aalipour MD); Department of Nursing (H Aalruz PhD), Al Zaytoonah University of Jordan, Amman, Jordan; Department of Radiation Oncology (H S Ababneh MD), Cardiovascular Research Center (S Abohashem MPH), Department of Orthopaedic Surgery (M Ilyas MBBS), Department of Anesthesia, Critical Care and Pain Medicine (Prof J Kang PhD), Department of Radiology (Xiao Liu PhD), Department of Orthopaedics (O Subasi PhD), Massachusetts General Hospital, Boston, MA, USA (A Eighaei Sedeh MD, M Kim MD); Menzies Institute for Medical Research (B J Abafita MSc, F Pan PhD), University of Tasmania, Hobart, TAS, Australia; School of Health & Life Scinces (U O Abaraogu PhD), University of the West of Scotland, Paisley, UK; Department of Medical Rehabilitation (U O Abaraogu PhD), Department of Pharmacology and Therapeutics (Prof O E Onwujekwe PhD), University of Nigeria Nsukka, Enugu, Nigeria; Department of Legal and Economic Studies (Prof C Abbafati PhD), Department of Public Health and Infectious Diseases (M S Cattaruzza PhD), La Sapienza University, Rome, Italy; Infectious and Tropical Research Center (M Abbasi PhD), Department of Health Policy and Management (Prof L Doshmangir PhD), Department of Immunology (F Jadidi-Niaragh PhD), Social Determinants of Health Research Center (S Karimi PhD), Faculty of Nursing and Midwifery (Prof M Mirghafourvand PhD), Neurosciences Research Center (NSRC) (R Mosaddeghi Heris MD), Student Research Committee (R Mosaddeghi Heris MD), Department of Geriatric Health (F Naddafi PhD), Molecular Medicine Research Center (S Pirouzpanah PhD), Iranian Research Center for Evidence-based Medicine (F Sadeghi-Ghyassi PhD), Drug Applied Research Center (H Samadi Kafil PhD), Women's Reproductive Health Research Center (R Sattarpour MD), Tabriz University of Medical Sciences, Tabriz, Iran; Department of Medicine (F Abbaspour MD), Department of Global Health Sciences (S Ghasemi Assl MD), Center for Tobacco Control Research and Education (P Kodali PhD), Division of Cardiology (J Noubiap MD), Department of Neurosurgery (A Orscelik MD, Y Senol MD), School of Nursing (J Ouner PhD), University of California San Francisco, San Francisco, CA, USA; Advanced Diagnostic and Interventional Radiology Research Center (H Abbastabar PhD), Research Center for Immunodeficiencies (H Abolhassani PhD, Prof Ni Rezaei PhD, A Saghazadeh MD), Tehran University School of Medicine (T Adl Parvar MD), Urology Research Center (Prof S Aghamir PhD, A Mohammadi MD), School of Medicine (N S Ahmadi MD, A Azarboo MD, H Farrokhpour MD, N Kazemi Rad MD, S Khanmohammadi MD, D Salabat MD), Orthopedic Department (N Anaraki MD), Department of Health Information Management (S Ayyoubzadeh PhD), Department of Medicine (A Azimi MD), Rheumatology Research Center (A Azizan PhD), School of Public Health (N Bahmanziari PhD, F Hosseini PharmD, A Sheidaei PhD), Non-communicable Diseases Research Center (M Bastan MD, M Malekpour MD, M Rashidi MD, Na Rezaei MD), Pastor Institute (M Bayat MD), Department of Scientific Research (F Chichagi MD), Department of Neurosurgery (P Delbari MD), Department of Radiology (R Elahi MD, A Teymouri MD), Digestive Diseases Research Institute (DDRI) (S Fahimi MD, Prof R Malekzadeh MD, P Parhizkar Roudsari MD, S G Sepanlou MD), Pediatric Infectious Disease Research Center (M Farahmand PhD), Dentistry Research Institute (F Farshad DDS), Obesity and Eating Habits Research Center (F Farsi MD), Department of Ophthalmology (Prof F Ghassemi MD, A Mahmoudi MD), Sina Trauma and Surgery Research Center (M Hassan Zadeh Tabatabaei MD, M Jalloh MD, Prof V Rahimi-Movaghar MD, Z Ramezani MD, Prof P Salamati MD, S Shool MD), Department of Pharmacoeconomics and Pharmaceutical Administration (F Hosseini PharmD), Department of Immunology (J Karami PhD), Cardiac Primary Prevention Research Center (S Kazemian MD), Department of Cardiac Electrophysiology (S Kazemian MD), Health Equity Research Center (A Khosravi PhD), Children's Medical Center (Prof F Kompani MD), Department of Epidemiology and Biostatistics (M Mansournia PhD, E Sanjari PhD), Urology Department (A Mohammadi MD), Department

of Psychiatry (M Motavvef MD), Tehran Heart Center, Cardiovascular Diseases Research Institute (A Mousavi MD, S Shojaei MD), Department of Physiotherapy (Prof N Nakhostin Ansari PhD), Research Center for War-affected People (Prof N Nakhostin Ansari PhD), Family Health Research Institute (M Noorafrooz MD), Cardiac Research Center (P Parhizkar Roudsari MD), Department of Bioinformatics (M Piroozkhkah MD), Department of Infectious Diseases and Tropical Medicine (E Rajabi MD), Non-Communicable Diseases Research Center (NCDRC) (D Salabat MD), Maternal, Fetal, and Neonatal Research Center (R Sattarpour MD), Department of Medical Education (A Sedigh PhD), Department of Neurology (M Shafie MD), Department of Endocrinology and Metabolism Population Sciences (A Sheikhy MD), Cancer Research Center (R Shirkoohi PhD), Cancer Biology Research Center (R Shirkoohi PhD), Student Scientific Research Center (S Shojaei MD), Tehran University of Medical Sciences, Tehran, Iran (A Azizan PhD); College of Pharmacy (A H A Abd Al Magied MSc), College of Pharmacy and Health Sciences (A Al Amiry MS, H Yasin PhD), Centre of Medical and Bio-allied Health Sciences Research (A Al Amiry MS, Prof G Gupta PhD), College of Medicine (Su Dutta PhD), Nonlinear Dynamics Research Center (NDRC) (Prof S Momani PhD), Center for Medical and Bio-Allied Health Sciences Research (Prof M J Shahwan PhD, A Shamsi PhD), Ajman University, Ajman, United Arab Emirates (Prof N Hassan PhD); Department of Epidemiology (S Abd ElHafeez DrPH), Pediatric Dentistry and Dental Public Health Department (Prof O A A Elmeligy PhD), Tropical Health Department (R M Ghazy PhD), Department of Pathology (Prof I M Talaat PhD), Alexandria University, Alexandria, Egypt; College of Pharmacy (Prof A N Abdalla PhD), Umm Al-Qura University, Makka, Saudi Arabia; Hull York Medical School (M A Abdalla PhD), University of Hull, Hull, UK; Department of Biology (Prof E M Abdallah PhD), College of Applied Medical Sciences (Prof F A Alhumaydhi PhD), Department of Medical Laboratories, College of Applied Medical Sciences (Prof K Allemailem PhD), Department of Health Informatics (Q Jamal PhD), Qassim University, Buraydah, Saudi Arabia; Department of Health and Nutrition (B A Abdeeq MSc), Save the Children, Hargeisa, Somalia; School of Nursing (Prof N M I Abdel Razeq PhD), School of Pharmacy (S M Aleidi PhD, Prof Y Bustanji PhD), The School of Medicine (M Al-Iede MD), Department of Mathematics (Prof S Momani PhD), Department of Pathology, Microbiology and Forensic Medicine (M Sallam PhD), Department of Clinical Laboratories and Forensic Medicine (M Sallam PhD), Department of Movement Sciences and Sports Training (K Trabelsi PhD), The University of Jordan, Amman, Jordan (Prof L A Dardas PhD); College of Pharmacy (A Abdelgalil PhD), Pediatric Intensive Care Unit (A Al-Eyadhy MD, Prof M Temsah MD), Pediatric Department (Prof K A Alhasan MD), Department of Computer Engineering (K Aurangzeb PhD), Section of Adult Hematology (Prof G M T ElGohary MD), Department of Physiology (Prof S Meo PhD), University Diabetes Center (A Sultan Meo MPH), Research Chair for Evidence-Based Health Care and Knowledge Translation (Prof M Temsah MD), Department of Pharmaceutical Chemistry College of Pharmacy (Prof T A Wani PhD), King Saud University, Riyadh, Saudi Arabia; Basic Science Department (Prof R Abdel-Hameed PhD, N M Abourashed PhD), Department of Biology (Prof M Adnan PhD, Prof M Saeed PhD), Department of Public Health (Prof F D Algahtani PhD, M G M Zeariya PhD), College of Applied Medical Science (S Ashraf PhD), Department of Medical Laboratory Sciences (N K Binsaleh PhD), Department of Chemistry (A Haque PhD), College of Medicine (Y S Khan MD), Department of Biochemistry (Prof M Kuddus PhD), Medical and Diagnostic Research Centre (Prof C T Sreeramareddy MD), Department of Basic Science (G Yunus PhD), Family and Community Medicine Department (M Zafar PhD), Department of Medical-Surgical Nursing (R M Zrieq PhD), University of Hail, Hail, Saudi Arabia; Chemistry Department (Prof R Abdel-Hameed PhD), Department of Zoology and Entomology (A I Hasaballah PhD, M G M Zeariya PhD), Al-Azhar University, Cairo, Egypt; Department of Surgery (M Abdelmasseh MD), Marshall University, Huntington, WV, USA; Department of Cardiovascular Medicine

(M Abdelnabi MBBCh, Ra Ibrahim MD), Department of Pulmonary Critical Care Medicine (S Li MD), Mayo Clinic, Phoenix, AZ, USA; Department of Medical Laboratory Science (Prof W M Abdel-Rahman PhD), Clinical Sciences Department (Prof E Abu-Gharbieh PhD, H J Barqawi MPhil, Prof R Halwani PhD, Prof M M Ramadan PhD, Prof M M Saber-Ayad PhD, N Saheb Sharif-Askari PhD, Prof I M Talaat PhD), Department of Nursing (Prof S Abuhammad PhD), Department of Pharmacy Practice and Pharmacotherapeutics (A Y Abuhelwa PhD), Department of Restorative Dentistry (A B Acharya PhD), College of Pharmacy (S M Aleidi PhD, H Y Alniss PhD, Prof M H Semreen PhD), College of Medicine (Prof A Amin PhD, Prof R Halwani PhD, Prof M A Saleh PhD), Department of Physiotherapy (A Arumugam PhD), Center of Excellence of Cancer Research (Prof R Bendardaf PhD), Department of Basic Biomedical Sciences (Prof Y Bustanji PhD), Department of Basic Medical Sciences (M A Eladl PhD, Prof W El-Huneidi PhD), Sharjah Institute for Medical Research (N M Elemam PhD), Sharjah Institute of Medical Sciences (F Saheb Sharif-Askari PhD), Research Institute of Medical & Health Sciences (Prof M H Semreen PhD), Department of Medicinal Chemistry (S S M Soliman PhD), University of Sharjah, Sharjah, United Arab Emirates (K A Altirkawi MD); Faculty of Veterinary Medicine (A Abdous MD), Islamic Azad University, Karaj, Iran; Department of Cardiovascular Medicine (M M Abdrabou PhD), Faculty of Veterinary Medicine (Prof N Abu-Elala PhD), Public Health and Community Medicine Department (Prof T T Amin MD), Department of Clinical and Chemical Pathology (Prof M A Elmonem PhD), Department of Neurology (Prof A Hassan MD), Faculty of Pharmacy (H M Mohamed PhD), Medical Microbiology and Immunology Department (H L Mowafy MD), Cairo University, Cairo, Egypt; Komar University of Science and Technology, Sulaymaniyah, Iraq (J M Abdul Aziz MSc); Baxshin Hospital (J M Abdul Aziz MSc), Baxshin Research Center, Sulaymaniyah, Iraq; Community and Maternity Nursing Unit (D M Abdulah MPH), Department of Pathology and Microbiology (Meq S Ahmed PhD), Duhok Research Centre (Z M Taha PhD), University of Duhok, Duhok, Iraq; Department of Physiotherapy (A Abdullahi PhD, A W Awotidebe PhD, J S Usman PhD), Department of Community Medicine (Prof M A Gadanya MD), Department of Nursing Science (M Ladan PhD), Bayero University Kano, Kano, Nigeria; Department of Physiotherapy (A Abdullahi PhD), Federal University Wukari, Wukari, Nigeria; Department of Research (T Abdul-Rahman MD), Toufik's World Medical Association, Sumy, Ukraine; Department of Epidemiology and Biostatistics (H Abebe Getahun MSc), Department of Health Systems and Policy (M B Alemu MSc), Department of Reproductive Health (Z N Azene MPH), Department of Pharmacology (Z D Kifle MSc), School of Nursing (H B Netsere MSc), Department of Pediatrics and Child Health (N W Teshager MD), Department of Pharmacy (M Worku MSc), University of Gondar, Gondar, Ethiopia (A Mohammed MSc); Department of Neurosurgery (Ai Abedi MD), Keck School of Medicine (Ai Abedi MD), Department of Radiology (M Fotouhi MD), University of Southern California, Los Angeles, CA, USA; Department of Emergency Medicine (Ar Abedi MD), Department of Food Safety and Hygiene (M Aminzare PhD), School of Medicine (M Ashrafi MD, H Nasiri MD), Department of Immunology (S Athari PhD), Department of Critical Care and Emergency Nursing (N Hanifi PhD), Zanjan University of Medical Sciences, Zanjan, Iran; Yale School of Medicine (P Abedi MD), Department of Radiology and Biomedical Imaging (Xiao Liu PhD, M Mayeli MD, S Rahmani MD), Department of Genetics (Shr Pawar PhD), Department of Psychiatry (T Rhee PhD), Yale University, New Haven, CT, USA; Neuroendocrine Unit (P Abedi MD), Department of Radiology (S Abohashem MPH), Department of Global Health and Population (B Bhandari PhD), Division of Cardiovascular Medicine (G Chi MD), Nutrition Department (G Dalla Costa MD), Department of Medicine (M Kokkorakis BSc, F Tabatabaei MD), Department of Health Policy and Oral Epidemiology (Z S Natto DrPH), Department of Global Health and Social Medicine (S Onie PhD), Cardiovascular Division (J W Ostrominski MD), Department of Physical Medicine and Rehabilitation (K Pacheco-Barrios MD),

Division of Global Health Equity (P Rohloff MD), Department of Ophthalmology (M Shayan MD), Joslin Diabetes Center (S Tye PhD), Harvard Medical School (A Zhong MA), Harvard University, Boston, MA, USA; School of Pharmacy (A Abejew MSc), Department of Midwifery (A Abie MSc, B A Alemayehu MSc, F Alemnew MSc, W F Balcha MSc, A T Nega MSc, F W Sendeku MSc, A A Tesfu MSc, T H Wassie MSc), Department of Emergency and Critical Care Nursing (O Adal MSc, A G Belayneh MSc, Y M Wubie MPH), Department of Medical Laboratory Science (G M Aregu MSc, A Melese MSc), School of Public Health (M A Asemahagn PhD), Department of Public Health (M Belayneh PhD), Department of Nursing (A Y Berhie MSc), Department of Health Promotion and Behavioural Science (E K Bogale MPH), Department of Environmental Health (T S Bunare MPH), Department of Nutrition and Dietetics (H A Derseh MPH), College of Medicine and Health Science (K Y Gete MD), Department of Medical Microbiology (A D Habteyohannes PhD), Department of Adult Health Nursing (E A Mengistie MSc), Department of Epidemiology and Biostatistics (K M Mihretie MPH), Department of Pediatrics and Child Health Nursing (S Mulatu MSc), Department of Pharmacy (C T Negesse MSc, M Yismaw MSc), College of Medicine and Health Sciences (H B Netsere MSc), Department of Anatomy, Histology, and Embryology (D H Woldeyes MSc), Department of Pharmacology (Y E Yismaw MSc), Bahir Dar University, Bahir Dar, Ethiopia; Postgraduate Department (Prof R Abeldaño Zuñiga PhD), University of Sierra Sur, Miahuatlan de Porfirio Diaz, Mexico; Yhteiskuntadatatieteen keskus (Centre for Social Data Science) (Prof R Abeldaño Zuñiga PhD), Department of Public Health (Prof M Kivimäki PhD, Prof T Lallukka PhD), University of Helsinki, Helsinki, Finland (T J Meretoja MD); Nuffield Department of Population Health (S Abid MSc), Oxford Vaccine Group (O Akeju MPH), Nuffield Department of Surgical Sciences (O Almidani MSc, S Bandyopadhyay MPH), Nuffield Department of Orthopaedics, Rheumatology, and Musculoskeletal Sciences (S Graham PhD), Big Data Institute, Nuffield Department of Population Health (Zha Guo PhD), Nuffield Department of Medicine (Prof R J Maude PhD, B Sartorius PhD), Centre for Global Epilepsy (M Romoli MD), Department of Psychiatry (V Suresh MBBS), University of Oxford, Oxford, UK; National Heart Foundation Hospital and Research Institute, Dhaka, Bangladesh (S Abid MSc); Department of Biomedical Sciences (S Abidi PhD), Department of Medicine (R Akhmedullin MPH), Nazarbayev University School of Medicine, Astana, Kazakhstan; Department of Internal Medicine (O O Abiodun FWACP), Federal Medical Centre, Abuja, Nigeria; Department of Family and Community Health (R G Aboagye MPH), Department of Epidemiology and Biostatistics (L A Adzigbli BSc, S A Bosoka MPhil, R K Dowou MPhil), School of Medicine (R S Maalman PhD), Department of Nursing (F K Nyande PhD), Department of Microbiology and Immunology (Prof V N Orish PhD), University of Health and Allied Sciences, Ho, Ghana; School of Population Health (R G Aboagye MPH, Z Dai PhD, V Keshri PhD, Prof B A Saddik PhD, Prof A E Schutte PhD, X Xu PhD), Centre for Social Research in Health (I Y Addo PhD, S R Okeke PhD), St George and Sutherland Clinical School (H Akbarialiabad MD), The Graduate School of Biomedical Engineering (Prof H Alinejad Rokny PhD), Transport and Road Safety (TARS) Research Centre (S Boufous PhD), National Drug and Alcohol Research Centre (Prof L Degenhardt PhD), The George Institute for Global health (F Haghdoost PhD), Centre for Healthy Brain Ageing (M J Lennon PhD), International Centre for Future Health Systems (J Lin PhD), School of Medicine (Prof P K Maulik PhD), Discipline of Psychiatry and Mental Health (Prof P B Mitchell MD), Kirby Institute (G Perez Chacon PhD), School of Optometry and Vision Science (Prof K Pesudovs PhD), Centre for Healthy Brain Ageing (CHeBA) (S Röhr PhD, S Röhr PhD), School of Psychiatry (Prof P S Sachdev MD), The George Institute for Global Health (P Ye PhD), University of New South Wales, Sydney, NSW, Australia; Department of Medical Biochemistry and Biophysics (H Abolhassani PhD), Department of Global Public Health (Prof P Allebeck MD), Department of Neurobiology, Care Sciences and Society (Prof J Ärnlöv PhD, B Bizzozero-Peroni

PhD), Department of Medical Epidemiology and Biostatistics (Prof J J Carrero PhD), Department of Neurobiology, Care Sciences, and Society (S Fereshtehnejad PhD), Karolinska Institutet Campus Solna (A Javanmardi MD), Department of Molecular Medicine and Surgery (Prof J H Kauppila MD), Karolinska Institutet (Karolinska Institute), Stockholm, Sweden; Department of Sport, Exercise and Rehabilitation (U S Abonie PhD), Northumbria University, Newcastle, UK; Zoology Department (N M Abourashed PhD), Benha University, Benha, Egypt; Department of Physical Pharmacy and Pharmacokinetics (M Abouzid PharmD), Chair and Department of Medical Microbiology (Prof T M Karpiński DDS), Poznan University of Medical Sciences, Poznan, Poland; Department of Cardiovascular Disease (D Abramov MD), Loma Linda University Medical Center, Loma Linda, CA, USA; Department of Pediatric Dentistry (Prof L Abreu PhD), Department of Internal Medicine (Prof L C Brant PhD, Prof A P Ribeiro MD), Department of Maternal-Child Nursing and Public Health (Prof D C Malta PhD, E J S Prates BS), Department of Clinical Medicine (Prof B R Nascimento PhD), Clinical Hospital (Prof B R Nascimento PhD), Centre of Telehealth (Prof A P Ribeiro MD), Escola de Enfermagem da UFMG (Prof T M Ribeiro da Silva PhD), Vaccination Research Observatory (T Rodrigues da Silva PhD), Department of Infectious Diseases and Tropical Medicine (B P Sao Jose PhD), Universidade Federal de Minas Gerais (Federal University of Minas Gerais), Belo Horizonte, Brazil (M A Sousa PhD); Clinical Pharmacy and Therapeutics Department (Prof R K Abu Farha PhD), Applied Science Research Center (A B Al-Tammemi MPH, R M Zrieq PhD), Department of Clinical Nutrition and Dietetics (Prof M E M Faris PhD), Faculty of Nursing (E H Othman PhD), Applied Science Private University, Amman, Jordan; Community Health Nursing Department (F H A Abuadas PhD), Preventive Dentistry Department (Prof M K Alam PhD), College of Medicine (R Basri PhD), Jouf University, Sakaka, Saudi Arabia; Graduate School of Public Health (A K Abubakar MPH, H Jamil MD), St. Luke's International University, Tokyo, Japan; Division of Population Data Science (A K Abubakar MPH, H Jamil MD), National Cancer Center, Tokyo, Japan; Faculty of Veterinary Medicine (Prof N Abu-Elala PhD), King Salman International University, Rus Sudr, Egypt; Department of Biopharmaceutics and Clinical Pharmacy (Prof E Abu-Gharbieh PhD), College of Pharmacy (Prof S Aburuz PhD), The University of Jordan School of Medicine (Prof M A Al-Abbadi MD), University of Jordan, Amman, Jordan; Department of Maternal and Child Health Nursing (Prof S Abuhammad PhD), Jordan University of Science and Technology, irbid, Jordan; Medical Research Center (H J Abukhadijah MPH), Department of Pharmacy (D Abushanab MSc), Department of Surgery (A Alansari MD), Surgical Research Section (A R Al-Qudimat MPH, A EL Omri PhD), Nursing & Midwifery Research Department (NMRD) (G Joy MSc, J Kunjavara PhD, A J Nashwan PhD), Research Department (K Singh PhD), Hematology Section (Prof M A Yassin MD), Hamad Medical Corporation, Doha, Qatar; College of Health Sciences (Prof N M Abu-Rmeileh PhD), College of Nursing (Prof F N Alhalaiqa PhD), Rehabilitation Sciences Department (Prof N A Almasri PhD), Department of Pharmaceutical Sciences (Prof K H Alzoubi PhD), Department of Population Medicine (Prof G Babu PhD), Department of Rehabilitation Sciences (S F Kanaan PhD), College of Medicine (Prof Y Kinfu PhD, Prof M A Yassin MD), Social and Economic Survey Research Institute (SESRI) (Prof A Perianayagam PhD), Qatar University, Doha, Qatar; Birzeit University, Ramallah, Palestine (Prof N M Abu-Rmeileh PhD); Department of Pharmacology and Therapeutics (Prof S Aburuz PhD), Institute of Public Health (Prof L A Ahmed PhD, Prof M Grivna PhD), College of Medicine and Health Sciences (Prof M Z Allouh PhD, Prof G Khan PhD, J Nauman PhD), Department of Veterinary Medicine (H O Khalifa PhD), Department of Computer Science and Software Engineering (Prof N Zaki PhD), United Arab Emirates University, Al Ain, United Arab Emirates (B Aden PhD); Department of Disease Control (M M K Accrombessi PhD), Department of Infectious Disease Epidemiology (J M Azam PhD), Faculty of Epidemiology and Population Health (Prof Sh Chen PhD), Epidemiology Programme (A Hafiz PhD),

Department of Non-Communicable Disease Epidemiology (M Iwagami PhD), Department of Health Services Research and Policy (Prof M McKee DSc), Department of Medical Statistics (Prof N Pearce PhD), London School of Hygiene & Tropical Medicine, London, UK; Department of Clinical Research (M M K Accrombessi PhD), Clinical Research Institute of Benin (IRCB), Abomey-Calavi, Benin; Department of Forensic Medicine and Toxicology (A Acharya MD), Department of Clinical Microbiology (C Thakur PhD), Karnali Academy of Health Sciences (KAHS), Jumla, Nepal; Department of Diagnostic and Interventional Radiology (L C Adams PhD), School of Medicine and Health (F Busch MD), Technical University of Munich, Munich, Germany; Department of Cardiothoracic Surgery (D N Kamtam MS), Division of Pediatric Hospital Medicine (R P Mediratta MD), School of Medicine (Ji Zhou PhD), Stanford University, Palo Alto, CA, USA (L C Adams PhD); Department of Global Health (A A Adamu PhD, Prof C S Wiysonge MD), Department of Psychiatry (Prof S Seedat PhD), Department of Epidemiology (J L Tamuzi MSc), Stellenbosch University, Cape Town, South Africa; Cochrane South Africa (A A Adamu PhD, Prof C S Wiysonge MD), South African Medical Research Council, Cape Town, South Africa; School of Medicine (I Y Addo PhD), Faculty of Medicine and Health (D B Anderson PhD, R Cairns PhD, M M Kamal MPH), Sydney Musculoskeletal Health (D B Anderson PhD, S Mathieson PhD), School of Health Science (A Carvalho-e-Silva PhD), School of Pharmacy and Charles Perkins Centre (Z Dai PhD), School of Public Health (Prof T R Driscoll PhD), Institute for Musculoskeletal Health (M Jamshidi PhD), Asbestos and Dust Diseases Research Institute (J Leigh MD), Central Clinical School, Faculty of Medicine and Health (S Mitra PhD), School of Veterinary Science (B B Singh PhD), University of Sydney, Sydney, NSW, Australia (S Mathieson PhD, S R Okeke PhD); Department of Health Promotion, Education and Behavior (O A Adeagbo PhD), Department of Epidemiology and Biostatistics (A M Alfalki MPH), University of South Carolina, Columbia, SC, USA; Department of Public Health (O A Adeagbo PhD), School of Nursing and Public Health (A W Awotidebe PhD), University of KwaZulu-Natal, Durban, South Africa; Department of Microbiology (T A Adebisi BSc), Ladoke Akintola University, Osogbo, Nigeria; NMC Healthcare (T A Adebisi BSc), Independent Consultant, Sharjah, United Arab Emirates; Department of Sociology (I A Adedeji PhD), Olabisi Onabanjo University, Ago-Iwoye, Nigeria; Department of Immunology (K A Adedokun MSc), Roswell Park Comprehensive Cancer Center, Buffalo, NY, USA; Graduate Program Division (K A Adedokun MSc), University at Buffalo, Buffalo, NY, USA; Department of Pediatrics (O E Adegbile MD), East Tennessee State University, Johnson City, TN, USA; Center for Cardiovascular Risk Research, Johnson City, TN, USA (O E Adegbile MD); Translational Research Team (N A Adegoke PhD), Melanoma Institute Australia (N A Adegoke PhD), The University of Sydney, Sydney, NSW, Australia; Department of Family Medicine (O T Adeleke MD), College of Health Sciences (O I Olabisi PhD), Bowen University, Iwo, Nigeria; Department of Family Medicine (O T Adeleke MD), Bowen University Teaching Hospital, Ogbomoso, Nigeria; Department of Nursing (B G Adema MSc), Wolaita Sodo University, Woliata Sodo, Ethiopia; Institute of Public Health (B Aden PhD), Walden University, Al Ain, United Arab Emirates; Department of Microbiology (I A Adesina PhD), University of Medical Sciences, Ondo, Ondo City, Nigeria; Slum and Rural Health Initiative Research Academy (M A Adesina BPT), Slum and Rural Health Initiative, Ibadan, Nigeria; Department of Physiotherapy (M A Adesina BPT), Department of Educational Counselling and Developmental Psychology (H O Adewuyi PhD), Department of Veterinary Medicine (T E Adeyeoluwa PhD), Department of Epidemiology and Medical Statistics (R F Afolabi PhD, A F Fagbamigbe PhD), Department of Periodontology and Community Dentistry (O F Fagbule FWACS), Department of Health Promotion and Education (S Ibitoye PhD, A Ogunkoya MPH), Counselling and Human Development Studies (D O Okeke-Obayemi BSc), College of Medicine (A P Okekunle PhD, O I Olabisi PhD), Department of Medicine (O V Olalusi MD, Prof M O Owolabi DrM), University of Ibadan,

Ibadan, Nigeria; Department of Biochemistry (J B Adetunji PhD), Osun State University, Osogbo, Nigeria; Department of Educational Psychology (H O Adewuyi PhD), Department of Mathematics, Science and Technology Education (O A G Opesemowo PhD), University of Johannesburg, Johannesburg, South Africa; Department of Pharmacology and Therapeutics (T E Adeyeoluwa PhD), Department of Environmental and Occupational Health (B S Anuoluwa MPH), Department of Microbiology (I A Anuoluwa PhD, O O Bello PhD, T C Ekundayo PhD), Department of Biosciences and Biotechnology (O T Oyeyemi PhD, A J Udoakang PhD), Mathematical and Computer Sciences (O Peter PhD), University of Medical Sciences, Ondo, Ondo, Nigeria; School of Public Health (M T Adhana PhD), Mekelle University, Mekelle, Ethiopia; Department of Fisheries and Marine Bioscience (R K Adhikary PhD), Jashore University of Science and Technology, Jashore, Bangladesh; Research School of Population Health (R K Adhikary PhD), School of Medicine and Psychology (D Ahmad PhD), National Centre for Epidemiology and Population Health (R A Burns PhD), Australian National University, Canberra, ACT, Australia; Apollo Institute Of Medical Sciences & Research Chittoor (Prof U Adiga PhD), Apollo Hospital, Chittoor, India; Department of Public Health (Q Adnani PhD), Universitas Padjadjaran (Padjadjaran University), Bandung, Indonesia; Department of Health Administration and Education (P O Adoma PhD), University of Education Winneba, Winneba, Ghana; School of Public Health (D Adzrago PhD), University of Texas Health Science Center at Houston, Houston, TX, USA (D T Araki MPH); Department of Public Health and Preventive Medicine (G Affinito PhD), Department of Public Health (C Fiorilla MD, R Palladino MD), University of Naples "Federico II", Naples, Italy; Department of Surgery (A M Afifi MD), University of Toledo, Toledo, OH, USA; Australian Centre for Health Services Innovation (C Afoakwah PhD, Prof S M McPhail PhD), Queensland University of Technology, Kelvin Grove, QLD, Australia; Jamieson Trauma Institute (C Afoakwah PhD), Metro North Health, Herston, QLD, Australia; Technical Services Directorate (A A Afolabi MPH), MSI Nigeria Reproductive Choices, Abuja, Nigeria; Medical Oncology Department (V Afrăsânie PhD), University of Medicine and Pharmacy '' Gr. T. Popa'' Iași in Romania, Iasi, Romania; Medical Oncology Department (V Afrăsânie PhD), Regional Institute of Oncology, Iasi, Romania; Department of Community Medicine (Prof S Afzal PhD), King Edward Memorial Hospital, Lahore, Pakistan; Department of Public Health (Prof S Afzal PhD), Public Health Institute, Lahore, Pakistan; Department of Public Health (G B Agafari PhD, M H Nunemo MPH), Department of Health Education and Health Promotion (F D Agide PhD), Wachemo University, Hossana, Ethiopia; Department of New Initiatives (Prof S B Agampodi MD), International Vaccine Institute, Seoul, South Korea; Department of Community Medicine (T C Agampodi PhD, N Wickramasinghe MD), Department of Family Medicine (Prof D Rathish PhD), Rajarata University of Sri Lanka, Anuradhapura, Sri Lanka; MM College of Pharmacy (N Aggarwal PhD), Maharishi Markandeshwar (Deemed to be University), Ambala, India; Department of Orthopedic Surgery and Sports Medicine (M Aghaalikhani MD), Boston Children's Hospital, Boston, MA, USA; Department of Neurosurgery (S Aghajanian MD), Research Center for Health, Safety and Environment (Prof L Salehi PhD), School of Medicine (M Shams-Beyranvand MSc), Alborz University of Medical Sciences, Karaj, Iran; Neuroscience Research Center (S Aghajanian MD), Health Management and Economics Research Center (J Arabloo PhD, H Ayatollahi PhD), Department of Health Information Management (H Ayatollahi PhD), School of Medicine (M Bastan MD), Department of Medical Laboratory Sciences (F Dorostkar PhD), Department of Medicine (M Fotouhi MD), Department of Cardiology (A Ghaffari Jolfayi MD), Department of Ophthalmology (H Hasani MD), Department of Biostatistics (M Imani MSc), Minimally Invasive Surgery Research Center (A Kabir MD), Endocrine Research Center (A Karimi Behnagh MD), Department of Echocardiography (A Karimi Behnagh MD), Department of Obstetrics & Gynecology (P Khalili MD), Gastrointestinal and Liver Diseases Research Center (Prof M

Moradi-Lakeh MD), Preventive Medicine and Public Health Research Center (Prof M Moradi-Lakeh MD), Antimicrobial Resistance Research Center (K Mozahheb Yousefi MD), Hazrat-e Rasool General Hospital (K Mozahheb Yousefi MD), Breast Health and Cancer Research Center (Y Pakbaz MD), Physiology Research Center (H Pazoki Toroudi PhD), Department of Physiology (H Pazoki Toroudi PhD), Colorectal Research Center (A Sarveazad PhD), Center for Technology and Innovation in Cardiovascular Informatics (S Shool MD), The Five Senses Health Institute (F Taghizadeh-Hesary MD), Iran University of Medical Sciences, Tehran, Iran (F Eghbali MD); Health Research and Innovation Sciences Center (C Agostinis Sobrinho PhD, R C D Espírito Santo PhD), Klaipeda University, Klaipeda, Lithuania; SPRINT Sport Physical Activity and Health Research & Innovation Center (C Agostinis Sobrinho PhD), Sport Physical Activity and Health Research & Innovation Center (SPRINT) (Prof L M L R Silva PhD), Polytechnic Institute of Guarda, Guarda, Portugal; Trivedi School of Biosciences (Prof A Agrawal PhD), Ashoka University, Sonipat, India; Department of Public Health Sciences (W Agyemang-Duah PhD), Queen's University, Kingston, ON, Canada; Rajaie Trauma Research Center (M Ahadi MD), Health Policy Research Center (B Amidi MD, R Khademi MD, M Nouri PhD, Y Sarikhani PhD), Student Research Committee (A Faramarzi MD), Department of Otolaryngology (A Faramarzi MD), Trauma Research Center (P Fazeli MSc, M Yadollahi MD), Department of Medical Immunology (P Fazeli MSc), Research Center for Traditional Medicine and History of Medicine (Prof M Hashempur PhD), Poostchi Ophthalmology Research Center (M Heydari PhD), Shiraz Neuroscience Research Center (M Jafarinia PhD), Non-communicable Disease Research Center (Prof R Malekzadeh MD), Department of Occupational Health and Safety Engineering (R Pourbabaki PhD), Department of Health Services Management (Prof R Ravangard PhD), Department of Biostatistics (Er Sadeghi PhD), Shiraz University of Medical Sciences, Shiraz, Iran (S Mousavi Kiasary DVM); School of Public Health (B O Ahinkorah MPhil), School of Nursing and Midwifery (M Chutiyami PhD), Discipline of Physiotherapy (P Stubbs PhD), University of Technology Sydney, Sydney, NSW, Australia; College of Medicine (A Ahmad PhD, M Tabish MPharm, H Ullah FCPS), Shaqra University, Shaqra, Saudi Arabia; Health Research Institute (D Ahmad PhD, Prof N Bagheri PhD), University of Canberra, Canberra, NSW, Australia; Biological Production Unit (F Ahmad PhD), National Institute of Health, Islamabad, Pakistan; World Health Organization (WHO), Islamabad, Pakistan (F Ahmad PhD); College of Veterinary Sciences (I Ahmad PhD), The University of Agriculture, Peshawar, Peshawar, Pakistan; Department of Research (Prof Kha Ahmad PhD), King Khaled Eye Specialist Hospital & Research Center, Riyadh, Saudi Arabia; Department of Health Informatics (Khu Ahmad PhD), Qassim University, Buraidha, Saudi Arabia; Department of Health and Biological Sciences (S Ahmad PhD), Abasyn University, Peshawar, Pakistan; Department of Natural Sciences (S Ahmad PhD), Gilbert and Rose-Marie Chagoury School of Medicine (Prof L Roever PhD), Lebanese American University, Beirut, Lebanon; School of Public Health (T Ahmad PhD), Department of Sociology (S Shan PhD), Department of Epidemiology and Biostatistics (Jia Zhao MD), Zhejiang University, Hangzhou, China; Department of Community Health Sciences (T Ahmad PhD), Sohail University, Karachi, Pakistan; College of Medicine (W Ahmad PhD), University of Cincinnati, Cincinnati, OH, USA; Department of Pharmacy Practice (Al Ahmed PhD), Riphah Institute of Pharmaceutical Sciences, Islamabad, Pakistan; Division of Infectious Diseases and Global Public Health (IDGPH) (Al Ahmed PhD), Moores Cancer Center (S Luo PhD), University of California San Diego, San Diego, CA, USA; Institute of Endemic Diseases (Ay Ahmed MSc), Department of Oral Rehabilitation (N T Hashim PhD), Department of Medicine (I Hassan MD), Faculty of Medicine (K A H Mohamed Ahmed MD), Unit of Basic Medical Sciences (E E Siddig MD), University of Khartoum, Khartoum, Sudan; Swiss Tropical and Public Health Institute (Ay Ahmed MSc), Department of Ophthalmology (Prof Z Gatzioufas PhD), Department of Epidemiology and Public Health (N Zepro MSc),

University of Basel, Basel, Switzerland; Medical Laboratory Science Department (G S Ahmed MSc, H M Rahim MSc), University of Human Development, Sulaymaniyah, Iraq; Department of Biosciences (H Ahmed PhD), COMSATS Institute of Information Technology, Islamabad, Pakistan; Manipal College of Dental Sciences, Mangalore (Prof J Ahmed MDS), Department of General Medicine (J Jeganathan MD), Department of Community Medicine (N Joseph MD, Nith Kumar MD, R Thapar MD), Kasturba Medical College (G A Menezes PhD), Department of Forensic Medicine and Toxicology (Prof J Padubidri MD, Prof B K Shetty MD), Department of Conservative Dentistry and Endodontics (M S Thomas MDS), Manipal Academy of Higher Education, Mangalore, India; Department of Health Sciences and Informatics (M Ahmed BSc), Bangladesh Institute of Innovative Health Research, Dhaka, Bangladesh; College of Nursing (Meh S Ahmed MSc), Majmaah University, Al Majmaah, Saudi Arabia; College of Medicine and Public Health (M B Ahmed PhD, B Kaambwa PhD, G R Naik PhD, J Opio MPH), Flinders Health and Medical Research Institute (N B Bulamu PhD), Health Economics Unit (B Kaambwa PhD), Department of Nursing and Health Sciences (S Shorofi PhD), Flinders University, Adelaide, SA, Australia; Faculty of Public Health (M B Ahmed PhD), Department of Health Behavior and Society (L A A Ayana MPH), School of Pharmacy (H K Kebede MSc), Institute of Health Science (A I Mohamed MSc), Department of Epidemiology (D Shiferaw MPH, A Workicho PhD), Jimma University, Jimma, Ethiopia (B Feyisa MPH); Department of Medicine (M Ahmed MBBS), Rawalpindi Medical University, Rawalpindi, Pakistan; Maternal and Child Health Division (MCHD) (S Ahmed MDS, L Hossain MPH, S Noor MS, N Saha MSc, A Sayeed MSc), International Centre for Diarrhoeal Disease Research, Bangladesh, Dhaka, Bangladesh; Department of Public Health Epidemiology (S M Ahmed MSc), Debre Berhan University, Deberbirhan, Ethiopia; Menelik II Medical and Health Science College (S M Ahmed MSc), EpiMetrics, Inc., Addis Ababa, Ethiopia; Brody School of Medicine (S Ahmed PhD), East Carolina University, Greenville, NC, USA; School of Medicine (G Aimagambetova PhD, D Galiyeva PhD, Y Semenova PhD, K A Yergaliyev DrPH), Department of Biomedical Sciences (M Aljofan PhD), Department of Medicine (J U Almazan PhD), Nazarbayev University, Astana, Kazakhstan; Clinical Academic Department of Women's Health (G Aimagambetova PhD), University Medical Center, NU Medicine, Astana, Kazakhstan; Faculty of Medicine and Public Health (B Aji DrPH), Jenderal Soedirman University, Purwokerto, Indonesia; Department of Water Engineering (S Akbarifard PhD), Graduate University of Advanced Technology, Kerman, Iran; Department of Physiology (R E Akhigbe PhD), Department of Medical Laboratory Science (A Busari MSc), Department of Medicine (A O Shitu MBBS), Ladoke Akintola University, Ogbomoso, Nigeria; School of Veterinary Medicine (O A Akinkuotu PhD), Texas Tech University, Amarillo, TX, USA; Department of Cardiology (M Akkaif PhD), Fudan University, Shanghai, China; Faculty of Health and Behavioural Sciences (W Akosile PhD), Centre for Sensorimotor Performance (D Anderlini MD), School of Public Health (A J Ferrari PhD, Md Shar Islam MSc, J C Maravilla PhD, J V Rowlands MPH, D F Santomauro PhD), Centre for the Business and Economics of Health (I Koomson PhD, A Pak PhD), UQ Centre for Clinical Research (H L S Lawford PhD), Faculty of Medicine (B Sartorius PhD), The University of Queensland, Brisbane, QLD, Australia; Chicago College of Osteopathic Medicine (A E Akrami BS), Midwestern University, Downers Grove, IL, USA; Feinberg School of Medicine (A E Akrami BS, D B Srivastava BA), Department of Microbiology and Immunology (O Ebohon MPH), Medical Scientist Training Program (S Marzouk MA), Department of Radiology (A Shafieioun MD), Department of Preventive Medicine (M Teramoto MD), Northwestern University, Chicago, IL, USA (M D Szeto MS); Centre for Academic Primary Care (R K Akyea PhD), Institute of Applied Health Research (N Bhala PhD), Division of Ophthalmology & Visual Sciences (Prof G D Panos MD), University of Nottingham, Nottingham, UK; Department of Communicable Diseases (S Al Awaidy MSc), Ministry of Health, Muscat, Oman; Middle East, Eurasia, and Africa Influenza Stakeholders

Network, Muscat, Oman (S Al Awaidy MSc); Division of Public Health Sciences (S Al Hasan PhD), Department of Research and Development (Z Al-Aly MD), Department of Surgery (S Azadnajafabad MD), Department of Psychiatry (Z Li BA), Washington University in St. Louis, St. Louis, MO, USA; Department of Urology (A Al Homsi MD, O Almidani MSc), Research Department (N Dababo MD), Department of Cardiac Surgery (Prof L Göbölös PhD), Cleveland Clinic Abu Dhabi, Abu Dhabi, United Arab Emirates; The University of Jordan (M K Al Nawayseh PhD), Jordanian Public Health Society, Amman, Jordan; American University in the Emirates, Dubai, United Arab Emirates (M K Al Nawayseh PhD); Fundamentals and Administration Department (Prof O Al Omari PhD), Department of Adult Health and Critical Care (O A M Al Zaabi PhD), Department of Geography (W Ali PhD), College of Nursing (E Lazarus PhD), Sultan Qaboos University, Muscat, Oman; Jordan Medical Association, Amman, Jordan (Z Al Ta'ani MD); Faculty of Pharmacy (Y Al Thaher PhD), Philadelphia University, Amman, Jordan; School of Pharmacy (Y Al Thaher PhD), Division of Population Medicine (A Sha'aban PhD), Cardiff University, Cardiff, UK; School of Public Health (M A M Al Zoubi PhD), Management Policy and Community Health (J A Atta MPH, Me Iqbal MPH), MD Anderson Cancer Center Department of Plastic Surgery (R Elmorsi MD), Department of Plastic Surgery (A M Hassan MD), University of Texas, Houston, TX, USA; Department of Biology (T A Alalwan PhD), College of Health and Sport Sciences (A G Vaithinathan MSc), University of Bahrain, Zallaq, Bahrain; Clinical Epidemiology Center (Z Al-Aly MD), US Department of Veterans Affairs (VA), St. Louis, MO, USA; Murdoch Business School (K Alam PhD), Murdoch University, Perth, WA, Australia; Department of Bioengineering (Ma Alam PhD), Department of Nutrition and Food Studies (S Tyrovolas PhD), George Mason University, Fairfax, VA, USA; School of Nursing (R M Al-Amer PhD), Department of Basic Sciences (Z Altaany PhD), Department of Basic Medical Sciences (R A Karasneh PhD, Prof M M Khatatbeh PhD), Faculty of Nursing (H Khatatbeh PhD), Faculty of Medicine (M Tanashat MD), Yarmouk University, Irbid, Jordan; School of Nursing and Midwifery (R M Al-Amer PhD), Western Sydney University, Sydney, NSW, Australia; Department of Nursing and Midwifery (A Alamrew MSc), College of Health Sciences (T Kitaw MSc), College of Health Science (C Mulugeta MSc), Woldia University, Woldia, Ethiopia; Department of Health Information Management and Technology (Prof T M Alanzi PhD), Deanship of Preparatory Year and Supporting Studies (Prof S El-Ashker PhD), Department of Pathology - Forensic Medicine Division (Prof R G Menezes MD), Imam Abdulrahman Bin Faisal University, Dammam, Saudi Arabia; Department of Clinical Pharmacy (F Y Al-Ashwal PhD), Al-Ayen Iraqi University, Thi-Qar, Iraq; Department of Clinical Pharmacy and Pharmacy Practice (F Y Al-Ashwal PhD), University of Science and Technology, Sana'a, Yemen; Department of Community and Mental Health (Prof M Albashtawy PhD), Al al-Bayt University, Mafraq, Jordan; Division of Gastroenterology and Hepatology (W A Aldhaleei MD), Department of Radiology (G Belge Bilgin MD, F Nugen PhD), Department of Physiology and Biomedical Engineering (Z Khashim PhD, F Pourghazi MD), Department of Cardiovascular Medicine (H Pham MD), Department of Endocrinology (M Salehi MD), Mayo Clinic, Rochester, MN, USA; General Directorate of Research and Studies (M S Aldossary MClinDent), Ministry of Health, Riyadh, Saudi Arabia; Institute of Health Informatics (R W Aldridge PhD), Department of Health Informatics (S-C Chung PhD), Department of Brain Sciences (Prof M Kivimäki PhD), Division of Medicine (T Oyelade PhD), Department of Population Health Sciences (D Sunkersing PhD), Department of Epidemiology and Public Health (V Tseriotis MSc), Center for Clinical Microbiology (Prof A Zumla PhD), University College London, London, UK; Curtin School of Population Health (M B Alemu MSc), Faculty of Health Sciences (K A Alene PhD), School of Public Health (E K Chowdhury PhD), School of Population Health (Prof P W Gething PhD, S Nyadanu PhD, G A Tessema PhD, D J Weiss PhD), enAble Institute (Prof B C M Stephan PhD), Curtin University, Perth, WA, Australia; Geospatial and Tuberculosis Research Team (K A Alene PhD), Child

Health Analytics Research Program (Prof P W Gething PhD, F Sanna PhD, D J Weiss PhD), Geospatial Health and Development Team-Child Health Analytics (J Lubinda PhD), The Malaria Atlas Project (M A McPhail PhD, S F Rumisha PhD, T L Symons PhD), Telethon Kids Institute, Perth, WA, Australia; Department of Bacteriology, Immunology, and Mycology (Prof A M Algammal PhD), Faculty of Veterinary Medicine (M Mabrok PhD), Suez Canal University, Ismailia, Egypt; College of Medicine and Health Sciences (K Al-Habbal MD), Department of Public Health and Epidemiology (Prof B A Saddik PhD), Khalifa University, Abu Dhabi, United Arab Emirates; Africa Center of Excellence for Mycotoxin and Food Safety, Minna, Nigeria (N B Alhaji PhD); Faculty of Health Sciences, Epidemiology and Population Health Department (Prof S Al-Hajj PhD), American University of Beirut, Beirut, Lebanon; British Columbia Injury Research Prevention Unit (Prof S Al-Hajj PhD), British Columbia Children's Hospital Research Institute, Vancouver, BC, Canada; Department of Health Services and Hospital Administration (M K Al-Hanawi PhD), Health Economics Research Group (M K Al-Hanawi PhD), Department of Respiratory Therapy (M A Althobiani PhD), Respiratory Therapy Unit (M A Althobiani PhD), Pediatric Dentistry Department (K K Baghlaf PhD), Department of Family and Community Medicine (Prof N S Butt PhD), Department of Physical Therapy (F Khan PhD), Rabigh Faculty of Medicine (Prof A Malik PhD), Department of Dental Public Health (Z S Natto DrPH), Department of Community Medicine (S Samargandy PhD), King Abdulaziz University, Jeddah, Saudi Arabia; Kidney and Pancreas Health Center (Prof K A Alhasan MD), Liver, Digestive, and Lifestyle Health Research Section (S A Alqahtani MD), Biostatics, Epidemiology, and Science Computing Department (S Yezli PhD), King Faisal Specialist Hospital & Research Center, Riyadh, Saudi Arabia; Faculty of Dentistry (A Alhumaidi DDS), Ibn Al-Nafis University for Medical Sciences, Sana'a, Yemen; Department of Biotechnology and Genetic Engineering (A Ali PhD, M Waqas PhD), Hazara University Mansehra, Mansehra, Pakistan; Department of Biotechnology (H M Ali MS), University of Malakand, Chakdara, Pakistan; Department of Statistics and Operations Research (I Ali PhD), Aligarh Muslim University, Aligarh, India; Department of Biological Sciences (L Ali PhD, S Naz PhD), National University of Medical Sciences (NUMS), Rawalpindi, Pakistan; School of Food and Agricultural Sciences (M Ali PhD), Department of Life Sciences (Prof M Umair PhD), University of Management and Technology, Lahore, Pakistan (A Latif BS); Department of Pharmacy (M Ali PhD), Mohammed Al-Mana College for Medical Sciences, Dammam, Saudi Arabia; Department of Medical Rehabilitation (Physiotherapy) (M U Ali PhD), Department of Microbiology (M A Isa PhD), University of Maiduguri, Maiduguri, Nigeria; Nethersole School of Nursing (M U Ali PhD), The Nethersole School of Nursing (Y Chong PhD, Jia Li PhD), Faculty of Medicine (J Huang MD), School of Public Health and Primary Care (L Yao MSc), Jockey Club School of Public Health and Primary Care (C Zhong PhD), The Chinese University of Hong Kong, Hong Kong, China; Department of Biosciences (R Ali PhD), Centre for Interdisciplinary Research in Basic Sciences (CIRBSc) (S Anwar PhD, Su Khan MSc), Centre For Interdisciplinary Research In Basic Sciences (CIRBSc) (T Mohammad PhD, A Shamsi PhD), Jamia Millia Islamia, New Delhi, India; Center for Biotechnology and Microbiology (S Ali PhD), University of Swat, Charbagh, Pakistan; Center for Biotechnology and Microbiology (S S Ali PhD, M Suleman PhD), University of Swat, Swat, Pakistan; Department of Pathophysiology and Transplantation (G Alicandro PhD), Department of Pharmacological and Biomolecular Sciences (Prof A L Catapano PhD), Department of Clinical Sciences and Community Health (Prof C La Vecchia MD), Department of Food, Environmental and Nutritional Sciences (Prof D Martini PhD), Università degli Studi di Milano (University of Milan), Milan, Italy; Cystic Fibrosis Center (G Alicandro PhD), Pediatric Emergency Department (A La Vecchia MD), Fondazione IRCCS Ospedale Maggiore Policlinico (IRCCS "Ca' Granda Maggiore Policlinico" Hospital Foundation), Milan, Italy; Institute of Health and Wellbeing (S M Alif PhD), Federation University Australia, Melbourne, VIC,

Australia; School of Public Health and Preventive Medicine (S M Alif PhD, Prof M Asghari-Jafarabadi PhD), Faculty of Medicine, Nursing, and Health Sciences (S Aslani PhD), Department of Epidemiology and Preventative Medicine (E K Chowdhury PhD), Department of Infectious Diseases (M J Loftus MBBS), Department of General Practice (S Melwani PhD), School of Primary and Allied Health Care (F Sousa PhD), Monash University, Melbourne, VIC, Australia; Biomedical Physics Group (M Alipour BSc), University of Hamburg, Hamburg, Germany; Department of Clinical and Community Pharmacy (Prof S W Al-Jabi PhD, Prof S H Zyoud PhD), Department of Pharmacy (F Amer PhD), Department of Chemistry (Prof A H Zyoud PhD), An-Najah National University, Nablus, Palestine; Family and Community Medicine Department (M S Aljohani MD), Qassim University, Al Qassim, Saudi Arabia; Department of Public Health and Community Medicine (Prof S M Aljunid PhD, Prof C T Sreeramareddy MD), International Medical University, Kuala Lumpur, Malaysia; International Centre for Casemix and Clinical Coding (Prof S M Aljunid PhD), National University of Malaysia, Bandar Tun Razak, Malaysia; College of Life Sciences (Prof A Alkhatib PhD, C C T Clark PhD, Md Shah Islam PhD), Birmingham City University, Birmingham, UK; Cardiovascular Divivsion (M Alkhawam MD), University of Alabama, Birmingham, AL, USA; Faculty of Medicine (Prof M Z Allouh PhD, Prof M S I Alyahya PhD, R M Odat MD), Department of Physical Therapy and Rehabilitation Sciences (Prof M A Alomari PhD), Department of Rehabilitation Sciences and Physical Therapy (Prof M A Alomari PhD), Department of Rehabilitation Sciences (M Al-Wardat PhD), Department of Clinical Pharmacy (Prof K H Alzoubi PhD), Department of Public Health (Prof Y S Khader PhD, Prof K A Kheirallah PhD), Jordan University of Science and Technology, Irbid, Jordan; Faculty of Nursing (W T Almagharbeh PhD), Nursing Faculty (K A Alnawafleh PhD), Prince Fahad bin Sultan Chair for Biomedical Research (S Muthupandian PhD), University of Tabuk, Tabuk, Saudi Arabia (S Muthupandian PhD); Independent Consultant, Amman, Jordan (S Al-Marwani MSc); Department of Parasitology (Prof H M Al-Mekhlafi PhD), University of Malaya, Kuala Lumpur, Malaysia; Department of Parasitology (Prof H M Al-Mekhlafi PhD), Department of Biochemistry and Molecular Biology (Prof A S Al-Zubairi PhD), Sana'a University, Sana'a, Yemen; Ophthalmology Department (A Almobayed MD), University of Miami, Miami, FL, USA; School of Public Health (M Alocious Sukumar MPH), SRM Institute of Science and Technology, Chennai, India; Faculty of Nursing (M R Alosta PhD), Department of Nursing (A H Khalifeh PhD), Zarqa University, Zarqa, Jordan; Department of Respiratory Care (J S Alqahtani PhD), Prince Sultan Military College of Health Sciences, Dammam, Saudi Arabia; Division of Gastroenterology and Hepatology (S A Alqahtani MD), Weill Cornell Medicine, New York, NY, USA; American University of the Middle East, Egaila, Kuwait (M R Alqudimat PhD); Department of Nursing (I Alrimawi PhD), Georgetown University, Washington, DC, USA; Macro-Fiscal Policy Department (S M Alrousan PhD), Ministry of Finance, Dubai, United Arab Emirates; Department of Surgery (S K Al-Sabah MD), Kuwait University, Kuwait, Kuwait; Jaber Al Ahmad Al Sabah Hospital (S K Al-Sabah MD), Ministry of Health, Kuwait, Kuwait; Department of Emergency Medicine (M A Alsabri MD), Sana'a University, Sanaa, Yemen; Pediatric Emergency Medicine Department (M A Alsabri MD), Drexel Dornsife School of Public Health (E Ezenwankwo MPH), School of Biomedical Engineering, Science and Health Systems (M Noroozi BSc), Drexel University, Philadelphia, PA, USA; Health Science Division (Z Altaany PhD), Higher Colleges of Technology, Sharjah, United Arab Emirates; Institute of Molecular Biology and Biotechnology (A Altaf PhD, M Ume Khan PhD, T Maqbool PhD, S Shahid PhD), University College of Medicine & Dentistry (Prof M Arooj PhD), University Institute of Food Science and Technology (S Bashir PhD), University Institute of Radiological Sciences and Medical Imaging Technology (Prof Z Fatima PhD, M Latif PhD), University Institute of Diet and Nutritional Sciences (A Khalil PhD), University Institute of Public Health (F Malik PhD, S Nargus PhD), Research Centre for Health Sciences (RCHS) (S Shahid PhD, M Umar‡ MBA), Lahore Business School (M Umar‡

MBA), Faculty of Sciences (Prof A B Waqar PhD), The University of Lahore, Lahore, Pakistan; Faculty of Health Sciences (A Altaf PhD), Equator University of Science and Technology, Uganda, Masaka, Uganda; Research, Policy, and Training Directorate (A B Al-Tammemi MPH), Jordan Center for Disease Control, Amman, Jordan; Department of Specialty Internal Medicine (Prof J A Al-Tawfiq MD), Johns Hopkins Aramco Healthcare, Dhahran, Saudi Arabia; Department of Medicine (Prof J A Al-Tawfiq MD), Indiana University School of Medicine, Indianapolis, IN, USA; Faculty of Health Sciences (J Alvarez-Galvez PhD), University of Cadiz, Cadiz, Spain; Research Group in Health Economics (Prof N Alvis-Guzman PhD), Universidad de Cartagena (University of Cartagena), Cartagena, Colombia; Research Group in Hospital Management and Health Policies (Prof N Alvis-Guzman PhD), Department of Economic Sciences (N J Alvis-Zakzuk MSc), Universidad de la Costa (University of the Coast), Barranquilla, Colombia; National Health Observatory (N J Alvis-Zakzuk MSc), National Institute of Health, Bogota, Colombia; Department of Clinical Pharmacology and Toxicology (H Alwafi PhD), Department of Microbiology and Parasitology (A Hafiz PhD), Institute of Center and Research Studies (F U Rehman PhD), Umm Al-Qura University, Makkah, Saudi Arabia; Department of Medical Sciences (Prof Y M Al-Worafi PhD), Azal University for Human Development, Sana'a, Yemen; Department of Clinical Sciences (Prof Y M Al-Worafi PhD), University of Science and Technology of Fujairah, Fujairah, United Arab Emirates; Department of Pediatrics (Prof H Aly MD), Lerner College of Medicine (M Balkis MD), Lerner Research Institute (Prof Xue Liu PhD), Department of Internal Medicine (A Mushtaq MD), Department of Cardiovascular Medicine (J Rajendran MD), Cleveland Clinic, Cleveland, OH, USA; Department of Physiotherapy (H Alzahrani PhD), Taif University, Taif, Saudi Arabia; Laboratory Medicine Department (Prof A S Al-Zubairi PhD), Al-Baha University, Al-Aqiq, Saudi Arabia; London School of Hygiene and Tropical Medicine (E J Amafah MSc), UCL Institute for Global Health (Prof S Jaffar PhD), University of London, London, UK; Global Health Advocacy Incubator (GHAI) (J Amafah MPH), University of Central Nicaragua, Washington, DC, USA; Isfahan Cardiovascular Research Institute, Heart Failure Research Center. (R Amani-Beni MD), Ophthalmology Department (P Bolourinejad MD), Heart Failure Research Center (B Darouei MD, D Narimani Davani MD), Department of Epidemiology and Biostatistics (Prof M Mansourian PhD), Department of Health Services Management (M Mohseni PhD), Family and Prevention Medicine (R Rouzbahani MD), Isfahan University of Medical Sciences, Isfahan, Iran; Student Research Committee (B Amidi MD), Lorestan University of Medical Sciences, Khorramabad, Iran; Summer Program (Prof A Amin PhD), Biological Science Division (M Bayat Tork MD), Department of Public Health Sciences (A Jamal BS), Pritzker School of Medicine (Prof H Yao PhD), University of Chicago, Chicago, IL, USA (Prof J Wan PhD); Department of Radiology and Radiological Science (A Amindarolzarbi MD), Department of Medicine (U Khan MD), University of Maryland, Baltimore, MD, USA; Laboratory Science Department (J Karami PhD), Operating Room Department (A Sedigh PhD), Khomein University of Medical Sciences, Khomein, Iran (S Amini PhD); Gastrointestinal and Liver Diseases Research Center (E Amini-Salehi MD, S Hassanipour PhD, N Letafatkar MD, F Sheida MD), Regenerative Medicine, Organ Procurement and Transplantation Multi-disciplinary Center (S Anvari MD), School of Health (S Doaei PhD), Gastrointestinal and Liver Disease Research Center (B Eftekhari MD, N Eslami MSc), Department of Social Medicine and Epidemiology (A Feizkhah MD), Cardiovascular Diseases Research Center (Z Ghorbani PhD), Caspian Digestive Disease Research Center (S Hassanipour PhD), Department of Environmental Health Engineering (J Jaafari PhD), Department of Medicine (A Khalili MD), Burn and Regenerative Medicine Research Center (Prof M Mobayen MD), Department of Biology (M SobhZahedi PhD), Medical Biotechnology Research Center (M YektaKooshali PhD), Guilan University of Medical Sciences, Rasht, Iran; Spiritual Health Research Center (S Amiri PhD), Nephrology and Urology Research Center (K Hushmandi PhD), Baqiyatallah University of

Medical Sciences, Tehran, Iran; Department of Health and Wellbeing (D A Amugsi PhD), African Population and Health Research Center, Nairobi, Kenya; Department of Medicine (G A Amusa MD), Department of Chemical Pathology (L C Imoh MPH), University of Jos, Jos, Nigeria; Department of Internal Medicine (G A Amusa MD), Department of Chemical Pathology (L C Imoh MPH), Jos University Teaching Hospital, Jos, Nigeria; Center for Biomedical Image Computing & Analytics (F Anagnostakis MD), Department of Pathobiology (U M Femoe PhD), Penn Medicine (S K Khokhar PhD), Population Studies Center (Weil Li PhD), Center for Global Health, Perelman School of Medicine (K Ma DDS), Department of Biostatistics, Epidemiology, and Informatics (J Puvvula PhD), University of Pennsylvania, Philadelphia, PA, USA; Dipartimento di Scienze Mediche e Chirurgiche (M Bergami PhD), Department of Medical and Surgical Sciences (Prof R Bugiardini MD, Prof A F G Cicero PhD, F Fogacci MDc, M Sassano MD, Prof F S Violante MD), Department of Biomedical and Neuromotor Sciences (S Guicciardi MD, A Mazzotti PhD, F Sanmarchi MD), Department of Medicine and Surgery (I Papadimopoulos MD), University of Bologna, Bologna, Italy (F Anagnostakis MD); Department of General Medicine (R A Ananda MD), Eastern Health, Box Hill, VIC, Australia; Faculty of Pharmacy (Prof R Ancuceanu PhD), Department of Internal Medicine (M Hostiuc PhD), Department of Legal Medicine and Bioethics (Prof S Hostiuc PhD), Department of General Surgery (I Negoi PhD), Department of Anatomy and Embryology (R I Negoi PhD), Department of Diabetes, Nutrition and Metabolic Diseases (Prof A Pantea Stoian PhD), Department of Dermatology (M Tampa PhD), Carol Davila University of Medicine and Pharmacy, Bucharest, Romania; Neurology Department (D Anderlini MD), Royal Brisbane and Women's Hospital, Brisbane, QLD, Australia; Department of General Medicine (N Anh MD), Thai Binh University of Medicine and Pharmacy in Vietnam, Thai Binh City, Viet Nam; Department of Microbiology (A A Anjorin PhD), Lagos State University, Ojo, Nigeria; Department of Management (S E Ankomah PhD, S E Ankomah PhD), Department of Population and Health (J K Oduro PhD), University of Cape Coast, Cape Coast, Ghana; Department of Public Health (K Annadurai PhD), The Apollo University, Chittoor, India; Department of Physiotherapy (S Ansari PhD), Galgotias University, Greater Noida, India; Department of Epidemiology and Biostatistics (Prof A Ansari-Moghaddam PhD), Department of Health Promotion (A Nazri-Panjaki MSc), Health Promotion Research Center (H Okati-Aliabad PhD), Zahedan University of Medical Sciences, Zahedan, Iran; Agribusiness Study Program (E Antriyandarti DrAgrSc), Sebelas Maret University, Surakarta, Indonesia; School of Chemical and Life Sciences (SCLS) (S Anwar PhD), Jamia Hamdard, New Delhi, India; Department of Surgery (S Anwar PhD), Department of Pharmacology (I Fitriana PhD), Gadjah Mada University, Yogyakarta, Indonesia; Department of Pathology (R Anwer PhD), Department of Pharmacology (T Jawaid PhD), Imam Mohammad Ibn Saud Islamic University, Riyadh, Saudi Arabia; Department of Rehabilitation Sciences (S Anwer PhD), Hong Kong Polytechnic University, Kowloon, Hong Kong, China; Rural Health Research Institute (A E Anyasodor PhD, S A I Mahmood PhD, Prof J Sun PhD), Charles Sturt University, Orange, NSW, Australia; Department of Social Sciences (F Appiah MPhil), Berekum College of Education, Berekum, Ghana; School of Public Health (F Appiah MPhil), Kwame Nkrumah University of Science and Technology, Kumasi, Ghana; Division of Gastroenterology, Hepatology, and Nutrition (J Arab MD), Division of Infectious Diseases (P R Ching MD), Virginia Commonwealth University, Richmond, VA, USA; Gastroenterology Department (J Arab MD), Department of Gastroenterology (L Diaz MD), School of Government (E A Undurraga PhD), Pontificia Universidad Catolica de Chile (Pontifical Catholic University of Chile), Santiago, Chile; Geneva University Hospital (H Arabi PhD), University of Geneva, Geneva, Switzerland; College of Pharmacy (M Arafat PhD), Al Ain University, Abu Dhabi, United Arab Emirates; College of Art and Science (D Areda PhD), Ottawa University, Surprise, AZ, USA; School of Life Sciences (D Areda PhD), Arizona State University, Tempe, AZ,

USA; Care in Long Term Conditions Research Division (J Arias de la Torre PhD), Institute of Psychiatry, Psychology & Neuroscience (D Urso MD), School of Life Course and Population Sciences (Prof Y Wang PhD), King's College London, London, UK; CIBER Epidemiology and Public Health (CIBERESP), Madrid, Spain (J Arias de la Torre PhD); Department of Cardiovascular, Endocrine-Metabolic Diseases and Aging (B Armocida MD), Istituto Superiore di Sanità (ISS), Rome, Italy; School of Health and Social Studies (Prof J Ärnlöv PhD), Dalarna University, Falun, Sweden; Department of Biotechnology (Prof J Arockiaraj PhD, T Sundaram PhD), SRM Medical College Hospital and Research Centre (J James MD), Division of Medical Research (R Janardhanan PhD), Sri Ramaswamy Memorial Institute of Science and Technology, Kattankulathur, India; Institute for Biomedical Problems (A A Artamonov PhD), Russian Academy of Sciences, Moscow, Russia; Department of Physiotherapy (A Arumugam PhD, Prof V K PhD, M K Sinha PhD), Manipal College of Pharmaceutical Sciences (S Dharmagadda PhD, J Manikkath PhD), Department of Pharmacology (S Gangachannaiah MD, R R Shenoy PhD), Prasanna School of Public Health (R Kamath MHA), Department of Community Medicine (S Kini B MD, C R Rao MD), Kasturba Medical College, Manipal (P L C MD, J P Raj DM, Prof S Shastry MD), Manipal College of Dental Sciences (Prof A I Narayana PhD, Prof R A Radhakrishnan PhD), Manipal College of Nursing (S Nayak PhD), Department of Forensic Medicine (Prof V C Nayak MD), Kasturba Medical College Mangalore (M Rao MD, Prof B Unnikrishnan MD), Department of Health Information Management (B Reshmi PhD), Kasturba Medical College (D Upadhya PhD), Manipal Academy of Higher Education, Manipal, India (Prof V Jha MD); Department of Periodontics (D Arumuganainar PhD), Department of Physiology (E Dilipan PhD), Department of Prosthodontics (D Ganapathy PhD), Department of Oral Medicine and Periodontology (Prof R D Jayasinghe MS), Saveetha Medical College and Hospital (Prof M Karobari PhD), Saveetha Dental College and Hospitals (G Minervini PhD, M Selvamani PhD, M Tovani-Palone PhD), Department of Biomaterials (N Rabiee PhD), Department of Biochemistry (P Royapuram Parthasarathy PhD), Center for Global Health Research (Prof A Sahebkar PhD), Department of Microbiology (S Sankar PhD), Saveetha University, Chennai, India; Department of Research (U R Aryal PhD), Research Department (M Dhimal PhD, B P Marasini PhD), Nepal Health Research Council, Kathmandu, Nepal; Department of Public Health (N Aryntayeva MSPH, A Kuttybayev MSc), Atchabar Scientific Research Institute (B Assembekov PhD), Atchabarov Scientific-Research Institute of Fundamental and Applied Medicine (D Davletov MD, A Zhumagaliuly MD), Population Health Research Center (Prof K Davletov PhD), Director of the Scientific and Technological Park (I R Fakhradiyev PhD), Science and Technology Park (A Ibrayeva PhD), Department of Urology (Y Ismoldayev PhD), Department of General Medical Practice No. 2 (Prof S Kamenova DMedSc), Scientific and Educational Center for Neurology and Applied Neuroscience (A Kondybayeva PhD), Research and Publication Activity Division (M Kulimbet MSc), Department of Research (B Lakanova MD), Scientific Laboratory "Center for Collective Use" (A S Oradova PhD), Department of Science (A Shamsutdinova MD, A Y Tazhiyeva PhD), Department of Prosthetic Dentistry (M Tleshev MSc), Department of Internal Disease (Prof S B Zhangelova PhD), Kazakh National Medical University, Almaty, Kazakhstan; Department of Clinical Disciplines (N Aryntayeva MSPH), Department of Clinical Subjects (A Kurmanova MD), Al Farabi Kazakh National University, Almaty, Kazakhstan; College of Medicine (M Asadi Anar MD), Department of Internal Medicine (H Pham MD), University of Arizona, Tucson, AZ, USA; Department of Community Medicine and Global Health (M Asaduzzaman MPH), University of Oslo, Oslo, Norway; Department of Pharmacy Practice (Prof S Asdaq PhD), College of Medicine (M Fareed PhD), AlMaarefa University, Riyadh, Saudi Arabia (Prof A Dutta PhD); Department of Public Health (M T Asemu MSc), College of Health Science (D Belay MSc), Debre Tabor University, Debre Tabor, Ethiopia; National Agency for Strategic Research in Medical Education (NASRME) (Prof S Asgary

MSc), Deputy for Public Health (A Khosravi PhD), Ministry of Health and Medical Education, Tehran, Iran; Cabrini Research (Prof M Asghari-Jafarabadi PhD), Cabrini Health, Malvern, VIC, Australia; Pioneer Journal of Biostatistics and Medical Research (PJBMR), Pakistan, Pakistan (T Ashraf PhD); Department of Radiation Oncology (M Ashrafizadeh DVM), Shandong University, Shandong, China; Deakin Health Economics/School of Health and Social Development (B K Y Asiamah-Asare PhD), School of Exercise and Nutrition Sciences (N Subedi PhD), Deakin University, Melbourne, VIC, Australia; Nursing Department (Y Asri PhD), Faculty of Health Science (Y Asri PhD), Institute of Technology and Health Science RS dr Soepraoen, Malang, Indonesia; Department of Forensic Medicine (A Atreya MD), Department of Community Medicine (S Nepal MD), Lumbini Medical College, Palpa, Nepal; College of Medicine (Prof Z A Atwan PhD), University of Basrah, Basrah, Iraq; School of Business (Prof M Ausloos PhD), Department of Health Sciences (Prof T Brugha MD, S J Tromans PhD), Diabetes Research Centre (E Vounzoulaki PhD), University of Leicester, Leicester, UK; Department of Statistics and Econometrics (Prof M Ausloos PhD, A Mirica PhD, A Otoiu PhD, I Petcu PhD), Management Department (Prof I Popa PhD), Bucharest University of Economic Studies, Bucharest, Romania; Robarts Research Institute (A Avan MD), School of Physical Therapy (A Lawan PhD), The University of Western Ontario, London, ON, Canada; Departament of Physiotherapy (N C P Avelar DSc), Federal University of Santa Catarina, Araranguá, Brazil; Institute of Molecular Biology and Biotechnology (S J Awan PhD), The University of Lahore, lahore, Pakistan; Department of Public Health (L A A Ayana MPH), Department of Pharmacy (Gi Fekadu PhD), Department of Nursing (G Fetensa MSc), Institute of Health Sciences (B Feyisa MPH), School of Public Health (L K Solbana MPH), Wollega University, Nekemte, Ethiopia; Medicinal Chemistry Unit (Y O Ayipo PhD), Kwara State University, Malete, Ilorin, Nigeria; Centre for Drug Research (Y O Ayipo PhD), Universiti Sains Malaysia, Pinang, Malaysia; Research and Technology Deputy (A Azadnia PhD), Department of Epidemiology and Biostatistics (Y Moradi PhD), Epidemiology and Biostatistics (M Rasouli PhD), Kurdistan University of Medical Sciences, Sanandaj, Iran; Department of Applied Mathematics (J M Azam PhD), Stellenbosch University, Stellenbosch, South Africa; The World Bank, Washington, DC, USA (G S Azhar PhD); Department of Psychiatry (F Azimi MD), Iranian Research Center on Aging (V Rashedi PhD), University of Social Welfare and Rehabilitation Sciences, Tehran, Iran; Advanced Medical & Dental Institute (M Aziz PhD), Universiti Sains Malaysia, Penang, Malaysia; Department of Anesthesia (S A Aziz PhD), Research Center (N H Mahmood PhD), College of Health Sciences (H H R Najmuldeen PhD), Cihan University -Sulaimaniya, Sulaymaniyah, Iraq; Department of Basic Sciences (S A Aziz PhD), College of Science (Prof K H Hama Aziz PhD, F M Rahman PhD), University of Sulaimani, Sulaymaniyah, Iraq (H H R Najmuldeen PhD); ASIDE Healthcare, Lewes, DE, USA (A Y Azzam MD); Faculty of Medicine (A Y Azzam MD), The Orthopaedic Department (A M Makram MD), Department of Cardiology (O M Makram MD), October 6 University, 6th of October City, Egypt; Department of Precision Medicine (Y Bae MD), Sungkyunkwan University, Seongnam, South Korea; Department of Pediatrics (Prof A Bagga DSc, Prof S Gulati MD, J Meena DM), Centre for Community Medicine (P Halder MD), Department of Biophysics (T Mohammad PhD), Centre for Dental Education and Research (H Priya MDS), Department of Psychiatry (Prof R Sagar MD), Department of Laboratory Medicine (A Singh PhD), Medical Oncology Lab (M Singh PhD), All India Institute of Medical Sciences, New Delhi, India; Dental Material Research Center (S Baghizadeh DDS), Islamic Azad University, Tehran, Iran (M Zaghampour MD); Clinical Research Center (Prof R Bai MD), Nanjing Children's Hospital, Nanjing, China; Faculty of Medicine (M I Baklola BS), Clinical Pathology Department (Prof M El Sayed Zaki PhD), Department of Anatomy and Embryology (M A Eladl PhD), Department of Clinical Pathology (Prof M Elshaer PhD), Department of Cardiology (Prof M M Ramadan PhD), Faculty of Pharmacy (Prof M A Saleh PhD), Rheumatology and Immunology Unit (Prof S

Tharwat MD), Faculty of Nursing (M Zoromba PhD), Mansoura University, Mansoura, Egypt; TIRR Memorial Hermann, Houston, TX, USA (A T Bako PhD); Chen Senior Medical Center, Tamarac, FL, USA (M Balkis MD); Anahuac Business School (J Balmori-de-la-Miyar PhD), Universidad Anahuac Mexico, Mexico City, Mexico; Department of Epidemiology and Biostatistics (M Balooch Hasankhani PhD), Department of Biostatistics and Epidemiology (P Dehesh PhD), Department of Immunology (Prof A Jafarzadeh PhD), Kerman University of Medical Sciences, Kerman, Iran; College of Medicine (Prof O Baltatu PhD), Alfaisal University, Riyadh, Saudi Arabia; Center of Innovation, Technology and Education (CITE) (Prof O Baltatu PhD), Anhembi Morumbi University, São José dos Campos, Brazil; Department of Neurosurgery (S Bandyopadhyay MPH), School of Psychology (Prof S Cortese PhD), Centre for Innovation in Mental Health (M Garcia-Argibay PhD), Southampton Clinical Trials Unit (P H Lee PhD), Faculty of Medicine (R Thayakaran PhD), Department of Surgery (G Verras MSc), University of Southampton, Southampton, UK; Department of Non-communicable Diseases (P C Banik MPhil, L Barua MPH), Bangladesh University of Health Sciences, Dhaka, Bangladesh; Department of Translational Medicine (N C Barengo PhD), Robert Stemple College of Public Health and Social Work (S Chowdhury MPH), Department of Epidemiology (Si Roy MPH), Florida International University, Miami, FL, USA; School of Psychology (Prof S L Barker-Collo PhD), University of Auckland, Auckland, New Zealand; Department of Public and Environmental Health (A Barrow MPH), University of The Gambia, Banjul, The Gambia; Department of Epidemiology (A Barrow MPH, D Braithwaite PhD), College of Medicine (K T Root BS), Department of Health Services Research, Management and Policy (R Wang PhD), University of Florida, Gainesville, FL, USA; Heidelberg Institute of Global Health (HIGH) (S Barteit PhD), Heidelberg University Hospital, Heidelberg, Germany; Department of Community and Family Medicine (M Bashar MD, V Rajendran MD), Department of Community Medicine and Family Medicine (S G MD, V J MD), All India Institute of Medical Sciences, Gorakhpur, India; Department of General Surgery and Medical-Surgical Specialties (Prof G Basile MD, Prof G Isola PhD), Department of Medical and Surgical Sciences and Advanced Technologies "GF Ingrassia" (Prof E D'Amico MD, Prof M Veroux PhD), Department of Biomedical and Biotechnological Sciences (L Falzone PhD), Department of Clinical and Experimental Medicine (Prof C Ledda PhD), University of Catania, Catania, Italy; Barcelona Institute for Global Health (Prof Q Bassat MD), NCDs and Environment Programme (L Delgado-Ortiz MSc), ISGlobal Instituto de Salud Global de Barcelona (Barcelona Institute for Global Health), Barcelona, Spain; Catalan Institution for Research and Advanced Studies (ICREA), Barcelona, Spain (Prof Q Bassat MD); Faculty of Nursing (Prof A Batiha PhD), King Khalid University, Mahyil Asir, Saudi Arabia; Department of Medical Education (K Batra PhD), Department of Social and Behavioral Health (Prof M Sharma PhD), University of Nevada Las Vegas, Las Vegas, NV, USA; Department of Health Sciences (DISSAL) (Prof M Bauckneht PhD), University of Genoa, Genova, Italy; The George Institute for Global Health (T Beaney PhD, Prof S Yaya PhD), Department of Brain Sciences (L D'Anna PhD), UK Dementia Research Institute Care Research & Technology Centre (Y Hbid PhD), School of Public Health (Y Hbid PhD, A M Makram MD), WHO Collaborating Centre for Public Health Education and Training (Q Lin MPH, D L Rawaf MD), Department of Surgery and Cancer (Prof E Mossialos PhD), Department of Primary Care and Public Health (R Palladino MD, C Tabche MSc, L Tudor Car PhD), Imperial College London, London, UK; School of Public Health (Prof N Bedi MD), Dr. D. Y. Patil University, Mumbai, India; Clinical Nutrition Department (R M Chandika PhD), Department of Public Health (S Dohare MD, P Rajpoot PhD, W Rehman MS, J Varghese PhD), Epidemiology Program (M Khan MD), College of Nursing and Health Sciences (M Shanawaz MD), Department of Clinical Practice (A Shoaib PhD), Department of Nursing and Applied Health Sciences (M Wahid PhD), Jazan University, Jazan, Saudi Arabia (Prof N Bedi MD); Department of Human Anatomy and Histology (Prof N Beeraka PhD),

Department of Epidemiology and Evidence-Based Medicine (V A Korshunov PhD, P D Lopukhov PhD, R V Polibin PhD), I.M. Sechenov First Moscow State Medical University, Moscow, Russia; Department of Mental Health (M Beghi MD), AUSL Romagna, Ravenna, Italy; Milken Institute of Public Health (B K Bekele MPH), Department of Global Health (R S Bernstein MD), George Washington University, Washington, DC, USA; College of Medicine and Health Science (A N Belay MSc), Bahir Dar University, Bahir dar, Ethiopia; Menzies School of Health Research (D Belay MSc), Charles Darwin University, Darwin, NT, Australia; Department of Public Health (M Belayneh PhD), University of South Africa, Pretoria, South Africa; Department of Midwifery (A C Belete MSc, T S Hadaro MSc), Department of Nursing and Midwifery (A Haile MSc), Department of Public Health (T Mekene Meto MPH), Department of Biomedical Sciences (H T Wada MSc), Arba Minch University, Arba Minch, Ethiopia; Infectious Disease Research Department (M B Bello PhD), Medical Genomics Research Department (Prof M Umair PhD), King Abdullah International Medical Research Center, Riyadh, Saudi Arabia; Department of Veterinary Microbiology (M B Bello PhD), Department of Veterinary Public Health and Preventive Medicine (B Garba PhD, A Shittu MSc), Medical Microbiology Department (Prof Y Mohammed FWACP), Clinical Pharmacy and Pharmacy Practice (S Shuaibu PhD), Usmanu Danfodiyo University, Sokoto, Sokoto, Nigeria; Department of Physiotherapy and Paramedicine (U M Bello PhD), Glasgow Caledonian University, Glasgow, UK; Department of Biological Sciences (Prof L Belo PhD), Research Unit on Applied Molecular Biosciences (UCIBIO) (Prof L Belo PhD, Prof F Carvalho PhD, Prof D Dias da Silva PhD, J Silva PhD), Research Centre for Physical Activity, Health, and Leisure (L Bohn PhD), Associated Laboratory for Green Chemistry (LAQV) (M Carvalho PhD), Institute for Research and Innovation in Health (i3S) (Prof N Cruz-Martins PhD), Department of Community Medicine Information and Decision-Making in Health (MEDCIDS) (A Freitas PhD), Faculty of Medicine (J R Rocha-Gomes MD), University of Porto, Porto, Portugal; Department of Biomedical Sciences (Prof A Beloukas PhD), National AIDS Reference Center of Southern Greece (Prof A Beloukas PhD), University of West Attica, Athens, Greece; Department of Internal Medicine (I M Bensenor PhD, I S Santos PhD), Department of Psychiatry (Prof J Castaldelli-Maia PhD, Y-P Wang PhD), Department of Epidemiology (Prof A C Goulart PhD), Center for Clinical and Epidemiological Research (A B Oliveira PhD), Faculty of Medicine (J M F Zhang MD), Universidade de São Paulo (University of São Paulo), São Paulo, Brazil; BRAC James P Grant School of Public Health (S Bente Kamal Tune MPH), School of Pharmacy (M R Islam PhD), BRAC University, Dhaka, Bangladesh; School of Public Health (A A Berihun MA, M T Negassa MD), Department of Anesthesia and Critical Care Medicine (S Boppana MD), Department of Biostatistics (A Columbus MS), Russell H. Morgan Department of Radiology and Radiological Science (A Kamireddy MD), Department of Neurosurgery (F Kazemi MD), Department of Epidemiology (R Olum MD), Division of Minimally Invasive Surgery (N Woldehana MD), Department of Psychiatry (E M Zeru MPH), Department of International Health (H Zhang PhD), Johns Hopkins University, Baltimore, MD, USA; Department of Epidemiology and Biostatistics (A C Bermudez PhD), Department of Neurosciences (Prof R G Jamora PhD), University of the Philippines Manila, Manila, Philippines; Hubert Department of Global Health (R S Bernstein MD), Department of Cardiothoracic Imaging (A Siddiqi MD), Rollins School of Public Health (Prof D A Sleet PhD, J W Ward MD), Emory University, Atlanta, GA, USA; Department of Public Health, Experimental and Forensic Medicine (P Bertuccio PhD), University of Pavia, Pavia, Italy; Faculty of Medicine (P J G Bettencourt PhD), Universidade Católica Portuguesa (Catholic University of Portugal), Sintra, Portugal; Center for Interdisciplinary Research in Health (CIIS) (P J G Bettencourt PhD), Universidade Católica Portuguesa (Catholic University of Portugal), Lisbon, Portugal; Department of Community and Family Medicine (A S Bhadoria MD), All India Institute of Medical Sciences, Rishikesh, India; Community Health Department (A

S Bhadoria MD), University of South Wales, South Wales, UK; Department of Public Health (A S Bhagavathula PhD), North Dakota State University, Fargo, ND, USA; Institute of Applied Health Research (N Bhala PhD, R Thayakaran PhD), Department of Applied Health Sciences (Prof J S Chandan PhD), NIHR Global Health Research Unit on Global Surgery (S K Kamarajah MD), Department of Metabolism and Systems Science (S Tariq PhD), University of Birmingham, Birmingham, UK; Dwyer School of Health Sciences (B Bhandari PhD), Indiana University South Bend, South Bend, IN, USA; Department of Internal Medicine (C Bhanushali MD), Saint Vincent Hospital, Worcester, Worcester, MA, USA; Department of Anatomy (N Bhardwaj MD), Department of Community Medicine and Family Medicine (Prof P Bhardwaj MD), School of Public Health (Prof P Bhardwaj MD), Department of Pharmacology (M Shamim MBBS, Surj Singh MD), Department of Biochemistry (S Tomo MD), All India Institute of Medical Sciences, Jodhpur, India; Department of Internal Medicine (A Bhargava MD), Wayne State University, Gross Pointe Woods, MI, USA; Global Health Neurology Lab (S Bhaskar MD), NSW Brain Clot Bank, Sydney, NSW, Australia; Division of Cerebrovascular Medicine and Neurology (S Bhaskar MD), National Cerebral and Cardiovascular Center, Suita, Japan; Manipal College of Health Professions (A Bhat MSPT), Department of Gastroenterology and Hepatology (Prof S Shetty MD), Manipal Academy of Higher Education, Udupi, India; Translational and Clinical Research Institute (P Bhattacharjee MD), Newcastle University, Newcastle upon Tyne, UK; School of Sport & Health Sciences (S Bhattacharjee MPH), University of Brighton, Brighton, UK; Department of Public Health Research (S Bhattacharjee MPH), Bengal Rural Welfare Service (BRWS), Kolkata, India; Department of Medical Lab Technology (Prof G K Bhatti PhD), University Centre for Research and Development (S Kalra DM), Chandigarh University, Mohali, India; Laboratory of Translational Medicine and Nanotherapeutics (Prof J S Bhatti PhD), Department of Human Genetics and Molecular Medicine (Prof A Munshi PhD, U Sharma PhD), Department of Microbiology (A Singh PhD, M Yadav PhD), Department of Biochemistry (B Singh PhD), Department of Computer Science & Engineering (Prof S Singh PhD), Central University of Punjab, Bathinda, India; Department of Pharmacy (Prof M A Bhuiyan PhD), University of Asia Pacific, Dhaka, Bangladesh; Centre for Global Child Health (Prof Z A Bhutta PhD), Division of Neurology (S Fereshtehnejad PhD), University Health Network (S Mirshahvalad MD), University of Toronto, Toronto, ON, Canada; Institute for Global Health & Development (Prof Z A Bhutta PhD), Aga Khan University, Karachi, Pakistan; Department of Health Administration (S S Bhuyan PhD), Rutgers University, New Brunswick, NJ, USA; School of Public Health (H Bi PhD, Ye Huang PhD, Z Huang PhD, Wei Li PhD, Prof Z Qi PhD, Prof W Song PhD, Fa Wang PhD, Q Wang PhD, L Xiao PhD, T Zhan PhD), Department of Biostatistics, School of Public Health (J Fu MD), Department of Biostatistics (Prof X Gao PhD), Key Lab of Environment and Health (Prof X Gao PhD), Department of Epidemiology (D Yin DrPH), Xuzhou Medical University, Xuzhou, China (M Jiang PhD); Independent Consultant, Addis Ababa, Ethiopia (S K Biadgilign PhD); Fondazione Banca Degli Occhi Del Veneto (R Bievel-Radulescu MD), Carol Davila University of Medicine and Pharmacy, Venice, Italy; Department of Neurology (Prof A Biswas DM), Institute of Post-Graduate Medical Education and Research and Seth Sukhlal Karnani Memorial Hospital, Kolkata, India; Department of Community Medicine and Family Medicine (B Biswas MD), Department of Physiology (H Mondal MD), Department of Pharmacology (S T Y MD), All India Institute of Medical Sciences, Deoghar, India; Department of Biochemistry and Biotechnology (M Biswas PhD), University of Science and Technology Chittagong, Chittagong, Bangladesh; Department of Clinical Pharmacy (A Bitar PhD), Universiti Sultan Zainal Abidin, Besut, Malaysia; Health Biotechnology Directorate at Bio and Emerging Technology Institute (M Bitew PhD), School of Public Health (K Deribe PhD), College of Health Sciences (F S Gebre MD, A M Zenebe MSc), Department of Medical Physiology (H T Wada MSc), Addis Ababa University, Addis Ababa,

Ethiopia; Department of Physical Education and Health (B Bizzozero-Peroni PhD), Universidad de la República, Rivera, Uruguay; Department of Global Public Health and Primary Care (Prof T Bjørge PhD), Bergen Center for Ethics and Priority Setting (M K Mirutse PhD), Department of Psychosocial Science (Prof D Sagoe PhD), University of Bergen, Bergen, Norway; Cancer Registry of Norway, Oslo, Norway (Prof T Bjørge PhD); School of Business Administration (Prof V Bodolica PhD), American University of Sharjah, Sharjah, United Arab Emirates; Faculty of Psychology, Education, and Sport (L Bohn PhD), University Lusofona, Porto, Portugal; Global healthcare management (O A Bolarinwa PhD), York University, London, UK; Demography and Population Studies (O A Bolarinwa PhD), School of Public Health (C Dare PhD), University of the Witwatersrand, Johannesburg, South Africa; Faculty of Medicine and Pharmaceutical Sciences (Prof A Bonny MD), University of Douala, Douala, Cameroon; Department of Cardiology (Prof A Bonny MD), Centre Hospitalier Montfermeil (Montfermeil Hospital Center), Montfermeil, France; Department of Internal Medicine (M Borran PharmD), Community-Oriented Nursing Midwifery Research Center (M Heidari PhD), Modeling in Health Research Center (A Mohammadian-Hafshejani PhD), Department of Epidemiology and Biostatistics (H Raeisi Shahraki PhD), Shahrekord University of Medical Sciences, Shahrekord, Iran (S JamshidiRastabi MSc); College of Human and Social Futures (S Bose PhD), University of Newcastle, Sydney, NSW, Australia; Disease Surveillance Department (S A Bosoka MPhil), Ghana Health Service, Ho, Ghana; Facultad de Salud (Faculty of Health) (Prof A Botero Carvajal PhD), Universidad Santiago de Cali, Cali, Colombia; Department of Earth, Environment, and Equity (C Boxe PhD), Howard University, Washington, DC, USA; Cancer Population Sciences Program (D Braithwaite PhD), University of Florida Health Cancer Center, Gainesville, FL, USA; School of Population and Public Health (Prof M Brauer DSc, M Hossain PhD, I O Iyamu MD), Department of Medicine (J L Stubbs PhD), University of British Columbia, Vancouver, BC, Canada; Department of Psychiatry and Behavioral Health (Prof N J K Breitborde PhD), Department of Psychology (Prof N J K Breitborde PhD), Division of Cardiovascular Medicine (A Guha MD), Ohio State University, Columbus, OH, USA; Institute for Medical Information Processing, Biometry, and Epidemiology (S Breitner DSc), LMU Munich, Neuherberg, Germany; Institute of Epidemiology (S Breitner DSc), Helmholtz Zentrum München (German Research Center for Environmental Health), Neuherberg, Germany; Division of Clinical Epidemiology and Aging Research (Prof H Brenner MD), German Cancer Research Center, Heidelberg, Germany; Joint China-Cuba Lab for Neurotechnology (Prof M L Bringas Vega PhD), University of Electronic Sciences and Technology of China UESTC, Chengdu, China; Neuroinformatics Department (Prof M L Bringas Vega PhD), Cuban Neuroscience Center, Havana, Cuba; Department of Injury (J Brown PhD), Global Women's Health Program (P Cullen PhD), The George Institute for Global Health, Newtown, NSW, Australia; Faculty of Medicine (J Brown PhD), School of Population Health (P Cullen PhD, A E Peden PhD), University of New South Wales, Kensington, NSW, Australia; The Malaria Atlas Project (A J Browne DPhil), Telethon Kids Institute, Nedlands, WA, Australia; Department of Woman and Child Health and Public Health (D Buonsenso MD), Fondazione Policlinico Universitario A. Gemelli IRCCS (Agostino Gemelli University Polyclinic IRCCS), Rome, Italy; Global Health Research Institute (D Buonsenso MD), Department of Life Science and Public Health (M Di Pumpo DrPH), Department of Health Science and Public Health (Prof L Villani MD), Università Cattolica del Sacro Cuore (Catholic University of the Sacred Heart), Rome, Italy; Department of Radiology (F Busch MD), Department of Public Health and Primary Care (M Dalakoti MPH, Fanc Shi PhD, Prof P Willeit PhD), University of Cambridge, Cambridge, UK; School of Public Health Sciences (Z A Butt PhD), University of Waterloo, Waterloo, ON, Canada; Al Shifa School of Public Health (Z A Butt PhD), Al Shifa Trust Eye Hospital, Rawalpindi, Pakistan; JSS Dental College & Hospital (S C J MDS), Department of Respiratory Medicine (Prof M P A DNB), Department of

Forensic Medicine and Toxicology (S Rani MD), Jagadguru Sri Shivarathreeswara University, Mysore, India; Department of Sociology (Prof T Cai PhD), Faculty of Health Sciences (J Wang BPharm), University of Macau, Macau, China; The Children's Hospital at Westmead (R Cairns PhD), New South Wales Poisons Information Centre, Sydney, NSW, Australia; Faculty of Health Sciences Healthcare Management Department (M Çakmak Barsbay PhD), Ankara University, Ankara, Türkiye; Department of Clinical Pharmacy (Prof D Calina PhD), University of Medicine and Pharmacy of Craiova, Craiova, Romania; Department of Internal and Geriatric Medicine (Prof L A Cámera MD), Hospital Italiano de Buenos Aires (Italian Hospital of Buenos Aires), Buenos Aires, Argentina; Board of Directors (Prof L A Cámera MD), Argentine Society of Medicine, Buenos Aires, Argentina; Center of Innovation, Technology and Education (CITE) (Prof L A Campos PhD), Anhembi Morumbi University, Sao Jose dos Campos, Brazil; Center for Nutrition and Health Research (I Campos-Nonato PhD), Public Health Intelligence Unit (Prof D Diaz PhD), Infectious Disease Research Center (Prof V Pando-Robles PhD), Center for Health Systems Research (E Serván-Mori PhD), National Institute of Public Health, Cuernavaca, Mexico; Department of Ophthalmology (F Cao MD), Beijing Institute of Ophthalmology, Beijing, China; Department of Anesthesiology (S Cao MD), Third Xiangya Hospital of Central South University, Changsha, China; Unit of Hygiene and Public Health (A Capodici MD), Romagna Local Health Authority, Forlì-Cesena, Italy; Interdisciplinary Research Center for Health Science (A Capodici MD), Sant'Anna School of Advanced Studies, Pisa, Italy; Department of Health Care (Prof R Cárdenas DSc), Metropolitan Autonomous University, Mexico City, Mexico; Institute for Cancer Research, Prevention and Clinical Network, Florence, Italy (G Carreras PhD); Department of Medicine and Surgery (A Carugno PhD), University of Insubria, Varese, Italy; IMPInstitute for Mental and Physical Health and Clinical Translation (IMPACT) (A F Carvalho MD), Deakin University, Geelong, VIC, Australia; Faculty of Health Sciences (M Carvalho PhD), University Fernando Pessoa, Porto, Portugal; Education Center of Australia (A Carvalho-e-Silva PhD), Health Science College, Sydney, NSW, Australia; Public Health Department (C A Castañeda-Orjuela PhD), Epidemiology and Public Health Evaluation Group (C A Castañeda-Orjuela PhD), Department of Public Health (Prof F P De la Hoz PhD), National University of Colombia, Bogota, Colombia; Division of Country Health Policies and Systems (CPS) (G Castelpietra PhD), World Health Organization (WHO), -, Italy; Mental Health Flagship (G Castelpietra PhD), World Health Organization (WHO), Copenhagen, Denmark; Institute of Public Goods and Policies (IPP) (F Catalá-López PhD), Spanish National Research Council, Madrid, Spain; Centre for Biomedical Research in Mental Health Network (CIBERSAM) (F Catalá-López PhD), National School of Public Health (A Padron-Monedero PhD), Institute of Health Carlos III, Madrid, Spain; MultiMedica Sesto San Giovanni IRCCS, Sesto San Giovanni, Italy (Prof A L Catapano PhD); Department of Medical, Surgical, and Health Sciences (Prof L Cegolon PhD, Prof M D'Oria MD), University of Trieste, Trieste, Italy; Public Health Unit (Prof L Cegolon PhD), University Health Agency Giuliano-Isontina (ASUGI), Trieste, Italy; Department of Nutrition (Prof F Cembranel DSc), Department of Physical Education (Prof D A S Silva PhD), Federal University of Santa Catarina, Florianópolis, Brazil; College of Public Health, Medical, and Veterinary Sciences (M Cenderadewi MPHTM, A E Peden PhD), College of Medicine, Dentistry and Public Health (Prof R C Franklin PhD), James Cook University, Townsville, QLD, Australia; Department of Public Health (M Cenderadewi MPHTM), University of Mataram, Mataram, Indonesia; Mary MacKillop Institute for Health Research (Prof E Cerin PhD), Australian Catholic University, Melbourne, VIC, Australia; School of Public Health (Prof E Cerin PhD, C J P Zhang PhD), Department of Medicine (H Chou MSc), Department of Urban Planning and Design (Prof C Guo PhD), Centre for Suicide Research and Prevention (Prof P Yip PhD), Department of Social Work and Social Administration (Prof P Yip PhD), Department of Surgery (Y Zhan PhD), University of Hong Kong,

Hong Kong, China; Posgrado de Medicina, Facultad de Ciencias de la Salud (P Chacón-Uscamaita DDS), Dirección General de Investigación, Desarrollo e Innovación (DGIDI) (W Mendoza MD), Universidad Científica del Sur (University of the South), Lima, Peru; Department of Biotechnology (Prof C Chakraborty PhD), Adamas University, Kolkata, India; Institute for Skeletal Aging & Orthopedic Surgery (Prof C Chakraborty PhD), Hallym University, Chuncheon, South Korea; State Disease Investigation Laboratory (S Chakraborty MVSc), Animal Resources Development Department, Agartala, India; Department of Psychiatry (Prof M Chandradasa MD), Department of Pharmacology (Prof C D K Mettananda PhD), Department of Paediatrics (Prof S Mettananda DPhil), University of Kelaniya, Ragama, Sri Lanka; University Psychiatry Unit (Prof M Chandradasa MD), Clinical Medicine Department (Prof C D K Mettananda PhD), University Paediatrics Unit (Prof S Mettananda DPhil), Colombo North Teaching Hospital, Ragama, Sri Lanka; Manipal College of Health Professions (B Chandrasekaran PhD), Manipal Academy of Higher Education, Karnataka, India; Department of Epidemiology and Biostatistics (V Chattu PhD), Semey Medical University (SMU), Semey, Kazakhstan; Department of Community Medicine (V Chattu PhD), Datta Meghe Institute of Medical Sciences, Sawangi, India; Department of Endocrinology (V Chatzimavridou-Grigoriadou MD), Department of Cardiovascular Science (F Mannan MD), Division of Immunology, Immunity to Infection and Respiratory Medicine (A G Mathioudakis PhD), Division of Psychology and Mental Health (F Mughal FRCGP, M R Radojčić PhD), University of Manchester, Manchester, UK; Department of Endocrinology (V Chatzimavridou-Grigoriadou MD), Christie Hospital NHS Foundation Trust, Manchester, UK; Department of Biology (A A Chaudhary PhD), Al-Imam Mohammad Ibn Saud Islamic University, Riyadh, Saudi Arabia; Department of Public Health (S Chaudhuri MD), Indian Institute of Public Health, Hyderabad, India; Department of Oral Medicine and Radiology (Prof A Chaurasia MD), Department of Psychiatry (S K Kar MD), Department of Neurology (Prof H S Malhotra DM), Internal Medicine Department (J Tewari MBBS), King George's Medical University, Lucknow, India; Peking Union Medical College Hospital (A Chen PhD), Chinese Academy of Medical Sciences, Beijing, China; Hospital of Stomatology (G Chen PhD), Sun Yat-sen University, Guangzhou, China; Clinical Project Management Office (Hai Chen MPH), National Clinical Research Centerfor Infectious Diseases, Shenzhen, Shenzhen, China; Faculty of Humanities and Health Sciences (Han Chen MSc), Curtin University, Miri, Malaysia; Clinical Research Center (Hao Chen PhD), Zhujiang Hospital of Southern Medical University, Guangzhou, China; Science and Technology Department (Hu Chen MMed), Northern Jiangsu People's Hospital, Yangzhou, China; School of Public Health (Prof R Chen PhD), Zhejiang Chinese Medical University, Hangzhou, China; Heidelberg Institute of Global Health (HIGH) (Si Chen DSc, Prof S Mohammed PhD), Department of Ophthalmology (S Panda-Jonas MD), Heidelberg University, Heidelberg, Germany; Department of Computer, Electrical and Mathematical Sciences and Engineering (X Chen MSc), Computer, Electrical, and Mathematical Sciences and Engineering Division (P Moraga PhD), King Abdullah University of Science and Technology, Thuwal, Saudi Arabia; School of Chinese Medicine (Teaching and Research Division) (H Cheng BSc), Hong Kong Baptist University, Hong Kong, China; Department of Rehabilitation Sciences (K Cheung MSc, J S Usman PhD), Department of Biomedical Engineering (A Jor MSc), School of Nursing (S Tyrovolas PhD), Hong Kong Polytechnic University, Hong Kong, China; Yong Loo Lin School of Medicine (N W Chew MD, L Goh PhD, M Ng PhD, Prof H Z Sun PhD, Prof N Venketasubramanian MSc), Department of Medicine (B Chong MBBS), Cardiovascular Metabolic Translational Research Program (M Dalakoti MPH), Department of Surgery (J Lau MPH, K Tan PhD), Saw Swee Hock School of Public Health (Prof S Yi PhD), National University of Singapore, Singapore, Singapore; Department of Public Health and Health Policy (O Chimed-Ochir PhD, A Fukunaga PhD), Hiroshima University, Hiroshima, Japan; Department of Public Health, Administration,

and Social Sciences (J L Chirinos-Caceres DrPH), Cayetano Heredia University, Lima, Peru; Division of Plastic Surgery (D Y Cho MD), Department of Ophthalmology and Visual Sciences (A Roshanshad MD), University of Wisconsin–Madison, Madison, WI, USA; Department of Clinical Oncology (W C S Cho PhD), Queen Elizabeth Hospital, Hong Kong, China; Bispebjerg Hospital (Prof H Christensen DMSci), University of Copenhagen, Copenhagen, Denmark; Department of Molecular Parasitology and Tropical Diseases (Prof T Chuang PhD), School of Pharmacy (B Iskandar PhD), School of Nursing (M Kurniasari PhD, I Rohmah MSN), Department of Global Health and Health Security (K Latief PhD), International Ph.D. Program in Biotech and Healthcare Management (M Muhtar MBA), College of Nursing (Y A Rias MNS), Taipei Medical University, Taipei, Taiwan; Department of Paediatric Surgery (I S Chukwu BMedSc), Federal Medical Centre, Umuahia, Nigeria; Health Data Research UK, London, UK (S Chung PhD); Department of Health Behavior (S Chung MPH), Texas A&M University, College Station, TX, USA; The David S. and Ruth L. Gottesman Center for Headache Treatment and Translational Research (F Cohen MD), Department of Medicine (F Cohen MD), Department of Cardiology (P Devarakonda MD), Department of Psychiatry (S Gunturu MD), Icahn School of Medicine at Mount Sinai, New York, NY, USA; Nova Medical School (Prof J Conde PhD), Ecological Economics and Environmental Management (C S E S Farinha PhD), NOVA University of Lisbon, Lisbon, Portugal; Department of Medicine (S E Congly MD, Prof M Tonelli MD), Cumming School of Medicine (M I Olatubi PhD), Department of Clinical Neurosciences (Prof S Wiebe MD), Department of Community Health Sciences (Prof S Wiebe MD), University of Calgary, Calgary, AB, Canada; Department of Cardiovascular Sciences (N Conrad PhD, A Schuermans BSc, J Van den Eynde BSc), Faculty of Medicine (A Schuermans BSc), Department of Abdominal Surgery (A Teymouri MD), Katholieke Universiteit Leuven, Leuven, Belgium; Department of Cardiovascular Medicine (L T Cooper MD), Division of Gastroenterology and Hepatology (Yu Huang MD, Yi Wang MD), Mayo Clinic, Jacksonville, FL, USA; Department of Respiratory Medicine and Allergology (Prof A Corlateanu PhD), Nicolae Testemitanu State University of Medicine and Pharmacy, Chisinau, Moldova; Department of Child and Adolescent Psychiatry (Prof S Cortese PhD), School of Global Public Health (S Jaka MD, E K Peprah PhD), Rory Meyers College of Nursing (X Qi PhD), New York University, New York, NY, USA; Research Center on Public Health (CESP) (P Cortesi PhD), Center for Public Health Research (P Ferrara PhD), School of Medicine and Surgery (Prof L G Mantovani DSc), University of Milan Bicocca, Monza, Italy; Laboratory of Public Health (P Cortesi PhD), Instituto Auxologico Italiano IRCCS (Italian Auxological Institute), Milan, Italy; Department of Health Sciences (C Cosma MD), University of Florence, Florence, Italy; Department of Family Medicine and Public Health (Prof M H Criqui MD), University of California San Diego, La Jolla, CA, USA (L Diaz MD); Life and Health Sciences Research Institute (ICVS) (Prof N Cruz-Martins PhD), University of Minho, Braga, Portugal; School of Medicine (X Cui PhD), School of Data Science (Ju Zhou PhD), The Chinese University of Hong Kong, Shenzhen, Shenzhen, China; Faculty of Medicine (N Dababo MD), University of Aleppo, Aleppo, Syria; Research Center for Child Psychiatry (O Dadras PhD), Heart Center (V Kytö MD), University of Turku, Turku, Finland; Health Statistics and Informatics, Public Health Division (O Dadras PhD), Northern Territory Government, Darwin, WA, Australia; Department of Community Medicine (Prof T Dahiru MA, A A Olorukooba MD, S S Umar FWACS), Health Systems and Policy Research Unit (Prof S Mohammed PhD), Ahmadu Bello University, Zaria, Nigeria; Institute for Health Sciences (Prof K Dalal PhD), Mid Sweden University, Sundsvall, Sweden; Department of Community Medicine (R A Daniel MD, S Rajaa MD), Employees' State Insurance Model Hospital, Chennai, India; Department of Internal Medicine (P Danpanichkul MD), Texas Tech University, Lubbock, TX, USA; Ga East Municipal Hospital (S E Danso MPH), Ghana Health Service, Accra, Ghana; Department of Public Health (S D Darcho MPH), School of Nursing (Ge Fekadu MSc), Department

of Clinical Pharmacy (M D Gudeta MSc), School of Pharmacy (A S Mohammed BA), School of Public Health (A Oumer PhD), Department of Epidemiology and Biostatistics (B S Tusa MPH), Department of Psychiatry (M T Walde MSc), Haramaya University, Harar, Ethiopia; Department of Environmental Health (R Darvishi Cheshmeh Soltani PhD), Department of Nursing (A Jadidi PhD), Arak University of Medical Sciences, Arak, Iran; Department of Pharmacology (S K Das MD), Department of Biochemistry (S Vasishta PhD), Apollo Institute of Medical Sciences and Research, Chittoor, India; Department of Population and Development (C A Dávila-Cervantes PhD), Latin American Faculty of Social Sciences Mexico, Mexico City, Mexico; Department of Legal Medicine, Psychiatry and Pathology (A de la Torre-Luque PhD), Universidad Complutense de Madrid (Complutense University of Madrid), Madrid, Spain; Memorial Sloan Kettering Cancer Center, New York, NY, USA (E Dee MD); Department of Pediatrics (S Deekonda MD), Brookdale University Hospital Medical Center, Brooklyn, NY, USA; Department of Experimental and Health Sciences (L Delgado-Ortiz MSc), Pompeu Fabra University, Barcelona, Spain; Ophthalmology Department (M Delsoz MD), Department of Ophthalmology (A Nabavi MD), University of Tennessee, Memphis, TN, USA; Department of Neurosurgery (A K Demetriades MD), Postgraduate School (U A Eze MD), College of Medicine and Veterinary Medicine (G Verras MSc), University of Edinburgh, Edinburgh, UK; Department of Neurosurgery (A K Demetriades MD), National Health Service (NHS) Scotland, Edinburgh, UK; Dirección de Nutrición (E Denova-Gutiérrez DSc), Department of Infectious Diseases (B A Martinez-Guerra MSc), Instituto Nacional de Nutrición Salvador Zubirán (Salvador Zubiran National Institute of Medical Sciences and Nutrition), Mexico City, Mexico; Research and Training Directorate (T N Derese MPH), Eka Kotebe General Hospital, Addis Ababa, Ethiopia; Department of Biological Sciences (I Dergaa PhD), University of Manouba, Manouba, Tunisia; Department of Social Sciences (I Dergaa PhD), University of Jendouba, El Kef, Tunisia; Wellcome Trust Brighton and Sussex Centre for Global Health Research (K Deribe PhD), Brighton and Sussex Medical School, Brighton, UK; St Paul's Eye Unit (N Dervenis MD), Royal Liverpool University Hospital, Liverpool, UK; Department of Forensic Medicine (E Dervišević PhD), University of Sarajevo, Sarajevo, Bosnia and Herzegovina; Clinical and Public Health Research (H Desai MBBS), Independent Consultant, Ahmedabad, India; Department of Statistics, Computer Science, Applications "G. Parenti" (DiSIA) (A Desta MSc), University of Florence and University of Palermo, Florence, Italy; Chettinad Hospital & Research Institute (Prof V Devanbu MD), Chettinad Academy of Research and Education, Chennai, India; Department of Pharmacy (S Dewan PhD), United International University, Dhaka, Bangladesh; Pharmacology Division (S Dewan PhD), Center for Life Sciences Research Bangladesh, Dhaka, Bangladesh; Sheffield Teaching Hospitals NHS Foundation Trust, Sheffield, UK (A Dhali MBBS); Division of Pathology (K Dhama PhD), ICAR-Indian Veterinary Research Institute, Bareilly, India; Research Department (M L Dhimal PhD), Planetary Health Research Centre (PHRC), Kathmandu, Nepal (C L Ranabhat PhD); Institute of Occupational, Social and Environmental Medicine (M L Dhimal PhD, M Dhimal PhD), Goethe University Frankfurt, Frankfurt am Main, Germany; Population Interventions Unit (B Dhungel DrPH), School of Health Sciences (A Meretoja MD), Melbourne School of Population and Global Health (L Reifels PhD), Nossal Institute for Global Health (A A Tareke MPH), University of Melbourne, Melbourne, VIC, Australia; Escola Superior de Saúde (Higher School of Health) (Prof D Dias da Silva PhD), Instituto Politécnico do Porto (Polytechnic Institute of Porto), Porto, Portugal; Department of Anesthesiology (K Didehvar MD), Rutgers University, Newark, NJ, USA; Department of Otolaryngology - Head and Neck Surgery (L K Dillard PhD, J R Dubno PhD), Medical University of South Carolina, Charleston, SC, USA; Joe C. Wen School of Population & Public Health (X Ding MA), University of California Irvine, Irvine, CA, USA; Department of Social Medicine and Health Care Organisation (Prof K G Dokova PhD), Medical University of Varna, Varna, Bulgaria; Cardio-Thoraco-

Vascular Department (Prof M D'Oria MD), Azienda Sanitaria Universitaria Giuliano Isontina, Trieste, Italy; School of Medicine (Prof S Xu PhD), University of Rochester, Rochester, NY, USA (E Dorsey MD); Independent Consultant, Bridgewater, NJ, USA (O P Doshi MS); Department of Epidemiology (M Dresse MD), University of Pittsburgh, Pittsburgh, PA, USA; Department of Psychiatry (M Dresse MD), University of Pittsburgh Medical Center, Pittsburgh, PA, USA; Department of Medicine (A C Dsouza MBBS), Bangalore Medical College and Research Institute, Bangalore, India; Department of Pathology (Prof J Du PhD), China Medical University, Liaoning, China; Office of Institutional Analysis (J Dube MA), University of Windsor, Windsor, ON, Canada; School of Sociology (E W Dumbili PhD), UCD Centre for Disability Studies (C Linehan PhD), University College Dublin, Dublin, Ireland; Postgraduate Program in Health Sciences (S C Dumith PhD), Federal University of Rio Grande do Sul, Rio Grande, Brazil; Postgraduate Program in Epidemiology (Prof B B Duncan MD, Prof M I Schmidt MD), Department of Social Medicine (R Mattiello PhD), Federal University of Rio Grande do Sul, Porto Alegre, Brazil; School of Medicine (Prof A R Duraes PhD), Institute of Collective Health (Prof D Rasella PhD), Federal University of Bahia, Salvador, Brazil; Department of Internal Medicine (Prof A R Duraes PhD), Escola Bahiana de Medicina e Saúde Pública (Bahiana School of Medicine and Public Health), Salvador, Brazil; Department of Infection and Tropical Medicine (O C Durojaiye MPH), School of Medicine and Population Health (N S George MPH), University of Sheffield, Sheffield, UK; Department of Pharmacology (Si Dutta MD), All India Institute of Medical Sciences, Rajkot, India; Department of Biological and Chemical Sciences (O Ebohon MPH), Michael and Cecilia Ibru University, Delta State, Nigeria; Department of Psychiatry (E Eboreime PhD, E Tsermpini PhD), Dalhousie University, Halifax, NS, Canada; Department of Psychiatry (E Eboreime PhD), Department of Medicine (E Lytvyak MD), Division of Preventive Medicine (Prof S Straube DPhil), School of Public Health (Prof S Straube DPhil), Faculty of Nursing (U Yunusa PhD), University of Alberta, Edmonton, AB, Canada; Histology Department (L L M Ebraheim PhD), Department of Animal Medicine (I Elsohaby PhD), Department of Pathology (Prof M M M Metwally PhD), Cardiovascular Department (Prof A M A Saad MD), Department of Microbiology and Immunology (G Yahya PhD), Zagazig University, Zagazig, Egypt (Prof M I Husseiny PhD); Fred Hutchinson Cancer Research Center, Seattle, WA, USA (A Ebrahimi MD); Environmental and Occupational Health Research Center (M Ebrahimi MD), Shahroud University of Medical Sciences, Shahroud, Iran; Higher School of Technology (Prof A Ed-Dra PhD), Sultan Moulay Slimane University, Beni Mellal, Morocco; School of Nursing and Midwifery (Prof D Edvardsson PhD), La Trobe University, Melbourne, VIC, Australia (Prof F Efendi PhD); Advanced Nursing Department (Prof F Efendi PhD), Department of Epidemiology Population Biostatistics and Health Promotion (A Hargono PhD), Faculty of Public Health (Prof S Martini PhD, D A Syaiful MGPH), Department of Fundamental Nursing (R Pradipta MS), Department of Health Policy and Administration (Prof R D Wulandari DrPH), Universitas Airlangga (Airlangga University), Surabaya, Indonesia; Isenberg School of Management (A Eighaei Sedeh MD), University of Massachusetts Amherst, Amherst, MA, USA; Centre for Global Health Inequalities Research (CHAIN) (Prof T Eikemo PhD, H Hoven DrPH), Department of Circulation and Medical Imaging (J Nauman PhD), Norwegian University of Science and Technology, Trondheim, Norway; Private Orthodontist, Ahvaz, Iran (E Eini MSD); Faculty of Science and Health (M Ekholuenetale PhD), University of Portsmouth, Hampshire, UK; Almoosa College of Health Sciences, Al Ahsa, Saudi Arabia (R A El Arab PhD); College of Medicine (Prof R Elbeshbeishy PhD), Department of Periodontics (N T Hashim PhD), Department of Pharmacology (S Srinivasamurthy MD), RAK Medical and Health Sciences University, Ras Al Khaimah, United Arab Emirates; Faculty of Medicine (Prof R Elbeshbeishy PhD), Department of Internal Medicine (Prof G M T ElGohary MD), Department of Neuropsychiatry (Prof G ELNahas MD), Department of Entomology (A M Samy PhD), Medical Ain Shams

Research Institute (MASRI) (A M Samy PhD), Neurology Department (Prof A S Shalash PhD), Ain Shams University, Cairo, Egypt; College of Medicine (M Elhadi MD, Prof S Jeong PhD), Department of Medicine (I R Fakhradiyev PhD), School of Health and Environmental Science (Prof J Kang PhD), Department of Health Policy and Management (Prof J Kim PhD), Department of Preventive Medicine (Prof Y Lee PhD, Prof S Yoon PhD), Korea University, Seoul, South Korea (Prof M Shin PhD); Houston Methodist Hospital, Houston, TX, USA (M Elhadi MD); National Institute of Public Health Research (M Elhoumed PhD), Ministry of Health, Nouakchott, Mauritania; School of Pharmacy and Pharmaceutical Sciences (M Elnaem PhD), Ulster University, Coleraine, UK; Executive Committee (Prof G ELNahas MD), International Association for Women Mental Health, Potomac, MD, USA; Department of Infectious Diseases and Public Health (I Elsohaby PhD, Gi Fekadu PhD, Prof W Ming MD), Department of Biomedical Sciences (W Jin MD), Department of Infectious Disease and Public Health, Jockey Club College of Veterinary Medicine and Life Sciences (T Mok MD), City University of Hong Kong, Hong Kong, China; Faculty of Veterinary Medicine (Prof A S Eltahawy PhD), Damanhour University, Damanhur, Egypt; Department of Midwifery (T Emagneneh MSc), Woldia University, Addis Ababa, Ethiopia; Health Research and Technology Transfer Directorate (M Endriyas MSc), South Ethiopia Region Public Health Institute, Jinka, Ethiopia; Department of Public Health (M Endriyas MSc), Department of Pathology (A M Nisro MD), Hawassa University, Hawassa, Ethiopia; Evidence-Based Medical Research Institute of Mongolia, Ulaanbaatar, Mongolia (R Erkhembayar MD); Department of Paediatrics (C I Esezobor MB), University of Lagos, Lagos, Nigeria; Department of Paediatrics (C I Esezobor MB), Lagos University Teaching Hospital, Lagos, Nigeria; Goba College of Medicine and Health Sciences (D Eshetu MSc), Madda Walabu University, Robe, Ethiopia; Department of Bacteriology and Virology (M Eslami PhD), Cancer Research Center (M Eslami PhD), Research Center of Physiology (H Yaribeygi PhD), Semnan University of Medical Sciences, Semnan, Iran; Faculty of Health (O Eyawo PhD), York University, Toronto, ON, Canada; Department of Ophthalmology (U A Eze MD), Federal Medical Centre, Asaba, Nigeria; Department of Electrical and Computer Engineering (ECE) (H Fadavian MSc, Prof D Fathi PhD), Department of Biostatistics (K Gohari MS), Department of Bacteriology (M Mohammadi MSc), Department of Hematology (B Razi PhD), Tarbiat Modares University, Tehran, Iran; Research Centre for Healthcare and Community (A F Fagbamigbe PhD), Faculty of Health and Life Sciences (O P Kurmi PhD), Centre for Intelligent Healthcare (H Liu PhD), Coventry University, Coventry, UK; School of Public Health (O F Fagbule FWACS), University of Nevada Reno, Reno, NV, USA; Department of Oral Biology (A Fahim PhD), Riphah International University, Islamabad, Pakistan (Z Z Piracha PhD); Key Laboratory of Computer-Aided Drug Design (A Fahira PhD, M Waqas PhD), Guangdong Medical University, Dongguan, China; Department of Food Hygiene and Quality Control (A Fakhri-Demeshghieh PhD), School of Biotechnology (M Yeganeh PhD), University of Tehran, Tehran, Iran; Epidemiology and Biostatistics Unit (L Falzone PhD), IRCCS Pascale, Naples, Italy; Department of Public Health Sciences (Q Fan DrPH), Clemson University, Clemson, SC, USA; Saveetha Medical College and Hospital (M Fareed PhD, M Tabish MPharm), Department of Public Health Dentistry (Prof G Mini PhD), Saveetha Institute of Medical and Technical Sciences (SIMATS), Chennai, India; Division of Statistics (Z Farhana MS), Bangladesh Bank, Sylhet, Bangladesh; School of Human and Social Sciences (FCHS) (L Faria PhD), University of Algarve, Faro, Portugal; University Research Center in Psychology, Faro, Portugal (L Faria PhD); Environmental Statistics Unit (C S E S Farinha PhD), National Institute of Statistics, Lisbon, Portugal; Department of Psychology (A Faro PhD), Federal University of Sergipe, São Cristóvão, Brazil; Department of Radiography and Imaging Technology (S Farooq PhD), Green International University, Lahore, Pakistan; Endocrinology and Metabolism Research Institute (H Farrokhpour MD), Quantitative Department (K Gohari MS, A Sheidaei PhD), Department of Epidemiology

(S Khanmohammadi MD, S Rashedi MD), Department of Epidemiology and Biostatistics (S Khosravi MD), School of Medicine (A Sheikhy MD), Non-Communicable Diseases Research Center (NCDRC), Tehran, Iran (A Golestani MD); Department of Veterinary Tropical Diseases (Prof F O Fasina PhD), University of Pretoria, Pretoria, South Africa; Animal Production and Health Division (EMPRES) (Prof F O Fasina PhD), Food and Agriculture Organization of the United Nations, Rome, Italy; Institute of Public Health (F Fischer PhD), Charité Universitätsmedizin Berlin (Charité University Medical Center Berlin), Berlin, Germany (M M Fasina MSc); School of Engineering (A Fatehizadeh PhD), Edith Cowan University, Joondalup, WA, Australia; Biostatistics Unit (Prof M Fayaz PhD), Shahed University, Tehran, Iran; National Institute for Stroke and Applied Neurosciences (Prof V L Feigin PhD), The National Institute for Stroke and Applied Neurosciences (I Rautalin PhD), Auckland University of Technology, Auckland, New Zealand; Research Center of Neurology, Moscow, Russia (Prof V L Feigin PhD); Department of Microbiology and Physiology (U M Femoe PhD), Department of International Health and Sustainable Development (Prof A Kisa PhD), Department of Environmental Health Sciences (S P Sherchan PhD), Tulane University, New Orleans, LA, USA (E Lytvyak MD); Department of Biomedical Engineering (T Ferdous MSc), Department of Decision and Information Sciences (M Hossain DrPH), Department of Biology and Biochemistry (S Ullah MSc), University of Houston, Houston, TX, USA; Cardiovascular Health and Imaging Laboratory (R Fernandez-Jimenez PhD), Centro Nacional de Investigaciones Cardiovasculares (CNIC) (National Centre for Cardiovascular Disease Research), Madrid, Spain; Department of Cardiology (R Fernandez-Jimenez PhD), Hospital Clinico San Carlos, IdISSC, Madrid, Spain; Laboratory of Public Health (P Ferrara PhD, Prof L G Mantovani DSc), IRCCS Istituto Auxologico Italiano, Milan, Italy; Queensland Centre for Mental Health Research, Wacol, QLD, Australia (A J Ferrari PhD, D F Santomauro PhD); Department of Social Sciences (Prof N Ferreira PhD, Prof M J M Sullman PhD), Department of Life and Health Sciences (Prof M J M Sullman PhD), University of Nicosia, Nicosia, Cyprus; Medical School (A Finnemore Dipl), Universidad de Navarra, Pamplona, Spain; Department of Child Dental Health (Prof M O Folayan PhD), Obafemi Awolowo University, Ile-Ife, Nigeria; Clinical Science Department (Prof M O Folayan PhD), Department of Biochemistry and Nutrition (K Oyebola PhD), Nigerian Institute of Medical Research, Lagos, Nigeria; Department of Cell Biology and Biotechnology (A A Fomenkov PhD), K.A. Timiryazev Institute of Plant Physiology, Moscow, Russia; Department of Cardiac, Thoracic, Vascular Sciences and Public Health (M Fonzo MD), University of Padova, Italy, Padova, Italy; Division of Pediatric Hematology-Oncology (L M Force MD), St. Jude Children's Research Hospital, Seattle, WA, USA; Innovation in Healthcare and Social Services Department (D Fortuna MSc), Emilia-Romagna Region, Bologna, Italy; Department of Neuroscience (M Foschi MD), Multiple Sclerosis Research Center, Ravenna, Italy; Department of Biotechnological and Applied Clinical Sciences (M Foschi MD, R Ornello PhD), University of L'Aquila, L'Aquila, Italy; Clinical Epidemiology Division (KEP) (K R Fowobaje PhD), Karolinska Institutet (Karolinska Institute), Stockholms, Sweden; Center for Health Technology and Services Research (CINTESIS), Porto, Portugal (A Freitas PhD); Department of Dermatology (Prof T Fukumoto PhD), Kyoto Prefectural University of Medicine, Kyoto, Japan; Health Services Management Training Centre (Prof P A Gaal PhD, T Joo PhD, J Lám PhD, T Palicz MD), Semmelweis University, Budapest, Hungary; Department of Applied Social Sciences (Prof P A Gaal PhD), Sapientia Hungarian University of Transylvania, Târgu-Mureş, Romania; Department of Community Medicine (Prof M A Gadanya MD), Aminu Kano Teaching Hospital, Kano, Nigeria; School of Public Health (D Gadeka PhD), School of Pharmacy (Prof I A Kretchy PhD), West African Center for Cell Biology of Infectious Pathogens (I A Owusu PhD), University of Ghana, Legon, Ghana; Hypertension in Africa Research Team (L F Gafane-Matemane PhD), North-West University, Potchefstroom, South Africa;

Department of Public Health (M Gajdács PhD), University of Szeged, Szeged, Hungary; Department of Food Technology (Y Galali ResM, B A Sadee PhD), Department of Statistics (Prof D H Kadir PhD), Salahaddin University-Erbil, Erbil, Iraq; Department of Nutrition and Dietetics (Y Galali ResM, B A Sadee PhD), Department of Business Administrations (Prof D H Kadir PhD), Cihan University-Erbil, Erbil, Iraq; Department of Medical Epidemiology (S Gallus PhD, A Lugo PhD), Mario Negri Institute for Pharmacological Research, Milan, Italy; School of American Education (B Ganesan PhD), Institute of Health & Management, Australia, Melbourne, VIC, Australia; Swinburne University of Technology (B Ganesan PhD), School of Engineering, Melbourne, VIC, Australia; Department of Joint Surgery and Sports Medicine (Y Gao MD), Institute of Science Tokyo, Tokyo, Japan; Department of Public Health (B Garba PhD), SIMAD University, Mogadishu, Somalia; School of Medicine (M Garcia-Argibay PhD), Orebro University, Orebro, Sweden; Department of Medicine (Prof D Garcia-Azorin MD), University of Valladolid, Valladolid, Spain; Department of Neurology (Prof D Garcia-Azorin MD), Hospital Universitario Rio Hortega, Valladolid, Spain; Human Nutrition Laboratory (W P Gastélum Espinoza MSc), Autonomous University of Sinaloa, Culiacán, Mexico; Department of Health Sciences (W P Gastélum Espinoza MSc), Autonomous University of Occident, Culiacán, Mexico; Professional Services Division (P Gautam PhD), Texas State Board of Pharmacy, Austin, TX, USA; Department of Pharmacology (Prof R K Gautam PhD), IES Institute of Pharmacy, Bhopal, India; Institute of Health and Development (ISED) (Prof B Gaye PhD), Alliance for Medical Research in Africa (AMedRA), Dakar, Senegal (Prof B Gaye PhD); School of Public Health (H Ge MD), Shandong First Medical University and Shandong Academy of Medical Sciences, Jinan, China; Department of Midwifery (M W Gebregergis MSc), Department of Epidemiology (M Mehari MPH), Department of Medical Laboratory Sciences (H N Meles MSc), Adigrat University, Adigrat, Ethiopia; Environmental Pollution Monitoring and Study Desk (M Gebrehiwot DSc), Ethiopian Environmental Protection Authority, Addis Ababa, Ethiopia; School of Public Health (M Gelchu MPH), Bule Hora University, Bule Hora, Ethiopia; Neurology and Stroke Unit (S Gelibter MD, G Schwarz MD), ASST Grande Ospedale Metropolitano Niguarda, Milan, Italy; Institute of Public Health (N S George MPH), Department of Health Economics and Social Security (K Kissimova-Skarbek PhD), Jagiellonian University Medical College, Krakow, Poland; Department of Public Health (L Getacher PhD), Debre Berhan University, Debre Berhan, Ethiopia; Department of Public Health (G K Getahun MPH), Menelik II Medical and Health Science College, Addis Ababa, Ethiopia; Infectious Disease Research Center (Prof K Ghadiri MD), Pediatric Department (Prof K Ghadiri MD), Department of Physiotherapy, School of Rehabilitation Sciences (M Mirzaei MSc), Research Center for Environmental Determinants of Health (Prof Eh Sadeghi PhD), Department of Speech Therapy (A Shiani PhD), Department of Infectious Disease (Prof S Vaziri MD), Kermanshah University of Medical Sciences, Kermanshah, Iran; Research Committee of Qom University of Medical Sciences (A Ghamkhar BSc), Qom University of Medical Sciences, Qom, Iran; Family and Community Medicine Department (R M Ghazy PhD), King Khalid University, Abha, Saudi Arabia; Department of Physics (Z Gholami PhD), University of Zanjan, Zanjan, Iran; Department of Dermatology (N Gholizadeh MD, G Rahmatpour Rokni MD), Invasive Fungi Research Center (J Javidnia PhD), Department of Medical Mycology (J Javidnia PhD), Department of Biostatistics (Prof A Khalilian PhD), Department of Medical-Surgical Nursing (S Shorofi PhD), Mazandaran University of Medical Sciences, Sari, Iran; Department of Biology (A Ghuge MPhil), Government Institute of Science, Nagpur, India; Department of Clinical Research (A Ghuge MPhil), National Institute For Research In Reproductive and Child Health, Mumbai, India; Department of Epidemiology and Prevention (A Gialluisi PhD), IRCCS Neuromed, Pozzilli, Italy; GBD Collaborating Unit (K Giannakis, Prof S E Vollset DrPH), Centre for Disease Burden (A S Knudsen PhD), Norwegian Institute of Public Health, Bergen, Norway; Department of

Biological Sciences and Chemistry (DBSC) (S A Gilani PhD, Prof J Hussain PhD), Natural and Medical Sciences Research Center (A Khan PhD, S Shahid MPhil, A Ullah MS), School of Pharmacy (A K Philip PhD), University of Nizwa, Nizwa, Oman; Adelaide Medical School (T K Gill PhD), School of Public Health (G A Tessema PhD), University of Adelaide, Adelaide, SA, Australia; Department of Zoology (B R Giri PhD), KKS Women's College, Balasore, India; Department of Nursing (A A Girmay MSc), Aksum University, Aksum, Ethiopia; Department of Anesthesiology and Critical Care Medicine (A Girombelli MD), Ospedale SS Annunziata Savigliano, Savigliano, Italy; Lerner College of Medicine (Prof L Göbölös PhD), Harrington Heart and Vascular Institute (A Guha MD), Department of Quantitative Health Science (Prof Xue Liu PhD), University Hospitals (O A Oyebanji MD), Department of Endocrinology (A Sood MD), Case Western Reserve University, Cleveland, OH, USA; Department of Radiation Oncology (Prof A K Goel MD), Department of Medicine (A Goel MBBS), Department of Human Anatomy (A Patra MD), Department of Dentistry (S S Rana MDS), Department of Radiodiagnosis (Pa Singh MD), Department of Community Medicine and Family Medicine (Me Verma MD), Department of Pediatrics (A Wander MD), All India Institute of Medical Sciences, Bathinda, India; Department of Pharmaceutical Sciences and Drug Research (Prof R K Goel PhD), Department of Human Genetics (Pu Singh PhD), Punjabi University Patiala, Patiala, India; Department of Health Systems and Policy Research (Prof M Golechha PhD), Department of Health Policy, Management and Behavioural Sciences (A Pandya PhD), Indian Institute of Public Health, Gandhinagar, India; Department of Life Sciences, Health and Healthcare Professions (Prof D Golinelli MD), Link Campus University, Rome, Italy; Health Services Research, Evaluation and Policy Unit (Prof D Golinelli MD), AUSL della Romagna, Ravenna, Italy; Research Institute for Endocrine Sciences, Tehran, Iran (M Golmohammadi MD); Senior Department of Tuberculosis (Prof W Gong PhD), The Eighth Medical Center of PLA General Hospital, Beijing, China; Department of Dermatology (A Grada MD), Case Western Reserve University, Libertyville, IL, USA; Liverpool Orthopaedic and Trauma Service (S Graham PhD), University of Liverpool, Liverpool, UK; Department of Public Health and Preventive Medicine (Prof M Grivna PhD), Charles University, Prague, Czech Republic; Department of Epidemiology and Biostatistics (S Guan MD), Department of Urology (C Mao MSc), Anhui Medical University, Hefei, China; Health Directorate (G Guarducci MD), Local Health Authority of Ferrara, Ferrara, Italy; Department of Clinical Science (M I M Gubari PhD), University of Sulaimani, Sulaimani, Iraq; Department of the Health Directorate (S Guicciardi MD), Local Health Authority of Bologna, Bologna, Italy; Department of Psychiatry (S Gunturu MD), Bronxcare Health System, Bronx, NY, USA; Department of Geriatric Neurology (X Guo PhD), Shaanxi Provincial People’s Hospital, Xi'an, China; Nanyang Maternal and Child Health Care Hospital (Zhi Guo MPH), Nanyang Central Hospital, Nanyang, China; Department of Public Health (B Gupta PhD), Department of Health and Education (F Hanna PhD), Torrens University Australia, Melbourne, VIC, Australia; Department of Pharmacology (Prof G Gupta PhD), Centre for Research Impact & Outcome (O Hassan Ahmed PhD), Chitkara University, Rajpura, India; Department of Anaesthesia (Prof L Gupta MD), Maulana Azad Medical College, New Delhi, India; Department of Preventive Cardiology & Medicine (Prof R Gupta MD), Eternal Heart Care Centre & Research Institute, Jaipur, India; Department of Medicine (Prof R Gupta MD), Mahatma Gandhi University Medical Sciences, Jaipur, India; Department of Epidemiology and Psychosocial Research (R A Gutiérrez PhD), Ramón de la Fuente Muñiz National Institute of Psychiatry, Mexico City, Mexico; Doctoral Program in Biomedical Gerontology (R S Gutiérrez-Murillo PhD), Pontifical Catholic University of Rio Grande do Sul, Porto Alegre, Brazil; Research Unit in Epidemiology Clinic (J Guzman-Esquivel DSc), Mexican Institute of Social Security, Colima, Mexico; College of Health Science (A Habteyes MPH), Dilla University, Dilla, Ethiopia; Department of Clinical Pharmacology and Medicine (Prof N R Hadi PhD), University of Kufa, Najaf, Iraq; Malaria Atlas Project,

Perth, WA, Australia (S Hafsia PhD); School of Medicine (A Haghtalab MD, S Pourasghary MD, S Soraneh MD), Maternal and Childhood Obesity Research Center (A Mokari-Yamchi PhD), Urmia University of Medical Sciences, Urmia, Iran; School of Medicine (A Haghtalab MD), Hamedan University of Medical Sciences, Hamedan, Iran; Department of Liver Tumor, Cancer Center (N Hai Nam PhD), Liver Transplant Unit (N Hai Nam PhD), Vascular Surgery Department (D Le PhD), Cho Ray Hospital, Ho Chi Minh City, Viet Nam; Department of Community Medicine (P Halder MD, A KM MD), Department of Pharmacology (A K Kakkar MD), Department of Pediatrics (J Kumar MD), Post Graduate Institute of Medical Education and Research, Chandigarh, India; Department of Infectious Disease Epidemiology (S Haller MD), Robert Koch Institute, Berlin, Germany; Department of Public Health (S Haller MD), Charité Insitute of Public Health, Berlin, Germany; Department of Pharmacy (Prof I M Hamad PhD), American University of Madaba, Amman, Jordan; Department of Family and Community Medicine (Prof R R Hamadeh PhD), College of Medicine and Health Sciences (H Jahrami PhD), Arabian Gulf University, Manama, Bahrain; School of Health and Environmental Studies (Prof S Hamidi DrPH), Hamdan Bin Mohammed Smart University, Dubai, United Arab Emirates; Department of Medical and Technical Information Technology (A Hammoud PhD), Bauman Moscow State Technical University, Moscow, Russia; Sakarya University, Sakarya, Türkiye (A Hanif PhD); Centre for Neuromuscular and Neurological Disorders (Perron Institute) (Prof G J Hankey MD), School of Biomedical Sciences (Prof L Wang PhD), The University of Western Australia, Perth, WA, Australia; Stroke Research Centre (Prof G J Hankey MD), Perron Institute for Neurological and Translational Science, Perth, WA, Australia; Department of Population Science and Human Resource Development (Prof M Haque PhD, Prof Md M Rahman PhD, Prof Mo Rahman DrPH), Department of Physics (A Hossain PhD), University of Rajshahi, Rajshahi, Bangladesh; Department of Medicine (O I Haque MD), MedStar Health, Baltimore, MD, USA; Directorate General of Health Human Resources (A M A Hariandja DrPH), Ministry of Health, Jakarta, Indonesia; Research Unit (J M Haro MD), Parc Sanitari Sant Joan de Deu, Barcelona, Spain; Department of Mental Health (J M Haro MD), Carlos III Health Institute (Prof R Tabarés-Seisdedos PhD), Biomedical Research Networking Center for Mental Health Network (CiberSAM), Madrid, Spain; Faculty of Nursing (F Hasan PhD, D S Romadlon PhD), Center of Excellence in Precision Medicine and Digital Health (T Porntaveetus PhD), Chulalongkorn University, Bangkok, Thailand; Department of Health Research Methods, Evidence, and Impact (M Hasan MPH), Department of Medicine (O P Kurmi PhD), Population Health Research Institute (PHRI) (F Mannan MD), McMaster University, Hamilton, ON, Canada; Department of Biochemistry and Molecular Biology (M Hasan MPH), Tejgaon College, Dhaka, Bangladesh; Department of Food Technology and Nutrition Science (T Hasan PhD), Noakhali Science and Technology University, Noakhali, Bangladesh; Department of Medical Surgical (Prof A Hasanpour- Dehkordi PhD), Shahroud University of Medical Sciences, Shahrekord, Iran; Department of Biotechnology (A Hasnain PhD), Lahore University of Biological and Applied Sciences, Lahore, Pakistan; Department of Community Medicine (I Hassan PhD), Federal University Teaching Hospital, Lafia, Nigeria; Department of Epidemiology and Community Medicine (I Hassan PhD), Federal University of Lafia, Lafia, Nigeria; Institute of Research and Development (O Hassan Ahmed PhD), School of Engineering and Technology (Prof M Hosseinzadeh PhD), Duy Tan University, Da Nang, Viet Nam; Department of Health Policy and Financing (Y Hassan Wada MPH), Society for Family Health, Abuja, Nigeria; Department of Paediatrics (L W Hathagoda MD, Prof S Rajindrajith PhD), Department of Anatomy, Genetics and Biomedical Informatics (Y Mathangasinghe PhD), Postgraduate Institute of Medicine (Prof S N K Navaratna MD, J Samaranayake MBBS), Department of Surgery (D P Wickramasinghe MD), University of Colombo, Colombo, Sri Lanka; Paediatric Professorial Unit (L W Hathagoda MD), Lady Ridgeway Hospital for Children, Colombo, Sri Lanka;

Institute of Diagnostic and Interventional Radiology and Neuroradiology (Prof J Haubold MD, M Opitz MD), Institute of Artificial Intelligence in Medicine (Prof J Haubold MD), University Hospital Essen, Essen, Germany; Skaane University Hospital (R J Havmoeller PhD), Skaane County Council, Malmö, Sweden; Faculty of Kinesiology (Prof J J Hebert PhD), University of New Brunswick, Fredericton, NB, Canada; School of Allied Health (Prof J J Hebert PhD), Murdoch University, Murdoch, WA, Australia; Independent Consultant, Santa Clara, CA, USA (G Heidari MD); Department of Microbiology (K Hezam PhD), Taiz University, Taiz, Yemen; School of Medicine (K Hezam PhD), Nankai University, Tianjin, China; Graduate School of Medicine (Y Hiraike PhD), Department of Global Health Policy (S K Rauniyar PhD), University of Tokyo, Tokyo, Japan; Department of Pulmonology (N Horita PhD), Yokohama City University, Yokohama, Japan; National Human Genome Research Institute (NHGRI) (N Horita PhD), Center for Translation Research and Implementation Science (G A Mensah MD), National Institutes of Health, Bethesda, MD, USA; Centre for Advancing Health Outcomes, Vancouver, BC, Canada (M Hossain PhD); Public Health Research Group (M Hossain DrPH), Nature Study Society of Bangladesh, Khulna, Bangladesh; Department of Statistics (M Hossain MSc, S Noor MS), Shahjalal University of Science and Technology, Sylhet, Bangladesh; Department of Population Sciences (Prof M B Hossain PhD), University of Dhaka, Dhaka, Bangladesh; Jadara Research Center (Prof M Hosseinzadeh PhD), Jadara University, Irbid, Jordan; Department of Clinical Legal Medicine (Prof S Hostiuc PhD), National Institute of Legal Medicine Mina Minovici, Bucharest, Romania; National School of Tropical Medicine (Prof P J Hotez PhD), School of Medicine (Prof J A Singh MD), Baylor College of Medicine, Houston, TX, USA; Internal Medicine Department (P Hotwani MD), Parkview Health, Fort Wayne, IN, USA; Department of Medicine (P Hotwani MD), Liaquat University Of Medical and Health Sciences, Jamshoro, Pakistan; Institute for Occupational and Maritime Medicine (ZfAM) (H Hoven DrPH), University Medical Center Hamburg-Eppendorf (UKE), Hamburg, Germany; Department of Psychological and Cognitive Sciences (C Hu PhD), Vanke School of Public Health (J S Ji DSc), School of Clinical Medicine (Xiaoh Sun PhD), Department of Neurology (Xiaoh Sun PhD), Tsinghua University, Beijing, China; Maternal Care and Child Health Department (Prof Y Hu PhD), Department of Neurosurgery (S Wang MD), Capital Medical University, Beijing, China; Department of Otorhinolaryngology Head and Neck Surgery (W Huang PhD), School of Medicine (Z Li PhD), Shanghai Mental Health Center (Prof M R Phillips MD), Renji Hospital (Fanc Shi PhD), Ruijin Hospital (S Xu MPH), Shanghai Jiao Tong University, Shanghai, China; Pediatric Nursing department (M H Huda PhD), Faculty of Public Health (D Kusuma DSc, Prof I Trihandini PhD), Centre for Family Welfare (K Latief PhD), University of Indonesia, Depok, Indonesia; Department of Public Health and Community Medicine (Prof A Humayun PhD), Shaikh Zayed Postgraduate Medical Institute, Lahore, Pakistan; Department of Humanities (W Husain PhD), COMSATS University Islamabad, Islamabad, Pakistan; Department of Biomolecular Sciences (Prof N R Hussein PhD), Department of Biology (K S Ibrahim PhD), Department of Biomedical Sciences (Prof I A Naqid PhD), University of Zakho, Zakho, Iraq; Artur Riggs Diabetes & Metabolism Research Institute (Prof M I Husseiny PhD), Cancer Prevention and Research Institute, Duarte, CA, USA; Department of Biomedical, Metabolic, and Neural Science (L F Iannone MD), University of Modena and Reggio Emilia, Modena, Italy; Genetics and Molecular Biology Department (Re Ibrahim PhD), Abu Dhabi University, Abu Dhabi, United Arab Emirates; Faculty of Pharmacy (U I Ibrahim PhD), Sultan Zainal Abidin University, Terengganu, Malaysia; Pharmacoepidemiology Department (F Ida PhD), Sanofi, Cambridge, MA, USA; Division of Infectious Diseases (K S Ikuta MD), Veterans Affairs Greater Los Angeles, Los Angeles, CA, USA; West Africa RCC (O S Ilesanmi PhD), Africa Centre for Disease Control and Prevention, Abuja, Nigeria; Department of Community Medicine (O S Ilesanmi PhD), Department of Neurology (O V Olalusi MD), Department of

Medicine (Prof M O Owolabi DrM), University College Hospital, Ibadan, Ibadan, Nigeria; Faculty of Medicine (I M Ilic PhD, Prof M M Santric-Milicevic PhD, A Stevanović MD, I S Vujcic PhD), School of Public Health and Health Management (Prof M M Santric-Milicevic PhD), University of Belgrade, Belgrade, Serbia; Faculty of Medical Sciences (Prof M D Ilic PhD), University of Kragujevac, Kragujevac, Serbia; Department of Clinical Pharmacy (M Imam PhD), College of Pharmacy (M Kamal PhD), Department of Health and Rehabilitation Sciences (Prof G Nambi PhD), Prince Sattam bin Abdulaziz University, Al Kharj, Saudi Arabia; Faculty of Health and Life Sciences (A Inok PhD), University of Exeter, Exeter, UK; Department of Psychology (Mu Iqbal PhD), Department of Orthopedics (W Jin MD), Center for Evidence-Based and Translational Medicine (L Luo MPH), Department of Epidemiology and Biostatistics (Prof S Mubarik PhD, Prof C Yu PhD), Wuhan University, Wuhan, China; Faculty of Pharmacy (L M Irham PhD), Faculty of Public Health (Prof S Solikhah DrPH), Universitas Ahmad Dahlan, Yogyakarta, Indonesia; Department of Biotechnology (M A Isa PhD), Sharda University, Greater Noida, India; Department of Pharmaceutical Technology (B Iskandar PhD), Sekolah Tinggi Ilmu Farmasi Riau, Pekanbaru, Indonesia; Independent Researcher, Cairo, Egypt (T R Iskander BSc); Journal of Biosciences and Public Health, Published by 4-Green Research Society (Md Shah Islam PhD), Journal of Biological Sciences and Public Health, Dhaka, Bangladesh; Institute for Physical Activity and Nutrition (Prof S Islam PhD), Deakin University, Burwood, VIC, Australia; Department of Surveillance and Health Equity Science (F Islami PhD), American Cancer Society, Atlanta, GA, USA; Clinical Laboratory Department (F Ismail PhD), Tobruk University, Tobruk, Libya; Department of Blood Transmitted Diseases (F Ismail PhD), National Centre for Disease Control (NCDC), Tobruk, Libya; Department of Clinical Pharmacy & Pharmacy Practice (Prof N E Ismail PhD), Asian Institute of Medicine, Science and Technology, Bedong, Malaysia; Malaysian Academy of Pharmacy, Puchong, Malaysia (Prof N E Ismail PhD); Department of Health Services Research (M Iwagami PhD), Department of Public Health Medicine (Prof K Yamagishi MD), University of Tsukuba, Tsukuba, Japan; Knowledge Translation Program (I O Iyamu MD), Centre for Health Evaluation and Outcome Sciences, Vancouver, BC, Canada; Department of Physical Medicine and Rehabilitation (L Jacob MD), Université Paris Cité, Paris, France; Research and Development Unit (L Jacob MD), Biomedical Research Networking Center for Mental Health Network (CiberSAM), Barcelona, Spain; Department of Health Studies (K H Jacobsen PhD), University of Richmond, Richmond, VA, USA; School of Medicine (M Jafari MD), Volgograd State Medical University, Volgograd, Russia; Department of Immunology (Prof A Jafarzadeh PhD), Department of Epidemiology and Biostatistics (Prof M Rezaeian PhD), Rafsanjan University of Medical Sciences, Rafsanjan, Iran; Government Hospitals, Manama, Bahrain (H Jahrami PhD); Department of Health and Safety (A A Jairoun PhD), Dubai Municipality, Dubai, United Arab Emirates; Department of Research and Academic Affairs (V Jaiswal MD), Larkin Community Hospital, South Miami, FL, USA; Department of Medicine (V Jaiswal MD), AMA School of Medicine, Makati, Philippines; Department of Behavioral Health (S Jaka MD), Nassau University Medical center, East Meadow, NY, USA; UNESCO-TWAS Section of Economic & Social Sciences, Humanities & Arts (Prof M Jakovljevic PhD), The World Academy of Sciences UNESCO-TWAS, Trieste, Italy; Shaanxi University of Technology, Hanzhong, China (Prof M Jakovljevic PhD); Department of Environmental Engineering (Prof R Jalilzadeh Yengejeh PhD), Islamic Azad University, Ahvaz, Iran; Department of Neurosurgery (M Jalloh MD), Division of Hematology and Oncology (J F Wu MD), Medical College of Wisconsin, Milwaukee, WI, USA; Department of Primary Care Medicine (J Jamaluddin MMed), Universiti Malaya, Kuala Lumpur, Malaysia; Department of Public Health (S Jamil MPH, M Shimul MPH), Department of Development Studies (M Sohel MPH), Daffodil International University, Dhaka, Bangladesh; Department of Public and Community Health (S Jamil MPH), Frontier University Garowe, Puntland, Somalia; Institute for

Neurosciences (Prof R G Jamora PhD), St. Luke's Medical Center, Bonifacio Global City, Philippines; Department of Internal Medicine (C T Jani MD), Harvard T.H. Chan School of Public Health (D Nguyen BS), Harvard Extension School (S G Saxena DrPH), Department of Orthopaedics (O Subasi PhD), Harvard Kennedy School (K J Uzor MD), Harvard University, Cambridge, MA, USA; Department of Stem Cells and Developmental Biology (E Jarrahi MSc, M Piroozkhkah MD), Royan Institution, Tehran, Iran; Health Informatics Lab (T Javaheri PhD), Department of Computer Science (R Rawassizadeh PhD), Boston University, Boston, MA, USA; Department of Medicine (S Javaid MD), University of Mississippi Medical Center, Jackson, MS, USA; Department of Medicine (S Javaid MD), Jinnah Sindh Medical University, Karachi, Pakistan; Department of Nursing (Q Jawell Odah Abed PhD), Middle Technical University of Kut Technical Institute, Baghdad, Iraq; The Medical City for Military and Security Services School (S Jayapal PhD), The Medical City for Military and Security Services School, Oman, Muscat, Oman; Department of Biochemistry (Prof S Jayaram MD), Government Medical College, Mysuru, India; Department of Oral Medicine and Periodontology (Prof R D Jayasinghe MS, Y A Jayasinghe BSc), University of Peradeniya, Peradeniya, Sri Lanka; Department of Research (Y A Jayasinghe BSc, Prof K K Kanmodi MPH), University of Puthisastra, Phnom Penh, Cambodia; Department of Epidemiology and Health Promotion (Prof S Jee PhD), Institute for Global Engagement & Empowerment (Prof S Oh PhD), Yonsei University, Seoul, South Korea; Graphic Era Deemed to be University (D Jena PhD), Department of Biotechnology (B Sharma PhD), Graphic Era (Deemed to be University), Dehradun, India; Department of Internal Medicine (B M Jeswani MBBS), GCS Medical College, Hospital & Research Centre, Ahmedabad, India; Research Division (Prof P K Maulik PhD), The George Institute for Global Health, New Delhi, India (Prof V Jha MD); Department of Microbiology, Faculty of Medicine (N Jomehzadeh PhD), Nursing Care Research Center in Chronic Diseases (Prof K Zarea PhD), Ahvaz Jundishapur University of Medical Sciences, Ahvaz, Iran; Department of Microbiology (N Jomehzadeh PhD), Abadan School of Medical Sciences, Abadan, Iran; Rothschild Foundation Hospital (Prof J B Jonas MD), Institut Français de Myopie, Paris, France; Singapore Eye Research Institute, Singapore, Singapore (Prof J B Jonas MD); Hungarian Health Management Association, Budapest, Hungary (T Joo PhD, T Palicz MD); Department of Gastroenterology and Hepatology (A Joseph MD), Management Science and Engineering (Y Ling MS), Department of Biomedical Data Science (S Park MD), Department of Radiology (S Ramasamy MD), Stanford University, Stanford, CA, USA; Department of Management (M Joshi PhD), Indira Gandhi Delhi Technical University for Women, Delhi, India; Department of Family Medicine and Public Health (J J Jozwiak PhD), University of Opole, Opole, Poland; Institute of Family Medicine and Public Health (M Jürisson PhD), University of Tartu, Tartu, Estonia; Research Department (Z Kabir PhD), TobaccoFree Research Institute Ireland, Dublin, Ireland; School of Public Health (Z Kabir PhD), University College Cork, Cork, Ireland; Policy, Research, and International Development Directorate (R Kadel MPH), Public Health Wales, Cardiff, UK; Independent Consultant, Pune, India (P V Kakodkar MDS); Department of Health (K Kalavani PhD), Khoy Medical Sciences, Khoy, Iran; Department of Dermatology (F Kaliyadan MD), King Faisal University, Hofuf, Saudi Arabia; Department of Endocrinology (S Kalra DM), Bharti Hospital Karnal, Karnal, India; Canberra Business School (M M Kamal MPH), University of Canberra, Hawker, ACT, Australia; Care and Public Health Research Institute (CAPHRI) (R Kamath MHA), Maastricht University, Maastricht, Netherlands; Department of Public Health (R T Kamorudeen MPH), South Wales University, Treforest, UK; Department of Biostatistics and Epidemiology (N Kamyari PhD), Abadan University of Medical Sciences, Abadan, Iran; Microbiology, Virology and Immunology Department (Prof O Kamyshnyi DSc), I. Horbachevsky Ternopil National Medical University, Ternopil, Ukraine; Department of Health Sciences (Prof M Kanaan PhD), University of York, York, UK; Office of the Executive Director (Prof K K Kanmodi

MPH), Cephas Health Research Initiative Inc, Ibadan, Nigeria; Department of Community Medicine (S Kannan S MD), ESIC Medical College and Hospital Chennai, Chennai, India; The Hansjörg Wyss Department of Plastic and Reconstructive Surgery (R S Kantar MD), NYU Langone Health, New York, NY, USA; Cleft Lip and Palate Surgery Division (R S Kantar MD), Global Smile Foundation, Norwood, MA, USA; Community and Primary Care Research Group (D Kar MD), Plymouth University, Plymouth, UK; 2nd Department of Cardiology (P Karakasis MD), First Department of Ophthalmology (Prof G D Panos MD), Second Propedeutic Department of Internal Medicine (Prof D Patoulias PhD), Laboratory of Clinical Pharmacology (V Tseriotis MSc), Aristotle University of Thessaloniki, Thessaloniki, Greece; Department of Medicine (A Katamreddy MD), Jacobi Medical Center, New York, NY, USA; Surgery Research Unit (Prof J H Kauppila MD), Oulu Business School (I Shiue PhD), Martti Ahtisaari Institute (I Shiue PhD), University of Oulu, Oulu, Finland; Department of Clinical Research and Epidemiology (K Kaushal MD), Institute of Liver and Biliary Sciences, New Delhi, India; Department of Internal Medicine (Y T Kebede MD), Yale New Haven Health—Bridgeport Hospital, Bridgeport, CT, USA; Institute of Biological Chemistry and Nutrition (T S Keflie PhD), University Hohenheim, Stuttgart, Germany; Department of Biochemistry (S N Kempegowda PhD), JSS Medical College, Mysuru, India; Center of Global Child Health (S Kerai PhD), The Hospital for Sick Children, Toronto, ON, Canada; Centre for Adolescent Health (J A Kerr PhD), Department of Critical Care and Neurosciences (Prof R G Weintraub MB), Murdoch Childrens Research Institute, Parkville, VIC, Australia; Department of Psychological Medicine (J A Kerr PhD), University of Otago, Christchurch, New Zealand; Jindal School of Public Health and Human Development (V Keshri PhD), O. P. Jindal Global University, Sonipat, India; Department of Biomedical Informatics (K Keshtkar BSc), Arizona State University, Phoenix, AZ, USA; Department of Human Nutrition of INRAE (E Kesse-Guyot PhD), National Research Institute for Agriculture, Food and Environment, Paris, France; Department of Health, Medicine and Human Biology (M Touvier PhD), Sorbonne Paris Nord University, Bobigny, France (E Kesse-Guyot PhD); Faculty of Medicine (R Khademi MD, F Shahrahmani MD), Department of Medical Genetics and Molecular Medicine (G Khalili-Tanha PhD), Biotechnology Research Center (Prof A Sahebkar PhD), Department of Medical Informatics (S Tabatabaei PhD), Applied Biomedical Research Center (S Tabatabaei PhD), Department of Medicine (A Yarahmadi PhD), Mashhad University of Medical Sciences, Mashhad, Iran; Research and Development Department (Prof R Mehboob PhD), Lahore Medical Research Center, Lahore, Pakistan (S Khalid PhD); Faculty of Veterinary Medicine (H O Khalifa PhD), Kafrelsheikh University, Kafrelsheikh, Egypt; Department of Public Health (Prof M Khalis PhD), Mohammed VI Center for Research and Innovation, Rabat, Morocco; Higher Institute of Nursing Professions and Health Techniques, Rabat, Morocco (Prof M Khalis PhD); Food and Drug Research Center (F Khamesipour PhD), Iran Food and Drug Administration, Tehran, Iran; Internal Medicine Department (I Khan MD), Reading Hospital Tower Health, Reading, PA, USA; Department of Community Medicine (M A S Khan MPH), Department of Biostatistics (Prof Mo M Rahman MS), National Institute of Preventive and Social Medicine, Dhaka, Bangladesh; BDStatistics Center for Research, Dhaka, Bangladesh (M J Khan MPH); Karachi Medical and Dental College, Karachi, Pakistan (M H Khan MBBS); Center for Atmospheric Particle Studies (CAPS) (M M Khan MSc), Department of Mechanical Engineering (MechE) (M M Khan MSc), Carnegie Mellon University, Pittsburgh, PA, USA; Aston Pharmacy School (M Uma Khan PhD), Aston University, Birmingham, UK; Joint Doctoral School (S A Khan MSc), Silesian University of Technology, Gliwice, Poland; Dr. Panjwani Center for Molecular Medicine & Drug Research (S A Khan MSc), International Center for Chemical and Biological Sciences (F Mansoor MS, S Ullah MSc), H.E.J. Research Institute of Chemistry (M Shahbaz MPH), University of Karachi, Karachi, Pakistan; International Center for Chemical and Biological Sciences, Karachi, Pakistan (Se Khan PhD); Department

of Cardiology (Z Khan MD), University of South Wales, Treforest, UK; Department of Cardiology (Z Khan MD), University of Buckingham, Buckingham, UK; Department of Health (V Khanal PhD), Nepal Development Society, Chitwan, Nepal; Department of Preventable Non Communicable Disease (V Khanal PhD), Menzies School of Health Research, Alice Springs, NT, Australia; Department of Pharmacology (S U Khasbage MD), All India Institute of Medical Sciences, Raipur, India; College of Health, Wellbeing and Life Sciences (Prof K Khatab PhD), Sheffield Hallam University, Sheffield, UK; College of Arts and Sciences (Prof K Khatab PhD), Ohio University, Zanesville, OH, USA; Global Consortium for Public Health Research (Prof M Khatib PhD, Prof Z Quazi PhD), Datta Meghe Institute of Higher Education and Research, Wardha, India; Department of Orthopaedics (K Khatri MS), Postgraduate Medical Institute, Sangrur, India; University of Sulaimani College of Medicine (N S H Khoshnaw PhD), Sulaimani Polytechnic University, Sulaymaniyah, Iraq; Department of Internal Medicine (A A Khosla MD), Corewell Health East William Beaumont University Hospital, Royal Oak, MI, USA; Department of Medical Oncology (A A Khosla MD), Department of Medical and Surgical Oncology (A Pon Avudaiappan MD), Miami Cancer Institute, Miami, FL, USA; Department of Clinical Research (S Khosravi MD), Icahn School of Medicine at Mount Sinai, New York City, NY, USA; Research Department (M Khosrowjerdi PhD), University of Inland Norway, Elverum, Norway; Ashok & Rita Patel Institute of Physiotherapy (P Khuman PhD, K D Vyas MPH), Charotar University of Science and Technology, Changa, India; Department of Biomedical Sciences (H Kim MS), Department of Psychiatry (W Myung PhD), Department of Food and Nutrition (A P Okekunle PhD), Seoul National University, Seoul, South Korea; School of Medicine (Prof K Kim PhD), Creighton University, Omaha, NE, USA; Cardiovascular Disease Initiative (M Kim MD), Broad Institute of MIT and Harvard, Cambridge, MA, USA; School of Traditional Chinese Medicine (Y Kim PhD), Xiamen University Malaysia, Sepang, Malaysia; Health and Healing Research, Education, and Service, Inc., Boston, MA, USA (R W Kimokoti MD); Millennium Prevention, Inc., Westwood, MA, USA (R W Kimokoti MD); Department of Nursing (T Kinati MSc), Salale University, Fitche, Ethiopia; The Pacific Community, Noumea, New Caledonia (Prof Y Kinfu PhD); School of Health Sciences (Prof A Kisa PhD), Kristiania University College, Oslo, Norway; Department of Nursing and Health Promotion (S Kisa PhD), Faculty of Health Sciences (Prof A W Wolf PhD), Oslo Metropolitan University, Oslo, Norway; Department of Public Health Dentistry (Prof S KM PhD), Krishna Vishwa Vidyapeeth (Deemed to be University), Karad, India; Endocrinology Department (Prof N Kobyliak DSc), Bogomolets National Medical University, Kyiv, Ukraine; Scientific Department (Prof N Kobyliak DSc), Medical Laboratory CSD, Kyiv, Ukraine; Global Healthcare Consulting, New Delhi, India (S Kochhar MD); Department of Public Health and Community Medicine (P Kodali PhD), Central University of Kerala, Kasaragod, India; Mycobacteriology Unit (D Kolieghu Tcheumeni MSc), Center for Health Promotion and Research, Bamenda, Cameroon; Australian Institute for Suicide Research and Prevention (Prof K Kolves PhD), Griffith University, Mount Gravatt, QLD, Australia; Department of Population and Behavioural Sciences (J Komesuor PhD), University of Health and Allied Sciences, Hohoe, Ghana; Copernicus Institute of Sustainable Development (G Koren PhD), Utrecht University, Utrecht, Netherlands; Department of Science and Environmental Studies (T Kormoker PhD), The Education University of Hong Kong, Tai Po, New Territories, Hong Kong, China; Department of General Practice and Family Medicine (Prof O Korzh DSc), Kharkiv National Medical University, Kharkiv, Ukraine; Independent Consultant, Jakarta, Indonesia (S Kosen MD); Department of Epidemiology (Prof K Kostev PhD), IQVIA, Frankfurt am Main, Germany; University Hospital Marburg, Marburg, Germany (Prof K Kostev PhD); Department of Internal and Pulmonary Medicine (Prof P A Koul MD), Sheri Kashmir Institute of Medical Sciences, Srinagar, India; Department of Public Health (J Kretchy PhD), Central University, Accra, Ghana (J Kretchy PhD);

Department of Anthropology (Prof K Krishan PhD), Institute of Forensic Science & Criminology (V Sharma PhD), Panjab University, Chandigarh, India; School of Applied Science (C Kua PhD), Republic Polytechnic, Singapore, Singapore; Centre for Biotechnology (A Kuanar PhD, S K Panda PhD, S Patel PhD, P Verma PhD), Siksha 'O' Anusandhan Deemed to be University, Bhubaneswar, India; Department of Demography (Prof B Kuate Defo PhD), Department of Social and Preventive Medicine (Prof B Kuate Defo PhD), University of Montreal, Montreal, QC, Canada; Department of Pediatrics (I Kuitunen PhD), Kuopio University Hospital, Kuopio, Finland; Institute of Clinical Medicine (I Kuitunen PhD), University of Eastern Finland, Kuopio, Finland; Center of Medicine and Public Health (M Kulimbet MSc), Director of Central Asia Research Collaboration Group (Prof F Rahim PhD), Asfendiyarov Kazakh National Medical University, Almaty, Kazakhstan; Amity Centre for Water Studies and Research (S Kulshreshtha PhD), Amity Institute of Biotechnology (E Upadhyay PhD), Amity University Rajasthan, Jaipur, India; Department of Community Medicine (De Kumar MD), Department of Anaesthesiology (Tu Kumar DNB), Rajendra Institute of Medical Sciences, Ranchi, India; SRM Centre for Clinical Trials and Research (CCTR) (Dh Kumar PhD), Sri Ramaswamy Memorial Institute of Science and Technology, Chennai, India; Department of Mathematics (Prof K Kumar PhD), Amity University Haryana, Gurugram, India; Department of Community Medicine (M Kumar MD), Vardhman Mahavir Medical College and Safdarjung Hospital, Delhi, India; Department of Pharmacology and Toxicology (Nite Kumar PhD), National Institute of Pharmaceutical Education and Research, Hajipur, Hajipur, India; Department of Pharmacology (Ta Kumar MD), Regional Institute of Medical Sciences, Imphal, India; Department of Economics (Vij Kumar PhD), Manipal University, Jaipur, Jaipur, India; Department of Gastroenterology & Hepatology (Vik Kumar MD), Creighton University, Phoenix, AZ, USA; IITM Pravartak Technologies Foundation, Chennai, India (S Kumaran PhD); Section of Cardiology (Prof S K Kunutsor PhD), University of Manitoba, Winnipeg, MB, Canada; Department of Translational Health Sciences (Prof S K Kunutsor PhD), Bristol Medical School (Y Nartey PhD), Integrative Epidemiology Unit (A Suhag PhD), University of Bristol, Bristol, UK; Faculty of Medicine and Health Science (M Kurniasari PhD), Universitas Kristen Satya Wacana (Satya Wacana Christian University), Salatiga, Indonesia; Division of Cardiology (K Kurpad MD), University of Illinois, Champaign, IL, USA; Research Center for Public Health and Nutrition (B Rachmat MPH), National Research and Innovation Agency (BRIN), Jakarta, Indonesia (A Kusnali MA, I U Tarigan PhD); Institute for Health Sciences (C Kustanti PhD), STIKES Bethesda Yakkum Yogyakarta Indonesia, Yogyakarta, Indonesia; Department of Public Health and Epidemiology (D Kusuma DSc), Khalifa University of Science and Technology, Abu Dhabi, United Arab Emirates; Department of Pediatric Oncology (Prof T Kutluk MD), Medicana Health International, Istanbul, Türkiye; Department of Pediatric Oncology (Prof T Kutluk MD), Hacettepe University, Ankara, Türkiye; Department of Nursing (E F Kyei PhD, G K Kyei BSc), University of Massachusetts Boston, Boston, MA, USA; Department of Environment and Public Health (F Kyei-Arthur PhD), University of Environment and Sustainable Development, Somanya, Ghana; Clinical Research Center (V Kytö MD), Turku University Hospital, Turku, Finland; Department of Medicine and Surgery (A La Vecchia MD), University of Milano - Bicocca, Milan, Italy; Department of Medicine (A Lachi PhD), UniCamillus University, Rome, Italy; Genetic Resource Program (A K Lagat MSc), International Maize and Wheat Improvement Center (CIMMYT), Nairobi, Kenya; Department of Basic Sciences (A K Lagat MSc), Statistics and Actuarial Sciences Department (D F Mulwa PhD), Department of Animal and Human Health (D F Mulwa PhD), Jomo Kenyatta University of Agriculture and Technology, Nairobi, Kenya; Division of Evidence Synthesis (C Lahariya MD), Foundation for People-centric Health Systems, New Delhi, India; Division of Lifestyle Medicine (C Lahariya MD), Centre for Health: The Specialty Practice, New Delhi, India; School of Digital Science (D Lai PhD), Institute

of Applied Data Analytics (D Lai PhD), Faculty of Science (E Leong PhD), Universiti Brunei Darussalam (University of Brunei Darussalam), Bandar Seri Begawan, Brunei; Department of Chemistry (Prof A Lakhani PhD), Dayalbagh Educational Institute, Agra, India; NEVES Society for Patient Safety, Budapest, Hungary (J Lám PhD); Unidad de Genética y Salud Pública (Prof I Landires MD), Instituto de Ciencias Médicas, Las Tablas, Panama; Ministry of Health (Prof I Landires MD), Hospital Joaquín Pablo Franco Sayas, Las Tablas, Panama; Department of Psychiatry and Psychotherapy (B Langguth PhD), University of Regensburg, Regensburg, Germany; Department of Behavioural Sciences and Learning (Prof A Laplante-Lévesque PhD), Linköping University, Linköping, Sweden; Department of Otorhinolaryngology (S Lasrado MS), Father Muller Medical College, Mangalore, India; International Society of Doctors for the Environment, Arezzo, Italy (P Lauriola MD); Centre for Clinical Trials, Research, and Implementation Science (CCTRIS), Lagos, Nigeria (T Lawanson MD); Nam Can Tho University (D Le MD), Faculty of Medicine, Can Tho, Viet Nam; Department of Thoracic and Vascular Surgery (D Le PhD), Nam Can Tho University, Vietnam, Can Tho City, Viet Nam; Department of General Medicine (V T Nguyen MD), Department of Internal Medicine (T H Tran MD), University of Medicine and Pharmacy at Ho Chi Minh City, Ho Chi Minh City, Viet Nam (T T T Le MD); STEM (Prof I Lee PhD), University of South Australia, Adelaide, SA, Australia; Department of Precision Medicine (Prof S Lee MD), Sungkyunkwan University, Suwon-si, South Korea; Department of Cardiothoracic and Vascular Surgery (V Leivaditis PhD), Westpfalz Klinikum, Kaiserslautern, Germany; Department of Cardiothoracic Surgery (V Leivaditis PhD), University of Patras, Patras, Greece; SC Neurologia, Salute Pubblica e Disabilità (Neurology, Public Health, Disability Unit) (M Leonardi MD), Fondazione IRCCS Istituto Neurologico Carlo Besta (IRCCS Foundation Carlo Besta Neurological Institute), Milan, Italy; Nutrition & Health Innovation Research Institute (C Li MPH), Edith Cowan University, Perth, WA, Australia; Department of Rheumatology and Immunology (H Li MD), The People's Hospital of Baoan Shenzhen, Shenzhen, China; Global Health Research Center (Prof Jie Li PhD), Guangdong Academy of Medical Sciences and General Hospital, Guangzhou, China; Department of Health Promotion and Health Education (M Li PhD), National Taiwan Normal University, Taipei, Taiwan; Discipline of Physiology (Y Lian MA), National University of Ireland - Galway, Galway, Ireland (D Shan MD); Cardiovascular Medicine Department (C Liao MD), The Second Affiliated Hospital of Nanchang University, NanChang, China; Department of Food Science and Human Nutrition (Q Lin MPH), Iowa State University, Ames, IA, USA; The Center for Drug Safety and Policy Research (S Lin PhD), Xi'an Jiaotong University, Xi'an, China; Department of Medical Sciences (D Lindholm MD, Prof J Sundström PhD), Uppsala University, Uppsala, Sweden; Department of Medicine (D Lindholm MD), Norrtälje Hospital (Tiohundra), Norrtälje, Sweden; School of Public Health (Prof S Linn DrPH), Zefat Academic College, Haifa, Israel; Department of Epidemiology and Biostatistics (Prof J Liu PhD), Peking University First Hospital (H Luo PhD), National Institute of Health Data Science (W Wang PhD), School of Public Health (Yo Wang MD, H Zhang PhD), Institute of Child and Adolescent Health (Prof Z Zou MD), Peking University, Beijing, China; School of Nursing and Health Sciences (Xian Liu PhD), Hong Kong Metropolitan University, Hong Kong, China; College of Mathematics and Computer (Prof Zhe Liu PhD), Xinyu University, Xinyu, China; Department of Urology (Zhen Liu PhD), Department of Orthopaedics (F Wei PhD), General Hospital of Central Theater Command, Wuhan, China; School of Medicine (Zhen Liu PhD), School of Public Health (Y Zhang PhD), Hubei Province Key Laboratory of Occupational Hazard Identification and Control (Y Zhang PhD), Wuhan University of Science and Technology, Wuhan, China; Department of Molecular Epidemiology (E Llanaj PhD), German Institute of Human Nutrition Potsdam-Rehbrücke, Potsdam, Germany; German Center for Diabetes Research (DZD), München-Neuherberg, Germany (E Llanaj PhD); Department of Infectious Diseases (M J Loftus MBBS), Alfred Health,

Melbourne, VIC, Australia; Department of Cardiology (V Lohner PhD), University of Cologne, Cologne, Germany; School of Medicine (J López-Gil PhD), Universidad Espíritu Santo, Samborondón, Ecuador (J Sharifi Rad PhD); Vicerrectoría de Investigación y Postgrado (J López-Gil PhD), Universidad de Los Lagos, Osorno, Chile; Institute of Nutritional Sciences (Prof S Lorkowski PhD), Friedrich Schiller University Jena, Jena, Germany; Competence Cluster for Nutrition and Cardiovascular Health (nutriCARD), Jena, Germany (Prof S Lorkowski PhD); School of Medicine (Prof R Lozano MD), National Autonomous University of Mexico, Mexico City, Mexico; Department of Spine Surgery (S Luan MD), Qingdao Municipal Hospital Group, Qingdao, China; Scientific Research and Surveillance Systems (J Lubinda PhD), Macha Research Trust, Choma, Zambia; School of Medicine (Prof G Lucchetti PhD), Federal University of Juiz de Fora, Juiz de Fora, Brazil; Department of Emergency General and Trauma Surgery (Prof R Lunevicius DSc), NHS University Hospitals of Liverpool Group, Aintree Hospital, Liverpool, UK; The Third Department of Hepatic Surgery (S Luo PhD), Eastern Hepatobiliary Surgery Hospital, Shanghai, China; Department of Clinical Data Science and Evidence (L Lv PhD), Novo Nordisk, Plainsboro, NJ, USA; College of Engineering (Prof M D Lytras PhD), Effat University, Jeddah, Saudi Arabia; Management of Information Systems Department (Prof M D Lytras PhD), The American College of Greece, Aghia Paraskevi, Greece; Centre for Public Health and Wellbeing (Z Ma PhD), University of the West of England, Bristol, UK; Department of Microbiology and Parasitology (M Mabrok PhD), King Salman International University, South of Sinai, Egypt; 2nd Department of Propaedeutic Surgery (N Machairas PhD), Department of Biophysics (Prof P Papadopoulou PhD), 3rd Department of Cardiology (M Spartalis PhD), University of Athens, Athens, Greece; Department of Periodontology (Prof M Machoy PhD), Department of Propedeutics of Internal Diseases & Arterial Hypertension (Prof T Miazgowski MD), Pomeranian Medical University, Szczecin, Poland; Associate Laboratory i4HB (A M Madureira-Carvalho PhD), University Institute of Health Sciences - CESPU, Gandra, Portugal; UCIBIO Research Unit on Applied Molecular Biosciences (A M Madureira-Carvalho PhD), University Institute of Health Sciences, Gandra, Portugal; School of Infection & Immunity (Prof P Maffia PhD), School of Cardiovascular and Metabolic Health (F E Petermann-Rocha PhD), University of Glasgow, Glasgow, UK; Department of Pharmacy (Prof P Maffia PhD), University of Naples Federico II, Naples, Italy; Department of Emergency Medicine (S Mahalingam MD), Sri Lakshmi Narayana Institute of Medical Science, Puducherry, Pondicherry, India; Department of One Health in Tropical Infectiousness Diseases (S A Mahamed MSc), Department of Public Health (A Yousuf PhD), Jigjiga University, Jigjiga, Ethiopia; College of Health Science (S A Mahamed MSc), Amoud University, Borama, Somalia; Institute of Health Science (M T Mai MD), Faculty of Medicine (H T Nguyen MD, T T T Truyen MD), International Medical Faculty (N P Nguyen MD), Nam Can Tho University, Can Tho, Viet Nam; Smart Healthcare Management (H Mai Xuan MSc), National Taipei University, New Taipei City, Taiwan; Department of Pharmacology (Prof R Maiti MD, Ar Mishra DM), Department of Psychiatry (A Parmar DM), All India Institute of Medical Sciences, Bhubaneswar, India; Department of Public Health (M Majdan PhD, J Pekarcikova PhD), Trnava University, Trnava, Slovakia; Department of Medicine (O M Makram MD), Medical College of Georgia at Augusta University, Augusta, GA, USA; University of Kansas Medical Center (M Mangdow MSc), A.T. Still University, Kansas City, KS, USA; Internal Medicine Department (Y Manla MD), Eisenhower Health, Palm Desert, CA, USA; Biomedical Engineering Research Center (CREB) (Prof M Mansourian PhD), Automatic Control Department (H Marateb PhD), Universitat Politècnica de Catalunya (Barcelona Tech - UPC), Barcelona, Spain; Department of Biotechnology (B P Marasini PhD), Tribhuvan University, Kathmandu, Nepal; Department of Biomedical Engineering (H Marateb PhD, M Noroozi BSc), University of Isfahan, Isfahan, Iran; Far Eastern University, Manila, Philippines (J C Maravilla PhD); Faculty of Human Kinetics (Prof A Marques PhD), Research Institute for

Medicines (Prof J Perdigão PhD), Universidade de Lisboa (University of Lisbon), Lisbon, Portugal; Department of Non-communicable Diseases and Mental Health (R Martinez-Piedra BSc), Department of Evidence and Intelligence for Action in Health (O J Mujica MD), Pan American Health Organization, Washington, DC, USA; Indonesian Public Health Association, Surabaya, Indonesia (Prof S Martini PhD); Campus Fortaleza (F R Martins-Melo PhD), Federal Institute of Education, Science and Technology of Ceará, Fortaleza, Brazil; Department of Nutrition and Dietetics (M Martorell PhD), Centre for Healthy Living (M Martorell PhD), University of Concepción, Concepción, Chile; Clinical Institute of Medical and Chemical Laboratory Diagnostics (Prof W März MD), Medical University of Graz, Graz, Austria; Medical Clinic V (Prof W März MD), Department of Experimental Pharmacology (Y Wibowo MD), Heidelberg University, Mannheim, Germany; Faculty of Humanities and Health Sciences (Prof R R Marzo MD), Curtin University, Sarawak, Malaysia; Jeffrey Cheah School of Medicine and Health Sciences (Prof R R Marzo MD), School of Pharmacy (Y Wong PhD), Monash University, Subang Jaya, Malaysia; Department of Nursing (S Mashudi PhD), Muhammadiyah University of Surakarta, Ponorogo, Indonesia; Department of Clinical and Experimental Medicine (Prof S Masi PhD, Prof N Pugliese PhD, D Trico MD), University of Pisa, Pisa, Italy; Department of Anatomy and Developmental Biology (Y Mathangasinghe PhD), Monash University, Clayton, VIC, Australia; North West Lung Centre (A G Mathioudakis PhD), Manchester University NHS Foundation Trust, Manchester, UK; Department of Community Medicine (M Mathur MD), Geetanjali Medical College and Hospital, Udaipur, India; Department of Community Medicine (N Mathur MD, P Varma MD, S Yahoo (Syed) MD), Apollo Institute of Medical Sciences and Research, Hyderabad, India; Department of Epidemiology (Prof R J Maude PhD), Mahidol-Oxford Tropical Medicine Research Unit, Bangkok, Thailand; Nuffield Department of Population Health (M Mazidi PhD), University of Oxford, London, UK; Orthopedic Trauma Pathology Department (A Mazzotti PhD), IRCCS, Bologna, Italy; Department of Obstetrics and Gynaecology (Prof I I Mbachu FWACS), Department of Paediatrics (C A Nri-Ezedi PhD), Nnamdi Azikiwe University, Awka, Nigeria; Digital Health and Informatics Directorate (Prof S M McPhail PhD), Queensland Health, Brisbane, QLD, Australia; National Heart, Lung and Blood Institute (Prof R Mehboob PhD), National Heart, Lung, and Blood Institute, Bethesda, MD, USA; Centre for Health Innovation and Policy, Noida, India (Prof R Mehrotra PhD); Department of Dental Research Cell (Prof V Mehta PhD), Dr. D. Y. Patil University, Pune, India; Health Care Authority, Olympia, WA, USA (A Memetova MA); Department of Medical Microbiology and Immunology (G A Menezes PhD), Trinity Medical Sciences University, St. Vincent, Saint Vincent and the Grenadines; Department of Medicine (G A Mensah MD, Prof L J Zuhlke PhD), Technical Department (C A Nnaji PhD), School of Public Health and Family Medicine (C A Nnaji PhD), Institute of Infectious Disease and Molecular Medicine (O G Oluwole PhD), SAMRC Unit on Risk and Resilience in Mental Disorders (Prof D J Stein FRCPC), Department of Paediatrics and Child Health (Prof H J Zar PhD, Prof L J Zuhlke PhD), University of Cape Town, Cape Town, South Africa; Department of Public Health (M Mercogliano MD), University "Federico II" of Naples, Naples, Italy; General Administration Department (A Meretoja MD), Comprehensive Cancer Center (T J Meretoja MD), Department of Neurosurgery (I Rautalin PhD), Helsinki University Hospital, Helsinki, Finland; University Centre Varazdin (T Mestrovic PhD), University North, Varazdin, Croatia; Stritch School of Medicine (A Mhlanga PhD), Loyola University Chicago, Chicago, IL, USA; Department of Pathology (I Michalek PhD), Maria Sklodowska-Curie National Research Institute of Oncology, Warsaw, Poland; Dermatology Unit (A Michelerio PhD), Fondazione IRCCS Policlinico San Matteo, Pavia, Italy; Department of Oncology (H S Mideksa MD), Addis Ababa University, Addis Abeba, Ethiopia; College of Human Medicine (T R Miller PhD), Michigan State University, Flint, MI, USA; Multidisciplinary Department of Medical-Surgical and Dental Specialties (G Minervini PhD), University of Campania Luigi Vanvitelli,

Naples, Italy; Global Institute of Public Health (Prof G Mini PhD), Ananthapuri Hospitals and Research Institute, Trivandrum, India; Department of Radiology (S Mirshahvalad MD), Health Sciences North, Sudbury, ON, Canada; Department of Forensic Medicine and Toxicology (As Mishra MD), Rohilkhand Medical College, Bareilly, India; Thumbay College of Management and AI in Healthcare (V Mishra PhD), Department of Biomedical Sciences (P Sengupta PhD), Gulf Medical University, Ajman, United Arab Emirates; Research and Development Department (V Mishra PhD), Panacea Institute of Interdisciplinary Research and Education, Varanasi, India; Department of Forensic Medicine and Toxicology (C Mittal MD), All India Institute of Medical Sciences, Patna, India; Department of Internal Medicine (S Modi MD), Albert Einstein Hospital, Philadelphia, PA, USA; College of Health Science (A I Mohamed MSc), College of Applied and Natural Science (J Mohamed MSc), University of Hargeisa, Hargeisa, Somalia; Higher Colleges of Technology-Health Sciences Division-Pharmacy Program (H M Mohamed PhD), Higher Colleges of Technology, Dubai, United Arab Emirates; RAK College of Nursing (M Mohamed PhD), RAK Medical and Health Sciences University, Ras Alkhima, United Arab Emirates; Nursing College (M Mohamed PhD), Sohag University, Sohag, Egypt; Molecular Biology Unit (N S Mohamed MSc), Bio-Statistical and Molecular Biology Department (N S Mohamed MSc), Sirius Training and Research Centre, Khartoum, Sudan; Department of Public Health (H Mohammed PhD, A Oumer PhD), Dire Dawa University, Dire Dawa, Ethiopia; Department of Medicine (O Mohammed MBBS), Government Medical College Kozhikode, Kozhikode, India; Department of Health Sciences, Azare (S Mohammed MSc), National Institute for Research in Tribal Health, Bauchi, Nigeria; Medical Microbiology Department (Prof Y Mohammed FWACP), Usmanu Danfodiyo University Teaching Hospital, Sokoto, Nigeria; Department of Health Services Management (M Mohseni PhD), Iran University of Medical Sciences, Iran, Iran; Institute of Clinical Physiology (S Molinaro PhD), National Research Council, Pisa, Italy; Department Medical-Surgical Nursing (A Mollaei PhD), Department of Nursing and Midwifery (F - Ranjbar Noei MSc), Golestan University of Medical Sciences, Gorgan, Iran; Clinical Epidemiology and Public Health Research Unit (L Monasta DSc, L Ronfani PhD, G Zamagni MSc), Burlo Garofolo Institute for Maternal and Child Health, Trieste, Italy; Department of Sport Physiology (A Monazzami PhD), Razi University, Kermanshah, Iran; Department of Collective Prevention and Public Health (M Montalti MD), General Directorate for Personal Care, Health, and Welfare, Bologna, Italy; Social Determinants of Health Research Center (M Moradi-Joo PhD), Yasuj University of Medical Sciences, Yasuj, Iran; International Laboratory for Air Quality and Health (Prof L Morawska PhD), Faculty of Science (M Sarmadi MSc), Queensland University of Technology, Brisbane, QLD, Australia; Department of Public Health (Prof R S Moreira PhD), Oswaldo Cruz Foundation, Recife, Brazil; Department of Public Health (Prof R S Moreira PhD), Federal University of Pernambuco, Recife, Brazil; Faculty of Medicine (M Morsy MD), October 6 University, Giza, Egypt; Department of Health Policy (Prof E Mossialos PhD), London School of Economics and Political Science, London, UK; Faculty of Biotechnologies (BioTech) (Prof A Mousavi Khaneghah PhD), ITMO University, Saint Petersburg, Russia; Department of Physical and Environmental Sciences (S Mousavi Kiasary DVM), Texas A&M University, Corpus Christi, TX, USA; René Rachou Institute (M Mrejen PhD), Oswaldo Cruz Foundation, Belo Horizonte, Brazil; PMAS Arid Agriculture University Rawalpindi, Rawalpindi, Pakistan (R Mubarak MSc); Unit of Pharmacotherapy, Epidemiology and Economics (Prof S Mubarik PhD), Rijksuniversiteit Groningen (University of Groningen), Groningen, Netherlands; Department of Pharmacy (S Mudenda PhD), University of Zambia, Lusaka, Zambia; School of Medicine (F Mughal FRCGP, T Oyelade PhD), Keele University, Keele, UK; Institute of Molecular Biology and Biotechnology (S Muhammad PhD), Bahauddin Zakariya University Multan, Multan, Pakistan; Department of Biochemistry (Suk Mukherjee PhD), Department of Dentistry (Prof A Singh MDS), All India Institute of Medical

Sciences, Bhopal, India; Knowledge Management Department (Sum Mukherjee PhD), Prahlad Omkarwati Foundation (POF), Mumbai, India; Changescape Consulting (Sum Mukherjee PhD), Independent Consultant, New Delhi, India (P Sood PhD); Department of Medicine (A Mukhopadhyay MD), National University Health System, Singapore, Singapore; Department of Mechanical Engineering (M Muktadir PhD), North Carolina Agricultural and Technical State University, Greensboro, NC, USA; Department of Surgery (F Mulita PhD), General University Hospital of Patras, Patras, Greece; Faculty of Medicine (F Mulita PhD), Department of Emergency Medicine (Prof I Pantazopoulos PhD), University of Thessaly, Larissa, Greece; Epidemiology, Biostatistics and Prevention Institute (EBPI) (J Muñoz Laguna MSc), University of Zürich, Zurich, Switzerland; Clinical Epidemiology Research Unit (E Murillo-Zamora PhD), Mexican Institute of Social Security, Villa de Alvarez, Mexico; Postgraduate in Medical Sciences (E Murillo-Zamora PhD), Universidad de Colima, Colima, Mexico; Operational Research Center in Healthcare (M T Mustapha PhD), Near East University, Nicosia, Cyprus; Department of Research Methods (S Muthu PhD), Orthopaedic Research Group, Coimbatore, India; Central Research Laboratory (S Muthu PhD), Meenakshi Medical College Hospital and Research Institute, Chennai, India; Director General (Prof C M Muvunyi PhD), Rwanda Biomedical Centre, Kigali, Rwanda; College of Medicine and Health Sciences (Prof C M Muvunyi PhD), University of Rwanda, Kigali, Rwanda; Department of Neuropsychiatry (W Myung PhD), Seoul National University Bundang Hospital, Seongnam, South Korea; Department of Health Education & Promotion (F Naddafi PhD), School of Medicine (M Rostamian PhD), Gonabad University of Medical Sciences, Gonabad, Iran; Elderly Health Research Center (A Nafei PhD), Research and Academic Institution, Tehran, Iran; Research and Analytics Department (A J Nagarajan MTech), Initiative for Financing Health and Human Development, Chennai, India; Department of Research and Analytics (A J Nagarajan MTech), Bioinsilico Technologies, Chennai, India; Department of Computer Science and IT (G R Naik PhD), Centre for Health Policy Research (Prof P Ward PhD), Torrens University Australia, Adelaide, SA, Australia; Department of Health Services Research (G Naik MPH), Department of Internal Medicine (A Rehman MD), Department of Psychology (D C Schwebel PhD), University of Alabama at Birmingham, Birmingham, AL, USA; Faculty of Pharmacy (Prof F Nainu PhD), Hasanuddin University, Makassar, Indonesia; Department of Pulmonary Medicine (Prof S Nair MD), Government Medical College, Thrissur, Thrissur, India; Health Action by People, Trivandrum, India (Prof S Nair MD); Suraj Eye Institute, Nagpur, India (V Nangia PhD); Department for the Control of Disease, Epidemics, and Pandemics (J Nansseu MD), Ministry of Public Health, Yaoundé, Cameroon; Department of Public Heath (J Nansseu MD), Department of Public Health (G Nguefack-Tsague PhD), University of Yaoundé I, Yaoundé, Cameroon; National Dental Research Institute Singapore (G G Nascimento PhD), Duke-NUS Medical School, Singapore, Singapore; Department of Applied Pharmaceutical Sciences and Clinical Pharmacy (A Y Naser PhD), Isra University, Amman, Jordan; Division of Endocrinology and Diabetes (M Nassar PhD), University of Vermont, South Burlington, VT, USA; Department of Community Medicine (Prof S N K Navaratna MD), University of Peradeniya, Kandy, Sri Lanka; Amity Institute of Forensic Sciences (B P Nayak PhD), Amity Institute of Public Health and Hospital Administration (P S Singh PhD), Amity University, Noida, India; Department of Research (G Nchanji PhD), TroDDIVaT Initiative, Buea, Cameroon; Department of Microbiology and Parasitology (G Nchanji PhD), University of Buea, Buea, Cameroon; School of Pharmacy (S O Nduaguba PhD), West Virginia University, Morgantown, WV, USA; Department of General Surgery (I Negoi PhD), Emergency University Hospital of Bucharest, Bucharest, Romania; Department of Cardiology (R I Negoi PhD), Cardio-Aid, Bucharest, Romania; Department of Cardiology (A G Negru PhD), University of Medicine and Pharmacy "Victor Babes", Timisoara, Romania; Rocordis Heart Center (A G Negru PhD), Cardiology and Cardiovascular Surgery

Hospital, Timisoara, Romania; Euromed Research Center (Prof C Nejjari MD), Euromed University of Fes, Fez, Morocco; Faculty of Medicine, Pharmacy, and Dentistry (Prof C Nejjari MD), University Sidi Mohammed Ben Abdellah, Fez, Morocco; Department of Biological Sciences (J W Ngunjiri PhD), University of Embu, Embu, Kenya; Institute for Global Health Innovations (C T Nguyen MPH), Duy Tan University, Hanoi, Viet Nam; Department of Medical Engineering (D Nguyen BS), University of South Florida, Tampa, FL, USA; Cardiovascular Research Department (N P Nguyen MD), Methodist Hospitals, Merrillville, IN, USA; Hitotsubashi Institute for Advanced Study (HIAS) (T Nguyen DrPH), Hitotsubashi University, Tokyo, Japan; Institute for Cancer Control (T Nguyen DrPH), National Cancer Center, Chuo-ku, Japan; Tuberculosis Group (V T Nguyen MD), Oxford University Clinical Research Unit, Vietnam, Ho Chi Minh City, Viet Nam; Department of Public Health (A M Ngwa MSc), University of Bamenda, Bamenda, Cameroon; International Islamic University Islamabad, Islamabad, Pakistan (R K Niazi PhD); Department of Humanities and Social Science (L Nieddu PhD), University for International Studies in Rome, Rome, Italy; Institute for Mental Health Policy Research (Y T Nigatu PhD), Centre for Addiction and Mental Health, Toronto, ON, Canada; School of Medicine (V Niranjan PhD), University of Limerick, Limerick, Ireland; Department of Public Health (V Niranjan PhD), UNICAF, Larnaca, Cyprus; Global Research Institute (Prof S Nomura PhD), Keio University, Tokyo, Japan; Department of Microbiology and Molecular Genetics (M Noreen PhD), The Women University Multan, Multan, Pakistan; Health Research Institute (M Nouri PhD), School of Medicine (S Soraneh MD), Babol University of Medical Sciences, Babol, Iran; Internal Medicine Department (V C Nriagu MD), Maimonides Medical Center, Brooklyn, NY, USA; Global Health Department (J Nshimiyimana MPH), Euclid University, Banqui, Central African Republic; School of Information (F Nugen PhD), University of California Berkeley, Berkeley, CA, USA; Midwifery Department (N Nurfatimah MPH), Poltekkes Kemenkes Palu, Palu, Indonesia; Department of Public Health (D Nurrika PhD), Banten School of Health Science, South Tangerang, Indonesia; Ministry of Research, Technology and Higher Education (D Nurrika PhD), Higher Education Service Institutions (LL-DIKTI) Region IV, Bandung, Indonesia; Department of Applied Economics and Quantitative Analysis (Prof B Oancea PhD), University of Bucharest, Bucharest, Romania; Bioinformatics Department (Prof B Oancea PhD), National Institute of Research and Development for Biological Sciences, Bucharest, Romania; Department of Biomedicine and Prevention (F Oddi PhD), University of Rome "Tor Vergata", Rome, Italy; Department of Veterinary Public Health and Preventive Medicine (I A Odetokun PhD), University of Ilorin, Ilorin, Nigeria; Department of Community Health and Primary Care (Prof O O Odukoya MSc), University of Lagos, Idi Araba, Nigeria; Department of Family and Preventive Medicine (Prof O O Odukoya MSc), Department of Biomedical Informatics (D Villarreal-Zegarra MPH), University of Utah, Salt Lake City, UT, USA; PSSM Data Sciences, Pfizer Research & Development (M Oduro PhD), Pfizer Inc., Groton, CT, USA; Technical Unit (O T Ogundeko-Olugbami MSc), Malaria Consortium, London, UK; Department of Physiology (O E Ogunmiluyi MSc), University of Medical Sciences, Ondo, Nigeria; Department of Preventive Medicine (Prof I Oh MD), University of Ulsan, Seoul, South Korea; Faculty of Medicine (O J Okesanya MPH), University of Thessaly, Volos, Greece; Department of Medical Laboratory Science (O J Okesanya MPH), Federal Neuropsychiatric Hospital, Abeokuta, Nigeria; School of Pharmacy (O C Okonji MSc), University of the Western Cape, Cape Town, South Africa; Department of Psychiatry (Prof B A Ola PhD), School of Medicine and Dentistry (M N Wanjau PhD), Griffith University, Gold Coast, QLD, Australia; Department of Nursing Science (M I Olatubi PhD), Bowen University Iwo, Iwo, Nigeria; Associação Brasileira de Cefaleia em Salvas e Enxaqueca (ABRACES), São Paulo, Brazil (A B Oliveira PhD); Cardiology Department (Prof G M M Oliveira PhD), Federal University of Rio de Janeiro, Rio de Janeiro, Brazil; School of Health and Life Sciences (O O Oludoye PhD), Teesside University, Middlesbrough, UK;

School of Public Health (R Olum MD), Makerere University, Kampala, Uganda; Research Policy & Administration (J O Olusanya MBA), Centre for Healthy Start Initiative, Lagos, Nigeria (B O Olusanya PhD); Department of Pharmacology and Therapeutics (O G Oluwole PhD), Olabisi Onabanjo University, Sagamu, Nigeria; Institute of Chemistry (F Omage PhD), Universidade Estadual de Campinas (State University of Campinas), Campinas, Brazil; Department of Computational Biology (F Omage PhD), Brazilian Agricultural Research Institute (EMBRAPA), Campinas, Brazil; Surgery Department (G L Omer MD), Sulaimani University, Sulaimani, Iraq; ENT Department (G L Omer MD), Tor Vergata University of Rome, Rome, Italy; Department of Anatomic Pathology (A E Omonisi FWACP), Ekiti State University, Ado- Ekiti, Nigeria; Department of Anatomic Pathology (A E Omonisi FWACP), Ekiti State University Teaching Hospital, Ado Ekiti, Nigeria; Wellspring Research (S Onie PhD), Wellspring Center Indonesia, Jakarta, Indonesia; Department of Health (J Opio MPH), Lira District Local Government, Lira, SA, Australia; Department of Pharmacotherapy and Pharmaceutical Care (M Ordak PhD), Department of Biochemistry and Pharmacogenomics (M Zielińska MPharm), Medical University of Warsaw, Warsaw, Poland; Sickle Cell Unit (Prof V N Orish PhD), Ho Teaching Hospital, Ho, Ghana; Department of Neurology (R Ornello PhD), ASL Avezzano-Sulmona-L'Aquila, L'Aquila, Italy; Department of Nephrology and Hypertension (Prof A Ortiz MD), IIS-Fundacion Jimenez Diaz, Madrid, Spain; Department of Medicine (Prof A Ortiz MD), Hospital Universitario de La Princesa (Prof J B Soriano MD), Universidad Autónoma de Madrid (Autonomous University of Madrid), Madrid, Spain; One Health Global Research Group (Prof E Ortiz-Prado PhD), Universidad de las Americas (University of the Americas), Quito, Ecuador; Department of Biological Sciences (A Osborne MSc), Njala University, Freetown, Sierra Leone; School of Medicine (U L Osuagwu PhD), Western Sydney University, Bathurst, NSW, Australia; Department of Optometry and Vision Science (U L Osuagwu PhD), University of KwaZulu-Natal, KwaZulu-Natal, South Africa; Department of Biological Sciences (O Osuolale PhD), Elizade University, Ilara-Mokin, Nigeria; Faculty of Medicine (Prof A Ouyahia PhD), University Ferhat Abbas of Setif, Setif, Algeria; Division of Infectious Diseases (Prof A Ouyahia PhD), University Hospital of Setif, Setif, Algeria; Department of Biological Sciences (K A Oyeniran PhD), Bamidele Olumilua University of Education Science & Technology, Ikere-Ekiti, Nigeria; Plant Systems Biology (K A Oyeniran PhD), International Center for Genetic Engineering & Biotechnology (ICGEB), Cape Town, South Africa; Operational Research Center in Healthcare (I Ozsahin PhD, Prof U Saeed PhD), Near East University, Nicosia, Türkiye; Department of Mathematical Sciences (I Ozsahin PhD), Saveetha School of Engineering (SIMATS), Chennai, India; Escuela de Posgrado (C Reategui-Rivera MD), Universidad San Ignacio de Loyola, Lima, Peru (K Pacheco-Barrios MD); Ashok & Rita Patel Institute of Physiotherapy (D M Paija MPT, S Sunny PhD), Charotar University of Science and Technology, Anand, India; Department of Neurology (Prof P K Pal DM), National Institute of Mental Health and Neurosciences, Bangalore, India; Primary Health Center (T Paluvai MBBS), Directorate of Public Health and Family Welfare, Eluru District, India; Amity Institute of Biotechnology (Prof D Pande Katare PhD), Centre for Medical Biotechnology, Amity University Uttar Pradesh, Noida, India; Centre for Research and Development (Prof S R Pandi-Perumal MSc), Department of University Institute of Biotechnology (R Sharma PhD), Chandigarh University, Punjab, India; Division of Research and Development (Prof S R Pandi-Perumal MSc), Lovely Professional University, Phagwara, India; National Institute of Health Research and Development (H U Pangaribuan MSc), Ministry of Health Indonesia, Jakarta, Indonesia; Department of Neurology (L D Panos MD), University of Bern, Biel/Bienne, Switzerland; Department of Neurology (L D Panos MD), University of Cyprus, Nicosia, Cyprus; Department of Emergency Medicine (Prof I Pantazopoulos PhD), Division of Neurological Science, VETSUISSE (F Shams DVM), University of Bern, Bern, Switzerland; Unit of Dermatology (G Paolino PhD),

IRCCS Ospedale San Raffaele, Milano, Italy; University of Padua, Padua, Italy (M Papa MD); Medical University of Vienna, Vienna, Austria (I Papadimopoulos MD); Department of Science and Mathematics (Prof P Papadopoulou PhD), Deree-The American College of Greece, Athens, Greece; Division of Health Policy and Management (R R Parikh MD), Department of Surgery (J Rickard MD), University of Minnesota, Minneapolis, MN, USA; Department of Sociology, Anthropology, and Public Health (C Park DrPH), University of Maryland, Baltimore County, Baltimore, MD, USA; Department of Medical Sciences (R Passera PhD), University of Torino, Torino, Italy; Department of Imaging (R Passera PhD), AOU Città della Salute e della Scienza di Torino (AOU City of Health and Science of Turin), Torino, Italy; Faculty of Medicine and Health (J Patel MChD), University of Leeds, Leeds, UK; Marwadi University Research and Development Cell (M Patel PhD), Marwadi University, Rajkot, India; Department of Cardiovascular Medicine (N N Patel MD), University of Tennessee, Nashville, TN, USA; Department of Research and Training (S K Patel PhD), Population Council Institute, New Delhi, India; Mahatma Gandhi Institute of Medical Sciences, Sevagram (B S U Patil MD), Maharashtra University of Health Sciences, Wardha, India; College of Dental Medicine (Prof S Patil PhD), Roseman University of Health Sciences, South Jordan, UT, USA; Department of Internal Medicine (V Patthipati MD), Advent Health, Palm Coast, FL, USA; Department of Hospital Medicine (V Patthipati MD), Sound Physicians, Palm Coast, FL, USA; Department of Interventional Cardiology (Shu Pawar MD), Cedars Sinai Medical Center, Los Angeles, CA, USA; IRCCS Fondazione Don Carlo Gnocchi, Milan, Italy (P Pedersini MSc); Department of Clinical and Experimental Sciences (P Pedersini MSc), University of Brescia, Brescia, Italy; Center for Research and Innovation (V F Pepito MSc), Ateneo De Manila University, Pasig City, Philippines; Australian Institute of Health Innovation (P Peprah MSc), Macquarie University, Sydney, NSW, Australia; School of Population Health (Prof G Pereira PhD), Curtin University, Bentley, WA, Australia; Centre for Fertility and Health (Prof G Pereira PhD), Norwegian Institute of Public Health, Oslo, Norway; Mario Negri Institute for Pharmacological Research, Bergamo, Italy (N Perico MD, Prof G Remuzzi MD); Department of Food, Environmental and Nutritional Sciences (Prof S Perna PhD), Department of Biomedical Sciences for Health (S Villa MD), Università degli Studi di Milano (University of Milan), Milano, Italy; Department of Biochemistry and Pharmacology (P Petakh PhD), Uzhhorod National University, Uzhhorod, Ukraine; Facultad de Medicina (Faculty of Medicine) (F E Petermann-Rocha PhD), Universidad Diego Portales (Diego Portales University), Santiago, Chile; School of Medicine (W A Petri MD), University of Virginia, Charlottesville, VA, USA; Department of Internal Medicine (H Pham MD), Weiss Memorial Hospital, Chicago, IL, USA; College of Health Sciences (T T Pham PhD), College of Health Sciences (CHS) (Prof D Poddighe PhD), College of Health Science (Q X N Truong PhD), VinUniversity, Hanoi, Viet Nam; Research Advancement Consortium in Health, Hanoi, Viet Nam (T T Pham PhD); Departments of Psychiatry and Epidemiology (Prof M R Phillips MD), Columbia University, New York, NY, USA (D Shan MD); International Center of Medical Sciences Research, Islamabad, Pakistan (Z Z Piracha PhD, Prof U Saeed PhD); Department of Promoting Health, Maternal-Infant, Excellence and Internal and Specialized Medicine (PROMISE) G. D'Alessandro (E Pirera MD), University of Palermo, Palermo, Italy; Air and Climate Unit (E Pisoni PhD), European Commission, Ispra, Italy; Mental Health Research Institute (E Plotnikov PhD), Tomsk National Research Medical Center, Tomsk, Russia; Siberian State Medical University, Tomsk, Russia (E Plotnikov PhD); Department of Dermatology (I Podder MD), College of Medicine and Sagore Dutta Hospital, Kolkata, India; Department of Data Management and Analysis (R Poluru PhD), The INCLEN Trust International, New Delhi, India; Department of Ortopedics and Traumatology (V T Ponkilainen PhD), University of Tampere, Tampere, Finland; Academy of Romanian Scientists, Bucharest, Romania (Prof I Popa PhD); Department of Internal Medicine (D S Popovic PhD),

University of Novi Sad, Novi Sad, Serbia; Clinic for Endocrinology, Diabetes and Metabolic Disorders (D S Popovic PhD), Clinical Center of Vojvodina, Novi Sad, Serbia; Non-communicable Diseases Research Center (N Pourtaheri PhD), Bam University of Medical Sciences, Bam, Iran; Centro de Investigaciones Clinicas (Clinical Research Center) (S I Prada PhD), Fundación Valle del Lili (Valle del Lili Foundation), Cali, Colombia; Centro PROESA (S I Prada PhD), Departamento de Ciencias Básicas Médicas (E Rubagotti PhD), Universidad ICESI, Cali, Colombia; Department of Humanities and Social Sciences (Prof J Pradhan PhD), National Institute of Technology Rourkela, Rourkela, India; Research Center in Advancing Community Healthcare, Surabaya, Indonesia (R Pradipta MS); Department of Biochemistry (Prof A Prashant PhD), JSS Academy of Higher Education and Research, Mysuru, India; Department of Medical Instrumentation Techniques Engineering (N H Qasim DSc), Al-Rafidain University College, Baghdad, Iraq; Department of Cybersecurity (N H Qasim DSc), Kyiv National University of Construction and Architecture, Kyiv, Ukraine; Department of Neonatology (I Qattea MD), Case Western Reserve University, Akron, OH, USA; Department of Epidemiology (Y Qiao MD), School of Nursing and Rehabilitation (N Xiao BS), Shandong University, Jinan, China; Oman Dental College, Oman (Prof R A Radhakrishnan PhD); Department of Medical Oncology (Prof V Radhakrishnan MD), Cancer Institute (W.I.A), Chennai, India; Deputy of Health (L Rafati PhD), Hamadan University of Medical Sciences, Hamadan, Iran; Health Research Institute (HRI) (I Rafique PhD), National Institutes of Health, Islamabad, Pakistan; Department of Epidemiology (P Raghuveer MD), National Institute of Mental Health and Neurosciences, Bengaluru, India; Osh State University, Osh, Kyrgyzstan (Prof F Rahim PhD); Department of Environmental Health Engineering (S Rahimi PhD), Health Science Research Centre (S Rahimi PhD, M Sarmadi MSc), Torbat Heydariyeh University of Medical Sciences, Torbat Heydariyeh, Iran; Faculty of Health Sciences (F M Rahman PhD), Qaiwan International University, Sulaymaniyah, Iraq; Department of Epidemiology (Ma Rahman PhD), Institute of Epidemiology, Disease Control and Research (IEDCR), Dhaka, Bangladesh; Department of Pathobiology and Population Sciences (PPS) (Ma Rahman PhD), Royal Veterinary College (RVC), London, UK; College of Medicine and Health Sciences (M H U Rahman PhD), National University of Science and Technology, Sohar, Oman; Future Technology Research Center (A Rahmani PhD), National Yunlin University of Science and Technology, Yunlin, Taiwan; Health Service Research and Quality of Life Center (CEReSS) (Prof M Rahmati PhD), Aix-Marseille University, Marseille, France; Faculty of Medicine (H Rahmoune PhD), LIRSSEI Research Lab (H Rahmoune PhD), University of Setif Algeria, Setif, Algeria; Division of Gynecology and Human Reproduction Physiopathology (D Raimondo PhD), IRCCS Azienda Ospedaliero-Universitaria di Bologna, Bologna, Italy; Department of Medical, Surgical and Experimental Sciences (I Raimondo MD), University of Sassari, Sassari, Italy; Gynecology and Breast Care Center (I Raimondo MD), Mater Olbia Hospital, Olbia, Italy; Dr. Rajendra Prasad Government Medical College, Tanda, Kangra, India (Prof S K Raina MD); Department of Cardiology (A Raja MD), Dow University of Health Sciences, Karachi, Pakistan; Emergency Medicine Department (G Rajendran MD), Sri Manakula Vinayagar Medical College and Hospital, Puducherry, India; Centre for Chronic Disease Control, New Delhi, India (P Rajput PhD); Department of Population Health (M Ramadan DrPH), King Saud bin Abdulaziz University for Health Sciences, Jeddah, Saudi Arabia; Department of Midwifery (K Ramadhan MPH), Ministry of Health of the Republic of Indonesia, Palu, Indonesia; Department of Anatomy (C Ramasamy MD), Govt. Siddhartha Medical College, Vijayawada, India; Department of Biological Science and Bioengineering (M Ramezani Farani PhD), Inha University, Incheon, South Korea; The Navarra Medical Research Institute (IdiSNA) (R Ramírez-Vélez PhD), Universidad Pública de Navarra (Public University of Navarra), Pamplona, Spain; South Asian Institute for Social Transformation (SAIST), Dhaka, Bangladesh (J Rana MPH); Department of Epidemiology, Biostatistics and Occupational Health (J Rana

MPH), McGill University, Montreal, QC, Canada; Department of Community Medicine (K Rana MD), NKP Salve Institute of Medical Sciences and Research Centre, Nagpur, India; Department of Research (C L Ranabhat PhD), Eastern Scientific LLC, Richmond, KY, USA; Centre for Clinical Pharmacology (N Rancic PhD), University of Defence in Belgrade, Belgrade, Serbia; Centre for Clinical Pharmacology (N Rancic PhD), Medical College of Georgia at Augusta University, Belgrade, Serbia; Department of Oral Medicine and Radiology (K Rao PhD), NITTE (Deemed to be University), Mangalore, India; Barcelona Institute for Global Health, Barcelona, Spain (Prof D Rasella PhD); Brigham and Women's Hospital (S Rashedi MD), Harvard Medical School, Boston, MA, USA; Unit for Public Health Science (M Rashid PhD), University of Gävle, Sweden, Stockholm, Sweden; Department of Geography (A Rasul PhD), Soran University, Soran, Iraq; University of Swabi, Swabi, Pakistan (A Rauf PhD); Inovus Medical, St Helens, UK (D L Rawaf MD); School of Health, Medical and Applied Sciences (L Rawal PhD), CQ University, Sydney, NSW, Australia; Department of Hematology (B Razi PhD), North Khorasan University of Medical Sciences, Bojnurd, Iran; Department of Biological Sciences (Prof E Redwan PhD), King Abdulaziz University, Jeddah, Egypt; Department of Protein Research (Prof E Redwan PhD), Research and Academic Institution, Alexandria, Egypt; Department of Internal Medicine (A Rehman MD), King Edward Medical University, Lahore, Pakistan; Human Capability Building (F U Rehman PhD), Saudi Authority for Data and Artificial Intelligence, Riyadh, Saudi Arabia; The School of Pharmaceutical Sciences (W Rehman MS), University of Science Malaysia, Penang, Malaysia; Department for Epidemiology and Biostatistics (R Reile PhD), National Institute for Health Development, Tallinn, Estonia; Department of Obstetrics and Gynecology (S Restaino MD), Azienda Sanitaria Universitaria Friuli Centrale, Udine, Italy; Unisabana Center for Translational Science (L Reyes PhD), Universidad de La Sabana (Savannah University), Chia, Colombia; Critical Care Department (L Reyes PhD), Clinica Universidad De La Sabana (Savannah University Clinic), Chia, Colombia; School of Environment (M Rezaei PhD), Tehran University, Tehran, Iran; Network of Immunity in Infection, Malignancy and Autoimmunity (NIIMA) (Prof Ni Rezaei PhD), Universal Scientific Education and Research Network (USERN), Tehran, Iran; Rasoul Akram Hospital (D Rezazadeh Eidgahi MD), Islamic Azad University, Iran, Iran; Department of Public Health Sciences (T Rhee PhD), University of Connecticut, Farmington, CT, USA; College of Nursing (Y A Rias MNS), Institut Ilmu Kesehatan Bhakti Wiyata Kediri (Bhakti Wiyata Kediri Institute of Health Sciences), Kediri, Indonesia; Department of Surgery (J Rickard MD), University Teaching Hospital of Kigali, Kigali, Rwanda; Department of Physiology and Physiotherapy (Prof M R Rizvi PhD), DIT University, Delhi, India; Community Health Department (Prof H A L Rocha PhD), Federal University of Ceará, Fortaleza, Brazil; Department of Geography and Demography (M Rodrigues PhD), University of Coimbra, Coimbra, Portugal; Department of Nursing in Women's Health (T Rodrigues da Silva PhD), Federal University of São Paulo, São Paulo, Brazil; Department of Pharmacology and Toxicology (Prof J A B Rodriguez PhD), University of Antioquia, Medellin, Colombia; Warwick Medical School (Prof J A B Rodriguez PhD), University of Warwick, Coventry, UK; Department of Clinical Research (Prof L Roever PhD), Universidade de São Paulo (University of São Paulo), Ribeirão Preto, Brazil; Center for Indigenous Health Research (P Rohloff MD), Wuqu' Kawoq Maya Health Alliance, Tecpan, Guatemala; Department of Environmental and Radiological Health Sciences (Prof D Rojas-Rueda PhD), Colorado State University, Fort Collins, CO, USA; Department of Anesthesiology (M L Rolfzen MD), Department of Environmental, Agricultural and Occupational Health (J Taiba PhD), University of Nebraska Medical Center, Omaha, NE, USA; Department of Neurosciences (M Romoli MD), Maurizio Bufalini Hospital, Cesena, Italy; Department of Pharmacy Services (K Rotimi MSc), Alberta Health Services, Edmonton, AB, Canada; West African Postgraduate College of Pharmacists, Lagos, Nigeria (K Rotimi MSc); Department of Analytical and Applied Economics

(Prof H Rout PhD, C Swain MPhil), RUSA Centre of Excellence in Public Policy and Governance (Prof H Rout PhD), UGC Centre of Advanced Study in Psychology (Prof M Satpathy PhD), Utkal University, Bhubaneswar, India; Isfahan University of Medical Sciences (H Rouzbahani MD), Islamic Azad University, Isfahan, Iran; Department of Community Medicine (A Roy MD), RVM Medical College and Research Centre, Hyderabad, Hyderabad, India; Achutha Menon Centre for Health Science Studies (A Roy MD), Sree Chitra Tirunal Institute for Medical Sciences and Technology, Thiruvananthapuram, India; Faculty of Medicine (B Roy PhD), Quest International University Perak, Ipoh, Malaysia; Department of Labour (P Roy PhD), Government of West Bengal, Kolkata, India; Department of Public Health (Sha Roy MD), New Mexico State University, Las Cruces, NM, USA; Research Department (Shu Roy MSc), Indian Institute of Public Health, Delhi, India; Department of Health Statistics (S F Rumisha PhD), National Institute for Medical Research, Dar es Salaam, Tanzania; Department of Cardiology (M Russo PhD), SS. Annunziata Hospital - ASL2 Abruzzo, Chieti, Italy; Department of Internal Medicine (G M Rwegerera MD), Muhimbili University of Health and Allied Sciences, Dar es Salaam, Tanzania; Department of Internal Medicine (G M Rwegerera MD), University of Botswana, Gaborone, Botswana; Department Infectious Diseases (M Sabbatucci PhD), National Institute of Health, Rome, Italy; Department for Health Prevention (M Sabbatucci PhD), Ministry of Health, Rome, Italy; Department of Medical Pharmacology (Prof M M Saber-Ayad PhD), Public Health and Community Medicine Department (M R Salem MD), Cairo University, Giza, Egypt; Neuropsychiatric Institute (Prof P S Sachdev MD), Prince of Wales Hospital, Randwick, NSW, Australia; Department of Computer (T Sadegh MSc), University of Science and Culture, Tehran, Iran; Department of Nursing and Midwifery (M Saeedi PhD), Saveh University of Medical Sciences, Saveh, Iran; Department of Neurology (M Safdarian MD), Christian-Doppler University Hospital, Salzburg, Austria; Spinal Cord Injury and Tissue Regeneration Center Salzburg (SCI-TReCS) (M Safdarian MD), Paracelsus Medical University, Salzburg, Austria; Faculty of Medicine, Bioscience and Nursing (S Safi PhD), MAHSA University, Selangor, Malaysia; Interdisciplinary Research Centre in Biomedical Materials (IRCBM) (S Safi PhD), COMSATS Institute of Information Technology, Lahore, Pakistan; ICMR - National Institute for Research in Bacterial Infections (Prof I Saha PhD), Indian Council of Medical Research, Kolkata, India; Canadian Red Cross (K Sahu PhD), Red Cross, Ottawa, ON, Canada; Department of Psychiatry (Z Saif MBA), Ministry of Health, Manama, Bahrain; College of Pharmacy (Prof S Sajadi PhD), Al-Hadba University, Mosul, Iraq; Department of Health and Kinesiology (M Sajib BDS), University of Illinois, Urbana-Champaign, IL, USA; Department of Statistics (M R Sajid PhD), University of Gujrat, Gujrat, Pakistan; Department of Integrated Health Education (Prof L B Salaroli PhD), Federal University of Espirito Santo, Vitória, Brazil; Department of Health Education & Promotion (Prof L Salehi PhD), A.C.S. Medical College and Hospital, Karaj, Iran; Student Research Committee (M Salehi MD), Kashan University of Medical Sciences, Kashan, Iran; Technology Management Department (Prof M Z Y Salem PhD), University College of Applied Sciences, Gaza, Palestine; School of Economics and Management (Prof M Z Y Salem PhD), Department of Theory and Empiricism of Healthcare (D T Worede MSc), Universität Kassel (University of Kassel), Kassel, Germany; Department of Biochemistry (A J Salemcity PhD), University of Medical Sciences, Ondo, Ondo city, Nigeria; College of Nursing (D Salihu PhD), Jouf University, Jouf, Saudi Arabia; Surgical Department (J Samaranayake MBBS), North Colombo Teaching Hospital, Ragama, Sri Lanka; College of Nursing (W Sami PhD), Qatar University, Lusail, Qatar; Institute of Epidemiology and Preventive Medicine (Y L Samodra PhD), National Taiwan University, Taipei, Taiwan; Benang Merah Research Center (BMRC), Minahasa Utara, Indonesia (Y L Samodra PhD); Department of Forensic Biology (S G Sangle PhD), Government Institute of Forensic Science Chhatrapati Sambhajinagar, Chhatrapati Sambhajinagar, India; Primary Healthcare Department (F Sanmarchi MD),

Azienda USL di Bologna, Bologna, Italy; Center for Clinical and Epidemiological Research (I S Santos PhD), University of São Paulo, São Paulo, Brazil; University of São Paulo City, São Paulo, Brazil (L H C C Santos MSc); Department of Osteopathic Medicine (Prof A Sanyaolu PhD), D'Youville University, Buffalo, NY, USA; Department of Sociology and Gerontology (K P Sapkota MSc), Miami University, Oxford, OH, USA; Independent Consultant, Thiruvananthapuram, India (S Y I Saraswathy PhD); Department of Public Health (Y Sarikhani PhD), Jahrom University of Medical Sciences, Jahrom, Iran; Botany Department (H Sarma PhD), Bodoland University, Kokrajhar, India; Department of Oral Pathology and Microbiology (Prof G S Sarode PhD, Prof S C Sarode PhD), Dr. D. Y. Patil Vidyapeeth, Pune (Deemed to be University), Pune, India; Department of Epidemiology (M Sathya Narayanan MBBS), National Institute for Research in Tuberculosis, Chennai, India; Udyam-Global Association for Sustainable Development, Bhubaneswar, India (Prof M Satpathy PhD); IRCCS Istituti Clinici Scientifici Maugeri (IRCCS Maugeri Scientific Clinical Institute), Milan, Italy (D Sattin PsyD); Precision Medicine Department (M Savabi Far MD, S Tajabadi MSc), Università degli studi della Campania Luigi Vanvitelli (University of Campania Luigi Vanvitelli), Naples, Italy; Department of Public Health Sciences (M Sawhney PhD), University of North Carolina at Charlotte, Charlotte, NC, USA; Department of Public Health Sciences (S G Saxena DrPH), Coastal Carolina University, Conway, SC, USA; Department of Preventive and Social Medicine (G Saya MD), Jawaharlal Institute of Postgraduate Medical Education and Research, Puducherry, India; Department of Post-Harvest Technology and Marketing (A Sayeed MSc), Patuakhali Science and Technology University, Patuakhali, Bangladesh; Faculty of Business and Computing (Prof C Schinckus PhD), University of the Fraser Valley, Abbotsford, BC, Canada; Graduate School of Business (Prof C Schinckus PhD), ESAN University, Lima, Peru; Chief Data Officer Directorate (J C Schmidt MSc), UK Department of Health and Social Care, London, UK; Cardiovascular Program (X Xu PhD), The George Institute for Global Health, Sydney, NSW, Australia (Prof A E Schutte PhD, Prof J Sundström PhD); Clinic for Conservative Dentistry and Periodontology (Prof F Schwendicke PhD), University Hospital of the Ludwig-Maximilians-University Munich, Munich, Germany; Augusta Health, Fishersville, VA, USA (S Sebastian MD); Department of Medical Statistics (M Šekerija PhD), University of Zagreb, Zagreb, Croatia; Department of Epidemiology and Prevention of Chronic Noncommunicable Diseases (M Šekerija PhD), Croatian Institute of Public Health, Zagreb, Croatia; Department of Applied Mechanics and Biomedical Engineering (V Selvaraj PhD), Indian Institute of Technology Delhi, Chennai, India; Emergency Department (S Senthilkumaran PhD), Manian Medical Centre, Erode, India; Department of Medicine (Y Sethi MD), Swami Vivekanand Subharti University, Meerut, India; National Heart, Lung, and Blood Institute (A Seylani MD), National Institutes of Health, Rockville, MD, USA; School of Health Sciences (S Shaharudin PhD), Universiti Sains Malaysia, Kota Bharu, Malaysia; Department of Biotechnology (S Shahid MPhil), Quaid-i-Azam University Islamabad, Islamabad, Pakistan; Gastroenterology Unit (E Shahini MD), IRCCS, Castellana Grotte (Bari), Italy; Department of Chemistry (H R Shahsavari PhD), Institute for Advanced Studies in Basic Sciences (IASBS), Zanjan, Iran; Independent Consultant, Karachi, Pakistan (M A Shaikh MD); Noncommunicable Diseases Research Center (A Shakerimoghaddam PhD), Neyshabur University of Medical Sciences, Neyshabur, Iran; Department of Statistics (S Shan PhD), Harbin Institute of Technology, Harbin, China; Department for Evidence-based Medicine and Evaluation (A Sharifan PharmD), University for Continuing Education Krems, Krems, Austria; Amity Institute of Biotechnology (A Sharma PhD), Amity University Rajasthan, Rajasthan, India; Department of Forensic Science (Prof B K Sharma PhD, M Walia MPhil), Department of Chemistry (K Sharma PhD), Faculty of Medicine and Health Sciences (Prof N P Singh MD), Shree Guru Gobind Singh Tricentenary University, Gurugram, India; Department of Biotechnology (G Sharma PhD), Indian Institute of Technology Hyderabad, Kandi, India; UN Mehta Institute of Cardiology and Research

Center (Prof K Sharma MD), B.J. Medical College, Ahmedabad, India; Department of Cardiology (Prof K Sharma MD), Government Medical College, Ahmedabad, India; Physiotherapy Department (B Shehu Bappah MSc), Federal University of Health Sciences, Azare, Nigeria; Department of Biology (S P Sherchan PhD), Morgan State University, Baltimore, MD, USA; Department of Epidemiology and Health Statistics (Fang Shi PhD), The Second Affiliated Hospital (Prof A Wu MD), Wenzhou Medical University, Wenzhou, China; Department of HIV/AIDS Prevention and Control (B F Shibesh MPH), Amahara Regional Sate Health Bureau, Bahir Dar, Ethiopia; Tokyo Foundation for Policy Research, Tokyo, Japan (Prof K Shibuya MD); Department of Public Health (D Shiferaw MPH), Dambi Dollo University, Dembi Dollo, Ethiopia; Department of Pharmacology (T Shimels MSc), Saint Paul's Hospital Millennium Medical College, Addis Ababa, Ethiopia; Finnish Institute of Occupational Health, Helsinki, Finland (R Shiri PhD); Department of Experimental Research (V Shivarov PhD), Medical University Pleven, Pleven, Bulgaria; Department of Genetics (V Shivarov PhD), Sofia University "St. Kliment Ohridiski", Sofia, Bulgaria; Department of Neurosurgery (N A Shlobin MD), Columbia University Medical Center, New York, NY, USA; Department of Research and Academics (S Shrestha PhD), Kathmandu Cancer Center, Bhaktapur, Nepal; Person-Centered Research (S Shrestha PhD), Monash University, Box Hill, VIC, Australia; Kenneth H. Cooper Institute (Prof K Shuval PhD), Texas Tech University Health Sciences Center, Dallas, TX, USA; Advanced Materials Division (N R S Sibuyi PhD), Mintek, Randburg, South Africa; Department of Biotechnology (N R S Sibuyi PhD), University of the Western Cape, Bellville, South Africa; Department of Medical Microbiology and Infectious Diseases (E E Siddig MD), Erasmus University, Rotterdam, Netherlands; RISE Health (Prof L M L R Silva PhD), University of Beira Interior, Covilhã, Portugal; School of Human and Health Sciences (Prof P P Simkhada PhD), University of Huddersfield, Huddersfield, UK; Department of Law, Economics, Management and Quantitative Methods (Prof B Simonetti PhD), University of Sannio, Benevento, Italy; WSB University in Gdańsk, Gdańsk, Poland (Prof B Simonetti PhD); School of Public Health & Zoonoses (B B Singh PhD), Guru Angad Dev Veterinary & Animal Sciences University, Ludhiana, India; Department of Agriculture and Environmental Sceinces (Prof B P Singh PhD), National Institute of Food Technology Entrepreneurship and Management-Kundli (NIFTEM-K), Sonipat, India; Department of Pharmacology (Harm Singh DM), Government Medical College and Hospital, Chandigarh, India; School of Pharmaceutical Sciences (Harp Singh PhD), IFTM University, Moradabad, India; Department of Medicine Service (Prof J A Singh MD), US Department of Veterans Affairs (VA), Houston, TX, USA; Department of Psychiatry (J Singh MD), All India Institute of Medical Sciences, Punjab, India; Department of Biochemistry (Prof R K Singh PhD), Institute of Medical Sciences (Sam Singh PhD), Banaras Hindu University, Varanasi, India; Department of Community Medicine (Sure Singh MD), Veer Chandra Singh Garhwali Government Institute of Medical Science and Research, Srinagar Garhwal, India; Department of Internal Medicine (R Sinto MD), University of Indonesia, Jakarta, Indonesia; Department of Internal Medicine (R Sinto MD), Dr. Cipto Mangunkusumo National Hospital, Jakarta Pusat, Indonesia; Department of Anesthesiology (D Siyoum MD), New York Medical College, Passaic, NJ, USA; Global and European Health Education and Study Institute (Prof N Skhvitaridze PhD), University of Georgia, Tbilisi, Georgia; National Center for Disease Control and Public Health, Tbilisi, Georgia (Prof N Skhvitaridze PhD); Department of Infectious Diseases and Epidemiology (A A Skryabina MD), Pirogov Russian National Research Medical University, Moscow, Russia; Division of Injury Prevention (Prof D A Sleet PhD), The Bizzell Group, Atlanta, GA, USA; Department of Pharmacy and Pharmaceutical Sciences (M Soheili MD), Western New England University, Springfield, MA, USA; Department of Biochemistry (S Solanki MD), American University of Integrative Sciences, Bridgetown, Barbados; Centro de Investigación Biomédica en Red Enfermedades Respiratorias (CIBERES) (Center for Biomedical Research in Respiratory Diseases

Network), Madrid, Spain (Prof J B Soriano MD); Hull York Medical School (I N Soyiri PhD), University of Hull, Hull City, UK; Doheny Eye Institute (C Soylu MD), University of California Los Angeles, Pasadena, CA, USA; College of Health and Public Service (S Sriram PhD), University of North Texas, Denton, TX, USA; Manipal College of Health Professions (P Srivastav PhD), Manipal Academy of Higher Education, manipal, India; Department of Public Health (M Stanikzai MPH), Kandahar University, Kandahar, Afghanistan; Department of Health Professions (Prof N Steckling-Muschack DrPH), DHGS German University for Health and Sports, Berlin, Germany; Department of Medicine (P Steiropoulos MD), Democritus University of Thrace, Alexandroupolis, Greece; Occupational and Environmental Medicine Department (L Stockfelt PhD), Institute of Health and Care Sciences (Prof A W Wolf PhD), University of Gothenburg, Gothenburg, Sweden; Research Department (N Subedi PhD), Nepal Development Society, Kathmandu, Nepal; Clinical Research Unit (H Sujon MSc), Projahnmo Research Foundation, Dhaka, Bangladesh; Praboromarajchanok Institute (T Sukaew PhD), Ministry of Public Health, Nonthaburi, Thailand; Department of Physiotherapy (S K Sulaiman PhD), Tishk International University, Erbil, Iraq; Department of Community Medicine (A G Suleiman MPH), Ahmadu Bello University, Kaduna State, Nigeria; Department of Human Anatomy (M Suleiman Odidi PhD), Federal University, Dutse, Dutse, Nigeria; School of Life Sciences (M Suleman PhD), Xiamen University, Xiamen, China; School of Medicine, Medical Sciences and Nutrition (A Sultan Meo MPH), University of Aberdeen, Aberdeen, UK; Yusuf Hamied Department of Chemistry (Prof H Z Sun PhD), University of Cambridge, Cambridgeshire, UK; Institute of Integrated Intelligence and Systems (Prof J Sun PhD), Griffith University, Brisbane, QLD, Australia; The First Hospital of China Medical University (M Sun MM), China Medical University, Shenyang, China; Department of Endocrinology and Metabolism (Xiaod Sun PhD), Affiliated Hospital of Shandong Second Medical University, Weifang, China; Department of Biomedical Sciences (Zho Sun PhD), Universiti Putra Malaysia (University of Putra Malaysia), Selangor, Malaysia; High-Quality Development Evaluation Research Institute (Zhu Sun PhD), Nanjing University of Posts and Telecommunications, Nanjing, China; Gandhi Medical College (S Sundaragiri MD), Kaloji Narayana Rao University of Health Sciences (KNRUHS), Secunderabad, India; Department of Clinical Pathology (H Susianti PhD), Brawijaya University, Malang, Indonesia; Hospital Central Laboratory (H Susianti PhD), Dr Saiful Anwar General Hospital, Malang, Indonesia; School of Population Health (T L Symons PhD), Curtin University, Perth, VIC, Australia; Department of Clinical Research and Development (Prof L Szarpak PhD), LUXMED Group, Warsaw, Poland; Collegium Medicum (Prof L Szarpak PhD), John Paul II Catholic University of Lublin, Lublin, Poland; Department of Neurology (P Tabaee Damavandi MD), Neurocenter of Southern Switzerland (NSI), Lugano, Switzerland; Department of Medicine (Prof R Tabarés-Seisdedos PhD), University of Valencia, Valencia, Spain; Department of Basic Medical Sciences (S Tabatabaeizadeh PhD), Department of Internal Medicine (S Tabatabaeizadeh PhD), Islamic Azad University, Mashhad, Iran; Division of Epidemiology (T Tabuchi MD), Tohoku University, Sendai, Japan; Department of Internal Medicine, Oncology Unit (G F Tadesse PhD), St Paul's Hospital Millenium Medical College, Addis Ababa, Ethiopia; Department of Dermato-Venereology (M Tampa PhD), Dr. Victor Babes Clinical Hospital of Infectious Diseases and Tropical Diseases, Bucharest, Romania; Department of Medicine (J L Tamuzi MSc), Northlands Medical Group, Omuthiya, Namibia; State Key Laboratory of Numerical Modeling for Atmospheric Sciences and Geophysical Fluid Dynamics (LASG) (H Tang PhD), Chinese Academy of Sciences, Beijing, China; Department of Computer and Software Engineering (M Tanveer PhD), NUST School of Health Sciences (Prof Y Waheed PhD), National University of Science and Technology (NUST), Islamabad, Pakistan; Department of Psychology (S Taridashti MA), Montclair State University, Montclair, NJ, USA; Department of Public Health (M K Tariku MPH), Debre Markos University, Debre Markos,

Ethiopia; Department of Pharmacology and Therapeutics (S Tariq PhD), The University of Faisalabad, Faisalabad, Pakistan; Department of Pharmaceutical Health Outcomes and Policy (T Temedie-Asogwa MSc), University of Houston College of Pharmacy, Houston, TX, USA; Amrita Vishwa Vidyapeetham (Prof K R Thankappan MD), Amrita Institute of Medical Sciences, Kochi, India; Department of Economics (I Tharwat PhD), The American University in Cairo, Cairo, Egypt; Department of Applied Bioscience (Prof M Thiruvengadam PhD), Konkuk University, Seoul, South Korea; School of Public Health (W Tian PhD, G Yan MD), Harbin Medical University, Harbin, China; Faculty of Public Health (J H V Ticoalu MPH), Universitas Sam Ratulangi (Sam Ratulangi University), Manado, Indonesia; Interdisciplinary Health Data Center (R Topor-Madry PhD), Jagiellonian University Medical College, Kraków, Poland; Nutritional Epidemiology Research Team (EREN) (M Touvier PhD), National Institute for Health and Medical Research (INSERM), Paris, France; High Institute of Sport and Physical Education of Sfax (K Trabelsi PhD), University of Sfax, Sfax, Tunisia; Second Department of Internal Medicine (Q T H Tran MD), Kansai Medical University, Hirakata, Japan; John T. Milliken Department of Medicine (T Q M Tran MSc), Washington University in St. Louis, Saint Louis, MO, USA; Department of Business Analytics (T H Tran MD), University of Massachusetts Dartmouth, Dartmouth, MA, USA; Molecular Neuroscience Research Center (N Tran Minh Duc MD), Shiga University of Medical Science, Shiga, Japan; ALS Vietnam Research and Advocacy Initiative (N Tran Minh Duc MD), ALS Vietnam, Quang Ngai, Viet Nam; Department of Neurology (Prof M Tripathi MD), All India Institute of Medical Sciences, Delhi, India; Department of Studies in Economics and Planning (T Tripathi PhD), Central University of Gujarat, Gandhinagar, India; Adult Learning Disability Service (S J Tromans PhD), Leicestershire Partnership National Health Service Trust, Leicester, UK; Department of Cardiology (Prof G Tse PhD), Tianjin Medical University, Tianjin, China; Kent and Medway Medical School, Canterbury, UK (Prof G Tse PhD); Department of Internal Medicine (M Tumurkhuu PhD), Wake Forest University, Winston-Salem, NC, USA; Department of Urology (Z Tuo MS), The Second Hospital of Tianjin Medical University, Tianjin, China; Hayatabad Medical Complex (H Ullah FCPS), Postgraduate Medical Institute, Peshawar, Pakistan; Department of Human Anatomy (H Umar MSc), Federal University Dutse, Dutse, Nigeria; Department of Physiotherapy (L Umar PhD), Federal Ministry of Health, Azare, Nigeria; Federal University of Health Sciences Teaching Hospital Azare, Azare, Nigeria (L Umar PhD); Department of Medicine (M Umar§ MBBS), Khairpur Medical College, Khairpur, Pakistan; Department of Oncology (S S Umar FWACS), Federal Medical Centre, Gusau, Nigeria; Section of Advanced Heart Failure and Transplant (D Uppal MD), Northwell Health / North Shore University Hospital, Manhasset, NY, USA; Center for Neurodegenerative Diseases and the Aging Brain (D Urso MD), University of Bari, Tricase, Italy; Department of Orthodontics (H Uzunçıbuk PhD), University of Trakya, Edirne, Türkiye; Johnson & Johnson (P Vadagam MS), Duquesne University, Pittsburgh, PA, USA; Sociedad Argentina de Medicina, Buenos Aires, Argentina (Prof P R Valdez PhD); Hospital Vélez Sarsfield, Buenos Aires, Argentina (Prof P R Valdez PhD); Department of Biomedical Sciences (M Valenti MD), Humanitas University, Milan, Italy; Dermatology Unit (M Valenti MD), IRCCS Humanitas Research Hospital, Milan, Italy; Department of Psychology (Z Vally PhD), Zayed University, Abu Dhabi, United Arab Emirates; Faculty of Sciences (J Varasteh MSc), University of Guilan, Rasht, Iran; UKK Institute, Tampere, Finland (Prof T J Vasankari PhD); Faculty of Medicine and Health Technology (Prof T J Vasankari PhD), Tampere University, Tampere, Finland; Department of Otolaryngology Head and Neck Surgery (S Vasudevan MS), Louisiana State University Health Sciences Center, Shreveport, LA, USA; Biomedical Engineering Department (A Vaysi MSc), University of Texas, Arlington, TX, USA; Raffles Neuroscience Centre (Prof N Venketasubramanian MSc), Raffles Hospital, Singapore, Singapore; Department of Neurology (S Vidale MD), Infermi Hospital, Rimini, Italy; Department of Neurology & Stroke Unit (S

Vidale MD), Sant'Anna Hospital, Como, Italy; Department of Community Medicine (M Vijayageetha MD), All India Institute of Medical Sciences, Nagpur, India; Department of Physiotherapy (J H Villafañe PhD), Universidad Europea de Madrid (European University of Madrid), Villaviciosa de Odón, Spain; Saint Camillus International University of Health Sciences - UniCamillus, Rome, Italy (Prof L Villani MD); Digital Health Research Center (D Villarreal-Zegarra MPH), Instituto Peruano de Orientación Psicológica, Lima, Peru; Occupational Medicine Unit (Prof F S Violante MD), Sant'Orsola Malpighi Hospital, Bologna, Italy; Department of Bioengineering, School of Chemical and Biotechnology (S Visaga Ambi PhD), SASTRA Deemed to be University, Thanjavur, India; Faculty of Medicine of Itajubá, Brazil, Itajubá, Brazil (Prof L M Vitorino PhD); Department of Health Care Administration and Economics (Prof V Vlassov MD), National Research University Higher School of Economics, Moscow, Russia; Department of Environmental Health Engineering (M Vosoughi PhD), Ardabil University of Medical Science, Ardabil, Iran; Faculty of Public Health (L Vu PhD), International Institute for Training and Research (INSTAR) (L Vu PhD), VNU University of Medicine and Pharmacy, Hanoi, Viet Nam; Széchenyi István University, Gyor, Hungary (Prof Y Waheed PhD); Research Organization for Health (M Wahidin PhD), National Research and Innovation Agency (BRIN), Bogor, Indonesia; School of Chinese Medicine (Prof J Wan PhD), School of Traditional Chinese Medicine (Prof H Yao PhD), Beijing University of Chinese Medicine, Beijing, China; West China Hospital, Chengdu, China (Fu Wang DrPH); Department of Laboratory Medicine (Prof L Wang PhD), Guangdong Provincial People's Hospital, Guangzhou, China; Department of Neurosurgery (S Wang MD), Beijing Tiantan Hospital, Beijing, China; College of Agriculture (Xi Wang PhD), Northwest A&F University, Xianyang City, China; Enze Medical Health Academy (Xu Wang PhD), Taizhou Hospital of Zhejiang Province, Taizhou, China; School of Public Health (Ya Wang MPH), Zhengzhou University, Zhengzhou, China; Division of Life Sciences and Medicine (Prof Z Wang PhD), University of Science and Technology of China, Heifei, China; School of Nursing Sciences (M N Wanjau PhD), University of Nairobi, Nairobi, Kenya; Coalition for Global Hepatitis Elimination (J W Ward MD), Task Force for Global Health, Decatur, GA, USA; Institute of Health and Wellbeing (I Weerasekara PhD), Federation University, Melbourne, VIC, Australia; University of Adelaide, North Terrace, NSW, Australia (I Weerasekara PhD); Fourth Military Medical University, Xi'an, China (F Wei PhD); Department of Geriatrics (X Wei MS), The Eighth Affiliated Hospital of Sun Yat-sen University, Shenzhen, China; Cardiology Department (Prof R G Weintraub MB), Royal Children's Hospital, Melbourne, VIC, Australia; Demographic Change and Aging Research Area (A Werdecker PhD), Competence Center of Mortality-Follow-Up of the German National Cohort (R Westerman DSc), Federal Institute for Population Research, Wiesbaden, Germany; Department of Physical Therapy (T Wiangkham PhD), Naresuan University, Phitsanulok, Thailand; Department of Nursing (A Wilandika PhD), Universitas Aisyiyah Bandung, Bandung, Indonesia; Institute of Clinical Epidemiology (Prof P Willeit PhD), Medical University Innsbruck, Innsbruck, Austria; Research Organisation (A Wireko MD), Inter-Continental Omni-Research in Medicine Collaborative, Berlin, Germany; Department of Public Health (A T Woday MPH), Samara University, Samara, Ethiopia; Department of Population Health Monitoring and Analysis (B Wojtyniak DSc), National Institute of Public Health, Warsaw, Poland; Department of Surgery (N Woldehana MD), MyungSung Medical College, Addis Ababa, Ethiopia; Faculty of Health (T E Wonde MPH), University of Technology Sydney, Australia, NSW, Australia; Global Health Research Center (C Wu PhD), Duke Kunshan University, Kunshan, China; Duke Global Health Institute (C Wu PhD), Duke University, Durham, NC, USA; Department of Food Science and Human Nutrition (Prof F Wu PhD), Michigan State University, East Lansing, MI, USA; Department of Public Health (Prof J Wu MPH), Wuhan Fourth Hospital, Wuhan, China; Shenzhen Institute of Advanced Technology (P Wu PhD), Chinese Academy of Sciences, Shenzhen, China; Division of Gastroenterology

(Prof Z Wu PhD), Tongji Medical College (G Xiao MD), Huazhong University of Science and Technology, Wuhan, China; Western Institute of Digital-Intelligent Medicine (Z Xia MD), Chongqing Medical University, Chongqing, China; Department of Intelligent Medical Engineering (Prof W Xie DrPH), Anhui Medical University, Anhui, China; Department of Surgery (Prof W Xie DrPH), The First Affiliated Hospital of Anhui Medical University, Hefei, Anhui, China; Department of Endocrinology (Prof S Xu PhD), University of Science and Technology of China, Hefei, China; Department of Environmental Health and Epidemiology (V Yadav MD), National Institute for Research in Environmental Health, Bhopal, India; Department of Cells and Tissues (G Yahya PhD), Molecular Biology Institute of Barcelona, Barcelona, Spain; Department of Public Health (Prof K Yamagishi MD, Prof N Yonemoto PhD), Faculty of Medicine (Y Yano MD), Juntendo University, Tokyo, Japan; Department of Public Health Administration (H Yang MD), Linyi People's Hospital, Linyi, China; Department of Medicine (A Yarahmadi PhD), Thomas Jefferson University, Philadelphia, PA, USA; Department of Biostatistics and Data Science (Y Yasufuku MSc), The University of Osaka, Suita, Japan; National Center for Chronic and Noncommunicable Disease Control and Prevention (P Ye PhD), Chinese Center for Disease Control and Prevention, Beijing, China; Department of Public Health (A Yekdeş MD), Trakya University, Edirne, Türkiye; Department of Family Medicine (S A Yesuf MSc), St. Paul's Hospital Millennium Medical College, Addis Ababa, Ethiopia; Family Medicine Department (S A Yesuf MSc), St. Peter's Specialized Hospital, Addis Ababa, Ethiopia; KHANA Center for Population Health Research, Phnom Penh, Cambodia (Prof S Yi PhD); Pharmacy Department (Y E Yismaw MSc), Alkan Health Science, Business and Technology College, Bahir Dar, Ethiopia; Department of Pediatrics (Prof D Yon MD), Kyung Hee University, Seoul, South Korea; Department of Biostatistics (Prof N Yonemoto PhD), University of Toyama, Toyama, Japan; Department of Health Policy and Management (Prof M Z Younis PhD), Jackson State University, Jackson, MS, USA; School of Business & Economics (Prof M Z Younis PhD), Universiti Putra Malaysia (University of Putra Malaysia), Kuala Lumpur, Malaysia; Department of Public Health (S Yousefi PhD), Sirjan School of Medical Sciences, Sirjan, Iran, Sirjan, Iran; School of Public Health (Prof Y Yu MS), Hubei University of Medicine, Shiyan, China; Southeast University Affiliated Xuzhou Central Hospital (H Yuan PhD), Clinical Hospital, Xuzhou, China; Department of Nursing Science (U Yunusa PhD), Bayero University, Kano, Nigeria; Association for Socially Applicable Research (ASAR), Pune, India (S Zadey MS); Department of Emergency Medicine (S Zadey MS), Global Emergency Medicine Innovation and Implementation (GEMINI) Research Center, Durham, NC, USA; Epidemiology and Cancer Registry Sector (Prof V Zadnik PhD), Institute of Oncology Ljubljana, Ljubljana, Slovenia; Department of Environmental and Occupational Health (E Zainal Abidin PhD), Universiti Putra Malaysia (University of Putra Malaysia), UPM Serdang, Malaysia; Faculty of Medicine and Health Sciences (F Zakham PhD), Hodeidah University, Hodeidah, Yemen; Health Investigation Center (N Zamora MD), Universidad Católica Boliviana San Pablo, Tarija, Bolivia; San Pablo Catholic University Tarija Bolivia, Tarija, Bolivia (N Zamora MD); The Heller School for Social Policy and Management (H Zandam PhD), Brandeis University, Waltham, MA, USA; Sant'Elia Hospital (A Zanghì MD), University of Catania, Caltanissetta, Italy; Unit on Child & Adolescent Health (Prof H J Zar PhD), Medical Research Council South Africa, Cape Town, South Africa; Department of Clinical Practice (M Zawiah PhD), Northern Border University, Rafha, Saudi Arabia; Institute of Diagnostic and Interventional Radiology and Neuroradiology (S Zensen MD), University of Duisburg-Essen, Essen, Germany; Department of Nursing (N Zepro MSc), Samara University, Semera, Ethiopia; Department of Cardiology (B Zhang PhD), Zhongshan Hospital, Shanghai, China; Department of Endocrinology and Metabolism (K Zhang MD), Shandong Second Medical University, Weifang, China; Medical Oncology Department of Gastrointestinal Cancer (L Zhang MS), Cancer Hospital of Dalian University of Technology, Shenyang,

China; School of Biomedical Engineering (L Zhang MS), Dalian University of Technology, Dalian, China; Department of Internal Medicine (X Zhang MD), Jacobi Medical Center, Bronx, NY, USA; Department of Internal Medicine (X Zhang MD), Albert Einstein College of Medicine, Bronx, NY, USA; Burn Surgery Department (X-H Zhang PhD), The First Hospital of Jilin University, Changchun, China; Tianjin Medical University General Hospital (Z Zhang MD), Tianjin Centers for Disease Control and Prevention, Tianjin, China; College of Traditional Chinese Medicine (H Zhao MD), Hebei University, Baoding, China; The First Affiliated Hospital of Guizhou University of Traditional Chinese Medicine, Guiyang, China (Jie Zhao PhD); The First Affiliated Hospital of Jinan University (Y Zhao MD), Jinan University, Guangzhou, China; Department of Health Management (Z Zhao PhD), Shengjing Hospital of China Medical University, Shenyang, China; School of Public Health and Emergency Management (B Zhu PhD), Southern University of Science and Technology, Shenzhen, China; Endocrinology and Metabolism Research Center (G Zoghi MD), Hormozgan University of Medical Sciences, Bandar Abbas, Iran; College of Nursing (M Zoromba PhD), Prince Sattam bin Abdulaziz University, Al-Kharj, Saudi Arabia; Department of Public Health (L Zuhriyah PhD), Universitas Brawijaya, Malang, Indonesia; NIHR-Biomedical Research Centre (NIHR-BRC) (Prof A Zumla PhD), University College London Hospitals, London, UK; Clinical Research Centre (Prof S H Zyoud PhD), An-Najah National University Hospital, Nablus, Palestine; Department of Building Engineering and Environment (S H Zyoud PhD), Civil Engineering and Sustainable Structures (S H Zyoud PhD), Palestine Technical University (Kadoorie), Tulkarem, Palestine

Authors' Contributions

Managing the overall research enterprise

Robert W Aldridge, Catherine S Chen, Amanda Deen, Kara Estep, Lisa M Force, Erin B Hamilton, Ashley Ann Harris, Simon I Hay, Stephen S Lim, Miranda L May, Ali H Mokdad, Christopher J L Murray, Mohsen Naghavi, Olivia D Nesbit, Emily Rosenblad, Caitlyn Steiner, Stein Emil Vollset, and Eve E Wool.

Writing the first draft of the manuscript

Gregory J Bertolacci, Matthew Cunningham, Nicole Davis Weaver, Hmwe Hmwe Kyu, Vincent Mougin, Mohsen Naghavi, Emily Rosenblad, and Eve E Wool.

Primary responsibility for applying analytical methods to produce estimates

Gregory J Bertolacci, Michael Brauer, Ewerton Cousin, Matthew Cunningham, Lisa M Force, Demewoz Haile, Chieh Han, Hannah Han, Madeline E Moberg, Ali H Mokdad, Vincent Mougin, Mohsen Naghavi, Taylor Noyes, Natalie Pritchett, Sarah Brooke Sirota, Lauryn K Stafford, Jeffrey D Stanaway, Jaimie D Steinmetz, Avina Vongpradith, Yvonne Yiru Xu, Faith H Yuh, and Meixin Zhang.

Primary responsibility for seeking, cataloguing, extracting, or cleaning data; designing or coding figures and tables

Andrew Crist, Matthew Cunningham, Lisa M Force, Sama Ghoba, Chieh Han, Ali H Mokdad, Vincent Mougin, Mohsen Naghavi, Taylor Noyes, Louise Penberthy, Hannah Elizabeth Robinson-Oden, Lauryn K Stafford, and Yvonne Yiru Xu.

Providing data or critical feedback on data sources

Bhoomadevi A, Mohammad Amin Aalipour, Cristiana Abbafati, Hedayat Abbastabar, Abdallah H A Abd Al Magied, Samar Abd ElHafeez, Mohammed Altigani Abdalla, Barkhad Aden Abdeeq, Nadin M I Abdel Razeq, Jeza Muhamad Abdul Aziz, Auwal Abdullahi, Toufik Abdul-Rahman, Aidin Abedi, Armita Abedi, Roberto Ariel Abeldaño Zuñiga, Olugbenga Olusola Abiodun, Richard Gyan Aboagye, Shady Abohashem,

Hassan Abolhassani, Lucas Guimarães Abreu, Sawsan Abuhammad, Hana J Abukhadijah, Niveen ME Abu-Rmeileh, Salahdein Aburuz, Dina Abushanab, Anirudh Balakrishna Acharya, Apurba Acharya, Oluwafemi Atanda Adeagbo, Tajudeen Adesanmi Adebisi, Kamoru Ademola Adedokun, Olumide Thomas Adeleke, Bulcha Guye Adema, Habeeb Omoponle Adewuyi, Mohd Adnan, Qorinah Estiningtyas Sakilah Adnani, Leticia Akua Adzigbli, David Adzrago, Saira Afzal, Gizachew Beykaso Agafari, Mahdi Aghaalikhani, Feleke Doyore Agide, César Agostinis Sobrinho, Williams Agyemang-Duah, Bright Opoku Ahinkorah, Aqeel Ahmad, Khurshid Ahmad, Sajjad Ahmad, Tauseef Ahmad, Waqas Ahmad, Ali Ahmed, Ayman Ahmed, Gasha Salih Ahmed, Haroon Ahmed, MD Faisal Ahmed, Mehrunnisha Sharif Ahmed, Muktar Beshir Ahmed, Mushood Ahmed, Sindew Mahmud Ahmed, Gulzhanat Aimagambetova, Budi Aji, Hossein Akbarialiabad, Roland Eghoghosoa Akhigbe, Salah Al Awaidy, Ammar Al Homsi, Omar Al Omari, Zain Al Ta'ani, Yazan Al Thaher, Omar Ali Mohammed Al Zaabi, Mostafa Alam, Rasmieh Mustafa Al-Amer, Abebaw Alamrew, Turki M Alanzi, Mohammed Albashtawy, Robert W Aldridge, Kefyalew Addis Alene, Abdelazeem M Algammal, Nma Bida Alhaji, Ashraf Alhumaidi, Liaqat Ali, Shahid Ali, Syed Shujait Ali, Waad Ali, Montaha Al-Iede, Sheikh Mohammad Alif, Hamid Alinejad Rokny, Morteza Alipour, Mohamad Aljofan, Syed Mohamed Aljunid, Mustafa Alkhawam, Peter Allebeck, Khaled S Allemailem, Wesam Taher Almagharbeh, Sabah Al-Marwani, Joseph Uy Almazan, Hesham M Al-Mekhlafi, Omar Almidani, Amr Almobayed, Khaldoon Aied Alnawafleh, Hasan Yaser Alniss, Jaber S Alqahtani, Saleh A Alqahtani, Ahmad Rajeh Al-Qudimat, Intima Alrimawi, Salman Khalifah Al-Sabah, Awais Altaf, Alaa B Al-Tammemi, Nelson Alvis-Guzman, Nelson J Alvis-Zakzuk, Hassan Alwafi, Mohammad Al-Wardat, Hany Aly, Reza Amani-Beni, Amr Amin, Alireza Amindarolzarbi, Saeed Amini, Ehsan Amini-Salehi, Majid Aminzare, Dickson A Amugsi, Filippos Anagnostakis, Nazanin Anaraki, Deanna Anderlini, Nguyen Hoang Anh, Abdul-Azeez Adeyemi Anjorin, Samuel Egyakwa Ankomah, Kabilan Annadurai, Sumbul Ansari, Ernoiz Antriyandarti, Saeid Anvari, Saleha Anwar, Sumadi Lukman Anwar, Francis Appiah, Jalal Arabloo, Daniel T Araki, Jorge Arias de la Torre, Benedetta Armocida, Jesu Arockiaraj, Mahwish Arooj, Anton A Artamonov, Deepavalli Arumuganainar, Umesh Raj Aryal, Nurila Aryntayeva, Mahsa Asadi Anar, Mulusew Andualem A Asemahagn, Tahira Ashraf, Mitra Ashrafi, Saeed Aslani, Yuni Asri, Seyyed Shamsadin Athari, Alok Atreya, Khursheed Aurangzeb, Marcel Ausloos, Núbia Carelli Pereira Avelar, Sana Javaid Awan, Arian Azadnia, Amirali Azimi, Farya Azimi, Mohd Yusmaidie Aziz, Amin Azizan, Ahmed Y Azzam, Giridhara Rathnaiah Babu, Youngoh Bae, Arvind Bagga, Sana Baghizadeh, Abdulaziz T Bako, Ovidiu Constantin Baltatu, Palash Chandra Banik, Amadou Barrow, MD Abu Bashar, Shahid Bashir, Mohammad-Mahdi Bastan, Abdul-Monim Batiha, Narasimha M Beeraka, Jina Behjati, Melesse Belayneh, Olorunjuwon Omolaja Bello, Apostolos Beloukas, Riyad Bendardaf, Samiun Nazrin Bente Kamal Tune, Robert S Bernstein, Gregory J Bertolacci, Akshaya Srikanth Bhagavathula, Buna Bhandari, Kayleigh Bhangdia, Charmi Bhanushali, Pankaj Bhardwaj, Sonu Bhaskar, Anup Bhat, Priyadarshini Bhattacharjee, Gurjit Kaur Bhatti, Jasvinder Singh Bhatti, Zulfiqar A Bhutta, Sibhatu Kassa Biadgilign, Raluca Bievel-Radulescu, Bijit Biswas, Ahmad Naoras Bitar, Molalegne Bitew, Lucimere Bohn, Obasanjo Afolabi Bolarinwa, Sri Harsha Boppana, Hamed Borhany, Dejana Braithwaite, Luisa C Brant, Michael Brauer, Nicholas J K Breitborde, Edmond D Brewer, Annie J Browne, Traolach Brugha, Danilo Buonsenso, Felix Busch, Nadeem Shafique Butt, Sanjay C J, Luciana Aparecida Campos, Juan Jesus Carrero, Joao Mauricio Castaldelli-Maia, Carlos A Castañeda-Orjuela, Ferrán Catalá-López, Francieli Cembranel, Vijay Kumar Chattu, Sirshendu Chaudhuri, Akhilanand Chaurasia, Guangjin Chen, Hui Chen, Haojin Cheng, Nicholas WS Chew, William C S Cho, Bryan Chong, Ting-Wu Chuang, Sunghyun Chung, Fred Cohen, Alyssa Columbus, Joao Conde, Nathalie Conrad, Paolo Angelo Cortesi, Ewerton Cousin, Michael H Criqui, Andrew Crist, Natalia Cruz-Martins, Matthew Cunningham, Tukur Dahiru, Xiaochen Dai, Mayank Dalakoti, Gloria Dalla Costa, Pojsakorn Danpanichkul,

Samuel E Danso, Samuel Demissie Darcho, Chengetai Dare, Fernando Pio De la Hoz, Alejandro de la Torre-Luque, Edward Christopher Dee, Sindhura Deekonda, Louisa Degenhardt, Pouria Delbari, Andreas K Demetriades, Ismail Dergaa, Kebede Deribe, Hunegnaw Almaw Derseh, Nikolaos Dervenis, Hardik Dineshbhai Desai, Abraham Aregay Desta, Vinoth Gnana Chellaiyan Devanbu, Pradeep Kumar Devarakonda, Sreedhar Dharmagadda, Mandira Lamichhane Dhimal, Meghnath Dhimal, Marcello Di Pumpo, Diana Dias da Silva, Kimia Didehvar, Elangovan Dilipan, Xueting Ding, Klara Georgieva Dokova, Ojas Prakashbhai Doshi, Leila Doshmangir, Robert Kokou Dowou, Tim Robert Driscoll, Jiang Du, Judy R Dubno, Emeka W Dumbili, Bruce B Duncan, Andre Rodrigues Duraes, Lamiaa Labieb Mahmoud Ebraheim, Alireza Ebrahimi, Mohammad Hossein Ebrahimi, Foolad Eghbali, Ashkan Eighaei Sedeh, Ebrahim Eini, Michael Ekholuenetale, Temitope Cyrus Ekundayo, Rabie Adel El Arab, Abdelfatteh EL Omri, Maysaa El Sayed Zaki, Mohamed A Elmonem, Mohammed Elshaer, Abdelgawad Salah Eltahawy, Ryenchindorj Erkhembayar, Derese Eshetu, Majid Eslami, Narges Eslami, Ugochukwu Anthony Eze, Heidar Fadavian, Adeniyi Francis Fagbamigbe, Ayesha Fahim, Saman Fahimi, Ildar Ravisovich Fakhradiyev, Aliasghar Fakhri-Demeshghieh, Luca Falzone, Qiping Fan, Zaki Farhana, Carla Sofia e Sá Farinha, Syed Muhammad Yousaf Farooq, Hossein Farrokhpour, Fatemeh Farshad, Ali Fatehizadeh, Davood Fathi, Mohammad Fayaz, Alireza Feizkhah, Ginenus Fekadu, Ulrich Membe Femoe, Seyed-Mohammad Fereshtehnejad, Alexander Finnemore, Morenike Oluwatoyin Folayan, Artem Alekseevich Fomenkov, Lisa M Force, Matteo Foschi, Maryam Fotouhi, Richard Charles Franklin, Takeshi Fukumoto, John E Fuller, Peter Andras Gaal, Muktar A Gadanya, Yaseen Galali, Silvano Gallus, Balasankar Ganesan, Yijie Gao, Bashiru Garba, William M Gardner, Zisis Gatzioufas, Nsikakabasi Samuel George, Lemma Getacher, Kalab Yigermal Gete, Peter W Gething, Keyghobad Ghadiri, Arin Ghamkhar, Shakiba Ghasemi Assl, Ramy Mohamed Ghazy, Sama Ghoba, Zainab Gholami, Elena Ghotbi, Arun Ghuge, Syed Abdullah Gilani, Alem Abera Girmay, Laszlo Göbölös, Archit Goel, Rajesh Kumar Goel, Kimiya Gohari, Mahaveer Golechha, Davide Golinelli, Wenping Gong, Ayman Grada, Shi-Yang Guan, Avirup Guha, Sasidhar Gunturu, Zhaoyu Guo, Bhawna Gupta, Gaurav Gupta, Rajeev Gupta, Awoke Derbie Habteyohannes, Tesfahun Simon Hadaro, Najah R Hadi, Abdul Hafiz, Sarah Hafsia, Arian Haghtalab, Nguyen Hai Nam, Addisalem Haile, Demewoz Haile, Pritam Halder, Sebastian Haller, Kosar Hikmat Hama Aziz, Islam M Hamad, Chieh Han, Hannah Han, Nasrin Hanifi, Obaid I Haque, Andy Martahan Andreas Hariandja, Josep Maria Haro, Faizul Hasan, Ali Hasanpour- Dehkordi, Omed Hassan Ahmed, Yusuf Hassan Wada, Mahgol Sadat Hassan Zadeh Tabatabaei, Soheil Hassanipour, Lasanthi Wathsala Hathagoda, Rasmus J Havmoeller, Simon I Hay, Jiawei He, Jeffrey J Hebert, Golnaz Heidari, Nobuyuki Horita, Md Mahbub Hossain, Mohammad Bellal Hossain, Mehdi Hosseinzadeh, Priya Hotwani, Chengxi Hu, Weijun Huang, Nawfal R Hussein, Mohamed Ibrahim Husseiny, Luigi Francesco Iannone, Segun Emmanuel Ibitoye, Ramzi Ibrahim, Kevin S Ikuta, Olayinka Stephen Ilesanmi, Muhammad Hamza Ilyas, Lalu Muhammad Irham, Teresa R Iskander, Md Shahinul Islam, Sheikh Mohammed Shariful Islam, Nahlah Elkudssiah Ismail, Gaetano Isola, Jalil Jaafari, Kathryn H Jacobsen, Mohammadsadegh Jafari, Morteza Jafarinia, Haitham Jahrami, Vikash Jaiswal, Sanobar Jaka, Mihajlo Jakovljevic, Mohamed Jalloh, Armaan Jamal, Jazlan Jamaluddin, Jerin James, Safayet Jamil, Rajiv Janardhanan, Tahereh Javaheri, Syed Sarmad Javaid, Qassim Jawell Odah Abed, Sathish Kumar Jayapal, Shubha Jayaram, Yovanthi Anurangi Jayasinghe, Bijay Mukesh Jeswani, John S Ji, Wenyi Jin, Jost B Jonas, Tamas Joo, Abel Joseph, Meha Joshi, Jacek Jerzy Jozwiak, Mikk Jürisson, Billingsley Kaambwa, Zubair Kabir, Rajendra Kadel, Dler H Hussein Kadir, Ashish Kumar Kakkar, Pradnya Vishal Kakodkar, Khalil Kalavani, Md Moustafa Kamal, Sivesh Kathir Kamarajah, Saltanat Kamenova, Ramat T Kamorudeen, Devanish Narasimhasanth Kamtam, Oleksandr Kamyshnyi, Rami S Kantar, Salah Eddin Karimi, Arman Karimi Behnagh, Mohmed Isaqali Karobari, Tomasz M

Karpiński, Adarsh Katamreddy, Kanica Kaushal, Foad Kazemi, Nastaran Kazemi Rad, Vikash Ranjan Keshri, Yousef Saleh Khader, Anas Husam Khalifeh, Anees Ahmed Khalil, Pantea Khalili, Faham Khamesipour, Ajmal Khan, Iman Waheed Khan, Maseer Khan, Muhammad Mueed Khan, Muhammad Umer Khan, Ubaid Khan, Zahid Khan, Sameer Uttamaro Khasbage, Khaled Khatab, Mahalaqua Nazli Khatib, Khalid A Kheirallah, Atulya Aman Khosla, Ardeshir Khosravi, Farbod Khosravi, Zemene Demelash Kifle, Jinho Kim, Yun Jin Kim, Tadele Kinati, Yohannes Kinfu, Sanjay Kini B, Mary Kirk, Adnan Kisa, Sezer Kisa, Tegene Atamenta Kitaw, Shivakumar KM, Ann Kristin Skrindo Knudsen, Jonathan M Kocarnik, Michail Kokkorakis, Diana Gladys Kolieghu Tcheumeni, Kairi Kolves, Aida Kondybayeva, Gerbrand Koren, Tapos Kormoker, Oleksii Korzh, Soewarta Kosen, Irene Akwo Kretchy, James-Paul Kretchy, Kewal Krishan, Chong-Han Kua, Barthelemy Kuate Defo, Shweta Kulshreshtha, Dewesh Kumar, Dhasarathi Kumar, Jogender Kumar, Kamal Kumar, Tarun Kumar, Vijay Kumar, Vikash Kumar, Subramanian Kumaran, Maria Dyah Kurniasari, Asep Kusnali, Dian Kusuma, Assylkhan Kuttybayev, Ville Kytö, Hmwe Hmwe Kyu, Pallavi L C, Muhammad Awwal Ladan, Chandrakant Lahariya, Tea Lallukka, Savita Lasrado, Kamaluddin Latief, Areeba Latif, Mahrukh Latif, Aliyu Lawan, Duc Tin Le, Thao Thi Thu Le, Caterina Ledda, Seung Won Lee, Yo Han Lee, James Leigh, Vasileios Leivaditis, Matthew J Lennon, Hui Li, Jiaying Li, Si Li, Weilong Li, Zhengrui Li, Yanxue Lian, Stephen S Lim, Queran Lin, Jue Liu, Xuefeng Liu, Zhe Liu, Erand Llanaj, Michael J Loftus, Stefan Lorkowski, Rafael Lozano, Jailos Lubinda, Miltiadis D Lytras, Ellina Lytvyak, Kevin Sheng-Kai Ma, Zheng Feei Ma, Monika Machoy, Seyed Ataollah Madinezad, Aurea Marilia Madureira-Carvalho, Sasikumar Mahalingam, Samatar Abshir Mahamed, Nozad Hussein Mahmood, Shakeel Ahmed Ibne Mahmood, My Tra Mai, Rituparna Maiti, Omar M Makram, Mohammad-Reza Malekpour, Hardeep Singh Malhotra, Ahmad Azam Malik, Deborah Carvalho Malta, Jyothsna Manikkath, Fahmida Mannan, Mohammad Ali Mansournia, Lorenzo Giovanni Mantovani, Changkun Mao, Joemer C Maravilla, Bernardo Alfonso Martinez-Guerra, Francisco Rogerlândio Martins-Melo, Winfried März, Roy Rillera Marzo, Sammer Marzouk, Sugeng Mashudi, Stefano Masi, Alexander G Mathioudakis, Medha Mathur, Ikechukwu Innocent Mbachu, Michael A McPhail, Steven M McPhail, Jitendra Meena, Medhin Mehari, Vini Mehta, Tesfahun Mekene Meto, Addisu Melese, Aishe Memetova, Walter Mendoza, Godfred Antony Menezes, Ritesh G Menezes, Atte Meretoja, Tomislav Mestrovic, Chamila Dinushi Kukulege Mettananda, Sachith Mettananda, Irmina Maria Michalek, Andrea Michelerio, Hiwot Soboksa Mideksa, Kebadnew Mulatu Mihretie, Ted R Miller, Wai-kit Ming, Seyed Ali Mirshahvalad, Archana Mishra, Chaitanya Mittal, Mohammadreza Mobayen, Shivani Modi, Heba M Mohamed, Mona Gamal Mohamed, Nouh Saad Mohamed, Khabab Abbasher Hussien Mohamed Ahmed, Mohammad Reza Mohammadi, Abdollah Mohammadian-Hafshejani, Abdulwase Mohammed, Shafiu Mohammed, Yahaya Mohammed, Ali H Mokdad, Lorenzo Monasta, Amirabbas Monazzami, Yousef Moradi, Mohammad Moradi-Joo, Mahmoud M Morsy, Jonathan F Mosser, Maha Motavvef, Vincent Mougin, Seyed Mohamad Sadegh Mousavi Kiasary, Rabia Mubarak, Sumaira Mubarik, Steward Mudenda, Faraz Mughal, Syed Aun Muhammad, Muhammad Solihuddin Muhtar, Oscar J Mujica, Sukhes Mukherjee, Sumoni Mukherjee, Francesk Mulita, Chalie Mulugeta, Efren Murillo-Zamora, Christopher J L Murray, Ali Mushtaq, Saravanan Muthupandian, Ahamarshan Jayaraman Nagarajan, Mohsen Naghavi, Ganesh R Naik, Gopal Nambi, Shumaila Nargus, Yvonne Nartey, Bruno Ramos Nascimento, Abdallah Y Naser, Hamide Nasiri, Zuhair S Natto, Biswa Prakash Nayak, Vinod C Nayak, Ionut Negoi, Ruxandra Irina Negoi, Henok Biresaw Netsere, Georges Nguefack-Tsague, Josephine W Ngunjiri, Cuong Tat Nguyen, Dang Nguyen, Nghia Phu Nguyen, Van Thanh Nguyen, Ambe Marius Ngwa, Robina Khan Niazi, Luciano Nieddu, Chukwudi A Nnaji, Shuhei Nomura, Mamoona Noreen, Masoud Noroozi, Jean Jacques Noubiap, Mehran Nouri, Valentine C Nriagu, Fred Nugen, Nurfatimah Nurfatimah, Dieta Nurrika, Bogdan Oancea, Ismail A Odetokun, Michael

Safo Oduro, Oluwafunmbi Ebenezer Ogunmiluyi, Sarah Oh, Olalekan John Okesanya, Oladotun Victor Olalusi, Matthew Idowu Olatubi, Ronald Olum, Bolajoko Olubukunola Olusanya, Jacob Olusegun Olusanya, Folorunsho Bright Omage, Abidemi E Omonisi, Kanyin Liane Ong, Sandersan Onie, Obinna E Onwujekwe, John Nelson Opio, Marcel Opitz, Aksoltan Shyhdurdyevna Oradova, Atakan Orscelik, Alberto Ortiz, Samuel M Ostroff, Uchechukwu Levi Osuagwu, Adrian Otoiu, Abdu Oumer, Amel Ouyahia, Mayowa O Owolabi, Oladayo Ayobami Oyebanji, Kehinde Adewole Oyeniran, Mahesh P A, Jagadish Rao Padubidri, Dimpal Manilal Paija, Feng Pan, Sujogya Kumar Panda, Songhomitra Panda-Jonas, Seithikurippu R Pandi-Perumal, Apurvakumar Pandya, Anca Pantea Stoian, Mario Virgilio Papa, Paraskevi Papadopoulou, Romil R Parikh, Chulwoo Park, Seoyeon Park, Mitesh Patel, Neel Navinkumar Patel, Sangram Kishor Patel, Shankargouda Patil, Apurba Patra, Shrikant Pawar, Shubhadarshini Pawar, Neil Pearce, Paolo Pedersini, Veincent Christian Filipino Pepito, Emmanuel K Peprah, Prince Peprah, Gavin Pereira, Arokiasamy Perianayagam, Simone Perna, William A Petri, Hoang Nhat Pham, Hoang Tran Pham, Zahra Zahid Piracha, Moein Piroozkhkah, Ramesh Poluru, Arjun Pon Avudaiappan, Thantrira Porntaveetus, Sajjad Pourasghary, Naeimeh Pourtaheri, Jalandhar Pradhan, Rifky Octavia Pradipta, Elton Junio Sady Prates, Natalie Pritchett, Harsh Priya, Nicola Riccardo Pugliese, Jagadeesh Puvvula, Xiang Qi, Zahiruddin Syed Quazi, Navid Rabiee, Venkatraman Radhakrishnan, Maja R Radojčić, Negar Radpour, Pracheth Raghuveer, Fakher Rahim, Hawbash Mohammed-Amin Rahim, Sajjad Rahimi, Vafa Rahimi-Movaghar, Mohammad Meshbahur Rahman, Amir Masoud Rahmani, Masoud Rahmati, Ghasem Rahmatpour Rokni, Diego Raimondo, Sunil Kumar Raina, Jeffrey Pradeep Raj, Adarsh Raja, Sathish Rajaa, Erta Rajabi, Judah Rajendran, Mahmoud Mohammed Ramadan, Kadar Ramadhan, Chitra Ramasamy, Shakthi Kumaran Ramasamy, Robinson Ramírez-Vélez, Shailendra Singh Rana, Chhabi Lal Ranabhat, Nemanja Rancic, Smitha Rani, Chythra R Rao, Mithun Rao, Sina Rashedi, Vahid Rashedi, Mohammad Aziz Rasouli, Santosh Kumar Rauniyar, Lal Rawal, Reza Rawassizadeh, Elrashdy Redwan, Wajiha Rehman, Bhageerathy Reshmi, Stefano Restaino, Luis Felipe Reyes, Mina Rezaei, Nima Rezaei, Donya Rezazadeh Eidgahi, Taeho Gregory Rhee, Yohanes Andy Rias, Antonio Luiz P Ribeiro, Moattar Raza Rizvi, Hermano Alexandre Lima Rocha, João Rocha Rocha-Gomes, Mónica Rodrigues, Jefferson Antonio Buendia Rodriguez, Leonardo Roever, Peter Rohloff, Debby Syahru Romadlon, Michele Romoli, Luca Ronfani, Kevin T Root, Amirhossein Roshanshad, Gregory A Roth, Kunle Rotimi, Hanieh Rouzbahani, Reza Rouzbahani, Priyanka Roy, Sharmistha Roy, Shubhanjali Roy, Parameswari Royapuram Parthasarathy, Enrico Rubagotti, Susan Fred Rumisha, Godfrey Mutashambara Rwegerera, Aly M A Saad, Perminder S Sachdev, Seyed Kiarash Sadat Rafiei, Basema Ahmad Saddik, Bashdar Abuzed Sadee, Tarannom Sadegh, Ehsan Sadeghi, Umar Saeed, Maryam Saeedi, Mahdi Safdarian, Sher Zaman Safi, Rajesh Sagar, Mastooreh Sagharichi, Fatemeh Saheb Sharif-Askari, Narjes Saheb Sharif-Askari, Kirti Sundar Sahu, Zahra Saif, S Mohammad Sajadi, Mirza Rizwan Sajid, Leili Salehi, Mahdi Salehi, Marwa Rashad Salem, Malik Sallam, Waqas Sami, Abdallah M Samy, Sathish Sankar, Francesca Sanna, Damian F Santomauro, Lucas H C C Santos, Milena M Santric-Milicevic, Hemen Sarma, Mohammad Sarmadi, Mukesh Kumar Sathya Narayanan, Maheswar Satpathy, Monika Sawhney, Christophe Schinckus, Jurgen Carlo Schmidt, Maria Inês Schmidt, Ghil Schwarz, David C Schwebel, Sneha Annie Sebastian, Muthamizh Selvamani, Vimalraj Selvaraj, Yigit Can Senol, Subramanian Senthilkumaran, Edson Serván-Mori, Yashendra Sethi, Allen Seylani, Mahan Shafie, Muhammad Shahbaz, Samiah Shahid, Endrit Shahini, Masood Ali Shaikh, Muhammad Aaqib Shamim, Farzane Shams, Mehran Shams-Beyranvand, Anas Shamsi, Alfiya Shamsutdinova, Dan Shan, Shan Shan, Amin Sharifan, Javad Sharifi Rad, Avimanu Sharma, Gaurav Sharma, Ravi Kumar Sharma, Vishal Sharma, Shamee Shastry, Maryam Shayan, Ali Sheidaei, Ali Sheikhy, Samendra P Sherchan, B Suresh Kumar Shetty, Md Monir Hossain Shimul, Aminu Shittu, Ivy Shiue,

Nathan A Shlobin, Ambreen Shoaib, Shayan Shojaei, Sunil Shrestha, Suleiman Adeiza Shuaibu, Zahra Siavashpour, Ahmed Kamal Siddiqi, Luís Manuel Lopes Rodrigues Silva, Padam Prasad Simkhada, Abhinav Singh, Amit Singh, Baljinder Singh, Harmanjit Singh, Harpreet Singh, Jasvinder A Singh, Jawahar Singh, Kalpana Singh, Narinder Pal Singh, Paramdeep Singh, Rakesh K Singh, Samer Singh, Surendra Singh, Anna Aleksandrovna Skryabina, Mahdieh SobhZahedi, Lencho Kajela Solbana, Aayushi Sood, Soroush Soraneh, Reed J D Sorensen, Fernando Sousa, Marco Aurelio Sousa, Ireneous N Soyiri, Michael Spartalis, Chandrashekhar T Sreeramareddy, Prateek Srivastav, Devin Bailey Srivastava, Muhammad Haroon Stanikzai, Nadine Steckling-Muschack, Muhammad Suleman, Haitong Zhe Sun, Xiaohui Sun, Zhuanlan Sun, Suraj Sundaragiri, Thanigaivel Sundaram, Johan Sundström, David Sunkersing, Chandan Kumar Swain, Dayinta Annisa Syaiful, Tasmin L Symons, Lukasz Szarpak, Mindy D Szeto, Sree Sudha T Y, Rafael Tabarés-Seisdedos, Fatemeh Sadat Tabatabaei, Seyed Shahaboddin Tabatabaei, Seyyed Mohammad Tabatabaei, Shima Tabatabai, Celine Tabche, Takahiro Tabuchi, Zanan Mohammed-Ameen Taha, Jabeen Taiba, Mircea Tampa, Ker-Kan Tan, Mohammad Tanashat, Haosu Tang, Mohsan Tanveer, Gizachew A Tessema, Jay Tewari, Chandan Kumar Thakur, Manuel Sebastian Thomas, Madi Tleshev, Mathilde Touvier, Marcos Roberto Tovani-Palone, Quynh Thuy Huong Tran, Tam Quoc Minh Tran, Nguyen Tran Minh Duc, Domenico Trico, Indang Trihandini, Tulika Tripathi, Quynh Xuan Nguyen Truong, Gary Tse, Munkhtuya Tumurkhuu, Sok Cin Tye, Aniefiok John Udoakang, Himayat Ullah, Muhammad Umair, Hauwa Onozasi Umar, Muhammad Umar,[§] Bhaskaran Unnikrishnan, Dinesh Upadhya, Era Upadhyay, Pascual R Valdez, Jef Van den Eynde, Joe Varghese, Pavani Varma, Tommi Juhani Vasankari, Sampara Vasishta, Srivatsa Surya Vasudevan, Narayanaswamy Venketasubramanian, Georgios-Ioannis Verras, Mathavaswami Vijayageetha, David Villarreal-Zegarra, Luciano Magalhães Vitorino, Vasily Vlassov, Theo Vos, Linh Vu, Krishna Dhavan Vyas, Yasir Waheed, Mugi Wahidin, Mandaras Tariku Walde, Megha Walia, Jin-Yi Wan, Junshi Wang, Liang Wang, Qingzhi Wang, Shu Wang, Xing Wang, Youxin Wang, Tanveer A Wani, Stefanie Watson, Daniel J Weiss, Andrea Werdecker, Ronny Westerman, Taweewat Wiangkham, Dakshitha Praneeth Wickramasinghe, Angga Wilandika, Peter Willeit, Andrew Awuah Wireko, Charles Shey Wiysonge, Bogdan Wojtyniak, Nathnael Abera Woldehana, Tewodros Eshete Wonde, Yen Jun Wong, Daniel Tarekegn Worede, Minichil Chanie Worku, Felicia Wu, Peng Wu, Zenghong Wu, Lishun Xiao, Na Xiao, Site Xu, Suowen Xu, Yvonne Yiru Xu, Kazumasa Yamagishi, Haibo Yang, Haiqiang Yao, Laiang Yao, Amir Yarahmadi, Haya Yasin, Sanni Yaya, Pengpeng Ye, Mohammad Hossein YektaKooshali, Siyan Yi, Malede Berihun Yismaw, Yazachew Engida Yismaw, Naohiro Yonemoto, Mustafa Z Younis, Abdilahi Yousuf, Chuanhua Yu, Yong Yu, Faith H Yuh, Siddhesh Zadey, Vesna Zadnik, Mubashir Zafar, Emilia Zainal Abidin, Hussaini Zandam, Kourosh Zarea, Sebastian Zensen, Beijian Zhang, Julio Min Fei Zhang, Xiaoyi Zhang, Xiu-Hang Zhang, Sholpan Bolatovna Zhangelova, Jiefeng Zhao, Zhongyi Zhao, Anthony Zhong, Juexiao Zhou, Abzal Zhumagaliuly, and Magdalena Zielińska.

Developing methods or computational machinery

Daniel T Araki, Aleksandr Y Aravkin, Gregory J Bertolacci, Michael Brauer, Kelly M Cercy, Emma Johnson Cowart, Andrew Crist, Jessica A Cruz, Garland T Culbreth, Matthew Cunningham, Xiaochen Dai, Lisa M Force, William M Gardner, Sama Ghoba, Erin B Hamilton, Chieh Han, Simon I Hay, Jiawei He, Kevin S Ikuta, Jonathan M Kocarnik, Stephen S Lim, Kelsey Lynn Maass, Ali H Mokdad, Jonathan F Mosser, Vincent Mougin, Christopher J L Murray, Mohsen Naghavi, Kanyin Liane Ong, Austin E Schumacher, Reed J D Sorensen, Jeffrey D Stanaway, Caitlyn Steiner, Vivianne M Swart, Megan Verma, Eli J Weiss, Shadrach Wilson, Eve E Wool, and Faith H Yuh.

Providing critical feedback on methods or results

Bhoomadevi A, Mohammad Amin Aalipour, Hazim S Ababneh, Bedru J Abafita, Ukachukwu O Abaraogu, Cristiana Abbafati, Madineh Abbasi, Faezeh Abbaspour, Hedayat Abbastabar, Abdallah H A Abd Al Magied, Samar Abd ElHafeez, Ashraf Nabiel Abdalla, Mohammed Altigani Abdalla, Emad M Abdallah, Barkhad Aden Abdeeq, Nadin M I Abdel Razeq, Ahmed Abdelrahman Abdelgalil, Reda Abdel-Hameed, Michael Abdelmasseh, Mahmoud Abdelnabi, Wael M Abdel-Rahman, Arman Abdous, Mostafa M Abdrabou, Jeza Muhamad Abdul Aziz, Deldar Morad Abdulah, Auwal Abdullahi, Toufik Abdul-Rahman, Habtamu Abebe Getahun, Armita Abedi, Parisa Abedi, Asrat Agalu Abejew, Roberto Ariel Abeldaño Zuñiga, Shehab Uddin Al Abid, Syed Hani Abidi, Alemwork Abie, Olugbenga Olusola Abiodun, Richard Gyan Aboagye, Shady Abohashem, Hassan Abolhassani, Ulric Sena Abonie, Nagah M Abourashed, Mohamed Abouzid, Lucas Guimarães Abreu, Dariush Abtahi, Rana Kamal Abu Farha, Fuad Hamdi A Abuadas, Nermeen Abu-Elala, Eman Abu-Gharbieh, Sawsan Abuhammad, Ahmad Y Abuhelwa, Hana J Abukhadijah, Niveen ME Abu-Rmeileh, Salahdein Aburuz, Dina Abushanab, Manfred Mario Kokou Accrombessi, Anirudh Balakrishna Acharya, Apurba Acharya, Ousman Adal, Lisa C Adams, Abdu A Adamu, Isaac Yeboah Addo, Oluwafemi Atanda Adeagbo, Tajudeen Adesanmi Adebisi, Kamoru Ademola Adedokun, Oluwatobi E Adegbile, Nurudeen A Adegoke, Olumide Thomas Adeleke, Bulcha Guye Adema, Bashir Aden, Isaac Ayodeji Adesina, Miracle Ayomikun Adesina, Habeeb Omoponle Adewuyi, Temitayo Esther Adeyeoluwa, Mache Tsadik Adhana, Ripon Kumar Adhikary, Mohd Adnan, Qorinah Estiningtyas Sakilah Adnani, Leticia Akua Adzigbli, David Adzrago, Giuseppina Affinito, Ahmed M Afifi, Aanuoluwapo Adeyimika Afolabi, Rotimi Felix Afolabi, Saira Afzal, Gizachew Beykaso Agafari, Suneth Buddhika Agampodi, Thilini Chanchala Agampodi, Navidha Aggarwal, Mahdi Aghaalikhani, Sepehr Aghajanian, Seyed Mohammad Kazem Aghamir, Feleke Doyore Agide, César Agostinis Sobrinho, Anurag Agrawal, Williams Agyemang-Duah, Mahsa Ahadi, Bright Opoku Ahinkorah, Aqeel Ahmad, Danish Ahmad, Faisal Ahmad, Ijaz Ahmad, Khabir Ahmad, Khurshid Ahmad, Sajjad Ahmad, Tauseef Ahmad, Waqas Ahmad, Negar Sadat Ahmadi, Ali Ahmed, Ayman Ahmed, Gasha Salih Ahmed, Haroon Ahmed, Junaid Ahmed, Luai A Ahmed, MD Faisal Ahmed, Mehrunnisha Sharif Ahmed, Muktar Beshir Ahmed, Mushood Ahmed, Shabbir Ahmed, Sindew Mahmud Ahmed, Syed Anees Ahmed, Gulzhanat Aimagambetova, Budi Aji, Hossein Akbarialiabad, Saeid Akbarifard, Oluwasefunmi Akeju, Roland Eghoghosoa Akhigbe, Ruslan Akhmedullin, Olufemi Ambrose Akinkuotu, Mohammed Ahmed Akkaif, Wole Akosile, Ashley E Akrami, Ralph Kwame Akyea, Alaa Al Amiry, Salah Al Awaidy, Syed Mahfuz Al Hasan, Ammar Al Homsi, Mohammad Khaled Al Nawayseh, Omar Al Omari, Zain Al Ta'ani, Yazan Al Thaher, Omar Ali Mohammed Al Zaabi, Mohammad Ahmmad Mahmoud Al Zoubi, Mousa Ali Al-Abbadi, Ziyad Al-Aly, Khurshid Alam, Manjurul Alam, Mohammad Khursheed Alam, Mostafa Alam, Rasmieh Mustafa Al-Amer, Abebaw Alamrew, Amani Alansari, Turki M Alanzi, Fahmi Y Al-Ashwal, Mohammed Albashtawy, Robert W Aldridge, Shereen M Aleidi, Bezawit Abeje Alemayehu, Fentahun Alemnew, Melaku Birhanu Alemu, Kefyalew Addis Alene, Ali M Alfalki, Fahad D Algahtani, Abdelazeem M Algammal, Khairat Al-Habbal, Nma Bida Alhaji, Mohammed Khaled Al-Hanawi, Khalid A Alhasan, Ashraf Alhumaidi, Fahad A Alhumaydhi, Haroon Muhammad Ali, Irfan Ali, Liaqat Ali, Maratab Ali, Mohammad Daud Ali, Mohammed Usman Ali, Rafat Ali, Shahid Ali, Syed Shujait Ali, Waad Ali, Gianfranco Alicandro, Montaha Al-Iede, Sheikh Mohammad Alif, Hamid Alinejad Rokny, Morteza Alipour, Samah W Al-Jabi, Mohamad Aljofan, Moath Saleh Aljohani, Syed Mohamed Aljunid, Mustafa Alkhawam, Khaled S Allemailem, Mohammed Z Allouh, Wesam Taher Almagharbeh, Sabah Al-Marwani, Joseph Uy Almazan, Hesham M Al-Mekhlafi, Omar Almidani, Amr Almobayed, Khaldoon Aied Alnawafleh, Hasan Yaser Alniss, Margret Beaula Alocious Sukumar, Mahmoud A Alomari, Mohammad R Alosta, Jaber S Alqahtani, Saleh A Alqahtani,

Mohammad R Alqudimat, Intima Alrimawi, Salman Khalifah Al-Sabah, Mohammed A Alsabri, Zaid Altaany, Awais Altaf, Alaa B Al-Tammemi, Jaffar A Al-Tawfiq, Malik A Althobiani, Khalid A Altirkawi, Javier Alvarez-Galvez, Nelson Alvis-Guzman, Nelson J Alvis-Zakzuk, Hassan Alwafi, Mohammad Al-Wardat, Yaser Mohammed Al-Worafi, Hany Aly, Mohammad Sharif Ibrahim Alyahya, Hosam Alzahrani, Karem H Alzoubi, Adel Sharaf Al-Zubairi, Ekiyor Joseph Amafah, Joy Amafah, Reza Amani-Beni, Faten Amer, Amr Amin, Tarek Tawfik Amin, Alireza Amindarolzarbi, Saeed Amini, Majid Aminzare, Sohrab Amiri, Dickson A Amugsi, Ganiyu Adeniyi Amusa, Filippos Anagnostakis, Roshan A Ananda, Nazanin Anaraki, Robert Ancuceanu, Deanna Anderlini, David B Anderson, Nguyen Hoang Anh, Abdul-Azeez Adeyemi Anjorin, Samuel Egyakwa Ankomah, Kabilan Annadurai, Sumbul Ansari, Alireza Ansari-Moghaddam, Ernoiz Antriyandarti, Boluwatife Stephen Anuoluwa, Iyadunni Adesola Anuoluwa, Saeid Anvari, Saleha Anwar, Sumadi Lukman Anwar, Razique Anwer, Shahnawaz Anwer, Anayochukwu Edward Anyasodor, Francis Appiah, Juan Pablo Arab, Jalal Arabloo, Mosab Arafat, Demelash Areda, Getnet Mesfin Aregu, Jorge Arias de la Torre, Ghazal Arjmand, Benedetta Armocida, Jesu Arockiaraj, Mahwish Arooj, Anton A Artamonov, Ashokan Arumugam, Deepavalli Arumuganainar, Umesh Raj Aryal, Mahsa Asadi Anar, Muhammad Asaduzzaman, Syed Mohammed Basheeruddin Asdaq, Mulusew Andualem A Asemahagn, Mulu Tiruneh Asemu, Saeed Asgary, Mohammad Asghari-Jafarabadi, Syed Amir Ashraf, Tahira Ashraf, Mitra Ashrafi, Bernard Kwadwo Yeboah Asiamah-Asare, Yuni Asri, Batyrbek Assembekov, Seyyed Shamsadin Athari, Alok Atreya, Julie Alaere Atta, Khursheed Aurangzeb, Marcel Ausloos, Abolfazl Avan, Núbia Carelli Pereira Avelar, Sana Javaid Awan, Adedapo Wasiu Awotidebe, Lemessa Assefa A Ayana, Haleh Ayatollahi, Yusuf Oloruntoyin Ayipo, Sina Azadnajafabad, Arian Azadnia, James Mba Azam, Alireza Azarboo, Zelalem Nigussie Azene, Gulrez Shah Azhar, Amirali Azimi, Farya Azimi, Mohd Yusmaidie Aziz, Sadat Abdulla Aziz, Amin Azizan, Ahmed Y Azzam, Giridhara Rathnaiah Babu, Youngoh Bae, Arvind Bagga, Nasser Bagheri, Sara Bagheri, Elahe Baghizadeh, Fereshteh Baghizadeh, Sana Baghizadeh, Khlood K Baghlaf, Najmeh Bahmanziari, Ruhai Bai, Abdulaziz T Bako, Wondu Feyisa Balcha, Maher Balkis, Jose Balmori-de-la-Miyar, Mohammadreza Balooch Hasankhani, Ovidiu Constantin Baltatu, Palash Chandra Banik, Noel C Barengo, Hiba Jawdat Barqawi, Amadou Barrow, Sandra Barteit, Lingkan Barua, MD Abu Bashar, Shahid Bashir, Guido Basile, Rehana Basri, Quique Bassat, Mohammad-Mahdi Bastan, Abdul-Monim Batiha, Kavita Batra, Matteo Bauckneht, Mahdis Bayat, Mohammad Amin Bayat Tork, Thomas Beaney, Narasimha M Beeraka, Jina Behjati, Bezawit K Bekele, Almaz Nibret Belay, Demeke Mesfin Belay, Asnake Gashaw Belayneh, Melesse Belayneh, Abel Cherkos Belete, Gokce Belge Bilgin, Olorunjuwon Omolaja Bello, Umar Muhammad Bello, Apostolos Beloukas, Riyad Bendardaf, Isabela M Bensenor, Samiun Nazrin Bente Kamal Tune, Maria Bergami, Alemshet Yirga Berhie, Abiye Assefa Berihun, Amiel Nazer C Bermudez, Robert S Bernstein, Gregory J Bertolacci, Paola Bertuccio, Paulo J G Bettencourt, Ajeet Singh Bhadoria, Akshaya Srikanth Bhagavathula, Neeraj Bhala, Buna Bhandari, Kayleigh Bhangdia, Charmi Bhanushali, Nikha Bhardwaj, Pankaj Bhardwaj, Ashish Bhargava, Sonu Bhaskar, Anup Bhat, Priyadarshini Bhattacharjee, Shuvarthi Bhattacharjee, Gurjit Kaur Bhatti, Jasvinder Singh Bhatti, Mohiuddin Ahmed Bhuiyan, Zulfiqar A Bhutta, Haoran Bi, Sibhatu Kassa Biadgilign, Raluca Bievel-Radulescu, Naif Kandash Binsaleh, Bijit Biswas, Mohammad Shahangir Biswas, Ahmad Naoras Bitar, Molalegne Bitew, Bruno Bizzozero-Peroni, Virginia Bodolica, Eyob Ketema Bogale, Lucimere Bohn, Obasanjo Afolabi Bolarinwa, Paria Bolourinejad, Aime Bonny, Sri Harsha Boppana, Hamed Borhany, Mina Borran, Sudipta Bose, Samuel Adolf Bosoka, Alejandro Botero Carvajal, Soufiane Boufous, Christopher Boxe, Dejana Braithwaite, Luisa C Brant, Nicholas J K Breitborde, Hermann Brenner, Maria L Bringas Vega, Julie Brown, Annie J Browne, Traolach Brugha, Raffaele Bugiardini, Norma B Bulamu, Tsion Samuel Bunare, Richard A Burns, Felix Busch, Yasser Bustanji, Nadeem Shafique Butt, Zahid A Butt,

Sanjay C J, Tianji Cai, Rose Cairns, Mehtap Çakmak Barsbay, Luis Alberto Cámera, Luciana Aparecida Campos, Ismael Campos-Nonato, Fan Cao, Si Cao, Angelo Capodici, Rosario Cárdenas, Juan Jesus Carrero, Andre F Carvalho, Márcia Carvalho, Ana Paula Carvalho-e-Silva, Joao Mauricio Castaldelli-Maia, Giulio Castelpietra, Ferrán Catalá-López, Luca Cegolon, Francieli Cembranel, Muthia Cenderadewi, Ester Cerin, Pamela Roxana Chacón-Uscamaita, Chiranjib Chakraborty, Joht Singh Chandan, Rama Mohan Chandika, Vijay Kumar Chattu, Victoria Chatzimavridou-Grigoriadou, Sirshendu Chaudhuri, Akhilanand Chaurasia, An-Tian Chen, Guangjin Chen, Haiyan Chen, Hana Chen, Haowei Chen, Hui Chen, Rucheng Chen, Shanquan Chen, Xiang Chen, Haojin Cheng, Nicholas WS Chew, Gerald Chi, Fatemeh Chichagi, Izumi Chihara, Odgerel Chimed-Ochir, Jesus Lorenzo Chirinos-Caceres, William C S Cho, Bryan Chong, Yuen Yu Chong, Hou In Chou, Enayet Karim Chowdhury, Sreshtha Chowdhury, Hanne Christensen, Ting-Wu Chuang, Isaac Sunday Chukwu, Erin Chung, Sheng-Chia Chung, Sunghyun Chung, Muhammad Chutiyami, Arrigo Francesco Giuseppe Cicero, Cain C T Clark, Fred Cohen, Alyssa Columbus, Joao Conde, Stephen E Congly, Nathalie Conrad, Leslie Trumbull Cooper, Alexandru Corlateanu, Samuele Cortese, Paolo Angelo Cortesi, Ewerton Cousin, Michael H Criqui, Natalia Cruz-Martins, Xiaolin Cui, Garland T Culbreth, Patricia Cullen, Matthew Cunningham, Nour Dababo, Ali Dabbagh, Omid Dadras, Tukur Dahiru, Xiaochen Dai, Zhaoli Dai, Mayank Dalakoti, Koustuv Dalal, Gloria Dalla Costa, Lucio D'Anna, Pojsakorn Danpanichkul, Samuel E Danso, Samuel Demissie Darcho, Latefa Ali Dardas, Chengetai Dare, Bahar Darouei, Reza Darvishi Cheshmeh Soltani, Nicole Davis Weaver, Dimash Davletov, Kairat Davletov, Fernando Pio De la Hoz, Alejandro de la Torre-Luque, Edward Christopher Dee, Sindhura Deekonda, Louisa Degenhardt, Paria Dehesh, Pouria Delbari, Laura Delgado-Ortiz, Mohammad Delsoz, Andreas K Demetriades, Tadios Niguss Derese, Ismail Dergaa, Hunegnaw Almaw Derseh, Nikolaos Dervenis, Emina Dervišević, Hardik Dineshbhai Desai, Abraham Aregay Desta, Vinoth Gnana Chellaiyan Devanbu, Pradeep Kumar Devarakonda, Arkadeep Dhali, Kuldeep Dhama, Sreedhar Dharmagadda, Mandira Lamichhane Dhimal, Meghnath Dhimal, Bibha Dhungel, Marcello Di Pumpo, Diana Dias da Silva, Daniel Diaz, Kimia Didehvar, Elangovan Dilipan, Lauren K Dillard, Xueting Ding, Saeid Doaei, Klara Georgieva Dokova, Mario D'Oria, Fariba Dorostkar, E Ray Dorsey, Ojas Prakashbhai Doshi, Leila Doshmangir, Robert Kokou Dowou, Jiang Du, Judy R Dubno, Emeka W Dumbili, Oyewole Christopher Durojaiye, Ashit Kumar Dutta, Siddhartha Dutta, Sulagna Dutta, Osamudiamen Ebohon, Ejemai Eboreime, Lamiaa Labieb Mahmoud Ebraheim, Alireza Ebrahimi, Mohammad Hossein Ebrahimi, Abdelaziz Ed-Dra, David Edvardsson, Ferry Efendi, Foolad Eghbali, Ashkan Eighaei Sedeh, Terje Andreas Eikemo, Ebrahim Eini, Michael Ekholuenetale, Temitope Cyrus Ekundayo, Rabie Adel El Arab, Abdelfatteh EL Omri, Maysaa El Sayed Zaki, Mohamed Ahmed Eladl, Reza Elahi, Said El-Ashker, Noha Mousaad Elemam, Muhammed Elhadi, Mohamed Elhoumed, Waseem El-Huneidi, Omar Abdelsadek Abdou Elmeligy, Mohamed A Elmonem, Rami Elmorsi, Mohamed Hassan Elnaem, Gihan ELNahas, Mohammed Elshaer, Ibrahim Elsohaby, Abdelgawad Salah Eltahawy, Tadele Emagneneh, Misganu Endriyas, Ryenchindorj Erkhembayar, Derese Eshetu, Majid Eslami, Narges Eslami, Ugochukwu Anthony Eze, Heidar Fadavian, Adeniyi Francis Fagbamigbe, Omotayo Francis Fagbule, Ayesha Fahim, Saman Fahimi, Aamir Fahira, Ildar Ravisovich Fakhradiyev, Aliasghar Fakhri-Demeshghieh, Luca Falzone, Qiping Fan, Mohammad Farahmand, Ali Faramarzi, Mohammad Fareed, Zaki Farhana, Liliana Faria, Carla Sofia e Sá Farinha, Andre Faro, Syed Muhammad Yousaf Farooq, Hossein Farrokhpour, Fatemeh Farshad, Farima Farsi, Folorunso Oludayo Fasina, Modupe Margaret Fasina, Ali Fatehizadeh, Davood Fathi, Zareen Fatima, Mohammad Fayaz, Pooria Fazeli, Valery L Feigin, Alireza Feizkhah, Gelana Fekadu, Ginenus Fekadu, Ulrich Membe Femoe, Talukdar Raian Ferdous, Seyed-Mohammad Fereshtehnejad, Rodrigo Fernandez-Jimenez, Pietro Ferrara, Alize J Ferrari, Getahun Fetensa, Bikila Regassa Feyisa, Alexander Finnemore, Claudio Fiorilla, Florian

Fischer, Federica Fogacci, Artem Alekseevich Fomenkov, Lisa M Force, Matteo Foschi, Maryam Fotouhi, Kayode Raphael Fowobaje, Richard Charles Franklin, Alberto Freitas, Takeshi Fukumoto, Sridevi G, Peter Andras Gaal, Muktar A Gadanya, Lebo Francina Gafane-Matemane, Márió Gajdács, Yaseen Galali, Dinara Galiyeva, Dhanraj Ganapathy, Balasankar Ganesan, Xiang Gao, Yijie Gao, Bashiru Garba, Miguel Garcia-Argibay, David Garcia-Azorin, William M Gardner, Wendy Paola Gastélum Espinoza, Zisis Gatzioufas, Rupesh K Gautam, Bamba Gaye, Hong-Han Ge, Feven Sahle Gebre, Miglas Welay Gebregergis, Mesfin Gebrehiwot, Miesa Gelchu, Stefano Gelibter, Nsikakabasi Samuel George, Lemma Getacher, Genanew K Getahun, Kalab Yigermal Gete, Amir Ghaffari Jolfayi, Arin Ghamkhar, Shakiba Ghasemi Assl, Fariba Ghassemi, Ramy Mohamed Ghazy, Sama Ghoba, Maryam Gholamalizadeh, Zainab Gholami, Zeinab Ghorbani, Elena Ghotbi, Arun Ghuge, Alessandro Gialluisi, Konstantinos Giannakis, Syed Abdullah Gilani, Tiffany K Gill, Bikash Ranjan Giri, Alem Abera Girmay, Alessandro Girombelli, Laszlo Göbölös, Anil Kumar Goel, Archit Goel, Rajesh Kumar Goel, Lay Hoon Goh, Kimiya Gohari, Mahaveer Golechha, Ali Golestani, Melika Golmohammadi, Wenping Gong, Alessandra C Goulart, Ayman Grada, Simon Matthew Graham, Michal Grivna, Shi-Yang Guan, Mohammed Ibrahim Mohialdeen Gubari, Mesay Dechasa Gudeta, Avirup Guha, Stefano Guicciardi, Sasidhar Gunturu, Cui Guo, Xingzhi Guo, Zhaoyu Guo, Zhifeng Guo, Bhawna Gupta, Lalit Gupta, Reyna Alma Gutiérrez, Roberth Steven Gutiérrez-Murillo, Jose Guzman-Esquivel, Abrham Tesfaye Habteyes, Awoke Derbie Habteyohannes, Tesfahun Simon Hadaro, Najah R Hadi, Zahra Hadian, Abdul Hafiz, Arian Haghtalab, Nguyen Hai Nam, Addisalem Haile, Demewoz Haile, Pritam Halder, Sebastian Haller, Rabih Halwani, Kosar Hikmat Hama Aziz, Islam M Hamad, Randah R Hamadeh, Samer Hamidi, Ahmad Hammoud, Hannah Han, Asif Hanif, Nasrin Hanifi, Fahad Hanna, Ashanul Haque, Md Nuruzzaman Haque, Obaid I Haque, Ahmed I Hasaballah, Faizul Hasan, Md Kamrul Hasan, Towhid Hasan, Hamidreza Hasani, Ali Hasanpour- Dehkordi, Mohammad Hashem Hashempur, Nada Tawfig Hashim, Ammarah Hasnain, Abbas M Hassan, Ibrahim Nagmeldin Hassan, Ikrama Hassan, Omed Hassan Ahmed, Yusuf Hassan Wada, Mahgol Sadat Hassan Zadeh Tabatabaei, Soheil Hassanipour, Lasanthi Wathsala Hathagoda, Johannes Haubold, Rasmus J Havmoeller, Simon I Hay, Youssef Hbid, Jeffrey J Hebert, Golnaz Heidari, Mohammad Heidari, Mojtaba Heydari, Kamal Hezam, Yuta Hiraike, Nobuyuki Horita, Alamgir Hossain, Lubna Hossain, Md Belal Hossain, Md Mahbub Hossain, Md Sabbir Hossain, Mohammad Bellal Hossain, Fatemeh Sadat Hosseini, Mehdi Hosseinzadeh, Mihaela Hostiuc, Peter J Hotez, Priya Hotwani, Hanno Hoven, Chengxi Hu, Yifei Hu, Weijun Huang, Yefei Huang, Yuting Huang, Zhenyao Huang, Mega Hasanul Huda, Ayesha Humayun, Waqar Husain, Kiavash Hushmandi, Javid Hussain, Nawfal R Hussein, Mohamed Ibrahim Husseiny, Luigi Francesco Iannone, Segun Emmanuel Ibitoye, Khalid S Ibrahim, Ramzi Ibrahim, Reem Ibrahim, Umar Idris Ibrahim, Fidelia Ida, Kevin S Ikuta, Olayinka Stephen Ilesanmi, Irena M Ilic, Milena D Ilic, Muhammad Hamza Ilyas, Mohammad Tarique Imam, Masoud Imani, Lucius Chidiebere Imoh, Arit Inok, Meesha Iqbal, Mujahid Iqbal, Lalu Muhammad Irham, Mustafa Alhaji Isa, Benni Iskandar, Teresa R Iskander, Md Rabiul Islam, Md Shahinul Islam, Sheikh Mohammed Shariful Islam, Farhad Islami, Nahlah Elkudssiah Ismail, Gaetano Isola, Masao Iwagami, Ihoghosa Osamuyi Iyamu, Vinothini J, Jalil Jaafari, Louis Jacob, Kathryn H Jacobsen, Ali Jadidi, Farhad Jadidi-Niaragh, Mohammadsadegh Jafari, Morteza Jafarinia, Shabbar Jaffar, Haitham Jahrami, Ammar Abdulrahman Jairoun, Vikash Jaiswal, Sanobar Jaka, Mihajlo Jakovljevic, Reza Jalilzadeh Yengejeh, Mohamed Jalloh, Armaan Jamal, Jerin James, Hasan Jamil, Safayet Jamil, Roland Dominic G Jamora, Masoud Jamshidi, Shaghayegh JamshidiRastabi, Rajiv Janardhanan, Chinmay T Jani, Esmaeil Jarrahi, Tahereh Javaheri, Syed Sarmad Javaid, Anita Javanmardi, Javad Javidnia, Talha Jawaid, Qassim Jawell Odah Abed, Sathish Kumar Jayapal, Shubha Jayaram, Ruwan Duminda Jayasinghe, Yovanthi Anurangi Jayasinghe, Sun Ha Jee, Jayakumar Jeganathan, Diptismita Jena, Seogsong Jeong, Bijay Mukesh Jeswani, Vivekanand Jha, John S

Ji, Min Jiang, Wenyi Jin, Nabi Jomehzadeh, Jost B Jonas, Tamas Joo, Abu Jor, Abel Joseph, Nitin Joseph, Jacek Jerzy Jozwiak, Mikk Jürisson, Billingsley Kaambwa, Ali Kabir, Zubair Kabir, Rajendra Kadel, Dler H Hussein Kadir, Ashish Kumar Kakkar, Pradnya Vishal Kakodkar, Rizwan Kalani, Khalil Kalavani, Feroze Kaliyadan, Md Moustafa Kamal, Mehnaz Kamal, Sivesh Kathir Kamarajah, Rajesh Kamath, Saltanat Kamenova, Ramat T Kamorudeen, Devanish Narasimhasanth Kamtam, Naser Kamyari, Oleksandr Kamyshnyi, Mona Kanaan, Jiseung Kang, Kehinde Kazeem Kanmodi, Suthanthira Kannan S, Rami S Kantar, Debasish Kar, Sujita Kumar Kar, Paschalis Karakasis, Jafar Karami, Mohammad Amin Karimi, Salah Eddin Karimi, Arman Karimi Behnagh, Mohmed Isaqali Karobari, Tomasz M Karpiński, Adarsh Katamreddy, Joonas H Kauppila, Kanica Kaushal, Foad Kazemi, Nastaran Kazemi Rad, Sina Kazemian, Hafte Kahsay Kebede, Yabets Tesfaye Kebede, Tibebeselassie S Keflie, Salima Kerai, Jessica A Kerr, Vikash Ranjan Keshri, Kamyab Keshtkar, Emmanuelle Kesse-Guyot, Reza Khademi, Yousef Saleh Khader, Hazim O Khalifa, Anas Husam Khalifeh, Anees Ahmed Khalil, Pantea Khalili, Alireza Khalilian, Ghazaleh Khalili-Tanha, Mohamed Khalis, Faham Khamesipour, Ajmal Khan, Fayaz Khan, Iman Waheed Khan, Maseer Khan, Md Abdullah Saeed Khan, Mohammad Jobair Khan, Muhammad Hamza Khan, Muhammad Mueed Khan, Muhammad Umair Khan, Muhammad Umer Khan, Serab Khan, Sumaiya Khan, Ubaid Khan, Yusuf Saleem Khan, Zahid Khan, Vishnu Khanal, Shaghayegh Khanmohammadi, Sameer Uttamaro Khasbage, Zenith Khashim, Khaled Khatab, Haitham Khatatbeh, Moawiah Mohammad Khatatbeh, Mahalaqua Nazli Khatib, Khalid A Kheirallah, Sunil Kumar Khokhar, Najmaddin Salih Husen Khoshnaw, Atulya Aman Khosla, Ardeshir Khosravi, Farbod Khosravi, Mahmood Khosrowjerdi, P Ratan Khuman, Zemene Demelash Kifle, Hye Jun Kim, Jinho Kim, Kwanghyun Kim, Min Seo Kim, Yun Jin Kim, Ruth W Kimokoti, Tadele Kinati, Yohannes Kinfu, Sanjay Kini B, Adnan Kisa, Sezer Kisa, Katarzyna Kissimova-Skarbek, Tegene Atamenta Kitaw, Mika Kivimäki, Abdul Basith KM, Shivakumar KM, Ann Kristin Skrindo Knudsen, Jonathan M Kocarnik, Sonali Kochhar, Prakash Babu Kodali, Michail Kokkorakis, Ali-Asghar Kolahi, Diana Gladys Kolieghu Tcheumeni, Kairi Kolves, Farzad Kompani, Aida Kondybayeva, Isaac Koomson, Gerbrand Koren, Tapos Kormoker, Oleksii Korzh, Irene Akwo Kretchy, James-Paul Kretchy, Kewal Krishan, Chong-Han Kua, Barthelemy Kuate Defo, Mohammed Kuddus, Ilari Kuitunen, Shweta Kulshreshtha, Dewesh Kumar, Dhasarathi Kumar, Jogender Kumar, Kamal Kumar, Mukesh Kumar, Nitesh Kumar, Nithin Kumar, Tushar Kumar, Vijay Kumar, Vikash Kumar, Subramanian Kumaran, Jibin Kunjavara, Setor K Kunutsor, Om P Kurmi, Maria Dyah Kurniasari, Krishna Prasad Kurpad, Asep Kusnali, Christina Yeni Kustanti, Dian Kusuma, Tezer Kutluk, Evans F Kyei, Grace Kwakyewaa Kyei, Frank Kyei-Arthur, Ville Kytö, Hmwe Hmwe Kyu, Pallavi L C, Adriano La Vecchia, Carlo La Vecchia, Alessio Lachi, Muhammad Awwal Ladan, Abraham K Lagat, Chandrakant Lahariya, Daphne Teck Ching Lai, Anita Lakhani, Tea Lallukka, Judit Lám, Iván Landires, Ariane Laplante-Lévesque, Laura Lara-Castor, Savita Lasrado, Kamaluddin Latief, Areeba Latif, Mahrukh Latif, Jerrald Lau, Paolo Lauriola, Aliyu Lawan, Teniola Lawanson, Harriet L S Lawford, Duc Tin Le, Thao Thi Thu Le, Caterina Ledda, Ivan Lee, Seung Won Lee, Yo Han Lee, James Leigh, Vasileios Leivaditis, Matthew J Lennon, Matilde Leonardi, Elvynna Leong, Chengfeng Li, Hui Li, Jiaying Li, Jie Li, Ming-Chieh Li, Si Li, Wei Li, Weilong Li, Zhaolong Adrian Li, Zhengrui Li, Yanxue Lian, Chen Liao, Stephen S Lim, Jialing Lin, Queran Lin, Shuzhi Lin, Daniel Lindholm, Yuewei Ling, Shai Linn, Haipeng Liu, Jue Liu, Xianliang Liu, Xiaofeng Liu, Xuefeng Liu, Zhe Liu, Zhenyu Liu, Erand Llanaj, Valerie Lohner, José Francisco López-Gil, Stefan Lorkowski, Rafael Lozano, Shanjie Luan, Jailos Lubinda, Taraneh Lucas, Giancarlo Lucchetti, Lisha Luo, Susu Luo, Lei Lv, Miltiadis D Lytras, Ellina Lytvyak, Kevin Sheng-Kai Ma, Zheng Feei Ma, Raymond Saa-Eru Maalman, Kelsey Lynn Maass, Mahmoud Mabrok, Nikolaos Machairas, Monika Machoy, Seyed Ataollah Madinezad, Aurea Marilia Madureira-Carvalho, Pasquale Maffia, Sasikumar Mahalingam, Samatar Abshir Mahamed, Nozad Hussein Mahmood, Alireza Mahmoudi, My Tra Mai, Hao

Mai Xuan, Rituparna Maiti, Omar M Makram, Reza Malekzadeh, Hardeep Singh Malhotra, Ahmad Azam Malik, Farihah Malik, Deborah Carvalho Malta, Mustapha Mangdow, Jyothsna Manikkath, Yosef Manla, Fahmida Mannan, Farheen Mansoor, Marjan Mansourian, Mohammad Ali Mansournia, Lorenzo Giovanni Mantovani, Changkun Mao, Tahir Maqbool, Bishnu P Marasini, Hamid Reza Marateb, Joemer C Maravilla, Adilson Marques, Bernardo Alfonso Martinez-Guerra, Ramon Martinez-Piedra, Daniela Martini, Santi Martini, Francisco Rogerlândio Martins-Melo, Miquel Martorell, Winfried März, Roy Rillera Marzo, Sammer Marzouk, Sugeng Mashudi, Stefano Masi, Yasith Mathangasinghe, Stephanie Mathieson, Alexander G Mathioudakis, Medha Mathur, Neeta Mathur, Rita Mattiello, Richard James Maude, Mahsa Mayeli, Mohsen Mazidi, Antonio Mazzotti, Ikechukwu Innocent Mbachu, Martin McKee, Michael A McPhail, Steven M McPhail, Rishi P Mediratta, Jitendra Meena, Medhin Mehari, Riffat Mehboob, Ravi Mehrotra, Vini Mehta, Tesfahun Mekene Meto, Hadush Negash Meles, Addisu Melese, Satish Melwani, Walter Mendoza, Godfred Antony Menezes, Ritesh G Menezes, Emiru Ayalew Mengistie, Sultan Ayoub Meo, Michelangelo Mercogliano, Atte Meretoja, Tuomo J Meretoja, Tomislav Mestrovic, Chamila Dinushi Kukulege Mettananda, Sachith Mettananda, Mohamed M M Metwally, Adquate Mhlanga, Tomasz Miazgowski, Irmina Maria Michalek, Andrea Michelerio, Hiwot Soboksa Mideksa, Kebadnew Mulatu Mihretie, Ted R Miller, Giuseppe Minervini, Wai-kit Ming, GK Mini, Andreea Mirica, Alireza Mirkheshti, Seyed Ali Mirshahvalad, Mizan Kiros Mirutse, Maryam Mirzaei, Archana Mishra, Vinaytosh Mishra, Sayan Mitra, Chaitanya Mittal, Mohammadreza Mobayen, Shivani Modi, Ahmed Ismail Mohamed, Heba M Mohamed, Jama Mohamed, Mona Gamal Mohamed, Nouh Saad Mohamed, Khabab Abbasher Hussien Mohamed Ahmed, Taj Mohammad, Abdolreza Mohammadi, Mohammad Reza Mohammadi, Abdollah Mohammadian-Hafshejani, Ibrahim Mohammadzadeh, Abdulwase Mohammed, Ammas Siraj Mohammed, Omer Mohammed, Shafiu Mohammed, Suleiman Mohammed, Yahaya Mohammed, Mohammad Mohseni, Tsz-ngai Mok, Amin Mokari-Yamchi, Ali H Mokdad, Sabrina Molinaro, Amirabbas Mollaei, Shaher Momani, Lorenzo Monasta, Amirabbas Monazzami, Himel Mondal, Marco Montalti, Yousef Moradi, Mohammad Moradi-Joo, Maziar Moradi-Lakeh, Paula Moraga, Lidia Morawska, Rafael Silveira Moreira, Mahmoud M Morsy, Reza Mosaddeghi Heris, Jonathan F Mosser, Elias Mossialos, Maha Motavvef, Vincent Mougin, Asma Mousavi, Seyede Zohre Mousavi, Amin Mousavi Khaneghah, Seyed Mohamad Sadegh Mousavi Kiasary, Hagar Lotfy Mowafy, Kimia Mozahheb Yousefi, Rabia Mubarak, Sumaira Mubarik, Steward Mudenda, Faraz Mughal, Syed Aun Muhammad, Muhammad Solihuddin Muhtar, Oscar J Mujica, Sukhes Mukherjee, Sumoni Mukherjee, Amartya Mukhopadhyay, M A Muktadir, Sileshi Mulatu, Francesk Mulita, Chalie Mulugeta, Damaris Felistus Mulwa, Javier Muñoz Laguna, Anjana Munshi, Efren Murillo-Zamora, Christopher J L Murray, Ali Mushtaq, Mubarak Taiwo Mustapha, Sathish Muthu, Saravanan Muthupandian, Claude Mambo Muvunyi, Woojae Myung, Amin Nabavi, Fatemehzahra Naddafi, Ayoub Nafei, Ahamarshan Jayaraman Nagarajan, Mohsen Naghavi, Ganesh R Naik, Gurudatta Naik, Firzan Nainu, Sanjeev Nair, Hastyar Hama Rashid Najmuldeen, Gopal Nambi, Ni Gusti Ayu Nanditha, Vinay Nangia, Jobert Richie Nansseu, Ibrahim A Naqid, Aparna Ichalangod Narayana, Shumaila Nargus, Delaram Narimani Davani, Yvonne Nartey, Bruno Ramos Nascimento, Abdallah Y Naser, Abdulqadir J Nashwan, Hamide Nasiri, Mahmoud Nassar, Zuhair S Natto, Javaid Nauman, Samidi Nirasha Kumari Navaratna, Biswa Prakash Nayak, Shalini Ganesh Nayak, Vinod C Nayak, Shumaila Naz, Athare Nazri-Panjaki, Amanuel Tebabal Nega, Meti T Negassa, Chernet Tafere Negesse, Ionut Negoi, Ruxandra Irina Negoi, Chakib Nejjari, Henok Biresaw Netsere, Marie Ng, Josephine W Ngunjiri, Cuong Tat Nguyen, Dang Nguyen, The Phuong Nguyen, Van Thanh Nguyen, Ambe Marius Ngwa, Robina Khan Niazi, Luciano Nieddu, Yeshambel T Nigatu, Ali Nikoobar, Vikram Niranjan, Abebe Melis Nisro, Chukwudi A Nnaji, Shuhei Nomura, Syed

Toukir Ahmed Noor, Mamoona Noreen, Masoud Noroozi, Jean Jacques Noubiap, Mehran Nouri, Taylor Noyes, Valentine C Nriagu, Chisom Adaobi Nri-Ezedi, Jean Claude Nshimiyimana, Fred Nugen, Mengistu H Nunemo, Nurfatimah Nurfatimah, Dieta Nurrika, Sylvester Dodzi Nyadanu, Felix Kwasi Nyande, Bogdan Oancea, Ramez M Odat, Ismail A Odetokun, Joseph Kojo Oduro, Michael Safo Oduro, Oluwafunmilayo Tosin Ogundeko-Olugbami, Oluwafunmbi Ebenezer Ogunmiluyi, Sarah Oh, Hassan Okati-Aliabad, Akinkunmi Paul Okekunle, Olalekan John Okesanya, Osaretin Christabel Okonji, Oluwaseyi Isaiah Olabisi, Oladotun Victor Olalusi, Matthew Idowu Olatubi, Gláucia Maria Moraes Oliveira, Abdulhakeem Abayomi Olorukooba, Oluseye Olalekan Oludoye, Ronald Olum, Bolajoko Olubukunola Olusanya, Jacob Olusegun Olusanya, Oluwafemi G Oluwole, Folorunsho Bright Omage, Goran Latif Omer, Abidemi E Omonisi, Kanyin Liane Ong, Sandersan Onie, Obinna E Onwujekwe, Oluwaseyi Aina Gbolade Opesemowo, Marcel Opitz, Aksoltan Shyhdurdyevna Oradova, Michal Ordak, Atakan Orscelik, Samuel M Ostroff, John W Ostrominski, Uchechukwu Levi Osuagwu, Olayinka Osuolale, Elham H Othman, Adrian Otoiu, Abdu Oumer, Jerry John Ouner, Amel Ouyahia, Mayowa O Owolabi, Irene Amoakoh Owusu, Oladayo Ayobami Oyebanji, Kolapo Oyebola, Tope Oyelade, Kehinde Adewole Oyeniran, Oyetunde T Oyeyemi, Ilker Ozsahin, Mahesh P A, Kevin Pacheco-Barrios, Alicia Padron-Monedero, Jagadish Rao Padubidri, Dimpal Manilal Paija, Anton Pak, Pramod Kumar Pal, Tamás Palicz, Raffaele Palladino, Tejasri Paluvai, Feng Pan, Sujogya Kumar Panda, Songhomitra Panda-Jonas, Deepshikha Pande Katare, Seithikurippu R Pandi-Perumal, Apurvakumar Pandya, Georgios D Panos, Leonidas D Panos, Ioannis Pantazopoulos, Anca Pantea Stoian, Giovanni Paolino, Mario Virgilio Papa, Ilias Papadimopoulos, Paraskevi Papadopoulou, Peyvand Parhizkar Roudsari, Romil R Parikh, Chulwoo Park, Seoyeon Park, Arpit Parmar, Roberto Passera, Jay Patel, Mitesh Patel, Neel Navinkumar Patel, Sangram Kishor Patel, Satyananda Patel, Shankargouda Patil, Dimitrios Patoulias, Apurba Patra, Venkata Suresh Patthipati, Shrikant Pawar, Shubhadarshini Pawar, Amy E Peden, Paolo Pedersini, Jarmila Pekarcikova, Emmanuel K Peprah, Prince Peprah, Gavin Pereira, Arokiasamy Perianayagam, Simone Perna, Konrad Pesudovs, Pavlo Petakh, Ionela-Roxana Petcu, Olumuyiwa James Peter, Fanny Emily Petermann-Rocha, William A Petri, Hoang Nhat Pham, Hoang Tran Pham, Tung Thanh Pham, Anil K Philip, Michael R Phillips, Zahra Zahid Piracha, Moein Piroozkhkah, Saeed Pirouzpanah, Enrico Pisoni, Evgenii Plotnikov, Roman V Polibin, Ramesh Poluru, Arjun Pon Avudaiappan, Ville T Ponkilainen, Ion Popa, Thantrira Porntaveetus, Sajjad Pourasghary, Reza Pourbabaki, Farzad Pourghazi, Naeimeh Pourtaheri, Sergio I Prada, Jalandhar Pradhan, Rifky Octavia Pradipta, Akila Prashant, Elton Junio Sady Prates, Harsh Priya, Nicola Riccardo Pugliese, Jagadeesh Puvvula, Nameer Hashim Qasim, Ibrahim Qattea, Xiang Qi, Zhipeng Qi, Yanan Qiao, Zahiruddin Syed Quazi, Navid Rabiee, Reza Rabiei, Basuki Rachmat, Raghu Anekal Radhakrishnan, Venkatraman Radhakrishnan, Maja R Radojčić, Negar Radpour, Hadi Raeisi Shahraki, Pracheth Raghuveer, Fakher Rahim, Hawbash Mohammed-Amin Rahim, Sajjad Rahimi, Vafa Rahimi-Movaghar, Fryad Majeed Rahman, Mahbubur Rahman, Md Mosfequr Rahman, Mohammad Meshbahur Rahman, Mosiur Rahman, Amir Masoud Rahmani, Saeed Rahmani, Masoud Rahmati, Ghasem Rahmatpour Rokni, Hakim Rahmoune, Diego Raimondo, Sunil Kumar Raina, Jeffrey Pradeep Raj, Adarsh Raja, Sathish Rajaa, Erta Rajabi, Gunaseelan Rajendran, Judah Rajendran, Vinoth Rajendran, Shaman Rajindrajith, Pushp Lata Rajpoot, Prashant Rajput, Mahmoud Mohammed Ramadan, Majed Ramadan, Kadar Ramadhan, Chitra Ramasamy, Shakthi Kumaran Ramasamy, Zahra Ramezani, Marzieh Ramezani Farani, Robinson Ramírez-Vélez, Juwel Rana, Kirtan Rana, Shailendra Singh Rana, Nemanja Rancic, Smitha Rani, Chythra R Rao, Kumuda Rao, Mithun Rao, Sina Rashedi, Vahid Rashedi, Mohammad-Mahdi Rashidi, Mohammad Aziz Rasouli, Ashkan Rasouli-Saravani, Azad Rasul, Devarajan Rathish, Abdur Rauf, Santosh Kumar Rauniyar, Ilari Rautalin, Ramin Ravangard, David Laith Rawaf, Lal Rawal, Reza Rawassizadeh, Bahman Razi, C

Mahony Reategui-Rivera, Elrashdy Redwan, Aqeeb Ur Rehman, Faizan Ur Rehman, Wajiha Rehman, Lennart Reifels, Rainer Reile, Bhageerathy Reshmi, Stefano Restaino, Luis Felipe Reyes, Mina Rezaei, Nazila Rezaei, Nima Rezaei, Mohsen Rezaeian, Donya Rezazadeh Eidgahi, Taeho Gregory Rhee, Yohanes Andy Rias, Antonio Luiz P Ribeiro, Tércia Moreira Ribeiro da Silva, Jennifer Rickard, Moattar Raza Rizvi, Hermano Alexandre Lima Rocha, João Rocha Rocha-Gomes, Mónica Rodrigues, Thales Philipe Rodrigues da Silva, Jefferson Antonio Buendia Rodriguez, Leonardo Roever, Peter Rohloff, Iftitakhur Rohmah, Susanne Röhr, Megan L Rolfzen, Debby Syahru Romadlon, Kevin T Root, Amirhossein Roshanshad, Gregory A Roth, Kunle Rotimi, Himanshu Sekhar Rout, Hanieh Rouzbahani, Reza Rouzbahani, Adrija Roy, Priyanka Roy, Sharmistha Roy, Shubhanjali Roy, Simanta Roy, Parameswari Royapuram Parthasarathy, Enrico Rubagotti, Susan Fred Rumisha, Michele Russo, Godfrey Mutashambara Rwegerera, Aly M A Saad, Michela Sabbatucci, Maha Mohamed Saber-Ayad, Siamak Sabour, Seyed Kiarash Sadat Rafiei, Basema Ahmad Saddik, Bashdar Abuzed Sadee, Tarannom Sadegh, Ehsan Sadeghi, Erfan Sadeghi, Fatemeh Sadeghi-Ghyassi, Mohd Saeed, Umar Saeed, Maryam Saeedi, Mahdi Safdarian, Sare Safi, Sher Zaman Safi, Rajesh Sagar, Mastooreh Sagharichi, Amene Saghazadeh, Dominic Sagoe, Indranil Saha, Nondo Saha, Fatemeh Saheb Sharif-Askari, Narjes Saheb Sharif-Askari, Kirti Sundar Sahu, Zahra Saif, S Mohammad Sajadi, Md Refat Uz Zaman Sajib, Mirza Rizwan Sajid, Dorsa Salabat, Payman Salamati, Mohamed A Saleh, Leili Salehi, Mahdi Salehi, Marwa Rashad Salem, Aanuoluwa James Salemcity, Sohrab Salimi, Malik Sallam, Hossein Samadi Kafil, Jayami Eshana Samaranayake, Saad Samargandy, Waqas Sami, Yoseph Leonardo Samodra, Abdallah M Samy, Sandeep G Sangle, Elaheh Sanjari, Sathish Sankar, Francesco Sanmarchi, Damian F Santomauro, Itamar S Santos, Lucas H C C Santos, Milena M Santric-Milicevic, Krishna Prasad Sapkota, Sivan Yegnanarayana Iyer Saraswathy, Yaser Sarikhani, Hemen Sarma, Mohammad Sarmadi, Gargi Sachin Sarode, Sachin C Sarode, Benn Sartorius, Arash Sarveazad, Michele Sassano, Mukesh Kumar Sathya Narayanan, Maheswar Satpathy, Reza Sattarpour, Davide Sattin, Mehrdad Savabi Far, Monika Sawhney, Sangeeta Gopal Saxena, Ganesh Kumar Saya, Abu Sayeed, Christophe Schinckus, Art Schuermans, Austin E Schumacher, Aletta Elisabeth Schutte, Ghil Schwarz, David C Schwebel, Falk Schwendicke, Sneha Annie Sebastian, Amin Sedigh, Soraya Seedat, Mario Šekerija, Muthamizh Selvamani, Vimalraj Selvaraj, Yuliya Semenova, Mohammad H Semreen, Fikadu Waltengus Sendeku, Pallav Sengupta, Yigit Can Senol, Subramanian Senthilkumaran, Sadaf G Sepanlou, Edson Serván-Mori, Yashendra Sethi, Seyed Mohammad Seyed Alshohadaei, Abubakar Sha'aban, Mahan Shafie, Arezoo Shafieioun, Shazlin Shaharudin, Muhammad Shahbaz, Samiah Shahid, Syed Ahsan Shahid, Endrit Shahini, Fatemeh Shahrahmani, Hamid R Shahsavari, Masood Ali Shaikh, Muhammad Aaqib Shamim, Farzane Shams, Mehran Shams-Beyranvand, Anas Shamsi, Dan Shan, Shan Shan, Mohd Shanawaz, Amin Sharifan, Javad Sharifi Rad, Avimanu Sharma, Bhoopesh Kumar Sharma, Bunty Sharma, Gaurav Sharma, Kamal Sharma, Ravi Kumar Sharma, Ujjawal Sharma, Vishal Sharma, Shamee Shastry, Maryam Shayan, Babangida Shehu Bappah, Fateme Sheida, Ali Sheidaei, Ali Sheikhy, Rekha Raghuveer Shenoy, Samendra P Sherchan, Shiran Shetty, Fanchao Shi, Fang Shi, Amir Shiani, Belayneh Fentahun Shibesh, Kenji Shibuya, Desalegn Shiferaw, Tariku Shimels, Md Monir Hossain Shimul, Min-Jeong Shin, Rahman Shiri, Aminu Shittu, Ivy Shiue, Nathan A Shlobin, Ambreen Shoaib, Shayan Shojaei, Sina Shool, Seyed Afshin Shorofi, Sunil Shrestha, Suleiman Adeiza Shuaibu, Kerem Shuval, Zahra Siavashpour, Nicole Remaliah Samantha Sibuyi, Emmanuel Edwar Siddig, Ahmed Kamal Siddiqi, Diego Augusto Santos Silva, Luís Manuel Lopes Rodrigues Silva, Padam Prasad Simkhada, Biagio Simonetti, Abhinav Singh, Amit Singh, Balbir Bagicha Singh, Baljinder Singh, Bhim Pratap Singh, Harmanjit Singh, Harpreet Singh, Jasvinder A Singh, Kalpana Singh, Mayank Singh, Narinder Pal Singh, Paramdeep Singh, Puneetpal Singh, Rakesh K Singh, Samer Singh, Satwinder Singh, Surendra Singh, Mukesh Kumar Sinha, Natia Skhvitaridze,

Anna Aleksandrovna Skryabina, David A Sleet, Mahdieh SobhZahedi, Marzieh Soheili, MdSalman Sohel, Somaye Sohrabi, Lencho Kajela Solbana, Solikhah Solikhah, Sameh S M Soliman, Weiyi Song, Aayushi Sood, Prashant Sood, Soroush Soraneh, Reed J D Sorensen, Joan B Soriano, Fernando Sousa, Marco Aurelio Sousa, Ireneous N Soyiri, Ceren Soylu, Michael Spartalis, Chandrashekhar T Sreeramareddy, Shyamkumar Sriram, Prateek Srivastav, Devin Bailey Srivastava, Jeffrey D Stanaway, Muhammad Haroon Stanikzai, Nadine Steckling-Muschack, Blossom Christa Maree Stephan, Aleksandar Stevanović, Sebastian Straube, Jacob L Stubbs, Peter Stubbs, Omer Subasi, Alisha Suhag, Hasnat Sujon, Muritala Suleiman Odidi, Muhammad Suleman, Mark J M Sullman, Anusha Sultan Meo, Haitong Zhe Sun, Jing Sun, Mao-ling Sun, Xiaodong Sun, Xiaohui Sun, Zhong Sun, Zhuanlan Sun, Suraj Sundaragiri, Thanigaivel Sundaram, Johan Sundström, David Sunkersing, Sumam Sunny, Vinay Suresh, Hani Susianti, Chandan Kumar Swain, Dayinta Annisa Syaiful, Lukasz Szarpak, Mindy D Szeto, Sree Sudha T Y, Payam Tabaee Damavandi, Rafael Tabarés-Seisdedos, Fatemeh Sadat Tabatabaei, Seyed Shahaboddin Tabatabaei, Seyyed Mohammad Tabatabaei, Seyed-Amir Tabatabaeizadeh, Shima Tabatabai, Celine Tabche, Mohammad Tabish, Takahiro Tabuchi, Getu Ferenji Tadesse, Farzad Taghizadeh-Hesary, Zanan Mohammed-Ameen Taha, Jabeen Taiba, Shima Tajabadi, Iman M Talaat, Mircea Tampa, Jacques Lukenze Tamuzi, Ker-Kan Tan, Mohammad Tanashat, Haosu Tang, Mohsan Tanveer, Abiyu Abadi Tareke, Sarvenaz Taridashti, Ingan Ukur Tarigan, Mengistie Kassahun Tariku, Saba Tariq, Aigul Yelgondiyevna Tazhiyeva, Tarilate Temedie-Asogwa, Mohamad-Hani Temsah, Masayuki Teramoto, Azimeraw Arega Tesfu, Nahom Worku Teshager, Gizachew A Tessema, Jay Tewari, Alireza Teymouri, Kavumpurathu Raman Thankappan, Rekha Thapar, Ismaeel Tharwat, Rasiah Thayakaran, Muthu Thiruvengadam, Manuel Sebastian Thomas, Wei Tian, Jansje Henny Vera Ticoalu, Madi Tleshev, Marcello Tonelli, Roman Topor-Madry, Mathilde Touvier, Marcos Roberto Tovani-Palone, Khaled Trabelsi, Quynh Thuy Huong Tran, Tam Quoc Minh Tran, Nguyen Tran Minh Duc, Domenico Trico, Indang Trihandini, Tulika Tripathi, Samuel Joseph Tromans, Quynh Xuan Nguyen Truong, Gary Tse, Vasilis-Spyridon Tseriotis, Evangelia Eirini Tsermpini, Lorainne Tudor Car, Munkhtuya Tumurkhuu, Zhouting Tuo, Biruk Shalmeno Tusa, Sok Cin Tye, Stefanos Tyrovolas, Aniefiok John Udoakang, Atta Ullah, Himayat Ullah, Saeed Ullah, Muhammad Umair, Hauwa Onozasi Umar, Muhammad Umar,[‡] Muhammad Umar,[§] Shehu Salihu Umar, Bhaskaran Unnikrishnan, Era Upadhyay, Dipan Uppal, Jibrin Sammani Usman, Kelechi Julian Uzor, Hande Uzunçıbuk, Pascual R Valdez, Mario Valenti, Zahir Vally, Jef Van den Eynde, Joe Varghese, Pavani Varma, Sampara Vasishta, Srivatsa Surya Vasudevan, Alireza Vaysi, Siavash Vaziri, Narayanaswamy Venketasubramanian, Madhur Verma, Massimiliano Veroux, Georgios-Ioannis Verras, Simone Vidale, Mathavaswami Vijayageetha, Simone Villa, Jorge Hugo Villafañe, David Villarreal-Zegarra, Francesco S Violante, Senthil Visaga Ambi, Luciano Magalhães Vitorino, Stein Emil Vollset, Avina Vongpradith, Theo Vos, Mehdi Vosoughi, Elpida Vounzoulaki, Linh Vu, Isidora S Vujcic, Krishna Dhavan Vyas, Henok Toga Wada, Yasir Waheed, Mohd Wahid, Mugi Wahidin, Mandaras Tariku Walde, Megha Walia, Jin-Yi Wan, Fang Wang, Fulin Wang, Junshi Wang, Liang Wang, Qingzhi Wang, Ruixuan Wang, Shu Wang, Wanzhou Wang, Xing Wang, Xuequan Wang, Yan Wang, Yanzhong Wang, Yichen Wang, Youxin Wang, Yuan-Pang Wang, Zhihua Wang, Tanveer A Wani, Mary Njeri Wanjau, Ahmed Bilal Waqar, Muhammad Waqas, John W Ward, Paul Ward, Toyiba Hiyaru Wassie, Ishanka Weerasekara, Fei-Long Wei, Xueying Wei, Andrea Werdecker, Ronny Westerman, Taweewat Wiangkham, Yohanes Cakrapradipta Wibowo, Dakshitha Praneeth Wickramasinghe, Nuwan Darshana Wickramasinghe, Samuel Wiebe, Angga Wilandika, Peter Willeit, Shadrach Wilson, Andrew Awuah Wireko, Charles Shey Wiysonge, Abay Tadesse Woday, Nathnael Abera Woldehana, Axel Walter Wolf, Tewodros Eshete Wonde, Yen Jun Wong, Eve E Wool, Daniel Tarekegn Worede, Abdulhalik Workicho, Minichil Chanie Worku, Ai-Min Wu, Chenkai Wu, Felicia

Wu, James Fan Wu, Jinyi Wu, Peng Wu, Yihun Miskir Wubie, Ratna Dwi Wulandari, Zhijia Xia, Guangqin Xiao, Lishun Xiao, Na Xiao, Wanqing Xie, Site Xu, Suowen Xu, Xiaoyue Xu, Mukesh Kumar Yadav, Vikas Yadav, Mahnaz Yadollahi, Saba Yahoo (Syed), Galal Yahya, Guangcan Yan, Haibo Yang, Yuichiro Yano, Haiqiang Yao, Laiang Yao, Amir Yarahmadi, Habib Yaribeygi, Haya Yasin, Mohamed A Yassin, Sanni Yaya, Pengpeng Ye, Meghdad Yeganeh, Ali Cem Yekdeş, Mohammad Hossein YektaKooshali, Kuanysh A Yergaliyev, Subah Abderehim Yesuf, Saber Yezli, Siyan Yi, Dehui Yin, Paul Yip, Malede Berihun Yismaw, Dong Keon Yon, Naohiro Yonemoto, Seok-Jun Yoon, Mustafa Z Younis, Saideh Yousefi, Abdilahi Yousuf, Chuanhua Yu, Yong Yu, Hui Yuan, Faith H Yuh, Ghazala Yunus, Umar Yunusa, Siddhesh Zadey, Vesna Zadnik, Mubashir Zafar, Manijeh Zaghampour, Emilia Zainal Abidin, Fathiah Zakham, Nazar Zaki, Nelson Zamora, Hussaini Zandam, Kourosh Zarea, Mohammed Zawiah, Mohammed G M Zeariya, Sebastian Zensen, Nejimu Biza Zepro, Eyael M Zeru, Tiansong Zhan, Yongle Zhan, Beijian Zhang, Casper J P Zhang, Haijun Zhang, Julio Min Fei Zhang, Kexin Zhang, Liqun Zhang, Xiaoyi Zhang, Xiu-Hang Zhang, Yunquan Zhang, Zhiqiang Zhang, Sholpan Bolatovna Zhangelova, Hanqing Zhao, Jianhui Zhao, Jiefeng Zhao, Yang Zhao, Zhongyi Zhao, Anthony Zhong, Claire Chenwen Zhong, Jiayan Zhou, Juexiao Zhou, Bin Zhu, Magdalena Zielińska, Mohamed Ali Zoromba, Zhiyong Zou, Rafat Mohammad Zrieq, Liesl J Zuhlke, Lilik Zuhriyah, Alimuddin Zumla, Ahed H Zyoud, Sa'ed H Zyoud, and Shaher H Zyoud.

Drafting the work or revising it critically for important intellectual content

Bhoomadevi A, Mohammad Amin Aalipour, Hasan Aalruz, Hazim S Ababneh, Ukachukwu O Abaraogu, Cristiana Abbafati, Madineh Abbasi, Faezeh Abbaspour, Hedayat Abbastabar, Abdallah H A Abd Al Magied, Samar Abd ElHafeez, Ashraf Nabiel Abdalla, Emad M Abdallah, Barkhad Aden Abdeeq, Nadin M I Abdel Razeq, Ahmed Abdelrahman Abdelgalil, Reda Abdel-Hameed, Michael Abdelmasseh, Wael M Abdel-Rahman, Arman Abdous, Mostafa M Abdrabou, Jeza Muhamad Abdul Aziz, Auwal Abdullahi, Toufik Abdul-Rahman, Aidin Abedi, Armita Abedi, Roberto Ariel Abeldaño Zuñiga, Shehab Uddin Al Abid, Olugbenga Olusola Abiodun, Shady Abohashem, Hassan Abolhassani, Ulric Sena Abonie, Nagah M Abourashed, Mohamed Abouzid, Dmitry Abramov, Lucas Guimarães Abreu, Rana Kamal Abu Farha, Fuad Hamdi A Abuadas, Aminu Kende Abubakar, Nermeen Abu-Elala, Eman Abu-Gharbieh, Sawsan Abuhammad, Ahmad Y Abuhelwa, Hana J Abukhadijah, Niveen ME Abu-Rmeileh, Salahdein Aburuz, Dina Abushanab, Anirudh Balakrishna Acharya, Apurba Acharya, Lisa C Adams, Isaac Yeboah Addo, Oluwafemi Atanda Adeagbo, Isaac Akinkunmi Adedeji, Kamoru Ademola Adedokun, Oluwatobi E Adegbile, Nurudeen A Adegoke, Olumide Thomas Adeleke, Bulcha Guye Adema, Isaac Ayodeji Adesina, Juliana Bunmi Adetunji, Habeeb Omoponle Adewuyi, Usha Adiga, Tanin Adl Parvar, Mohd Adnan, Qorinah Estiningtyas Sakilah Adnani, Prince Owusu Adoma, David Adzrago, Ahmed M Afifi, Clifford Afoakwah, Aanuoluwapo Adeyimika Afolabi, Rotimi Felix Afolabi, Vlad-Adrian Afrăsânie, Saira Afzal, Gizachew Beykaso Agafari, Suneth Buddhika Agampodi, Thilini Chanchala Agampodi, Mahdi Aghaalikhani, Sepehr Aghajanian, Feleke Doyore Agide, César Agostinis Sobrinho, Mahsa Ahadi, Bright Opoku Ahinkorah, Danish Ahmad, Ijaz Ahmad, Khabir Ahmad, Tauseef Ahmad, Waqas Ahmad, Negar Sadat Ahmadi, Ali Ahmed, Ayman Ahmed, Gasha Salih Ahmed, Haroon Ahmed, Junaid Ahmed, Luai A Ahmed, Mehrunnisha Sharif Ahmed, Meqdad Saleh Ahmed, Muktar Beshir Ahmed, Shabbir Ahmed, Sindew Mahmud Ahmed, Syed Anees Ahmed, Gulzhanat Aimagambetova, Marjan Ajami, Hossein Akbarialiabad, Roland Eghoghosoa Akhigbe, Mohammed Ahmed Akkaif, Wole Akosile, Ashley E Akrami, Alaa Al Amiry, Salah Al Awaidy, Ammar Al Homsi, Mohammad Khaled Al Nawayseh, Omar Al Omari, Zain Al Ta'ani, Yazan Al Thaher, Omar Ali Mohammed Al Zaabi, Mohammad Ahmmad Mahmoud Al Zoubi, Tariq A Alalwan, Khurshid Alam, Mostafa Alam, Rasmieh Mustafa Al-Amer, Abebaw Alamrew, Amani Alansari, Fahmi Y Al-Ashwal, Mohammed Albashtawy, Wafa A Aldhaleei, Mohammed S Aldossary, Shereen M

Aleidi, Fentahun Alemnew, Ayman Al-Eyadhy, Fahad D Algahtani, Abdelazeem M Algammal, Khairat Al-Habbal, Nma Bida Alhaji, Samar Al-Hajj, Fadwa Naji Alhalaiqa, Mohammed Khaled Al-Hanawi, Khalid A Alhasan, Ashraf Alhumaidi, Fahad A Alhumaydhi, Amjad Ali, Mohammed Usman Ali, Shahid Ali, Syed Shujait Ali, Waad Ali, Gianfranco Alicandro, Montaha Al-Iede, Morteza Alipour, Samah W Al-Jabi, Moath Saleh Aljohani, Ahmad Alkhatib, Mustafa Alkhawam, Peter Allebeck, Mohammed Z Allouh, Wesam Taher Almagharbeh, Nihad A Almasri, Hesham M Al-Mekhlafi, Omar Almidani, Amr Almobayed, Khaldoon Aied Alnawafleh, Hasan Yaser Alniss, Mahmoud A Alomari, Mohammad R Alosta, Jaber S Alqahtani, Saleh A Alqahtani, Ahmad Rajeh Al-Qudimat, Intima Alrimawi, Sahel Majed Alrousan, Awais Altaf, Alaa B Al-Tammemi, Jaffar A Al-Tawfiq, Malik A Althobiani, Nelson J Alvis-Zakzuk, Hassan Alwafi, Mohammad Al-Wardat, Yaser Mohammed Al-Worafi, Hany Aly, Mohammad Sharif Ibrahim Alyahya, Hosam Alzahrani, Karem H Alzoubi, Reza Amani-Beni, Faten Amer, Bardia Amidi, Amr Amin, Tarek Tawfik Amin, Alireza Amindarolzarbi, Saeed Amini, Ehsan Amini-Salehi, Majid Aminzare, Sohrab Amiri, Dickson A Amugsi, Ganiyu Adeniyi Amusa, Filippos Anagnostakis, Roshan A Ananda, Nazanin Anaraki, Robert Ancuceanu, Deanna Anderlini, David B Anderson, Nguyen Hoang Anh, Abdul-Azeez Adeyemi Anjorin, Samuel Egyakwa Ankomah, Kabilan Annadurai, Sumbul Ansari, Boluwatife Stephen Anuoluwa, Iyadunni Adesola Anuoluwa, Saeid Anvari, Saleha Anwar, Sumadi Lukman Anwar, Shahnawaz Anwer, Anayochukwu Edward Anyasodor, Francis Appiah, Juan Pablo Arab, Hossein Arabi, Jalal Arabloo, Jorge Arias de la Torre, Ghazal Arjmand, Benedetta Armocida, Johan Ärnlöv, Jesu Arockiaraj, Mahwish Arooj, Ashokan Arumugam, Umesh Raj Aryal, Mahsa Asadi Anar, Muhammad Asaduzzaman, Syed Mohammed Basheeruddin Asdaq, Mulu Tiruneh Asemu, Saeed Asgary, Mitra Ashrafi, Milad Ashrafizadeh, Bernard Kwadwo Yeboah Asiamah-Asare, Yuni Asri, Seyyed Shamsadin Athari, Alok Atreya, Zeenah A Atwan, Marcel Ausloos, Abolfazl Avan, Núbia Carelli Pereira Avelar, Adedapo Wasiu Awotidebe, Lemessa Assefa A Ayana, Yusuf Oloruntoyin Ayipo, Seyed Mohammad Ayyoubzadeh, Sina Azadnajafabad, Arian Azadnia, James Mba Azam, Alireza Azarboo, Zelalem Nigussie Azene, Gulrez Shah Azhar, Amirali Azimi, Farya Azimi, Mohd Yusmaidie Aziz, Sadat Abdulla Aziz, Ahmed Y Azzam, Giridhara Rathnaiah Babu, Youngoh Bae, Arvind Bagga, Elahe Baghizadeh, Fereshteh Baghizadeh, Sana Baghizadeh, Khlood K Baghlaf, Ruhai Bai, Mohamed Ibrahem Baklola, Abdulaziz T Bako, Jose Balmori-de-la-Miyar, Mohammadreza Balooch Hasankhani, Ovidiu Constantin Baltatu, Soham Bandyopadhyay, Noel C Barengo, Suzanne Lyn Barker-Collo, Hiba Jawdat Barqawi, Amadou Barrow, MD Abu Bashar, Shahid Bashir, Guido Basile, Quique Bassat, Mohammad-Mahdi Bastan, Abdul-Monim Batiha, Matteo Bauckneht, Mahdis Bayat, Thomas Beaney, Neeraj Bedi, Massimiliano Beghi, Jina Behjati, Bezawit K Bekele, Demeke Mesfin Belay, Melesse Belayneh, Muhammad Bashir Bello, Olorunjuwon Omolaja Bello, Umar Muhammad Bello, Luis Belo, Apostolos Beloukas, Isabela M Bensenor, Samiun Nazrin Bente Kamal Tune, Maria Bergami, Gregory J Bertolacci, Paulo J G Bettencourt, Ajeet Singh Bhadoria, Akshaya Srikanth Bhagavathula, Neeraj Bhala, Buna Bhandari, Pankaj Bhardwaj, Ashish Bhargava, Sonu Bhaskar, Anup Bhat, Priyadarshini Bhattacharjee, Shuvarthi Bhattacharjee, Gurjit Kaur Bhatti, Jasvinder Singh Bhatti, Mohiuddin Ahmed Bhuiyan, Soumitra S Bhuyan, Raluca Bievel-Radulescu, Naif Kandash Binsaleh, Catherine Bisignano, Atanu Biswas, Bijit Biswas, Mohammad Shahangir Biswas, Molalegne Bitew, Bruno Bizzozero-Peroni, Tone Bjørge, Virginia Bodolica, Lucimere Bohn, Obasanjo Afolabi Bolarinwa, Paria Bolourinejad, Sri Harsha Boppana, Hamed Borhany, Mina Borran, Sudipta Bose, Samuel Adolf Bosoka, Alejandro Botero Carvajal, Soufiane Boufous, Christopher Boxe, Dejana Braithwaite, Luisa C Brant, Nicholas J K Breitborde, Susanne Breitner, Hermann Brenner, Maria L Bringas Vega, Julie Brown, Traolach Brugha, Raffaele Bugiardini, Norma B Bulamu, Danilo Buonsenso, Akeem Olayinka Busari, Felix Busch, Yasser Bustanji, Sanjay C J, Rose Cairns, Mehtap Çakmak Barsbay, Daniela Calina, Luciana Aparecida Campos, Ismael

Campos-Nonato, Angelo Capodici, Giulia Carreras, Juan Jesus Carrero, Andrea Carugno, Andre F Carvalho, Felix Carvalho, Márcia Carvalho, Ana Paula Carvalho-e-Silva, Joao Mauricio Castaldelli-Maia, Carlos A Castañeda-Orjuela, Giulio Castelpietra, Ferrán Catalá-López, Alberico L Catapano, Maria Sofia Cattaruzza, Luca Cegolon, Francieli Cembranel, Muthia Cenderadewi, Ester Cerin, Pamela Roxana Chacón-Uscamaita, Chiranjib Chakraborty, Sandip Chakraborty, Joht Singh Chandan, Rama Mohan Chandika, Miyuru Chandradasa, Baskaran Chandrasekaran, Vijay Kumar Chattu, Victoria Chatzimavridou-Grigoriadou, Anis Ahmad Chaudhary, Sirshendu Chaudhuri, Akhilanand Chaurasia, An-Tian Chen, Hana Chen, Haowei Chen, Simiao Chen, Ka Ching Cheung, Nicholas WS Chew, Fatemeh Chichagi, Patrick R Ching, Jesus Lorenzo Chirinos-Caceres, Daniel Youngwhan Cho, William C S Cho, Bryan Chong, Yuen Yu Chong, Hou In Chou, Sreshtha Chowdhury, Hanne Christensen, Sunghyun Chung, Muhammad Chutiyami, Arrigo Francesco Giuseppe Cicero, Cain C T Clark, Fred Cohen, Alyssa Columbus, Joao Conde, Stephen E Congly, Nathalie Conrad, Samuele Cortese, Paolo Angelo Cortesi, Claudia Cosma, Michael H Criqui, Natalia Cruz-Martins, Patricia Cullen, Matthew Cunningham, Zhaoli Dai, Mayank Dalakoti, Koustuv Dalal, Gloria Dalla Costa, Emanuele D'Amico, Roy Arokiam Daniel, Lucio D'Anna, Samuel E Danso, Samuel Demissie Darcho, Latefa Ali Dardas, Chengetai Dare, Sayan Kumar Das, Claudio Alberto Dávila-Cervantes, Nicole Davis Weaver, Dimash Davletov, Alejandro de la Torre-Luque, Edward Christopher Dee, Sindhura Deekonda, Louisa Degenhardt, Paria Dehesh, Andreas K Demetriades, Edgar Denova-Gutiérrez, Tadios Niguss Derese, Ismail Dergaa, Kebede Deribe, Hunegnaw Almaw Derseh, Nikolaos Dervenis, Emina Dervišević, Hardik Dineshbhai Desai, Abraham Aregay Desta, Pradeep Kumar Devarakonda, Syed Masudur Rahman Dewan, Arkadeep Dhali, Mandira Lamichhane Dhimal, Meghnath Dhimal, Marcello Di Pumpo, Diana Dias da Silva, Daniel Diaz, Luis Antonio Diaz, Kimia Didehvar, Lauren K Dillard, Xueting Ding, Sushil Dohare, Klara Georgieva Dokova, Mario D'Oria, Ojas Prakashbhai Doshi, Leila Doshmangir, Menayit Tamrat Dresse, Tim Robert Driscoll, Ashel Chelsea Dsouza, Jiang Du, John Dube, Judy R Dubno, Emeka W Dumbili, Samuel C Dumith, Bruce B Duncan, Oyewole Christopher Durojaiye, Siddhartha Dutta, Osamudiamen Ebohon, Lamiaa Labieb Mahmoud Ebraheim, Mohammad Hossein Ebrahimi, David Edvardsson, Behrad Eftekhari, Foolad Eghbali, Ashkan Eighaei Sedeh, Ebrahim Eini, Michael Ekholuenetale, Rabie Adel El Arab, Abdelfatteh EL Omri, Maysaa El Sayed Zaki, Reza Elahi, Rana Elbeshbeishy, Noha Mousaad Elemam, Ghada Metwally Tawfik ElGohary, Muhammed Elhadi, Mohamed Elhoumed, Omar Abdelsadek Abdou Elmeligy, Mohamed A Elmonem, Rami Elmorsi, Mohamed Hassan Elnaem, Gihan ELNahas, Mohammed Elshaer, Abdelgawad Salah Eltahawy, Christopher Imokhuede Esezobor, Derese Eshetu, Rafaela Cavalheiro do Espírito Santo, Oghenowede Eyawo, Elochukwu Ezenwankwo, Heidar Fadavian, Adeniyi Francis Fagbamigbe, Omotayo Francis Fagbule, Ayesha Fahim, Saman Fahimi, Aamir Fahira, Aliasghar Fakhri-Demeshghieh, Luca Falzone, Ali Faramarzi, Mohammad Fareed, Zaki Farhana, Liliana Faria, MoezAlIslam Ezzat Mahmoud Faris, Andre Faro, Syed Muhammad Yousaf Farooq, Hossein Farrokhpour, Fatemeh Farshad, Farima Farsi, Folorunso Oludayo Fasina, Modupe Margaret Fasina, Ali Fatehizadeh, Davood Fathi, Zareen Fatima, Mohammad Fayaz, Gelana Fekadu, Ulrich Membe Femoe, Talukdar Raian Ferdous, Seyed-Mohammad Fereshtehnejad, Rodrigo Fernandez-Jimenez, Pietro Ferrara, Nuno Ferreira, Getahun Fetensa, Alexander Finnemore, Claudio Fiorilla, Florian Fischer, Ida Fitriana, Federica Fogacci, Morenike Oluwatoyin Folayan, Marco Fonzo, Lisa M Force, Daniela Fortuna, Matteo Foschi, Maryam Fotouhi, Alberto Freitas, Jinming Fu, Takeshi Fukumoto, Ami Fukunaga, Peter Andras Gaal, Muktar A Gadanya, Dominic Dormenyo Gadeka, Márió Gajdács, Yaseen Galali, Silvano Gallus, Balasankar Ganesan, Shivaprakash Gangachannaiah, Xiang Gao, Bashiru Garba, Miguel Garcia-Argibay, David Garcia-Azorin, Zisis Gatzioufas, Prem Gautam, Rupesh K Gautam, Bamba Gaye, Miglas Welay Gebregergis, Miesa Gelchu, Stefano Gelibter, Nsikakabasi Samuel

George, Lemma Getacher, Kalab Yigermal Gete, Fataneh Ghadirian, Arin Ghamkhar, Shakiba Ghasemi Assl, Fariba Ghassemi, Ramy Mohamed Ghazy, Sama Ghoba, Zainab Gholami, Nasim Gholizadeh, Elena Ghotbi, Arun Ghuge, Alessandro Gialluisi, Syed Abdullah Gilani, Tiffany K Gill, Bikash Ranjan Giri, Alem Abera Girmay, Alessandro Girombelli, Laszlo Göbölös, Anil Kumar Goel, Archit Goel, Rajesh Kumar Goel, Lay Hoon Goh, Ali Golestani, Davide Golinelli, Melika Golmohammadi, Wenping Gong, Alessandra C Goulart, Ayman Grada, Simon Matthew Graham, Michal Grivna, Shi-Yang Guan, Giovanni Guarducci, Avirup Guha, Stefano Guicciardi, Sheffali Gulati, Sasidhar Gunturu, Cui Guo, Zhifeng Guo, Bhawna Gupta, Lalit Gupta, Rajeev Gupta, Reyna Alma Gutiérrez, Roberth Steven Gutiérrez-Murillo, Awoke Derbie Habteyohannes, Tesfahun Simon Hadaro, Najah R Hadi, Zahra Hadian, Abdul Hafiz, Faraidoon Haghdoost, Arian Haghtalab, Nguyen Hai Nam, Addisalem Haile, Pritam Halder, Rabih Halwani, Kosar Hikmat Hama Aziz, Islam M Hamad, Randah R Hamadeh, Ahmad Hammoud, Chieh Han, Nasrin Hanifi, Graeme J Hankey, Fahad Hanna, Ashanul Haque, Obaid I Haque, Arief Hargono, Josep Maria Haro, Ahmed I Hasaballah, Faizul Hasan, Md Kamrul Hasan, Towhid Hasan, Ali Hasanpour- Dehkordi, Mohammad Hashem Hashempur, Nada Tawfig Hashim, Abbas M Hassan, Amr Hassan, Ibrahim Nagmeldin Hassan, Nageeb Hassan, Yusuf Hassan Wada, Mahgol Sadat Hassan Zadeh Tabatabaei, Lasanthi Wathsala Hathagoda, Johannes Haubold, Rasmus J Havmoeller, Simon I Hay, Jeffrey J Hebert, Golnaz Heidari, Kamal Hezam, Yuta Hiraike, Nobuyuki Horita, Alamgir Hossain, Lubna Hossain, Md Mahbub Hossain, Md Sabbir Hossain, Mohammad Bellal Hossain, Fatemeh Sadat Hosseini, Sorin Hostiuc, Priya Hotwani, Hanno Hoven, Chengxi Hu, Junjie Huang, Weijun Huang, Waqar Husain, Nawfal R Hussein, Mohamed Ibrahim Husseiny, Luigi Francesco Iannone, Segun Emmanuel Ibitoye, Khalid S Ibrahim, Ramzi Ibrahim, Reem Ibrahim, Umar Idris Ibrahim, Anel Ibrayeva, Fidelia Ida, Olayinka Stephen Ilesanmi, Irena M Ilic, Milena D Ilic, Muhammad Hamza Ilyas, Masoud Imani, Lucius Chidiebere Imoh, Arit Inok, Mujahid Iqbal, Lalu Muhammad Irham, Benni Iskandar, Teresa R Iskander, Md Rabiul Islam, Md Shariful Islam, Sheikh Mohammed Shariful Islam, Farhad Islami, Faisal Ismail, Nahlah Elkudssiah Ismail, Yerlan Ismoldayev, Gaetano Isola, Ihoghosa Osamuyi Iyamu, Louis Jacob, Kathryn H Jacobsen, Mohammadsadegh Jafari, Morteza Jafarinia, Abdollah Jafarzadeh, Haitham Jahrami, Vikash Jaiswal, Sanobar Jaka, Mihajlo Jakovljevic, Mohamed Jalloh, Armaan Jamal, Qazi Mohammad Sajid Jamal, Jazlan Jamaluddin, Jerin James, Hasan Jamil, Safayet Jamil, Roland Dominic G Jamora, Masoud Jamshidi, Shaghayegh JamshidiRastabi, Rajiv Janardhanan, Chinmay T Jani, Esmaeil Jarrahi, Syed Sarmad Javaid, Anita Javanmardi, Sathish Kumar Jayapal, Shubha Jayaram, Ruwan Duminda Jayasinghe, Diptismita Jena, Seogsong Jeong, Bijay Mukesh Jeswani, Vivekanand Jha, John S Ji, Wenyi Jin, Nabi Jomehzadeh, Jost B Jonas, Tamas Joo, Abel Joseph, Nitin Joseph, George Joy, Jacek Jerzy Jozwiak, Mikk Jürisson, Vaishali K, Ali Kabir, Rajendra Kadel, Ashish Kumar Kakkar, Pradnya Vishal Kakodkar, Feroze Kaliyadan, Md Moustafa Kamal, Sivesh Kathir Kamarajah, Rajesh Kamath, Saltanat Kamenova, Arun Kamireddy, Ramat T Kamorudeen, Devanish Narasimhasanth Kamtam, Oleksandr Kamyshnyi, Mona Kanaan, Saddam Fuad Kanaan, Jiseung Kang, Kehinde Kazeem Kanmodi, Suthanthira Kannan S, Rami S Kantar, Debasish Kar, Paschalis Karakasis, Reema A Karasneh, Mohammad Amin Karimi, Mohmed Isaqali Karobari, Tomasz M Karpiński, Adarsh Katamreddy, Joonas H Kauppila, Kanica Kaushal, Foad Kazemi, Sina Kazemian, Swetha N Kempegowda, Salima Kerai, Jessica A Kerr, Vikash Ranjan Keshri, Emmanuelle Kesse-Guyot, Yousef Saleh Khader, Sidra Khalid, Hazim O Khalifa, Anas Husam Khalifeh, Anees Ahmed Khalil, Anita Khalili, Pantea Khalili, Ajmal Khan, Fayaz Khan, Gulfaraz Khan, Iman Waheed Khan, Maseer Khan, Md Abdullah Saeed Khan, Mohammad Jobair Khan, Muhammad Hamza Khan, Muhammad Mueed Khan, Muhammad Umair Khan, Muhammad Umer Khan, Salman Ali Khan, Sumaiya Khan, Yusuf Saleem Khan, Zahid Khan, Vishnu Khanal, Shaghayegh Khanmohammadi, Sameer Uttamaro Khasbage, Zenith Khashim, Khaled

Khatab, Haitham Khatatbeh, Moawiah Mohammad Khatatbeh, Mahalaqua Nazli Khatib, Kavin Khatri, Hamid Reza Khayat Kashani, Khalid A Kheirallah, Sunil Kumar Khokhar, Atulya Aman Khosla, Sepehr Khosravi, Mahmood Khosrowjerdi, Hye Jun Kim, Kwanghyun Kim, Min Seo Kim, Sanjay Kini B, Adnan Kisa, Sezer Kisa, Katarzyna Kissimova-Skarbek, Mika Kivimäki, Abdul Basith KM, Shivakumar KM, Ann Kristin Skrindo Knudsen, Nazarii Kobyliak, Jonathan M Kocarnik, Sonali Kochhar, Michail Kokkorakis, Diana Gladys Kolieghu Tcheumeni, Kairi Kolves, Joyce Komesuor, Aida Kondybayeva, Vladimir Andreevich Korshunov, Oleksii Korzh, Karel Kostev, Parvaiz A Koul, Irene Akwo Kretchy, James-Paul Kretchy, Kewal Krishan, Chong-Han Kua, Ananya Kuanar, Barthelemy Kuate Defo, Mohammed Kuddus, Ilari Kuitunen, Mukhtar Kulimbet, Shweta Kulshreshtha, Dewesh Kumar, Jogender Kumar, Tarun Kumar, Tushar Kumar, Vikash Kumar, Jibin Kunjavara, Setor K Kunutsor, Almagul Kurmanova, Om P Kurmi, Maria Dyah Kurniasari, Krishna Prasad Kurpad, Asep Kusnali, Christina Yeni Kustanti, Dian Kusuma, Tezer Kutluk, Frank Kyei-Arthur, Ville Kytö, Pallavi L C, Adriano La Vecchia, Carlo La Vecchia, Muhammad Awwal Ladan, Chandrakant Lahariya, Daphne Teck Ching Lai, Balzhan Lakanova, Anita Lakhani, Tea Lallukka, Judit Lám, Iván Landires, Berthold Langguth, Ariane Laplante-Lévesque, Savita Lasrado, Kamaluddin Latief, Areeba Latif, Mahrukh Latif, Paolo Lauriola, Aliyu Lawan, Harriet L S Lawford, Eilean Rathinasamy Lazarus, Dai Quang Le, Duc Tin Le, Thao Thi Thu Le, Caterina Ledda, Paul H Lee, Vasileios Leivaditis, Matthew J Lennon, Matilde Leonardi, Elvynna Leong, Negin Letafatkar, Hui Li, Jiaying Li, Jie Li, Si Li, Wei Li, Zhaolong Adrian Li, Zhengrui Li, Yanxue Lian, Stephen S Lim, Queran Lin, Daniel Lindholm, Christine Linehan, Yuewei Ling, Jue Liu, Xianliang Liu, Zhenyu Liu, Erand Llanaj, Michael J Loftus, Valerie Lohner, José Francisco López-Gil, Platon D Lopukhov, Stefan Lorkowski, Giancarlo Lucchetti, Alessandra Lugo, Raimundas Lunevicius, Huaxia Luo, Susu Luo, Miltiadis D Lytras, Ellina Lytvyak, Kevin Sheng-Kai Ma, Zheng Feei Ma, Raymond Saa-Eru Maalman, Mahmoud Mabrok, Nikolaos Machairas, Monika Machoy, Seyed Ataollah Madinezad, Aurea Marilia Madureira-Carvalho, Sasikumar Mahalingam, Samatar Abshir Mahamed, Shakeel Ahmed Ibne Mahmood, My Tra Mai, Rituparna Maiti, Marek Majdan, Abdelrahman M Makram, Omar M Makram, Mohammad-Reza Malekpour, Reza Malekzadeh, Ahmad Azam Malik, Farihah Malik, Deborah Carvalho Malta, Mustapha Mangdow, Jyothsna Manikkath, Marjan Mansourian, Lorenzo Giovanni Mantovani, Changkun Mao, Hamid Reza Marateb, Adilson Marques, Bernardo Alfonso Martinez-Guerra, Ramon Martinez-Piedra, Daniela Martini, Francisco Rogerlândio Martins-Melo, Miquel Martorell, Winfried März, Roy Rillera Marzo, Sammer Marzouk, Sugeng Mashudi, Stefano Masi, Yasith Mathangasinghe, Stephanie Mathieson, Alexander G Mathioudakis, Medha Mathur, Rita Mattiello, Pallab K Maulik, Mahsa Mayeli, Antonio Mazzotti, Ikechukwu Innocent Mbachu, Steven M McPhail, Rishi P Mediratta, Medhin Mehari, Riffat Mehboob, Vini Mehta, Hadush Negash Meles, Satish Melwani, Walter Mendoza, Godfred Antony Menezes, Ritesh G Menezes, George A Mensah, Sultan Ayoub Meo, Michelangelo Mercogliano, Atte Meretoja, Tuomo J Meretoja, Tomislav Mestrovic, Chamila Dinushi Kukulege Mettananda, Sachith Mettananda, Mohamed M M Metwally, Tomasz Miazgowski, Irmina Maria Michalek, Andrea Michelerio, Ted R Miller, Giuseppe Minervini, Wai-kit Ming, Mojgan Mirghafourvand, Alireza Mirkheshti, Archana Mishra, Ashim Mishra, Philip B Mitchell, Sayan Mitra, Chaitanya Mittal, Heba M Mohamed, Mona Gamal Mohamed, Nouh Saad Mohamed, Khabab Abbasher Hussien Mohamed Ahmed, Taj Mohammad, Abdulwase Mohammed, Hussen Mohammed, Omer Mohammed, Shafiu Mohammed, Suleiman Mohammed, Yahaya Mohammed, Tsz-ngai Mok, Ali H Mokdad, Sabrina Molinaro, Amirabbas Mollaei, Lorenzo Monasta, Amirabbas Monazzami, Himel Mondal, Marco Montalti, Yousef Moradi, Mohammad Moradi-Joo, Maziar Moradi-Lakeh, Paula Moraga, Rafael Silveira Moreira, Mahmoud M Morsy, Maha Motavvef, Asma Mousavi, Amin Mousavi Khaneghah, Seyed Mohamad Sadegh Mousavi Kiasary, Hagar Lotfy Mowafy, Kimia Mozahheb Yousefi, Matías

Mrejen, Steward Mudenda, Faraz Mughal, Syed Aun Muhammad, Oscar J Mujica, Sukhes Mukherjee, Amartya Mukhopadhyay, Francesk Mulita, Chalie Mulugeta, Damaris Felistus Mulwa, Javier Muñoz Laguna, Efren Murillo-Zamora, Christopher J L Murray, Ali Mushtaq, Sathish Muthu, Claude Mambo Muvunyi, Amin Nabavi, Fatemehzahra Naddafi, Ayoub Nafei, Ahamarshan Jayaraman Nagarajan, Mohsen Naghavi, Noureddin Nakhostin Ansari, Gopal Nambi, Jobert Richie Nansseu, Ibrahim A Naqid, Shumaila Nargus, Bruno Ramos Nascimento, Gustavo G Nascimento, Abdallah Y Naser, Abdulqadir J Nashwan, Hamide Nasiri, Mahmoud Nassar, Zuhair S Natto, Javaid Nauman, Samidi Nirasha Kumari Navaratna, Biswa Prakash Nayak, Vinod C Nayak, G Takop Nchanji, Sabina Onyinye Nduaguba, Chernet Tafere Negesse, Ionut Negoi, Ruxandra Irina Negoi, Alina Gabriela Negru, Samata Nepal, Georges Nguefack-Tsague, Josephine W Ngunjiri, Cuong Tat Nguyen, Dang Nguyen, Huong-Dung Thi Nguyen, Nghia Phu Nguyen, Van Thanh Nguyen, Ambe Marius Ngwa, Robina Khan Niazi, Luciano Nieddu, Yeshambel T Nigatu, Vikram Niranjan, Abebe Melis Nisro, Mohammadamin Noorafrooz, Mamoona Noreen, Masoud Noroozi, Jean Jacques Noubiap, Valentine C Nriagu, Chisom Adaobi Nri-Ezedi, Jean Claude Nshimiyimana, Fred Nugen, Nurfatimah Nurfatimah, Sylvester Dodzi Nyadanu, Bogdan Oancea, Ramez M Odat, Fabio Massimo Oddi, Ismail A Odetokun, Oluwakemi Ololade Odukoya, Abiola Ogunkoya, Oluwafunmbi Ebenezer Ogunmiluyi, In-Hwan Oh, Sarah Oh, Sylvester Reuben Okeke, Deborah Oluwatosin Okeke-Obayemi, Akinkunmi Paul Okekunle, Olalekan John Okesanya, Osaretin Christabel Okonji, Bolanle Adeyemi Ola, Oladotun Victor Olalusi, Matthew Idowu Olatubi, Arão Belitardo Oliveira, Abdulhakeem Abayomi Olorukooba, Oluseye Olalekan Oludoye, Bolajoko Olubukunola Olusanya, Jacob Olusegun Olusanya, Folorunsho Bright Omage, Abidemi E Omonisi, Sandersan Onie, Obinna E Onwujekwe, John Nelson Opio, Marcel Opitz, Aksoltan Shyhdurdyevna Oradova, Michal Ordak, Verner N Orish, Raffaele Ornello, Atakan Orscelik, Alberto Ortiz, Esteban Ortiz-Prado, Augustus Osborne, John W Ostrominski, Uchechukwu Levi Osuagwu, Olayinka Osuolale, Elham H Othman, Adrian Otoiu, Jerry John Ouner, Amel Ouyahia, Mayowa O Owolabi, Irene Amoakoh Owusu, Oladayo Ayobami Oyebanji, Kolapo Oyebola, Tope Oyelade, Ilker Ozsahin, Mahesh P A, Kevin Pacheco-Barrios, Alicia Padron-Monedero, Jagadish Rao Padubidri, Anton Pak, Yeganeh Pakbaz, Tamás Palicz, Raffaele Palladino, Tejasri Paluvai, Feng Pan, Sujogya Kumar Panda, Songhomitra Panda-Jonas, Deepshikha Pande Katare, Seithikurippu R Pandi-Perumal, Victoria Pando-Robles, Helena Ullyartha Pangaribuan, Leonidas D Panos, Ioannis Pantazopoulos, Anca Pantea Stoian, Giovanni Paolino, Mario Virgilio Papa, Ilias Papadimopoulos, Paraskevi Papadopoulou, Romil R Parikh, Roberto Passera, Jay Patel, Mitesh Patel, Neel Navinkumar Patel, Satyananda Patel, Bharat Smita Umakant Patil, Shankargouda Patil, Dimitrios Patoulias, Venkata Suresh Patthipati, Shrikant Pawar, Shubhadarshini Pawar, Hamidreza Pazoki Toroudi, Amy E Peden, Paolo Pedersini, Veincent Christian Filipino Pepito, João Perdigão, Gavin Pereira, Gladymar Perez Chacon, Arokiasamy Perianayagam, Norberto Perico, Simone Perna, Konrad Pesudovs, Pavlo Petakh, Ionela-Roxana Petcu, Fanny Emily Petermann-Rocha, Hoang Nhat Pham, Hoang Tran Pham, Michael R Phillips, Zahra Zahid Piracha, Edoardo Pirera, Moein Piroozkhkah, Saeed Pirouzpanah, Indrashis Podder, Dimitri Poddighe, Ville T Ponkilainen, Ion Popa, Djordje S Popovic, Sajjad Pourasghary, Reza Pourbabaki, Sergio I Prada, Jalandhar Pradhan, Akila Prashant, Elton Junio Sady Prates, Harsh Priya, Nicola Riccardo Pugliese, Jagadeesh Puvvula, Ibrahim Qattea, Xiang Qi, Zahiruddin Syed Quazi, Navid Rabiee, Raghu Anekal Radhakrishnan, Venkatraman Radhakrishnan, Maja R Radojčić, Negar Radpour, Hadi Raeisi Shahraki, Lida Rafati, Ibrar Rafique, Fakher Rahim, Hawbash Mohammed-Amin Rahim, Sajjad Rahimi, Vafa Rahimi-Movaghar, Mahbubur Rahman, Mohammad Hifz Ur Rahman, Mohammad Meshbahur Rahman, Masoud Rahmati, Ghasem Rahmatpour Rokni, Hakim Rahmoune, Diego Raimondo, Ivano Raimondo, Sunil Kumar Raina, Jeffrey Pradeep Raj, Sathish Rajaa, Gunaseelan Rajendran, Judah

Rajendran, Vinoth Rajendran, Shaman Rajindrajith, Prashant Rajput, Mahmoud Mohammed Ramadan, Kadar Ramadhan, Chitra Ramasamy, Shakthi Kumaran Ramasamy, Zahra Ramezani, Shailendra Singh Rana, Nemanja Rancic, Smitha Rani, Fatemeh - Ranjbar Noei, Chythra R Rao, Kumuda Rao, Mithun Rao, Davide Rasella, Sina Rashedi, Vahid Rashedi, Mamunur Rashid, Mohammad-Mahdi Rashidi, Mohammad Aziz Rasouli, Ashkan Rasouli-Saravani, Devarajan Rathish, Santosh Kumar Rauniyar, Ilari Rautalin, Ramin Ravangard, David Laith Rawaf, Lal Rawal, C Mahony Reategui-Rivera, Elrashdy Redwan, Aqeeb Ur Rehman, Rainer Reile, Giuseppe Remuzzi, Bhageerathy Reshmi, Stefano Restaino, Luis Felipe Reyes, Mina Rezaei, Nima Rezaei, Donya Rezazadeh Eidgahi, Yohanes Andy Rias, Antonio Luiz P Ribeiro, Tércia Moreira Ribeiro da Silva, Jennifer Rickard, Moattar Raza Rizvi, Hermano Alexandre Lima Rocha, João Rocha Rocha-Gomes, Mónica Rodrigues, Thales Philipe Rodrigues da Silva, Jefferson Antonio Buendia Rodriguez, Leonardo Roever, Peter Rohloff, Susanne Röhr, David Rojas-Rueda, Megan L Rolfzen, Debby Syahru Romadlon, Michele Romoli, Luca Ronfani, Kevin T Root, Emily Rosenblad, Amirhossein Roshanshad, Morteza Rostamian, Gregory A Roth, Kunle Rotimi, Hanieh Rouzbahani, Reza Rouzbahani, Jemma V Rowlands, Bedanta Roy, Sharmistha Roy, Shubhanjali Roy, Simanta Roy, Enrico Rubagotti, Susan Fred Rumisha, Michele Russo, Godfrey Mutashambara Rwegerera, Aly M A Saad, Michela Sabbatucci, Maha Mohamed Saber-Ayad, Siamak Sabour, Perminder S Sachdev, Seyed Kiarash Sadat Rafiei, Basema Ahmad Saddik, Bashdar Abuzed Sadee, Tarannom Sadegh, Mohd Saeed, Umar Saeed, Maryam Saeedi, Rajesh Sagar, Mastooreh Sagharichi, Dominic Sagoe, Indranil Saha, Fatemeh Saheb Sharif-Askari, Amirhossein Sahebkar, Kirti Sundar Sahu, Zahra Saif, Md Refat Uz Zaman Sajib, Mirza Rizwan Sajid, Luciane B Salaroli, Leili Salehi, Mahdi Salehi, Marwa Rashad Salem, Mohammed Z Y Salem, Dauda Salihu, Malik Sallam, Jayami Eshana Samaranayake, Saad Samargandy, Abdallah M Samy, Sandeep G Sangle, Elaheh Sanjari, Sathish Sankar, Francesco Sanmarchi, Itamar S Santos, Lucas H C C Santos, Milena M Santric-Milicevic, Adekunle Sanyaolu, Bruno Piassi Sao Jose, Krishna Prasad Sapkota, Yaser Sarikhani, Mohammad Sarmadi, Gargi Sachin Sarode, Sachin C Sarode, Arash Sarveazad, Michele Sassano, Maheswar Satpathy, Reza Sattarpour, Sangeeta Gopal Saxena, Ganesh Kumar Saya, Abu Sayeed, Maria Inês Schmidt, Art Schuermans, Aletta Elisabeth Schutte, Ghil Schwarz, David C Schwebel, Falk Schwendicke, Mario Šekerija, Muthamizh Selvamani, Vimalraj Selvaraj, Yuliya Semenova, Mohammad H Semreen, Yigit Can Senol, Sadaf G Sepanlou, Edson Serván-Mori, Yashendra Sethi, Seyed Mohammad Seyed Alshohadaei, Allen Seylani, Abubakar Sha'aban, Mahan Shafie, Shazlin Shaharudin, Muhammad Shahbaz, Samiah Shahid, Endrit Shahini, Fatemeh Shahrahmani, Moyad Jamal Shahwan, Alireza Shakeri, Ali Shakerimoghaddam, Ali S Shalash, Muhammad Aaqib Shamim, Mehran Shams-Beyranvand, Anas Shamsi, Alfiya Shamsutdinova, Dan Shan, Shan Shan, Mohd Shanawaz, Amin Sharifan, Javad Sharifi Rad, Avimanu Sharma, Bunty Sharma, Gaurav Sharma, Kamal Sharma, Kamlesh Sharma, Manoj Sharma, Ujjawal Sharma, Vishal Sharma, Shamee Shastry, Babangida Shehu Bappah, Rekha Raghuveer Shenoy, Samendra P Sherchan, Fang Shi, Md Monir Hossain Shimul, Reza Shirkoohi, Aminu Shittu, Abdul-karim Olayinka Shitu, Velizar Shivarov, Nathan A Shlobin, Ambreen Shoaib, Shayan Shojaei, Sina Shool, Seyed Afshin Shorofi, Sunil Shrestha, Suleiman Adeiza Shuaibu, Kerem Shuval, Zahra Siavashpour, Emmanuel Edwar Siddig, Ahmed Kamal Siddiqi, Diego Augusto Santos Silva, João Pedro Silva, Luís Manuel Lopes Rodrigues Silva, Padam Prasad Simkhada, Abhinav Singh, Balbir Bagicha Singh, Harmanjit Singh, Jasvinder A Singh, Jawahar Singh, Paramdeep Singh, Poornima Suryanath Singh, Rakesh K Singh, Samer Singh, Satwinder Singh, Surendra Singh, Surjit Singh, Robert Sinto, Dagne Feleke Siyoum, Natia Skhvitaridze, Anna Aleksandrovna Skryabina, David A Sleet, Mahdieh SobhZahedi, MdSalman Sohel, Shipra Solanki, Lencho Kajela Solbana, Solikhah Solikhah, Aayushi Sood, Prashant Sood, Soroush Soraneh, Joan B Soriano, Fernando Sousa, Marco Aurelio Sousa, Ireneous N Soyiri, Ceren Soylu, Michael

Spartalis, Chandrashekhar T Sreeramareddy, Suresh Kumar Srinivasamurthy, Prateek Srivastav, Muhammad Haroon Stanikzai, Nadine Steckling-Muschack, Dan J Stein, Paschalis Steiropoulos, Blossom Christa Maree Stephan, Aleksandar Stevanović, Leo Stockfelt, Sebastian Straube, Peter Stubbs, Omer Subasi, Narayan Subedi, Alisha Suhag, Hasnat Sujon, Thitiporn Sukaew, Surajo Kamilu Sulaiman, Auwal Garba Suleiman, Muritala Suleiman Odidi, Muhammad Suleman, Mark J M Sullman, Anusha Sultan Meo, Mao-ling Sun, Zhuanlan Sun, Suraj Sundaragiri, Thanigaivel Sundaram, Johan Sundström, David Sunkersing, Chandan Kumar Swain, Lukasz Szarpak, Sree Sudha T Y, Payam Tabaee Damavandi, Rafael Tabarés-Seisdedos, Fatemeh Sadat Tabatabaei, Seyed Shahaboddin Tabatabaei, Seyed-Amir Tabatabaeizadeh, Shima Tabatabai, Celine Tabche, Takahiro Tabuchi, Farzad Taghizadeh-Hesary, Zanan Mohammed-Ameen Taha, Iman M Talaat, Mircea Tampa, Jacques Lukenze Tamuzi, Ker-Kan Tan, Mohammad Tanashat, Abiyu Abadi Tareke, Saba Tariq, Aigul Yelgondiyevna Tazhiyeva, Mohamad-Hani Temsah, Azimeraw Arega Tesfu, Alireza Teymouri, Chandan Kumar Thakur, Ismaeel Tharwat, Samar Tharwat, Manuel Sebastian Thomas, Wei Tian, Sojit Tomo, Marcello Tonelli, Roman Topor-Madry, Mathilde Touvier, Marcos Roberto Tovani-Palone, Khaled Trabelsi, Tam Quoc Minh Tran, Thang Huu Tran, Nguyen Tran Minh Duc, Domenico Trico, Manjari Tripathi, Samuel Joseph Tromans, Thien Tan Tri Tai Truyen, Gary Tse, Vasilis-Spyridon Tseriotis, Evangelia Eirini Tsermpini, Lorainne Tudor Car, Sok Cin Tye, Stefanos Tyrovolas, Himayat Ullah, Muhammad Umair, Hauwa Onozasi Umar, Lawan Umar, Shehu Salihu Umar, Eduardo A Undurraga, Bhaskaran Unnikrishnan, Dinesh Upadhya, Era Upadhyay, Dipan Uppal, Daniele Urso, Jibrin Sammani Usman, Hande Uzunçıbuk, Pratyusha Vadagam, Asokan Govindaraj Vaithinathan, Mario Valenti, Zahir Vally, Jef Van den Eynde, Javad Varasteh, Joe Varghese, Pavani Varma, Tommi Juhani Vasankari, Sampara Vasishta, Srivatsa Surya Vasudevan, Alireza Vaysi, Narayanaswamy Venketasubramanian, Madhur Verma, Poonam Verma, Massimiliano Veroux, Georgios-Ioannis Verras, Simone Vidale, Simone Villa, Jorge Hugo Villafañe, Leonardo Villani, David Villarreal-Zegarra, Luciano Magalhães Vitorino, Vasily Vlassov, Stein Emil Vollset, Theo Vos, Mehdi Vosoughi, Linh Vu, Yasir Waheed, Mohd Wahid, Mugi Wahidin, Mandaras Tariku Walde, Arvinder Wander, Fang Wang, Liang Wang, Ruixuan Wang, Shu Wang, Wanzhou Wang, Xing Wang, Xuequan Wang, Yan Wang, Yanzhong Wang, Yichen Wang, Youxin Wang, Yuan-Pang Wang, Tanveer A Wani, Mary Njeri Wanjau, Ahmed Bilal Waqar, Paul Ward, Ishanka Weerasekara, Fei-Long Wei, Robert G Weintraub, Andrea Werdecker, Ronny Westerman, Taweewat Wiangkham, Dakshitha Praneeth Wickramasinghe, Nuwan Darshana Wickramasinghe, Samuel Wiebe, Peter Willeit, Shadrach Wilson, Andrew Awuah Wireko, Charles Shey Wiysonge, Abay Tadesse Woday, Nathnael Abera Woldehana, Dawit Habte Woldeyes, Axel Walter Wolf, Tewodros Eshete Wonde, Yen Jun Wong, Eve E Wool, Minichil Chanie Worku, Ai-Min Wu, James Fan Wu, Jinyi Wu, Peng Wu, Na Xiao, Site Xu, Suowen Xu, Mukesh Kumar Yadav, Vikas Yadav, Saba Yahoo (Syed), Galal Yahya, Kazumasa Yamagishi, Guangcan Yan, Haibo Yang, Laiang Yao, Amir Yarahmadi, Haya Yasin, Yuichi Yasufuku, Sanni Yaya, Pengpeng Ye, Meghdad Yeganeh, Ali Cem Yekdeş, Mohammad Hossein YektaKooshali, Kuanysh A Yergaliyev, Saber Yezli, Siyan Yi, Malede Berihun Yismaw, Dong Keon Yon, Naohiro Yonemoto, Abdilahi Yousuf, Faith H Yuh, Ghazala Yunus, Umar Yunusa, Vesna Zadnik, Mubashir Zafar, Manijeh Zaghampour, Emilia Zainal Abidin, Fathiah Zakham, Giulia Zamagni, Nelson Zamora, Aurora Zanghì, Heather J Zar, Kourosh Zarea, Mohammed Zawiah, Mohammed G M Zeariya, Abay Mulu Zenebe, Sebastian Zensen, Nejimu Biza Zepro, Beijian Zhang, Casper J P Zhang, Haijun Zhang, Julio Min Fei Zhang, Kexin Zhang, Xiaoyi Zhang, Xiu-Hang Zhang, Zhiqiang Zhang, Jianhui Zhao, Jiefeng Zhao, Yang Zhao, Anthony Zhong, Claire Chenwen Zhong, Bin Zhu, Magdalena Zielińska, Ghazal Zoghi, Mohamed Ali Zoromba, Rafat Mohammad Zrieq, Liesl J Zuhlke, Alimuddin Zumla, and Sa'ed H Zyoud.

Managing the estimation or publications process

Joanne O Amlag, Catherine M Antony, Catherine S Chen, Nicole Davis Weaver, Lisa M Force, Ashley Ann Harris, Simon I Hay, Hmwe Hmwe Kyu, Stephen S Lim, Miranda L May, Madeline E Moberg, Ali H Mokdad, Amanda Movo, Christopher J L Murray, Mohsen Naghavi, Olivia D Nesbit, Kanyin Liane Ong, Emily Rosenblad, Gregory A Roth, Rachel D Schneider, Caitlyn Steiner, Vivianne M Swart, Katherine M Wells, and Eve E Wool.
